
JOURNAL INFORMATION
==============================
NLM Title Abbreviation: Intensive Care Med Exp
Journal ID: Intensive Care Med Exp
NLM Journal ID: 101645149

Intensive Care Medicine Experimental

EISSN: 2197-425X
Publisher: Springer

ARTICLE INFORMATION
==============================
PMCID: PMC6195497
PMID: 30341496
DOI: 10.1186/s40635-018-0201-6
Article ID: 201
Article version: 1
Subjects: Meeting Abstracts

ESICM LIVES 2018

Paris, France. 20-24 October 2018

Electronic publication date: 2018 Oct 19
Publication date: 2018 Oct
Volume: 6
Issue: Suppl 2
Issue sponsor: Publication of this supplement has not been supported by sponsorship.
Electronic Location ID: 40
Copyright: © The Author(s). 2018
License: Open AccessThis article is distributed under the terms of the Creative Commons Attribution 4.0 International License (http://creativecommons.org/licenses/by/4.0/), which permits unrestricted use, distribution, and reproduction in any medium, provided you give appropriate credit to the original author(s) and the source, provide a link to the Creative Commons license, and indicate if changes were made.
License URL: https://creativecommons.org/licenses/by/4.0/

Conference name: 31st ESICM Annual Congress
Conference Acronym: ESICM 2018
Conference location: Paris, France
Conference date: 20-24 October 2018
issue-copyright-statement: © The Author(s) 2018

==============================

### Acute respiratory failure, clinical studies

#### 0001

#### Prediction model for nosocomial pulmonary aspiration in patients needing ICU admission

E. Festic[1], V. Bansal[2], M. Zuberi[2], R. Kashyap[2], R. Frank[3], A. Lee[2], O. Gajic[3]
[1]Mayo Clinic, Critical Care, Jacksonville, United States; [2]Mayo Clinic, Jacksonville, United States; [3]Mayo Clinic, Rochester, United States
**Correspondence:** E. Festic
*Intensive Care Medicine Experimental* 2018, **6(Suppl 2):**0001

**INTRODUCTION.** Pulmonary aspiration syndrome is the principal cause of ARDS and nosocomial pneumonia, which increase hospital morbidity, mortality and healthcare costs. Previous attempts to identify the risk factors for overt nosocomial aspiration were not systematic.

**OBJECTIVES.** To derive and validate comprehensive prediction model for overt nosocomial pulmonary aspiration. We hypothesized that such prediction model will show adequate discrimination and calibration in historical patient cohorts.

**METHODS.** A retrospective case-control study of consecutive adults admitted to Mayo Clinic hospital in Rochester, MN from 2006 through 2015. Nosocomial aspiration was defined using a novel electronic medical record-based diagnostic algorithm (termed "aspiration sniffer") based on natural language processing of clinical notes[1]. The patients who screened positive for aspiration and who were either in the ICU within 48 hours before aspiration or required ICU care within 48 hours after aspiration, were enrolled and split into the derivation and the validation cohorts. An equal number of controls were selected from among those who screened negative for aspiration, matching for age, sex, and year of hospitalization. A blinded manual chart review was performed on a random sample to assess the sniffer's accuracy for aspiration/non-aspiration. Predictor variables included a comprehensive list of demographic characteristics, comorbidities, prognostic scores (Charlson, SOFA, APACHE), and clinical variables (all present or have occurred before aspiration). We performed LASSO regression analysis to select the variables for the model. All variables with P< 0.15 contributed to the final multivariable conditional logistic regression model. The model's discrimination was assessed with the receiver operating characteristic curve.

**RESULTS.** Out of 45,115 patients, 917 fulfilled the aspiration enrollment criteria and after adding matched controls were split into the derivation (458 pairs) and validation (459 pairs) datasets. The random-sample manual chart review showed 100% accuracy of the sniffer-based aspiration/non-aspiration assignment. Variables selected for the final model are presented in Table 1. This aspiration prediction model showed very good discrimination. The concordance statistic using the point score for the derivation and validation models were 0.777 (95% CI 0.74, 0.82) and 0.78 (95% CI 0.74, 0.82). Using the best discrimination cutoff of 4.5, sensitivity and specificity were 71.2/72.3 and 72.5/69.9 in the derivation/validation models, respectively.

**CONCLUSIONS.** We derived and validated electronic prediction model for overt nosocomial pulmonary aspiration, programmable for automated identification of hospitalized patients' at risk, in whom potential prevention strategies can be developed and tested.

**REFERENCE(S)**

1. Bansal et al. Retrospective Derivation and Validation of a Computable Phenotype of Nosocomial Aspiration. Crit Care Med 2016;44(12):343.

**Table 1 (abstract 0001).** Conditional Logistic Regression Model of the Selected Model

Conditional Logistic Regression Model of the Selected Model

| Characteristic | Controls N=458 | Cases N=458 | Odds Ratio (95% CI) | p-value |
|---|---|---|---|---|
| **Cardiac Arrhythmia** | | | | **0.086** |
| No | 194 (60.2%) | 128 (39.8%) | 1.00 (ref) | |
| Yes | 264 (44.4%) | 330 (55.6%) | 1.42 (0.95, 2.11) | |
| **Charlson Index** | | | | **<.001** |
| Per 1 unit increase | 3.1 (3.1) | 4.7 (3.7) | 1.12 (1.06, 1.18) *** | |
| **Depression** | | | | **0.048** |
| No | 309 (55.3%) | 250 (44.7%) | 1.00 (ref) | |
| Yes | 149 (41.7%) | 208 (58.3%) | 1.42 (1.00, 2.01) * | |
| **SOFA** | | | | **<.001** |
| Per 1 unit increase | 3.6 (3.0) | 5.6 (3.4) | 1.19 (1.13, 1.25) *** | |
| **Delirium** | | | | **0.007** |
| No | 454 (51.3%) | 431 (48.7%) | 1.00 (ref) | |
| Yes | 4 (12.9%) | 27 (87.1%) | 5.59 (1.61, 19.37) ** | |
| **NG OG Tubes Preceding Aspiration** | | | | **0.006** |
| No | 449 (52.1%) | 413 (47.9%) | 1.00 (ref) | |
| Yes | 9 (16.7%) | 45 (83.3%) | 4.12 (1.51, 11.22) ** | |
| **Schizophrenia and other psychotic disorders** | | | | **0.001** |
| No | 429 (53.0%) | 380 (47.0%) | 1.00 (ref) | |
| Yes | 29 (27.1%) | 78 (72.9%) | 2.56 (1.44, 4.55) ** | |
| | | | | **0.001** |
| **Pneumonia** | | | | |
| No | 415 (48.9%) | 433 (51.1%) | 1.00 (ref) | |
| Yes | 43 (63.2%) | 25 (36.8%) | 0.33 (0.17, 0.64) ** | |
| **Endotracheal tube** | | | | **<.001** |
| No | 345 (46.2%) | 401 (53.8%) | 1.00 (ref) | |
| Yes | 113 (66.5%) | 57 (33.5%) | 0.28 (0.17, 0.47) *** | |
| **Non-invasive Vent** | | | | **0.432** |
| No | 438 (51.3%) | 415 (48.7%) | 1.00 (ref) | |
| Yes | 20 (31.7%) | 43 (68.3%) | 1.38 (0.62, 3.09) | |
| **Pneumonia/Endo Tube Interaction** | | | | **0.015** |
| Not both | 452 (49.9%) | 453 (50.1%) | 1.00 (ref) | |
| Both | 6 (54.5%) | 5 (45.5%) | 8.49 (1.52, 47.32) * | |
| **Endo tube/Non-invasive interaction** | | | | **0.014** |
| Not both | 455 (50.7%) | 442 (49.3%) | 1.00 (ref) | |
| Both | 3 (15.8%) | 16 (84.2%) | 8.44 (1.53, 46.66) * | |

Numbers indicate N (%) for categorical variables and mean (SD) for continuous variables
Multivariable conditional logistic regression model contained all covariates listed in the table.
* P <.05 ** P <.01 *** P <.001

#### 0002

#### Protocol driven management improves outcomes in acute respiratory distress syndrome

A. Duggal[1], A. Panitchote[2], S. Krishnan[2], E. Mireles-Cabodevila[2]
[1]Cleveland Clinic, Critical Care, Cleveland, United States; [2]Cleveland Clinic, Cleveland, United States
**Correspondence:** A. Duggal
*Intensive Care Medicine Experimental* 2018, **6(Suppl 2):**0002

**INTRODUCTION.** Acute respiratory distress syndrome (ARDS) remains under-recognized, under-treated and is associated with a high mortality. There is a potential for improvement in the management of these patients.

Springer Open

**OBJECTIVES.** To study the impact of an evidence based ARDS management on outcomes (28 and 90-day mortality, Ventilator Free days, use of adjunctive).

**METHODS.** A prospective before-after study for patients with ARDS admitted to 5 intensive care units (ICU´s) from 2012-2017 at Cleveland Clinic. An ARDS management protocol addressing multiple domains (adherence to lung protective strategy: tidal volume< 8; Plateau Pressure< 30; PEEP titrated to ARDSnet PEEP: FiO2 ; volume conservative strategies; sedation, analgesia and neuromuscular blocker protocol; prone position ventilation protocol) was developed and launched in 2016 in all the participating ICU´s.

**RESULTS.** 546 patients with ARDS were admitted during the study period. After the implementation of the protocol clinician recognition of ARDS improved significantly (23% vs 9%; P< 0.0001 of mechanically ventilated patients). There was no difference in the demographics, causes of ARDS, co-morbidities, severity of illness score (APACHE III and SOFA) in the before and after group. After the implementation of the protocol there was no difference in the median tidal volume over the first 72 hours (7.4 vs 7.6; p 0.11), but the Plateau Pressure (25 vs 29; p < 0.001) and driving pressure (14 vs 15, P0.004) were significantly lower. Similarly, after the implementation of the protocol PEEP discrepancy from the ARDSnet protocol was lower (-6 vs -7.2; P0.03), minute ventilation was lower (10.7 vs 11.3; p 0.002). Use of adjunctive therapies decreased significantly after the implementation of the protocol (40% vs 46%; p0.01). After the implementation of the Protocol the ventilator free days increased (4.5 vs 0 p< 0.001) and both 28-day (42% vs 54% p0.009) and 90-day (52% vs 66% p 0.002) mortality decreased significantly. We also developed a sub-group analysis for patients with moderate-severe ARDS (PF< 150), and there was significantly higher adherence with lung protective ventilation (p0.001), and PEEP based on ARDSnet recommendations (p0.02). The cumulative fluid balance was significantly lower (4 vs 6.5 liter p0.04) after the implementation of the protocol. Use of adjunctive therapies, and 28 and 90-day mortality remained significantly lower after the implementation of the protocol. A cox proportional hazard model was developed and after adjustment for Charlson index, APACHE III, PF ratio, PEEP and volume status on day 7, the mortality remained significantly lower after the implementation of the ARDS protocol (HR 0.54 (0.38-0.78), P< 0.001).

**CONCLUSIONS.** Implementation of an evidence based protocol improves clinician recognition of ARDS, and is associated with a significant decrease in 28 and 90-day mortality. This approach also decreases the use of adjunctive therapy.

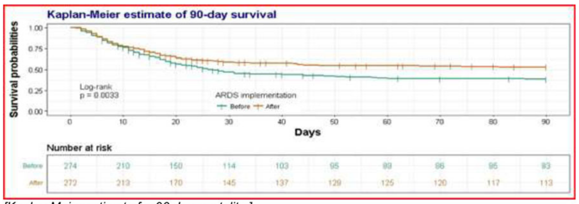

[Kaplan Meier estimate for 90 day mortality ]

**Fig. 1 (abstract 0002).** Kaplan Meier estimate for 90 day mortality

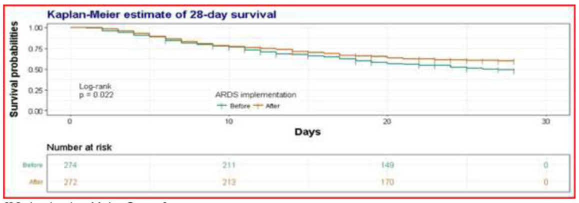

[28 day kaplan Meier Curve ]

**Fig. 2 (abstract 0002).** 28 day kaplan Meier Curve

**Table 1 (abstract 0002).** Baseline characteristics of patients before and after the implementation of ARDS protocol

| Baseline Characteristics | All patients n-546 | Before Protocol n-274 | After Protocol n-272 | p-value |
|---|---|---|---|---|
| APACHE III | 117 (95-142) | 115 (94-142) | 117 (98-142) | 0.53 |
| Charlson comorbidity index | 4 (2-6) | 3 (2-6) | 4 (2-6) | 0.89 |
| Pneumonia | 409 (74.9) | 204 (74.5) | 205 (75.4) | 0.81 |
| Septic shock | 308 (56.5) | 158 (57.7 | 150 (55.4) | 0.59 |
| Acute Kidney Injury | 415 (76) | 214 (78.1) | 201 (73.9) | 0.25 |
| Use of Adjunctive therapies | 233 (42.7 | 125 (45.6) | 108 (39.7) | 0.016 |
| Ventilator-free days to day 28 | 0 (0-16) | 0 (0-13) | 4.5 (0-18) | <0.001 |
| Day 28 Mortality | 245 (48) | 137 (54) | 108 (42) | 0.009 |
| Day 90 Mortality | 285 (59) | 161 (66) | 124 (52) | 0.002 |

**Table 2 (abstract 0002).** Characteristics and outcomes after implementation of ARDS protocol in moderate- severe ARDS

| | All patients (314) | Before (172) | After (142) | P-value |
|---|---|---|---|---|
| APACHE III, median (IQR | 120.5 (31.5) | 119.4 (32) | 121.9 (31) | 0.49 |
| Charlson comorbidities index, median (IQR) | 3 (2-5) | 3 (2-5) | 3 (1-5) | 0.35 |
| Pneumonia | 240 (76.4) | 131 (76.2) | 109 (76.8) | 0.9 |
| AKI | 242 (77.1) | 142 (82.6) | 100 (70.4) | 0.01 |
| Use of Adjunctive Therapy | 163 (51.9) | 92 (53.5) | 71 (50) | 0.54 |
| Ventilator-free days to day 28 | 0 (0-15) | 0 (0-10) | 6 (0-17) | 0.001 |
| Day 28 Mortality | 139 (47) | 89 (56) | 50 (37) | 0.001 |
| Day 90 Mortality | 163 (60 | 104 (68) | 59 (48) | <0.001 |

### 0003

#### Respiratory system compliance, plateau pressure and driving pressure during spontaneous assisted breathing: association with clinical outcomes

S. Sosio[1], A. Grassi[1], S. Gatti[1], M. Polo Friz[1], M. Viganò[1], C. Cunsolo[1], D. Celsi[1], G. Valsecchi[1], G. Bellani[1,2], G. Foti[1,2]
[1]Università degli Studi di Milano Bicocca, Monza, Italy; [2]ASST Monza, Anesthesiology and Intensive Care Medicine, Monza, Italy
**Correspondence:** A. Grassi
*Intensive Care Medicine Experimental* 2018, **6(Suppl 2):**0003

**INTRODUCTION.** Airway Driving Pressure (DP),Plateau Pressure (Pplat) and Respiratory System Compliance (Crs) are known to correlate with patients' outcome during Controlled Mechanical Ventilation (CMV)[1].Monitoring patient respiratory mechanics during Assisted Mechanical Ventilation is more challenging but could help avoiding lung injury.The measurement of DP, Pplat and Crs during Pressure Support Ventilation (PSV) is feasible[2] and routinely performed in our clinical practice but correlation with clinical outcomes has not been investigated yet.

**OBJECTIVES.** We aimed to understand the reliability of respiratory system mechanical properties (DP, Pplat and Crs) measured during PSV and their association with clinical outcomes.

**METHODS.** We conducted a retrospective study on patients undergoing invasive mechanical ventilation for at least 5 days. We included all subjects who spent >24 hours in CMV followed by >24 hours in PSV. We collected from the Electronic Medical record data of the first and last CMV day and averaged the first three days of PSV.We analyzed a) the correlation between measurements taken during CMV and PSV and b) the association between DP, Pplat and Crs values during PSV and clinical outcomes (mortality in ICU and weaning duration in survivors). Data are expressed as mean±SD.

**RESULTS.** We screened 373 patients, 193 of which met the inclusion criteria. Crs measured during the last day of CMV and during the first three days of PSV were correlated ($R^2$=0.63). Crs and DP collected during PSV were significantly different between survivors and non survivors (Crs 54

±17 vs 42±16 ml/cm$H_2O$, DP 9.9±2.2 vs 11.5±3 cm$H_2O$ during PSV in survivors vs non survivors, p< 0.05 for all comparisons). When DP and Crs were partitioned into quartiles, increasing DP and decreasing Crs were correlated with increasing mortality (Fig.1). No correlation with mortality was found for Peak Pressure and Pplat values. We further stratified survivors into two groups according to the weaning duration; higher PEEP (9.5±3 vs 11.6±3.6 cm$H_2O$), Pplat (19.4±3.8 vs 21.6±4.4 cm$H_2O$), Ppeak (Ppeak 17.4 ±4.5 vs 21±4.7 cm$H_2O$) and PS level (7.9±2.5 vs 9.4±2.6 cm$H_2O$) and lower Crs (58±17 vs 52±16 ml/cm$H_2O$) were associated with weaning longer than 7 days (p< 0,05 for all comparisons).

**CONCLUSIONS.** The measurement of DP, Crs and Pplat during PSV is feasible and clinically meaningful. Both DP and Compliance of the respiratory system are strongly associated with mortality in patients undergoing spontaneous assisted breathing.

**REFERENCE(S)**

1. Amato MB et al., *Driving pressure and survival in the acute respiratory distress syndrome.*,N Engl J Med. 2015 Feb 19;372(8):747-55.
2. Bellani G et al., *Do spontaneous and mechanical breathing have similar effects on average transpulmonary and alveolar pressure? A clinical crossover study.*, Crit Care. 2016 Apr 28;20(1):142.

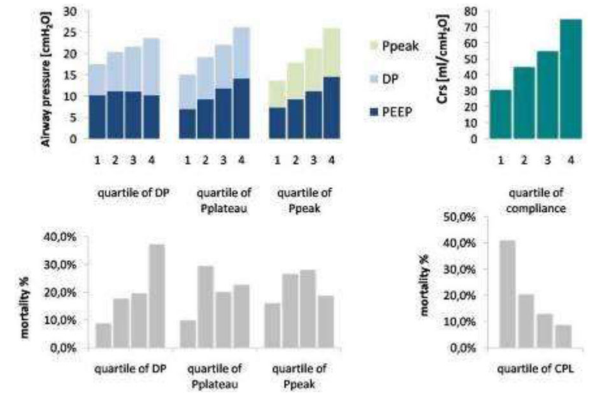

**Fig. 1 (abstract 0003).** Quartiles of DP, Pplat, Ppeak and Crs during PSV and correlation with mortality

### 0004

#### The effect of using airway pressure release ventilation (APRV) mode on physiological dead space in comparison to Biphasic positive airway pressure (BiPAP) mode. A randomized controlled trial

A. Kahwagy, W. Hamimy, M. Elayashy, A. Lotfy, S. Farouk
Faculty of Medicine, Cairo University, Cairo, Egypt
**Correspondence:** A. Kahwagy

*Intensive Care Medicine Experimental* 2018, **6(Suppl 2):**0004

**INTRODUCTION.** APRV is a relatively new mode of mechanical ventilation which can be used in treatment of patients with impaired oxygenation, but this mode is not widely used by clinicians. Physiological dead space (VDphys.) is an important parameter to assess respiratory function which is equal to the the volume of airway plus ventilated non- perfused alveoli. We conducted this study to compare APRV and BiPAP modes regarding VDphys. and other respiratory variables (pressures and blood gases). Our hypothesis is that the long inspiratory phase in APRV may increase VDphys. (by impairing perfusion) and may affect other respiratory variables.

**OBJECTIVES.** To compare APRV and BiPAP modes regarding VDphys., respiratory pressures and blood gases parameters.

**METHODS.** Mechanically ventilated patients with PO2/FiO2 ratio< 300 were randomized into two groups: Group A (APRV group) :APRV mode was used with high pressure (Phi) 20 cmH2O , low pressure (Plo) 5 cmH2O with I:E ratio ( Phi phase: Plo phase ratio ) 4:1 for 3 hours. Group B(BiPAP group) : BiPAP mode was used with high pressure (Phi) 20 cmH2O, low pressure (Plo) 5 cmH2O with I: E ratio (Phi phase: Plo phase ratio) 1:1 for 3 hours. Then all patients were left for ventilation according to the preference of the physician in charge. VDphys. was measured using the metabolic module of GE ventilators. Respiratory variables were also recorded. All measurements were done at 30 minutes and at the end of the intervention period (3 hours).

**RESULTS.** Sixty patients were available for final analysis (30 in each group). There was no statistically significant difference in age (51.7±15.2 vs 54.3 ±16.1, p=0.52) and weight (75.80±13.28 vs 76.57±15.93, p=0.840) between the two groups. VDphys/tidal volume (VT) at 30 minutes was (0.47 ±0.09 vs 0.46 ± 0.08, P=0.61) and at 3 hours was (0.44 ± 0.10 vs 0.43 ± 0.11 , p=0.67) in the two groups respectively. there was a reduction in VDphys/VT within each group at 3 hours but it was not statistically significant (p=0.11 for group A and 0.13 for group B). There was improvement of dynamic compliance within each group (36.17 ± 9.1 vs 39.7±8.33, p=0.002 for group A) and (37.87 ± 7.84 vs 42.37 ± 7.84, p=0.001 for group B). $PCO_2$ was increased in group B (39.17 ± 8.72 vs 42.67 ± 7.73, p=0.013) but it was not clinically significant.

**CONCLUSIONS.** APRV mode didn't increase physiological dead space compared to BiPAP mode. Both modes improved dynamic compliance.

### 0005

#### Nebulisation during nasal high flow therapy: a randomized clinical trial

F. Réminiac[1], V. Gissot[2], L. Vecellio[3], L. Plantier[4], S. Ehrmann[5]
[1]CHRU de Tours, Université de Tours, Médecin Intensive Réanimation, Centre d'Etude des Pathologies Respiratoires INSERM U1100, Tours, France; [2]CHRU de Tours, CIC 1415 INSERM, Tours, France; [3]Université de Tours, Centre d'Étude des Pathologies Respiratoires, INSERM U1100, Tours, France; [4]CHRU de Tours, Université de Tours, Pneumologies et Explorations Fonctionnelles Respiratoires, Centre d'Étude des Pathologies Respiratoires INSERM U1100, Tours, France; [5]CHRU de Tours, Université de Tours, Médecin Intensive Réanimation, Centre d'Etude des Pathologies Respiratoires INSERM U1100, CIC 1415 INSERM, Tours, France
**Correspondence:** S. Ehrmann

*Intensive Care Medicine Experimental* 2018, **6(Suppl 2):**0005

**INTRODUCTION.** *In vitro* and animal studies of nebulisation via nasal high flow (NHF) therapy circuits showed theoretical delivery of drug amounts to patients compatible with a significant bronchodilatatory effect.

**OBJECTIVES.** To compare bronchodilatory effect observed after beta-2-adrenergic agonist nebulisation via a NHF circuit and standard facemask nebulisation in adult patients.

**METHODS.** Patients with recently (< 3 weeks) documented reversible airflow obstruction received 2.5 mg albuterol nebulised via 3 methods. 1. NHF circuit (vibrating mesh nebuliser placed upstream of the humidification chamber of a Fisher & Paykel Airvo®2 device, 30 L/min gas flow); 2. a standard facemask jet nebuliser and 3. sham nebulisation via NHF (no drug nebulised), in a blinded random order. Pulmonary function tests were performed before and after each session.

**RESULTS.** Twenty four patients were included. No side effects were observed, and tolerance was good in both active and sham nebulisation delivery. 15 patients (62%) showed significant reversibility after nebulisation via the NHF circuit (forced expiratory volume in one second ($FEV_1$) increase > 12% and/or > 200 mL): median increase 340 mL (110; 380), 18% (7; 23). Of the remaining 9 patients; two patients showed significant reversibility to standard facemask nebulisation only and 7 did not show reversibility to either active nebulisation. Seventeen patients (70%) showed no reversibility after sham nebulization via the NHF circuit. $FEV_1$ improvement after active nebulization via the NHF circuit and after standard facemask nebulisation were well correlated (rho 0.73, *p*=.0005).

**CONCLUSIONS.** Nebulisation of 2.5 mg albuterol via NHF is feasible and may produce clinically significant bronchodilation among patients with reversible airflow obstruction similar to the effects observed after standard facemask nebulisation.

**REFERENCE(S)**

1. Réminiac F, Vecellio L, Loughlin RM, Le Pennec D, Cabrera M, Vourc'h NH, Fink JB, Ehrmann S. Nasal high flow nebulization in infants and toddlers: an in vitro and in vivo scintigraphic study. Pediatr Pulmonol 2017;52:337-344

3. Réminiac F, Vecellio L, Heuzé-Vourc'h N, Petitcollin A, Respaud R, Cabrera M, Le Pennec D, Diot P, Ehrmann S. Aerosol therapy in adults receiving high flow nasal cannula oxygen therapy. J Aerosol Med Pulm Drug Deliv 2016;29:134-141

## Cardiovascular experimental studies

### 0006

#### Comparison of global enddiastolic volume index (GEDVI), extravascular lung water index (EVLWI) and cardiac index (CI) derived from transpulmonary thermodiltion (TPTD) during high flow vs. low/no flow ECMO: the HEUREKA-III animal study

W. Huber, M. Konrad, S. Kammerzell, R. Schmid, A. Herner
Klinikum rechts der Isar; Technical University of Munich, Medizinische Klinik und Poliklinik II, Munich, Germany
**Correspondence:** W. Huber
*Intensive Care Medicine Experimental* 2018, **6(Suppl 2):**0006

**INTRODUCTION.** Accurate measurement of CI to optimize the choice of cannulas and ECMO-flow might increase the effectiveness of ECMO. Furthermore, markers of preload (GEDVI) and pulmonary oedema (EVLWI) might help to improve patient selection for ECMO and fluid management. However, there are concerns regarding a potential loss of indicator (LOI) into the ECMO-circuit during indicator injection into right atrium for TPTD and pulmonary arterial thermodilution.

**OBJECTIVES.** We hypothesized that in particular the derivation of GEDVI and EVLWI might be prone to loss of indicator (LOI). We expected the highest risk of LOI during *high* ECMO-flows, whereas previous data suggest that there is little risk of LOI at low flow (≤0.5L/min). Therefore, the HEUREKA-III-study compared (primary endpoint) GEDVI, EVLWI and CI during maximum ECMO-flow vs. low or no ECMO-flow (GEDVI_max, GEDVI_base etc.).

**METHODS.** 8 pigs (49.4±4.2kg) with a jugular CVC and femoral artery PiCCO catheter (Pulsion, Germany). Veno-venous ECMO with CARDIOHELP (Maquet/Getinge; Rastatt, Germany) via a femoral drainage and a jugular return cannula. In each animal several sessions with increasing and decreasing ECMO-flow (0-3.5L/min) were performed.

Due to different endpoints, several modifications were performed for the HEUREKA-studies I-IV including variations in the catheter positions, injectate sites and pharmacological modulations of CI by ß-blockers or dobutamine. For the HEUREKA-III study only the first session with increasing ECMO-flow up to the maximum and only sessions with constant conditions except the ECMO-flow were included.

Finally, a total of 13 sessions could be analyzed.

All indicator injections for TPTD were performed via a jugular CVC. Triplicate TPTDs with 15mL of ice cold saline 0.9mL were performed during no/low-flow and during maximum ECMO-flow.

Wilcoxon-test for paired samples. Spearman correlation. SPSS 4

**RESULTS.** ECMO-flow was significantly (p=0.001) increased from 0.12 ±0.15 (0-0.5) L/min to a maximum flow of 3,0±0.56 (2.0-3.5) L/min. GEDVI_max and GEDVI_base were not significantly different (411±76 vs. 423±49mL/m²; p=0.507). Changes in GEDVI ranged from -111 to +65mL/m². Similarly, EVLWI did not change during maximum ECMO-flow (8.6±1.4 vs. 8.5±1.5mL/kg; p=0.589) with differences of ±2mL/kg. Finally, there were no changes in CI (3.69±1.25 vs. 3.58±1.44L/min/m²; p=0.638) with differences between -1.68 and +1,80L/min/m². Changes in GEDVI, EVLWI and CI were neither associated with baseline values of GEDVI, EVLWI and CI nor with the maximum ECMO-flow.

**CONCLUSIONS.**

1.) Our data suggest that in pigs equipped with a femoral drainage and a jugular vein return cannula for ECMO with a maximum flow of 3.5L/min does not result in LOI, if a jugular CVC and a femoral arterial catheter are used for TPTD.
2.) Further studies and techniques are required to detect and quantify a potential LOI in case of other catheter settings and higher ECMO flow-rates.

### 0007

#### Performance comparison of central and peripheral $Ea_{dyn}$ for the evaluation of the coupling between left ventricle and arterial system

M.I. Monge García[1], J. Zhongping[2], F. Hatib[2], J. Settels[2], M. Cecconi[3], M.R. Pinsky[4]
[1]Hospital SAS de Jerez, Intensive Care Unit, Jerez de la Frontera, Spain; [2]Edwards Lifesciences, Irvine, United States; [3]St. George's Healthcare NHS Trust, Department of Intensive Care Medicine, London, United Kingdom; [4]University of Pittsburgh School of Medicine, Department of Critical Care Medicine, Pittsburgh, United States
**Correspondence:** M.I. Monge García
*Intensive Care Medicine Experimental* 2018, **6(Suppl 2):**0007

**INTRODUCTION.** The relationship between arterial pressure and stroke volume changes during a respiratory cycle (pulse pressure variation, PPV; and stroke volume variation, SVV; respectively) has been termed dynamic arterial elastance ($Ea_{dyn}$)[1]. Since, $Ea_{dyn}$ describes the interaction between the flow generated by the heart and the properties of the arterial system, it has been postulated that this parameter could represent an index of the coupling between the heart and the arterial circulation[1].

**OBJECTIVES.** We aimed to confirm that $Ea_{dyn}$ is a ventriculo-arterial coupling (VAC) index, affected by contractility and the arterial system properties. For this purpose, we use the gold-standard as reference for defining the contractility and afterload, and for investigating VAC.

**METHODS.** Experimental study in 11 pigs. LV pressure-volume data was obtained with a conductance catheter and peripheral arterial pressures were measured via a fluid-filled catheter in the aorta and radial artery. The slope of the end-systolic pressure volume relationship or Ees (a load independent-indicator of myocardial contractility), was calculated during a transient occlusion of the inferior vena cava. Effective arterial elastance (Ea, a global index of afterload) was calculated as the ratio LV end-systolic pressure / stroke volume. VAC was defined as the ratio Ea/Ees. LV efficiency was defined as the ratio between stroke work (SW) and the pressure-volume area (PVA). $Ea_{dyn}$ was calculated as the ratio between the aortic PPV and conductance-derived SVV, and radial $Ea_{dyn}$ as the ratio between arterial pressure waveform analysis-derived SVV and radial PPV. The experimental protocol consisted in three consecutive phases with two opposite interventions each: changes in afterload (phenylephrine and nitroprusside), preload (bleeding and fluid bolus), and contractility (esmolol and dobutamine). Measurements were obtained before and after each hemodynamic intervention. The impact of different components of VAC (Ees, Ea, Ea/Ees) and LV efficiency on central $Ea_{dyn}$ and radial $Ea_{dyn}$ was assessed using a mixed-effect regression analysis and reported as their estimated fixed effect.

**RESULTS.** VAC, LV efficiency, and $Ea_{dyn}$ variations and throughout the study are shown in Figure 1. The estimated fixed effects for all components of VAC and LV efficiency on central and radial $Ea_{dyn}$ are detailed in Table 1. Dynamic arterial elastance was inversely related with VAC and directly related with LV efficiency. The higher the Eadyn, the higher the LV efficiency and the lower the VAC.

**CONCLUSIONS.** Eadyn is a ventriculo-arterial coupling parameter that is directly related to LV efficiency and inversely related to VAC.

**REFERENCE(S)**

1. Pinsky MR. Protocolized cardiovascular management based on ventricular-arterial coupling. In: Pinsky MR, Payen D, editors. Functional hemodynamic monitoring. Update in Intensive Care Medicine. Berlin: Springer-Verlag; 2006. p. 381-95.

**Table 1 (abstract 0007).** Estimated values of fixed effects on central $Ea_{dyn}$ and radial APWA-derived $Ea_{dyn}$ according to a linear mixed analysis

| | Central $Ea_{dyn}$ | | APWA-derived $Ea_{dyn}$ | |
|---|---|---|---|---|
| Fixed effect | Estimate (SE) | P value | Estimate (SE) | P value |
| Ea, mmHg/ml | -1.15 (0.16) | <0.0001 | -0.20 (0.09) | 0.025 |
| Ees, mmHg/ml | 1.81 (0.29) | <0.0001 | 0.25 (0.20) | 0.137 |
| VAC | -0.34 (0.05) | <0.0001 | -0.06 (0.02) | 0.014 |
| LV mechanical efficiency | 3.04 (0.36) | <0.0001 | 0.42 (0.20) | 0.038 |

Estimates reflect the average change in $Ea_{dyn}$ per unit increase of fixed effect.
$Ea_{dyn}$: dynamic arterial elastance; APWA: arterial pressure waveform analysis Ea: effective arterial elastance; Ees: end-systolic elastance; VAC: ventriculo-arterial coupling; LV: left ventricle.

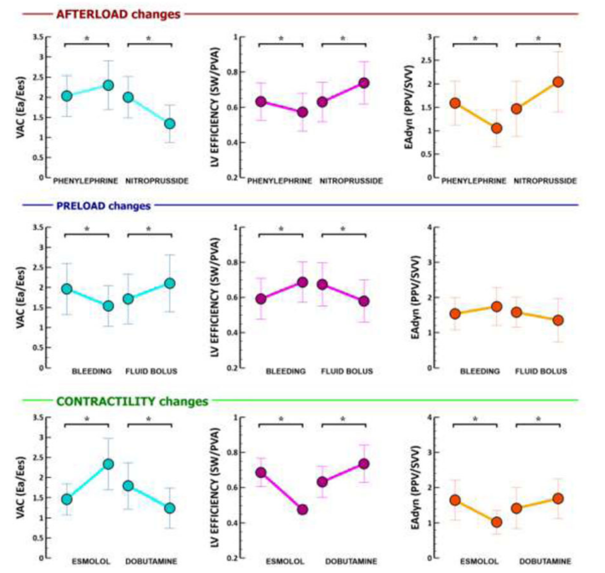

**Fig. 1 (abstract 0007).** See text for description.

### 0008

#### Implication of Vasopressin in the vascular response to myocardial infarction and cardiogenic shock in a rat model

P. Gaudard[1,2], H. David[1,2], P. Sicard[2], P. Bideaux[2], S. Richard[2], P. Colson[1], A. Virsolvy[2]
[1]CHU Montpellier Arnaud de Villeneuve, Anaesthesiology and Critical Care Medicine, Montpellier, France; [2]University of Montpellier, PhyMedExp INSERM U 1046 CNRS UMR 9214, Montpellier, France
**Correspondence:** P. Gaudard
*Intensive Care Medicine Experimental* 2018, **6(Suppl 2):**0008

**INTRODUCTION.** Acute heart failure (AHF) due to acute myocardial infarction (AMI) is likely to involve cardiogenic shock (CS), with neurohormonal activation, when early revascularization is not efficient. An increase in circulating vasopressin (VP) has been associated with poor prognosis in AHF (1). Thus, in CS, vasopressin system may be involved in microcirculation alteration leading to inadequate tissue oxygenation.

**OBJECTIVES.** The aim of the experimental study was to investigate the implication of vasopressin in the alterations of circulation and tissue oxygenation observed in AMI with suspected cardiogenic shock.

**METHODS.** This animal study was approved by the local ethics committee (CEEA-LR). Surgical left coronary artery ligation was performed (AMI, n=39) or not (Sham, n=21) on male Wistar rats treated (AMI-SR, n=6 and Sham-SR, n=6) or not with the VP antagonist SR-49059. Animals were sacrificed one day after surgery. Echocardiography was performed to determine ejection fraction (EF) and to validate AHF. Mesenteric oxygenation (MO) was measured by percutaneous abdominal near infrared spectroscopy to estimate tissue oxygenation (INVOS™ 5100C, Medtronic) and was expressed as absolute values or normalized to the basal MO measured before surgery. Plasma lactate and copeptin levels were determined on the day of sacrifice. The contractile responses to VP were evaluated *ex vivo* on aortic rings by measuring variations of isometric tensions. Results expressed as mean ±SEM were compared between AMI with EF< 40% and Sham; 14 AMI animals had an EF< 40%.

**RESULTS.** In Sham and AMI groups, mortality rates were respectively 4 and 36%. The treatment with SR-49059 prevented mortality in AMI as no death was noticed in this group (AMI-SR). We observed that MO dropped immediately after surgery in all groups and fully or partially recovered except in AMI as illustrated in Fig1. MO absolute values increased from 31 ±1.1 to 38±2.4% in Sham (p=0.02), from 30±0.5 to 38±2.2% in Sham-SR (p=0.01) and from 30±1.4 to 38±2.0% in AMI-SR (p=0.01) while in AMI MO remained low (29±0.9 to 32±1.4%, p=0.16). Lactate and copeptin levels were respectively higher in AMI group when compared to Sham (2.9±0.3 vs 2.0±0.2 mmol/l, p=0.01; 637±94 vs 384±47 pmol/l, p=0.01). Furthermore, the contractile response of aorta to VP was impaired in AMI (p=0.04) with a decreased sensitivity consistent with a reduced availability of receptors (Fig2).

**CONCLUSIONS.** CS after AMI, associated with an increase in plasma lactate level, was characterized by a high mortality rate and a prolonged decrease in mesenteric oxygenation which were prevented by treatment with a vasopressin antagonist (SR-49059). A higher level of copeptin reflected an acute increase in VP secretion in severe AMI that may explain the alteration of arterial contractile response to vasopressin and indicate a critical role of vasopressin system in CS.

**REFERENCE(S)**

1. Khan SQ, et al. Circulation 2007; 115:2103-10.

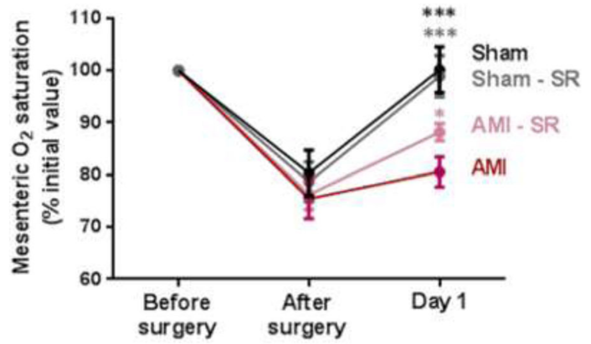

**Fig. 1 (abstract 0008).** Effect of AMI on mesenteric oxygenation (% of basal value)

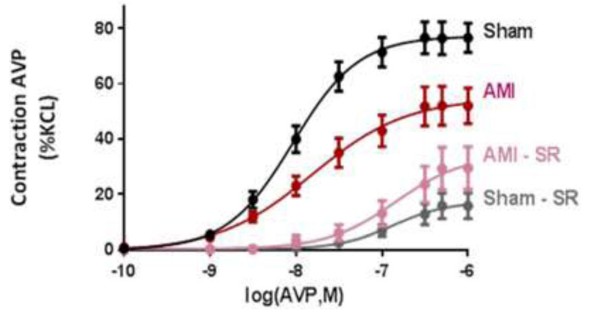

**Fig. 2 (abstract 0008).** Vasopressin-induced contractile response of aorta (% of maximal response to KCl)

### 0009

#### Acute heart failure and renal resistive index assessed by Doppler: application to a murine model

A. Blet[1], B. Deniau[1], P.-R. Koundé[2], P. Bonnin[1], J.-L. Samuel[1], A. Mebazaa[1]
[1]U942 INSERM, Paris, France; [2]Paris Diderot University, Paris, France
**Correspondence:** A. Blet
*Intensive Care Medicine Experimental* 2018, **6(Suppl 2):**0009

**INTRODUCTION.** Heart failure (HF) is a common condition that affects more than 10% of people over the age of 65[1]. Whatever the form, acute or chronic, kidney injury is common during HF[1]. An increase in the renal resistance index (RRI) is correlated with the evolution, severity and mortality of HF[2].

**OBJECTIVES.** The objective of this study is to describe, for the first time, the change of RRI in an acute heart failure (AHF) model in mice.

**METHODS.** The study was approved by the local ethics committee (S84/CNREEA#9). AHF was induced by 4 injections of subcutaneous isoproterenol for 2 consecutive days[3] and was defined by a decrease in the shortening fraction of $\geq$ 10% compared to the baseline value. The RRI was defined by the formula: RRI=(systolic peak - diastolic peak)/ systolic peak. The RRI was measured on the right renal artery 3 times and averaged. A first RRI assessment was performed before the induction of the AHF, then a second 24 hours after the last isoproterenol injection. RRI assessments were compared by a paired Wilcoxon test (GraphPad Prism 5.0). A p value < 0.05 was considered significant.

**RESULTS.** 17 C57Bl6 mice were included. 24 hours after the last isoproterenol injection, all mice had AHF with systolic failure. The RRI was unchanged after AHF induction (0.73 ± 0.12 vs 0.70 ± 0.16, p=0.89) as well as mean velocity (16.68 ± 5.98 vs 17.56 ± 2.51 cm/s, p=0.84). The systolic and diastolic peaks of the right renal artery were unchanged (67.82 ± 26.55 vs 53.90 ± 18.33 cm/s, p=0.34 and 16.42 ± 6.18 vs 13.73 ± 4.76 cm/s, p=0.10). Cardiac output was significantly increased (17.73 ± 13.61 vs 26.61 ± 14.97 ml/min, p< 0.05). Heart rate was significantly decreased after induction of AHF (560 ± 73 vs 445 ± 78 bpm, p< 0.05). Table 1 summarizes the change of these parameters after induction of AHF by isoproterenol in mice.

In our study, the RRI was not altered after induction of AHF by isoproterenol injection in mice. The RRI assessed by Doppler during the experimental AHF could be an additional tool to study renal injury in mice. The initial objective of our study was to describe the feasibility of this technique in this model.

**CONCLUSIONS.** Kidney injury is one of the most common organ dysfunctions during AHF. The RRI could be an additional tool in small animals. Our study shows that this technique is feasible in mice with acute heart failure. Further studies are needed to demonstrate the validity of this marker during acute AHF after isoproterenol injection.

**REFERENCE(S)**

1. European Heart Journal ; 2016 ; 37(27) : 2129-2200
2. Nephrol Dial Transplant ; 2011 ; 26(12) : 3908-13
3. Hypertension ; 2016 ; 67 :606-12

**GRANT ACKNOWLEDGMENT**

**Table 1 (abstract 0009).** Change of main cardiac and renal hemodynamics parameters after acute heat failure induction by isoproterenol in WT mice. ns=non-significant

| | Evolution of hemodynamic parameter after AHF induction | p |
|---|---|---|
| Renal resistive index | ↘ | ns |
| Left renal artery peak systolic velocity | ↘ | ns |
| Left renal artery peak diastolic velocity | ↘ | ns |
| Left renal artery mean velocity | ↘ | ns |
| Cardiac output | ↗ | <0.05 |
| Heart rate | ↘ | <0.05 |

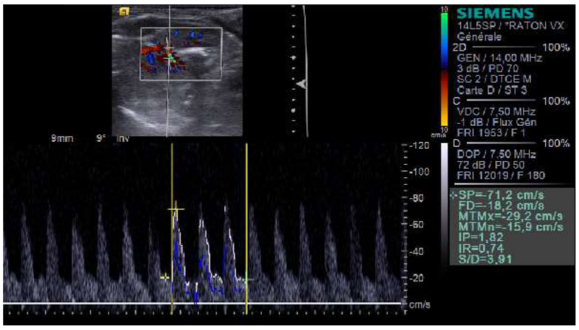

**Fig. 1 (abstract 0009).** A. Left renal artery pulsed Doppler in WT mouse

### 0010

#### Effects of hyperoxia on vascular tone in animal models: systematic review and Meta-analysis

B. Smit[1], Y. Smulders[1], E. Eringa[2], H. Oudemans - van Straaten[1], A. Girbes[1], K. Wever[3], C. Hooijmans[3], A. Spoelstra - de Man[1]
[1]VU University Medical Center, Intensive Care, Amsterdam, Netherlands;
[2]VU University Medical Center, Physiology, Amsterdam, Netherlands;
[3]Radboudumc, Health Evidence, Nijmegen, Netherlands
**Correspondence:** B. Smit
*Intensive Care Medicine Experimental* 2018, **6(Suppl 2):**0010

**INTRODUCTION.** Arterial hyperoxia may induce vasoconstriction and reduce cardiac output, which is particularly undesirable in patients with already compromised perfusion of vital organs. Due to the inaccessibility of vital organs in humans, vasoconstrictive effects of hyperoxia have primarily been studied in animal models. However, the results of these studies vary substantially. Here, we investigate the variation in magnitude of the hyperoxia effect among studies and explore possible sources of heterogeneity, such as vascular region and animal species.

**OBJECTIVES.** The objective of this systematic review and meta-analysis is to provide an overview of the characteristics, quality and outcomes of all animal studies on the effect of hyperoxia on arteriolar tone, both *in vivo* and *ex vivo*. In addition, we investigate the variation in magnitude of the hyperoxia effect among studies and explore sources of heterogeneity.

**METHODS.** Pubmed and Embase were searched for studies up to November 2017. *In vivo* and *ex vivo* animal studies investigating changes in vascular tone induced by local or systemic normobaric hyperoxia were included. Data of lung, brain or fetal vasculature or the ductus arteriosus were not included. We extracted the following data: species, vascular region, blood vessel characteristics and method of hyperoxia induction. Overall effect sizes were estimated with a standardized mean difference (SMD) random effects model.

**RESULTS.** We identified a total of 60 studies, which reported data of 67 *in vivo* and 18 *ex vivo* experiments. In *in vivo* studies, hyperoxia caused vasoconstriction with an SMD of -1.42 (95%CI -1.65 to -1.19). *Ex vivo*, the overall effect size was SMD -0.56 (95%CI -1.09 to -0.03). Between-study heterogeneity ($I^2$) was high for *in vivo* (72%, 95%CI 62% to 85%) and *ex vivo* studies (86%, 95%CI 78% to 98%). *In vivo*, in comparison to the overall effect size, hyperoxic vasoconstriction was less pronounced in the intestines and skin (*P*=0.03) but enhanced in the cremaster muscle region (*P*< 0.001). Increased constriction was seen in vessels 15-25µm in diameter. Hyperoxic constriction appeared to be directly proportional to oxygen concentration. For *ex vivo* studies, heterogeneity could not be explained with subgroup analysis.

**CONCLUSIONS.** The effect of hyperoxia on vascular tone is substantially higher *in vivo* than *ex vivo*, suggesting that extravascular mechanisms play a role in hyperoxic vasoconstriction. The magnitude of the constriction is most pronounced in vessels ~15-25µm in diameter and is proportional to the level of hyperoxia. Relatively increased constriction was seen in muscle vasculature, while reduced constriction was seen in the skin and intestines. *Ex vivo*, the response of the coronary vasculature to hyperoxia is highly variable.

**GRANT ACKNOWLEDGMENT**
The authors received funding from ZonMW (project 114024107)

## Trauma care

### 0011

#### Reporting the largest long term experience with diaphragm pacing for traumatic spinal cord injury: successfully decreasing mechanical ventilation

R. Onders[1], M. Elmo[2], B. Katirji[2], R. Schilz[2]
[1]University Hospitals Cleveland Medical Center, Surgery, Cleveland, United States; [2]University Hospitals Cleveland Medical Center, Cleveland, United States
**Correspondence:** R. Onders
*Intensive Care Medicine Experimental* 2018, **6(Suppl 2):**0011

**INTRODUCTION.** Cervical spine injury (SCI) can result in catastrophic respiratory failure requiring invasive mechanical ventilation (MV) which is a leading cause of mortality and cost. Diaphragm Pacing (DP) was developed to replace/decrease mechanical ventilation.

**OBJECTIVE.** Report the largest long term analysis of DP in a subgroup of traumatic SCI.

**METHODS.** A retrospective review of prospective IRB approved protocols. All patients underwent laparoscopic diaphragm mapping and implantation of electrodes. DP electrodes were characterized and diaphragm strengthening with mechanical ventilator weaning was initiated immediately post op.

**RESULTS.** March 2000 through June 2017 there have been a total of 486 DP implants at this single site. Within this group, 155 had spinal cord damage and 92 of those patients had SCI secondary to trauma. The manner of injury: MVA 44, Sports 22, Falls 12, GSW 7, Crush 3, Forceps Delivery 2, Assault 1, and electrocution 1. The age at time of injury ranged from birth to 74 years old with the average of 27.3 years and median age of 23 years. Time on mechanical ventilation prior to DP was an average of 47.5 months (6 days to 25 years with median of 1.58 years). Patients' highest level of injury: 27 C1, 36 C2, 14 C3, 7 C4, 5 C5 and 3 C6. Twelve patients had internal permanent cardiac pacemakers. There was no device to device interactions between DP and cardiac pacemakers. A total of 83% of patients achieved 4 consecutive hours of pacing with fifty-six patients (60.8%) being full time and an additional 13% using DP >12 hours. DP decreased the need and risk of cuffed tracheostomy with 60% of patients going to cuffless tracheostomy and 7 decannulations. One patient with early DP implant avoided tracheostomy. Five patients were not successful in weaning off MV. Five patients had full recovery of automatic breathing with subsequent DP removal. Two pediatric patients have had growth spurts of 10 inches or greater without need of electrode replacement. There have been 23 deaths with no primary respiratory origin. Subgroup analysis showed that earlier DP implantation leads to greater 24 hour use of DP and no need for any MV. This group also had the greater proportionate recovery of breathing.

**CONCLUSIONS.** DP can successfully decrease need for mechanical ventilation for a significant number of SCI patients. There is a correlation of early implantation to greater weaning success, recovery of independent breathing and has the potential to obviate need for tracheostomy. Liberation from MV can substantially improve SCI rehabilitation. To minimize MV days and complications, DP should be considered early after injury even in those who may re-gain independent breathing.

**REFERENCE(S)**
Posluszny JA, Onders R, Kerwin AJ, et al. Multicenter Review of Diaphragm Pacing in Spinal Cord Injury: Successful not only in weaning from ventilators but also in bridging to independent respiration. J Trauma Acute Care Surg 2014;76:303-310.

### 0012

#### Copeptin improve the outcome prediction of blunt trauma patients by trauma triage scores

C.-C. Lee[1], Y.-C. Hsieh[2], T.-C. Hsu[1], C.C.-Y. Liu[3], M. Lee[1]
[1]National Taiwan University Hospital, Emergency Medicine, Taipei, Taiwan, Province of China; [2]National Taiwan University Hospital Yunlin Branch, Laboratory Medicine, Douliou, Taiwan, Province of China; [3]School of Health, BPP University, McTimoney College of Chiropracti, Abingdon, Oxfordshire, United Kingdom
**Correspondence:** C.-C. Lee
*Intensive Care Medicine Experimental* 2018, **6(Suppl 2):**0012

**BACKGROUND.** Existing prognostic models for trauma patients are limited. Copeptin is a sensitive surrogate marker for AVP release indicating the individual stress response. The prognostic value of copeptin in trauma patients has not been evaluated.

**OBJECTIVE.** The aim of the current study is twofold. First, we seek to evaluate the prognostic significance of copeptin in patients with trauma. Second, we aim to investigate whether the addition of copetin to the existing trauma triage score, specifically TRISS (Trauma and Injury Severity Score) and MGAP (Mechanism Glasgow scale and Arterial Pressure) score, could improve their predictive power.

**METHODS.** Trauma patients presented to the emergency department of a level 1 trauma center between 2016 June to 2016 December were prospectively enrolled. Copeptin levels were measured at admission with the commercial assay (Kryptor, Thermo Fisher Scientific) and trauma triage scores including TRISS and MGAP scores were calculated for each patient. The association of admission copeptin level with mortality or composite outcome of death and ICU admission was tested using receiver operating characteristics curve. Incremental value of copeptin toTRISS or MGAP score was evaluated by logistic regression and reclassification analysis.

**RESULTS.** One hundred and thirty one trauma patients were enrolled with a median injury severity score of 9 (Interquartile range 4-26) and 30-day mortality of 16%. After exclusion of patients presenting with out-of-hospital cardiac arrest, copeptin showed a significant correlation with 30-day in-hospital mortality. Median copeptin admission levels were significantly higher in non-survivors as compared with survivors ( 495 IQR 75-761 pmol/L versus 35.7 IQR 16.5-73.0 pmol/L; $p < 0.0001$). The area under the ROC curve in predicting in hospital mortality was 0.901 for MGAP score, 0.892 for copeptin, and 0.734 for TRISS. Adjusted for TRISS or MGAP in the regression model, copeptin remains significantly associated with the death outcome, indication an incremental value of copetin to the two trauma triage scores. The risk prediction improvement was also reflected by a net reclassification improvement of 37% ( $p = .006$) for TRISS and 16% ($P < 0.001$) for MGAP score. Copetin-augmented MGAP score could achieved the best discrimination (AUC 0.96, 95% CI 0.87-1.00) with a sensitivity of 94.4% (95%CI: 72.7%-99.9%) and a specificity of 99.1% (95%CI: 95.0%-100.0%). Copeptin alone could reach a sensitivity of 92.6 % (85.3%- 97.0%) and a specificity of 92.55 % (85.3%-97.0%) to predict death among trauma patients at a cutoff of 125 pmol/L.

**CONCLUSION.** Copeptin is a highly accurate biomarker that can be used for rapid risk stratification of polytrauma patients. Combining the information of copeptin and MGAP score will further increase the mortality prediction accuracy. Prospective multicenter studies are needed to validate our findings.

### 0013

#### Serial analysis of the immune cells in severe trauma patients

D.W. Kim, I.S. Jeong
Chonnam National University Hospital, Thoracic and Cardiovascular Surgery, Gwangju, Korea, Republic of
**Correspondence:** D.W. Kim
*Intensive Care Medicine Experimental* 2018, **6(Suppl 2):**0013

**INTRODUCTION.** Trauma-induced injury actives innate immune responses to produce pro- and antiinflammatory cytokines.These immune cells are thought to have a significant impact on the prognosis of the patient.Therefore, we tried to analyze the effect of serial measurement of immune cells in severe trauma patients.

**OBJECTIVES.** Immune function is very important in the severe trauma patients, but it is usually overlooked. We examined the progression of immune function in trauma patients.

**METHODS.** From January to December 2017, we studied 21 patients who were admitted to Chonnam National University Hospital, Regional Trauma Center with chest trauma.

Blood sample tests were performed on the day of admission, day1, day3, day7, and day30 of admission. Granulocyte (CD66b), helper T-cell (CD4), cytotoxic T cell (CD8), Gamma delta T cell (CD3+TCRrd+), regulatory T cell (CD25+Foxp3+), B-cell (CD19), NK cell subtypes (CD56brightCD16±, CD56dimCD16+, CD56-CD16+), Monocyte subtypes (CD14+CD16-, CD-14CD16+, CD14+CD16+) level were measured in the peripheral blood by flow cytometry.

**RESULTS.** Mean age was 55.3±16.3 yearls old and male were 16 patients (76.2%), The mean ISS was 17.3±6.9, SOFA score 1.6±2.3. The CD56 +CD16+ NK cell wea significantly decreased in severe trauma patients (ISS>15, N=6), compared to patietns with ISS < 15 ( p=0.04). and CD4/CD8 was also decreased in severe trauma patients (p< 0.01). Most of the immune cells in severe trauma patients were not recovered within 1 month.

**CONCLUSIONS.** This study confirmed that the immune function of patients with severe trauma was not recovered within one month. Further studies are needed to confirm the association of immune function with clinical outcomes.

##### REFERENCE(S)

1. Valparaiso AP, et al. Modeling acute traumatic injury. J Surg Res. 2015;194(1):220-32.
2. Dilektasli E, et al. The prognostic value of neutrophil-to-lymphocyte ratio on mortality in critically ill trauma patients. J Trauma Acute Care Surg. 2016;81(5):882-888.
3. Imtiaz F, et al. Neutrophil lymphocyte ratio as a measure of systemic inflammation in prevalent chronic diseases in Asian population. Int Arch Med. 2012;5(1):2.
4. Fan W, et al. Neutrophil-to-lymphocyte and platelet-to-lymphocyte ratios as predictors of survival and metastasis for recurrent hepatocellular carcinoma after transarterial chemoembolization. PLoS One. 2015;10(3):e0119312.
5. Lord JM, et al. The systemic immune response to trauma: an overview of pathophysiology and treatment. Lancet. 2014;384(9952):1455-65.
6. Abe R,et al. Up-regulation of interleukin-10 mRNA expression in peripheral leukocytes predicts poor outcome and diminished human leukocyte antigen-DR expression on monocytes in septic patients. J Surg Res. 2008;147(1):1-8.

##### GRANT ACKNOWLEDGMENT

The authors declare that they have no competing interests.

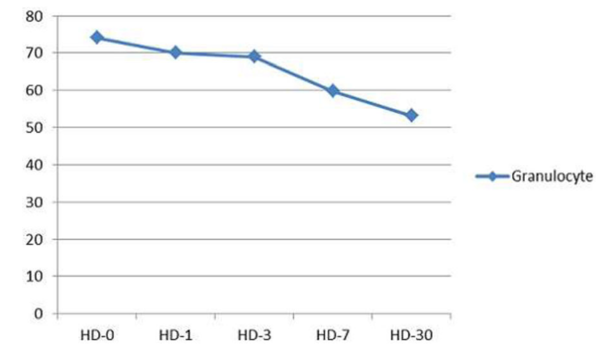

**Fig. 1 (abstract 0013).** serial measurement of granulocyte

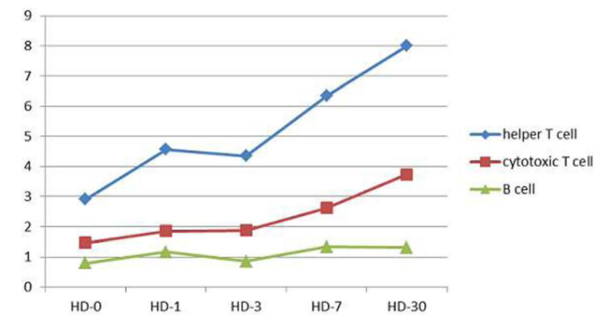

**Fig. 2 (abstract 0013).** serial measurement of cytokine cells

**Table 1 (abstract 0013).** serial comparison of immune cells (hospital day - 30)

| | Trauma group (N=21) ,% | Healthy control group (N=20),% | p-value |
|---|---|---|---|
| Granulocyte (CD66b+) | 53.2 ± 13.4 | 38.3 ± 5.9 | 0.01 |
| Helper T ell (CD4+) | 8.2 ± 4.7 | 8.8 ± 2.6 | 0.61 |
| Cytotoxic T cell (CD8+) | 3.8 ± 2.1 | 5.9 ± 1.8 | <0.01 |
| Gamma delta T cell (CD3 +TCRrd+) | 1.7 ± 1.5 | 2.2 ± 1.2 | 0.29 |
| Regulatory T cell (CD25 +Foxp3+) | 0.2 ± 0.1 | 0.5 ± 0.3 | <0.01 |
| B-cell (CD19+) | 1.3 ± 1.1 | 1.3 ± 1.1 | 0.03 |
| Monocyte subtypes | | | |
| Classic type (CD14+CD16-) | 4.1 ± 1.8 | 2.5 ± 0.7 | 0.01 |
| Non-classic type (CD14CD16+) | 0.3 ± 0.1 | 0.2 ± 0.1 | 0.01 |

### 0014

### Infection after gunshot wound trauma in ICU: preliminary results from the EpiTAF study

S. Tanaka[1,2], L. Falque-Pierrotin[1], A. Tran-Dinh[1,3], D. Bouzid[1], B. Lortat-Jacob[1], E. Guivarch[1], A. Snauwaert[1], P. Tashk[1], S. Jean-Baptiste[1], Y. Castier[4], E. Atchade[1], P. Montravers[1,5]

[1]Département d'Anesthesie-Réanimation CHU Bichat Claude-Bernard, Paris, France; [2]INSERM, UMR 1188, DéTROI (Diabète Athérothrombose Thérapies Réunion Océan Indien), Université de la Réunion, Saint-Denis de La Réunion, France; [3]Inserm U 1148, Paris, France; [4]Chirurgie Vasculaire et Thoracique, CHU Bichat Claude-Bernard, Paris, France; [5]Inserm U 1152, Paris, France

**Correspondence:** S. Tanaka

*Intensive Care Medicine Experimental* 2018, **6(Suppl 2):**0014

**INTRODUCTION.** Over the last decade, the incidence of gunshot wound trauma (GWT) globally increased in France (1,2). Few studies have characterized the infectious complications in this setting (3), and no international guidelines are available for the antibiotic prophylaxis (ABX) in these cases.

**OBJECTIVES.** The goal of this present study is to assess the risk factors of infection after GWT.

**METHODS.** Retrospective study(2011-2017) of all GWT admitted in our ICU. Results are presented in median (IQR) and proportions. The associated factors of infections were analysed in univariate analysis followed by an exact logistic regression (variables with a p-value< 0.15).

**RESULTS.** 50 patients were analysed; 98% male, median age 26 (22-34) years, ISS score 9 (5-12), SAPSII score 15 (8-20). The entry anatomical region of the projectile was chest (n=29), abdomen (n=22), lower extremity (n=23), upper extremity (n=17) and head (n=1). At the time of admission, 25% of the patients were in haemorragic shock and

18% had a vascular effraction with active bleeding. 44 (88%) were admitted in operative room during the first 24h. 46 (92%) of the patients were treated by an ABX for a duration of 2 (2-5) days by amoxicilline-clavulanate (100%). 9 (18%) of the patients developed a wound infection in a delay of 5 (3-8) days : 2 chest wall abscesses (*E. faecalis*), 1 pulmonary abscess (*E. coli, Bacteroides fragilis, Clostridium spp, Pediococcus spp*), 2 abdominal abscesses (*S. aureus*, and one negative culture), 1 lower limb infection (*E. coli*) and 3 homolateral pneumonia (2 *Enterobacter spp, P. aeruginosa)*. One patient died after a *P. aeruginosa* pneumonia. Table represents the risk factors of GWT infections (univariate analysis). In multivariate analysis, mechanical ventilation (MV) was the only risk factor identified for GWT infection (OR = 10.7, p=0.013).

**CONCLUSIONS.** In this cohort of GWT, 18% of the patients developed an infection. Except MV, no associated factors were identified. A prolonged duration of ABX≥48h was not associated with a decreased incidence of these infections (OR 5.7 (0.8-37), p=0.07).

**REFERENCE(S)**

1. Taichman et al. Plos One Medicine 2017;
2. Hirsch et al. Lancet 2015 ; Omoke et al. World J Surg 2016

**Table 1 (abstract 0014).** Risk factors of GWT infections (univariate analysis)

| Risk factors | OR (95% IC) | p | Risk factors | OR (95% IC) | p |
|---|---|---|---|---|---|
| Limb trauma | 0.5 (0.1-2.2) | 0.47 | Transfusion | 11.5 (1.3-101) | 0.02 |
| Chest trauma | 1.7 (0.4-7.2) | 0.7 | MV | 9.4 (2-47) | 0.006 |
| Abdominal trauma | 1.2 (0.3-5.4) | 1 | ISS≥10 | 2.9 (0.6-12.8) | 0.24 |
| Norepinephrine | 0.7 (0-7) | 1 | SAPSII≥20 | 4.3 (0.9-32) | 0.09 |
| Vascular effraction | 0.2 (0-1.4) | 0.18 | ABX≥48h | 5.4 (0.9-32) | 0.09 |
| Lactates≥3mmol/l | 3.2 (0.6-15) | 0.23 | Haemorragic wound | 0.9 (0.2-5.5) | 1 |
| Surgery ≥ 2 | 4.1 (0.7-21) | 0.11 | Fractures | 0.8 (0.2-3.5) | 1 |

#### 0015

#### Incidence, risk factors and outcome of late hypercoagulability in trauma as measured by viscoelastic haemostatic assays

M.R. Wirtz[1,2], M.C.A. Muller[1], P.I. Johansson[3], K. Brohi[4], S.J. Stanworth[5], M. Meagele[6], C. Gaarder[7], J.C. Goslings[2,8], N.P. Juffermans[1]
[1]Academic Medical Center, Department of Intensive Care Medicine, Amsterdam, Netherlands; [2]Academic Medical Center, Department of Trauma Surgery, Amsterdam, Netherlands; [3]Rigshospitalet University of Copenhagen, Section for Transfusion Medicine, Capital Region Blood Bank, Copenhagen, Denmark; [4]Barts and the London School of Medicine and Dentistry, Queen Mary University of London, Centre for Trauma Sciences, London, United Kingdom; [5]Oxford University Hospitals NHS Trust, John Radcliffe Hospital, Department of Haematology, Oxford, United Kingdom; [6]Cologne-Merheim Medical Centre (CMMC), Witten/Herdecke University, Department of Traumatology, Orthopaedic Surgery and Sports Traumatology, Cologne, Germany; [7]Oslo University Hospital, Department of Traumatology, Oslo, Norway; [8]Onze Lieve Vrouwe Gasthuis, Department of Trauma Surgery, Amsterdam, Netherlands
**Correspondence:** M.R. Wirtz
*Intensive Care Medicine Experimental* 2018, **6(Suppl 2):**0015

**INTRODUCTION.** Trauma can induce hypocoagulability, emanating from imbalances between procoagulant factors, anticoagulant factors and fibrinolysis. Early hypocoagulability may convert into a hypercoagulable state(1), possibly as an endogenous response to trauma or due to therapeutic intervention. Whether late hypercoagulability predisposes patients to organ failure is unknown.

**OBJECTIVES.** We aimed to study the incidence, risk factors and outcome of late hypercoagulability in trauma patients, as assessed by viscoelastic hemostatic assays (VHA).

**METHODS.** A substudy of the prospective multicenter study 'Activation of Coagulation and Inflammation in Trauma II' (ACIT II) was performed in patients recruited between January 2008 and January 2015 in 6 level-1 trauma centers in Europe. Clot strength, as measured by the G-value on thromboelastometry (ROTEM) or thromboelastography (TEG), was used to define hypercoagulability (TEG>12400 dynes/cm$^2$, ROTEM>11700 dynes/cm$^2$). Blood samples were taken immediately in the emergency department and 72 hours after injury. Multiple Organ Failure (MOF) was defined as failure of at least two organ systems (SOFA score ≥6). Logistic regression models were used to determine the drivers of hypercoagulable VHA tracings and the association with organ failure.

**RESULTS.** A total of 819 patients were included with a median ISS of 21 (IQR: 10-29). At baseline, hypercoagulability was present in 8% of all patients. Hypercoagulable patients were less likely to have traumatic brain injury (AIS head score ≥3, 29.1% vs 36.1, p=0.039) and were less shocked, (Base Deficit, -2.6±4.6 vs -3.5±5.7 mmol/L, p=0.022). After 72 hours, 41% of patients were hypercoagulable. Multivariable modeling showed that tranexamic acid (TXA) was the only risk factor for the development of late hypercoagulability (OR 1.9, 95%CI 1.3-2.6, p< 0.001). TXA also was an independent risk factor for the development of MOF (OR 1.9, 95%CI 1.3-2.8, p=0.001). However, late hypercoagulability itself showed no association with the development of MOF (OR 1.3, 95%CI 0.9-2.0, p=0.146).

**CONCLUSIONS.** Late hypercoagulability is present in nearly half of this severely injured cohort 3 days after trauma. This was associated with early TXA administration, but not with the development of MOF.

### Optimising the management of severe infections in the ICU

#### 0016

#### Effect on ICU-mortality of early therapy with oseltamivir in critically ill patients with severe influenza pneumonia

G. Moreno[1], A. Rodríguez[1], L.F. Reyes[2], J. Sole-Violan[3], E. Díaz[4], M. Bodí[1], S. Trefler[1], J.C. Yebenes[5], I. Martin-Loeches[6], M.I. Restrepo[7]
[1]Hospital Universitari Joan XXIII/URV/IISPV/CIBERES, Critical Care, Tarragona, Spain; [2]University of Texas Health, San Antonio, United States; [3]Hospital Dr. Negrín, Critical Care, Gran Canaria, Spain; [4]Hospital Parc Tauli, Critical Care, Sabadell, Spain; [5]Hospital de Mataró, Critical Care, Mataró, Spain; [6]St James's University Hospital, Trinity Centre for Health Sciences, Department of Anaesthesia and Critical Care, Dublin, Ireland; [7]South Texas Veterans Health Care System, Respiratory Critical Care, San Antonio, United States
**Correspondence:** G. Moreno
*Intensive Care Medicine Experimental* 2018, **6(Suppl 2):**0016

**INTRODUCTION.** Seasonal as well as pandemic influenza are a major cause of serious illness and deaths worldwide each year. Early antiviral treatment of the disease might improve outcomes.

**OBJECTIVES.** To study the effect on ICU-mortality of early treatment with oseltamivir in patients with severe pneumonia due to influenza.

**METHODS.** Secondary analysis of a prospective study in critically ill patients with severe pneumonia due to confirmed influenza, admitted to 148 Spanish ICUs between June 2009 and April 2014. Demographic, clinical and treatment data were collected. All patients who required mechanical ventilation were analysed. Patients who received early oseltamivir (EO) therapy (within ≤ 48h of onset of symptoms) were compared with those who received late oseltamivir (LO) therapy (> 48h). To evaluate the effect of oseltamivir on mortality, a multivariate analysis and a Propensity score (PS) analysis were performed.

**RESULTS.** The total population comprises 1814 ventilated patients. The median APACHE II and SOFA scores were 17.2 (SD ± 6.9) and 7.5 (± 3.5) respectively. The EO group (412 patients, 22.7%) was compared with LO group (1402 patients, 77.3%). Patients in EO group were older (median, 54.3 vs 52.3 years, p=0.03), had higher APACHE II score (18.2 vs 17.0, p=0.03) and they were more asthmatic than patients in the LO group (14.6% vs 7.9%, p=0.001). In contrast, patients with LO were more obese (36.2% vs. 29.6%, p=0.01), had a longer hospitalization delay (5 vs. 3.2 days, p=0.001) and had more bacterial coinfection rate (22.1% vs. 17.7%, p=0.05). The overall mortality was 28.2% (511 patients) and ICU-mortality rate was higher in the LO group (29.6% vs 23.3%, p = 0.012). The multivariate analysis showed that early therapy with oseltamivir was associated with better survival rates (OR=0.70, 95% CI 0.51-0.95, p=0.025). Furthermore, these results were confirmed after performing the PS analysis (OR=0.75, 95% CI 0.56-1.00, p=0.05) (Figure). Finally, we also found that the number needed to treat (NNT) was only 15 to prevent one additional bad outcome, even greater impact was observed (NNT = 8) in patients who did not have comorbidities (OR=0.5, 95% CI 0.2-0.9, p=0.03).

**CONCLUSIONS.** Our results suggest that the early administration of oseltamivir in ventilated patients with severe influenza pneumonia is associated with better outcomes.

**REFERENCE(S)**

Alejandro Rodríguez, Emili Díaz, Ignacio Martín-Loeches, et al. Impact of early oseltamivir treatment on outcome in critically ill patients with 2009 pandemic influenza A. J Antimicrob Chemother 2011 May;66:1140-9.

**GRANT ACKNOWLEDGMENT**

Thankfully to the H1N1 SEMICYUC working group

### 0017

#### Continuous infusion piperacillin in critically ill patients: predicting concentrations using published population pharmacokinetic models

S. Dhaese[1], A. Farkas[2], P.J. Colin[3,4], J.A. Roberts[5,6,7], J. Lipman[5,6], A.G. Verstraete[8,9], V. Stove[8,9], J.J. De Waele[1]

[1]Ghent University Hospital, Department of Intensive Care Medicine, Ghent, Belgium; [2]Mount Sinai West Hospital, Department of Pharmacy, New York, United States; [3]University Medical Center Groningen, Department of Anaesthesiology, Groningen, Netherlands; [4]Ghent University, Laboratory of Medical Biochemistry and Clinical Analysis, Ghent, Belgium; [5]University of Queensland, Burns, Trauma and Critical Care Research Centre, Brisbane, Australia; [6]Royal Brisbane and Women's Hospital, Department of Intensive Care Medicine, Brisbane, Australia; [7]Royal Brisbane and Women's Hospital, Department of Pharmacy, Brisbane, Australia; [8]Ghent University Hospital, Department of Laboratory Medicine, Ghent, Belgium; [9]Ghent University, Department of Clinical Chemistry, Microbiology and Immunology, Ghent, Belgium

**Correspondence:** S. Dhaese

*Intensive Care Medicine Experimental* 2018, **6(Suppl 2):**0017

**INTRODUCTION.** Critical illness may profoundly alter the pharmacokinetics (PK) of antimicrobial drugs[1]. Dosing regimens based on PK data from healthy volunteers may therefore not be accurate. Several population PK models have been constructed based on data from critically ill patients. The population PK model with the lowest bias and imprecision should be implemented in dosing software designed for dose-optimisation[2].

**OBJECTIVES.** To compare the accuracy of seven piperacillin population PK models to predict concentrations in an independent validation cohort of critically ill patients receiving continuous infusion piperacillin.

**METHODS.** All population PK models included were constructed with data from critically ill patients receiving either intermittent, extended or continuous infusion. A statistical evaluation was undertaken to compare imprecision (root mean square error, RMSE and ΔRMSE) and bias (mean error, ME and ΔME) of predictions by these seven PK models for an independent dataset containing piperacillin concentrations of 45 critically ill patients receiving continuous infusion piperacillin. The application used to predict serum piperacillin concentrations was ID - ODSTM (Individually Designed Optimum Dosing Strategies) (http://www.optimum-dosing-strategies.org).

**RESULTS.** Absolute imprecision (RMSE) ranged from 20.82 mg/L to 135.70 mg/L and absolute bias (ME) ranged from -76.85 mg/L to 93.13 mg/L. The approached that ranked best with regards to absolute bias showed an ME of 7.04 mg/L (95% CI -3.68; 17.77) and the model that ranked best with regards to absolute impression showed an RMSE of 20.82 mg/L (95% CI 54.79; 75.29)

**CONCLUSIONS.** All evaluated PK models demonstrated a low to moderate prediction accuracy of the independent dataset of critically ill patients receiving continuous infusion piperacillin. Whilst software-guided dosing is likely better than product information dosing, we recommend supplementing dosing software with TDM using an adaptive feedback approach to maximize accuracy.

**REFERENCE(S)**

1 Roberts J, Abdul-Aziz M, Lipman J, *et al.* Individualised antibiotic dosing for patients who are critically ill: challenges and potential solutions. *The Lancet Infectious Diseases* 2014; **14**: 498-509.

2 Felton TW, Roberts JA, Lodise TP, *et al.* Individualization of piperacillin dosing for critically ill patients: dosing software to optimize antimicrobial therapy. *Antimicrob Agents Chemother* 2014; **58**: 4094-102.

**GRANT ACKNOWLEDGMENT**

Sofie Dhaese is funded by a CRE grant (APP1099452) from the NHMRC awarded to Jason A. Roberts.

**Table 1 (abstract 0017).** Absolute prediction bias and imprecision versus observed levels of piperacillin

| Model | Mean prediction error | | | Root mean square prediction error | | |
|---|---|---|---|---|---|---|
| | Mean | 95% CI | Rank | Mean | 95% CI | Rank |
| *Alobaid* | 7.04 | -3.68 to 17.77 | 1 | 78.43 | 60.60 to 90.16 | 2 |
| *Felton* | -43.99 | -52.97 to 103.78 | 4 | 80.57 | 64.68 to 93.29 | 3 |
| *Öbrink-Hansen* | 93.13 | 82.48 to 103.78 | 7 | 121.19 | 114.99 to 127.51 | 6 |
| *Roberts* | -76.85 | -87.80 to -65.89 | 5 | 110.75 | 94.58 to 124.85 | 5 |
| *Dhaese* | 78.19 | 62.96 to 93.43 | 6 | 135.70 | 112.78 to 491.05 | 7 |
| *Tsai* | 11.18 | 2.27 to 20.09 | 2 | 20.82 | 54.79 to 75.29 | 1 |
| *Udy* | 30.44 | 18.46 to 42.42 | 3 | 91.81 | 75.48 to 106.59 | 4 |

### 0018

#### Efficacy and pharmacokinetics of aerosolized vancomycin in methicillin-resistant *Staphylococcus aureus* pneumonia under mechanical ventilation

J.Y. Cho[1], S. Hong[2], K.S. Kim[2], S.H. Shin[2], H.-S. Kim[3], H.J. Yang[1], Y.J. Lee[1], S.-H. Choi[4], J.-J. Yim[5], H.B. Kim[6], Y.-J. Cho[1]

[1]Seoul National University Bundang Hospital, Division of Pulmonary and Critical Care Medicine, Department of Internal Medicine, Seongnam, Korea, Republic of; [2]Seoul National University Bundang Hospital, Department of Nursing, Medical Intensive Care Unit, Seongnam, Korea, Republic of; [3]Seoul National University Bundang Hospital, Department of Pharmacy, Seongnam, Korea, Republic of; [4]Semyung University, Department of Nursing, Jecheon, Korea, Republic of; [5]Seoul National University Hospital, Division of Pulmonary and Critical Care Medicine, Department of Internal Medicine, Seoul, Korea, Republic of; [6]Seoul National University Bundang Hospital, Department of Internal Medicine, Seongnam, Korea, Republic of

**Correspondence:** J.Y. Cho

*Intensive Care Medicine Experimental* 2018, **6(Suppl 2):**0018

**INTRODUCTION.** Pneumonia due to Methicillin-resistant *Staphylococcus aureus* (MRSA) in the intensive care unit treated with intravenous vancomycin or linezolid still showed lower clinical response rate. However, there were few studies evaluating the efficacy of aerosolized vancomycin in MRSA pneumonia.

**OBJECTIVES.** This study was prospective, non-comparative, two-staged trial to investigate the efficacy and pharmacokinetics of aerosolized vancomycin in mechanically ventilated patients with MRSA pneumonia. (ClinicalTrials.gov Identifier NCT01925066).

**METHODS.** More than 48 hours mechanically ventilated patients on intravenous vancomycin treatment of MRSA pneumonia were screened. Enrolled patients received aerosolized vancomycin (250 mg every 12 hours) via a nebulizing device for five days. The primary outcome was the clinical response at the end day of treatment. Secondary outcomes included microbiological response, bronchoscopic response, ICU and hospital mortality. Pharmacokinetics of aerosolized vancomycin and were monitored in selected patients. Treatment-related adverse events such as bronchospasm, desaturation, systemic toxic effect, and the emergence of vancomycin-resistant microbe were also evaluated.

**RESULTS.** Total 20 patients (nosocomial pneumonia, n=18; community-acquired pneumonia, n=2) were included. 13 patients (65.0%) showed clinical cure or improvement at the end day of treatment, which was satisfied with the completion of the first stage. Microbiologic eradication of MRSA also identified in 14 patients (70.0%). ICU and hospital mortality were 30.0% and 35.0%, respectively. Some patients with tracheobronchitis showed an improvement in airway inflammation. The peak concentration of aerosolized vancomycin was 105.31 mcg/mL at 4-5 hour after nebulization. No significant treatment-related adverse events were reported except asynchrony to the ventilator.

**CONCLUSIONS.** In conclusion, additional aerosolized vancomycin showed acceptable clinical and microbiological outcomes in mechanically ventilated MRSA pneumonia patients. This strategy might be safety and alternative treatment option.

**REFERENCE(S)**

1) Dhand, R., *The role of aerosolized antimicrobials in the treatment of ventilator-associated pneumonia.* Respiratory care, 2007. **52**(7): p. 866-884.
2) Kalil, A.C., et al., Management of adults with hospital-acquired and ventilator-associated pneumonia: 2016 clinical practice guidelines by the Infectious Diseases Society of America and the American Thoracic Society. Clinical Infectious Diseases, 2016. 63(5): p. e61-e111.
3) Ehrmann, S., et al., Aerosol therapy during mechanical ventilation: an international survey. Intensive care medicine, 2013. 39(6): p. 1048-1056.
4) Palmer, L.B., et al., Aerosolized antibiotics and ventilator-associated tracheobronchitis in the intensive care unit. Critical care medicine, 2008. 36(7).

**GRANT ACKNOWLEDGMENT**

This study was conducted by SNUBH Research Fund No.11-2012-022).

**Table 1 (abstract 0018).** Baseline characteristics

| Variables | Total (N=20) |
|---|---|
| Median age, years (range) | 75.5 (47-87) |
| Sex, male | 13 (65) |
| Pneumonia type | |
| CAP | 2 (10) |
| HCAP | 3 (15) |
| HAP | 11 (55) |
| VAP | 4 (20) |
| Combined MRSA bacteremia | 4 (20) |
| MRSA MIC, μg/ml | 1.1 ± 0.3 |
| Combined respiratory infection due to gram negative bacteria | 10 (50) |
| Bronchoalveolar lavage | 14 (70) |
| APACHE II | 21.8 ± 7.0 |
| SOFA | 7.0 ± 3.6 |
| Clinical Pulmonary Infection Score (CPIS), Day 1 | 7.8 ± 1.2 |
| Total course of systemic vancomycin | 17.5 ± 10.4 |
| Extracorporeal membrane oxygenation | 3 (15) |
| Renal replacement therapy | 7 (35) |
| Tracheostomy | 17 (85) |

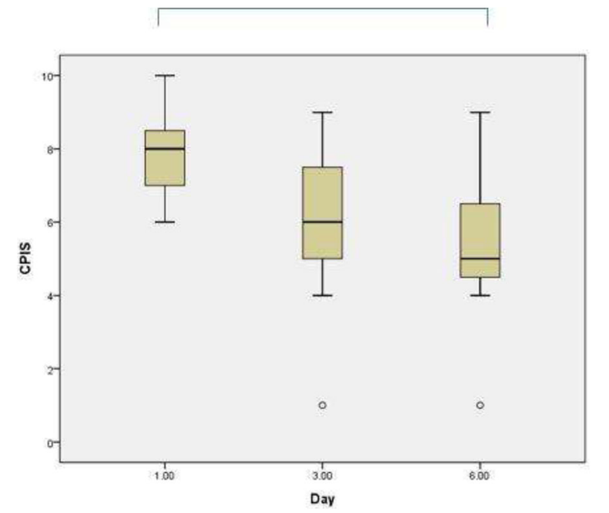

**Fig. 1 (abstract 0018).** CPIS change at D1, D3, and the end of treatment

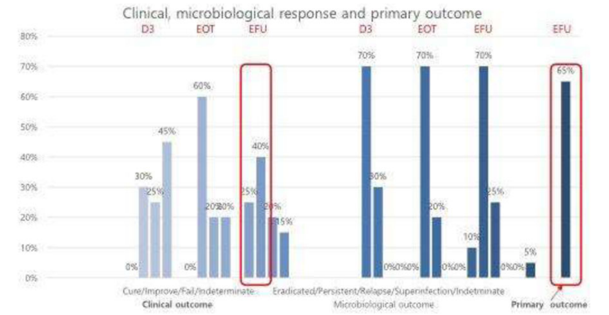

**Fig. 2 (abstract 0018).** Clinical and microbiological response

### 0019

#### Elaboration of consensus endpoints to evaluate antimicrobial treatment efficacy in future HABP/VABP clinical trials

E. Weiss[1,2,3], J.-R. Zahar[4,5,6], A. Shorr[7], A. Torres[8], C.M. Luna[9], G. Dimopoulos[10], G. Poulakou[11], G.H. Talbot[12], I. Martin-Loeches[13], J. De Waele[14], J. Chastre[15], J. Alder[16], J. Lipman[17], J. Rello[18], J. Rex[19], K. Asehnoune[20], L. Pagani[21], L. Palmer[22], L. Papazian[23], M.J.M. Bonten[24], M. Bassetti[25], M. Engelhardt[26], M. Kollef[27], P. Eggimann[28], P. Prokocimer[29], R. Wunderink[30], S. Ewig[31], V. Thamlikitkul[32], J.-F. Timsit[2,6,33]

[1]APHP, Beaujon, Anesthesiology and Critical Care, Clichy, France; [2]Paris Diderot University, Paris, France; [3]Inserm U 1149, Centre for Research on Inflammation, Paris, France; [4]APHP, Avicennes, Clinical Microbiology, Bobigny, France; [5]Paris 13 University, Paris, France; [6]Inserm UMR 1137 IAME, Paris, France; [7]Medstar Washington Hospital Center, Washington, DC, Pulmonary and Critical Care Medicine, Washington, United States; [8]Hospital Clínic de Barcelona, Universitat de Barcelona and IDIBAPS, CIBERES, Pulmonology, Barcelona, Spain; [9]Universidad de Buenos Aires, Hospital de Clínicas 'José de San Martin', Buenos Aires, Argentina; [10]Medical School, University Hospital Attikon, National and Kapodistrian University of Athens, Critical Care Medicine, Athens, Greece; [11]Attikon University General Hospital, Athens University School of Medicine, 4th Department of Internal Medicine, Athens, Greece; [12]Tufts Medical Center and Tufts University School of Medicine and U.S. Food and Drug Administration, Anna Maria, United States; [13]Wellcome Trust - HRB Clinical Research Facility, St James's Hospital, Trinity College, Clinical Medicine, Dublin, Ireland; [14]Ghent University Hospital, Critical Care Medicine, Ghent, France; [15]APHP, Pitié-Salpêtrière, Service de

Réanimation Médicale, Institut de Cardiologie, Paris, France; [16]Bayer HealthCare, Whippany, United States; [17]Royal Brisbane and Womens' Hospital, The University of Queensland, Brisbane, Australia; [18]CIBERES, Vall d'Hebron Barcelona Campus Hospital, Barcelona, Spain; [19]Tufts Medical Center, Infectious Diseases, Boston, United States; [20]University Hospital of Nantes, Anesthesia and Critical Care, Nantes, France; [21]Bolzano Central Hospital, Bolzano, Italy; [22]SUNY at Stony Brook, Pulmonary, Critical Care and Sleep Division, Stony Brook, United States; [23]Hospital Nord, Respiratory Intensive Care Unit, Marseille, France; [24]University medical Centre Utrecht, Microbiology, Utrecht, Netherlands; [25]Presidio Ospedaliero Universitario Santa Maria della Misericordia, Infectious Diseases Clinic, Department of Medicine University of Udine and Azienda Sanitaria Universitaria Integrata, Udine, Italy; [26]Basilea Pharmaceutica International Ltd, Basel, Switzerland; [27]Washington University School of Medicine, Pulmonary and Critical Care Medicine, St Louis, United States; [28]University Hospital, Hospital Preventive Medicine, Lausanne, Switzerland; [29]Merck & Co., Inc, Kenilworth, United States; [30]Northwestern University Feinberg School of Medicine, Pulmonary and Critical Care Medicine, Chicago, United States; [31]Evangelic Hospital in Herne and Augusta Hospital in Bochum, Respiratory Medicine and Infectious Diseases CAPNETZ Stiftung and Thorax Centre in the Ruhr Area, Bochum, Germany; [32]Faculty of Medicine Siriraj Hospital, Mahidol University, Microbiology, Bangkok, Thailand; [33]APHP, Bichat, Medical and Infectious Diseases ICU, Paris, France
**Correspondence:** E. Weiss
*Intensive Care Medicine Experimental* 2018, **6(Suppl 2):**0019

**INTRODUCTION.** Randomized Clinical Trials (RCTs) on Hospital-Acquired (HABP) and Ventilator-Associated Bacterial Pneumonia (VABP) are common methods to investigate antimicrobials seeking new drug approval. However, the heterogeneity in endpoints used in these RCTs is striking (1).

**OBJECTIVES.** To reach a consensus on possible clinical endpoints for future clinical trials evaluating the efficacy of antimicrobial treatment for HABP/VABP.

**METHODS.** Twenty-six international experts with intensive care (n=15) and infectious disease (n=7) departments background and from the pharmaceutical industry (n=4) were polled using the Delphi method (four successive rounds) from January 2016 to January 2017. To achieve consensus, a similarity greater than 70% of the answers to a question was required.

**RESULTS.** The panel recommended a hierarchical composite endpoint (Figure 1) including, by priority order, for VABP (i) all-cause mortality, (ii) mechanical ventilation (MV)-free-days and (iii) clinical cure; and for HABP (i) all-cause mortality and (ii) clinical cure. Clinical cure was defined by consensus as the association of 3 criteria: resolution at end of therapy of signs and symptoms present at enrolment, no further antimicrobial treatment requirement, and improvement or lack of progression of radiological signs. No consensus timepoint was achieved for clinical cure and MV-free days assessment, due to the variability in expert answers. However, more than 70% of them finally agreed to assess mortality, MV-free days at Day 28 and clinical cure within the first seven days following end of treatment. Hierarchy between endpoint components (especially clinical cure and MV-free days) was also difficult to obtain, requiring 3 Delphi rounds to achieve an agreement of only 72% of the experts. Indeed, expert opinions varied according to their specialty and their nationality: all-cause mortality was more frequently chosen as most desirable among non-European experts (55% vs. 7%, p=0.02) and from industry (75% vs. 18%, p=0.04).

**CONCLUSIONS.** Based on a Delphi process, we provide separate hierarchical composite endpoints for VABP and for HABP, and a consensus definition of clinical cure that could be considered as a component of the efficacy measurements of antimicrobial agents used in future HABP/VABP clinical trials.

#### REFERENCE(S)

(1) Weiss E. et al. Crit Care 2017

#### GRANT ACKNOWLEDGMENT

The research leading to these results has received support from the Innovative Medicines Initiative Joint Undertaking under grant agreement n° 115523 [Combatting Bacterial Resistance in Europe – COMBACTE], resources of which are composed of financial contribution from the European Union´s 7th Framework Programme (FP7/2007-2013) and EFPIA companies´ in kind contribution.

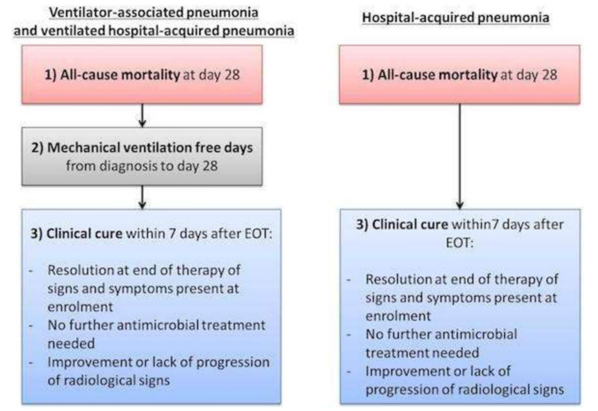

**Fig. 1 (abstract 0019).** Final hierarchical composite endpoint proposed

### 0020

### Does dosing of piperacillin-tazobactam in critically ill patients on ECMO need to change? A pharmacokinetics study

V. Cheng[1,2], K. Shekar[2], M.H. Abdul-Aziz[1], H. Buscher[3], A. Corley[2], A. Diehl[4], E. Gilder[5], P. Jarrett[2], I. Lye[2], S. McGuinness[5], R. Parke[5], V. Pellegrino[4], C. Reynolds[3], S.C. Wallis[1], S. Welch[3], D. Zacharias[6], J.F. Fraser[2], J.A. Roberts[1], ASAP ECMO Investigators
[1]University of Queensland, Faculty of Medicine, Brisbane, Australia; [2]Critical Care Research Group-The Prince Charles Hospital, Adult Intensive Care Services, Brisbane, Australia; [3]St Vincent's Hospital, Intensive Care Services, Sydney, Australia; [4]Alfred Hospital, Intensive Care Services, Melbourne, Australia; [5]Auckland City Hospital, Cardiothoracic and Vascular Intensive Care Unit, Auckland, New Zealand; [6]Inselspital, Universitätsklinik für Intensivmedizin, Bern, Switzerland
**Correspondence:** V. Cheng
*Intensive Care Medicine Experimental* 2018, **6(Suppl 2):**0020

**INTRODUCTION.** The growing adoption of extracorporeal membrane oxygenation (ECMO) into adult intensive care units (ICUs) has lead clinicians to re-evaluate the use of conventional dosing regimens in this patient population. Neonatal studies have suggested modified antimicrobial dosing regimens are necessary in the presence of ECMO to attain PK/PD targets for optimised bacterial kill and clinical cure. However, due to significant differences in physiology, these findings cannot be safely transferred to the adult population. Empirical antibiotics such as piperacillin-tazobactam have no defined titratable end-point, leading to possible sub- or supra- therapeutic management in this group of critically ill patients.

**OBJECTIVES.** The aim of this study is to describe the pharmacokinetics (PK) of piperacillin in critically ill patients receiving ECMO. Through the improved understanding of ECMO-induced PK effects, propose dosing strategies that are more likely to attain the PK/PD targets of 50% $fT_{>MIC}$ to optimise therapeutic outcomes.

**METHODS.** This study was a multi-national, open-label study designed to describe the PK of piperacillin over one dosing interval. Critically ill adults with severe cardiac and/or pulmonary dysfunction from six intensive care units in Australia, New Zealand and

Switzerland were eligible for recruitment. Serial blood samplings were taken over pre-defined time-points. Centralised bioanalysis occurred to determine plasma piperacillin concentration through validated chromatographic methods. The concentration-time data were then used to generate pharmacokinetic parameters through non-compartmental methods.

**RESULTS.** Eight patients were recruited to the study [Table 1]. One patient was excluded from analysis due to incorrect labelling. Non-compartmental analysis yielded a median (IQR) of 12.76 (3.28-22.37) L/h for total clearance and 32.76 (22.82-68.70) L for volume of distribution at steady state. Refer to Figure 2 for the total plasma concentration time graph.

**CONCLUSIONS.** The PK parameters generated by this study were generally consistent with published parameters of critically ill patients not receiving ECMO. It appears that conventional dosing of piperacillin in the critically ill population may provide the target attainment of 50% $fT_{>MIC}$. It is important to acknowledge the heterogeneity within the results and that further analysis and recruitment is required to produce robust recommendations in dosing antibiotics in adults on ECMO.

**REFERENCE(S)**

1. Shekar K, Roberts JA, Ghassabian S, Mullany DV, Wallis SC, Smith MT, et al. Altered antibiotic pharmacokinetics during extracorporeal membrane oxygenation: cause for concern? The Journal of antimicrobial chemotherapy. 2013;68(3):726-7.
2. Sherwin J, Heath T, Watt K. Pharmacokinetics and Dosing of Anti-infective Drugs in Patients on Extracorporeal Membrane Oxygenation: A Review of the Current Literature. Clinical therapeutics. 2016;38(9):1976-94.

**Table 1 (abstract 0020).** Patient demographics and clinical characteristics presented as median (IQR) or number (percentage)

| | |
|---|---|
| Age in years | 58 (53.0 - 67.0) |
| Male, n (%) | 5 (71.4) |
| Weight (kg) | 90.0 (75.0 - 112.0) |
| BMI (kg/m^2) | 27.8 (23.1- 36.7) |
| APACHE II | 23.5 (15.3 - 36.3) |
| SOFA | 7.5 (5.3 - 9.8) |
| VV modality, n (%) | 3 (42.9) |
| Serum creatinine (µmol/L) | 71.0 (46.0 - 122.0) |
| Blood urea nitrogen (mmol/L) | 14.4 (10.5 - 18.6) |
| Albumin (g/L) | 29.0 (28.0 - 40.0) |

**Table 2 (abstract 0020).** Pharmacokinetic parameter estimates presented as median (IQR)

| | |
|---|---|
| AUC0-t (mg/L·h) | 310.96 (162.41 - 769.09) |
| AUC0-inf (mg/L·h) | 313.52 (178.80 - 1220.96) |
| Cmax (mg/L) | 164.09 (94.36 - 222.99) |
| Ke (h^(-1)) | 0.33 (0.10 - 0.41) |
| t1/2 (h) | 2.13 (1.68 - 6.93) |
| CL (L/h) | 12.76 (3.28 - 22.37) |
| CL/kg (L/h/kg) | 0.12 (0.06 - 0.20) |
| Vd (L) | 32.76 (22.82 - 68.70) |
| Vd/kg (L/kg) | 0.29 (0.28 - 0.61) |

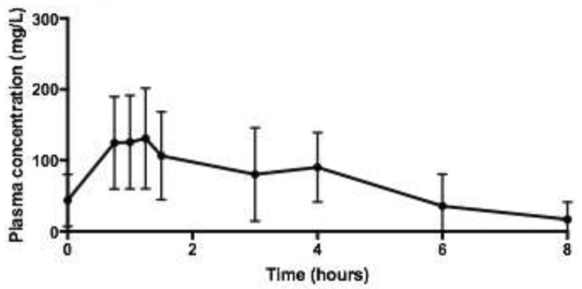

**Fig. 1 (abstract 0020).** Mean +/- standard deviation of piperacillin plasma concentration over time

## Sepsis pathobiology

### 0021

#### Cytokine profile of pediatric sepsis patients: preliminary results of a cohort study

V.S. Lanziotti[1,2], P. Póvoa[3], R.T. Amâncio[4], V.C. Souza-Dantas[2,5], L.B. Pulcheri[6], F.A. Bozza[2,4], A. Prata-Barbosa[7], J.R. Lapa e Silva[8], M. Soares[2], J.I.F. Salluh[2]

[1]Federal University of Rio de Janeiro, Pediatric Intensive Care Unit, Rio de Janeiro, Brazil; [2]D'Or Institute for Research and Education, Intensive Care Research, Rio de Janeiro, Brazil; [3]Nova Medical School (Universidade Nova de Lisboa), Intensive Care Unit, Lisboa, Portugal; [4]Oswaldo Cruz Foundation (FIOCRUZ), Instituto Nacional de Infectologia Evandro Chagas, Rio de Janeiro, Brazil; [5]Federal University of Rio de Janeiro, Intensive Care Unit, Rio de Janeiro, Brazil; [6]Rio's D'Or Hospital, Pediatric Intensive Care Unit, Rio de Janeiro, Brazil; [7]D'Or Institute for Research and Education, Pediatric Research, Rio de Janeiro, Brazil; [8]Federal University of Rio de Janeiro, Internal Medicine Department- Medical School, Rio de Janeiro, Brazil

**Correspondence:** V.S. Lanziotti

*Intensive Care Medicine Experimental* 2018, **6(Suppl 2):**0021

**INTRODUCTION.** Clinical judgment is insufficient to an early identification of outcomes in septic children and studies of biomarkers in this scenario are relatively scarce.

**OBJECTIVES.** We aimed to describe the plasma cytokine profile at the admission at Pediatric Intensive Care Unit (PICU) and its association with outcomes.

**METHODS.** We prospectively included children with community-acquired sepsis, from 3 PICUs in tertiary hospitals in Rio de Janeiro, Brazil. Patients' plasma samples were analyzed, using a multiplex system simultaneously measuring 17 cytokines (IL-1 beta, IL-2, IL-4, IL-5, IL-6, IL-7, IL-8, IL-10, IL-12, IL-13, IL-17, interferon-gamma, granulocyte colony-stimulating factor [G-CSF], granulocyte-macrophage colony-stimulating factor, monocyte chemoattractant protein [MCP]-1, macrophage inflammatory protein-1 and tumour necrosis factor-alpha).Outcomes of interest were PICU mortality, use of vasopressors and invasive mechanical ventilation (IMV).

**RESULTS.** We studied 93 septic children (median age: 2 years, 55% male; infectious focus 65% respiratory, 13% central nervous system). Overall PICU mortality rate was 13%.We considered for analysis in this study cytokines whose detection percentage was at least 50% (G-CSF, IL-7, IL-8, MCP-1, MP-1 and TNFα). The cytokine profile at admission was comparable in survivors and non-survivors. When we considered organ failures and the use of invasive support, MIP-1 and TNFa levels were higher in children with shock requiring vasopressors and use of IMV, respectively. These results were even more evident when we analysed a more homogeneous subgroup with pulmonary infections.

**CONCLUSIONS.** In this exploratory analysis, plasma cytokine values on admission were not able to discriminate survivors and non-survivors. However, we observed higher levels of of MIP-1 in patients with shock and higher levels of TNFα in those requiring IMV. With the current preliminary results, we conclude that single-point evaluation of cytokines at ICU admission are not useful prognostic tools and can't be used in the decision-making process.

### 0022

#### Blood genomic profile in hypothermic sepsis differs from febrile sepsis patients

M. Harmon, M. Wiewel, B. Scicluna, J. Horn, M. Schultz, T. van de Poll, N. Juffermans
Amsterdam Medical Center, Amsterdam, Netherlands
**Correspondence:** M. Harmon
*Intensive Care Medicine Experimental* 2018, **6(Suppl 2):**0022

**INTRODUCTION.** Spontaneous hypothermia in sepsis is associated with increased mortality compared to patients presenting with fever. Studies have failed to illicit a biological mechanism for the hypothermic response. Thereby, it is unknown whether hypothermia merely reflects disease severity or that specific pathophysiologic pathways are involved.
**OBJECTIVES.** In this prospective cohort study we aimed to characterize the blood genomic response and associated cellular biological pathways in patients presenting with hypothermic sepsis compared to patients with febrile sepsis.
**METHODS.** This study prospectively included septic patients in two tertiary intensive care units from January 2011 to June 2012. Blood was collected in PAXgene tubes within 24 hours of ICU admission. Total RNA was isolated and genome-wide blood gene expression profiles of sepsis patients with hypothermia (defined as a body temperature < 36°C in the first 24 hours of admission) were compared to febrile patients (body temperature >38,3°C in the first 24 hours of admission). Analysis was performed between unmatched patient groups as well as between groups matched for Acute Physiology And Chronic Health Evaluation (APACHE)-IV score and/or the presence of shock (hypotension requiring treatment with vasopressors).
**RESULTS.** In total, 67 patients with hypothermic sepsis and 101 patients with febrile sepsis were included in this study. Patients with hypothermic sepsis had significantly increased 30-day mortality compared to febrile septic patients (43% vs 11%, p=0.001). Blood microarray analysis of unmatched patients revealed 6 elevated and 12 reduced gene expression indices (adjusted p< 0.05). The matched analysis for APACHE-IV included 60 patients in both groups and the APACHE-IV + shock analysis included 55 patients in both groups. In the comparison matched for APACHE-IV score, 6 gene pathways were over-expressed in the hypothermia group, including genes involved in dopamine, noradrenaline and adrenaline degradation pathways. These cellular biological pathways appeared to be related to shock as these were no longer significant in the analysis corrected for shock. The analysis matched for APACHE-IV + shock revealed 3 over-expressed gene pathways in hypothermic patients, including; tryptophan degradation X, putrescine degradation III and phenylaline degradation IV (adjusted p< 0.05).
**CONCLUSIONS.** Hypothermic septic patients show marked differences in blood genomic response compared to febrile septic patients. Several pathways remain upregulated after correcting for disease severity and shock. Given the high mortality associated with hypothermia during sepsis, these pathways warrant further investigation.

##### GRANT ACKNOWLEDGMENT

This work was supported by the Center for Translational Molecular Medicine (CTMM) (www.ctmm.nl), project MARS (grant 04I-201).

### 0023

#### Composition of alveolar myeloid cells is altered and their phagocytic capacity is decreased in septic shock patients

T. Skirecki[1,2,3], G. Hoser[1], M. Pirożyński[2], B. Adamik[4], J. Śmiechowicz[4], U. Zielińska-Borkowska[2]
[1]Centre of Postgraduate Medical Education, Laboratory of Flow Cytometry, Warsaw, Poland; [2]Centre of Postgraduate Medical Education, Department of Anesthesiology and Intensive Therapy, Warsaw, Poland; [3]Nalecz Institute of Biocybernetics and Biomedical Engineering, PAS, Warsaw, Poland; [4]Wroclaw Medical University, Department of Anaesthesiology and Intensive Therapy, Wrocław, Poland
**Correspondence:** T. Skirecki
*Intensive Care Medicine Experimental* 2018, **6(Suppl 2):**0023

**INTRODUCTION.** Sepsis evokes multiple maladaptive changes in the immune system but most of them are investigated in the peripheral blood (PB). However, local tissue milieu can specifically influence the activity of immune cells. Despite systemic immunosuppression, sepsis often generates an inflammatory response in the lungs. Therefore, we hypothesized that lung myeloid cells can be differently affected during sepsis, then their circulating counterparts.
**OBJECTIVES.** To analyze the composition of myeloid cells in the bronchoalveolar lavage fluid (BAL) of septic patients and compare the phagocytic activity of these cells to the circulating cells.
**METHODS.** BAL and PB were collected from 10 patients with peritonitis derived septic shock on the 1$^{st}$ day. Flow cytometry was applied to analyze the phenotype of the myeloid cells populations and the FITC-labeled *E. coli* assay (Phagotest, BD) was used to evaluate the phagocytic capacity of monocytes and macrophages. Median values are presented.
**RESULTS.** BAL analysis revealed that CD206+CD169+CD14- alveolar macrophages constituted 48.8% of CD45+ cells, CD16+CD24+ neutrophils constituted 21.9% of CD45+ cells and frequency of CD14+CD206-CD24- monocytes was 1.1%. The main subpopulation of monocytes were the CD14+CD16+ cells (57.6%) followed by CD14+CD16- (34.1%) and CD14-CD16+ (1.2%). Phagocytosis of *E. coli* was executed by almost 100% of monocytes and granulocytes from the PB. However, among BAL cells, only 5% of CD14+ monocytes and 5% of all myeloid cells were able to engulf bacteria. Incubation of BAL cells in the plasma from the same patient increased the phagocytosis rate almost 10-fold.
**CONCLUSIONS.** BAL from septic shock patients contains an increased proportion of neutrophils and reduced frequency of macrophages. Interestingly, alveolar monocytes and macrophages showed reduced phagocytic activity in comparison to PB cells. Furthermore, this discrepancy seems to be partially an intrinsic feature of the alveolar cells. Activation status of the alveolar cells differs from their circulating counterparts and its monitoring should be considered in the trials with immunomodulatory agents.

##### REFERENCE(S)

J Endotoxin Res. 2006; Compartmentalization of the inflammatory response in sepsis and SIRS.Cavaillon JM, Annane D.

##### GRANT ACKNOWLEDGMENT

National Science Centre UMO-2013/11/N/NZ6/02581

### 0024

#### Metabolic profiles in sepsis evolve over time

D.B. Antcliffe, F. Al-Beidh, A.C. Gordon
Imperial College London, Section of Anaesthetics, Pain Medicine and Intensive Care, Department of Surgery and Cancer, London, United Kingdom
**Correspondence:** D.B. Antcliffe
*Intensive Care Medicine Experimental* 2018, **6(Suppl 2):**0024

**INTRODUCTION.** Despite hundreds of trials investigating novel treatments for sepsis, there are no new therapies in routine clinical use. In part this reflects the huge heterogeneity of this syndrome and that some therapies may benefit some, but not all, patients. New methods to stratify patients and track response to treatment are needed to identify subgroups who may benefit from specific therapies.

**OBJECTIVES.** To analyse metabolic profiles in serum samples collected from patients with septic shock to identify clinically useful groups and to track metabolic changes over time.

**METHODS.** Serum was collected from 162 patients enrolled into the VANISH (1) clinical trial at enrolment and up to three subsequent time points. Metabolic profiles were measured using ultra performance liquid chromatography -mass spectroscopy (UPLC-MS) and analysed using a combination of unsupervised and supervised multivariate statistics.

**RESULTS.** Multivariate analysis was able to successfully distinguish abdominal from respiratory infection ($R^2$ 0.73, $Q^2$ 0.29, p< 0.001, AUROC 0.86) and those who had surgical intervention for abdominal sepsis from those who did not ($R^2$ 0.94, $Q^2$ 0.58, p< 0.001, AUROC 0.97). Metabolic trajectories over time were different between those with uneventful recovery from sepsis and those with liver failure ($R^2$ 0.41, $Q^2$ 0.31, p< 0.001).

**CONCLUSIONS.** Metabolic profiling shows promise both to detect meaningful subgroups of patients with septic shock at the point of admission and to track clinical trajectories over time. This has promise to determine subgroups who may respond to specific sepsis therapies and to track response to treatment over time.

#### REFERENCE(S)

Gordon AC et al. Effect of Early Vasopressin vs Norepinephrine on Kidney Failure in Patients With Septic Shock: The VANISH Randomized Clinical Trial. JAMA 2016;316(5):509-18

#### GRANT ACKNOWLEDGMENT

This abstract is independent research funded by the National Institute for Health Research (NIHR) Imperial Biomedical Research Centre (BRC). ACG is an NIHR Research Professor (RP-2015-06-018)

Figure: PLS-DA scores plot ($R^2$ 0.41, $Q^2$ 0.31, p< 0.001) showing the different metabolic trajectories over the first 60h after the onset of septic shock in patients with and without liver failure (Liver SOFA >1)

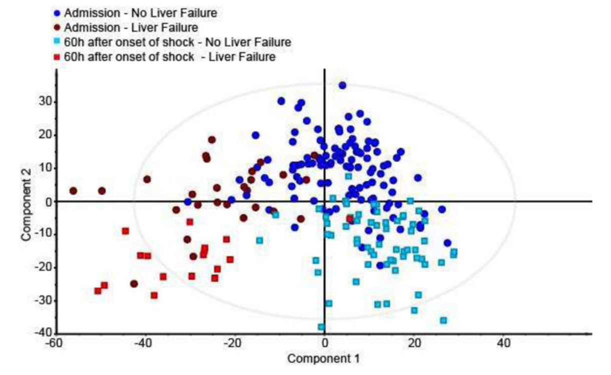

**Fig. 1 (abstract 0024).** Metabolic trajectories

### 0025

#### Blockade of beta-3 adrenergic receptor ameliorates septic cardiomyopathy through the improvement of cardiac fatty acid oxidation

S. Kawaguchi[1], M. Okada[1], E. Ijiri[1], N. Hasebe[2], S. Fujita[1]
[1]Asahikawa Medical University, Department of Emergency Medicine, Asahikawa, Japan; [2]Asahikawa Medical University, Cardiovascular, Respiratory and Neurology Division Department of Internal Medicine, Asahikawa, Japan
**Correspondence:** S. Kawaguchi
*Intensive Care Medicine Experimental* 2018, **6(Suppl 2):**0025

**INTRODUCTION AND OBJECTIVES.** Septic cardiomyopathy is a severe complication of sepsis and leads to poor prognosis (1). Recently, septic cardiomyopathy is thought to be associated with mitochondrial dysfunction and impaired metabolism in myocardium. The beta-3 adrenergic receptor (β3AR) has a lipolysis effect and the expression is enhanced in failing heart (2). Several studies have reported β3AR stimulation has a beneficial effect for heart failure, however the role of β3AR in septic heart is unknown.

**METHODS AND RESULTS.** We made a sepsis model by intraperitoneal injection of lipopolysaccharide (LPS) of 10 mg/kg into C57Bl/6 mice. We divided these subjects into 3 groups as 1mg/kg of the β3AR agonist CL316243 (CL), 1mg/kg of the β3AR selective antagonist SR59230A (SR) and 200μl of normal saline (NS). Kaplan-Meier analysis showed that 24 hour-survival rate was significantly improved in the SR as compared to the CL and the NS (the survival rate was 100% of the SR, 60% of the NS and 20% of the CL, n=10 of each). Echocardiography revealed decreased LVEF was observed at 6 hours after LPS and gradually restored within 24 hours. LVEF did not reduce in the SR (62.3±9.7%), whereas it got worse in the CL(32.5 ±6.4%) than that in the NS (47.1±6.2%). Myocardial ATP was preserved in the SR, on the other hand it was significantly decreased in the NS and the CL. Quantitative PCR analysis revealed that gene expressions associated with fatty acid oxidation (FAO) and mitochondrial function was improved in the SR; *Cpt-1*, an enzyme for free fatty acid intake to mitochondria, and *Ppar*α, a nucleus transcriptional factor to promote FAO, were upregulated in the SR. In addition, *Pgc1a* and *Erra*, which are transcriptional factors associated with mitochondrial biogenesis, were also improved in the SR. In contrast, those expressions were downregulated in the NS and the CL. Interestingly, *Il-6*, a major inflammatory cytokine, was significantly decreased in the SR, whereas it was significantly increased in the CL. Oil red O staining of the septic heart showed that the deposition of lipid droplets which had not been utilized for energy in mitochondria was markedly increased in the CL, but significantly reduced in the SR. These results suggested the β3AR antagonist improved metabolic pathway of FAO.

**CONCLUSIONS.** Blockade of the β3AR spared cardiac energy by improving FAO in a septic heart. The β3 AR is a novel metabolic target for septic cardiomyopathy.

#### GRANT ACKNOWLEDGMENT

The present study was supported by Japan Society for the Promotion of Science, Grant Number JP15K10965.

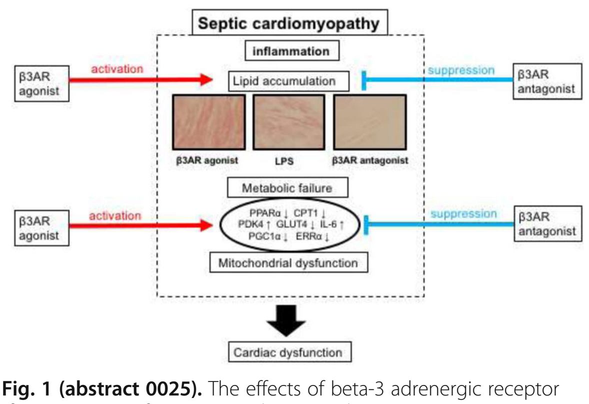

**Fig. 1 (abstract 0025).** The effects of beta-3 adrenergic receptor (β3AR) reagents for septic cardiomyopathy

## ECMO

### 0026

#### The train ECMO international survey: hemoglobin trigger and approach to RBC transfusions during veno-venous extracorporeal membrane oxygenation

G. Martucci[1], G. Grasselli[2], K. Tanaka[3], G. Panarello[1], F. Tuzzolino[4], G. Bellani[5], A. Arcadipane[1]
[1]Ismett, Department of Anesthesia and Intensive Care, Palermo, Italy; [2]Fondazione IRCCS Ca' Granda - Ospedale Maggiore Policlinico, Milano, Italy; [3]University of Maryland, Department of Anesthesiology, Baltimore, United States; [4]Ismett, Research Office, Palermo, Italy; [5]University of Milan-Bicocca, Department of Emergency and Intensive Care, Monza, Italy
**Correspondence:** G. Martucci
*Intensive Care Medicine Experimental* 2018, **6(Suppl 2):**0026

**INTRODUCTION.** Optimal transfusion practice in VV-ECMO patients is still under debate. Traditionally, the threshold for transfusions of packed red blood cells (PRBC) in ECMO aims to keep hemoglobin (Hb) in the near-normal range (12-14 g/dL). Since blood management practices in critically ill patients have changed over the years, we hypothesize that practice may vary also in the treatment of the ECMO patients
**OBJECTIVES.** To assess Hb triggers worldwide, and parameters considered, in addition to Hb, for transfusion during VV-ECMO.
**METHODS.** Web-based (SurveyMonkey) descriptive, semi-structured and voluntary, anonymous, non-funded, self-administered cross-sectional survey, approved and endorsed by the European Society of Intensive Care Medicine (ESICM) Research Committee. Organized into four domains:

1) participant experience and ECMO program characteristics;
2) institution-specific blood product transfusion trigger;
3) transfusion trigger in VV-ECMO;
4) other parameters taken into account in case of uncertainty.

**RESULTS.** In total, 447 respondents worldwide answered the questionnaire: Europe 277 (61.9%), North America 99 (22.1), Asia-Oceania 36 (8.2%), Central and South America 35 (7.8), working mainly in teaching hospitals (79.4%). According to volume of the center, 59.2% manage < 12 VV-ECMO run/year, 19.4% between 12 and 24 run/year, and 21.4% > 24 run/year. The circuit adopted is more frequently "tip-to-tip" heparin coated (75.6%).
In terms of transfusion policy, most centers do not have a fixed Hb trigger for critically ill patients (56.4%) or for ECMO patients (54.4%), and hematocrit is not considered a trigger in critically ill (80.6%) and in ECMO (79.9%) patients. For centers that do have a defined trigger, it was higher in ECMO patients (9.1 g/dl) than in critically ill patients (8.3 g/dl) (p value < 0.01). The adoption of a definite Hb trigger was higher among respondents from higher volume centers: < 12 run/year 37.6%, 12-24 run/year 48.5%, >24 run/year 63.6% (p value: < 0.01). Moreover the mean Hb value was inversely associated with the center´s volume: < 12 run/year 9.6 g/dl, 12-24 run/year 8.9 g/dl, >24 run/year 8.4 g/dl (p value < 0.01).
The main parameters considered when there is uncertainty about a transfusion are SvO2, lactates, peripheral perfusion, hemodynamic stability, and bleeding.
**CONCLUSIONS.** The survey evidenced that there is no consensus on the Hb trigger among ECMO practitioners, but a higher Hb target is considered plausible, and higher-volume centers seem more frequently to adopt a protocol of transfusion practice, and tolerate lower Hb levels during VV-ECMO. Prospective observational studies are needed to define current triggers for transfusion during VV-ECMO. Transfusion practice may be one of the reasons that centralization of ECMO patients in high volume centers carries benefits, as recent position papers have highlighted.

**GRANT ACKNOWLEDGMENT**
This survey was endorsed by the European Society of Intensive Care Medicine

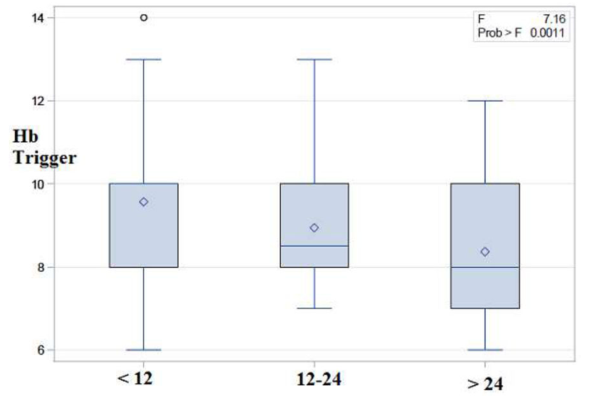

**Fig. 1 (abstract 0026).** Adopted Hemoglobin Triggers according to the ECMO volume center

### 0027

#### Combination of prone position during veno venous extracorporeal membrane oxygenation: effects on extra corporeal membrane oxygenation weaning and ICU survival

C. Guervilly[1,2,3], E. Prud'homme[2], S. Hraiech[2,3], V. Pauly[4], J. Bourenne[3,5], M. Adda[2,3], R. Rambaud[2], B. Coiffard[2,3], F. Daviet[2,3], A. Roch[2,3], J.M. Forel[2,3], L. Papazian[2,3]
[1]Hôpital Nord, Marseille Cedex, France; [2]Aix-Marseille Université, Assistance Publique - Hôpitaux de Marseille, Hôpital Nord, Réanimation des Détresses Respiratoires et Infections Sévères, Marseille, France; [3]Centre d'Études et de Recherche sur les Services de Santé et la Qualité de Vie, CEReSS, Aix-Marseille Université, Faculté de Médecine, Marseille, France; [4]Laboratoire de Santé Publique EA 3279, Centre d'Évaluation de la Pharmacodépendance-Addictovigilance (CEIP-A) de Marseille (PACA Corse) Associé, Faculté de Médecine, Aix-Marseille Université, Marseille, France; [5]Assistance Publique - Hôpitaux de Marseille, Hôpital Timone, Service de Médecine Intensive Réanimation, Réanimation des Urgences, Marseille, France
**Correspondence:** C. Guervilly
*Intensive Care Medicine Experimental* 2018, **6(Suppl 2):**0027

**INTRODUCTION.** Prone position (PP) is a life-saving therapy for severe ARDS[1] although widely underused in the largest recent observational study[2]. Veno venous extracorporeal membrane oxygenation (vvECMO) is proposed for severe hypoxemia unresponsive to PP. The combination of both therapies has been investigate in only small cohorts for feasibility and safety issues[3].
**OBJECTIVES.** We report herein a large retrospective cohort study of consecutive severe ARDS patients treated with vvECMO and compared patients exposed to at least one PP session during ECMO run with patients in the supine position during all their ECMO run.
**METHODS.** Retrospective analysis of medical charts of patients > 18 years supported by vvECMO in two ICU in Marseille, France, between January 2012 and April 2017. Patients supported by vvECMO after lung transplantation were not included. The decision to perform or not PP during ECMO belonged to the intensivist staff in charge of the patient.
**RESULTS.** During the studying period 235 consecutive patients were supported with vvECMO. Among them, 66 were lung transplant recipients and therefore not studied. We compared 77 patients in the supine position during their ECMO run (supine ECMO group) to 92 patients with at least one PP session during their ECMO run (prone ECMO group). Among the 169 patients, 103 (61%) were weaned alive from vvECMO and 80 (47%) were discharged from the ICU. Median ECMO duration was 12 IQR (6;18) days and ICU length of stay 25 IQR (12;35) days. In the prone ECMO group, a median of two sessions of PP was performed IQR (1;4) totalizing 269 sessions. Patients from the prone ECMO group were more frequently weaned alive from vvECMO than patients in the supine ECMO group 70% vs 51 %, p=0.017. ICU survival rate was greater in the prone ECMO group as compared with the supine ECMO group 55% vs 38 %, p=0.03. Concerning potential confounding factors, patients in the prone ECMO group were more frequently postured on PP before cannulation as compared with the supine ECMO group, 76% vs 49 %, p< 0.01, were hospitalized more frequently during years 2015 to 2017, 64 % vs 38%, p< 0.01 and were less critically ill, SAPS II score of 46 (39;54) vs 52 (41;64) in the supine ECMO group, p=0.045.

**CONCLUSIONS.** Combination of PP during vvECMO for severe ARDS is potentially associated with a higher rate of ECMO weaning and ICU survival. However, confounding factors need to be analyzed in a competing risks approach model.

**REFERENCE(S)**

1. Guérin C. N Engl J Med. 2013, 368:2159-68. doi: 10.1056/NEJMoa1214103. PubMed PMID: 23688302.
2. Bellani G. JAMA. 2016 315:788-800. doi: 10.1001/jama.2016.0291. PubMed PMID: 26903337.
3. Guervilly C. Minerva Anestesiol. 2014 80:307-313. PubMed PMID: 24257150.

**GRANT ACKNOWLEDGMENT**

None

### 0028

#### Cardiac index during ECMO derived from conventional transpulmonary thermodilution vs. transpulmonary thermodilution with pulmonary arterial indicator injection: the HEUREKA-I animal study

W. Huber, M. Konrad, S. Kammerzell, R. Schmid, A. Herner

Klinikum rechts der Isar; Technical University of Munich, Medizinische Klinik und Poliklinik II, Munich, Germany

**Correspondence:** W. Huber

*Intensive Care Medicine Experimental* 2018, **6(Suppl 2):**0028

**INTRODUCTION.** Among the attempts to improve the effectiveness of extracorporeal membrane oxygenation (ECMO) are less invasive devices as well as optimization of patient selection, anticoagulation and treatment set-up. Since the extra-corporeal flow should exceed 40-60% of cardiac output (CO), accurate measurement of CO is important before the onset of ECMO (choice of cannulas) and during the extracorporeal procedure to adjust the ECMO-flow and to optimize haemodynamics. Indicator dilution techniques such as the pulmonary arterial catheter (PAC) and transpulmonary thermodilution (TPTD) are considered as gold standard to measure CO. However, due to a potential loss of indicator into the extra-corporeal circuit there are concerns regarding both PAC and TPTD during ECMO. One option to avoid a loss of indicator could be a combination of PAC and TPTD with an injection of the indicator into the distal lumen of the PAC and detection of the signal with a TPTD catheter in the femoral artery (CI_PATPTD). A similar option has been suggested two decades ago using trans-pulmonary *dye* dilution. To the best of our knowledge it has not been validated combined with single indicator transpulmonary *thermo*dilution.

**OBJECTIVES.**

1.) To validate CI_PATPTD vs. CI_TPTD in a subset with ECMO-flow ≤0.5L/min (no risk for loss of indicator).
2.) Comparison of CI_TPTD vs. CI_PATPTD during higher ECMO-flows up to 3.5L/min which might result in loss of indicator for CI_TPTD, but not for CI_PATPTD.

**METHODS.** 8 pigs (49.4±4.2kg) instrumented with a PAC and a CVC (both via jugular vein) and a PiCCO catheter (Pulsion, Germany) in the femoral artery. Veno-venous ECMO with CARDIOHELP (Maquet/Getinge; Rastatt, Germany) via a femoral drainage and a jugular return cannula.

Haemodynamic measurements were recored before and during ECMO with increasing and decreasing extracorporeal flow (0.5, 1.0, 2.0 and maximum flow of 3.5L/min). Each dataset consisted of triplicate measurements of CI_TPTD and CI_PATPTD.

**RESULTS.** In 18 datasets with an ECMO-flow ≤0.5 (mean 0.21 ±0.22) L/min CI_TPTD and CI_PATPTD (3.36±1.06 vs. 3.46±1.07L/min/m²; n.s.) were comparable and showed an excellent bias (-0.01±0.17L/min/m²) and percentage error PE (9.6%). Therefore, we considered CI_PATPTD as gold-standard for all measurements. In all 64 datasets (ECMO-flow of 1.66±1.17 L/min), CI_TPTD correlated with CI_PATPTD (r=0.982; p< 0.001). CI_TPTD and CI_PATPTD were slightly different (3.45±1.13 vs. 3.55±1.23L/min/m²; p=0.042). This resulted in a low bias of -0.08±0.26L/min/m² and an acceptable PE of 14.7%. In multivariate regression analysis the bias (CI_TPTD - CI_PATPTD) was neither associated with high ECMO-flow nor with low CI_PATPTD.

**CONCLUSIONS.**

1.) Measurement of CI_PATPTD combining PAC with TPTD is feasible, accurate and precise.
2.) Measurement of CI using conventional TPTD (CI_TPTD) did not result in a substantial bias and PE compared to CI_PATPTD even during high ECMO flows.

### 0029

#### A systematic review of the effectiveness of extracorporeal membrane oxygenation (ECMO) to treat severe acute respiratory failure in adults

E.C. Camacho, A.A. da Silva, G.F.J.d. Matos

Hospital Albert Einstein, Critical Care Unit, São Paulo, Brazil

**Correspondence:** E.C. Camacho

*Intensive Care Medicine Experimental* 2018, **6(Suppl 2):**0029

**INTRODUCTION.** Acute respiratory failure (ARF) has been associated with poor outcomes in intensive care unit (ICU). Extracorporeal membrane oxygenation (ECMO) has been introduced to increase survival in patients with ARF.

The ECMO in many western countries is one of the main issues regarding the different modes and types of cannula. This is because of its strong impact on the organisation of the ICU in terms of the number of personnel, their expertise, the infrastructure and costs.

**OBJECTIVES.** To provide an up-to-date assessment of the effectiveness of ECMO on mortality, length of stay (LOS) in intensive care unit (ICU) and hospital and adverse events in ICU patients with ARF.

**METHODS.** We have combined highly sensitive searches utilizing text words with hand searching and acquisition and review of cited references searched the Cochrane Central Register of Controlled Trials (CENTRAL) (The Cochrane Library 2017), PUBMED (1966 to November 2017), LILACS (2005 to January 2018), Scopus (2004 to January 2018). In an effort to identify further published, unpublished and ongoing trials we searched trials registers (November 2012) and reference lists.

We included any study that examined ECMO versus NON-ECMO treatment for ARF conducted in adults ICU.

Data from the studies were independently selected and extracted by two reviewers utilizing the online tool COVIDENCE. The disagreement was resolved with a third reviewer consensus.

**RESULTS.** See the attached figures

**CONCLUSIONS.** The mortality in ICU has not shown big differences between ECMO and mechanical ventilation in this SR. The high heterogeneity between studies can influence the results.

ECMO could be effective for ARDS but some design changes must be required. A development ECMO program brings all staff in the ICU to a higher level so they all have similar skills and knowledge.

**REFERENCE(S)**

1: Roch A, Lepaul-Ercole R et al. Extracorporeal membrane oxygenation for severe influenza A (H1N1) acute respiratory distress syndrome: a prospective observational comparativestudy. Intensive Care Med. 2010 Nov;36(11):1899-905.

2: Pham T, Combes A et al. Extracorporeal membrane oxygenation for pandemic influenza A(H1N1)-induced acute respiratory distress syndrome: a cohort study and propensity-matched analysis. Am J Respir Crit Care Med. 2013 Feb 1;187(3):276-85.

3: Peek GJ, Mugford M et al. Efficacy and economic assessment of conventional ventilatory support versus extracorporeal membrane oxygenation for severe adult respiratory failure (CESAR): a multicentre randomised controlled trial. Lancet. 2009 Oct 17;374(9698):1351-63.

**GRANT ACKNOWLEDGMENT**

**Table 1 (abstract 0029).** Mortality between ECMO and NON ECMO patients

| Study or Subgroup | ECMO Events | ECMO Total | NON ECMO Events | NON ECMO Total | Weight | Risk Difference M-H, Random, 95% CI |
|---|---|---|---|---|---|---|
| Bein 2013 | 7 | 40 | 6 | 39 | 12.2% | 0 .02 [-0.14, 0.18] |
| ANZ ECM 2009 | 13 | 61 | 17 | 133 | 1 5. 3% | 0 .09 [- 0.03, 0. 20] |
| Morris 1994 | 14 | 21 | 11 | 19 | 6.2% | 0 .09 (-0.21, 0.39] |
| Noah 2011 | 18 | 75 | 38 | 75 | 13.2% | -0.27 (-0.42, -0.12] |
| Peek 2009 | 33 | 90 | 45 | 90 | 13.5% | - 0 .13 [- 0.28 , 0.01] |
| Pham 2013 | 26 | 52 | 21 | 52 | 10.6 % | 0 . 10 (-0.09, 0.29] |
| Zapol 1989 | 34 | 42 | 41 | 48 | 12.8% | - 0 .0 4 [-0.20, 0.11] |
| Roch 2010 | 5 | 56 | 5 | 56 | 16.2% | 0 .00 [-0.11, 0.11] |
| **Total (95% CI)** | | **437** | | **512** | **100.0%** | **-0.03 (-0.12, 0.06]** |
| Total events | 150 | | 184 | | | |

Heterogeneity : $Tau^2 = 0.01$ ; $Chi^2 = 18.75$, df = 7 (P = 0.009); $I^2 = 63$ %

Test for overall effect : Z = 0.61 (P = 0.54)

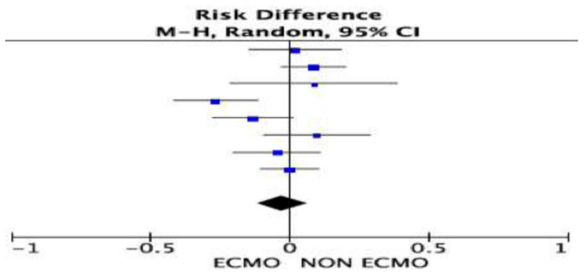

**Fig. 1 (abstract 0029).** Forest plot

### 0030

#### How much airway pressure to apply while resting the lungs in severe ARDS supported with ECMO?

J. Araos[1,2], L. Alegria[1], D. Soto[1], P. Garcia[3], A. Garcia[1], S. Dubo[1,4], T. Medina[5], B. Erranz[6], T. Salomon[7], P. Cruces[5,8], J. Retamal[1], G. Bugedo[1], A. Bruhn[1]

[1]Pontificia Universidad Catolica de Chile, Departamento de Medicina Intensiva, Santiago, Chile; [2]Université de Montréal, Faculté de Médecine Vétérinaire, Centre Hospitalier Universitaire Vétérinaire, Saint-Hyacinthe, Canada; [3]Pontificia Universidad Catolica de Chile, Departamento de Ciencias de la Salud, Carrera de Kinesiología, Santiago, Chile; [4]Universidad de Concepción, Departamento de Kinesiología, Concepción, Chile; [5]Hospital El Carmen de Maipú, Unidad de Pacientes Críticos, Santiago, Chile; [6]Facultad de Medicina, Clinica Alemana Universidad del Desarrollo, Centro de Medicina Regenerativa, Santiago, Chile; [7]Clínica Alemana de Santiago, Unidad de Pacientes Críticos Pediatrica, Santiago, Chile; [8]Universidad Andres Bello, Centro de investigación de Medicina Veterinaria, Santiago, Chile

**Correspondence:** A. Bruhn

*Intensive Care Medicine Experimental* 2018, **6(Suppl 2):**0030

**INTRODUCTION.** A lung rest strategy has been recommended during ECMO for severe ARDS. This strategy is based on the rationale of preventing ventilator-induced lung injury by minimizing dynamic strain. However, the optimal approach to PEEP in this context is unclear.

**OBJECTIVES.** To compare the effects of low, intermediate and high airway pressures on lung injury, while applying a near-apneic ventilation, in a severe ARDS model supported with ECMO.

**METHODS.** Domestic pigs (27-35 kg) were anesthetized, mechanically ventilated (Vt 10 ml/kg, PEEP 5, O2 1.0) and invasively monitored. Lung injury was induced by repeated lavages with 30 ml/kg of warm saline until PaO2/FiO2 dropped below 250, followed by a 2-hour injurious ventilation with PEEP = 0, Pinsp = 40 cmH2O, RR = 10/min, I:E = 1:1. After completing lung injury (time 0, T0) animals were connected to ECMO through an AVALON 23F double-lumen cannula at a blood flow of 60-70 ml/kg/min (1). Animals were randomized into one of three groups (n=6 each):

i) Low:PEEP: 0, Pinsp 10 cmH2O, RR 5/min, I:E 1:1,
ii) Intermediate:PEEP 10, Pinsp 20 cmH2O, RR 5/min, I:E 1:1,
iii) High:PEEP 20, Pinsp 30 cmH2O, RR 5/min, I:E 1:1, and ventilated according to randomization for the following 24 hours. Respiratory and hemodynamic data were collected during the 24h study period. After euthanizing animals tissue samples were extracted from the lungs and histological injury evaluated by microscopy. Total lung water content was estimated by the wet-dry weight ratio.

**RESULTS.** All animals developed severe respiratory failure at T0 with profound hypoxemia and low compliance, without differences between groups. Oxygenation improved throughout the 24-hour study period in the Intermediate and High groups, but not in the Low group (Figure 1). During the study period most animals required hemodynamic support with Noradrenaline but this effect was more marked in the High group. Lung wet-dry weight ratio was 9.9±1.0 in the Low group, versus 7.4±1.0 and 7.5±0.5, in the Intermediate and High groups, respectively (p< 0.05). Histological lung injury was lower in the Intermediate group compared to the other 2 groups (Figure 2).

**CONCLUSIONS.** Applying low airway pressures, associated to near-apneic ventilation, favors more lung edema and impedes recovery of gas exchange during ECMO. On the other hand, high airway pressures favor more hemodynamic compromise. An intermediate level of airway pressure is associated to less lung injury while allowing the best balance between gas exchange, lung edema and hemodynamic tolerance.

**REFERENCE(S)**

1. Araos J, et al. Am J Transl Res. 2016; 8(6): 2826-2837.

**GRANT ACKNOWLEDGMENT**

CONICYT, Fondecyt 1130428 & 1161556

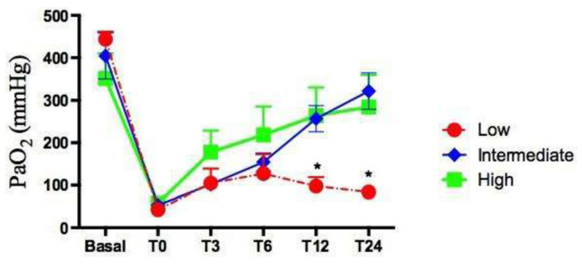

**Fig. 1 (abstract 0030).** Evolution of PaO2 levels in the 3 study groups. p<0.05 vs Intermediate and High groups

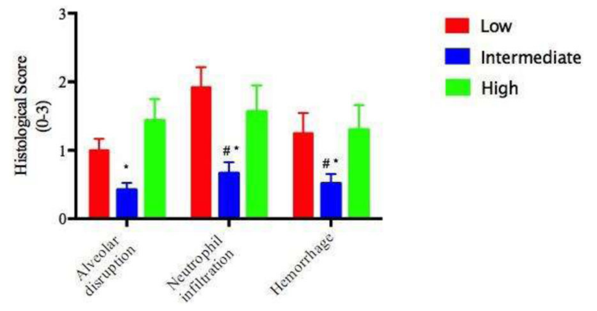

**Fig. 2 (abstract 0030).** Histological scores. 0=Normal; 3=maximal injury. #p<0.05 vs Low, *p<0.05 vs High

## Sedation & delirium

### 0031

#### Preoperative cognitive dysfunction and the incidence of postoperative delirium in elderly oncologic patients

I. Ristescu[1], D. Rusu[1], G. Pintilie[2], M. Jitca[2], I. Grigoras[1]
[1]Grigore T Popa University of Medicine and Pharmacy, Regional Institute of Oncology, Anesthesia and Intensive Care, Iasi, Romania; [2]Grigore T Popa University of Medicine and Pharmacy, Anesthesia and Intensive Care, Iasi, Romania
**Correspondence:** I. Ristescu
*Intensive Care Medicine Experimental* 2018, **6(Suppl 2):**0031

**INTRODUCTION.** The development of postoperative delirium (POD) is associated with major postoperative complications, cognitive decline, longer hospital stay, higher medical costs and mortality (1). Preoperative identification of predisposing factors has a major role in POD prevention.

**OBJECTIVES.** We aimed to investigate the incidence of preoperative cognitive dysfunction and its relation with POD in elderly patients submitted to oncologic surgery.

**METHODS.** After approval by the ethics committee, an observational prospective study was performed in consecutive oncologic patients aged 65 and older, scheduled for elective surgery. For the evaluation of pre-existing cognitive impairment, Mini-Cog test was applied during the preoperative assessment. Nursing Delirium Screening Scale (NU-DESC) was used for the detection of delirium during the postoperative period. We collected data on education, ASA score, preoperative medications, substance use, comorbidities, sensorial deficits, surgery type and duration, type of anaesthesia, anaesthetic drugs, Mini-Cog score, postoperative pain, NU-DESC score. Statistical analysis was performed using Student's t test for continuous data and Chi-square test for nonparametric data. A probability value < 0.05 was considered statistically significant.

**RESULTS.** Of 131 patients enrolled, 68 (51.9%) were diagnosed with preoperative cognitive dysfunction, defined as a Mini-cog score between 0 and 3. 113 patients received general anaesthesia and 26 patients (23%) developed delirium in the 48 hours after surgery as assessed using the NU-DESC. In bivariate analysis, predictive factors for delirium were Mini-Cog score (p< 0.01), clock draw (p< 0.001), word recall (p< 0.001), surgery type (p< 0.009) and duration (p< 0.001) and postoperative pain score (p< 0.001). The probability of developing POD was 50% in patients with a preoperative Mini-Cog score of 0 to 1 and 13.4% in patients with a score of 4 to 5.

**CONCLUSIONS.** In this sample of elderly surgical oncologic patients, preoperative cognitive dysfunction had a high incidence and predicted the emergence of postoperative delirium. Incorporating Mini-Cog as a screening tool for the cognitive assessment, into the preoperative evaluation of elderly patients seems valuable and feasible.

**REFERENCE(S)**

1. Aldecoa C. et al. European Society of Anesthesioogy evidence and consensus-based guidelines on postoperative delirium. Eur J Anaesthesiol 2017; 34:192-214.

### 0032

#### Effectiveness and safety of a flexible family visitation model for delirium prevention in adult intensive care units: a cluster-randomized, crossover trial (The ICU Visits Study)

R.G. Rosa[1], M. Falavigna[2], C.C. Robinson[2], R. Kochhann[3], M.M.S. Santos[2], D. Sganzerla[2], R.M. de Moura[2], M.G. Barbosa[2], D.B. da Silva[4], T.D.S.R. Haack[4], C.S. Eugênio[5], D. Schneider[2], D. Mariani[2], R.W. Jeffman[2], F. Bozza[6], A.B. Cavalcanti[7], L.C.P. Azevedo[8], F.R. Machado[9], J.I. Salluh[10], J.A.S. Pellegrini[11], R.B. Moraes[11], A. Giannini[12], N. Brandão[13], C. Teixeira[4], The ICU Visits Study Group investigators and BRICnet
[1]Hospital Moinhos de Vento, Intensive Care Unit, Porto Alegre, Brazil; [2]Hospital Moinhos de Vento, Research Projects Office, Porto Alegre, Brazil; [3]Hospital Moinhos, Research Projects Office, Porto Alegre, Brazil; [4]Hospital Moinhos de Vento, Intensive Care, Porto Alegre, Brazil; [5]Hospital Moinhos, Intensive Care, Porto Alegre, Brazil; [6]Instituto D'OR de Pesquisa e Ensino, Critical Care, Rio de Janeiro, Brazil; [7]HCor, Research Institute, São Paulo, Brazil; [8]Hospital Sírio Libanês, Intensive Care, São Paulo, Brazil; [9]Universidade Federal de São Paulo (UNIFESP), Department of Anesthesiology, Pain and Intensive Care, São Paulo, Brazil; [10]Instituto D'Or de Pesquisa e Ensino, Critical Care, Rio de Janeiro, Brazil; [11]Hospital de Clínicas de Porto Alegre, Intensive Care, Porto Alegre, Brazil; [12]Ospedale dei Bambini - ASST Spedali Civili, Unit of Pediatric Anesthesia and Intensive Care, Brescia, Italy; [13]Universidade Federal de Ciências da Saúde de Porto Alegre, Internal Medicine, Porto Alegre, Brazil
**Correspondence:** R.G. Rosa
*Intensive Care Medicine Experimental* 2018, **6(Suppl 2):**0032

**INTRODUCTION.** Flexible adult ICU visiting hours have been suggested as a potential intervention for the prevention of delirium, yet most adult ICUs worldwide adopt restrictive visitation policies. Randomized trials evaluating the impact of flexible family visitation models (FFVMs) on patients, family members and ICU professionals are scarce.

**OBJECTIVES.** To compare the effectiveness and safety of an FFVM versus a restrictive family visitation model (RFVM) on delirium prevention among ICU patients, as well as to analyze its potential effects on family members and ICU professionals.

**METHODS.** A cluster-randomized crossover trial, which compares a FFVM (12 h/day) with a RFVM (< 4.5 h/day), has been conducted in 40 mixed adult ICUs in Brazil since March 2017 [1]. Participant ICUs with restrictive visiting policies (< 4.5h/day) are randomly assigned to either a FFVM or a RFVM in a 1:1 ratio. After enrollment and follow-up of 25 patients, each ICU is crossed over the other visitation model, until 25 more patients per site are enrolled and followed. The primary outcome is the cumulative incidence of delirium measured using the Confusion Assessment Method for the ICU. Secondary outcomes include daily hazard of delirium, ventilator-free days, any ICU-acquired infections, ICU length of stay, and hospital mortality among the patients; symptoms of anxiety and depression and satisfaction among the family members; and prevalence of burnout syndrome among the ICU professionals. Tertiary outcomes include need for antipsychotic agents and/or mechanical restraints, coma-free days, unplanned loss of invasive devices, and ICU-acquired pneumonia, urinary tract infection, or bloodstream infection among the patients; self-perception of involvement in patient care among the family members; and satisfaction among the ICU professionals.

**RESULTS.** The results of the present study will be presented in the 31st ESICM annual congress. Currently, 40 ICUs representative of the Brazilian geopolitical territory were included in the study (figure). Until April 2018, 1620 patients, 1208 family members and 829 ICU professionals were included and followed. We expect that this study will be concluded in May 2018.

**CONCLUSIONS.** The results of the ICU Visits study will allow health care professionals, researchers, and policymakers to drawn conclusions about the effectiveness and safety of a flexible family visitation model for delirium prevention in adult ICUs.

[1] Rosa RG, Falavigna M, Robinson CC, et al. Study protocol to assess the effectiveness and safety of a flexible Family visitation model for delirium prevention in adult intensive care units: a cluster-randomized, crossover trial (The ICU Visits Study). BMJ Open 2018. doi: 10.1136/bmjopen-2017-021193.

**GRANT ACKNOWLEDGMENT**
The present study was funded by the Brazilian Ministry of Health through the Program of Institutional Development of the Brazilian Unified Health System (PROADI-SUS).

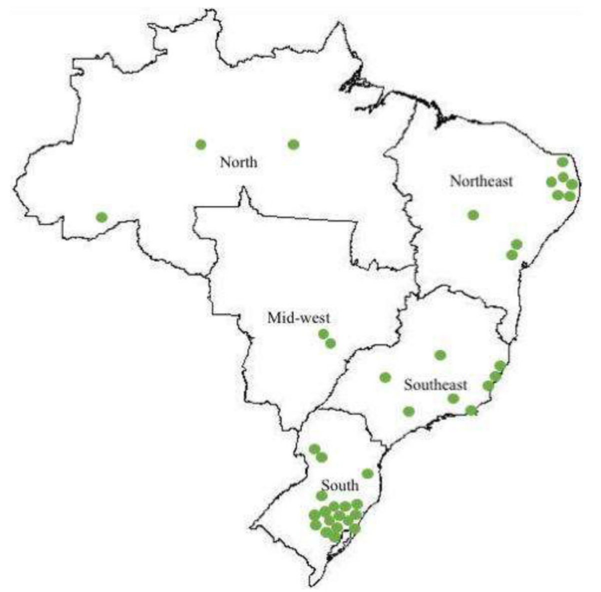

**Fig. 1 (abstract 0032).** Geographical distribution of participating ICUs

### 0033
### Delirium, expectation and mortality
A. Al-Hindawi[1,2], M. Vizcaychipi[1,2]
[1]Chelsea and Westminster Hopsital, London, United Kingdom; [2]Imperial College London, London, United Kingdom
**Correspondence:** A. Al-Hindawi
*Intensive Care Medicine Experimental* 2018, **6(Suppl 2):**0033

**INTRODUCTION.** The development of delirium in patients can be thought of in terms of organ failure. This metaphor has implications as new data has suggested that patients who develop delirium have increased mortality compared to patients who do not 1-3. Thus the screening, diagnosis and treatment of delirium is of paramount importance 4.
Recently, the PRE-DELIRIC score has been developed; a multivariate linear regression model that aims to predict the development of delirium 5.
**OBJECTIVES.**

1. Calculate the Expected Delirium Rate (EDR) using PRE-DELIRIC and visualise how that has changed overtime in our local ICU.
2. Explore the relationship between Mortality and PRE-DELIRIC.
3. Calculate the Standardised Delirium Index (SDI) to benchmark how 'deliriogenic' our practice is.

**METHODS.** Our Electronics Health Record (EPR) was interrogated for variables that are required for the calculation of PRE-DELIRIC and Mortality. ICNARC primary admission diagnosis was used for admission category. Acidosis was defined as a Base Excess < 2. Coma, Morphine and Sedatives use were not available; the minimum coefficients were used instead thus forming a 'lower' bound.
Expected Delirium Rate was calculated as the mean PRE-DELIRIC score multiplied by the number of patients; akin to the calculation of Standardised Mortality Index.
Diagnosis of delirium was not available on our EPR and thus the calculation of the SDI was not performed.
**RESULTS.** A strong correlation between PRE-DELIRIC and Mortality was found (p < 0.0001) despite controlling for severity of disease using APACHE-II.
**CONCLUSIONS.** Expected Delirium Rate is increasing in recent times with a statistically significant correlation with mortality.
The Standarised Delirium Index was developed; a new index that encompasses both patient and environmental factors that benchmark's the local unit's 'deliriogenic' practices.
We aim to prospectively collect targetted data aimed at calculating the SDI as well as compare our local data with publically available datasets.

**REFERENCE(S)**
1. Lin, S.-M. et al. The impact of delirium on the survival of mechanically ventilated patients. Crit. Care Med. 32, 2254-9 (2004).
2. Ouimet, S., Kavanagh, B. P., Gottfried, S. B. & Skrobik, Y. Incidence, risk factors and consequences of ICU delirium. Intensive Care Med. 33, 66-73 (2007).
3. McCusker, J., Cole, M., Abrahamowicz, M., Primeau, F. & Belzile, E. Delirium Predicts 12-Month Mortality. Arch. Intern. Med. 162, 457 (2002).
4. Meagher, D. J. Delirium: optimising management. BMJ 322, 144-9 (2001).
5. van den Boogaard, M. et al. Development and validation of PRE-DELIRIC (PREdiction of DELIRium in ICu patients) delirium prediction model for intensive care patients: observational multicentre study. BMJ 344, e420 (2012).

**GRANT ACKNOWLEDGMENT**
None

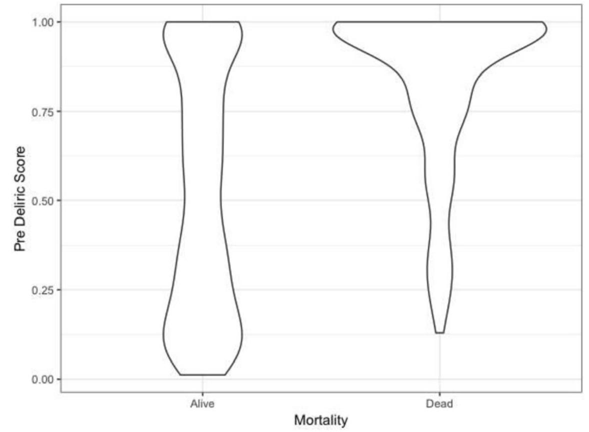

**Fig. 1 (abstract 0033).** Violin plot of Mortality VS Pre-Deliric Score. PRE-DELIRIC scores were bimodal; the two modes are hypothesised to be post-operative patients and emergency admissions

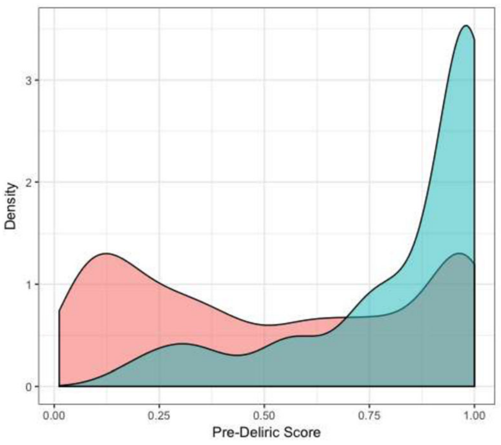

**Fig. 2 (abstract 0033).** Density graph of Pre-Deliric score VS Mortality. EDR was found to be increasing in the last 3 years without a clear explanation

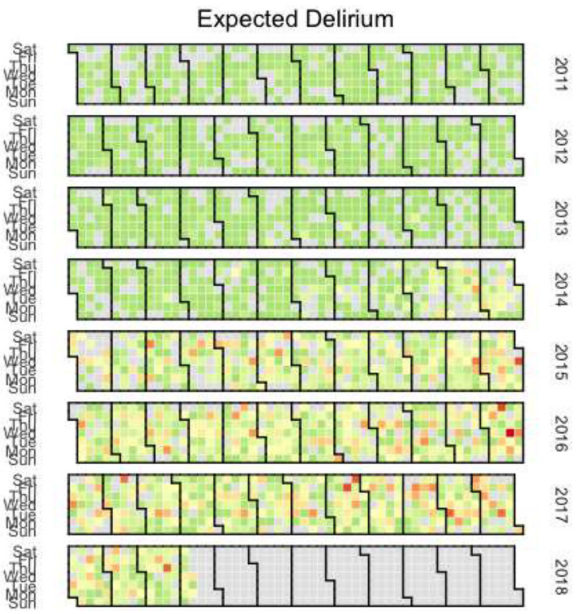

**Fig. 3 (abstract 0033).** Calendar Heatmap

### 0034

#### Calculating PRE-DELIRIC model with SAPS II for early detection of delirium in ICU Patients

M. Sampaio[1], T. Cardoso[1], M.L. Bastos[2], I. Aragão[1]
[1]Centro Hospitalar do Porto, Porto, Portugal; [2]Faculdade de Farmácia da Universidade do Porto, Porto, Portugal
**Correspondence:** M. Sampaio
*Intensive Care Medicine Experimental* 2018, **6(Suppl 2):**0034

**INTRODUCTION.** Delirium is a common and serious disorder, prevalent in Intensive Care Units (ICU) patients, associated with negative clinical outcomes, namely higher morbidity and mortality. Accurate diagnosis is limited. General preventive measures in all ICU patients are time and resources consuming without consensual results. Predicting delirium enables physicians to focus on high-risk patients with non-pharmacological preventive measures and drug interventions resulting in better outcomes. The risk of delirium can be assessed through PRE-DELIRIC model which stratifies patients as having low, moderate, high and very high-risk of developing delirium. It uses APACHE II as one of its parameters. Although most European ICUs use SAPS II instead of APACHE II as physiological severity score to estimate death risk, there are no studies using SAPS II for delirium risk assessment.

**OBJECTIVES.** The purpose of this study was to validate PRE-DELIRIC model using SAPS II score in sedated patients.

**METHODS.** Prospective cohort study performed for six months in adult patients admitted at a Tertiary Care Hospital twelve-bed mixed ICU, for a minimum period of 48 hours and sedated for at least 24 hours. Our main outcome measure was to compare PRE-DELIRIC scores calculated with APACHE II and SAPS II. Results generally presented as median (IQR) and Intraclass Correlation Coefficient (ICC).

**RESULTS.** One-hundred patients (71 male) were included from a total of 219 admissions. Mean age was 59 years old (SD±15). Satisfying PRE-DELIRIC criteria, 52 patients were medical, 25 neurological or neurosurgical, 15 surgical, and eight trauma at ICU admission. ICU length of stay (LOS) was 10.5(6-16) days and patients were sedated for 88.5 hours (19.5-158.8). APACHE II and SAPS II scores were 20.5(15-26) and 48.0(41-58), respectively. APACHE II scored for patient was compared with APACHE II score calculated through a mathematical model from SAPS II and results presented an ICC of 0.93(0.890-0.950, *p*=0.000). PRE-DELIRIC calculated with APACHE II and PRE-DELIRIC calculated from SAPS II scored a median of 87 and presented an ICC of 0.99(0.986-0.994, *p*=0.000) between them. Delirium was not evaluated in 31 patients (due to patient´s death or discharge to other unit with deep alterations of conscience before evaluation). Among remaining 69 patients delirium was observed in 23 patients, an incidence of 33%. ICU LOS for patients with delirium was 16(10-23) days *vs.* 11(8-16) days in patients without delirium (*p*=0.074).

**CONCLUSIONS.** APACHE II score correlation with SAPS II score presents a coincident agreement with other studies. Use of SAPS II to calculate PRE-DELIRIC score has an excellent correlation with the one calculated with APACHE II. It is possible to use SAPS II in the calculi of risk of delirium through PRE-DELIRIC score, allowing identification of high-risk patients and early institution of preventive measures.

**GRANT ACKNOWLEDGMENT**
No conflict of interests.

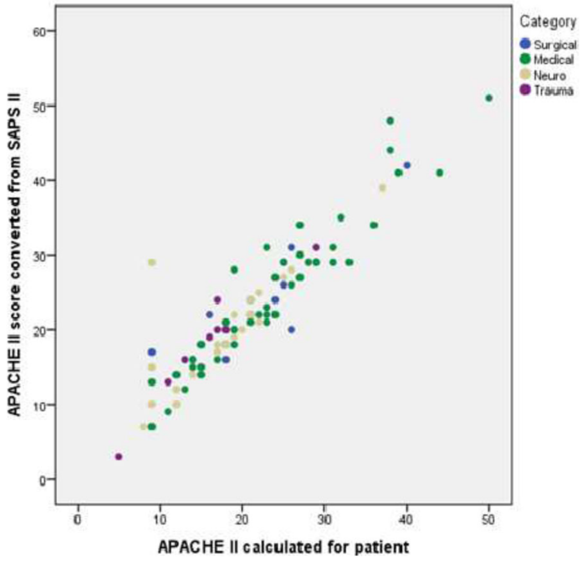

**Fig. 1 (abstract 0034).** ICC SAPS II - APACHE II

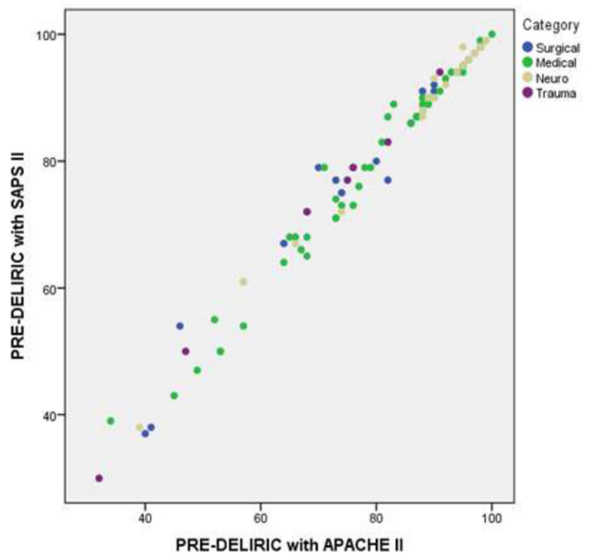

**Fig. 2 (abstract 0034).** ICC PRE-DELIRIC SAPS II - PRE-DELIRIC APACHE II

### 0035

#### Conscious sedation versus general anesthesia for thrombectomy in acute ischemic stroke. Does it really make a difference?

S. Alcántara Carmona, L. Esteban García, N. Martínez Sanz, P. Matía Almudevar, A. Naharro Abellán, I. Fernández Simón, A. Pérez Lucendo, M. Pérez Redondo

Hospital Universitario Puerta de Hierro Majadahonda, Majadahonda, Spain

**Correspondence:** S. Alcántara Carmona

*Intensive Care Medicine Experimental* 2018, **6(Suppl 2):**0035

**INTRODUCTION.** Thrombectomy (TRB) for acute ischemic stroke (AIS) has become a well-recognized and widely available treatment. Whether it should be performed under conscious sedation (CS) or general anesthesia (GA) remains controversial.

**OBJECTIVE.** To compare six-month mortalities as well as variations in treatment times and neurological improvement for both strategies.

**METHODS.** Retrospective study (October 2013-June 2017). We included all AIS patients treated with TRB admitted to our ICU. Data collected are shown in Tables 1 and 2. Complete angiographic reperfusion was defined as a IIb/III punctuation in the Modified Treatment in Cerebral Infarction Score (mTICI) and neurological improvement as a difference ≥4 points between the initial and before discharge NIHSS scores. Duration of mechanical ventilation and ICU and hospital length of stays where also registered. Univariate analysis: Mann-Whitney (numerical) and $X^2$or Fishers test (categorical). Multivariate analysis: logistic regression.

**RESULTS.** Out of the 297 ICU admissions for AIS, 129 (43,4%) underwent TRB. Systemic thrombolysis previous to TRB was administered to 63 (48,8%). CS was applied in 55 cases and GA in 74. Reasons for GA were: elective (51), GCS< 8 (12), agitation (9) and respiratory impairment (2). In three patients TRB was started with CS but changed to GA due to patient restlessness during the procedure. These patients were analyzed within the GA group.

APACHE II and initial NIHSS scores were significantly higher in the GA group (Table 1). We found no differences in onset to reperfusion times. The GA group had longer arrival to puncture intervals but puncture to reperfusion times were shorter. Angiographic reperfusion was similar for both groups. Although there were no differences regarding neurological improvement (NIHSS≥4), modified Rankin scales scores at six-months were significantly better for the CS group even after adjusting by initial NIHSS [OR for Rankin 3-6 for GA: 2.3 (IC95 1.00-4.34;p=0.048)](Table 2). Median extubation time was 12h (5-28.7). Patients undergoing GA had longer ICU stays [2 days (2-5) vs 1 day (1-2);p< 0.001] but hospital length of stay was similar [13 days (8-28) vs. 12 days (9-27);p>0.05].

In the unadjusted logistic regression analysis for six-month mortality, the OR for the GA group was 2.5 (IC95 0.93-7.03;p=0.068). After adjusting for age, APACHE II and initial NIHSS scores the OR dropped to 1.3 (IC95 0.36-4.89;p=0.66).

**CONCLUSION.** In our series the use of GA did not lead to an increase in six-month mortality even though this group had higher initial APACHE II and NIHSS scores.

GA was related with a shorter TRB procedure, but onset to reperfusion times were similar. Although no differences in reperfusion rates or neurological improvement (NIHSS≥4) were found, the CS group performed neurogically better at the six-month mark.

According to our results the use of GA or CS should be a customized election based on TRB feasibility and patient's safety.

**Table 1 (abstract 0035).** Demographics and clinical characteristics

**TABLE 1: Demographics and clinical characteristics**

| | CS (n = 55) | GA (n = 74) |
|---|---|---|
| Age (years) | 65 (59 – 76) | 73 (60 – 78)' |
| Sex (male) | 32 (58.2) | 45 (60.8) |
| Nº comorbidities | 2 (1 – 3) | 2 (2 – 3) |
| APACHE II | 10 (8 – 14) | 13 (10 – 17)* |
| Previous ischemic stroke | 4 (7.3) | 7 (9.5) |
| Previous hemorrhagic stroke | 1 (1.8) | 0 (0.0) |
| Initial NIHSS | 17 (12 – 18) | 20 (16 – 22)* |
| Vascular territory involved | LMCA: 19 (34.5)<br>RMCA: 22 (40.0)<br>Basilar: 0 (0)<br>ICA: 2 (3.6)<br>More than 1: 12 (21.9) | LMCA: 30 (40.5)<br>RMCA: 17 (23.0)<br>Basilar: 7 (9.5)<br>ICA:2 (2.7)<br>More than 1: 18 (24.3) |
| ASPECTS | 8 (7 – 10) | 8 (7 -10) |

Data are shown as median (q25 – q75) or absolute values (%). NIHSS: National Institute of Health Stroke Scale. LMCA: left middle cerebral artery; RMCA: right middle cerebral artery; ICA: internal carotid artery. ASPECTS: Alberta Stroke Program Early CT Score.
'p = 0.051; *p < 0.001

**Table 2 (abstract 0035).** Treatment characteristics and evolution

| Treatment characteristics and evolution | | |
|---|---|---|
| | **Conscious sedation** | **General anesthesia** |
| Reason for thrombectomy | Thrombolysis failure: 29 (53.7)<br>Thrombolysis contraindication: 19 (35.2)<br>Symptoms for > 4.5 h: 6 (11.1) | Thrombolysis failure: 34 (46.6)<br>Thrombolysis contraindication: 23 (31.5)<br>Symptoms for > 4.5 h: 16 (21.9) |
| Symptoms to thrombolysis (min) | 120 (105 – 140) | 107 (90 – 145) |
| Onset to arrival to TRB center (min) | 159 (73 – 240) | 176.5 (66.5 – 230) |
| Arrival to puncture (min) | 55 (35 – 99) | 68.5 (50 – 103)* |
| Puncture to reperfusion (min) | 70.5 (47 – 106.5) | 55 (40 – 73)* |
| Onset to reperfusion (min) | 315 (270 – 382) | 300 (241 – 360) |
| Type of endovascular treatment | Stent retriever: 24 (52.2)<br>Direct aspiration: 6 (13)<br>Retriever + aspiration: 16 (34.8) | Stent retriever: 34 (57.6)<br>Direct aspiration: 8 (13.6)<br>Retriever + aspiration: 17 (28.8) |
| mTICI IIb/III | 42 (80.8) | 62 (84.9) |
| Thrombectomy complications | 3 (5.5) | 5 (6.8) |
| Incidence of bronchoaspiration | 4 (7.3) | 7 (9.5) |
| Vasoactive drugs | 4 (7.3) | 15 (20.3)* |
| Hypotensive drugs | 6 (10.9) | 13 (17.6) |
| $p_aCO_2$ 2h upon ICU arrival (mmHg) | 39.1 (36.6 – 42.7) | 38.1 (34.7 – 44.2) |
| Hemorrhagic transformation | 11 (20) | 21 (28.4) |
| Change in NIHSS score | < 4: 15 (28.9)<br>≥ 4: 37 (71.1) | < 4: 10 (17.5)<br>≥ 4: 47 (82.5) |
| Destination upon discharge | Home: 30 (62.5)<br>Rehabilitation facility: 18 (37.5) | Home: 37 (66.1)<br>Rehabilitation facility: 19 (33.9) |
| Modified Rankin Scale at 6 months | 0 – 2: 35 (71.4)<br>3 – 6: 14 (28.6) | 0 – 2: 29 (43.9)*<br>3 – 6: 37 (56.1)* |

Data are shown as absolute values (%) or as median (q25 – q75). mTICI: modified treatment in cerebral infarction score. Modified Rankin Scale score range: 0 to 6 (0 = symptom free to 6 = dead). NIHSS: National Institute of Health Stroke Scale. *p < 0.05

## Information systems in the ICU

### 0036

#### Reducing length of stay in ICU patients and workload of the care team members by implantation of information system and care bundle in ICU

C.-C. Chao[1,2], W.-F. Kao[1]
[1]Taipei Medical University Hospital, Emergency Department, Taipei, Taiwan, Province of China; [2]Taipei Medical University, Emergency Department, Taipei, Taiwan, Province of China
**Correspondence:** C.-C. Chao
*Intensive Care Medicine Experimental* 2018, **6(Suppl 2):**0036

**INTRODUCTION.** Clinical situations in intensive care unit (ICU) change rapidly and may result in alarm activation; however false alarm and alarm fatigue may potentially increase error rates _ and workload in ICU. By introducing information system into daily care for patient can reduce workload and improve quality of care.

**OBJECTIVES.** This prospective study aims to detect the effect of implantation of information system and care bundle in ICU on length of stay and work load for the ICU team member.

**METHODS.** Taipei Medical University Hospital is a community based teaching hospital with 736 beds capacity (includes 58 general ICU beds). We build up electronic ICU (e-ICU) dashboard to automatically upload and calculate physiologic information and clinical data within critical care unit to provide real time information to the care team start on 1th Feb, 2017. We use e-ICU software with automatic collect clinical information and calculation of SOFA (Sequential Organ Failure Assessment) score every 48 hours. If SOFA score decrease more than 2 points, e-ICU system will automatic show reminder for transferring patient to ordinary ward. We prospective collected data from intensive care unit (ICU) before and after implantation of e-ICU. Patients who admitted to our ICU from July 2016 through July 2017 were collected for basic information, time-points of intervention and cost for analysis.

**RESULTS.** There are 1857 patients who admitted to our ICU during this period (915patients before e-ICU and 942 after). There is no significant difference in background and mortality rate between the two group, and length of stay is reduced from 7.2 days to 6.5 days (p=0.01) after automatic reminder for patient with decrease SOFA score.
Additional effect:

1. 187.9 work hours/per month of documentation by simplify the work flow through automatic uploads.
2. 887.4 work hours/per month of nursing handovers by incorporating clinical information and data.
3. Response time to abnormal laboratory and physiologic information alarm of the patient can decrease 0.6 minutes /episode (8.6 minutes to 8 minutes).
4. Actual paper print-out saves 600 USD/ year.
5. Accuracy of vital signs input can improve from 85.9% (manual entry) to 100 %( automatic upload).

The cost for implantation of the e-ICU is 122,000 USD (117,000 USD for hardware set up, and 5,000 for software development).

**CONCLUSIONS.** Implantation of information system (e-ICU) with incorporated clinical information and automatic care bundle in ICU can help reduce length of stay in ICU and decrease workload of the ICU team members.

**REFERENCE(S)**

Lilly, Craig M., et al. "Five-Year Trends of Critical Care Practice and Outcomes." CHEST 152.4 (2017): 723-735.

**GRANT ACKNOWLEDGMENT**
Nil

**Table 1 (abstract 0036).** Effect before and after e-ICU implantation

| | Before (n=915) (95% CI) | After (n=942) (95% CI) | P value |
|---|---|---|---|
| Male | 546 (59.7%) | 549(58.2%) | 0.40 |
| Age | 68.0 ± 0.6 (66.9~69.1) | 67.5 ± 0.7 (66.5~68.4) | 0.12 |
| APACH II | 11.9 ± 0.3 (11.3~ 12.3) | 11.8 ± 0.3 (11.3~ 12.4) | 0.20 |
| Turn-over rate (per month) | 4.32 | 4.53 | 0.09 |
| Mortality | 207(22.6%) | 207(21.9%) | 0.62 |
| Length of stay (days) | 7.2 ± 1.4 (5.6~8.7) | 6.5 ± 1.6 (4.8~8.2) | 0.01 |

### 0037

#### Factors associated to poor outcome and management in critically ill elderly patients

A. Marculeta Juanicorena[1], I. Arbelaiz Beltran[1], L. Costa Quintela[1], M.I. Calvo Monge[1], G. Rodriguez Calero[1], N. Mas Bilbao[1], U. Aguirre Larracoechea[2], P.M. Olaechea Astigarraga[1]
[1]Hospital de Galdakao-Usansolo, Intensive Care Unit, Galdakao-Usansolo, Spain; [2]Hospital de Galdakao-Usansolo, Research Unit, Galdakao-Usansolo, Spain
**Correspondence:** A. Marculeta Juanicorena
*Intensive Care Medicine Experimental* 2018, **6(Suppl 2):**0037

**INTRODUCTION.** During the last decades our society is growing older, and so do the patients admitted to Intensive Care Units (ICU)[1]. Thus, resources management acquires a strategic importance, due to limited means. We need studies to determine which patients will benefit from being admitted to an ICU in order to provide a better care.

**OBJECTIVES.** To describe the population of patients over 79 years old admitted to our ICU and the applied level of invasiveness in management. To search for mortality related factors in the elderly.

**METHODS.** Retrospective monocentric study based on data collection and analysis from electronic clinical records in those patients older than 79 years old admitted in our ICU between 2006-2016. We excluded patients admitted only for programmed procedures and had no complications. We collected variables concerning demographic data, comorbidity, clinical course and procedures. We used logistic regression to describe factors associated with in-hospital mortality. Results expressed in mean value and standard deviation (SD) for numerical variables and percentage over total patients for categorical variables.

**RESULTS.** 1360 elderly patients between 80 and 99 years were admitted to ICU during the studied period, 11.9% of the total ICU patients. After exclusions, 84.0% were analysed. The mean age was 83.2 years old and 61.9% of the patients were male. The most common comorbidities were diabetes mellitus (30.5%), isquemic cardiomyopathy (24.0%) and peripheric vasculopathy (17.4%). The mean Charlson index score was 5.8 points (SD 1.6 points). In 87.0% of the patients the admission cause was medical and only 30.0% of them were due to coronary heart disease. Vasoactive drugs were used in 26.4% of the cases, mechanic ventilation in 9.7% and renal replacement therapy in 2.5%. Delirium in ICU was registered in 12.7% of the patients. Limitation of life-sustaining treatment (LST) occurred in 12.2%. Mortality in ICU was 9.5% and in-hospital mortality 14.0%. In the multivariant analysis, in-hospital mortality was associated with limitation of LST (OR 122.8; CI 95% 47.8-315.4), urgent surgery as ICU admission cause (OR 6.0; CI 95% 2.0-18.5), use of vasoactive drugs (OR 6.0; CI 95% 2.6-13.9), renal replacement therapy (OR 5.5; CI 95% 1.1-27.0) and mechanic ventilation (OR 4.8; CI 95% 1.9-11.7).

**CONCLUSIONS.** Elderly patients are a very significant population in our ICU. No relation has been found between mortality and previous comorbidity; mortality and ICU admission cause are associated. The outcome is worse when the management is more aggressive.

**REFERENCE(S)**

1 Olaechea PM et al. Characteristics and outcomes of patients admitted to Spanish ICU: A prospective observational study from the ENVIN-HELICS registry (2006-2011). Med Intensiva. 2016 May;40(4):216-29. doi: 10.1016/j.medin.2015.07.003.

### 0038

#### ABO blood group and short-term and long-term mortality in a large cohort of ICU patients

S.P. Kevenaar, M.W.N. Nijsten
UMCG, Intensive Care, Groningen, Netherlands
**Correspondence:** M.W.N. Nijsten
*Intensive Care Medicine Experimental* 2018, **6(Suppl 2):**0038

**INTRODUCTION.** With the exception of sex, genetic markers have not been widely used in ICU cohorts. A very common recorded genetic feature in medicine is ABO blood group. It is known that populations with different genetic backgrounds may display different ABO blood group distributions [1]. Recent research showed that a non-O blood group was associated with higher risk of arterial and venous thromboembolic events [2], possibly related to higher circulating levels of von Willebrand factor [3]. Whether a non-O blood group is associated with outcome has not been studied in ICU patients.

**OBJECTIVES.** The objective of this study was to assess the association of blood group non-O with in-hospital mortality and long-term outcome in ICU patients.

**METHODS.** This study was a retrospective cohort study. Over a period of 24 years (1992-2015) laboratory findings were recorded in an anonymized database of patients admitted to the ICU as previously described [4]. Amongst these laboratory findings ABO blood group was determined. The presence or absence of blood group O was used as the independent variable. In-hospital mortality and long-term survival after ICU admission were the main outcomes. The chi-square test and Kaplan-Meier survival analysis with the log-rank test were used for statistical analysis

**RESULTS.** The database contained more than 48,000 patients with ABO blood group data, of these patients, 63% were male and 37% female with a mean ±SD age 60 ±15years. The prevalence of blood groups O, A, B and AB was 46%, 42%, 8.7% and 3.4% respectively. Rhesus positivity and negativity were 83.6% and 16.4% respectively . Hospital mortality was 12.4%. For blood group O versus non-O, hospital mortality was 12.3% and 12.5% respectively (P= 0.66;chi-square test). Kaplan-Meier curves with a follow up of up to 20 years showed similar survival for blood groups O and non-O (P=0.215; with log-rank test).

**CONCLUSIONS.** In our large cohort of ICU patients from the northern part of the Netherlands, ABO blood group non-O was not associated with outcome.

### 0039

#### Association of the Modified Frailty Index (MFI) with resource use and short-term outcomes in 129,680 critically ill patients

F. Zampieri[1], L. Taniguchi[2], J. Salluh[3], F. Bozza[4], M. Soares[3], ORCHESTRA Study Investigators
[1]HCor-Hospital do Coração, São Paulo, Brazil; [2]University of São Paulo School of Medicine, Intensive Care Unit, Sao Paulo, Brazil; [3]D´Or Research Institute, Rio de Janeiro, Brazil; [4]Fiocruz, Rio de Janeiro, Brazil
**Correspondence:** F. Zampieri
*Intensive Care Medicine Experimental* 2018, **6(Suppl 2):**0039

**INTRODUCTION.** The MFI is a composite summary instrument widely used to assess frailty and is associated with outcomes in hospitalized patients. However, studies evaluating MFI use in ICU patients are still scarce.

**OBJECTIVES.** To evaluate the association of the MFI hospital mortality and resource use in a large cohort of critically ill patients.

**METHODS.** Retrospective cohort study in 129,680 admitted to 93 Brazilian ICUs during 2014 and 2015. MFI encompasses 11 domains related to frailty (comorbidities, previous acute complications and functional capacity) in a 0-11 scale. Using MFI, we classified patients in no-frail (0), pre-frail (1-2) and frail (≥3). Primary outcome was hospital mortality. Secondary outcomes were use of organ support during ICU stay, ICU and hospital LOS, and hospital discharge needing nursing care. We used multivariable mixed regression modeling to assess the association between MFI and the outcomes adjusting for confoudersn.

**RESULTS.** There were 40,779 (32%) no-frail, 64,407 (50%) pre-frail and 24,494 (18%) frail patients. Frail patients were older, more frequently admitted for non-surgical reasons and had higher illness severity. Frail patients had higher hospital mortality (OR 2.41, 95% CI 1.89-3.08); this association was more pronounced in patients with lower illness severity (Figure). Frail patients were more frequently discharged in need of nursing care (OR 1.86, 95% CI 1.26-2.75), required more often mechanical ventilation (OR 1.36, 95% CI 1.05-1.75), dialysis (OR 3.56 , 95% CI 2.76-4.61), vasopressors (OR 1.84, CI 95% 1.45-2.35) and blood transfusion (OR 1.69, CI 95% 1.43-2.01) during the ICU stay. Frail patients had higher ICU and hospital LOS, with lower hazard for discharge (hazard for ICU and hospital discharge of 0.94; 95% CI 0.92-0.96 and 0.79; 95% CI 0.78-0.81).

**CONCLUSIONS.** The MFI seems an interesting surrogate marker for frailty in large studies in ICU patients. Using this instrument, frailty was independently associated with short term outcomes and resource use in these patients.

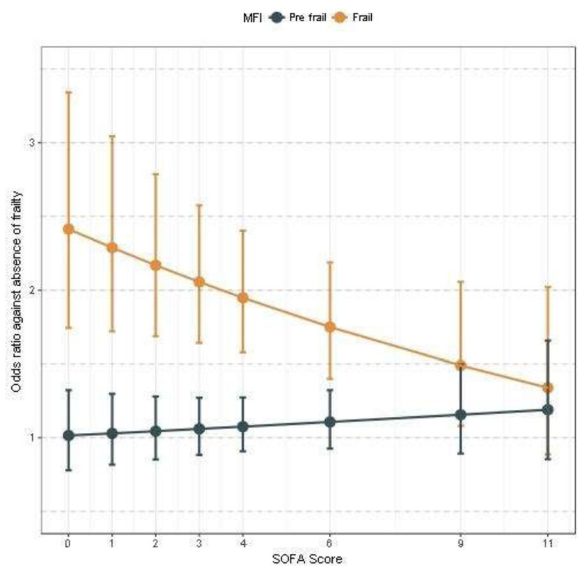

**Fig. 1 (abstract 0039).** Odds ratio for frailty and pre-frailty at increasing admission SOFA scores

### 0040

#### Validity of a modified sequential organ failure assessment using the richmond agitation-sedation scale in an international multicenter cohort

S. Rakhit[1,2,3], L. Wang[4], C.J. Lindsell[4], M.A. Hosay[1,5], J.W. Stewart[1,6], G.D. Owen[7], F. Frutos-Vivar[8,9], O. Peñuelas[8,9], A. Esteban[8,9], A.R. Anzeuto[10], E.W. Ely[2,3,11], E.E. Vasilevskis[2,11,12], M.B. Patel[1,2,3]

[1]Vanderbilt University Medical Center, Surgery, Nashville, United States; [2]Vanderbilt University School of Medicine, Nashville, United States; [3]Vanderbilt Critical Illness, Brain Dysfunction, and Survivorship Center, Nashville, United States; [4]Vanderbilt University Medical Center, Biostatistics, Nashville, United States; [5]Baylor University, Waco, United States; [6]Meharry Medical College, Nashville, United States; [7]Vanderbilt University Medical Center, Pharmacy, Nashville, United States; [8]Hospital Universitario de Getafe, Madrid, Spain; [9]Centro de Investigación Biomédica en Red de Enfermedades Respiratorias, Madrid, Spain; [10]University of Texas Health Science Center at San Antonio, Medicine, San Antonio, United States; [11]Vanderbilt University Medical Center, Medicine, Nashville, United States; [12]Vanderbilt Center for Health Services Research, Nashville, United States

**Correspondence:** S. Rakhit

*Intensive Care Medicine Experimental* 2018, **6(Suppl 2):**0040

**INTRODUCTION.** ICU mortality can be predicted using the Sequential Organ Failure Assessment (SOFA) score, whose neurologic subscore is based on the Glasgow Coma Scale (GCS). But, GCS is not reliably measured in all ICUs, and many use other neurologic assessments, such as the reliable and validated Richmond Agitation-Sedation Scale (RASS). Single center data suggested a RASS-based SOFA (mSOFA) predicted ICU mortality.

**OBJECTIVES.** In a prospective, multicenter, international ICU cohort, we aimed to validate a mSOFA using RASS, which we hypothesized would be comparable to the GCS-based SOFA.

**METHODS.** In our nested cohort within the 2016 Fourth International Study of Mechanical Ventilation, we found ICU subjects with both RASS and GCS assessments. We transformed daily RASS into GCS via a proportional odds logistic regression model adjusting for neurologic injury (stroke, traumatic brain injury) and daily exposure to propofol, dexmedetomidine, benzodiazepines, and/or opiates. ICU mortality logistic regression models and c-statistics were constructed using SOFA (GCS-based) and mSOFA (RASS-transformed), adjusted for age, sex, body-mass index (BMI), region (Europe [reference], North America, South America, Africa, Asia, Australia-New Zealand), and post-operative status (medical/surgical).

**RESULTS.** In 4,120 patients, representing 20,023 patient-days across 32 countries, the mean SOFA=9.4+/- 2.8 and mean mSOFA=10.0 +/-2.3, with ICU mortality=31%. Mean SOFA and mSOFA similarly predicted ICU mortality (SOFA: AUC=0.784, 95%CI=0.769-0.799; mSOFA: AUC=0.778, 95%CI=0.763-0.793, P=0.139). Across models, other strong independent predictors of mortality included higher age, female sex, medical patient, and African region (all P< 0.001).

**CONCLUSIONS.** We present the first mSOFA model in an externally generalizable multicenter international clinical cohort. RASS transformed into GCS preserves predictive validity for ICU mortality. ICU mortality may be influenced by sex, post-operative status, and world region. Alternative neurologic measurements can be viably integrated into severity of illness scoring systems.

**GRANT ACKNOWLEDGMENT**

Supported by Vanderbilt and NIH (UL1 TR000445, R01 GM120484, R01 AG035117, R01 HL111111, R01 AG053264).

## Nutrition in specific conditions

### 0041

#### Metabolomic basis for clinical response to high dose vitamin D in critical illness

K. Amrein[1], J. Lasky-Su[2], K. Christopher[3]

[1]Medical University of Graz, Division of Endocrinology and Metabolism, Graz, Austria; [2]Brigham and Women's Hospital, Channing Division of Network Medicine, Boston, United States; [3]Brigham and Women's Hospital, Boston, United States

**Correspondence:** K. Christopher

*Intensive Care Medicine Experimental* 2018, **6(Suppl 2):**0041

**INTRODUCTION.** The relationship among the disruption of metabolic homeostasis in critical illness and the effects of treatment remains obscure.

**OBJECTIVES.** We conducted an ancillary study on plasma samples from the VITdAL-ICU trial to determine whether vitamin $D_3$ alters the metabolic landscape of critical illness over time. The VITdAL-ICU trial randomized 475 critically ill adult patients with 25(OH)D< 20 ng/mL to 540,000 IU vitamin $D_3$ or placebo given orally.

**METHODS.** 1255 plasma samples from 453 patients at randomization, day 3 and day 7 were analyzed using four ultra high-performance liquid chromatography/tandem accurate mass spectrometry methods identifying 769 metabolites. We removed metabolites with the lowest interquartile range of variability, leaving 576 metabolites. The data was normalized with cube root transformation and Pareto scaling.

Response to vitamin $D_3$ was defined as an increase of 25(OH)D> 10 ng/mL between baseline and day 3. Correlations between individual metabolites and response to vitamin $D_3$ over time were determined via linear mixed models correcting for age, gender, baseline 25(OH)D, SAPS II score, admission diagnosis and plasma day. Mixed effects logistic regression was used to estimate the odds of 28-day mortality of individual metabolites adjusted for response to vitamin $D_3$, age, gender, baseline 25(OH)D, SAPS II score, admission diagnosis and plasma day. A multiple test-corrected threshold of $P< 8.68 \times 10^{-5}$ was used to identify all significant associations.

**RESULTS.** 23.8% of the cohort demonstrated a response to vitamin $D_3$. 28-day mortality was 24.3%. 28-day mortality in responders to vitamin $D_3$ was 11.7%. In non-responders the 28-day mortality was 28.3%. In responders to vitamin $D_3$, the odds for 28-day mortality was decreased by 68% [OR 0.32 (95%CI 0.16-0.64) P=0.001] relative to non-responders following adjustment for age, gender, baseline 25(OH)D level, SAPS II score, and admission diagnosis.

44 metabolites had significant positive associations with response to vitamin $D_3$ dominated by increases in Sphingomyelins, Plasmalogens, Lysoplasmalogens and Lysophospholipids. Increases in 28 of these metabolites were significantly associated with decreases in 28-day mortality. 112 metabolites had significant negative associations with response to vitamin $D_3$ primarily by decreased Acylcarnitines, Phosphatidylethanolamines and Tryptophan metabolites. Decreases in 96 of these metabolites were significantly associated with decreases in 28-day mortality.

**CONCLUSIONS.** Our observations have biological plausibility and relevance to the observed improved outcomes in responders to vitamin $D_3$. The changes in Sphingomyelins, Plasmalogens, Lysophospholipids and Acylcarnitines are reflective of stress response, antioxidants, immunomodulation and mitochondrial dysfunction respectively. Dynamic metabolomics can illuminate complex mechanisms associated with response to interventions in critical illness.

**GRANT ACKNOWLEDGMENT**

NIH R01GM115774

### 0042

#### Prognostic relationship between the Maximum Acute Gastrointestinal Injury score and caloric intake in critically ill patients requiring vasopressors: a multicenter cohort study from the Sociedad Argentina de Terapia Intensiva (SATI)

C.I. Loudet[1,2], M. Perman[3], M.C. Marchena[1], L.I. Tumino[1], R. Gimbernat[4], M.L. Cabana[5], G. Capurro[6], P. Astegiano[7], M.A. Velásquez[8], M. Casanova[9], M.C. Roth[10], G. Roda[11], P. Okursati[12], Y. Balmaceda[4], J. Rodríguez Bugueiro[9], R. Reina[1], E. Estenssoro[1], and other investigators from the CALNUCI group

[1]Hospital San Martín, La Plata, Argentina; [2]Universidad Nacional de La Plata, La Plata, Argentina; [3]Asociación Argentina de Nutrición Enteral y Parenteral (AANEP), Buenos AIres, Argentina; [4]Centro de Cuidados Intensivos, San Juan, Argentina; [5]Hospital Pablo Soria, Jujuy, Argentina; [6]Hospital Interzonal General de Agudos Oscar Alende, Mar del Plata, Argentina; [7]Hospital José María Cullen, Santa Fe, Argentina; [8]Sanatorio Nuestra Señora del Rosario, Santa Fe, Argentina; [9]Hospital El Cruce, Florencio Varela, Argentina; [10]Hospital San Juan de Dios, La Plata, Argentina; [11]Hospital Municipal Eva Perón, Merlo, Argentina; [12]Casa Hospital San Juan de Dios, Ramos Mejía, Argentina

**Correspondence:** C.I. Loudet

*Intensive Care Medicine Experimental* 2018, **6(Suppl 2):**0042

**INTRODUCTION.** Whether dose or route of nutritional support in patients with different grades of gastrointestinal dysfunction and requiring vasopressors affects the outcomes remains unclear.

**OBJECTIVES.**

1. To evaluate the prognostic relationship between the Maximum Acute Gastrointestinal Injury Score($AGI_{max}$) and the mean Kcal/kg received in patients requiring vasopressors, and the role of supplemental parenteral nutrition.
2. To identify hospital mortality determinants in this same population.

**METHODS.** Prospective multicenter cohort study carried out in 10-ICUs in Argentina. Consecutive patients admitted to the ICU on mechanical ventilation ≥48 hr, requiring vasopressors (>0.1μ/kg/min) and receiving enteral nutrition (EN) were included. Epidemiological data, APACHE II, $SOFA_{24}$, NUTRIC scores, days on vasopressors, mean kcal/kg/day intake, and use of supplemental parenteral nutrition were collected. Acute gastrointestinal Injury (AGI) score was calculated daily; the maximum AGI ($AGI_{max}$) was identified, and patients were classified in 5 categories (0 to 4) according to AGI score*. Comparisons of clinical and outcome variables were performed in 3 predetermined groups, according to the mean amount of calories received via EN: < 10 kcal/kg/d, ≥10 to < 20 kcal/kg/d, or ≥20 kcal/kg/d. During episodes of shock, the decision to maintain, decrease or withhold EN was taken by attending physicians. Hospital mortality was the main endpoint. A Kaplan-Meier curve was built to assess hospital mortality according to the 3-different kcal/kg groups. Differing variables from survivors and nonsurvivors ($p< 0.20$) were entered into a logistic regression model. A p value ≤0.05 was considered significant. STATA 14 software was used for statistical analysis.

**RESULTS.** Out of 800 patients, 494 (62%) required vasopressor therapy. Hospital mortality was 53%. Tables 1 and 2 show characteristics and outcomes of the 3 subgroups of patients on vasopressors. Figure 1 shows a Box-plot graph of $AGI_{max}$ and the 3 caloric subgroups, as well as the K-M curve of hospital mortality according to the 3 caloric subgroups. Table 3 shows the multiple logistic rogression model for hospital mortality.

**CONCLUSIONS.**

1. Patients who received < 10 kcal/kg were more severely ill, had more organ failures, recorded more $AGI_{max}$ and hospital mortality. However, patients who received ≥10kcal/kg, presented lower $AGI_{max}$ and hospital mortality.
2. Determinats of hospital mortality were age, Charlson score, type of admission, and $AGI_{max}$ .
3. $AGI_{max}$ shows a direct relationship with hospital mortality and an inverse relationship with caloric intake. Therefore, kcal intake cannot be considered a protective factor for hospital mortality in patients receiving vasopressors, but it reflects/evidences this lower mortality.
4. Supplemental Parenteral Nutrition in this population might be beneficial, but its role needs to be evaluated further.

*Reintam et al, ESICM Working Group on Abdominal Problems, ICM 2012;38:384.*

**Table 1 (abstract 0042).** Characteristics of the 3 subgroups of patients on vasopressors

| Patients 494 | Group1 Kcal/kg received <10 | Group2 Kcal/kg received ≥ 10/ < 20 | Group3 Kcal/kg received ≥20 | p |
|---|---|---|---|---|
| Patients (N,%) | 85 (17) | 305 (62) | 104 (21) | |
| Age (mean, SD) | 47±19 | 47±19 | 47±20 | 0.75 |
| Male gender (N, %) | 55/85(67) | 198/305(65) | 66/104(64) | 0.80 |
| BMI (kg/m2) (mean, SD) | 23±8 | 22±9 | 22±6 | 0.10 |
| Charlson score (mdn, IQR) | 0 [0-1] | 1 [0-2] | 0 [0-1] | 0.24 |
| Type of admission (Medical/emergency /elective surgery) (%) | 43/51/6 | 54/39/7 | 56/35/9 | 0.20 |
| APACHE II (mean, SD) | 22±6 | 18±6 | 18±6 | 0.000 |
| SOFA score (mdn, IQR) | 10 [8-11] | 8 [6-10] | 7 [5-9] | 0.000 |
| Days of vasopressors (mdn, IQR) | 5 [3-7] | 5 [3-7] | 3 [2-6] | 0.002 |

**Table 2 (abstract 0042).** Nutritional and outcome variables of the 3 subgroups of patients on vasopressors

| Patients 494 | Group 1 Kcal/kg received <10 | Group 2 Kcal/kg received ≥ 10/< 20 | Group 3 Kcal/kg received ≥20 | p |
|---|---|---|---|---|
| Patients (N, %) | 85(17) | 305(62) | 104 (21) | |
| NUTRIC score (mdn,IQR) | 6 [5-6] | 5 [3-5] | 4 [3-5] | 0.000 |
| AGImax (0/1/2/3/4) (%) | 0/1/11/61/27 | 0/16/36/38/10 | 0/64/33/0/0 | 0.000 |
| AGImax (mdn, IQR) | 3 [3-4] | 2 [2-3] | 1 [1-2] | 0.000 |
| Early EN (N, %) | 23/85 (27) | 163/305(53) | 24/104(23) | 0.000 |
| Supplemental Parenteral Nutrition (N, %) | 9/85(11) | 37/305 (12) | 2/104 (2) | 0.000 |
| ICU length of stay (mdn, IQR) | 8 [5-13] | 16 [10-26] | 22 [13-33] | 0.000 |
| Length of MV (mdn, IQR) | 7 [5-11] | 12 [8-20] | 16 [9-26] | 0.000 |
| Hospital Mortality (N, %) | 74/85(87) | 163/305(53) | 24/104(23) | 0.000 |

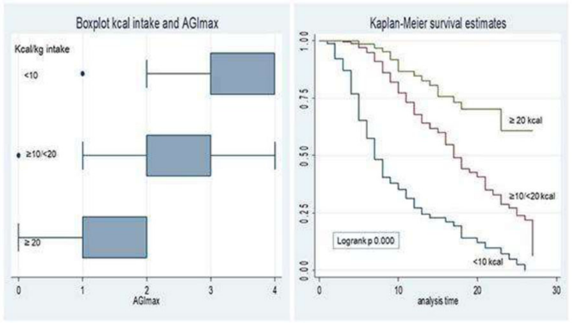

**Fig. 1 (abstract 0042).** Boxplot AGImax and caloric intake and K-M for hospital mortality and caloric intake

**Table 3 (abstract 0042).** Multiple logistic regression model for hospital mortality

| Variable | Odds Ratio | 95% Confidence Interval | p value |
|---|---|---|---|
| Age | 1.02 | 1.01-1.03 | 0.008 |
| Charlson score | 1.24 | 1.06-1.45 | 0.006 |
| Days of vasopressors | 1.07 | 1.01-1.13 | 0.039 |
| Type of admission | 7.07 | 2.30-21.7 | 0.001 |
| Kcal/kg ≥ 10/< 20 | 0.27 | 0.11-0.63 | 0.003 |
| Kcal/kg ≥20 | 0.17 | 0.06-0.50 | 0.001 |
| AGImax | 2.25 | 1.61-3.16 | 0.000 |
| Supplemental Parenteral Nutrition | 0.30 | 0.11-0.78 | 0.014 |

### 0043

#### Liver steatosis in brain-dead donors, progressing pattern and affecting factors

K. Kyoung[1], H. Suk-Kyung[2], L. Sung-Gyu[2], S. Hwang[2], K.-H. Kim[2]
[1]University of Ulsan College of Medicine/Ulsan University Hospital, General Surgery/Trauma Center, Ulsan, Korea, Republic of; [2]University of Ulsan College of Medicine/Asan Medical Center, General Surgery, Seoul, Korea, Republic of
**Correspondence:** K. Kyoung
*Intensive Care Medicine Experimental* 2018, **6(Suppl 2):**0043

**INTRODUCTION.** Steatotic liver grafts reduce the success rates of liver transplantation. Diabetes and obesity have been known as risk factors for liver steatosis (LS). Insulin resistance is frequently associated with critical illness and LS is a common phenomenon in the ICU patients. But there has been no interest the links between acute insulin resistance and LS in the area of intensive care.

**OBJECTIVES.** We hypothesized that acute insulin resistance in brain-dead donors (BDDs) may promote the development of LS. The purpose of the current study was to evaluate the effect of acute insulin resistance on LS and its progressing pattern in BDDs.

**METHODS.** A retrospective cohort study was conducted between January 2003 and December 2017. The liver samples were classified into a mild (< 30%) and a moderate-to-severe (≥ 30%) (MTS) steatotic group. Serum glucose level was adopted to reflect insulin resistance. According to the recommendation of the Western Pacific Regional Office of the WHO, BDDs with body mass index (BMI) < 18.5 kg/m$^2$ and ≥ 30 kg/m$^2$ were excluded to avoid a bias by extreme values.

**RESULTS.** A total of 179 BDDs were included in the study. MTS was found in 29 BDDs (15.1%). The length of predonation period was negatively correlated with steatosis ($r$ = -0.379, $P$ < 0.001). BMI ($r$ = 0.176, $P$ = 0.019) and the serum glucose level ($r$ = 0.267, $P$ < 0.001) were also correlated with steatosis significantly. The incidence of MTS was 25.2% in the group of predonation period less than 4 days and 10.9% more or equal than 4 days ($P$ = 0.014). The procurement rate of the liver reached 94.7% in mild steatotic group and 77.8% in MTS group ($P$ = 0.009).

**CONCLUSIONS.** Liver steatosis can be developed acutely in critically ill condition and is related to blood glucose level, reflecting insulin resistance. Therapeutic intervention that maintains proper glucose level and reduces both severity and duration of systemic inflammation is essential, not only to recovery of critically ill patients but also to improvement of graft quality by reducing liver steatosis.

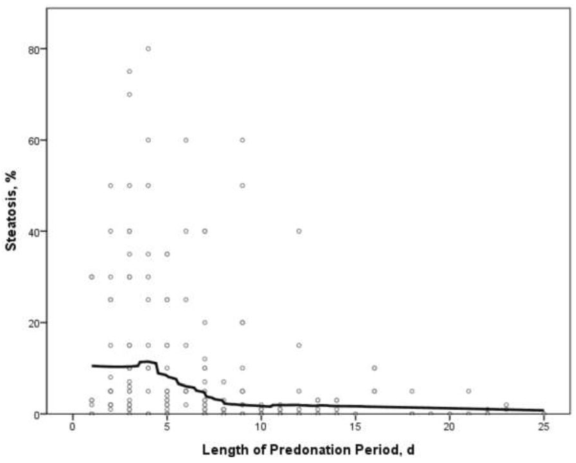

**Fig. 1 (abstract 0043).** Distribution of steatosis according to the length of predonation period

### 0044

#### Prevalence, nutrition risk evaluation and resource use of frail critically ill patients undergoing nutrition support therapy in a Brazilian tertiary hospital

L. Taniguchi[1,2], I.A.O. Souza[1,3], E.M.P. Siqueira[1], P.C. Ribeiro[3], Nutrition Support Team Hospital Sírio-Libanês
[1]Hospital Sirio Libanes, Sao Paulo, Brazil; [2]Hospital das Clínicas da Universidade de São Paulo (HC-FMUSP), Emergency Medicine Discipline, São Paulo, Brazil; [3]Hospital Sirio Libanes, Nutrition Support Team, Sao Paulo, Brazil
**Correspondence:** L. Taniguchi
*Intensive Care Medicine Experimental* 2018, **6(Suppl 2):**0044

**INTRODUCTION.** Frailty has recently been described as an independent predictor of short- and long-term outcomes in critically ill patients. Since nutrition might be associated with frailty, further characterization of nutritional status in critically ill patients with frailty is warranted.
**OBJECTIVE.** To describe the prevalence, nutrition risk scores and resource use associated with frailty among critically ill patients who required nutrition support therapy in a Brazilian tertiary hospital.
**METHODS.** We conducted a prospective single-center cohort study of patients admitted to the Intensive Care Unit (ICU) of Hospital Sírio-Libanês (São Paulo, Brazil), between October 2015 and March 2017. Frailty syndrome was classified using the Clinical Frailty Scale (CFS). Patients were considered to be frail if they had a score>4[1].
**RESULTS.** We studied 449 patients during the study period (mean age was 69.4 years, 64.8% were admitted for non-surgical reasons, 49.9% had cancer, 56.6% were frail and 27.2% died during hospitalization). Frail patients were more severely ill compared to non-frail patients (SAPS 3 of 52 [44 - 60] versus 37 [32 - 47] respectively; p< 0.001), were older, more frequently came from the emergency room due to non-surgical reasons, and had higher hospital mortality (36.6% vs 14.9% respectively, p< 0.001). Frailty was associated with lower body mass index (p=0.045) and higher NUTRIC scores (p< 0.001), but with a lower prevalence of significant weight loss (p=0.04) and lower MUST scores (p=0.012). Frail patients stayed longer in the ICU (p=0.03), and more frequently required enteral nutrition for a longer period (p< 0.001), although invasive organ support during ICU stay was not different comparing frail with non-frail patients.
**CONCLUSION.** Frailty is very common in our cohort of critically ill patients undergoing nutrition support therapy, and it was associated with higher hospital mortality. Although nutrition risk assessment performed differently, frail patients required enteral nutrition more frequently.

### 0045

#### Prospective observational study on vitamin D levels in liver transplantation recipients. DLiverX study

G. Martucci[1], R. Volpes[2], G. Panarello[1], D. Di Carlo[3], C. Ricotta[4], F. Tuzzolino[5], S. Gruttadauria[4], M. Piazza[1], G. Burgio[1], G. Vizzini[2], K. Amrein[6], A. Arcadipane[1]
[1]Ismett, Department of Anesthesia and Intensive Care, Palermo, Italy; [2]Ismett, Hepatology Unit, Palermo, Italy; [3]Ismett, Department of Laboratory Medicine, Palermo, Italy; [4]Ismett, Abdominal Surgery and Organ Transplantation Unit, Palermo, Italy; [5]Ismett, Research Office, Palermo, Italy; [6]Medical University of Graz, Department of Internal Medicine, Division of Endocrinology, Graz, Austria
**Correspondence:** G. Martucci
*Intensive Care Medicine Experimental* 2018, **6(Suppl 2):**0045

**INTRODUCTION.** Vitamin D has pleiotropic effects that extend beyond the muskuloskeletal system, with a strong association between a poor vitamin D status and excess morbidity and mortality. Vitamin D deficiency is common in patients with chronic liver disease, and has been associated with increased morbidity and mortality, but has not been extensively evaluated in patients undergoing orthotopic liver transplantation (OLT).
**OBJECTIVES.** Explore the level of Vitamin D before and after OLT in the first 28 days. Explore if vitamin D level is a marker of disease in OLT recipients and a marker of good graft function.
**METHODS.** Prospective observational study. Explorative analyses of vitamin D levels in 68 consecutive OLT recipients during the first 28 days after OLT in a liver transplantation center in southern Italy from September, 2016 to July, 2018.
**RESULTS.** Sixty-eight (68) OLT recipients were enrolled: Age 57 years ±7, BMI, 26 ±4, Child-Pugh 8 ±2, clinical MELD 15 ±8, HCC n. 43, Charlson Comorbidity Index 6 ±1.4, creatinine 0.9 mg/dl (0.72-1.27), MDRD 86 ±31, total calcium 8.5 mg/dl ±0.59, phosphate 3.30 mg/dl ±0.75, parathyroid hormone 51 pg/ml (35-80). The baseline vitamin D value was 8.7 ng/ml (6.2-12-7), indicating a severe deficiency. On Spearman correlation analysis, vitamin D was inversely associated with Child-Pugh and MELD (p< 0.01), and was significantly higher in the presence of hepatocarcinoma and when chronic kidney disease was not present.
During the first week after OLT the vitamin D value decreased further, while there was a consistent increase at 28 days (p< 0.01) (Table 2, Figure 1), In a multiple generalized linear model this increase was not influenced by the trend in creatinine or MDRD.
The baseline value of vitamin D was associated with the risk of a diagnosed invasive infection in the first 28 days after OLT: OR 0.87, 95% CI 0.78-0.97, p=0.01, and it was associated with increased ICU length of stay (LOS) (Spearman Correlation Coefficient -0.29; p=0.01).
The vitamin D value on day 7 was associated with hospital Lenght Of Stay (Spearman Correlation Coefficient -0.31; p=0.01).
The lower vitamin D levels at POD 28 were associated with an incomplete graft function (defined as INR>1.5 and/or bilirubin >2 mg/dl and/or large volume of ascites): 8.1 ng/mg (6.0 - 10.4) vs. 13.7 (10.9 - 16.4) p< 0.01.
**CONCLUSIONS.** The baseline vitamin D level was very low in a large population of adult OLT recipients, showed a strong correlation with pre-OLT disease severity, and was highly associated with ICU LOS and with the risk of acquiring invasive infection in the first 28 days after OLT. Moreover, at 28 days after OLT vitamin D showed a relevant association with graft function. Being a possibly modifiable factor, further studies should shed light on the role of vitamin D supplementation in this select population.

**Table 1 (abstract 0045).** Association of Vitamin D levels with baseline covariates

| | | |
|---|---|---|
| Male 9.8 (8.4-11.4) | Female 9.8 (6.8-12.9) | 0.96 |
| Hepatocarcinoma 11.4 (9.6-13.1) | No Hepatocarcinoma 7.3 (5.6-8.9) | <0.01 |
| Coronary Artery Dis. 14.2 (7.9-20) | No Coronary Artery Dis. 9.6 (8.2-10.9) | 0.10 |
| Diabetes 9.3 (6.8-11.7) | No Diabetes 10.2 (8.6-11.8) | 0.49 |
| Chronic Kidney Dis. 6.3 (3.1-9.5) | No Kronic Kidney Dis. 10.4 (8.9-11.8) | 0.03 |
| COPD 11.3 (7.8-14.7) | No COPD 9.5 (8-10.9) | 0.25 |
| Encephalopathy 7.8 (5.4-10.3) | No Encephalopathy 10.6 (9-12.2) | 0.07 |
| Bed Hospitalization >7 days 4.7 (1.1-10) | Bed Hospitalization<7 days 10.2 (8.8-11.5) | 0.05 |

**Table 2 (abstract 0045).** Mean Vitamin D values ± Standard Deviation at baseline and Post Operative Day (POD) 1,3, 7 and 28 after Orthotopic Liver Transplantation

| Baseline | POD 1 | POD 3 | POD 7 | POD 28 |
|---|---|---|---|---|
| 9.89 ± 5.51 | 5.97 ± 3.97 | 6.83 ± 4.05 | 6.55 ± 3.50 | 12.14 ± 8.58 |

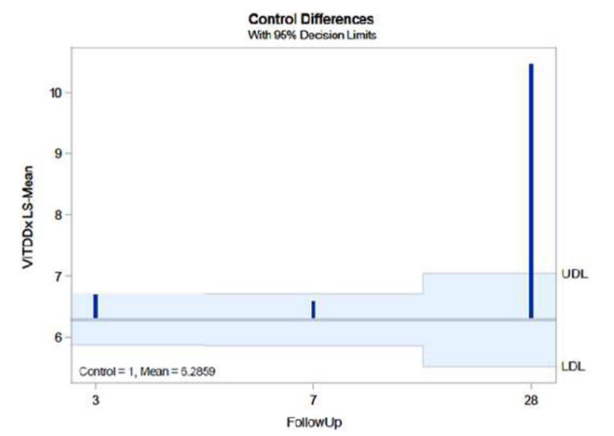

**Fig. 1 (abstract 0045).** Postoperative variation of vitamin D level

## End-of-life practices

### 0046

#### Ethical decision-making climate and intent to jobleave in the ICU

B. Van den Bulcke[1], R. Piers[2], H.I. Jensen[3], J. Malmgren[4], V. Metaxa[5], A.K. Reyners[6], M. Darmon[7], K. Rusinova[8], D. Talmor[9], A.-P. Meert[10], L. Cancelliere[11], L. Zubek[12], P. Maia[13], A. Michalsen[14], J. Decruyenaere[1], E. Kompanje[15], S. Vanheule[16], E. Azoulay[17], J. Roels[18], D. Benoit[1]
[1]Ghent University Hospital, Department of Intensive Care, Gent, Belgium; [2]Ghent University Hospital, Department of Internal Medicine, Gent, Belgium; [3]Lillebaelt Hospital Denmark, University of Southern Denmark, Institute of Regional Health Research, Vejle, Denmark; [4]Sahlgrenska University Hospital, Department of Anaesthesiology and Intensive Care, Gothenburg, Sweden; [5]King's College Hospital, Critical Care and Major Trauma, London, United Kingdom; [6]University Medical Center, Department of Internal Medicine, Groningen, Netherlands; [7]Saint-Etienne University Hospital, Saint-Etienne, France; [8]General University Hospital Charles University, Department of Anaesthesia and Intensive Care Medicine, Prague, Czech Republic; [9]Beth Israel Deaconess Medical Center and Harvard Medical School, Department of Anesthesia, Critical Care and Pain Medicine, Boston, United States; [10]Institut Jules Bordet, Service des Soins Intensifs et Urgences Oncologiques, Bruxelles, Belgium; [11]Azienda Ospedaliero Universitaria Maggiore della Carità, Novara, Italy; [12]Semmelweis University, Budapest, Hungary; [13]Hospital S.António, Intensive Care Department, Porto, Portugal; [14]Tettnang Hospital, Tettnang, Germany; [15]Erasmus Hospital, Department of Intensive Care, Rotterdam, Netherlands; [16]Ghent University, Department of Psychoanalysis and Clinical Consulting, Ghent, Belgium; [17]Hôpital Saint Louis, Service de Réanimation Médicale, Paris, France; [18]Ghent University, Department of Applied Mathematics and Computer Science and Statistics, Ghent, Belgium
**Correspondence:** B. Van den Bulcke
*Intensive Care Medicine Experimental* 2018, **6(Suppl 2):**0046

**INTRODUCTION.** ICUs are challenged to retain clinicians and therefore need to understand why they are leaving their career prematurely. Beside organizational issues, the quality of interprofessional collaboration during ethical decision-making (EDM) may further affect the intent to jobleave.

**OBJECTIVES.** To determine the relationship between the EDM climate and the intent to job leave in the ICU.

**METHODS.** During April-May 2014, perceptions of EDM climate among clinicians of 68 adult ICUs in 13 European countries and the US were measured, using a self-assessment questionnaire, together with the intent to jobleave (1). The EDM climate questionnaire (EDMCQ) was validated and determined 7 important factors (2): mutual respect within the interdisciplinary team, open interdisciplinary reflection, ethical awareness, self-reflective physician leadership, active decision-making by physicians, more specifically at end-of-life, and involvement of nurses at end-of-life. We used hierarchical mixed effect models to assess associations between these 7 factors and the intent to job leave in clinicians within ICUs, within hospitals and within countries. Results are expressed in odds ratios together with 95% confidence intervals.

**RESULTS.** Of the 3610 nurses and 1137 physicians providing ICU bedside care, 63.1% and 62.9% participated respectively. Of the 2992 participating clinicians, 782 (26.1%) had the intention to leave their current job, of which 615 (78.6%) were nurses, 74 (9.5%) junior and 93 (11.9%) senior physicians. Factors significantly associated with a higher intent to jobleave were the Big Mac index of the country (OR 1.59 [95% CI 1.006- 2.53]), the numbers of hours working per week (1.01 [1.001-1.02]) and the mortality in the ICU (1.03 [1.003-1.05]). ´Mutual respect within the interdisciplinary team' (0.77 [0.66-0.90]), ´open interdisciplinary reflection' (0.73 [0.62-0.86]) and 'active decision-making by physicians' (0.87 [0.75-1.006]) more specifically at end-of-life (0.87 [0.77-0.98]) were associated with a lower intent to jobleave together with the clinicians' older age (0.98 [0.97-0.99]).

**CONCLUSIONS.** This is the first large multicenter study showing an independent association between intent to jobleave and the quality of the EDM climate. Interventions to reduce intent to jobleave should mainly focus on improving mutual respect, open interdisciplinary reflection and active decision-making by physicians at end-of-life in the ICU.

##### REFERENCE(S)

[1] Benoit D et al. Outcome in patients perceived as receiving excessive care across different ethical climates: a prospective study in 68 Intensive Care Units in Europe and the US. (Forthcoming 2018).
[2] Van den Bulcke B et al. Ethical decision-making climate in the ICU: theoretical framework and validation of a self-assessment tool. BMJ Qual Saf 2018

##### GRANT ACKNOWLEDGMENT

Supported by a ESCIM/ECCRN clinical research award and a FWO senior clinical investigators grant (1800513N) obtained in 2012 by DDB.

### 0047

#### Timing is everything: early DNR in the ICU matters for patient outcomes

D. Ouyang[1], D. Russell[2], D. Berlin[1,3], A. Su[1], Z. Cooper[4], P. Maciejewski[1,5,6], H. Prigerson[1,5], L. Lief[1,3]
[1]Weill Cornell Medicine, Center for Research on End of Life Care, New York, United States; [2]Appalachian State University, Department of Sociology, Boone, United States; [3]Weill Cornell Medicine, Division of Pulmonary and Critical Care, New York, United States; [4]Brigham and Women's Hospital, Center for Surgery and Public Health, Boston, United States; [5]Weill Cornell Medicine, Department of Medicine, New York, United States; [6]Weill Cornell Medicine, Department of Radiology, New York, United States
**Correspondence:** L. Lief
*Intensive Care Medicine Experimental* 2018, **6(Suppl 2):**0047

**INTRODUCTION.** Do-Not-Resuscitate (DNR) orders are intended to prevent cardiopulmonary resuscitation (CPR) of patients to avoid suffering at the end of life and allow a natural death. Nevertheless, little is known about the timing of DNR orders in patients' final intensive care unit (ICU) stay and its impact on patients' quality of death.

**OBJECTIVES.** To determine the relationship between the timing of DNR order placement in the ICU and patients' perceived suffering, peacefullness, and quality of death.

**METHODS.** 200 ICU patients at New York Presbyterian Hospital in New York and Brigham and Women's Hospital in Boston were assessed by the ICU nurses (n=100) who cared for them during their last week of life. Trained research staff interviewed the ICU nurses about their perceptions of the patients' experience using validated measures to assess the patients´ quality of death, extent of peacefulness, and suffering. Data from charts were abstracted in order to assess the timing of DNR orders in the patients´ terminal ICU stay. A

mixed-effects model that adjusted for the nurse as a random effect was used to model DNR timing and length of stay as fixed effects.

**RESULTS.** 77.5% (155/200) of enrolled patients had a DNR order at the time of death. 18% (36/200) of patients were admitted to the ICU with a pre-existing DNR order and 59.5% (119/200) had a DNR order placed during their ICU stay. 13% (26/200) of patients had a DNR placed within 48 hours of ICU admission (early DNR) and 46.5% (93/200) had DNR orders placed after 48 hours of ICU admission (late DNR). 42.5% (85/119) of patients had a DNR order placed within 24 to 48 hours of their death. Compared to those without a DNR order, patients with an early DNR order were much less likely to have a poor quality of death (27.6% vs. 54.5%; $p< 0.05$) and more likely to be "at peace" (56.0% vs. 38.8%; $p< 0.05$). [Table 1] Results from a mixed effects regression model adjusting for length of stay indicated that those with late DNR were significantly more likely than those with early DNR to experience suffering in the last week of life ($p< 0.05$).

**CONCLUSIONS.** At the time of death in the ICU, many patients have DNR orders in place, though most of these were placed after admission to the ICU and many in the last 1 to 2 days of life. Placement of a DNR order prior to ICU admission and earlier in the terminal ICU stay is associated with an improved quality of death. Ensuring earlier conversations about DNR among patients dying in the ICU may improve patients' quality of death and reduce suffering at the end of life.

**GRANT ACKNOWLEDGMENT**

This study was supported by a grant to Dr. Prigerson from the National Cancer Institute (CA197730 and CA218313).

**Table 1 (abstract 0047).** Patient Quality of Death by DNR Status

| | Early DNR | Late DNR (Within 48 Hours) | No DNR (After 48 Hours) | p-value |
|---|---|---|---|---|
| Poor Overall Death | 27.6% | 31.5% | 54.5% | 0.01 |
| Not at Peace | 14% | 27.1% | 38.5% | 0.03 |

### 0048

#### A quarter of cardiac arrest survivors admitted to an ICU in Greece should not have been resuscitated. The need for a Do Not Attempt Resuscitation (DNAR) policy

I. Andrianopoulos, A. Papathanasiou, G. Papathanakos, V. Salma, X. Zikou, V. Koulouras

University Hospital of Ioannina, Intensive Care Unit, Ioannina, Greece

**Correspondence:** I. Andrianopoulos

*Intensive Care Medicine Experimental* 2018, **6(Suppl 2):**0048

**INTRODUCTION.** DNAR orders are employed in cases where CPR is considered futile,or it will prolong patients´ suffering.Lack of DNAR policy in Greece probably results in an increased frequency of CPR performance in cardiac arrest cases with a questionable outcome.

**OBJECTIVES.** The purpose of this study was to identify those patients that survived a cardiac arrest and in whom CPR would have been deemed inappropriate and assess their final outcome.

**METHODS.** We retrospectively collected all data and recorded the outcome of all consecutive patients that were admitted to our ICU after surviving cardiac arrest during a 4 year-period.We reviewed each case separately and discussed whether CPR was appropriate in terms of patients´comorbodities,QoL and wishes.

**RESULTS.** 56 patients that survived a cardiac arrest and were admitted to our ICU were identified.26 out of 56(46%) survived to exit ICU.-When we retrospectively assessed each case separately we found that in 14/56(25%)CPR was inappropriate.From these 14 patients 13 passed away in ICU, and one was discharged home in coma receiving mechanical ventilation after a 3 month hospital admission.Inappropriateness was justified for the following reasons.7 patients had advanced dementia requiring assistance with most of their daily needs while 4 of them were bed-bound. 2 had advanced metastatic cancer not responding to treatment, 2 had end-stage disease(decompensated cirrhosis with hepatorenal syndrome not suitable for transplant, end-stage pulmonary fibrosis), one was elderly(88) with NYHAIV heart failure, one(bed bound for years with ependymomas of the spine) had expressed in the past that did not wish to receive CPR and a bed-bound patient with Duchenne´s muscular dystrophy was the only one to be discharged from hospital.Unfortunately there is neither DNAR regulation nor laws regarding treatment futility in Greece setting doctors in an area of medicolegal vulnerability.Doctors do need to practice medicine with the principle "first do no harm" and discussion among the caring physician, the patient as well as its NOK regarding the patient´s prognosis/outcome should take place well before the patient becomes moribund, allowing time for the patient and its family to decide on further treatment and CPR.This will reduce the cases of inappropriate CPR.

On the other hand medical societies in Greece need to campaign in favor of DNAR policy, demanding improvement in palliative care and to push-on for legislation amendments.It would be of great assistance if European medical societies were also involved in this process by providing counseling and making appeals for EU legislation on these matters.

**CONCLUSION.** CPR was retrospectively considered inappropriate in a quarter of patients that actually achieved return of spontaneous circulation resulting only in postponing patients´ death by a few hours/days.These findings underline the need for a DNAR policy and improvements in end-of-life care.

### 0049

#### Identifying improvement opportunities for patient- and family-centered care in the ICU: using qualitative methods to understand family perspectives

H.I. Jensen[1,2], A.S. Ågård[3], J.G.M. Hofhuis[4], M. Koopmans[5], R.T. Gerritsen[5], P.E. Spronk[4,6], R.A. Engelberg[7,8], J.R. Curtis[7,8], J.G. Zijlstra[9]

[1]Vejle and Middelfart Hospitals, Department of Anaesthesiology and Intensive Care, Vejle, Denmark; [2]University of Southern Denmark, Institute of Regional Health Research, Odense, Denmark; [3]Aarhus University Hospital, Department of Surgery and Intensive Care, Head and Heart Centre, Aarhus, Denmark; [4]Gelre Hospitals Apeldoorn, Department of Intensive Care Medicine, Apeldoorn, Netherlands; [5]Medisch Centrum Leeuwarden, Center of Intensive Care, Leuuwarden, Netherlands; [6]University of Amsterdam, Academic Medical Center, Amsterdam, Netherlands; [7]Harborview Medical Center, University of Washington, Division of Pulmonary, Critical Care and Sleep Medicine, Seattle, United States; [8]University of Washington, Cambia Palliative Care Center of Excellence, Seattle, United States; [9]University of Groningen, University Medical Center, Gronningen, Netherlands

**Correspondence:** M. Koopmans

*Intensive Care Medicine Experimental* 2018, **6(Suppl 2):**0049

**INTRODUCTION.** The illness and recovery of ICU survivors or the loss of a loved one impacts fundamental aspects of everyday family life. Therefore, high quality ICU care must also meet the needs of family members.

**OBJECTIVES.** To use free-text responses of family members on a family-experience questionnaire to provide richer context for families' quantitative assessments of the quality of ICU care, describe further quality areas of importance for families, and compare experiences of Danish and Dutch family members.

**METHODS.** Written comments from a total of 1,077 euroQ2 (euroFS-ICU and euroQODD) questionnaires (1, 2) focusing on family evaluation of the quality of care in the ICU completed in Denmark and The Netherlands were analyzed by content analysis.

**RESULTS.** More than half of participants provided comments describing a wide range of patient- or relative-related issues and providing a varied description of experiences in the ICU. Four themes emerged as important to families: information, clinician skills, ICU environment, and discharge from the ICU. Families highlighted the importance of receiving information that was accessible, understandable and honest. They indicated that quality care was ensured by having clinicians who were both technically and interpersonally competent. The ICU

environment and the circumstances of the transfer out of the ICU were described as contributing to experiences of quality of care. The comments identified room for improvement within all four themes. These analyses did not reveal differences between Danish and Dutch comments.
A total of 7% of family members (n=77) provided strongly negative comments, and of those 87% (n=67) had scored 7-10 in the quantitative assessment of overall quality of care (on a scale from 0-10) suggesting an important disconnect between the quantitative and qualitative results.
**CONCLUSIONS.** Family members identified information, clinician skills, ICU environment, and discharge from the ICU as issues that contributed significantly to their perception of the quality of ICU care. The study suggests the important role for incorporating both qualitative and quantitative sources of data to enable a more complete picture of family experiences in the ICU.

##### REFERENCE(S)

1. Jensen HI, Gerritsen RT, Koopmans M, Downey L, Engelberg RA, Curtis JR, Spronk PE, Zijlstra JG, Ørding H. Satisfaction with quality of ICU care for patients and families: the euroQ2 project. Crit Care 2017;21:239
2. Gerritsen RT, Jensen HI, Koopmans M, Curtis JR, Downey L, Hofhuis JGM, Engelberg RA, Spronk PE, Zijlstra JG. Quality of dying and death in the ICU. The euroQ2 project. J Crit Care 2017;44:376-382.

##### GRANT ACKNOWLEDGMENT

The study was supported by The Region of Southern Denmark, The Novo Nordic Foundation, Denmark (11415), The Augustinus Foundation, Denmark (14-2421), and The Frisian ICU Research Fund, The Netherlands

#### 0050

#### Ethical decisions in patients with severe acute respiratory failure: determining factors, time of decision and impact on outcome: the eDecide project An ancillary analysis of LUNG SAFE study

M. Berleur[1], T. Pham[2], J.-D. Ricard[1,3], M. Uchida[4], B.T. Thompson[5], J. Messika[1,3], D. Dreyfuss[1,3], D. Roux[1,3], LUNG SAFE Investigators.
[1]Assistance Publique Hopitaux de Paris - Hopital Louis Mourier, Intensive Care Unit, Colombes, France; [2]University of Toronto, Interdepartmental Division of Critical Care Medicine, Toronto, Canada; [3]Paris Diderot University, INSERM UMR 1137 IAME, Paris, France; [4]Dokkyo medical university, Department of Emergency and Critical Care Medicine, Tochigi, France; [5]Massachusetts General Hospital, Department of Medicine, Division of Pulmonary and Critical Care, Boston, United States
**Correspondence:** M. Berleur
*Intensive Care Medicine Experimental* 2018, **6(Suppl 2):**0050

**INTRODUCTION.** Withholding and withdrawing life-sustaining therapy (LST) are complex ethical decisions made in many critically ill patients. End of life decisions (EOL) are part of intensive care daily practice and they may be influenced by many factors beyond patient characteristics and severity.
**OBJECTIVES.** We aimed to describe EOL decisions in patients with acute respiratory failure and their impact on patients' prognosis.
**METHODS.** The centers participating in the international observational LUNG SAFE study included all patients with acute hypoxemic respiratory failure over a one-month period. More than 450 ICUs from 50 countries were involved in the project. Demographic, clinical and biological data were compared between patients with and without decisions of LST limitation. We also compared LST decisions according to the model of physician/patient relationships. Countries were classified as directed physician decisions or patient autonomy based on published data. This classification was validated by the countries coordinators.
**RESULTS.** A total of 4041 hypoxemic patients were included. Overall mortality was 37.2%. A decision of withholding or withdrawing LST was reported in 948 patients (23.4%). Mortality in patients with EOL decisions was 84.4% (N=800).
In bivariate analysis, patients with a decision of LST limitation were older, had a lower body weight, a higher SOFA score, were more frequently hospitalized for a medical condition; they also presented with more frequent active neoplasia/immunosuppressionor chronic liver failure (p< 0.001 for all). Patients admitted for non-cardiogenic shock had more LST limitation decisions (p=0.018). Patients admitted after trauma, drug overdose orpulmonary contusion were less likely to have a LST limitation decision (p< 0.001). EOL decisions were less frequent in lower-middle income countries as compared to high and middle-high income countries (p< 0.001) and less frequent in countries with physician directed relationship model than in countries with patient autonomy model.
Almost half of the 1503 patients who died during their hospital stay did not receive a decision of LST limitation (46.8%, N=703).
**CONCLUSIONS.** Decisions of LST limitation are frequent in the ICU, concerning almost one quarter of hypoxemic patients. Not only patients' characteristics but also health system (patient-physician relationship, gross domestic product) seem to impact these decisions. Almost half of the patients who eventually died in hospital had not been the subject of a decision of LST limitation.

##### REFERENCE(S)

Bellani G, Laffey JG, Pham T, Fan E, Brochard L, Esteban A, et al. Epidemiology, patterns of care, and mortality for patients with acute respiratory distress syndrome in intensive care units in 50 countries. JAMA. 2016;315:788-800.

### Spontaneous breathing: Good or bad?

#### 0051

#### Spontaneous breathing in early acute respiratory distress syndrome: insights from the LUNG SAFE study

F. van Haren[1], T. Pham[2], L. Brochard[3], G. Bellani[4], J. Laffey[3], M. Dres[3], E. Fan[5], E. Goligher[6], L. Heunks[7], J. Lynch[8], H. Wrigge[9], D. McAuley[10], LUNG SAFE Investigators
[1]The Canberra Hospital, Woden, Australia; [2]Hôpital Tenon, Paris, France; [3]St Michael's Hospital, Toronto, Canada; [4]University of Milan-Bicocca, Monza, Italy; [5]Mount Sinai Hospital, Toronto, Canada; [6]University of Toronto, Toronto, Canada; [7]VU University Medical Centre, Amsterdam, Netherlands; [8]Liverpool Hospital, Sydney, Australia; [9]University of Leipzig, Leipzig, Germany; [10]Queen's University Belfast, Belfast, United Kingdom
**Correspondence:** F. van Haren
*Intensive Care Medicine Experimental* 2018, **6(Suppl 2):**0051

**BACKGROUND.** Ventilatory modes that rely on patient spontaneous breathing (SB) activity are increasingly used in patients with acute respiratory distress syndrome (ARDS). The effects of SB during invasive mechanical ventilation on relevant patient outcomes are controversial and largely unknown.
**METHODS.** Planned secondary analysis of the LUNG SAFE study[1], to describe the characteristics and outcomes of patients with or without SB. Because inspiratory effort was not measured in LUNG SAFE, SB was defined by actual respiratory rate > set respiratory rate during the first 48 hours of mechanical ventilation.
**RESULTS.** SB was present in 67% (344/515) of patients with mild ARDS, 58% (488/844) of patients with moderate ARDS, and 46% (184/397) of patients with severe ARDS. Patients with SB were older and had significantly lower ARDS severity, SOFA scores, ICU and hospital mortality, and were less likely to be diagnosed with ARDS by clinicians. Mean tidal volumes (VT) were higher in patients with SB (7.9 ±1.7 vs. 7.5±1.5 ml/kg PBW, p< 0.001). In adjusted analysis, SB during the first 2 days was not associated with an effect on ICU or hospital mortality (respectively 33% vs. 37%, OR 1.18 [0.92-1.51], p=0.19, and 37 vs. 41%, OR 1.18 [0.93-1.50], p=0.196). SB was associated with increased ventilator free days (13 [0-22] vs. 8 [0-20], p=0.014) and shorter duration of ICU stay (11 [6-20] vs. 12 [7-22], p=0.04).
**CONCLUSIONS.** SB is common in patients with ARDS during the first 48 hours of ventilation. SB was not associated with worse outcomes and may hasten liberation from the ventilator and from ICU. Further studies incorporating the magnitude of inspiratory effort are required.

##### REFERENCE(S)

1 Epidemiology, Patterns of Care, and Mortality for Patients With Acute Respiratory Distress Syndrome in Intensive Care Units in 50 Countries.

Bellani G, Laffey JG, Pham T, Fan E, Brochard L, Esteban A, Gattinoni L, van Haren F, Larsson A, McAuley DF, Ranieri M, Rubenfeld G, Thompson BT, Wrigge H, Slutsky AS, Pesenti A, for the LUNG SAFE Investigators and the ESICM Trials Group. JAMA 2016, 315: 788-800

GRANT ACKNOWLEDGMENT

**Funding/Support: This work was funded and supported by the European Society of Intensive Care Medicine (ESICM), Brussels, Belgium, by St Michael's Hospital, Toronto, Canada, and by the University of Milan-Bicocca, Monza, Italy.**

### 0052

### The effect of neurally adjusted ventilatory assist on sedation score and sedation load

D. Hadfield[1,2], P. Hopkins[1], N. Hart[2,3], C. Finney[1], B. Penhaligon[1], J. Molai[1], F. Reid[4], G. Rafferty[2]

[1]King's College Hospital, Anaesthetics, Critical Care, Emergency Medicine and Trauma (ACET) Research Group, London, United Kingdom; [2]King's College London, Centre for Human and Aerospace Physiological Sciences, London, United Kingdom; [3]Guy's and St. Thomas' NHS Foundation Trust, Lane Fox Respiratory Unit, London, United Kingdom; [4]King's College London, Primary Care & Public Health Sciences, London, United Kingdom

**Correspondence:** D. Hadfield

*Intensive Care Medicine Experimental* 2018, **6(Suppl 2):**0052

**INTRODUCTION.** Sedation load is strongly associated with morbidity and mortality in mechanically ventilated, critically ill patients [1]. Neurally adjusted ventilatory assist (NAVA) may help reduce sedation requirement by improving patient-ventilator synchronisation and the ability to monitor and optimise neural respiratory drive [2].

**OBJECTIVE.** To compare the effect of NAVA versus Pressure Support Ventilation (PSV) on Richmond Agitation Scale (RASS) scores and sedation load.

**METHODS.** Secondary a priori outcomes including RASS and sedation load, were analysed from a pilot RCT (NCT01826890) in 78 patients at risk of prolonged invasive ventilation conducted in a London hospital between 2012 and 2017. Patients were randomised to NAVA or PSV for weaning. The primary outcome was protocol compliance (time in NAVA mode). Mean hourly RASS, proportion of time at, above and below target RASS (-2 to 0) and mean sedation dose per infusion day were analysed in the following sub-groups:

1. All ventilation modes via endo-tracheal tube or tracheostomy
2. All modes excluding time with tracheostomy
3. NAVA or PSV mode
4. NAVA or PSV in ***allocated mode***. Compliant data with no cross-over
5. Mandatory ventilation modes (to assess impact of diaphragmatic monitoring in the acute phase)

**RESULTS.** At randomisation, groups were well matched with no differences in age, APACHE II, RASS or sedation load (Table 1). A trend towards improved RASS was observed in the NAVA group in all data (p=0.086) and all sub-groups, which reached significance when compliant to allocated mode (p=0.020) (Figure 1). A trend towards greater time at target RASS (Figure 2), and significantly less time below target RASS (p=0.043) (Figure 3) was observed in the NAVA group. Sedation doses, number of bolus doses per infusion day and unplanned extubations were similar between groups (Table 2).

**CONCLUSIONS.** These results suggest that compared to PSV, NAVA was associated with better RASS and less time in deeper sedation. The differences observed during mandatory mode ventilation suggest a possible additional effect of diaphragmatic monitoring. These data are consistent with a potential benefit from NAVA. Further research is needed to better understand mechanism and establish clinical effectiveness.

REFERENCE(S)

Kollef M, et al. (1998). The use of continuous iv sedation is associated with prolongation of mechanical ventilation. Chest. 114(2):541-548

Kallio, M, et al. (2015). Neurally adjusted ventilatory assist (NAVA) in pediatric intensive care–a randomized controlled trial. Pediatr Pulmonol. 50(1): 55-62

FUNDING

This report is independent research supported by the National Institute for Health Research (NIHR/HEE CAT Clinical Doctoral Research Fellowship, Mr Daniel Hadfield, CDRF-2014-05-056). Additional funding received from The Moulton Foundation and Guy's and St Thomas' Biomedical Research Centre. KCH acknowledges the support of the NIHR Clinical Research Network (NIHR CRN).

**Table 1 (abstract 0052).** Baseline characteristics

| Variable | NAVA (n=39) | Control (n=38) |
| --- | --- | --- |
| Age | 67.0 (13.9) | 67.0 (12.9) |
| APACHE II | 20.5 (6.0) | 20.1 (6.1) |
| RASS | -3.6 (1.1) | -3.3 (1.7) |
| Propofol dose day 1, g | 1.7 (0.6 - 2.9) n=30 | 1.5 (0.8 - 2.3) n=29 |
| Fentanyl equivalent dose day 1, mg | 2.6 (1.6 - 3.5) | 2.6 (1.7 - 4.3) |
| Midazolam dose day 1, mg | 54 (27 - 71) n=11 | 23 (12 - 45) n=7 |

Data are median (IQR) or mean (SD). Drug doses are per infusion day. Fentanyl equivalent conversion factors. 1mg fentanyl = 0.5mg Remifentanyl = 100mg morphine = 50 mg diamorphine

**Table 2 (abstract 0052).** Sedation load, number of neuromuscular blocker infusions and events associated with increased arousal and anxiety

| Variable | NAVA (n = 39) | Control (n = 38) |
| --- | --- | --- |
| Propofol dose, g | 1.7 (1.1 - 2.6) n=32 | 1.8 (1.0 - 2.8) n=33 |
| Midazolam dose, mg | 50.3 (29.3 - 84.9) n=14 | 54.0 (26.0 - 95.3) n=15 |
| Fentanyl equivalents dose, mg | 2.1 (1.3 - 3.4) n=39 | 2.2 (1.6 - 3.5) n=36 |
| Patients with NMB infusion | 7 (18.0) | 4 (10.5) |
| Bolus doses per infusion day, mg | 2.4 (0.7 - 4.2) | 2.7 (1.6 - 4.3) |
| Unplanned extubation | 2 (5.1) | 1 (2.6) |

Data are median (IQR) or number (%). Drug doses are per infusion day. NMB = neuromuscular blockers. Fentanyl equivalent conversion factors. 1mg fentanyl = 0.5mg Remifentanyl = 100mg morphine = 50 mg diamorphine

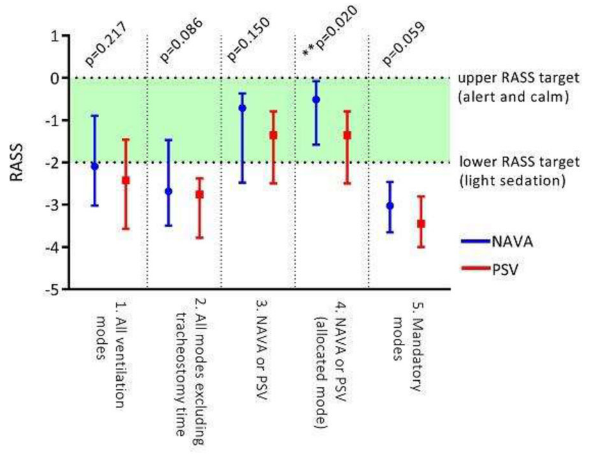

**Fig. 1 (abstract 0052).** Median (IQR) of hourly RASS score in different phases of ventilatory support

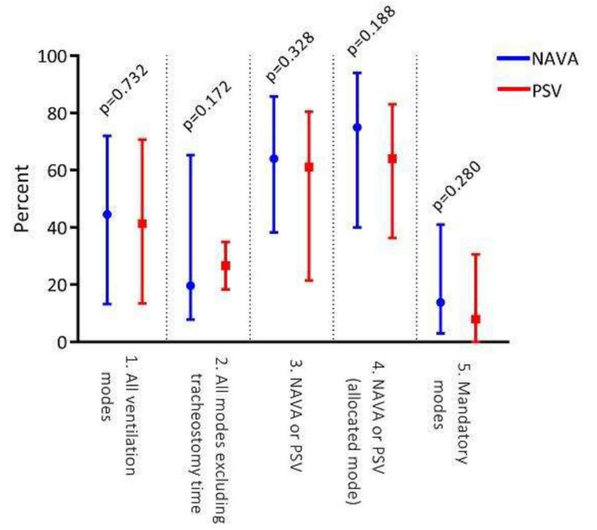

**Fig. 2 (abstract 0052).** Median (IQR) of time within target RASS score of -2 to 0

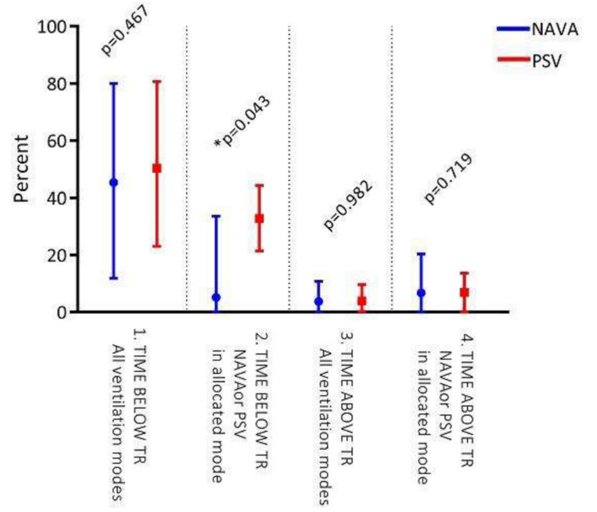

**Fig. 3 (abstract 0052).** Median (IQR) of time below and above target RASS of -2 to 0

### 0053

#### Integrated ultrasound protocol in predicting weaning success and failure: a prospective observational study

R. Kundu[1], R. Subramanium[1], R. Anand[1], D. Baidya[1], K. Soni[2]
[1]All India Institute of Medical Sciences, Anaesthesiology and Critical Care Medicine, New Delhi, India; [2]All India Institute of Medical Sciences, Trauma Critical Care, New Delhi, India
**Correspondence:** R. Kundu
*Intensive Care Medicine Experimental* 2018, **6(Suppl 2):**0053

**INTRODUCTION.** Difficulty in weaning from mechanical ventilation is encountered in approximately 20% of patients in the intensive care unit.Recent investigations focusing on lung, diaphragmatic and cardiac ultrasound highlights the potential role of ultrasonography in prediction of outcome following extubation. The present study attempted to assess the ability of a combined lung, diaphragmatic and cardiac ultrasound protocol to predict weaning success and failure following extubation.
**OBJECTIVES.**

**Primary:**

1. To assess the ability of an Integrated USG protocol during weaning to predict weaning success or failure.

**Secondary:**

2. To assess the role of lung, diaphragmatic and cardiovascular dysfunction to failed weaning.
3. To assess the changes in Lung ultrasound Score (LUS) following SBT.

**METHODS.** All patients scheduled to undergo SBT were evaluated by USG to measure LUS, Diaphragmatic Thickness fraction(DTF) and changes in Velocity Time Integral (VTI) to passive leg raise at the beginning of SBT. Changes in LUS following SBT was recorded. Patients extubated following SBT were classified as weaning success if non invasive or invasive mechanical ventilator support was not required in the next 48 hours.
**RESULTS.** 60 patients who underwent extubation following a successful SBT were included in the study.27 patients(45%) required either non invasive or invasive mechanical ventilator support during the next 48 hours and were classified as weaning failure (Group F). Rest 33 patients were designated as weaning success (Group S).Patients in Group F had significantly longer ICU length of stay, higher LUS change during SBT($p< 0.001$),lower DTF ($p< 0.05$) and showed lower VTI increment to PLR($p< 0.001$).Using a binary logistic regression model,DTF< 26% (odds ratio 6.20,95% CI 1.06-36.04) and VTI change to PLR< 10.2% (odds ratio 6.16,95%CI 1.14-33.13) were found to be significant predictors of weaning failure($p< 0.05$).The AUROC for VTI and DTF for predicting weaning failure were 0.79 and 0.64 respectively(Figure 1).
**CONCLUSIONS.** An integrated Ultrasound protocol using a combination of ultrasonographic parameters of lung, diaphragm and cardiac sonography was a reliable predictor of weaning failure. This study highlights the role of diaphragmatic dysfunction and preload independence of the heart in contributing to failed weaning, earlier detection of which may lead to better tailoring of therapy.

**REFERENCE(S)**

1) Dres M, Teboul J-L, Anguel N, Guerin L, Richard C, Monnet X. Passive leg raising performed before a spontaneous breathing trial predicts weaning-induced cardiac dysfunction. Intensive Care Medicine. 2015 Mar;41(3):487-94.
2) Giovanni Ferrari, Giovanna De Filippi, Fabrizio Elia, Francesco Panero, Giovanni Volpicelli and Franco Aprà: Diaphragm ultrasound as a new index of discontinuation from mechanical ventilation. Critical Ultrasound Journal 2014, 6:8.

**GRANT ACKNOWLEDGMENT**
NO GRANTS RECEIVED FROM ANY SOURCE

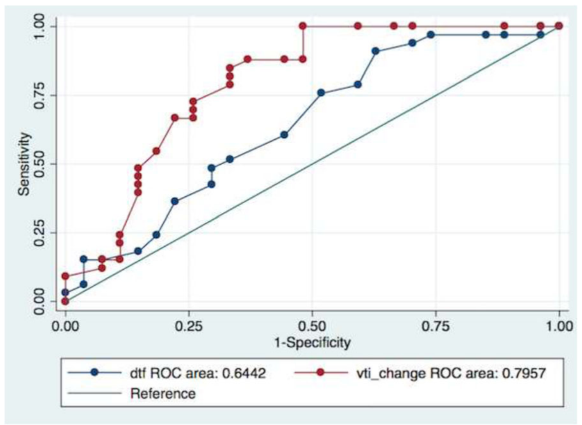

**Fig. 1 (abstract 0053).** AUROC curve for Diaphragmatic Thickness Fraction and VTI change to PLR

### 0054

#### Evaluation of diaphragmatic function using Tissue Doppler Imaging (TDI) - correlation with transdiaphragmatic (Pdi) parameters

E. Soilemezi, A. Vemvetsou, K. Kosmidis, C. Dimitroulakis, M. Tsagourias, D. Matamis
Papageorgiou General Hospital, ICU, Thessaloniki, Greece
**Correspondence:** E. Soilemezi
*Intensive Care Medicine Experimental* 2018, **6(Suppl 2):**0054

**INTRODUCTION.** Tissue Doppler Imaging (TDI) is an ultrasonographic technique extensively used in cardiology to evaluate cardiac systolic and diastolic function. It uses Doppler to quantify the velocity of tissue rather than blood, and it, therefore, allows direct measurement of the velocity of moving tissue. TDI of the diaphragm has never being used so far to assess the characteristics of diaphragmatic contraction and relaxation.
**OBJECTIVES.** This study attempts to correlate TDI-derived parameters with transdiaphragmatic (Pdi)-derived parameters, which are already evaluated and used in clinical practice.
**METHODS.** In twelve patients undergoing a T-piece weaning trial (9 weaning success and 3 weaning failures) a TDI examination of the diaphragm was performed. High quality images of diaphragmatic TDI were very easily obtained in all patients with a cardiac 4 MHz sector transducer. In all patients a nasogastric tube, equipped with two balloons for measuring esophageal and gastric pressures, was previously inserted for feeding purposes allowing for Pdi recording. Simultaneous Pdi and TDI tracings were recorded for 207 breaths in total (approximately 15 breaths/patient). For every breath, the Pdi signal was analyzed to measure the following parameters (fig 1): maximum Pdi ($Pdi_{max}$), pressure time product (PTP), and the slope of the initial steepest part of the descending part of the Pdi curve, ie the diaphragmatic Maximum Relaxation Rate (Pdi-MRR). These Pdi-derived parameters were respectively correlated with the following TDI-derived parameters (fig.2): TDI peak contraction velocity, the velocity-time integral during inspiration (VTI), and the slope of the initial steepest part of the velocity curve of diaphragmatic motion during relaxation, ie, the TDI-derived maximum relaxation rate. The level of significance and the correlation coefficient were calculated for each comparison.
**RESULTS.** A highly significant correlation was found between the Pdi-MRR and the TDI-derived maximum relaxation rate (r=0.93, y=1.7x+30, p< 0.001). Furthermore, TDI peak contraction velocity was also highly correlated with PTP and $Pdi_{max}$, (r=0.91 and r=0.89 respectively, p< 0.001). On the contrary, a weak but significant correlation (r=0,54, y=2x+3.2, p< 0.01) was found between the PTP and the VTI.
**CONCLUSIONS.** Our results suggest that TDI represents an ultrasonographic technique that can be used to assess features of the contraction and relaxation of the diaphragm, until now thought to be acquired only with Pdi recordings; it bears the additional advantages of all ultrasonographic techniques, ie, it is non-invasively performed on the bedside, compared to the cumbersome method of acquiring and interpreting Pdi tracings. Further studies are needed to demonstrate the potential utilities of this new ultrasonographic examination of the diaphragm in the everyday ICU practice.

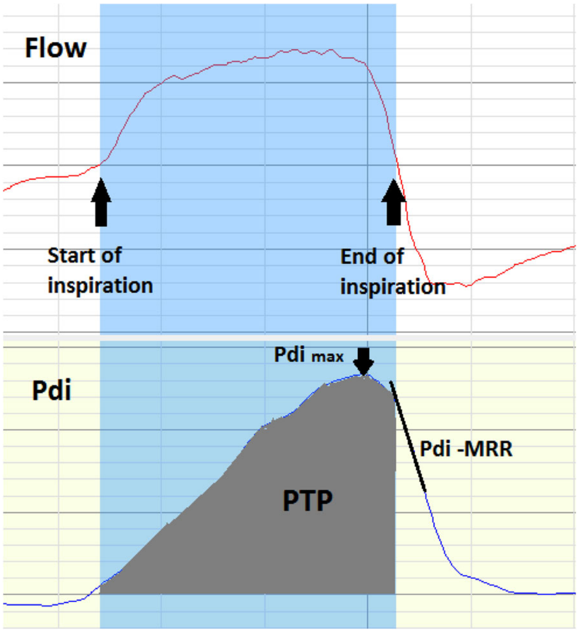

**Fig. 1 (abstract 0054).** Parameters measured from the Pdi tracing

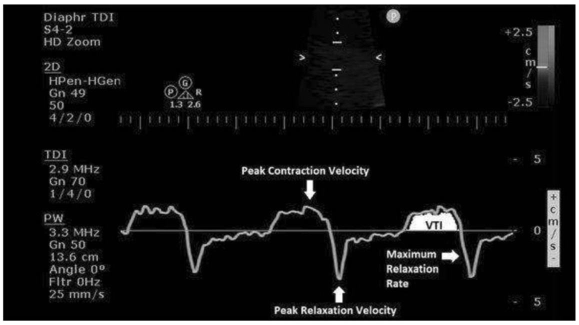

**Fig. 2 (abstract 0054).** Parameters measured from the TDI waveform

### 0055

#### Using ultrasound of heart, lungs and diaphragm to predict weaning success: a prospective observational study

M.E. Haaksma, J. Smit, B. Hilderink, L. Atmowihardjo, E. Lim, A. Jonkman, L. Heunks, A. Girbes, P.R. Tuinman
VU Medical Center, Amsterdam, Netherlands
**Correspondence:** M.E. Haaksma
*Intensive Care Medicine Experimental* 2018, **6(Suppl 2):**0055

**BACKGROUND.** In ICU patients deciding the optimal timing for extubation is challenging and clinical predictors are not very accurate. Ultrasonographic assessment of the diaphragm function has been used to predict successful extubation.[1] However, cardiorespiratory function also greatly impacts a patient's ability to wean from mechanical ventilation.

**OBJECTIVE.** To assess if a combination ultrasound measurements of the diaphragm, heart and lungs could predict extubation success more accurately than using single-organ ultrasonography assessment.

**METHODS.** This prospective observational study in the Intensive Care Unit of a tertiary academic hospital included adult patients who were invasively ventilated for > 72 hrs. Exclusion criteria included paraplegia, tracheostomy or planned non-invasive ventilation (NIV) after extubation. Ultrasound measurements of heart (left ventricular function (LVF)), lungs (number of B-lines) and diaphragm (thickening fraction (TFdi%)) were performed within 6 hours before extubation during spontaneous breathing trial. Patients not needing reintubation or NIV within 48 hrs after extubation were recorded as successful extubation. A logistic regression prediction model using backward selection was made.

**RESULTS.** In this interim analysis, 39 patients were included of which 77% were male, with a mean age of 61 (±17) years and a median of 126 [95-207] and 96 [84-185] hours on mechanical ventilation in the successful and failed group, respectively. Of these patients, 7 (18%) required reintubation within 48 hours. Patients in need of reintubation compared to patients who were extubated successfully had no significant difference in TFdi% (21.2% vs. 25.5%; p=0.36), left ventricular function (72% good vs. 71% good, p=.94) or B- lines (17 [9-24] vs. 7 [3-15] p=.072), although a trend for more B-lines was observed in the extubation failure group. Multivariable regression analysis showed that addition of B- lines and classic parameters such as $PaO_2$ and $FiO_2$ to the thickening fraction has added value in predicting extubation (Table 1). This was not the case for LVF.

**CONCLUSION.** The results of this study suggest that addition of lung ultrasound to diaphragm ultrasound might have added benefit in predicting extubation success, while for LVF this does not seem to be the case.

Variables included:

Age, Gender, Ventilation Time (VT), FiO2, PaO2, SOFA-score, Thickening fraction, BLUE-Profile, B-lines, Whitebloodcellcount, CRP, Hemoglobin (Hb), LVF, Creat, Breathing Frequency (BF), Pressure Support (PS), Tidal Volume (TV)

Variables removed:

Age, Gender, VT, SOFA, BLUE-Profile, WBC, CRP, Hb, LVF, BF, PS, TV

N=39

Nagelkerke $R^2$=.651

**Table 1 (abstract 0055).** See text for description.

| Parameters in equation | B | SE | Odds-Ratio | P.value |
|---|---|---|---|---|
| TFdi% | .37 | .2 | 1.5 | .06 |
| B-lines | -.43 | .25 | .65 | .08 |
| PaO2 | .12 | .08 | 1..13 | .12 |
| FiO2 | .26 | .18 | 1.3 | .14 |
| Creat | .09 | .05 | 1.1 | .09 |
| Constant | -16.4 | 10.3 | | .11 |

### 0056

#### Prolonged ICU stays and difficult-to-wean-patients: first year of experience in a french Post ICU Rehabilitation Center

M. Faure, J. Delemazure, M. Dres, J. Mayaux, A. Demoule, T. Similowski, E. Morawiec
Assistance Publique Hôpitaux de Paris, Respiratory Care and Medical ICU, Paris, France
**Correspondence:** M. Faure
*Intensive Care Medicine Experimental* 2018, **6(Suppl 2):**0056

**INTRODUCTION.** In February 2016, we opened a 12 beds-post ICU rehabilitation center (Service de Rééducation Post Réanimation, « SRPR»), dedicated to weaning from mechanical ventilation and global post ICU rehabilitation.

**OBJECTIVES.** To describe the characteristics and main outcomes of patients admitted over the first year of activity.

**METHODS.** Retrospective analysis of prospectively collected data

**RESULTS.** Ninety patients were admitted (100 admissions) in the unit over 12 months from 34 different ICUs (median duration of stay in the ICU 38,5 days (IQR 29 -61)). 86 % were ventilated (11% with NIV). 85% had a tracheostomy. 64% had ICU acquired weakness ; 5% were able to walk. An underlying chronic respiratory disease was present in 53% of cases. 18% were obese. Difficult weaning was found to have one or several respiratory components in 77% of cases (including 20 post surgery diaphragmatic paralysis), cardiac in 34%, neurologic in 18%. Significant complications occured in 60% of cases. Median duration of stay was 19,5 (12-29,5) days. Ten patients died in the unit, 13 patients were re-transferred in the ICU, where 6 of them died. Over half of the patients were discharged at home, in a rehabilitation unit or in a hospital ward awaiting a rehabilitation program. The remaining 24%, that still needed some form of medical or surgical care were discharged in the ward.

In intention to treat, successful weaning from *invasive* ventilation was obtained in 70% of patients. Of the patients discharged alive from the unit after completing the rehabilitation program (n= 74), 60% were completely weaned from mechanical ventilation, 35% were discharged with NIV or CPAP ; 4 patients (5%) were considered not weanable from invasive ventilation ; decanulation of tracheostomy was obtained in 82% of cases; 87% of the patients could walk.

**CONCLUSIONS.** SRPRs offer a new concept of care for difficult to wean patients, with promising results.

### 0057

#### Lung and diaphragm ultrasound in post-surgical patients to predict weaning failure

A. Colombo[1,2], M. Luperto[1,2], M. Bouard[1], S. Mongodi[2], O. Ellouze[1], F. Mojoli[2], B. Bouhemad[1]
[1]CHU Dijon Bourgogne, Anesthesia and Intensive Care, Dijon, France; [2]IRCCS Policlinico San Matteo, Anestesia e Rianimazione, Pavia, Italy
**Correspondence:** A. Colombo
*Intensive Care Medicine Experimental* 2018, **6(Suppl 2):**0057

**INTRODUCTION.** Weaning failure (WF) in post-surgical patients may be due to lung and diaphragm dysfunction; they can both be assessed by ultrasound (LUS and DUS), helping in predicting WF.[1]

**OBJECTIVES.** To assess lung aeration and diaphragm activity in post-surgical patients to early identify those at risk of WF.

**METHODS.** Prospective observational monocentric study. We enrolled ICU post-surgical adult patients within 24 hours after extubation. They were in spontaneous breathing and had no neuromuscular diseases. LUS score for aeration assessment was computed: sternum, anterior and posterior axillary lines define anterior, lateral and posterior regions, each divided in superior and inferior to have 12 areas per side; in each area a score from 0 (normal aeration) to 3 (complete loss of aeration) was given.[2] Global and anterolateral scores corresponded to the sum of all and anterolateral areas' score respectively. DUS analyzed right thickening fraction (TF=(expiratory - inspiratory)/inspiratory thickness*100) and caudal displacement (CD).[1] WF was defined as NIV/reintubation within 48 hours after extubation.

**RESULTS.** We enrolled 74 patients (60 males, age 69.0±10.0yrs, BMI 26.4±5.1, mechanical ventilation length in ICU 272.0±125.6 mins, ICU stay 2[1.0-3.0] days). Surgery was performed by laparotomy (17), sternotomy (47) and right thoracotomy (10). WF occurred in 21 patients (28.4%); WF patients differed by BMI only (30.3±5.4 vs. 24.9±4.1; p< 0.0001). WF wasn't significantly different among surgical approaches; a trend in higher WF is observed in thoracotomies (50% vs. laparotomy 23.5% and sternotomy 25.5%, p=0.284). Overall global and anterolateral LUS scores were 11.0[8.0-14.0] and 3.0[1.0-6.0] respectively. No significant differences were remarked among surgical approaches. WF patients had higher scores (global 13.0[11.0-17.0] vs. 10.5 [7.5-13-0], p=0.0089; anterolateral 5.0[3.0-8.0] vs. 3.0[1.0-4.0], p=0.0019). Relative risk of WF if anterolateral LUS≥8 was 2.9 (95% CI 1.5-5.5; p=0.0341). Anterolateral LUS alone predicted WF with AUC 0.7405 [95%CI 0.60519-0.87572] (fig. 1). Overall CD was 1.5[1.1-1.74] and 2.5[1.7-3.1] cm and TF was 17.7[8.5-29.6] and 23.1[11.0-37.3] in normal and forced inspiration respectively. No significant differences were remarked among different surgical approaches and in patients with/without WF.

**CONCLUSIONS.** Preliminary data suggests LUS score, but not DUS, is useful to predict WF in post-surgical extubated patients. Data need to be confirmed in a wider population.

1 P. Mayo, Intensive Care Med, 2016
2 S. Mongodi, Ultraschall Med, 2017

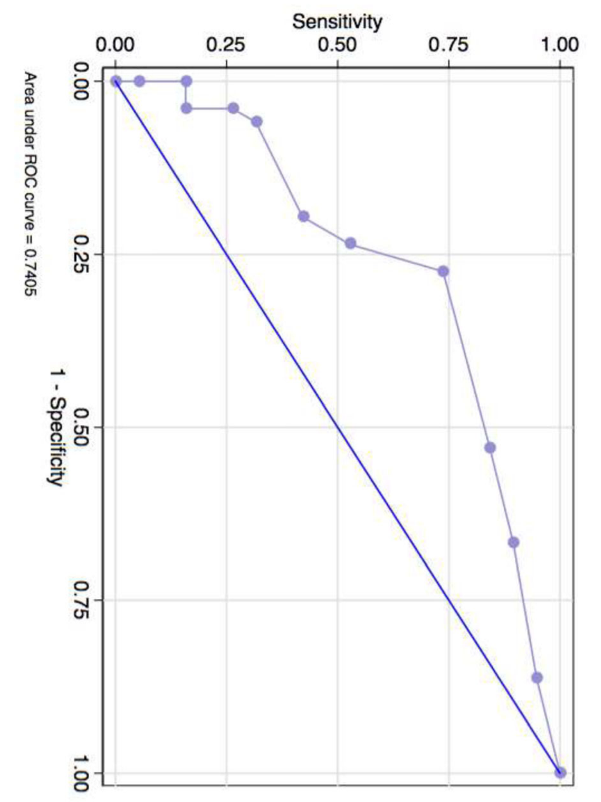

**Fig. 1 (abstract 0057).** See text for description

### 0058

#### Application of the SpO2/FiO2 index as an indicator of the necessity and failure of Non-Invasive mechanical Ventilation (NIV) in the critical care setting

J. Gonzalez Londoño, S. Cuenca Barrero, C. Lorencio Cardenas, C. Murcia Gubianas, J. Morales Pedrosa, J.M. Sirvent
Josep Trueta Hospital, Girona, Spain
**Correspondence:** J. Gonzalez Londoño
*Intensive Care Medicine Experimental* 2018, **6(Suppl 2):**0058

**OBJCTIVES.**Non-invasive mechanical ventilation (NIV) is a therapy that has gained relevance during the last decades in the treatment of patients with acute respiratory failure(ARF) and is currently part of the first line treatment of critical care patients. Our objective was to evaluate if the SpO2/FiO2 index (SaFi) could be a useful tool to indicate the need of NIV and to predict the success or failure of the NIV in critical patients with ARF.

**MATERIALS AND METHODS.** We performed a prospective observational study from August 2017 to March 2018, during which we calculated the SaFiand the PaO2/FiO2 (PaFi) indexes of all patients that required NIV in the ICU of a tertiary hospital. Measures were taken before NIV was started, and after 1 hour of treatment.

**RESULTS.** 74 patients were included, of which 59.5%were men with a median age of 61.3years (SD13.6). The median APACHE II upon intake was 18.8 (SD8.2).The main reasons for intake were respiratory tract pathology in 52.7% of the cases, sepsis in 12.2% and other causes in 35.1%. The type of ARF was of hypoxic origin in 62.2% of the patients, hypercapnic in 4.1% and mixed in 33.8%. In 32.4% NIV was unsuccessful and the patient required intubation.

In the multiple linear regression, there was a significant association between the values of pre NIV SaFi and pre NIV PaFi (p < 0.001). When comparing failure and success of NIV, the values of post NIV PaFi (p 0.26) and SaFi (p 0.09) were lower in patients in whom NIV failed, even though there was no statistical significance.

**CONCLUSIONS.** In our series, the values of pre NIV SaFi were positively and significantly associated with the pre NIV PaFi values and were useful as an indicator of ARF and necessity to start NIV. In patients in who NIV failed, the SaFi values had a tendency to be a better indicator of NIV failure than the PaFi values, even though there was no statistical significance.

The SaFi index is easy to obtain and is useful to evaluate if NIV is unsuccessful and determine the need for intubation and mechanical ventilation.

### 0059

#### Gas conditioning during noninvasive ventilation with helmet interface

V. Raggi, F. Bongiovanni, G.M. Anzellotti, D.L. Grieco, T. Michi, S. D'Arrigo, A.M. Dell'Anna, B. Mura, D. Eleuteri, M.G. Bocci, M.A. Pennisi, R. Maviglia, M. Antonelli
IRCCS Fondazione Policlinico Universitario 'A. Gemelli', Roma, Italy
**Correspondence:** V. Raggi
*Intensive Care Medicine Experimental* 2018, **6(Suppl 2):**0059

**INTRODUCTION.** Noninvasive ventilation (NIV) is among first line treatments for acute respiratory failure. Helmet is proposed to optimize tolerability, deliver higher PEEP and foster NIV success[1, 2]. No data clarify the optimal humidification strategy during Helmet NIV.

**OBJECTIVES.** Cross-over study to compare four different humidification strategies during helmet NIV in patients with hypoxemic respiratory failure.

**METHODS.** Non-hypercapnic patients with hypoxemic respiratory failure underwent 1-hour helmet pressure-support ventilation (PEEP 10cm$H_2O$, pressure support≥15 cm$H_2O$, maximum raise time) with all the following humidification settings in a random order: heating and humidification (Fisher and Paykel, MR 850) with humidification chamber temperature set at 34°C(HH34) and 37°C(HH37); bitube circuit with y-piece and heat and moisture exchanger(HME); bitube circuit with no humidification (NoH). Patients' discomfort, arterial blood gases, ventilator performance, asynchrony index(AI), helmet temperature, absolute (AH) and relative humidity (RH) were measured. Results are expressed as medians [interquartile range].
**RESULTS.** Seven patients were enrolled (male 71%, age 66 [52-66] years, SAPSII 34 [30-38], duration of ICU stay before enrolment 43 [14-144] hours). Blood gases were similar in the four study steps.
Patients' comfort and perception of dyspnea were optimal during NoH and they worsened during HH34 and HH37 but not during HME (VAS discomfort p=0.004; VAS dyspnea: p=0.02).
Gas temperature, RH and AH were higher during HH34, HH37 and HME vs. NoH: RH (%): respectively 100[93-100], 100[100-100], 96[45-100], 45[37-75]; p=0.005; AH (mgH2O/l): respectively 29[24-32], 32[27-32], 24[13-29], 12[11-19]; p=0.001.
Asynchrony index was lower during NoH and HME (NoH 7,5 [4-20]%, HME 5[3-21]%, HH34 15[13-24]%,.HH37 18[10-23]%; p=0.001), mainly due to a higher autotrigger rate during HH34 and HH37 (p=0.002).
Ventilator performance was worsened with the use of HME and HH as compared to NoH (p=0.05).
**CONCLUSIONS.** In hypoxemic patients undergoing helmet PSV, a bitube circuit with no humidification allows adequate gas conditioning, optimizes ventilator performance, patient's comfort and dyspnea. HHs significantly worsens patient-ventilator interaction due to higher autotrigger rates.
These results are useful to optimize helmet pressure support ventilation in the clinical setting and in future randomized studies on the topic.

**REFERENCE(S)**

1. Antonelli M, Conti G, Pelosi P, et al (2002) New treatment of acute hypoxemic respiratory failure: noninvasive pressure support ventilation delivered by helmet–a pilot controlled trial. Crit Care Med 30:602-8. doi: 10.1097/00003246-200203000-00019
2. Patel BK, Wolfe KS, Pohlman AS, et al (2016) Effect of Noninvasive Ventilation Delivered by Helmet vs Face Mask on the Rate of Endotracheal Intubation in Patients With Acute Respiratory Distress Syndrome: A Randomized Clinical Trial. JAMA 315:2435-41. doi: 10.1001/jama.2016.6338

#### 0060

#### Current use of pain and sedation management on noninvasive mechanical ventilation in a pilot Korean multi-center observational study

J. Cho[1], J. Kim[2], Y. Shin[3], Y. Shim[4], J. Ha[5], J. Moon[6], W. Choi[7], S. Park[8], Korean Noninvasive Ventilation Study
[1]Yonsei University College of Medicine, Pulmonology and Critical Care, Seoul, Korea, Republic of; [2]Inha University, Incheon, Korea, Republic of; [3]Choongbuk University, Choongju, Korea, Republic of; [4]Hallym University Kangnam Sacred Heart Hospital, Seoul, Korea, Republic of; [5]Catholic University School of Medicine, Incheon, Korea, Republic of; [6]Chonnam National University Hospital, Daejeon, Korea, Republic of; [7]Keimyung University, Daegu, Korea, Republic of; [8]Hallym University Sacred Heart Hospital, Anyang, Korea, Republic of
**Correspondence:** J. Cho
*Intensive Care Medicine Experimental* 2018, **6(Suppl 2):**0060

**INTRODUCTION.** Noninvasive ventilation (NIV) has been used frequently in the intensive care unit. In Korea a nationwide survey of NIV usage was not done yet. The Korean noninvasive mechanical ventilation study group has proposed and collected NIV use data from nationwide ICUs.
**OBJECTIVES.** we analyzed status and safety in the management of pain, sedation on NIV therapy in Korea.
**METHODS.** The eleven ICU intensivists in nationwide hospitals were participated and collected data of NIV from May 2017 to October 2017. Demographic date of patients, clinical parameters of NIV, hospital mortality were included. We analyzed using chi-square test on categorical variables and Mann-Whitney U test on continuous variables.
**RESULTS.** The seventy-three patients were included during those periods. Demographic characteristics and reasons of NIV were shown as table 1. We divided the intervention group that who received management of pain, sedation and control group. The pH, PaCO2 and PaO2/FoiO2 ration before NIV and 30 minutes after NIV were not different between intervention and control group. The number of failure were 5 (50%) in intervention and 21 (33%) in control (p=0.51). The hospital mortality rate was 30% in intervention and 21% in control (p=0.581).
**CONCLUSIONS.** The management of pain, sedation was performed in 14 percent of NIV patients. Small numbers of patients are limited. The control of pain, sedation might be safe during NIV.

**GRANT ACKNOWLEDGMENT**
This work was supported by research grant of The Korean Academy of Tuberculosis and Respiratory Diseases and grant of Yonsei University College of medicine.

**Table 1 (abstract 0060).** Demographics of patients and reasons of noninvasive ventilation

| | intervention (n=10) | control (n=63) | p value |
|---|---|---|---|
| male (n) | 5 | 39 | 0.475 |
| age (years) | 71±6 | 73±10 | 0.694 |
| BMI (kg/m2) | 22.4±8.0 | 21.8±5.2 | 0.760 |
| SOFA score | 3.9±2.1 | 4.1±2.2 | 0.732 |
| Diabetes melitus (n) | 1 | 16 | 0.285 |
| Hypercapnic RF (n) | 3 | 37 | 0.502 |
| Hypoxemic RF (n) | 2 | 13 | |
| Postextubation (n) | 5 | 10 | |
| Heart failure and other (n) | 0 | 3 | |

### AKI in different clinical scenarios

#### 0061

#### The incidence and associations of acute kidney injury in trauma patients admitted to critical care: a systematic review and meta analysis

R. Haines[1,2], A. Fowler[1,2], J. Prowle[1,2]
[1]Queen Mary, University of London, Adult Critical Care Unit, London, United Kingdom; [2]Queen Mary, University of London, William Harvey Institute, London, United Kingdom
**Correspondence:** R. Haines
*Intensive Care Medicine Experimental* 2018, **6(Suppl 2):**0061

**INTRODUCTION.** More patients are surviving their initial traumatic injury and are subsequently exposed to complications of critical illness including acute kidney injury (AKI). Studies suggest trauma associated AKI has unique risk factors and contributes to worse outcomes[1,2].
**OBJECTIVES.** To establish the incidence of AKI in patients admitted to critical care after major trauma, explore any specific risk factors and evaluate the association of AKI with outcomes.
**METHODS.**
*Data Sources:* Systematic search of MEDLINE, EMBASE and Cochrane library from January 2004 to January 2018.
*Study Selection:* Studies of adult major trauma patients admitted to intensive care units that applied recent consensus AKI criteria (RIFLE, AKIN or KDIGO) and reported outcomes of interest were included.
*Data Extraction and Synthesis:* Studies were assessed independently by two reviewers and extracted data included study design and setting, population characteristics, AKI definition and outcomes. The pooled incidence of AKI and relative risk of death were estimated using random effects models. Results are provided with a 95% confidence interval (CI) and associated p-value. Study quality was assessed using the Newcastle-Ottawa score.

*Main Outcomes and Measures:* Incidence of AKI was the primary outcome. The secondary outcome was study defined mortality.

**RESULTS.** We included 16 articles describing AKI outcomes in 24267 patients admitted to intensive care after major trauma. In a meta-analysis of proportion using a random effects model, the pooled incidence of AKI was 20.4% (95% CI 16.5-24.9, $I^2$=98%). Twelve studies reported the breakdown of stages of AKI with 55.7% of patients classified as RIFLE-R or Stage 1, 30.3% as RIFLE-I or Stage 2 and 14.0% as RIFLE-F or Stage 3. Studies focused on predominantly blunt trauma had the highest pooled incidence of AKI, 31.9 % (CI 22.4-43.3%, $I^2$=97% p < 0.001). In a random effects model the pooled relative risk for hospital, 30 day or 90 day mortality for patients with AKI compared to patients with no AKI was 3.6 (95% CI 2.4-5.3, $I^2$=96%, p < 0.01).

In addition, among five studies that adjusted for multiple variables including illness severity, there was an increased odds of death compared to patients with no AKI (adjusted OR 1.6 [95% CI 1.3-1.9]; $I^2$=82%, n=15, 539, p< 0.01),

**CONCLUSIONS.** Acute kidney injury is common after major trauma and associated with increased mortality. Future research into early identification and targeted interventions is warranted to help reduce the potential for harm associated with AKI.

**REFERENCE(S)**

1. Shashaty MG, et al. African American race, obesity, and blood product transfusion are risk factors for acute kidney injury in critically ill trauma patients. *J Crit Care.* 2012;27(5):496-504.
2. Bagshaw SM, et al. A multi-center evaluation of early acute kidney injury in critically ill trauma patients. *Ren Fail.* 2008;30(6):581-589.

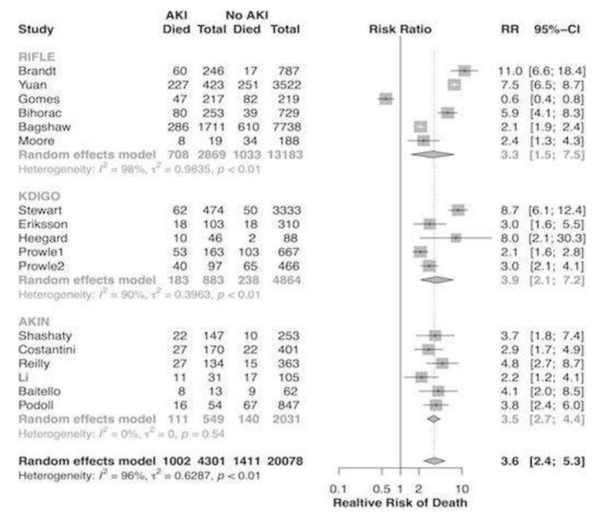

**Fig. 1 (abstract 0061).** Random-effects meta analysis of the effect of acute kidney injury diagnosis on short-term survival

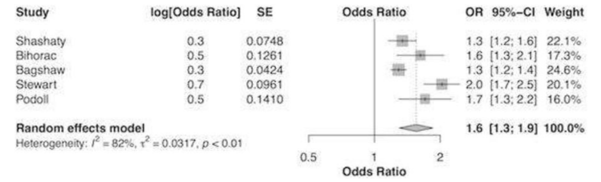

**Fig. 2 (abstract 0061).** Effect of acute kidney injury diagnosis on survival in studies with multivariable adjustment

### 0062

#### Doppler-based renal resistive index is independently associated with acute kidney injury stages and outcome in ICU patients

G. Fotopoulou[1], I. Poularas[2], G. Adamos[1], A. Giannopoulos[1], E. Brountzos[3], S. Zakynthinos[1], C. Routsi[1]

[1]Medical School, National and Kapodistrian University of Athens, 1st Department of Intensive Care, Athens, Greece; [2]General Hospital of Athens, Athens, Greece; [3]Medical School, National and Kapodistrian University of Athens, 2nd Radiology Department, Athens, Greece

**Correspondence:** G. Fotopoulou

*Intensive Care Medicine Experimental* 2018, **6(Suppl 2):**0062

**INTRODUCTION.** Acute kidney injury (AKI) is common in intensive care unit (ICU) patients and is associated with severe morbidity and mortality (1). Early prediction and diagnosis are essential to guide management. The Doppler-based Renal Resistive Index (RRI) [(peak systolic velocity - end-diastolic velocity)/peak systolic velocity] is advanced as a useful, non-invasive technique for quantifying the alterations in renal blood flow that may occur with renal disease (1).

**OBJECTIVES.** The objective of this study was to evaluate the correlation between RRI and ongoing deterioration of renal function, represented by AKI stages as defined by the Kidney Disease Improving Global Outcomes (KDIGO) Clinical Practice Guidelines (2) in ICU patients.

**METHODS.** We performed a prospective observational study in our multidisciplinary university ICU. Patients consecutively admitted in the ICU requiring mechanical ventilation, without pre-existing renal failure, with an expected length of stay for more than 48 hours, were enrolled. Illness severity scoring systems (APACHE II and SOFA) were calculated on the first ICU day. The RRI was measured within the first 24 hours of ICU admission, after initial hemodynamic stabilization. Clinical data including serum creatinine and urine output were collected in order to define AKI and AKI stages.

**RESULTS.** One hundred and sixty-eight mechanically ventilated, critically ill patients were included (median age 64 years, 58% males). Median values for APACHE II and SOFA scores were 19 and 9, respectively. AKI developed in 82 patients (52%) during the first 48h post-ICU admission; 23 patients (30%) had AKI stage I, 13 patients (14%) had AKI stage II and 46 patients (56%) had AKI stage III. Continuous renal replacement therapy (CRRT) was applied in 29% of patients with AKI. Patients with AKI stage III had statistically significantly higher RRI [0.84 (0.79-0.90) vs 0.79 (0.75-0.81), p=0.004] compared to patients with AKI stage I/II. Mortality was significantly associated with RRI (p=0.001), AKI (p=0.001) as well as AKI stage (p=0.001). RRI was statistically significantly associated with the probability of developing AKI (p< 0.001). More specifically, an increase in RRI values multiples over 50 times the probability of developing AKI (OR >50, p< 0.001 crude regression). After adjusting for potential confounders, OR remained high and statistically significant (OR >50, p< 0.001 adjusted regression). Also, ROC analysis shown that RRI could be considered as a good prognostic factor for AKI and for death (both AUCs >0.7).

**CONCLUSIONS.** RRI is independently associated with AKI. RRI calculation in the ICU setting enables prediction of AKI and anticipation of its severity.

**REFERENCE(S)**

(1) Boddi M et al. 2016;46:242-51
(2) Kidney Intl. Suppl 2012; 2:1-138

### 0063

#### The value of clinical examination and cardiac ultrasonography to forecast acute kidney injury in critically ill patients: a sub-study of the Simple Intensive Care Studies-I

R. Wiersema, J. Koeze, F. Keus, I.C.C. van der Horst, SICS Study Group

University of Groningen, University Medical Center of Groningen, Departement of Critical Care, Groningen, Netherlands

**Correspondence:** R. Wiersema

*Intensive Care Medicine Experimental* 2018, **6(Suppl 2):**0063

**INTRODUCTION.** Acute Kidney Injury (AKI) occurs in up to 50% of all critically ill patients and its severity is associated with increased mortality. There is a paucity of data on the role of hemodynamics in the development of AKI. Recent studies suggest the role of venous congestion, but to our best knowledge no studies investigated left versus right ventricular cardiac function objectified with ultrasonography in relation to AKI.

**OBJECTIVES.** The objective of this study was to investigate the association between simple hemodynamic variables, left and right cardiac function and AKI in critically ill patients.

**METHODS.** This study describes one of the outcomes of the Simple Intensive Care Studies - I (SICS-I: NCT02912624; Hiemstra et al. BMJ open 2017). This prospective cohort study included all consecutive adults who were acutely admitted with an intensive care unit (ICU) stay expected to last beyond 24 hours. For this substudy, we excluded patients with chronic kidney disease (CKD) defined by the National Intensive Care Evaluation criteria. Cardiac index was used as reflection of left ventricular function, tricuspid annular plane systolic excursion (TAPSE) and right ventricular systolic excursion (RV S') were used to reflect right ventricular function. AKI was defined by the KDIGO criteria within 72 hours after ICU admission. Both variables with univariate association and with known association with AKI were included in the multivariate logistic regression models.

**RESULTS.** From 27 March 2015 until 22 July 2017, 1075 patients were included in the SICS-I of which 72 had CKD. Of the remaining 1003 patients, 541 patients (54%) had at least AKI stage 1 within 72 hours after ICU admission (Table 1). The final multivariate model showed that higher age (OR 1.02, 95% CI 1.01-1.03 per year increase), lower mean arterial pressure (MAP) (OR 0.98, 95% CI 0.97-0.99 per mmHg), lower central temperature (OR 0.84, 95% CI 0.70-0.99 per °C), use of vasoactive medication (OR 1.46, 95% CI 1.04-2.05) and lower TAPSE (OR 0.96, 95% CI 0.93-0.98 per mm) were associated with AKI.

**CONCLUSIONS.** In all acutely admitted ICU patients, higher age, decreased MAP, lower central temperature, the use of vasoactive medication and lower TAPSE were independently associated with the development of AKI, whereas cardiac index was not. The former variables confirm previous findings and the association of TAPSE suggests that right ventricular function and possibly venous congestion may contribute to the high rate of AKI in critically ill patients.

#### REFERENCE(S)

Hiemstra B, Eck RJ, Koster G, et al. Clinical examination, critical care ultrasonography and outcomes in the critically ill: cohort profile of the Simple Intensive Care Studies-I. BMJ Open 2017;e017170

#### ACKNOWLEDGMENT

Currently analysis are being perfored to refine the outcomes of the SICS-I. In case of admission, enhanced results will be presented.

**Table 1 (abstract 0063).** General patient characteristics and CCUS variables

| Variable | No AKI | AKI | P value |
|---|---|---|---|
| N | 462 | 541 | |
| Age in years, mean (± SD) | 59 (16) | 64 (13) | <0.001 |
| Gender male, N (%) | 168 (36) | 233 (39) | 0.12 |
| SAPS II score (± SD) | 40.7 (14.8) | 50.7 (16.7) | <0.001 |
| Vasoactive medication, yes (%) | 186 (40) | 355 (59) | <0.001 |
| MAP, mmHg (± SD) | 81 (14) | 76 (14) | <0.001 |
| Central temperature, °C (± SD) | 37.1 (0.9) | 36.9 (0.9) | <0.001 |
| Cardiac index, L/min/m$^2$ (IQR) | 2.6 (2.1 - 3.2) | 2.5 (1.9 - 3.1) | 0.005 |
| TAPSE, mm (± SD) | 20.6 (5.6) | 18.3 (6.4) | <0.001 |

### 0064

### Effects of perioperative dexmedetomidine infusion on microcirculation and kidney and intestinal injury in kidney transplant recipients

Y.-C. Wang[1], Y.-C. Yeh[1], A. Chao[1], M.-K. Tsai[2], C.-Y. Lee[2], T.-J. Wei[1]
[1]National Taiwan University Hospital, Department of Anesthesiology, Taipei, Taiwan, Province of China; [2]National Taiwan University Hospital, Department of Surgery, Taipei, Taiwan, Province of China
**Correspondence:** Y.-C. Wang
*Intensive Care Medicine Experimental* 2018, **6(Suppl 2):**0064

**INTRODUCTION.** During kidney transplantation, microcirculation in implanted kidney might be affected by surgical stress, ischemia-perfusion injury, and pain stimulation. Dexmedetomidine is reported to improve intestinal microcirculation in surgical and pain animal model. [1]

**OBJECTIVES.** This study aims to determine whether continuous infusion of dexmedetomidine during kidney transplantation surgery can improve microcirculation and the post-operative kidney function.

**METHODS.** This is a single-blind randomized controlled trial. Patients who received kidney transplantation were included and randomized into the control or dexmedetomidine groups. Cardiac index and stroke volume index were recorded using bioimpedance technique. At specific time points, sublingual microcirculation was recorded by Cytocam, a third generation video microscope, and analyzed using a software, AVA 3.0. [2] Arterial blood gas analysis, blood urea nitrogen (BUN), creatinine, lactate, neutrophil gelatinase-associated lipocalin (NGAL), and diamine oxidase (DAO) levels were examined. Repeated measures ANOVA and t-test were used to compare the differences between the two groups. A p-value $< 0.05$ was considered significant. The error bars in all figures represent the 95% confidence intervals of means.

**RESULTS.** Among the 24 enrolled patients, baseline characteristics, post-operative BUN and creatinine levels did not differ significantly between the two groups. Perioperative slower heart rate, lower cardiac index (Figure 1) and lower lactate level (Figure 2) were noted in the dexmedetomidine group comparing to the control. There was a trend that total small vessel density and perfused small vessel density on day 2 and day 7 were higher in the dexmedetomidine group than in the control group (Table 1 and 2). NGAL and DAO did not differ significantly due to limited preliminary sample size.

**CONCLUSIONS.** Continuous infusion of dexmedetomidine might be related to slower heart rate and lower cardiac index. Meanwhile, dexmedetomidine is associated with lower lactate level, and has a trend to improve microcirculation.

#### REFERENCE(S)

1. Yeh YC, Sun WZ, Ko WJ, Chan WS, Fan SZ, Tsai JC, Lin TY: Dexmedetomidine prevents alterations of intestinal microcirculation that are induced by surgical stress and pain in a novel rat model. *Anesthesia and analgesia* 2012, 115(1):46-53.
2. De Backer D, Hollenberg S, Boerma C, Goedhart P, Buchele G, Ospina-Tascon G, Dobbe I, Ince C: How to evaluate the microcirculation: report of a round table conference. *Crit Care* 2007, 11(5):R101.

#### GRANT ACKNOWLEDGMENT

This work was supported, in part, by a grant from the Taiwan Ministry of Science and Technology (MOST 105-2314-B-002-037-M) and a grant from the National Taiwan University Hospital (NTUH 105-A125).

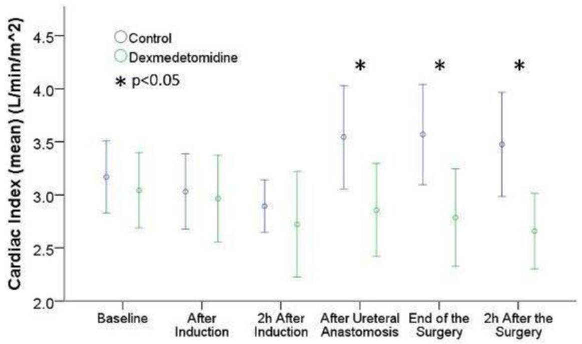

**Fig. 1 (abstract 0064).** Cardiac index in the two groups at different time points

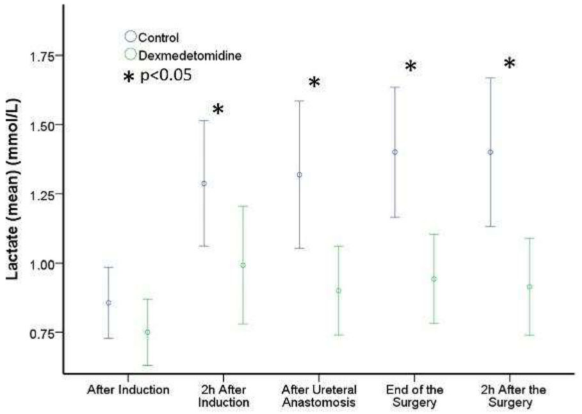

**Fig. 2 (abstract 0064).** Lactate level in the two groups at different time points

**Table 1 (abstract 0064).** Total small vessel density (mm/mm2). Repeated-measure ANOVA revealed time p=0.004, group p=0.805 and time x group p=0.382

| Group | Control (n=14) | Dexmedetomidine (n=10) | t-test p-value |
|---|---|---|---|
| Baseline | 24.7±2.6 | 25.2±2.5 | 0.661 |
| Post-operative 2 hours | 23.4±4.5 | 23.6±2.7 | 0.891 |
| Post-operative day 2 | 21.9±3.4 | 23.9±3.4 | 0.186 |
| Post-operative day 7 | 24.0±2.7 | 26.0±2.6 | 0.104 |

**Table 2 (abstract 0064).** Perfused small vessel density (mm/mm2). Repeated-measure ANOVA revealed time p=0.005, group p=0.831 and time x group p=0.502

| Group | Control (n=14) | Dexmedetomidine (n=10) | t-test p-value |
|---|---|---|---|
| Baseline | 24.4±2.5 | 24.9±2.2 | 0.647 |
| Post-operative 2 hours | 23.2±4.4 | 23.2±2.4 | 0.987 |
| Post-operative day 2 | 21.6±3.3 | 23.5±3.2 | 0.198 |
| Post-operative day 7 | 23.7±2.6 | 25.5±2.6 | 0.125 |

### 0065

#### Modification of sequential organ failure assessment score using acute kidney injury classification

Y. Kotani[1,2], T. Fujii[3,4], S. Uchino[5], K. Doi[6]
[1]Kameda Medical Center, Department of Intensive Care Medicine, Kamogawa, Japan; [2]Japanese Red Cross Society Wakayama Medical Center, Department of Critical Care Medicine, Wakayama, Japan; [3]Kyoto University Graduate School of Medicine, Department of Epidemiology and Preventive Medicine, Kyoto, Japan; [4]Japan Society for the Promotion of Science, Tokyo, Japan; [5]Jikei University School of Medicine, Intensive Care Unit, Department of Anaesthesiology, Tokyo, Japan; [6]University of Tokyo, Department of Emergency and Critical Care Medicine, Tokyo, Japan
**Correspondence:** Y. Kotani
*Intensive Care Medicine Experimental* 2018, **6(Suppl 2):**0065

**INTRODUCTION.** Although Sequential Organ Failure Assessment (SOFA) score is one of the most important severity scoring systems in intensive care unit (ICU), validated for predicting mortality, a change in clinical practice over time raised a problem. Renal component of SOFA score is defined by absolute serum creatinine level. However, renal dysfunction of critically ill patients in current clinical practice is commonly evaluated by acute kidney injury (AKI) defined by Kidney Disease Improving Global Outcomes (KDIGO) classification, which adopted a relative change in serum creatinine level from the baseline. Even a small increase of serum creatinine is associated with mortality and the risk for mortality increases with advancing stage of AKI. We hypothesized that modification of SOFA score using AKI classification would have a better predictive performance of mortality than the original SOFA score.

**OBJECTIVES.** To evaluate the prognostic validity of the SOFA score using KDIGO classification compared with the original SOFA score.

**METHODS.** This was a post-hoc analysis of the Japan AKI Database study, which was a multicenter prospective cohort study on AKI of all consecutive adult patients who stayed for over 24 hours in the 13 mixed ICUs in Japan from July through December in 2016. We applied KDIGO classification of AKI to determine the renal component of KDIGO-based SOFA score, with converting 3 stages of AKI in KDIGO definition to 4 stages.
The primary outcome was hospital mortality. The secondary outcome was ICU mortality. We first evaluated the construct validity of the KDIGO-based SOFA score by assessing the correlation with Acute Physiology and Chronic Health Evaluation (APACHE) II score at ICU admission by Spearman rank correlation. We then assessed the predictive validity of the original and KDIGO-based SOFA score for hospital and ICU mortality by logistic regression analysis. We compared the discriminative performance of the two scores using the areas under the curve (AUC) of the receiver operating characteristic curve.

**RESULTS.** Among 2292 patients eligible for the analysis, hospital mortality was 11.6% (266 patients) and ICU mortality was 5.1% (117 patients). A moderate correlation was observed between the KDIGO-based SOFA score and APACHE II score (Spearman rho = 0.476). The AUC for hospital mortality of the KDIGO-based and original SOFA score were 0.749 and 0.745, respectively (p = 0.393). The AUC for ICU mortality of the KDIGO-based and original SOFA score were 0.790 and 0.791, respectively (p = 0.892).

**CONCLUSIONS.** The prognostic validity of the KDIGO-based SOFA score was not superior to that of the original SOFA score.

##### REFERENCE(S)

1) Vincent et al. Intensive Care Med (1996) 22:707-710.
2) Ferreira et al. JAMA (2001) 286:1754-1758.
3) KDIGO. Kidney Int. 2012;Suppl 2(1):1-138.

**Table 1 (abstract 0065).** The renal component of the KDIGO-based and original SOFA score

| Score | | 1 | 3 | 4 | |
|---|---|---|---|---|---|
| KDIGO-based | 1.5-1.9 x baseline or ≥ 0.3 mg/dL increase | 2-2.9 x baseline | ≥ 3.0 x baseline or Increase in serum creatinine to ≥ 4.0 mg/dL | Initiation of RRT | |
| | UO | < 0.5 mL/kg/h for 6-12 h | < 0.5 mL/kg/h for ≥ 12 h | < 0.3 mL/kg/h for ≥ 24 h or Anuria ≥ 12 h | |
| Original | Cre | 1.2-1.9 mg/dL | 2.0-3.4 mg/dL | 3.5-4.9 mg/dL | > 5.0 mg/dL |
| | UO | | | < 500 mL/day | < 200 mL/day |

**Table 2 (abstract 0065).** Area under the receiver operating characteristic curves of the KDIGO-based and original SOFA score for hospital and ICU mortality

| | KDIGO-based SOFA score (95% CI) | Original SOFA score (95% CI) | p value |
|---|---|---|---|
| Hospital mortality | 0.749 (0.717-0.780) | 0.745 (0.713-0.777) | 0.39 |
| ICU mortality | 0.790 (0.749-0.831) | 0.791 (0.751-0.831) | 0.89 |

### 0066

#### Frailty is an important confounder of creatinine levels when studying acute kidney injury in the ICU

P.J. Gleeson[1], I.A. Cripppa[1], D. Sexton[2], V. Fontana[1], F. Taccone[1], J.-L. Vincent[1], J. Creteur[1]
[1]Erasme Hospital,Université Libre de Bruxelles, Bruxelles, Belgium; [2]Trinity College, Dublin, Ireland
**Correspondence:** P.J. Gleeson
*Intensive Care Medicine Experimental* 2018, **6(Suppl 2):**0066

**INTRODUCTION.** Retrospective observations and prospective randomized controlled trials, using creatinine based determinants, have reported contradictory results regarding the best time to start CRRT[1]. Creatinine levels are related to muscle mass, which in-turn is an index of frailty. Frailty is known to be an important predictor of ICU outcome[2].

**OBJECTIVES.** To determine if muscle mass interacts significantly with creatinine levels with respect to ICU mortality.

**METHODS.** We previously reported results (ESICM Berlin 2015) from a retrospective analysis of patients (n=157) requiring CRRT between January 2012-December 2013 in our medical-surgical ICU however, creatinine measurements were not adjusted for muscle-mass. To assess individual muscle mass, we analysed available CT-abdomen images that were taken between 1 year pre- and 5 days post-initiation of CRRT. Psoas muscle surface area was measured on axial sections at the level of mid-L3 and the vertical height of L3 vertebral body was measured on coronal sections[3]. A muscle mass index (MMI) was calculated as (Psoas Muscle Surface Area)/(L3 Height^2). Binary logistic regression analysis was performed. Multiple collinearity was tested for (VIF cut-off = 5) and eliminated using centred variables.

**RESULTS.** CT-images were available for 88/157 patients (56%). Muscle Mass Index (MMI) correlated significantly with creatinine level at the start of CRRT (start-creat) (R=0.35; p=0.001) (Fig. 1). Start-creat was an independent predictor of ICU mortality (OR 2.9 95%CI 1.6 - 5.2; p< 0.001) however, MMI also predicted ICU mortality (OR 3.6 95%CI 1.6-10.2; p=0.02) and the interaction between MMI and start-creat was a significant predictor of ICU mortality (p=0.03). When MMI was included in the model, start-creat lost its predictive power (OR 1.4 95%CI 0.6 - 3.1; p=0.38) and MMI remained the only significant predictor of ICU mortality (OR 3.3 95%CI 1.1-10; p=0.04).

**CONCLUSIONS.** Muscle mass, which is an index of frailty[3], is an important confounder of creatinine levels in studies of AKI and CRRT in the ICU. This should be considered in the planning and analysis of clinical studies. A bio-marker of renal function that is not dependent on muscle mass, such as cystatin C, could be more appropriate when studying AKI in the ICU.

**REFERENCE(S)**

1 Bagshaw et al. Strategies for the optimal timing to start renal replacement therapy in critically ill patients with acute kidney injury. *Kidney Int*91(2017)
2 Muscedereet *al.*The impact of frailty on intensive care unit outcomes: a systematic review and meta-analysis. *Intensive Care Med*43, 1105-1122 (2017).
3 Dahyaet *al.*Computed tomography-derived skeletal muscle index: A novel predictor of frailty and hospital length of stay after transcatheter aortic valve replacement. *Am Heart J*182, 21-27 (2016).

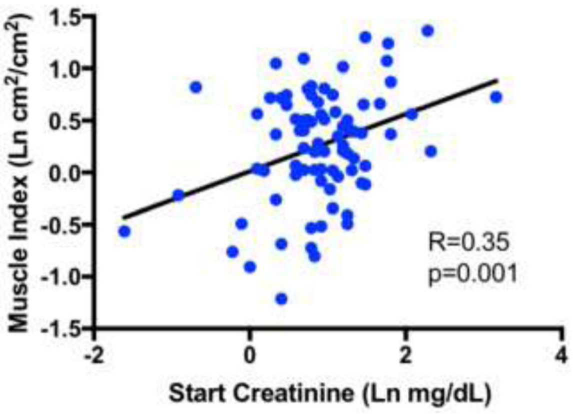

**Fig. 1 (abstract 0066).** Correlation Between Creatinine and Muscle Mass Index

### 0067

#### Association between use of mannitol and acute kidney injury in patients with moderate to severe traumatic brain injury

M. Skrifvars[1,2], M. Bailey[3], J. Mårtensson[4], E. Moore[5], C. French[3], J. Presneill[6], A. Nichol[7], L. Little[3], J. Duranteau[8], O. Huet[9], S. Haddad[10], Y. Arabi[10], C. McArthur[11], D.J. Cooper[3], J. Anstey[12], R. Bellomo[13]
[1]University of Helsinki and Helsinki University Hospital, Department Anaesthesiology, Intensive Care and Pain Medicine, Helsinki, Finland; [2]University of Helsinki and Helsinki University Hospital, Department of Emergency Medicine, Helsinki, Finland; [3]Australian and New Zealand Intensive Care Research Centre, Melbourne, Australia; [4]Karolinska Institute, Department of Physiology and Pharmacology, Stockholm, Sweden; [5]Monash University;, Melbourne, Australia; [6]Royal Melbourne Hospital, Department of Intensive Care, Melbourne, Australia; [7]University College Dublin, School of Medicine and Medical Sciences, Dublin, Ireland; [8]Hopitaux Universitaires Paris Sud, Department of Anesthesia and Intensive Care, Paris, France; [9]Hopital de la Cavale Blanche, Département d'Anesthésie-Réanimation, Brest, France; [10]King Saud Bin Abdulaziz University for Health Sciences and King Abdullah International Medical Research Center, Riyadh, Saudi Arabia; [11]Auckland City Hospital, Department of Critical Care Medicine, Auckland, New Zealand; [12]St Vincent's Hospital, Department of Intensive Care, Melbourne, Australia; [13]Austin Hospital, Intensive Care, Melbourne, Australia
**Correspondence:** M. Skrifvars
*Intensive Care Medicine Experimental* 2018, **6(Suppl 2):**0067

**INTRODUCTION.** Mannitol is commonly used for the treatment of raised intracranial pressure in patients with traumatic brain injury (TBI). However, mannitol has side effects such as polyuria and hypovolemia possibly causing renal failure.

**OBJECTIVES.** To study the effect of mannitol use during the first three days of TBI on the incidence of acute kidney injury (AKI) during intensive care unit stay.

**METHODS.** We studied patients included in the Erythropoietin in TBI (EPO-TBI) trial who had not developed AKI or died before day 3. AKI was defined according to the creatinine criteria of the Kidney Disease Improving Global Outcome (KDIGO) staging. Severity of TBI was categorized according to the International Mission for Prognosis and Analysis of Clinical Trials (IMPACT) model. We included the amount of mannitol and hypertonic saline (HTS) administered during the first three days. Cox regression analysis was used to predict time to AKI beyond day three until discharge from the ICU.

**RESULTS.** Among 568 survivors without AKI at day, 72 patients developed AKI later during ICU stay. The mean time to development of AKI after day 3 was 4 days (95% confidence interval [CI] 3-5 days). Of the patients that developed AKI 19% received mannitol compared to 5% in those who did not develop AKI (p< 0.001). The corresponding figures for HTS was 50% compared to 26% (p< 0.001). The mean amount of mannitol administered during the first 2 days with and without AKI after day 3 was 64 ml (95% CI 20-108 ml) and 12 ml (95% CI 7-18 ml) respectively. The corresponding figures for HS was 359 ml (95% CI 212-507 ml) and 134 ml (98-170 ml). Independent predictors of time to AKI beyond day 3 were proportion of hours of ICP higher than 20 mmHg (HR 5.7 95% CI 2.0-16.1, p=0.001), IMPACT TBI severity (per 10% increase of poor outcome (HR 1.2 95% 1.1-1.4, p=0.007), increasing weight (per kg) (HR 1.02 95% CI 1.02- 1.03, p=0.32) and amount of administered mannitol (per 100 ml increase) (HR 1.2 95% CI 1.0-1.5, p=0.049).

**CONCLUSIONS.** The amount of administered mannitol during the first 3 days was independently associated with time to AKI beyond day 3. The role of mannitol in moderate to severe TBI should be studied further.

**REFERENCE(S)**

Nichol A, French C, Little L, et al. The EPO-TBI Investigators and the ANZICS Clinical Trials Group. Erythropoietin in Traumatic Brain Injury. Lancet. 2015 Dec 19;386(10012):2499-506.

**GRANT ACKNOWLEDGMENT**

National Health and Medical Research Council of Australia (grant 545902), Transport Accident Commission of Victoria (grant D162). Markus Skrifvars has received personal research funding from Medicinska Understodsforeningen Liv och Halsa, Finska Lakaresallskapet, Svenska Kulturfondenand Instrumentarium Tiedesätiö.

### 0068

### Heme oxygenase 1 repeat polymorphism in septic acute kidney injury

L.M. Vilander[1], M.A. Kaunisto[2], S.T. Vaara[1], K.M. Donner[2], V. Pettilä[1], FINNAKI Study Group

[1]University of Helsinki and Helsinki University Hospital, Division of Intensive Care Medicine, Department of Anesthesiology, Intensive Care and Pain Medicine, Helsinki, Finland; [2]University of Helsinki, Institute for Molecular Medicine Finland (FIMM), Helsinki, Finland

**Correspondence:** L.M. Vilander

*Intensive Care Medicine Experimental* 2018, **6(Suppl 2):**0068

**INTRODUCTION.** Acute kidney injury (AKI) is a syndrome that frequently affects the critically ill. However, its pathophysiology and the predisposing factors are not completely understood. The role of genetic variability in iron metabolism pathway has been associated with AKI. Recently, a repeat length polymorphism in the *HMOX1* gene promoter sequence was associated with development of AKI in patients undergoing cardiac surgery (1). This gene encodes heme oxygenase 1 enzyme, which is inducible and expressed in response to stress.

**OBJECTIVES.** We investigated the association of *HMOX1* repeat length polymorphism with AKI in a Finnish cohort of critically ill septic patients (N=653).

**METHODS.** This prospective, observational, multicenter study was part of the FINNAKI study conducted in 17 Finnish intensive care units (ICU) in years 2011 to 2012. We defined AKI according to KDIGO criteria, with the phenotype of interest being severe AKI (KDIGO stage 2 or 3) in comparison to no AKI (KDIGO stage 0) in septic patients. We genotyped 300 patients with severe AKI and 353 without AKI, using fragment analysis. According to the number of dinucleotide (GTn) repeats in the promoter region of *HMOX1* gene, these alleles were grouped into either short, S (n< 27), or long alleles, L (n≥27).

**RESULTS.** The allele calling resulted in similar distribution than previously published, with peaks at 23 and 30 repeats (range 16 to 39 repeats). 85 patients had the SS, 323 patients the SL and 245 patients the LL allele combination. No significant difference in AKI incidence between these groups was observed. Moreover, the risk of the LL group compared to the SS group was 0.63-fold, suggesting a protective role of increasing number of GTn repeats (*p* value 0.077, Figure 1). In allelic analyses, the risk addition of an individual S-allele remained insignificant in additive, recessive and dominant models.

**CONCLUSIONS.** In septic patients, we did not detect association between the length of repeat polymorphism in *HMOX1* gene and AKI risk. Contrary to the previous findings in a cardiac surgery cohort, it appears that carriers of the short allele rather than the long one have an increased risk of AKI in this phenotype although the difference was not statistically significant.

**REFERENCE(S)**

1. Leaf DE, Body SC, Muehlschlegel JD, McMahon GM, Lichtner P, Collard CD, Shernan SK, Fox AA, Waikar SS: Length polymorphisms in heme oxygenase-1 and AKI after cardiac surgery. J Am Soc Nephrol 2016

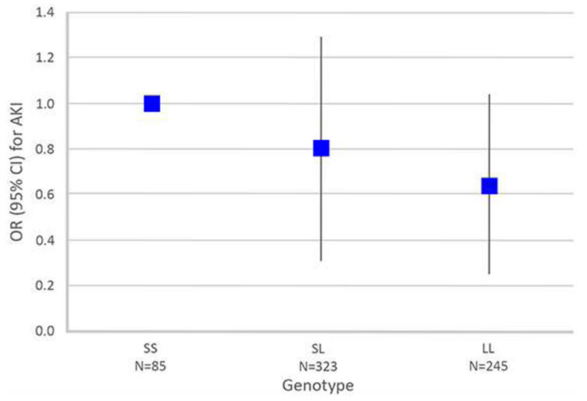

**Fig. 1 (abstract 0068).** Risk of AKI according to genotype

**GRANT ACKNOWLEDGMENT**
This study was supported by a grant form Munuaissäätiö Foundation (LV).

### 0069

#### Citrate versus heparin lock for non-tunneled hemodialysis catheters in critically ill patients: a multicentre randomized double-blind controlled trial (the VERROU-REA study)

R. Bruyère[1], A. Bourredjem[2], F. Meziani[3], A. Dargent[4], J. Helms[3], J. Badie[5], G. Blasco[6], G. Piton[7], G. Capellier[7], C. Mezher[5], J.-M. Rebibou[8], A. Nadji[9], T. Crepin[10], S.D. Barbar[11], C. Fleck[12], A. Cransac[13], M. Boulin[13], C. Binquet[2], A. Soudry-Faure[14], J.-P. Quenot[4], and the VERROU-REA Trial Investigators Group
[1]CH de Bourg-en-Bresse, Service de Réanimation, Bourg-en-Bresse, France; [2]Université Bourgogne Franche Comté, INSERM CIC 1432 Epidémiologie Clinique, Dijon, France; [3]CHU Strasbourg, Nouvel Hôpital Civil, Service de Réanimation Médicale, Strasbourg, France; [4]CHU Dijon Bourgogne, Médecine Intensive et Réanimation, Dijon, France; [5]CH de Belfort-Montbéliard, Service de Réanimation Polyvalente, Belfort, France; [6]CHU de Besançon, Service de Réanimation Chirurgicale, Besançon, France; [7]CHU de Besançon, Service de Réanimation Médicale, Besançon, France; [8]CHU Dijon Bourgogne, Service de Néphrologie, Dijon, France; [9]CHU Dijon Bourgogne, Service de Réanimation Neuro-Traumatologique, Dijon, France; [10]CHU de Besançon, Service de Soins Intensifs Néphrologiques, Besançon, France; [11]CHU de Nimes, Service de Réanimation Médicale, Nimes, France; [12]CHU Dijon Bourgogne, Délégation à le Recherche Clinique et à l'Innovation, Dijon, France; [13]CHU Dijon Bourgogne, Département de Pharmacie, Dijon, France; [14]CHU Dijon Bourgogne, Unité de Support Méthodologique, Dijon, France
**Correspondence:** R. Bruyère
*Intensive Care Medicine Experimental* 2018, **6(Suppl 2):**0069

**INTRODUCTION.** Hemodialysis non-tunneled catheters are currently used for critically ill patients with acute kidney injury requiring extracorporeal renal replacement therapy. Strategies to prevent catheter dysfunction and infection with catheter lock remain controversial.

**OBJECTIVES.** The objective is to compare the duration of event-free survival of the first non-tunneled hemodialysis catheter between trisodium citrate 4% and heparin as the catheter lock solution.

**METHODS.** In a multicenter, randomized, controlled, double-blind trial, we compared two strategies for catheter locking of non-tunneled hemodialysis catheters, namely trisodium citrate at 4% (intervention group) vs unfractionated heparin (control group). The primary endpoint was length of event-free survival of the first non-tunneled hemodialysis catheter. Secondary endpoints were : rate of fibrinolysis, incidence of catheter dysfunction and incidence of catheter-related bloodstream infection (CRBSI), all per 1000 catheter-days, number of hemorrhagic events requiring transfusion, length of stay in intensive care and in hospital, 28-day mortality.

**RESULTS.** In total, 396 patients were randomized : 199 to the citrate group and 197 to the heparin group. There was no significant different in baseline characteristics between groups. The duration of event-free survival of the first non-tunneled hemodialysis catheter was not significantly different between groups: respectively 7 days (IQR 3-10) in the citrate group and 5 days (IQR 3-11) in the heparin group (p=0.51). Rates of catheter thrombosis, CRBSI, and adverse events were not statistically different between groups.

**CONCLUSIONS.** In conclusion, trisodium citrate 4% as a catheter lock solution does not yield any benefit compared to heparin in the duration of event-free survival of a first non-tunneled hemodialysis catheter in critically ill patients.

**REFERENCE(S)**

1. Zhao Y, Li Z, Zhang L, Yang J, Yang Y, Tang Y, et al. Citrate versus heparin lock for hemodialysis catheters: a systematic review and metaanalysis of randomized controlled trials. Am J Kidney Dis. 2014;63(3):479-90.
2. Weijmer MC, van den Dorpel MA, Van de Ven PJ, ter Wee PM, van Geelen JA, Groeneveld JO, et al. Randomized, clinical trial comparison of trisodium citrate 30% and heparin as catheterlocking solution in hemodialysis patients. J Am Soc Nephrol. 2005;16(9):2769-77.
3. Hermite L, Quenot JP, Nadji A, Barbar SD, Charles PE, Hamet M, et al. Sodium citrate versus saline catheter locks for nontunneled hemodialysis central venous catheters in critically ill adults: a randomized controlled trial. Intensive Care Med. 2012;38(2):279-85.

**GRANT ACKNOWLEDGMENT**
This study was supported by a grant from the CHU Dijon Bourgogne (AAP Dijon Besançon 2012)

### 0070

#### Impact of renal replacement therapy in patients with acute respiratory distress syndrome: insights from the LUNG SAFE study

B.A. McNicholas[1,2], E. Rezoagli[2,3,4], T. Pham[5], F. Madotto[6], V. Fanelli[7], E. Guiard[8], M. Griffin[9,10], G. Bellani[3], M. Ranieri[11], J.G. Laffey[4,12,13], LUNG SAFE Investigators and the ESICM Trials Group
[1]National University of Ireland Galway, School of Medicine, Discipline of Medicine, Galway, Ireland; [2]Galway University Hospitals, Department of Anaesthesia and Intensive Care Medicine, Galway, Ireland; [3]University of Milano-Bicocca, School of Medicine and Surgery, Monza, Italy; [4]National University of Ireland Galway, School of Medicine, Department of Anaesthesia, Galway, Ireland; [5]St Michael's Hospital, Keenan Research Centre for Biomedical Science, Department of Critical Care Medicine, Toronto, Canada; [6]University of Milano-Bicocca, School of Medicine and Surgery, Research Centre on Public Health, Monza, Italy; [7]University of Turin, AOU Città della Salute e della Scienza di Torino - Ospedale Molinette, Department of Anesthesia and Critical Care, Torino, Italy; [8]Toronto General Hospital, Division of Nephrology, Toronto, Canada; [9]National University of Ireland Galway, Regenerative Medicine Institute at CÚRAM Centre for Research in Medical Devices, School of Medicine, College of Medicine, Nursing and Health Sciences, Galway, Ireland; [10]Galway University Hospitals, Saolta University Health Group, Department of Nephrology, Galway, Ireland; [11]Sapienza University, Policlinico Umberto I, Department of Anesthesia and Intensive Care Medicine, Roma, Italy; [12]St Michael's Hospital, Critical Illness and Injury Research Centre, Keenan Research Centre for Biomedical Science, Departments of Anesthesia and Critical Care Medicine, Toronto, Canada; [13]University of Toronto, Departments of Anesthesia, Physiology and Interdepartmental Division of Critical Care Medicine, Toronto, Canada
**Correspondence:** B.A. McNicholas
*Intensive Care Medicine Experimental* 2018, **6(Suppl 2):**0070

**INTRODUCTION.** The impact of the development of acute renal failure (ARF) requiring renal replacement therapy (RRT) on the ventilatory management and outcome of patients with Acute Respiratory Distress Syndrome (ARDS) is relatively poorly understood. We wished to address these issues in this secondary analysis of the LUNG SAFE patient cohort (1-3).

**OBJECTIVES.** Our primary objective was to determine the effect of developing stage 3 ARF requiring RRT (4) on the outcome of patients with ARDS. A secondary objective was to examine the effect of ARF on the ventilatory management of patients with ARDS.

**METHODS.** The large observational study to understand the global impact of severe acute respiratory failure (LUNG SAFE) was an international, multicenter, prospective cohort study of patients with severe respiratory failure. LUNG SAFE was conducted during 4 consecutive weeks in winter 2014, in a convenience sample of 459 ICUs in 50 countries across six continents. This study examined the association between the presence of stage 3 ARF requiring RRT and demographics, management, and outcome of patients with ARDS. The study population was restricted to patients that fulfilled the Berlin criteria for ARDS on day-1 or day-2 of acute hypoxaemic respiratory failure, who did not have chronic kidney disease (i.e. creatinine clearance< 60 mL/min) at intensive-care unit (ICU) admission.

**RESULTS.** 2153 patients fulfilled inclusion criteria, of whom 409 (19%) patients required RRT during the 28-day follow-up period. Patients that developed ARF were more likely to have moderate-severe ARDS (78% versus 69%, RRT versus non-RRT, p< 0.001), and to have higher Sequential Organ Failure Assessment (SOFA) scores (mean ±SD, 12±4 versus 9±4, RRT versus non-RRT, p< 0.001). In regard to ventilatory management, tidal volume was similar, while PEEP was higher (mean±SD, 9.1±3.7 versus 8.2±3.2 $cmH_2O$, RRT versus non-RRT, p< 0.001), in patients with ARF requiring RRT. Patients requiring RRT during 28-day follow up had an increased duration of ICU stay and higher crude ICU and hospital mortality rate compared to patients without RRT (see Table).

**CONCLUSIONS.** One fifth of patients with ARDS develop ARF requiring dialysis. These patient have greater ARDS and systemic illness severity, while mortality is substantially increased, with over half of these patients dying in hospital.

**REFERENCE(S)**

1. Bellani G, Laffey JG, Pham T, et al. *JAMA.* 2016;315(8):788-800.
2. Madotto F, Pham T, Bellani G, et al. *Intens Care Med.* 2018; doi: 10.1007/s00134-018-5152-6.
3. Laffey JG, Bellani G, Pham T, et al. *Intens Care Med.* 2016; 42(12):1865-1876.
4. http://kdigo.org/guidelines/acute-kidney-injury/

**GRANT ACKNOWLEDGMENT**

This work was funded and supported by the European Society of Intensive Care Medicine (ESICM), Brussels, Belgium, by St Michael's Hospital, Toronto, Canada, and by the University of Milan-Bicocca, Monza, Italy

**Table 1 (abstract 0070).** Association among RRT and outcomes in patients with ARDS. Differences among groups were tested using a Mann-Whitney U test and a Chi-Square test

| Parameter | RRT n=409 | Non RRT n=1744 | P value |
|---|---|---|---|
| Invasive ventilation free days to Day 28, median (IQR), days | 0.0 (0.0;15.0) | 16.0 (0.0;24.0) | <0.001 |
| Duration of invasive mechanical ventilation, median (IQR), days | 12.0 (5.0;20.0) | 7.0 (4.0;14.0) | <0.001 |
| Duration of ICU Stay, median (IQR), days | 14.0 (7.0;24.0) | 10.0 (5.0;18.0) | <0.001 |
| ICU Mortality, n (%) | 207 (50.6) | 539 (30.9) | <0.001 |
| Duration of Hospital Stay, median (IQR), days | 19.0 (8.0;38.0) | 16.0 (8.0;32.0) | 0.062 |
| Hospital Mortality, n (%) | 226 (55.3) | 616 (35.3) | <0.001 |

## Infections

### 0071

#### Healthcare associated infections (HAI) in critically ill cardiac patients (CICP): a categorical principal components analysis (CATPCA) study in Spanish ICU based on ENVIN-HELICS National Registry

E. Renes Carreño[1], A. Escribá Bárcena[2], M. Catalan Gonzalez[1], F. Alvarez Lerma[3], M. Palomar Martinez[4], S. Otero Romero[5], S. Uriona Turma[5], P. Olaechea Astigarraga[6], I. Seijas Betazola[7], J.C. Montejo Gonzalez[1]
[1]Hospital Universitario 12 de Octubre, Servicio de Medicina Intensiva, Madrid, Spain; [2]Hospital Universitario de Fuenlabrada, Servicio de Medicina Intensiva, Fuenlabrada, Spain; [3]Hospital del Mar, Servicio de Medicina Intensiva, Barcelona, Spain; [4]Hospital Universitario Arnau de Vilanova, Servicio de Medicina Intensiva, Lerida, Spain; [5]Hospital Universitario Vall de Hebron, Servicio de Medicina Preventiva, Barcelona, Spain; [6]Hospital Galdakao-Usansolo, Servicio de Medicina Intensiva, Galdakao, Spain; [7]Hospital Universitario de Cruces, Servicio de Medicina Intensiva, Baracaldo, Spain
**Correspondence:** E. Renes Carreño
*Intensive Care Medicine Experimental* 2018, **6(Suppl 2):**0071

**INTRODUCTION.** Traditionally, CICP have been considered as low risk patients for the development of HAI. However, when HAI take place, it is associated with a large increase in mortality and ICU length of stay.

**OBJECTIVES.** To obtain a model that takes into account the multiple factors that intervene inthe development of HAIs in CICP admitted to the ICU, such as different diagnostic groups,the presence of invasive devices, the type of infection or the systemic inflammatory response (SIR).

**METHODS.** Retrospective study based on the national registry ENVIN-HELICS in the period 2006-2015. Those patients whose cause of admission was a cardiac process were selected. After data exploration (bivariate and stratified analysis) 5 nominal variables (origin of the patient, admission code, diagnoses, type of infection, and death) and 5 ordinal variables (Hospital size, APACHE 2 at 24h after admission, days of vascular catheter, artificial airway and ICU stay) were included. A first CAPTCA model was made with all the cases. A second CATCPA model was made including only infected cases in which the SIR was introduced. The study was conducted through the SPSS 20 program.

**RESULTS.** 67266 cases were included in the analysis. The first CATPCA generated a spatial model with 2 dimensions (dim), with an Crombach alpha value of 0,899 which guarantees the adequacy of the model. The eigenvalue was 4,1 for the first dim and 2,4 for the dim 2. The first dim was conditioned by the duration of the invasive devices and to the severity of the disease. The second has to do with the size and complexity of the hospital and the process. The diagnostic groups with less complexity (uncomplicated coronary acute syndrome) and no infection were grouped around low or negative values of both dim. At the opposite side was the cardiac arrest (CRP) and most serious infections as ventilation associated pneumonia (VAP) or CLABSI. Diagnostic groups of high complexity and low mortality, such as postoperative cardiac surgery patients were located in positive values of the first dim and negatives of the second. The second CATPCA model had an Crombach alpha value of 0,855 The eigenvalue was 2,9 for the first dim and 1,5 for the second dim. The first dim was conditioned by the duration of the devices and the second by the severity of the disease, especially by the inflammatory response and mortality, and to a lesser extent by the APACHE 2 scale. The groupings were similar to the first model: Most of the serious infections were grouped into very positive values of the first dim and positive of the second. However, a greater degree of overlap was observed in the groupings of the diagnoses.

**CONCLUSIONS.** The CATPCA analysis allows explaining the relationship of different clinical aspects with the HAI in cardiac patients admitted to the ICU, providing a model with an excellent fit and intuitive in its spatial representation.

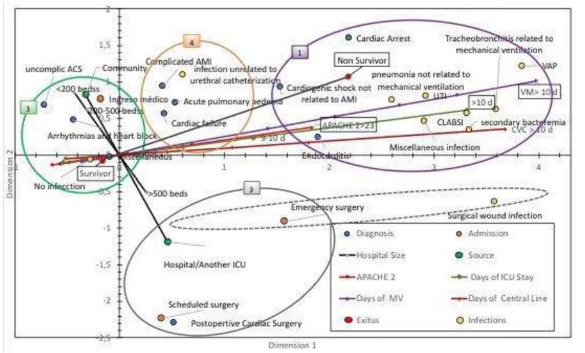

**Fig. 1 (abstract 0071).** CAPTCA Model 1:Variables at admission , diagnosis,infections,lenght of devices and mortality

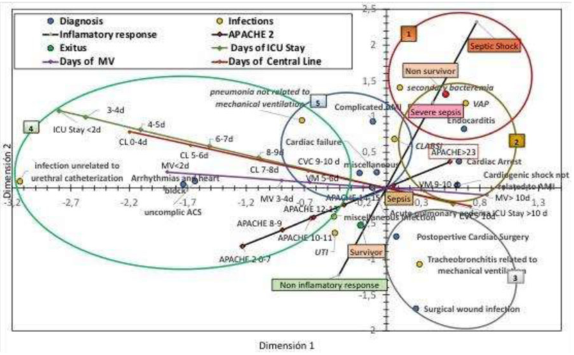

**Fig. 2 (abstract 0071).** CATPCA Model 2. Only patients with infection.The inflammatory response has been incorporated

### 0072

#### Development of a clinical prediction rule to exclude catheter-related bloodstream infections in patients with new-onset fever during central venous catheter placement

W.S. Oh[1], S.-B. Chon[2], H. Kwon[3], Y. Kang[1]

[1]Kangwon National University School of Medicine, Infectious Diseases, Chuncheon, Korea, Republic of; [2]CHA Bundang Medical Center, CHA University, Emergency Medicine, Gyeonggi, Korea, Republic of; [3]Kangwon National University School of Medicine, Internal Medicine, Chuncheon, Korea, Republic of

**Correspondence:** W.S. Oh

*Intensive Care Medicine Experimental* 2018, **6(Suppl 2):**0072

**INTRODUCTION.** Catheter-related bloodstream infection (CRBSI) is a leading cause of nosocomial infection. Non-tunnelled central venous catheters (CVCs) are most commonly used in critically ill patients and account for >90% of all CRBSIs. Because CRBSI lacks specific clinical features, its definite diagnosis depends entirely on microbiological techniques, i.e. catheter-sparing methods versus methods requiring catheter removal.

**OBJECTIVES.** Sterile non-tunneled central venous catheters are frequently removed under circumstances where only methods requiring catheter removal are available. To avoid the unnecessary removal of CVCs, a clinical prediction rule (CPR) was developed to identify CRBSI cases among the patients with new-onset fever.

**METHODS.** A retrospective study included patients with new-onset fever during CVC placement at one university hospital between January 2011 and December 2014. The primary outcome was the occurrence of CRBSI, which was defined as isolation of the same organism simultaneously from catheter tip and peripheral blood cultures. A multiple logistic regression analysis identified predictors associated with CRBSI to develop CPR. Its calibration and discrimination performance was estimated by generating a receiver operating characteristic curve, focusing on sensitivity and negative predictive values (NPVs) to help exclusion of CRBSI.

**RESULTS.** A total of 426 patients were included: 39 CRBSI (9.2%) and 387 non-CRBSI (90.8%) cases. The CPR comprised age of >65 years (one point), hospital stay prior to CVC insertion of >14 days (one point), repeated CVC placements (one point), absence of pneumonia on chest radiography (two points), and duration of CVC placement of >7 days (one point). Its *c* statistic was 0.826 (95% confidence interval [CI]: 0.759–0.892). The sensitivity and NPV were 76.9% (95% CI: 60.3–88.3%) and 97.1% (95% CI: 94.3–98.6%) at the score of >3. Using this CPR, 71.8% (306/426) of all cases were stratified into the low-risk group (CPR score: 0–3) and 97.0% (297/306) of low-risk patients did not have a CRBSI.

**CONCLUSIONS.** Prevalence of CRBSI is relatively low (9.2%) in patients with new-onset fever during CVC placement. Our CPR has a good discriminatory power for identifying CRBSI cases among febrile patients with a CVC. Because of its high NPV, the CPR scoring $\leq 3$ may support clinical exclusion of CRBSI and thus obviate the unnecessary removal of CVCs.

##### REFERENCE(S)

1. Mermel LA, Farr BM, Sherertz RJ. Guidelines for the management of intravascular catheter-related infections. Clin Infect Dis 2001;32:1249–1272.
2. Safdar N, Fine JP, Maki DG. Meta-analysis: methods for diagnosing intravascular device-related bloodstream infection. Ann Intern Med 2005;142:451–466.
3. Deliberato RO, Marra AR, Corrêa TD. Catheter related bloodstream infection in intensive care units: making the decision to remove or not to remove the central venous catheter. PLoS One 2012;7:e32687.

### 0073

#### Nebulized amikacin as adjunctive therapy for multi-drug resistant *P. aeruginosa* right-sided pneumonia: an experimental study

G. Li Bassi[1,2,3], H. Yang[1], L. Fernández-Barat[1,2,3], A. Motos[1], A. Meli[1,4], J. Bobi[1], D. Battaglini[1,5], F. Pagliara[5], M.L. Yang[1], E. Aguilera-Xiol[1], J. Miller[1], G. Frigola[6], M. Rigol[1,2,3], A. Ferrer[1], R. Cabrera[1,2], A. San José[1], V. Rosario[1], D. Chiumello[4,7], P. Pelosi[5], J. Ramirez[6], A. Torres[1,2,3]

[1]Hospital Clinic, Division of Animal Experimentation, Department of Pulmonary and Critical Care Medicine, Barcelona, Spain; [2]IDIBAPS/CIBERES, Barcelona, Spain; [3]University of Barcelona, Barcelona, Spain; [4]University of Milan, Milan, Italy; [5]University of Genoa, Department of Surgical Sciences and Integrated Diagnostics, San Martino Policlinico Hospital, IRCCS for Oncology, Genoa, Italy; [6]Hospital Clinic, Department of Pathology, Barcelona, Spain; [7]ASST Santi Paolo e Carlo, SC Anestesia e Rianimazione, Milan, Italy

**Correspondence:** G. Li Bassi

*Intensive Care Medicine Experimental* 2018, **6(Suppl 2):**0073

**INTRODUCTION.** Intravenous antibiotics may exert limited therapeutic efficacy in severe pneumonia caused by multi-drug resistant (MDR) pathogens, due to incomplete pulmonary penetration. The use of nebulized antibiotics could potentially enhance bactericidal efficacy.

**OBJECTIVES.** The effects of nebulized amikacin combined with intravenous meropenem on lung bacterial burden and clinical parameters were tested in an animal model of mono-lateral pneumonia.

**METHODS.** Eighteen pigs (32.4±1.8 Kg) were anesthetized and mechanically ventilated for 102 hours. Right-sided pneumonia was induced through bronchoscopic instillation of MDR *P. aeruginosa* (1), resistant to amikacin and susceptible to meropenem. Following clinical diagnosis of pneumonia, animals were randomized to receive the following treatments: nebulized and intravenous saline (control); nebulized saline and 25 mg/kg of intravenous meropenem (IV-MER); 450 mg of nebulized amikacin (BAY41-6551, Bayer AG, Germany) and 25 mg/kg of intravenous meropenem (AMK-MER). Nebulization was performed every 12 hours through a vibrating mesh nebulizer (Nektar Lpt, Nektar Therapeutics, USA), while intravenous meropenem was administered every 8h. Every 12h, white blood cells, creatinine, arterial blood gases, lung elastance and ventilatory settings were assessed. The primary outcome was *P.aeruginosa* lung tissue burden. Tracheal secretions and bronchoalveolar lavage (BAL) fluids were cultured upon clinical diagnosis of pneumonia, and every 24h thereafter.

**RESULTS.** Table 1 reports primary and secondary outcomes. Of note, AMK-MER only augmented bactericidal effects in tracheal secretions; nonetheless, creatinine was higher in the AMK-MER group. In the control, IV-MER and AMK-MER groups, white blood cell count was 21.0±16.4, 17.9±10.3 and 19.1±14.8 $10^9$/L, respectively (p=0.682). In addition, lung elastance was 32.4±10.6, 28.8±7.1 and 32.4±12.8 $cmH_2O/L$, respectively (p=0.414). Finally, $PaO_2/FIO_2$ was 319.9±83.9, 340.3±53.3 and 340.5±61.5 (p=0.202) and PEEP was 10±1.1, 8.6±1.3 and 9.1±1.0 $cmH_2O$, respectively (p< 0.001).

**CONCLUSIONS.** In an animal model of MDR *P.aeruginosa* right-sided pneumonia, the use of inhaled amikacin as adjuvant treatment only reduced proximal airways bacterial burden, while increasing risks of renal injury.

##### REFERENCE(S)

(1) Luna C et al. Chest 132, 2007: 523-31.

##### GRANT ACKNOWLEDGMENT

Bayer AG, Germany

**Table 1 (abstract 0073).** (*p<0.05 vs. IV-MER and AMK-MER groups; $ p<0.05 vs. control and IV-MER groups)

| | Control (6 animals) | IV Meropenem (6 animals) | Nebulized Amikacin and IV Meropenem (6 animals) | P-value |
|---|---|---|---|---|
| Primary Outcome | | | | |
| P.aeruginosa right-lung tissue concentration (cfu/gr) | 4.8±2.1* | 2.5±2.3 | 3.0±2.4 | p<0.001 |
| Secondary Outcomes | | | | |
| Tracheal secretions P. aeruginosa concentration (cfu/ml) | 6.0±0.9 | 4.8±1.0 | 3.2±2.3$ | p<0.001 |
| Right Lung BAL P. aeruginosa concentration (cfu/gr) | 4.5±0.8* | 3.4±1.1 | 3.5±1.0 | p<0.001 |
| Left Lung P.aeruginosa dissemination (%) | 83.3 | 16.7 | 16.7 | 0.038 |
| Creatinine (mg/dL) | 0.9±0.1 | 1.1±0.3 | 1.6±1.4 | 0.001 |

### 0074

#### Evaluation of steady-state pharmacokinetic and pharmacodynamic properties of intravenous colistimethate sodium in cystic fibrosis and critically ill patients

A.A. Morris[1], M. Saavedra[2], M.F. Wempe[3], J.A. Nick[2], S.W. Mueller[3], R. Maclaren[3], D.N. Fish[3], T.H. Kiser[3]
[1]University of California San Francisco Medical Center, San Francisco, United States; [2]National Jewish Health, Denver, United States; [3]University of Colorado Anschutz Medical Campus, Aurora, United States
**Correspondence:** T.H. Kiser
*Intensive Care Medicine Experimental* 2018, **6(Suppl 2):**0074

**INTRODUCTION.** The incidence of multi-drug resistant (MDR) Pseudomonas aeruginosa infections amongst patients with cystic fibrosis is growing and frequently requires ICU admission. Colistin has an emerging role in the treatment of these infections due to its sustained activity against MDR bacteria. However, a major obstacle to its use is the paucity of pharmacokinetic (PK) and pharmacodynamic (PD) data in this patient population.
**OBJECTIVES.** We aimed to evaluate colistin PK and PD in patients being treated for resistant gram negative infections.
**METHODS.** This study was a mult-center prospective cohort study. Cystic fibrosis patients ≥ 18 years old receiving intravenous colistimethate sodium for the treatment of infection were eligible for inclusion. Colistin was dosed according to current standards of practice: 3-5 mg/kg/day in 2-3 divided doses with adjustment for renal dysfunction. Steady-state plasma concentrations were obtained at 5 time points. Concentrations for colistin were determined via LC-MS/MS. Steady-state PK parameters were determined for each patient. Pharmacodynamic target attainment was considered acceptable if the fAUC0-24/MIC was > 35. Monte carlo simulation was utilized to determine the probability of PD target attainment.
**RESULTS.** Thirty patients were enrolled in this study, with 28 completing the PK analysis. Patients were 33 ± 13 years old, 50% female, 56 ± 9 kg, and had a creatinine clearance of 102 ± 35 ml/min. The mean ± SD Cmax, Cmin, half-life, Vd, Cl, and AUC0-24 were: 2.2 ± 3 mg/L, 0.9 ± 1.4 mg/L, 8.9 ± 5.4 hours, 1.9 ± 1.4 L/kg, 8.7 ± 4.0 L/hr, and 42 ± 59 mg*hr/L. Colistin A, on average, accounted for 75% of the total active colistin concentration, with colistin B representing 25% of the total concentration. Colistin total daily doses of 2.5 mg/kg/day were effective at achieving a fAUC/MIC > 35 for lower MIC organisms, but total daily doses of 6.85 mg/kg/day were necessary to reliably achieve pharmacodynamics targets for MICs up to 0.5 mg/L. Much higher total daily doses of 14.5 mg/kg and 29.5 mg/kg every 8 hours were needed to have > 90% target attainment for MICs of 1 and 2 mg/L respectively. Clinical cure or improvement was observed in 94% of patients, despite microbiologic persistence in 56% of patients. Nephrotoxicity and neurotoxicity were frequent, occurring in 38% and 19% of patients, respectively. No statistically significant difference was observed in Cmax or AUC0-24 between patients with and without nephrotoxicity.
**CONCLUSIONS.** Colistin exhibits considerable interpatient PK variability when administered to critically ill cystic fibrosis patients. Usual dosages of 3-5 mg/kg/day were only adequate to reliably obtain pharmacodynamics targets when treating gram negative infections with an MIC < 0.5 mg/L. We were unable to demonstrate a strong concentration effect relationship with either clinical cure or adverse effects (nephrotoxicity or neurotoxicity).

**GRANT ACKNOWLEDGMENT**
Cystic Fibrosis Foundation

### 0075

#### The prevalence of suspected ventilator-associated pneumonia in Scottish intensive care units: a prospective analysis

R. Hart[1], S. Maclean[2], S. McNeill[3], J. Hornsby[1], S. Ramsay[1]
[1]Queen Elizabeth University Hospital, Anaesthesia and Critical Care, Glasgow, United Kingdom; [2]Ninewells Hospital, Department of Anaesthesia and Critical Care, Dundee, United Kingdom; [3]Royal Infirmary of Edinburgh, Department of Anaesthesia and Critical Care, Edinburgh, United Kingdom
**Correspondence:** R. Hart
*Intensive Care Medicine Experimental* 2018, **6(Suppl 2):**0075

**INTRODUCTION.** Ventilator-associated pneumonia (VAP) is the most common healthcare associated infection (HAI) in mechanically ventilated patients[1]. Given there are many modifiable risk factors, VAP rates may be viewed as a quality indicator. The diagnostic criteria applied to detect VAP must therefore be robust and standardised[2]. There is concern that our existing diagnostic tool (HELICS) has become outdated. A sub-group of patients may therefore exist, who are being treated for VAP but not positively diagnosed or declared through our HAI screening process.
**OBJECTIVES.** We aim to determine the magnitude and characteristics of patients in Scottish ICU´s who are being treated for suspected VAP but not be detected using the HELICS criteria.
**METHODS.** A prospective national sprint audit was conducted over a one-month period. Local data collectors in Scottish ICUs were recruited and co-ordinated by the Scottish Intensive Care Society (SICS) Trainee Committee. All patients ventilated for more than 48-hours were included. Baseline HAI data was gathered for each eligible patient on a daily basis. Further collection of physiological data occurred if a new lower respiratory tract infection was suspected in order to determine whether that episode could be diagnosed as a VAP according to the HELICS definition.
**RESULTS.** This study included 227 patients and 1751 days of data collection. We successfully recruited 14 ICU sites from 11 Scottish NHS Health Boards. 32 patients were suspected of having a VAP (S-VAP). By applying the HELICS criteria, only 13/32 (40.6%) patients were positively diagnosed with VAP (H-posVAP). Of the 19 patients with S-VAP who failed to achieve a positive HELICS VAP diagnosis, 12 patients (63.2%) were due to the lack of radiological evidence alone. Median duration of ventilation was increased in patients with suspected VAP (14 days) compared to baseline (8 days). Hospital mortality was higher in patients with suspected VAP (42.8%) compared to baseline (33.7%). Patients who were HELICS negative had a higher hospital mortality than the HELICS positive patients (47.1% vs 36.4%).
**CONCLUSIONS.** We have demonstrated the existence of a sub-group of patients who are treated for suspected VAP but not diagnosed by the HELICS criteria. These patients would therefore be missed by our national HAI screening process. This population demonstrates a raised hospital mortality regardless of the HELICS screen. We believe the HELICS criteria is not compatible with modern ICU practice and an alternative tool should be sought.

**REFERENCE(S)**

1. Kalanuria, Zai and Mirski. Ventilator-associated pneumonia in the ICU. Critical Care 2014 18:208.
2. Ego, Preiser, Vincent. Impact of diagnostic criteria on the diagnosis of ventilator-associated pneumonia. Chest 2015 Feb; 147(2):347-55.

### 0076

#### Avian anti-*Pseudomonas aeruginosa* IgY-antibodies can reduce tracheal colonization with *P. aeruginosa* in mechanically ventilated piglets

A. Otterbeck, K. Hanslin, E. Lidberg Lantz, A. Larsson, J. Stålberg, M. Lipcsey

Uppsala University, Akademiska Hospital, Uppsala, Sweden

**Correspondence:** A. Otterbeck

*Intensive Care Medicine Experimental* 2018, **6(Suppl 2):**0076

**INTRODUCTION.** *P. aeruginosa* is a pathogen frequently resistant to antibiotics and a common cause of ventilator-associated pneumonia (VAP), VAP caused by *P. aeruginosa* has a mortality of 30-40% (1,2). Non-antibiotic strategies to treat or prevent VAP are therefore of great interest. Specific polyclonal avian IgY-antibodies have previously been shown to be effective against pneumonia caused by *P. aeruginosa* in rodents (3).

**OBJECTIVES.** To study the effect of *P. aeruginosa* specific polyclonal avian IgY-antibodies (IgY) on colonization of the airways in a porcine model.

**METHODS.** The pigs were anesthetized, mechanically ventilated and subject to invasive hemodynamic monitoring and allocated to either receive $10^9$ CFU nebulized *P. aeruginosa* (n=6) or $10^9$ CFU nebulized *P. aeruginosa* + 200 mg IgY antibodies (n=6). Physiological measurement, blood samples and tracheal cultures were then secured regularly for 27 hours after which the pigs were sacrificed and lung biopsies were analyzed for microbial growth.

**RESULTS.** After nebulization, tracheal growth of *P. aeruginosa* increased in both groups but with lower growth in the IgY-treated group (p = 0.02, mixed linear models). There was no difference between groups in respiratory function, physiological parameters or inflammatory markers during the observation period.

**CONCLUSIONS.** Our conclusion is that specific polyclonal avian IgY-antibodies can lessen bacterial colonisation of the airways. Further studies into the use of IgY-antibodies as a treatment for VAP are warranted.

**REFERENCE(S)**

1. Fujitani S, Sun H-Y, Yu VL, Weingarten JA. Pneumonia due to Pseudomonas aeruginosa: part I: epidemiology, clinical diagnosis, and source. Chest. 2011 Apr;139(4):909-19.
2. Chen X-C, Yang Y-F, Wang R, Gou H-F, Chen X-Z. Epidemiology and microbiology of sepsis in mainland China in the first decade of the 21st century. Int J Infect Dis IJID Off Publ Int Soc Infect Dis. 2015 Feb;31:9-14.
3. Thomsen K, Christophersen L, Bjarnsholt T, Jensen PØ, Moser C, Høiby N. Anti-Pseudomonas aeruginosa IgY antibodies augment bacterial clearance in a murine pneumonia model. J Cyst Fibros Off J Eur Cyst Fibros Soc. 2016 Mar;15(2):171-8.

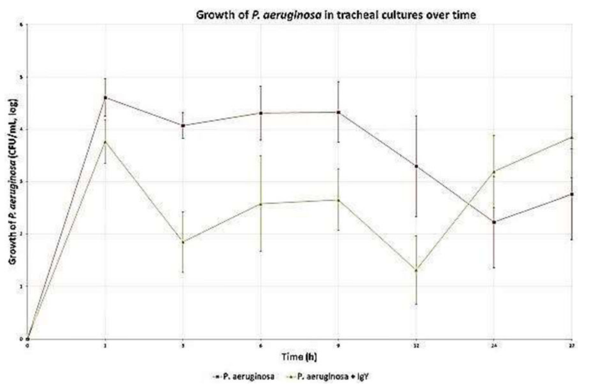

**Fig. 1 (abstract 0076).** Growth of P. aeruginosa in tracheal cultures over time

### 0077

#### Accuracy of the Clinical Pulmonary Infection score to differentiate ventilator-associated tracheobronchitis from ventilator-associated pneumonia: a retrospective analysis from the TAVeM study

A. Gaudet[1], I. Martin-Loeches[2], P. Povoa[3], A. Rodriguez[4], J. Salluh[5], S. Nseir[1]

[1]Lille University Hospital, Department of Intensive Care, Lille, France; [2]Trinity College, Dept of Clinical Medicine, Dublin, Ireland; [3]Hospital de São Francisco Xavier, Polyvalent ICU, Lisbon, Portugal; [4]Joan XXIII University Hospital, Tarragona, Spain; [5]D'Or Institute for Research and Education, Department of Critical Care, Rio de Janeiro, Brazil

**Correspondence:** A. Gaudet

*Intensive Care Medicine Experimental* 2018, **6(Suppl 2):**0077

**INTRODUCTION.** Ventilator-Associated Pneumonia (VAP) is the most frequent infectious complication in Intensive Care Units. Its natural history is tightly linked with that of Ventilator-Associated Tracheobronchitis (VAT), which has been described as an intermediate process between tracheobronchial colonisation and VAP (1). The management of VAP currently relies on antibiotic therapy, in contrast with VAT, for which antibiotic treatment is not currently recommended (2). The Clinical Pulmonary Infection Score (CPIS) has been widely described as an early predictor of VAP (3). However, its efficiency to differentiate VAP from VAT has never been studied.

**OBJECTIVES.** To assess the accuracy of CPIS in differentiating VAT from VAP.

METHODS: This was a retrospective study based on the data from the large prospective multinational TAVeM study (1). Patients with a microbiologically confirmed diagnostic of VAT or VAP were systematically included in our analysis. A sensitivity analysis was performed and ROC curves were computerized. The best-cut-off point was assessed through calculation of the Youden Index.

**RESULTS.** ROC analysis of CPIS showed an Area Under Curve of 0.757 (95% CI 0.722 - 0.793) for the diagnostic of VAP in our population. A CPIS value $\geq$ 7 was associated with the best Youden index, and showed a sensitivity (Se) of 0.51 (95% CI 0.45 - 0.56), a specificity (Sp) of 0.88 (95% CI 0.84 - 0.91), a Positive Predictive Value (PPV) of 0.83

(95% CI 0.76 - 0.88), a Negative Predictive Value (NPV) of 0.61 (95% CI 0.57 - 0.64), a Positive Likelihood Ratio (LR+) of 4.13 (95% CI 2.77 - 6.3) and a Negative Likelihood Ratio (LR-) of 0.56 (95% CI 0.48 - 0.65). In this cohort, a CPIS value ≥ 6, usually considered as the best cut-off for the diagnostic of VAP (3), was associated with a Se of 0.68 (95% CI 0.63 - 0.73), a Sp of 0.68 (95% CI 0.63 - 0.74), a PPV of 0.71 (95% CI 0.66 - 0.76), a NPV of 0.65 (95% CI 0.6 - 0.7), a LR+ of 2.17 (95% CI 1.72 - 2.77) and a LR- of 0.46 (95% CI 0.37 - 0.58).

**CONCLUSIONS.** CPIS exhibited moderate accuracy for the diagnostic of VAP among patients with microbiologically confirmed ventilator associated infections. Adjunctions of supplemental variables should be tested before considering its application to initiate preemptive antimicrobial treatments in ventilated patients with criteria of ventilator associated infections.

**REFERENCE(S)**

1. Martin-Loeches I, Povoa P, Rodríguez A, et al.: Incidence and prognosis of ventilator-associated tracheobronchitis (TAVeM): a multicentre, prospective, observational study. *Lancet Respir Med* 2015; 3:859-868
2. Torres A, Niederman MS, Chastre J, et al.: International ERS/ESICM/ESCMID/ALAT guidelines for the management of hospital-acquired pneumonia and ventilator-associated pneumonia. *Eur Respir J* 2017; 50
3. Shan J, Chen H-L, Zhu J-H: Diagnostic accuracy of clinical pulmonary infection score for ventilator-associated pneumonia: a meta-analysis. *Respir Care* 2011; 56:1087-1094

### 0078

#### Patients with digestive colonization with Extended-Spectrum β-Lactamase-producing *Enterobacteriaceae* (ESBLE): how to predict the risk of ESBLE infection following liver transplantation

E. Weiss[1,2,3], E. Logre[1], S. Janny[1], M. Giabicani[1,2], B. Grigoresco[1], A. Toussaint[1], M. Hachouf[1], L. Khoy-Ear[1], F. Bert[3,4], C. Paugam-Burtz[1,2,3]
[1]APHP, Beaujon, Anesthesiology and Critical Care, Clichy, France; [2]Paris Diderot University, Paris, France; [3]Inserm U 1149, Centre for Research on Inflammation, Paris, France; [4]APHP, Beaujon, Microbiology, Clichy, France
**Correspondence:** E. Weiss
*Intensive Care Medicine Experimental* 2018, **6(Suppl 2):**0078

**INTRODUCTION.** After liver transplantation (LT),40% of patients develop an infection of which 5% are related to Extended-Spectrum β-Lactamase-Producing Enterobacteriaceae (ESBLE) (1). EBLSE colonization is a recognized risk factor for ESBLE infection. However, only 15% of ESBLE carriers develop an infection caused by these pathogens and various studies have shown a poor positive predictive value of colonization to predict infection (2). Identifying risk factors of ESBLE infection in carriers is therefore important to rationalize the choice of empirical antimicrobial therapy in case of infection suspicion.

**OBJECTIVES.** To identify risk factors for ESBLE-related infection after LT in patients with prior ESBLE rectal colonization.

**METHODS.** Retrospective observational study from a prospective database after ethics committee agreement. All patients transplanted between 2010 and 2016 were screened and those with ESBLE in systematic preoperative rectal swabs were included. Postoperative infections were followed for 3 months and those related to colonizing ESBLE identified. Risk factors for ESBLE infections in carriers were investigated by univariate analysis (non parametric tests) and then multivariate analysis (logistic regression). Results are expressed as number (percentage) and median [interquartile range].

**RESULTS.** 749 LT were performed during the study period. 100 (13.3%) patients were colonized with ESBLE (68 *E. Coli*, 28 *K. pneumoniae* (KP), 10 *E. cloacae*) before TH. 45 (45%) patients developed an ESBLE-related infection including 39 (39%) with the same species (13 E. Coli (33.3%), 22 KP (56.4%), 4 E. cloacae (10.3%)) within 11 (6.5-18) postoperative days. Sites of infection were pulmonary (n=10), intra-abdominal (n=10), urinary (n=14), systemic (isolated blood culture, n=5) and biliary (n=2). The factors associated with ESBLE infection in carriers in univariate analysis are described in Table 1. In multivariate analysis, preoperative antibiotic prophylaxis of spontaneous bacterial peritonitis (SBP) (OR=5.4, p=0.02), colonization with ESBL-producing KP (OR=14.1, p< 0.001) and MELD score (OR=1.1, p< 0.001) were predictive of infection. Intraoperative administration of antibiotic prophylaxis active against colonizing ESBLE was associated with a reduced risk of infection (OR=0.2, p=0.04).

**CONCLUSIONS.** In liver transplant patients with preoperative digestive colonization with ESBLE, postoperative infections caused by these pathogens are common. Colonization with ESBL-producing KP, preoperative antibiotic prophylaxis against SBP and MELD score are predictive factors for postoperative ESBLE-related infection in these patients. Conversely, intraoperative antibiotic prophylaxis was protective when it was active against colonizing ESBLE. These data argues in favour of preoperative screening and tailoring antibiotic prophylaxis to colonization.

**REFERENCE(S)**

1. Bert et al. Emerg Inf Dis 2012
2. Carbonne et al. Ann Intensive Care 2017

**Table 1 (abstract 0078).** Factors associated with ESBLE-related infections in patients with prior ESBL rectal colonization

| Variables | Postoperative ESBLE-related infection (n=39) | No postoperative ESBLE-related infection (n=61) | p |
|---|---|---|---|
| MELD score | 24,5 [16,75-35,25 | 18 [12-27] | 0 ,021 |
| Long-term SBP antibiotic prophylaxis (%) | 12 (30,8) | 8 (13,1) | 0,042 |
| Colonization with ESBL-producing K.pneumoniae (%) | 21 (53,8) | 18 (29,5) | <0,001 |
| Antibiotic free-days within the 28 days before LT | 24 [20-28] | 28 [28-28] | <0,001 |
| Perioperative antibiotic prophylaxis active against colonizing ESBLE (%) | 30 (76%) | 52 (91,2%) | 0,07 |
| Intraoperative blood loss (ml) | 2000 [1100-3000] | 1000 [725-2000] | 0,008 |

### 0079

#### Accuracy of T2MR in diagnosing candidemia in critically ill patients: a prospective observational study

O. Pouly, A. Rouze, B. Voisin, C. Marjorie, N. Francois, B. Sendid, S. Nseir
Lille University Hospital, Lille, France
**Correspondence:** O. Pouly
*Intensive Care Medicine Experimental* 2018, **6(Suppl 2):**0079

**INTRODUCTION.** Early antifungal therapy is associated with better outcomes in patients with candidemia. Blood cultures and serological monitoring are gold standard for IC diagnosis. T2 Magnetic Resonance (T2MR) is a new diagnostic test that is able to detect in 3 to 5 hours the five clinically relevant species of Candida directly from the whole blood at a density as low as 1 colony forming unit (CFU) per ml. No study to date has demonstrated the clinical relevance for this new tool in the ICU.

**OBJECTIVES.** We conducted a monocentric prospective study to determine the accuracy of T2MR in diagnosing candidemia in ICU patients.

**METHODS.** In all patient with suspected candidemia, fungal blood culture, T2MR assay and fungal biomarkers were performed. The definite diagnosis of candidemia was based on standard fungal blood cultures.

**RESULTS.** Between November 1st, 2017 and February 1st 2018, 62 patients were hospitalized in our 10 beds ICU. 69 fungal blood samples were ordered in 38 patients. Median SOFA score was 10 [9-14], median SAPSII score was 54.5 [44-67] and median candida score was 2 [1,25-2]. 7 fungal blood cultures were positive in 5 patients, and 12 T2MR were positive in 9 patients with a sensitivity of 100%, a specificity of 91.8%, a

PPV of 58,3% and a NPV of 100%. All patients with positive fungal blood culture had at least one T2MR positive assay. 3 patients had a positive T2MR assay without positive blood culture. A positive manan antigen was found in 2 out of the 3 patients with no matching between blood culture and T2MR, suggesting that T2MR might be more sensitive than standard fungal blood cultures. Median time for positive culture was 5h, and 72 hours for standard fungal blood cultures and T2MR; respectively.

**CONCLUSIONS.** T2MR could be an interesting tool to early diagnose candidemia in ICU patients.

**REFERENCE(S)**

1. Hasan M. Al-Dorzi et al. Invasive Candidiasis in Critically Ill Patients: A Prospective Cohort Study in 2 Tertiary Care Centers. J Intensive Care Med. 2018 Jan 1:885066618767835.
2. Zacharioudakis IM et al. T2 Magnetic Resonance Assay: Overview of Available Data and Clinical Implications. J Fungi (Basel). 2018 Apr 4;4(2).
3. Clancy CJ et al. Detecting Infections Rapidly and Easily for Candidemia Trial, Part 2 (DIRECT2): A Prospective, Multicenter Study of the T2Candida Panel. Clin Infect Dis. 2018 Feb 9.

#### 0080

#### Risk factors of being a carrier of multiresistant bacteria upon admission to ICU

M. Palomar[1], F. Alvarez Lerma[2], M. Catalan[3], S. Uriona[4], I. Seijas[5], R.M. Granada[6], B. Balsera[7], J.C. Pozo[8], X. Nuvials[4], P. Olaechea[9], ENVIN

[1]I Recerca Biomedica de LLeida, Lleida, Spain; [2]Parc del Mar, Barcelona, Spain; [3]H Doce de Octubre, Madrid, Spain; [4]H Vall Hebron, Barcelona, Spain; [5]H Cruces, Bilbao, Spain; [6]H Bellvitge, Barcelona, Spain; [7]H Arnau de Vilanova, Lleida, Spain; [8]H Reina Sofia, Cordova, Spain; [9]H Galdakao, Galdakao, Spain

**Correspondence:** M. Palomar

*Intensive Care Medicine Experimental* 2018, **6(Suppl 2):**0080

**INTRODUCTION.** Bacterial resistance has become widespread both in hospitals and the community. It is not uncommon for patients, to be carriers of MRB at admission in the ICU

**OBJECTIVES.** To identify risk factors (RF) that can predict which patients are carriers of multiresistant bacteria (BMR) upon admission to the ICU, including infection and colonization

**METHODS.** Prospective, multicenter cohort study (ENVIN registry) that included patients admitted> 24 h in ICU from 2014 to 2016. The MRB studied were S aureus meticillin resistant (MRSA), P aeruginosa (Paer), A baumannii (Acin), Enterobacteria producers of extended beta-lactamases (ESBL) and carbapenemase-producing Enterobacteriaceae (CBP). We included as intrinsic data age, gender, severity (APACHE II, GCS and neutropenia), origin (community, another ICU, ward, and long-stay centers), pathology (medical, trauma, coronary, scheduled and emergency surgery), comorbidities (diabetes, chronic renal insufficiency, COPD, Cirrhosis, Immunosuppression, neoplasia, solid organ transplantation (SOT) and malnutrition. The chi square test and a multivariate analisys were applied.

**RESULTS.** Among the 69,741 patients admitted> 24h in the ICU, the presence of some MRB was diagnosed in 4.830 (6.4% of the total). Of these, in 3024 patients (4.3%) the identification was on admission. The BMR upon admission were 1,508 ESBL, 1020 MRSA, 448 P aer, 227 CBP and 154 Acin.Almost all risk factors were associated with a higher proportion of patients with MRB. In the multivariate analysis, the risk factors that remained significant were: APACHE ≥15: OR (95% CI) 1, 9 (1.8-2.0); hospital stay before ≥10 days: OR (95% CI) 3.7 (3.5-4.0); long-stay center: OR (95% CI) 3.4 (2.7-4.4); coronary disease: OR (95% CI) 0.2 (0.2-0.2); Neutropenia: OR (95% CI) 3.4 (2.8-4.2), C renal faliure: OR (95% CI) 2.3-2.1-2.6), Cirrhosis: OR (95% CI) %) 1.9 (1.6-2.2), COPD: OR (95% CI) 1.8 (1.7-2.0), malnutrition: OR (95% CI) 3.5 (3, 1-3,8) and TOS OR (95% CI) 3.3 (2.8-4.0).

The most significant RFs for **ESBL** were TOS: 4.0 (3.2-5.1), Pre-stay 3.7 (3.3-4.1), Malnutrition 3.6 (3.1-4.1) , origin LSC 3.3 (2,3-4,1) and neutropenia 3,2 (2,4-4,2). **MRSA** origin LSC 5,9 (4.2-8.2), Pre-stay 3.4 (3.0-3.9), malnutrition 3.3 (2.7-3.9), neutropenia 2.9 ( 2.0-4.2) and IRC 2.5 (2.2-2.9); ***P aeruginosa***: hospital stay 7.4 (6.0-9.1), TOS 6.2 (4.3-8.8), neutropenia 5.9 (4.0-8.7), malnutrition 4.9 (3.9-6.1) and IRC 4.7 (3.8-5.8). **CBP**: hospital stay 7.0 (5.3-9.3), TOS 5.7 (3.5-9.4), neutropenia 5.3 (3.0-9.4), malnutrition 4.2 (3.0-5.9) and chronic renal faliure 4.2 (3.1-5.7); ***Acinetobacter***: malnutrition 9.3 (6.7-13.0), stay h previous 8.9 (6.2-12.7), origin LSC 4.2 (1.8-9.7), neutropenia 2, 9 (1,2-7,2) and immunosuppression 2,7 (1,8-7,2)

**CONCLUSIONS.** The previous hospital stay, the origin from a LSC, malnutrition, neutropenia and TOS were the RFs with the greatest significance. The MRB showed some differences between them.

### New insights into post resuscitation care

#### 0081

#### Targeting normoxia vs. mild hyperoxia after cardiac arrest and resuscitation: a randomized pilot trial (NCT02698917)

P. Jakkula[1], J. Hästbacka[1], M. Reinikainen[2], P. Loisa[3], M. Tiainen[1], V. Pettilä[1], M. Lähde[3], M. Okkonen[1], J. Toppila[1], T. Birkelund[4], S. Bendel[5], A. Pulkkinen[6], J. Heinonen[1], M. Skrifvars[1], COMACARE Study Group

[1]University of Helsinki and Helsinki University Hospital, Helsinki, Finland; [2]North Karelia Central Hospital, Joensuu, Finland; [3]Päijät-Häme Central Hospital, Lahti, Finland; [4]Aarhus University Hospital, Aarhus, Denmark; [5]Kuopio University Hospital, Kuopio, Finland; [6]Central Finland Central Hospital, Jyväskylä, Finland

**Correspondence:** P. Jakkula

*Intensive Care Medicine Experimental* 2018, **6(Suppl 2):**0081

**BACKGROUND.** Mild hyperoxia has been associated with better neurological recovery and improved organ function after cardiac arrest and resuscitation. [1] In unconscious, mechanically ventilated patients, arterial oxygen tension ($PaO_2$) can be modified by changing the fraction of inspired oxygen ($FiO_2$) and the level of end-expiratory pressure (PEEP) but the optimal target level remains unknown. [2]

**OBJECTIVES.** We conducted a randomized pilot trial to assess the feasibility of targeting normal or mildly elevated $PaO_2$ in comatose, mechanically ventilated patients after out-of-hospital cardiac arrest. Also, we wanted to evaluate the effect of normal or mildly elevated $PaO_2$ on serum concentration of neuron-specific enolase (NSE) and cerebral oxygenation as measured by near-infrared spectroscopy (NIRS).

**METHODS.** In the Carbon dioxide, Oxygen and Mean arterial pressure After Cardiac Arrest and REsuscitation (COMACARE) trial, we used a $2^3$ factorial design to randomly assign patients after OHCA and resuscitation to normal or mildly elevated levels of $PaO_2$, low-normal or high-normal arterial carbon dioxide tension and low-normal or high-normal mean arterial pressure. The results of normal (10-15 kPa) vs. mildly elevated (20-25 kPa) $PaO_2$ comparison are reported here.

The primary outcome was the serum concentration of neuron-specific enolase (NSE) at 48 h after cardiac arrest. The main feasibility outcome was the difference in $PaO_2$ between the groups during the first 36 h after ICU admission. Secondary outcomes included cerebral oxygenation measured with NIRS.

**RESULTS.** 120 patients were included in the final analysis. Although we achieved a good separation in $PaO_2$ between the groups (Figure 1), there was no significant difference in serum NSE concentration at 48 h after cardiac arrest (for normal $PaO_2$ group, 22.4 μg/l [IQR, 14.8-28.3 μg/l] and for mildly elevated $PaO_2$ group 20.6 μg/l [IQR, 14.2-34.9 μg/l], p = 0.649). Median cerebral oxygen saturation was significantly higher in the mild hyperoxia group as compared with the normal $PaO_2$ group (Figure 2).

**CONCLUSIONS.** Targeting a specific range of $PaO_2$ is feasible in comatose, mechanically ventilated patients during post-resuscitation care. Mild hyperoxia did not affect the levels of NSE at 48 h after OHCA when compared with normoxia. However, mild hyperoxia resulted in higher cerebral oxygen saturation suggesting better oxygen delivery to the brain.

**REFERENCE(S)**

1. Elmer J, Scutella M, Pullalarevu R, et al. The association between hyperoxia and patient outcomes after cardiac arrest: analysis of a high-resolution database. Intensive Care Med 2014;41:49-57.
2. Nolan JP, Soar J, Cariou A, et al. European Resuscitation Council and European Society of Intensive Care Medicine Guidelines for Post-Resuscitation Care. Resuscitation 2015;95:202-22.

**GRANT ACKNOWLEDGMENT**

University of Helsinki three year project grant (H3702,WBS73702705) and Helsinki University Hospital governmental grant (TYH2018227).

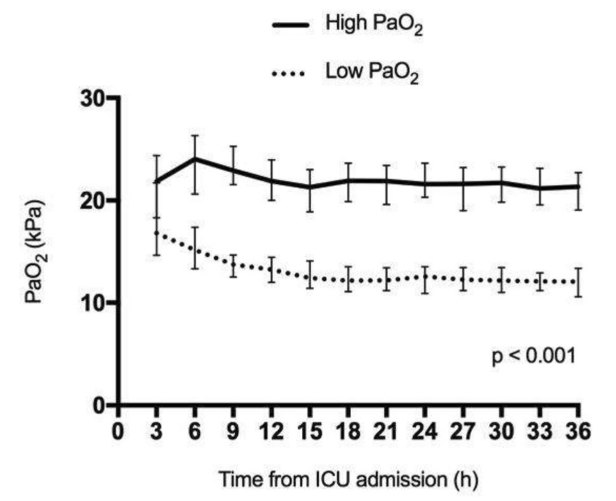

**Fig. 1 (abstract 0081).** Median (IQR) arterial oxygen tension in the different intervention groups

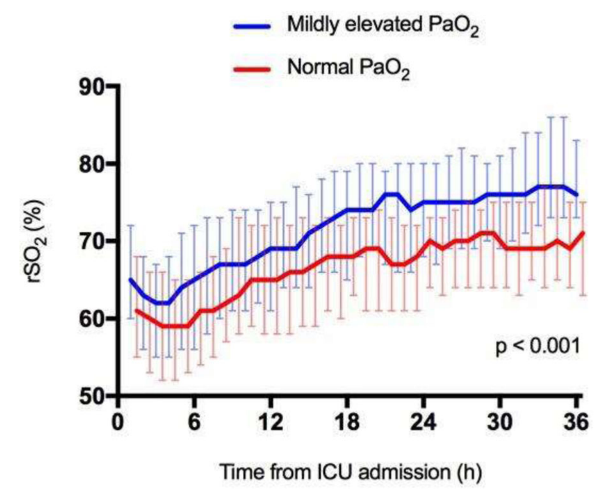

**Fig. 2 (abstract 0081).** Median (IQR) frontal cerebral oxygen saturation in the different intervention groups

### 0082

#### Comparison of two sedation regimens during targeted temperature management after cardiac arrest

M. Paul[1], W. Bougouin[2], F. Dumas[3], G. Geri[4], B. Champigneulle[5], L. Guillemet[6], O. Ben Hadj Salem[7], S. Legriel[8], J.D. Chiche[1], J. Charpentier[1], J.P. Mira[9], C. Sandroni[10], A. Cariou[9]

[1]Cochin, Medical ICU, Paris, France; [2]Paris Sudden-Death-Expertise-Center, Paris, France; [3]Cochin, Emergency Department, Paris, France; [4]Ambroise Pare Hospital, ICU, Boulogne Billancourt, France; [5]Georges Pompidou European Hospital, Surgical & Trauma Intensive Care Unit, Paris, France; [6]Argenteuil Hospital, ICU, Argenteuil, France; [7]Poissy Hospital, ICU, Poissy, France; [8]Mignot Hospital, ICU, Le Chesnay, France; [9]Cochin Hospital, Medical ICU, Paris, France; [10]Catholic University School of Medicine, Department of Anaesthesiology and Intensive Care, Rome, Italy

**Correspondence:** M. Paul

*Intensive Care Medicine Experimental* 2018, **6(Suppl 2):**0082

**INTRODUCTION.** Guidelines on post-resuscitation care recommend the use of short-acting agents for sedation during targeted temperature management (TTM) after cardiac arrest (CA), the potential advantages of this strategy have not been clinically demonstrated.

**OBJECTIVES.** Compare short and long-acting-drugs sedation regimen after CA and report proportion of patients with delayed awakening.

**METHODS.** We compared two sedation regimens (propofol-remifentanil, period P2, vs midazolam-fentanyl, period P1) among comatose TTM-treated CA survivors. Management protocol, apart from sedation and neuromuscular blockers use, did not change between the two periods. Baseline severity was assessed with Cardiac-Arrest-Hospital-Prognosis (CAHP) score. Time to awakening was measured starting from discontinuation of sedation at the end of rewarming. Awakening was defined as delayed when it occurred after more than 48 hours.

**RESULTS.** 460 patients (134 in P2, 326 in P1) were included. CAHP score did not significantly differ between P2 and P1 (P=0.93). Sixty percent of patients awoke in both periods (81/134 vs. 194/326, P=0.85). Median time to awakening was 2.5 (IQR 1-9) hours in P2 vs. 17 (IQR 7-60) hours in P1. Awakening was delayed in 6% of patients in P2 vs. 29% in P1 (p< 0.001). After adjustment, P2 was associated with significantly lower odds of delayed awakening (OR 0.08, 95% CI 0.03-0.2; P< 0.001). Patients in P2 had significantly more ventilator-free days (25 vs. 24 days; P=0.007), and lower catecholamine-free days within day 28. Survival and favorable neurologic outcome at discharge did not differ across periods.

**CONCLUSIONS.** During TTM following resuscitation from CA, sedation with propofol-remifentanil was associated with significantly earlier awakening and more ventilator-free days as compared with midazolam-fentanyl.

### 0083

#### Extracorporeal cardiopulmonary resuscitation for cardiac arrest patients: a multi-centric cohort study

L. Calabro[1,2], D. Lunz[3], M. Belliato[4], E. Contri[4,5], L.M. Broman[6], M. Malfertheiner[7], J. Creteur[1], F. Pappalardo[2], F.S. Taccone[1]

[1]Hopital Erasme, Intensive Care, Bruxelles, Belgium; [2]Vita Salute University, San Raffaele Hospital, Cardiothoracic ICU, Advanced Heart Failure and Mechanical Circulatory Support Program, Milan, Italy; [3]University Medical Center Regensburg, Department of Anesthesiology, Regensburg, Germany; [4]Fondazione IRCCS Policlinico San Matteo, UOS Advanced Respiratory Intensive Care Unit, UOC Anestesia e Rianimazione 1, Pavia, Italy; [5]AREU Lombardia, 6 AAT 118, Pavia, Italy; [6]Karolinska University Hospital, ECMO Centre Karolinska, Stockholm, Sweden; [7]University Medical Center Regensburg, Department of Internal Medicine II, Cardiology and Pneumology, Intensive care, Regensburg, Germany

**Correspondence:** L. Calabro

*Intensive Care Medicine Experimental* 2018, **6(Suppl 2):**0083

**INTRODUCTION.** Several case-series report controversial results of extracorporeal cardiopulmonary resuscitation (ECPR) for patients with refractory in-hospital (IHCA) and out-of-hospital cardiac arrest (OHCA); however, few data on large cohorts are available.

OBJECTIVES: To assess the survival and neurologic outcome following ECPR in five European centers.

**METHODS.** Retrospective analysis of database from a prospective observational cohort of patients undergoing ECPR patients in five University hospitals (Brussels, Regensburg, Pavia, Stockholm and Milan) from January 2012 to December 2016. The primary outcomes were survival to ICU discharge and 3-month favorable neurologic outcome, assessed by the Cerebral Performance Categories (CPC) of 1-2.

**RESULTS.** A total of 423 CA patients treated with ECPR were included (median age: 57 [48-65] years; male gender 78%) over the study period. Cause of arrest was presumed cardiac in 307 (73%) of patients and ECPR was initiated for OHCA in 258 (61%) patients. Time from arrest to ECMO implementation was 65 [48-84] minutes. ICU mortality was 76% (n=321); main causes of death were cardiovascular or multiple organ failure. A total of 80 patients (19%) had favorable neurological outcome. Median time from arrest to ECMO was significantly lower in IHCA than OHCA (42 [30-60] vs. 74 [60-90] minutes; p< 0.01). ICU mortality was significantly higher (219/258, 85% vs. 102/165, 62%, p< 0.01) and good neurological outcome rate lower (24/258, 9% vs. 56/165, 34%, p< 0.01) in ECPR for OHCA when compared to IHCA. A significant decrease in good neurological outcome rate was observed from shorter to longer time from arrest to ECMO (< 30 minutes: 55%; 31-60 minutes: 20%; 61-90 minutes: 9%, >90 minutes; 7% - p< 0.01).

**CONCLUSIONS.** ECPR was associated with intact neurological recovery in 19% of CA victims. Time from arrest to ECMO implementation is a significant determinant of clinical success.

### 0084

#### EEG reactivity for prognosis in patients after cardiac arrest

M. Admiraal[1], A.-F. van Rootselaar[2], J. Hofmeijer[3], C.W.E. Hoedemaekers[4], M.J.A.M. van Putten[5], J. Horn[6]

[1]Academic Medical Center Amsterdam, Intensive Care, Amsterdam, Netherlands; [2]Academic Medical Center, Neurology/Clinical Neurophysiology, Amsterdam, Netherlands; [3]Rijnstate Hospital, Neurology, Arnhem, Netherlands; [4]Radboud University Medical Centre, Intensive Care, Nijmegen, Netherlands; [5]Medisch Spectrum Twente, Neurology/Clinical Neurophysiology, Enschede, Netherlands; [6]Academic Medical Center, Intensive Care, Amsterdam, Netherlands

**Correspondence:** J. Horn

*Intensive Care Medicine Experimental* 2018, **6(Suppl 2):**0084

**INTRODUCTION.** EEG reactivity testing is often suggested to have prognostic value in patients after cardiac arrest. EEG reactivity testing regimens, however, vary and definitions for reactivity are poorly described (Admiraal et al., Eur J Neurol. 2016).

OBJECTIVE. We investigate the prognostic value of standardized EEG reactivity testing in a cohort of patients after cardiac arrest.

**METHODS.** This is a multicenter prospective observational study. We aim to enroll and monitor 160 patients, using continuous (c) EEG, from < 24 hours after cardiac arrest to day 3 or until the patient is awake, whatever comes first. EEG reactivity is tested twice a day according to a strict stimulation protocol comprising of five different stimuli, repeated three times for five seconds at an interval of at least 30 seconds. Three blinded experts score background activity and reactivity (yes/no) per stimulus for all EEGs. An EEG is scored as 'reactive' if at least one type of stimulus repeatedly elicits a change. The prognostic value of EEG reactivity for good and poor outcome is calculated as sensitivity and specificity. Furthermore, added prognostic value of EEG reactivity besides other known prognostic markers is given as the increased accuracy of a random forest classifier if EEG reactivity is included compared to excluded. Outcome at 6 months is defined as 'good' when the best score on the Cerebral Performance Category scale is 1-2. A score of 3-5 is considered 'poor'.

**RESULTS.** All 160 patients are enrolled; follow-up data is expected to be available May 2018. In an interim analysis of 80 patients an intra-class correlation coefficient of 0.78 is found for scoring an EEG as 'reactive'. Of these 80 patients, 39 (49%) have a good outcome. Mean age is 59 (±15) years in the good outcome group and 61 (±14) years in the poor outcome group. Time to return of spontaneous circulation is shorter in the good outcome group, 15 (±12) min versus 25 (±12) min in respectively good and poor outcome groups.

**CONCLUSIONS.** We conduct a dedicated prospective trial to determine the prognostic value of EEG reactivity and place this modality in the context of other known prognostic markers. An interim analysis showed a good correlation between raters when scoring EEG reactivity and a representative distribution of patient characteristics. Final results on the prognostic value of EEG reactivity will be presented at the conference.

### 0085

#### Long-term changes in quality of life after out-of-hospital cardiac arrest

H. Wimmer[1], J. Henriksen[2], T. Drægni[3,4], H. Stær-Jensen[3], D. Jacobsen[1], G.Ø. Andersen[5], C. Lundqvist[6,7,8], K. Sunde[7,9], E. Nakstad[1]

[1]Oslo University Hospital, Acute Medicine, Oslo, Norway; [2]Oslo University Hospital, Neurology, Oslo, Norway; [3]Oslo University Hospital, Anesthesiology, Oslo, Norway; [4]Oslo University Hospital, Research and Development, Oslo, Norway; [5]Oslo University Hospital, Cardiology, Oslo, Norway; [6]Akershus University Hospital, Neurology, Lørenskog, Norway; [7]University of Oslo, Clinical Medicine, Oslo, Norway; [8]Akershus University Hospital, Health Services Research Unit, Lørenskog, Norway; [9]Oslo University Hospital Ullevaal, Anesthesiology, Oslo, Norway

**Correspondence:** H. Wimmer

*Intensive Care Medicine Experimental* 2018, **6(Suppl 2):**0085

**INTRODUCTION.** Out-of-hospital cardiac arrest (OHCA) may lead to hypoxic brain injury. Cognitive impairment, with anxiety and depression, are common symptoms even in survivors with good neurological outcome. Little is known how cognitive impairment and related quality of life (QoL) change in the years after CA

**OBJECTIVES.** We therefore investigated, in a sub study of the Norwegian Cardio-Respiratory Arrest Study (NORCAST), how cerebral performance, QoL and psychological symptoms change from six months to four years after CA in adult OHCA survivors.

**METHODS.** The observational NORCAST cohort (NCT 01239420) evaluated a multimodal approach for early prognostication of the outcome in 259 comatose OHCA survivors. All patients were treated according to a standard treatment protocol, including targeted temperature management at 33°C, for 24 hours. In the present sub study, survivors eligible for follow-up investigations at both six months (T1) and 4 years or later (T2), were included. Cerebral performance categorisation (CPC) and a detailed examination protocol including cognitive and functional assessment were performed at both time points. Levels of depression and anxiety and QoL were assessed by the EuroQol EQ-5D-3L questionnaire. The questionnaire at T2 was supplemented with a visual analogue scale reflecting self-experienced health status (SEHS) over time. For the EQ-5D-3L and SEHS over time, time-series analyses using linear mixed models (LMM) were used. In addition, descriptive results were analysed using t-tests for paired samples and Wilcoxon test as appropriate. For all analyses SPSS 25 (IBM Corp, Armonk, NY, USA) was used. P-values < 0,05 were defined as statistically significant.

**RESULTS.** Among 259 OHCA patients (81% cardiac cause, median age 63 years, 83 % male), 137 (53%) survived to T1 and 115 (44 %) to T2. Altogether 108 patients were eligible for follow-up. So far, 83 patients have been investigated at T2. Among these, 74 (89 %) had CPC 1 at T1 and 77 (93 %) at T2 (NS). Self-experienced health status improved significantly from year to year after the 6 months control with greatest improvement over the first three years (Figure 1, p < 0,001). Time series analyses of EQ-5D results will be presented. In addition, significantly fewer patients were depressed or anxious at T2 vs T1 (Figure 2, p = 0,002).

**CONCLUSIONS.** Overall survival years after OHCA is good, with 93% of the patients having CPC 1 after four years. Self-experienced health status improves further even after 6 months, and levels of depression and anxietysignificantly decrease over time. Thus, a longer time horizon and more detailed assessment for cognitive impairment might to be needed to evaluate final outcome after OHCA.

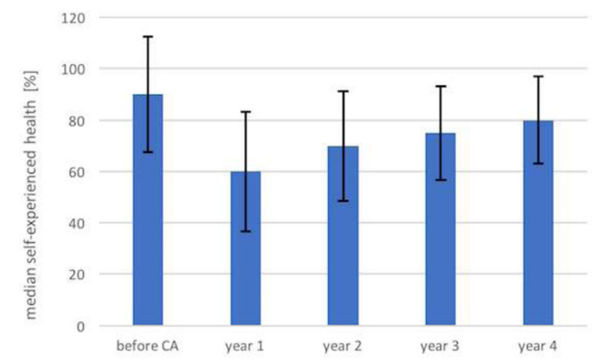

**Fig. 1 (abstract 0085).** Self-experienced health from before until 4-years after CA

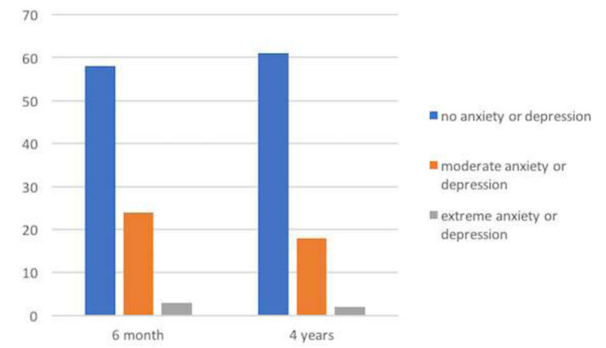

**Fig. 2 (abstract 0085).** Anxiety and depression at 6 month and 4 years after cardiac arrest

### 0086

#### Platelet dysfunction post cardiac arrest. Results from the Platelets in out of Hospital Cardiac ARrest (PoHCAR) study

A. Skorko[1], M. Thomas[1], A. Mumford[2], A. Pickering[3], J. Benger[4]
[1]University Hospitals Bristol NHS Foundation Trust, Bristol, United Kingdom; [2]University Hospitals Bristol NHS Foundation Trust, Department of Haematology, Bristol, United Kingdom; [3]University of Bristol, School of Physiology, Pharmacology & Neuroscience, Bristol, United Kingdom; [4]University Hospitals Bristol NHS Foundation Trust, Academic Department of Emergency Care, Bristol, United Kingdom
**Correspondence:** A. Skorko
*Intensive Care Medicine Experimental* 2018, **6(Suppl 2):**0086

**INTRODUCTION.** Out of hospital cardiac arrest (OHCA) is most commonly as a result of coronary disease, the mainstay treatment of which is prompt recanalization with percutaneous coronary intervention (PCI). Post PCI antiplatelet drugs are given for vessel patency. There is limited evidence as to what strategy should be employed post OHCA. Current practice is extrapolated from cardiology literature. Pertinently it is not clear as to what, if any, derangement of platelet function exists immediately post OHCA before antiplatelet drugs are delivered.
**OBJECTIVES.** The primary aim of this study was to assess platelet function and coagulation in Utstein cohort patients admitted to hospital with return of spontaneous circulation (ROSC) following OHCA before the administration of antiplatelet drugs.
**METHODS.** Any adult with ROSC following OHCA meeting Utstein criteria (witnessed, shockable arrest of presumed cardiac origin) was eligible for enrollment. A control group of patients admitted with acute ST elevation myocardial infarction (STEMI) who underwent primary PCI were also recruited.
Immediately at admission, patients in both groups had a blood sample drawn and analyzed using the ROTEM® platelet and delta modules. Data regarding preadmission antiplatelet and anticoagulant use was collected. Patients were followed up for 30 days to look for bleeding complications.
**RESULTS.** 30 patients were recruited to each arm and were well matched (median age 65.8 years OHCA and 65.4 in STEMI groups, 22 men in each group). Immediately at admission to hospital thrombin receptor function was reduced in OHCA as compared to STEMI patients. 32% of OHCA patients demonstrated thrombin receptor activation below the lower limit of normal.
Maximum clot formation time (MCFt) was prolonged in the OHCA group. Clotting time (CT) and clot formation time (CFT) showed a tendency to prolongation post OHCA, however maximum clot firmness was the same in both groups.
Hyperfibrinolysis (maximum lysis >=15%) was common in both groups (56% in STEMI; 52% in OHCA) and was correlated to markers of hypoperfusion In the OHCA cohort.
**CONCLUSIONS.** Immediately post OHCA, platelet thrombin receptor activity is reduced. Coagulation suggests a prolongation in the time taken to achieve maximum clot formation but, as compared to patients post STEMI, the same clot size is eventually produced. The implications on post cardiac arrest management require further study.

##### GRANT ACKNOWLEDGMENT

Dr Skorko is an NIHR academic clinical fellow. Grants from the David Telling charity and Resuscitation council UK are assisting with sample processing.

**Table 1 (abstract 0086).** ROTEM (R) parameter results by study group

| Parameter (ROTEM® reference range) | OHCA group Mean (95% confidence interval) | STEMI group Mean (95% confidence interval) | Mean difference (95% confidence interval) | P value |
|---|---|---|---|---|
| AUC of TRAP (61-156) | 75.0 (59.9 - 90.2) | 101.6 (87.4 -115.8) | 26.5 (6.2 - 46.8) | 0.01 |
| AUC of ADP (38-113) | 49.9 (36.3 - 63.4) | 58.5 (49.9 - 67.1) | 8.6 (-6.5 - 23.8) | 0.26 |
| Clotting Time (s) (38-79) | 70.1 (63.8 - 76.5) | 64.6 (62.0 - 67.2) | 5.6 (-0.5 - 11.6) | 0.07 |
| Maximum Clot Firmness (mm) (50-72) | 66.2 (65.6 - 68.7) | 67.9 (66.4 - 69.4) | - 1.7 (-4.9 - 1.5) | 0.28 |
| Maximum Clot Firmness time (s) | 1764 (1605 - 1923) | 1541 (1451 - 1632) | 223 (55 - 391) | 0.01 |

### 0087

#### Late awakening in survivors of post-anoxic coma: early neurophysiological predictors and association with ICU and long-term neurological recovery

A. Rey[1], A.O. Rossetti[2], J.-P. Miroz[1], P. Eckert[1], M. Oddo[1]
[1]Lausanne University Hospital, Intensive Care Medicine, Lausanne, Switzerland; [2]Lausanne University Hospital, Neurology, Lausanne, Switzerland
**Correspondence:** A. Rey
*Intensive Care Medicine Experimental* 2018, **6(Suppl 2):**0087

**INTRODUCTION.** Up to one-third of cardiac arrest (CA) survivors might experience late awakening (L-AW) from coma, defined as a time to awakening of more than 48 hours following targeted temperature management (TTM) and sedation stop. No study has sought to examine - using early neurological and neurophysiological assessment - whether late awakening is a clinical marker of increased post-anoxic cerebral injury. Furthermore, it is unclear whether late awakening may be associated with worse neurological impairment in CA survivors.

**OBJECTIVES.** To identify early neurophysiological predictors of L-AW in comatose CA survivors and examine its association with ICU and long-term recovery.

**METHODS.** Single-center observational cohort study of comatose subjects resuscitated from CA who underwent early prognostication, including Glasgow Coma Scale (GCS) motor response, brainstem reflexes, EEG and serum neuron specific enolase (NSE). Baseline demographic variables, CA characteristics and SOFA score at day 1 were analyzed. Sedation administered until first sedation stop was recorded. Awakening was identified retrospectively in medical records. Time to awakening was defined as the time from sedation stop to the first documentation of a GCS-M=6. L-AW was defined as an awakening time of more than 48 hours. Hyperactive delirium was defined as the presence of a Richmond Agitation Sedation Scale (RASS) > 2 with repeated use of anti-psychotic agents after initial sedation stop. Recovery was assessed prospectively at 3 months using the Glasgow-Pittsburgh Cerebral Performance Categories (CPC).

**RESULTS.** 418 patients were included : 229 (55%) awoke, of whom 214 completed 3-month outcome follow-up. L-AW occurred in 71 patients (33%). Patient demographics and CA characteristics did not differ between patients with early awakening (E-AW) and L-AW. Patients with L-AW had a higher rate of absent motor response (38 vs. 9%), absent brainstem reflexes (21 vs. 4%), discontinuous EEG (46 vs. 21% at day 1; 20 vs. 5% at day 2) and NSE > 33 ng/mL (23 vs. 8%) (all *p*< 0.01). L-AW was also associated with higher SOFA score at day 1 and greater midazolam dose during TTM. By multivariable analysis - after adjusting for cumulative midazolam dose during TTM and SOFA score - absence of motor response (odds ratio [OR] 3.35 [confidence interval 1.22-9.18], *p*=0.019), presence of a discontinuous EEG (OR 2.63 [1.10-6.29], *p*=0.029) and elevated NSE > 33 ng/mL (OR 4.71 [1.39-15.99], *p*=0.013), within 3 days from CA, were independent predictors of L-AW. Patients with L-AW had higher rate of hyperactive delirium (61 vs. 38% in E-AW, *p*=0.001) and 3-month unfavorable recovery (CPC 3-5 25 vs. 11% in E-AW, *p*=0.008).

**CONCLUSIONS.** Late awakening from post-anoxic coma is common and correlates with neurophysiological signs of increased cerebral damage and worse ICU and 3-month neurological outcomes.

**GRANT ACKNOWLEDGMENT**

This work was supported by the Swiss National Science Foundation.

### 0088

#### Intravascular versus surface cooling for targeted temperature management after out-of-hospital cardiac arrest: an analysis of the TTH48 study

C. De Fazio[1], M. Skrifvars[2], E. Søreide[3], J. Creteur[1], A.M. Grejs[4], H. Kirkegaard[5], F.S. Taccone[1], TTH48 Investigators

[1]ULB, Hopital Erasme, Brussels, Belgium; [2]University of Helsinki and Helsinki University Hospital, Division of Intensive Care, Department of Anesthesiology, Intensive Care and Pain Medicine, Helsinki, Finland; [3]Stavanger University Hospital, Stavanger, Norway; [4]Aarhus University Hospital, Department of Anaesthesiology and Intensive Care Medicine, Aarhus, Denmark; [5]Research Center for Emergency Medicine, Aarhus University Hospital, Department of Emergency Medicine and Department of Clinical Medicine, Aarhus, Denmark

**Correspondence:** C. De Fazio

*Intensive Care Medicine Experimental* 2018, **6(Suppl 2):**0088

**INTRODUCTION.** Different cooling devices can be used to achieve targeted temperature management (TTM) after out-of-hospital cardiac arrest.

**OBJECTIVES.** The aim of this study was to explore the performance and outcomes for intravascular versus other devices for TTM after out-of-hospital cardiac arrest.

**METHODS.** A retrospective analysis of data from the TTH48 study (NCT01689077), which compared whether TTM at 33°C for 48 hours results in better neurologic outcomes compared with standard 24-hour duration. Devices were assessed for speed of cooling and rewarming rates. Precision was assessed by measuring temperature variability (TV), i.e. the standard deviation (SD) of all temperature measurements in the cooling phase. Main outcomes were overall mortality, and poor neurological outcome, including death, severe disability or vegetative status (i.e. Cerebral Performance Category of 3-5).

**RESULTS.** A total of 355 patients were included in the study; of those, 217 (61%) were managed with intravascular catheter, while the others were treated with other devices, mainly surface cooling. A total of 114 (53%) patients treated with endovascular devices and 62 (42%) with other devices were cooled for 48 hours (p=0.19). Time to target (≤34°C) was significantly shorter for patients treated with endovascular devices (2.3 [1.1-4.0] vs. 4.3 [2.8-6.7] hours, p< 0.001), but temperature was also lower on admission (35.0 [34.2-35.6] vs. 35.3 [34.5-35.8] °C; p=0.02) and cooling rate was similar between groups (endovascular, 0.4 [0.2-0.8] °C/hour vs. others, 0.4 [0.2-0.6]°C/hour; p=0.14). Temperature variability was significantly lower in the endovascular device group when compared to other methods (0.6 [0.4-0.9] °C/hour vs. 0.7 [0.5-1.0]°C/hour; p=0.007), as was rewarming rate (0.3 [0.2-0.4] °C/hour vs. 0.4 [0.3-0.5]°C/hour; p=0.02). There was no statistically significant difference in mortality (endovascular 65/217, 30% vs. others 43/155, 27%; p = 0.72) or poor neurological outcome (endovascular 69/217, 32% vs. others 50/155, 32%; p = 1.00) between type of devices.

**CONCLUSIONS.** Endovascular cooling devices were more precise and had a slower rewarming rate than other methods in patients cooled at 33°C after out-of-hospital cardiac arrest. Main outcomes were similar with regard of the cooling methods.

### 0089

#### Functional recovery and quality of life after in-hospital cardiac arrest - Preliminary results. Australia and New Zealand Cardiac Arrest Outcome Determinants and ECMO suitability study (ANZ-CODE): a multi-centre prospective observational study of in-hospital cardiac arrests

G. Pound[1,2], D. Jones[1,3], G. Eastwood[1,3], C. Hodgson[1,2]

[1]Australian and New Zealand Intensive Care Research Centre, Department of Epidemiology and Preventive Medicine; Monash University, Melbourne, Australia; [2]Alfred Hospital, Department of Physiotherapy, Melbourne, Australia; [3]Austin Hospital, Intensive Care Department, Melbourne, Australia

**Correspondence:** G. Pound

*Intensive Care Medicine Experimental* 2018, **6(Suppl 2):**0089

**INTRODUCTION.** Reported frequency of in-hospital cardiac arrest (IHCA) in Australia and New Zealand ranges from 1.3-6.0 per 1000 admissions with an overall in-hospital mortality of 74.6%[1]. Little is known about the long-term functional outcome and health-related quality of life (HRQoL) of survivors of IHCA. It is recommended that multiple impairment and disability measures with longer term end-points are used to characterise recovery among cardiac arrest survivors[2].

**OBJECTIVES.** To determine functional recovery and quality of life of survivors of IHCA at 6-months.

**METHODS.** A multi-centre prospective cohort study. Emergency calls were screened and data collected for all IHCA. Patients were included if aged >18 years, unresponsive with no respiratory effort and cardiac compressions commenced. Patients were excluded if a pre-existing NFR order was in place or if the emergency call did not fit the criteria for IHCA. Patients were contacted by telephone if they survived to 6-months post IHCA. Measurements included discharge destination, re-admission to hospital, return to work, functional status (modified Rankin Scale (mRS) and Barthel Index (BI)) and HRQoL (EQ5D-5L) at 6-months.

**RESULTS.** There were 52 IHCA across five sites between July and November 2017 (male 61.4%, median age 76.5 years (IQR 20.0)). 15 patients (28.8%) survived to hospital discharge of which 10 were discharged home. 14 patients (26.9%) survived to 6-months. Of the 10 survivors who consented to follow-up interview (71.4%), 1 patient remains in hospital and 9 patients are residing at home. Three patients had subsequent hospital admissions, 2 were then discharged to rehabilitation. Median mRS and BI at 6 months were 3/6 (IQR 4.5) and 19/20 (IQR 6.5) respectively. In the EQ5D-5L domains there were no problems reported by 3 patients (33.3%) for mobility and self-care, 2 (22.2%) for usual activities, 1 (11.1%) for pain and 4 (44.4%) for anxiety/depression. The median EQ5D VAS score was 70/100 (IQR 30). Of the 4 patients working prior to IHCA, 2 (50%) had returned to work. Those who did not return to work reported this was due to health post IHCA.

**CONCLUSIONS.** Functional recovery and HRQoL at 6 months post IHCA is varied. Two thirds of patients report some level of functional disability and few patients report 'no problems' with HRQoL domains at 6 months. Only half of patients previously employed returned to work.

**GRANT ACKNOWLEDGMENT**

Felice Rosemary Lloyd Physiotherapy Grant

### 0090

#### Combination of NSE and Synek score to predict poor neurological outcome in post anoxic comatose patients treated with induced hypothermia

D. Titeca Beauport[1], P. Merle[2], B. Perin[2], Y. Zerbib[1], C. Brault[1], A. Sagnier[1], L. Kontar[1], B. De Cagny[1], T. Soupison[1], M. Slama[1], J. Maizel[1]
[1]CHU Amiens-Picardie, Medical ICU, Amiens, France; [2]CHU Amiens-Picardie, Neurophysiology, Amiens, France
**Correspondence:** D. Titeca Beauport
*Intensive Care Medicine Experimental* 2018, **6(Suppl 2):**0090

**INTRODUCTION.** Neurological prognostication from cardiac arrest (CA) survivors is a current concern. The last decade was marked by the widespread use of electroencephalography (EEG) and Neuron specific enolase (NSE) dosage, improving the evaluation of brain injury in usual practice. Nevertheless, there is a wide degree of variability in the NSE serum threshold observed in different studies, limiting the generalization of reliable cut-off value. EEG patterns can be classified in five grades according to the Synek classification; but few data are available on the specific performances of this classification since generalization of induced hypothermia.

**OBJECTIVES.** We aimed to assess the performances of the combination of NSE dosage performed within the 48-72h and the Synek score to predict hard outcome defined by a CPC 3-5, in post anoxic comatose patients treated with induced hypothermia.

**METHODS.** We conducted a prospective monocentric study in our medical intensive care unit, between November 01, 2013 and November 01, 2017. All comatose patients having at least one early standard EEG and H48-72 NSE dosage available were included. Were excluded moribund patients, dead from post cardiac arrest shock, awoke within the first 48 hours of admission, neurological causes of CA, and patients with missing data. The primary outcome was the CPC score at 3 months or at death time.

**RESULTS.** Among 240 cardiac arrest survivors, 101 were analysed. All patients received sedation with midazolam and sufentanil and 33° mild therapeutic hypothermia for 24 h. Thirty-one patients had a good neurologic outcome (CPC 1-2) and 70 had a poor neurologic outcome (CPC $\geq$ 3). A NSE serum level > 46 µg/l was associated with a poor neurological outcome, with an AUC of 0.92 95%CI [0.85 to 0.97], a sensitivity of 77%, 95%CI [66 to 86] and a specificity of 97% 95%CI [83 to 100]. The Synek score $\geq$3 was also strongly associated with poor neurological outcome, with a sensitivity of 90%, 95%CI [81 to 96], a specificity of 87% 95%CI [70 to 96] and a AUC of 0.95 95%CI [0.89 to 0.98]. The multivariable log-binomial regression retained NSE (Odd ratio [OR], 2.10; 95% CI, 1.04 to 4.29; p=0.039) and the Synek grade (OR, 8.27; 95% CI, 2.63 to 25.96; p=0.0003) as independent predictors. The combination of these two parameters achieved an AUC of 0.96 95% CI [0.96 to 1.00] to predict a poor neurological outcome.

**CONCLUSIONS.** In the limit of this study, a NSE serum concentration > 46 µg/l at H48-72 and a Synek grade $\geq$3 one the early post sedation standard EEG are highly predictive of a poor neurological outcome, in post anoxic comatose patients treated with induced 33° C hypothermia. Their combination improved the discriminant capacity, independently to other prognostic factors. Nevertheless, these results should not exempt the clinician to apply a multimodal approach and need to be confirmed by a multicenter prospective study.

## Cardiovascular dynamics

### 0091

#### Biological scoring system for early prediction of acute bowel ischemia after cardiac surgery: the PALM score

E. Zogheib[1], C. Cosse[2], A. Vecten[3], S. Marx[3], J. Nader[4], M. Henry[3], M. Bernasinski[3], F. Trojette[3], C. Sabbagh[2], T. Caus[4], J.M. Regimbeau[2], H. Dupont[5]
[1]CHU Amiens - Picardie, Amiens, France; [2]Digestive Surgery Department, CHU Amiens, Amiens, France; [3]Cardiothoracic and Vascular Intensive Care Department, CHU Amiens, Amiens, France; [4]Cardiac Surgery Department, CHU Amiens, Amiens, France; [5]Cardiothoracic and Vascular Intensive Care Department, Amiens, France
**Correspondence:** E. Zogheib
*Intensive Care Medicine Experimental* 2018, **6(Suppl 2):**0091

**INTRODUCTION.** Bowel ischemia is a life-threatening emergency defined as an inadequate vascular perfusion leading to bowel inflammation resulting from impaired colonic/small bowel blood supply. Main issue for physicians regarding bowel ischemia diagnosis lies in the absence of informative and specific clinical or biological signs leading to delayed management, resulting in a poorer prognosis, especially after cardiac surgery.

**OBJECTIVES.** The aim of the present series was to propose a simple scoring system based on biological data for the diagnosis of bowel ischemia.

**METHODS.** In a retrospective monocentric study, patients admitted in cardiac ICU, after cardiovascular surgery, were screened for inclusion. According to a 1:2 ratio (case: control), matching between two groups was based on sex, type of cardiovascular surgery and the operative period (per month). Patients were divided into two groups: "ischemic group" corresponds to patients with confirmed bowel ischemia and "non-ischemic group" corresponds to patients without bowel ischemia. Primary objective was the conception of a scoring system for the diagnosis of bowel ischemia. Secondary objectives were to detail the post-operative morbidity and the diagnostic features for the distinction between acute mesenteric ischemia and ischemic colitis.

**RESULTS.** Forty-eight patients (1.3%) had confirmed bowel ischemia (“ischemic group”). According to the 2:1 matching, 96 patients were included in the “non-ischemic group”. Aspartate aminotransferase >449 UI/L, lactate >4 mmol/L, procalcitonin >4.7 μg/L and myoglobin >1882 μg/L were found to be independently associated with bowel ischemia. Based on their respective odds ratios, points were assigned to each item ranging from 4 to 8. AUROCC [95% confidence interval] of the scoring system to diagnose bowel ischemia was 0.93 [0.91-0.95], p< 0.001. The optimal threshold after bootstrapping was ≥14 points; this yielded a sensitivity of 85.4%, a specificity of 94.8%, a positive likelihood ratio of 16.42, a negative likelihood ratio of 0.15, a Youden´s index of 0.802 and a diagnostic odds ratio of 106.62.

**CONCLUSIONS.** A biological scoring system based on PCT, ASAT, lactate and myoglobin measurement allows the diagnosis of bowel ischemia after cardiac surgery with high accuracy. This score could help clinician to propose an early diagnosis and an early treatment in this high mortality disease.

##### REFERENCE(S)

Biological scoring system for early prediction of acute bowel ischemia after cardiac surgery: the PALM score.

Zogheib E, Cosse C, Sabbagh C, Marx S, Caus T, Henry M, Nader J, Fumery M, Bernasinski M, Besserve P, Trojette F, Renard C, Duhaut P, Kamel S, Regimbeau JM, Dupont H.

Ann Intensive Care. 2018 Apr 18;8(1):46. doi: 10.1186/s13613-018-0395-5. PMID: 29671149.

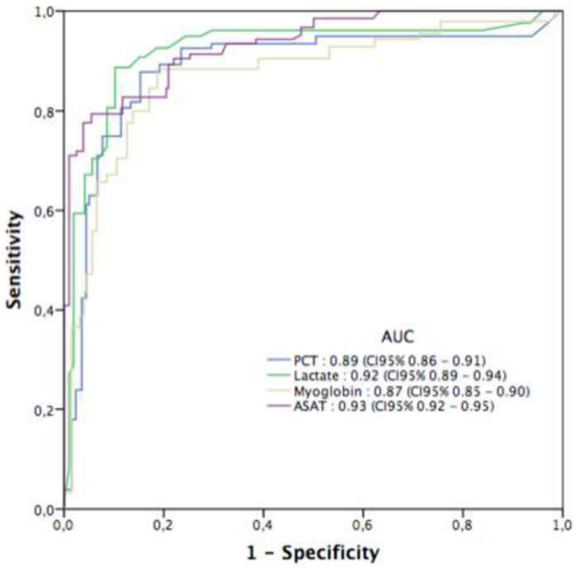

**Fig. 1 (abstract 0091).** Comparison of the ROC curves showing the Se and Sp of each marker (PCT, ASAT, last, Myog)

### 0092

#### Clinical and epidemiological characteristics of acute heart failure in the critically ill patient (ICAUCI study group)

M.C. Guia[1], L. Zapata[2], R. Gomez[3], E. Rodriguez[4], E. Portugal Rodruiguez[5], I. Leache[6], M. García[7], F. Roche-Campo[8], M. Lafuente[9], A. Palazón[10], ICAUCI (Insuficiencia Cardíaca Aguda en Unidades de Cuidados Intensivos)
[1]Corporació Sanitària Parc Taulí en Sabadell, Intensive Care, Sabadell, Spain; [2]Hospital de la Santa Creu i Sant Pau, Intensive Care, Barcelona, Spain; [3]Hospital Quironsalud Miguel Dominguez, Intensive Care, Pontevedra, Spain; [4]Hospital General de Castellón en Castellón, Castellon, Spain; [5]Hospital Universitario Lucus Augusti, Lugo, Spain; [6]Hospital Joan XXIII, Tarragona, Spain; [7]Hospital Universitari Vall d'Hebron, Barcelona, Spain; [8]Hospital Verge de la Cinta, Tortosa, Spain; [9]Hospital Nuestra Sra. de Gracia, Zaragoza, Spain; [10]Hospital General Universitario de Murcia, Murcia, Spain
**Correspondence:** M.C. Guia
*Intensive Care Medicine Experimental* 2018, **6(Suppl 2):**0092

**OBJECTIVES.** To assess the epidemiology and clinical characteristics of acute heart failure (AHF) in the Spanish Intensive Care Units (ICU).

**METHODS.** Multicenter prospective observational study that analized patients older than 18 years admitted to the ICU for more than 24 hours between April and June 2017. Demographic, physiological and clinical variables, including complementary explorations of those who suffered AHF during ICU stay, were collected. Statistical analysis was made using ANOVA or chi-square test as appropriate. The multivariate study was performed by Cox regression for survival analysis and logistic regression. Values of p ≤ 0.05 were considered statistically significant.

**RESULTS.** During the study period 32 ICU treat a total of 4419 patients. Of these, 2971 patients met the inclusion criteria. Five hundred and eighty-seven (19.9%) patients suffered AHF, 318 (54.2%) as the main diagnosis at admission (Adm) (190 first AHF episode (AHFnew) and 128 chronic heart failure exacerbation (CHFex)) and 269(45.8%) as a complication (Com) during ICU stay for another reason (182 AHFnew and 87 CHFex). Patients with AHF were older (69 12.9 vs 60.6 16.5, P < 0.001) and presented higher severity-of- illness (SAPS III 58.6 16.4 vs 48.9 30, P < 0.001), higher BMI ( 29.19 vs 27.76.2, P < 0.001) and higher comorbidity (Charlson Index 3.7 2.4 vs 2.3 2.3, P < 0.001) in terms of hypertension, diabetes, dyslipidemia, ischemic heart disease, peripheral arteriopathy, anemia and chronic renal failure. There were no significant differences in gender distribution. The Adm-ICAnew patients have shorter length of stay in hospital (18 days, 95% CI 15-21) than Com-ICAnew patients (25 days, 95% CI 21-30, P = 0.021).

The most frequent type of AHF at admission was pulmonary congestion (85.6%) followed by tissue hypoperfusion (45.7%). Ischemic heart disease (36.5%) was the main associated diagnosis, followed by atrial fibrillation (23.7%). In 47.7% of the cases, no precipitating factor was found, while in the 28% AHF was associated with infection and in the 21.1% to overhydration. ICU and hospital mortality were higher in patients with AHF (16.7% vs 9.4% and 20.6% vs 11.8%, p < 0.001), although in the multivariate study only the Com-CHFex group showed a higher mortality OR 2.99 (95% CI 1.48-6.07, p = 0.002).

**CONCLUSIONS.** AHF is frequent in critically ill patients, in terms of cause of admission and as a complication. The decompensation of a CHF during admission to the ICU is a factor independently related to mortality.

##### GRANT ACKNOWLEDGMENT

ICAUCI study group is part of the Cardiac Intensive Care Task Force of the Spanish Society of Intensive Care Medicine (SEMICYUC)

### 0093

#### Effects of adjustment of blood flow of venoarterial extracorporeal membrane oxygenation life support on microcirculation

T.J. Wei[1], C.-H. Wang[2], C.-H. Lai[2], A. Chao[3], Y.-C. Wang[3], Y.-C. Yeh[3], NCMMR
[1]National Taiwan University Hospital, Anesthesiology, Taipei, Taiwan, Province of China; [2]National Taiwan University Hospital, Department of Surgery, Taipei, Taiwan, Province of China; [3]National Taiwan University Hospital, Taipei, Taiwan, Province of China
**Correspondence:** T.J. Wei
*Intensive Care Medicine Experimental* 2018, **6(Suppl 2):**0093

**INTRODUCTION.** xtra-corporeal membrane oxygenation (ECMO) can maintain cardiopulmonary function in patient with heart failure waiting for recovery or heart transplant. A study shows that microcirculatory dysfunction is severer in the nonsurvivors than in the survivors with venoarterial ECMO (VA-ECMO) [1]. In our previous study, we found that hemodynamic parameters did not differ significantly between survivors and non-survivors, but lower perfused small vessel density (PSVD) within 12 hours after venoarterial ECMO (VA-ECMO) placement is associated with 28 days mortality.

**OBJECTIVES.** This study aims to investigate the effects of adjustment of VA-ECMO flow on microcirculation.

**METHODS.** Patients with VA-ECMO placement were enrolled in this study. Hemodynamic parameters and laboratory data were recorded. Sublingual microcirculation were obtained by using a third generation of video microscope (Cytocam) before and after the adjustment of VA-ECMO blood flow by the ECMO team within 24 hrs and 48 hrs after placement of VA-ECMO.

**RESULTS.** The difference of sublingual microcirculation after adjustment of VA ECMO blood flow did not correlate with any hemodynamic parameter within 24 hrs after VA-ECMO placement. At 48 hrs after VA-ECMO placement, both the differences of total small vessel density (TSVD) and PSVD after adjustment of VA-ECMO blood flow negatively correlated to systolic blood pressure (SBP) before adjustment of blood flow (TSVD-difference and SBP: Pearson correlation -0.760, P=0.018; PSVD-difference and SBP: Pearson correlation -0.678, P=0.045).

The difference of TSVD after adjustment of VA-ECMO blood flow negatively correlated to mean arterial pressure (MAP) before adjustment of VA-ECMO blood flow (TSVD-difference and MAP: Pearson correlation: -0.795, P=0.01). The difference of PSVD after adjustment of VA-ECMO blood flow had a trend to negatively correlate to MAP and pulse pressure (PP) before adjustment of VA-ECMO blood flow (PSVD-difference and MAP: Pearson correlation: -0.531, p=0.141; PSVD-difference and PP: Pearson correlation: -0.602, p=0.086).

**CONCLUSIONS.** At 48 hrs after V-A ECMO placement, the differences of sublingual microcirculatory parameters after adjustment of VA-ECMO blood flow have negative correlations with SBP before adjustment of VA-ECMO blood flow.

##### REFERENCE(S)

1. Kara A, Akin S, Dos Reis Miranda D, et al. Microcirculatory assessment of patients under VA-ECMO. Crit Care 2016;20:344.

**Table 1 (abstract 0093).** Correlations between hemodynamic and microcirculation parameters

| Within 48 hrs after ECMO insertion | | SBP-before (mm Hg) | DBP-before (mm Hg) | MAP-before (mm Hg) | PP-before (mm Hg) | TSVD-before ($mm/mm^2$) | TSVD-difference ($mm/mm^2$) | PSVD-before ( $mm/mm^2$) | PSVD-difference ($mm/mm^2$) |
|---|---|---|---|---|---|---|---|---|---|
| SBP-before (mm Hg) | Correlation Significance | 1.000 0.000 | 0.429 0.188 | 0.837 0.001 | 0.766 0.006 | 0.314 0.377 | -0.760 0.018 | 0.397 0.290 | -0.678 0.045 |
| DBP-before (mm Hg) | Correlation Significance | 0.429 0.188 | 1.000 0.000 | 0.821 0.002 | -0.252 0.455 | 0.175 0.628 | -0.516 0.155 | -0.331 0.384 | -0.164 0.674 |
| MAP-before (mm Hg) | Correlation Significance | 0.837 0.001 | 0.821 0.002 | 1.000 0.000 | 0.313 0.349 | 0.208 0.564 | -.795 0.010 | 0.035 0.929 | -0.531 0.141 |
| PP-before (mm Hg) | Correlation Significance | 0.766 0.006 | -0.252 0.455 | 0.313 0.349 | 1.000 0.000 | 0.219 0.544 | -0.431 0.247 | 0.665 0.051 | -0.602 0.086 |
| TSVD-before ($mm/mm^2$) | Correlation Significance | 0.314 0.377 | 0.175 0.628 | 0.208 0.564 | 0.219 0.544 | 1.000 0.000 | -0.358 0.345 | 0.195 0.615 | 0.088 0.821 |
| TSVD-difference ($mm/mm^2$) | Pearson Significance (2-tailed) | -0.760 0.018 | -0.516 0.155 | -0.795 0.010 | -0.431 0.247 | -0.358 0.345 | 1.000 0.000 | -0.094 0.811 | 0.544 0.130 |
| PSVD-before ($mm/mm^2$) | Correlation Significance | 0.397 0.29 | -0.331 0.384 | 0.035 0.929 | 0.665 0.051 | 0.195 0.615 | -0.094 0.811 | 1.000 0.000 | -0.701 0.035 |
| PSVD-difference ($mm/mm^2$) | Correlation Significance | -0.678 0.045 | -0.164 0.674 | -0.531 0.141 | -0.602 0.086 | 0.088 0.821 | 0.544 0.130 | -0.701 0.035 | 1.000 0.000 |

### 0094

#### Dynamic assessment of fluid challenge in critically ill patients

A. Messina[1], M. Romano[2], G. Cammarota[1], R. Tarquini[1], P. Persona[3], A. Graziano[3], A. Kundakci[4], S. Romagnoli[5], M. Cecconi[6], D. Payen[1]
[1]AOU Maggiore della Carità, Novara, Italy; [2]Unit of Internal Medicine and Cardiology, Department of Experimental and Clinical Medicine, Firenze, Italy; [3]Azienda Ospedaliera Universitaria, Department of Anesthesia and Intensive Care, Padova, Italy; [4]Baskent University, Department of Anesthesia and Intensive Care, Ankara, Turkey; [5]University of Florence, Azienda Ospedaliero-Universitaria Careggi, Department of Anesthesia and Intensive Care, Firenze, Italy; [6]IRCCS Humanitas, Milano, Italy
**Correspondence:** A. Messina
*Intensive Care Medicine Experimental* 2018, **6(Suppl 2):**0094

**INTRODUCTION.** The conditions to use adequately the pulse pressure variation (PPV) to predict fluid response are rarely all present in critically ill patients (1).

In a previous paper including elective neurosurgical patients, we demonstrated that the assessment of responder/non responder outcome of a fluid challenge (FC) could be dynamically evaluated, considering the adaptive responses of both cardiac efficiency and peripheral response to fluid administration (2).

**OBJECTIVES.** To investigate:

1- whether the changes in baseline PPV are associated with cardiac cycle efficiency (CCE), stroke volume index (SVI), arterial elastance (Ea) and systolic-dicrotic pressure (SYS-DIC) variations during FC infusion.
2- whether a combined model could predict the response at 10 and 30 minute to FC.

METHODS: Prospective multicentric study (Novara University Hospital; Padova University Hospital; Ankara University Hospital) enrolling hemodynamically unstable rhythmic critically ill patients. Hemodynamic measurements were performed by means of MOSTCARE$^{TM}$ system (Vygon, Ecouen, Fr). FC challenge consisted in 500 ml infusion of crystalloids in 10 minutes. Responders were defined on a ≥ 10% increase in SVI. Variations in all hemodynamic variables were compared by analysis of variance (ANOVA). The multivariate analyses and the logistic regression were used to create a composite model to predict fluid responsiveness at 10 minutes. This was also used to compare the subgroup of responders at 10 and 30 minutes in parallel with the non-responders.

**RESULTS.** 87 patients having FC were analysed, with 2 excluded because of supraventricular arrhythmia during the study. The baseline classic PPV cut-off at 14% predicted fluid responsiveness with a sensitivity at 70.0% and specificity at 67.4%. The model with PPV, Ea and CCE correctly classified 90% of FC response after 5 min of infusion (7 patients misclassified). In a subgroup of 65 patients (30 responders at both 10 and 30 minutes and 35 non responders), increased CCE, SVI and SYS-DIC and reduced PPV and Ea at any measuring time as compared to the baseline in responders. In non-responders, none of these variables changed, except Ea that increased at 10 and 30 minutes (see Fig 1 and 2). The composite model included the variations of SYS-DIC, PPV and CCE at 5 minutes and Ea at 10 minutes correctly classified the changes in SVI at 30 minutes of the 93.8% of this subgroup (4 of 65 patients were misclassified, area under the curve: 0.94). Of note, Ea significantly increased at 5 10 and 30 minutes in non-responders and decreased in responders and, when evaluated at 10 minutes, this parameter alone was able to predict the 30-minute response with 80% of sensitivity and specificity.

**CONCLUSIONS.** The changes in PPV, Ea, CCE and SYS-DIC reliably predict the effect of FC on SVI in responders vs non-responders after 10 and 30 minutes.

##### REFERENCE(S)

1) Biais et al. Critical Care 2014

2) Messina et al. Eur J Anaesthesiol. 2017

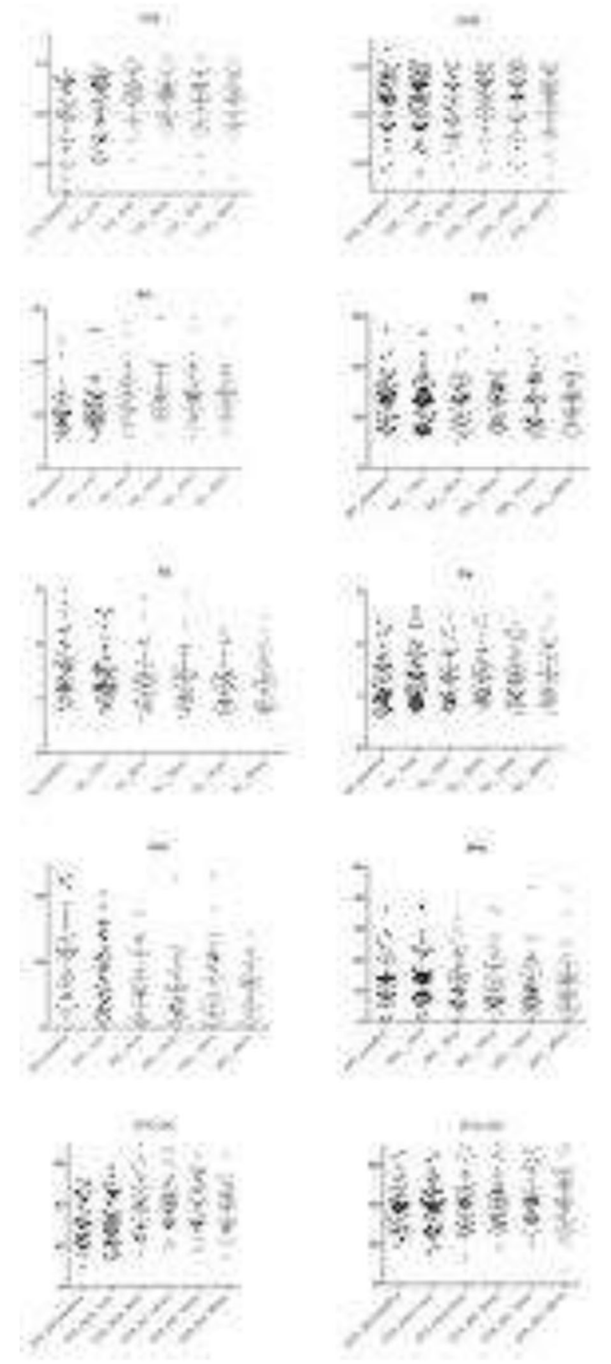

**Fig. 1 (abstract 0094).** Hemodynamic variations in responders at 10 and 30 minutes and non-responders

| RESPONDERS | | | | | NON RESPONDERS | | | | |
|---|---|---|---|---|---|---|---|---|---|
| Test details | Mean 1 | Mean 2 | Mean Diff, | p value | Test details | Mean 1 | Mean 2 | Mean Diff, | p value |
| CCE_baseline vs. CCE__5min | -0,0147 | 0,1716 | -0,1863 | p < 0,01 | CCE_baseline vs. CCE__5min | 0,1587 | 0,08718 | 0,07153 | ns |
| CCE_baseline vs. CCE__10min | -0,0147 | 0,1553 | -0,17 | p < 0,01 | CCE_baseline vs. CCE__10min | 0,1587 | 0,1585 | 0,0001951 | ns |
| CCE_baseline vs. CCE__15min | -0,0147 | 0,1421 | -0,1568 | p < 0,01 | CCE_baseline vs. CCE__15min | 0,1587 | 0,1663 | -0,007625 | ns |
| CCE_baseline vs. CCE__30min | -0,0147 | 0,132 | -0,1467 | p < 0,01 | CCE_baseline vs. CCE__30min | 0,1587 | 0,1303 | 0,02843 | ns |
| SVI_baseline vs. SVI__5min | 21,63 | 27,19 | -5,557 | p < 0,001 | SVI_baseline vs. SVI__5min | 27,7 | 26,63 | 1,066 | ns |
| SVI_baseline vs. SVI__10min | 21,63 | 29,04 | -7,411 | P < 0,0001 | SVI_baseline vs. SVI__10min | 27,7 | 26,69 | 1,014 | ns |
| SVI_baseline vs. SVI__15min | 21,63 | 28,31 | -6,673 | P < 0,0001 | SVI_baseline vs. SVI__15min | 27,7 | 26,68 | 1,015 | ns |
| SVI_baseline vs. SVI__30min | 21,63 | 28,58 | -6,942 | P < 0,0001 | SVI_baseline vs. SVI__30min | 27,7 | 27,12 | 0,5829 | ns |
| PPV_baseline vs. PPV__5min | 23,21 | 14,94 | 8,265 | p < 0,001 | PPV_baseline vs. PPV__5min | 13,95 | 13,5 | 0,4507 | ns |
| PPV_baseline vs. PPV__10min | 23,21 | 11,89 | 11,32 | P < 0,0001 | PPV_baseline vs. PPV__10min | 13,95 | 13,15 | 0,8014 | ns |
| PPV_baseline vs. PPV__15min | 23,21 | 14,73 | 8,48 | p < 0,01 | PPV_baseline vs. PPV__15min | 13,95 | 12,19 | 1,755 | ns |
| PPV_baseline vs. PPV__30min | 23,21 | 13,18 | 10,02 | p < 0,001 | PPV_baseline vs. PPV__30min | 13,95 | 12,65 | 1,299 | ns |
| SYS_DICbaseline vs. SYS_DIC 5min | 31,6 | 38,93 | -7,333 | p < 0,01 | SYS_DICbaseline vs. SYS_DIC 5min | 39,91 | 41,4 | -1,486 | ns |
| SYS_DICbaseline vs. SYS_DIC 10min | 31,6 | 40,63 | -9,033 | P < 0,0001 | SYS_DICbaseline vs. SYS_DIC 10min | 39,91 | 41,66 | -1,743 | ns |
| SYS_DICbaseline vs. SYS_DIC 15min | 31,6 | 40,13 | -8,533 | p < 0,001 | SYS_DICbaseline vs. SYS_DIC 15min | 39,91 | 43,09 | -3,171 | ns |
| SYS_DICbaseline vs. SYS_DIC 30min | 31,6 | 38,03 | -6,433 | p < 0,01 | SYS_DICbaseline vs. SYS_DIC 30min | 39,91 | 42,03 | -2,114 | ns |
| Ea_baseline vs. Ea__5min | 1,638 | 1,425 | 0,2134 | p < 0,001 | Ea_baseline vs. Ea__5min | 1,274 | 1,363 | -0,08894 | ns |
| Ea_baseline vs. Ea__10min | 1,638 | 1,372 | 0,2658 | p < 0,01 | Ea_baseline vs. Ea__10min | 1,274 | 1,391 | -0,1167 | p < 0,05 |
| Ea_baseline vs. Ea__15min | 1,638 | 1,396 | 0,2419 | p < 0,01 | Ea_baseline vs. Ea__15min | 1,274 | 1,408 | -0,1338 | ns |
| Ea_baseline vs. Ea__30min | 1,638 | 1,339 | 0,2994 | P < 0,0001 | Ea_baseline vs. Ea__30min | 1,274 | 1,411 | -0,1374 | p < 0,05 |

**Fig. 2 (abstract 0094).** Anova analysis of the considered variables in responders and non responders

### 0095

#### Systemic and tissue response to fluid challenge

P. De Santis, C. De Fazio, O. Bond, J.-L. Vincent, J. Creteur, F.S. Taccone
Université Libre de Bruxelles, Department of Intensive Care, Brussels, Belgium
**Correspondence:** P. De Santis
*Intensive Care Medicine Experimental* 2018, **6(Suppl 2):**0095

**INTRODUCTION.** Fluid responsiveness is defined by a relative increase in cardiac output after fluid administration. The relation with microvascular perfusion has not been adequately studied.

**OBJECTIVES.** To assess the microvascular response to a fluid challenge (FC) in critically ill patients.

**METHODS.** We prospectively collected data in patients and requiring a FC between February and June 2016, whilst cardiac index (CI) was monitored. The sublingual microcirculation was assessed using the incident dark field (IDF) CytoCam device (Braedius Medical, Huizen, The Netherlands). The proportion of small perfused vessels (PSPV) was calculated using the De Backer score, according to standard formulas. Fluid responders were defined by at least 10% increase in CI during FC. Responders according to changes in microcirculation were defined by at least 10% increase in PSPV at the end of FC. Cohen´s kappa coefficient was measured to assess agreement to categorize patients as "responders" to FC according to CI and PSPV.

**RESULTS.** A total of 41 FC were performed in 38 patients, after a median time of 1 [0-1] days after ICU admission. Most of the fluid challenges (39/41, 95%) were performed using crystalloids and the median total amount of fluid was 500 [500-500] mL. The main raisons for fluid challenge were oliguria (n=22) and hypotension (n=10). After FC, CI significantly increased in 24 (58%) cases; a total of 19 (46%) FCs resulted in an increase in PSPV. Both CI and PPV increased in 13 responders and neither in 11; the coefficient of agreement was only 0.19. We found no correlation between absolute changes in CI and PPV after fluid challenge.

**CONCLUSIONS.** Microvascular response to FC cannot be predicted by changes in cardiac output.

### 0096

#### Differences in EVLWi´s behaviour among survivors and not survivors critically ill patients in a mexican intensive care unit

M.A. Carrasco Flores[1], S.E. Zamora Gomez[1], M. Hernández Romero[1], M.A. Toledo Rivera[1], J.O. Montoya Rojo[1], M.A. Amezcua Gutierrez[1], I.M. Lima Lucero[1], J. Garduño López[1], K. Castillo Medrano[1], J.C. Gasca Aldama[1], N.I. Medveczky Ordoñez[1], H.A. Morales Morales[1], M.L. Pacheco Rivera[1], S. Sosa Santos[1], S. Orozco Ruiz[2], J.A. Castañon González[1]
[1]Hospital Juarez de México, Intensive Care Unit, Mexico City, Mexico;
[2]Hospital Juárez de México, General Medicine, Mexico City, Mexico
**Correspondence:** M.A. Carrasco Flores
*Intensive Care Medicine Experimental* 2018, **6(Suppl 2):**0096

**INTRODUCTION.** Extravascular Lung water (EVLW) has been identified as an independent mortality risk factor in several scenarios in critically ill patients.

**OBJECTIVES.** Describe the behaviour of extravascular lung water during the first 72 hours from admission in intensive care unit among survivors and not survivors critically ill patients.

**METHODS.** A descriptive retrospective case study was carried out. We analyzed the files of patients in wich continuous hemodynamic monitoring with the transpulmonary thermodilution technique (TPTD) was performed during janurary 2016 to March 2018. Extravascular Lung Water Index (EVLWi) and Pulmonary Vascular Permeability Index (PVPi) were analyzed at the beginning of the monitoring, 24 and 72 hours. The patients were classified according with otucome in survivors and not survivors; EVLWi and PVPi behaviour was described and analyzed in both groups.

**RESULTS.** 15 patients were included in the study, 7 females and 8 males, with an average age of 45 years, and 6.9 days average of mechanical ventilation. TPTD monitoring used for advanced monitoring in patientes with infectious diseases in more than 75% and only in one patient with severe acute pancreatitis. The global mortality rate during ICU stay was of 33%. The EVLWi average was of 11.1 ml/kg and PVPi of 2.4. In survivors EVLWi average was of 9.7 ml/kg and PVPi of 2.4 in contrast with non survivors were EVLWi average was 13.8 and PVPi average of 2.5. EVLWI´s behaviour during the 72 hours of the study was 4 ml/kg average higher among not survivors in comparation with survivors; meanwhile there was not significant difference regarding PVPi among both groups.

**CONCLUSIONS.** In our study, persistent values of EVLWi in critically ill patients above 13 ml/kg during the first 72 hours of ICU stay were associated with poor prognosis and mortality.

**REFERENCE(S)**

1- Brown LM, Calfee CS, Howard JP, Craig TR, Matthay MA, McAuley DF. Comparison of thermodilution measured extravascular lung water with chest radiographic assessment of pulmonary oedema in patients with acute lung injury. Annals of Intensive Care. 2013;3:25.

2- Tagami T, Nakamura T, Kushimoto S, Tosa R, Watanabe A, Kaneko T, Fukushima H, Rinka H, Kudo D, Uzu H, et al. Early-phase changes of extravascular lung water index as a prognostic indicator in acute respiratory distress syndrome patients. Ann Intensive Care. 2014;4:27. 3-Monnet X, Teboul J-L. Transpulmonary thermodilution: advantages and limits. Critical Care. 2017;21:147.

### 0097

#### Acute heart failure treatment in critically ill patients

R. Gomez[1], C. Guía[2], T. García Paredes[3], A. Barros Perez[4], A. Fernandez Ferreira[5], M.A. Solla Buceta[6], D.P. Rodriguez Giardini[7], B. Besteiro Grandio[8], I. Keituqwa[9], L. Zapata[10], ICAUCI (Insuficiencia Cardíaca Aguda en Unidades de Cuidados Intensivos)

[1]Hospital Quironsalud Miguel Dominguez, Pontevedra, Spain; [2]Corporació Sanitària Parc Taulí en Sabadell, Sabadell, Spain; [3]Hospital Regional Universitario de Málaga, Malaga, Spain; [4]Hospital Universitario de la Ribera, Alzira, Spain; [5]Complejo Hospitalario Universitario de Vigo, Vigo, Spain; [6]Complejo Hospitalario Universitario de A Coruña, A Coruña, Spain; [7]Consorci Sanitario de Terrassa, Terrassa, Spain; [8]Clínica Juaneda Menorca, Ciutadella, Spain; [9]Hospital Rafael Mendez, Lorca, Spain; [10]Hospital de la Santa Creu i Sant Pau, Barcelona, Spain

**Correspondence:** R. Gomez

*Intensive Care Medicine Experimental* 2018, **6(Suppl 2):**0097

**OBJECTIVES.** To assess the treatment of acute heart failure (AHF) in spanish Intensive Care Units (ICU).

**METHODS.** Multicenter prospective observational study that included patients older than 18 years admitted to the ICU for more than 24 hours between April and June 2017. Demographic, physiological and clinical variables, including complementary explorations of those who suffered AHF during their ICU stay were collected. Comparisons were made among the different subgroups of patients according to the timing of AHF: AHF as main reason for admission vs. complication during ICU stay (Adm-AHF Vs Com-AHF) or first episode of AHF vs. exacerbation of chronic heart failure (AHFnew Vs CHFex). Statistical analysis was made using ANOVA or chi-square test as appropriate. The multivariate study was performed by Cox regression for survival analysis and logistic regression. Values of $p \leq 0.05$ were considered statically significant.

**RESULTS.** We analyzed 587 patients with AHF admitted in 32 ICUs. The most used drugs during the first 24 hours after diagnosis were diuretics (83.1%), morphine (52.5%), noradrenaline (38%), intravenous nitrates (30.4%), amiodarone (23.5%), dobutamine (22.3%), betablockers (18.2%) and ACE inhibitors (9.8%). Early use of diuretics was lower in Adm-ICAnew patients (75% vs Com-ICAnew 88.5%, Adm-CHFex 85.2% and Com-CHFex 85.1%, $P < 0.05$). 93% of the patients required oxygen therapy, 13.3% CPAP, 28.4% non-invasive ventilation for an average of 2.55±3.37 days and 35.8% invasive mechanical ventilation (MV) for 10.2± 14.7 days, without significant differences between subgroups.

Coronary angiography was performed in 31.2% of the patients, requiring coronary revascularization in 18.7%, most frequently in first AHF episodes. Mechanical circulatory support was used in 3.1% of cases and extracorporeal renal replacement in 9.4%, without differences between subgroups. After adjusting for severity, age and confounding factors, MV was the only treatment independently associated with mortality (OR 8.29 CI 3.7-18.5, $p < 0.001$).

**CONCLUSIONS.** There are small differences between subgroups of AHF. Diuretics are the most commonly used drugs, except in patients with AHFnew, in which more coronariographies are performed. In our population, MV was independently associated with mortality.

**GRANT ACKNOWLEDGMENT**

ICAUCI study group is part of the Cardiac critical care Task Force of the Spanish Society of Intensive Care (SEMICYUC)

### 0098

#### The values from continual monitoring of arterial dP/dt max correlate with left ventricular contractility assessed by echocardiography in patients with advanced heart failure

D. Vondrakova, A. Krüger, M. Janotka, J. Naar, P. Neužil, P. Ostadal

Na Homolce Hospital, Prague, Czech Republic

**Correspondence:** D. Vondrakova

*Intensive Care Medicine Experimental* 2018, **6(Suppl 2):**0098

**INTRODUCTION.** Continuous reliable evaluating of left ventricular (LV) contractile function in patients with advanced heart failure requiring intensive care remains challenging. Recently, continual monitoring of dP/dt max from arterial line became available for hemodynamic monitoring. However, the relation between arterial dP/dt max and standard echocardiographic LV dP/dt measurement is not fully understood.

**OBJECTIVES.** The aim of our study was to determine the relation of arterial dP/dt max and LV dP/dt assessed by echocardiography in patients with advanced heart failure.

**METHODS.** Twenty patients with advanced heart failure requiring intensive care and hemodynamic monitoring were recruited into the study (mean age 63 years, 90% were males). Hemodynamic variables including dP/dt max were continually monitored using arterial line pressure waveform analysis. Left ventricular dP/dt was assessed using continuous-wave Doppler analysis of mitral regurgitation flow.

**RESULTS.** The values from continual arterial dP/dt max monitoring significantly correlated with the LV dP/dt max assessed by echocardiography (r=0.94, 95% confidence interval [CI] 0.82-0.98, P˂0.0001). Linear regression revealed that (LV dP/dt) = 1.073×(arterial dP/dt), P˂0.0001. Arterial dP/dt max significantly correlated also with the stroke volume (r=0.67, P˂0.0059), cardiac output (r=0.65, P˂0.0083), mean arterial blood pressure (r=0.60, P˂0.017) and systolic blood pressure (r=0.85, P˂0001). On the other hand arterial dP/dt did not correlate with the systemic vascular resistance, heart rate, dynamic arterial elastance, diastolic blood pressure or central venous pressure

**CONCLUSIONS.** Our results revealed that arterial dP/dt max values tightly and highly significantly correlate with LV dP/dt. Arterial dP/dt could be, therefore, used for continual monitoring of LV contractility.

**GRANT ACKNOWLEDGMENT**

The study was supported by an Institutional grant MH CZ - DRO (Nemocnice Na Homolce - NNH, 00023884), IG150501

### 0099

#### Fluid responsiveness depend on arterial load and vasopressor use in ICU patients

M. Nguyen[1], M.-O. Fischer[2], J. Mallat[3], J. Marc[4], E. Lorne[4], B. Bouhemad[1], P.-G. Guinot[1]

[1]CHU Dijon Bourgogne, Dijon, France; [2]CHU de Caen, Caen, France; [3]CH de Lens, Lens, France; [4]CHU d'Amiens, Amiens, France

**Correspondence:** M. Nguyen

*Intensive Care Medicine Experimental* 2018, **6(Suppl 2):**0099

**INTRODUCTION.** Fluid responsiveness represents the ability of the cardiovascular system to increase cardiac output in response to fluid expansion (FE). Most studies have focused on the interaction between preload, cardiac output and blood pressure. Arterial load represents a force developed by the vascular system opposed to the ventricular ejection. Because arterial load depends on FE and norepinephrine (NE) use, it may have an effect on fluid responsiveness defined by an increase of stroke volume.

**OBJECTIVES.** To assess the impact of norepinephrine over the relationship between arterial load, FE and NE.

**METHODS.** We analysed the relation between arterial load, NE and SV changes following FE in 282 patients hospitalised in three ICU, monitored with a PICCO device, and for whom a 500 ml FE was performed. Arterial load was estimated by arterial elastance ($E_A$) : $E_A$ = 0.9 x systolic arterial pressure (SAP) / stroke volume (SV). Arterial compliance ($C_A$) was calculated as pulse pressure / stroke volume (mmHg $ml^{-1}$), total vascular resistance (TVR) as mean arterial pressure - central venous pressure / cardiac output (mmHg $ml^{-1}$). Hemodynamic, arterial load and cardiac parameters were measured before and immediately after FE.

**RESULTS.** Among the patients, 61% were treated with NE, and 44% had sepsis. In the overall population, FE increased blood pressure, SV, cardiac output, and decreased TVR and $E_A$. At baseline blood pressure, SV, $E_{A,}$ $C_A$ did not differ between patients with NE and those without NE. SV and heart rate changes were similar in patients with and those without NE (-3 (-7 to 0) % vs -3 (-8 to 1) %, p=0.87, and 22 (13 to 37) % vs 20 (7 to 33) %, p=0.09). $E_A$ changes with FE differed between patient with and those without NE (-4 (-12 to 6) % vs -10 (-18 to -1) %; p< 0.01). TVR changes differed between patient with and those without NE (-6 (-11 to 5) % vs -10 (-16 to -4) %, p < 0.01). $C_A$ increased in patient without NE, and did not change in patient treated with NE (5 (-6 to 15) %, p< 0.01 vs -2 (-13 to 9) %, p=0.15) (Figure1). Baseline $E_A$ was correlated with SV changes only in the absence of NE infusion (r = 0.32, p < 0.01 vs r = 0.12; p = 0.12). By using a multivariate analysis adjusted to the presence of sepsis, SV changes with FE were associated to only baseline $E_A$ (β = 10.9 [5.2; 16.7]), NE infusion, and interaction between $E_A$ and NE (β = -9.1 [-15.8; -2.5]). The model confirmed an effect of NE on the association between arterial load and SV changes induced by FE.

**CONCLUSIONS.** Those results suggest that arterial load, NE and their interactions exert an effect on fluid responsiveness. Under NE infusion, changes of arterial load with FE are mainly driven by resistive component (i.e TVR). NE fixed the pulsatile component ($C_A$) of arterial load. These changes were independent of sepsis.

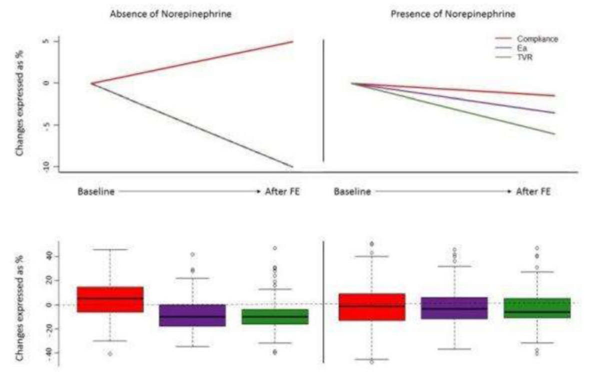

**Fig. 1 (abstract 0099).** Changes of arterial elastance and its determinant with fluid expansion

### 0100

#### Model based estimation of instability severity level in continuously monitored patients

L. Chen[1], A.W. Dubrawshi[1], G. Clermont[2], T. Pellathy[3], A. Wertz[1], M.R. Pinsky[2], M. Hravnak[3]

[1]Carnegie Mellon University, Auton Lab. Computer Science, Pittsburgh, United States; [2]University of Pittsburgh, School of Medicine, Pittsburgh, United States; [3]University of Pittsburgh, School of Nursing, Pittsburgh, United States

**Correspondence:** M. Hravnak

*Intensive Care Medicine Experimental* 2018, **6(Suppl 2):**0100

**INTRODUCTION.** Bedside alerts are issued whenever patient vital signs (VS) exceed a threshold but do not indicate potential severity of the emerging instability. This limits their clinical utility.

**OBJECTIVES.** We propose a model-based approach to differentiate cardiorespiratory instability (CRI) manifested by VS derangement that is mild and in need solely of nurse awareness or intervention (MinCRI), versus more severe cases that require escalation of care (SevCRI).

**METHODS.** We collected continuous noninvasive VS monitoring data (heart rate, respiratory rate, blood pressure, $SpO_2$) from 326 step-down unit (SDU) patients and reviewed data time-plots for 1087 real alerts across normal VS thresholds defining CRI. Two clinical experts (RN and MD) independently labeled those CRI events with increasing severity (level 1: mild, nurse needs to be aware; level 2: in need of nurse encounter and intervention; level 3: in need of routine care escalation to an MD; level 4: in need of immediate care escalation to a Medical Emergency Team). We fit a multi-class random forest model to the data using statistical features extracted during the alert periods and evaluated it via cross-validation. We also tested the model on a separate sample of 85 alerts from a different patient cohort. We compared the estimated CRI severity levels with those independently provided by the experts.

**RESULTS.** The overall accuracy of predicting CRI severity level is 72.8% as measured with leave-one-patient-out cross validation (Figure 1). The vast majority (89.9%) of classification errors are made with respect to neighboring severity levels. By grouping levels 1 with 2 (to form MinCRI, requiring nurse awareness/intervention) and 3 with 4 (SevCRI requiring escalation of care; the positive class), the accuracy of the resulting binarized prediction model increases to 91.6% with a true positive rate of 77.9% and false positive rate of 2.3%. If, however, we increase the tolerance level of the false positive rate to 10%, the true positive rate is 86.0%. When testing the model on the external validation set, among the 53 MinCRI identified by the model, 90.6% were correctly classified, as well as 80.3% of the 32 model-identified SevCRI cases. The overall inter-rater agreement between two experts for the severity labels is 0.83 (Weighted Cohen's kappa).
**CONCLUSIONS.** A model trained from annotated historical VS data can discern between instability severity patterns in need of nurse attention versus those requiring care escalation. Such models, if embedded in real-time monitoring systems, could assist bedside clinical decision making regarding care escalation and resource allocation.

**GRANT ACKNOWLEDGMENT**
NIH R01NR013912

| | | | TRUE ANNOTATED LABELS | | | | |
|---|---|---|---|---|---|---|---|
| | | | Nurse Intervention MinCRI | | Care Escalation SevCRI | | |
| | | | Level 1 | Level 2 | Level 3 | Level 4 | Total |
| PREDICTED LABELS | Nurse Intervention MinCRI | Level 1 | **403** | 35 | 19 | 3 | **460** |
| | | Level 2 | 86 | **211** | 43 | 9 | **349** |
| | Care Escalation SevCRI | Level 3 | 1 | 15 | **88** | 50 | **154** |
| | | Level 4 | 0 | 1 | 34 | **89** | **124** |
| | | Total | **490** | **262** | **184** | **151** | **1087** |

**Fig. 1 (abstract 00100).** See text for description

## Endocrine disorders - Liver failure

### 0101

#### Hyperglycemia was not a risk factor for mortality in malnourished critically ill patients

M. Viana[1], A.L. Tavares[2], L.A. Gross[2], V.L. Costa[2], T.A. Tonietto[1], M.J.d. Azevedo[2], L.V. Viana[2]
[1]Hospital de Clinicas de Porto Alegre, Porto Alegre, Brazil; [2]Universidade Federal do Rio Grande do Sul, Porto Alegre, Brazil
**Correspondence:** M. Viana
*Intensive Care Medicine Experimental* 2018, **6(Suppl 2):**0101

**INTRODUCTION.** Malnourished critically ill patients may derive more benefit from nutritional support. Optimizing nutrition support can improve their outcomes but this is frequently associated with hyperglycemia, a condition linked to an increased morbi-mortality. They also have altered glucose homeostasis characterized by decreased fasting blood glucose levels despite below-normal insulin levels[1]. The aim of this study was to evaluate the relationship of glycemic control with nutritional support and mortality in malnourished critical ill patients.
**METHODS.** In this two-center prospective cohort, malnourished critically ill patients (BMI < 20kg/$m^2$) had their caloric and protein intakes and their glycemic status estimated by capillary glucose (CG) evaluated in two occasions: between day 2-3 and between day 5-7 of ICU admission. CG was performed as prescribed by the assistant physician, at minimum every 6 hours. Maximum and minimum registered CG values were used to classify hyperglycemic (CG>180 mg/dl), hypoglycemic (CG< 54 mg/dl) events. The cohort was followed until death or hospital discharge.
**RESULTS.** Prevalence of low BMI in 4236 screened patients was 16%. Therefore, 342 patients were included (age 54.0±17.3 years, 59.9% males, BMI 17.5±2.1 kg/$m^2$, mortality 58.5%) and followed during 21 [11-38.3] days. In the first evaluation, median caloric/protein intakes were 864.0 [447-1223] kcal/day and 41.8 [14.0-65.0] g/day and their maximum and minimum CG (mean) were 164.2±55.9 mg/dl and 108.4±33.3 mg/dl, respectively. In the second evaluation, median caloric/protein intakes were 1197.0 [911-1500] kcal/day and 65 [45.0-80.0] g/day and their maximum and minimum CG (mean) were 160.0±57.1 mg/dl and 107.8±32.4 mg/dl, respectively. Hyperglycemia occurred in 36.3% and hypoglycemia in 3.8% of patients. Neither in the first nor in the second evaluation caloric intake correlated with hyperglycemia. In multivariate analyses, hyperglycemia event was associated with age (RR 1.02, 95%CI 1.01-1.04), previous diabetes (RR 3.70 95% CI 1.80 - 7.64) but not with severity of illness (SAPS3) nor BMI. Mortality was not associated with hyperglycemia or hypoglycemia.
**CONCLUSION.** In critically ill malnourished patients, different from eutrophic or overweight patients, hyperglycemia or hypoglycemia were not associated with caloric intake nor mortality.

**REFERENCE(S)**
1. Rao RH. Adaptations in glucose homeostasis during chronic nutritional deprivation in rats: hepatic resistance to both insulin and glucagon. Metabolism: clinical and experimental. 1995;44(6):817-24.

**GRANT ACKNOWLEDGMENT**
Fundação de Amparo a Pesquisa do Rio Grande do Sul (FAPERGS) and Fundo de Incentivo a Pesquisa Hospital de Clínicas de Porto Alegre (FIPE-HCPA).

### 0102

#### The association of age and steroid use with insulin resistance in ICU patients: a cohort study

I.E. Drogt-Bilaseschi[1], P.J.W.H. Kappelle[1], P.F.J. Freire Jorge[2], I.C.C. van der Horst[1], M.W.N. Nijsten[1]
[1]University Medical Center of Groningen, Department of Critical Care, Groningen, Netherlands; [2]University of Groningen, University Medical Center of Groningen, Department of Critical Care, Groningen, Netherlands
**Correspondence:** I.E. Drogt-Bilaseschi
*Intensive Care Medicine Experimental* 2018, **6(Suppl 2):**0102

**INTRODUCTION.** Insulin resistance (IR) in patients without diabetes increases with age.[1] In the Intensive Care Unit (ICU), adaptive glucose-control systems target equal glucose levels irrespective of age. Whether the association of steroid use on IR varies with age in patients at the ICU is unknown.
**OBJECTIVES.** Determine the association of IR - as derived from glucose control data - with age in ICU patients and examine the role of disease severity, body mass index (BMI) and steroid use.
**METHODS.** All adult patients admitted for >12h to the ICU over a 4 year period were examined. Age (< 45,46-55,56-65,66-75,>75), sex, diabetes status (type I or II), APACHE-IV and steroid use within 18h of ICU admission were recorded. Steroid doses were typically not weight or age adjusted. During the first 24h the ICU mean glucose ($G_{mean}$) in mmol/L and mean insulin administration ($I_{mean}$)rate in U/h were determined. IR was estimated as $G_{mean}$ · ($I_{mean}$+1), where the term 1 was introduced as an estimate of endogenous insulin production as well as to avoid zero values.

**RESULTS.** In 9074 patients had a mean±SD age of 61±15 year and an APACHE-IV score of 53±27. Diabetes prevalence was 17% and 13% received steroids. The hospital mortality was 12%. IR was 15.0 in non-diabetics and 29.4 mmol·U·L ·h in diabetics. IR steadily increased in non-diabetics from 12.3 in patients < 45 year to 15.6 mmol·U·L ·h in patients of 66-75 year (P< 0.001). In diabetics who received steroids IR was 19.5±13.9 compared to 14. ±11.7 mmol·U·L ·h in non-diabetics who received steroids (P< 0.001). Also within this latter group an age-related increase of IR from the ≤45year group to the 66-75year group was observed (P< 0.001). In non-diabetics multivariable linear regression analysis with IR as dependent variable, age group, sex, BMI, APACHE-IV and steroid use were all independently related with IR (all P≤0.001).

**CONCLUSIONS.** In ICU patients, insulin resistance as estimated from computerized glucose control was independently associated with disease severity, steroid use and age. Since the combination of higher age and steroid use is associated with an increased IR, adjusted steroid doses could be considered in older patients.

**REFERENCE(S)**

[1] Fink R et al. Mechanisms of insulin resistance in aging. *J Clin Invest.* 1983 Jun;71(6):1523-35.

[2] Vogelzang M et al. Design and implementation of GRIP: a computerized glucose control system at a surgical intensive care unit. *BMC Med Inform Decis Mak.* 2005;5:38

### 0103

#### Features and monitoring of liver transplants from donation after cardiac death in a Spanish hospital

E. Trujillo García, A. Valverde Montoro, H. Molina Díaz, M.J. Furones Llorente, M. Lebrón Gallardo, E. Banderas Bravo

H.R.U de Málaga, Intensive Care, Málaga, Spain

**Correspondence:** E. Trujillo García

*Intensive Care Medicine Experimental* 2018, **6(Suppl 2):**0103

**OBJECTIVES.** Describing the main features of the liver transplant in Controlled Donation after Cardiac Death (CDCD), in which Super Rapid Extraction (SRE) and Normothermic Regional Perfusion (NRP) with premortem cannulation techniques were used, with the postoperative receptor course.

**METHODS.** An observational, retrospective and descriptive in patients ho received a liver transplant from CDCD were performed in our hospital from January 2014 to January 2017. Dates about surgical intervention, basal characteristics of the recipients and donors, and the recipients evolution until the end of the data collect were analyzed. Recipients had been followed-up for 1 year.

**RESULTS.** Twenty-nine transplants were performed in this time. Donors were mostly males (68%). Average age was 52 years. The causes wich motivate the withdrawal of the life-sustaining medical support were 14 cerebrovascular accident, 11 anoxic encephalopathy, 3 traumatic brain injury and one chronic respiratory disease. Donoros average ICU stay was 7.5 days. Average surgery duration were 40.1 min of functional warm ischemia, 46.2 min of total warm ischemia and 323.9 min of cold ischemia. In 22 cases SRE was used and in the other 7 NRP was the chosen technique.

Recipients were mostly males too, (79%), and average age was 57.9 years. The enzymatic peak of aspartate aminotransferase (AST) was 4373.47U/L and the alanine aminotransferase (ALT) one 947.03U/L. Average factor V on the first day after transplantation was 33.8% and reached 97% on the third day. Average ICU stay was 3.8 days and hospitalization stay was 11.5 days.

Regarding evolution of this recipients, there were 3 deaths (10.3%): One on the day +80, another on the day +5, both because of liver failure and acute respiratory distress syndrome, and the last one on the first day after the surgery due to humoral rejection. Regarding complications, two patients were affected by an stenosis of the bile duct, one of them was retransplanted, and the other one had to be operated with a Roux-en-Y Hepaticojejunostomy. Finally, the other 24 patients have an adequate follow-up in liver specialist consultation.

**CONCLUSIONS.** Our experience with liver transplantation from CDCD is positive. The most frequent complication described on de literature (bile duct stenosis) has been presented in two patients in our series. The majority of transplant were performed with Super Rapid Extraction (SRE) technique so we consider that this technique should not be reject at all until more data its available.

### 0104

#### Prediction of outcome in severe acute pancreatitis: a prospective comparison of the BISAP-, APACHE-II- and Ranson-Score with the "quick-BISAP-score"

S. Hage, J. Höllthaler, S. Mair, V. Phillip, A. Beitz, T. Lahmer, R. Schmid, W. Huber

Klinikum rechts der Isar; Technical University of Munich, Medizinische Klinik und Poliklinik II, Munich, Germany

**Correspondence:** W. Huber

*Intensive Care Medicine Experimental* 2018, **6(Suppl 2):**0104

**INTRODUCTION.** Up to 15% of the patients with acute pancreatitis (AP) develop severe AP (SAP) which results in organ-failure and a mortality of up to 42%. Early identification of patients with SAP is crucial to otimize therapy. The lack of a single parameter with outstanding sensitivity and specificity resulted in a number of prognostic scores including the Ranson- and APACHE-II-score. These scores are well validated, but their calculation is cumbersome and requires data of the first 24-48h. The acronymic BISAP-score includes blood urea nitrogen (BUN), impaired mental state, SIRS, age and pleural effusion.

**OBJECTIVES.** Considering that SIRS is composed of 4 components, the BISAP-score includes a total of 8 parameters. Therefore, also the BISAP-score is cumbersome. Despite several studies validating the complete BISAP-score with given cut-offs, these thresholds are poorly validated and might be improved. Our study aimed to evaluate single components of the BISAP and to establish a simplified "quick-BISAP"-score.

**METHODS.** Analysis of a prospectively maintained database including 442 patients with AP screened for the EAGLE-study (NCT00894907). Statistics: ROC-analysis; binary regression analysis. Youden-index. IBM SPSS 24.

**RESULTS.** n=442 (175 f, 267 m); age 54±18 years; aetiology: 32% biliary, 27% alcoholic, 15% idiopathic, 10% post-ERCP, 3% drug-related, 13 % various. In-hospital-mortality was 13/442 (2.9%).ICU-transfer was required in 71/442 (16.1%) patients.

All five BISAP criteria BUN (AUC=0.844; p< 0.001; best cut-off 25.5mg/dl), impaired mental status (AUC=0.604; p=0.005), SIRS (AUC=0.869; p< 0.001; cut-off 2 of 4 criteria), age (AUC=0.661; p=0.047; best cut-off 63.4 years) and pleural effusion (AUC=0.663; p< 0.001) significantly predicted in-hospital mortality. Among the four SIRS criteria, heart rate >90/min had the highest sensitivity (0.846) and second highest specificity (0.834) regarding mortality. This was confirmed in binary regression analysis which demonstrated that "heart rate >90/min" was the only SIRS criterion which was independently associated with mortality. Combined binary regression analysis demonstrated an independent association of BUN>25mg/dl, pleural effusion and heart rate >90/min with mortality. A simplified model giving one point each for these criteria was termed "quick-BISAP" which provided an excellent prediction (AUC 0.946, p< 0.001) of in-hospital mortality as did APACHE-II (0.928, p< 0.001), BISAP (0.924, p< 0.001) and SIRS (0.869, p< 0.001).

**CONCLUSIONS.** BISAP, APACHE-II and SIRS predict mortality in SAP. Among the BISAP criteria BUN>25mg/dl, pleural effusion and heart rate>90beats/min were the best predictors of in-hospital mortality. A simplified model consisting of these 3 parameters ("quick BISAP") was a strong predictor of in-hospital mortality. Finally our data confirm the cut-offs for age and BUN of the original BISAP-score.

**GRANT ACKNOWLEDGMENT**

Deutsche Forschungsgemeinschaft HU1707/2-1

### 0105

#### Glycemic variability and infections in critically ill patients

S. Mulet Mascarell[1], M.D.M. Juan Diaz[2], B. Furquet lopez[2], J.M. Segura Roca[2], N. Carbonell Monleon[2], M. Rodriguez Gimillo[2], C. Sanchis Piqueras[2], A. Serrano Lazaro[2], A. Gil Santana[2], F. Rosa Rubio[2], R. Company Pont[2], A. Gonzalez Diaz[2], M. Garcia Simon[2], J. Ferreres Franco[2], M.L. Blasco Cortes[2]
[1]Hospital Clinico Universitario, Intensive Care, Valencia, Spain; [2]Hospital Clinico Universitario, Valencia, Spain
**Correspondence:** S. Mulet Mascarell
*Intensive Care Medicine Experimental* 2018, **6(Suppl 2):**0105

**INTRODUCTION.** It has been demonstrated that high Glycemic Variability(GV) is associated with increased mortality among critically ill patients and it is not yet determined its relationship with other factors such as nosocomial infections.

**OBJECTIVES.** The aim of the study is to investigate high GV as a predisposing factor to present nosocomial infections in critically ill patients.

**METHODS.** The observational prospective study is based on a sample of critically ill patients receiving artificial nutrition(first 48h). Our exclusive criteria were patients presenting diabetic ketoacidosis or are not receiving artificial nutrition during the first 48 hours. The study was carried out at a Medical ICU, from Nov'15-Dec'18.

Data on admission: demographic, clinical characteristics, APACHEII, SOFA. Nutritional parameters during the first and seventh day. GV during the first 7 days. Infectious complications, days of mechanical ventilation(MV), ICU length of stay(LOS) and mortality.

We calculated GV as VC=SD/Mx100. We performed a univariate statistical analysis considering the significance $p< 0.05$. T-student for quantitative independent group variables and $\chi 2$ for qualitative variables. We also employed linear regression for continuous variables and binary logistic for dichotomous variables.

**RESULTS.** The sample consists of 77 patients, 63.6% men with a mean age of 61±12.9 years, BMI: 29.5±19.6, mean of ICU LOS 16.1 ±14.2 and days of MV 12.48±13.43.

On admission: APACHEII 20±7.8 and SOFA 8.25±3.7.

19.5% of the patients were diabetics, 74.7% requiring Noradrenaline,90% presented stress hyperglycemia(SHG),92% received enteral nutrition(EN) and 8% parenteral nutrition(PN).

38.5% of patients had infectious complications: Ventilated-associated pneumonia(VAP) 16.3%, tracheobronchitis 35%, bacteremia 4.7%, catheter related bacteremia 2.3% and urinary tract infection(UTI) 4.7%.

We divided our sample into quartiles depending on theGV: (1)11-16.61;(2)16.62-21.41; (3)21.42-27.41; (4)27.42-43.6. GV mean22.67±7.9. The results are presented in Table 1.

**CONCLUSIONS.**

- It is observed that patients with increasing GV have increasing requirements of noradrenaline
- We observe higher GV among patients receiving PN.
- Patients with higher illness severity scores (APACHEII on admission and SOFA 7th day) shows higher GV.

##### REFERENCE(S)

Mechanick JI. Metabolic mechanisms of stress hyperglycemia. JPEN2006
Montori VM, Bristian BR, McMahon MM. Hyperglycemia in acutely ill patients. JAMA 2002
Mesejo A. Tratamiento nutricional del paciente crítico hiperglucémico. Actualidad y controversias de la nutricion en el paciente crítico. La opinión de los expertos, nº16. Ed. Glosa. 2002
Waeschle RM, Moerer O, Hilgers R et al The impact of the severity of sepsis on the risk of hypoglycaemia and glycaemic variability. Crit Care 2008
Krinsley JS et al. Glycemic variability: A strong independent predictor of mortality in critically ill patients. Crit Care Med 2008
Vincent JAMA 2009; Barcenilla Cap libro: Infecciones en MI

**Table 1 (abstract 0105).** See text for description.

| | Quartile1(n19) | Quartile2(n20) | Quartile3(n19) | Quartile4(n19) | p |
|---|---|---|---|---|---|
| Noradrenaline needed (factor) | 3.78 | 1.78 | 0.94 | **5.42*** | ***p=0.02** |
| PNn(%) | 2(10.5%) | 3(15%) | 2(10.5%) | **6(31.5%)*** | ***p=0.03** |
| APACHEII (mean) | 18.4±1.8 | 19.75±1.6 | 21.03±.04 | **25±1.6*** | ***p=0.03** |
| SOFA 7thDay (mean) | 6.4±0.75 | 6.6±1 | 6.9±0.9 | **10.5±0.9*** | ***p=0.005** |
| Days MV (mean) | 9.6±2 | 14.2±3 | 8.5±1.8 | 17±4.2 | NS |
| PCT 7thday(mean) | 0.6±0.2 | 1.8±0.8 | 1.34±0.9 | 2.7±0.8 | NS |
| Mortality n(%) | 5(26.3%) | 4(20%) | 11(57.9%) | 12(63.1%) | NS |
| IMC (mean) | 26.4±1.4 | 28.3±1.1 | 27.5±0.7 | 35.7±8.9 | NS |
| Infection(n) | 4(21%) | 8(40%) | 7(36.8%) | 5(26.3%) | NS |

### 0106

#### Impact of chloride level on outcome in critically ill patients

Z. Abdelhalim[1], M. Megahed[2], D. Zidan[2]
[1]Faculty of Medicine, Alexandria University, Critical Care Department, Alexandria, Egypt; [2]Alexandria University, Critical Care Department, Alexandria, Egypt
**Correspondence:** Z. Abdelhalim
*Intensive Care Medicine Experimental* 2018, **6(Suppl 2):**0106

**INTRODUCTION.** Chloride abnormalities in the intensive care unit (ICU) have received considerable attention, especially hyperchloremia as a cause of metabolic acidosis and hypochloremia as a cause of metabolic alkalosis However; Cl abnormalities themselves have not been studied sufficiently.

**OBJECTIVES.** The aim of this study was to understand the nature of Cl abnormalities in critically ill patients.

**METHODS.** Retrospective study was conducted on 375 critically ill adult patients admitted to Alexandria University hospitals. The following data was collected from the patient's files. Demographics, diagnosis and cause of admission, relevant medical history, calculated APACHE-II score and laboratory data ($1^{st}$ 4 days after admission) including serum electrolyte level, ABG analysis (anion gap - gap/gap ratio - corrected anion gap) and serum albumin level.

According to the recorded Cl levels at days 1 and 3 from ICU admission, patients were defined as normo (99-110 mEq/L), hypo (< 99) or hyperchloremic (>110) patients. Outcome measures were all-cause mortality and length of stay (LOS) in ICU.

**RESULTS.** On admission 55 (14.6%) patients were hyperchloremic, 259 (69%) patients were normochloremic and 61 (16.2%) patients were hypochloremic.

There were no any statistically significant differences between the 3 groups regarding demographic data, sodium and potassium level or diagnosis.

The mean of the measured PH values was significantly higher in Hypo (7.46) than in Normo (7.41) and Hyperchloremic group (7.36) ($p< 0.001$).

Cl abnormalities at day 1 were associated with increased mortality. The mortality rate was significantly higher in hyperchloremic (67.3%) and hypochloremic (72.1%) than normochloremic patients (36.3%) ($p< 0.001$).

Cl abnormalities (at days 1 and 3) were associated with increased ICU length of stay. the mean of ICU length of stay in days was significantly higher in hyperchloremic (21.29) and hypochloremic (16.38) than normochloremic patients (13.81) ($p< 0.001$)

**CONCLUSIONS.** In a mixed general intensive care population, after careful controlling for confounders, Cl abnormalities (outside 99-110 mEq/L) were independent predictors for poorer outcomes as ICU mortality and length of stay.

##### REFERENCE(S)

1. Neyra JA, Canepa-Escaro F, Li X, Manllo J, Adams-Huet B, Yee J, et al. Association of hyperchloremia with hospital mortality in critically ill septic patients. Criti Care Med 2015; 43(9):1938.

2. Constable PD. Hyperchloremic acidosis: the classic example of strong ion acidosis. Anesth Analg 2003; 96(4):919-22.
3. Thongprayoon C, Cheungpasitporn W, Cheng Z, Qian Q. Chloride alterations in hospitalized patients: Prevalence and outcome significance. PloS One 2017; 12(3):e0174430.
4. Boniatti MM, Cardoso PR, Castilho RK, Vieira SR. Is hyperchloremia associated with mortality in critically ill patients? A prospective cohort study. J Criti Care 2011; 26(2):175-9.

### 0107

#### Evaluation of glycemic status in critically ill Indian patients: a prospective observational study

V. Sundarsingh S, B. Poddar, M. Gurjar, R.K. Singh, A. Azim, A.K. Baronia
Sanjay Gandhi Post Graduate Institute of Medical Sciences, Critical Care Medicine, Lucknow, India
**Correspondence:** V. Sundarsingh S
*Intensive Care Medicine Experimental* 2018, **6(Suppl 2):**0107

**INTRODUCTION.** There is enough evidence that hyperglycemia and hypoglycemia increase morbidity and mortality in critically ill patients.[1] Recently, glycemic variability and time in target blood glucose (BG) range(TIR) have also been recognized as important domains of glycemic control in the critically ill.[2]

**OBJECTIVES.** In critically ill patients requiring insulin infusion to maintain blood glucose control a) Evaluation of hypoglycemic and hyperglycemic episodes b) Evaluation of glycemic lability index (GLI) c) Evaluation of TIR; i.e. between 100-180 mg/day.

**METHODS.** The study was approved by the Institute Ethics committee. Setting: Mixed ICU of a teaching Institute in North India. Consecutive patients > 18 years requiring insulin infusion for glycemic control included. Hyperglycemia was defined as blood glucose(BG) ≥180mg/dl and hypoglycemia as BG< 70 mg/dl.

Insulin infusion initiated when 2 consecutive BG levels ≥ 180 mg/dl and titrated according to the modified Yale insulin infusion protocol. BG measurements were done using a standard hospital glucometer (Freestyle Optium H, Abbott Ltd).

The first 7 days' glucose values were noted and the following were calculated: Median BG, mean BG, standard deviation of BG (SD), episodes of hypoglycemia and hyperglycemia, GLI and TIR. These were correlated with secondary outcomes, i.e., mortality, length of ICU stay, ventilator days, vasopressor days. All values are expressed in median (interquartile range, IQR).

**RESULTS.** 60 patients included between May 2017 to March 2018. 27 were female. Median age was 56 years (45-67). Median APACHE II was 18 (15.5-23.5) and SOFA score 9 (6-11). Episodes of hypoglycemia was 0.04% (15/3439) and hyperglycemia 42% (1463/3439). 28-day mortality was 25% (15/60). Median length of ICU stay was 22.5 days (13.5-36.5). Median duration of ventilation was 13 days (7-22). Median duration of vasopressor support 7.5 days (4-14)

Patients were divided into two groups based on median TIR and quartiles based on GLI. No statistical difference in any of the secondary outcomes seen in these groups but a trend was observed of better outcomes in patients with the least glycemic variability. 31 were diabetic; diabetics had worse glycemic metrics when compared to non-diabetics (SD:42 vs 37 mg/dL, p = 0.004 and TIR of 46 vs 60%, p =0.032)

**CONCLUSIONS.** The use of Yale insulin infusion in a set of critically ill Indian patients is feasible, safe and effective and certain modifications are suggested for the Indian setup. Patients with the least glycemic variability showed a trend of better outcomes and diabetics showed worse glucose metrics.

**REFERENCE(S)**

1. Freire AX, Bridges L, Umpierrez GE, Kuhl D, Kitabchi AE. Admission hyperglycemia and other risk factors as predictors of hospital mortality in a medical ICU population. Chest 2005; 128:3109-16.
2. Clain, Jeremy, Kannan Ramar, and Salim R. Surani. Glucose control in critical care. World journal of diabetes 6.9 (2015): 1082.

**Table 1 (abstract 0107).** Blood glucose(BG) values and glycemic metrics

| Parameter | Value |
|---|---|
| Total no of BG readings | 3439 |
| Average no of readings per patient/ day | 8.3 |
| Median BG (for all readings) | 172 mg/dL(167-176) |
| Mean BG (for all readings) | 177.7 mg/dL |
| SD | 39.03 mg/dL |
| Median GLI | 1093.3 [(mg/dL)2/h] (567.7 - 1368.2) |
| Median TIR | 50% (44-62) |

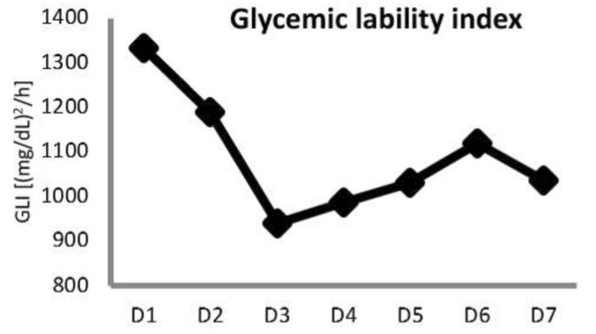

**Fig. 1 (abstract 0107).** Trends of GLI over D1-D7

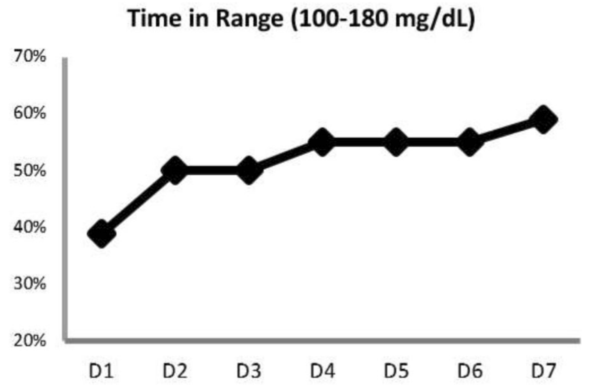

**Fig. 2 (abstract 0107).** Trends of TIR over D1-D7

### 0109

#### Acute pancreatitis in critical care area: epidemiology of 127 cases in a Spanish hospital

A. Nieto García[1], N. Mejuto Montero[1], M. Mourelo Fariña[1], M. Gestal Vázquez[2], I. Suarez Paul[3], H. Triguero San Cristobal[3], D. Freire Moar[1]
[1]Complexo Hospitalario Universitario de A Coruña, Intensive Care Unit, A Coruña, Spain; [2]Complexo Hospitalario Universitario de A Coruña, Anesthesia and Reanimation, Spain; [3]Complexo Hospitalario Universitario de A Coruña, A Coruña, Spain
**Correspondence:** A. Nieto García
*Intensive Care Medicine Experimental* 2018, **6(Suppl 2):**0109

**INTRODUCTION.** Acute pancreatitis (AP) is a common acute condition associated with high morbidity and mortality in severe cases
**OBJECTIVES.** The aim of this study is to describe the demographics and outcomes of these patients managed in critical care area (CCA) at the Complexo Hospitalario Universitario de A Coruña.
**METHODS.** A descriptive, retrospective and single-center observational study of 127 patients. Period study: January 1st, 2010 to December 31st, 2017. Inclusion criteria: diagnosis of AP and admission in CCA. Variables: age, gender, severity scores in the first 24 hours, need for percutaneous drainage or surgery, CCA length of stay (LOS), hospital LOS and hospital mortality. A p-value of 0.05 was considered statistically significant
**RESULTS.** 127 patients. 67,7% were male. The mean age of 59,9±15,6 years. 26,6±3,7 was the mean body mass index (BMI). Most frecuent comorbidities were alcohol abuse (27%), tobacco abuse (26,8%) and diabetes (16,7%). Most common etiologies included biliary (48%) and alcoholic (12,6%). According to the revised Atlanta classification of acute pancreatitis, severe pancreatitis was presented in 57,1% of cases, while moderate and mild pancreatitis were presented in 22,2% and 20,6% respectively. We collected data from organ failure (haemodynamic, kidney and respiratory) and the most common disfunction was transitory. Median Charlson Comorbidity Index was 3 (2,99 ± 2,2). Severity scores were: APACHE II score 13,8±6,6 and SOFA at the day of admission8,7±4,2. 44,8% of the patients presented necrosis collections and 46,2% of them were infected. 50% of the overall developed sepsis and the most common microorganism isolated was *E. coli* (27,8%) while 28,4% was polymicrobial. Previous antibiotherapy was used in 43,7%, antibiotic was appropriate in 94% of cases. 31 patients (24,6%) presented complications and the most prevalent included haemorrhage (51,6%), fistula (25,8%) and perforation (19,3%). 21,1% of the patients developed abdominal compartment syndrome. Different treatments were performed: ERCP-cholecystostomy (13,7%), percutaneous radiologic drainage (18,7%) and surgery (56,3%). In those who underwent surgery procedure, necrosectomy was performed in 37,9%, cholecystectomy in 57,6%, abdominal cavity cleaning in 62,3% and vacuum-assisted closure (VAC) therapy in 33,3%. Mortality rate was 34,9% and the main cause was initial SIRS (30,2%). Median CCA stay was 5 days (15,77 ± 24,64) and overall hospital stay was 27,5 days (42,79 ± 48,4).
**CONCLUSIONS.** Severe acute pancreatitis represents 57,1% of CCA acute pancreatitis admissions. Its clinical course is frequently complicated by infections or haemorrhage and it is associated with a high mortality rate. This epidemiological observational study may be used for calculating sample size for future multi-center interventional therapeutic studies.

**GRANT ACKNOWLEDGMENT**
This study received no financial support.

### 0110

#### Fucoidan, a natural sulfated polysaccharide, attenuates uptake of advanced glycation end-products by macrophages

S. Hamasaki[1], A. Kitaura[1], T. Kobori[2], Y. Yamazaki[2], T. Nishinaka[2], A. Niwa[2], H. Takahashi[2], S. Nakao[1]
[1]Kindai University, Anesthesiology, Osakasayama, Japan; [2]Kindai University, Pharmacology, Osakasayama, Japan
**Correspondence:** S. Hamasaki
*Intensive Care Medicine Experimental* 2018, **6(Suppl 2):**0110

**INTRODUCTION.** Advanced glycation end-products (AGEs), which are formed by nonenzymatic glycation reaction of reducing sugar and proteins, accumulate in the body due to the persistence of hyperglycemia and produce reactive oxygen species and inflammatory cytokines when they bind to their receptor, RAGE (receptor for AGEs)[1]. Thus, AGEs reduction is considered to be an important treatment for hyperglycemia-induced cytotoxicity. On the other hand, macrophage is capable of eliminating AGEs possibly via its phagocytic activity, and an accumulation of AGEs-containing macrophages on the surface of blood vessels is involved in the progression of arteriosclerosis and inflammation, resulting in ischemic coronary diseases or a stroke [2]. Fucoidan are a type of highly sulfated polysaccharides found mainly in the extracellular matrix of brown seaweeds and significantly attenuates atherosclerotic plaque formation and enhances plaque stability by decreasing serum lipids and inhibiting macrophage infiltration[3]. It remains unclear, however, whether fucoidan affects uptake of AGEs by macrophages.
**OBJECTIVES.** We investigated whether fucoidan influences macrophage phagocytosis of AGEs.
**METHODS.** RAW264.7 cells, the mouse macrophage-like cell line, were incubated with AGE-modified bovine serum albumin (BSA), glyceraldehyde-derived AGE and glycolaldehyde-derived AGE at different concentrations ranging at 200 μg/ml and fucoidan at concentrations ranging from 1-1,000 μg/ml for 1 h. The uptake level of AGEs and the expression levels of surface scavenger receptors in RAW264.7 cells were analyzed by flow cytometry. The intracellular localization of AGEs phagocytosed by RAW264.7 cell were visualized by immunofluorescence microscopy.
**RESULTS.** Fucoidan, at 100-1000 μg/ml, markedly inhibited AGEs uptake into RAW264.7 cells. AGEs significantly enhanced the surface expression levels of CD204, macrophage scavenger receptor type I, in RAW 264.7 cells, but the expression was markedly inhibited by fucoidan.
**CONCLUSIONS.** Fucoidan inhibited the facilitation of AGEs uptake into macrophages possibly by inhibiting AGEs-induced CD204 expression on their surface. The results suggest a vasculoprotective potential of fucoidan for the clinical treatment of atherosclerosis.

**REFERENCE(S)**

1. Takeuchi M, Takino J, Yamagishi S. Involvement of the toxic AGEs (TAGE)-RAGE system in the pathogenesis of diabetic vascular complications: a novel therapeutic strategy. Curr. Drug Targets 11, 1468-82, 2010.
2. Nagai R, Fujiwara Y, Mera K. Investigation of pathways of advanced glycation end-products accumulation in macrophages. Mol. Nutr. Food Res. 51, 462-7, 2007.
3. Yokota T, Nomura K, Nagashima M. Fucoidan alleviates high-fat diet-induced dyslipidemia and atherosclerosis in ApoE(shl) mice deficient in apolipoprotein E expression. J. Nutr. Biochem. 32, 46-54, 2016.

**GRANT ACKNOWLEDGMENT**
This work was supported by Japan Society for the Promotion of Science Grants-in-Aid for Young Scientists (Grant Numbers 26861254).

### 0111

#### Sodium input in intensive care

N. Samal[1], E. O'Neill[2], M. Carpenter[2]
[1]Newcastle University, Newcastle upon Tyne, United Kingdom; [2]City Hospitals Sunderland NHS Foundation Trust, Integrated Critical Care Unit, Sunderland, United Kingdom
**Correspondence:** N. Samal
*Intensive Care Medicine Experimental* 2018, **6(Suppl 2):**0111

**INTRODUCTION.** Sodium is a key electrolyte that is crucial in maintaining homeostasis through the regulation of blood pressure, blood volume, and pH levels.
Administration of fluids containing sodium is an essential part of resuscitation and ongoing care. However, sodium overload is thought to be common in intensive care patients with hypernatraemia occurring in up to 25% of patients during their stay [1], potentially adding further complications to care.
**OBJECTIVES.** To collect the individual daily sodium intake data of all patients on a general UK ICU over a 14-day period. The sodium contents of all fluids, drugs, and nutritional sources was calculated and divided into source categories. Daily weights were recorded to calculate the recommended sodium intake for each patient according to current National Institute for Health and Care Excellence (NICE) guidelines [2]. Serum sodium results, and fluid balance data were also collected.
**METHODS.** Prospective audit of all patients admitted to the ICU, during a 14-day period in January 2018. Data was collected from daily charts, and e-records at the end of a 24-hour period. Sodium content of all fluids, drugs, and nutrition was calculated using electronic Medicines Compendium, Institute of Food Research/Public Health England, and British Dietetic Association resources.
Patient days were divided into 2 phases; resuscitation and maintenance. Resuscitation was defined as administration of vasoactive therapy at any stage during the 24-hour period. All individual fluid, drug, or nutritional sources totalling less than 1mmol of sodium were excluded. Daily weights were monitored using bed scale readings.
**RESULTS.** 28 patients were reviewed, totalling 158 bed days. Maintenance days: n=116, resuscitation days: n=42.
2/3 of all patients were over the NICE recommended guidelines for sodium intake.
The mean sodium intake for the maintenance phase was x1.4 NICE recommendations, with a median of 1.1 (IQR 0.8-1.7). In the resuscitation phase mean average was x2.1, with a median of 1.7 (IQR 1.1-2.3).
18% of all patients were hypernatraemic (≥146 mmol: n=29, ≥155 mmol: n=11), and 4/5 of these patients had received more than their recommended daily sodium intake.
In the maintenance group 24% of sodium came from fluids, whilst 64% was from nutrition. In comparison, 41% of the sodium in the resuscitation group was from fluid, and 38% from nutrition. In both groups nearly 20% came from other sources with the potential to reduce input.
**CONCLUSIONS.** The majority of intensive care patients receive more than their recommended sodium intake. It may be possible to reduce sodium input from background fluids and nutrition whilst maintaining fluid homeostasis, and protein calorie intake.

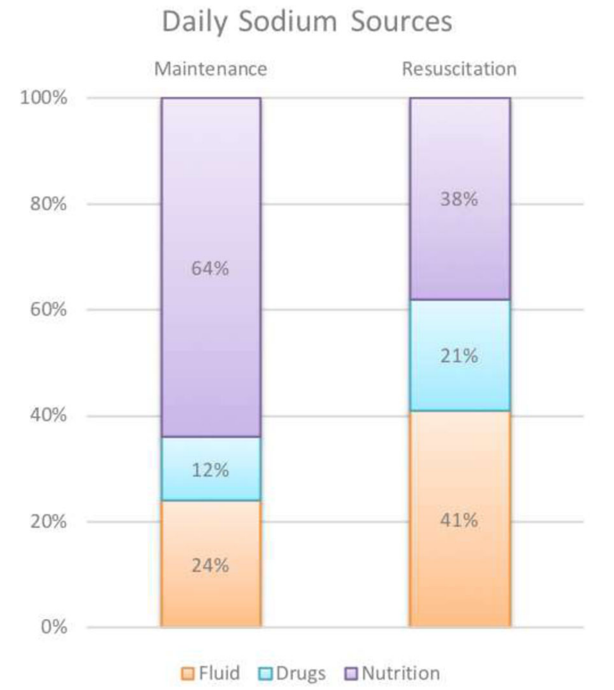

**Fig. 1 (abstract 0111).** Daily Sodium Sources

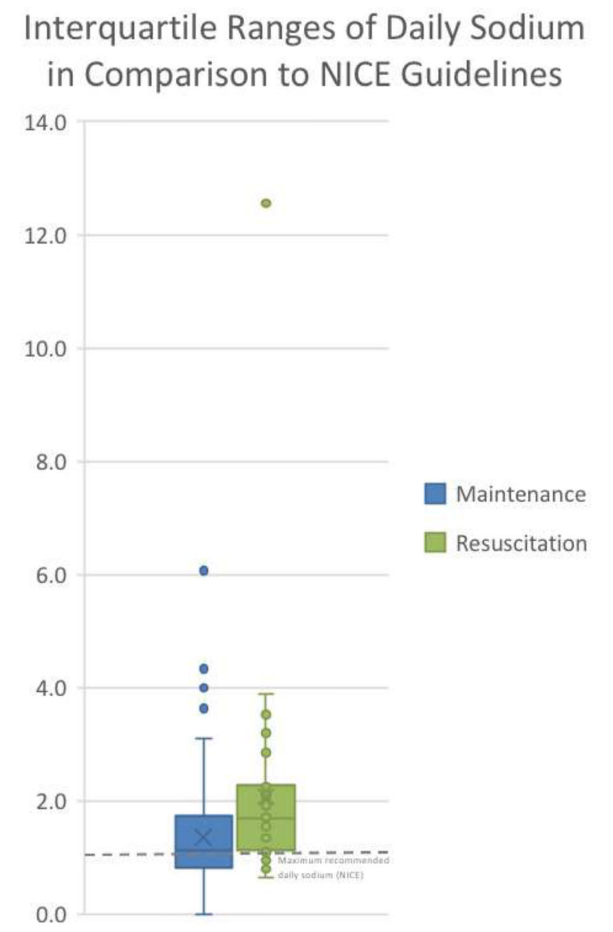

**Fig. 2 (abstract 0111).** Daily Sodium in Comparison to NICE Guidelines

### 0112

#### Influence of complications of diabetic ketoacidosis treatment on length of stay in intensive care unit

D. Adukauskienė, L. Jazokaitė
Hospital Kaunas Clinics of Lithuanian University of Health Sciences, Intensive Care Unit, Kaunas, Lithuania
**Correspondence:** L. Jazokaitė
*Intensive Care Medicine Experimental* 2018, **6(Suppl 2):**0112

**INTRODUCTION.** Diabetic ketoacidosis (DKA) is an acute metabolic complication of diabetes. Hyperglycaemia and ketoacidosis is controlled by insulin, so use of sodium bicarbonate for treatment of metabolic acidosis is not suppose to be indicated, especially if hemodynamic is stable. Permanent monitoring of level of serum glucose and potassium also pH as an adequate treatment may prevent life-threatening complications of treatment of DKA.
**OBJECTIVES.** To estimated rate of hypokalemia, hypoglycaemia and relate these DKA treatment complications due to use of insulin also sodium bicarbonate use with length of stay in Intensive Care Unit (ICU).
**METHODS.** This is a single-centre at the largest Lithuanian University hospital Kaunas Clinics pilot retrospective study carried out during 2012 - 2018. Inclusion criteria: 1. patients (pt) with DKA; 2. age ≥ 18; 3. stay at ICU. Serum kalemia, glycaemia; hypokalemia, hypoglycaemia episodes; rate of insulin interruption; use of sodium bicarbonate, and length of stay (LOS) in ICU were analysed. SPSS 25.0 was used for statistic calculations. Traits evaluated as significant as $p < 0.05$.

**RESULTS.** During the treatment of DKA obvious hypokalemia (2.1 - 3.4 (3.1 ± 0.3 mmol/l)) was recorded in 48/91 (52.7 %) pt, LOS in ICU was 51.9 ± 32.2 vs 32.8 ± 18.7 hr, $p < 0.05$. Also due to disregarding of blood pH (6.8 - 7.3 (7.1 ± 0.1)) it was misinterpreted as "normokalemia" or "hyperkalemia" (3.5 - 6.1 (2.5 ± 0.7 mmol/)) in 49/91(53.8 %) pt, and obvious hypokalemia occured in 20/49 (40.8 %) pt. Insulin use has caused hypoglycaemia (1.2 - 3.3 (2.5 ± 0.7 mmol/l)) in 18/91 (19.8 %) pt, LOS in ICU 63.1 ± 41.3 vs 37.9 ± 21.5 hr, $p < 0.05$. Insulin use was interrupted in case of normo - and hypoglycaemia still with persisting ketoacidosis in 31/91 (34.1 %) pt, LOS in ICU was found to be 56.6 ± 32.9 vs 35.8 ± 22.7 hr, $p < 0.05$. Sodium bicarbonate was given for symptomatic treatment of acidosis during first 10 hrs of DKA in 19/91 (20.9 %) pt with stable hemodynamic: $HCO^-_3$ buffer has increased (4.2 ± 3.3 - 7.4 ± 3.2 mmol/l), $p < 0.05$, but ketoacidosis has still persisted, LOS in ICU was 54.6 ± 30.4 vs 39.7 ± 26.9 hr, $p < 0.05$.

**CONCLUSIONS.** Obvious hypokalemia occured in half of all pt, also due to disregarding of blood pH value in half of pt hypokalemia has been interpreted as normo- or hyperkalemia. Due to insuline use hypoglycaemia was recorded in 1/5 pt. Insulin use was interrupted in case of hypo- and normoglycaemia despite of ketoacidosis almost in 1/3 pt, it's prolonged length of stay in ICU. In case of hypokalemia or hypoglycaemia length of stay in ICU for DKA treatment has increased. Sodium bicarbonate was used in 1/4 of pt and it did not control ketoacidosis, length of stay in ICU has been prolonged.

**REFERENCE(S)**

1. AR Gosmanov, et al. Management of adult diabetic ketoacidosis. Diabetes, Metabolic Syndrome and Obesity: Targets and Therapy 2014;7:255-264.

**GRANT ACKNOWLEDGMENT**

None.

### 0113

#### Nutrition in pancreatitis: a tertiary centre experience

S. Milton-White[1], R. Mullhi[2]

[1]Sandwell and West Birmingham Hospitals NHS Trust, Birmingham, United Kingdom; [2]University Hospitals Birmingham NHS Foundation Trust, Birmingham, United Kingdom

**Correspondence:** S. Milton-White

*Intensive Care Medicine Experimental* 2018, **6(Suppl 2):**0113

**INTRODUCTION.** Patients with acute pancreatitis (AP) are at high risk of malnutrition. Current evidence supports early enteral nutrition[1] which maintains gastrointestinal mucosa integrity and is associated with reductions in mortality, organ failure, sepsis and surgical intervention[2]. NCEPOD (National Confidential Enquiry into Patient outcome and Death) found 67% of patients admitted with AP were screened for malnutrition with adequate management in 85%[3]. This recommends all patients should be assessed for risk of malnutrition using Malnutrition Universal Screening Tool (MUST) on admission. International Association of Pancreatology (IAP) and American Pancreatic Association (APA) guidelines in 2013 recommend enteral feeding with TPN reserved for failure of post-pyloric feeding[4].

**OBJECTIVES.** To assess compliance with NCEPOD and IPA/APA recommendations in a tertiary centre and identify reasons for delayed enteral nutrition.

**METHODS.** This is a retrospective audit of patients admitted to Intensive Care (ICU) between January 2015 and December 2017 with AP. Patients were identified by Intensive Care National Audit and Research Centre (ICNARC) coding and electronic patient records were used to collect data.

**RESULTS.** 43 patients were admitted with AP accounting for 50 admission episodes: 11 were inter-hospital transfers. ICU mortality was 11.8% with median organ support required of 2.

MUST scoring occurred during 46 (92%) admissions with 21 (42%) at ICU admission. Median MUST score was 1 and 50% were referred to dieticians. Initial nutrition on ITU was enteral for 34 (68%), nasojejunal (NJ) for 8 patients and TPN for 3 patients. Nutrition was commenced in 70% of patients within 24 hours of admission. Reasons cited for delay included pending surgical review and patient instability. 9 patients (19%) were commenced on TPN after failure of enteral feeding.

**CONCLUSIONS.** Enteral nutrition was the most common form of nutrition in ITU and was commenced within 24 hours in 70%. Less than 50% of patients were screened for malnutrition on ICU admission.

In this setting, NCEPOD and IAP/APA standards are not being achieved. This is in part due to concerns regarding commencing nutrition prior to surgical review. The low conversion rate to TPN provides good evidence to support enteral nutrition. We are developing a departmental guideline on nutrition in AP to guide nutritional assessment and reinforce the role of early nutrition.

2 Al-Omran et al. Cochrane Database Syst Rev 2010;20(1):CD002837

3 NCEPOD. http://www.ncepod.org.uk/2016report1/downloads/TreatTheCause_fullReport.pdf

### 0114

#### Risk factors for raised glycosylated hemoglobin at ICU admission

D. Prevedello, A. Khaldi, J.-C. Preiser

Erasme Hospital,Université Libre de Bruxelles, Intensive Care, Brussels, Belgium

**Correspondence:** D. Prevedello

*Intensive Care Medicine Experimental* 2018, **6(Suppl 2):**0114

**INTRODUCTION.** Individualizing the glycemic target requires the knowledge of the level of glycosylated hemoglobin HbA1c at the time of ICU admission (1,2). In the critically ill, risk factors for raised HbA1c, regardless of the preexistence of diabetes mellitus (DM), are currently not well characterized.

**OBJECTIVES.** The aim of this study was to assess whether the prevalence of HbA1c > 6.5% was influenced by demographic variables, type of admission, preexisting co-morbidities including DM, severity of disease, and outcome variables.

**METHODS.** Consecutive patients admitted over a 4- month period in a medical/surgical 32-bed ICU were included. HBA1c, age, gender, admission type, Charlson co-morbidity score, knowledge of DM, severity scores (SAPS II and SOFA) were collected at admission. ICU and hospital length of stay (LOS) and mortality were recorded. Patients were categorized according to the HbA1c admission value (< 6.5 % and ≥ 6.5%) and subcategorized according to the pre-existence of DM. A chi-square test was calculated to compare categorical variables, T-student test for age and non-parametrical tests for other variables.

**RESULTS.** A total of 572 patients were included (mean age 61 ±16 years). Admission HbA1C was ≥6.5% in 16% of the patients. As compared with patients whose HbA1C was < 6.5%, patients with raised HbA1C were older, more often males admitted for medical reasons, had a lower prevalence of unknown DM, and had more organ failures (table). There was no difference in LOS or mortality between the two groups.

**CONCLUSIONS.** In a mixed population of critically ill patients, elevated HbA1c was frequent even in patients without DM. A systematic determination of HbA1C at the time of admission could be particularly valuable in older males with several organ failures, even in the absence of known diabetes.

**REFERENCE(S)**

1. Dodson C, Simpson J, D F. Glycemic control in a medical intensive care setting: revision of an intensive care unit nurse-driven hyperglycemia protocol. Crit Care Nurs Q. 2014;37(2):170-81.

2. Hoang Q, Pisani M, Inzucchi S, Hu B, S H. The prevalence of undiagnosed diabetes mellitus and the association of baseline glycemic control on mortality in the intensive care unit: a prospective observational study. J Crit Care. 2014;29(6):1052-6.

**Table 1 (abstract 0114).** Summary of results

| | HbA1c < 6.5% (n=481) | HbA1c ≥ 6.5% (n=91) | p-value |
|---|---|---|---|
| Age (years), mean ± SD | 60 ±16 | 65 ±12 | < 0.05 |
| Proportion of males, (percentage) | 267 (55.5) | 66 (72.5) | < 0.05 |
| Medical admission, n (percentage) | 189 (39.3) | 47 (51.6) | < 0.05 |
| Charlson score, median (IQR) | 2 (0-3) | 3 (0-4) | 0.7 |
| Unknown DM, n (percentage) | 397 (83) | 22 (25) | < 0.001 |
| SOFA, median (IQR) | 2 (0-5) | 3 (1-7) | < 0.05 |
| SAPS II, median (IQR) | 42 (31-56) | 46 (34-61) | 0.1 |
| ICU LOS (days), median (IQR) | 1 (1-3) | 1 (1-4) | 0.76 |
| ICU mortality (percentage) | 2.3 | 2.6 | 0.7 |

### 0115

#### Serum S100B is associated with the severity of liver cirrhosis, rather than the severity of hepatic encephalopathy

J.W. Park[1], C. Kim[2]
[1]Hallym University Sacred Heart Hospital, Department of Internal Medicine, Anyang-si, Korea, Republic of; [2]Jeju National University Hospital, Department of Internal Medicine, Jeju-si, Korea, Republic of
**Correspondence:** C. Kim
*Intensive Care Medicine Experimental* 2018, **6(Suppl 2):**0115

**INTRODUCTION.** S100B, a $Ca^{2+}$-modulated protein, is predominantly found in astroglia and Schwann cells, plays important roles in central nervous system development and recovery after injury. There are very few studies that have suggested serum S100B as a diagnostic marker for hepatic encephalopathy (HE) in the patients with cirrhosis or fulminant hepatic failure.
**OBJECTIVES.** This study was conducted to investigate the association between serum level of S100B and the severity of liver cirrhosis and HE.
**METHODS.** The patients with liver cirrhosis were enrolled to the study, and their serum levels of S100B were measured by the enzyme-linked immunosorbent assay. We classified the patients into two groups based on the level of S100B and labeled those at or above the 25$^{th}$ percentile (51.0 pg/ml) as high S100B group and those below the 25$^{th}$ percentile as non-high S100B group. The patients were also divided into three groups, without HE, with minimal HE and with overt HE. The psychometric hepatic encephalopathy score was used for the diagnosis of minimal HE.
**RESULTS.** A total of 95 subjects were included, 70 (73.7%) males and 25 (26.3%) females. The mean age was 52.7 years. A positive linear correlation was observed between serum S100B level and the Model for End-Stage Liver Disease (MELD) score (r = 0.385, p < 0.001). High S100B was detected in 24 patients (25.3%). The MELD score was significantly higher in high S100B group compared with non-high S100B group (16.39 ± 7.91 vs 11.61 ± 6.22, p = 0.003). However, no significant differences were observed for age, BMI, platelet count, serum levels of ammonia, cholesterol, bilirubin, and albumin, protrombin time, and the Child-Pugh score. Of the 95 patients, 22 (23.2%) was diagnosed with minimal HE and 25 (26.3%) with overt HE. The mean levels of serum S100B of each group were 38.1 ± 17.2 (without HE), 36.5 ± 13.9 (with MHE), and 43.5 ± 19.7 (with overt HE) pg/ml, respectively. One-way analysis of variance revealed that serum S100B levels did not differ among these three groups (p = 0.208).
**CONCLUSIONS.** Cirrhotic patients with high serum S100B are associated with an increased risk of severe liver disease. However, serum S100B levels could not well discriminate the severity of HE.

**REFERENCE(S)**

1. Donato R, et al. Functions of S100 proteins. Curr Mol Med. 2013;13(1):24-57.
2. Isobe-Harima Y, et al. Serum S100b (astrocyte-specific protein) is a useful marker of hepatic encephalopathy in patients with fulminant hepatitis. Liver Int. 2008;28(1):146-7.
3. Duarte-Rojo A, et al. Clinical scenarios for the use of S100beta as a marker of hepatic encephalopathy. World J Gastroenterol. 2016;22(17):4397-402.
4. Amodio P, et al. Detection of minimal hepatic encephalopathy: normalization and optimization of the Psychometric Hepatic Encephalopathy Score. A neuropsychological and quantified EEG study. J Hepatol. 2008;49(3):346-53.

**GRANT ACKNOWLEDGMENT**
The authors have no conflicts of interest to disclose.

### 0116

#### Efficacy of coupled plasma filtration adsorption (CPFA) in patients with liver disease

M.D. Arias-Verdu, J. Barrueco-Francioni, G. Seller-Perez, G. Quesada-Garcia, M.J. Diez de los Rios, M. Herrera-Gutierrez
Hospital Regional Universitario Carlos Haya, Intensive Care Unit, Málaga, Spain
**Correspondence:** M.D. Arias-Verdu
*Intensive Care Medicine Experimental* 2018, **6(Suppl 2):**0116

**OBJECTIVE.** Reports exist of the possible usefulness of coupled plasma filtration adsorption (CPFA) for the purification of hepatic patients but current evidence is just based on isolated clinical cases. We aim to examine the efficacy of CPFA in this situation compared with a standard treatment, MARS.
**METHODS.** This exploratory, case series involved four patients, two admitted for acute liver failure and two for acute on chronic liver failure. The indication for use was adjusted in all cases to the protocol used in our ICU. Alternative sessions were given with MARS (6 sessions) and CPFA (4 sessions) to compare between the patients. The duration of the sessions was 12 hours and anticoagulation was done using a mixed technique; heparin 4 U/Kg/h + epoprostenol 5 mcg/Kg/min; venous access was made with a 24 cm, 13.5F catheter via the right femoral vein. The statistical analysis was done with non-parametric tests for analysis of paired samples (Wilcoxon), with significance set at 0.05. The data are shown as the median (quartiles).
**RESULTS.** Of the two patients with acute liver failure, one had Wilson disease and MELD 38, and underwent transplantation; and the other HAV infection with MELD 24 and was discharged alive. Of the two with acute on chronic failure, one had autoimmune disease and MELD 25, and died; and the other NASH and MELD 34, receiving a transplant.
Technical difficulties encountered included one assembly problem in a MARS session and early coagulation in a MARS session. The reduction in platelets for MARS was 4.1% (-4.2- 7.3[IKJ1] ) and for CPFA 20.7% (-3.3-20.7). We detected no bleeding problems in any patient.
Concerning the purification capacity, the reduction in creatinine was similar with both techniques: MARS 32.4 (25.9-45.9), CPFA 30.5 (7.1-30.5), p ns; as was sodium: MARS 0.7 (-2.2-1.1); CPFA 0.7 (-10.7-0.7), p ns. Direct bilirubin fell with both: MARS 23.9 (15.3-44.1), CPFA 41.1 (37.1-41.1), p ns; but indirect bilirubin only with CPFA: MARS 8.9 (-9.5-16.6); CPFA[IJ2] (-20.1-44.6), p < 0.05).
**CONCLUSION.** In our patients CPFA had a similar elimination profile to MARS in hepatic patients. The elimination of indirect bilirubin was only effective with CPFA but the reduction in platelets was greater with CPFA. Studies specifically addressing this disease are needed to assess the indication for CPFA as a liver purification technique.
[IKJ1]Vendría bien separar los extremos con un signo como una coma o dos puntos (:). Hay que tener cuidado cuando hay valores negativos, para distinguir entre el signo de menos y el guion de separación. Este comentario va para todos los valores en el texto.

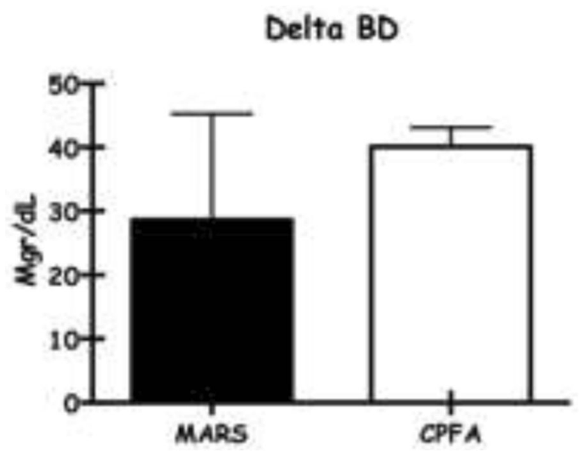

**Fig. 1 (abstract 0116).** Difference in elimination of direct bilirubin

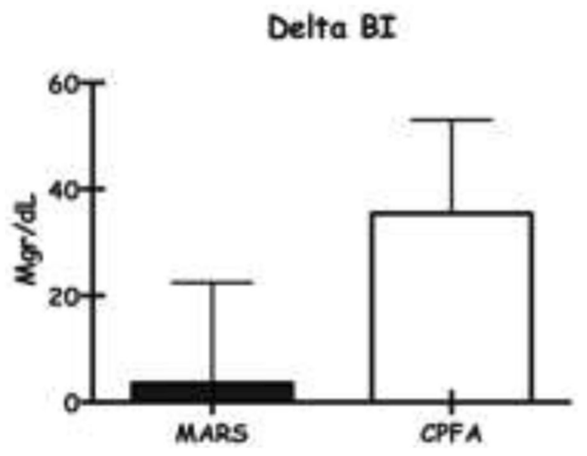

**Fig. 2 (abstract 0116).** Difference in elimination of indirect bilirubin

#### 0117

#### Severe acute liver injury: etiologies and outcome

N. Foudhaili, S. Khedher, A. Khaled, K. Ben Ismail, M. Salem
Charles Nicolle Hospital, Digestive Intensive Care Unit, Tunis, Tunisia
**Correspondence:** N. Foudhaili
*Intensive Care Medicine Experimental* 2018, **6(Suppl 2):**0117

**INTRODUCTION.** Severe acute liver injury is characterized by an acute liver damage without underlying chronic liver disease. It is a critical condition as it may lead to acute liver failure.

**OBJECTIVES.** The aim of this work was to provide epidemiological and prognostic features of this condition.

**METHODS.** It was a retrospective one year study. We included all patients admitted for severe acute liver injury (ALI).

**RESULTS.** Forty-six patients were included with a sex-ratio at 0.5 and a mean age of 48±19 years (16-87). The main comorbidities were diabetes mellitus (31%) and hypertension (28%). Principal etiologies of ALI were hepatitis (46%) and drugs (28%). Hepatitis type B was incriminated in 11 patients (24%), type A in 8 patients and type E in 2 patients. Acute liver failure (ALF) occurred in 7 patients. ALF was classified as hyperacute in 2 patients, acute in 3 patients and subacute in 2 patients. Six patients died (13%) because of ALF.Patients with unfavorable outcome (death or ALF), in whom demographic characteristics were similar to patients with favorable outcome, had lower platelet count ($198.10^3$/ml vs 110 $.10^3$/ml, p =0.025), lower prothrombin time ratio (22±11% vs 45 ±22%, p< 0.001), higher INR level (1.52±2.1 vs 3.6±0.44 p < 0.001), lower hemoglobin amount (9.5±3.4 g/dl vs 12±2.2 g/dl, p = 0.029), lower diastolic blood pressure (58±8 mmHg vs 72±13 mmHg, p = 0.015) and lower mean blood pressure (76±9 mmHg vs 89±13 mmHg, p =0.026). The results of the logistic regression analysis showed that only diastolic blood pressure level was significantly related to unfavorable outcome. ROC curve analysis showed that a diastolic blood pressure level < 60 mmHg was a predictive factor of unfavorable outcome with a sensibility of 75% and specificity of 95% (AUR=0,802, 95% IC 0,627-0,977).

**CONCLUSIONS.** ALI is associated with a non-negligible mortality mainly due to ALF. A diastolic blood pressure level less than 60 mmHg may help to predict unfavorable outcome.

### Biomarkers in sepsis

#### 0118

#### The role of hepcidin in innate immune response and survival during septic shock in critically ill adults

R. Fuchs[1,2], S. Noel[3], L. Zheng[4], A. Navas-Acien[5], Z.L. Harris[6]
[1]Hauora Tairawhiti, Anaesthesia, Gisborne, New Zealand; [2]The University of Auckland, Anaesthesiology, Auckland, New Zealand; [3]Johns Hopkins University, Division of Nephrology, Department of Medicine, Baltimore, United States; [4]Johns Hopkins University, Bloomberg School of Public Health, Baltimore, United States; [5]Columbia University, Environmental Health Sciences, New York City, United States; [6]Northwestern University Feinberg School of Medicine, Department of Pediatrics Division of Critical Care Medicine, Chicago, United States
**Correspondence:** R. Fuchs
*Intensive Care Medicine Experimental* 2018, **6(Suppl 2):**0118

**INTRODUCTION.** Sepsis is characterized by an inappropriate host immune-inflammatory response including altered iron homeostasis. Increased hepatic hepcidin production leads to hypoferremia. Hepcidin inhibits macrophage iron release and intestinal iron absorption, and has been suggested to be an important link between host defense, infection, and iron metabolism.

**HYPOTHESIS.** We hypothesize that increased gene expression of hepcidin is protective and could be correlated with an improved survival outcome.

**METHODS AND STUDY DESIGN.** Prospective cohort. Settings: Adult patients with septic shock in medical and surgical ICUs. Sample: 106 patients were enrolled between January 2008 and March 2010 within 24-48 h of diagnosis. Exposure measure: hepcidin gene expression. Hepcidin was measured in PBMC by RT-PCR. Outcome measures: 60-day mortality. Statistics: To assess the prospective association of hepcidin levels with mortality, we used Cox proportional hazard models. With 60-day mortality as the outcome and hospital days as the time metric, crude models for the outcome and log2 transformed hepcidin gene expressions were run. Statistical analyses were adjusted as follows: 1) crude model; 2) model adjusted for age, gender, race, body mass index (BMI), and baseline APACHE (Acute Physiology and Chronic Health Evaluation) II score. Hazard ratios are presented.

**RESULTS.** At 60 days 58/106 (54.7%) patients were alive and 48/106 (45.3%) patients were dead. The hazard ratio for doubling the hepcidin gene expression was significant in the crude model (HR = 1.12; C.I. 1.00, 1.24): for each doubling in hepcidin gene expression there is a 12% increased instant threat of dying; adjusted for age, gender, race, and body mass index (BMI): HR = 1.14; C.I. 1.02,1.28). Once adjusted for severity of illness (Apache 2 score) the effect was not anymore statistically significant: HR = 1.08; C.I. 0.95, 1.22. Ventilator days and days of vasopressor support were not statistically different between hepcidin mRNA levels.

**CONCLUSIONS.** Higher hepcidin mRNA expression at enrollment is associated with higher 60-day mortality based on the crude hazard ratio for hepcidin gene expression level and adjusted for age, gender, race, and BMI. Since this effect is not present once adjusted for severity of illness, we postulate that hepcidin gene expression level is a mediator in the pathophysiological cascade of sepsis that leads to increased mortality. Therefore, while we must reject our hypothesis, we are able to further corroborate a link between altered iron metabolism and sepsis, and speculate the increase in hepcidin correlates with the disease burden.

**GRANT ACKNOWLEDGMENT**

International Anesthesia Research Society (IARS) and institutional funds by the Department of Anesthesiology and Critical Care Medicine at The Johns Hopkins University.

### 0119

#### Lower serum kallistatin level is associated with 28-day mortality in patients with septic shock

T. Kim[1], G.J. Suh[1], W.Y. Kwon[1], K.S. Kim[1], Y.S. Jung[1], S.M. Shin[2], H. Jeong[1]
[1]Seoul National University Hospital, Department of Emergency Medicine, Seoul, Korea, Republic of; [2]Seoul National University Hospital, Division of Critical Care Medicine, Department of Emergency Medicine, Seoul, Korea, Republic of
**Correspondence:** H. Jeong
*Intensive Care Medicine Experimental* 2018, **6(Suppl 2):**0119

**INTRODUCTION.** Kallistatin has anti-inflammatory effect by inhibiting tumor necrosis factor-alpha (TNF-α) and high mobility group box-1 (HMGB1) (1, 2), and it also attenuates endothelial dysfunction through TNF-α- and endothelial nitric oxide synthase (eNOS)-associated pathways (2-4).

**OBJECTIVES.** We performed this study to investigate whether serum levels of kallistatin, vascular cell adhesion molecule-1 (VCAM-1), and E-selectin are associated with 28-day mortality in patients with septic shock.

**METHODS.** Serum levels of kallistatin, VCAM-1, and E-selectin were measured by enzyme-linked immunosorbent assay using blood samples obtained at admission and at 24 h after admission from the patients with septic shock and from healthy volunteers. The primary outcome was 28-day mortality.

**RESULTS.** Serum kallistatin levels were significantly lower and serum VCAM-1 and E-selectin levels were significantly higher both in the survivors and the non-survivors compared with the healthy volunteers both at admission and at 24 h. Serum kallistatin level at 24 h was significantly higher in the survivors compared with the non-survivors (4.3 μg/mL [3.3-5.2] vs. 2.8 μg/mL [2.2-3.8], *P* = 0.002). The levels of VCAM-1 and E-selectin showed no differences between the survivors and the non-survivors at either time point. In a multivariable logistic regression analysis with stepwise selection, the serum kallistatin level at 24 h was independently associated with 28-day mortality (odds ratio 0.45, 95% confidence interval 0.21-0.82, *P* = 0.025).

**CONCLUSIONS.** The lower serum kallistatin level at 24 h was independently associated with 28-day mortality in patients with septic shock.

**REFERENCE(S)**

1. Li P et al.: Human kallistatin administration reduces organ injury and improves survival in a mouse model of polymicrobial sepsis. *Immunology* 142(2):216-226, 2014.
2. Yin H et al.: Kallistatin inhibits vascular inflammation by antagonizing tumor necrosis factor-alpha-induced nuclear factor kappaB activation. *Hypertension* 56(2):260-267, 2010.
3. Shen B et al.: Kallistatin attenuates endothelial apoptosis through inhibition of oxidative stress and activation of Akt-eNOS signaling. *Am J Physiol Heart Circ Physiol* 299(5):H1419-1427, 2010.
4. Shen B et al.: Kruppel-like factor 4 is a novel mediator of Kallistatin in inhibiting endothelial inflammation via increased endothelial nitric-oxide synthase expression. *J Biol Chem* 284(51):35471-35478, 2009.

**GRANT ACKNOWLEDGMENT**

This study was funded by Seoul National University Hospital (grant number 25-2016-0040). There are no conflicts of interest to be declared.

**Table 1 (abstract 0119).** Serum levels of kallistatin, VCAM-1, and E-selectin among 28-day survivors, non-survivors, and healthy volunteers

| | Survivors (n = 58) | Non-survivors (n = 14) | Healthy volunteers (n = 6) | P value |
|---|---|---|---|---|
| At admission | | | | |
| Kallistatin, μg/mL | 4.4 (2.9-6.1) | 2.5 (2.1-5.0) | 13.3 (11.7-13.9) | < 0.001 |
| VCAM-1, μg/mL | 2.1 (1.5-3.0) | 1.8 (1.2-2.7) | 0.3 (0.3-0.4) | < 0.001 |
| E-selectin, ng/mL | 199.6 (83.0-327.2) | 97.9 (51.6-178.9) | 22.2 (21.3-24.2) | < 0.001 |
| At 24 h | | | | |
| Kallistatin, μg/mL | 4.3 (3.3-5.2) | 2.8 (2.2-3.8) | 13.3 (11.7-13.9) | < 0.001 |
| VCAM-1, μg/mL | 2.0 (1.3-3.1) | 1.9 (1.2-3.3) | 0.3 (0.3-0.4) | < 0.001 |
| E-selectin, ng/mL | 141.0 (82.5-257.3) | 109.6 (49.8-214.5) | 22.2 (21.3-24.2) | 0.001 |

**Table 2 (abstract 0119).** Logistic regression analysis for 28-day mortality

| | Unadjusted OR | 95% CI | P value | Adjusted OR | 95% CI | P value |
|---|---|---|---|---|---|---|
| Serum lactate level at admission | 1.10 | 0.97-1.25 | 0.139 | | | |
| Serum lactate level at 24 h | 1.40 | 1.10-1.85 | 0.009 | | | |
| SOFA score at admission | 1.18 | 0.96-1.47 | 0.131 | | | |
| SOFA score at 24 h | 1.51 | 1.20-2.02 | 0.002 | 1.40 | 1.10-1.86 | 0.012 |
| Serum kallistatin level at admission | 0.71 | 0.48-0.97 | 0.052 | | | |
| Serum kallistatin level at 24 h | 0.38 | 0.18-0.69 | 0.004 | 0.45 | 0.21-0.82 | 0.025 |

### 0120

#### The effects of septic state on plasma orexin concentrations in human: a preliminary study

M. Akaishi[1], E. Hashiba[2], T. Suganuma[1], H. Niwa[2], T. Kushikata[3], K. Hirota[3]
[1]Hirosaki University Hospital, Hirosaki-shi, Japan, [2]Hirosaki University Hospital, ICU, Hirosaki-shi, Japan, [3]Hirosaki University Postgraduate School of Medicine, Hirosaki-shi, Japan
**Correspondence:** M. Akaishi
*Intensive Care Medicine Experimental* 2018, **6(Suppl 2):**0120

**INTRODUCTION.** Orexinergic system plays an important role not only on sleep-awake cycle, but also on analgesia and autonomic function (1). In animal study orexinergic activity was reduced during sepsis and exogenous administration of orexin restored the hemodynamic depressions (2). However, few study have focused on the interaction between septic state and orexinergic activity in septic patients.

**OBJECTIVES.** The aim of this study was to investigate the effects of septic state or non-septic inflammatory state on plasma orexin A (OXA) concentrations in human.

**METHODS.** This study was approved by our university ethical committee and registered in the Japan Primary Registries Network (UMIN000018427). The written informed consents were taken from patients and adult volunteers. Septic patients (S group) and non-septic surgical patients (NS group) who were treated in our ICU and adult healthy volunteers (V group) were enrolled. Plasma concentrations of OXA for all groups and septic markers of procalcitonin(PCT), presepsin(P-SEP) and C-reactive protein (CRP) for S- and NS- groups were measured. The chronological changes in those parameters through the treatments were also investigated in S group on ICU-admission day 0, 1, 2, 3, 5, 7 and 14 and in NS group before the anesthesia induction, on ICU day 1, 2, 3. The correlations between plasma OXA concentrations and the 3 biomarkers were analyzed. Statistical analysis was performed with Kruskal Wallis test followed by Dunn's multiple comparison test. Data were expressed as median (interquartile range, IQR) and a p value less than 0.05 was considered as significant.

**RESULTS.** Twenty-six septic patients, 31 non-septic surgical patients and 6 volunteers were enrolled.Median (IQR) values of plasma OXA concentrations in S-, NS- and V- groups were 0.087(0.052-0.122)ng/ml, 0.09(0.061-0.146)ng/ml and 0.059(0.048-0.084) ng/ml, respectively (figure 1). There was no statistical significance among them. In S group P-SEP, PCT and CRP markedly increased on ICU day 0 and PCT and CRP gradually and significantly decreased through the treatments (figure 2A). In NS group CRP and PCT significantly increased, compared with preoperative values, but the median values of PCT and P-SEP did not become more than those cut-off values which suggested infections, 0.5 ng/ml and 500 pg/ml, respectively (figure 2B). Orexin A did not show significant changes through the treatments in S group and NS group (Figure 3A&B).

CONCLUSION. It was suggested that plasma concentrations of OXA in septic patients were not different from those of healthy volunteers and preoperative awake patients. Furthermore, non-septic inflammation did not affect plasma concentrations of OXA.

##### REFERENCE(S)

1) Palomba M. et al. Neurosci Lett. 2014; 570: 47-52.
2) Deutschman CS. et al. Crit Care Med. 2013; 41(11): e368-75.

##### GRANT ACKNOWLEDGMENT

This study was supported by Grant-in-Aid for challenging Exploratory Research of Japan Society for the Promotion of Science (No. 15K15561)

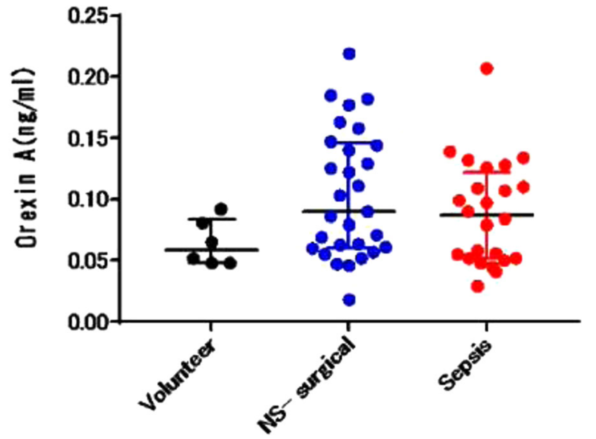

**Fig. 1 (abstract 0120).** Comaparison of plasma concentrations of orexin A among three groups. Data of non-septic surgical group and septic group were those before the operation and on ICU day 0, respectively.

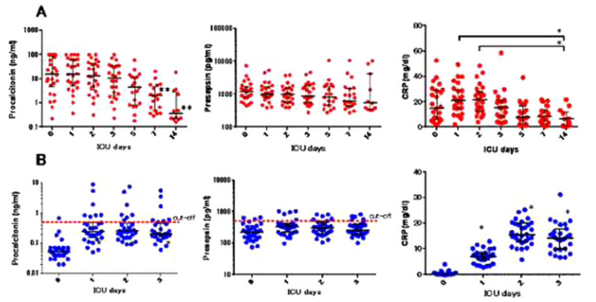

**Fig. 2 (abstract 0120).** Chronological changes in procalcitonin, presepsin and CRP in septic group and non-septic surgical group. A: Data of septic group, B: Data of non-septic surgical group.

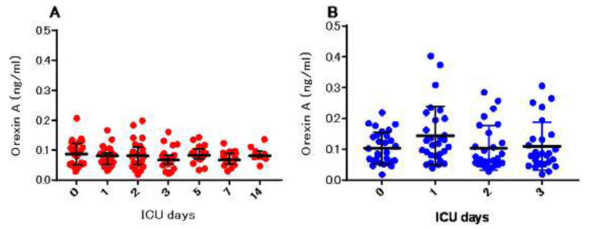

**Fig. 3 (abstract 0120).** Chronological changes in plasma concentrations of orexin A in Septic patients and in non-septic surgical patients. A: Septic group, B: Non-septic surgical group

### 0121

#### Presepsin as a predictor of sepsis outcome in comparison to procalcitonin and CRP

A. Mahmoud[1], H. Sherif[2], H. Saber[1], K. Taema[2]
[1]Beni Seuif University, Critical Care Medicine Department, Beni Seuif, Egypt, [2]Cairo University Hospitals, Critical Care Medicine Department, Cairo, Egypt
**Correspondence:** K. Taema
*Intensive Care Medicine Experimental* 2018, **6(Suppl 2):**0121

**Withdrawn**

### 0122

#### Study of the changes and roles of BMP-2 and BMP-4 in early phase of sepsis

Y. Wen, M. Zhang
The First Affiliated Hospital of Xiamen University, ICU, Xiamen, China
**Correspondence:** Y. Wen
*Intensive Care Medicine Experimental* 2018, **6(Suppl 2):**0122

**INTRODUCTION.** Endothelial dysfunction is confirmed to be involved in the development of sepsis[1]. In vivo and in vitro experiments have shown that bone morphogenetic protein (BMP)-2 and BMP-4 (BMP-2/4 subgroup is one of BMPs' subgroups) both exist in human microvascular endothelial cells (ECs) and indeed modulate EC migration and capillary tube formation[2, 3]. However, the roles of BMP-2/4 on the development of endothelial dysfunction in sepsis have not been determined.

**OBJECTIVES.** To study the changes and roles of serum BMP-2 and BMP-4 in the development of sepsis.

**METHODS.** Blood was collected for determining the levels of serum BMP-2 and BMP-4 from patients with strictly defined sepsis (group sepsis, n=28), patients without sepsis admitted in intensive care unit (ICU) (group non-sepsis, n=25), and healthy volunteers (group control, n=24). ELISA was used to determine the values of serum BMP-2 and BMP-4.

**RESULTS.** Compared with the controls, the serum BMP-2 and BMP-4 were significantly increased in early phase of sepsis (BMP-2: 568.3 ± 193.6 pg/ml *vs*. 23.94 ± 3.366 pg/ml, *P*=0.0122; and BMP-4: 81.67 ± 21.6 pg/ml *vs*. 24.53 ± 3.412 pg/ml, *P*=0.0191, Fig 1), and the serum BMP-2 was significantly higher in group sepsis than those of group non-sepsis (568.3 ± 193.6 pg/ml *vs*. 78.52 ± 33.56 pg/ml, *P*= 0.0221, Fig 1). Pearson correlation analysis showed that BMP-2 of group sepsis was significantly associated with serum lactate (r= 0.481, *P*= 0.013) within 24 hours after admitted in ICU, and counts of platelet on the third day after admitted in ICU (r= -0.459, *P*=0.018). The serum BMP-2 showed good diagnostic performance (area under ROC=0.95, *P*< 0.0001, cutoff value=50.30 pg/ml, sensitivity=89.29 % and specificity=95.83 %, Fig 2 and Fig 3) in separating sepsis from healthy volunteers, and also showed good diagnostic performance in separating sepsis from non-sepsis in ICU (area under ROC= 0.83, *P*< 0.0001, cutoff value=54.57 pg/ml, sensitivity=89.29 % and specificity= 72 %, Fig 2-3), but the level of serum BMP-4 did not show good diagnostic performance in separating sepsis from healthy volunteers (area under ROC= 0.68, sensitivity=57.14 % and specificity= 83.33 %, Fig 2-3) or sepsis from non-sepsis in ICU (area under ROC= 0.54, sensitivity=60.00 % and specificity= 53.57 %, Fig 2-3).

**CONCLUSIONS.** The level of serum BMP-2 was significantly increased and might be a potential diagnostic marker in early phase of sepsis.

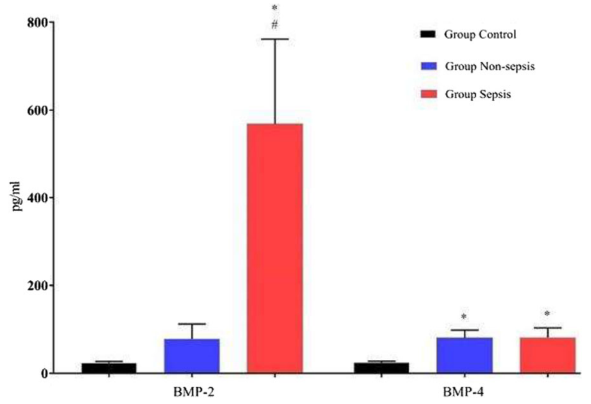

**Fig. 1 (abstract 0122).** Comparisons of BMP-2 and BMP-4 between group sepsis, group non-sepsis and group control

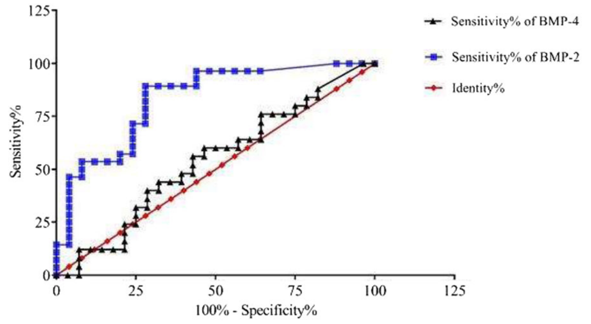

**Fig. 3 (abstract 0122).** ROC curves for BMP-2 and BMP-4 in separating sepsis from non-sepsis in ICU

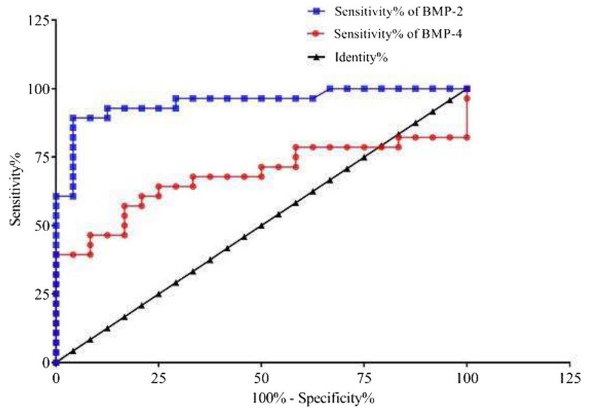

**Fig. 2 (abstract 0122).** ROC curves for BMP-2 and BMP-4 in separating sepsis from healthy volunteers

### 0123

#### Prognostic value of serum cholinesterase activity in septic shock

S. Bradai, M. Bahloul, O. Turki, M. Dlela, M. Bouaziz
Habib Bourguiba University Hospital, Intensive Care Departement, Sfax, Tunisia
**Correspondence:** S. Bradai
*Intensive Care Medicine Experimental* 2018, **6(Suppl 2):**0123

**INTRODUCTION.** Although biomarkers could help clinicians in the early diagnosis of septic shock and immediate onset of antibiotics, there are always questions to be answered about their usefulness in the prognosis of septic shock.

**OBJECTIVE.** Recently, it was demonstrated that Serum Cholinesterase activity (SChEA) can be useful in the diagnosis of septic shock related to bacterial infection. We aimed to analyse it kinetic in patients with septic shock in order to explore if it has a prognostic value.

**METHODS.** It is a prospective analytical study, conducted at the intensive care unit (ICU) of Habib Bourguiba university hospital, Sfax, Tunisia, between January 01, 2017, and December 31, 2017. All Patients who developed septic shock due to bacterial infection during the study period were enrolled. The SChEA was measured on day of admission, next on days 1, 3 and 5 of the shock. Then, we compare it kinetic on patients who survived and those who died.

**RESULTS.** There were 60 cases of septic shock, admitted to the ICU during the study. Sex ratio (male/female) was 3.3. The mean age was 47.7±19 years with extremes of 12 and 83 years. Mean SAPS II on ICU admission was 40.7±16.1 (median: 40). Mean SOFA on ICU admission was 16±4 (median: 7). Mean onset delay of shock was 6.3±9.2 days. The main infection was pneumonia in 36 patients (60%). The most identified pathogen was *Klebsiella pneumoniae* in 12 patients (20%).

The analysis of SChEA's kinetic showed that in survivors the value of this biomarker increase in the third day then in the 5$^{th}$ day, in comparison with it value on the day of the shock. However in dead patients, there was a decrease in SChEA in comparison with it value on the day when septic shock was settled (**Figure1**). A SChEA > 3270 UI /l on the 5$^{th}$ day predicted a good prognosis with sensitivity of 71%, specificity of 91% and an area under the curve =0.83 (**Figure2**).

**CONCLUSIONS.** SChEA can be used as a biomarker to predict the prognosis of patients with septic shock due to bacterial infection. Other studies are needed on this subject.

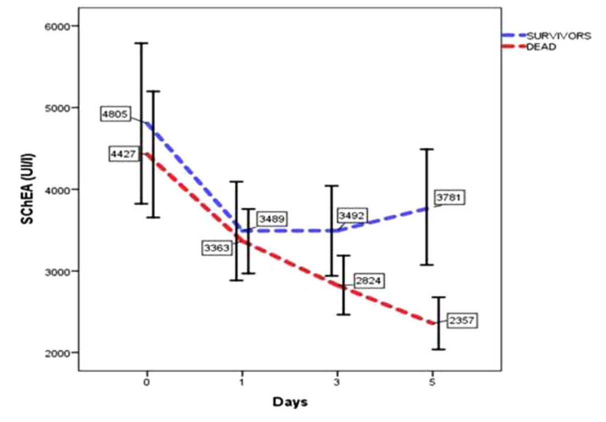

**Fig. 1 (abstract 0123).** SChEA's kinetic in dead and survivor patients on the day of admission then on 1st, 3rd and 5th days of septic shock

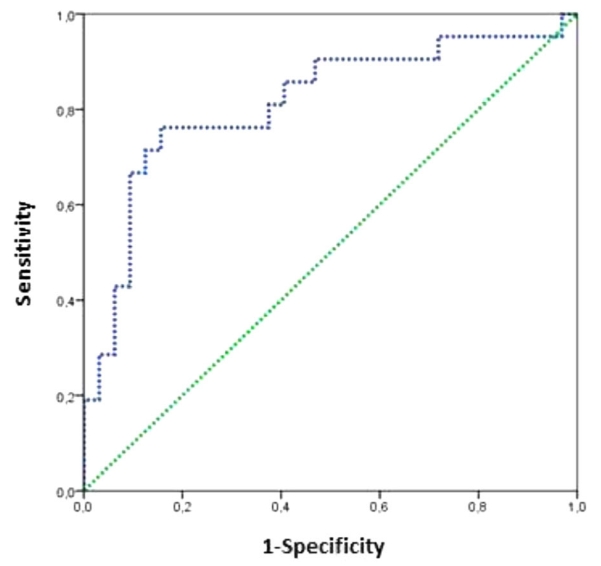

**Fig. 2 (abstract 0123).** Prognostic value of SChEA on the 5th day of septic shock (area under the curve =0.83)

### 0124

#### MR proadrenomedulin, procalcitonin, C-reactive protein as prognostic biomarkers of mortality in abdominal sepsis after surgery admitted to the ICU

B. Valenzuela-Méndez[1], F. Valenzuela-Sánchez[2], L. Fernandez Ruiz[2], J.F. Rodríguez Gutiérrez[2], R. Bohollo de Austria[2], A. Estella García[2], J. Rello[3]
[1]Hospital Universitario Germans Trias i Pujol, Badalona, Spain; [2]Hospital Universitario del SAS de Jerez, Jerez de la Frontera, Spain; [3]CIBERES. Vall d´Hebron Institut of Research, Barcelona, Spain
**Correspondence:** F. Valenzuela-Sánchez
*Intensive Care Medicine Experimental* 2018, **6(Suppl 2):**0124

**INTRODUCTION.** Secondary abdominal sepsis has an important incidence and high mortality rate. It is more dramatic when it is in context of an inadvertent perforation or suture dehiscence after a schedule surgery. Finding a biomarker that anticipate early severity in this patients is very important for their prognosis.

**OBJECTIVES.** The objective of this study is to evaluate the utility of MR-proadrenomedullin (MR-ProADM), Procalcitonin (PCT) and C-reactive protein (CRP) levels in the manage and prognosis of severe sepsis after a abdominal surgery admitted to the ICU.

**METHODS.** Prospective observational study. A total of 262 consecutive surgical patients with suspected abdominal sepsis were recruited in the ICU. Epidemiological, clinical, clinical, laboratory and MR-proADM, Procalcitonin (PCT), C-reactive protein (CRP) and lactate levels were collected at the time of admission, at 48 hours, on the 5th day and the day of discharge from the UCI. The values are expressed in Median (Confidence Intervals at 95%).

**RESULTS.** We included 262 patients in the study. Mortality was 24% (63/262). Upon admission to the ICU, the levels of biomarkers in the survivors were statistically higher in the septic patients who died in the ICU: CRP 31.5 (24-35.5) vs 26 (23.2-28.8) mg/dl (p = 0.0230); PCT 11 (6.6- 8.15) vs 5.9 ( 3.9-9.25) ng/ml (p=0.0229);MR-proADM 5.24 (4.11-7.05; ) vs 2.94 (2.58-3.95) nmol/l (p=0.0011). At 48 hours only MR-proADM levels were significantly higher in thenon survivors: 3.76 (2.5-5.1) vs 1.8 (1.42-2.29) (p = 0.0002). The incidence of intestinal complications as dehiscence and perforation after abdominal surgery was 34%.The area under the ROC curve (AUC) for the prediction of mortality at admission was CRP of 0.660, PCT of 0.705 and MR-proADM of 0.713 (Figure 1), and at 48 hours the AUC for the MR pro ADM was 7.24. The optimal cut-off point to predict the mortality of the MR-proADM levels at admission was 3.15 and at 48 hours of 1.9, both with a sensitivity of 80% and a specificity of 53%. In patients with surgical complications, the AUC-ROC at admission was 0.792 for the MR pro ADM and 0.578 for the PCT (Figure 2). In the multivariate analysis (Cox proportional hazards models) MR-proADM levels at admission, were statistically significant predictive factors for mortality in the ICU and at 90 days, in the total group and in the subgroup of surgical complications (Figure 3).

**CONCLUSIONS.** High levels of CPR, PCT and proADM on admission are related to poor prognosis in septic patients due to abdominal origin , but the levels of MR-proADM are the most effective to determine the unfavorable evolution and risk of mortality, especially followed a scheduled surgery.

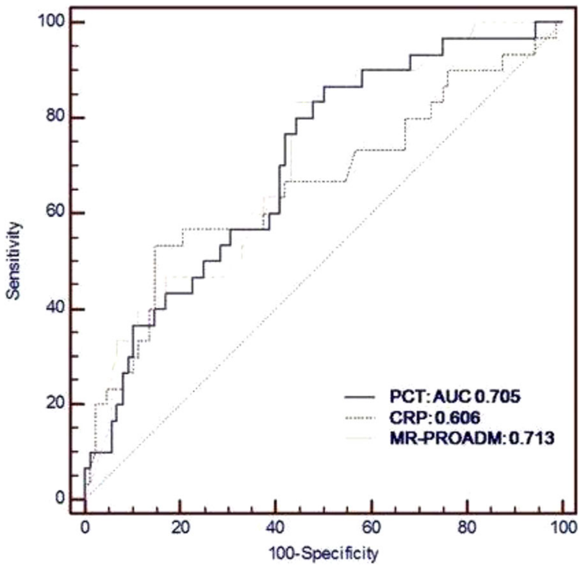

**Fig. 1 (abstract 0124).** Comparison of ROC curves of biomarkers to ICU admission

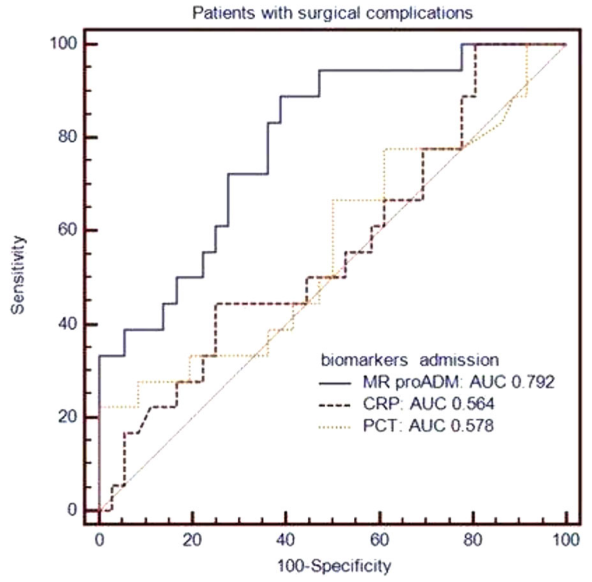

**Fig. 2 (abstract 0124).** Comparison of ROC curves of biomarkers to ICU admission in patients with surgical complications

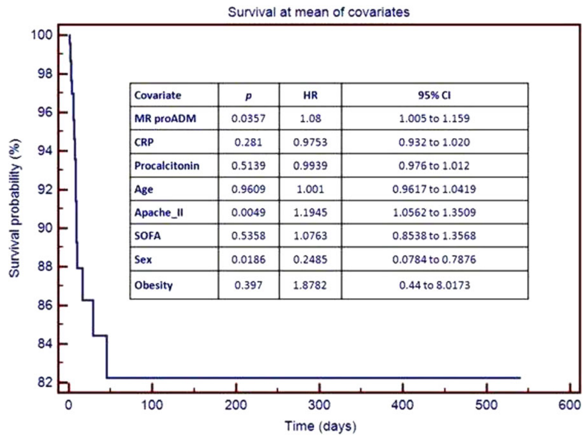

**Fig. 3 (abstract 0124).** multivariate analysis (Cox proportional hazards models)

### 0125

#### N-terminal pro-B-type natriuretic peptide is a good predictor of mortality in patients with sepsis admitted to the ICU

F. Valenzuela-Sánchez[1], L. Fernandez Ruiz[1], B. Valenzuela-Méndez[2], R. Bohollo de Austria[1], J.F. Rodríguez Gutiérrez[1], J. Rello[3]
[1]Hospital Universitario del SAS de Jerez, Jerez de la Frontera, Spain; [2]Hospital Universitario Germans Trias i Pujol, Badalona, Spain; [3]CIBERES. Vall d´Hebron Institut of Research, Barcelona, Spain
**Correspondence:** F. Valenzuela-Sánchez
*Intensive Care Medicine Experimental* 2018, **6(Suppl 2):**0125

**INTRODUCTION.** The identification of septic patients at high risk of death remains a challenge. Early and aggressive resuscitation is an important factor in reducing sepsis mortality. The role of prognosis of N-terminal pro-B-type natriuretic peptide (NT-proBNP) in septic patients remains controversial

**OBJECTIVES.** The objective was to determine NT-proBNP values of septic patients admitted to the ICU and relate them to mortality, presence of cardiac dysfunction and severity parameters.

**METHODS.** Prospective observational single-center study. 621 consecutive septic patients were admitted to the ICU. Epidemiological, clinical and laboratory data and NT-proBNP, MRproADM, Procalcitonin (PCT), C-reactive protein (CRP) and lactate levels were collected at the time of admission, at 48 hours, on the 5th day and the day of discharge from the ICU

**RESULTS.** During 5 years, 621 patients were admitted to the ICU with the diagnosis of severe sepsis. The mortality rate was 33%. The origin of the patients was mainly from the emergency room (50%), followed by the medical department in 22%. The biomarker levels are shown in Figure 1. The average levels of NT-proBN P on admission were 2700 (2025.8-3334.1) pg / ml (median and 95% CI) , at 48 hours these values decrease to 2268 (1776.7- 2817.7 pg / ml (ns) , coinciding with the highest incidence of heart failure and with the highest volume replacement;On the 5th day, a significant decrease in the levels was observed: 1535 (1169.3-2108.8) pg / ml (median and 95% CI) ($p < 0.05$), which were kept to discharge: 1188.5 (789.4-1705 , 5) pg / ml. The incidence at discharge of elevated levels of NT-proBNP (> 400 pg / ml) was 86% and they were discharged without signs of cardiac dysfunction or positive balance. These values are similar to the maximum showed during the acute phase of a control group studied in patients with acute ischemic heart disease, which was 1847 ± 3733 pg / ml. In the survival groups there was a significant increase in the levels in the non survivors compared to the survivors at admission 3980 pg / ml (3195-6150.3) vs 1818 pg / ml (1541-2691) (median and 95% CI) ($p < 0.0001$) . In the ROC curve as a predictor of mortality, the AUC was 0.650 on admission and 0.660 on the 2nd day.

In the multivariate analysis (Cox proportional hazards models) high levels of NT-proBNP were significantly associated with increased mortality, together with the Apache II score and lactate levels.

**CONCLUSIONS.** The determination of NT-proBNP is a useful initial prognostic biomarker in septic patients but is not related to the diagnosis of cardiac dysfunction in sepsis.

| Biomarker / Score | Median | 95% CI |
|---|---|---|
| Lactate mMol/l | 1.8 | 1.7 – 1.98 |
| NT-ProBNP pg / ml at admission | 2700 | 2025.8 – 3334.15 |
| NT-ProBNP pg / ml at 48 hours | 2268 | 1776.74 – 2817.75 |
| NT-ProBNP pg / ml at 5º day | 1535 | 1169.38 – 2108.82 |
| NT-ProBNP pg / ml at discharge | 1188.5 | 789.44 – 1705.52 |
| CRP (mg/dl) admission | 23.4 | 21.8 – 24.6 |
| PCT (µg/l) admission | 4.1 | 3.1- 5.9 |
| MR-proADM (nmol/l) admission | 2.67 | 2.53 – 3.01 |
| Apache II | 22 | 21 - 23 |
| SAPS II | 63 | 58 - 65 |
| SOFA | 9 | 9 - 10 |

**Fig. 1 (abstract 0125).** Levels of biomarkers and severity scores

### 0126

### Role of novel biomarker MR-proADM in diagnosis and prognosis of sepsis

R. Cicuéndez[1], L. Nogales[1], C. Andrés[2], E. Zarca[2], D. Calvo[2], D. Andaluz Ojeda[1]

[1]Hospital Clínico Universitario Valladolid, Adult Intensive Care Medicine Department, Valladolid, Spain; [2]Hospital Clínico Universitario Valladolid, Clinical Analysis Department, Valladolid, Spain

**Correspondence:** R. Cicuéndez

*Intensive Care Medicine Experimental* 2018, **6(Suppl 2):**0126

**INTRODUCTION.** Early diagnosis and severity assessment in sepsis is crucial, increasing the possibility of starting specific treatment and adequate management. Novel biomarker mid regional proadrenomedullin (MR-ProADM) has been used successfully to assess prognosis in patients with pneumonia. Here we evaluate its utility as sepsis diagnosis and prognosis biomarker.

**OBJECTIVES.** Evaluate the ability of MR-ProADM to predict mortality and facilitate diagnosis of sepsis and septic shock in a cohort of critically ill patients admitted to the ICU.

**METHODS.** 170 patients with sepsis or septic shock (SS) and 69 patients with SIRS without sepsis criteria admitted to ICU were evaluated. Clinical data, SOFA, APACHE II, leucogram data and levels of PCR, PCT, lactate, MR-proADM, were measured at admission. $\chi 2$ test for categorical and Mann Withney U- test for continuous variables were used. To evaluate accuracy of sepsis diagnosis and ICU mortality, the area under the ROC curve (AUROC) was employed. A p-value of < 0.05 was considered significant.

**RESULTS.** Data from 239 adult patients were collected. Patients were 63-year-old on average and 63.4% males. It showed a SOFA score of 7.4 and APACHE II of 20.2 at admission with 25.4% of ICU mortality. When all clinical variables and biomarkers were analyzed in global cohort for mortality ICU accuracy, only MR-proADM, SOFA, and APACHE showed statistical significance: AUROC (IC 95%, p)= 0.72 (0.62-0.81, 0.04); 073 (0.64-0.82, 0.04); 0.70 (0.60-0.8, 0.05) respectively. When ICU mortality was analyzed in sepsis subgroup alone, only SOFA, APACHE, and two biomarkers, MR-ProADM and lactate; showed significative accuracy: AUROC (IC 95%, p): 0.74 (0.60-0.88, 0.004); 0.75 (0,62-0.89, 0.002); 0.74 (0.59-0,88, 0.002); 0.69 (0.56-0.86, 0.02) respectively. When patients were classified as septic or SIRS, only PCT, PCR and MR-ProADM showed diagnosis accuracy to distinguish both diseases with AUROC (IC 95%, p): 0,83 (0.75-0.91, < 0.01); 0.79 (0.68-0.89, < 0.01); 0.75 (0.68-0.83, < 0.01) respectively. Neither the lactate nor other parameters of the leucogram like such as white blood cell or neutrophils count showed diagnostic utility.

**CONCLUSIONS.** Our results suggest that MR-ProADM is better prognostic marker than the rest of the biomarkers used routinely, not only in septic patients but also in overall critically ill patient. It also behaves as a good diagnosis marker of sepsis, although other molecules such as PCR or PCT have equal or better diagnostic accuracy in this field. Its usefulness in the future must be confirmed by new larger studies.

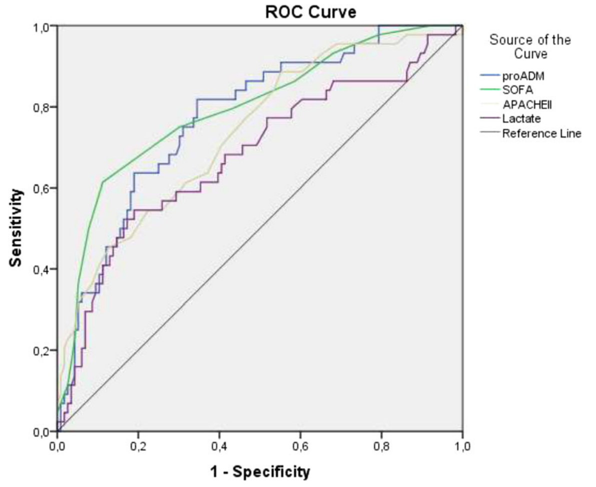

**Fig. 1 (abstract 0126).** AUROC for ICU mortality in overall population

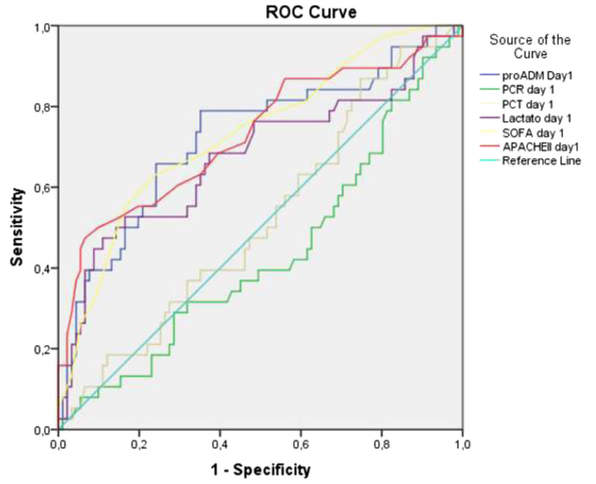

**Fig. 2 (abstract 0126).** AUROC for ICU mortality in septic subpopulation

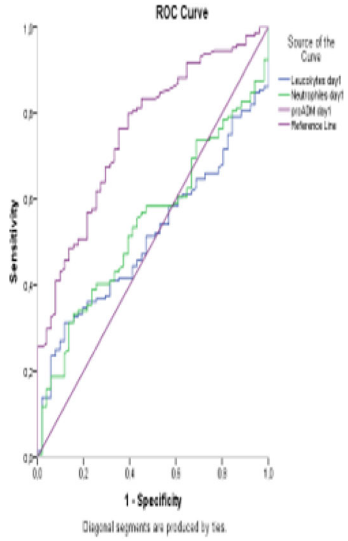

**Fig. 3 (abstract 0126).** AUROC for sepsis diagnosis and SIRS diagnosis in critical patients cohort

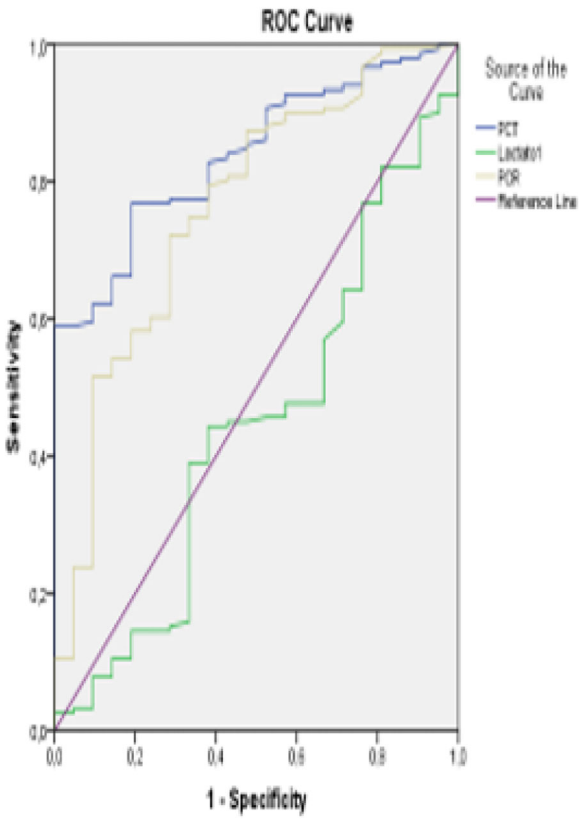

**Fig. 3 (abstract 0126)**. AUROC for sepsis diagnosis and SIRS diagnosis in critical patients cohort

### 0127

#### Apelin-13 and ELABELA-induced metabolic signature during experimental sepsis

E. Delile[1], D. Coquerel[1], L. Dumont[1], F. Chagnon[1], C. Alcindor[1], R. Dumaine[1], P. Sarret[1], E. Marsault[1], D. Salvail[2], M. Auger-Messier[1], O. Lesur[1]
[1]University of Sherbrooke, Depts of Medicine & Pharmacology-Physiology, Sherbrooke, Canada; [2]IPS Therapeutic, Sherbrooke, Canada
**Correspondence:** E. Delile
*Intensive Care Medicine Experimental* 2018, **6(Suppl 2):**0127

**INTRODUCTION.** ELABELA (ELA) and Apelin-13 (APL-13) bind to the apelin receptor (APJ), a GPCR highly expressed in the cardiovascular system, and are of the strongest inotropic and dominant apelinergic (APL) ligands in human heart which improve heart performance during sepsis. Apelins have been also clearly committed in improving insulin sensitivity and glucose handling, including in the heart.

**OBJECTIVES.** To establish the metabolic impact of APL infusion in an experimental model of rat septic shock *(i.e.* cecal ligation and puncture: CLP).

**METHODS.** Sepsis-related myocardial dysfunction was induced by CLP with APL infusion (APL-13 15μg/kg/hr, ELA 39μg/kg/hr) and saline resuscitation (2.5mL/kg/hr) started afterwards for 24hrs. Outcomes as well as indirect calorimetric assays (Promethion) were recorded and arterial lactates measured at the endpoint. The major cellular glucose transporter GLUT4 expression and dynamic membrane translocation under these APL infusion(s)/exposure(s) were measured by Western blotting (in whole hearts) and by confocal fluorescence microscopy in rat cardiomyocytes (CM) (neonatal ventricular: RNVC, trans-RA phenotypically differentiated H9C2) -with or without induced human APJ overexpression-, respectively.

**RESULTS.** Arterial lactates (mmol/L) rose in CLP + saline (NS) at 24 hrs (2.5 vs. 1.7 ± 0.2mmol/L, p< 0.05 vs. Sham), but less with APL infusions (CLP + APL 13 1.9 ±, CLP + ELA 2.2 ± 0.20.17mmol/L, NS vs CLP + saline). CLP induced a significant decrease of the respiratory quotient (RQ) in the dark phase, as defined by the ratio of C02 production (VC02)/02 consumption (V02) (Sham 0.90 ± 0.003, CLP + NS 0.72 ± 0.001, CLP + ELA 0.73 ± 0.001, p< 0.001 vs Sham), and of energy expenditure (EE) (kcal/hr) (Sham 3.40 ± 0.02, CLP + NS 1.83 ± 0.01, CLP + ELA 1.99 ± 0.01, p< 0.001 vs Sham). APL-13 infusion enhanced RQ (CLP + APL-13 0.72 ± 0.001, p< 0.01 vs CLP + NS), and EE energy (CLP + APL-13 2.50 ± 0.01, p< 0.001 vs CLP + NS). Substrates utilization calculated from the RQ after CLP induction revealed a metabolic switch from a major use of fatty acids to a higher use of carbohydrates with APL infusions (Sham 67% carbohydrates - 33% fat, CLP + NS 4.76% - 95.24%, CLP + ELA 8.4% - 91.6%, CLP + APL-13 15.6% - 84.4%). GLUT4 myocardial expression slightly down-regulated 24hrs after CLP was not affected by APL infusions. However, APL-13 but not ELA incubation triggered a 1.75-to-2 fold enhanced GLUT4 translocation to APJ-overexpressing RNVC membranes at 2 hrs (p< 0.05 vs control and APJ-overexpressing cells).

**CONCLUSIONS.** CLP induced major metabolic changes with increased arterial lactates and decreased RQ & EE. APL infusions modified substrate utilization. APL-13 over ELA administration/exposure promoted the use of carbohydrates and improved the availability of membrane GLUT4 location in CM.

##### REFERENCE(S)

29347994, 28777197, 27571457, 26986036.

##### GRANT ACKNOWLEDGMENT

Fellowship Program of Medicine Department, University of Sherbrooke

### 0128

#### NanoSepsis: nanoparticle based biomarker discovery in sepsis

A. Claxton[1,2], L. Papafilippou[1], M. Hadjidemetriou[1], P. Dark[2,3], K. Kostarelos[1]
[1]University of Manchester, Nanomedicine Lab, Manchester, United Kingdom; [2]Salford Royal NHS Foundation Trust, Critical Care Department, Salford, United Kingdom; [3]University of Manchester, Division of Immunity, Infection and Respiratory Medicine, Manchester, United Kingdom
**Correspondence:** A. Claxton
*Intensive Care Medicine Experimental* 2018, **6(Suppl 2):**0128

**INTRODUCTION.** Accurately diagnosing sepsis remains a challenge. The absence of specific diagnostic markers can result in delays in recognition and treatment, or over-diagnosis and over use of broad-spectrum antibiotics.
Abundant proteins such as albumin reduce the resolution of mass spectroscopy in high-throughput biomarker discovery.[1] Elimination of these proteins ahead of analysis is key to uncovering less abundant proteins that are more likely to represent potential biomarkers. Current state-of-the-art immunoaffinity depletion techniques eliminate both abundant and scarce proteins indiscriminately.[1]
A complex bioshell known as the 'protein corona' forms around nanoparticles when immersed in plasma.[2] This corona is comparatively enriched with less abundant proteins, and as such may be a source of novel candidate biomarkers.
We hypothesise that the composition of the protein corona that forms around AmBisome (a liposomal nanoparticle), which has been incubated in *ex vivo* plasma from patients with sepsis will differ to that which forms in plasma from patients with sterile inflammation or healthy control plasma, revealing potential biomarkers.
**OBJECTIVES.**

- Characterize the protein corona formed around AmBisome following ex vivo incubation with human plasma.
- Identify potential candidate biomarkers for future clinical validation.

**METHODS.** AmBisome was incubated in plasma samples from patients with sepsis, sterile inflammation or healthy control plasma for one hour. A two stage purification process separated the corona coated AmBisome and ensured no contamination from free unbound proteins. The protein corona was quantitatively and qualitatively characterized using dynamic light scattering, gel electrophoresis, and mass spectroscopy.
**RESULTS.** AmBisome that was incubated with plasma samples from sepsis patients had a significantly higher concentration of protein within the corona when compared to healthy control plasma (figure 1). Gel electrophoresis demonstrated the protein corona eliminating the effect of 'albumin masking' (figure 2), increasing the number of proteins identified on mass spectroscopy. Identified proteins were analysed to provide a panel of novel potential biomarkers.
**CONCLUSIONS.** New technologies are urgently needed to provide rapid diagnostic information in sepsis. This study provides evidence for the potential exploitation of the protein corona in aiding comprehensive analysis of the blood proteome, and facilitating the discovery of sepsis-specific proteins that may represent novel diagnostic biomarkers.

**REFERENCE(S)**

1. Tu C, Rudnick PA, Martinez MY, *et al*. Depletion of abundant plasma proteins and limitations of plasma proteomics. *J Proteome Res* 2010;9(10):4982-91
2. Hadjidemetriou M, Kostarelos K. Nanomedicine: Evolution of the nanoparticle corona. *Nat Nanotechnol* 2017;12(4):288-90

**GRANT ACKNOWLEDGMENT**
This work was funded by a grant from the Manchester Molecular Pathology Innovation Centre (MMPathIC).

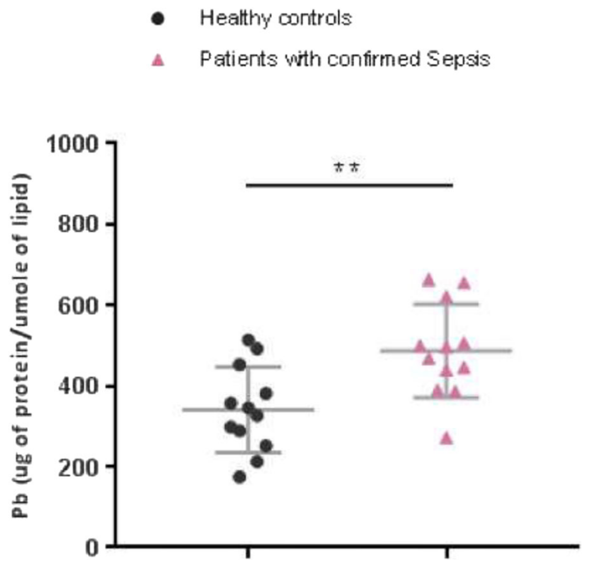

**Fig. 1 (abstract 0128).** More protein is adsorbed onto AmBisome incubated in plasma from sepsis patients (p=0.004)

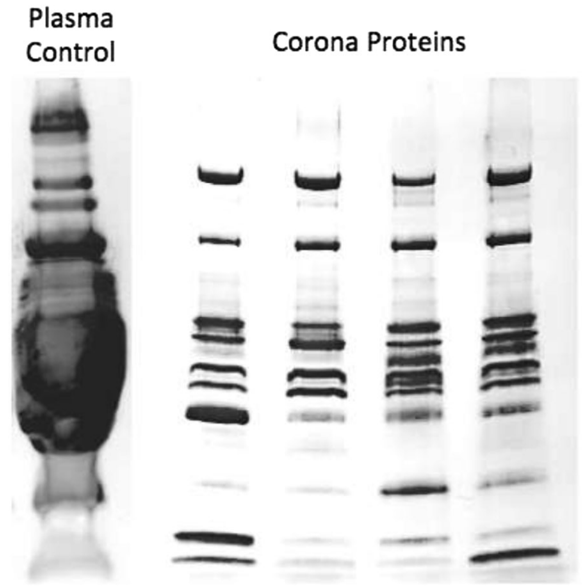

**Fig. 2 (abstract 0128).** SDS-PAGE Gel electrophoresis of protein corona compared to plasma

### 0129

#### Cholesterol oxidation in sepsis

A. Kleyman[1], M. Singer[2]
[1]UCL, London, United Kingdom; [2]UCL, London, United Nations Interim Administration Mission in Kosovo
**Correspondence:** A. Kleyman
*Intensive Care Medicine Experimental* 2018, **6(Suppl 2):**0129

**INTRODUCTION.** Hypocholesterolaemia is both a hallmark of sepsis and a prognosticator of poor outcome. However, mechanisms underlying this fall and any impact on tissue cholesterol levels are unknown. As cholesterol has well-recognized antioxidant properties, we hypothesized that increased ROS production in sepsis leads to cholesterol oxidation and decreases in plasma and tissue cholesterol levels.

**OBJECTIVES.** We used a well-characterized rat faecal peritonitis model and an H9C2 rat cardiomyocyte cell line to assess

(i) concentrations of cholesterol in plasma and different tissues taken from sham and septic animals at 24 hours post-sepsis, and
(ii) the effect of hydrogen peroxide on cholesterol levels in H9C2 cells.

**METHODS.** Awake, instrumented male Wistar rats (325±15g) received an i.p. injection of faecal slurry with i.v. fluid resuscitation commencing from 2h. Control animals were treated identically other than not receiving slurry. A heart rate cut-off of 460 bpm at 6h was used to classify animals into predicted survivors or non-survivors (1). [SM1] At 24h post-sepsis animals were killed and organs were immediately collected into liquid nitrogen. Cholesterol in plasma, organs and cells was measured either with Amplex Red assay or by mass spectrometry. In H9C2 cell studies, cells were either incubated with $H_2O_2$ or septic plasma (pooled from 6 rats after 24h sepsis) for 2h. Results are presented as mean ± SE, analysed using Student's t-test and considered statistically significant when $p < 0.05$.

**RESULTS.** A marked, severity-dependent decrease in cholesterol concentration was seen in plasma at 6h and 24h and membrane fraction of heart at 24h following induction of sepsis. Treatment of H9C2 cells with either septic plasma or hydrogen peroxide resulted in marked falls in cell cholesterol after 2h or 5 minutes correspondingly.

**CONCLUSIONS.** Cholesterol levels in both plasma and tissue fall rapidly after induction of sepsis in this rat peritonitis model, the magnitude of which is dependent on illness severity. This fall could be reproduced in a cardiomyocyte cell line incubated with either $H_2O_2$ or septic plasma. This suggests oxidation of cholesterol by intracellular ROS production. Further investigations of cholesterol oxidation and the influence of altered cholesterol membrane content on cellular processes in sepsis are ongoing.

##### REFERENCE(S)

(1) Rudiger A. et al. Clin Sci 2013; 124:391-401

**Table 1 (abstract 0129).** See text for description

| Cholesterol | Sham | Predicted survivor | Predicted non-survivor |
|---|---|---|---|
| Plasma 6h mg/ml | 2.34 ± 0.1 | 2.27 ± 0.05 | 1.95 ± 0.08b |
| Plasma 24h mg/ml | 2.5 ± 0.13 | 2.03±0.03 | 1.91 ± 0.03b |
| Heart membrane fraction µg/mg protein | 0.027± 0.002 | 0.023a± 0.0017 | 0.019 a,b± 0.0009 |

**Table 2 (abstract 0129).** See text for description

| | Control | H2O2 5 min | Septic serum 2h |
|---|---|---|---|
| H9C2 cells | 0.048±0.012 | 0.024±0.004a | |
| H9C2 cells | 0.0178± 0.001 | | 0.009004± 0.0015 |

### 0130

#### The effects of epinephrine and propranolol on the expression of tumor necrosis factor-α mRNA induced by lipopolysaccharide in RAW 264.7 cells

Y. Fujiwara, G.-G. Feng, K. Tanaka, R. Ishihara, N. Hatakeyama, A. Hashimoto, Y. Fujita
Aichi Medical University, Department of Anesthesiology, Nagakute, Japan
**Correspondence:** Y. Fujiwara
*Intensive Care Medicine Experimental* 2018, **6(Suppl 2):**0130

**INTRODUCTION.** Catecholamine has been reported to precipitate immune dysfunction and modulate inflammatory respose after infection (1). Recent studies suggested that β-blockers may improve outcome after sepsis by attenuating the deleterious effect of catecholamine on immune and inflammatory system (2).

**OBJECTIVES.** The objective of this study was to determine the effects of epinephrine on the expression of tumor necrosis factor-α (TNF-α) mRNA induced by lipopolysaccharide (LPS) in RAW 264.7 cells. We also tried to find out if propranolol attenuates the effect of epinephrine on TNF-α expression.

**METHODS.** RAW 264.7 cells were cultured in growth media Dulbecco's Modified Eagle Medium supplemented with 10% fetal bovine serum (FBS), 100 U/mL penicillin, and 100 mg/mL streptomycin in a 5% CO2 atmosphere at 37°C. Expression of TNF-a mRNA levels were determined by real-time polymerase chain reaction after each treatment as follows. First, cumulative dose of epinephrine (0.01, 0.1, 1, 10, 100 μM) were added to the medium without LPS. Then, after the application of LPS (1μg/ml) , the same doses of epinephrine were added. Finally, 100 and 200 μM of propranolol were added after LPS and 1 μM epinephrine were administered.

**RESULTS.** TNF-a mRNA induction after each treatment was shown in Fig 1-3.

**CONCLUSIONS.** Epinephrine decreased basal TNF-α mRNA expression of RAW 264.7 cells. In contrast to the results of previous studies, epinephrine attenuated the expression of TNF-α mRNA induced by LPS. Propranolol reversed the effect of epinephrine on the expression of TNF-α mRNA.

##### REFERENCE(S)

(1) Rough et al. Surgery 2009;145:235-42
(2) Morelli et al. JAMA 2013;310:1683-91.

##### GRANT ACKNOWLEDGMENT

This study was not supported by any grants.

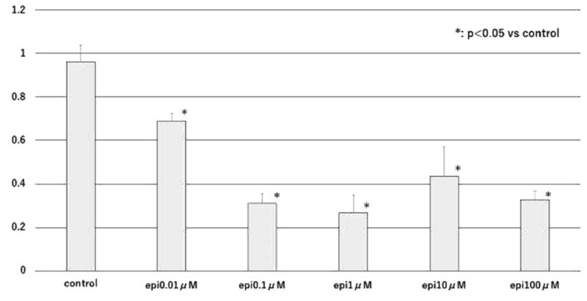

**Fig. 1 (abstract 0130)** . The effects of epinephrine on basal TNF-α expression

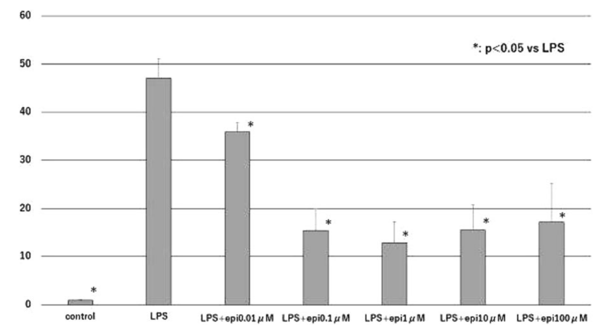

**Fig. 2 (abstract 0130)** . The effects of LPS and epinephrine on TNF-α expression

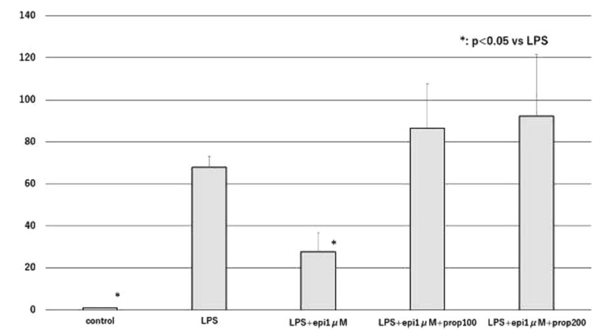

**Fig. 3 (abstract 0130)** . The effects of propranolol on TNF-α expression after LPS and epinephrine(1μM)

### 0131

#### Inflammatory markers and lung injury in an ovine model of 0.9% saline resuscitated endotoxemic shock

M. Passmore[1], L. Byrne[1,2], N. Obonyo[1,3], L. See Hoe[1], A.-C. Boon[1], S. Diab[4], K. Dunster[4,5], K. Bisht[1], J.-P. Tung[4,6], M. Fauzi[7], M. Narula[1], S. Pedersen[4], A. Esguerra-Lallen[4], J. Millar[1], G. Simonova[1,6], A. Sultana[6], C. Anstey[8], K. Shekar[1], K. Maitland[3,9], J. Fraser[1,4]
[1]University of Queensland, Chermside, Brisbane, Australia; [2]Australian National University, Canberra, Australia; [3]KEMRI-Wellcome Trust Research Programme, Kilifi, Kenya; [4]Prince Charles Hospital, Brisbane, Australia; [5]Queensland University of Technology, Brisbane, Australia; [6]Australian Red Cross Blood Service, Brisbane, Australia; [7]University Sains Malaysia Health Campus, Kelantan, Malaysia; [8]Nambour General Hospital, Nambour, Australia; [9]Imperial College London, London, United Kingdom
**Correspondence:** M. Passmore
*Intensive Care Medicine Experimental* 2018, **6(Suppl 2):**0131

**INTRODUCTION.** Sepsis is a multi-system syndrome that remains the leading cause of mortality and critical illness worldwide. Hemodynamic support is one of the cornerstones of the acute management of sepsis however, the ideal strategy remains under debate. Acute lung injury and ARDS are common complications of sepsis. It is unclear if the observed incidence of ARDS in sepsis is wholly attributable to the response to infection, or partially due to iatrogenic injury from fluid resuscitation.

**OBJECTIVES.** We used an ovine model of endotoxemic shock to determine if 0.9% saline resuscitation contributes to lung inflammation and injury in ARDS, and investigated the potential role of cytokines and matrix metalloproteinases in this process.

**METHODS.** Sheep were randomly divided into no endotoxemia (control) (n=5) or endotoxemia groups (n=16) with an escalating dose of lipopolysaccharide (LPS) up to 4 μg/kg/hr. Endotoxemia sheep received either no saline resuscitation (n=8) or a 0.9% saline bolus (40 mL/kg over 60 minutes) (n=8). Control animals underwent fluid resuscitation with a 0.9% bolus of saline. Lung tissue, bronchoalveolar lavage fluid and plasma were analysed by real-time PCR and ELISA to assess cytokines and matrix metalloproteinases.

**RESULTS.** Quantitative real-time PCR studies showed higher expression of IL-6 (p=0.03), IL-8 (p=0.01), IL-1β (p=0.02), MMP-1 (p=0.03) and TIMP-1 (p=0.003) in lung tissue of endotoxemia + saline animals compared to controls, while the level of TIMP-2 was decreased with endotoxemia (p< 0.05). BAL and tissue homogenate levels of IL-6 (p< 0.01), IL-8 (p< 0.05) and IL-1β (p< 0.05) were also elevated.

**CONCLUSIONS.** Endotoxemia results in inflammation and lung injury. Saline resuscitation augments this dysfunctional physiology with increased intra-pulmonary levels of inflammatory cytokines, and up-regulation of matrix metalloproteinases. These results suggest fluid resuscitation may be an independent contributor to lung injury in sepsis and a potentially modifiable factor in the prevention of ARDS in septic patients.

**GRANT ACKNOWLEDGMENT**
Queensland Emergency Medicine Research Foundation (EMPJ-358R25-2016)

### 0132

#### Comparison between procalcitonin and C-reactive protein as sepsis diagnostic marker

S. Zampieri, K. Donadello, V. Schweiger, L. Gottin, E. Polati
University of Verona, Department of Surgery, Dentistry, Pediatrics and Gynaecology, Verona, Italy
**Correspondence:** S. Zampieri
*Intensive Care Medicine Experimental* 2018, **6(Suppl 2):**0132

**INTRODUCTION.** The ideal sepsis biomarker still has to be born. The need of a useful and reliable clinical tool, nourish clinical and experimental research.

**OBJECTIVE.** Retrospective analysis of prospectively collected data to compare the clinical informative value of Procalcitonin (PCT) and C-Reactive protein (CPR) plasma concentrations in the detection of infection and sepsis and in the assessment of severity of sepsis in a medico-sukrgical intensive care unit (ICU).

**METHODS.** We collected all clinical data, drug administration, scores and PCT values of 927 consecutive adult patients who were admitted to the intensive care unit for an expected stay >48hours. PCT and PCR plasma concentrations were measured daily during the intensive care unit stay. Each patient was examined daily for signs and symptoms of infection and was classified daily in one of the following six categories: negative, localized infection, systemic inflammatory response syndrome (SIRS), sepsis, severe sepsis or septic shock. The severity of sepsis-related organ failure was assessed by the sepsis-related organ failure assessment (SOFA) score.

**RESULTS.** A total of 8538 patient days were classified into the six categories. The median plasma PCT concentrations in each class was respectively: negative 0.62 ng/ml, localized infection: 0.69 ng/ml, SIRS: 2.29 ng/ml, sepsis: 4.34 ng/ml, severe sepsis 17.30 ng/ml and septic shock 26.80 ng/ml. The median plasma PCR concentrations in each class were negative 49.6 mg/dl, localized infection 68.6 mg/dl, SIRS 86.3 mg/dl, sepsis 125.6 mg/dl, severe sepsis 154.4 mg/dl and septic shock 183.5 mg/dl. The linear correlation between PCT plasma concentrations and the six categories was much stronger than in the case of PCR (Spearman´s rho, 0.668 vs. 0.489; p< 0.0001). The area under the receiver operating characteristic curve was 0.846 for PCT (95% CI, 0.838-0.854), compared with 0.751 for PCR (95% CI 0.742-0.760) (p < 0.0001).

**CONCLUSIONS.** PCT is a better marker of sepsis than PCR. The course of PCT shows a closer correlation than PCR with the severity of infection and organ dysfunction.

#### 0133

**Association between plasma cell-free hemoglobin and mortality in patients with sepsis**

D.Y. Kim, H.L. Chang, Y.H. Jo, J.H. Lee, J.E. Hwang
Seoul National University Bundang Hospital, Department of Emergency Medicine, Seongnam-si, Gyeonggi-do, Korea, Republic of
**Correspondence:** D.Y. Kim
*Intensive Care Medicine Experimental* 2018, **6(Suppl 2):**0133

**INTRODUCTION.** Plasma cell-free hemoglobin (PHb) has been reported to be associated with poor outcomes in acute kidney injury, pulmonary hypertension, acute respiratory distress syndrome and myocardial infarction.[1-3] In sepsis, alterations in red blood cell membrane occur and PHb could be elevated in sepsis.[4-5]

**OBJECTIVES.** This study was performed to investigate the association between PHb and mortality in sepsis and septic shock.

**METHODS.** We performed a retrospective analysis of prospective collected data of patients with sepsis and septic shock. Patients were divided into 4 groups according to the PHb concentrations using an adjusted cubic spline: PHb ≤ 20 mg/dL, 20 < PHb ≤ 40 mg/dL, 40 < PHb ≤ 60 mg/dL and PHb > 60 mg/dL. The primary outcome was the 28-day mortality and a multivariate Cox proportional hazard regression method was used.

**RESULTS.** A total of 372 patients were included and the overall 28-day mortality rate was 16.7%. The median PHb concentration in the overall patients was 24.8 mg/dL (reference range, 0 - 5). The mortality rate did not increase in proportion to the PHb concentration and was the lowest in the 20 < PHb ≤ 40 mg/dL group (20.3%, 11.0%, 16.3% and 26.7% in the PHb ≤ 20 mg/dL, 20 < PHb ≤ 40 mg/dL, 40 < PHb ≤ 60 mg/dL and PHb > 60 mg/dL groups, respectively). In the Cox proportional hazard regression analysis, the PHb ≤ 20 mg/dL group was independently associated with 28-day mortality compared with the 20 < PHb ≤ 40 mg/dL group (hazard ratio, 2.19; 95% confidence interval, 1.18 - 4.07). The PHb > 60 mg/dL group was associated with 28-day mortality, but it was not statistically significant (hazard ratio, 2.17; 95% confidence interval, 0.85 - 5.56).

**CONCLUSIONS.** The low concentration as well as high concentration of PHb was associated with 28-day mortality in patients with sepsis and septic shock.

**REFERENCE(S)**

1. Brittain EL, Janz DR, Austin ED, Bastarache JA, Wheeler LA, Ware LB, Hemnes AR. Elevation of plasma cell-free hemoglobin in pulmonary arterial hypertension. Chest. 2014;146(6):1478-1485.
2. Quaye IK. Extracellular hemoglobin: the case of a friend turned foe. Front Physiol. 2015;6:96
3. Janz DR, Ware LB. The role of red blood cells and cell-free hemoglobin in the pathogenesis of ARDS. J Intensive Care. 2015;3:20.
4. Janz DR, Bastarache JA, Peterson JF, Sills G, Wickersham N, May AK, Roberts LJ 2nd, Ware LB. Association between cell-free hemoglobin, acetaminophen, and mortality in patients with sepsis: an observational study. Crit Care Med. 2013;41(3):784-90.
5. Adamzik M, Hamburger T, Petrat F, Peters J, de Groot H, Hartmann M. Free hemoglobin concentration in severe sepsis: methods of measurement and prediction of outcome. Crit Care. 2012;16(4):R125

**GRANT ACKNOWLEDGMENT**
none

#### 0134

**Time-dependent changes of soluble CD14 subtype (presepsin) after uncomplicated major surgery: comparison with procalcitonin, CRP, and leukocytes**

M. Protuš[1], A. Jabor[2], J. Franeková[2], E. Kieslichová[1], P. Sečník[2], Z. Kubíček[2], A. Březina[3]
[1]Institute for Clinical and Experimental Medicine, Anaesthesiology, Resuscitation and Intensive Care, Prague, Czech Republic; [2]Institute for Clinical and Experimental Medicine, Department of Laboratory Methods, Prague, Czech Republic; [3]Institute for Clinical and Experimental Medicine, Department of Anesthesiology and Resuscitation, Prague, Czech Republic
**Correspondence:** M. Protuš
*Intensive Care Medicine Experimental* 2018, **6(Suppl 2):**0134

**BACKGROUND.** Concentrations of respective biomarkers are time-dependent, even under uncomplicated conditions; therefore, a suitable method should be used for the evaluation of serial measurements. Nonparametric quantile regression can be used for such purposes instead of simple point reference values.

The aim of our work was to monitor the response of the studied biomarkers (presepsin, procalcitonin, CRP, and leukocytes) to postoperative SIRS after major cardiac and abdominal surgery without systemic infectious complications and to define acceptable time-dependent reference limits for the respective biomarkers during the postoperative course (up to 7 days after surgery).

**METHODS.** A group of 24 patients (19 men and 5 women) was retrospectively evaluated. The median of age of the patients was 66 years, and the interquartile range was 63.0-73.0 years (minimum 52 years, maximum

The median total length of surgery was 225 minutes (interquartile range of 180-263 minutes).

43 patients underwent cardiopulmonary bypass surgery (median CPB of 83 minutes, interquartile range of 70.0-100.0 minutes). Multiple comorbidities were present in all patients. All (large or small intestine surgery, resection of the liver or stomach, pancreatectomy, or multi-organ surgery) were performed at the Institute for Clinical and Experimental Medicine, Prague, Czech Republic (IKEM).

All patients survived 30 days, and there were no cases of sepsis, septic shock, peritonitis or other infectious complication during this period. Only one patient was treated with immunosuppressants (cyclosporine and prednisone). None of the patients needed renal replacement therapy in the postoperative course. There were three cases of transient postoperative left ventricle insufficiency and one of decompensation of diabetes mellitus. Blood samples were taken immediately before the surgery, at the 3 hour, and on the 1 , 3 and 7 days following the surgery.

**RESULTS.** Early peaks were found in presepsin and leukocyte concentrations (median concentrations of 819 ng/L and 11.4 x 109/L with 95th percentiles of 2017 and 17.8, respectively, both within the first 24 hours postoperatively). Procalcitonin peaked on the 1st day after surgery (median 0.54 μg/L, 95th percentile 4.76), while CRP peaked on the 3rd day after surgery (median of 133.3 mg/L, 95th percentile of 287.5). The peak values were 1.5, 9, 66, and 1.9 times higher than the basal values for presepsin, procalcitonin, CRP, and leukocytes, respectively.

**CONCLUSIONS.** Presepsin is less influenced by postoperative SIRS than procalcitonin or CRP and may therefore act as a valuable biomarker for the detection of infectious complications in patients after major surgery.

**GRANT ACKNOWLEDGMENT**
Supported by Ministry of Health of the Czech Republic (AZV MZ 15-27579A) and VZ IKEM G9045.

### Weaning from mechanical ventilation

#### 0135

**Epidemiology of weaning outcomes according to the WIND classification**

R.-E. Ko[1], B.-H. Jeong[2], K. Jeon[2]
[1]Samsung Medical Center, Sungkyunkwan University School of Medicine, Department of Critical Care Medicine, Seoul, Korea, Republic of; [2]Samsung Medical Center, Sungkyunkwan University School of Medicine, Division of Pulmonology and Critical Care Medicine, Department of Medicine, Seoul, Korea, Republic of
**Correspondence:** R.-E. Ko
*Intensive Care Medicine Experimental* 2018, **6(Suppl 2):**0135

**INTRODUCTION.** Weaning from mechanical ventilation (MV) is an important issue for all patients who undergo MV. A previous simple classification known as the International Consensus Conference classification (ICC) has several limitations, which include that it ignores weaning failure, gives unclear approach with tracheostomy patients, and requires the performance of spontaneous breathing trials (SBT). In 2017, the WIND Study Group and the REVA Network proposed a new definition that groups patients according to the number of days between the first separation attempt (SA) from MV and successful weaning.

**OBJECTIVES.** This study aimed to evaluate whether the WIND classification (WIND) is able to predict patient outcomes more accurately than the ICC in clinical practice.

**METHODS.** We retrospectively reviewed 1,601 patients who received MV in medical intensive care units (ICU) (Jul 2010 to Sep 2013). Patients who transferred-in from another hospital more than 48 hours after intubation (n=42) or who succeeded on non-invasive ventilation (n=71) were excluded. The applicability of the ICC and the WIND was assessed in 1488 patients. After using both the ICC and the WIND to classify patients, we compared the weaning outcomes of each group to determine which classification better reflected the weaning outcome.

**RESULTS.** First, tracheostomized patients (n=33) and patients who did not undergo SBT (n=680) were not classifiable under the ICC. In contrast, all patients were able to be included in the WIND. Second, according to the ICC, 744 patients were grouped as simple weaning (n=505), difficult weaning (n=145), and prolonged weaning (n=94). Patients with a repeated SBT or increase in the number of days, the patients were more likely to have higher ICU and hospital mortality, and they also have an increased length of stay (LOS) in both the ICU and hospital (P< 0.001). According to the WIND, 1488 patients were grouped as Group 1 (short, n=593), Group 2 (difficult, n=187), Group 3 (prolonged, n=204), and no weaning (n=504). As the days after the first SA from MV increased, the patients were more likely to have higher ICU and hospital mortality, as well as, increased LOS in both the ICU and hospital (P< 0.001). Group 1 and Group 2 by the WIND showed a clearer difference in ICU mortality (2.7% vs 16.0%, P < 0.001) and hospital mortality (19.9% vs 34.2%, P < 0.001) compared to simple weaning group and difficult weaning group in the ICC (5.1% vs 5.5%, P = 0.860 in ICU mortality; 22.4% vs 25.5%, P = 0.429 in-hospital mortality).

**CONCLUSIONS.** Our study suggests that the WIND could be a good classification tool for predicting patient weaning outcomes because it is applicable to all weaning situations and identifies the clear difference in outcomes in patients who tolerate early weaning.

**REFERENCE(S)**

Beduneau G, Pham T, Schortgen F, et al. Epidemiology of Weaning Outcome according to a New Definition. The WIND Study. *Am J Respir Crit Care Med.* 2017;195(6):772-783.

**GRANT ACKNOWLEDGMENT**

None

### 0136

#### The use of the WIND classification for weaning from mechanical ventilation applied to Brazilian critically ill patients

A.F. Lago[1,2], A.A. Silva[1], V.B. Tanaka[1], V.C. Siansi[1], I.S. Reis[1], A.C. Gastaldi[2], A. Basile-Filho[1]

[1]Ribeirão Preto Medical School, University of São Paulo, Division of Intensive Care Medicine, Department of Surgery and Anatomy, Ribeirão Preto, Brazil; [2]Ribeirão Preto Medical School, University of São Paulo, Department of Physiotherapy, Ribeirão Preto, Brazil

**Correspondence:** A.F. Lago

*Intensive Care Medicine Experimental* 2018, **6(Suppl 2):**0136

**INTRODUCTION.** Weaning from mechanical ventilation (MV) is an important clinical issue in the care of critically ill patients. The knowledge of this period is fundamental to understanding the causes and consequences of the prolonged weaning. In April 2005 an International Consensus Conference (ICC) defined a classification of the weaning. However, in 2016 a new definition for the weaning (WIND) was suggested.

**OBJECTIVES.** To compare the weaning from MV in a Brazilian intensive care unit according to the ICC and the WIND classification.

**METHODS.** This study was a retrospective cohort study in an intensive care unit (ICU) in a tertiary University Hospital. Patient data such as population characteristics, MV duration, weaning classification, SAPS III and death probability were obtained from a medical records database of all patients, who were admitted at this ICU between January, 2016 and July, 2017. The analyses were made with statistics software R (Foundation for Statistical Computing, Austria) and SAS Statistical Software (version 9.3; SAS Institute, USA). ANOVA was used for multiples comparisons of quantitative variables. Equal letters on the top right side of the numbers express evidence of no differences between the groups and different letters show evidence of differences between them. P values lower than 0.05 express evidence that at least one group differs from the others. The logistic regression analysis was used to identify variables that are associated with extubation failure.

**RESULTS.** A total of 327 patients under MV were enrolled. The patients characteristics are shown in table 1. The number of patients belonging to ICC and WIND classification is shown in table 2. By applying the WIND classification it was possible to classify 100% of the patients and to identify that the probability of death and the number of days of MV increased significantly from patients of group 1 to patients of groups 3a, 3b and no weaning, p< 0.0001 (table 3). The logistic regression analysis identified the days of MV as being associated with extubation failure (OR= 1.23; 95% CI= 1.11-1.38).

**CONCLUSIONS.** The present study demonstrated that the WIND classification allows categorising all patients into 4 groups, different from ICC classification, in which only 17% of the whole cohort could be categorised. The probability of death and the number of days of MV increased according to the difficulty of the weaning. Finally the number of days of MV was associated with extubation failure.

**GRANT ACKNOWLEDGEMENT**

FAEPA- Clinics Hospital of Ribeirão Preto, SP, Brazil.

**Table 1 (abstract 0136).** Patient characteristics

| Characteristics | n(%) or mean ±SD |
|---|---|
| Age(years) | 56±16 |
| SAPS III | 74±18 |
| Probability of death | 63±27 |
| Days of MV | 10.6±9.7 |
| Lenght of stay in the ICU, days | 10.8±9.3 |
| Female gender | 165 (51%) |
| Admission type, Medical | 196(60%) |
| Status at ICU discharge, Dead | 173(53%) |
| Extubation | 75 (22%) |

**Table 2 (abstract 0136).** Number of patients belonging to ICC and WIND classification

| Classification n=327 | n(%) |
|---|---|
| ICC Group 1 | 33 |
| ICC Group 2 | 16 |
| ICC Group 3 | 9 |
| No classification | 269 |
| WIND Group 1 | 36 |
| WIND Group 2 | 18 |
| WIND Group 3a | 26 |
| WIND Group 3b | 59 |
| WIND Group no weaning | 188 |

**Table 3 (abstract 0136).** Multiple comparisons between groups. Equal letters on the top right side of the numbers express no differences between groups

| WIND classification | Group 1 | Group 2 | Group 3a | Group 3b | Group no weaning | p-Value |
|---|---|---|---|---|---|---|
| SAPS III | 60.4 ±18.7 $^{C}$ | 67.2 ±19.6$^{B,C}$ | 66.3 ±19.3$^{B,C}$ | 75.0±16.8$^{B,C}$ | 78.6±17.5$^{A}$ | 0.0001 |
| Probability of death (%) | 41.9 ±29.3$^{C}$ | 53.7 ±29.4$^{B,C}$ | 55.85 ±28.1$^{B}$ | 63.1±25.5$^{A,B}$ | 69.8±24.6$^{A}$ | 0.0001 |
| Days of MV | 4.8 ±4.2$^{D}$ | 6.8 ±4.2$^{C,D}$ | 13.2 ±7.7$^{B}$ | 17.5±10.6$^{A}$ | 9.4±9.5$^{B,C}$ | 0.0001 |
| Lenght of stay in the ICU, days | 7.3 ±4.5$^{C}$ | 10.8 ±5.5$^{B,C}$ | 14.4 ±8.6$^{A,B}$ | 17.7±10.8$^{A}$ | 8.7±8.6$^{C}$ | 0.0001 |
| ICC Classification | Group 1 | Group 2 | Group 3 | No classification | | p-Value |
| SAPS III | 59.6 ±18.9$^{B,C}$ | 66.8 ±15.0$^{A,B}$ | 53.4 ±17.5$^{C}$ | 77.3±17.4$^{A}$ | | 0.0001 |
| Probability of death (%) | 40.9 ±29.2$^{B}$ | 54.2 ±27.6$^{A,B}$ | 38.4 ±25.4$^{B}$ | 67.8±25.2$^{A}$ | | 0.0001 |
| Days of MV | 4.2 ±3.2$^{B}$ | 6.9 ±4.2$^{A,B}$ | 11.8 ±4.2$^{A}$ | 11.5±10.3$^{A}$ | | 0.0001 |
| Lenght of stay in the ICU, days | 6.8 ±3.9$^{B}$ | 10.8 ±5.2$^{A,B}$ | 14.5 ±5.0$^{A}$ | 11.1±9.9$^{B}$ | | 0.0477 |

### 0137

#### Age and mental status influence on extubation success

C. Aluwihare, K. Gerrard, A. Jethudasan, V. Metaxa
King's College Hospital, London, Critical Care Medicine, London, United Kingdom
**Correspondence:** C. Aluwihare
*Intensive Care Medicine Experimental* 2018, **6(Suppl 2):**0137

**INTRODUCTION.** Weaning is the process by which a patient is liberated from mechanical ventilation and extubation is the process of liberation from endotracheal tube[1]. Extubation failure is defined as the need for reintubation within a specific time period : either within 24 - 72 hours or up to 7 days[1,2]. Failed extubation, is common in intensive care units (ICU). This can lead to higher morbidity and mortality, higher cost and increased length of hospital stay. There is limited literature available on weaning/extubation predictors and outcomes. Main objective of the study was to identify the rate, causes and predictors of extubation failure and to identify the rate of reintubation across 3 Intensive care units(ICU) at King's College Hospital, London.

**OBJECTIVES.**

1. To determine the reintubation rate.
2. To determine the causes and predictors of reintubation.

**METHODS.** This was a prospective study. A standard questionnaire was filled by the auditors by direct observation, observation of daily chart or by direct questioning from the person who decided to extubate. The study was carried out over 1 month across 3 ICU s. All elective extubations during this period was analysed. Patients over 18 years of age, intubated and mechanically ventilated for more than 24 hours and extubated electively were included in the study. Patients less than 18 years of age and all self extubations were excluded. The results were statistically analysed.

**RESULTS.** A total of 49 (male 77.6%) elective extubations were analysed. 49% were more than 61 years of age. Patient with cardiovascular diseases were the commonest indication for intubation (24.5%). The mean heart rate, systolic and diastolic blood pressure, $PaO_2$ and $FiO_2$ were 86.2 ± 20.64p/min, 138.9 ± 23.77mmHg, 87.72 ± 26.59mmHg, 11.28 ± 1.90mmHg, 27.27 ± 7.26% respectively prior to extubation. 36 (73.4%) patients were extubated successfully and 13(26.5%) were re-intubated. Acute respiratory failure/ respiratory distress was the commonest indication for re-intubation (33.3%). 10 (76.9 %) of reintubations took place within 48 hours of extubation and the rest within 72 hours.

Based on logistic regression analysis (adjusted) patients who were agitated had higher risk of re-intubation than non (EXP (B) =4.195, p=0.0.015). Higher the age there was 16% more risk of re-intubation (EXP (B) =1.159, p=0.042).

**CONCLUSIONS.** Extubation success was significantly influenced by the age of the patient and the mental status.

**REFERENCE(S)**

1. Arnaud W. T, Jean-C. M. Richard, Laurent B. The Decision to Extubate in the Intensive Care Unit. American journal of respiratory and critical care medicine 2013;108: 1294-1300.
2. James S K, Praveen K R, Abid I. What is the optimal rate of failed extubation? Critical Care 2012: 16:2-5.

### 0138

#### Thoracic fluid content: a novel parameter for predicting failure of weaning from mechanical ventilation

M. Raafat, A. Hasanin, S. Fathy, A. Fouad, A. El-adawy, M. Beshara, H. Kamal
Cairo University, Anesthesia and Critical Care Medicine, Cairo, Egypt
**Correspondence:** M. Raafat
*Intensive Care Medicine Experimental* 2018, **6(Suppl 2):**0138

**INTRODUCTION.** Weaning of mechanically ventilated patients is a daily decision in intensive care practice. Failure of weaning is associated with poor outcome; thus, several tools are used to predict successful liberation from the ventilator.

**OBJECTIVES.** The aim of this work is to evaluate total fluid content (TFC) as a predictor for failure of weaning from mechanical ventilation.

**METHODS.** A prospective observational study was conducted in surgical intensive care unit (ICU), Cairo University hospital including 37 mechanically ventilated patients scheduled for weaning. During spontaneous breathing trial (SBT), Electrical cardiometry (ICON$^{R}$ monitor) device was used to measure TFC. Other hemodynamic variables (arterial blood pressure, ventral venous pressure, and heart rate) were also recorded. Successful weaning was defined as passing a 48-hour period without the need for re-connection to the ventilator. Predictive properties for TFC for failed weaning were estimated using area under receiver operating characteristic (AUROC) curve. Subgroup analysis for patients with impaired cardiac contractility was also performed.

**RESULTS.** The incidence of weaning failure was 38%. TFC showed good predictive ability for weaning failure {AUROC: 0.767, sensitivity: 50, specificity: 96%, cutoff value >63 $Kohm^{-1}$}. In the subgroup of cardiac patients, TFC showed excellent predictive ability for weaning failure {AUROC: 0.917, sensitivity: 83%, specificity: 89%, cutoff value >48 $Kohm^{-1}$}.

**CONCLUSIONS.** TFC measured by electrical cardiometry during SBT is a useful predictor for failure of weaning from mechanical ventilation. TFC showed excellent predictive properties in the subgroup of patients with impaired cardiac contractility. The best cutoff value for predicting weaning failure is >63 $Kohm^{-1}$ in general patients and >48 $Kohm^{-1}$ in cardiac patients.

### 0139

#### A pilot study to develop the feasibility of patient-controlled ventilatory weaning using neutrally adjusted ventilatory assist

D. Hadfield, J. Smith, C. Donegan, H. Noble, L. Grieg, E. Corcoran, E. Clarey, C. Bell, C. Harris, P. Hopkins
King's College Hospital, London, King's Critical Care, London, United Kingdom
**Correspondence:** D. Hadfield
*Intensive Care Medicine Experimental* 2018, **6(Suppl 2):**0139

**INTRODUCTION.** Neurally-adjusted ventilatory assist (NAVA) utilises diaphragmatic monitoring and a proportional, neurally triggered ventilatory support (1). Small efficacy studies have demonstrated the benefit of NAVA on patient ventilator interactions and prevention of lung over-inflation, however clinical effectiveness has not been demonstrated, Here we describe novel pilot data examining the feasibility of 'patient-controlled' weaning using this technology.

**OBJECTIVES.** To test the feasibility of patient-controlled ventilatory support weaning using NAVA technology.
**METHODS.** HRA/ethics approval was obtained Adult patients with prolonged ventilatory weaning on NAVA mode were screened for the ability to trial patient-controlled NAVA gain selection. Patients were excluded if they had cognitive impairment, psychological injury, treatment limitation, delirium, neuromuscular weakness of upper limbs, significant visual impairment or were part of another research study. Following consent, patients received a 10-minute explanation of the technology including a 1 minute period at reduced NAVA gain (50% reduction) which was clinician led. This was followed by a one-hour trial of patient-controlled NAVA gain setting with medical and nursing supervision throughout. Patients were encouraged to set NAVA gain at a level they would be happy to breathe at for at least two hours. NAVA gain, neural drive (EAdi) and dyspnea by visual analogue scale was assessed at baseline and at the end of the patient control hour. Following, one hour of patient controlled ventilation, the patient was returned to baseline support levels.
**RESULTS.** 10/634 patients with prolonged weaning screened, met the inclusion criteria. All 10 patients reported better understanding of NAVA versus pressure support ventilation and support for the concept of patient control as a component of the weaning strategy. Patients set NAVA gain at lower levels than critical care staff (Figure 1), associated with a small rise in neural drive (Mean EAdi 18 vs 20.1) and respiratory rate (26.4 vs 29.5 BPM). No adverse incidents were reported during the patient control phase or 24 hours post the patient control hour. Despite slightly elevated EAdi and respiratory rate, levels of dyspnea were not increased during the patient-control hour.
**CONCLUSIONS.** We have reported the novel use of patient controlled ventilatory support using NAVA technology. Patients found NAVA understandable; the introduction of patient control was safe and we have established the potential for acceleration of weaning through selection and tolerance of reduced support compared to that set by healthcare professionals.

**REFERENCE(S)**

Spahija J et al. 2010. Crit Care Med 38: 518-26.

**GRANT ACKNOWLEDGMENT**

DH is supported by an NIHR Clinical Fellowship Award and RESTUS is an NIHR Portfolio Study (Critical Care Specialty Group)

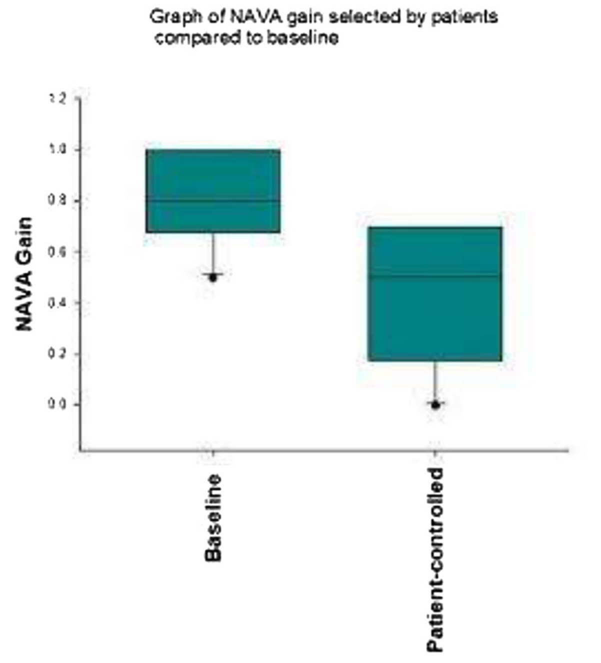

**Fig. 1 (abstract 0139).** NAVA Gain Levels set by patients compared to baseline

### 0140

#### Role of lung aeration pattern using ultrasonography in predicting failure of spontaneous breathing trial in mechanically ventilated patients

A. Fayed, W. Mohamed, M. Osman
Alexandria University, Critical Care Medicine, Alexandria, Egypt
**Correspondence:** A. Fayed
*Intensive Care Medicine Experimental* 2018, **6(Suppl 2):**0140

**INTRODUCTION.** Discontinuing Mechanical Ventilation (MV) is a time when the load associated with breathing is returned from the ventilator to the patient´s respiratory muscles. Failure of weaning from MV occurs in up to 25-30% of patients. It is associated with high mortality rates. Indexes predicting success can be clinically helpful. However, the predictive capacity may be low and there are a lot of controversies about which ones should be used.
**OBJECTIVE.** This study evaluated the role of lung ultrasound (LUS) in predicting the failure of spontaneous breathing trials (SBTs) in critically ill patients on MV using LUS aeration score (LAS) 30 minutes before and after the start of SBT. It was calculated according to numeric rating scale ranging from 0 to 32 (low =0 to10, moderate = 11 to 20 and high = 21+).
**METHODS.** This prospective study was conducted on 55 adult MV patients whom were eligible for SBT during their stay in the critical care units of Alexandria main university hospital. LUS was performed using a 2- to 4-MHZ convex probe (Mindray DP-3300). LAS was recorded. Then, all patients were followed up for success or failure of their SBT.
**RESULTS.** Results after follow up showed a high successful rate of SBTs performed. 60% (33) of all enrolled patients showed successful SBT, 40% (22) showed failed one. Regarding demographic data, 32 males and 23 females were enrolled. There was no difference in sex between both groups wasn't statistically significant ($p$ = 0.911). The mean age of all enrolled patients was 56.91 years. in group A ranged between 19 and 84 with a mean of 53.82 ± 20.91 years. There were no significant differences in demographics between patients of successful SBT group and failed group.
Regarding LUS assessment, the mean of LAS 30 minutes before SBT was significantly higher in the group of failed SBT (16.77 Vs 6.06, $p$< 0.001). The mean of LAS 30 minutes after start of SBT was higher than before in both groups but it was still significantly higher in the group of failed SBT (20.73 Vs 8.67, $p$< 0.001).
Data analysis showed that LAS after SBT was associated with higher sensitivity in predicting failure of SBT than the LAS before SBT (90.91 Vs 81.82%) but with lower specificity (69.70 Vs 78.79). LAS before SBT was associated with higher accuracy (80%) than after SBT (78.18%).
**CONCLUSIONS.** To date, there are no easy clinical indices can be powerful predictors of weaning failure. LUS may predict weaning success or failure through detection of aeration pattern before SBT through LUS scoring system.

**GRANT ACKNOWLEDGMENT**

Not applicable.

### 0141

#### Clinical outcome of difficult-to-wean patients with ventilator dependency at intensive care unit discharge

N.E. Kim[1], J.M. Lee[2], K.S. Chung[1], M.S. Park[1], Y.S. Kim[1], J.H. Song[1]
[1]Institute of Chest Disease, Severance Hospital, Yonsei University College of Medicine, Pulmonology, Seoul, Korea, Republic of; [2]National Health Insurance Service Ilsan Hospital, Pulmonology, Goyang, Korea, Republic of
**Correspondence:** J.H. Song
*Intensive Care Medicine Experimental* 2018, **6(Suppl 2):**0141

**INTRODUCTION.** Patients who are difficult to wean from invasive mechanical ventilator (IMV) in intensive care unit (ICU) with ventilator dependency have been increasing.1-2 However, specialized ventilator weaning unit is not available in the tertiary hospitals in Korea, and data on the clinical outcome of difficult-to-wean patients is lacking.

**OBJECTIVES.** We aimed to evaluate clinical outcome in patients who were discharged from ICU with tracheostomy and ventilator dependency.
**METHODS.** We retrospectively investigated clinical course and survival in patients requiring home mechanical ventilator (HMV) with tracheostomy for difficult weaning from IMV during medical ICU admission between September 2013 and Augustin 2016 at Severance Hospital, Yonsei University.
**RESULTS.** Of 84 difficult-to-wean patients who started on HMV in medical ICU, a total of 72 patients who survived and discharged from the ICU were identified and included for this analysis. HMV was initiated after a median of 23 days of IMV, and successful weaning rate was 46% (n = 33): all but one were successfully weaned at hospital discharge after a median of 31 days of HV. In-hospital mortality rate was significantly lower in successfully weaned group (0% vs. 23.1%; P = 0.003). Weaning rate was similar according to the main diagnosis. After a median follow-up of 4.6 months (range, 1 - 27) for survivors, 3- (n = 57) and 6-(n=54) months survival rates were 82.5% and 72.2%, respectively. The respective survival rates were better for successfully weaned group at 3 months (96.4% vs. 69.0%; P = 0.012) and 6-months (84.0% vs. 62.1%; P = 0.073) following ICU discharge.
**CONCLUSIONS.** Post-ICU survival for the difficult-to-wean patients with ventilator dependency was moderate with the use of HMV as a bridge to liberation from ventilator.

### 0142

#### Changes over time in the liberation from mechanical ventilation 1998-2016

F. Frutos-Vivar[1], O. Peñuelas[1], J. Mancebo[2], K. Raymondos[3], M. González[4], A.W. Thille[5], S.M. Maggiore[6], L. del Sorbo[7], A. Anzueto[8], A. Esteban[1], VENTILA Group
[1]Hospital Universitario de Getafe, Getafe, Spain; [2]Hospital Santa Creu i Sant Pau, Barcelona, Spain; [3]Medizinische Hochschule Hannover, Hannover, Germany; [4]Clinica Universidad de Navarra, Clínica Medellín & Universidad Pontificia Bolivariana, Medellin, Colombia; [5]Hospital of Poitiers, Poitiers, France; [6]Università degli Studi G. d'Annunzio Chieti e Pescara, Pescara, Italy; [7]Interdepartmental Division of Critical Care Medicine, Toronto, Canada; [8]South Texas Veterans Health Care System and University of Texas Health Science Center, San Antonio, United States
**Correspondence:** O. Peñuelas
*Intensive Care Medicine Experimental* 2018, **6(Suppl 2):**0142

**INTRODUCTION.** Liberation from mechanical ventilation is a common procedure in intensive care units. Several studies published in the last decades have change the way to perform weaning.
**OBJECTIVES.** To evaluate the changes observed in the liberation from mechanical ventilation from 1998 to 2016.
**METHODS.** Analysis of four prospective, observational, multicenter international studies on mechanical ventilation, in 1998, 2004, 2010 and 2016. For the purpose of this secondary analysis we selected patients who were successfully weaned and scheduled extubated. The onset of weaning was the time that the physician in charge considered the patient likely to resume and sustain spontaneous breathing after a patient met standard criteria for weaning readiness. We registered the method of weaning: spontaneous breathing trial (T-tube circuit, pressure support ventilation of 7 cm of water, continuous positive airway pressure of 5 cm of water, other mode) or gradual reduction of support (pressure support, synchronized intermittent mandatory ventilation with or without pressure support, other mode), days of weaning and reintubation. Patients were classified into one of three weaning groups: simple weaning group, difficult weaning group and prolonged weaning group.
**RESULTS.** In the table 1 and Figure 1 are showed the changes observed over the time. In the table 2 the comparison of outcomes.
**CONCLUSIONS.** These data showed a significant clinical variability over the time, however it is possible to observe that in last study there is a change toward a higher use of gradual reduction of pressure support to start the liberation of mechanical ventilation and to wean difficult/prolonged-weaning patients.

**Table 1 (abstract 0142).** Methods used for liberation from mechanical ventilation

| | 1998 N = 2858 | 2004 N = 2714 | 2010 N = 4151 | 2016 N = 3993 | P value |
|---|---|---|---|---|---|
| Method to perform first attempt, no. of patients (%) | | | | | |
| Spontaneous breathing trial | 1789(63) | 1780(66) | 3124(75) | 1904(48) | < 0.001 |
| Gradual reduction of support | 1069(37) | 934(34) | 1027 (25) | 2089(52) | < 0.001 |
| Failure in first attempt, no. of patients (%) | 1470(51) | 1212(45) | 1557(37.5) | 1163(29) | < 0.001 |
| Method for liberation from mechanical ventilation in patients who failed first attempt, no. of patients (%) | | | | | |
| Spontaneous breathing trial | 765 (52) | 343 (28) | 862 (55) | 447 (38) | < 0.001 |
| Gradual reduction of support | 705 (48) | 869(72) | 695 (45) | 716 (62) | < 0.001 |

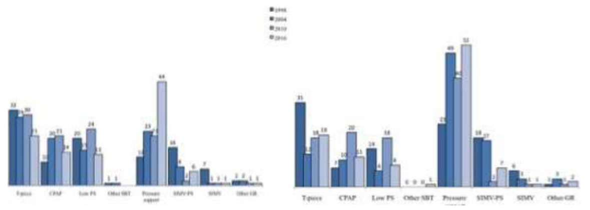

**Fig. 1 (abstract 0142).** Methods use for first attempt (left) and weaning (right)

**Table 2 (abstract 0142).** Comparison of outcomes

| | 1998 N = 2858 | 2004 N = 2714 | 2010 N = 4151 | 2016 N = 3993 | P value |
|---|---|---|---|---|---|
| Weaning group, % | | | | | < 0.001 |
| Simple | 49 | 55 | 62 | 71 | |
| Difficult | 44 | 39 | 31 | 25 | |
| Prolonged | 7 | 6 | 7 | 4 | |
| Weaning days (median, IQR) | 2 (1,3) | 1 (1,2) | 1 (1,2) | 1(1,2) | < 0.001 |
| In Difficult/Prolonged weaning | 3 (2,5) | 3 (2,4) | 3 (2, 5) | 3 (2,4) | < 0.001 |
| Reintubation 48h, % | 12 | 10 | 8 | 8 | < 0.001 |
| ICU mortality, % | 6 | 7 | 8 | 10 | < 0.001 |

### 0143

#### Heart rate variability evaluation during weaning from mechanical ventilation

B. Simon, C. Fasquel, V. Pateau, E. Lher
Brest University Hospital, MICU, Brest, France
**Correspondence:** B. Simon
*Intensive Care Medicine Experimental* 2018, **6(Suppl 2):**0143

Determinants of weaning or extubation failure are difficult to assess and are not solely related to respiratory condition. Heart rate variability is presumed to be an indicator of the patients' clinical status [1].
**OBJECTIVES.** The main purpose of this study was to evaluate whether heart rate variability evaluation before, during and after a weaning procedure may enable to discriminate the determinants of either weaning and extubation success.

**METHODS.** This project is based on a prospective physiological tracing data-warehousing program (Rea STOC, clinicaltrials.gov #NCT02893462) that aims to record 1500 consecutive ICU patients, over a 3-years period. This study was approved by our local ethical committee.

All ICU patients that were considered as candidates for a T-tube testing could be included. First continuous ECG recording was performed at 126 Hz for half an hour prior to the test itself. Same recordings were performed during the entire test duration (max 30 min), and after extubation if considered positive. Heart rate variability was assessed either in the time, frequency and Pointcare domain using Kubios.

**RESULTS.** 34 patients were included (age 62 ±11 yr; 25 male/ 9 female; SAPS II 48±17), and 40 weaning tests were recorded. Main associated diseases were cardiopathy (n=11), COPD (n=13), and neurological disease (epilepsy, stroke, multiple sclerosis). Main diagnoses related to mechanical ventilation requirement were acute respiratory failure (56%) and neurological failure (35%).

Most significant difference in between groups was a mean initial HRV value (prior to the beginning of the test) difference in between success and failure of the T-tube test, whatever the domain of consideration.

**CONCLUSIONS.** HRV evaluation, prior to a T-tube test, may help discriminate patients that will be considered as a success and deemed to be extubated.

**Table 1 (abstract 0143).** HRV values in the different domains

| RMSSD | | SDNN | | SD1/SD2 | | VLF | | TI | |
|---|---|---|---|---|---|---|---|---|---|
| Success | Failure | Success | Failure | Success | Failure | Success | Failure | Success | Failure |
| 34 ± 36,4 | 74,1 ± 77,2 | 32 ± 34,6 | 73,9 ± 77,9 | 1,5 ± 0,5 | 1,6 ± 0,4 | 313 ± 719 | 1897 ± 3628 | 4,7 ± 3,3 | 11,4 ± 12,2 |

### 0144

#### Long-term mechanical ventilation-dependent patients in ICU transferred to a home mechanical ventilation unit

O. Moreno Romero, M. Muñoz Garach, M.E. Poyatos Aguilera
Hospital Universitario San Cecilio, Granada, Spain
**Correspondence:** O. Moreno Romero
*Intensive Care Medicine Experimental* 2018, **6(Suppl 2):**0144

**OBJECTIVES.** To analyse the transfer of the long-term mechanical ventilation-dependent patients from an intensive carte unit to a home mechanical ventilation unit (HMVU).

**PATIENTS AND METHODS.** Observational restrospectively study of all patients who underwent mechanical ventilation (MV) therapy in our intensive care unit of 18 beds and became ventilatory dependent and were transfered to our HMVU, situated in a different hospital in the same city, in a 22 year period (1995-2017). Variables analyzed: age, sex, cause for prolonged MV, type of IMV dependency (full-time, parcial), NIMV, if they were still mechanically dependent at the time of discharge from HMVU, ICU and HMVU length of stay, overall survival.

**RESULTS.** In 22 years of study, 41 patients ventilatory dependent were transfered from ICU to our HMVU as a long term weaning process. 24 were male (58.56%), mean age 53 year, and 17 female (41.46%), mean age 54 years. Cause for prolonged MV: 25 (60.9%) neuromuscular disease, 8 (19.5%) chronic pulmonary disease, 6 (14.6%) complicated surgery, 2 (4.8%) neurovascular disease. ICU length of stay of 75 days (6-322).

At the time to transfer to HMVU: 38 (92%) patients with IVM, from these, 25 (65.7%) were full-time ventilated and 13 (34.3%) only parcially ventilated (12 hours at night). 3 (8%) patients with NIMV. At the time of discharged from the HMVU and thuss the length of stay was, depending of the type of ventilation: 9 months (93-144) in full-time IMV, 134 days (3-2880) in parcially IMV and 15 days (10-22) in NIMV.

Overall survival: 33 (80.5%) of the patients outlive more than 2 years and 20 (48.8%) of them outlive 5 years (2-16). 13 (31.7%) of the patients still alive today, with a mean time of 4.5 years.

**CONCLUSIONS.** The HMVU has become part of the process for treating mechanical ventilation dependent patients and thuss offloads intensive care unit longterm patients. From the ICU income until discharge from the HMVU it takes from 15 days in patients with NIMV to 134 days in those depending of IMV parcially and extends until the 9 months in the ones totally dependent. 80% of the patients survive more tan 2 years, and half of them outlive 5 years.

### 0145

#### Evaluation of lung ultrasound examination for predicting failure of weaning from mechanical ventilation

M. Raafat, A. Hasanin, S. Fathy, A. Fouad, A. El-adawy, H. Kamal
Cairo University, Anesthesia and Critical Care Medicine, Cairo, Egypt
**Correspondence:** S. Fathy
*Intensive Care Medicine Experimental* 2018, **6(Suppl 2):**0145

**Withdrawn**

### 0146

#### A comparison between five predicting tests; the search for an ideal weaning predictor continues

C. Dimitroulakis, C. Spyridonidou, M. Georgiadis, M. Vasileiou, E. Soilemezi, D. Matamis
Papageorgiou General Hospital, ICU, Thessaloniki, Greece
**Correspondence:** C. Spyridonidou
*Intensive Care Medicine Experimental* 2018, **6(Suppl 2):**0146

**INTRODUCTION.** Difficult weaning from mechanical ventilation remains a major challenge for the ICU physicians. The ideal weaning prediction test remains to be defined. Many weaning predictors have been proposed since the introduction of rapid shallow breathing index (RSBI), but their usefulness or superiority remains unclear.

**OBJECTIVES.** In a prospective observational study, we compared the performance of four already known weaning prediction tests, and a new one, the peak relaxation velocity of the diaphragm, acquired sonographically using Tissue Doppler Imaging (TDI).

**METHODS.** Seventy-one ICU patients ventilated for more than 48 hours were screened for weaning success or failure while performing a T-piece trial for 20 min.The following weaning prediction tests, with their respective cutoff points for weaning failure, were studied: RSBI>80 breaths/lit, diaphragmatic displacement < 1cm, diaphragmatic thickness ratio < 25%, diaphragmatic - rapid shallow breathing index (D-RSBI) > 1.3 breaths/mm of diaphragmatic displacement, and TDI-acquired diaphragmatic peak relaxation velocity < 4cm/sec. Diaphragmatic ultrasound measurements were performed on the right hemi-diaphragm. Weaning success was defined as no need for re-intubation or noninvasive ventilation for 48 hours after extubation. Receiver operator characteristic (ROC) curves and logistic regression were used to evaluate the diagnostic accuracy of these indices.

**RESULTS.** Mean duration of mechanical ventilation was not different between the weaning success and weaning failure groups (6.4±3 and 5.8±3 days respectively).Concerning the ROC curves, RSBI, D-RSBI and peak relaxation velocity performed more or less equally (0.7). Logistic regression confirmed the above mentioned data: Odds ratio for RSBI was 0.98 (95% CI 0.96-0.99), for peak relaxation velocity was 0.11 (95% CI 0.06-0.08) and for D-RSBI was 0.54 (95%CI 0.3-9.97); the significance level was similar (p< 0.04) for all three indices. Diaphragmatic displacement and thickness ratio were not found to be statistically significant predictors of weaning outcome.

**CONCLUSIONS.** Our study demonstrates that RSBI remains the simplest weaning predictor test to perform; D-RSBI and TDI -acquired diaphragmatic peak relaxation velocity could also be used in everyday ICU practice for the same purpose. Surprisingly, the current study did not establish significance for diaphragmatic displacement and thickness ratio.

### 0147

#### The effect of total thoracic content assessed by cadiometery on anticipating the deterioration of trial of weaning from mechanical ventilation

A. Wahdan
Cairo University Hospitals, Anesthesia, SICU and Pain Management, Cairo, Egypt
*Intensive Care Medicine Experimental* 2018, **6(Suppl 2):**0147

**INTRODUCTION.** Weaning from mechanical ventilation is a serious and challenging process in critical care practice. Weaning failure is associated with poor patient outcomes. I Fluid overload and positive cumulative fluid balance is a risk factor of weaning failure.

**PATIENT AND METHOD.** A prospective cohort study. : The study was conducted in24-bed trauma and surgical ICU Cairo University hospital. : Patients scheduled for a 2-h spontaneous weaning trial (SBT) . During the weaning trial , Total thoracic fluid content will be measured using electrical cardiometry connected to the patient before starting the weaning trial, during the trial at 30 minutes and 2 hours and at 30 minutes, 2, 6, 12, 24 and 48 hours after the end of the trial however success or failure. Also, cardiac output, cardiac index, stroke volume and ICON . The use of diuretics or nitrates will be considered and recorded. These data will be measured with the first weaning trial and with each subsequent trial

*Primary outcome* TFC (Total thoracic fluid content): absolute TFC value before SBT and the change in TFC by the end of SBT.

*Secondary outcome(s)* Any adverse events. and Cardiometry variables: stroke volume (SV), cardiac output (CO) and ICON ( Index of contractility). and Lung Ultrasound: To validate that the total thoracic fluid content measured by cardiometry is a good predictor of weaning outcome from mechanical ventilation in comparison to lung ultrasound.

**RESULT.** Hemodynamic variables, ultrasound variables, and cardiometry variables for patients with impaired cardiac contractility: In the subgroup of patients with impaired cardiac contractility , TFC was higher in the group of failed weaning patients compared to the group of successful weaning patients. CVP was also higher in the group of failed weaning compared to the group of successful weaning.

**DISCUSSION.** Weaning induced pulmonary edema (WIPE) has been reported in both cardiac and non-cardiac patients.Many tools for prediction of weaning induced cardiac edema have been reported. The use of electrical velocimetry in measurement of TFC has the advantage of being non-invasive with no need for advanced skills. So , In this work we tried to validate the use of TFC measured by electrical velocimetry in prediction of weaning success. Total thoracic fluid content was measured using electrical cardiometry connected to the patient before starting the weaning trial, during and after the trial. Lung ultrasound was performed, patients was investigated in a supine position before the start, during and after the trial. We found that total thoracic fluid content measured by electrical cardiometry and Lung ultrasound score showed good predictive ability for weaning failure especially in the subgroub of patients with impaired cardiac contractility. Positive fluid balance showed moderate predictive value for weaning failure.

### 0148

#### Physiologic comparison of the diaphragm behavior on spontaneous breathing (SB), pressure support ventilation (PSV) and proportional assisted ventilation with load-adjustable gain factor (PAV +) on acute increase of the ventilatory demand

P. Nery de Souza, M. Henrique Claro dos Santos, L. Pastore Junior, S. Elaine Uhlig, R. Florentina Scardua, E. Leite Costa
Hospital Sírio Libanes, São Paulo, Brazil
**Correspondence:** P. Nery de Souza
*Intensive Care Medicine Experimental* 2018, **6(Suppl 2):**0148

**INTRODUCTION.** Pressures support ventilation (PSV) is an assisted modality in which the airway pressure level is independent of the system impedance and patient effort. Conversely, Proportional assisted ventilation (PAV+) uses load gain factor to deliver a level of assistance that depends on both the mechanical properties of the respiratory system and on patient effort.

**OBJECTIVES.** To asses the effect of increased demand on patient effort and on diaphragmatic neuromechanical coupling in healthy volunteers during spontaneous breathing (SB) in PSV or PAV+ mode.

**METHODS.** This study was conducted at Sírio Libanês Hospital Education and Research Institute. The hospital ethics committee approved the study and Informed consent was obtained from all subjects.

Airway pressure (Paw) and flow were recorded at the mouth. Esophageal (Pes) and gastric (Pgas) pressures were measured with balloon-tiped catheters. Transdiaphragmatic pressure (Pdi) was obtained as Pgas -ΔPes, and transpulmonary pressure (PL), as Paw-Pes. Diaphragm electrical activity (EAdi) was recorded using electrodes attached to a nasogastric tube (NAVA, Maquet). Proper positioning of the esophageal and gastric balloons and NAVA electrodes was verified using standard tests [1]. Electrical impedance tomography was used to determine end-expiratory lung impedance (ELIC), as a surrogate for lung volume [2]. Amplitude of the diaphragmatic excursion (DE) was evaluated with the ultrasonography by a blinded investigator [3].

Six subjects were studied in semi-recumbent position breathing with a nose clip through a mouthpiece. Baseline measurements were obtained with unassisted breathing. PAV+ with 50% of assistance was adjusted in ZEEP, followed by PSV set to the same level of ΔPdi obtained in the PAV+. The level of assistance in PAV+ or PSV remained unaltered throughout the study. Thereafter, in random order, the volunteers were ventilated in SB, PAV+ or PSV with the increase of workload with sandbags over the abdomen (load1) or 150mL of dead space(load2)[1].

**RESULTS.** The ΔPDI and $\Delta P_L$ was lower on PAV+ while the ΔPes was higher compared to PSV(Tab.1). PAV+ resulted in a greater reduction in ΔEAdi and DE as compared with PSV, especially with the addition of dead space (Fig. 1).

**CONCLUSIONS.** PAV+ compared to SB and PSV was more efficient in terms of unloading the respiratory muscles. Neuromechanical coupling was better in PAV+ compared to PSV.

##### REFERENCE(S)

1- E. Akoumianaki et al. Physiologic comparison of neurally adjusted ventilator assist, proportional assist and pressure support ventilation in critically ill patients. Respiratory Physiology & Neurobiology 2014; 203:82-89.
2- J. Hinz et al. End-expiratory lung impedance change enables bedside monitoring of end-expiratory lung volume change. Intensive Care Med 2003; 29:37-43.
3- E.Cohen et al. Excursion- volume relation of the right hemidiaphragm measured by ultrasonography and respiratory airfllow measurements. Thorax 1994; 49: 885-889.

**Table 1 (abstract 0148).** Patient effort indices

| Variable | Modality | Load1 | Load2 | ρ |
|---|---|---|---|---|
| ΔPL | SB | 7,46 [4,23;11,05] | 8,47 [4,09;11,37] | 1 |
| | PAV+ | 5,58 [5,22;6,12] | 5,14 [4,46;5,99]" | 0,98 |
| | PSV | 6,34 [5,96;7,51] | 8,76 [8,30;9,115] | 0,03 |
| ΔPes | SB | -7,46[-11,05;-4,23] | -8,47[-11,37;-4,09] | 1 |
| | PAV+ | -1,36[-2,75;-1,05] | -1,48[-2,47;-1,05]" | 1 |
| | PSV | -2,99[-3,59;-1,68] | -4,01[-4,74;-3,64] | 0,56 |
| ΔPDI | SB | 15,06[10,03;18,53] | 13,17[9,93;13,49] | 0,98 |
| | PAV+ | 8,21 [5,83;9,55]* | 4,67[3,98;5,85]"# | 0,05 |
| | PSV | 8,58[6,81;10,39]° | 8[6,02;9,9,05] | 0,99 |

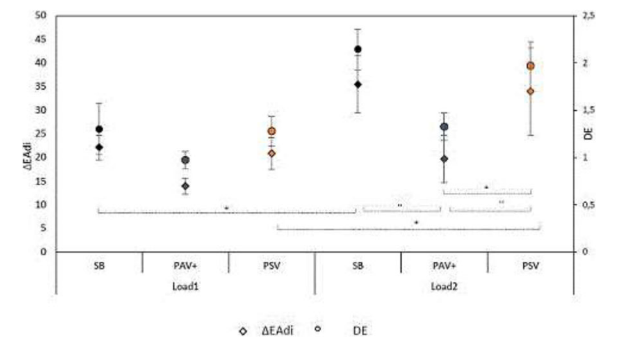

**Fig. 1 (abstract 0148).** Changes in ΔEAdi and DE

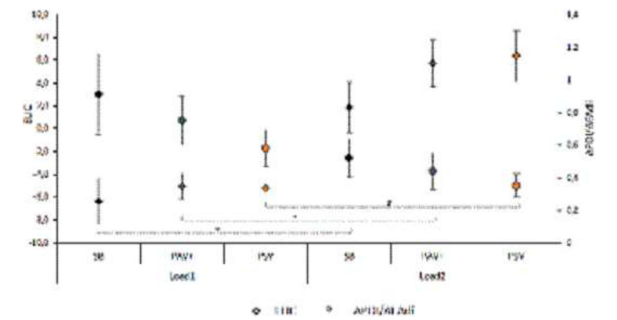

**Fig. 2 (abstract 0148).** Changes in ELIC and ΔPDI/ ΔEAdi

### 0149

#### Liberation from the ventilator: an observational study of extubation failure in a district general hospital in Scotland

J. Neil[1], G. Fletcher[2], R. Sundaram[1]
[1]Royal Alexandra Hospital, Intensive Care, Paisley, United Kingdom; [2]Royal Alexandra Hospital, Anaesthetics, Paisley, United Kingdom
**Correspondence:** J. Neil
*Intensive Care Medicine Experimental* 2018, **6(Suppl 2):**0149

**INTRODUCTION.** Extubation failure is an important quality indicator and is independently associated with mortality and increase in length of stay in mechanically ventilated critically ill patients.[1] The introduction of standardised protocols that include spontaneous breathing trials (SBT) on a T-piece are associated with a reduction in adverse outcomes. [2] In 2014, a standardised weaning protocol was introduced in our critical care unit which included an SBT on a T-piece for 2 hours in 2014.

**OBJECTIVES.** We aimed to study the factors that may influence the success of extubation in patients that passed an SBT in their first attempt compared to those that failed.

**METHODS.** We performed a retrospective observational study looking at patients admitted to our 7 bedded Level 3 critical care unit (370 patients a year) for a period of 9 months between January and September 2016. We included patients over 16 years of age who were mechanically ventilated and length of stay was greater than 48 hours. Data was collected from WardWatcher, a SICSAG database and electronic patient records. Data included fluid balance, haemoglobin, neurological status at time when an SBT was performed. Statistical analysis of data was performed using the Mann-Whitney U test.

**RESULTS.** There were 84 patients who met the eligibility criteria. Of those, 55 patients underwent an SBT prior to assessment for extubation. 38 patients passed their first SBT and 17 failed. Between these two groups there was no significant difference in median haemoglobin, Richmond Agitation-Sedation Scale (RASS), fluid balance at time of SBT and time from intubation to 1st SBT. However, median time from intubation to extubation was significantly greater in those who failed their first SBT, 5.49 days vs. 8.13 days ($p < 0.007$). 16% of those extubated after passing their 1st SBT required reintubation. No patients were reintubated after planned extubation in those who failed their 1st SBT.

**CONCLUSIONS.** Many factors contribute to extubation failure in critically ill patients. This study failed to show differences in a few of these factors that may have affected their ability to wean from mechanical ventilation. The SBT, although not perfect in predicting a successful extubation, does appear to highlight those patients who require more time intubated.

**REFERENCE(S)**

[1] Rothaar RC et al. Curr Opin Crit Care. 2003 Feb;9(1):59-66
[2] Kollef MH et al. Crit Care Med. 1997 Apr;25(4):567-74

**Table 1 (abstract 0149).** Patient Characteristics

| | Passed 1st SBT | Failed 1st SBT | p-value |
|---|---|---|---|
| Total Patients | 38 | 17 | |
| Median Age / yrs | 59.5 | 59 | |
| Median Hb / g/L | 98 | 92 | 0.84 |
| Median RASS | 0 | -1 | 0.32 |
| Median Fluid Balance / ml | 3401.5 | 5015 | 0.34 |
| Median Time Intubation to Extubation / Days | 5.49 | 8.13 | 0.007* |
| Median Time Intubation to 1st SBT / Days | 5.43 | 5.28 | 0.43 |
| Median Time 1st SBT to Extubation / Days | 0.06 | 2.04 | 0.00* |
| Reintubation after Extubation | 6 | 0 | |

### 0150

#### Carbon dioxide production and ventilatory inefficiency along a T-piece spontaneous breathing trial are associated with difficult weaning

R. López[1,2], R. Pérez[1,2], I. Caviedes[2,3], J. Graf[1,2]
[1]Clínica Alemana de Santiago, Departamento de Paciente Crítico, Santiago, Chile; [2]Facultad de Medicina Clínica Alemana - Universidad del Desarrollo, Santiago, Chile; [3]Clínica Alemana de Santiago, Unidad de Enfermedades Respiratorias, Santiago, Chile
**Correspondence:** R. López
*Intensive Care Medicine Experimental* 2018, **6(Suppl 2):**0150

**INTRODUCTION.** Ventilatory monitoring along a T-piece spontaneous breathing trial (SBT) is limited. Carbon dioxide ($CO_2$) monitoring with time capnography has been used to identify hypoventilation along the weaning process, but with high number of false positives [1]. Volumetric capnography (VC) can provide direct or derivate physiological variables that may be associated with difficult weaning, such as $CO_2$ production ($VCO_2$) and ventilatory inefficiency calculated from the slope between minute ventilation and VCO2 (VE/VCO2). Furthermore a good correlation between VE/VCO2 and physiological dead space has been recently reported in mechanically ventilated patients [2].

**OBJECTIVES.** To evaluate if VCO2 and/or VE/VCO2 along a T-piece SBT are associated with difficult weaning.

**METHODS.** We prospectively performed a one-hour SBT with a CO2/flow sensor between the endotracheal tube and the T-piece. Data was continuously recorded on a personal computer connected to a VC monitor (NICO2, Wallinford, CT, USA). Mean VCO2

and VE/VCO2 slopes were calculated. Difficult weaning included 3 categories: SBT failure (SBT-F) defined as inability to complete the trial or to extubate upon its completion, post extubation respiratory failure (PERF) defined as the need of non-invasive ventilation within 48 hours after extubation and extubation failure (EF) defined as the need for reintubation within 48 hours after extubation. Associations between VC variables and weaning outcomes were explored with t-test and ROC curves.

**RESULTS.** Twenty seven T-piece SBT were performed on 24 patients mechanically ventilated for 6 ± 5 days with an APACHE II of 17 ± 11 and a SOFA score 8 ± 3 points. 46% had sepsis/shock, 33% had acute respiratory failure and 21% were trauma/surgical. Sixteen trials presented difficult weaning; 5 with SBT-F, 7 with PERF and 4 with EF.

Patients with difficult weaning had a higher mean VCO2 or VE/VCO2 slope (table 1).

The AUC of the ROC curves of meanVCO2 and VE/VCO2 slope for difficult weaning were 0.73 [0.54-0.92] and 0.75 [0.55-0.94], respectively (figure 1).

Patients with SBT-F only had a higher mean VCO2 (table 2) while patients with PERF only had a higher VE/VCO2 slope (table 2).

**CONCLUSIONS.** Mean carbon dioxide production and ventilatory inefficiency are associated to difficult weaning; specifically a higher VCO2 is related to inability to tolerate a SBT and ventilatory inefficiency to the need for non-invasive ventilation after extubation.

**REFERENCE(S)**

[1] Saura P, et al. Use of capnography to detect hypercapnic episodes during weaning from mechanical ventilation. Intensive Care Med 1996; 22:374-381

[2] López R, et al. Minute ventilation to carbon dioxide production ratio is a simple and non-invasive index of ventilatory inefficiency in mechanically ventilated patients: proof of concept. Intensive Care Med 2017;4 3(10):1542-1543.

**GRANT ACKNOWLEDGMENT**

None.

**Table 1 (abstract 0150).** Association between difficult weaning and physiological variables from volumetric capnography

| | Difficult weaning = Yes | Difficult weaning =Not | Significance |
|---|---|---|---|
| VCO2 ml/min | 274±22 | 212±12 | 0,020 |
| VE/VCO2 slope | 33±3 | 25±2 | 0,021 |

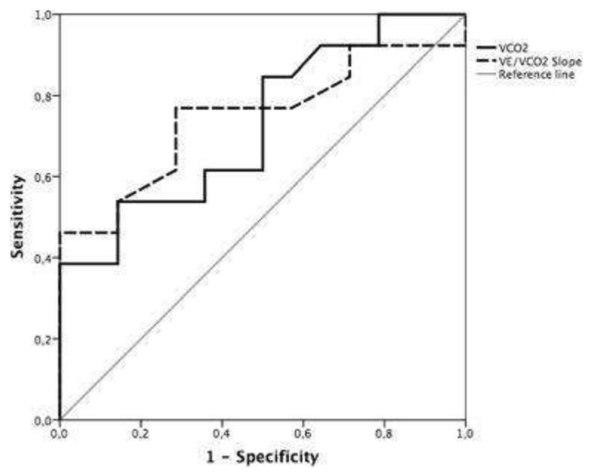

**Fig. 1 (abstract 0150).** ROC curves between physiological variables from VC and difficult weaning

**Table 2 (abstract 0150).** Association among volumetric capnography variables and SBT-F, PERF or EF

| | SBT-F=Yes | SBT-F=Not | Significance | PERF=Yes | PERF=Not | Significance | EF=Yes | EF=Not | Significance |
|---|---|---|---|---|---|---|---|---|---|
| VCO2 ml/min | 303 ±35 | 228 ±13 | 0,030 | 235±21 | 212±13 | NS | 254 ±29 | 211 ±12 | NS |
| VE/VCO2 slope | 32±7 | 28±7 | NS | 35±4 | 25±2 | 0,019 | 34±7 | 27±2 | NS |

## Acute Kidney Injury, clinical studies

### 0151

#### Influence of contrast media on patient with septic acute kidney injury

Y. Goto[1,2], S. Katayama[1], K. Koyama[1], T. Koinuma[1], K. Tonai[1], J. Shima[1], S. Nunomiya[1]

[1]Jiichi Medical University, School of Medicine, Tochigi, Japan; [2]Sapporo Medical University, Sapporo, Japan

**Correspondence:** Y. Goto

*Intensive Care Medicine Experimental* 2018, **6(Suppl 2):**0151

**INTRODUCTION.** Recent study has raised questions about the existence of contrast-induced kidney injury in the condition of relatively normal renal function. However, it is undetermined whether contrast media induce additional deterioration of renal function in patients with acute kidney injury (AKI). Furthermore, its influence on septic AKI patients is unclear. In this study, we examined the effect of contrast media on patients with septic AKI, compared with patients not receiving contrast media.

**OBJECTIVES.** This retrospective observational cohort study aimed to explore the outcomes for sepsis patient on our ICU over a 7-year period and determine the effect of contrast media.

**METHODS.** Using our database of sepsis patient records from 2011 to 2017 were investigated. Patients with septic AKI (according to the Sepsis-3 and KDIGO criteria) were divided into two groups (Group C, who administered contrast media within 24 hours of admission) and Group NC, not receiving contrast media and underwent propensity score analysis. The primary outcome was deterioration of kidney function (DRF) defined as elevation of serum Cre level (>0.5mg/dL or 1.25-fold from baseline) or induction of renal replacement therapy.

**RESULTS.** A total of 354 septic AKI patients were included. After propensity score adjustment, matching 80 couples of patients were analyzed. The rates of DRF were similar (37.5 vs 33.8%, OR 0.84 (95% CI 0.44-1.62), *P*= 0.870) between group C and group NC. ICU length of stay (8(5-15) vs 7(5-12)days, *P* = 0.220), ventilation days (7(5-13.5) vs 6(4-10.5)days, *P*=0.139), 7-day-mortality and 28-day-mortality (2.5 vs 6.3%, OR 0.39 (95%CI 0.08-1.78), *P* =0.443 and 8.7 vs 19.4% OR 0.40 (95%CI 0.15-1.09) *P* =0.086, respectively) were similar between two groups.

**CONCLUSIONS.** This study suggests that administration of contrast media was not associated with deterioration of AKI and poor outcome of septic AKI patient.

**REFERENCE(S)**

McDonald JS, et al. ICM 2017, 43:774-84.

### 0152

#### Impact of dexmedetomidine on acute kidney injury following living donor liver transplantation: a randomized controlled pilot study

K.-W. Jung, H.-M. Kwon, Y.-J. Moon, I.-G. Jun, J.-G. Song, G.-S. Hwang

Asan Medical Center, University of Ulsan College of Medicine, Seoul, Korea, Republic of

**Correspondence:** K.-W. Jung

*Intensive Care Medicine Experimental* 2018, **6(Suppl 2):**0152

**INTRODUCTION.** The incidence of acute kidney injury (AKI) has shown a wide range of 51% and 94% following liver transplantation (LT), [1-3] and is associated with increased morbidity, and mortality. [4] Dexmedetomidine has been noted as a highly selective agonist of α2-adrenergic receptors with a potential protective anti-inflammatory effect. [5,6] However, the impact of dexmedetomidine on AKI has not been determined yet after living donor LT (LDLT).

**OBJECTIVES.** The primary aim of the present study was to assess the relationship between the use of dexmedetomidine and the incidence of AKI in patients undergoing LDLT. The secondary aim was to assess the incidence of early graft dysfunction (EAD).

**METHODS.** Forty two recipients were randomly assigned into two groups: Group I (Control, n=20) and Group II (Treatment, n=22). The treatment group was given dexmedetomidine by constant intravenous (IV) infusion at a rate of 0.4mcg/kg/hour. The incidence of AKI was defined by Kidney Disease Improving Global Outcomes (KDIGO) classification.

**RESULTS.** There was no difference in demographic data such as age, sex, underlying disease and MELD score between the two groups. Of 42 patients, 40.5% developed AKI following LT. The incidence of AKI was lower in the treatment group (50.0% vs. 31.8), although there was no statistical significance (*P*=0.377). In addition, no difference was found in the incidence of EAD between the two groups.

**CONCLUSION.** Our demonstrated that dexmedetomidine may not have protective effects on AKI and EAD in patients after LDLT.

**REFERENCE(S)**

1. Rimola A, Gavaler JS, Schade RR, el-Lankany S, Starzl TE, Van Thiel DH. Effects of renal impairment on liver transplantation. *Gastroenterology*. 1987;93:148-156.
2. McCauley J, Van Thiel DH, Starzl TE, Puschett JB. Acute and chronic renal failure in liver transplantation. *Nephron*. 1990;55:121-128.
3. Bilbao I, Charco R, Balsells J et al. Risk factors for acute renal failure requiring dialysis after liver transplantation. *Clin Transplant*. 1998;12:123-129.
4. Wyssusek KH, Keys AL, Yung J, Moloney ET, Sivalingam P, Paul SK. Evaluation of perioperative predictors of acute kidney injury post orthotopic liver transplantation. *Anaesth Intensive Care*. 2015;43:757-763.
5. Cho JS, Shim JK, Soh S, Kim MK, Kwak YL. Perioperative dexmedetomidine reduces the incidence and severity of acute kidney injury following valvular heart surgery. *Kidney Int*. 2016;89:693-700.
6. Liu Y, Sheng B, Wang S, Lu F, Zhen J, Chen W. Dexmedetomidine prevents acute kidney injury after adult cardiac surgery: a meta-analysis of randomized controlled trials. *BMC Anesthesiol*. 2018;18:7.

**GRANT ACKNOWLEDGMENT**

Author's own work

### 0153

#### Is CKD-EPI equation appropriate for the estimation of glomerular filtration in critically ill patients with augmented renal clearance?

V. Philibert[1], Y. Rovira[1], S. Triginer[1], M. Sánchez-Satorra[1], A. Campos[1], J.M. Manciño[1], P. Marcos[1], J. Roca[2], T.M. Tomasa[1]

[1]Germans Trias i Pujol Hospital, Intensive Care, Badalona, Spain; [2]Germans Trias i Pujol Hospital, Epidemiology, Badalona, Spain

**Correspondence:** Y. Rovira

*Intensive Care Medicine Experimental* 2018, **6(Suppl 2):**0153

**INTRODUCTION.** There are several formulas to estimate glomerular filtration rate (eGFR). These formulas, however, are questioned under certain conditions: clinically unstable patients, acute renal failure, severe hepatopathy, generalized edema or ascites, pregnancy, significant alterations in muscle mass or the presence of body mass index < 19 Kg / $m^2$ or> 35 Kg / $m^2$ and liver kidney donors. In such conditions these equations are inadequate and glomerular filtration should be measured by the gold standar collection of urine (mGFR).

We investigated whether Augmented Renal Clearance (ARC) (mGFR > 130 ml / min/1.73 $m^2$) could be another condition in which these formulas should be reconsidered.

**OBJECTIVES.** To compare the validity of the Chronic Kidney Disease Epidemiology Collaboration equation (CKD-EPI) with the gold standard (mGFR) in 24-hour urine collection: 1) In critically ill patients with ARC and 2) In all critically ill patients.

**METHODS.** Observational study in a tertiary ICU hospital during 15 months period (January 2016 / March -September 2017). We studied the Chronic Kidney Disease Epidemiology Collaboration equation (CKD-EPI) as eGFR and the gold standard (mGFR) in 24-hour urine collection.

**RESULTS.** A total of 595 samples of 329 patients were studied. Pearson correlation coefficient was used for the statistical tests.

1) When we analized all the samples we obtained a good correlation (r = 0.78) between the eGFR and mGFR.
2) However, when we analized separately only the samples of critically ill patients with ARC (mGFR> 130 ml / min) a very bad correlation (r = 0.20) was obtained.

**CONCLUSIONS.** The CKD-EPI equation is not valid for the estimation of eGFR in critically ill patients with ARC, while it seems appropriate to estimate GFR in the general ICU population.

**REFERENCE(S)**

Levey AS, Inker LA, Coresh J. GFR estimation: from physiology to public health. Am J Kidney Dis. 2014;63(5):820-834.

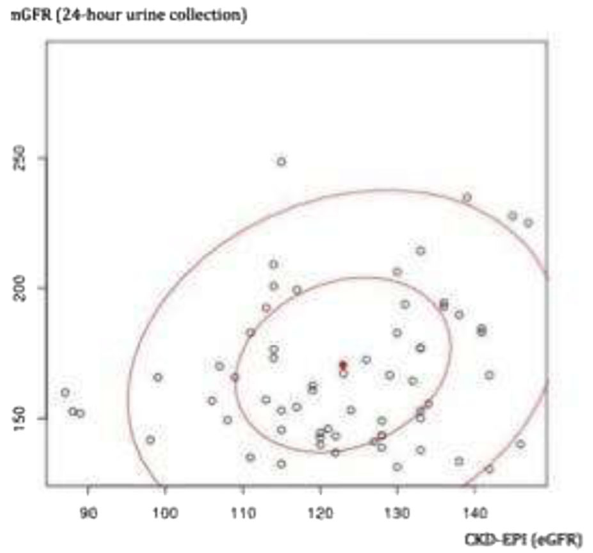

**Fig. 1 (abstract 0153).** mGFR and eGFR correlation (r= 0.20) in patients with ARC

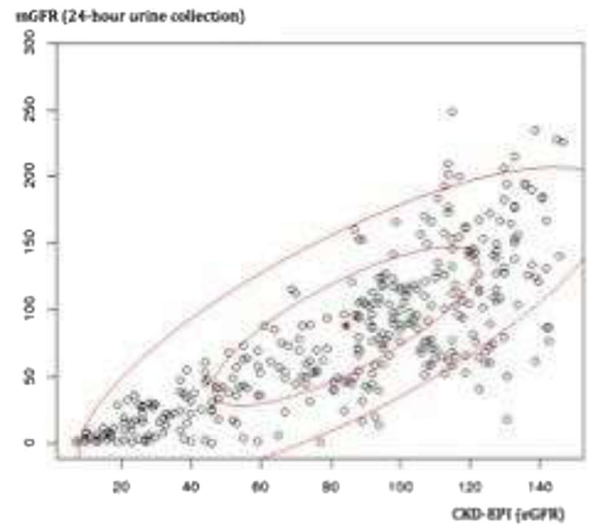

**Fig. 2 (abstract 0153).** mGFR and eGFR correlation (r= 0.78) in all patients.]

**REFERENCE(S)**

1. Myburgh JA, Finfer S, Bellomo R, Billot L, Cass A, Gattas D, Glass P, Lipman J, Liu B, McArthur C *et al*: Hydroxyethyl Starch or Saline for Fluid Resuscitation in Intensive Care. *New Engl J Med* 2012, **367**(20):1901-1911.
2. Guidet B, Martinet O, Boulain T, Philippart F, Poussel JF, Maizel J, Forceville X, Feissel M, Hasselmann M, Heininger A *et al*: Assessment of hemodynamic efficacy and safety of 6% hydroxyethylstarch 130/0.4 vs. 0.9% NaCl fluid replacement in patients with severe sepsis: The CRYSTMAS study. *Crit Care* 2012, **16**(3).
3. Perner A, Haase N, Guttormsen AB, Tenhunen J, Klemenzson G, Aneman A, Madsen KR, Moller MH, Elkjaer JM, Poulsen LM *et al*: Hydroxyethyl Starch 130/0.4 versus Ringer´s Acetate in Severe Sepsis. *New Engl J Med* 2012, **367**(2):124-134.

### 0154

#### Perioperative use of 6% balanced hydroxyethyl starch following cardiopulmonary bypass: perspectives of renal function

J.Y. Lim[1], P.J. Kang[2], J.B. Kim[2]
[1]Anam Hospital Korea University College of Medicine, Seoul, Korea, Republic of; [2]Asan Medical Center, University of Ulsan College of Medicine, Seoul, Korea, Republic of
**Correspondence:** J.Y. Lim
*Intensive Care Medicine Experimental* 2018, **6(Suppl 2):**0154

**INTRODUCTION.** As patients who undergo cardiac surgery under cardiopulmonary bypass (CPB) experience increased capillary leakage in the immediate postoperative period, 6% hydroxyethyl starch (HES) may be very effective volume expander in addition to crystalloids with less pulmonary fluid accumulation. Main concerns about the use of HES were potential harmful effects on renal function and coagulation. However, there was lack of reliable evidence.
**OBJECTIVES.** We aimed to evaluate the effect of limited volume of HES administration on postoperative renal function in patients undergoing cardiac surgery under CPB.
**METHODS.** Consecutive 1,657 patients undergoing cardiac surgery over two years were evaluated retrospectively. The patients were divided into high dose HES (n = 418, 18.8%) and low dose HES (n = 1,239, 81.2%) groups during the first two days after cardiac surgery. Outcomes were compared by using propensity scores and inverse probability weighting (IPTW) to adjust bias.
**RESULTS.** Incidence of AKI, the primary outcome, was higher in the high dose HES group (p=0.02). However, new renal replacement therapy (RRT) (P=0.30) and early mortality (p=0.97) was similar between the groups. When the baseline characteristics were adjusted by IPTW model, the high dose HES use was associated with increased risks of AKI (odds ratio, 1.66; 95% confidence interval (CI), 1.12-2.44; p = 0.01), but did not increase the risk of new RRT (OR, 1.27; 95% CI, 0.71-2.18; p = 0.40) or early mortality (Hazard ratio, 0.73; 95% CI, 0.29-1.81; p = 0.50), and these results were also validated by further adjustments for significant covariates.
**CONCLUSIONS.** The high dose administration of HES (≥20ml/kg) in the postoperative period following cardiac surgery may increase the risk of AKI. However, it did not increase the risk of new RRT or mortality. Benefits and risks should be weighed in the administration of HES. Further randomized controlled studies are needed to validate study results.

### 0155

#### Augmented renal clearance: an epidemiological multicentre study

T.M. Tomasa[1], A. Betbesé[2], P. Ortiz[3], S. Barbadillo[4], Y. Díaz[5], F.J. González de Molina[6], A.M. Navas[7], M. Rodriguez[8], J. Xirgu[9], C. Rovira[10], I. Oliva[11], R.M. Catalan[12], E. Vendrell[13], M. Ibarz[14], M. Pérez[15], J. Sabater[16], S. Cano[17], M. Miralbés[18], Y. Rovira[19], A. Campos[19], P. Marcos[19], Investigators of the Blood Purification Team Group of the Catalan Society of Intensive Care Medicine (GTDE-SOCMIC)
[1]Germans Trias i Pujol Hospital, Barcelona, Spain; [2]Santa Creu i Sant Pau Hospital, Barcelona, Spain; [3]Josep Trueta Hospital, Girona, Spain; [4]General de Catalunya Hospital, Sant Cugat del Vallès, Spain; [5]Hospital del Mar, Barcelona, Spain; [6]Hospital Mutua de Terrassa, Terrassa, Spain; [7]Parc Taulí University Hospital, Sabadell, Spain; [8]Moises Borggi Hospital, Sant Joan Despí, Spain; [9]Hospital General de Granollers, Intensive Care, Granollers, Spain; [10]Sant Joan de Reus Hospital, Reus, Spain; [11]Joan XXIII University Hospital, Intensive Care, Tarragona, Spain; [12]General de Vic Hospital, Vic, Spain; [13]Mataró Hospital, Mataró, Spain; [14]Sagrat Cor Hospital, Intensive Care, Barcelona, Spain; [15]Vall d´Hebron Hospital, Intensive Care, Barcelona, Spain; [16]Bellvitge Hospital, Intensive Care, L'Hospitalet de Llobregat, Spain; [17]Althaia Xarxa Assistencial Manresa, Intensive Care, Manresa, Spain; [18]Arnau de Vilanova Hospital, Intensive Care, Lleida, Spain; [19]Germans Trias i Pujol Hospital, Intensive Care, Badalona, Spain
**Correspondence:** Y. Rovira
*Intensive Care Medicine Experimental* 2018, **6(Suppl 2):**0155

**INTRODUCTION.** Augmented renal clearance (ARC) incidence varies from 20-80% in critically ill patients. ARC is considered if GFR is greater than 130 mL / min / 1.73 $m^2$. ARC is associated with drug under dosage.
**OBJECTIVES.** To analyze the prevalence of ARC in our environment and determine the characteristics of these patients.
**METHODS.** An Epidemiological Multicenter Study was conducted in Catalonia, Spain. GFR was measured by 24-hour urine collection. Patients were followed for 3 months. We compared ARC patients with non-ARC patients (GFR lower than 130 mL / min / 1.73 $m^2$). Preliminary data is shown from Phase 1 of the study.
**RESULTS.** 128 patients were included. Median age was 64.25 years old. SOFA score at ICU admission was 5.36. ICU length of stay (ICU LOS) was 23.03 days; hospital length of stay (HOSP LOS) was 35.37 days. We found ARC in 38.46% of the patients, with an average of GFR of 163.92 mL / min.
ARC patients were less severe (SOFA 3 vs. 6, p 0.0004) and younger (57.5 vs. 70.5 years, p 0.0000) and than non-ARC. Male sex was 73.91% in ARC group vs. 59.26% in non-ARC, p 0.070.
We found less septic patients at admission in ARC patients (30.43% vs. 48.15%, p 0.039). ARC patients were treated with fewer vasoactive agents (13.04% vs. 29.63%, p 0.026) and fewer diuretics (21.74% vs. 39.51%, p 0.031) than in non-ARC. Mechanical ventilation was used in 44.44% ARC patients vs. 30.43%, p 0.086 in non-ARC. The neurological cause of admission had a tendency to be higher in ARC patients (32.61% vs. 22.22%, p 0.142) and the same happened with anti epileptic drugs (23.91% vs. 16.05%, p 0.196), however it was not statistically significant different, probably because of the sample size. ICU LOS was lower in ARC patients (8 vs. 18.5 days, p 0.0014) so as ICU mortality (2.17% vs. 14.81%, p 0.019) than in non-ARC. HOSP LOS was not statistically significant different (21 vs. 25 days, p 0.0614).

**CONCLUSIONS.** ARC was recorded in 38.46% of patients with a median measured GFR of 163.92 mL / min. ARC patients were younger and less severe, and ICU LOS and ICU mortality were lower.

**REFERENCE(S)**

Udy, A.A., Roberts, J.A., Boots, R.J. et al. Augmented Renal Clearance. Implications for Antibacterial Dosing in the Critically Ill. Clin Pharmacokinet. 2010; 49: 1-16.

Udy, Andrew A.; Baptista, João P.; Lim, Noelle L.; Joynt, Gavin M; Jarrett, Paul; Wockner, Leesa; Boots, Robert J.; Lipman, Jeffrey. Augmented Renal Clearance in the ICU: Results of a Multicenter Observational Study of Renal Function in Critically Ill Patients with Normal Plasma Creatinine Concentrations*.Critical Care Medicine. 2014; 42 (3): 520-527.

### 0156

#### The use of renal doppler ultrasonography in critically ill patients with acute kidney injury

R. Passos, É.B. Melo, L.V. Freire, J.G.R. Ramos, J. Caldas, P. Batista, E. Mendonça, M. Peixoto, M. Dutra
Hospital Portugues da Bahia, Salvador, Brazil
**Correspondence:** R. Passos
*Intensive Care Medicine Experimental* 2018, **6(Suppl 2):**0156

**INTRODUCTION.** Acute kidney injury is one of the major organic dysfunctions that occur in critically ill patients. Ultrasonography of the kidneys and urinary tract is always a tool used by nephorologists as a way of evaluating this patology. Despite this practice, its clinical utility in determining the cause of acute kidney injury is poorly established.

**METHODS.** Prospective cohort study of critically ill patients that underwent renal ultrasound for acute kidney injury over a 5-year period at Hospital Português da Bahia. Renal Resistive Index was determined by the doppler sonography.

**RESULTS.** Over the 5-year period, 350 renal ultrasounds were perfomed for evaluation of acute kidney injury. Renal ultrasound was normal in 76% of patients. Hydronephrosis was detected in only 6% of studies and in only 2% of the cases was obstructive uropathy considered the cause of acute kidney injury. Less than 1% of patients had urinary tract obstruction on ultrasound without a suggestive medical history. Median renal RIs were 0,68 (o,62-o,75) in patients with sepsis related AKI and 0,74 (0,72-0,80) in patients with non sepsis related AKI (P=0,001). RIs were 0,72 (0,70-0,79) in transient AKI and 0,76 (0,70-0,80) in persistent AKI (P=0,64).

**CONCLUSIONS.** The role of renal ultrasonography in every critically ill patients with acute renal injury still needs to be determined in patients with history suggestive of urinary tract obstruction renal ultrasound for evaluation of acute kidney injury should be indicated. The use renal resistive index could to be a tool to reflect vasomotor tone in patients with AKI related to sepsis.

### 0157

#### Base deficit is a predictive factor of AKI in major trauma patients

J.Y. Lee[1], S.H. Lee[2]
[1]Chungbuk National University Hospital, Department of Trauma Surgery, Cheongju-si, Korea, Republic of; [2]Yonsei University College of Medicine, Department of Surgery, Seoul, Korea, Republic of
**Correspondence:** J.Y. Lee
*Intensive Care Medicine Experimental* 2018, **6(Suppl 2):**0157

**INTRODUCTION.** Acute kidney injury (AKI) is common in trauma patients and associated with poor clinical outcomes. Base deficit (BD) are well known marker of global tissue acidosis and correlated with the magnitude of blood loss. Hence, persistent elevations in BD might be associated with a prelude to organ failure in trauma patients. The purpose of this study was to determine whether initial BD was associated with AKI development in patients who had sustained major trauma.

**OBJECTIVES.** This study enrolled 284 major trauma patients by retrospective chart review, from January 2011 to December 2015. Patients were older than 16 years; were admitted to an intensive care unit; survived more than 48 hours; and had sustained major trauma, which defined as an injury severity score ≥ 16. Acute kidney injury (AKI) was defined according to the consensus Risk, Injury, Failure, Loss and End-Stage Renal Disease (RIFLE) definition. BD was categorized with level ≤ -7 mmol/L

**METHODS.** Clinical and laboratory variables were compared between AKI group (n = 87) and non-AKI group (n = 197). A multivariate logistic regression analysis was performed to assess the association between initial BD and AKI.

**RESULTS.** AKI developed in 87 (31%) patients, and significantly associated with higher mortality (33% vs. 5%, p < 0.001). Multivariate logistic analysis identified BD (adjusted odds ratio [OR] = 2.326, 95% confidence interval [CI] = 1.207 - 4.482, *p* = 0.012) with Age (OR, 1.020; CI, 1.002 - 1.039; p = 0.032), transfusion (OR, 3.554; CI, 1.746 - 7.234; *p* < 0.001), and use of vasopressor (OR, 2.217; CI, 1.082 - 4.390; *p* = 0.029) as independent predictive factors for AKI development in major trauma patients.

**CONCLUSIONS.** BD (initial base deficit ≤ -7 mmol/L) was independently associated with AKI development in major trauma patients, and it may be a useful predictive marker.

**REFERENCE(S)**

1. Eriksson M, Brattström O, Mårtensson J, Larsson E, Oldner, A Acute kidney injury following severe trauma: Risk factors and long-term outcome. J Trauma Acute Care Surg. 2015 Sep; 79(3):407-12.
2. Ostermann M, Chang RW , Acute kidney injury in the intensive care unit according to RIFLE., Crit Care Med. 2007 Aug; 35(8):1837-43.
3. Hodgman EI, Morse BC, Dente CJ, Mina MJ, Shaz BH, Nicholas JM, Wyrzykowski AD, Salomone JP, Rozycki GS, Feliciano DV., Base deficit as a marker of survival after traumatic injury: consistent across changing patient populations and resuscitation paradigms., J Trauma Acute Care Surg. 2012 Apr; 72(4):844-51.
4. Cheddie S, Muckart DJ, Hardcastle TC., Base deficit as an early marker of coagulopathy in trauma., S Afr J Surg. 2013 Jul 31; 51(3):88-90.

**GRANT ACKNOWLEDGMENT**

The authors have no funding or conflicts of interest to disclose

### 0158

#### Description of nephrotoxicity and infections associated with colistin in a intensive care unit

M.S. Costantini[1], N. Pires[1,2], C. Lovesio[1]
[1]Sanatorio Parque, Rosario, Argentina; [2]Universidad Nacional de Rosario, Rosario, Argentina
**Correspondence:** M.S. Costantini
*Intensive Care Medicine Experimental* 2018, **6(Suppl 2):**0158

**INTRODUCTION.** The use of colistin for the treatment of multidrug resistant (MDR) infections has led to an increase of colistin-associated acute kidney injury (AKI) and mortality.

**OBJECTIVES.** The aim of this study is to describe the colistin treated infections and the AKI colistin associated use onset of in the intensive care unit.

**METHODS.** A prospective study in a surgical and medical intensive care unit (ICU) carried out from March to November 2017. Patients on 48 hours colistin treatment (DC 300 UI and DM: 150 UI/12hs) and normal serum creatinine (Scr< 1,5mg/dl) at the beginning of the treatment, were included. Patients with chronic renal failure were excluded. Serum Creatinine was measured daily and AKI was defined as an SCr increase greater than or equal to 1,5 in baseline SCr.

**RESULTS.** Forty three patients aged 61 years old (DE: 16) were included, thirty-three (76,3%) of them were male.The basal Scr was 0,98 mg/dl (DE: 0,49 mg/dl) and the treatment´s day media was 8 days.Ten patients died (23,6%). Twenty six (60,47%) developed AKI and in nineteen of them (73%) was between the 1st and the 4th days of antibiotic (table 3). None of them required hemodialysis and all recovered complete renal function.
Tables 1 and 2 show the most frequent infection focus and bacteria founded. The exposure to drugs which could cause AKI was analyzed by a chi square test, none of them was associated with the AKI. Also, the relationship between AKI, the cultivated microorganisms, the infection focus and mortality was analyzed. Both, the microorganism and infections focus, have been associated with AKI: PS (OR: 1,22), KN (OR: 1,33), SW (OR: 2,40) and UC (OR: 2,90). On the other hand, PS (OR: 1,81) and KN (OR: 1,92) were associated with death and all the infection focus, except UC, have been associated with higher mortality. In addition, we found than renal failure was associated with a higher mortality (OR: 3,33). Dispite of this, none of them has a statistical significance.
**CONCLUSIONS.** Although we obtained results similar to those demonstrated in other studies about the incidence of renal failure in the use of colistin, in our population, AKI occurs in the first days of treatment. This could mean that patients require more control at the beginning of treatment and dose adjustments in that period could prevent future kidney damage.
Both the microorganism and the infections focus have been associated to renal fail and death. PS and KN seems to be wich have assosiattion with AKI and mortality in our population. About infections foci, HQ is associated with both renal failure and mortality of patients studied. On the other hand, UC is related with AKI but is not assosiated to mortality. However, none of this was statistically significant.

**Table 1 (abstract 0158).** The most frequent infection focus founded

| Infections focus | Frequency | % |
|---|---|---|
| BC | 5 | 11,63% |
| SW | 23 | 53,48% |
| TA | 8 | 18,60% |
| UC | 7 | 16,28 % |
| Total | 43 | 100,00% |

**Table 2 (abstract 0158).** The most frequent bacteria founded

| Microorganism | Frequency | % |
|---|---|---|
| AB | 22 | 51,16% |
| EC | 3 | 6,98% |
| KN | 8 | 18,60% |
| PS | 6 | 13,95% |
| OTHER | 4 | 9,30% |
| Total | 43 | 100% |

**Table 3 (abstract 0158).** Day of antibiotic in which AKI was develop

| Day of antibiotic in which AKI was develop | Frequency | % | Cum. Percent |
|---|---|---|---|
| 1 | 4 | 15,38% | 15,38% |
| 2 | 4 | 15,38% | 30,77% |
| 3 | 5 | 19,23% | 50,00% |
| 4 | 6 | 23,08% | 73,08% |
| 5 | 2 | 7,69% | 80,77% |
| 6 | 1 | 3,85% | 84,62% |
| 8 | 2 | 7,69% | 92,31% |
| > 10 | 2 | 7,70% | 100,00% |
| Total | 26 | 100,00% | 100,00% |

### 0159

#### Renal ischemia-reperfusion injury in cardiac arrest survivors: a retrospective, observational study

S. De Rosa[1,2], A. Rigobello[1], G. Villa[3], S. Marcante[1], E. Boni[1], S. Samoni[4], E. Roman-Pognuz[5], A. Messina[6], S. Bazzano[2], F. D'Ippoliti[2], R. Bonato[1], C. Ronco[4]
[1]St Bortolo Hospital of Vicenza, Anesthesia and Intensive Care, Vicenza, Italy; [2]San Bortolo Hospital, International Renal Research Institute of Vicenza, Vicenza, Italy; [3]Azienda Ospedaliera Universitaria, Anesthesia and Intensive Care, Firenze, Italy; [4]San Bortolo Hospital, Nephrology, Dialysis and Transplantation, Vicenza, Italy; [5]Azienda Sanitaria Universitaria Integrata di Trieste, Trieste, Italy; [6]Azienda Ospedaliera Universitaria Maggiore della Carità, Novara, Italy
**Correspondence:** S. De Rosa
*Intensive Care Medicine Experimental* 2018, **6(Suppl 2):**0159

**INTRODUCTION.** Ischemia and reperfusion during the stages of cardiac arrest, resuscitation and post-resuscitation can particularly affect the kidney. It causes incomplete repair of tubular cells and the development of fibrosis.
**OBJECTIVES.** To evaluate the impact of Ischemia-Reperfusion Injury on incidence of Acute Kidney Injury (AKI) and renal outcome in Cardiac Arrest (CA) Survivors treated with TTM 35°C versus no treatement
**METHODS.** We performed a retrospective study of consecutive comatose patients resuscitated from CA and admitted to our ICU from March 2016 to March 2017. The surface cooling device used for patient with GCS< 8 and treated TTM 35° was Arctic Sun® 5000. Patients with GCS> 8 did not performed TTM 35 °C treatment. Data obtained at baseline and at 24,48 and 72 hours were: temperature trend and rate, serum creatinine,diuretic use, urine output, fluid balance. AKI was defined according to KDIGO criteria.
**RESULTS.** We included 44 patients out of 95 ICU admissions. Twenty-one patients were treated with TTM 35°, while 23 patients did not performed the treament beacuse of GCS >8. In TTM 35° group, according to KDIGO classification, 10 (48%) had no evidence of AKI while 11 (52%) presented AKI during the treatment. In particular, the incidence of AKI was 52% at 24 h, 29% at 48 h and 25% at 72 h from the treatment. In the no treated group, according to KDIGO classification, 11 (49%) had no evidence of AKI while 12 (51%) presented AKI during the observation period. In particular, the incidence of AKI was 21% at 24 h, 16% at 48 h and 28% at 72 h. In overall populaziont, only one patient required renal replacement therapy (2.3%) and he was part of TTM 35° group. Median cumulative FB was -1100 [-6859-3517] ml for all patients; 1120 [-6859-2209] and -443 [-6796-3517] specifically for TTM 35° and not-treated patients. In overall population, ICU mortality was 45 %, and specifically 47.6 % in TTM 35° group and 43.4 % in no TTM group.
**CONCLUSIONS.** Treatment with a target temperature of 35 degrees can be affected by difficult temperature control and cause damage due to temperature increase. Unfortunately, the beneficial effects of the treatment could be canceled and lead to a worsening of renal function. In those who did not undergo temperature control, a deterioration in renal function is present with a similar mortality rate compared to those treated with TTM 35 degrees and with worse GCS.

**REFERENCE(S)**

1: De Rosa S, De Cal M, Joannidis M, Villa G, Pacheco JLS, Virzì GM, Samoni S,D´ippoliti F, Marcante S, Visconti F, Lampariello A, Zannato M, Marafon S, Bonato R, Ronco C. The effect of whole-body cooling on renal function in post-cardiac arrest patients. BMC Nephrol. 2017 Dec 29;18(1):376.
2: De Rosa S, Antonelli M, Ronco C. Hypothermia and kidney: a focus on ischaemia-reperfusion injury. Nephrol Dial Transplant. 2017 Feb 1;32(2):241-247.

### 0161

#### Evaluation of high oxygen affinity of bovine PEGylated Carboxyhemoglobin (PEG-COHb) on the renal oxygenation and function in septic rats

B. Ergin[1], P. Guerci[2], C. Ince[1]
[1]Academic Medical Center, Department of Translational Physiology, Amsterdam, Netherlands; [2]University Hospital of Nancy, Department of Anesthesiology and Critical Care Medicine, Nancy, France
**Correspondence:** B. Ergin
*Intensive Care Medicine Experimental* 2018, **6(Suppl 2):**0161

**INTRODUCTION.** Sepsis is defined as a life-threatening organ dysfunction accompanied with systemic hypotension, microcirculatory disturbance, tissue hypoxia and an unregulated systemic inflammation in response to infection. Hemoglobin-based oxygen carrier's (HBOC's) might be able to provide an extra oxygen carrying capacity as well as fluid replacement in septic patients.

**OBJECTIVE.** The primary outcome for this study is to improve in microcirculatory perfusion and oxygenation by use of hemoglobin-based oxygen carrier additional to fluid, and provide a better organ perfusion, oxygenation and function in sepsis induced renal microcirculatory disturbance.

**METHODS.** 36 fully instrumented, mechanically ventilated and anesthetized male *Wistar albino* rats were subjected 6 groups as sham, LPS (25mg/kg LPS i.v), LPS + Ringer acetate (RA, 30ml/kg/h, 1 hours), LPS+ RA+ Norepinephrine (NE, 5mg/kg/min), LPS + RA in addition to 320 mg/kg/h bovine PEG-COHb, and LPS + RA with 320 mg/kg/h PEG-COHb + NE. Systemic and renal hemodynamic, renal oxygenation (Pd-porphyrine phosphorescence life-time technique), blood gas and electrolites, and biochemistry of PEG-COHb were assessed.

**RESULTS.** The fluid supplemented with PEG-COHb caused an increase in MAP and CVP levels compared to LPS+RA ($p < .05$) and LPS+RA+NE ($p < .05$) groups during late resuscitation phase (Fig 1).

Despite an improvement of the urine output ($p < .05$), PEG-COHb did not correct the depletion of renal oxygenation and renal blood flow (Fig 2).

Lactate level was depleted by RA+PEG-CO-Hb resuscitation compared to RA alone ($p < .05$) in late resuscitation (Fig 3).

$SvrO_2$ was enhanced after PEG-COHb ($p < .05$) and NE ($p < .05$) treatments but not RA alone. Base excess, $HCO_3^-$, pH and anion gab $K^+$ were improved by the fluid resuscitation supplemented with PEG-COHb ($p < .05$) and NE ($p < .05$).

**CONCLUSION.** To conclude, high oxygen affinity of PEG-COHb appears to have a systemic and peripheral vasoactive property, in oppose to general believe it did not release its oxygen in hypoxic renal tissue and also reduce the oxygen bioavailability in renal cortex. Therefore, we suggested that blood substitute with low oxygen affinity would provide a better microcirculation and re-oxygenation in hypoxic organs.

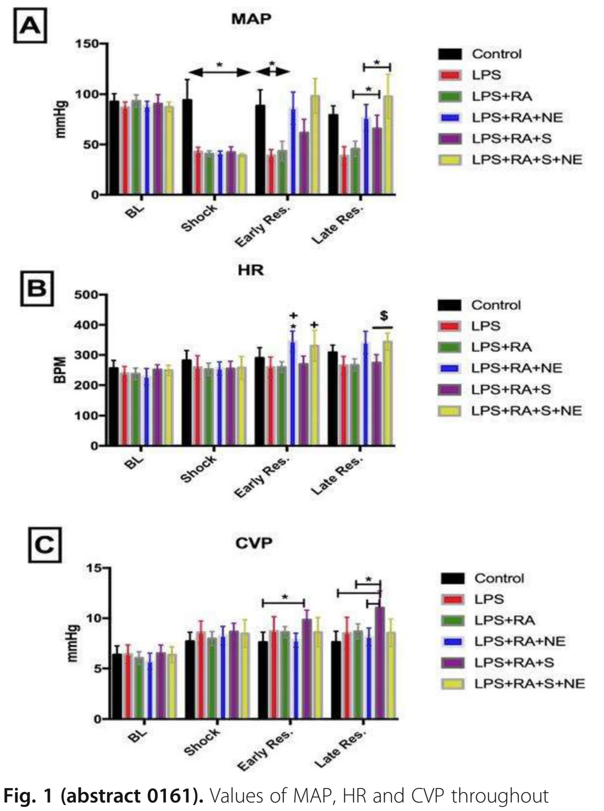

**Fig. 1 (abstract 0161).** Values of MAP, HR and CVP throughout experiments

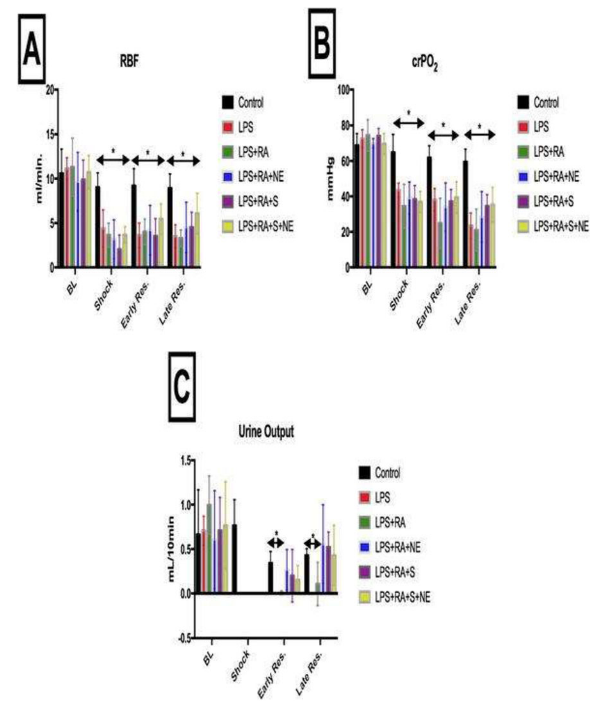

**Fig. 2 (abstract 0161).** Renal blood flow (RBF), urine output and renal cortical oxygenation (CrPO2) values

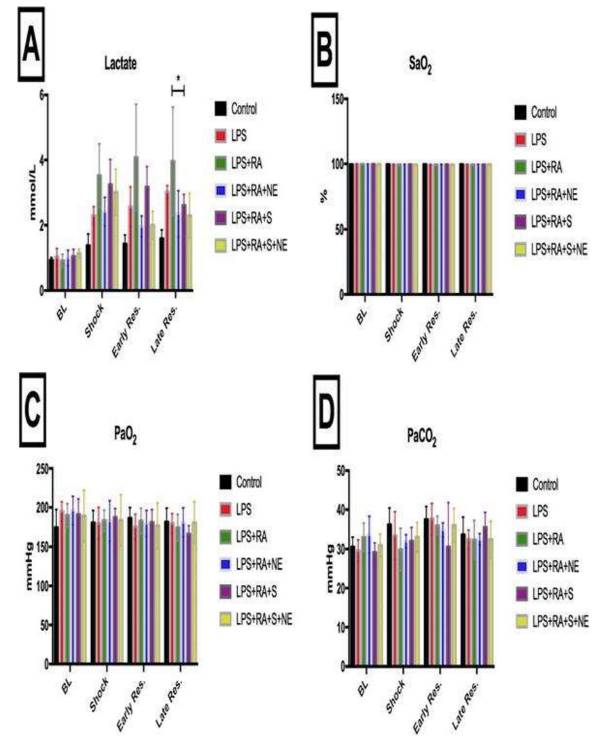

**Fig. 3 (abstract 0161).** Lactate, SaO2, PaO2 and PaCO2 levels

### 0162

#### Exploring early diagnostic markers of acute kidney injury in sepsis by metabolomics methods

F. Ping[1,2], Y. Cao[1], W. Huang[1], Y. Guo[1], L. Wang[2], Y. Li[1]
[1]Shanghai Jiaotong University affiliated the Sixth People's Hospital, Critical Care Medicine, Shanghai, China; [2]Shanghai Jiaotong University affiliated the Sixth People's Hospital, Anesthesiology, Shanghai, China
**Correspondence:** Y. Li
*Intensive Care Medicine Experimental* 2018, **6(Suppl 2):**0162

**INTRODUCTION.** Sepsis-induced acute kidney injury (AKI) causes high mortality in critical ill patients owing to elevated levels of endotoxin. This situation might be partially attributed to the delayed recognition of AKI when the current diagnosis is based on either an elevation of serum creatinine levels or the detection of oliguria. Metabolomics, possessing diverse detection platforms and multiple analysis methods, is a potential tool for identifying small molecule biomarkers of renal diseases.

**OBJECTIVES.** This study aimed to explore new early diagnostic markers of septic AKI in rats by metabolomics methods.

**METHODS.** A rat model of septic AKI was established by intraperitoneal injection of Lipopolysaccharide (LPS, 5 mg/kg). Concentrations of serum creatinine(Scr), blood urea nitrogen(BUN), alanine aminotransferase(ALT), aspartate aminotransferase(AST) and histopathological changes of kidney were determined at 2h and 6h after LPS administration respectively. At the same time, serum metabolites were detected and analyzed by gas chromatography-time-of-flight /mass spectrometry (GC-TOF/MS) to find early diagnostic markers of septic AKI.

**RESULTS.** 2h after LPS intraperitoneal injection, serum levels of ALT and AST stayed normal, indicating serum metabolites were not affected by liver function and the histopathological analysis revealed renal damage and tubular injury score also increased significantly while Scr and BUN levels had no significant change until 6h after LPS treatment. Meanwhile, at the time of 2h post LPS injection, 24 statistically altered metabolites were detected by GC-TOF/MS, mainly from amino acid metabolism and organic acid metabolism. Among them, 9 metabolites having a larger fold change such as malic acid, glycolic acid, ratio of asparagine/ aspartic acid, acetylglycine, alanine, urea, α-aminobutyric acid, aminomalonic acid and methionine sulfoxide were more sensitive than Scr and BUN.

**CONCLUSIONS.** Our findings demonstrated that 9 representative differential metabolites above are expected to be candidates for early diagnosis of sepsis-induced AKI in rats.

##### REFERENCE(S)

1. Mir SM, et al. Ferulic acid protects lipopolysaccharide-induced acute kidney injury by suppressing inflammatory events and upregulating antioxidant defenses in Balb/c mice. *Biomedicine & pharmacotherapy = Biomedecine & pharmacotherapie* 2018, 100:304-315.
2. Pozzoli S, et al. Predicting acute kidney injury: current status and future challenges. *J Nephrol* 2017.
3. Kellum JA, Lameire N. Diagnosis, evaluation, and management of acute kidney injury: a KDIGO summary (Part 1). *Crit Care* 2013, 17(1):204.
4. Barrios C, Spector TD, Menni C. Blood, urine and faecal metabolite profiles in the study of adult renal disease. *Arch Biochem Biophys* 2016, 589:81-92.

##### GRANT ACKNOWLEDGMENT

This work was supported by the National Nature Science Foundation of China (No. 81272145).

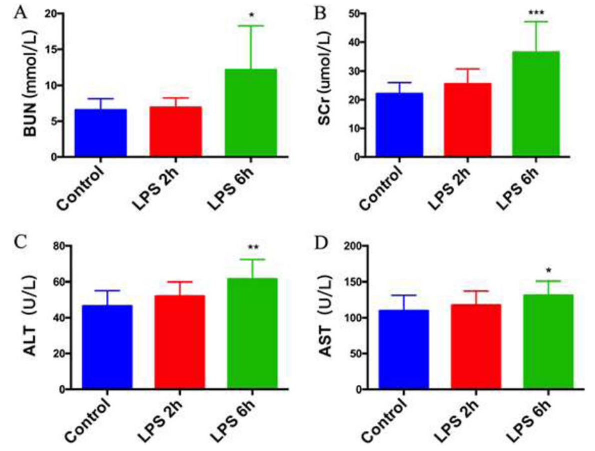

**Fig. 1 (abstract 0162).** effect of PBS or LPS treatment on renal function and liver function in rats

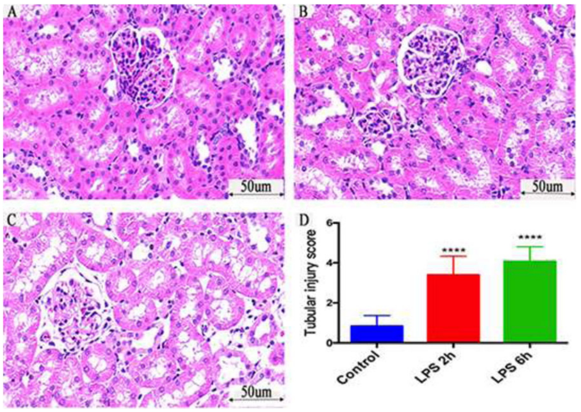

**Fig. 2 (abstract 0162).** Histological assessment of renal injury of PBS- or LPS-treated rats by HE-based staining

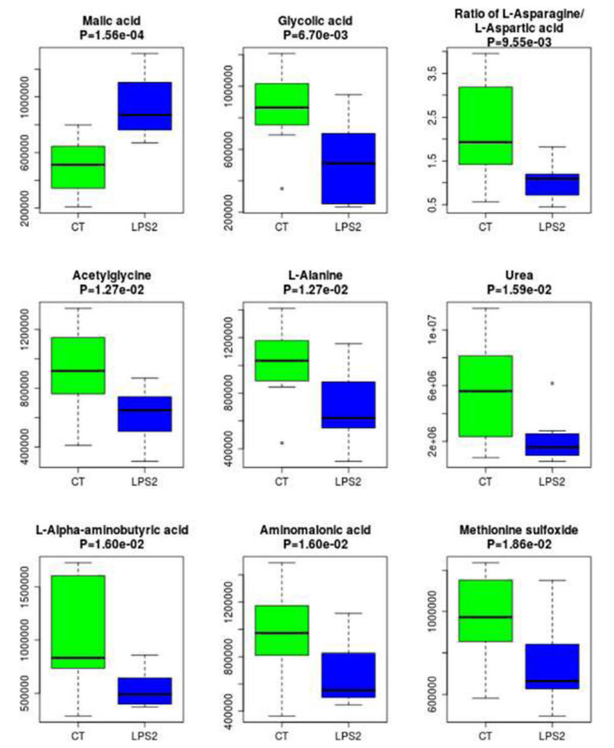

**Fig. 3 (abstract 0162).** Top ranked differential metabolites between control (CT) group and LPS-2hour (LPS2) group

### 0163

#### How is the kidney of the survivors after a sepsis-associated AKI?

A.M.A. Liberatore, M.N. Nakamae, Y.R. Kang, R.B. Souza, C.J.F. Vieira, I.H.J. Koh
Universidade Federal de São Paulo, Surgery, São Paulo, Brazil
**Correspondence:** I.H.J. Koh
*Intensive Care Medicine Experimental* 2018, **6(Suppl 2):**0163

**Withdrawn**

### 0164

#### Metabolomic characteristics and expression of urinary biomarkers in patients developing acute kidney injury after cardiopulmonary bypass

B. Fohlen[1,2], T.M. Huynh[1,2], N. Pallet[1,2], B. Cholley[1,2]
[1]Paris Descartes University, Paris, France; [2]Hopital Europeen Georges Pompidou, Paris, France
**Correspondence:** B. Fohlen
*Intensive Care Medicine Experimental* 2018, **6(Suppl 2):**0164

**OBJECTIVE.** Acute kidney injury (AKI) is a major complication that affects 20 to 30% of patients undergoing cardiac surgery with cardiopulmonary bypass (CBP). None of the current serum biomarkers (creatinine, NGAL) appears capable to predict AKI following CBP. An Analysis of the urinary metabolomic signatures of patients undergoing CPB using proton-based nuclear magnetic resonance (1H-NMR) spectroscopy could provide a rapid diagnostic approach to AKI. Our goal was to analyze urinary metabolomics and biomarkers variations in patients after CBP and to check their association with postoperative AKI.

**MATERIAL AND METHODS.** All consecutive patients undergoing elective cardiac surgery with CBP between 17/02/17 and 26/04/17 were studied. Exclusion criteria were: an eGFR < 30 ml/min/1.73 $m^2$and a preoperative left ventricular ejection fraction < 40%. AKI was determined according to the KDIGO classification. Continuous mean arterial pressure (MAP) was digitally acquired during CPB. Urine samples were collected at 3 different time points: before the start of CPB, at the end of the CPB procedure, and on the day after surgery in the ICU.

The urinary metabolites changes were analyzed by magnetic resonance spectrometry. Urinary NGAL was assayed by ELISA. A French ethical committee approved this study. All patients provided written consent for study participation and for the biological analysis before inclusion.

**RESULTS.** 42 patients were included. AKI occurred in 28% (n = 12) of patients. The main characteristics are shown in Table 1.The mean CPB duration was 99 ± 36 min and the mean aortic cross-clamp duration was 75 ± 30 min. There was no correlation between MAP during CBP and KDIGO class after surgery. Baseline renal function (before CPB) influenced the occurrence of AKI. The mean estimated glomerular filtration rate was 78 ± 24 ml/min/1.73 $m^2$in individuals without AKI compared with 63 ± 13, 78 ± 24 ml/min/1.73 $m^2$in individuals with AKI (p = 0.01). None of the other parameters that could impact renal function after CBP did influence AKI occurrence. Analysis of the urinary metabolomes of the 42 patients before CPB, at the end of the CPB, and on the day after shows that CPB promotes renal metabolic reprogramming and profoundly alters the urinary metabolome (figure 1). We observed 46% of AKI in patients who did not modify their urinary metabolites vs. 10% in those who did, p = 0.02). Urinary NGAL did not vary during the CBP, urinary NGAL (pg/l) was 12±30 before CBP, 16±31 at the end of CBP and 22±27 on the day after CBP (p=0.6).

**CONCLUSIONS.** A change in urinary metabolomics, indicating renal adaptation, characterized patients whose renal function did not deteriorate after CBP. Neither the per-CBP MAP level nor the post-CBP urinary NGAL values were associated with the occurrence of an AKI.

**Table 1 (abstract 0164).** Description of the cohort undergoing cardiac surgery

| | All Patients (n = 42) | AKI (n = 12) | No AKI (n =30) | p |
|---|---|---|---|---|
| Age (years) | 62.1 ± 15.3 | 64.3 ± 4.4 | 61.3 ± 2.8 | 0.56 |
| eGFR (ml/min/1.73 m2) | 73.9 ± 22.9 | 63 ± 6.3 | 78.3±4 | 0.49 |
| Ejection fraction (%) | 60.6 ± 9.4 | 62 ± 2.7 | 61.1 ± 1.7 | 0.57 |
| Euroscore 2 | 2.7±2.8 | 3.2 ± 0.8 | 2.5 ± 0.5 | 0.47 |
| CBP length (min) | 99.3 ± 22.9 | 109 ± 10.4 | 95 ± 6.5 | 0.25 |
| Aortic cross-clamp length (min) | 75.1 ± 30.7 | 84 ± 8.8 | 71.6 ± 5.6 | 0.25 |
| ICU stay (days) | 3.5 ± 3.7 | 5.3±1 | 2.8 ± 0.6 | 0.04 |
| Death, n (%) | 2 (5) | 2 (16) | 0 (0) | 0.02 |

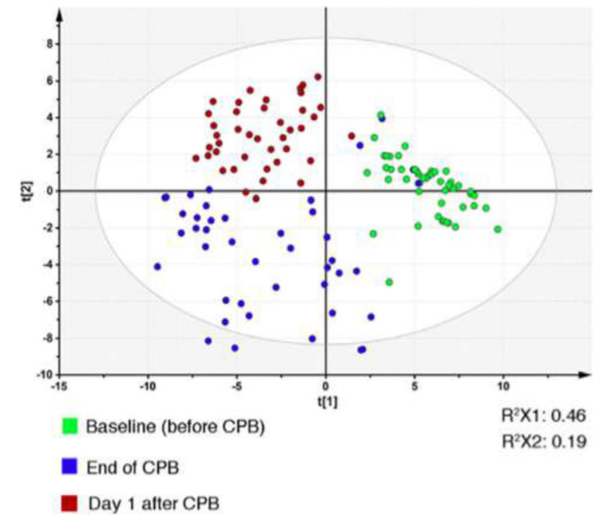

**Fig. 1 (abstract 0164).** Analysis of 126 urine samples collected from 42 individuals

### 0165

#### Calculating kinetic glomerular filtration rate in the intensive care unit as an early way to detect acute kidney injury

E. Nájera[1,2], A. Contreras[3], A. Aisa[4], J. Franco[1], J. Aguirre[1], G. Camarena[3]
[1]Centro Médico ABC, Critical Care, Mexico City, Mexico; [2]Universidad Nacional Autonoma de México, Mexico City, Mexico; [3]Centro Médico ABC, Mexico, Mexico; [4]Centro de Investigación Biomédica en Red de Enfermedades Respiratorias, Mexcio, Mexico
**Correspondence:** E. Nájera
*Intensive Care Medicine Experimental* 2018, **6(Suppl 2):**0165

**INTRODUCTION.** Calculating Kinetic Glomerular Filtration Rate (GFR) In the Intensive Care Unit (ICU) could improve early detection of acute kidney injury (AKI), as compared to KDGO (kidney improving Global outcomes) classification that only takes into account creatinine and urine output. Creatinine increase could take as much as 36 hrs and urine output can be variable in a non steady state as in ICU patients. Different formulas have been elaborated to provide a mathematical description of the GFR in a non steady state.

**OBJECTIVES.** To compare different kinetic GFR formulas in critically ill patients with AKI as a way to detect AKI earlier.

**METHODS.** We enrole 84 patients over 18 years old who developed AKI 3 during their hospitalization and calculate three different kinetic formulas of GFR in a non steady state and compare their results to the GFR calculated by the MDRD (modification in diet in renal desase) and the CKD EPI (Chronic Kidney Disease Epidemiology Collaboration) formulas of the 24 and 48 hours posterior to the day of the kinetic calculation.

We search the electronic records of the patients the maximum value of creatinine (Cr) during their hospitalization in the intensive care, from which, retrospectively, we took the Cr value at the previous 72 h (Cr1) and at the previous 48 h (Cr2) to calculate the Kinetic GFR.

**RESULTS.** We use Pearson´s r to search for a correlation between kinetic GFR to CKD EPI formula at 24 hrs with an r: 0.637 p=0.001 and at 48 hrs r:0.465, p: 0.001, and to the MDRD formula with an r:0.688, p:0.001 at 24 hrs, and at 48 hrs r:0.526, p=0.001. The best correlation found was the formula from Jelliffe with an r at 24 hrs of 0.696; p= 0.001 and at 48 hrs with and r: 0.501; p=0.001 to MDRD and to CKD EPI at 24 hrs with an r: 0.648; p=0.001 and at 48 hrs with an r: 0.463; p=0.001.

**CONCLUSIONS.** The results we find in our study and the correlation of the formula from Jelliffe and S. Cheng (Kinetic GFR) with MDRD and CKD EPI make both of them a good option to estimate GFR in patients in the ICU, plus the calculation of the GFR whit a kinetic formula on a daily basis could improve the time of the diagnosis of AKI in patients in the ICU.

**REFERENCE(S)**

Bragadottir G, Assessing glomerular filtration rate in critically ill patients with acute kidney injury true GFR versus urinary creatinine clearance and estimating equations. Critical Care 2013, 17:R108

Masatomo Y. Evaluation of estimated creatinine clearance before steady state in acute kidney injury by creatinine kinetics. clinical Exp Nephrol 2012 16:570-579

Sheldon Cheng. Retooling the creatinine clearance equation to estimate Kinetic GFR when the plasma creatinine is changing acutely. J Am Soc Nephrol 24: 877-888, 2013

Jelliffe R; Estimation of creatinine clearance in patients with unstable renal function without a urine specimen. Am J Nephrol 22:320-324, 2002

**Table 1 (abstract 0165).** Demographic personal characteristics of the patients

| Characteristics | n=84 |
|---|---|
| Age - (years) | 69.5 ± 13.65 |
| Height - (cm) | 166.2 ± 7.07 |
| Weight - (kg) | 72.55 ± 16.08 |
| APACHE II | 23.49 ± 7.86 |
| SOFA at admission | 12.7 ± 2.81 |
| Days of mechanical ventilation | 9 (4 - 17) |
| Length of stay | 17.5 (9.25 - 28.25) |
| Mortality (%) | 51 (60.7%) |
| Basal GFR MDRD formula | 76.06 (52.24 - 94.24) |

**Table 2 (abstract 0165).** GFR calculated with the creatinine values of the first 48 hrs

| | |
|---|---|
| Masatomo | 89.54 (49.85 - 136.44) |
| Kinetic GFR | 37.18 (25.70 - 56.18) |
| Jelliffe | 53.32 (38.76 - 72.52) |

### 0166

#### Using serum creatinine to estimate glomerular flitration rate (GFR) may be inadecuate in patients with rapidly-changing renal function

M. Herrera-Gutiérrez[1], M.D. Arias-Verdu[1], J. Barrueco-Francioni[1], M. Monsalud Arrebola[2], M.J. Diez de los Rios[3], G. Seller-Perez[1]
[1]Hospital Regional Universitario Carlos Haya, Intensive Care Unit, Málaga, Spain; [2]Hospital Regional Universitario Carlos Haya, Nanotecnologia, Málaga, Spain; [3]Hospital Regional Universitario Carlos Haya, Málaga, Spain
**Correspondence:** M.D. Arias-Verdu
*Intensive Care Medicine Experimental* 2018, **6(Suppl 2):**0166

**OBJECTIVE.** To assess whether estimation of the GFR using the CKD-EPI (Chronic Kidney Disease Epidemiology Collaboration)in patients with rapid changes in thefiltration rate can be considered adequate for monitoring.

**METHODS.** We undertook a retrospective exploratory study of a series of patients undergoing kidney-pancreas transplantation. After admission to the ICU we determined the GFR by creatinine clearance (CrCl) in a one-hour urine collection and compared it with the GFR reported by the laboratory (CKD-EPI). We selected these patients as they showed rapid recovery of the GFR after transplantation, requiring adjustment of drugs (immunosuppressants, antivirals, etc.) based on their GFR and because they had their CrCl measured during follow-up in our unit. For the statistical analysis we used paired sample tests (Wilcoxon) for a significance of 0.05. The data are shown as mean (standard error of the mean). Signed informed consent was obtained accepting use of the data obtained during this process.

**RESULTS.** We studied 16 patients (43.8% women, mean age 41.2±4.3 years) with a total of 37 determinations of CrCL in the immediate post-operative transplant period. On admission the average APACHE II was 21.7±1.01 and SOFA 4.9±0.3. Median ICU stay was 4 days (interquartile range 4).

The mean GFR using CrCl on day 1 was 39.9±7.5 mL/min versus CKD-EPI 20.1±2.1, p 0.029; day 2: 58.3±8.9 versus 37.4±5.2, p 0.002; day 3: 74.1±16.9 versus 52.6±11.9, p 0.016; and day 4: 63.9±20.9 versus 59.6±14.2, p ns. Figure 1.

**CONCLUSION.** Creatinine kinetics makes estimation of the GFR erroneous when there are rapid changes in the filtration rate. Contrary to what is generally accepted, we propose that in this setting the use of serum creatinine or formulas derived from it prove inadequate and that estimation by creatinine clearance with short time intervals is the only means that can reflect in a practical way the changes in GFR. This conclusion may be relevant for adjusting drug dosing according to the GFR.

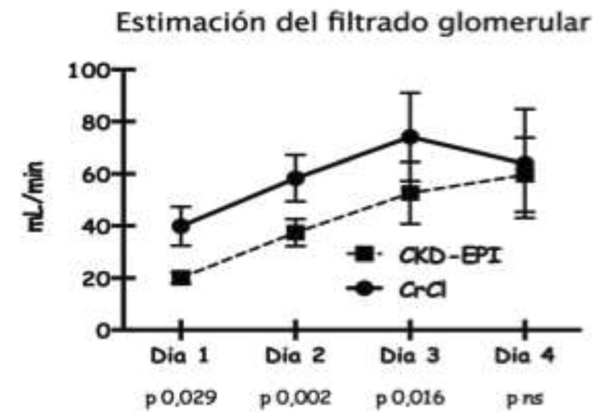

**Fig. 1 (abstract 0166).** Estimated GFR and day

### 0167

#### Right dose, right now: volume-based urine collection to assess creatinine clearance in the critically ill

L. Fleuren[1], H. Lemmens[1], H.J. de Grooth[1], L. Roggeveen[1], T. Guo[1,2], P. Thoral[1], K. Jorna[1], H. Oudemans-van Straaten[1], P.W.G. Elbers[1]
[1]VU University Medical Center, Department of Intensive Care Medicine, Research VUmc Intensive Care (REVIVE), Amsterdam Cardiovascular Sciences (ACS), Amsterdam Infection and Immunity Institute (AI&II), Amsterdam, Netherlands; [2]Amsterdam Medical Center, Department of Pharmacy, Amsterdam, Netherlands
**Correspondence:** L. Fleuren
*Intensive Care Medicine Experimental* 2018, **6(Suppl 2):**0167

**INTRODUCTION.** Glomerular filtration rate (GFR) is a pivotal parameter for guiding antibiotic dosing to prevent toxic and subtherapeutic concentrations in ICU patients. Plasma creatinine-based formulas to estimate GFR, however, are unreliable in the critically ill. Additionally, the clinical gold standard of 24-hour creatinine clearance (CrCl) is error prone and delays decision making. Shorter time windows have shown reasonable agreement with 24-hour CrCl but require strong nursing commitment. Lastly, shorter time windows potentially introduce sampling errors by yielding low or very low volumes, especially in renal dysfunction. Therefore, we propose the concept of volume-based urine collection to improve sampling accuracy, ease of collection, and readiness to serve clinical decision making. We hypothesize that this concept could improve personalized antibiotic dosing.

**OBJECTIVES.** The volume based urine collection concept involves nurses to collect a predetermined volume of urine and record its collection time in order to calculate CrCl. Our main goal was to identify patients with very low and augmented CrCl as they are likely to benefit most from dosing adaptations.

**METHODS.** We included non-anuric adult patients that were admitted to our 24-bed tertiary ICU. For each patient, total urine produced in a two-hour time window was collected. At midpoint, i.e. after one hour, serum creatinine was determined. At that same time point, collection of a 10mL sample started and its collection time was recorded. Subsequently, the additional time it took to fill up the initial sample to a total of 30mL was recorded. Predictive performance of CrCl in the 10mL and 30mL samples to identify very low and augmented renal function was determined and compared to the two-hour samples. In addition, correlation and agreement between methods was assessed.

**RESULTS.** Data were obtained from 95 patients. A positive correlation was observed for CrCl in 10mL and 30mL samples compared to the 2-hour samples, although Bland-Altman analysis showed a percentage of error greater than 30%. The positive predictive value for patients with CrCl < 30mL/min/1.73m$^2$ was 88% in the 10mL and 73.8% in the 30mL sample. For CrCl >130mL/min/1.73m$^2$, the positive predictive values were 85.7% and 79.1% for the 10mL and 30mL sample, respectively.

**CONCLUSIONS.** The concept of volume-based urine collection for determination of creatinine clearance is feasible and helpful. Using a volume as low as 10 mL, very low as well as augmented creatinine clearance could be predicted. This could benefit bedside adjustment of antibiotic dosing. Interestingly, the volume-based method does not provide the same information as the time-based method in our sample. Possible explanations include inaccuracies in sample collection, but also rapid fluctuations in renal function.

**GRANT ACKNOWLEDGMENT**
Partially funded by the ZonMw Rational Pharmacotherapy Program.

## Managing ICU infections

### 0168

#### Important changes in clinical presentation and outcomes of patients treated for severe malaria in a referral French university hospital from 2004 to 2017

J. Lebut[1], B. Mourvillier[2], C. Vinclair[2], A. Radjou[2], S. Houze[3], M. Neuville[2], N. Argy[3], S. Ruckly[4], R. Sonneville[2], L. Bouadma[2], M. Wolff[2], J.-F. Timsit[2,5]
[1]APHP Bichat, Infectious Intensive Care Medicine, Paris, France; [2]APHP, Infectious Intensive Care Medicine, Paris, France; [3]APHP Bichat, Mycology-Parasitology, Paris, France; [4]ICURESEARCH SA, Statistics, Paris, France; [5]Inserm université Paris-Diderot, U 1137 IAME Team 5, Paris, France
**Correspondence:** J. Lebut
*Intensive Care Medicine Experimental* 2018, **6(Suppl 2):**0168

**INTRODUCTION.** The incidence of severe malaria increased since early 2000. Patients at risk, clinical characteristics and prognosis changed during the last decade with the approval of artesunate in 2012.

**OBJECTIVES.** To describe the epidemiology of patients admitted in a referral Intensive Care Unit (ICU) in France for severe malaria and to analyze changes in clinical presentation and outcomes since artesunate became first-choice treatment.

**METHODS.** Retrospective observational single-center study in the medical ICU of a referral university hospital conducting on patients admitted for severe malaria over a 14-year period (2004-2017). Demographic variables, severity scores, WHOs severity criteria on admission, treatment, ICU and hospital lengths of stay were collected. Patients' characteristics and outcomes were compared between two periods, namely 2004-2012 and 2013-2017 when artesunate has become first-choice treatment. A poor outcome was defined as the composite endpoint of death, or ICU length of stay >2 days, or requirement for vasopressors, invasive mechanical ventilation and/or renal replacement therapy started after the first day in ICU. Univariate analysis and stepwise multivariate logistic regression stratified by period were performed to identify factors associated with a poor outcome. Data are presented as median (interquartile range) or numbers (%).

**RESULTS.** 189 patients were included, 98 before and 91 after 2013. Infection was acquired mainly in countries from West and Central Africa (93.6%). Main epidemiological and clinical characteristics are on Table. Even if the number of WHO criteria for severe malaria was comparable in both groups, SAPS II, SOFA and ICU length of stay were significantly higher before 2013. Treatment started before ICU admission was similar between periods. Patients visiting friends or relatives (VFR) in their home country or living in endemic areas were more frequent after 2013 (p = 0.07).

Poor outcome occurred in 63 cases before 2013 and 32 cases after 2013 (p< .01). Risk factors of poor outcome were impaired consciousness (adjOR = 3.25 ; 95%CI(1.50-7.07), p = 0.003), shock (adjOR = 3.46 ; 95%CI(1.36-8.85), p = 0.01) and creatinine >265 μmol/L (adjOR = 14.16 ; 95%CI(4.95-40.48), p< .001). Patients VFR or living in endemic areas were associated with a better outcome (adjOR = 0.34 ; 95%CI(0.15-0.77), p = 0.01). In the final model, artesunate therapy did not significantly improve the outcome as compared to quinine-based therapy (adjOR = 0.53 ; 95%CI(0.16-1.71), p = 0.28).

**CONCLUSION.** Patients with malaria admitted in our ICU after 2013 were less severe than those before 2013. These trends could be partially explained by the increasing proportion of immune patients VFR or living in endemic areas. After adjustment on severity and stratification by period, artesunate was not significantly associated with a better outcome than IV quinine. Other factors such as changes in virulence of plasmodium should be studied.

**Table 1 (abstract 0168).** Patients' demographics and outcome according to periods

| Characteristics | All patients | Before 2013 | After 2013 | p value |
|---|---|---|---|---|
| Gender, male | 119 (63) | 65 (66,3) | 54 (59,3) | 0.32 |
| Age, median [IQR] | 45.3 [31.1 ; 56.3] | 44.6 [29.4 ; 55.6] | 45.4 [32.7 ; 56.8] | 0.33 |
| Visiting friends or relatives/Living in endemic areas | 136 (72) | 65 (66.3) | 71 (78) | 0.07 |
| Europeans traveling for work or tourism | 53 (28) | 33 (33.7) | 20 (22) | . |
| Complete anti-malarial chemoprophylaxis | 12 (6.3) | 6 (6.1) | 6 (6.6) | 0.89 |
| Treatment started before ICU admission | 98 (51.9) | 51 (52) | 47 (51.6) | 0.96 |
| ICU length of stay, median [IQR] | 2 [2 ; 4] | 3.5 [2 ; 7] | 2 [2 ; 3] | <.01 |
| Hospital length of stay, median [IQR] | 7 [5 ; 13] | 8 [6 ; 15] | 7 [5 ; 10] | 0.05 |
| SAPS II, median [IQR] | 25 [18 ; 36] | 27.5 [20 ; 36] | 22 [16 ; 35] | 0.03 |
| SOFA, median [IQR] | 6 [4 ; 8] | 6 [5 ; 8] | 5 [4 ; 7] | <.01 |
| Criteria leading to classification as severe malaria at admission | | | | |
| Hyperparasitaemia > 4% | 112 (59.3) | 59 (60.2) | 53 (58.2) | 0.78 |
| Impaired consciousness | 68 (36) | 35 (35.7) | 33 (36.3) | 0.94 |
| Multiple convulsions | 8 (4.2) | 6 (6.1) | 2 (2.2) | 0.18 |
| Shock | 36 (19) | 12 (12.2) | 16 (17,6) | 0.31 |
| Respiratory failure | 11 (5.8) | 5 (5.1) | 6 (6.6) | 0.66 |
| Spontaneous bleeding | 3 (1.6) | 2 (2) | 1 (1.1) | 0.60 |
| Creatinine > 265 μmol/L | 45 (23.8) | 29 (29.6) | 16 (17.6) | 0.05 |
| Bilirubine > 50 μmol/L | 73 (38.6) | 42 (42.9) | 31 (34.1) | 0.21 |
| Hemoglobin < 7 g/dL | 22 (11.6) | 15 (15.3) | 7 (7.7) | 0.10 |
| Number of criteria, median [IQR] | 2 [1 ; 4] | 2 [1 ; 4] | 2 [1 ; 3] | 0.28 |
| Treatment | | | | |
| Artesunate | 96 (50.8) | 12 (12.2) | 84 (92.3) | <.01 |
| Quinine | 93 (49.2) | 86 (87.8) | 7 (7.7) | <.01 |
| Outcome | | | | |
| Death | 7 (3.7) | 5 (5.1) | 2 (2.2) | 0.29 |
| ICU length of stay > 2 days | 93 (49.2) | 62 (63.3) | 31 (34.1) | <.01 |
| Need of vasopressors after Day 1 | 5 (2.6) | 4 (4.1) | 1 (1.1) | 0.20 |
| Need of mechanical ventilation after Day 1 | 11 (5.8) | 10 (10.2) | 1 (1.1) | <.01 |
| Need of renal replacement therapy after Day 1 | 18 (9.5) | 13 (13.3) | 5 (5.5) | 0.07 |
| Bad outcome | 95 (50.3) | 63 (64.3) | 32 (35.2) | <.01 |

| Univariate analysis | Good outcome | Poor outcome | OR [95%CI] | p value |
|---|---|---|---|---|
| Hyperparasitaemia > 4% | 48 (51.1) | 64 (67.4) | 2.0 [1.1 ; 3.8] | 0.02 |
| Impaired consciousness | 25 (26.6) | 43 (45.3) | 2.5 [1.3 ; 4.7] | <.01 |
| Shock | 11 (11.7) | 25 (26.3) | 4.3 [1.8 ; 10.0] | <.01 |
| Respiratory failure | 1 (1.1) | 10 (10.5) | 13.8 [1.7 ; 113.7] | 0.01 |
| Creatinine > 265 μmol/L | 6 (6.4) | 39 (41.1) | 9.6 [3.7 ; 24.5] | <.01 |

### 0169

#### Diagnostic and prognostic value of HMGB1 for patients with late-onset ventilator-associated pneumonia

L. Dong, L. Li

Wuxi People's Hospital affiliated to Nanjing Medical University, Intensive Care Unit, Wuxi, China

**Correspondence:** L. Dong

*Intensive Care Medicine Experimental* 2018, **6(Suppl 2):**0169

**INTRODUCTION.** The diagnostic and prognostic value of HMGB1 for patients with late-onset ventilator-associated pneumonia (VAP) remains unclear

**OBJECTIVES.** To explore the diagnostic and prognostic value of high mobility group box-1(HMGB1) for patients with late-onset VAP.

**METHODS.** A total of 164 late-onset VAP patients were enrolled from June 2013 to May 2015 in the ICU of Wuxi People's Hospital affiliated to Nanjing Medical University. The basic characteristics and clinical data were collected, while serum HMGB1, procalcitonin (PCT), C-reactive protein(CRP), clinical pulmonary infection score(CPIS) and acute physiology and chronic health evaluation II(APACHEII) were measured. Patients were divided into the death group and the survival group according to 28 d survival status. The basic characteristics and clinical data between groups were compared. The values of sTREM-1, PCT, CPIS and APACHEIIfor predicting 28 d death were evaluated by receiver operating curves(ROC). The surviving curve was drawn by Kaplan-Meier method. The possible prognostic factors were analyzed by univariate and logistic multivariate analysis.

**RESULTS.** There were 88 patients in the survival group and 76 patients in the death group, the basic characteristics between groups exhibited no difference($P$>0.05). The serum s HMGB1, PCT, CPIS and APACHEII were higher in the death group[(289.50±18.45) pg/ml, (123.86±12.74) pg/ml, (17.20±1.74) and (16.58±3.43)] than in the survival group[(154.11±12.61) pg/ml, (79.81±193.45) pg/ml, (11.79 ±1.93) and (7.23±1.12), all $P$< 0.05].The areas under the ROC of HMGB1, PCT, CPIS and APACHEII for predicting 28 d death were 0.84 ±0.04(95%CI:0.75-0.92, $P$< 0.01), 0.65±0.05(95%CI:0.55-0.74, $P$=0.49), 0.67±0.06(95%CI:0.55-0.79, $P$< 0.01), 0.79±0.04(95%CI:0.70-0.87, $P$=0.03) respectively. Patients were assigned into two groups by the best cutoff point of HMGB1=175.00 pg/ml, Kaplan-Meier survival analysis showed that 28 d survival rate in the low HMGB1 group was significantly higher than that in the high HMGB1 group (82.5% vs 63.4%, $\chi^2$=3.96, $P$< 0.05). Multivariate logistic regression analysis showed that HMGB1 (OR=1.08,95%CI:1.04-1.13,$P$< 0.01) and APACHEII (OR=1.39,95%CI:1.15-1.67,$P$< 0.01) were both risk factors associated with 28 d death.

**CONCLUSIONS.** Serum HMGB1 can be used as a reliable predictor for the outcome of patients with late-onset VAP.

**GRANT ACKNOWLEDGMENT**

This work was financially supported by the National Natural Science Foundation of China (No.81400054), the Natural Science Foundation of Jiangsu Province (No.BK20140122), and the Talented Youth Program of Jiangsu Province (No.QNRC2017179).

### 0170

#### Catheter associated urinary tract infections in an intensive care unit of a teaching hospital in Istanbul

A. Inan[1], A. Ozgultekin[2], S. Aksaray[3]

[1]University of Health Sciences , Haydarpasa Numune Training and Research Hospital, Infectious Diseases, Istanbul, Turkey; [2]University of Health Sciences , Haydarpasa Numune Training and Research Hospital, Anaesthesiology and Intensive Care, Istanbul, Turkey; [3]University of Health Sciences , Haydarpasa Numune Training and Research Hospital, Microbiology, Istanbul, Turkey

**Correspondence:** A. Inan

*Intensive Care Medicine Experimental* 2018, **6(Suppl 2):**0170

**INTRODUCTION.** Catheter associated-urinary infections (CAUTI) are one of the most frequent hospital acquired infections in ICU. The infections increase morbidity, mortality, length of hospital stay, and cost. Therefore, early and effective empiric antimicrobial therapy are essential and unit-spesific CAUTI rates and CAUTI causing organisms should be determined.

**OBJECTIVES.** The aim of the study was to evaluate the rate of hospital acquired (CAUTI) and the change of profiles causative microorganisms in an intensive care unit (ICU) of a teaching hospital.

**METHODS.** CAUTI was diagnosed by using the clinical criteria of the Centers for Disease Control and Prevention. Isolated strains was identified and antibiotic resistance of species was determined by the disk diffusion method according to the The European Committee on Antimicrobial Susceptibility Testing criteria and Vitek 2 system. Fisher exact test was used for the statistical analysis.

**RESULTS.** Between January 2010 and December 2017, a total of 8756 patients hospitalized for 59,317 patient days in ICU and acquired 184 urinary infection (2.10 per 100 patients, and 3.13 per 1,000 patient days). The most frequently isolated microorganisms were *Candida spp*, *Enterococcus spp*. and *Enterobacteriacea spp*. The incidence densities of infections decreased from 5.79 to 0.81 per 1,000 device-days (p< 0,05) for catheter-associated urinary tract infection (CAUTI) during the study period. It was found that an increase in the proportion of *Enterococcus spp*. isolated from CAUTI (from 17.0 % to 20.0 %, p=0.716), and a decrease in the proportion of C*andida spp*. isolated from CAUTI (from 45.5% to 20.0%, p< 0.05).

**CONCLUSIONS.** In conclusion, CAUTI rates are decreasing in our ICU. An increasing trend of infections caused by *Enterococcus spp*. and a decrease in the proportion of *Candida* isolates is the striking findings of this study.

### 0171

#### Diagnostic and prognostic value of HMGB1 for patients with abdominal infection of severe acute pancreatitis

L. Dong, L. Li
Wuxi People's Hospital affiliated to Nanjing Medical University, Intensive Care Unit, Wuxi, China
**Correspondence:** L. Dong
*Intensive Care Medicine Experimental* 2018, **6(Suppl 2):**0171

**INTRODUCTION.** The diagnostic and prognostic value of HMGB1 for patients with abdominal infection of severe acute pancreatitis (SAP) remains unclear

**OBJECTIVES.** To explore the diagnostic and prognostic value of high mobility group box-1(HMGB1) for patients with abdominal infection of SAP.

**METHODS.** A total of 103 SAP patients were enrolled from June 2013 to May 2015 in the ICU of Wuxi People's Hospital affiliated to Nanjing Medical University. The basic characteristics and clinical data were collected, while serum HMGB1, procalcitonin (PCT), Ranson score and acute physiology and chronic health evaluation II(APACHEII) were measured. Patients were divided into the death group and the survival group according to 28 d survival status. The basic characteristics and clinical data between groups were compared. The values of HMGB1, PCT, Ranson and APACHEIIfor predicting 28 d death were evaluated by receiver operating curves(ROC). The surviving curve was drawn by Kaplan-Meier method. The possible prognostic factors were analyzed by univariate and logistic multivariate analysis.

**RESULTS.** There were 76 patients in the survival group and 27 patients in the death group, the basic characteristics between groups showed no difference(*P*>0.05). The serum HMGB1, PCT, Ranson and APACHEII were higher in the death group[(289.50±18.45) pg/ml, (823.86±182.74) pg/ml, (7.20±1.74) and (19.58±3.43)] than in the survival group[(154.09±12.71) pg/ml, (579.81±193.45) pg/ml, (4.79±1.93) and (17.23±3.12), all *P*< 0.05]. The areas under the ROC of HMGB1, PCT, Ranson and APACHEII for predicting 28 d death were 0.84±0.04 (95%CI:0.75-0.92, *P*< 0.01), 0.65±0.05(95%CI:0.55-0.74, *P*=0.49), 0.67 ±0.06(95%CI:0.55-0.79, *P*< 0.01), 0.79±0.04(95%CI:0.70-0.87, *P*=0.03) respectively. Patients were assigned into two groups by the best cutoff point of HMGB1=200.00 pg/ml, Kaplan-Meier survival analysis showed that 28 d survival rate in the low HMGB1 group was significantly higher than that in the high HMGB1 group (82.5% vs 63.4%, $\chi^2$=3.96, *P*< 0.05). Multivariate logistic regression analysis showed that HMGB1 (OR=1.08,95%CI:1.04-1.13,*P*< 0.01) and APACHEII (OR=1.39,95%CI:1.15-1.67,*P*< 0.01) were both risk factors associated with 28 d death.

**CONCLUSIONS.** Serum HMGB1 can be used as a reliable predictor for the diagnosis and outcome of patients with abdominal infection of SAP.

**GRANT ACKNOWLEDGMENT**
This work was financially supported by the National Natural Science Foundation of China (No.81400054), the Natural Science Foundation of Jiangsu Province (No.BK20140122), and the Talented Youth Program of Jiangsu Province (No.QNRC2017179).

### 0172

#### Outcome of influenza related intensive care unit admissions in 2017- 2018 flu season: a multicenter study from Turkey

E. Ortac Ersoy[1], B. Er[1], S. Eyupoglu[1], F. Ciftci[2], A. Gulleroglu[3], K. Suner[4], B. Arpinar[5], G. Aygencel[6], S. Akpinar[7], H. Sungurtekin[8], K. Rollas[9], S. Turan[10], A. Topeli[1]
[1]Hacettepe University Faculty of Medicine, Medical Intensive Care Unit, Ankara, Turkey; [2]Ankara University Faculty of Medicine, Chest Diseases Intensive Care Unit, Ankara, Turkey; [3]Baskent University, Intensive Care Unit, Ankara, Turkey; [4]Konya Education and Research Hospital, Konya, Turkey; [5]Yedikule Chest Diseases and Chest Surgery Training and Research Hospital, Intensive Care Unit, Istanbul, Turkey; [6]Gazi University Faculty of Medicine, Medical Intensive Care Unit, Ankara, Turkey; [7]Dışkapı Yıldırım Beyazıt Education and Research Hospital, Medical Intensive Care Unit, Ankara, Turkey; [8]Pamukkale University Faculty of Medicine, Intensive Care Unit, Denizli, Turkey; [9]Zonguldak State Hospital, Intensive Care Unit, Zonguldak, Turkey; [10]Türkiye Yüksek İhtisas Education and Research Hospital, Intensive Care Unit, Ankara, Turkey
**Correspondence:** E. Ortac Ersoy
*Intensive Care Medicine Experimental* 2018, **6(Suppl 2):**0172

**INTRODUCTION.** Influenza is an acute respiratory disease which can cause local outbreaks or seasonal epidemics. Although the clinical presentation is mostly asymptomatic or mild, it can be fulminant requiring intensive care.

**OBJECTIVES.** To evaluate outcome of patients admitted to intensive care unit (ICU) due to influenza in 2017-2018 flu season in Turkey.

**METHODS.** This is the preliminary results of a retrospective multicenter study conducted in 10 medical ICUs. From October 1st 2017 to April 5th 2018, all adult patients with confirmed influenza infection admitted to ICUs were included and demographic and clinical features and ICU outcome were evaluated.

**RESULTS.** A total of 99 patients were included. The mean (±SD) age was 64.2±16.8 years and 51.4% were male. The mean body mass index (BMI) was 27.3±7.4 kg/m$^2$. Chronic cardiac disease (56.6%), lung disease (45.5%), and diabetes (33.3%) were the most common underlying diseases. The mean APACHE II, first day SOFA scores and $PaO_2/FiO_2$ ratio were 19.7±8.1, 6.0±4.1 and 132.4±80.8, respectively. Of them, 70.7% received noninvasive mechanical ventilation, 29.0% received high flow nasal oxygen and 52.5% required invasive mechanical ventilation. 56.6% of the patients had sepsis, 44.4% shock and 40.5% acute renal failure. 65.7% patients were infected with Influenza A, 30.3% with Influenza B and 4% with both. 20.7% of the patients had concomitant positive viral serology for RSV and rhinovirus. 29.3% of patients had positive respiratory bacterial cultures within 72 hours of admission. The mean ICU and hospital length of stay were 13.8 ±13.9 and 23.4±21.8 days, respectively. ICU mortality rate was 31.3%.

Hospital mortality rate was 33.3%. In bi-variate analysis, increased age, APACHE II and SOFA scores; presence of sepsis, shock, chronic renal failure and malignancy; and invasive mechanical ventilation were associated with ICU mortality. However, in logistic regression analysis, presence of malignancy and invasive mechanical ventilation were the only factors affecting mortality (OR [95% CI] 6.57 [1.18-36.43]; p=0.031 and 50.03 [5.34-468.60]; p=:0.001, respectively).
**CONCLUSIONS.** ICU and hospital mortality rates in patients admitted to 10 ICUs in Turkey due to Influenza in 2017-2018 flu season were 31.3% and 33.3%. Presence of malignancy and invasive mechanical ventilation were related with increased ICU mortality.

**REFERENCE(S)**

1. Paules, C. and K. Subbarao, *Influenza.* Lancet, 2017. **390**(10095): p. 697-708.

**GRANT ACKNOWLEDGMENT**
None

### 0173

#### Intensive care in tetanus: management, complications and mortality in 13 cases

S. Bradai, O. Turki, M. Dlela, K. Bouchaala, M. Bahloul, M. Bouaziz
Habib Bourguiba University Hospital, Intensive Care Departement, Sfax, Tunisia
**Correspondence:** S. Bradai
*Intensive Care Medicine Experimental* 2018, **6(Suppl 2):**0173

**INTRODUCTION.** Tetanus is an acute infectious disease caused by toxigenic strains of *Clostridium tetani*. The spores enter the body through contaminated skin wounds. This disease is associated with poor outcomes.
**OBJECTIVES.** To study the demographic and clinical features, outcome, complications and treatment of tetanus patients requiring intensive care unit (ICU) admission in Habib Bourguiba University Hospital, Sfax, Tunisia.
**METHODS.** It is a retrospective, descriptive and observational study conducted at ICU between January 01, 2006 and December 31, 2017. All Patients suffering from tetanus, admitted during the study period, were enrolled.
**RESULTS.** There were 13 cases of tetanus. The Sex ratio (male/female) was 1.6. The mean age was 62.1±11.3 years (range 46-82). Regarding the vaccination status, 11 patients were either unvaccinated or didn't know their immunization status. The incubation period was on average 10 days (range 3-20). The period of onset was on average 2 days (range1-5). All patients developed lock jaw, neck stiffness, dysphagia and muscle rigidity. Ten patients developed spasms. Only one patient hadn't developed autonomic instability. All patients needed mechanical ventilation and sedation with a mean duration of 20±15 days (range 0-57). Ten patients needed tracheotomy. The APACHEII score ranged between 3 and 19 with an average of 8. The mean DAKAR score was 2. The mean PHILIP score was 15 (range 7-19). The mean M.Thwaites score was 3 (range 0-11). According to Ablett classification, the majority of our patients (6 patients) belonged to grade II, 1 patient had grade I, 2 patients had grade III and 3 patients had grade IV. Pulmonary complications were dominated by ventilator-associated pneumonia (9 patients). Eight patients had autonomic cardiovascular disturbances. Other systemic complications were observed: gastrointestinal stasis in 3 patients, acute kidney failure in 3 patients, myopathies and neuropathies in 3 patients. All patients received the following specific treatment. All wounds were cleaned and debrided. Immunotherapy and vaccination were administrated by intramuscular injections in two different sites. The association of Penicilline G and Metronidazole remains the antibiotic most frequently used (11 patients). To control muscle spasm, Benzodiazepines were used with mean dosage of 200mg and mean period of 12 days. Mean ICU stay was 26.6±15.8 days. Five patients died. The mortality rate was 38.5%.
**CONCLUSIONS.** Tetanus is fortunately a rare disease in Tunisia and is entirely preventable by vaccination. Major therapeutic challenges lie in the control of spasms and muscular rigidity, autonomic dysfunction and the prevention of complications associated with prolonged critical illness.

**REFERENCE(S)**

1. Mahieu R, Reydel T et al. Admission of tetanus patients to the ICU: a retrospective multicentre study. Ann Intensive Care 7 nov 2017
2. Thwaites C.L., Farrar J.J. Preventing and treating tetanus. BMJ.2003;326:117-118.

### 0174

#### Multidrug-resistant pattern in a tertiary hospital ICU in Spain

M.C. Molina de la Torre, M.I. Ruiz Garcia, M.M. Gordillo Resina, R.M. Vela Colmenero, J.F. Machado Casas
Complejo Hospitalario de Jaen, Unidad de Cuidados Criticos, Jaén, Spain
**Correspondence:** M.C. Molina de la Torre
*Intensive Care Medicine Experimental* 2018, **6(Suppl 2):**0174

**INTRODUCTION.** The increasing problem of antimicrobial resistence (AMR) in the intensive care units, constitutes a serius threat to public health of global reach,
**OBJECTIVES.** To identify and to describe the MDR map in our ICU and its evolucion among the last six years (from 2012 to 2017), as well as analyce several epidemiological and clinical factors associated.
**METHODS.** Retrospective study including all patients admitted from 2012 to 2017 in the ICU in a tertiary hospital. Data concerning demographic characteristics and clinical history were collected from each patient. We analysed the MDR bacteria infection and colonization cases in our ICU. We used the MDR pathogens cataloged as such by "Resistencia-Zero" project. We do also analysed the percentage use of several antibiotics, as well as its possible association with resistance map changes.
**RESULTS.** There were 269 patiens with infection by MDROs in these period, of these, 68.57% were male. The length of stay in ICU was 8.11 days. The average age was 62.73 years, with an APACHE II average of 13.48. The global mortality in these patiens was 18.83%.
A situation of a very high rate of Imipenem-Resistant *Acinetobacter* was detected, we could say this is an *endemic* infection. This situation changes in 2016 when the number of cases decreases, although a high percentage of cases still persists in comparation with the nationally data . In the same year there was a increase in the appearance of *Enterobacteriaceae ESBL*, being similar to the national registry. A decrease of more than 50% in carbapenem use is also detected this year.
Our *MRSA* cases are anecdotal. Also noteworthy is the absence of Vancomicin resistant *Enterococcus*.
In 2017 *Carbapenem-R GN* makes its appearance on stage, a fact that we intent to analyse later.
**CONCLUSIONS.** This study shows a turnaround in the pattern of antimicrobial resistance in our intensive care unit, which consists in a progreive decrease in cases of *Acinetobacter* R infection, and an increase in infections by *Enterobacteriaceae ESBL* (the same data were found also in the national registry). We think it could be related to a decrease use of carbapenem, as well as the aplication of selective decontamination of the digestive tract (SDD). The frecuency of infections by the rest of the multidrug-resistant microorganisms analysed remains practically unchanged or with minimal changes over the years. It is remarkable the appereance in 2017 of *Carbapenem-R GN*. This is an alarming fact, wich we should analyse in the future. We consider that knowing own patterns, their changes and relationed factors is esential to prevent and control AMR, and may help to reduce the burden of MDR in the ICUs.

**REFERENCE(S)**

What's new in multidrug-resistant pathogens in the ICU. Gabor Zilahi, Antonio Artigas and Ignacio Martin-Loeches Intensive Care (2016)
The rising problem of antimicrobial resistance in the intensive care unit. Nele Brusselaers Annals of Intensive Care 2011, 1:47

#### 0175

**MDR colonization: what´s the matter in the perioperative setting?**

A. Lerose, K. Donadello, D. Cigolini, L. Gottin, E. Polati
University of Verona, Department of Surgery, Dentistry, Pediatrics and Gynaecology, Verona, Italy
**Correspondence:** A. Lerose
*Intensive Care Medicine Experimental* 2018, **6(Suppl 2):**0175

**INTRODUCTION.** Multi-Drug-Resistant (MDR) colonization is an actual major issue in intensive care units (ICUs) and perioperative medicine, frequently resulting in life-threatening infections.

**OBJECTIVES.** The aim of this study was to evaluate the post-operative course of patients requiring ICU admission after surgery and to compare MDR colonized patients (pts) undergoing abdominal surgery to non colonized ones.

**METHODS.** We retrospectively analyzed all consecutive patients undergoing surgery in our hospital for a three-month period (July-September 2017) who required admission to our 12-bed-medico-surgical ICU on post-operative day (POD) 0 (Dept Anesthesia and Intensive Care B, Policlinico GB Rossi). We then compared MDR colonized (rectal and/or pharyngeal swab) patients (MDR+) to non colonized patients (MDR-) for post operative complications, ICU and hospital length of stay (LOS) and mortality. We also investigated the impact of administered targeted perioperative antibiotic therapy compared to empiric antibiotic prophylaxis.

**RESULTS.** 70 pts were included in the study (47 men), median age 67(55-79) years; they underwent either elective (n=57, 81.4%) or emergency (n= 13, 18.6%) surgery; 33 pts (47.1%) were submitted to pancreatic surgery, 15 (21.4%) to intestinal resection, 13 (18.6%) to hepato-biliary surgery, and the rest to other kind of abdominal surgery; they were admitted to ICU on POD 0 for scheduled (n =52, 74,3%) or unscheduled (n= 18, 25,7%) intensive PO monitoring. These latter were admitted because of surgical length (10, 4.9%), septic episode (4, 5.7%), intraoperative hypotension (2, 2.9%) , intraoperative hemorrage (2, 2.9%).

11 pts were MDR+ pre operatively (15,7%), 4 of whom were either Klebsiella Pneumoniae Carbapenemasis Producer or Vancomycin Resistant Enterococcus (VRE) colonized and 7 pts were Extended Spectrum Beta Lactamase (ESBL) Escherichia Coli colonized. Both ICU and hospital LOS were significantly higher in MDR+ compared to MDR- (16±23 vs 2±3 and 37±25 vs 22±16, respectively, $p < 0.05$). Hospital mortality occured in 4 patients, all MDR+ ($p < 0.001$). Postoperative complication incidence and type did not significantly differ between the two groups.

4 MDR+ patients (5.7%) received targeted pre-operative antibiotic therapy compared to standard prophylaxis but this did not influence their outcome (small number).

**CONCLUSIONS.** Pre-operative surveillance swab positivity correlates with both ICU and hospital LOS and post-operative mortality, no matter swap and surgical sites. Targeted antibiotic therapy may be routinely used to improve patients' outcome. More studies are needed to further investigate the possibile therapeutic options in MDR+ patients undergoing surgery.

#### 0176

**A point prevalence study of ventilator associated pneumonia (VAP) across four London ICUs**

N. Shah[1,2], J. Hadley[1,3], P. Zolfaghari[1,3], C. Hinds[1,4]
[1]Queen Mary, University of London, London, United Kingdom; [2]Queen Elizabeth Hospital, Department of Chest Medicine, London, United Kingdom; [3]Royal London Hospital, Intensive Care Medicine & Anaesthesia, London, United Kingdom; [4]St Bartholomew's Hospital, Intensive Care Medicine, London, United Kingdom
**Correspondence:** N. Shah
*Intensive Care Medicine Experimental* 2018, **6(Suppl 2):**0176

**INTRODUCTION.** There are few studies describing the incidence and prevalence of VAP in the UK. This may partly be due to the complexity of the various definitions of VAP in current use. We describe a one-day point-prevalence study of VAP using a pragmatic definition and a specially adapted electronic case report form.

**OBJECTIVES.** To determine the point prevalence of VAP with 30-day follow-up using an electronic data collection tool in 4 ICUs at Barts Health NHS Trust: The Royal London (RLH-trauma/neuro/general), St. Bartholomew's Cardiac ICU (SBH cardiac), St.Bartholomew's general/oncology (SBH), and Whipps Cross University Hospital (WXH-general).

**METHOD.** This was a multi-centre 24-hour cross-sectional observational study. Data was collected for all patients receiving Advanced Respiratory Support over a 24-hour period on the 13 April 2016 with 30-day follow-up on the 13 May 2016. Study data was collected using a purpose built REDCap™ (Research Electronic Data Capture) portal hosted at Queen Mary University of London (UK). Additional data were collected for patients with a study definition of VAP (commenced on antibiotics for chest sepsis 48 hours or more after initiation of invasive ventilation). This audit was registered with the clinical effectiveness unit.

**RESULTS.** Data was submitted for 35 patients across 4 ICUs on study entry: mean age 54.7 years (std±18.1,17-83); male 28 (80%) and female 7 (20%); mean ICU length of stay 18.8 days (range 1-126); duration of ventilation 17.3(std±26.6,1-126); mean $FiO_2$ 0.4 (std±0.1,0.21-0.90). Seven patients (20%) met the study definition for a current VAP: mean age 60.3 years (std±19.0,34-82); mean initial APACHEII score 21.1 (std±8.6,13-34); mean ICNARC score (5 of 7 patient data) 17.8 (std±4.0,12-22) and mean initial SOFA score (5 of 7 patient data) 7.2 (std±3.9,3-13).

30-day follow-up for those who met study definition of VAP revealed: mean ventilator days 23.7 (±19.7,8-65); mean ICU LOS (days) 26.4 (±17.8,13-65); 2 patients (28.6%) died on ICU; 3 patients (42.9%) were discharged from ICU; 2 patients (28.6%) remained as ICU inpatients; mean hospital LOS (days) 38.4 (std±14.8, 21-65) and tracheostomy was performed in 4 (57%) patients. Pathogens were identified in 6 cases (85.7%), *E.coli* was isolated most frequently (3/7, 42.9%); Proteus spp. in 2 cases (28.6%); *S.aureus* (MSSA) in 2 cases (28.6%); with single cases of Klebsiella spp., unidentified *S.aureus* and Candida spp. Antibiotics for VAP included piperacillin tazobactam (4); vancomycin (2); meropenem (2); amikacin (1) and fosfomycin (1).

**CONCLUSIONS.** This pilot study of an electronic data collection portal was successfully used to determine a point prevalence of 20% (7/35) of VAP across 3 ICUs with a varied case-mix. The methodology used showed good feasibility to carry out a wider national VAP study to identify the epidemiology of VAP.

**GANt ACKNOWLEDGMENT**
Support from Barts Charity Clinical Research Training Fellowship Grant.

#### 0177

**Hospital-acquired infections due to Enterobacteriaceae in critical care**

M.A. Boujelbèn[1], H. Kallel[1], M. Tobich[1], D. Sakis[1], I. Fathallah[1], S. Habacha[1], N. Ben Salah[2], N. Kouraichi[1]
[1]Yasminette Regional Hospital, Medical Intensive Care Unit, Ben Arous, Tunisia; [2]Yasminette Regional Hospital, Clinical Laboratory, Ben Arous, Tunisia
**Correspondence:** M.A. Boujelbèn
*Intensive Care Medicine Experimental* 2018, **6(Suppl 2):**0177

**INTRODUCTION.** Hospital acquired infections (HAI) due to gram negative bacteria are oppose tremendous challenges in the spectrum of critically ill patients. These infections are associated with higher morbidity, mortality and healthcare costs. Besides *Acinetobacter baumannii* and *Pseudomonas aeruginosa,* the *Enterobacteriaceae* family is increasingly responsible for nosocomial infections in the recent years.

**OBJECTIVES.** To determine the frequency, the microbiological characteristics, risk factors and outcomes of HAI due to *Enterobacteriaceae* in a 6 separate beds MICU in Tunisia.

**METHODS.** A retrospective analysis of chart reviews of all patients admitted more than 48H to the 6-bed tertiary MICU of Yasminette regional Hospital since the inauguration of the unit on October 17th,

2016 to July 17th, 2017.Were assessed the patients' characteristics, bacteriological aspects of HAI and outcomes. Univariate and multivariate regression analyses were performed to identify factors independently associated to *Enterobacteriaceae´* HAI.

**RESULTS.** Of the 97 admitted patients during the 9 months of the study, 90 (92.7%) were eligible and they acquired 57 nosocomial infections. The main characteristics of the patients were : mean age, 55.9±20.6 ; male gender, 63(70%) ; SAPS II, 41.7±20.4 ; invasive mechanical ventilation, 49(50.5%) ; median mechanical ventilation duration, 5[3-17] days ; length of stay, 8[4-15.5] days ; mortality, 28(28.9%). 22(38.6%) HAI episodes were due to *Enterobacteriaceae* and they were detected in 14(15.5%) patients. The identified *Enterobacteriaceae* were responsible for 8 (36.4%) central venous catheter acquired infections (CVC-AI), 7 (31.8%) ventilator associated pneumonias (VAP) and 7 (31.8%) urinary tract infections (UTI). 2 (28.5%) VAPs, 2 (25%) CVC-AIs and 1 (14.2%) UTIs were poly-microbial (associated with *Pseudomonas aeruginosa*). 13(59.1%) of the isolated bacteria were Extended-spectrum β- lactamases (ESBL) producers and 6(27.2%) were carbapenemases producers. *Klebsiella pneumoniae* was the most frequently isolated microorganism: 40.9% (n = 9). Table 1 describes the type of nosocomial infection according to the responsible *Enterobacteriaceae*. The independent risk factors for Enterobacterial infection were acute renal failure upon admission (OR=6.23, CI95[1.06; 36.56], p=0.043) and a length of stay ≥ 12 days (OR=8.36, CI95[1.26; 55.31], p=0.028).

**CONCLUSIONS.** More than the third (38.6%) of nosocomial infections in our MICU are due to *Enterobacteriaceae* with a recurrent presence of *Klebsiella pneumoniae*. The alarming emergence of carbapenem-resistant strains requires improved measures of infection control and prevention.

**Table 1 (abstract 0177).** Type of nosocomial infection according to the responsible Enterobacteriaceae

| | Central venous catheter acquired infection | Ventilator associated pneumonia | Urinary tract infection | Total |
|---|---|---|---|---|
| Klebsiella pneumoniae | 4 | 1 | 4 | 9 |
| Escherichia coli | 1 | 3 | 2 | 6 |
| Enterobacter cloacae | 1 | 0 | 1 | 2 |
| Providencia rettgeri | 1 | 1 | 0 | 2 |
| Pantoea spp | 1 | 1 | 0 | 2 |
| Proteus vulgaris | 0 | 1 | 0 | 1 |
| Total | 8 | 7 | 7 | 22 |

### 0178

#### The rate of the catheter-related urinary system infection after constructive training to the staff in the intensive care unit

S. Yarimoglu[1], S. Erdinc[1], C. Kaymak[2], N. Tulek[1], H. Basar[2], G. Ertem[1], S. Kinikli[1]

[1]Ministry of Health, Ankara Training ang Research Hospital, Infectious Diseases and Clinical Microbiology, Ankara, Turkey; [2]Health Science University, Ankara Heealth Application and Research Center, Anaesthesiology and Reanimation, Intensive Care Unit, Ankara, Turkey

**Correspondence:** C. Kaymak

*Intensive Care Medicine Experimental* 2018, **6(Suppl 2):**0178

**INTRODUCTION.** Healthcare-related infections are controllable and avoidable infectious diseases.

**OBJECTIVES.** The aim of this study is to reduce catheter associated urinary tract infection (CA-UTI) rates by carrying out a constructive training programme for the staff working in Intensive Care Units (ICU) of the Hospital.

**METHODS.** This study is a retrospective and prospective interventional thesis study. There are three Anesthesiology and Reanimation ICU in our hospital. The health staff of these three units consists of 62 people. There are 38 nurses, 17 assistant health personnels and 7 doctors. Constructive training interviews were conducted with all ICU staffs between 1-31 December 2016 to prevent CA-UTI. Before the constructive training programme, CA-UTI data of ICUs from 1 June to 30 November were obtained from the infection control committee (ICC) of the hospital. After the constructive training programme, the surveillance data were obtained from 1 January to 30 June and the cases were evaluated with ICC. In the hospital, National Hospital Infection Surveillance Standards are used according to CDC´s 2008 diagnostic criteria, as diagnostic criteria for health-care related infections by ICC. Data was compared before and after constructive training, statistical analysis of the study was done by SPSS version 15.0 program. A four-question survey was conducted by the staff to assess the constructive training programme. The result of this survey is also evaluated.

**RESULTS.** When the 6-month CA-UTI rates of the ICUs before and after the constructive training programme were compared, pre-training and post-training CA-UTI rates were 8.3 and 8.7 (p=0,837) in ICU-1 respectively; 7.0 and 8.1 (p=0,745) in ICU-2; In ICU-3 it is calculated as 7.3 and 9.9 (p=0,495). There was no statistically significant difference found between pre-training and post-training data for these three ICUs. According to the assessment questionnaire, 92% of nurses (35/38) and 88% of assistant health personnels (15/17) were positively effected from the training programme but among them, 63% of nurses and 33% of assistant health personnel were able to apply what they learned.

**CONCLUSIONS.** In all three ICUs, the intervention of constructive training did not significantly reduce the CA-UTI rates alone. However, the awareness of the staff about CA-UTIs were increased and also improvable, but time-consuming problems are identified. As a suggestion, the constructive training programme which aims to prevent CA-UTIs should be improved.

**GRANT ACKNOWLEDGMENT**

None

### 0179

#### Clinical outcome, microbiological spectrum and antibiotic susceptibility of clinical isolates from adult intensive care unit in a tertiary care hospital in India

A. Sardar, A.M. Kumar, L. Kashyap, V. Darlong, P. Khanna

All India Institute of Medical Sciences, Anaesthesiology Pain and Critical Care, New Delhi, India

**Correspondence:** A. Sardar

*Intensive Care Medicine Experimental* 2018, **6(Suppl 2):**0179

**INTRODUCTION.** Incidence and prevalence of hospital acquired infections are increasing alarmingly over the years throughout the world.

**OBJECTIVE.** To find out microbiological spectrum, antibiotic susceptibility of the organisms prevalent in the intensive care unit (ICU) and to determine the cause of ICU mortality, clinical outcome & organ dysfunction due to nosocomial infection.

**METHODS.** The study was conducted in a 12 bedded mixed medical and surgical ICU of the All India Institute of medical Sciences, New Delhi, India. A cross sectional retrospective analysis were carried out based on ICU chart review of microbiological data, clinical parameters of all the patients whose clinical sample's culture came out to be positive during the ICU stay from 1st May 2014 to 30th April 2016.

**RESULTS.** Total 1243 patients were admitted in the specific time period. Out of which 172 (100 alive and 72 dead) patients were found to be microbiological culture positive of their clinical samples and included in the analysis. Total 207 organisms were isolated from the clinical samples. Only 17 samples had grown gram positive infection where as rest 190 samples had gram negative infection. Most common organisms were Pseudomonas, Acinetobacter, Klebsiella and E.coli. Most of the gram negative organisms were multidrug resistant. Risk factors associated with overwhelming gram negative infections were diabetes mellitus, hypotension, cardiovascular support >7days, respiratory support >15 days, renal support >2days, haematological support >2days, pre admission placement of arterial catheter, pre admission hemodialysis therapy and immunosuppressive drugs, whereas presence of COPD was found to be associated with survival.

**CONCLUSIONS.** Most of the hospital acquired infections are fatal, multidrug resistant and carries poor prognosis to the patients.

**REFERENCE(S)**

1. Vincent J-L, Rello J, Marshall J, Silva E, Anzueto A et al. International study of the prevalence and outcomes of infection in intensive care units. JAMA. 2009;302(21):2323-9.

**GRANT ACKNOWLEDGMENT**

None.

**Table 1 (abstract 0179).** RESISTANCE PATTERN OF THE MOST COMMON ORGANISMS

| | Pseudomonas N=63 N(%) | Acinetobacter N=40 N(%) | Klebsiella N=38 N(%) | E.coli N=25 N(%) |
|---|---|---|---|---|
| Third generation cephalosporins | 50 (79.4) | 34 (85) | 28 (73.7) | 15 (60) |
| BL+BLI combinations | 33 (52.4) | 31 (77.5) | 25 (65.8) | 9 (36) |
| Carbapenems | 27 (42.9) | 22 (55) | 8 (21) | 7 (28) |
| Aminoglycosides | 41 (65) | 24 (60) | 26 (68.4) | 11 (44) |
| Fluoroquinolones | 37 (58.7) | 28 (70) | 28 (73.3) | 12 (48) |
| Resistant to all Five classes (XDR) | 21 (33.3) | 12 (30) | 6 (15.8) | 3 (12) |

**Table 2 (abstract 0179).** REGRESSION MODEL FOR RISK FACTORS FOR MORTALITY WITH INFECTION

| Parameter | P value | Odd Ratio (95% CI) |
|---|---|---|
| Diabetes Mellitus | 0.001 | 6.42 (2.06-20.03) |
| COPD | 0.05 | 0.20 (0.04-1.00) |
| Hypotension | 0.005 | 4.20 (1.54-11.43) |
| CVS support >7days | 0.003 | 3.88 (1.56- 9.61) |
| Ventilator > 15 days | 0.001 | 5.52 (2.15- 14.15) |
| Dialysis > 2 sessions | 0.001 | 3.5 (1.68- 9.36) |
| Blood products >2 days | 0.002 | 4.43 (1.75- 11.20) |
| Arterial Catheter | 0.025 | 4.79 (0.90- 25.34) |
| Immunosupression | 0.011 | 5.06 (1.45- 17.54) |

### 0180

#### Nosocomial bacterial meningitis (NBM) in the ICU: a two year retrospective study

M.P. Almyroudi[1], M. Rizos[1], E. Paramythiotou[1], F. Frantzeskaki[1], A. Vasilakopoulou[2], S. Pournaras[2], I. Tsangaris[1], A. Armaganidis[1], G. Dimopoulos[1]

[1]National and Kapodistrian University of Athens, Medical School / Attikon University Hospital, Second Department of Critical Care, Athens, Greece; [2]National and Kapodistrian University of Athens, Medical School / Attikon University Hospital, Microbiology Department, Athens, Greece

**Correspondence:** M.P. Almyroudi

*Intensive Care Medicine Experimental* 2018, **6(Suppl 2):**0180

**INTRODUCTION.** Nosocomial infection of the central nervous system is related to neurosurgical procedures, trauma, or rarely to systemic infection[1]. Risk factors include increased drainage duration, intraventricular hemorrhage, surgical technique and CSF leak[2].

**OBJECTIVES.** Retrospective analysis of NBM treated in the ICU in a 2 year period.

**METHODS.** During a 2 year period (1/1/2016 - 31/12/2017), 15 patients, managed as NBM and treated in the ICU were identified. Lumbar puncture(LP), CSF analysis, GRAM stain, CSF cultures were performed in all patients(pts). Cellular pleocytosis was consistent with the diagnosis of NBM.

**RESULTS.** The analyzed sample included 15 non-immunocomprised pts (50±20y and APACHE II score 19±6): 7 had traumatic brain injury, 5 intracerebral hemorrhage, 1 hydrocephalus, 1 tumor and 1 no history of neurosurgery or trauma. 7/15 pts underwent craniectomy, 2/15 aneurysm embolization, 1/15 tumor resection. External ventricular drain(EVD) or intraventricular catheter for intracranial pressure(ICP) monitoring was inserted in 12/15pts.

NBM was diagnosed 12±6 days after ICU admission while ICP catheter/EVD was retained for 8±4 days. All pts were febrile, 6/15 experienced new onset seizures and 2/15 developed septic shock. Meningeal enhancement or brain abscess was found on CT in 7pts(47%).

NBM was confirmed by a positive CSF culture in 5(33%)pts. In pts with positive and negative CSF cultures, there was no statistically significant difference regarding CRP (117-167mg/L p=0.51), procalcitonin (0.69-0.82ng/ml p=0.87), WBC ($15.69x10^9$-$15.10x10^9$/L p=0.80). Differences in CSF WBC (8.394-2.332/μl p=0.12), CSF RBC (34.218-31.837/μl p=0.94), CSF protein (643-250mg/dl p=0.08) and CSF/serum glucose ratio (0,38-0,49 p=0.37) between the two groups were not statistically significant. Isolated microorganisms in CSF cultures were multidrug resistant(MDR) *Klebsiella pn.* and *Acinetobacter baumanii*. In 2/15 pts the EVD/ICP catheter tip culture was positive for *Klebsiella KPC* and MDR *Acinetobacter baumanii*. One patient had a positive blood culture. All pts had previously received broad spectrum antibiotics and 13(87%) were colonized with MDR pathogens. Specific individualized therapy for NBM was administered according to overall clinical and laboratory findings of each patient and despite negative cultures. 2 pts(13%) received intraventricular antibiotics combined with intravenous therapy. Meningitis attributed mortality was 20%.

**CONCLUSIONS.** Neurosurgical procedure or head trauma must raise a high level of suspicion for intracranial infections. Negative CSF culture should not preclude the diagnosis, given the wide use of broad spectrum antibiotics. Additional clinical, laboratory and imaging characteristics should be co-evaluated in order to avoid treatment delays.

**REFERENCE(S)**

1. Beer R.et al.J Neurol.2008
2. van de Beek D.et al.N Engl J Med.2010

### 0181

#### The unrecognized in-ICU days of TB patients without isolation in South Korea

E. Lee, J.S. Kim, H.W. Kim

The Catholic Medical Center,St. Mary's Hospital, Internal Medicine, Pulmonology, Incheon, Korea, Republic of

**Correspondence:** E. Lee

*Intensive Care Medicine Experimental* 2018, **6(Suppl 2):**0181

**INTRODUCTION.** Delay in dignosing Mycobacterium tuberculosis (TB) aggravates the nosocomial transmission in a tertiary care hospital, especially in ICU (intensive care unit). Backgrounds: The aim of the study is to estimate the unrecognized in-ICU days of TB patients without isolation in South Korea, using national health insurance claim data.

**METHODS.** Total patients who had claim data of drug susceptibility test for TB during 2014~2016, which means culture-proven tuberculosis were included. Among them, patients with admission records of ICU during infectious periods were selected, and their in-ICU days were calculated after subtracting periods of isolation. Infectious periods were defined according to the CDC's guideline for contact investigation. The first day of infectious periods was simply defined as 3 months before doctor's suspicion of TB in patients who received the prescription for respiratory medications, which means he had respiratory symptoms, and 1 month before doctor's suspicion without prescription records of respiratory medications. The last day of infectious periods was defined as the day of first prescription of anti TB medications.

**RESULTS.** From 2014 to 2016, 1282 cases were identified as having ICU care records during their infectious periods. Among them, 1174 cases had records of staying ICU for at least one day without isolation. Total sum of in-ICU days without isolation were 18,584 person-days during 3 years. Among the 1174 cases of unrecognized in-ICU TB patients, 374 cases underwent bronchoscopy, and 56 cases had records of laryngoscopy during their infectious periods. There were 201 cases of endotracheal intubation, 19 cases of cardiopulmonary resuscitation, 602 cases of nebulizer treatment, 63 cases of of high flow nasal cannula oxygen therapy, and 350 cases of mechanical ventilation during infectious periods in ICU.

**CONCLUSIONS.** The burden of nosocomial transmission of TB in ICU is still substantial in South Korea.

### 0182

#### Evolution of the microbial flora in patients of a Greek ICU over two decades

P. Kontou[1], I. Tsioulis[1], C. Giannaki[1], E. Sourla[1], A. Lavrentieva[1], M. Bitzani[1], I. Kioumis[2]

[1]G. Papanikolaou Hospital, A' ICU, Thessaloniki, Greece; [2]G. Papanikolaou Hospital, Pulmonology Department, Thessaloniki, Greece

**Correspondence:** A. Lavrentieva

*Intensive Care Medicine Experimental* 2018, **6(Suppl 2):**0182

**INTRODUCTION.** The continuously rising antimicrobial resistance in the ICU is a major problem worldwide. Although the widespread use of antibiotics appears to be the main cause, the relevant studies aren't always conclusive.

**OBJECTIVES.** The purpose of this study was to evaluate the change of the microbial flora and the level of antimicrobial resistance of bacteria in ICU patients in a timeframe of two decades.

**METHODS.** This is a retrospective study of the findings of cultures of patients in a general ICU of a tertiary hospital in Greece at the years 1995 and 2016. The cultures were from respiratory samples, blood, tips from central venous catheters, urine, cerebrospinal fluid and wound exudates. The microorganisms evaluated were staphylococci, enterococci, Enterobacteriaceae and non-fermenting Gram-negative bacilli. The antibiotics tested were oxacillin, trimethoprim-sulfamethoxazole, imipenem, ciprofloxacin, ceftazidime, cefoxitin, amikacin, gentamicin and ampicillin-sulbactam.

**RESULTS.** A similar number of Gram-positive and Gram-negative microbes was identified in 1995, whereas there was a clear benefit in favor of the latter in 2016, which tripled over time. In total, there was a decrease in Gram-positive isolates from 48% in 1995 to 33% in 2016 and a corresponding increase in Gram-negative isolates from 52% in 1995 to 67% in 2016. The most common Gram-positive pathogens were *Staphylococcus aureus* in 1995 and *Staphylococcus epidermidis* in 2016. The 3 most frequently isolated Gram-negative pathogens were *Acinetobacter baumannii*, *Klebsiella pneumoniae* and *Pseudomonas aeruginosa*, with a small difference in their ranking at the two time points.Finally, some microbes were isolated in 2016 (*Proteus mirabilis*, *Klebsiella oxytoca*, *Morganella morganii* and *Stenotrophomonas maltophilia*), that were not reported in 1995.

Regarding antimicrobial resistance, there was a fall in MRSA percentage in our ICU by 50% during these years. On the other hand, an increased resistance to all classes of antibiotics was noted concerning *A. baumannii* and *K. pneumoniae* in the more recent year. Interestingly, *P. aeruginosa* showed higher sensitivity to cephalosporins, quinolones and aminoglygocides but not to carbapenems. A major concern was found to be multi-drug resistant (MDR) *A. baumannii* (sensitive only to colistin and tigecycline) that appeared in 2016 in a great percentage (47% of strains). Current rates of *K. pneumoniae* carbapenemase-producing isolates (KPC) are also alarming (68% of strains).

**CONCLUSIONS.** The differentiation of the microbial flora in ICU patients with time is important. MRSA and other resistant Gram-positive pathogens are not the worst threat in our ICU. The urgent issue is the uncontrollable spread of MDR Gram-negative microbes, which are endemic in our ICU. Nevertheless, the development of antimicrobial resistance due to the widespread use of antibiotics isn't always a fact and is of unpredictable direction.

### 0183

#### Acinetobacter baumannii patterns and course in a tertiary-care mixed-type ICU

Y. Durmus, C. Ates, O. Mert, I. Kurt, F. Yilmaz, M. Reis, Z. Ademoglu, H. Aggul, A.S. Uyar, S. Efe, V. Inal

Trakya University Medical Faculty Research Hospital, ICU, Edirne, Turkey

**Correspondence:** V. Inal

*Intensive Care Medicine Experimental* 2018, **6(Suppl 2):**0183

**INTRODUCTION.** *Acinetobacter baumannii* is an increasingly common nosocomial pathogen and multidrug-resistant strains (MDR-AB) have emerged, most frequent in ICUs.

**OBJECTIVES.** This study was conducted to review the annual-2017 data of A. baumannii patterns and course in our University based tertiary-care mixed-type ICU, as a part of quality management implementations.

**METHODS.** The annual data (2017) of patients with isolated *A. baumannii* were retrospectively analyzed at respect of antibiogram/susceptibility and survival patterns.

**RESULTS.** The overall 445 patients, invasive mechanic ventilator (IMV) and VAP rates were 71% and 12.7%, in addition, central venous catheter (CVC) and CVC related BCI rates were 12.7% and 82% at respect. A. baumannii was isolated in 32 (7%), 24 in endotracheal and eight in central venous catheter of all. We compared A. baumannii to all-patients data as; the mean age was 65±18.3 to 65±16 (ns); LOS was 35.7±7.5 to 8.9±6 days ($p < 0.01$); IMV length 10.2±3.7 to 6.5±3 days ($p < 0.05$), mortality rate was 72% to 44% ($p < 0.05$), at respect. On the other hand, additional gram negatives (87%) and fungal agents (60%) were co-isolated with in A. baumannii positive patients. According to MIC data, the mean stream therapy was required colistin+carbapenem combinations. However, de novo ARF required CVVHD, determined and heralded to colistin in 68% of patients after 1.8±0.6 days of treatment (KDIGO).

**CONCLUSIONS.** Take home messages; (1)Infections caused by A. baumannii were associated with adverse clinical outcomes, including high rates of morbidity and mortality, prolonged hospital stay, and substantial health care expenses, yet. (2)In addition, colistin-assumptive high ARF rates were also a matter of debate. (3)At last, concomitant MDROs (gr-) and occult IFIs were quite frequent, should be precluded and revised for preemptive or empiric treatments.

**Hidron, A. I., J. R. Edwards, J. Patel, T. C. Horan, D. M. Sievert, D. A. Pollock, and S. K. Fridkin.** NHSN annual update: antimicrobial-resistant pathogens associated with healthcare-associated infections: annual summary of data reported to the National Healthcare Safety Network at the Centers for Disease Control and Prevention, 2006-2007. Infect. Control Hosp. Epidemiol. 2008, 29:996-1011.

**Abbo, A., Y. Carmeli, S. Navon-Venezia, Y. Siegman-Igra, and M. J. Schwaber.** Impact of multi-drug-resistant Acinetobacter baumannii on clinical outcomes. Eur. J. Clin. Microbiol. Infect. Dis. 2007, 26:793-800.

**Bassetti, M., E. Righi, S. Esposito, N. Petrosillo, and L. Nicolini.** Drug treatment for multidrug-resistant Acinetobacter baumannii infections.Future Microbiol. 2008, 3:649-660.

##### GRANT ACKNOWLEDGMENT

We thank to Prof.Dr.Zerrin Yulugkural and Infection Control and Prevention Committee of Trakya University Medical Faculty Research Hospital, for theirs kind assistance and cooperation.

### 0184

#### Association of ABO Rh blood group with dengue fever & thrombocytopenia

K. Borawake[1], R. Khasne[2], S. Dixit[3], K. Khatib[4]
[1]VishwaRaj Hospital, Critical Care Department, Pune, India; [2]Apollo Hospital, Critical Care Department, Nashik, India; [3]Sanjeevan & MJM Hospital, Critical Care Department, Pune, India; [4]Prayag Hospital, Critical Care Department, Pune, India
**Correspondence:** K. Borawake
*Intensive Care Medicine Experimental* 2018, **6(Suppl 2):**0184

**INTRODUCTION.** Infection with dengue virus is a serious emerging health threat and has commanded considerable medical and public-health concern worldwide in terms of morbidity and mortality. The platelet count is key in management of dengue fever. A relationship between blood groups and disease was first hypothesized by Kaipainen and Vuorinen during 1960, and the gene involved in ABO blood groups was discovered in 1990. We hypothesized association between distribution of ABO Rh blood groups and dengue fever and thrombocytopenia.
**OBJECTIVES.** The aim of study was to evaluate association between ABO Rh blood groups and dengue fever with respect to thrombocytopenia.
**METHODS.** Prospective observational study was performed in the ICU of tertiary care hospital with mixed population from June 2017 to Dec 2017. Serum samples were collected from patients with acute febrile illnesses. Acute dengue virus infections were confirmed serologically by enzyme linked immunosorbent assay for immunoglobulin (Ig)M/IgG (four-fold rise) and NS 1 antigen either of one. Standard hemagglutination assays were used to type the blood groups in all patients. The platelet count was done on each day for all patients. We compared ABO and Rh blood-group frequencies in dengue disease.
Statistical analysis was performed with SPSS 17 windows. Comparison between categorical data was performed using Chi Square test.
**RESULTS.** Results were as per table 1 and 2 given below.
**CONCLUSIONS.** The frequencies of each blood group in the dengue disease were consistent with those in the general Indian population EXCEPT we observed that dengue infections were higher in individuals with O positive blood group (42.8%). The severe thrombocytopenia was more common in AB blood group (62.5%). These data present further evidence for the association of the blood groups to susceptibility of dengue infection. Further studies are needed to answer why thrombocytopenia was more severe in AB blood group. Thus, from this study we propose that blood group analysis should be done in all patients with dengue fever, this will help us to predict subgroup of patients likely to develop profound thrombocytopenia which can have impact on morbidity and mortality.

##### REFERENCE(S)

1. World Health Organization. Dengue. Geneva: World Health Organization; 2004.
2. Greenwell P. Blood group antigens: Molecules seeking a function? Glycoconj J 1997;14:159-73.
3. Das PK, Nair SC, Harris VK, Rose D, Mammen JJ, Bose YN, et al. Distribution of ABO and Rh-D blood groups among blood donors in a tertiary care centre in South India. Trop Doct 2001;31:47-8.
4. Kalayanarooj S, Gibbons RV, Vaughn D, Green S, Nisalak A, Jarman RG, et al. Blood Group AB is associated with increased risk for severe dengue disease in secondary infections. J Infect Dis 2007;195:1014-7.

##### GRANT ACKNOWLEDGMENT

No funding was associated with this study

**Table 1 (abstract 0184).** ABO and Rh blood group distribution in dengue disease (Sex: p=0.873, blood group: p=0.043, Rh p=0.497)

| SEX | SEX | BLOOD GROUP | BLOOD GROUP | BLOOD GROUP | BLOOD GROUP | Rh | Rh |
|---|---|---|---|---|---|---|---|
| MALE | FEMALE | A | B | AB | O | POSITIVE | NEGATIVE |
| 130 | 108 | 50 | 78 | 8 | 102 | 224 | 14 |
| 54.6% | 45.3% | 21% | 32.7% | 3.3% | 42.8% | 94% | 5.8% |

**Table 2 (abstract 0184).** The Platelet count below 25,000

| A | B | AB | O |
|---|---|---|---|
| 10/50 | 14/78 | 5/8 | 21/102 |
| 20% | 17.9% | 62.5% | 20.5% |

### 0185

#### Epidemiology and prognosis of ICU nosocomial ventilator-associated pneumonia

N. Foudhaili, M. Fatnassi, H. Maamouri, S. Habacha, N. Brahmi
Poison Control Center, Intensive Care Unit, Tunis, Tunisia
**Correspondence:** N. Foudhaili
*Intensive Care Medicine Experimental* 2018, **6(Suppl 2):**0185

**INTRODUCTION.** Ventilator-associated pneumonia (VAP) is an important cause of morbid-mortality.
**OBJECTIVES.** The aim of the present study was to report epidemiological aspects of VAP and to evaluate its prognosis.
**METHODS.** It was a 1-year prospective study from April 2015 to May 2016 including all patients undergoing mechanical ventilation for longer than 48 hours. Patients, in whom VAP was clinically suspected with positive direct examination of a blind protected specimen (BPS), were eligible.
**RESULTS.** Two hundred sixty eight patients were enrolled. Fifty-four VAP episodes were diagnosed in thirty four patients, which represent 1.58±0.85 episode/patient and a frequency of 12%. They were 12 males and 22 females aged of 39±19 years with a mean SOFA score of 5.2±1.8. The delay between admission and VAP suspicion was 5.9 ±2.7 days and between two VAP episodes was a 17±20 day (4-39 days). ICU acquired VAP were microbiologically documented in 32 patients (11%) : *Acinetobacter baumannii* (35%), *Pseudomonas aeruginosa* (33%), *Klebsiella pneumonia* (22%) and methicillin-resistant *Staphylococcus aureus* (7%). Multidrug resistant isolates were extended- spectrum β-lactamases (ESBL) in 10% of cases and carbapenemases in 12%. Initial antibiotherapy included imipenem and colistin according to local ecology and was revised in 33 of cases. The main complications were septic shock (28%), ARDS (24%) and second VAP episode (26%). The mean length of stay was 62.5±10 days (7-180). Favorable outcome was observed in 34 patients (63%). VAP related death in our study are the direct result of the infection , caused by *Pseudomonas aeroginosa* in 7 second VAP episodes (5%) and imipenem-resistant *Acinetobacter baumannii* in 63% of cases.
**CONCLUSIONS.** Morbi-mortality in nosocomial VAP is often due to respiratory or hemodynamic complications and also resistant strains of pathogens.

### 0186

#### Ventilator-associated pneumonia in an intensive care unit of a teaching hospital in Istanbul

A. Ozgultekin[1], A. Inan[2], S. Erol[2], O. Ekinci[1]
[1]University of Health Sciences , Haydarpasa Numune Training and Research Hospital, Anaesthesiology and Intensive Care, Istanbul, Turkey; [2]University of Health Sciences , Haydarpasa Numune Training and Research Hospital, Infectious Diseases, Istanbul, Turkey
**Correspondence:** A. Ozgultekin
*Intensive Care Medicine Experimental* 2018, **6(Suppl 2):**0186

**INTRODUCTION.** Ventilator-associated pneumonia (VAP) is the most frequent hospital-acquired infection among intensive care unit (ICU) patients and is a leading cause of morbidity and mortality. Therefore, early diagnosis and appropriate antibiotic treatment are essential.
**OBJECTIVES.** The aim of the study was to determine the rate of VAP and the change of profiles causative microorganisms in an intensive care unit (ICU) of a teaching hospital.
**METHODS.** An active, targeted prospective surveillance was performed by infectious disease specialists and infection control nurses in the unit. VAP was diagnosed by using the clinical criteria of the

Centers for Disease Control and Prevention. Isolated strains was identified and antibiotic resistance of species was determined by the disk diffusion method according to the The European Committee on Antimicrobial Susceptibility Testing criteria and Vitek 2 system. Fisher exact test was used for the statistical analysis.

**RESULTS.** Between January 2010 and December 2017, a total of 8756 patients hospitalized for 59.317 patient days in ICU and acquired 749 VAP (8.5 per 100 patients and 13.2 per 1,000 patient days). The most frequently isolated microorganisms were *Acinetobacter baumannii, Pseudomonas aeruginosa* and *Enterobacteriacea spp*. The incidence densities of infections decreased from 31.5% to 7.7 % per 1,000 device-days (p< 0,05) for VAP during the study period. It was found that an increase in the proportion of *Klebsiella spp*.isolated from VAP (from 6.0% to 13.2 %, p=0.14), and a significant decrease in the proportion of *Staphylococcus aureus* isolated from VAP(from 7,8 % to 1.8 , p=0.05).

**CONCLUSIONS.** In conclusion, VAP rates are decreasing in our ICU. An increasing trend of infections caused by *Klebsiella spp*. and a decrease in the proportion of *S.aureus* isolates is the remarkable findings of this study.

## Cardiovascular epidemiology & outcome

### 0187

#### Sex-related differences in patients who suffered an acute coronary syndrome

A. Ubeda, I. Fernandez, R. Torcuato, A. Alvarez, A. Fregosi, P. Cobo
Hospital Punta de Europa, Intensive Care Unit, Algeciras, Spain
**Correspondence:** A. Ubeda
*Intensive Care Medicine Experimental* 2018, **6(Suppl 2):**0187

**INTRODUCTION.** Despite remarkable advances in the treatment of acute coronary syndrome, substantial early patient mortality remains. Individual patients reflect a combination of clinical features that influence prognosis, and these factors must be appropriately weighted to produce an accurate assessment of risk.

**OBJECTIVES.** To analyze the differences according to gender in patients with acute coronary syndrome (ACS) admittedto the intensive care unit (ICU) of a regional hospital using the ARIAM database.

**METHODS.** Retrospective analysis performed on a prospective cohort in a 12-bed ICU during 8 years (2009-2017). Demographic variables, comorbidities, risk factors, Killip, location and degree of obstruction, risk scores (TIMI, GRACE) and bleeding score (CRUSADE). Statistical analysis: categorical variables (frequencies and percentages) and numerical variables (mean and standard deviation or medians and interquartile range). Comparisons: X2 test (percentages), Student test (means) and Mann-Whitneytest (medians).Statistical significance with p< 0.05.

**RESULTS.** 1009 patients were included, 751 (74.43%) male, 258 (25.65) female. Most frequent risk factors: arterial hypertension (AH) (52.6%), dyslipidemia (40.3%), smoking (37.5%), diabetes mellitus (DM) (37.2%), STEMI 466 (46.2%), NSTEMI 543 (53.8%). Complications: shock 96 (9.5%), cardiac arrest 80 (7.9%). Discharge diagnosis: AMI 671 (66.5%), unstable angina 316 (31.1%). Location: inferior 288 (42.9%), anterior 235 (35%), undeterminated 100 (14.9%). ICU length of stay (LOS): 4.1 ± 3.8 days. Hospital LOS: 10.5 ± 9.7 days. ICU mortality: 76 (7.5%). Hospital morality: 97 (9.6%).

Men vs. women (bivariant analysis): age (63.02 [±12.62] vs. 69.58 [± 12.60], p< .001), smoking (43.9% vs. 18.6%, p< .001), previous smoker (23.4% vs. 5.4%, p< .001), diabetes mellitus (DM) (34.4% vs. 45.3%, p=.002), arterial hypertension (AH) (48.9% vs. 63.6%, p< .001), congestive heart failure (CHF) (3.6% vs. 8.5%, p< .001), chronic obstructive pulmonary disease (COPD) (7.9% vs. 1.2%, p< .001), elevated ST (51.8% vs. 59.7%, p=.028), TIMI (2.71 [±2.05] vs. 3.30 [±2.36]), p< .001), CRUSADE (28.11 [±15.34] vs. 44.71 [±16.35], p< .001). Body mass index (26.56 ± 4.09 vs. 27.87 ± 5.00, p< .001). ICU mortality (6.1% vs. 11.6%, p=.004), hospital mortality (8.4% vs. 13.2%, p=.024).

**CONCLUSIONS.** Women who suffered ACS were older and had higher prevalence of DM, AH and CHF such as higher TIMI, CRUSADE and GRACE. ICU and hospital mortality were significantly higher among women.

### 0188

#### Trends and outcome of patients with complicated infective endocarditis in intensive care unit; a 20 years retrospective study

F. Ahmad[1], S. Cox[2], C. Soon Thim[3], E. Abdul Rahman[4], I. Zainal Abidin[1], M.A. Sadiq[5]
[1]Universiti Malaya Medical Centre, Kuala Lumpur, Malaysia; [2]Healthcare Partners, Cardiology, Brisbane, Australia; [3]Putrajaya Hospital, Kuala Lumpur, Malaysia; [4]Universiti Teknologi Mara, Selangor, Malaysia; [5]Sultan Qaboos University, Muscat, Oman
**Correspondence:** F. Ahmad
*Intensive Care Medicine Experimental* 2018, **6(Suppl 2):**0188

**INTRODUCTION.** Despite improvement of diagnosis and treatment, the mortality for patients for complicated infective endocarditis (IE) is still high. Unfortunately, data about IE-patients with the need for Intensive Care Unit treatment are sparse.

**OBJECTIVES.** This study investigates predictors of mortality and the short term, intermediate and long term outcome in patients with complicated IE admitted to MICU/CCU for medical treatment.

**METHODS.** A single - centre retrospective analysis was conducted among patients with complicated infective endocarditis that fulfilled the modified Duke Criteria requiring admission to MICU between 1998 and 2017. All patients undergo both transthoracic echocardiography (TTE) within 3 days on presentation to the hospital and then followed by transesophageal echocardiography (TOE) within 3 days prior to transfer to MICU or on the day of transfer itself.

**RESULTS.** 110 patients, with a mean age of 59.7 ± 4.1 years, were diagnosed with complicated IE, in which, 76 (69%) of them were male. IE survivors were defined as patients who survived after being discharged from MICU and at 6 months follow up with a documented diagnosis of IE. A total of 51 survivors from total patients of 110 were identified. 104 patients required inotropic support and 94 patients developed multi-organ failure. 42% of the patients developed mobitz type II heart block or complete heart block. 23 patients of the mobitz type II heart block progressed to complete heart block requiring temporary/ permanent pacemaker. Microbiology laboratory investigation showed 93% of the patients grew gram-positive cocci in their blood cultures. Surgical intervention was conducted in 54% of the patients and the remaining was medically treated. Biomarkers of acute infection, APACHE II as well as echocardiographic findings were analyzed. Using multivariate analysis, patients who developed acute kidney injury prior transfer to ICU (OR 9 95% CI 1.18-23.00, P=0.03) or echocardiographic evidence of vegetation size > 15mm (OR 7 95% CI 1.09-17.77 P = 0.03) were identified as predictors for in-patient death.

**CONCLUSIONS.** In contrast to general IE populations, infectious agents and the type of infected impaired valve are not the main predictors of survival in critically ill IE patients. Our study concludes that markers for severity of illness (scores and organ failure) as well as echocardiographic findings of vegetations size >15 mm are independent risk factors for poor prognosis and mortality.

### 0189

#### Diagnostic process of acute heart failure in the ICU, impact on mortality (ICAUCI study group)

L. Zapata[1], R. Gómez[2], M.C. Guia[3], I. Rodado[4], J. Cabrera[5], I. Torrejón[6], C. Tarancón[7], MJ. De la Torre Fernandez[8], M.L. Martinez[9], L. Colinas[10], ICAUCI (Insuficiencia Cardíaca Aguda en Unidades de Cuidados Intensivos)
[1]Hospital de la Santa Creu i Sant Pau, Barcelona, Spain; [2]Hospital Quironsalud Miguel Dominguez, Intensive Care, Pontevedra, Spain; [3]Corporació Sanitària Parc Taulí en Sabadell, Sabadell, Spain; [4]Hospital Universitario de Torrejon, Torrejon de Ardoz, Spain; [5]Hospital Universitario de Canarias, Santa Cruz de Tenerife, Spain; [6]Hospital del Henares, Coslada (Madrid), Spain; [7]Hospital Virgen de la Concha, Zamora, Spain; [8]Complexo Hospitalario Universitario de Ourense, Ourense, Spain; [9]Hospital General de Catalunya, Sant Cugat del Valles., Spain; [10]Hospital Universitario de Toledo, Toledo, Spain
**Correspondence:** L. Zapata
*Intensive Care Medicine Experimental* 2018, **6(Suppl 2):**0189

**OBJECTIVES.** To analyze the diagnostic process of acute heart failure (AHF) in the Spanish Intensive Care Units (ICU) and its impact on mortality.

**METHODS.** Multicenter prospective observational study that included patients older than 18 years admitted to the ICU for more than 24 hours between April and June 2017. Demographic, physiological and clinical variables, including complementary explorations of those who suffered AHF during their ICU stay were collected.

Comparisons were made among the different subgroups of patients according to the timing of AHF: AHF as main reason for admission vs. complication during ICU stay (Adm-AHF Vs Com-AHF) or first episode of AHF vs. exacerbation of chronic heart failure (AHFnew Vs CHFex). Statistical analysis was made using ANOVA or chi-square test as appropriate. The multivariate study was performed by Cox regression for survival analysis and logistic regression. Values of $p \leq 0.05$ were considered statically significant.

**RESULTS.** We analyzed 587 patients with AHF admitted in 32 ICUs. In 98.6% of patients a chest x-ray was performed and in 98.8% an EKG. In all the patients, at least a blood sample test was extracted, the 78% included troponin and 37.5% natriuretic peptide concentrations. In the 24.6% of the episodes an invasive or minimally invasivehemodynamic monitoring system was used (5.4% pulmonary artery catheter, 4.9% transpulmonary thermodilution, 14.3% other systems) In the 81.2% of patients an echocardiography (ECHO) was performed: 61.1% during the first 24h, 10.1% between the 24-48h and 8.5% > 48h after the diagnosis. In 91.2% of ECHOs, the LVEF data was reported, and showed a 57.9% of systolic dysfunction (LVEF < 50%). Diastolic function were reported in the 17% of ECHOs, with an incidence of diastolic dysfunction of 29.1%. Right ventricular function was reported in the 52.8% of ECHOs, with a 43.8% of right ventricular dysfunction. A significant delay in the diagnosis of AHF was observed in the 9.3% of the episodes. The existence of delay was associated with an 30.8% (p = 0.015) and 46.2% (p = 0.007) absolute risk increase in mortality in the Com-AHFnew and in Com-AHFexarcebation subgroups respectively.

**CONCLUSIONS.** In patients with AHF, ECHO is the most used advanced monitoring system. Diastolic function and right ventricle function are reported in less than half of the ECHO. Natriuretic peptides are underused. The diagnostic delay of AHF when it appears as a complication in patients admitted to the ICU for another reason, is related to a significant increase in mortality.

**GRANT ACKNOWLEDGMENT**

ICAUCI istudy group is part of the Cardiac Intensive Care Task Force of the Spanis Society of intensive care medicine (SEMICYUC)

### 0190

#### Predictive model for shock in patients with acute coronary syndrome

A. Ubeda, I. Fernandez, R. Torcuato, A. Alvarez, A. Fregosi, P. Cobo
Hospital Punta de Europa, Algeciras, Spain
**Correspondence:** A. Ubeda
*Intensive Care Medicine Experimental* 2018, **6(Suppl 2):**0190

**INTRODUCTION.** Despite remarkable advances in the treatment of acute coronary syndrome, substantial early patient mortality remains. Individual patients reflect a combination of clinical features that influence prognosis, and these factors must be appropriately weighted to produce an accurate assessment of risk.

**OBJECTIVES.** To analyze the factors related to the develpment of shock in patients who suffered acute coronary syndrome (ACS) and were admitted to the intensive care unit (ICU) of a regional hospital using the ARIAM database.

**METHODS.** Retrospective analysis performed on a prospective cohort in a 12-bed ICU during 8 years (2009-2017). Demographic variables, comorbidities, risk factors, Killip, location and degree of obstruction, risk scores (TIMI, GRACE) and bleeding score (CRUSADE). Statistical analysis: categorical variables (frequencies and percentages) and numerical variables (mean and standard deviation or medians and interquartile range). Comparisons: X2 test (percentages), Student test (means) and Mann-Whitney test (medians). *Multiple logistic regression*. Statistical significance with $p < 0.05$.

**RESULTS.** 1009 patients were included, 751 (74.4%) male. 96 patients (9.51%) suffered shock during their ICU stay. Those who suffered shock: Univariable *logistic regresion*: age (OR 1.04, CI 95% [1.02-1.06], p< .001), diabetes mellitus (OR 1.63, CI 95% [1.07-2.49], p=0.023), previous ACS (OR 1.89, CI 95% [1.13-3.16], p=0.015), congestive heart failure (OR 8.85, CI 95% [4.79-16-34], p< .001), chronic renal failure (OR 2.06, CI 95% [1.04-4.09], p=0.039), Killip II (OR 13.25, CI 95% [4.4-39.83], p< .001), Killip III (OR 91.48, CI 95% [32.51-257.44], p< .001), rescue percutaneous coronary intervention (OR 2.84, CI 95% [1.15-6.98], p=0.23), anterior vs. inferior acute myocardial infarction (OR 2.27, CI 95% [1.45-3.55], p< .001), left main coronary artery (OR 7.22, CI 95% [1.48-35.17], p=.014), TIMI (OR 1.55, CI 95% [1.41-1.70], p< .001), GRACE (OR 1.04, CI 95% [1.03-1.05], p< .001), ICU LOS (OR 1.13, CI 95% [1.08-1.18], p< .001), ICU mortality (OR 47.69, CI 95% [26.78-84.95], p< .001), hospital mortality (OR 39.46, CI 95% [23.27-66.91], p< .001). Multivariable regresion model: Killip II (OR 19.66, CI 95% [4.40-87.86], p< .001), Killip III (OR 191.16, CI 95% [44.38-823.46], p< .001), Q infarction (OR 3.44, CI 95% [1.55-7.61], p=.002). AUROC 0.92 (CI 95% 0.89-0.95).

**CONCLUSIONS.** Q infarction and Killip II-III were predictive factors for shock in patients admitted to ICU who suffered from ACS.

### 0191

#### A retrospective observational cohort study of patients admitted to a mixed medical and surgical intensive care unit in the United Kingdom with atrial fibrillation: incidence, risk factors and treatment efficacy

A. Hampden-Martin[1], N. Miller[1], B. Johnston[1,2], I. Welters[1,2]
[1]University of Liverpool, Institute of Ageing and Chronic Disease, Liverpool, United Kingdom; [2]Royal Liverpool and Broadgreen University Hospital Trust, Intensive Care Department, Liverpool, United Kingdom
**Correspondence:** B. Johnston
*Intensive Care Medicine Experimental* 2018, **6(Suppl 2):**0191

**INTRODUCTION.** Atrial Fibrillation (AF) is the most common arrhythmia in ICU.[1] New onset AF (NoAF) is estimated to occur in 5-15% of patients.[1] Development of atrial fibrillation is associated with increased length of hospital stay, increased ICU length of stay and increased mortality.[1] It has been suggested that NoAF is likely to have a separate aetiology and respond differently to treatment compared to chronic AF. It remains unclear what risk factors are associated with new onset AF and the most effective management of NoAF in ICU patients.[1]

**OBJECTIVES.**

1. To determine the incidence and risk factors associated with NoAF
2. Assess the efficacy of first and second line anti-arrhythmic agents used in NoAF
3. Assess outcomes associated with NoAF

**METHODS.** A retrospective observational cohort study was conducted at a large mixed medical and surgical intensive care unit in Northwest England. Patients admitted to ICU that developed AF during their admission were enrolled in the study. Data was extracted retrospectively from daily ICU nursing sheets, online patient notes system and online patient investigation system. Demographic data and co-morbidities were recorded. Data regarding first and second line treatment was recorded. First and second line anti-arrhythmic were assessed for efficacy. Outcomes included, mortality, length of ICU stay, length of organ support, length of hospital stay.

**RESULTS.** 1185 patients were admitted to ICU of which 115 had AF during their admission. 42% had permanent AF, 46% had NoAF and 11% had paroxysmal AF. Patients with permanent AF compared to NoAF were older (75 vs 67) and had more hypertension (70 vs 53), cerebrovascular disease (35 vs 14.3), structural heart disease (62.5 vs 36), diabetes (40 vs 12) and chronic kidney disease (25 vs 8). Patients with NoAF had higher CRP, white cell count and lactate. Amiodarone was the most commonly used anti-arrhythmic, followed by beta blockers then digoxin in NoAF. Digoxin was the most commonly used anti-arrhythmic in permanent AF. Patients with NoAF had longer length of ICU stay (13d vs 6d), longer hospital stay (21d vs 16d). Amiodarone more often achieved heart rate control compared to beta blockade (64 vs 58).

**CONCLUSIONS.** In our study the overall incidence of AF was 6% of all ICU admissions. In keeping with previous studies, we have shown that NoAF is associated with increased inflammatory markers and sepsis.[1] Permanent AF is more associated with chronic medical co-morbidity and structural heart disease. Amiodarone was more effective compared to beta blockers for the management of acute AF. AF leads to worse outcomes for patients admitted to ICU.

##### REFERENCE(S)

Yoshia T, Fuji T, Uchino S, Takinami M. (2015) Epidemiology, prevention, and treatment of new-onset atrial fibrillation in critically ill: a systematic review. Journal of intensive care;3(9):2-11.

### 0192

#### Combination copeptin with troponin for early exclusion of myocardial infarction in the emergency department: a systematic review and meta-analysis

C.-C. Lee[1], P.-Y. Tsou[2]
[1]National Taiwan University Hospital, Emergency Medicine, Taipei, Taiwan, Province of China; [2]Bloomberg School of Public Health, Johns Hopkins University, Department of Epidemiology, Baltimore, United States
**Correspondence:** C.-C. Lee
*Intensive Care Medicine Experimental* 2018, **6(Suppl 2):**0192

**INTRODUCTION.** Recent studies have shown a mixed results on the accuracy of combining copeptin with different cardiac troponin assays for rapid rule out of acute myocardial infarction (AMI).

**OBJECTIVES.** We aimed to perform a systematic review and meta-analysis to evaluate the diagnostic accuracy of copeptin in conjunction with different troponin assays for early rule-out of AMI.

**METHODS.** Primary studies evaluating the diagnostic accuracy for copeptin with conventional or high sensitivity troponin assays in patients with symptoms suggestive of AMI were eligible for inclusion. Eligible studies were identified by searching PubMed and EMBASE from inception to March 2018. QUADAS-II was used to appraise the quality of included studies. A random-effect bivariate model and a hierarchical summary receiver operating curve were used to summarize the performance characteristics of different combination of copeptin and troponin assays.

**RESULTS.** A total of 31 studies consisting of 15,661 patients were included for final analysis. The sensitivity for early rule out AMI was similar for combining coopetin with conventional troponin T assay (summary sens: 0.90; 95%CI: 0.78-0.96) or troponin I assay (summary sens: 0.90; 95%CI: 0.84-0.94). There was no significant increase in sensitivity when combining copeptin with high sensitivity troponin T assay (summary sens: 0.92; 95%CI: 0.86-0.96) or high sensitivity troponin I (summary sens: 0.92; 95%CI: 0.86-0.96). For all combinations, the summary specificity estimates were between o.63 to 0.67.

**CONCLUSION.** In conclusion, combining copeptin with conventional troponin assays could achieve a comparable sensitivity and negative likelihood ratio to combining copeptin with high sensitivity troponin assay for early exclusion of AMI.

### 0193

#### Effect of diabetes mellitus on heart rate variability and blood pressure variability in patients with acute ischemic stroke

E. Hasen
Faculty of Medicine, Minia University, Neurology, Minia, Egypt
*Intensive Care Medicine Experimental* 2018, **6(Suppl 2):**0193

**BACKGROUND.** Diabetes mellitus is frequently characterized by autonomic nervous system (ANS) dysfunction. Analysis of heart rate variability (HRV) has become a popular noninvasive tool for assessing the activities of ANS.

**OBJECTIVES.** We aimed to evaluate possible changes of heart rate variability (HRV) and blood pressure variability (BPV) parameters in acute ischemic stroke patients with diabetes mellitus.

**SUBJECT AND METHODS.** 15 diabetic and 15 non-diabetic patients with first-ever acute ischemic stroke were studied. All patients were subjected to Brain C-T scan, assessment of stroke severity by the National Institute of Health Stroke Scale (NIHSS). Heart rate and blood pressure variability were evaluated by 24 hour monitoring for electrocardiogram (ECG) and blood pressure. The ANS activity was quantified by means of frequency and time domain analysis of R-R interval variability in electrocardiogram. Systolic and diastolic blood pressure variability parameters were measured.

**RESULTS.** In diabetic group, both sympathetic and parasympathetic parameters were significantly reduced. Diurnal variability of the sympathetic tone was with higher sympathetic level in the morning. The diurnal changes of the BPV parameters were higher in diabetic group especially the systolic parameters.

**CONCLUSION.** In diabetic patients, HRV parameters were reduced as well as the daily variability of the sympathetic fluxes which was predominant in the late night (at 2 am). There were more changes in the blood pressure levels especially the systolic blood pressure parameters**.** This highlights the importance of considering these alterations in diabetic patients.

**KEYWORDS.** Heart rate variability, blood pressure, acute ischemic stroke, diabetes

### 0194

#### Predictive model of mortality for patients with acute coronary syndrome

A. Ubeda Iglesias, I. Fernandez, A. Alvarez, A. Fregosi, R. Torcuato, P. Cobo
Hospital Punta de Europa, Algeciras, Spain
**Correspondence:** A. Ubeda Iglesias
*Intensive Care Medicine Experimental* 2018, **6(Suppl 2):**0194

**INTRODUCTION.** Despite remarkable advances in the treatment of acute coronary syndrome, substantial early patient mortality remains. Individual patient risk factors reflect a combination of clinical features that influence prognosis, and these factors must be appropriately weighted to produce an accurate assessment of risk.

**OBJECTIVES.** To analyze the factors related to mortality of patients who suffered an acute coronary syndrom (ACS) and were admitted to the ICU of a regional hospital using the ARIAM database.

**METHODS.** Retrospective analysis performed on a prospective cohort in a 12-bed ICU during 8 years (2009-2017). Demographic variables, comorbidities, risk factors, Killip, location and degree of obstruction, risk scores (TIMI, GRACE) and bleeding score (CRUSADE). Statistical analysis: categorical variables (frequencies and percentages) and numerical variables (mean and standard deviation or medians and interquartile range). Comparisons: X2 test (percentages), Student test (means) and Mann-Whitney test (medians). *Multiple logistic regression.* Statistical significance with p< 0.05.

**RESULTS.** 1009 patients were included, 74.4% male. Global mortality 7.5%. Those who died: female (39.5% vs. 24.4%, p=.004), age (72.13 ±11.47 vs. 64.09 ± 12.86; p< .001), no risk factors (10.5% vs. 4.5%, p=.020), smoking (25% vs.38.5%, p=.020), dyslipidemia (25% vs. 41.6%, p=.005), previous ACS (27.6% vs. 13.4%, p=.001), known coronary artery lesión (21.1% vs. 11%, p=.009), *cardiac insufficiency* (23.7% vs. 3.3%, p< .001), cerebrovascular accident (11.8% vs. 5.9%, p=.041), chronic obstructive pulmonary desease (11.8% vs. 5.7%, p=.031), chronic renal failure (14.5% vs. 5.8%, p=.003), shock as a complication (69.7% vs. 4.6%, p< .001), cardiac arrest (52.6% vs. 4.3%, p< .001), anterior acute myocardial infarction (52.1% vs. 33.1%, p=.001), TIMI (4.84 ± 2.66 vs. 2.70 ± 2.02; p< .001). *Multiple logistic regression*: female (OR 2.27, 95%CI [1.09-4.74] p=.029), age (OR 1.05, 95%CI [1.01-1.08] p=.005), Killip III (OR 3.70, 95%CI [1.27-10.81] p=.017), Killip IV (OR 5.98, 95%CI [1.25-28.46] p=.034), shock as a complication (OR 31.16, 95%CI [12.78-76.02] p< .001), cardiac arrest (OR 23.68, 95%CI [10.25-54.66] p< .001), ICU length of stay (OR 0.93, 95%CI [0.88-0.99] p=.002). AUROC 0.95 (95%CI 0.92-0.98).

**CONCLUSIONS.** Age, female sex, Killip III, IV and complications such as shock and cardiac arrest were statistically related to mortality in patients who suffered an ACS.

### 0195

#### Prognostic value of admission blood glucose level in critically ill patients admitted to cardiac intensive care unit according to the presence or absence of diabetes mellitus

S. Kim[1], S.J. Na[2], J.H. Yang[2]
[1]Korea University School of Medicine / Ansan Hospital, Ansan, Korea, Republic of; [2]Sungkyunkwan University School of Medicine / Samsung Medical Center, Seoul, Korea, Republic of
**Correspondence:** S. Kim
*Intensive Care Medicine Experimental* 2018, **6(Suppl 2):**0195

**INTRODUCTION.** Admission blood glucose (BG) level is a predictor of mortality in critically ill patients with various conditions. However, limited data are available in this relationship in critically ill patients with cardiovascular diseases according to their diabetic state.

**OBJECTIVES.** We sought to investigate the association between baseline BG level and clinical outcomes in critically ill patients with various cardiovascular diseases admitted to cardiac intensive care unit (CICU) and whether prognostic role of admission BG level may differ according to the presence or absence of diabetic mellitus.

**METHODS.** A total of 1780 patients (595 diabetic patients) who admitted cardiac intensive care unit (CICU) were enrolled from a single center registry. Admission BG level was defined as their maximal serum glucose level within 24 hours of admission. Patients were divided by their admission BG level; Group 1 < 7.8 mmol/L, Group 2 7.8 - 10.9 mmol/L, Group 3 11.0 - 16.5 mmol/L, Group 4 > 16.5 mmol/L.

**RESULTS.** One hundred and five patients died in CICU (62 non-diabetic patients [5.2%] and 43 diabetic patients [7.9%], p = 0.105). CICU mortality rate increased with admission BG (1.7%, 4.8 %, 10.3%, and 18.8% from Group 1 to Group 4, p < 0.0001). In non-diabetic patients, hypertension, mechanical ventilator, continuous renal replacement therapy, APACHE II score and admission BG level (Group 1 versus Group 3, hazard ratio [HR] 3.31, 96% confidence interval [CI] 1.47 - 7.44, p = 0.004; Group 1 versus Group 4, HR 6.56, 95% CI 2.76 - 15.58, p < 0.0001) significantly influenced CICU mortality. However, in diabetic patients, continuous renal replacement therapy and APACHE II score influenced on CICU mortality, but not admission BG level.

**CONCLUSIONS.** Admission BG level was associated with an increased CICU mortality in critically ill non-diabetic patients admitted to CICU but not diabetic patients.

##### REFERENCE(S)

Stress hyperglycaemia. *Lancet.* 2009;373(9677):1798-1807.
Prognostic value of admission blood glucose level in patients with and without diabetes mellitus who sustain ST segment elevation myocardial infarction complicated by cardiogenic shock. *Crit Care.* 2013;17(5):R218.

##### GRANT ACKNOWLEDGMENT

None.

### 0196

#### Advanced age as a risk factor for postoperative bleeding in the intensive care unit after left ventricular assist device implantation

R. Sakaguchi, R. Abe, A. Matsumoto, Y. Horiguchi, A. Tanaka, A. Uchiyama, Y. Fujino
Osaka University, Anesthesiology and Intensive Care Medicine, Suita, Japan
**Correspondence:** R. Sakaguchi
*Intensive Care Medicine Experimental* 2018, **6(Suppl 2):**0196

**INTRODUCTION.** Left ventricular assist device (LVAD) is a treatment of choice for patients with end stage heart failure (1). Shortage of donors is a serious problem in Japan. LVAD plays a pivotal role for patients waiting for heart transplantation. However, post-operative bleeding after LVAD implantation is a common adverse event (2). Few reports are available on risk factors of early postoperative bleeding in the intensive care unit (ICU).

**OBJECTIVES.** The aim of our study was to investigate the incidence and risk factors of early postoperative bleeding in the ICU.

**METHODS.** A retrospective medical review was performed on 91 patients who underwent continuous flow type of LVAD (CF-LVAD) implantation from January 2012 to June 2016 at Osaka University Hospital. Patients under 18 years of age were excluded. In this study, postoperative bleeding was defined as bleeding that needed surgical intervention and cerebral hemorrhage in the ICU. Univariate and multivariate analysis were performed to clarify the risk factors.

**RESULTS.** The rate of postoperative bleeding was 26% (24 of 91 patients); 14 (58%) of the bleeding events occurred within 4 days postoperatively and the main site was the thorax (70.1%). Compared with patients without postoperative bleeding, those with postoperative bleeding had longer ICU stay [23 days (interquartile range, 14-33 days) vs. 8 days (interquartile range, 4-16 days), p< 0.01] and shorter duration of ventilator-free days within 28 days postoperatively (10 days [interquartile range, 0-22 days] vs. 25 days [interquartile range, 18-27 days], p < 0.01). Univariate analyses showed that advanced age, re-sternotomy, total bilirubin, and longer operation time were significantly associated with postoperative bleeding. Multivariate analysis of the preoperative factors showed that advanced age was associated with postoperative bleeding (odds ratio: 1.05, 95% CI: 1.0-1.1).

**CONCLUSIONS.** Advanced age was the risk factor for early postoperative bleeding after LVAD implantation in the ICU. Older patients warrant careful monitoring after operation.

##### REFERENCE(S)

1. Rose EA, Gelijns AC, Moskowitz AJ, Heitjan DF, Stevenson LW, Dembitsky W et al. Long-term use of a left ventricular assist device for end-stage heart failure. N. Engl. J. Med.2001;345(20):1435-43.

2. Slaughter MS, Rogers JG, Milano CA, Russell SD, Conte JV, Feldman D et al. Advanced heart failure treated with continuous-flow left ventricular assist device. N. Engl. J. Med.2009;361(23):2241-51.

**GRANT ACKNOWLEDGMENT**
Not applicable.

### 0197

#### Atrial fibrillation incidence and prophylaxis adherence post cardiac surgery

H. Burns, N. Velev, M. Ahmed
St Georges Hospital, CTICU, London, United Kingdom
**Correspondence:** H. Burns
*Intensive Care Medicine Experimental* 2018, **6(Suppl 2):**0197

**INTRODUCTION.** Post- operative Atrial Fibrillation (AF) is common post cardiac surgery, with an incidence of 33% post Coronary Artery Bypass Graft (CABG) surgery, higher post valve surgery. There is clear guidance from National Institute of Clinical Excellence (NICE) for the prophylaxis and management of perioperative AF.
"In patients undergoing cardiothoracic surgery reduce the risk of postoperative atrial fibrillation by offering 1 of the following: Amiodarone or beta blocker"
**OBJECTIVES.** To review our incidence of AF, post cardiac surgery and adherence to prophylaxis guidance.
**METHODS.** Retrospective review of all patients who had cardiac surgery and were discharged from our Cardiothoracic Intensive Care Unit (CTICU) from 01/03/2017 to 30/06/2017. We included all patients that had Cardiac Surgery which involved either a CABG and/or valve Surgery. Basic demographic data was collected. The patient CTICU charts, online notes, electronic results and online prescriptions were interrogated to identify those with documented AF. Data was collected regarding the strategies for the prevention and management of AF following up from CTICU to ward to clinic.
**RESULTS.** Data was collected from 296 patients who had cardiac surgery over 4 months. Pre-existing AF was present in 49 patients (16.5%). Amongst the 247 patients with no history of AF, 76 (30%) had an episode of AF during their admission, peak onset on day 2-3 post op. Incidence of AF amongst patients having a CABG, single Valve Surgery or combined procedures was 25%, 46% and 35% respectively.
Beta blocker prophylaxis was poor across all patients, and when comparing new onset AF with no AF, there was a statistical significance on Day 2.
Noradrenaline peak doses (mcg/kg/min) were different on Day 1 in those developing AF 0.034 vs. 0.021 (p=0.021). Those that developed AF were more likely to be paced post operatively 48% vs. 31% (p=0.002).
Patients who were paced were much less likely to be given beta blockers on Day 1 (p=0.0011) and Day 2 (p=0.000021). For management of AF 60 patients (79%) were treated with amiodarone during their inpatient stay. Three patients (4%) remained in AF on 6 week follow up.
**CONCLUSIONS.** Our incidence of Post-operative AF was similar to previous studies. Adherence to perioperative beta blocker prophylaxis was poor immediately post surgery. Patients who develop AF were more likely to have had valve surgery, have high noradrenaline levels and be paced on day 1. Those on noradrenaline are more likely not to be given prophylaxis early and develop AF. This has helped inform guidance within our department which stipulate introducing beta blockade on day 1 post surgery, which is not dependent on noradrenaline dose and will aim to reduce of incidence of post-operative AF and the potential long term sequelae of long-term AF.

**REFERENCE(S)**
National Institute for Health and Care Excellence. (2014). Atrial Fibrillation: Management. (CG180) https://www.nice.org.uk/guidance/cg180

**Table 1 (abstract 0197).** Percentage of patients recieving Beta Blockers by day

| Day | AF (%) | No AF (%) | P Value |
|---|---|---|---|
| 0 | 38 | 41 | 0.62 |
| 1 | 19 | 25 | 0.31 |
| 2 | 35 | 55 | 0.005 |
| 3 | 63 | 73 | 0.14 |
| 4 | 69 | 77 | 0.18 |

### 0198

#### Presence of atrial fibrillation in patients with acute myocardial infarction undergoing percutaneous coronary intervention predicts contrast-induced nephropathy

J. Wi
Severance Hospital, Yonsei University College of Medicine, Cardiology, Seoul, Korea, Republic of
*Intensive Care Medicine Experimental* 2018, **6(Suppl 2):**0198

**PURPOSE.** Atrial fibrillation (AF) is known as one of factors that negatively influence kidney function. Contrast-induced nephropathy (CIN) is associated with poor clinical outcomes in patients with acute myocardial infarction (AMI) undergoing percutaneous coronary intervention (PCI). We investigated whether AF could predict CIN in AMI patients treated with PCI.
**METHODS.** We analyzed clinical data from 1041 AMI patients treated with PCI. CIN was defined as an increase in serum creatinine level (>25% or >0.5 mg/dL) within 48 hours after PCI. AF was divided into AF with prior history or new-onset AF developed during AMI without previous history.
**RESULTS.** Atrial fibrillation was observed in 70 patients (6.7%) and CIN in 148 patients (14.2%). Patients with AF exhibited higher incidence of CIN than those without AF (31.4 vs. 13.0%, p < 0.001). Multivariate analysis showed that AF was the second most significant independent predictor of CIN [odds ratio (OR) 3.48, 95% confidence interval (CI) 1.69 - 7.15, p = 0.001], following impaired renal function (serum creatinine level >1.5 mg/dL) at baseline (OR 3.50, 95% CI 1.89 - 6.49, p < 0.001). Each new-onset AF (OR 3.94, 95% CI 1.55 - 9.99, p = 0.004) and AF with prior history (OR 2.98, 95% CI 1.04 - 8.57, p = 0.043) was also a significant predictor of CIN, compared to non-AF group. Left ventricular systolic dysfunction (ejection fraction < 40%, OR 2.01, 95% CI 1.25 - 3.23, p = 0.004), age >75 years (OR 1.97, 95% CI 1.16 - 3.34, p = 0.012), contrast volume >300 mL (OR 1.89, 95% CI 1.13 - 3.17, p = 0.015), diabetes (OR 1.83, 95% CI 1.17 - 2.88, p = 0.009), female gender (OR 1.71, 95% CI 1.09 - 2.70, p = 0.020) were other clinical predictors of CIN. Variables including heart rate, LV filling pressure (E/E`), low body mass index (< 24 kg/m2), anemia, hypertension, and multi-vessel disease were not statistically significant in multivariate analysis, although significant in univariate analysis.
**CONCLUSION.** Presence of AF predicts CIN in patients with AMI undergoing PCI, regardless of pre-existing AF or new-onset AF.

### 0199

#### High sensitive troponin T predicts myocardial ischemia and mortality in medical-surgical ICU

G. Kotnik[1], M. Mežnar[1,2], G. Voga[1,2], M. Golmajer[3], M. Podbregar[1,4]
[1]University of Ljubljana, Medical Faculty, Ljubljana, Slovenia; [2]General and Teaching Hospital Celje, Department for Internal Intensive Care, Celje, Slovenia; [3]General and Teaching Hospital Celje, Department for Anesthesiology, Intensive Care and Pain Treatment, Celje, Slovenia; [4]UMC Ljubljana, Clinical Department of Cardiology, Ljubljana, Slovenia
**Correspondence:** G. Kotnik
*Intensive Care Medicine Experimental* 2018, **6(Suppl 2):**0199

**INTRODUCTION.** Elevated cardiac troponin (cTn) level is detected in up-to 84% of critically ill and is negative predictor of survival. (1,2,3) cTn alone is not sufficient for diagnosis of myocardial ischemia or myocardial infarction and must be interpreted along with other diagnostic procedures (i.e. ECG, echocardiography, angiography). (1,2)

**OBJECTIVES.** High sensitive cTn (cTn-hs) enables detection of more patients at risk for myocardial ischemia, it has high prognostic value for cardiac events in patients with renal failure and shortens the time to diagnosis by almost 3 hours. A protocol-guided cTn-hs with admission echocardiography was compared to retrospective cohort of critically ill to assess the prognostic value and outcome of patients.

**METHODS.** In this prospective, observational study, 100 consecutive patients, hospitalized in a level 3 medical-surgical ICU at General and Teaching Hospital Celje in 2017 for more than 48 hours, were enrolled. 12-lead ECG and cTn-hs (Elecsys® Troponin T high sensitive, Roche Diagnostics; 99th percentile upper reference limit: 14 ng/L) for detecting myocardial ischemia were screened at admission, twice daily for the first week and then weekly until discharge/death. An echocardiographic exam was also performed at admission and during acute events. At events indicating the presence of myocardial ischemia (hypotension, acute heart failure, chest pain...) ECG, cTn-hs and echo were repeated. Myocardial ischemia was diagnosed according to Third universal definition of myocardial infarction. Results were available to the medical team for further therapeutic decisions. The prospective group was compared to case-matched retrospective group treated in 2016.

**RESULTS.** In the prospective group, 95% patients had at least one elevated cTn-hs level, 21% had myocardial ischemia, 74% had elevated cTn-hs only. All patients with myocardial ischemia in the prospective group had cTn-hs >100 ng/L. Maximal level cTn-hs, but not admission level, was associated with ICU mortality (ROC: AUC 0.80, p< 0.01, cut-off 150ng/L, sensitivity 80.0% specificity 72.2%). More patients with myocardial ischemia were detected in prospective compared to retrospective study (21/100 vs. 5/100, p=0.006). ICU-mortality mortality was lower in protocol base population (10/100 vs 27/100, p=0.01).

**CONCLUSION.** Elevated cTn-hs is detected in majority of ICU-patients. cTn-hs >100ng/L indicates myocardial ischemia. Maximal level of cTn-hs predicts ICU-mortality. It is possible to detect more patients with myocardial ischemia with protocol based approach.

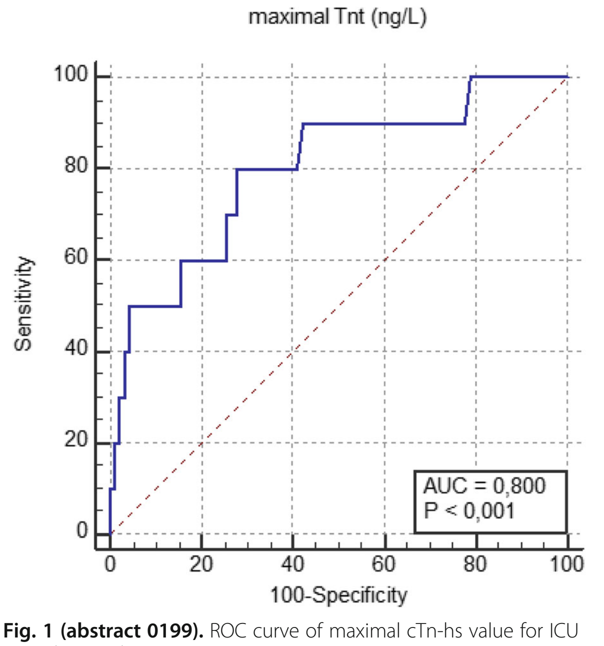

**Fig. 1 (abstract 0199).** ROC curve of maximal cTn-hs value for ICU mortality prediction

### 0200

#### LOS and mortality evolution in the acute myocardial infarction in a polyvalent ICU across 15 years

J. Ruiz, M. Pujol, B. Sánchez, S. Quintana, J. Trenado
Hospital Universitario Mutua de Terrassa, Terrassa. Barcelona, Spain
**Correspondence:** J. Ruiz

*Intensive Care Medicine Experimental* 2018, **6(Suppl 2):**0200

**OBJECTIVE.** To analyze the in Hospital and ICU (intensive care unit) LOS (long of stay) and mortality in patients affected by acute myocardial infarction (AMI) attended in a polyvalent ICU of a university hospital.

**METHOD.** Unicentric, observational and descriptive study between January 2000 and June 2015. Three study periods were established. The first period covered from January 2000 to December 2004. The second period covered from January 2005 to May 2009 (Start of Hemodynamic Service in the hospital). The last period covered from June 2009 to June 2015 (involves the implementation of the Infarct Code in Catalonia).

**STATISTICS.** Qualitative variables are expressed as percentages and compared using the X2-test; quantitative ones are expressed as means and standard deviations (± S.D), and analyzed using Student ́s t-test. Analysis of variance (ANOVA) was performed for intergroup comparison The level of significance was placed at p < 0.05. The statistical analysis was performed using specific software (IBM SPSS Statistics for Window s, Version 19.0. Armonk, NY: IBM Corp).

**RESULTS.** 11445 patients were included, 1645 (14.4%) presented an AMI as the main diagnosis. The severity of these patients measured by the MPM 0 scales, MPM 24, SAPS 2 and APACHE III were respectively 15.16 (14.37-15.95), 10.35 (9.70-11.00), 26.64 (25.97-27.31) and 39.29 (38.10-40.49) with no inter-period differences observed. The age of the patients remains stable over the years without inter-period differences 64.14 years (63.51-64.76).

A decrease was observed in both ICU LOS 3.04 days (2.84-3.24), 2.40 days (1.94-2.86) and 1.51 days (1.32-1.69) (ANOVA 422, 05 p < 0.001) and in HOSPITAL LOS 14.04 days (13.29-14.78), 12.59 days (11.34-13.85) 5.66 days (5.01-6) , 30) (ANOVA 25484, 45 P < 0.001) respectively throughout the three periods.

Mortality in the same periods also decreased. ICU mortality 6.79% (4.67-8.91), 4.86% (2.53-7.20) and 2.98% (1.78-4.19) (ANOVA 4649.18) p = 0.005). Hospital mortality 9.17% (6.74-11.61), 8.21% (5.23-11.19), 4.54% (3.07-6.01) ( ANOVA 7662.75 p = 0.002) in the three periods.

**CONCLUSIONS.** Related to the improvement in the treatment of AMI, both, mortality and LOS in ICU and HOSPITAL have decreased over the past 15 years without change in other aspects such as age or severity of patients.

### 0201

#### Intensive care outcome of left main stem disease surgery

A. Omar[1,2,3], P. Sivadasan[1], S. Hanoura[1], H. Osman[1], S. Sudarsanan[1], Y. Shouman[1], R. Singh[4], A. AlKhulaifi[1]
[1]Hamad Medical Corporation, Cardiothoracic Surgery, Doha, Qatar; [2]Beni Suef University, Critical Care, Beni Suef, Egypt; [3]Weill Cornell Medical College in Qatar, Clinical Medicine, Doha, Qatar; [4]Hamad Medical Corporation, Medical Research Center, Doha, Qatar
**Correspondence:** A. Omar

*Intensive Care Medicine Experimental* 2018, **6(Suppl 2):**0201

**INTRODUCTION.** Defining the intensive care unit (ICU) outcome predictors after cardiac surgery remains an optimum goal. [1] Left main coronary artery (LMCA) disease is a disease of the main coronary branch that gives more than 80% of blood supply to the left ventricle, it carries high mortality without surgical intervention; [2] however the influence of LMCA surgery on morbidity ICU measures needs to be explored.

**OBJECTIVES.** To determine whether LMCA is a risk factor for prolonged ICU stay as a primary outcome and whether LMCA is definitive risk factor for early morbidity.

**METHODS.** Retrospective descriptive study with purposive sampling analyzing 398 patients underwent isolated coronary artery bypass surgeries (CABG). Patients were divided into 2 groups those with LMCA disease as group 1 (75 patients) and those with coronary artery disease requiring surgery but without LMCA disease as group 2 (324 patients) then we did correlations with ICU outcome parameters including ICU stay length, post-operative atrial fibrillation, acute kidney injury, re-exploration, perioperative myocardial infarction, post operative bleeding and early mortality.

**RESULTS.** Patients with LMS had significantly higher diabetes prevalence (43.3% vs 29%, p=0.001). However, we did not find a statistical significant difference regarding ICU stay, or other morbidity and mortality outcome measures

**CONCLUSIONS.** Diabetes was more prevalent in patients with LMS. The latter group showed similar outcome as those without LMS in this study; these findings may help in guiding decision making for future practice and stratifying the patients care.

##### REFERENCE(S)

1) Ryan TA, Rady MY, Bashour CA, Leventhal M, Lytle B, Starr NJ. Predictors of outcome in cardiac surgical patients with prolonged intensive care stay. Chest. 1997 Oct 31;112(4):1035-42.
2) Göl MK, Özsöyler I, Şener E, Göksel S, Saritaş A, Taşdemír O, Bayazit K. Is left main coronary artery stenosis a risk factor for early mortality in coronary artery surgery?. Journal of cardiac surgery. 2000 May 1;15(3):217-22.

##### GRANT ACKNOWLEDGMENT

To all members of the cardiothoracic surgery, and academic health system Hamad medical corporation

### 0202

#### The association of circadian mean arterial pressure variation with mortality: an retrospective cohort study

J. Li[1], R. Li[1], J. Zhang[1], Y. Gao[1], D. Yao[2], J. Huang[3], X. Mei[4], Y. Zhao[5], Y. Chai[6], S. Pan[7], G. Wang[1]
[1]The Second Affiliated Hospital of Xi'an Jiaotong University, Department of Emergency Medicine, Xi'an, China; [2]The Second Hospital of Hebei Medical University, Department of Emergency Medicine, Shijiazhuang, China; [3]Chongqing Emergency Medical Center, Department of Emergency Medicine, Chongqing, China; [4]Beijing Chaoyang Hospital, Capital Medical University, Department of Emergency Medicine, Beijing, China; [5]Zhongnan Hospital of Wuhan University, Department of Emergency Medicine, Wuhan, China; [6]Tianjin Medical University General Hospital, Department of Emergency Medicine, Tianjin, China; [7]Xinhua Hospital, Shanghai Jiao Tong University School of Medicine, Department of Emergency Medicine, Shanghai, China
**Correspondence:** G. Wang
*Intensive Care Medicine Experimental* 2018, **6(Suppl 2):**0202

**INTRODUCTION.** The mean arterial pressure (MAP) is routinely monitored in intensive care unit (ICU) as a hemodynimic parameter for assessing tissue perfusion. The relationship between circadian MAP variation and mortality in critically ill patients remains unclear.

**OBJECTIVE.** The purpose of this study was to investigate the influence of circadian MAP variation on ICU mortality and long term prognosis.

**METHODS.** This retrospective cohort study was performed in all adult ICUs using the Multiparameter Intelligent Monitoring in Intensive Care II (MIMIC II) database with authorised access. All patients with complete MAP recordings at the first 24 hours in ICU and complete data of their age, gender, ethnicity, first SOFA score, first SAPS-I score, vasoactive and sedative medications, major comorbidities such as congestive heart failure, hypertension, diabetes and delirum were recruited for analysis. Participants were excluded if they had: multiple ICU admissions, diagnosis of shock, ICU stay of < 1 day. The circadian variation of MAP was calculated as mean night time MAP/mean day time MAP according to our previous publications. Then the patients were divided into 2 groups based on the value of circadian MAP variation: group A (≤1) and group B (>1). The associations of circadian MAP variation and ICU mortality, hospital mortality, 30-day mortality and 1-year mortality were evaluated in multivariable logistic regression models and cox proportional hazards models. Two sensitivity analyses were performed on those with or without receiving vasoactive medications, and with first SOFA score ≤ 8 or > 8.

**RESULTS.** A total of 7,492 patients were eventually included. The median age of the population was 65 years, and 61% were male. Patients in group B (n=2584) had higher ICU mortality than those in group A (n=4908; odds ratio [OR], 1.37; 95% confidence interval [CI], 1.16-1.61; P< 0.001). Following multivariable adjustments, the circadian variation of MAP >1 was significantly associated with ICU mortality (odds ratio [OR], 1.29; 95% confidence interval [CI], 1.09-1.54; P=0.004) and hospital mortality (odds ratio [OR], 1.33; 95% confidence interval [CI], 1.14-1.56; P< 0.001), which was also found in the subgroups regardless of the use of vasoactive medications or first SOFA scores. During the follow-up observation, the survival analysis revealed that patients with MAP variation >1 had higher 30-day mortality (hazard ratio [HR], 1.22; 95% confidence interval [CI], 1.07-1.40; P=0.003) and 1-year mortality (hazard ratio [HR], 1.19; 95% confidence interval [CI], 1.06-1.32; P=0.002) after adjustment for multiple variables.

**CONCLUSION.** The circadian MAP variation may associate with ICU mortality, hospital mortality, 30-d and 1-year mortality in critically ill patients.

##### GRANT ACKNOWLEDGMENT

The MIMIC-II database was supported by the National Institute of Biomedical Imaging and Bioengineering of the National Institutes of Health (grant number R01 EB001659).

### 0203

#### Autonomic system evaluation in critically ill patient and its relation with ICU mortality

C. Hernandez Cardenas, L.F. Jurado Camacho, L. Goytia
Instituto Nacional de Enfermedades Respiratorias, Unidad de Cuidados Intensivos Respiratorios, Tlalplan, Mexico
**Correspondence:** C. Hernandez Cardenas
*Intensive Care Medicine Experimental* 2018, **6(Suppl 2):**0203

**INTRODUCTION.** Human organism has a complex system of physiologic interactions; disease-driven compromise of this mechanisms significantly reduces the ability for adaptation and survival. The application of non-lineal dynamic system analysis in prognosis of critically ill septic patient is considerably unexplored. We hypothesized that the heart rate viability (HRV) has a pivotal role in the mortality of the critically ill.

**OBJECTIVES.** Define the relationship between HRV measured within the first day of ICU and mortality in critically ill mechanical-ventilated subjects.

**METHODS.** Prospective cohort in a pulmonary academic hospital admitted to the respiratory intensive care unit with invasive mechanical ventilation. Finapres Medical Systems|Finometer was used to measure RR intervals; HRV, its standard deviation (SDNN), asymmetry index and Poincaré plots SD2/SD1 were used to evaluate the Autonomous Nervous System (ANS) within the first 24 hours of R-ICU admittance. ROC curves were used to infer the ability to predict death before RICU discharge.

**RESULTS.** Patients included (115) had mean (±SD) age and SOFA score of 46 (±10) and 7.1 (±3.7) respectively; there is a significant statistical relation between HRV, SDNN and Poincaré plot with mortality before RICU discharge (p=.003). We found similar AU ROC for SDNN, HRV and SOFA (AU ROC=0.7).

**CONCLUSIONS.** We found significant relation between HRV, SDNN and mortality; both having a similar area under the ROC with SOFA score (0.7); Poincaré plot appears to have predictive value for mortality in the RICU Patient. We are currently working to explore the predictive value of HRV analysis and mortality at R-CU discharge

##### REFERENCE(S)

Bolton C, Thompson J, Bernardi L, Voll C, Young B. The cardiac R-R variation and Sympathetic skin response in the intensive care unit. Can Journ Neurol Science.2007;34:313-315.

Schmidt H, Müller-Werdan U, Hoffman T, etal. Autonomic Dysfunction predicts mortality in patients with multiple organ dysfunction syndrome of different age goups. Crit Care Med 2005;33:1994-2002.
Garrard C, Kontoyannis D, Piepoli M. Spectral analysis of heart rate variability in the sepsis syndrome. Clinical Autonomic Research; 1993:5-13.
Kovatchev BP, Farhy LS, aso H. Sample asymmetry analysis of heart rate characteristics with application to neonatal sepsis and systemic inflammatory response síndrome Pediatr Res;2003:892-898.
Tulen JH, Boomsma F, Man int`veld AJ Cardiovascular control and plasma catecholamines during rest and mental stress: effects of posture. Clin Sc; 1999: 567-576
Kleiger RE, Bigger JT, Bosner MS etal. Stability over time of variables measuring heart rate variability in normal subjects. Am J Cardiol; 1991, 626-630.
Karim N, Ara Hasan J, Sanowar S. Heart Rate Variability- A review. Journal of Basic and Applied Sciences 2011;7:71-77.
Kitlas A. Poincaré plots in analysis of selected biomedical signals. Studies in Logic , Grammar and rhetoric ;201·:117-127.

**GRANT ACKNOWLEDGMENT**
None.

## Outcomes of the ICU

### 0204

#### Outcome of NIV in critical care setting: 5-year experience from a tertiary care center

M. Esteves Brandão[1], S. Xavier Pires[2], A.P. Dias[3], N. Barros[3], F. Esteves[3]
[1]Centro Hospitalar de Trás-os-Montes e Alto Douro, Respiratory Medicine, Lordelo, Vila Real, Portugal; [2]Hospital da Senhora da Oliveira, Guimarães EPE, Internal Medicine, Guimarães, Portugal; [3]Centro Hospitalar de Trás-os-Montes e Alto Douro, Intensive Care Medicine, Vila Real, Portugal
**Correspondence:** M. Esteves Brandão
*Intensive Care Medicine Experimental* 2018, **6(Suppl 2):**0204

**INTRODUCTION.** Non-invasive ventilation (NIV) it is strongly recommended in the management of acute respiratory failure (ARF) due to COPD exacerbation and cardiogenic pulmonary oedema[1]. However there is a lower certainty of evidence for its safety and effectiveness in other specific clinical settings.

**OBJECTIVE.** To describe the use and outcomes of NIV in adults presenting with ARF to an Intensive Care Unit (ICU) of a tertiary hospital.

**METHODS.** Retrospective cohort study including all adult ICU admissions for ≥ 24 hours from 1 January 2010 to 31 December 2015. Demographic data, admitting diagnosis, severity of illness (APACHE II, SAPS II and SOFA), duration of mechanical ventilation (MV), intubation rate, mortality rate and ICU length-of-stay (ICU-LOS) were recorded. Logistic regression was performed to identify predictors of adverse outcome (defined as need for endotracheal intubation or death).

**RESULTS.** Among 3.384 subjects admitted to ICU during the study period, 810 (57.8% male; mean age 68 ± 14 years) received NIV as the initial ventilatory support for the treatment of ARF. The most frequent admitting diagnosis was pneumonia (25.8%), followed by post-operative respiratory failure (15.3%).Baseline mean PaO2/FiO2 ratio was 207.6 ± 113.1 mmHg and mean APACHE II was 20.1 ± 7.9.Three hundred and four patients (37.5%) had sepsis and 311 (38.4%) evolved to septic shock. The mean duration of MV was 7±6 days and the mean ICU-LOS was 9±8 days. Orotracheal intubation rate was 46.7% and in-ICU mortality rate was 7.2% (composite adverse outcome in 53.9% of admissions). Patients who avoided intubation had significantly (p < 0.001) shorter ICU-LOS and lower mechanical ventilation duration (p< 0.001) but no different in-ICU mortality rate. NIV failure rates varied significantly across ARF etiologies with lower rates in COPD exacerbation (38.5%) and cardiogenic pulmonary oedema (36.5%) and higher rates in ARF in immunocompromised patient (52%), pneumonia (60.3%), and post-operative respiratory failure (63.7%). In multivariate analysis, APACHE II (p=0.04, OR 0.94, 95%CI 0.88-1.0), SAPS II (p< 0.001, OR 1.07, 95%CI 1.04-1.11) and SOFA (p< 0.001, OR 1.29, 95%CI 1.16-1.44) scores at admission, the occurrence of ARDS (p=0.04, OR 1.87, 95%CI 1.02-3.42) and nosocomial infection (p< 0.001, OR 3.08, 95%CI 1.79-5.31) were independent predictors of NIV failure.

**CONCLUSIONS.** Even in highly monitored setting like ICU, significant NIV failure rates can be observed in critically-ill patients with ARF caused by conditions other than COPD exacerbation and cardiogenic pulmonary oedema. APACHE II, SAPS II and SOFA scores at admission were independent predictors of NIV failure and can be useful in patient selection. Odds for NIV failure was three times higher in the presence of nosocomial infection.

### 0205

#### Factors predicting mortality for acute exacerbation of chronic obstructive pulmonary disease (AE/COPD) in a Tunisian ICU

K. Meddeb[1], A. Khedher[1], I. Ben Saida[1], M.A. Boujelbèn[1], N. Fraj[1], W. Zarrougui[1], A. Azouzi[1], M. Boussarsar[1,2]
[1]Farhat Hached University Hospital, Medical Intensive Care Unit, Sousse, Tunisia; [2]Ibn Al Jazzar Faculty of Medicine, Research Laboratory N° LR12SP09 Heart Failure, Sousse, Tunisia
**Correspondence:** M. Boussarsar
*Intensive Care Medicine Experimental* 2018, **6(Suppl 2):**0205

**INTRODUCTION.** AECOPD are a leading cause of admissions in ICU. Significant heterogeneity of clinical presentation and disease progression exists defining phenotypes of AE/COPD and classified according to aetiology, inflammatory biomarkers, clinical manifestation, comorbidity and the frequency of exacerbations.

**OBJECTIVES.** To identify independent risk factors of ICU mortality in a cohort of AE/COPD admitted to ICU.

**METHODS.** It is a retrospective study including consecutive patients admitted for AE/COPD in a 9-bed Tunisian medical ICU going from 2008 to 2017. Baseline characteristics were collected on admission. Univariate and multivariate analyses were performed comparing patients according to their vital status.

**RESULTS.** During the study period, 1090 episodes fulfilled criteria for COPD exacerbation. Patients characteristics on admission were: median age, 68[61-76] years old; sex ratio, 5/1; Sleep obstructive apnea syndrome, 74(6.8%); obesity, 165(15.1%); mMRC score ≥ III, 459(42.1%); severe acute respiratory failure, 630(57.8%); shock, 290(26.6%); SAPSII, 31[24-40]; pH, 7.31[7.25-7.37]; PaCO2, 61[49-76] mmHg; PaO2, 89[59-168]mmHg; PaO2/FiO2, 230[180-273]mmHg; initial NIV, 368(33.8%); success rate, 199(54%); intubation delay, 2[1-4]days; mechanical ventilation duration, 5[2-10] days; tracheostomy, 88(8.1%); VAP occurrence, 180(16.5%); length of stay, 8[5-14]days; mortality rate 433(39.7%).
Univariate then multivariate logistic regression analyses identified the following factors as independently associated to ICU mortality: age (OR=1.033, 95%CI, [1.019-1,048]; p=0.0001), obesity (OR=0.46, 95%CI, [0.28-0.75]; p=0.002), shock on admission (OR=1.66, 95%CI, [1.18-2.33]; p=0.003), NIV success (OR=0.048, 95%CI, [0.022-0.108]; p=0.0001), mechanical ventilation duration (OR=1.18, 95%CI, [1.14-1.23]; p=0.0001), VAP occurrence (OR=34.7, 95%CI, [17.4-69.1]; p=0.0001).

**CONCLUSION.** The present study confirmed underlying comorbidities, severity on ICU admission, prolonged mechanical ventilation and ventilator acquired pneumonia to be independently associated to ICU mortality in AE/COPD patients.

### 0206

#### Economic impact of an outbreak of extensively drug-resistant bacteria in an intensive care unit

E. Atchade[1], V. Goldstein[2], S. Meslem[1], S. Jolivet[2], B. Lortat-Jacob[1], P. Tashk[1], N. Zappella[1], A. Tran-dinh[1], L. Armand-Lefèvre[3], J.-C. Lucet[2], S. Tanaka[1], P. Montravers[1]
[1]Bichat Claude Bernard Hospital, Département d'Anesthésie Réanimation, Paris, France; [2]Bichat Claude Bernard Hospital, Unité d'Hygiène et de Lutte contre les Infections Nosocomiales, Paris, France; [3]Bichat Claude Bernard Hospital, Laboratoire de Microbiologie, Paris, France
**Correspondence:** E. Atchade
*Intensive Care Medicine Experimental* 2018, **6(Suppl 2):**0206

**INTRODUCTION.** Between October 2016 and November 2017, an outbreak of Extensively Drug-Resistant (XDR) bacteria occurred in our surgical Intensive Care Unit (ICU). A specific organization was implemented to avoid cross-transmission, including restriction of ICU admission/discharge, cohorting of the colonized/infected patients (pts) and dedicated nursing staff (DNS) [1].

**OBJECTIVES.** The aim of this study was to assess the consequences of these procedures in terms of bed-days unavailability [2] and to estimate their financial impact.

**METHODS.** This retrospective monocentric study compared the total number of ICU admissions (TNA) and mean ICU length of stay (LS) in 2017 to the activity reported in 2016 before the outbreak. The attributable cost of bed-days unavailability was estimated as follows: (number of beds unavailable x number of days)/(ICU LS to December, 2017 x Average income (in euros) for the treated cases balanced to end of December, 2017). Number of XDR-positive pts and specific procedures implemented for their management were collected by the Hygiene Operational Team. TNA and ICU LS were collected by the Medical Information Department.

**RESULTS.** On a routine basis in our 20 beds ICU, the nursing staff consists of 44 nurses (N) and nurse-assistants (NA). The N/pts and NA/pts ratios are respectively 1/2.5 and 1/4 pts. From January to December 2017, 747 pts were admitted in ICU, including 19 XDR-positive pts (NDM-producing *Enterobacteriaceae* (NDM-E): 16 pts for 19 stays representing 470 days of ICU stay; OXA-48-producing *Enterobacteriaceae* (OXA-E): 3 pts for 3 stays representing 23 days of ICU stay). The overall ICU LS was 6.1 days in 2017 versus (vs) 6.5 days in 2016. For NDM-E and OXA-E, ICU LS was 25 and 8 days respectively. The total number of unavailable bed-days in 2017 was 547. Total attributable cost of bed unavailability was € 1 870 229. Nursing staff was reinforced for 165 days (NA and N for respectively 164 and 1 days). The TNA dropped dramatically in 2017 (366 vs 450 in 2016). The cost calculation based on bed-unavailability days under-estimated the financial burden of this outbreak, as it did not include costs linked to staff reinforcement, loss of income due to iterative interruptions of admissions, decreased bed occupancy, temporary cessation of the transplantation program, decreased surgical activities, increased ICU LS in ICU linked to temporary suspension of contact patients and XDR-positive patients transfers.

**CONCLUSIONS.** Management of an XDR outbreak in ICU is associated with a significant number of bed-days unavailability and with a huge financial burden for the Unit and the Institution. Costs linked to prolongation of hospital stay of colonized pts, staff reinforcement, and decreased TNA have to be added to this direct cost calculation.

**REFERENCE(S)**

[1] https://www.hcsp.fr/Explore.cgi/avisrapportsdomaine?clefr=372
[2] BMJ open 2016;6:e009029

### 0207

#### Analysis of patients older than 80 years admitted in intensive care unit

M.D. Pola-Gallego-de-Guzmán[1], M. Guerrero-Marin[1], E. Aguilar-Alonso[2], C. Lopez-Caler[3], L. Perez-Borrero[4], P. Lara-Aguayo[2], E. Morán-Fernández[2], R. Montoiro-Allué[5], J.L. Flordelís-Lasierra[6], D. Iglesias-Posadilla[7]
[1]Complejo Hospitalario, Intensive Care Unit, Jaen, Spain; [2]Hospital Infanta Margarita, Intensive Care Unit, Cabra, Spain; [3]Hospital Regional Universitario Carlos Haya, Intensive Care Unit, Malaga, Spain; [4]Hospital de la Serrania, Intensive Care Medicine, Ronda, Spain; [5]Hospital Clínico Universitario Lozano Blesa, Intensive Care Unit, Zaragoza, Spain; [6]Hospital Severo Ochoa, Intensive Care Unit, Madrid, Spain; [7]Hospital Universitario, Intensive Care Unit, Burgos, Spain
**Correspondence:** M.D. Pola-Gallego-de-Guzmán
*Intensive Care Medicine Experimental* 2018, **6(Suppl 2):**0207

**INTRODUCTION.** In recent years, the number of patients older than 80 years admitted to the Intensive Care Unit (ICU) has increased exponentially.

OBJECTIVES: To analyze the characteristics of patients with age over 80 years admitted in ICU of Spain.

**METHODS.** Multicentric prospective observational study in the ICU of 7 Spanish hospitals (Carlos Haya in Malaga, Cabra (Córdoba), Jaén, Burgos, Arnau Vilanova in Valencia, Clinic of Zaragoza, Ronda).

Data were expressed as the mean and standard deviation for quantitative variables and percentages for qualitative variables. For the comparison of two means we used the Student´s t-test and the chi- squared test was used to compare proportions. Statistically significant differences $p < 0.05$.

**RESULTS.** N=2008 patients. 181 (9%) were over 80 years. 57 patients (3%) were between 85-90 years old and only 4 patients older than 90 years.

The type of pathology of patients with more than 80 years: 62% were medical patients and 38% surgical.

Of the medical patients, acute coronary syndrome (20% of all patients, 12% with ST-elevation myocardial infarction - STEMI), arrhythmias, especially bradycardias related to the need or surveillance of pacemaker (17% of the total), 5% for sepsis and a 4% for cardiac or respiratory arrest. 38% were surgical patients, of them 10% only for central venous catheterization, and 7% for emergency surgery. The most frequent surgeries were cardiac and maxillofacial surgery.

Previous functional situation (normal, self-sufficient but dysfunction and not self-sufficient): 50%, 36% and 14% respectively.

The SAPS-3 at admission was 53.83 ±12.71 points and the mortality was 23%, with mortality in the ICU of 14%. At admission, 26% required mechanical ventilation, with mortality of ventilated patients of 40% and non-ventilated patients of 17% ($p = 0.001$).

**CONCLUSIONS.** Of the patients admitted to the ICU, 9% are older than 80 years, the majority are self-sufficient. Rhythm disorders and ischemic heart disease are the most frequent causes. Mortality is higher than 20%, and mortality was specially high in patients required mechanical ventilation at admission.

### 0208

#### Factors related to delayed ICU admission from emergency department

M. Aitavaara[1,2], J. Liisananti[1,2], P. Ohtonen[3], T. Ala-Kokko[1,2]
[1]Oulu University Hospital, Department of Anesthesiology, Division of Intensive Care Medicine, Oulu, Finland; [2]Oulu University Medical Research Center, Research Group of Intensive Care Medicine, Oulu, Finland; [3]Oulu University Hospital, Department of Operative Care, Oulu, Finland
**Correspondence:** M. Aitavaara
*Intensive Care Medicine Experimental* 2018, **6(Suppl 2):**0208

**INTRODUCTION.** The delays in patient transfer from emergency department (ED) to Intensive care unit (ICU) are known to be linked in several adverse events including prolonged ICU stay and hospital mortality (1,2,3,7,9). The factors associated to delayed ICU admission include shortage of ICU beds (4,6) organisational factors (5), ED overcrowding (2,8) and patient related factors including sepsis as admission diagnosis (2).

**OBJECTIVES.** Aim for this study was to examine ED related factors associated to prolonged ED stay.

**METHODS.** The study population consisted of every adult patient admitted from ED to ICU between May 31, 2016 and March 19, 2017 in Oulu University hospital. The total number of patients was 479.

**RESULTS.** The majority of the patients (380, 79.3%) were admitted within three hours of hospital admission. The median ED length of stay (LOS) was 92 [25th- 75th PCT 60-162] minutes. In the multivariate analysis, OR for delayed EDLOS was higher, if patient had GCS above 9, thrombocytes less than 100 x 10ˆ9, absence of pre-arrival notification, arrival time during office hours, use of vasoactive medication or use of radiological examinations. (Table 1).

**CONCLUSIONS.** In conclusion, OR for delayed EDLOS was higher, if there were absence of pre-arrival notification, use of vasoactive medication, arrival time during office hours (between 8am and 4pm), GCS 9 or more, low thrombocytes or use of radiological examinations.

**GRANT ACKNOWLEDGMENT**

**Table 1 (abstract 0208).** Multivariate analysis

| | OR (95% CI) | P-value |
|---|---|---|
| GCS <9 | 2.73 (1.41-5.29) | 0.003 |
| Platelets <100 mmol/l | 6.12 (2.22-16.84) | <0.001 |
| Pre-arrival notification | 4.61 (2.71-7.82) | <0.001 |
| Vasoactive | 1.80 (1.00-3.27) | <0.001 |
| ED arrival time between 8am and 4pm | 1.925 (0.98-3.77) | 0.056 |
| ED arrival time between 4pm and 12 am | 1.81 (0.88-3.72) | 0.110 |

### 0209

#### Readmissions in ICU during same hospitalisation: risk factors, reasons and outcome

K. Zirpe[1], S. Gurav[2], R.N. Yellapu[1], A. Deshmukh[2], S. Yadav[1]
[1]Ruby Hall Clinic, Neurotrauma Unit, Pune, India; [2]Same As Above, Neurotrauma Unit, Pune, India
**Correspondence:** K. Zirpe
*Intensive Care Medicine Experimental* 2018, **6(Suppl 2):**0209

**INTRODUCTION.** Readmission in ICU results in increased consumption of medical resources, costs and increased mortality. So in resource limited countries like India it is important to decrease health care costs and have to utilize the available resources wisely.

**OBJECTIVES.** To identify patients at risk for ICU readmission, to know readmission rate , reasons for readmission, average length of stay in ICU and mortality after readmission during period from Jan 1$^{st}$ 2015 to June 30 Th 2017.

Readmission episodes with in 72 hrs were analyzed for risk factors, cause of readmission, length of stay and mortality.

**RESULTS.** Total 3290 patients admitted in ICU during study period. Total patients transferred out (2472), discharged (246), 47patients readmitted in ICU. Readmission rate in ICU 1.90%. Males constitutes 72.3 % (34/47), females constitute 27.65 % (13/47). Mean and standard deviation for age of readmitted patients is 52.77 (+/-14.98) Out of all readmitted patients 72.3% (34/47) patients are < 60 yrs age group.27.6% (13/47) are > 60 years age group. Majority of patients are in decade of 50-60 years age group (19/47). 63.82 % (30/47) of readmitted patients had associated co-morbidity. 59.57 % (28/47) patients readmitted within 24 hours, 25.53 % (12/47) patients admitted between 24-48 hrs. 14.89 % (7/47) patients admitted after 48 hrs.

Primary diagnoses are analysed. Neurotrauma 51.06%(24/47), oncology 10.6% (5/47), hepato biliary system 8.5% (4/47), postop 6.38% (3/47), respiratory 4.25% (2/47), others 8.5% (4/47).

Causes of readmission were due to respiratory system 34.04% (16/47), neurological 31.83% (13/47), cardiac 23.40% (11/47), GI causes 4.2% (2/47), and Renal causes 4.2% (2/47) and Drug reaction 2.1% (1/47).

Mortality rate for readmission patients was 27.65% (13/47). Main causes for death in readmitted patients were cardio pulmonary 46.15% (6/13), neurologic 38.46% (5/13), gastric 7.69% (1/13) and renal 7.69% (1/13).Average length of stay for our icu readmitted patients (6.79 days).

**CONCLUSIONS.** Readmission rate of our ICU was 1.9%. Mortality rate of readmitted patients compared with non readmitted patients was not statistically significant (27.64% vs21.05%) p value calculated was 0.35 by chi square test.

The probable reason for our promising readmission(1.9% Vs 5-6%) as compared to standard bench mark was due toMortality rate of readmitted patients was more than mortality rate of shifted out patients (27.64% vs21.05%) p value calculated was 0.35 by chi square test.That means higher mortality rate in readmitted patients is not stastically significant. increased length of stay (6.63 days Vs 4.5days).

**REFERENCE(S)**

1) Tracy R. McMillan, MD; Robert C. Hyzy, Bringing quality improvement into the intensive care unit Crit Care Med 2007;35[Suppl.]:S59-S65).
2) Maria Cruz Martin Delgado, Lluis Cabre Pericas, Javier Ruiz Moreno et al. Quality indicators in critically ill patients. SEMICYUC work groups. First edition May 2005. ISBN 609- 5974

### 0210

#### Retrospective study of use of glucometrics to compare glucose control in medical and surgical intensive care units

S. Dixit[1], K. Khatib[2]
[1]Apka Clinic, Pune, India; [2]SKN Medical College, Medicine, Pune, India
**Correspondence:** S. Dixit
*Intensive Care Medicine Experimental* 2018, **6(Suppl 2):**0210

**INTRODUCTION.** Glucometrics (defined as the analysis of blood glucose data) can be used as a quality indicator of glycemic control in a patient care unit of the hospital. Metrics generated are utilized to compare the quality of care provided to patients and different units of the hospitals can be compared. We studied the use of glucometrics to compare the glucose control metrics in the medical (MICU) and surgical (SICU) Intensive Care Unit of our hospital over one month.
METHODS: Indoor records of all patients admitted to MICU and SICU of our hospital from 1st July 2017 to 31st July 2017 were evaluated. Data regarding blood glucose level (BSL), its timing, its relation to feeding, as well as insulin dose and type given at that time, along with patient characteristics were noted. All this data was entered into a spreadsheet and then analyzed to generate metrics. A Glucometrics Report was prepared for the period of assessment. The glucometrics data was compared between the ICUs.
**RESULTS.** A total of 700 BSLs were studied. Of these 325 were in MICU and 275 were in SICU. The mean BSL in MICU and SICU was160 mg/dl and 145 mg/dl (p=0.45) and the Median BSL was 180 mg/dl and 166 mg/dl (p=0.75).
1.5% and 2% of BSL were in the hypoglycemic range (BSL< 70mg/dl) in MICU and SICU respectively (p=0.865). 56% and 54% of BSL were in the acceptable range (BSL in 100-180mg/dl range) in MICU and SICU respectively (p=0.65).
When patients were taken as unit of analysis, 15 and 12 % (p=0.35) of patients had hypoglycemia and 25 and 30 % (p=0.44) of patients had extreme hyperglycemia (BSL>300mg/dl) in MICU and SICU respectively.
**CONCLUSIONS.** There was no statistical difference in the BSL control in the two ICUs over the period of study. Glucometrics can be used to compare glycemic control between units of hospitals and hence can be used for quality assessment and benchmarking of ICU units.

**REFERENCE(S)**

1. Thompson BM, Cook CB. Glucometrics and Insulinometrics. Curr Diab Rep (2017) 17:121.
2. Maynard G, et al. How sweet is it? The use of benchmarking to optimize inpatient glycemic control. Diabetes Spectr. 2014;27(3):212-7.
3. Khatib K, Borawake K. Glucometrics of diabetic patients admitted to intensive care unit in hospitals with limited information technology support: is it possible? J Diabetes Sci Technol. 2014;8(5):1055-6.

**GRANT ACKNOWLEDGMENT**
NIL

### 0211

#### Patient selection and attendance/non-attendance at critical care rehabilitation clinic

E. Morino, L. Ma, M. Allen, M. Dabliz
Kingston Hospital NHS Foundation Trust, Critical Care, Kingston-upon-Thames, United Kingdom
**Correspondence:** E. Morino
*Intensive Care Medicine Experimental* 2018, **6(Suppl 2):**0211

**INTRODUCTION.** Large investments are made during a patient's stay on critical care and their care can only be sustained with good quality care following discharge. Rehabilitating patients discharged from a critical care setting has been recommended by the National Institute for Health and Clinical Excellence (NICE) in 2009 (Clinical Guidance 83)[1]. Non-attendance at these clinics may negatively impact the health of this often vulnerable patient group.
**OBJECTIVES.** Our aims were to review our patient selection methods and processes leading to invitation to our newly-developed multidisciplinary intensive care rehabilitation clinic over a 6-month period between October 2017 to March 2018. Our purpose was to analyse the frequency and reasons for non-attendance in order to devise strategies to reduce 'Did Not Attend' (DNA) rates in the future.
**METHODS.** Our study took place at Kingston Hospital, Surrey, UK. We retrospectively reviewed the cohort of patients for selection to our clinic based on length of stay in critical care, discharge destination and time since discharge. We reviewed their suitability for invitation to clinic via electronic medical records and contact via general practitioners. We recorded attendance/non-attendance rates and reasons for the latter if these were known if patients informed us prior to clinic or via telephone conversation.
**RESULTS.** When developing the clinic, it was decided that patient would be selected for invitation to clinic if they had been on critical care for longer than 5 days and discharged from hospital. In total, we identified 57 patients eligible for invitation to clinic. Of these, 14 patients (25%) were deemed unsuitable. There were 3 deaths (5%), 2 moved out of area on discharge (4%), and 9 patients (16%) had moved to a nursing home or were too unwell or were hospitalised at the time of the clinic appointment. 43 patients were invited to clinic with a DNA rate of 13 (30%). Reasons given for non-attendance were unknown (41%), did not feel they needed to attend (23%), frailty (12%), transport (12%), and language (12%).
**CONCLUSIONS.** Non-attendance at clinic appointments is a multifactorial problem. In our study it appeared that a significant proportion of patients eligible for invitation to rehabilitation clinic after discharge home were unsuitable due to worsening ill health and frailty. The DNA rate was considerable, and caused by many reasons. Strategies to reduce DNAs in the future may rely on increasing awareness and understanding of the purposes of critical care rehabilitation via leaflets/posters/communication from outreach teams prior to discharge home/social media, as well as administrative processes including electronic text reminders, phone-calls to confirm attendance prior to clinic date, and auditing patient feedback.

**REFERENCE(S)**

1. NICE. Rehabilitation after critical illness NICE Clinical Guideline 83. London, UK: National Institute for Health and Clinical Excellence, 2009

### 0212

#### Measurement and analysis of noise levels in ICU

G. Vasileiadou[1], V. Vasilopoulos[2], C. Giannaki[1], C. Sevastiadis[2], G. Papanikolaou[2], I. Rodini[1], M. Bitzani[1]
[1]General Hospital G.Papanicolaou Thessaloniki, A ICU, Thessaloniki, Greece; [2]Aristotle University of Thessaloniki, School of Electrical and Computer Engineering, Thessaloniki, Greece
**Correspondence:** G. Vasileiadou
*Intensive Care Medicine Experimental* 2018, **6(Suppl 2):**0212

**INTRODUCTION.** Good professional care includes the establishment of an appropriate surrounding environment that promotes rest, sleep and wellbeing that probably contribute to better outcomes. Noise levels exert an important impact on the establishment of such a physical environment. According to World Health Organization (WHO) the recommended Equivalent Continuous Sound Level ( $L_{Aeq}$ ) in hospitals should not exceed 30 dBA while maximum noise levels ( $L_{AFmax}$ ) during the night should not exceed 40 dBA . However, it is not clear whether these recommendations can be applied in the high tech environment of an ICU .
**OBJECTIVES.** The aim of the study was the assessment of noise in a general ICU of a tertiary hospital on a 24hour basis, during weekdays.
**METHODS.** The measurements were conducted in a big 10- bed area (223m$^2$ ). The equipment used was: a class 1NTi XL2 sound level meter and an acoustic analyzer with a class 2 microphone (Type M4260) . The microphone was placed in 4 different, discrete positions, about 2 meters above the floor and 50 cm away from the patient's head. The installation took place 5 days prior to the commecement of actual data collection.Data collection ran over 28 consecutive days. The measurement and data analysis procedures strictly followed the International Standard (ISO1996-1, ISO1996-2 ) guidelines.
**RESULTS.** The spatial average of the Continuous Equivalent Sound Pressure ( $L_{Aeq}$ ) in the 10-bed area room was 63.5 dBA, with maxima reaching 97.5 dBA (table 1).
There was no significant difference between nurse shifts or between the week days concerning the $LA_{eq}$ (table 2,3).The night shift is less noisy compared to the morning one because the medical and nurse personnel is less and fewer interventions are performed.The main source of noise was staff activity, especially during urgent events, followed by the alarms of the multimodal monitoring used in ICUs, while the impact of the air-conditioning system noise was very low.
**CONCLUSIONS.** The noise levels within the ICU do not conform to the acceptable range defined by WHO.The results of this study are similar to the findings of other studies and underline the necessity to implement measures aiming to reduce noise caused by personnel and instrumentation in the ICU.Reduction of sound levels to the recommended level of 45 dBA is a desirable target.

**REFERENCE(S)**

1. Berglund B, Lindvall T, Schwela DH. Guidelines for Community Noise. World Health Organization, 1999.
2. Darbyshire JL, Young JD. An investigation of sound levels on intensive care units withreference to the WHO guidelines. *Crit Care* 2013;17:R187.
3. Konkani A, Oakley B. Noise in hospital intensive care units—a critical review of a critical topic.J Crit Care 2012;27:522.e1-9. doi:10.1016

**Table 1 (abstract 0212).** Maximum (LAFmax), minimum (LAFmin), equivalent continuous (LAeq) and spatial average over all positions continuous equivalent (LAeq,spavg) soundlevels

| dBA | Position 1 | Position 2 | Position 3 | Position 4 |
|---|---|---|---|---|
| LAFmax | 97.5 | 94.2 | 94.5 | 91.3 |
| LAFmin | 43.5 | 46.2 | 46.8 | 38.5 |
| LAeq (dBA) | 60.5 | 67.3 | 61.6 | 60.6 |
| LAeq,spavg over all positions | 63.5 | | | |

**Table 2 (abstract 0212).** Continuous equivalent sound pressure (LAeq,pershift) during shifts

| | Morning shift | Evening shift | Night shift |
|---|---|---|---|
| LAeq,pershift (dBA) | 65.2 | 63.6 | 60.2 |

**Table 3 (abstract 0212).** Continuous equivalent sound pressure ( LAeq,perday ) during weekdays

| | Monday | Tuesday | Wednesday | Tuesday | Friday | Saturday | Sunday |
|---|---|---|---|---|---|---|---|
| LAeq,perday (dBA) | 63.5 | 62.8 | 61.6 | 63.3 | 61.9 | 61.9 | 60.1 |

### 0213

#### How much does sepsis cost? Economic impact of sepsis in Catalonia

C. Lorencio[1], J.C. Yébenes[2], J. Gonzalez Londoño[1], M. Cleriès[3], E. Vela[3], L. Espinosa[4], J.C. Ruiz[5], A. Rodriguez[6], E. Esteban[7], R. Ferrer[5], A. Artigas[8]
[1]Josep Trueta Hospital, Girona, Spain; [2]Mataró Hospital, Mataro, Spain; [3]Unitat d'Informació i Coneixement de CatSalut, Barcelona, Spain; [4]Oficina Tècnica de l'Àrea Integral de Salut Barcelona Dreta, Barcelona, Spain; [5]Vall d´Hebron Hospital, Barcelona, Spain; [6]Joan XXIII University Hospital, Tarragona, Spain; [7]Sant Joan de Deu Hospital, Barcelona, Spain; [8]Parc Taulí University Hospital, Barcelona, Spain
**Correspondence:** C. Lorencio

*Intensive Care Medicine Experimental* 2018, **6(Suppl 2):**0213

**OBJECTIVE.** To measure the economic impact of sepsis in Catalonia during the years 2015-2016
**MATERIALS AND METHODS.** The CatSalut morbidity database was used, this database integrates exhaustive information of the resident population in Catalonia. Sepsis cases in Catalonia (54.671) between the years 2015 and 2016 were identified through the methodology described by Angus [Crit Care Med 2011; 29: 1303-101] and the economic costs of these cases was analyzed.
**RESULTS.** The average cost per patient during the first year since the diagnosis of sepsis was €15,334. In addition, we observed that during the year prior to the sepsis diagnose, patients showed an increase in the average annual health expenditure (€ 6,738 ppy). These costs were especially at the expense of an increase in hospital admissions, emergency consults, pharmacy expenditure and non-urgent medical transport use and took place predominantly during the 3 months prior to the sepsis diagnose.
The average cost per patient during the 2nd year after the sepsis episode continues to be higher (€ 7,411 ppy) than the global average patient cost (€ 968 ppy). These costs involve hospital admission expenses, use of healthcare resources, pharmacy consumption, urgent consultations and the use of non-urgent medical transport.
**CONCLUSIONS.** Sepsis represents an important economic cost for Catalan Health, not only during the active episode of the disease, but also in the months before the diagnosis and up to two years after the diagnosis. Any intervention to improve the detection, diagnosis or treatment of sepsis could directly affect the economic impact of this pathology.

### 0214

#### Hyperoxia effects on intensive care unit mortality: a retrospective pragmatic cohort study

M. Ruggiu[1,2], N. Aissaoui[1,2], S. Ortuno[1,2], A. Lancelot[1,2], A. Couteau-Chardon[1,2], D. Vimpère[1,2], B. Hermann[1,2], A. Novara[1,2], J.-L. Diehl[1,2], C. Bailleul[1,2], E. Guérot[1,2]
[1]Hopital Europeen Georges Pompidou, Réanimation Médicale, Paris, France; [2]Paris Descartes University, Paris, France
**Correspondence:** M. Ruggiu

*Intensive Care Medicine Experimental* 2018, **6(Suppl 2):**0214

**Introduction**
Supplementary oxygen is frequent in management of critically ill patients with supra-normal values of partial arterial pressure in oxygen (PaO2) being frequently achieved. However, some studies have demonstrated deleterious effects of hyperoxia in critically ill patients presenting brain injury, cardiac arrest, acute distress respiratory syndrome and/or septic shock[1-3]. These studies focused on 48 first hours of admission.
**Objectives**
Our objective was to evaluate the impact of hyperoxia exposure at any moment in ICU stay on ICU mortality and on overall survival.
**Methods**
It was an observational, retrospective, and single-centre study conducted in the department of critical care of George Pompidou European Hospital, in Paris. All patients admitted in ICU between November and December 2017 were included. Hyperoxia was defined as PaO2 >100 mmHg. Outcomes were compared between patients presenting at least one hyperoxia episode and patients who did not. A multivariate analysis was performed to assess factors associated with ICU mortality.
**Results**
130 patients, median age 68 years [57-79], were included. Median SAPS II was 45 [35-56]. 83 patients (64%) had mechanical ventilation during their stay during a median time of 5 days [2-11.5]. The median time of ICU stay was 4 days [2-10]. 1.450 arterial blood gas (ABG) were assessed with a median number of AGB for each stay of 4 [2-13]. Eighty patients (62%) presented at least one episode of hyperoxia.
During their ICU stay, 35 patients died (27%). Overall survival was significantly lower in patients that presented at least one episode hyperoxia during ICU stay, p=0.0047 (Figure). In univariate analysis, maximal PaO2 and time of hyperoxia exposure are significantly higher in the deceased group.
In multivariate analysis including age, sex, SAPS II and admission for respiratory cause, to have at least one hyperoxia episode during ICU stay was independent risk factor of ICU mortality, OR=5.18, $_{95}$CI=[1.53-21.71], p=0.013 (Table).
**Conclusion**
Presenting at least one hyperoxia episode at any moment of ICU stay decreased overall survival and is an independent risk factor of ICU mortality regardless the severity of the patients or the cause of ICU admission.

**Table 1 (abstract 0214).** Mortality risk factor in medical ICU, multivariate analysis

| | Deceased (n=35) | Alive (n=95) | Odd ratio [95CI] | p |
|---|---|---|---|---|
| Age - mean, 95[CI] (years) | 70 [66-75] | 65 [61-65] | | 0.17 |
| Male sex | 23 (65.7%) | 62 (65.2%) | 1.02 [0.33 - 2.74] | 0.9 |
| SAPS II - mean, 95[CI] | 64 [56-72] | 41 [37 - 45] | | < 0.001 |
| Respiratory disease | 18 (51.4%) | 42 (44.2%) | 1.87 [0.70-5.27] | 0.22 |
| At least one PaO2 > 100 mmHg | 31 (89%) | 49 (52%) | 5.18 [1.53-21.71] | 0.013 |

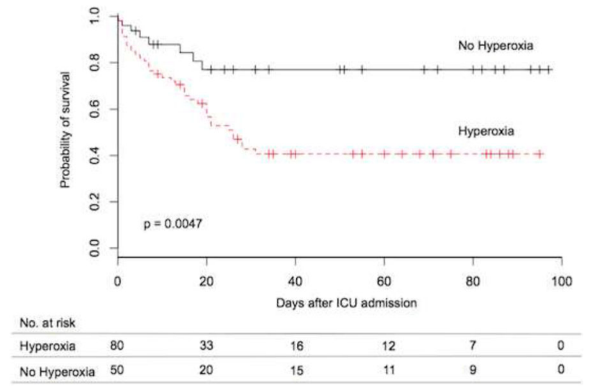

**Fig. 1 (abstract 0214).** Kaplan-Meier estimation of overall survival

### 0215

#### Elderly patients admitted to intensive care units: descriptive analysis and mortality related factors. Data from the 2013-2016 ENVIN-HELICS Registry

N. Mas Bilbao[1], U. Aguirre Larracoechea[2], F. Álvarez Lerma[3], M. Palomar Martínez[4], X. Nuvials Casals[5], M. Catalán Gonzalez[6], P.M. Olaechea Astigarraga[1], ENVIN-UCI Group

[1]Hospital de Galdakao-Usansolo, Intensive Care Unit, Galdakao, Spain; [2]Hospital de Galdakao-Usansolo, Research Unit, Galdakao, Spain; [3]Hospital del Mar, Intensive Care Unit, Barcelona, Spain; [4]Hospital Universitari Arnau de Villanova, Intensive Care Unit, Lleida, Spain; [5]Hospital vall d`Hebron, Intensive Care Unit, Barcelona, Spain; [6]Hospital 12 de Octubre, Intensive Care Unit, Madrid, Spain

**Correspondence:** N. Mas Bilbao

*Intensive Care Medicine Experimental* 2018, **6(Suppl 2):**0215

**INTRODUCTION.** The elderly are a growing population among patients admitted to Intensive Care Units (ICU) in the industrialized world, who will most probably influence in a capital way on the future development of the critical medicine. To delve in the features and prognosis of these patients will provide reliable information to help the clinicians manage the ICU resources properly and offer an adjusted care to older patients.

**OBJECTIVES.** To describe characteristics and clinical course of patients over 65 years old admitted to Spanish ICU during the period from 2013 to 2016. To identify factors related to in-ICU mortality.

**METHODS.** Data gathered from a prospective multicentric registry (ENVIN-HELICS) that compiles information from every region in Spain. Variables regarding demographics, comorbidities, clinical course and applied devices were studied. Multivariate analysis was employed to determine factors related to in-ICU mortality. Predictive power of the model measured by the AUC. Categorical variables are expressed as percentages; for continuous variables mean value and standard deviation (SD) are provided.

**RESULTS.** 37,172 patients over 65 years old were registered, 62.5% males and 8.0% over 85 years old. 80.0% of the patients had comorbidities: diabetes was the most frequent one (32.6%), followed by malignancies (18.0%). The mean value of the APACHE II scale was 11.9 points (SD 8.9 points). Pre-ICU length of stay (LOS) was 3.3 days (SD 10.0 days) and mean ICU LOS 6.3 days (SD 9.4 days). In 8.6% of the patients multidrug resistant pathogens (MRP) were isolated, more frequently acquired before ICU admission (OR 1.97; IC95% 1.63-2.38). The most frequent in-ICU acquired infections were catheter related urinary tract infections (2.3%). 4,260 patients died in ICU (11.5%). The variables that showed association to mortality were: to be admitted for a non-surgical pathology (OR 1.44; IC95% 1.19-1.74), pre-ICU LOS over 7 days (OR 1.30; IC95% 1.09-1.58); chronic kidney disease (OR 1.22; IC95% 1.04-1.44), previous immunosuppression (OR 1.74; IC95% 1.39-2.19); use of renal replacement therapies (OR 4.56; IC95% 3.73-5.58), artificial nutrition (enteral OR 1.33; IC95% 1.10-1.61 and parenteral OR 1.27; IC95% 1.03-1.57), arterial, central venous and urinary catheter insertion (OR 1.40; IC95% 1.22-1.62, OR 3.60; IC95% 2.89-4.48 and OR 3.04; IC95% 2.25-4.10 respectively); and among in-ICU acquired infections, ventilator associated pneumonia (OR 2,44; IC95% 1.76-3.40) and secondary bacteremia (OR 2.20; IC95% 1.43-3.40). The predictive power of the model was adequate, AUC 0.82 (IC95% 0.80-0.83).

**CONCLUSIONS.** Characteristics and clinical course of the elderly admitted in ICU are described. MRP are more frequently acquired pre-ICU. In-ICU mortality was affected by various factors such as comorbidities, previous clinical course or in-ICU acquired infections. The predictive model obtained out of the sum of prognosis factors had an adequate discriminatory power.

### 0216

#### Implementing specialized Intensivist lead multidisciplinary critical care in allogeneic hematopoietic stem cell transplantation patients improves ICU survival

A. Tzalavras[1], A. Turki[1], W. Lamm[2], N. Steckel[1], D. Beelen[1], T. Liebregts[1]

[1]University of Duisburg-Essen, University Hospital Essen, West German Cancer Center, Department of Bone Marrow Transplantation, Essen, Germany; [2]Medical University of Vienna, Division of Oncology, Department of Medicine I, Vienna, Austria

**Correspondence:** T. Liebregts

*Intensive Care Medicine Experimental* 2018, **6(Suppl 2):**0216

**INTRODUCTION.** Despite improved survival of critically ill cancer patients hematopoietic stem cell transplantation (HSCT) patients remain a subgroup with poor prognosis. Consequently, ICU management is still seriously debated. ICU structure and organization is known to affect patient outcome and quality of critical care in general ICU but data on implementing specialized oncological ICU structures are lacking.

**OBJECTIVES.** We therefore aimed to elucidate the effect of implementing an intensivist lead multidisciplinary care team in the highly-specialized field of critically ill HSCT patients on ICU survival.

**METHODS.** We compared ICU mortality of HSCT patients admitted to the ICU in 2011/2012 (before an intensivist model was established) with those in the years 2013/2014 and 2015/2016 (after the model was established). Organizational change implemented in 2012 consisted of high intensity staffing according to the Leapfrog model and daily rounds between intensivist and hematologist. We allowed for 1

year of transition (2012) to implement the changes required. In 2015, a respiratory therapist was added to secure ventilation and weaning protocol adherence. We assessed the effect of staffing on ICU outcome by accounting for patient and illness-related confounders using multivariate logistic regression analysis.

**RESULTS.** A total of 182 patients were included. In 2011/2012 (n=43) overall ICU, hospital, 6 months and 1 year - survival was 20.9%, 20.9%, 18.6% and 9.3%. In patients with IMV (83.7%) ICU, hospital, 6 months and 1 year - survival was 8.3%, 8.3%, 5.5% and 2.7%. In 2013/2014 (n=71) overall ICU, hospital, 6 months and 1 year - survival increased to 32.4%, 31.0%, 31.0% and 26.8%. In patients with IMV (61.9%) ICU, hospital, 6 months and 1 year - survival increased to 18.2%, 18.2%, 18.2% and 15.9%. In 2015/2016 (n=68) overall ICU, hospital, 6 months and 1 year - survival further increased to 51.4%, 42.6%, 32.4% and 30.9% while IMV patients (66.2%) ICU, hospital, 6 months and 1 year - survival increased to 31.1%, 24.4%, 17.8% and 17.8%. The following variables were associated with ICU survival; CRRT (OR 0.12 [95%CI 0.01-0.34]; p< 0.01), IMV (OR 0.03 [95%CI 0.01-0.10], p< 0.01) and study period (p=0.04; 2011/2012, OR 1; 2013/2014, OR 1.28 [95%CI 0.4-4.07], p=0.68; 2015/2016, OR 3.6 [95%CI 1.17-11.1], p=0.03).

**CONCLUSION.** Implementing a specialized intensivist lead multidisciplinary critical care team significantly improves ICU and long-term survival of hematopoietic stem cell transplantation patients.

### 0217

#### A tailored multicomponent program for discomfort reduction in the ICU decreases the incidence of PTSD at one year: a case-control multicenter study

P. Kalfon[1], M. Alessandrini[2], M. Boucekine[2], M.-A. Geantot[3], S. Renoult[4], S. Deparis-Dusautois[5], O. Mimoz[6], C. Vigne[7], J. Amour[8], N. Revel[9], E. Azoulay[10], M. Garrouste-Orgeas[11], T. Sharshar[12], K. Baumstarck[2], P. Auquier[2], The IPREA Study Group

[1]CH de Chartres, Chartres, France; [2]Aix Marseille Université, Marseille, France; [3]CHU Dijon Bourgogne, Département d'Anesthésie Réanimation, Dijon, France; [4]Clinique Ambroise Paré, Réanimation, Neuilly/Seine, France; [5]CH Troyes, Réanimation, Troyes, France; [6]CHU La Miletrie, Réanimation Chirurgicale, Poitiers, France; [7]CHU Hôpital Nord Assistance Publique Hôpitaux de Marseille, Réanimation Polyvalente et Traumatologique, Marseille, France; [8]Groupe Hospitalier Pitie Salpetrière, Réanimation de chirurgie cardiaque, Paris, France; [9]CHU Nice, Réanimation Polyvalente, Nice, France; [10]CHU Saint Louis Assistance Publique Hôpitaux de Paris, Réanimation Médicale, Paris, France; [11]Groupe Hospitalier Paris Saint Joseph, Médecine Intensive et Réanimation, Paris, France; [12]CHU Raymond Poincaré, Réanimation, Garches, France

**Correspondence:** P. Kalfon
*Intensive Care Medicine Experimental* 2018, **6(Suppl 2):**0217

**INTRODUCTION.** Critically ill patients in intensive care units (ICUs) are exposed to stressful conditions and experience discomfort from multiple sources with potential short-term and long-term consequences, such as various degrees of anxiety and/or depression or post-traumatic stress disorder (PTSD), which may affect their quality of life and lead to increasing healthcare utilization.

**OBJECTIVES.** The aim of this study was to assess the impact of the implementation of a tailored multicomponent program (TMCP) for discomfort reduction on the occurrence of post-traumatic stress disorder (PTSD) 12 months after ICU discharge.

**METHODS.** After carrying out the IPREA3 study (a multicenter, cluster-randomized, two-arm parallel, controlled study involving 34 French adult ICUs to assess the efficacy of our TMCP for discomfort reduction in the ICU), we conducted, in 30 of the 34 ICUs, the AQVAR study consisting of 1-year follow-up of the patients already included in IPREA3. The TMCP consisted of assessment of ICU-related self-perceived discomforts by using the IPREA questionnaire, immediate and monthly feedback to healthcare teams, and tailored site-targeted measures under control of a duo of local champions.The main objectives of the AQVAR study were to assess anxiety and depressive symptoms and PTSD using resp. the Hospital Anxiety and Depression Scale (HAD-S) and the Impact of Event Scale-revised (IES-R). Based on a design of a case-control study, the cases were patients hospitalized in the ICUs which implemented the TMCP and the controls were patients hospitalized in the ICUs which did not implement the TMCP. HAD-S and IES-R were administered either by telephone or returned by patients after mailing. Sociodemographic data, clinical data related to the ICU stay, discomfort´s levels assessed the day of ICU discharge, life situation (home/care center) were collected at 12 months after ICU discharge, in addition to IES-R and HAD-S.

**RESULTS.** From the 617 eligible cases and 847 eligible controls, 344 cases and 475 controls were included. A total of 6.1% of the cases and 12.0 % of the controls presented certain symptoms of PTSD at 12 months (*p*=0.004). After adjustment for age, gender, IPREA score, McCabe score, use of invasive devices and considering anxiety-depression symptoms at 12 months, cases are less likely to have PTSD symptoms than controls.

**CONCLUSIONS.** A tailored multicomponent program for discomfort reduction in the ICU may significantly reduce the incidence of PTSD assessed at 12 months. Diffusion of such a program should be enhanced in the ICUs paving the way for a new strategy in care management.

**REFERENCE(S)**

1. Kalfon P, Baumstarck K, Estagnasie P et al.: A tailored multicomponent program to reduce discomfort in critically ill patients: a cluster-randomized controlled trial. *Intensive Care Med* 2017, 43; 1829:40.

**GRANT ACKNOWLEDGMENT**

French Ministry of Health

### 0218

#### Can we predict weaning outcome in multiple trauma patients by using diaphragmatic rapid shallow breathing index (D-RSBI)?

G. Sabetian[1,2], F. Zand[3], M. Masjedi[3], M. Mackie[2]

[1]Trauma Research Center, Shiraz University of Medical Sciences, Shiraz, Iran, Islamic Republic of; [2]Shiraz University of Medical Sciences, Shiraz, Iran, Islamic Republic of; [3]Anesthesiology and Critical Care Research Center, Shiraz University of Medical Sciences, Shiraz, Iran, Islamic Republic of

**Correspondence:** G. Sabetian
*Intensive Care Medicine Experimental* 2018, **6(Suppl 2):**0218

**INTRODUCTION.** Predicting weaning outcome from mechanical ventilation in multiple trauma patients is very important. The rapid shallow breathing index (RR/TV) is one of most widely used index to predict weaning outcome.As diaphragm plays a fundamental role in generating TV ,substitution of Tv with diaphragmatic displacement measuring by ultrasonography and calculate the ratio between respiratory rate and diaphragmatic displacement could predict weaning outcome.

**OBJECTIVES.** The aim of this study was showing the ability of D-RSBI to predict weaning outcome in multiple trauma patients .

**METHODS.** In this study fifty patients in the trauma intensive care unit who required mechanical ventilation over 48 hrs and met the criteria for weaning were assessed. Patients with a history of diaphragmatic disseases before trauma or cervical cord injury were excluded. During weaning, each hemidiaphragm was evaluated by M-mode ultrasonography using the liver and spleen as windows with the patient in supine position.

**RESULTS.** Most of the patients 26(52%) were successfully weaned from mechanical ventilation and 24(48%) were failed. Right D-RSBI and Left D-RSBI were successfully predict weaning outcome with pvalue (right=0.007) and (left=0.02). The cut of point for RD-RSBI is 22.2250 rr/min/cmn with sensitivity 87% ans specifity 93%. The cut of point for LD-RSBI is 18.97 rr/min/cm with sensitivity 87.5% and specifity 89%.

**CONCLUSIONS.** This study shows that, the assessment of D-RSBI by ultrasonography may predict weaning outcom in multiple trauma patients

**REFERENCE(S)**

1. El Morsy A, Ibrahim M, Sakr M. Serial ultrasonographic evaluation of diaphragm thickness during mechanical ventilation in ICU patients.
2. Ali ER, Mohamad AM. Diaphragm ultrasound as a new functional and morphological index of outcome, prognosis and discontinuation from mechanical ventilation in critically ill patients and evaluating the possible protective indices against VIDD. Egyptian Journal of Chest Diseases and Tuberculosis. 2017;66(2):339-51.
3. Doorduin J, van Hees HW, van der Hoeven JG, Heunks LM. Monitoring of the respiratory muscles in the critically ill. American journal of respiratory and critical care medicine. 2013;187(1):207.
4. Grosu HB, Im Lee Y, Lee J, Eden E, Eikermann M, Rose KM. Diaphragm muscle thinning in patients who are mechanically ventilated. Chest. 2012;142(6):1455-60.

**GRANT ACKNOWLEDGMENT**

This study was funded by a grant from Vice_Chancellery of Research and Technology in Shiraz University of Medical Sciences, Shiraz,Iran

### 0219

#### Agreement between chest radiography and lung ultrasound in the existance of concomitant pneumia and pleural effusion in ICU patients

G. Gursel[1], N. Demirci[1], S. Eyüpoğlu[2], B.S. Kalın[3], M. Çimen[4]

[1]Gazi University School of Medicine, Department of Pulmonary Critical Care Medicine, Ankara, Turkey; [2]Gazi University School of Medicine, Department of Anaesthesiology Division of Critical Care Medicine, Ankara, Turkey; [3]Gazi University School of Medicine, Department of Internal Medicine Division of Critical Care Medicine, Ankara, Turkey; [4]Gazi University School of Medicine, Department of Anaesthesiology Critical Care Medicine, Ankara, Turkey

**Correspondence:** G. Gursel

*Intensive Care Medicine Experimental* 2018, **6(Suppl 2):**0219

**INTRODUCTION.** Sensitivity of chest radiograph(CXR) is as low as 49% especially in ICU patients taken in supine position. Lung ultrasound (LUS) has a number of advantages in the intensive care unit (ICU). For example, it can be used in real time at the bedside, radiation-free, and it more accurately defines the causes of undifferentiated opacities on CXR. In mechanically ventilated patients, it has been found that lung ultrasound (LUS) is more accurate than chest radiograph (CXR) in diagnosing and distinguishing different types of consolidations and dynamic air-bronchograms(DAB) has good sensitivity and specificity (>90%) to diagnose pneumonia.

**OBJECTIVES.** We aimed to investigate the agreement of chest radiograph and LUS when concomitantly performed in the evaluation of pleural effusion and pneumonia and pneumonia under the pleural effusion at the same site which can be happen frequently in ICU patients and can be hardly detected with CXR.

**METHODS.** The presence of pleural effusion, signs of pulmonary congestion and/or consolidation was evaluated on the CXR by an independent expert pulmonologist. Bedside LUS was performed in the same day with CXR while patients in the supine position by the experienced intensivists. The Vividq(GE) ultrasound system and linear and phase array probes were used for the data collection. The LUS exam was required to identify the lung sliding, lung point, A-lines, B-lines, consolidation/atelectasis and pleural effusion, dynamic and static air bronchograms associated with a pleural effusion. The lung consolidation accompanied by an dynamic air bronchogram was accepted as main feature of pneumonia on LUS. Kappa statistics were used for agreement analysis.

**RESULTS.** The study included 57 cases that admitted to ICU with in one year period. According to CXR there were pneumothoracis in 2 patients but LUS and thorax CT were not confirm this finding. There were poor aggrement between LUS and CXR(less than moderate(Kappa:0.39, p 0.001) in the comparison of pleural effusion and there were no aggrement in the comparison of pneumonia(kappa: 0.21,p: 0.09). Agreement was better in patients with COPD (kappa>0.5,p< 0.05). CXR underestimated bilateral pleural effusions (9% versus 25%). 17% of patients had DAB+Pleural effusion in the left side and 21% of them had on the right side with LUS but CXR detected only 8% of them in both side.

**CONCLUSIONS.** These results suggest that in the suspicion of pleural effusion and concomitant pneumonia which is very frequent in ICU patients, LUS is better than CXR to detect them.

Winkler MH, Touw HR, Van de Ven PM, Twisk J, Tuinman PR, Diagnostic Accuracy of Chest Radiograph, and When Concomitantly Studied Lung Ultrasound, in Critically Ill Patients With Respiratory Symptoms: A Systematic Review and Meta-Analysis Crit Care Med DOI: 10.1097/CCM.0000000000003129

**GRANT ACKNOWLEDGMENT**

### 0220

#### Relationship between sonographic measurement of rectus femoris and vastus intermedius muscles with conventional biochemical parameters to assess the nutritional status in the intensive care unit

A.E. Hernández-Plata, M.N. Gómez-González, A. Hernández-Gutiérrez, R. Soriano-Orozco, P.L. González-Carrillo

Instituto Mexicano del Seguro Social, Unidad de Cuidados Intensivos, León, Mexico

**Correspondence:** A.E. Hernández-Plata

*Intensive Care Medicine Experimental* 2018, **6(Suppl 2):**0220

**INTRODUCTION.** The muscle loss described in the critical patient has been related to a poor prognosis. This process is conventionally identified through biochemical parameters. The muscle reserves can reflect the body protein reserve, muscle ultrasound has a strong correlation with magnetic resonance ($r = 0.91$, $p < 0.001$). The depletion of myofibers can be assessed the Heckmatt scale (Table 1). There may be a correlation between muscle loss and protein decrease reflected in nutritional assessment.

**OBJECTIVE.** The goal of this study was to determine the correlation of the sonographic measurement of the anterior muscles of the thigh with the conventional biochemical parameters to assess the nutritional status in the ICU.

**METHODS.** This longitudinal, prospective, observational study, was conducted at the Intensive Care Unit, Centro Médico Nacional del Bajío UMAE 1, in León Guanajuato, between December 2017 to March 2018. Patients were admitted over 18 years with medical, traumatic and surgical diagnoses, who received enteral diet in the first 24 hrs after admission, with a minimum protein intake of 0.8mg / kg / day. Nitrogen Balance, Prealbumin, Albumin and simultaneously sonographic parameters at the level of the Rectus Femoris (RF) and Vastus intermedius (VI) (Muscle wasting percentage, AP diameter, Lateral diameter and Cross sectional area and Muscular Ecogenicity) were measured at their admission and 7 later days. We excluded patients with any pathology that affects the muscular area to evaluate or amputation of pelvic limbs. Statistical analysis was performed using MDCalcX version 18.

**RESULTS.** We included 8 patients, mean age of 36 years, Female Gender: 37.5% Male: 62.5%. 50% with normal Body Mass Index, 37.5% with overweight and 12.5% with Obesity grade I. The average protein intake per early enteral diet was 1.28 gr / kg /day. The mean APACHE II and SOFA were 18 and 10 points respectively; The most frequent diagnosis was of traumatic origin (62.5%). We found a significant difference between the biochemical and sonographic values between days 0 and 7 (Table 2). A correlation test was performed for the most significant parameters (cumulative nitrogen balance and the cross-sectional area VI, however, was not positive ($r = 0.14$) (Figure 1), whereas the Heckmatt scale on day 0 had an average and on day 7 of 2.8 , showing deterioration in the quality of the myofibrils throughout the stay in the ICU.

**CONCLUSION.** Although biochemical parameters such as the nitrogen balance is used to assess the nutritional status of critical patients, there is no positive correlation with muscle sonographic assessment.

**Table 1 (abstract 0220).** HECKMATT SCALE: VISUAL GRADING SCALE TO CLASSIFY MUSCLE ECHO INTENSITY

| GRADE | ULTRASOUND APPEAREANCE |
|---|---|
| GRADE 1 | Normal |
| GRADE 2 | Increased muscle echo intensity with distintc bone echo |
| GRADE 3 | Marked increased muscle echo intensity with a reduced bone echo |
| GRADE 4 | Very strong muscle echo and complete loss of bone echo |

**Table 2 (abstract 0220).** Characteristics of Biochemical and Sonographic Measurements

| VARIABLE | DAY 0 | DAY 7 | p |
|---|---|---|---|
| NITROGEN BALANCE (g/24 hrs) | 3.6 | -5.22 | 0.01 |
| PREALBUMIN (mg/dL) | 21.3 | 10.3 | 0.03 |
| ALBUMIN (g/L) | 3.3 | 2.06 | 0.02 |
| CROSS SECTIONAL AREA RECTUS FEMORIS (CM2) | 2.85 | 2.11 | 0.01 |
| CROSS SECTION AREA VASTUS INTERMEDIUS (CM2) | 3.56 | 3.18 | 0.03 |
| REDUCTION PERCENTAGE RECTUS FEMORIS ‰ | — | 23.48 | — |
| REDUCTION PERCENTAGE VASTUS INTERMEDIUS ‰ | — | 10.5 | — |
| HECKMATT SCALE RECTUS FEMORIS | 1 | 2.8 | — |
| HECKMATT SCALE VASTUS INTERMEDIUS | 1 | 2.5 | — |

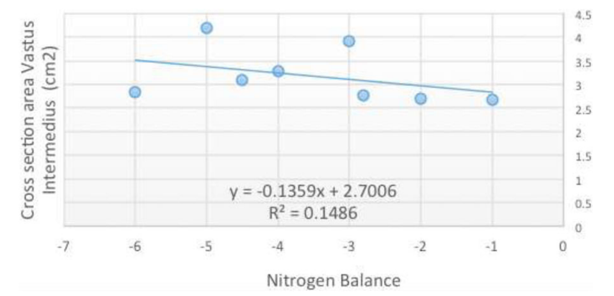

**Fig. 1 (abstract 0220).** Correlation between Nitrogen Balance and cross section area Vastus Intermedius

## Acute brain injury

### 0221

#### Evolution of the quality of life after traumatic brain injury between two monitoring periods of 1 and 3-4 years

M. Guerrero-Marin[1], M.D. Pola-Gallego-de-Guzmán[1], M.I. Ruiz-Garcia[1], M. Delange-Van Der Kroft[2], M.D. Arias-Verdu[3], E. Curiel-Balsera[3], A. Muñoz-Lopez[3], J.F. Fernandez-Ortega[3], M.A. Prieto-Palomino[3]
[1]Complejo Hospitalario, Intensive Care Unit, Jaen, Spain; [2]Hospital Comarcal de la Axarquia, Intensive Care Unit, Velez Málaga, Spain; [3]Hospital Regional Universitario Carlos Haya, Intensive Care Unit, Malaga, Spain
**Correspondence:** M. Guerrero-Marin

*Intensive Care Medicine Experimental* 2018, **6(Suppl 2):**0221

**INTRODUCTION.** Many instruments have been developed to evaluate hospital mortality, but less attention has been paid to the long-term functional status and quality of life (QOL) of traumatic brain injury (TBI) patients.

**OBJECTIVES.** To study the QOL in two follow-up period between 1 year and 3-4 years and the related variables in TBI admitted to the intensive care unit at the University Clinical Hospital (Malaga) between 2004 to 2008.

**METHODS.** Prospective cohort study of all TBI admitted to the Carlos Haya Hospital (Málaga) between 2004-2008.

Data were expressed as the mean and standard desviation for quantitative variables and percentages for qualitative variables.

We used ANOVA and Newman Keuls test to compare means, Pearson coefficient for correlation and linear regression for multivariate analysis. Statistically significant differences $p < 0.05$.

**RESULTS.** 531 patients. Mean age 40.35±19.75 years, APACHE-II 17.94 ±6.97, admission GCS 7.53±3.83 points. Computerized tomography (CT) on admission by Marshall score was: diffuse injury type I (10.4%), type II (28.1%), type III (24.5%), type IV (8.3%), mass evacuated (22.6%), mass not evacuated (6.2%). Hospital mortality 28.6%. 171 patients died at first year (32.2%) (6.6% missing) and at 4 years 175 died (33%) (16.2% missing). Imputation with statistical techniques the 86 patients were classified as alive.

Normal functional status or with dysfunction but self-sufficient: 1st year 65.3% of the 325 patients alive and 3-4 years: 75.76% of the 264 alive and followed.

The evaluation of the QOL was performed by PAECC (Project for the Epidemiological Analysis of Critical Care Patients) questionnaire (0 points: normal QOL, 29 points: worst score). Mean score at first year follow-up (n = 324) 9.44±8.73 points, indicating high deterioration in QOL. At 3-4 years 6.77±7.70 points (n=238). An improvement in QOL was observed between 3-4 years (-2.39±3.71 points, N=238) ($p < 0.001$).

The evolution of QOL between 1 and 3-4 years is associated with age ((r=0.235, $p < 0.001$), age < 35 years have greater improvement (-3.07± 3.58 vs -1.16± 3.64 points, $p < 0.001$), with QOL 1° year ((r = -0.378, $p < 0.001$) ≤ 1 point present lower improvement than the rest (-0.16± 0.37 vs -315± 4.02 points, $p < 0.001$), stay in ICU (r = - 0.245, $p < 0.001$), admission GCS (r = 0.252, $p < 0.001$), days of mechanical ventilation (r = -0.239, $p < 0.001$) and admission TC.

Multivariate analysis by multiple regression found association between the QOL evolution with the quality of life at 1°year and age. If only variables evaluated during hospitalization are included, the model included: age, stay in ICU and CT at admission by Marshall scale.

**CONCLUSIONS.** Approximately 75% of TBI that survive are self-sufficient at three years. Between 1st and 3rd year, QOL modifies favorably, being higher in young people and with worst QOL 1° year.

### 0222

#### Safety of a neuromuscular electrostimulation protocol in patients with traumatic brain injury submitted to invasive mechanical ventilation

L. Nunes, Lara Pereira, Vinícius Maldaner, Priscilla Flávia Melo, João Luiz Durigan, Alexandra Miranda, Lara Rocha, Paulo Eugênio Silva, Monalisa Santos
Universidade de Brasilia, Brasília, Brazil
**Correspondence:** L. Nunes

*Intensive Care Medicine Experimental* 2018, **6(Suppl 2):**0222

**INTRODUCTION.** The traumatic brain injury (TBI) requires critical management of the patient in an intensive manner to avoid secondary damage to the trauma. The use of continuous sedation, strict control of mean arterial pressure (MAP), and the definitive airway are important interventions used in this population. Hemodynamic monitoring offers valuable information on cardiovascular performance in the critically ill. The literature points to neuromuscular electrostimulation (NMES) as an alternative to intensive care unit-acquired weakness (ICU-AW) combat, with positive results in critically ill patients, but there is a need to evaluate the safety of this intervention.

**OBJECTIVES.** The present study aimed to verify the hemodynamic safety of a NMES protocol in patients with TBI.

**METHODS.** We performed an experimental prospective trial in the trauma intensive care unit (ICU). It was included traumatic brain injury patients, aged from 18 to 65 years, in the first day or until 72 hours of mechanical ventilation. Electrical stimulation was applied daily in the muscles: quadriceps, tibialis anterior, gastrocnemius and hamstrings for 14 days. The protocol (pulse width of 400 μs, frequency of 100 Hz, rise and fall times of 1 second each, 5 second flow time and rest time of 25 seconds) lasted 25 minutes in the anterior (quadriceps and tibialis anterior) muscles and 25 minutes in the posterior ones (hamstrings and gastrocnemius). Hemodynamic variables were assessed before and immediately after the end of each NMES session. Mean arterial pressure (MAP) was measured in invasive arterial line and heart rate (HR), using a five-lead electrocardiogram.

**RESULTS.** We enrolled 18 patients totaling 226 interventions and 109 were finished 14 days of follow up. 266 predicted sessions, 52% were lost due to hemodynamic instability, deaths, ICU discharge and protocol discontinuity. There were no statistically significant differences between MAP (P = 0.81) and HR (p = 0.63); pre and post intervention

**CONCLUSION.** Considering the findings of this study, we can say that NMES is safe for this population because not producing variations in MAP and HR.

### 0223

#### Tracheostomy in patients with acute brain injury: a prospective observational study

E. Picetti, F. Minardi, C. Sacchetti, A. Oppici, G. Migliavacca, G. Cappé, A. Farina, I. Rossi, S. Rossi
I Servizio Anestesia Rianimazione - AOPR, Parma, Italy
**Correspondence:** E. Picetti
*Intensive Care Medicine Experimental* 2018, **6(Suppl 2):**0223

**INTRODUCTION.** Tracheostomy is an operative procedure frequently utilized in acute brain injury (ABI) patients admitted to the Intensive Care Unit (ICU). This maneuver is associated with several complications (1).

**OBJECTIVES.** To analyze complications associated with tracheostomy during the ICU stay in patients with acute brain injury (ABI). In particular, to evaluate: 1) acute intraoperative problems, 2) perioperative respiratory, hemodynamic and cerebral effects and 3) postoperative complications during ICU stay.

**METHODS.** A prospective single-center observational study was performed from January to December 2017 including all ICU patients with acute brain injury undergoing tracheostomy.

**RESULTS.** n the study period, 515 patients were admitted to our ICU (50% with ABI]. We enrolled 104 patients [69 (66%) males]. The main diagnosis was traumatic brain injury [35 (33%)]. Median Glasgow Coma Scale (GCS) score at ICU admission was 7 (IQR 5-9). All tracheostomies were performed at the bedside by ICU physicians [surgical technique in 88 (85%) cases and percutaneous technique in 16 (15 %) cases]. Tracheostomy was performed on average 5 day (± 2.8) after ICU admission. The mean duration of the procedure was 22 (± 10.7) minutes. Intracranial pressure (ICP) monitoring was performed in 62 (60%) patients. Acute intraoperative problems requiring interventions were: increase in arterial blood pressure (ABP) [71 (68%)], increase in ICP [5 (5%)], simultaneous increase in ABP and ICP [14 (14%)] and surgical site bleeding [1 (1%)] requiring maxillo-facial surgeon intervention. Postoperative complications were: stoma bleeding [8 (8%)] (not needing surgery), stoma infection [8 (8%)] and cannula obstruction [1 (1%)] (needing urgent change without clinical consequences). Perioperative respiratory, hemodynamic and cerebral effects were reported in Table 1. Ninety-eight (94%) patients were discharged alive from ICU [median GCS 10 (IQR 8-11)]. Mean length of stay in ICU was 21.5 (± 10.8). Tracheostomy was present at discharge in 89 (85%) patients.

**CONCLUSIONS.** Tracheostomy seems to be a safe procedure in ABI patients. However, some complications (mainly intraoperative), requiring a rapid and appropriate management, may be present.

**REFERENCE(S)**

1) Engels PT et Al. Tracheostomy: from insertion to decannulation. Can J Surg. 2009 Oct; 52(5): 427-33.

**Table 1 (abstract 0223).** Perioperative respiratory, hemodynamic and cerebral effects

| | START | END | P |
|---|---|---|---|
| HR (bpm) | 79.0 ± 17.8 | 86.5 ± 17.7 | <0.001 |
| MAP (mmHg) | 89.1 ± 18.0 | 102.5 ± 17.9 | <0.001 |
| ICP (mmHg) | 12.8 ± 4.3 | 17.0 ± 7.4 | <0.001 |
| CPP (mmHg) | 77.1 ± 18.5 | 87.5 ± 19.1 | 0.000 |
| SpO2 (%) | 98.8 ± 1.6 | 99.4 ± 1.3 | 0.003 |
| | PRE (24 hrs before) | POST (24 hrs after) | P |
| ICP (mmHg) | 10.7 ± 3.0 | 10.9 ± 6.1 | 0.686 |
| CPP (mmHg) | 79.3 ± 10.9 | 78.9 ± 11.5 | 0.729 |
| PaO2/FiO2 | 297.4 ± 88.4 | 269.5 ± 92.2 | 0.000 |

### 0224

#### Association between use of mannitol and hypertonic saline on mortality in patients with moderate to severe traumatic brain injury

M. Skrifvars[1,2], M. Bailey[3], J. Mårtensson[4], E. Moore[3], C. French[5], J. Presneill[6], A. Nichol[7], L. Little[3], J. Duranteau[8], O. Huet[9], S. Haddad[10], Y. Arabi[10], C. McArthur[11], J. Anstey[12], R. Bellomo[13]
[1]University of Helsinki and Helsinki University Hospital, Department Anaesthesiology, Intensive Care and Pain Medicine, Helsinki, Finland; [2]University of Helsinki and Helsinki University Hospital, Department of Emergency Medicine, Helsinki, Finland; [3]Australian and New Zealand Intensive Care Research Centre, Melbourne, Australia; [4]Karolinska Institute, Department of Physiology and Pharmacology, Stockholm, Sweden; [5]Western Health, Department of Intensive Care, Melbourne, Australia; [6]Royal Melbourne Hospital, Melbourne, Australia; [7]University College Dublin, School of Medicine and Medical Sciences, Dublin, Ireland; [8]Hopitaux Universitaires Paris Sud, Department of Anesthesia and Intensive Care, Paris, France; [9]CHU La Cavale Blanche, Department of Anesthesia and Intensive Care, Brest, France; [10]King Saud Bin Abdulaziz University for Health Sciences and King Abdullah International Medical Research Center, Riyadh, Saudi Arabia; [11]Auckland City Hospital, Department of Critical Care Medicine, Auckland, New Zealand; [12]St Vincent's Hospital, Department of Intensive Care, Melbourne, Australia; [13]Austin Hospital, Intensive Care, Melbourne, Australia
**Correspondence:** M. Skrifvars
*Intensive Care Medicine Experimental* 2018, **6(Suppl 2):**0224

**INTRODUCTION.** Mannitol and hypertonic saline (HTS) are commonly used for the treatment of elevated intracranial pressure (ICP) in patients with traumatic brain injury (TBI). However, their effects on mortality and neurological outcome are unclear.

**OBJECTIVES.** To study the effect of mannitol and HTS during the first three days on mortality in TBI patients treated in the intensive care unit.

**METHODS.** We studied patients included in the Erythropoietin in TBI (EPO-TBI) trial, excluding patients who died before day three. We included the amount of mannitol and HTS administered during the first three days. Severity of TBI was categorized according to the International Mission for Prognosis and Analysis of Clinical Trials (IMPACT) model. Cox regression analysis was used to predict time to death beyond day three until discharge from the ICU.

**RESULTS.** Out of 600 survivals at day three, 76 (13%) later died and 264 (44%) died or survived with poor neurological recovery. Patients who died received on average 67 ml (95% confidence interval [CI] 20-114 ml) of mannitol and those who survived received 18 ml (95% CI 10-26 ml) (p=0.006). The corresponding figures of patients with poor outcome were 18 ml (95% CI 7-29 ml) and 34 ml (95% 18-51 ml) (p=0.04). Patients who died received 277 ml (95% CI 156-399 ml) of HTS and survivors received 226 ml (95% CI 170-282 ml) (p=0.06). Patients who survived with poor outcome received 258 ml (187-329 ml) compared to 207 ml (136-278 ml) in those with good outcome (p=0.44). Independent predictors of time to mortality were proportion of hours of ICP higher than 20 mmHg during the first two days (per 100%) (HR 6.7 95% CI 2.9-15.6, p< 0.001), IMPACT TBI severity (per 10% increase of risk of poor outcome) (HR 1.3 95% 1.2-1.5, p< 0.001), APACHE II score (per point) (HR 1.07 95% CI 1.03- 1.1, p=0.001) and amount of administered mannitol (per 100 ml increase) (HR 1.2 95% CI 1.0-1.4, p=0.02). The administered amount of HTS was not associated with mortality (HR 1.0 95% 1.0-1.001, p=0.21)

**CONCLUSIONS.** The amount of administered mannitol during the first two days was independently associated with time to survival beyond day three when controlling for several factors of TBI severity. The role of mannitol in moderate to severe TBI should be studied further.

**GRANT ACKNOWLEDGMENT**

National Health and Medical Research Council of Australia (grant 545902), Transport Accident Commission of Victoria (grant D162). Markus Skrifvars has received personal research funding from Medicinska Understodsforeningen Liv och Halsa, Finska Lakaresallskapet and Svenska Kulturfonden

### 0225

#### Barbiturate coma vs secondary decompressive craniectomy in traumatic intracranial hypertension treatment: a retrospective 6-year trial in a tertiary hospital

J.F. Martins[1], J. Rato[2], E. Sousa[1], P. Martins[1]
[1]Centro Hospitalar e Universitário de Coimbra, Serviço de Medicina Intensiva, Coimbra, Portugal; [2]Centro Hospitalar e Universitário de Coimbra, Serviço de Neurocirurgia, Coimbra, Portugal
**Correspondence:** J.F. Martins
*Intensive Care Medicine Experimental* 2018, **6(Suppl 2):**0225

**INTRODUCTION.** Last-tier treatments benefit on traumatic Intracranial Hypertension remains unclear. Recent randomised controlled trial suggest that there is a significant impact on survival in surgical versus medical therapy.[1]

**OBJECTIVES.** Characterization of the population, aged 18 years to 75 years, diagnosed with traumatic brain injury and refractory elevated intracranial pressure, submitted to last-tier intervention (secondary decompressive craniectomy vs. barbiturate coma) admitted to an intensive care unit, of a tertiary hospital, between 2011 and 2017.

**METHODS.** Retrospective, descriptive and inferential trial of 37 patients, evaluating: age; sex; Glasgow Come Scale (GCS), paO2, pupillary symmetry and hypotension at admission; time to Intracranial pressure monitoring, start and duration of last-tier treatment, ventilatory support, length of stay in ICU, in-hospital mortality, and systematic analysis of primary clinical outcome at 6 months using the Extended Glasgow Outcome Scale (GOS-E). Secondary outcomes were also evaluated, namely imaging pattern of ICP elevation, duration of barbiturate coma, extracranial lesions and duration of intracranial hypertension.

**RESULTS.** A total of 37 patients, 81,1% male, with mean age of 43.6 years (+/- 15.6). GCS on admission to the emergency room was similar in both groups. The time to the last-tier intervention was higher in the surgical group (137h 6min vs. 69h 34min). The Barbiturate coma patients had longer length of stay in the ICU (18days 6h vs. 14days 16h). In-hospital mortality rates were 19% in the medical group and 18% in the surgical group. At 6 months, the GOS-E in surgical group vs. barbiturate coma were: 25% vs. 47,1 % death; vegetative state, 35% versus 17,6%; moderate disability, 15% versus 17,6%; and good recovery, 20% versus 11,8%, respectively.

**CONCLUSIONS.** Despite the better prognosis associated with surgical therapy, with lower mortality rates at 6 months, there was a negative impact on the functional status and unfavorable neurological results compared to medical therapy.

**REFERENCE(S)**

1. Hutchinson PJ et al. Trial of Decompressive Craniectomy for Traumatic Intracranial Hypertension. N Engl J Med 2016

### 0226

#### Effect of antibiotic-impregnated external ventricular drains versus standard ones, in increasing number of free infection days in patients of a general intensive care unit

A. Tsirogianni, G. Kyriazopoulos, F. Tsimpoukas, C. Georgiadis, D. Sfyras
General Hospital of Lamia, ICU, Lamia, Greece
**Correspondence:** A. Tsirogianni
*Intensive Care Medicine Experimental* 2018, **6(Suppl 2):**0226

**OBJECTIVES.** To assess whether implementation of antibiotic impregnated (AI) external ventricular drains (EVD) catheters, instead of the ordinary silastic ones, can eliminate the burden of prophylactic catheter exchange for the critically ill patients of general ICUs.

**METHODS.** A retrospective cohort study of all the EVD cases that were treated in the general ICU of a regional tertiary hospital in Greece during the decade 2006 - 2016. Comparisons and statistical analyses were performed between two groups of patients (with AI-EVDs or common EVDs) and two groups of EVD catheters (AI-EVDs versus ordinary ones). Data collected from 49 patients (median age 67 years), 81 EVD catheters (AI = 43,ordinary =38) and 1080 days of drainage. The analysis referred mainly to duration of catheterization, in relation to confirmed ventricular assosiated infections (VAI), catheter colonizations, predisposing to VAI factors, handling and catheter exchange protocols, clinical and laboratory findings and patients' outcome.

**RESULTS.** We found that median duration of catheterization with AI-EVD catheters was more than double the duration with ordinary ones, 17 and 7.5 days respectively, p < .0001, with the rate of colonization being unaffected (p = .4). In addition, per 1000 days of EVD the colonization rate for the ordinary catheters was threefold the AI catheters one (OR = 0.65). The accumulative median level of white blood cell count in CSF samples was significantly higher for the ordinary catheters (p < .05) whereas number of EVD manipulations varied inversely (median number 7 for AI and 3 for ordinary catheters, p < .001). Our colonization (and confirmed VAI) flora, for both types of catheters, was resistant Gram (-) bacilli and median per patient, EVD duration was 17 days.

**CONCLUSIONS.** Antibiotic impregnated EVD catheters seem to last much longer than ordinary silastic ones in general ICU settings, by means of colonization, hence they do not require a predetermined exchange protocol. They are also more resistant, colonization-wise, to increased number of manipulations. The mechanism of their efficacy towards Gram (-) bacteria needs to be investigated further with clinical and laboratory studies.

### 0227

#### Does physical therapy care provided within 24 hours improve outcomes compared to physical therapy provided within 18 hours in trauma intensive care unit? A cohort study

L. Rocha[1], V. Maldaner[2], D. Andrade[3], J.A. Neto[4], M. Priscilla[3], M. Lucena[1], G. Cipriano Jr[1]
[1]Universidade de Brasilia, Brasilia, Brazil; [2]Fundação de Ensino e Pesquisa em Ciências da Saúde, Braslia, Brazil; [3]IHBDF, Brasilia, Brazil; [4]Hospital Santa Luzia, Brasilia, Brazil
**Correspondence:** L. Rocha
*Intensive Care Medicine Experimental* 2018, **6(Suppl 2):**0227

**OBJECTIVE.** To verify if physiotherapy care (PTc) provided within 24 h/day for Trauma patients in the Intensive Care Unit (ICU) reduces the length of stay, mechanical ventilation (MV) support and mortality compared to a physiotherapy care provided within 18 h/day.

**METHODS.** A longitudinal study was designed to assess differences between first three months of the year (2016) hospital where patients were given physiotherapy care for 18 h/day and last 3 months of 2016 with 24 h/day. We considered the following as outcome measurements: clinical diagnosis, Acute Physiology and Chronic Health disease Classification System II (APACHE II), ICU and mechanical ventilation length of stay and survival. T test of student was made to compare differences between two groups.

**RESULTS.** 118 patients were enrolled in this study (70% male, age 45 ± 16 years, APACHE II 18 (15-23). Patients admitted during 24h/day of PTc showed a lower length of stay in ICU (17 ± 3.1 days X 21.1 ± 3.9 days respectively, p = 0.03) and mortality rate (71% versus 79% respectively, p =0.04). No difference was found for APACHE II score (p = 0.8) and MV length of stay (11.5 ± 3.4 X 13.5 ± 3.3 days, p > 0.05) between two groups.

**CONCLUSION.** The presence of a PTc contributes decisively to reduce number of hospitalization days and mortality of ICU trauma patients.

##### REFERENCE(S)

11. Finfer SR, Cohen J. Severe traumatic brain injury. Resuscitation 2001;48:77-90.
12. Rutland-Brown W, Langlois JA, Thomas KE, Xi YL. Incidence of traumatic brain injury in the United States, 2003. J Head Trauma Rehabil. 2006;21:544.
13. Cardoso LGDS, Chiavone PA. The APACHE II measured on patients´ discharge from the Intensive Care Unit in the prediction of mortality. Revista latino-americana de enferma-gem. 2013;21(3):811-9.
14. Chiavone PA, Sens YAS. Evaluation of APACHE II system among intensive care patients at a teaching hospital. São Paulo Med J. 2003;121(2):53-7
15. Sakr Y, Krauss C, Amaral AC, Réa-Neto A, Specht M, Reinhart K, et al. Comparison of the performance of SAPS II, SAPS 3, APACHE II, and their customized prognostic models in a surgical intensive care unit. Br J Anaesth. 2008;101(6):798-803.
16. Freitas ERFS. Profile and severity of the patients of intensive care units: prospective application of the APACHE II index. Rev. Latino-Am. Enferma-gem. 2010;18(3):317- 23.
17. Chen YC, Lin MC, Lin YC, Chang HW, Huang CC, Tsai YH. ICU discharge APACHE II scores help to predict post-ICU death. Chang Gung Med J. 2007;30(2):142-50.
18. Joe BH, Jo U, Kim HS, Park CB, Hwang HJ, Sohn IS, et al. APACHE II score, rather than cardiac function, may predict poor prognosis in patients with stress-induced cardio myopathy. J Korean Med Sci. 2012;27(1):52-7.

### 0228

#### Glycemic variability in patients admitted to the ICU due to TBI and its relationship with morbidity and mortality

J.F. Martínez Carmona, F.A. Hijano Muñoz, E. Lopez Luque, J.M. Mora Ordoñez, M. Delgado Amaya, G. Quesada
H. R. U de Málaga, Intensive Care, Málaga, Spain
**Correspondence:** J.F. Martínez Carmona
*Intensive Care Medicine Experimental* 2018, **6(Suppl 2):**0228

**INTRODUCTION.** Critical patients, diabetic or not, present glycaemic alteration, due to an increase in glucose production (glycogenolysis / gluconeogenesis), as well as the presence of insulin resistance. Dysglicemia leads to an increase in morbidity and mortality. Glycemic variability has been shown to be an independent risk factor for mortality in critically ill patients.

OBJETIVES. The main objective of the study is to assess the glycemic variability in patients admitted to the ICU by TBI, assessing the possible relationship with mortality and morbidity.

**MATERIAL AND METHODS.** We studied a sample of 17 patients admitted to the ICU due to TBI. It is a retrospective study, we analyzed the glycemic controls during admission to the ICU, with at least 4 determinations per day of glycemia. The following variables are collected: age, sex, diabetes mellitus, APACHE II, SOFA, stay in ICU and hospital, mortality in ICU and hospital.

**RESULTS.** In our sample, 82.4% were male, and only 29.4% were diabetic. The average stay was 13.94 days. The ICU mortality was 17.6%. We did not find significant differences in glycemic variability between diabetic and non-diabetic patients. When we analyze the Standard Deviation (SD) of the standard deviation of glycemic variability during admission to the ICU, we observe a statistically significant relationship with mortality, using Chi2 test (p 0.02), as well as a linear relationship using the Coefficient of Pearson correlation (p 0.019). We found a cut-off SD in 20.13 (Sensitivity 66.7% and Specificity 85.7%). We found a relationship between APACHE II on admission and SD glycemic, but without statistical relationship (p 007).

**CONCLUSIONS.** Glycemic variability in critically ill patients is an independent risk factor for mortality. Strict control of blood glucose levels has proven to be detrimental to the patient. In our sample, patients with Standard Deviation below 20.13 presented higher survival in ICU, as well as lower in-hospital mortality.

- Matsushima et al. Glucose variability negatively impacts Long-term functional outcome in patients with traumatic brain injury. J Crit Care 2012; 27(2): 125 - 31.
- Bhattachargee et al. Perioperative glycemic status of adult traumatic brain injury patients undergoing craniotomy: a prospective observational study. J Neurosurg Anesthesiol 2014; 26(4): 313-319.
- Krinsley et al. Glucose control, diabetes status, and mortality in critically ill patients: the continuum from intensive care Unit admission to Hospital discharge. Mayo Clin Proc 2017; 92(7): 1019 - 1029.

### 0229

#### Milrinone as a rescue therapy for refractory cerebral vasospasm after subarachnoid hemorrhage: a 5 years review

F. Alves[1], F. Brás Monteiro[2], I. Militão[3], A. Costa[4], C. Dias[5]
[1]Hospital São Sebastião, Intensive Care Unit, Santa Maria da Feira, Portugal; [2]Hospital Egás Moniz, Intensive Care Unit, Lisboa, Portugal; [3]Hospital de Vila Real, Intensive Care Unit, Vila Real, Portugal; [4]Hospital de São João, Porto, Portugal; [5]Hospital Sao João, Intensive Care Unit, Porto, Portugal
**Correspondence:** F. Alves
*Intensive Care Medicine Experimental* 2018, **6(Suppl 2):**0229

**INTRODUCTION.** Subarachnoid hemorrhage (SAH) is a devastating form of stroke. The patients present approximately a 50% mortality rate, mostly occurring before hospital admission, and another 30% have permanent disability. Cerebral vasospasm (CVS) is a major complication in these patients and may lead to delayed cerebral ischemia (DCI). The improvement of outcome is limited by the reversal of arterial narrowing and improvement of perfusion. Several agents and endovascular therapy have been used in patients with SAH with consistent benefit in relieving vasospasm. Milrinone is an inotropic and vasodilatory drug proven safe for use in the treatment of cerebral vasospasm.
**OBJECTIVES.** We aimed to review the success rate of aggressive management with milrinone for severe and refractory CVS used in our center from 2013 to 2018.
**METHODS.** A total of 11 patients who received milrinone treatment for CVS following SAH between 2013 and 2018 were included in the study. Refractory vasospasm was defined as patients with CVS refractory to therapies requiring ≥3 endovascular interventions. Overall, 8 patients meet criteria for refractory CVS. WFNS, Fisher, APACHE II e SAPS II score were assessed. The protocol management of these patients included nimodipine and control of arterial blood pressure according to autoregulation evaluation, decrease of blood viscosity and normal body temperature.
**RESULTS.** All patients had aneurysmal subarachnoid hemorrhage. The median WFNS was IV (min: I, Max: V), Fisher: 4 (min: 3, Max: 4), APACHE II: 14 (min: 7, max: 24) and SAPS II score: 27 (min: 8, max: 52). Nimodipine was given to all patients (11) and daily transcranial Doppler was performed. When severe vasospasm occured (3 men and 5 women), an endovascular intervention was made with verapamil (25) or milrinone (2) intra-arterial injection. Afterwards, an IV perfusion of milrinone was added (15 days, min: 9, Max: 18) and a progressive reduction of CVS was observed.
**CONCLUSIONS.** A combination therapy including milrinone seems to be a useful approach as a rescue therapy to avoid DCI related to hypoperfusion secondary to severe refractory vasospasm.

### 0230

#### Effects of acute subdural hematoma on cerebral nitrosative stress in a porcine resuscitated model

T. Merz[1], S. Dumitrescu[2], A. Weidinger[2], A.V. Kozlov[2], F. Zink[1], T. Kapapa[3], S. Röhrer[3], M. Wepler[1,4], P. Radermacher[1], O. McCook[1], S. Unmuth[1]
[1]Universitätsklinikum Ulm, Anesthesiological Pathophysiology, Ulm, Germany; [2]Ludwig Boltzmann Institute for Experimental and Clinical Traumatology, Wien, Austria; [3]Universitätsklinikum Ulm, Neurosurgery, Ulm, Germany; [4]Universitätsklinikum Ulm, Anesthesiology, Ulm, Germany
**Correspondence:** T. Merz
*Intensive Care Medicine Experimental* 2018, **6(Suppl 2):**0230

**INTRODUCTION.** Acute subdural hematoma (ASDH) assumes major importance in traumatic brain injury (TBI), in particular in the elderly and/or vascular co-morbid patient (1). Increased levels of reactive oxygen and nitrogen species (ROS/RNS) are reported to promote TBI-related neuro-inflammation (2). In TBI, enhanced formation of nitric oxide (NO) may lead to peroxynitrite production due to reaction with the superoxide anion ($O^{2-}$), thus aggravating oxidative and nitrosative stress (3,4). Peroxynitrite is capable of protein nitrosylation of tyrosine residues, thus leading to nitrotyrosine formation (3).
**OBJECTIVES.** The aim of this experiment was to investigate cerebral nitrosative stress in a resuscitated porcine model of ASDH.
**METHODS.** Anesthetized, mechanically ventilated and surgically instrumented pigs underwent ASDH (trepanation above the left parietal cortex and subdural injection of 20 ml of blood). Resuscitation was performed according to the guidelines of TBI management (5) and comprised fluid and catecholamine administration to maintain mean arterial pressure at pre-ASDH levels and cerebral perfusion pressure >75 mmHg. Fifty four hours after ASDH induction, animals were sacrificed and the brains were harvested. Tissue nitrite ($NO_2^-$) levels were assessed on homogenized ipsi- and contralateral specimens using a chemiluminescent assay (N=5). Immunohistochemistry for nitrotyrosine was performed on formalin-fixed cortical paraffin sections (N=7).
**RESULTS.** $NO_2^-$ levels, as an indicator for tissue NO production, were consistently elevated in the ipsilateral hemisphere (1.3 fold ipsi- vs. contralateral). Nitrotyrosine formation was increased in the ipsilateral hemisphere, in particular in areas of hematoma and tissue injury.
**CONCLUSIONS.** This study revealed increased rate of NO synthesis in ipsilateral specimens accompanied by increased oxidative and nitrosative stress in a porcine resuscitated model of ASDH. These data suggest that inhibition of NO production or ROS/RNS scavenging can help to attenuate TBI-induced neuro-inflammation.

**REFERENCE(S)**
1: Lee 2017: J Neurotrauma 128(1):236-249,
2: Abdul-Muneer 2013: Free Radic Biol Med. 60:282-91,
3: Pacher 2007: Physiol Rev. 87(1):315-424.
4: Kozlov 2016: BBA - Molecular Basis of Disease 1863(10):2627-32,
5: Carney 2018: J Neurotrauma 35(1):64-72.

**GRANT ACKNOWLEDGMENT**
supported by the DFG, CRC1149; DAAD

### 0231

#### Role of $H_2S$ enzymes in a porcine resuscitation model of traumatic brain injury

S. Unmuth[1], O. McCook[1], M. Wepler[1], T. Datzmann[1], D. Yilmazer-Hanke[2], T. Kapapa[1], S. Roehrer[1], P. Radermacher[1], T. Merz[1]
[1]University Ulm, Institute of Anesthesiologic Pathophysiology and Process Development, Ulm, Germany; [2]University Ulm, Clinical Neuroanatomy, Neurology, Ulm, Germany
**Correspondence:** S. Unmuth
*Intensive Care Medicine Experimental* 2018, **6(Suppl 2):**0231

**INTRODUCTION.** Traumatic brain injury (TBI) is characterized by increased intracranial pressure (ICP) associated with hypoxia, microvascular collapse, ischemia, blood-brain barrier (BBB) dysfunction, and swelling (1,2). Over 40% of all TBI cases develop an acute subdural hematoma (ASDH) resulting from rupture of the bridging veins (3,4). We recently showed in septic shock that reduced tissue expression of the $H_2S$-producing enzyme cystathionine-γ-lyase (CSE) was associated with kidney vascular barrier dysrupture (5) and impaired coronary artery integrity and left ventricular dysfunction (6). Exogenous $H_2S$ was protective in TBI (7), but little is known on the role of CSE in TBI.
**OBJECTIVES.** To study brain CSE expression after long-term, resuscitated porcine ASDH-induced TBI.
**METHODS.** Anesthetized mechanically ventilated pigs (n=9) were surgically instrumented for bilateral multimodal brain monitoring, ASDH was induced by injection of 20mL blood into the subdural space at the left parietal cortex. TBI management was performed according to TBI guidelines for up to 54h (8). Post mortem cortical brain samples were analysed by immunohistochemistry for CSE and albumin expression as a marker of vascular leakage. All arteries in frontal cortex samples of the injured (ASDH) and non-injured (neurosurgical instrumentation for monitoring alone) were analysed using a scale from 0-3, averaged and then expressed as fold increase injured.
**RESULTS.** ASDH caused a several-fold higher CSE expression than the neurosurgical instrumentation alone (100(40;100) vs. 8(7;50)$\cdot 10^6$ densitometric units). Vascular expression of CSE was present in the media as expected and increased 1.5-fold in the injured region. The increased CSE expression coincided with higher tissue albumin extravasation.
**CONCLUSION.** In a long-term, resuscitated porcine model of ASDH-induced TBI CSE was upregulated as a response to injury, more than likely as an adaptive stress mechanism. The non-injured hemisphere, although instrumented, showed little albumin extravasation and reduction of CSE expression.

**REFERENCE(S)**
1: Veenith 2016: JAMA Neurol. 73(5):542-50,
2: Abdul Muneer 2015: Mol Neurobiol. 51(3):966-79,
3: Harvey 2012: Injury.;43(11):1821-6,
4: Meissner 2011: Eur Surg Res. 47(3):141-53,
5: Stenzel 2016: Shock 46(4):398-404,

6: Merz 2017: Intensive Care Med Exp 5:17,
7: Zhang 2013, 2014: Neurochem Res. 38(4):714-25,
8: Cairns 2017: Neurosurgery.;80(1):6-15

**ACKNOWLEDGEMENT**
Supported by the DFG CRC1149, IGradU

### 0232

#### Intracraneal pressure monitoring with a ventricular device is not gold standard due to the zero reference point

J. Cabrera-Arrocha[1], M. Unnerbäck[2], K. Hesselgard[3], N. Marklund[3], E.L. Bloomfield[4], P. Reinstrup[3]
[1]Hospital Universitario de Gran Canaria Dr Negrin, Intensive Care Unit, Las Palmas de Gran Canaria, Spain; [2]Skanes University Hospital, Intensive & Perioperative Care, Malmö, Sweden; [3]Skanes University Hospital, Neurosurgery, Lund, Sweden; [4]Mayo Clinic, Anesthesiology/CCM, Rochester, United States
**Correspondence:** J. Cabrera-Arrocha
*Intensive Care Medicine Experimental* 2018, **6(Suppl 2):**0232

**INTRODUCTION.** In 1959 Lundberg presented a system to monitor ICP continuously which is used worldwide and regarded as gold standard. The zero reference point for the external pressure gauge has to use anatomical structures on the head. These structures vary according to the literature and may cause deviation from the true ICP values at different head positions.
**OBJECTIVES.** We wanted to evaluate some of the set ups for placing the zero reference point of the ICP device and their relation to Midbrain, Monroe and brain surface by looking at 3D MRI scans.
**METHODS.** Patients with MRI scanning's, intact cranial cavity and no space occupying lesions or hydrocephalus at the Neuroradiologic Dept. of Lund, was selected. Length and height for the brain were measured. Different structures, Monroe, Meatus, Orbit and Midbrain were identified (Fig 1) and distance between them measured. The pictures were placed like the patients were lying supine, with $45^0$ head elevations (Fig 2), upright and $45^0$ on the side (Fig 3). The skin to Midbrain and Monroe were measured at the same positions of the head. All values are presented as Mean ± SD. Distance in mm is converted to mmHg by multiplying with 0,074.
**RESULTS.** MR scanning's from 10 females 40 ± 10, 10 males 42 ± 9 were examined. A zero point at meatus was ideal when the patients was supine with no head elevation, resulting in a minor deviation from Midbrain of -1 to + 0,5 mmHg. Elevating the head overestimates the ICP by up to 6 mmHg. Head on the side, the glabella results in an ideal Midbrain zero point, but if turned $45^0$ an underestimation up to 6 mmHg was found. Monroe was situated at a point 39 to 56 mm up from the mid-OM line. This point follows head elevation, but cannot be used when the head is turned.The highest point of the head deviated from Midbrain by maximum1,8 mmHg, depending on the position of the head, if the ICP reading was corrected by adding 6 mmHg. The shortest distance is to vertex and the longest from midpoint to the frontal skin resulting in a difference of 2 to 3 mmHg when the head is repositioned from lying to upright. Monroe is, compared to Midbrain situated ≈ 12 mm in front, and ≈ 25 mm below Midbrain, making the skin to Monroe distance more equal in all clinical positions of the head. If the ICP reading at the highest point of the head is corrected by adding 6,3 mmHg, the deviation from Monroe would be ≤ 0,9 mmHg.
**CONCLUSIONS.** ICP pressure device placed at Meatus and nose tip-glabella are well defined structures and represent Midbrain ICP when the head is strict supine or strict on the side, but in all other positions they result in an up to 6 mmHg deviation up and down from ideal values. The highest point on the head underestimates the ICP at Midbrain as well as Monroe, but this underestimation is fairly constant and can be corrected providing the most exact ICP under all clinical head positions.

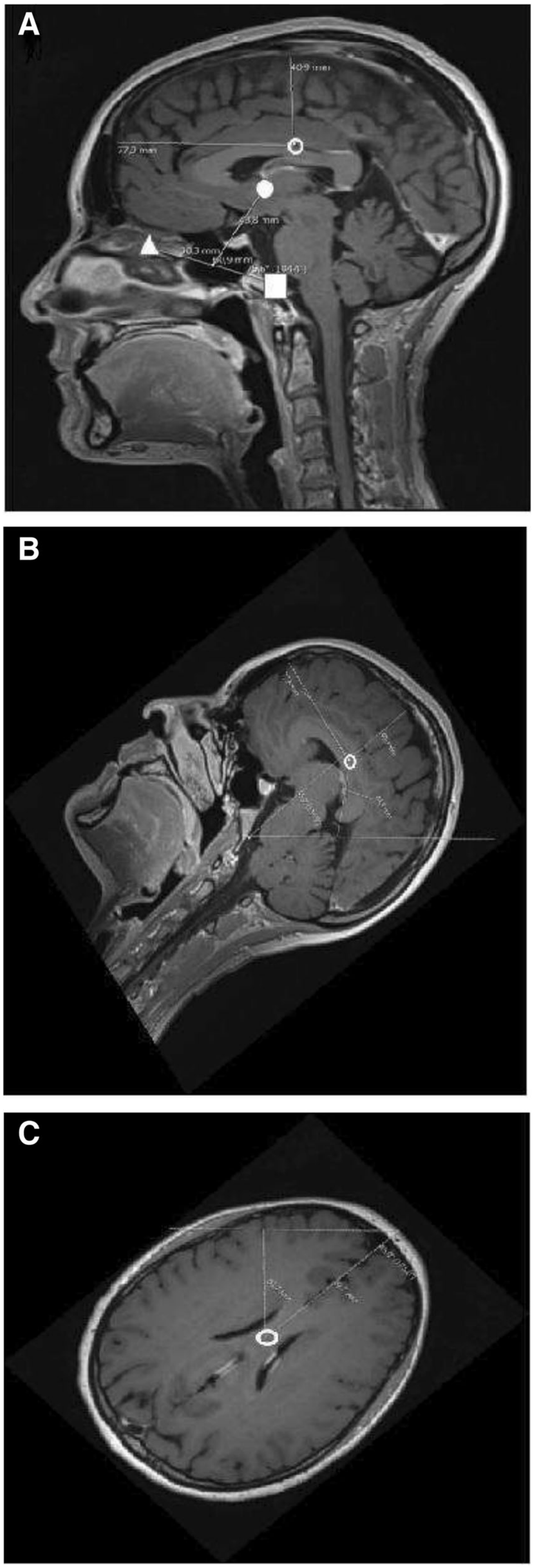

**Fig. 1 (abstract 0232).** Symbols: ○ midpoint; ■ meatus; • Monroe; ▲ Orbit

### 0233

#### Acute energy metabolism in patients after severe traumatic brain injury

J.J. Lenstra[1], M.W.N. Nijsten[2], J. van der Naalt[1]
[1]University Medical Center Groningen, Neurology, Groningen, Netherlands; [2]University Medical Center Groningen, Intensive Care Medicine - Adults, Groningen, Netherlands
**Correspondence:** J.J. Lenstra
*Intensive Care Medicine Experimental* 2018, **6(Suppl 2):**0233

**INTRODUCTION.** Little is known about the predictive value of early systemic lactate and glucose levels in patients after severe traumatic brain injury (TBI), in particular in relation to in-hospital mortality. The role of lactate in the ischemic brain is topic for debate since there is growing evidence for a neuroprotective role.[1,2,3,4]

**OBJECTIVES.** In this study we evaluated the association of arterial lactate and glucose levels in the acute phase after severe TBI in relation with in-hospital mortality. We investigated the predictive value of arterial lactate and glucose levels in patients with severe TBI from the Emergency Department (ED) to the first 48 hours (h) after hospital admission (HA).

**METHODS.** This study is a retrospective analysis of an observational follow-up study of 298 patients (age ≥16 years) with severe TBI admitted to the Intensive Care Unit (ICU) of the University Medical Center Groningen (UMCG) in the period of 2000-2014. Median age at injury was 34.0 years (interquartile range (IQR) 21.0-54.0) and Glasgow Coma Scale at admission 6 (IQR 5-7). Outcome was defined as in-hospital mortality.

**RESULTS.** The in-hospital mortality was 30%. From the ED to 48h after HA mean ±SD glucose was 7.2 ± 1.7 and lactate was 1.6 ± 1.1. Concerning in-hospital mortality, a significant difference from ED to 48h after HA was present respectively for survivors (mean ±SD glucose 7.0 ± 1.5 and lactate 1.5 ± 1.0) and for non-survivors (mean ±SD glucose 7.7 ± 2.1 and lactate 1.9 ± 1.2), (both p< 0.001) (see figure 1).

**CONCLUSIONS.** Both the arterial glucose and lactate course in patients with severe traumatic brain injury showed an increase from the Emergency Department to the first 48 hours after hospital admission. This data suggests that dysregulation from the glycometabolism in the early phase after injury are valuable predictors for in-hospital mortality.

#### GRANT ACKNOWLEDGMENT

N.A.

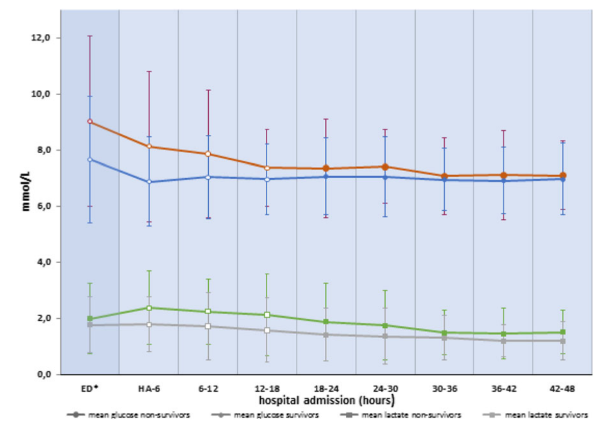

**Fig. 1 (abstract 0233).** Early arterial glucose and lactate course in patients during hospital admission after severe TBI

### 0234

#### New prognostic marker in traumatic brain injury patients: pBtO2/paO2 ratio

R. Badenes, B. Monleón, S. Martínez-Castro, A. Serrano, M.L. García
Hospital Clinic Universitari Valencia, Anesthesiology and Surgical-Trauma Intensive Care, Valencia, Spain
**Correspondence:** R. Badenes

*Intensive Care Medicine Experimental* 2018, **6(Suppl 2):**0234

**INTRODUCTION.** Traumatic brain injury (TBI) is a common cause of morbidity and mortality worldwide. Neuromonitoring have improved considerably in recent years in TBI patients. Brain tissue oxygenation ($pBtO_2$) is an important variable in TBI patients treatment.

**OBJECTIVES.** The main goal is to examine whether clinical scales (Glasgow Coma Scale [GCS], Injury Severity Score [ISS], radiographic scales based on admission computed tomography (Marshall), Intra Cranial Pressure [ICP], $pBtO_2$and $pBtO_2/paO_2$ratio are associated with clinical outcome after severe TBI.

**METHODS.** This is prospective observational study with institutional review board approval. TBI patients with a GCS ≤8 were identified on admission. GCS, ISS, Marshall score, ICP, $pBtO_2$and $pBtO_2/paO_2$ratio during first 24 hours were recorded. Patient outcome was determined as favorable (patient had no, little, or moderate disability) or unfavorable (patient died, or was in a vegetative state or had severe disability) using the Glasgow Outcome Scale by medical record review at 6 months after TBI. We considered an ordinal analysis with proportional odds methodology. A logistic regression model was used to calculate the 95% CI for the odds of unfavorable outcome.

**RESULTS.** Over a 4-year period, 44 patients were entered into the study. The mean age was 48 ±28.4 years, ISS was 27.7±10.7, and GCS was5.2±3.4. At 6 months, 8 (18.18%) patients had died, and 28 (63.63%) had a favorable outcome.Favorable outcome consistently demonstrated higher $pBtO_2/paO_2$ratio compared with non-survivors including age as a covariate ($p < 0.001$). Factors associated with unfavorable outcome (univariate analysis): ISS>50 (OR 1.47, CI 1.22-1.68), Marshall ≥ 3 (OR 1.88, CI 1.44-2.20), ICP > 20 mmHg (OR 2.88, CI 2.52-3.24), GCS < 5 (OR 2.11, CI 1.94-2.30), PtiO2 < 23 mmHg (OR 2.16, CI 2.05-2.27) and $pBtO_2/paO_2$ratio < 0.2 (OR 3.88, CI 3.48-4.27).

**CONCLUSIONS.** The first 24 hours of $pBtO_2/paO_2$ ratio < 0.2 predicts mortality and outcome. When $pBtO_2/paO_2$ ratio remains below 0.2 in the first 24 hours of monitoring, mortality and unfavorable outcome is increased. This study challenges the brain oxygenation threshold of 23 mm Hg that has been used conventionally and delineates a time for monitoring $pBtO_2/paO_2$ ratio that is predictive of outcome.

#### REFERENCE(S)

J Neurotrauma 2017;34:3153-3157.
J Cereb Blood Flow Metab 2017;37:762-775.

### 0235

#### Leucocyte profiles as simple immunomonitoring tools and their association with outcome in subarachnoid hemorrhage: a retrospective cohort study

C.L. Francoeur[1,2], A. Najjar[3], R. Frédéric[3], B. Michel[3], J.-F. Cailhier[4,5]
[1]CHU de Québec - Université Laval Research Centre, Population Health and Optimal Health Practices Research Unit (Trauma-Emergency-Critical Care Medicine), Québec, Canada; [2]CHU de Québec - Université Laval, Critical Care and Medicine, Quebec, Canada; [3]Centre Hospitalier de l'Université de Montréal, Surgery, Neurosurgery Division, Montreal, Canada; [4]Centre Hospitalier de l'Université de Montréal, Medicine, Montreal, Canada; [5]Centre de Recherche du CHUM, Montreal, Canada
**Correspondence:** C.L. Francoeur

*Intensive Care Medicine Experimental* 2018, **6(Suppl 2):**0235

**INTRODUCTION.** Early Brain Injury is believed to be a major component of poor outcomes after aneurysmal subarachnoid haemorrhage (aSAH), leading to delayed cerebral ischemia (DCI) . The underlying mechanisms, however, are poorly understood and probably include complex interactions between the immunologic and inflammatory systems. Leucocyte profiles, in the form of various ratios, are considered to reflect immune function and have been associated with outcome in numerous pathologies, including admission neutrophil to lymphocyte (NLR) and platelet to lymphocyte (PLR) ratios in subarachnoid hemorrhage.

**OBJECTIVES.** To explore the association of other components of the leucocyte profiles to outcome in subarachnoid patients and validate NLR and PLR association with unfavorable outcome.

**METHODS.** We retrospectively reviewed 100 consecutive aSAH patients admitted to the Centre Hospitalier Universitaire de Montréal from February 2011 to June 2014. We selected 50 patients treated with endovascular approach and 50 with a surgical approach to control for its potential effect on immuno-inflammatory indices. Clinical, radiological, laboratory and follow-up data were extracted from patient records. Functional outcome was assessed using the modified Rankin scale with a good neurological outcome defined as a score of 0-3. Logistic regression was used to measure the association between NLR, monocyte to lymphocytes ratio (MLR), eosinophil to lymphocytes ratio and PLR at admission, day 5 and day 10 with odds of poor outcomes.

**RESULTS.** Included patients had a mean age of 57 years with 30% presenting as poor grades (WFNS $\geq$ 4). Mean follow-up was 16 months. After adjustment for age, poor neurological grade at admission and the presence of IVH, the results are as follows: 1) at admission, there is no association between leucocyte profiles and outcome 2) at day 5, higher NLR and higher MLR are associated with lower odds of poor outcomes ( OR 0.88 [0.78, 0.96] and 0.34 [0.11, 0.98] respectively) and 3) at day 10, there is no association between leucocyte profiles and outcome. It is worth noting that there was no difference in inflammatory parameters between treatment approaches.

**CONCLUSIONS.** Our data does not support the association between admission PLR or NLR with outcome, contrary to a previously published report. The immunological signal emerging from the leucocyte profiles suggests a potential role of neutrophils and monocytes at a later time point in aSAH patient outcomes. The positive association with good prognosis implies that the phenotype of these cells might be important to the patient natural inflammatory history. The discrepancy with a previous study underscores the limitations of retrospective data and the urgency of prospective and comprehensive immunomonitoring in SAH patients to unravel the contribution of immune cells in the complex events leading to DCI.

**GRANT ACKNOWLEDGMENT**
Claude-Bertrand Neurosurgery Chair, Université de Montréal.

### 0236

#### Preserve encephalus in surgery of trauma: online survey (P.E.S.T.O.)

E. Picetti[1], S. Rossi[1], A.W. Kirkpatrick[2], W. Biffl[3], P.F. Stahel[4], Y. Kluger[5], L. Ansaloni[6], V. Agnoletti[7], F. Catena[8]
[1]I Servizio Anestesia Rianimazione - AOPR, Parma, Italy; [2]General, Acute Care, Abdominal Wall Reconstruction and Trauma Surgery, Foothills Medical Centre, Calgary, Canada; [3]Trauma, Acute Care Surgery and Trauma Critical Care, Scripps Memorial Hospital, La Jolla, United States; [4]Rocky Vista University, College of Osteopathic Medicine, Parker, United States; [5]Rambam Health Campus, Department of General Surgery, Haifa, Israel; [6]Bufalini Hospital, Department of General and Emergency Surgery, Cesena, Italy; [7]Bufalini Hospital, Department of Anesthesia and Intensive Care, Cesena, Italy; [8]Parma University Hospital, Department of Emergency Surgery, Parma, Italy
**Correspondence:** E. Picetti
*Intensive Care Medicine Experimental* 2018, **6(Suppl 2):**0236

**INTRODUCTION:** Traumatic brain injury (TBI) is a leading cause of morbidity and mortality worldwide (1). Extra-cranial lesions needing emergency surgery, especially if associated with hemorrhage, worsen further the outcome of TBI (2).

**OBJECTIVES:** To survey acute phase management practices in polytrauma patients with severe TBI. The main endpoints of the survey were the evaluation of: a) intracranial pressure (ICP) monitoring during extracranial emergency surgery (EES), b) hemodynamic management without ICP monitoring during EES c) coagulation management, and d) utilization of simultaneous multisystem surgery (SMS).

**METHODS:** A questionnaire including 24 items was available on the World Society of Emergency Surgery (WSES) website (https://docs.google.com/forms/d/1Y7-L1ZNJWIlb9e4ea9QQkGqQZYZ2Byr2-uNcHXZI4nxI/edit) between December 2017 and February 2017. The survey was endorsed and promoted by WSES and restricted to emergency surgeons.

**RESULTS:** Respondents (RSP) were 122 [70 (57%) from Europe]; mainly working in a Level I Trauma Center [66 (54%)]. ICP probes were inserted mostly by neurosurgeons [117 (96%)]. ICP monitoring was utilized in 10-30% of patients, at risk of intracranial hypertension undergoing EES, by the majority of respondents [35 (29%)]. Few respondents utilized an ICP monitoring protocol in this setting [48 (39%)]. However, 102 (84%) respondents considered ICP monitoring during EES important, very important or mandatory. Hemodynamic and coagulation management issues are reported in Table 1. SMS was performed in 5-19% of patients, needing both an emergency neurosurgical operation and EES, by the majority of respondents [49 (40%)]. Few respondents have a protocol for SMS [33 (27%)]. Nevertheless, 112 (92%) respondents considered the ability to perform SMS important, very important or mandatory. In polytrauma patients with ICP monitoring and intracranial hypertension, extra-cranial pressures (such as intrathoracic pressure and abdominal pressure) were monitored by the majority of respondents [93 (76%)].

**CONCLUSIONS:** A great variability in practices during the acute phase management of severe TBI patients with polytrauma was identified in this survey. These findings may be helpful to define future investigations in this topic and for educational purposes.

**REFERENCE(S)**
1) Maas AIR et Al. Traumatic brain injury: integrated approaches to improve prevention, clinical care, and research. Lancet Neurol. 2017 Dec; 16(12): 987-1048.
2) Galvagno SM Jr et Al. Outcomes after concomitant traumatic brain injury and hemorrhagic shock: A secondary analysis from the Pragmatic, Randomized Optimal Platelets and Plasma Ratios trial. J Trauma Acute Care Surg. 2017 Oct; 83(4): 668-674.

**Acknowledgements**
The Survey was endorsed and promoted by WSES.

**Table 1 (abstract 0236).** Hemodynamic and coagulation parameters issues

| COAGULATION | | |
|---|---|---|
| | ICP PLACEMENT | CRANIOTOMY |
| SAFE PLTs COUNT | > 50.000 mm [57 (46.7%)] | > 100.000 mm3 [67 (54.9%)] |
| PT-aPTT | 1.5 times [73 (59.8%)] | 1.5 times [76 (62.3%)] |
| | POLYTRAUMA | POLYTRAUMA-TBI |
| RBCs/Plasma/PLTs | 1/1/1 [51 (41.8%)] | 1/1/1 [53 (43.4%)] |
| HEMODYNAMIC | | |
| | EES without ICP | |
| SYSTOLIC Art. Press. | 90-110 mmHg [70 (57.4%)] | |
| MEAN Art. Press. | > 70 mmHg [44 (36.1%)] | |

### 0237

#### The role of brain tissue $P_{tiO2}$ monitoring after severe subarachnoid haemorrhage (SAH)

C. Iasonidou, E. Lazoudi, S. Papoti, E. Siomos, A. Kosmas, N. Kapravelos
G. Papanikolaou Hospital, Thessaloniki, Greece
**Correspondence:** E. Lazoudi
*Intensive Care Medicine Experimental* 2018, **6(Suppl 2):**0237

**INTRODUCTION.** SAH is a complex neurovascular syndrome with high disability and mortality. Pour outcomes are usually secondary to early brain injury or delayed cerebral ischemia (DCI). Recent clinical data suggest that cerebral hypoxia may occur despite normal levels of ICP and CPP in poor grade SAH. Therefore the use of multimodal neuromonitoring, including PtiO2 in an early reversible state may be a good practice to improve management and prognosis.

**OBJECTIVES.** To investigate the utility of PtiO2 monitoring in detecting cerebral hypoxic events and the relation between them, the outcome and the development of DCI.

**METHODS.** This observational study evaluated 19 ICU patients after severe SAH with Hunt- Hess grade 4 or with secondary deterioration to this grade. Monitoring of ICP, CPP, $P_{ti}O_2$ was performed. Patients were divided in non-survivor and survivor group. The second group was further divided in infraction and non-infraction group. Critical values of $P_{ti}O_2 \leq$ 15mmHg, CPP≤70mmHg and ICP>20mmHg were derived from data sets for different time intervals (first 24h, 48h and >48h). We investigated the relation between pts outcome and the appearance and duration of hypoxic events.

**RESULTS.** 19pts were enrolled, mean age 51.52 (± 9.8), mean APACHE II 17.15 (± 5.5). In 14 pts an external ventricular drain was inserted. Survivor pts were 11 ( 57.89%) and non survivor 8 (42.1%). 5 pts developed DCI (45.4%). Critical $P_{ti}O_2$ on first 24h was detected in 14pts (73.6%), but this frequency was decreased later. After 48h brain hypoxia was detected in 10pts (52.6%). Long lasting and persistent low $P_{ti}O_2$ was detected in 9pts (48.3%). Among these pts only 4 were associated with low CPP (21.05%). In the survivor group low $P_{ti}O_2$ in first 24h was detected in 9pts (81.8%), but in only 3 of them (27%) after 48h. In non-survivor this proportion was 5 (62.5%) and 7 (87.5%) respectively . Moreover, persistent brain hypoxia was detected in 1 survivor (9%) and 7 non-survivor (87.5%). From the DCI group all pts developed long-lasting brain hypoxia mostly after 48h.

**CONCLUSIONS.** Monitoring of PtiO2 provides additional information in detection of cerebral hypoxic events. Therefore the use of multimodal monitoring may be a good complement to ICP/ CPP monitoring to detect cerebral oxygen in an early reversible state and to guide therapy, in order to improve outcome.

**REFERENCE(S)**

1. Monitoring of brain tissue oxygenation following severe subarachnoid haemorrhage, Meixensberger et al. Neurol Res. 2003 Jul 25(5):445-50
2. The Critical Care Management of poor-grade subarachnoid haemorrhage, AL de Oliviera Manoel et al. Crit Care 2016; 20:21

## Outcome in perioperative medicine

### 0238

#### Usefulness of presepsin for the early detection of infectious complications after elective colorectal surgery, compared with C-reactive protein and procalcitonin

E. Amanai[1], K. Nakai[2], J. Saito[3], E. Hashiba[1], T. Ono[2], H. Niwa[2], K. Hirota[3]
[1]Hirosaki University Hospital, Division of Intensive Care Unit, Hirosaki, Japan; [2]Hirosaki University Hospital, Department of Anesthesiology, Hirosaki, Japan; [3]Hirosaki University Graduate School of Medicine, Department of Anesthesiology, Hirosaki, Japan
**Correspondence:** E. Amanai
*Intensive Care Medicine Experimental* 2018, **6(Suppl 2):**0238

**INTRODUCTION.** Infectious complications remain a major clinical problem in colorectal surgery, contributing to prolonged hospital stays, additional costs and significant postoperative mortality. Presepsin has been reported to be a useful marker to diagnose sepsis, similar or superior to C-reactive protein (CRP) and procalcitonin (PCT),[1] and plasma presepsin concentrations are associated with the severity of sepsis and its outcome.[2]

**OBJECTIVES.** The aim of this study was to assess the diagnostic value of presepsin in the early detection of infectious complications after elective colorectal surgery, compared with CRP and PCT.

**METHODS.** This study was a prospective observational study. The protocol was approved by our university ethics committee and registered in the Japan Primary Registries Network (UMIN000025313). After written informed consent was obtained, patients of age >18 who underwent elective colon resections were enrolled from January to December 2017. Patients with end-stage kidney disease were excluded. Blood samples were collected just before surgery and on postoperative day (POD) 1, 2, 3, 4, and 6. Infectious complications were diagnosed by surgeons who were not involved in this study. Statistical analyses were performed with two-way RM ANOVA followed by Bonferroni's multiple comparison test. Predictive performance was assessed by the area under the ROC curves. A p value < 0.05 was accepted as significant.

**RESULTS.** A total of 114 patients were examined, and 27 patients (23.7%) developed infectious complications: 11 anastomotic leaks, 13 intra-abdominal infections, and 3 wound infections. The median interval between surgery and the diagnosis of infection was 5 days. The changes in biomarkers are shown in the Figure. CRP and PCT markedly increased from POD1 to POD3 and then gradually decreased toward POD6 in both groups, but the trends of the decrease in the infected group were blunt, compared with those in the non-infected group. On the other hand, presepsin did not show major changes just after surgery, but it increased on POD4 and POD6, when the complications occurred. ROC analysis to predict infectious complications revealed that the best accuracy was obtained on POD 6 for all biomarkers. The cut-off values, sensitivities, specificities and AUCs on POD6 are shown in the Table. CRP showed excellent predictability and presepsin showed good predictability. However, the cut-off values of all biomarkers were relatively lower than expected.

**CONCLUSIONS.** The trends of change in presepsin following colorectal surgeries were distinct from those of CRP and PCT. Monitoring the presepsin trends after colorectal surgeries could be helpful to detect postoperative infectious complications.

**REFERENCE(S)**

1. Endo S, et al. J Infect Chemother 2012; 18: 891-7.
2. Masson S, et al. Crit Care 2014; 18: R6.

**GRANT ACKNOWLEDGMENT**
This study was supported by grants from the Hirosaki University Work-Life Balance Support Program for Researchers.

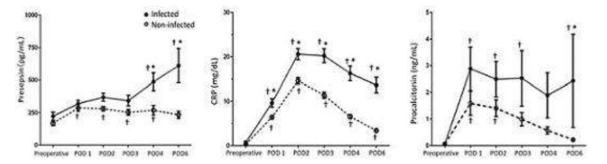

**Fig. 1 (abstract 0238).** Changes in Presepsin, CRP, and Procalcitonin There were significant differences between groups of presepsin, CRP, and procalcitonin concentrations at P < 0.05 y two-way repeated measrures analysis of variance (ANOVA). Error bars show SEM. *$p < 0.05$ between groups, +$p < 0.05$ versus preoperative

**Table 1 (abstract 0238).** ROC curve predictions of presepsin, CRP and procalcitonin (PCT) values

**Table: ROC curve predictions of presepsin, CRP and procalcitonin (PCT) values based on the presence or absence of infectious complications**

| | Cut off value | Sensitivity (%) | Specificity (%) | AUC(95%CI) |
|---|---|---|---|---|
| Presepsin | 299 pg/ml | 73.1 | 81.0 | 0.82(0.73-0.91) |
| CRP | 7.394 mg/dl | 81.5 | 88.4 | 0.91(0.83-0.98) |
| PCT | 0.09 ng/ml | 92.3 | 43.5 | 0.75(0.65-0.86) |

### 0239

#### The impact of goal-directed fluid therapy during major abdominal surgery on postoperative oxygenation and extravascular lung water

A. Hasanin, K. Hussein, I. Farouk, S. Abdelraouf, H. Ezzat
Cairo University, Anesthesia and Critical Care Medicine, Cairo, Egypt
**Correspondence:** A. Hasanin
*Intensive Care Medicine Experimental* 2018, **6(Suppl 2):**0239

**INTRODUCTION.** Proper fluid management during major abdominal surgery is essential for good patient outcome. Goal-directed fluid therapy (GDFT) was previously investigated during major abdominal surgery; however, its impact on extravascular lung water (EVLW) was not evaluated.

**OBJECTIVES.** To investigate the impact of GDFT during major abdominal operations on postoperative EVLW.

**METHODS.** We recruited 120 patients undergoing major abdominal operations in Cairo University hospital in a randomized controlled trial. Patients were divided into GDFT group (n=60) and control group (n=60). In the control group, fluids were infused at a rate of 6 mL/Kg/hour; whilst, in GDFT group, fluids were infused at a rate of 2 mL/Kg/hour. Fluid boluses were administered in GDFT group to optimize stroke volume variation (measured by electrical cardiometry) less than 10%. In both groups, mean arterial pressure (MAP) of 65 mmHg or more was targeted. Both groups were compared in demographic data, hemodynamic variables (MAP and heart rate), infused fluids, and post-operative P/F ratio. lung ultrasound score (LUS) was evaluated using 12-region lung ultrasound technique.

**RESULTS.** One-hundred and twenty patients were available for final analysis with mean age of 49±13 years. Seventy-four (62%) of our patients were males. Mean surgical duration was 4.5±0.7 hours. MAP and heart rate were comparable between both study groups; however, GDFT group received less fluids compared to control group {1350(1125-1700) mL versus 3000(2500-3475) mL, P< 0.001}. Post-operative LUS was lower in GDFT compared to the control group {2(0-4) versus 4(3-6)} mL, P< 0.001}. P/F ratio was slightly higher in the GDFT group compared to the control group (368 ±37 versus 353±39 P=0.04). The number of patients who showed significant postoperative increase in LUS was higher in the control group {52(87%) patients versus 31(52%) patients, P< 0.001}.

**CONCLUSIONS.** Using GDFT in major abdominal surgery was associated with less total fluid intake, lower EVLW, and slightly higher P/F ratio without compromising patient hemodynamics.

### 0240

#### Lactate levels in predicting in-hospital mortality after surgery for intestinal perforation

S.-Y. Oh[1], H. Lee[2], H.G. Ryu[2]
[1]Seoul National University College of Medicine, Critical Care Center and Department of Surgery, Seoul, Korea, Republic of; [2]Seoul National University College of Medicine, Department of Anesthesiology, Seoul, Korea, Republic of
**Correspondence:** S.-Y. Oh
*Intensive Care Medicine Experimental* 2018, **6(Suppl 2):**0240

**INTRODUCTION.** Although there is a scoring system predicting prognosis of patients with intestinal perforation, the existing system was established too long ago and many new prognostic factors have been reported since then.

**OBJECTIVES.** The aims of this study are to investigate the risk factors for in-hospital mortality after surgery for intestinal perforation and evaluate the effect of lactate level on in-hospital mortality.

**METHODS.** Among the patients (≥ 18 years) who underwent surgery for intestinal perforation from 2013 to 2017, only those patients whose lactate levels were checked before surgery and within 12 hours after surgery were retrospectively analyzed. We evaluated risk factors for in-hospital mortality after surgery for intestinal perforation using univariable and multivariable analyses.

**RESULTS.** A total of 105 patients were enrolled and 17 patients (16.2%) died before discharge. There were no significant differences in Charlson Comorbidity Index score (5.8 vs. 4.7, *P* = 0.114), preoperative C-reactive protein level (14.3 vs. 16.9, *P* = 0.468), proportion of emergency surgery (82.4% vs. 72.0%, *P* =1.000), and proportion of large bowel as perforation site (35.3% vs. 54.5%, *P* = 0.146). The patients in the mortality group had higher preoperative maximum lactate level (6.3 vs. 3.4, *P* = 0.036), higher proportion of dirty and fecal contaminated ascites (94.1% vs. 68.2%, *P* = 0.036), lower albumin level (2.5 vs. 2.9, *P* =0.022), and lower delta lactate level between pre and postoperative check (-1.6 vs. -0.2, *P* = 0.026) than the patients who survived after the surgery. Multivariable analyses revealed that the preoperative maximum lactate level (OR 1.272, 95% CI 1.094~1.479, *P* = 0.002) and delta lactate level (OR 0.732, 95% CI 0.576~0.942, *P* = 0.015) had significant effects on the in-hospital mortality after surgery for intestinal perforation.

**CONCLUSIONS.** Preoperative maximum lactate level and delta lactate level between pre and postoperative period were predictive risk factors for the in-hospital mortality after surgery for intestinal perforation.

**REFERENCE(S)**

1. H. Wacha, M.M. Linder, U. Feldmann, G. Wesch, E. Gundlach, R.A. Steifensand, Mannheim peritonitis index-prediction of risk of death from peritonitis: construction of a statistical and validation of an empirically based index, Theor. Surg. 1 (1987) 169e177.
2. A. Billing, D. Fr€ohlich, F.W. Schildberg, Prediction of outcome using the Mannheim peritonitis index in 2003 patients, Br. J. Surg. 81 (1994) 209e213.
3. Rhodes A, Evans LE, Alhazzani W, Levy MM, Antonelli M, Ferrer R et al. Surviving Sepsis Campaign: International Guidelines for Management of Sepsis and Septic Shock: 2016. Crit Care Med. 2017 Mar;45(3):486-552.

### 0241

#### Indications and outcomes of emergency laparotomy in patients on extracorporeal membrane oxygenation

C. McCann[1], K. Adams[2], A. Schizas[2], M. George[2], N. Barrett[1], D. Wyncoll[1], L. Camporota[1]
[1]Guys and St Thomas' NHS Foundation Trust, Department of Critical Care, London, United Kingdom; [2]Guys and St Thomas' NHS Foundation Trust, Department of Colorectal Surgery, London, United Kingdom
**Correspondence:** C. McCann
*Intensive Care Medicine Experimental* 2018, **6(Suppl 2):**0241

**INTRODUCTION.** Between 200 and 300 patients per year currently receive extracorporeal membrane oxygenation (ECMO) at our tertiary referral service. A proportion of these patients develop indications for emergency laparotomy which might have previously been considered a futile undertaking in this potentially more unstable population in multiple organ failure, however it is now regarded as a feasible treatment strategy in our institution. There is no other comparable service in the United Kingdom and a paucity of literature on the subject[1].

**OBJECTIVES.** The study objectives were to evaluate our current service, identify the indications and outcomes of emergency laparotomy in this population and inform future service planning with regards to the co-location of ECMO services in general surgical centres.

**METHODS.** A single-centre, retrospective, observational cohort study of adult patients admitted to a university teaching hospital severe respiratory failure service between December 2011 and January 2017.

**RESULTS.** Of 355 patients established on ECMO, 13 patients required emergency laparotomy, 11 on veno-venous and 2 on veno-arterial circuits. Mean time from cannulation to surgical referral was 5.23 days, 11 of 13 for increasing abdominal distension, 4 with rising lactate and 2 for rising noradrenaline requirements. Mean lactate immediately pre-operatively was 7.62 with 3 patients having a normal lactate. 11 of 13 had pre-operative CT scanning with features of bowel ischaemia the most common feature. P-POSSUM over-predicted mortality, with group predicted mortality 83.38% (SD 14.64), versus actual mortality 69.2%, weighted mortality ratio 0.83. Most patients required a bowel resection (53.8%) and similar number had planned re-look laparotomy or laparostomy (61.5%). Operative mortality was 0% but 6 patients required an emergency change of ECMO circuit post-operatively. Hospital mortality for ECMO patients not requiring laparotomy was 25.1% and 69.2% for those undergoing emergency laparotomy (*p*=0.001).

There was no significant difference in age, SOFA score on admission, days on ECMO or total Length of Stay on ICU between groups.

**CONCLUSIONS.** Emergency laparotomy can be successfully undertaken in patients on ECMO with 30% surviving to hospital discharge. Emergency requirement for ECMO circuit change is a post-operative risk. Traditional methods of diagnosis and risk assessment such as CT scans, lactate levels and P-POSSUM scoring, are not wholly applicable in this niche population and the decision to operate is best made on the clinical judgement of the intensive care and general surgical teams. There is a substantial need for general surgical speciality input into patients on ECMO and this should be recognised as one of the requirements of the service.

##### REFERENCE(S)

1. Taghavi S, Jayarajan SN, Mangi AA, et al (2015) Examining Noncardiac Surgical Procedures in Patients on Extracorporeal Membrane Oxygenation. ASAIO J 61(5):520-525.

### 0242

#### Intraoperative positive end-expiratory pressure setting guided by esophageal pressure measurement during laparoscopic gynecologic surgery: a pilot study

A. Piriyapatsom, S. Phetkampang
Faculty of Medicine Siriraj Hospital Mahidol University, Department of Anesthesiology, Bangkok, Thailand
**Correspondence:** A. Piriyapatsom
*Intensive Care Medicine Experimental* 2018, **6(Suppl 2):**0242

**INTRODUCTION.** Setting of positive end-expiratory pressure (PEEP) has an important role in respiratory management during laparoscopic surgery. However, there is no consensus on the optimal PEEP level or the best method to set PEEP during laparoscopic surgery.

**OBJECTIVE.** To investigate whether setting of PEEP guided by measurement of esophageal pressure would affect oxygenation and compliance of respiratory system ($C_{rs}$) during laparoscopic surgery.

**METHODS.** Ten patients undergoing laparoscopic gynecologic surgery were randomly divided into the intervention and the control groups. In the intervention group, PEEP was set according to esophageal pressure measured to maintain transpulmonary pressure during expiration ($P_{TP,\ exp}$) between 0 and 5 cm $H_2O$. In the control group, PEEP was constantly set at 5 cm $H_2O$. Gas exchange, lung mechanics, and hemodynamic parameters were recorded after induction and intubation (T0) and at 15 and 60 minutes after gas insufflation (T1 and T2, respectively) and data were analyzed.

**RESULTS.** There were no significant differences regarding age, BMI, ASA physical status, anesthetic time, insufflation time, and intraabdominal pressure applied during gas insufflation between two groups. During gas insufflation, plateau pressure ($P_{plat}$), PEEP and $P_{TP,\ exp}$ were significantly higher in the intervention group compared to the control group ($P_{plat}$; T1, 26.0±2.0 vs. 19.0±1.6 cm $H_2O$, p< 0.001; T2, 26.8±1.3 vs. 19.0±0.7 cm $H_2O$, p< 0.001; PEEP; T1, 13.4±2.3 vs. 5.0 ±0.0 cm $H_2O$, p=0.001; T2, 13.2±2.2 vs. 5.0±0.0 cm $H_2O$, p< 0.001; and $P_{TP,\ exp}$; T1, 0.3±2.4 vs. -6.2±5.3 cm $H_2O$, p=0.04; T2, 1.0±2.1 vs. -6.7±5.1 cm $H_2O$, p=0.03). $PaO_2/FiO_2$ ratio and $C_{rs}$ were decreased from T0 but these change did not reach statistical significance neither within group nor between groups (change in $PaO_2/FiO_2$ ratio; T1, -24.2±23.2 in the intervention group vs. -64.3±74.6 in the control group; T2, -34.1±34.1 vs. -19.2±69.0 and change in $C_{rs}$; T1, -12.0±9.3 vs. -21.4±2.9 mL/cm $H_2O$; T2, -15.2±6.2 vs. -21.6±6.1 mL/cm $H_2O$). There were no significant changes in hemodynamic parameters during gas insufflation neither within group nor between groups. There was one patient in the control group required oxygen supplement after discharge from the recovery room.

**CONCLUSIONS.** This pilot study showed that PEEP setting guided by measurement of esophageal pressure during laparoscopic surgery was feasible. However, a larger number of subjects may be required to investigate any potential benefits on oxygenation or Crs.

##### GRANT ACKNOWLEDGMENT

This study was supported by Faculty of Medicine Siriraj Hospital, Mahidol University, Thailand.

### 0243

#### Electrolytes disorders and kidney complications in immediate postoperative after cytoreductive surgery and hyperthermic intraperitoneal chemotherapy

A. Acha[1], M.-C. Pintado[1,2], A. Pardo[1], B. Gracia[1], R. de Pablo[2,3]
[1]Hospital universitario Principe de Asturias, Intensive Care Medicine Unit, Alcala de Henares, Spain; [2]Universidad de Alcalá, Alcala de Henares, Spain; [3]Hospital universitario Ramón y Cajal, Intensive Care Medicine Unit, Madrid, Spain
**Correspondence:** A. Acha
*Intensive Care Medicine Experimental* 2018, **6(Suppl 2):**0243

**INTRODUCTION.** In select patients with peritoneal carcinomatosis, cytoreductive surgery (CRS) and hyperthermic intraperitoneal chemotherapy (HIPEC) is an optional treatment to improve survival. The procedure is lengthy and involves extensive surgical debulking, needing aggressive resuscitation with fluids required intraoperatively due to blood losses and fluid shifts. Several complications are described in postoperative as acute renal failure, hypovolemia and electrolytes disorders.

**OBJECTIVES.** to determine the electrolytes disorders and kidney complications during intensive care unit (ICU) stay after CRS and HIPEC.

**METHODS.** prospective observational study during 5 years. All patients admitted to ICU postoperatively after CRS and HIPEC therapy. Data recorded were age, sex, type of cancer, severity at ICU admission (APACHE II and SOFA scores), laboratory data (creatinine and electrolytes), volume of diuresis and ICU mortality.

We defined normal range in plasma of potassium as 3.5- 5 mmol/l, magnesium as 1.5-2.3 mg/dl, sodium as 135-145 mmol/l and phosphorus as 2.4-5.1 mgr/dl. Acute kidney injury (AKI) as increase of serum creatinin e x1.5 from baseline (serum creatinine at hospital admission). Polyuria as diuresis $\geq$3 ml/kgr/h.

Qualitative variables are described as number and percentages, quantitative variables with normal distribution as mean ± S.D. and compared with chi-square test, quantitative variables with non-normal distribution as median (interquartile range).

**RESULTS.** we included 73 patients. 40 (55%) were males. Mean age was 60.3±9.9 years. (86%) had a digestive cancer (52% colorectal, 44% gastric cancer). Mean APACHE II and SOFA scores at ICU admission were 9.6±4.2 and 2 (1-3), respectively. 53 patients (73%) developed electrolytes disorders. 19(26.0%) had hyponatremia, 2 (2.7%) hypernatremia, 28 (38.4%) hypokalemia, 1 (1.4%) hyperkalemia, 21 (28.8%) hypomagnesemia, 5 (6.8%) hypermagnesemia and 19 (26.0%) hypophosphatemia. Only 1 patient with hypomagnesemia suffers a hemodynamically stable ventricular tachycardia, treated with intravenous magnesium.

4 (5,5%) patients had AKI. 20 (27,4%) had polyuria.

ICU mortality was 1.36%. There were not a statistically significant difference in ICU mortality between patients without this complications and patients who suffers electrolytes disorders (1.9% vs. 0.0%, p=1.00), polyuria (1.9% vs. 0.0%, p=1.00) or AKI (4.4% vs. 0.0%, p=1.00).

**CONCLUSIONS.** Electrolytes disorders are frequent in patients in inmediate postoperative after CRS and HIPEC), most of them are asyntomatic. The development of kidney damage in these patients is rare and not related to mortality.

**REFERENCE(S)**

Rieff EA et al. Anaesth Intensive Care. 2015 Jul;43(4):532-3 / Kapoor S et al. World J Crit Care Med 2017 May 4; 6(2):116-123 / Wallet F et al. Eur J Surg Oncol. 2016 Jun;42(6):855-60.

**GRANT ACKNOWLEDGMENT**

This work was not supported.

### 0244

#### Outcome of extreme oncology digestive surgery at intensive care unit

O.V. Báez-Pravia[1], C. Pey Richter[1], I. De Sousa[1], J.A. Gallardo Alvarez[1], M. Díaz-Cámara[1], B. Morato-Bellido[1], J.E. Guerrero Sanz[1,2], P. Cardinal-Fernández[3,4]
[1]Hospital Universitario HM Sanchinarro, Intensive Care Unit, Madrid, Spain; [2]Universidad CEU San Pablo, Madrid, Spain; [3]Hospital Universitario HM Sanchinarro, Madrid, Spain; [4]Fundación de Investigación HM Hospitales, Madrid, Spain
**Correspondence:** O.V. Báez-Pravia
*Intensive Care Medicine Experimental* 2018, **6(Suppl 2):**0244

**INTRODUCTION.** Extreme digestive oncology surgery (EDOS) is considered when 2 or more solid organs and/or the peritoneum are totally or partially removed as a cancer treatment. Due to the risks of the surgery, its indications are very restrictive and only skilled facilities can provide a reasonable benefit-to-risk ratio.
**OBJECTIVES.**

(a) To identify clinical variables associated to mortality.
(b) To develop a predictive model for hospital mortality.

**METHODS.** We conducted a retrospective study that include all patients

(a) older than 18 years old,
(b) undergoing EDOS and
(c) admitted to the intensive care unit (ICU) of the Hospital Universitario HM Sanchinarro during the period 1/01/14 to 10/31/16.

Day 0 was the day of ICU admission. All data are expressed as means (±SD) or proportions. Alive vs death patients at hospital discharge were compared. The proportions and qualitative data were compared using the Chisquare test and quantitative data using the unpaired Student's t test for normally distributed variables or the Kruskal-Wallis test. The maximum logistic regression model include all variables with a $p$ value < 0.1 in the univariate analysis. $P$ values less than 0.05 were considered significant. The discriminatory capability of the model calculated using the area under receiver operation curve (AUROC).
**RESULTS.** There were 164 consecutive patients admitted to the ICU after an EDOS. The following variables recorded the day 0 were: age 63±11 y.o., male 106, Charlson comorbidity index 6.31±2.2; Nutritional Risk Screening 2002 (NRS-2002) 4.02±0.88 and APACHE-II punctuation 11.2±4.4; length of ICU stayed 6±10 and days undergoing invasive mechanical ventilation 6±9. Site of the cancer: esophagus (n=8), gastric (n=11), yeyuneum-ileon (n=3); colon-rectal (n= 91); pancreas (n=38); liver (n=5), gallbladder and bile duct (n=8). Cancer stage: stage II (26), stage III (56) and stage IV (82). Hospital mortality n=22 (13.4%). The maximum logistic regression model for hospital mortality included: NRS-2002, APACHE II, Charlson score, invasive mechanical ventilation at ICU admission, and Easter Cooperative Oncology Group Scale - Performance Status (ECOG-PS). Variables associated to mortality were: Charlson comorbidity index (OR 1.37; IC 95% 1.07; 1.80; p 0.012), APACHE II (OR 1.13; IC 95% 1.01; 1.27; p 0.032) and NRS-2002 (OR 1.98; IC95% 1.10; 3.72; p 0.02). The AUROC for the regression model was 0.79 (95% CI: 0.67-0.92) and for the APACHE II at day 0 was 0.66 (95% CI: 0.51-0.81). The difference was statistically significant (DeLong test p < 0.01)
**CONCLUSIONS.** In this wide cohort of patients undergoing and EDOS the mortality was acceptable. Type and stage of the cancer does not seem to influence hospital outcome. The predictive model we have developed predicted the outcome better than the APACHE II score. Future studies should validate this new tool.

(1) Taccone et al. Crit Care 2009 (2) Azoulay, et al. ICM 2000.
(3) Caruso et al. Eur J Cancer Care 2010.

### 0245

#### Norton score: a useful tool for identifying high risk patients prior to emergency surgery

I. Trostchansky[1], V. Vigoria[2], T. Ezri[3,4], A. Nimrod[5], G. Karp[6]
[1]Kaplan Medical Center, Department of General Surgery, Rehovot, Israel; [2]University Hospital Complex of Vigo Alvaro Conquieiro Hospital, Department of General and Digestive Surgery, Vigo, Spain; [3]Wolfson Medical Center, Department of Anesthesia, Holon, Israel; [4]Tel Aviv University, Tel Aviv, Israel; [5]Kaplan Medical Center, Intensive Care Unit, Rehovot, Israel; [6]Kaplan Medical Center, Rehovot, Israel
**Correspondence:** G. Karp
*Intensive Care Medicine Experimental* 2018, **6(Suppl 2):**0245

**INTRODUCTION.** Emergency surgery in elderly patients (> 80 years) is associated with high mortality rates. Various scoring systems may be valuable to predict mortality and morbidity rates. It has lately been suggested that the Norton Score (NS) could be used to indirectly quantify frailty, thus, it may be employed to predict surgical patients outcome.
**OBJECTIVES.** We hypothesized that NS could be a useful tool for identifying high risk patients prior to emergency/urgent surgeries.
**METHODS.** A retrospective study was conducted in the Department of Surgery at Kaplan Medical Center, Rehovot, Israel. Inclusion criteria were: age ≥ 50 years, American Society of Anesthesiologists (ASA) physical status score ≥ 3 and urgent/emergency laparotomy (no trauma surgery).
Four hundred patients who underwent emergency/ urgency surgeries were identified, of which 150 (37.5%) matched the inclusion criteria.
**RESULTS.** Our study included 150 patients, ASA 3-5, who underwent emergent/urgent laparotomy not for trauma, from 1.1.2011 through 31.1.2013. Age was 77 ± 9,7 years. Mortality rate at 1 month was 44% (66 patients) and at 1 year was 54.7% (82 patients). A higher ASA score was significantly associated with mortality (p< 0,001). Survivors had lower frailty scores and presented significantly higher pre-operative NS (Modified Frailty Index 2.45 vs 3.06, p< 0,05; NS 16.09 vs 12.94, p< 0,01). Preoperative NS was the most significant variable that predicted poor patient prognosis (OR,0,84; 95% CI, 0.73-0.96). In addition, for ASA 3 and ASA 4 patient independence showed positive correlation with survival (p< 0.001).
**CONCLUSIONS.** Norton Score can be a very useful and quick tool to evaluate surgical risk in emergency surgery. Our study supports the use of NS in the perioperative evaluation prior to emergency surgery.

**REFERENCE(S)**

1. Partridge JS, Harari D, Martin FC, Dhesi JK. The impact of pre-operative comprehensive geriatric assessment on postoperative outcomes in older patients undergoing scheduled surgery: a systematic review. *Anaesthesia*. 2014 Jan;69 Suppl1:8-16.
2. Morse BC, Cobb WS 4th, Valentine JD, Cass AL, Roettger RH. Emergent and elective colon surgery in the extreme elderly: do the results warrant the operation? *Am Surg*. 2008 Jul;74(7):614-8; discussion 618-9 .
3. Zerbib P, Kulick JF, Lebuffe G, Khoury-Helou A, Plenier I, Chambon JP. Emergency major abdominal surgery in patients over 85 years of age.-*World J Surg*. 2005 Jul;29(7):820-5.
4. Partridge JS, Harari D, Dhesi JK. Frailty in the older surgical patient: a review. *Age Ageing* 2012 Mar;41(2):142-7.
5. Gold A, Sever R, Lerman Y, Salai M, Justo D. Admission Norton scale scores (ANSS) and postoperative complications following hip fracture surgery in the elderly. *Arch GerontolGeriatr*. 2012 Jul-Aug;55(1):173-6.

### 0246

#### Effects of perioperative high inspired oxygen fraction on surgical site infection in the patients with gastrointestinal surgery

A. Kumasaka, T. Hayasaka, N. Momose, M. Ogura, M. Yoshioka, M. Kudo
Nihonkai General Hospital, Anesthesiology, Sakatashi, Yamagata, Japan
**Correspondence:** A. Kumasaka

*Intensive Care Medicine Experimental* 2018, **6(Suppl 2):**0246

**INTRODUCTION.** Surgical Site Infection (SSI) in the general surgical population is serious health issue[1)].

There is evidence that optimized blood flow to the surgical incision decreases SSI rates through avoidance of hypothermia, hypoxia and decreased perfusion[2)].

In the WHO surgical site infection prevention guidelines, use of high fractions of inspired oxygen concentration ($FiO_2$) during the perioperative period is recommended to reduce SSI. Because additional oxygen contribute less to arterial oxygen content of blood and high $FiO_2$ has been reported to cause atelectasis[3)] and pulmonary toxicity[4)], use of high $FiO_2$ is still controversial.

**OBJECTIVES.** The aim of this study is to assess whether the use of high $FiO_2$ perioperatively reduce the SSI, and to investigate postoperative complications.

**METHODS.** 586 patients older than 18 years who were undergoing elective and emergency operation of gastrointestinal surgery were included. Patients who were hemodynamically unstable and had an American Society of Anesthesiology physical status 4 or 5 were excluded.

We compared patients who were supplied high $FiO_2$ with patients who were supplied lower inspired oxygen. Patients with the use of high $FiO_2$ were given 80% of oxygen during surgery and 6 hours after the surgery.

Surgical site infection within 30 days after surgery was determined by clinical assessment.

As the secondary outcome, respiratory complications and mortality were determined.

**RESULTS.** The overall incidence of SSI was 23.4%. The incidence of infection in the group with high $FiO_2$ had tendency to lower than the group with the patients with conventional treatment. (17% vs. 24%; P=0.09)

There were no significant differences between the groups in the postoperative complications.

**CONCLUSIONS.** Administration of high perioperative $FiO_2$ for the patients with gastrointestinal surgery might have some effect to reduce the risk of SSI.

The frequencies of pulmonary complications were not significantly different by the $FiO_2$.

**REFERENCE(S)**

1) Coello R et al, J Hosp Infect. 2005; 60:93-103.
2) Greif R et al, N Engl J Med. 2000;342:161-167.
3) Wanger PD et al, J Clin Invest. 1974;54:54-68.
4) Winter PM et al, Anesthesiology. 1972;37:210-241.

**GRANT ACKNOWLEDGMENT**

None reported.

### 0247

#### Prevalence and risk factors of preoperative sarcopenia and malnutrition in elderly cancerous patients

O. Chaiwat, W. Muangpaisan
Siriraj Hospital Mahidol University, Bangkok, Thailand
**Correspondence:** O. Chaiwat

*Intensive Care Medicine Experimental* 2018, **6(Suppl 2):**0247

**INTRODUCTION.** The aging population is associated with an increasing prevalence of frailty and comorbidities. In combination with the diagnosis of cancer, the incidence of perioperative adverse outcomes is considerably increased. Sarcopenia and malnutrition have been reported as the major risk factors for adverse outcomes during perioperative period[1]

**OBJECTIVES.** To identify the prevalence of sarcopenia and malnutrition in preoperative elderly cancerous patients who scheduled to have elective surgeries at Siriraj Preanesthesia Assessment Center (SiPAC) and to determine the risk factors and outcomes of sarcopenia patients

**METHODS.** The assessment of sarcopenia according to the Asia working group for sarcopenia[2] and malnutrition by the Mini Nutrition Assessment -Short Form[3] were performed at SiPAC. The inclusion criteria were the patients aged over 60 years old and had the diagnosis of cancer that required surgery

**RESULTS.** 226 patients were enrolled. Sarcopenia was found in 71 patients (31%). 31 patients (13%) were diagnosed presarcopenia. The incidence of malnutrition was 3-times lesser (9.3%). Older age [adjusted odd ratio (AOR) 1.14, CI (1.08-1.20)], lower scores of preoperative Barthel index [AOR 0.94, CI (0.91-0.98)] and malnutrition [AOR 5.01, CI (2.54-9.88)] were identified as independent risk factors for sarcopenia. Barthel index scores was significantly lower (p value = 0.01) and mortality rate was significantly higher (p value = 0.01) at 3 months after discharge from the hospital in sarcopenia patients compared to non-sarcopenia patients

**CONCLUSIONS.** The incidence of sarcopenia was high in preoperative elderly cancerous patients. Older age, impaired functional status and malnutrition were the risk factors for preoperative sarcopenia in this population

**REFERENCE(S)**

1. Correia MIT, Waitzberg DL. The impact of malnutrition on morbidity, mortality, length of hospital stay and costs evaluated through a multivariate model analysis. Clinical Nutrition. 2003;22(3):235-9
2. Chen LK, Liu LK, Woo J, Assantachai P, Auyeung TW, Bahyah KS, et al. Sarcopenia in Asia: consensus report of the Asian Working Group for Sarcopenia. J Am Med Dir Assoc. 2014;15(2):95-101.
3. Kaiser MJ, Bauer J, Ramsch C, Uter W, Guigoz Y, Cederholm T, et al. Validation of the Mini Nutritional Assessment Short-Form (MNA®-SF): A practical tool for identification of nutritional status. JNHA-The Journal of Nutrition, Health and Aging. 2009;13(9):782-8

**GRANT ACKNOWLEDGMENT**

The faculty of Medicine Siriraj Hospital, Bangkok, Thailand

### 0248

#### Regional cerebral oxygen saturation changes monitored during spinal neurosurgery in prone position and postoperative cognitive dysfunction evaluation

S. Murniece[1,2], B. Mamaja[1,3], I. Vanags[3,4]
[1]Riga East Clinical University Hospital, Riga, Latvia; [2]Riga Stradins University, Doctoral Studies, Riga, Latvia; [3]Riga Stradins University, Riga, Latvia; [4]Pauls Stradins Clinical University Hospital, Riga, Latvia
**Correspondence:** S. Murniece

*Intensive Care Medicine Experimental* 2018, **6(Suppl 2):**0248

**INTRODUCTION.** Significant numbers of patients present postoperative cognitive decline after non-cardiac surgery. Postoperative cognitive dysfunction (POCD) has a tendency to improve over a certain period of time, but some patients suffer from permanent cognitive impairment. Adequate intraoperative cerebral oxygen supply is one of the inciting causes for postoperative cognitive disturbances. Prone position, used during spinal neurosurgery, causes important physiological changes in a human body and can lead to impaired brain oxygen supply. Regional cerebral oxygen saturation monitoring devices are non-invasive and provide continuous, real time monitoring of brain tissue oxygenation.
**OBJECTIVES.**

1. To identify regional cerebral oxygen saturation (rScO2) intraoperative parameters in patients undergoing spinal neurosurgery in prone position.
2. To evaluate intraoperative rScO2 relationship with POCD.
3. To evaluate POCD in patients undergoing spinal neurosurgery in prone position but not receiving intraoperative rScO2 monitoring (control group).

**METHODS.** 48 patients were scheduled for spinal neurosurgery in prone position. In the study group (n=38) rScO2 was intraoperatively continuously monitored using INVOS 4100 NIRS device. The control group (n=10) didn't receive intraoperative rScO2 monitoring. All patients received standard general anaesthesia. During the surgery every 5 min in both groups MAP, HR, SpO2, EtCO2 was fixed. We also fixed intraoperative blood loss, duration of the operation. Cognitive function was assessed in both groups using Montreal - Cognitive Assessment (MoCA) scale before surgery and 2 days after. MoCA scores range between 0-30. 26 or over is considered to be normal.
**RESULTS.** We didn't observe any significant changes in our calculated medium rScO2 intraoperative values. During the whole surgery when patients were lying supine rScO2 was 72±9%, in prone position rScO2 was 73± 9%. We didn't observe differences in medium MoCA scores when comparing study and control group. MoCA score before surgery in study group was 24±3 points and 23±4 points in control group. MoCA 2 days after the surgery was 25 ±3 points in study group, 23±3 points in control group.
In our study group 1 patient showed POCD-from MoCA 27 points before surgery to MoCA 23 points after the surgery. We didn't observe any significant changes in other patients' measurements that would differ from average in the study group.
In control group in 6 from 10 patients we observed MoCA score decrease for 1-2 points after the surgery.
**CONCLUSIONS.** No significant changes were observed in medium MoCA scores between patients who intraoperatively received non-invasive cerebral oxygen saturation monitoring and patients who did not receive it.
Intraoperative regional cerebral oxygen saturation monitoring can help to obviate cerebral desaturation that can lead to postoperative cognitive decline.

### 0250
### Genetic polymorphism of pleiotrophine is associated with severity of cancer pain but not postoperative pain

Y. Sudo[1], M. Sumitani[2], D. Nishizawa[3], K. Ikeda[3], H. Abe[2], R. Inoue[1], J. Hozumi[1], M. Sumitani[4], Y. Yamada[1]
[1]University of Tokyo Hospital, Department of Anesthesiology and Pain Relief Center, Tokyo, Japan; [2]University of Tokyo Hospital, Department of Pain and Palliative Medicine, Tokyo, Japan; [3]Tokyo Metropolitan Institute of Medical Science, Department of Psychiatry and Behavioral Science, Tokyo, Japan; [4]Keiyu ENT Clinic, Tokyo, Japan
**Correspondence:** Y. Sudo
*Intensive Care Medicine Experimental* 2018, **6(Suppl 2):**0250

**INTRODUCTION.** Pleiotrophin (PTN) is one of nerve growth factors, which particularly promotes neurite growth. PTN is higher expressed in dorsal root ganglions and the dorsal horn of the spinal cord in a neuropathic pain model involving with improvement of allodynia and nerve recovery. Further, gene mutation of PTN reportedly affects nociceptive transmission and opioid sensitivity in the spinal cord. However, clinical significance of PTN has not yet been investigated.
**OBJECTIVES.** We examined the relationship between polymorphism of PTN and individual variation of cancer pain severity.
**METHODS.** Patients with cancer pain (n=89) and patients with postoperative pain after laparotomy (n=57) participated. Regarding 53 single nucleotide polymorphisms (SNPs) in the PTN gene, each patient-group was categorized into into three sub-groups, major allele homozygosity (mAajor), heterozygosity (hetero), and minor allele homozygosity (mInor). We analyzed associations among respective SNPs and pain intensity, and opioid consumptions (translating into intravenous fentanyl-equivalent dosages: mcg/kg/day). We used non-parametric comparison tests and corrections if necessary.
**RESULTS.** One of SNPs of the PTN gene (rs11764598) was associated with cancer pain; mAjor demonstrated significantly stronger pain intensity than other groups (mAjor 6.3+/-1.7, hetero 4.6+/-1.4, mInor 3.0+/-0.7; K-W test, p< 0.0001). There was no defference in opioid consumptions among three sub-groups of cancer pain. No patients which had mInor SNPs in postoperative pain patients, and no difference in both pain intensity and opioid consumptions between mAjor and hetero subgroups.
**CONCLUSIONS.** PTN is known to have relationships with nociceptive transmission and opioid analgesia, and cancer pain severity followed these in the present study. On the other hand, postoperative pain is considered as nociceptive pain, but postoperative pain did not demonstrate such relationships. PTN contributes to recovery from a neuropathic pain condition. Different pathophysiological mechanisms of cancer pain group and postoperative pain in the present patient groups might affect the findings because many cancer pain patients suffered from neuropathic cancer pain.

**REFERENCE(S)**

1) E Gramage, et al. "The heparin binding growth factors midkine and pleiotrophin regulate the antinociceptive effects of morphine through α2-adrenergic independent mechanisms" Pharmacol Biochem Behav. 2012 May;101(3):387-93
2) E Gramage, et al. "Genetic deletion of pleiotrophin leads to disruption of spinal nociceptive transmission: evidence for pleiotrophin modulation of morphine-induced analgesia" Eur. J. Pharmacol. 2010;647, 97-102

**GRANT ACKNOWLEDGMENT**
We thank to MHLW TR-Cancer Pain Reseach Group.

### 0251
### Frequency of occurrence of arterial hypotension in anesthesia, including alpha2-adrenoagonists in neurooncological patients

M. Rumiantceva, R. Nazarov, A. Kondratiev
Russian Polenov Neurosurgical Institute, Saint Petersburg, Russian Federation
**Correspondence:** M. Rumiantceva
*Intensive Care Medicine Experimental* 2018, **6(Suppl 2):**0251

**INTRODUCTION.** An integral part of neuroregulatory systems of the brain stem are opioid and adrenergic antinociceptive systems. The use of anesthesia, including a combined effect on opioid and adrenergic antinociceptive systems, creates favorable conditions for performing operations on brain tumors. Arterial hypotension is the most common side effect of alpha2-adrenoagonists.
**OBJECTIVE.** Frequency of use of vasopressors with anesthesia, including alpha2-adrenoagonists in neurosurgical operations.

**METHODS.** The study included 118 patients (mean age 50.5±13.6) who underwent planned surgical treatment at the RNHI named after prof. A.L.Polenov about brain tumors (supratentorial localization - 25% and subtentorial - 75%).

In all patients induction of anesthesia included: muscle relaxants (pipecuronium 0.1mg/kg or rocuronium 0.6mg/kg), hypnotics (propofol 2mg/kg), opioid analgetic (fentanyl 4.8±0.6mcg/kg) + alpha2-adrenoagonist (clonidine or dexmedetomidine). Maintenance of anesthesia: hypnotic (propofol 4.5±0.9mg/kg/h), opioid analgetic (fentanyl 1.3±0.4mcg/ kg/h) + alpha2-adrenoagonist (clonidine or dexmedetomidine).

All patients were divided into three groups, depending on the alpha2-adrenoagonist used and its dosage. In group I (26 patients) induction of anesthesia: clonidine 1.5±0.4mcg/kg; maintenance of anesthesia: clonidine 0.4±0.15mcg/kg/h. In group II (58 patients) induction of anesthesia: dexmedetomidine 1.5±0.4mcg/kg; maintenance of anesthesia: dexmedetomidine 0.4±0.2mcg/kg/h. In group III (34 patients) induction of anesthesia: dexmedetomidine 0.7±0.1mcg/kg; maintenance of anesthesia: dexmedetomidine 0.2±0.1 mcg/kg/h. All three groups are statistically comparable by sex, age, initial blood pressure, initial heart rate, position on the operating table and localization of the brain tumor.

**RESULTS.** In group I, 5 patients (19%) had arterial hypotension, which required the use of vasopressors (mezaton). Two out of five patients immediately after induction of anesthesia, and in three patients after shrinkage on the operating table (operations in the sitting position). In two out of five cases, the use of vasopressors was required before the end of the operation. In group II only one patient (1.7%) had arterial hypotension. Arterial hypotension was noted after shrinkage on the operating table (operation in the sitting position), the need for the use of a vasopressor was short-lived. In group III, 3 patients (9%) had arterial hypotension. In all three cases, vasopressors were used after shrinkage on the operating table. In two of the three cases, the need remained until the tumor was removed. In one of three cases, the application was short-lived.

**CONCLUSIONS.** The combined use of opioid analgesic and alpha2-adrenoagonist mutually reduces the frequency of side effects and at the same time creates optimal conditions for performing neurosurgical operations. When using dexmedetomidine, arterial hypotension is much less common than with clonidine.

### 0252

#### Sedatives for use during gastrointestinal endoscopic procedures

Y. Seo[1], H. Kim[2], J. Kim[1]

[1]Yonsei University College of Medicine, Department of Anesthesiology and Pain Medicine, Seoul, Korea, Republic of; [2]Ajou University School of Medicine, Department of Anesthesia and Pain Medicine, Suwon, Korea, Republic of

**Correspondence:** Y. Seo

*Intensive Care Medicine Experimental* 2018, **6(Suppl 2):**0252

**INTRODUCTION.** Gastrointestinal endoscopy is gradually expanding the diagnostic capacity and therapeutic field of the gastroenterologist. Appropriate patient tolerance is essential to successfully complete a safe endoscopic procedure and subsequent follow-up. Key aspects of proper sedation include selection of the optimal sedative and continuous monitoring of the patient during the procedure. Traditional sedation is performed using intravenous benzodiazepines such as midazolam (gamma-aminobutyric acid [GABA] agonists) and short-acting opiates, resulting in moderate sedation. Recently, the use of alternative sedatives such as propofol (phenolic derivative) and dexmedetomidine (selective alpha-2 agonist) has increased.

**OBJECTIVES.** We compared the safety of sedatives (Midazolam, propofol, and dexmedetomidine) for use during gastrointestinal endoscopic procedures.

**METHODS.** We searched for all relevant randomized controlled trials published until September 2017, and examined the incidence of adverse events (desaturation, bradycardia, and hypotension) according to the type of sedative used during gastrointestinal endoscopy. A network meta-analysis was performed using the Review Manager 5 and R packages.

**RESULTS.** Fifty-four studies and 7,057 patients were included in this network meta-analysis. The risks of desaturation when using propofol and propofol with midazolam were 2.56 and 3.46 times higher than that of dexmedetomidine (odds ratio [OR], 2.56; 95% confidence interval [CI], 1.14-5.74 vs. OR, 3.46; 95% CI, 1.47-8.12, respectively). In terms of hemodynamics, the risks of bradycardia using midazolam and propofol were 0.22 times and 0.25 times lower than when using dexmedetomidine (OR, 0.22; 95% CI, 0.10-0.50 vs. OR, 0.25; 95% CI, 0.11-0.59, respectively). Hypotension was more common when using propofol and propofol with midazolam than when using midazolam (OR, 2.40; 95% CI, 1.95-2.95 vs. OR, 2.35; 95% CI, 1.63-3.38, respectively).

**CONCLUSIONS.** Our network meta-analysis provided a better understanding of the comparative safety of sedatives used during gastrointestinal endoscopy. Dexmedetomidine had less of a respiratory depression effect and increased the risk of bradycardia compared to other sedatives. Patients who received dexmedetomidine had a similar risk of development of hypotension compared to midazolam, and propofol use was twice as likely to cause hypotension as midazolam in gastrointestinal endoscopy.

**REFERENCE(S)**

1. Wadhwa V, Issa D, Garg S, et al. Similar Risk of Cardiopulmonary Adverse Events Between Propofol and Traditional Anesthesia for Gastrointestinal Endoscopy: A Systematic Review and Meta-analysis. Clin Gastroenterol Hepatol 2017;15:194-206.
2. Tsai HC, Lin YC, Ko CL, et al. Propofol versus midazolam for upper gastrointestinal endoscopy in cirrhotic patients: a meta-analysis of randomized controlled trials. PLoS One 2015;10:e0117585.

### 0253

#### Effect of transfusion ratio on mortality in patients undergoing massive transfusion during cytoreductive surgery: a retrospective cohort study

S. Pawar[1], K. Deshpande[1], D. Morris[2]

[1]St George Hospital, Intensive Care, Sydney, Australia; [2]St George Hospital, Liver Surgery, Sydney, Australia

**Correspondence:** S. Pawar

*Intensive Care Medicine Experimental* 2018, **6(Suppl 2):**0253

**INTRODUCTION.** The evidence suggests favorable effects of blood transfusion with high plasma and platelet to RBC ratios on the outcomes of patients receiving MT. Our local massive transfusion (MT) protocol recommends that red blood cells (RBC), plasma (FFP), and platelets should be administered in 1:1:1 ratio. The adherence to the recommendation, and its impact on the clinical outcome f peritonectomy patients has not been described.

**OBJECTIVES.**

1. To describe characteristics and outcomes of patients undergoing peritonectomy with heated intraperitoneal chemotherapy (HIPEC) who received MT.
2. To determine the ratios of blood products transfused and to evaluate the predictors of mortality.

**METHODS.** This was a retrospective cohort study involving patients who received MT during peritonectomy from January 2009 to December 2017 at St George Hospital. Data on patient demographics, diagnosis, surgery, blood products transfused, length of stay, morbidity and mortality were collected from peritonectomy database. Appropriate descriptive statistics were used to describe patients' baseline characteristics. The distribution of patient characteristics and clinical outcome were compared using chi-square test or fisher exact test for categorical parameters, and independent t-test, non-parametric Wilcoxon test, and Mann-Whitney U test for continuous variables. A multiple logistic regression analysis was performed to determine predictors of mortality.

**RESULTS.** Out of 896 patients a total of 338 (38%) patients (mean age 55.8±12.7 years, 43.2% males, were included in the analysis. Of the 338 patients who underwent surgery 126 (37.3%) were diagnosed with appendicular carcinoma, 22.5% with colorectal cancer, 20.7% with DPAM and mesothelioma and ovarian cancer constituted 8% and 5.9% each. No patient received transfusion with blood products using the RBC: FFP: Platelets ratio of 1:1:1. Compliance with 1:1 ratio of FFP: RBC and Platelets: RBC was 70.5 % and 6.5% respectively. The overall hospital mortality rates 3.9 %, while 46.1 % patients died during 8 years follow up. Patients who received FFP: RBC ratio > 1 had survival benefit of 2.5 years with hazard ratio of 0.62. On multivariate analysis, ratio FFP: RBC transfusion > 1, was an independent predictor of decreased mortality, while completeness of cytoreduction score (CC2/3), diagnosis of ovarian Ca were independent predictors of increased mortality.

**CONCLUSIONS.** The compliance with the recommended 1:1:1 ratio of blood products was poor. There was significant survival benefit with more FFP transfusion compared to packed red blood cells. Large multicenter randomized controlled trial is warranted to confirm the benefit of higher FFP and Platelet ratios during massive transfusion.

**REFERENCE(S)**

Saxena A et al.Effectiveness of early and aggressive administration of fresh frozen plasma to reduce massive blood transfusion during cytoreductive surgery. J Gastrointest Oncol 2013;4(1):30-39

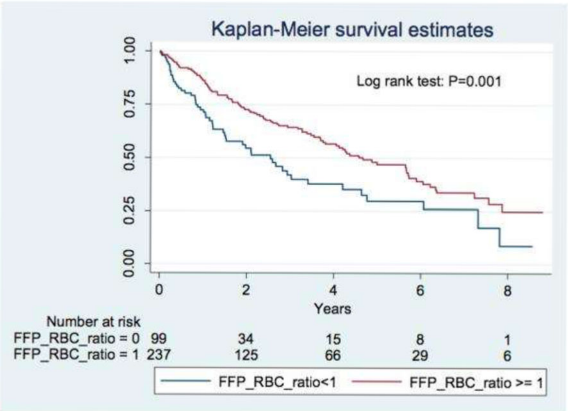

**Fig. 1 (abstract 0253).** Kaplan Meier Curve demonstrating survival benefit with FFP:RBC ratio >1:1

### 0254

#### Do statins help prevent VTE in haemorrhagic stroke during the acute period?

K. Jo
Bucheon St. Mary's Hospital, The Catholic University of Korea, Neurosurgery, Bucheon, Korea, Republic of
*Intensive Care Medicine Experimental* 2018, **6(Suppl 2):**0254

**INTRODUCTION.** Venous thromboembolism (VTE) is a common disease affecting more than one per 1000 persons each year. Patients with stroke have a relatively high risk of VTE because of immobility and increased prothrombotic activity. Statins represent a mainstay in the prevention of arterial events. However, two recent meta-analyses suggested that statins may also reduce the incidence of VTE by 10-20%. The ability to decrease tissue factor expression offers a biological rational for statin's anti-thrombotic effect. These studies showed that statin medication for more than 3 months helps prevent VTE.

**OBJECTIVES.** The effect of statins on venous thromboembolism (VTE) especially pulmonary embolism (PE) is debatable. This study investigated whether statin medication can decrease the occurrence of VTE in patients with haemorrhagic stroke during the acute period.

**METHODS.** This is a retrospective study of patients with haemorrhagic stroke between March 2011 and December 2013. Patients with newly diagnosed haemorrhagic stroke were observed during 6 weeks of hospitalisation. Occurrence determined using Doppler ultrasound and computed angiography was used to assess risk factors of VTE in patients with haemorrhagic stroke during the acute period. The difference and incidence of the VTE among acute haemorrhagic stroke was analysed in patients who did not receive no statin medication, who received statin medication after the current haemorrhagic stroke, and who received statin medication after previous haemorrhagic stroke.

**RESULTS.** Among 98 patients, 9 (9.2%) patients representing 3 from each group had VTE (6 for deep vein thrombosis and 3 for PE) during the follow-up. Each incidence of VTE was 6.4%, 13.6%, and 10.3% in patients who did not receive statins, who received statin medication after the current haemorrhagic stroke, and who received statin medication after previous haemorrhagic stroke, respectively (p>0.05).

**CONCLUSIONS.** Average dose of statin use was not associated with a reduced risk of VTE in patients with haemorrhagic stroke, especially in the acute period.

## Antibiotic therapy in septic patients

### 0255

#### Clinical impact of the use of procalcitonin-based algorithms for duration of antibiotic treatment in critical adult patients: a meta analysis of randomized clinical trials

A. Gutiérrez-Pizarraya[1], M.C. León-García[2], R. de Juan-Idígoras[2], J. Garnacho-Montero[1]
[1]Hospital Virgen Macarena, Intensive Care Unit, Seville, Spain; [2]Hospital Virgen Macarena, Seville, Spain
**Correspondence:** J. Garnacho-Montero
*Intensive Care Medicine Experimental* 2018, **6(Suppl 2):**0255

**OBJECTIVES.** To assess the impact on 28-day mortality and ICU stay as well as antimicrobial therapy duration of a strategy based on an algorithm based on the sequential measurement of procalcitonin compared to usual clinical practice.

**METHODS.** Two independent reviewers performed a systematic search in PubMed, Embase, ISI Web of Knowledge, BioMed Central, ScienceDirect and the Cochrane Central Register of Controlled Trials. Eligible studies had to be clinical trials comparing a procalcitoninguided antimicrobial therapy strategy with the standard of care in patients with sepsis admitted to the ICU. The search was restricted to articles in English and published before December 31th, 2017. Studies in the pediatric population were excluded. Heterogeneity and inconsistency were evaluated using the test with the $I^2$ parameter, the variance between Tau2 studies and the Cochrane Q test. The publication bias was also evaluated with the Egger method.

**RESULTS.** Ten clinical trials met the inclusion criteria with a total of 4249 patients (2122 in the PCT-guided therapy arm and 2127 in the control group). The control group of a clinical trial included an algorithm based on C-reactive protein. All the studies included patients with severe sepsis except one that was performed in patients with ventilation-associated pneumonia. The antimicrobial therapy guided with PCT was a protective factor of 28-day mortality (RR = 0.88, 95% CI 0.79-0.99, p = 0.040). Q test = 5.4; p = 0.788. There was no heterogeneity among studies (variance between studies Tau2 = 0, Q test = 5.4, p = 0.788). This strategy also decreased the duration of antimicrobial therapy (-1.97 days, IC95% -1.19, -2.76; p< 0.001) as well as the ICU length of stay (-1.2 days 95%CI -4.15, 1.74; p< 0.001).

**CONCLUSION.** Our meta-analysis produced evidence that, in critically ill adults with sepsis, a procalcitonin-guided strategy is associated with a significant reduction in mortality and also in the duration of antimicrobial therapy as well as in the length of ICU stay.

### 0256

#### Delayed antibiotic therapy and deterioration of organ dysfunction in critically-ill septic patients in the emergency department

M.G. Kim[1], S.Y. Hwang[1,2], J.K. Shin[1], I.J. Jo[1], H. Yoon[1,2], J.H. Park[1,2], W.C. Cha[1], M.S. Sim[1], T.G. Shin[1], J.-M. Kim[1]

[1]Samsung Medical Center, Sungkyunkwan University School of Medicine, Department of Emergency Medicine, Seoul, Korea, Republic of; [2]Kangwon National University School of Medicine, Department of Emergency Medicine, Gangwon, Korea, Republic of

**Correspondence:** J.-M. Kim

*Intensive Care Medicine Experimental* 2018, **6(Suppl 2):**0256

**INTRODUCTION.** Sepsis and septic shock are life-threatening conditions that arise when the dysregulated host response to microbial infection injures their own organ system (1). It affects millions of people worldwide, and one of the leading causes of morbidity and mortality (2). The SCC guidelines recommend broad-spectrum antibiotic therapy to be initiated as soon as possible after recognition and within 1 hour of for both sepsis and septic shock (3).

**OBJECTIVES.** We investigated the effect of timing of antibiotic therapy on outcomes assessed by the changes in surrogate markers of organ failure including platelet, serum bilirubin, and serum creatinine, which are components of SOFA score.

**METHODS.** This was a single-center, retrospective observational study of patients with critically-ill septic patients who presented to the emergency department (ED). The study period was from August 2008 to September 2016. The primary outcome was 48 hours changes in platelet, serum bilirubin, and serum creatinine (δ platelet=platelet count at ED enrolled - platelet count after 48 hours, δ serum bilirubin=serum bilirubin at ED enrolled - serum bilirubin after 48 hours, δ serum creatinine=serum creatinine at ED enrolled - serum creatinine after 48 hours, respectively). A multivariable linear regression model was developed to assess variables related to outcomes (δ platelet, δ serum bilirubin, and δ serum creatinine).

**RESULTS.** We analyzed 1,784 patients who met the inclusion criteria. Of the eligible patients, the overall 28-day mortality was 14% (n =256/1784). In multivariable linear regression analysis, hourly delay of antibiotic therapy was significantly associated with an increase in the δ platelet (coefficient, 1636.468; standard error, 749.786; P=0.029), and decrease in the δ serum bilirubin (coefficient, -0.055; standard error, 0.021; P=0.008). hourly delay of antibiotic therapy was not associated with δ creatinine (coefficient, -0.008; standard error, 0.010; P=0.437)

**CONCLUSIONS.** Hourly delay of antibiotic therapy was associated with decreased platelet count and increased serum bilirubin concentration in critically-ill septic patients during the first 48 hours after ED arrival.

**REFERENCE(S)**

1. Singer M, Deutschman CS, Seymour CW, Shankar-Hari M, Annane D, Bauer M, et al. The Third International Consensus Definitions for Sepsis and Septic Shock (Sepsis-3). Jama. 2016;315(8):801-10.
2. Martin GS, Mannino DM, Eaton S, Moss M. The epidemiology of sepsis in the United States from 1979 through 2000. The New England journal of medicine. 2003;348(16):1546-54.
3. Rhodes A, Evans LE, Alhazzani W, Levy MM, Antonelli M, Ferrer R, et al. Surviving Sepsis Campaign: International Guidelines for Management of Sepsis and Septic Shock 2016. Critical care medicine. 2017;45(3):486-552.

**Grant Acknowledgment**

Not applicable

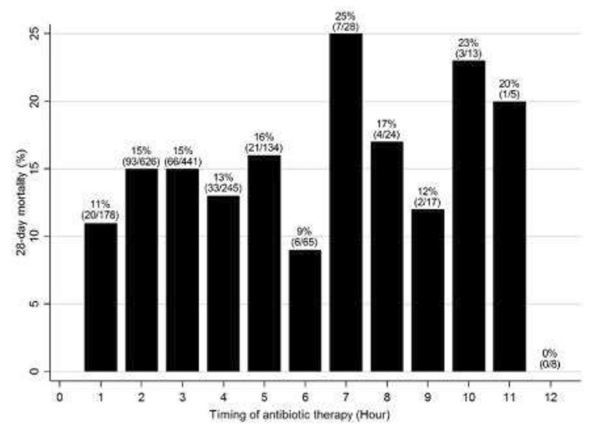

**Fig. 1 (abstract 0256).** Anti-time and mortality

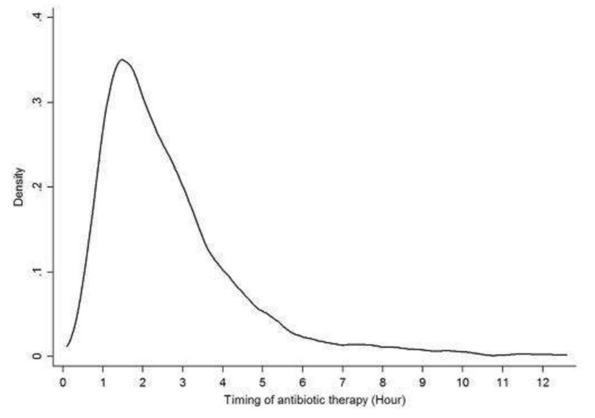

**Fig. 2 (abstract 0256).** Timing of antibiotic therapy

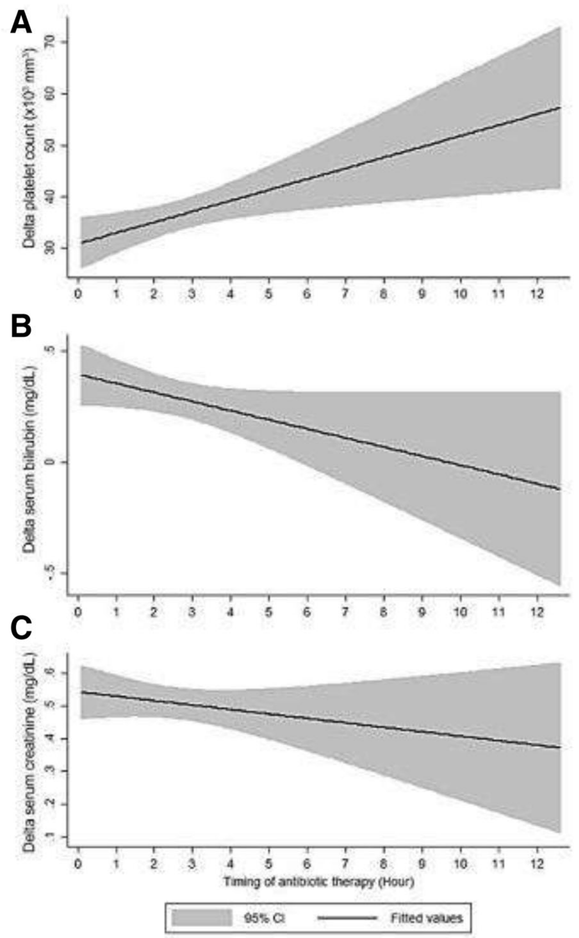

**Fig. 3 (abstract 0256).** Results of delta platelet, serum bilirubin, serum creatinine

### 0257

#### Relationship between antibiotic desescalation and microbiological documentation. Influence on outcome of sepsis patients detected by a sepsis team in a teaching hospital

R. Zaragoza, V. Ramirez, S. Sancho, R. González, C. Ibañez, C. Martínez, C. López, J.J. Camarena
Hospital Universitario Dr. Peset, Valencia, Spain
**Correspondence:** R. Zaragoza
*Intensive Care Medicine Experimental* 2018, **6(Suppl 2):**0257

**INTRODUCTION.** Although threre is positive evidence for desescalation in ICU patiens, no enough data about this strategy in sepsis patients and its relationship with microbiological documentation (MD) has been provided.

**OBJECTIVES.** The aim of this study were to asses the principal factors associated to desescalation strategy (DS) in patients with severe sepsis and septic shock, to describe the relationship of DS and MD, to know their updated prognosis and specially to analyze the influence on global mortality of antibiotic desescalation of these septic patients.

**METHODS.** During a four years and a half period ( October-2012-March 2017), 2405 severe sepsis and septic shock patients detected by a sepsis unit form ICU using an electronic automatic alert in a teaching hospital were prospectively evaluated. Clinical and microbiological variables were recorded. Any patient with a confirmed sepsis was potentially eligible through the whole hospital. DS was defined as narrowing of the antimicrobial spectrum based on the susceptibility of the pathogen or based in clinical criteria of good evolution. Several multivariate analysis was performed to describe independently factors associated to global mortality in these patients using SPSS package (16.0).

**RESULTS.** Among 2405 electronic activations 667 of them corresponded to septic shock (27.7%). Only 27.1% of patients were admitted to ICU. Their mean APACHE II and SOFA score were 17.9±6.06 and 5.18±3.3 respectively. The most frequent sources of infections were the respiratory focus (39.5%), urinary (26.6%) and abdominal (18.5%). Global mortality was 20.7%. The majority of episodes were community adquired (56.4%). Associated bacteremia was present in 34.2% of episodes. MD was achieved in 52.6 %. DS was present in 29.1% of the episodes. Statistical significant factors associated to DS in univariate analysis ($p < 0.05$) were: MD (39,7% vs. 19%), ICU admission (40% vs. 23.7%), septic shock (34,4% vs. 26.8%), bacteremia (45.2% vs. 24.8%) and adequate empirical antibiotic treatment (31.4% vs. 15.1%). DS was associated to a lower global mortality (13% vs 28,4%; p= =0.0001) There were no differences in mortality rates among DS in patients with MD or not. (12.2% vs 14.4%) Multivariate analysis did not confirm DS (OR 1.7; CI95% 0.59-4.94) as a independently factor associated to global mortality.

**CONCLUSIONS.** DS was achieved in almost one of three patients with sepsis detected by a sepsis team in a teaching hospital. MD, ICU admission, bacteremia, adequate empirical antibiotic treatment and septic shock were associated to DS. DS with no MD was nor associated with higher mortality. DS had no any influence on global mortality.

##### REFERENCE(S)

1. Garnacho-Montero J, Gutiérrez-Pizarraya A, Escoresca-Ortega A et al. De-escalation of empirical therapy is associated with lower mortality in patients with severe sepsis and septic shock. Intensive Care Med. 2014 Jan;40(1):32-40.

### 0258

#### Impact of appropriate empirical antibiotics on clinical outcome in *Klebsiella pneumoniae* bacteraemia in the intensive care unit

M.Y. Man, K.C. Li, H.P. Shum, W.W. Yan
Pamela Youde Nethersole Hospital, Department of Intensive Care, Hong Kong, Hong Kong, China
**Correspondence:** K.C. Li
*Intensive Care Medicine Experimental* 2018, **6(Suppl 2):**0258

**INTRODUCTION.** Klebsiella Pneumoniae (KP) is a common Gram-negative bacterium causing a myriad of infections such as pneumonia, liver abscess, urinary tract infection and other nosocomial infections. Treatment options have been reduced due to the emergence of multi-drug resistant strains worldwide. Inappropriate empirical antibiotics in septic shock are associated with poor outcome, yet the impact on confirmed bacteremia is not well studied.

**OBJECTIVES.** We aim to evaluate the impact of appropriate empirical antibiotics on clinical outcomes in KP bacteraemia in critical care setting.

**METHODS.** Adults admitted to intensive care unit of a regional hospital in Hong Kong with KP bacteraemia from January 2009 to June 2017 were retrospectively reviewed. Demographics, antibiotics, microbiology and outcomes were identified and analyzed. Cox regession analysis was performed to determine independent predictors for 90-day mortality and logistic regression analysis was used to identify independent predictors for patients to have received inappropriate empirical antibiotics.

**RESULTS.** During the study period, a total of 205 patients with KP bacteraemia were identified, in which 148 received appropriate empirical antibiotics (72.20%) while 57 did not (27.80%). Inappropriate or no empirical antibiotics was associated with significantly higher 90-day mortality (HR 3.001; 95% CI 1.856-4.854; $p < 0.001$). Other independent predictors include: patient with concomitant congestive heart failure (HR 2.281; 95% CI 1.141-4.561; p=0.02), medical patients (HR 1.944; 95% CI 1.132-3.340; p=0.016), higher total SOFA score (HR 1.169; 95% CI 1.101-1.242; $p < 0.001$). Patients with chronic kidney disease or end stage renal failure (OR 4.802, 95% CI 1.563-14.759, p=0.006), infection caused by ESBL or carbapenam resistance strain (OR 4.339, 95% CI 1.667-11.259, p=0.003), patients who were mechanically ventilated (OR 4.066, 95% CI 1.831-9.030, p=0.001) were more likely to have received inappropriate empirical antibiotics.

**CONCLUSIONS.** Appropriate antibiotics improve mortality of patients with KP bacteraemia. Patients with pre-existing chronic kidney disease or end stage renal failure, infection by ESBL or carbepenam resistance strain and mechanically ventilated patients are more likely to have received inappropriate antimicrobials treatment.

### 0259

#### Early empirical antimicrobial therapy does not prevent sepsis development in critically ill surgical patients with suspected nosocomial infection

E. Bassi[1,2], B.M. Tomazini[1], A.R.d.O. Siqueira[1], S.R.d.O. Siqueira[1], B.V. Carneiro[1], P.F.G.M.M. Tierno[1], F.M. Cadamuro[1], R.A.G. de Oliveira[1], T. Guimarães[3], F.F. Novo[1], E.M. Utiyama[1], L.M.S. Malbouisson[4]
[1]Hospital das Clínicas da Universidade de São Paulo (HC-FMUSP), Surgery and Trauma Discipline, São Paulo, Brazil; [2]Hospital Alemão Oswaldo Cruz, Critical Care, São Paulo, Brazil; [3]Hospital das Clínicas da Universidade de São Paulo (HC-FMUSP), Infectious Diseases, São Paulo, Brazil; [4]Hospital das Clínicas da Universidade de São Paulo (HC-FMUSP), Anesthesiology, São Paulo, Brazil
**Correspondence:** B.M. Tomazini
*Intensive Care Medicine Experimental* 2018, **6(Suppl 2):**0259

**INTRODUCTION.** Indiscriminate antibiotic use is a significant concern in critical care medicine and is known to be associated with increased drug-induced bacterial resistance among other adverse effects. Although evidence shows that early administration of antibiotics in patients with septic shock decreases in-hospital mortality[1], there is a lack of studies evaluating the role of early empirical antibiotics in patients with suspected nosocomial infection without sepsis.

**OBJECTIVES.** To compare early empiric antibiotic treatment with a conservative approach in critically ill surgical patients with suspected nosocomial infection without sepsis.

**METHODS.** A retrospective cohort of adult patients admitted to a Surgical ICU of a tertiary university hospital between 2012-2016. Early empiric antibiotic group was defined by immediate initiation of antibiotic therapy once infection was suspected, while in the conservative group, antibiotics were initiated only if a clear infectious focus, positive cultures, or sepsis/septic shock (Sepsis 3.0 definition) developed. The primary outcome was a composite of death, sepsis or septic shock in 14 days. A logistic regression model, including variables with p-value < 0.2 in the univariate regression and other clinical relevant variables, was used to estimate the association of risk factors and primary outcome.

**RESULTS.** From 2007 ICU admissions, 342 patients with suspected nosocomial infection without an obvious source or sepsis were included. Patients characteristics and outcomes are shown in table 1. Trauma represented 70% of the study population. There was no difference in age, SOFA at inclusion, SAPS 3, mechanical ventilation at inclusion, or in-hospital mortality between groups. Only 57% of patients in the conservative group had received antibiotics by day 14.

The multivariate analysis (table 2) showed that early antibiotic therapy, mechanical ventilation, and age were associated with the primary outcome. Vasopressors at inclusion, SAPS 3, SOFA, gender, SIRS, traumatic brain injury, and cause of ICU admission were not associated with the outcome.

**DISCUSSION.** Our results show that early empirical antimicrobial therapy does not decrease the incidence of sepsis, septic shock or death within 14 days. The concept of giving early empirical antibiotic treatment for critically ill patients with suspected infection is extrapolated from data of patients with sepsis or septic shock. However, our study shows that early empirical antibiotic treatment does not prevent sepsis in critically ill surgical patients with suspected nosocomial infection and might be harmful. A conservative approach can decrease antibiotic use by 43% and might be associated with better outcomes.

1. Seymour CW, Gesten F, Prescott HC et al. Time to Treatment and Mortality during Mandated Emergency Care for Sepsis N Engl J Med. 2017; 376(23):2235-2244.

**Table 1 (abstract 0259).** See text for description

| | Conservative group | Early empiric antibiotic group | p |
|---|---|---|---|
| N, % | 212 (62) | 130 (38) | |
| Age, years mean, (SD) | 44.3 (18.3) | 44 (17.7) | 0.89 |
| SOFA mean, (SD) | 5.24 (3.15) | 5.65 (3.08) | 0.235 |
| Admission SAPS 3 mean, (SD) | 54.9 (14.3) | 55.7 (12.15) | 0.61 |
| SIRS, % | 74 | 86 | 0.006 |
| Mechanical ventilation, % | 54 | 59 | 0.324 |
| Received antibiotics, % | 57 | 100 | <0.001 |
| ICU length of stay median, (IQR) | 18 (13-29) | 23 (24-37) | 0.025 |
| In-hospital mortality, % | 33 | 41 | 0.164 |

**Table 2 (abstract 0259).** See text for description

| | Multivariate analysis | | |
|---|---|---|---|
| | Odds ratio | 95% CI | p |
| Early empiric antibiotic group | 1.845 | 1.15-2.94 | 0.01 |
| Age | 1.014 | 1.001-1.027 | 0.032 |
| Mechanical ventilation | 1.835 | 1.15-2.92 | 0.011 |

### 0260

#### Time to antibiotic as a mortality factor in patients with sepsis and septic shock: a multicentric, point prevalence study

G. Merinos-Sánchez[1,2], L.A. Gorordo-Delsol[2,3], N. Medveczky-Ordoñez[3], S.E. Uribe-Moya[4], R. Estrada-Escobar[1], M.A. Amezcua-Gutiérrez[3], M.A. Morales-Segura[5], RENASE - Urgencias 2017
[1]Hospital General de México 'Dr. Eduardo Ligeaga', Adults Emergency Service, Cuauhtémoc, Mexico; [2]Fundación Sepsis México, Research Committee, Ciudad de México, Mexico; [3]Hospital Juárez de México, Adults Intensive Care Unit, Gustavo A. Madero, Mexico; [4]Hospital Juárez de México, Cardiology, Gustavo A. Madero, Mexico; [5]Instituto Politécnico Nacional, Escuela Superior de Medicina - Sección es Estudios de Posgrado e Investigación, México City, Mexico
**Correspondence:** G. Merinos-Sánchez
*Intensive Care Medicine Experimental* 2018, **6(Suppl 2):**0260

Within the goals of the management of sepsis, it has been pointed out that the shorter time to antibiotic has a positive impact on survival, although the exact time of greatest impact has not been defined (1-3). In Mexico it is estimated that close to 50 to 60% of emergency admissions were related to sepsis, of which 10% were admitted with septic shock (4).

**OBJECTIVES.** To establish the risk of 30-day mortality associated with the time of administration of the first dose of antibiotic in patients with sepsis and septic shock in the Emergency Department.

**METHOD.** A multicentre, single-day study was conducted (September 13, 2017), where all patients diagnosed with sepsis or septic shock were included, according to the Sepsis-3 criteria (5), admitted to the Emergency Department during the 24 hours a day, they were recorded on a digital platform (www.sepsismexico.org) and followed up for 30 days. The data analysis was performed in SPSS v.21.

**RESULTS.** 68 hospitals participated, 307 patients were included (56% women), of which 287 received antibiotic during their stay in the Emergency Room, the common odds ratio was estimated by the Mantel-Haenzel method to compare by hour, among the first six hours of treatment, to obtain the risk of mortality, days of stay> 5 days and to develop shock during the following 30 days Risk of mortali... Risk of length ... Risk of develop...

**DISCUSSION.** We observed a tendency to increase mortality and the risk of progressing from sepsis to septic shock to longer time to antibiotic, however, it is not a significant result, this can be explained by other elements of the treatment such as quantity and type of intravenous solutions employees, accumulated fluid balance, time of hypotension before vasopressors, time to surgical resolution and others. We found a significant risk to increase the days of stay in relation to time to antibiotic.

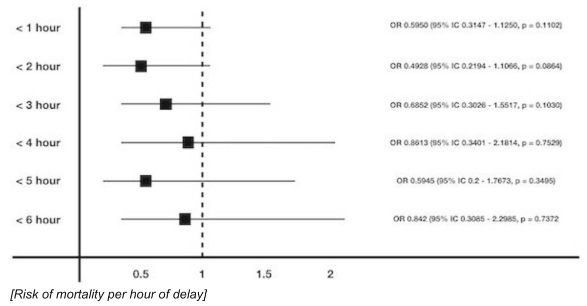

**Fig. 1 (abstract 0260).** Risk of mortality per hour of delay

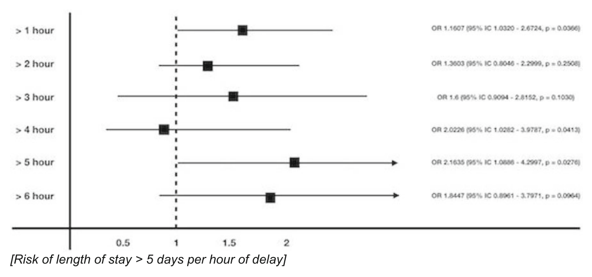

**Fig. 2 (abstract 0260).** Risk of length of stay > 5 days per hour of delay

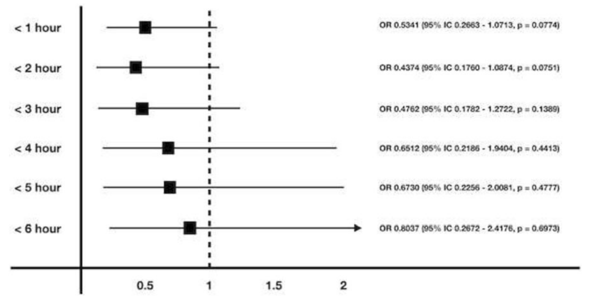

**Fig. 3 (abstract 0260).** Risk of developing septic shock during the following 30 days per hour of delay

### 0261

#### Association of antibiotic pharmacodynamic indices with survival in human septic shock

G. Vázquez-Grande[1,2], K. Meghairbi[2,3], A. Kumar[1,2]

[1]University of Manitoba, Section of Critical Care, Winnipeg, Canada; [2]University of Manitoba, Medical Microbiology, Winnipeg, Canada; [3]Dr. Soliman Fakeeh Hospital, Jeddah, Saudi Arabia

**Correspondence:** G. Vázquez-Grande

*Intensive Care Medicine Experimental* 2018, **6(Suppl 2):**0261

**INTRODUCTION.** Septic shock and sepsis associated multiple organ failure are a major cause of morbidity and mortality in intensive care units (ICUs) globally. Despite major improvements in sepsis treatment, septic shock remains a major challenge to ICU clinicians, with a mortality of 30-50%. Source control and an early administration of appropriate antimicrobial therapy remain the most important and powerful clinical interventions for sepsis treatment.

Animal studies suggest that the key for improving septic shock outcomes relay on the appropriate delivery of existing antimicrobials. Thus, understanding the pharmacodynamic indices (PDI) may be the cornerstone to improve sepsis outcomes.

**OBJECTIVE.** To assess the association of β-lactam pharmacokinetic indices and outcome in human septic shock in a wide range of bacterial pathogens.

**METHODS.** Retrospective analysis of a septic shock cohort treated with a b-lactam as the only effective antimicrobial, from a local validated high-quality database.

We collected patient, pathogen and treatment characteristics. And calculated b-lactam pharmacodynamic indices (PDI) during the first 24 hours of treatment, including: time above MIC; time above four times the MIC; peak drug concentration over MIC; and area under the drug concentration curve divided by MIC (AUC/MIC).

We applied logistic regression to examine survival to hospital discharge as a function of b-lactam PDI. And performed a multivariate regression model adjusting for age, time to appropriate antibiotic and APACHE II score.

**RESULTS.** 342 patients with septic shock treated with b-lactam as only effective antibiotic. 59.6% were males, with an average age of 63.8(±15.6) years, and a mean APACHE II score of 26.97(±8.8) points. The median time to appropriate drug administration following documentation of hypotension was 3.67 (1.0-7.5 IQR) hours.

Logistic regression analysis demonstrated that, during the first 24 hours, time above MIC (OR 1.17, 95%CI 1.07-1.28 per 10% increment, p=.0005) and time above 4xMIC (OR 1.12, 95%CI 1.05-1.20, p=.0003) were both strongly associated with an improved survival in septic shock patients.

In a multivariate regression model (controlling for age, time to appropriate antibiotic and APACHE II score) the relationship between time above the MIC (OR 1.12, 95%CI 1.04-1.29, p=.006) and time above four times the MIC (OR 1.13, 95%CI 1.04-1.22, p=.002) and hospital survival remained significant.

**CONCLUSIONS.** Our study shows that for β-lactams time-related PDI are associated with improved survival in a broad range of human septic shock; even after adjusting for time to appropriate antimicrobial therapy, age and severity of illness.
This suggests that there is potential value in using PDI to guide optimal dosing regimens of b-lactam therapy for the treatment of septic shock patients.

**Table 1 (abstract 0261).** Cohort characteristics (n=342 patients)

| Parameters | Units | |
|---|---|---|
| Age | years (mean, SD) | 63.9 (+/-15.6) |
| Sex | male (%) | 202 (59.1%) |
| APACHE II | score (SD) | 27.0 (+/-8.8) |
| IBW | Kg (median, IQR) | 70.2 (53.5-73.3) |
| Serum creatinine at shock | µmol/L (median, IQR) | 165 (113-273) |
| Serum creatinine at 24 hours post shock | µmol/L (median, IQR) | 171(98-271) |
| Creatinine clearance estimated from serum creatinine closest to 24 hours post shock onset | µmol/L/min (median, IQR) | 37.1 (23.3-63.1) |
| Time to appropriate antibiotic | hours (median, IQR) | 3.67 ( 1-7.5) |
| Microbiologically inappropriate initial empiric therapy | n (%) | 52 (15.2%) |

### 0262

#### The implementation of antibiotic therapy as part of sepsis six bundle: a twelve month single centre study of compliance with antibiotic therapy and outcomes

R. Mothukuri[1], H. John[2], M. Kakollu[1], H. King[1], M. Matthews[1], M. Thompson[1]
[1]Morriston Hospital, Swansea, Emergency Department, Swansea, United Kingdom; [2]Swansea University, Medical School, Swansea, United Kingdom
**Correspondence:** R. Mothukuri
*Intensive Care Medicine Experimental* 2018, **6(Suppl 2):**0262

**INTRODUCTION.** Sepsis is a life-threatening medical emergency which requires fast management to provide the best patient outcome.[1] Patients with an National Early Warning Score (NEWS) of 3 should be screened for sepsis, and if diagnosis is probable, the 'sepsis six' care bundle should be started.[2] The most important intervention to improve patient outcomes is administration of antibiotics within 1 hour.[1]
**OBJECTIVES.** This study aimed to assess whether patients in Morriston Hospital Emergency Department who are diagnosed with sepsis are receiving antibiotic therapy within 1 hour of diagnosis.
**METHODS.** The study was done in a single centre University Teaching Hospital which sees approximately 100,000 patients annually. Data was collected retrospectively between August 2017-November 2017. Information obtained using the sepsis screening tool filled in by ED staff, was inputted onto an excel spread sheet. The data collected included: age, sex, date of admission, admission time, NEWS, SIRS criteria, septic (Yes/No), severe sepsis identified time, presenting complaint, Sepsis 6 elements completed (yes/no), sepsis six completion time, sepsis six completed within 1 hour, sepsis six not completed, septic shock screening criteria, diagnosis, discharge outcome.
**RESULTS.** The antibiotic administration within 1 hour for all septic patients improved from 32% to 41% during the study period. Focussing specifically in patients with NEWS ≥ 6 the 1-hour antibiotic compliance rose from 42% in June 2017 to 47% in February 2018.
**CONCLUSIONS.** There was a steady improvement in antibiotic compliance for all septic patients and also for patients with a NEWS ≥ 6 during the study period. There is more room for improvement and further changes are being implemented to achieve this.

**REFERENCE(S)**

<ol style="list-style-type: decimal; direction: ltr;">
- Kumar, A., Roberts, D., Wood, KE., Light, B., Parrillo, JE., Sharma, S., et al. Duration of hypotension before initiation of effective antimicrobial therapy is the critical determinant of survival in human septic shock. Critical Care Medicine. 2006;34(6)
- The UK Sepsis Trust [Internet]. Birmingham: Sepsis Enterprises Ltd; year of publication unknown. Available from: https://sepsistrust.org/education/
- Rhodes, A., Evans, L., Alhazzani, W., Levy, MM., Antonelli, M., Ferrer, R. Surviving Sepsis Campaign: International Guidelines for Management of Sepsis and Septic Shock: 2016. Intensive Care Medicine. 2017;43:304-377

**GRANT ACKNOWLEDGMENT**
Not applicable

### 0263

#### Adherence to antibiotic guidelines in patients admitted to ICU with severe sepsis and septic shock: does it affect clinical outcome?

O. Lindberg
Linköping University Hospital, Intensive Care, Linköping, Sweden
*Intensive Care Medicine Experimental* 2018, **6(Suppl 2):**0263

**Background:** Sepsis remains associated with high morbidity and mortality and is a common reason for requiring intensive care. Several landmark studies have demonstrated the benefit of prompt antibiotic usage and the Swedish Society of Infectious Disease Medicine has since 2008 provided national guidelines for the treatment of sepsis in Sweden. Compliance with the Swedish national guidelines is largely unknown and further it is unknown if compliance to guidelines improves patient outcome. The aim of this study is to assess the current compliance to Swedish guidelines and secondly to investigate whether compliance to guidelines improves outcome for patients admitted to a mixed intensive care unit for severe sepsis and septic shock.
**METHODS.** A retrospective, journal audit was conducted on all patients admitted to the general ICU at Linköping University hospital from the 1st of January 2011 to the 31st of December 2015 with ICD-10(SE) diagnosis codes 65: 1 (Severe Sepsis) and 57: 2 (Septic Shock) identified through the Swedish ICU Registry (SIR).
**RESULTS.** Full compliance to national antibiotic guidelines was observed in 35.3 % of patients, partial compliance in 45.7%, and no compliance in 18.9%. Partial compliance was significantly associated with a 2-fold increase in risk of 30-day mortality (OR 2.155 [CI 1,19-3,87], p=0.01). Furthermore no significant association was observed between time to appropriate antibiotics and patient outcome. No compliance to guidelines was surprisingly not associated with an increased risk compared to full compliance.
**CONCLUSIONS.** Only 35 % of the most critically ill patients receive antibiotics in line with Swedish national guidelines for severe sepsis and septic shock. Full compliance with guidelines was associated with decreased mortality. Efforts should be made to increase the awareness and compliance to the current guidelines.

### 0264

#### Ceftazidime-avibactam to treat severe deep-seated infections due to KPcp in the critically ill

P. Mandelli, I. Cigada, A. Veronese, S. Silvia, F. Cantarero, C. Soru, A. Corona
University of Milano, Milano, Italy
**Correspondence:** A. Corona
*Intensive Care Medicine Experimental* 2018, **6(Suppl 2):**0264

**INTRODUCTION.** In the critially ill patients, carbapenemases producing (cp) *Klebsiella pneumoniae* (KP) infection rate ranges between 5-50% and is associated with a high mortality (19-51%). In our hospital ICU, KP strains are resistant to carbapenems in 45% of isolates.

**OBJECTIVES.** The use of ceftazidime-avibactam - a 3-generation cephalosporin plus a new inhibitor of class A (KPC), C (AmpC) and D (OXA48) ESBL, may resolve life-threatening conditions (1).

**METHODS.** Observational audit of 18 patients undergoing compassionate treatment with ceftazidime-avibactam after local Ethic Committee authorization.

**RESULTS.** From April 2017 to February 2018, 18 patients (pts) (13 M and 5 F), median age 59.5 (IQR = 42.5-70.5) were given ceftazidime/avibactam for major KP-cp (meropenem MIC > 16ng/ml) infections: 10 bacteramia (B) (3 abdominal sepsis, 2 mediastinitis, 2 CVC, 3 primary); 6 secondary peritonitis and 2 complicated UTI. 13/18 (72.2%) pts, developed a septic shock [median (IQR) SOFA score 10 (8-17)) and needed mechanical ventilation [median (IQR) 8 (4-17) days], norepinephrine infusion [median (IQR) 3 (2-5) days] and lactate values [median (IQR) 3.5 (2.4-7.3) mMol/l]; 5 patients underwent renal replacement therapy. The median treatment duration (IQR) was 14 (13-14) days. In 7/18 of cases, ceftazidime/avibactam was given in combination [fosfomycin + colistin (3 pts.), fosfomycin + aztreonam (2 pts.), fosfomycin + gentamicin (1 pt), colistin (1 pt)]. All the patients experienced a clinical response and improved by 72/96 hours from the ceftazidime/avibactam commencing. In 9/10 bacteraemic patients negativization of blood culture occurred by 96 hours, as well as of the rectal swab in 6/18 patients. A (B) recurred twice in the same patients and two further treatments were necessarily given. 12/18 (66.7%) patients survived. Only 4 out of 6 deaths (2 patients died because of cerebrovascular accident) were directly or indirectly related to KP-cp severe infections, therefore we recorded a likely attributable death of 22.3%. The susceptibility test of strains showed sensitivity to ceftazidime/avibactam, whereas (i) 100% of resistance to carbapenems (meropenem/imipenem/ertapenem), quinolones and III/IV generation cephalosporin, tigecycline and piperacillin/tazobactam. 62.5% of strains were susceptible to fosfomycin and colistin and less than 50% to main aminoglicosides (amikacin and gentamicin).

**CONCLUSIONS.** The 18 strains of KP-cp were susceptible to ceftazidime-avibactam despite the high carbapenem-resistance recorded in our ICU, because od rare identification of KP-cp VIM / NDL +. Our data seems to confirm the efficacy and clinical utility of this antibiotic for the critically ill patients.

**REFERENCE(S)**

(1) Rodríguez-Baño J, Gutiérrez-Gutiérrez B, Machuca I, Pascual A. Treatment of Infections Caused by Extended-Spectrum-Beta-Lactamase-, AmpC-, and Carbapenemase-Producing Enterobacteriaceae. Clin Microbiol Rev. 2018 Feb 14;31(2).

**GRANT ACKNOWLEDGMENT**
None

### 0265

#### High-speed antibiotic susceptibility testing of blood-culture isolates of septicemia with surface-enhanced Raman spectroscopy

Y.-Y. Han[1,2], W.-C. Cheng[3], Y.-C. Lin[3], C.-T. Wang[4], L.-J. Teng[5], J.-K. Wang[3,6], Y.-L. Wang[3,7]
[1]National Taiwan University Hospital, Trauma, Taipei, Taiwan, Province of China; [2]National Taiwan University, College of Medicine, Anesthesia, Taipei, Taiwan, Province of China; [3]Institute of Atomic and Molecular Sciences, Academia Sinica, Taipei, Taiwan, Province of China; [4]National Taiwan University Hospital, Internal medicine, Taipei, Taiwan, Province of China; [5]National Taiwan University, Clinical Laboratory Sciences & Medical Biotechnology, Taipei, Taiwan, Province of China; [6]National Taiwan University, Center for Condensed Matter Sciences, Taipei, Taiwan, Province of China; [7]National Taiwan University, Physics, Taipei, Taiwan, Province of China
**Correspondence:** Y.-Y. Han
*Intensive Care Medicine Experimental* 2018, **6(Suppl 2):**0265

**INTRODUCTION.** Timely effective antibiotic usage is crucial for survival of patients with septicemia. However, cultured-based microbiological diagnosis are time consuming, leading to poor clinical outcomes and rising antibiotic resistance.

**OBJECTIVES.** This study aimed to establish a rapid antibiotic susceptibility test (AST) protocol based on surface-enhanced Raman spectroscopy (SERS), which was a sensitive, specific and high-speed optical analytic technique.

**METHODS.** The samples from 32 patients with bacteremia of *S. aureus* and 43 of *E. coli* were aspirated from the positive-culture bottles. Sample pretreatment with cell lysing agent and repetitive wash procedure recovered the blood-culture isolates from complex blood-broth septic mixture. The SERS measurements of the samples with and without 2-hr antibiotic treatment were performed on SERS-active substrates made of Ag nanoparticle array. The Raman peaks at 730 and 724 $cm^{-1}$, signifying the metabolites released from *S. aureus* and *E. coli* respectively, were designated as their SERS biomarkers. Their signal ratios (r730 and r724 ) obtained from the antibiotic-treated sample and the untreated one were used to determine AST.

**RESULTS.** r730 of susceptible *S. aureus* decreased with the treated oxacillin concentration, while that of resistant *S. aureus* remained relatively invariant for all drug concentrations. Similar character was observed for *E. coli* treated with cefotaxime. The sensitivity and specificity of AST were 0.94 and 0.93 for *S. aureus* and 0.97 and 1.00 for *E. coli*. The antimicrobial activity of antibiotics was accurately indicated by SERS biomarkers.

**CONCLUSIONS.** A rapid AST protocol based on SERS was successfully demonstrated for positive blood culture of *S. aureus* and *E. coli*. This first proof-of-concept study lights the potential of a new high-speed microbiological analytic method assisting in fighting sepsis. The rapid and reliable AST results yielded by SERS would be of vital importance not only to guide the early antibiotic therapy but also reduce the emergence of antimicrobial-resistant strains.

**REFERENCE(S)**

1. Dellinger RP, et al. Crit Care Med. 2013; 41(2): 580-637.
2. Ferrer R, et al. Crit Care Med. 2014; 42:1749-1755.
3. Liu TT, et al. PLoS ONE. 2009; 4(5):e5470.
4. Liu CY, et al. Sci Rep. 2016;6:23375.
5. Boardman AK, et al. Anal. Chem. 2016;88(16):8026-8035.
6. Premasiri WR, et al. Anal Bioanal Chem. 2016;408(17):4631-47.

**GRANT ACKNOWLEDGMENT**
National Science Council

### 0266

#### Molecular regulation of acute Tie2 suppression in sepsis

T. Idowu[1], C. Schrimpf[2], J. Retzlaff[1], M. van Meurs[3], S.M. Parikh[4], S. David[1]
[1]Medical School Hannover, Hannover, Germany; [2]Medical School Hannover, Cardiothoracic, transplantation and vascular surgery, Hannover, Germany; [3]University Medical Center Groningen, Critical Care, Groningen, Netherlands; [4]Beth Israel Deaconess Medical Center, Harvard Medical School, Boston, United States
**Correspondence:** S. David
*Intensive Care Medicine Experimental* 2018, **6(Suppl 2):**0266

**INTRODUCTION.** Tie2 is a tyrosine kinase receptor expressed by endothelial cells (ECs) that maintains vascular barrier function. We recently reported that diverse critical illnesses acutely decrease Tie2 expression and that experimental Tie2 reduction suffices to recapitulate cardinal features of the septic vasculature.

**OBJECTIVES.** Here we investigated molecular mechanisms driving Tie2 suppression in settings of critical illness.

**METHODS.** Molecular biology assays (western blot, qPCR) + flow and transendothelial electrical resistance (TER) experiments in human umbilical vein endothelial cells (HUVECs); murine cecal ligation & puncture (CLP) and LPS administration. Kidney biopsies from deceased septic AKI patients (n=16) and controls (n=12) & serum from septic shock patients (n=57) and controls (n=22).

**RESULTS.** We observed rapid reduction of both Tie2 mRNA and protein in mice following CLP. In cultured ECs exposed to TNFa, suppression of Tie2 protein was more severe than Tie2 mRNA, suggesting distinct regulatory mechanisms. Evidence of protein-level regulation was found in TNFa-treated ECs, septic mice, and septic humans, all three of which displayed elevation of the soluble N-terminal fragment of Tie2. The matrix metalloprotease MMP14 was both necessary and sufficient for N-terminal Tie2 shedding. Since clinical settings of Tie2 suppression are often characterized by shock, we next investigated the effects of laminar flow on Tie2 expression. Compared to absence of flow, laminar flow induced both Tie2 mRNA and the expression of GATA3. Conversely, septic lungs exhibited reduced GATA3, and knockdown of GATA3 in flow-exposed ECs reduced Tie2 mRNA. Postmortem tissue from septic patients showed a trend toward reduced GATA3 expression that was associated with Tie2 mRNA levels.

**CONCLUSIONS.** Tie2 suppression is a pivotal event in sepsis that may be regulated both by MMP14-driven Tie2 protein cleavage and GATA3-driven flow regulation of Tie2 transcript.

**GRANT ACKNOWLEDGMENT**

DFG to S.D. (DA1209/4-3)

### 0267

#### Right dose, right now. developing autokinetics for personalised, real time, big data driven, antibiotic dosing

L.F. Roggeveen[1], T. Guo[1], B. van Dijk[1], R. Driessen[1], P. Thoral[1], L. Fleuren[1], A.R.J. Girbes[1], P.H.J. van der Voort[2], R. Bosman[2], P.W.G. Elbers[1]

[1]VU Medical Center, Intensive Care, Amsterdam, Netherlands; [2]OLVG Oost, Intensive Care, Amsterdam, Netherlands

**Correspondence:** L.F. Roggeveen

*Intensive Care Medicine Experimental* 2018, **6(Suppl 2):**0267

**INTRODUCTION.** Antibiotic dosing in critically ill patients is difficult because of altered pharmacokinetics[1]. Most intensive care units rely on standard dosing. Pharmacokinetic targets are only reached in about half of cases[2]. Several dosing tools exist for guiding antibiotic dosing. However, these require laborious manual data entry and pharmacometric knowledge and support and may be impractical, error prone and time consuming. Thus, truly personalized antibiotic dosing is currently lacking. We set out to change this by integrating large amounts of data from electronic patient records (EPRs) with complex pharmacometric models at the bedside for instantaneous advice.

**OBJECTIVES.** To develop real time antibiotic dosing software that interacts with common EPRs and can be implemented for personalised antibiotic dosing in critically ill patients in septic shock.

**METHODS.** We created AutoKinetics, a clinical decision support system for real time antibiotic dosing advice. We integrated AutoKinetics with two EPRs. We use a SQL database for backend support. We created a Windows- and Web-based front-end application.

In a separate study, we identified or developed models for 5 commonly used antibiotics, namely meropenem, ciprofloxacin, ceftriaxone, cefotaxime and vancomycin. Model performance was validated on retrospectively and prospectively collected data and were calibrated on prospectively collected plasma levels. Selected models were incorporated into AutoKinetics.

**RESULTS.** We successfully developed AutoKinetics. The software calculates predicted serum concentrations on a minute to minute basis. Upon request, it provides the treating physician and other healthcare professionals with personalized graphical advice on optimal antibiotic loading and maintenance dose at any given moment. AutoKinetics can handle numerous pharmacokinetic model structures and therefore potentially all antibiotics. While not required to function, AutoKinetics does provide automatic personalized Bayesian maximum a posteriori correction if plasma level are available.

**CONCLUSION.** We developed a clinical decision support system for real time personalised antibiotic dosing in the critically ill. We hypothesize that our solution may contribute to improved antibiotic dosing in the critically ill. Therefore, we recently started a multi-centre randomised control trial to validate this concept in 420 patients with severe sepsis and septic shock. EudraCT-number: 2017-002478-37.

**GRANT ACKNOWLEDGEMENT**

This work is has been partially funded by the ZonMw Rational Pharmacotherapy program.

### 0268

#### Variability of plasma concentrations of meropenem and piperacillin / tazobactam in standard doses in patients with severe infections

A. Fernández Galilea[1], M.M. Pinilla de torre[1], V. Merino Bohorquez[2], M. De Cueto[3], Á. Arenzana Seisdedos[1], M.Á. Calleja[2], J. Garnacho Montero[1]

[1]Hospital Universitario Virgen Macarena, Medicina Intensiva, Sevilla, Spain; [2]Hospital Universitario Virgen Macarena, Farmacología Clínica, Sevilla, Spain; [3]Hospital Universitario Virgen Macarena, Microbiología Clinica, Sevilla, Spain

**Correspondence:** A. Fernández Galilea

*Intensive Care Medicine Experimental* 2018, **6(Suppl 2):**0268

**OBJECTIVES.** To know if standard dosing of meropenem (MER) and piperacillin / tazobactam (PTZ) administered to critically ill patients including subjects on continuous renal replacement techniques (TCRR) achieve optimal levels to treat severe infections caused by Gram-negative bacilli including “difficult-to treat” pathogens.

**METHODS.** Prospective study including patients with septic shock under treatment with standard doses of MER (1g / 8h in extended infusion) and PTZ (4gr / 6h in extended infusion). Levels were monitored before the administration of the antibiotic (T0) and in 50% of the administration interval (T50), in equilibrium state (after the fourth dose) by liquid chromatography coupled to an ultraviolet detector. The creatinine clearance (CrCl) was determined in three-hour urine. Patients with a creatinine clearance ≥ 130 ml /min were considered patients with hyperfiltration. CRRT was started for oliguric renal failure. Optimal concentrations of the drug are considered if 100% of the time exceeds four times the minimum inhibitory concentration (MIC). The target is set at T0> 8 mg /L for MER and T0> 64 mg /dL for PTZ. Normally distributed continuous variables were expressed as means with standard deviations and compared with Student´s test for independent samples and compared with Mann-Whitney U test for non-normally distributed continuous variables.

**RESULTS.** In 26 patients treated with MER (11 on CRRT), there were no statistically significant differences in the T0 and T50 values between patients with or without CRRT (20.6 ± 4.5 mg / dL in patients with TCRR vs 17.2 ± 2.4 mg / dL in patients without CRRT for determination values at T0, with p = 0.52; 32.6 ± 4.3 mg / dL in patients with CRRT vs 38.2 ± 6.4 mg / dL in patients without CRRT for values in T50, p = 0.47). Among patients without CRRT (15/26), only one presented hyperfiltration and id not reach T0> 8mg / dL (7.54 mg / L).In 16 patients treated with PTZ, there were statistically significant differences in T0 values between patients who received standard doses (4 g / 6 h) and patients with non-standard doses (4 g / 8 h): 120.1 ± 33.6 mg/L in patients with standard doses vs 28 ± 11.4 mg/L in patients with non-standard doses for determination values at T0, with p=0,025. Otherwise, there were no statistically significant differences in T50 values between the groups: 161.2 ± 29.1 in patients with standard dose vs 102.6 ± 22.1 in patients with non-standard dose for determination values at T50, with p=0,13. Among patients with PTZ 3 were on TCRR (1 with standard dosing), only the patient on TCRR with standard regimen of antibiotic reached MIC>64 mg/L.

**CONCLUSIONS.** Given the variability of the concentrations and the different pharmacodynamic objectives that can be marked, it is recommended to measure the levels of MER and PTZ in critically ill patients with serious infections.

### 0269

#### Prevalence of insufficient plasma concentration of β-lactam antibiotics in ICU patients: a one-year retrospective single centre study

S. Serfati, F. Lamoureux, L. Imbert, E. Desmarest-Durand, S. Skallil, T. Clavier, S. Grange, F. Tamion, B. Misset
CHU de Rouen, Rouen, France
**Correspondence:** S. Serfati
*Intensive Care Medicine Experimental* 2018, **6(Suppl 2):**0269

**INTRODUCTION.** β-lactam antibiotics effectiveness is time-dependent and requires that their plasmatic concentration is constantly over the Minimal Inhibiting Concentration (MIC) of the targeted bacterial strain. Doses recommended by the pharmaceutical companies are based on data derived from patients with non-severe diseases and may not be appropriate for ICU patients with sepsis, due to variable alteration of clearance or distribution volume.
**OBJECTIVES.** To assess the prevalence of insufficient plasma concentrations of β-lactam antibiotics in ICU septic patients.
**METHODS.** A retrospective analysis of therapeutic drug monitoring using liquid chromatography-mass spectrometry (LC-MS/MS) was performed between December 2016 and November 2017 for 6 molecules. Dosing and frequency of beta-lactams and their adjustment to renal function were based on the recommendations of the French Vidal database of pharmaceutical drugs. We selected those assessments performed between 4 and 8 hours after IV administration for amoxicillin (AMX); 3 and 6 hours for cloxacillin (CLO), 6 and 12 hours for piperacillin (PIP), 6 and 12 hours for meropenem (MEM), 8 and 12 hours for cefepime (FEP), and 12 and 24 hours for ceftriaxone (CRO). EUCAST inferior breakpoint was used for each molecule to approach usual MICs.
**RESULTS.** 178 samples were assessed, of which 125 were residual concentrations (AMX = 24, CLO = 7, PIP = 61, MEM = 14, FEP = 6, CRO = 13) in 78 patients. Age = 61 ± 14, SAPS 2 = 50 ± 19, weight = 75 ± 22 kg, body mass index = 28 ± 6 kg/m$^2$, shock = 56 %, renal replacement therapy = 17%, LOS in ICU = 15.2 ± 12.8 d, mortality = 25 %. 30/125 plasma concentrations (24%) were below four-time the EUCAST inferior breakpoint: AMX = 4 (17 %), CLO = 1 (14 %), PIP = 15 (24.6%), MEM 8 (57.1%), FEP = 1 (16.7%), and CRO = 1 (7.7%). Except for MEM and CRO, plasma concentrations were poorly correlated with the time from infusion to sample. Patients with insufficient concentrations were younger (52 ± 15 versus 64 ± 13, p 0.006) and had longer ICU stays (23 ± 19 versus 12 ± 9 d, p = 0.02), while they had similar sex ratio, SAPS 2 score, weight, BMI, shock, RRT use, and mortality.
**CONCLUSIONS.** In our ICU population, dosing and frequency of β-lactam antibiotics based on the recommendations of the French Vidal database of pharmaceutical drugs led to low β-lactam exposure in 24 % of cases, particularly for antibiotics with short half-lives and in younger patients. Insufficient concentrations were associated with longer LOS in the ICU.

### 0270

#### What is the most important clinical determinant of procalcitonin?

S. Zampieri, K. Donadello, V. Schweiger, L. Gottin, E. Polati
University of Verona, Department of Surgery, Dentistry, Pediatrics and Gynaecology, Verona, Italy
**Correspondence:** S. Zampieri
*Intensive Care Medicine Experimental* 2018, **6(Suppl 2):**0270

**INTRODUCTION.** Procalcitonin (PCT) has been studied as sepsis marker in different clinical scenarios and its values are higher in gram-negative infections compared to gram-positive ones. PCT raise is more evident in bacterial then in invasive fungal infections. PCT values were higher in surgical than in medical patients, and in abdominal and urinary infections than others.
**OBJECTIVES.** The aim of our study was to identify the most important clinical determinant that influences PCT values in a population of medico-sukrgical ICU patients.
**METHODS.** Retrospective analisi of a prospective 4 year-data collection. 927 consecutive patients were analyzed and we collected clinical parameters, laboratory, microbiological and radiographic exams, antibiotics and vasopressors administration, C-Reactive Protein (CRP) and PCT values, in order to recognize etiology and site of infections. We daily classified the clinical states of patients into six diagnostic classes and the intensive care admission as medical or surgical. An optimal scaling analysis was performed in order to assess the most important clinical determinant of PCT.
**RESULTS.** PCT values were lower in fungal sepsis than in gram-negative and gram-positive ones ($p < 0.05$). We analyzed peak values of PCT during septic events: gram-negative sepsis have higher mean peak value than gram-positive and fungal ones ($p < 0.05$). PCT values of abdominal (3.84 ng/ml - IC95% 3.35-4.36) and urinary tract (6.63 ng/ml - IC95% 4.71-11.52) septic events were higher than in soft tissues (4,03 ng/ml - IC95% 3.42-4.81), lung (2.31 ng/ml - IC95% 2.09-2.68), and BSI (1.78 ng/ml - IC95%1.22 -2.35) ones ($p < 0.05$). PCT mean peak value was higher in abdominal than in lung sepsis ($p < 0.05$). There was no statistical significance for the other sites of infection in PCT peak means values.We found higher PCT values in surgical patients than in medical ones, anyway there's a difference due to the site of infection: actually there is prevalence of abdominal infection in surgical patients (61.6%), and prevalence of lung infection (73.2%) in medical patients. At optimal scaling analysis we found that septic events were the strongest determinant of PCT values (partial correlation=0.432 and importance=0.702), site of infection presents a high importance (=0.22), while type of patient (medical/surgical) (=0.08) and etiology (=0.02) importance were similar. Analyzing PCT peak values we found that the septic event was the most important clinical factor of the septic events PCT peak value (partial correlation=0.28, importance=0.8) than other ones.
**CONCLUSIONS.** Our study identified that PCT moves in different ways according to etiology, site of infection and type of patient admission (medical/surgical), anyway the most important clinical determinant factor of PCT peak value during a septic event is etiology.

1. Charles PE et al. BMC Infect Dis. 2008 Mar 26;8:38. .
2. Martini A et al. J Infect. 2010 Jun;60(6):425-30.

### 0271

#### Use of procalcitonin levels to guide the efficacy and safety in reducing the duration of antibiotic treatment in critically ill patients

S. Fernández Vilches[1], E. Diaz[1], A. Morón[2], D. Fontanals[2], J. Vallés[1]
[1]Hospital Parc Tauli, Intensive Care Unit, Sabadell, Barcelona, Spain;
[2]Hospital Parc Tauli, Pharmacy Department, Sabadell, Barcelona, Spain
**Correspondence:** S. Fernández Vilches
*Intensive Care Medicine Experimental* 2018, **6(Suppl 2):**0271

**INTRODUCTION.** Excessive duration of antibiotic therapy in critically ill patients can result in antibiotic overuse,increasing the risk of emergence and spread of multi drug resistant microorganisms in hospitals.In the last years,the utility of serial measurement of procalcitonin (PCT) for reduction the duration of antibiotic treatment has been reported.
**OBJECTIVES.** The objective of the study was to evaluate the usefulness of PCT in reducing the duration of antibiotic treatment compared with the standard treatment in patients admitted in ICU.
**METHODS.** A descriptive and prospective study was carried out in our 30-bed polyvalent ICU.Inclusion criteria:patients aged ≥ 18 years admitted to the ICU for a proven infection,and who received their first dose of antibiotics no longer than 24h before inclusion in the study.The patients included in the study received a minimum of five days of antibiotic treatment,and after the treatment was stopped if clinical signs of infection improved and the PCT values was either < 0.5 ng/L or decreased by 80% or more of its peak value.Patients with infections,which require prolonged antibiotic therapy, viral or parasitic infections and those immunocompromised or pregnant were excluded.For this study,data were collected for the period 1 June to 31 December 2017 and compared with the same period of the previous year,in which duration of antibiotic treatment was according to standard care.

**RESULTS.** A total of 43 intensive care patients receiving antibiotic therapy after confirmed infections were enrolled in this study,mostly due to community-acquired infections.Pneumonia was the most frequent.In the PCT-guided period the duration of antibiotic therapy was shorter (5.8 +/-0.9 days in community-acquired infections and 7.6 +/- 3.2 days in nosocomial infections)than compared to standard-of-care period in which the duration of treatment was according to local antibiotic protocols. Median consumption of antibiotics was reduced by 6.5 % according to daily defined doses(DDD)and 5.45% according to days of therapy(DOT)in the PCT-guided period compared to the standard-of-care period,with a reduction of antibiotic treatment costs of 8.9%.The number of patient-stays and the incidence of community-acquired infections and nosocomial infections reported during both periods was not different.

**CONCLUSIONS.** Our results suggest that a protocol based on serial PCT measurements may contribute to reduce antibiotic treatment duration and contribute to an antibiotic stewardship program in our intensive care unit.

- Bouadma L,Luyt CE,Tubach F, et al.Use of procalcitonin to reduce patients' exposure to antibiotics in intensive care units (PRORATA trial): a multicentre randomised controlled trial. Lancet 2010; 375:463-74
- de Jong,Evelien et al.Efficacy and safety of procalcitonin guidance in reducing the duration of antibiotic treatment in critically ill patients: a randomised, controlled, open-label trial. Lancet Infect Dis.2016; 16: 819-827

## Pneumonia, COPD and other causes of acute respiratory failure

### 0272

#### Pneumonia determines readmissions and mortality of lung transplant patients in the ICU: beyond the perioperative period

C. Mazo[1], A. Sandiumenge[1], T. Pont[1], M. Ballesteros[2], E. López[3], L. Rellan[4], J. Robles[5], J. Rello[6]
[1]Vall d´Hebron Hospital, Transplant Coordination, Barcelona, Spain; [2]Marques de Valdecilla Hospital, Intensive Care Department, Santander, Spain; [3]12 de Octubre Hospital, Intensive Care Department, Madrid, Spain; [4]A Coruña University Hospital, Intensive Care Department, A Coruña, Spain; [5]Reina Sofia University Hospital, Intensive Care Department, Cordoba, Spain; [6]Vall d´Hebron Hospital, Barcelona, Spain
**Correspondence:** A. Sandiumenge
*Intensive Care Medicine Experimental* 2018, **6(Suppl 2):**0272

**INTRODUCTION.** Lung transplant (LT) is the therapy of choice for a number of end-stage lung diseases. Although post-transplantation survival has steadily improved, long-term survival is still low with a median survival of 5.8 years [1]. As the number of LT and survival has been increasing, it is expected that ICU readmissions (ICUr) will continue going up [2]. Factors influencing the UCIr of this unique subgroup of immunocompromised patients after the post-operative period have been described for few studies, mainly retrospective. In the general population, there are clinical variables and scoring systems that can predict the outcome of patients that are admitted to the ICU. It is of paramount importance to study causes of admission, outcomes and potential predictors of ICU mortality in lung transplant patients. Better knowledge of these factors could be useful to patients, their families and their physicians.

**OBJECTIVES.** To identify causes of ICUr in LT patients, report outcome at ICU discharge, to determine the independent risk factors associated with ICU mortality and suggest interventions that may modify outcome. We hypothesised that pneumonia influences UCI mortality, over the need of invasive mechanical ventilation (IMV) or vasoppressors.

**METHODS.** Multicentre prospective study of all lung transplant patients readmitted to ICU after perioperative ICU discharge between 2012 and 2016 in five tertiary hospitals. Patients were followed until hospital discharge or death. Logistic regression analysis was performed to determine which factors were independently associated with ICU mortality.

**RESULTS.** There were 174 ICUr after a median of 7 month of LT. The main diagnosis at ICUr was pneumonia 67/174(38.5%). ICU mortality was 41.8%, with multi-organ failure (48.4%) and hypoxemia (45.4%) as the most common causes of death. On multivariate analysis the independent predictors of death were invasive mechanical ventilation >72h (OR =4.7, 95% CI, 2.1-10.4), SOFA score >6 (OR =2.9, 95% CI, 2.1-9.9), time after LT at ICUr >1 month (OR =3.4, 95% CI, 1.2-10.9), history of acute rejection (OR =2.8, 95% CI, 1.2-7.1), vasopressors (OR =2.3 95% CI, 1.1-6.5) and pneumonia (OR =2, 95% CI, 1.1-7.1). P < 0.05.

**CONCLUSIONS.** It is critical to recognize pneumonia in LT patients undergoing ICUr, because besides being the main etiology of ARF, sepsis and septic shock, it portends lower ICU-survival (independently of ARF, shock or associated organ dysfunction during ICUr or previous conditions prior to ICUr as CLAD or FEV1). In view of this, prompt pneumonia recognition and pathogen detection for targeted therapy, implies the need for development of rapid diagnostic tests at the patient`s bedside for both microbiology identification and resistance detection.

**GRANT ACKNOWLEDGMENT**

Supported in part by CIBERES & FISS PI14/1296

### 0273

#### Etiology and prognosis of acute respiratory failure in patients with primary malignant brain tumors admitted to the intensive care unit

M. Decavèle[1,2], I. Rivals[3], C. Marois[4], N. Weiss[4], A. Idbaih[5], T. Similowski[1,2], A. Demoule[1,2]
[1]APHP, Pitié-Salpêtrière Charles Foix, Service de Pneumologie et Réanimation Médicale (Département 'R3S'), Paris, France; [2]Sorbonne Université, INSERM, UMRS_1158 Neurophysiologie Respiratoire Expérimentale et Clinique, Paris, France; [3]Equipe de Statistique Appliquée, ESPCI, PSL Research University, UMRS 1158 Neurophysiologie Respiratoire Expérimentale et Clinique, Paris, France; [4]APHP, Pitié-Salpêtrière Charles Foix, Unité de Réanimation Neurologique, Paris, France; [5]APHP, Pitié-Salpêtrière Charles Foix, Sorbonne Université, Inserm, CNRS, UMRS_1127, ICM, Service de Neurologie 2-Mazarin, Paris, France
**Correspondence:** M. Decavèle
*Intensive Care Medicine Experimental* 2018, **6(Suppl 2):**0273

**INTRODUCTION.** Acute respiratory failure (ARF) is a common event in patients with primary malignant brain tumors (PMBT) [1]. Even if many factors (corticosteroid therapy, swallowing disorders) suggest a specific etiologic spectrum, few data are available regarding its precipitating factors.

**OBJECTIVES.** Our first aim was to compare the causes of ARF between patients with PMBT and those with other type solid tumors. Our second aim was to identify, among PMBT, the factors influencing survival in ICU.

**METHODS.** Bicentric case-control study from March 1996 to May 2014. Patients with PMBT (cases, primary central nervous system lymphoma included) admitted for ARF were compared to patients with other kind of solid tumors (controls). The causes of ARF was determined by three experienced respiratory physicians and were required for inclusion: a respiratory rate > 20 cycles/min and a $PaO_2/FiO_2 < 300$ for patients in spontaneous breathing and only a $PaO_2/FiO_2 < 300$ for patients under mechanical ventilation. In both groups were excluded patients with metastatic solid tumors, benign tumors or tumors with more than 5 years of complete remission, recent post-operative patients, and patients with other immunodeficiency.

**RESULTS.** A total of 84 cases and 133 controls were included. Acute infectious pneumonia was the leading cause of ARF in both groups but was more frequent among cases (77 vs. 36%, p< 0.001). Cardiogenic pulmonary edema and exacerbation of chronic respiratory diseases were more frequents in controls (10 vs. 37%, p< 0.001). Pulmonary embolism was similar between the two groups (10 vs. 4%, p = 0.143). Among acute infectious pneumonia, pneumocystis pneumonia (PCP) and aspiration pneumonia were more frequent in cases (19 vs. 2%, p < 0.001 and 19 vs. 8%, p< 0.001 respectively). The date of admission did not influence the incidence of PCP (p=0.950). ICU (24% vs. 24%, p=0.966) and hospital (46% for cases vs. 49%, p=0.687) mortality were similar in both groups. In multivariate analysis cancer progression (OR 7.25 95%CI [1.13-46.45], p=0.034), need for intubation (OR 7.01 95%CI

[1.29-38.54], p=0.022) and respiratory rate (OR 1.17 95%CI [1.05-1.30], p= 0.003) independently predicted ICU mortality of PMBT patients.
**CONCLUSIONS.** In PMBT patients, the causes of ARF differ significantly from other cancer patients. Up to 30% of the admissions was related to preventable causes (pulmonary embolism, PCP) and a curable cause was identified in the majority of cases. Our results suggest that PMBT alone is not a relevant criterion for ICU recusal.

**REFERENCE(S)**

Decavèle M., Weiss N., Rivals I. et al. Prognosis of patients with primary malignant brain tumor admitted to the intensive care unit: a two decade experience. J Neurol. 2017;264:2303-2312.

**GRANT ACKNOWLEDGMENT**

none

### 0274

#### Distribution of pulmonary ventilation and inflammation before and after intubation in acute hypoxemic respiratory failure patients: a preliminary report

M.C. Bachmann[1], R. Basoalto[2], D. Soto[1], P. García[3], R. Lagos[2], B. Fuenzalida[2], C. Labra[2], G. Bugedo[1], A. Bruhn[1], J. Retamal[1]
[1]Pontificia Universidad Católica de Chile, Departamento de Medicina Intensiva, Santiago, Chile; [2]Hospital Clínico Universidad Católica, Unidad de Paciente Crítico, Santiago, Chile; [3]Pontificia Universidad Catolica de Chile, Carrera de Kinesiología, Santiago, Chile
**Correspondence:** J. Retamal
*Intensive Care Medicine Experimental* 2018, **6(Suppl 2):**0274

**INTRODUCTION.** Spontaneous breathing efforts in acute respiratory distress syndrome patients on mechanical ventilation, has been associated with strong diaphragmatic activity and the generation of excessive strain and high regional pleural pressures in the dependent lung regions [1]. However, these mechanisms may will be present before intubation [2]. We hypothesize that the dependent lung regions are subjected to high levels of strain and subsequent inflammation in patients with acute hypoxemic respiratory failure breathing spontaneously, before intubation.
**OBJECTIVES.** To determine the effects of spontaneous breathing, during the early stage of acute hypoxemic respiratory failure, on strain and inflammation in lung dependent regions.
**METHODS.** Prospective observational clinical and physiological study. Patients with acute hypoxemic respiratory failure were included before intubation. An electrical impedance tomography belt was placed and physiological data recorded at baseline and then hourly after intubation for the first 4 hours of mechanical ventilation. A bronchoalveolar lavage was performed immediately after intubation both in dependent and nondependent lung regions.
**RESULTS.** Five patients were included (age 73 ± 9.9 y.o). After connection to mechanical ventilation, respiratory rate and minute ventilation decreased (36.8 ± 15.1 to 24.4 ±1.51 bpm, p< 0.05; and 21.9 ± 5.2 to 8.7 ± 1.1 L/min, p< 0.05, respectively) and $PaO_2/FiO_2$ ratio increased from 110.4 ± 44.9 to 252.2 ± 106.8 (p = 0.01). Also, after connecting to mechanical ventilation, ventral to dorsal impedance ratio increased from 0.75 ± 0.25 to 1.27 ± 0.53, p= 0.03 (Fig. 1 and 2). No differences were observed for the other electrical impedance tomography indices (tidal variation of impedance, end-expiratory lung volume and global inhomogeneity index). Regional cytokine quantification showed a tendency to greater inflammation in the dependent lung regions (3850 ± 2802 vs 643 ± 907.9 ng/ml, p= 0.06), before connection to mechanical ventilation, but without statistical significance (Fig. 3).
**CONCLUSIONS.** Connection to mechanical ventilation changes the distribution of ventilation from dorsal to ventral regions. In the pattern of pre-intubation ventilation, a tendency to greater inflammation can be observed in the dorsal regions of the lung.

**REFERENCE(S)**

1. Yoshida T, Roldan R, Beraldo MA, Torsani V, Gomes S, De Santis RR, et al. Spontaneous Effort During Mechanical Ventilation. Crit. Care Med. 2016;44:e678-88.
2. Mascheroni D, Kolobow T, Fumagalli R, Moretti MP, Chen V, Buckhold D. Acute respiratory failure following pharmacologically induced hyperventilation: an experimental animal study. Intensive Care Med. 1988;15:8-14.

**GRANT ACKNOWLEDGMENT**

This study was supported by a grant of Fondo Nacional de Desarrollo Científico y Tecnológico (FONDECYT) 1171810.

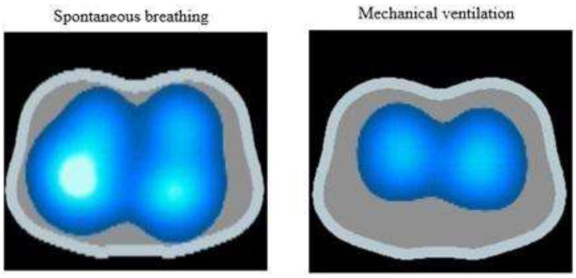

**Fig. 1 (abstract 0274).** Ventilation distribution images delivered by EIT in spontaneous breathing and mechanical ventilation

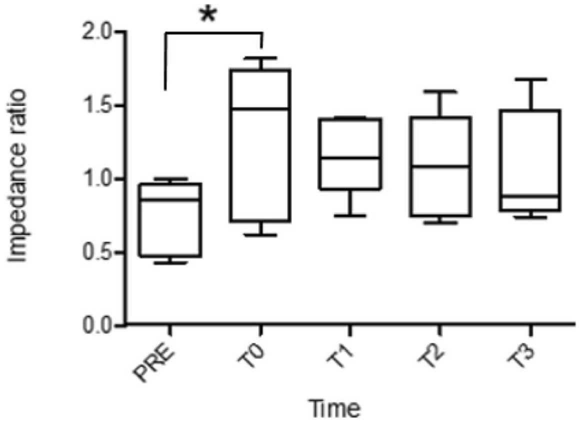

**Fig. 2 (abstract 0274).** Impedance Ratio. Changes between spontaneous breathing (PRE) and hours 0,1,2 and 3 post intubation. *p<0.05

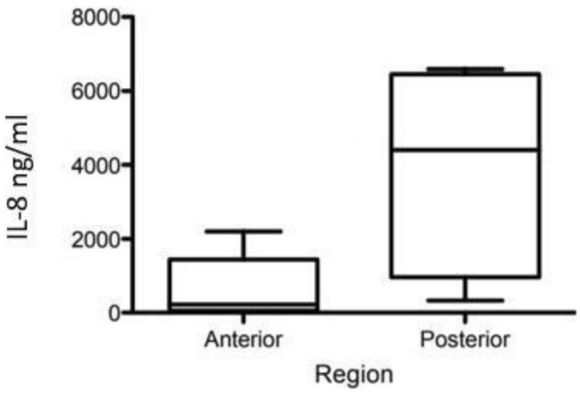

**Fig. 3 (abstract 0274).** Concentration of interleukin-8 (BAL) in the anterior and posterior regions of the lung

### 0275

#### Six-minute walk test as a predictor of changes in basic functional dyspnea assessed by the modified medical research council scale in ICU COPD survivors

W. Zarrougui[1], A. Khedher[1], I. El Meknassi[1], I. Ben Saida[1], M.A. Boujelbèn[1], S. Kortli[1], A. Azouzi[1], K. Meddeb[1], M. Boussarsar[1,2]
[1]Farhat Hached University Hospital, Medical Intensive Care Unit, Sousse, Tunisia; [2]Ibn Al Jazzar Faculty of Medicine, Research Laboratory N° LR12SP09 Heart Failure, Sousse, Tunisia
**Correspondence:** M. Boussarsar
*Intensive Care Medicine Experimental* 2018, **6(Suppl 2):**0275

**INTRODUCTION.** The 6-minute walk test (6MWT) is an established surrogate outcome measure in COPD due to its correlation with baseline functional dyspnea for what Modified Medical Research Council (mMRC) scale is commonly used to determine both the stage of the disease and the appropriate medical treatment.
**OBJECTIVES.** To explore the discriminative properties of the 6MWT in the prediction of changes in mMRC dyspnea grade and the delay to the return to baseline level of dyspnea for COPD survivors after ICU stay
**METHODS.** A prospective observational cohort study was performed in a Tunisian ICU between February 2017 and January 2018, including all consecutive survivors of AE/COPD. The 6MWT is performed the day of ICU discharge according to the international guidelines. The walked and predicted distances were calculated. Patients' severity of dyspnea is rated on mMRC scale ranging from 0 to 4 recorded at admission and at 3 months of ICU discharge via phone calls. Receiver operating characteristic (ROC) curves and the area under the curve (AUC) with 95% confidence intervals (CI), sensitivity, specificity, predictive values (PV) and likelihood ratios (LR) were used to assess test performance to predict return to baseline level of dyspnea at 3 months after ICU discharge.
**RESULTS.** Among 90 patients admitted for AECOPD during the study period, 62(69%) were included. General characteristics were: age, 66.4 ±9.5 years; sex ratio, 4,5/1; Charlson index, 3.9±1.8; COPD GOLD D, 55(88%); 34(55%) patients in classes III and IV of mMRC scale. SAPSII score, 29.8±9.9; initial invasive mechanical ventilation, 17 (27.4%); mean length of stay, 13.6±10.1days.
Mean 6-min walked distance, 223±96m; mean walked/predicted (W/P) distance ratio, 0.41±0.17. Three months after discharge, 58(93%) patients could be interviewed via phone, among them 44(77%) patients were in classes III and IV of mMRC scale. Median[IQR] delta mMRC (at 3months-at admission) was 0[0,1].
There was a significant difference in distance walked between those with mMRC class 0, I and II, compared with those classified as III or IV (257m vs 201m; p=0.03).
Return to baseline level of dyspnea prior to ICU stay was shown in 28(48.2%) patients and the mean delay to return was 65±29.2days. 6MWD was significantly correlated with return to level of dyspnea prior to ICU admission (p=0.012). ROC curve of W/P distance ratio and the return to basic functional dyspnea identified interesting AUC (AUC=0.7, 95%CI, [0.56-0.83], p=0.009); the optimal cut-point for the W/P distance ratio was 0.41 with sensitivity 69%[49-84], specificity 72.4%[52.5-86.6]. Positive and negative PV were respectively 71.4%[51.1-86] and 70%[50.4-84.6], positive and negative LR were respectively 2.46 and 0.43.
**CONCLUSIONS.** In this cohort, walked/predicted Six-Minute Walk Test distance in AE/COPD at ICU discharge seems to demonstrate interesting discriminative properties to predict the return to baseline level of dyspnea for COPD survivors.

### 0276

#### Pulmonary embolism among ICU population: prevalence, delay of onset, diagnostic features and prognosis

O. Turki, M. Dlela, S. Bradai, K. Bouchala, K. Regaieg, M. Bahloul, M. Bouaziz
CHU Habib Bourguiba Sfax, Intensive Care Unit, Sfax, Tunisia
**Correspondence:** O. Turki
*Intensive Care Medicine Experimental* 2018, **6(Suppl 2):**0276

**INTRODUCTION.** Pulmonary embolism (PE) is a well-established complication of ICU stay. Although well documented in the general population, there is little written about PE peculiarities in ICU, where it is more difficult to diagnose and to treat.
**THE AIM OF THIS STUDY** was to analyze the prevalence, delay of onset, diagnostic features and prognosis of PE among ICU population.
**METHODS.** This was a prospective cohort including all patients admitted to our ICU between the 01/06/2015 and the 30/06/2016. Patients with positive of PE at any point during ICU stay were included.Patients were classified according to their outcome. Factors associated with poor prognosis were identified using both univariate and multivariate analysis.
**RESULTS.** During the study period's, 75 patients (10.1 % ) with positive diagnosis of PE, were included, among which, 40 (53.3%) cases were admitted for post traumatic injuries.The average age was 53 ± 18,1 years and mean SAPS III was 43,4 ± 12,43.All patients had DVT prophylaxis with a mean delay of 24hours after admission.
PE diagnosis was revealed by hypoxemia in 73(97.3%) patients and fever in 31(41.3%).Twenty-two (29.3%) presented with circulatory failure and thirty-four (45.3%) were found to haveechographic signs of right ventricular (RV) dysfunction. Clinical assessment showed that 55 patients (73.3%) had sinus tachycardia and 38 had an increased shock index. Diagnosis was confirmed using CT-scan in 98.7% of the cases. PE was associated to a deep venous thrombosis in 8(23%) cases. Moreover, in this study, PE occurred within the first week in 60% of patients and within 5 days in 46.6% of them.
Although 92% of patients had anticoagulant therapy, there were 4 cases of recurrent PE. Among patients sustaining a PE, overall mortality was 38.6%.
Comparing to the survivors, non-survivors had older age, higher SAPSIII score, lower Glasgow-coma-scale and a higher Shock-index. Howevercirculatory failure and RV dysfunction were found to be the only predictive factors of mortality on multivariate analysis.
**CONCLUSIONS.** PE is a life-threatening complication of deep venous thrombosis.Based on this single-center data set, it appears to be associated witha high mortality rate among ICU patients. Consequently, prevention is highly warranted. Additionally, further study and understanding of thromboembolism in ICU patients is needed to improve our evaluation, prophylaxis, and treatment of this complication.

**REFERENCE(S)**

Pulmonary embolism following severe traumatic brain injury: incidence, risk factors and impact outcome.Bahloul M, Chelly H, Regaieg K, Rekik N, Bellil S, Chaari A, Bouaziz W, Chabchoub I, Haddar S, Hamida CB, Bouaziz M. Intensive Care Med. 2017 Sep;43(9)

### 0277

#### Factors associated with intra-abdominal hypertension in mechanically ventilated patients with acute exacerbation of chronic obstructive pulmonary disease (AE/COPD): aretrospective study

I. El Meknassi[1], M.A. Boujelbèn[1], S. Kortli[1], N. Fraj[1], S. Rouis[1], H. Zorgati[1], A. Khedher[1], A. Azouzi[1], I. Ben Saida[1], K. Meddeb[1], M. Boussarsar[1,2]
[1]Farhat Hached University Hospital, Medical Intensive Care Unit, Sousse, Tunisia; [2]Ibn Al Jazzar Faculty of Medicine, Research Laboratory N° LR12SP09 Heart Failure, Sousse, Tunisia
**Correspondence:** M. Boussarsar
*Intensive Care Medicine Experimental* 2018, **6(Suppl 2):**0277

**INTRODUCTION.** Intra-abdominal hypertension (IAH) and abdominal compartment syndrome (ACS) could be associated to severe AE/COPD. But little is known about its etiopathogenetic mechanisms.
**OBJECTIVES.** To identify risk factors of IAH in severe AE/COPD in a Tunisian medical ICU.
**METHODS.** Retrospective observational study conducted in a 9bed Tunisian medical ICU during 3 years from January 2015 to December 2017. Were included all consecutive patients admitted for AE/COPD who were mechanically ventilated (MV). Were compared, underlying conditions, circumstances, onset and natural history of IAH, the presence of deep and sustained episode of hypoxemia, clinical characteristics at ICU admission, management and outcomes. Univariate and multivariate analyses were performed to identify factors independently associated to IAH. AUC/ROC curves were used to check for the discriminative properties of the significant factors.
As reported by the World Society of the Abdominal Compartment Syndrome (WSACS)(1), IAH was defined by a sustained or repeated pathologic elevation of IAP ≥ 12mmHg.
**RESULTS.** Among the total of 717 admissions within the 3 years study period, 195(27%) patients presented with AE/COPD, 103(52.8%) patients were mechanically ventilated. 27(26.2%) presented an associated IAH at ICU admission. They were 68[61-77] years aged, 89(86,4%) male. 47(45,6%) had mMRC≥3. Median SAPSI, 32[26-42]. 63(61,2%), severe hypercapnic encephalopthy. Mean pH and PaCO2 were respectively 7,28[7.22-7.34] ; 62[48-82]mmHg. Median length of stay and mortality were respectively 14[6-25]days and 52,4%.
Univariate analysis identified the following to be associated with IAH, BMI≥30, 9(33.3%) vs 6(7.9%), p=0.001 ; diabetes mellitus, 10(37%) vs 7(9.2%), p=0.001 ; episode of deep and prolonged hypoxemia 21(77.8%) vs 19(25%), p=0.000 ; consultation delay, 17(63%) vs 31(40.8%), p=0.045 ; inadequate ventilation 16(59.3%) vs 25(32.9%), p=0.016 ; and severe obstructive ventilator disorder, 20(74.1%) vs 31(40.8%), p=0.003.
Multivariate analysis identified the male sex (OR=51.7, 95%CI, [3.89-688], p=0.003), severe obstructive ventilator disorder (OR=16.2, 95%CI, [2.57-101], p=0.003), severe hypercapnic encephalopathy at the admission (OR=0.12, 95%CI, [0.02-0.69], p=0.018) and an episode of deep prolonged hypoxemia (OR=42.1, 95%CI, [6.68-265], p=0.000) as independent risk factors of IAH in COPD patients.
**CONCLUSIONS.** The present study identified an at-risk-profile of severe AE/COPD patients presenting with IAH. They are male, presenting with severe hypercapnic encephalopathy associated with the highest resistive mechanics and experienced an episode of prolonged and deep hypoxemia.

**REFERENCE(S)**

1. Malbrain ML, De Laet IE, De Waele JJ, Kirkpatrick AW. Intra-abdominal hypertension: definitions, monitoring, interpretation and management. Best practice & research Clinical anaesthesiology. 2013;27(2):249-70.

### 0278

#### Evaluation of inflammatory markers with a routine blood test, for the identification of patients with severe exacerbation of chronic obstructive pulmonary disease, in intensive care unit

A.M. Bueno-Gonzalez[1], M. Portilla-Botelho[1], M. Sanchez-Casado[2], J. Monserrat[3], A. Ambros-Checa[1], C. Martin[1], M. Alvarez-Mon[3]
[1]Hospital General Universitario de Ciudad Real, Intensive Care Unit, Ciudad Real, Spain; [2]Hospital Virgen de la Salud, Intensive Care Unit, Toledo, Spain; [3]Universidad de Alcalá, Internal Medicine and Inmunology, Alcalá de Henares, Spain
**Correspondence:** M. Portilla-Botelho
*Intensive Care Medicine Experimental* 2018, **6(Suppl 2):**0278

**INTRODUCTION.** Nowadays, only few validated markers are available to evaluate exacerbation of chronic obstructive pulmonary disease (COPD) and to assess the efficacy of treatment. New markers are required to better characterize the entire clinical spectrum of the disease and to guide the development and evaluation of new more effective treatments
**OBJECTIVES.** 1º / To know the clinical characteristics of COPD patients admitted to ICU and their prognosis. 2º / To evaluate inflammatory markers and determine the optimal cut-off point associated with COPD exacerbation
**METHODS.** Patients with diagnosis of exacerbated COPD upon admission to Intensive Care Unit (ICU) were included, and different inflammatory markers were analyzed to determine their association with COPD exacerbation. Patients with COPD in stable phase, were obtained from the follow-up one year after discharge.
The differences between stable and exacerbated groups were analyzed using the chi squared test for categorical variables and the Student t test for quantitative variables. Sensitivity, specificity and ROC curves were calculated, using the diagnosis of exacerbated COPD performed by the intensive care physician upon admission to ICU, as a standard reference.
The optimal cut-off point was the value that minimizes the sum of false positives, maximizes sensitivity, specificity and AUC ROC. Statistical analysis with STATA software 13.0
**RESULTS.** 55 patients (p) were analyzed, with a mean age of 68.5 years, mean severity APACHE of 26 and mean stay in ICU of 6 days. 48 p (87.5%) required non-invasive mechanical ventilation and 16 p (29.1%) required orotracheal intubation during their admission. The overall mortality was 17.7% and 29% had annual readmission to hospital.
In the comparative study of serum inflammatory markers in acute and stable phase, significant differences were obtained in fibrinogen (510.8 vs 314.5: p < 0.001), C-reactive protein (CRP) (7.8 vs 0.9, p: 0.004), Leukocytes (13.4 vs 9.2, p: 0.012), neutrophils (11.26 vs 6.5, p: 0.007) and lactic (17.5; vs 10.2; p: 0.005). There were no significant differences in eosinophils, platelets and hematocrit.
The comparison of predictability made by analysis with ROC curves of CRP, fibrinogen, leukocyte and lactic variables shows that CRP presents the best prediction of COPD exacerbation (ROC area: 0.85 CI 95%: 0.74-0.95). The moderate elevation of CRP (1 to 10mg / dl) obtained a Sensitivity: 32- 68% and Specificity: 85-100%
**CONCLUSIONS.** Although the diagnosis of COPD exacerbation is clinical, the CRP value is the inflammatory marker that can help us the most when diagnosing COPD exacerbation. According to published data, being CRP a marker of inflammation in the respiratory tract, these patients should be treated with anti-inflammatory drugs

**REFERENCE(S)**

Agassandian, Shurin, Ma et al. C-reative protein and lung diseases. IntJ BiochemCellBiol. 2014;53:77-88

**GRANT ACKNOWLEDGMENT**
Fundación Sociosanitaria de Castilla-La Mancha

### 0279

#### Prone positioning in severe acute respiratory failure not fulfilling Berlin definition ARDS criteria: a case series

S. Kortli[1], A. Khedher[1], M.A. Boujelbèn[1], D. Ben Braiek[1], W. Zarrougui[1], N. Fraj[1], A. Azouzi[1], I. Ben Saida[1], K. Meddeb[1], M. Boussarsar[1,2]
[1]Farhat Hached University Hospital, Medical Intensive Care Unit, Sousse, Tunisia; [2]Ibn Al Jazzar Faculty of Medicine, Research Laboratory N° LR12SP09 Heart Failure, Sousse, Tunisia
**Correspondence:** M. Boussarsar
*Intensive Care Medicine Experimental* 2018, **6(Suppl 2):**0279

**INTRODUCTION.** Prone positioning is a recommended intervention for refractory hypoxemia in patients with ARDS. Diffuse atelectasis commonly occurs in the lung dependent regions in ventilated patients.
**OBJECTIVES.** To demonstrate the potential benefit of prone positioning in severe non-ARDS acute respiratory failure.
**METHODS.** A retrospective case series study of all consecutive patients within a period of 2 years (2016-2017). Were included patients who developed within their ICU stay a severe hypoxemic acute respiratory failure related to diffuse atelectasis without pulmonary edema or inflammation context and received at least one session of prone positioning.
**RESULTS.** 12 patients were collected within the study period among a total of 486 ICU admissions. Nine patients presented with AE/COPD. Median Charlson index, 5[3-7]; Median SAPSII, 35[31-40]; median P/F ratio, 87[71-97]mmHg ; median Plateau pressure, 23[19-27]cmH2O; median PEEP, 9[5.25-11.5]cmH2O. Median duration of prone positioning, 12.5[8-16]H ; median number of prone positioning sessions 1[1-2]. Post - prone positioning P/F ratio, 312[248-374]mmHg. Favorable ICU outcome was only achieved in 5(42%) patients.
**CONCLUSIONS.** In this case series of severe hypoxemic acute respiratory failure patients related to dependent lung regions atelectasis, prone positioning may improve oxygenation.

### 0280

#### Is the routine use of antipseudomonal antibiotics as empiric treatment of AECOPD in ICU indicated? A retrospective study

F. Marcy, T. Schroeder, C. Storm, J.M. Kruse
Charité - Universitätsmedizin Berlin, Medical Department, Division of Nephrology and Internal Intensive Care Medicine, Berlin, Germany
**Correspondence:** F. Marcy
*Intensive Care Medicine Experimental* 2018, **6(Suppl 2):**0280

**NTRODUCTION.** Chronic Obstructive Pulmonary Disease (COPD) is one the most frequent causes of death worldwide.[1] Patients benefit from an empiric antibiotic treatment when admitted to intensive care units (ICU) for acute exacerbations (AECOPD).[2] There is not sufficient evidence justifying the regular empiric treatment with antibiotics against *Pseudomonas aeruginosa*. However, their use seems to have increased over the last decade.[3]

**OBJECTIVES.** As antibiotic resistance remains a major challenge to modern medicine, proper antibiotic choice is crucial. This is especially important in pathogens with pronounced resistance mechanisms like *P. aeruginosa*. The aim of this study was to question a beneficial outcome in the routine use of antipseudomonal antibiotics in the empiric treatment of AECOPD in ICU.

**METHODS.** From January 2009 to June 2017, all patients with AECOPD admitted to two medical ICUs of Charité - Universitätsmedizin Berlin from outpatient care were retrospectively analyzed. The antibiotic treatment given within 72 hours of admission was documented considering its activity against *P. aeruginosa*. Samples taken from sputum, bronchoalveolar lavage and blood cultures were analyzed. The influence of SOFA score at admission and time of mechanical ventilation upon survival was investigated by a multivariate analysis.

**RESULTS.** A total of 437 patients were included in the study. Mean age was 68 years, 46,5% were female. 271 patients (62%) were initially treated with antipseudomonal antibiotics (PAA), 166 (38%) were given antibiotics inactive against *P. aeruginosa* (PIA). Age, sex, comorbidities and median length of stay in ICU did not differ significantly between the groups. The initial median SOFA score was 3 in the PIA group and 5 in the PAA group (p=0,002). Median time of mechanical ventilation was 66 hours in patients with PAA vs. 22 hours in PIA (p=0,003). Positive samples were found in 107 patients (24,5%). *P. aeruginosa* was positive in one patient with PIA (0,6%) vs. 15 patients in the PAA group (5,5%) (p=0,008). There was no significant difference in 30-day ICU mortality (20,4% ± 11,6 in patients with PIA vs. 29,3% ± 10,8 in patients with PAA, p=0,113).

**CONCLUSIONS.** In this study, antibiotics active against *P. aeruginosa* showed no benefit in the empiric treatment of AECOPD in ICU regarding ICU mortality. However, antibiotic choice should still be guided by an individual risk stratification.

### 0281

#### Compared prognosis of acute exacerbation of chronic obstructive pulmonary disease (AE/COPD) with and without intra-abdominal hypertension in mechanically ventilated patients

I. El Meknassi[1], S. Kortli[1], D. Ben Braiek[1], N. Fraj[1], W. Zarrougui[1], M.A. Boujelbèn[1], A. Khedher[1], A. Azouzi[1], I. Ben Saida[1], K. Meddeb[1], M. Boussarsar[1,2]
[1]Farhat Hached University Hospital, Medical Intensive Care Unit, Sousse, Tunisia; [2]Ibn Al Jazzar Faculty of Medicine, Research Laboratory N° LR12SP09 Heart Failure, Sousse, Tunisia
**Correspondence:** M. Boussarsar
*Intensive Care Medicine Experimental* 2018, **6(Suppl 2):**0281

**INTRODUCTION.** Intra-abdominal hypertension (IAH) and abdominal compartment syndrome (ACS) in critically ill patients are frequent and are reported to be associated with poor outcomes. Little is known about the association between AE/COPD and IAH.

**OBJECTIVES.** To compare the prognosis of AE/COPD patients with and without IAH in a Tunisian medical ICU.

**METHODS.** A retrospective observational study conducted in a 9bed Tunisian medical ICU during 3 years from January 2015 to December 2017. Were included all consecutive patients admitted for AE/COPD who were MV. Were assessed, underlying condition, clinical characteristics and outcomes. Were compared the length of stay (LOS), mechanical ventilation duration, ventilator free days and a composite outcome (LOS>14days and/or death).

As reported by the World Society of the Abdominal Compartment Syndrome (WSACS)(1), IAH was defined by a sustained or repeated pathologic elevation of IAP ≥ 12 mmHg.

**RESULTS.** Among the total of 717 admissions within the 3 years study period, 195(27%) patients presented with AE/COPD, 103(52.8%) patients were mechanically ventilated. 27(26.2%) presented an associated IAH at ICU admission. They were 68[61-77] years aged ; COPD GOLD D, 97(94.2%) ; mMRC dyspnea scale≥3, 47(45.6%) ; long term oxygen therapy, 22(21.4%) and home NIV use, 13(12.6). Median SAPSII was 32[26-42]. 63(61.2%) presented with a severe hypercapnic encephalopathy. Mean pH and PaCO2 were respectively 7,28[7.22-7.34] and 62[48-82]mmHg. 17(62.9%) patients presented with severe obstructive ventilatory disorder with high airway pressures (mean peak pressure, autoPEEP respectively 36.5[30-42.75] and 7[4-10]cmH2O).

Univariate analysis identified the following respectively for IAH+ and IHA : LOS, 20.78±17.03 days vs 16.93±13.94 days, p=0.198 ; MV duration, 18.63±16.92 vs 14.34±13.95 days, p=0.198 ; ventilator free days, 6(22.2%) vs 25(32.9%), p=0.299 ; composite outcome, 23(85.2%) vs 61(80.3%), p=0.571 and mortality, 14(51.9%) vs 40(52.6%), p=0.944.

**CONCLUSIONS.** In the present study with a small sample and highly selected severe AE/COPD patients, the association with IAH did not alter any of these studied outcomes.

**REFERENCE(S)**

1. Malbrain ML, De Laet IE, De Waele JJ, Kirkpatrick AW. Intra-abdominal hypertension: definitions, monitoring, interpretation and management. Best practice & research Clinical anaesthesiology. 2013;27(2):249-70.

### 0282

#### Management of intra-abdominal hypertension in acute exacerbation of chronic obstructive pulmonary disease (AE/COPD): experience of a medical Tunisian ICU

I. El Meknassi[1], N. Fraj[1], S. Kortli[1], M. Zghidi[1], M.A. Boujelbèn[1], W. Zarrougui[1], A. Khedher[1], A. Azouzi[1], I. Ben Saida[1], K. Meddeb[1], M. Boussarsar[1,2]
[1]Farhat Hached University Hospital, Medical Intensive Care Unit, Sousse, Tunisia; [2]Ibn Al Jazzar Faculty of Medicine, Research Laboratory N° LR12SP09 Heart Failure, Sousse, Tunisia
**Correspondence:** M. Boussarsar
*Intensive Care Medicine Experimental* 2018, **6(Suppl 2):**0282

**INTRODUCTION.** Intra-abdominal hypertension (IAH) is frequent in critically ill-patient. It is classified into primary (related to intra-abdominal disease or injury) and secondary IAH. Therefore, it is important to understand different therapeutics and their efficiencies.

**OBJECTIVES.** To describe our experience in the management of AE/COPD patients who presented with a secondary IAH.

**METHODS.** A retrospective observational study conducted in a 9bed Tunisian medical ICU during 3 years from January 2015 to December 2017. Were included all consecutive patients admitted for AE/COPD associated with IAH. Were assessed, frequency of IAH, patient´s characteristics, underlying condition, circumstances, natural history, clinical characteristics at ICU admission, management and outcomes. As reported by the World Society of the Abdominal Compartment Syndrome (WSACS), IAH was defined by a sustained or repeated pathologic elevation of IAP ≥ 12 mmHg. The abdominal compartment syndrome (ACS) was defined as a sustained IAP≥20 mmHg (with or without an abdominal perfusion pressure < 60 mmHg) that is associated with new organ dysfunction/failure.

**RESULTS.** Among the 717 patients admitted within the 3 years study period, 195(27%) presented with AE/COPD. Thirty-Two patients (16.4%) were diagnosed with IAH. They were 68.5[58.5-75.75]years aged ; COPD GOLD D, 30(93.8%). SPASII, 33[28-43.5]. 14(43.8%), severe hypercapnic encephalopathy. Median pH and PaCO2 were respectively 7.32[7.22-7.35] and 56[42-79]mmHg. 17(62.9%) presented with severe obstructive ventilatory disorder with high airway pressures (median peak pressure, autoPEEP and plateau respectively 40[35.5-47], 9.5[7-10.75] and 22[20-26]cmH2O).

First line noninvasive ventilation (NIV) was used in 19(59.4%) with NIV failure in 14(73.7%) within a median delay of 24[5.5-48] hours. Overall mechanical ventilation use was 27(84.3%) with VFD in 6 (22.2%). Median length of stay was 11.5[5-28.5] days. Overall mortality rate was 43.8%. In all patients IAH was present at admission. Median intra-abdominal pressure was 23.5[20.5-28.7]. 19(59.4%) patients had evolved to ACS.

Management in all patients with IAH was when appropriate: sedation cessation, mobilization, weaning of vasopressors, hydration with avoiding excessive fluid resuscitation, feeding, reduction of patient ventilator asynchrony and electrolyte disorders correction mainly hypokalemia and hypomagnesemia (n=5). All these interventions were systematic. When insufficient, other procedures were performed : nasogastric and rectal decompression (n=27), purgative drugs (n=5), lactulose (n=22), prostigmin (n=5), erythromycin as prokinetic, evacuation enema (n=16) and coloexsufflation (n=2). No surgical procedure was performed.

**CONCLUSIONS.** In the present study, the management of IAH in severe AE/COPD patients was stuttering. Yet, it went along with latest recommendations of WSACS.

### 0283

#### Factors affecting cost of patients with severe community-acquired pneumonia in intensive care unit

A. Gumus[1], A. Cilli[1], O. Cakın[1], M. Cengiz[1], Z. Karakurt[2]
[1]Akdeniz University Hospital, Antalya, Turkey; [2]Saglık Bilimleri University, Ankara, Turkey
**Correspondence:** A. Gumus
*Intensive Care Medicine Experimental* 2018, **6(Suppl 2):**0283

**OBJECTIVES.** The aim of this study is to investigate the factors affecting cost in patients with severe community-acquired pneumonia (CAP) who admitted to the intensive care unit.

**METHODS.** This retrospective cohort study was conducted at Süreyyapaşa Göğüs Hastalıkları Eğitim Araştırma Hospital, Dokuz Eylül University Hospital, Ankara University Hospital, Akdeniz University Hospital intensive care unit between January 2013 - December 2016. A total of 291 patients with severe CAP were included in the study. Demographic and clinical data were obtained from medical records of all cases.

**RESULTS.** Of 291 patients, mean age was 68.4 ± 16.8yrs, 61% were female. Median length of intensive care unit stay was 7 days (1st - 3rd percentiles: 4-11 days). Forty-six percent of patient had chronic obstructive pulmonary disease (COPD). Hypertension is the second comorbidity of the patients (42%). Mean cost was found as 2722 $ (5578 TL). The highest cost was found in the group of patients aged 50-59, lowest cost was found in the < 50 aged patients. Statistically significant relationship was found between PSI, APACHE II, CURB-65 scores and health cost (p=0.001, p=0.001, p=0.009 respectively). The cost of patients in PSI class V, APACHE II (>20 points) and CURB-65 score ≥3 points were higher. The presence of concomitant COPD (p=0.009), atrial fibrillation (p=0.046), congestive heart failure (p< 0.042), hypoalbuminemia (p=0.001), mental status disorder (p=0.013), in-hospital mortality (p=0.014), severe sepsis (p< 0.001), septic shock (p< 0.001), mechanical ventilation requirement (p< 0.001), use of haloperidol (p< 0.001) and vasopressor (p< 0.001) were associated with higher cost; while use of florokinolon (p=0.033) was associated with lower cost.

**CONCLUSIONS.** IMV and NIMV requirement, presence of sepsis, atrial fibrillation, COPD, congestive heart failure were associated with increased cost in patients with severe CAP. Hypoalbuminemia and impaired mental status were also associated with higher cost. Patients with high mortality rates were are also associated with higher cost.

**Table 1 (abstract 0283).** Statistics

**Tablo 3.** PSI, CURB-65, SOFA ve Quick SOFA ile maliyet arasındaki ilişkinin değerlendirilmesi

| | Maliyet Median (Q.1-Q.3) | p değeri |
|---|---|---|
| (PSI) | | |
| Grup I-II (n=16) | 1649 (1179-2532) | * 0.001 |
| Grup III (n=22) | 2339 (1576-3289) | |
| Grup IV (n=84) | 3249 (2069-5026) | |
| Grup V (n=169) | 3964 (2181-7004) | |
| APACHE II | | |
| <20 puan (n=144) | 3039 (1625-4541) | **0.001 |
| >20 puan (n=147) | 4129 (2230-6900) | |
| Quick SOFA | | |
| <2 puan (n=205) | 3198 (1980-4916) | **0.09 |
| ?2 puan (n=86) | 4071 (1710-8021) | |
| SOFA skoru | | |
| <3 (n=29) | 3069 (1470-4281) | **0.06 |
| ?3 (n=262) | 3414 (2061-5952) | |
| CURB-65 | | |
| 0 (n=12) | 3121 (2275-4393) | *0.009 |
| 1 (n=65) | 2477 (1480-3746) | |
| 2 (n=73) | 3592 (2269-5252) | |
| ?3 (n=141) | 4040 (2138-7472) | |

### 0284

#### Frequency, characteristics and prognosis of acute exacerbation of chronic obstructive pulmonary disease (AE/COPD) patients presenting with intra-abdominal hypertension in a Tunisian medical ICU: a retrospective study

I. El Meknassi[1], S. Kortli[1], S. Rouis[1], M.A. Boujelbèn[1], N. Fraj[1], W. Zarrougui[1], A. Khedher[1], A. Azouzi[1], I. Ben Saida[1], K. Meddeb[1], M. Boussarsar[1,2]
[1]Farhat Hached University Hospital, Medical Intensive Care Unit, Sousse, Tunisia; [2]Ibn Al Jazzar Faculty of Medicine, Research Laboratory N° LR12SP09 Heart Failure, Sousse, Tunisia
**Correspondence:** M. Boussarsar
*Intensive Care Medicine Experimental* 2018, **6(Suppl 2):**0284

**INTRODUCTION.** Intra-abdominal hypertension (IAH) and abdominal compartment syndrome (ACS) are increasingly recognized in the critically-ill and may seriously impede the respiratory mechanics. Various clinical conditions are associated with this syndrome and include mainly surgical ones. We were recently challenged by a growing frequency of IAH in the setting of severe AE/COPD patients.

**OBJECTIVES.** To determine the frequency of IAH in AE/COPD patients during ICU stay and to establish a clinical and prognostic profile.

**METHODS.** Retrospective observational study conducted in a 9bed Tunisian medical ICU during 3 years from January 2015 to December 2017. Were included all consecutive patients admitted for AE/COPD. Were assessed, frequency of IAH, patient´s characteristics, underlying condition, circumstances, natural history, clinical characteristics at ICU admission, management and outcomes.

As reported by the World Society of the Abdominal Compartment Syndrome (WSACS)(1), IAH was defined by a sustained or repeated pathologic elevation of IAP ≥ 12 mmHg. ACS was defined as a sustained IAP≥20 mmHg (with or without an abdominal perfusion pressure < 60 mmHg) that is associated with new organ dysfunction/ failure.

**RESULTS.** Among the 717 patients admitted within the 3 years study period, 195(27%) presented with AE/COPD. 32 (16.4%) patients were diagnosed with IAH. They were 68.5[58.5-75.75] years aged ; COPD GOLD D, 30(93.8%) ; mMRC dyspnea scale≥3, 12(37.5%) ; long term oxygen therapy, 7(21.9%) ; home NIV use, 4(12.5%) and median SPA-SII, 33[28-43.5]. Tracheobronchitis, 17(53.1%) ; left heart failure 6(18.8%) and pneumonia 14(12.5%) were among the most common triggering factors. 14(43.8%) presented with a severe hypercapnic encephalopathy. Median pH and PaCO2 were respectively 7.32[7.22-7.35] and 56[42-79] mmHg. 17(62.9%) presented with severe obstructive ventilator disorder with high airway pressures (median peak pressure and autoPEEP respectively 40[35.5-47] and 9.5[7-10.75] cmH2O). Firstline noninvasive ventilation (NIV) was used in 19(59.4%) patients with NIV failure in 14(73.7%) within a median delay of 24[5.5-48] hours. Overall mechanical ventilation use was 27(84.3%). Median length of stay was 11.5[5-28.5] days. In all patients, IAH was present on admission. Median intra-abdominal pressure was 23.5[20.5-28.7]. Median abdominal distension duration was 12.2 ±9.8days. 15(62.5%) patients had difficulty of weaning requiring tracheostomy in 28.1%. 19(59.4%) patients evolved to ACS. Only purgative drugs were used and no surgery was performed. Overall mortality rate was 43.8%.

**CONCLUSIONS.** IAH and ACS seem to be common in this selected setting of severe AE/COPD. It is associated to therapeutic challenges and poor outcomes.

**REFERENCE(S)**

1. Malbrain ML, De Laet IE, De Waele JJ, Kirkpatrick AW. Intra-abdominal hypertension: definitions, monitoring, interpretation and management. Best practice & research Clinical anaesthesiology. 2013;27(2):249-70.

### 0285

#### Severe Acute Respiratory Infection (SARI): frequency and microbiology in a Tunisian medical ICU

K. Meddeb[1], M.A. Boujelbèn[1], S. Rouis[1], N. Fraj[1], W. Zarrougui[1], D. Ben Braiek[1], A. Khedher[1], A. Azouzi[1], I. Ben Saida[1], I. Chouchene[1], M. Boussarsar[1,2]

[1]Farhat Hached University Hospital, Medical Intensive Care Unit, Sousse, Tunisia; [2]Ibn Al Jazzar Faculty of Medicine, Research Laboratory N° LR12SP09 Heart Failure, Sousse, Tunisia

**Correspondence:** M. Boussarsar

*Intensive Care Medicine Experimental* 2018, **6(Suppl 2):**0285

**INTRODUCTION.** Severe Acute respiratory infections occurring seasonally are a major health problem. Surveillance protocols aim to detect pandemics and any new viruses' species responsible for SARI cases. Intensive care unit of Farhat Hached university hospital is one of six Tunisian centres involved in national and international SARI surveillance program.

**AIM.** To assess frequency and microbiology, severity and outcome in patients admitted for SARI in Farhat Hached ICU.

**METHODS.** This is a retrospective study, including all patients admitted in ICU for SARI, over two seasons, going from September 2016 to May 2017 and from September 2017 to March 2018. SARI was defined according to the World Health Organisation (WHO) Global Epidemiological Surveillance Standards for Influenza 2014 [1], where patient report a fever or an objective measurement of temperature ≥ 38°C, associated to a cough, appearing ten days prior to consultation and requiring hospitalization.

**RESULTS.** Among 344 admissions, 77(22.4%) patients met SARI definition and were included in the study. They were 56±19 years old mean aged, 40(59%) AECOPD. Symptoms' onset was at a median delay of 4[1-7] days prior to consultation. ICU hospitalization occurred at a median of 4[1-5] days from first medical contact. On admission, SAPS II, 30±12; Acute Respiratory Distress Syndrome, 21(27,3%); invasive mechanical ventilation, 36(46.8%); vasopressors, 21(27.3%). Nasal swabs for viral polymerase chain reaction (PCR) were performed for 25(32,5%), 6(24%) were positive, AH1N1 (n=5), and Coronavirus OC43 (n=1). A bacterial pathogen was isolated in 3 patients *(Pseudomonas aeruginosa, Acinetobacter baumannii and staphylococcus aureus)* among 27(35,1%) sampled patients. 36(46,8%) patients did not have PCR or any other microbiology sampling. Mortality was estimated at 45,5%.

**CONCLUSION.** SARI patients were relatively frequent. PCR sampling for viral identification was performed in only one-third of cases. It can be considered insufficient in terms of surveillance system performance to identify viral species involved in SARI.

[1]. World Health Organization. Global Epidemiological Surveillance Standards for Influenza. 2014

### 0286

#### The effect of the ventilator care bundle on ventilator acquired pneumonia, length of stay and mortality rate: a systematic review and meta-analysis

L. Dolman

Imperial College Healthcare Trust, London, United Kingdom

*Intensive Care Medicine Experimental* 2018, **6(Suppl 2):**0286

**BACKGROUND.** Ventilator associated pneumonia (VAP), has the highest incidence rate of all healthcare-associated infections within critical care (Vincent et al 2006). VAP can increase mortality rate by 30% (Kollef et al 2006). The ventilator care bundle aims to reduce a patient's risk of these by grouping five elements of best practice: elevation of head of the bed, daily sedation interruption and daily assessment for readiness to extubate, peptic ulcer, deep vein thrombosis and gastrointestinal bleed prophylaxis. Previous reviews have been performed in this area looking at the effect of the ventilator care bundle on VAP rates, however these were unable to conclude a causal relationship due to the poor methodological quality of previous research and high risk of bias. New evidence has since to come to light that have hitherto not been systematically reviewed.

**AIM.** To investigate the effect of the ventilator care bundle on VAP rate, mortality rate and length of ICU stay, through a systematic review and meta-analysis.

**METHODS.** Following the Cochrane Handbook for systematic reviews, a comprehensive search of CINAHL, EMBASE and Medline was performed from 2004 - January 2017. The review included all study designs comparing the ventilator care bundle to a control group (no ventilator care bundle) in mechanically ventilated patients. Data was extracted and risk of bias evaluated independently by a single reviewer. The meta-analysis was performed using Review Manager (RevMan 5.3) following a random-effects model.

**RESULTS.** A total of fourteen studies were included within this review. The analysis showed that the ventilator care bundle decreases the risk of VAP by 55% (RR=0.45, 95% CI=0.33-0.61, p < 0.00001). The ventilator care bundle showed to decrease mortality rate (RR 0.80, 95% CI=081-0.9, p= 0.007) and also a decrease on length of stay (MD=-2.67 days, 95% CI = -5.88-0.55, p = < 0.00001).

**CONCLUSIONS.** Based on a very low quality body of evidence, this study revealed statistically and clinically significant reductions in VAP rate, mortality and length of stay resulting from the adoption of the ventilator care bundle. The low quality nature of the evidence do not allow for causal conclusions to be confidently drawn. Further methodologically robust research needs to be conducted. In the meantime, and in the absence of any adverse effects, the ventilator care bundle should continue to be supported in practice.

### 0287

#### Influence of hypophosphatemia at ICU admission on the duration of mechanical ventilation in sepsis

J. Shima, S. Katayama, K. Koyama, K. Tonai, Y. Goto, T. Koinuma, S. Nunomiya
Jichi Medical University School of Medicine, Division of Intensive Care, Department of Anesthesiology and Intensive Care Medicine, Shimotsuke, Japan
**Correspondence:** J. Shima
*Intensive Care Medicine Experimental* 2018, **6(Suppl 2):**0287

**INTRODUCTION.** Hypophosphatemia occurs in 30% of intensive care unit (ICU) patients and up to 70% in septic patients. Hypophosphatemia induces muscle weakness, arrhythmia, decreased myocardial contractility, acute respiratory failure, impairment of diaphragmatic contractility and death at worst. It is also reported that hypophosphatemia prolonged liberation from mechanical ventilation (MV) in ICU patients, but its influence in septic patients is not clear.

**OBJECTIVES.** The objective of this study was to evaluate the association between hypophosphatemia and duration of MV in patients with sepsis.

**METHODS.** A single-center, retrospective observational study was performed. Consecutive septic patients, diagnosed with Sepsis-3 criteria under MV admitted to a general ICU from June 2011 to December 2017, were included. Exclusion criteria were patients who received hemodialysis, with deep neck infection and who died within twenty-four hours from ICU admission. Included patients were allocated to two groups: hypophosphatemia group who had hypophosphatemia at ICU admission, defined as serum phosphorus concentrations < 2.0mg/dL, and non-hypophosphatemia group. Demographic data, infection sites, comorbidities, use of steroids, APACHE II score and SOFA score, hospital mortality, lengths of ICU stay and duration of MV were collected and analyzed from patients' records. The primary outcome was duration of MV (defined as the number of days from ICU admission to liberation from MV or discharge from ICU, whichever came first) and analyzed using the t-test. Time to liberation from MV was compared using log-rank tests and presented as Kaplan-Meier curves. To evaluate the relationship between duration of MV and hypophosphatemia, a multivariable logistic regression model was performed.

**RESULTS.** Five-hundred and sixty-six patients were included and 40 patients (7.5%) had hypophosphatemia at ICU admission. Hypophosphatemia group had longer duration of MV (median [IQR], 10 [7-17] vs. 7 [5-11] days; *P*=0.003). Lengths of ICU stay was also longer in the hypophosphatemia group. The severity of illness was not statistically different between two groups. Time to liberation from MV was compared and shown by Kaplan-Meier curves (Figure). Hypophosphatemia was one of the independent factors of prolonged duration of MV in the multivariable logistic regression model (Table).

**CONCLUSIONS.** This study revealed that hypophosphatemia at ICU admission in septic patients was one of the independent risk factors of prolonged duration of MV.

##### REFERENCE(S)

1) Am J Med 2005;118:1094-101.
2) Ann Clin Lab Sci 2010;40:144-8.
3) Crit Care Clin 2007;23:135-148.
4) NEJM 1971;285:763-768.

##### GRANT ACKNOWLEDGMENT

None.

**Table 1 (abstract 0287).** Multivariate analysis for duration of mechanical ventilation (shown only significantly different confounders)

| | Hazard ratio (95% CI) | *P*-value |
|---|---|---|
| hypophosphatemia | 1.53(1.10-2.19) | 0.010 |
| thoracic infection | 1.55(1.27-1.92) | <0.001 |
| use of steroid | 1.37(1.14-1.65) | <0.001 |
| APACHE II score | 1.04(1.02-1.05) | <0.001 |

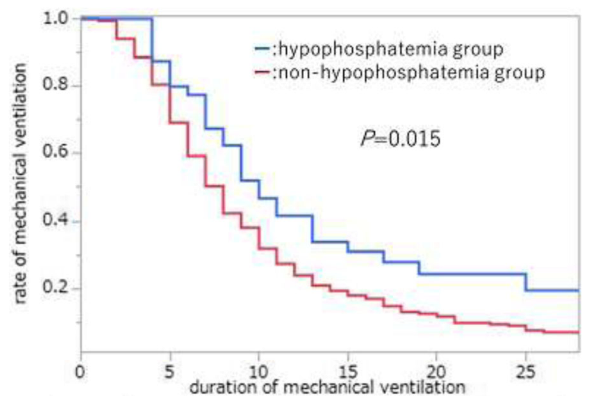

**Fig. 1 (abstract 0287).** Kaplan-Meier curves of time to liberation from MV

### 0288

#### Association between time to intubation and outcomes in a unselected population of mechanically ventilated ICU patients

R. Jiménez, S. Rebollo, A. Ortín, S. Sánchez, J. Batllés, A. Ojados, L. Herrera, S. Moreno, M. Galindo, A. Fernández, V. Serrano, C. Navarro, A. Ruiz, M.D. Rodríguez
Hospital General Universitario Santa Lucía, Servicio Medicina Intensiva, Cartagena, Spain
**Correspondence:** R. Jiménez
*Intensive Care Medicine Experimental* 2018, **6(Suppl 2):**0288

**INTRODUCTION.** To study the delay between hospital and ICU admission and the initiation of invasive ventilatory support and its association with patients outcomes.

**OBJECTIVES.** We performed a retrospective analysis of a prospective database of those patients admitted to our polivalent ICU in last 7 years and that required mechanical ventilation during their stay. We excluded patients admitted due to planned surgery. For statistical analysis we used Chi-square, T-Sudent or Fisher tests as appropriate.

**METHODS.** We performed a retrospective analysis of a prospective database of those patients admitted to our polivalent ICU in last 7 years and that required mechanical ventilation during their stay. We excluded patients admitted due to planned surgery. For statistical analysis we used Chi-square, T-Sudent or Fisher tests as appropriate.

**RESULTS.** During the study period, 1312 patients received invasive mechanical ventilation due to unplanned reason. Mean age was 61.9 (CI 95% 61-62.8), APACHE II 22.5 (22.1-22.9), SAPS II 51.6 (50-52.9), Glasgow at admission 10.8 (10.5-11.1). 54.3 % of patients were admitted from community and 75.3 % were medical. 43.1 % had some kind of comorbidity (20.9% diabetes, 9.5% inmunodepression, 9% malignancy, 7.9% COPD, 7.5% chronic kidney disease, 3% hepatopathy)

Mean duration of mechanical ventilation was 8.2 days (7.4-8.9) 8.4 % of patients had been under non-invasive ventilation (NIV) before intubation. In 14.9 % of patients, tracheostomy was performed and 8.3 % required subsequent intubation.

Mean length of ICU stay was 11.9 days (11-12.7) and hospital 22.3 days (20.9-23.6) 385 patients (29.3 %) died in ICU and 481 (36.7%) in hospital.

ICU non-survivors were significantrly older, had higher severity scores and had higher incidence of inmunodeppression, kidney disease and hepatopathy. Medical condition (31.6 vs 22.5 %, p 0.003) and admission from hospital (32.1 vs 27.1 %, p 0.048)showed higher mortality (31.6 vs 22.5 %, p 0.003)

No association was observed between duration of mechanical ventilation, subsequent intubation or tracheostomy and mortality. Non-survivors showed longer time from ICU admission to intubation (0.8

vs 0.45 days, p 0.003, and use of NIV before intubation was associated to higher mortality (41.2 vs 26 %, p < 0.001) Reintubation, tracheostomy, prior NIV and any kind of delay to intubation resulted in significant longer ICU and hospital stay.
**CONCLUSIONS.** In a general ICU population of mechanically ventilated patients, delay to intubation and prior use of NIV involved worse outcomes. The unselected nature of the sample may limit our conclusions.

## CRRT & RRT for AKI

### 0289

#### Acute kidney injury and renal replacement therapy in patients on ECMO: a single center experience

R. Avila, N. Carrizo, A. Fernandez, M.M. Filippi, C. Zuchela, H. Alassia, R. Schmuck, M. Peralta, H. Molina, M. Irazusta
Hospital Cullen, Santa Fe, Argentina
**Correspondence:** R. Avila
*Intensive Care Medicine Experimental* 2018, **6(Suppl 2):**0289

**INTRODUCTION.** Extracorporeal life support (ECMO) has proven to be effective in pulmonary and cardiovascular failure when standard therapies have failed. The incidence of AKI in this population is very high and the initiation of renal replacement therapy (RRT) is a critical situation. According to the literature, these patients have a high mortality rate despite the extracorporeal support offered.
**OBJECTIVES.** To evaluate the incidence and outcomes of ECMO patients who developed AKI and required RRT in our center.
**METHODS.** Our internal database was tapped into looking for patients who underwent ECMO at Jose Maria Cullen Hospital, a tertiary-level referral public health center, from June 2015 to March 2018. Statistical analysis was performed with STATA 14.
**RESULTS.** Over a 2-year period, 20 ECMO therapies were performed (18 ECMO V-V and 2 ECMO V-A), mean age was 23 years, and most patients were males (80%). The survival rate was 45% (50% ECMO V-A y 44% ECMO V-V). Of the 20 patients analyzed, 10 (50%) developed some AKI stage; one presented renal failure and the need for RRT 48 hours before the start of ECMO, while the remaining 9 developed AKI on 5-day average of ECMO run. ( 3 AKI patients in stage 1 and 6 AKI patients in stage 3 ). Seven patients required RRT (4 independent vascular access and 3 connected to the ECMO circuit). The most frequent indication was oligo-anuria (100%), followed by fluid overload (57%), metabolic acidosis (28%) and rhabdomyolysis (14%). The most frequent RRT modality was CVVH (5 patients 71%), followed by SLED 3 patients (42%) and SCUF 1 patient (14%). The average negative daily balance was: CVVH 660 ml/day, SLEED: 653 ml/day, and SCUF: 3200 ml/day. Survival of patients in ECMO who did not develop AKI was 50%, but for those who presented some degree of AKI 30% and much lower (15%) in those who needed RRT.
**CONCLUSIONS.** In our study, the incidence of AKI was high (50%) with a significant reduction in survival rate compared to those patients who did not develop AKI (30% vs. 50%). The implementation of some RRT did not improve the results of those with more severe renal failure. CVVH was the most frequent modality of RRT.

##### REFERENCE(S)

Askenazi, D. J., Selewski, D. T., Paden, M. L., Cooper, D. S., Bridges, B. C., Zappitelli, M., & Fleming, G. M. (2012). Renal replacement therapy in critically Ill patients receiving extracorporeal membrane oxygenation. Clinical Journal of the American Society of Nephrology, 7(8), 1328-1336.
Kilburn DJ, Shekar K, Fraser JF. The complex relationship of extracorporeal membrane oxygenation and acute kidney injury: causation or association? Biomed Res Int. 2016;1094-296.

### 0290

#### Urinary liver-type fatty acid-binding protein point-of-care kit for the prediction of renal replacement therapy requirement in critically ill patients with acute circulatory failure

K. Ishimura, A. Tsuruoka, R. Hiroshi
Osaka City General Hospital, Emergency and Critical Care Medical Center, Osaka, Japan
**Correspondence:** K. Ishimura
*Intensive Care Medicine Experimental* 2018, **6(Suppl 2):**0290

**INTRODUCTION.** Acute kidney injury (AKI) requiring renal replacement therapy (RRT) is common in critically ill patients with acute circulatory failure. RRT requirement is difficult to predict. Some AKI biomarkers can predict RRT requirement. However, they are associated with problems with respect to convenience and quickness of use. Recently, the liver-type fatty acid-binding protein point-of-care kit (L-FABP POC Kit, CMIC Holdings Co., Ltd., Tokyo, Japan), which can rapidly measure urinary L-FABP, was developed.
OBJECTIVE. The purpose of this study was to evaluate the usefulness of the L-FABP POC kit.
**METHODS.** This was an observational retrospective study that included patients admitted in our intensive care unit who required any vasoactive agents between December 2017 and February 2018. Urinary L-FABP level was measured using the L-FABP POC kit adopting the immunochromatography technique. Urine was added to the kit, and the L-FABP concentration in the urine sample was visually measured in 15 minutes. The results were classified as negative (< 12.5 ng/ml), 1+ (≥12.5 and < 100 ng/ml), or 2+ (≥100 ng/ml).
**RESULTS.** Thirty-five patients were included in our study (males: 66%; median age: 71 years). The median sequential organ failure assessment and acute physiology and chronic health evaluation II scores were 8 and 22, respectively. All intensive care unit admissions were emergencies. The main reasons of admission were sepsis in 19 patients (54%), acute respiratory failure in 7 patients (20%), and other in 9 patients (26%). The median lactate level was 1.9 mmol/l. Noradrenaline or dopamine, adrenaline, and vasopressin were used in 31 (89%), 5 (14%), 5 (14%), and 4 (11%) patients, respectively. Dobutamine and milrinone were used in 10 (29%)and 2 (6%) patients, respectively. Fourteen patients required RRT (40%), and the 28-day mortality was 26% (9 patients). In this study, the patients were divided into two groups according to the results of the L-FABP POC kit measurements. In the L-FABP POC-positive (1+ or 2+) group, renal replacement therapy was more frequently required than in the L-FABP POC-negative group (54.5% vs 15.4%, p = 0.034). The RRT duration was 3 days in the L-FABP POC-positive group and 5 days in the L-FABP POC-negative group (p = 0.19). The sensitivity and specificity of the L-FABP POC kit to predict RRT requirement were 85% and 52%, respectively.
**CONCLUSIONS.** This retrospective study shows that positive results from the L-FABP POC kit are associated with RRT requirement. The L-FABP POC kit may serve as a novel tool to predict RRT requirement.

##### REFERENCE(S)

1. Susantitaphong P, Siribamrungwong M et al (2013) Performance of urinary liver-type fatty acid-binding protein in acute kidney injury: a meta-analysis. Am J Kidney Dis 61:430-9.

##### GRANT ACKNOWLEDGMENT

L-FABP POC kit was provided by CMIC Holdings Co., Ltd.

### 0291

#### Urinary L-FABP level predicts initiation of renal replacement therapy and prognosis in patients with septic shock

A. Tsuruoka[1,2], Y. Inoue[1], A. Kawamoto[1], H. Rinka[2]
[1]Kyoto Min-iren Chuo Hospital, Kidney and Cardiovascular Center, Kyoto, Japan; [2]Osaka City General Hospital, Emergency and Critical Care Medical Center, Osaka, Japan
**Correspondence:** A. Tsuruoka
*Intensive Care Medicine Experimental* 2018, **6(Suppl 2):**0291

**INTRODUCTION.** Acute kidney injury (AKI) in sepsis patients is associated with high morbidity and mortality. Despite improvements in intensive care medicine and renal replacement therapy (RRT), patients with AKI have much higher morbidity and mortality rates than patients without AKI. In recent years, novel AKI biomarkers, such as NGAL, KIM-1, IL-18, L-FABP, IGFBP7, TIMP2 have been discovered and validated to improve early detection for progressive renal failure, need for RRT, or death.

**OBJECTIVES.** This study was to evaluate whether urinary L-FABP at intensive care unit admission can predict initiation of RRT and mortality in septic shock patients.

**METHODS.** This was a single-center retrospective observational study of patients with septic shock admitted in our intensive care unit from December 2015 to December 2016. A total of 26 patients with septic shock were screened and 21 patients were included, 5 patients with maintenance dialysis and anuria were excluded. We examined the relation of urinary L-FABP level at ICU admission and initiation of RRT, 28-day mortality and 90-day mortality.

**RESULTS.** A total of 21 patients were included. Median age was 82 years (IQR 70-84) and 8 (38%) were male. 6 patients (28.6%) had chronic kidney disease and 15 patients (71.4%) acquired AKI based on KDIGO criteria. Median urinary L-FABP(ng/ml) level was 31.51 (IQR 13.9-125.8). SOFA score and APACHE2 score were 9 and 21(median), respectively. 18 patients (85.7%) had received mechanical ventilation and 6 patients (28.6%) had received RRT. 28-day and 90-day mortality was 28.6% and 38.1%, respectively. The sensitivity and specificity of urinary L-FABP level for predicting initiation of RRT were 73.3 and 66.7% (area under the ROC curve 0.711, 95% CI: 0.447-0.975) at 54.0 ng/ml. The patients were divided into 2 groups according to the urinary L-FABP(ng/ml) quartiles (Q1, Q2, and Q3 = low group [L] vs. Q4 = high group [H]).Kaplan-Meier curves showed 28-day and 90-day mortality were significantly higher in the H group than in the L group (p = 0.014 and 0.000675, respectively).

**CONCLUSIONS.** Urinary L-FABP level is an effective biomarker for predicting initiation of RRT and mortality in septic shock patients.

**REFERENCE(S)**

(1) Intensive Care Med (2018) 44:323-336
(2) Kidney Int. 2015 Mar;87(3):640-8.

### 0292

#### Predictors of 90 day survival in CRRT patients

J.Y. Koh[1], H.K. Chua[1], C.H. Loh[2]
[1]Singhealth, Internal Medicine, Singapore, Singapore; [2]Changi General Hospital, Respiratory and Critical Care Medicine, Singapore, Singapore
**Correspondence:** C.H. Loh
*Intensive Care Medicine Experimental* 2018, **6(Suppl 2):**0292

**INTRODUCTION.** Survival rates for patients requiring CRRT are generally around 40-50%. The ATN study demonstrated 60 day all-cause mortality around 50%, while 90 day mortality in the RENAL study was 44%.

**OBJECTIVES.** To study the outcomes of our cohort of patients in our institution requiring CRRT and determine factors contributing to 90 day mortality in our MICU.

**METHODS.** We performed a retrospective chart review of patients requiring CRRT in our institution over 3 months between 1 April 2017 to 30 Jun 2017. This observational retrospective audit was part of the multicentre study by SICM-NICER and is approved by the NHG DSRB Ethics Review Board. Variables such as age, gender, comorbidities, ICU diagnosis, inotrope score, APACHE II score, fluid balance 24 hours prior to CRRT and D1-5 of ICU admission, modality of CRRT, dialysis dose were recorded. Univariate analyses and binary logistic regression with these variables were performed to determine factors contributing to 90 day survival.

**RESULTS.** 71 patients were included in this study. The mean age was 70.6 +/- 11.8. 63.4% of patients had preexisting CKD, of whom 60% had CKD stage 3-4. Mean inotrope score 2.5 and APACHE score was 27.2. Mean ICU days 3.8 +/- 4.1. Majority of patients were admitted to ICU for sepsis (44%), 16% for cardiovascular events, 13% for fluid overload.
CVVHDF was prescribed 96.4% of the time. Mean prescribed dose was 30.4 +/- 3.0 ml/kg/h. Citrate anticoagulation was used in 86.8%, followed by nil (9%) and heparin (3.8%). 70.6% patients survived upon ICU discharge while 90 day survival was 60%. 38% of survivors continued to require RRT on discharge. Among the survivors who did not have preexisting CKD, 50% who required RRT in ICU developed CKD at 90 days.
CRRT was stopped due to resolution of AKI (30.8%), death (27.7%), but renal replacement continued in 27% due to ESRF.
Cardiogenic shock was associated with lowest survival at 90 days at 30%, compared to 60.6% for sepsis. In a univariate analysis, survivors had significantly lower APACHE II scores (mean 23.9 +/- 6.8) compared to non survivors (33.4 +/- 10.1, p=0.009). They were also younger (68.4 +/- 12.3 compared to 73.0 +/- 11.5 in non survivors), had more negative fluid balance (-275ml versus 1424ml in non survivors), higher prescribed dose (31.1 +/- 3.2 compared to 30.0 +/- 2.6), but these were not clinically significant (p>0.05).
90 day survival was not significantly associated with age, APACHE or inotrope score, ICU fluid balance, prescribed dialysis dose in our regression model.

**CONCLUSIONS.** 90 day mortality for patients requiring CRRT was high at 40% and comparable to prior studies. APACHE II score was significantly associated with 90 day survival in a univariate analysis, but no significant factors were associated with 90 day survival in our regression model.

### 0293

#### Comparison of hemodynamic parameters among continuous, intermittent and hybrid renal replacement therapy in acute kidney injury: a systematic review of randomized clinical trials

D. Russo[1], S. Vieira Rios[2], R. Rosa Goulart[3], A. Stein[3], I. George Balestrin[3], D. Barbosa da Silva[3]
[1]Hospital Moinhos de Vento, ICU, Porto Alegre, Brazil; [2]Hospital de Clínicas de Porto Alegre, UTI, Porto Alegre, Brazil; [3]Hospital Moinhos de Vento, Porto Alegre, Brazil
**Correspondence:** D. Russo
*Intensive Care Medicine Experimental* 2018, **6(Suppl 2):**0293

**PURPOSE.** The use of renal replacement therapy (RRT) in acute kidney injury (AKI) patients in the intensive care unit (ICU) is associated with high hemodynamic instability leading to an in hospital mortality of about 50%. The aim of this study was to compare hemodynamic parameters among continuous, intermittent and hybrid renal replacement therapy in acute kidney injury.

**METHODS.** Systematic review conducted in accordance with the PRISMA and registered at the PROSPERO Database. Randomized clinical trials involving patients with AKI in the ICU submitted to continuous, intermittent or hybrid RRT will be included. We will investigate the electronic databases: PubMed, Embase and Cochrane. Two reviewers will independently carry out study selection, evaluation of methodological quality and data extraction.

**RESULTS.** Most of the studies did not find differences in hemodynamic parameters across different RTT modalities, except a CVVH group heart rate decrease after 1 and 4 hours in comparison with IHD group, an increase in systolic blood pressure after 0.5 and 2h of CVVH in contrast with IHD, and doses significant higher of Dobutamine in patients from the CVVHDF group, when compared to IHD. Lower baseline MAP, greater MAP variation on dialysis, higher number of pressors at baseline, and increase in pressor dose during dialysis were associated with shorter survival time; and greater MAP variation on dialysis negatively was correlated to renal recovery.

**CONCLUSIONS.** We believe that this study can provide important insights for further clinical trials designed specifically to evaluate hemodynamic parameters across different types of RRT.

### 0294

#### Antibiotic dosage in the critically ill patients in continuous renal replacement therapy: are we sure that a lesser posology is the best for the patient?

A. Corona[1], S. Santini[1], A. Veronese[1], D. Cattaneo[2], D. Scorza[3], S. Antinori[4], A. Castelli[1]
[1]University of Milan, ICU PO Luigi Sacco, Milano, Italy; [2]University of Milan, Pharmacology - PO Luigi Sacco, Milano, Italy; [3]University of Milan, Nephrology - PO Luigi Sacco, Milano, Italy; [4]University of Milan, Infectious DIseases Division - PO Luigi Sacco, Milano, Italy
**Correspondence:** A. Corona
*Intensive Care Medicine Experimental* 2018, **6(Suppl 2):**0294

**INTRODUCTION.** A reduction of antibiotic dosage is recommended in patients with acute kidney injury (AKI) who are undergoing continuous renal replacement therapy (CRRT). However, CRRT may impact on the antimicrobials pharmacokinetic/pharmacodynamic (PK/PD) causing both the antibiotic underdosing and the consequent likely therapeutic failure and microorganism resistance (1).

**OBJECTIVES.** Prospective observational study to verify CRRT removal of more frequently used antimicrobial agents and their PK/PD parameter changes in the critically ill patients experiencing AKI.

**METHODS.** Since January 2017, we recruited all the patients admitted to our ICU experiencing anuric AKI and the consequent needing of CRRT. On all patients demographics, clinical data and ICU parameters were collected. Rifle and AKIN criteria were used to define renal failure levels. Samples of each patient's blood and ultrafiltrate were taken (trough and peak levels) at 48 hrs. from the CRRT commencing to investigate PK/PD of mainly used antibiotics [vancomycin, linezolid, piperacillin/tazobactam, ciprofloxacin, levofloxacin and meropenem; (no cephalosporin TDM is determined in our hospital)]. Posology of the single antibiotic was standard as suggested by Sanford Antimicrobial Guide for patients with severe AKI. CRRT was based on only convective (standard effluent total dose of 25 ml/kg and M150 haemofilter) technique, using Prismaflex® SW 5.XX system set with anticoagulant citrate protocol.

**RESULTS.** Over the study period a total of 21 patients (M:F=3:1) has been recruited. We recorded respectively a median (IQR):

(i) age 64 yrs. (54-68);
(ii) 1st 24 hrs. SAPS II: 49 (38-52) and SOFA at the admission: 10 (7-11); Rifle: 3 (2.5-3) and AKIN: 2.8 (2.25-3), indicating severe AKI; length of CRRT: 5 (4-7), ICU-los 14 (10-18) and hospital-los 30 (22-34); albumin plasmatic levels: 22.5 (21.5-25.5).

The percentage of patient in septic shock was about 65% and the recorded hospital mortality was 33.3%. The ultrafiltrate/plasma concentration mean (SD) ratios were respectively: ciprofloxacin: 0.89 (0.11); vancomycin: 1.1 (0.33); meropenem: 0.89 (0.02); piperacillin/tazobactam: 0.79 (0.05); linezolid: 0.75 (0.1). The mean (SD) antibiotic clearances of CRRT (ml/min) were respectively: ciprofloxacin: 25.5 (6.4); vancomycin: 33.5 (13.1); meropenem: 20.1 (3.1) ; piperacillin/tazobactam: 22.3 (4.9); linezolid: 20,4 (0.39). Drugs were significantly cleared during CVVHF, with a sieving coefficient >0,8 for every antibiotics.

**CONCLUSIONS.** The high-flux membranes used in CRRT seems to make no filtration barrier to all studied antibiotics. Further progression of the study is warranted.

**REFERENCE(S)**

Shaw AR, Mueller BA. Antibiotic Dosing in Continuous Renal Replacement Therapy. Adv Chronic Kidney Dis. 2017 Jul;24(4):219-227.

### 0295

#### Renal replacement therapy with citrate in different doses (CiDoSes) study

A. Baptista[1,2], B. Banheiro[1], A. Ávila[1], G. Campello[1]
[1]Centro Hospitalar Universitário do Algarve, Intensive Care Unit, Portimão, Portugal; [2]Hospital Particular do Algarve, Intensive Care Unit, Faro, Portugal
**Correspondence:** A. Baptista
*Intensive Care Medicine Experimental* 2018, **6(Suppl 2):**0295

**INTRODUCTION.** Acute Kidney Injury (AKI) affects up to 60% of patients admitted to the intensive care unit (ICU), with continuous renal replacement therapy (CRRT) as one of the most common modalities. In the last decade regional anticoagulation with citrate (RAC) has gained its space as an anticoagulant for the extracorporeal circuit.

**OBJECTIVES.** The main aim of this study was to evaluate different levels of citrate concentration on a nurse driven protocol. Although PrismaFlex has an institutional protocol that advises a 3.0 mmol/L concentration of citrate, the authors design a prospective multicenter observational study in which lower concentrations of citrate were administered, not only reducing the odds of citrate accumulation but also allowing to understand what was the lowest level that could be achieved, without increasing the circuit coagulation.

**METHODS.** Citrate concentration was reduced, every 6-8 months, in 0.2 mmol/L. Several data were collected in order to evaluate the impact of different citrate doses in filter patency, citrate accumulation, duration of dialysis, outcomes and technical complications, for which patients were divided in citrate classes and compared either by the ANOVA One-way or by Kruskall-Wallis test. The study was approved by hospital's ethical committee.

**RESULTS.** Between 1 April 2016 and 31 December 2017, 58 patients required CRRT with citrate (325 days total) with 93 periods of continuous dialysis. The 2.6 mmol/L citrate group had a lower number of randomized patients (17 patients) compared with the 2.8 mmol/L and the 3.0 mmol/L group (respectively 39 and 41 patients). There was a predominance of men (70.7 vs 29.3%), mean APACHE of 27,2 ±7,8, with a mortality of 55.2%. Mean duration of filter circuit lifespans was 3±2,0 days, mean duration of CRRT of 5,8 days and mean length of stay in the ICU of 14±21,1 days.

There was a statistical significant association between the prescribed and the administered citrate concentration (p< 0,001).

There were no differences between the 3 groups concerning percentage of coagulated filters and filter lifespan, although there was a trend to higher total-to-ionized calcium in the 3.0 mmol/L group. Mean post-filter calcium (0,42±0,05), calcium compensation (95,9±17,1%) and ionized calcium (1,15±0,6) was similar in all groups. The depuration was also similar in all groups, as well as the electrolytes and acid-base balance. There were no significant differences concerning length of stay in ICU, renal replacement therapy time or mortality.

**CONCLUSIONS.** Our study showed that a citrate-RRT nurse driven protocol is safe with low levels of complications, extends circuit lifespans and limits the development of citrate accumulation.

The authors showed that a lower citrate concentration has a trend to lower citrate acumulation, without any significant impact on filter lifespan or coagulation.

**GRANT ACKNOWLEDGMENT**
None

### 0296

#### Renal replacement therapy under extracorporeal membrane oxygenation

N. Tsekouras, J.B. Bouchet, J. Morel
CHU Saint Etienne, Saint Etienne, France
**Correspondence:** N. Tsekouras
*Intensive Care Medicine Experimental* 2018, **6(Suppl 2):**0296

Different methods are described to perform renal replacement therapy(RRT) on patients under ECMO with even the possibility of connecting directly the RRT machine on the ECMO system.

The aim of this study is to present our experience using this type of connection.

This study was performed retrospectively in the Intensive Care Unit of the University Hospital in Saint Etienne from February 2014 until August 2016. All adult patients under ECMO venoarterial ( VA) and venovenous (VV) who had at least one session of RRT were included. The connection between the RRT machine and the ECMO circuit was elaborated as follows: the dialysis inflow was connected on the oxygenator (purge line) and the outflow directly onto the arterial cannula on a Luer port downstream of the oxygenator. We performed an analysis per patient and per dialysis sessions.
16 ECMO VA and 9 ECMO VV were evaluated, thus 69 filters (34 ECMO VA and 35 ECMO VV). The median lifespan of a filter was 39 IQR [15-65] hours. 74% of all filters were ceased prematurely (< 72hours), mainly due to excessive pressure (24%) among which transmembrane pressure (3%), filter pressure (20%), outlet pressure (1%). The median rate the RRT's blood flow and of dialysis dose for all ECMO's combined was respectively: 150[140-181]ml/min, 34[23-39]ml/kg/h. Patients presented mostly hemorrhagic complications (n=10). No case of air embolism was noticed
The combination of RRT and ECMO as described in our study may be efficient. Further comparative studies seem necessary in order to recommend such practice.

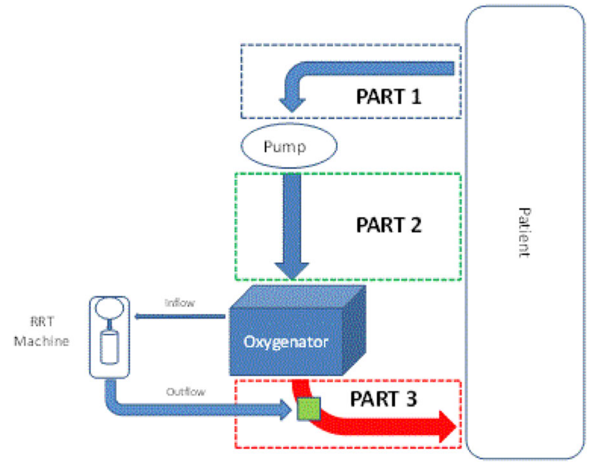

**Fig. 1 (abstract 0296).** RRT on ECMO according to our protocole

**Table 1 (abstract 0296).** Population of the study

| Patients | Total n=25 | ECMO VA n=16 | ECMO VV n=9 |
|---|---|---|---|
| Age (years) | 64[54-71] | 67[62-72] | 54[49-56] |
| Gender (male)(n(%)) | 14(56) | 8(50) | 4(44) |
| IGS 2 on admission | 60[49-67] | 61[53-70] | 52 [43-59] |
| Length of stay in ICU (days) | 15[5-33] | 8[3-18] | 39[15-52] |
| Survival in ICU (%) | 52 | 38 | 66 |

### 0297

#### Extracorporeal chloride removal to treat acidemia: in vitro evaluation of three techniques

E. Rezoagli[1], S. De Falco[2], J. Fumagalli[2], M. Busana[2], A. Rigoli[2], I. Protti[2], A. Tomaselli[2], M. Battistin[3], L. Castagna[3], S.A.E.A.E.S. Deab[4], M. Giani[4], A. Pesenti[2,3], A. Zanella[2,3]
[1]University of Milano-Bicocca, School of Medicine and Surgery, Monza, Italy; [2]Università degli Studi di Milano, Dipartimento di Fisiopatologia Medico-Chirurgica e dei Trapianti, Milano, Italy; [3]Ca' Granda Policlinico di Milano, IRCCS, Milano, Italy; [4]San Gerardo Hospital, Monza, Italy
**Correspondence:** E. Rezoagli
*Intensive Care Medicine Experimental* 2018, **6(Suppl 2):**0297

**INTRODUCTION.** Acidemia is a frequent disorder in critically ill patients. Blood chloride removal may increase blood pH. Extracorporeal chloride removal may be achieved through: 1. Electrodialysis (ED), a technique that selectively move anions from one solution to another through ion-exchange membranes using electricity (1); 2. ultrafiltration (UF) and postdilution with hypochlorous reinfusate; 3. UF through anion Exchange Resin (*a-ER*) which replaces chloride with bicarbonate ions.

**OBJECTIVES.** To evaluate, in-vitro, the chloride removal efficacy of these three different strategies.

**METHODS.** A standard reinfusate solution (CB 32, Novaselect) (technique 1) and a bicarbonate-based solution (Multibic®) (technique 2 and 3) were pumped through an hemodiafilter at 150 mL/min. The following strategies have been studied:

1. "ED group": the UF entered an ED chamber where chloride ions were replaced by hydroxide ions (OH-). Subsequently OH- ions were combined with CO2 to form bicarbonate within a membrane lung and the solution was reinfused in the main stream;
2. "Hypochlorous group": the UF was discarded and the same volume was reinfused in postdilution as sodium bicarbonate 140 mEq/L;
3. "a-ER group": the UF was pumped through an a-ER and then reinfused in postdilution.

In Hypochlorous and aER groups, UF flows of 11.4, 22.7 and 34.1 mL/min were tested. In the ED group, 3 different UF flows 15, 30 and 45 mL/min were tested, with a fixed amperage (4 Amp) and recirculating flow was set to equalize the UF tested, to achieve a theoretical removal of 1.25, 2.5, 3.75 mEq/min of chloride, respectively. Before the hemodiafilter and downstream after reinfusion the solution was sampled for UF gas analysis and then wasted. The change in chloride among the two sampling sites was calculated to quantify chloride removal. The experiment was repeated three times. Data are reported as mean±SD.

**RESULTS.**

1. In the "ED group", chloride removal was 0.47±0.06, 0.96±0.11 and 1.22±0.17 mEq/min at 15, 30 and 45 mL/min of UF flow, respectively.
2. In the "Hypochlorous group", chloride removal was 1.45±0.09, 2.65±0.09 and 4.50±0.15 mEq/min at 11.4, 22.7 and 34.1 mL/min of UF flow, respectively.
3. In the "a-ER group", chloride removal was 1.20±0.15, 2.05±0.09 and 3.35±0.09 at 11.4, 22.7 and 34.1 mL/min of UF flow, respectively (see Figure).

**CONCLUSIONS.** The three different extracorporeal techniques effectively removed chloride. Chloride removal proportionally increased with the rise of the UF flow. Further investigations will be required to confirm these findings and to prove safety and feasibility studies in-vivo.

**REFERENCE(S)**

1. Zanella A, et al. AJRCCM 2015;192(6):719-26.

**Grant acknowledgment**
This study was funded by the Italian Ministry of Health for the Project Code: GR-2013-02356711 (Young Researcher) awarded to Alberto Zanella.

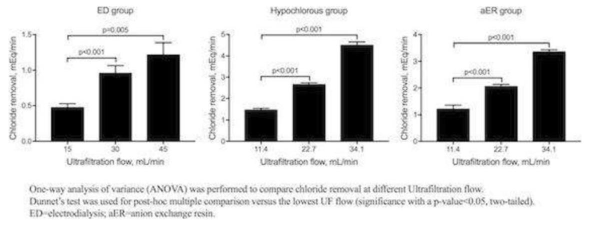

**Fig. 1 (abstract 0297).** Chloride removal by 3 different extracorporeal techniques

### 0298

#### Continuous renal replacement therapy during veno-venous ECMO: a retrospective study on regional citrate anticoagulation

M. Giani[1], F. Stefanini[2], M. Bombino[1], G. Valsecchi[2], C. Cunsolo[2], L. Bastia[2], R. Rona[1], G. Bellani[1,2], A. Pesenti[3,4], G. Foti[1,2]
[1]ASST Monza, Monza, Italy; [2]University of Milan-Bicocca, Monza, Italy; [3]University of Milan, Milano, Italy; [4]Ospedale Maggiore Policlinico, Milano, Italy
**Correspondence:** M. Giani
*Intensive Care Medicine Experimental* 2018, **6(Suppl 2):**0298

**INTRODUCTION.** Acute kidney injury (AKI) is common in patients treated with veno-venous extracorporeal membrane oxygenation (vv-ECMO) and continuous renal replacement therapy (CRRT) is required in up to 50% of cases(1). Systemic heparinization is considered the standard anticoagulation technique(2), as it is suitable both for CRRT and ECMO circuit. However, in the last few years, we opted for regional citrate as additional anticoagulation for CRRT during ECMO(3), as systemic heparinization often fails to avoid CRRT circuit clotting. The CRRT system (Fresenius multiFiltrate®) was directly connected to the ECMO circuit, a solution chosen by only a minority of the ECMO centers(1).
**OBJECTIVES.** To assess efficacy, safety and advantages of regional citrate anticoagulation compared to systemic heparin anticoagulation in patients undergoing RRT during ECMO.
**METHODS.** We performed a retrospective chart review (2009-2018) on patients treated with renal support therapy during ECMO. We evaluated filter lifespan, rate of CRRT circuit clotting, platelet consumption and the safety profile of citrate anticoagulation.
**RESULTS.** Among 110 adult vv-ECMO patients, 49 were treated with RRT between 2009 and 2018. A total of 259 RRT circuits were used, 171 anticoagulated with regional citrate (RCA group) and 88 with systemic heparin only (UFH group). Median circuit duration was 53 ± 23h for RCA circuits and 51±27h for UFH circuits. Filter clotting was the main cause to circuit change in the UFH group (50% vs 10% in the RCA group, p< 0,01). Most of the CRRT circuits in the RCA group were changed after 48-72h even in the absence of signs of clotting, following the manufacturer recommendation. A significant reduction of the platelet count was observed in the UFH group (104 ± 72 vs 122 ± 76 in the RCA group). No complication ascribable to citrate anticoagulation was recorded, total to ionized calcium ratio was less than 2.5 in all patient samples and no severe electrolyte imbalance occurred.
**CONCLUSIONS.** Regional citrate seems a feasible, safe and effective technique to anticoagulate CRRT circuits connected to ECMO. Compared to systemic heparinization alone, this technique may provide some advantages in terms of reduction of circuit clotting and platelet consumption.

### 0299

#### The relationship between base excess unmeasured anions and mortality in patients with acute kidney injury receiving continuous renal replacement therapy

B. Gucyetmez
Acibadem Mehmet Ali Aydınlar University School of Medicine, Department of Anesthesiology and Reanimation, Istanbul, Turkey
*Intensive Care Medicine Experimental* 2018, **6(Suppl 2):**0299

**INTRODUCTION.** It is known that acute kidney injury (AKI) is a reason for increased unmeasured anion (UA) and it can be corrected with continuous renal replacement therapy (CRRT) (1). Furthermore, it is demonstrated that increased UA is associated with higher mortality in critical ill patients (2). Base excess unmeasured anion (BEua), which leads to effect of UA on standard base excess (SBE), is a component of SBE and it can be calculated by using partitioned BE approach (3).
**OBJECTIVES.** The aim of this study is to investigate the association between increased negative BEua value and mortality in patients with AKI receiving CRRT.
**METHODS.** This study was designed as prospective observesional in 2017. Sixty three patients with AKI who were diagnosed in accordance with KDIGO Guideline required CRRT were included. ABL-800 blood gase analyser was used to measure arterial blood gas samples. BEua was calculated in accordance with partitioned BE approach (3). Demographic data and blood gas parameters, serum albumin, creatinine, blood urea nitrogen (BUN) and thrombocyte count ($T_C$) in the pre and 24$^{th}$ hour of CRRT and outcomes were recorded. Mann-Whitney U and Pearson correlation tests were used for statistical analysis. For likelihood of mortality, sofa score, systolic ejection fraction (SEF), 24$^{th}$ hour $PaCO_2$, $HCO_3$, lactate, SBE, BEua levels and $T_C$ were added to the logistic regression model.
**RESULTS.** In this study, mortality rate was 36.5%. In non-survivors, BEua levels in pre and 24$^{th}$ hour of CRRT were significantly lower than survivors. There was a positive correlation between the timing of initiation of CRRT after detecting negative BEua value and lenght of ICU stay ($r^2$=0.28 p< 0.001). In logistic regression analysis, likelihood of mortality was increased 3.8-fold (1.1-6.9) and 4.7-fold (1.4-8.1) by 24$^{th}$ hour lactate level>2.3mmol/L and 24$^{th}$ hour BEua≤-8.5mmol/L respectively (p=0.016 and p=0.005 respectively).
**CONCLUSIONS.** Increased negative BEua value is associated with higher mortality in patients with AKI receiving CRRT. Moreover, in patients with AKI, it should be discussed whether it may be an indicator together with known traditional indications for CRRT.

##### REFERENCE(S)

1. Rocktaschel J et al. Int J Artif Organs 2003; 26: 19-25
2. Masevicius FD et al. Crit Care Med 2017; 45:e1233-1239.
3. O'Dell E et al. Crit Care 2005; 9:R464-70

### 0300

#### Regional citrate anticoagulation for CRRT in critical pacients with liver failure

M. Sánchez Satorra, A. Campos Gómez, Y. Rovira Vallès, T.M. Tomasa Irriguible, V. Philibert, E. Mor Marco, L. Bielsa Berrocal, J. Manciño Contreras, M. Ayala Gallardo, O. Plans Galvan, P. Ricart Martí
Hospital Universitari Germans Trias i Pujol, Badalona, Spain
**Correspondence:** M. Sánchez Satorra
*Intensive Care Medicine Experimental* 2018, **6(Suppl 2):**0300

**INTRODUCTION.** Impaired liver function was initially a contraindication to the use of citrate in continuous renal replacement therapy (CRRT). However, increasingly, new articles defend the use and safety of this regional anticoagulation in critical patients with liver failure.
**OBJECTIVES.** To analyze the safety of a regional citrate anticoagulation protocol (RCA) for CRRT in critical patients with acute liver failure and decompensated cirrhosis.
**METHODS.** Prospective observational study from January to December 2017 in a intensive care unit (ICU). Patients with acute liver failure and any degree of decompensated liver cirrhosis were included during their admission in ICU. In all of them CRRT was indicated. Standard RCA protocol was applied in all patients. RCA protocol provides for a periodic analytical control to determine possible metabolic complications, among which toxicity stands out for the accumulation of citrate. Accumulation of citrate was detected indirectly by total serum calcium/ionised serum calcium (tCa/iCa) ratio. Statistical data are expressed through the median (minimum-maximum).

**RESULTS.** 8 patients (5 men and 3 women) with an age of 62 (41-70) years and APACHE II 26.5 (21-41) points were included. The sample consisted of 2 patients with acute liver failure in the context of a multi-organ dysfunction for septic shock and 6 chronic patients with decompensated liver cirrhosis during admission to ICU: 4 septic, 1 cardiac arrest and 1 postoperative cardiac surgery. Of these 6 chronic patients, they were able to stratify 2 in each stage of the Child-Pugh Classification and presented a CLIF-C ACLF Score 12.5 (8-14) points, corresponding to a grade 3 (1-3) of severity in the decompensation of liver disease. At the beginning of CRRT, MELD Score was 29.5 (19-33) points, Protrombin Time 53.5 (29-88) %, INR 1.64 (1.09-2.97), Platelets 55.5 x $10^9$/L (19-118 x$10^9$/L), Fibrinogen 364 (84-555) mg/dL, Serum lactate 3,05 (1.3-11.5) mmol/L and Bilirubin 3.18 (1.6-11.6) mg/dL. No patient obtained a tCa/iCa > 2.5 that, due to toxicity, would have forced to suspend the RCA. Only 2 patients presented tCa/iCa > 2.2 that could be corrected by modifying the flow of therapy. Nor were other complications with clinical relevance in the acid-base balance motivated to stop RCA. The duration of the filters was 2.4 (1-5) days. The stay at the ICU was 14.5 (4-21) days with a mortality of 62.5%.

**CONCLUSIONS.** The use of citrate for CRRT in critical patients with acute liver failure or decompensated cirrhosis appears to be safe, regardless of the degree of liver disease. However, it's mandatory to practice strict metabolic controls in order to detect, control and correct a possible toxicity by accumulation of citrate.

**REFERENCE(S)**

1. Klingele et al. *Long-term continuous renal replacement therapy and anticoagulation with citrate in critically ill patients with severe liver dysfunction*. Crit Care. 2017 Nov 29;21(1):294.

### 0301

#### Hemodinamic monitoring during continuous renal replacement therapy (CRRT) with oXiris filter in septic shock patients

M. Tengattini, F. Prato, U. Colageo, C. Pissaia
ASL BI - Ospedale Degli Infermi, Ponderano, Italy
**Correspondence:** M. Tengattini
*Intensive Care Medicine Experimental* 2018, **6(Suppl 2):**0301

**INTRODUCTION.** The immune dysfunction that characterizes sepsis is complex, affecting multiples pathways and it is still not well known.
The association of CRRT treatment with the oXiris filter, designed to adsorb endotoxin, remove cytokines and manage fluids, may positively affect the inflammatory cascade and it is safely used in clinical practice.
Optimizing hemodynamic status plays a key role in the treatment of sepsis-induced cardiovascular disturbances([1]).
The combination of oXiris Continuous veno-venous haemodiafiltration (CVVHDF) with functional hemodynamic monitoring maintain an adequate tissue perfusion by regulating the immunomodulation process to overcome the consequences of multiorgan failure.

**OBJECTIVES.** The aim of this study is to retrospectively evaluate the patients' hemodynamic response during CRRT with oXiris membrane and its impact on tissue perfusion.

**METHODS.** A total of 10 septic shock patients (6 women, 4 men), with mean age 70,9 yr. (range 63-75), were treated and monitored during 2017.
Average baseline SOFA was 12,4.
oXiris was used as adjunctive therapy in combination with Standard of Care according to 2016 Surviving Sepsis Campaign guidelines in order to modulate immune patient response and improve hemodynamics.
CVVHDF was performed within the first 24 hours from ICU admission, using Prismaflex together with citrate anticoagulation.
Dosages of vasopressors, evaluation of hemodynamic status (tab.1) and blood lactates was monitored at T0 (admission in ICU) and subsequently every 12 hours until 192 hours.
Haemodynamic variables was obtained intermittently using transpulmonary thermodilution with fully calibrated pulse contour Picco technique (PulsioFlex, Maquet, Germany).

**RESULTS.** 6 treated patients survived and were discharged from ICU.
Neither CRRT nor citrate-related adverse events were observed.
During the treatment a rapid and progressive reduction in vasopressor demand was registered in all patients: norepinephrine mean dose significantly decreased from 0,84 to 0,13 mcg/kg/min (p< 0,05) (fig. 1).
Trends of haemodynamics (median values) values are reported in fig. 2; CI started from 2,72 and raised up to 3,54 l/min/$m^2$.
A sustained reduction in lactate blood levels (from 5,6 to 1,4 mg/dl - mean values) and in CRP (from 30,7 to 15 mg/dl - mean values) was observed. (fig. 3).

**CONCLUSIONS.** The timely application of oXiris-CVVHDF was accompanied by the reduction of vasoactive drugs and the improvement of tissue perfusion, as shown by the decline in lactate levels. A rapid stabilization of hemodynamics parameters, consistent with the reduction of vasoactive drugs need, was shown by the sequential evaluation of haemodynamic status.

**Table 1 (abstract 0301).** See text for description.

| Hemodinamics parameters | |
|---|---|
| Cardiac Index | CI |
| Intrathoracic blood volume | ITBI |
| Global end diastolic volume | GEDI |

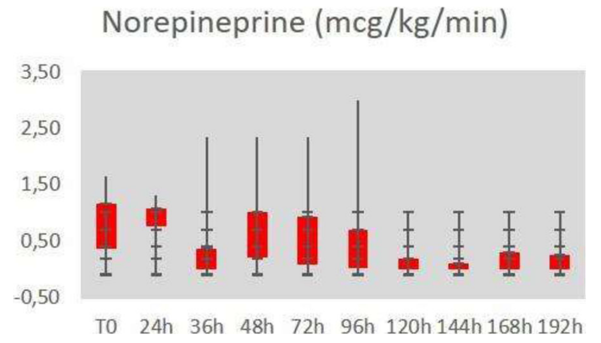

**Fig. 1 (abstract 0301).** See text for description

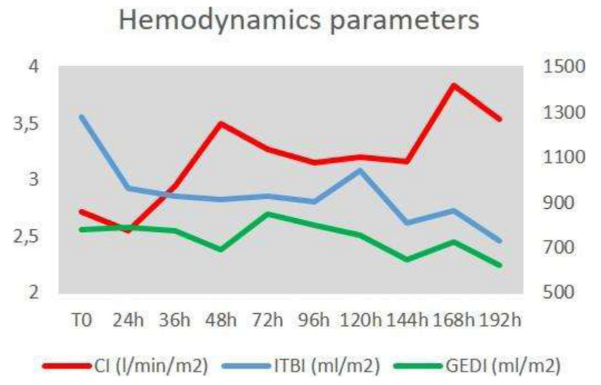

**Fig. 2 (abstract 0301).** See text for description

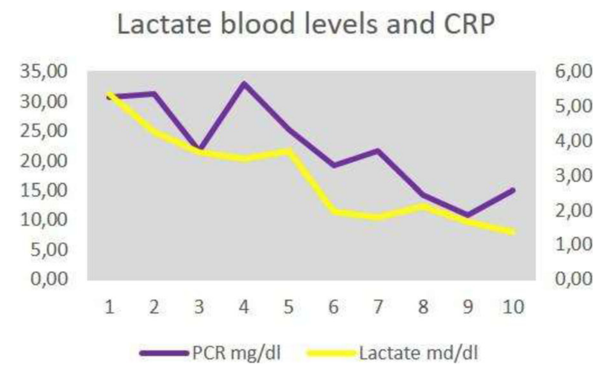

**Fig. 3 (abstract 0301).** See text for description

### 0302

#### Citrate anticoagulant does not influence CO2 extraction during CRRT

J. Jonckheer[1], H. Spapen[1], J. Demol[2], M. Diltoer[1], O. Costa[3], K. Lanckmans[3], T. Oshima[4], M. Malbrain[1], E. De Waele[1]
[1]University Hospital of Brussels, Intensive Care, Jette, Belgium; [2]University Hospital of Brussels, Nutrition, Jette, Belgium; [3]University Hospital of Brussels, Clinical Biology, Jette, Belgium; [4]Chiba University Graduate School of Medicine, Emergency and Critical Care Medicine, Chiba City, Japan
**Correspondence:** J. Jonckheer
*Intensive Care Medicine Experimental* 2018, **6(Suppl 2):**0302

**BACKGROUND.** Citrate is the preferred anticoagulation as it is associated with less complication during CRRT in a critical care setting. Citrate circulates through the body bound to calcium and in this state it is a weak acid. The resulting pH change will influence the balance between CO2 and bicarbonate. The use of CRRT is associated with CO2 extraction and thus citrate might alter their removal by CRRT. We studied the difference in CO2 removal between a citrate and a non-citrate setting of CRRT.

**MATERIALS AND METHODS.** In a prospective clinical trial in 8 patients receiving standard CVVH under citrate anticoagulation, total CO2 ( tCO2) was measured by blood gas analysis before and after the filter. Citrate was then replaced by NaCl 0.9% and sampling was repeated. CO2 flow at the sample points was calculated by multiplying measured tCO2 with blood flow. CO2 extraction was calculated by subtracting CO2 flow after the filter from CO2 before the filter. Transmembrane tCO2 was calculated by subtracting tCO2 after the filter from the tCO2 before the filter. Values during citrate predilution were compared with values with NaCl predilution.

**RESULTS.** There is no statistical difference ( p= 0,20) between mean CO2 extraction with citrate (26,3 +- 6,6 ml/min) versus NaCl predilution ( 25,9 +- 5,9 ml/min) during CRRT. tCO2 before and after the filter remained constant whether citrate or NaCl predilution was used (p=0,56 and p=0,63). Transmembrane tCO2 with citrate predilution ( 0,5 +- 1,4 mmol/l) did not differ significantly from NaCl predilution (0,4 +- 1,3 )mmol/l ( p=0,21)

**CONCLUSION.** Citrate has no impact on CO2 removal by CRRT.

### 0303

#### Retrospective analysis of different CVVH-substitution fluids and their effects on the acid-base-metabolism

D. Schwärzler, S.J. Klein, M. Joannidis
Medical University Innsbruck, Division of Intensive Care and Emergency Medicine, Department of Internal Medicine, Innsbruck, Austria
**Correspondence:** S.J. Klein
*Intensive Care Medicine Experimental* 2018, **6(Suppl 2):**0303

**INTRODUCTION.** CVVH is one of the major modalities of continuous renal replacement therapy (CRRT). During CVVH, it is recommended to apply anticoagulation [1], either through systemic anticoagulation (heparin) or through regional citrate anticoagulation (RCA). Most CVVH substitution solutions were developed for use with heparin anticoagulation with relatively high bicarbonate ($HCO_3^-$) levels. When applying RCA, $HCO_3^-$ is additionally originating from citrate metabolism, often leading to metabolic alkalosis.

**OBJECTIVES.** Recently, a substitute solution with lower $HCO_3^-$ levels (22 mmol/l; Biphozyl, Gambro) has become available. The goal of this study was to compare this solution to one with higher $HCO_3^-$ levels (30 mmol/l; Phoxilium, Gambro) with regard to their effects on the acid-base-metabolism in patients with metabolic alkalosis.

**METHODS.** Retrospective cohort analysis including all patients between 09/2016 and 05/2017 who have been treated with CVVH using RCA and switched from Phoxilium to Biphozyl because of persistent metabolic alkalosis. Patients were deemed eligible for inclusion, if they had at least 12 hours CVVH treatment time with Phoxilium as substitution fluid, a blood $HCO_3^-$ concentration $\geq$ 26 mmol/l and if a change of solutions was performed.

**RESULTS.** 26 patients and a total of 28 CVVH-cycles were analyzed. During the 48 hours prior to the change of solutions, $HCO_3^-$, base excess (BE) and $pCO_2$ levels increased. Those levels decreased significantly (p value for all < 0.05) after changing from Phoxilium to Biphozyl. No significant changes were observed for pH levels (p=0.827). Data is presented in Figure 1 and 2.

**CONCLUSIONS.** In this retrospective analysis of patients receiving two types of CVVH substitution fluids, we found an association between the fluid's $HCO_3^-$ concentration and blood levels of $HCO_3^-$ and BE, indicating, that in alkalotic patients, substitution fluids with lower $HCO_3^-$ concentrations may be beneficial. Interestingly, also the $pCO_2$ appears to be directly affected by the $HCO_3^-$ concentration of the substitution fluids. Although we cannot exclude other influences on these levels, due to the nature of this study. A prospective evaluation of different substitution fluids with standardized settings for CVVH and respiratory measures seams warranted.

| | | | | | | | |
|---|---|---|---|---|---|---|---|
| pH | 7.436 ± 0.06 | 7.419 ± 0.05 | 7.417 ± 0.09 | 7.440 ± 0.05 | 7.437 ± 0.05 | 7.434 ± 0.05 | 7.430 ± 0.05 |
| BE | 1.60 ± 1.97 | 2.32 ± 2.76 | 3.30 ± 1.88 | 3.91 ± 1.40 | 2.64 ± 1.56 | 2.08 ± 1.82 | 1.06 ± 1.39 |

**Fig. 1 (abstract 0303).** Time course of pH and base excess (BE)

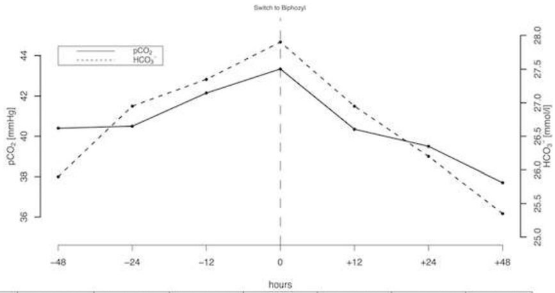

| | | | | | | | |
|---|---|---|---|---|---|---|---|
| $pCO_2$ | 39.59 ± 7.05 | 42.23 ± 7.42 | 42.89 ± 7.58 | 42.78 ± 6.8 | 39.99 ± 6.05 | 40.23 ± 6.06 | 38.85 ± 7.58 |
| $HCO_3^-$ | 25.84 ± 1.6 | 26.38 ± 2.26 | 27.25 ± 1.64 | 27.86 ± 1.26 | 26.75 ± 1.45 | 26.17 ± 1.72 | 25.42 ± 1.16 |

**Fig. 2 (abstract 0303).** Time course of pCO2 and HCO3-

### 0304

**Quality improvement project on efficacy of haemo-filters in intensive care care (ITU) by analysing various factors influencing the prescription in patients requiring RRT (renal replacement therapy) at Northampton General Hospital, ITU**

A. Parthasarathy, P. David
Northampton General Hospital, ITU, Northampton, United Kingdom
**Correspondence:** A. Parthasarathy
*Intensive Care Medicine Experimental* 2018, **6(Suppl 2):**0304

**INTRODUCTION.** RRT is widely used for Acute Kidney Injury (AKI) in the intensive care units (ICU). Having multiple individual approaches between CRRT prescribers leads to wide variability and increases the possibility for errors in medication dosing, machine set-up, and nursing operations. This was overcome by an existing protocol within our ITU designed by a multidisciplinary team.Our project was to monitor the quality of CRRT by analyzing key components such as the modality, vascular access, anticoagulation and dose of effluent prescribed.
**OBJECTIVES.** By August 2018 to reduce the downtime of our filter in ITU by maintaining necessary Filtration fraction.
**METHODS.** A Prospective study was done to analyse the factors involved in prescription CRRT using a preformed template. Our study included 10 patients with 30 episodes. Each time the filter was started it was considered as single episodes. We analysed the various factors that could affect the prescription and this led to education being delivered to the nurses and caretakers involved with CRRT. We are currently trying to improvise the existing prescription for effective survival of our filters.
**RESULTS.** 8 patients required CVVH Central veno venous hemofiltration and 2 of them required CVVHDF Central veno venous hemodialfiltarion. (RFV) right femoral vein access was used in 6 patients, 2 of them required (LFV) left femoral vein and 2 required (RIJ) Right internal jugular vein as access. Among the 30 episodes, the most frequent dose of effluent prescribed was 35ml/kg/hr., which constituted around 17 episodes, and 13 of them were prescribed 25ml/kg/hr. The replacement fluid prescription for majority of the study was 30:70 in 24 episodes, 2 of them had ratio adjusted according FF and 4 of them had 50.50 ratios in view of access pressures. On analysing the filtration fraction only 10 of them were found to have < 20, 14 episodes had FF>30, 6 of them had FF 21-29.The filter failed 12 times overall, among which 4 times it failed twice in a day, Access pressure being the most common cause of failure constituting to 6 out of 8 times the filter failed.
**CONCLUSIONS.** 20 out of 30 episodes the filtration fraction was above 20. Higher fractions are associated with increased circuit clotting [3]. Filter life of CRRT is influenced by volume of blood processed. Maximum volume of blood is achieved by high blood flow rate, which can increase pressure alarms leading clotting. In our study it was found to be the most common cause for filter failure. Blood flows are consistently higher with RIJ catheters compared with left-sided catheters [1], due to additional resistance to flow from the two necessary bends and longer length of the catheter for left-sided access. In our study the most common access was RFV, followed by LFV and RJV. After analysing the above we decided to add in few more details to the existing renal prescription and is currently in progress.

### 0305

**Can plasma nucleoproteins be removed with continuous renal replacement therapy?**

M. Crespo Gómez[1], J. Beltrán-Garcia[2], C. Romá-Mateo[2], J.L. Garcia-Gimenez[2], S. Borrás Pallé[1], E. Perez[3], E. Lopez[1], A. Navarro[1], D. Valera[1], R. Zaragoza[1], F. Pallardó[2]
[1]University Hospital Dr Peset, Intensive Care, Valencia, Spain; [2]University of Valencia, Physiology, Valencia, Spain; [3]University Hospital Dr Peset, Biochemistry, Valencia, Spain
**Correspondence:** M. Crespo Gómez
*Intensive Care Medicine Experimental* 2018, **6(Suppl 2):**0305

**INTRODUCTION.** Septic shock is one of the leading causes of mortality in the ICU. It has been proved that nucleoproteins, resulting from neutrophil production of extracellular DNA and protein traps, and from cell lysis, are increased in septic patients, contributing to create the endothelial injury underlying hyperinflammatory processes (1-2).
**OBJECTIVES.** To analyze the influence of continuous renal replacement therapy (CRRT) within the first 24 hours, in serum and effluent levels of nucleoproteins, procalcitonine (PCT), C reactive protein (CRP), and interleucine 6 (IL6).
**METHODS.** Prospective observational study. Patients with diagnostic of septic shock according to Sepsis-3 criteria who underwent CRRT were included. We excluded inmunodeficient, polytraumatized and patients with neoplasms. For CRRT we used : biocompatible semipermeable polysulfone filters 1.8 m2 and anticoagulation with trisodium citrate. Initiation of CRRT was based according to unit protocol. Nucleoprotein levels were analyzed using a home made ELISA in plasma and effluent samples at different times:

- T0: 1 hour before starting CRRT
- T1: after 1 hour of CRRT, both prefilter (T1 pre) and postfilter (T1 post) fractions, as well as effluent ones;
- T2: 24 hours after starting CRRT, prefilter.

We also analyzed biomarkers PCT CRP and IL6 at T0 and at T2 . Results are expressed in median and interquartile range, analysis were made using non-parametric tests; statistical significance of p-value 0.05.
**RESULTS.** 11 patients were enrolled during 1year. Patients baseline characteristics: APACHE II of 29 (19-37),SOFA at admission 10(7-15), age 66 years (50-78).
No nucleoproteins were detected in effluent samples.
**CONCLUSIONS.** There were no statistical differences between nucleoproteins, nor biomarkers, at different times in the small cohort of patients assayed. These preliminary data suggest that nucleoproteins are not removed by CRRT, but we identify a trend to reduction in plasmatic concentration after 24h.

**Table 1 (abstract 0305).** Nucleoproteins (a.u: arbitrary units) and biomarker levels are shown

| | Median | IQR | p (Wilcoxon test) |
|---|---|---|---|
| NP T0 (a.u) | 1.51 | 0.63-2.09 | |
| NP T2 (a.u) | 0.96 | 0.66-2.27 | 1 |
| NP T1 Prefilter (a.u) | 1.45 | 0.93-2.71 | |
| NP T1 Postfilter (a.u) | 1.58 | 0.92-2.19 | 0.484 |
| PCT T0 (ng/ml) | 9.40 | 3.92-26.09 | |
| PCT T2 (ng/ml) | 15.78 | 1.36-25.21 | 0.386 |
| IL6 T0 (pg/ml) | 467.80 | 191-21574 | |
| IL6 T2 (pg/ml) | 144.20 | 98.9-750.7 | 0.062 |

## Cardiac arrest and post resuscitation care

### 0306

**Interaction effects between targeted temperature management and hypertension on survival outcomes after out-of-hospital cardiac arrest**

H. Ryu[1], H. Kim[2]
[1]Chonnam National University Hospital, Gwangju, Korea, Republic of; [2]Chosun University Hospital, Gwangju, Korea, Republic of
**Correspondence:** H. Ryu
*Intensive Care Medicine Experimental* 2018, **6(Suppl 2):**0306

**BACKGROUND.** Targeted temperature management (TTM) currently represents the most efficacious treatment to improve neurological recovery in comatose patients after out-of-hospital cardiac arrest (OHCA). The effects of TTM with hypertension have not been reported. This study aims to investigate whether the effect of TTM on neurological recovery after OHCA differed between patients with or without hypertension.

**METHODS.** This study was conducted with the use of the national cardiac arrest registry of OHCA patients with presumed cardiac etiology who survived to hospital admission between 2009 and 2016. The primary exposure was TTM. The endpoint was cerebral performance category (CPC) 1 and 2 at discharge and survival to discharge. We compared outcomes between the TTM and non-TTM groups using multivariable logistic regression with an interaction term between TTM and hypertension for calculating adjusted odd ratios (AORs) and 95% confidence intervals (CIs) after adjusting for confounding factors.

**RESULTS.** Among 25,985 patients following OHCA that survived till hospital admission with presumed cardiac etiology, TTM was performed on 12.2%. The TTM group showed better outcomes than the non-TTM group: 28.1% vs. 15.5% for good neurologic recovery (P< 0.01). The AOR (95% CI) of TTM for good neurological recovery for all study groups was 1.65 (1.47-1.85). In interaction model, the AOR (95% CI) of TTM for good neurological recovery was 1.87 (1.26-2.76) in patients without hypertension vs. 0.87 (0.75-1.02) in patients with hypertension.

**CONCLUSIONS.** Hypertension modified the effect of TTM on neurological outcomes for OHCA patients. TTM is associated with good neurological recovery in the non-hypertension group, but is not a significant effect in the hypertension group.

### 0307

#### Prediction of neurologic outcome after return of spontaneous circulation in out-of-hospital cardiac arrest: retrospective analysis using fast and frugal tree

S.M. Shin, G.J. Suh, Y.S. Jung, T. Kim, K.S. Kim
Seoul National University Hospital, Emergency Medicine, Seoul, Korea, Republic of
**Correspondence:** S.M. Shin
*Intensive Care Medicine Experimental* 2018, **6(Suppl 2):**0307

**INTRODUCTION.** Prediction of neurological prognosis in out-of-hospital cardiac arrest (OHCA) patients remains difficult. We wanted to develop a simple decision rule to predict the neurologic outcome after the return of spontaneous circulation (ROSC) in OHCA patients using Fast and Frugal Tree (FFT) analysis.

**OBJECTIVES.** Prediction of neurological prognosis in out-of-hospital cardiac arrest (OHCA) patients remains difficult. We wanted to develop a simple decision rule to predict the neurologic outcome after the return of spontaneous circulation (ROSC) in OHCA patients using Fast and Frugal Tree (FFT) analysis.

**METHODS.** Prospective post-cardiac arrest registry including 3 hospitals were retrospectively analyzed. Among 1609 patients in registry, 456 patients were enrolled after excluding 132 patients transferred from other hospitals, 133 patients with baseline cerebral performance category (CPC) 3 or 4, and 721 patients without sustained return of spontaneous circulation. A good neurologic outcome was defined as having CPC 1 or 2 at hospital discharge. Variables used for FFT analysis included age, gender, witnessed cardiac arrest, bystander CPR, initial shockable rhythm, prehospital defibrillation, prehospital ROSC, no flow time, low flow time, prompt light reflex and GCS after ROSC. Enrolled patients were randomly split into a training set and a test set (228 patients in each set).

**RESULTS.** Among 456 patients enrolled, 83 (18.2%) patients had a good neurologic outcome. Prehospital ROSC (True = good), prompt light reflex after ROSC (False = bad) and Cardiac cause (True = good, False = bad) were selected for the nodes constructing decision tree. Sensitivity, specificity, and accuracy of the decision tree to predict a good neurologic outcome were 96% (44/46), 89.6% (163/182), and 90.8% (207/228) in a training set and 94.6% (35/37), 80.6% (154/191), and 82.9% (189/228) in a test set.

**CONCLUSIONS.** A simple decision rule developed by FFT analysis can predict the neurologic outcome after ROSC in OHCA patients.

**REFERENCE(S)**

Carter BG, Butt W. Review of the use of somatosensory evoked potentials in the prediction of outcome after severe brain injury. Critical Care Medicine. 2001;29.

Beesems SG, Blom MT, van der Pas MHA, Hulleman M, van de Glind EMM, van Munster BC, et al. Comorbidity and favorable neurologic outcome after out-of-hospital cardiac arrest in patients of 70 years and older. Resuscitation.94:33-9.

Adrie C, Cariou A, Mourvillier B, Laurent I, Dabbane H, Hantala F, et al. Predicting survival with good neurological recovery at hospital admission after successful resuscitation of out-of-hospital cardiac arrest: the OHCA score. European Heart Journal. 2006;27:2840-5.

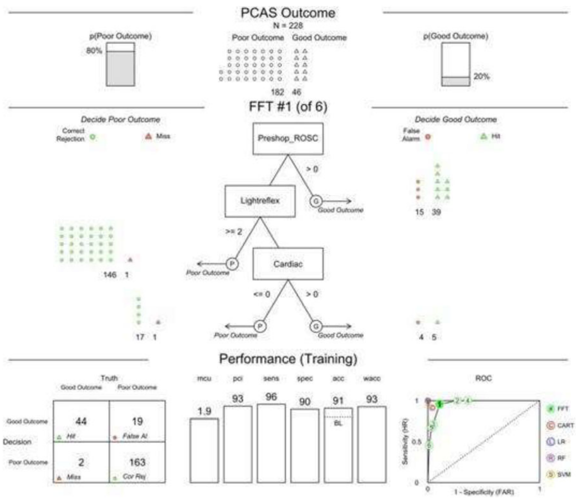

**Fig. 1 (abstract 0307).** Training FFT for Prediction of Neurologic Outcome

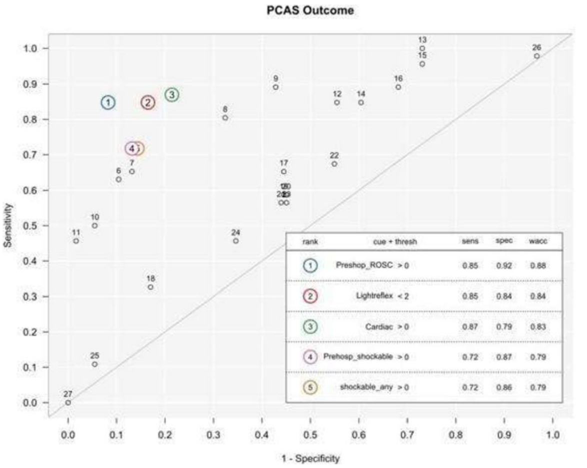

**Fig. 2 (abstract 0307).** Testing FFT for Prediction of Neurologic Outcome

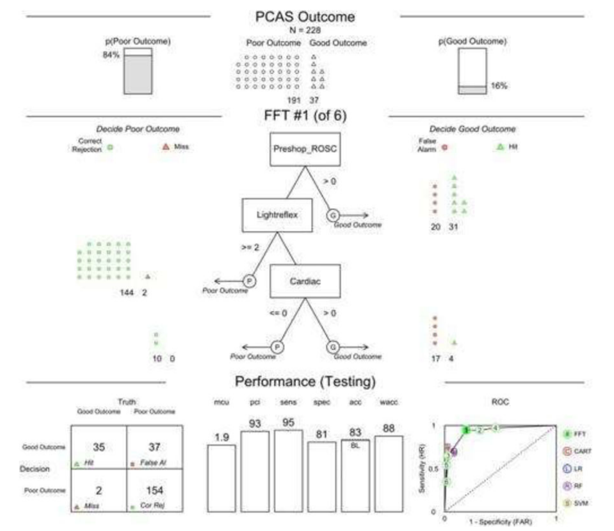

**Fig. 3 (abstract 0307).** Visualization of cue accuracies shown by the plot (what="cues", data="train") function

### 0308

#### Optimum chest compression point for cardiopulmonary resuscitation in children revisited using a three-dimensional coordinate system imposed on computed tomography: a retrospective, cross-sectional study

S.-B. Chon[1], M. Park[1], W.S. Oh[2], S. Cho[1]
[1]CHA Bundang Medical Center, CHA University, Emergency Department, Seongnam, Korea, Republic of, [2]Kangwon National University School of Medicine, Department of Internal Medicine, Chuncheon, Korea, Republic of
**Correspondence:** S.-B. Chon
*Intensive Care Medicine Experimental* 2018, **6(Suppl 2):**0308

**INTRODUCTION.** The optimum chest compression site (P_optimum) in children aged 1 y to puberty is debated: the European Resuscitation Council (ERC) recommends one finger breadth above the xiphisternal joint, while the American Heart Association (AHA) proposes a wider range, the lower sternal half.

**OBJECTIVES.** Using a 3-dimensional coordinate system imposed on computed tomography (CT), we aimed to discover the pediatric P_optimum to maximize stroke volume (SV) while minimizing hepatic injury.

**METHODS.** A retrospective, cross-sectional study was performed in children aged 1-15 y who underwent chest CT. We defined zero point (0, 0, 0) as the center of the xiphisternal joint and designated leftward, upward, and into-the-thorax directions as positive on each axis. The ultimate target, P_optimum (x_max. left ventricle (LV), y_max.LV, z_max.LV) was defined as the center of the maximum diameter of the LV, compression of which was assumed to produce maximum SV, the key point for successful CPR. We defined P_aorta (x_aorta, y_aorta, z_aorta), the reference structure separating the LV from the aorta, as the center of the aortic annulus. To compress the LV (vs. aorta) and not to compress the liver, we confined the compression range above the y coordinate of highest hepatic dome (y_liver_dome) and below y_aorta. Within this range, y_max.LV was located. To apply to pediatric CPR regardless of age, y coordinates were converted into relative ones with unit of sternal top (UST): 1 UST was the height from (0, 0, 0) to the sternal top. Data were presented as median (interquartile range) and compared among 3 age groups using Kruskal-Wallis test: 1.0-5.0, 5.1-10.0, and 10.1-15.0 y. As x_max.LV differed according to age, we investigated whether it could be expressed in terms of height by performing a linear regression analysis.

**RESULTS.** A total of 163 patients were enrolled, aged 8.8 (4.2-14.3) y; 49 (30.1%) were female. No significant difference was observed in the y_max.LV, relative y_max.LV, y_aorta, and y_liver_dome among age groups: 1.0 (0.1-1.9) cm, 0.10 (0.01-0.18), 0.39 (0.30-0.47), and −0.14 (−0.25-−0.03) UST, respectively (all P>0.05). The probability that neither the aorta nor the liver was compressed was ≥96% when placing hand at 0.05-0.20 UST, which embedded the y_max.LV centrally. X_max.LV (mm) was estimated as '0.173×(height, cm)+13' (n=106, P< 0.001, R2=0.278).

**CONCLUSIONS.** The study demonstrates that the optimum chest compression point is located 1 cm (or 0.1 UST) above the xiphisternal joint in children aged 1 y to puberty, supporting ERC and refuting AHA guidelines.

#### GRANT ACKNOWLEDGMENT

None.

### 0309

#### Impact of ischemic etiology-based difference on extracorporeal cardiopulmonary resuscitation in patients with refractory out-of-hospital cardiac arrest underlying cardiac diagnosis

K. Takeshige, Y. Kashima, H. Imamura
Shinshu University, Department of Emergency and Critical Care Medicine, Matsumoto, Japan
**Correspondence:** K. Takeshige
*Intensive Care Medicine Experimental* 2018, **6(Suppl 2):**0309

**INTRODUCTION.** Several studies have examined the impact of underlying causes of cardiac arrest on extracorporeal cardiopulmonary resuscitation (ECPR) survival. The ELSO registry demonstrated superior survival among pediatric patients with underlying cardiac pathology vs. non-cardiac reason for cardiac arrest.[1] However, it remains unclear whether the effectiveness of ECPR for refractory out-of-hospital cardiac arrest (OHCA) differ in patients with cardiac etiologies, such as acute coronary syndrome (ACS) vs. non-ACS.

**OBJECTIVES.** We investigated the prognostic effect of ECPR on refractory OHCA due to cardiac origin in patients with ACS or non-ACS.

**METHODS.** A consecutive patients with refractory OHCA due to cardiac cause who underwent ECPR at 7 tertiary centers were enrolled in this study. The exclusion criteria were as follows: aged < 20 years; non-cardiac cause, including pulmonary embolism, aortic dissection, trauma; successful return of spontaneous circulation before ECPR implantation; hypothermia (< 32°C); and not performed coronary angiography. A refractory cardiac arrest was defined as an absence of return to spontaneous circulation after continuous CPR for at least 30 min. ACS was diagnosed according to previous symptoms of cardiac arrest, laboratory data, 12-lead ECG, and coronary angiography. Patients with ACS were defined as the ACS patients, while the remaining patients formed the non-ACS patients. We assessed survival and neurological outcomes at 30 days after cardiac arrest. Neurological outcomes were assessed using the Cerebral Performance Category scale (CPC) and CPC1 and CPC2 were classified as a favorable neurological outcome.

**RESULTS.** We enrolled a total of 110 patients, including 71 ACS patients and 39 non-ACS patients. No significant intergroup differences were observed for age, sex, time from collapsed to ER arrival, the percentages of patients with shockable rhythm on arrival, witnessed, received countershocks during transportation, or administered CPR by a bystander. 30-day survival rate was significantly higher in the ACS patients than in the non-ACS patients (19.7%, n = 14 vs. 5.1%, n = 2, $P < 0.05$). The multivariate analysis showed that ACS was found to be an independent predictor of 30-day survival after cardiac arrest (odds ratio=7.15; 95% confidence intervals 1.04-48.94; $P < 0.05$). Kaplan-Meier analysis showed that patients with ACS had a significantly lower 30-day mortality rate compared to those with non-ACS patients ($P < 0.05$). However, no difference was noted in the neurological intact survival rate between the 2 groups (12.7%, n = 9 vs. 5.1%, n = 2, $P = 0.24$).

**CONCLUSIONS.** ECPR might be more effective for improving a survival outcome, but not a neurological outcome, in refractory OHCA patients due to cardiac origin with ACS than in those with non-ACS.

**GRANT ACKNOWLEDGMENT**

None.

### 0310

#### Gray matter to the white matter ratio for predicting neurological outcomes in patients treated with target temperature management after cardiac arrest: a systematic review and meta analysis

W. Kim[1], M.K. Na[2], T.H. Lim[3], B. Jang[4], Y. Cho[5], K.-S. Choi[6], H.-G. Shin[3], C. Ahn[3], J. Lee[3], J.G. Kim[1], G.-S. Jun[1]

[1]Hallym University Kangnam Sacred Heart Hospital, Emergency Medicine, Seoul, Korea, Republic of; [2]Yonsei University College of Medicine, Neurosurgery, Seoul, Korea, Republic of; [3]Hanyang University College of Medicine, Emergency Medicine, Seoul, Korea, Republic of; [4]Kyung Hee University College of Korean Medicine, Preventive Medicine, Seoul, Korea, Republic of; [5]Hallym University Kangdong Sacred Heart Hospital, Emergency Medicine, Seoul, Korea, Republic of; [6]Hanyang University College of Medicine, Neurosurgery, Seoul, Korea, Republic of

**Correspondence:** W. Kim

*Intensive Care Medicine Experimental* 2018, **6(Suppl 2):**0310

**INTRODUCTION.** Cerebral edema in cranial computed tomography, after the return of spontaneous circulation (ROSC), can be a marker of hypoxic brain injury for poor neurological outcome prediction; this appears as a loss of differentiation between the gray matter (GM) and the white matter (WM) on the cranial CT scans. Several studies demonstrated that low GM/WM ratio (GWR) values could predict poor neurological outcomes in cardiac arrest survivors. Nevertheless, the prognostic accuracy of the GWR varied by the location of the measurement on the cranial CT scans, among studies.

**OBJECTIVES.** This study aimed to evaluate the prognostic accuracy of the gray matter to the white matter ratio (GWR) in predicting the neurological outcomes in post-cardiac arrest patients treated with target temperature management.

**METHODS.** We systematically searched MEDLINE and EMBASE (Search date: 13 Sep 2017). Included studies were those evaluating neurological outcomes using the cerebral performance categories scale. We performed a subgroup analysis based on the location of the measurement. The Quality Assessment of Diagnostic Accuracy Studies-2 tool was used to assess the risk of bias of the included studies.

**RESULTS.** A total of 1150 patients from 10 observational studies were included in the meta-analysis. In evaluating the prognostic accuracy using area under the curve, the GWR of basal ganglia average showed the highest value (area under the curve 0.96, SE 0.02, Q 0.90) compared with the putamen/ posterior limb of internal capsule (area under the curve 0.93, SE 0.05, Q 0.87), overall average (area under the curve 0.91, SE 0.02, Q 0.85), and the cerebrum (area under the curve 0.89, SE 0.05, Q 0.82). Furthermore, the pooled diagnostic odd ratio values of GWR for predicting poor neurological outcomes also showed the highest value in the basal ganglia average (21.00, 95% confidence interval 6.85 - 64.40) followed by the overall average (20.71, 95% confidence interval 9.53 - 44.98), putamen / posterior limb of internal capsule (16.08, 95% confidence interval 4.36 - 59.23), and the cerebrum (13.96, 95% confidence interval 4.26 - 45.76).

**CONCLUSIONS.** The GWR in cranial computed tomography has a high prognostic value in predicting poor outcomes in post-cardiac arrest patients. In particular, it was easier to measure the GWR of basal ganglia average, which showed a higher prognostic accuracy, than the other cerebral locations.

**REFERENCE(S)**

1. Bernard SA, Gray TW, Buist MD, Jones BM, Silvester W, Gutteridge G, et al. Treatment of Comatose Survivors of Out-of-Hospital Cardiac Arrest with Induced Hypothermia. New England Journal of Medicine. 2002;346(8):557-63.
2. Gentsch A, Storm C, Leithner C, Schroeder T, Ploner CJ, Hamm B, et al. Outcome prediction in patients after cardiac arrest: a simplified method for determination of gray-white matter ratio in cranial computed tomography. Clinical neuroradiology. 2015;25(1):49-54.

### 0311

#### Survival following witnessed out-of-hospital cardiac arrests during nights and weekends

H. Ryu[1], H. Kim[2]

[1]Chonnam National University Hospital, Gwangju, Korea, Republic of; [2]Chosun University Hospital, Gwangju, Korea, Republic of

**Correspondence:** H. Ryu

*Intensive Care Medicine Experimental* 2018, **6(Suppl 2):**0311

**BACKGROUND.** The relationship between neurological outcomes following out-of-hospital cardiac arrests (OHCAs) and time of day or day of week is unknown.

**METHODS.** A nationwide, prospective, population-based observational studies of witnessed adult OHCAs (>18 years) with resuscitation attempts was conducted from January 2009 to December 2016. Days were defined as 9:00 am to 5:59 pm, nights as 6:00 pm to 8:59 am, weekdays as Mondays to Fridays, and weekends as Saturdays, Sundays, and national holidays. Primary outcome was survival to discharge and secondary outcome was survival with favorable neurologic outcome, defined as cerebral performance category 1 or 2.

**RESULTS.** A total of 11,748 bystander-witnessed adult OHCAs were included. Survival to discharge was significantly lower during nights than days (11.4% [95% CI: 9.4%-13.4%] vs. 22.8% [95% CI: 20.3%-25.3%]; P< 0.0001 and during weekends/holidays (15.7% [95% CI: 13.6%-18.0%] than weekdays (20.4$ [95% CI: 18.7%-22.2%]; P=0.0001. Survival rate with favorable neurologic outcome was substantially lower during night 7.5% [95% CI:6.3%-8.8%] than days (12.2% [95% CI: 10.6%-14.1%]; P< 0.0001), and during weekends/holidays (7.7% [95% CI: 6.2%-9.5%] than weekdays (10.4% [95% CI: 9.2%-11.8%]; P=0.012). After adjusting for potential confounding factors, survival to discharge remained significantly lower during nights compared to days (0dds ratio 0.79; 95% CI, 0.65-0.97).

**CONCLUSIONS.** Survival to discharge rate following bystander-witnessed adult OHCAs was lower during nights and weekends/holidays than days and weekdays, even when adjusted for potentially confounding factors.

### 0312

#### Chest compression-only vs conventional CPR by laypersons for unwitnessed out-of-hospital cardiac arrest

K. Maekawa, M. Hayakawa, T. Yoshida, Y. Itagaki, T. Tsuchida, S. Kawahara, A. Tomita, A. Mizugaki, H. Murakami, T. Oyasu, T. Saito, K. Katabami, T. Wada, A. Sawamura

Hokkaido University Hospital, Sapporo, Japan

**Correspondence:** K. Maekawa

*Intensive Care Medicine Experimental* 2018, **6(Suppl 2):**0312

**INTRODUCTION.** Cardiopulmonary resuscitation (CPR) by laypersons plays a key role in improving outcomes from out-of-hospital cardiac arrest (OHCA). Chest compression-only CPR has the potential physiological advantage of fewer compression interruptions as compared with conventional CPR, although at a possible cost to oxygen. In observational studies of bystander-initiated CPR for witnessed OHCA, two CPR approaches led to similar outcomes. However, it remains uncertain whether chest compression-only CPR should be applied for unwitnessed OHCA.

**OBJECTIVES.** To investigate whether chest compression-only CPR is non-inferior to conventional CPR in patients with unwitnessed OHCA.

**METHODS.** We performed retrospective analysis using data from Japan's nationwide OHCA registry from January 2005 through December 2012, in which patients had unwitnessed OHCA and chest compression-only or conventional CPR by laypersons, were transported to the hospital by EMS personnel. The primary endpoint was neurologically intact 30-day survival. We created well-balanced two groups (compression-only CPR group and conventional CPR group) using propensity score-matching. Non-inferiority of the proportion of patients with good functional status was analysed by Farrington and Manning test (significance level 5%, non-inferiority margin 0.35%).

**RESULTS.** Among the 210752 patients, compression-only CPR was performed in 154352 patients and conventional CPR was in 56400 patients. The propensity-score matching process selected 53863 patients each from both groups. Compared with conventional CPR group, compression-only CPR group incurred a lower rate of return of spontaneous circulation before hospital arrival (3.8% vs. 4.5%) and a lower survival rate (2.4% vs. 2.9%), and a similar rate of good functional status (1.1% vs. 1.2%). The non-inferiority of compression-only CPR was verified (percentage difference, -0.15%; 95%CI, -0.28% to -0.02%, p=0.0014 for non-inferiority). In multivariable logistic regression analysis, no association was observed between compression-only CPR and functional status (adjusted odds ratio, 0.96; 95%CI, 0.84 to 1.09).

**CONCLUSIONS.** Among patients with unwitnessd OHCA, chest compression-only CPR compared with conventional CPR did not result in an inferior rate of good functional status. Chest compression-only CPR may be a reasonable alternative to routine CPR approach for unwitnessed OHCA.

**REFERENCE(S)**

Rea TD, et al. CPR with chest compression alone or with rescue breathing. N Engl J Med. 2010 29;363:423-33.

Zhan L, et al. Continuous chest compression versus interrupted chest compression for cardiopulmonary resuscitation of non-asphyxial out-of-hospital cardiac arrest. Cochrane Database Syst Rev. 2017 Mar 27;3:CD010134.

**GRANT ACKNOWLEDGMENT**
none

### 0313

#### Effect of selenium supplementation on oxidative stress in patient after cardiac arrest

M. Janotka[1], D. Vondrakova[1], A. Kruger[2], P. Neuzil[1], P. Ostadal[2]
[1]Hospital Na Homolce, Prague, Czech Republic; [2]Hospital Na Homolce, Cardiology, Prague, Czech Republic
**Correspondence:** M. Janotka
*Intensive Care Medicine Experimental* 2018, **6(Suppl 2):**0313

**INTRODUCTION.** Prognosis of patients after cardiac arrest (CA) is still unsaatisfactory despite better care (5-20% survival to discharge).The anoxic phase of injury is followed by the reperfucion phase leading to postcardiac arrest syndrom.The oxidative stress is one of the main causes of postcardiac arrest syndrome.There is a data showing that increasing of the antioxidative protection by the supplementation of selenium in patients in septic shock or severe sepsis may have some impact on prognosis. But there is scarce data on the effect of this approach in patients after CA.

**OBJECTIVES.** Due to similarity between circulation impairment in sepsis and postcardiac arrest syndrome and because the most vulnerable system is the brain that consists of great amount of lipids easilly targeted by oxidative stress,we anticipated that we could decrease the damage by selenium substitution in the similar way as in septic shock

**METHODS.** We randomized 35 patients in 2 groups.The first group was treated by standart therapy.The second group received selenium intravenously (sodium selenite) in addition to the standart therapy. 3mg of selenium was administered as initial bolus (the first day) in 30 minutes infusion followed by continuous infusion of 2mg of selenium per day for the next 5 days.We measured the products of reactive oxygen species reactions (ROM test and 8-hydroxy-guanosin as marker of the damage of RNA),serum antioxidative capacity (BAP test),markers of inflammation and tissue injury and parameters of clinical outcome .

**RESULTS.** Patients treated by selenium had less pronounced oxidative stress.The results of ROM test showed significantly lower oxidative stress in the selenium group in the 2$^{nd}$ (p=0,005), 3$^{rd}$ (p=0,001), 4$^{th}$ (p=0,004) and 5$^{th}$ day (p=0,02). The results of BAP test implied significantly higher serum antioxidative capacity in the selenium treated group in the 2$^{nd}$ (p=0,04),3$^{rd}$ (p=0,008) and 4$^{th}$ day (p=0,01). There was significantly lower concentration of 8-hydroxy-guanosin in selenium treated group in the 3$^{rd}$ day (1,1 vs. 2,3 ng/ml, p=0,03).There was no difference in the levels of the markers of renal, hepatal or cerebral (neuron specific enolase) injury and no difference in inflamation markers (leukocytes,CRP,procalcitonin,incidence of infection). There was insignificantly better neurological outcome in the selenium treated group in 30day follow up (average CPC 2.1 vs. 3.1, p=0,06) and significantly more ventilator free days in 30day follow up in the selenium treated group (p=0,03).

**CONCLUSIONS.** Our data imply that intravenously administered selenium in patients after CA is safe and may decrease the oxidative stress and increase the antioxidative capacity. Although numerical trend to better neurological outcome and more ventilator free days do not mean necessarily positive effect on prognosis it shows that it might be the adjunctive way to influence prognosis of patients after CA.

**GRANT ACKNOWLEDGMENT**
IG 150501

### 0314

#### Outcomes of in-Hospital cardiac arrest in a British District General Hospital

E. Landymore, J. McLoughlin, P. Morgan
Surrey and Sussex Healthcare NHS Trust, Anaesthetics / Intensive Care, Redhill, United Kingdom
**Correspondence:** E. Landymore
*Intensive Care Medicine Experimental* 2018, **6(Suppl 2):**0314

**INTRODUCTION.** Return of spontaneous circulation following cardiac arrest in the adult population is a common reason for admission into intensive care. It is known that there is a substantial mortality rate following admission even if the patient survived the initial arrest.

**OBJECTIVES.** To compare the outcome of adult in-hospital cardiac arrests at East Surrey Hospital to a national Cardiac Arrest Audit database and to also assess outcome by age.

**METHODS.** A retrospective search of a critical care data base of 3576 patients from 03/10/2010 until 02/11/2017 revealed 151 in-hospital adult cardiac arrests that were subsequently admitted to intensive care.

**RESULTS.** The overall unadjusted discharge rate from the intensive care unit (ICU) to the wards was 42.4%. This does not reflect the number that were discharged home or later died on the wards. This rate falls within the rates of survival to discharge from hospital of 10.5% to 49.0% as per a 2014 UK National Cardiac Arrest Audit database [1]. Table 1 shows the discharges to back to the ward following ICU admission categorized by age.

**CONCLUSION.** These results show that the greatest number of ICU admissions post in hospital cardiac arrests peaks in the 8$^{th}$ decade of life. The proportion of patients who survive following in-hospital cardiac arrest decreases with each subsequent decade. This retrospective analysis allows us to better prognosticate and to be able to provide more accurate information to relatives of these patients.

**REFERENCES. 1. Nolan JP et al. Resuscitation 85(8): 987-92, 2014**

**Table 1 (abstract 0314).** Breakdown of patients being discharged back to the ward post in-hospital cardiac arrest

| Age Range | Survived (%) | Died (%) | Total (%) | Median # of days in ICU (range) |
|---|---|---|---|---|
| 18-29 | 1 (100) | 0 (0) | 1 (0.07) | 6.1 (6.1) |
| 30-39 | 3 (60) | 2 (40) | 5 (3.3) | 2.9 (0.2-5.6) |
| 40-49 | 6 (54.5) | 5 (46.5) | 11 (7.3) | 5.5 (0.1-53) |
| 50-59 | 8 (61.5) | 5 (38.5) | 13 (8.6) | 4.5 (0.2-47) |
| 60-69 | 20 (54.1) | 17 (45.9) | 37 (24.5) | 2.7 (0.1-97) |
| 70-79 | 16 (39.0) | 25 (61.0) | 41 (27.2) | 3.1 (0.1-41) |
| 80-89 | 15 (37.5) | 25 (62.5) | 40 (26.5) | 2.5 (0.1-53) |
| 90-99 | 1 (33.3) | 2 (66.7) | 3 (2.0) | 1.5 (0.1-2.9) |
| Totals | 70 (46.4) | 81 (53.6) | 151 (100) | |

### 0315

#### Changes in cardiac arrest profile after rapid response team implementation

M.V. Viana[1], J.S. Brauner[2], W.L. Nedel[2], H. Muller[2], J. França[2], S.R.R. Vieira[2], T.C.D. Butteli[2], P. Berto[2], P. Schwarz[2], M. Conceição Prestes[2], D.S.N. Leite[2], L.A. Zorzi[2], M.C. dos Santos[2], M.M. Boniatti[2]
[1]Hospital de Clinicas de Porto Alegre, Porto Alegre, Brazil; [2]Hospital de Clinicas de Porto Alegre, Intensive Care Unit, Porto Alegre, Brazil
**Correspondence:** M.V. Viana
*Intensive Care Medicine Experimental* 2018, **6(Suppl 2):**0315

**INTRODUCTION.** Rapid response teams (RRT) assess patients at early stage of clinical deterioration, preventing serious adverse events in hospitalized patients[1]. Previous studies showed a reduction in cardiac arrest after the implementation of RTT[1]. However, there is no data to assess if the profile of in-hospital cardiac arrest changes after implementation of RRT.

**OBJECTIVES.** To evaluate changes in the characteristics of in-hospital cardiac arrest after the implementation of RRT.

**METHODS.** Prospective observational study that included patients that presented a cardiac arrest from January 2013 to December 2017. Exclusion criteria was cardiac arrest in intensive care unit, emergence department and operating room. Rapid response team started in July 2014. Patients were classified in two groups pre-RRT (cardiac arrest before RRT implementation) and post-RRT (cardiac arrest after RRT implementation). Patients were followed until hospital discharge or death.

**RESULTS.** We had total of 308 cardiac arrest (64.6 ± 15.2 years, 60.3% were men, 13.9% had a shockable initial rhythm, 61% were post-RRT). Cardiac arrest reduced from 4.2/1000 to 2.5/1000 admissions after implementation of RRT and we have approximately 124 calls/ 1000 admission. Pre-RRT patients had more hypoxia (29.4% vs 14.3; p=0.006) and tachypnea (14.7% vs 4.2%; p=0.004) before cardiac arrest compared to post-RRT. There was no difference between groups regarding initial rhythm, return of spontaneous circulation (ROSC), and mortality between groups (Table 1). However, the main reason for cardiac arrest was different between pre-RRT and post-RRT groups (Table 2). In multivariate logistic regression [OR, 95% CI], ROSC was positively associated with shockable rhythm [0.36 (0.16-0.78)], but not with age [1.01 (0.99 -1.02)] and post-RRT [0.74(0.46-1.21)]. In multivariate logistic regression [OR, 95% CI], in-hospital survival was associated with age [1.03 (1.01-1.05)] shockable rhythm [0.187 (0.080 -0.439)] but not post-RRT [0.893(0.395-2.02)].

**CONCLUSION.** Even though implementation of RRT is associated with reduction of in hospital cardiac arrest, it does not change the mortality of in-hospital cardiac arrest victims. There was a significant reduction in cardiac arrest due to respiratory causes after RTT implementation.

##### REFERENCE(S)

1. Boniatti MM, Azzolini N, Viana MV, Ribeiro BS, Coelho RS, Castilho RK, et al. Delayed medical emergency team calls and associated outcomes. Critical care medicine. 2014;42(1):26-30

##### GRANT ACKNOWLEDGMENT

No grants for this study.

**Table 1 (abstract 0315).** Cardiac arrest profile before and after rapid response team implementation

| | pre-RRT (n=122) | Post-RRT (n=188) | p |
|---|---|---|---|
| Age (years) | 64.5±15.9 | 64.6±14.8 | 0.963 |
| Male sex (%) | 69 (53.9) | 122 (64.6) | 0.104 |
| Shockable rhythm | 19 (15.3) | 25 (13.4) | 0.642 |
| Cardiac arrest at night | 64 (52.5) | 100 (52.9) | 0.938 |
| Deterioration signs before cardiac arrest | 39 (57.4) | 86 (45.5) | 0.094 |
| ROSC | 42 (33.9) | 81 (43.1) | 0.103 |
| Mortality | 75 (87.2) | 153 (86.9) | 0.950 |

**Table 2 (abstract 0315).** Main reason for cardiac arrest before and after rapid response team implementation

| | Pre-RRT (n=122) | Post-RRT (n=188) | p |
|---|---|---|---|
| Hypovolemia (%) | 4 (3.9) | 11 (6.3) | <0.001 |
| Hypoxia (%) | 63 (61.2) | 67 (38.1) | <0.001 |
| Acidosis (%) | 9(8.7) | 12 (6.8) | <0.001 |
| Hyperkalemia (%) | 0 (0) | 6 (3.4) | <0.001 |
| Pulmonary thromboembolism (%) | 6 (5.8) | 4 (2.3) | <0.001 |
| Acute coronary syndrome (%) | 7 (6.8) | 18 (10.2) | <0.001 |
| Others or unknown (%) | 14 (13.6) | 58 (33) | <0.001 |

### 0316

#### Extracorporeal cardiopulmonary resuscitation for cardiac arrest patients: a propensity-matched study

D. Patricio, L. Peluso, A. Brasseur, O. Lheureux, J.-L. Vincent, C. Jacques, T. Fabio Silvio
Université Libre de Bruxelles, Intensive Care Unit, Bruxelles, Belgium
**Correspondence:** D. Patricio
*Intensive Care Medicine Experimental* 2018, **6(Suppl 2):**0316

**INTRODUCTION.** The potential benefit of extracorporeal cardiopulmonary resuscitation (ECPR) for patients with refractory cardiac arrest (CA) remains unsettled.

**OBJECTIVES.** To compare the survival and neurologic outcome following ECPR or conventional cardiopulmonary resuscitation (CCPR) in CA victims.

**METHODS.** Retrospective analysis of database from our prospective observational cohort of CA patients. All consecutive adult patients referred to the Department of Intensive Care with CA between January 2012 and December 2017. The decision to initiate ECPR was dependent on the attending physician and initiated by the ECPR team, which is composed of ICU physicians. A propensity score was derived using a logistic regression model, including characteristics that varied between groups with a p < 0.10 and others that potentially related to outcome. The primary outcomes were survival to ICU discharge and 3-month favorable neurologic outcome, assessed by the Cerebral Performance Categories (CPC) of 1-2.

**RESULTS.** On a total of 635 CA patients admitted over the study period (ECPR, n =120), 80 patients with ECPR were matched to 80 patients who underwent CCPR (median age: 57 years - out-of-hospital CA, 62%). Time from arrest to termination of CPR (i.e. return of spontaneous circulation - ROSC, ECMO initiation or death) was 54±22 and 54±19 min in the ECPR and CCPR groups, respectively. ROSC rates were 77/80 (96%) and 30 (38%) in the two groups ($p< 0.001$). Survival at ICU discharge was 18/80 (23%) vs. 14/80 (18%; $p=0.55$), respectively. Also, 3-month favorable outcome was higher, although not statistically significant, in ECPR (15/80, 19%) than CCPR (9/80, 11%; $p=0.26$)

**CONCLUSIONS.** ECPR after cardiac arrest may be associated with improved outcomes.

**GRANT ACKNOWLEDGMENT**

On behalf of the authors, huge thank you to the Erasme Hospital´s staff for making this study possible.

### 0317

#### Evaluation of ECLS management in cardiogenic shock after cardiac arrest in myocardial infarction

R. Zunarelli, L. Gaide Chevronnay, M. Durand, P. Albaladejo, J.-F. Payen
Grenoble Alpes University Hospital, Anesthesiology and Intensive Care, La Tronche, France
**Correspondence:** R. Zunarelli
*Intensive Care Medicine Experimental* 2018, **6(Suppl 2):**0317

**BACKGROUND.** The mortality rate of cardiogenic shock (CS) following acute myocardial infarction (AMI) is still high nowadays. Patients with cardiac arrest post AMI turning into CS has never been studied specifically. The aim of this study was to compare the mortality rate, with or without extra corporeal life support (ECLS), of CS post cardiac arrest following an AMI. Secondary objectives were factors associated with mortality.

**METHODS.** In this retrospective study conducted in Grenoble's university hospital, we compared patients with CS post cardiac arrest, due to AMI, implanted or not with ECLS. Demographical, clinical and biological datas were recorded. Univariate and multivariate statistical analyses have been performed looking for mortality risk factors at one month.

**RESULTS.** From January 2008 to December 2015, 85 patients have been included: 42 in the ECLS group versus 43 in the group without ECLS. Survival rate was 45%. There was no statistical difference of mortality rate between ECLS group (n=24, 57%) and non-ECLS group (n=23, 53%) ($p=0.74$). This lack of difference was confirmed on multivariate analysis (OR=1.43 IC $_{95\%}$ [0.44-4.69]). Other risk factors associated with mortality were age, lactates on arrival and no-flow duration. Intra-aortic balloon pump was a protective factor.

**DISCUSSION-CONCLUSION.** Only patients with refractory CC were implanted by ECLS. They were in a worse condition without an increase of mortality rate. In conclusion, ECLS was an efficient assistance in the CS post cardiac arrest due to AMI.

### 0318

#### Routine use of continuous insufflation of oxygen without tracheal intubation improves quality of cardio-pulmonary resuscitation

A. Gaillard[1], C. Ricard[1], G. Berthet[1], O. Baptiste[1], V. Peigne[1,2]
[1]Haute Savoie Fire Department, Meythet, France; [2]CH Métropole Savoie, Chambéry, France
**Correspondence:** V. Peigne
*Intensive Care Medicine Experimental* 2018, **6(Suppl 2):**0318

**INTRODUCTION.** Experimental studies suggest that continuous insufflation of oxygen (CIO) could improve cardio-pulmonary resuscitation (CPR). Since 2017, our fire department uses a specific device for CIO without tracheal intubation for prehospital CPR.

**OBJECTIVES.** To evaluate if CIO improves the quality of CPR

**METHODS.** All CPR performed by the firemen are monitored prospectively since 2011. Rate, duration and depth of the chest compressions (CC) are extracted from the automated external defibrillator. For each CPR, rates of CC with depth and rhythm respecting the guidelines and CPR fraction (ratio of the duration of CC on the duration of CPR) were measured. Results before and after implantation of CIO were compared.

**RESULTS.** CPR was performed by the firemen in 1270 patients (mean age 66.6 years) before and in 296 (mean age 67.3 years) after spreading of CIO. The proportion of CC with depth respecting the guidelines was similar (35.9% before, 38.9% after (p 0.07)). The proportion of CC with rate respecting the guidelines increased from 57.2% to 76.6% ($p < 0.001$) with CIO and the CPR fraction increased from 75.2% to 81.6% ($p < 0.001$).

**CONCLUSIONS.** Routine use of CIO without tracheal intubation by firemen was associated with an improvement of CPR quality.

**REFERENCE(S)**

None

**GRANT ACKNOWLEDGMENT**

None

### 0319

#### Is NIRO Pulse a useful monitor for cardiopulmonary resuscitation?

K. Sakaguchi[1], S. Takada[1], M. Takada[1], K. Takahashi[1], Y. Onodera[2], T. Kobayashi[1], K. Kawamae[2], M. Nakane[1]
[1]Yamagata University Faculty of Medicine, Department of Emergency and Critical Care Medicine, Yamagata, Japan; [2]Yamagata University Faculty of Medicine, Department of Anesthesiology, Yamagata, Japan
**Correspondence:** K. Sakaguchi
*Intensive Care Medicine Experimental* 2018, **6(Suppl 2):**0319

**INTRODUCTION.** Cerebral tissue $O_2$ saturation measured by near infra-red spectroscopy (NIRS) is a novel method for monitoring the response to cardiopulmonary resuscitation (CPR). There is evidence that higher cerebral tissue $O_2$ saturation during CPR after cardiac arrest (CA) is associated with return of spontaneous circulation (ROSC). The NIRO Pulse mode in the NIRO-200NX system (Hamamatsu Photonics, Japan) can measure the tissue oxygenation index (TOI) and $O_2$ saturation of the pulse wave ($SnO_2$). TOI reflects hemoglobin oxygenation in the arteries, capillaries, and veins. $SnO_2$ reflects hemoglobin oxygenation in the pulse wave flowing into the brain. $SnO_2$ is an important feature of the NIRO Pulse mode and may be a useful monitor during CPR. However, there are few studies on the association of ROSC with $SnO_2$.

**OBJECTIVES.** This was a single-center pilot study that investigated the ability of $SnO_2$ during CPR to predict ROSC in patients with CA.

**METHODS.** This study was conducted from October 2017 to March 2018. We included subjects who arrived in CA at the emergency department of Yamagata University Hospital and had a probe placed on their forehead for NIRS. Patients were treated according to the Japan Resuscitation Council Resuscitation Guidelines 2015. We calculated the average $SnO_2$ and TOI over 1 minute after the start of NIRS monitoring (Initial $SnO_2$, Initial TOI), the average TOI from the start of NIRS monitoring to the end of resuscitation (Mean TOI), and the maximum $SnO_2$ and TOI during the period of resuscitation (Highest $SnO_2$, Highest TOI).

**RESULTS.** The study included 24 cases with a median age of 83.5 years. CA was witnessed in 41.7%, bystander CPR was started before the arrival of emergency medical services (EMS) in 83.3%, and ROCS cases were 25%. An automatic external defibrillator was used in 2 cases (8%), and the initial ECG waveform when EMS arrived was pulseless electrical activity (25%) or asystole (67%). The causes of CA were acute coronary syndrome (21%), other cardiac etiology (17%), or foreign body airway obstruction (17%). The median Initial $SnO_2$ was significantly higher in the ROSC group (41.2, IQR 33.7-69.7) than the Non-ROSC group (31.0, IQR 15.2-38.7, p=0.005). The median Highest $SnO_2$ was significantly greater in the ROSC group (78.9, IQR, 70.3-100.3) than the Non-ROSC group (59.0, IQR 50.7-71.5, p=0.006). The median Mean TOI and Highest TOI were significantly larger in the ROSC group, but the Initial TOI was not significantly different between the groups.
**DISCUSSION.** In this study, the Initial $SnO_2$ was significantly higher in CA patients with than without ROSC. Cerebral tissue oxygenation and cerebral blood flow pulse wave oxygenation were improved when ROSC was achieved. These results suggest that the Initial $SnO_2$ can predict ROSC in CA patients; however, more CA cases are needed since this was a pilot study.
**CONCLUSIONS.** The Initial $SnO_2$ was significantly higher in CA cases with than without ROSC.

**GRANT ACKNOWLEDGMENT**
None

### 0320

#### Analysis of relationship between body temperature and survival rate of patients of in-hospital cardiac arrest

E. Choi[1], J.M. Lee[1], Y. Shin[1], J.W. Huh[2], S.-B. Hong[2]
[1]Asan Medical Center, Medical Emergency Team, Seoul, Korea, Republic of; [2]Asan Medical Center, Division of Pulmonary and Critical Care Medicine, Department of Internal Medicine, Seoul, Korea, Republic of
**Correspondence:** E. Choi
*Intensive Care Medicine Experimental* 2018, **6(Suppl 2):**0320

**INTRODUCTION.** Survival to hospital discharge after in-hospital cardiac arrest (IHCA) is poor. There are some reports about target temperature management (TTM) improve survival in patients with out-of-hospital cardiac arrest (OHCA). However the association of body temperature and survival rate of IHCA is uncertain.
**OBJECTIVES.** We aimed to analyze the relationship between body temperature and survival discharge of IHCA.
**METHODS.** This retrospective observational study of IHCA was performed during 2010 to 2015 at a tertiary care hospital in South Korea. We included patients who admitted to an ICU after IHCA at the general ward. We reviewed the medical records of 270 adult patients who survived for longer than 72h after IHCA. The patients were divided into four groups by the highest ($T_{max}$) and lowest body temperature ($T_{min}$) of 72 hours after return of spontaneous circulation (ROSC): normothermia (BT=36.0-37.9°C), hypothermia ($T_{min}$ < 36.0°C and $T_{max}$ = 36.0-37.9°C), hyperthermia ($T_{min}$= 36.0-37.9°C and $T_{max}$ ≥ 38.0°C), and hypo-hyperthermia ($T_{min}$ < 36.0°C and $T_{max}$ ≥ 38.0°C). The primary outcome was survival to discharge. Multivariate logistic regression models were used to examine differences in survival to discharge.
**RESULTS.** A total of 270 patients were enrolled, the survival rate at hospital discharge was 61.5% (166 cases). Normothermia had a significantly better survival rate than the other groups (74.4%, p=0.006). In hypothermic group, no induced hypothermia had a better survival rate than induced hypothermia (57.6% vs 26.1%, p=0.009). In the multivariate analysis, survival at hospital discharge was associated with defibrillation (OR 2.72, 95% CI 1.22-6.06, p=0.015), duration of CPR (min) (OR 0.96, 95% CI 0.93-0.99, p=0.037), and severity of disease(SOFA1)(OR 0.80, 95% CI 0.73-0.87, P< 0.001). Induced hypothermia was not associated with survival to discharge (OR 0.45, 95% CI 0.19-1.10, p=0.079).
**CONCLUSIONS.** Normothermia is associated with improved survival to discharge in IHCA. Survival to discharge in IHCA was associated to defibrillation, shorter time of CPR, and lesser severity of disease (SOFA1).

**REFERENCE(S)**
Suffoletto B, Peberdy MA, van der Hock T, Callaway C (2009) Body temperature changes are associated with outcomes following in-hospital cardiac arrest and return of spontaneous circulation. Resuscitation 80:1365-1370. http://dx.doi.org/10.1013/j.resuscitation.2009.08.020
The hypothermia after cardiac arrest study group (2002) Mild therapeutic hypothermia to improve the neurologic outcome after cardiac arrest. N Engl J Med 346:549-556. http://dx.doi: 10.1056/NEJMoa012689

### 0321

#### Long term follow up after out of hospital cardiac arrest: survival and performance status comparison between therapeutic hypothermia and targeted temperature management

T. Akbari[1], S.J. Brett[2], R. Stümpfle[3]
[1]Imperial College Healthcare NHS Trust, General Intensive Care Unit, London, United Kingdom; [2]Imperial College London, Directorate of Anaesthetics and Critical Care, London, United Kingdom; [3]Imperial College Healthcare NHS Trust, Directorate of Anaesthetics and Critical Care, London, United Kingdom
**Correspondence:** T. Akbari
*Intensive Care Medicine Experimental* 2018, **6(Suppl 2):**0321

**INTRODUCTION.** Cerebral performance category (CPC) scores (1) have been widely used to determine outcomes post cardiac arrest. However, there is less research into CPC scores on long term follow up.
**OBJECTIVES.** This study investigated the change in CPC scores on long term follow up in a cohort of patients admitted to the general intensive care unit after suffering out of hospital cardiac arrest (OOHCA). We also investigated the effect on mortality of Targeted Temperature Management (TTM) which was adopted in our unit towards the end of 2014, prior to which Therapeutic Hypothermia (TM) was used.
**METHODS.** Patients who had OOHCA and were admitted to the general intensive care unit between 25 July 2010 and 28 July 2017 were included. CPC on primary hospital discharge was determined by case note review; CPC of 1-2 was classed as a favourable outcome and 3-4 was classed as unfavourable. Follow up CPC was determined by telephone consultation in Mar 2018 using a questionnaire. Kaplan-Meier analysis was used to demonstrate survival over time. The data was then split into two cohorts; data up to and including 2014 vs data from 2015 to 2017, to investigate the effects of TTM adoption.
**RESULTS.**
· A total of 370 patients were included. The majority of the patients were male (80%) and mean age was 62.4 (SD 14.9). Average length of hospital stay was 22.7 days (SD 47.3, range 0 to 649). 177 were discharged alive from hospital giving overall mortality of 52.1%. 8 patients were excluded. 169 notes were reviewed retrospectively. Of these, 139 (82%) patients were CPC 1-2 and 30 (18%) were CPC 3-4.
· Follow up CPC status was available for 132 patients. Of these there were 19 deaths (14%), 99 (75%) were CPC 1-2 and 14 (11%) were CPC 3-4.
· 6 patients improved from CPC 3-4 on discharge to 1-2 on follow up whereas 3 patients changed from favourable (CPC 1-2) to unfavourable (CPC 3-4).
· There was no statistically significant difference in mortality before and after TTM adoption (mortality 2010-2014 vs Mortality 2015-17, p = 0.50). A somewhat higher proportion of patients were discharged with CPC 1-2 after 2014 (44%) as compared to the period up to and including 2014 (32.4%) although this did not reach statistical significance (p=0.45).
**CONCLUSIONS.** Our results suggest the majority of patients had a favourable outcome on discharge which was maintained on follow up, once they survived the initial insult and hospital admission. A small proportion of patients switched CPC classes. A switch to TTM did not have a significant effect on mortality but seems to have increased favourable outcomes on discharge. More research is needed to investigate the link between TTM and long term neurological outcomes in patients who survive out of hospital cardiac arrest.

**REFERENCE(S)**
1) Jennett B, Bond M. Assessment of outcome after severe brain damage. Lancet. 1975;1:480-4.

**GRANT ACKNOWLEDGMENT**
None

## Microcirculation & organ injury

### 0322

#### Cardiac hyaluronan may be associated with the persistence of atrial fibrillation

Y. Kashima, K. Takeshige, H. Imamura
Shinshu University, Department of Emergency and Critical Care Medicine, Matsumoto, Japan
**Correspondence:** Y. Kashima
*Intensive Care Medicine Experimental* 2018, **6(Suppl 2):**0322

**INTRODUCTION.** Hyaluronan (HA), a primary component of the extracellular matrix (ECM), is associated with several cardiovascular diseases. HA is a key ECM regulator of inflammation, cell migration and proliferation, and fibroblast activity during normal response to injury and inflammatory diseases.[1] Recent studies have shown that atrial fibrosis and diffuse left ventricular fibrosis are associated with atrial fibrillation (AF) and that fibrosis represents an excessive deposition of ECM components, that play a critical role in the pathogenesis of AF by interrupting cardiomyocyte-bundle continuity.[2,3] AF is the most common arrhythmia encountered clinically and the presence of AF has a negative impact on the mortality in critically ill patients. However, irrespectively of whether HA is associated with AF, the precise cardiac source of HA, and its role in AF patients remain unclear.

**OBJECTIVES.** We investigated chamber-specific HA levels and its relationship with oxidative stress, brain natriuretic peptide (BNP) levels in patients with paroxysmal AF (PAF) and persistent AF (PSAF) to further elucidate the pathophysiology of AF.

**METHODS.** We investigated chamber-specific HA levels in patients with PAF (n = 50) or PSAF (n = 35). Peripheral blood was drawn from the left cubital vein via a 20-gauge peripheral venous catheter before radiofrequency catheter ablation. Before ablation, a luminal coronary sinus (CS) catheter was used for plasma sampling from (1) the CS and advance transseptal guiding sheaths, (2) the right ventricle and atrium before transseptal puncture, and (3) the left atrium immediately after transseptal puncture. The levels of HA and a diacron-reactive oxygen metabolite (dROM) as a marker for oxidative stress at different cardiac sites, and peripheral brain natriuretic peptide (BNP) levels were measured.

**RESULTS.** HA levels in the CS (CS-HA) were significantly higher than those at other sites, in both PAF and PSAF patients, and CS-HA levels were significantly higher in PSAF patients than in PAF patients (37.1 [interquartile range, 31.2-48.3] vs. 30.6 [23.7-40.2] pg/mL, $P < 0.01$). CS-HA levels correlated with CS-dROM levels and peripheral BNP levels in PSAF patients ($r = 0.417$, $P = 0.03$ and $r = 0.579$, $P < 0.001$, respectively), but not in PAF patients ($r = -0.115$, $P = 0.421$ and $r = 0.048$, $P = 0.740$, respectively).

**CONCLUSIONS.** Our data suggests that cardiac HA could either be a cause or consequence of AF. Cardiac HA levels positively correlate with cardiac oxidative stress and peripheral BNP levels in PSAF patients, although this was not the case in PAF patients. These results indicate that cardiac HA may be associated with the persistence of AF.

##### REFERENCE(S)

1. Laurent TC et al. FASEB J 1992;6:2397-2404
2. Burstein B et al. J Am Coll Cardiol 2008;51:802-809
3. Ling LH et al. J Am Coll Cardiol 2012;60:2402-2408

##### GRANT ACKNOWLEDGMENT

None.

### 0323

#### A reduction in skin perfusion can reliably predict tissue hypoperfusion during ultrafiltration

W. Mongkolpun, J.-L. Vincent, J. Creteur
Erasme Hospital, Intensive Care Department, Brussels, Belgium
**Correspondence:** W. Mongkolpun
*Intensive Care Medicine Experimental* 2018, **6(Suppl 2):**0323

**INTRODUCTION.** After hemodynamic stabilization, a number of critically ill patients need continuous veno-venous hemofiltration (CVVH) with ultrafiltration (UF) to reduce fluid overload and tissue edema. Excessive fluid removal may impair tissue perfusion so that careful monitoring is necessary to guide the UF.

**OBJECTIVES.** To determine whether change in skin blood flow (ΔSBF) is related to tissue hypoperfusion and could be used to guide UF during CVVH.

**METHODS.** We included 30 patients who required UF with CVVH. All were hemodynamically stable after resuscitation from circulatory shock (septic (24), cardiogenic (6)). Sequential organ failure assessment (SOFA) and APACHE II scores were calculated at baseline. UF parameters were set by the attending physician and unchanged during the study.

SBF was measured (Periflux 5000, Perimed; Perfusion unit: PU) on the same finger for 3 minutes at the start of the study (H0), and 1 (H1) and 6 (H6) hours after start of UF.

Hemodynamic parameters and blood lactate levels were obtained at each time point.

ΔSBF was defined as the relative (%) change in SBF during the first hour of UF.

Tissue hypoperfusion was defined as an increase in blood lactate concentration (ΔL) of at least 10% at H6.

Receiver operating characteristic (ROC) curves were constructed and were reported as areas under the curve (AUC) with 95% confidence intervals. Data are reported as medians with 25$^{th}$ and 75$^{th}$ percentiles.

**RESULTS.** Median APACHE II and SOFA scores were 24 (17-26) and 11 (8-13), respectively. Mean arterial pressure (MAP) and cardiac index (CI) were 94 (92-112) mmHg and 3.0 (2.8-4.0) L/min/m$^2$, respectively, and the median norepinephrine (NE) dose was 0.2 (0.1-0.5) mcg/kg/min. UF rate was 100 (100-200) ml/hour. At baseline, there were no significant differences in hemodynamic variables, NE dose, or lactate concentrations in patients with and without ΔL ≥10% (Table 1). UF rate was similar in patients with and without ΔL ≥10% (150 (100-200) vs 100 (100-200) ml/hour, p=0.3). CI and MAP remained similar in patients with and without ΔL% ≥10% throughout the study, but NE dose was higher in patients with ΔL% ≥10 at H6 (p=0.03) (Table 1). Patients with ΔL% ≥10 had lower SBF at all time points and a higher ΔSBF at H1 than the other patients (Table 1). ΔSBF and baseline SBF predicted a ΔL ≥10% at H6 with AUCs of 0.96 and 0.83, respectively, and cut-off points of ΔSBF ≥ -46% (sensitivity 90%, specificity 99%) and SBF ≤ 57 PU (sensitivity 78%, specificity 87%) (Figure 1).

**CONCLUSION.** Baseline SBF and change in SBF at 1 hour represent early indicators of tissue hypoperfusion during UF.

**Table 1 (abstract 0323).** Hemodynamic and skin blood flow variables during the study period

| | Baseline | | | H 1 | | | H 6 | | |
|---|---|---|---|---|---|---|---|---|---|
| | ΔL<10% (N=11) | ΔL%≥10 (N=19) | p | ΔL<10% (N=11) | ΔL%≥10 (N=19) | p | ΔL<10% (N=11) | ΔL%≥10 (N=19) | p |
| Mean arterial pressure (mmHg) | 94 (92-106) | 95 (90-130) | 0.8 | 94 (92-101) | 96 (91-131) | 0.8 | 104 (83-119) | 103 (94-115) | 0.7 |
| Cardiac index (L/min/m2) (N=27) | 3.1 (2.6-4.1) | 3.0 (2.9-3.0) | 0.6 | 3.1 (2.9-3.2) | 3.0 (2.7-4.1) | 0.7 | 3.0 (2.6-3.2) | 3.1 (2.8-4.0) | 0.5 |
| Norepinephrine dose (mcg/kg/min) (N=30) | 0.2 (0.1-0.3) | 0.2 (0.1-0.6) | 0.3 | 0.2 (0.1-0.2) | 0.2 (0.1-0.5) | 0.3 | 0.2 (0.2-0.3) | 0.3 (0.2-0.5) | 0.04 |
| Central venous pressure (mmHg) | 11 (9-13) | 10 (7-15) | 0.9 | 11 (10-13) | 10 (9-12) | 0.9 | 11 (7-15) | 12 (8-15) | 0.7 |
| Lactate (mmol/L) | 1.7 (1.4-2.3) | 1.4 (1.2-2.4) | 0.3 | 1.9 (1.5-2.8) | 2.0 (1.5-2.3) | 0.2 | 1.5 (1.2-2.1) | 2.2 (1.8-2.7) | 0.03 |
| Central venous oxygen saturation (ScvO2) (%) | 74 (72-79) | 70 (68-71) | 0.2 | 75 (73-80) | 71 (65-72) | 0.1 | 71 (70-76) | 69 (66-71) | 0.1 |
| SBF (PU) | 118 (51-126) | 51 (31-77) | 0.04 | 74 (42-95) | 29 (16-52) | 0.02 | 79 (31-103) | 24 (14-43) | 0.04 |
| ΔSBF (%) | - | - | - | -23 (-32--12) | -54 (-68--38) | 0.01 | - | - | - |

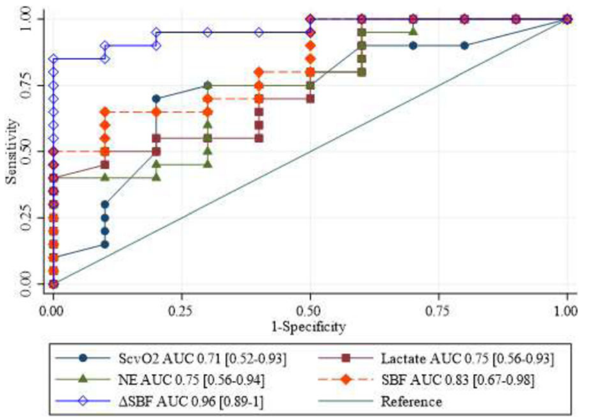

**Fig. 1 (abstract 0323).** Baseline variables and ΔSBF at H1 to predict ΔL% ≥10 during UF

### 0324
### Cardiac function in critically ill patients with traumatic brain injury: preliminary results of a prospective study
V.M. Moles, R.A. Avila, C. Valli, C.A. Zucchella, M.M. Filippi, N.I. Carrizo, A.G. Bailardo
Hospital Cullen, Santa Fe, Argentina
**Correspondence:** V.M. Moles
*Intensive Care Medicine Experimental* 2018, **6(Suppl 2):**0324

**INTRODUCTION.** The brain-heart interaction has been previously described. Cardiac dysfunction is a well-documented phenomenon that can occur during significant brain injury -such as subarachnoid hemorrhage and stroke- and is associated with increased morbidity and mortality. Currently, scant evidence exists about heart function during traumatic brain injury (TBI) and its impact in outcomes.

**OBJECTIVES.** We sought to describe the cardiac function in critically ill patients who sustained a moderate or severe TBI.

**METHODS.** We prospectively collected data on patients admitted to our Intensive Care Unit (ICU) with a diagnosis of moderate or severe TBI. An echocardiogram was obtained within 24 hours of admission and repeated at 72 hours. Parasternal and apical views were used to obtained the following measures: left ventricular (LV) diameters and volumes, left atrium (LA) volume, right ventricular (RV) diameter and area, mitral and tricuspid transvalvular Doppler velocities and mitral and tricuspid valve annulus tissue Doppler velocities. Routine laboratory blood studies, including troponin T and pro NT-BNP, were obtained with each echocardiogram. Epidemiologic and clinical data were obtained from our ICU database. Patients were treated according to their underlying medical condition.

**RESULTS.** We collected data from 21 patients with moderate or severe TBI. They were mostly young adults with a mean age 26.4 ± 9.6 years old, predominantly males (76.2%) and were severely ill with a mean APACHE II score 15.9 and ISS score > 18. At admission, the incidence of LV systolic dysfunction was 14.3% and RV systolic dysfunction 14.3%. During follow up, the incidence of LV systolic dysfunction was 9.5% and there was no evidence of RV systolic dysfunction. Of note, a hyperdynamic LV function (EF > 75%) was noted in 19% of the patients at admission and in 42.9% during follow up. We did not find any patients with echocardiographic signs of elevated LV filling pressures (mean e' septal 0.12 ± 0.03 m/seg, mean e' lateral 0.17 ± 0.05 m/seg, mean E/e' 4 ± 0.92 with no patients with E/e' > 14) or elevated RV filling pressures (mean e' 0.15 ± 0.03 m/seg, mean E/e' 4 with no patients with E/e' > 6).

**CONCLUSIONS.** In critically ill patients with moderate or severe TBI, the LV systolic function is predominantly normal, followed by a hyperdynamic pattern and less frequently LV systolic dysfunction. We did not find any evidence of LV diastolic dysfunction in this population.

**REFERENCE(S)**

1. Krishnamoorthy, V., Rowhani-Rahbar, A., Gibbons, E. F., Rivara, F. P., Temkin, N. R., Pontius, C., Luk, K., Graves, M., Lozier, D., Chaikittisilpa, N., Kiatchai, T., Vavilala, M. S. (2017). Early Systolic Dysfunction Following Traumatic Brain Injury: A Cohort Study. *Crit Care Med, 45*(6), 1028-1036.
2. Chen, Z., Venkat, P., Seyfried, D., Chopp, M., Yan, T., & Chen, J. (2017). Brain-Heart Interaction: Cardiac Complications After Stroke. *Circ Res, 121*(4), 451-468.

### 0325
### Non-invasive administration of inhaled nitric oxide in critically ill adults - a retrospective cohort study
J.-A. Tremblay[1], É. Couture[1], M. Albert[2], W. Beaubien-Souligny[3], M. Elmi-Sarabi[4], Y. Lamarche[2,5], A. Denault[4,6]
[1]Université de Montréal, Critical Care, Montreal, Canada; [2]Hopital Sacré-Coeur de Montréal, Critical Care, Montreal, Canada; [3]Université de Montréal, Nephrology, Montreal, Canada; [4]Institut de Cardiologie de Montréal, Anesthesia and Critical Care, Montreal, Canada; [5]Institut de Cardiologie de Montréal, Cardiac Surgery and Critical Care, Montreal, Canada; [6]Centre Hospitalier de l'Université de Montréal, Critical Care, Montréal, Canada
**Correspondence:** J.-A. Tremblay
*Intensive Care Medicine Experimental* 2018, **6(Suppl 2):**0325

**INTRODUCTION.** The importance of right ventricular (RV) failure in critically ill patients is increasingly recognized as its occurrence is associated with significant mortality and morbidity[1-3].

**OBJECTIVES.** We aimed to assess the hemodynamic effects of non-invasively administering inhaled nitric oxide (iNO) in critically ill patients with acute RV failure, as well as demonstrate the feasibility and explore the safety profile of this approach.

**METHODS.** This is a retrospective cohort study in which we evaluated the clinical course of all patients in whom iNO was initiated without invasive mechanical ventilation in the intensive care unit (ICU) of two tertiary care centers. Primary outcome was acute variation in RV function parameters after starting iNO.

**RESULTS.** Eighteen patients were included in the analysis. Median (IQR) iNO concentration was 20 (20; 20) ppm and therapy duration was 24 (12; 46) hours. Most patients received iNO through nasal prongs (66.7%) or high flow nasal cannula (27.8%). Comparing the hemodynamic parameters just before the initiation of therapy and one hour after, iNO reduced pulmonary vascular resistance (PVR) from 219.1 to 165.4 dyn·s/cm$^5$ ($P$< 0.001), mean pulmonary artery pressure (PAP) from 28.4 to 25.3 mmHg ($P$=0.01) and central venous pressure (CVP) from 17.5 to 13.1 mmHg ($P$=0.001). Indexed cardiac output increased from 2.0 to 2.6 l/min/m$^2$ ($P$=0.004). ICU mortality was 27.78% and median ICU length of stay was 7 (5; 9) days. Two significant bleeding episodes and one acute kidney injury occurred during iNO therapy. No headache was reported.

**CONCLUSIONS.** Non-invasively administered iNO was associated with favorable hemodynamic effects in ICU patients with acute RV failure. Our results suggest the safety and feasibility of this therapy for which further prospective study is warranted.

### 0326

#### Prognostic factors are useful in pulmonary thromboembolism in a intensive care unit?

X. Taboada Fraga[1], E. Alemparte Pardavila[1], E. Moreno López[2], S.D.J. Domínguez Pazos[3], I. Esmorís Arijón[1], N. Mejuto Montero[1], Á. Nieto García[1]
[1]Universitary Hospital Complex of A Coruña, Intensive Care, A Coruña, Spain; [2]Hospital Arquitecto Marcide, Anesthesiology, Ferrol, Spain; [3]Universitary Hospital Complex of A Coruña, Pulmonology, A Coruña, Spain
**Correspondence:** X. Taboada Fraga
*Intensive Care Medicine Experimental* 2018, **6(Suppl 2):**0326

**INTRODUCTION.** Pulmonary thromboembolism (PE) is a cardiovascular disease with a high incidence in Europe. In a number of cases, it is not diagnosed leading to a high mortality or disability in chronic phases.
**OBJECTIVES.**

- To describe the patients suffering from PE in our ICU.
- To assess the associated risk factors, the mortality and their prognosis in a medium term.

**METHODS.** Retrospective analysis of patients admitted in our Unit after being diagnosed with PE, as well as those who developed it during the admission, between 2010 and 2014.
We analysed different variables like risk factors, symptoms, diagnosis methods and long term prognosis. The connection between mortality, risk scores and treatment modality was evaluated by Pearson's Chi-square test and Fisher's exact test.
**RESULTS.** From 2010 to 2014, 55 patients were diagnosed with PE, 13 of them were diagnosed while admitted to the ICU.
We observed that 100% of patients treated with fibrinolysis had higher PESI score (p< 0,02); the mortality was also higher in this group (not statistically significant).
We have not found statistically significant correlation between mortality and poor prognosis in the available data, nor have discovered important morbidity in survivors.
**CONCLUSIONS.**

- The rates of mortality were similar to other series (29,1%), 12,7% of deaths were due to obstructive shock.
- In our study, we did not find data to predict prognosis properly.
- According to recommendations of the current guidelines, in our serie fibrinolysis was used in patients with higher severity rates: 100% patients whose PESI score is ≥ III (p < 0,02).

- V. Konstantinides; A. Torbicki; G. Agnelli. ESC Guidelines on the diagnosis and management of acute pulmonary embolism. European Heart Journal 2014;35:3033-3080.
- F.A. Klok; T. van der Hulle; P.L. den Exter. The post-PE syndrome: a new concept for chronic complications of pulmonary embolism. Blood Reviews 2014;28:221-226.
- Murray J F, Nadel J. Textbook of Respiratory Medicine, 2016 (6thEdition). Philadelphia, PA: Elsevier Saunders.

**Table 1 (abstract 0326).** Demografic data and risk factors

| | |
|---|---|
| Age | 56,6% (SD 13,8) |
| Women | 56,4 % |
| Length of ICU stay | 10,2 (SD 15,5) |
| PESI ≥ III | 81.8% |
| High risk (ESC guidelines) | 43.1% |
| Immobilisation | 45.5% |
| Smokers | 41.8% |
| Obesity | 34.5% |
| Antipsychotic treatment | 20% |
| Cancer | 18.2% |

**Table 2 (abstract 0326).** Symptoms and diagnostic test

| | |
|---|---|
| Dyspnea | 61.5% |
| Cardiorespiratory arrest | 21% |
| Asintomatic | 9% |
| Dimer D | Mín 1024-Máx 40000 |
| Troponin I | Mín <0,002-Máx 24 |
| Extensive affectation CT angiography | 79.6% |
| Right ventricle dilatation | 82.9% |

**Table 3 (abstract 0326).** Treatment, complications and prognosis

| | |
|---|---|
| Systemic fybrinolisis | 30.9% |
| Acenocoumarol at discharge | 83.8% |
| Local complications: pulmonary stroke | 11.1% |
| Systemic complications: haemorrhage | 11.1% |
| 30-day-mortality | 29.1% |
| Obstructive shock as cause of death | 12.7% |
| 2-years-recurrence | 2 patients |
| 1-year-TTE-control: pulmonary hypertension | 2 patients |
| 5-years-control-of-symtoms: exercise dyspnea | 3 patients |

### 0327

#### Systemic and tissue response to fluid challenge

P. De Santis, C. De Fazio, O. Bond, J.-L. Vincent, J. Creteur, F.S. Taccone
ULB, Hopital Erasme, Brussels, Belgium
**Correspondence:** P. De Santis
*Intensive Care Medicine Experimental* 2018, **6(Suppl 2):**0327

**INTRODUCTION.** Fluid responsiveness is defined by a relative increase in cardiac output after fluid administration. The relation with microvascular perfusion has not been adequately studied.
**OBJECTIVES.** To assess the microvascular response to a fluid challenge (FC) in critically ill patients.
**METHODS.** We prospectively collected data in patients and requiring a FC between February and June 2016, whilst cardiac index (CI) was monitored. The sublingual microcirculation was assessed using the incident dark field (IDF) CytoCam device (Braedius Medical, Huizen, The Netherlands). The proportion of small perfused vessels (PSPV) was calculated using the De Backer score, according to standard formulas. Fluid responders were defined by at least 10% increase in CI during FC. Responders according to changes in microcirculation were defined by at least 10% increase in PSPV at the end of FC. Cohen´s kappa coefficient was measured to assess agreement to categorize patients as "responders" to FC according to CI and PSPV.
**RESULTS.** A total of 41 FC were performed in 38 patients, after a median time of 1 [0-1] days after ICU admission. Most of the fluid challenges (39/41, 95%) were performed using crystalloids and the median total amount of fluid was 500 [500-500] mL. The main raisons for fluid challenge were oliguria (n=22) and hypotension (n=10). After FC, CI significantly increased in 24 (58%) cases; a total of 19 (46%) FCs resulted in an increase in PSPV. Both CI and PPV increased in 13 responders and neither in 11; the coefficient of agreement was only 0.19. We found no correlation between absolute changes in CI and PPV after fluid challenge.
**CONCLUSIONS.** Microvascular response to FC cannot be predicted by changes in cardiac output.

### 0328

#### Renal involvement during cardio-renal syndrome type 2: predictive factors and prognosis

E. Rachdi[1], B. Ben Kaab[2], S. Antit[3], B. Menjour[4], M.H. Douggui[2], L. Zakhama[3], S. Ben Youssef[3]
[1]Faculty of Medicine of Tunis, FSI Hospital, Intensive Care Unit, La Marsa, Tunisia; [2]Faculty of Medicine of Tunis, FSI Hospital, Intern Medecine, La Marsa, Tunisia; [3]Faculty of Medicine of Tunis, FSI Hospital, Cardiology, La Marsa, Tunisia; [4]Faculty of Medicine of Tunis, FSI Hospital, La Marsa, Tunisia
**Correspondence:** E. Rachdi
*Intensive Care Medicine Experimental* 2018, **6(Suppl 2):**0328

**INTRODUCTION.** Cardiorenal syndrome Type 2 (CRS) is defined by the occurrence of chronic progressive renal disease (CKD) in patients with chronic congestive heart failure (CHF) [1]. Given the increasing incidence of CHF [2], the resulting increase in SCR type 2 is of concern.
**OBJECTIVES.** To define the different predictive factors leading to the occurrence of CKD disease in patients with chronic abnormalities in cardiac function and its prognosis.
**METHODS.** Our work was a retrospective study of 70 patients with CHF included in the cardiology department of the Marsa FSI Hospital. It compared between two groups: G1 group control: patients with renal clearance> 60 ml / min (N=30) and G2 group CRS: patients with renal clearance ≤ 60ml / min (N=40). For all the patients we recorded epidemiological, clinical, biological, echocardiographic and therapeutic data as well as major events during the follow-up. The search for predictive factors was done by calculating the odds ratio.
**RESULTS.** The mean age of our patients was 58 and it appears as a predictive factor of the occurrence of SCR type 2 given that it was lower in group control (p=0,001), as well as arterial hypertension (p=0,001). Our results were consistent with the data found in several studies [3].
We concluded that the presence of CRS type 2 worsens the clinical features of CHF. In fact, dyspnea NYHA VI was more severe (68% versus 38%, p = 0.03) and blood pressure was higher for systolic (141 versus 122, p = 0.002) and diastolic (86% versus 75%, p = 0.01) in the CRS group type 2. Biologically, Hb was significantly lower in group 2 (p=0, 01).
As for the evolution of our patients, the occurrence of relapses of CHF as well as the mortality were significantly higher in the group SCR type 2 (OR were respectively 1, 33 and 1, 19), a result confirmed by the study of Campbell at al[4]. However, both groups were similar for duration of follow-up, number and duration of hospitalization, as well as for the occurrence of cardiovascular and thromboembolic complications.
**CONCLUSIONS.** Renal damage in the course of CHF worsens the prognosis and is associated with the most pejorative results. Thus, the prevention of cardiovascular risk factors remains the most effective way to limit the incidence of CHF and consequently the SCR.

##### REFERENCE(S)

1. Claudio Ronco et al. Cardiorenal Syndrome. Journal of the American College of Cardiology Vol. 52, No. 19, 2008.
2. Benjamin, E.J., et al., *Heart Disease and Stroke Statistics-2017 Update: A Report From the American Heart Association*. Circulation, 2017. **135**(10): p. e146-e603.
3. S Goursaud, D.d.C., *Le syndrome cardiorénal : diagnostic, physiopathologie et prise en charge*. Reanimation, 2014. **23**: p. 585-594.
4. Campbell, R.C., et al., *Association of chronic kidney disease with outcomes in chronic heart failure: a propensity-matched study.* Nephrol Dial Transplant, 2009. **24**(1): p. 186-93.

### 0329

#### MINOCA (myocardial infarction with nonobstructive coronary aterosclerosis)

E. Trujillo García, J. Muñoz-Bono, V. Olea Jiménez, J.A. Arboleda, E. Curiel Balsera, E. Banderas Bravo, M.D. Fernández Zamora
H.R.U de Málaga, Intensive Care, Málaga, Spain
**Correspondence:** E. Trujillo García
*Intensive Care Medicine Experimental* 2018, **6(Suppl 2):**0329

**INTRODUCTION.** MINOCA (Myocardial Infarction with NonObstructive Coronary Aterosclerosis) is a well-known pathology in Cardiac Care Units. Its knowledge is important to decide how to treat these patients.
**MATERIAL AND METHODS.** Observational, retrospective and multicentre study of all MINOCA patients in the Andalusian hospitals taking part in the ARIAM Project. The ARIAM Cardiac Infarction Register is a multicentre database including the 11 public and private hospitals in Andalusia taking part in the ARIAM Project since its creation in 1998.
**RESULTS.** A total number of 1021 MINOCA patients from 34 Andalusian hospitals since 1998 were analyzed. Average age was 54 years (15-95 years), 64% being males. 63% of these patients showed no respiratory cardiac-related problem upon admission (Killip I), and 2% were admitted in ICU in Killip IV situation. MINOCA hospitalization death rate was 2.3% (no = 24 cases), ICU death rate was 1.5% (no = 15 patients), and the most frequently death cause was cardiogenic shock.
Table 1 shows average age, gender and death rate (both for ICU and hospital) for each subtype of diagnosis in the MINOCA patients analyzed.
**CONCLUSIONS.** Myopericardial affectation was the most prevalent cause among MINOCA patients. Etiologic causes remain unproved in a high percentage of these patients, who also show higher hospitalization death rates, probably because no suitable etiologic treatment was applied.

**Table 1 (abstract 0329).** MINOCA

| DIAGNOSIS | n | % | Average age | % male | % ICU death | % Hospitalization death |
|---|---|---|---|---|---|---|
| Pericarditis | 306 | 30 | 53 | 81 | 0,3 | 0 |
| Miopericarditis | 177 | 17,3 | 39,6 | 83 | 0,6 | 0 |
| Tako-Tsubo Synd. | 166 | 16,3 | 48,5 | 74 | 1,4 | 1,4 |
| Miocarditis | 69 | 6,8 | 66,6 | 11,5 | 3,6 | 1,2 |
| Vasoespastic Angina | 140 | 13,7 | 56,2 | 74,3 | 0 | 0 |
| SCA related Synd. | 163 | 16 | 59,6 | 60,7 | 3,7 | 3,7 |

*[MINOCA]*

### 0330

#### Acute systemic microcirculatory effects of high frequency neuromuscular electrical stimulation in critically ill patients

A. Ozgultekin, Y. Konur, O. Ekinci
University of Health Sciences, Haydarpasa Numune Training and Research Hospital, Anaesthesiology and Intensive Care, Istanbul, Turkey
**Correspondence:** A. Ozgultekin
*Intensive Care Medicine Experimental* 2018, **6(Suppl 2):**0330

**INTRODUCTION.** Neuromuscular electrical stimulation(NMES) is a technique that the beneficial effects were shown on acquired muscular weakness by creating passive muscular contractions in ICU patients, as well as on pain therapy.
There are studies showing that the NMES induce skeletal muscle microcirculatory function and may create changes in periferic circulation either locally or systemically.
**OBJECTIVES.** In our study, we aimed to show the systemic effects of high frequency NMES on tissue microcirculation as assessed with near-infrared spectroscopy (NIRS).
**METHODS.** The study protocol was approved by the Scientific Committee.
Selection of patients: 65 ICU patients(Age18-85), COPD and/or pneumonia, LOS>48 hours; entubated, mechanically ventilated, all minimally sedated, APACHE score< 25, hemodynamically stable (no signs of sepsis -DIC or vasoconstrictor use or any problem interfering with the assesment of the microcirculation); no conditions that limit lower limb mobility.
A single 30-minute high frequency mode NMES session(80-120 Hz) was applied to the lower limbs bilaterally using current of increasing intensity. Current intensity was initially set to the lower limit that was 80 Hz, then increased to the maximal tolerated level, or until the visible contractions of the quadriceps muscles with or without the minimal knee extention were seen. Peripheral microcirculation was monitored at the thenar eminence of the appropriate hand using near-infrared spectroscopy (NIRS) to obtain tissue $O_2$ saturation ($StO_2$), tissue hemoglobin level(tHb), as well as systolic and diastolic blood pressure (SBP, DBP) and heart rate(HR).The recordings were taken before and after the application of NMES.

Comparisons were made using Paired Samples t test for dependent samples and Wilcoxon's signed rank test for independent samples.

**RESULTS.** High frequency mode was well tolerated in all patients.

Mean age: 63,7±16,9; female/male: 18/47; APACHE II on admission:19±7

**StO2:** 79,8±8,9 (Before NMES) / 81,7±9,4(After NMES); p=0,012

**tHb:** 9,7±2,9 / 10,4±2,9 ; p=0,048

**SpO2:** 96±2,6 / 96,1±2,3; p=0,396

**SAB:** 135,8±23,9 /134,1±19,9mmHg; p=0,177

**DAB:**71,1±16,3 / 76,3±13,1 mmHg; p=0,024

**HR:** 88±15,4 / 94,3±19,1/min; p=0,014

**CONCLUSIONS.** In our study, a single session of NMES-induced exercise using high frequency current (80-120 Hz) was shown to acutely induce systemic changes in skeletal muscle microcirculation. Although these changes were not clinically remarkable, it may have some beneficial effects in severe ICU patients with serious microcirculatory derengaments

**REFERENCE(S)**

Gerovasili V, Short-term systemic effect of electrical muscle stimulation in critically ill patients. Chest. 2009;3:1249-1256.

Angelopoulos E, Acute microcirculatory effects of medium frequency versus high frequency neuromuscular electrical stimulation in critically ill patients - a pilot study.Ann Intensive Care. 2013 Dec 19;3(1):39.

### 0331

### Eight years building bridges in cardiogenic shock

I. Martín Badía, P. Pagliarani Gil, J.L. Pérez Vela, M.A. Corres Peiretti, L. Orejón García, M. Valiente Fernández, E. Renes Carreño, J.C. Montejo González

Hospital Universitario 12 de Octubre, Intensive Care Unit, Madrid, Spain

**Correspondence:** I. Martín Badía

*Intensive Care Medicine Experimental* 2018, **6(Suppl 2):**0331

**INTRODUCTION.** Extracorporeal membrane oxygenation (ECMO) is nowadays a proven therapy strategy for refractory cardiogenic shock (CS) to maximum standard treatment efforts. It can keep the patient alive until the recovery of the disease or the arrival of another definitive therapy, such as heart transplant (HT). However, this invasive mechanical support is not free from complications.

**OBJECTIVES.** To describe our experience in the management of refractory CS that required venoarterial ECMO (VA-ECMO).

**METHODS.** We made a retrospective observational study based on a prospectively collected database, which included 65 patients suffering CS from different etiologies admitted in our cardiac ICU from March 2010 to March 2018.

**RESULTS.** Among the 65 patients, 61% were men, with a median age of 53 (IQR: 43-61). Median APACHE-II score was 15 (IQR: 9-19) and SAPS-II 35 (IQR: 29-49). The most prevalent causes of CS were: refractory end-stage heart failure (35.4%), acute myocardial infarction (24.6%), graft failure after HT (12.2%), postcardiotomy (10.8%), right ventricular dysfunction added to a refractory hypoxemia after pulmonary endarterectomy (10.8%, 1 of which had previously a VV-ECMO) and non-ischemic acute miocardiopathy (6.2%). However, 7 patients required the implantation of a second ECMO: 3 due to graft failure after receiving HT, 3 conversions from VA to VV (2 due to Harlequin Syndrome and 1 to arterial cannula-related bleeding) and 1 patient with a second VA-ECMO after failing the implant of a left ventricular assistance device; thus making a total of 69 VA-ECMOs (Fig. 1). The most frequent support objective was the bridge to recovery in 45% (n=31), of which 58% survived (16 recovered and 2 received a HT); followed by the bridge to transplant in 33.3% (n=23), of which 74% survived (16 that were transplanted and 1 recovered). About the 15 (21.7%) patients bridge to decision; 9 (60%) died, 3 (20%) were transplanted, 2 (13.3%) achieved recovery and 1 (6.7%) received another assistance (Fig. 2). The median time of ECMO assistance was 190 hours (IQR: 127-312) with a maximum of 960. Peripheral access was chosen in 82,6% of the cases. In total, 49 patients (68%) had complications related to the mechanical support. The most common one was cannula-related bleeding (20), followed by lower limb ischemia (17, of whom two patients required amputation of the ischemic limb), stroke (8), thrombosis (7), and accidental decannulation (1). The median time of mechanical ventilation was 312 hours (IQR: 126-564), being the median length of stay in ICU of 19 days (IQR: 11-36) and in the hospital of 34 (IQR: 17-68). The overall survival during the VA-ECMO support was 61.6% and at the ICU of 46.2%.

**CONCLUSIONS.** The use of VA-ECMO as a rescue support in patients with refractory cardiogenic shock is an effective therapeutic alternative, although not exempt from complications. In our case series, survival is greater when it is placed as bridge to heart transplantation.

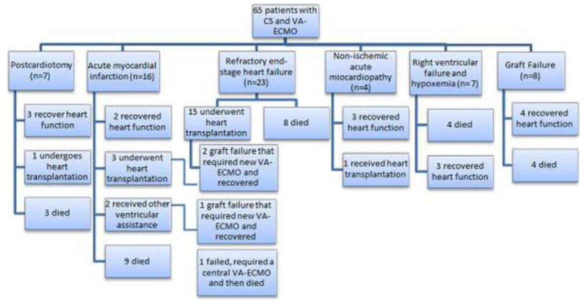

**Fig. 1 (abstract 0331).** Different cardiogenic shock etiologies

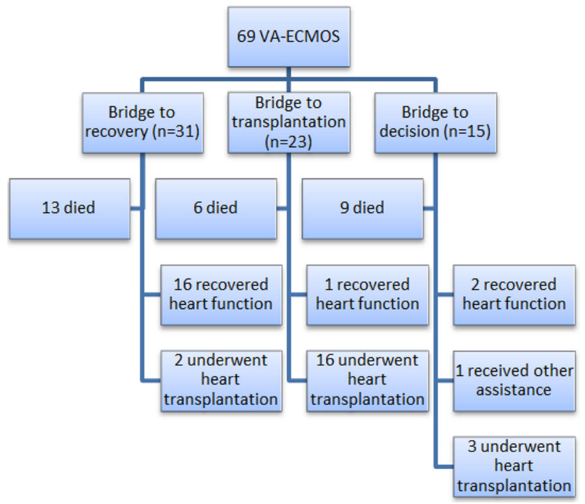

**Fig. 2 (abstract 0331).** VA-ECMOs bridges

### 0332

#### Veno-arterial extracorporeal membrane oxygenation blood flow affects left ventricular contractility in cardiogenic shock

P. Ostadal[1], M. Mlcek[2], P. Hala[1], A. Kruger[1], D. Vondrakova[1], M. Janotka[1], J. Naar[1], P. Neuzil[1], O. Kittnar[2]
[1]Na Homolce Hospital, Dept. of Cardiology, Prague, Czech Republic; [2]Charles University in Prague, First Faculty of Medicine, Dept. of Physiology, Prague, Czech Republic
**Correspondence:** P. Ostadal
*Intensive Care Medicine Experimental* 2018, **6(Suppl 2):**0332

**INTRODUCTION.** It has been repeatedly shown that extracorporeal membrane oxygenation blood flow (EBF) affects left ventricular (LV) functions in cardiogenic shock by changes in LV preload and especially LV afterload. However, the effect of EBF on LV contractility is not fully understood.

**OBJECTIVES.** The aim of our study was to assess LV contractility at different EBF levels in porcine model of acute cardiogenic shock.

**METHODS.** Eight female swine (sus scrofa domestica, 45 kg) underwent implantation of femoro-femoral veno-arterial extracorporeal membrane oxygenation (VA-ECMO). Acute cardiogenic shock with signs of tissue hypoperfusion was then developed using global myocardial hypoxia. Hemodynamic variables including LV dP/dt max as a parameter of contractility were measured using conductance catheter at different levels of EBF ranging from 4.5 L/min to 1.0 L/min.

**RESULTS.** Decreasing EBF was associated with increasing of LV stroke volume and reduction of end-diastolic volume, end-systolic volume and systolic blood pressure. Left-ventricular dP/dt max was gradually increasing with the reduction of EBF from 833.9 ± 80.8 mmHg/s at EBF 4.5 L/min reaching maximal values of 1119.0 ± 89.3 mmHg/s at EBF 1.5 L/min (P˂0.0001) with significant linear trend (slope 42.1, P˂0.0001).

**CONCLUSION.** The changes of EBF in cardiogenic shock treated with VA-ECMO influence not only LV preload and afterload of severely impaired left ventricle but directly affect also LV contractility.

**GRANT ACKNOWLEDGMENT**
The study was supported by an Institutional grant MH CZ - DRO (Nemocnice Na Homolce - NNH, 00023884), IG150501.

### 0333

#### A software tool to quantify capillary blood volume and absolute red blood cell velocity in sublingual incident dark field microscopy video clips

M.P. Hilty[1], S. Arend[1], M. Van Assen[1], F. Toraman[2], C. Ince[1]
[1]Academic Medical Center, Department of Translational Physiology, Amsterdam, Netherlands; [2]Acıbadem Mehmet Ali Aydinlar University School of Medicine, Department of Anesthesiology and Reanimation, Istanbul, Turkey
**Correspondence:** M.P. Hilty
*Intensive Care Medicine Experimental* 2018, **6(Suppl 2):**0333

**INTRODUCTION.** The second consensus statement on microcirculatory analysis [1] proposes the development of parameters related to physiological function of the microcirculation. We have thus developed a software tool employing advanced computer vision to objectively quantify capillary blood volume and absolute red blood cell velocity (RBCv). Our hypothesis was that (I) advanced computer vision reliably quantifies functional capillary density (FCD) compared to manual analysis while enabling estimation of functional capillary volume (FCV) and RBCv, (II) change in FCV reflects volume contraction of the capillary bed following microcirculatory derecruitment caused by cardiopulmonary bypass (CPB) similar to change in FCD, and (III) RBCv is sensitive to change in microcirculatory convective function induced by CPB that may not infulence microcirculatory flow index (MFI).

**METHODS.** In 11 patients undergoing coronary artery bypass surgery 3 sublingual capillary microscopy videos were recorded before and after initiation of CPB primed with HES 130/0.4. FCD and MFI were manually assessed using AVA 3.2 software, as well as using a novel advanced computer vision algorithm employing contrast limited histogram equalization and principal curvature-based region detection (Fig 1). FCV was calculated as volume of the microvessels per area (mm3 mm-2) by per-pixel summation of capillary diameter along the centerline.

**RESULTS.** 5760 capillaries in 66 video clips were analyzed (Fig. 1). Using the software 99357 individiual red blood cells were tracked across 322000 frames. A good correlation was found between algorithm determined and manual FCD (r=0.60, p< 0.001, Fig. 2). CPB resulted in an decrease in FCD from (mean±SEM) 25.6±1.4 to 20.6±0.7 and 35.3±0.7 to 30.9±1.4mm-1 (manual and algorithm), as well as in FCV from 23.8±0.9 to 19.6±1.2mmE-4 (all p< 0.01). An increase in RBCv from 245±7 to 270±7 μm min-1 (p< 0.01) was detected using the algorithm, while MFI remained unchanged at 3.0. An upward shift in RBC velocity distribution before and after initiation of CPB is visualized in Fig. 3.

**CONCLUSION.** Accurate measurement of FCD is possible using advanced computer vision. It enables quantification of FCV by adding information on capillary volume, reflecting volume contraction of the capillary bed after initiation of CPB similar to FCD. Quantitative estimation of RBCv is sensitive to changes in convective state of capillary perfusion induced by CPB which did not affect MFI. Combined with quantification of capillary hematocrit as recently demonstrated using similar techniques [2] direct insight is given into capillary delivery of oxygen.

**GRANT ACKNOWLEDGMENT**
This research was supported by a University of Zurich Walter und Gertrud Siegenthaler Foundation grant issued to MPH.

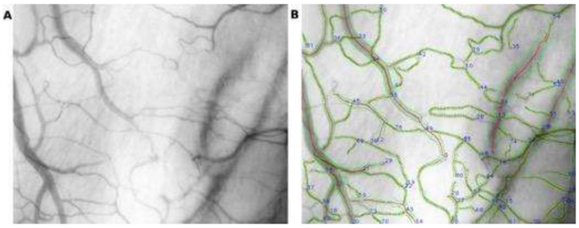

**Fig. 1 (abstract 0333)** Capillary detection (B) in an averaged video frame (A) using advanced computer vision.

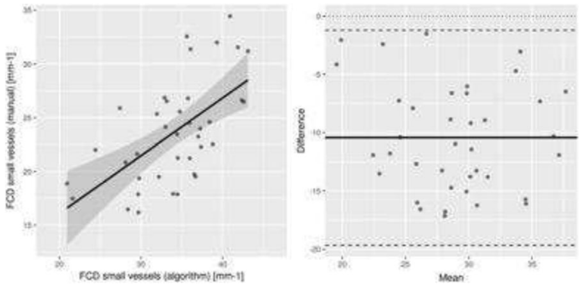

**Fig. 2 (abstract 0333)** Correlation of per video FCD as determined manually and using advanced computer vision.

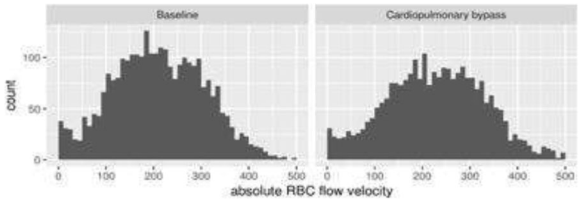

**Fig. 3 (abstract 0333).** Absolute RBC velocity frequency histograms before and after initiation of CPB.

### 0334

#### A method to derive local blood flow and local vascular resistance from tissue haemoglobin oxygen saturation

C. Smart[1], G. Ward[2], M. Singer[1]
[1]University College London, Bloomsbury Institute for Intensive Care Medicine, London, United Kingdom; [2]In Tandem Designs Pty Ltd, Warambatool, Australia
**Correspondence:** C. Smart
*Intensive Care Medicine Experimental* 2018, **6(Suppl 2):**0334

**INTRODUCTION.** Tissue haemoglobin saturation ($StO_2$) is distinct from pulse oximetry in that all compartments of the local blood volume are interrogated. Maintenance of the $StO_2$ depends on the arterial oxygen saturation, and the inflow of blood to the tissue. Higher values of $StO_2$ require exponentially more flow to maintain as the $StO_2$ approachs the unsurpassable asymptote of the arterial saturation. If the flow is abruptly occluded, there is a rapid decay in the $StO_2$ obeying predictable kinetics. Modelling these kinetics allows back-calculation of the arterial inflow. This estimate of flow may be combined with the systemic blood pressure to derive the local vascular resistance. Flow is expressed as an exchange percentage: volume of arterial inflow per second/interrogated blood volume (%.s$^{-1}$). Resistance is expressed in mmHg.s.% $^{-1}$.

**OBJECTIVES.** To validate the algorithm by incremental infusion of noradrenaline, and contrast to the pattern observed in stepwise haemorrhagic shock in an anaesthetised rat model.

**METHODS.** A bespoke fibreoptic $StO_2$ needle probe was implanted into the vastus lateralis muscles of the thigh in anaesthetised instrumented Wistar rats. One rat received central noradrenaline in incrementing infusion from 0.1-300 ug/kg/minute increased in half log order steps every 15 minutes. Four rats underwent progressive haemorrhage. Ten percent of initial estimated blood volume (EBV, 66ml/Kg) was withdrawn via an indwelling carotid line every 15 minutes until cardio-respiratory arrest. At this point the withdrawn blood volume was returned, and the animals observed for 20 minutes then euthanased. Tissue saturation and arterial blood pressure were recorded continuously and ABG sampling was performed at intervals.

**RESULTS.** During incremental noradrenaline infusion computed flow fell stepwise from 70 %.s$^{-1}$ at baseline to 20 %.s$^{-1}$ at 30 ug/kg/minute noradrenaline. Resistance rose stepwise from 1.2 to 34 mmHg.s.% $^{-1}$. Complete flow cessation with infinite resistance occurred beyond 100ug/kg/min.

In the Haemorrhage group, baseline computed flow varied from 40-75%.s$^{-1}$ with baseline resistance from 1.7-3.7 mmHg.s.% $^{-1}$. Animals suffered cardiac arrest between 40-60% loss of EBV. In the penultimate step before arrest, flow slowed to 1-6 %.s$^{-1}$ with resistance from 8.7-48 mmHg.s.% $^{-1}$. Immediately pre-arrest, flow was universally near zero, and resistance >100 mmHg.s.% $^{-1}$.

**CONCLUSIONS.** Imputing tissue blood flow unmasks significant early drops in blood flow that may only produce small decrements in $StO_2$. Resistance increments in a saw-tooth pattern, with initial vasoconstriction then relaxation. Persistently elevated resistance heralds imminent cardiac collapse. Animals surviving the resuscitation renormalized local vascular resistance to baseline.

**GRANT.** MRC industrial collaborative studentship.

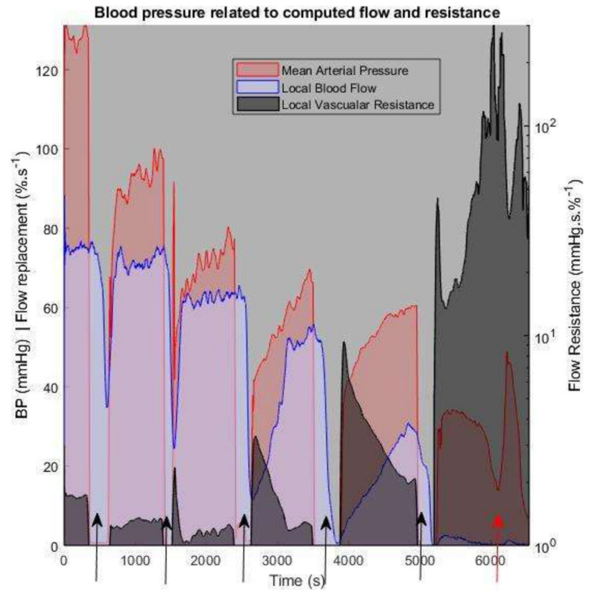

**Fig. 1 (abstract 0334).** Serial haemorrhage: non-survivor. 10% EBV drawn at black arrows. Blood returned at red arrow

### 0335

#### Microvascular reactivity at the early stage of ischemic cardiogenic shock: a preliminary observational study

T. Jouitteau[1], H. David[1,2], F. Roubille[2,3], J. Eliet[1], M. Mourad[1], N. Zeroual[1], P. Colson[1], P. Gaudard[1,2]
[1]CHU Montpellier Arnaud de Villeneuve, Anaesthesiology and Critical Care Medicine, Montpellier, France; [2]University of Montpellier, PhyMedExp INSERM U 1046 CNRS UMR 9214, Montpellier, France; [3]CHU Montpellier Arnaud de Villeneuve, Cardiology Department, Montpellier, France
**Correspondence:** T. Jouitteau
*Intensive Care Medicine Experimental* 2018, **6(Suppl 2):**0335

**INTRODUCTION.** Cardiogenic shock (CS) complicating acute coronary syndrome (ACS) is associated with a mortality rate ranging from 40 to 50% despite improvement of early revascularization. The impairment of microcirculation in CS is associated with poor outcome. Early variations of microcirculation reactivity under maximal hemodynamic support including mechanical circulatory support remain unknown in CS.

**OBJECTIVES.** To describe the hemodynamic, the microcirculation status and outcome in a selected population of severe CS over the first 48h and to evaluate the accuracy of a bed-side estimation of post-ischemic recovery slope (RS).

**METHODS.** This single-centre prospective observational study, approved by an ethics committee, included patients admitted consecutively the last 2 years to ICU for CS complicating ACS. Inclusion was performed within 24 h of CS onset. Patients with refractory cardiac arrest, older than 75 years or with therapeutic limitation were excluded. The InSpectra™ near infrared spectrometer was used to assess the effect of a vascular occlusion test on thenar eminence tissue oxygenation (StO2). The RS was estimated at bed-side using the average increments per second for the first two values of StO2 after reperfusion. StO2 monitoring data were recorded and analysed using a software for exact calculation of RS. Relevant data were recorded at H0, H12 and H48 and mortality rate calculated at 90 days. We compared survivors (SV) versus non-survivors (NSV) at 90 days using nonparametric tests with a p< 0.05 considered significant. A Pearson correlation test and Bland-Altman analysis were performed to compare estimated RS (eRS) to calculated RS (cRS).
**RESULTS.** 14 patients were included (median [interquartile range]: age 63 [59-67] y; SAPS II 62 [58-77]). All patients received catecholamine infusion and required mechanical ventilation under sedation, 10 (71%) were supported by an ECLS and/or Impella CP™ and 2 others by an intra-aortic balloon pump. The 90 days mortality rate was 50%. At H0, lactate (3.2 [2.4-4.3] vs 2.1 [1.5-9.4] mmol/l, p=0.689), RS (3.1 [2.8-3.4] vs 2.9 [1.3-3.1] %/s, p=0.224) and SOFA (9 [8-10] vs 12 [11-14], p=0.077) were not significantly different between SV and NSV, respectively. Unlike lactate, the H12 RS reached a significant difference between groups (4.8 [3.4-5.4] vs 1.9 [1.4-2.7] %/s (p=0.005), which was maintained at H48. The H48 SOFA was lower in SV (6 [5-8] vs 13 [12-15], p=0.022]. The mortality was 83% vs 14% for patients with a RS < 3%/s at H12 (p=0.013). The eRS was correlated with cRS (r=0.929, p< 0.001) with a bias at +0.5 %/s (95% CI: 0.3-0.7] between the 2 values.
**CONCLUSIONS.** The early improvement of microvascular reactivity indicates a good response to treatment with quick decrease of organs failure and better survival. The bed-side assessment of RS overestimates the slope but is well correlated to cRS and may provide an easy monitoring of microcirculation to physicians.

### 0336

#### Ivabradine effect on tissue perfusion in critical patients

E.I. Zamarron Lopez[1], A.E. Ramirez Gutierrez[2], C. Cruz Lozano[3], J.R. Sanchez Medina[3], O.R. Perez Nieto[4]
[1]Hospital Angeles Tampico, Intensive Care Unit, Tampico, Mexico; [2]Hospital CEMAIN, Cardiology, Tampico, Mexico; [3]Hospital PEMEX Madero, Intensive Care Unit, Tampico, Mexico; [4]Hospital General San Juan del Rio, Intensive Care Unit, San Juan del Río, Qro, Mexico
**Correspondence:** E.I. Zamarron Lopez
*Intensive Care Medicine Experimental* 2018, **6(Suppl 2):**0336

**INTRODUCTION.** An important part in the treatment of the critical patient is based on establishing adequate tissue perfusion to the organs, a therapeutic goal described has been the optimization of cardiac output (Q) whose components are stroke volume (SV) and heart rate (HR), alterations in any of the components can generate an inadequate cardiac output. Tachycardia is common in critically ill patients, and can cause a decrease in stroke volume by preventing adequate ventricular filling due to a decrease in diastolic time, which can generate ventricular-arterial decoupling and decrease in tissue perfusion.
**OBJECTIVE.** To observe the changes in tissue perfusion of patients in shock and tachycardia with the use of Ivabradine.
**METHODS.** Retrospective observational and analytical study conducted in the intensive care unit of the PEMEX Madero Hospital, Tamps. Mexico, records of patients in shock were reviewed and Ivabradina was prescribed for any medical indication, information on hemodynamics and tissue perfusion was collected in a data collection sheet, dividing values prior to the use of Ivabradine and after application.
**RESULTS.** n: 23. Diagnoses: septic shock (43.5%), cardiogenic shock (21.7%), mixed shock (21.7%), acute coronary ischemic syndrome (4.3%), high-risk pulmonary thromboembolism (4.3%) and severe pancreatitis (4.3 %), 34.8% were under mechanical ventilation. The following average values were reported prior to the use of Ivabradine: HR: 119, mean arterial pressure (MAP): 88 mmHg, dose of Norepinephrine: 24 mcg / min, uresis: 61 ml / h, central venous oxygen saturation (ScvO2) : 67%, lactate: 4.1 mmol / L, cardiac output (Q): 4.1 L / min, systemic vascular resistance (SVR): 1824 dynes and oxygen extraction index (O2EI): 30%. The data reported after the use of Ivabradine were the following: HR: 94, MAP: 83 mmHg, dose of Norepinephrine: 13 mcg / min, uresis 65 ml / h, ScvO2 of 70%, lactate: 1.3 mmol / L, Q: 3.7 L / min, SVR: 2018 dynes and O2Ei of 27%. There was a significant decrease in CR of 21% (p: < 0.0001), a decrease in norepinephrine dose of 46%, an increase in urine of 6.2%, an increase in ScvO2 of 4.3%, a decrease in lactate of 48%, a decrease of Q of 9.8%, an increase of the SVR of 9.7% and a decrease of O2Ei of 10%.

**CONCLUSIONS.** An improvement in the clinical and biochemical parameters of tissue perfusion was observed with the use of Ivabradine in patients in shock and tachycardia, independently of changes in cardiac output.

**GRANT ACKNOWLEDGMENT**

Without acknowledgment.

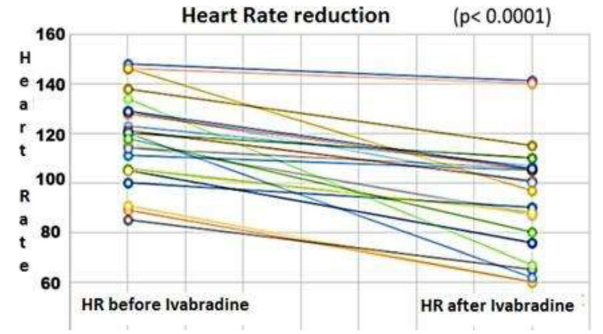

**Fig. 1 (abstract 0336).** Heart rate before and after Ivabradine administration

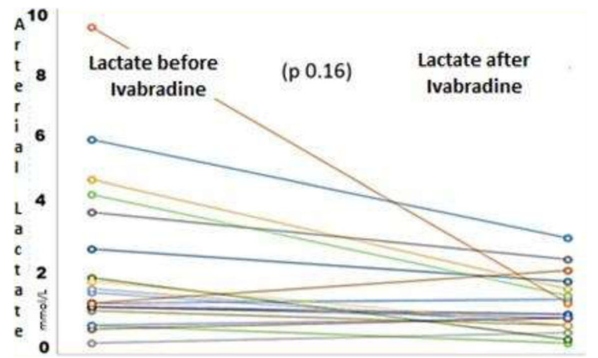

**Fig. 2 (abstract 0336).** Arterial lactate levels before and after Ivabradine administration

### 0337

#### Calculating cardiac output during veno-venous ECMO using indirect calorimetry and blood gas analysis

M.C. Müller[1], T. Wollersheim[1,2], N.M. Carbon[1], S. Frank[1], V. Skrypnikov[1], S. Weber-Carstens[1,2]
[1]Charité Universitätsmedizin Berlin, Department of Anesthesiology and Operative Intensive Care Medicine, Berlin, Germany; [2]BIH, Berlin Institute of Health, Berlin, Germany
**Correspondence:** M.C. Müller
*Intensive Care Medicine Experimental* 2018, **6(Suppl 2):**0337

**INTRODUCTION.** Veno-venous extracorporeal membrane oxygenation (vvECMO) is increasingly used for carbon dioxide removal and the treatment of severe hypoxemia. For optimal therapy the knowledge of the cardiac output (CO) is crucial. Conventional methods measuring the CO under vvECMO are either prone to errors by temperature changes (thermodilution), invasive (pulmonal artery catheter) or user-dependent (transesophageal echocardiography).

**OBJEKTIVES.** Calculate cardiac output (CO) with a new non-invasive method using a new formula including indirect calorimetry and blood gas analysis.
**METHODS.** We used a new simple equation, a modification of the Fick equation[1], based on basic physiological assumptions to calculate the cardiac output (CO):

$CO_{modfick} = (Q_{ECMO} \times cO2_{postECMO} - cO2_{preECMO} + VO2)/(cO_{2Art} - cO2_{preECMO})$

CO: cardiac output; $Q_{ECMO}$: ECMO blood flow; $cO2_{postECMO}$: Oxygen ($O_2$) content efferent from the ECMO, $cO2_{preECMO}$: $O_2$ content afferent to the ECMO; $V_{O2}$: $O_2$ consumption by the lung, $cO_{2ART}$: arterial O2 content

We investigated the equation as part of the MEEP-trial[2] (Measuring Energy Expenditure in ECMO Patients; Charité ethics EA/1/293/13), here shown preliminary data of 22 patients with ARDS and vvECMO and compared the CO results to the CO simultaneously measured by transesophageal echocardiography (TEE). For determination of $VO_2$ we used indirect calorimetry (Quark RMR, Cosmed), blood gas analysis was done according to clinical routine and $O_2$ content calculated with a formula by Dash[3].

**RESULTS.**

**$CO_{TEE}$: Mean 6,34 (SD 2,09), Median 6,11 l/min**

**$CO_{modfick}$: Mean 7,49 (SD 2,40), Median 7,07 l/min**

Relative Error (median, %): 25,1% (IQR -6%/+67%)

Correlation (Pearson): r=0,426*; p=0,048

**CONCLUSIONS.** While in some cases our new equation shows excellent correlation with the TEE values, other values differed up to 80%. This is a not acceptable error for a clinically relevant parameter. A median error of +21% with and IQR -6%/+67% indicates systemic over estimation and an additional individual error of the CO calculation.

Further research on the cause of these errors and their possible avoidance is necessary.

**REFERENCE(S)**

1 Fick A. Uber die Messung des Blutquantums in den Herzventrikeln. Wurzburg Physikalische Medizinische Gesellschaft. 1870 Jun;

2 Wollersheim T, Frank S, Müller MC, Skrypnikov V, Carbon NM, Pickerodt PA, et al. Measuring Energy Expenditure in extracorporeal lung support Patients (MEEP) - Protocol, feasibility and pilot trial. Clin Nutr. 2017 Jan 16;

3 Dash RK, Bassingthwaighte JB. Erratum to: Blood HbO2 and HbCO2 Dissociation Curves at Varied O2, CO2, pH, 2,3-DPG and Temperature Levels. Annals of Biomedical Engineering. 2010;38(4):1683-701.

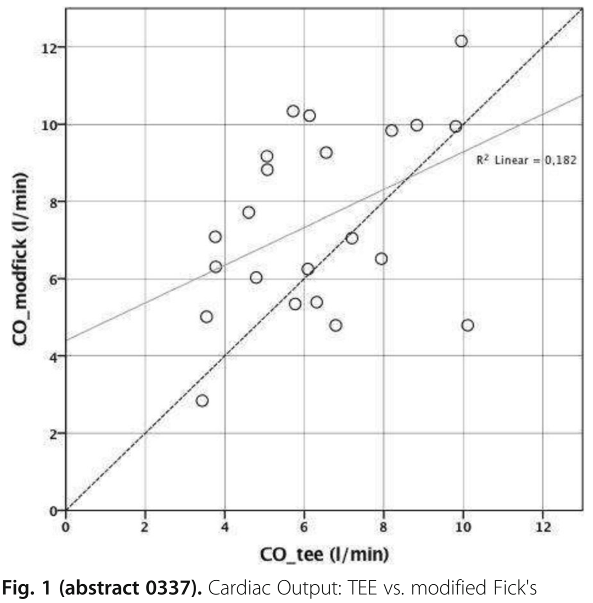

**Fig. 1 (abstract 0337).** Cardiac Output: TEE vs. modified Fick's principle on ECMO

### 0338

### Does airway pressure cause right ventricular dysfunction in septic patients undergoing mechanical ventilation?

V. Fraile-Gutiérrez[1], D. Pérez-Torres[1], J.M. Ayuela-Azcarate[2], A. Mayo-Iscar[3], A.I. Rodríguez-Villar[1], P. Blanco-Schweizer[1], E. Prol-Silva[1], J.Á. Ayala-Fernández[1], C. Díaz-Rodríguez[1], J. Blanco-Varela[1]

[1]Hospital Universitario Río Hortega, Intensive Care Department, Valladolid, Spain; [2]Complejo Asistencial Universitario de Burgos, Intensive Care Department, Burgos, Spain; [3]University of Valladolid, Department of Statistics and Operations Research, Valladolid, Spain

**Correspondence:** D. Pérez-Torres

*Intensive Care Medicine Experimental* 2018, **6(Suppl 2):**0338

**INTRODUCTION.** The right ventricle is often subject to an afterload increase in sepsis, mainly due to high pulmonary vascular resistance, leading to and adaptive dilation of the cavity and paradoxical septal motion (acute cor pulmonale). Airway pressures have been described to play a role in these changes, which reflect impairment of the right ventricular systolic function and are associated with worse clinical outcomes. Current guidelines for the assessment of the right heart in adults recommend more reproducible parameters to evaluate right ventricular systolic function.

**OBJECTIVES.** We aim to assess the impact of airway pressure on the right ventricular systolic function, by using transthoracic echocardiography (TTE), in septic patients undergoing mechanical ventilation.

**METHODS.** We conducted a single-centre prospective observational cohort study. Patients admitted to the ICU with sepsis over a 20-month period were enrolled, excluding those with ischemic or valvular heart disease and pacemakers. Tricuspid annular plane systolic excursion (TAPSE) and peak systolic velocity at the lateral tricuspid annulus (St) were analysed by comprehensive TTE on Days 1, 3 and 7 of evolution. Right ventricular systolic dysfunction (RVSD) was defined as recommended on ASE/EACVI Guidelines. Positive end-expiratory pressure (PEEP) and plateau pressure (Pplat) were recorded at the same time that TTE were performed. Ventilatory protocol targeted tidal volumes ≤8 ml/Kg predicted body weight, Pplat ≤30 $cmH_2O$ and PEEP adjusted to optimize gas exchange. We explored the effect of Pplat on TAPSE, and the impact of PEEP level on TAPSE and St. Variables are expressed as mean ± standard deviation. Student's T or ANOVA tests were applied as appropriate.

**RESULTS.** 94 patients were enrolled, 55% male, aged 63±12, APACHE-II 23, 67% mechanically ventilated, ICU mortality 23%, 90-day mortality 29%. 217 TTE were performed, 146 in mechanically ventilated patients. Ventilatory parameters on Day 1: Vt=8.2±1.3, Pplat=22.2±5.0, PEEP 8.6±2.9. Ventilatory parameters on Day 3: Vt=8.0±0.8, Pplat=22.6±5.6, PEEP 9.4±3.1. Ventilatory parameters on Day 7: Vt=7.9±1.1, Pplat=22.8±6.4, PEEP=8.9±2.9. We did not find any difference in $PaO_2$, $PaCO_2$ and Vt between patients with and without RVSD (p>0.05). The results are shown in the Tables.

**CONCLUSIONS.** We found high plateau pressure (>30 $cmH_2O$) in septic patients was significantly associated to RVSD. However, PEEP level did not influence the presence of RVSD, as assessed by TAPSE and St.

**Table 1 (abstract 0338).** Differences in TAPSE, based on Pplat level

| | Pplat ≥30 (n=13) | Pplat <30 (n=133) | p |
|---|---|---|---|
| TAPSE (mm) | 16.0±6.7 | 20.5±7.1 | 0.03 |
| | Pplat ≥27 (n=23) | Pplat <27 (n=123) | p |
| TAPSE (mm) | 18.9±5.9 | 20.5±7.3 | 0.32 |

**Table 2 (abstract 0338)** . Differences in TAPSE and St, based on PEEP level

| | No mechanical ventilation | PEEP 5-10 | PEEP ≥10 | p |
|---|---|---|---|---|
| Day 1 | n=31 | n=34 | n=27 | |
| TAPSE (mm) | 21.3±4.5 | 17.3±3.9 | 20.6±4.5 | 0.001 |
| St (cm/s) | 13.1±2.4 | 13.1±4.6 | 13.1±3.6 | 0.99 |
| Day 3 | n=24 | n=26 | n=28 | |
| TAPSE (mm) | 20.9±3.2 | 18.6±3.8 | 20.4±4.9 | 0.13 |
| St (cm/s) | 13.7±3.6 | 12.8±3.5 | 12.5±3.2 | 0.53 |
| Day 7 | n=16 | n=16 | n=15 | |
| TAPSE (mm) | 19.7±3.9 | 20.6±3.9 | 22.8±4.1 | 0.12 |
| St (cm/s) | 16.1±3.9 | 19.9±3.3 | 15.1±2.5 | 0.22 |

## Quality improvement

### 0339

#### The quality improvement of unplanned extubation among patients with mechanical ventilator in a medical center

C.-M. Chen[1,2,3]
[1]Chi Mei Medical Center, Intensive Care Medicine, Tainan, Taiwan, Province of China; [2]Society of Emergency and Critical Care Medicine, Taipei, Taiwan, Province of China; [3]Chia Nan University of Pharmacy and Science, Recreation and Health-Care Management, Tainan, Taiwan, Province of China
*Intensive Care Medicine Experimental* 2018, **6(Suppl 2):**0339

**INTRODUCTION.** It has been considered as an important quality indicator of care in the intensive care unit (ICU) among mechanically ventilated (MV) patients experienced unplanned extubation (UE). It is recognized as the most common airway adverse event in the ICU and might be associated with increased morbidity and mortality.

**OBJECTIVES.** A continuous quality improvement (CQI) concept has been promoted in reducing the incidence of UE since the year 2001 in a Medical center in Southern Taiwan.

**METHODS.** The CQI concept was initiated since year 2001 and was promoted in all 6 ICUs (96 beds) in Chi-Mei Medical center in southern Taiwan. The CQI concept, focusing on serial intervention tools including standardization of procedures, improvement of communication, revision of sedation and weaning protocols, changing strategy for physical restriction, establishment of task force for identification and management of high-risk patients, implementation of quality improvement models including Breakthrough Series (BTS) and Team Resource Management (TRM), was launched to reduce the incidence of UE in MV patients.

**RESULTS.** With the promotion of this CQI concept, the overall incidence rate of unplanned endotracheal extubation (defined as number of new UEs per total amount of MV patients during the same period) decreased gradually from 6.82% (188/2785) in the year 2001 to 0.61% (14/2309) in the year 2017 during a 17-year period.

**CONCLUSIONS.** The promotion of CQI concept in the ICU is effective in reducing the overall incidence of UE and subsequent reducing the morbidity and mortality, as well as evidenced in other literatures.

**REFERENCE(S)**

1. Krinsley JS, Barone JE. The drive to survive: unplanned extubation in the ICU. Chest. 2005;128:560-566
2. Epstein SK, Nevins ML, Chung J. Effect of unplanned extubation on outcome of mechanical ventilation. Am J Respir Crit Care Med. 2000;161:1912-1916.
3. Tanios M, Epstein S, Grzeskowiak M, Nguyen HM, Park H, Leo J. Influence of sedation strategies on unplanned extubation in a mixed intensive care unit. Am J Crit Care. 2014;23:306-314

### 0340

#### Collecting family feedback by digital survey

J.H. Coobs, A.M.M. Duijn, T. van Galen
VU University Medical Center, Intensive Care, Amsterdam, Netherlands
**Correspondence:** J.H. Coobs
*Intensive Care Medicine Experimental* 2018, **6(Suppl 2):**0340

**INTRODUCTION.** The ICU handles protocols, guidelines and standards to maintain quality of care[1]. With feedback from family or close contacts it is possible to create insights in the level of satisfaction and it can be used for improving ICU care[2, 3, 4]. Until 2016 we evaluated experienced care through a phone interview, performed by an ICU nurse[5, 6]. This post hospital interview took up to 30 minutes at a time. In 2017 it was decided to gather information a different way, using a digital survey.

**OBJECTIVES.** Is it more effective to collect feedback by digital survey compared to a phone based interview?

**METHODS.** An adjusted version of the Consumer Quality Index for family at the ICU is used[7]. The questions are gathered by a digital survey. This is send by mobile text link to all first contacts of ICU patients that were admitted for more than 48 hours 4 weeks after the patient is discharged. To increase response rate, no more than 20 questions are asked. The list of names of the first contacts is derived from our PDMS. This procedure of searching and sending takes approx. 2 ½ hours a month and is performed by the ward clerk.

**RESULTS.** In one year 243 surveys were sent with a 28% response rate (N=68). This is 35% higher than the response rate of the general hospital survey. It provides us information about the level of satisfaction. Alternate connections can be made. For example: does the time spent in the ICU influences the level of satisfaction? Are partners more or less satisfied than children of the patient? It takes approx. 4 minutes to fill in the survey.

No differences were found between respondents based on relation, age, length of stay or other. 80% is satisfied with current visiting hours. Clear communication with nurses and doctors is also rated positive. Handing out ICU information leaflets and diaries still deserves attention. The results are shared frequently with the team through presentations causing a higher level of involvement to improve overall quality care.

**CONCLUSIONS.** Sending the digital survey by text message improved effectiveness. It increases our chances to achieve a high response rate and creates an objective profile of experienced care. The effort is minimal for ICU and the respondent. Due to the results, we have insights in the level of satisfaction and areas that need attention or improvement.

**REFERENCE(S)**

1. *Quality standard, Organization of Intensive Care*. Zorginstituut Nederland 2016.
2. *Using Patient feedback*, Guide for Healthcare services. Picker Institute, 2009.
3. *Assessing Family satisfaction*. Roberti SM, Fitzpatrick JJ, Critical Care Nurse 2010; volume 30.
4. *Patient satisfaction with nurse-led telephone consultation*. Shaida N, PCP Dis. 2007; 10(4):369-373.
5. Communicatie kenniscentrum, interview of enquête.
6. *Using phone surveys for gathering post ICU feedback*. Krull J, Evelein-Brugman SFM, Galen T van, ESICM 2014.
7. *Consumer Quality Index*. Hogeschool Arnhem/Nijmegen. 2015.

**GRANT ACKNOWLEDGMENT**
None

### 0341

#### Geographic distribution of intensive care audit in Europe

J.K. Adamski[1], W. Weigl[2]
[1]Satakunta Central Hospital, Department of Anaesthesiology and Intensive Care, Pori, Finland; [2]Uppsala University, Akademiska Hospital, Anaesthesiology and Intensive Care, Department of Surgical Sciences, Uppsala, Sweden
**Correspondence:** J.K. Adamski
*Intensive Care Medicine Experimental* 2018, **6(Suppl 2):**0341

**INTRODUCTION.** The multiple-center quality improvement projects appear to be a useful mechanism to facilitate progress amongst individual institutions. Collection and analyzing of relevant data can determine why some units are used more efficiently than others.

**OBJECTIVES.** The aim of this work was to present the geographical distribution of organizations and initiatives appointed to conduct audits of intensive care units (ICUs) in Europe.

**METHODS.** We updated the list of national ICU registries listed on the Swedish Intensive Care registry (SIR) website (http://www.icuregswe.org/en/About-Intensive-Care/registries/) with other registries and initiatives that were found in articles on ICU outcomes available in PubMed. In our search, we used the combinations of terms ("intensive care unit" or "ICU") and ("mortality" or "outcome") published in articles, in all languages until December 31, 2017. The databases were analyzed in terms of accessibility, language and used ICU definitions.

**RESULTS.** Countries gathering nationwide ICU data in Europe are presented in Figure 1. Most of the ICU registries are located in Western Europe. The definition of an ICU patient and an ICU bed differed considerably among countries. Consequently, types of collected data, the way in which they were analyzed, and finally, the scope of publicly available information were not uniform. A deeper comparison of ICU outcomes between different countries was difficult [1,2]. Only a part of annual reports, which are usually published in native languages, has open access. No national ICU registries were found in eastern part of Europe. However, new initiatives are emerging: regional registry in Poland operates since 2010 [3], Italien project PROSAFE gathers a few participants from Greece, Cyprus, Hungary, Slovenia and Poland. Likewise, 1 unit from Estonia participates in Finnish registry run by Tieto Company and some units from Portugal participate in Austrian registry (ASDI) [4].

**CONCLUSIONS.** Large part of European countries does not have a national ICU registry. For international comparisons, the introduction of a uniform system of data collection and analysis is required.

##### REFERENCE(S)

1. Adamski J, Goraj R, Onichimowski D, Gawlikowska E, Weigl W, (2015) The differences between two selected intensive care units located in central and northern Europe - preliminary observation. Anaesthesiol Intensive Ther 47: 117-124
2. Weigl W, Adamski J, Gorynski P, Kanski A, Hultstrom M, (2017) Mortality rate is higher in Polish intensive care units than in other European countries. Intensive Care Med 43: 1430-1432
3. Knapik P, Krzych L, Weigl W, Adamski J, Hultstom M, (2017) Mortality rate in Polish intensive care units is lower than predicted according to the APACHE II scoring system. Intensive Care Med 43: 1745-1746
4. Walther S, Karlström G (2016) National ICU registries. In: Quality management in intensive care: a practical guide. Cambridge University Press, pp. 195-203

##### GRANT ACKNOWLEDGMENT

None.

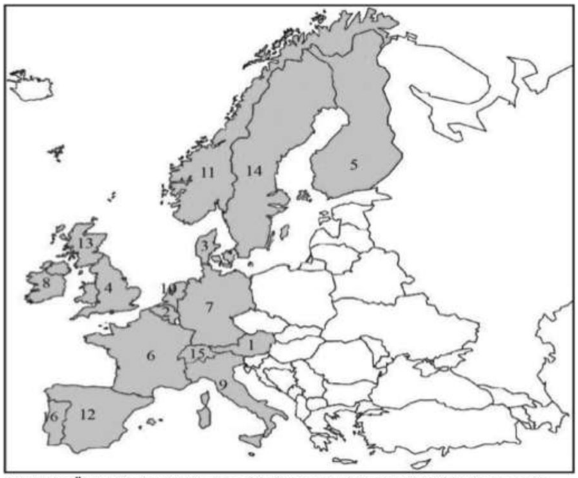

1) Verein Österreichisches Zentrum für Dokumentation und Qualitätssicherung in der Intensivmedizin; 2) Belgian Intensive Care Society; 3) Dansk Intensiv Database; 4) Intensive Care National Audit and Research Center; 5) Finnish Intensive Care Consortium; 6) Société de Réanimation de Langue Française; 7) Register Versorgungsforschung Intensivmedizin; 8) National office of clinical audit; 9) Gruppo Italiano per la Valutazione degli Interventi in Terapia Intensiva; 10) Nationale Intensive Care Evaluatie; 11) Norsk intensivregister; 12) La Sociedad Española de Medicina Intensiva, Crítica y Unidades; 13) Scottish Intensive Care Society Audit Group; 14) Svenska Intensivvårdregistret; 15) The Swiss ICU registry; 16) Centro de Controlo e Monitorização

**Fig. 1 (abstract 0341).** National ICU registeries and respective organizations in Europe. The updated map is based on previous publication (with permission) [2].

### 0342

#### Care beyond the ICU - using a novel level of care protocol for critical care support of the terminally ill patient

R. Gupta[1], J. Wang[1], S. Ahmed[1], S. Connolly[1], S. Huprikar[2], R. Kohli-Seth[1]
[1]Mount Sinai Hospital, Institute for Critical Care Medicine, New York, United States; [2]Mount Sinai Hospital, New York, United States
**Correspondence:** R. Gupta
*Intensive Care Medicine Experimental* 2018, **6(Suppl 2):**0342

**INTRODUCTION.** Traditionally, critical care has been intrinsically linked to the Intensive Care Unit (ICU) due to the need for close monitoring and titration of therapies. This can create unique challenges for health care systems, staff, patients and families especially when outcomes are anticipated to be poor.[1] In this regard, respect for a patient's desire for critical care can be met through a protocoled approach, where bedside critical care support is provided outside of the physical ICU.

**OBJECTIVES.** We developed a level of care (LOC) protocol to identify and manage critical care for terminally ill patients outside the ICU. We analyzed the impact of our LOC protocol used by the rapid response team (RRT) on the delivery of critical care at our 1171 bed quarternary care center.

**METHODS.** The RRT is an intensivist led critical care consult service designed to evaluate, manage, and triage acutely ill patients. We integrated our LOC protocol into the RRT consult, to help identify patients that have a terminal illness, who were now critically ill and were anticipated to pass away despite ICU support. Once identified, anticipated prognosis and goals of care discussion were held with patient and family. For patients requesting supportive care, comfort care plan were instituted in collaboration with the palliative service. If patients desired critical care, care was initiated at the bedside independent of triage to the ICU. A critical care plan was devised within defined limits anticipating vasopressor, ventilator, sedation and vascular access requirements. Operational details and concerns were discussed with floor staff and the care plan was distributed amongst hospital management, nursing and palliative services. The RRT team continued to provide close support for the primary team and family members. Over 12 months, we reviewed the performance of this protocol and its impact on observed mortality, hospital triage and hospice discharges.

**RESULTS.** Over 12 months RRT saw 10234 consults, of which 908 patients (8.9%) were triaged to the ICU and 619 patients (6%) were triaged to the stepdown unit. 185 consults were identified as level of care consults and 84 unique patients received bedside critical care support. Amongst these, 46 patients (54.8%) expired during their hospital stay, 15 patients (17.9%) were discharged to hospice, 13 patients (15.4%) were discharged to nursing facilities and 10 patients (11.9%) were discharged to home.

**CONCLUSIONS.** Our level of care protocol enabled critical care support to be provided to the terminally ill patient outside the ICU in a manner that balanced expectations, resources and end-of-life care. Through close cooperation of all stakeholders including families, nursing and support teams, it helped service patient autonomy, improve care and enhance resource utilization.

**REFERENCE(S)**

1. Huynh,T.N. *et al* (2014).The Opportunity Cost of Futile Treatment in the ICU.*Critical Care Medicine,42*(9),1977-1982.

**GRANT ACKNOWLEDGMENT**

None

### 0343

#### Specialized group implementation for in-hospital transport

M. Cenacchi Garcia Pereira[1,2], P. Pereira Travassos[1], R. Telles[1], E. Gadelha[1], W. Geres da Costa[1], R. Gonçalves de Lima[1], L. Junqueira[1], V. Cordeiro Veiga[1], S. Soriano Ordinola Rojas[1]

[1]Hospital BP - A Beneficência Portuguesa de São Paulo, Neurocriticalcare Unit, São Paulo, Brazil; [2]Universidade Cidade de São Paulo - UNICID, São Paulo, Brazil

**Correspondence:** M. Cenacchi Garcia Pereira

*Intensive Care Medicine Experimental* 2018, **6(Suppl 2):**0343

**INTRODUCTION.** Intra-hospital transport is associated with worse outcomes and adverse events, especially on critically ill patients.

**OBJECTIVES.** Our study's objective was to evaluate safeness profile of transport after specific group implementation for this purpose.

**METHODS.** In a period from October 2016 to September 2017 all critical patients transported were assessed, inside a large hospital, after implementation of specific group consisted by intensive care specialist physician, one nurse, and one physiotherapist. Complications, clinical and non-clinical, were evaluated to establish relation to transport.

**RESULTS.** The institute performed 1,559 transports, being 54.7% males and 60.9% were hospitalized patients. Use of vasoactive drugs was present in 19.8% and mechanical ventilation in 10.6%. Clinically stable patients comprehended 78.8% of our sample.

Clinical complications happened in 7.5% of the sample, more frequently in hemodynamically unstable patients (n=43) and respiratory failure (n=21). Eight percent of the sample (n=125) presented non-clinical complications. Occurrences were attributed to communication failures in 79.2% of cases.

**CONCLUSIONS.** Specialized group implementation to in-hospital transport made safer the process with lower rates of complication, including current literature data and guaranteed quality of care.

### 0344

#### Early rehabilitation in the ICU - reality in a large neurocritical care unit

M. Cenacchi Garcia Pereira[1,2], P. Pereira Travassos[1], R. Telles[1], R. Gonçalves de Lima[1], E. Gadelha[1], W. Geres da Costa[1], L. Junqueira[1], V. Cordeiro Veiga[1], S. Soriano Ordinola Rojas[1]

[1]Hospital BP - A Beneficência Portuguesa de São Paulo, Neurocritical Care Unit, São Paulo, Brazil; [2]Universidade Cidade de São Paulo - UNICID, São Paulo, Brazil

**Correspondence:** M. Cenacchi Garcia Pereira

*Intensive Care Medicine Experimental* 2018, **6(Suppl 2):**0344

**INTRODUCTION.** The Early Mobilization program during intensive care hospitalization has a number of benefits related to the outcome of the patient, including delirium reduction incidence, PICS, IMV and NIV time, risk of infections, vascular and length of hospital stay, and improved functional status at hospital discharge.

Progressive therapeutic activities, with application of additional facilitating resources, started immediately after hemodynamic and neurological stabilization. It requires adequate evaluation to determine the functional diagnosis, listing in the order of priority and its interrelation with the function to be worked.

**OBJECTIVES.** To evaluate the safety of implementing an Early Mobilization protocol within the first 24 hours of admission and the impact on the functional status of ICU discharge.

**METHODS.** Total patients admitted to the neurological ICU (Mar / 13 to May / 17). N = 11.219 Patients enrolled in PMP < 24 h of admission. N = 9,873

Excluded: Contraindication for PMP application < 24h; Impossibility to determine GFM at admission; Deaths; Clinical worsening after onset of monitoring; Non-adherence to PMP. N = 1.346

The patients were submitted to sessions with a mean duration of 30 minutes, 2x day, according to the tolerance, from admission serially monitoring hemodynamic, respiratory and neurological parameters.

**RESULTS.** There were 296 adverse events (3%), 97 of which were temporarily suspended. All events were reversible after discontinuation, with no clinical impact and resumed therapeutic activities within 24 hours.

**CONCLUSIONS.** The application of an Early Mobilization program within 24 hours of patient admission was shown to be safe, positively influencing the rehabilitation of neurological patients. Knowledge of the effect of postural changes and exercise, physiological and hemodynamic stability, type and intensity of activity need to be considered for the implementation of a PMP.

Effective communication, teamwork, planning and transparency in the therapeutic goal are essential for the implementation of the Early Rehabilitation program.

### 0345

#### The effects of music therapy on the level of depression and anxiety in family caregivers of critically ill patients

K. Jeong Min, N. Sungwon

Yonsei University College of Medicine, Department of Anesthesiology and Pain Medicine, Seoul, Korea, Republic of

**Correspondence:** K. Jeong Min

*Intensive Care Medicine Experimental* 2018, **6(Suppl 2):**0345

**INTRODUCTION.** Families of seriously ill patients are reported to experience severe caregiving and financial burdens, as well as serious emotional and psychological distress including anxiety, depression, and post-traumatic stress disorder. It has been studied that these experiences of negative emotions would affect caregivers in a long term after the ICU patients are discharged from their hospital. Recently, family centered care has received much attention in intensive care medicine, and there is an increasing interest in practical strategy of how to reduce the psychological difficulties of family caregivers.

Music has been applied in many clinical situations and its effect on pain, sleep, anxiety, stress response is well documented. It can be adapted to an individualized way at different levels in various musical activities. Thus, music therapy can help reduce the emotional burden of caregivers with varying degrees of depression and anxiety.

**OBJECTIVES.** The purpose of this study is to examine the influence of music therapy on depression, anxiety, and emotional states of family caregivers of ICU patients.

**METHODS.** Intervention: Subjects completed a consent form to participate in the study and completed a questionnaire on basic emotional state. Then, the family participated in an individual music therapy for 1 hour. Single session music therapy was composed of the following sequence (1) progressive muscle relaxation with utilizing music, (2) listening to participants' preferred music and confirming the emotional states, (3) emotional changes through singing, (4) confirming changed emotional states and discussing the way to adapt in everyday life. After the session, the subjects had an interval of 10 minutes and then completed the post - emotional state questionnaire.

Measures

Depression, anxiety, and emotional states are assessed using Center for Epidemiologic Studies Depression Scale(CES-D), State Anxiety Inventory(STAI), 100mm Visual Analogue Scale for emotional states (happiness, comfort, sad, anger, fear). Before and after variables are analyzed by repeated measures ANOVA, using Subjective Caregiving Burden Scale as a covariate.

**RESULTS.** A total of 24 caregivers enrolled in this study (13 spouses, 9 children, 1 parent, 1 sibling). After intervention, there were statistically significant differences in depression, anxiety, happiness, sadness, comfort items. On the other hand, music therapy interventions did not have statistically significant differences in fear and anger.

**CONCLUSIONS.** This study has identified that music therapy has an emotional improvement effect to family caregivers of critically ill patients. The main weakness of this study is this is a pilot study with a relatively small number of subjects. Another limitation is that music therapy is only a single session, and its long term effects are unknown. In spite of its limitations, this study showed the potentiality that music therapy can be used in family-centered care in ICU.

### 0346

#### Factors affecting consultant review of patients after admission to intensive care

N. Dobson[1], J. Robertson[2], H. Ahmed[2], Y. Jiang[2], T. Ali[1]

[1]Stoke Mandeville Hospital, Anaesthetics and Critical Care, Aylesbury, United Kingdom; [2]Stoke Mandeville Hospital, Aylesbury, United Kingdom

**Correspondence:** N. Dobson

*Intensive Care Medicine Experimental* 2018, **6(Suppl 2):**0346

**INTRODUCTION.** Guidelines for the Provision of Intensive Care Services (GPICS), compiled by the Faculty of Intensive Care and Intensive Care Society state, 'Patients must be reviewed in person by a consultant in Intensive Care medicine within 12 hours of admission to intensive care (ICU).[1]' Increasing comorbidities and complex medical health needs of the population highlight the importance of meeting these targets to ensure safe patient care.

**OBJECTIVES.** Ascertain whether the use of standardised admission clerking pro formas led to an improved number of patients receiving consultant review within 12 hours of admission to ICU across two moderate-sized ICUs in district general hospitals. Comparison was made to assess whether medical or surgical patients were more likely to meet the above guidelines.

**METHODS.** A retrospective analysis of patient admission notes across two ICUs was performed over a total of 4 months. Patient admission notes were reviewed before and after the introduction of an 'admission pro forma', which provided written prompts for staff to note when patient admission and consultant review occurred.

**RESULTS.** In total 262 patient notes were analysed; from February 2017 before admission pro formas were introduced and from September-November 2017, after their introduction. There were 131 medical patients and 131 surgical patients in total. Before introduction of admission pro formas, 46% of patients received consultant review within 12 hours of admission to ICU, compared to 51.8% of patients after pro forma introduction. 25.8% of surgical patients received a consultant review within 12 hours of admission, compared to 35% after pro forma introduction. 65.6% of medical patient were reviewed before pro formas were introduced, compared to 66.7% afterwards. Subjectively, data gathering was easier after pro forma introduction.

**CONCLUSIONS.** The use of admission pro formas correlated with a moderate improvement in the proportion of patients being reviewed by a consultant within 12 hours of admission to ICU. Medical patients were more likely to be reviewed compared to surgical patients, regardless of whether an admission pro forma was used. The pro forma had no effect on improving consultant reviews of medical patients but more surgical patients were reviewed within 12 hours after the introduction of the pro forma. This indicates there are other factors contributing to consultants being able to review patients. We speculate that these factors could include the detail of discussion between consultant and junior before admitting a patient and the level of care the patient required upon admission to ICU. This audit has highlighted that the use of standardised admission pro formas when clerking patients in ICU improves compliance with GPICS standards, as well as opportunities for further development.

**REFERENCE(S)**

1. Faculty of Intensive Care Medicine, Intensive Care Society. Guidelines for the Provision of Intensive Care Services, Edition 1.1, 2016

### 0347

#### Influence of an open-door policy on the noise generated in an intensive care unit (ICU)

A. Touceda, M. Segura, B. Porral, A. García, E.M. Menor, M.J. Rodríguez, D. Vila

Hospital Alvaro Cunqueiro, Intensive Care Unit, Vigo, Spain

**Correspondence:** A. Touceda

*Intensive Care Medicine Experimental* 2018, **6(Suppl 2):**0347

**INTRODUCTION.** The policy of visits in ICUs of our country have been generally restricted so far, only 3.8% have an open visiting schedule (24 hours a day), 9.8 % open only daytime, although in the majority the duration remains equal to or less than one hour (95.7%) (1). On the other hand, noise pollution in an ICU whose usual average levels (52-84 dB) are above those recommended by WHO (35-40 dB), represents an important problem with negative consequences for the staff and for the patients (2).

**OBJECTIVES.** Study if an open-door policy, allowing families to be present between 12:00 and 23:00 may have contributed to reduce the noise level of our Unit.

**METHODS.** Descriptive and retrospective study, analyzed the data of a Class 1 sound level meter placed in a polyvalent unit, with 15 beds, during a pre and post open-doors period (January and February 2017 and 18, respectively). We analyzed the number and characteristics of admissions (age, average stay and pathology) in these periods to know if the degree of activity could be comparable. The data have been analyzed with SPSS statistics.

**RESULTS.** The activity and characteristics of admissions in the two periods was similar: 89 patients entered in 2017 and 93 in 2018, without significant differences with respect to age (Mean 58.2±16.0 and 60.6±14.6 years respectively) nor in their average stay (8.8 vs 7.6 days) or type of pathology. When analyzing the data of the sound level meter, we have observed an overall decrease of the noise generated in ICU during the months in which it has been most permissive in the visiting schedule (Median 59.9±3.7 dB in 2018 vs 60.7±3.5 dB in 2017), which although it has been statistically significant, for sound effects, represents a very slight decrease. When comparing the data according to the time slot, we see that this decrease is maintained even in the period in which the families are found within the Units, from 12 to 23h (60.4±1.6 dB in 2018 vs. 61.2±1.7 dB in 2017; IC 95% -0.85;-0.60).

**CONCLUSIONS.** According to the existing literature, it is known that high levels of noise in ICU have a direct impact on the evolution of patients, modifying their circadian rhythm and directly influencing the sleep-wake cycle, in addition to a higher incidence of delirium and an increase in the need for sedation. Besides the intrinsic noise to the different machines and devices existing in an ICU, the main cause of this is that derived from human activities (2). Different strategies such as opening doors to families with a more permissive schedule, as we have seen in our study, seems to be able to contribute, although it is paradoxical, to reduce the noise in ICU.

1.D. Escudero et al. Política de visitas, diseño y confortabilidad en las unidades de cuidados intensivos españolas.Rev Calid Asist.2015;30:243-50.
2.AP
Garrido Galindo et al. Nivel de ruido en unidades de cuidado intensivo de un hospital público universitario en Santa Marta (Colombia).Med Intensiva.2016;40:403-10.

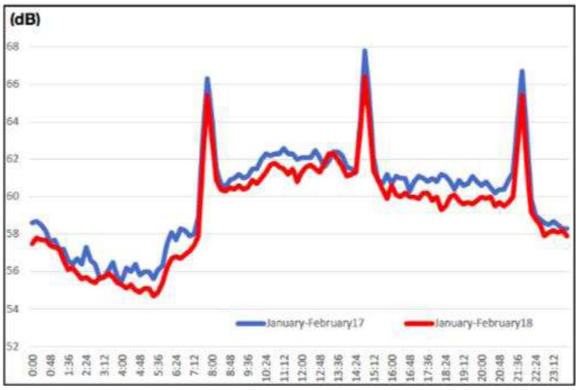

**Fig. 1 (abstract 0347).** Relationship between noise level and time in the same period of 2017 and 2018

### 0348

#### Social media in critical care is the hype worth the effort: a 3-year analysis and modelling for the future

A. Wong[1], M. Malbrain[2], J. Strachan[1], O. Olusanya[3], D. Lyness[4], International Fluid Academy Social Media Collaborative
[1]Oxford University Hospitals NHS Trust, Adult Intensive Care Unit, Oxford, United Kingdom; [2]Brussels University Hospital (UZB), Brussels, Belgium; [3]St Bartholomew's Hospital, London, United Kingdom, [4]Mater Infirmorum Hospital, Belfast, United Kingdom
**Correspondence:** A. Wong
*Intensive Care Medicine Experimental* 2018, **6(Suppl 2):**0348

**INTRODUCTION.** The use of social media (SoMe) in modern society has expanded tremendously. Platforms such as Twitter, Facebook and Instragram have revolutionized communication. This change has extended into medical education, including conferences, where it has become part of the way we learn and keep abreast of the latest developments. Healthcare professionals and societies have responded by having dedicated teams promoting the utilization of such platforms as a way of disseminating information and encouraging discussion beyond physical boundaries. Sceptics argue that this is an unsustainable fad and does not translate to meaningful educational content and discussion. The quality of content placed online has also been questioned.

**OBJECTIVES.** To quantify and qualify the use of SoMe at critical care conference in the last 3 years.

**METHODS.** The largest ICM conferences were identified by group consensus. Hashtags unique to the individual conferences were isolated and analysed using proprietary Symplur algorithms. Quantitative/qualitative measures were collated, with comparisons made across the various conferences (2015 - 2017).

**RESULTS.** The annual conferences of five main societies/organisations were identified between 2015 to 2017. These were the Society of Critical Care Medicine's Critical Care Symposium, European Society of Intensive Care Medicine's LIVES Congress, the International Symposium on Intensive Care and Emergency Medicine and the UK's Intensive Care Society Congress. Compared to 2015, the number of tweets and Twitter users during conferences increased in 2017. The average increase was 149.4% (range 63.5 - 267.7%). Analysis of the tweets during the conference showed an increase in the utilisation of media such as images and video clips. There was also a trend for tweets to contain hyperlinks so that contents/references could be accessed easily. There were more tweets with replies, confirming that such online platforms have the ability to generate online discussions.

**CONCLUSIONS.** This is the first attempt to quantify and qualify the educational content generated on SoMe over a prolonged period at critical care conferences. The analysis shows that the increasing use of SoMe has been sustained across international conferences examined. The increase use of multimedia content, users and interactions confirms that SoMe has matured as a platform and has gained credibility amongst healthcare professionals. The use of multimedia content has been shown to increase the educational value and impact to learners[1]. In conclusion, the use of SoMe at conferences has shown a sustained quantitative and qualitative increase at critical care conferences. It has a role in 21$^{st}$ century medical education but like all tools, has its benefits and limitations.

**REFERENCE(S)**

Rinaldo SR et al. Learning by Tweeting: Using Twitter as a Pedagogical Tool. https://doi.org/10.1177/0273475311410852

**GRANT ACKNOWLEDGMENT**

International Fluid Academy grant

### 0349

#### Evaluation in the hospitalary management of the high commitment human resource practices from the perspective of the ICU healthcare personnel

P. Vega Ocaña[1], J.D. Martín Santana[2], L. González Bautista[1], C. García del Rosario[3], L. Santana Cabrera[1], R. Lorenzo Torrent[1]
[1]Hospital Universitario Insular de Gran Canaria, Intensive Care Unit, Las Palmas de Gran Canaria, Spain; [2]Universidad de Las Palmas de Gran Canaria, Las Palmas de Gran Canaria, Spain; [3]Hospital Universitario Insular de Gran Canaria, Quality Unit, Las Palmas de Gran Canaria, Spain
**Correspondence:** P. Vega Ocaña
*Intensive Care Medicine Experimental* 2018, **6(Suppl 2):**0349

**INTRODUCTION.** Proper management of human resources is critical in providing a high quality of health care. The staff's perception of good high-commitment human resource practices and organizational management affects directly to their job and satisfaction.

**OBJECTIVE.** To evaluate the level of commitment of the management team in a Spanish public hospital towards the high performance work practices (HPWPs) from the perspective of the healthcare personnel in the Intensive Care Unit (ICU) and to analize the differences in the evaluation according to different profesional categories.

**METHODS.** Information was collected from August to November 2017 through a survey adapted to each professional category. The questionnaire included a 7-score Likert scale including 24 items aimed at assesing different aspects related to high-commitment human resources management.

The multidimensional nature of the scale required the completion of a Confirmatory Factorial Analysis (CFA) to corroborate the dimensions traditionally published in the literature on high-commitment human resource practices. In spite of the statistical significance of the model [$\chi^2$=107.87, *p*=0.000], results showed that othat other adjustment indicators were within the value ranges recommended in the literature (CFI=0.97, NFI=0.94, RMSEA=0.08). Regarding internal consistency measurements of every dimesion composite reliability (CR) reached values over 0.70 and average variance extracted (AVE) reached values over 0.50. The Cronbach's alpha values verifies those observed in composite reliability. These results indicated that the measurement model was valid and reliable.

To analyze the existence of differences of averages in the evaluation of the HPWPs according to the professional category of the ICUs healthcare personnel, an ANOVA analysis was used.

**RESULTS.** Based on the results of the CFA, we proceeded to create four new variables, which correspond to the weighted average of the items that shape each dimension, weighted by the regression weights of each of them in the CFA. The following table shows the average values for each dimension according to the assesments of the ICU healthcare personnel, globally and according to the professional category.

**CONCLUSIONS.** We may conclude that the ICU healthcare personnel evaluates, in a global way, with low score the high-commitment human resource practices. Internal promotion is the worst valued aspect by the staff, and on the other hand, participation is the aspect that has received the best assessment.

There are statistically significant differences in the evaluations according to the different professional categories. Nursing assistants has been the category with the best perception of HPWPs and, on the contrary, doctors award the worst assessment.

##### GRANT ACKNOWLEDGMENT

None.

**Table 1 (abstract 0349).** Descriptive analysis of the factors of the high-commitment human resource scale according to the professional category

| DIMENSIONS | Average (T.D.) | | | | *F (p)* |
|---|---|---|---|---|---|
| | Global | Doctors | Nurses | Assistants | |
| Training | 3.80 (1.64) | 2.65 (1.28) | 3.39 (1.54) | 4.99 (1.21) | 21.857 (0.000) |
| Participation | 4.33 (1.58) | 3.27 (1.36) | 4.07 (1.51) | 5.33 (1.27) | 15.915 (0.000) |
| Acknowledgment | 3.65 (1.76) | 2.75 (1.39) | 3.24 (1.71) | 4.78 (1.46) | 14.532 (0.000) |
| Internal Promotion | 3.55 (1.88) | 2.40 (1.47) | 3.03 (1.72) | 5.03 (1.40) | 23.960 (0.000) |

#### 0350

#### Change of hospital and critical care unit - does it influence outcome?

M. Strube, J. Oras, C. Rylander
Sahlgrenska University Hospital, Anaesthesia & Critical Care, Göteborg, Sweden
**Correspondence:** M. Strube
*Intensive Care Medicine Experimental* 2018, **6(Suppl 2):**0350

**INTRODUCTION.** Centralization and increased subspecialisation of care result in an increased need for interhospital transports during ongoing intensive care. The majority of these patients are transferred with continuous need of organ support but in a stable condition. Studies have repeatedly shown that interfacility transport has no significant impact on morbidity and mortality. However, a preliminary report has shown that change of hospital is associated with an increased risk of adverse outcomes for ICU patients (1).

**OBJECTIVE.** The aim of this study was to explore if the change of hospital and critical care unit influenced health care results. Our hypothesis was that interhospital transfer to another intensive care unit does not affect patient outcomes.

**METHODS.** This was a retrospective case-control study. After ethical approval, data from local and national ICU-registries was obtained regarding patient characteristics, ICU length of stay and survival.

All patients >18 years being transported from the critical care unit at Sahlgrenska University Hospital, an 18-bed tertiary care ICU, to other intensive care units during 2012 to 2016 were matched to non-transported patients as to ICU days before transfer, age, diagnosis and illness severity according to SAPS 3. Ninety-day mortality was chosen as the primary outcome.

**RESULTS.** 827 patients were transferred to other ICUs, with diagnoses predominantly cardiovascular (24%), pulmonary (16%) and traumatic (11%). The majority of patients were transported within the Region of Western Sweden (95%), but almost half of them to other hospitals in Gothenburg (46%). Mortality at 90-days was lower for patients transported vs controls (31,1% vs 36,1%, respectively; p=0.02). Patients moved within Gothenburg had no significant difference in 90-day mortality in comparison to the control group (39,7% vs 36,1% respectively; p=0.60). Compared to all non-transported and non-matched patients treated in our ICU during the study period, 90-day mortality was higher in patients exposed to interhospital transportation (p< 0.001).

**CONCLUSIONS.** Interhospital transfer had no negative impact on 90-day mortality compared to non-transported matched patients. The lower mortality in patients moved to hospitals outside Gothenburg may be explained by the fact that such transports are initiated after the termination of need for tertiary care, while patients moved within Gothenburg presumably represent a general ICU population transferred in times of limited ICU-bed availability. Still, transfer within Gothenburg was not associated with increased mortality as opposed to what has been reported earlier (1). This may be explained by our use of a matched control group and we conclude that interhospital transfer between ICUs in the Swedish Western Region appear safe.

##### REFERENCE(S)

1. SIR Annual Report 2013 (swedish)
*http://www.icuregswe.org/Documents/Annual%20reports/2013/Analyserande_2013.pdf*

##### GRANT ACKNOWLEDGMENT

none

#### 0351

#### Challenges associated with critically ill patients admitted to the intensive care unit following international repatriation: retrospective case study and analysis

D. Johnpillai, C.J. Fong, A.J. Cadamy, F. O'Sullivan
Queen Elizabeth University Hospital, NHS Greater Glasgow & Clyde, Intensive Care Unit, Glasgow, United Kingdom
**Correspondence:** D. Johnpillai
*Intensive Care Medicine Experimental* 2018, **6(Suppl 2):**0351

**INTRODUCTION.** The Queen Elizabeth University Hospital (QEUH) in Glasgow hosts Scotland's largest critical care unit and accepts patients who have developed critical illness abroad. As in other UK units such repatriations are infrequent, but they can be associated with particular logistical and clinical challenges. There is a perceived need to develop systems, at a local, national, and international level, which optimise care and mitigate the inherent risks to critically ill patients from international transfer.

**OBJECTIVES.** This project describes local workload and identifies common issues affecting level 3 patients admitted following international repatriation. It aims to inform protocols and governance systems at a local and national level.

**METHODS.** A retrospective case review was conducted of level 3 patients internationally repatriated to the QEUH intensive care unit (ICU) from June 2015 to January 2018. Cases were identified by a systematic search of the local Wardwatcher database. Clinical notes, imaging, and laboratory results were reviewed, including those provided from the originating hospital where available. Challenges surrounding transfer were recorded as 'errors' and coded by category: information, pressure sores, tracheostomy or other clinical. These were further described by established definitions designed to illustrate type, severity and preventability. Microbiology tests were also reviewed.

**RESULTS.** In the 31 months since opening, QEUH ICU has admitted 10 level 3 patients as a result of international repatriation: 7 from Spain, and 1 each from France, Italy, and Romania. The most common presenting diagnoses were trauma and pneumonia. Patients spent between 4-77 days in hospital before repatriation, with discharged after 11-20 days in ICU. 9 out of 10 admissions experienced errors related to transfer, most commonly due to information handover. No severe errors were identified, although 2 patients required a tracheostomy change, as extra inner tubes had not been provided. All errors were probably or definitely preventable. 2 of 10 patients grew carbapenem resistant organisms with 2 further growing intermediately-resistant organisms resulting in prolonged isolation. 6 were transferred with officially translated documentation, while only 2 were transferred with electronic imaging compatible with UK systems.

**CONCLUSIONS.** Every 3 months, the QEUH ICU admits a level 3 patient repatriated from overseas. Many transfers are complicated by poor information handover, complex microbiology, or incompatible imaging. Complications could potentially be avoided by developing clear guidance at a local, national or international level.

**REFERENCE(S)**

CCNNW London, "Memorandum of Understanding (MOU) between NHS and Assistance Companies for Critical Care Admission into the UK from Abroad Responsibilities of Assistance Companies," 2010.

J. Eddleston, D. Goldhill, and J. Morris, "Levels for Critical Care of Adult Patients," 2009.

### 0352

#### Analysis of unplanned transferences from intermediate care units to intensive care units: a cohort study

J.G. Rosa Ramos, G. Machado Naus dos Santos, M. Chetto Coutinho Bispo, R.C. De Almeida, G.M. Lopes Santos de Carvalho Junior, R. Da Hora Passos, M. Brito Teixeira, A.L. Nunes Gobatto, J. Caldas Ribeiro Bittencourt, S. Farias Da Guarda, M. Pordeus Ribeiro, P. Benigno Pena Batista

Hospital São Rafael, Salvador, Brazil

**Correspondence:** J.G. Rosa Ramos

*Intensive Care Medicine Experimental* 2018, **6(Suppl 2):**0352

**INTRODUCTION.** Guidelines on ICU admission define that potentially unstable patients could be admitted to intermediate care units (IMCUs) with later transference to intensive care units (ICUs), if necessary (step-up transferences). However, the safety of such approach is not well established in the literature.

**OBJECTIVES.** We aimed to analyze factors associated to such step-up transferences.

**METHODS.** Retrospective cohort in a tertiary hospital in Brazil. We analyzed consecutive unplanned step-up transferences from January 2013 to December 2015. Relationship between reason for admission in the IMCU and reason for transference to the ICU was assessed as a categorical variable.

**RESULTS.** In the study period, there were 815 admissions to the IMCU, of which 99 (12.1%) were transferred to the ICU (step-up transferences). Overall mortality was 10.7%, with a mortality of 4.4% for patients that were not transferred to the ICU and of 45.5% for step-up patients (p< 0.001).

Of the 99 step-up patients, 93 (93%) were admitted for monitoring, while 6 (6%) were admitted for active intervention. Median number of days from admission in the IMCU to step-up transfer was 2 (1-6). Transferences were rated as probably or definitively related to original admission in 81 (82.6%) cases.

**CONCLUSIONS.** Step-up transference to the ICU occurred frequently in this cohort and was associated to increased mortality. Most transferences were early and were rated as related to the reason for the original admission.

**GRANT ACKNOWLEDGMENT**

This work has no funding

### 0353

#### Audit of oral cavity assessment for patients with endotracheal tube in Neuro ICU

A. Kwan[1], V. Ventura[1], S. Shah[1]

[1]St. Georges University Hospitals NHS Foundation Trust, Neuro ICU, London, United Kingdom

**Correspondence:** A. Kwan

*Intensive Care Medicine Experimental* 2018, **6(Suppl 2):**0353

**INTRODUCTION.** Mouth assessment represents a significant role in standardising and implementing effective oral care (Baker and Shipway, 2017). A thorough assessment must be carried out as this provides the staff with accurate baseline information regarding the condition of the patient's oral cavity. It also enables the healthcare professional and wider multi-disciplinary team members (MDT) to observe the progress or deterioration of the patient's oral condition hence, allowing for appropriate treatment plans (Wilson, 2011). An application of tool is important for an adequate mouth assessment and therefore the delivery of appropriate and efficient holistic care.

**OBJECTIVES.** To determine how beside nurses review the state of the oral cavity, particularly for those individuals with EndoTracheal Tube (ETT) and ensure documentation correlates with the TONGUES tool assessment (Neuro Intensive Care Unit, 2008).

**STANDARD.** 100% of individuals with ETT should receive an appropriate mouth care assessment at the start of the shift.

**METHODS.** A retrospective analysis was initially done on 24 individuals with ETT. 98 nursing care plans were reviewed to a mouth condition checklist.

A questionnaire survey was given to trained nurses to determine the type of assessment they used in evaluating the oral cavity and there were 45 respondents.

**RESULTS.** Data shows that 96% (n=94) of care plans had forms of written mouth condition assessment and 4% (n=4) had none. The survey results revealed 42% (n=19) of respondents stated that they follow the ICU guideline in assessing the patient's mouth condition, whilst 58% (n=26) reported that they use their own system or unsure which guideline to follow.

**CONCLUSIONS AND ACTIONS.** The audit had shown nurses have consistently assessed the mouth condition of patients with ETT. However, most of the assessments were not thorough to cover the whole area of the oral cavity. In order to enhance the practice with minimal disruption to work routine, a standardised mouth condition pre-assessment checklist was designed in the care plan to easily carry out initial and on-going oral evaluations. The guide will prompt the staff to report the result to the rest of the MDT and provide best evidence based practice on mouth care in which will result to patient's having best outcome.

**Figure 1. Mouth assessment section of ICU care plan.**

Mouth condition
tongue: pink moist dry cracked coated
lips: pink moist dry ulceration sores
teeth: clean missing caries dentures
halitosis: yes no
gums: pink moist bleeding oedematous

St. Georges University Hospital NHS Trust `TONGUES Assessment, Neuro ICU' (2008)

### 0354

#### Yeast in the urine predicts longer hospitalization in liver transplant patients

Q.G. Chen[1], Q.C. Chen[2], W.M. He[1], L.S. Zhang[1], S.H. Ge[1], M. Zeng[1]

[1]First Affiliated Hospital of Sun Yat-sen University, Medical Intensive Care Unit, Guangzhou, China; Sun Yat-sen University, Zhongshan School of Medicine, Guangzhou, China

**Correspondence:** Q.G. Chen

*Intensive Care Medicine Experimental* 2018, **6(Suppl 2):**0354

**INTRODUCTION.** Little information is available about the association between yeast detected in urine culture and clinical outcomes of liver transplant patients.

**OBJECTIVES.** The aim of this study is to evaluate whether there is an association between yeast cultured from urine and poorer clinical outcomes for liver transplant patients.

**METHODS.** We performed a retrospective cohort study using a modifiable data mining technique applied to the publicly available Medical Information Mart for Intensive Care III (MIMIC III) database (v1.4). The primary endpoint was 90-day mortality. Hospital mortality and length of hospitalization were secondary outcomes. Propensity score matching (PSM) was performed and multivariate regression analysis was used to adjust for potential confounders. Survival curves were constructed according to Kaplan-Meier.

**RESULTS.** 273 liver transplant patients were included finally with 29 patients isolated yeast from urine. 21 patients with yeast-positive culture were successfully matched with 63 yeast-negative controls using PSM and no statistically significant difference was found between the two groups on age, gender, Elixhauser Comorbidity Index (SID30), and Simplified Acute Physiology Score II (SAPS II) (p> 0.05). Kaplan-Meier curves analysis of the PSM cohort found no difference on 90-day survival between the two groups (log-rank test, p= 0.43). The median length of hospitalization of yeast-positive patients was significantly longer than that of the yeast-negative controls (55.92 days versus 20.25 days, p< 0.001) but there is no difference on hospital mortality between the two groups (p= 0.259). Results of multivariate regression analysis indicated that isolated yeast from urine was independent risk factor of longer hospitalization (difference 36.64 days, 95% confidence interval [CI] 27.24- 46.04, p < 0.0001) but not for 90-day mortality (hazard Ratio 1.79, 95% CI 0.43-7.50, p= 0.4242) and hospital mortality (odds ratio 2.47, 95% CI 0.59-10.34, p= 0.2154). Analysis of all patients showed similar results.

**CONCLUSIONS.** Isolated yeast from urine might predict longer hospitalization for liver transplant patients, but whether this was a causal or associational finding cannot be determined.

**REFERENCE(S)**

1. Johnson A. E., et al. SCI DATA. 2016;3:160035.
2. Silveira FP, et al. Med Mycol. 2007;45(4):305-20
3. Chen LY, et al. J Microbiol Immunol Infect. 2015;48(4):425-30

**GRANT ACKNOWLEDGMENT**

This work was supported by National Natural Science Foundation of China (No.81670066), and Major Science and Technology Planning Project of Guangdong Province, China (No.2016A020216009).

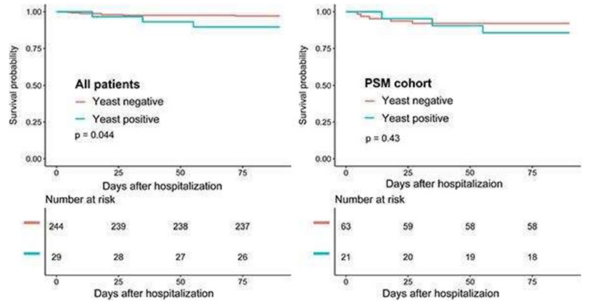

**Fig. 1 (abstract 0354).** Kaplan-Meier Survival Curve

**Table 1 (abstract 0354).** Association of isolated yeast from urine with clinical outcomes of liver transplant patients

Association of isolated yeast from urine with 90-day mortality, hospital mortality, and length of hospitalization in liver transplant patients

| All patients | HR/OR/β | 95% CI | p | PSM cohort | HR/OR/β | 95% CI | p |
|---|---|---|---|---|---|---|---|
| **90-day mortality** | | | | **90-day mortality** | | | |
| Non-adjusted | 3.67 | 0.95-14.19 | 0.0597 | Non-adjusted | 1.77 | 0.42-7.41 | 0.4340 |
| Adjusted | 2.69 | 0.67-10.79 | 0.1626 | Adjusted | 1.79 | 0.43-7.50 | 0.4242 |
| **hospital mortality** | | | | **hospital mortality** | | | |
| Non-adjusted | 5.42 | 1.48-19.79 | **0.0106** | Non-adjusted | 2.24 | 0.56-8.85 | 0.2520 |
| Adjusted | 2.70 | 0.60-12.11 | 0.1950 | Adjusted | 2.47 | 0.59-10.34 | 0.2154 |
| **length of hospitalization (days)** | | | | **length of hospitalization (days)** | | | |
| Non-adjusted | 34.52 | 26.77-42.27 | **<0.0001** | Non-adjusted | 37.33 | 27.69-46.96 | **<0.0001** |
| Adjusted | 29.01 | 20.94-37.09 | **<0.0001** | Adjusted | 36.64 | 27.24-46.04 | **<0.0001** |

### 0355

#### Compliance of the initial resuscitation bundle in patients with sepsis and septic shock in a catalan tertiary hospital

V.D. Gumucio Sanguino[1], L. Anguela Calvet[1], H. Alanez Saavedra[1], P. Sastre Perez[1], P. Malchair[2], F. Llopis Roca[2], X. Palom Rico[2], R. Gimenez[3], E.P. Plata Menchaca[1,4], C. Marroquin Guerrero[5], J. Sabater Riera[1], X.L. Pérez Fernández[1], Siraki Group

[1]Bellvitge Hospital, Intensive Care Medicine, Hospitalet de Llobregat, Spain; [2]Bellvitge Hospital, Emergency Department, Hospitalet de Llobregat, Spain; [3]Universitat de Barcelona, Medicine College, Hospitalet de Llobregat, Spain; [4]Institut d´Investigació Biomèdica de Bellvitge, Hospitalet de Llobregat, Spain; [5]Bellvitge Hospital, Emergency Department - Nursery, Hospitalet de Llobregat, Spain

**Correspondence:** V.D. Gumucio Sanguino

*Intensive Care Medicine Experimental* 2018, **6(Suppl 2):**0355

**INTRODUCTION.** In 2015, Catalonia began to require hospitals to follow protocols for the identification and early treatment of sepsis. However, there is controversy over whether a faster treatment of sepsis improves outcomes in patients.

**OBJECTIVES.** To evaluate the early recognition of patients with sepsis and septic shock in a Spanish university hospital.

To evaluate the compliance of the resuscitation bundle in the first 3h from the activation of the system and its association with hospital mortality.

**METHODS.** Prospective observational study in a tertiary hospital in Catalonia. The data were collected from December 2015 to December 2017 of patients with criteria of sepsis and septic shock at all levels of the emergency department (ED). Initial 3 hour bundle (lactate, cultures, and antibiotics) completion was evaluated as well as fluid administration within the first 6 hours in those patients who presented hypotension or lactate >4 mmol/L.

**RESULTS.** 295 patients were collected with the activation system, 59.1% were male; 63.4% were activated at the advanced medical, 6.1% at the advanced surgical level, and 31.2% at the basic ED level. Patients were mostly medical (67.3%), respiratory sepsis as the first cause with 40%, followed by urinary sepsis 26.3%. 87.8% of patients required hospital admission, overall 22% at the intensive care unit (ICU). The average SOFA score was 8±2. Septic shock was developed in 22.5% of patients. Regarding compliance with the 3-hour bundle, in 80% of the patients lactate was obtained within the first 3 hours (overall mean time 174±422 minutes (min)), cultures were obtained within the first 3h (and previous to antibiotics) in 73% (overall mean time 314±1820 min), and antibiotics were started within the first hour in 27.7% with a (overall mean time 213±408 min). The complete bundle (3 measures) was completed in 82% of patients (mean time 307±830 min) and was performed in less than 3h in 61.4% of them. 64% had hypotension±lactate> 4mmol/L, 51% of them receiving correct fluid therapy (30mL/kg), with 32% of them finally requiring vasoactive support. Among patients who had the 3-hour Bundle completed within the first 12 hours from emergency admission, a longer time to complete the Bundle was not associated with higher in-hospital mortality. A linear association was observed between the delay in lactate determination within the first 12 hours and in-hospital mortality (p < 0.02), as well as an association between higher crystalloid administered within the first 6h (p < 0.001) and in-hospital mortality.

**CONCLUSIONS.** The measurement of early serum lactate seems to improve in-hospital survival. Patients with higher amounts of crystalloids administered with the first 6 hours seem to present a worst outcome. No survival improvement was observed in those patients who early completed the 3 hour bundle.

**REFERENCE(S)**

(doi:
10.1007/s00134-017-4683-6

## Acute cerebrovascular diseases

### 0356

#### Impact of assignment of stroke specialist to the stroke care system on reducing in-hospital delay in reperfusion therapy

H.-K. Park[1], H.-J. Bae[2]
[1]Inje University Ilsan Paik Hospital, Goyang-si, Korea, Republic of; [2]Seoul National University Bundang Hospital, Seongnam, Korea, Republic of
**Correspondence:** H.-K. Park
*Intensive Care Medicine Experimental* 2018, **6(Suppl 2):**0356

**INTRODUCTION.** Efficacy of reperfusion therapies for acute ischemic stroke patients diminishes with the delay of time from symptom onset. However, the effect of the intervention with highly-skillful stroke specialist throughout the acute stroke treatment process on reducing the time delays in the hospital has not been evaluated yet.

**OBJECTIVES.** We evaluated the association of the assignment of the stroke specialist to the stroke care system with time intervals in stroke patients receiving reperfusion therapy.

**METHODS.** From a total of 7,358 patients who admitted to single tertiary referral stroke center in Korea between July 2007 and September 2015, we selected the subjects who arrived at hospital within 4.5 hours from symptom onset (N=2,100) with image-documented ischemic stroke cases (N=1360). We excluded cases who received reperfusion therapy at local hospitals and transferred to our hospital (N=10). The study hospital was designated as the regional cardiocerebrovascular center by the Ministry of Health and Welfare of Korea since January 2008. In addition to the pre-existing Stroke Critical Pathway Protocol that has been used for the quality improvement of stroke care, a stroke specialist was arranged to the acute stroke patients for immediate assessment and acceleration of whole process for stroke treatment. Included patients were divided into two groups according to the admission date [before 2008 (B2008) versus from 2008 (F2008)], and time intervals of study groups were compared.

**RESULTS.** Among the 1,350 included patients, 546 (40%) patients were treated with any reperfusion therapy [230 for intravenous thrombolysis (IVT), 73 for endovascular therapy (EVT), and 243 for both IVT and EVT (CMT)]. Compared with the B2008 group, the median door-to-needle time was shorter in the F2008 group in both patients receiving IVT alone (50 [40-73] vs. 31 [25-47], p< 0.01) and patients receiving CMT (50 [38-61] vs. 29 [24-39], p< 0.01). Door-to-puncture times were also significantly shorter in the F2008 group regardless of the use of IVT prior to EVT (116 [89-151] vs. 85 [66-110] in patients treated with EVT alone, and 113 [95-133] vs. 82 [68-98] in patients with CMT, all p-values< 0.01). Needle-to-puncture time in CMT showed 14 minutes reduction in the F2008 group (65 [48-81] vs. 51 [30-65], p< 0.01).

**CONCLUSIONS.** With intervention via immediate arrangement of stroke specialist and acceleration of the treatment process, it is possible to reduce the in-hospital waste of time.

**REFERENCE(S)**

Stroke. 2011;42:2983-2989

**GRANT ACKNOWLEDGMENT**

None.

### 0357

#### Stroke in elderly population: a descriptive study of patients admitted for reperfusion treatment in the ICU

F.I. Pino Sánchez[1], D. García Huertas[1], E. García Bautista[2], F. Guerrero López[1], P. Navarrete Navarro[1], E. Fernández Mondéjar[1]
[1]Hospital Universitario Virgen de las Nieves, Intensive Care Unit, Granada, Spain; [2]Hospital Universitario Virgen de las Nieves, Interventional Neuroradiology, Granada, Spain
**Correspondence:** D. García Huertas
*Intensive Care Medicine Experimental* 2018, **6(Suppl 2):**0357

**PURPOSE.** Analyze clinical profile, complications and results of elderly patients, age equal to or older than 80 years, admitted to our unit with an acute ischemic stroke receiving reperfusion treatment.

**MATERIAL AND METHODS.** Prospective observational study of patients aged 80 years or older, admitted to our unit with an acute ischemic stroke with reperfusion treatment (thrombolysis, endovascular therapy or both) from December 2016 to December 2017.

Demographic data, cardiovascular risk factors, previous Rankin, baseline NIHSS, baseline ASPECTS, reperfusion treatment and times will be collected. If there is endovascular therapy, sedation management will be used, endovascular technique, TICI score, will be collected. As results: NIHSS at 24 hours and at hospital discharge, RANKIN at hospital discharge, symptomatic intracranial hemorrhage and in-hospital mortality. Statistical analysis with a basic descriptive with absolute and relative frequency, qualitative, mean and standard deviation, quantitative ones of normal distribution, and median and interquartile range otherwise; Comparisons with ANOVA.

**RESULTS.** We included 206 patients with stroke and reperfusion treatment in our unit, of which 61 (29%) were 80 or older. 74% of this elderly population were women, age 84.14 ± 3.28 years, cardiovascular risk factors in 96%. Basal NIHSS 14.31 ± 6.5. Basal ASPECTS $\geq$ 7 in 89.4%. Reperfusion treatment was only thrombolysis in 39%, thrombolysis plus thrombectomy in 20%, and only thrombectomy in 41%. The thrombectomy was performed in 62% under mechanical ventilation; combination of aspiration plus stent retriever was the most common technique used (58%), with a good flow as result in 80% (TICI 2B or 3). Symptoms / thrombolysis time 3 h ± 1 h 30 min and symptoms / reperfusion by thrombectomy 4 h 55 min ± 1 h 46 min. NIHSS at 24 hours after ICU admission was 7 [2, 12]. Length of stay in ICU was 1 day [1, 2], and hospital stay 13 [7, 19]. Results at discharge: RANKIN $\leq$ 2 in 11 patients (18%), RANKIN 3, 13 patients (21%), RANKIN 4-5, 30 (49%), mortality of 11% (7 patients). Symptomatic intracerebral hemorrhage occurred in 4 patients (6 %). There is a relationship between RANKIN at discharge, baseline NIHSS and NIHSS at 24 hours; thus, patients with RANKIN at discharge 0-2 had baseline NIHSS of 8.9 ± 4.3 and at 24 hours 3.36 ± 5.8; Patients with RANKIN

at discharge 3 had 10.6 ± 5.8 and 3.8 ± 3.15, respectively; patients with RANKIN at discharge 4-5, had 16.5 ± 5 and 10.9 ± 6.12, respectively; the deceased had 20.14 ± 7.4 and 20.6 ± 3.5, respectively. There is no relationship between RANKIN at discharge and time between onset of symptoms and administration of fibrinolysis or reperfusion with thrombectomy.

**CONCLUSIONS.** The results of ischemic cerebrovascular disease in the elderly population are still poor, although the introduction of endovascular treatment techniques offers an alternative treatment that allows to rescue and improve results in some of them.

### 0358

#### Selective cerebral hypothermia is an effective component of neuroprotection in patients with ischemic stroke

B. Torosyan, A.V. Butrov, O.A. Shevelev, D.V. Cheboxarov, O.P. Artukov
RUDN University, Moscow, Russian Federation
**Correspondence:** B. Torosyan
*Intensive Care Medicine Experimental* 2018, **6(Suppl 2):**0358

**INTRODUCTION.** Ischemic stroke (IS) is one of the main causes of death and disability of the Russian working population and at present there are no known drugs with a proven neuroprotective effect.

**OBJECTIVES.** The purpose of our study was to evaluate the clinical efficacy of selective cerebral hypothermia(SCH) in the complex therapy of IS and its effect when used in long-term therapy.

**METHODS.** 113 patients were treated, which were divided into the experimental group (SCH administered) and control group (without SCH). Special helmets were used, which provides selective cooling of the head, while the core temperature did not decrease below 36 °C. To evaluate the temperature of the brain, a non-invasive microwave thermometer was used that could record the average temperature in a given tissue volume. The degree of neurologic deficiency was evaluated using the NIHSS scale, the degree of disability on the 90th day from the onset of the disease - according to the modified Rankin scale.

**RESULTS.** In the experimental group, there was a marked decrease in the level of neurologicaldeficit in the acute period after 24 hours of CCH ($p < 0.01$). Also, there was a decrease in the degree of disability by 0.6-0.9 points in groups with CCH. The mortality on the 90th day from the onset of the disease in the group of patients with severe and extremely severe neurologic deficit decreased by 28% ($p < 0.01$), in the group of patients with a deficit of less than 13 points according to the NIHSS scale, there were no significant differences. SCH has a pronounced therapeutic effect when applied in combination withstandard treatment. It can be assumed that this effect is a consequence of the decrease in brain edema and the protection of the penumbra zone.

**CONCLUSIONS.** SCH can be considered as an effective technique for treating patients with IS and isrecommended for use in its complex therapy.

### 0359

#### Elevated cardiac troponin I is associated with lower blood pressure on the first admission day after aneurysmal subarachnoid hemorrhage

M. Hravnak[1], E. Crago[1], Y. Chang[2], K. Mahmoud[1], K. Yousef[3], T. Lagatutta[1], A. Fisher[1], M.R. Pinsky[2], R.M. Friedlander[2]
[1]University of Pittsburgh, School of Nursing, Pittsburgh, United States; [2]University of Pittsburgh, School of Medicine, Pittsburgh, United States; [3]University of Jordan, School of Nursing, Amman, Jordan
**Correspondence:** M. Hravnak
*Intensive Care Medicine Experimental* 2018, **6(Suppl 2):**0359

**INTRODUCTION.** Aneurysmal subarachnoid hemorrhage (aSAH) patients may be exhibit neurocardiac injury (NCI) evidenced by elevated cardiac troponin I (cTnI), but the association between NCI and impact on early hemodynamics as evidenced by blood pressure (BP) monitoring parameters over the first day after admission is not well established. Such information could help to inform about the consequences of elevated cTnI in these patients.

**OBJECTIVES.** We aimed to determine blood pressure trajectory groups in the first 24 hours after aSAH admission, and the relationship between systolic BP (SBP) and cTnI.

**METHODS.** Prospective analysis of 65 patients with aSAH. Inclusion criteria: age 21-75 years, spontaneous aneurysm rupture, Fisher grade ≥ 2 and/or Hunt and Hess grade ≥ 3, cTnI levels and recorded blood pressures in the first 24 hours after aSAH. Exclusion: traumatic SAH, and recent myocardial infarction. All blood pressures recorded in the medical record at the type (peripheral or intra-arterial) and frequency recorded were collected. The hourly peak (maximum) and hourly nadir (minimum) SBP obtained for each patient in each of their first 24 hours of admission was used in the trajectory analyses, performed with Proc Traj in SAS. The peak cTnI obtained on the first day of admission was utilized. The relationship between SBP and cTnI was performed using mixed model linear regression for the independent variable of cTnI to predict the dependent variable of hourly peak or nadir SBP.

**RESULTS.** From the 65 patients, two trajectory groups for each of hourly peak and nadir SBP were identified. The lower SBP trajectory group (Group 1) for both peak SBP (n=31) and nadir SBP (n=38) had higher mean cTnI levels (1.09±2.8ng/ml and 1.01±2.6ng/ml) respectively, while the higher SBP trajectory group (Group 2) for both peak SBP (n=34) and nadir SBP (n=31) had lower mean cTnI levels (0.47 ±1.1ng/ml and 0.49±1.1ng/ml respectively). The results of the mixed model linear regression revealed an inverse relationship between cTnI and SBP, with higher cTnI predicting both a lower hourly peak SBP (estimate -1.52, SE 0.6, p=0.24) and hourly nadir SBP (estimate -1.90, SE 0.62, p=0.003).

**CONCLUSIONS.** There is an inverse relationship between cTnI levels and SBP in the first day of aSAH admission, and patients with higher cTnI exhibit lower SBP trajectories. Clinicians should closely observe patients with elevated troponin to evaluate for potentially impaired perfusion.

**GRANT ACKNOWLEDGMENT**
NIH R01NR014221

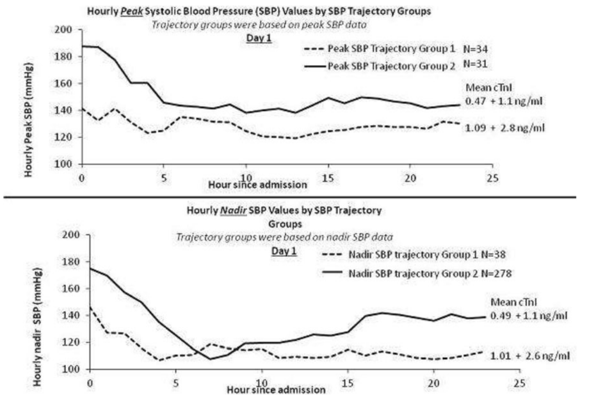

**Fig. 1 (abstract 0359).** Blood pressure trajectory groups after subarachnoid hemorrhage

### 0360

#### Morbidity and mortality after acute ischemic stroke: differences upon age

P. Matía Almudévar, A. Naharro Abellán, S. Alcántara Carmona, N. Martínez Sanz, A. Pérez Lucendo, J. Palamidessi Domínguez, P. Rodríguez Villamizar, J. Veganzones Ramos, L. Pérez Pérez, J.M. Mateos Pérez
Hospital Universitario Puerta de Hierro Majadahonda, Madrid, Spain
**Correspondence:** P. Matía Almudévar
*Intensive Care Medicine Experimental* 2018, **6(Suppl 2):**0360

**INTRODUCTION.** Acute ischemic stroke (AIS) is one of the leading causes for both death and disability worldwide, and its prevalence increases with age.

**OBJECTIVES.** To compare mortality and neurological status at 6 months of three different age groups (≤65, 66-79 and >80 years) of acute ischemic stroke (AIS) patients and to study if there are any disparities regarding thrombectomy application and destination upon discharge.

**METHODS.** Retrospective and observational study in a Spanish tertiary university hospital (October 2013 to September 2016). We included all AIS patients admitted to our ICU who received either intravenous thrombolysis (IVT), endovascular thrombectomy (TRB) or both. Neurological status was assessed at six months with the modified Rankin scale (mRS), defining good outcome as a mRS of 0-2, and functional dependency as a mRS ≥ 3.

Statistical analysis: $\chi^2$ test (categorical) and logistic regression or ANOVA (multivariate analysis).

**RESULTS.** During the period of study, a total of 164 patients with an AIS diagnosis and treated either with IVT and/or TRB were admitted to our ICU. After subdividing by age, 42% (69) were ≤65, 37% (60) between 66-79 and 21% (35) ≥80 years old. *Table 1* describes demographic data and general characteristics.

Crude six-month mortality according to age group was 6.7% (4), 25% (14) and 21% (7) respectively (p = 0.02), an association that was lost after adjusting by APACHE II (OR 1.6; IC95 0.81-3.22; p=0.179)

When analysing the neurological outcome at six-months, 70% (42) of our younger patients had a good outcome (mRS 0-2), decreasing to 54% (30) in the 66-79 years-old group and to 29% (10) in those > 80 years (p=0.01; *Graph 1*). This association was maintained after adjusting by initial NIHSS and previous mRS [(OR 2.5; IC95 1.52-4.32; p< 0.001).

We found no differences in TRB rates regardless of age group (p=0.154).

The percentage of patients who required an admission in a rehabilitation facility upon discharge was significantly higher for the ≥ 80 years-old group than for the ≤ 65 [55% (17) vs. 25% (16); p=0.011].

Results are summarized in *Table 2*

**CONCLUSIONS.** In our series, after adjusting by severity index, age did not act as a risk factor for mortality. However, it was associated with a poorer neurological outcome at six-months, a higher disability rate and, consequently, an increased need for admittance in a rehabilitation facility upon hospital discharge.

According to our results we think that age should be taken into account as a prognostic factor when treating patients with AIS, especially those above 80 years of age.

**Table 1 (abstract 0360).** See text for description

| | ≤ 65 (n= 69) | 66-79 (n=60) | ≥ 80 (n=35) |
|---|---|---|---|
| Male | 43 (62) | 34 (56) | 16 (45) |
| APACHE II * | 9 (6-11) | 12 (10-15) | 12 (10-14) |
| No. Comorbidities * | 2 (1-3) | 2 (2-3) | 2 (2-4) |
| Previous mRS | 0 (0-0) | 0 (0-0) | 0 (0-0) |
| Initial NIHSS | 16 (11-19) | 17 (10-20) | 16 (12-20) |
| ASPECTS | 8 (7-10) | 9 (7-10) | 9 (6-10) |
| Time: onset - hospital adm. | 79 (56-104) | 73 (60-120) | 91 (60-113) |
| Location | | | |
| LMCA | 31 (45) | 25 (42) | 16 (46) |
| RMCA | 13 (19) | 19 (32) | 9 (26) |
| Others | 25 (36) | 15 (26) | 10 (28) |

* p< 0,05

Data are shown as median (q25-q50) or absolute values (%)

mRS: modified Rankin Scale; NIHSS: National Institute of Health of Health Stroke Scale; ASPECTS: Alberta Stroke Program CT Score; RMCA: Right Middle Cerebral Artery; LMCA: Left Middle Cerebral Artery

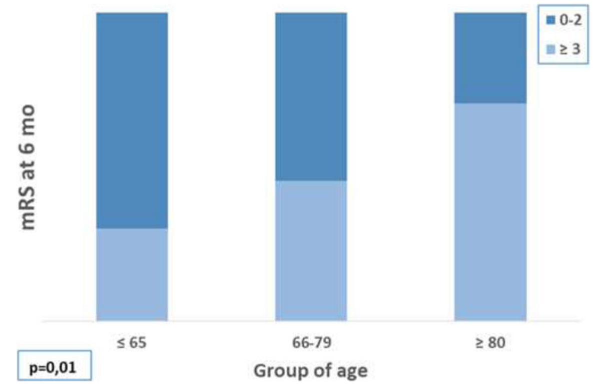

**Graph 1 (abstract 0360).** See text for description

**Table 2 (abstract 0360).** See text for description

| | ≤ 65 (n= 69) | 66-79 (n=60) | ≥ 80 (n=35) |
|---|---|---|---|
| Treatment | | | |
| IVF | 19 (28) | 18 (30) | 16 (46) |
| TRB | 18 (26) | 21 (35) | 10 (29) |
| Both | 32 (46) | 21 (35) | 9 (26) |
| Reason for TRB | | | |
| IVT failure | 32 (64) | 21 (50) | 9 (47) |
| Contraindication | 11 (22) | 16 (38) | 5 (26) |
| Out of time | 7 (14) | 5 (12) | 5 (26) |
| Neurological outcome at 6mo* | | | |
| mRS 0-2 | 42 (70) | 30 (54) | 10 (29) |
| mRS ≥ 3 | 18 (30) | 25 (46) | 24 (71) |
| Crude mortality at 6 mo* | 4 (6,7) | 14 (25) | 7 (21,2) |
| Destination upon discharge* | | | |
| Home | 47 (75) | 34 (72) | 14 (45) |
| Rehabilitation facility | 16 (25) | 13 (28) | 17 (55) |

* p< 0,05

Data are shown as absolute values (%)

IVF: IV trombolysis; TRB: endovascular thrombectomy; mRS: modified Rankin Scale

### 0361

### Frequency of brain death in acute cerebrovascular disease related mortality

M.A. Qureshi, M.R. Afzal, R. Khatri, P. Piriyawat, A. Maud, G.J. Rodriguez, S. Cruz-Flores, A.R. Vellipuram

Texas Tech University Health Sciences Center, Neurology, El Paso, United States

**Correspondence:** A.R. Vellipuram

*Intensive Care Medicine Experimental* 2018, **6(Suppl 2):**0361

**BACKGROUND.** Despite the frequent occurrence of brain death in intensive care unit, the prevalence rate of brain death in ischemic stroke (IS), intracranial hemorrhage (ICH) and subarachnoid hemorrhage (SAH) is not known at national level. In addition the prevalence of cardiac arrest in these patients is not established.

**DESIGN/METHODS.** From 2012, ICD-9-CM includes a specific code for brain death (348.82). Brain death patients were identified from the nationwide in-patient sample database for the years 2012 to 2014. Associated in-hospital cardiac arrest patients were also identified under each category. Demographics, medical comorbidities, in-hospital complications, procedures, length of stay and hospital charges were compared.

**RESULTS.** Rate of brain death was highest in SAH related mortality (21.8%, 3190 brain deaths/ 14620 in-hospital mortality), followed by ICH,

(11.5%, 6950 brain deaths/ 60475 in-hospital mortality) and ischemic stroke (3%, 1790 brain death/62285 in-hospital mortality). Majority of the patients had brain death secondary to neurological insult (ICH n=5260, 91.3%; SAH 2625 (80.6%) and IS n=1640, 89.1%) without any secondary diagnosis of in-hospital cardiac arrest.Length of stay was shorter in patients with SAH and ICH (mean 2.92 ± 4.1 days and 2.57 ± 4.2 days respectively) compared to IS patients (mean 4.93 ± 10.2 days, p < 0.001) suggesting brain death is an early event in hemorrhagic stroke.

**CONCLUSION.** Brain death in the majority of cerebrovascular patients occurs as a result of primary neurological insult without associated cardiac arrest. It occurs much earlier in hemorrhagic stroke compared to ischemic stroke.

### 0362

#### Impact of hyperoxia and hypocapnia on neurological outcome in patients with aneurysmal subarachnoid hemorrhage

K.C. Li, C.W.Y. Tam, H.P. Shum, W.W. Yan

Pamela Youde Nethersole Hospital, Department of Intensive Care, Hong Kong, Hong Kong, China

**Correspondence:** K.C. Li

*Intensive Care Medicine Experimental* 2018, **6(Suppl 2):**0362

**INTRODUCTION.** Aneurysmal subarachnoid hemorrhage (SAH) accounts for 80% of non-traumatic SAH and has high rates of death and complications.Hyperoxia and hypocapnia are associated with poor outcomes in critically ill patients with brain injuries or post-cardiac arrest status due to the independent cerebral vasoconstrictive effect. Yet, the impact of hyperoxia and hypocapnia on neurological outcome in patients with aneurysmal SAH has not been well studied.

**OBJECTIVES.** We aim to evaluate the impact of hyperoxia and hypocapnia on neurological outcome in patients with aneurysmal SAH.

**METHODS.** Patients with aneurysmal SAH who were admitted to intensive care unit from January 2011 to December 2016 were included into this retrospective single center study in a regional hospital in Hong Kong. Patients' demographics, comorbidities, radiological findings and clinical grading of SAH, $PaO_2$ and $PaCO_2$ level, Glasgow Outcome Scale (GOS) at 3 months were recorded. Logistic regression analysis was performed to assess independent predictors for poor neurological outcome, which was defined as GOS 1-3 at 3 months after initial insult.

**RESULTS.** Among 244 patients with aneurysmal SAH, 50% (122 patients) had poor neurological outcome at 3 months. Compared with those patients with good neurological outcome (GOS 4-5 at 3 months), patients with poor neurological outcome were older, had higher APACHE IV score, WFNS grade and Fisher grade, lower Glasgow Coma Scale (GCS) on day 1 and more likely to have intracranial or intraventricular hemorrhage (ICH/IVH). Hyperoxia ($PaO_2$ >200mmHg) and hypocapnia ($PaCO_2$ < 30mmHg) were more common among those with poor neurological outcome at 3 months. Logistic regression analysis indicated that hyperoxia (OR 3.698, 95%CI 1.167-11.718, p=0.026), APACHE IV score > 50 (OR 5.172, 95%CI 2.348-11.393, p< 0.001), age > 55 (OR 4.113, 95%CI 1.871-9.039, p< 0.001), presence of ICH/ IVH (OR 3.812, 95%CI 1.658-8.764, p=0.002), aneurysm involving posterior circulation (OR 3.643, 95%CI 1.454-9.172, p=0.006), Fisher grade > 2 (OR 3.555, 95%CI 1.472-8.586, p=0.005), and WFNS grade > 3 (OR 2.986, 95%CI 1.282-6.955, p=0.011) independently predicted poor neurological outcome at 3 months. On the other hand, patients who had underwent interventional radiological procedure treatment was shown to have better outcome (OR 0.375, 95% CI 0.167-0.843, p=0.018).

**CONCLUSION.** Hyperoxia but not hypocapnia was associated with poor neurological outcomes at 3 months in patients with aneurysmal SAH.

### 0363

#### Clinical predictors of outcome of acute stroke patients requiring invasive mechanical ventilation

E. de Montmollin[1,2], R. Sonneville[3,4], C. Schwebel[5], F. Philippart[6], E. Azoulay[7], C. Vinclair[3], F. Bruneel[8], Y. Cohen[9], M. Darmon[10], G. Marcotte[11], S. Siami[12], M. Gainnier[13], B. Sztrymf[14], J. Reignier[15], S. Ruckly[1], J.-F. Timsit[1,3], OUTCOMEREA Study Group

[1]UMR 1137; IAME; Université Paris Diderot, Paris, France; [2]Intensive Care Unit, Delafontaine Hospital, Saint-Denis, France; [3]Medical and Infectious Diseases Intensive Care Unit, Bichat-Claude Bernard Hospital, AP-HP, Paris, France; [4]UMR 1148; LVTS; Université Paris Diderot, Paris, France; [5]Medical Intensive Care Unit; Grenoble University Hospital, La Tronche, France; [6]Intensive Care Unit, Saint Joseph Hospital Network, Paris, France; [7]Medical Intensive Care Unit, Saint-Louis Hospital, AP-HP, Paris, France; [8]Intensive Care Unit, André Mignot Hospital, Le Chesnay, France; [9]Intensive Care Unit, Avicenne Hospital, AP-HP, Bobigny, France; [10]Medical Intensive Care Unit, Saint Etienne University Hospital, Saint-Etienne, France; [11]Surgical Intensive Care Unit, Edouard Herriot Hospital, Lyon, France; [12]Intensive Care Unit, Etampes-Dourdan Hospital, Etampes, France; [13]Intensive Care Unit, La Timone Hospital, AP-HM, Marseille, France; [14]Intensive Care Unit, Antoine Béclère Hospital, AP-HP, Clamart, France; [15]Medical Intensive Care Unit, Nantes University Hospital, Nantes, France

**Correspondence:** E. de Montmollin

*Intensive Care Medicine Experimental* 2018, **6(Suppl 2):**0363

**INTRODUCTION.** Mortality in acute stroke patients requiring ICU admission is high and seems to occur in the short-term (1, 2). Recent studies have limitations that may impact accurate identification of indicators of outcome, including single-center design and lack of adjustment for early decisions to forgo life-sustaining treatment (DFLST).

**OBJECTIVES.** To determine the predictive value of clinical parameters upon ICU admission for 30-day mortality in acute stroke patients admitted to the ICU and requiring invasive mechanical ventilation.

**METHODS.** We conducted a retrospective analysis of the French prospective multicenter OUTCOMEREA database over a 20-year period (1997-2016). We included all adult stroke patients admitted to the ICU who required invasive mechanical ventilation at any time during ICU stay. Patients with traumatic brain injury or cerebral thrombophlebitis were exluded. Primary outcome was mortality at 30 days after ICU admission. Clinical predictors of 30-day mortality were investigated using a Cox proportional hazard model. Data are presented as median (interquartile range) or numbers (percentages).

**RESULTS.** We identified 444 patients (age 68 (58-76) years, male gender 264 (59.5%) patients) from 14 ICUs. On ICU admission, the Glasgow coma scale score (GCS) was 6 (4-9) and the Simplified Acute Physiology score 2 (SAPS 2) score was 58 (46-71). Overall, 425 (95.7%) patients were intubated during the first 48 hours. Patients required vasopressors in 211 (47.5%) cases, and renal replacement therapy in 28 (6.3%) cases. DFLST occurred 3 (1-8) days after ICU admission in 152 (34.2%) patients. Withholding of care and withdrawal of care were observed in 69 (15.5%) and 83 (18.7%) cases, respectively.

At day 30, 285 (64.2%) patients had died, 133 (46.7%) of which had had a prior DFLST. Day-30 mortality according to stroke subtype was 34/52 (65,4%) for SAH, 139/187 (74.3%) for hemorrhagic stroke and 112/205 (54.6%) for ischemic stroke.

Univariate and multivariate analyses of factors associated with mortality are presented in Table 1. Factors independently associated with 30-day mortality were hemorrhagic stroke and SAH as compared to ischemic stroke, a lower GCS score on admission and a higher modified SAPS 2 (calculated without the neurological component).

**CONCLUSIONS.** In this large cohort of mechanically ventilated patients with acute stroke, prognosis was poor, with one third of the patients alive at day 30. Early mortality seems mainly driven by the type of stroke, neurological presentation and the intensity of non-neurologic organ failure, irrespective of DFLST. Functional outcomes in survivors should investigated in further studies.

**Table 1 (abstract 0363).** Prognostic factors of 30-day mortality

| Variable n (%) or median (IQR) | Total n=444 | Univariate analysis: Status at day 30 Alive n=159 | Dead n=285 | p | Multivariate model HR [95% CI] |
|---|---|---|---|---|---|
| Stroke subtype | | | | <.01 | |
| SAH | 52 (11.7) | 18 (11.3) | 34 (11.9) | . | 1.52 [1.17 - 1.99] |
| Hemorrhagic | 187 (42.1) | 48 (30.2) | 139 (48.8) | . | 1.78 [1.2 - 2.65] |
| Ischemic | 205 (46.2) | 93 (58.5) | 112 (39.3) | . | Ref |
| Reason for ICU admission | | | | <.01 | |
| Impaired consciousness | 307 (69.1) | 92 (57.9) | 215 (75.4) | | - |
| Respiratory failure | 47 (10.6) | 25 (15.7) | 22 (7.7) | | - |
| Seizure | 37 (8.3) | 23 (14.5) | 14 (4.9) | | - |
| other | 53 (11.9) | 19 (11.9) | 34 (11.9) | | - |
| GCS on admission | | | | <.01 | |
| 3-7 | 346 (77.9) | 98 (61.6) | 248 (87) | | 4.90 [2.49 - 9.65] |
| 8-12 | 57 (12.8) | 29 (18.2) | 28 (9.8) | | 2.68 [1.26 - 5.71] |
| 13-15 | 41 (9.2) | 32 (20.1) | 9 (3.2) | | Ref |
| SAPS 2 without GCS | 38 (30.5-48) | 34 (27-42) | 41 (32-52) | <.01 | 1.03 [1.02 - 1.04] |
| DFLST during first 48h | 67 (15.1) | 5 (3.1) | 62 (21.8) | <.01 | 2.30 [1.69 - 3.13] |
| Glycaemia>11mmol/L during first 48h | 117 (26.4) | 33 (20.8) | 84 (29.5) | 0.05 | 1.26 [0.97 - 1.64] |

Abbreviations: CI, confidence interval; SAH, subarachnoid hemorrhage; GCS, Glasgow coma scale score; SAPS 2, Simplified Acute Physiologic score; DFLST, decisions to forgo life-sustaining treatment

### 0364

#### Incidence, diagnosis and burden of sepsis on subarachnoid haemorrhage

B. Gonçalves[1], R. Turon[1], N. Melo[1], P. Lacerda[1], T. Santos[1], M. Prazeres[1], F. Miranda[1], P. Kurtz[1], F. Bozza[2], C. Righy[1]
[1]Paulo Niemeyer State Brain Institute, Rio de Janeiro, Brazil; [2]Fiocruz, Rio de Janeiro, Brazil
**Correspondence:** F. Bozza

*Intensive Care Medicine Experimental* 2018, **6(Suppl 2):**0364

**INTRODUCTION.** Aneurysmal subarachnoid haemorrhage (SAH) is an acute, and often catastrophic, cerebrovascular event, with high mortality and morbidity. One characteristic of the disease is the intense systemic inflammatory response, occurring in up to 83% of patients. There are few studies regarding the prevalence of systemic inflammatory response syndrome (SIRS) and sepsis and its association with outcomes.

**OBJECTIVES.** Our goal is to describe the outcomes on septic and non-septic group of patients with SAH, and the role of biomarkers on the diagnosis of sepsis.

**METHODS.** From April 2016 to January 2018, all consecutive patients admitted to the Neuro ICU of Paulo Niemeyer State Brain Institute with aneurysmal SAH were enrolled in our cohort. Clinical and laboratory data were collected during the first 14 days of hospital stay. The primary endpoint was mortality, dichotomized functional outcome (poor outcome defined as Modified Rankin Scale 4-6) at hospital discharge, and the diagnosis of SIRS and sepsis, according to the most recent criteria (Sepsis 3). We also analyzed the incidence of sepsis discarding the neurological component of the SOFA score, since SAH itself can be the cause of neurological dysfunction. Numeric were assessed using Mann-Whitney U-test and endpoints assessed with Chi-square test.

**RESULTS.** A total of 128 patients were enrolled in the study. Demographic characteristics are detailed on table 1, and baseline characteristics on table 2.

Among all patients, 106 patients (85%) developed SIRS. The incidence of infection was 82% (102 patients).

Using the current definitions (Sepsis 3), 65 (51%) patients had the diagnosis of sepsis. Excluding neurologic SOFA, the incidence of sepsis was 44%.

Mortality was 25% on the septic group and 5% on the non-septic group (p=0.001). Poor outcome occurred on 71% of the septic group and 10% of the non-septic group (p=0.00001).

Both analyzed biomarkers, procalcitonin and C reactive protein, were higher on the septic group on days one and three after admission. Results are detailed on table 3, expressed as mean (range).

**CONCLUSIONS.** Our data confirm the hypothesis that SAH is a highly inflammatory disease, with a large proportion of patients developing SIRS. In this cohort of patients with SAH, mortality, and mainly poor-outcome were more frequent in the sepsis group. Both procalcitonin and CRP were higher in septic patients, which could aid the differential diagnosis and prompt early treatment of patients at high risk of sepsis.

**REFERENCE(S)**

Festic E, Siegel J, Stritt M, Freeman WD. The utility of serum procalcitonin in distinguishing systemic inflammatory response syndrome from infection after aneurysmal subarachnoid hemorrhage. Neurocrit Care 2014;20:375-381.

Oconnor E, Venkatesh B, Mashongonyika C, et al. Serum procalcitonin and C-reactive protein as markers of sepsis and outcome in patients with neurotrauma and subarachnoid haemorrhage. Anaesth Intensive Care. 2004;32:465-470.

**Table 1 (abstract 0364).** Demographic characteristics

| | N = 128 |
|---|---|
| Median age | 55 (22-79) |
| Sex | 94 (73%) |

**Table 2 (abstract 0364).** Baseline characteristics

| | |
|---|---|
| Modified Fisher Scale 0 | 19 (15%) |
| 1 | 15 (12%) |
| 2 | 15 (12%) |
| 3 | 39 (30%) |
| 4 | 40 (31%) |
| WNFS 1 | 59 (46%) |
| 2 | 25 (20%) |
| 3 | 10 (8%) |
| 4 | 19 (15%) |
| 5 | 15 (12%) |

**Table 3 (abstract 0364).** Biomarkers on septic and non-septic patients

| Biomarker | Day | Septic | Not septic | p |
|---|---|---|---|---|
| Procalcitonin (ng/ml) | 1 | 0.34 (0.02 to 6.98) | 0.06 (0.02 to 0.18) | 0.001 |
| | 3 | 0.50 (0.02 to 11.32) | 0.31 (0.02 to 8.43) | 0.003 |
| C Reactive Protein (mg/L) | 1 | 74.7 (0.06 to 351) | 45 (0.66 to 250.2) | 0.01 |
| | 3 | 86.8 (0.7 to 350.5) | 52.3 (0.7 to 180) | 0.001 |

### 0365

#### The effect of daily fluid balance on mechanical ventilation in the subarachnoid hemorrhage patient population in the ICU

A.B. Kumar[1], O.K. Katan[1], S.R. Mehr[1], Y. Shi[2], M. Shotwell[2]
[1]Vanderbilt University Medical Center, Anesthesiology and Critical Care, Nashville, United States; [2]Vanderbilt University Medical Center, Biostatistics, Nashville, United States
**Correspondence:** A.B. Kumar
*Intensive Care Medicine Experimental* 2018, **6(Suppl 2):**0365

**INTRODUCTION.** A net positive fluid balance is associated with less ventilator free days and worse outcomes in intensive care patients especially in ARDS. The fluid prescription in post intervention aneurysmal subarachnoid hemorrhage (SAH) deviates from fluid management strategies of the general postoperative patient. Hypovolemia is a known risk factor for severe symptomatic vasospasm and frequently hypothalamic dysfunction and cerebral salt wasting syndromes compound fluid management in SAH patients.

**OBJECTIVES.** To evaluate the effect of daily fluid balance on pulmonary status and MV in the SAH population.

**METHODS.** This is an IRB approved retrospective cohort study of 726 ICU admissions for subarachnoid hemorrhage at Vanderbilt University Medical Center, a level-1 trauma and stroke referral center, between Jan 06 -Dec, 2012. We followed the patients for 14 days (vasospasm risk window) or discharge, whichever came earlier. The majority of patients had aneurysmal SAH of all Hunt and Hess Classifications. The primary outcome was the effect of fluid balance in the past 24 hours on mechanical ventilation (MV) the following day. We fitted a transition model using logistic regression to examine the association between MV and net fluid balance from the previous day conditional on patient age, gender, body mass index, diabetes mellitus, daily minimum sodium level and mechanical ventilation. All analyses were implemented using R 3.3.0 (R Foundation for Statistical Computing, Vienna, Austria).

**RESULTS.** Out of 726 admissions with SAH, 399 (55%) required MV. 275 (69%) of patiets presented on MV at admission. Those requiring MV were more likely to have traumatic SAH at presentation (22% vs 13%, p< 0.002), were more likely to have a lower Glasgow Coma Score (11 vs 15, p< 0.001), and were more likely to have acute kidney injury (12% vs 5%, p < 0.001). The cumulative fluid intake for those requiring MV was significantly higher than for those not requiring MV (33,665mL vs 20,518mL, p< 0.001), although the net fluid balance was lower (-680mL vs 0mL, p=0.03). For patients that were not on MV, each additional 1000mL of fluid loss was associated with 25.8 percent (95% CI: 17.4 - 33.4, p < 0.001) reduction in the odds of needing mechanical ventilation the following day. For patients already requiring MV, there was no significant effect of fluid loss on continuing to require MV the following day, likely highlighting the neurological status as a key determinant in the evaluation of extubation.

**CONCLUSIONS.** The effect of fluid balance is more pronounced in the non-intubated SAH patient with a negative fluid balance decreasing the odds of requiring MV the following day.

##### REFERENCE(S)

1. Fluid management in ALI and ARDS. Annals of Intensive Care. 2011; 1:16.
2. Guidelines for the Management of Aneurysmal SAH. Stroke. 2012; 43:1711-1737.
3. Management of delayed cerebral ischemia after SAH. Crit Care. 2016; 20: 277.

##### GRANT ACKNOWLEDGMENT

Departmental funding only

### 0366

#### Perimesencephalic subarachnoid haemorrhage: is there enough evidence to change our attitude?

S. Dias[1], R. Moreno[1,2]
[1]Hospital de São José, Centro Hospitalar Lisboa Central, Unidade de Cuidados Intensivos Polivalentes Neurocríticos, Lisboa, Portugal; [2]Nova Medical School (Universidade Nova de Lisboa), Lisboa, Portugal
**Correspondence:** S. Dias
*Intensive Care Medicine Experimental* 2018, **6(Suppl 2):**0366

**INTRODUCTION.** The heterogeneity of angiogram negative subarachnoid haemorrhage (SAH) patients is well established after Van Gijn et al (1985) first described the "benign" perimesencephalic (PM) form. Since then several studies have shown distinct prognoses for different forms of SAH. Nevertheless, guidelines focused on angiogram negative SAH are still missing and we treat all SAH patients roughly the same way.

**OBJECTIVES.** To analyse the outcomes and neurologic complications of PM-SAH and to assess the possibility of differentiated treatment.

**METHODS.** A systematic literature review was performed using PubMed to search for clinical studies describing outcomes and neurologic complications of PM-SAH patients in the last 6 years. Search terms included "subarachnoid haemorrhage", "subarachnoid hemorrhage", "perimesencephalic", "unknown cause", "unknown origin", "unknown etiology", "nonaneurysmal", "idiopathic", "angiogram negative".

**RESULTS.** We found 16 relevant studies (repeated cohorts and works that did not analyse separately the subgroups of angiogram negative SAH were excluded), all retrospective, that collected data since 1980, with a total of 727 PM-SAH patients.

The proportion of PM-SAH (*versus* diffuse) patients is highly variable (graph 1) which may be explained by inconsistency in establishing the diagnosis in different centres.

All studies report at least 95% of World Federation of Neurological Surgeons grading of I-III or 83,3% of Hunt and Hess I-II for PM-SAH patients.

Different definitions of vasospasm are used: 3 studies use Doppler measured velocity, 6 studies use angiography with variable and subjective criteria, and the others did not clarify the criteria used. 10 studies add clinical criteria but these are also variable. Prat *et al* reported the largest proportion of patients with radiological vasospasm (42,9%) but 0% of clinical vasospasm. Konczalla *et al* reported the largest proportion of patients with clinical vasospasm (12%) but only 1% of vasospasm dependent infarction.

Hydrocephaly was diagnosed using clinical criteria in some studies and radiological criteria in others. The largest proportion of patients with hydrocephaly requiring treatment is reported by Sahin *et al* (20,8%).

None of the studies reports seizures in PM-SAH patients.

The only two studies (Lin et al, Sprenker et al) that focused on the protocol of treatment of these patients suggest early discharge of the ICU.

**CONCLUSIONS.** Although several studies address the outcomes of PM-SAH patients, they lack consistency and neurologic complications may be overestimated. Nevertheless, in most studies the PM form of SAH seems to be a benign subtype and thus it could probably be treated outside the ICU. Prospective multicentric studies are needed to establish the correct prognosis of this disease to avoid medical complications in these patients and for a better use of limited resources.

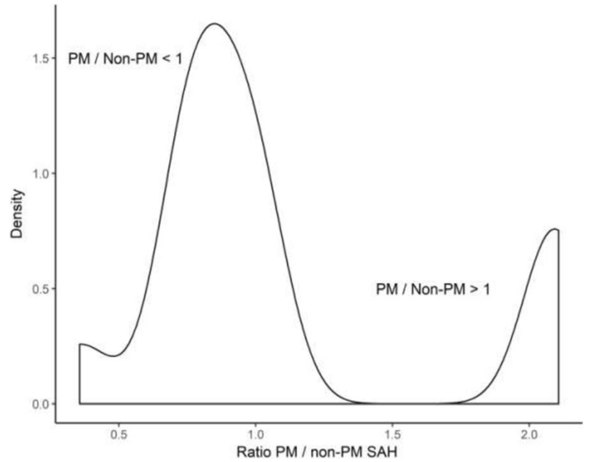

**Graph 1 (abstract 0366).** Ratio of patients with PM / non-PM angiogram negative SAH

### 0367

#### In-hospital complications following acute neurological injuries

A.R. Vellipuram, M.A. Qureshi, M.R. Afzal, A. Maud, R. Khatri, G.J. Rodriguez, P. Piriyawat, S. Cruz-Flores
Texas Tech University Health Sciences Center, Neurology, El Paso, United States
**Correspondence:** A.R. Vellipuram
*Intensive Care Medicine Experimental* 2018, **6(Suppl 2):**0367

**OBJECTIVE.** In-hospital complications following acute neurological injury has been a topic of extensive research to help reduce the morbidity and mortality among the patients. However the incidence and prevalence of in-hospital infections following acute neurological injury at the national level has never been studied. The aim of our study is to determine the frequency and prevalence of in-hospital complications among different patient groups admitted following acute neurological injury.

**DESIGN/METHODS.** We identified patients with primary diagnosis of ischemic stroke (IS), subarachnoid stroke (SAH), intracerebral hemorrhage (ICH), status epilepticus (SE), meningitis, encephalitis and traumatic brain injury (TBI) from nationwide inpatient database (2011-2014) through using the respective ICD- 9 codes. Common in-hospital complications among the above mentioned diagnoses through using their respective ICD-9 codes

**RESULTS.** Patients with primary diagnoses of IS (n=1855297), SAH (n=101576), ICH (n=254758), SE (n=190701), Meningitis (n=46067), Encephalitis (n=23839), TBI (n=1142155) were identified. In-hospital events such as myocardial infarction (MI), sepsis, pneumonia, deep venous thrombosis (DVT), pulmonary embolism (PE), urinary tract infections (UTI), and GI bleed were identified and compared among different patient groups. Patients with SE were noted to experience higher systemic complications, MI (3.6%), sepsis (11.2%), pneumonia (8.6%), DVT(1.7%), UTI (16.2%), GI bleed (0.41%). Patients admitted with meningitis had higher incidence of sepsis (20.5%), pneumonia (9.0%), DVT (2.2%), PE (1.5%) and UTI (12.7%) compared to the other groups. UTI was the most common in-hospital complication observed.

**CONCLUSIONS.** Based on our analysis, we report higher incidence of urinary tract infections among all patients admitted following acute neurological injuries. Patients with primary diagnosis of status epilepticus experienced more systemic complications compared to the other diagnoses.

### 0368

#### Some characteristics of hormonal status in patients in the acute period after spontaneous intracerebral hemorrhage

L. Tsentsiper, N. Dryagina, A. Kondratyev
Russian Polenov Neurosurgery Institute, Aneasthesiology and Intensive Care, St Petersburg, Russian Federation
**Correspondence:** L. Tsentsiper
*Intensive Care Medicine Experimental* 2018, **6(Suppl 2):**0368

**INTRODUCTION.** The incidence of spontaneous intracranial hemorrhage (SICH) in the world according to different authors is between 1.5 and 2 million a year. SICH is associated with an increased risk of endocrine dysfunction. In our opinion, the determination of neuroendocrine disorders in the acute period of various Genesis of SICH will allow to assess the adequacy of the therapy, including aimed at neurovegetative stabilization, timely correction of hormonal insufficiency and improve the quality of life of patients.

**OBJECTIVES.**

1. To assess hormonal disorders in patients in the acute phase of spontaneous intracranial hemorrhage.
2. A comparative evaluation of hormonal status during and post sedation.
3. To identify their prognostic significance revealed neuroendocrine changes.
4. Comparative analysis of hormonal status in patients after rupture of arterial aneurysm, AVM genesis and spontaneous hypertensive intracerebral hemorrhage.

**METHODS.** This study included 58 patients aged 13 to 72 years. We studied the levels of ACTH and cortisol in the morning and the evening, TSH, FT3 and FT4, prolactin, GH, men determined LH, FSH, testosterone. GCS; rating on a scale of Hunt and Hess patients with arterial aneurysm rupture were consistent with points 2-4, 2-4 class Fischer scale. All patients were subjected to sedation and analgesia (further in this article “sedation”) as therapeutic narcosis - opioid analgesic fentanyl 0.5-1 μg/kg/ h, alpha 2-adrenergic agonist clonidine 0.2-0.5 μg/kg/ h, sodium thiopental 2.4 μg/kg/ h. for the duration of 3 to 9 days.

**RESULTS.** The endocrine status of the patient was estimated depending on the functional state of the patient at the sedation and post sedation period. Our results indicate lower activity of the PAS, PTS for patients under sedation. Our studies conducted within 28 days after a SICH revealed no signs of hormonal deficiency requiring correction in the acute period.

Therapeutic sedation limits the severity of the stress response indicating that patient in sedation period had ACTH and cortisol levels lower than in post- sedation period.

**CONCLUSIONS.**

· The hormone response to intracerebral hemorrhage is different in patients after aneurysmal SAH and intracerebral hemorrhages of hypertensive genesis and caused by AVM rupture, the latter had more pronounced HP activation.

· Patients with acute spontaneous intracerebral hemorrhage were diagnosed with pituitary-adrenal system hyperfunction with daily secretion rhythm irregularities.

· Patients with negatiTve outcome in both groups during post-sedation period had more evident pituitary-adrenal system hyperfunction than those with positive outcome.

· Patients with negative outcome in both groups had FT3 level reduction (34-45%).

· After SICH men developed hypogonadotropic hypogonadism.

### 0369
### Glycaemia in patients with ischemic stroke
A. Gritsan[1], N. Dovbysh[1], A. Danilovich[2], D. Kurnosov[1], E. Korchagin[3]
[1]Krasnoyarsk State Medical University, Krasnoyarsk Regional Clinical Hospital, Anaesthesiology and Intensive Care, Krasnoyarsk, Russian Federation; [2]Krasnoyarsk Regional Clinical Hospital, Anaesthesiology and Intensive Care, Krasnoyarsk, Russian Federation; [3]Krasnoyarsk Regional Clinical Hospital, Krasnoyarsk, Russian Federation
**Correspondence:** A. Gritsan
*Intensive Care Medicine Experimental* 2018, **6(Suppl 2):**0369

**INTRODUCTION.** The development of stress hyperglycemia is one of the manifestations of the severity of the critical state. It is also known that the presence of diabetes mellitus is one of the aggravating factors affecting the course of ischemic stroke and its outcome.

**OBJECTIVES.** To assess the incidence of 1 and 2 types of diabetes mellitus and the frequency of development of stress hyperglycemia in patients with ischemic stroke.

**METHODS**. A prospective observational study was conducted in patients with ischemic stroke, who were admitted to the NICU of the Krasnoyarsk Regional Hospital during one month (the month was randomly selected). The 88 patients were included in the study. the patients were divided into 2 groups: 1 group - surviving patients (78 patients) and 2 group - died patients (10 patients). The age of patients in 1 group was 66.1 ± 10.9 years and 2 group 71.5 ± 14.8 years ($p \leq 0.05$). Patients were assessed the presence and type of diabetes mellitus in history, the level of glycemia determined by an emergency team of Ambulance, in the 1st, 3rd and 5th day. Control of blood sugar was carried out at least 6 times a day with its correction by the introduction of Insulin.

Data are given in the form M [25%; 75%], absolute values and percentages. A statistical check for the parametric data was carried out using the Mann-Whitney criterion, non-parametric χ-test data using the IBM STSS v.20 program. The changes were considered statistically significant at $p \leq 0.05$.

**RESULTS.** Patients of the 1st group had a history of diabetes of type 2 in 18 patients (23%), there was no data for type 1 diabetes. In the 2nd group of patients, there was not history of the presence of diabetes so 1 type as 2 type ($p \leq 0.05$). The number of patients with elevated blood sugar levels than the reference values was higher 1 in group - 25 (32%) patients and in 2 group - 4 (40%) patients ($p \leq 0.05$). The values of blood sugar at the study stages are presented in the table (Table 1).

The patients in 2 group had a statistically significant higher level of blood sugar at all stages of the study compared with patients in 1 group, although there were no patients in the 2nd group who had a history of diabetes.

**CONCLUSIONS.** The development of stress hyperglycemia is typical for patients with severe stroke. At the same time, we did not find the effect of having a history of diabetes mellitus on the outcome of the disease.

**Table 1 (abstract 0369).** Blood sugar level, mmol/l, M [25%; 75%

| | Ambulance | 1 day | 3 day | 5 day |
|---|---|---|---|---|
| 1 group | 8.3 [4.1; 10.3] | 4.1 [3.9; 5.1] | 5.1 [4.8; 5.6] | 5.2 [4.8; 6.8] |
| 2 group | 10.2 [5.8; 12.1] | 5.3 [4.5; 7.4] | 7.6 [5.9; 9.1] | 7.7 [6.1; 9.5] |
| p | <0.05 | <0.05 | <0.05 | <0.05 |

### 0370
### Neutrophil-to-lymphocyte ratio as a predictor of poor prognosis after intracerebral haemorrhage
A. Ramos[1], A. Dogliotti[2], D. Latasa[1], M. Perezlindo[1], J. Toledo[3], J.F. Gerber[1], C. Lovesio[1]
[1]Sanatorio Parque, Critical Care, Rosario, Argentina; [2]Grupo Oroño, Estadística y Epidemiología Clínica, Rosario, Argentina; [3]Sanatorio Parque, Neurosurgery, Rosario, Argentina
**Correspondence:** D. Latasa
*Intensive Care Medicine Experimental* 2018, **6(Suppl 2):**0370

**INTRODUCTION.** Inflammation plays a central role in secondary brain injury. Neutrophil-to-lymphocyte ratio (NLR) is an inflammation marker.

**OBJECTIVES.** The aim was to assess the relationship between NLR and poor outcome.

**METHODS.** Between January 2007 and October 2017, 102 patients with supra-tentorial ICH within 24 hours of onset of symptoms were admitted. On admission we recorded patient characteristics, clinical and laboratory findings, radiologic features and functional neurologic outcome. Poor outcome was defined as a Glasgow Outcome Scale (GOS) ≤3 at hospital discharge.The logistic regression model was used to identify if NLR was independently associated with poor outcome, including variables which showed a p value ≤0.2 and NLR regardless of the value of p.

**RESULTS.** From 102 patients admitted to intensive care unit due to acute ICH, 71 presented a poor outcome. Multivariate model was constructed by: APACHE II (p = 0.01), age (p = 0.05), Glasgow Coma Scale (p< 0.001), white blood cells counts elevated (p= 0.02), hyperglycaemia (p< 0.001), hipomagnesemia (p= 0.17), NLR (p= 0.34) and normal pupils (p= 0.04). There was no relation between NLR and poor outcome (p=0.55) in the multivariate model. Instead, we found an association between hyperglycaemia and older age with the end point (p=0.02 and 0.04, respectively).

**CONCLUSIONS.** We found no relationship between NLR and poor outcome in patients with ICH.

### 0371
### Frequency of severe hypocapnia in patients in the acute period of non-traumatic subarachnoid hemorrhages with various clinical outcomes
A. Solodov[1], E. Mekhia Mekhia[1], S. Petrikov[1], V. Krylov[2]
[1]Sklifosovsky Research Institute, Neurosurgical ICU, Moscow, Russian Federation, [2]Sklifosovsky Research Institute, Neurosurgery Department, Moscow, Russian Federation
**Correspondence:** A. Solodov
*Intensive Care Medicine Experimental* 2018, **6(Suppl 2):**0371

**INTRODUCTION.** Hyperventilation and hypocapnia lead to a cerebral vasospasm. Severe hypocapnia may be accompanied by a disturbance of cerebral blood flow in critically ill patients with non-traumatic subarachnoid hemorrhages (SAH)

**OBJECTIVES.** To determine the effect of severe hypocapnia on the outcomes in critically ill patients in acute period of non-traumatic SAH after cerebral aneurisms rupture

**METHODS.** 85 patients in acute period of SAH who required invasive mechanical ventilation for more than 6 hours after operations enrolled in the study. Mean age was 51±12 years, male/female - 44/41. The patients with grade II on Hunt-Hess scale were 18 (21.2%), grade III - 47 (55.3%), grade IV - 16 (18.8%), grade V - 4 (4.7%). 17 patients (20%) were with grade III on the Fisher scale, 68 patients (80%) - grade IV. Good outcomes on Glasgow Outcome Scale (GOS 1-2) were in 43 patients (50.6%), severe neurologic deficit (GOS 3) - in 7 patients (8.2%), poor outcomes (GOS 4-5) - in 35 patients (41.2%).

**RESULTS.** Medians of the $PaCO_2$ levels did not differ between the groups of patients: GOS 1-2 - 32 (28.1;36.2) mmHg, GOS 3 - 33.1 (28.8;34.5) mmHg, GOS 4-5 - 32.2 (28.6;36.4) mmHg. Episodes of $PaCO_2$ reduction to 33 mmHg and less were detected in 29 patients (67.4%) in the GOS 1-2 group, in 5 patients (71.4%) in the GOS 3 group, in 26 patients (74.3%) in the GOS 4-5 group. Severe hyperventilation ($PaCO_2$ < 30,1 mmHg) in 1-2 days after surgery were most often observed in patients with poor outcomes (GOS 4-5), in 21 patients (60%). Severe hypocapnia was noted in 18 patients (41.9%) in the GOS 1-2 group and in 3 patients (42.9%) in the GOS 3 group.

**CONCLUSIONS.** Frequency of severe hypocapnia ($PaCO_2$ < 30.1 mmHg) in 1-2 days after surgery in patients with SAH with poor outcomes (GOS 4-5) higher on 18.1% compared patients with good outcomes (GOS 1-2). Severe hypocapnia in acute period of SAH is one of predictors of poor outcomes in patients with high risk of cerebral vasospasm.

#### 0372

#### Maintenance of cerebral perfusion pressure (CPP) determines neurology - a traumatic brain injury (TBI) model in swine

T. Datzmann[1], T. Kapapa[2], S. Röhrer[2], S. Unmuth[1], R. Mathieu[3], S. Mayer[3], C. Hartmann[1], U.M. Mauer[3], T. Merz[1], O. McCook[1], P. Radermacher[1], B. Nussbaum[4], M. Wepler[1]
[1]University Hospital, Institute for Anesthesiological Pathophysiology and Process Engineering, Ulm, Germany; [2]University Hospital, Department of Neurosurgery, Ulm, Germany; [3]Federal Army Hospital, Department of Neurosurgery, Ulm, Germany; [4]University Hospital, Department of Anesthesiology, Ulm, Germany
**Correspondence:** M. Wepler
*Intensive Care Medicine Experimental* 2018, **6(Suppl 2):**0372

**INTRODUCTION.** A major cause of TBI in particular in the elderly and/or cardiovascular co-morbid patient is acute subdural hematoma (ASDH) [1]. Therefore, we established a long-term, resuscitated porcine model of ASDH-induced TBI in human-sized pigs comprising brain multimodal monitoring.

**METHODS.** Eleven adult, anesthetized, mechanically ventilated and surgically instrumented pigs (body weight 65±8 kg) with human-like coagulation [2] underwent TBI induced by trepanation above the left parietal cortex and subdural injection of 20 mL of blood [3]. After induction of ASDH swine underwent intensive care therapy according to the guidelines of TBI management [4] for up to 54h. Monitoring included measurement of carotid flow (Doppler ultrasound probe), intracranial (ICP) and cerebral perfusion pressure (CPP), cerebral tissue $O_2$ partial pressure ($PtcO_2$) and intracerebral tissue metabolites (micro-dialysis for glutamate, lactate, pyruvate, glucose) together with systemic hemodynamics, gas exchange, acid-base status, and metabolism. Crystalloid fluid resuscitation and continuous i.v. norepinephrine was titrated to maintain mean arterial pressure (MAP) at pre-ASDH levels and CPP > 75 mmHg. Every 12h, after reduction of anesthetic depth, the veterinary modified Glasgow Coma Score (MGCS, range 3-18) [5] was used for neurological assessment. Immediately post-mortem, the brain was harvested for immune blotting, histology, and immunohistochemistry.

**RESULTS AND CONCLUSION.** In n = 9 of n = 11 swine, experiments could be carried out for 54 h and maintenance of target MAP and CPP was associated with normal tissue metabolite concentrations and, ultimately, normal MGCS (13 ± 2). In 2 animals, secondary cardiovascular failure unrelated to the neurosurgical instrumentation caused a marked drop of CPP resulting in neurological failure, ultimately necessitating termination of the experiments.

##### GRANT ACKNOWLEDGMENT

Supported by the DFG (CRC 1149 GEROK Martin Wepler).

### Rehabilitation & outcomes

#### 0373

#### Hygiene and safety for both your patient and yourself: how to improve the compliance with three of the five moments of hand hygiene on the ICC unit of the VU Medical Centre?

R. Al, A. van Wijk, S. Evelein-Brugman, T. van Galen
VU University Medical Center, Intensive Care, Amsterdam, Netherlands
**Correspondence:** R. Al
*Intensive Care Medicine Experimental* 2018, **6(Suppl 2):**0373

**INTRODUCTION.** Healthcare related infections have a severe negative influence on morbidity and mortality rates in intensive and Critical Care(ICC) units. The most simple and effective way to prevent cross-infections is by practicing hand hygiene[1]. Guidelines[2]for hand hygiene have been available for years, yet compliance remains poor. Recent studies [3]show that healthcare workers practice hand hygiene in less than 40% of the 5 moments for hand hygiene. This suggests that healthcare related infections can be lowered by 40-50% if compliance improves. Over the years '14-'17 we conducted surveys on healthcare workers in the ICC unit of the VUmc where the mean compliance with the 5 moments rose from 39%('14) up to 54%('16). The goal of VUmc is a mean compliance of >80%.

**OBJECTIVE.** Which barriers are present for achieving > 80% hand hygiene compliance with the 5 moments at our ICC?

**METHODS.** We performed a multimodal approach. Firstly, we trained the healthcare workers regarding the 5 moments for hand hygiene. We organized an Hand Hygiene Day(HHD) on the ICC unit, in which we made the healthcare workers aware of the 5 moments for hand hygiene. We monitored the compliance with 3 of the 5 moments by scoring on an application and giving direct feedback. In follow-up compliance of hand hygiene was measured by one hour of weekly floor surveillance. In addition to the HHD and surveillance, we also conducted quantitative research of the population of intensive care nurses by handing out surveys regarding their opinion and work flow on hand hygiene.

**RESULTS.** We found that even with an optimum in support and coaching, compliance with the 3 surveyed moments for hand hygiene during the Hand Hygiene Day was 89% among the healthcare workers in the ICC unit. The ongoing monitoring after the HHD indicates that compliance with moment five is the best, whereas moment one is poor and hand hygiene before a clean or aseptic procedure and requires attention.

The mean compliance with the five moments a month after is 64%, which is a 10% increase compared to the results obtained prior to the HHD.

**CONCLUSIONS.** Improving compliance with the 5 moments of hand hygiene can be achieved by implementing multimodal strategies[4,5] such as actively addressing compliance, increased awareness by education, instruction and coaching. These to help healthcare workers gain insight into workflow barriers and imperfections in compliance. A striking observation is the impossibility for achieving 100% hand hygiene even with 1 to 1 supervision. We would suggest additional likely beneficial measures. These include easier access to proper material as well as using a combination of general hygiene measures and isolation guidelines per patient.

The question remains, can we maintain this compliance with our strategy?

1. Knot, R. 2010.Theory of Planned Behavior
2. WHO. 2009.Hand Hygiene
3. Sax. 2007.Five moments for hand hygiene
4. Huis, 2012.HH improvement strategies
5. Doronina, 2016 Compliance

#### 0374

#### Effects on oral intake by supporting HDU patients with a nutrition assistant

A.J.M. Dobber-Lambalk, A.P. Bouterse-Dekker, T. van Galen
VU University Medical Center, Intensive Care, Amsterdam, Netherlands
**Correspondence:** A.J.M. Dobber-Lambalk
*Intensive Care Medicine Experimental* 2018, **6(Suppl 2):**0374

**INTRODUCTION.** Malnutrition is a problem for 25% up to 40% of patients. Therefore a good nutritional status is important for reducing complications, LOS and mortality[1, 2]. At the intensivist led HDU we agreed all (ICU step down) patients are at risk of malnutrition due to disease, metabolic need, muscle atrophy and reduced oral intake or disturbed appetite. Although feeding guidelines are clear, achieving an optimum nutritional status in daily practice is difficult[3, 4]. Due to a busy workflow HDU nurses are not always able to prioritize oral nutrition above other tasks.

**OBJECTIVES.** To clarify if oral intake improves by embedding a nutritional assistant (NA) at the HDU ward.

**METHODS.** Most HDU patients start with tube feeding at the ICU. Often HDU patients receive tube feeding balanced with oral intake. HDU nurses perform daily screenings and evaluate this with the doctor and dietitian. The NA makes multiple rounds per day, provide meals/snacks that are as close to the patients flavor and appetite and stimulates the patient to eat or drink. Before the NA started, oral nutrition was served by the HDU nurse, but usually without structured stimulus or checking of actual intake.

Two periods were selected: one year before and after start of the NA. Registration methods stayed the same. Because the NA only works during daytime (8:00-16:00), we only included meals and snacks that were consumed during these hours. The collected data was cleared up from all with exception of protein containing meals or snacks with a minimum of 5 grams of protein. Providing at least 5 grams of protein per meal or snack is one of our basic nutritional goals. For all energy and/or protein deficit that remains, there is supplemental (nightly) tube feeding.

**RESULTS.** With no major changes in HDU casemix and no major changes in staffing as well as nursing workflow, the oral protein intake increased with almost 30% after embedding the NA.

**CONCLUSIONS.** We believe embedding a nutrition assistant contributed strongly to our increase in oral protein intake. Although most of the energy intake is provided by tube feeding, the increase in oral intake is tangible. With an increased protein intake, we think it's plausible that the overall oral energy intake increased, although in this study we only focused on protein intake. We did not measure an improved nutritional status for all admitted HDU patients, but this study showed us that a dedicated assistant is able to stimulate patients to eat more and/or better in a way that also supports nurse care.

**REFERENCE(S)**

[1] Carlos Seron-Arbeloa et al. *Enteral Nutrition in Critical Care*. Clin Med Res. 2013 Feb; 5(1): 1-11.

[2] Dvir, D et al. *Computerized energy balance and complications in critically ill patients: an observational study*. Clin Nutr. 2006 Feb;25(1):37-44.

[3] Guideline *Early recognition of malnutrition*. National document. www.stuurgroepondervoeding.nl/ziekenhuis

[4] Guideline ICU nutrition. Local document.

**GRANT ACKNOWLEDGMENT**

None.

### 0375

#### Short-term and long-term impact of training for ICU nurses on incompatibilities and drug interactions

M.S. Kochat[1], F. Ferhi[2], M. Hedhili*[3], M. Mokni*[3], M. Sboui[1], K. Ben Jazia[2], C. Bachraoui[1]

[1]Faculty of Medicine Ibn Al Jazar Sousse, Sousse, Tunisia; [2]Farhat Hached Hospital, Anesthesia, Sousse, Tunisia; [3]Pharmacological Faculty of Monastir, Monastir, Tunisia

**Correspondence:** M.S. Kochat

*Intensive Care Medicine Experimental* 2018, **6(Suppl 2):**0375

**INTRODUCTION.** Patients in the intensive care unit are at increased risk of potential drug interactions due to the complexity of pharmacotherapy and the restriction of routes of administration.

**OBJECTIVES.** This study aimed to determine whether immediate improvements in knowledge of incompatibilities and drug interactions in ICU are maintained at 3 and 6 months after training for nurses with different levels of experience.

**METHODS.** Twenty-six ICU nurses from different levels of experience completed a training course on incompatibilities and drug interactions in intensive care by resuscitators and pharmacists. This training included lectures, experimental lab sessions and clinical case analysis. Each nurse was assessed before, after, at 3 and 6 months using a previously validated scale. The clinical impact of this training was judged from the register of pharmacovigilance declarations.

**RESULTS.** Nurses´ knowledge scores improved after training (42% vs. 94%, $p < 0.05$). Three months after the training there was an apparent decline in knowledge but an overall improvement over the baseline test (42% vs. 79%, $p < 0.05$). The group of novice nurses showed the greatest improvement in knowledge after training. At 6 months, the rate of correct answers was stable compared to the evaluation performed at 3 months (79% vs. 76% $p = 0.4$). No pharmacovigilance declaration was made after the training and during the study period.

**CONCLUSIONS.** Nurses´ knowledge of incompatibilities and drug interactions in ICU improved immediately after our training program. Although decreasing 3 months after it remains stable at 6 months. The novice nurses had the greatest benefit.

**REFERENCE(S)**

1. Kanji S, Lam J, Johanson C, Singh A, Goddard R, Fairbairn J, et al. Systematic review of physical and chemical compatibility of commonly used medications administered by continuous infusion in intensive care units. Crit Care Med. 2010;38:1890-8.
2. Kanji S, Lam J, Goddard RD, Johanson C, Singh A, Petrin L, et al. Inappropriate medication administration practices in Canadian adult ICUs: a multicenter, cross-sectional observational study. Ann Pharmacother. 2013 ; 47:637-43.
3. Boehne M, Jack T, Köditz H, Seidemann K, Schmidt F, Abura M, et al. In-line filtration minimizes organ dysfunction: new aspects from a prospective, randomized, controlled trial. BMC Pediatr. 2013; 13:21.

### 0376

#### Predictors and outcomes of delirium within a multi-specialty United kingdom based ICU: a single centre retrospective cohort study

D. McWilliams[1], F. Howroyd[1], C. Snelson[2]

[1]Queen Elizabeth Hospital NHS FT, Birmingham, United Kingdom, [2]Queen Elizabeth Hospital NHS FT, Critical Care, Birmingham, United Kingdom

**Correspondence:** D. McWilliams

*Intensive Care Medicine Experimental* 2018, **6(Suppl 2):**0376

**INTRODUCTION.** Incidence of delirium ranges from 45-87% in ICU, with higher levels seen in specialised ICU's and patients mechanically ventilated [1]. When present, ICU related delirium is an independent predictor of mortality and morbidity and is associated with a prolonged hospital and ICU length of stay [2]. Early, structured rehabilitation in the ICU has been found to improve delirium status, functional mobility and length of stay [3]. We recently completed a trial of early and structured rehabilitation within our ICU demonstrating a greater intensity of physical rehabilitation than that provided as standard care in other previous trials [4].

**OBJECTIVES.** To evaluate the incidence of delirium in a critical care unit with an established and effective early rehabilitation service and identify associated risk factors and short term outcomes.

**METHODS.** We performed a retrospective analysis of patients recruited to a previous trial of early and structured rehabilitation between June 2016 and September 2017 and surviving to hospital discharge. We defined delirium as having a positive CAM-ICU assessment at any point during the ICU stay. Data was analysed using the students t-test.

**RESULTS.** 87 patients were included in the analysis, of which 32 (34%) developed delirium within the ICU. Age and sedation were significantly associated with the incidence of delirium (see table. 1) and patients who developed delirium were slower to mobilise (10.8 vs 8.9 days, $p< 0.05$), ventilated for longer (15.5 vs 11.4 days, $P< 0.05$) and had lower Barthel scores at hospital discharge (76.6 vs 89.6, $p=0.015$). (table. 2).

**CONCLUSIONS.** Despite an established and effective rehabilitation service, delirium remains a common complication in our ICU. Older age and longer periods of sedation are significantly associated with development of delirium. When present, delirium is associated with restrictions to early mobilisation, prolonged periods of mechanical ventilation and reduced function at the point of hospital discharge. Further work is required to explore strategies to prevent or minimise the impact of delirium within rehab active ICU´s.

REFERENCE(S)

1. Cavallazzi et al (2012) Delirium in the ICU: an overview. Ann Intensive Care 2(49)
2. Elliott, S. R. (2014) ICU delirium: A survey into nursing and medical staff knowledge of current practices and perceived barriers towards ICU delirium in the intensive care unit. Intensive and Critical Care Nursing, 30 (6), pp 333 - 338.
3. Needham, D. M. and Korupolu, R. (2015) Rehabilitation Quality Improvement in an Intensive Care Unit Setting: Implementation of a Quality Improvement Model. Topics in Stroke Rehabilitation, 17 (4) pp. 271 - 281.
4. McWilliams et al (2018) Earlier and enhanced rehabilitation of mechanically ventilated patients in critical care: A feasibility randomised controlled trial. Journal of critical care. 44: pp. 407-412

GRANT ACKNOWLEDGMENT

n/a.

**Table 1 (abstract 0376).** Baseline

| | No delirium (n=55) | Delirium (n=32) | p |
|---|---|---|---|
| Age (years) | 54.2 | 63/1 | 0.009 |
| SOFA | 8.8 | 10.3 | 0.148 |
| APACHE II | 16.2 | 18.1 | 0.173 |
| Charlson | 0.8 | 1.1 | 0.21 |
| Sedation Days | 7.1 | 9.7 | 0.0139 |

**Table 2 (abstract 0376).** Outcomes

| | No Delirium (n=55) | Delirium (n=32) | p |
|---|---|---|---|
| Time to mobilise (days) | 8.9 | 10.8 | 0.049 |
| Median Manchester Mobility Score at ICU d/c | 6 | 6 | 0.90 |
| Vent days | 11.4 | 15.5 | 0.035 |
| ICU LOS (days) | 19.9 | 23.9 | 0.18 |
| Hospital LOS (days) | 32.1 | 40.1 | 0.096 |
| Barthel hospital d/c | 89.6 | 72.6 | 0.014 |

### 0377

### Current situation of rehabilitation in a polyvalent ICU before the implementation of a program of early mobilization

M.D.M. Fernandez[1], M. Alvarado[2], A. García[2], N. Carrasco[2], A. Girbau[2], M. Salamero[2], J. Trenado[2], I. Sandalinas[2]

[1]Hospital Universitari Mútua Terrassa, ICU, Terrassa, Spain; [2]Hospital Universitari Mútua Terrassa, Terrassa, Spain

**Correspondence:** M.D.M. Fernandez

*Intensive Care Medicine Experimental* 2018, **6(Suppl 2):**0377

**INTRODUCTION.** Survivors of critical illness frequently experience muscle weakness and relevant functional impairment after intensive care unit (ICU) discharge. This study aimed to evaluate current of mobilization practice in ICU patients with more than 72 hours of mechanical ventilation.

**METHODS.** Unicenter prospective observational study from September 2016 to July 2017. We enrolled patients under MV longer than 72h and measure the baseline functional level, ICU-acquired weakness (ICUAW) and functional status at hospital discharge. We recorded the mobilization related adverse events.

**RESULTS.** We recruited 30 patients (63±12 years; 70% men), mean Acute Physiology and Chronic Health Evaluation II (APACHE II) 16±5. Mean time of ICU stay and hospital stay were 23 and 32 days respectively. Mean time to ask for rehabilitation evaluation was 8±4 days after ICU admission. Finally, rehabilitation began 10±5 days after admission to the ICU. 11 (37%) patients were mobilized with an endotracheal tube and 19 (63%) with endotracheal tube initially and after with tracheostomy tube. Strength was assessed at ICU discharge and 23 patients (80%) had ICU-acquired weakness (Medical research Council Manual Muscle Test Sum Score (MRC-SS) score 48< 60).

The functional level obtained during sessions was sitting at the edge of the bed (n= 24, 80%), standing at the bedside (n=10, 33%) and walking (n=5, 17%). Not adverse effects during rehabilitation therapy were reported. ICU and hospital mortality were 27% and 33% respectively.

The myopathic patients discharged from the ICU had higher mortality at day 90 ($p$ = 0.002).

**CONCLUSIONS.** Early mobilization was uncommon in our ICU. Most of our patients discharged from the ICU had developed ICU-acquired weakness with higher mortality at day-90 in most severe myopathic ones. Implementation of an early mobilization protocol is urgent and requires a cultural change.

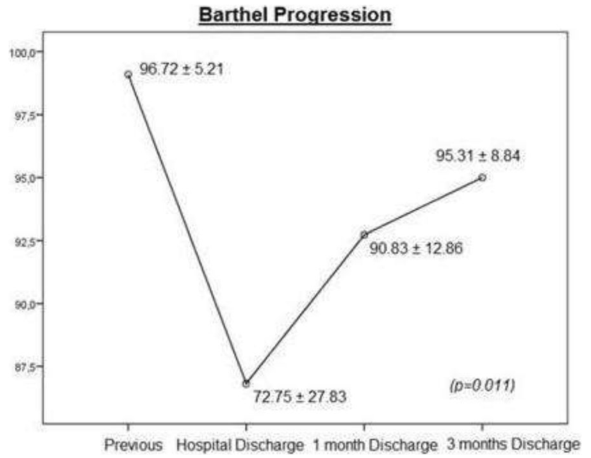

**Fig. 1 (abstract 0377).** Barhel Progression]

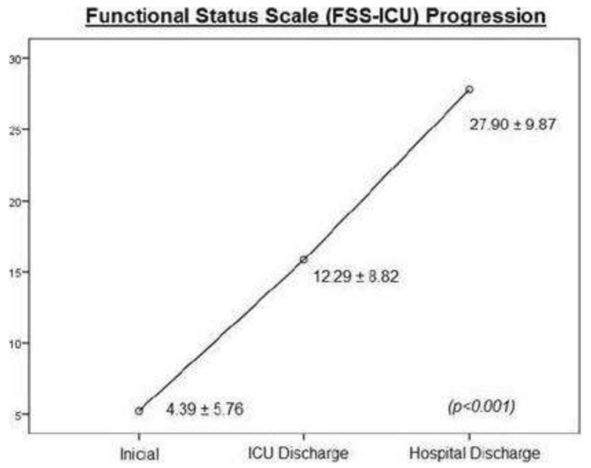

**Fig. 2 (abstract 0377).** FSS-ICU Progression

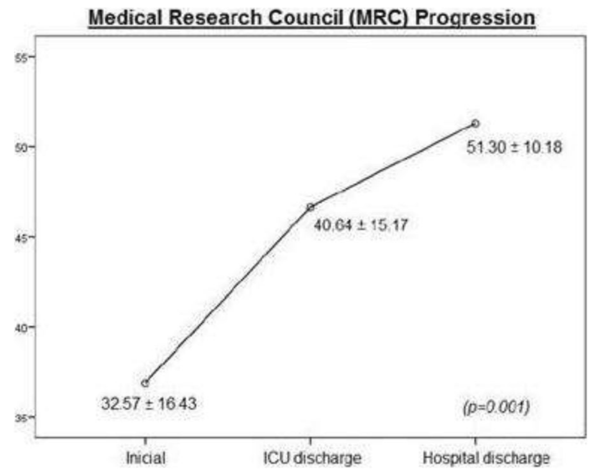

**Fig. 3 (abstract 0377).** MRC-Progression

### 0378

#### Worldwide qualitative investigation of nursing after death rituals

J. Benbenishty[1], M.B. Bennun[2], R. Lind[3]
[1]Hadassah Hebrew University Medical Center, Jerusalem, Israel, [2]Belinson Medical Center, ICU, Petuch Tikva, Israel, [3]Norges Arktiske Universitet, Campus Harstad Institutt for helse- og omsorgsfag, Health Sciences, Tromso, Norway
**Correspondence:** J. Benbenishty
*Intensive Care Medicine Experimental* 2018, **6(Suppl 2):**0378

**INTRODUCTION.** Worldwide, both religious and non-religious groups have developed rituals to help provide structure and support to those who mourn and to mark the death of the deceased. Care of the dying is a significant component of nursing practice particularly in hospitals. Nurses who work in certain areas like oncology, intensive care unit (ICU) face the care of the dying, more so than other units. No literature was found regarding country multi culture review of nursing care in the after death practices.

**OBJECTIVES.** Worldwide investigation of ICU nursing care during after death rituals

**METHODS.** Multi country nurse investigators researched by using Prospective qualitative interviews of ICU nurses describing the after death care of their patients and delivery of bereavement care to family as well as the reasoning behind the ritual.

**RESULTS.** 15 nurses from 18 countries from Scandinavia through Mediterranean to African regions were interviewed. Themes found included much about the physical care of the bodies, emphasis on dignity; Family presence and composure of nurse regulating the ritual;Follow up care was described in Northern European ICUs; Spiritual care was provided in diverse fashions including bible reading, insuring a religious leader was present and others. Protocols directing specific care policies were discussed;

Introspective feelings and beliefs were exposed.

**CONCLUSIONS.** This is the first examination of after death rituals investigated in Europe. Nurses can learn from other cultures in order to be empathetic and sensitive to others at this challenging time. This work can demonstrate several models of after death care

##### REFERENCE(S)

none

##### GRANT ACKNOWLEDGMENT

none

### 0379

#### Freedom from physical restraints in ICU: result from a scoping review

E. Mattiussi, M. Danielis, A. Froncillo, A. Palese
Università degli Studi di Udine, Udine, Italy
**Correspondence:** E. Mattiussi
*Intensive Care Medicine Experimental* 2018, **6(Suppl 2):**0379

**INTRODUCTION.** Physical restraint (PR) has been defined as "any action or procedure that prevents a person's free body movement to a position of choice and/or normal access to his/her body by the use of any method that is attached or adjacent to a person's body and that he/she cannot control or remove easily". To date several studies have been performed in acute and chronic settings but little data is available on ICUs.

**OBJECTIVES.** To map all the relevant literature focusing on the use, prevention ad avoidability of PR inside ICU setting.

**METHODS.** A systematic scoping review was performed.

1) The following databases were examinated: PubMed, Scopus, the Cochrane Library, CINHAL and TripDatabase.
2) Different key words were matched to find any other relevant literature.
3) All references were considered to find other studies of interest.
4) Two independent reviewers screened all eligibile articles to meet inclusion criteria.
5) All studies were individually analysed and all data extracted by using a semi-structured form. 6) Literature selected was summarised with both qualitative and quantitative method to find out cathegories and themes.

**RESULTS.** On a total of 44 studies identified the main areas explored by literature were:

a) *prevalence* of PR in ICU;
b) *policies and guidelines* focusing on actual strategies aiming at preventing the use of PR in ICU;
c) the *determinants* and the *consequences* of PR use;
d) patients, caregivers and healthcare professionals' *perceptions*.

The prevalence ranges from 0% to 100% depending on the definition of PR considered and on organizational aspects of ICU (i.e. nurse/patient ratio). There were few policies across countries guiding the use of PR in ICUs. The main determinants of the PR use aimed at assuring patients safety: the risk of device removal, the cognitive state of patients and the risk of fall. The consequences of PR were: direct damages (i.e. skin sores) and indirect damages (i.e. patient's agitation and device removal). Most of patients admitted in ICU reported that PR determined anxiety and frustration; some others did not remember or understood its use. Personnel's perception varied on their knowledge and skills about PR use.

**CONCLUSIONS.** As a novel approach for mapping relevant literature on a topic, we focused in performing a broad study of literature concerning the use, prevention and avoidability of PR in ICUs. The results confirmed that there should be a continuous updating of knowledge about PR in ICU, both to ensure patients' outcomes and to improve knowledge and skills of health care personnel.

##### REFERENCE(S)

Arksey, H, O'Malley, L (2005). Scoping studies: towards a methodological framework. International Journal of Social Research Methodology, 8:19-32.

### 0380

#### The effect of an informatics system on use of informal/ad hoc critical care nursing notes as an adjunct to formal systems of communication, work flow support and documentation in a central London teaching hospital

H. Rook, R. West, C. Bell, F. Master, R. Mehta, I. Salako, S. Shah, P. Hopkins
King's College Hospital, London, King's Critical Care, London, United Kingdom
**Correspondence:** H. Rook
*Intensive Care Medicine Experimental* 2018, **6(Suppl 2):**0380

**INTRODUCTION.** We have conducted a mixed methods observational analysis of communication, work flow and information recording within an adult critical care service before and after the deployment of a 'best of breed' informatics system (Intelliview Critical Care and Anaesthesia-ICCA, Philips HealthCare). Here, we describe the impact of ICCA on the format and content of informal transient hand written notes by critical care nurses outside the formal medical record and the attitude of nurses to such notes.

**OBJECTIVES.** To assess the impact of a clinical information system on the use of informal notes made by critical care nurses.

**METHODS.** Centralized HRA approval for non-participant observation, surveys and recording of anonymised data was obtained ICU bedspaces were reviewed sequentially on each of three critical care units until 250 had been reached, recording the presence of informal notes and asking the allocated nurse whether they were utilising such notes. Notes were analysed for content and nurses surveyed to record their attitude to such notes. On two units, the exercise was repeated 6 months after deployment of the ICCA system, with a third ´control´ unit remaining on paper.

**RESULTS.** 1500 bed spaces were successfully reviewed at handover on three intensive care units before and after the deployment of an informatics system. The commonest form of informal documentation were a paper hand towel (52%), paper medical continuation sheets (24%), the back of other paper documentation or blood gas slips (20%) or by personal note books (8%). Common types of information recorded included task lists; listing questions and information for handover (table 1). Patient identifiers were present on 8% of informal notes. Informal notes were present in 72.7% of bed spaces before ICCA implementation and in 49.2% of bed spaces afterwards. The content of informal notes also changed after ICCA deployment, with less recording of medication/administration reminders; information for handover and metadata, In a survey of attitudes to informal notes, nurses listed speed of notation, immediate availability of informal notes, deficiencies in paper based/ electronic systems; control of visibility and personal reassurance as the main benefits of informal notes. The main disadvantages were risk of breach of confidentiality; risks of wrong patient identification; and invisibility of information in the investigation of adverse events or errors.

**CONCLUSIONS.** To our knowledge, the use of informal and transient notes for communication and work flow support has not previously been reported. Further work on the use of informal communication systems is needed to better understand deficiencies in more formal methods of documentation and workflow support whether electronic or paper-based and to assess the risk-benefit through use of such notes.

GRANT ACKNOWLEDGMENT
PH, RW, HR were supported by an NHS Digital Award.

**Table 1 (abstract 0380).** Content Analysis of Informal Nursing Notes

| Information | Before ICCA | After ICCA | Control |
|---|---|---|---|
| Task listing/support | 407/545 (74.6%) | 179/246 (72.7%) | 143/179 (79.9%) |
| Queries to ask NIC/TL | 211/545 (38.7%) | 107/246 (43.5%) | 89/179 (49.7%) |
| Queries to ask other professions | 366/545 (67.2%) | 185/246 (75.2%) | 122/179 (68.2%) |
| Medication/administration support | 108/545 (19.8%) | 11/246 (4.5%) | 47/179 (26.3%) |
| New information pending formal documentation | 42/545 (7.7%) | 3/246 (1.2%) | 41/179 (22.9%) |
| Key metadata | 56/545 (10.3%) | 12/246 (4.9%) | 23/179 (12.8%) |
| Handover | 301/545 (55.2%) | 92/246 (37.4%) | 122/179 (68.2%) |
| Other | 29/545 (5.3%) | 22/246 (8.9%) | 19/179 (10.6%) |

### 0381

#### Sedation assessment and management in ICUs in Australia and England

C. Green[1], A. Jackson[2], A. Macey[1], C. Boulanger[2], V. Plummer[3,4], R. Endacott[3,5]
[1]Peninsula Health, Intensive Care Unit, Frankston, Australia; [2]Royal Devon and Exeter Hospital, Intensive Care Unit, Exeter, United Kingdom; [3]Monash University, School of Nursing and Midwifery, Melbourne, Australia; [4]Peninsula Health, Frankston, Australia; [5]Plymouth University/ Royal Devon and Exeter Hospital, Clinical School, Exeter, United Kingdom
**Correspondence:** R. Endacott
*Intensive Care Medicine Experimental* 2018, **6(Suppl 2):**0381

**INTRODUCTION.** The goal of sedation, with regular orientation, is often not achieved in ICU (1,2) despite evidence that sedation stops are safe (3). Careful assessment and management of sedation is essential in order to enable timely weaning and extubation. Responsibility for sedation decisions is different in England and Australia; it is unclear whether this influences achievement of optimal sedation.

**OBJECTIVES.**

1. To examine documentation of sedation and extubation plans in similar ICUs in England and Australia
2. To explore factors associated with assessment and management of sedation in ICU patients.

**METHODS.** Point prevalence survey to review sedation plans for all adult patients (n=234) in two ICUs at 1600hrs 3 days per week in January, April, July and October. Patients with a tracheostomy, receiving short-term ventilation, intubated less than 24 hrs previously or paralysed for therapeutic cooling were excluded. Data collection instrument was designed to capture workload, skill mix and sedation management. The primary outcome measure was sedation hold.

**RESULTS.** Mean age was 59.09 years (SD 16.38); mean ICU length of stay was 6.34 days (SD 5.97) and mean ventilation time was 118.19

hours (SD 136.97).A RASS target was documented for 54.7% of patients (n=128) and 46.6% of patients (n=109) did not have a sedation hold. Sedation hold was significantly associated with site ($x^2$ 58.3, df 1, *p*=0.00), and presence of an extubation plan ($x^2$ 66.20, df 2, *p*=0.00). Sedation hold was significantly correlated with ventilation hours (*r*=+0.27, *p*=0.00) and ICU length of stay (*r* =+0.13, *p*=0.00), with sedation hold more likely for patients who had longer ventilation hours and longer ICU stay. There were incidents of sub-optimal sedation in both countries.

**CONCLUSIONS.** Pilot study using the instrument with patients in both countries has demonstrated utility in detecting factors associated with sub-optimal sedation. The next stage of the work is to refine the instrument to detect more subtle differences, particularly relating to nurse skill mix. The final stage will be to use outcomes to plan an intervention study.

**REFERENCE(S)**

1. Borkowska, M., Labeau, S., Schepens, T., Vandijck, D., Van de Vyver, K., Christiaens, D., Lizy, C., Blackwood, B. and Blot, S.I., 2018. Nurses' Sedation Practices During Weaning of Adults From Mechanical Ventilation in an Intensive Care Unit. *American Journal of Critical Care*, *27*(1): 32-42.
2. Richards-Belle, A., Canter, R.R., Power, G.S., Robinson, E.J., Reschreiter, H., Wunsch, H. and Harvey, S.E., 2016. National survey and point prevalence study of sedation practice in UK critical care. *Critical Care*, *20*(1): 355.
3. Strom T, Martinussen T, Toft P: 2010 A protocol of no sedation for critically ill patients receiving mechanical ventilation: a randomized trial. *Lancet* 375: 475-480.

**GRANT ACKNOWLEDGMENT**

None

### 0382

#### Early mobilisation of critically ill patients in Singapore - a quality improvement project

D. Sani[1], K.K. Myint[2], J. Taculod[3], A.Y. Li[4,5], H.S. Yip[4,5]
[1]National University Hospital, Department of Rehabilitation, Singapore, Singapore; [2]National University Hospital, Department of Nursing, Singapore, Singapore; [3]National University Hospital, Division of Critical Care, Respiratory Therapy, Singapore, Singapore; [4]National University Hospital, Division of Respiratory and Critical Care Medicine, University Medicine Cluster, Singapore, Singapore; [5]National University of Singapore, Department of Medicine, Yong Loo Lin School of Medicine, Singapore, Singapore
**Correspondence:** D. Sani
*Intensive Care Medicine Experimental* 2018, **6(Suppl 2):**0382

**INTRODUCTION.** Survivors of critical illness may experience post-intensive care syndrome (PICS) [1], where physical, cognitive and/or psychological impairments persist after discharge. Early mobilisation (EM) in intensive care unit (ICU) has been shown to be safe, feasible and may help to ameliorate the incidence of PICS [2]. Well-designed studies have shown that EM improves quality of life, physical function, muscle strength, increase ventilator-free days, and decrease length of stay (LOS) [2]. In Singapore, the mobilisation practice in ICU and its effects on clinical outcomes are unknown.

**OBJECTIVES.** This is a six month pre-post quality improvement (QI) pilot project in a single-centre medical ICU (MICU) to (1) investigate the feasibility of EM, (2) hasten mobility and (3) evaluate effects on clinical outcomes.

**METHODS.** Patients admitted to MICU were recruited if they were >18years old, admitted for ≥48 hours and premorbidly able to ambulate ≥10m independently. Patients were excluded if they had lower limb amputation or fractures limiting mobility, neurological conditions or actively dying.

Prior to QI implementation, MICU operated on a blanket-referral system for physiotherapy. Suitability for EM was not clear.
A multidisciplinary team implemented multifaceted changes which included

1) improving visibility on the mobility status of patients,
2) implementing a sedation and mobilisation protocol,
3) training staff to assess suitability for EM, and
4) implementing functional outcome measures. Waiver of informed consent was granted by the institutional review board.

**RESULTS.** 107 and 92 patients were recruited in the pre-QI and post-QI group respectively.
Compared to patients in the pre-QI project, patients in the post-QI group had a reduction in sedation (11.2 vs 18 hours, p=.029), intubation duration (38 vs 73 hours, p< .001), sat out of bed earlier (2 vs 4 days, p= .013), and ambulated earlier (4 vs 6 days, p< .001). There was no significant difference in ICU delirium days. ICU and hospital LOS decreased in the post-QI group (4 vs 5 days, p=.079) and (13.5 vs 15 days, p=.12) respectively, but this did not reach statistical significance. ICU Mobility Scale (IMS) scores achieved at ICU discharge were higher in the post-QI group, with 56.5% of patients in the post-QI group ambulating (IMS ≥ 7) at ICU discharge compared to 28% in the pre-QI group (p< .001).

**CONCLUSION.** EM in ICU is feasible and improves clinical outcomes and functional status. Impact on patient's long-term outcomes is unclear, and warrants further research.

**GRANT ACKNOWLEDGEMENT**

NA

### 0383

#### Trauma outcome - quality of life, Injury perception, dream recall and symptom severity over time trajectory from 2 weeks to 5 years post hospital discharge

J. Benbenishty[1], L. Churi[2]
[1]Hadassah Medical Organization, Jerusalem, Israel; [2]Hadassah Hebrew University Medical Center, Orthopedics, Jerusalem, Israel
**Correspondence:** J. Benbenishty
*Intensive Care Medicine Experimental* 2018, **6(Suppl 2):**0383

**INTRODUCTION.** Injury is a leading cause of preventable mortality and morbidity worldwide, with approximately 10% of trauma patients admitted to intensive care. Trauma patients experience significant ongoing physical and psychological burden after discharge; however, the patterns of recovery and the subgroups of intensive care patients who experience the greatest burden are not described. Prospective studies have not succeeded in proving enough long-term follow up data, therefore a 6-month prevalence design was chosen.

**OBJECTIVES.** Understand the relationship between symptom severity, injury perception, quality of life, ICU dreams and health status in revered trauma patients over trajectory of time.

**METHODS.** Prevalence study; 6-month follow up clinic of all trauma patients in one tertiary referral hospital city of 1million. After IRB approval, all trauma patients in follow up clinic were invited to join study. Questionnaires included quality of life, injury perception, symptom severity, dream recollection, health status and demographics.

**RESULTS.** 223 participants. 11% of patients recovered from ICU, 20% were more than 1-year post trauma, 20% were over 60 years of age. Range of injuries included multiple trauma multi-organ injuries to limb fractures, solitary rib fractures and soft tissue damage. Length of Hospitalization ranged from 1-90 days. The trend of symptom severity changed significantly over time ranging from severe pain and mobility limitations immediately post hospital discharge to Distress/ feeling upset and insomnia severity after 1 year. Over 60 year olds described their health status as very low, but their quality of life as very high. Dreams ranged from recalling psychedelic experience to feelings of calmness and floating sensations. Those injured over 6 months scored highest in feeling that injury affected emotional stability and the perception that the injury will continue for the rest of their life. Women scored their health status and QOL significantly lower than men. No association was found between injury severity, ICU stay and longevity or severity of symptoms. More than 10% had not year returned to work 1-year post injury; 15% returned part time. Those recovering from complex ankle fracture share the same perception of injury severity as those after multi-trauma.
**CONCLUSIONS.** It is important that we raise awareness about the healing process of recovery among the post trauma population. Patients do not forget their ICU dreams. The complexity and severity of trauma injuries does not affect the long-term perception of this life-changing event. Nurses can instruct patients and the families of the trajectory journey of post trauma.

**GRANT ACKNOWLEDGMENT**
1000$ grant was provided by the study hospital

### 0384
### Better estimation of height in ICU is essential for effective lung protective ventilation

R.S. Millington, V. Edwards, C. Soanes, A. Wong, B. Borgatta
[1]Oxford University Hospitals NHS Trust, Adult ICU, Oxford, United Kingdom
**Correspondence:** V. Edwards
*Intensive Care Medicine Experimental* 2018, **6(Suppl 2):**0384

**INTRODUCTION.** Inaccurate height recording negatively impacts delivery of lung protective ventilation as tidal volume (TV) targets are derived from patient height. In our unit, a 1m tape is expected to be used to measure recumbent patient height. This can be challenging and error prone[1]. Furthermore we suspected it was often not done. Validated anthropometric methods for height estimation in ICU include patient measurement with a 2m tape or ulna-length measurement. The latter is a simple method and amongst the most accurate[12].
**OBJECTIVES.**

1. Evaluate how height is recorded in our ICU.
2. Assess the impact of discrepancies on TV targets between recorded heights (from the electronic record) and predicted heights (via the ulna-length method).
3. Adopt ulna-length as standard if significant discrepancies exist.

**METHODS.** Prospective data collection included height entered into the electronic record and measured ulna-length using the Malnutrition Universal Screening Tool from the British Association for Parenteral and Enteral Nutrition[3]. Significant discrepancies in TV were defined as ±0.2 ml/kg. After finding a high rate of visual estimation, a nursing staff survey was used to identify barriers to accurate height recording in ICU.
**RESULTS.** 50 patients were included. 56% were female and 50% < 65years. 78% were unplanned admissions and 50% were postoperative. Height recording methods: 26% visually estimated, 36% from medical records, 8% GP/pre-admission, 24% self-reported and 6% other.
In 22% of patients recorded-height TV matched the ulna-length derived TV of 6±0.2 ml/kg with 52% below and 26% above. The difference in recorded and predicted height ranged from -17 to 20cm (IQR: -5 to 5cm), equating to TV difference ranging from -2.16 to 1.6 ml/kg (IQR: -0.48 to 0.23 ml/kg). Certain subgroups showed greater differences: females, age < 65years, non-postoperative, unplanned admissions, visual height estimation.
37 nurses were surveyed. 30% visually estimated >60% of the time despite 75% viewing accurate height measurement as very/ extremely important. Key barriers to physical measurement were: needing help (24%); time taken locating a tape (19%); perceived difficulty (13%); task prioritisation during admission (22%). Nursing preference was equal for both ulna-length and 2m tape measurement.
**CONCLUSIONS.** There is no consistent method for recording height on ICU and there is a high rate of visual estimation. This negatively impacts TV targets as compliance to 6ml/kg ventilation is < 25%. To improve this, where erect height is unavailable, we recommend ulna-length measurement as the standard for height prediction as it can be done without help and more easily than the 2m tape. All bed spaces should have a 1m tape. Mandatory completion of height recording should be within the admitting shift rather than immediately on admission.

**REFERENCE(S)**
1. Bojmehrani et al. Respir Care 2014:59:1025
2. L'her et al. Ann Intensive Care 2016:6:55
3. www.bapen.org.uk

**GRANT ACKNOWLEDGMENT**
None

### 0385
### Physiotherapy outcome measure use on adult intensive care units in the United Kingdom (UK)

C. Purkiss
Royal Brompton and Harefield NHS Trust, Rehabiltation and Therapies, London, United Kingdom
*Intensive Care Medicine Experimental* 2018, **6(Suppl 2):**0385

**INTRODUCTION.** Standardised, validated outcome measures are an essential tool when measuring quality and efficacy of therapy interventions.
NICE guideline CG83 recommends assessment with an outcome measure to assess physical function at regular intervals during critical illness, however does not recommend a specific tool with which to do so.
A 2015 review identified inconsistency in tools used to measure physical function in survivors of critical illness and there is currently no consensus regarding which tools best assess physical impairment in this cohort.
**OBJECTIVES.** To review outcome measure use throughout Adult Intensive Care Units (AICU's) in the UK, and benchmark the use of outcome measures at the Royal Brompton Hospital against other UK AICU´s.
**METHODS.** A questionnaire regarding outcome measure use was posted to 247 AICU's across the UK taken from the Intensive Care National Audit Research Centre (ICNARC) in 2016.
**RESULTS.** 36.8% (n=91/247) of surveys were returned. 79.1% (n=72/ 91) of responding trusts were using outcome measures, with a range of 27 different outcome measures reported.
Of those using measures the most commonly used was the Chelsea critical care physical assessment tool (66.7% n=48/72), followed by the Manchester Mobility Scale (25% n=18/72), Functional Independence Measure (11.1% n=8/72), Barthel Index (11.1% n=8/ 72), and the Goal Attainment Scale (6.9% n=5/72). 22.2% (n=16/72) of trusts were using a combination of several measures, hence the sum total of outcomes used exceeds the response rate of the survey.
The most sited reasons for choosing a measure were ´ease of use´ (23.61% n=17/72), ´evidence base´ (22.22% n=16/72) and ´ appropriateness of the tool´ (20.83% n=15/72).

33.3% (n=24/72) of units completed measures with all patients on AICU, others used set time frame criteria. 12.5% (n=9/72) assessed after >48hrs AICU stay, 11.1% (n=8/72) after >48 hrs ventilated. The longest time frame until patients were assessed was a stay of >2 weeks on AICU (1.4% n=1/72).

The frequency of assessment varied widely with a range from daily, to on admission and discharge from AICU only. The most common intervals for assessment were weekly (22.2% n=16/72) and daily (19.4% n=14/72).

On discharge from AICU, 66.7% (n=48/72) of the trusts using measures continued to do so on high dependency units and the ward.

**CONCLUSIONS.** The scope of this survey is limited due to the response rate, however it shows a wide disparity of UK AICU outcome measure use. This variance includes the tools being used, which patients are assessed, and the timing and frequency of assessment. This may be due to heterogeneous patient cohorts, staffing levels, patient acuity and a lack of consensus regarding the best outcomes to use. Further investigation into specific outcome measures would be beneficial to provide best quality, standardised care and the creation of a core outcome set to make outcomes translatable for research purposes.

### 0386

#### Does functional ability improve for patients in the intensive care unit (ICU) with a structural physiotherapy indication using the patient mobility framework (PMF) protocol?

N.L. O'Sullivan[1], B.I. Cleffken[2]

[1]Maasstad Hospital, Department of Physiotherapy, Rotterdam, Netherlands; [2]Maasstad Hospital, Department of Intensive Care Medicine, Rotterdam, Netherlands

**Correspondence:** N.L. O'Sullivan

*Intensive Care Medicine Experimental* 2018, **6(Suppl 2):**0386

**INTRODUCTION.** There are numerous negative side effects associated with an ICU stay, which include immobility, deconditioning and weakness. It is well documented that early mobilization in the ICU is safe and aids in reducing the negative effects of an ICU hospitalization. The aim of this study is to evaluate the effect of the patient mobility framework (PMF) developed by Castelijn (2012), as a tool to increase patient activity through increased cooperation amongst the intensive care team. The PMF algorithm is divided into 3 alphabetic and 3 numerical categories and offers corresponding mobility advice for nurses and physiotherapists. Each patient receives a single alphabetic and numerical score from the supervising consultant and authorization for therapy daily.

**OBJECTIVES.** The primary objective was to investigate if the protocol has a beneficial effect on patients' functional ability. Furthermore, the possible difference in mean number of delirium days per cohort was examined.

**METHODS.** The study is a single center observational study in a Level 3, ICU. It is a retrospective, medical records, cohort study comprising of two cohorts. The PMF protocol was introduced in 2015. The primary cohort included patients admitted between January-July 2014 and was compared to patients after the implementation from January-July 2016. Patients were included 48 hours after ICU admission. Patients were excluded if they categorized as receiving end of life care, thrombolysis, burns patients and multi-trauma patients. All patient's daily activities are recorded in their electronic patient dossier. The measurement point for functional activity is an active transfer from the bed to a chair on discharge from the Intensive Care/Medium Care departments. Early mobilization is a key component in the prevention and treatment of delirium. The primary objective was tested with a chi-square analysis in the study population. The mean number of delirium days in 2014 and 2016 was assessed by a Wilcoxon rank sum test.

**RESULTS.** The number of patients included in the 2014 cohort was N=174 and in 2016 N=170. There was no statistically significant difference in baseline characteristics in the study population. There was a 19% increase in the ability to perform an active transfer in the PMF group, however, chi-squared tests revealed no significant distinctions between cohorts. There was a 37% decrease in the mean number of delirium days from 2.5 in 2014 to 1.82 in 2016, however, there was no significant difference between cohorts.

**CONCLUSIONS.** In order to ease the psychological and physical comorbidities, associated with an ICU stay, a complete multidisciplinary, multi-facet approach to physical activity is needed to reduce the facilitation of a sedentary ICU stay. There is a positive shift in functional ability but further focus on enabling factors such as mobility protocols, the ABCDE bundle and mobility teams are vital for early rehabilitation in the ICU.

### 0387

#### The benefits of a specialist physiotherapist in ICU rehabilitation clinic

V. Robinson[1], E. Orme[2], M. Allen[2], M. Dabliz[2], E. Morino[2], L. Ma[2]

[1]Kingston Hospital NHS Foundation Trust, Physiotherapy, Kingston Upon Thames, United Kingdom; [2]Kingston Hospital NHS Foundation Trust, Kingston Upon Thames, United Kingdom

**Correspondence:** V. Robinson

*Intensive Care Medicine Experimental* 2018, **6(Suppl 2):**0387

**INTRODUCTION.** Intensive care acquired weakness is a common condition in critically ill patients who have been admitted to ICU for a prolonged period of time (Hodgson and Tippin, 2016) and many patients fail to recover fully within one year (Herridge et a, 2013). As a result the NICE guidelines recommend patients are followed up 2-3 months after discharge. In October 2017, Kingston Hospital was given funding from the CCG (clinical commissioning group) to start an Intensive Care Unit (ICU) rehabilitation clinic. The funding was for a clinic one day a month to include a consultant, a senior nurse, a psychologist and a physiotherapist. There are few clinics in the UK that have a funded physiotherapist at every clinic.

**OBJECTIVES.** To analyse the number of patients who attended ICU rehabilitation clinic that required specialist physiotherapy assessment, treatment, advice and follow up.

**METHODS.** Patients were invited to attend the ICU rehabilitation clinic if they had been admitted to the ICU for five days or more and all obstetric patients.

A retrospective study was performed, analysing the notes of all patients who attended clinic from October 2017 to March 2018. The physiotherapy needs of the patients in ICU rehabilitation clinic were recorded following specialist physiotherapy assessment.

**RESULTS.** Twenty six patients attended ICU rehabilitation clinic between October 2017 and March 2018. Seventeen patients (70%) needed physiotherapy input with ten requiring ongoing physiotherapy input due to significant muscle loss and balance impairment. Three patients were already attending community physiotherapy programmes (one at a hospice and two since discharge from a rehabilitation hospital). Two patients refused referral onto community services. Direct physiotherapy input in clinic included taking patients to the physiotherapy gym, teaching of balance and strengthening exercises and advice on exercise at home and education on the effects of ICU acquired weakness.

**CONCLUSIONS.** The majority of patients who attended the Kingston Hospital ICU rehabilitation clinic had physiotherapy needs which were appropriately assessed and treated by the physiotherapist at the clinic and ongoing physiotherapy input provided. The waiting time for outpatient or community physiotherapy assessment is more than four months. A physiotherapist in ICU rehabilitation is vital for patients' care following ICU stay.

**REFERENCE(S)**

Herridge, MS.,Tansey CM, Matté A, Tomlinson G, Diaz-Granados N,Cooper N,, Guest CB, Mazer CD, Mehta S, Stewart TE, Kudlow P, Cook D M.D, N Engl J Med 2011; 364:1293-1304

**GRANT ACKNOWLEDGMENT**

None.

### 0388

#### Interactive gaming for evaluating dysphagia in ICU patients

J. Lut[1], M. Jansen[1], R. Hemler[2], E. Dekker[3], A. Kröner[1], P. Spronk[1]
[1]Gelre Hospitals Apeldoorn, Intensive Care, Apeldoorn, Netherlands, [2]Gelre Hospitals Apeldoorn, ENT, Apeldoorn, Netherlands, [3]Gelre Hospitals Apeldoorn, SLP, Apeldoorn, Netherlands
**Correspondence:** J. Lut
*Intensive Care Medicine Experimental* 2018, **6(Suppl 2):**0388

**INTRODUCTION.** Swallowing disorders, dysphagia, occur frequently in patients admitted to the intensive care unit (ICU). Dysphagia in the ICU is caused by many factors including drugs, muscle wasting, neuromyopathy, delirium and the presence of nasogastric tubes and tracheostomies. Dysphagia is associated with insufficient feeding and an increased chance of aspiration and associated pneumonia. These effects may cause prolonged length of stay in the ICU and the hospital with inherent worse outcome. We present two cases in which we used surface electromyographic (sEMG) signals as input for an interactive biofeedback game (Rephagia®) to evaluate and train swallowing strength.

**CASE DESCRIPTION.** Case 1 is a 67 year old woman admitted to the ICU because of abdominal sepsis due to strangulated and perforated stomach. Case 2 is an 82 year old male admitted to the ICU because of abdominal sepsis due to a complicated hemicolectomy because of a sigmoid carcinoma. Both patients suffered from multiple organ failure and severe muscle weakness and received a tracheostomy. Clinical signs of dysphagia (abnormal water swallow-test) were present. Using FEES, both demonstrated severe dysphagia, i.e. they scored a Murray Secretion Score of 3 (Most severe rating - Secretions seen in the laryngeal vestibule. Pulmonary secretions were included if they were not cleared by swallowing or coughing.) and a Rosenbek Penetration-Aspiration Scale (PAS) of 8 for thin ánd for thickened liquid (Material enters the airway, passes below the vocal folds, and no effort is made to eject).

**RESULTS.** The patients welcomed the interactive training exercise and were actively involved in the game.

Patient 1 trained 8 times in 12 days before she was discharged from the ICU. At the start of the training she had no oral intake (Functional Oral Intake Scale 1). At discharge she had a functional oral intake scale (FOIS) of 3 (tube dependent with consistent oral intake). The average peak swallow values on day 1 increased from mean 33.2μV (SD 8.5) to 59.6μV (SD 11.4) on day 8.

Patient 2 trained 25 times in 47 days. Days of clinical deterioration and/or delirium made daily exercising impossible. Unfortunately he deteriorated further due to a recurrence of his carcinoma and he died at the ICU. During training his FOIS improved from 1 to 3. The average peak swallow values increased from mean 34.6μV (SD 3.8) on day 1 to 74.6μV (SD 8.7) on day 25 (see graph).

**CONCLUSION.** The swallowing strength and oral intake improves while training with sEMG biofeedback. However studies should compare the use of sEMG biofeedback training with standard optimal care to evaluate its additional value in regaining swallow function.

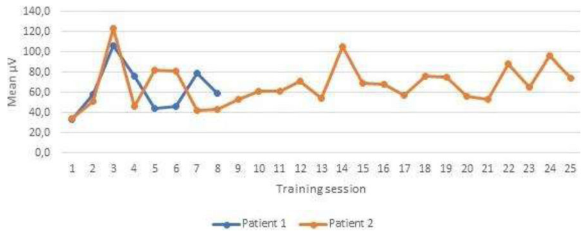

**Fig. 1 (abstract 0388).** Average peak swallow values per training session]

## Making the best of antibiotic use

### 0389

#### Impact of AST meetings on antimicrobial therapy decisions in the ICU

S. Dhaese[1], L. De Bus[1], J. Boelens[2], L. Coorevits[2], B. Verhasselt[2], P. Depuydt[1], J.J. De Waele[1]
[1]Ghent University Hospital, Department of Intensive Care Medicine, Ghent, Belgium; [2]Ghent University Hospital, Department of Laboratory Medicine, Ghent, Belgium
**Correspondence:** S. Dhaese
*Intensive Care Medicine Experimental* 2018, **6(Suppl 2):**0389

**INTRODUCTION.** Antimicrobial stewardship (AST) aims to improve outcome and reduce resistance through better use of antimicrobials. Its success relies on collaboration between microbiologists, pharmacists, infectious diseases specialists and treating physicians[a]. It is unclear whether AST meetings impact antibiotic therapy decisions.

**OBJECTIVES.** To assess whether AST meetings impact antimicrobial therapy decisions (initiation, switch and discontinuation of antibiotics) in the ICU.

**METHODS.** Start and end date of all antibiotic prescriptions for surgical (SICU) and medical (MICU) ICU patients admitted between Jan 2013 and Dec 2017 were recorded using the COSARA software package[b]. Days were categorized based on the availability of results from surveillance cultures (SC), routine microbiological service (weekdays versus weekend) and AST meetings (Table 1, '+' = available and '-' = not available). Based on this classification we assessed the impact of AST meetings by comparing the proportion of antibiotic prescriptions changed (start, switch or end) on days with and without AST meetings and days with and without SC results available.

**RESULTS.** We reviewed 8848 antibiotic prescriptions, 5487 (62%) and 3361 (38%) in SICU and MICU respectively. Overall, significantly more prescriptions were changed during weekdays ($p < .001$, proportions not shown). Also, significantly more changes in antibiotic prescriptions were noted on days when both SC results were available and AST meetings were organised, when compared to days when SC were available but no AST meetings were organised ($p < .001$). Vice versa, significantly more changes in antibiotic prescriptions were made on days when both AST meetings were held and SC were available when compared to days when AST meetings were held in the absence of SC results ($p < .001$). Of note, we did not find a significant difference in the proportion of changed antibiotic prescriptions when we compared AST+/SC- versus AST-/SC- (p=0.191). Also, we did not find a significant difference between AST-/SC- and AST-/SC+ (Table 2). The absence of a difference between AST-/SC- and AST-/SC+ could be explained by the fact that microbiology results (first day reading) of SC may be reported to the treating physician on days that are classified as SC-.

**CONCLUSIONS.** Antimicrobial stewardship meetings significantly influence antibiotic therapy decisions when results of surveillance cultures are available.

**REFERENCE(S)**

a Patel R, Fang FC. Diagnostic Stewardship: Opportunity for a Laboratory-Infectious Diseases Partnership. *Clin Infect Dis* 2018; published online March 3.

b Steurbaut K, Colpaert K, Gadeyne B, *et al.* COSARA: integrated service platform for infection surveillance and antibiotic management in the ICU. *J Med Syst* 2012; **36**: 3765-75.

**GRANT ACKNOWLEDGMENT**

Sofie Dhaese is funded by a CRE grant (APP1099452) from the NHMRC awarded to Jason A. Roberts

**Table 1 (abstract 0389).** Timing of AST meetings, availability of surveillance cultures and routine microbiological services

| Day of the week | SICU | MICU |
|---|---|---|
| *Monday* | AST + / SC - | AST - / SC - |
| *Tuesday* | AST - / SC - | AST - / SC - |
| *Wednesday* | AST + / SC + | AST - / SC + |
| *Thursday* | AST - / SC - | AST - / SC - |
| *Friday* | AST + / SC - | AST + / SC - |
| *Saturday* | AST - / SC + / on call + | AST - / SC + / on call + |
| *Sunday* | AST - / SC - / on call + | AST - / SC - / on call + |

**Table 2 (abstract 0389).** Proportion of changed antibiotic prescriptions according to the availability of SC and AST meetings

| | AST+ | AST- |
|---|---|---|
| SC- | 15.5% | 14.8% |
| SC+ | 26.5% | 15.8% |

### 0390

#### Bactericidal effects of telavancin in comparison with linezolid for the treatment of severe MRSA pneumonia: an experimental study

D. Battaglini[1,2], G. Li Bassi[2,3,4], L. Fernández-Barat[2,3,4], A. Motos[2], M.L. Yang[2], A. Meli[2,5], H. Yang[2], F. Pagliara[1], J. Bobi[2], E. Aguilera-Xiol[2], T. Senussi[1], J. Miller[2], E. Barbeta Viñas[2], P. Pelosi[1,6], D. Chiumello[5,7], A. Torres[2,3,4]

[1]University of Genoa, Department of Surgical Sciences and Integrated Diagnostics, San Martino Policlinico Hospital, IRCCS for Oncology, Genoa, Italy; [2]Hospital Clinic, Division of Animal Experimentation, Department of Pulmonary and Critical Care Medicine, Barcelona, Spain; [3]IDIBAPS/CIBERES, Barcelona, Spain; [4]University of Barcelona, Barcelona, Spain; [5]University of Milan, Milan, Italy; [6]University of Genoa, Genoa, Italy; [7]University of Milan, ASST Santi Paolo e Carlo, SC Anestesia e Rianimazione, Milan, Italy

**Correspondence:** A. Meli

*Intensive Care Medicine Experimental* 2018, **6(Suppl 2):**0390

**INTRODUCTION.** Methicillin-resistant *Staphylococcus aureus* (MRSA) is a common causative pathogen of nosocomial pneumonia. Although current guidelines recommend vancomycin and linezolid as first-line agents against MRSA pneumonia, in a recent clinical trial[1], telavancin achieved higher curative rates in comparison with vancomycin, specifically in a subset of patients with monomicrobial *MRSA* pneumonia. Nevertheless, studies comparing telavancin with linezolid are lacking.

**OBJECTIVES.** To compare the bactericidal efficacy of telavancin vs. linezolid in a porcine model of severe MRSA pneumonia.

**METHODS.** Eighteen pigs (32.11±1.18 kg) were anesthetized and mechanically ventilated for 76 hours. Pigs were challenged with 15 mL of $10^{7-8}$ cfu/mL of MRSA into each lobe via bronchoscopic inoculation. After onset of pneumonia, pigs were randomized to receive the following intravenous treatments: saline solution (control), 22.5 mg/kg q24h of telavancin, or 600mg q12h of linezolid. At the end of the study, animals were euthanized and tissue samples were harvested from ventral and dorsal sections of each lobe. Microbiology studies then quantified MRSA pulmonary burden.

**RESULTS.** All animals completed the study. A total of 180 lung tissue samples, 90 from the ventral and 90 from the dorsal pulmonary regions were analysed. Overall, MRSA was found in 46.7, 43.3, and 26.7% of all lung tissue samples treated with control, linezolid and telavancin, respectively (p=0.013), yielding mean±SD MRSA lung concentration of 1.86±2.30, 1.58±2.38 and 0.79±1.60 (p=0.015). Figure 1 depicts MRSA colonization of the ventral and dorsal lung samples, corroborating augmented bactericidal effects of telavancin, specifically in the dorsal regions.

**CONCLUSIONS.** In a porcine model of severe MRSA pneumonia, telavancin, in comparison with linezolid, exerts higher bactericidal efficacy, specifically hindering colonization of the dorsal pulmonary regions. We are currently evaluating in our animal model pulmonary distribution of telavancin and linezolid to draw inferences on the above mentioned pulmonary colonization. Comparative clinical studies are needed to confirm the benefits of telavancin in the treatment of severe MRSA pneumonia.

**REFERENCE(S)**

[1] Rubinstein E *et al. Clin Infect Dis* 2011; 52: 31-4.

**GRANT ACKNOWLEDGMENT**

Theravance Biopharma R&D, Inc. George Town, Cayman Islands, which is the manufacturer of telavancin, funded this study.

### 0391

#### Clinical impact of an Antimicrobial Stewardship Program in critical patients based on early antibiotic adequacy

F.S. Amado, A.M. Silva, M.R.G. Freitas, N.S. Silva, F. Spada, L.H.B. Gonçalves, J.R.A. de Azevedo, R.P. de Azevedo

Sao Domingos Hospital, Intensive Care, São Luís, Brazil

**Correspondence:** F.S. Amado

*Intensive Care Medicine Experimental* 2018, **6(Suppl 2):**0391

**INTRODUCTION.** The main goal in treatment of infections in critically ill patients is to provide an effective, early and proper antibiotic therapy and, at the same time, minimize future bacterial resistance, especially in an era of rising antibiotic resistance and lack of new antimicrobial development. Reducing microbiologic time results has been aimed to minimize costs, improve antibiotic adequacy and patient's outcomes. Antibiotic stewardship in the intensive care unit (ICU) encompasses rapid identification of patients with bacterial infections, better empirical treatment selection, de-escalation once culture results become available, shortening therapy duration, and reducing the numbers of patients treated unnecessarily [1].

**OBJECTIVES.** To evaluate the clinical impact and feasibility of the implementation of an Antimicrobial Stewardship Program (A.S.P.) based on early antibiotic adequacy in critical care patients.

**METHODS.** All patients, aged above 18 years, admitted to a 45-bed ICU of a tertiary hospital, with positive cultures from November 2016 to August 2017, were included. This program is based on rapid germ identification, effective communication and proper antibiotic adequacy. The germ identification started with gram-staining followed by mass spectrometry (MALDI-TOF MS) and results were sent by an on-line instant messages software *(WhatsApp®)* to ICU care team. The team should answer (closed-loop communication) and adequate the therapy in 2 hours after the message was sent. The antibiotic therapy was chosen in compliance with recommendations aimed to isolated germ by MALDI-TOF MS. Then, final adequacy occurred as soon as susceptibility tests and MIC by E-TEST were available. These final results were sent by daily electronic reports.

**RESULTS.** We included 209 patients (601 positive cultures). The information sent was confirmed in 2 hours in 88.2% of all exams. The median time to administration of proper antibiotic therapy since the delivery of information was 90 (56 -185) minutes. Median time to MALDI TOF germ identification was 41 (24-49) hours. The recommendations for empirical antibiotic therapy had accuracy of 98,1% and final adequacy rate after susceptibility tests was 99%. This A.S.P. enabled a de-escalation rate of 76.5%. The early antibiotic therapy had impact on ICU mortality rate (34,2% in the early group against 56% in the late adequacy group; p = 0,034), on hospital mortality rate (35,9% in the early group against 60% in the late adequacy group; p = 0,02) and also on ICU and hospital length of stay.

**CONCLUSIONS.** As showed by this study results, the adoption of an Antimicrobial Stewardship Program organizes information and consequently facilitate the early and proper antibiotic adequacy, improving outcomes.

**REFERENCE(S)**

1. Luyt C-E, Bréchot N, Trouillet J-L, Chastre J. Antibiotic stewardship in the intensive care unit. Crit Care. 2014;18:480. doi:10.1186/s13054-014-0480-6.

**Table 1 (abstract 0391).** Mortality Rates according to early antibiotic adequacy

| Patient characteristics | Early Group n = 184 | Late Group n = 25 | p-value |
|---|---|---|---|
| ICU Mortality n (%) | 63 (34,2) | 14 (56) | 0,03 |
| Hospital Mortality n (%) | 66 (35,9) | 15 (60) | 0,02 |

**Table 2 (abstract 0391).** Lenght of stay according to early antibiotic adequacy

| Lenght of stay (LOS) | Early Group n = 184 | Late Group n = 25 | p-value |
|---|---|---|---|
| ICU LOS Median (IQR), days | 13 (6 - 27) | 19 (7,5 - 67,5) | 0,049 |
| Hospital LOS Median (IQR), days | 29 (14 - 59,5) | 51 (19 - 97,5) | 0,049 |

### 0392

#### Postoperative intra-abdominal infections with positive cultures of staphylococci: epidemiology, clinical relevance and prognosis

P. Montravers, K. Boussion, E. Kantor, S. Tanaka, N. Grall, L. Ribeiro, A. Tran-Dinh
CHU Bichat, Paris, France
**Correspondence:** P. Montravers
*Intensive Care Medicine Experimental* 2018, **6(Suppl 2):**0392

**INTRODUCTION.** Prevalence of staphyloccoci (Staph) in surgical samples of postoperative intra-abdominal infections (PIAI) varies from 4 up to 16% of the cases. Few studies mention these organisms in microbiological samples and their pathogenicity is debated, especially coagulase-negative Staph (CNS).

**OBJECTIVES.** Our purpose was to compare in a prospective cohort of PIAI patients those with positive cultures of Staph (PIAIS) in surgical samples versus patients without Staph (PIAInS), then to differentiate cases with coagulase-positive Staph (PIAICPS) and CNS (PIAICNS).

**METHODS.** Between 1999 and 2017, we prospectively included all patients (pts) admitted in ICU for the management of PIAI. Clinical characteristics, severity scores (SAPS II and SOFA on admission), microbiological data (blood cultures and surgical operative samples), empirical antibiotic therapy and outcome (mortality, reoperation, ICU stay) were collected. Results are presented in means and DS or proportions. Statistical analysis used Fisher exact test, Mann Whitney and Kruskall Wallis tests(significance $p< 0.05$).

**RESULTS.** 380 pts were analysed, including 87 PIAIS (23%). Clinical and demographic characteristics, were similar in both groups (age, gender, fatal underlying diseases, immunosuppression, cancer, diabetes, initial surgery). Severity scores on ICU admission were lower for PIAIS : SAPS II 44±15 versus 49±18 ($p$=0.03), and SOFA 7±3 vs 8±4 ($p$=0.04) for PIAIS and PIAInS respectively (without any difference between PIAICPS and PIAICNS). Frequency of bacteraemia was similar in both groups while increased proportions of polymicrobial peritoneal samples were reported in PIAIS cases (94% vs 80% in PIAInS ($p$=0.001)). *Escherichia coli* and *Enterococcus faecium* were less frequently cultured from peritoneal samples in PIAIS vs PIAInS ((36% vs 47% ($p$=0.007) and 8% vs 20% ($p$=0.01), respectively). Proportions of fungi in surgical samples were similar in both groups. Adequate empirical anti-infective therapy targeting all the organisms was only obtained in 54% PIAIS and 72% PIAInS cases ($p$=0.0025), mainly explained by methicillin-resistant Staph. Similar mortality and morbidity (re-operation, duration of mechanical ventilation) rates were observed between PIAIS vs PIAInS and between PIAICPS and PIAICNS.

**CONCLUSIONS.** Our data suggest that PIAIS marginally differ from PIAInS cases. The prognosis of PIAIS cases is similar to what is reported from other patients. While it is not possible to fully assess staphylococci pathogenicity in the course of PIAI, our data suggest that these organisms should be considered cautiously and probably as authentic pathogens in a majority of the cases.

**REFERENCE(S)**

J Antimicrob Chemother 2010;65:342 ; Crit Care 2010;14:R20

**GRANT ACKNOWLEDGMENT**
None

### 0393

#### Incidence and time dependence of inadequate and supratherapeutic vancomycin levels in critically ill: a retrospective study

J.E. van der Mast[1], M.W.N. Nijsten[2], D.J. Touw[1], W. Bult[1]
[1]University Medical Center Groningen, Clinical Pharmacy and Pharmacology, Groningen, Netherlands; [2]University Medical Center Groningen, Critical Care, Groningen, Netherlands
**Correspondence:** J.E. van der Mast[1]
*Intensive Care Medicine Experimental* 2018, **6(Suppl 2):**0393

**INTRODUCTION.** Timely adequate antibiotic treatment is paramount in critically ill patients with severe infection. In case of suspected or proven infection with Gram-positive bacteria, vancomycin is often the appropriate first line treatment. Since 2011 our protocol in is to use continuous vancomycin infusion with therapeutic drug monitoring (TDM) with daily or less frequent measurements. We evaluated how effective our policy was in achieving the required concentrations, i.e. 20 to 25 mg/L.

**OBJECTIVES.** Determine the incidence and time dependence of inadequate or supratherapeutic vancomycin levels.

**METHODS.** Retrospective analysis of vancomycin levels in all adult patients admitted to our ICU over a 64 month interval who were treated with vancomycin bolus followed by continuous infusion. Levels were classified compared with the desired range of 20 to 25 mg/L. Only vancomycin levels determined within 30 days of ICU admission were included.

**RESULTS.** We included 472 patients with a mean ±SD age of 61 ±14, 62% male and an acute physiology and chronic health evaluation score (APACHE-IV) of 76 ±30. Hospital mortality was 32%.

The mean number of vancomycin measurements was 5.0 ±4.8. Per patient the percentage of vancomycin levels < 15, < 20, within range, >25 and >30 was 22%, 43%, 26%, 31% and 12%, respectively. The percentage of within range levels did not differ between survivors and non-survivors, although non-survivors had a higher fraction of levels >25 mg/L (< 0.001). Moreover, only at two days after ICU-admission more than 50% of the vancomycin levels were in the therapeutic range.

**CONCLUSIONS.** Timely target attainment vancomycin is difficult with our current TDM-protocol. Protocol changes and more frequent vancomycin sampling appear to be mandatory.

**REFERENCE(S)**

1. Bakke V, Sporsem H, Von der Lippe E, Nordøy I, Lao Y, Nyrerød HC, et al. Vancomycin levels are frequently subtherapeutic in critically ill patients: a prospective observational study. Acta Anaesthesiol Scand. 2017;61(6):627-35.
2. Reardon J, Lau TTY, Ensom MHH. Vancomycin Loading Doses: A Systematic Review. Annals of Pharmacotherapy. 2015;49(5):557-565.

**GRANT ACKNOWLEDGMENT**
None.

### 0394

#### Prevalence and role of respiratory viruses in ICU patients with community acquired-pneumonia (CAP): a one-year retrospective single center study

M. Giry, A. Chefdeville, M. Gueudin, D. Boyer, A. Baron, G. Beduneau, S. Skallil, D. Carpentier, C. Girault, B. Misset
CHU de Rouen, Rouen, France
**Correspondence:** M. Giry
*Intensive Care Medicine Experimental* 2018, **6(Suppl 2):**0394

**BACKGROUND.** Pneumonia is the most frequent community-acquired infection responsible for ICU admission. Multiplex PCR enables early diagnosis of viral infection in daily practice. Few series have been described since this technique has been made available in routine.

**OBJECTIVES.** We aimed at assessing the prevalence and distribution of viruses among ICU patients with CAP and their relationship with severity and outcome.

**METHODS.** Retrospective analysis of the consecutive viral multiplex PCR (Eplex™, Genmark Dx) between November 2016 and October 2017 in a French 21 bed medical ICU admitting around 1,000 patients per year. Patients' nasopharynx was sampled within 72 hours following their ICU admission. We selected those patients with a diagnosis of CAP and split them into 4 groups according to causal agent: none, viruses, bacteria, combination of both. Comparisons were made with non-parametric Kruskall-Wallis and Fischer's exact tests.

**RESULTS.** 223 patients were sampled, of whom 109 had CAP, 38 aspiration or opportunistic pneumonia, 22 non-pulmonary infections, 11 pulmonary edema, 19 exacerbations of chronic lung disease, and 24 other diagnoses.

Patients with CAP had the following characteristics: age 59± 16 y, male sex 60%, SAPS 2 score 40 ± 18, ICU length of stay (LOS) 8.7±9.0 d, mortality 11 %. No infectious agent was found in 33 (30%), a virus in 27 (25%), bacteria in 33 (30%) and both a virus and bacteria in 16 (15%). The main bacteria were *S. pneumonia* (23%) and *H. influenza* (6.5%). The main viruses were Rhinovirus/Enterovirus (13.8 %), Influenzae A (11 %) and Parainfluenzae (4.6%). The most frequent virus-bacteria association was *S. pneumoniae* and Influenzae A (3%) and *S. pneumoniae* and Rhinovirus/Enterovirus (3%).

The SAPS 2 score was higher in the mixed group (p = 0.02). The ICU-LOS was 5.2 (no agent), 7.8 (virus), 10.1 (bacteria) and 14.8 (mixed) days respectively (p = 0.04). Mortality was similar among groups.

**CONCLUSIONS.** In our ICU population, respiratory viruses were present in 39.5 % of CAP. Patients with mixed infection had higher severity at admission and longer ICU-LOS.

### 0395

#### Risk factors for multiresistant bacterias in critical patients. Are there differences between the previous acquisition or during the stay in ICU?

M. Palomar[1], F. Alvarez Lerma[2], M. Catalan[3], S. Uriona[4], X. Nuvials[4], R. Gimeno[5], P. Olaechea[6], C. Aragon[7], M.P. Gracia[2], M. Vallverdu[8], ENVIN
[1]Institut Recerca Biomedica Lleida, Lleida, Spain; [2]Parc del Mar, Barcelona, Spain; [3]Hospital Doce de Octubre, Madrid, Spain; [4]H Vall Hebron, Barcelona, Spain; [5]H La Fe, Valencia, Spain; [6]H Galdakao, Galdakao, Spain; [7]H Regional de Malaga, Malaga, Spain; [8]H Arnau de Vilanova, Lleida, Spain
**Correspondence:** M. Palomar
*Intensive Care Medicine Experimental* 2018, **6(Suppl 2):**0395

**INTRODUCTION.** Bacterial resistance is a global problem. It can influence the outcome of critical patients.

**OBJECTIVES.** To find out the risk factors (RF) that can predict which patients are carriers of multiresistant bacteria (MRB) on admission or during their stay in the ICU, including infection and colonization as well as the type of BMR.

**METHODS.** Prospective, multicenter cohort study (ENVIN registry) that included patients admitted> 24 h in ICU from 2014 to 2016. The MRB studied were: Meticillin resistant *S aureus* (MRSA), *P aeruginosa A baumannii*, Enterobacteria producers of extended beta-lactamases (ESBL) and carbapenemase-producing Enterobacteriaceae (CBP). We included as intrinsic data: age, gender, severity (APACHE II, GCS and neutropenia), origin (community, another ICU, ward, and long-stay centers), pathology (medical, trauma, coronary, scheduled and emergency surgery), comorbidities (diabetes, chronic renal insufficiency, COPD, cirrhosis, immunosuppression, neoplasia, solid organ transplantation (SOT) and malnutrition); previous hospital stay (before ICU admission).

The chi square test was applied and the Odd ratio (95% IC) was calculated. The $p < 0.05$ and the OR $\geq 1.1$ were considered significant.

**RESULTS.** Among the 69,741 patients registered, the presence of some MRB was diagnosed in 4.830 (6.4% of the total). Of these, in 3024 patients (4.3%) the identification was on admission and in 1.940 (2.8%) during the stay.

The MRB at admission / during ICU stay were 1,508 / 870 ESBL, 1020/ 265 MRSA, 448/505 *P aeruginosa,* 227/281 CBP and 154/297 *Acinetobacter*.

When comparing the RF of the pts with BMR at admission or during ICU stay, some differences were observed: APACHE ≥15: OR (95% IC) 1.8 (1.7-2.0) vs 2.6 (2.4- 2.9); previous hospital stay ≥10 days: OR (95% IC) 3.7 (3.5-4.0) vs 34.2 (29.1-40.2); long-stay center: OR (95% IC) 3.4 (2.7-4.4) vs 0.5 (0.0-3.0); coronary disease: OR (95% IC) 0.2 (0.2-0.2) vs 0.2 (0.2-0.3); traumatic: OR (95% CI) 0.5 (0.4-0.6) vs 1.8 (1.5-2.1); Emergency surgery: OR (95% IC) 1.4 (1.3-1.6) vs 4.1 (3.7-4.5); Neutropenia: OR (95% CI) 3.4 (2.8-4.2) vs 2.5 (1.9-3.3), Chronic Kidney Ins: OR (95% IC) 2.3-2 , 1-2.6) vs1.19 (1.0-1.3), Cirrhosis: OR (95% IC) 1.9 (1.6-2.2) vs 1.4 (1.2-1) , 8), COPD: OR (95% IC) 1.8 (1.7-2.0) vs 1.5 (1.3-1.7), malnutrition: OR (95% IC) 3.5 ( 3.1-3.8) vs 2.5 (2.2-2.9) and ORT (95% IC) 3.3 (2.8-4.0) vs 1.2 (0.8-7.0) 1,7)

In addition, the stay in ICU> 10 d had the highest OR in the acquisition of MRB 43, 4 (37.9-49.6) and also the exposure to extrinsic RF such as MV, CVC, UC

**CONCLUSIONS.** RFs vary when considering the acquisition prior to admission or during the stay in the ICU. The type of MRB also varies, predominantly ESBL and MRSA in the previous ones, and *P aeruginosa*, CBP and *Acinetobacter* during.

### 0396

#### Diagnostic performance of a rapid detection test (PCR) for multiresistant bacteria (MRB) in patients admitted to the ICU

A. Carranza Pinel, O. Moreno Romero, I. Cruz Valero, J. Tejero Aranguren, M.E. Yuste Ossorio, M. Colmenero Ruiz, M. Muñoz Garach
Hospital Universitario San Cecilio Granada., Intensive Care Unit, Granada, Spain
**Correspondence:** A. Carranza Pinel
*Intensive Care Medicine Experimental* 2018, **6(Suppl 2):**0396

**OBJECTIVES.** To determine the diagnostic performance of a rapid detection test for multiresistant bacteria by PCR in patients admitted to the ICU. The performance of the test is measured in terms of accuracy and agreement with the bacteria isolated in the culture.

**METHODS.** Prospective observational cohort study during a seven-month period. Patients admitted to a mixed ICU who underwent the PCR test in rectal and pharyngeal swabs and cultures (rectal swab and tracheal aspirate if under mechanical ventilation) at the time of admission, considering this last one as the reference test. Patients screened were those who had at least one risk factor of the six proposed in the "Resistencia Zero" program of Spanish Intensive Care Society for preventive isolation. These are:

1. Hospitalization of more than five days in the three previous months.
2. Institutionalized patients (prison, social health centers, nursing homes, etc.)
3. Colonization or known infection by multiresistant bacteria (MRB)
4. Antibiotherapy >7 days in the previous month (especially cephalosporins of 3rd-4th generation, quinolones and carbapenems)
5. Patients with chronic kidney failure undergoing hemodialysis or continuous ambulatory peritoneal dialysis
6. Patients with chronic pathology and high incidence of colonization / infection by MRB: cystic fibrosis, bronchiectasis, chronic ulcers, etc.

We calculated the accuracy parameters of the test and the concordance values of the PCR test were determined by the Kappa value.

**RESULTS.** There were 86 patients who met criteria for preventive isolation. There were 28 patients had a positive PCR, 22 of them had also a positive culture, showing concordance between the multiresistant bacteria isolated in culture and the PCR in all cases. There were 58 patients who presented negative PCR at their admission to the ICU. In this group, only 3 showed discrepancy between negative PCR and positive culture. All three had only one risk factor. The values of diagnostic precision were: Sensitivity: 0.88; Specificity: 0.9; PPV: 0.79; NPV: 0.95; LR +: 8.8; LR-: 0.13. The Kappa concordance index was 0.754 with a 95% CI of 0.965-0.544.

**CONCLUSIONS.** The PCR technique for rapid detection of multiresistant bacteria presents a good agreement with conventional cultures. Preventive isolation could be withdrawn for patients who only had a single risk factor.

### 0397

#### Bronchial colonization from multidrug-resistant pathogens and ventilator-associated pneumonia in ICU during a 3-years period. Preventive measures and innovation

A. Karathanou[1], E. Kouskouni[2], P. Efstathiou[2], Z. Manolidou[2], S. Tsikriki[1], T. Topalis[1], A. Antoniou[1], A. Pentheroudaki[1], E. Vaitsi[1], S. Pamouki[1], M. Zigra[3], I. Kokoris[1,3]

[1]General Hospital of Volos, Intensive Care Unit, Volos, Greece; [2]National and Kapodistrian University of Athens, Aretaeion Hospital, Microbiology Laboratory, Athens, Greece; [3]General Hospital of Volos, Comittee for Prevention of Hospital Acquired Infections, Volos, Greece

**Correspondence:** A. Karathanou

*Intensive Care Medicine Experimental* 2018, **6(Suppl 2):**0397

**INTRODUCTION.** Multidrug-resistant (MDR) bacteria (*Klebs spp., Pseud. aerog.,Acinet b.,Staph.MRSA*) account for a significant percentage of health care associated infections in Greek ICU setting .

**OBJECTIVES.** To establish the incidence of lower inspiratory tract colonization by MDR pathogens and ventilator-associated pneumonia (VAP) , in a 3-years period (2015-2017) before and after implementation of care bundle and furthermore, after incorporation of antimicrobial copper ($Cu^{++}$) in a general 8-bed ICU.

**METHODS.** All intubated patients admitted in ICU from 1/1/2015 to 31/12/2017 were examined for MDR colonization, on admission day, every Monday and any day there was a clinical or laboratory deterioration.Non-invasive tracheal aspiration and semiquantitative or qualitative cultures were performed.CPIS score was used for VAP definition. Patients with positive culture on admission were excluded. 2015 was observation year.In 2016 care bundle was implemented including: hand hygiene, elevation of head of bed 30-45$^o$, no scheduled ventilator circuit changes, oral hygiene/8hrs, subglottic secretions drainage.In 2017 $Cu^{++}$ was incorporated in key touch surfaces in ICU as an additional antimicrobial measure.

Colonizations ( in the same patient different pathogen was a different colonization),VAP, APACHE II score on admission, total days on ventilator,total days in ICU and day to first detected colonization were recorded.Results were analyzed by SPSS vs.20 using Mann-Whitney U test for median values,Shapiro-Wilk test of normality and Pearson Chi-square for correlation, with significance level p< 0,05.

**RESULTS.**

**CONCLUSIONS.** Care bundle for VAP prevention resulted in significant reduction to MDR VAP occurrence during the second semester in 2016 and in 2017 when $Cu^{++}$ was added to standard care.Prolongation to 1$^{st}$ detected colonization was significant. Decrease in pharmaceutical expense was noticable.

**Table 1 (abstract 0397).** Results

| | 2015 | 2016 | 2017 | |
|---|---|---|---|---|
| patients | 186 | 176 | 170 | |
| Colonizations total/ • Acinet - • Klebs- • Pseud.aerog- • Staph.MRSA | 106/ 46- 28- 29- 3 | 74/ 43- 11- 14- 6 | 80/ 40- 28- 6- 6 | |
| VAP | 17 | 16 | 4 | p=0,01 |
| Days on ventilator (dmv) Total/ year Median/patient | | 1671 / 5 | 1778 / 4 | NS |
| Days to 1st detected colonization mean median | | 10,27±6,71 / 9 | 14,27±8,3 / 12 | p=0,004 |
| Total days in ICU (los) | 2354 | 1988 | 2318 | |
| dmv/los | | 84% | 76% | |
| Pharmaceutical expense | | 429.093E 218/per day | 378.621E 163/per day | |
| APACHE II score | 17,33 ±8,3 | 20,63±8,9 | 20,38±8,1 | |

### 0398

#### Does dosing of ceftriaxone in critically ill patients on ECMO need to change? A pharmacokinetics study

V. Cheng[1,2], K. Shekar[2], M.H. Abdul-Aziz[1], H. Buscher[3], A. Corley[2], A. Diehl[4], E. Gilder[5], P. Jarrett[2], I. Lye[2], S. McGuinness[5], R. Parke[5], V. Pellegrino[4], C. Reynolds[3], S.C. Wallis[1], S. Welch[3], D. Zacharias[6], J.F. Fraser[2], J.A. Roberts[1], ASAP ECMO Investigators

[1]University of Queensland, Faculty of Medicine, Brisbane, Australia; [2]Critical Care Research Group-The Prince Charles Hospital, Adult Intensive Care Services, Brisbane, Australia; [3]St Vincent's Hospital, Intensive Care Services, Sydney, Australia; [4]Alfred Hospital, Intensive Care Services, Melbourne, Australia; [5]Auckland City Hospital, Cardiothoracic and Vascular Intensive Care Unit, Auckland, New Zealand; [6]Inselspital, Universitätsklinik für Intensivmedizin, Bern, Switzerland

**Correspondence:** V. Cheng

*Intensive Care Medicine Experimental* 2018, **6(Suppl 2):**0398

**INTRODUCTION.** The growing adoption of extracorporeal membrane oxygenation (ECMO) into adult intensive care units (ICUs) has lead clinicians to re-evaluate the use of conventional dosing regimens in this patient population. Neonatal studies have suggested modified antimicrobial dosing regimens are necessary in the presence of ECMO to attain PK/PD targets for optimised bacterial kill and clinical cure. However, due to significant differences in physiology, these findings cannot be safely transferred to the adult population. Empirical antibiotics such as ceftriaxone have no defined titratable end-point, leading to possible sub- or supra- therapeutic management in this group of critically ill patients.

**OBJECTIVES.** The aim of this study is to describe the pharmacokinetics (PK) of ceftriaxone in critically ill patients receiving ECMO. Through the improved understanding of ECMO-induced PK effects, propose dosing strategies that are more likely to attain the PK/PD target of 60-70% $fT{>}_{MIC}$ to optimise therapeutic outcomes.

**METHODS.** This study was a multi-national, open-label study designed to describe the PK of ceftriaxone over one dosing interval. Critically ill adults with severe cardiac and/or pulmonary dysfunction from six intensive care units in Australia, New Zealand and Switzerland were eligible for recruitment. Serial blood samplings were taken over pre-defined time points over one dosing interval. Centralised bioanalysis occurred at the University of Queensland to determine plasma ceftriaxone concentration through validated chromatographic methods. The concentration-time data were then used to generate pharmacokinetic parameters through non-compartmental methods.

**RESULTS.** Nine patients were recruited to the study [Table 1]. One patient was excluded from analysis due to incorrect documentation. Non-compartmental analysis yielded a median (IQR) of 0.67 (0.5-0.85) L/h for total clearance and 12.19 (8.72-15.88) L for volume of distribution. Refer to Figure 1 for the total plasma concentration time graph.

**CONCLUSIONS.** The PK parameters generated by this study were generally consistent with published parameters of critically ill patients not receiving ECMO. It appears that conventional dosing of ceftriaxone in the critically ill population may provide the target attainment of 60-70% $fT{>}_{MIC}$. It is important to acknowledge the heterogeneity within the results and that further analysis and recruitment is required to produce robust recommendations in dosing antibiotics in adults on ECMO.

**REFERENCE(S)**

1. Shekar K, Roberts JA, Ghassabian S, Mullany DV, Wallis SC, Smith MT, et al. Altered antibiotic pharmacokinetics during extracorporeal membrane oxygenation: cause for concern? The Journal of antimicrobial chemotherapy. 2013;68(3):726-7.
2. Sherwin J, Heath T, Watt K. Pharmacokinetics and Dosing of Anti-infective Drugs in Patients on Extracorporeal Membrane Oxygenation: A Review of the Current Literature. Clinical therapeutics. 2016;38(9):1976-94.

**Table 1.1 (abstract 0398).** Patient demographic and clinical characteristics presented as median (IQR) or number (percentage)

| | |
|---|---|
| Age in years | 61.0 (34.0 - 74.0) |
| Male, n (%) | 6 (75.0) |
| Weight (kg) | 84.0 (75.0 - 90.0) |
| BMI (kg/m2) | 27.8 (23.1 - 28.7) |
| APACHE II | 19.5 (14.8 - 37.8) |
| SOFA | 7.0 (5.0 - 9.0) |
| VV-ECMO modality, n (%) | 4 (50.0) |
| Serum creatinine (μmol/L) | 99.0 (85.0 - 180.0) |
| Blood urea nitrogen (mmol/L) | 9.0 (4.4 - 14.1) |
| Albumin (g/L) | 29.0 (27.0 - 31.0) |

**Table 1.2 (abstract 0398).** Pharmacokinetic parameter estimates presented as median (IQR)

| | |
|---|---|
| AUC0-t (mg/L·h) | 1159.98 (764.33 - 1368.01) |
| AUC0-inf (mg/L·h) | 1812.66 (1388.34 - 2446.86) |
| Cmax (mg/L) | 136.0 (116.40 - 185.99) |
| Ke (h^(-1)) | 0.05 (0.05 - 0.08) |
| t1/2 (h) | 13.51 (9.28 - 14.97) |
| CL (L/h) | 0.67 (0.50 - 0.85) |
| CL/kg (L/h/kg) | 0.008 (0.006 - 0.012) |
| Vd (L) | 12.19 (8.72 - 15.88) |
| Vd/kg (L/kg) | 0.16 (0.11 - 0.19) |

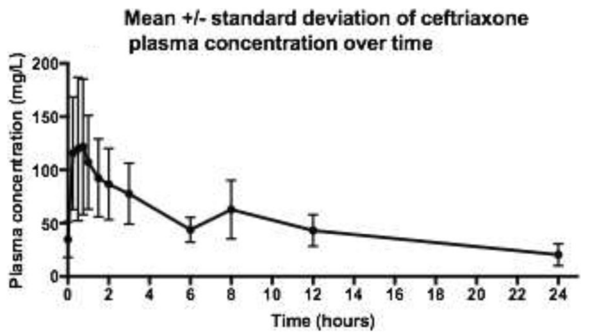

**Fig. 1 (abstract 0398).** Mean +/- standard deviation of ceftriaxone plasma concentration over time

### 0399

#### The first experience of targeted antibiotics for the regulation of the metabolic activity of the gut microbiota (MAGM) in critically ill patients with pneumonia or abdominal infection

N. Beloborodova, Y. Sarshor

Federal Research and Clinical Center of Intensive Care Medicine and Rehabilitology, Negovsky Research Institute of General Reanimatology, Moscow, Russian Federation

**Correspondence:** N. Beloborodova

*Intensive Care Medicine Experimental* 2018, **6(Suppl 2):**0399

**INTRODUCTION.** We suggest that antimicrobial therapy in critically ill patients should be aimed not only at the death of bacteria in the focus of infection, but also at reducing the metabolic activity of gut microbiota (MAGM). The gut is the main reservoir of bacteria and the main source of bacterial metabolites. Serum levels of some aromatic microbial metabolites (AMM) reflect MAGM and closely correlated with the severity of organ dysfunction and mortality: this has been shown previously for phenyllactic (PhLA), p-hydroxyphenylacetic (p-HPhAA), p-hydroxyphenyllactic (p-HPhLA) acids [1, 2].

**OBJECTIVES.** Try to correct the metabolic activity of microbiota and evaluate its effectiveness.

**METHODS.** For research purposes, we use enteral antibiotics that prevent protein synthesis in bacteria and/or possess anti-biofilm activity: gentamicin (GEN) 240 mg 2/day plus clarithromycin (CLAR) 500 mg 1/day. Adult patients (pts) with pneumonia or abdominal infection were divided into two groups: group 1 with GENT+CLAR in addition to standard treatment, and group 2 - only standard treatment. The levels of AMM were measured in blood serum using GC-FID [3]. Data of patients, the SOFA scales, serum levels of AMM, their sum (∑3AMM) were evaluated at admission to ICU and in dynamics, than analyzed by nonparametric methods of statistics (Statistica 10).

**RESULTS.** The study included 56 pts: 26 pts in 1st group, 30 pts in the 2nd group. On admission, patients of both groups were comparable in age and clinical state (APACHE II and SOFA scales).

The next day, patients of the 1st group had an improvement in the state - a decrease in the SOFA score by 29% (p< 0,05), and tendency towards lower p-HPhLA and p-HPhAA, whereas in the 2nd group were no positive dynamics in SOFA score and tendency towards higher p-HPhLA and p-HPhAA when comparing to baseline (Fig. 1). The correlation between SOFA and AMM exceeded r=0.6 (p< 0.05). The survival rate in 1st group was higher than in in the 2nd group (57% vs 46%, p> 0.05).

Conclusions. Despite the absence of significant differences, the downward trend of mortality in group 1 by 11% is encouraging. Enteric correction of metabolic activity of gut microbiota contributes to the improvement of indicators of organ dysfunction (SOFA). This means that it is advisable to further explore the possibilities of targeted antibiotic use to regulate the MAGM.

**GRANT ACKNOWLEDGMENT**

This study is supported by the Russian Science Foundation, Grant Number: 15-15-00110-П

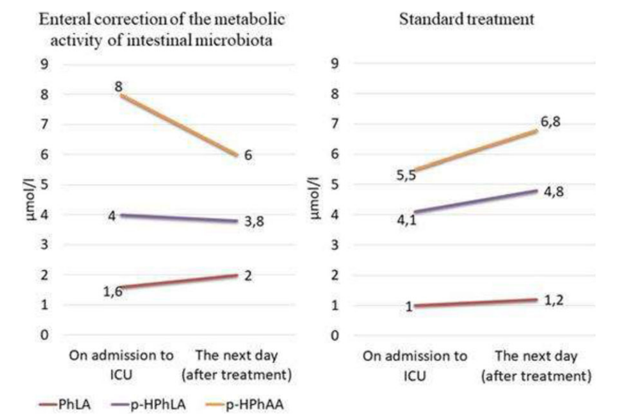

**Fig. 1 (abstract 0399).** Dynamics of AMM in groups (1 vs 2)

### 0400

#### Appropriate empirical antibiotic treatment rate in multirresistant ESKAPE bacteria in a critical care unit

O. Moreno Romero, M. Muñoz Garach, J. Tejero Aranguren, A. Carranza Pinel, I. Cruz Valero, M.E. Yuste Ossorio

Hospital Universitario San Cecilio, Granada, Spain

**Correspondence:** O. Moreno Romero

*Intensive Care Medicine Experimental* 2018, **6(Suppl 2):**0400

**INTRODUCTION.** Multiresistant bacteria (MRB) are an important Public Health issue as it increases the patient´s morbimortality rates, particularly in the critically ill. The choice of a correct empirical antibiotic is relevant as it may reduce the impact of these infections and the disseminations in our units.

**OBJECTIVES.** First, to describe the frequencies of MRB infection and colonization from ESKAPE microorganisms in our intensive care unit (ICU) in a twelve-month period. Second, to define our appropiate empirical antibiotic rate for MRB infections.

**METHODS.** Analysis of a prospectively collected database (ENVIN-HELICS registry of Spanish Society of Intensive Care) during 12 months (year 2017) in our ICU, 20 bed, mostly neurotraumatologic patients. We registered all positive cultures with ESKAPE germs (Enterobacteriae, SARM, Klebsiella, Acinetobacter, Pseudomona, Enterococcus). Cultures were divided into 2 categories. The first one, "colonization culture", as part of a surveillance program at the admission of patients at risk of harboring MRB (Zero Resistance). The second, "suspected infection", obtained when an infection was clinically suspected at admission or during ICU stay. From all “suspected infection”, we registered the empirical antibiotic therapy used. When the microorganism was identified, we analysed if the antibiotic was either maintained, changed or added a second one. Finally, with the antibiogram, we defined whether the empirical treatment was appropriate or not.

**RESULTS.** 1398 patients admitted to ICU in 2017. 61% male, 49% female. Mean age of 57±11. There were a total of 498 positive cultures, 111 (22.2%) with MRB-ESKAPE in 75 different patients. From these, 78 (70.3%) were “colonization cultures” and 33 (29.7%) “suspected infection”. The localization of these infections were: 25 (60%) respiratory, 8 (19%) urine tract, 5 (12%) bacteremia and 4 (9%) other infections.

In these suspected infections (33) an empirical antibiotic was initiated. After the identification of the microorganism: in 22 (66.66%) there were no modifications in treatment, in 7 (21.21%) the antibiotic was changed and in 4 (12.12%) we added a second one. After knowing the antibiogram: in 26 (78.78%) first empirical treatment was appropriate, in 3 (9%) we were right at the second election, and in 4 (12.12%) none of the antibiotic were correct. ESKAPE percentages were: 29% Enterobacteria, 25% SARM, 19% Klebsiella, 16% Pseudomona, 11% Acinetobacter and no vancomicine-resistant enterococcus.

**CONCLUSIONS.** Most frequent ESKAPE bacteria in our unit is the Enterobacteriae group. Along with SARM they represent more than half of MRB infections. Our ESKAPE MRB infection rate is 7.8% and the appropriate empirical antibiotic rate for these were 79%. In half of the 21% of inappropriate empirical antibiotic treatment was due to an unusual early infection with klebsiella MR in patients with no risk factors for developing infection or colonization with MRB.

### 0401

#### Effects of antibiotic prophylaxis on ventilator-associated pneumonia in severe traumatic brain injury. A post hoc analysis of two trials

F. Reizine[1], K. Asehnoune[2], A. Roquilly[2], B. Laviolle[3], C. Rousseau[3], C. Dayot-Fisselier[4], P. Seguin[1]

[1]CHU de Rennes, Département d'Anesthésie-Réanimation, Rennes, France; [2]CHU de Nantes, Département d'Anesthésie-Réanimation, Nantes, France; [3]CHU de Rennes, Centre d'Investigation Clinique, Rennes, France; [4]CHU de Poitiers, Département d'Anesthésie-Réanimation, Poitiers, France

**Correspondence:** F. Reizine

*Intensive Care Medicine Experimental* 2018, **6(Suppl 2):**0401

**INTRODUCTION.** Ventilator-associated pneumonia (VAP) is one of the most frequent nosocomial infections in intensive care patients, particularly in traumatic brain injury (TBI), with an incidence ranging from 28 to 67% (1-2). Although its attributable mortality in trauma seems close to zero, reports have emphasized the potential deleterious effect of VAP in TBI patients (3-4).

**OBJECTIVES.** We aimed to investigate the role of antibiotic prophylaxis (AP) on the incidence of VAP in patients suffering from severe TBI.

**METHODS.** This post hoc analysis of 2 multicentre double studies extracted and pooled data on severe TBI (Glasgow coma scale ≤8). Patients were differentiated into 2 groups: those who received AP before the occurrence of VAP (AP group) and those who did not receive prophylaxis (control group). The main objective was to evaluate the impact of AP on the incidence of VAP (early VAP (>2 days and ≤5 days) and late VAP (>5 days)).
**RESULTS.** A total of 295 patients were included in the analysis (AP group, n=146 and control group, n=149). The global incidence of VAP was 145 (49%), early VAP was 62 (43%) and late VAP was 83 (57%). The rate of VAP was significantly lower in the AP group than in the control group (n=57 [39%] vs n=88 [59%], Relative Risk=0.33, 95% CI, 0.19-0.56, p=0.001) and time to VAP occurrence was significantly reduced (hazard ratio=0.50, 95% CI 0.36-0.69, p< 0.001). The rate of early VAP was significantly lower in the AP group (15 [10%] vs 47 [32%]; p< 0.001), whereas that of late VAP did not differ between the 2 groups (42 [29%] in the AP group vs 41 [28%] in the control group; p=0.811).
The total duration of antibiotic exposure was significantly lower in the AP group. ICU length of stay and mortality in the ICU and at day 28 were not significantly different between the 2 groups.
**CONCLUSIONS.** In a large population of severe TBI, AP decreased the incidence of early VAP. It remains to be seen whether this treatment has a significant impact on bacterial ecology and late neurologic functional outcomes.

**REFERENCE(S)**

1. Sirvent JM, Torres A, El-Ebiary M, et al. Protective effect of intravenously administered cefuroxime against nosocomial pneumonia in patients with structural coma. *Am J Respir Crit Care Med*. 1997; **155**: 1729-34.
2. Vallés J, Peredo R, Burgueño MJ, et al. Efficacy of single-dose antibiotic against early-onset pneumonia in comatose patients who are ventilated. *Chest*. 2013; **143**: 1219-25.
3. Melsen WG, Rovers MM, Groenwold RH, et al. Attributable mortality of ventilator-associated pneumonia: a meta-analysis of individual patient data from randomised prevention studies. *Lancet Infect Dis*. 2013; **13**: 665-71.
4. Esnault P, Nguyen C, Bordes J, et al. Early-onset ventilator-associated pneumonia in patients with severe traumatic brain injury: incidence, risk factors, and consequences in cerebral oxygenation and outcome. *Neurocrit Care*. 2017 . doi: 10.1007/s12028-017-0397-4.

### 0402

#### The effects of intervention by a ward pharmacist on vancomycin blood level control in the emergency medical center

K. Takahashi, T. Tatsumichi, K. Yamaguchi, H. Tanaka, S. Kosaka, H. Hochi
Kagawa University Hospital, Kita, Japan
**Correspondence:** K. Takahashi

*Intensive Care Medicine Experimental* 2018, **6(Suppl 2):**0402

**INTRODUCTION.** Vancomycin (VCM) has a narrow therapeutic range, therefore, therapeutic drug monitoring is necessary for effective and safe use. At Kagawa University Hospital, starting from April 2016, we assigned a ward pharmacist to the emergency medical center and began intervening for blood level control for VCM administered patients. This research will report what effects have been confirmed through the process of these interventions.
**OBJECTIVES.** To research the effects of intervention by a ward pharmacist on VCM blood concentration control in the emergency medical center.
**METHODS.** We compared two sets of data, patients who received VCM in intravenous infusions during a period in which we had not assigned a ward pharmacist (April to October 2015), and during a period in which we had assigned a ward pharmacist (April to October 2016). The data collected included the implementation rate of dosing regimen initiated by a pharmacist, retention rate of blood concentration maintained in the therapeutic range (10-20 μg/mL) and in the toxic range (over 20 μg/mL), duration of administration, survival rate on 28th day after the start of administrations, the number of days stayed in the life emergency medical center, incidence of renal dysfunction and compare them to before and after assigning a ward pharmacist.
**RESULTS.** There were 15 subjects before the assignment of a ward pharmacist and 12 subjects after the assignment. The implementation rate of dosing regimen initiated by a pharmacist had significantly increased after assignment (20.0% vs 83.3%, p = 0.002). The days spent in the emergency medical center had significantly decreased after assignment (median: 19.0 days vs 9.5 days, p = 0.032). The retention rate of blood concentration maintained in the toxic range decreased after significant resident (45.1% vs 9.1%, p = 0.003).
**CONCLUSIONS.** Up until now, there was no pharmacist stationed in the emergency medical center of our hospital, and we had only limited intervention in VCM blood concentration management. However, as a ward pharmacist has been assigned, it has become possible to participate in designing proactive administration schedules from an early stage of VCM administration in almost all cases, which is thought to have led to a decrease in the rate of intoxication. In addition, decrease of intoxication rates leads to prevention of adverse events and contributes to the shortening of days in which patients stay within the ward. It is expected that continued active intervention in the blood concentration control of VCM by ward pharmacists will lead to a good therapeutic effect and treatment outcome in the future.

**REFERENCE(S)**

Zhe Han, et al. Impact of Pharmacy Practice Model Expansion on Phrmacokinetic Services: Optimization of Vancomycin Dosing and Improved Patient Safety. Hospital Pharmacy. 2017; 52(4), 273-279.

**GRANT ACKNOWLEDGMENT**
Nothing

### 0403

#### Antifungal treatment in peritonitis: do we need it?

P. Bernardo[1], S. Fernandes[1,2], D. Pinto[2], G. Nobre Jesus[1,3], D. Silva[1,2], Á. Pereira[2], A. Alvarez[2], J. Gouveia[2]
[1]Faculdade de Medicina da Universidade de Lisboa, Lisboa, Portugal; [2]Hospital de Santa Maria - Centro Hospitalar de Lisboa Norte, EPE, Lisboa, Portugal; [3]Hospital de Santa Maria - Centro Hospitalar de Lisboa Norte, EPE, Serviço de Medicina Intensiva, Lisboa, Portugal
**Correspondence:** S. Fernandes

*Intensive Care Medicine Experimental* 2018, **6(Suppl 2):**0403

**Background:** Pre-emptive fungus coverage in patients with secondary peritonitis and sepsis is still controversial. Expert opinion recommends its use in upper gastro-intestinal lesion and in patients with septic shock (1, 2). However, there is still no accurate score to predict fungal infection or when to decide to provide antifungal.
**OBJECTIVE.** We aimed to evaluate antifungal usage in patients with peritonitis admitted to the ICU and its impact on mortality, as well as to identify factors related to positive culture to fungus.
**METHODS.** We performed a retrospective study of patients submitted to urgent abdominal surgery due to peritonitis. We analyzed global mortality and its relationship with empirical antifungal treatment and the presence of positive culture for fungus in the blood or products collected during surgery. Statistical analysis was performed using STATA.
**RESULTS.** We included 188 patients between 2016 and 2017 admitted after gastro-intestinal urgent surgery, in which 77% was the first intervention. Patients were predominantly male (53.7%), with a mean age of 69.1 (±15.2), a mean SAPSII of 51.6 ( ±19.3), and an admission SOFA of 7.6 (±4.3). Importantly 53.7% had septic shock.
Fungus were isolated in only 8.5% of patients (n=16) during the first 4 days of ICU admission, with a median length of stay in hospital of 4.5 days until isolation. 81% of isolates were Candida albicans or Candida glabrata. All the fungal identification were done in products collected during surgery excepted in one patient that had candidemia. Empirical fungal coverage was provided to 20.7% (n=39), 8 of which had fungal infection documented later on, but mortality was not related to antifungal prescription.
ICU mortality in patients without fungal isolation was 26.1%, and 31.3% in patients with fungal isolation (p=0.66). Significantly, SAPSII, time in hospital, number of previous surgeries, gender, age and segment of gastrointestinal lesioned were not linked with a high risk for fungal isolation. Of note, there was a trend for septic shock patients to have increased risk of fungal infection (odds ratio 2.8, P=0.08), even when adjusted for SAPSII.

**CONCLUSION.** There is a need to better define indication for fungal coverage in patients with peritonitis(1). Although limited by the low number of patients with positive cultures, we propose that antifungal coverage is not needed in peritonitis in the first event that leads to ICU admission. These results will be further confirmed in a larger dataset.

#### 0404

#### Target controlled infusion for dose optimization of cefepime in critically ill patients

A. Grigoryan[1], S. Jonckheere[2,3,4], N. De Neve[1], K. De Decker[1], J. Verbeke[1], I. Brandt[2], A. Boel[2], J. Van Bocxlaer[4], M. Struys[3], P. Colin[3,4]
[1]OLV Aalst, Anesthesiology, Aalst, Belgium; [2]OLV Aalst, Microbiology, Aalst, Belgium; [3]Univeristy Medical Center Groningen, Anesthesiology, Groningen, Netherlands; [4]Ghent University, Laboratory for Medical Biochemistry and Clinical Analysis, Faculty of Pharmaceutical Sciences, Ghent, Belgium
**Correspondence:** A. Grigoryan
*Intensive Care Medicine Experimental* 2018, **6(Suppl 2):**0404

**BACKGROUND.** Target Controlled Infusion (TCI) is a technique of infusing IV drugs to quickly achieve a user-defined drug concentration in a specific body compartment. TCI systems have been widely implemented in the field of anesthetics, but have never been applied for antibiotic dosing. As there is growing evidence that pharmacokinetic/pharmacodynamic (PK/PD) target attainment is related to an improved clinical outcome, TCI seems an attractive tool to accurately achieve target concentrations. In this study we have used TCI to administer cefepime in critically ill patients.

**MaATERIAL/METHODS.** A previously developed population pharmacokinetic (PopPK) model for cefepime was integrated in Rugloop II software (Demed, Belgium). Rugloop II calculated the amount of cefepime required to quickly achieve and maintain target concentrations. A Fresenius Orchestra syringe pump was connected to Rugloop II for drug delivery. The estimated creatinine clearance (eCrCl) based on the Cockcroft-Gault formula was used as an input variable to calculate cefepime clearance. A cefepime blood concentration of 16 mg/L was targeted and a total of 21 ICU patients were treated using TCI for a median time of 4.5 days. During therapy there were no adaptations based on changes in eCrCl or cefepime concentration measurements. To evaluate the measured versus predicted plasma concentrations, blood samples were taken (median number of samples per patient: 13) and total cefepime concentrations were measured using UPLC-MS/MS. Performance of the TCI system was characterized using the Varvel criteria.

**RESULTS.** The median prediction error (MdPE) was 21.1%, median absolute prediction error (MdAPE) was 32.0%, wobble was 11.4%, and divergence was -3.72%.$h^{-1}$. Clinical trial simulation showed that the results for MdAPE, wobble and divergence were as expected by the TCI model. The positive bias (MdPE) was caused by an overestimation of the volume of the central compartment in the TCI model. A combined fit of the original and the new dataset showed that the volume of distribution for cefepime is 12.2 L and not 18.3 L as previously estimated.

**CONCLUSIONS.** Measured cefepime concentrations were close to those predicted using TCI in critically ill ICU patients. TCI is a promising tool for individualized dose optimization of antibiotics.

## Epidemiology & diagnosis of AKI

#### 0405

#### Hospital-acquired and community-acquired acute kidney injury in critically ill patients with renal replacement therapy requirements: an observational study

E.P. Plata-Menchaca[1], H. Alanez-Saavedra[2], L. Anguela-Calvet[2], V.D. Gumucio-Sanguino[2], A. Farré-Estebe[2], K. Maisterra-Santos[2], D. Berbel-Franco[2], G. Muñóz-del Río[2], J. Sabater-Riera[2], X.L. Pérez-Fernández[2], J.A. Kellum[3]
[1]Institut d´Investigació Biomèdica de Bellvitge, Research, L'Hospitalet de Llobregat, Spain; [2]Hospital Universitari de Bellvitge, Critical Care Medicine, L'Hospitalet de Llobregat, Spain; [3]University of Pittsburgh Medical Centre, Critical Care Medicine, Pittsburgh, United States
**Correspondence:** E.P. Plata-Menchaca
*Intensive Care Medicine Experimental* 2018, **6(Suppl 2):**0405

**INTRODUCTION.** Patients developing an AKI episode in the community (known as *community-acquired AKI*, CA-AKI) have been described as having different clinical features and better outcomes from *hospital-acquired* AKI patients (HA-AKI). However, previous studies have included mixed populations of AKI patients. If better outcomes still could be expected once CA-AKI has progressed to severe AKI with renal replacement therapy (RRT) requirements is unclear.

**OBJECTIVES.** To compare the aetiologies, clinical characteristics, severity and outcomes of critically ill patients with CA-AKI and HA-AKI requiring RRT during ICU stay.

**METHODS.** From 2006 to 2016, 889 adult critically ill patients older than 18 years diagnosed with severe acute kidney injury with RRT requirements were included and classified into CA-AKI and HA-AKI according to prespecified criteria. Clinical characteristics, RRT dependence, inhospital mortality, hospital and ICU length of stay, and other secondary outcomes were registered. Comparison of baseline and follow-up serum creatinine values was done using analysis of variance (ANOVA) test. Time-to-event distributions (survival curves) were estimated using the Kaplan-Meier method. We performed a Cox regression model to analyse risk factors for RRT dependence and mortality. Robustness of the results was assessed using a sensitivity analysis.

**RESULTS.** Of the 889 patients with severe AKI, 56.2% (500 patients) had CA-AKI, from which 78% had AKI stage 3 before RRT; 43.7% (389 patients) had HA-AKI and 66% AKI stage 3 before RRT. HA-AKI patients had a higher number of pre-existing medical conditions (Charlson Comorbidity Index) (3.1 ± 2 *vs* 1.8 ± 2; $P < 0.0001$), than CA-AKI, including chronic kidney disease (CKD) (9.5% vs 4.4%, p< 0.0001) and chronic liver failure (90(28%) vs 81(17%), p=0.001). CA-AKI patients had worse Acute Physiology And Chronic Health Evaluation II (APACHE II) scores and higher baseline sCr values (70 vs 66 μmol/L, p< 0.0001). Peak sCr levels during the first 24 hours of ICU admission were 305 mmol/L vs 207 mmol/L (p< 0.0001). sCr at discharge did not differ between the groups, however, hospital LOS was significantly longer for HA-AKI compared with CA-AKI (63±54 vs 43±37 days, p< 0.0001). Overall in-hospital, 30-day and 90-day mortality was 30.7% vs 23.3% (p= 0.216) in CA-AKI and HA-AKI respectively. There were no differences neither in duration of CRRT, nor in mechanical ventilation free days. Sensitivity analyses were applied in order to confirm the replicability of these findings (p < 0.001).

**CONCLUSIONS.** This study found no differences on mortality rates between CA-AKI and HA-AKI in a population of critically ill patients with severe AKI with RRT requirements.

**REFERENCE(S)**

Inokuchi R, Hara Y, Yasuda H, Itami N, Terada Y, Doi K. Differences in characteristics and outcomes between community- and hospital-acquired acute kidney injury: A systematic review and meta- analysis. Clin Nephrol 2017;88:167-182.

### 0406

#### Usefulness of NephroCheck™ test for the therapeutic approach of acute kidney injury in critically ill patients

G. Echarri[1], C.I. Alfaro[2], P. Duque-Sosa[1], M. Izaguirre[3], M. Sagardoy[1], O.J. González-Aróstegui[2], P. Monedero[1], N. García-Fernández[2]
[1]Clinica Universidad de Navarra, Anesthesiology, Pamplona, Spain; [2]Clinica Universidad de Navarra, Nephrology, Pamplona, Spain; [3]Clinica Universidad de Navarra, Biochemistry, Pamplona, Spain
**Correspondence:** G. Echarri
*Intensive Care Medicine Experimental* 2018, **6(Suppl 2):**0406

**INTRODUCTION.** Acute kidney injury (AKI) has a high incidence in critical ill patients[1]. Recently, biomarkers have been developed to detect AKI earlier and their implementation could be able to contribute to an early therapeutic approach leading to better outcomes[1].

**OBJECTIVES.** To evaluate the predictive value of biomarkers TIMP2 and IGFBP7 (NephroCheck™) for early detection of renal replacement therapy (RRT) requirements. METHODS A retrospective study was performed. We enrolled adult patients admitted in the intensive care unit (ICU) whom the NephroCheck™ was tested in a period between February 2015 and June 2017. The predictive value of NephroCheck™ was assessed by a receiver operating characteristic curve (AUC).

**RESULTS.** 123 patients were analyzed. Agree to KDIGO criteria, 24.4% had AKI I, 46.3% AKI II and 29.3% AKI III. 50.4% patients required RRT.

According to the previous publications, the NephroCheck™ was classified into three risk categories[1]. Table 1 shows the comparison of the variables analyzed by the three groups.

There were no differences in baseline renal function based on estimation of glomerular filtration rate for the three groups. The high-risk patients, had more oliguria 12, 24 and 48 h (p< 0.001) after admission and they took more time to recover di-uresis (p< 0.001). The AUC of NephroCheck™ for urine output at 12, 24 and 48h was 0.728, 0.703 and 0.724 (p < 0.001) re-spectively. And the AUC of NephroCheck™ for predicting RRT was 0.719 (p< 0.001).

CONCLUSIONS: NephroCheck™ is able to predict oliguria and RRT requirements. In the future, this test could be recommended to contribute to therapeutic decisions in critically ill patients.

**REFERENCE(S)**

1. Vijayan A, et al. Clinical Use of the Urine Biomarker [TIMP-2] × [IGFBP7] for Acute Kidney Injury Risk Assessment. Am J Kidney Dis. 2016 Jul;68(1):19-28.

**GRANT ACKNOWLEDGMENT**

The authors thank the Clinic-University of Navarra for their support for the research.

**Table 1 (abstract 0406).** Comparison of the variables analyzed by the three groups. Continuous variables are given as mean (standard deviation); categorical, as number (%)

| | Low-risk <0.3 (ng/ml)2/1000 N=44 | Intermediate-risk 0.3-2 N=43 | High-risk >2 N=36 | P |
|---|---|---|---|---|
| Urine output coincident NephroCheck ™ (ml/kg/h) | 1.0 (0.89) | 0.77 (0.75) | 0.25 (0.44) | <0.001 |
| Urine output after 12h to the test | 1.99 (1.69) | 1.95 (1.74) | 0.55 (0.89) | <0.001 |
| Urine output after 24h | 1.32 (1.23) | 1.03 (0.96) | 0.34 (0.57) | <0.001 |
| Urine output after 48h | 1.3 (0.96) | 1.17 (0.97) | 0.46 (0.82) | <0.001 |
| Diuresis recovery time: <12h 12-24h >24h No recovery | 26 (29.1) 7 (15.9) 10 (22.7) 1 (2.3) | 17 (39.5) 11 (25.6) 12 (27.9) 3 (7) | 7 (19.4) 2 (5.6) 20 (55.6) 7 (19.4) | <0.001 |
| Outcomes | | | | |
| RRT | 13 (29.5) | 22 (51.2) | 27 (75) | <0.001 |
| Length of ICU stay | 5.8 (7.08) | 7.49 (6.01) | 11.56 (14.46) | 0.027 |
| Number of days of CRRT | 1.6 (3.4) | 1.6 (2.7) | 4.6 (4.7) | <0.001 |

### 0407

#### Preoperative prediction of acute kidney injury requiring renal replacement therapy after left ventricular assist device implantation: comparison of three clinical risk scores

K. Pilarczyk[1], H. Carstens[2], J. Heckmann[2], A. Koch[2], H. Jakob[2], M. Kamler[2], N. Pizanis[2]
[1]imland Klinik Rendsburg, managed by Sana Kliniken AG, Department of Intensive Care Medicine, Rendsburg, Germany; [2]West German Heart and Vascular Center Essen, University Hospital Essen, Department of Thoracic and Cardiovascular Surgery, Essen, Germany
**Correspondence:** K. Pilarczyk
*Intensive Care Medicine Experimental* 2018, **6(Suppl 2):**0407

**INTRODUCTION.** Postoperative acute kidney injury (AKI) is a prominent problem in patients undergoing LVAD implantation and associated with increased morbidity and mortality. Although several risk models for the prediction of postoperative renal replacement (RRT) have been widely used in cardiothoracic patients, none of these scoring systems have been validated in patients after LVAD implantation.

**OBJECTIVE.** Validation of three clinical risk scores for the prediction of AKI requiring RRT after LVAD-implantation.

**METHODS.** A retrospective analysis of all patients undergoing LVAD-implantation at our institution between 09/2013 and 07/2016 was performed. Primary outcome measure was AKI requiring RRT within 14 days after LVAD implantation. The predictive capacity of the Cleveland Clinic Score (CCS), the Society-of-Thoracic-Surgeons-Score (STS) and the simplified renal index score (SRI) were evaluated using receiver-operator characteristic curve analyses and Hosmer-Lemeshow test.

**RESULTS.** During the observation period, 76 patients underwent LVAD-implantation, 19 patients were excluded due to preoperative RRT. RRT was associated with a prolonged ventilation time (2.41±5.86 vs. 14.13±18.88 d, p< 0.001), LOS on the ICU (6.12±7.49 vs. 26.78 ±

27.07 d, p< 0.001) and 180 day mortality (14(60.9%) vs. 6(17.6%), p< 0.01).

Patients with postoperative RRT were characterized by a higher serum creatinine (1.86±1.3 vs. 1.36±0.4 mg/dl, p=0.04), a higher Alanine transaminase/ Aspartate aminotransferase (De Ritis-Ratio) (1.45±1.05 vs. 0.89±0.56, p= 0.01), and a higher INR (1.59±1.05 vs. 1.19±0.4, p=0.04).

Whereas the CCS (7.43±1.75 vs. 6.44±1.44, p=0.02) and the STS (28.12±15.08 vs. 21.53±5.43,p=0.02) were significantly higher in patients with RRT than in those without RRT, the SRI did not differ between these groups (3.96±1.15 vs. 3.44±1.05, p =0.08). Using ROC-analyses, CCS, STS and SRI showed moderate predictive capacity for AKI with need for RRT with an AUC of 0.661 ± 0.073 (95%CI 0.518-0.804, p =0.040), 0.637 ±0.079 (95%CI 0.082- 0.482, p =0.792) and 0.618±0.075 (95%CI 0.135 -0.471,p =0.764) respectively with comparable accuracy in the Delong test. Using univariate logistic regression analysis, only the De Ritis-Ratio (OR 2.67, 95% CI 1.08-6.62, p = 0.034) and MELD (OR 1.11, 95% CI 1.01-1.22, p= 0.028) were identified as predictors of postoperative RRT.

**CONCLUSION.** Our data suggest that risk scores derived from general cardiac surgery cannot predict RRT in patients after LVAD implantation. Therefore, it seems to be necessary to develop a specific risk score for this patient population.

### 0408

#### Acute kidney injury after Kasai portoenterostomy for biliary atresia

M.S. Song, J.H. Park
Yonsei University College of Medicine, Department of Anaesthesiology and Pain Medicine, Seoul, Korea, Republic of
**Correspondence:** J.H. Park
*Intensive Care Medicine Experimental* 2018, **6(Suppl 2):**0408

**INTRODUCTION.** Acute kidney injury (AKI) after surgery is associated with high morbidity and mortality in children. Biliary atresia (BA) is a progressive inflammatory destructive process of the bile ducts. Children with BA are at risk of AKI because of their young age at the time of surgery, long operation time and liver failure.

**OBJECTIVES.** The aim of this study was to evaluate the incidence and risk factors for postoperative AKI after Kasai operation.

**METHODS.** We performed a retrospective investigation of 100 children undergoing elective Kasai operation between November 2005 and December 2015. The incidence of AKI was assessed on the basis of Acute Kidney Injury Network criteria. Children were divided into 2 groups depending on the occurrence of AKI. Multivariable logistic regression models were developed to analyze risk factors for AKI.

**RESULTS.** AKI occurred in 34 of 100 children (34 %). Age older than 30 days of age at the time of surgery was the leading cause for AKI. Lower pre-operative serum creatinine and hemoglobin levels were independent risk factors for AKI. Children born before 37 weeks of gestation had lower incidence of AKI.

**CONCLUSIONS.** Increased age at the time of surgery had progressive and deleterious effects on postoperative AKI in children undergoing Kasai operation. These finding indicate the importance of early diagnosis and treatment for BA.

##### REFERENCE(S)

1. Basu RK, Devarajan P, Wong H, Wheeler DS. An update and review of acute kidney injury in pediatrics. Pediatr Crit Care Med 2011;12:339-47.
2. Andreoli SP. Acute kidney injury in children. Pediatr Nephrol 2009;24:253-63.
3. Altman RP, Lilly JR, Greenfeld J, Weinberg A, van Leeuwen K, Flanigan L. A multivariable risk factor analysis of the portoenterostomy (Kasai) procedure for biliary atresia: twenty-five years of experience from two centers. Ann Surg 1997;226:348-53; discussion 53-5.

##### GRANT ACKNOWLEDGMENT

This study was supported by a new faculty research seed money grant of Yonsei University College of Medicine for 2017 (2017-32-0028).

### 0409

#### Predicting factors of mortality during the first 24 hours of continous renal replacement therapy in patients with acute renal injury stage 3 in an intensive care unit

N.E. Carrillo Molina[1], J. Franco Granillo[1], J.S. Aguirre Sanchez[1], G. Camarena Alejo[2]
[1]American British Cowdray Medical Center I.A.P., Critical Care, México, Mexico; [2]American British Cowdray Medical Center I.A.P., Intensive Care Unit, México, Mexico
**Correspondence:** N.E. Carrillo Molina
*Intensive Care Medicine Experimental* 2018, **6(Suppl 2):**0409

**INTRODUCTION.** AKI refers to an abrupt decrease in kidney function, established in hours to days. Several consensus definitions of AKI have been developed in order to provide a uniform definition. AKI occurs in more than 50% of critically ill patients, 23% need renal replacement therapy, preferring continuous therapies. However mortality seems not to change with this technology.

**OBJECTIVES.** Identify factors thay may predict mortality in AKI patients in the first 24 hours of the star of continuos renal replacement therapy (CRRT).

**METHODS.** Restrospective cohort study in a single center. Included patients with AKI 3 under CRRT. We estimate the global mortality, total free time of CRRT and risk factors for mortality.

**RESULTS.** 48 patients were included 56.3% were masculine with an average age of 69 years (SD±14.7). Overall mortality during ICU stay was 68% of all patients and the average length of stay was 14 days (IQR 6-28). 85% of all patients required CRRT with 140 hours free of CRRT (IQR 63-221). Creatinine and blood urea nitrogen at the time of diagnosis of AKI was 2.7 mg/dl (SD ±1.6) and 62.6 mg/dl (SD ±31). The average scores severity scores at the time of admission were APACHE II 24 points (SD ±8), SOFA 13 points (SD ±2), SAPS III 70 points (SD ±12). Median lactate levels at the time of admission were 2.3 mEq/L (IQR 1.2-2.7). The variables with best capacity to predict mortality where creatinine and BUN at the time of admission with an AUC of 0.59 (p=0.07) and AUC 0.73 (p=0.01) respectively. Severity scores $AUC_{APACHEII}$=0.56, $AUC_{SOFA}$=0.51, $AUC_{SAPSIII}$=0.55.

**CONCLUSIONS.** The variables predictive capacity for mortality at the time of admission or at the time of diagnosis of AKI were creatinine >2.0 mg/dl HR = 2.5 (IC 95% 1.13 - 5.9, p=0.024), APACHE II >= 20 points HR = 2.7 (IC 95% 1.17-6.2, p=0.019). The rest of variables had no predictive capacity at the time of admission and where not associated as independent risk factors at the time of admission for mortality.

##### REFERENCE(S)

1. Zarbock A,JAMA. 2016;315:2190-9.
2. Gaudry S,N Engl J Med. 2016;375:122-33.
3. Prasad B,Canadian Journal of Kidney Health and Disease. 2016;3:36. doi:10.1186/s40697-016-0124-7.
4. Kawarazaki H,Hemodial Int. 2013 Oct;17(4):624-32.

### 0410

#### Acute kidney injury in a medical ICU: a prospective observational study

M. Simsek, E. Ortac Ersoy, E.K. Kaya, S. Ocal, A. Topeli
Hacettepe University Faculty of Medicine, Medical Intensive Care Unit, Ankara, Turkey
**Correspondence:** M. Simsek
*Intensive Care Medicine Experimental* 2018, **6(Suppl 2):**0410

**INTRODUCTION.** Acute kidney injury (AKI) is a common and a serious complication among patients admitted to ICUs.

**OBJECTIVES.** The aim of this study was to describe the incidence, severity and outcome of AKI among patients admitted to a medical ICU; and to compare the characteristics of AKI and non-AKI patients.

**METHODS.** This is the preliminary report of patients admitted to the medical ICU of Hacettepe University Faculty of Medicine between 1st February and 1st April 2018. All adult patients admitted to ICU except those who died within first 24 hours of admission were included. Re-admitted patients and patients on chronic hemodialysis were excluded, as well. Diagnosis and staging of AKI was based on KDIGO (Kidney Disease Improving Global Outcomes) criteria as any of the following: increase in serum creatinine (Cr) by >0.3 mg/dl within 48 hours, increase in Cr to 1.5 times from baseline which is known or presumed to have occurred within the prior 7 days or urine volume < 0.5 ml/kg/hr for 6 hours. Positive fluid balance was calculated as total fluid intake minus total fluid output divided by body weight (x100) and more than 5% is accepted as positive fluid balance. Variables during the first 15 days after admission are reported.

**RESULTS.** Fourty-two patients were included. Twenty (47.6%) patients were male and mean age (±SD) was 64.8±19.5 years. Mean APACHE II and SOFA scores were 21.5±8.3 and 6.8±4.5, respectively. Major reasons for ICU admission were respiratory failure (n=16; 38.1%) and sepsis (n=15; 35.7%). Twenty eight (66.7%) patients received invasive mechanical ventilation and total of 25 (59.5%) patients had sepsis during ICU stay. AKI was present in 24 (57.1%) patients according to KDIGO classification (22 AKI cases on admission, 2 during ICU stay). Stage 1 AKI was present in 12 (50.0%) patients, stage 2 in 2 (8.3%) and stage 3 in 10 (41.7%) patients. Mean ICU length of stay was 10.3±12 days. ICU mortality and hospital mortality were 23.8% and 26.2%, respectively. Renal replacement therapy was admitted to 13 (54.2%) patients with AKI. Of them, 11 patients received intermittent hemodialysis, 1 received continuous renal replacement therapy and 1 received both. Statistically significant variables in the comparison of AKI and non-AKI patients are shown in the Table. AKI patients had higher APACHE II and SOFA scores, basal and peak creatinine levels, peak BUN levels, sepsis, shock and positive fluid balance, and they received mechanical ventilation more than non-AKI patients, whereas they had lower albumin levels. AKI patients had higher ICU and hospital mortality rates than non-AKI patients.

**CONCLUSIONS.** AKI was present in 57.1% of critically-ill patients admitted to the medical ICU. In 91.7% of patients AKI was present on admission. ICU and hospital mortality rates were higher in AKI than non-AKI patients.

**Table 1 (abstract 0410).** Comparison of AKI and non-AKI patients

| | ALL n=42 | AKI n=24 | Non- AKI n=18 | P |
|---|---|---|---|---|
| APACHE II | 21.5±8.3 | 24.9±7.4 | 17.3±7.7 | 0.002 |
| SOFA | 6.8±4.9 | 9.4±3.6 | 3.2±2.9 | <0.001 |
| Invasive mechanical ventilation | 28 (66.7%) | 19 (79.2%) | 9 (50%) | 0.047 |
| Sepsis during ICU stay | 25 (59.5%) | 19 (79.2%) | 6 (33.3%) | 0.003 |
| Shock during ICU stay | 21 (50.0%) | 7 (29.2%) | 14 (77.8%) | 0.002 |
| ICU Mortality | 10 (23.8%) | 9 (37.5%) | 1 (5.6%) | 0.017 |
| Hospital Mortality | 11 (26.2%) | 9 (37.5%) | 2 (11.1%) | 0.014 |

**Table 2 (abstract 0410).** Comparison of Laboratory variables and fluid balance between AKI and non-AKI patients

| | ALL n=42 | AKI n=24 | Non- AKI n=18 | p |
|---|---|---|---|---|
| Basal Creatinine, mg/dl | 0.9±0.6 | 1.1±0.6 | 0.6±0.2 | <0.001 |
| Peak Creatinine during ICU stay, mg/dl | 2.16±2.0 | 3.2±2.0 | 0.7±0.2 | <0.001 |
| Peak BUN during ICU stay, mg/dl | 57.2 ±35.7 | 79.9 ±29.2 | 27.0±15.1 | <0.01 |
| Median albumin during ICU stay, g/dl | 2.51±0.5 | 2.3±0.5 | 2.7±0.4 | 0.043 |
| Positive fluid balance during ICU stay, >5% * | 26 (61.9%) | 19 (79.2%) | 7 (38.9%) | 0.014 |

### 0411

#### Epidemiology study of patients with advanced chronic kidney disease admitted in intensive care

M.A. Martinez Arcos[1], M. Guerrero Marin[1], E. Merino Garcia[2]
[1]Complejo Hospitalario de Jaen, Unidad de Cuidados Intensivos, Jaen, Spain; [2]Complejo Hospitalario de Jaen, Nefrologia, Jaen, Spain
**Correspondence:** M. Guerrero Marin
*Intensive Care Medicine Experimental* 2018, **6(Suppl 2):**0411

**INTRODUCTION.** It is known that the main reasons for admission in different intensive care units are shock followed by cardiac disease, neurologic, respiratory failure and septic conditions. The main studies published so far date from the general population.

**OBJECTIVES.** To analyze the epidemiology in patients with stage 3-4 chronic kidney disease admitted to intensive care.

**METHODS.** A single-center retrospective descriptive study. Inclusion period of six years. Qualitative variables are expressed in frequencies, and quantitative variables using mean, median, range and standard deviation, according to the probability distribution. Five groups of patients were established:

1. Cardiac (arrhythmia, acute pulmonary edema, acute coronary syndrome, cardiac tamponade),
2. Septic (endocarditis, abdominal post-operative, others),
3. Cerebrovascular disease(CVD),
4. Hemorrhagic Shock (post-surgery urological, vascular, gastrointestinal bleeding, ...) and
5. Others (pancreatitis, respiratory insufficiency, hepatic failure and cholelithiasis).

**RESULTS.** 99 patients, 59% female and 41% male, were analyzed. Mean age was 70 ± 12 years old. APACHE II 22.8 ± 9.2 points. The average stay was 7.3 ± 13.4 days. Analyzing these three variables as a cause of admission to the age differences between the septic vs cardiac (73.3 ± 10.3 in cardiology, P = 0.003) were found, and the APACHE between cardiac vs CVD,(31 ± 8.3 in CVD, P = 0.004). In males, the acute coronary syndrome was more frequent (p = 0.012) and in women the acute pulmonary edema (p = 0.058). The main cause of admission was cardiology with 57.6%, followed by 17.2% in septic, 12.1% for stroke, hemorrhagic shock in 6.1% and 7.1% in others. The overall mortality of the population was 34.3% (being higher in CVD group with 91.7% and lower in cardiology in 21.1%, P ≤ 0.001). Mortality was similar between men and women. When we analyzed by age, APACHE and stay, significant differences were observed for APACHE, being greater in deceased ($p \leq 0.001$), and stay more days for dead ($p = 0.083$).In the cardiac group, those admitted for increased incidence of arrhythmia had died (19.4%, P = 0.018). The rest did not reach statistical significance. In the septic group no differences were observed.

Regarding substitutive techniques depurative was observed that 78.9% (n = 79) did not perform any technique in 15.2% (n = 15) and they were performed hemofiltration and 5.1% (n = 5) hemodialysis. Septic were performed more frequently these techniques (52.9%, P = 0.007)

**CONCLUSIONS.** Cardiac's group was the main reason for admission and presented a high age in admission and APACHE was higher in CVD group. Mortality in cardiac´s group was lower compared to CVD. Deceases presented greater APACHE and average hospital stay. Techniques extrarenal depuration were higher in the septic´s group.

##### REFERENCE(S)

Crit Care. 2017 Dec 28;21(1):326; Rev Bras Ter Intensiva. 2017 Oct-Dec;29(4):444-452; Intern Med J. 2017 Jan;47(1):62-67.

##### GRANT ACKNOWLEDGMENT

No

### 0412

#### Mortality prediction in patients with severe dialysis - dependent acute kidney injury

V. Balciuviene[1], I. Skarupskiene[1,2], D. Adukauskiene[3], E. Ziginskiene[1,2], A. Adukauskaite[4], I.A. Bumblyte[2,5]

[1]Hospital of Lithuanian University of Health Sciences Kauno Klinikos, Department of Nephrology, Kaunas, Lithuania; [2]Medical Academy, Lithuanian University of Health Sciences, Department of Nephrology, Kaunas, Lithuania; [3]Medical Academy, Lithuanian University of Health Sciences, Department of Intensive Care, Kaunas, Lithuania; [4]Hospital of Innsbruck Medical University, Department of Cardiology and Angiology, Innsbruck, Austria; [5]Hospital of Lithuanian University of Health Sciences Kauno klinikos, Department of Nephrology, Kaunas, Lithuania

**Correspondence:** D. Adukauskiene

*Intensive Care Medicine Experimental* 2018, **6(Suppl 2):**0412

**INTRODUCTION.** Despite improvements in the quality of medical care, the incidence and mortality of acute kidney injury (AKI) continues to rise. Every year about 2 mln. people worldwide die due to AKI. Such high mortality persists for already several decades, and stimulates to search for the possibilities to reduce it.

**OBJECTIVES.** The aim of our study was to establish the predictive factors of mortality of the patients with severe dialysis - dependent AKI (stage 3).

**METHODS.** Single-centre of the largest university Lithuanian hospital Kaunas Clinics within a 2-year period retrospective pilot study was carried out. Inclusion criteria: 1) severe dialysis - dependent AKI stage 3; 2) age ≥ 18.

**RESULTS.** In 573 patients hospital mortality was found to be 51.7% (n=296). For 32.5% (n=186) of the investigated patients the renal function improved, and almost 16% (n=91) of patients remained dialysis - dependent after their discharge from the hospital. Significant prognostic factors of mortality were found to be: age > 85 years (OR 2.356, 95% CI [1.105-5.021], p=0.026), chronic kidney disease before AKI (OR 1.734, 95% CI [1.121-2.682], p=0.013), AKI after a cardiosurgery (OR 3.358, 95% CI [1.564-7.210], p=0.002), sepsis (OR 3.048, 95% CI [2.128 - 4.367] p< 0.001), requirement of vasopressors (OR 17.164, 95% CI [11.306 - 26.056], p< 0.001), mechanical ventilation (OR 7.732, 95% CI [5.268 - 11.350], p < 0.001), administration of aminoglycosides (OR 2.402, 95% CI [1.345 - 4.291], p=0.003), oliguria (OR 3.504, 95% CI [2.430 - 5.054], p< 0.001), alveolar pulmonary oedema (OR 2.018, 95% CI [1.391 - 2.926], p< 0.001), systolic blood pressure (BP) ≤ 120 mmHg (OR 8.452, 95% CI [5.784 - 12.352], p< 0.001), diastolic BP ≤ 65 mmHg (OR 5.892, 95% CI [4.099 - 8.470], p< 0.001), SOFA score ≥ 7.5 (OR 13.149, 95% CI [8.812 - 19.619], p< 0.001), serum sodium > 140 mmol/l (OR 2.206, 95% CI [1.546 - 3.148], p< 0.001), requirement for the combination of renal replacement therapy (RRT) methods (OR 3.410, 95% CI [2.238-5.196] p< 0.001), and multiple organ failure (MOF) (especially when function of 3 and more organs is affected) (OR 19.885, 95% CI [12.956-30.519], p< 0.001). Model of multifactorial logistic regression analysis predicting lethal outcome was significantly forecasted by age ≥ 65 yrs, systolic BP ≤ 120 mmHg, oliguria, requirement of vasopressors and MOF.

**CONCLUSIONS.** More than half of patients with severe dialysis - dependent acute kidney injury has died. The prognosis of lethal outcome especially strongly relates with elderly, some limits of systolic blood pressure, oliguria, requirement of vasopressors and multiple organ failure.

##### REFERENCE(S)

1. Malhotra R et al. Nephrol Dial Transplant 2017,32(5):814-822.
2. Bouchard J et al. Kidney Dis (Basel) 2016,2(3):103-110.
3. Pakula AM et al. J Intensive Care Med 2016,31(5):319-324.

##### GRANT ACKNOWLEDGMENT

None.

### 0413

#### Early detection of acute kidney injury in politrauma patients who admitted to the Emergency Department of Dr. Hasan Sadikin Hospital, Bandung, Indonesia

T.T. Maskoen[1], D. Purnama[1], I. Fuadi[1], H. Nagarasyid[2], A. Adam[3], T. Bisri[1], Neuromuscular

[1]University of Padjadjaran - Dr. Hasan Sadikin Hospital, Anesthesiology and Intensive Therapy, Bandung, Indonesia; [2]University of Padjadjaran - Dr. Hasan Sadikin Hospital, Orthopedic, Bandung, Indonesia; [3]University of Padjadjaran - Dr. Hasan Sadikin Hospital, Neurosurgeon, Bandung, Indonesia

**Correspondence:** T.T. Maskoen

*Intensive Care Medicine Experimental* 2018, **6(Suppl 2):**0413

**INTRODUCTION.** Poly-trauma is a condition with ≥ 2 trauma in the body that life threatening with Injury Severity Score (ISS) ≥ 16[1]. Ischemic-reperfusion injury can happen in these patients that can be caused by hypovolemia, hypoxia and release of inflammatory mediators and cytokines [2,3,4]. These conditions may cause acute kidney injury (AKI) that can be detected by measurement of creatinine and cystatin-C serum. Cystatin-C is cysteine protease inhibitor that filtrated freely by renal tubule and absorbed completed, so it can be used to diagnose AKI[5,6]

**OBJECTIVES.** This study was to assess that AKI can be detected earlier using cystatin-C than creatinine measurements.

**METHODS.** Cross sectional study in poly-trauma adult patients, 18-65 year old, with ISS ≥ 16 who admitted to Emergency Department. Patients with history of chronic kidney disease, hemodialysis, cardiac disease and malignancy from hetero anamnesis were excluded from this study.

As soon as admitted to the Emergency Department, the patients were given fluid resuscitation and then blood samples were withdrawal for creatinine and cystatin-C measurements. The data were analyzed using chi-square, Kolmogorov Smirnov and Exact Fisher.

**RESULTS.** In this study we found that the incidence of AKI using cystatin-C more frequent than creatinine (7/23 than 5/23). It can be detected that 11,1% patients non-AKI in creatinine group were AKI in cystatin-C group. Cut off point for creatinine is > 1.3 mg/dl and Cystatin C >354.98 ng/ml. We also found that Sensitivity, Specificity, Positive Predicted Value, Negative Predictive Value and Accuracy of Cystatin C were 100%, 88.9%, 71.4%, 100% and 91.3%

**CONCLUSIONS.** Cystatin-C can detect AKI earlier than creatinine and accuracy was good

##### REFERENCE(S)

1. World Health Organization. Injuries and violence: the fact. 2014

2. Butcher NE, Enninghost N, Sisak K, Balogh ZJ. The definition of polytraum: variable interrater versus intrater agreement a prospective international study among trauma surgeons. J Trauma Acute Care Surg.2013;73(3):884-9
3. De Abreu KS, Silva GB, Barreto AG, Melo FM, Oliveira BB, Mota RM, Rocha MA, Silva SL, Araujo SM, Daher EF. Acute Kidney Injury after trauma: Prevalence, clinical characteristics and RIFLE classification. Indian J Crit Care Med. 2010;14:121-8
4. Basile D, Anderson M, Sutton T. pathophysiology of acute kidney injury. Compr Physiol. 2012;2(2):13031-353
5. Soto K, Coelho S, Rodrigues B, Martins H, Frade F, Lopes S, Cunhs L, Pspoils AL, Devarajan P. Cystanin C as marker of acute kidney injury in the emergency department:1745-54.2010;5(1)
6. Nicholas TL, Barasch J, Devarajan P. Biomarker in acute and chronic kidney disease. Curr Opin Nephrol Hypertens. 2008;23:3737-43

**GRANT ACKNOWLEDGMENT**

Academic Leadership Grand

### 0414

#### Prospective study of patients with acute kidney injury with co-morbidities with respect to their outcomes

S. Dixit[1], K. Khatib[2]

[1]Sanjeevan Hospital, Critical Care, Pune, India; [2]SKN Medical College, Medicine, Pune, India

**Correspondence:** S. Dixit

*Intensive Care Medicine Experimental* 2018, **6(Suppl 2):**0414

**INTRODUCTION AND OBJECTIVES.** To compare clinical profile and outcome in patients with Acute Kidney Injury (AKI) with respect to underlying co-morbidities.

**METHODS.** This is prospective study of 60 patients with AKI admitted to our hospital from July 2017 to December 2017.

Inclusion criteria:

i) Patients age >18 years.
ii) All admitted patients with AKI as defined by KDIGO guidelines.

Exclusion criteria: Known cases of CKD.

**RESULTS.** Of the total 120 patients with AKI, 88 had underlying comorbidity (Group A) and 32 had no co-morbidity (Group B). Of the patients with co-morbidities (Group A), 41% had diabetes mellitus, 31% had hypertension, and 23% had chronic liver disease.

Of the patients in Group A, 91% patients were more than 50 yrs old, male: female ratio was 1.5:1, 72.8% were oliguric, 54.5% required dialysis, and 86.4% required ICU management. Of the patients in Group B, 25% were more than 50 yrs old, male: female ratio was 1:1, 87.5% were oliguric, 37.55 required dialysis, and 37.5% required ICU management.

When comparing patients according to the cause of renal failure, 54.5% patients in group A and 75% patients in group B, had pre-renal cause of AKI(p= 0.311); while 36.5% patients in group A & 25% patients in group B, had renal cause of AKI(p= 0.311). 9% patients in group A & 0% patients in group B had post renal cause of AKI. (p= 0.311).

Morbidity (residual renal disease on discharge) and mortality was 50 % in Group A and 12.5% in Group B .

Patients more than 50yrs of age with underlying co-morbidities had significant risk of developing AKI (p= 0.0003). . There was significant association between presence of comorbidies and requirement of ICU management (p= 0.007). The outcome was worse in patients with underlying co-morbidities though not statistically significant (p=0.06).

**CONCLUSION.** Patients with AKI and co-morbidities require more ICU care and have worse outcomes than AKI patients without associated comorbidities.

**REFERENCE(S)**

1. Bhadade R, De'Souza R, Harde MJ, Mehta KS, Bhargava P. A Prospective Study of Acute Kidney Injury According to KDIGO Definition and its Mortality Predictors J Assoc Physicians Ind 2016;64:22-27
2. Monseu M, Gand E, Saulnier PJ, Ragot S, Piguel X, Zaoui P, Rigalleau V. Acute Kidney Injury Predicts Major Adverse Outcomes in Diabetes: Synergic Impact With Low Glomerular Filtration Rate and Albuminuria. Diabetes Care 2015;38:2333-2340

**GRANT ACKNOWLEDGMENT**

NIL

**Table 1 (abstract 0414).** Results

Parameters Pts with comorbidities Pts without Comorbidities P value AGE GRS <50 2 (9%) 6(75) 0.000306 Significant >50 20(91%) 2(25) GENDER M 13(59%) 4(50%) 0.6567 NS F 9(41%) 4(50%) CAUSES PRE-R 12(54.5%) 6(75%) 0.3118* NS RENAL 8(36.5%) 2(25%) POST-R 2(9%) 0 ICU REQUIRE YES 19(86.4%) 3(37.5%) 0.0074 Significant NO 3(13.6%) 5(62.5%) DIALYSIS REQUIRE YES 12(54.5%) 3(37.5%) 0.4089 NS NO 10(45.5%) 5(62.5%) OUT-COME CURED 11(50%) 7(87.5%) 0.3975* NS RRD 5(22.8%) 0 DEATH 6(27.8) 1(12.5%) URINE OUTPUT OLIGUR 16(72.8%) 7(87.5%) 0.3975 NS NONOLI 6(27.2%) 1(12.5%)

### 0415

#### Performance of two scales in the detection of renal failure progression and need for renal replacement therapy

J. Cruz Lopez[1], E. Monares Zepeda[1], C.A. Galindo Martín[2]

[1]Hospital San Angel Inn Universidad, Intensive Care Unit, Mexico, Mexico; [2]Hospital San Angel Inn Universidad, Nutrition, Mexico, Mexico

**Correspondence:** J. Cruz Lopez

*Intensive Care Medicine Experimental* 2018, **6(Suppl 2):**0415

**INTRODUCTION.** Acute kidney injury (AKI) is one of the most common entities in the intensive care unit (ICU), it can be presented in up to 50% of the admitted patients and shows an independent correlation with higher morbidity and mortality. The usual criteria to diagnose AKI relies on serum levels of creatinine and urine output both in function of time. New methods for early detection of AKI risk have been developed (Discriminant value of Erdfelder et al. 2015 and the AKI predictor of Fletcher et al. 2017) pointing out the relevance of detecting renal angina.

**OBJECTIVES.** To analyze the performance of two scales in the detection of renal damage progression and renal replacement therapy (RRT) in critically ill adult patients.

**METHODS.** Retrospective analysis of 95 adult patients admitted to the ICU. Two scales where calculated, Discriminant value at 24 hours from admission and AKI predictor at 0 and 24 hours from admission. Demographic, biochemical, severity and fluid status including urine output also were collected. Receiver Operating Characteristics curves where constructed in order to analyze the performance of the two scales in detecting the need for RRT (decided by the care team) and renal damage progression (increase of ≥0.3mg/dL in 24 hours and/or urine output < 0.5mL/Kg/h at 48 hours).

**RESULTS.** The AKI predictor at 0 hours showed an area under the curve (AUC) of 0.87 (Sen: 86, Sp: 69.3, p< 0.05) for detecting RTT and AUC of 0.74 (Sen:60.9, Sp: 73.6, p< 0.05) for detecting renal damage progression. The discriminant value showed an AUC of 0.87 (Sen: 85.7, Sp: 94.6, p< 0.05) for detecting RTT and AUC of 0.83 (Sen:673.9, Sp: 83.3, p< 0.05) for detecting renal damage progression.

**CONCLUSIONS.** Both scales showed promising results for the detection of renal angina by using AKI predictor at admission and discriminant value at 24 hours.

**REFERENCE(S)**

1. J Clin Monit Comput. 2017 Feb;31(1):195-204. doi: 10.1007/s10877-015-9814-4. Epub 2015 Dec 19. Dynamic prediction of the need for renal replacement therapy in intensive care unit patients using a simple and robust model. Erdfelder F(1), Grigutsch D(1), Hoeft A(2), Reider E(3), Matot I(3), Zenker S(4)
2. Sheffield Kidney Institute, Northern General Hospital, Sheffield, UK. KDIGO clinical practice guidelines for acute kidney injury.2012.

3. Intensive Care Med. 2017 Jun;43(6):764-773. doi: 10.1007/s00134-017-4678-3. Epub 2017 Jan 27. AKI predictor, an online prognostic calculator for acute kidney injury in adult critically ill patients: development, validation and comparison to serum neutrophil gelatinase-associated lipocalin. Flechet M(1), Güiza F(2), Schetz M(1), Wouters P(1), Vanhorebeek I(1), Derese I(1), Gunst J(1), Spriet I(3), Casaer M(1), Van den Berghe G(1), Meyfroidt G(1)

**GRANT ACKNOWLEDGMENT**
No grant was received for the development of the present study.

### 0416

#### Urinary TIMP2 and IGFBP7 identifies high risk patients of progression from mild and moderate to severe acute kidney injury during septic shock

J. Maizel[1], D. Daubin[2], L.V. Vong[3], D. Titeca Beauport[1], M. Wetzstein[1], L. Kontar[1], M. Slama[1], K. Klouche[1], C. Vinsonneau[3]
[1]CHU Amiens-Picardie, Amiens, France; [2]CHU Montpellier, Lapeyronie, Montpellier, France; [3]CH Melun, Melun, France
**Correspondence:** J. Maizel
*Intensive Care Medicine Experimental* 2018, **6(Suppl 2):**0416

**INTRODUCTION.** Septic shock is one of the leading causes of death in patients admitted to the intensive care unit (ICU). Acute kidney injury (AKI) occurs in almost 50% of septic patients and is associated with significant mortality.
**OBJECTIVES.** To examine whether the new urinary biomarkers TIMP2 and IGFBP7 can predict progression within 24 hours and 72 hours from mild and moderate (KDIGO 1 or 2) to severe AKI (KDIGO 3) in patients with septic shock.
**METHODS.** Prospective, multicentre observational study performed in three French ICUs. The urinary biomarkers TIMP2*IGFBP7 were analyzed at the early phase (< 6 hours) of patients admitted for septic shock with mild and moderate AKI.
**RESULTS.** Among the 112 patients included, 45 (40%) progressed to KDIGO3 level 24 hours after inclusion (KDIGO3 H24) and 47 (42%) 72 hours after inclusion (KDIGO3 H72). The median urinary TIMP2*IGFBP7 at inclusion (baseline) were higher in the KDIGO3 group than in the KDIGO< 3 group at H24 and H72. All covariates with a p value < 0.2 in the univariate analysis were included in a stepwise multiple logistic regression models to identify factors independently associated with the risk of KDIGO3 at H24 and H72. TIMP2*IGFBP7 remained independently associated with KDIGO3 only at H24 but not at H72. Baseline posology of norepinephrine, baseline urine output and baseline serum creatinine remained also significantly associated with progression to KDIGO3 at H24. Baseline TIMP2*IGFBP7 and baseline urinary output had the best AUC ROC. A baseline TIMP2*IGFBP7 >2.0 $(ng/ml)^2$/1,000 identified the population at high risk of KDIGO 3 H24 (relative risk 4.19 (1.7-10.4)) with a sensitivity of 76% (60-87) and a specificity of 81% (69-89).
**CONCLUSIONS.** The urinary TIMP2*IGFBP7 concentration and the urine output at the early phase of septic shock is an independent factor to identify high risk population of progression from mild and moderate to severe AKI over the next 24 but not 72 hours. A TIMP2*IGFBP7 concentration > 2.0 $(ng/ml)^2$/1,000 quadruple the risk of KDIGO3 AKI within 24 hours.

### 0417

#### Bioelectrical impedance measurements for assessment of kidney function in critically ill patients

L.A.A. van Gendt - de Jong[1], A.G. Otten - Helmers[1], P.E. Spronk[2], H.J.M. van Kan[1]
[1]Gelre Hospitals Apeldoorn, Clinical Pharmacy, Apeldoorn, Netherlands; [2]Gelre Hospitals Apeldoorn, Intensive Care, Apeldoorn, Netherlands
**Correspondence:** L.A.A. van Gendt - de Jong
*Intensive Care Medicine Experimental* 2018, **6(Suppl 2):**0417

**INTRODUCTION.** Acute kidney injury, renal impairment and augmented renal clearance are common conditions in critically ill patients making appropriate dosing of renally excreted drugs a challenging task on the intensive care unit (ICU) (1). Close and accurate monitoring of kidney function in daily clinical practice is important to ensure medication safety (2). More than 50 mathematical formulas using biomarkers have been developed to give an estimate of the GFR (eGFR) as a measure for kidney function (3). However, these equations suffer from relevant limitations when applied for patient populations with low muscle mass or otherwise altered body composition like the critically ill (4, 5).
**OBJECTIVES.** To evaluate the use of Multifrequency Bioelectrical Impedance Analysis (MBIA) to predict 24-hour creatinine-urea clearance (24h CrUCL). A practical formula was developed and its performance was compared with that of established formulae such as Cockcroft-Gault, MDRD and Jelliffe's.
**METHODS.** An open-label prospective observational cohort study was performed in a twelve-bed intensive care unit at Gelre hospitals. All patients were included with an expected length of stay on the ICU of at least 48 hours. Each patient's body composition was assessed using a validated Quadscan 4000 analyzer (Bodystat® Ltd, UK, CE 0120) by placing 4 electrodes on wrists and ankles. A 24-hour urine specimen was collected and laboratory parameters in serum including creatinine, urea and albumin were obtained at the beginning and end of the collection period.
**RESULTS.** 151 patients were enrolled in the study over a 2-year period. An equation to predict 24h CrUCL was developed using stepwise linear regression using a training data set (n = 75). This equation was subsequently tested and compared with other relevant predictive equations using a validation data set (n = 76). Serum creatinine ranged from 40 μmol/l to 446 μmol/l. With the predictive model based on estimated body cell mass and a so called "prediction marker" more than 71% of the observed variance could be explained (Fig. 1). Predictive performance was superior to eight other evaluated models ($R^2$: 0.39 - 0.55) and demonstrated to be homoscedastic (Fig. 2).
**CONCLUSIONS.** MBIA measurements can be used to predict creatinine clearance with superior performance than currently established prediction models. This rapid, non-invasive method enables correction for influences of a patient's actual body composition and may prove very valuable in daily clinical practice, specifically to ensure adequate dosing of renally excreted drugs.

**REFERENCE(S)**
1. Udy AA, Roberts JA, Shorr AF, et al.: *Crit Care* 2013; 17:1-9
2. Warlé-van Herwaarden MF, Kramers C, Sturkenboom MC, et al.: *Drug Saf* 2012; 35:245-59
3. Luis-Lima S, Porrini E: *Nephron* 2017; 136:287-291
4. Kees MG, Hilpert JW, Gnewuch C, et al.: *Int J Antimicrob Agents* 2010; 36:545-8
5. Hoste E a J, Damen J, Vanholder RC, et al.: *Nephrol Dial Transplant* 2005; 20:747-753

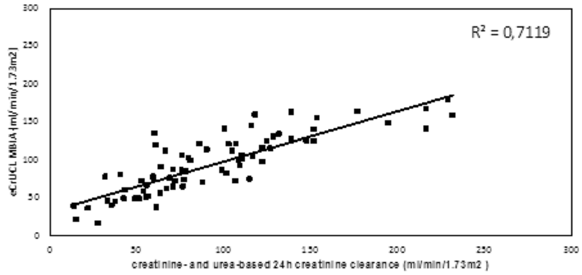

**Fig. 1 (abstract 0417).** correlation observed vs MBIA-predicted 24h CrUCL (ml/min/1.73 m2)

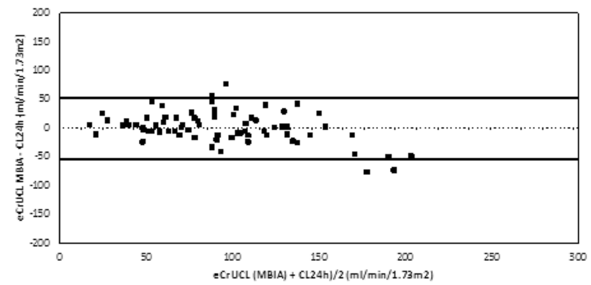

**Fig. 2 (abstract 0417).** Bland-Altman plot observed vs MBIA-predicted 24h CrUCL (ml/min/1.73 m2)

### 0418

#### Postoperative level of urine neutrophilic gelatinase-associated lipocalin (u-NGAL) can predict acute renal injury, MODS and outcome in cardiovascular surgery patients

O. Dymova[1], A. Eremenko[2], N. Minbolatova[2]
[1]Federal State Budgetary Scientific Institution «Petrovsky National Research Centre of Surgery», Scientific and Clinical Laboratory, Moscow, Russian Federation; [2]Federal State Budgetary Scientific Institution «Petrovsky National Research Centre of Surgery», ICU, Moscow, Russian Federation
**Correspondence:** A. Eremenko
*Intensive Care Medicine Experimental* 2018, **6(Suppl 2):**0418

**INTRODUCTION.** As a rule acute kidney injury after cardiovascular surgery in the patients with normal preoperatively renal function occurs as a component of multiorgan failure.

**The goal** of the study was to evaluate diagnostic and prognostic value of the u-NGAL to detect AKI, MOSF and predict the mortality in patients after cardiac surgery.

**METHODS.** 117 patients undergoing cardiac and aortic surgery with CPB were included into the study. All of them had no signs of renal failure and MODS preoperatively. 43 of the patients had complicated postoperative period and multiorgan failure (mortality rate among them was 23.3%). The control group consisted from 74 patients. Blood biochemical, hematological and coagulation tests were analyzed before and on the first and third days after operation. Studies of u-NGAL were performed on Abbott Architect i1000sr.

**RESULTS.** Preoperative concentrations of NGAL in the urine do not allow predicting the risk of developing MODS and the outcome after surgery (AUC 0,636, 95% confidence interval 0,477-0,794).

The main causes of MODS in cardiosurgical patients in the early postoperative period were: prolonged cardiopulmonary bypass, aortic cross-clamp time and a large volume of blood loss.

The most informative for predicting the development MODS and death was the values of the uNGAL in the first postoperative day (AUC 0.957, 95% confidence interval 0.921-0.992).

Interpretation of the results obtained in the first postoperative day, using reference u-NGAL values (130 ng/ml), allows to predict the risk of severe AKI and death in patients with postoperative MODS (sensitivity of 100% and a specificity of 91%).

The levels lower than the reference values more effectively predict uncomplicated postoperative period after cardiac surgery. The cut-off u-NGAl level in the first postoperative day of 23,75 ng/ml had the sensitivity of 74.4%, and specificity of 56.8%, while in case of the level 76,45 ng/ml, the sensitivity was 48.8%, and the specificity was 74.3%.

**CONCLUSION.** The level of u-NGAL over 130 ng/ml in the first postoperative day after cardiovascular surgery can be used as a predictor of acute kidney injury, MODS and poor outcome.

### 0419

#### Investigating the association between in-hospital mortality and age in patients receiving continuous veno-venous haemofiltration during their hospital admission

G. Amaratunga, T. Samuels, J. McLoughlin
East Surrey Hospital, Redhill, United Kingdom
**Correspondence:** G. Amaratunga
*Intensive Care Medicine Experimental* 2018, **6(Suppl 2):**0419

**INTRODUCTION.** We assessed whether increasing age is associated with higher in-hospital mortality in patients receiving continuous veno-venous haemofiltration (CVVHF) on the intensive care unit. In other patient groups receiving CVVHF it has been shown that older age is a predictor of both 30-day and late mortality[1]. Acute renal failure is often part of multi organ failure and CVVHF is the most common mode of renal replacement therapy used in intensive care units.[2]

**METHODS.** From an 8 year period, complete data for 913 patients was analysed. Mean age was 66.3 years (SD 14.8 years) with ages ranging from 16 to 93 years. Patients were separated into two groups based upon age, above and below the age of 75. Data was analysed using the program R 3.4.1 to perform a Pearson's Chi-squared test and produce the fourfold plot.

**RESULTS.** The association between age and in-hospital mortality was found to be significant (p < 0.001). The odds ratio [95% CI] is 0.52 [0.40 - 0.69] suggesting that the odds of a patient over the age of 75 surviving if they receive CVVH is reduced by nearly 50%.

**CONCLUSIONS.** Our results demonstrate that patients over the age of 75 that received CVVH are less likely to survive their admission. Limitations include unbalanced group sizes, single centre study and confounding variables. The results do warrant further research into age and its association with in-hospital mortality in patients commenced on CVVH.

**REFERENCE(S)**

1. Haanschoten, MC et al, Journal of Cardiac Surgery, 29:464-469, 2014
2. Saqib, A et al, Critical Care Medicine, 43: 246, 2015

### 0420

#### Descriptive analysis of patients with AKI admitted in ICU

N. Ramon Coll[1,2], J.J. Prados Chica[1,2], J. Codina Calero[1,2], M. Miralbés Torner[1,2], G. Jiménez Jiménez[1,2], S. Carvalho Brugger[1,2], E. Val Jordán[1,2], T. Pérez González[1,2], X. Castillo Peña[1,2], M. Escarp Sans[1,2], E. Juan Pérez[1,2], M. Cots Begué[1,2], M. de la Llana Chavarria[1,2], E. García Puvia[1,2], J. Caballero López[1,2]
[1]Hospital Arnau de Vilanova, Intensive Care Department, Lleida, Spain; [2]IRBLleida, Lleida, Spain
**Correspondence:** N. Ramon Coll
*Intensive Care Medicine Experimental* 2018, **6(Suppl 2):**0420

**INTRODUCTION.** In 2011, the Spanish Society of Intensive Care Medicine (SEMICYUC) published an actualization of quality indicators in critical patients.

**OBJECTIVES.** To analyze the accomplishment of two continuous renal replacement therapy (CRRT) quality indicators that weren´t accomplished in our ICU since then: the correct stratification of acute kidney injury (AKI) and the CRRT monitoring. In addition we analyzed the duration of the filters and the complications of CRRT.

**METHODS.** Descriptive analysis including all patients admitted from November 2016 to October 2017 in the ICU of a referral hospital for a population of 450000 inhabitants. AKI was diagnosed according to the Acute Kidney Injury Network (AKIN) criteria.

**RESULTS.** During this period, 913 patients were admitted in the ICU. Mean APACHE-II score was 14.79 and mean days of admission were 4.89.

AKI was present in 27% of the patients. CRRT was needed in 14% of them and 68% were hemodynamically unstable. The reason for admission was mainly medical (50%), followed by surgical (31%) and traumatic (19%). Mean days of admission of patients with AKI was 6.1, mean age was 63 years and 46% of them were men. Mean APACHE-II was 18.5, and 21.9 in those who needed CRRT. 11% of these patients had medical history of chronic kidney disease.

The most frequent indication of CRRT was anuria (80%) followed by hypervolemia (62.9%) and non-responsive acidosis (54.3%). More than a half of our patients presented the combination of anuria and non-responsive acidosis. The main prescribed ultrafiltration dose was 29 ml/kg/h and the real administrated dose was 26 ml/kg/h. With this prescription the blood concentration of urea and creatinine decreased to less than a half of the baseline value and there was no evidence of dialytrauma caused by hyperdyalisis.

The mean duration of the filters was 22 hours, taking into account that only 45.7% of the patients with CRRT received anticoagulation, and only 35% of these patients achieved an optimal range of rPTTA (1.5-1.9). In all cases the filtration fraction was under 25%. Citrate was used in only 2 patients.

The CRRT associated complications were: hypothermia (83%), electrolytic alterations (43%) and anaemia that required transfusion (34%). The main electrolytic alterations were hypokalemia (70%) and hypophosphataemia (21%).

Patients with CRRT recovered an urine output higher than 0.5 ml/kg/h in 30 days. 15% died during their admission in ICU and 37% died after a 30 days follow up.

**CONCLUSIONS.** Quality indicators have been accomplished. All our patients with AKI have been stratified following the AKIN criteria and, in those requiring TRRC, monitoring was performed following the SEMICYUC recommendations. Mean duration of the filters is similar to that described by other groups, although only 35% of heparin-anticoagulated patients achieved a correct rPTTA. The most frequent adverse effect was hypothermia.

**Table 1 (abstract 0420).** AKIN of patients with AKI

| AKIN | At admission | At discharge |
|---|---|---|
| 0 | - | 34% |
| 1 | 50% | 25% |
| 2 | 22% | 31% |
| 3 | 28% | 10% |

### 0421

#### The comparison of urinary kidney injury molecule 1 as a biomarker of renal function to creatinine in critically ill patients under colistin treatment at intensive care units in two referral teaching hospitals

A. Vazin[1], I. Karimzade[2], M. Malek[2]
[1]Shiraz University of Medical Sciences, Clinical Pharmacy, Shiraz, Iran, Islamic Republic of; [2]Shiraz University of Medical Sciences, Shiraz, Iran, Islamic Republic of
**Correspondence:** A. Vazin
*Intensive Care Medicine Experimental* 2018, **6(Suppl 2):**0421

**INTRODUCTION.** Nephrotoxicity is the most important side effect of intravenous colistin. With growing incidence of multi drug resistant bacteria and increasing the use of colistin in the intensive care units (ICUs), early recognition of colistin induced acute kidney injury (AKI) in ICUs setting remains a critical problem. Kidney injury molecule-1 (KIM-1) elevations do occur within hours of acute tubular necrosis; then, among the potential advantages of this biomarker are its apparent specificity for ischemic renal injury and the availability of a rapid assay. Using serum creatinine levels to detect renal toxicity has several limitations in clinical practice.

**OBJECTIVES.** To compare the patterns and the accuracy of urine KIM-1 with that of serum and urine creatinine in critically ill patients receiving colistin.

**METHODS.** A longitudinal study was performed during 13 months at ICUs of two Hospitals in Shiraz, Iran. Patients > 18 years with no documented history of acute kidney injury or chronic kidney disease scheduled to receive colistin for at least 1 week were recruited. Using the pre-designed form, the clinical information of the population under study was collected and recorded. Urinary and serum levels of creatinine, urea, KIM-1 were measured at 4 time intervals including zero, three, five, seven days of Colistin treatment.

**RESULTS.** During the study period,18.18% of patients developed nephrotoxicity, with a mean ± standard deviation onset of 7.33 ± 2.25 days.Furosemide co-administration (odds ratio = 0.07, 95% confidence interval = 0.07-0.707, P = 0.024) was significantly associated with colistin nephrotoxicity. The changes in KIM-1 levels during colistin treatment in either group with or without renal toxicity (P < 0.001) and between two groups with and without renal toxicity (P = 0.402) showed a decreasing trend.The accuracy of KIM-1 in renal toxicity was significantly lower than serum creatinine on days 3, 5 and 7.

**CONCLUSION.** In this study, serum creatinine was more accurate than urine KIM1 in detecting colistin induced nephrotoxicity. More studies with adequate sample size and more frequent sampling times are warranted to elucidate the role of new biomarkers of renal function in the detection of colistin nephrotoxicity.

**GRANT ACKNOWLEDGMENT**

We would also like to thank staffs of ICUs of Nemazee and Rajaee hospitals for support to accomplish this study

## ARDS management

### 0422

#### Bedside ultrasound assessment of lung reaeration in patients receiving veno-venous extracorporeal membrane oxygenation

L. Xiao[1], L. Qin[2]
[1]Second Affiliated Hospital, Zhejiang University School of Medicine, Department of Emergency Medicine, Hangzhou, China; [2]La Pitié-Salpêtrière Hospital, Assistance-Publique-Hôpitaux-de-Paris, UPMC Univ Paris 06, Department of Anesthesiology and Critical Care Medicine, Paris, France
**Correspondence:** L. Xiao
*Intensive Care Medicine Experimental* 2018, **6(Suppl 2):**0422

**INTRODUCTION.** Deciding when and how to wean the ECMO from the patients represents a significant challenge. The usual weaning process is to switch off the fresh gas flow of the oxygenator for a few hours when pulmonary function is improved.;if the patient can be ventilated with plateau pressure < 30 $cmH_2O$, tidal volume < 6 ml/kg, and PEEP 8 to 12 $cmH_2O$ without acute corpulmonale, the ECMO cannula can be pulled out. However, among these weaning criteria, lung function recovery attested by lung reaeration is not taken into consideration. It has been reported that assessment of lung aeration changes during a spontaneous breathing trial could predict postextubation distress.Lung ultrasound has been validated to assess alveolar recruitment induced by PEEP for patients with ARDS, antibiotic-induced lung reaeration in ventilator-associated pneumonia (VAP) as well as loss of lung aeration after fluid loading in patients with ARDS. To date, however, the changes of lung aeration in patients with ARDS requiring VV -ECMO support have never been evaluated by lung ultrasound

**OBJECTIVES.** Bedside Ultrasound assessment of lung reaeration in patients receiving veno-venous extracorporeal membrane oxygenation.

**METHODS.** Thirty seven patients with acute respiratory failure who received VV-ECMO treatment of intensive care unit were included in the study.Thirty two patients assessed by transthoracic lung ultrasound at 4 time points as before receiving VV-ECMO (T0),24 hours after the VV-ECMO treatment (T1),before weaning off the VV-ECMO (T2) and the mechanical ventilation(T3). Transthoracic lung ultrasound was performed by an experienced investigator with level-3 certification, using a Siemens Acuson CV70 echograph and a 2- to 4-MHz round-tipped or convex probe were used.

**RESULTS.** There were 24 patients weaning from the VV-ECMO and ventilation successfully, lung ultrasound score (LUS) of the whole lung decreased 45.6% from T0 to T2 ($P < 0.05$),and 52% from T0 to T3 ($P < 0.05$) ;but there was no significantly decreased of LUS after 24 hours VV-ECMO treatment (T0 to T1) and from T2 to T3 ($P > 0.05$). Lung ultrasound score on right and left lung distubrion from T0 to T2 also had significantly decreased trend ($P < 0.05$).It is proved there is a close relation between the changes of $PaO_2$/ $FiO_2$ and LUS from T0 to T2 was to be positive.

**CONCLUSIONS.** The transthoracic lung ultrasound may serve as a useful tool for lung aeration assessment on adult patients with severe acute respiratory distress syndrome receiving veno-venous extracorporeal membrane oxygenation. Further studies are needed to evaluate the impact of this screening strategy on the weaning process and patients outcome.

**GRANT ACKNOWLEDGMENT**

none

### 0423

#### The predictive factors for success of high flow nasal cannula in acute hypoxemic respiratory failure patients in a regional hospital in Hong Kong

C.-T. Lun[1], C.-K. Leung[1], H.-P. Shum[2], S.-O. So[1]

[1]Alice Ho Miu Ling Nethersole Hospital, Medicine and ICU, Hong Kong, Hong Kong, China; [2]Pamela Youde Nethersole Hospital, Hong Kong, Hong Kong, China

**Correspondence:** C.-T. Lun

*Intensive Care Medicine Experimental* 2018, **6(Suppl 2):**0423

**INTRODUCTION.** The predictive factors for success of high flow nasal cannula in acute respiratory failure are inconsistent in different studies.

**OBJECTIVES.** We conducted a retrospective study evaluating the predictive factors for success of high flow nasal cannula in acute hypoxemic respiratory failure.

**METHODS.** This retrospective study was conducted in the Intensive Care Unit (ICU) of Pamela Youde Nethersole Eastern Hospital in Hong Kong. The hospital records of patients admitted to ICU between May 2012 to April 2017 were retrospectively evaluated, and patients were included if they were given high flow nasal cannula in acute respiratory failure. Patients were excluded if they were

(1) given high flow nasal cannula (HFNC) as a tool to wean from mechanical ventilation,
(2) given HFNC as a palliative management in malignancies,
(3) considered not suitable for enrolment by the investigators.

**RESULTS.** Among the 124 eligible patients, the majority (77.4%) suffered from pneumonia. Before commencement of high flow nasal cannula, 72 patients (58.1%) , 35 patients (28.2%), 14 patients (11.3%) and 1 patient (0.8%) received nonrebreathing mask, nasal cannula, noninvasive ventilation, and Hudson mask respectively.

Fifty-five patients (44.35%) were defined as success which is defined as no escalation to non-invasive ventilation, mechanical ventilation, extra-corporeal membrane oxygenation or death within 28 days after commencement of HFNC.

*Primary Endpoints*

Compared with the 69 patients with failure, the 55 patients defined as success with HFNC had higher APACHE IV Score and GCS score upon ICU admission. (p=0.002, 0.024) They had lower heart rate and respiratory rate 1 hour before HFNC high flow nasal cannula (p= 0.011, 0.032) and lower heart rate 1 hour after HFNC ($p< 0.001$). They had higher platelet count (p=0.012) and serum sodium level (p=0.011) upon ICU admission and lower pH on day of HFNC (p=0.029).

The Respiratory rate-oxygenation (ROX) index which is defined as ratio of SpO2/ FiO2 to respiratory rate was significantly higher in the success group at 1 hour and 12 hours after HFNC.

By multivariate binary logistic regression, success of HFNC is only associated with higher ROX index at 12 hours after HFNC (p=0.01 OR 0.802).

The Receiver's operating curve has its largest area under curve if ROX index at 12 hours is used to predict the success of HFNC in our patients. (AUC=0.653). The sensitivity and specificity would be 0.88 and 0.41 if cut-off value for ROX 12 hour would be 5.626.

**CONCLUSIONS.** By multivariate analysis, success of HFNC is only associated with higher ROX index at 12 hours after HFNC. Clinical monitoring in the first 12 hours after commencement of HFNC is necessary for prediction of success of HFNC.

### 0424

#### High-flow nasal cannula in adults with acute respiratory failure and after extubation: aSystematic review and meta analysis

Z. Xu[1], Y. Li[1], J. Zhou[1], X. Li[1], Y. Huang[1], X. Liu[1], K.E.A. Burns[2], N. Zhong[1], H. Zhang[2]

[1]State Key Laboratory of Respiratory Diseases, National Clinical Research Center for Respiratory Disease, Guangzhou Institute for Respiratory Health, Guangzhou, China; [2]Interdepartmental Division of Critical Care Medicine, University of Toronto, Toronto, Canada

**Correspondence:** Z. Xu

*Intensive Care Medicine Experimental* 2018, **6(Suppl 2):**0424

**INTRODUCTION.** High-flow nasal cannula (HFNC) can be used as an initial support strategy for patients with acute respiratory failure (ARF) and after extubation. However, no clear evidence exists to support HFNC use in practice.

**OBJECTIVES.** We summarized the effects of HFNC compared to conventional oxygen therapy (COT) and noninvasive ventilation (NIV) on important outcomes in adults presenting with ARF and after extubation.

**METHODS.** We searched four electronic databases (Pubmed, EMBASE, Scopus and Web of Science) to identify randomized clinical trials (RCTs) involving adults, comparing HFNC with COT or NIV, and reporting clinically important outcomes. Primary outcomes were treatment failure, and intubation/reintubation rates.

**RESULTS.** Eighteen RCTs were included for analysis. Compared to COT-treated patients, HFNC treatment resulted in significantly lower risk of treatment failure (OR, 0.65; 95%CI, 0.43-0.98; $p$=0.04; $I^2$=32%), but had no effect in preventing intubation (OR, 0.74; 95%CI, 0.45-1.21; $p$=0.23; $I^2$=0%). For post extubation patients, HFNC (vs. COT) significantly reduced reintubation rate (OR, 0.47; 95%CI, 0.29-0.76; $p$=0.002; $I^2$=30%) and extubation failure (OR, 0.43; 95%CI, 0.25-0.73; $p$=0.002; $I^2$=66%). In contrast, when compared to NIV-treated patients, HFNC significantly reduced intubation rates (OR, 0.57; 95%CI, 0.36-0.92; $p$=0.02; $I^2$=0%) in patients with ARF. However, HFNC was not superior over NIV on treatment failure and on reintubation rates for patients after extubation. There was no difference between HFNC vs. COT or NIV on both ICU and hospital mortality or length of stay.

**CONCLUSIONS.** HFNC is superior to COT in reducing treatment failure as an initial support strategy and in reducing extubation failure and reintubation rates after extubation. HFNC could reduce intubation rates as an initial use but had similar effects in patients after extubation compared to NIV treatment.

**REFERENCE(S)**

1. Roca O, Hernandez G, Diaz-Lobato S, Carratala JM, Gutierrez RM, Masclans JR, et al. Current evidence for the effectiveness of heated and humidified high flow nasal cannula supportive therapy in adult patients with respiratory failure. Crit Care. 2016;20(1):109.

2. Papazian L, Corley A, Hess D, Fraser JF, Frat J-P, Guitton C, et al. Use of high-flow nasal cannula oxygenation in ICU adults: a narrative review. Intensive care medicine. 2016:1-14.

**GRANT ACKNOWLEDGMENT**

The study was supported by National Natural Science Foundation of China (Grant Nos.81361128003 (NZ and YL), 81490534(NZ and HZ), 81490530(NZ and HZ) and 81370177(HZ) and by Natural Science Foundation of Guangdong Province, China (Grant Nos. 2015A030313480, YL and 2015A030313456, XQL), and by the Chief Scientist Project of Yangcheng Scholar in Guangzhou (Grant No. 1201541642, HZ).

### 0425

#### Switch to spontaneous breathing during nearly total extracorporeal $CO_2$ removal: a novel test to assess severity of ARDS patients on ECMO

E. Spinelli[1], T. Mauri[1,2], M. Albanese[3], D. Tortolani[3], S. Colombo[2], D. Tubiolo[1], A. Lissoni[1], S. Alongi[1], G. Grasselli[1,2], A. Pesenti[1,2]

[1]IRCCS Ca' Granda Ospedale Maggiore Policlinico Milano, Milano, Italy; [2]Università degli Studi di Milano, Milano, Italy; [3]Università degli Studi di Ferrara, Ferrara, Italy

**Correspondence:** E. Spinelli

*Intensive Care Medicine Experimental* 2018, **6(Suppl 2):**0425

**INTRODUCTION.** Few data exist on the control of respiratory drive and effort through extracorporeal $CO_2$ removal in spontaneously breathing early ARDS patients on ECMO [1]. Uncontrolled drive and effort could result in injurious transpulmonary pressure despite ECMO support and this response might be correlated with ARDS severity and clinical outcome [2].

**OBJECTIVES.** To investigate the spontaneous breathing pattern during nearly total extracorporeal $CO_2$ removal and its correlation with severity and clinical outcome in early severe ARDS patients on ECMO.

**METHODS.** Patients with severe ARDS on ECMO since 2-7 days undergoing ultraprotective mechanical ventilation were included. $CO_2$ excretion from the native lung ($VCO_2$_NL) was continuously monitored by volumetric capnography. After awakening (Richmond Agitation Sedation Scale value between -2 and 0), patients were switched to pressure support ventilation. Sweep gas flow was increased until reaching minimal $VCO_2$_NL, so that extracorporeal $CO_2$ removal ($VCO_2$_ECMO) corrensponded to nearly total $CO_2$ production ($VCO_2$_tot). The waveforms of esophageal (Pes) and airway pressure, flow and volume were continuously recorded to obtain: tidal volume (Vt), respiratory rate (RR), esophageal pressure swings (ΔPes) and driving transpulmonary pressure ($\Delta P_L$). According to the value of RR (below or above the median value), patients were categorized as “responders” and “non-responders”.

**RESULTS.** Median $VCO_2$_NL was 0 [1-6] ml/min with a $VCO_2$_ML of 290 [266-321] ml/min (98 ± 3% of VCO2_tot). Responders and non-responders did not differ in terms of baseline controlled mechahical ventilation settings and indexes of lung and systemic severity (Table 1). Despite similar low values of Vt and ΔPes, higher RR was associated with higher $\Delta P_L$ in non-responders vs. responders (Table 2). Moreover, time to successful switch to assisted ventilation was shorter in responders, with similar positive trends for ECMO duration and mortality (Table 2).

**CONCLUSIONS.** In early ARDS on ECMO, failure to decrease respiratory drive and effort during a spontaneous breathing test with “maximized” extracorporeal $CO_2$ removal might help identifying more severe patients requiring longer controlled mechanical ventilation and ECMO course and with higher mortality.

**REFERENCE(S)**

[1] Crotti et al. Anesthesiology 2017; 126(4):678-687.

[2] Mauri et al. Int Care med 2016; 42(12):2101-2103.

**Table 1 (abstract 0425).** Baseline characteristics

| Variable | Responders (n=8) | Non-responders (n=7) | p |
|---|---|---|---|
| PEEP (cmH2O) | 15 ± 4 | 16 ± 6 | 0.470 |
| Pplateau (cmH2O) | 26 ± 3 | 27 ± 4 | 0.693 |
| Driving pressure (cmH2O) | 11 ± 2 | 10 ± 3 | 0.238 |
| End-expiratory esophageal pressure (cmH2O) | 11 ± 5 | 11 ± 4 | 0.795 |
| Respiratory system compliance (ml/cmH2O) | 28 [22-39] | 30 [19-58] | 0.694 |
| Shunt (%) | 42 ± 13 | 47 ± 13 | 0.479 |
| SOFA | 7 ± 4 | 9 ± 2 | 0.319 |

**Table 2 (abstract 0425).** Spontaneous breathing test

| Variable | Responders (n=8) | Non-responders (n=7) | p |
|---|---|---|---|
| Arterial pH | 7.42 ± 0.05 | 7.41 ± 0.06 | 0.874 |
| PaCO2 (mmHg) | 43 [40-48] | 43 [39-47] | 0.955 |
| RR (bpm) | 8 ± 5 | 24 ± 8 | < 0.001 |
| Vt (ml/kg) | 2.5 ± 1.4 | 3.1 ± 1.9 | 0.482 |
| ΔPes (cmH2O) | 2.4 ± 1.4 | 2.1 ± 1.1 | 0.747 |
| ΔPL (cmH2O) | 6 ± 5 | 13 ± 8 | 0.039 |
| Time to successful switch to assisted ventilation (days) | 7 [4-17] | 31 [14-61] | 0.021 |
| ECMO free days at day 28 | 14 ± 9 | 7 ± 11 | 0.194 |
| Mortality n (%) | 0 (0%) | 3 (43%) | 0.077 |

### 0426

#### High flow oxygen therapy for the treatment of acute respiratory failure outside the intensive care unit: feasibility, safety and efficacy

G. Grasselli[1,2], N. Corcione[1], S. Colombo[3], A. Guzzardella[3], A. Galazzi[1], P. Tagliabue[1], F. Binda[1], T. Mauri[1,2], M. Macrì[3], R. Russo[1], A. Pesenti[1,2]

[1]IRCCS (Institute for Treatment and Research) Ca' Granda Maggiore Policlinico Hospital Foundation, Department of Anesthesia, Critical Care and Emergency, Milan, Italy, [2]University of Milan, Department of Pathophysiology and Transplantation, Milan, Italy, [3]University of Milan, Milan, Italy

**Correspondence:** G. Grasselli

*Intensive Care Medicine Experimental* 2018, **6(Suppl 2):**0426

**INTRODUCTION.** Non-invasive ventilation and continuous positive airway pressure are commonly used outside the Intensive Care Unit (ICU) for the treatment of respiratory failure due to chronic obstructive pulmonary disease or heart failure. Conversely, the use of these “advanced” respiratory supports in general wards for acute hypoxemic respiratory failure (AHRF) is debated, since delaying intubation results in increased mortality. The use of High flow oxygen therapy (HFOT) outside the ICU is rare. We hypothesized that early application of HFOT in general ward could be safe and could reduce the ICU admission rate.

**OBJECTIVES.** To assess the feasibility and safety of HFOT for the treatment of AHRF or mixed (hypoxemic and hypercapnic) respiratory failure in patients hospitalized in general wards, under the strict supervision of an intensivist.

**METHODS.** An ICU physician and an ICU nurse performed 12 educational meetings in 9 wards of Ospedale Maggiore Policlinico in Milan, Italy. The functioning of HFOT was illustrated to both nurses and medical staff members. HFOT was started in case of: a. AHRF with $PaO_2/FiO_2$ ≤300 and ≥150 mmHg; b. Hypoxemia defined as above and respiratory acidosis (pH < 7.35 and ≥7.30 + $PaCO_2$ >45 mmHg). Patients had to be hemodynamically stable and with preserved mental status. Doctors informed the ICU Outreach Team whenever HFOT was started. Data collected in each patient immediately before HFOT application, after 2 hrs of treatment and then once daily, were: respiratory rate (RR), arterial blood gas, $SpO_2$, blood pressure, Borg scale and comfort (visual scale). (*Fig 1*)

**RESULTS.** From January 1$^{st}$ to April 15$^{th}$, 34 patients were treated with HFOT in the following Hospital Departments: Haematology (n= 14, 41.2%), General Medicine (n= 6, 17.6%), Neurology (n= 2, 5.9%), Pulmonology (n= 4, 11.8%), Emergency (n= 8, 23.5%). Mean patients age was 64.3 and 18 were male (53%). Patients had a SOFA score of 3.31±1.97. Causes of respiratory failure were: pneumonia 26 pts (76.4 %), sepsis 2 pts (5.9 %), COPD and asthma 2 pts (5.9 %) and others 4 pts (11.8 %).Duration of HFOT was 4.35±1.95 days. After 2 hrs of HFOT, we observed a significant decrease in RR (26.2±7.0 Vs. 23.0±5.2) and Borg scale value (3.8±1.9 Vs. 2.3±1.2) and a significant increase in comfort score (2.5±0.8 Vs. 3.5±0.7). No differences in gas exchange was detected (*Table 1*). After 24 hrs, data confirmed an improvement of RR, dyspnea, comfort. No worsening of $PaCO_2$ or acidosis was observed in hypercapnic pts; there was a trend toward $PaCO_2$ reduction, but it did not reach statistical significance. After 24 hrs, only one pt needed ICU-admission.

**CONCLUSIONS.** HFOT in patients hospitalized in general wards with acute hypoxemic or mixed respiratory failure is safe and effective. The patients should be daily monitored by an intensivist, to avoid delayed recognition of clinical deterioration.

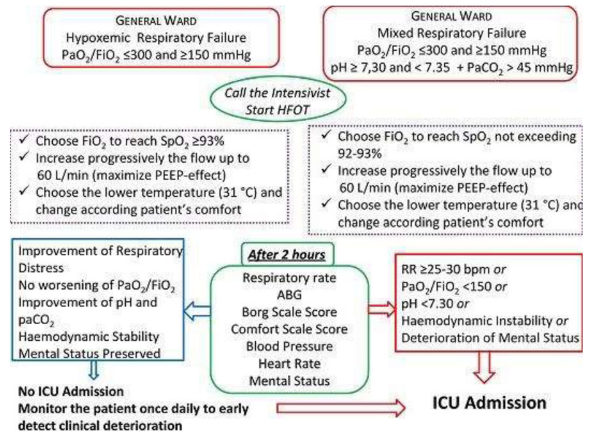

**Fig. 1 (abstract 0426).** Our algorithm for HFOT application in patients hospitalized in general wards

**Table 1 (abstract 0426).** Data are reported as mean ± SD. one-way ANOVA for repeated measurement with a post hoc Tukey's correction was used.

| | Pre HFOT | Post 2 hrs HFOT | Post 24 hrs HFOT | *p-value* |
|---|---|---|---|---|
| pH | 7.43 ± 0.07 | 7.43 ± 0.07 | 7.47 ± 0.05 | 0.1125 |
| $PaCO_2$ (mmHg) | 44.9 ± 16.4 | 44.7 ± 15.0 | 39.7 ± 9.5 | 0.4772 |
| $PaO_2/FiO_2$ | 168.6 ± 71.4 | 141.9 ± 47.8 | 165.6 ± 60.4 | 0.2573 |
| Respiratory Rate (breath/min) | 26.2 ± 7.0 | 23.0 ± 5.2 | 22.3 ± 6.2 | ***0.0452*** |
| Borg Scale | 3.8 ± 1.9 | 2.3 ± 1.2 | 2.3 ± 1.1 | < ***0.0001*** |
| Comfort Scale | 2.5 ± 0.8 | 3.5 ± 0.7 | 3.5 ± 0.9 | < ***0.0001*** |
| HFOT - Flow (L/min) | - | 55.6 ± 6.6 | 53.0 ± 7.8 | 0.1588 |
| HFOT - $FiO_2$ | - | 51.7 ± 17.3 | 52.1 ± 17.5 | 0.9260 |
| HFOT - Temperature (°C) | - | 34.8 ± 2.6 | 34.3 ± 2.5 | 0.5017 |

### 0427

#### Bedside ultrasound assessment of lung reaeration in patients with blunt thoracic injury receiving high-flow nasal cannula therapy: a retrospective study

L. Xiao, M. Zhang
Second Affiliated Hospital, Zhejiang University School of Medicine, Department of Emergency Medicine, Hangzhou, China
**Correspondence:** L. Xiao

*Intensive Care Medicine Experimental* 2018, **6(Suppl 2):**0427

**INTRODUCTION.** The purpose of this retrospective study is to describe the use of HFNC in a population of patients with blunt thoracic injury to examine if HFNC was associated with positive patient outcomes such as reduced rates of intubation and decreased ICU days,and use the lung transthoracic ultrasound to assess if the HFNC could improve the lung reaeration in these patients.

**OBJECTIVES.** The study aims to observe the evolution of lung aeration by "Lung Ultrasound Score" in a chest-injured population treated with HFNC, and to assess the benefit of the HFNC in trauma patients.

**METHODS.** A retrospective study examined trauma patients with moderate to severe thoracic injury admitted to the ICU at a tertiary hospital between October 2015 and March 2017. The decision to initiate HFNC was made at the discretion of the trauma surgeon and respiratory therapist when supplemental oxygen delivery was required. All patients assessed by transthoracic lung ultrasound everyday after admit into the ICU,we retrospective analysised 3 time points for this study: initial EICU presentation within 12 hours (T1), 24-48 hours after including (T2) and 72-96 hours after including (T3). Transthoracic lung ultrasound was performed by an experienced investigator with level-3 certification, using a Mindray M9 echograph and a 2- to 4-MHz round-tipped were used. Primary outcomes were the need for intubation after HFNC for respiratory failure during the treatment, length of ICU during and mortality.

**RESULTS.** During the study period, 34 patients with blunt chest trauma were admitted to the study; 16 patients received HFNC therapy and 18 received Conventional oxygen therapy (COT);There was no significantly different of the baseline clinical characteristics between two groups.The length of ICU stay and intubation rate for respiratory failure was significantly different between the 2 groups

(P< 0.05), but without difference for the in-hospital mortality (P=0.78).The lung ultrasound score of COT group had no significantly different from T1 to T2,neither from T2 to T3 (P >0.05).However,the lung ultrasound score decreased significantly by 25% at T2 (P < 0.05) and by 31% between at T3 (P < 0.05) compared to T1 in the HFNC therapy group. LUS of the patients intubated for respiratory failure in the COT group increased from T1 (17±3) to T3 (21±3 ),and the LUS (21±3 ) were much higher than the patients not intubated (11±3) at T3;LUS of HFNC group were all above 15 which had no significantly different from T1 to T2,neither from T2 to T3 (P >0.05).

**CONCLUSIONS.** HFNC may be considered as an initial respiratory therapy for trauma patients with blunt chest injury. HFNC could improve lung aeration through the transthoracic lung ultrasound assessment, it may help the attending physicians identify the useful of HFNC,and decide if continue use the HFNC or have the intubation.

**GRANT ACKNOWLEDGMENT**

none

### 0428

#### High flow nasal oxygen therapy in patients with acute exacerbations of COPD

P. Hancı, S. Öcal, Ş. Eyüpoğlu, E. Ortaç Ersoy, A. Topeli

Hacettepe University Faculty of Medicine, Medical Intensive Care Unit, Ankara, Turkey

**Correspondence:** A. Topeli

*Intensive Care Medicine Experimental* 2018, **6(Suppl 2):**0428

**INTRODUCTION.** Noninvasive mechanical ventilation (NIMV) has been proven to reduce intubation rate and mortality and is being widely used to support ventilation in COPD acute exacerbations. The rate of NIMV failure in COPD patients with acute respiratory failure ranges from 5-40%.[1] Some of these patients cannot tolerate mask or high inspiratory pressure during NIMV.

**OBJECTIVES.** We investigated the efficacy of high flow nasal oxygen therapy (HFNOT) in addition to standard medical treatment in patients admitted to intensive care unit (ICU) because of acute exacerbation of COPD. Our primary endpoints were changes in respiratory rate and visual analogue scale (VAS) for assessment of dyspnea; secondary endpoints were changes in $PaCO_2$ and $PaO_2/Fi0_2$ within 48 hours.

**METHODS.** Patients were eligible for the study if they were admitted with an acute exacerbation of COPD with respiratory rate > 20/min, signs of respiratory distress, pH > 7.30 or increase in $PaCO_2 \geq 5$ mmHg from the basal value.

HFNOT was administered on the ICU with Optiflow™ (Fisher & Paykel Healthcare, New Zealand) by using a predefined protocol. Vital signs, VAS, signs of respiratory distress, arterial blood gas parameters were recorded at the beginning and at the 1st, 6th, 12th, 24th and 48th hours of therapy. If deterioration in patient's general condition, vital signs and dyspnea severity were detected, it was recorded as treatment failure and NIMV or IMV was commenced as a rescue therapy. Results are presented using median (25-75 percentile). Friedman test was conducted to test whether there is a significant change in the vital signs, VAS and ABG parameters.

**RESULTS.** Nine patients have been enrolled between October 2017 and February 2018. The median age was 71 (61-75) and 8 patients were male. Patients GOLD[2] COPD stages were D (n=8) and C (n=1). HFNOT was applied for a median of 100 hrs (50-120). There was not any significant improvement in respiratory rate, pH, $PaCO_2$, $PaO_2$, $PaO_2/FiO_2$ ratio within 48 hours. However, there was a decrease in VAS (p=0.010) and heart rate (p=0.024). Seven patients were weaned from HFNOT. HFNOT failure was observed in 2 patients; one of them was intubated and NIMV was applied to the other one. One of the 6 patients was transferred to the ward, but was lost due to cardiac arrest during follow-up. 28th and 60th-day mortality rates were 11%.

**CONCLUSIONS.** The preliminary results of this ongoing study revealed that HFNOT decreased VAS and heart rate. Therefore, HFNOT might be feasible and reliable for patients with acute exacerbations of COPD.

**REFERENCE(S)**

1. M. Moretti, C. Cilione, A. Tampieri et al. Incidence and causes of non-invasive mechanical ventilation failure after initial success. Thorax. 2000 Oct; 55(10): 819-825.
2. Vogelmeier CF, Criner GJ, Martinez FJ et al. Global Strategy for the Diagnosis, Management, and Prevention of Chronic Obstructive Lung Disease 2017 Report. Am J Respir Crit Care Med. 2017 Mar 1;195(5):557-582.

### 0429

#### Pulmonary vasodilators use before ecmo initiation in lung transplantation affects mortality?

M. López Sánchez[1], M.I. Rubio López[1], M.A. González-Gay Mantecón[2], F.J. Llorca Díaz[3]

[1]University Hospital Marqués de Valdecilla, Intensive Care Unit, Santander, Spain; [2]University Hospital Marqués de Valdecilla, Rheumatology Service, Santander, Spain; [3]Cantabria University, Preventive Medicine and Public Health, Santander, Spain

**Correspondence:** M. López Sánchez

*Intensive Care Medicine Experimental* 2018, **6(Suppl 2):**0429

**INTRODUCTION.** Indications for Extracorporeal Membrane Oxygenation (ECMO) use in lung transplantation (LT) are: bridge to transplantation, intraoperative extracorporeal respiratory and/or circulatory support and treatment of primary graft dysfunction (PGD) in postoperative period. Several mortality risk factors pre-ECMO initiation has been investigated in ARDS, but not in LT patients. Presence of pulmonary hypertension (PH) before LT is common. If pulmonary vasodilators use pre-ECMO initiation can affect mortality has not been studied.

**OBJECTIVES.** To estimate if pulmonary vasodilators use before ECMO initiation in patients waiting LT, in the intraoperative phase or for PGD treatment affects ICU mortality.

**METHODS.** Retrospective, descriptive and observational study between January 2009 to December 2016 in 12 beds intensive care unit (ICU). Inclusion criteria for ECMO entry: bridge to PT, intraoperative support and in PGD. ECMO systems: centrifugal pump and polymethylpentene membrane oxygenation with Bioline® coated circuits and cannulas. Demographic data, APACHE II and SOFA, ECMO indications, type of ECMO support, PH grade, cardiac output (CO), type of vasodilator and inotrope/vasopressor use were collected at the time of ECMO initiation. Pulmonary vasodilators and inotropes/vasopressors use were compared between survivors and non-survivors. Continuous variables, reported as mean ± standard deviation (SD) were compared using the Student t-test. Categorical variables were compared using the $X^2$ test.

**RESULTS.** 35 patients (p), 23 males (65.71%) were included with a median age of 49.57±13.14 years. Median APACHE II was 19.85±7.73 and median SOFA was 7.11±3.02. VA ECMO was used in 22 patients (62.85%) and VV ECMO in 13 patients (37.14%). Intraoperative support was used in 54.28% (19 p); bridge to LT in 20% (7 p) and PGD was the indication in 25.71% (9 p). Median CO was 4.74±1.95. Interstitial lung diseases disease (ILD) were the principal indication for LT in 13 p (37.14%), and idiopathic pulmonary fibrosis was the most frequent disease. PH was present in 88.5% (severe in 48.5%). No significant statistical differences were observed between survivors and non-survivors in inhaled nitric oxide use (45% vs 46%; p=0.596), inhaled iloprost (30% vs 33.3%; p=0.560). Continuous intravenous epoprostenol use was significantly higher in survivors than in non-survivors (35% vs 0%; p=0.012). There were not statistical differences in noradrenalin, adrenalin, dopamine, dobutamine and milrinone use between survivors and non-survivors, but a higher use of milrinone was observed in survivors than in non-survivors (50% vs 20%; p=0.07).

**CONCLUSIONS.** In this retrospective review about ECMO in LT patients, intravenous epoprostenol use before ECMO initiation was higher in survivors than in non-survivors (p=0.012).

REFERENCE(S)

1. Abrams D, Brodie D, Arcasoy SM. Extracorporeal life support in lung transplantation. Clin Chest Med 2017;384:655-6.

### 0430

#### Management of venovenous extracorporeal membrane oxygenation with moderate-target anticoagulation: a retrospective study

L. Bastia[1], M. Bombino[2], M. Giani[2], C. Cunsolo[1], F. Stefanini[1], G. Valsecchi[1], D. Albiero[2], R. Rona[2], G. Bellani[1,2], A. Pesenti[3,4], G. Foti[1,2]
[1]University of Milan-Bicocca, Monza, Italy; [2]ASST Monza, Monza, Italy, [3]University of Milan, Milano, Italy, [4]Ospedale Maggiore Policlinico, Milano, Italy
**Correspondence:** L. Bastia
*Intensive Care Medicine Experimental* 2018, **6(Suppl 2):**0430

**INTRODUCTION.** Extracorporeal membrane oxygenation (ECMO) requires systemic anticoagulation. Despite technological improvement (i.e. coating of extracorporeal tubing and membranes) international guidelines still recommend full anticoagulation, with a recommended aPTT ratio target of 1.5-2 and ACT target of 180-220 sec. Previous studies by Panigada et al. (1,2) showed that pursuing these targets may lead to excessive anticoagulation, resulting in a high prevalence of "flat line" thromboelastography (TEG®) traces (defined as kaolin R-time >90min), ranging from 38 to 46%. At our institution we manage these patients with moderate anticoagulation, targeting an aPTT ratio of 1.3-1.7 and a ACT of 160-180.
**OBJECTIVES.** The aim of our study was to retrospectively assess the TEG R-time and other coagulation parameters in a 10-years ECMO patients cohort. We hypothesized that lower anticoagulation target may correspond to a more adequate anticoagulation level when assessed by TEG.
**METHODS.** Electronic charts, TEG database and a prospective ECMO database were reviewed to assess the level of anticoagulation in ECMO patients.
**RESULTS.** 110 adult patients were treated with vv-ECMO between 2009 and 2018. Median ECMO duration was 16 days [IQR 10-25], for a total of 2650 ECMO days. Average circuit duration was 7±5 days. Mean aPTT was 1.48±0.24, heparin daily dose was 24,400±11,100 IU. A total of 973 K-TEGs were collected. Median TEG kaolin R-time was 21.6 min [IQR11.8-40 min]. The fraction of TEG tracings with r>90 min ("flat line TEG") was 8.6%.
**CONCLUSIONS.** An anticoagulation strategy based on moderate aPTT and ACT targets resulted in an adequate level of anticoagulation when assessed by kaolin R-time.

### 0431

#### Use of high flow nasal cannula therapy for acute hypoxemic respiratory failure: a review of predictors of success and outcomes

H.Z. Chai, K.J.Y. Goh, V.P.V. Lagutap, G.C. Phua, T.H. Ong, Q.L. Tan
Singapore General Hospital, Respiratory and Critical Care Medicine, Singapore, Singapore
**Correspondence:** K.J.Y. Goh
*Intensive Care Medicine Experimental* 2018, **6(Suppl 2):**0431

**INTRODUCTION.** Heated and humidified high flow nasal cannula (HFNC) therapy is a form of non-invasive respiratory support for patients with non-hypercapnic acute hypoxemic respiratory failure[1]. Common indications include pneumonia and respiratory support post-extubation. Studies on outcomes describe the duration of HFNC use before intubation and the ROX index (ratio of pulse oximetry/ fraction of inspired oxygen to respiratory rate) for patients with pneumonia as significant predictors of survival and success[2, 3].
**OBJECTIVES.** A review of the factors that predict HFNC success in patients with hypoxemic respiratory failure from pneumonia.
**METHODS.** We conducted a retrospective analysis of our unit's HFNC registry from February 2017 to 2018, for patients initiated with pneumonia. Patient demographics, co-morbidities, ROX index at 12 hours, ICU severity scores (APACHE and SOFA) and mortality outcomes were analyzed.
**RESULTS.** In total, 22 patients were initiated on HFNC (mean age, 61 ± 16, 68.1% male), of which 50% were immunocompromised hosts (ICH). Twelve patients (54.5%) were successfully weaned off HFNC while 10 patients (45.5%) required intubation and mechanical ventilation. There was no significant difference in baseline characteristics between the two groups, including Charlson Comorbidity Index, severity of hypoxemia based on PF ratio, ICU severity scores and mortality. There was a trend towards a higher ROX index at 12 hours for those who were successfully weaned off HFNC (9.16 ± 4.43 vs 5.97 ± 1.65, p = 0.145). The duration of HFNC was also significantly longer in the successful group (73.7 ± 58.3 hours vs 27.4 ± 29.6 hours, p = 0.034). Between the ICH and non-ICH group, there was no significant difference in baseline characteristics, duration of HFNC and outcomes of successful termination and mortality. The overall ICU mortality and in-hospital mortality was 13.6% and 22.7% respectively.
**CONCLUSIONS.** In our cohort of patients, HFNC success was associated with a higher ROX index at 12 hours, consistent with the predictive utility of ROX as described in literature[3]. A longer duration of HFNC treatment was also observed in patients who were successfully weaned off HFNC and was not associated with increased mortality. This differs from other studies that have a 48 hour limit to the duration of HFNC usage[1], with concerns that failure of HFNC might cause delayed intubation and worse outcomes[2]. We did not observe any significant difference in HFNC usage and outcomes between the ICH and non-ICH patients. Our findings may be the result of our small sample size, a larger study is needed to further evaluate predictors of HFNC success and to identify specific groups of patients who may benefit from a longer duration of HFNC usage.

REFERENCE(S)

1 Hernández G. 2017:1-11. doi:10.1186/s13054-017-1640-2.
2 Kang BJ. 2015:623-632. doi:10.1007/s00134-015-3693-5.
3 Roca O. 2017;35(2016):200-205. doi:10.1016/j.jcrc.2016.05.022.

GRANT ACKNOWLEDGMENT
NA

### 0432

#### Use of prone positioning and airway pressure release ventilation (APRV) for patients with acute respiratory distress syndrome (ARDS) in intensive care units in London and the South-East of England

R.D. Lakhani, A.F. Nwamarah, R. Kumar, A.E. Myers, S. Ranjan, T.L. Samuels
Surrey and Sussex Healthcare NHS Trust, Intensive Care Unit, Redhill, United Kingdom
**Correspondence:** R.D. Lakhani
*Intensive Care Medicine Experimental* 2018, **6(Suppl 2):**0432

**INTRODUCTION.** ARDS carries significant morbidity and mortality (35%-46% in mild to severe forms)[1]. There is good evidence to support prone positioning in the management of ARDS[2]. Evidence is also emerging to support APRV[3]. Despite this, use of both remains inconsistent, with literature suggesting prone positioning is only used for between 8-33% of patients with ARDS[1,2].
**OBJECTIVES.** To review the use of prone positioning and APRV in the treatment of ARDS in critical care units within one region of the UK.
**METHODS.** An online survey consisting of 25 questions on ICU demographics, prone positioning and the use of APRV was distributed to ICUs in the South-East Coast and London Critical Care Networks.

**RESULTS.** There were 18 responses, mainly from mixed medical & surgical ICUs (67%), with a median size of 13.5 beds (total range 2-68). 50% (n=9) reported proning patients on a regular basis, with reasons for not proning being lack of a protocol, lack of experience, risk of complications, the procedure being too labour intensive or rarely clinically required. The median number of patients proned by these units in the last 12 months was 4 (IQR 2-6). 33% (n=6) of respondents had a protocol for when to prone patients. P:F ratio ($PaO_2/FiO_2$), $PaO_2$, and $FiO_2$ were the most common variables used to determine when to prone patients. 33% had a protocol for how to prone patients, with common factors including the use of neuromuscular blockade, five or more staff required, and pronation during the daytime, with durations of 12-18 hours. 56% (n=10) used APRV infrequently, with only 17% (n=3) using it frequently. There was a protocol for when to commence APRV in 22% (n=4), and how to start it in 33% (n=6).

**CONCLUSIONS.** In spite of compelling evidence, only half of the ICUs in this region use prone positioning regularly. The main impediments appear to involve human factors, such as risk of complications or labour intensiveness. However, where pronation does take place, there appears to be some consistency regarding when and how this should be done. APRV is used infrequently and tends to be initiated based upon clinician decision. This may be due to the lack of a large multi-centre trial supporting its use. However, if more convincing evidence does emerge, APRV may represent a valuable alternative rescue technique for ARDS which avoids the risks and difficulties of prone positioning.

**REFERENCE(S)**

1. Bellani G, et al. Epidemiology, Patterns of Care, and Mortality for Patients With Acute Respiratory Distress Syndrome in Intensive Care Units in 50 Countries. JAMA. 2016;315(8):788-800
2. Guérin C, et al. A prospective international observational prevalence study on prone positioning of ARDS patients: the APRONET (ARDS Prone Position Network) study. Int Care Med. 2018;44(1):22-37
3. Zhou Y, et al. Early application of airway pressure release ventilation may reduce the duration of mechanical ventilation in acute respiratory distress syndrome. Int Care Med. 2017;43(11):1648-1659

### 0433

#### A retrospective observational study of prone positioning practices in the management of acute respiratory distress syndrome (ARDS) over a 2 year period in a single adult district general hospital (DGH) intensive care unit (ICU)

A.F. Nwamarah, R.D. Lakhani, A.E. Myers, S. Ranjan, T.L. Samuels
Surrey and Sussex Healthcare NHS Trust, Intensive Care, Surrey, United Kingdom
**Correspondence:** A.F. Nwamarah
*Intensive Care Medicine Experimental* 2018, **6(Suppl 2):**0433

**INTRODUCTION.** Mechanical ventilation in ARDS may contribute to further lung damage due to high ventilatory pressures/volume delivered to a poorly compliant lung. Although various strategies for improving outcomes in ARDS have been tried, the only technique with good supporting evidence is prone positioning [1]. However, it is labour intensive, requires staff training and has potential complications.

**OBJECTIVES.** To review prone positioning practices within one DGH ICU in terms of demographics, reasons for initiation and outcomes.

**METHODS.** The electronic patient record database for a single ICU was interrogated for the keywords 'prone', 'proned', 'proning' and 'ECMO' for the period 03/2016 to 03/2018. Cases were reviewed to find the PaO2/FiO2 (PF) ratio and ventilation modes at instigation of prone positioning. Outcomes were recorded in terms of in-hospital and ICU mortality, and whether extra corporeal membrane oxygenation (ECMO) was required. Demographics and Clinical Frailty Scores (CFS) were recorded.

**RESULTS.** Of the 14 patients placed in prone position, 64.3% met the classification for severe ARDS with a PF ratio of < 13.3 kPa. The mean age was 61 years (SD 19.3 years), 4 males and 10 females. We observed 50% survival. A higher CFS was associated with a worse outcome. Although 6 patients were referred to an ECMO centre, only 2 were transferred and none received ECMO. 2 of the patients referred for ECMO were not deemed candidates due to increased bleeding risks. The median number of proning episodes was 2 (IQR 1- 2.25) with a mean duration of 14hr10mins (SD 4hrs10mins). There appears to be no correlation between the number of proning episodes and PF ratios. Patients with a lower CFS were found to tolerate a lower PF ratio in comparison to those with a higher CFS. 28.6% deemed CFS 1, were all classified as severe ARDS. 57.1% of patients received a trial of airway pressure release ventilation (APRV) prior to proning; and had a better outcome with 75% survival. 2 patients had documented complications secondary to proning: re-intubation; and facial oedema.

**CONCLUSIONS:** In this small group, reduced frailty and a trial of APRV, may both be associated with better outcomes in prone positioning for ARDS. Further work should be done to investigate the relationship between CFS and PF ratios, in terms of impact upon survival with proning. It is likely that, during this 2 year study period, other patients with ARDS may have benefited from prone positioning. The duration of proning was intended to be 16 hrs, but there was considerable variation. Introduction of an evidence based protocol for the initiation of prone positioning in ARDS may lead to more frequent instigation of proning in ICU.

**REFERENCE(S)**

1. Guérin C, et al. Prone Positioning in Severe Acute Respiratory Distress Syndrome. N Engl J Med.2013;368(23):2159-2168

### 0434

#### Effects of set flow rate on the ROX index in acute hypoxemic respiratory failure patients undergoing high flow therapy

E. Carlesso[1], T. Mauri[1], E. Spinelli[1], A. Galazzi[1], F. Binda[1], D. Tortolani[2], C. Turrini[2], L. Alban[2], M. Lazzeri[2], C. Abbruzzese[1], P. Tagliabue[1], S. Spadaro[2], G. Grasselli[1], O. Roca[3], A. Pesenti[1]
[1]University of Milan, Department of Anesthesia and Critical Care, Fondazione IRCCS Ca' Granda Ospedale Maggiore Policlinico, Milan, Italy; [2]University of Ferrara, Ferrara, Italy; [3]Universidad Autonoma de Barcelona, Val d'Hebron Univerity Hospital, Val d'Hebron Research Institute, Critical Care Department, Barcelona, Spain
**Correspondence:** D. Tortolani
*Intensive Care Medicine Experimental* 2018, **6(Suppl 2):**0434

**INTRODUCTION.** Interest in High-flow nasal cannula (HFNC), a non-invasive support for acute hypoxemic respiratory failure (AHRF) patients, is rapidly growing. A major clinical challenge is to avoid delayed intubation by recognition of more severe AHRF patients on HFNC. The ROX (Respiratory rate-OXygenation) index was recently introduced as predictor of the need for mechanical ventilation in patients with AHRF treated with HFNC. However, the variables included in the ROX index can vary significantly with set flow rate.

**OBJECTIVES.** We analysed the effects of two set HFNC flow rates on the ROX index value in a population of AHRF patients.

**METHODS.** Fifty-seven non-intubated AHRF patients with $PaO_2/FiO_2$ ≤300 supported by HFNC, previously included in two prospective randomized cross-over studies (2, 3) were analyzed. All underwent two steps for 15-20 minutes in randomized order with HFNC delivered at 30 and 60 l/min, leaving $FiO_2$ unchanged. Towards the end of each phase, we collected $SatO_2$ ($SO_2$ in 17 patients (2) and $SpO_2$ in 40 patients (3)), $FiO_2$, respiratory rate (RR), Borg dyspnea scale, heart rate and mean arterial pressure. The ROX index was then calculated as ($SatO_2/FiO_2$)/RR. Demographics, number of lung quadrants involved and SOFA score were collected, too.

**RESULTS.** Patients were 59±14 years old, 25 (44%) were female, clinical $PaO_2/FiO_2$ was 199±54.

Increasing set HFNC flow rate from 30 to 60 l/min led to a significant increase of the ROX index values (10.21 [7.15 - 13.33] vs. 11.14 [8.81 -

13.93], median [interquartile range, IQR] p=0.003). Only 4 patients had ROX index < 4.88.

Forty patients showed a change in ROX index between the study phases >0 (+1.04 [0.49 - 2.22]), while 17 had unchanged or decreased ROX index at higher flow (-1.22 [-2.05 - -0.29]); p< 0.001 vs. patients with increased ROX) (Figure 1). The two subgroups showed significant differences in terms of severity of AHRF, SatO2 and RR measured at 30 l/min flow rate, with patients responding to the "flow challenge" showing worse conditions (Table 1).

**CONCLUSIONS.** Set HFNC flow significantly impacts the ROX index. Increase of this index at higher flow seem to occur in more severe patients.

**GRANT ACKNOWLEDGMENT**

Ricerca corrente Fondazione IRCCS Ca' Granda Ospedale Maggiore Policlinico, Milan, Italy.

**Table 1 (abstract 0434).** Physiological variables divided according to ROX index change from baseline (30 l/min).Values are reported as means±std or medians [IQR]

| Baseline variables | Switch from 30 to 60 l/min ("flow challenge") | | P value |
|---|---|---|---|
| | Unchanged/decreased ROX index (N=17) | Increased ROX index (N=40) | |
| SOFA | 4 [3-6] | 6 [3-9] | 0.102 |
| Borg dyspnea scale | 2 [1-3] | 3 [2-6] | 0.126 |
| FiO2 | 40 [40-40] | 50 [40-55] | **0.001** |
| RR bpm | 18.0 [18.0-21.0] | 23.5 [19.0-30.0] | **0.007** |
| SatO2 | 97.0 [96.0-99.0] | 96.0 [93.5-97.8] | **0.044** |
| SatO2/FiO2 | 243 [238-248] | 195 [171-239] | **<0.001** |
| ROX index | 13.61 [11.43-14.29] | 8.64 [6.30-11.61] | **<0.001** |

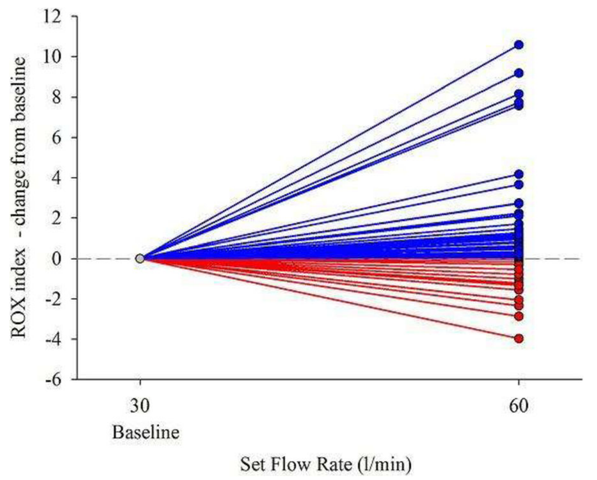

**Fig. 1 (abstract 0434).** See text for description.

### 0435

#### Effects of spontaneous breathing during ECMO in a severe ARDS experimental model

S. Dubo[1,2], V. Oviedo[3], A. Garcia[3], L. Alegria[3], P. Garcia[4], D. Valenzuela[3], F. Damiani[3], J. Araos[5], T. Medina[6], M.C. Bachmann[3], D. Soto[3], P. Cruces[6,7], J. Retamal[3], R. Cornejo[8], G. Bugedo[3], A. Bruhn[3]

[1]Universidad de Concepción, Departamento de Kinesiología, Concepción, Chile; [2]Universidad de La Frontera, Programa de Doctorado en Cs. Médicas, Temuco, Chile; [3]Pontificia Universidad Catolica de Chile, Departamento de Medicina Intensiva, Santiago, Chile; [4]Pontificia Universidad Catolica de Chile, Departamento de Ciencias de la Salud, Carrera de Kinesiología, Santiago, Chile; [5]Université de Montréal, Faculté de Médecine Vétérinaire, Centre Hospitalier Universitaire Vétérinaire, Saint-Hyacinthe, Canada; [6]Hospital El Carmen de Maipú, Unidad de Pacientes Críticos, Santiago, Chile; [7]Universidad Andres Bello, Centro de investigación de Medicina Veterinaria, Santiago, Chile; [8]Universidad de Chile, Hospital Clínico, Unidad de Pacientes Críticos, Santiago, Chile

**Correspondence:** A. Bruhn

*Intensive Care Medicine Experimental* 2018, **6(Suppl 2):**0435

**INTRODUCTION.** Although a lung rest strategy is recommended during ECMO in severe ARDS, observational studies show that alternative ventilatory approaches are used, including allowing spontaneous breathing (SB). SB may have some theoretical benefits, but it has been suggested to induce further lung injury in severe ARDS. However, this risk associated to SB may decrease with ECMO due to the possibility of modulating ventilatory drive.

**OBJECTIVES.** To compare the effects of spontaneous breathing versus near-apneic ventilation with neuromuscular blockade (NMB), on lung injury, in a porcine severe ARDS model assisted by ECMO.

**METHODS.** Ten pigs (30±5kg) were anesthetized, mechanically ventilated and monitored. Lung Injury was induced by repeated lavages (30 ml/kg of warm saline) until $PaO_2/FiO_2$ dropped below 100, followed by a 2-hour injurious ventilation (PEEP 0 $cmH_2O$, PInsp 40 $cmH_2O$, RR 10/min, I:E 1:2). After completing lung injury (time 0, $T_0$) animals were connected to an ECMO circuit by inserting an AVALON 23F double-lumen cannula through the right jugular vein. Blood flow was set at 60-70 ml/kg/min and sweep gas flow adjusted to $PaCO_2$ 30-50 mmHg. During the first 3 hours, all animals were ventilated with near-apneic ventilation (PEEP 10, driving pressure 10, RR 5/min, I:E ratio 1:2) and NMB. After $T_3$, animals were randomized into 2 groups: i) Near-apneic ventilation, which continued with the same settings used before, and ii) SB: in this group NMB were stopped and sweep gas flow decreased until regaining respiratory efforts. Thereafter, ventilation was switched to Pressure support mode (PS 10 $cmH_2O$, PEEP 10 $cmH_2O$) and sweep gas flow adjusted to keep $PaCO_2$ 30-50 mmHg while preserving SB at a respiratory rate of 30 to 70 bpm. Respiratory and hemodynamic data, as well as regional lung impedance, assessed by EIT (EIT-Pioneer Set, Swisstom, Switzerland), were collected at baseline and times 0, 3, 6, 12 and 24h. After 24 hours, animals were euthanized and lungs extracted for tissue analysis. Lung edema was estimated by the wet-dry weight ratio and lung injury by histological analysis (alveolar disruption, neutrophil infiltration or hemorrhage). Preliminary results of this study are presented here.

**RESULTS.** SB group exhibited a rapid shallow breathing pattern (Table 1). Median sweep gas flow was 7 (5.5-10) L/min in the Near-apneic ventilation group vs 10 (7-12) L/min in the SB group, with no differences in $PaCO_2$. No hemodynamic differences were observed. Regional impedance was similar between groups (Fig.1), as well as global end-expiratory lung impedance. No differences were observed in lung wet/dry weight ratio or in the histological lung injury score (Fig.2).

**CONCLUSIONS.** In this preliminary analysis, we found no differences between SB and Near-apneic ventilation in lung injury, lung function, or in regional lung impedance.

**GRANT ACKNOWLEDGMENT**
CONICYT, FONDECYT 1161556 and PFCHA/Doctorado Nacional/2018/21181376

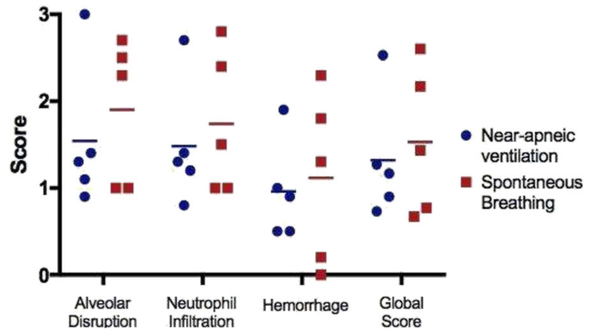

**Fig. 1 (abstract 0435).** Histological Lung Injury Score. Scored from 0= no alteration to 3= maximal injury

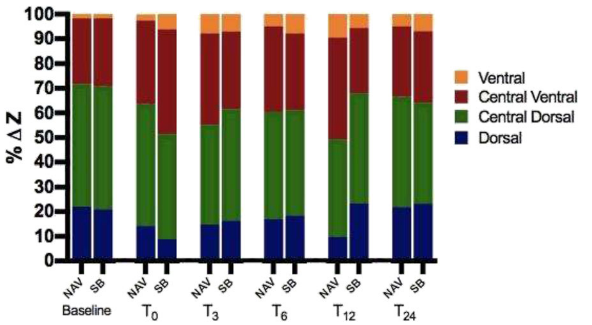

**Fig. 2 (abstract 0435).** Relative regional impedance distribution. NAV: near-apneic ventilation; SB: spontaneous breathing

### 0436

#### Initial $ECCO_2R$ experience in the great Paris area: the REXECOR observatory

J.L. Augy[1], N. Aissaoui[1], C. Richard[2], E. Maury[3], M. Fartoukh[4], A. Mekontso-Dessap[5], R. Paulet[6], N. Anguel[7], C. Blayau[8], Y. Cohen[9], B. Megarbane[10], J.-D. Chiche[11], S. Gaudry[12], S. Voicu[10], A. Demoule[13], A. Combes[13], E. Charpentier[14], S. Haghighat[15], M. Panczer[15], J.-L. Diehl[1]
[1]Hopital Europeen Georges Pompidou, Paris, France; [2]Bicetre Hospital, Le Kremlin-Bicêtre, France; [3]Saint-Antoine University Hospital, Paris, France; [4]Hopital Tenon, Paris, France; [5]Hopital Henri Mondor, Créteil, France; [6]Centre Hospitalier Général de Longjumeau, Longjumeau, France; [7]Hopital Bicêtre, Le Kremlin Bicêtre, France; [8]hopital Tenon, Paris, France; [9]Hopital Avicennes, Bobigny, France; [10]Lariboisière Hospital, Paris, France; [11]Cochin Hospital, Paris, France; [12]Hopital Louis-Mourier, Colombes, France; [13]La Pitié-Salpêtrière Hospital, Assistance-Publique-Hôpitaux-de-Paris, UPMC Univ Paris 06, Paris, France; [14]DRCI-APHP, Paris, France, [15]AGEPS-APHP, Paris, France
**Correspondence:** J.L. Augy
*Intensive Care Medicine Experimental* 2018, **6(Suppl 2):**0436

**INTRODUCTION.** Veno-venous extracorporeal CO2 removal (ECCO2R) is a promising new therapeutic option in the critical care setting. We conducted a prospective observational study of the use of ECCO2R in selected voluntary centers during two years aiming to assess the prevalence of the ECCOR2 use mainly among COPD and ARDS patients.

**METHODS AND PATIENTS.** Two medical devices: Hemolung (Alung Technologies, Pittsburgh, USA) and iLA Activve (Xenios Novalung, Heilbronn, Germany) were selected after literature and medico-economic evaluations. A specific medical and nurses training was provided in each center. Data were collected on a dedicated form and were centralized by the coordinating center. Primary outcome was the number of patients treated per month and per center during the 2-years study period. Secondary outcomes were ICU and hospital-mortality and adverse events/complications related to device use.

**RESULTS.** We present results from 70 patients recruited in 10 centers (41 men, 29 women, median age 65 years (IC 25-75: 61-74)). The utilization rate was of 0.19 (min: 0.04, max: 1.20) patient/month/center. Fifty-nine patients were under invasive and 11 under noninvasive mechanical ventilation. Hemolung was used in 53 patients (60% in jugular site, cannula size: 15.5 F) and iLA Activve in 19 (56% in jugular site, cannula size: 18 F, 24F for femoral site). Main indications were COPD AE (n=30) and ARDS (n=24). Twenty-one were treated as a part of a clinical trial and 49 were treated as decided by the physician in charge according to current practice. Mean duration of ECCO2R was 5days (IC25-75: 3-8). Thirty-two ECCO2R treatments were discontinued because of clinical condition improvement, 12 because of complications, 9 because of death and 16 for futility. Twenty-one hemolysis (either biological: free Hb > 100µmol/L or clinical), 17 hemorrhagic complications, 11 thrombosis, 1 cannula disinsertion, and 1 local hematoma occurred. Thirty-five deaths occurred during ICU stay, 36 during the hospitalization, 3 of which in relation with ECCO2R.

**CONCLUSION.** Our data indicate a preferential use of veno-venous ECCO2R devices in very severe (as illustrated by the overall high mortality) COPD and ARDS patients; with a lower than expected rate of utilization. Safety remains a major concern, indicating the need for further technological improvements as well of for optimization of anticoagulation regimen. Ultimately, RCTs will help to delineate clinical indications in these 2 main settings (COPD and ARDS).

**GRANT ACKNOWLEDGMENT**
None

**Table 1 (abstract 0436).** Respiratory variables. Values are expressed as median (range). *p<0.05 comparing groups

| VARIABLE | Group | Baseline | $T_0$ | $T_3$ | $T_6$ | $T_{12}$ | $T_{24}$ |
|---|---|---|---|---|---|---|---|
| Respiratory rate | Near-apneic | 18 (18-20) | 22 (18-26) | 5 (5-5) | 5 (5-5) | 5 (5-5) | 5 (5-5) |
| (bpm) | SB | 18 (18-22) | 18 (18-22) | 5 (5-5) | 51 (40-88)* | 60 (34-64)* | 44 (32-85)* |
| Tidal volume | Near-apneic | 10.8 (10.1-11.3) | 10.2 (10.1-12.0) | 3.0 (2.3-3.5) | 2.9 (2.6-3.5) | 3.5 (2.4-4.0) | 2.9 (2.2-4.6) |
| (ml/kg) | SB | 10.5 (10.0-10.7) | 10.2 (10.2-11.4) | 3.0 (1.4-4.4) | 2.1 (1.5-3.7) | 1.7 (1.4-3.4) | 2.4 (1.3-3.7) |
| Minute volume | Near-apneic | 6.0 (3.0-8.9) | 6.7 (4.6-8.2) | 1.2 (0.7-1.7) | 1.1 (0.7-1.5) | 1.2 (0.7-1.4) | 0.9 (0.7-1.3) |
| (L/min) | SB | 6.3 (5.9-7.2) | 6.2 (5.6-7.7) | 1.3 (0.9-1.6) | 4.1 (2.5-6.1)* | 3.3 (2.5-6.3)* | 3.2 (3.1-4.6)* |
| $PaO_2/FiO_2$ | Near-apneic | 337 (319-444) | 70 (52-95) | 115 (69-146) | 126 (59-198) | 167 (55-230) | 212 (65-306) |
| | SB | 394 (342-509) | 55 (51-276) | 91 (72-201) | 159 (88-314) | 189 (108-317) | 143 (54-305) |

### 0437

#### Physiological evaluation of high flow oxygen therapy versus standard oxygen therapy effects in tracheostomized patients

R. Di Mussi[1], S. Spadaro[2], G. Garribba[1], G. Frasso[1], F. Sechi[3], C.A. Volta[2], F. Bruno[1], T. Stripoli[4], S. Grasso[1]
[1]University 'Aldo Moro', Department of Emergency and Organ Transplant, Bari, Italy; [2]Ferrara University, Ferrara, Italy; [3]Sassari University, Sassari, Italy; [4]Bari, Policlinico, Department of Emergency and Organ Transplant, Bari, Italy
**Correspondence:** R. Di Mussi
*Intensive Care Medicine Experimental* 2018, **6(Suppl 2):**0437

**INTRODUCTION.** High flow nasal cannula (HFNC) has been shown to decrease the reintubation rate in critically ill patients after planned extubation. As compared to conventional oxygen therapy, HFNC decreases work of breathing and respiratory rate and improves oxygenation. Recently, a novel interface made it possible to deliver high flow oxygen therapy through the tracheostomy cannula.
**OBJECTIVES.** To evaluate if high flow oxygen therapy delivered through tracheotomy cannula has the same physiological effects of HFNC in tracheostomized patients at risk of reconnection after disconnection from the ventilator.
**METHODS.** After a planned disconnection from mechanical ventilation, 11 tracheostomized patients underwent a cross-over study alternating high flow and standard oxygen therapy (HF TRACH and LF TRACH), respectively, according to an ON-OFF design:

1) HF TRACH;
2) LF TRACH;
3) HF TRACH.

Heated and humidified HF TRACH oxygen therapy was delivered at flow rates between 50 and 70 L/min (F&P, Auckland, New Zealand). The diaphragmatic electrical activity (Eadi) signal was continuously recorded (Maquet, Solna Sweden) to obtain the mean Eadi peak ($Eadi_{PEAK}$). Work of breathing, respiratory rate and gas exchange parameters were assessed at the end of each study step.
**RESULTS.**
**CONCLUSIONS.** Our data show that, differently than HFNC oxygen therapy, the application of high flows to the tracheostomy cannula does not improve oxygenation and work of breathing. Our finding that, as compared to LF TRACH, HF TRACH does not decrease the $Eadi_{PEAK}$ suggests that it has no impact on the neural ventilatory drive.

**REFERENCE(S)**

Hernández G, Vaquero C, González P, Subira C, Frutos-Vivar F, Rialp G, Laborda C, Colinas L, Cuena R, Fernández R. -Effect of Postextubation High-Flow Nasal Cannula vs Conventional Oxygen Therapy on Reintubation in Low-Risk Patients: A Randomized Clinical Trial. - JAMA. 2016 Apr 5;315(13):1354-61.
Mauri T, Turrini C Eronia N, Grasselli G, Volta CA, Bellani G, Pesenti A. - Physiologic Effects of High-Flow Nasal Cannula in Acute Hypoxemic Respiratory Failure. - Am J Respir Crit Care Med. 2017 May 1;195(9):1207-1215.

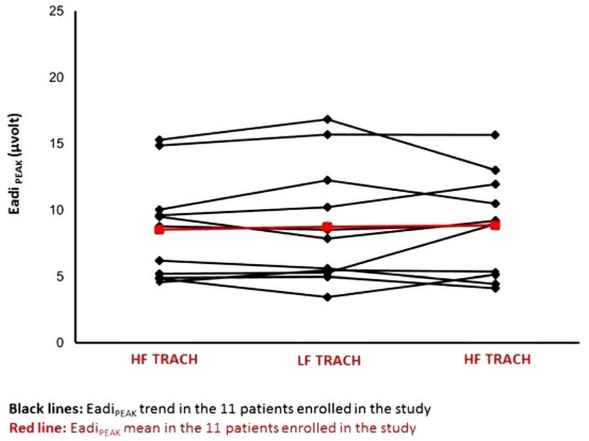

**Fig.1 (abstract 0437).** Diaphragmatic electrical acticity (Eadi) trend in the 3 hours of the study

| | HF TRACH | LF TRACH | HF TRACH | p |
|---|---|---|---|---|
| PaO2 (mmHg) | 111 ± 27.3 | 92.6 ± 18 | 124 ± 50 | NS |
| PaCO2 (mmHg) | 43 ± 7 | 43.6 ± 7 | 42 ± 6 | NS |
| P/F | 218 ± 62 | 188.5 ± 56 | 238 ± 63 | NS |
| Respiratory rate (breaths/min) | 21.7 ± 5 | 21.8 ± 6 | 21.8 ± 6.3 | NS |

**Fig. 2 (abstract 0437).** Gas exchange parameters and respiratory rate trend in the patients studied

## Cardiac arrest - Aspects before and after ROSC

### 0438

#### Effect of hypertension across the age group on survival outcomes in out-of-hospital cardiac arrest

R. Hyunho[1,2], H. Kim[2]
[1]Chonnam National University Hospital, Gwangju, Korea, Republic of; [2]Chosun University Hospital, Gwangju, Korea, Republic of
**Correspondence:** R. Hyunho

*Intensive Care Medicine Experimental* 2018, **6(Suppl 2):**0438

**OBJECTIVE.** There are few studies on the effects hypertension has on survival outcomes in out-of-hospital-cardiac arrest (OHCA) patients, although hypertension is a major risk factor for the incidence of cardiac arrest. This study aims to investigate whether hypertension is associated with survival outcomes in cardiac arrest patients across age groups.
**METHODS.** This study was conducted using the national cardiac arrest registry of OHCA patients who survived to hospital admission from 2012 to 2016. The clinical histories of hypertension were obtained from patients´ medical records. The endpoint was cerebral performance category (CPC) 1 and 2 (good CPC) and survival to discharge. Multivariable logistic regression analysis was performed on the data collected. The final model with an interaction term was evaluated to compare the effects of hypertension across age groups.
**RESULTS.** A total 11,610 patients (61.0% hypertensive patients and 39.0% non-hypertensive patients) were included. The group over 80 years old with hypertension were more likely to have good neurologic recovery (AOR 2.53 [1.43-4.50]) and those under 65 years old with hypertension were more likely to survive to hospital discharge with statistical significance (AOR 1.19 [1.04-1.35]).
**CONCLUSIONS.** Hypertension does not adversely affect the survival outcomes of OHCA patients across all age groups of this study. In the group over 80 years old, HTN is significantly associated with survival with favorable neurologic recovery.

### 0439

#### Perioperative cardiac arrest & major non cardiac surgery admitting to the general surgical ICU

S. Kongsayreepong[1], A. Piriyapatsom[2]
[1]Siriraj Hospital, Mahidol University, Anesthesiology & Critical Care, Bangkok, Thailand; [2]Siriraj Hospital, Mahidol University, Deaprtment of Anesthesiology, Bangkok, Thailand
**Correspondence:** S. Kongsayreepong

*Intensive Care Medicine Experimental* 2018, **6(Suppl 2):**0439

**INTRODUCTION.** Perioperative cardiac arrest (CA) is one of the worse unfavorable outcome associated with noncardiac surgery. Knowing about predictors & outcome may help improve quality care of these patients.

**OBJECTIVES.** To study incidence, characteristics, predictors & outcome of perioperative CA in patients undergoing major noncardiac surgery & admitting to the general SICU.

**METHODS.** This prospective observational study was done in perioperative (pre, intra & 24 hours postoperative) CA patients undergoing major noncardiac surgery & admitting to the general SICU of the 3rd referral university hospital (2,400 beds, 50 ORs, > 34,000 noncardiac surgery/yr) from Jan 2013-Dec 2017. Patients < 18 yrs, undergoing cardiac, neurosurgical, traumatic & transplant surgery were excluded from this study. Study data including: patient demographic data, co-morbidities, ASA physical status, detail of the event associated with CA, type of CA, epinephrine dose, time to ROSC, TTM, 90 days neurological outcome & mortality.

**RESULTS.** There were 132 CA (77.37: 100,000 operation. 4.5% of ICU admission), as 10, 98 & 24 patients with ROSC time of 8$\pm$ 18, 4 $\pm$ 26 & 14 $\pm$ 37 mins & 90 days mortality of 68%, 40% & 78% for pre, intra & postoperative CA respectively. Late & inadequate hemodynamic resuscitation were the main causes of preoperative CA especially in septic shock, most of the EKG were PEA which ¼ also had intraoperative CA & 38% had unfavorable neurological outcome at 90 days. Severe hypovolemic shock (41%) either from severe bleeding or septic shock, airway complications (19%), perioperative MI (12%), massive PE (10%), serious cardiac arrhythmia (8%) & the rest were from anaphylaxis, invasive procedure (eg, line placement), air embolism, LAST & MH were the main causes of intraoperative CA which most of the EKG were PEA, VF & asystole. Two patients had immediate ECPR with good outcome & 6 patients had ECMO supports which 4 patients had good outcome. Most of the causes of postoperative CA were hypoxia either from airway obstruction or pulmonary aspiration, almost half occurred in the recovery room which most EKG were asystole. Postoperative CA that occurred on the floor had worst neurological outcome & highest mortality. Three fourth of the patient did not have hypothermic (33C) TTM because of unfavorable surgical conditions but there wasn´t significant different of the neurological outcome between TTM of 33 C & 36 C (p< 0.05). Data from multivariate analysis of poor neurological outcome at 90 days were hypoxic arrest, older, higher ASA physical status, more initiate time of CPR & ROSC time, higher vasopressor, lower Hb & lower MAP in the first 48 Hr & not have TTM.

**CONCLUSIONS.** Perioperative CA was significant associate with poor perioperative outcome. Efforts should be made to prevent this catastrophic event especially in sick surgical patient undergoing major noncardiac surgery. Early identifying, immediate and effective CPR & TTM may help improve neurological outcome.

### 0440

#### Oxygen and carbon dioxide in patients after cardiac arrest

L. Peluso, I. Belloni, L. Calabro, J. Creteur, J.-L. Vincent, F.S. Taccone
Hopital Erasme, Intensive Care, Brussels, Belgium
**Correspondence:** L. Peluso
*Intensive Care Medicine Experimental* 2018, **6(Suppl 2):**0440

**INTRODUCTION.** The relationship between oxygen or carbon dioxide levels with outcome after cardiac arrest (CA) has yielded conflicting results. Few studies have combined the analysis of $PaO_2$ and $PaCO_2$ in this setting.

**HYPOTHESIS.** High $PaO_2$ and low $PaCO_2$ values are associated with better outcomes after CA.

**METHODS.** We reviewed all comatose patients admitted after successful resuscitation from CA to our Department between January 2009 and December 2017. Inclusion criteria were age ≥18 years, non-traumatic CA and survival ≥24 hours after admission. We analyzed all arterial blood gas data for the first 24 hours after admission, and we calculated the maximum and minimum values of $PaO_2$ and $PaCO_2$. Combination of $PaCO_2$ and $PaO_2$ values were analysed per quartiles. We assessed ICU mortality as well as neurological outcome. Poor neurological outcome was defined as a Cerebral Performance Category (CPC) score of 3-5 at 3-months.

**RESULTS.** A total of 412 patients met the inclusion criteria; of those, 355 had complete $PaO_2$ and $PaCO_2$ values (10 [8-12] blood gas analyses per patient over the first 24 hours) and were included in the final analysis (median age: 65 [52-73] years). Overall, 208 (59%) patients died during the ICU stay and 241 (68%) had poor neurological outcome. Survivors and non-survivors had similar maximum and minimum $PaO_2$ values, as well as maximum and minimum $PaCO_2$ values. Results were similar in patients with good and poor neurological outcome. There were no differences in outcomes in relation to quartiles of maximum $PaO_2$ while the lowest (i.e. ≤41mmHg) and the highest (i.e. ≥55mmHg) quartiles of maximum $PaCO_2$ had the highest rates of ICU mortality (P=0.001) and poor outcome (p=0.02). The worst outcomes (87%) were found in the lowest combination of $PaO_2$ and $PaCO_2$ quartiles (i.e. ≤140mmHg + ≤41mmHg), and the best outcomes in patients with $PaO_2$ of 200-299 mmHg and $PaCO_2$ of 49-55 mmHg (29% mortality and 41% of poor CPC).

**CONCLUSIONS.** In this study, extreme $PaCO_2$ values were associated with poor outcome after CA. Outcomes were the worst in patients with concurrently low $PaO_2$ and $PaCO_2$ values.

### 0441

#### Urine biomarkers may early predict acute kidney injury and outcome after out-of-hospital cardiac arrest

S. Beitland[1,2], E.R. Nakstad[2], J.P. Berg[1,2], A.M.S. Trøseid[2], B.S. Bruslettto[2], C. Brunborg[2], K. Sunde[1,2]
[1]University of Oslo, Oslo, Norway; [2]Oslo University Hospital, Oslo, Norway
**Correspondence:** S. Beitland
*Intensive Care Medicine Experimental* 2018, **6(Suppl 2):**0441

**INTRODUCTION.** Acute kidney injury (AKI) is a common complication after out-of-hospital cardiac arrest (OHCA) associated with increased morbidity and mortality.

**OBJECTIVES.** The aim of the study was to evaluate if biomarkers measured in urine early after OHCA could predict AKI and patient outcome.

**METHODS.** \This was a prospective observational a priori planned substudy of the Norwegian Cardiorespiratory Arrest Study (NORCAST) in resuscitated, comatose OHCA patients admitted to Oslo University Hospital. Urine samples were collected at admission and day three post arrest, and analysed for β-2-microglobulin (β2M), osteopontin and trefoil factor 3 (TFF3). Outcomes were AKI according to the KDIGO criteria within three days, in addition to six-month mortality and poor neurological outcome defined as a cerebral performance category 3-5. Patients with known chronic kidney disease, or who died within 24 hours of ICU stay, or for some reason did not receive active treatment, were excluded.

**RESULTS.** Among 195 included patients (85 % males, mean age 60 years), 88 (45 %) developed AKI, 88 (45 %) died and 96 (49 %) had poor neurological outcome. In univariate analysis, increased urine β2M, osteopontin and TFF3 levels sampled at admission and day three were independent risk factors for AKI, mortality and poor neurological outcome. Exceptions were that β2M measured at day three did not predict any of the outcomes, and TFF3 at admission did not predict AKI. In multivariate analysis, combining clinical parameters and biomarker concentrations, the area under the receiver operating characteristic curve (95 % confidence interval) were 0.729 (0.658-0.800), 0.797 (0.733-0.861) and 0.812 (CI 0.750-0.874) for AKI, mortality and poor neurological outcome, respectively.

**CONCLUSIONS.** In comatose OHCA patients, urine levels of β2M, osteopontin and TFF3 at admission and day three were independent risk factors for AKI, mortality and poor neurological outcome.

**GRANT ACKNOWLEDGMENT**
Financial support was provided solely from institutional sources.
Trial registration: Clinicaltrials.gov NCT01239420.

### 0442

#### Transcranial doppler flow pattern during and after target temperature management for outcome prediction after out-of-hospital cardiac arrest

A. Reichenbach[1], L. Alteheld[2], J. Henriksen[2], E.R. Nakstad[3,4], H. Stær-Jensen[5], G.Ø. Andersen[6], D. Jacobsen[3], K. Sunde[5,7], C. Lundqvist[1,8,9], NORCAST Study Group
[1]Akershus University Hospital, Dept of Neurology, Lørenskog, Norway; [2]Oslo University Hospital Ullevaal, Dept of Neurology, Oslo, Norway; [3]Oslo University Hospital Ullevaal, Dept of Emergency Medicine, Oslo, Norway; [4]Oslo University Hospital Ullevaal, Norwegian National Unit for CBRNE Medicine, Oslo, Norway; [5]Oslo University Hospital Ullevaal, Dept of Anaesthesiology, Oslo, Norway; [6]Oslo University Hospital Ullevaal, Dept of Cardiology, Oslo, Norway; [7]University of Oslo, Faculty of Medicine, Institute of Clinical Medicine, Oslo, Norway; [8]Akershus University Hospital, HOKH, Research Centre, Lørenskog, Norway; [9]University of Oslo, Campus Akershus Universitiy Hospital, Dept of Clinical Medicine, Lørenskog, Norway
**Correspondence:** A. Reichenbach
*Intensive Care Medicine Experimental* 2018, **6(Suppl 2):**0442

**INTRODUCTION.** Monitoring of cerebral function and prognostication of clinical outcome in comatose, sedated patients after out of hospital cardiac arrest (OHCA) is challenging. Currently, a multimodal approach is recommended, including clinical neurological examination, imaging, biomarkers and neurophysiological tests.

**OBJECTIVES.** The aim of the present study was to examine whether consecutive bedside transcranial doppler (TCD) examinations during the first week after OHCA could reflect pathophysiological mechanisms of brain damage, and whether flow patterns were associated with clinical outcome six months later.

**METHODS.** This was a sub-study of the Norwegian Cardiorespiratory Arrest Study (NORCAST). Altogether 259 primary successfully resuscitated comatose adult OHCA patients admitted to ICU between 2010 and 2014 were included. Patients underwent standardised post-resuscitation care including target temperature management (TTM) to 33$^{o}$ C for 24 hours. We aimed to perform bedside TCD, blinded to the treating physician, both during TTM within the first 24 hours, at day three after rewarming and at day seven in those still alive. Primary endpoint was Cerebral Performance Category (CPC) at six months, dichotomized into good (CPC 1-2) and poor outcome (CPC 3-5). Predictive values of TCD at day seven were analysed by logistic regression, and time series analysis using linear mixed methods (LMM) was used for the whole time-series data.

**RESULTS.** TCD was performed in 53, 44 and 109 patients during TTM, after rewarming and at day seven, respectively. Peak systolic velocity (PSV) in the middle cerebral artery (MCA) at day seven was significantly associated with late outcome as was LMM time-series data. PSV was lowest during TTM, increased after rewarming at day three and thereafter normalised at day seven for patients with good outcome. For patients with poor outcome peak flow remained high (figure). Resistance index and pulsatility index were not associated with clinical outcome.

**CONCLUSIONS.** TCD is a simple method for bedside monitoring of intracranial blood flow in OHCA patients. Specific dynamic changes of TCD-parameters were observed both during and after TTM. However, only late TCD seven days after cardiac arrest was associated with clinical outcome at six months.

### 0443

#### The clinical outcome of patients with refractory cardiac arrest treated with extracorporeal cardiopulmonary resuscitation

A. Krüger, P. Ostadal, M. Janotka, D. Vondrakova, J. Naar, P. Neuzil
Homolka Hospital, Cardiology, Prague, Czech Republic
**Correspondence:** A. Krüger
*Intensive Care Medicine Experimental* 2018, **6(Suppl 2):**0443

**INTRODUCTION.** Extracorporeal cardiopulmonary resuscitation (ECPR) has been introduced as a potentially life-saving procedure in refractory cardiac arrest.

**METHODS.** Eligible patients for this analysis had to undergo ECPR after unsuccessful cardiopulmonary resuscitation with a minimum of three defibrillation attempts. For extracorporeal life support (ECLS) we used Cardiohelp system (Maquet-Cardiopulmonary-AG, Hirrlingen, Germany) or Levitronix CentriMag blood pump (Levitronix LLC, Waltham, MA, USA). LUCAS II (Physio-Control, Lund, Sweden) system was used for chest compressions during ECPR insertion. The relations of blood lactate and pH levels, measured before ECPR insertion and after 24 hours as well as comorbities (diabetes, hypertension, BMI) to the clinical outcomes at 3, 6 and 12 months were evalueated.

**RESULTS.** We analyzed data from 36 patients treated with ECPR for refractory cardiac arrest. The mean age of our patients was 53 years. Out-of-hospital cardiac arrest (OHCA) occurred in 22 patients, 14 patients suffered from in-hospital arrest (IHCA). Baseline value of lactate was 12.52 ± 4.63 mmol/l, initial pH 6.97 ± 0.33. In comparison with survivors, patients who died had significantly higher initial lactate levels (16.05 ± 1.56 vs. 10.01 ± 1.03; $P< 0.05$) and lower baseline pH (6.77 ± 0.06 vs 7.06 ± 0.04; $P< 0.05$). Moreover, survivors had significantly lower lactate levels after 24 hours and lower BMI (26.4 vs 33.4; $P< 0.05$). Diabetes or hypertension in our group have no influence on the mortality. The difference of mortality in the group of OHCA or IHCA was also not significant. With good neurological outcome (CPC 1-2) survive 34% three months, 28% six months and one year 24% of the patients.

**CONCLUSIONS.** ECPR give the last chance to survive refractory cardiac arrest. The initial levels of blood lactate and pH are significantly associated with clinical outcomes of ECPR.

**GRANT ACKNOWLEDGMENT**
IG 150501

### 0444

#### ICU characteristics and outcome after cardiac arrest in patients ≥ 90 years old

K. Roedl, D. Jarczak, S. Becker, V. Fuhrmann, S. Kluge, J. Müller
University Medical Center Hamburg-Eppendorf, Department of Intensive Care Medicine, Hamburg, Germany
**Correspondence:** K. Roedl
*Intensive Care Medicine Experimental* 2018, **6(Suppl 2):**0444

**INTRODUCTION.** The world's population is ageing rapidly, thus the proportion of elderly patients suffering from cardiac arrest (CA) is expected to increase markedly. However, only little is known about long term neurological outcome and characteristics during intensive care unit (ICU) stay of patients ≥ 90 years suffering from CA.

**OBJECTIVES.** The aim was to evaluate ICU-characteristics and outcome after CA in patients ≥ 90 years.

**METHODS.** We conducted a single-center retrospective study and included all adult patients ≥ 90 years after CA and return of spontaneous circulation (ROSC) treated at the Department of Intensive Care Medicine of the University Medical Center Hamburg-Eppendorf. Patient characteristics, admission diagnosis, severity of disease, course of the disease and ICU mortality were assessed.

**RESULTS.** Overall we could identify 48 patients ≥ 90 years suffering from CA and which had ROSC, and were treated at our centre. Of these 27 (56%) were female and had a median age of 91.7 (90.7 - 92.6) years. 41 (85%) patients suffered from in-hospital CA. Cardiac events leading to CA were observed in 19 (40%) patients. Initial rhythm was shockable (VT/VF) in 12 (25%), non-shockable (PEA/Asystole) in 30 (63%) and unknown in 6 (13%) patients. Time to ROSC was median 4 (1.6 - 9.5) minutes. 39 (81%) patients needed mechanical ventilation, 44 (92%) received catecholamine support, 4 (8%) received renal replacement therapy and 17 (35%) received transfusion of red blood cells during ICU-stay. ICU non-survivors had significantly higher SAPS II score and lower pH on admission (both $p < 0.05$). Highest lactate levels were observed in ICU non-survivors (6.2 (4 - 9) mmol/l vs. 2.8 (1.9 - 4.3) mmol/l, $p < 0.001$). Need for mechanical ventilation and red blood cell transfusion was significantly more common in ICU non-survivors. 26 (54%) of patients died during ICU stay, of patients surviving ICU-stay 3 (14%) had bad neurological outcome (CPC III/IV) at ICU-discharge.

**CONCLUSIONS.** ICU survival of patient's ≥ 90 years after CA is low. However, resuscitation and post-CA care in this age group seems to be not futile.

### 0445

#### Dynamic changes in coagulation and fibrinolysis during percutaneous cardiopulmonary support in patients after out-of-hospital cardiac arrest

T. Tsuchida[1], T. Wada[1], S. Gando[2]
[1]Hokkaido University, Sapporo-shi, Hokkaido, Japan; [2]Sapporo Higashi Tokushukai Hospital, Sapporo-shi, Hokaido, Japan
**Correspondence:** T. Tsuchida
*Intensive Care Medicine Experimental* 2018, **6(Suppl 2):**0445

**INTRODUCTION.** Disseminated intravascular coagulation (DIC) with the fibrinolytic phenotype during the early phase of post cardiopulmonary resuscitation (CPR) affects the outcome of out-of-hospital cardiac arrest (OHCA) patients.

**OBJECTIVES.** We evaluated changes in coagulation and fibrinolysis during percutaneous cardiopulmonary support (PCPS) in OHCA patients.

**METHODS.** A review of the computer-based medical records of OHCA patients was retrospectively conducted and included 431 patients who were divided into to two groups; patients who underwent PCPS and those did not undergo PCPS during the early phase of post CPR. Pre-hospital factors, platelet count, coagulation and fibrinolysis markers and lactate levels within 24 hours after CPR and resuscitation were evaluated. DIC was diagnosed by Japanese Association of Acute Medicine scoring system. Organ dysfunction was assessed by sequential organ failure assessment (SOFA) score. Hyperfibrinolysis was defined as an FDP level of ≥ 100 μg/mL, and the FDP/D-dimer ratio was used as a surrogate marker of fibrin (ogen) olysis. The outcome measure was all-cause hospital mortality.

**RESULTS.** Patients who underwent PCPS exhibited significantly lower platelet counts, prolonged prothrombin time ratio, decreased levels of fibrinogen and antithrombin, extreme increases in the levels of FDP, D-dimer and FDP/D-dimer ratios associated with higher prevalence of DIC than those who did not undergo PCPS (95.3% vs. 53.6%). Incidence of hyperfibrinolysis was significantly higher in patients with PCPS than those without PCPS (55.8% vs. 17.8%). Patients who underwent PCPS more frequently developed multiple organ dysfunction syndrome (48.9% vs. 14.4%) with increased levels of lactate. There were no differences in survival probability (Log Rank, p=0.130) and outcome (Death 55.8% vs. 40.5%) between the two groups.

**CONCLUSION.** Patients who underwent PCPS showed dynamic changes in platelet counts, coagulation and fibrinolysis during early phase of post CPR and were associated with high prevalence of DIC, hyperfibrinolysis and organ dysfunctions.

##### GRANT ACKNOWLEDGMENT

No. 17H04361. Grant-in-Aid for Scientific Research B 2017, Japan Society of the Promotion of Science.

### 0446

#### Neuron specific enolase as a prognostic biomarker after cardiac arrest

J. Worthy, F. Baldwin, R. Gray
Royal Sussex County Hospital, Brighton, United Kingdom
**Correspondence:** J. Worthy
*Intensive Care Medicine Experimental* 2018, **6(Suppl 2):**0446

**INTRODUCTION.** Neuron Specific Enolase (NSE) with a cut off value greater than 33μg/L at 1-3 days post cardiac arrest (CA) is a highly predictive biomarker of poor neurological outcome [1]. This cut off value was based on studies with patients who were not treated with therapeutic hypothermia (TH). Since this recommendation in 2006 there have been many studies to assess the cut off value for patients treated with TH. Some have found unacceptable false positive rates (FPR) and have suggested cut off levels of 78.9μg/L (0% FPR) at 48 hours[2].

**OBJECTIVES.** To assess the usefulness of NSE along with clinical assessment in our ICU to prognosticate on post CA patients.

**METHODS.** An observational retrospective study was carried out on all patients who had an NSE requested post CA over the time period 1st April 2017-1st April 2018. The NSE result, TH, survival to ICU discharge and GCS on ICU discharge were recorded.

**RESULTS.** A total of 43 patients with an NSE result were identified. The results of the patients who died on ICU are shown in table 1 and the survivors to ICU discharge are shown in table 2.

The average time of NSE testing was 75 hours post admission. Our results found 5 patients with NSE levels between 33μg/L and 78.9μg/L who survived to ICU discharge and went to a rehabilitation ward. 100% of patients with an NSE of > 78.9μg/L had a poor outcome resulting in eventual death.

**CONCLUSIONS.** Our data supports that using a NSE cut off level of 33μg/L has an unacceptable FPR to predict a poor neurological outcome and that a cut off level of > 78 μg/L at 72 hours post admission is highly predictive of a poor outcome with a 0% FPR in all patients.

##### REFERENCE(S)

1. Wijdicks E et al, 2006. Practice Parameter: Prediction of outcome in comatose survivors after cardiopulmonary resuscitation (an evidence-based review) Report of the Quality Standards Subcommittee of the American Academy of Neurology. Neurology, 67(2), pp.203-210.
2. Steffen I et al, 2010. Mild therapeutic hypothermia alters neuron specific enolase as an outcome predictor after resuscitation: 97 prospective hypothermia patients compared to 133 historical non-hypothermia patients. Critical care, 14(2), p.R69.

##### GRANT ACKNOWLEDGMENT

Nil

**Table 1 (abstract 0446).** Patients who died on ICU

| | No. of patients | Average NSE μg/L (range) | Average time NSE sent from ICU admission hrs (range) |
|---|---|---|---|
| TH | 9 | 176.2 (35-346) | 93.3 (23-192) |
| No TH | 20 | 87 (14-191) | 67.8 (1-168) |

**Table 2 (abstract 0446).** Patients who survived to ICU discharge

| | No. of patients | Average NSE μg/L (range) | Average time NSE sent from ICU admission hrs (range) |
|---|---|---|---|
| TH | | | |
| GCS ≥ 14 | 6 | 20.6 (10-36) | 84 (24-120) |
| GCS ≥ 8-13 | 4 | 49 (38-55) | 102 (72-192) |
| GCS < 8 | 2 | 76.5 (64-89) | 120 (96-144) |
| No TH | | | |
| GCS ≥ 14 | 2 | 26 (25-27) | 36 (24-48) |

### 0447

#### Clinical neurological examination in prediction of late outcome after cardiac arrest - the Neuro-NORCAST sub-study

J. Henriksen[1,2], A. Reichenbach[3], L. Alteheld[1], E. Nakstad[4,5], H. Stær-Jensen[6], G.Ø. Andersen[7], D. Jacobsen[4], J. Šaltytė Benth[2,8], K. Sunde[6,9], C. Lundqvist[2,3,8]
[1]Oslo University Hospital, Ullevaal, Dept of Neurology, Oslo, Norway; [2]Akershus University Hospital, HØKH Health Services Research Unit, Lørenskog, Norway; [3]Akershus University Hospital, Dept of Neurology, Lørenskog, Norway; [4]Oslo University Hospital, Ullevaal, Dept of Acute Medicine, Oslo, Norway; [5]Oslo University Hospital, Ullevaal, Norwegian National Unit for CBRNE Medicine, Oslo, Norway; [6]Oslo University Hospital, Ullevaal, Dept of Anaesthesiology, Oslo, Norway, [7]Oslo University Hospital, Ullevaal, Dept of Cardiology, Oslo, Norway; [8]University of Oslo, Campus Akershus University Hospital, Institute of Clinical Medicine, Lørenskog, Norway; [9]University of Oslo, Institute of Clinical Medicine, Oslo, Norway
**Correspondence:** J. Henriksen
*Intensive Care Medicine Experimental* 2018, **6(Suppl 2):**0447

**INTRODUCTION.** Prognosis of patients with out-of-hospital cardiac arrest (OHCA) has improved due to better post-resuscitation care, but irreversible brain damage is still frequent. As prognostication is challenging, a multimodal approach is recommended, including clinical neurological examination. A major concern is self-fulfilling prophecy due to withdrawal of life sustaining therapy (WLST) in patients assumed to have poor prognosis but with an unrecognised potential for good recovery.

**OBJECTIVE.** To clarify the predictive accuracy of clinical neurological examinations performed by neurologists or intensive care unit (ICU) staff (blinded to the treating physician), and elucidate the optimal time point for making predictions.

**METHODS.** Prospective inclusion of all successfully resuscitated adult comatose OHCA patients admitted to the ICU in 2010 - 2014. Patients underwent standardised post-resuscitation care including targeted temperature management (TTM) at 33°C for 24 hours. A structured examination by a neurologist including brain stem reflexes, Glasgow Coma Score (GCS), cortical responses and other focal neurological tests was done 72 hours after rewarming and sedation stop. Patients were also examined daily by ICU staff using a simplified protocol. The main outcome was survival with good vs poor Cerebral Performance Category scores (CPC 1 - 2 vs CPC 3 - 5) at 6 months. Descriptive statistics with predictive power and logistic regression analyses were performed. Significance limits were set at $p< 0.05$. Chance corrected k and Bland- Altman analyses were used for comparison of evaluations by ICU staff vs. neurologists.

**RESULTS.** We included 259 patients, median age 61 years, and 49.5% of all had CPC 1-2 at 6 months. 72 h after sedation stop 185 patients were alive. Among 49% that were still in coma (GCS < 9) at this time point, 32% had CPC 1-2 at 6 months. Of the 76 patients examined by a neurologist, 66% achieved CPC 1-2. The strongest neurological predictors for poor outcome were GCS, absent pupillary/corneal reflex, voluntary/reflex eye movements and early myoclonic state. All predictors had low sensitivities. Gag reflex, startle response, autonomic pain response, tendon reflexes and Babinski's sign were also associated with prognosis. WLST (20%) was absolutely predictive of poor outcome. Agreement between evaluations by ICU staff and neurologists were moderate-weak (k=0.69 to 0.21) with only absence of pupillary response and ventilator requirement as significant ICU predictors. GCS scores showed good agreement without significant bias between neurologists and ICU staff.

**CONCLUSIONS.** Neurological examination alone (72 h after sedation stop), is not accurate enough in predicting outcome in comatose OHCA patients, and should be interpreted with caution. As late awakening to good outcome occurs in 32% comatose patients 72 hours after sedation stop, early WLST should be avoided. Prognostication may be blunted by TTM and sedation.

### 0448

#### Optic nerve sheath diameter measured using early unenhanced brain computed tomography shows no correlation with neurological outcomes in patients undergoing targeted temperature management after cardiac arrest

D.H. Lee[1], S.H. Lee[2], J.H. Oh[1], I.S. Cho[3], Y.H. Lee[4], C. Han[5], W.J. Choi[6], Y.D. Sohn[7], Korean Hypothermia Network
[1]Chung-Ang University Hospital, Emergency Medicine, Seoul, Korea, Republic of; [2]Sanggye Paik Hospital, Emergency Medicine, Seoul, Korea, Republic of; [3]Hanil General Hospital, Emergency Medicine, Seoul, Korea, Republic of; [4]Soonchunhyang University Bucheon Hospital, Emergency Medicine, Bucheon, Korea, Republic of; [5]Ewha Woman's University, Emergency Medicine, Seoul, Korea, Republic of; [6]University of Ulsan College of Medicine, Emergency Medicine, Ulsan, Korea, Republic of; [7]Hallym University Sacred Heart Hospital, Emergency Medicine, Anyang, Korea, Republic of
**Correspondence:** D.H. Lee
*Intensive Care Medicine Experimental* 2018, **6(Suppl 2):**0448

**INTRODUCTION.** Previous studies indicated that the optic nerve sheath diameter (ONSD) measured using brain computed tomography (CT) is a prognostic factor for poor neurological outcome after cardiac arrest. However, these studieswere conducted involving PCAS patients, and in these studies limitations existed owing to the single-centred and retrospective nature of the studies, and the small sample sizes. Additionally, TTM and the time at which the CT scan was performed were not controlled.

**OBJECTIVES.** We performed a prospective multi-centre observational study to investigate the correlation between the ONSD on early brain CT and neurological outcomes in patients undergoing targeted temperature management (TTM).

**METHODS.** This study used data from the Korean Hypothermia Network prospective registry between November 2015 and October 2016. Out-of-cardiac arrest patients who underwent brain CT within 2 h after return of spontaneous circulation (ROSC) were included. The primary endpoint was neurological outcomes at 6 months (cerebral performance category; CPC); the secondary outcome was hospital mortality. The ONSD was measured using unenhanced brain CT images.

**RESULTS.** In total, 374 patients were included from 18 hospitals, and 329 underwent CT within 2 h after ROSC. Six months after cardiac arrest, good (CPC 1-2) and poor (CPC 3-5) neurological outcomes were observed in 99 (30.09%) and 230 (69.91%) patients, respectively. There was no significant difference in the ONSD between groups (good outcome group: 5.61 ± 0.59 mm, poor outcome group: 5.69 ± 0.79 mm; p = 0.275), nor between discharged patients who survived and those with hospital mortality (5.63 ± 0.64 mm and 5.70 ± 0.67 mm, respectively, p = 0.399).

**CONCLUSIONS.** The ONSD on initial brain CT after ROSC was not correlated with neurological outcome at 6 months in patients who underwent TTM.

**GRANT ACKNOWLEDGMENT**
The authors have no financial relationships relevant to this study to disclose.

### 0449

#### Prolonged transport of patients after out-hospital cardiac arrest (OHCA) primarily to regional cardiac arrest center (CAC) doesn´t affect initial hemodynamic parameters and outcomes

J. Karasek[1,2], J. Seiner[1], M. Renza[2], F. Salanda[2], M. Moudry[2], M. Strycek[1], J. Lejsek[3], R. Polasek[1], P. Ostadal[4]
[1]Hospital Liberec, Cardiology, Liberec, Czech Republic; [2]Charles University, 3rd Medical Faculty, Prague, Czech Republic; [3]EMS Region Liberec, Liberec, Czech Republic, [4]Hospital Na Homolce, Cardiology, Prague, Czech Republic
**Correspondence:** J. Karasek
*Intensive Care Medicine Experimental* 2018, **6(Suppl 2):**0449

**INTRODUCTION.** Systematic care of patients after OHCA and CAC developement is recommended by the guidelines, but important „contra" argument is prolonged transport of often hemodynamicly unstable patients in limited prehospital emergency care.

**AIM.** To determine if prolonged primarily transport of patients after OHCA to regional CAC influence initial hemodynamic parameters after admission, mortality a neurological outcome.

**METHODS.** Analysis from prospective OHCA Registry of regional CAC 2013 - 2017. Data were divided into 2 datasets: 1) INSIDE - when CAC is the nearest hospital and 2) OUTSIDE - patients transfered to CAC, but in past would be transferred to one of the 7 another closer hospitals in the region. We observed duration of transport, baseline characteristics,hemodynamic parameters on arrival to hospital and final outcomes (30-day/in-hospital mortality, length of ICU stay, artificial ventilation days, 1 year CPC).

**RESULTS.** 232 patients were enrolled after OHCA in years 2013 to 2017, 27 were excluded for insuficient data and 19 for secondary transfer to CAC. We analyzed 186 patients, 93 in both groups. We observed no differences in baseline characteristics in both groups: men (66,7% vs. 80,6%, p= 0.29), age (64,51±1.324 years vs. 61.25± 1.443 years , p= 0.1), shockable rhythm (65,6% vs. 74,2%, p= 0.26), bystander CPR (68,8% vs. 72%, p= 0.75), ROSC (median, IQR) :17 (11-26) min vs. 20 (15-30) minutes, p= 0.29, ACS ( 44,1% vs. 48,4%, p= 0.66) and catecholamine administration during transfer (80% vs. 70%, p= 0.18). We observed no differences in initial hemodynamic parameters in time of admission in both groups. Systolic blood pressure: *(median,IQR): 103 (82-120) vs. 105(82-124)mm Hg, p= 0.6,* serum lactate level (m*edian,IQR): 4.6 (2-8.1) vs. 3.5 (2-6.75) mmo/l, p= 0.372, pH (median,IQR): 7.242 (7.122-7.322) vs. 7.286 (7.177-7.318), p=0.159,* body temperature: *(median,IQR): 35.95 (35.08-36.5) vs. 36 (35.5-36.5), p=0.218* and oxygen saturation (SpO2):*(median,IQR): 95 (91-100) vs. 98 (94-100), p= 0.14.*

We observed no differences in catecholamins dosages. Norepinephrine (7.54±+1.75 vs. 5.98±1.17 mcg/min), p= 0.46 and dobutamine ( 66.31±45.81 vs. 38.6±15.62 mcg/min),p=0.56. There was no significant difference in in-hospital/30-day mortality ( 44.1% vs. 42.3%, p= 0.88). 1-year good neurological outcome (CPC 1,2) was identical ( 54,2% vs 54.2%, p= 0.999). Median of artificial ventilation duration without significant difference: (median/IQR) 3 (1-8) vs.5 (1-7.75) days, p= 0.36 and median of lenght of ICU stay without significant difference: (median/IQR) 6 (2-14.75) vs. 7 (3-12) days, p= 0.74.

**CONCLUSION.** Strategy of primary transport of patients after OHCA to CAC significantly prolonged time of transport, but didn´t affect hemodynamic parameters and outcome of patients.

### 0450

#### "School of relatives", basic CPR training for laymen. Where do we start?

J. Priego Sanz[1], A. Fernández Ferreira[2], P. Fernández Ugidos[1], A.I. Tizón Varela[1], L. del Río Carbajo[1], D.M. Combarros Méndez[1], V.J. López Ciudad[1]
[1]Complexo Hospitalario Universitario de Ourense, Intensive Care Unit, Ourense, Spain; [2]Hospital Alvaro Cunqueiro, Intensive Care Unit, Vigo, Spain
**Correspondence:** J. Priego Sanz
*Intensive Care Medicine Experimental* 2018, **6(Suppl 2):**0450

**INTRODUCTION.** We have identified a lack of Knowledge in CPR skills in our population. We want to improve this with an educational workshop in basic CPR directed to the relatives of our patients

**OBJECTIVES.** To describe the demographic characteristics and knowledge before a workshop in Basic Cardiopulmonary Resuscitation (BCPR) for relatives (potential bystanders) of patients admitted to the Intensive Care Unit (ICU). Assess the relationship between educational level, age and previous CPR training.

**METHODS.** We make an initial anonymous survey in each workshop for relatives (laymen) of patients admitted to a 22 beds ICU between November 2016 and November 2017. The workshop was carried out by the medical staff of the ICU (Instructors in advanced life support). We ask for 9 items that included: demographic characteristics (age, sex and educational level) and previous knowledge in CPR (facing an emergency situation, recognition of cardiac arrest, knowledge in CPR skills, previous training, belief in the importance of education in CPR and fear to use them) of attendants. The results are expressed as average for the continuous variables and percentages for the categorical variables. For hypothesis contrast we compare proportions using chi-square test.

**RESULTS.** A total of 90 people with an average age of 48.77 ± 17.83 years (oldest 83). A 65.2% were women. Attendants educational levels were a 26.1% Primary Education, a 36.4% had a General Certificate of Secondary Education and a 37.5% had an University degree. Only a 26.1% had witnessed an emergency situation and the 31.4% believed that they knew how to recognize a cardiac arrest. The 78.4% did not know CPR skills and the 76.1% had never received CPR training. There is not significant relationship between educational level (p = 0.177) nor age ≤ 65 years (p = 0.209) and have received BCPR training. The 62.9% of the attendants would not be afraid to deliver BCPR if they had the opportunity. Finally, 100% of our attendants believed that the training was useful.

**CONCLUSIONS.**

- We identified a loss formation in the CPR skills in our population. Almost the 80% of our population did not know them.
- In our population we did not find a relationship between age or educational level and having received training in CPR.
- We can complete our daily assistance work, performing relatives of our patients in BCPR and helping them to feel more secure.
- We can also minimize the hard experience of having a family member in the ICU, turning the ICU more human.

##### GRANT ACKNOWLEDGMENT

We have not grant acknowledgemnt to make this abstract.

### 0451

#### "School of relatives", basic CPR training for laymen. How much and what do our attendants remain?

J. Priego Sanz[1], A. Fernández Ferrerira[2], P. Fernández Ugidos[1], A.I. Tizón Varela[1], L. de Río Carbajo[1], D.M. Combarros Méndez[1], V.J. López Ciudad[1]
[1]Complexo Hospitalario Universitario de Ourense, Intensive Care Unit, Ourense, Spain; [2]Hospital Alvaro Cunqueiro, Intensive Care Unit, Vigo, Spain
**Correspondence:** J. Priego Sanz
*Intensive Care Medicine Experimental* 2018, **6(Suppl 2):**0451

**INTRODUCTION.** Training in CPR is a gap in our population. We start a project to diffuse basic CPR skills to relatives of our ICU patients. Is important for us to evaluate the ability to remember of our attendants 6 months after the workshop and detect improvement points.

**OBJECTIVES.** To describe the demographic characteristics and assimilated knowledge of the attendants (laymen) 6 months after receiving a workshop in BCPR. Assess the association between age and educational level with the retention of knowledge.

**METHODS.** Phone survey at 6 months of workshop for relatives of patients admitted to a 22-beds ICU from November 2016 to November 2017. The workshop was carried out by the medical staff of the ICU (Instructors in advanced life support). Attendants were asked for: chain of survival correct sequence, frequency of compressions, use of CPR skills, fear to use CPR skills and workshop valuation. The results are expressed as averages for the continuous variables and percentages for the categorical variables. For hypothesis contrast we compare proportions using chi-square test.

**RESULTS.** Of a total of 45 surveyed, answered 33 (73.33%). The average age was 50.97 ± 17.20 years. 66.7% were women. The workshop average evaluation was 9.47 ± 0.73 out of 10 points.

**Could you recognize a PCR?** Yes: 97%/No: 3%

**Chain of survival correct sequence**: Airway-Help-CPR: 36.4%/Help-Airway-CPR: 45.5%/Help-CPR-Airway: 3%/Airway-CPR-Help: 15.2%

**Chest compressions rate**:60: 29%/80: 25.8%/100: 45.2%

**Have you used CPR in this 6 months?** No: 100%

**Would you be afraid to use CPR?** Yes: 12.1%/No: 87.9%

**Would you go back to the workshop?** Yes: 100%

Of the answered, 28.9% had Primary education, 33.3% had General Certificate of Secondary Education and 37.8% had an University degree. There is no significant relationship between age ≤ 65 years (p = 0.071) or educational level (p = 0.509) and the chain of survival correct sequence retention. We also didn´t find significant relationship between the age ≤ 65 years (p = 0.703) or educational level (p = 0.616) and the correct compressions frequency.

**CONCLUSIONS**

- The workshop evaluation of our attendants is satisfactory.
- After the workshop almost all respondents, know how to recognize a CPR.
- The retention of the correct sequence and compressions frequency at 6 months is improvable and we did not find significant relationship between age ≤ 65 years or the educational level of studies and better knowledge of CPR skills.

### 0452

### Streamlined infrastructure reduces time-to-extracorporeal life support for in-hospital cardiac arrest

M.M. Suverein[1], A. Bruekers[1], T. Delnoij[1,2], M.E. Bol[1], R. Lorusso[3], J.W. Sels[1,2], P. Weerwind[3], P. Roekaerts[1], J. Maessen[3], M. van de Poll[1,4]
[1]Maastricht UMC+, Intensive Care, Maastricht, Netherlands; [2]Maastricht UMC+, Cardiology, Maastricht, Netherlands; [3]Maastricht UMC+, Cardiothoracic Surgery, Maastricht, Netherlands, [4]Maastricht UMC+, Surgery, Maastricht, Netherlands
**Correspondence:** M.M. Suverein
*Intensive Care Medicine Experimental* 2018, **6(Suppl 2):**0452

**INTRODUCTION.** Since 2013, extracorporeal cardiopulmonary resuscitation (ECPR) has been used as a treatment option for refractory cardiac arrest at our university hospital. ECPR requires rapid activation of a multidisciplinary team and a well-organized ECPR infrastructure [1, 2]. In 2017, we started optimising our ECPR infrastructure for out-of-hospital cardiac arrest by introducing regular training sessions with simulation cases, evaluating all procedures and introducing a dedicated ECPR-cart. This infrastructure is also used for in-hospital cardiac arrest (IHCA).

**OBJECTIVES.** The objective of this retrospective analysis was to determine the effect of the regular training and the introduction of a dedicated ECPR-cart on the ECPR procedures. We hypothesize that this led to a reduction in time needed to initiate extracorporeal life support (ECLS) in IHCA.

**METHODS.** A single-centre, retrospective review was conducted a year after the start of training and introduction of the ECPR-cart. All adult patients (≥18 years) with a witnessed, refractory IHCA who received ECPR from 2013 to 2018 were included. The primary outcome was time-to-ECLS, defined as time from start of arrest to adequate flow on ECLS. Data are presented as median [IQR]. Statistical analysis was performed using a Mann-Whitney U test.

**RESULTS.** 13 patients received ECPR for a witnessed, refractory IHCA from 2013 to April 2017. The median age was 62 [60-67], most were male (69,2%) and the Charlson Comorbidity Index (CCI) was 2,5 [2,0-3,0]. The most common causes of arrest were cardiogenic shock (30,8%) and myocardial infarction (23,1%) (Table 1).

After start of training and introduction of the ECPR-cart in May 2017, 4 patients received ECPR, with median age of 53 [33-60] and CCI of 1,5 [0,5-1,5]. The time-to-ECLS was significantly reduced from 40 minutes [25-45] to 27 minutes [22-33] (p = 0,019) (Figure 1). Successful weaning of mechanical circulatory support remained similar for both time periods (pre 46,2 % vs. post 50%).

**CONCLUSIONS.** Optimisation of the ECPR infrastructure significantly reduced time-to-ECLS. It can be imagined that this ultimately will improve survival with good neurological outcome of ECPR for IHCA.

**Table 1 (abstract 0452).** Baseline characteristics and causes of arrest.

| | Pre ECPR infrastructure (n = 13) | Post ECPR infrastructure (n = 4) |
|---|---|---|
| Age (years) | 62 [60-67] | 53 [33-60] |
| Sex (male %) | 69,2% (9) | 50% (2) |
| Charlson Comorbidity Index | 2,5 [2,0-3,0] | 1,5 [0,5-1,5] |
| Causes of arrest (%): Cardiogenic shock | 30,8% (4) | 25% (1) |
| Myocardial Infarction | 23,1% (3) | 50% (2) |
| Hypoxia | 7,7% (1) | 25% (1) |
| Pulmonary Embolism | 15,4% (2) | - |
| Post CTC | 15,4% (2) | - |
| Sepsis | 7,7% (1) | - |

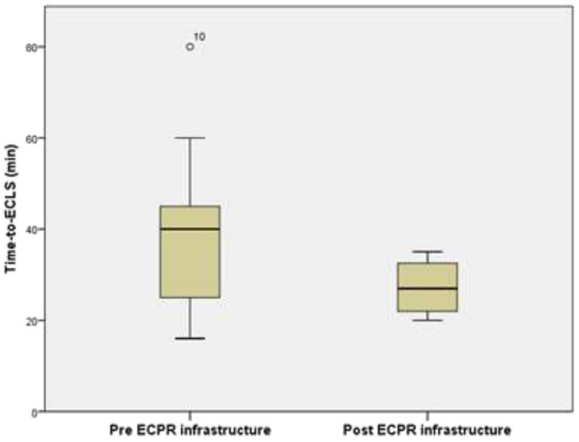

**Fig. 1 (abstract 0452).** Boxplot of time-to-ECLS (min) pre- and post-optimisation of the ECPR infrastructure (p = 0,019).

### 0453

#### What happens after cardiorespiratory arrest? Description of the neurological population and prognosis

E.A. Ines, A.F. Vanessa, M.F. Mónica, T.F. Xiana, M.M. Natalia, T.S.C. Helena, F.M. David
University Hospital of A Coruña, Intensive Care, A Coruña, Spain
**Correspondence:** E.A. Ines
*Intensive Care Medicine Experimental* 2018, **6(Suppl 2):**0453

**INTRODUCTION.** The prognosis for postcardiac arrest patients remains very bleak, not only because of anoxic-ischemic neurological damage, but also because of the postcardiac arrest syndrome,a phenomenon often severe enough to cause death before any neurological evaluation.

**OBJECTIVES.** To describe patients recovered from cardiorespiratory arrest (CRA) and analyze variables that are related to neurological prognosis.

**METHODS.** Retrospective observational study of 2015-2017 CRA who were admitted to the ICU. 32 patients who died without neurological evaluation are excluded. We collect demographic variables, comorbidity, related to CRA (location, attention times, first rhythm), complementary tests, stays and prognosis at discharge and at one year (Cerebral Performance Categories Scale (CPC) and exitus). Statistical analysis by contrast of hypothesis and comparison of means with SPSS.19.0

**RESULTS.** 70 patients, median age of 62 years and 70% men. Charlson index mean4.0 ± 2.5.52.9% extrahospitalar CRA. FV / TV without pulse in66.7%. Mean time to advanced CRA2.6 ± 3.4min; Total CRA time10 min. Most frequent cause was AMI54.3%, primary arrhythmia10% and IC5.7%. MEDA of SOFA6. Hypothermia was performed in 34.3%. Initial neurological examination in 41.4%, TAC at admission with edema42.9%. In neurological evaluation in ICU average GCS 10.9 ± 4.7 and 11.5 ± 5.8 on the FOUR scale. Hyperthermia in first 72 hours 2 patients (31.4%). In the evaluation of anoxic encephalopathy, EEG was performed in 24 patients (3.4% status), NMR in 4 (1diagnostic), evoked potentials in 2patients, and mioclonias5.7%. The median stay in the ICU was 4 days and hospital16.5. At discharge and at one year the mean CPC was 2.9 ± 1.7 and 1.7 ± 1.2, with mortality of 40%. Differences of worse neurological prognosis at discharge (CPC> 2) were identified in: cause of CRA (41% vs70%) p = 0.05), myoclonus (14.3% vs.0%, p = 0.03), tracheostomy at discharge (100% vs. 10.3%, p = 0.003), SOFA (8.3%). 2.6 vs.5.0 ± 3.3, p < 0.01), total CRA time (21.8 ± 17.5 vs 11.9 ± 10.7, p < 0.001), GCS (6.9 ± 4.5vs13.7 ± 1.6, p < 0.001) and FOUR scale (1.7 ± 1.7 vs.3.6 ± 0.8, p < 0.001). Multivariate analysis showed that lower GCS at admission ( HR: 0.08, 95% CI: 0.06-0.1, p = 0.008) and a worse FOUR value (HR: 0.02, 95% CI: 0.00-0.05, p = 0.049) are associated to worse forecast at discharge.

**CONCLUSIONS.** Comparison of patients according to the neurological prognosis at discharge, a predictive capacity was found in the GCS and FOUR scale. No association with other variables was probably influenced by the sample size.

**GRANT ACKNOWLEDGMENT**
This study received no financial support.

## Advanced haemodynamic monitoring

### 0454

#### Follow-up evaluation of patients with heart failure: prognostic role of ambulatory blood pressure monitoring

A.M. González González[1], A.M. Garcia Bellon[2], A.A. Rojas Sanchez[2], M. De Mora Martin[2]
[1]Regional Hospital of Malaga, Malaga, Spain; [2]Regional Hospital of Malaga, Cardiology, Malaga, Spain
**Correspondence:** A.M. González González
*Intensive Care Medicine Experimental* 2018, **6(Suppl 2):**0454

**INTRODUCTION.** Ambulatory blood pressure monitoring (ABPM) is routinely used in patients with high blood pressure. However, its use in patients with heart failure (HF) has been poorly reported.

**OBJECTIVES.** Our aim was to evaluate circadian variation in blood pressure in this group of patients and their prognostic role.

**METHODS.** We included 184 patients followed-up in a Heart Failure Unit. They underwent an echocardiogram, laboratory tests and a 24 hours ambulatory blood pressure monitoring. All patients were followed-up for 1 year. Primary event was defined as hospitalization or death due to HF.

**RESULTS.** Mean age 63 ± 10; 75% males. BMI 30.1 ± 6 Kg /m2. Cardiovascular risk factors: Hypertension 56.5%; diabetes 34.7%; dyslipidemia 40%; obesity 29.3%. Smokers 22.9%. Etiology of HF: Hypertensive 19.5%; ischemic 12%; ischemic-hypertensive 27.1%; enolic 9,8%; myocarditis 4.3%; valvulopathy 5.2%; idiopathic 21%. Therapeutic regimen: SRA blockers 93.4%; beta blockers 85.7%; diuretics 81%; spironolactone 42.3%; statins 68,4%; antiplatelets/anticoagulants 89%. Patients presented optimal blood pressure values (systolic BP: 112.63 ± 15.4, diastolic BP: 65.1 ± 7.9), however, the vast majority (80.4%) had an altered circadian pattern. Table 1.

**Table 1 (abstract 0454).** See text for description

| Circadian patterns | Global |
|---|---|
| Dipper n(%) | 36 (19,6) |
| No-dipper n(%) | 94 (51,1) |
| Riser n(%) | 54 (29,3) |
| Dipper extremo n(%) | 0(0) |

In 20 patients (10.9%) a major event ocurred at 1 year follow-up, with higher proportion of events among patients with altered circadian pattern (non-dipper or riser: 12.2% vs dipper: 5.6%; 0.2).
**CONCLUSIONS.** In our area, patients with HF despite an optimal BP control, most of them presented an altered circadian pattern. In addition, this group of patients presents a tendency to a greater proportion of hospitalization and/or death due to HF. Longer-term studies are needed to confirm that finding.

### 0455

#### Study of left ventricular diastolic dysfunction in critically chronic obstructive pulmonary disease patients with and without corpulmonale

O. Momtaz
Fayoum University, Faculty of Medicine, Critical Care, Fayoum City, Egypt
**BACKGROUND.** There is growing data reporting increased risk of developing left ventricular diastolic dysfunction (LVDD) in patients with COPD though classically linked to presence and severity of corpulmonale.
*Intensive Care Medicine Experimental* 2018, **6(Suppl 2):**0455

**OBJECTIVE.** To evaluate the possible burden of left ventricular diastolic dysfunction and other left sided cardiac changes associated with COPD with and without corpulmonale and their relation to COPD severity and right sided cardiac affection.
**PATIENTS AND METHODS.** This study included 35 patients with severe and very severe COPD admitted to the medical ICU and 25 selected healthy volunteers. Pulmonary function tests (PFTs), echocardiography and tissue Doppler imaging (TDI) studies were performed for patients and control to obtain comparative data.
**RESULTS.** LVDD was detected in 31(88.6%) COPD patients compared to 6(24%) control patients with risk ratio 24.5.There was significant increase in left atrial diameter and LVDD parameters among COPD patients. Increased PASP was encountered in 23(65.7%) of the COPD patients; 20(86.9%) patients had LVDD whereas 64.5% of LVDD patients had increased PASP. There were negative correlations between PASP and LVEDD, LVESD and E/e´ ratio. PASP at 31 mmHg and different PFTs´ parameters were predictive of LVDD.
**CONCLUSION.** COPD patients have higher risk of LVDD, proportional to the disease severity and usually occurs earlier than evident RV dysfunction. TDI is a better tool than the conventional echocardiography in LVDD assessment. We recommend the routine use of echocardiography and TDI in ICU-COPD admitted patients for focusing LVDD and selecting patients for optimizing heart failure therapy.

### 0456

#### Thyrotoxic burden in the ICU and the value of bedside thyroid ultrasound in the diagnosis of thyrotoxicosis and thyrotoxic cardiac emergencies in critically-ill patients

O. Momtaz
Faculty of Medicine, Fayoum University, Egypt, critical care, fayoum city, Egypt
*Intensive Care Medicine Experimental* 2018, **6(Suppl 2):**0456

**BACKGROUND.** Hyperthyroidism, second to diabetes, is the second common endocrinal disorder in the general population. The most common causes of hyperthyoidism are diffuse toxic goiter (Graves' disease) (GD) (1%), solitary toxic nodules (STN) (0.1%) and toxic multinodular goiter (TMG) (0.53%) [1]. Thyroid ultrasound (US) has been used as an adjunctive tool to assess different thyroid diseases in specialized centers and outpatient clinics. This test is rapid, safe, feasible, easily available and informative in many cases if performed by expert personnel [2-4]. However, its use in the ICU to diagnose thyrotoxicosis has not been studied.
**AIM OF THE STUDY.** This study aimed to:

(1) Assess the thyrotoxic burden in critically ill patients
1) admitted in ICU.
(2) Evaluate the value of thyroid ultrasound as a rapid and feasible bedside diagnostic tool in early evaluation of suspected thyroid disease in critically ill patients especially those with life threatening cardiac emergencies.

**PATIENTS AND METHODS.** 854 patients admitted in 2 general intensive care units from Jan to November 2011 were evaluated. All patients suspected to have thyrotoxicosis were assessed by thyroid ultrasound and by measurement of serum levels of Ft3,Ft4,TSH, echocardiography and continuous ECG monitoring. Radioisotope scanning was done in selected cases.
**RESULTS.** Out of 854 admitted patients, 113 (13.2%) were suspected to have thyrotoxicosis. Thyrotoxicosiswas proved in 28 patients (24.7% of suspected cases and 3.3% of all admitted cases):
15% of all suspected cases had Graves' disease and 9.7% had toxic nodules. Most diagnosed patients (No: 16, 59.3%) had masked thyrotoxicosis. Seventy-six patients (67.3% of suspected patients) were admitted as critically ill cardiac patients and presented mainly by cardiac arrhythmias, heart failure, acute chest pain and malignant hypertension and 21 of these patients(27.6%) were proved to be thyrotoxic.
**CONCLUSION.** Thyrotoxicosis and thyrotoxic cardiac emergencies should be thoroughly and rapidly investigated as most cases are easily overlooked. Thyroid ultrasound is a feasible, rapid, accurate, noninvasive bedside tool useful in diagnosis of thyrotoxic emergencies in ICU.

### 0457

#### Fluid challenge definitions and fluid responsiveness: an epidemiological study on passive leg raising

A. Messina[1], G. Sotgiu[2], S. Laura[2], L. Capuano[1], E. Garofalo[3], L. Muratore[1], A. Bruni[3], G. Cammarota[1], M. Cecconi[4], P. Navalesi[3]
[1]AOU Maggiore della Carità, Novara, Italy; [2]Clinical Epidemiology and Medical Statistics Unit, Dept of Biomedical Sciences, Sassari, Italy; [3]Anesthesia and Intensive Care, Magna Graecia University, Dept of Medical and Surgical Sciences, Catanzaro, Italy; [4]IRCCS Humanitas, Humanitas University, Milano, Italy
**Correspondence:** A. Messina
*Intensive Care Medicine Experimental* 2018, **6(Suppl 2):**0457

**INTRODUCTION.** Fluid challenge (FC) evaluates hemodynamic effects on stroke volume index (SVI) or cardiac index (CI) following the administration of a small aliquot of fluid in a defined time period (1). Patients can be considered FC responder based on predefined SVI and CI thresholds and time-points.The FC, which is actually not standardized in the literature (2), is deemed the gold standard to assess the reliability of the hemodynamic test passive leg raising (PLR).
**OBJECTIVES.** The primary study aim was to assess whether changing FC definition would affect the assessment of fluid responsiveness. Secondary aim was to evaluate the impact of different FC definitions on PLR reliability.

**METHODS.** Hemodynamically unstable critically ill patients requiring fluid administration were randomly recruited. PLR was used according to the decision of the attending intensivist. FC consisted in 500 ml of crystalloids infused over 10 minutes. The fluid responsiveness was evaluated considering a) SVI or CI as hemodynamic targets; b) a SVI or CI increase of ≥10% or ≥15%; 10, 15, and 30 minutes as time-points. Receiving operator characteristics (ROC) curves of PLR were compared with the following gold standards: 10-minute response in CI or SVI ≥10%.

**RESULTS.** 85 FCs and 30 PLRs were analyzed. The rate of fluid responsiveness ranged from 27.1% (CI evaluated at 15' threshold 15%) to 48.2% (SVI evaluated at 10' threshold 10%). A mean (SD) of 19.7% (3.2%), 10.9% (5.1%), and 20.3% (6.0%) of patients changed responder/non-responder classification at 10, 15, and 30 minutes, respectively. A mean (SD) of 8.8% (2.6%) patients changed responder/non-responder classification after increasing the threshold from 10% to 15% (Tables 1-2). The effect on PLR reliability of different FC definitions is summarized in Figure 1. PLR sensitivity and specificity ranged from 54.1% to 81.1% and from 79.5% to 100.0%, respectively, whereas the area under the curve (95% CI) from 0.7 (0.6-0.8) to 0.9 (0.8-1.0) (see. Figure 1) FC subgroups evaluated after 30 minutes for both CI and SVI showed a significantly smaller area under the curve.

**CONCLUSIONS.** Rates of responsiveness and classification as responder or non-responder are affected by FC definitions. The reliability of PLR could be questioned when the assessment of the effect of FC administration is performed after 15 minutes.

**REFERENCE(S)**

(1) Cecconi M. et al. Curr Opin Crit Care. 2011 Jun;17(3):290-5
(2) Messina et al. Anesth Analg. 2017 Nov;125(5):1532-1543

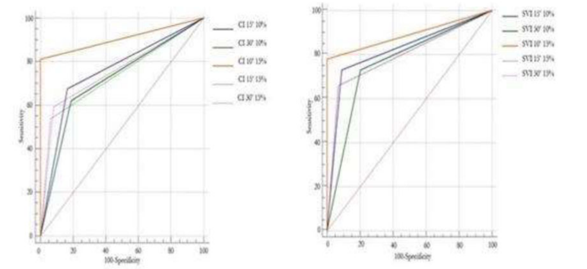

| | Sensitivity (95% CI) | Specificity (95% CI) | ROC area (95% CI) | PPV (95% CI) | NPV (95% CI) |
|---|---|---|---|---|---|
| CI 15' 10% | 67.6 (50.2-82.0) | 83.3 (69.8-92.5) | 0.8 (0.7-0.9) | 75.8 (57.7-88.9) | 76.9 (63.2-87.5) |
| CI 30' 10% | 62.2 (44.8-77.5) | 81.3 (67.4-91.1) | 0.7 (0.6-0.8) | 71.9 (53.3-86.3) | 73.6 (59.7-84.7) |
| CI 10' 15% | 81.1 (64.8-92.0) | 100 (92.6-100) | 0.9 (0.8-1.0) | 100 (88.4-100) | 87.3 (75.5-94.7) |
| CI 15' 15% | 54.1 (36.9-70.5) | 93.8 (82.8-98.7) | 0.7 (0.7-0.8) | 87.0 (66.4-97.2) | 72.6 (59.8-83.1) |
| CI 30' 15% | 59.5 (42.1-75.2) | 91.7 (80.0-97.7) | 0.8 (0.7-0.9) | 84.6 (65.1-95.6) | 74.6 (61.6-85.0) |

| | Sensitivity (95% CI) | Specificity (95% CI) | ROC area (95% CI) | PPV (95% CI) | NPV (95% CI) |
|---|---|---|---|---|---|
| SVI 15' 10% | 73.2 (57.1-85.8) | 90.9 (78.3-97.5) | 0.8 (0.7-0.9) | 88.2 (72.5-96.7) | 78.4 (64.7-88.7) |
| SVI 30' 10% | 73.2 (57.1-85.8) | 79.5 (64.7-90.2) | 0.8 (0.7-0.9) | 76.9 (60.7-88.9) | 76.1 (61.2-87.4) |
| SVI 10' 15% | 78.0 (62.4-89.4) | 100.0 (92.0-100.0) | 0.9 (0.8-1.0) | 100 (89.1-100.0) | 83.0 (70.2-91.9) |
| SVI 15' 15% | 65.9 (49.4-79.9) | 93.2 (81.3-98.6) | 0.8 (0.7-0.9) | 90.0 (73.5-97.9) | 74.4 (61.0-85.3) |
| SVI 30' 15% | 65.9 (49.4-79.9) | 93.2 (81.3-98.6) | 0.8 (0.7-0.9) | 90.0 (73.5-97.9) | 74.4 (61.0-85.3) |

**Fig. 1 (abstract 0457).** PLR-ROC curve comparison. Gold standards: CI increase at 10 minutes ≥ 10% (left) and SVI increase at 10 minutes ≥ 10% (right).

**Table 1 (abstract 0457).** Change in the number of responders at the different timepoints

| TEST | 10' VS. 15' | 15' VS. 30' | 10' VS. 30' |
|---|---|---|---|
| *CI 10%, n (%)* | 20 (23.5) | 5 (5.9) | 23 (27.1) |
| *CI 15%, n (%)* | 15 (17.7) | 7 (8.2) | 12 (14.1) |
| *SVI 10%, n (%)* | 15 (17.7) | 15 (17.7) | 20 (23.5) |
| *SVI 15%, n (%)* | 18 (21.2) | 10 (11.8) | 14 (16.5) |

**Table 2 (abstract 0457).** Change in the number of responders at different thresholds

| TEST | 10% VS. 15% |
|---|---|
| *CI 10', n (%)* | 7 (8.2) |
| *CI 15', n (%)* | 10 (11.8) |
| *CI 30', n (%)* | 6 (7.1) |
| *SVI 10', n (%)* | 9 (10.6) |
| *SVI 15', n (%)* | 4 (4.7) |
| *SVI 30', n (%)* | 9 (10.6) |

### 0458

### Changes in central venous pressure during passive leg raising do not allow predicting preload unresponsiveness in critically ill patients

O. Hamzaoui[1], C. Gouëzel[1], M. Jozwiak[2], M. Millereux[1], B. Sztrymf[1], D. Prat[1], F. Jacobs[1], X. Monnet[2], C. Richard[2], P. Trouiller[1], J.-L. Teboul[2]
[1]Hôpital Antoine Béclère, Hôpitaux Universitaires Paris-Sud, Assistance Publique-Hôpitaux de Paris, Service de Réanimation Polyvalente, Clamart, France; [2]Hôpital Bicêtre, Hôpitaux Universitaires Paris-Sud, Assistance Publique-Hôpitaux de Paris, Service de Réanimation Médicale, Kremlin-Bicêtre, France
**Correspondence:** C. Gouëzel

*Intensive Care Medicine Experimental* 2018, **6(Suppl 2):**0458

**INTRODUCTION.** Passive leg raising (PLR) is considered as a reliable test to predict fluid responsiveness in critically ill patients. However, it requires either a continuous monitoring of cardiac output (CO) or repeated transthoracic echocardiography measurement of CO derived from the velocity-time integral (VTI) of the flow in the left ventricular outflow tract (1). As critically ill patients are routinely equipped by a central venous catheter, central venous pressure (CVP) can be measured. By analogy with the CVP rules to stop a fluid challenge (2), we suggested that an increase in CVP by more than 5 mmHg during PLR could predict preload unresponsiveness and avoid any CO measurement during the PLR test.

**OBJECTIVES.** We aimed to assess whether a high increase in CVP during PLR can predict preload unresponsiveness diagnosed by the absence of increase of VTI by more than 10% (3).

**METHODS.** We prospectively included critically ill patients equipped with a central venous catheter and for whom the physician in charge decided to test preload responsiveness by a PLR test. For each patient hemodynamic parameters were recorded before and during PLR such as mean arterial pressure (MAP), CVP and VTI.

**RESULTS.** Twenty-four measurements were obtained in 18 patients. Their age was 73 ± 10, their SAPSII was 52 ± 21. At baseline, norepinephrine was already administered in 22 measurements, its mean dose was 0.40 ± 0.45 µg/kg/min, the lactate concentration was of 1.6 ± 0.5 mmol/L, the MAP was 77 ± 9 mmHg and their mean CVP was 8 ± 4 mmHg. After the PLR test, 11 cases were defined as preload responders with an increase in CVP of 5 mmHg in one of them, and 13 cases were defined as preload non-responders with only two cases with an increase in CVP of 5 and 6 mmHg. There was no significant difference of the mean changes in CVP between responders and non-responders: 2.7 ± 1.9 mmHg and 2.3 ± 1.7 mmHg (p=0.7) respectively. There was no correlation between the changes in CVP and the changes in VTI (r=-0.08; p=0.7) (fig 1). Neither the change in CVP, nor its baseline value predicted preload unresponsiveness with areas under the receiver operating characteristic curves (with 95% CIs) being 0.56 (0.34-0.76) and 0.52 (0.31-0.73) respectively (fig 2).

**CONCLUSIONS.** Assessing the changes in CVP during PLR is not accurate to predict preload unresponsiveness in critically ill patients.

**REFERENCE(S)**

1. Monnet X, Marik P, Teboul JL: Passive leg raising for predicting fluid responsiveness: a systematic review and meta-analysis. Intensive Care Med 2016.
2. Weil MH, Henning RJ. New concepts in the diagnosis and fluid treatment of circulatory shock. Thirteenth annual Becton, Dickinson and Company Oscar Schwidetsky Memorial Lecture. Anesth Analg 1979; 58:124-132.
3. Vignon P, Begot E, Mari A, et al. Hemodynamic Assessment of Patients With Septic Shock Using Transpulmonary Thermodilution and Critical Care Echocardiography: A Comparative Study. Chest. 2018; 153:55-64.

**GRANT ACKNOWLEDGMENT**

None

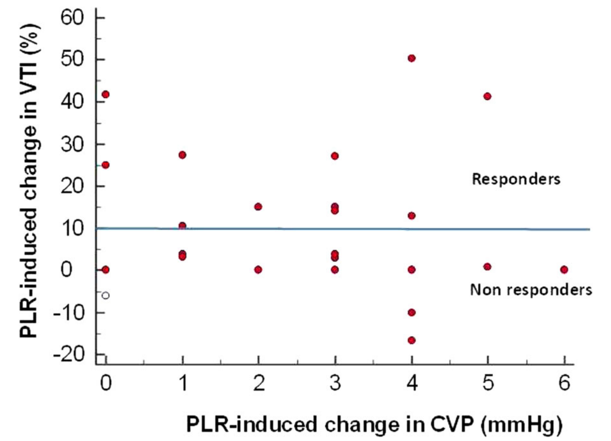

**Fig. 1 (abstract 0458).** PLR-induced change in VTI (%) as a function of PLR-induced change in CVP (mmHg)

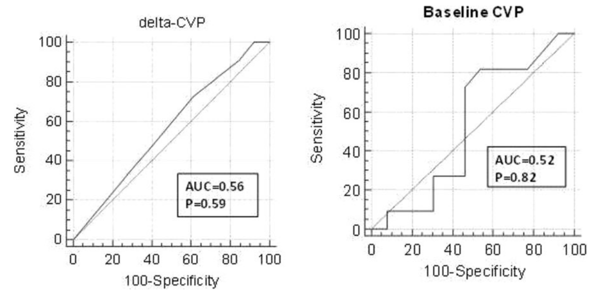

**Fig. 2 (abstract 0458).** Preload unresponsiveness prediction of delta-CVP and CVP baseline value (AUC ROC)

### 0459

#### Optimal atrio-ventricular (AV) interval election in dual-chamber pacemaker implantation by two bedside echocardiographic technics in an intensive care unit

O. Moreno Romero[1], C. Salazar Ramirez[2], M. Carballo Ruiz[2]

[1]Hospital Universitario San Cecilio, Granada, Spain; [2]Hospital Virgen de la Victoria, Cardiac Electroestimulation Unit, Intensive Care Unit, Malaga, Spain

**Correspondence:** O. Moreno Romero

*Intensive Care Medicine Experimental* 2018, **6(Suppl 2):**0459

**INTRODUCTION.** In the last decades the use of dual-chamber pacemakers has become relevant as it allows the physician to ensure the best dyastolic function and filling time by calculating the optimal atrioventricular interval to achieve a right systolic function for improving the clinical status of the patients with AV disorders.

**OBJECTIVE.** To select the optimal AV interval between two technics measured by a routine echocardiography in patients with acute AV block after the implantation of a dual-chamber pacemaker in our cardiac estimulation unit in intensive care.

**METHODS.** Prospectively study in a two-month period (1 november - 31 december 2017) in which we registered all patients with acute AV block admitted in our ICU of 18 beds and with an indication of dual-chamber pacemaker implantation. A routinary echocardiography was performed by our cardiac estimulation unit physicians (intensivists) in the next 6 hours after the implantation (maximum). Two technics were applied (image 1): First, calculating the AV by the Ritter's Method, using a short-AV interval of 50 ms and a long-AV interval of 200 ms. In all patient we assured using the mitral doppler that there was neither E-A fusion nor truncated A wave. Second, measuring the Time Velocity Integral (TVI) of the Left Ventriclular Outflow Tract (LVOT) at different AV intervals selected (100, 150, 180 and 200 ms), making the average of 3 cardiac cycles of each interval. The average time in applying both methods were noted.

**RESULTS.** In the two month-period, 28 patients with cardiac arrhythmia were admitted in our unit. 18 (64.3%) with AV disorders. 13 (46.4%) with dual-chamber pacemaker implantation. Only 7 (25%) patients fullfill the inclusión criteria (AV block and US in next 6 hours maximmum) and were admitted in our study. 5 male (71.4%), mean age 72±8.5 years. Average times applied: with Ritter's method, 7.5 min (IC 95:5.2-9.8) and with TVI method, 9.7 min (IC95:7.5-11.9). Complete results on image 2.

**CONCLUSIONS.** In our study we demonstrate the high variability of AV interval amongst patients. The use of bedside echocardiography in ICU in these patients is an assumable procedure, technicaly and temporarily, and it may facilitate the selection of the optimal AV interval after a dual chamber pacing implantation, although our sample is small and we need the long-term follow up to evaluate the clinical improvement.

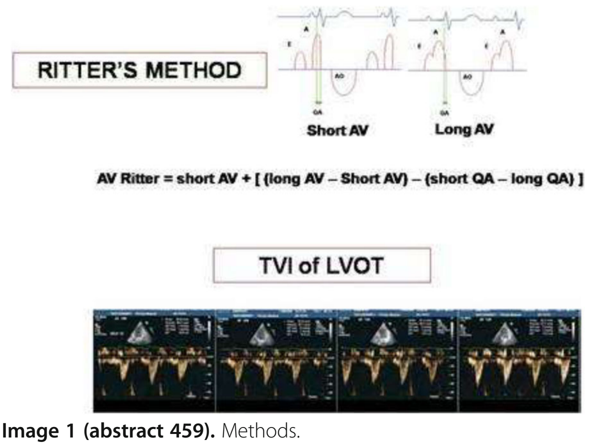

**Image 1 (abstract 459).** Methods.

**RESULTS**

| PATIENT | 1 | 2 | 3 | 4 | 5 | 6 | 7 |
|---|---|---|---|---|---|---|---|
| AV RITTER (ms) | 166 | 136 | 162 | 93 | 127 | 180 | 150 |
| BEST TVI (cm) at interval AV (ms) | 16 cm at 180 ms | 27.6 cm at 150 ms | 21.6 cm at 150 ms | 23 cm at 100 ms | 20 cm at 150 ms | 19.8 cm at 180 ms | 25 cm at 150 ms |
| AV SELECTED (ms) | 180 | 150 | 160 | 120 | 150 | 180 | 150 |

**Image 2 (abstract 459).** Results.

### 0460

#### A modified intracavitary electrocardiographic method to localise the tip of central venous access devices in atrial fibrillation

M. Calabrese[1], L. Montini[2], G. Arlotta[1], A. La Greca[3], D. Biasucci[4], F. Bevilacqua[1], E. Antoniucci[1], A. Scapigliati[2], F. Cavaliere[1], M. Pittiruti[3]
[1]"Agostino Gemelli" University Hospital Foundation, Catholic University of the Sacred Heart, Department of Cardiovascular Sciences, Institute of Anesthesia and Intensive Care, Rome, Italy; [2]"Agostino Gemelli" University Hospital Foundation, Catholic University of the Sacred Heart, Department of Intensive Care Medicine and Anesthesiology, Rome, Italy; [3]"Agostino Gemelli" University Hospital Foundation, Catholic University of the Sacred Heart, Department of Surgery, Rome, Italy; [4]"Agostino Gemelli" University Hospital Foundation, Catholic University of the Sacred Heart, Department of Intensive Care Medicine and Anesthesiology, Rome, Italy
**Correspondence:** D. Biasucci
*Intensive Care Medicine Experimental* 2018, **6(Suppl 2):**0460

**INTRODUCTION.** The intracavitaryECGmethod (ICECG) is recommended as accurate, safe and cost-effective for assessing the proper location of the tip of central VenousAccessDevices (VADs)(1). This method is considered applicable when there is a identifiable P wave.

**OBJECTIVES.** We hypothesised that *f* waves resulting from chaotic depolarisation in atrial fibrillation (AF) may be utilised as a surrogated of P waves.

**METHODS.** Eighteen patients with atrial fibrillation undergoing cardiac surgery were enrolled in the study. They all underwent central VAD positioning and intraoperative transesophageal echocardiography (TEE). After general anaesthesia induction, a TEE probe was placed so to obtain a mid-esophageal bicaval view, with visualization of the superior vena cava (SVC), of the cavo-atrial junction (CAJ) and of the right atrium (RA). Then a VAD was insered into the internal jugular vein, the guidewire was removed and the catheter filled with normal saline. The proximal end of the catheter was connected to the ECGmonitor in order to have an ICECGtracing. Under TEE visualization, the trace was recorded with the catheter tip in three different positions: in the SVC (approximately 2 cm above the CAJ), at the crista terminalis (i.e. at the CAJ), and in the upper part of the RA (2 cm below the CAJ). The tip of the catheter was finally left in corrispondence of the CAJ. The *f* wave pattern was evaluated in all traces by three different criteria of measurement: 1) the mean height of *f* waves visible in the TQtract, considering only the waves higher than one mm (method A); 2) the height of the highest f wave visible in the TQtract (method B); 3) the difference between the highest point (most positive peak) and the lowest point (most negative peak) of the *f* waves visible in the TQtract (method C).

**RESULTS.** When the tip was placed at the CAJ, the mean value of the *f* waves was significantly higher than in the other two positions (SVC and RA), regardless of the measurement method. All three methods were effective in discriminating the tip position at the CAJ. According to the ROCanalysis, method B proved to be the most effective in predicting the tip position at the CAJ if compared to the position in SVC and in RA (AUC±SE 0.84±0.02 and 0.86±0.02, respectively).

**CONCLUSIONS.** Our study demonstrates that ICECG technique can be used for detecting the location of the tip of central VADs in AF patients: the highest activity of the f waves is an accurate indicator of the tip location at the CAJ. Though each method was significantly accurate in detecting the correct tip location at the CAJ, the ROCanalysis suggests that method B was the most accurate.

**REFERENCE(S)**

(1) Pittiruti M et al Intracavitary ECG method for positioning the tip of central venous catheters: results of an Italian multicenter study. J Vasc Access 2012, 13(3):375-65.

### 0461

#### Routine versus triggered echocardiography in the intensive care unit- an audit of clinical practice

O.O. Olusanya[1], R. Spiritoso[2], S. Bhattacharyya[2]
[1]Barts Heart Centre, Reading, United Kingdom; [2]Barts Heart Centre, London, United Kingdom
**Correspondence:** O.O. Olusanya
*Intensive Care Medicine Experimental* 2018, **6(Suppl 2):**0461

**INTRODUCTION.** Focused echocardiography is a recognised clinical tool in intensive care practice (1). its use in a general ICU population has been associated with clinically relevant changes in practice (2). In our unit, focused echocardiography is used in a triggered fashion to answer specific clinical questions; the impact of daily "non-triggered" scans is unknown to us.

**OBJECTIVES.** To assess the impact of routine focused echocardiography, as opposed to triggered echocardiography, on the care of patients in the ICU

**METHODS.** A prospective audit of "routine" focused echocardiography of patients in a tertiary cardiac ICU. Each patient on the unit was scanned at least once a week in a standard focused echocardiographic sequence (3).

**RESULTS.** Over a one month period from 1st of March 2018 to 31st of March 2018, 22 routine scans were performed. 18 (82%) of these scans revealed clinical abnormalities. 1 scan (4.5%) led to a rapid change in management (institution of cardiac output monitoring and subsequent VA ECMO) which led to a subsequent improvement of the patient.

**CONCLUSIONS.** In a one month audit of our practice, the use of routine focused echocardiography demonstrated a large amount of pathology. Only one patient had changes in their management plan due to unexpected findings on echocardiography.

**REFERENCE(S)**

1. Farkas, JD, Anawati, MK. Bedside ultrasonography evaluation of shock. Hospital Med Clin 2015; 4: 135-149
2. Hall DP, Jordan H, Alam S, Gillies MA. The impact of focused echocardiography using the Focused Intensive Care Echo protocol on the management of critically ill patients, and comparison with full echocardiographic studies by BSE-accredited sonographers*Journal of the Intensive Care Society* Vol 18, Issue 3, pp. 206 - 211
3. Focused Intensive Care Echocardiography. https://www.ics.ac.uk/ICS/fice.aspx (last checked 18/4/2018)

### 0462

#### Estimation of thermodilution derived cardiac index (CI_TD) by combined information from the uncalibrated devices ClearSight, FloTrac and a completely non-invasive assessment of CI (CI_CNI) derived from surface temperatures and biometrics: a prospective observational study

W. Huber, J. Mangold, T. Lahmer, A. Herner, U. Mayr, G. Batres-Baires, R. Schmid, M. Heilmaier
Klinikum rechts der Isar; Technical University of Munich, Medizinische Klinik und Poliklinik II, Munich, Germany
**Correspondence:** W. Huber
*Intensive Care Medicine Experimental* 2018, **6(Suppl 2):**0462

**INTRODUCTION.** Cardiac Index CI is a key target of haemodynamic monitoring. Clinical assessment based on body surface temperature BST provides a rough estimate of CI ("cold shock" vs. "warm shock").

Non-contact infrared thermometers provide a more accurate estimate of BST than clinical examination. In a recent study, a complete non-invasive estimate of CI (CI_CNI) based on BSTs and biometric data provided at least comparable estimation of the gold-standard CI derived from thermodilution (CI_TD) compared to two uncalibrated devices based on pulse contour analysis (ClearSight (CI_CS) and FloTrac (CI_FT); both Edwards Lifesciences).

**OBJECTIVES.** We hypothesized that bias and percentage error of CI_CS and CI_FT could be improved by a combination of the data from both devices or by a combination with CI (CI_CIN). We investigated the agreement of these combinations with CI_TD and compared their predictive capacities with those of CI_CS, CI_FT and CI_CNI alone.

**METHODS.** In 22 patients (15m; 7f; APACHE-II 28±6) with TD-Monitoring (PiCCO; Pulsion; Germany) a total of 176 datasets were recorded (8 datasets per patient within 24h). Immediately before TD we measured BST on the forehead, forearm (middle and distal), finger and great toe with a non-contact infrared thermometer (Thermofocus; Tecnimed) to derive CI_CNI. Calculation of CI_CNI is also based on age, gender, weight and height.

Simultaneously, we recorded CI_CS and CI_FT.

These estimates of CI were compared to CI_TD.

Statistics: Multiple regression analysis; Bland-Altman Analysis (Primary endpoint); ROC-AUCs regarding CI_TD≤2.5 and CI_T≥5L/min*m$^2$.

Derivation and validation of CI_CIN from this dataset have been presented at the ISICEM 2018.

**RESULTS.** Among the un-combined devices, CI_CIN provided better bias (-0.039 L/min/$^2$) and percentage error (PE; 49%) values compared to CI_CS (-0.85 L/Min/m$^2$; 60%) and CI-FT (-0.097 L/Min/m$^2$; 65%). Combination of CI_CS with CI_FT (CI_CS_FT) markedly improved the bias (0.0L/min/m$^2$), whereas the PE (49%) for this combination was not lower than for CI_CNI alone. CI_CS_CNI combining CI_CS and CI_CNI had a markedly improved bias (-0.001 L/min/m$^2$) and PE (47.5%) compared to CI_CS alone, but not compared to CI_CNI. The best values for bias (0.0003 L/min/m$^2$) and PE (43.7%) were found for CI_FT_CNI derived from CI_FT and CI_CNI. The largest ROC_AUC regarding CI_TD ≤2.5 was found for CI_CNI alone (0.793; p< 0.001 except as indicated). The AUCs were 0.627 for CI_FT (p=0.060), 0.679 (p=0.005) for CI_CS, 0.772 for CI_FT_CNI and 0.775 for CI_CS_CNI.

ROC_AUCs regarding CI_TD≥5 were 0.794 (CI_FT), 0.888 (CI_CS), 0.906 (CI_CS_FT), 0.920 (CI_CNI), 0.924 (CI_CS_CNI) and 0.944 (CI_FT_CNI).

**CONCLUSIONS.** Combination with CI_CNI markedly improved the estimate of CI_TD by CI_CS and CI_FT.

Un-combined CI_CNI provides at least comparable estimates of CI_TD as CI_CS and CI_FT.

### 0463

#### Effects of inspiratory flow waveforms on arterial pulse pressure variation and inferior vena cava distensibility in mechanically ventilated patients: a randomized cross-over study

Z. Hajjej, M.R. Yazidi, W. Sellami, O. Yengui, R. Bousselmi, M. Ferjani
Université de Tunis El Manar, Faculté de Médecine de Tunis, Department of Anesthesiology and Critical Care Medicine, Military Hospital of Tunis, Tunis, Tunisia
**Correspondence:** Z. Hajjej
*Intensive Care Medicine Experimental* 2018, **6(Suppl 2):**0463

**INTRODUCTION.** The clinical usefulness of inspiratory flow pattern manipulation remains unclear.

**OBJECTIVES.** The purpose of this study was to compare the effects of flow patterns (sinusoidal, constant and decelerating) on dynamic measurements of cardiac preload dependence such as arterial pulse pressure variation (ΔPP) and distensibility index of the inferior vena cava (dIVC).

**METHODS.** Over four months (May 1$^{st}$, 2013 to August 31$^{th}$, 2013.) a prospective, crossover, observational study was performed in an 18-bed medical surgical intensive care unit at Tunis military hospital. Inclusion criteria were predefined as follows: mechanical ventilation with volume assist-control mode, full adaptation to ventilator and sinus rhythm. The study design was a 30 minutes crossover with 60 minutes washout during which time the patients received usual ventilator with decelerating inspiratory flow pattern. Each inspiratory flow waveform was maintained for 30 min. During the last 5 min of this period the physiological signals were collected and measures (ΔPP and dIVC) were performed. This trial is registered with Clinicaltrials.gov, number NCT01892696

**RESULTS.** Among 82 patients admitted in the ICU during the study period, 60 patients were included. The decelerating waveform was significantly associated with a lower mean of Peak pressure (Ppeak) and Plateau pressure (Pplat) compared to both constant and sinusoidal flow patterns (p< 0.001).Although the decelerating waveform was associated with a slightly higher mean airway pressure (Pmean) than the other two flow patterns, this difference did not achieve statistical significance. There was no difference between sinusoidal and constant waveforms. In contrast the decelerating waveform produced significantly lower values of ΔPP and dIVC than did both constant and sinusoidal waveforms.

**CONCLUSIONS.** Our findings suggest that decelerating waveform was significantly associated with a lower mean of Ppeak and Pplat and produced significantly lower values of ΔPP and dIVC compared to both constant and sinusoidal flow patterns

##### REFERENCE(S)

Niranjan S C, Bidani A, Ghorbel F, Zwischenberger JB, Clark JW. Theoretical Study of Inspiratory Flow Waveforms during Mechanical Ventilation on Pulmonary Blood Flow and Gas Exchange. Computers and Biomedical Research 1999; 32:355-390

##### GRANT ACKNOWLEDGMENT

No one

### 0464

#### Assessment of 24-hour ambulatory blood pressure monitoring in nonhypertensive patients with chronic heart failure

A.M. González González, A.M. Garcia Bellon, C. Lara Garcia, M. De Mora Martin
Regional Hospital of Malaga, Cardiology, Malaga, Spain
**Correspondence:** A.M. González González
*Intensive Care Medicine Experimental* 2018, **6(Suppl 2):**0464

**INTRODUCTION.** Ambulatory blood pressure monitoring (ABPM) permites the evaluation of 24 hours blood pressure pattern. It is well defined the prognosis value of the abnormalities in the circadian variation in hypertensive patients. In the phisiopathology of heart failure, neurohumoral mechanism plays an important role. Nevertheless, the circadian variation in nonhypertensive heart failure patients has not been well evaluated.

**OBJECTIVES.** To evaluate 24 hours monitoring blood pressure in nonhypertensive heart faire patients.

**METHODS.** We studied 80 patients with a clinical diagnosis of nonhypertensive CHF. They were followed-up by the Heart Failure Unit. We permormed a 24-h ambulatory blood pressure monitoring as well as an echocardiogram and anaytical test.

**RESULTS.** 80 patients. Mean age: 62,7 ± 12. Males: 72,5%. Mean BMI: 29,6 ± 5 Kg/m$^2$. Mean time of follow-up of CHF: 69 ± 66 months. Associated risk factors: dyslipidemia 25% Diabetes 20% obesity 45% active smoking 30%, ex-smoking 27,5%.

Therapeutic regimen applied: RAS blockers 85%; betablockers 92,5%; loop diuretic 75%; spironolactone 35%; statins 55%; antiplatelet/anticoagulant drugs 75%, nitrates 20%, digoxin 30%

The 24 h ABPM measurements are in Table 1.

The majority of CHF patients (77,5%) have an abnormal pattern of ABPM, as it is shown on on Table 2.

**CONCLUSIONS.** Nonhypertensive heart failure patients had, in majority, an abnormal circadian rhythm of blood pressure. It is well known the prognosis values of this alterations. This highlights the high cardiovascular risk and the neurhumoral alterations of this patients with chronic heart failure, whether they are hypertensive or not.

**Table 1 (abstract 0464).** See text for description

| Mean ABPM (mmHg) | 24 hours | Daytime | Nightime |
|---|---|---|---|
| Sysolic BP | 107,7 ± 13,8 | 109,6 ± 14,2 | 104,5 ± 14,5 |
| Diastolic BP | 64,4 ± 7,8 | 66,4 ± 8,8 | 60,4 ± 7,6 |

**Table 2 (abstract 0464).** See text for description

| Patterns of ABPM | Global |
|---|---|
| Dipper n(%) | 18 (22,5) |
| Non-dipper n(%) | 46 (57,5) |
| Riser n(%) | 16 (20) |
| Extreme dipper n(%) | 0 (0) |

### 0465

#### Guiding ultrafiltration with echocardiography

S. Patel[1], R. Vashisht M.D[2], E. Fleig[2], S. Demirjian M.D[3]
[1]Cooper University Hospital, Critical Care, Philadelphia, United States;
[2]Cleveland Clinic, Respiratory Institue, Cleveland, United States;
[3]Cleveland Clinic, Kidney Institute, Cleveland, United States
**Correspondence:** S. Patel
*Intensive Care Medicine Experimental* 2018, **6(Suppl 2):**0465

**INTRODUCTION.** Patient's requiring hemodialysis have high mortality rates. Overly rapid UF rates can lead to intradialytic hypotension leading to poor outcomes. Conversely, fluid overload may also be a predictor of mortality[1,2]. There is a lack of literature on methods to individualize UF rates for optimal fluid removal. The left ventricular outflow tract velocity time integral (LVOT VTI) is a validated non-invasive echocardiographic surrogate for stroke volume. We hypothesize that lack of VTI proven volume responsiveness is suggestive preload independence, therefore, may have a lower propensity for intradialytic hypotension.

**PURPOSE.** We propose the use of bedside echocardiography proven preload dependence as a predictor of intradialytic hypotension.

**METHODS.** We conducted a prospective observational study within a tertiary medical center from September 2016 to June 2017. We included patients with the need for intermittent hemodialysis with a minimum UF rate of 5cc/kg/hr, adequate echocardiography windows, patient consent and catheter dialysis access. Patients with age < 18 years of age, pregnancy, AV fistula/graft access, more than one vasopressor/inotrope, hemorrhagic/hypovolemic shock and inability to consent were excluded. Baseline vitals obtained before dialysis. We obtained an apical five-chamber view; three VTI measurements were recorded on end expiration pre and post a 50 cc normal saline bolus. Fig 1,2 Percentage increase in VTI was deemed to be delta VTI (dVTI). The primary outcome was intradialytic hypotension, which was defined as a systolic or MAP drop of > 30 or > 10 mmHg respectively.

**RESULTS.** 37 patients were recruited for the study. For analysis , we used logistic regression and ROC curves for the statistical analysis. We also found predictive values. Using a dVTI cutoff of 9% or higher, the odds ratio (OR) for developing intradialytic hypotension was 17 (CI 2.19-99). The PPV and NPV were 66.7 and 89.5% respectively. The sensitivity and specificity were 85.7 and 73.9 respectively. The AUROC was 0.776 (CI 0.600-0.952). Fig 3 The regression yielded an OR of 1.2 ( CI 1.08-1.4) for every percent increase in dVTI.

**CONCLUSIONS.** Evidence of preload dependence through the use of bedside echocardiography may predict intradialytic hypotension. Conversely, a lack of response had an excellent negative predictive value. Preload dependence is a predictor of intradialytic hypotension and should be studied in more extensive clinical trials.

**REFERENCE(S)**

1. Assimon MM, Wenger JB, Wang L, Flythe JE. Ultrafiltration Rate and Mortality in Maintenance Hemodialysis Patients. *American Journal of Kidney Diseases.* 2016;68(6):911-922.
2. Wu Y, Zhou S, Zhou Z, Liu B. A 10-second fluid challenge guided by transthoracic echocardiography can predict fluid responsiveness. *Critical care (London, England).* 2014;18(3):R108.

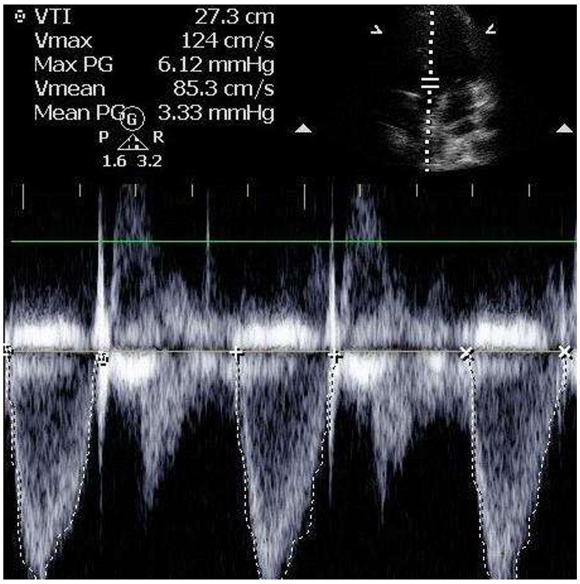

**Fig. 1 (abstract 0465).** See text for description.

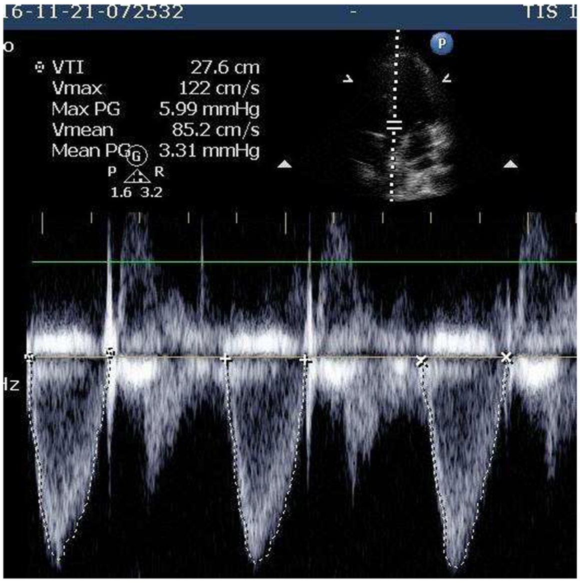

**Fig. 2 (abstract 0465).** See text for description.

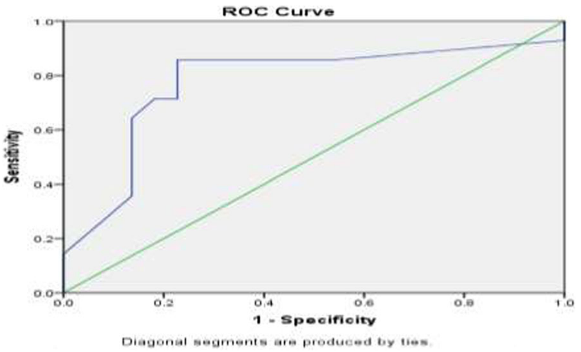

**Fig. 3 (abstract 0465).** See text for description.

supporting clinicians in critical care, although further improvements are still advocated, towards even more accurate outcomes.

**REFERENCE(S)**

[1] Moody, George B., and L-W. H. Lehman. "Predicting acute hypotensive episodes: The 10th annual physionet/computers in cardiology challenge." Computers in Cardiology, 2009. IEEE, 2009.

[2] Chen, Zhe, et al. "Dynamic assessment of baroreflex control of heart rate during induction of propofol anesthesia using a point process method." Annals of biomedical engineering 39.1 (2011): 260-276.

**GRANT ACKNOWLEDGMENT**

Progetto Roberto Rocca, MIT-Italy Program, NIH grant R01GM104987, and European Commission LINK project (H2020-692023)

### 0466

#### Predictive assessment of Baroreflex in the ICU

R. Barbieri[1], G. Angelotti[2], P. Morandini[2], L.-W. Lehman[3], R.G. Mark[3]
[1]Politecnico di Milano, Electronics, Informatics and Bioengineering, Milano, Italy; [2]Politecnico di Milano, DEIB, Milano, Italy; [3]Massachusetts Institute of Technology, Cambridge, United States
**Correspondence:** R. Barbieri
*Intensive Care Medicine Experimental* 2018, **6(Suppl 2):**0466

**INTRODUCTION.** In the intensive care. unit, Acute Hypotensive Episodes (AHE) represent a phenomenon of high clinical relevance. If not promptly treated, AHEs may lead to critical conditions, and they are associated with a more than double mortality rate compared to normopressive subjects and side effects of late treatment (e.g irreversible organs damage) [1].

AHE prediction has been the subject of several previous studies, showing it is possible to predict such events. We present a method that, by means of machine learning and a statistical model framework, predicts AHE onset by several minutes using information from ECG and Arterial Blood Pressure (ABP) recordings.

Additionally, we proved that by introducing variables related to baroreflex sensitivity (BRFX) in the previous model we were able to improve the quality of the
prediction (AUC) by 9.8%.

**OBJECTIVES.** The aim of this study is to predict the onset of AHE using a mathematical framework applied on ECG and ABP waveforms from bedside Monitor recordings of ICU patients.

**METHODS.** We define an AHE by the 2009 Physionet Challenge definition:

30 minutes interval in which 90% of MAP is below 60mmHg [1].

Data were pulled from the MIMIC-II Waveforms Database.

The final cohort counted 83 records, of which 32 (38.55%) hypotensive.

The first model (Model A) we deployed was based solely on blood pressure variables, whereas in the second (Model B) we introduced BRFX Features.

BRFX was estimated using a bivariate point process framework based on a closed-loop Heart Rate (HR) - Systolic Blood Pressure (SBP) model [2].

Following several processing stages, we extracted indicators describing BRFX activity.

The prediction was performed 10 minutes ahead of the AHE onset, using 10 minutes worth of data (time windows starting 20 minutes before the AHE onset and ending
10 minutes prior) using Linear Discriminant Analysis.

**RESULTS.** Model A obtained an AUC of .74, while Model B further improved results (AUC) by as much as 9.8%.

**CONCLUSIONS.** Results from this study show that it is possible to extract relevant information from a beat-to-beat analysis, able to generate a prediction by using only 10 minutes of data previous to the event. Additionally, the inclusion of BRFX further improves the outcome. Indeed, machine Learning is proving to be an extremely useful tool for

### 0467

#### Correlation between the measurement of cardiac output by magnetic cardiac pulsepresiometer (PPC) and fick method in critical patients

A. Hernández-Gutiérrez[1], M.N. Gómez-González[1], A.E. Hernández-Plata[1], R. Soriano-Orozco[1], T. Córdova-Fraga[2], M.A. Hernández-González[3], P.L. González-Carrillo[1], A. Hernández-Rayas[2]
[1]Instituto Mexicano del Seguro Social, Unidad de Cuidados Intensivos, León, Mexico; [2]Instituto Mexicano del Seguro Social, León, Mexico; [3]Instituto Mexicano del Seguro Social, Enseñanza, León, Mexico
**Correspondence:** A. Hernández-Gutiérrez
*Intensive Care Medicine Experimental* 2018, **6(Suppl 2):**0467

**INTRODUCTION.** The hemodynamic monitoring is fundamental for the diagnostic approach and therapeutic guide in the Intensive Care Unit (ICU). A continuous non-invasive monitoring of cardiovascular function is required. The magnetic cardiac pulsepresiometer (PPC) is a device of regional invention, inexpensive, non-invasive, easy application to patient's bedside which in healthy volunteers is able to record the amplitude of the arterial pulse continuously, obtaining the measurement of cardiac output by means of electromagnetism

**OBJECTIVE.** The goal of this study was to determine the correlation between the measurement of cardiac output by PPC and Fick method in the ICU.

**METHODS.** This prospective, observational, longitudinal study was conducted at the ICU, Centro Médico Nacional del Bajío UMAE 1, in León Guanajuato between December 2017 and February 2018. Patients were admitted over 18 years with a functional central venous with any diagnosis, regardless of the hemodynamic status. The cardiac output following the Fick method and with the PPC was measured at the first 4 hrs from admission to the unit. Patients who had a tracheostomy or a disfunctional central venous catheter dysfunction were excluded. The statistical analysis was performed with the statistical package MedCal V 19.1

**RESULTS.** We included 13 patients, with a mean age of 36.3 years, 7 women (53.84%), 6 men (46.15%), the mean APACHE II and SOFA were 25.3 and 7.6 points respectively; more frequent admission diagnosis was subarachnoid hemorrhage.

Finding a positive correlation between the non-invasive measurement of cardiac output measured by the PPC and that calculated by the Fick method using the samples obtained from the central venous catheter, r = 0.91 (fig. 1). With a concordance by kappa coefficient of 0.67, which is considerable.

**CONCLUSION.** The magnetic cardiac pulsopresiometer is a low-cost device that can be used at the patient´s bedside to perform a non-invasive monitoring of cardiac output in critical patients.

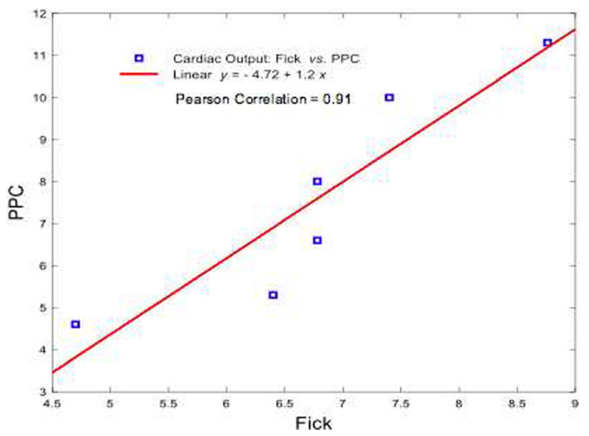

**Fig. 1 (abstract 0467).** Correlation between the measurement of cardiac output by PPC and Fick method

CVP vs SVV (BioZ®); r = -0.199, P=0.027, SVV (the FloTrac®/Vigileo®) vs SVV (BioZ®); r = 0.048, P=0.597). And there was no agreement between central venous pressure and SVVs measured by the FloTrac®/Vigileo® system and BioZ® system (CVP vs. SVV (the FloTrac®/Vigileo®); kappa = 0.057 (95% CI -0.010 ~ 0.214 ), CVP vs SVV (BioZ®); kappa = 0.039 (95% CI -0.009 ~ 0.087), SVV (the FloTrac®/Vigileo®) vs SVV (BioZ®) ; kappa = 0.005 (95% CI -0.103 ~ 0.112).

**CONCLUSIONS.** Our preliminary study showed disagreement the hemodynamic parameters measured by one of the two devices and it should be interpreted with caution before therapeutic interventions.

### 0468

#### Disagreement between FloTrac®/Vigileo® system and BioZ® system for stroke volume variation measurement for determining of fluid administration: a preliminary study

S. Park[1], C. Kim[2], D. Jeong[2], J. Lee[3], J. Jeong[1], Y. Choe[1], S. Kim[1], S. Park[1], Y. Lee[1], H. Lee[1]

[1]Chonbuk National University Medical School, Internal Medicine, Jeonju, Korea, Republic of; [2]Chonbuk National University Hospital, Nursing, Jeonju, Korea, Republic of; [3]Chonbuk National University Medical School, Preventive Medicine, Jeonju, Korea, Republic of

**Correspondence:** S. Park

*Intensive Care Medicine Experimental* 2018, **6(Suppl 2):**0468

**INTRODUCTION.** For patients with sepsis and septic shock, we recommend the infusion of intravenous fluids (30mL/kg) within the first three hours of presentation, rather than vasopressors, inotropes, or red blood cell transfusions.

Static or dynamic predictors of fluid responsiveness should be employed in order to determine further fluid management. Guidelines state a preference for dynamic measures (eg, SVV, PPV etc) since they are more accurate than static measures (eg, CVP) at predicting fluid responsiveness.

**OBJECTIVES.** The objective of this study is to determine the agreement between stroke volume variation (SVV) measured simultaneously by the FloTrac®/Vigileo® system and BioZ® system to assess fluid status in patients with sepsis or septic shock.

**METHODS.** In this retrospective observational study, we evaluated the medical records of patients who monitored by stroke volume variance parameters measured simultaneously by the FloTrac®/Vigileo® system and BioZ® system in patients with sepsis or septic shock in the medical intensive care unit at a tertiary university-affiliated hospital, from June 2017 to January 2018. Patients with arrhythmia or extracorporeal therapy or without mechanical ventilation were excluded.

**RESULTS.** Total 14 patients with sepsis or septic shock were analyzed. The mean age of subjects was 72.4 ± 10.4 and 4 patients (28.6%) were female. Each parameters were measured average 9 times in one patient (total 125 times). The mean RASS score was -3.14 ± 0.86 and the mortality rate was 28.6% (4 patients). All patients was treated with vasoactive drugs and 4 patients had the heart failure.

There were no significant correlations between central venous pressure and SVVs measured by the FloTrac®/Vigileo® system and BioZ® system (CVP vs. SVV (the FloTrac®/Vigileo®); r = -0.063, P=0.488,

### 0469

#### Choices of ultrasonograhic fluid evaluation measurement methods and their applicability in medical ICU patients

B.S. Kalın [1], S. Eyüpoğlu[2], M. Çimen[2], G. Gürsel[3]

[1]Gazi University School of Medicine, Department of Internal Medicine Division of Critical Care Medicine, Ankara, Turkey; [2]Gazi University School of Medicine, Department of Anaesthesiology Critical Care Medicine, Ankara, Turkey; [3]Gazi University School of Medicine, Department of Pulmonary Critical Care Medicine, Ankara, Turkey

**Correspondence:** B.S. Kalın

*Intensive Care Medicine Experimental* 2018, **6(Suppl 2):**0469

**INTRODUCTION.** Deciding fluid status of ICU patients is very important and difficult issue. Recently critical care USG has been investigating for this aim as an easy, quick and noninvasive tool. Most frequently investigating parameters are cardiac output(CO), stroke volume(SV), LVOT Vmax variability, inferior vena cava variability index (IVCVI), and carotis blood flow(CBF) and carotis Vmax. However all of them have their own limitations in image acqusition and measurements.

**OBJECTIVES.** Aim of this study is to investigate applicability of CO and other parameters in the evaluation of fluid status in the medical ICU patients.

**METHODS.** All adult ICU patients regardless of fluid status included in the study and tried to measure CO, SV, LVOT Vmax, IVC variability, CBF and carotis Vmax. If heart visualized which windows were visualized(apical 4 chamber(A4C), apical 5 chamber( A5C), parasternal long axis(PLAX), parasternal short axis (PSAX)) were also recorded. If any obstacles for measurement of them were exist they recorded. Why they could not visualize or why measurements could not be performed, if patient has severe aortic or mitral regurgitation(AR, MR), atrial fibrillation, COPD(chronic obstructive pulmonary disease) or receiving mechanical ventilation. If they are obese or have abdominal distention were also recorded. Experienced intensivist were performed all measurements, color, pulse wave continue way doppler and M and 2D modes were used for the measurements.

**RESULTS.** 61 patients were included in the study their mean age was 72±13 years, APACHE II: 24±11, 41% of them were on mechanical ventilation, 53% of them had COPD, and 17 had atriall fibrillation. In 2, 18, and 20% of the patients carotis artery, IVC and heart could not be visualized respectively. Among 49 patients whose heart could be seen in 47% of them PSAX, 35% PLAX, 31% A4C, 45% A5C windows could not be visualized enough to measurements. 16 and 10 of the patients had severe MR and AR respectively. At the end of the study valuable cardiac measurements(CO(mean± SD: 5.4±2.3 l/min, SV(268 ±91 ml, LVOT Vmax:1.1±0.3 m/sec) could be performed in only 56% of the patients. Mean CBF of the patients: 547±238 ml/min, carotis Vmax:0.75±0.87 m/s and IVC VI : 40±22%)

**CONCLUSIONS.** These results suggest that availability of measurements of CO and SV may be low in some ICU populations and if there is no TEE chance trying to standardize measurement protocol for IVC and carotis parameters would be wise for the noninvasive evaluation of fluid status. Further studies are necessary to explore applicability of these methods and compare their performances.

**REFERENCE**
Miller A, Mandeville J. Predicting and measuring fluid responsiveness with echocardiography. Echo Res Pract. 2016 Jun;3(2):G1-G12. doi: 10.1530/ERP-16-0008.

**GRANT ACKNOWLEDGMENT**
Study was supported by Gazi University Scientific Reserch Projects Foundation(Project No: 01/2015-48).

## Can we improve our ICU system?

### 0470

#### Platelets count and serum calcium could be two important parameter for aritificial intelligent decisions in fluid resuscitation

Z. Qin
Ruijin Hospital School of Medicine, Shanghai Jiaotong University, Burn Center, Shanghai, China
*Intensive Care Medicine Experimental* 2018, **6(Suppl 2):**0470

**INTRODUCTION.** Fluid resuscitation in initial phase for post-burn patients is considered as a model for researching the hypo-volemic shock in critical care. Artificial intelligence decision system in critical care would be an up-and-coming and revolutionary work in near future.
**OBJECTIVES.** The present study try to explore on which indicators are needed to be parameters for AI decision-making
**METHODS.** A study involving adult patients with extremely severe burn admitted to our hospital from January 2010 to July 2015. The relationship between initial serum calcium and influence factors was assessed. The dynamic changes in platelet counts and serum calcium of first 10 days post-burn injury were analyzed.
**RESULTS.** The incidence rates of hypocalcemia of patients were 97.2% (138/142), and severe hypocalcemia accounted for 36.62% (52/142). It was found that the initial serum calcium was correlation with total burn area, admission time after injury and initial albumin (r respectively -0.195, -0.273, 0.564, p respectively 0.020,0.001,0.000). Initial pH and albumin were the independent risk factors for severe hypocalcemia of patients with extremely severe burn (with OR respectively 0.000,1.199,95% CI respectively 0.000-1.081,1.092-1.315, p respectively 0.011,0.000). The platelet count was decreased from 1st to 4th day post-burn and gradully increased between 4th and 10th day post-burn in both the survival and no survive (no survival) groups. However, in the survival group the platelet count returned to normal within 1 week after injury but not in the no survive group. The mortality was significantly higher in patients with platelet counts that did not return to normal within 1 week compared to those when the platelet count returned to normal (27.2% vs 6.1%, $P < 0.01$). Multi-variable Cox regression analysis showed that a platelet count that did not return to normal within 1 week after burn was an independent risk factor for mortality in severely burned patients (hazard ratio (HR): 2.249; 95% CI: 0.993 to 5.096), $P = 0.041$). The sensitivity and specificity were measured as 0.714 and 0.710, respectively using a ROC curve. Multi-factor logistic regression analysis suggested that the reason for the burn, the area of full-thickness burn, and the time from the injury to being admitted to hospital were independent factors correlated with early recovery of the platelet count.
**CONCLUSIONS.** Platelet count and serum calcium in the early stage post burn of patients with extremely severe burn was significantly associated with causes of injury, total burn area, admission time after injury, with inhalation injury and receiving mechanical ventilation, pH and albumin were the independent risk factors for severe hypocalcemia. They could be two important parameters for aritificial intelligent decisions in near future.

### 0471

#### Identification of patients with septic shock using natural language processing on electronic health records

J. Vermassen[1], W. Dupre[2], D. Van Hyfte[2], T. Vermassen[3], L. De Bus[1], P. Depuydt[1,4], J. Decruyenaere[1,4]
[1]Ghent University Hospital, Intensive Care Medicine, Ghent, Belgium; [2]Intersystems BV, Vilvoorde, Belgium; [3]Ghent University Hospital, Ghent, Belgium; [4]Ghent University, Ghent, Belgium
**Correspondence:** J. Vermassen
*Intensive Care Medicine Experimental* 2018, **6(Suppl 2):**0471

**INTRODUCTION.** Retrospective identification of patients with septic shock where the digital documentation is done in natural language may be time-consuming since all patient records have to be manually reviewed. This approach is potentially further complicated since there may be underreporting if the terminological description "septic shock" is not explicitly mentioned in healthcare records.
**OBJECTIVES.** This study aims to investigate whether patients who have had septic shock, can be reliably identified from clinical notes in electronic health records using NLP.
**METHODS.** Electronic health records were collected from all adult patients admitted to the intensive care unit of the Ghent University Hospital from 2010 until 2014. Four search strategies were used to detect patients who had septic shock. The first one was to use hospital clinical coding data for "septic shock" (ICD-9 785.52). Using Intersystems iKnow text analytic technology (1), we developed an explicit strategy ("septic shock" literally stated in the records), an implicit strategy (mention of the combination of "infection", "need for vasopressors" and "rise in lactate") and a combined explicit-implicit strategy. The Ghent University Hospital COSARA-database, which is prospectively capturing every septic shock in near real-time, is considered our gold standard. Pairwise comparison between search strategies were calculated and statistical significance was attained at $p < 0.05$.
**RESULTS.** Of the 9202 patients admitted during the defined period, 653 (7.1%) suffered from septic shock according to the COSARA-database. Hospital clinical coding data identified 602 (6.5%) patients. The explicit, implicit and combined strategy identified 572 (6.1), 818 (8.9%) and 1117 (12.1%) patients respectively. The implicit strategy and the combined explicit-implicit strategy proved significantly inferior and superior, respectively, to all other strategies (all $p < 0.0001$). When compared to the COSARA gold standard, the explicit strategy had the highest specificity (97.5% [95% CI: 97.2% - 97.9%]) compared to the implicit (94.1 % [95% CI: 93.6% - 94.6%]) and the combined explicit/implicit strategy (92.5% [95% CI: 91.9% - 93.1%]). The highest sensitivity was found for the combined strategy (72.9% [95% CI: 69.3% - 76.3%]) vs. the explicit (55.3% [95% CI: 51.4% - 59.1%]) and the implicit strategy (47.6% [95% CI: 43.7% - 51.5%]). The combined strategy also had the highest negative predictive value (97.8 [95% CI: 97.5% - 98.1%]) vs. the explicit (96.6% [95% CI: 96.3% - 96.9%]) and the implicit strategy (95.9% [95% CI: 95.6% - 96.2%]). Positive predictive value was low for all search strategies.
**CONCLUSIONS.** NLP is able to filter clinical notes in electronic health records and identify patients with septic shock, which may save substantial research time.

**REFERENCE(S)**
1. Bronselaer A, Debergh S, Van Hyfte D, De Tré G. Text clustering based on concept-relational decomposition. ICL 2010 proceedings 357-359

### 0472

#### Association between postoperative 30-day mortality and night and weekend postoperative admissions to the surgical intensive care unit in a setting with day-time intensivist coverage

T.K. Oh[1], I.-A. Song[2], J. Hong[1]
[1]Seoul National University Bundang Hospital, Anesthesiology and Pain Medicine, Seongnam, Korea, Republic of; [2]Seoul National University Bundang Hospital, Seongnam, Korea, Republic of
**Correspondence:** J. Hong
*Intensive Care Medicine Experimental* 2018, **6(Suppl 2):**0472

**INTRODUCTION.** The time of admission is important to consider in the care of postoperative patients in the surgical Intensive Care Unit (ICU).
**OBJECTIVES.** This study aimed to investigate the correlation between postoperative surgical ICU admission time and 30-day mortality after ICU admission.
**METHODS.** This was a retrospective observational study in a tertiary care hospital ICU, with daytime intensivist coverage. We collected the electronic medical records for all patients who were admitted to the postoperative ICU after undergoing a surgery between January 1,

2007 and December 31, 2016. The primary outcome was to examine the differences in 30-day mortality after ICU admission stratified by the ICU admission time interval: office hours (08:00-15:59), evening (16:00-23:59), and nighttime (00:00-07:59).

**RESULTS.** Over the 10-year study period, there were 16,615 postoperative ICU admissions; 6846 (41.2%), 8,186 (49.3%), and 1,583 (9.5%) were during office hours, evening, and nighttime, respectively. The 30-day mortality after ICU admission during the nighttime was higher than that after ICU admission during office hours (odds ratio: 1.77, 95% confidence interval: 1.59-1.97, P< 0.001). Compared to Sunday, 30-day mortality was significantly lower for ICU admission from Monday to Friday [Sunday versus Monday (OR: 0.72), Tuesday (OR: 0.56), Wednesday (OR: 0.58), Thursday (OR: 0.52), Friday (OR: 0.54), P< 0.001].

**CONCLUSIONS.** We found an increase in postoperative 30-day mortality when the postoperative ICU admission occurred during the nighttime as opposed to during office hours. Additionally, postoperative 30-day mortality was significantly higher when postoperative ICU admission occurred during the weekend rather than weekdays.

**GRANT ACKNOWLEDGMENT**

None

### 0473

#### Visit of minors in an intensive care unit: what do the health personnel believe?

E. Calvet Gonzalez, J. Valls Matarín, R.M. Peradejordi Torres, A. Jorge Castillo, S. Calvo Alonso, I. Sandalinas Mulero

Hospital Universitari Mútua Terrassa, ICU, Terrassa, Spain

**Correspondence:** E. Calvet Gonzalez

*Intensive Care Medicine Experimental* 2018, **6(Suppl 2):**0473

**INTRODUCTION.** The visit of minors aren´t get used planning in the organization of the Intensive Care Unit (ICU), although they belong to the family group[1,2].

**AIMS.** Determine the beliefs and compare the attitude of health staff in the presence of minors in an intensive care unit of adults.

**METHODS.** A transversal descriptive research in a polyvalent ICU with 62 professionals, between September and December 2017. Each professional was given a translated Knutsson[3] questionnaire with 10 closed questions and 2 open ones.

Descriptive statistics with absolute frequencies and percentages. Chi-squared test or Fisher´s exact test to determine the relation between profession and the different variables. Significance p < 0.05.

**RESULTS.** 61 questionnaires were collected (98.4% of the population): 18 physicians (29.5%) and 43 nurses (70.5%).

50% of physicians would restrict the entrance of 0 to 6-year old minors versus 76.2% of nurses (p=0.04).

Likewise, if the minor was between 7 to 12 years old, 16.7% of medical staff would restrict such visits versus 46.5% of nurses (p=0.02). Only 8.2% of professionals would prohibit it if the child was between 13 to 17 years old (p=0.06).

75% of health staff thought that minors´ access could entail in risk of infection for the children and 55% for the patient. 60% of survey respondents (69.1% nurses and 38.9% physicians, p=0.03) believed that the environment could intimidate the minor, and 66.7% (74.4% nurses and 47.1% physicians, p=0.04) thought that the patient´s condition could affect the child. Nevertheless, they would permit 0 to 6-year-old minors to access the ICU if the patient was dying (70.6% physicians/ 41% nurses, p= 0.04), awake and alert (57,5% nurses/ 77,8% physicians) and close relatives (parents) (60% nurses/ 66,7% physicians).

**CONCLUSIONS.** The health personnel isupports minors' visiting in a ICU of adults if they are >6 years old. Staff show a more positive attitude towards visits in special circumstances such as close relatives, awake patient and death regardless of their age. The main reasons for the restriction them are: environment, patient´s condition and risk of infection.

Nurse staff show a less positive attitude in relation to minors´ visits.

**REFERENCE(S)**

1.McAdam

JL, Dracup KA, White DB, Fontaine DK, Puntillo KA. Symptom experiences of family members of intensive care unit patients at high risk for dying. Crit Care Med.2010;38(4):1078-85.

2. Clarke CM. Children visiting family and friends on adult intensive care units:the nurses´ perspective. J Adv Nurs. 2000;31(2):330-8
3. Knutsson S, Bergbom I. Nurses´ and physicians´ viewpoints regarding children visiting/not visiting adult ICUs. Nurs Crit Care. 2007;12(2):64-73.

### 0474

#### Are machine learning techniques better suited for outcome prediction than classic statistical methods?

N. Keller[1], M. Jenny[2], C. Spies[1], F. Kork[3], F. Balzer[1]

[1]Charité - Universitätsmedizin Berlin, Berlin, Germany; [2]Max-Planck-Institut für Bildungsforschung, Harding-Zentrum für Risikokompetenz, Berlin, Germany; [3]Universitätsklinikum Aachen, Klinik für Anästhesiologie, Berlin, Germany

**Correspondence:** N. Keller

*Intensive Care Medicine Experimental* 2018, **6(Suppl 2):**0474

**INTRODUCTION.** Besides ASA as the gold standard for the assessment of patients' physical status prior to anaesthesia, morphometric scores such as the Charlson Comorbidity Score, have been proposed to evaluate postoperative risk stratification. Though techniques of machine learning have been successfully applied in similar contexts, little is known whether they perform generally better than classic statistical methods in predicting mortality in low-frequency populations.

**OBJECTIVES.** Aim of this study was to compare different classes of statistical and machine learning models for predictive accuracy regarding postoperative mortality.

**METHODS.** Secondary analysis of a retrospective study at a university hospital in Germany (clinicaltrials.gov identifier NCT01810133). The study was approved by the federal data protection officer and the hospital ethics commission (EA1/007/13). All patients with a digitalized electronic anaesthesia record between January 2006 and December 2011 who were not treated in the intensive care unit were eligible for inclusion. Emergency and ambulatory cases were excluded, and only cases with complete data were analysed. The dataset included the following information categories: ASA PS, surgical discipline, priority of surgery (elective or urgent/emergency), localization of surgery, age, gender, ICD-coded diagnoses, and in-hospital death. If the patient underwent >1 surgical procedure during his hospital stay, only the data of the first surgical intervention was included in the analysis. RStudio version 1.0.153 was used for data analysis. Model classes included in the comparative analyses were logistic regressions (LR), simple decision trees (FFT), Charlson Comorbidity Score (CSS), classification and regression trees (CART), ASA PS (ASA), and random forests (RF).

**RESULTS.** Figures 1a and 1b show the predictive accuracy of the best performing models in the holdout set compared to ASA and the CCS. The median predictive accuracy (AUC) over all models was 0.93 (range = 0.89 to 0.98, mean = 0.93). Logistic regression (AUC = 0.98) and random forests (AUC=0.96) performed best, followed by a simple FFT (AUC=0.91), the CCS (AUC=0.91) and the more complex CART (0.89) decision trees. All models outperformed anaesthesiologists' intuitive judgment of how ill a patient was (AUC = 0.80).

**CONCLUSIONS.** Gains in predictive accuracy beyond those of anaestheiologists' assessment by means of ASA can be achieved using statistical and machine learning approaches. Which model is best suited to a particular decision problem, however, is an empirical question that cannot be answered a priori. Both simpler and more complex models may outperform each other in different situations.

**GRANT ACKNOWLEDGMENT**
No external funding received.

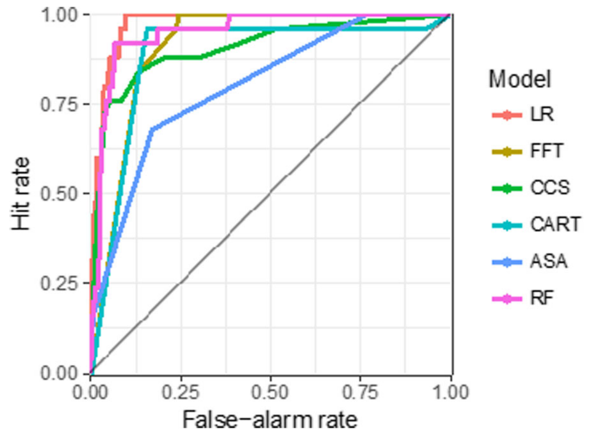

**Fig. 1 (abstract 0474).** See text for description.

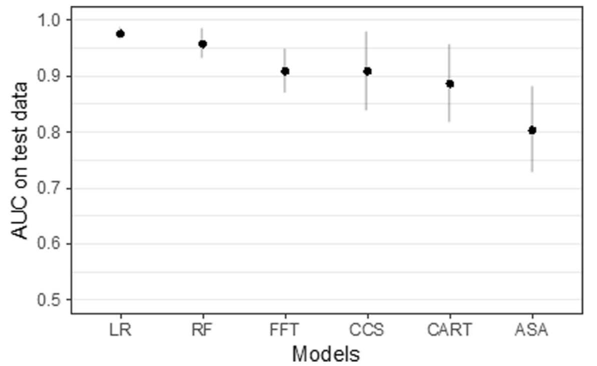

**Fig. 2 (abstract 0474).** See text for description.

### 0475

#### Can quadriceps femoris muscle thickness measured by ultrasonography predict fluid and malnutrition status in critically ill patient?

U. Özdemir[1], M. Özdemir[2], B. Kaya[1], M. Türkoğlu[1], G. Aygencel[1]
[1]Gazi University Medical Faculty Hospital, Medical Intensive Care Unit, Ankara, Turkey; [2]Baskent University, Nutrition and Dietetics, Ankara, Turkey
**Correspondence:** U. Özdemir
*Intensive Care Medicine Experimental* 2018, **6(Suppl 2):**0475

**INTRODUCTION.** Skeletal muscle loss due to immobilization, systemic inflammation, sepsis and malnutrition in critically ill patients is the major cause of morbidity and mortality(1). Quadriceps femoris muscle thickness(QFMT) measured by ultrasonography(USG) can successfully predict lean body mass(2). However, it is not known how the maximal compressed QFMT(MCQFMT) and uncompressed QFMT (UCQFMT) are affected in different fluid and malnutrition conditions in medical intensive care unit(MICU) patients.
OBJECTIVES: The purpose of this study is to measure QFMT in MICU patients by USG and to determine the malnutrition status independently from the volume status of the patients.

**METHODS.** All patients older than 18 years admitted to Gazi University Hospital MICU between August 1, 2017 and March 1, 2018 were included in this study. Within first 24 hours left and right MCQFMT, UCQFMT were measured in all patients by USG(GE, S7 model USG device and 4C-RS 2-5 MHz convex probe). Concurrently, NUTRIC2 and NRS2002 scores were recorded.
RESULTS: 56 patients were included in the study. The median age of the patients was 71.5[56.2-82.7] and 55.4% were male. 23(41%) patients died. There was a significant difference between survivors and non-survivors for thickest MCQFMT(1.03 [0.62-1.28] vs 0.70[0.5-0.96] cm, p=0.035). There was also a significant difference between thickest UCQFMT in normovolemic and hypervolemic patients (2.02[1.26-2.65] vs 2.70[1.8-3.2] cm, p=0.028) but there is no difference in MCQFMT(p=0.192). In 20 patients without malnutrition according to NUTRIC2 score, right, left, thickest MCQFMT were 1.00[0.57-1.27], 0.95[0.61-1.28], 1.09[0.63-1.38] cm respectively. In 36 malnourished patients, right, left, thickest MCQFMT were 0.62[0.46-0.97], 0.65[0.46-1.01] and 0.73[0.50-1.11] cm respectively. There were significant differences between right, left, thickest MCQFMT according to NUTRIC2 score (p values: 0.048, 0.040, 0.044 respectively) but no difference found in UCQFMT(p values: 0.225, 0.090, 0.151 respectively). We didn't compare QFMT according to NRS2002, because all patients were malnourished according to this score.
**CONCLUSIONS.** Volume status of MICU patients effects UCQFMT measurement and should not be used as a marker of malnutrition in this patient group. MCQFMT measurements, independent from volume status of MICU patients, are significantly related with nutritional status according to NUTRIC2 score. Further studies are needed to determine the use of MCQFMT measured by USG in the detection of malnutrition.

**REFERENCE(S)**
1: Griffiths RD, Hall JB. Exploring intensive care unit-acquired weakness. Preface. Crit Care Med. 2009 Oct;37(10 Suppl):S295.
2: Thomaes T, Thomis M, Onkelinx S, Coudyzer W, Cornelissen V, Vanhees L.Reliability and validity of the ultrasound technique to measure the rectusfemoris muscle diameter in older CAD-patients. BMC Med Imaging. 2012 Apr 2;12:7.

**GRANT ACKNOWLEDGMENT**
No financial support or grant was received for this study.

### 0476

#### Prospective study of optic nerve sheath diameter by ocular ultrasonography in adult Indian patients admitted to ICU

S. Dixit[1], K. Khatib[2], A. Baviskar[3]
[1]MJM Hospital, ICU, Pune, India; [2]SKN Medical College, Medicine, Pune, India; [3]Global Hospital, ICU, Mumbai, India
**Correspondence:** S. Dixit
*Intensive Care Medicine Experimental* 2018, **6(Suppl 2):**0476

**INTRODUCTION.** Optic nerve sheath diameter (ONSD) determined by ocular ultrasonography (OU) has been associated with elevated intracranial pressure (EICP). However, most studies have analyzed ONSD and its optimal cut-off point for EICP in Caucasian populations. Considering that ONSD may differ according to race and ethnicity, it is important to determine ONSD cut-off reflecting EICP in Indians.
**OBJECTIVES.** To investigate normal range of ONSD and its optimal threshold for detecting EICP in adult Indian ICU patients.
**METHODS.** Prospective, single-center study was performed in adult (age>18 years)patients with suspected EICP admitted to ICU, after institutional ethical committee approval and consent from patients/caregivers. ONSD was measured 3mm behind the globe using a 13-MHz US probe over a closed eyelid. EICP was defined as significant brain edema &/or midline shift, with/without compression of ventricle or basal cistern, effacement of sulci, and transfalcine herniation by Computed Tomography (CT) of Brain. The person performing the ONSD was unaware of the history and examination findings of the patients and the radiologist reporting the CT brain were aware of the history of the patients. The results of the ONSD are described as the median (25th-75th percentile). A receiver operator characteristic (ROC) curve was generated to determine the optimal cut-off point for identifying EICP.

**RESULTS.** A total of 188 patients were enrolled. The patients were divided into 2 groups as follows: patients with EICP, n=129 (68.5%); patients without EICP, n=59 (31.5%). ONSD in patients with EICP (5.8mm [5.5-6.2]) is significantly higher than those without EICP (5.1mm [4.8-5.4]) (P< 0.05). ONSD >5.5mm yielded a sensitivity of 98.01% (95% CI: 93.3%-100%) and a specificity of 86.16% (95% CI: 66.3%-95.8%).

**CONCLUSIONS.** The optimal cut-off point of ONSD for identifying EICP was 5.8mm. ONSD seen on OU is a feasible method for detection and serial monitoring of ICP in Indian adult patients.

**REFERENCE(S)**

1. Girisgin AS, Kalkan E, Kocak S, et al. The role of optic nerve ultrasonography in the diagnosis of elevated intracranial pressure. Emerg Med J 2007;24:251-4.
2. Moretti R, Pizzi B. Ultrasonography of the optic nerve in neurocritically ill patients. Acta Anaesthesiol Scand 2011;55:644-52.
3. Wang L, Feng L, Yao Y, et al. Optimal optic nerve sheath diameter threshold for the identification of elevated opening pressure on lumbar puncture in a Chinese population. PloS One 2015;10:e0117939.

**GRANT ACKNOWLEDGMENT**

NIL

### 0477

#### UltraNurse: teaching bedside ultrasound to ICU nurses

A. Morreale-Tulleken, T. Smits, H. Gelissen, E. Lust, T. van Galen, A.R.J. Girbes, P.R. Tuinman, P.W.G. Elbers

[1]VU University Medical Center Amsterdam, Department of Intensive Care Medicine, Research VUmc Intensive Care (REVIVE), Amsterdam Cardiovascular Sciences (ACS), Amsterdam Infection and Immunity Institute (AI&II), Amsterdam, Netherlands

**Correspondence:** A. Morreale-Tulleken

*Intensive Care Medicine Experimental* 2018, **6(Suppl 2):**0477

**INTRODUCTION.** Bedside ultrasound is now extensively used by intensivists worldwide to optimize patient management at the bedside [1]. However, monitoring is discontinuous and relies on intensivist availability. Therefore, teaching bedside ultrasound to ICU nurses holds great promise, as they typically spend far more time at the bedside.

**OBJECTIVES.** To assess the feasibility of teaching bedside ultrasound to ICU nurses.

**METHODS.** We enrolled 8 ICU nurses into our UltraNurse training program that relied on a focused 3-phase approach [2] within our 24-bed university hospital ICU. First, these UltraNurses attended two 4-hour theory and practice sessions led by an ultrasound-certified intensivist. Our UltraNurse scanning protocol consisted of anterior and posterolateral lung ultrasound (LUS) imaging as well as measuring the left ventricular outflow tract flow velocity time integral (LVOT-VTI) using pulsed wave Doppler as a proxy for stroke volume. UltraNurses then performed 5 bedside ultrasound examinations under direct supervision. Finally, they continued performing bedside ultrasound examinations until full proficiency was reached. Every examination was supervised by ultrasound-certified intensivists and graded on dexterity, image quality and accuracy of interpretation, using Likert scales. A priori, full proficiency was defined as 5 consecutive examinations performed without assistance and interpreted correctly. Using structured questionnaires, UltraNurses were interviewed frequently to identify barriers and facilitators for our program. Data are given as medians and interquartile range.

**RESULTS.** All UltraNurses reached full proficiency status. This required 13 [12-18] examinations for LUS and 13 [11-16] for LVOT-VTI. Total time required to become fully proficient was 21 [14-26] weeks for LUS and 26 [19-29] weeks for LVOT-VTI. Figures 1 and 2 depict UltraNurse progress. Table 1 shows UltraNurse characteristics. Total duration of the program was 7 months in which 123 patients underwent 230 examinations. Varying support from intensivists and perceived high workload were barriers for our program while facilitators included intensivist enthusiasm, increased work satisfaction and improved perceived valuable contribution to the treatment team.

**CONCLUSION.** This is the first report to show that teaching bedside ultrasound to ICU nurses is feasible. The number of examinations to reach full proficiency for our UltraNurse protocol was small, although this took many weeks. Teaching bedside ultrasound to ICU nurses deserves widespread consideration. It holds great promise for increasing ICU nurse work satisfaction but most importantly for further improving management of our critically ill patients. We are currently evaluating the clinical impact of our UltraNurse program.

**REFERENCE(S)**

1. Mayo et al. The ICM research agenda on critical care ultrasonography. ICM 2017; 43:1257
2. Grol et al. Improving Patient Care. 2013, Wiley-Blackwell

**Table 1 (abstract 0477).** UltraNurse Characteristics

| | |
|---|---|
| Age | 42.5 [27-48] |
| Female gender (n, %) | 5 (62.5%) |
| ICU experience (years) | 9 (3-12) |
| Prior ultrasound experience (n, %) | 0 (0%) |

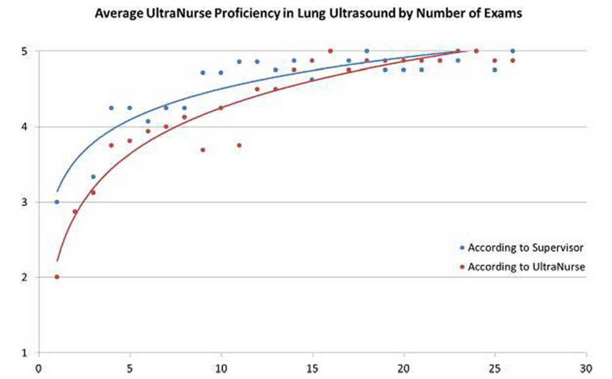

**Fig. 1 (abstract 0477).** Lung Ultrasound

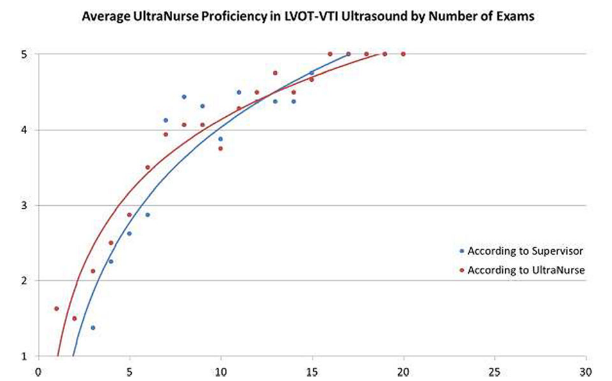

**Fig. 2 (abstract 0477).** LVOT-VTI

### 0478

#### The additional value of strain imaging to detect right ventricular dysfunction in critically ill patients: a sub-study of the Simple Intensive Care Studies-I

M.E. Vos[1], Y.M. Hummel[2], F. Keus[1], G. Koster[1], I.C.C. van der Horst[1], SICS Study Group
[1]University of Groningen, University Medical Center of Groningen, Department of Critical Care, Groningen, Netherlands; [2]University of Groningen, University Medical Center of Groningen, Groningen Imaging Core Laboratory, Groningen, Netherlands
**Correspondence:** M.E. Vos
*Intensive Care Medicine Experimental* 2018, **6(Suppl 2):**0478

**INTRODUCTION.** Right ventricular (RV) dysfunction is common in the critically ill and associated with adverse clinical outcomes. Critical care ultrasonography (CCUS) evaluates RV function with conventional measurements such as Tricuspid Annular Plane Systolic Excursion (TAPSE) and peak systolic tissue velocity at the tricuspid annulus (RV s'). Myocardial deformation imaging is a relative new technique to assess regional systolic function and is expressed in strain. Several studies suggest that strain imaging has an additional diagnostic and prognostic value compared to CCUS. In patients with heart failure and pulmonary hypertension, RV strain detected RV dysfunction when conventional CCUS measurements were preserved. However, the diagnostic value of RV strain has never been evaluated in a large unselected population of critically ill patients.

**OBJECTIVES.** The objective of this study was to investigate the association between TDI-derived RV strain and conventional CCUS measurements to evaluate RV function in an unselected population of critically ill patients.

**METHODS.** This sub-study was part of the Simple Intensive Care Studies-I (SICS-I: NCT02912624; Hiemstra et al. BMJ open 2017). This prospective cohort study included all consecutive adults who were acutely admitted with an intensive care unit (ICU) stay expected to last beyond 24 hours. Patients with atrial arrhythmias were excluded. Within 24 hours after admission transthoracic ultrasonography was preformed, recording TAPSE, RV 's and color tissue Doppler images of the RV free wall and septum in the AP4CH view. Offline strain analysis was preformed to calculate global longitudinal RV peak strain (GLPS) and RV free wall longitudinal peak strain (RVLPS). TAPSE < 17 mm, RV s' < 9,5 cm/s, GLPS > -17% and RVLPS > -20% were considered as reduced RV function.

**RESULTS.** From March 2017 until July 2017, 238 patients were included in the SICS-I of which 51 patients were included in this sub-study. Baseline characteristics are displayed in table 1 in the appendix. Moderate correlations were found between TAPSE & GLPS (r= -0,65, p=0.00) and TAPSE & RVLPS (r= -0.58, p=0.00). The same applies for RV s' & GLPS (r= -0.57, p=0.00) and RV s' & RVLPS (r= -0.56, p=0.00). GLPS and RVLPS detected RV dysfunction in 8 out of 32 patients with normal TAPSE and/or RV s'. When examining the profile of these patients, a common feature was myocardial ischemia.

**CONCLUSIONS.** This pilot study provides additional arguments for the application of strain imaging in the critically ill. RV strain seems to have an added value for detecting RV dysfunction when conventional measurements of RV function are preserved, especially in patients with underlying cardiac pathologies.

**ACKNOWLEDGEMENT**

Currently more patients are analyzed to elucidate and refine the outcomes of this study. In case of admission, enhanced results will be presented.

**Table 1 (abstract 0478).** General patient characteristics

| Variable | Value |
|---|---|
| N | 51 |
| Age, years (IQR) | 64 [53, 71] |
| Gender, male (%) | 59% |
| Admission reason(%): Medical; Cardiovascular; Acute surgery / trauma; Neurological; Sepsis | 27%; 33%; 20%; 14%; 6% |
| SAPS II score (SD) | 47 (16) |
| Myocardial infarction, yes (%) | 18% |

**Table 2 (abstract 0478).** Critical care ultrasonography - and strain variables

| Variable | Value |
|---|---|
| N | 51 |
| Cardiac output (L/min) (IQR) | 4.48 [3.41, 5.54] |
| Cardiac index (L/min/m2) (IQR) | 2.25 [1.76, 2.95] |
| TAPSE (mm) (SD) | 19.8 (5.7) |
| RV s' (cm/s) (SD) | 13.0 (4.4) |
| Strain (Ɛ) RV free wall: Basal (%) (SD); Mid (%) (SD); Apical (%) (SD) | -28.46 (9.60); -24.94 (9.98); -23.76 (10.02) |
| Strain (Ɛ) septum: Basal (%) (IQR); Mid (%) (SD); Apical (%) (IQR) | -22.14 [-25.75, -18.92]; -17.13 (6.33); -18.46 [-21.91, -12.55] |
| Global RV longitudinal peak strain (%) (SD) | -22.82 (5.99) |
| RV free wall longitudinal peak strain (%) (SD) | -25.75 (7.78) |

### 0479

#### Diagnostic performance of the BLUE-lung ultrasound protocol in critically ill patients undergoing thoracic CT: a prospective observational study

M.E. Haaksma, B. Hilderink, L. Arts, M. Winkler, J. Smit, A.R.J. Girbes, L. Heunks, P.W.G. Elbers, P.R. Tuinman
VU Medical Center, Amsterdam, Netherlands
**Correspondence:** M.E. Haaksma
*Intensive Care Medicine Experimental* 2018, **6(Suppl 2):**0479

**BACKGROUND.** The BLUE protocol was developed to match artifacts of lung ultrasound (LUS) with the final diagnosis in patients in respiratory distress presenting to the emergency department. Since then, it has found its way into routine use on the intensive care unit (ICU) to assess respiratory failure [1]. However, the diagnostic accuracy of this protocol has never been verified in an ICU setting.

**OBJECTIVE.** We set out to determine diagnostic performance of the BLUE protocol in ICU patients undergoing thoracic CT-scan.

**METHODS.** This prospective single center observational study included patients admitted to our 24-bed university hospital ICU who underwent thoracic CT scan. LUS was performed within 3 hours of the CT scan according to the BLUE protocol without venous analysis [1]. The ultrasound operator was blinded for the result of the CT scan. Additionally, an intensivist, blinded for the result of the CT scan, provided a BLUE-protocol diagnosis (pulmonary edema, pneumothorax, obstructive lung disease and pneumonia) based on the LUS

findings in combination with clinical information. The reference test was the diagnosis made by one radiologist based on the CT scan.
**RESULTS.** 61 patients, of which 41 were male, were included with a median age of 61 (IQR 25). Of all diagnoses, signs for pneumothorax yielded the highest effective accuracy with 89%. Overall diagnostic accuracy of the BLUE protocol was 48%. Intensivists diagnosis based on LUS and clinical findings yielded an accuracy of 62%. Sensitivity, specificity, PPV and NPV for the four final diagnoses are shown in Table 1.
**CONCLUSION.** In ICU patients undergoing thoracic CT, the highest accuracy of the BLUE protocol without venous analysis is 89% for pneumothorax, while overall accuracy is 48%. When LUS findings are combined with clinical findings its accuracy increases to 62%.

**REFERENCES**
[1] Lichtenstein D. Relevance of Lung Ultrasound in the Diagnosis of Acute Respiratory Failure. The BLUE Protocol. Chest. 2008.

**GRANT ACKNOWLEDGMENT**
No grants were received for this study.

**Table 1 (abstract 0479).** Diagnostic accuracy of BLUE protocol

| BLUE-diagnosis | Signs used | Accuracy (%) | Sensitivity (%) | Specificity (%) | PPV (%) | NPV (%) |
|---|---|---|---|---|---|---|
| Cardiogenic Pulmonary Edema (n=15) | Predominant bilateral B profile with lungsliding | 81 | 77 | 82 | 53 | 91 |
| Pneumothorax (n=8) | Absence of lungsliding with A profile or a lungpoint | 89 | 71 | 96 | 100 | 96 |
| Obstructive disease (n=2) | A profile with present lungsliding | 93 | 0 | 91 | - | 93 |
| Pneumonia (n=36) | Unilateral B profile or A profile with + PLAPS or bilateral B profile without lung sliding | 56 | 71 | 48 | 50 | 71 |
| Overall BLUE protocol | | 48 | | | | |
| Intensivists 'diagnosis based on LUS | | 62 | | | | |

### 0481
## Development of an intensive care advanced echo course

A. Blackstock, S. Morton, J. Powell-Tuck, J. Aron
St George's Hospital, Intensive Care Unit, London, United Kingdom
**Correspondence:** J. Aron
*Intensive Care Medicine Experimental* 2018, **6(Suppl 2):**0481

**INTRODUCTION.** The use of echocardiography in intensive care (ICU) is increasing and has been shown to have a positive impact on patient care.[1] The Focussed Intensive Care Echo (FICE) has been developed as an entry level course in ICU echo with a curriculum developed around 2D scanning.[2,3] However, the use of echocardiography as a haemodynamic monitor requires further competency development to use effectively and reliably.
**OBJECTIVES.** The aim was to develop an advanced ICU echo course that builds on the FICE curriculum to include more advanced techniques such as continuous/pulse wave Doppler, colour flow Doppler and M-mode.
**METHODS.** A one-day advanced echo course was developed and taught at St George's Hospital, London in March 2018. The course was tutorial-based with small groups rotating through stations scanning live models, simulators and reporting videos. These consisted of a step-wise increase in techniques used, starting from advanced 2D views, and progressing through 2D and M Mode measurement, the use of colour flow to assess haemodynamically significant valvular pathology and the use of spectral analysis to assess haemodynamic response to interventions. This culminated in case-based discussions compromising clinical decision making based on echocardiography findings (Figure 1).
An online questionnaire was administered to all participants before and after the course to gain an understanding of previous experience and confidence in advanced echo techniques
**RESULTS.** All participants (n=20) completed the questionnaire pre and post; 5 senior house officers, 12 registrars and 3 consultants. 75% had previously completed a FICE course; only 25% of people had performed 50 cases or more to become accredited.
Figure 2 shows the change in confidence following the course. On average, there was an improvement in confidence in every technique taught on the advanced echo course. Prior to the course participants felt it was highly likely (50%) or likely (50%) that the course would change their practice; following the course 63% felt it was highly likely, 32% likely and 5% felt it would perhaps change their practice (one participant did not answer).
**CONCLUSIONS.** A one-day advanced echo course in a tutorial based structure allows participants to develop confidence and knowledge in echo skills that are beyond the FICE curriculum. It is hoped that this can then be incorporated in participants' clinical practice. We anticipate this course can be expanded nationally to allow more clinicians to include echo assessment in their clinical practice on ICU.

**REFERENCE(S)**
1. Roscoe A, Strang T. Echocardiography in intensive care. *Continuing Education in Anaesthesia, Critical Care and Pain.* 2008:8;46-9.
2. Fletcher SN, Grounds RM. Critical care echocardiography: cleared for take up. *Br J Anaesth.* 2012:109;490-92.
3. Intensive Care Society. FICE. 2016. [Online] Available at: www.isc.ac.uk/ICS/FICE.aspx [Accessed 29th March 2018]
No grant received

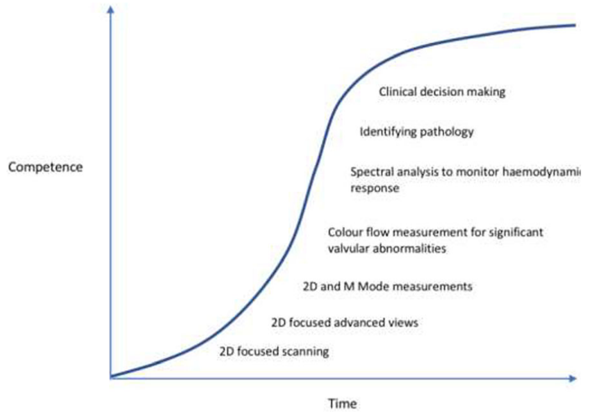

**Fig. 1 (abstract 0481).** Development of competence on ICU advanced echo course

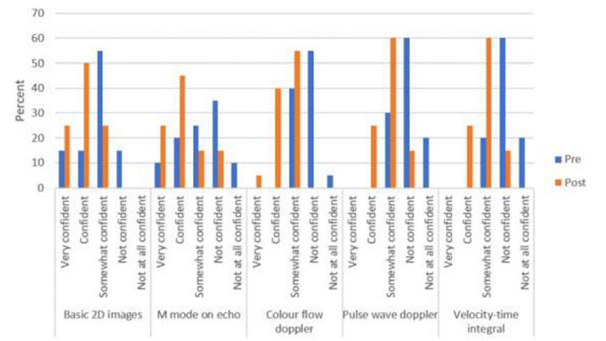

**Fig. 2 (abstract 0481).** Change in Confidence Pre- and Post-ICU advanced echo course

### 0482

#### Comparison of bedside ultrasound with Chest X-ray to detect CVC related mechanical complications: a prospective observational study (COMBUX-study)

J.M. Smit[1], M.E. Haaksma[1], E.H.T. Lim[1], T. Steenvoorden[1], M.J. Blans[2], F.H. Bosch[2], M. Petjak[3], H.R.W. Touw[1], P.R. Tuinman[1]

[1]VU University Medical Centre, Intensive Care, Amsterdam, Netherlands; [2]Rijnstate Hospital, Intensive Care, Arnhem, Netherlands; [3]Groene Hart Ziekenhuis, Intensive Care, Gouda, Netherlands

**Correspondence:** J.M. Smit

*Intensive Care Medicine Experimental* 2018, **6(Suppl 2):**0482

**INTRODUCTION.** Complications arising from central venous catheter (CVC) placement are mostly due to pneumothorax or malposition . Correct position is currently confirmed by chest X-ray (CXR), while ultrasound (US) might be a more suitable option.

**OBJECTIVES.** We investigated whether CVC malposition and iatrogenic pneumothorax could be accurately detected by US compared to CXR.

**METHODS.** Design - Single-center prospective, observational study conducted at the VU University Medical Center in the Netherlands. The study population consisted of critically ill adult patients who underwent central venous cannulation of the internal jugular vein (IJV) or subclavian vein (SV). The US evaluation of pneumothorax and CVC malposition consisted of three parts; vascular US of the IJV and SV bilaterally, lung-US to detect pneumothorax and a cardiac US examination combined with contrast enhanced ultrasound. Primary outcome was the feasibility and accuracy of bedside ultrasound in detecting CVC misplacement. Regarding pneumothorax, the overall agreement percentage and Cohen's kappa coefficient between US and CXR were secondary outcomes.

**RESULTS.** Between April 2016 and June 2017, 450 CVCs in 427 patients were included, with a median age of 68.5 (±IQR: 13.0). 70.2% of the population was male. Malposition occurred in 2.3% of all central line placements and pneumothorax in 0.9% - 2.2%. Vascular ultrasound of the IJV and SV bilaterally and lung-US were feasible in 100% of the cases, whereas cardiac US examination was feasible in 88.9% of the cases. Sensitivity of US to detect CVC malposition was 0.727 (95% CI: 0.435 - 0.924) and specificity was 0.995 (95% CI: 0.984 - 0.999). In 442 out of 450 cases (98.2%) US and CXR showed agreement for pneumothorax with a Cohen's κ-coefficient of 0.421 (95% CI: 0.255 - 0.587).

**CONCLUSIONS.** To our knowledge this is the largest study so far investigating the feasibility and accuracy of US to detect CVC malposition and associated pneumothorax. In conclusion, US is a suitable diagnostic modality to detect CVC malposition and iatrogenic pneumothorax with an excellent specificity and moderate sensitivity. Furthermore, US has multiple advantages over CXR in the critical care setting; it is performed faster and does not subject patients to radiation.

**REFERENCE(S)**

- Parienti, J.J., et al., Intravascular Complications of Central Venous Catheterization by Insertion Site. N Engl J Med, 2015. 373(13): p. 1220-9.
- Zanobetti, M., et al., Verification of correct central venous catheter placement in the emergency department: comparison between ultrasonography and chest radiography. Intern Emerg Med, 2013. 8(2): p. 173-80.
- Hourmozdi, J.J., et al., Routine Chest Radiography Is Not Necessary After Ultrasound-Guided Right Internal Jugular Vein Catheterization. Crit Care Med, 2016. 44(9): p. e804-8.

**GRANT ACKNOWLEDGMENT**

**Funding was departmental.**

### 0483

#### Comparison between percutaneous drainage and open surgery for acute pancreatitis in intensive care medicine. Which is the best option?

N. Mejuto Montero[1], A. Nieto García[1], M. Mourelo Fariña[2], M. Gestal Vázquez[2], X. Taboada Fraga[2], A. Novo[2], D. Freire Moar[2]

[1]Complexo Hospitalario Universitario de A Coruña, Intensive Care Unit, A Coruña, Spain; [2]Complexo Hospitalario Universitario de A Coruña, A Coruña, Spain

**Correspondence:** N. Mejuto Montero

*Intensive Care Medicine Experimental* 2018, **6(Suppl 2):**0483

**INTRODUCTION.** The techniques of image-guided percutaneous abscess drainage (IGPAD) are relatively simple and minimally invasive option of treatment for AP (acute pancreatitis). IGPAD has a significant impact on patient care, and can affect hospital length of stay, the development of sepsis, organ failure and reducing the cost of treatment.

**OBJECTIVES.** To compare approach by percutaneous drainage or open surgery in a sample of patients with acute pancreatitis admitted to Intensive are Units (ICU).

**METHODS.** Observational and prospective study. Period study: January 1, 2016 to December 31, 2016. Inclusion criteria: diagnosis of AP and admission in ICU. Variables: age, gender, APACHEII and SOFA in the first 24 h, organ failure, need for percutaneous drainage or surgery, ICU length of stay (LOS), hospital LOS and hospital mortality. A p-value of 0.05 was considered Statistically significant.

**RESULTS.** 126 patients were admitted. 67,7% of the patient were male and mean of age was 59,9+15,6 years. The most common aetiologies were gallstones (48%) and alcohol (12,6%). SOFA in admision was 9,3+4,0. Mortality 34,9%. In the case that drainage percutaneous of the collections were made, the patients were: younger (51,7±14,4 vs 61,7±15,4; p=0,02) and female (80,5% vs 18,3; p=0,8). Severity scores in the first 24 hours were not significant (APACHE II 12,2 vs 14,1; p=0,3 and SOFA 9,3 vs 9,2; p=0,8). Patients who had IGPAD showed a statistically lower rate of hemodinac failure 25% vs 75% (p=0,03), renal failure 7,1% vs 92,9% (p=0,05), sepsis 31% vs 69% (p< 0,05), mechanical ventilation 22,5% vs 77,5% (p=0,08), abdominal compartment syndrome was significantly less frequent (41,7% vs 58,3%; p=0,01), unlike the rest of complications collected, and higher rate of lengh of stay in ICU (30,1±28,2 vs 12,8 ±23,2 (p< 0,05). No significant differences were found in terms of mortality depending on whether percutaneous drainage was performed or not (11,9% vs 88,1%; p=0,2)

**CONCLUSIONS.** In our population patients undergoing this treatment had better results in terms of organ failure (hemodynamic, renal), less lengh of mechanical ventilataion, less rate of abdominal compartment syndrome and intracavity lavage. IGPAD is a safe and effective procedure for the treatment of abdominal abscess and fluid collections in patients admitted to ICU.

early and proactively in the course of necrotizing pancreatitis. J Vas Interv Radiol 2016; 27: 418-25

**GRANT ACKNOWLEDGMENT**

This study received no financial support.

#### 0484

#### Chest sonography in intensive care unit (ICU): comparison of computerized scanning findings with sonographing findings performed by intensivist and radiologist in patients with pleural effusion

B. Maghsoudi[1], M.R. Sasani[2], F. Zand[2,3], M. Masjedi[3], G. Sabetian[3], Central and General ICU patients of Namazi Hospital ,Shiraz Iran

[1]Shiraz University of Medical Sciences, Shiraz, Iran, Islamic Republic of; [2]Shiraz University of Medical Sciences, Radiology, Shiraz, Iran, Islamic Republic of; [3]Shiraz University of Medical Sciences, Anesthesia and Intensive Care Resarch Center, Anesthesia and Intensive Care Department, Shiraz, Iran, Islamic Republic of

**Correspondence:** B. Maghsoudi

*Intensive Care Medicine Experimental* 2018, **6(Suppl 2):**0484

**INTRODUCTION.** Pleural effusion is a common finding in patients admitted in ICU. It could be due to a pulmonary problem (primary) or accompanying other medico-surgical conditions (secondary).

**OBJECTIVES.** Assessing accurately the amount of pleural effusion may be helpful in weaning from mechanical ventilation by draining problematic pleural effusions.

**METHODS.** During one year period (September 1st 2016 to August 30 2017) patients admitted to a Referral-University hospital general ICU, were considered for inclusion. Enrolling criteria were mechanical ventilation ,chest X ray indicating possible pleural effusion , no primary pulmonary involvement , no progressive organ involvement and planned respiratory weaning.

Chest sonography was performed by a radiology senior(last year) resident and an ICU fellowship and the results compared with CT scan findings.

**RESULTS.** 67 patients were eligible. Pleural effusion rated as nil-minimal-moderate; severe without a strict milliliter definition.Accuracy of the results with the same day CT scan were 54% in radiologist as compared with 74% in intensivists assessment, differing significantly.

**CONCLUSIONS.** Chest sonography for evaluation of pleural effusion is more accurate when performed by intensive care fellow as compared with a senior radiology senior.

**REFERENCE(S)**

1. Expert Agreement in the Interpretation of Lung Ultrasound Studies Performed on Mechanically Ventilated Patients. Millington SJ, Arntfield RT, Guo RJ, Koenig S, Kory P, Noble V, Mallemat H, Schoenherr JR. J Ultrasound Med. 2018 Apr 15. doi: 10.1002/jum.14627. [Epub ahead of print].
2. The diagnosis of **pleural** effusions.Porcel JM et al. Expert Rev Respir Med. (2015)

**GRANT ACKNOWLEDGMENT**

Shiraz university of medical sciences , intensive care division of anesthesia department,and radiol ogy department.

#### 0485

#### Monobacterial versus polibacterial multidrug-resistant A.baumannii ventilator-associated pneumonia: prognostic factors of mortality

A. Ciginskiene[1], D. Adukauskiene[2], A. Dambrauskiene[3]

[1]Lithuanian University of Health Science, Medicine academy, Kaunas, Lithuania; [2]Lithuanian University of Health Science, Intensive Care Clinic, Kaunas, Lithuania; [3]Lithuanian University of Health Science, Infection Control Service, Kaunas, Lithuania

**Correspondence:** A. Ciginskiene

*Intensive Care Medicine Experimental* 2018, **6(Suppl 2):**0485

**INTRODUCTION.** Clinical studies of VAP (e.g. Acinetobacter strain) use to be carried out despite of mono- or polibacterial origin [1]. It is not suppose to be constructed methodologically correctly if the aim of clinical issues is addressed to just one strain of polibacterial VAP.

**OBJECTIVES.** To compare mortality prognostic factors of VAP due to multidrug-resistant A.baumannii (MDRAB) in case of mono- versus (vs) polibacterial origin.

**METHODS.** Ongoing retrospective cohort study of patients (pts) treated in ICUs with MDRAB strains as pathogens of VAP during 2014-2016.

**RESULTS.** Data of 164 pts were evaluated. Mono- vs polibacterial MDRAB VAP cases were found to be 77 (47 %) vs 87 (53%). In-hospital mortality of VAP due to MDRAB was 63,6% in mono- and 50,6% in polibacterial origin (p=0.115). Univariate analysis of survivors vs nonsurvivors in monobacterial MDRAB VAP: differences were found in medians of length of stay (LOS) after onset of VAP 26 days (d) (interquartile range (IQR) 19-61.5) vs 7 d (IQR 3-17), p< 0.001, SOFA score at the onset of VAP 5 (IQR 4-7) vs 8 (IQR 6-11), p=0.015, length of definitive antibacterial treatment 14 d (IQR 10-18) vs 7 d (IQR 4-13), p< 0.001, in means of oxygenation index 171.67 (SD 47.33) vs 145.56 (SD 60.65), p=0.042, SAPS II 41.27 (SD 12.24) vs 50.84 (SD 16.66), p=0.021 at ICU admission, and between proportions of previous hospitalization (≥2 d) in the preceding 90 d 11/46 (23.9%) vs 35/46 (76.1%), p=0.008, shock 17/58 (29.3%) vs 41/58 (70.7%), p=0.031, multiple organ dysfunction syndrome (MODS) 18/61 (29.5%) vs 43/61 (70.5%), p=0.019. Polibacterial MDRAB VAP: differences were found in medians of LOS after VAP diagnosis 29 d (IQR 14-39) vs 10.5 d (IQR 3.25-25.5), p< 0.001, SOFA score 5 (IQR 2-8) vs 8 (IQR 4-10), p=0.005 at the onset of VAP, length of definitive antibacterial treatment 14 d (IQR 9-17) vs 11.5 d (IQR 5-16), p=0.009, and between proportions of sepsis 36/79 (45.6%) vs 43/79 (54.4%), p=0.03, shock 20/57 (35.1%) vs 35/47 (74.5%), p< 0.001, MODS 20/57 (35.1%) vs 37/57 (64.9%), p=0.019, renal replacement therapy 6/23 (26.1%) vs 17/23 (73.9%), p=0.014.

In multivariate analysis odds ratio (OR) for in-hospital mortality: 9.43 for previous hospitalization (≥2 d) in the preceding 90 d (95% CI 1,68-57.94) for MDRAB VAP of monobacterial origin, but 7.45 for shock (95% CI 2.5-22.14) for polibacterial one.

**CONCLUSIONS.** Differences found in this study for prognostic factors of mortality due to monobacterial vs polibacterial MDR A.baumannii VAP discourage to use polibacterial VAP studies of MDR A.baumannii strains in case of correlation of this only pathogen with clinical issues as methodologically correct ones. Further studies are required to confirm our results.

**REFERENCE(S)**

1. Inchai J, et al. Prognostic factors associated with mortality of drug-resistant *Acinetobacter baumannii* ventilator-associated pneumonia. J Inten Care. 2015;3:9. doi: 10.1186/ s40560-015-0077-4.

### Pearls in Neurocritical Care

#### 0486

#### Using of dexmedetomidine in term neonates with hypoxic-ischemic encephalopathy

D. Surkov

Regional Children's Hospital, NICU, Dnipro, Ukraine

*Intensive Care Medicine Experimental* 2018, **6(Suppl 2):**0486

**INTRODUCTION.** Hypoxic - ischemic encephalopathy (HIE) remains the one of most frequent causes of cerebral palsy and other severe neurological disorders in infants. There is compelling clinical evidence that therapeutic hypothermia in term babies with moderate to severe HIE improves neurodevelopmental outcome. Despite this, the challenge for the future is to find ways of improving the effectiveness of treatment [1, 4, 5].

**OBJECTIVES.** To evaluate possible neuroprotective effect of dexmedetomidine (DEX) in term neonates with moderate to severe HIE.

**METHODS.** Prospective single-center randomized clinical study in 52 term neonates with HIE Sarnat stage II-III was performed in NICU Level III in 2014-2016. All intensive care included therapeutic hypothermia, respiratory and hemodynamics support, antibiotics, TPN etc. as usual. Babies in control group received morphine sedation 10-20 mcg/kg/hour vs. dexmedetomidine 0.1-0.5 mcg/kg/hour in study group [3, 4]. End-points included Glasgow Coma Scale (GCS), aEEG, transfontanel Doppler, NIRS and serum neuronal biomarkers (neuron-specific enolase, protein S-100).

**RESULTS.** 52 term neonates were randomized in DEX (n=34, 65.4%) and standard care group (n=18, 34.6%). The average gestational age was 39.6 (37-42) weeks vs. 38.9 (37-41) weeks, the birth weight was 3533.8±859.2 vs. 3390.0± 510.4 grams respectively. There was no difference in initial Apgar score (4.3 [1-7] vs. 4.2 [1-7]). No significant differences between two groups in Doppler cerebral blood flow indexes, serum lactate level, NSE and S-100 protein as well as aEEG pattern were found. A significant difference between two groups was found in dobutamine dose (Estimating Variance, EV -1.87, 95% CI -3.25 to -0.48, p=0.009), mean blood pressure (EV 2.9, 95% CI -0.27 to 6.08, p=0.072). Also there was difference in GCS (EV 1.36, 95% CI -0.36 to 3.09) but not significant (p=0.118).

**CONCLUSIONS.** Dexmedetomidine is a safe sedative agent with stable hemodynamics profile, no adverse cerebral influence and possible neuroprotective effect in HIE, additional to standard therapeutic hypothermia.

**REFERENCE(S)**

1. Douglas-Escobar M, Weiss MD. Hypoxic-ischemic encephalopathy: a review for the clinician. JAMA Pediatr. 2015; 169: 397-403.
2. Estkowski LM, Morris JL, Sinclair EA. Characterization of Dexmedetomidine Dosing and Safety in Neonates and Infants. J Pediatr Pharmacol Ther. 2015; 20(2): 112-118.
3. O´Mara K, Gal P, Wimmer J et al. Dexmedetomidine Versus Standard Therapy with Fentanyl for Sedation in Mechanically Ventilated Premature Neonates. J Pediatr Pharmacol Ther. 2012; 17(3): 252-262.
4. Wassink G, Gunn ER, Drury PP et al. The mechanisms and treatment of asphyxial encephalopathy. Front Neurosci. 2014; 8: 40-51.
5. Zanelli SA, Stanley DP. Hypoxic-ischemic encephalopathy. 2018. https://emedicine.medscape.com/article/973501-overview#a8

**GRANT ACKNOWLEDGMENT**

NSE and protein S-100 lab evaluation was granted by Orion Corporation.

### 0487

#### The use of automated pupillometry to assess cerebral autoregulation

A.A. Quispe-Cornejo, I.A. Crippa, L. Peluso, L. Calabrò, J.-L. Vincent, J. Creteur, F.S. Taccone

Université Libre de Bruxelles, Department of Intensive Care, Erasme Hospital, Brussels, Belgium

**Correspondence:** A.A. Quispe-Cornejo

*Intensive Care Medicine Experimental* 2018, **6(Suppl 2):**0487

**INTRODUCTION.** Automated pupillometry (AP) can be used to quantify pupil light reflex (PLR) in critically ill patients. However, the complexity of sympathetic and parasympathetic pathways involved in the PLR may expand the use of AP to quantify other phenomena related to nervous system, such as the regulation of vascular tone and regional blood flow.

**OBJECTIVES.** To assess the association between AP and autoregulation of cerebral blood flow (CAR) in non-neurological critically ill patients.

**METHODS.** Observational ongoing study including critically ill patients admitted to the Intensive Care Unit. Exclusion criteria were: ocular diseases; intracranial disease; arrhythmias; extracorporeal membrane oxygenation; supra-aortic arteriopathy. Quantitative pupillometry was performed using the NPi®-200 pupillometer (Neuroptics®), which calculates the Neurological Pupil Index (NPI), pupillary contraction, latency, constriction velocity and dilation velocity. The mean value of these variables measured on each eye was calculated. Transcranial Doppler (DWL, Germany) was performed insonating the left middle cerebral artery (LMCA) with a 2MHz probe. LMCA blood flow velocity (FV) and arterial blood pressure (BP) signals were simultaneously recorded; Pearson´s correlation coefficient between BP and FV (Mxa) was calculated using MATLAB (MathWorks, USA). Impaired CAR was defined as Mxa>0.3.

**RESULTS.** We studied 35 patients (median age 61 [52-69] years and median APACHE II score on admission 16 [9-25]), including 20 (57%) with sepsis. Median NPI was 4.5 (4.1-4.8) and median Mxa 0.28 (0.13-0.50); 14 (40%) patients had altered CAR. We observed a significant correlation between Mxa and NPI (r=-0.51; p=0.001) but not with other variables measured by the AP. NPI was significantly lower in patients with altered CAR than others (4.0 (3.6-4.6] vs. 4.6 [4.4-4.8]; p=0.03). NPI values had an AUC of 0.72 [0.53-0.90] to predict altered CAR. A NPI < 4.0 had a sensitivity of 44% and a specificity of 95% to predict altered CAR.

**CONCLUSIONS.** The Neurological Pupil Index measured with automated pupillometry allows to assess cerebral autoregulation in critically ill patients.

### 0488

#### N-terminal pro-brain natriuretic peptide as a bio-marker of the acute brain injury

L. Tsentsiper, A. Kondratyev, N. Dryagina

Russian Polenov Neurosurgery Institute, Aneasthesiology and Intensive Care, St Petersburg, Russian Federation

**Correspondence:** L. Tsentsiper

*Intensive Care Medicine Experimental* 2018, **6(Suppl 2):**0488

**INTRODUCTION.** Recently along with neuro-visual and neurophysiological methods of study of brain injuries a lot of attention is drawn to the laboratory diagnostics methods. The detection of biomarkers levels facilitates an early diagnosis of brain tissues damage, allows to assess the prognosis of the disease and its outcome.

**OBJECTIVES.** Evaluate the significance of changes in the level of N-terminal Pro-Brain natriuretic peptide in patients with acute brain injury

**METHODS.** We studied 64 patients between 2008 and 2013, 36 male and 28 female patients. 1st group comprised 12 patients with severe brain trauma: 1a - survivors with good outcome (on Glasgow scale groups I-II) (n=8), 1b - dead or severely disabled (on Glasgow scale groups III-V) (n=4). 2nd group comprises 37 patients with intracranial and sub-arachnoid hemorrhages: Assignment to groups 2a (n=14), 2b (n=22) was done using the same criteria as group 1. 3rd group comprises 16 patients operated in conjunction with brain tumor. Assignment to groups 3a (n=6) and 3b (n=10) was done using the same criteria as groups 1 and 2. We tested the level of N-terminal Pro-Brain natriuretic peptide in blood we use immunochemiluminescent analyzer Immulite (normal range - 0-125 pg/ml). Testing was conducted between 1st and 3rd days after severe head injury, brain blood flow disruption of hemorrhagic type or brain tumor related surgery, and then every 2-12 days for the total duration of 21 days. For assessing the significance of differences we utilized Student's t-test.

**RESULTS.** Statistical analysis failed to demonstrate noticeable difference in the level of NTproBNP between groups 1,2,3. We detected the differences between subgroups (p< 0.01). Patients from groups 1a,2a,3a (n=28) NTproBNP level stayed below 700 pg/ml in 20 cases (71%), in the 8 cases (29%) the level was above 700 pg/ml, but by 14-21th day decreased to the normal values. For patients in subgroups 1b,2b,3b (n=36) in 28 cases (78%) NTproBNP level was above 700 pg/ml at least once, in 8 cases (12%) level stayed below 700 pg/ml but remain high for the entire duration of the study without significant decrease.

**CONCLUSIONS.**

1. All the patients with acute brain injury showed the increased level of NTproBNP above normal values, irrespective of ethiology of injury
2. In case when NTproBNP level increases above 700 pg/ml and/or does not decrease to the normal values it is possible to predict a negative outcome of the treatment.

### 0489

#### Multicenter retrospective study of early in-ICU anesthetic drugs' management in convulsive status epilepticus

S. Zeidan[1], B. Rohaut[1], H. Outin[2], F. Bolgert[1], V. Navarro[3], S. Demeret[1]
[1]Pitié Salpêtrière Hospital, Neuro ICU, Paris, France; [2]Poissy Hospital, Medical ICU, Poissy, France; [3]Pitié Salpêtrière Hospital, Department of Clinical Neurophysiology and Epileptology, Paris, France
**Correspondence:** S. Zeidan
*Intensive Care Medicine Experimental* 2018, **6(Suppl 2):**0489

**INTRODUCTION.** Patients with convulsive status epilepticus (CSE) often require mechanical ventilation (MV), either for general anesthesia (GA) in case of refractory CSE, or for airway protection. Guidelines for the management of refractory generalized CSE recommend to maintain GA during 24-48h, followed by a gradual withdrawal. Our objective was to evaluate the incidence of refractory generalized CSE among patients who required MV during pre-hospital management of SE, and to describe the management of GA in the ICU.

**PATIENTS AND METHODS.** This multicenter retrospective observational study was conducted in 4 French ICUs. All mechanically ventilated patients diagnosed with status epilepticus (SE) admitted in the ICU between 01/01/2014 and 12/31/2016 were included. Exclusion criteria were: age < 18 years, post anoxic SE, acute traumatic brain injury, initiation of MV in the ICU, transfer from another ICU, inclusion in a therapeutic trial on SE, non-convulsive SE. Collected data included type, duration and pre hospital treatment of SE, reason for MV, dosage and duration of GA drugs, EEG monitoring, relapse of SE, MV duration, in-ICU length of stay, complications (i.e. sepsis, acquired ventilation pneumonia, etc.) and mortality.

**RESULT.** Among the 251 medical files reviewed, 101 met the inclusion criteria and were analyzed. Most patients (n=74, 73,3%) had a non-refractory generalized CSE; only 14 (13,9%) had a refractory generalized CSE. The main reason for intubation was coma (n=59, 58,4%). Among patients intubated for persisting convulsions (n=28), two thirds (n=17) had received first and second line antiepileptic drugs at correct dose and 14 had actual refractory SE (2 had psychogenic seizures, 1 refractory partial CSE); the others (n=11) were intubated either directly after the first line, or before the end of the second line infusion. The median duration of GA before weaning was 12h (IQR [5-18]) and was similar in refractory CSE patients (p=0,79). Only 7 patients (6,9%) had a relapse of SE in ICU; they had similar refractory SE occurrence (n=2(12,8%), p=0,91) and GA duration (p=0,39), and none had history of epilepsy. GA duration >12h before weaning was associated with a higher rate of in-ICU complications (n=37 (74%) vs. n=27 (54%), p= 0,04), without decreasing the rate of SE relapse (n=4 vs n=3, p=0,72).

**CONCLUSIONS.** These data show that most patients admitted in ICU while receiving MV for CSE do not have a refractory status, but mainly coma without persisting convulsions. The mean duration of GA before weaning was < 24h, and thus in discrepancy with guidelines, but did not seem associated with a frequent relapse of SE. Patients with a duration of GA ≤12h had less complications in ICU, without increasing the rate of SE relapse. If this low rate of refractory SE and the safety of rapid GA withdrawal are confirmed, the recommended 24-48h duration of GA in ICU may be challenged.

### 0490

#### Validation of a new clinical tool to help diagnose Minimally Conscious State patients: the Disorders of Consciousness [DoC] - feeling scale

B. Hermann[1,2,3], G. Goudard[1], K. Courcoux[1], M. Valente[2,4], S. Labat[1], L. Despoix[1], J. Bourmaleau[1], L. Richard-Gilis[1,2], F. Faugeras[2], S. Demeret[1], J. Sitt[2], L. Naccache[2,3,4], B. Rohaut[1,2,5]
[1]Pitié-Salpêtrière Hospital, Neurointensive Care Unit, Neurology Department, Paris, France; [2]Brain and Spine Institute, Inserm U1127, Paris, France; [3]Sorbonne Université, Paris, France; [4]Pitié-Salpêtrière Hospital, Neurophysiology Department, Paris, France; [5]Columbia University Medical Center, Neurology Department, New York, United States
**Correspondence:** B. Hermann
*Intensive Care Medicine Experimental* 2018, **6(Suppl 2):**0490

**INTRODUCTION.** Accurate diagnosis of consciousness level (CL) is a prerequisite to elaborate a prognosis and appropriate medical plan for patients suffering from a disorder of consciousness (DoC) after an acute brain injury (ABI). The clinical distinction between unresponsive wakefulness syndrome (UWS) and minimally conscious state (MCS) is a key step with a huge impact on decision making. However, this assessment is often challenging and may require specialized expertise. We hypothesized that pooling subjective reports of CL of a given patient across independent nursing staff members can help improving clinical diagnosis of MCS.

**OBJECTIVES.** To evaluate the diagnostic accuracy of a new clinical scale (DoC-Feeling) that capitalizes on the nursing staff subjective perception of patient's CL.

**METHODS.** All brain injured patients referred to our neurointensive care unit for consciousness assessment were prospectively screened between February 2016 and October 2017. Consents were obtained from patients' relative. UWS and MCS were defined according to the reference standard Coma Recovery Scale - Revised (CRS-R[1]). Nurses and nursing assistants were asked to rate their subjective perception of the best CL observed during their shift on a 100 mm visual analog scale. The index test DoC-feeling consisted in the median value of these multiple ratings obtained from the staff members in charge of the patient during the hospitalisation. Caregivers were blinded from other ratings as well as from expert evaluation. We evaluated the association between individual ratings and CS using a linear mixed model and the diagnostic accuracy of DoC-feeling using area under the receiver operating characteristic curve (AUC), sensitivity and specificity metrics.

**RESULTS.** 692 ratings performed by 83 nursing staff members were collected from 47 patients. 20 were in a UWS and 27 in a MCS. Main ABI etiologies were anoxic (53%) and traumatic brain injury (17%), with a median delay of 134 [IQR: 40-762] days. 20 patients (43%) were mechanically ventilated. Individual ratings were reliably associated with CL (t-value=6.47, df=45, $p=10^{-7}$). DoC-feeling scores were significantly greater in MCS than in UWS patients (59 mm [27-77] vs. 7 mm [2-11]; $p=10^{-7}$). Diagnostic accuracy of DoC-feeling evaluation revealed an AUC of 0.92 (95%CI: 0.84-0.99), yielding a sensitivity of 89% (71-98) and a specificity of 85% (62-97) for MCS diagnosis, using a 16.7mm cut-off value.

**CONCLUSIONS.** We propose a new clinical tool called DoC-feeling that capitalizes on the expertise of nursing staff to evaluate patients' consciousness. Together with the CRS-R as well as with brain imaging, DoC-feeling might improve diagnostic and prognostic accuracy of DoC patients.

**REFERENCE(S)**

1. Giacino et al. Neurology, 2002

**GRANT ACKNOWLEDGMENT**

We thank the members of the Neuro-ICU and the PICNIC-lab.
This work was supported by Inserm; AAIHP-SCCAHP, AP-HP and Philippe Foundation; Sorbonne Université, JSMF and Académie des Sciences.

### 0491

#### Is impairment similar between upper and lower limbs measured using ultrasonography in critically ill traumatic brain injury patients

V. Maldaner[1], P. Melo[2], J.A. Neto[3], S.M. Parry[4], J.L. Durigan[2], G. Cipriano[2], L. Vieira[1]
[1]IHB, Brasília, Brazil; [2]Universidade de Brasilia, Brasília, Brazil; [3]Hospital Santa Luzia, Brasília, Brazil; [4]University of Melbourne, Melbourne, Australia
**Correspondence:** V. Maldaner
*Intensive Care Medicine Experimental* 2018, **6(Suppl 2):**0491

**PURPOSE.** To document patterns of muscle changes in upper and lower limbs using ultrasound imaging and to determine if there is a difference between upper and lower limb musculature over the first 7 days of ICU admission in individuals with an acute traumatic brain injury.

**METHODS.** Adults with traumatic brain injury mechanically ventilated >48 hours were prospectively assessed. Patients underwent muscle ultrasound (US) of biceps Brachii (BB), tibialis anterior (TA) and rectus femoris (RF) to obtain measurements of muscle thickness and echointensity during the first 7 days of hospitalization. The change in muscle thickness (ΔTh) and echogenicity (ΔEcho) for all the three muscles (TA, RF and BB) between day 1 and day 7 was analyzed.

**RESULTS.** 25 patients were enrolled (age 35 ± 14 years; APACHE II median [IQR] 17 [14-22]). The following statistically significant changes were noted over time from baseline to day 7: the mean thickness reduced for all muscles: RF 22,35%; TA 19,70%; and BI 15,01%. The echointensity analysis increased for all muscles: RF 28.90%; TA 20.75% and BI 12.90% (suggesting deterioration in muscle quality). The changes to muscle thickness and echointensity were less affected in the upper limbs ($p < 0.001$).

**CONCLUSION.** Preferential reduction in muscle thickness and deterioration in muscle quality (echogenicity) was observed in the lower limb muscle groups over the first xxx days of ICU admission.

Raj R, Bendel S, Reinikainen M, Hoppu S, Luoto T, Ala-Kokko T, et al. Traumatic brain injury patient volume and mortality in neurosurgical intensive care units: a Finnish nationwide study. Scand J Trauma Resusc Emerg Med 2016;24(1):133.

[2] Ghroubi S, Alila S, Feki I, Elleuch MH. Quality of life after traumatic brain injury. Ann Phys Rehabil Med 2016;59S:e135.

[3] Azouvi P, Ghout I, Bayen E, Darnoux E, Azerad S, Ruet A, et al. Disability and health-related quality-of-life 4 years after a severe traumatic brain injury: A structural equation modelling analysis. Brain Inj 2016:1-7.

[4] Salottolo K, Carrick M, Stewart Levy A, Morgan BC, Slone DS, Bar-Or D. The epidemiology, prognosis, and trends of severe traumatic brain injury with presenting Glasgow Coma Scale of 3. J Crit Care 2016;38:197-201.

[5] de Almeida CE, de Sousa Filho JL, Dourado JC, Gontijo PA, Dellaretti MA, Costa BS. Traumatic Brain Injury Epidemiology in Brazil. World Neurosurg 2016;87:540-7.

[6] Stocchetti N, Taccone FS, Citerio G, Pepe PE, Le Roux PD, Oddo M, et al. Neuroprotection in acute brain injury: an up-to-date review. Crit Care 2015;19:186.

[7] Dirks ML, Hansen D, Van Assche A, Dendale P, Van Loon LJ. Neuromuscular electrical stimulation prevents muscle wasting in critically ill comatose patients. Clin Sci (Lond) 2015;128(6):357-65.

[8] Mourtzakis M, Parry S, Connolly B, Puthucheary Z. Skeletal Muscle Ultrasound in Critical Care: A Tool in Need of Translation. Ann Am Thorac Soc 2017.

### 0492

#### A comparison of mortality with hypertonic saline versus mannitol in severe traumatic brain injury - The TBI Collaborative

J. Anstey[1], F. Taccone[2], A. Udy[3], G. Citerio[4], J. Duranteau[5], C. Ichai[6], R. Badenes[7], J. Prowle[8], A. Ercole[9], M. Oddo[10], A. Schneider[10], M. van der Jagt[11], S. Wolfe[12], R. Helbok[13], D. Nelson[14], M. Skrifvars[15], J. Presneill[16], J. Cooper[17], M. Bailey[18], R. Bellomo[16,19]
[1]Royal Melbourne Hospital, Intensive Care Unit, Melbourne, Australia; [2]Erasme Hospital, ICU, Brussels, Belgium; [3]Alfred Hospital, Intensive Care Unit, Melbourne, Australia; [4]San Gerardo Hospital, Monza, Italy; [5]Bicetre Hospital, Paris, France; [6]CHU Nice, Nice, France; [7]Hospital Clinico Universitario, Valencia, Spain; [8]Royal London Hospital, London, United Kingdom; [9]University of Cambridge, Cambridge, United Kingdom; [10]Lausanne University Hospital, Lausanne, Switzerland; [11]Erasmus University Rotterdam, Rotterdam, Netherlands; [12]Charité - Universitätsmedizin Berlin, Berlin, Germany; [13]Innsbruck Medical University, Innsbruck, Austria; [14]Karolinska Institute, Solna, Sweden,; [15]Helsinki University Hospital, Helsinki, Finland; [16]Royal Melbourne Hospital, Melbourne, Australia; [17]Alfred Hospital, Melbourne, Australia; [18]University of Melbourne, Melbourne, Australia; [19]Austin Hospital, Melbourne, Australia
**Correspondence:** J. Anstey
*Intensive Care Medicine Experimental* 2018, **6(Suppl 2):**0492

**INTRODUCTION.** Hypertonic saline and mannitol are both widely used as osmotherapy in traumatic brain injury (TBI) patients in the management of intracranial hypertension. However, clinical outcome data comparing the two treatments are still lacking.

**OBJECTIVES.** We compared the association of mannitol and hypertonic saline with mortality in an international cohort of severe TBI patients.

**METHODS.** We performed a retrospective study involving 14 tertiary Intensive Care Units (ICUs) in Australia and Europe, that contributed data from the first 20 severe TBI patients in 2015 (GCS < = 8 post resuscitation) with an intracranial pressure (ICP) monitor in-situ for at least 72 hours. Baseline demographic details, illness severity (APACHE-II, Injury Severity Scores, and IMPACT-TBI Scores), ICP management strategies for up to 7 days of ICP monitoring, and outcome data were collected for each patient.

Those patients who received osmotherapy with mannitol only or hypertonic saline only during the first 96 hours of their ICU stay were compared with regards to mortality.

Hazard ratios (95% CI) comparing time to death between the two groups were derived using raw and multivariable Cox proportional hazards regression adjusting for treatment centre, Injury Severity Score (ISS), extended IMPACT-TBI score, and mean ICP over the first 96 hours.

**RESULTS.** Data on 262 patients was collected, with primary analysis performed on the 251 who survived the first 96 hours. Overall, 46 patients received mannitol only, and 46 patients received hypertonic saline only during this period.

HTS versus mannitol patients had a mean age of 40.5 (SD 16.8) versus 49.2 (19.2) years respectively (p=0.02), and 71.7% were male versus 76.1% (p=0.64). Injury Severity Score, APACHE-II and extended IMPACT-TBI for the HTS and mannitol groups respectively were 32.1 (11.3) versus 42 (15.9) (p=0.001), 20 [15-23] versus 22 [17-32] (p=0.1), and 25% [13-38] versus 34.5% [23-46] (p=0.023). There was no significant difference between groups with regards to ICPs and noradrenaline requirements in the first 96 hours.

Treatment with decompressive craniotomy (23.9% versus 21.7%, p=0.8), CSF drainage (45.7% versus 43.5%, p=0.83), barbiturate coma (13% versus 19.6%, p=0.4) and hypothermia (no patients) was similar between groups.

ICU and hospital mortality were significantly lower in the HTS than in the mannitol group (ICU mortality 15.2% versus 47.8%, p=0.001; hospital mortality 21.7% versus 52.2% p = 0.002). The raw hazard ratio for death with mannitol compared to HTS was 3.39 (95% CI 1.4-8.2, p=0.007).

After adjustment for centre, Injury Severity Score, extended IMPACT-TBI score, and mean ICP, the hazard ratio for death with mannitol rather than HTS use was 2.64 (95% CI 0.96-7.30, p=0.061).

**CONCLUSIONS.** These data raise concerns about mannitol when used as osmotherapy in TBI patients. Further studies are needed to understand whether choice of osmotherapy agents affects clinical outcomes.

**GRANTS**
n/a

### 0493

#### Inspiratory muscular traning in non-collaborative patients using mechanical ventilation in the ICU

P. Melo[1], D. Pereira[2], V. Maldaner[3], M. Freres[2], R. Mendes[2], L. Santos[2], L. Gomes[2], L. Rocha[2], G. Cipriano[4]

[1]Universidade de Brasilia, Brasilia, Brazil; [2]Fundação de Ensino e Pesquisa em Ciências da Saúde, Brasilia, Brazil; [3]IHB, Brasilia, Brazil; [4]Universisade de Brasilia, Brasilia, Brazil

**Correspondence:** P. Melo

*Intensive Care Medicine Experimental* 2018, **6(Suppl 2):**0493

**INTRODUCTION.** Patients with neurological disorders require continuous and multiprofessional surveillance, and are sometimes admitted to the ICU (Intensive Care Unit), who spend a lot of time under in mechanical ventilation, which can lead to Ventilator-Induced Diaphragmatic Dysfunction (VIDD). To avoid such a change, assessments and treatments on the risk of developing muscle weakness are employed. There are no studies that show the efficacy of inspiratory muscle training (IMT) in non-collaborative neurocritical patients.

**OBJECTIVES.** Demonstrate that IMT is able to increase respiratory muscle strength in non-collaborative patients using mechanical ventilation in the ICU compared to patients who did not perform the training.

**METHODS.** This was a randomized clinical trial. The sample consisted of 27 non-collaborative patients of both sexes, older than 18 years old, using mechanical ventilation, who were divided into two groups: In trainig Group (TG) and Control Group (CG), who performed the MIP (Maximum Inspiratory Pressure) and the IMT (inspiratory muscular training for a period of 14 days. To analyze the data, the Shapiro Wilk test and the K-S distance test were used to see the distribution of the studied population. For the comparison of the pre and post ΔIPmax measurements (variation of the pre and post inspiratory pressure), the unpaired Student´s t-test was used, being considered significant values of $p < 0.05$.

**RESULTS.** There was a higher MIP variation in TG (59.2 ± 18.2 cm H2O) than in CG (44 ± 17 cm H2O).

**CONCLUSIONS.** IMT was able to increase MIP of non-collaborative neurocritical patients.

##### REFERENCE(S)

MARINI, J. J.; SMITH, T.C.; LAMB, V. Estimation of inspiratory muscle strength in mechanically ventilated patients: the measurement of maximal inspiratory pressure. Journal of Critical Care, v. 1, n. 1, p. 32-38, 1986.

NEDER, J. A. *et al*. Reference values for lung function tests: II. Maximal respiratory pressures and voluntary ventilation. Brazilian journal of medical and biological research, v. 32, n. 6, p. 719-727, 1999.

SAAD, I. A. B. *et al*. A new device for inspiratory muscle training in patients with tracheostomy tube in ICU: A randomized trial. European Respiratory Journal, v. 44, n. Suppl 58, p. P4297, 2014.

SOUZA, L. C. *et al*. Mechanical ventilation weaning in inclusion body myositis: feasibility of isokinetic inspiratory muscle training as an adjunct therapy. Case reports in critical care, v. 2014, 2014..

TONELLA, R. M. *et al*. Inspiratory Muscle Training in the Intensive Care Unit: A New Perspective. Journal of clinical medicine research, v. 9, n. 11, p. 929, 2017

### 0494

#### A prognostic prediction model for critically ill patients with status epilepticus

J. Cedeño Mora, S. Casanova Prieto, P. García-Olivares, J.C. Barrios, N. Fernández, C. Delgado, A. Moni, J.C. Sotillo, J. Peral, J.M. Gómez

H.G.U Gregorio Marañón, Intensive Care Unit, Madrid, Spain

**Correspondence:** J. Cedeño Mora

*Intensive Care Medicine Experimental* 2018, **6(Suppl 2):**0494

**INTRODUCTION.** Status epilepticus (SE) is a emergency that requires prompt diagnosis and treatment. The identification of factors associated with a poor prognosis could be useful to guide and improve treatment.

**OBJECTIVES.** The aim of this study was to identify the risk factors for unfavourable prognosis and to obtain a prognostic prediction model for critically ill patients with SE.

**METHODS.** Retrospective study performed on patients admitted to ICU with SE between 2015 and 2016. We collected epidemiological and relevant clinical data related to SE. The prognosis was measured by the Rankin scale (severe disability or death).

Descriptive data were described as means (SD) for normally distributed continuous variables, medians (IQR) for non-normally distributed variables, and percentages for categorical data. The continuous variables were compared using a simple logistic regression analysis (OR), and the categorical data by the Chi-square test (RR). The factors associated with a poor prognosis were identified, and using a multiple logistic regression analysis a predictive model was obtained.

**RESULTS.** Fifty-eight patients were included, 53% were male, mean age was 60 yrs (40-70). Charlson Index 1 pts (1-3). The epileptic seizures occurred at the hospital in 43% of patients, and were more frequently generalized (83%). Persistence of seizures on first evaluation by the ICU team was found in 31% of cases (refractory SE 10%), 35% presented a structural lesion in CT and 38% alterations in the EEG. The antiepileptic drugs most frequently used were benzodiazepines (78%), Levetiracetam (36%) and 43% of patients required deep sedation (26% Propofol and 22% Midazolan associated with Propofol). Severity scores: APACHE II 17±10 pts, SOFA 5±3 pts, GCS 6±3 pts, and 76% of patients required mechanical ventilation (2 days, 1-9). ICU stay was 3 days (2-7) and hospital stay was 11 days (6-18). The prognosis was unfavourable for 29% of patients, with 14% mortality.

In the **univariate analysis**, the variables related to a poor prognosis were: age (OR 1,05; 95% CI 1,01-1,09), **Charlson Index** (OR 1,45; 95% CI 1,04-1,20), **GCS** (OR 0,82; 95% CI 0,67-0,99), **APACHE II** (OR 1,12; 95% CI 1,03-1,23), **SOFA** (OR 1,27; 95% CI 1,03-1,57), **in-hospital seizures** (RR 3,54; 95% CI 1,08-11,57), **seizures on first ICU evaluation** (RR 4.01; 95% CI 1,19-13,36), **structural lesion** (RR 3,01; 95% CI 1,01-9,95) and **refractory SE** (RR 12,31; 95% CI 1,26-120,19).

Using **multiple logistic regression,** we created a model including the following variables: **APACHE II** (OR 1,22; 95% CI 1,06-1,39), **structural lesion** (OR 11,79; 95% CI 1,86-74,84) and **refractory SE** (OR 44,61; 95% CI 2,26-881,51). The **model obtained** had a good prognosis discriminatory ability: AUROC 0.85, CI 0.73-0.96; Hosmer-Lemeshow test $X^2$ 3.94, p=0.86.

**CONCLUSIONS.** The risk factors for unfavourable prognosis were APACHE II, structural lesion and refractory SE. The predictive model obtained was able to discriminate the prognosis of patients with SE.

### 0495

#### Effects of acute subdural hematoma on cerebral mitochondrial respiration in a porcine resuscitated model

T. Merz[1], S. Unmuth[1], M. Wepler[1,2], T. Datzmann[1], T. Kapapa[3], S. Röhrer[3], P. Radermacher[1], E. Calzia[1], O. McCook[1]
[1]Universitätsklinikum Ulm, Anesthesiological Pathophysiology, Ulm, Germany; [2]Universitätsklinikum Ulm, Anesthesiology, Ulm, Germany; [3]Universitätsklinikum Ulm, Neurosurgery, Ulm, Germany
**Correspondence:** T. Merz
*Intensive Care Medicine Experimental* 2018, **6(Suppl 2):**0495

##### Introduction

Acute subdural hematoma (ASDH) assumes major importance in traumatic brain injury (TBI), in particular in the elderly and/or vascular co-morbid patient (1). Mitochondria assume importance during secondary TBI: they play a crucial role for neuronal survival (2), but may also be a source of detrimental reactive oxygen species (ROS, 3). Mitochondrial ROS formation is directly related to the degree of coupling of the respiratory chain (4), whereas limited uncoupling through expression of uncoupling protein 2 (UCP2) reduces tissue oxidative stress (5), however at the cost of ATP production (6).

##### Objectives

The aim of this experiment was to investigate cerebral mitochondrial respiration and uncoupling in a resuscitated porcine model of ASDH.

##### Methods

Anesthetized, mechanically ventilated and surgically instrumented pigs underwent ASDH (trepanation above the left parietal cortex and subdural injection of 20 ml of blood). Resuscitation, according to the guidelines of TBI management (7), comprised fluid and catecholamine administration to maintain mean arterial pressure at pre-ASDH levels and cerebral perfusion pressure >75 mmHg. 54 hours after ASDH, mitochondrial respiration on fresh homogenized ipsi- and contralateral frontal cortex was determined via high-resolution respirometry (Oroboros Oxygraph 2K; N=6): maximum capacity in the coupled (OxPhos) and uncoupled state (ETS) and leak compensation flux were determined. UCP2 was analyzed *in situ* in fixed brain sections.

##### Results

Two out of six animals died early (6 and 18h after TBI induction) due to cardiovascular failure unrelated to induction of ASDH. In these animals, maximum mitochondrial respiration in the coupled and uncoupled state were reduced (p=0.008 and p=0.004) and consequently OxPhos/ETS (p=0.004) and LEAK/ETS ratios were increased (p=0.002) in comparison to hemodynamically stable animals. The increased LEAK/ETS was associated with higher UCP2 expression in the hemodynamically unstable group, most pronounced in the ipsilateral hemisphere.

##### Conclusions

This study revealed dysregulated cerebral mitochondrial respiration in hemodynamically unstable animals after TBI in a long-term, resuscitated porcine model of ASDH-induced TBI. Increased UCP2 expression might be an adaptive response to reduce ROS formation as a consequence of stronger coupling of the mitochondrial respiratory chain (higher OxPhos/ETS ratio). Cerebral mitochondrial dysregulation seemed to be a crucial factor in TBI-related mortality.

1: Lee 2017: J Neurotrauma 128(1):236-249 2: Nicholls 2000: Physiol Rev. 80(1):315-60, 3: Bains 2012: Biochim Biophys Acta. 1822(5):675-84, 4: Zorov 2014: Physiol Rev. 94(3):909-50, 5: Nègre-Salvayre 1997: FASEB J. 11(10):809-15, 6: Busiello 2015: Front Physiol. 2015; 6: 36. 7: Carney 2018: J Neurotrauma 35(1):64-72

##### Acknowledgement

Supported by the DFG, CRC1149

### 0496

#### Neurophysiological fluctuations in patients with disorders of consciousness impact conscious processing

P. Perez[1,2], F. Raimondo[3], L. Naccache[3,4], J. Sitt[2]
[1]Pitié Salpêtrière Hospital, Paris, France; [2]Institut du Cerveau et de la Moelle épinière, Paris, France; [3]Institut du Cerveau et de la Moelle Epinière, Paris, France; [4]Pitié Salpêtrière Hospital, Neurophysiology, Paris, France
**Correspondence:** P. Perez
*Intensive Care Medicine Experimental* 2018, **6(Suppl 2):**0496

**INTRODUCTION.** The disorders of consciousness frequently follow after severe head injury. The non-communicating patients are categorized into three groups: comatose, vegetative state (VS) and minimally conscious state (MCS). Patients in VS have a loss of awareness and patients in MCS either have fluctuating awareness with non-reflex behavior (MCS minus) or response to command (MCS plus). The clinical fluctuations of awareness constitute an important part of MCS definition.

**OBJECTIVES.** The aim of this study is to quantified periods with high or poor treatment of information and to study the response evoked to better classify states of consciousness.

**METHODS.** We investigated whether the EEG counterpart of these rapid clinical fluctuations (fluctuations in alpha power band, complexity and information exchange (1,2)) modulated conscious access to external stimuli during an auditory task probing conscious access to global regularities (the 'local global paradigm' (3)). We identified time-windows characterized by high or low levels of postulated markers and studied the evoked responses of an auditory paradigm (Local-global) during the same corresponding periods. The local-global paradigm is designed to elicit early potentials post-stimulus, related to unconscious processing, and late potentials, related to conscious processing. We used a method of generalization analysis, named decoding, consisting in training a classifier to quantify the level of early and late responses.

**RESULTS.** 96 patients underwent high density EEG recording with a 256-electrode geodesic sensor net (EGI, Oregon, USA) in intensive care unit.

The level of automatic and conscious responses, were higher in the rich periods than in poor periods (p-value=0.02 and 0.03 respectively, Wilcoxon paired test).There was no significant difference, in either low or high states, between VS patients and MCS- patients. In contrast, we found in MCS+ patients a specific increase, compared to the other clinical groups, of the decoding performance for the late responses (global effect).

**CONCLUSIONS.** These results support the idea that conscious process is not continuous and highlights the differences of global states of consciousness between different clinical diagnoses.

##### REFERENCE(S)

1. King, J.-R., Sitt, J. D., Dehaene, S. (2013). Information sharing in the brain indexes consciousness in noncommunicative patients. Current Biology : CB, 23(19), 1914-9. .
2. Sitt, J. D., King, J.-R., Naccache, L. (2014). Large scale screening of neural signatures of consciousness in patients in a vegetative or minimally conscious state. Brain : A Journal of Neurology, 137(Pt 8), 2258-70.
3. Bekinschtein, T. A., Dehaene, S., Naccache, L. (2009). Neural signature of the conscious processing of auditory regularities. Proceedings of the

National Academy of Sciences of the United States of America, 106(5), 1672-7.

**GRANT ACKNOWLEDGMENT**
FRM has supported this work.

### 0497

#### Intra- and postoperative effects of remifentanil and fentanyl infusion in patients with elective brain tumour surgery

D. Loncar Stojiljkovic[1], M.P. Stojiljkovic[2]
[1]SGH Jevremova, Belgrade, Serbia; [2]University of Banja Luka, Faculty of Medicine, Department of Clinical Pharmacology, Banja Luka, Bosnia and Herzegovina
**Correspondence:** D. Loncar Stojiljkovic
*Intensive Care Medicine Experimental* 2018, **6(Suppl 2):**0497

**INTRODUCTION.** Patients undergoing brain tumour surgery require proper level of analgesia and cardiovascular stability during operation and immediately thereafter. Contemporary anaesthetic techniques use for that purpose continuous *iv*infusion of opioid analgesic (1).

**OBJECTIVES.** Goal of this randomized clinical study was to compare the effects of two opioid analgesics, remifentanil and fentanyl, administered as continuous iv infusion during neurosurgery.

**METHODS.** A total of 32 patients, ASA II-III, with frontal region brain tumour were included in this single blind, controlled clinical study.-They were randomised in two similar groups.

Remifentanil (RF) group received RF 1-2 μg/kg of body weight *iv*bolus and continuously *iv*at the average infusion rate of 0.44 μg/kg/min, while the fentanyl (F) group received 100 μg *iv* bolus, followed by a continuous *iv* infusion at an average rate of 0.012 μg/kg/min. The infusion rates were adjusted according to the sympathetic response to operational stimuli. Postoperative infusion rates were 0.05μg/kg/min in RF group and 1-2 μg/kg/h in F group. At critical points of anaesthesia and operation (0 - baseline values;

1 - Intubation;
2 - brain manipulation,
3 - end of operation;
4 - 1 h postoperatively;
5 - 12 h postoperatively;
6 - 24 h postoperatively) haemodynamic parameters were recorded.

Analgesic consumption and adverse effects of opioids were recorded during one day after the end of surgery.

Length of the operation in RF and F group were 227.5 (165-340) and 212 (175-298) min, respectively.

**RESULTS.** In almost all the critical points of operation and immediate postoperative course cardiovascular stability was similar. RF group showed tendency to lower, yet statistically not significant values. The only significant difference in heart rate was found during induction to anesthesia, when average values were lower in F than in RF group (72 vs 100/min). In RF group in 2 patients there was need for increase in the analgesic dose and in F group in only one patient. Adverse effects were found in 7 RF patients (43.75%) and in 2 F patients (12.5 %) Shivering was most frequent adverse effect found in the RF group, and it was registered in 4 patients.

**CONCLUSIONS.** There are no significant differences between the groups in cardiovascular stability. Postoperative pain control was similar in both groups. Adverse effects were more frequent in RF than in the F group (7 vs 2). Postoperative shivering was present in 4 RF patients, which is in accordance with literature data (2). Shivering can be explained by the development of acute opioid tolerance after long infusions of a very potent opioid, remifentanil.

**REFERENCE(S)**

1) Guy J et al. Comparison of remifentanil and fentanyl in patients undergoing craniotomy for supratentorial space-occupying lesions. Anesthesiology 1997;86:514-24.
2) Naksui M et al. Intraoperative high dose remifentanil increases postanesthetic shivering. Br J Anaesth 2010;105(2):162-7.

### 0498

#### Assessment of quality measures after implementation of treatment guidelines with an electronic health record order set for the treatment of status epilepticus: a single center experience

K.S. Smetana[1], D. Harper[2], M. Sidner[3], C.C. May[1], M.T. Torbey[4]
[1]Wexner Medical Center at The Ohio State University, Specialty Practice Pharmacist - Neurocritical Care, Columbus, United States; [2]Wexner Medical Center at The Ohio State University, Central Quality and Education - Critical Care/Neurosciences, Columbus, United States; [3]Wexner Medical Center at The Ohio State University, Quality Program and Outcomes Manager, Columbus, United States; [4]Wexner Medical Center at The Ohio State University, Neurology and Neurosurgery, Neurovascular Stroke Center, Cerebrovascular and Neurocritical Care Division, Neurology for Hospital Affairs, Columbus, United States
**Correspondence:** K.S. Smetana
*Intensive Care Medicine Experimental* 2018, **6(Suppl 2):**0498

**INTRODUCTION.** Guidelines "assist practitioner and patient decisions about appropriate health care for specific clinical circumstances."[1] Status Epilepticus (SE) requires appropriate emergent therapy to reduce mortality and morbidity. However, patients continue to receive inadequate initial doses of antiepileptic's (AED) which is exacerbated by a hesitancy to escalate care aggressively.[2] Thus institutional guidelines and order sets within the Electronic Health Record (EHR) are necessary and highly encouraged to help manage these complexities.[3]

**OBJECTIVES.** To assess quality measures in SE before and after the implementation of institutional guidelines and an order set in the EHR.

**METHODS.** We conducted a retrospective analysis of adults with SE discharged from 2012 - 2017 at a 1,382 bed academic medical center. The following quality measures were evaluated: continuous electroencephalogram (cEEG) use, mechanical ventilation (MV), benzodiazepine (BZD) administration, number of AED administered, length of stay (LOS), and mortality/hospice.

**RESULTS.** 353 patients were included. After guideline and order set implementation in October 2015 (n=86) more patients received cEEG (79.1% vs 36%; $p < 0.05$), MV (65.1% vs 21.3%; $p < 0.05$), and a second (37.2% vs 19.9%; $p < 0.05$) or third AED (47.7% vs 13.1%; $p < 0.05$) compared to the pre-implementation group (n=267). There was no difference in the amount of patients that received a BZD (88.4% vs 86.5%; $p = 0.657$). After implementation patients LOS was 8 (IQR 4, 15.5) vs 4 days (IQR 2, 6) ($p < 0.05$), and higher rates of death/hospice were observed (11.6% vs 3.4%; $p = 0.003$).

**CONCLUSIONS.** Our preliminary results reveal that after implementation, SE patients were more likely to receive cEEG monitoring, more AEDs, LOS doubled, and had a three-fold increase in mortality. These findings may have overlapping explanations: 1) the post-implementation group may have had higher rates of refractory SE and therefore more likely to receive additional treatment and monitoring, 2) the institutional guidelines increased awareness to the emergent nature of the disease which was followed by more aggressive pharmacologic interventions and cEEG monitoring. The rise in mortality observed aligns with previous literature reporting a mortality increase of nearly three times for refractory SE.[4]

**GRANT ACKNOWLEDGMENT**
None.

### 0499

#### Piperacillin concentrations and neurological toxicity in septic patients

F. Montanaro[1,2], B. Moro Salihovic[3], L. Peluso[1], L. Calabrò[1], N. Gaspard[4], M. Hites[5], F. Cotton[6], J.-L. Vincent[1], F. Jacobs[5], J. Creteur[1], F.S. Taccone[1]
[1]Erasme Hospital,Université Libre de Bruxelles, Department of Intensive Care, Bruxelles, Belgium; [2]Università degli studi di Ferrara, Anestesia, Rianimazione, Terapia Intensiva e del Dolore, Ferrara, Italy; [3]Erasme Hospital, Department of Intensive Care, Bruxelles, Belgium; [4]Erasme Hospital,Université Libre de Bruxelles, Department of Neurology, Bruxelles, Belgium; [5]Erasme Hospital,Université Libre de Bruxelles, Department of Infectious Diseases, Bruxelles, Belgium; [6]Erasme Hospital,Université Libre de Bruxelles, Department of Clinical Biochemistry, Bruxelles, Belgium
**Correspondence:** F. Montanaro
*Intensive Care Medicine Experimental* 2018, **6(Suppl 2):**0499

**INTRODUCTION.** Although high piperacillin concentrations have been associated with clinical neurological deterioration in septic patients, no data have been reported on whether trough or peak drug concentrations are both associated with neurotoxicity.
**OBJECTIVES.** The aim of this study was to assess the association between piperacillin concentrations and EEG abnormalities in ICU patients with sepsis.
**METHODS.** We reviewed all ICU patients over a 5-year period (2012-2017) who were treated with piperacillin-tazobactam and in whom at least two β-lactam concentrations (trough, $C_{min}$; concentration 2 hours after the bolus injection, $C_{2H}$) and a concomitant EEG monitoring were available. Drug levels were measured using high-performance liquid chromatography. An "altered" EEG was defined when at least one between ictal EEG pattern, generalized periodic discharges (GPDs) or burst suppression was identified.
**RESULTS.** We collected 442 piperacillin concentrations (221 at $C_{min}$ and 221 at $C_{2H}$) over the study period. Altered EEG was found in 62 (28%) of cases, with ictal EEG pattern in 38 of them. Piperacillin trough concentrations were higher in patients with an altered EEG than others (61 [33-98] vs. 33 [11-63] mg/L; p< 0.001). Also, $C_{2H}$ were higher in patients with an altered EEG than others (96 [59-141] vs. 139 [98-174] mg/L; p< 0.001). The occurrence of altered EEG significantly increased over different ranges of $C_{min}$ (from 3% for $C_{min}$ < 10 mg/L to 50% for $C_{min}$ >80; p=0.001); similarly, the occurrence of altered EEG significantly increased over different ranges of $C_{2H}$ (from 0% for $C_{min}$ < 60 mg/L to 48% for $C_{min}$ >160; p=0.001).
**CONCLUSIONS.** There was a significant association between both minimal and maximal piperacillin concentrations and an increased occurrence of EEG abnormalities in ICU patients with sepsis. Monitoring of piperacillin levels should be considered when neurological and/or EEG alterations occur in patients with sepsis

**REFERENCE(S)**
None.

**GRANT ACKNOWLEDGMENT**
None.

### 0500

#### Retention of knowledge on external ventricular drain care among nurses

R.C. Souza[1], E. Pires[2], L. Meira[3], G. Araujo[2], M. Bersaneti[2]
[1]Hospital Sírio Libanês, Nucleo de Pós Graduação, São Paulo, Brazil; [2]Hospital Sírio Libanês, Unidade de Terapia Intensiva, São Paulo, Brazil; [3]Hospital Sírio Libanês, Desenvolvimento de Enfermagem, São Paulo, Brazil
**Correspondence:** R.C. Souza
*Intensive Care Medicine Experimental* 2018, **6(Suppl 2):**0500

**INTRODUCTION.** Hydrocephalus is one of the most frequent complications in patients in neurosurgery units. In acute cases, hydrocephalus is treated by placing an external ventricular drain (EVD); however, an EVD increases the risk of infections and adverse events, resulting in increased morbidity and mortality[1,2].
**OBJECTIVE AND METHODS.** This quasi-experimental study was based on the training of nurses from an Intensive Care Unit (ICU) on EVD care guidelines. The study was conducted between November 2015 and March 2016 with the objective of evaluating the nurses' knowledge at three different times: before, one week after and three months after training.
The intervention was based on an expository class on the subject and a video that contained information on the techniques of dressing and handling of the system. It was performed during the participants´ work shift.
**RESULTS.** The sample comprised 38 nurses with a mean age of 33.7 ±4.8 years. Nurses from 20-29 and 30-39 years of age represented 21.1% and 65.8% of the sample, respectively. The nurses were predominantly female (33 women; 86.8%).
For the sampled nurses, an average of 9.5±5.0 years had passed since graduation. Time since graduation was more than 10 years for 50% of the sample. A total of 13 participants (34.2%) reported having prior experience in a neurological ICU. Overall, 94.7% of the nurses in the sample had a specialization; 73% of these nurses had ICU specialization, and 29.7% had two specializations.
The nurses with ICU specialization showed better retention for issues related to handling of the EVD system, with higher retention among 30- to 39-year-old nurses than among 20- to 29-year-old nurses. Although knowledge retention regarding treatment specificities did not differ significantly among the study phases, median retention was higher for this topic than for other topics.
**CONCLUSIONS.** In conclusion, in this study, although there was no significant knowledge retention among nurses three months after the intervention, the most experienced nurses with Intensive Care Unit (ICU) expertise performed better than the other nurses in the sample.

**REFERENCE(S)**
1- Lwin S, low SW, Choy DKS, Yeo TT, Chou N. External ventricular drain infections: successful implementation of strategies to reduce infection rate. Singapore Med J. 2012; 53(4):255-9.
2- American Association of Neuroscience Nurses. Care of the Patient Undergoing Intracranial Pressure Monitoring/ External Ventricular Drainage or Lumbar Drainage. http://www.aann.org/uploads/AANN11_ICPEVDnew.pdf

### 0501

#### Evaluation of the diameter of the optic nerve in patients with TBI and its correlation with intracranial pressure

J.F. Martínez Carmona[1], E. Lopez Luque[2], F.A. Hijano Muñoz[2], M. Delgado Amaya[2], J.M. Mora Ordoñez[2], G. Quesada[2]
[1]H. R. U de Málaga, Málaga, Spain; [2]H. R. U de Málaga, Intensive Care, Málaga, Spain
**Correspondence:** J.F. Martínez Carmona
*Intensive Care Medicine Experimental* 2018, **6(Suppl 2):**0501

**INTRODUCTION.** Neuromonitoring in patients with TBI is a fundamental pillar for the correct management of this pathology. There are different devices in neuromonitoring, however, not all are available in most intensive care units. The evaluation of the diameter of the optic nerve by CT is a useful tool and available in all units.
OBJETIVES. The main objetive is to correlate the diameter of the optic nerve measured in cranial CT with the values of Intracranial pressure (ICP) recorded by a pressure sensor, and its relationship with mortality.
**METHODS.** Our study includes patients admitted to the ICU due to Traumatic Brain Injury (TBI), who need neuromonitoring using an intracranial pressure sensor. Epidemiological data are collected, Glasgow Coma Scale, mortality, diameter of the optic nerve in cranial CT and previously registered intracranial pressure.
**RESULTS.** Our sample includes 20 patients, with average age: 45.65 years. 50% males. APACHE II average on admission: 19.68. 70%

needed urgent surgery upon admission. 65% had GCS < 8 at first assistance. 75% had GCS >12 at discharge. Mortality 25%.
We observed a statistically significant relationship between the diameter of the optic nerve and the recorded ICP, using Chi2 test (p 0.005), with good correlation using the Spearman test (p 0.003). Those patients with ICP < 15mmHg and optic nerve diameter < 4.8mm had better evolution (GCS >12 at discharge), and higher survival. If we perform a COR curve relating ICP and mortality, we obtain AUC 0.827 (95% CI 0.636 - 1) p 0.032. We plotted a cut-off at 15.5mmHg (Sensitivity 80% and Specificity 66%)
**CONCLUSIONS.** The management of patients with TBI is complex, neuromonitoring is essential for optimal management.
We observed good correlation between the diameter of the optic nerve and intracranial pressure, as well as mortality. It is a useful tool for the management of these patients.

**REFERENCE(S)**

- Lee et al. Optic nerve sheath diameter based on preoperative brain computed tomography and intracranial pressure are positively correlated in adults with hydrocephalus. Clin Neurol Neurosurg 2018; 167: 31-35.
- Bekerman et al. The quantitative evaluation of intracranial pressure by optic nerve sheath diameter/ese diameter CT Measurement. Am J Emerg Med 2016; 34 (12): 2336 - 2342.
- Bekerman et al. Monitoring of Intracranial Pressure by CT-Defined Optic Nerve Sheath Diameter. J Neuroimaging 2016; 26 (3): 309 - 14.
- Sehkon et al. Optic nerve sheath diameter on computed tomography is correlated with simutaneously measured intracranial pressure in patients with severe traumatic brain injury. Intensive Care Med 2014; 40(9): 1267 - 1274.

### 0502

#### Systemic hemodynamics and lung mechanics during increased positive end-expiratory pressure levels in critically ill neurosurgical patients with different baseline intracranial pressure levels

A. Solodov[1], S. Petrikov[1], V. Krylov[2]
[1]Sklifosovsky Research Institute, Neurosurgical ICU, Moscow, Russian Federation; [2]Sklifosovsky Research Institute, Neurosurgery Department, Moscow, Russian Federation
**Correspondence:** A. Solodov

*Intensive Care Medicine Experimental* 2018, **6(Suppl 2):**0502

**INTRODUCTION.** Positive final expiratory pressure (PEEP) is one of the main parameters determine gas exchange during mechanical ventilation. The use of high PEEP levels can lead to increase of intracranial pressure (ICP).
**OBJECTIVES.** To assess the systemic hemodynamics and lung mechanics during increased PEEP levels in neurosurgical patients with different baseline ICP levels.
**METHODS.** 22 critically ill patients with intracranial hemorrhages with ICP-monitoring, systemic hemodynamics and lung mechanics monitoring enrolled in the study. Mean age was 49±13 years, male/female - 9/13. In 12 patients (group 2) increase PEEP to 15 and 20 $cmH_20$ was accompanied by an increase ICP, in 10 patients (group 1) ICP were stable during of the study.
**RESULTS.** The increase of PEEP was accompanied by tendency to a decrease cardiac index and preload, an increase stroke volume variation and systemic vascular resistance index in both groups (Table 1, 2). In patients with unstable ICP (group 2, Table 2) was noted a more pronounced decrease in the global end diastolic index with an increase PEEP to 15 and 20 cmH20. Negative cerebral and hemodynamic effects were due to different states of lung mechanics (static compliance of respiratory system) in patients of both groups (Table 1, 2).
**CONCLUSIONS.** Patients with good lung compliance have a greater negative impact of increased PEEP levels on cardiac output and preload, which causes an increase in ICP, compared with patients with reduced lung compliance (Table 1).

**Table 1 (abstract 0502).** Dynamics parameters of systemic hemodynamics and lungs mechanics during increased PEEP levels in patients with STABLE ICP levels

| Parameters | PEEP 5 cmH2O | PEEP 10 cmH2O | PEEP 15 cmH2O | PEEP 20 cmH2O |
|---|---|---|---|---|
| Intracranial pressure, mmHg | 11,8±4,7 | 12,1±4,6 | 12,0±4,2 | 12,9±4,2 |
| Cardiac index, litres/min/m2 | 3,44±0,71 | 3,5±0,9 | 3,35±0,74 | 2,92±0,55 |
| Global end diastolic index, ml/m2 | 738±191 | 731±230 | 735±228 | 718±251 |
| Systemic vascular resistance index, dyn*s*cm-5*m2 | 2334±651 | 2382±645 | 2547±851 | 2596±549 |
| Stroke volume variation, % | 10,7±8 | 13,5±7,8 | 17,9±8,8 | 23,2±11,1 |
| Extravascular lung water index, ml/kg | 6,9±2,8 | 7,2±2,9 | 7,2±3 | 7,6±4,0 |
| Compliance static, ml/mmH20 | 54 (51,25; 61,25) | 55 (52; 67) | 53 (48; 58) | 48,5 (44,5; 52,75) |

**Table 2 (abstract 0502).** Dynamics parameters of systemic hemodynamics and lungs mechanics during increased PEEP levels in patients with UNSTABLE ICP levels

| Parameters | PEEP 5 cmH2O | PEEP 10 cmH2O | PEEP 15 cmH2O | PEEP 20 cmH2O |
|---|---|---|---|---|
| Intracranial pressure, mmHg | 13,8±3,2 | 14,6±3,6 | 16,2±3,3 | 17,1±3,8 |
| Cardiac index, litres/min/m2 | 3,9±0,7 | 3,6±0,74 | 3,49±1,1 | 3,4±1,0 |
| Global end diastolic index, ml/m2 | 773±140 | 707±118 | 698±117 | 649±112 |
| Systemic vascular resistance index, dyn*s*cm-5*m2 | 2114±606 | 2337±590 | 2470±845 | 2498±761 |
| Stroke volume variation, % | 10,3±8,4 | 12,2±10,2 | 14,8±9,3 | 17,8±10,4 |
| Extravascular lung water index, ml/kg | 7,7±2,4 | 7,3±1,6 | 7,1±1,6 | 7,5±1,4 |
| Compliance static, ml/mmH20 | 61,85 (52; 67,13) | 54 (50, 59,5) | 49,2 (46,5; 56) | 42,5 (39,25; 47) |

## Abstract award winning session

### 0503

#### Comparison of two opposite strategies of weaning: high vs. low work of breathing: a multicentre randomised controlled trial

C. Subirà[1], L. Ventura[1], G. Hernández[2], A. Vázquez[3], R. Rodríguez[4], A. Castro[5], C. García[6], E. Keough[7], V. Arauzo[8], M.M. Fernández[9], M. de la Torre[10], C. Hermosa[11], C. Sánchez[12], A. Tizón[13], A. López[14], S. Cabañes[15], V. Lacueva[16], C. Laborda[17], E. Tenza[18], F. Rafael[1]
[1]Althaia Xarxa Assistencial Manresa, Critical Care Department, Manresa, Spain; [2]Complejo Hospitalario de Toledo, Critical Care Department, Toledo, Spain; [3]Hospital del Mar, Critical Care Department, Barcelona, Spain; [4]Hospital Central de Asturias, Critical Care Department, Oviedo, Spain; [5]Hospital Marqués de Valdecilla, Critical Care Department, Santander, Spain; [6]Hospital Universitario de Canarias, Critical Care Department, Tenerife, Spain; [7]Hospital Gregorio Marañón, Critical Care Department, Madrid, Spain; [8]Consorci Sanitari de Terrassa, Critical Care, Terrassa, Spain; [9]Hospital Mútua de Terrassa, Critical Care Department, Terrassa, Spain; [10]Hospital de Mataró, Critical Care Department, Mataró, Spain; [11]Hospital de Henares, Critical Care Department, Coslada, Spain; [12]Hospital de Cáceres, Critical Care Department, Cáceres, Spain; [13]Complexo Hospitalario Universitario de Ourense, Critical Care Department, Ourense, Spain, [14]Hospital General de Granollers, Critical Care Department, Granollers, Spain; [15]Hospital Universitario de Araba, Critical Care Department, Araba, Spain; [16]Hosptial de Sagunt, Critical Care Department, Sagunt, Spain; [17]Hospital de la Vall d'Hebron, Critical Care Department, Barcelona, Spain, [18]Hospital d'Elx, Critical Care Department, Elx, Spain
**Correspondence:** C. Subirà

*Intensive Care Medicine Experimental* 2018, **6(Suppl 2):**0503

**INTRODUCTION.** A daily spontaneous breathing trial (SBT) is the best approach to wean patients from the ventilator, but mode and duration of SBT are still controversial.

**DESIGN.** Multicentre randomised controlled trial, comparing two SBTs with very different work of breathing: T-piece for 2 hours vs. PSV 8 cmH2O for 30 minutes.

**OBJECTIVE.** To determine whether a low demanding SBT could improve extubation rate.

**METHODS.** During January 2016 and April 2017, in 18 Spanish ICUs, we recruited patients >18 yr. old after >24 hours of mechanical ventilation and ready for weaning. We excluded patients with tracheostomy or orders of non-reintubation. Patients were randomised to perform a SBT with T-piece for 2 hours or PSV 8 cmH2O for 30 minutes. The primary outcome was successful extubation rate, defined as remaining extubated 72 hours after the first SBT. Secondary objectives were reintubation rate, ICU and hospital length of stay, and ICU and hospital mortality. Variables associated with successful extubation were examined by multivariable logistic analysis.

**RESULTS.** We studied 578 patients in the 2-h T-piece SBT and 575 patients in the PSV 8 SBT group. Both groups were comparable at randomization. Successful SBT was higher in the PSV group than in the T-piece group (92.7% vs 84.1%; $p < 0.001$), whereas reintubation in patients who tolerated the SBT was similar in both groups (12.9% PSV group vs. 12.6% T-piece group; p = 0.9). Then, successful extubation was higher in the PSV group than in the T-piece group (80.7% vs. 73.5%; p = 0.004). The ICU mortality was not different (5% in PSV vs. 6.6% in T-piece; p= 0.3), but the hospital mortality was higher in the T-piece group (14.9% vs 10.4%, p= 0.022). The mortality at 90 days of randomisation was also higher in the T piece group (log Rank 0.033). The variables associated with successful extubation by logistic multivariable analysis were the length of MV before weaning (OR 0.96 in days of MV, p =0.02) and the SBT with PSV for 30 minutes (OR 1.5, p = 0.002).

**CONCLUSIONS.** The SBT using PSV 8 cmH2O for 30 minutes results in a better successful extubation rate compared with T-piece for 2 hours. The benefit was mostly related to a better tolerance of the PSV trial, without increasing reintubation. Unexpectedly, T-piece group experienced higher hospital and 90 days mortality.

**REFERENCE(S)**

- Burns K, Soliman I, Adhikari M, Swein A, Wong J, Gomez-Builes C, Pellegrini JA et al. Trials directly comparing alternative spontaneous breatghing trials techniques: a systematic review and meta-analysis. Crit Care 2017 (21): 127.
- Schmidt G, Girard T, Kress J, Morris P, Oullette D, Alhazzani W, Burns S et al. Liberation from mechanical ventilation in citically ill adults. Chest 2017 (151);1: 160-165.

### 0504

#### Targeting low-normal vs. high-normal carbon dioxide after cardiac arrest and resuscitation: a randomized pilot trial (NCT02698917)

P. Jakkula[1], M. Reinikainen[2], J. Hästbacka[1], P. Loisa[3], M. Tiainen[1], V. Pettilä[1], J. Toppila[1], M. Lähde[3], M. Bäcklund[1], S. Bendel[4], T. Birkelund[5], A. Pulkkinen[6], T. Tikka[1], M. Skrifvars[1], COMACARE Study Group
[1]University of Helsinki and Helsinki University Hospital, Helsinki, Finland; [2]North Karelia Central Hospital, Joensuu, Finland; [3]Päijät-Häme Central Hospital, Lahti, Finland; [4]Kuopio University Hospital, Kuopio, Finland; [5]Aarhus University Hospital, Aarhus, Denmark; [6]Central Finland Central Hospital, Jyväskylä, Finland
**Correspondence:** P. Jakkula
*Intensive Care Medicine Experimental* 2018, **6(Suppl 2):**0504

**INTRODUCTION.** Arterial carbon dioxide tension ($PaCO_2$) affects cerebral blood flow (CBF) and cerebral oxygen delivery and may influence the course of developing brain injury after cardiac arrest. [1] The level of $PaCO_2$ can be modified by different ventilator settings in unconscious patients during post-resuscitation care but optimal targets remain unknown. [2]

**OBJECTIVES.** We conducted a randomized pilot trial to assess the feasibility of targeting low-normal or high-normal $PaCO_2$ in comatose, mechanically ventilated patients after out-of-hospital cardiac arrest (OHCA) and to evaluate the effect of low- or high-normal $PaCO_2$ on neuron-specific enolase (NSE) and cerebral oxygenation as measured by near-infrared spectroscopy (NIRS).

**METHODS.** In the Carbon dioxide, Oxygen and Mean arterial pressure After Cardiac Arrest and REsuscitation (COMACARE) trial, we used a $2^3$ factorial design to randomly assign patients after OHCA and resuscitation to low-normal or high-normal levels of $PaCO_2$, arterial oxygen tension and mean arterial pressure. The results of low-normal (4.5-4.7 kPa) vs. high-normal (5.8-6.0 kPa) $PaCO_2$ comparison are reported here.

The primary outcome was serum concentration of NSE at 48 h after cardiac arrest. The main feasibility outcome was the difference in $PaCO_2$ between the groups during the first 36 h after ICU admission. Secondary outcomes included cerebral oxygenation measured with continuous NIRS.

**RESULTS.** A total of 123 patients were enrolled in the trial and 120 patients included in the final analysis. Despite a clear separation in $PaCO_2$ between the low-normal and high-normal groups (Figure 1), there was no significant difference in the median NSE concentration at 48 h after cardiac arrest (for low-normal $PaCO_2$, 18.8 μg/l [IQR, 13.9-28.3 μg/l] and for high-normal $PaCO_2$, 22.6 μg/l [IQR, 14.8-34.9 μg/l], p = 0.290). Median regional cerebral oxygen saturation ($rSO_2$) was significantly higher in the high-normal $PaCO_2$ group as compared with the low-normal group (Figure 2).

**CONCLUSIONS.** Targeting a specific narrow range of $PaCO_2$ is feasible in comatose, mechanically ventilated patients during post-resuscitation care. The level of $PaCO_2$, when within the normal range, did not affect the level of NSE at 48 h after OHCA. High-normal $PaCO_2$ resulted in better regional cerebral oxygen saturation indicating higher CBF and oxygen delivery.

**REFERENCE(S)**

1. Vaahersalo J, Bendel S, Reinikainen M, et al. Arterial blood gas tensions after resuscitation from out-of-hospital cardiac arrest: associations with long-term neurologic outcome. Crit Care Med 2014;42:1463-70.
2. Nolan JP, Soar J, Cariou A, et al. European Resuscitation Council and European Society of Intensive Care Medicine Guidelines for Post-resuscitation Care. Resuscitation 2015;95:202-22.

**GRANT ACKNOWLEDGMENT**
University of Helsinki project grant (H3702,WBS73702705) and Helsinki University Hospital governmental grant (TYH2018227).

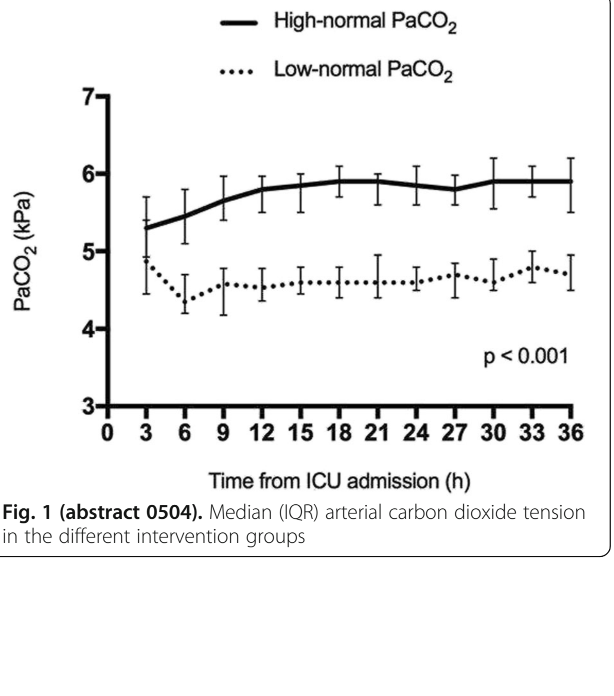

**Fig. 1 (abstract 0504).** Median (IQR) arterial carbon dioxide tension in the different intervention groups

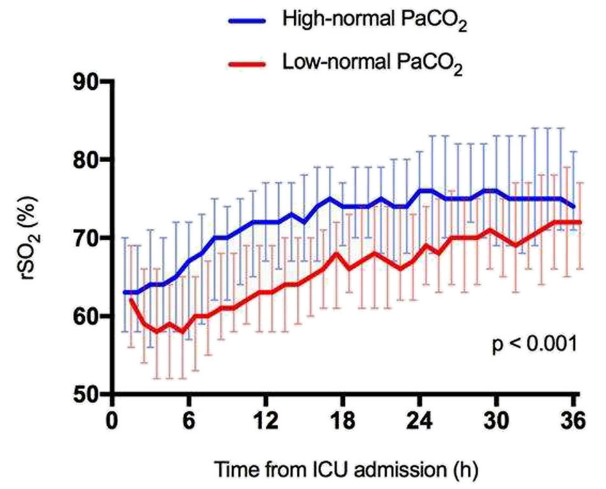

**Fig. 2 (abstract 0504).** Median (IQR) frontal cerebral oxygen saturation in the different intervention groups

### 0505

#### Target attainment of empirically dosed continuous infusion of piperacillin/tazobactam and meropenem in critically ill patients

S. Dhaese[1], A. Thooft[2], A. Farkas[3], J.A. Roberts[4,5,6], J. Lipman[4,5], A.G. Verstraete[7,8], V. Stove[7,8], J.J. De Waele[1]
[1]Ghent University Hospital, Department of Intensive Care Medicine, Ghent, Belgium; [2]Ghent University, Ghent, Belgium; [3]Mount Sinai West Hospital, Department of Pharmacy, New York, United States; [4]University of Queensland, Burns, Trauma and Critical Care Research Centre, Brisbane, Australia; [5]Royal Brisbane and Women's Hospital, Department of Intensive Care Medicine, Brisbane, Australia; [6]Royal Brisbane and Women's Hospital, Department of Pharmacy, Brisbane, Australia; [7]Ghent University Hospital, Department of Laboratory Medicine, Ghent, Belgium; [8]Ghent University, Department of Clinical Chemistry, Microbiology and Immunology, Ghent, Belgium
**Correspondence:** S. Dhaese
*Intensive Care Medicine Experimental* 2018, **6(Suppl 2):**0505

**INTRODUCTION.** Continuous infusion has proven to optimize beta-lactam antibiotic pharmacokinetic/pharmacodynamic (PK/PD) properties by increasing the time above the minimal inhibitory concentration ($\%fT_{>MIC}$)[1]. There is no consensus on the optimal PK/PD target but experts have recommended beta-lactam levels as high as 4 times the MIC [2]. It is largely unknown whether standard, empirical dosing of piperacillin/tazobactam (TZP) and meropenem (MER) reach adequate plasma levels when infused continuously in critically ill patients.

**OBJECTIVES.** To describe target attainment rates (%) of empirical, standard dosing of continuous infusion TZP and MER in critically ill patients.

**METHODS.** Daily sampling of patients admitted to the ICU receiving continuous infusion TZP or MER was attempted. Standard dosing for TZP was 16/2 g per day, while MER was dosed at 3g per day. Dosing was adapted based on renal function. Total plasma concentrations of TZP and MER were determined using ultra high-performance liquid chromatography coupled to mass spectrometry (UPLC-MS/MS). A level of protein binding of 30% and 2% was adopted for piperacillin and MER respectively. Target attainment was calculated for $100\%fT_{>4xMIC}$ for the micro-organism with the highest clinical breakpoint as published by EUCAST, i.e. 16 mg/L for TZP and 2 mg/L for MER.

**RESULTS.** 233 and 65 patients were included for TZP and MER analysis respectively. Over the entire antibiotic course, 137 (59%) and 16 (25%) of the patients receiving TZP, respectively MER in continuous infusion had one or more concentrations below the PK/PD target of 4 times the MIC. When only the first 48 hours of therapy were considered, 79 out of 153 (52%) and 7 out of 35 (20%) patients did not achieve target concentrations for TZP and MER respectively. The observed piperacillin, respectively meropenem concentrations and the number of samples per day over the first 10 days of therapy are shown in figure 1.

**CONCLUSIONS.** ICU patients receiving empirical, standard doses of piperacillin/tazobactam (16/2g /24h) in continuous infusion tend to have plasma concentrations below the proposed PK/PD target level of 4 times the MIC while ICU patients receiving empirical, standard doses of meropenem (3g /24h) in continuous infusion are likely to achieve target concentrations.

##### REFERENCE(S)

1 Abdul-Aziz MH, Lipman J, Mouton JW, Hope WW, Roberts JA. Applying pharmacokinetic/pharmacodynamic principles in critically ill patients: optimizing efficacy and reducing resistance development. 2015; **36**: 136-153.
2 Delattre IK, Taccone FS, Jacobs F, *et al.* Optimizing β-lactams treatment in critically-ill patients using pharmacokinetics/pharmacodynamics targets: are first conventional doses effective? *Expert Rev Anti Infect Ther* 2017; **15**: 677-688.

##### GRANT ACKNOWLEDGMENT

Sofie Dhaese is funded by a CRE grant (APP1099452) from the NHMRC awarded to Jason A. Roberts.

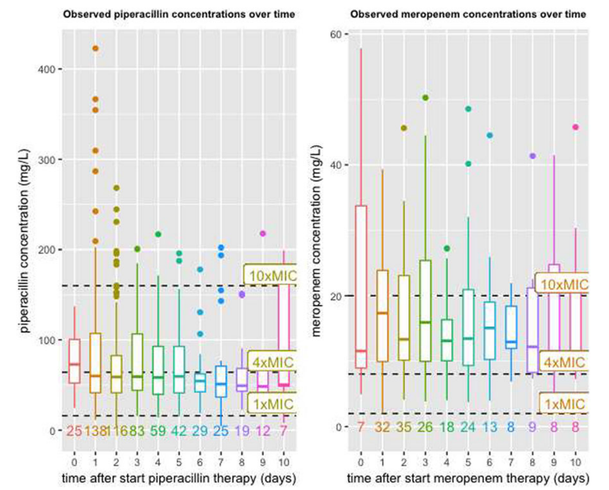

**Fig. 1 (abstract 0505).** Observed piperacillin (left) and meropenem (right) concentrations over time

### 0506

#### Translational studies into the immunomodulatory effects of norepinephrine and alternative vasopressors

R. Stolk[1,2,3], J. Schouwstra[1,2], S. Bressers[1,2], J. Gerretsen[1,2], H. van Leeuwen[3], P. Pickkers[1,2], M. Kox[1,2]
[1]Radboud University Medical Centre, Intensive Care, Nijmegen, Netherlands; [2]Radboud Center for Infectious Diseases, Nijmegen, Netherlands; [3]Rijnstate Hospital, Intensive Care, Arnhem, Netherlands
**Correspondence:** R. Stolk
*Intensive Care Medicine Experimental* 2018, **6(Suppl 2):**0506

**INTRODUCTION.** Sepsis-induced immunoparalysis, a phenomenon characterized by a severely suppressed state of the immune system, is increasingly recognized as the overriding immune dysfunction in sepsis. Norepinephrine represents the cornerstone vasopressor treatment for patients with septic shock, yet, *in vitro* evidence indicates it exerts anti-inflammatory effects. Therefore, norepinephrine could inadvertently compromise host immunity. Alternative vasopressors, such as phenylephrine or vasopressin, might hold an advantage in this respect. However, *in vivo* data on the immunomodulatory effects of vasopressors are lacking. We assessed the effects of norepinephrine and alternative vasopressors on the systemic immune response *in vivo* in mice. Furthermore, we performed a proof-of-principle study in healthy volunteers to translate these findings to the human setting.

**METHODS.** C57BL/6J mice received continuous intravenous infusion of norepinephrine (5 µg/kg/min), phenylephrine (50 µg/kg/min), vasopressin (0.0006 IU/kg/min) or placebo (PBS) for 3 or 24 hours via micro-osmotic pumps. Thereafter, they were challenged with 5 mg/kg bacterial lipopolysaccharide (LPS) i.v. to elicit a systemic immune response, or saline, and were sacrificed 90 minutes later. Additionally, 40 healthy male volunteers were randomized to a 5-hour i.v. infusion of either norepinephrine (0.05 µg/kg/min), phenylephrine (0.5 µg/kg/min), vasopressin (0.04 IU/min), or saline (n=10 per group). One hour into the start of vasopressor/saline infusion, subjects were intravenously challenged with 2 ng/kg LPS.

**RESULTS.** In mice, both norepinephrine and phenylephrine profoundly enhanced LPS-induced plasma levels of the anti-inflammatory cytokine IL-10, while attenuating concentrations of virtually all pro-inflammatory mediators. Furthermore, norepinephrine reduced neutrophilic ROS production. Vasopressin exerted no effects on inflammatory parameters. In humans, norepinephrine and phenylephrine increased blood pressure before LPS administration, but did not prevent LPS-induced hypotension. Similar to the findings in mice, norepinephrine and phenylephrine profoundly enhanced LPS-induced plasma concentrations of the anti-inflammatory cytokine IL-10, while attenuating levels of pro-inflammatory cytokines IP-10, MCP-1, IL-8, and GM-CSF. Vasopressin neither affected blood pressure, nor cytokine response.

**CONCLUSIONS.** Norepinephrine and phenylephrine cause a distinct shift towards an anti-inflammatory cytokine profile during systemic inflammation in mice and men, which was not observed for vasopressin. Furthermore, norepinephrine attenuated ROS production, an important effector mechanism of neutrophils. These results may signify that norepinephrine contributes to the development of immunoparalysis in septic shock patients, and that vasopressin might represent an alternative agent with less untoward immunologic effects.

**GRANT ACKNOWLEDGEMENT**

This work was supported by a Rijnstate-Radboudumc PhD grant.

### 0507

### Painful intensive care unit (ICU) stay reported by ICU survivors: influence of chest tube removal, intrahospital transport, and other events occurring during the stay in the ICU

P. Kalfon[1], M. Boucekine[2], P. Estagnasie[3], M.-A. Geantot[4], A. Berric[5], G. Simon[6], B. Floccard[7], T. Signouret[8], M. Fromentin[9], M. Nyunga[10], J. Audibert[11], A. Ben Salah[11], B. Mauchien[12], A. Sossou[13], M. Venot[14], R. Robert[15], A. Follin[16], A. Renault[17], M. Garrouste-Orgeas[18], O. Collange[19], Q. Levrat[20], I. Villard[21], D. Thevenin[22], J. Pottecher[23], R.-G. Patrigeon[24], N. Revel[25], C. Vigne[26], E. Azoulay[14], O. Mimoz[27], P. Auquier[2], K. Baumstarck[2], The IPREA Study Group

[1]CH de Chartres, Chartres, France; [2]Université Aix Marseille, Marseille, France; [3]Clinique Ambroise Paré, Réanimation, Neuilly/Seine, France; [4]CHU Dijon Bourgogne, Département d'Anesthésie Réanimation, Dijon, France; [5]Centre Hospitalier Intercommunal Toulon La Seyne, Réanimation polyvalente, Toulon, France; [6]CH Troyes, Réanimation, Troyes, France; [7]CHU Edouard Herriot, Hospices Civils de Lyon, Réanimation Polyvalente, Lyon, France; [8]Hôpital Européen de Marseille, Réanimation, Marseille, France; [9]CHU Cochin, Assistance Publique Hôpitaux de Paris, Réanimation Chirurgicale, Paris, France; [10]CH Victor Provo, Réanimation Polyvalente, Roubaix, France; [11]CH Chartres, Réanimation Polyvalente, Chartres, France; [12]CH Chartres, Réanimation, Chartres, France; [13]CH Emile Roux, Réanimation, Le Puy-en-Velay, France; [14]CHU Saint Louis Assistance Publique Hôpitaux de Paris, Réanimation Médicale, Paris, France; [15]CHU La Miletrie, Réanimation Médicale, Poitiers, France; [16]Hôpital Européen Georges Pompidou, Réanimation Chirurgicale, Paris, France; [17]CHU La Cavale Blanche, Réanimation Médicale, Brest, France ; [18]Groupe Hospitalier Paris Saint Joseph, Médecine Intensive et Réanimation, Paris, France; [19]CHU Strasbourg, Nouvel Hôpital Civil, Réanimation Chirurgicale Polyvalente, Strasbourg, France; [20]Groupe Hospitalier de La Rochelle-Ré-Aunis, Réanimation, La Rochelle, France; [21]CHU Beaujon, Anesthesie Réanimation, Clichy, France; [22]CH Lens, Réanimation, Lens, France; [23]CHU Strasbourg, Hôpital Hautepierre, Réanimation Chirurgicale, Strasbourg, France; [24]CH Auxerre, Réanimation, Auxerre, France; [25]CHU Nice, Réanimation Médico-Chirugicale, Nice, France; [26]CHU Hôpital Nord Assistance Publique Hôpitaux de Marseille, Réanimation Polyvalente et Traumatologique, Marseille, France; [27]CHU La Miletrie, Réanimation Chirurgicale, Poitiers, France

**Correspondence:** P. Kalfon

*Intensive Care Medicine Experimental* 2018, **6(Suppl 2):**0507

**INTRODUCTION.** Chest tube removal and turning in bed were reported as the most painful procedures performed in a multinational study of procedural pain in the ICU[1]. Little is known of the short-term consequences of such procedures, particularly patient´s perceptions at the end of the ICU stay.

**OBJECTIVES.** The objective of the study was to search an association between chest tube removal and other potential painful events that may occur in the ICU and self-reported pain at the end of the ICU stay when the patient is interviewed about the entire ICU stay

**METHODS.** We have conducted a multicenter, cluster-randomized, two-arm parallel, 3-period trial involving 34 adult ICUs to test the efficacy of a tailored multicomponent program (TMCP) for discomfort reduction in critically ill patients[2]. To assess the primary endpoint, we used the French 16-item questionnaire on discomforts in ICU patients (IPREA) including the item pain, each item discomfort score ranging from 0, the lowest possible level, to 10 the highest. Since pain was the only item of IPREA whose score was not reduced by the TMCP, we included in the analysis all the patients regardless of the arm or the period. The associations of main patient characteristics at ICU admission, events or treatments that occurred in the ICU with the pain score (PS) were tested using Kruskal-Wallis tests or Spearman´s rank-correlation coefficients as appropriate.

**RESULTS.** During the 3 periods, 2130 patients with complete IPREA questionnaire were included. PS was correlated, negatively with age (ρ=-0.051; *p*=0.019) and positively with the duration of the pre-ICU hospitalization (ρ=0.068; *p*=0.002) and the length of stay (ρ=0.046; *p* =0.034) but not with SAPS 2 or gender. Surgical patients reported a higher PS than medical patients (3.11±2.87 vs 2.85±3.02, *p*=0.007). Among procedures or treatments occurring in the ICU, PS was correlated with chest tube removal, chest tube insertion, tracheostomy, complex dressing, intrahospital transport, central venous catheter insertion, gastric tube, and bladder catheter (table 1), but not with mechanical ventilation, non invasive ventilation, use of vasopressors, renal replacement therapy, extra corporeal membrane oxygenation, bronchial fibroscopy, upper gastrointestinal endoscopy, transesophageal echography, arterial catheter insertion, or lumbar puncture.

**CONCLUSIONS.** ICU survivors experiencing chest tube removal or intrahospital transport during the ICU stay reported at discharge a more painful ICU stay than patients not experiencing these events.

**REFERENCE(S)**

1. Puntillo K et al. Determinants of procedural pain intensity in the intensive care unit. The Europain® study. *Am J Respir Crit Care Med* 2014 189:39-47
2. Kalfon P, Baumstarck K, Estagnasie P et al. A tailored multicomponent program to reduce discomfort in critically ill patients: a cluster-randomized controlled trial. *Intensive Care Med* 2017, 43; 1829:40.

**GRANT ACKNOWLEDGMENT**

French Ministry of Health.

**Table 1 (abstract 507).** See text for description

| | N (no event / ≥1event) | PS (no event) | PS (≥1event) | p |
|---|---|---|---|---|
| Chest tube removal | 1732 / 398 | 2.90 ± 3.01 | 3.36 ± 2.58 | <0.001 |
| Chest tube insertion | 1980 / 150 | 2.95 ± 2.94 | 3.43 ± 2.95 | 0.037 |
| Tracheostomy | 2078 / 52 | 2.97 ± 2.94 | 3.90 ± 2.89 | 0.015 |
| Central venous catheter insertion | 1340 / 789 | 2.86 ± 2.87 | 3.21 ± 3.06 | 0.014 |
| Complex dressing | 1711 / 419 | 2.92 ± 2.91 | 3.28 ± 3.06 | 0.032 |
| Intrahospital transport | 1520/ 610 | 2.84 ± 2.91 | 3.36 ± 3.00 | <0.001 |
| Gastric tube | 1199 / 931 | 2.82 ± 3.05 | 3.23 ± 2.70 | 0.006 |
| Bladder catheter | 367 / 1763 | 2.54 ± 2.84 | 3.08 ± 2.96 | 0.001 |

## Sepsis identification & prognosis

### 0508

#### Association of septic shock definitions and standardized mortality ratio in a contemporary cohort of critically Ill patients

R. Kashyap, T. Singh, H. Rayes, J. O'Horo, G. Wilson, O. Gajic,
Multidisciplinary Epidemiology and Translational Research in Intensive Care - METRIC
Mayo Clinic, Rochester, United States
**Correspondence:** R. Kashyap
*Intensive Care Medicine Experimental* 2018, **6(Suppl 2):**0508

**INTRODUCTION.** The newly proposed septic shock definition has provoked a substantial controversy in the emergency and critical care communities.

**OBJECTIVES.** Our objective was to compare new (SEPSIS-III) versus old (SEPSIS-II) definitions for septic shock in a contemporary cohort of critically ill patients.

**METHODS.** Retrospective cohort of consecutive patients, age ≥18 years admitted to intensive care units at the Mayo Clinic between January 2009 and October 2015. We excluded patients without research authorization on file, and those admitted after elective surgery. We extracted demographics, clinical characteristics and outcomes from the validated electronic medical record database and compared patients who met the old, new, both, or neither definition of sepsis shock. Standardized mortality ratios were calculated using Acute Physiology and Chronic Health Evaluation (APACHE) IV predicted mortality.

**RESULTS.** The initial cohort consisted of 16720 patients who had suspicion of infection, of whom 7463 required vasopressor support. The median (interquartile range) age was 65(54-75) years and 4167(55.8%) were male. A total of 2690(36%) patients met both definitions, 3256(44%) patients met only the criteria for the old definition, 361(4.8%) met only the criteria for new definition, and 1156(15%) patient had infection and shock but met neither old nor new criteria. Compared to patients who met only old definition, the patients who met new definition had higher APACHE III score; (84[66-106] vs. 70[56-89], p< 0.01); Sequential Organ Failure Assessment day-1 score; (9[6-11]) vs. 6[4-9], p< 0.01), were older; (65[54-76] versus 64[54-75] years, p=0.03), and were more likely to have limited resuscitation preferences; (555[18%] versus 383[12%], p< 0.01). They also had higher hospital mortality (27% vs. 13%, p< 0.01) and a higher standardized mortality ratio (0.69 vs. 0.45, p< 0.01). Patients meeting both definitions faired worst, while the patients meeting neither of the definitions had better outcomes.

**CONCLUSIONS.** Compared to SEPSIS-II, SEPSIS-III definition of septic shock identifies patients further along disease trajectory with higher likelihood of poor outcome. This finding has implications for quality improvement and performance measures. A significant number of patients with infection and shock meet neither definition.

**REFERENCE(S)**

1. Singer M, Deutschman CS, Seymour CW, Shankar-Hari M, Annane D, Bauer M, et al. The Third International Consensus Definitions for Sepsis and Septic Shock (Sepsis-3). JAMA. 2016;315(8):801-10.
2. Mikkelsen ME, Miltiades AN, Gaieski DF, Goyal M, Fuchs BD, Shah CV, et al. Serum lactate is associated with mortality in severe sepsis independent of organ failure and shock. Crit Care Med. 2009;37(5):1670-7.

**GRANT ACKNOWLEDGMENT**

This project is supported by intramural funding from the Critical Care Independent Multidisciplinary Program (IMP) at Mayo Clinic, Rochester, MN, USA

### 0509

#### Hospital pre-alerting in a cohort of potentially septic patients brought into the emergency department by ambulance, is associated with more rapid delivery of antibiotics and improvement in physiological parameters at hospital admission

D. Hargreaves[1], J. de Carvalho[2], L. Hodgson[1], L. Smith[2], G. Picton[1], R. Venn[1]

[1]Western Sussex Hospitals Foundation NHS Trust, Worthing, United Kingdom; [2]University of Brighton and Sussex Medical School, Brighton, United Kingdom

**Correspondence:** D. Hargreaves

*Intensive Care Medicine Experimental* 2018, **6(Suppl 2):**0509

**INTRODUCTION.** Although prompt administration of antibiotics are associated with improved outcomes in sepsis (1), it is unclear whether using a pre-hospital sepsis screening tool provides significant clinical benefit (2). In 2015 our regional ambulance service introduced a prehospital sepsis screening tool (coined 'Code Yellow'), which combines physiological parameters and symptoms and signs of compromised perfusion, to be used when paramedics suspected an acute infection. Positive cases were given initial resuscitation, pre-alerted and rapidly delivered to the local emergency department (ED).

**OBJECTIVES.** To evaluate whether outcomes and processes of care were improved with use of sepsis pre-alert tool in a cohort of potentially septic patients brought by ambulance to two UK EDs. Mortality risk was assessed by the UK National Early Warning Score (NEWS) in the pre- and hospital environments.

**METHODS.** A dual-centre, prospective cohort (2015-16) of ED patients brought in by ambulance (n=1232), identified as potentially septic at triage, was analysed for mortality (death at 30 days) and change and trend in mortality risk (NEWS score pre-hospital, in the ED, and at first recording on admission to the medical ward). Cases where the ED had been pre-alerted (Code Yellow positive) were compared with those identified on ED arrival.

**RESULTS.** 58% (714/1232) of the cohort were Code Yellow. There was no difference in 30-day mortality (19% vs 18%, p=0.515), age, gender, previous medical history, admission time and day, AKI or lactate between the groups. **[Fig 1]**

Mortality risk, indicated by first pre-hospital NEWS, was greater in the Code Yellow group (9 [IQR] vs 7 [IQR], p< 0.001). This difference was maintained in first ED NEWS score also (7 vs 6, p=0.04) but by admission to a medical ward there was no significant difference in NEWS between the groups (3 [IQR] vs 3 [IQR], p=0.125). **[Fig 2]**

Repeat measure factorial analysis of variance of the differences in scores and the trend in change of score were very significantly different (p < 0.0001). **[Fig 3]**

The Code Yellow group was significantly more likely to receive antibiotics within 1 hour (72% vs 49%) p< 0.0001, and a completed sepsis resuscitation bundle (34% vs 21%) p< 0.0001.

**CONCLUSIONS.** A prehospital sepsis tool with hospital pre-alert identified a cohort with a higher risk of death. The group identified had improved delivery of antibiotics and significant improvement in trend for mortality risk (assessed by NEWS score) by the time of admission to a hospital ward. They had similar in-patient outcomes to those not pre-alerted. This equivalent outcome and mortality risk on admission to the ward could be interpreted as an overall mortality benefit.

##### REFERENCE(S)

1. Seymour CW, et al. Time to Treatment and Mortality during Mandated Emergency Care for Sepsis. *NEJM*. 2017;376(23):2235-44.
2. Smyth MA, et al. Identification of adults with sepsis in the prehospital environment: a systematic review. *BMJ Open*. 2016;6(8):e011218-11.

**Patient Characteristics Pre-alert vs Not Pre-alert**

| Characteristic | Pre-alerted (Code Yellow) n=714 | Not pre-alerted n= 519 | Odds ratio (95% CI) | p-value |
|---|---|---|---|---|
| *Demographics* | | | | |
| Male | 380 (53.2%) | 289 (55.7%) | 0.91 (0.72-1.14) | 0.424 |
| Female | 334 (46.8%) | 230 (44.3%) | | |
| Age | 76.5 (±13.5) | 74.8 (±16.5) | | 0.672 |
| Site Worthing (n=547) | 300 (42.0%) | 247 (47.6%) | 0.80 (0.64-1.00) | 0.059 |
| Site St Richards (n=686) | 414 (58.0%) | 272 (52.4%) | | |
| Weekday admission | 513 (72.2%) | 374 (72.1%) | 1.00 (0.78-1.29) | 0.972 |
| Weekend admission | 198 (27.8%) | 145 (27.9%) | | |
| *Past Medical History* | | | | |
| Hypertension | 245 (36.0%) | 153 (30.7%) | 1.27 (0.99-1.63) | 0.057 |
| CCF | 117 (17.2%) | 85 (17.1%) | 1.01 (0.74-1.37) | 0.951 |
| Diabetes | 171 (25.1%) | 136 (27.3%) | 0.89 (0.69-1.16) | 0.404 |
| Vascular Disease | 193 (28.4%) | 115 (23.1%) | 1.32 (1.01-1.72) | 0.042* |
| Liver Disease | 17 (2.5%) | 20 (4.0%) | 0.61 (0.32-1.18) | 0.144 |
| Indwelling Urinary Catheter | 43 (6.0%) | 31 (6.0%) | 1.01 (0.63-1.63) | 0.957 |
| Indwelling Vascular Device | 9 (1.3%) | 2 (0.4%) | 3.31 (0.71-15.40) | 0.126 |
| AKI in ED | 43 (8.6%) | 20 (5.8%) | 1.53 (0.88-2.64) | 0.132 |
| *Mortality Outcomes & Predictors* | | | | |
| ED Lactate | 2.16 (±1.72) | 2.18 (±1.75) | | 0.059 |
| Mortality at 30 days | 137 (19.2%) | 92 (17.7%) | 1.10 (0.82-1.48) | 0.515 |
| Pre-Hospital first NEWS | 9 [6-10] | 7 [5-9] | | <0.001* |
| ED first NEWS | 7 [5-8] | 6 [4-8] | | 0.037* |
| Ward first NEWS | 3 [2-6] | 3 [2-5] | | 0.125 |

Data are presented as either count (%), mean ±SD, or median [IQR]. * = statistically significant difference between groups.
Statistical tests used: Mann-Whitney, Chi-Square, Fisher's and Mantel-Haenszel.
CCF, Congestive Cardiac Failure; CNS, Central Nervous System; ENT, Ear Nose & Throat; NEWS, National Early Warning Score; ED, Emergency Department; AKI, Acute Kidney Injury, NEWS.

**Fig. 1 (abstract 0509).** Description of Prehospital Cohort

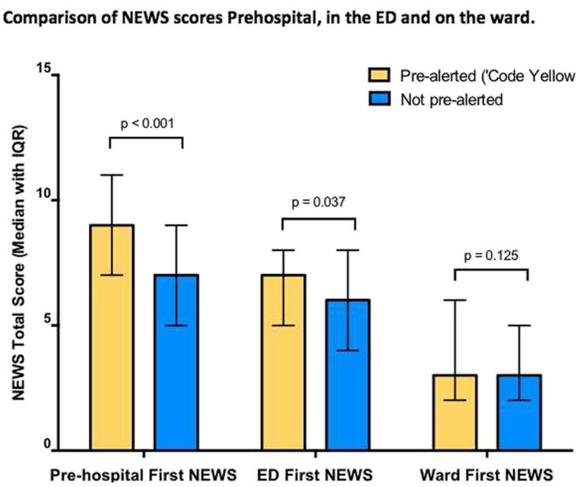

**Fig. 2 (abstract 0509).** Trend of NEWS scores Prehospital, in the ED and on the ward [Tails = IQR] and p-values

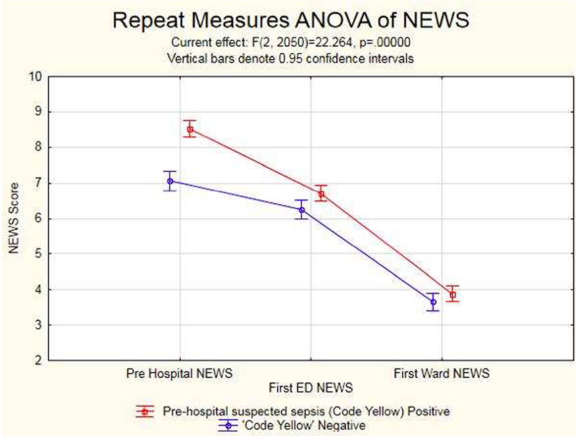

**Fig. 3 (abstract 0509).** Repeat Measure Factorial ANOVA of NEWS Scores and trend from Prehospital to the Ward

### 0510

#### Diagnostic accuracy of a novel multiplex real-time PCR on whole blood for pathogen identification in critically ill patients with presumed sepsis

K. van de Groep[1,2], M.R.J. Varkila[1,2], M.P. Bos[3], P.H.M. Savelkoul[3,4,5], D.S.Y. Ong[1,6], N.P. Juffermans[7], T. van der Poll[8,9], M.J.M. Bonten[1,10], O.L. Cremer[2], on behalf of the MARS Consortium
[1]UMC Utrecht, Epidemiology, Julius Center for Health Sciences and Primary Care, Utrecht, Netherlands; [2]UMC Utrecht, Intensive Care Medicine, Utrecht, Netherlands; [3]Microbiome, Amsterdam, Netherlands; [4]VU Medical Center, Medical Microbiology and Infection Control, Amsterdam, Netherlands; [5]Maastricht University Medical Center, Medical Microbiology, Maastricht, Netherlands; [6]Franciscus Gasthuis & Vlietland, Medical Microbiology and Infection Control, Rotterdam, Netherlands; [7]Academic Medical Center, Intensive Care, Amsterdam, Netherlands; [8]Academic Medical Center, Center of Experimental and Molecular Medicine, Amsterdam, Netherlands; [9]Academic Medical Center, Infectious Diseases, Amsterdam, Netherlands; [10]UMC Utrecht, Medical Microbiology, Utrecht, Netherlands
**Correspondence:** K. van de Groep
*Intensive Care Medicine Experimental* 2018, **6(Suppl 2):**0510

**INTRODUCTION.** Rapid pathogen identification is essential for optimizing antimicrobial treatment (AMT) during sepsis. Culture based methods are slow and their sensitivity is impaired by concurrent AMT. A novel multiplex real-time PCR has recently been developed (BSI-PCR, Microbiome), enabling detection of microbial DNA of common pathogens directly in blood.

**OBJECTIVES.** To determine the diagnostic accuracy of BSI-PCR results in sepsis patients acutely admitted to the Intensive Care Unit (ICU).

**METHODS.** Patients were enrolled as part of the Molecular Diagnosis and Risk Stratification of Sepsis (MARS) cohort in two tertiary ICUs in the Netherlands from 2012 to 2014. We selected subjects presenting with presumed sepsis and related organ failure or septic shock, in whom a blood culture was ordered within 24h from start of AMT. Whole blood specimens (5 mL) were simultaneously collected, in which enrichment and isolation of microbial DNA (Polaris method) and BSI-PCR runs were performed. Likelihood of infection and potential causative microorganism(s) were prospectively recorded by trained observers based on a post-hoc assessment of available clinical and microbiological evidence. Accuracy of BSI-PCR was then determined using these causal pathogens as reference test (RT).

**RESULTS.** Out of 791 sepsis patients, 325 (41%) patients had a PCR sample available at ICU-admission. Pneumonia was the most common source of infection (53%), followed by abdominal infections (19%). BSI-PCR identified one or more pathogens in 169 (52%) patients (Table 1), with a median of 2 (IQR 1-2) positive results per patient. In 99 (30%) patients, at least one identified pathogen was in accordance with the RT. In comparison, performed clinical blood cultures revealed a presumed causative pathogen in 63 (19%) patients.Discordant positive PCR results were observed in 101 (31%) patients, but for 29 patients these were related to a RT classifying the pathogen as unknown. In 156 (48%) patients BSI-PCR did not identify any pathogen. However, for 79 (51%) of these subjects this could likely be a true negative finding, since their RT post-hoc infection likelihood was classified as only possible or none. The multiplex set up of BSI-PCR consists of both species-specific and generic PCRs (Table 2). The positive predictive value (PPV) per individual PCR varied from 16-82% and the overall PPV of BSI-PCR results was 54% (Table 2). False positive rate per individual PCR varied from 0-8%. Ct-values for true positive results were significantly lower than for false positive results (median 32.0 (IQR 27.7-35.8) versus median 37.7 (IQR 35.3-40.0); $p < 0.001$).

**CONCLUSIONS.** BSI-PCR identified a causative pathogen in 30% of patients with presumed sepsis and the overall positive predictive value was 54%.

##### REFERENCE(S)

1. Klein Kouwenberg PM et al. 2013 Crit Care Med.

##### GRANT ACKNOWLEDGMENT

Center for Translational Molecular Medicine, MARS (grant 04I-201) and MARS&MORE (grant 09P-107).

**Table 1 (abstract 0510).** Patient-level BSI-PCR performance (*n*=325)

| Finding | Number of patients (%) | |
|---|---|---|
| BSI-PCR identified ≥1 pathogen: | 169 | (52%) |
| - BSI-PCR identified ≥1 true positive pathogen | 99 | (30%) |
| - BSI-PCR identified ≥1 positive pathogen and unknown pathogen as RT | 29 | (9%) |
| - BSI-PCR identified ≥1 false positive pathogen | 72 | (22%) |
| BSI-PCR identified no pathogen | 156 | (48%) |

RT: reference test. As a consequence of the multiplex set-up of BSI-PCR, results consisted of 11 individual PCR results per patient. Thus a single patient could have true positive as well as false positive results.

**Table 2 (abstract 0510).** Evaluation of positive BSI-PCR results in comparison to the presumed causative pathogens indentified by the reference test in critically ill patients with sepsis

| Pathogen or target (*n* positive) | | True positive | | False positive | |
|---|---|---|---|---|---|
| | | *n* | (PPV %) | *n* | (FPR %) |
| **A. Species-specific PCRs** | | | | | |
| *E. faecalis* | (16) | 7 | (44%) | 9 | (3%) |
| *E. faecium* | (11) | 9 | (82%) | 2 | (1%) |
| *S. aureus* | (34) | 15 | (44%) | 19 | (6%) |
| *S. pneumoniae* | (17) | 11 | (65%) | 6 | (2%) |
| *A. baumannii* | (1) | - | | 1 | (0%) |
| *E. coli* | (59) | 32 | (54%) | 27 | (8%) |
| *Klebsiella* species | (11) | 7 | (64%) | 4 | (1%) |
| *P. aeruginosa* | (32) | 5 | (16%) | 27 | (8%) |
| *C. albicans* | (0) | - | | - | |
| *C. glabrata* | (0) | - | | - | |
| *C. krusei* | (0) | - | | - | |
| **Total A.** | **(181)** | **86** | **(48%)** | **95** | **(3%)** |
| **B. Generic PCRs** | | | | | |
| *Enterococcus* genus | (25) | 12 | (48%) | 13 | (4%) |
| *Staphylococcus* genus [1] | (24) | 11 | (46%) | 13 | (4%) |
| Gram-positive bacteria | (30) | 24 | (80%) | 6 | (2%) |
| Gram-negative bacteria | (33) | 27 | (82%) | 6 | (2%) |
| 3*Candida* [2] | (2) | - | | 2 | (1%) |
| Pan-*Aspergillus* | (1) | - | | 1 | (0%) |
| **Total B.** | **(115)** | **74** | **(64%)** | **41** | **(2%)** |
| **Total** | **(296)** | **160** | **(54%)** | **136** | **(2%)** |

PPV: positive predictive value (= true positive / (true positive + false positive)).
FPR: false positive rate (= False positive/total *n*).
*n*: number of positive BSI-PCR results (multiple positive results possible per patient)

[1] Inferred from positivity in the *mecA* and/or *Staphylococcus* genus PCR.
[2] 3*Candida* PCR detects *C. albicans*, *C. tropicalis*, and *C. parapsilosis*.

### 0511

#### Role of Interleukin-6 on critically ill patients outcome: a post-hoc analysis of the FROG-ICU study

B.G. Chousterman[1,2], E. Feliot[1], A. Mebazaa[1,3], E. Gayat[1,3], FROG-ICU study group
[1]Hôpital Lariboisière, AP-HP, Département d'Anesthésie-Réanimation, Paris, France; [2]INSERM, U1160, Paris, France, [3]INSERM, U942, Paris, France
**Correspondence:** B.G. Chousterman
*Intensive Care Medicine Experimental* 2018, **6(Suppl 2):**0511

**INTRODUCTION.** Interleukin-6 (IL-6) is a pro-inflammatory cytokine known to increase during acute conditions such as those observed in intensive care units (ICUs). Elevated levels of IL-6 at ICU discharge were shown to be associated with increased mortality after sepsis or pneumonia (1).

**OBJECTIVES.** We aimed to study the impact of IL-6 levels on outcomes of ICU patients from a large multicenter cohort.

**METHODS.** We conducted a post-hoc study of the FROG-ICU cohort (NCT01367093) (2), a prospective, observational, multicenter cohort study conducted in 21 ICUs in France and Belgium. All consecutive adult patients admitted to ICU and requiring mechanical ventilation and / or inotropes were included. Clinical and demographic data were collected prospectively. Plasma and urine samples were collected at admission and at ICU discharge. The prognosis of the patients was followed up to 1 year. We included all patients for whom IL-6 measurements were performed. Correlations with the occurrence of organ failure or prognosis were performed using univariate and multivariate analyzes. A value of $p < 0.05$ was considered significant.

**RESULTS.** 2076 patients were included in the study, 65% were males. Patients had a median age of 63 (51-74) years, an SAPS II score of 49 (36-63), and IL-6 level at admission was 100.9 [43.5 - 261.7] pg / mL and 31.5 [15.3 - 61.5] pg / mL at ICU discharge. The level of IL-6 at admission is not correlated with the number of circulating leukocytes The admission level of IL-6 varies with (a) the admission diagnosis ($p < 0.001$), the highest rates being observed in patients with sepsis and pancreatitis, (b) the SOFA score (rho = 0.32, $p < 0.001$), (c) mortality in ICU (survivors: 89.34 [38.67 - 226.50] vs non-survivors: 164.35 [77.50 - 528.93], $p < 0.01$) The IL-6 level at ICU discharge is correlated with mortality at day 90 and at one year (J90: 28.4 [13.6 - 54.0] vs 53.9 [30.5 - 89.4] pg / mL and J365: 28.2 [13.7 - 53.8] vs 50.7 [25.0 - 91.9] pg / mL for survivors vs deceased, $p < 0.001$ for all). The AUC of IL-6 for predicting mortality at day 90 is 0.726 (0.684-0.768). An IL-6 level> 31 pg / mL is associated with a hazard ratio of 4.2 (2.8-6.3) and 2.7 (2.0-3.5) for mortality at day 90 and at one year respectively. These associations persist after adjustment on SAPS II score.

**CONCLUSIONS.** The levels of IL-6 at admission and at ICU discharge are confirmed to be associated with morbidity and mortality in ICU and after discharge. The monitoring of inflammation at ICU discharge may allow to identify patients at risk of mortality.

##### REFERENCE(S)

(1) Yende et al. AJRCCM 2008
(2) Gayat et al. Crit Care 2018

##### GRANT ACKNOWLEDGMENT

The FROG-ICU study was funded by the Programme Hospitalier de la Recherche Clinique (AON 10-216) and by a research grant from the Société Française d'Anesthésie—Réanimation

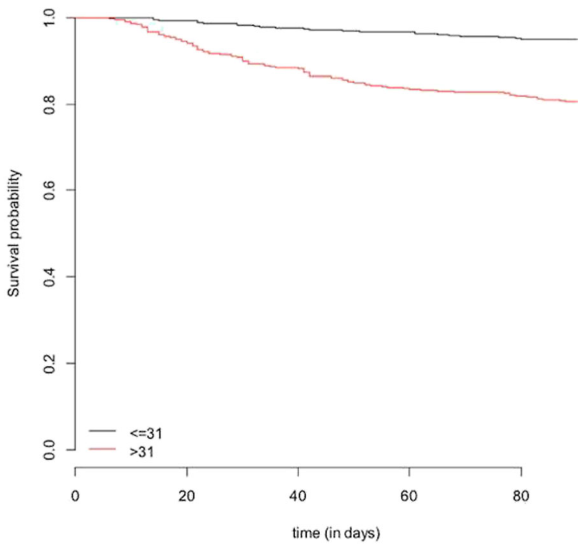

**Fig. 1 (abstract 0511).** Mortality at day 90 depending on the level of IL-6 at ICU discharge

### 0512

#### Global trends in sepsis mortality between 1985 and 2015: a temporal analysis of the WHO mortality database

J. Salciccioli[1], M. Komorowski[2], J. Shalhoub[2], P. Clardy[1], D. Marshall[3]
[1]Mount Auburn Hospital, Medicine, Cambridge, United States; [2]Imperial College London, Surgery and Cancer, London, United Kingdom; [3]Oxford University Hospitals NHS Trust, Oxford, United Kingdom
**Correspondence:** J. Salciccioli
*Intensive Care Medicine Experimental* 2018, **6(Suppl 2):**0512

**INTRODUCTION.** Sepsis consistently ranks among the most fatal disease syndromes globally. Various efforts have been made to improve standards for diagnosis, management and outcome reporting. Understanding the burden of disease is essential for monitoring the effectiveness of international strategies to improve management of sepsis.

**METHODS.** We assessed 30-year temporal trends in sepsis mortality globally using the WHO Mortality Database. Sepsis was defined by International Classification of Disease (ICD) versions 9 and 10 as previously described (1). We obtained sepsis-related, sex-specific mortality data from countries with "high usability data" from the WHO Mortality database and computed Age-Standardised Death Rates (ASDR). We used Joinpoint regression analysis to describe trends in sepsis morality.

**RESULTS.** A total of 34 countries were included in Europe, Australasia, and North America. In men, the median ASDR for all countries decreased from 36.2 / 100 000 (IQR 29.2 - 48.9) in 1985 to 27.1 /100 000 (IQR 21.4 - 40.3) in 2015. For women, the median ASDR for all countries decreased from 23.2 / 100 000 (IQR 17.2 - 31.0) in 1985 to 19.6 / 100 000 (IQR 14.0 - 15.3) in 2015. For men, the countries with the greatest percentage change over the observation period include Finland (- 80.9%), Iceland (-76.0%), New Zealand (-70.2%) and Ireland (-68.9%) while increases in mortality were observed in Denmark (+5.8%), Israel (+15.9%), Greece (+16.3%), and

Lithuania (+39.2%). For women, the greatest percentage decreases were observed in Finland (-79.4%), Iceland (-76.6%), Bulgaria (-70.0%), and Ireland (-62.3%) and increases were observed in Denmark (+20.4%), Lithuania (+23.0%), Greece (+33.5%), and Malta (+43.0%). The results of Joinpoint trend analysis are shown in Figure 1.

**CONCLUSION.** Overall, we observed a decrease in reported sepsis-related mortality across the majority of analysed nations between 1985 and 2015. However, there remains significant variability between health systems with respect to trends in sepsis-related mortality and between sexes in some countries. System-level and population-level factors may contribute to these differences and additional investigations are necessary to further explain these trends.

**REFERENCE(S)**

1. Angus D, Linde-Zwirble W, Lidicker J, Clermont G, Carcillo J, Pinsky M. Epidemiology of severe sepsis in the United States: analysis of incidence, outcome, and associated costs of care. Crit Care Med. 2001;29:1303-10

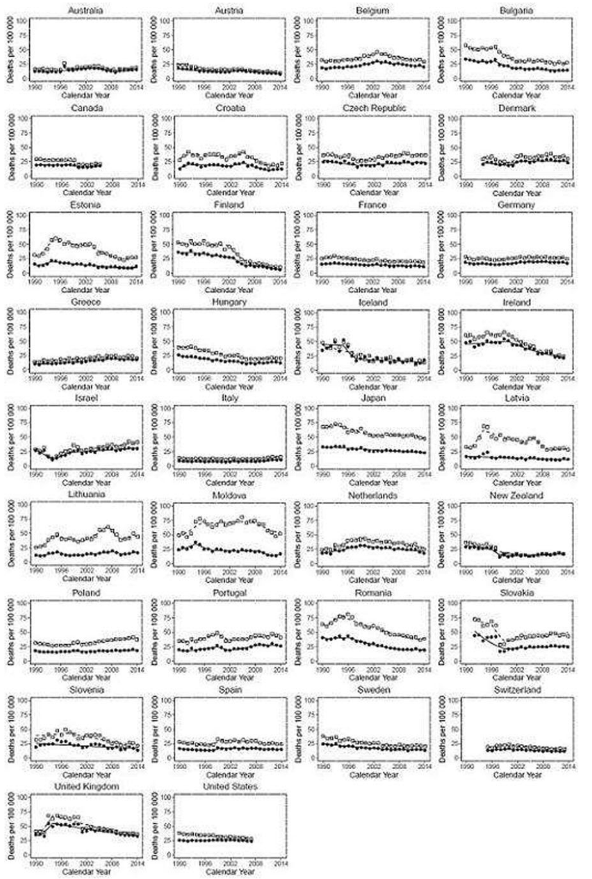

**Fig. 1 (abstract 0512).** Sepsis-related Age-Standardised Death Rates (ASDR) per 100,000. Boxes represent men, circles women

## Mechanical ventilation monitoring

### 0513

#### Transpulmonary pressures measurements as a mean to optimize mechanical ventilation in an unique series of patients combining ARDS and very severe morbid obesity

D. Vimpere, E. Guerot, A. Novara, N. Aissaoui, J.-L. Diehl
Hopital Europeen Georges Pompidou, Paris, France
**Correspondence:** D. Vimpere
*Intensive Care Medicine Experimental* 2018, **6(Suppl 2):**0513

**INTRODUCTION.** Morbid obesity and ARDS both affect respiratory mechanics mainly through their respective impacts on chest wall and lung elastances. We present a unique series of patients combining very severe morbid obesity and moderate to severe Acute Respiratory Distress Syndrome (ARDS).

**OBJECTIVES.** We describe the use of trans-pulmonary pressures (TPP) measurements for optimization of external PEEP setting.

**METHODS.** The monocentric observational study was performed in 10 morbidly obese patients admitted for moderate to severe ARDS. We performed an incremental PEEP trial (5 cm $H_2O$ steps) with TPP measurement (NutriVent probe, Sidam, Italy) in a semi-recumbent position as previously described (ref 1). A decremental PEEP trial after a recruitment maneuver was not performed since the safety of such a maneuver in this specific population is largely unknown. We defined two ways for determination of external PEEP setting: (1) PEEP necessary to obtain a positive expiratory TPP and (2) PEEP necessary to obtain a plateau pressure between 28 and 30 cm$H_2O$ (ref 2, maximal alveolar recruitment Express strategy). Data are expressed as numbers (%) and medians (interquartile range). Statistical analysis was made using the XLSTAT software.

**RESULTS.** We enrolled during 5 years 10 morbidly obese patients (62 (46-66))) admitted for a moderate to severe ARDS. Clinical characteristics are displayed in Table 1. The Express strategy indicated a PEEP setting of 17,5 cm$H_20$ (IR 15-20) whereas TPP-guided PEEP was 30 cm$H_20$ (IR 20-30), p=0,003. Driving pressure was higher in the Express strategy PEEP setting (10 cm $H_20$ (IR 9-12)) than in the TPP-guided PEEP (8 cm $H_20$ (IR 6-10)), p=0,78 . TPP-guided PEEP setting was higher than indicated by the Express strategy in all but one patient. Two patient suffered from transient hypotension when external PEEP was set at 25 and 30 cm$H_2O$, while no patient displayed an inspiratory TPP higher than 25 cmH20. Additional data will be provided during the meeting: pressure-volume curve at ZEEP (8 patients), CRF measurements (6 patients) and ABG and capnometry values at each PEEP level (7 patients).

**CONCLUSIONS.** In our ARDS patients with extremely severe obesity, an incremental PEEP trial with TPP measurements appeared to be safe and indicated a PEEP setting significantly higher than for the commonly-used ARDS strategies. Such an approach deserves further comparisons with other modalities of monitoring, such as CRF measurements, EIT studies, etc.

**REFERENCE(S)**

1. Diehl JL & Al. Paradoxical effects of positioning in patients with ARDS and preexisting pulmonary vasculitis or similar disorders. Am J Respir Crit Care Med. 2013;188:875-7.
2. Mercat A & Al. Positive end-expiratory pressure setting in adults with acute lung injury and acute respiratory distress syndrome: a randomized controlled trial. Expiratory Pressure (Express) Study Group. JAMA. 2008; 299(6): 646-55.

**Table 1 (abstract 0513).** Caracteristics of the 10 morbidly obese patients admitted for moderate to severe ARDS

| | |
|---|---|
| Age (years), median (IR) | 56 (48-64) |
| Weight (kg), median (IR) | 176 (145-197) |
| BMI, median (IR) | 62 (46-66) |
| Tidal volume (ml/kg IBW), median (IR) | 6 (6-8) |
| ARDS etiology (n) | |
| Pneumopathy | 5 |
| Secondary (sepsis) | 3 |
| Aspiration | 2 |
| PEEP for positive expiratory transplumonary pressure | 30 (20-30) |
| PEEP for Plateau oressure 28-30 cmH20 | 17,5 (15-20) |

### 0514

#### Early changes of end expiratory lung volume and lung strain predict lung collapse after PEEP decrement in recovering ARDS patients

N. Kongpolprom, M. Praipruksaphan
Chulalongkorn University King Chulalongkorn Memorial Hospital, Thai Red Cross, Bangkok, Division of Pulmonary and Critical Care Medicine, Department of Medicine, Faculty of Medicine, Pathumwan, Thailand
**Correspondence:** N. Kongpolprom
*Intensive Care Medicine Experimental* 2018, **6(Suppl 2):**0514

**INTRODUCTION.** Currently, there is limited evidence of PEEP titration techniques and predictors of the alveolar de-recruitment in recovering ARDS patients.

**OBJECTIVES.** To evaluate predictors of lung collapse after PEEP decrement in recovering ARDS patients.

**METHODS.** We conducted a prospective observational study in ARDS patients undergoing PEEP reduction. Eligible criteria were adult patients with ARDS by Berlin definition 2010, in recovery phase defined as PF ratio > 100 and using stable ventilator setting for > 24 hours with $FiO_2$ < 65% and PEEP ≥ 8 $cmH_2O$, $SpO_2$ >90% and no hemodynamic instability. All eligible patients were ventilated with Engstorm ventilators. Respiratory parameters including end expiratory lung volume(EELV) measured by nitrogen washout/washin technique, lung strain, respiratory system compliance($C_{RS}$) at the baseline and 5 minutes after PEEP reduction were recorded. PEEP was decreased by 2 $cmH_2O$ when primary physicians decided to perform decremental PEEP titration. Lung collapse events, defined as >2% decrease in $SpO_2$ or >10% decrease in $PaO_2$ from the baseline, were continuously observed during a 2 hour period after PEEP reduction.

**RESULTS.** Totally, 25 decremental PEEP procedures were performed in 16 ARDS patients. The majority of the patients were male with the median age of 65.5, median APACHE II of 15, median PF ratio of 260 and median PEEP of 10. The median baseline $C_{RS}$ was 35.63 ml/$cmH_2O$. There were 8 lung collapse events occurring after PEEP reduction. Most of the events(75%) appeared within 30 minutes after the procedures. The decreased EELV and increased lung strain at 5 minutes were associated with the lung collapse events with the thresholds of 10% (AUC 0.808, 95% CI 0.592 to 1.000, p = 0.017) and 24% (AUC 0.817, 95% CI 0.611 to 1.000, p = 0.014), respectively. However, the change of $C_{RS}$ was not associated with the lung collapse.

**CONCLUSIONS.** The 10% reduction in EELV and 24% increase in lung strain at 5 minutes after PEEP reduction predicted alveolar de-recruitment in 2 hours after the procedure, while the change of respiratory system compliance did not predict the event. A protocol of PEEP titration guided by the changes of these 2 parameters should be further studied in recovering ARDS patients to confirm our results.

### 0515

#### Diaphragmatic Tissue Doppler Imaging: a new technique to evaluate diaphragmatic dysfunction? A preliminary study

P. Sotiriou, E. Soilemezi, K. Kosmidis, A. Vemvetsou, M. Tsagourias[1], D. Matamis
Papageorgiou General Hospital, ICU, Thessaloniki, Greece
**Correspondence:** E. Soilemezi
*Intensive Care Medicine Experimental* 2018, **6(Suppl 2):**0515

**INTRODUCTION.** Tissue Doppler Imaging (TDI) is a useful echocardiographic technique extensively used to evaluate global and regional myocardial systolic and diastolic function. TDI uses Doppler to quantify the velocity of tissue rather than blood, and it can, therefore, be used to estimate the velocity of any moving tissue, including the diaphragm. However, TDI of the diaphragm has never being attempted before, meaning that no information so far exists regarding the values of diaphragmatic velocity during contraction and relaxation of the muscle even in healthy subjects. In this study, diaphragmatic motion was investigated using TDI in healthy individuals, in patients who succeeded a weaning trial and in weaning failure patients.

**METHODS.** Diaphragmatic TDI was easily recorded in 10 healthy individuals (5males and 5 females), in 9 weaning failure and in 23 weaning success patients. Weaning success or failure was identified using the generally accepted criteria at the end of a 20 min T-piece trial. In all subjects the following diaphragmatic TDI-derived parameters were measured: TDI peak contraction and peak relaxation velocity, velocity time integral (VTI) during inspiration, and the slope of the initial steepest part of the velocity waveform of diaphragmatic motion during relaxation, ie, the TDI-derived maximum relaxation rate (fig). We recorded 15 breaths for each individual, analyzing a total of 600 breaths. All the above mentioned diaphragmatic TDI-derived parameters in the three groups of participants were compared for differences between groups. Statistical analysis was performed with Kruskal-Wallis ANOVA after a Kolmogorov-Smirnov test for normal distribution.

**RESULTS.** TDI peak contraction velocities were no different between healthy volunteers and weaning success patients (1,3 ±0,2 $cm.sec^{-1}$ vs 1,5±0,6 $cm.sec^{-1}$ ); on the contrary, weaning failure patients exhibited significantly higher peak contraction velocities (4±3 $cm.sec^{-1}$, p< 0.01). Furthermore, similar differences between the three groups were found in peak relaxation velocities (1,32±0,3 $cm.sec^{-1}$ vs 1,7±0,6 $cm.sec^{-1}$ vs 2,4 ±2 $cm.sec^{-1}$ , p< 0.01) and in the TDI-derived relaxation rate (5,3±1,9 $cm.sec^{-2}$ vs 12,4±4 $cm.sec^{-2}$ vs 45±29 $cm.sec^{-2}$, p< 0.01). VTI was not found statistically different in the three groups.

**CONCLUSIONS.** Our results suggest that diaphragmatic TDI may be a valuable technique for the evaluation of the diaphragmatic contraction and relaxation. More studies are necessary to validate the normal values in males and females, the influence of aging on these values and to confirm the possible alterations of these values in diaphragmatic fatigue and in patients with difficult weaning.

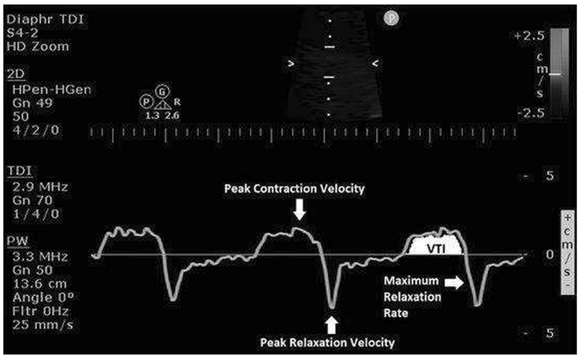

**Fig. 1 (abstract 0515).** Tissue Doppler Image (TDI) of the diaphragm]

### 0516

#### Effects of timing of tracheostomy on mortality in a heterogeneous population of critically ill patients admitted to Italian ICUs

M. Greco, M. Cecconi
Humanitas Research Hospital, Anesthesia and Intensive Care Department, Rozzano, Italy
**Correspondence:** M. Greco
*Intensive Care Medicine Experimental* 2018, **6(Suppl 2):**0516

**INTRODUCTION.** Tracheostomy is commonly performed to reduce complications of prolonged mechanical ventilation (MV). Delayed tracheostomy has been associated with increased mortality. However, evidence on timing of tracheostomy is still conflicting, and may vary according to the reason for ICU admission. (1)

**OBJECTIVES.** To identify whether early tracheostomy (performed within 9 days of MV) reduces mortality compared to delayed tracheostomy according to ICU admission diagnosis. We identified four admission diagnosis groups: trauma (not limited to brain injury), admission after surgery without known infection, admission due to infection, and other reasons.

**METHODS.** This is a secondary analysis from the GiVITI Prosafe prospective cohort study,(2) including tracheostomized patients aged ≥16 admitted to 261 Italian ICU between 2011 and 2013.

We performed bivariate analyses to check for association between mortality, tracheostomy timing, and covariates. Logistic regression was used to estimate odds ratio for hospital mortality, controlling for patient comorbidities, patient clinical condition at ICU admission and ventilation characteristics, and ICU admission reason. We tested for interaction between tracheostomy timing and ICU admission diagnosis.

**RESULTS.** From a total of 14,949 included patients, 10,075 had early and 4,874 late tracheostomy.

There were 2,949 (29.3%) deaths in early tracheostomy and 1,968 (40.4%) deaths in late tracheostomy group. The effect of tracheostomy timing on mortality depended on ICU admission reason. Late tracheostomy increased odds for mortality in patients admitted after surgery (31% [13-53%]) or for infection (23% [8-39%]), while it had no effect on trauma patients (OR 0.99, 95% CI 0.80-1.23) or patients admitted for other reasons (OR 0.97 [0.81-1.16]). With interaction between timing and admission diagnosis, late tracheostomy resulted in an 81% (53%-213%) increase in odds for mortality in surgical patients, in a 64% (40-92%) increase after infection, and in a 73% (44-207%) increase in patients admitted for other reasons. There was no association with trauma.

**CONCLUSION.** Using a large sample of critically ill patients undergoing tracheostomy, this study determined that the effect of timing of tracheostomy on mortality depends on patients' reason for ICU admission. Early tracheostomy is beneficial in patients admitted to ICU for infection or after surgery, while no clear effect was found in patients with trauma (not limited to brain trauma). Results of this study should be confirmed by further trials.

**ACKNOWLEDGMENT**

This is a secondary analysis of GIVITI Prosafe cohort study. We thank the GiViTI who collected and provided the data for this study.

### 0517

#### Assessment of reverse triggering during mechanical ventilation: validation of an automatic detection software

T. Pham[1,2], J. Montanya[3], T. Piraino[4], R. Magrans Nicieza[5], R. Mellado Artigas[1,2], F. Damiani[1,2], L. Chen[1,2], J. Gu[2], I. Telias[1,2], M. Dres[1,2,6], M. Rauseo[1,2,7], D. Junhasavasdikulj[1,2], L. Melo[1,2], P. Greco[4], H. Every[4], G. Sandhu[2], L. Blanch[5,8,9], L. Brochard[1,2]
[1]University of Toronto, Interdepartmental Division of Critical Care, Toronto, Canada; [2]Li Ka Shing Knowledge Institute, Keenan Research Centre for Biomedical Science, St. Michael's Hospital, Toronto, Canada; [3]Institut d'Investigació i Innovació Parc Taulí I3PT, Better Care SL, Sabadell, Spain; [4]St Michael's Hospital, Department of Respiratory Therapy, Toronto, Canada; [5]Institut d'Investigació i Innovació Parc Taulí I3PT, Sabadell, Spain; [6]CHU Pitié Salpêtrière, Réanimation Médicale et Surveillance Continue, Paris, France; [7]Università degli Studi di Foggia, Anestesia, Timaniazione e Terapia Intensiva, Foggia, Italy; [8]Universitat Autònoma de Barcelona, Hospital Universitari Parc Taulí, Critical Care Center, Sabadell, Spain; [9]Instituto de Salud Carlos III, Ciber Enfermedades Respiratorias (CIBERES), Madrid, Spain
**Correspondence:** T. Pham
*Intensive Care Medicine Experimental* 2018, **6(Suppl 2):**0517

**INTRODUCTION.** Reverse triggering (RT) is a diaphragmatic contraction triggered by an insufflation initiated by the ventilator [1]. This type of patient-ventilator dyssynchrony is often missed in clinical settings and can have important clinical impact such as increased tidal volume, breath stacking or eccentric diaphragm contraction. The Better Care system® (Sabadell, Spain) uses patient's flow and airway pressure signals to detect different types of dyssynchrony [2]. As part of the BEARDS study (NCT03447288) of the PLUG group, an algorithm to detect RT was recently developed based on tracings with either electrical activity of the diaphragm (EAdi) or esophageal pressure.

**OBJECTIVES.** We aimed at validating the accuracy of an automatic detection of reverse triggering with a software using flow and airway pressure only.

**METHODS.** Three researchers visually reviewed the airway pressure, flow and electrical activity of the diaphragm (EAdi) recordings of 9 patients included in a physiologic study (DIVIP study: NCT02434016). These patients were selected based on recent intubation (within 24h), use of a controlled mode and expected to stay on the ventilator at least another 24h; they had a one hour continuous recording of all signals.

Reverse triggering was defined as an EAdi starting after a machine triggered breath. To detect events with a potential clinical relevance we only kept events with peak>5μV. Discrepancies were solved by consensus. We then compared expert assessment with the results of the automatic detection of RT on the same breaths. Sensitivity, specificities, predictive values, accuracy and kappa for inter-rater agreement were calculated using standard formulas.

**RESULTS.** The 9 patients included had a mean±SD age of 62±19, 67% were male, 78% had a medical cause of admission, and APACHE II score of 24±10. Their median [IQR] length of mechanical ventilation was 5 [4-10] days and 89% were discharged alive from the ICU.

Among the 9 recordings, 7 were performed during pressure or volume control ventilation and 2 during pressure regulated volume control. A total of 1621 breaths were assessed of which 177 breaths (11%) were considered as RT>5 μV by experts. RT occurred mostly during the insufflation phase more rarely during the exhalation phase, and sometimes induced breath stacking (Figure). The automatic detection had an accuracy of 97%: a sensitivity of 89%, a specificity of 98%, a positive predictive value of 84%, a negative predictive value of 99% and a Kappa index of 85% [$_{95\%}$CI=81%; 89%].

**CONCLUSIONS.** Automatic detection of RT having potential clinical relevance seems reliable and could allow easy bedside continuous assessment and quantification. Future studies will determine the impact of this patient-ventilator dyssynchrony.

**REFERENCE(S)**

1. Akoumianaki E,et al (2013) Chest 143:927-938
2. Blanch L, et al (2012) Intensive Care Med 38:772-780 .

**GRANT ACKNOWLEDGMENT**

SRLF: Mobility Grant 2015
ESICM: Young investigator Award 2016
SCCM: Discovery Grant 2018

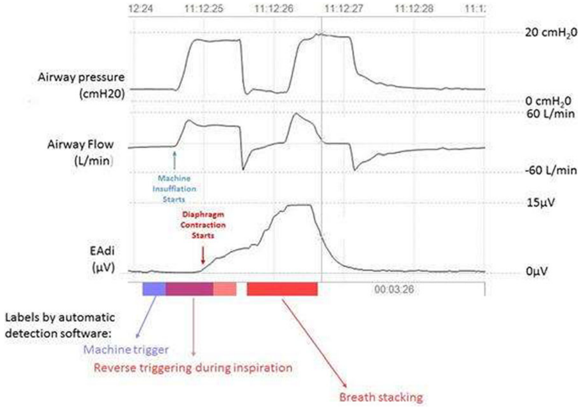

**Fig. 1 (abstract 0517).** Reverse triggering inducing breath-stacking

## Neurocritical Care

### 0518

#### Cerebral ketone metabolism at the acute phase of traumatic brain injury in humans: implications for glycemic and nutritional control

A. Bernini[1], M. Masoodi[2], D. Solari[3], L. Carteron[3], N. Christinat[2], P. Morelli[3], M. Beaumont[4], J.-P. Miroz[3], S. Abed-Maillard[5], M. Hartweg[4], F. Foltzer[4], B. Cuenoud[6], M. Oddo[3]
[1]Neuroscience Critical Care Research Group, Department of Intensive Care Medicine, CHUV-University Hospital and Faculty of Biology and Medicine, SMIA, Lausanne, Switzerland; [2]Nestlé Institute of Health Science, Ecole Polytechnique Fédérale (EPFL), Lausanne, Switzerland; [3]University of Lausanne - CHUV-Neuroscience Critical Care Research Group, Department of Intensive Care Medicine, CHUV-University Hospital and Faculty of Biology and Medicine, Lausanne, Switzerland; [4]Nestlé Research Center, Clinical Development Unit, Lausanne, Switzerland; [5]Neuroscience Critical Care Research Group, Department of Intensive Care Medicine, CHUV-University Hospital and Faculty of Biology and Medicine, Lausanne, Switzerland; [6]Nestlé Health Science, Lausanne, Switzerland
**Correspondence:** A. Bernini
*Intensive Care Medicine Experimental* 2018, **6(Suppl 2):**0518

**INTRODUCTION.** Adaptive metabolic response to injury includes utilization of alternative energy substrates, such as ketones, to protect the brain against further damage. Here, we investigated cerebral ketone metabolism at the acute phase of traumatic brain injury (TBI) in humans, focusing on the impact of caloric intake, glycemic targets and endogenous ketosis.
**METHODS.** This was an observational cohort study conducted in TBI subjects (N=34; mean age 49 years, mean GCS 7) who underwent cerebral microdialysis for the measurement of total brain interstitial tissue ketone bodies (KBs). We first analyzed (Study 1; N=24) changes of brain KBs from fasting (0 Kcal; mean 37 h from hospital admission) to initial caloric intake (500 Kcal; mean 16 h from nutrition start) and the influence of glycemic targets (categorized as tight [4-6 mmol/L] vs. moderate [6.1-10 mmol/L]) in this setting. We further examined (Study 2; N=10) the relationship between brain and endogenous circulating plasma KBs.
**RESULTS.** Initial caloric intake was associated with a significant decrease in total fasted brain KBs (from 32.9 [IQR 19.8-45.1] to 13.3 [8.8-25.9] μmol/L, p=0.0002, paired t-test), which was paralleled by a concomitant increase in brain and blood glucose (both p< 0.01). Brain KBs were higher at tight vs. moderate glycemic targets (41.2 [32.3-83.7] vs. 17.3 [9.4-32.5] μmol/L, p=0.001). Endogenous circulating plasma KBs reached markedly elevated levels (mean 1.14 [range 0.17-3.82] mmol/L), and correlated strongly with brain KBs (r=0.83, p< 0.0001).
**CONCLUSIONS.** Our data at the acute phase of TBI demonstrate increased endogenous production and transfer of KBs to the injured human brain, where they may act as supplemental cerebral energy substrate in conditions of limited carbohydrate availability. These unprecedented clinical findings might have implications for glycemic and nutritional control in neurointensive patients.

**GRANT ACKNOWLEDGMENT**
Supported by research grants from the Swiss National Science Foundation and Nestlé Health Science, Switzerland.

### 0519

#### Results of the implementation of a protocol and clinical pathway for the cerebral reperfusion of the acute ischemic stroke

J. Trenado Alvarez[1], F.J. González De Molina Ortiz[1], J. Krupinski Bielecki[2], G. Muñoz Gamito[3], Comissió Codi Ictus Hospital Universitari Mutua Terrassa
[1]Hospital Universitari Mutua Terrassa, Intensive Care Department, Terrassa, Spain; [2]Hospital Universitari Mutua Terrassa, Neurology Department, Terrassa, Spain; [3]Hospital Universitari Mutua Terrassa, Emergency Department, Terrassa, Spain
**Correspondence:** J. Trenado Alvarez
*Intensive Care Medicine Experimental* 2018, **6(Suppl 2):**0519

**INTRODUCTION.** Until the development of the SITS-MOST study, treatment with recombinant plasminogen activator (rt-PA) was only given in large centers. Treatment with rt-PA within the first 4.5 hours from the onset of the symptoms is beneficial .In our center, with no previous experience, we propose the treatment of intravenous cerebral reperfusion in the scope of the ICU, through a protocol based on a clinical pathway.
**OBJECTIVES.**

1) To describe the population affected by an ischemic stroke and treated with intravenous fibrinolysis within the first 3-4.5 hours of the onset of the symptoms.
2) To analyze the factors that influence the latency times and the variables of efficacy and safety.
3) To describe and compare the populations treated with rtPA i.v. in the pre and post periods ECAS III
4) To compare the population treated in our center in the first 4.5 hours with those of the SITS-MOST registry.

**METHODS.** All patients between March 2006 and December 2015 who had received fibrinolytic treatment were included. Demographic data, baseline status, intensity of pre-treatment neurological deficit, times of transfer and activation of stroke code, latency times, efficacy and safety variables were collected.
**RESULTS.** Fibrinolysis was performed in 493 patients; 52.1% were male, mean age 75 years old; 95.5% were previously independent. NIHSS score of 10.86 (SD 6.55). Latency times were as follows: start-ED 81.56 min, ED-CT 25.11 min, ED-needle 48.28 min and start-needle time 129.22 min. The ED-needle time decreased from the year 2010 (p < 0.001). The onset of symptoms-ED was lower in the in-hospital patients group and in those who arrived through the EMs with higher neurological impairments (p < 0.05). Mortality at 3 and 12 months was of 13.2 and 16.5%. The hemorrhagic transformation was symptomathic in the 3.9%, and the clinical presentation associated was TACI form (p < 0.001). When the pre and post ECASS III periods were compared, in 2010-2015 period, the patients were older (72.78 vs 66.39 years old). Time of latency, start-activation and ED-needle times decreased (p < 0.01). No differences were detected in the efficacy and safety variables. Patients at our center were older than the SITS registry (67 vs 77 years old). ED-needle and onset of simptoms-needle times were much lower in our patients (36 vs. 65 min and 105 vs 140 min, respectively). Although the number of symptomatic intracraneal haemorrhages was slightly higher (1.7% vs 3.6%), they did not present higher mortality and their functional prognosis was better (mRS 0-1 40.8% vs 51.2%).
**CONCLUSIONS**

1) Our latency times were short; and clinical and functional outcome were good.
2) In-hospital activation and transfer through EMS were associated with short latency times.
3) Treatment in the first 4.5 hours was as safe and effective as in less than 3 hours.4) Compared to the SITS-MOST register, our latency times were shorter and the functional prognosis was better.

### 0520

#### Imaging predictors of early recovery of consciousness after intracerebral hemorrhage

B. Rohaut[1], A.S. Reynolds[1], K. Igwe[2], K.W. Doyle[1], C. Couch[1], A. Matory[1], B. Rizvi[2], D. Roh[1], A. Velasquez[1], M. Megjhani[1], S. Park[1], S. Agarwal[1], C.M. Mauro[3], G. Li[3], V. Perlbarg[4], E.S. Connolly[5], A.M. Brickman[2], J. Claassen[1]
[1]Columbia University, Department of Neurology, Critical Care Neurology, New York, United States; [2]Columbia University, Department of Neurology, The Taub Institute, Gertrude H. Sergievsky Center, New York, United States; [3]Columbia University, Mailman School of Public Health, Department of Biostatistics, New York, United States; [4]Institut du Cerveau et de la Moelle Epinière, Bioinformatics and Biostatistics Core Facility, iCONICS, IHU-A-ICM, Paris, France; [5]Columbia University, Department of Neurosurgery, New York, United States
**Correspondence:** B. Rohaut
*Intensive Care Medicine Experimental* 2018, **6(Suppl 2):**0520

**INTRODUCTION.** Mechanisms underlying recovery of consciousness implicates brainstem, subcortical and cortical regions as well as their connecting neuronal pathways.[1,2] Intracerebral hemorrhage (ICH) may cause both focal injury to specific brain regions and also diffuse injury (i.e., from edema) affecting connecting neuronal pathways.[3]

**OBJECTIVE.** To determine the significance of focal structural lesions and diffuse injury in impairment and recovery of consciousness following ICH.

**METHODS.** We studied a consecutive series of spontaneous ICH patients that underwent MRI within 7 days of the bleed. We assessed consciousness by testing for command following during daily interruption of sedation at the time of MRI acquisition and ICU discharge, and determined functional outcomes using the Glasgow Outcome Scale-Extended (GOS-E) at 3 months. ICH and edema volumes and location, and midline shift (MLS) were quantified. A machine learning approach using logistic regression with elastic net regularization was applied to identify MRI parameters that best predict consciousness.

**RESULTS.** From 158 ICH patients that underwent MRI, 66% (N=105) were conscious and 34% (N=53) were unconscious at the time of MRI. Almost half of the unconscious patients (49%, N=26) recovered consciousness by ICU discharge. Consciousness at ICU discharge was best predicted when combining focal lesions within the anterior-forebrain-mesocircuit with measures of MLS, and ICH and edema volumes (AUC: 0.74, 95%-CI: 0.73 to 0.76). Patients predicted to recover consciousness at discharge had better 3-month functional outcomes (GOS-E $\geq$ 4: 35% vs 0%; p-value=0.02).

**CONCLUSION.** Focal lesions in key structures within previously described circuit models of consciousness together with measures related to mass effect of the hemorrhage (ICH and edema volume, MLS) best predict early recovery of consciousness. MRI within one week of injury might be helpful to identify patients that will recover consciousness early and predict 3-month functional outcomes.

**REFERENCE(S)**

1 Adams, J. H., Graham, D. I. & Jennett, B. The neuropathology of the vegetative state after an acute brain insult. Brain. 123 ( Pt 7), 1327-1338 (2000).

2 Schiff, N. D. Recovery of consciousness after brain injury: a mesocircuit hypothesis. Trends Neurosci. 33, 1-9 (2010).

3 Qureshi, A. I., Mendelow, A. D. & Hanley, D. F. Intracerebral haemorrhage. Lancet. 373, 1632-1644 (2009).

**GRANT ACKNOWLEDGMENTS**

This work was supported by the NIH National Library of Medicine (R01LM011826, J.C.), DANA Foundation, McDonell Foundation, « Amicale des Anciens Internes des Hôpitaux de Paris & Syndicat des Chefs de Cliniques et Assistants des Hôpitaux de Paris » (B.R.), Assistance Publique - Hôpitaux de Paris (B.R.), Philippe Foundation (B.R.). We thank the nurses, attending physicians, fellows, neurology and neurosurgery residents of the Neuroscience ICU and Epilepsy Division for their overall support of this project.

### 0521

#### Neurological symptoms in cirrhotic patients in ICU: hepatic or drug-induced encephalopathy? a metabolomic study

N. Weiss[1], P. Barbier Saint-Hilaire[2], A. Schaefer[1], M. Rudler[1], S. Mouri[1], F. Sedel[1], F. Fenaille[2], B. Colsch[2], C. Junot[2], D. Thabut[1], Brain Liver Pitié-Salpêtrière (BLIPS) Study Group

[1]Sorbonne Université, Paris, France; [2]CEA, iBiTec-S, Service de Pharmacologie et d'Immunoanalyse, Laboratoire d'Etude du Métabolisme des Médicaments, MetaboHUB-Paris, Gif sur Yvette, France

**Correspondence:** N. Weiss

*Intensive Care Medicine Experimental* 2018, **6(Suppl 2):**0521

**INTRODUCTION.** Hepatic encephalopathy is a complication of liver disease and/or portosystemic shunts. Its pathophysiology is incompletely understood; mechanisms include hyperammonia in association with systemic inflammation. An impairment of blood-brain barrier (BBB) permeability is also hypothesized. Thus, most frequently neurological symptoms in cirrhotic patients are attributed to hepatic encephalopathy. Metabolomics enables to detect a wide range of metabolites without any a priori. In a recent metabolomic study including patients who underwent cerebrospinal fluid (CSF) collection, our group outlined that drugs that usually don't cross BBB were retrieved in the CSF, suggesting a potential neurological toxicity of drugs. CSF collection is invasive.

**OBJECTIVES.** Hence, we aimed to describe the drugs present in the plasma of cirrhotic patients, using the same metabolomic approach.

**METHODS.** We conducted a retrospective study of plasma samples in the Hepatological ICU. Plasma samples from cirrhotic patients displaying neurological symptoms were compared to plasma from cirrhotic patients without neurological symptoms, and to plasma from healthy controls. Liquid chromatography coupled to high-resolution mass spectrometry was performed and then after the metabolic fingerprints were compared to database and between the different groups.

**RESULTS.** Plasma samples were obtained from 12 cirrhotic patients with neurological symptoms (age 59 [40-68], MELD 20 [16-31], alcohol 58%), 13 cirrhotic patients without encephalopathy (age 56 [55-64], MELD 17 [14-29], alcohol 38%) and 9 healthy controls. Among 495 identified metabolites, 25 corresponded to xenobiotics or its derivatives. Fluoxetine was detected with a more than 200 fold increase, aminosalicylic acid with a more than 10 fold increase and benzyl alcohol (present in cough pills and antiseptics) with a 3 fold increase in cirrhotic patients with neurological symptoms as compared to cirrhotic patients. In cirrhotic patients with or without neurological symptoms, propranolol was detected with a more than 8500 fold increase, acetaminophen with a 40 fold increase, penicillamine and ampicillin both with a 2 fold increase as compared to healthy controls. Interestingly, several substances which were not expected to have systemic diffusion were detected in cirrhotic patients and in healthy controls: eugenol, isoeugenol (used in mouth bathing solution), triethanolamine (trolamin, used in cutaneous creams) and resorcinol monoacetate (used in mouth bathing solution and in cutaneous creams).

**CONCLUSIONS.** Cirrhotic patients, especially those with neurological symptoms, display dramatically increased levels of several xenobiotics in plasma. These results confirm that PK/PD parameters of commonly used drugs are highly modified in those patients. This suggests a potential role of xenobiotics in the pathophysiology of encephalopathy in cirrhotic patients.

### 0522

#### Impact of tolvaptan on osmolar efficacy of hypertonic saline in severe traumatic brain injury: a single centric, prospective, double blind, investigator initiated, randomized controlled clinical trial

B.P. Das[1], S. Kamath[2], U. Rao[2], G. Yadav[1]

[1]IMS-BHU, Anaesthesia and Critical Care, Varanasi, India; [2]NIMHANS, Neuroanesthesia & Neurocritical Care, Bengaluru, India

**Correspondence:** B.P. Das

*Intensive Care Medicine Experimental* 2018, **6(Suppl 2):**0522

**INTRODUCTION.** The recent Brain Trauma Foundation guidelines did not carry forward its earlier recommendations on osmotherapy in severe traumatic brain injury (TBI) because of insufficient high quality evidence from comparative studies. HTS is no more advantageous than mannitol in maintaining hyperosmolar state and controlling ICP, which might be due to urinary sodium loss, which curtails its superior osmolar effect.

**OBJECTIVES.** The current study attempts to explore the impact of tolvaptan when added to HTS on osmolar efficacy in patients with severe TBI.

**METHODS.** After ethics committee approval and written informed consent from the guardians, a single-centric, prospective, doubleblind, investigator-initiated clinical comparative trial was conducted in between october 2016 to october 2017 at Trauma ICU, IMS-BHU, Varanasi, in which 76 adult patients with severe TBI belonging to age group 15-70years, receiving aliquots of 2.5ml/kg of 3% HTS for refractory-ICH, were randomised to receive tolvaptan (Group A) or placebo (Group B) to achieve target ICP < 22 mmHg and CPP >60mmHg. We evaluated ICP, along with serum and urine sodium and osmolarity over 6 days. Chi-square and student-t test were used for qualitative and quantitative data, respectively. Statistical significant difference was declared when p-value ≤0.05.

**RESULTS.** The demographic variables were comparable. A total of 372 and 502 boluses of HTS, respectively, were administered during the study period (p=0.01). Addition of tolvaptan increased serum sodium and osmolality significantly in a sustained manner throughout the study period (p=0.03 and 0.02, respectively) (Fig 1), which translated into better ICP control with lesser boluses of HTS (p=0.01). The outcome parameters (duration of hospital stay 22 ± 10 vs 42 ± 11 days; p = 0.04) and unfavorable GOS (GOS = 1 to 3) at 6 months (10/40 vs 18/36; p = 0.02) and mortality at 6 months (8/40 vs 14/36; p = 0.03) were better with tolvaptan use (Group A) (Fig 2).

**CONCLUSIONS.** Addition of tolvaptan to HTS not only curtails the effect of urinary sodium loss, thereby increasing osmolar efficacy but also leads to better ICP control in severe TBI, translating into better GOS with mortality benefit.

#### REFERENCE(S)

(1) Mangat HS, Chiu Y-L, Gerber LM, Alimi M, Ghajar J, Härtl R: Hypertonic saline reduces cumulative and daily intracranial pressure burdens after severe traumatic brain injury. J Neurosurg 2015; 122:202-10

(2) Jagannatha AT, Sriganesh K, Devi BI, Rao GSU: An equiosmolar study on early intracranial physiology and long term outcome in severe traumatic brain injury comparing mannitol and hypertonic saline. J Clin Neurosci 2016; 27:68-73

#### GRANT ACKNOWLEDGMENT

The authors declare no conflicts of interest and did not receive any grant for this study.

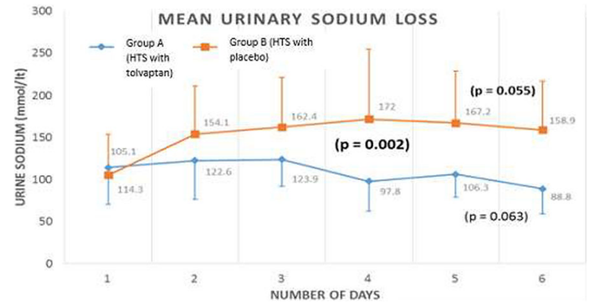

**Fig. 1 (abstract 0522).** Mean urinary sodium from day 1 to day 6 with tolvaptan use

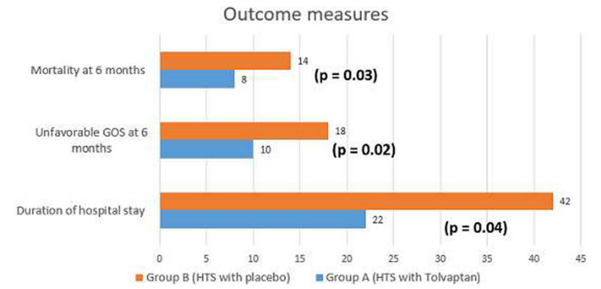

**Fig. 2 (abstract 0522).** Outcome measures with tolvaptan use

## Delirium biomarkers and treatment

### 0523

#### Reduction effect of ramelteon, a melatonin receptor agonist, for delirium in ICU patients: a subgroup analysis from MELIt trial

Y. Yasuda, M. Nishikimi, M. Higashi, T. Yamamoto, A. Numaguchi, N. Matsuda
Nagoya University Graduate School of Medicine, Department of Emergency & Critical Care Medicine, Nagoya city, Japan
**Correspondence:** Y. Yasuda

*Intensive Care Medicine Experimental* 2018, **6(Suppl 2):**0523

**INTRODUCTION.** We reported that ramelteon, a selective melatonin receptor agonist, decreased the occurrence rate and duration of delirium in ICU patients (1), but a subgroup who has more benefit from ramelteon for the prevention of delirium among ICU patients remains unknown.

**OBJECTIVES.** To evaluate the different effects of ramelteon for the delirium in ICU patients according to a degree of illness severity.

**METHODS.** This subgroup study was performed by using data from our previously published, Melatonin Evaluation of Lowered Inflammation in ICU (MELIt) Trial (1). Eligible patients in this trial were ICU patients who could take medicines orally or through a nasogastric tube during the first 48 hours of admission, and received a ramelteon (8 mg/d) or placebo during their ICU stay. In the subgroup analysis, we divided all patients into two groups by the levels of Acute Physiology and Chronic Health Evaluation (APACHE) II score, and evaluated the effect of ramelteon for delirium occurrence in the two groups, respectively.

**RESULTS.** In all 88 patients, 47 subjects (ramelteon: 26 cases, placebo: 21) were divided into high APACHE II score (≥23), while 41 (ramelteon: 19, placebo: 22) were into low APACHE II score group (< 23). The mean APACHE II score were 30.0 ± 4.7 and 17.1 ± 4.3, respectively. The number of intubated patients was 31 (66%) in the high score group and 7 (17%) in the low score group. In the high score group, the use of ramelteon was associated with a statistically significant decrease in the incidence of delirium (30.8% vs. 71.4%; p < 0.01). On the other hands, in the low score group, no statistical significance was observed (15.8% vs. 22.7%; p = 0.70).

**CONCLUSIONS.** Ramelteon statistically significantly decreased the occurrence rate of delirium in the patients with APACHE II sore ≥23, while not in those with APACHE II sore < 23. Ramelteon reduced delirium in highly severe ICU patients.

#### REFERENCE(S)

1. Nishikimi M, et al., Effect of Administration of Ramelteon, a Melatonin Receptor Agonist, on the Duration of Stay in the ICU: A Single-Center Randomized Placebo-Controlled Trial. Crit Care Med. 2018 Mar 27. PMID: 29595562.

### 0524

#### Serum orexin-A concentration and acute brain dysfunction in critically ill patients

J. Oto, N. Nakanishi, Y. Ueno, T. Itagaki
Tokushima University Hospital, Tokushima, Japan
**Correspondence:** J. Oto

*Intensive Care Medicine Experimental* 2018, **6(Suppl 2):**0524

**INTRODUCTION.** Acute brain dysfunction is common in critically ill patients. Orexin is one of the neuronal bioactive substances and may play an important role in several neuro-physiological functions such as sleep-wake cycle and cognitive function. Several experimental studies suggest that degeneration of orexinergic (OXergic) neurons occurs during acute critical illness, i.e., sepsis or traumatic brain injury, and the decline of OXergic activity may partly characterize the symptoms of acute brain dysfunction such as delirium or coma, in critical illness (1). However, there are few clinical data that validated this hypothesis.

**OBJECTIVES.** To investigate the relationship between serum orexin-A concentration and the occurrence of delirium and coma in critically ill patients.

**METHODS.** In this prospective cohort study, adult patients admitted to ICU at the Tokushima University Hospital (general ICU: 10 beds) for more than 24 hours were recruited. Delirium was assessed with the confusion assessment method for the ICU (CAM-ICU) performed every 2 to 3 hours during the ICU stay. Coma was defined as Richmond Agitation-Sedation Scale of -4 to -5. Serum orexin-A were measured at once daily during daytime (6:00 AM to 9:00 PM) for up to 7 days post-ICU admission by commercial enzyme linked immunosorbent assay. We compared the serum orexin-A concentration among the patients with delirium (D Group), or with coma (C group) or without delirium/coma (Non-D/C group).

**RESULTS.** Sixty-four patients were included and 176 samples were collected. The incidence of delirium and/or coma at the orexin-A concentration measurement was 59.7%. In patients who did not develop delirium/coma, serum orexin-A levels were significantly higher than in patients with delirium or coma [median: inter-quartile range: Non-D/C group: 31.0 (22.0, 49.3) pg/mL, D group: 23.1 (14.2, 33.3) pg/mL, C group: 22.6 (12.8, 32.8) pg/mL, p=.003, respectively].

**CONCLUSIONS.** To our knowledge, this is the first study to investigate the serum orexin-A levels and acute brain dysfunction in critically ill patients. Low serum orexin-A levels are associated with the occurrence of delirium and coma.

##### REFERENCE(S)

1. Hirota K. J Anesth 2016; 30: 919-22.

##### GRANT ACKNOWLEDGMENT

This work was supported by Japan Society for the Promotion of Science (JSPS KAKENHI) Grant Number: 16K11406

### 0525

#### Haloperidol serum concentrations in critically ill patients included in the REDUCE study

A. van Schijndel[1], E. Fransen[2], M. van den Boogaard[3], P. van der Voort[4]
[1]OLVG Oost, ICU, Amsterdam, Netherlands; [2]OLVG Oost, Clinical Pharmacy, Amsterdam, Netherlands; [3]Radboud UMC Nijmegen, ICU, Nijmegen, Netherlands; [4]OLV Aalst, ICU, Amsterdam, Netherlands
**Correspondence:** A. van Schijndel
*Intensive Care Medicine Experimental* 2018, **6(Suppl 2):**0525

**INTRODUCTION.** ICU delirium occurs frequently and is associated with worse outcomes. A recent delirium prevention study, the REDUCE study, has assessed the prophylactic use of haloperidol [1]. This study showed no effect for any group for all outcomes. Therapeutic serum levels of haloperidol for treatment of schizophrenia are reported to be 3-20 μg/L [2,3]. The pharmacokinetics in ICU patients are known to be variable.

**OBJECTIVES.** To determine haloperidol serum concentrations in ICU patients receiving prophylactic haloperidol.

**METHODS.** Subgroup analysis of all included patients in the REDUCE study in one participating ICU. A blood sample was drawn immediately before the third dose of the study medication. All results were blinded until the study was ended. Patients received placebo or 1 mg or 2 mg haloperidol q8hrs intravenously. Non-parametric tests were used (Kruskal-Wallis; Mann-Whitney).

**RESULTS.** We included 139 patients for the REDUCE study of whom in 100 blood was drawn to determine serum haloperidol concentration. 39 patients were discharged early or did not give consent. Baseline characteristics showed no differences between subgroups (Table 1). The median serum level of haloperidol was 0 mg/l for placebo,1.1 mg/L (IQR 0.1-1.6) in the 1mg group and2.6 mg/L (IQR 1.8-3.2) in the 2 mg group (figure 1), p=0.001 for 1 vs 2 mg haloperidol. Two patients had a serum level above 20 mg/L as an indication for poor metabolism.

**CONCLUSIONS.** In this subset of patients included in the REDUCE study we found a linear dose-concentration relation of haloperidol trough concentrations. The observed haloperidol serum levels were lower compared to those reported when haloperidol was given in a treatment setting for patients with schizophrenia and other psychotic disorders.

**Table 1 (abstract 0525).** Patient characteristics, median and inter quartile range

| Baseline characteristic | Placebo (n=31) | 1 mg haloperidol (n=40) | 2 mg haloperidol (n=29) | P-value |
|---|---|---|---|---|
| Creatinine day 3 | 148 (101-195) | 133 (92-176) | 144 (92-197) | 0.65 |
| ALT day 3 | 339 (22-900) | 297 (36-558) | 116 (53-285) | 0.81 |
| APACHE II | 20 (17-22) | 22 (19-26) | 22 (18-25) | 0.55 |
| Age | 71 (65-77) | 66 (61-71) | 69 (63-75) | 0.51 |

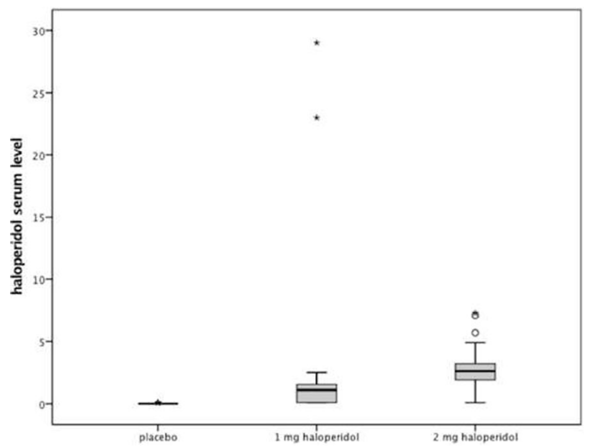

**Fig. 1 (abstract 0525).** See text for description

### 0526

#### Pharmacological interventions for the treatment of delirium in critically ill patients: a network meta analysis

L. Burry[1,2], B. Hutton[3], D. Williamson[4], S. Mehta[1], N. Adhikari[5], I. Egerod[6], E.W. Ely[7], W. Cheng[3], D. Fergusson[3], L. Rose[8]
[1]Sinai Health System, Toronto, Canada; [2]University of Toronto, Toronto, Canada; [3]Ottawa Hospital Research Institute, Ottawa, Canada; [4]University of Montreal, Montreal, Canada; [5]Sunnybrook Health Sciences, Toronto, Canada; [6]University of Copenhagen, Copenhagen, Denmark; [7]Vanderbilt University, Nashville, United States; [8]Sunnybrook Health Sciences Centre, Toronto, Canada
**Correspondence:** L. Burry
*Intensive Care Medicine Experimental* 2018, **6(Suppl 2):**0526

**INTRODUCTION.** The relative effectiveness of different interventions for treatment of ICU delirium remains unclear. To address this knowledge gap, we conducted a systematic review (SR) and network meta-analysis (NMA) to evaluate the comparative benefits and harms of interventions from published trials.

**OBJECTIVES.** To assess 1) effects of drug interventions on delirium duration and other clinical outcomes (delirium-free and coma-free days, duration of mechanical ventilation, length of stay, mortality) in ICU patients with confirmed or at high risk of delirium; and 2) drug safety.

**METHODS.** We used well established Cochrane SR and NMA methods. We searched the following databases (inception-April 2018): MEDLINE, Embase, PsychINFO, Cochrane Library, CINAHL, Web of Science, and grey literature. We included all randomized controlled trials (RCTs) of critically ill adults with confirmed or high risk of delirium treated with any drug and compared to any control group (other drug, or placebo).Treatment effects for continuous outcomes were summarized as ratios of means (RoM: mean[expt]/ mean[ctrl]) and binary endpoints were summarized as OR. All estimates were reported along with 95% CrI. We performed conventional pairwise meta-analyses and NMA within a Bayesian framework, both using random-effects (RE) models; the reference treatment for all analyses was placebo. This approach enabled the estimation of each drug's probability of being at each possible rank (e.g., $1^{st}$,...,$n^{th}$ for n drugs) for each outcome. For NMAs, we estimated the corresponding probability of each drug to be the best, surface under the cumulative ranking curve (SUCRA) values, and the mean rank of each drug.

**RESULTS.** We screened 4996 papers and included 13 RCTs (n=1278 subjects). Nine RCTs were placebo-controlled; 4 were comparisons of different drugs. Drugs included antipsychotics (n=9), $alpha_2$-agonists (n=3), statins (n=2), opioid (n=1), serotonin antagonist (n=1), and cholinesterase (CHE) inhibitors (n=1). Nine studies (n=964) reported on duration of delirium. The forest plots from pairwise meta-analyses on duration of delirium are shown in figure 1; only $alpha_2$-agonists were significantly better compared to placebo (mean difference -0.53, 95% CI -0.84 to -0.22, P=0.0007). Figure 2 displays the RoM estimates of pairwise comparisons from NMA. The smallest RoM vs. placebo was associated with alpha-2 agonists (mean rank 2.13), followed by atypical antipsychotics (mean rank 2.37), although 95% CrIs of all interventions included 1. None of the secondary outcomes identified differences between therapies. Adverse events were rarely reported, with rates similar between drugs and placebo where available.

**CONCLUSION.** Secondary effect measures from our NMAs suggest $alpha_2$-agonists and atypical antipsychotics may be most effective in reducing delirium duration. However, corresponding pair-wise comparisons from NMA could not rule out a possible null difference between drugs.

**FUNDING**
CIHR

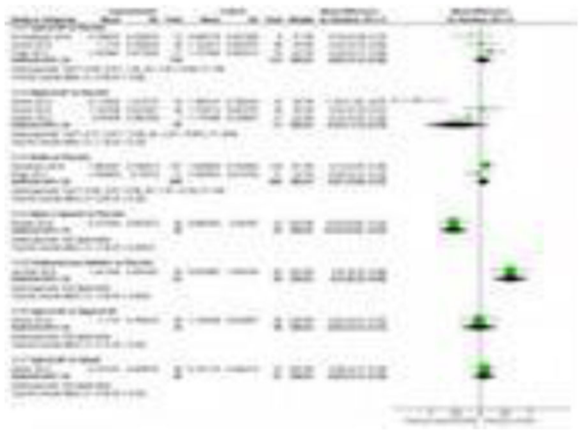

**Fig. 1 (abstract 0526).** Forest plot of Duration of Delirium

| | | | | | | |
|---|---|---|---|---|---|---|
| **α-2 Agonist** | | | | | | |
| 0.85 (0.24, 3.40) | **Atypical AP** | | | | | |
| 0.67 (0.12, 3.89) | 0.79 (0.19, 3.11) | **Opioid** | | | | |
| 0.61 (0.17, 2.39) | 0.72 (0.30, 1.62) | 0.92 (0.30, 2.87) | **Typical AP** | | | |
| 0.56 (0.14, 2.33) | 0.66 (0.22, 1.80) | 0.84 (0.18, 3.82) | 0.92 (0.32, 2.52) | **Statin** | | |
| 0.32 (0.06, 1.67) | 0.38 (0.09, 1.38) | 0.48 (0.08, 2.71) | 0.52 (0.13, 1.95) | 0.57 (0.14, 2.35) | **CHE Inhibitor** | |
| 0.59 (0.19, 1.87) | 0.69 (0.33, 1.28) | 0.88 (0.23, 3.19) | 0.96 (0.48, 1.79) | 1.04 (0.46, 2.32) | 1.83 (0.57, 5.92) | **Placebo** |

Caption: For each comparison, the lower right-most intervention is the reference group. Values < 1 favor the comparator while values>1 fav the control group.

**Fig. 2 (abstract 0526).** League table of posterior median pairwise RoM and CrI

### 0527

#### Efficacy of dexmedetomidine in reducing duration of mechanical ventilation in patients with severe burn injury

A. Lavrentieva[1], M. Papaioannou[1], I. Tsioulis[1], E. Sourla[1], C. Giannaki[1], G. Vasileiadou[1], S. Tsotsolis[2], M. Bitzani[1]
[1]Papanikolaou Hospital, Burn-ICU, Thessaloniki, Greece; [2]Aristotle University of Thessaloniki, Thessaloniki, Greece
**Correspondence:** A. Lavrentieva
*Intensive Care Medicine Experimental* 2018, **6(Suppl 2):**0527

**INTRODUCTION.** Although causal relation between the level of sedation and the patient outcomes is difficult to be determined, many studies suggest that the use of sedatives could account for the prolonged duration of mechanical ventilation in patients with severe burn injury.

**OBJECTIVES.** The goal of the study was to determine the effectiveness of dexmedetomidine vs. midazolam in reducing duration of mechanical ventilation in patients with severe burn injury.

**METHODS.** Forty nine burn patients admitted during 2015-2017 in four-bed burn ICU receiving sedation and mechanical ventilation for more than 96 hours were included in the study (mean total body surface area burned 42±23%, mean SOFA score at admission 5.8±4). After four days of mechanical ventilation patients were randomly assigned to 1 of 2 treatment groups: receiving dexmedetomidine (20 patients), titrated to rates between 0.5 and 1.5 μg/kg/h and receiving midazolam (19 patients), titrated to rates between 0.03-0.09 mg/kg/hr) to achieve sedation goals. Sedation was continued until no longer require or up to 5 days. Ventilator-free hours and time to extubation in the 5 days following randomization were assessed and compared between two groups of patients.

**RESULTS.** Dexmedetomidine increased ventilator-free hours at 5 days compared with midazolam (median, 89 hours vs. 65 hours, respectively; median difference between groups 23.0 hours [95% CI, 6.0 to 29.4 hours]; $p < 0.01$). Patients in dexmedetomidine group had reduced time to extubation (median, 29.5 hours vs. 51.6 hours with midazolam; median difference between groups, 20 hours [95% CI, 8.9 to 32 hours]; $p = 0.01$) and lower rate of delirium (15 % vs. 31.6%, $p <$

0.01). Using Cox modeling to adjust for imbalanced baseline characteristics, allocation to dexmedetomidine was significantly associated with earlier extubation (hazard ratio, 0.5 [95% CI, 0.29-0.83]; *p*= 0.002). Bradycardia was detected in 4 patients (20%) receiving dexmedetomidine. No unplanned extubations were observed in both groups.

**CONCLUSIONS.** Among patients with severe burn injury receiving prolonged mechanical ventilation the addition of dexmedetomidine to standard burn care compared with midazolam resulted in more ventilator-free hours, lower rate of delirium and reduced time to extubation. Dexmedetomidine seems to be safe and effective for sedation of burn patients on prolonged mechanical ventilation however close cardiovascular monitoring should be used to detect bradycardia.

## Weaning from mechanical ventilation: New ideas

### 0528

#### The Weaning score to predict duration of weaning from readiness to extubation

M. Dres[1,2], E. Goligher[3,4], A. Kiss[5], A. Amaral[6], E. Fan[3,6], L. Brochard[3,7]
[1]Assistance Publique Hôpitaux de Paris, Respiratory and ICU, Paris, France; [2]Saint Michael Hospital, Mixed ICU, Toronto, Canada; [3]Interdepartmental Division of Critical Care Medicine, Toronto, Canada; [4]Mount Sinai Hospital, Intensive Care Unit, Toronto, Canada; [5]Institute for Clinical Evaluative Sciences, Toronto, Canada; [6]Toronto General Hospital, Intensive Care Unit, Toronto, Canada; [7]Saint Michael Hospital, Medical-Surgical ICU, Toronto, Canada
**Correspondence:** M. Dres
*Intensive Care Medicine Experimental* 2018, **6(Suppl 2):**0528

**INTRODUCTION.** The duration of weaning from mechanical ventilation is strongly associated with clinical outcomes. Predicting the duration of weaning may be useful to identify high-risk patients for intervention and for quality assurance benchmarking.

**OBJECTIVES.** We aimed to develop a prognostic score to predict the risk of prolonged weaning based on readily available clinical characteristics at the onset of weaning readiness.

**METHODS.** Clinical data were collected twice daily in 5197 invasively mechanically ventilated patients included in the Toronto Intensive Care Observational Registry (iCORE) from April 2014 to October 2016. The onset of weaning readiness was defined as the first day when patients were receiving inspired fraction of oxygen < 50% with positive end expiratory pressure < 10 $cmH_2O$ without vasopressor infusions. The two primary outcomes were the proportion of patients requiring 1) > 4 days and 2) > 6 days to be liberated from mechanical ventilation. Multivariable logistic regression modelling with bootstrapping for internal validation was used to create the Weaning score. Then, the score was tested into a validation cohort (n=862) issued from the iCORE registry from November 2016 to July 2017.

**RESULTS.** After exclusion of patients with missing or inconsistent data, 2814 patients with mechanical ventilation for at least 24h were evaluated. Among them, 673 (28%) never reached readiness criteria. Eventually, 1163 (41%) patients presented simultaneously all three readiness criteria during the ICU stay. From them, 219 patients (19%) required > 4 days and 109 (9%) > 6 days to be liberated from mechanical ventilation. Of more than 30 pre-selected potential predictors, logistic regression identified 3 variables measured on the day of readiness that were independently associated with a duration of weaning > 4 days:

1) time from ICU admission to weaning readiness;
2) lowest Sedation-Agitation Scale (SAS) score on the day of readiness; and
3) lowest respiratory rate on the day of readiness.

The model had good predictive discrimination (c = 0.75; 95% CI 0.72-0.79). Variables independently associated with weaning duration > 6 days were:

1) time from ICU admission to weaning readiness;
2) trauma as a primary reason for acute respiratory failure; and
3) lowest respiratory rate on the day of readiness.

This model also had good predictive discrimination (c = 0.80; 95% CI 0.76-0.84). External validation performed on 862 additional patients confirmed the performance of the score to predict duration of weaning longer than 4 days (c = 0.69; 95% CI 0.64-0.74) and duration of weaning longer than 6 days (c = 0.74; 95% CI 0.67-0.81).

**CONCLUSIONS.** The Weaning score is a new clinical score employing simple variables available at the time of weaning readiness that predicts the subsequent duration of weaning with acceptable performance.

### 0529

#### The effects of pressure support and proportional assist ventilation on metabolic parameters during weaning

M. Arslan, A. Kuntman, K. Demirag, I. Cankayali, M. Uyar
Ege University Hospital, Anesthesiology and Intensive Care, Izmir, Turkey
**Correspondence:** M. Arslan
*Intensive Care Medicine Experimental* 2018, **6(Suppl 2):**0529

**INTRODUCTION.** Weaning refers to the process of gradually withdrawing invasive mechanical ventilation. Various spontaneous breathing modes may be used for weaning. The patients respond to these modes by different metabolic and respiratory patterns.

**OBJECTIVES.** The aim of this study is to compare pressure support (PS) and proportional assist ventilation (PAV+) modes regarding these parameters.

**METHODS.** After obtaining consent from local ethics committee, 50 intensive care patients who were mechanically ventilated for at least 24 hours, hemodynamically stable and met the criteria for weaning were included. The study was designed as a prospective, cross-over study.

The patients were randomized to start on either PSV or PAV+ mode by Puritan Bennett 980 ventilator and measurements were obtained after an adaptation period of 30 minutes with a $PaCO_2$ value between 35-45 mmHg and $PaO_2$ above 60 mmHg. Patients were then switched to the other mode and the same measurements were repeated. Ventilatory parameters were adjusted during the study period to maintain the blood gas values within clinically acceptable limits. Indirect calorimetry was performed during each ventilatory mode for a period of 30 minutes. Energy expenditure (EE), oxygen consumption ($VO_2$), and $CO_2$ production ($VCO_2$) were measured. Also respiratory rate, pulse rate, mean arterial pressure, oxygen saturation, tidal volume, peak and mean airway pressures and arterial blood gas values were recorded at 10 min intervals during the study period.

**RESULTS.** The parameters did not differ significantly between the two ventilatory modes, regardless of the patient´s randomization except $VO_2$. All patients tolerated both ventilatory modes without signs of discomfort.

**CONCLUSIONS.** PSV and PAV+ modes produced clinically similar results in terms of metabolic and other variables. The difference regarding $VO_2$ between PSV and PAV+ was not considered as clinically significant.

**REFERENCE(S)**

1. Thille AW, Cortes-Puch I, Esteban A. Weaning from the ventilator and extubation in ICU. Curr Opin Crit Care 2013;19(1):57-64.

**Table 1 (abstract 0529).** Demographic data (n=50) (Mean±SD)

| | |
|---|---|
| Gender (M/F) | 25 / 25 |
| Age (yrs) | 61,5±18,9 |
| APACHE II | 19,4±6,2 |
| BMI (kg/m2) | 27,2±4,8 |

**Table 2 (abstract 0529).** Metabolic data (Mean±SD) *p<0,05

| | PSV | PAV+ |
|---|---|---|
| EE (kcal) | 1431,1±485,7 | 1464,1±481,7 |
| VO2 (ml/min) | 206,2±76,7 | 215,2±68,3* |
| VCO2 (ml/min) | 193,4±66,4 | 196,3±69,05 |

### 0530

#### Sleep depth before a weaning test from mechanical ventilation

M. Dres[1,2], M. Younes[3], N. Rittayamai[2], T. Kendzerska[4], I. Telias[2], D. Luca Grieco[2], T. Pham[2], D. Junhasavasdikul[2], E. Chau[2], S. Mehta[5], E. Wilcox[6], R. Leung[4], X. Drouot[7], L. Brochard[2]
[1]Assistance Publique Hôpitaux de Paris, Respiratory and ICU Department, Paris, France; [2]Saint Michael Hospital, Medical-Surgical ICU, Toronto, Canada; [3]YRT Ltd., Winnipeg, Canada; [4]Saint Michael Hospital, Respirology and Sleep Laboratory, Toronto, Canada; [5]Mount Sinai Hospital, Intensive Care Unit, Toronto, Canada; [6]Toronto Western Hospital, Department of Medicine (Critical Care Medicine), Toronto, Canada; [7]CHU de Poitiers, Neurophysiologie clinique et Explorations fonctionnelles, Poitiers, France
**Correspondence:** M. Dres
*Intensive Care Medicine Experimental* 2018, **6(Suppl 2):**0530

**INTRODUCTION.** Sleep disruption and poor sleep quality are common in critically ill patients. An association between sleep quality and weaning outcome has never been studied.

**OBJECTIVES.** To investigate whether the level of sleep depth assessed the night before a spontaneous breathing trial (SBT) would be associated with different subsequent weaning outcomes.

**METHODS.** Mechanically ventilated patients from three ICUs were enrolled if the clinical team planned to conduct a SBT the following day. On the night before, polysomnography (PSG) was performed using an unattended portable sleep diagnostic device (Alice PDx, Philips, Murrysville, USA). Alice PDx includes six electrodes and two bands (thorax and abdomen) and records electroencephalography (EEG), electrooculography, electromyography, electrocardiography, and chest and abdominal effort.

The odds ratio product (ORP) is a new continuous index for evaluating the level of sleep depth: it is calculated from the power of 4 EEG frequency band (delta, theta, alpha-sigma, and beta) relative to each other (1). Its range extends from full wakefulness (2.5) to the deepest sleep (0). ORP was calculated off-line as well as the intra-class correlation between right(R) and left(L) hemispheres' ORP (ICC R/L). Normal ICC R/L is greater than 90%.

The SBT was performed under continuous positive airway pressure set at 0 cm$H_2O$. Average ORP and ICC were compared between patients depending of their weaning outcome (Kruskall-Wallis test): passed SBT followed by extubation, passed SBT without extubation and failed SBT without extubation. Data are given as mean (standard deviation) or median (25$^{th}$-75$^{th}$ percentiles).

**RESULTS.** 37 patients (34 PSGs) were enrolled: 11 patients passed the SBT and were extubated, 8 passed the SBT but were not extubated (clinical decision) and 15 failed the SBT.

ORP was significantly higher in patients who passed the SBT as compared both to those who passed the SBT but were not extubated and to those who failed the SBT (Figure 1). Sixty-seven percent of patients who passed SBT and were extubated spent >20% of recording time with ORP >2.0 (meaning alert); this contrasted with 16% of patients who passed SBT but were not extubated and 13% of patients who failed SBT (Chi2=0.02). The ICC R/L was 0.83 (0.77-0.91), 0.82 (0.75-0.86) and 0.62 (0.49-0.65) in patients who passed the SBT and were extubated, in patients who passed the SBT but were not extubated and in patients who failed the SBT and respectively (p< 0.01).

**CONCLUSIONS.** On the night before weaning, spending little or no time with a high ORP (full wakefulness) is predictive of weaning failure. In addition, hemispheric correlation was higher in patients who passed the SBT. This new analysis shed a new light on the weaning process.

**GRANT ACKNOWLEDGMENT**

"Mitacs Globalink Research Award - Campus France- for research in Canada"

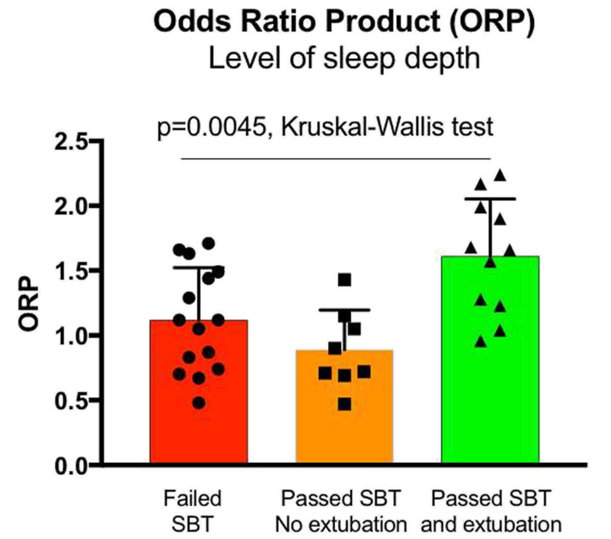

**Fig. 1 (abstract 0530).** Values of ORP (odds ratio product) in patients who failed the spontaneous breathing trial

### 0531

#### Indices of diaphragm ultrasound during voluntary cough and extubation outcome: an interim report

T. Santanda[1], S. Tomita[1], M. Nabeshima[1], Y. Fujimoto[1], S. Saito[1], J. Kataoka[1], Y. Norisue[1], S. Fujitani[2]
[1]Tokyo Bay Urayasu/Ichikawa Medical Center, Urayasu, Japan; [2]St Marianna University, Kawasaki, Japan
**Correspondence:** T. Santanda
*Intensive Care Medicine Experimental* 2018, **6(Suppl 2):**0531

**INTRODUCTION.** Assessing the ability to clear secretions and protect airway with effective cough is an important part of pre-extubation evaluation in patients who have passed spontaneous breathing trial (SBT). We hypothesized that cough strength and extubation outcome can be predicted by diaphragm ultrasound.

**OBJECTIVES.** The aim of this study is to investigate the predictive values of diaphragm excursion and max velocity during voluntary cough measured by ultrasound for cough peak expiratory flow (CPEF) measured by ventilator graphics and extubation outcome.

**METHODS.** All mechanically ventilated patients who passed SBT were enrolled in the study. In the supine position, diaphragm excursion and max velocity were measured during cough by ultrasound. CPEF was also measured using the ventilator flow waveforms. The primary outcome of this study was extubation failure. Patients who require re-intubation more than 72 hours after extubation were classified as having a failure extubation.

**RESULTS.** A total of 196 mechanically ventilated patients were included. Both excursion and max velocity of the diaphragm during voluntary cough were significantly correlated with CPEF (r=0.276; p< 0.01 and r=0.464; p< 0.01respectively). The areas under the curves (AUCs) of excursion and max velocity of the diaphragm during voluntary cough for extubation failure are 0.656 (95% Cl 0.389 - 0.924) and 0.517 (95% Cl 0.25 - 0.784) , respectively.

**CONCLUSIONS.** Excursion of the diaphragm during voluntary cough is a potential predictor of extubation outcome in patients who passed SBT.

### 0532

#### Ventilator-induced diaphragm dysfunction in brain-dead and Maastricht III donors

J. Marin-Corral[1,2], I. Dot Jordana[1,2], J. Boncompte[2,3,4], M. Bogunyà[2,5], L. Cecchini[6], A. Zapatero[1,2,3], M.P. Gracia-Arnillas[1,2,3], Y. Díaz[1,2], S. Pascual-Guardia[7,8], C. Vilà[1,2], A. Castellví[1,2], J.R. Masclans[1,2,3]

[1]Hospital del Mar, Intensive Care Department, Barcelona, Spain; [2]Institut Hospital del Mar d'Investigacions Mèdiques (IMIM), Research Group in Critical Care Disorders (GREPAC), Barcelona, Spain; [3]Universitat Autònoma de Barcelona, Barcelona, Spain; [4]Universitat Pompeu Fabra, Barcelona, Spain; [5]Universitat de Barcelona, Barcelona, Spain; [6]Hospital del Mar, Urology Department, Barcelona, Spain; [7]Hospital del Mar, Respiratory Department, Barcelona, Spain; [8]CIBER de Enfermedades Respiratorias (CIBERES), Barcelona, Spain

**Correspondence:** I. Dot Jordana

*Intensive Care Medicine Experimental* 2018, **6(Suppl 2):**0532

**INTRODUCTION.** Atrophy of diaphragm(DF) fibers is correlated with mechanical ventilation(MV) duration and it is associated with ventilatior-induced diaphragm dysfunction(VIDD). In animal models it typically appears after periods of controlled MV(CMV). Human data are basically available from mechanically ventilated brain-dead organ donors(BD) in controlled modalities. This is the first study to evaluate DF histological characteristics of Maastricht III donors(MS) who can tolerate spontaneous MV(SMV).

**OBJECTIVE.** To compare DF and quadriceps(QD) histological characteristics of BD donors(ventilated with CMV), MS donors(who can tolerate SMV modalities) and control patients(CTL, ventilated during a thoracic surgery).

**METHODS.** Unicentric, prospective and interventionist cohort study was conducted in a polyvalent ICU(IRB2017/7183/I). Biopsies from DF and QD were obtained from ICU donor patients(MS and BD) and CTL. Tissue was preserved immediately in alcohol-formol bath to be thereafter embedded in paraffin. Immunohistochemical and morphometric analyses were done:cross-sectional area(CSA), and proportions of type I and II fibers were assessed. Demographic, clinical and relevant mechanical ventilation data(days MV, modality, time in CMV, time in SMV, time with DF stimuli) were collected. Significance was set to p< 0.05.

**RESULTS.** A total of 35 mechanically ventilated patients(14BD,16MS and 5CTL) were included in the study. BD were younger than MS(56(17)years vs.69(12)years;p=0.03) without differences with CTL(63(6)years). There were no significant differences in other demographic variables among the three groups. MS donors were more hours on MV than BD, and as a result, the number of hours in CMV were higher than in BD. MS donors spent a higher percentage of time in SMV and the percentage with DF stimulus trend to statistical significance(table1).

The size of type I and II fibers were significantly reduced in the DF and QD of both donor groups in comparison with CTL. Although MS donors were significantly more hours under MV, there were no differences in the DF CSA between BD and MS. Size of QD type II fibers were significantly decreased in MS compared to BD. Proportions of type I and II fibers did not differ between the three groups.

**CONCLUSIONS.** BD and MS donors developed both DF and QD atrophy in comparison to CTL patient. More sever QD atrophy was observed in MS donor as a consequence of more days in MV and more muscle inactivity. Despite more time spent in MV, MS and BD presented no difference in grade of DF atrophy. Our observations suggest that patients who maintain diaphragm stimulus (MS) could present a protector factor for the development of VIDD, and we speculate that an early switch to an assisted mode or spontaneous mode seems clearly desirable in critical patients to attenuate the progressive instauration VIDD.

**GRANT ACKNOWLEDGMENT**

Spanish Foundation of Critical Patient(FEEC 2017) and the Catalan Society of Intensive Care(SOCMIC 2018).

**Table 1 (abstract 0532).** Ventilation data from organ donors

| | Brain-dead n=14 | Maastricht III n=16 | p-value |
|---|---|---|---|
| Days of Mechanical Ventilation,days, median (IQR) | 3 (1.75-5.25) | 8.5 (5.25-15.75) | 0.001 |
| Hours of mechanical ventilation,hours, median (IQR) | 71 (42-125) | 202 (126-343) | 0.001 |
| Hours of controlled modalities, hours, median (IQR) | 68 (41-96) | 122 (67.5-180.75) | 0.034 |
| Ratio in controlled modalities, %, mean (SD) | 93 (11) | 61 (36) | 0.009 |
| Hours of assisted modalities,hours, median (IQR) | 0 (0-23.5) | 73 (0-133.75) | 0.034 |
| Ratio in assisted modalities, %, mean (SD) | 6 (11) | 38 (36) | 0.009 |
| Hours with diaphragm stimuli,hours, median (IQR) | 0.5 (0-28.2) | 116 (1.5-192.5) | 0.020 |
| Ratio of time with diaphragm stimuli, %, mean (SD) | 13 (19) | 39.6 (34.3) | 0.06 |

## Preventive strategies in critical care infections

### 0533

#### Impact of enteral nutrition on microaspiration of gastric contents in intubated critically ill patients with shock: a planned ancillary study of a randomized controlled trial

S. Nseir[1], A. Le Gouge[2], J.-B. Lascarrou[3], J.-C. Lacherade[4], E. Jaillette[1], J.-P. Mira[5], E. Mercier[6], P.-L. Declercq[7], M. Sirodot[8], G. Piton[9], F. Tinturier[10], A. Ait Hssain[11], S. Gaudry[12], M. Djibré[13], D. Thévenin[14], F. Zerimech[15], J. Reignier[3]

[1]CHU de Lille, ICU, Lille, France; [2]Tours Univ, Statistics Department, Tours, France; [3]CHU de Nantes, ICU, Nantes, France; [4]CHD de Vendée, ICU, La Roche sur Yon, France; [5]Hôpital Cochin-APHP, ICU, Paris, France; [6]CHU de Tours, ICU, Tours, France; [7]CH de Dieppe, ICU, Dieppe, France; [8]Centre Hospitalier Annecy-Genevois, Epagny Metz-Tessy, France; [9]CHRU Besançon, ICU, Besançon, France; [10]CHU d'Amiens, Surgical ICU, Amiens, France; [11]CHU Clermont Ferrand, ICU, Clermont Ferrand, France; [12]Hôpital Louis Mourier, APHP, ICU, Colombes, France; [13]Hôpital Tenon, APHP, ICU, Paris, France; [14]Ch de Lens, ICU, Lens, France; [15]CHU de Lille, Biochemistry Department, Lille, France

**Correspondence:** S. Nseir

*Intensive Care Medicine Experimental* 2018, **6(Suppl 2):**0533

**INTRODUCTION.** Microaspiration of contaminated gastric and oropharyngeal secretions is the main mechanism of entry of bacteria into the lower respiratory tract in intubated critically ill patients.

**OBJECTIVES.** The aim of this study is to determine the impact of enteral nutrition, as compared with parenteral nutrition, on microaspiration of gastric contents.

**METHODS.** Planned ancillary study of the randomized controlled multicenter Nutri Réa2 study. Primary objective was to determine the impact of enteral nutrition on abundant microaspiration of gastric contents. Secondary objective was to determine the impact of enteral nutrition on abundant microaspiration of oropharyngeal secretions. Patients with shock receiving invasive mechanical ventilation were randomized to receive early enteral or parenteral nutrition. All tracheal aspirates were collected during the 48h following randomization. Abundant microaspiration of gastric contents and oropharyngeal secretions were defined as the presence of significant levels of pepsin (>200 ng/ml), and salivary amylase (>1685 UI/ml) in >30% of tracheal aspirates, respectively.

**RESULTS.** A total of 151 patients were included (78, and 73 patients in enteral and parenteral nutrition groups, respectively), and 1074 tracheal aspirates were quantitatively analyzed for pepsin and amylase. Although vomiting rate was significantly higher (31% vs 15%, p = 0.016, HR 2.33 (95% CI 1.17; 4.62)), constipation rate was significantly lower (6% vs 21%, p = 0.010) in patients with enteral than in patients with parenteral nutrition. No significant difference was found regarding other patient characteristics, including the use of subglottic secretion drainage, cuff pressure, or tracheal tube size.

The percentage of patients with abundant microaspiration of gastric contents was significantly lower in enteral than in parenteral nutrition groups (14% vs 36%, p = 0,004; unadjusted OR 0.80 (95% CI 0.69, 0,93), adjusted OR 0.79 (0.76, 0,94)). The percentage of patients with abundant microaspiration of oropharyngeal secretions was significantly higher in enteral than in parenteral nutrition groups (74% vs 54%, p = 0,026; unadjusted OR 1.21 (95% CI 1.03, 1,44), adjusted OR 1.23 (1.01, 1,48)). No significant difference was found in percentage of patients with ventilator-associated pneumonia between enteral (8%) and parenteral (10%) nutrition groups (HR 0.78 (0.26; 2.28)).

**CONCLUSIONS.** Enteral nutrition, as compared with parenteral nutrition, is associated with significantly lower rates of abundant microaspiration of gastric contents and significantly higher rates of microaspiration of oropharyngeal secretions. Our results suggest that the route of nutrition should probably not be determined based on the risk of abundant microaspiration.

**REFERENCE(S)**

Reignier J, et al. Lancet. 2018;391:133-143.

**GRANT ACKNOWLEDGMENT**

French Ministry of Health

### 0534

#### Impact of selective digestive decontamination on nosocomial infections and multiresistant bacteria in two intensive care units: one with versus another without selective digestive decontamination

C. Sánchez Ramírez[1], A.L. Balán Mariño[2], L. Caipe Balcázar[1], S. Hípola Escalada[1], M.A. Hernández Viera[1], F. Iglesias Llaca[2], M. Cabrera Santana[1], R.E. Morales Sirgado[1], P. Saavedra Santana[3], S. Ruiz Santana[1]
[1]University Hospital of Gran Canaria Dr Negrín, Intensive Care Unit, Las Palmas de Gran Canaria, Spain; [2]University Hospital San Agustín of Avilés, Intensive Care Unit, Avilés, Spain; [3]University of Las Palmas de Gran Canaria, Mathematics and Informatics Deparment, Las Palmas de Gran Canaria, Spain
**Correspondence:** C. Sánchez Ramírez
*Intensive Care Medicine Experimental* 2018, **6(Suppl 2):**0534

**OBJECTIVES.** To analyze the impact of SDD on ICU nosocomial infections (NI) and multiresistant bacteria (MRB) compared to another ICU without SDD.

**METHODS.** Observational, analytical, cohort study, in ICUs of two different hospitals [Hospital Universitario Dr. Negrín, University of Las Palmas (HUDN): 30 mixed beds] and University Hospital San Agustín de Avilés (HUSA)], with 10 mixed beds. The aim was, to compare the incidence on NI in patients that received SDD (HUDN) with patients whose did not receive SDD (HUSA). In order to compare the NI rates we assessed three groups of patients, the group with SDD (HUDN) from October 1, 2011 to September 30, 2016; the group without SDD (HUDN) from October 1, 2010 to 30 / September / 2011 and the HUSA group, without SDD, from April 1, 2012 to September 30, 2016. SDD was applied in HUDN to patients in whom mechanical ventilation was considered for more than 48 hours. It consists of an oropharyngeal paste, and a solution of tobramycin, colistin and nystatin and intravenous cefotaxime for 4 days. We analyzed demographic variables, type of admission, comorbidities, according to the ENVIN-Helics database. A multivariate statistical analysis of SDD and MRB was performed, as well as an incidence of IN / 1000 days of exposure for each group and were compared with the Poisson model.

**RESULTS.** Of the 517 NI patients treated in both ICUs, 342 were given SDD (HUDN) and of the remaining 175 patients without SDD, 110 belonged to HUDN and 65 belonged to HUSA. Regarding the analysis of patients with one or more MRB, *Acinetobacter spp.* was more frequent in HUSA (p < 0.001) while extended spectrum betalactamases (ESBLs) were significantly more frequent in HUDN (p = 0.012), although both of them decreased significantly after SDD (from 34.5 % to 23.7%) (Table 1).

In the multivariate analysis (Table 2)

The proportion of patients without SDD in HUDN was almost 5 times higher compared to those of SDD, OR 4.802 (95% CI 1951-11819) In HUSA, without SDD, the risk of *Acinetobacter* infection was 38.73 times higher, OR 38,773 (95% CI 10.812-139.037). In the multivariate logistic regression analysis it can be observed that, a patient without SDD in the HUDN, the OR of developing *Acinetobacter spp.* infection was OR = 10.072 (3.142; 32.291). Also, a patient without SDD in the HUDN, the OR of developing MRB-GNB (gram negative bacteria) infection was OR = 4.8 (1.951; 11.819).In the multivariate analysis of MRB the risk of acquiring a MRB infection was significantly reduced after the application of SDD: OR 0.438 (95% CI 0.299-0.640) and increased in the presence of septic shock. Moreover, ICU NI (ventilator-associated pneumonia, secondary bacteremias and urinary infections), also decreased after SDD (Table 3).

**CONCLUSIONS.** The SDD decreases the NIs in an environment of high-level of bacteria resistance, as a protective factor for the acquisition of MRB.

**Table 1 (abstract 0534).** Univariate analysis SSD

| | Selective Digestive Decontamination | | | |
|---|---|---|---|---|
| | Total N = 532 | No N = 110 | Yes N = 422 | P |
| *Age*, years | 60.7 ± 15.4 | 59.5 ± 15.8 | 61.0 ± 15.3 | 0.376 |
| Male / Female, n (%) | 355 (66.8) | 74 (67.3) | 281 (66.8) | 0.917 |
| Apache-II | 22.0 ± 7.6 | 21.2 ± 7.7 | 22.3 ± 7.6 | 0.201 |
| Immunossupression, n (%) | 17 (3.2) | 3 (2.7) | 14 (3.3) | 1 |
| Neutropenia, n (%) | 5 (0.9) | 3 (2.7) | 2 (0.5) | 0.062 |
| Parenteral nutrition, n (%) | 161 (30.3) | 26 (23.6) | 135 (32.0) | 0.089 |
| *Patient type* | | | | 0.113 |
| Medical | 380 (71.6) | 79 (71.8) | 301 (71.5) | |
| Scheduled surgical | 74 (13.9) | 10 (9.1) | 64 (15.2) | |
| Urgent surgical | 77 (14.5) | 21 (19.1) | 56 (13.3) | |
| Traumatic patient, n (%) | 68 (12.8) | 17 (15.4) | 51 (12.1) | 0.351 |
| *Acinetobacter*, n (%) | 18 (3.4) | 13 (11.8) | 5 (1.2) | < .001 |
| SAMR, n (%) | 11 (2.1) | 4 (3.6) | 7 (1.7) | 0.251 |
| ESBL, n (%) | 132 (24.8) | 38 (34.5) | 94 (22.3) | 0.008 |
| *PSEUDOMONAS MR*, n (%) | 43 (8.1) | 10 (9.1) | 33 (7.8) | 0.758 |
| MR-GNB, n (%) | 28 (5.3) | 12 (10.9) | 16 (3.8) | 0.003 |
| Renal Replacement Therapy, n (%) | 194 (36.5) | 34 (30.9) | 160 (37.9) | 0.174 |
| VAP, n (%) | 210 (39.5) | 59 (53.6) | 151 (35.8) | < .001 |
| Urinary infection, n (%) | 139 (26.1) | 29 (26.4) | 110 (26.1) | 0.950 |
| Catheter related Bacteremia, n (%) | 190 (35.7) | 26 (23.6) | 164 (38.9) | 0.003 |
| Secondary bacteremia, n (%) | 134 (25.2) | 31 (28.2) | 103 (24.4) | 0.417 |
| Inflammatory response | | | | < .001 |
| Non sepsis | 23 (4.3) | 2 (1.8) | 21 (5.0) | |
| Sepsis | 174 (32.7) | 23 (20.9) | 151 (35.8) | |
| Severe Sepsis | 81 (15.2) | 34 (30.9) | 47 (11.1) | |
| Septic Shock | 254 (47.7) | 51 (46.4) | 203 (48.1) | |
| Renal failure, n (%) | 126 (23.7) | 40 (36.4) | 86 (20.4) | < .001 |
| COPD, n (%) | 77 (14.5) | 9 (8.2) | 68 (16.1) | 0.035 |
| Diabetes mellitus, n (%) | 170 (31.9) | 34 (30.9) | 136 (32.2) | 0.792 |
| Days in ICU, days | 30 (17 ; 48) | 28 (16 ; 45) | 30 (17 ; 49) | .227 |
| Death, n (%) | 164 (36.7) | 36 (32.7) | 128 (38.0) | .321 |

MRSA: methicillin resistant Sthaphyloccocus aureus; ESBL: extended spectrum belalactamase; MR: multiresistant; GNB: gram negative bacteria; VAP: ventilator associated pneumonia; COPD: Chronic Obstructive Pulmonary Disease

**Table 2 (abstract 0534).** Multivariate analysis SDD

| | P | OR (95% CI) |
|---|---|---|
| *Inflammatory response* | < .001 | |
| Non sepsis | - | 1 |
| Sepsis | 0.479 | 0.573 (0.123 - 2.678) |
| Severe Sepsis | 0.014 | 0.143 (0.030 - 0.672) |
| Septic Shock | 0.434 | 0.545 (0.119 - 2.493) |
| *VAP* | 0.006 | 0.517 (0.324 - 0.827) |
| *Renal failure* | 0.001 | 0.446 (0.271 - 0.734) |
| *Acinetobacter infection* | < .001 | 0.099 (0.032 - 0.304) |

SDD: Selective Digestive Decontamination; VAP: ventilator associated pneumonia

**Table 3 (abstract 0534).** Nosocomial.Infection rates

| | | SDD | | P | RR |
|---|---|---|---|---|---|
| | | No | Yes | | (95% CI) |
| VAP /MV | VAP/1000 days of MV | 10.31 | 4.36 | | 0.423 (0.316 - 0.565) |
| *Urinary infections* | Infections/1000 days of catheter | 3.79 | 2.46 | .026 | 0.649 (0.443 - 0.951) |
| *Bacteremia related to catheter* | Bacteremias/1000 days of CVC | 3.59 | 3.85 | .733 | 1.074 (0.712 - 1.621) |
| *Secondary bacteremias* | Bacteremias./1000 ICU days | 4.69 | 1.98 | < .001 | 0.422 (0.296 - 0.599) |
| *ARB* | ARB /1000 ICU days | 9.59 | 2.83 | < .001 | 0.295 (0.227 - 0.383) |

SDD: Selective Digestive Decontamination; VAP: ventilator associated pneumonia;MV: mechanical ventilation; CVC: central venous catéter; ARB: Antibiotic resistant bacteria

### 0535

#### Influence of tracheostomies, sub-glottic suction endotracheal tubes and routine chlorhexidine on the rate of suspected ventilator-associated pneumonia in Scottish intensive care units

R. Hart[1], S. Maclean[2], S. McNeill[3], J. Hornsby[1], S. Ramsay[1]
[1]Queen Elizabeth University Hospital, Anaesthesia and Critical Care, Glasgow, United Kingdom; [2]Ninewells Hospital, Department of Anaesthesia and Critical Care, Dundee, United Kingdom; [3]Royal Infirmary of Edinburgh, Department of Anaesthesia and Critical Care, Edinburgh, United Kingdom
**Correspondence:** R. Hart
*Intensive Care Medicine Experimental* 2018, **6(Suppl 2):**0535

**INTRODUCTION.** Ventilator-associated pneumonia (VAP) is the most common healthcare associated infection (HAI) in mechanically ventilated patients. VAPs are independently associated with an increased length of intensive care unit (ICU) stay, duration of mechanical ventilation and mortality. For this reason it is important that our ICU´s implement strategies to prevent this serious complication of mechanical ventilation. The influence of tracheostomies and routine chlorhexidine are the subject of debate. The use of sub-glottic suction endotracheal tubes (SGT) is not universal.

**OBJECTIVES.** We aim to determine whether the presence of tracheostomy, sub-glottic suction endotracheal tube or routine chlorhexidine has an influence on the rate of suspected VAP.

**METHODS.** A prospective national audit was conducted over a one-month period. Local data collectors were recruited and coordinated by the Scottish Intensive Care Society (SICS) Trainee Committee. All patients ventilated for more than 48-hours were included. Baseline HAI data was gathered for each patient on a daily basis. Additional relevant data, such as the presence of a subglottic tube, tracheostomy or regular chlorhexidine administration was also recorded.

**RESULTS.** This study included 227 patients and 1751 days of data collection. We successfully recruited 14 ICU sites from 11 Scottish Health Boards. 32 patients were suspected of having a VAP (S-VAP). The incidence of suspected VAP was reduced in patients with a subglottic drainage ET tube in place (5.7% vs 11%, RR 0.36, p=0.12). S-VAP was significant increased in patients with a tracheostomy (35.1% vs 10%, RR 3.51, p< 0.0001). Regular chlorhexidine did not demonstrate a significant reduction in S-VAP (12.2% vs 16.7%, RR0.73, p=0.34).

**CONCLUSIONS.** VAP is a serious complication of mechanical ventilation therefore preventative strategies should be routine. Tracheostomy was associated with a significant increase in suspected VAP, therefore the decision to site a tracheostomy should be carefully considered. Our study showed a tend toward reduction in S-VAP using SGT and may have been significant if our population was larger. Although SGT are more expensive than standard endotracheal tubes we believe our study interpreted with the existing literature suggests that they are effective in reducing VAP rates. We therefore believe routine SGT use should be encouraged in our units, which is in line with existing guidance from the Intensive Care Society (ICS)[1,2]. We believe our results show there is no role for routine use of chlorhexidine therapy in the prevention of S-VAP.

**REFERENCE(S)**

1. Hellyer et al. The Intensive Care Society recommended bundle of interventions for the prevention of ventilator-associated pneumonia. Journal of the Intensive Care Society 2016 Vol 17(3) 238-242.
2. Damas et al. Prevention of ventilator-associated pneumonia and ventilator-associated conditions. Crit Care Med 2015; 43:22-30.

### 0536

#### The introduction of a policy of chlorhexidine washing is not associated with a reduction in rates of intensive care associated bloodstream infections, blood culture contamination, and colonisation or infection with multidrug-resistant microorganisms: an interrupted time series analysis

E. Thoonen[1], R. Kengen[1], K. Daveson[1], B. Loong[2], H. Rodgers[1], W. Beckingham[1], K. Kennedy[1], R. Suwandarathne[1], F. van Haren[1]
[1]Canberra Hospital, Canberra, Australia; [2]Australian National University, Canberra, Australia
**Correspondence:** F. van Haren
*Intensive Care Medicine Experimental* 2018, **6(Suppl 2):**0536

**BACKGROUND.** Healthcare-associated infections (HAIs) are a major cause of morbidity and mortality in intensive care patients. The effect of daily washing with chlorhexidine on HAIs is controversial.

**METHODS.** Interrupted time series (ITS) analysis in a 31-bed tertiary referral mixed ICU, comparing daily washing with water and soap (January 2011 - August 2013) with chlorhexidine washing (November 2013 - December 2015), following the introduction of a unit-level policy of chlorhexidine washing. All ICU patients were included in the study, except: if they were under 18 years of age; if their ICU stay was less than 24 hours, to ensure that all studied patients had at least one exposure to the daily wash intervention; if patients had a known allergy to chlorhexidine. Outcome measures included: clinically significant positive blood cultures attributable to the ICU stay; contaminated blood cultures; newly acquired multidrug-resistant microorganisms (MDRO): methicillin resistant staphylococcus aureus (MRSA), vancomycin resistant enterococcus (VRE), or multi-resistant gram-negative (MRGN) isolates attributable to ICU from clinical and screening cultures; and newly acquired Clostridium difficile (CDI) cases.Incidence rates of these outcomes were calculated per 1000 patient days. MDRO acquisition rates were corrected for background hospital period prevalence rates of MDRO.

**RESULTS.** A total of 6634 patients were included in the study. ITS analysis showed no significant level or slope changes in any of the outcome measures following implementation of chlorhexidine washing. The incidence rate of clinically significant positive blood cultures during the chlorhexidine period compared to the water and soap period was 3.6 vs. 4.7 (p=0.37); blood culture contamination rates were 11.8 vs. 9.5 (p=0.56); incidence rates of new ICU associated MDRO acquisitions were 3.22 vs. 3.69 (p=0.27); incidence rates of new CDI were 2.01 vs. 0.79 (p=0.16). Outcomes after adjustment for known and potential confounders were similar.

**CONCLUSIONS.** In this real-world long-term ICU study, implementation of a unit -level policy of daily washing with chlorhexidine impregnated cloths was not associated with a reduction in the rates of ICU associated clinically significant positive blood cultures, blood culture contamination, newly acquired MDRO isolates, and CDI.

### 0537

#### Value of semi-quantitative throat and rectal swabs to predict ESBL-VAP in ESBL carriers: a prospective cohort study

O. Andremont[1], L. Armand[2,3], M. Neuville[1], B. Mourvillier[1], J.-C. Lucet[3,4], R. Sonneville[1], C. Vinclair[1], E. Ruppé[3,5], L. Bouadma[1,3], J.-F. Timsit[1,3]
[1]APHP Bichat, Infectious Intensive Care Medicine, Paris, France; [2]APHP, Microbiology, Paris, France; [3]Inserm Université Paris-Diderot, IAME UMR 1137, Paris, France; [4]APHP Bichat, Infection Control, Paris, France; [5]APHP Bichat, Microbiology, Paris, France
**Correspondence:** O. Andremont
*Intensive Care Medicine Experimental* 2018, **6(Suppl 2):**0537

**INTRODUCTION.** Carriage of extended-spectrum betalactamase-producing Enterobacteriaceae (ESBL-E) is frequent in ICU patients.The knowledge of previous ESBL-E colonization makes clinicians more prone to empirically start carbapenems if a ventilator-associated pneumonia (VAP) is suspected, pending results of respiratory samples cultures (2,3). Studies suggested that previous ESBL-E carriage did not accurately predict an ESBL-E VAP(4). We hypothesized that the quantification of rectal and throat ESBL-E carriage will allow a better prediction of ESBL-E-associated VAP.

**OBJECTIVES.** To evaluate the impact of rectal and throat carriage of ESBL-E assessed semi-quantitatively to predict ESBL-E- associated VAP in a single medical ICU.

**METHODS.** Between May 2014 and May 2017, all patients were screened at admission and weekly for ESBL-E (ChromID ESBL, Biomérieux) throat and rectal carriage. Results were given semi-quantitatively (fFig). We recorded the last positive sample collected at least 48 hours before the VAP. VAP was diagnosed using BAL or PTC quantitative culture.

**RESULTS.** 1,319 patients were analyzed. 284 (21.5%) were ESBL-E carriers.

79 ESBL-E carriers (rectal only n=54; throat only n=2; both n=48 ) (SAPS 2, 48 [35-64]; age, 58 [49-70] years) acquired 104 VAP episodes after a median of 13 ICU-days. VAP was not due to ESBL-E in 56 (54%) cases (Klebsiella sp . 28 (50%) P. aeruginosa 5 (9%) E. cloacae, 5 (9%) P. mirabilis, 5 (9%) other). ESBL-E was recovered in 48 cases (28 (58%) Klebsiella, 11 (23%), E. coli, 9 (19%) E. cloacae). The duration of ICU stay after VAP (22 [11.5-41] and 15.5 [8.5-36.5] for no ESBL-E and ESBL-E respectively) was similar between both groups.

Empirical therapy contained carbapenems in 62 (61%) cases including 26 (48%) of the no ESBL-E VAP. Predictors of ESBL-E VAP were: lower albumin level on admission (24 vs 26 g/l, p=0.04), a higher ARDS rate (20 (42%) vs 13 (23%) p=0.04) and SOFA score (8 vs 10 p=0.04 ) at VAP onset. ESBL E coli carriers developed ESBL-E VAP less frequently (n=16 (34%)) than others (n=37 (67.3%, p< .01) Number of ESBL-E rectal carriers were similar in both groups (ESBL-E VAP 97.9% vs no ESBL-E VAP 98.2%; p=0.9) whereas ESBL throat carriage was more frequent in ESBL VAP (no ESBL VAP 13 (23.2%) vs ESBL VAP 37 (77.1%); p< .01).

When analysing throat and rectal samples semi-quantitatively, the rectal bacterial semi-quantitative load (fig1A, p=0.0079 test for trend) but not the throat bacterial load (fig 1B; p< .0001) were associated with ESBL VAP.

**CONCLUSIONS.** ESBL-E throat carriage and a high semi-quantitative ESBL-E rectal carriage predict ESBL-E VAP, and should be used to limit the empiric use of carbapenems in patients suspected of ESBL-E VAP.

**REFERENCE(S)**

1- Gbaguidi-Haore H et al- J Antimicrob Chemother. 2013 Feb;68(2):461-70
2- Barbier F et al - JAC. 2016 Apr;71(4):1088-97
3- Barbier F et al- Intensive Care Med 2018 april EPub
4- Razazi K et al - Intensive Care Med. 2012;38:1769-78

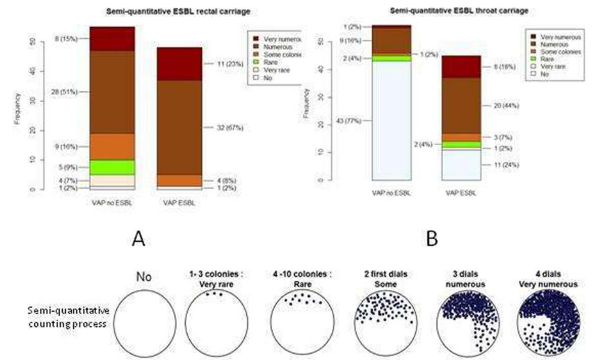

**Fig. 1 (abstract 0537).** Semi-quantitative throat and rectal ESBL-E swabs and VAP (n colonies observed after streaking)

## Monitoring in sepsis

### 0538

#### Early identification of the microorganism causing bacteremia by metabolomic study in urine of patients diagnosed with sepsis and septic shock

D.L. Aguillón[1], F.J. Rosa[1], M. García Simón[1], V. Gonzalez[2], D. Monleón Salvado[2], J.M. Morales[2], C. Gimeno Cardona[3]
[1]Hospital Clinico Universitario de Valencia, Critical Care, Valencia, Spain; [2]Universidad de Valencia, Central Unit of Research in Medicine, Valencia, Spain; [3]Universidad de Valencia, Department of Microbiology, Valencia, Spain
**Correspondence:** F.J. Rosa
*Intensive Care Medicine Experimental* 2018, **6(Suppl 2):**0538

**INTRODUCTION.** Sepsis is one of the main causes of death in ICU and, in almost half of the cases, bacteremia is detected. The early detection of bacteremia is valuable, because an adequate and early treatment is fundamental for the survival of the patient.

**OBJECTIVE.** To identify a metabolomic profile able to predict early the type of microorganism that causes bacteremia in patients admitted in ICU with a diagnosis of sepsis and septic shock, using H1-NMR spectroscopy in urine samples.

**MATERIAL AND METHODS.** Prospective observational study of cohorts in patients admitted in the ICU with a diagnosis of sepsis and septic shock during a period of one year. The urine of 62 patients was analyzed on H1-NMR spectroscopy the day of admission. NMR spectra were obtained for each sample in a Broker AVANCE III 600 spectrometer of 14.1 Teslas at 25ºC. These spectre were analyzed by multivariable analysis (PCA) and discriminatory (PLS-DA) and peak integration. The relevant spectral regions were identified and quantified in arbitrary units (relative spectral area). We calculated the means, standard deviations and statistical significance of them by T-Student between the group that tested positive for bacteremia and those that did not (according to microbiological results) and according to the type of microorganism identified (Gram positive / Gram negative / fungi).

**RESULTS.** 62 patients. 62.9% were men. Age 61.51(± 17.5). Overall Mortallity 28.6%. ICU stay of 5 days (1-61). Urine NMR spectra on admission show signs of more than 50 metabolites. The multivariate analysis PLS-DA relies from the admission, metabolic differences between the patients who present bacteremia and those who do not (TPR 87.5%, FPR 17.77%) (Figure 1) and depending on the type of microorganism causing the bacteremia (Figure 2). The metabolites that contribute to the separation of the most prevalent types of microorganisms are isocaproate, alanine, acetyl-lysine, hydroxyacetone, creatine, creatinine and N-oxide of Trimethylamine.

**CONCLUSIONS.** The metabolomic profile obtained in urine by H1-NMR spectroscopy is able to identify early type of microorganism causing bacteremia in the first hours of admission to the ICU, which may prove to be a crucial advantage in the evolution of the patient.

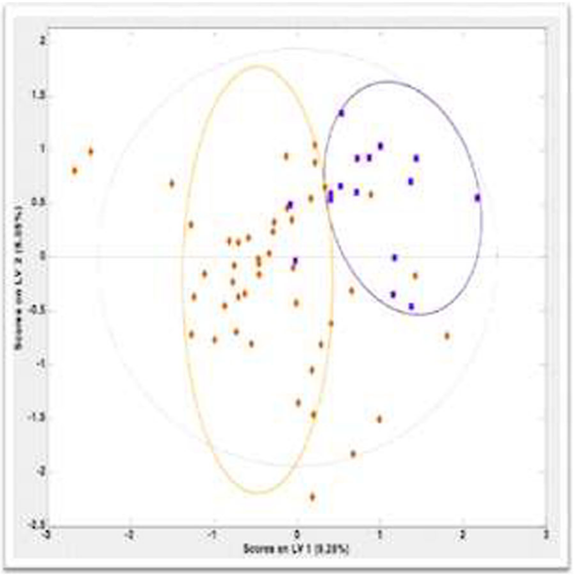

**Fig. 1 (abstract 0538).** PLS-DA load diagram of urine at admission. Bacteremia (Purple) and not Bacteremia (Orange)

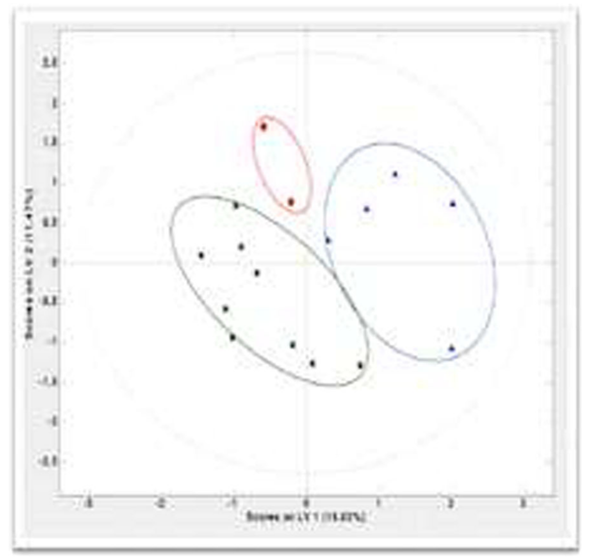

**Fig. 2 (abstract 0538).** PLS-DA load diagram of urine on admission. Gram Negatives (Green), Gram Positives (Blue) and Fungi (Red)

### 0539

#### Neuroimaging findings in sepsis-induced brain dysfunction: association with clinical and laboratory findings

G. Orhun[1], F. Esen[1], P. Ergin Ozcan[1], S.N. Sencer[2], B. Bilgic[3], C. Ulusoy[4], H. Noyan[4], M. Kucukerden[4], A. Ali[1], M. Barburoglu[2], E. Tuzun[4]
[1]Istanbul University, Istanbul Faculty of Medicine, Anesthesiology and Intensive Care, Istanbul, Turkey; [2]Istanbul University, Istanbul Faculty of Medicine, Deparment of Neuroradiology, Istanbul, Turkey; [3]Istanbul University, Istanbul Faculty of Medicine, Deparment of Neurology, Istanbul, Turkey; [4]Istanbul University, Aziz Sancar Institute of Experimental Medicine, Deparment of Neuroscience, Istanbul, Turkey
**Correspondence:** G. Orhun
*Intensive Care Medicine Experimental* 2018, **6(Suppl 2):**0539

**INTRODUCTION.** Acute brain dysfunction due to the pathologic neuro-inflammatory process associated with sepsis is common in the intensive care unit (1). One of the challenges in studying sepsis-induced brain dysfunction (SIBD) is that without a specific diagnostic test, it remains a syndrome diagnosed by exclusion (2). Brain imaging methods are diagnostic procedures which complement neurologic examination. Magnetic resonance imaging (MRI) provides important information on the primary pathologic mechanism of sepsis associated encephalopathy and structural alterations in the brain (3). Furthermore, morphometric analysis of the MRI data using voxel-based approaches has a potential of providing more detailed information about the structure of the brain structures.

**OBJECTIVES.** Our objective was to provide findings from MRI and VBM of the brain in patients showing neurological alterations due to sepsis and relate it to the inflammatory and disease severity markers of SIBD.

**METHODS.** In this prospective observational study, 93 SIBD patients (45 men, 48 women; 50.6±12.7 year-old) were enrolled. Patients underwent a neurological examination and brain MRI. Several severity-of-disease scoring systems were used for evaluation of patient outcome. Serum levels of a panel of mediators [IL-1 β, IL-6, IL-8, IL-10, IL-12, IL-17, IFN- γ, TNF- α, complement factor Bb, C4d, C5a, iC3b, amyloid-b peptides, total tau, phosphorylated tau (p-tau), S100b, neuron-specific enolase] were measured by ELISA. VBM was employed to available patients for assessment of neuronal loss pattern in SIBD.

**RESULTS.** MRI of SIBD patients were normal (n=27, 29%) or showed brain lesions (n=51, 54.9%) or brain atrophy (n=15, 16.1%). VBM analysis showed neuronal loss in the insula, cingulate cortex, frontal lobe, precuneus, and thalamus. Patients with abnormal MRI findings had worse APACHE II, SOFA, GOSE scores, increased prevalence of delirium and mortality. Presence of MRI lesions was associated with reduced C5a and iC3b levels and brain atrophy was associated with increased p-tau levels. Multivariate regression analysis identified an association between reduced IL-12 level and occurrence of coma.

**CONCLUSIONS.** Neuronal loss predominantly occurs in limbic and visceral pain perception regions of SIBD patients. Complement breakdown products, IL-12, and p-tau stand out as adverse neuroimaging and neurological outcome markers for SIBD.

**REFERENCE(S)**

1. Iacobone E, Bailly-Salin J, Polito A, Friedman D, Stevens RD, Sharshar T. Sepsis-associated encephalopathy and its differential diagnosis. Crit Care Med 2009; 37: S331- 336.
2. Gofton TE, Young GB. Sepsis-associated encephalopathy. Nat Rev Neurol 2012; 8: 557- 566.
3. Oddo M, Taccone FS. How to monitor the brain in septic patients? Minerva Anestesiol. 2015; 81: 776- 788.

**GRANT ACKNOWLEDGMENT**

This work was supported by Scientific Research Projects Coordination Unit of Istanbul University. Project number 35165 and 46960.

### 0540

#### Defining optimal dosing of ciprofloxacin in patients with septic shock

J. Roberts[1,2,3], A. Alobaid[1,4], S. Wallis[1], A. Perner[5], J. Lipman[1,2,6], F. Sjövall[7,8]

[1]The University of Queensland, Burns Trauma and Critical Care Research Centre, University of Queensland Centre for Clinical Research, Brisbane, Australia; [2]Royal Brisbane and Women's Hospital, Brisbane, Australia; [3]The University of Queensland, Centre for Translational Pharmacodynamics, School of Pharmacy, Brisbane, Australia; [4]King Saudi Medical City, Department of Pharmacy, Riyadh, Saudi Arabia; [5]University of Copenhagen, Department of Intensive Care, Copenhagen University Hospital Rigshospitalet, Copenhagen, Denmark; [6]Queensland University of Technology, Faculty of Health, Brisbane, Australia; [7]Skane University Hospital, Lund University, Intensive Care and Perioperative Medicine, Malmö, Sweden; [8]Lund University, Department of Clinical Sciences, Lund, Sweden

**Correspondence:** F. Sjövall

*Intensive Care Medicine Experimental* 2018, **6(Suppl 2):**0540

**INTRODUCTION AND OBJECTIVES.** Patients with septic shock undergo extensive physiological alterations that can alter drug pharmacokinetics. A growing body of evidence indicates that standard dosing of antibiotics is not sufficient in many of these patients. Ciprofloxacin is a fluoroquinolone with a wide spectrum of action and the objectives of this study were to use a population pharmacokinetic approach to define **recommendations for effective ciprofloxacin dosing in patients with septic shock.**

**PATIENTS AND METHODS.** Adult patients with septic shock treated with ciprofloxacin were eligible for inclusion. A drug concentration curve was created from seven blood samples collected during one dosing interval. Ciprofloxacin concentrations were measured by a validated HPLC-MS/MS method. Pharmacokinetic modelling was performed with Monte-Carlo simulations to define dosing regimens that optimised attainment of AUC:MIC ratio > 125 for different minimal inhibitory concentration (MIC) breakpoints as well as empirical or targeted therapy of Pseudomonas aeruginosa.

**RESULTS.** We included 48 patients with median SAPS II of 49 and 90-day mortality of 33%. Ciprofloxacin pharmacokinetics was best described by a two-compartment linear model including creatinine clearance (CrCL) and body weight as covariates. With a dose of 400 mg q8 and CrCL of 80 mL/min successful target attainment was reached for bacteria with MICs ≤ 0.25 mg/L. For empirical treatment of P. aeruginosa, 600 mg q8 (the maximal simulated dose) only reached a maximum of 68% target attainment. For directed therapy, against P. aeruginosa, a dose of 400 mg q8 reached a 95% successful target attainment only for patients with a low CrCL of 30 mL/min whereas for patients with normal CrCL of 80 mL/min, a dose of 600 mg q8 was needed to reach sufficient AUC:MIC ratios.

**CONCLUSIONS.** In patients with septic shock, standard ciprofloxacin dosing only achieves sufficient concentrations to successfully treat bacteria with MICs of ≤ 0.25 mg/L and then only in patients with normal or reduced CrCL. To cover bacterial isolates with higher MICs or in patients with augmented renal clearance, doses should be increased and/or administration should be tailored by therapeutic drug monitoring to optimise drug concentration, increase the likelihood of treatments success and reduce the potential of selection of resistant strains.

### 0541

#### Evaluating the response to volume administration in septic shock patients. Time to move from the Frank-Starling “Responder”?

E. Cortés, A. Pérez-Madrigal, S. Nogales, C. Espinal, G. Gruartmoner, J. Mesquida

Parc Taulí University Hospital, Critical Care Department, Sabadell, Spain

**Correspondence:** J. Mesquida

*Intensive Care Medicine Experimental* 2018, **6(Suppl 2):**0541

**INTRODUCTION.** Hemodynamic resuscitation of septic shock patients is based on optimization of cardiac output in order to improve oxygen delivery to the tissues. Microcirculatory evaluation of critically ill patients has stressed the possibility of discrepancy between the microcirculation and global flow response to a fluid challenge. On the other hand, some authors have shown that some metabolic markers might better reflect the microcirculatory status than traditional macrocirculatory parameters.

**OBJECTIVES.** To evaluate the coherence between the macrocirculatory and the metabolic response to a fluid challenge in septic shock patients in the early phase of resuscitation.

**METHODS.** Single-center, prospective, observational study. Septic shock patients within the first 24 hours of ICU admission, monitored with a cardiac output system ($PiCCO_2$), and receiving a fluid bolus (500 mL of crystalloids within 15 minutes) according to their medical team. We measured hemodynamic variables (trans-pulmonary thermo-dilution, TPTD) immediately before, and 15 minutes after volume infusion. Simultaneously to both TPTD, blood samples were obtained from an arterial and a central venous line. We determined cardiac index (CI), central venous oxygen saturation ($S_{cv}O_2$), lactate, central venous-to-arterial carbon dioxide difference ($P_{cva}CO_2$), PcvaCO2 corrected by the arterial-to-venous oxygen content difference ($P_{cva}CO_2/C_{av}O_2$), and the global oxygen consumption index ($iVO_2$) before and after the fluid challenge. Comparisons were performed using non-parametric tests.

**RESULTS.** .We studied 65 fluid boluses. The trigger for fluid administration was $S_{cv}O_2$ < 70% (43%) and/or persistence of elevated lactate values (79%). Only 34% of the patients showed an increase in CI > 10% (CI-Responders). Among CI-Responders, only 32% also increased $iVO_2$ values > 10% ($VO_2$-R). The presence of low $S_{cv}O_2$ values at inclusion was not associated with $VO_2$-R, regardless of the CI response. Baseline lactate, $P_{cva}CO_2$ and $P_{cva}CO_2/C_{av}O_2$ values were not different in $VO_2$-R and non-responders ($VO_2$-NR). In those patients whose CI increased (CI-R), changes in the metabolic parameters did not differ between $VO_2$-R and $VO_2$-NR: $S_{cv}O_2$ increased in 80% of $VO_2$-R vs 92% of $VO_2$-NR (*p* ns); lactate decreased in 20% of $VO_2$-R vs

58% of $VO_2$-NR (*p* ns); and $P_{cva}CO_2/C_{av}O_2$ did not decrease in any $VO_2$-R, while it decreased in 33% of $VO_2$-NR (*p* ns).

**CONCLUSIONS.** Increases in CI as results of a fluid challenge do not preclude a positive metabolic response, defined as the increase in $VO_2$, even in low $S_{cv}O_2$ situations. The current concept of "Fluid-Responder" might not be the most appropriate, since it does not take into account the evaluation of the $VO_2/DO_2$ dependence. No single metabolic parameter seems to accurately reflect the $VO_2$ response to a fluid challenge. Integration of metabolic markers informing of this $VO_2/DO_2$ dependence seems complex, but necessary in order to avoid potentially harmful interventions.

### 0542

#### Utility of brain natriuretic peptide levels to predict left ventricular systolic dysfunction in septic patients admitted to the intensive care unit

V. Fraile-Gutiérrez[1], D. Pérez-Torres[1], J.M. Ayuela-Azcarate[2], A. Mayo-Iscar[3], A.I. Rodríguez-Villar[1], P. Blanco-Schweizer[1], E. Prol-Silva[1], J.Á. Ayala-Fernández[1], C. Díaz-Rodríguez[1], J. Blanco-Varela[1]

[1]Hospital Universitario Río Hortega, Intensive Care Department, Valladolid, Spain; [2]Complejo Asistencial Universitario de Burgos, Intensive Care Department, Burgos, Spain; [3]University of Valladolid, Department of Statistics and Operations Research, Valladolid, Spain

**Correspondence:** D. Pérez-Torres

*Intensive Care Medicine Experimental* 2018, **6(Suppl 2):**0542

**INTRODUCTION.** Brain natriuretic peptide (BNP) is a well-studied biomarker of heart dysfunction, secreted by cardiomyocytes in response to stretching, in the presence of volume or pressure overload. BNP has been suggested to correlate with left ventricular systolic dysfunction (LVSD) and mortality in sepsis. However, the optimal cut-off level of BNP in the intensive care setting is still unclear.

**OBJECTIVES.** To determine the optimal cut-off level of BNP rule out the diagnosis of LVSD in septic patients admitted to the Intensive Care Unit (ICU). To create a model to predict LVSD after initial resuscitation in septic patients, based upon biomarkers and easy to obtain echocardiographic parameters.

**METHODS.** We conducted a single-centre prospective observational cohort study. Patients admitted to the ICU with sepsis over a 20-month period were enrolled, excluding those with ischemic or valvular heart disease and pacemakers. Admission levels of biomarkers were obtained and transthoracic echocardiogram (TTE) was performed within the first 24 hours. Multivariate logistic regression analysis was used to create predictive models. Clinical, echocardiographic and analytical variables with p < 0.1 on univariate analysis were considered for inclusion in the models.

**RESULTS.** In our analysis, a cut-off level of BNP >400 pg/ml for the diagnosis of LVSD in septic patients showed 82% sensitivity, 68% specificity and 95% negative predictive value (p< 0.0001). We created three predictive models to identify LVSD in the septic patient (see Table and Figure):

• Model 1: $Log_{10}$BNP, OR 7.57 (95%CI: 1.85-39.76) and TAPSE, OR 0.63 (95%CI: 0.47-0.78).

• Model 2: $Log_{10}$BNP, OR 11.72 (95%CI: 2.36-82.74), LVOT VTI OR 0.73 (95%CI: 0.54-0.93) and TAPSE, OR 0.75 (95%CI: 0.55-0.95).

• Model 3: BNP >400 pg/mL, OR 14.62 (95%CI: 3.08-105) and TAPSE, OR 0.62 (95%CI: 0.45-0.78).

**CONCLUSIONS.**

• Detection of BNP levels < 400 pg/mL after initial resuscitation excludes the presence of LVSD in the septic patient, with a high negative predictive value. Therefore, the optimal cut-off levels of BNP for the diagnosis of LVSD in septic patients should be considered higher than in general population.

• Combination of plasma BNP levels >400 pg/mL and TAPSE < 17 mm can predict the presence of sepsis-induced LVSD in the critically ill patient.

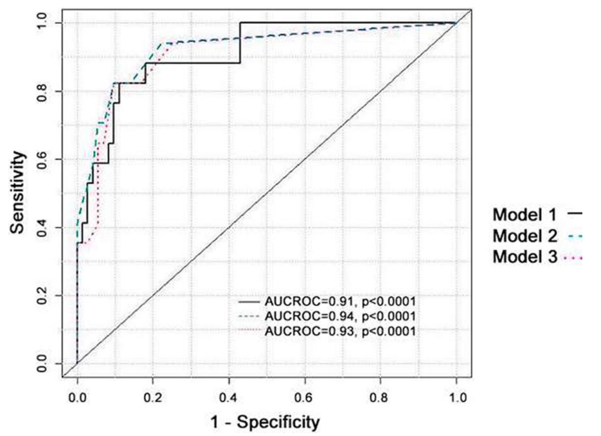

**Fig 1 (abstract 0542).** Predictive models to identify LVSD in the septic patient: AUCROC comparison

**Table 1 (abstract 0542).** Predictive models to identify LVSD in the septic patient

| | AUCROC | Standard error | 95%CI | p |
|---|---|---|---|---|
| Model 1 ($Log_{10}$BNP, TAPSE) | 0.9093 | 0.03 | 0.836-0.983 | <0.0001 |
| Model 2 ($Log_{10}$BNP, LVOT VTI, TAPSE) | 0.9403 | 0.02 | 0.892-0.989 | <0.0001 |
| Model 3 (BNP >400 pg/mL, TAPSE) | 0.9330 | 0.03 | 0.882-0984 | <0.0001 |

## Outcomes in critical illness

### 0543

#### Memories: what the patient remembers after ICU discharge

J.B. Costa[1], S. Taba[1], C.R.F. Lordani[2], K.C.B. Luzzi[3], S.T. Duarte[4], T.V.A. Lordani[5], I.R. Porto[3], M.M. Jorge[5], A.C. Jorge[3], P.A.D. Duarte[3]

[1]Hospital Universitário do Oeste do Paraná, Dept of Psychology, Cascavel, Brazil; [2]Hospital Universitário do Oeste do Paraná, Dept of Nutrition, Cascavel, Brazil; [3]Hospital Universitário do Oeste do Paraná, General ICU, Cascavel, Brazil; [4]Hospital Universitário do Oeste do Paraná, Dept of Speech Therapy, Cascavel, Brazil; [5]Hospital Universitário do Oeste do Paraná, Cascavel, Brazil

**Correspondence:** A.C. Jorge

*Intensive Care Medicine Experimental* 2018, **6(Suppl 2):**0543

**INTRODUCTION.** After surviving ICU, some patients may experience memories and recollections about his/ her ICU stay. The aim of this study was to report and analyze the memories and recollections of adult patients three months after ICU discharge.

**METHODS.** Retrospective cohort observational study. The study participants were all patients who were discharged alive from general ICU and from the hospital between february 2012 and November 2017, and who were evaluated by the psychology team in the Multiprofessional Office Clinic (MPOC) three months after ICU discharge. Inclusion criteria: patients > 24 h in ICU; >18 years old and attended the MPOC. The tool used to evaluate memory was a questionnaire applied by the MPOC psychologist. The types of memories were classified as memories of real events (memories related to actual facts regarding treatment, environment, emotional aspects) and illusion memories (related to illusory events such as nightmares, dreams and hallucinations, or which the patient believes was not real). All memories of any event related to the treatment, environment or emotional experience in which the patient was believed to have occurred during the period of consciousness recovery were accepted as memories.

**RESULTS.** A total of 749 patients were evaluated (62.8% male, mean age 43.5 years, mean ICU length of time 10.2 days, mean APACHE II 20.7, most common admission causes: trauma and medical).

More than half (56.0%) of the patients reported some type of ICU memory. Of these (n = 419), 61% reported memories of real events, 4% illusion memories and 35% presented a combination of real and illusory memories. As for memories of real events, the most frequent memories were: discharge to the ward (83.2%), family visit (76.4%); presence of confusion / agitation (67.2%), physical restraint (53.7%) and feeling thirsty (51.2%). Remarks related to the endotracheal tube, bronchial hygiene therapy and extubation were reported by only 24.7% of the patients. When comparing patients with or without illusory memories, patients with illusory memories had a lower incidence of male gender (42% x 57%), older age (44.2 x 41.4), higher APACHE II (22 x 17) and longer MV ( 7.0 x 4.9 days) and ICU (9.8 x 7.6 days) than patients without illusory memories.

**CONCLUSIONS.** The incidence of memories and recollections in post-ICU survivors is high, including illusory memories. The patient´s own perception of being agitated and confused is frequent. Patients with higher severity of acute disease and longer MV and ICU time tend to have a higher chance of presenting illusory memories after the ICU.

### 0544

#### Moral Distress, Burnout, and Coping among Intensive Care Professionals in Japanese ICUs

T. Fujii[1,2], S. Katayama[3], H. Nashiki[4], T. Niitsu[5], T. Takei[6], A. Utsunomiya[7], K. Miyazaki[8], P. Dodek[9], A. Hamric[10], T. Nakayama[8]

[1]Kyoto University Graduate School of Medicine, Kyoto, Japan; [2]Japan Society for the Promotion of Science, Tokyo, Japan; [3]Jichi Medical University School of Medicine, Intensive Care Unit, Tochigi, Japan; [4]Iwate Prefectural Central hospital, Intensive Care Unit, Morioka, Japan; [5]Saitama Children's Medical Center, Intensive Care Unit, Saitama, Japan; [6]Yokohama City Minato Red Cross Hospital, Intensive Care Unit, Yokohama, Japan; [7]St Luke's International University, Adult Nursing, Tokyo, Japan; [8]Kyoto University Graduate School of Medicine, Health Informatics, Kyoto, Japan; [9]University of British Columbia, Department of Medicine, Vancouver, Canada; [10]Virginia Commonwealth University, Richmond, United States

**Correspondence:** T. Fujii

*Intensive Care Medicine Experimental* 2018, **6(Suppl 2):**0544

**INTRODUCTION.** Moral distress occurs when health care professionals are constrained from practicing what they believe to be ethically appropriate actions. It causes feelings of frustration, guilt, anger, sadness, fear, and depression. Previous studies showed high levels of moral distress among intensive care nurses, and association with burnout and attrition. Recent reports on intensive care professionals´ well-being rarely focus on how they cope with this kind of stress.

**OBJECTIVES.** To examine moral distress among intensive care physicians and nurses, and to explore coping strategies in those who had high levels of moral distress.

**METHODS.** A survey was done in seven ICUs of five hospitals in Japan. Two of the hospitals were academic, and the other three were non-academic. All ICU physicians and nurses who were directly involved in patient care for more than one year were invited to participate during February and March 2018. The survey included Japanese translations of the Measure of Moral Distress for Healthcare Professionals (MMD-HP, a revised version of the Moral Distress Scale-R), Maslach Burnout Inventory General Survey (MBI-GS), Brief COPE (measures coping strategies), and demographics. We assessed correlations between MMD-HP scores and scores of each MBI domain. Among participants whose MMD-HP scores were greater than the mean, coping strategies were explored stratified by their intention to leave the position due to moral distress ever.

**RESULTS.** We distributed 274 questionnaires; 215 (79%) were returned, of which 27 (13%) were excluded because the MMD-HP questionnaire was not completed. The overall mean MMD-HP score was 91 (standard deviation 54). The MMD-HP score was correlated with the intention to leave the position due to moral distress (Odds ratio 1.14, 95% Confidence Intervals, 1.07 to 1.22). The mean MMD-HP score of physicians was 82 (SD 57), and that of nurses was 94 (SD 53, p = 0.22). Nurses also had higher MBI scores than physicians (Table1). The MMD-HP score correlated weakly with the MBI exhaustion domain (R = 0.28, p < 0.001) and the cynicism domain (R = 0.36, p < 0.001), but not with the professional efficacy domain (R = 0.005, p = 0.32) (Figure 1). Among those who had a high MMD-HP score, participants with intention to leave their position cope with stressful situations most frequently by behavioral disengagement, self-blame, and less frequently by positive reframing and humor (Table 2).

**CONCLUSIONS.** This study is the first to demonstrate the level of moral distress and burnout among ICU physicians and nurses in Japan. Moral distress was associated weakly with burnout. Those who had intention to leave their position due to moral distress cope with stressful situations less frequently in positive ways.

**REFERENCE(S)**

1. Dodek PM et al. J Crit Care. 2016;31:178-82.
2. Hamric A et al. the National Nursing Ethics Conference, 2018, LA (USA).

**GRANT ACKNOWLEDGMENT**

JSPS. Fujiwara Memorial Foundation.

**Table 1 (abstract 0544).** Participants demographic and scores of moral distress and burnout. N (%) or mean (standard deviation)

| | Physicians (N = 42) | Nurses (N = 146) | P value |
|---|---|---|---|
| Age, years | 38 (7) | 34 (8) | 0.01 |
| Female | 4 (10) | 130 (91) | <0.001 |
| Clinical experience, years | 12 (7) | 12 (8) | 0.62 |
| Work hours per month | 57 (25) | 44 (9) | <0.001 |
| Number of night shifts per month | 6 (2) | 5 (2) | 0.49 |
| MMD-HP score | 82 (57) | 94 (53) | 0.22 |
| MBI, exhaustion | 3 (2) | 4 (1) | <0.001 |
| MBI, professional efficacy | 3 (1) | 2 (1) | <0.001 |
| MBI, cynicism | 2 (1) | 2 (2) | 0.02 |

**Table 2 (abstract 0544).** Coping skills among intensive care professionals who have high MMD-HP scores. Mean (standard deviation)

| | No intention of leaving (N = 33) | Intention of leaving (N = 43) | P value |
|---|---|---|---|
| Substance use | 4 (2) | 4 (2) | 0.40 |
| Using emotional support | 6 (1) | 6 (1) | 0.91 |
| Using instrumental support | 6 (1) | 6 (1) | 0.19 |
| Behavioral disengagement | 4 (1) | 5 (1) | 0.01 |
| Positive reframing | 6 (1) | 5 (1) | 0.04 |
| Humor | 5 (2) | 4 (1) | 0.04 |
| Self-blame | 5 (2) | 6 (1) | 0.02 |

### 0545

#### Association between delirium in the ICU and post-ICU functional dependency status

M.M. Moraes[1], E.F. Osaku[1], C.R.L.M. Costa[1], B.A. Kanezawa[1], E.R. Penteado[1], P.D.S. Quadros[1], T.C. Schnaufer[1], A. Tomazelli[1], G. Antonelli[1], T.L. Cagnini[1], J.B. Anjos[1], M.A. Leite[1], M.M. Jorge[2], T.T. Chung[1], A.C. Jorge[1], P.A.D. Duarte[1]

[1]Hospital Universitário do Oeste do Paraná, General ICU, Cascavel, Brazil; [2]Hospital Universitário do Oeste do Paraná, Cascavel, Brazil

**Correspondence:** A.C. Jorge

*Intensive Care Medicine Experimental* 2018, **6(Suppl 2):**0545

**OBJECTIVE.** To analyze patients who survived the critical illness who presented delirium during ICU admission, and to evaluate the impact on functional independence three months after discharge from the ICU.

**METHODS.** Adult patients admitted to the general ICU who responded to CAM-ICU and survived to hospital discharge. The evaluation of Confusion Assessment Method scale (CAM-ICU) was performed three times a day until ICU discharge; the Functional Independency Measure (FIM) scale was evaluated at the ICU discharge and at the outpatient clinic (three months later). For the variables that presented normal distribution, the data were presented using mean ± standard deviation and the t student test was performed. For the variables that did not present normal distribution, the data were presented as a median ± interquartile range and the non-parametric Mann Whitney test was performed.

**RESULTS.** Eighty-one patients were included in the study, of whom 33% had delirium in the ICU. The delirious patients presented higher age, higher incidence of comorbidities, higher SOFA and APACHE II admission score, longer ICU and hospital stay, use and time of MV, time of sedation and total dose of midazolan and fentanyl than non-delirium patients. Regarding GCS and FIM, both were lower in the delirium patients than the non delusional group at ICU discharge and at the outpatient clinic; however, both groups showed improvement in functional Independence (FIM) three months later.

**CONCLUSION.** Delirium was more common in the ICU in more severe patients, with longer ICU and MV, and with greater use of sedatives. The delirium in the ICU negatively influences the functional independence in the discharge of the intensive care unit, and potentially affecting the recovery of the functionality of these patients three months after discharge.

**Table 1 (abstract 0545).** See text for description

| | Delirium (n=27) | No Delirium (n=54) | p-value |
|---|---|---|---|
| ICU length of stay (days), median (IQR) | 10 (6-15) | 4 (3-8) | <0.001 |
| Hospital length of stay (days), median (IQR) | 25 (20-41) | 15.5 (11-21) | 0.001 |
| GCS at ICU discharge, median (IQR) | 14 (11-15) | 15 (15-15) | <0.001 |
| FIM at ICU discharge, mean ± SD | 56 ± 26.9 | 76 ± 26.5 | 0.003 |
| GCS after 3 months, median (IQR) | 14 (11-15) | 15 (15-15) | 0.0009 |
| FIM after 3 months, median (IQR) | 122 (105-126) | 126 (123-126) | 0.023 |

### 0546

#### Evaluation of sleep architecture in patients recovering from critical care illness (CCI) in intensive care unit (ICU and step down unit (SDU)

B. Prajapat[1], A.S. Sandhya[2], D. Chaudhry[1]

[1]PGIMS, Pulmonary and Critical Care Medicine, Rohtak, India; [2]PGIMS, CHEST and TB, Rohtak, India

**Correspondence:** B. Prajapat

*Intensive Care Medicine Experimental* 2018, **6(Suppl 2):**0546

**INTRODUCTION.** Sleep disruption is a well known problem among critically ill patients in an intensive care unit (ICU) which persist during recovery and after transfer to step down unit (SDU) or wards.

Objective. To evaluate 1)patterns of sleep in patients recovering from CCI in ICU and SDU and 2) Effect of environmental factors (noise, light) on sleep.

**MATERIALS AND METHODS.** 24 hr polysomnography (PSG) study was done in patients recovering from CCI in ICU with simultaneous recording of noise, light and nursing care activities and repeated after shifting to step down unit (SDU).

**RESULTS.** 26 patients underwent PSG study in ICU and then in SDU. Mean APACHE II and SOFA Score were 19.38 ± 3.72 and 5.11± 1.27. Paired t-test showed reduction in N1% (28.80 ± 7.30, 21.8 ± 4.3: p = 0.0001) and N2% (60.3 ± 8.65, 55.8± 4.0p = 0.021) and improvement in N3% (6.66 ± 2.30, 14.34 ± 3.8 p < 0.0001) and REM% (4.20 ± 3.68, 8.2 ± 3.5 p = 0.01) . Day time sleep and arousal index were reduced (49.55 %, 37.71%; p< 0.0001) and (22.65 ± 10.49, 16.55 ± 5.15: p = 0.006) respectively. The difference in mean illuminance during day and night time in ICU (77.09 ± 3.66, 14.98 ± 2.04 lux) and HDU (160.52 ±6.07, 23.67 ±2.62lux) was significant (p< 0.0001). Mean Sound level was high (59.17±1.69 dB,59.12±1.49 dB:p = 0.904). Mean nursing care activities difference was significant (50.8± 6.34 , 33.96 ± 5.34: p< 0.0001)(table).

**REFERENCE(S)**

Sleep is highly disturbed and fragmented in ICU and SDU. However, the pattern of sleep shows trend towards recovery as patient recovers from critical care illness.

### 0547

#### Long-term developmental effects of withholding parenteral nutrition in PICU

S. Verstraete[1], S.C. Verbruggen[2], J.A. Hordijk[2], I. Vanhorebeek[1], K. Dulfer[2], F. Güiza[1], E. van Puffelen[2], A. Jacobs[1], S. Leys[1], A. Durt[1], H. Van Cleemput[1], R.D. Eveleens[2], G. Garcia Guerra[3], P.J. Wouters[1], K.F. Joosten[2], G. Van den Berghe[1]

[1]KU Leuven, Laboratory of Intensive Care Medicine, Leuven, Belgium; [2]Erasmus MC - Sophia Children's Hospital, Rotterdam, Netherlands; [3]University of Alberta - Stollery Children's Hospital, Edmonton, Canada

**Correspondence:** S. Verstraete

*Intensive Care Medicine Experimental* 2018, **6(Suppl 2):**0547

**INTRODUCTION.** The PEPaNIC-RCT has shown that withholding supplemental parenteral nutrition for up to one week in the PICU (late-PN) reduced infections and accelerated recovery from critical illness in children as compared with early-PN [1].

**OBJECTIVES.** The aim of this study was to investigate the impact of withholding supplemental parenteral nutrition early during pediatric critical illness on long-term physical and neurocognitive development.

**METHODS.** Two years after inclusion in the RCT, the 1440 patients were approached for possible assessment of physical/neurocognitive development as compared with 405 matched healthy children. Assessed outcomes comprised growth, health status, parent/caregiver-reported executive functions and emotional and behavioral problems, tests for intelligence, visual-motor integration, alertness, motor-coordination, inhibitory control, cognitive flexibility, and memory. To handle partial response rates among tested children, multiple data imputation by chained equations was performed prior to multivariable linear and logistic regression analyses adjusted for risk factors.

**RESULTS.** At 2-years follow-up, 60 late-PN children (8.4%) and 63 early-PN children (8.7%) had died (P=0.81), 68 late-PN patients (9.5%) and 91 early-PN patients (12.6%) were too disabled for neurocognitive assessment (P=0.05) and 786 patients consented for testing. Late-PN did not adversely affect growth, health status or neurological functioning, but improved executive functioning (beta-estimate late-PN versus early-PN -2.258; 95%CI[-4.012;-0.504], P=0.01), more specifically inhibition (-3.422; 95%CI[-5.171;-1.673], P< 0.001), working memory (-2.016; 95%CI[-3.761;-0.270], P=0.02) and meta-cognition

(-1.957; 95%CI[-3.694;-0.220], P=0.02). Also externalizing behavioral problems (-1.715; 95%CI[-3.325;-0.106], P=0.03) and visual-motor integration (0.468; 95%CI[0.087;0.850], P=0.01) were improved. After Bonferroni-correction for multiple comparisons, the effect on inhibitory control remained significant.
**CONCLUSIONS.** Withholding early-PN in the PICU did not negatively affect survival, growth or health status 2 years later and partially improved neurocognitive development. (clinicaltrials.gov-number, NCT01536275)

##### REFERENCE(S)

1. Fivez et al. NEJM. 2016

##### GRANT ACKNOWLEDGMENT

This work was supported by an ERC Advanced Grant (AdvG-2012-321670) from the Ideas Program of the European Union 7th framework program to GVdB; by the Methusalem program of the Flemish government (through the University of Leuven to GVdB, METH/08/07 and to GVdB and IV, METH14/06); by the Institute for Science and Technology, Flanders, Belgium (through the University of Leuven to GVdB, IWT/070695/TBM); by the Research Foundation-Flanders (FWO), Belgium (FWO fellowship to SV); by the Sophia Foundation (SSWO) to SCV; by the Stichting Agis Zorginnovatie to SCV; by the Erasmus Trustfonds to SCV; and by an ESPEN research grant to EvP and SCV.

## Improving quality and ICU outcomes

### 0548

#### Less is more: "Not to do" recommendations in the intensive care unit

M.D. Bosque, M.L. Martínez, O. Moreno, S. Barbadillo, R. Tomás, M. Irazábal, A. Olmo, J. Lema
Hospital Universitari General de Catalunya, Intensive Care Department, Sant Cugat del Vallés, Spain
**Correspondence:** M.D. Bosque
*Intensive Care Medicine Experimental* 2018, **6(Suppl 2):**0548

**INTRODUCTION.** In recent years there has been growing concern about the unnecessary application of certain interventions in critical patients that have proven to be ineffective, consume a significant amount of health resources and are not exempt from yatrogeny.
With the intention of improving the quality of health care and reducing the use of unnecessary interventions, the project Commitment for Quality coordinated by the Ministry of Health of Spain and scientific societies as the Spanish Society of Intensive Care Medicine and Coronary Units (SEMICYUC) established 5 "not to do" recommendations.
**OBJECTIVE.** The purpose of this study is to measure the extent of unnecessary interventions in our Intensive Care Unit (ICU).
**METHODS.** We conducted a preliminary observational study in a 16-bed polyvalent intensive care unit from April 2017 to January 2018. A randomized non-consecutive 8-day follow up of the 5 recommendations in all admitted patients to the ICU was carried out.
All attending physicians in the Intensive Care Unit were asked about the need for antibiotic therapy, clinical indications of scheduled blood tests and chest XR, the need to continue isolation measures initiated and the need to red blood cells (RBC) transfusion according to clinical status and hemoglobin (Hb) levels, following the 5 recommendations established for critical patients from the project Commitment for Quality, which are:
1. Do not continue with empirical antibiotic treatment without assessing daily its need and possible de-escalation
2. Do not perform blood tests routinely
3. Do not perform chest x-rays routinely
4. Do not maintain isolation measures when they are no longer needed
5. Do not transfuse RBC in hemodynamically stable, non-bleeding critically ill patients, without cardiological or central nervous system involvement with Hb greater than 7 gr / dl
Descriptive statistics include frequencies and percentages for categorical variables.
**RESULTS.** Six attending physicians provided input for a total of 114 patient days. Results are shown in the table.
The recommendation in which there was less compliance was not to perform routine blood tests (15% of the patients analyzed), followed by not to perform chest radiographs routinely(3.5%).
A total of 26 recommendations were not achieved from all the patients-day analyzed (23%).
**CONCLUSIONS.** A significant part of the interventions performed in our patients were not justified with the consequent patient discomfort, work overload and increase in costs.
Health care professionals should carefully strategize and optimize efforts to reduce unnecessary interventions and avoid wasteful clinical practice.

##### REFERENCE(S)

http://www.msc.es/organizacion/sns/planCalidadSNS/cal_sscc.htm

**Table 1 (abstract 0548).** Implementation of the recommendations. n: number of patients in which recommendations were achieved or unachieved

| Recommendation | Achieved n (%) | Unachieved n (%) |
|---|---|---|
| 1. Antibiotics | 112 (98%) | 2 (1,7%) |
| 2. Blood Tests | 97 (85,1%) | 17 (15%) |
| 3. Chest XR | 110 (96,5%) | 4 (3,5%) |
| 4. Isolation measures | 113 (99,1%) | 1 (0,9%) |
| 5. Transfusion | 112 (98%) | 2 (1,7%) |
| TOTAL | | 26 (23%) |

### 0549

#### Association between aging and the prevalence of systemic inflammatory response syndrome (SIRS) signs in patients admitted to the ICU with severe infection: a retrospective cohort study

F. Zampieri[1], F. Aguiar[2], J. Salluh[3], M. Soares[3], ORCHESTRA Study Group
[1]HCor-Hospital do Coração, São Paulo, Brazil; [2]Hospital Sírio-Libanês, São Paulo, Brazil; [3]D´Or Research Institute, Rio de Janeiro, Brazil
**Correspondence:** F. Zampieri
*Intensive Care Medicine Experimental* 2018, **6(Suppl 2):**0549

**INTRODUCTION.** SIRS has been long advocated as a prognosis and triage method to identify patients at high risk of dying. Aging has been suggested to impact host response to stressors such as trauma, surgery and infection and could therefore modulate the presence of abnormal laboratory and vital signs in patients with severe infection.
**OBJECTIVES.** To assess the association between age and presence of SIRS criteria in patients admitted to intensive care units with severe infection.
**METHODS.** We included critically ill patients admitted to 93 intensive care units (ICUs) in Brazil from January 2014 until December 2015 with suspected infection and admission SOFA score ≥2 points; we excluded patients with missing information on SIRS criteria. We build a Poisson regression model with number of SIRS criteria (0-4) as dependent variable using age, need for urgent surgery, admission source (ward or emergency room), SOFA score, diabetes and cancer as predictors. We also built linear models for each of the SIRS criteria components using the same variables as predictors. Finally, we built a logistic regression model for SIRS higher or lower than 2 points using the same approach.
**RESULTS.** Out of 18,184 with infection and a SOFA score ≥2 points, 15,971 (89%) had complete data for analysis. Mean age was 68 years (SD 19). 6,490 (40%) patients had less than 2 SIRS criteria. The number of SIRS criteria decreased with increasing age (Figure). Age was independently associated with lower number of SIRS criteria (reduction of 3% on mean SIRS value for each 10 years increase in age). Increasing age was also associated with lower heart rate (-2.25 beats per minute /10 years), respiratory rate (-0.1 breaths per minute/10 years) and temperature (-.05 $^{o}$C/10 years) at admission, but not with leukocyte count. In logistic regression, the odds for presence of at least 2 SIRS criteria was 0.90 for each 10-years increase in age.

**CONCLUSIONS.** Age was associated with lower number of SIRS criteria in patients admitted to ICUs with severe infection. Prospective studies are necessary to assess whether age may impact the use of SIRS criteria for severe infection screening.

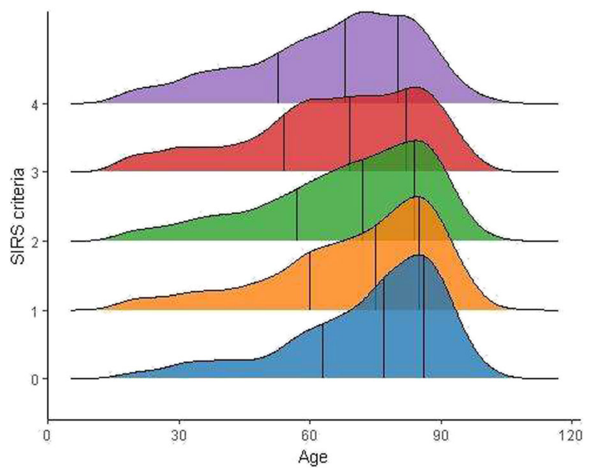

**Fig 1 (abstract 0549).** Ridge plot for density of age according to number of SIRS criteria

### 0550

#### A nudge to improve protective lung ventilation strategies

S. Fry[1], J. Nicholson[1], J. Hayward[1], L. Hodgson[1,2]
[1]Western Sussex Hospitals Foundation NHS Trust, Worthing, United Kingdom; [2]University of Southampton, Southampton, United Kingdom
**Correspondence:** L. Hodgson
*Intensive Care Medicine Experimental* 2018, **6(Suppl 2):**0550

**INTRODUCTION.** Randomised controlled trials have shown that low tidal volume venitlation (TV) (6-8 mL/kg ideal body weight (IBW)), is beneficial in ventilated patients on the intensive care unit (ICU) (1-2), but the adoption of this strategy has been inconsistent.(3-4).

**OBJECTIVES.** Assess compliance with protective lung ventilation strategies before and after the introduction of display monitors highlighting recommended ventilation in 2 UK general ICUs (total of 22 beds).

**METHODS.** A prospective observational before-after study was undertaken (2016-2017). The units use a clinical information system (CIS) that automatically collects information relating to patient care including ventilator data. Consecutive patients undergoing controlled mechanical ventilation were included. The intervention consisted of two large display screens at each ICU configured to display TVes derived from the CIS in mL/kg IBW. Warnings were produced when TVe >8ml/kg, which resulted in the TVe cell turning red. We included only patients with at least 24 hours of ventilator data. The first 12-month period acted as the control period. No protocols or quality improvement processes were changed, nor was additional training or education undertaken and ventilation equipment was the same in both the control and intervention period.

**RESULTS.** From 70,654 data points we included 36,197 from patients meeting the inclusion criteria. There was a small statistically significant fall in median VTe/Kg after the intervention (from 7.7 [interquartile range 6.6-9.0] to 7.5 [6.6-8.6], P< 0.001). Fewer ventilator episodes after the intervention were ≥8ml/kg (38% vs 42%, OR 0.84 95% CI 0.81-0.88, P< 0.001) or ≥10mls/kg (14% vs 18%, OR 1.37 95% CI 1.29-1.44, P< 0.001). PEEP levels were higher following the intervention (6 [5-8] pre vs 7 [5-10] post, P< 0.001) and were more often in the recommended range (79% vs 76%, OR 1.16, 95% CI 1.11-1.22, P< 0.001).

**CONCLUSIONS.** A simple prominent display improved compliance with best ventilation practice in two non-specialist general ICUs, maintained over a 1-year period. Effects on patient outcomes should be an avenue of future study.

**REFERENCE(S)**

1. Ventilation with lower tidal volumes as compared with traditional tidal volumes for acute lung injury and the acute respiratory distress syndrome. The Acute Respiratory Distress Syndrome Network. N Engl J Med. 2000;342(18):1301-8.
2. Petrucci N, De Feo C. Lung protective ventilation strategy for the acute respiratory distress syndrome. Cochrane Database Syst Rev. 2013(2):CD003844.
3. Weiss CH, Baker DW, Weiner S, Bechel M, Ragland M, Rademaker A, et al. Low Tidal Volume Ventilation Use in Acute Respiratory Distress Syndrome. Crit Care Med. 2016;44(8):1515-22.
4. Needham DM, Colantuoni E, Mendez-Tellez PA, Dinglas VD, Sevransky JE, Dennison Himmelfarb CR, et al. Lung protective mechanical ventilation and two year survival in patients with acute lung injury: prospective cohort study. BMJ. 2012;344:e2124.

**Table 1 (abstract 0550).** Summary of ventilation pre and post intervention

| | Pre-intervention | Post-intervention | Odds ratio (95% CIs), P-value |
|---|---|---|---|
| Median (IQR) VTe/Kg | 7.7 (6.6-9.0) | 7.5 (6.6-8.6) | P<0.001 |
| % of values VTe/Kg ≥8mls/Kg IBW | 42% | 38% | OR 0.84 (0.81-0.88), P<0.001 |
| Median (IQR) PEEP | 6 (5-8) | 7 (5-10) | P<0.001 |
| Appropriate PEEP level | 76% | 79% | OR 1.16, (1.11-1.22), P<0.001 |

### 0551

#### Clinical examination for estimating low cardiac output in critically ill patients: a SICS-I substudy

T. Kaufmann[1], P. Simons[2], B. Hiemstra[2], R. Wiersema[2], F. Keus[2], I.C.C. van der Horst[2], SICS Study Group
[1]University of Groningen, University Medical Center of Groningen, Department of Anesthesiology, Groningen, Netherlands; [2]University of Groningen, University Medical Center of Groningen, Department of Critical Care, Groningen, Netherlands
**Correspondence:** T. Kaufmann
*Intensive Care Medicine Experimental* 2018, **6(Suppl 2):**0551

**INTRODUCTION.** Each day we investigate measures of the circulation to guide diagnosis and treatment. The diagnostic accuracy of clinical examination in diagnosing shock as reflected by an inadequate cardiac output (CO) or cardiac index (CI) has been questioned [1].

**OBJECTIVES.** To determine whether students and trained clinicians can give an estimate of low cardiac output based on standardized clinical examination in patients acutely admitted to the Intensive Care Unit (ICU).

**METHODS.** This study describes one of the outcomes of the Simple Intensive Care Studies - I (SICS-I). This prospective cohort study included all consecutive adults who were acutely admitted with an ICU stay expected to last beyond 24 hours. We assessed accuracy scores of cardiac index (CI) estimates and compared them to CI measured by critical care ultrasonography. CI measures were independently scored by a core laboratory. Investigators were asked to estimate cardiac pump function as ´poor´, ´moderate´, ´reasonable´, or ´good´. For diagnostic test analyses ´poor´ and ´moderate´ estimates were grouped as ´low´, and ´reasonable´ or ´good´ estimates were grouped as ´high´. The CI measurements were categorized in two groups to match the estimate groups as follows: ´low´ CI ≤ 2.2 L/min/$m^2$ and ´high´ CI > 2.2 L/min/$m^2$.

**RESULTS.** From 27 March 2015 until 22 July 2017, 1075 patients were included in the SICS-I of which 828 patients had CI measurements. Diagnostic test analyses for estimating a low cardiac index is shown in table 1. Sensitivity varied from 47% to 67% and specificity varied from 64% to 76%. Positive likelihood ratio was 1.47 (1.22 - 1.78) and negative likelihood ratio was 0.77 (0.66 - 0.90) for all groups. Level of training had no influence on improvements of estimates.

**CONCLUSIONS.** The diagnostic accuracy as defined by the positive and negative likelihood ratio for estimation of cardiac pump function indicate that estimating of cardiac pump function in critically ill patients has value. The first step in gathering information on the circulation is clinical examination.

**REFERENCE(S)**

1. Hiemstra B, Eck RJ, Keus F et al. Clinical examination for diagnosing circulatory shock. Curr Opin Crit Care. 2017; 23(4): 293-301

**ACKNOWLEDGMENT**

Currently more data is being analyzed to elucidate and refine the outcomes of the SICS-I. In case of admission to the ESICM congress, revised results will be presented.

**Table 1 (abstract 0551).** Accuracy scores with 95% confidence intervals to detect a low cardiac index

| Variable | Students (n=602) | Interns (n=201) | Residents (n=25) | All Groups (n=828) |
|---|---|---|---|---|
| Sensitivity (%) | 49 (38 - 59) | 47 (35 - 60) | 67 (9 - 99) | 48 (41 - 56) |
| Specificity (%) | 65 (61 - 69) | 76 (68 - 83) | 64 (41 - 83) | 67 (63 - 71) |
| PPV (%) | 22 (18 - 26) | 50 (40 - 60) | 20 (9 - 40) | 28 (24 - 32) |
| NPV (%) | 86 (84 - 88) | 74 (69 - 78) | 93 (73 - 99) | 83 (81 - 85) |
| LR+ | 1.39 (1.10 - 1.75) | 1.96 (1.32 - 2.90) | 1.83 (0.69 - 4.85) | 1.47 (1.22 - 1.78) |
| LR- | 0.79 (0.65 - 0.97) | 0.70 (0.55 - 0.89) | 0.52 (0.10 - 2.68) | 0.77 (0.66 - 0.90) |
| Overall accuracy | 62 (58 - 66) | 66 (59 - 73) | 64 (43 - 82) | 63 (60 - 67) |

### 0552

### Lung ultrasound reliably predicts loss of aeration and ARDS morphology

I. Steinberg[1], A. Costamagna[2], P. Arina[1], E. Pivetta[1], S. Veglia[2], A.T. Mazzeo[1], O. Davini[2], V. Fanelli[1]

[1]University of Turin, Turin, Italy; [2]Città della Salute e della Scienza di Torino, Turin, Italy

**Correspondence:** I. Steinberg

*Intensive Care Medicine Experimental* 2018, **6(Suppl 2):**0552

**INTRODUCTION.** Computerized tomography (CT) is the gold standard imaging technique to quantify loss of aeration in ARDS patients and to determine the morphology of lung injury and the consequent response to recruitment manoeuvres. Transfers to radiology ward is time and resource-consuming and implies a significative risk for the patient. The role of LUS in evaluating aeration in comparison to CT scans has not been determined.

**OBJECTIVES.** The aim of our study was to assess if LUS can be a reliable method to quantify the loss of aeration and to predict lung injury morphology in ARDS patients compared to CT scan.

**METHODS.** LUS was performed bedside whenever a CT scan was available for comparison. Linear (15-12 Mhz) and convex (5-3 Mhz) probe were used to evaluate pleural line and lung parenchyma. Twelve sections, corresponding to 6 CT landmarks for hemithorax, were evaluated and a score from 0 (normal) to 3 (consolidation) was given. Total LUS score ($LUS_{tot}$) and regional cranio-caudal LUS score, derived as the sum of the 4 non-dependent ($LUS_{ND}$), the 4 intermediate ($LUS_{int}$) or the 4 dependent ($LUS_{dep}$) fields, were calculated. CT scans quantitative analysis was performed and expressed in Hounsfield Units and the percentage of air (Pair) was derived. According to CT scans, ARDS pattern was defined as diffuse/patchy or lobar. The optimal cut-offs values of global and regional LUS scores in identifying ARDS pattern were analysed by ROC curve analyses with Youden method for empirical cut-point estimation.

**RESULTS.** Forty-one LUS evaluations were performed on 26 patients. Study population, mechanical ventilation and extracorporeal support characteristics are described in **Table 1** and **2**. The agreement between LUS score for every field and CT scan evaluation in terms of Pair is shown in **Figure 1**. $LUS_{tot}$ was 24 (18 - 27). Regional LUS score was 7 (4 - 8), 8 (6 - 9) and 10 (7 - 10) for non-dependent, intermediate and dependent fields, respectively (p< 0.01 $LUS_{dep}$ VS $LUS_{ND}$ and $LUS_{dep}$ VS $LUS_{int}$). Regional $LUS_{ND}$ score could identify diffuse/patchy pattern with an $AUC_{ROC}$ of 0.95 (SE=91% and SP=86% for LUS cut-point=3 - **Figure 2**).

**CONCLUSIONS.** LUS can reliably quantify loss of aeration and can be used to identify ARDS morphology.

**REFERENCE(S)**

1. Constantin J-M, Grasso S, Chanques G, et al. Lung morphology predicts response to recruitment maneuver in patients with acute respiratory distress syndrome: *Critical Care Medicine* 2010;38(4):1108-1117.
2. Bouhemad B, Brisson H, Le-Guen M, Arbelot C, Lu Q, Rouby J-J. Bedside Ultrasound Assessment of Positive End-Expiratory Pressure-induced Lung Recruitment. *American Journal of Respiratory and Critical Care Medicine* 2011;183(3):341-347.
3. Lichtenstein DA, Lascols N, Mezière G, Gepner A. Ultrasound diagnosis of alveolar consolidation in the critically ill. *Intensive Care Medicine* 2004;30(2):276-281.

**GRANT ACKNOWLEDGMENT**

We have no grant to disclose.

**Table 1 (abstract 0552).** Study population

| Variables | Patients (N=26) |
|---|---|
| Age (years) | 57 (50 - 65) |
| BMI (kg m-2) | 26 (23 - 29) |
| Gender (M/F) | 18/8 |
| Risk factors for ARDS (N (%)) | |
| Pneumonia (bacterial/aspiration) | 13 (50) |
| Pneumonia (viral) | 5 (19) |
| Pneumonia (fungal/other) | 4 (15) |
| Abdominal sepsis | 1 (4) |
| Pancreatitis/Septic shock | 3 (12) |

**Table 2 (abstract 0552).** Characteristics of mechanical ventilation and extracorporeal support

| Variables | LUS evaluation (N=41) |
|---|---|
| Respiratory mechanics | |
| Tidal volume (mL) | 450 (375 - 500) |
| Respiratory rate (b min-1) | 24 (14 - 30) |
| PEEP total (cmH2O) | 12 (8 - 15) |
| Plateau pressure (cmH2O) | 26 (24 - 27) |
| Static compliance (ml cmH2O-1) | 31 (26 - 45) |
| FiO2 (%) | 51 (40 - 70) |
| Extra corporeal life support (N (%)) | |
| Venous - Venous ECMO | 8 (20) |

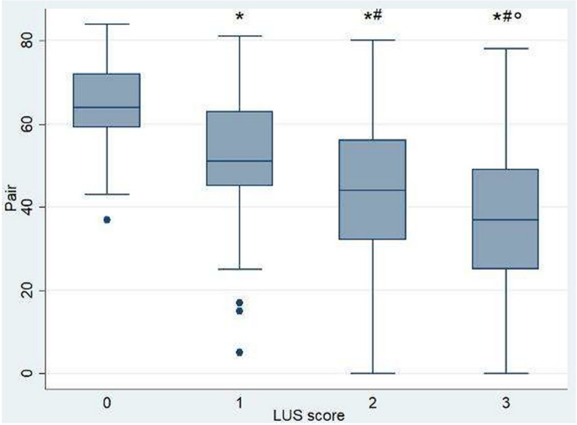

**Fig 1 (abstract 0552).** Comparison between overall TC's Pair grouped by LUS score * p<0.001vs0; #p<0.01vs1; °p<0.001vs2

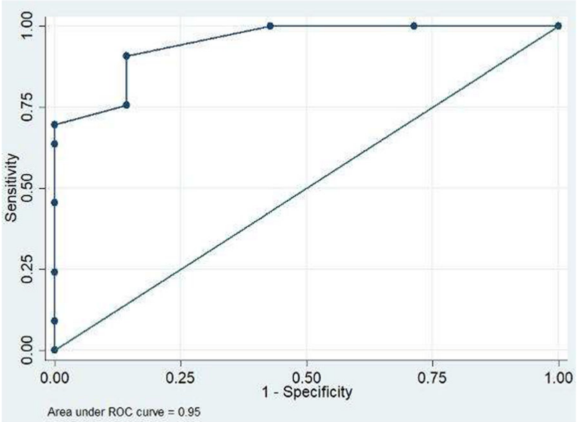

**Fig 2 (abstract 0552).** ROC curve for regional LUS score for diffuse pattern prediction. AUC for LUSND score is 0.95

## Sepsis: From genetics to therapeutics

### 0553

#### Vasopressin versus norepinephrine for the management of septic shock in cancer patients (VANCS II)

C. Zambolim[1], J.P. Almeida[1], F. Galas[2], C. Park[1], G. Oliveira[1], G. Landoni[3], S. Rizk[1], R. Nakamura[1], M. Mourao[1], T.F. Szeles[1], J. Fukushima[1], R. Kalil-Filho[1], J.O. Auler Jr.[1], L.A. Hajjar[2]
[1]Instituto do Cancer do Estado de Sao Paulo, Sao Paulo, Brazil; [2]Instituto do Câncer do Estado de São Paulo, Anesthesia and Critical Care, São Paulo, Brazil; [3]IRCCS San Raffaele Scientific Institute, Department of Anesthesia and Intensive Care, Milano, Italy
**Correspondence:** L.A. Hajjar
*Intensive Care Medicine Experimental* 2018, **6(Suppl 2):**0553

**INTRODUCTION.** Although arginine vasopressin has been used as an additional drug in refractory shock in worldwide clinical practice, there are no prospective studies using it as a first choice therapy in patients with cancer and septic shock.

**OBJECTIVES.** The aim of this study is to assess if the use of arginine vasopressin would be more effective on treatment of septic shock in cancer patients than norepinephrine regarding 28-day mortality and organ failure.

**METHODS.** We performed a single center, randomized, parallel-group, double-blind, controlled trial in Brazil. Patients with septic shock were randomized in a 1:1 ratio to receive either vasopressin or norepinephrine as vasopressor therapy. The vasopressor infusion was titrated to maintain a mean arterial pressure (MAP) of at least 65 mmHg. Eligible participants were all adult patients with cancer, aged 18 yr or more, with septic shock admitted to the intensive care unit (ICU). The primary outcome was defined as all-cause mortality rate at 28 days after randomization. The pre-specified secondary outcomes were defined as all-cause mortality rate at 90 days after randomization and Sequential Organ Failure Assessment (SOFA) score 96 hours after randomization.

**RESULTS.** A total of 250 patients were randomly assigned, 125 into the vasopressin group and 125 into the norepinephrine group. The duration of the vasopressor therapy was similar between groups. No significant difference was observed regarding hemodynamic data, urinary output and perfusion markers. All-cause mortality rate at 28 days was 56.8% in the vasopressin group and 52.8% in the norepinephrine group (P=0.525). All-cause mortality rate at 90 days was also similar between groups. There was no significant difference between groups in SOFA score within the first 96 hours after randomization.

**CONCLUSIONS.** In this randomized clinical, the use of arginine vasopressin did not reduced 28-day all-cause mortality in cancer patients with septic shock when compared with norepinephrine (NCT01718613).

##### REFERENCE(S)

1 - Russell JA, Walley KR, Singer J, Gordon AC, Hébert PC, Cooper DJ, Holmes CL, Mehta S, Granton JT, Storms MM, Cook DJ, Presneill JJ, Ayers D; VASST Investigators. Vasopressin versus norepinephrine infusion in patients with septic shock. N Engl J Med. 2008 Feb 28;358(9):877-87.

##### GRANT ACKNOWLEDGMENT

This study was sponsored by the Universidade de São Paulo, Brazil.

### 0554

#### Relationship between time to positivity of blood culture and mortality according to the site of infection

Y.W. Um, J.H. Lee, Y.H. Jo, J. Kim, Y.J. Kim, H. Kwon
Seoul National University Bundang Hospital, Emergency Medicine, Seongnam, Korea, Republic of
**Correspondence:** Y.W. Um
*Intensive Care Medicine Experimental* 2018, **6(Suppl 2):**0554

**INTRODUCTION.** Sepsis is a life-threatening condition resulting from a dysregulated host response to infection, and the incidence of sepsis is increasing and mortality rate still remains high.

Time to positivity of blood culture (TTP) could reflect the bacterial load of patients, and it was associated with severity and a prognostic factor in bloodstream infection according to previous studies. However, these studies mainly focused on the relationship between TTP and specific microorganisms in blood stream infection. In clinical settings, clinicians could not aware of specific microorganisms until the final report would be made. In addition, the rate of positivity of blood culture and the severity of sepsis were different according to the site of infection. Therefore, the relationship between TTP and the outcomes in sepsis might be different, but it has not been evaluated yet.

**OBJECTIVES.** This study was performed to evaluate the relationship between time to positivity of blood culture (TTP) and mortality rate in sepsis and septic shock according to the site of infection.

**METHODS.** We performed a retrospective cohort study of patients with sepsis and septic shock in a tertiary care, emergency department. The rates of positivity of blood culture and mortality and the relationship between TTP and 28-day mortality rate were compared between patients with different sites of infection of lung, abdomen, urogenital and others.

**RESULTS.** A total of 2668 patients were included and overall mortality rate was 21.6%. The rates of positivity of blood culture and mortality were different between the sites of infection (P < 0.001).

The relationship between TTP and mortality rate of total patients, patients with lung and urogenital infection did not have any relationship. Patients with abdominal infection showed negative correlation between TTP and 28-day mortality rate. In patients with abdominal infection, TTP < 20 h was independently associated with 28-day mortality compared with patients with negative blood culture (hazard ratio. 1.65; 95% confidence interval, 1.12-2.42). However, there was no difference in mortality rate between patients with TTP ≥ 20 h and negative blood culture.

**CONCLUSIONS.** The shorter TTP of the patients with abdominal infection was associated with 28-day mortality rate in patients with sepsis and septic shock.

**REFERENCE(S)**

1. Rello J, Lisboa T, Lujan M, Gallego M, Kee C, Kay I, et al. Severity of pneumococcal pneumonia associated with genomic bacterial load. Chest. 2009;136(3):832-40.
2. Ning Y, Hu R, Yao G, Bo S. Time to positivity of blood culture and its prognostic value in bloodstream infection. Eur J Clin Microbiol Infect Dis. 2016;35(4):619-24.
3. Leligdowicz A, Dodek PM, Norena M, Wong H, Kumar A, Kumar A, et al. Association between source of infection and hospital mortality in patients who have septic shock. Am J Respir Crit Care Med. 2014;189(10):1204-13.

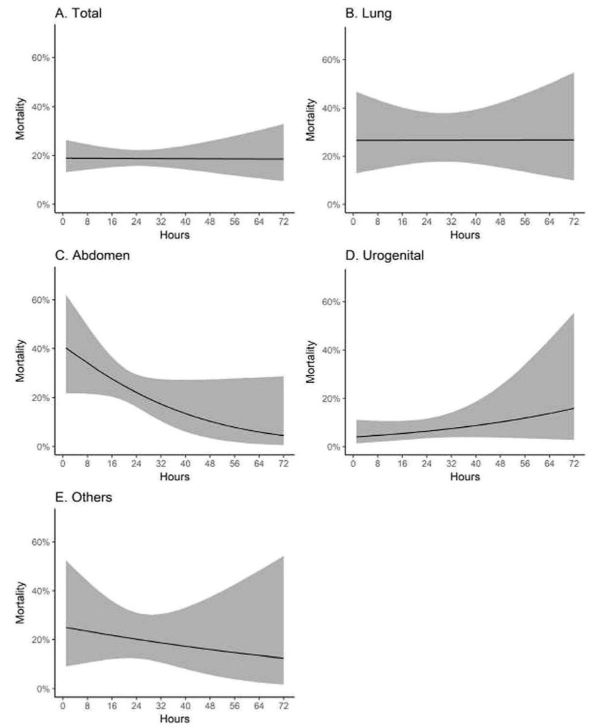

**Fig 1 (abstract 0554).** Mortality rate of patients over time to positivity of blood culture according to the different sites

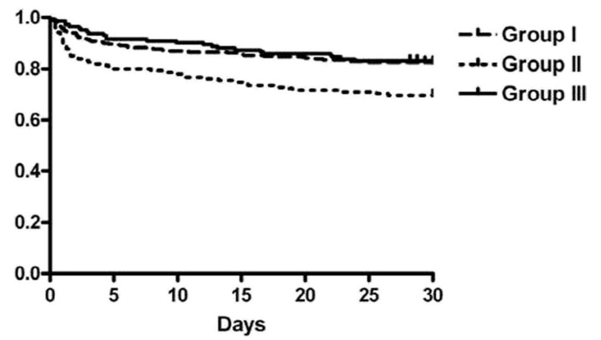

**Fig 2 (abstract 0554).** Kaplan-Meier survival curve of the patients

### 0555

#### Obstructive sleep apnea increases risk of sepsis, but with improved survival

V. Bansal, T. Weister, R. Kashyap, E. Festic, A. Lee
Mayo Clinic, Jacksonville, United States
**Correspondence:** A. Lee

*Intensive Care Medicine Experimental* 2018, **6(Suppl 2):**0555

**INTRODUCTION.** Obstructive sleep apnea (OSA) is a prevalent disorder with an estimated prevalence of 20% in the United States. Animal models have demonstrated sleep deprivation is associated with immune dysregulation and an increased susceptibility to infection, while hypoxic preconditioning may suppress inflammation and theoretically provide a protective effect.

**OBJECTIVES.** We aimed to investigate whether OSA would predispose patients to sepsis, and whether it would worsen sepsis-related outcomes.

**METHODS.** Adult residents of Olmstead County that required hospitalization for at least one day in 2013 were eligible. Validated electronic tools were used to identify OSA and sepsis by both sepsis-2 (systemic inflammatory response syndrome) and sepsis-3 (sequential organ failure assessment) definitions. Sepsis-related outcomes included need for invasive ventilation and hospital mortality. Multivariable logistic regression analysis was utilized to determine whether OSA was an independent risk factor for sepsis and hospital mortality; adjusting for age, sex, and the Charlson comorbidity index (CCI), and for those requiring ICU admission, the acute physiology and chronic health evaluation-III score (APACHE-3). Odds ratios (OR) with their 95% confidence intervals (CI) are reported as a measure of risk.

**RESULTS.** Of the 6131 eligible subjects (median age 56-years, 39% male), OSA was identified in 21% (n=1265), sepsis-2 in 17% (n=1052), and sepsis-3 in 5% (n=319). In univariate analyses, OSA was a significant risk factor for both sepsis-2 (OR=2.3, CI:2.0-2.6) and sepsis-3 (OR=1.4, CI:1.1-1.8). After adjustment for age, sex, and comorbidities, OSA remained a significant risk factor for sepsis-2 (OR=1.8, CI:1.5-2.1), but not sepsis-3 (OR=0.92, CI:0.70-1.2). As expected, sepsis-2 and sepsis-3 were associated with increased mortality, but OSA was independently protective against all-cause hospital mortality (OR=0.50, CI:0.27-0.92), including after adjustment for sepsis-3 (OR=0.50, CI:0.27-0.95) and sepsis-2 (OR=0.47, CI:0.25-0.87). However, for the subset of patients admitted to the ICU (17%) with sepsis, OSA was not significantly protective against death (OR=0.67, CI:0.025-1.8), nor invasive ventilation (OR=1.2, CI:0.70-2.2). OSA patients required less vasopressors, but this was not statistically significant (4% v. 7%, p=0.11).

**CONCLUSION.** In this large cohort of patients, OSA was found to be an independent risk factor for sepsis by the more sensitive sepsis-2 criteria, but not the more prognostic sepsis-3 definition. Paradoxically, OSA appeared protective against hospital mortality, even after adjustment for age, gender, comorbidities, and sepsis. The findings are consistent with pre-clinical work and hypotheses that OSA patients may be at an increased risk for infection, but may better endure its complications.

### 0556

#### The effect antibiotics duration on mortality and infectious complications in critically-ill septic patients- a meta-analysis

M. Khpal, A. Baheerathan, N. Arulkumaran, M. Singer
UCL, Bloomsbury Institute of Intensive Care Medicine, London, United Kingdom
**Correspondence:** M. Khpal
*Intensive Care Medicine Experimental* 2018, **6(Suppl 2):**0556

**OBJECTIVES.** To investigate the effect antibiotics duration on mortality and infectious complications in critically-ill septic patients (PROSPERO registration number: CRD42018091566).

**METHODS.** We carried out a systematic search of Pubmed, Embase, clinicaltrials.gov, and CENTRAL databases to identify randomized clinical studies investigating duration of antibiotics in intensive care units. The primary outcome was mortality. Secondary outcomes were treatment failure, secondary infections, and incidence of antimicrobial resistance. Statistics were performed using Review manager for Mac (version 5.1) software. Data on dichotomous outcomes are presented as (odds ratio (OR) (95% confidence intervals), p-values) and data on continuous outcomes are presented as mean difference (95% confidence intervals) and p-values.

**RESULTS.** 5018 titles were retrieved from the search. Of these, 20 studies (5373 patients) were included in the final analysis (Figure 1). Strategies to optimise antibiotic use included
(i) biomarker guidance (procalcitonin; PCT) (12 studies),
(ii) clinical algorithms (3 studies), or
(iii) pre-specified fixed antibiotic duration (5 studies).
PCT-guidance (-1.26 (-1.98 to -0.89); p< 0.001) resulted in shorter antibiotics duration, however, clinical-algorithms did not demonstrate a reduction (-3.7 (-11.7 to 4.5); p=0.38). The average number of days of antibiotics use in fixed-duration studies were 6.6±2.6 days and 12.4±3.7 in the short and long duration arms respectively (p=0.021). PCT-guided antibiotics therapy was associated with lower *mortality* (OR=0.85 (0.74-0.99); p=0.03), while clinical algorithm based treatment (OR=0.73 (0.46-1.16); p=0.18) and fixed-duration antibiotic treatment (OR= 1.28 (0.89-1.85); p=0.18) were not.
*Treatment failure* was not reported in the clinical algorithm studies. Neither fixed duration (OR=1.23 (0.89-1.70), p=0.21) nor PCT-guided antibiotic studies (OR=0.90 (0.78-1.04); p=0.15) were associated with treatment failure.
The incidence of*secondary infections* was reported in all 3 clinical-algorithm studies, 3 of 5 fixed-duration studies, and 4 of 12 PCT-guided studies; no difference was seen between treatment and control arm in any group. Reporting of incidence of antimicrobial resistance was only available in 3 studies.

**CONCLUSIONS.** Studies using PCT-guidance for antibiotics duration and fixed duration were associated with shorter duration of antibiotic use. The reduction in the number of days of antibiotics use was not associated with greater treatment failure. The fixed duration studies did not factor in biological response to treatment and were not associated with a mortality benefit. The reduction in antibiotics duration seen with PCT- guided treatment was associated with a mortality benefit. Further research is required to investigate the potentially negative impact of prolonged antibiotics duration on the immune cells of patients with sepsis.

**GRANT ACKNOWLEDGMENT**
M.K and N.A- Salary support from the UK NIHR

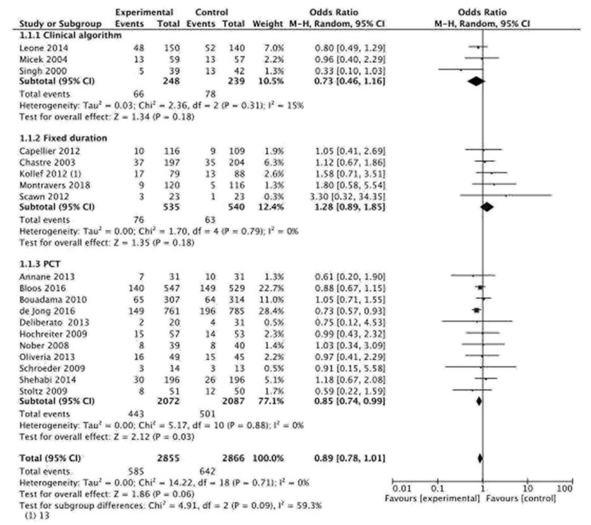

**Fig. 1 (abstract 0556).** Forest plot of mortality by method of reducing antibiotic duration among ICU patients

### 0557

#### A prospective and external validation of quick sequential organ failure assessment versus systemic inflammatory response syndrome criteria in patients with suspected infection in the emergency department

A. Shiraishi[1], S. Gando[2], S. Kushimoto[3], T. Abe[4,5], T. Mayumi[6], H. Ogura[7], D. Saitoh[8], S. Fujishima[9], A. Hagiwara[10], Y. Shiino[11], S.-I. Shiraishi[12], T. Iba[4], M. Ueyama[13], Y. Otomo[14], H. Ikeda[15], K. Okamoto[16], J. Kotani[17], J. Sasaki[9], K. Takuma[18], R. Tsuruta[19], T.-A. Nakada[20], T. Hifumi[21], K. Yamakawa[22], T. Masuno[23], N. Takeyama[24], Y. Mizushima[25], N. Yamashita[26], Japanese Association for Acute Medicine (JAAM) Sepsis Prognostication in Intensive Care Unit and Emergency Room (SPICE) Study Group
[1]Kameda Medical Center, Kamogawa, Japan; [2]Hokkaido University Graduate School of Medicine, Sapporo, Japan; [3]Tohoku University Graduate School of Medicine, Sendai, Japan; [4]Juntendo University, Tokyo, Japan; [5]Tsukuba University, Tsukuba, Japan; [6]University of Occupational and Environmental Health, Kitakyushu, Japan; [7]Osaka University Graduate school of Medicine, Osaka, Japan; [8]National Defense Medical College, Tokorozawa, Japan; [9]Keio University School of Medicine, Tokyo, Japan; [10]Center Hospital of the National Center for Global Health and Medicine, Tokyo, Japan, [11]Kawasaki Medical School, Kurashiki, Japan; [12]Aizu Chuo Hospital, Aizuwakamatsu, Japan; [13]Chukyo Hospital, Nagoya, Japan; [14]Tokyo Medical and Dental University, Tokyo, Japan; [15]Teikyo University School of Medicine, Tokyo, Japan; [16]Kitakyushu City Yahata Hospital, Kitakyushu, Japan; [17]Kobe University Graduate School of Medicine, Kobe, Japan; [18]Kawasaki Municipal Kawasaki Hospital, Kawasaki, Japan; [19]Yamaguchi University Hospital, Ube, Japan; [20]Chiba University Graduate School of Medicine, Chiba, Japan; [21]St Luke's International Hospital, Tokyo, Japan; [22]Osaka General Medical Center, Osaja, Japan; [23]Nippon Medical School, Tokyo, Japan; [24]Aichi Medical University Hospital, Nagakute, Japan; [25]Rinku General Medical Center, Izumisano, Japan; [26]Kurume University Hospital, Kurume, Japan
**Correspondence:** A. Shiraishi
*Intensive Care Medicine Experimental* 2018, **6(Suppl 2):**0557

**INTRODUCTION.** The quick Sequential Organ Failure Assessment (qSOFA) has been proposed for prediction of mortality in patients with suspected infection outside the intensive care unit.

**OBJECTIVES.** To test the hypothesis that the qSOFA can more accurately predict in-hospital mortality than systemic inflammatory response syndrome (SIRS) criteria in patients with suspected infection in the emergency department.

**METHODS.** This prospective, multi-center, diagnostic accuracy study, initiated in December 2017, included 37 emergency departments in Japan. Patients consecutively participated in the study if they were suspected of infection, received antibiotics, underwent cultivation of body fluids or imaging for detecting sites of infection, and were hospitalized in the study hospitals or died in the emergency department. Study index tests included the qSOFA and SIRS criteria based on information at the time of suspected infection. The study reference standard was defined as in-hospital death. Predefined statistical analysis plans included multiple imputation for all the study variables, comparison of area under the receiver-operating characteristic (AUROC) curve as the primary analysis, and net reclassification improvement (NRI) analysis as the secondary analysis. Interim analysis was planned to determine the final study sample size after the number of study participants exceeded 900, which was within the upper limit of 2,000.

**RESULTS.** This study was terminated in February 2018 after the number of participants reached 1,065. Of these, 407 (38%) and 915 (86%) participants were qSOFA >1 and SIRS criteria > 1, respectively. A total of 160 participants (15%) died in the hospitals. The primary analysis demonstrated greater diagnostic accuracy for in-hospital death with qSOFA than with SIRS criteria (AUROC 0.65 versus 0.51, difference +0.14 95% confidence interval (CI) [+0.08, +0.19]). Sensitivity and specificity to predict in-hospital mortality at the given thresholds (qSOFA >1 and SIRS criteria >1) were 0.58 and 0.65 with the qSOFA and 0.87 and 0.14 with SIRS criteria, respectively. The secondary analysis also demonstrated positive NRI between the qSOFA and SIRS criteria (+0.45 95% CI [+0.22, +0.62]).

**CONCLUSIONS.** In patients who visited the emergency department and were suspected of infection, this prospective, multi-center study for external validation demonstrated that the qSOFA improved diagnostic accuracy and reclassification in predicting in-hospital death compared to those with SIRS criteria. However, early prediction of subsequent in-hospital death with the qSOFA in those patients remained insufficient.

**GRANT ACKNOWLEDGMENT**

This study was supported by the Japanese Association for Acute Medicine.
**STUDY REGISTRATION.** UMIN000027452

### 0558

### Combining procalcitonin with the quick SOFA score improves sepsis mortality prediction

C.-C. Lee[1], H. Yu[2], L. Nie[3], A. Liu[2], D.W. Yen[4], T.-C. Hsu[1]
[1]National Taiwan University Hospital, Emergency Medicine, Taipei, Taiwan, Province of China; [2]Sichuan Provincial People's Hospital, Department of Laboratory Medicine, Chengdu, China; [3]The First People's Hospital of Foshan, Department of Laboratory Medicine, Foshan, China; [4]Washington University School of Medicine, Department of Medicine, Saint Louis, United States
**Correspondence:** C.-C. Lee
*Intensive Care Medicine Experimental* 2018, **6(Suppl 2):**0558

**INTRODUCTION.** Since the introduction of qSOFA, concerns have been raised. None of the elements in qSOFA are specific for the detection of infection, and subsequent validation studies showed suboptimal overall discrimination and sensitivity (reported sensitivity 32%) under the recommended cutoff.

**OBJECTIVES.** To investigate whether procalcitonin can improve the performance of quick SOFA score in sepsis mortality prediction.

**METHODS.** We conducted a multicenter retrospective cohort study with prospective validation in an independent cohort. Patients who presented to the emergency department or hospital floors with suspected severe infection and complete data on procalcitonin were eligible for inclusion. The derivation cohort include 1318 patients with presumed sepsis between June 1, 2015, and December 31, 2016 and the validation cohort included 493 prospectively collected patients in 2017. Serum procalcitonin levels were measured at admission with the commercial assay (VIDAS, bioMerieux, Marcy, France). Quick SOFA score and SIRS (Systemic Inflammatory Response Syndrome) criteria were calculated for each patient. PCT levels were assigned into 0, 1, 2 points for a serum level of < 0.25, 0.25-2, and >2 ng/mL and added to the qSOFA score. The incremental value of PCT to qSOFA was then evaluated by logistic regression, ROC curve, and reclassification analysis. Lastly, we calculated the sensitivity, specificity, positive predictive value, and negative predictive value of SIRS, qSOFA, and qSOFA_PCT in predicting in-hospital mortality.

**RESULTS.** A total of 1318 patients with presumed severe infection were enrolled with a 30-day mortality of 13.5%. Serum level of procalcitonin showed a high correlation with qSOFA score and 30-day in-hospital mortality. The area under the ROC curve in predicting mortality was 0.56 for SIRS criteria, 0.67 for qSOFA score, and 0.73 for qSOFA_PCT. The risk prediction improvement was reflected by a net reclassification improvement of 35% (17%-52%, p=0.00011). At a cut-off of 2 points, qSOFA has a sensitivity of 32.6% (95% CI: 25.8%-40.0%) and a specificity of 87.0% (87.3%-91.0%) to predict 30-day mortality. Incorporation of PCT into the qSOFA model could raise the sensitivity to 86.5% (95%CI: 80.6%-91.2%) with a specificity 47.5% (95%CI: 44.6%-50.5%). In the validation cohort, we confirmed qSOFA_PCT greatly improve the sensitivity to 90.9%.

**CONCLUSION.** A simple modification of qSOFA score by adding the ordinal scale of PCT could greatly improves the sensitivity of qSOFA. The highly sensitive qSOFA_PCT score can used as a quick screening tool for early identification of sepsis patients. Prospective multicenter studies are needed to validate our findings.

### 0559

### The effects of human chorionic gonadotropin hormone-derivative EA-230 on the systemic inflammatory response and renal function following on-pump cardiac surgery, a randomized double-blind placebo-controlled phase II study

R. Beunders[1], R. van Groenendael[2], M. Kox[1], J. Hofland[2], W. Morshuis[3], L. van Eijk[2], P. Pickkers[1]
[1]Radboud University Medical Center, Intensive Care Medicine, Nijmegen, Netherlands; [2]Radboud University Medical Center, Anesthesiology, Pain and Palliative Medicine, Nijmegen, Netherlands; [3]Radboud University Medical Center, Cardiothoracic Surgery, Nijmegen, Netherlands
**Correspondence:** R. van Groenendael
*Intensive Care Medicine Experimental* 2018, **6(Suppl 2):**0559

**INTRODUCTION.** The systemic inflammatory response following on-pump cardiac surgery is related to post-operative hemodynamic instability and impairment of renal function [1]. Pregnancy is associated with immunotolerance and an increased glomerular filtration rate (GFR).

EA-230, a linear tetrapeptide (AQGV), derived from the human chorionic gonadotropin hormone (hCG), has shown immunomodulatory and renal protective properties in several preclinical animal models of systemic inflammation. Also, an excellent safety profile of EA-230 was observed in phase 1 studies in humans. Furthermore, the immune modulating effects of EA-230 were recently demonstrated during human experimental endotoxemia.

**OBJECTIVES.** To investigate immunomodulatory and renal protective effects, as well as safety and tolerability of EA-230 in patients with systemic inflammation following on-pump cardiac surgery.

**METHODS.** We designed a prospective, double-blind, placebo-controlled, randomized trial in 180 patients undergoing elective CABG-surgery with or without valve surgery. Patients were randomized to receive either 90 mg/kg/hour EA-230 or placebo (1:1 ratio) during the surgical procedure. The primary endpoint is the modulation of the inflammatory response by EA-230, quantified as the change in IL-6 plasma concentrations. Key secondary endpoints are changes in glomerular filtration rate as measured with iohexol plasma clearance (iGFR) [2] and safety parameters.

The trial is conducted in two parts to enable an (unblinded) interim safety analysis by an independent data safety monitoring board (DSMB) at n=60. Second, an adaptive design is used to re-assess power at n=90 with intrinsic data. All study procedures are summarized in figure 1.

**RESULTS.** Last patient last visit was February 22 2018. 158 Male and 22 female patients were enrolled with an median [IQR] age of 68 [62-74] years and BMI of 25 [26-29] kg/m$^2$. During the DSMB interim analyses no safety concerns were raised. Following adaptive sample size re-assessment, the group size did not need adjustments.
Median [IQR] iGFR prior to surgery was 94 [77-104] mL/min/1.73m$^2$. Efficacy data will be available after database lock in May 2018.
**CONCLUSIONS.** A large randomized double-blind placebo-controlled phase II study in patients undergoing elective on-pump cardiac surgery was conducted with the hCG-derivative EA-230. It was designed to study the safety, tolerability and immunomodulatory and renal protective effects of EA-230 during systemic inflammation. Results will be available in May 2018.

**REFERENCE(S)**

1. Balk, R.A., Systemic inflammatory response syndrome (SIRS): where did it come from and is it still relevant today? Virulence, 2014. 5(1): p. 20-6.
2. Sterner, G., et al., Determining ´true´ glomerular filtration rate in healthy adults using infusion of inulin and comparing it with values obtained using other clearance techniques or prediction equations. Scand J Urol Nephrol, 2008. 42(3): p. 278-85.

**GRANT ACKNOWLEDGMENT**

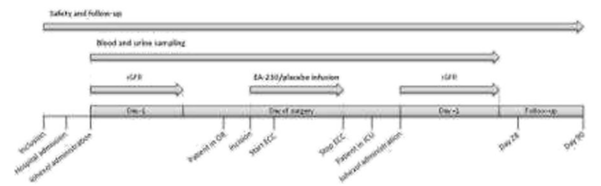

**Fig 1 (abstract 0559).** Study procedures for the primary and key-secondary endpoints of the EASI study

### 0560

#### Epigenome-wide DNA methylation profiling of sepsis

A. Binnie[1,2], C.J. Walsh[3,4], P. Hu[5], D. Dwivedi[6], A. Fox-Robichaud[6], P.C. Liaw[6], J.L.Y. Tsang[6,7], J. Batt[3,4], G. Carrasqueiro[1,8], P. Castelo-Branco[1,8], C.C. dos Santos[4,9], Canadian Critical Care Translational Biology Group (CCCTBG)
[1]University of Algarve, Departamento de Ciências Biomédicas e Medicina, Faro, Portugal; [2]William Osler Health System, Department of Critical Care, Toronto, Canada; [3]University of Toronto, Toronto, Canada; [4]Keenan and Li Ka Shing Knowledge Institute, Toronto, Canada; [5]University of Manitoba, Department of Biochemistry and Medical Genetics, Winnipeg, Canada; [6]McMaster University, Department of Medicine, Hamilton, Canada; [7]Niagara Health, Department of Critical Care, St Catherine's, Canada; [8]Algarve Biomedical Centre, Faro, Portugal; [9]University of Toronto, Institute of Medical Sciences and Department of Medicine, Toronto, Canada
**Correspondence:** A. Binnie

*Intensive Care Medicine Experimental* 2018, **6(Suppl 2):**0560

**INTRODUCTION.** Epigenetic alterations may provide important insights into the regulation of gene expression in sepsis and the genetic pathways that drive the critical illness phenotype.
**METHODS.** A nested case-control pilot study of whole blood DNA methylation profiling in 68 critically-ill septic ICU patients vs 66 critically-ill non-septic ICU patients with similar severity of illness and mortality.
**RESULTS.** Epigenome-wide DNA methylation profiling identified 668 differentially methylated regions (DMRs) corresponding to 443 unique differentially-methylated genes (DMGs). Analysis of transcriptomic data from human sepsis cohorts reveals that DMGs are overrepresented amongst genes showing differential expression in sepsis. Functional analysis of all DMGs showed enrichment for antigen processing and presentation, methyltransferase activity, cell adhesion proteins, cell junction proteins, and metal-binding proteins. A text-mining tool, the "SepsisScore", identified those DMGs most associated with sepsis in the literature including complement component 3, angiopoietin 2, myeloperoxidase, HLA-A, HLA-DRB1, HLA-C, and HLA-DQB1. Intriguingly, a large number of genes are both differentially-methylated and differentially-expressed in sepsis yet do not appear in the sepsis literature. Weighted gene correlation network analysis (WGCNA) was performed using the same patient dataset and identified gene methylation modules associated with important clinical traits including multiple organ dysfunction score, need for vasopressors, length of ICU stay and length of hospital stay.
**CONCLUSIONS.** Whole blood DNA methylation profiling of critically-ill patients reveals a "sepsis" phenotype corresponding to differential methylation of multiple sepsis-associated genes. We also identify DNA methylation modules that correlate with features of critical illness including multiple organ dysfunction score, need for vasopressors, length of ICU stay and length of hospital stay. DNA methylation profiling of whole blood is a powerful new method for characterizing critically ill patients and may have utility as both a diagnostic and prognostic tool.

**GRANT ACKNOWLEDGMENT**

McLaughlin Foundation Accelerator Grant in Genomic Medicine and Health Informatics (2015-2016); Canadian Institutes of Health Research (Grant # MOP-130331 to CCDS). Canadian Thoracic Society Lung Association Studentship award to CJW.

### 0561

#### Performance-based assessment of capillary leak index as mortality predictor in septic patients

P. Palacios Moguel[1], C. Gomez Moctezuma[2], A.J. Fuentes Gomez[2], O.E. Palacios Calderon[2], J.S. Aguirre Sanchez[2], J. Franco Granillo[2], G. Camarena Alejo[2]
[1]American British Cowdray Medical Center, Intensive Care Unit, Mexico City, Mexico; [2]American British Cowdray Medical Center, Intensive Care, Mexico City, Mexico
**Correspondence:** P. Palacios Moguel

*Intensive Care Medicine Experimental* 2018, **6(Suppl 2):**0561

**INTRODUCTION.** Capillary Leak Index (CLI) reflects endothelial damage most of the time associated with certain pathologies such as sepsis, severe trauma, extracorporeal circulation, etc. Described as systemic edema, hypoproteinemia, reduction of effective arterial blood volume and hemoconcentration.
Capillary Leak Index increases mortality in septic patients. Currently, there is not effective treatment, so early recognition and staging its crucial in medical prognosis. An objective score such as CLI, can reasonably infer the integrity and functionality of endothelial barrier. This index proposed by Condermans et al[1], is obtained as a ratio of C reactive protein (CRP) over albumin times 100, with normal range values between 2.5 to 25.
**OBJECTIVE.** Validate CLI as a tool in septic patients for severity and outcome. Determine a cut-off value related to mortality.
**METHODOLOGY.** Observational prospective trial conducted in two tertiary centers in Mexico which included ICU patients admitted with diagnosis of severe sepsis and septic shock. The study was divided in two phases: generation and validation. In first phase we analyzed CLI, SOFA, APACHE, CRP, albumin, hospital length of stay and mortality. Student´s t test was used to compare data and ROC curve analysis was performed to validate CLI as a prognostic factor. On second phase the cut-off point obtained from previous stage, was compared with all variables to validate it as a prognostic scale.
**RESULTS.** 116 patients included. ROC curve analysis on generation phase (n=62 patients), set a cut-off point (85.55, AUC 0.88) as mortality predictor. On phase two, based on cut-off point, complete group was analyzed obtaining a 100% sensibility, 89% specificity, PPV 67% and a NPV of 100%. Kaplan Meier survival curve and Log Rank test analysis compared 28 days mortality, based on CLI cut-off point, resulting in survival of 88.6% vs 11.4% (P< 0.001).

**CONCLUSION.** CLI is a useful tool to predict poor prognosis in septic shock patients. Cut-off point of 85.55 reflects better sensitivity and specificity. This scale is simple, accessible and low cost.

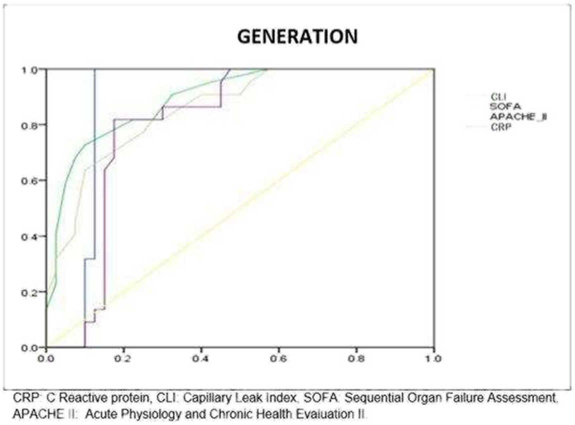

**Fig. 1 (abstract 0561).** ROC curve CLI, SOFA, APACHE II, CRP Vs Mortality (Generation Group phase 1)

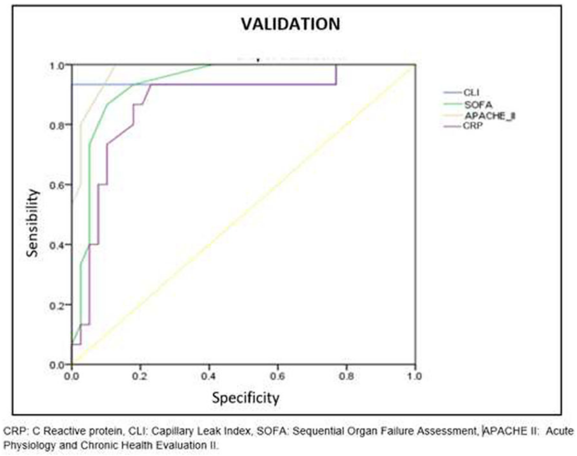

**Fig. 2 (abstract 0561).** ROC curve CLI, SOFA, APACHE II, CRP Vs Mortality ( Validation group, phase 2)

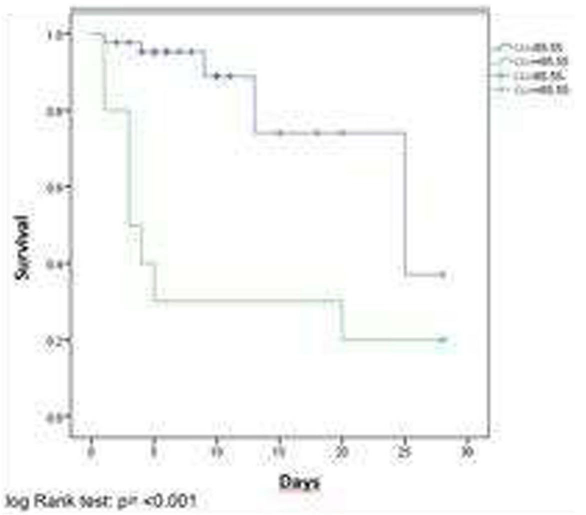

**Fig. 3 (abstract 0561).** Kaplan Meier curve, 28 days mortality (Validation group)

### 0562

#### Clinical usefulness of modified CRB-65 score compared to SIRS and qSOFA as a screening tool for sepsis in initial emergency department: a propensity score matching study

H. Oh, J. Lee
Jeju National University School of Medicine, Internal Medicine, Jeju-si, Korea, Republic of
**Correspondence:** H. Oh
*Intensive Care Medicine Experimental* 2018, **6(Suppl 2):**0562

**INTRODUCTION.** A revision of sepsis definitions proposed the Quick Sequential Organ Function Assessment (qSOFA) as a bedside indicator of sepsis. But practical application is not well established in the Emergency Department (ED).

**OBJECTIVES.** We aimed to elucidate modified CRB-65 (mCRB-65)score in recognizing sepsis compared to systemic inflammatory response syndrome (SIRS) and qSOFA.

**METHODS.** A retrospective chart review was performed among patients with suspected or documented infection between January 2014 and December 2015. We assigned each patient into the sepsis and the no-sepsis group by the presence of predefined organ dysfunction. The number of SIRS, qSOFA, mCRB-65 criteria was calculated for each patient. And we then calculated the sensitivity and specificity of predefined criteria for each score for prediction of sepsis in our data set, and compared an area under the curve (AUC) for each score. In addition, we performed the matched case-control study through using the propensity score matching method. More than 2 points in three tools was considered as positive for risk of sepsis.

**RESULTS.** A total of 4,253 patients with pneumonia were assessed for eligibility, and 2,441 of whom(933 with sepsis and 1,508 with no-sepsis) were included in the study. Of these patients, the rate of patients met mCRB-65, qSOFA, and SIRS positive criteria was 37.6%, 12.3%, and 52.3%, respectively. The crude AUC value of mCRB-65 positive criteria in predicting sepsis was significantly higher than that of qSOFA and SIRS positive criteria (0.728 vs. 0.633. vs. 0.608, respectively). After the propensity score matching process, the rate of patients met mCRB-65, qSOFA, and SIRS positive criteria was 49.2%, 15.8%, and 56.5%, respectively. With respect to mCRB-65, qSOFA, and SIRS positive criteria, the sensitivity for sepsis were 0.66 (95% CI, 0.63 to 0.69), 0.29 (95% CI, 0.26 to 0.32), and 0.66 (95% CI, 0.62 to 0.69), respectively. And for positive criteria of three tools, the specificity for sepsis were 0.72 (95% CI, 0.69 to 0.75), 0.96 (95% CI, 0.95 to 0.97), and 0.54 (95% CI, 0.50 to 0.57), respectively. And mCRB-65 positive criteria still showed higher discrimination for prediction sepsis compared to qSOFA and SIRS positive criteria (adjusted AUC 0.688 vs. 0.630 vs. 0.596, respectively).

**CONCLUSIONS.** Proposed mCRB-65 score seemed to provide better discrimination than SIRS and qSOFA for predicting sepsis. After adjustment of baseline variables, high discrimination of mCRB-65 was still maintained.This may have quality improvement implications in predicting sepsis. Our finding suggest that this simple screening tool might be helpful for physicians to identify sepsis promptly and to escalate therapy appropriately in initial ED.

**REFERENCE(S)**

1. Singer M, et al. JAMA 2016; 315:801-810
2. Liu V, et al. JAMA 2014; 312:90-92
3. Williams JM, et al. Chest 2017; 151:586-596

**GRANT ACKNOWLEDGMENT**

None

## Nutrition

### 0563

#### Predictor of developing type 2 diabetes mellitus: glycosylated hemoglobin in patients with coronary disease and without known diabetes

A.M. González González, A.M. Garcia Bellon, M. Cano García, M. De Mora Martin

Regional Hospital of Malaga, Cardiology, Malaga, Spain

**Correspondence:** A.M. González González

*Intensive Care Medicine Experimental* 2018, **6(Suppl 2):**0563

**INTRODUCTION AND OBJECTIVES.** To analize the value of glycosylated hemoglobin (HbA1c), determined at admission, as predictor of developing type 2 diabetes mellitus in non-diabetic patients, hospitalized for coronary disease.

**METHODS.** We collected 578 consecutive patients, admitted for Acute Coronary Syndrome (ACS) in our Cardiology department, from May 2016 to September 2017. Their levels of Hb1Ac were requested at admission systematically. Finally, 199 patients (34%) were included with diagnosed coronary disease and without known diabetes. We collected clinical data, cardiovascular risk factors (CVRF), anthropometric and laboratory values (fasting glucose and HbA1c).

Two months after hospital discharge, all patients were reevaluated clinically and analytically undergoing an oral glucose tolerance test (OGTT). Patients were grouped into four categories according to the criteria of the American Diabetes Association (ADA): a) normal fasting glucose < 110 mg / dL and OGTT < 140 mg / dL, b) altered basal glucose (ABG): fasting glucose 110 - 126 mg / dL, c) glucose intolerance (GI): a 2-h OGTT 140-200 mg / dL, d) ABG + IG, e) DM: fasting glucose> 126 mg / dL and 2 hours OGTT> 200 mg / dL

We conducted a descriptive analysis of the data. We determine the odds ratio (OR) of having DM or any disorder of carbohydrate metabolism in relation to the values of HbA1c at admission.

**RESULTS.** Mean age was 68.3 years. 69.2% were male and 30.8%, female. Mean body mass index (BMI): 28.4 kg / m$^2$. Regarding the prevalence of cardiovascular risk factors: 74.6% hypertensive, 34.7% dyslipidemic, 32.9% active smokers. Prior history of heart disease: 26.5% acute myocardial infarction (AMI), 19.3% prior stable angina, 41.6% with no history of heart disease. Mean fasting blood glucose 104 mg / dL ± 24, and 6% on HbA1c.

The evaluation at 2 months by fasting glucose and OGTT: normal result 24.1%, 3.5% GBA, IG 33.7%, 11.1% GBA + IG, 27.6% DM. The OR of having any disorder of carbohydrate metabolism in terms of HbA1c levels was 6.65 (95% CI, 1.25-35) (p < 0.029). The cutoff point that best discriminated Hb1Ac was Hb1Ac risk = 6.5%.

**CONCLUSIONS.** HbA1c level at admission is a good predictor for the risk of developing DM or other alterations in carbohydrate metabolism. This small measure would be useful for an early detection of DM, allowing to start a suitable dietary and pharmacological prevention.

### 0564

#### Feasibility of intervention delivery for a Phase 2 randomised clinical trial to investigate the effect of intermittent versus continuous enteral nutrition on muscle wasting in critical illness

A. McNelly[1,2], D. Bear[3], B. Connolly[3,4,5], G. Arbane[3], L. Allum[3], P. Hopkins[6], M. Wise[7], D. Brealey[8], K. Rooney[9], J. Cupitt[10], B. Carr[11], N. Hart[3,4], H. Montgomery[1,2], Z. Puthucheary[1,12]

[1]UCL, Dept. Medicine, London, United Kingdom; [2]University College London Hospitals NHS Foundation Trust/UCL, National Institute for Health Research Biomedical Research Centre, London, United Kingdom; [3]St Thomas' Hospital, Lane Fox Clinical Respiratory Physiology Research Centre, London, United Kingdom; [4]Guy's and St. Thomas' NHS Foundation and King's College London, National Institute for Health Research Biomedical Research Centre, London, United Kingdom; [5]King's College London, Centre for Human and Applied Physiological Sciences, London, United Kingdom; [6]King's College Hospital, Intensive Care Medicine, Major Trauma & Anaesthesia, London, United Kingdom; [7]University Hospital of Wales, Dept. Critical Care, Cardiff, United Kingdom; [8]University College London Hospitals, Dept. Critical Care, London, United Kingdom; [9]Bristol Royal Infirmary, Dept. Critical Care, Bristol, United Kingdom; [10]Blackpool Victoria Hospital, Dept. Critical Care, Blackpool, United Kingdom; [11]University Hospitals of North Midlands, Dept. Critical Care, Stoke-on-Trent, United Kingdom; [12]Royal Free London NHS Foundation Trust, Dept. Anaesthesia & Critical Care, London, United Kingdom

**Correspondence:** A. McNelly

*Intensive Care Medicine Experimental* 2018, **6(Suppl 2):**0564

**INTRODUCTION.** Loss of skeletal muscle during early critical illness can be profound[1], with major functional sequelae[2]. In contrast to continuous enteral feeding, which may inhibit muscle anabolism through the muscle-full effect, intermittent feeding may promote anabolism and preserve muscle mass. In the current study, we investigated the safety and feasibility of intermittent feeding.

**OBJECTIVES.** We sought to determine whether intermittent enteral feeding was safe and feasible during the first ten days of intensive care unit (ICU) stay.

**METHODS.** Mechanically ventilated adults with multi-organ failure were recruited within 24 hours of admission to one of 8 UK ICUs, and randomised to receive four-hourly intermittent feed (intervention) or continuous (control) enteral feed for 10 days.

For participants who received feed for $\geq$48 hours, we recorded the days during which feed was received, reasons for any missed days, average daily energy and protein intakes, and percent target energy and protein intakes received. Patients were excluded if extubated $\leq$48 hours or discharged from ICU $\leq$7 days. Data are reported descriptively and analysed using two sample t-tests or Mann-Whitney U-tests as appropriate.

**RESULTS.** Participants (mean ± standard deviation age 57.4 ± 15.8 years; 68%:32% male:female) received intermittent (n=52) or continuous (n=47) enteral feed on a mean of 77% and 74% of total possible feed days (10 days per patient), respectively; ICU discharge prior to Day 10 was the main reason for missed feed days. No intermittent feed-related serious adverse events were reported. Two participants transferred from intermittent to continuous feed, and one from intermittent to total parenteral nutrition, due to gastric residual volumes $\geq$300 mls or vomiting. There was significantly greater daily energy intake from intermittent feed (median ± interquartile range (IQR):1539 ± 427 kCal) versus that from continuous feed (median ± IQR: 1363 ± 384 kCal; p=0.02). Groups did not differ for median percent energy requirements met or protein intake (either percent daily requirements met or daily intake) (Table 1).

**CONCLUSIONS.** Delivery of intermittent enteral feed to ICU patients appears feasible and safe, and associated with greater median energy intake than is continuous enteral feeding.

**GRANT ACKNOWLEDGMENT**

AM: Moulton Foundation; National Institute for Health Research (NIHR) Biomedical Research Centre (BRC) UCL Hospitals NHS Foundation Trust and UCL; BC, NH: NIHR BRC Guy's and St Thomas' NHS Foundation Trust and King's College London. HM: NIHR BRC UCL Hospitals NHS Foundation Trust and UCL. ZP: ASPEN Rhoads Research Foundation. The research team acknowledges the support of the NIHR Clinical Research Network. The views expressed are those of the authors and are not necessarily those of the NHS, NIHR or the Department of Health.

**Table 1 (abstract 0564).** Energy and Protein Requirements Met, and Mean Daily Energy and Protein Intake. IQR: Interquartile range; CI: Confidence interval.

| | Energy Requirements Met, % | | | Daily Energy Intake, kCal | | |
|---|---|---|---|---|---|---|
| | Median | IQR | p value | Median | IQR | p value |
| Intermittent Feed, n=52 | 83.2 | 17.6 | 0.12 | 1539 | 427 | 0.02 |
| Continuous Feed, n=47 | 77.2 | 29.2 | | 1363 | 384 | |
| | Protein Requirements Met, % | | | Daily Protein Intake, g | | |
| | Mean | 95%CI | p value | Mean | 95%CI | p value |
| Intermittent Feed, n=52 | 77.9 | 83.1-72.8 | 0.12 | 70.66 | 75.06-66.26 | 0.58 |
| Continuous Feed, n=47 | 71.6 | 77.9-65.3 | | 68.65 | 74.55-62.75 | |

### 0565

#### Safety and efficacy of volume-based feeding in critically ill, mechanically ventilated adults: a before-and-after study

S. Brierley-Hobson[1], G. Clarke[2], V. O'Keeffe[3]
[1]Betsi Cadwaladr University Health Board, Dietetics, Rhyl, United Kingdom; [2]Bangor University, Healthcare Sciences, Bangor, United Kingdom; [3]Betsi Cadwaladr University Health Board, Anaesthetics, Rhyl, United Kingdom
**Correspondence:** S. Brierley-Hobson
*Intensive Care Medicine Experimental* 2018, **6(Suppl 2):**0565

**INTRODUCTION.** Underfeeding in critical illness is common and associated with poor outcomes. According to large prospective hospital studies, volume-based feeding (VBF) safely improves nutrition delivery to critically ill patients compared to traditional rate-based feeding (RBF) (Heyland et al. 2015), and confers the potential to improve patient outcomes.

**OBJECTIVES.** A before-and-after study was designed to evaluate the safety, efficacy and clinical outcomes associated with VBF compared to RBF in an Intensive Care Unit (ICU).

**METHODS.** The sample included consecutively admitted critically ill adults, mechanically ventilated for at least 72 hours and fed enterally for a minimum 48 hours. The first cohort (*n*=46) were fed using RBF, the second (*n*=46) using VBF, and observed for 7 days, or until extubation or death. Statistical comparison of percentage feed-volume, energy and protein delivered, plus indices of feed intolerance including diarrhoea, vomiting, gastric residual volume and mean morning blood glucose were the primary outcomes of interest. Secondary observations included ventilation period, mortality, and length of ICU stay (LOICUS).

**RESULTS.** Groups were comparable in baseline clinical and demographic characteristics, and nutrition practices. Volume delivered to the VBF group increased significantly by 11.2% ($p$=< 0.001) compared to the RBF group, and did so significantly every day of the study (Fig 1). Energy increased in the VBF group by 13.4% ($p$=< 0.001) and protein by 8.4% ($p$=0.02), compared to the RBF group. In the VBF group, patients meeting >90% of energy requirements increased significantly from 47.8% to 84.8% ($p$=< 0.001); those meeting >90% of protein requirements changed from 56.5% to 73.9% ($p$=0.134).

VBF did not increase symptoms of feed intolerance. Adjusted binomial logistic regression found each additional 1% of prescribed feed delivered decreased the odds of vomiting by 0.942 (5.8%), 95% CI [0.900-0.985], $p$=0.010.

There was no difference in mortality or LOICUS. Kaplan-Meier found a significantly increased extubation rate in patients receiving >90% of protein requirements compared to those meeting < 80%, ($p$=0.006) (Fig 2). Adjusted Cox regression found the daily probability of extubation tripled in patients receiving >90% of their protein needs compared to the group receiving < 80%, hazard ratio 3.473, $p$=0.021, 95% CI [1.205-10.014].

**CONCLUSIONS.** VBF safely and effectively increased the delivery of nutrition to critically ill patients. Increased protein delivery may improve extubation rate which has positive patient and financial implications, warranting larger confirmatory trials. This investigation adds weight to the ICU literature supporting VBF, and the growing evidence which advocates for enhanced protein delivery to improve patient outcomes.

**REFERENCE(S)**

Heyland DK, et al. (2015) Implementing the PEPuP protocol in critical care units in Canada: Results of a multicentre Quality Improvement study. JPEN, 39(6), 698-706.

No grant

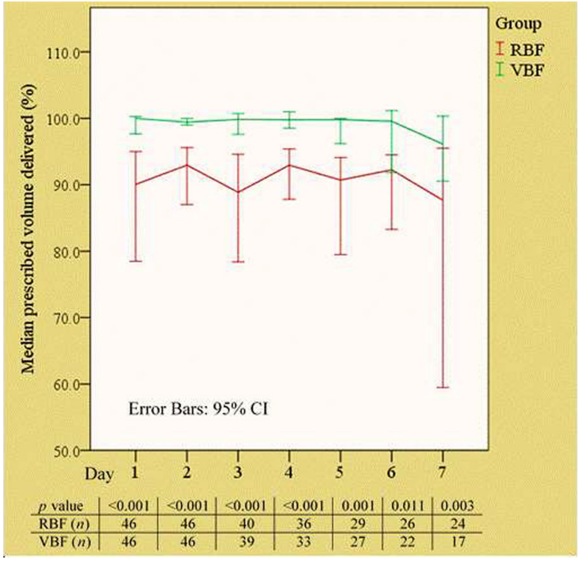

**Fig 1 (abstract 0565).** Daily median percentage feed-volume delivered to RBF and VBF groups: shows 95% CI error bars & n

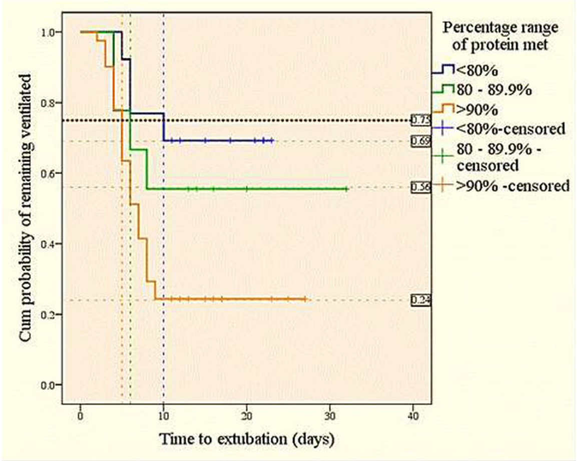

**Fig. 2 (abstract 0565).** Kaplan-Meier curves for time to extubation by percent protein delivered: shows 75th centile

### 0566

#### Glucocorticoid receptor expression in critically ill patients

A.G. Vassiliou[1], E. Jahaj[2], G. Stamogiannos[2], S. Gennimata[2], G. Floros[2], D.A. Vassiliadi[2], S. Tsagarakis[2], S.E. Orfanos[2], A. Kotanidou[2], I. Dimopoulou[2]
[1]Medical School of the National & Kapodistrian University of Athens, Evangelismos Hospital, 1st Department of Critical Care Medicine & Pulmonary Services, GP Livanos and M Simou Laboratories, ΑΘΗΝΑ, Greece; [2]Medical School of the National & Kapodistrian University of Athens, Evangelismos Hospital, ΑΘΗΝΑ, Greece
**Correspondence:** A.G. Vassiliou
*Intensive Care Medicine Experimental* 2018, **6(Suppl 2):**0566

**INTRODUCTION.** Critical illness-related corticosteroid insufficiency (CIRCI) is a condition in which endogenous cortisol levels are relatively low compared to illness severity. Cortisol has to bind to the glucocorticoid receptor (GCR) to be biologically active. Sepsis and septic shock are major healthcare problems with a very high mortality rate in critically ill patients. Hydrocorticosteroid administration is recommended in the Surviving Sepsis Campaign Guidelines 2016 if adequate fluid resuscitation and vasopressor therapy are unable to restore hemodynamic stability (Rhodes et al., 2017). However, each patient responds differently to steroids, and as yet there is no agreement as to who would benefit from their use and how to accurately identify these patients.

**OBJECTIVES.** To assess glucocorticoid receptor expression in peripheral polymorphonuclear cells of critically ill patients.

**METHODS.** Thirty two (32) critically ill patients suffering from medical, surgical and trauma-related pathologies were included in the study. Patients who received corticosteroids at ICU admission were excluded. GCR-α and GCR-β expression were measured in our critically ill cohort at four time points: at admission to the intensive care unit (ICU - baseline), and twice weekly for up to two weeks or until discharge from the ICU or death. Polymorphonuclear cells were isolated from the peripheral blood of the patients at all time points, total RNA was extracted, and GCR-α and GCR-β mRNA levels were quantified by real-time PCR (RT-PCR). At admission, demographics, APACHE II & SOFA scores were recorded.

**RESULTS.** Among the 32 critically ill adult patients studied, approximately one third were female. The median patient age in our sample was 54 years {interquartile range (IQR): 37-73}, the median acute physiology and chronic health evaluation (APACHE) II score was 17 (IQR: 14-22) and the median SOFA score was 8 (IQR: 7-10). The mean ICU stay was 26 days. GCR-α expression decreased from its baseline value at all three time points ($p < 0.05$), while GCR-β expression, which is expressed in lower copies at baseline compared to the α receptor, remained stable until the second measurement, and afterwards decreased ($p < 0.05$) during the remaining ICU stay.

**CONCLUSIONS.** Our results indicate that critically ill patients have a very variable expression of the glucocorticoid receptor that cortisol has to bind to in order to enter the nucleus and inhibit transcription of inflammatory genes. Therefore, the extent to which patients respond to cortisol treatment may be dependent on the levels of GCR receptors.

Rhodes et al., Surviving Sepsis Campaign: International Guidelines for Management of Sepsis and Septic Shock: 2016. Intensive Care Med. 43:304-77.

### 0567

#### Optimized caloric-protein nutrition in critically ill patients. Impact on short and long-term outcomes

J. Azevedo, H. Lima, W. Montenegro, S. Souza, I. Moreira, M. Silva, N. Muniz
Hospital São Domingos, ICU, São Luís, Brazil
**Correspondence:** J. Azevedo
*Intensive Care Medicine Experimental* 2018, **6(Suppl 2):**0567

**INTRODUCTION.** Studies and guidelines suggests that in the critically ill patient indirect calorimetry is the most appropriate form to establish adequate caloric intake and some observational studies suggests that a high protein intake could improve outcomes (1).

**OBJECTIVES.** To evaluate the effects of an optimized nutrition with caloric expenditure determined by indirect calorimetry and a high protein intake compared to standard of care nutrition, primarily on physical component (PCS) of quality of life after 3 and 6 months of randomization and secondarily on short term outcomes in critically ill adult patients.

**METHODS.** We randomized mechanically ventilated critically ill adult patients expected to stay in the ICU for at least 3 days. In the optimized caloric-protein nutrition (OCPN) group caloric intake was determined by indirect calorimetry and protein intake was established in 2.0 to 2.2 g/kg/day. The control group received 25 kcal/kg/day and 1.4 to 1.5 g/kg/day of protein.The primary outcome was the PCS score at 3 and 6 months. Secondary outcomes included handgrip strength at ICU discharge, duration of mechanical ventilation, ICU and hospital mortality.

**RESULTS.** We randomized 138 patients and 120 were included in the analysis. Demographic and clinical data were comparable between the 2 groups (Table 1). There was no difference between the two groups in relation to calories received ($p = 0.70$). On the other hand, the amount of protein received by the OCPN group was significantly higher compared to control group (1.69; 1.33 -1.80 vs. 1.13; 0.97 - 1.39, $p < 0.0001$) (Table 2).The PCS score at 3 and 6 months did not differ between the two groups, neither did handgrip strength at ICU discharge, ICU LOS, duration of mechanical ventilation, and ICU and hospital mortality (Table 3). However, after adjusting for demographic and nutrition covariates a negative protein balance was associated to a lower PCS score at 3 months (OR, 2.63, 95% CI, 1.02 - 6.76; p= 0.045), and 6 months (OR, 3.26, 95% CI, 1.21 - 8.80; p= 0.019) after randomization, while a negative caloric balance did not influencePCS score at 3 months (OR, 1.91, 95% CI, 0.63 - 5.78; p= 0.255), and 6 months (OR, 2.67, 95% CI, 0.86 - 8.24; p= 0.089).

**CONCLUSIONS.** In this study an optimized caloric and high protein strategy did not appear to improve physical quality of life and other important outcomes compared to a standard nutrition care. However, after adjusting for important covariates a negative protein balance was associated to a lower PCS score at 3 and 6 months after randomization.

##### REFERENCE(S)

1. Nicolo M, Heyland DK, Chittams J, Sammarco T, Compher C. Clinical outcomes related to protein delivery in a critically ill population: a multicenter, multinational observation study. JPEN J Parenter Enteral Nutr. 2016; 40(1):45-51.

##### GRANT ACKNOWLEDGMENT

**Table 1 (abstract 0567).** Baseline Characteristics and Short-Term Outcomes

| VARIABLE | OCPN Group n=57 | Control Group n=63 | p value |
|---|---|---|---|
| Age, yr (SD) | 65.0 (18.8) | 67.4 (18.9) | 0.49 |
| Female (%) | 23 (40.3) | 31 (49.2) | 0.33 |
| APACHE IV Score Mean (SD) | 81.1 (32.4) | 77.2 (30.7) | 0.50 |
| SOFA baseline Mean (SD) | 9.8 (14.6) | 6.8 (4.0) | 0.11 |
| Nutrition risk (NRS-2002) Mean (SD) | 3.9 (0.9) | 4.1 (1.0) | 0.22 |
| Admission category (%) Medical/ Surgical | 46 (80)/ 11 (20) | 46 (73)/ 17 (27) | |
| Primary ICU diagnosis (%) Cardiovascular/ Respiratory/ Neurological/ Gastrointestinal/ Sepsis/ Other | 17 (29.8)/ 9 (15.7)/ 11 (19.2)/ 4 (7.0)/ 12 (21.0)/ 4 (7.0)/ | 23 (36.5)/ 11 (17.4)/ 9 (14.2)/ 4 (6.3)/ 15 (23.8)/ 1 (1.5)/ | |

OCPN Optimized Caloric-Protein Nutrition ; APACHE IV Acute Physiology and Chronic Health Evaluation IV SOFA Sequential Organ Failure Assessment; NRS-2002 Nutrition Risk Score-2002

**Table 2 (abstract 0567).** Nutrition therapy

| Variable | OCPN Group N= 57 | Control Group N= 63 | p value |
|---|---|---|---|
| Energy requirement (kcal/day) / Measured (a)/ Calculated (b) Median (IQR) | 1554 (1383-1862) | 1450 (1300-1625) | 0.02 |
| Calculated protein, g/kg/day Median (IQR) | 2.1 (2.1-2.1) | 1.45 (1.45 -1.45) | < 0.0001 |
| Energy received, Kcal/day) Median (IQR) | 1139 (890-1278) | 1140 (889 -1331) | 0.70 |
| Protein received, g/kg/day Median (IQR) | 1.69 (1.33 - 1.80) | 1.13 (0.97 - 1.34) | <0.0001 |
| Energy balance (c), Kcal/d Median (IQR) | -488 (-895- -278) | -353.7 (-549.5 - -122.5) | 0.002 |
| Protein balance (c), g/d Median (IQR) | -0.41 (-0.77 - -0.3) | -0.32 (-0.48 - -0.11) | 0.001 |

(a) Measured by indirect calorimetry; (b) Calculated as 25 kcal/kg/day; (c) Energy and protein balances were calculated as measured requirements minus intake per day

**Table 3 (abstract 0567).** Primary and secondary outcomes measures

| Variable | OCPN Group N= 57 | Control Group N= 63 | P value |
|---|---|---|---|
| PCS score at 3 months Mean (SD) | n= 55 / 93.6 (126.1) | n= 59/ 85.2 (110.6) | 0.70 |
| PCS score at 6 months Mean (SD) | n =52/ 92.0 (133.4) | n=58 / 90.0 (120.6) | 0.93 |
| Handgrip at ICU discharge, Kgf Male Median (IQR) Female Median (IQR) | n=15 / 18 (15-25) n=9/ 8.0 (2-17) | n=14 / 23.5 (13.7-32.0) / n+13 / 14 (7.0-22.5). | 0.35/ 0.18 |
| ICU LOS , Median (IQR) | 21 (13-33) | 18 (10-35) | 0.56 |
| Duration of Mechanical Ventilation, Median (IQR) | 9 (5-14) | 9 (5-14) | 0.64 |
| ICU mortality, n (%) | 22 (38.5) | 28 (42.8) | 0.69 |
| Hospital mortality, n (%) | 26 (45.6) | 29 (46.0) | 0.88 |

PCS score Physical Component Summary score, ICU LOS Intensive Care Unit Length of Stay

### 0568

### Early vs. late parenteral nutrition in patients with cancer undergoing major gastrointestinal surgery: a randomized and clinical study

P. Camargo[1], J.P. Almeida[1], G. Landoni[2], J.T. Fukushima[1], C. Park[1], S. Rizk[1], G. Oliveira[1], R. Nakamura[1], U. Ribeiro Jr.[1], I. Roitman[1], M. Mourao[1], R. Kalil-Filho[1], J.O. Auler Jr.[1], L.A. Hajjar[1]
[1]Instituto do Cancer do Estado de Sao Paulo, Sao Paulo, Brazil; [2]IRCCS San Raffaele Scientific Institute, Milano, Italy
**Correspondence:** P. Camargo
*Intensive Care Medicine Experimental* 2018, **6(Suppl 2):**0568

**INTRODUCTION.** Patients undergoing major abdominal surgery for cancer treatment are at substantial risk for postoperative complications. The impact of a strategy based on early parenteral nutrition on postoperative outcomes is unknown.

**OBJECTIVES.** The objective of this study was to compare the incidence of postoperative complications in surgical patients submitted to different parenteral nutrition strategies: Early vs. Late.

**METHODS.** Phase III study of superiority, unicentric, prospective and randomized, performed at the Cancer Institute of the State of São Paulo, Faculty of Medicine, University of São Paulo, Brazil. We included adult patients with gastrointestinal neoplasia (esophagus, stomach and / or intestine), metastatic or not, submitted to non-palliative elective oncologic surgery. Patients were randomized into two groups: Early parenteral nutrition or Late parenteral nutrition. In the Early group, total parenteral nutrition was started on the second postoperative day, and in the Late group, patients received supplemental parenteral nutrition on the 7th postoperative day if necessary. Patients and outcome assessors were blinded to the treatment group. The analysis was performed according to intent to treat. The primary outcome was a composite endpoint of postoperative complications in 30 days including respiratory, cardiovascular, renal, neurological, infectious and surgical complications. Secondary outcomes were 30-day mortality, need for intensive care unit, total length of hospital and intensive care unit stay, intensive care unit readmission, duration of mechanical ventilation, duration of vasopressors, hepatic dysfunction and hospital readmission.

**RESULTS.** Between May 8, 2013 and October 7, 2017, 658 patients were assessed for eligibility and 335 patients were enrolled, 167 patrients randomized to the Early group and 168 patients to the Late group. At 30 days, the Early group had 46 complications (27.5%) and the Late group had 68 complications (40.5%) - [absolute difference, 95% Confidence Interval of the absolute difference -12.9 (-22.7 to -2.8), p = 0.013]. The secondary outcomes were not different between the groups.

**CONCLUSIONS.** In patients with cancer undergoing elective gastrointestinal surgery, early parenteral nutritional strategy was superior than the late strategy in avoiding postoperative complications. Registration: www.clinicaltrials.gov: NCT01839617.

**REFERENCE(S)**

1- Casaer MP, Mesotten D, Hermans G, Wouters PJ, Schetz M, Meyfroidt G, Van Cromphaut S, Ingels C, Meersseman P, Muller J, Vlasselaers D, Debaveye Y, Desmet L, Dubois J, Van Assche A, Vanderheyden S, Wilmer A, Van den Berghe G; Early versus late parenteral nutrition in critically ill adults. N Engl J Med. 2011 Aug 11;365(6):506-17.

**GRANT ACKNOWLEDGMENT**

This study was sponsored by the Universidade de São Paulo, Brazil.

### 0569

### Supplemental parenteral nutrition does not alter substrate metabolism but improves immunity: the SPN2 randomized trial

M.M. Berger[1], O. Pantet[2], N. Jacquelin[1], M. Charriere[1,3], S. Schmidt[4], F. Becce[4], R. Audran[5], F. Spertini[5], L. Tappy[6], C. Pichard[7]
[1]CHUV, Adult ICU and Burns, Lausanne, Switzerland; [2]CHUV, Lausanne, Switzerland; [3]CHUV, Nutrition Clinique, Lausanne, Switzerland; [4]CHUV, Radiology, Lausanne, Switzerland; [5]CHUV, Immunology & Allergy, Lausanne, Switzerland; [6]Physiology Institute, UNIL, Lausanne, Switzerland; [7]Geneva University Hospital, Nutrition Clinique, Geneva, Switzerland
**Correspondence:** M.M. Berger
*Intensive Care Medicine Experimental* 2018, **6(Suppl 2):**0569

**INTRODUCTION.** Individualized supplemental parenteral nutrition (SPN) improved clinical outcome in a cohort of 305 patients by reducing energy deficit and infectious complications (1). Importantly feeding targets were individually set by indirect calorimetry.

**OBJECTIVES.** We reproduced the conditions of the prior SPN-1 trial to investigate the metabolic and immune response underlying the results.

**METHODS.** Randomized controlled trial starting on day4 (D4) of ICU admission. Inclusion: receiving < 60% of energy target by EN. Patients were randomised to either continued EN or to SPN to cover the indirect calorimetry target. Protein and glucose metabolism were investigated on D4 and D9 of the ICU stay with $^{13}C$-Leucine, $^{2}H_2$ glucose and $^{13}CO_2$ HCO3. Immune response was investigated in stimulated peripheral blood mononuclear cells and by serum cytokines. Nosocomial Infections were recorded. Muscle mass was measured by ultrasound of the thigh cross-sectional area (CSA). Data as medians.

**RESULTS.** Twenty-three patients completed the trial: SPN patients were better fed (Table). Baseline data were similar (age 65 years, SAPS 48). Protein and glucose kinetics did not differ. Immune response differed significantly: on D9, compared to EN patients, TNFα (p=0.018) and IL-6 (p=0.024) decreased more in SPN patients, and response to phytohemagglutinin was stronger, with trend to less infections (p=0.12). Length of stay and mortality did not differ.

**CONCLUSIONS.** Feeding patients to cover an indivualised energy target with SPN from D4 improved the energy balance, and was associated with an improved immunity, attenuation of inflammation, and a lesser muscle mass loss, confirming the results of the SPN-1 trial.

##### REFERENCE(S)

Heidegger CP, Berger MM, Graf S, et al. Optimisation of energy provision with supplemental parenteral nutrition in critically ill patients. Lancet 2013;381:385.

##### GRANT ACKNOWLEDGMENT

Unrestricted grant from Nutrition2000 foundation, and Fresenius Kabi AG

**Table 1 (abstract 0569).** Results (medians)

| | EN (n=12) | SPN (n=11) | p |
|---|---|---|---|
| Energy during intervention (kcal/kg/d) | 16.1 | 24.3 | <0.001 |
| Protein during intervention (g/kg/day) | 0.67 | 1.16 | <0.001 |
| Cumulated energy balance Day 9 (kcal) | -6´050 | -3´415 | 0.002 |
| Muscle mass difference Day15-04 (% of CSA) | -21% | -16% | 0.06 |

### 0570

#### Diarrhea: interventions, consequences and epidemiology in the ICU (DICE-ICU) study

J. Dionne[1,2], K. Sullivan[3], L. Mbaugbaw[2], A. Takaoka[2], E. Duan[1], W. Alhazzani[1,2], J. Devlin[4], M. Duprey[4], P. Moayyedi[5], D. Armstrong[5], L. Thabane[2], J. Tsang[1], R. Jaeschke[1,2], C. Hamielec[1], T. Karachi[1], R. Cartin-Ceba[6], J. Muscedere[7], M. Ahshahrani[8], D. Cook[1,2], DICE Investigators
[1]McMaster University, Department of Medicine, Division of Critical Care Medicine, Hamilton, Canada; [2]McMaster University, Department Health Research Methods, Evidence, and Impact, Hamilton, Canada; [3]McMaster University, Department of Medicine, Hamilton, Canada; [4]Northeastern University, Department of Pharmacy and Health Systems Sciences, Boston, United States; [5]McMaster University, Department of Medicine, Division of Gastroenterology, Hamilton, Canada; [6]Mayo Clinic, Department of Pulmonary Medicine, Phoenix, United States; [7]Queen's University, Department of Critical Care Medicine, Kingston, Canada; [8]University of Dammam, College of Medicine, Dammam, Saudi Arabia
**Correspondence:** J. Dionne
*Intensive Care Medicine Experimental* 2018, **6(Suppl 2):**0570

**INTRODUCTION.** Diarrhea is a frequent concern in the Intensive Care Unit (ICU) and incidence varies from 2-95% in previous studies. This variation is due to lack of a consistent definition of diarrhea and scientific inattention to this clinical problem.

**OBJECTIVES.** The objectives of this study were to describe the epidemiology of diarrhea in critically ill patients including the incidence, risk factors, and consequences of diarrhea, and the incidence of *Clostridium difficile*-associated diarrhea.

**METHODS.** This prospective cohort study was undertaken over 10 weeks in 9 ICUs in Canada and the United States. We included all patients >18 years old who were admitted to the ICU for $\geq$24 hours and followed them daily until ICU discharge. The bedside nurse documented all bowel movements, classifying them using the Bristol and Bliss Stool Charts. We defined diarrhea 3 ways: 1) WHO Criteria of $\geq$3 liquid bowel movements per day (Bristol type 7), 2) any Bristol type 6 or 7 stool, and 3) any Bliss score of 4. We collected daily data on life support, laboratory values (e.g., electrolytes), treatment (e.g., medications, fecal management devices, nutrition), and outcomes (overall mortality).

**RESULTS.** Among 865 patients, the incidence of diarrhea varied based on the definition (WHO Criteria: 77.3%; 95% CI 774.4-80.0, Bristol Stool Chart definition: 56.8%; 95% CI 53.5-60.1, Bliss Stool Chart definition: 39.9%; 95% CI 36.7-43.2). Risk factors associated with diarrhea on multivariate analysis included total number of antibiotic days (p< 0.001), use of sorbitol-containing medications (p< 0.001) and enteral nutrition (< 0.001), while opiates were associated with a lower risk (OR 0.22, 95%CI 0.13-0.36, p< 0.001). Diarrhea often prompted cessation of enteral nutrition, prokinetics or stool softeners, or fecal management device insertion. Diarrhea was not associated with increased overall mortality (78.6% in those with diarrhea and 73.8%% in those without, p=0.184). *Clostridium difficile* toxin was identified in 56 patients but only 18 (2.0%) met the definition of *Clostridium difficile*-associated diarrhea concurrently requiring $\geq$3 liquid bowel movements per day.

**CONCLUSIONS.** In this multicenter cohort of critically ill patients, diarrhea occurred frequently, and the incidence varied based on the definition employed. *Clostridium difficile*-associated diarrhea was an uncommon cause for diarrhea. Antibiotics, hyperosmolar medications and enteral nutrition decreased the risk of diarrhea, while opiates decreased the risk. Further research is needed to identify feasible cost-effective interventions that prevent or reduce this problem.

##### GRANT ACKNOWLEDGEMENT

Hamilton Regional Medical Associates, Hamilton Health Sciences Department of Medicine, Physicians Services Incorporated of Ontario, Canadian Association of Gastroenterology, Canadian Institutes for Health Research.

### 0571

#### Does parenteral nutrition affect liver function in the critically ill patient: a retrospective study

A. Lunt[1,2], I. Ashurst[1], A. Avery[2], N. Walker[2]
[1]Royal Brompton Hospital, London, United Kingdom; [2]University of Nottingham, Nottingham, United Kingdom
**Correspondence:** A. Lunt
*Intensive Care Medicine Experimental* 2018, **6(Suppl 2):**0571

**INTRODUCTION.** Nutrition support in critical care is complex with a reported 15.6% of patients requiring parenteral nutrition (PN). Liver dysfunction has been observed in 30% of critical care patients receiving PN. This study sought to review current evidence and investigate the relationship between liver function, inflammation and PN in a cardiorespiratory tertiary critical care centre.

**METHODS.** There was a two phased approach comprising of a systematic review of published evidence, followed by a retrospective service evaluation. The systematic review of NICE Healthcare databases (AMED, BNI, CINAHL, Cochrane, EMBASE, HBE, HMIC, Medline, PsycINFO and Pubmed) found nine full text observational studies written in English, published in the past 20 years which assessed the relationship between critical care adults, PN and liver function. The retrospective service evaluation captured data of all adult critical care patients who received PN from April 2009 to July 2017. Biochemistry results, length of say, outcome on leaving intensive care and anthropometry data were collated before statistical analysis to establish significant associations.

**RESULTS.** The systematic review found observed liver function changes may be a result of PN or sepsis but was not able to clarify which, if either was responsible. The retrospective service evaluation found 147 patients, of which 62.6% were male, mean age was 56.8 years and 63.9% left intensive care alive. Biochemistry data were analysed for the initial 5 feeding days. An elevated Bilirubin was significantly associated with increased mortality (p=0.0001). Other biochemical markers analysed showed no significant difference associated with outcome. Despite no significant association, elevated Inflammatory and infection markers C reactive protein (64%) and white blood cells (76%) suggests that liver function is affected by sepsis rather PN. This lack of association is consistent with published systematic review evidence. Females were found to have a 41% lower risk of death compared to males. Underweight (BMI < 18.5kg/m$^2$) or obese (BMI >30 kg/m$^2$) patients, had a relative risk of death increased by 13% and 30% respectively. The risk of death increased by 0.3% with each increased year of age.

**CONCLUSION.** This review suggests that liver dysfunction and inflammation in adults receiving PN are a common occurrence and likely to be related to sepsis. Consequently, liver function should not affect the decision to initiate PN as no associated risk on liver function or mortality outcome has been seen in this or bigger studies for this patient group. For optimal outcome in this patient group the dietetic treatment should focus on minimising malnutrition and sepsis through PN formulation. Elevated Bilirubin is a predictor of mortality outcome, however more work should be done to assess the relationship with sepsis or severity scores.

### 0572

#### Postoperative hypometabolic state persists in non-survivors after major surgery

L. Füreder, M. Bernardi, B. Zapletal, A. Schiferer, M. Mouhieddine, A. Lassnigg, M. Hiesmayr
Medical University of Vienna, Department of Anesthesia, Critical Care and Pain Medicine, Division of Cardiothoracic and Vascular Anesthesia and Intensive Care, Vienna, Austria
**Correspondence:** L. Füreder
*Intensive Care Medicine Experimental* 2018, **6(Suppl 2):**0572

**INTRODUCTION.** Metabolic rate after major surgery is modulated by inflammation, metabolically active drugs[1] and patient factors. In small patient groups with a pulmonary artery catheter (PAC) the reversed Fick method was used to investigate the link between oxygen transport and consumption[2,3] despite concerns about mathematical coupling[4]. Multivariate modeling of the postoperative course of metabolic rate has not been done so far in a large cohort.

**OBJECTIVES.** We investigated the metabolic rate and its association with intraoperative hyperlactatemia within the first week after major cardiac surgery using continuous PAC measurements.

**METHODS.** We included all consecutive patients with a PAC after major surgery treated at our ICU between Jan. 2012 and Dec. 2015. Data was stored every 10 minutes in the patient data management system. Median oxygen consumption (VO2) was calculated using the Fick equation (VO2 = CO × (CaO2 - CvO2)) over 1-hour periods. Resting energy expenditure (REE) was calculated with VO2 × 7 assuming a RQ of 0.83. A multivariate regression model with patients as random factors was used to estimate the association between metabolic rate and patient characteristics, intraoperative lactate level and duration of surgery. Patient weight and height were centered on the overall mean and analyzed for 10 cm and 10 kg increments. Age was analyzed in 10-year increments with age 60-70 as reference.

**RESULTS.** We analyzed a total of 634 (475 male, 159 female) patients with median age 65a (IQR 56;72) and median SAPS2 score 35 (IQR 13;65). Hospital mortality was 9.31%. In total, 39044 hourly VO2 measurements were used. Median PAC monitoring lasted 66.5 hours (IQR 34;101). Median VO2 was 236 ml.min$^{-1}$ (IQR 199;272). Median REE was 1650 kcal.d$^{-1}$ (IQR 1391;1901) and 20.4% lower in women (1392 kcal.d$^{-1}$ (CI95 1330;1453)) than men (1751 kcal.d$^{-1}$ (CI95 1715;1787)). REE increased by 329 kcal.d$^{-1}$ (CI95 146;513) in patients aged 30-40 (p< 0.001) and decreased by 89 kcal.d$^{-1}$ (CI95 11;166) in patients aged 80-90 years (p=0.025). REE increased by 108 kcal.d$^{-1}$ (CI95 81;135) per 10 kg patient weight (p< 0.001) but not with height (p=0.368). REE decreased by 24 kcal.d$^{-1}$ (CI95 3;46) per 1 mmol/l increase in intraoperative lactate (p=0.028). An additional anesthesia hour decreased REE by 30 kcal.d$^{-1}$ (CI95 13;48) (p=0.001). REE was only decreased by 83 kcal.d$^{-1}$ (CI95 37;129) (p< 0.001) on the first postoperative day, but remained stable during further ICU stay (Fig.1). REE was more than 20% higher in survivors (Fig.2).

**CONCLUSIONS.** Postoperative metabolic rate is affected by patient characteristics, prolonged surgery and intraoperative hyperlactatemia. A slight hypometabolic state is commonly present early after ICU admission and persists in non-survivors.

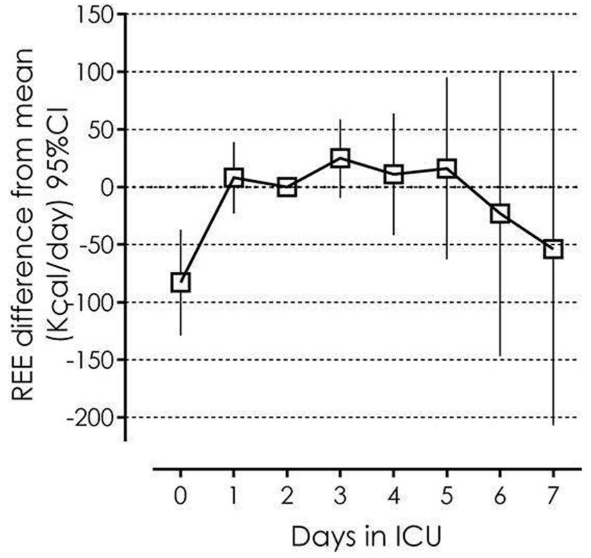

**Fig. 1 (abstract 0572).** REE difference from mean over the course of ICU stay

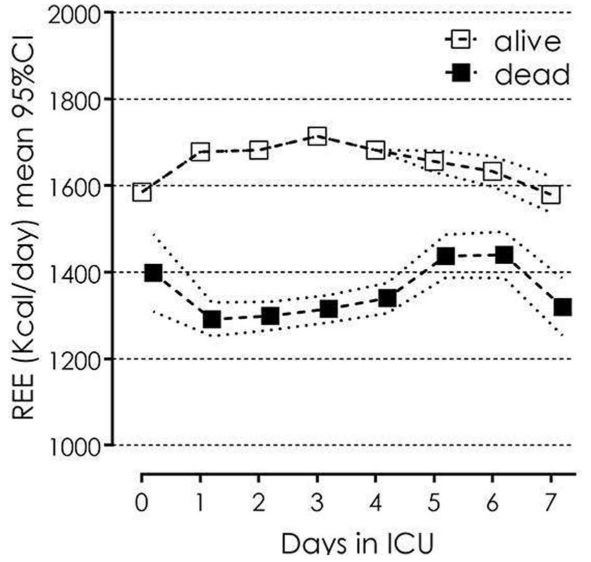

**Fig. 2 (abstract 0572).** REE over the course of ICU stay in survivors compared to non-survivors

## Insights into brain diseases

### 0573

#### Dysfunction of the blood-brain barrier in postoperative delirium patients, referring to the neuronal axonal damage biomarker (pNF-H)

K. Mietani[1], M. Sumitani[2], T. Ogata[3], N. Shimojo[4], R. Inoue[5], J. Hozumi[1], G. Kawamura[1], Y. Yamada[1]

[1]University of Tokyo Hospital, Department of Anesthesiology and Pain Relief Center, Tokyo, Japan; [2]University of Tokyo Hospital, Department of Pain and Palliative Medicine, Tokyo, Japan; [3]National Rehabilitation Center for Persons with Disabilities, Department of Rehabilitation for the Movement Functions, Research Institute, Saitama, Japan; [4]University of Tsukuba Hospital, Department of Emergency and Critical Care Medicine, Ibaraki, Japan; [5]JR Tokyo General Hospital, Department of Anesthesiology and Pain Relief Center, Tokyo, Japan

**Correspondence:** K. Mietani

*Intensive Care Medicine Experimental* 2018, **6(Suppl 2):**0573

**INTRODUCTION AND OBJECTIVES.** Delirium is the most common postoperative complication of the central nervous system (CNS). Its underlying mechanism is still unclear, but previous reports suggest its association with dysfunction of the blood-brain barrier (BBB).

By measuring serum levels of the neuronal axonal damage biomarker, phosphorylated neurofilament (pNF-H), we demonstrated objective CNS anatomical damage in moderate to severe delirium patients, but not in mild delirium patients. These findings indicate postoperative delirium can induce the irreversible CNS damage and pNF-H has a potential to objectively evaluate the severity of the CNS damage. Focusing serum biomarkers of the BBB, we investigated relationship between postoperative CNS damage and the BBB dysfunction in reference to pNF-H.

**METHODS.** After approval at the Ethics Committee, we measured following serum biomarkers: five BBB biomarkers (ICAM-1, PECAM-1, VCAM-1, E-selectin, P-selectin), 3 proinflammatory cytokines (TNF-alpha, IL-1beta, IL-6) and pNF-H. Blood samples were collected within 14 postoperative days after major surgery in 117 patients. Among these, postoperative delirium was clinically diagnosed by using CAM-ICU. At first, we performed a multilinear regression analysis on all of the participants to identify independent contributing biomarkers for pNF-H positive during postoperative periods. Next, we performed a multivariable logistic regression analysis in the stepwise manner to determine whether biomarkers contribute to severity of the pNF-H level in patients with pNF-H positive.

**RESULTS.** Of the 117 subjects, 41 were clinically diagnosed of delirium during postoperative periods, and 30 were pNF-H positive (delirium positive:23, negative:7). P-selectin was only revealed as the independent variable to associate with presence of pNF-H by the multilinear regression analysis ($p < 0.0001$). Further, PECAM-1 only was positively associated with serum levels of pNF-H ($p = 0.02$).

**DISCUSSION AND CONCLUSIONS.** Clinical diagnosis of delirium based on CAM-ICU or other conventional tools usually demonstrate high specificity but low sensitivity, and the hypoactive-type delirium is frequently misdiagnosed. In order to compensate such insufficient clinical diagnosis of delirium, we here used serum pNF-H as an objective CNS damage biomarker. In the process where immunocompetent cells invade into the central nervous system through the BBB and exert the CNS cytotoxicity, there are several steps. P-selectin is an adhesion molecule involved in the very early “capture” step, in which of the immunocompetent cells adhere to vascular endothelia of the BBB. PECAM-1, also known as an adhesion factor, works in the terminal “transmigration” step, in which the immunocompetent cells finally pass the BBB and invade into the CNS. Our findings suggest that P-selectin might be a potential target for preventing the CNS damage and that PECAM-1 should be referenced when treating delirium to protect the CNS.

### 0574

#### Pharmacokinetics of levetiracetam in neurosurgical ICU patients

J. Ong[1], M. Teo[2], J. Goh[2], X. Ang[1], C.P. Lim[1], O.F. Lai[3], G. Ong[3], N.H. Jamaludin[4], K. Lim[4], P.S. Wong[1], B.T. Ang[5], T.P. Lim[1], A. Kwa[1]

[1]Singapore General Hospital, Pharmacy, Singapore, Singapore; [2]Singapore General Hospital, Anesthesia, Singapore, Singapore; [3]Singapore General Hospital, Clinical Research, Singapore, Singapore; [4]Singapore General Hospital, Nursing, Singapore, Singapore; [5]Singapore General Hospital, Neurosurgery, Singapore, Singapore

**Correspondence:** J. Ong

*Intensive Care Medicine Experimental* 2018, **6(Suppl 2):**0574

**INTRODUCTION.** The pharmacokinetics (PK) of drugs is dramatically altered in critical illness. Augmented renal clearance (ARC), a phenomenon characterized by creatinine clearance (CrCl) greater than 130ml/min, is commonly described in critically ill patients. Levetiracetam, an antiepileptic drug commonly prescribed for seizure prophylaxis in the neurosurgical ICU, undergoes predominant elimination via the kidneys. Hence, we hypothesize that current dosing practice of intravenous (IV) levetiracetam 500mg twice daily is inadequate for patients with ARC due to enhanced drug elimination.

**OBJECTIVES.** The objectives of our study were to describe the population PK of levetiractam using a nonparametric approach and design an optimal dosing regimen for critically ill neurosurgical patients with and without ARC.

**METHODS.** his was a prospective, observational, population PK study. Serial blood samples were obtained from neurosurgical ICU patients who received at least one dose of IV levetiracetam. We used uHPLC to analyze these samples and Pmetrics software to perform PK analysis.

**RESULTS.** Nineteen subjects were included, with a median age of 57 years and CrCl of 99ml/min. A one-compartmental model with linear elimination adequately described the profile of levetiracetam. Mean clearance (CL) was 3.12 L/hr and volume of distribution (V) was 14.92 L. Significant covariates included in the final model were CrCl and body weight. Monte Carlo simulations showed a low probability of target attainment (PTA, trough at steady state of $\geq$ 6 mg/L) with a standard dose of 500mg twice daily. A dose of 1000mg every 8 hours and 1000mg twice daily is required for subjects with or without ARC respectively to achieve 80% PTA. Two subjects developed early onset seizures despite therapeutic trough concentrations.

**CONCLUSIONS.** We found that subjects with ARC required higher doses to achieve target levels. Our study was the first to provide recommendations for levetiracetam dosing in neurosurgical ICU patients with ARC.

##### REFERENCE(S)

1. Patsalos PN. Clinical pharmacokinetics of levetiracetam. Clin Pharmacokinet. 2004; 43 (11): 707-724
2. Smith BS, et al. Introduction to drug pharmacokinetics in the critically ill patient. Chest. 2012; 141(5):1327-36

##### GRANT ACKNOWLEDGMENT

Singapore General Hospital (SGH) Research Grant

### 0575

#### The behavior of immobility on the thickness and ecointensity muscle of the upper and lower limbs after traumatic brain injury in the intensive care unit

L. Nunes, V. Maldaner, G. Cipriano Junior, P.F. Melo, J.L. Durigan, L. Pereira, M. Santos, A. Miranda, L. Rocha, P.E. Silva

Universidade de Brasilia, Brasília, Brazil

**Correspondence:** L. Nunes

*Intensive Care Medicine Experimental* 2018, **6(Suppl 2):**0575

**INTRODUCTION.** Traumatic brain injury (TBI) is one of the main causes of morbidity and mortality. Immobilism generates acquired myopathy, neuropathy or a combination of both, called muscle weakness acquired in the intensive care unit (ICU-AW). Ultrasound (US) has been shown to be effective because it allows the identification of changes in muscle structure and morphology.

OBJECTIVE. To compare muscle thickness and echo intensity changes in upper and lower limbs of TBI patients submitted to mechanical ventilation on admission and after 14 days of ICU admission.

**METHODS.** The present study is observational prospective. TBI adults victims were admitted by automobile accident using mechanical ventilation (MV) at a trauma reference unit in the capital. Patients underwent muscle US (SonoSite, M-Turbo®) of biceps brachii (BB), tibialis anterior (TA) and rectus femoris (RF) to obtain measurements of muscle thickness and echogenicity for all the muscles refered between day 1 and day 14 was analyzed. Measurements were performed by us on the upper and lower limbs of patients in the RF, TA and BB muscles on the first day and 14 days thereafter. Quantitative and qualitative analyzes of the images were performed through the ImageJ program.

**RESULTS.** 9 patients were enrolled (age 34.22 ± 6.83 years; 89% male; 22,2% surgery after brain injury; 100% neuro protective). The BB muscle had a loss thickness significant (P = 0.0023) and the RF had a loss echo intensity significant (P= 0.0124) in 14 days. For the evaluation of variation between muscles, a significant difference was observed in echo intensity of BB was bigger RF and TA (P< 0.05).

**CONCLUSION.** The loss of muscle thickness in patients with TBI submitted to MV was higher in the upper limb than lower limb and the variation of the echo intensity was shown to be lower in the upper limbs.

### 0576

#### Outcome comparison of mechanically ventilated Guillain-Barre syndrome versus Myasthenia gravis in US hospitals

A.R. Vellipuram, M.R. Afzal, M.A. Qureshi, R. Khatri, A. Maud, G.J. Rodriguez, D. Kassar, S. Cruz-Flores
Texas Tech University Health Sciences Center, Neurology, El Paso, United States
**Correspondence:** A.R. Vellipuram
*Intensive Care Medicine Experimental* 2018, **6(Suppl 2):**0576

**BACKGROUND.** Myasthenia gravis (MG) crisis and Guillain-Barre Syndrome (GBS) are immune mediated diseases that may require mechanical ventilation as part of their management if severe. Comparative analysis of outcomes in terms of length of stay, disability and mortality between these two disease entities at national level is not reported.

**DESIGN/METHODS.** Mechanically ventilated patients with primary diagnosis of Guillain-Barre Syndrome and Myasthenia Gravis were identified from the nationwide in-patient sample database for the years 2012 to 2014.

**RESULTS.** Mechanically ventilated MG patients (n= 2330, mean 62.0 ±19.5 years) were older compared to GBS patients (n=2060, mean 55.9 ±20.0 years, p= 0.001). Medical Comorbidities were significantly higher in MG patients (diabetes mellitus, congestive heart failure, coagulopathy, chronic lung disease and dyslipidemia) whereas significantly higher nicotine dependence and alcohol abuse was noted in GBS. Significantly higher in hospital complications of pneumonia and urinary tract infection were noted in GBS. Disease severity measured by APDRG severity index and rate of treatment with intravenous immunoglobulin and plasma exchange were comparable. Length of stay (25.3 ± 18.2 days , p < 0.0001 ); hospital charges ( $353361.2 ± 293863.2 vs 232160.12 ± 222881.3 p = 0.001) ;moderate to severe disability ( 86.6% vs 46.2% p < 0.001) were significantly higher for GBS patient compared to MG. In-hospital mortality was comparable ( 8.7% GBS vs 8.6 % MG, p =0.93).In multivariate analysis after adjusting for confounders including treatment, myasthenia gravis patients had significantly less disability (OR 0.06 (95% CI 0.03-0.10) and shorter length of stay (OR 0.32, 95% CI 0.16-0.61).

**CONCLUSIONS.** Mechanically ventilated GBS patients have higher in-hospital complications, length of stay and disability compared to MG. This may reflect delay in diagnosis of GBS at admission and poor response to immunotherapy in certain GBS variants.

### 0577

#### Migraine and its relation to other risk factors in patients with acute ischemic stroke and acute coronary syndrome

H. Enas[1], O. Momtaz[2]
[1]Minia University, Neurology, Minia, Egypt; [2]Faculty of Medicine, Fayoum University, Critical Care, Fayoum City, Egypt
**Correspondence:** H. Enas
*Intensive Care Medicine Experimental* 2018, **6(Suppl 2):**0577

**BACKGROUND.** Cerebral ischemia and ischemic heart diseases are the main manifestation of circulatory diseases with shared identifiable risk factors. Migraine has been recently studied as a risk factor for ischemic stroke (IS) and a possible link to a broader range of ischemic vascular disorders including angina and myocardial infarction is suggested.

**OBJECTIVES.** We aimed to study migraine and its relation to other risk factors in patients with acute IS and acute coronary syndrome (ACS).

**METHODS.** We studied 200 patients, 114 patients had acute IS and 86 patients with ACS collected from the inpatient department of neurology and coronary care units of El-Minia Insurance Hospital in addition to 850 control participants. All patients were subjected to detailed clinical and laboratory evaluation including evaluation of traditional risk factors. All stroke patients were subjected to CT scan. Assessment of stroke severity was measured by National Institute of Health Stroke Scale (NIHSS). Diagnosis of acute coronary syndrome was established clinically as well as by ECG and cardiac specific enzymes. Migraine was diagnosed in all patients and control according to the international headache society and assessment of migraine severity was measured by the Migraine Disability Assessment (MIDAS) questionnaire.

**RESULTS.** Risk ratios (RR) of migraine were adjusted for gender, age, BMI, smoking, physical inactivity, hypertension (controlled and uncontrolled), diabetes mellitus and abnormal lipid profile in both cerebrovascular and cardiovascular groups. In patients with ischemic stroke, RR of migraine was 3.3 for all migrainous patients, 4.97 for MA and 2.14 for MO. In the cardiovascular group, RR of migraine was 2.75 in all migranous patients, 4.12 for MA and 1.88for MO. Other risk factors and their relation to migraine had been discussed. A positive correlation between migraine severity and both stroke severity and cardiac affection severity was found though non-significant in the cardiovascular group. There was no significant difference in hospital outcome in migrainous patients in both groups.

**CONCLUSION.** Patients with migraine (with and without aura) have higher risk than non-migrainous patients for both cerebrovascular and coronary vascular diseases. Risk is stronger in MA in both conditions. Considering migraine in risk stratification of cerebrovascular and cardiovascular diseases is recommended.

**KEYWORDS.** Migraine, risk factors, ischemic storke, coronary artery disease.

### 0578

#### Evolution of the functional situation after trauma brain injury between two monitoring periods of 1 and 3-4 years

M. Guerrero-Marin[1], M. Delange-Van Der Kroft[2], E. Aguilar-Alonso[3], M.D. Arias-Verdu[4], E. Curiel-Balsera[4], A. Muñoz-Lopez[4], J.F. Fernandez-Ortega[4], M.A. Prieto-Palomino[4]
[1]Complejo Hospitalario, Intensive Care unit, Jaen, Spain; [2]Hospital Comarcal de la Axarquia, Intensive Care Unit, Velez Málaga, Spain; [3]Hospital Infanta Margarita, Intensive Care Unit, Cabra (Cordoba), Spain; [4]Hospital Regional Universitario Carlos Haya, Intensive Care Unit, Malaga, Spain
**Correspondence:** M. Guerrero-Marin
*Intensive Care Medicine Experimental* 2018, **6(Suppl 2):**0578

INTRODUCTION: Many instruments have been developed to evaluate hospital mortality, but less attention has been paid to the long-term functional status and quality of life (QOL) of traumatic brain injury (TBI) patients.

OBJECTIVES: To study the evolution of functional situation in the follow-up period between 1 year and 3-4 years and the related variables in traumatic brain injury (TBI).

**METHODS.** Prospective cohort study of traumatic brain injury admitted to the Carlos Haya Hospital (Málaga) between 2004-2008. Evaluated functional situation with Glasgow Outcome Scale (GOS) .

Data were expressed as the mean and standard deviation for quantitative variables and percentages for qualitative variables. We used ANOVA and Newman Keuls test to compare means, Pearson coefficient for correlation and logistic regression for multivariate analysis. . Statistically significant differences p < 0.05.

**RESULTS.** 531 patients. Mean age 40.35±19.75 years, APACHE-II 17.94 ±6.97, admission GCS 7.53±3.83 points. Computerized tomography (CT) on admission by Marshall score was: diffuse injury type I (10.4%), type II (28.1%), type III (24.5%), type IV (8.3%), mass evacuated (22.6%), mass not evacuated (6.2%). Hospital mortality 28.6%.

171 patients died at first year (32.2%) (6.6% missing) and at 4 years 175 died (33%) (16.2% missing). Of the 264 live and followed up at 3-4 years, 10 patients (3.8%) were in vegetative state, 54 (20.5%) were not self-sufficient, 68 (25.8%) were self-sufficient but dysfunctional, and 132 (50% ) did not present dysfunction.

Normal functional status or with dysfunction but self-sufficient: 1st year 65.3% of the 325 patients alive and 3-4 years, 75.76% of the 264 alive and followed. Of these, 65 (24.6%) patients improved their functional situation, worsened 1 (0.4%) and 198 (75.3%) same.

The patients that improved their functional situation were younger (28.38±12.83 vs. 35.73±17.42 years, p=0.002), longer stay in ICU (15.43±12.55 vs. 12.56±12.88 days, p=0.038).

Multivariate analysis by multiple logistical regression found association between the improvement in functional situation(between 1 year and 3-4 years) with age OR: 0.97 (0.95-0.99) and with previous functional status (vegetative or not self-sufficient vs. without or with dysfunction but self-sufficient) OR 1.92 (1.06-3.50).

**CONCLUSIONS.** Approximately 75% of TBI that survive are self-sufficient at 3-4 years approx. Between 1 year and 3-4 years, the functional situation modifies favorably at 25% of the survivors, being greater in the youngest patients with functional situation dysfunction at first year.

**Table 1 (abstract 0578).** Improved situation functional (%)

| | | | |
|---|---|---|---|
| Injury Type | Type I | 11.6 | p = 0.017 |
| | Type II | 23.9 | |
| | Type III | 40 | |
| | Type IV | 21.4 | |
| | mass evacuated | 18.8 | |
| | Not evacuated | 0 | |
| Functional situation first year | vegetative | 9.1 | p<0.001 |
| | Not self-sufficient | 34.2 | |
| | dysfunction but self-sufficient | 46.2 | |

### 0579

#### Does physical therapy care provided within 24 hours improve outcomes compared to physical therapy provided within 18 hours in clinical cardiac intensive care unit? A cohort study

R. Lara, D. Andrade, V. Maldaner, A. Miranda, L. Nunes, A. Ultra, L. Pereira, P.F. Melo
Universidade de Brasilia, Brasília, Brazil
**Correspondence:** R. Lara
*Intensive Care Medicine Experimental* 2018, **6(Suppl 2):**0579

**INTRODUCTION.** The presence of the physiotherapist in the Intensive Care Unit (ICU) with the
extended assistance from 12 to 24 hours, reduces the number of respiratory complications and the time of the individuals´ hospitalization, as well as the financial costs to the instituition.

**OBJECTIVE.** To verify if physiotherapy care (PTc) provided within 24 h/day for Cardiac patients in the Intensive Care Unit (ICU) reduces the length of stay, mechanical ventilation (MV) support and mortality compared to a physiotherapy care provided within 18 h/day.

**METHODS.** A longitudinal study was designed to assess differences between first three months of the year (2016) hospital where patients were given physiotherapy care for 18 h/day and last 3 months of 2016 with 24 h/day. We considered the following as outcome measurements: clinical diagnosis, APACHE II, ICU and mechanical ventilation length of stay and survival. T test of student was made to compare differences between two groups.

**RESULTS.** 103 patients were enrolled in this study (60% male, age 55 ± 18 years, APACHE II 20 (17-26). Patients admitted during 24h/day of PTc showed a lower length of stay in ICU (7 ± 3.1 days X 11.1 ± 3.9 days respectively, p = 0.03) and mortality rate (31% versus 39% respectively, p =0.04) , No difference was found for APACHE II score (p = 0.8) and MV length of stay (4.5 ± 3.4 X 5.5 ± 3.3 days, p > 0.05) between two groups.

**CONCLUSION.** The presence of a PTc contributes decisively to reduce number of hospitalization days and mortality of ICU clinical cardiac patients.

1. Borges DL, de Almeida Arruda L, Rosa TRP, Costa MDAG, Baldez TEP, da Silva GDJP.
Influência da atuação fisioterapêutica no processo de ventilação mecânica de pacientes
admitidos em UTI no período noturno após cirurgia cardíaca não complicada. Fisiotera-
pia e Pesquisa. 2016;23(2):129-35.
2. Davison J, Velloso M. Importância da Fisioterapia pneumofuncional para a retirada de
ventilação mecânica dos pacientes submetidos à cirurgia de revascularização do miocár-
dio; Rev Soc Cardiol Estado de São Paulo. 2003;5(supl A):12-20.
3. Mendez-Tellez PA, Needham DM. Early physical rehabilitation in the ICU and ventilator
liberation. Respiratory Care. 2012;57(10):1663-9.

### 0580

#### Apnea test in brain death determination using oxygen diffusion method is safe

A. Daneshmand[1], C. Dudley[2], E. Wijdicks[1]
[1]Mayo Clinic, Division of Critical Care Neurology, Rochester, United States; [2]Mayo Clinic, Department of Information Technology, Rochester, United States
**Correspondence:** A. Daneshmand
*Intensive Care Medicine Experimental* 2018, **6(Suppl 2):**0580

**INTRODUCTION.** The determination of brain death is based on a comprehensive clinical assessment which includes catastrophic structural brain injury, absence of confounders, absence of motor response, absence of brainstem reflexes, and a formal apnea test. Several methods of apnea testing have been proposed, and each of them may have concerns. Physicians may be apprehensive about performing apnea test due to potential hypoxia, hypotension, and cardiac arrhythmias due to acidemia. Failure to perform the apnea test may lead to additional ancillary tests and possibly delay in organ procurement.

**OBJECTIVES.** To evaluate the safety profile of apnea test using oxygen diffusion method performed by neurointensivists.

**METHODS.** We reviewed 114 consecutive patients (16 years or older) who underwent an apnea test in the setting of brain death determination from 2008 to 2017 at Mayo Clinic.

**RESULTS.** 114 patients had a single apnea test to complete a brain death determination. Mean age was 48 years. Apnea test was terminated in only two patients (1 due to hypoxemia associated with pulmonary edema and one due to hypotension associated with chest trauma), and diagnosis of brain death was confirmed with a cerebral angiogram showing no intracranial circulation. In all but four patients (96 %), the apnea test was performed by a neurointensivist. The protocol included 10 minutes of pre-oxygenation and placement of the endotracheal catheter at the carina with 6 L/min. The baseline arterial blood gas showed median pCO2 of 41 mmHg, median pO2 of 302 mmHg and median pH of 7.38. After 8-12 minutes of disconnection from the ventilator, targets were reached with a median pCO2 of 72 mmHg, median pO2 of 306 mmHg and median pH of 7.19. Pretest mean arterial pressure of 90 mmHg (interquartile range: 78-100) decreased to 76 mmHg (interquartile range: 68.5-85) after the test. Median heart rate remained stable (91 to 94 beats/min). 100 patients (89 %) were on vasopressors before the apnea test, of which 39 patients required an increase in pressor dose during the test. None of the patients developed cardiac arrhythmia or cardiac arrest.

**CONCLUSIONS.** With preoxygenation and tracheal oxygen insufflation, less than 2% of the apnea tests are terminated. When compared with prior series of patients in our institution (1), without a consistent strict protocol, a marked decline (from 10%) in discontinuation of apnea tests was noted. Apnea testing using a predefined protocol and performed by neurointensivists is safe.

**REFERENCE(S)**

1. Wijdicks EF, Rabinstein AA, Manno EM, Atkinson JD. Pronouncing brain death: Contemporary practice and safety of the apnea test. Neurology. 2008; 71:1240-1244

**GRANT ACKNOWLEDGMENT**
None

### 0581

#### Analysis of factors associated with disability and mortality in patients with decompressive craniectomy six months after discharge, along five years in a neurotraumatic ICU

C. Sánchez Ramírez, C.F. Lübbe Vázquez, L.D.M. Díaz Suarez, C. Agüero Senovilla, S. Ruiz Santana
University Hospital of Gran Canaria Dr Negrín, Intensive Care Unit, Las Palmas de Gran Canaria, Spain
**Correspondence:** C. Sánchez Ramírez
*Intensive Care Medicine Experimental* 2018, **6(Suppl 2):**0581

**OBJECTIVES.** To evaluate the factors associated with disability and mortality six month after discharge in patients with decompressive craniectomy (DC) in the ICU.

**METHODS.** Prospective study of patients admitted from January 1, 2013 to march 2018 who required DC. DC was performed by intracranial hypertension (ICH) refractory to medical treatment. We analyzed: main diagnosis at admission; demographic data; neurological data (clinical examination and Glasgow Coma Score: GCS); hypotension: at the site, at hospital admission, in ICU and at hospital discharge; type of craniectomy and DC complications; Rankin scale, and Glasgow outcome scale (GOS) at 30 days after UCI admission, at ICU and 6 months after ICU discharge ; preoperative serum lactate levels and number of hours with intracranial pressure (ICP)> 20, 25 or 30 mmHg before DC; hypo and hyperglycemia; application of mannitol or hypertonic saline solution before and after DC; dose of catecholamines needed; leukocytes and platelets previous and after to DC and other factors related to prognosis. Categorical variables were summarized as frequencies and percentages and the continuous variables as medians and interquartile ranges (IQR) or means and standard deviations. Univariate analysis of disability (Rankin > 3) and mortality in ICU, 30 days after DC and 6 months after ICU discharge were performed. Statistical significance was set at $p \leq 0.05$.

**RESULTS.** Twenty two DC patients were collected. Demographic data and types of admission are shown in Table 1.

Most were subarachnoid haemorrhages (SAH) (59,1%) and 68,2% of DC were hemicranial. The most frequent complications were reoperation due to complications (54.5%), hydrocephalus (50%) and extraaxial hematoma (36,4%). Five patients died at the ICU discharge (22,7%), 4 (80%) of them were SAH. Rankin mean at ICU discharge was 4 and GOS was 3. Rankin mean 6 months after ICU discharge was 2 and GOS was 4. Abcess after DC, number of platelets post DC and GCS at admission was associated with disability 30 days after DC (Table 2).

Mortality 6 months after discharge was significantly associated with bilateral pupillary reactivity prior DC (Table 3).

We found no significant factors associated with mortality in ICU or disability in ICU and six months.after discharge.

**CONCLUSIONS.** Preliminary data show low mortality (22,5%) of DC patients. Rankin and GOS reflect moderately severe disability of DC patients at ICU discharge. Rankin and GOS showed an improvement in disability.six months after ICU discharge.The presence of abcess after DC, number of platelets post DC and GCS at admission were significantly associated with disabiliity thirty days after DC. The presence of bilateral pupillary reactivity prior DC (with lower mortality), was significantly associated with mortality six months after ICU discharge.

**Table 1 (abstract 0581).** Patient data

| Table 1. Patients data | N 22 |
|---|---|
| Age years, n (IQR) | 46 (34-55) |
| Male/Female, n (%) | 9 (39.1%) |
| Dyslipenia, % | 3 (13.6%) |
| Diabetes, n, (%) | 2 (9.13%) |
| Hypertension, n (%) | 2 (9.13%) |
| Antiplatelets treatment, n(%) | 1 (4.5%) |
| APACHE-II, n ,(±SD) | 23.19 ± 4.02 |
| TBI on admission, n (%) | 8 (36.4%) |
| **Diagnosis on admission** | |
| Subarachnoid hemorrhage, n (%) | 13 (59.1%) |
| Stroke Malignant middle cerebral artery, n (%) | 6 (27.3%) |
| Tumor, n (%) | 3 (13.6%) |
| Acute Subdural | 10 (45.5%) |
| **Type of craniectomy** | |
| Primary, n (%) | 12 (54.5%) |
| Secondary, n (%) | 8 (36.4%) |
| Bifrontal, n (%) | 3 (13.6%) |
| Hemicraneal, n (%) | 15 (68.2%) |
| Other, n (%) | 2 (9.1%) |
| **Mortality** | 8 (40%) |
| ICU, n (%) | 5 (22.7%) |
| Hospital, n (%) | 1 (4.5%) |
| Upon discharge from hospital, n (%) | 2 (9.1%) |
| ICU admission days, days | 24.5 (17.75-44.75) |
| Days with craniectomy, days | 53.5 (53-70) |
| ICU admission days until EXITUS, days | 51 (14-370) |
| Highest GCS in ICU | 11 (9-15) |
| GOS at ICU discharge | 3 (2-4) |
| GOS 30 days of ICU admission | 3 (2-4) |
| GOS 6 months | 4 (1.75-5) |
| Rankin at CU discharge | 4 (1.75-5) |
| Rankin 30 days of ICU admission | 4 (2.75-5) |
| Rankin 6 months | 2 (1-5) |

ICU Intensive Care unit, GCS Glasgow Coma Score, GOS Glasgow outcome score, TBI trauma brain injury

**Table 2 (abstract 0581).** Univariate analysis thirty days after DC

| Table 1. Patients data | N 22 |
|---|---|
| Age years, n (IQR) | 46 (34-55) |
| Male/Female, n (%) | 9 (39.1%) |
| Dyslipenia, % | 3 (13.6%) |
| Diabetes, n, (%) | 2 (9.13%) |
| Hypertension, n (%) | 2 (9.13%) |
| Antiplatelets treatment, n(%) | 1 (4.5%) |
| APACHE-II, n ,(±SD) | 23.19 ± 4.02 |
| TBI on admission, n (%) | 8 (36.4%) |
| **Diagnosis on admission** | |
| Subarachnoid hemorrhage, n (%) | 13 (59.1%) |
| Stroke Malignant middle cerebral artery, n (%) | 6 (27.3%) |
| Tumor, n (%) | 3 (13.6%) |
| Acute Subdural | 10 (45.5%) |
| **Type of craniectomy** | |
| Primary, n (%) | 12 (54.5%) |
| Secondary, n (%) | 8 (36.4%) |
| Bifrontal, n (%) | 3 (13.6%) |
| Hemicraneal, n (%) | 15 (68.2%) |
| Other, n (%) | 2 (9.1%) |
| **Mortality** | 8 (40%) |
| ICU, n (%) | 5 (22.7%) |
| Hospital, n (%) | 1 (4.5%) |
| Upon discharge from hospital, n (%) | 2 (9.1%) |
| ICU admission days, days | 24.5 (17.75-44.75) |
| Days with craniectomy, days | 53.5 (53-70) |
| ICU admission days until EXITUS, days | 51 (14-370) |
| Highest GCS in ICU | 11 (9-15) |
| GOS at ICU discharge | 3 (2-4) |
| GOS 30 days of ICU admission | 3 (2-4) |
| GOS 6 months | 4 (1.75-5) |
| Rankin at CU discharge | 4 (1.75-5) |
| Rankin 30 days of ICU admission | 4 (2.75-5) |
| Rankin 6 months | 2 (1-5) |

ICU Intensive Care unit, GCS Glasgow Coma Score, GOS Glasgow outcome score, TBI trauma brain injury

**Table 3 (abstract 0581).** Univariate analysis mortality 6 months after discharge

| | Death | | |
|---|---|---|---|
| Variables | Yes n 8 | No n 14 | P |
| Female/Male | 5/3 | 4/10 | 0.362 |
| Married | 6 (85.7) | 4 (30.8) | 0.066 |
| HBP | 1 (12.5) | 1 (7.1) | 1 |
| Dyslipemia | 2 (25) | 1 (7.1) | 0.537 |
| **Reason for admission** | | | |
| Tumor | 1 (14.3) | 2 (15.4) | 1 |
| SAH | 5 (71.4) | 8 (57.1) | .656 |
| Stroke Malignant middle cerebral artery | 3 (37.5) | 3 (21.4) | .624 |
| Acute subdural hematoma | 3 (42.9) | 7 (53.8) | 1 |
| Obliteration of the base cisterns | 2 (25) | 6 (46.2) | .400 |
| Focal contusion with edema and expansivity | 1 (14.3) | 8 (61.5) | .070 |
| Evacuated Injury | 3 (42.9) | 1 (7.7) | .249 |
| TBI | 2 (28.6) | 6 (42.9) | .656 |
| **Pre Pupillary reactivity** | | | |
| Both | 3 (37.5) | 12 (92.3) | .014 |
| None | 1 (12.5) | 1 (7.1) | 1 |
| **Endotracheal intubation** | | | |
| Pre Hospital | 2 (25) | 3 (21.4) | 1 |
| Emergency | 4 (50) | 4 (28.6) | .386 |
| Operating room | 2 (25) | 4 (28.6) | 1 |
| Nº Plateletsx $10^3$ Post, n (IQR) | 232 (162 ; 480) | 242 (208 | .183 |
| Previous transfusión | 3 (42.9) | 1 (7.1) | .101 |
| Midline shift on CT at admission | 8.5 (8 ; 18) | 10 (4 ; | 1 |
| GCS at admission | 7 (6 : 13) | 15(12 ; | .061 |
| Pre-craniectomy seizures | 3 (42.9) | 1 (7.1) | .249 |
| APACHE II | 25 (22; 28) | 19 (24 ; | 1 |

HBP High blood pressure; SAH subarachnoid hemorrhage;DC, decompresive craniectomy; ICU Intensive Care unit, GCS Glasgow Coma Score, GOS Glasgow outcome score, TBI trauma brain injury;, CT computer tomography,

### 0582

#### The frontal bone window for transcranial Doppler ultrasonography in intensive care units: a new approach

P. Sentenac[1,2], J. Charbit[1], C. Maury[1], P. Bory[2], G. Dagod[1], F. Greco[2], X. Capdevila[1], P.F. Perrigault[2]
[1]Lapeyronie University Hospital of Montpellier, Trauma ICU, Anesthesia and Critical Care Department, Montpellier, France; [2]Gui de Chauliac University Hospital of Montpellier, Neurological ICU, Anesthesia and Critical Care Department, Montpellier, France
**Correspondence:** P. Sentenac
*Intensive Care Medicine Experimental* 2018, **6(Suppl 2):**0582

**INTRODUCTION.** The standard temporal bone window (TBW) for transcranial Doppler (TCD) is most often inadequate to insonate the anterior cerebral artery (ACA) because of a unfavourable angle of insonation. By improving the insonation rate of the ACA [1], the frontal bone window (FBW) is a promising alternative approach but has never been evaluated in intensive care units (ICU).

**OBJECTIVES.** To determine the ability of the FBW to assess ACA velocities in comatose populations admitted to ICU and the reproducibility of the technique.

**METHODS.** A prospective study was conducted at the University Hospital of Montpellier (France). Patients 18 years of age or older, admitted to ICU for neurological disease with *GCS* score less than 13, were enrolled within the first three days after admission, provided that they were clinically stable. Brain deaths were excluded. According to a standardized protocol (*Fig1*), a *first TCD exam* was carried out bilaterally through the TBW and FBW by an expert examiner categorizing patients into two groups: the *FBW success* (unilaterally or bilaterally) or *FBW failure* group. A *second TCD exam* was performed 15 min later by the same examiner. A *third TCD exam* was performed 30 min later by a blinded non-expert examiner. Intra and interobserver concordance of FBW measurements were analysed according to the limits of agreement (LOA) method. The correlation between ACA measurements recorded by TBW and FBW was expressed using correlation coefficient (r) and its 95% confidence interval (CI). P-values < 0.05 were considered significant.

**RESULTS.** Between Nov 2014 and Sept 2016, 147 patients were included. The *first TCD exam* was able to measure ACA velocities through the TBW in 91 patients (62%, CI[54-70]), 70 bilaterally and 21 unilaterally, and through the FBW in 66 patients (45%, CI[37-53]), 45 bilaterally and 21 unilaterally (*Fig2*). For 16 out of the 147 patients included (11%), the FBW was the only acoustic window allowing to measure ACA velocities. By combining the TBW with the FBW, the overall proportion of patients with at least one-sided ACA velocities measurements increased from 62% CI(54-70) to 73% CI(65-79) (p< 0.05). No risk factors for *FBW failure* were found. Intra and interobserver mean bias and 95% LOA for ACA systolic velocity measurements were similar, 1 (-33 to 35) and 2 (-34 to 38) $cm.s^{-1}$ (*Fig3*). ACA systolic velocity was lower on average when measured through the FBW than TBW, 79±3 *vs.* 93±3 $cm.s^{-1}$ (p< 0.05). ACA systolic velocities recorded by the FBW were not well correlated with those measured in the same patient by the TBW, r=0.47 CI(0.28-0.62).

**CONCLUSIONS.** This study was the first to evaluate the FBW in ICU. About one in two patients had an adequate FBW. Reproducibility was good. By insonating the ACA in a more distal segment (A2) than the TBW (A1), the FBW may enhance the monitoring of cerebral vasospasms.

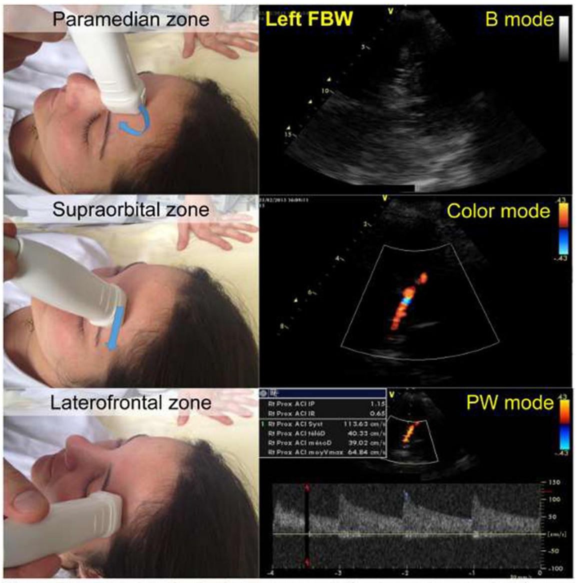

**Figure 1**. The transducer is positioned vertically at the *paramedian frontal zone* (index mark at the top), rotated 90° outwards and shifted (blue arrows) horizontally (index mark laterally) to the *supraorbital zone* at the top of the orbital arcade, up to the *laterofrontal zone*. Visualization of the brain parenchyma in *B-mode* (two-dimensional mode), insonation of the A2 segment of the anterior cerebral artery (ACA) in *color-mode*, and blood flow velocities measurements using the *Pulsed Doppler* (PW) mode. [FBW = frontal bone window].

**Fig. 1 (abstract 0582).** Technique of transcranial Doppler ultrasonography using the frontal bone window

**GRANT ACKNOWLEDGMENT**
None

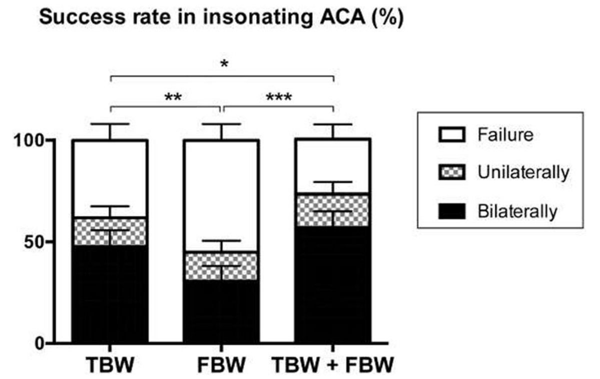

**Fig. 2 (abstract 0582).** TBW, FBW and combination of both techniques success rate in insonating the ACA

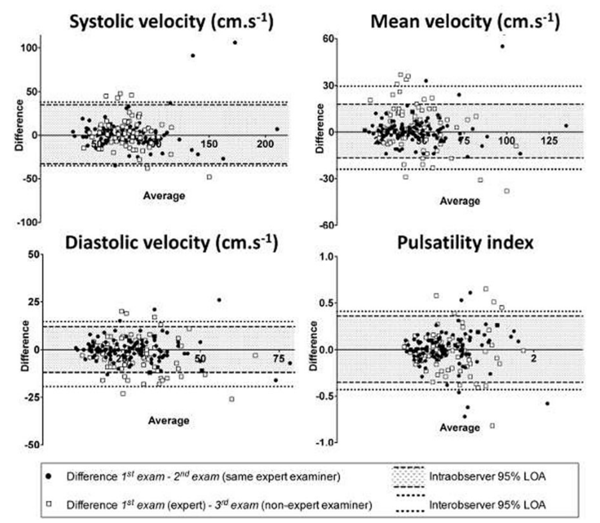

**Fig. 3 (abstract 0582).** Intra and interobserver limits of agreement for ACA measurements through the FBW

## Making a difference

### 0583

#### Social media at international intensive care medicine conference - are the voices of nurses and AHPs being heard? A three-year analysis across continents

A. Wong[1], M. Malbrain[2], O. Olusanya[3], J. Strachan[1], D. Lyness[4], O. Gustafson[1], International Fluid Academy Social Media Collaborative
[1]Oxford University Hospitals NHS Trust, Adult Intensive Care Unit, Oxford, United Kingdom; [2]Brussels University Hospital (UZB), Brussels, Belgium; [3]St Bartholomew's Hospital, London, United Kingdom; [4]Mater Infirmorum Hospital, Belfast, United Kingdom
**Correspondence:** O. Gustafson
*Intensive Care Medicine Experimental* 2018, **6(Suppl 2):**0583

**INTRODUCTION.** The use of social media (SoMe) such as Twitter and Facebook has become ubiquitous at medical conferences including that for intensive care medicine (ICM). The perceived benefits of SoMe include increase interaction, enhanced learning and networking opportunities beyond the physical boundaries of the conference. Critics of SoMe have often cited that such online discussions are often heavily biased and without meaningful educational benefit. In a multi-disciplinary specialty of ICM, where nurses and Allied Health Professionals (AHPs) make up a numerically larger proportion of the team, we sought to assess their contributions, engagement and impact to such online conversations.

**OBJECTIVES.** To analyse the SoMe engagement of Nurses and AHPs at ICM conferences.

**METHODS.** The largest ICM conferences were identified by group consensus. The hashtags unique to the individual conferences were identified and analysed using proprietary Symplur algorithms. Quantitative and qualitative measures were collected and comparisons made across the various conferences in 2015 to 2017.

**RESULTS.** The annual conference of five main societies/organisations were identified during 2015 to 2017. These were the Society of Critical Care Medicine congress, European Society of Intensive Care Medicine congress, the International Symposium on Intensive Care & Emergency Medicine, the Social Media and Critical Care (SMACC) Congress and the UK's Intensive Care Society Congress. The number of tweets at each conference increased annually between 2015 to 2017 with the exception of SMACC 2017; although this had the most number of tweets of all the other organisations. The increase ranged from -12.1 to 267.7%. Tweets from nurses/AHPs made up an average of 10.8% of tweets (4.9 - 21.7%). Quantitative measures in the form of a Healthcare Social Graph (HSG) score showed that that tweets from nurses/AHPs were of lower impact compared to physicians. Average HSG score for nurses/AHPs was 6.6 (1.9 - 14). The average physician score was 57.0 (30.8 - 82.9). There was a significant difference when comparing European and American conferences.

**CONCLUSIONS.** Although utilisation of SoMe in ICM conferences have increased, the online conversation is dominated by physicians. Despite the possibilities of improved discussion and accessibility that SoMe offers, nurses and AHPs lag behind their physician colleagues. There is a risk that their opinions are not heard and that conferences do not provide any meaningful educational attraction to them. ICM is a multidisciplinary specialty and it is important that this is addressed. Professional societies have dedicated Nurses/AHP committees with involvement in conference organisation. Dedicated conference SoMe teams should consider the composition of the team to be more reflective of the specialty.

**GRANT ACKNOWLEDGMENT**
International Fluid Academy Grant

### 0584

#### An evaluation of the role of occupational therapy within a United Kingdom multi speciality critical care unit

E. Felton[1], T. Downey[1], C. Hassan[1], D. McWilliams[2]
[1]Queen Elizabeth Hospital NHS FT, Therapy Services, Birmingham, United Kingdom; [2]Queen Elizabeth Hospital NHS FT, Birmingham, United Kingdom
**Correspondence:** E. Felton
*Intensive Care Medicine Experimental* 2018, **6(Suppl 2):**0584

**INTRODUCTION.** There is an increasing awareness of the benefits of Occupational Therapy (OT) within the intensive care setting, with a number of studies demonstrating the safety and feasibility of early OT input whilst patients are still invasively ventilated. When introduced at this early part of the patient pathway, OT has been associated with improved function at hospital discharge, reduced incidence and duration of delirium, reduced ICU and hospital length of stay, and reduced hospital costs (Pohlman et al, 2010; Schweickert et al, 2009). As these studies were completed predominantly in the United States, further research into the role of the OT within critical care in the UK is needed.

**OBJECTIVES.** To establish a general overview of the role and potential impact of the OT within a UK based critical care unit over a 6 month period.

**METHODS.** A new OT post was created on the 1$^{st}$ January 2017 to work within the already established critical care physiotherapy and rehabilitation team. Data was collected for a period of 6 months to evaluate the number of referrals received for OT assessment and any subsequent interventions provided.

**RESULTS.** A Total of 262 referrals were received from a range of specialities during the study period. Approximately half of these (n=135, 52%) were assessed by an OT prior to ward transfer, with reasons for a lack of OT assessment in critical care due to a lack of service capacity or patients being transferred to ward < 48 hours after referral. A range of different interventions were provided by the OT within critical care (See box 1), including the early assessment and diagnosis of Post Traumatic Amnesia (n=46) and earlier establishment of specialist seating and postural programmes (n=51).

**CONCLUSIONS.** A large number of referrals were received during the trial period highlighting a significant need for OT within critical care. When available, OT's can fulfil a range of positive roles for patients admitted to critical care including the earlier diagnosis of PTA which otherwise would remain undiagnosed until ward transfer. The OT was also able to assess and provide specialist seating where required to facilitate earlier mobilisation. The overall impact was limited however as a large number of patients were not assessed due to capacity issues. Further research should aim to review the impact of OT input on length of stay, delirium and enhancing patient experience.

**REFERENCE(S)**

Pohlman, M.C., et al. (2010). Feasibility of physical and occupational therapy beginning from initiation of mechanical ventilation. Critical Care Medicine, 38 (11), pp. 2089-2094.
Schweickert, W.D., et al. (2009). Early physical and occupational therapy in mechanically ventilated, critically ill patients: a randomised controlled trial. The Lancet, 373 (9678), pp.1874-1882

**GRANT ACKNOWLEDGMENT**
N/A

- Provision of specialist seating and postural programmes
- Pressure mapping and advice regarding pressure relief
- Cognitive assessments
- Delirium assessment, interventions and education.
- Splinting
- Assessment for and provision of over-bed aids
- Assessment and provision of communication devices
- Upper limb assessment/ rehab programmes
- Functional assessments and transfers
- Education of patients and carers
- Coordination of complex and palliative discharges directly from ITU

**Box 1 (abstract 0584).** Range of interventions provided by OT

### 0585

#### The impact of a 7 day working model on physiotherapy intervention and outcomes in the abdominal surgical patient group compared to a traditional 5 day service

P. Piper[1], O. Gustafson[1], J. Ede[2], S. Vollam[2]
[1]Oxford University Hospitals NHS Trust, Adult Intensive Care Unit, Oxford, United Kingdom; [2]University of Oxford, Nuffield Department of Clinical Neurosciences, Oxford, United Kingdom
**Correspondence:** P. Piper
*Intensive Care Medicine Experimental* 2018, **6(Suppl 2):**0585

**INTRODUCTION.** Physiotherapy services in critical care are traditionally delivered Monday-Friday, with the provision of an acute respiratory service at weekends and overnight. Early goal-directed rehabilitation has been shown to improve short term clinical outcomes in patients following abdominal surgery[1]. The effect of a 7 day physiotherapy critical care rehabilitation service on this patient group is unknown. This prompted a mixed methods evaluation of the implementation of 7 day working in critical care on patients following abdominal surgery, and the multidisciplinary staff involved in their care. This evaluation is part of a larger retrospective evaluation of 7 day working in critical care.

**OBJECTIVES.** To assess the impact of a limited 7 day model on the provision of physiotherapy and rehabilitation interventions to patients following abdominal surgery.

To assess the impact on the level of mobility achieved on ICU discharge, post-ICU length of stay and hospital discharge destination.

**METHODS.** A service change which included an additional 1.5 physiotherapists and an adjustment to the ICU physiotherapy team working patterns was instigated at a UK general tertiary adult ICU in July 2017. An emergency respiratory physiotherapy weekend service was replaced with a limited critical care physiotherapy service that provided both rehabilitation and respiratory interventions. Data was collected for all patients following abdominal surgery admitted to the ICU between September and December 2017, and was directly compared to all patients undergoing abdominal surgery in the same time period in 2016. The primary outcomes were the number of physiotherapy and rehabilitation interventions. Secondary outcomes were: post-ICU length of stay, level of mobility on ICU discharge and hospital discharge destination.

**RESULTS.** There were no statistically significant differences in patient characteristics between the two groups. Following the service change there was a significant improvement in: the number of rehabilitation interventions received by patients (p= 0.015), level of mobility on ICU discharge (p=0.042) and number of patients discharged home (p=0.014). There was a reduction in post-ICU length of stay of 2.5 days and an increase in the median number of physiotherapy interventions per patient. These did not reach statistical significance.

**CONCLUSIONS.** Following the instigation of a 7 day critical care physiotherapy model, patients undergoing abdominal surgery received more rehabilitation interventions. They achieved a higher level of mobility on ICU discharge with a greater number discharged home from hospital.

**REFERENCE(S)**

Schaller, S.J., Anstey, M., Blobner, M. et al 2016. Early, goal-directed mobilisation in the surgical intensive care unit: a randomised controlled trial. *The Lancet*, *388*(10052), pp.1377-1388.

**GRANT ACKNOWLEDGMENT**
The authors received no funding to undertake this work

### 0586

#### An Investigation into the use of ultrasound as a surrogate measure of respiratory muscle function

S. Holtzhausen, A. Lupton-Smith, M. Unger, S. Hanekom
Stellenbosch University, Cape Town, South Africa
**Correspondence:** S. Hanekom
*Intensive Care Medicine Experimental* 2018, **6(Suppl 2):**0586

**INTRODUCTION.** Sonographic measurements of diaphragm thickness may be a surrogate for interpreting diaphragm function in a mechanically ventilated population, where volitional measures of respiratory muscle function may not be possible. Given the effects of mechanical ventilation and critical illness on respiratory muscle function, early identification of "at risk" patients may help earlier, targeted interventions to optimise respiratory muscle function and improve outcomes. To understand what happens in illness, we first need to understand what happens in health.

**OBJECTIVE.** This study aimed to determine the correlation between respiratory muscle strength, fatigue, endurance and diaphragm thickness in a healthy population.

**METHODS.** A descriptive correlational study using a sample of convenience was conducted. University students were stratified according to three activity-levels (sedentary; endurance- and strength related sporting activities), gender and body mass index (BMI).

Diaphragm thickness was determined by sonographic measurement. Respiratory muscle strength, fatigue and endurance was determined using a mouth pressure manometer and calculating the maximal inspiratory pressure and fatigue resistance index and time to fatigue.

**RESULTS.** 55 subjects with a mean (SD) age 21.16±1.55 years were studied. The mean (SD) diaphragm thickness was 1.92 mm (0.61), mean fatigue resistance index was 0.93 (0.13) and median (IQR) time to fatigue was 3:00:00 minutes (12:28:51). Diaphragm thickness moderately correlated with strength (r=0.52; r2=0.27; p< 0.001) and BMI (r=0.57; r2=0.33; p< 0.001). There was a weak independent correlation between strength and BMI (r=0.37; $r^2$=0.14; p=0.01). Respiratory muscle fatigue was not correlated with thickness (r=-0.15; $r^2$=0.02; p=0.29) or strength (r=-0.19; $r^2$=0.04; p=0.16).

**CONCLUSION.** Diaphragm thickness was moderately correlated to strength but not to fatigue or endurance in healthy individuals. Sonography may be a surrogate measure of volitional respiratory muscle strength, and requires confirmation in the critically ill population. Whether the sonographic measures will facilitate early identification of patients at risk of poor respiratory muscle function and allow for earlier, more targeted interventions, needs further work.

### 0587

#### Spouse as continuity: a qualitative study of the experiences of patient and spouse following discharge from intensive care to home

J. Bruun Frandsen[1,2], E. Laerkner[1,2], L. Thrysoee[2,3]

[1]Odense University Hospital, Department of Anaesthesia and Intensive Care Medicine, Odense C, Denmark; [2]University of Southern Denmark, Odense, Denmark, [3]Odense University Hospital, Department of Cardiology, Odense, Denmark

**Correspondence:** J. Bruun Frandsen

*Intensive Care Medicine Experimental* 2018, **6(Suppl 2):**0587

**INTRODUCTION.** More patients survive critical illness in intensive care due to advanced medical technology. The last years there has been an increased focus on post intensive care. Patients and their family experience post intensive care symptoms, defined as PICS and PICS-F (1). The spouse is the primary caregiver and plays a significant role in helping and supporting the patient after discharge to home.

**OBJECTIVES.** The aim of this study was to describe experiences of patient and their spouse following discharge from intensive care to home.

**METHODS.** Within a phenomenological-hermeneutical framework, a qualitative interview study was conducted. The study had an inductive, explorative approach. Three patients (age 72-74) and their spouses, 3 men and 3 women, were recruited from a Danish general ICU. Patients had been mechanical ventilated between 93 and 140 hours, with APACHE II scores between 28-48. All the Patients were following a no sedation strategy during mechanical ventilation in the ICU. Patients and spouses have been married between 49-51 years. Semi-structured individual interviews were conducted 8-11 weeks after discharge from the ICU. Five interviews were conducted in private homes of the participants and one patient participant was interviewed at a community rehabilitation facility. Data was analysed with a thematic analyses based on Interpretative Phenomenological Analyses (IPA) approach.

**FINDINGS.** The findings give an insight to patients and spouses experiences and the post intensive care when recovering during and after hospital admission. Three themes have emerged: "Spouse as continuity", "Need to be accommodated" and "Turning point - Road to recovery". "Spouse as continuity" describes the spouse as a continuous figure in the patient's illness trajectory where illness has first priority and the spouse has a caregiver role. The second theme, "Need to be accommodated", describes patient and spouse experiences when meeting healthcare professionals, feelings of helplessness, post intensive symptoms and transitions in post intensive care. The third theme, "Turning point - Road to recovery" contributes descriptions into patient and spouse experiences of the patient getting better and more positive about the future in the illness trajectory and includes perspectives on rehabilitation and needs of professional follow-up.

**CONCLUSION.** Perspectives of patients and spouse's experiences gives healthcare professionals a unique insight, which can be used in future planning of supporting initiatives to patients and near relatives in the post intensive care.

### 0588

#### Negotiated mobilization: an ethnographic exploration of the nurse-patient interaction in the intensive care unit

E. Laerkner[1], I. Egerod[2], F. Olesen[3], P. Toft[4], H.P. Hansen[5]

[1]Odense University Hospital, University of Southern Denmark, Odense C, Denmark; [2]University of Copenhagen, Health & Medical Sciences, Rigshospitalet, Intensive Care Unit 4131, Copenhagen, Denmark; [3]Department of Aesthetics and Communication & Information Studies, University of Aarhus, Aarhus, Denmark; [4]Odense University Hospital, Odense C, Denmark; [5]University of Southern Denmark, Odense C, Denmark

**Correspondence:** E. Laerkner

*Intensive Care Medicine Experimental* 2018, **6(Suppl 2):**0588

**INTRODUCTION.** Nurses are present at the bedside 24/7 in the intensive care unit (ICU) and carry the responsibility for patient mobilization when other healthcare professionals are absent (1). Lighter sedation has enabled early mobilization of mechanically ventilated patients (2), but little is known regarding the nurses' role and interaction with critically ill patients in relation to mobilization.

**OBJECTIVES.** We aimed to explore the nurse-patient interaction in relation to mobilization of non-sedated and awake mechanically ventilated patients in the ICU.

**METHODS.** The study had a qualitative design using an ethnographic approach within the methodology of Interpretive Description. Data were generated in two intensive care units in Denmark where a strategy of no sedation was applied. Participant observation was conducted during nurse-patient interaction in relation to mobilization with nurses (n=44) and mechanically ventilated patients (n=25). We conducted individual, semi-structured interviews with nurses (n=16), and patients (n=13), who had been mechanically ventilated for at least three days. Data were analyzed using inductive, thematic analysis.

**RESULTS.** We identified three themes: *"Diverging perspectives on mobilization"* showed that nurses had a long-term and treatment oriented perspective on mobilization while patients had a short-term perspective and regarded mobilization as overwhelming in their present situation. *"Negotiation about mobilization"* demonstrated how patients actively negotiated the terms of mobilization with the nurse. *"Inducing hope through mobilization"* captured how nurses encouraged mobilization by integrating aspects of the patient's daily life as a way to instill hope for the future.

**CONCLUSIONS.** Exploring the nurse-patient interaction illustrated mobilization as more than physical activity. Mobilization is accomplished in nurse-patient collaboration as a negotiated, complex and meaningful achievement, driven by logic of care and leading to hope for the future. The study demonstrated the important role of nurses in achieving mobilization in collaboration and negotiation with mechanically ventilated patients in the intensive care unit.

##### REFERENCE(S)

1. Hickmann CE, Castanares-Zapatero D, Bialais E, Dugernier J, Tordeur A, Colmant L, et al. Teamwork enables high level of early mobilization in critically ill patients. Ann Intensive Care. 2016;6(1):80.
2. Kress JP. Sedation and mobility: changing the paradigm. Crit Care Clin. 2013;29(1):67-75.

##### GRANT ACKNOWLEDGMENT

University of Southern Denmark, Odense University Hospital and Danish Nursing Research Society.

### 0589

#### Comparative evaluation of the efficiency of using hygienic washcloths for skin care in severely ill patients

T. Beznosyuk, G. Bodunova, A. Pivkina[1], V. Gusarov
Federal State Public Institution «National Medical and Surgical Center named after N.I. Pirogov» of the Ministry of Healthcare of the Russian Federation, Moscow, Russian Federation
**Correspondence:** T. Beznosyuk
*Intensive Care Medicine Experimental* 2018, **6(Suppl 2):**0589

**INTRODUCTION.** The use of a specialized skin care devices reduces the risk of pressure ulcers in comparison with the traditional methods of skin cleansing using water and soap[1]. However it is unclear whether these methods are effective in the prevention microdamage of the patient´s skin, as well as in reducing the physical effort of medical personnel in the ICU.

**OBJECTIVES.** Comparison of the use of specialized washcloths and the traditional skin care method for ICU-patients in terms of efficacy and safety.

**METHODS.** We conducted a monocentric, open-label, randomized, controlled trial (Sept. 2016 - Jun. 2017) to compare two skin care regimes. The traditional method including cleansing with water, liquid soap, cotton napkins and towels was used in group I. In group II patients' skin was cleaned by hygienic single use washcloths pre-moistened with a hypoallergenic cleansing lotion with an extract of chamomile, aloe, glycerin, panthenol, vitamin E; the lotion did not contain irritants (soap, alcohol). After every skin cleaning procedure nurses filled in the form, where they had to rate the simplicity of the method, the intensity of the physical effort, the necessity of additional personnel and the duration of the procedure. We used 5-step scale - from 1 (very bad) to 5 (very good). Both groups were assessed for the frequency of skin damage (maceration, intertrigo, excessive dryness, microdamage, dermatitis) and pressure ulcers (stages I-IV).

**RESULTS.** Thirty patients were enrolled in each group. The groups were comparable in Waterlow scale 13,8±5,2 in group I vs. 14,9±5,2 in group II, p=0,18; use of mechanical ventilation 15 (50%) vs. 20 (66,7%), p=0,3, use of catecholamines 14 (46,6%) vs. 17 (56,7%), p=0,6. The significant differences were identified in SOFA scale: 3,4 ±2,4 in group I vs. 4,2±3,7 in group II, p=0,012. No statistically significant differences in skin damage 52 (34,7%) vs. 53 (35,3%) cases, p=0,998, in frequency of pressure ulcers of I-II stages 7 (23,3%) vs. 12 (40%), p=0,267 were detected. The pressure ulcers of III-IV stages were not recorded. Eighteen nurses took part in the survey, 676 forms were processed. The results of survey are reflected in the table.

Special skin care devices made it possible to reduce the time of skin care from 16 mins. to 9 mins.

**CONCLUSIONS.** The use of a hygienic pre-moistened washcloths is safe for skin care. It's time-saving and ergonomic effective while not negatively affecting the skin.

**Table 1 (abstract 0589).** Evaluation of methods of skin care

| Sing, % | Group I | | | | Group II | | | |
|---|---|---|---|---|---|---|---|---|
| 5-step scale | 1 | 2 | 3 | 4 & 5 | 1 | 2 | 3 | 4 & 5 |
| The simplicity of the method | - | 21.9 | 41.5 | 36.6 | - | - | 11.2 | 88.8 |
| The intensity of the physical effort | - | 26.7 | 34.8 | 38.5 | - | 5.4 | 24.9 | 69.7 |
| The necessity of additional personnel | 18.7 | 24.2 | 30.5 | 26.6 | 2.1 | 3.4 | 16.8 | 77.7 |

### 0590

#### Effect of institutional case volume on mortality after pediatric liver transplantation: analysis of cases of the last decade in Korea

H. Lee, H.G. Ryu, S.Y. Oh, B. Kim
Seoul National University Hospital, Seoul, Korea, Republic of
**Correspondence:** H. Lee
*Intensive Care Medicine Experimental* 2018, **6(Suppl 2):**0590

**INTRODUCTION.** The level of expertise with a surgical procedure are often proportional to the amount of experience. The effect of case volume on postoperative outcomes is well established for surgical procedures including cardiac surgery, pyloromyotomy, and inguinal hernia in pediatric patients.

**OBJECTIVES.** The purpose of this study was to evaluate whether institutional case volume affects clinical outcomes after pediatric liver transplantation.

**METHODS.** We conducted a nationwide retrospective cohort study using the database of Korean National Healthcare Insurance Service. Between January 2007 and December 2016, 521 pediatric liver transplantations were performed at 22 centers in Korea. Centers were categorized according to the average annual number of liver transplantations: >10, 1-10, and < 1.

**RESULTS.** In-hospital mortality rates in the high-, medium-, and low-volume centers were 5.8%, 12.5%, and 32.1%, respectively. After adjustment, in-hospital mortality was significantly higher in low-volume centers (adjusted odds ratio, 9.693; 95% CI, 4.636-20.268; *P*< 0.001) and medium-volume centers (adjusted odds ratio, 3.393; 95% CI, 1.980-5.813; *P*< 0.001) compared to high-volume centers. Long-term survival for up to 9 years was better in high-volume centers.

**CONCLUSIONS.** Centers with higher case volume (>10 pediatric liver transplantations/year) had better outcomes after pediatric liver transplantation, including in-hospital mortality and long-term mortality, compared to centers with lower case volume (≤10 liver transplantations/year).

##### REFERENCE(S)

Tracy, ET, Bennet KM, et al. low volume is associated with worse patient outcomes for pediatric liver transplant centers. J Ped Surg 2010;45:18-113
Rana A, Pallister Z, et al. pediatric liver transplant center volume and the likelihood of transplantation. Pediatrics 2015:136;e99-107

##### GRANT ACKNOWLEDGMENT

None.

### 0591

#### Subtherapeutic antimicrobial plasma concentrations with standard amoxicillin-clavulanate dosing regimens in paediatric cardiac surgery patients

E. Dhont[1], J. Willems[1], W. Vandenberghe[2], M. Carlier[3], A. Verstraete[3], J. Vande Walle[4], P. De Paepe[5], P. De Cock[1,6]
[1]Ghent University Hospital, Pediatric Intensive Care Department, Ghent, Belgium; [2]Ghent University Hospital, Cardiac Intensive Care Department, Ghent, Belgium; [3]Ghent University Hospital, Department of Clinical Chemistry, Microbiology and Immunology, Ghent, Belgium; [4]Ghent University Hospital, Pediatric Nephrology Department, Ghent, Belgium; [5]Ghent University, Heymans Institute of Pharmacology, Ghent, Belgium; [6]Ghent University Hospital, Pharmacy Department, Ghent, Belgium
**Correspondence:** E. Dhont
*Intensive Care Medicine Experimental* 2018, **6(Suppl 2):**0591

**INTRODUCTION.** Paediatric patients who underwent cardiac surgery are prone to postoperative infectious complications. For β-lactams, the time interval during which plasma concentration is above the minimal inhibitory concentration (fT>MIC) should be at least 50% of the dosing interval and ideally 100% to treat severe infections in critically ill patients [1,2]. Cardiac surgery with cardiopulmonary bypass, critical illness and intensive care therapies can significantly alter antibiotic disposition in the postoperative period.

**OBJECTIVES.** The aim of this study was to determine whether adequate antimicrobial plasma concentrations of amoxicillinclavulanate were obtained with a standard dosing regimen of 25 to 50 mg/kg every 6 hours in paediatric intensive care patients after cardiac surgery.

**METHODS.** Children treated with amoxicillin-clavulanate after cardiac surgery were included in this prospective, pharmacokinetic study. Mid-dose ($C_{MD}$) and trough ($C_{min}$) plasma amoxicillin (AMO) concentrations were determined after the first dose and in assumed steady-state (after $4^{th}$ dose or more) and compared to the MIC clinical breakpoint for Escherichia coli of 8 mg/L as defined by the European Committee of Antibiotic Susceptibility Testing (EUCAST).

**RESULTS.** 37 patients (age range: 7 days to 15 years) were included, a total of 53 mid-dose ($C_{MD}$ AMO) samples and 97 trough ($C_{min}$ AMO )samples were obtained. After the $1^{st}$ dose, median $C_{MD}$ AMO was 14.3 mg/L (range: 3.4-75.2 mg/L); median $C_{min}$ AMO was 4.6 mg/L (range: 0.4-35.7 mg/L). After the $1^{st}$ dose, $C_{MD}$ AMO was below 8 mg/L in 33% and $C_{min}$ AMO was below 8 mg/L in 64% of the samples.

In SS, median $C_{MD}$ AMO was 19.6 mg/L (range: 4.5-132.3 mg/L); median $C_{min}$ AMO was 6.6 mg/L (range: 0.6-75.1 mg/L). In steady-state, $C_{MD}$ AMO was below 8 mg/L in 18% and $C_{min}$ AMO was below 8 mg/L in 56% of the samples.

**CONCLUSIONS.** With standard amoxicillin-clavulanate dosing regimens, more than half of the patients did not achieve the ideal pharmacokinetic target of fT>MIC of 100%. In addition, 33% and 18% of the patients did not reach a minimum antimicrobial target of fT>MIC of 50% after the first dose or in steady-state conditions respectively. This could result in failure to treat severe postoperative infections after cardiac surgery in children, therefore evidence-based dosing regimens are needed for critically ill children.

**REFERENCE(S)**

1. Turnidge JD. The pharmacodynamics of beta-lactams. Clinical infectious diseases : an official publication of the Infectious Diseases Society of America 1998;27:10-22.
2. Carlier M, Noe M, Roberts JA, et al. Population pharmacokinetics and dosing simulations of cefuroxime in critically ill patients: non-standard dosing approaches are required to achieve therapeutic exposures. The Journal of antimicrobial chemotherapy 2014;69:2797-803.

### 0592

#### Focal status epilepticus hemispheric($r_cSO_2$ reading´s correlational analysis to seizure complexity, anticonvulsant therapy and possible prediction trends in a PED

T. Abramo[1], H. Hargrave[1], Z.L. Harris[2], N. Hobart-Porter[1], T. McCarty[1], C. Velasco Gonzalez[1]

[1]UAMS/ACHRI, Pediatrics, Little Rock, United States; [2]Lurie Children's Hospital Northwestern School of Medicine, Pediatrics, Chocago, United States

**Correspondence:** T. Abramo

*Intensive Care Medicine Experimental* 2018, **6(Suppl 2):**0592

Pediatric ED focal seizures have more subtle manifestation variability then generalized seizures. For every first-line anticonvulsant minute delay (> 5 minutes), a 10% increase in decreasing anticonvulsant efficacy, increase status epilepticus (SE) incidence and duration. PED non-epileptic focal seizure, focal seizure rcSO2 readings were < 60% or >80%, with interhemispheric rcSO2 discordance >10, focal seizures correlation to abnormal focal ipsilateral rcSO2 readings and returned to pre-seizure rcSO2 readings.[1]Comparison of PED hemispheric Focal SE rcSO2 readings to seizure severity and anticonvulsant has never been investigated.

**OBJECTIVES.** Correlational analysis of hemispheric focal SE seizure rcSO2 readings to seizure's complexity and anticonvulsants.

**METHODS.** Observational study comparing focal SE rcSO2 readings to seizure complexity and anticonvulsants in PED non-traumatic, neurologically normal, first-time focal SE patients.

**RESULTS.** TABLE 1

Focal seizures: more seizure rcSO2 readings < 60% then >80% p< 0.001, age p=0.5/EMS duration p=0.7 not significant. Comparing seizure rcSO2 readings: rcSO2 < 60%, had longer EMS p=0.0002, PED p=0.001 seizure times, more EMS p=0.001 & PED p=0.0009 anticonvulsants then rcSO2 >80%. All focal seizures had interhemispheric discordance > 14 p< 0.0001. Overall rcSO2 < 60% had longer seizures p=0.0002 and required more anticonvulsants p=0.003. Patient's < 2 years old had more focal seizures < 60% rcSO2 readings p=0.0005 then >2 yrs who had more seizure rcSO2 readings >80% p=0.003. However, age was not associated with anticonvulsant use p=0.08 or EMS seizure duration p=0.19. Correlation of seizure rcSO2 readings < 60% to >80% during seizure´s nonresponsive anticonvulsant, both rcSO2 readings had consistent readings: delta change < 5% (p< 0.0001). Focal seizure rcSO2 readings < 60% [left 48.3% 37.8, 58.2, p=0.0007, right 42.6% 35.7, 55.3, p=0.0005, and EMS seizure duration >23 minutes p=0.005 had the greatest significance for anticonvulsants. Age p=0.17 and Focal seizure side showed no significance p=0.6. Seizure rcSO2 reading rcSO2 > 80% changed earlier than rcSO2 < 60% p=0.001 and both changed earlier than EMR seizure cessation time p=0.001.

**CONCLUSION.** Focal SE seizures, rcSO2 readings < 60% with EMS seizure >23 minutes correlated to greater seizure complexity by longer PED seizure duration and more anticonvulsants compared to rcSO2 >80%. During seizure´s nonresponsive anticonvulsants, both seizure rcSO2 readings < 60%, >80 had < 5% change. Both rcSO2 readings < 60%, > 80% rcSO2 readings changed significantly sooner than clinical seizure cessation with rcSO2 readings > 80% changed sooner than rcSO2 < 60%. In PED focal SE seizure, hemispheric cerebral oximetry monitoring has shown its functionality for rapid seizure cerebral physiology and anticonvulsant response assessment while initial focal seizure rcSO2 readings has potential for predicting patient´s seizure complexity and anticonvulsant needs.

**Table 1 (abstract 0592).** See text for description.

| Table 1.A. Patients (N=100): AGE(yrs):2.2(1.1, 5)( 58.9% male) | |
|---|---|
| All Seizure Events N= 166<br>Left focal (patients N= 48, seizures events N = 80, Right Focal patients N=52, seizures events N= 86) | |
| Focal Left Seizure $r_cSO_2$ readings < 60% events | N=59 |
| Focal Left Seizure $r_cSO_2$ readings > 80% events | N=21 |
| Focal Right Seizure $r_cSO_2$ readings < 60% events | N=64 |
| Focal Right Seizure $r_cSO_2$ readings > 80% events | N=20 |
| All PED Seizure Duration: minutes (EMS seizure duration 23 (12,29)) | 40.2 (33.9,60.2) |
| PED & EMS Left Focal Seizure $r_cSO_2$ readings < 60% duration(minutes)(PED seizure duration: 32.5 (24.1,38.9)) | 42.9(30.1, 45.9.8) |
| PED & EMS Left Focal Seizure $r_cSO_2$ readings >80% duration (minutes)(PED seizure duration: (18.2,24.4)) | 31.2 (24.1,36.1) |
| PED & EMS Right Focal Seizure $r_cSO_2$ readings < 60% duration(minutes)(PED seizure duration: 39.1 (31.7,48.5)) | 49.3.9(34.7, 56.8) |
| PED & EMS Right Focal Seizure $r_cSO_2$ readings >80% duration(minutes)(PED seizure duration: 25.9 (19,34)) | 34.2 (27.1,40.1) |
| Anticonvulsant Doses: N= 553, PED dose 5(4,9) EMS 2(2,3) | N |
| Versed | 246 |
| Ativan | 203 |
| Keppra | 91 |
| Dilantin | 13 |
| Anticonvulsant dose per Patient: PED:4.9(3,6), EMS2(1,3) | N |
| 3-5 | 49 |
| 5-7 | 35 |
| 8 | 7 |

Table 1.B. Comparison of Hemispheric Left and Right Focal Seizure $r_cSO_2$ readings < 60% to > 80% rcSO2 readings over time for all seizure events

| | PATIENT'S SEIZURE STOP TIMES (seconds) Before Seizure Stopped After Seizure Stopped | Left Focal $r_cSO_2$ < 60% N=59 | | Group Comparison | | Left Focal $r_cSO_2$ >80% N= 21 | | Group Comparison | |
|---|---|---|---|---|---|---|---|---|---|
| | | Left Focal $r_cSO_2$ < 60% N=59 | Right $r_cSO_2$ < 60% Non-Focal N=59 | | | Left Focal $r_cSO_2$ > 80% N=21 | Right $r_cSO_2$ > 80% Non-Focal N= 21 | | |
| | | Median | Median | P-Value(1c) | Difference(2) | Median | Median | P-Value(1c) | Difference(2) |
| Left $r_cSO_2$ | 360-300 sec Prior | 36.6(35.4,44.3) | 70.5(68.7,72.3) | <0.0001 | -33.9(-33., -38) | 90.2(91.1,94.2) | 67.3(65.1,70.2) | <0.0001 | 22.9(26,24) |
| | 300-180 sec Prior | 37.6(35.5,47.3) | 70.4(68.3,72.3) | <0.0001 | -32.8(-33.5,-25) | 89.7(87.2,91.2) | 66.2(64.9,74.3) | <0.0001 | 23.5(22.3,16.9) |
| | 180-90 sec Prior | 36.9(35.3,45.3) | 69.2(67.5,72.4) | <0.0001 | -32.3(-32.2,-27.1) | 90.4(87.5,90.3) | 67.1(69.7,74.1) | <0.0001 | 23.3(17.8,16.2) |
| | 90-0 sec Prior | 37.1(35.1,48.2) | 70.5(68.7,72.3) | <0.0001 | -33.4(-33.6,-24) | 91.2(91.1,94) | 66.3(67.1,74.5) | <0.0001 | 24.9(24,20.5) |
| | 0-90 sec After | 66.3(63,70) | 70.4(68.3,72.3) | 0.72 | -4.1(-4.3,-2.3) | 68.3(63.5,71.2) | 66.2(67.9,74.3) | 0.64 | 2.1(-4.5,3.1) |
| | 90-180 sec After | 66.3(64.3,72.4) | 69.7(67.3,74.6) | 0.88 | -3.4(-3,-2.2) | 69.4(66.3,70.3) | 66.1(68.7,72.6) | 0.9 | 3.3(-3.4,2.3) |
| | 180-300 sec After | 68.5(64.6,71.1) | 69.6(67.6,72.5) | 0.87 | -1.1(-3,-1.5) | 69.3(67.2,70.1) | 67.5(68.2,72.3) | 0.89 | 1.8(-1,-2.2) |
| | 300-360 sec After | 68.4(64,70.3) | 71.8(67.2.72.8) | 0.96 | -3.4(-3.2,-2.8) | 70(66.6,71.1) | 67.3(68.9,74.3) | 0.82 | 2.7(-2.3,-3.2) |
| | P-Value 1b | <0.0001 | 0.49 | | <0.0001 | <0.0001 | 0.49 | | <0.0001 |
| Right $r_cSO_2$ | | Right Focal $r_cSO_2$ <60% N= 64 | Left $r_cSO_2$ < 60% Non-Focal N= 64 | P-Value(1c) | Difference (2) | Right Focal $r_cSO_2$ > 80% N= 20 | Left $r_cSO_2$ > 80% Non-Focal N= 20 | P-Value(1c) | Difference(2) |
| | 360-300 sec Prior | 33.1(30.4,45.4) | 73.2(66.1,75.3) | <0.0001 | -40.1(-56, -20) | 91.2(91.1,94) | 66.3(64.1,74) | <0.0001 | 24.9(27,20) |
| | 300-180 sec Prior | 33.7(34.5,45.2) | 72.4(68.3,73.3) | <0.0001 | -38.7(-12,-28.1) | 92.4(89.3,94.2) | 65.2(64.1,70.1) | <0.0001 | 27.2(25.2,24.1) |
| | 180-90 sec Prior | 34.5(32.3,43.9) | 72.2.2(67.5,74.9) | <0.0001 | -37.7(-35.2,-31) | 90.8(89.3,90.8) | 66.1(64.1,70.1) | <0.0001 | 24.7(24.2,20.7) |
| | 90-0 sec Prior | 33.2(32.2,45) | 72.5(68.7,75.3) | <0.0001 | -39.3(-36.5, -30.3) | 90.2(91.1,94.7) | 67.3(65.1,72.7) | <0.0001 | 22.9(26,22) |
| | 0-90 sec After | 67.3(64.5,70) | 70.4(68.3,74.3) | 0.76 | -3.1(-3.8,-4.3) | 68.3(63.5,71.1) | 66.2(62.2,73.1) | 0.68 | 2.1(1.3,-2) |
| | 90-180 sec After | 67.3(64.3,71.3) | 70.5(67.5,75.4) | 0.99 | -3.2(-3.2,-6.1) | 69(66.7,70.3) | 65.1(64.7,72.4) | 0.89 | 3.9(2,-2.1) |
| | 180-300 sec After | 67.1(64.6,70.3) | 69.2(68.1,76.4) | 0.90 | -2.1(-4.1,-6.1) | 69(67.2,70.4) | 67.5(67.2,72.5) | 0.79 | 2.5(0,-2.1) |
| | 300-360 sec After | 66.3(67.4,70.1) | 68.5(69.6,73.2) | 0.86 | -2.2(-2.2,-3.1) | 70(67.6,71.1) | 66.3(66.9,72.6) | 0.93 | 3.7(1.3,-2.5) |
| | P-Value (1b) | <0.0001 | 0.53 | | <0.0001 | <0.0001 | 0.56 | | <0.0001 |

(1a) test for interaction (Group x Period) term of repeated measures model. Ho: group median do not (1b) slice test by Group, Sidak-corrected p-values. Ho: medians for specific group do not differ over periods. (1c) slice test by Period, Sidak-corrected p-values. Ho: group medians do not differ for specific period. (2) Hodges-Lehman location shift and 95%CI.

## Transfusion & fluids in the perioperative period

### 0593

#### Perioperative number of transfused red blood cell units in coronary artery bypass surgery and outcome: the less is better and zero is the best

F. Ampatzidou, C. Mavromanolis, A. Dimaki, T. Asteri, E. Dalampini, M. Nastou, E. Voulioti, G. Drossos

G.Papanikolaou Hospital, Thessaloniki, Greece

**Correspondence:** F. Ampatzidou

*Intensive Care Medicine Experimental* 2018, **6(Suppl 2):**0593

**INTRODUCTION.** Cardiac surgery-related blood transfusions remain high while the relationship with the clinical outcome is unclear.

**OBJECTIVES.** To compare postoperative complications after elective coronary artery bypass surgery (CABG) with the use of cardiopulmonary bypass based on transfused Red Blood Cell (RBC) units.

**METHODS.** Patients who underwent isolated elective CABG from June 2012 to March 2018, in G.Papanikolaou Hospital Thessaloniki Greece, were investigated. Based on number of transfused RBC we compared patients without transfusion (Group A) with those transfused with at least 1 unit (Group B). The following prospectively collected data were analyzed: Age, Euro-score II, Prolonged mechanical ventilation (>24 hours),Acute Kidney Injury-AKI (defined by RIFLE criteria), renal replacement therapy (RRT) because of AKI, use of non invasive ventilation because of postoperative respiratory dysfunction (NIV), sternal wound infections, septicemia and pneumonia. Chi square test was used for the statistical analysis. Transfusion threshold was Hb ≤ 8.5 g/dl. Finally, we did a second statistical analysis between group A and group B after excluding, patients who have transfused with more than 3 RBC units creating Group C.

**RESULTS.** From a total of 1672 elective CABG patients, 349 (20,9%) didn't receive any RBC unit. In group A, mean aged was 65.6±8.8 vs 65.1±9.5, mean Euro score II was 1.2±0.8 vs 1.6±1.4. No statistical significance was found for septicemia(p=0.15),post-op pneumonia (p=0.08) and mortality (p=0.07). Factors with statistical correlation are shown in table 1.

**CONCLUSIONS.** Compared with patients without any transfusions, CABG patients with at least 1 RBC unit transfusion have higher incidence of prolonged mechanical ventilation, use of NIV, AKI, AKI-RRT and sternal wound infections. Prolonged ventilation, AKI, and sternal wound infections are more often in patients with only 1- 3 RBC units compared with no transfused patients.

**REFERENCE(S)**

C. David Mazer.et al Restrictive or Liberal Red-Cell Transfusion for Cardiac Surgery N Engl J Med 2017; 377:2133-2144

**Table 1 (abstract 0593).** Complications related with RBC transfusions

| | Group A-Bloodless n=349 | Group B RBC ≥1 n=1323 | p value | Group C RBC 1-3 N=1033 | p value |
|---|---|---|---|---|---|
| Prolonged vent n,(%) | 0(0%) | 50(3.8%) | <0.001 | 14(1.4%) | 0.03 |
| Sternal infection | 5(1.4%) | 54(4.1%) | 0.02 | 34(3.3%) | 0.07 |
| NIV | 14(4%) | 117 (8.8%) | 0.01 | 65(6.23%) | 0.112 |
| AKI | 18(5.2%) | 186(14.1%) | <0.001 | 104(10.1%) | 0.005 |
| AKI-RRT | 1(0.3%) | 17(1.3%) | 0.005 | 10(0.97%) | 0.215 |

### 0594

#### Use of tidal volume challenge to predict fluid responsiveness in operating room

A. Messina[1], C. Montagnini[1], G. Cammarota[1], F. Giuliani[1], T. Esposito[1], S. De Rosa[2], S. Baltieri[1], F. Della Corte[1], D. Payen[3], M. Cecconi[4]
[1]AOU Maggiore della Carità, Novara, Italy; [2]San Bortolo Hospital, Anesthesiology and Intensive Care, Vicenza, Italy; [3]Lariboisière Hospital, Department of Anesthesiology and Critical Care Medicine, Paris, France; [4]IRCCS Humanitas, Milano, Italy
**Correspondence:** A. Messina
*Intensive Care Medicine Experimental* 2018, **6(Suppl 2):**0594

**INTRODUCTION.** The reliability of dynamic indexes as the pulse pressure (PPV) and stroke volume (SVV) variations to predict fluid responsiveness are affected by tidal volume (Vt). To overcome this problem, functional hemodynamic tests have been proposed, which may reduce the risk of fluid overload. Among these, the end-expiratory occlusion test (EEO) was tested in 2 studies on patients having elective surgery and ventilated with a Vt higher than 6 ml/kg, showing conflicting results (1-2). The recent successful use of Vt challenge (VtC) by Myatra et al. in 20 critically ill patients stimulated the present clinical study (3).

**OBJECTIVES.** Primary aim: to test the VtC as a good predictor of fluid response in surgical patients ventilated with a 6 ml/kg Vt; secondary aim: to compare the reliability of VtC and EEO performed at 6 and 8 ml/kg.

**METHODS.** Patients having elective neurosurgery in supine position, having a continuous cardiac index (CI) monitoring by the MostCare$^{TM}$ device (Vygon Comp, Ecouen Fr). After general anesthesia and before fluid challenge (FC) decided by the attending anesthetist, the VtC was performed by increasing Vt from 6 mL/kg to 8 mL/kg of predicted body weight for one minute. The EEO was performed by interrupting the positive pressure ventilation for 30 seconds, and was repeated twice at 6 and 8 ml/kg-Vt ventilation ($EEO_6$ and $EEO_8$, respectively). The FC consisted in 250 mL saline infused over 10 minutes. Receiving operating characteristic (ROC) curve and the Area Under the Curve (AUC) were constructed comparing the variations before and after FC. The following parameters were collected: PPV and SVV for VtC and stroke volume index (SVI) and CI for EEO. To be responder to the FC, SVI had to increase ≥ 10%.

**RESULTS.** A cohort of the first 26 patients is presented. Nor PPV neither SVV at baseline could predict fluid responsiveness (AUC of 0.57 and 0.72, respectively). $EEO_6$ poorly predicted fluid responsiveness (AUC of CI 0.55; p = 0.66; AUC of SVI 0.52; p = 0.85). Both VtC and $EEO_8$ reliably predicted the responsiveness to FC (see Table 1). The AUCs of PPV and SVV after VtC application (p = 0.33) and the AUCs of SVI and CI after $EEO_8$ application (p = 0.42) were comparable.

**CONCLUSIONS.** The changes in PPV or SVV induced by VtC are more pronounced than in baseline helping to predict the FC responsiveness in anesthetized surgical patient under mechanical ventilation at 6 ml/kg. The EEO performed at 8 ml/kg and not at 6 ml/kg predicted well the fluid responsiveness. The reliability of both $EEO_8$ and VtC was comparable (see Table1).

**REFERENCE(S)**

1) Guinot PG et al. BJA 2014; 112: 1050-4.
2) Biais M et al. Anesth Analg. 2017 Dec;125(6):1889-1895.
3) Myatra et al. Crit Care Med. 2017;45:415.

**Table 1 (abstract 0594).** EEO and VtC prediction of fluid responsiveness

| Test | Variable | Cut-off (%) | AUC | 95% CI | Sensitivity (%) | Specificity (%) |
|---|---|---|---|---|---|---|
| EEO6 | CI | - | 0.55 | - | - | - |
| EEO6 | SVI | - | 0.52 | - | - | - |
| VtC | PPV | 19 | 0.85 | 0.70 - 1.01 | 90.9 | 80.0 |
| VtC | SVV | 5 | 0.73 | 0.53 - 0.96 | 63.6 | 80.0 |
| EEO8 | CI | 2 | 0.84 | 0.65 - 1.01 | 81.6 | 93.3 |
| EEO8 | SVI | 2 | 0.91 | 0.51 - 0.97 | 81.8 | 93.3 |

### 0595

#### Incidence, predictive factors and prognostic value of post-cardiac surgery vasoplegia: an observational prospective study

P. Gaudard[1,2], M. Ratie[1], H. David[1,2], T. Jouitteau[1], A.M. Dupuy[3], T. Mura[4], P. Colson[1]
[1]CHU Montpellier, Arnaud de Villeneuve, Anaesthesiology and Critical Care Medicine, Montpellier, France; [2]University of Montpellier, INSERM U1046, CNRS UMR 9214, Montpellier, France; [3]CHU Montpellier, Lapeyronie, Department of Biochemistry, Montpellier, France; [4]CHU Montpellier, La Colombière, Department of Medical Information, Montpellier, France
**Correspondence:** P. Gaudard
*Intensive Care Medicine Experimental* 2018, **6(Suppl 2):**0595

**INTRODUCTION.** Cardiac surgery under Cardio-Pulmonary Bypass (CPB) may lead to an inflammatory response with various clinical presentations including post-cardiac surgery vasoplegia (PCSV). Preoperative copeptin level, a biomarker of vasopressin secretion, may predict PCSV (1).

**OBJECTIVES.** Our objective was to describe the PCSV syndrome after CPB and assess its consequences on microcirculation, organ function and outcome.

**METHODS.** This single-centre prospective observational study, approved by an ethics committee, included patients with written informed consent and scheduled for cardiac surgery under CPB from January to June 2017, excluding age above 80 years, chronic renal failure, sepsis and postoperative cardiogenic or haemorrhagic shock. During the first 24 hours, PCSV was defined by the need of norepinephrine infusion to maintain a mean arterial pressure above 60mmHg without low cardiac output. The primary endpoint was the duration of postoperative hospital stay, secondary endpoints were ICU admission hemodynamic and microcirculatory data, severity scores and ICU length of stay. Quantitative data were expressed as median [interquartile range], qualitative data as number of patients (%) and patients with PCSV were compared to the other patients using non-parametric or chi-square tests. A p< 0.05 was considered significant. A logistic regression was used for multivariate analysis of preoperative predictive factors (odds ratio [confidence interval 95%]).

**RESULTS.** Among 178 patients included (age 67yo [60-73], Euroscore 4.8% [3.2-8.2]), 81 (46%) experienced a PCSV. The hospital stay of PCSV patients was significantly longer than for controls (respectively 9 [8-12] vs 8 [7-9] days, p< 0.0001). Preoperative copeptin level higher than 14pmol/l, combined cardiac surgery, and preoperative anaemia were associated with PCSV on multivariate analysis (adjusted OR: 3.1 [1.4-6.7], 3.0 [1.5-6.1], 2.4 [1.1-5.1] respectively). Macrocirculatory parameters were comparable but oxygenation recovery slope was lower in PCSV group (3.2 [2.6-3.9] vs 3.8 [2.8-4.4] %/s, p=0.048). PCSV patients had more organ failure (SOFA score 6 [5-7] vs 3 [2-4], p< 0.0001), higher maximal lactate level (2.0 [1.6-2.9], p=0.017) within the first 24 hours and longer ICU stay (26 [22-54] vs 23 [21-25] hours, p< 0.0001).

**CONCLUSIONS.** Post-cardiac surgery vasoplegia is a frequent syndrome following CPB. Preoperative anaemia or high copeptin may identify patients at higher risk of vasoplegia. PCSV is a relevant complication associated with more organ dysfunction and an extended stay in ICU and in hospital. Preventive strategies and early microcirculatory optimization should be studied in high risk population for cardiac surgery.

**REFERENCE(S)**

(1) Colson P, et al. Post cardiac surgery vasoplegia is associated with high preoperative copeptine plasma concentration. Crit Care 2011; 15:R255.

**GRANT ACKNOWLEDGMENT**

Thermo-Fisher Laboratory provided copeptine assay kits.

### 0596

#### Infections after cardiac surgery

P. Serra-Paya[1], J.C. Lopez-Delgado[1,2], R.E. Avila-Espinoza[1], G. Muñoz-del Río[1], D. Berbel-Franco[1], S.M. Luna-Solis[1], E. Farrero-Bayarri[1], H. Torrado-Santos[1], F. Esteve-Urbano[1,2]

[1]Hospital Universitari de Bellvitge, Intensive Care Medicine, Barcelona, Spain; [2]Institut d´Investigació Biomèdica de Bellvitge, L'Hospitalet de Llobregat, Spain

**Correspondence:** J.C. Lopez-Delgado

*Intensive Care Medicine Experimental* 2018, **6(Suppl 2):**0596

**INTRODUCTION.** Infection after cardiac surgery (CS) may be a very severe complication that worsens the prognosis of those patients.

**OBJECTIVES.** The aim of our study was to describe and evaluate infections after CS.

**METHODS.** Prospective, observational study at our institution from 2008 to 2012 at our institution. Baseline characteristics on admission, outcomes, infections and microbiological profile were analyzed. Statistical analysis was performed (SPSS 20.0).

**RESULTS.** 3031 patients were included. 63.9%(1937) were male, age was 64.7±11.6 years, APACHE II:12.5±4.9, with an infection rate of 6%(192). Pneumonia was the most frequent infection(34%) and those who suffer from mediastinitis(80%) developed bacteremia more frequently(P< 0.001). Urinay infection and catheter infection rate were 2.98 episodes/1000days of de urinary catheter and 3.35/1000days of catheter. Gram Negative Bacilli were predominant in all infections (75%). Factors associated with the development of infection after CS were longer CPB time (OR:1.004;95% IC:1.001-1.008;P=0.047), a higher drainage loss during first 12h (OR:1.002;95% IC:1.001-1.004;P< 0.001), lower levels of albumin at 24h after CS (OR:0.902;95% IC:0.860-0.946;P< 0.001) and lower PaO2/FiO2 24h after CS (OR:0.993;95% IC:0.990-0.995;P< 0.001). Patients who were infected required longer time on vasoactive drugs (OR:1.008;95% IC:1.006-1.010;P< 0.001), a longer ICU length of stay (OR:1.043;95% IC:1.021-1.065;P< 0.001) and a higher in-hospital mortality (33.3%) (OR:3.348;95% IC:1.700-6.595;P< 0.001). Those patients showed a worst long-term survival (Follow up 4.8±2.3years): 82.9%vs.52.9% (HR:3.583;95% IC:2.850-6.742;P=0.003).

**CONCLUSIONS.** Infections after CS are a severe complication with high impact in mortality, even in the long-term scenario. Inflammatory and nutritional factors may be associated with their occurrence. GNB and pneumonia were the most frequent isolated germ and type of infection respectively.

### 0597

#### Evaluation of nutritional status in cardiac surgery: the influence in outcomes

J.C. Lopez-Delgado[1,2], G. Muñoz-del Río[1], P. Serra-Paya[1], D. Berbel-Franco[1], S. Gonzalez-del Hoyo[1], N. Latorre-Feliu[1], M. Martinez-Medan[1], C. Sanz-Mellado[1], E. Farrero-Bayarri[1]

[1]Hospital Universitari de Bellvitge, Intensive Care Medicine, Barcelona, Spain; [2]Institut d´Investigació Biomèdica de Bellvitge, L'Hospitalet de Llobregat, Spain

**Correspondence:** J.C. Lopez-Delgado

*Intensive Care Medicine Experimental* 2018, **6(Suppl 2):**0597

**INTRODUCTION.** The presence of a preoperative malnutrition or a high nutritional risk influences outcomes after cardiac surgery (CS).

**OBJECTIVES.** The aim of our study was to evaluate the influence of preoperative nutritional status in patients who underwent CS.

**METHODS.** Prospective, observational study at our institution from 2012 to 2015 at our institution. Baseline characteristics, intraoperative variables and outcomes were analyzed. Nutritional status was evaluated by means of an screening tool for CONtrolling NUTritional status (CONUT). Statistical analysis was performed (SPSS 20.0).

**RESULTS.** 952 patients were included; age 65.5±10.5 years; 70.4%(670) were male; BMI:27.6±4.1Kg•m$^{-2}$; EuroSCORE:6.2±2.8; APACHE II:12.8±4.8; SAPS III:41.7±10.6. 42.6% were valvular and 43.7% were bypass surgeries. In-hospital mortality was 7.2%(69) and long-term mortality 21.1%(195). Based on CONUT screening, 45.9%(437) of the patients showed a preoperative normal status, whereas 43.2%(412) has low malnutrition and 10.9%(103) moderate-severe malnutrition. Univariate analysis showed more post-CS complications and mortality in those subgroups with worst nutritional status. Multivariate analysis showed higher perioperative transfusional needs (OR:1.419;95% IC:1.118-1.801;P=0.004), longer time in vasoactive drug support (OR:1.005;95% IC:1.001-1.008;P=0.011) and longer ICU length of stay (OR:1.004;95% IC:1.004-1.007;P=0.001) at the subgroup of patients with moderate-severe malnutrition compared with normal status subgroup. Long-term mortality showed a worst survival in those with worst nutritional (normal:84.8%, low:76.4%, moderate-severe:63.4%; P(LogRank)< 0.001). A higher absolute CONUT value was associated with worst survival (HR:1.153;95% IC:1.072-1.239;P< 0.001) whereas to be included in the normal CONUT subgroup was associated with better survival (HR:0.552;95% IC:0.345-0.884;P=0.013) .

**CONCLUSIONS.** A worst preoperative nutritional state evaluated by means of the screening tool CONUT was associated with higher morbidities and worst long-term survival. Physicians and surgeons should be aware about nutritional status of patients in order to avoid preoperative malnutrition and plan a nutritional approach previous CS.

### 0598

#### Hematological complications in immediate postoperative after cytoreductive surgery and hyperthermic intraperitoneal chemotherapy

A. Acha[1], M.-C. Pintado[1,2], B. Gracia[1], A. Pardo[1], R. De Pablo[3]
[1]Hospital Universitario Principe de Asturias, Intensive Care Medicine Unit, Alcala de Henares, Spain; [2]Universidad de Alcalá, Alcala de Henares, Spain; [3]Hospital Universitario Ramón y Cajal, Intensive Care Medicine Unit, Madrid, Spain
**Correspondence:** A. Acha
*Intensive Care Medicine Experimental* 2018, **6(Suppl 2):**0598

**INTRODUCTION.** Cytoreductive surgery (CRS) and hyperthermic intraperitoneal chemotherapy (HIPEC) are treatments for peritoneal carcinomatosis in select patients with cancer (gastric, colorectal, appendiceal, ovarian cancer and primary peritoneal malignancies). This treatment is associated whit a morbidity rate around 12% to 52%, and include coagulopathy, chemotherapy induced myelosuppression and need for blood transfusion.

**OBJECTIVES.** To determine the hematological complications during intensive care unit (ICU) stay after CRS and HIPEC.

**METHODS.** Prospective observational study during 5 years. All patients admitted to ICU postoperatively after CRS and HIPEC therapy. Data recorded were age, sex, type of cancer, severity at ICU admission (APACHE II and SOFA scores), daily blood count and coagulation times, bleeding complications, ICU mortality and lenth of stay. We defined leucopenia as < 4000/μL, thrombocytopenia as < 150.000/μL, anemia as hemoglobin < 10 gr/dL and prolongation of coagulation times as INR >1.3 and/or APTT >37seg, and hypofribrinogenemia as < 200 mgr/dl.

Qualitative variables are described as number and percentages, quantitative variables with normal distribution as mean ± S.D., quantitative variables with non-normal distribution as median (interquartile range).

**RESULTS.** We included 73 patients. 40 (55%) were males. Mean age was 60.3±9.9 years. 63 (86%) had a digestive cancer (52% colorectal, 44% gastric cancer). Mean APACHE II and SOFA scores at ICU admission were 9.6±4.2 and 2 (1-3), respectively. All patients had some type of hematological complications: 9 (12%) had leucopenia, 40 (55%) thrombocytopenia, 34 (47%) prolongation of coagulation times, 47 (64%) anemia and 10 (14%) hypofribrinogenemia. Only 11 (15%) patients had bleeding complications (45% bleeding from surgical drainages, 45% haematuria and 10% digestive hemorrhage), all were treated in a conservative manner. 14 (19%) patients needed blood transfusion and 1 (< 1%) platelet transfusion. Mean ICU stay was 5 (4-5) days. ICU mortality was 1.36%, but not related to bleeding. CONCLUSIONS: Although hematological complications in patients in early postoperative of CRS and HIPEC are frequent, the rate of severe complications is low and not related with mortality.

**REFERENCE(S).** Kapoor S et al. Critical care management of HIPEC patients. World J Crit Care Med 2017 May 4; 6(2): 116-123. / Wallet F et al. No impact on long-term survival of prolonged ICU stay and re-admission for patients undergoing cytoreductive surgery with HIPEC. Eur J Surg Oncol. 2016 Jun;42(6):855-60. / Falcón Araña L at al. Alterations in hemostasis during cytoreductive surgery and hyperthermic intraperitoneal chemotherapy in patients with peritoneal carcinomatosis. Cir Esp. 2015 Oct;93(8):496-501.

**GRANT ACKNOWLEDGMENT**
This work was not supported.

### 0599

#### Integration of psychological well-being service into critical care unit: stepped care model

P. Polgarova[1], L. Enoch[1], J. Preller[1], C. Lambert[2], Z. Martin[3], A. Newton[3], D. Christmas[3]
[1]Cambridge University Hospitals, JVF ICU, Cambridge, United Kingdom; [2]Cambridge University Hospitals, JVF ICU & NCCU, Cambridge, United Kingdom; [3]Cambridge University Hospitals, Psychology Department, Cambridge, United Kingdom
**Correspondence:** P. Polgarova
*Intensive Care Medicine Experimental* 2018, **6(Suppl 2):**0599

**INTRODUCTION.** The Intensive Care Unit (ICU) is often a disorientating and frightening environment for patients and relatives. Delirium, anxiety, depression and Post-Traumatic Stress Disorder (PTSD) (Ryu et al. 2016; Dimitry et al. 2010) often adversely affect short and long-term outcomes. The is an increasing focus on the quality of survival. The 2009 NICE guidance, 'Rehabilitation after Critical Illness (CG83)', stresses the need to address all areas of functioning: physical, psychological and cognitive in early rehabilitation (NICE 2009). We identified a significant care gap relating to psychological outcomes.

**OBJECTIVES.** To implement and assess a Stepped Care Psychological Well-being Model in order to improve psychological outcomes in ICU survivors and relatives while affecting culture change and improving staff resilience.

**METHODS.** A quality improvement initiative to integrate best psychological practice into routine multidisciplinary ICU care. New resources from February 2017 include 0.8 whole time equivalent (WTE) Senior Psychological Wellbeing Practitioner (PWP) and a 0.2 (WTE) Clinical Psychologist. Early focus was on service needs assessment, project development and validation of psychological tools while increasing targeted psychological interventions. During the first 12 months 235 patients were assessed by the PWP. 225 patients completed the Intensive care psychological assessment tool (IPAT) of which 108 (48%) scored 7 or more in keeping with psychological distress (Wade et al. 2014). 501 intervention sessions have been offered to patients and 53 to relatives as well as 32 1:1 sessions with staff members.

**RESULTS.**

Further subjective feedback from patient focus groups suggests that this service substantially contributes to improved psychological outcomes along the patient journey. The effect seems to be larger than actual interventions offered which suggest some background culture change in our ICU.

**CONCLUSIONS.** Initial results suggest that imbedding psychological services in the daily practice of our ICU MDT allows early identification and improved psychological outcomes. Within the MDT we experience integration of new knowledge and a culture of emotional resilience and well-being.

**REFERENCE(S)**

NICE, 2009. *Rehabilitation after Critical Illness (CG83)*, London. Available at: https://www.nice.org.uk/Guidance/CG83.

Ryu, J.H. et al., 2016. Post-Traumatic Stress Disorder in Survivors of Critical Illness. In C. R. Martin, V. R. Preedy, & V. B. Patel, eds. *Comprehensive Guide to Post-Traumatic Stress Disorders*. Cham: Springer International Publishing, pp. 263-280.

Wade, D.M. et al., 2014. Detecting acute distress and risk of future psychological morbidity in critically ill patients: Validation of the intensive care psychological assessment tool. *Critical Care*, 18(5), p.519.

**GRANT ACKNOWLEDGMENT**
Addenbrookes Charitable Trust.

**Table 1 (abstract 0599).** RESULTS. implementation of a Stepped Psychological Wellbeing Care Model

| | Pre-implementation (Dec 16 - Feb 17; n = 40) | Post-implementation (May 17 - July 17; n = 71) | Change |
|---|---|---|---|
| Anxiety | 35.9% | 26.2% | -9.7% |
| Depression | 26.8% | 18.0% | -8.8% |
| PTSD symptoms | 37.5% | 34.8% | -6.5% |

### 0600

#### Transfusion results in priming and adhesion of neutrophils in human endotoxemia as well as in a cohort of critically ill patients

M.E. van Hezel[1,2], M. Boshuizen[1,2], L. van Manen[1,2], A.P.J. Vlaar[1], M.W.T. Tanck[3], A.T.J. Tool[2], B. Beuger[2], N.P. Juffermans[1], R. van Bruggen[2]
[1]Academic Medical Center Amsterdam, Intensive Care, Amsterdam, Netherlands; [2]Sanquin Research, Blood Cell Research, Amsterdam, Netherlands; [3]Academic Medical Center, Deparment of Clinical Epidemiology, Biostatistics and Bioinformatics (KEBB), Amsterdam, Netherlands
**Correspondence:** M.E. van Hezel
*Intensive Care Medicine Experimental* 2018, **6(Suppl 2):**0600

**INTRODUCTION.** Red blood cell (RBC) transfusion is associated with adverse outcome, in particular in the critically ill. The mechanisms are unknown, but may involve an effect on immune cells.

**OBJECTIVES.** To investigate the effect of RBC transfusion in recipients suffering from inflammation on priming and adhesion capacity of neutrophils.

**METHODS.** Healthy male volunteers were injected with Escherichia coli lipopolysaccharide(2ng/kg), and after two hours transfused with an autologous RBC unit (n=6). Blood samples were taken at baseline, 2 hours after LPS but prior to transfusion and 4 hours after transfusion. In addition, as part of an observational study on the effects of RBC transfusion, 22 critically ill patients with sepsis and 25 non-septic patients in need of 1 RBC unit were consecutively included. Blood samples were taken prior to and 24hr after transfusion, centrifuged and plasma was stored at -80˚C until further use. Healthy volunteers served as a control group. Human neutrophils were isolated from other healthy volunteers and incubated with the plasma from both study cohorts and the control group. Neutrophil priming was assessed by measuring hydrogen peroxide production (in relative fluorescence units (RFU)), after formyl-Met-Leu-Phe stimulation and by Amplex Red conversion to resorufin. Adhesion was assessed by the fluorescence of absorbent calcein-labeled neutrophils after plasma incubation. Adhesion was determined as a percentage of the total input of calcein labeled cells (100%).

**RESULTS.** In the endotoxemia model, LPS alone did not increase the capacity of plasma to induce ex vivo neutrophil priming or adhesion. Following RBC transfusion, ex vivo neutrophil priming increased compared to pre-transfusion levels, albeit not reaching statistical significance[580 RFU (IQR 380-1388] vs 475 RFU[IQR 287-614], p=0.053). RBC transfusion also resulted in increased capacity of plasma to induce neutrophil adhesion when compared to healthy volunteers receiving saline (13,73% [IQR 11,65-24,86] vs 9,26% [IQR 9,26-12,0] in controls, p< 0.05). In the critically ill, prior to transfusion, the capacity of plasma to induce ex vivo neutrophil priming was already increased when compared to healthy controls (616 RFU (IQR 480-894) vs 429 RFU (IQR 337-624), P< 0.05) and was not further augmented following transfusion. Neutrophil adhesion was not increased prior to transfusion when compared to plasma of healthy controls. Following transfusion, the ability of plasma to induce neutrophil adhesion versus baseline increased in the septic patients when compared to the non-septic patients (+ 3,2% (IQR 0,9- 6,3) vs 0,8% (-1,3- 3,6), p< 0.05).

**CONCLUSIONS.** RBC transfusion results in an increased ability to induce ex vivo neutrophil adhesion in a model of human endotoxemia as well as in a cohort of critically ill patients with sepsis. Therefore, neutrophil adhesion may be a mechanism of inducing transfusion-associated effects in individuals suffering from systemic inflammation.

### 0601

#### Platelet trajectory phenotypes in the ICU and how they predict 60-day mortality

Y. Wei[1], G. Clermont[2], R. Parker[3], M. Neal[4], J. Yabes[5]
[1]University of Pittsburgh, Biostatistics, Pittsburgh, United States; [2]University of Pittsburgh, Critical Care Medicine, Industrial Engineering, and Mathematics, Pittsburgh, United States; [3]University of Pittsburgh, Chem/Petroleum Engineering, Bioengineering Department, Pittsburgh, United States; [4]University of Pittsburgh, Surgery, and Critical Care Medicine, Pittsburgh, United States; [5]University of Pittsburgh, Medicine, Biostatistics, and Clinical and Translational Science, Pittsburgh, United States
**Correspondence:** Y. Wei
*Intensive Care Medicine Experimental* 2018, **6(Suppl 2):**0601

**INTRODUCTION.** Platelets are involved in hemostasis and are a core component of the acute inflammatory response. Phenotypes of platelet trajectories may exist and may be associated with patient outcome.

**OBJECTIVES.** To identify data-driven phenotypes of long-term and short-term platelet trajectories in acutely ill patients, examine their association with clinical features and 60-day mortality, and identify predictors of phenotype membership.

**METHODS.** From a retrospective cohort of 164,788 ICU admissions, we focused on patients who stayed in hospital at least 7 days or at least 3 days after ICU admission. We used a two-step clustering method combining linear mixed modeling and hierarchical clustering to identify groups of trajectories. To study the association between platelet trajectory groups and 60-day mortality, logistic regression was used with age, sex, race, body-mass index, burden of chronic illness, baseline organ function, severity of disease and trauma status as additional covariates. To evaluate group membership prediction at baseline, multinomial logistic regression was used with platelet counts on days 1 and 2 of ICU admission, as well as baseline covariates. Model performance is calculated in a validation set of 33% of the study population.

**RESULTS.** 55,442 admissions and 108,128 admissions met 7-day and 3-day study criteria. We identified 7 groups of trajectories in the 7-day cohort and 4 groups in the 3-day cohort (Figure). Group membership was, independently associated with 60-day mortality (p< 0.001) in both cohorts. For 7-day trajectories, compared to group 1, 60-day mortality was lower in group 2 (odds ratio (OR)=0.66 95% confidence interval: (0.61-0.71)) and group 5 (OR=0.64 (0.53-0.77)), and higher in group 3 (OR=1.11 (1.03-1.21)), group 4 (OR=1.26 (1.13-1.41)) and group 7 (OR=1.68 (1.46-1.94)). AUROC was 0.74 (95% CI: 0.73, 0.75). For 3-day trajectories, compared to group 1', 60-day mortality was higher in group 3' (OR=1.08 (1.01-1.16)) and group 4' (OR=1.44 (1.35-1.54)). AUROC was 0.74 (95% CI: 0.73, 0.75). When second day platelet count was added in a model to predict group membership from baseline variables, accuracy increased from 47% to 57% for 7-day trajectories and from 68% to 79% for 3-day trajectories.

**CONCLUSIONS.** 7-day and 3-day platelet trajectory phenotypes were identified and were associated with 60-day mortality. First and second day platelet counts predicted trajectory phenotype with reasonable accuracy. In the 7-day cohort, a rebound shape was predictive of lower 60-day mortality. In both cohorts, consistently high platelet trajectory was predictive of higher mortality. Further analysis will focus on studying whether platelet trajectories are associated with morbidities such as late bleeding, embolism, and thrombosis.

**GRANT ACKNOWLEDGMENT**
Supported by National Institutes of Health (R21-HL133891)

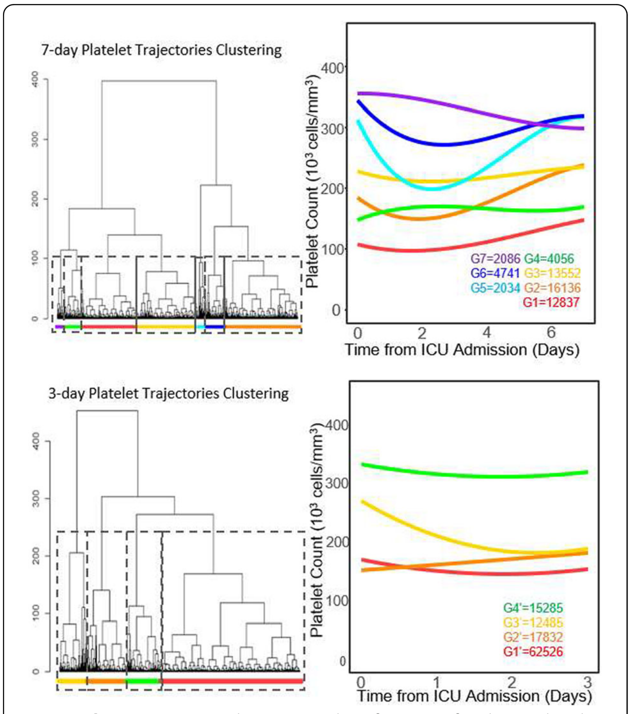

**Fig. 1 (abstract 0601).** Phenotype identification of 7-day and 3-day platelet trajectories

### 0602

#### Association of perioperative allogeneic red blood cell transfusion with cancer recurrence in patients undergoing abdominal surgery for cancer: a systematic review, meta-analysis, and meta-regression

O. Hunsicker[1], M. Körber[1], P. Milack[1], A. Krannich[2], D.N. Lobo[3], I. Braicu[4], O. Meyer[5], L. Kaufner[1], C. Spies[1], J. Sehouli[4], A. Pruß[5], A. Feldheiser[1]
[1]Charité - Universitätsmedizin Berlin, Department of Anesthesiology and Operative Intensive Care Medicine (CCM, CVK), Berlin, Germany; [2]Charité - Universitätsmedizin Berlin and Max Delbrueck Center for Molecular Medicine in the Helmholtz Association, Experimental and Clinical Research Center, Berlin, Germany; [3]Nottingham University Hospitals NHS Trust and University of Nottingham, Queen's Medical Centre, Gastrointestinal Surgery, Nottingham Digestive Diseases Centre, National Institute for Health Research (NIHR) Nottingham Biomedical Research Centre, Nottingham, United Kingdom; [4]Charité - Universitätsmedizin Berlin, Campus Virchow-Klinikum, Department of Gynecology, European Competence Center for Ovarian Cancer, Berlin, Germany; [5]Charité - Universitätsmedizin Berlin, Institute of Transfusion Medicine, Berlin, Germany
**Correspondence:** O. Hunsicker
*Intensive Care Medicine Experimental* 2018, **6(Suppl 2):**0602

**INTRODUCTION.** About 30% of patients undergoing abdominal surgery for cancer still require allogeneic red blood cell transfusion (RBCT) during hospitalization, but it is still debated whether perioperative RBCT increases risk for recurrent disease after curative surgery. Cohort studies comparing patients receiving and not receiving RBCT showed a strong bias regarding most baseline, clinicopathological and surgical characteristics indicating the need to adjust for the most important confounding variables to remove their effects on outcome.

**OBJECTIVES.** To assess the relation of perioperative RBCT with cancer recurrence in patients undergoing abdominal surgery for cancer, considering the most important confounding variables of cancer recurrence after surgery.

**METHODS.** This systematic review was registered prospectively with the PROSPERO database (CRD42017052026). MEDLINE, EMBASE, and the Cochrane Library were searched systematically from January 2000 to February 2017. Eligible studies were cohort studies investigating the association of perioperative RBCT with cancer recurrence in patients undergoing abdominal surgery for cancer. The main outcome measure was disease-free survival. Summary hazard ratios (HR) and 95% confidence intervals (95% CI) were calculated with generic inverse variance method using a random effects model. Meta-regression was used to assess the impact of adjusting confounding variables on outcome.

**RESULTS.** A total of 25 studies were eligible for analysis. To remove the effect of confounding variables on outcome, all studies used multivariable Cox proportional hazards regression (MV-CPH), while 7 studies additionally performed propensity score methods (PSM). In PSM analyses, RBCT did not increase the risk for cancer recurrence (HR 1.06 [95% CI, 0.89-1.25], P=0.51). In contrast, in MV-CPH analyses, RBCT was related to a higher risk of cancer recurrence (HR 1.42 [95% CI, 1.27-1.58], P< 0.001). However, the number of confounding variables considered in MV-CPH analyses was lower than in PSM analyses, and meta-regression indicated that a lower degree of adjustment for confounding variables was independently related with increased HRs (Figure 1+2). Selecting MV-CPH analyses with similar high degree of adjustment for confounding variables comparable to PSM analyses revealed that RBCT was no longer related to an increased risk of cancer recurrence (HR 1.07 [95% CI, 0.90-1.27], P=0.43).

**CONCLUSIONS.** Many studies did not sufficiently adjust their analysis for most important confounding variables when assessing the relation of RBCT with cancer recurrence. Considering high quality studies that sufficiently adjusted their analyses, perioperative RBCT did not affect cancer recurrence after abdominal surgery for cancer. Concerns about oncological outcomes should not be an issue in the decision-making process for perioperative RBCT.

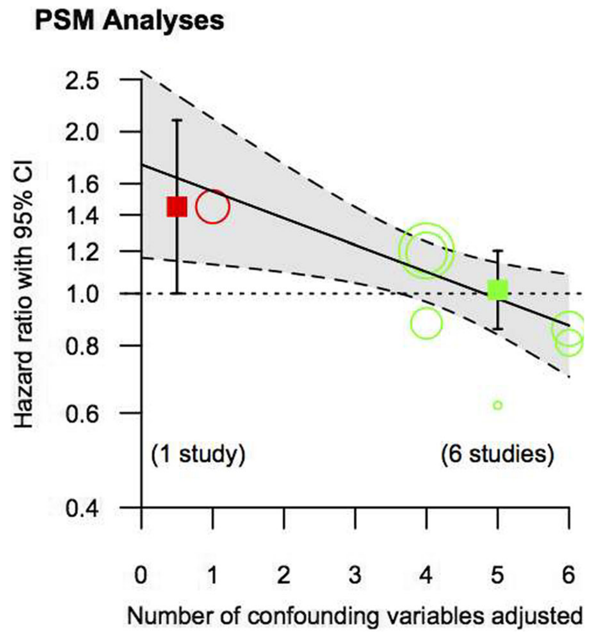

**Fig. 1 (abstract 0602).** Meta-regression plot: association of confounding adjustment with HR of RBCT for cancer recurrence

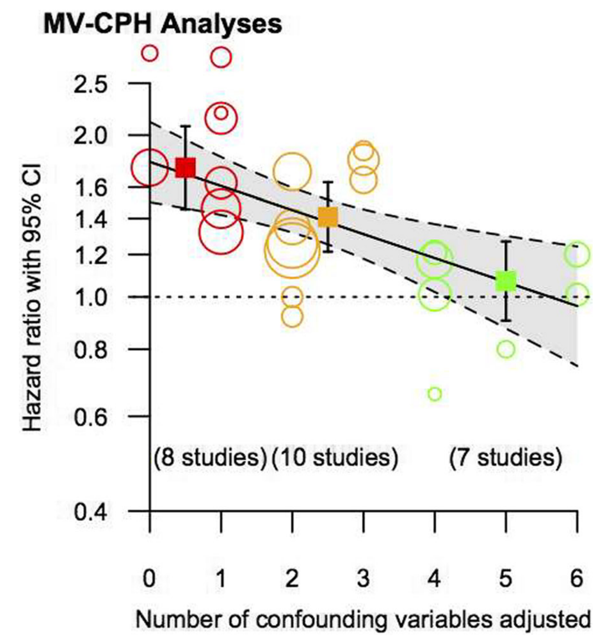

**Fig. 2 (abstract 0602).** Meta-regression plot: association of confounding adjustment with HR of RBCT for cancer recurrence

## "Food & thoughts"

### 0603

#### Long-term mechanical ventilation patients in intensive care units, what to do

M. Muñoz Garach, O. Moreno Romero, R. Ramierez Puerta
Hospital Universitario San Cecilio Granada, Granada, Spain
**Correspondence:** M. Muñoz Garach
*Intensive Care Medicine Experimental* 2018, **6(Suppl 2):**0603

**INTRODUCTION.** Patients with chronic mechanical ventilation-dependency should not stay all the time in our intensive care units. Ideally, if their personal situation allows, is to try a follow-up at home, for this it is necessary the support of a Home Mechanical Ventilation Unit where the patients can rehabilitate and adapt their family and personal needs for beeing home-ventilated.
**OBJECTIVE.** To describe and statistically analyse the population of a subgroup of patients with long-term mechanical ventilation in our ICU who are referred to our Home Mechanical Ventilation Unit (HMVU) and will be home-ventilated and followed up afterwards.
**METHODS.** Retrospectively four-year study (from 1/1/2014 to 12/31/2017) from our own data base. Variables analysed: sex, age, cause of admission to the ICU, cause of prolonged mechanical ventilation, origin hospital, dependence hours on mechanical ventilation (MV) at admission and discharge, stay days in ICU, stay in HMVU, evolution of the process and destination to discharge.
**RESULTS.** 12 patients, gender: men 8 (66.7%). Average age: 59.9 $\pm$7.4. Causes ICU admission: postoperative cardiac / vascular surgery / general 6 (50%) and reagudized chronic respiratory failure 6 (50%). Cause of prolonged mechanical ventilation (requires more than three tests of spontaneous breathing and / or the process lengthens during more than 7 days): 4 tetraparesis (33.3%), 5 multifactorial (weakness, COPD, obesity...) (41.7%), 2 bilateral diaphragmatic paralysis (16.7%), severe kyphoscoliosis 1 (8.4%). Origin hospital: San Cecilio 5 (41.7%), Virgen de las Nieves 6 (50%), Santa Ana-Motril 1 (8.4%). Hours dependence of MV on admission at HMVU: night-twelve hours: 5 (41.7%) and daily-twenty-four hours: 7 (58.3%). Median days stay in ICU: 93.5 (range 69-270). Median days of stay in HMVU: 176.2 (range 64-425). Evolution of the process: decanulation 6 (50%), nocturnal VM dependence 3 (50%) and daily dependence twenty-four hours 3 (25%), was impossible the weaning of VM patients with bilateral diaphragmatic paralysis and severe kyphoscoliosis. Destination at discharge: home 10 (83.3%) and exitus 2 (16.7%) due to cardiac arrest not recovered.
The beds occupancy in the ICU of the 12 patients was 1282 days, with an average stay of 106.8 ±58.2 days.
**CONCLUSIONS.** There is a short subgroup of patients who, frequently after aggressive surgeries, remain chronically dependent on MV, resulting in prolonged ICU stay. The HMVU created in the beginning for neuromuscular patients also allows the transfer of other kind of patients.

### 0604

#### End-of-life practices (EOLP) in worldwide intensive care units (ICUs): the Ethicus II study

A. Avidan[1], C. Sprung[1], B. Ricou[2], M. Weiss[3], A. Michalsen[4], C. Feldman[5], M. Anstey[6], M. Baras[7], H.H. Bulow[8], C. Hartog[9], Ethicus II investigators
[1]Hadassah Hebrew University Medical Center, Jerusalem, Israel; [2]Hôpital Cantonal Universitaire de Geneve, Geneva, Switzerland; [3]University Hospital Ulm, Ulm, Germany; [4]Tettnang Hospital, Tettnang, Germany; [5]University of the Witwatersrand, Johannesburg, South Africa; [6]Sir Charles Gairdner Hospital, Perth, Australia; [7]Hebrew University, Hadassah School Public Health, Jerusalem, Israel; [8]Holbæk Hospital, Holbæk, Denmark; [9]University Hospital Jena, Jena, Germany
**Correspondence:** A. Avidan
*Intensive Care Medicine Experimental* 2018, **6(Suppl 2):**0604

**INTRODUCTION.** Substantial variability in EOLP occurs around the world [1]. Differences in EOLP were previously reported in Europe in the Ethicus I study [2].
**OBJECTIVES.** Determine worldwide ICU EOLP.
**METHODS.** ICUs worldwide were invited to participate through their country societies. Consecutive admitted ICU patients who died or had treatments limitations during a 6 month period from 1.9.2015 to 30.9.16 were prospectively studied. Regions included North, Central and Southern Europe (NE, CE, SE), North and Latin America (NA, LA), Asia (As), Australia and New Zealand (Au) and Africa (Af). Previous EOLP definitions were used [2]. Mental competency was defined as having the capacity to make end of life decisions.
**RESULTS.** 199 ICUs in 36 countries participated enrolling 12,857 patients. Figure 1 shows differences in EOLP by region and Figure 2 patient competency and patient and family discussions by region.
**CONCLUSIONS.** Worldwide differences included more CPR in Af, LA & SE and less CPR in NE, Au & NA. There was more withdrawing (WD) in NE & Au and less WD in LA & Af. Interestingly, all regions had more withholding (WH) than WD except NE. Shortening of the dying process (SDP) was uncommon. More patients were competent in Au & NA and less were competent in Af, SE & LA. More discussions with patients occurred in NE, CE, NA & Au and more with families in NE, NA, As & Au.

#### REFERENCE(S)

1. Mark, NM et al. ICM 2015;41:1572-1585
2. Sprung CL et al. JAMA 2003; 290:790-797

#### GRANT ACKNOWLEDGMENT

None

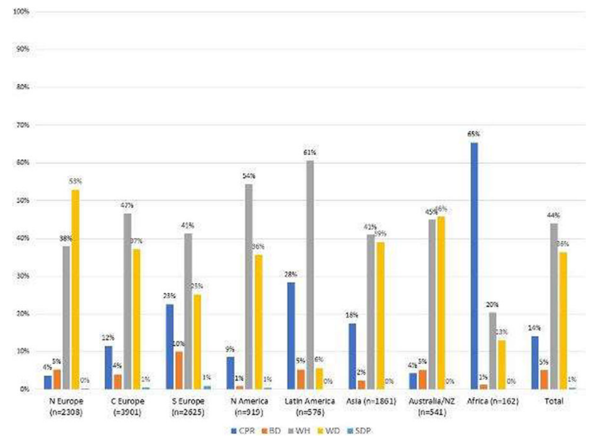

**Fig. 1 (abstract 0604).** See text for description.

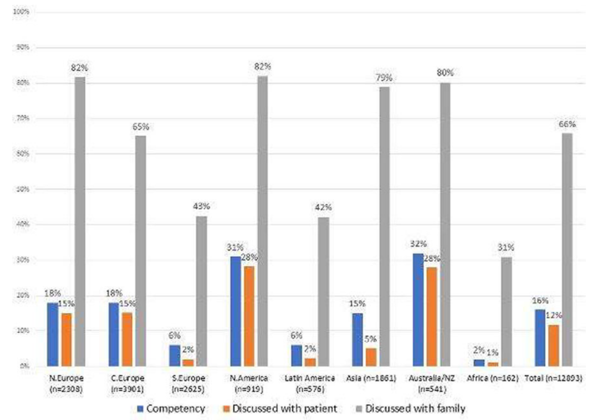

**Fig. 2 (abstract 0604).** See text for description.

### 0605

#### Don´t write off the liver! 20 year retrospective analysis of patients with decompensated alcoholic liver disease (ALD) admitted to a single district general hospital (DGH) intensive care unit (ICU)

N. Raeside, A. Myers, T. Samuels, R. Kumar
East Surrey Hosptial, Intensive Care Unit, Redhill, United Kingdom
**Correspondence:** N. Raeside
*Intensive Care Medicine Experimental* 2018, **6(Suppl 2):**0605

**INTRODUCTION.** ALD is associated with high mortality and admissions to ICU for patients with decompensated ALD are increasing (1). Whilst there is some research into prognostic markers for these patients, most published studies have originated from tertiary liver referral units (2).

**OBJECTIVES.** To assess mortality of patients admitted to a DGH ICU with decompensated ALD and whether dysfunction of other organ systems on admission affects mortality in decompensated ALD or acute liver failure (ALF) secondary to alcohol misuse.

**METHODS.** Data for 120 patients with decompensated ALD or ALF secondary to alcohol misuse were collected for the period January 1998 to March 2018. Additional information included organ dysfunction on admission and/or reason for admission, such as acute renal failure (ARF), chest involvement (e.g. chest infection or pleural effusion), encephalopathy, upper gastrointestinal bleed (UGIB) and requirement for vasopressor or inotropic support. The primary outcome was hospital mortality, with secondary outcomes including ICU mortality and survival to hospital discharge.

**RESULTS.** Of the total (n=120), 69% were male, with a mean age of 51.2 years (SD 10.9 years). Overall ICU and hospital mortality were 51% and 69% respectively. Patients admitted with additional renal impairment had the highest ICU mortality (78%), followed by patients admitted requiring inotropes (70%) and those with chest pathology (66%). Those admitted with encephalopathy had an ICU mortality of 48%, but the lowest overall hospital mortality (66%). Patients admitted following an UGIB had the lowest ICU mortality (35%). However, these patients had the highest in-hospital mortality of out of those who survived to ICU discharge (38%).

**CONCLUSIONS.** Patients admitted to ICU with decompensated ALD with additional ARF have the worst outcomes. Those admitted following an UGIB have the lowest ICU mortality, but similar hospital mortality. These results are similar to a 3 year retrospective study at a tertiary liver referral ICU where patients admitted with an UGIB or encephalopathy had the lowest ICU mortality (< 50%), compared to those admitted with severe renal impairment (75%) or requiring renal replacement therapy (81%) (2).

Patients must be reviewed on a case by case basis and, while, for example, the knowledge that ARF confers an increased risk of ICU mortality in patients with decompensated ALD, this alone should not preclude admission. It does, however, assist managing the expectations of the patient, relatives and healthcare team, should the patient be admitted to ICU.

##### REFERENCE(S)

1. Welch C, Harrison D, Short A, et al. The increasing burden of alcoholic liver disease on United Kingdom critical care units: secondary analysis of a high quality clinical database. J Health Res Policy2008; 13(Suppl. 2): 40-44
2. Mackle IJ, Swann DG, Cook B. One year outcome of intensive care patients with decompensated alcoholic liver disease. BJA 2006; 97: 496-498

### 0606

#### Acute-on-chronic liver failure score in acute decompensated cirrhosis: measurement and prognostic value

N. Foudhaili, S. Khadher, A. Khaled, K. Ben Ismail, M. Salem
Charles Nicolle Hospital, Digestive Intensive Care Unit, Tunis, Tunisia
**Correspondence:** N. Foudhaili
*Intensive Care Medicine Experimental* 2018, **6(Suppl 2):**0606

**INTRODUCTION.** Prognostic value of the North American Consortium for the Study of End-Stage Liver Disease definition of Acute-on-Chronic Liver Failure (NACSELD-ACLF score) in predicting 30-day survival was confirmed in a large recent study.

**OBJECTIVES.** The aim of this work was to analyze of NACSELD-ACLF score performance to predict intrahospital mortality in patients with decompensated cirrhosis.

**METHODS.** It was an 8-month retrospective study from March 2017 to October 2017. We enrolled patients with decompensated cirrhosis. Organ failures assessment were obtained upon admission according to NACSELD definition. Patients were analyzed in terms of prediction of 30-day using either a Chi-square test or Fisher's Exact test. Backwards elimination multi-variable logistic regression analyzing 30-day mortality was performed on all patients.

**RESULTS.** Fifty-seven patients were included with a sex-ratio at 0.9 and a mean age of 67±9 years (23-76). The main comorbidities were diabetes mellitus (35%) and hypertension (29%). Hepatitis C was the most common etiology of cirrhosis (44%). Child-Pugh score was mainly C (62%) and 12±2. Main MELD score was 14±8. Bacterial infection was the main cause of decompensating (52%). Patients with and without unfavorable outcome (death) had similar demographics characteristics. 30-day mortality rate was 16%. NACSELD-ACLF was ≥2 in 15% of patients. Patients who met criteria for NACSELD-ACLF had 20% 30-day survival vs 85% in patients without NACSELD-ACLF ($p < 0.001$) with a RR of 22 (95% IC :5.47 - 91.15).

**CONCLUSIONS.** NACSELD-ACLF might predict 30-day mortality in cirrhotics in decompensation with a good sensitivity and specificity. However, it concerns only 15% of patients admitted for acute decompensation of cirrhosis which makes it a very restrictive score.

### 0607

#### Prognostic value of albumin-to-bilirubin score in decompensated cirrhosis

N. Foudhaili, S. Khedher, A. Khaled, C. Abdennebi, M. Salem
Charles Nicolle Hospital, Digestive Intensive Care Unit, Tunis, Tunisia
**Correspondence:** N. Foudhaili
*Intensive Care Medicine Experimental* 2018, **6(Suppl 2):**0607

**INTRODUCTION.** Prognostic value of albumin-to-bilirubin (ALBI) score during liver diseases was reported in several studies.

**OBJECTIVES.** The aim of this work was to compare the performance of Child- Pugh score, MILD Score and ALBI score to predict intrahospital mortality in patients with decompensated cirrhosis.

**METHODS.** It was a 6-Month retrospective study from January 2017 to June 2017. We enrolled patients with decompensated cirrhosis. Biological markers were obtained upon admission. They were analyzed in terms of prediction of intrahospital mortality using the Receiver Operating Characteristic (ROC) curves analysis.

**RESULTS.** Ninety-seven patients were included with a sex-ratio at 0.95 and a mean age of 58±10 years (19-77). The main comorbidities were diabetes mellitus (42%) and hypertension (30%). Hepatitis C was the most common etiology of cirrhosis (45%). Child-Pugh score was mainly C (68%) and 10±2. Main MELD score was 13±7. Bacterial infection was the main cause of decompensating (55%). Intrahospital mortality was 13%. Demographics characteristics were similar in patients with and without unfavorable outcome (death). ALBI score was significantly higher in dead patients (-1.399±0.381 vs -0.842±0.456, p< 0.001). Comparing to Child-Pugh score and MELD score, ROC analysis of ALBI showed the highest AUC with a value of 0.810 (95% IC: 0.695-0.930, p< 0.001). The cut-off point was found to be -1.265 with a sensitivity of 85% and a specificity of 80%.

**CONCLUSIONS.** ALBI score might predict intra-hospital mortality in cirrhotics in decompensation with a good sensitivity and specificity.

### 0608

#### Metabolic differences during normothermic regional preservation in donation after circulatory death type II and type III

N. Palomo-López, S. Martin-Sastre, L. Martín-Villén, Z. Ruiz de Azúa-López, S. Escalona-Rodriguez, D. Solis-Clavijo, S. Caballero-Gálvez, J.M. Carballo-Caro, J.J. Egea-Guerrero
Hospital Universitario Virgen del Rocío, Seville, Spain
**Correspondence:** N. Palomo-López
*Intensive Care Medicine Experimental* 2018, **6(Suppl 2):**0608

**OBJECTIVES.** To analyze metabolic differences during normothermic regional perfusion (NRP) between the dissimilar types of donation after circulatory death (DCD): type II and type III.

**METHODS.** Observational, prospective and cohort study. We included patients from DCD type II and type III maintained with normothermic regional perfusion ( NRP) in 2017. Six consecutive blood gases were collected with determination of pH and lactic acid. Creatinine levels monitored the renal transplants at 24 hours and 3 months after transplant. Descriptive statiscal analysis was performed, presenting the qualitative variables as frequencies and percentages, and quantitative as mean ( +/- SD) or median ( interquartile range-ICR) depending its distribution. We use chi square for bivariate analysis of qualitative variables

**RESULTS.** We included 18 patients; 83,3%(15) were males ,the median age was 51 years ( 46-60). From the total of donors 38,9% ( 7 ) were DCD type II and 61,1 % ( 11) DCD type III. Of the blood gases we highlight the significant relation between the type of DCD and pH (pH 3: p=0,02, pH4 : p= 0,02 y pH 5 : p= 0,03.). There are also a significant relation with the lactic acid (Lac 1: p= 0,01, Lac 3: p=0,01, Lac 4: p= 0,016 y lac 5: 0,029). We found a significant relation between the creatinine values 24 hours after transplantation and DCD (type II 7.04 (6.2-7, 9) vs. type III 6.2 (5.3-6.5) vs p = 0.05), and 3 months after the transplant (type II 1.9 (1.9-3.8) Vs. type III 1.1 (1-1.3) p = 0.07).

**CONCLUSIONS.** There are more metabolic disorders in our series in DCD type II organ donation compared with DCD type III.
The recovery of the renal function of organs from DCD type II is slower than those from type III, however; tendency is towards normality.

**REFERENCE(S)**

1. Miñambres E, Suberviola B, Dominguez-Gil B, Rodrigo E, Ruiz-San Millan JC, Improving the outcomes of organs obtained from controlled donation after circulatory death donors using abdominal Normothermic regional perfusion.. Am J Transplant. 2017,17:2165-2172
2. Perez-Villares JM, Rubio JJ, Del Río F, Miñambres E. Validation of a new proposal to avoid donor resuscitation in controlled donation after circulatory death with normothermic regional perfusion. Resuscitation. 2017 ;117:46-49

**GRANT ACKNOWLEDGMENT**

**Table 1 (abstract 0608).** Comparative metabolic results from DCD type II and III. IR: interquartile range. Lac: lactic acid.

| VARIABLES | DCD type II ( 38,9%) | DCD type III ( 61,1%) | p |
|---|---|---|---|
| pH3 Median (IR) | 7,2 ( 6,98-7,3) | 7,49 ( 7,42-7,52) | 0,02 |
| pH4 Median (IR) | 7,34 ( 7,28-7,4) | 7,49 ( 7,47-7,5) | 0,02 |
| pH5 Median (IR) | 7,27 ( 7,23-7,32) | 7,49 ( 7,38-7,48) | 0,03 |
| Lac1 Median (IR) | 16 ( 15-26) | 3,8 ( 0,7-7,6) | 0,01 |
| Lac3 Median (IR) | 21 ( 17,8-23,5) | 4,2 ( 3,5-5) | 0,01 |
| Lac4 Median (IR) | 21 ( 15-24) | 3,1 ( 2,8-4,9) | 0,016 |
| Lac5 Median (IR) | 18 ( 11-22) | 3,1( 2,8-3,6) | 0,029 |

**Table 2 (abstract 0608).** Evolution of renal transplants according to the type of DCD: type II or type III. IR: interquartile range

| VARIABLES | DCD type II | DCD type III | p |
|---|---|---|---|
| Creatinine at discharge (ICU) Median(IR) | 7( 6,2 -7,9) | 6,2 ( 5,3-6,5) | 0,05 |
| Creatinine after 3 months Median (IR) | 1,9 ( 1,9-3,8) | 1,1 ( 1-1,3) | 0,07 |

### 0609

#### Actual and potential organ donation procedures after successfully resuscitated cardiac arrest in a French general ICU

Y. Arrahmane, L. Lainé, E. De Montmollin, M. Benaïs, L. Ferreira, N. Memain, D. Da Silva, V. Ioos, G. Preda
Centre Hospitalier Saint Denis, Service de Réanimation, Paris, France
**Correspondence:** Y. Arrahmane
*Intensive Care Medicine Experimental* 2018, **6(Suppl 2):**0609

**INTRODUCTION.** Considerable progress has been made in the management and prognostication of patients after successful resuscitation of a cardiac arrest. There are scarce data concerning the possibility of organ donation in patients after cardiac resuscitation. The French organ donation regulation authority (Agence de Biomédecine) reported in 2016 an increase of 2.5% of organ harvesting but also highlighted the number of unmet needs with 22.617 patients on waiting list[1]. Multimodal strategies to increase the number organ donation procedures are urgently needed.

**OBJECTIVES.** Describe the characteristics of patients admitted after cardiac resuscitation in the ICU of a community hospital with organ harvesting authorization over a period of 9 years.

**METHODS.** We performed a retrospective study from our hospital information system between January 2009 and December 2017, in a 19-bed ICU. All patients admitted after successful resuscitation of cardiac arrest were included. Patients with in-ICU cardiac arrest, admitted with the diagnosis of brain death and those transferred to a specialized center were excluded. Brain death is defined by French legislation and anoxic encephalopathy was defined as Cerebral Performance Category of (2-3-4) at ICU discharge. Data are presented as counts (%) and means (SD).
**RESULTS.** During the study period, 265 patients were included, of which 165 (62%) and 100 (38%) occurred after out of hospital and in-hospital cardiac arrests respectively. The mean age was 61 years (SD 16), and 159 (60%) were male. The mean SAPSII was 78 (SD 20). No flow time was less than 5 min in 136 (51%), and mean low flow was 20 min (SD 16). Thirty eight (14%) patients progressed to brain death in a mean of 3 days (SD 2). Among those patients with brain death 9 (24%) donated organ. Anoxic encephalopathy was diagnosed in 89 (34%), and among those 21 (24%) were discharged alive from the ICU. Their CPC score was of 2 in 12 (5%), 3 in 6 (2%) and 4 in 3 (1%) respectively. The other 68 (76%) patients with anoxic encephalopathy died after decision to withdrawal life support after a mean of 16 (SD 11) days post admission. Neurological prognostication could not be performed in 100 (38%) patients due to multiorgan failure.
**CONCLUSIONS.** After cardiac arrest, 14% of the patients sustained brain death and one fourth of those donated organs. Anoxic encephalopathy was diagnosed in 34% of the study population. Withdrawal of life support was decided in three fourth of those patients who may be eligible for a Maastricht III type organ donation procedure. This could potentially result in a significant increase in the organ harvesting activity of our center.

1.Activité
de greffe d'organes en France en 2016 ; Communiqué de presse 20 mars 2017. Agence de Biomédecine.

**GRANT ACKNOWLEDGMENT**

Not grant acknowledgment to declare.

### 0610

#### Uncontrolled donation after circulatory death (uDCD) in Donostia University Hospital, 2015-2018

M. De Almeida e Silva[1], L. Atutxa[1], R. Sebastián[1], I. García[1], N. González[1], B. Pagalday[2], H. Olasagasti[1]
[1]Hospital Universitario Donostia, Donostia - San Sebastian, Spain; [2]Emergencias Osakidetza, Donostia - San Sebastian, Spain
**Correspondence:** M. De Almeida e Silva
*Intensive Care Medicine Experimental* 2018, **6(Suppl 2):**0610

**INTRODUCTION.** Donostia University Hospital is a third-level Hospital without solid organ transplantation programme, neither cardiac surgery unit nor extracorporeal membrane oxygenation (ECMO) assistance programme. Our referral transplantation center is located 100km away. These particular features are an obstacle when it comes to organ donation. In 2015, we started two programmes in donation after circulatory death (DCD): both controlled (cDCD) and uncontrolled (uDCD) donation after circulatory death. During these 3 years, we've reached an average of 88 donors pmp.
**OBJECTIVES.** Analysis of the characteristics of uDCD donors in a three years period.
**METHODS.** Prospective observational study. Center: Donostia University Hospital, reference center for a population of 700.000 inhabitants. Period of study: from March 2015 to April 2018. Sample: All patients deceased after a witnessed cardiac arrest (CA), unsuccessfully resuscitated according to advanced cardio-pulmonary resuscitation (aCPR) protocols based on international guidelines, are transferred to the Hospital for uDCD. Study variables: sex, age, cause of CA, time of CA - beginning of aCPR, time of CA - time of Intensive Care Unit (ICU) Arrival, time of CA - time of ECMO cannulation, time of organs preservation, removed and transplanted grafts. Organ preservation technic: normothermic regional perfusion (nRP) with ECMO.
**RESULTS.** Total number of alerts for uDCD: 49, of which 71% were effective donors. 29% weren't effective donors due to: 8% family refusal for organ donation, 12.2% ECMO cannulation errors, 2% positive serology, 4.1% cancer and 2% increased times. CA took place during evening/night time in 73.5% of the cases, and 55% during weekends.
Thirty-six male and 13 female. Cause of CA: 42.9% STEMI, 28.6% sudden cardiac death and 28.5% other causes. Mean age: 48.5 years old [29-65]. CA - aCPR mean time: 6.8´ [0´-19´], CA - ICU mean time 61.7´[0´-123´], CA- nRP mean time 109.7´[69´-144´], nRP mean time 164.9´ [114-254´]. Total number of removed kidneys: 66. Transplanted kidneys 69.7% (46). Removed corneas 80. 26.5% donors of bone and tendons, with an average of 18 samples per donor [14-24].
**CONCLUSIONS.**
- Starting an uDCD program led to an increase in the number of organ donors.
- In our region, this represents up to 17.5 donors per million of population. 17.9% of the total donors.
- Our data shows that uDCD can be performed in a small city.
- If proper training is provided, all ICU staff might be able to complete the uDCD process successfully (including cannulation and ECMO).

**REFERENCE(S)**

E. Miñambres, B. Suberviola, C. Guerra, N. Lavid, M. Lassalle, A. González-Castro, M.A. Ballesteros. Experience of a Maastrich type II non heart beating donor program in a small city: Preliminary results. Medicina Intensiva (English Edition), Volume 39, Issue 7, October 2015, Pages 433-441.

**GRANT ACKNOWLEDGMENT**

None.

### 0611

#### Admission conditioned to organ donation in a hospital without on site neurosurgical facilities

N. Arriero Fernández[1], J.E. Romo Gonzales[1], A. Estrella Alonso[1], S. Saboya Sánchez[2], P. Gallardo Culebradas[1], J.A. Silva Obregón[1], Z. Eguileor Marín[1], C. Marián Crespo[1], E. Yáñez Parareda[1], P. Rojo Villar[1], E. Quirós Oyaguez[1]
[1]Guadalajara University Hospital, ICU, Guadalajara, Spain; [2]Hospital Universitario Puerta de Hierro, ICU, Mahadahonda, Spain
**Correspondence:** N. Arriero Fernández
*Intensive Care Medicine Experimental* 2018, **6(Suppl 2):**0611

**INTRODUCTION.** Brain death (BD) donors still remain the major source for organ procurement. Intensive care unit (ICU) admission conditioned to organ donation (COD) is a common practice to increase organ donation in hospitals without on site neurosurgical facilities.
**OBJECTIVE.** The aim of the study was to analyze patients with admission COD: time to BD, the conversion rate (CR) to effective donation (CRED) of those patients with admission COD (real CR) and the CRED of potential donors (successful CR).
**METHODS.** A descriptive, observational, retrospective study was undertaken from November/2013 to October/2017 in the ICU of the Guadalajara University Hospital (GUH).
We analyze patients with severe brain injury secondary to acute stroke (AS) or traumatic brain injury (TBI). All of them were refused as candidates for neurosurgical intervention, so their admission was COD.
Age, sex, diagnosis, development and time to BD, and procured organs were recorded. Qualitative variables are expressed as a percentage; quantitative variables are expressed as a median and interquartile range (IR).
**RESULTS.** A total of 36 patients with admission COD were included. Median age 70 years (IR, 61-80.8); 58.3% were male.
Diagnosis: AS 31 (86.1%): Ischemic 3 (8.3%) or hemorrhagic 28 (77.8%). TBI 5 (13.9%).

After ICU admission 7 patients (19.4%) were dismissed as donors; 5 rejected due to National Transplant Organization criteria (NTO) and the 2 remaining were transferred to a neurosurgery ICU after clinical reassessment.

Evolution to BD:

1) No BD: 10 patients (34.5%); 4 died in ICU after withdrawal of life support and 4 in hospital wards after ICU discharge. One patient was transferred to another center (family request) and the last one was discharged from hospital. Median ICU stay was 111.5 hours (IR, 79.9-119.7)

2) BD: 19 patients (65.5%), 15 of them (78.9%) in the first 72 hours from admission. Median time to BD was 28.7 hours (IR, 39.6-93.9).

Donation: real CR of 52.8% (19/36) and successful CR of 61.3% (19/31). Successful multi-organ procurement was achieved in 14 donors (73.68%). The most frequently procured grafts were kidney (31 grafts from 16 donors) and liver (16 donors). One double lung transplant donor and 3 heart donors.

**CONCLUSIONS.** In our series, 79% of patients suffered from BD in the first 72 hours. A successful CR of 61.3% is lower than published by the NTO (78%), most likely due to the characteristics of patients refused for neurosurgical intervention. Transplant coordinator 24 hours on site would help to improve real CR, decreasing economic and human costs.

### 0612

#### Ethical evaluation before living kidney donation: a pilot study

K. Hadaya[1], M. Lamuela Naulin[2], M. Escher Imhof[3], T. Berney[4], C. Iselin[5], P.-Y. Martin[6], B. Ricou[3]

[1]Geneva University Hospital, Nephrology and Transplantation, Geneva, Switzerland; [2]Geneva University Hospitals, Gynecology and Obstetrics, Geneva, Switzerland; [3]Geneva University Hospitals, Anesthesiology, Pharmacology and Intensive Care, Geneva, Switzerland; [4]Geneva University Hospitals, Transplantation, Geneva, Switzerland; [5]Geneva University Hospitals, Urology, Geneva, Switzerland; [6]Geneva University Hospitals, Nephrology, Geneva, Switzerland

**Correspondence:** K. Hadaya

*Intensive Care Medicine Experimental* 2018, **6(Suppl 2):**0612

**INTRODUCTION.** Faced with ever-increasing demand and far exceeding supply from deceased donors, many transplantation teams have focused their programs on encouraging living kidney donation. An independent living donor advocate team to adequately inform, ensure voluntariness of the decision, and judge about the acceptability of the risk-benefit for living donors is strongly encouraged but not always possible in the organization of transplant centers. At Geneva University Hospitals, such an independent team was partially built up with a dedicated nephrologist and surgeon.

**OBJECTIVES.** In April 2016, Geneva University Hospitals' medical direction commissioned the Clinical Ethics Committee (CEC) to evaluate all potential living kidney donors, as was already the case for living liver donors.

**METHODS.** From July 2016 until February 2018, on monthly dates pre-booked for the year, 29 potential living donors (women 65%) have been evaluated by 2 members of the CEC at the end of their assessment and in the absence of medical, surgical and psychiatric contra-indications for donation. One woman could not follow this path because of organizational difficulties. Conclusions of the CEC concerning the eligibility or not of the potential living donor were sent within 24 hours to the transplant and donor's teams.

**RESULTS.** 27 potential living donors had been deemed eligible for donation. The 2 unfavourable opinions delivered, in connection with a lack of information on the surgical plan and regarding financial support, respectively, lead to a second evaluation between 1 and 2.5 months, that was positive this time. Following the 29 ethical evaluations, 24 renal transplantations were performed, 3 are in the process of organization and 2 were cancelled after the recipient underwent a deceased donor transplantation. No delay in the organization of transplantations was attributed to the ethical evaluation. The transplant and donor's teams were reassured in their care and were able to improve their information to potential living donors, thanks to the expertise and feedback of the CEC.

**CONCLUSIONS.** To best of our knowledge, we report the first systematic ethical evaluation of kidney living donors in a transplant center. As complex ethical issues relate to all kind of living donation, with kidney donors making up 95% of all worldwide, we suggest to provide each potential living donor with an independent advocacy team including an ethical evaluation.

### 0613

#### Analysis of potential organ donors (explantation of abdominal organs) in ≥ 75-year-old brain dead patients in an intensive care unit (medical ICU) in a secondary-level university hospital

J.L. Martinez Melgar[1], E. Moreno Lopez[2], J. Bravo Doviso[3], J.I. Cenoz Osinaga[3], E. Sanmartin Mantiñan[3], A. Ortega Montes[3], T. Sanchez de Dios[3], A. Pais Almozara[3], P. Posada Gonzalez[3]

[1]Hospital Montecelo, Transplant Coordination Team, Pontevedra, Spain; [2]Hospital Arquitecto Marcide, Ferrol, Spain; [3]Hospital Montecelo, Intensive Care Unit, Pontevedra, Spain

**Correspondence:** E. Moreno Lopez

*Intensive Care Medicine Experimental* 2018, **6(Suppl 2):**0613

**OBJECTIVES.** To analyze the possibility of explantation of abdominal organs (AO), liver and kidneys, in our Unit ( Medical ICU- secondary-level Hospital) in ≥ 75-year-old brain dead patients who became real donors (RD) in a 10-year-period.

**METHODS.** restrospective and descriptive study carried out between 2008 and 2017 of patients admitted to our ICU who were declared brain dead (BD). The following parameters were analyzed in ≥75-year-old real donors: gender, age group, causes of brain dead, medical history, use of inotropic agents needed, liver and/or kidney alterations, organ explantation ( liver (Hex) and kidney (Rex)) and unexplanted organs (non viable, liver (Hne) and kidney (Rne)) and reason for this situation. Statistical analysis: quantitative variables are expressed using mean± standard deviation, and qualitative variables are expressed using %. The difference between qualitative variables are expressed using chi-square test and quantitative using an ANOVA analysis.

**RESULTS.** 111 patients were declared brain dead, being real donors 60 patients and 22 being ≥ 75-years-old. Gender: 11 women ( 11 men), age group: 14 patients ( between 75-79 years old), between 80-84: 5 patients, >85 years old: 3 patients. Causes of BD: acute cerebrovascular disease (ACVD) 20 patients ( hemorraghe 12, ischemic 6, subarachnoid hemorraghe 2) and anoxic encephalopathy: 2. Medical history: Hypertension:14, Diabetes: 9, alcoholism:6. Use of inotropic agents: 12. Kidney alterations: 7 and liver alterations: 4. 52 abdominal organs were explanted: 16 Hex ( group 75-79 years old: 10, group 80-84 years old: 3 and >85 years old: 3), and 36 Rex ( group 75-79 years old: 26, group 80-84 years old: 4, >85 years old: 4). 14 abdominal organs could not be explanted : 6 Hne ( group 75-79 : 4, group 80-84: 2) and 8 Rne ( group 75-79 :2 , group 80-84: 4 and group>85: 2). Reason for non explantation: Hne ( fatty liver:4 and anatomical alterations: 2) and Rne ( vascular abnormalities: 5 and macroscopic alterations: 3)

**CONCLUSIONS.** Patients in group ≥ 75 years old represent 19.8% of brain dead and 36.6% of real donors. In this group, 78.8% of the abdominal organs are viable for explantation (72.7% liver and 81.8% kidney), being dismissed for explantation 21.2% of the abdominal organs (27.3% liver and 18.2 kidneys). The most common reason for non explantation: fatty liver in Hne and vascular abnormalities in Rne.

### 0614

#### Iconographic (brain CT-scan) and clinical characteristics of patients with acute cerebrovascular disease (ACVD), hemorrhagic or ischemic, who were declared brain dead in a secondary-level university hospital in a six-year period

J.L. Martinez Melgar[1], E. Moreno Lopez[2], J.I. Cenoz Osinaga[3], J. Bravo Doviso[3], A. Ortega Montes[3], E. Sanmartin Mantiñan[3], T. Sanchez de Dios[3], A. Pais Almozara[3], P. Posada Gonzalez[3]
[1]Hosptal Montecelo, Transplant Coordination Team, Pontevedra, Spain; [2]Hospital Arquitecto Marcide, Ferrol, Spain; [3]Hospital Montecelo, Intensive Care Unit, Pontevedra, Spain
**Correspondence:** E. Moreno Lopez
*Intensive Care Medicine Experimental* 2018, **6(Suppl 2):**0614

**OBJECTIVES.** To analyze the iconographic (brain CT-scan (CTb)) and clinical characteristics on admission in a medical Intensive Care Unit (ICU) in a secondary-level University Hospital of patients with ACVD hemorrhagic ( HIC) or ischemic (ISC) who were declared brain dead in a six-year period.

**METHODS.** Prospective and descriptive study , from 2012 to 2017 of patients admitted to our ICU with the diagnose of ACVD (HIC) or (ISC) who were declared brain dead (BD). Patients with traumatic brain injury (TBI), subarachnoid hemorrhage (SAH), epidural or subdural hematoma or anoxic encephalopaty were excluded. The following parameters were analyzed: age, gender, risk factors ( hemorrhagic or embolic) for ACVD, characteristics of CT-scan according to the radiological report on ICU admission of patients with ACVD ( location, type of herniation (h), size (>60 ml), extension of the hemorrhage into the ventricles (ev), midline shift (MLS)(< or > 1cm), as well as neurological status on admission: Glasgow Coma Scale (GCS) and pupillary condition. Statistical analysis: quantitative variables are expressed using mean± standard deviation, and qualitative variables are expressed using %. The difference between qualitative variables are expressed using chi-square test and quantitative using an ANOVA analysis.

**RESULTS.** 83 patients were declared BD ( real donors 43, potential donors 40). 18 patients were excluded (TBI: 4, SAH:2, Epidural/subdural hematoma: 3 and anoxic encephalopaty: 9). 65 patients with ACVD were included : 51 HIC and 14 ISC. Gender: 38 male (27 female), age (66±17 years old, risk factors for hemorrhage or embolism ( antiaggregants:7, oral anticoagulants:15, thrombocytopenia:3). CTb iconographic characteristics in HIC: location ( cerebral hemisphere: 21, cerebellar:4, basal ganglia: 17, brain stem: 9), type of herniation ( subfalcine:6, uncal:9), size(>60 ml): 22, extension into ventricles:34, MLS 35 ( ≤ 1 cm:15, >1 cm : 20). In patients with ischemic ACVD: location ( cerebral hemisphere:9, cerebellar: 2, basal ganglia: 1 and brain stem: 2), type of herniation ( subfalcine:6, uncal: 2), extension into ventricles: 3 and MLS: 11 (≤1 cm:5, >1 cm: 6). Neurological status on ICU admission: GCS score 6-8: 17 ( HIC:18, ISC: 9), score 3-5: 38 (HIC:33, ISC: 5) and pupillary condition: anisocoria 39 (HIC: 30, ISC:9), mydriasis 16 (HIC:14, ISC: 2), isocoric 10 (HIC:7, ISC:3)

**CONCLUSIONS.** From 2012 to 2017 of 83 patients who were declared brain dead. ACVD was the reason in 78.3% ( HIC: 61.5% and ISC: 16.9%). Iconographic characteristics in CT-scan of HIC: cerebral hemisphere:41%, size (>60 ml):43%, subfalcine herniation:47%, ev: 66% and MLS: 68.6%. In ISC patients: cerebral hemisphere: 64%, subfalcine herniation: 42.8% and MLS: 78.5%. GCS on admission: score 3-5 (HIC:64.7%) and 6-8 (ISC: 64.2%) and the most common pupillar condition was anisocoria (HIC: 58.8% and ISC: 64.2%).

### 0615

#### Brain dead donors (BDD) following cerebral gunshot wounds: renal allografts" validity

B. Estébanez Montiel[1], M. Vaca Gallardo[2], C. Gómez Aragón[3], F. Merino Gómez[3], D. Gurria Sanz[3], A. Agrifoglio Rotaeche[4], E. Flores Cabeza[4], E. Herrero de Lucas[4], L. Cachafeiro Fuciños[4], P. Millán Estañ[4], M.S. Sánchez Sánchez[4], C. Jiménez Martín[2], A. García de Lorenzo y Mateos[4]
[1]Hospital Universitario La Paz, Intensive Medicine Department, Transplant Coordination, Madrid, Spain; [2]Hospital Universitario La Paz, Nephrology Department, Madrid, Spain; [3]Hospital Universitario La Paz, Transplant Coordination, Madrid, Spain; [4]Hospital Universitario La Paz, Intensive Medicine Department, Madrid, Spain
**Correspondence:** B. Estébanez Montiel
*Intensive Care Medicine Experimental* 2018, **6(Suppl 2):**0615

**INTRODUCTION.** Severe brain injury, after cerebral gunshot wounds, may release large amounts of tissue factor to the circulation, may develop disseminated intravascular coagulation (DIC) and it can lead to renal dysfunction.

**OBJECTIVES.** Analyse the validity for transplantation of kidneys harvested from BDD following cerebral gunshot wounds and the outcome of the transplanted renal grafts.

**METHODS.** Retrospective descriptive study of renal allografts harvested from BDD following cerebral gunshot wounds, at a single center, between January 1, 2007 and May 31, 2017. Donors´ data (patients´ characteristics, blood tests, associated injuries) and recipients (graft survival, rejection) were collected.

**RESULTS.** We analysed data from six BDD following cerebral gunshot wounds (age 40.3 ± 9.6 years) without any cardiovascular risk factors, except one patient who had hypertension. All patients presented brain death in the first 24 hours of hospital admission. None of them had any associated injuries. One patient suffered a cardiopulmonary arrest (during 20 minutes) prior to ICU admission. One of the patients had elevated creatinine levels in the blood tests when he was admitted to the hospital. But, during the ICU length of stay, up to 50% of the patients presented acute renal failure. Fifty percent of the patients presented compatible or suggestive criteria for DIC. Four kidney grafts were not valid for transplantation because: acute renal failure (two kidney grafts), poor graft perfusion (one case) and complex vascular anomalies (one case). Eight kidney grafts (66.67%) were transplanted. Seven kidney grafts were transplanted in our center. One of them was explanted, in the first 24 hours after transplantation, due to cortical necrosis (pathology exam: glomerular and vascular thrombotic microangiopathy lesions). Fig 1. CT scan. The donor´s contralateral kidney was not implanted due to poor perfusion. The pathology exam of this kidney showed multiple fibrin trombi in glomerular capillaries and small-vessels (Figure 2). The remaining 6 grafts presented adequate function one year after the transplantation, except in one case which suffered underlying disease recurrence.

**CONCLUSIONS.** An accurate evaluation of kidney grafts from BDD following cerebral gunshot wounds is required, because they are patients at risk for DIC and acute renal failure. Pre-transplant biopsy and reduction of ischemia time may be indicated. Almost all transplanted renal allografts from BDD, following cerebral gunshot wounds, had good results in our study. Further studies with larger simple sizes are needed.

(1) Sibulesky L, et al. Kidney Transplantation from Donors with Severe DIC. ISRN Transplantation 2013. http://dx.doi.org/10.5402/2013/646310.
(2) Pastural M, et al. Successful kidneys transplantation using organs from a donor with DIC and impaired renal function: case report and review of the literature. Nephrol Dial Transplant 2011,16:412-15

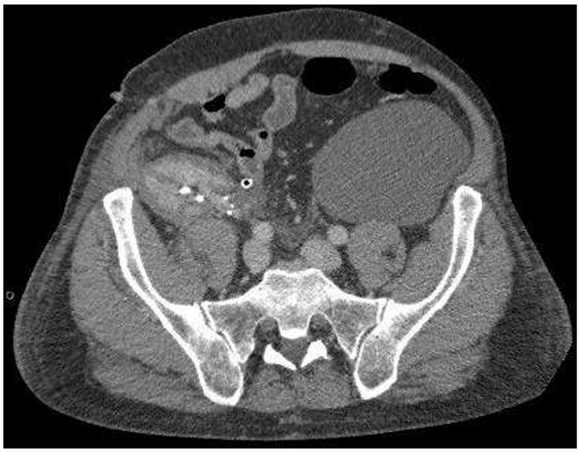

**Fig 1 (abstract 0615).** CT: Transplanted kidney with cortical necrosis

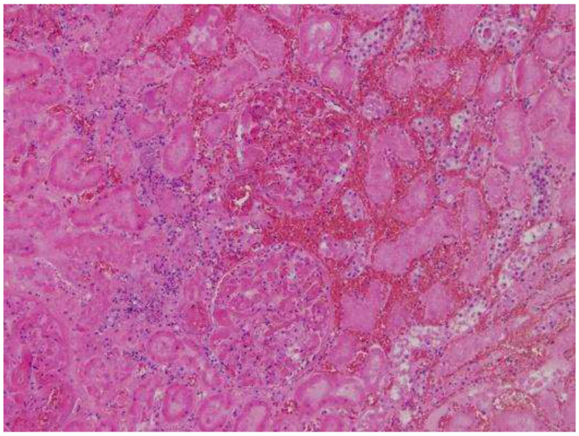

**Fig 2 (abstract 0615).** Pathology exam: glomerular and vascular thrombotic microangiopathy lesions

### 0616

#### Depression and anxiety symptoms among living donor liver transplantation patients' relatives

H.K. Atalan
Acibadem Mehmet Ali Aydinlar University School of Medicine, Anesthesiiology and Reanimation, Istanbul, Turkey
*Intensive Care Medicine Experimental* 2018, **6(Suppl 2):**0616

**INTRODUCTION.** Hospitals in general, and specific wards of the hospital such as intensive care units (ICU) are the places where emotions of anxiety and/or depression can be expressed by patients' relatives (1). It's been shown that half of the patients' relatives have difficulties in understanding doctors' information about the condition of the patient (2).

**OBJECTIVES.** The aim of this study was to identify the symptoms of anxiety and/or depression in relatives of living donor liver transplant patients admitted in SICU and correlation of such symptoms with the relatives age, gender, degree of relationship, educational level and the patients' status.

**METHODS.** 147 adult relatives of 147 different patients were enrolled in this study between january 2017 and january 2018. The questionnary, including the hospital anxiety and depression scale and the demographic data of the patients' relative, was given to the patients' relatives, in early postoperative period, after visiting the patient and being informed by the doctor.

**RESULTS.** We observed anxiety and/or depression symptoms (ADS) in 108 of 147 patients' relatives. The anxiety symptoms were significantly higher in patients' parents and spouses than children (p=0.004). Depression symptoms were significantly higher in relatives of patients who had prior ICU stay (p< 0.001). There wasn't any significant association between ADS and relatives age, gender, educational level and the patients' medical status.

**CONCLUSIONS.** Relatives of LDLT patients face symptoms of anxiety and/or depression. ICU staff need to recognize these symptoms and emotional/psychological professional support must be prioritized.

##### REFERENCE(S)

1- Carlet J et al. J Crit Care. 2010; 2:359.e9-359.e12.
2- Davidson JE et al. Crit Care Med. 2007; 2:605-622.

### 0617

#### Analysis of preconditioning factors influencing immediate graft function after deceased donor kidney transplantation

J.J. Kim, E.Y. Kim
Seoul St. Mary's Hospital, The Catholic University of Korea College of Medicine, Division of Trauma and Surgical Critical Care, Department of General Surgery, Seoul, Korea, Republic of
**Correspondence:** J.J. Kim
*Intensive Care Medicine Experimental* 2018, **6(Suppl 2):**0617

**INTRODUCTION.** The management of deceased donor in the intensive care unit (ICU) before organ transplantation would be important for the outcome of recipient. Herein, we analyze the preconditioning factors managed before procurement that could influence on the immediately postoperative graft function after deceased donor kidney transplantation (DDKT).

**OBJECTIVES.** A total of 271 DDKT which were performed at Seoul St. Mary's Hospital, Korea, from January 2009 to March 2016 was finally reviewed.

**METHODS.** We divided the patients into delayed graft function (DGF group) and no DGF (non-DGF group) and compared preoperative outcomes between groups. We also analyzed the predisposing factors of DGF using multivariate analysis.

**RESULTS.** A total of 36 cases developed DGF whereas 235 patients had no DGF and the demographic characteristics of recipients in each group had no significant difference. Regarding the preconditioning factors, the preoperative polyuria, the maximal level of serum sodium and BUN, and transfer time were significantly higher in DGF group ($p< 0.001$, respectively). In multivariable analysis, the preoperative polyuria (Odds Ratio 4.835, $p$=0.036), elevated preoperative level of sodium (Odds ratio 1.227, $p$=0.001) and the extended transfer time (Odds ratio 1.001, $p<$ 0.001) were the independent risk factors of donor in preconditioning management associated with DGF.

**CONCLUSIONS.** The polyuria and high level of sodium before the organ procurement and the prolonged transfer time would be independent risk factors for DGF after DDKT. The active intervention and early implementation of the intensivist could be helpful to manage these factors effectively resulting in the improved graft function, ultimately.

##### REFERENCE(S)

1. Port FK, Bragg-Gresham JL, Metzger RA et al. Donor characteristics associated with reduced graft survival: an approach to expanding the pool of kidney donors. Transplantation 2002: 74: 1281.
2. Jacobi J, Rebhan D, Heller K, Hilgers KF, Wullich B, Eckardt K-U. Donor acute kidney injury and short term outcome in renal transplantation. Clin Transplant 2014: 28: 1131.

3. Deroure B, Kamar N, Depreneuf H et al. Expanding the criteria of renal kidneys for transplantation: use of donors with acute renal failure. Nephrol Dial Transplant 2010: 25: 1980

**GRANT ACKNOWLEDGMENT**
The authors have nothing to disclose.

### 0618

#### DCD process in Italy: a growing experience

P. Fassini[1], M. Lucchelli[1], E. Borotto[1], G. Lanzillotti[1], A. Calzolari[2], D. Radrizzani[1]
[1]Ospedale di Legnano, Rianimazione, Legnano, Italy; [2]Università degli Studi di Milano, Scuola di Specializzazione Anestesia e Rianimazione, Milano, Italy
**Correspondence:** P. Fassini
*Intensive Care Medicine Experimental* 2018, **6(Suppl 2):**0618

**INTRODUCTION.** Ethical and end of life issues still represent a main and daily challenge in ICU. Withdrawal of life sustainig therapy (WLST) deemed futile is due, this arises the chance of donation. Because of the growing gap between recipients and available organs, today the chance of donation after cardiac death (DCD) has emerged. In Italy, despite a very conservative law about determination of death (20 min for "no touch")[1], normothermic regional perfusion (NRP) allows abdominal organs donation. However thoracic organs procurement still remain a challenge.

**OBJECTIVES.** Due to time constriction, DCD requires a series of coordinated actions organized in a specific pathway.

**METHODS.**

DCD protocol consists of DCD II-III-IV. DCD II algorithm (Fig. 1-2):

DCD III: WLST is decided before any proposal of donation. After checking patient's will and eligibility WLST could start providing comfort for patient and relatives, light sedation, extubation and prevention of stridor and dispnea. After determination of death (20 min 'no touch period') we arrange femoral vessels cannulation for NRP and orotracheal intubation following 'Valenza protocol'[2].

DCD IV: after determination of death on cardiac or neurologic standard, ECLS is converted into NRP.

**RESULTS.** We collected 2 patients from Oct. 2016 till now, both admitted for post anoxic coma after cardiac arrest (CA). Both patients showed very poor prognostic scores that led to WLST. In the first case (53 yo male) next of kin expressed willingness to donate organs. Unfortunately technical problems impeded NRP. In the second case (63 yo male) the patient had living will to donate. Progression to CA took 21 min. After "no touch period", we started NRP and provided reintubation for lungs preservation[2]. Time to reintubation was 22 min and warm ischemia time lasted 48 min. Lungs were ready for ex vivo lung perfusion (EVLP) in 207 min. NRP proceeded for 227 min, with decrease of ALT and AST and lactate (fig. 3).

Liver was recovered in 228 min from CA. Liver and kidneys were connected to machine perfusion. All the organs were suitable for transplantation. After five months, all transplanted organs have good function.

**CONCLUSIONS.** DCD protocol allows solid organ donation after cardiac death, preserving the dead donor rule and the italian law. Extubation doesn't preclude lung donation. Combined lung and abdominal organs procurement is still a challenge, however thanks to team synergy we succeded in. Hospitals without ECLS program could supply lung donors through reconditioning and EVLP.

**REFERENCE(S)**
1."Why
can´t I give you my organs after my heart has stopped beating?"
Giannini A et al, Minerva Anestesiol 2016 Mar; 82 (3): 359-68
2.Successful
Transplantation of Lungs From an uDCD Preserved In Situ by Alveolar Recruitment Maneuvers and Assessed by EVLP
Valenza F et al, Am J Transplant 2016 Apr; 16(4): 1312-8

**GRANT ACKNOWLEDGMENT**
None.

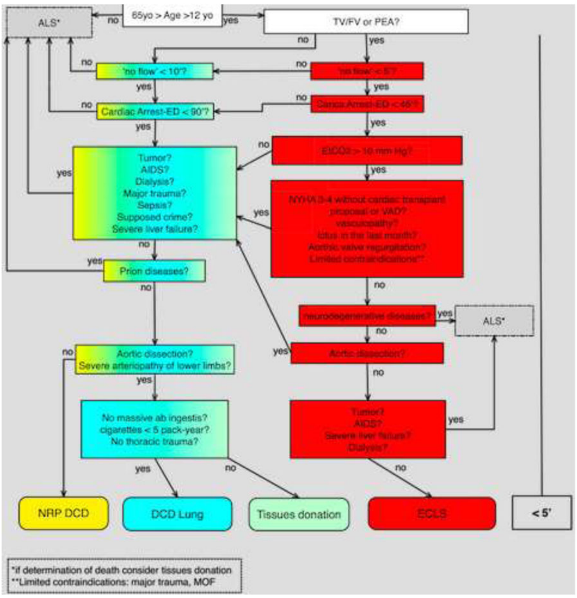

**Fig. 1 (abstract 0618).** ECLS-DCD-ALS

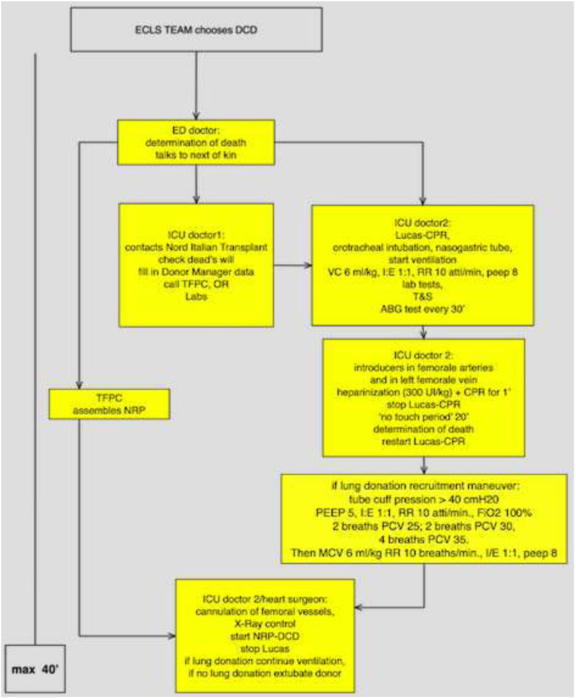

**Fig. 2 (abstract 0618).** From ED to NRP

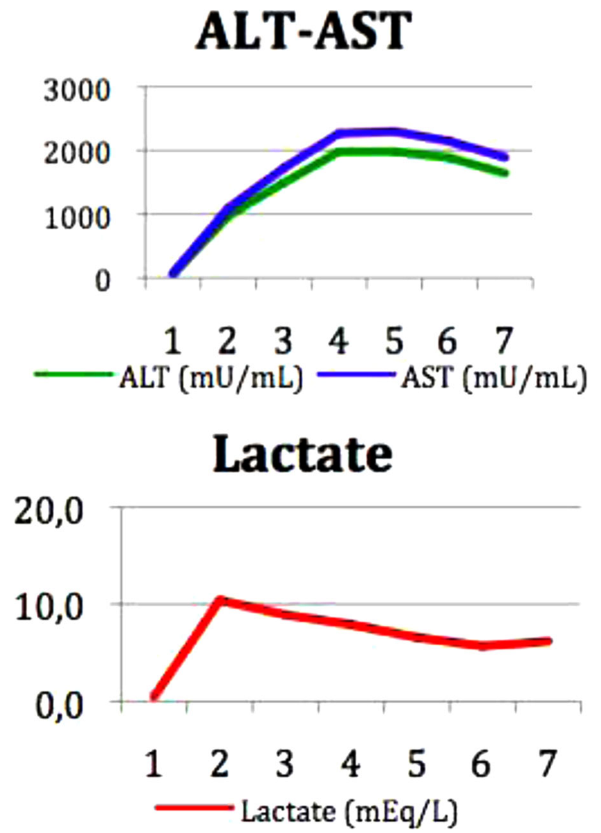

**Fig. 3 (abstract 0618).** ALT AST Lactate

### 0619

#### Causes of failure during the management process from identification of brain-dead potential organ donors to actual donation in Korea - 5-year data analysis (2012-2016)

J.-M. Lee[1], M.-I. Kim[2]
[1]Korea University Anam Hospital, Surgery, Seoul, Korea, Republic of; [2]Korea University Anam Hospital, Transplantation Center, Seoul, Korea, Republic of
**Correspondence:** J.-M. Lee
*Intensive Care Medicine Experimental* 2018, **6(Suppl 2):**0619

**OBJECTIVES.** This is a retrospective analysis of the causes of failure in the management process from identification of brain-dead potential organ donors to actual donation, in Korea over the past 5 years.

**METHOD.** The data used were from 8,120 potential brain deaths reported to the Korea Organ Donation Agency, including the information received at the time of reporting, the donation suitability evaluation by the coordinator after the report, and the data obtained from interviews of hospital medical staff and the donor's family.

**RESULTS.** From January 2012 to December 2016, the total number of brain-dead potential organ donors in Korea was 8,120, of which 2,348 (28.9%) had undergone organ procurement surgery. The number of transplanted donors increased over time, but the ratio of transplanted donors to medically suitable brain-dead donors decreased. The most common causes of donation failure included donation refusal (27.6%), non-brain-death (15.5%), and incompatible donation (11.6%), and 104 potential donors (7.8%) were unable to donate their organs because they were not pronounced brain dead.

**CONCLUSION.** The organ donation success rate can be increased by analyzing the major causes of failure in the management process from identification of brain-dead potential organ donors to actual donation and through various efforts to prevent failure.

## Endotoxaemia & blood purification in sepsis

### 0620

#### The effects of genistein supplementation to oral/enteral nutrition solutions on inflammatory cytokines in septic ICU patients: prospective, single-center, randomized controlled, a pilot study

K. Gundogan[1], G. Elay[2], I.G. Tatlimisir[3], H. Sipahioglu[1], M. Sungur[1], M. Guven[1], C. Yazici[3], O. Kucuk[4]
[1]Erciyes University, School of Medicine, Department of Medicine, Division of Intensive Care, Kayseri, Turkey; [2]Ministry of Health, Dr. Ersin Arslan Training and Research Hospital, Intensive Care Unit, Gaziantep, Turkey; [3]Erciyes University, School of Medicine, Department of Clinical Biochemistry, Kayseri, Turkey; [4]Emory University, Department of Hematology and Medical Oncology, Winship Cancer Institute, Atlanta, United States
**Correspondence:** M. Sungur
*Intensive Care Medicine Experimental* 2018, **6(Suppl 2):**0620

**INTRODUCTION.** Sepsis is a state develops as a response to severe infection with high mortality rate. There is exaggerated and irregular host response in sepsis. Cytokines such as IL-1, IL-6, IL-8, TNF-α, İnterferon-γ and HMGB-1 are released as response to invading microorganisms and they play a major role in sepsis pathogenesis. Soybean proteins are used for prevention and treatment of cardiovascular diseases, osteoporosis and different cancer types. Soy isoflavons such as genistein, daidzein and glycitein are the main components for cancer prevention. Genistein is the dominant isoflavons. The main mechanism for anti-inflammatory effect of genistein is related to transcription nuclear factor Kb (NF-κB) and inhibition of chemokine-8

**OBJECTIVES.** To evaluate effects of genistein supplementation to oral/enteral nutrition on inflammatory cytokines in sepsis patients in the intensive care unit.

**METHODS.** This prospective randomized study was conducted at a Medical Intensive Care Unit of Erciyes University Hospital. The study included patients with sepsis above 18 years of age, required to stay in ICU more than 48 hours and patients receiving oral/enteral nutrition. Patients divided into two groups each containing 16 patients. Intervention group received supplemental genistein (60 mg/day)added to enteral nutrition. Control group patients received only oral/enteral nutrition. Serum IL-6, IL1- β, TNFand HMGB-1 concentrations were measured within 24 hours of sepsis diagnosis and serially at baseline, 24 hours and 72hours after entry.

**RESULTS.** A total of 32 subjects were included (genistein group: 16 pts, control group: 16 pts). The mean age was 56±16 years. The most commonsources of sepsis were respiratory (56%) and gastrointestinal system (19%). All patients had an Acute Physiology and Chronic Health Evaluation II score (APACHE II) of 16.4±8.7. Genistein group's APACHE II score was 17.2±9.3,and control group's APACHE II score was 15.6±8.3 (p=0.606).Genistein group had higher serum levels of IL-6 at 72 hours compared to the control group (31.6±14.9, 10.8±5.3 respectively) (p=0.000).TNF levels were lower in genistein group at 72hours compared to the control group (86.3±15.1, 104.6±23.6 respectively) (p=0.014).There were no statistically significant differences of baseline, 24 hours and 72hoursIL1-β and HMGB-1 levels between the two groups.

**CONCLUSIONS.** Proinflamatory cytokines IL-6 serum level was higher and TNF serum level was lower at 72hoursin patients who received Genistein supplementation. There was no difference in anti-inflammatory cytokine HMGB-1 serum levels between the two groups.

##### REFERENCE(S)

1. Cinel I, Dellinger RP. Advances in pathogenesis and management of sepsis. Curr Opin Infect Dis. 2007 Aug;20(4):345-52.

##### GRANT ACKNOWLEDGMENT

This study was supported by Erciyes University Scientific Research Unit

### 0621

#### Multivariate analysis of cytokines in septic shock predicts outcome

D.B. Antcliffe, J.K. Ward, T.J. Marshall, F. Al-Beidh, K. O'Dea, A.C. Gordon
Imperial College London, Section of Anaesthetics, Pain Medicine and Intensive Care, Department of Surgery and Cancer, London, United Kingdom
**Correspondence:** D.B. Antcliffe
*Intensive Care Medicine Experimental* 2018, **6(Suppl 2):**0621

**INTRODUCTION.** Trials of novel treatments in sepsis have failed to provide new therapies, in part, likely due to the heterogeneity of this condition. The need for a more stratified approach to trial recruitment is recognised and to achieve this goal, novel methods of patient stratification are needed.
**OBJECTIVES.** To apply multivariate analysis to a panel of cytokines measured in the plasma of patients with septic shock to identify subgroups that can predict outcome and could influence treatment choices.
**METHODS.** Nineteen cytokines were measured using LEGENDplex kits (BioLegend, UK) in the plasma of 174 patients admitted to ICU at enrolment into the VANISH clinical trial (1). Unsupervised analysis was performed with principal component analysis (PCA) and hierarchical cluster analysis to identify patient subgroups based on profiles of inflammatory mediators. Univariate statistics were applied to compare individual mediators and outcome parameters.
**RESULTS.** PCA identified two groups of patients: one with a hyper-inflamed phenotype (n=31) compared to the other (n=143). Several cytokines were substantially higher in the hyper-inflamed group including G-CSF (32,898pg/ml vs 349pg/ml, p< 0.001), MCP-1 (12,491pg/ml vs 1,239pg/ml, p< 0.001), IL-6 (35,000pg/ml vs 617pg/ml, p< 0.001) and IL-8 (4,210pg/ml vs 59pg/ml, p< 0.001). This group was associated with a higher mortality at 28 days (OR 3.9 (1.7-8.7), p=0.001). No interaction was seen between inflammatory group and the choice of vasopressor (noradrenaline or vasopressin) (p=0.27) or the use of hydrocortisone (p=0.50) on day 28 survival. Clinical parameters had only a moderate ability to predict cytokine grouping with APACHE II score (AUROC 0.71 (0.61-0.80), misclassification rate 26%) and lactate (AUROC 0.74 (0.64-0.83) misclassification rate 26%) performing similarly.
**CONCLUSIONS.** Applying unsupervised analysis to a panel of inflammatory mediators detected a subgroup categorised by higher levels of circulating cytokines and worse clinical outcome. Using such information to stratify patients into clinical trials may overcome the effects of patient heterogeneity to improve the identification of treatment responsive subgroups.

### 0622

#### The clinical application of extracorporeal membrane oxygenation for sepsis related non pulmonary associated infection

T. Hong, H. Kim
Hallym University Sacred Heart Hospital, Surgery, Anyang-si, Korea, Republic of
**Correspondence:** T. Hong
*Intensive Care Medicine Experimental* 2018, **6(Suppl 2):**0622

**INTRODUCTION.** Application of Extracorporeal membrane oxygenation (ECMO) as a therapeutic option in critically ill patients is increasing. Recent studies found that using ECMO for pneumonia, asthma, trauma and burn were associated with better outcomes. However the role of ECMO as a potentially useful therapeutic option in case of sepsis or septic shock remains questionable. Besides, Role of ECMO in Sepsis induced by non-pulmonary associated infection was no clearly known, compared than pulmonary infection.
**OBJECTIVES.** The aim of this study is to analyze the clinical characteristics of ECMO in non-pulmonary associated infection, through the comparison of pulmonary infection group.
**METHODS.** The study was conducted on 32 patients who were clearly identified for infectious origin and septic shock among 154 patients who applied ECMO from a single center between January 2015 and December 2017. Medical records of these patients were reviewed. The patients divided in two groups, a pulmonary infection group and a non pulmonary associated infection group on the basis of a primary origin of infection. Patients under 18 years of age were excluded. The clinical variables related with patient were extracted and analyzed.
**RESULTS.** Total number of the included patients was 32. The number of pulmonary infection patients was 18 and the number of non pulmonary associated infection patients was 14. There were differences in hospital mortality, SAPS3 score, SOFA score at ECMO starting day, ICU stay, ECMO duration, time interval from ICU admission to ECMO start. The average of SAP3 score (74.5 vs 51.72) (p< 0.00) and SOFA score at ECMO starting day (14.36 vs 10.33) (p=0.001) was higher in the non pulmonary associated infection group. Also the non pulmonary associated infection group had higher mortality rate compare than the pulmonary infection group. (71.4% vs 50%) More veno-arterial ECMO applied to non pulmonary associated infection group. (78.6% vs 11.1%) The hospital stay (23.86 days vs 46.89 days) (p=0.042) and ICU stay (19.71 days vs 35.56 days) (p=0.026) of the non pulmonary associated infection group was shorter than the pulmonary infection group. Also Lactate level was higher in the non pulmonary associated infection group when ECMO was applied. (10.481 mmol/L vs 2.872 mmol/L) (p< 0.00).
**CONCLUSIONS.** The non pulmonary associated infection group had more severe aspect compared than the pulmonary infection group when ECMO was applied. Higher mortality of the non pulmonary associated infection group compare than that of the pulmonary infection group was cause of shorter hospital stay and ECMO duration. These results indicate that more consideration is needed when applying ECMO in non-pulmonary associated infection patient.

##### GRANT ACKNOWLEDGMENT

We would like to thank HY Kim, HS Kim, SH park for linguistic review.

### 0623

#### Effects of polymyxin B hemoperfusion on septic shock in patients after liver surgery: a pilot study

A.L. Lee[1], S.Y. Hong[2], B.W. Kim[2], H.J. Wang[2]
[1]University of Ulsan College of Medicine, Asan Medical Center, Division of Acute Care Surgery , Department of Surgery, Seoul, Korea, Republic of; [2]Ajou University School of Medicine, Department of Liver Transplantation and Hepatobiliary Surgery, Suwon, Korea, Republic of
**Correspondence:** A.L. Lee
*Intensive Care Medicine Experimental* 2018, **6(Suppl 2):**0623

**INTRODUCTION.** Polymyxin B direct hemoperfusion (PMX-DHP) can remove plasma endotoxins. It is considered an effective treatment for sepsis, particularly for intra-abdominal infection with gram-negative bacteria. Clinical data for the effect of PMX-DHP on septic shock in patients with liver disease are limited.
**OBJECTIVES.** To evaluate clinical effects of PMX-DHP treatment on septic shock in patients after hepatectomy or liver transplantation.
**METHODS.** From August 2016 to September 2017, 10 patients who underwent PMX-DHP treatment for septic shock after liver surgery (3 cases of hepatectomy and 7 cases of liver transplantation) in surgical intensive care unit were enrolled. PMX-DHP treatment was performed one or twice in each patient. Two patients who died within

24 hr after PMX-DHP were excluded. Changes in patient's hemodynamics and infection marker before and after PMX-DHP treatment (24hr, 48hr, 72hr, 7 day) were analyzed.

**RESULTS.** Most patients had Gram-negative bacteremia. Mortality in 28 days was 50%. Mean value of sequential organ failure assessment (SOFA) score and serum lactate level before PMX-DHP treatment were 17.6 (range, 13.0-22.0) and 8.3 mg/dL (range, 2.0-26.7mg/dL). After PMX-DHP treatment, serum lactate, vasoactive-inotropic scores (VIS), vasopressor dependency index (VDI), and $PaO_2/FiO_2$ ratio were significantly decreased (Table 1). Levels of C-reactive protein (CRP) as an infection marker were decreased after PMX-DHP treatment (Table 1).

**CONCLUSIONS.** Our pilot study suggests that PMX-DHP treatment can significantly improve hemodynamics in patients with septic shock after liver surgery.

**REFERENCE(S)**

1. Cruz DN, Antonelli M, Fumagalli R, et al. Early use of polymyxin B hemoperfusion in abdominal septic shock: the EUPHAS randomized controlled trial. JAMA 2009;301:2445-52.
2. Mitaka C, Tomita M. Polymyxin B-immobilized fiber column hemoperfusion therapy for septic shock. Shock 2011;36:332-8.
3. Davies B, Cohen J: Endotoxin removal devices for the treatment of sepsis and septic shock. Lancet Infect Dis 2011;11:65-71
4. Saito N, Sugiyama K, Ohnuma T, et al. Efficacy of polymyxin B-immobilized fiber hemoperfusion for patients with septic shock caused by Gram-negative bacillus infection. PLoS One. 2017;12(3):e0173633
5. Ruberto F, Pugliese F, D´Alio A, et al. Clinical effects of use polymyxin B fixed on fibers in liver transplant patients with severe sepsis or septic shock. Transplant Proc. 2007;39(6):1953-5.

**Table 1 (abstract 0623).** Hemodynamics of patients before and after PMX-DHP treatment

| Variables | before (n=8) | 24hr(n=8) | 48hr(n=7) | 72hr(n=7) | 7day(n=6) | P-value |
|---|---|---|---|---|---|---|
| CRP | 10.7 ± 5.6 | 9.6 ± 6.9 | 7.9 ± 5.3 | 8.9 ± 4.3 | 5.0 ± 3.5 | 0.009 |
| SOFA score | 17.6 ± 2.7 | 17.4 ± 2.9 | 17.4 ± 3.5 | 17.7 ± 4.7 | 15.2 ± 3.7 | 0.254 |
| Lactic acid (mg/dL) | 8.3 ± 8.0 | 6.7 ± 6.2 | 6.5 ± 5.8 | 5.5 ± 4.9 | 1.8 ± 0.6 | 0.003 |
| VIS | 46.5 ± 49.6 | 25.3 ± 30.6 | 20.7 ± 24.5 | 1.6 ± 2.1 | 1.7 ± 2.0 | <0.001 |
| VDI | 0.9 ± 1.0 | 0.4 ± 0.5 | 0.4 ± 0.5 | 0.3 ± 0.3 | 0.03 ± 0.04 | 0.003 |
| PaO2/FiO2 | 113.6 ± 39.0 | 141.4 ± 60.0 | 167.1 ± 80.3 | 186.6 ± 87.5 | 185.3 ± 127.4 | 0.026 |

### 0624

#### Endotoxin and cytokin reduction function of the oXiris filter in a prospective double-blinded cross-over setting in patients with critical Gram- septic shock and continuous renal replacement therapy requiring acute kidney injury

M.E. Broman, M. Bodelsson

Skåne University Hospital, Lund, Perioperative and Intensive Care, Lund, Sweden

**Correspondence:** M.E. Broman

*Intensive Care Medicine Experimental* 2018, **6(Suppl 2):**0624

**INTRODUCTION.** In Gram-negative sepsis endotoxin (lipopolysaccharide), a component from the outer bacterial wall, is released into the circulation, causing endothelial leakage and vasodilation. The septic cascade is further escalated by triggering a host immune response mediated by cytokines. The oXiris filter for continuous renal replacement therapy (CRRT) has a 3 times thicker endotoxin-adsorbing polyethylene-imine layer than a standard ST filter.

**OBJECTIVES.** We wanted to investigate whether CRRT with oXiris could affect lipopolysaccharide and cytokine levels in sepsis patients with acute kidney injury (AKI).

**METHODS.** Sixteen patients with Gram-negative septic shock, blood endotoxin levels >0.03 EU/ml and CRRT requiring AKI were randomized in a double-blinded cross-over regime to 24 h CRRT with oXiris followed by 24 h with a standard filter (ST), or vice versa. Blood samples were drawn at 0, 1, 3, 8, 16 and 24 hours of each treatment period for measurement of endotoxin levels (turbidimetric kinetic Limulus amoebocyte lysate assay) and tumor necrosis factor (TNF)-α, interleukin (IL)-1β, -2, -4, -6, -8, -10, interferon-γ and granulocyte-macrophage colony-stimulating factor (GM-CSF, Bio-Plex multiplex immunoassay).

**RESULTS.** Both filters lowered the endotoxin levels over 24 h. Total endotoxin balance was negative throughout the oXiris study periods, while mean levels were positive compared to baseline during the first 8 h of the ST study periods. Levels of IL-6, -8 and TNF-α decreased significantly during use of both filters, while interferon-γ was significantly lowered only in the oXiris group. The remaining cytokines did not significantly change.

**CONCLUSIONS.** Both filters lower the endotoxin levels, but the oXiris filter shows an enhanced effect during the first 8 h, which could come from a more active endotoxin adsorption. The filters were equally effective in reducing cytokine levels suggesting a similar binding capacity of these mediators and/or reducing the septic inflammatory response alike.

**REFERENCE(S)**

1. Danner RL, Elin RJ, Hosseini JM, Wesley RA, Reilly JM, Parillo JE. Endotoxemia in human septic shock. Chest 2009 Nov; 136 (5 Suppl): e30.

**GRANT ACKNOWLEDGMENT**

The study was funded by research grants from Baxter, USA and from Skåne Regional Council, Sweden

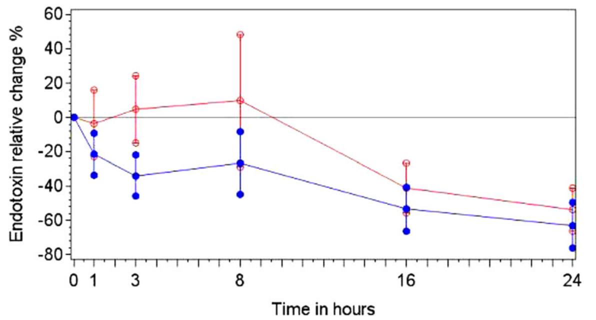

**Fig 1 (abstract 0624).** Endotoxin profiles for oXiris (blue) and for ST (red) filter treatment periods, means and SEM

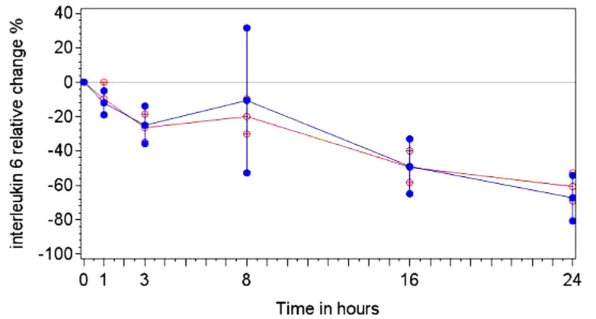

**Fig 2 (abstract 0624).** Interleukin 6 profiles for oXiris (blue) and for ST (red) filter treatment periods, means and SEM

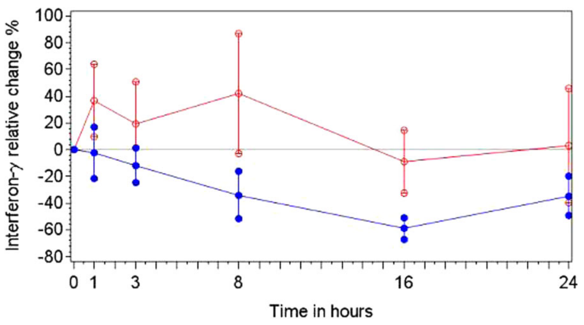

**Fig 3 (abstract 0624).** Interferon γ profiles for oXiris (blue) and for ST (red) filter treatment periods, means and SEM

### 0625

#### The affecting factors for EAA (Endtoxin Activity Assay) are important in safety and efficacy of polymyxin B hemoperfusion (PMX) for septic shock (EUPHRATES) trial

M. Kang, M. Tsunoda, M. Saito, T. Oshiro, M. Takeda, A. Yaguchi
Tokyo Women's Medical University, Critical Care and Emergency Medicine, Tokyo, Japan
**Correspondence:** M. Kang
*Intensive Care Medicine Experimental* 2018, **6(Suppl 2):**0625

**INTRODUCTION.** Since 1994, PMX-DHP (Polymyxin B-direct hemoperfusion) (Toraymyxin®, Toray medical Co., Tokyo) has been approved as a therapy for patients with endotoxic or Gram-negative infectious severe sepsis in Japan. Recently, randomized placebo-controlled trials (such as EUPHRATES trial) shows PMX-DHP treatment improved their mortality in some population in septic shock, although did not reach statistical significance of primary endopoint.

**OBJECTIVES.** The purpose of the present study is to compare between EUPHRATES trial and our institution's data, and to clarify the efficacy of PMX-DHP for septic shock.

**METHODS.** From November 2014 to December 2017, all adult patients treated with PMX-DHP and EAA (Endtoxin Activity Assay) was measured at pre-treatment of PMX-DHP in our medico-surgical ICU of university hospital were included in this retrospective study. And of those whom, patients met both of the inclusion and exclusion criteria of EUPHRATES trial were selected and compared with EUPHRATES trial results. The criteria of Patients' data were collected from medical archives. Values were expressed as mean ± SD.

**RESULTS.** There were fifty two patients who were treated with PMX-DHP in all (25 men, 27 women; age [mean 63.8 ± 14.5]; 28 day mortality 30.8%). Mean SOFA score, MOD score, and EAA values at pre-treatment of PMX-DHP were 9.6 ± 4.6, 7.6 ± 3.9 and 0.58 ± 0.19, respectively. Of 52 patients, there were 7 patients with pre-treatment EAA between 0.6 and 0.9, and MODS>9. And there were only 3 patients who had 2 session treatment of PMX-DHP. Mean age, SOFA score, MOD score, EAA values at pre-treatment of PMX-DHP and 28 day mortality in those 3 patients were 64 ± 14.5, 15.3 ± 2.3, 11.7 ± 2.9, 0.73 ± 0.10 and 33.3%, respectively. Of 3 patients, one non-survivor at 28 day were giving chemotherapy for peritoneal dissemination of appendiceal cancer and showed pre-treatment WBC count was 1010/ $mm^3$.

**CONCLUSIONS.** The limitations of this study were small numbers of patients and small results were published about EUPHRATRES trial. The 28 day mortality in our patients was 33.3%, while 26.1% of EUPHRATRES trial. Non-survivor in our patients had leukocytopenia due to chemotherapy. EAA values are using for inclusion criteria of that trial and that assay is based on the reaction of neutrophils to lipopolysaccharide. Therefore, EAA value could be influenced by leukocytopenia. Such participants might be included in EUPHRATES trial. Using EAA for criteria could be difficult to show the efficacy of PMX-DHP in those patients. The analysis of subgroup in that trial should be reconsidered to regard affecting factors of EAA.

### 0626

#### Hemoadsorption by extracorporeal cytokine adsorption therapy (CytoSorb®) in the management of sepsis: a retrospective observational study

Y.P. Singh[1], S.C. Chhabra[2], K. Lashkari[1], A. Taneja[1], A. Garg[1], A. Chandra[1], M. Chhabra[2], G.P. Singh[1], S. Jain[1], S.K. Singh[1]
[1]Max Super Specialty Hospital, Critical Care Medicine, New Delhi, India; [2]Max Super Specialty Hospital, Nephrology, New Delhi, India
**Correspondence:** Y.P. Singh
*Intensive Care Medicine Experimental* 2018, **6(Suppl 2):**0626

**INTRODUCTION.** Sepsis results in immunologic disturbances with the release of various inflammatory mediators including cytokines[1]. Cytokines are able to damage the cells and the continuous release of inflammatory mediators leads to impaired immunity. Therefore, the reduction in cytokine levels by hemoadsorption represents a new concept for blood purification. CytoSorb® is an Extracorporeal Cytokine Adsorption Device (ECAD) which is used to reduce toxic levels of cytokines. It is designed to directly capture and reduce mid-molecular weight inflammatory mediators (~10-55 kDa) in blood including pro and anti-inflammatory cytokines, chemokines, and bacterial exotoxins[2]. The current retrospective study was carried out to understand the inflammatory status as a result of evident immunomodulatory effect of CytoSorb therapy.

**METHODOLOGY.** This retrospective study was analyzed in Max Super Specialty Hospital, Patparganj, Delhi, India for the patients who were admitted between 2015 to 2017 in compliance with current ICH-GCP and ICMR guidelines. A total of 36 patients (range [24-84 year]) were included in the analysis.

**RESULTS.** In the present retrospective analysis, majority of the patients were admitted in medical & surgical ICU due to infection. After CytoSorb® therapy, a significant decrease of 91% in the PCT levels within 24 hours of the therapy was observed. The post-Cytosorb, procalcitonin (PCT) level was decreased to 3.16±2.10 ng/mL from the baseline value of 36.06±33.86 ng/mL. Similarly, a 44 % decrease (i.e. from 26.4± 15.28×109/L to 16.31±6.81×109/L) in TLC levels was observed after CytoSorb® therapy. The sepsis severity scores, namely, SOFA and APACHE II, reduced to 7.8±3.67 and 11.41± 3.14 from the initial value of 12.41± 4.23 and 24.4±6.01 respectively after CytoSorb® therapy. Within 24 hours after the CytoSorb® therapy, shock reversal was observed in eight (22.2%) patients. The predicted mortality for the included patients was more than 50%.

**CONCLUSIONS.** In the present study, most of the patients had shown significant reduction in PCT, TLC Counts & Sepsis Scores after CytoSorb® therapy. Also, the shock reversal was observed in few patients when treated with CytoSorb® therapy. This could be attributed to the known-immunomodulatory effect of CytoSorb as an adjuvant therapy. However, a larger prospective study is required to derive the above stated benefit of the ECAD therapy.

**REFERENCE(S)**

1. Tsertsvadze A, Royle P, Seedat F, Cooper J, Crosby R, McCarthy N. Community-onset sepsis and its public health burden: a systematic review. Systematic reviews. 2016; 5(1):81
2. Wiegele M, Krenn CG. Cytosorb™ in a Patient with Legionella Pneumonia-Associated Rhabdomyolysis: A Case Report. ASAIO Journal. 2015;61(3):e14-e6

**GRANT ACKNOWLEDGMENT**
The authors of this retrospective study did not receive any kind of grant or financial support. It was purely a academic / research study.

### 0627

#### Cytokine and DAMP adsorption in septic acute kidney injury

C.M. Laubach[1], K.-P. Janssen[2], W. Johannes[2], R. Bogdanski[1], B. Kapfer[1]
[1]Klinikum rechts der Isar, Technical University of Munich, Department of Anesthesiology, Munich, Germany; [2]Klinikum rechts der Isar, Technical University of Munich, Department of Surgery, Munich, Germany
**Correspondence:** C.M. Laubach
*Intensive Care Medicine Experimental* 2018, **6(Suppl 2):**0627

**INTRODUCTION.** Sepsis represents a life-threatening organ dysfunction caused by a dysregulated host response to infection with massive systemic cytokine storms. Sepsis-induced acute kidney injury (S-AKI) is frequently occurring in the early course of sepsis and increases mortality significantly.[1] However, the pathophysiological pathways associated with S-AKI remain poorly understood.
High-mobility group box-1 (HMGB1) is a damage-associated molecular pattern molecule, which is thought to stimulate proinflammatory responses in tubular kidney cells and to promote acute kidney damage via Toll-like receptor 4 (TLR4).[2]
Here, we investigated the role of cytokines and DAMPS in the pathogenesis of S-AKI, and report on a randomized, controlled, open-labeled clinical trial CASAKI (NCT02588794).
**OBJECTIVES.** We hypothesized that treatment of patients with S-AKI with an extracorporeal cytokine and DAMP adsorber (Cytosorb) decreases IL-6 and HMGB1 levels, leading to a better clinical outcome.
**METHODS.** 13 patients (=control group) with S-AKI were treated in accordance to guidelines with CVVHD. Treatment group (n=12) received an additional hemoadsorption therapy by Cytosorb until serum levels of IL-6 reached < 1000 pg/ml. Serum and urine samples were taken before CRRT start and after 24h, and IL-6 and HMGB1 levels were quantified by ELISA.
**RESULTS.** After 24 hours a significant reduction of IL-6 serum levels was found in the treatment group (median: 22% of initial baseline levels; interquartile range 8-31%), compared to an increase of IL-6 in the control group (median: 123%; IQR 29-300%; p=0,009).
HMGB1 in urine decreased significantly in the treatment group after 24 hours ($t_0$: mean 46ng/ml ±22; $t_{24}$: mean 16ng/ml ±5; p=0,004), as well as HMGB1 in serum ($t_0$: mean 37ng/ml ±14; $t_{24}$: mean 26ng/ml ±18; p=0,043). In contrast, HMGB1 levels remained essentially unchanged in the control group.
Of note, baseline pre-therapeutic urinary HMGB1 levels correlated strongly with 28-day kidney function (spearman |r|=0,725; p=0,003).
**CONCLUSIONS.** Hemoadsorption therapy efficiently removed HMGB1 and IL-6 within the first 24 hours. Tendencies to increased 28-day kidney function and 90-day survival in the treatment group were observed, although not attaining significance. To confirm these effects, more patients need to be included.
Furthermore, urinary HMGB1 seems to play a major role in the pathogenesis of S-AKI, with the potential as putative biomarker in S-AKI to predict long-term renal-function.

**REFERENCE(S)**

1. Bagshaw, S.M., et al., *Septic acute kidney injury in critically ill patients: Clinical characteristics and outcomes.* Clinical Journal of the American Society of Nephrology, 2007. 2(3): p. 431-439.
2. Zarbock, A., H. Gomez, and J.A. Kellum, *Sepsis-induced acute kidney injury revisited: pathophysiology, prevention and future therapies.* Current Opinion in Critical Care, 2014. 20(6): p. 588-595.

**GRANT ACKNOWLEDGMENT**
The authors declare no conflict of interests.

### 0628

#### Early therapeutic plasma exchange as rescue therapy in septic shock

K. Stahl[1], T. Idowu[1], M. Busch[1], O. Wiesner[1], T. Welte[1], M.M. Hoeper[1], J.T. Kielstein[2], S. David[1]
[1]Medical School Hannover, Hannover, Germany; [2]Teaching Hospital Brunswick, Brunswick, Germany
**Correspondence:** S. David
*Intensive Care Medicine Experimental* 2018, **6(Suppl 2):**0628

**INTRODUCTION.** The overwhelming host response in sepsis is a key driver of morbidity and mortality. The theoretical concept of therapeutic plasma exchange (TPE) in sepsis combines two major aspects in one intervention: 1) Removal of harmful circulating molecules - as part of the injurious cytokine storm - that directly contribute to the manifestation of the disease. 2) Replacement of protective plasma proteins that compensate for the loss of factors important for coagulation, fibrinolysis and counteract inflammation and vascular leakage to ultimately restore hemostasis.
**OBJECTIVES.** We explored the effects of early TPE in severely ill individuals with septic shock.
**METHODS.** Prospective single center, open-label, non-randomized pilot study enrolling 20 patients with septic shock (**onset< 12h**) requiring high doses of norepinephrine (**NE>0.4mg/kg/min**). Clinical and biochemical data were obtained before and after TPE and plasma samples were taken for *ex vivo* stimulation of human umbilical vein endothelial cells (HUVECs) to analyze barrier function (immunocytochemistry & transendothelial electrical resistance). Cytokines were measured by cytometric bead array and enzyme linked immunosorbent assays. An immediate response was defined as >20% NE reduction from baseline to the end of TPE.
**RESULTS.** TPE was well tolerated without the occurrence of any adverse events and resulted in rapid reduction in NE (0.82 (0.61-1.17) vs. 0.56 (0.41-0.78) mg/kg/min, p=0.002) to maintain mean arterial pressure (MAP) above 65 mmHg. The actual overall 28-day mortality was 65%, while the expected (APACHE-II) mortality was 91.1%. Key pro-inflammatory cytokines and permeability factors (e.g. IL-6, IL-1b, Angiopoietin-2) were significantly reduced after TPE while the anti-inflammatory cytokine IL-10 was not changed. *Ex vivo* stimulation of HUVECs with plasma obtained before TPE induced substantial cellular hyper-permeability, which was completely abolished with plasma obtained after TPE.
**CONCLUSIONS.** In this uncontrolled pilot study, early TPE was safe and resulted in rapid hemodynamic improvement and favorable changes in the cytokine profile in patients with septic shock. It has yet to be determined whether early TPE also improves outcomes in this patient cohort. An appropriately powered multicenter RCT is desirable.

### 0629

#### The combination of AN69ST-CRRT and PMX-DHP reduces the dosage of vasopressors needed and fluid infusion in patients with septic AKI

H. Narumiya[1], R. Iiduka[1], S. Hashimoto[2]
[1]Japanese Red Cross Society Kyoto Daini Hospital, Emergency and Critical Care, Kyoto, Japan; [2]Kyoto Prefectural University of Medicine, Intensive Care, Kyoto, Japan
**Correspondence:** H. Narumiya
*Intensive Care Medicine Experimental* 2018, **6(Suppl 2):**0629

**INTRODUCTION.** Continuous blood purification for the treatment of sepsis and septic acute kidney injury (AKI) is currently widely used. After the polymethylmethacrylate (PMMA) membrane, the AN69ST membrane is the latest hemofilter for septic shock. Polymyxin B immobilized direct hemoperfusion (PMX-DHP) is one of the adjuvant therapies for sepsis and septic shock; however, the optimal combination of blood purification and PMX-DHP remains unclear.
**OBJECTIVES.** This study aimed to clarify the additional effects of PMX-DHP administered alongside continuous renal replacement therapy (CRRT) for septic AKI patients.
**METHODS.** One-hundred-fourteen patients who received CRRT with or without PMX-DHP for septic shock during the study period were recruited. Patients were divided into the following four groups: the AN69ST-CRRT group (n=35), PMMA-CRRT group (n=29), AN69ST+PMX group (n=23), and PMMA-PMX group (n=27). The patients' baseline characteristics, including information on age, sex, APACHE II score, lactate levels, and infection details, were recorded. The primary outcome measure was 28-day all-cause mortality. Secondary outcomes included mortality after intensive care unit discharge, vasopressor dose, and total fluid dosage in 24 h.
**RESULTS.** While the baseline parameters were not significantly different across the groups, the lactate levels of the AN69ST+PMX group and PMMA+PMX group were significantly higher than other groups (4.0±2.4 mmol/L, 4.2±2.3 mmol/L, 5.1±2.7 mmol/L and 5.0 ±2.9mmol/L, respectively). Death occurred in in 9/35 patients in the AN69ST-CRRT group, 7/29 patients in the PMMA-CRRT group, 8/23 patients in the AN69ST+PMX group and 11/27 patients in the PMMA-PMX group. There were no significant differences in mortality between these four groups. The vasopressors significantly decreased during 24 h in the AN69ST group, with or without PMX-DHP, compared to the PMMA-CRRT group despite PMX-DHP therapy ($p< 0.05$). The total fluid dosage for 24 h was significantly lower in the AN69ST +PMX group (mean ± SD; 2922 ± 1499 mL). Univariate regression analysis showed that the combination of AN69ST-CRRT and PMX-DHP was independently associated with differences in fluid dosage.
**CONCLUSIONS.** The combination of AN69ST-CRRT and PMX-DHP reduces the dosage of vasopressors needed and fluid infusion in patients with septic AKI.

**REFERENCE(S)**

Yamada H, Tsukamoto T, Narumiya H, et al. J Intensive Care 2016,10;4:64.

### 0630

#### The selection criteria for blood purification as a mediator modulator in critical care: a single-center case-control study

Y. Hara[1], K. Shimizu[2], N. Kuriyama[1], T. Nakamura[1], Y. Kurimoto[1], C. Yamashita[1], J. Shibata[1], H. Komura[1], O. Nishida[1]
[1]Fujita Health University School of Medicine, Department of Anesthesiology and Critical Care Medicine, Toyoake, Japan; [2]Fujita Health University Hospital, Department of Clinical Engineering, Toyoake, Japan
**Correspondence:** Y. Hara
*Intensive Care Medicine Experimental* 2018, **6(Suppl 2):**0630

**INTRODUCTION.** High flow-volume intermittent hemodiafiltration (sustained high-efficiency daily diafiltration [SHEDD-fA] using a mediator-adsorbing membrane) or continuous hemofiltration (CHF) were indicated as mediator modulation therapy. We previously reported the efficacy of SHEDD-fA hemodynamics in septic patients and oxygenation in patients with acute respiratory distress syndrome [1]. Intensivists were responsible in deciding whether SHEDD-fA or CHF should be used at the initial administration in the intensive care unit (ICU) based on a daily multidisciplinary conference. However, the selection criteria in choosing between SHEDD-fA and CHF have not yet been determined.
**OBJECTIVES.** This preliminary case-control study aimed to retrospectively determine the selection criteria of both modalities and to associate with randomized controlled trials planned in the future.
**METHODS.** Patients who underwent blood purification to modulate mediators from August 2014 to December 2016 in the ICU at the Fujita Health University Hospital in Japan were selected. Parameters on infection, circulation, kidney function, respiration, coagulation, and inflammation, acute physiology and chronic health evaluation (APACHE) II score, and sequential organ failure assessment (SOFA) score during the first blood purification were evaluated. Data were presented as median and interquartile range (IQR). Chi-squared test or Mann-Whitney U-test was used for between-group comparisons. All statistical analyses were performed using EZR (Saitama, Japan), a graphical user interface for R (Vienna, Austria). This case-control study was approved by the ethics committee of the Fujita Health University School of Medicine in Japan (No. HM17-301).
**RESULTS.** A total of 73 patients were eligible in this retrospective study. The median (IQR) of age, APACHE II score, and SOFA score before blood purification were 68 (65-76) years, 26 (19-33), and 9.0 (6.8-10.3), respectively. About 42% (n=31) of the patients had no acute kidney injury. Among the enrolled patients, 61 underwent SHEDD-fA and 12 underwent CHF. The proportion of infectious cases was significantly higher in the SHEDD-fA group than that in the CHF group (75.4% vs. 33.3%, $p=0.004$). Catecholamine index (CI) tended to be higher in the SHEDD-fA group than that in the CHF group (5.0 vs. 0 μg/kg/min, $p=0.11$). The differences in other parameters, such as blood lactate concentration, $PaO_2/F_IO_2$ ratio, white blood cell count, C-reactive protein, procalcitonin, fibrin/fibrinogen degradation products, thrombin-antithrombin complex, and severity scores were statistically insignificant. SHEDD-fA was significantly selected for critically ill patients with both infections and CI 4 or others ($p=0.0022$).
**CONCLUSIONS.** We frequently selected SHEDD-fA for patients with infection and CI 4 or others at the first administration of blood purification in our ICU.

**GRANT ACKNOWLEDGMENT**
None.

### 0631

#### Polymyxin hemoperfusion in gramnegative septic shock

J.-M. Bonell Goytisolo[1], R. Vento Rehues[1], I. Jara Zozaya[1], J. Barceló Planas[1], J. Lago Rodríguez[2], M. Romero Carratalá[1]
[1]Hospital Quirón Palmaplanas, Intensive Care Unit, Palma de Mallorca, Spain; [2]Hospital Quirón Palmaplanas, General Surgery, Palma de Mallorca, Spain
**Correspondence:** J.-M. Bonell Goytisolo
*Intensive Care Medicine Experimental* 2018, **6(Suppl 2):**0631

**OBJECTIVE.** To study the evolution of patients with gramnegative septic shock treated with B-polymyxin (B-PMX) hemoperfusion in a 16-bed ICU.
**INTRODUCTION.** B-polymyxin hemoperfusion is a therapy that can be used in refractory septic shock after an adequate antibiotic, hemodinamic management and focus control.
**METHODS.** B-PMX has an strong bactericide activity against gramnegative bacilli (GNB) and a great affinity for the endotoxin (LPS) of their wall. The cartridge with B-PMX molecules linked to polystyrene fibers can be used to neutralize LPS molecules in gramnegative sepsis through an extracorporeal therapy (hemoperfusion) with less adverse effects than administered systemically. Our study is prospective with those patients who were treated with B-PMX hemoperfusion in our unit since 2012.
**RESULTS.** 14 patients have been treated since then, 12 with abdominal foci (9 peritonitis, 3 biliary), t2 of unknown origin. Growing of GNB was confirmed in 8 cases (4 polymicrobial, 3 Escherischia coli, 1 Klebsiella pneumoniae), 1 Candida albicans and 5

negative cultures. All the patients were in refractory shock (noradrenalin dose required > 1 mcg/kg/min to keep MBP > 60-65 mm Hg) as well as other recommended measures according to Surviving Sepsis Campaign (SCC) guidelines, including focus drain. Eight patients were treated only with one session and other six with two. No complications due to the procedure were observed. Hospital mortality was 50%. In patients who survived a dramatical decrease 24h after B-PMX of noradrenalin requirement (1,71 ± 1,04 mcg/kg/min vs 0,46 ± 0,52 mcg/kg/min) and lactate levels (3,9 ± 1,3 mmol/L vs 2 ± 0,7 mmol/L) were observed. Patients who died received a similar noradrenalin dosage (1,44 ± 0,4 mcg/kg/min) before B-PMX compared with survivors, but had higher lactate levels (10 ± 5,2 mmol/L), the focus control was considered as suboptimal or was not done in 5 cases (71%), in front of 100% of non-indicated removal or optimal control in survivors.

**CONCLUSIONS.** B-PMX therapy may offer some hemodynamic improvement in patients with gramnegative septic shock, always associated with other recommended SCC measures. Patients with suboptimal focus control and higher lactate levels had a greater risk for poor response and mortality.

**REFERENCE(S)**

Cruz DN et al. Early use of polymyxin B hemoperfusion in abdominal septic shock: the EUPHAS randomized controlled trial. JAMA. 2009;301:2445-2452.

Cutuli SL et al. Polymyxin-B hemoperfusion in septic patients: analysis of a multicenter study. Ann Intensive Care. 2016; 6: 77.

### 0632

#### Not only endotoxin values but also coagulopathy and renal failure could contribute to 28 day mortality in septic shock after PMX-DHP therapy

M. Tsunoda, M. Kang, M. Saito, T. Oshiro, A. Yaguchi, M. Takeda
Tokyo Women's Medical University, Critical Care and Emergency Medicine, Tokyo, Japan
**Correspondence:** M. Tsunoda
*Intensive Care Medicine Experimental* 2018, **6(Suppl 2):**0632

**INTRODUCTION.** The Endotoxin Activity Assay (EAA™; Spectral Diagnostics Inc., Toronto, Canada) is a rapid in vitro diagnostic test of the neutrophil's reaction to endotoxin and reflects the endotoxemia1). Recently, EAA is used to inclusion criteria for the EUPHRATES (Evaluating the Use of Polymyxin B Hemoperfusion in a Randomized controlled trial of Adults Treated for Endotoxemia and Septic shock) trial study2). And MOD score is also used as inclusion criteria in that trial.

**OBJECTIVES.** The purpose of the present study is to clarify which is more effective factor for 28 day mortality between EAA (Endtoxin Activity Assay) values and MOD score at pre-treatment Polymyxin B hemoperfusion (PMX-DHP).

**METHODS.** The present study is a single-center retrospective observational analysis. From November 2014 to December 2017, all adult patients treated with PMX-DHP in our ICU and with pre-treatment EAA between 0.6 and 0.9 were included. EAA values between immediately before and immediately after PMX-DHP therapy, and MOD score, SOFA score, mean arterial pressure (MAP)(mmHg), P/F ratio, platelet counts (x $10^4$/μL), GCS and creatinine (mg/dL) immediately before PMX-DHP therapy, age and gender were compared between survivors and non-survivors at 28 day after therapy. Values are expressed as mean ± SD. Data was analysed by chi-square test and unpaired Students t-test. P values less than 0.05 were considered significant.

**RESULTS.** Twenty one patients (12 men and 9 women; mean age 64.2 ± 13.4 years) were studied. There were 12 survivors (6 men and 6 women) and 9 non-survivors (6 men and 3 women) at 28 day after therapy. EAA values immediately before and immediately after PMX-DHP therapy, MOD score and SOFA score, platelet counts and creatinine were statistically significant differences between survivors and non-survivors (0.68 ± 0.10 vs. 0.76 ± 0.08, p=0.03, 0.38 ± 0.10 vs. 0.71 ± 0.25, p=0.04, 6.8 ± 3.7 vs. 10.4 ± 3.8, p=0.04, 8.2 ± 4.9 vs. 13.7 ± 4.2, p=0.006, 16.6 ± 12.8 vs. 8.1 ± 5.8, p=0.03, 1.6 ± 1.1 vs. 2.8 ± 1.8, p=0.04, respectively). MAP, P/F ratio, GCS, age and gender were no significant differences between survivors and non-survivors.

**CONCLUSIONS.** There were significant differences in EAA values, MOD score, SOFA score, platelet counts and creatinine between survivors and non-survivors. Not only endotoxin values but also organ dysfunction such as coagulopathy and renal failure could contribute to 28 day mortality.

**REFERENCE(S)**

1) JC. Marshall,D Foster, Jean-Louis Vincent et al. JID 2004; 190: 527-534,
2) DJ. Klein, D Foster, CA Schorr et al. Trials 2014; 11: 218,
3) M Singer, CS. Deutschman, CW Seymour et al. JAMA 2016; 315: 801-810

### 0633

#### Initial experience of using polymyxin B hemoperfusion in abdominal septic shock: things to consider for better outcome

J.J. Kim, E.Y. Kim
Seoul St. Mary's Hospital, The Catholic University of Korea College of Medicine, Division of Trauma and Surgical Critical Care, Department of General Surgery, Seoul, Korea, Republic of
**Correspondence:** J.J. Kim
*Intensive Care Medicine Experimental* 2018, **6(Suppl 2):**0633

**INTRODUCTION.** Polymyxin B hemoperfusion, which treats septic shock by removing endotoxin of gram-negative bacteria (GNB), has been proposed as one of the treatment modalities for intraabdominal sepsis. However, there is a lack of studies about the factors need to be taken into consideration for a better outcome. The aim of this article is to introduce our experience of using polymyxin B hemoperfusion in abdominal septic shock and assess the factors affecting the outcome of this therapy.

**OBJECTIVES AND METHODS.** We performed polymyxin B hemoperfusion in 6 patients who were diagnosed with abdominal septic shock. During the treatment, the change of Sequential Organ Failure Assessment (SOFA) scores and dose of inotropic agents used were analyzed to assess the effectiveness of this therapy. Surgical outcomes and clinical courses of the patients were analyzed while reviewing the case with worse outcome to identify the negative factors affecting the outcome and the literature review was performed for the prior studies reporting polymyxin B hemoperfusion use in abdominal sepsis patients.

**RESULTS.** Except for one case, total SOFA score decreased steadily from the time immediately after the completion of hemoperfusion. The significant decrement of inotropic agent dosage was also observed in all of the 6 patients after hemoperfusion. There was one case of death, in which the initiation of hemoperfusion was delayed and prior source control was insufficient.

**CONCLUSIONS.** Authors suppose that polymyxin B hemoperfusion could be an effective therapy for treating abdominal septic shock and early use of this modality with definite initial source control might be important for the better outcome.

**REFERENCE(S)**

1. Fujii, T., Ganeko, R., Kataoka, Y. et al. Polymyxin B-immobilized hemoperfusion and mortality in critically ill adult patients with sepsis/septic shock: a systemic review with meta-analysis and trial sequential analysis. Intensive Care Med (2018) 44: 167
2. Coudroy R, Payen D, Launey Y, ABDOMIX group et al. Modulation by polymyxin-B hemoperfusion of inflammatory response related to severe peritonitis. Shock. 47:93

**GRANT ACKNOWLEDGMENT**

The authors have nothing to disclose.

### 0634

#### Fever control reduces the severity of diffuse intravascular dissemination in human endotoxemia, associated with inhibition of endothelial activation

M. Harmon[1], N. Heijnen[1], S. de Bruin[1], N. Sperna Weiland[1], M. Schultz[1], J. Horn[1], J. Meijers[2], N. Juffermans[1]

[1]Amsterdam Medical Center, Amsterdam, Netherlands; [2]Sanquin Research, Amsterdam, Netherlands

**Correspondence:** M. Harmon

*Intensive Care Medicine Experimental* 2018, **6(Suppl 2):**0634

**INTRODUCTION.** Fever may adversely affect outcome in critically ill patients. In patients with septic shock, fever control significantly improved 14-day mortality and reduced vasopressor use compared to baseline. However, the underlying physiological mechanisms of any potential benefit of fever control have yet to be determined.

**OBJECTIVES.** In this study, we aimed to investigate the effect of fever control on markers of coagulation and endothelial activation during endotoxemia in healthy volunteers.

**METHODS.** Healthy volunteers were injected with LPS (from *E.coli*, 2ng/kg) and randomized to either fever control to normothermia (36,0°C-38,0°C) or no fever control (n=6 per group). Fever control was achieved using an external surface cooling device (Artic Sun©), and cooled IV fluids (4°C). To control shivering, buspirone, clonidine and magnesium sulphate were given. In the control group, temperature was not managed. Both groups received an equal amount of IV fluids.

Blood samples were taken prior to LPS-infusion and 1, 3, 6, and 8 hours thereafter. Platelet levels, von Willebrand factor (vWF), conventional coagulation tests, rotational thromboelastometry (ROTEM©) analysis and calculated disseminated intravascular coagulation (DIC)-scores were used to assess the coagulation state of the volunteers. Data are presented as median (interquartile range) or mean ± standard deviation at T=3. A mixed effects model was used to asses differences between groups.

**RESULTS.** Mean body temperature was significantly lower in the normothermia group compared to the control group (37.2°C (36.8 - 37.7) vs 38.7°C (38.7 - 38.8), p = < 0.0001) as was SIRS-score (0 ± (0-0) vs 2 ± (1.3-2), p = 0.002). Heart rate decreased from 94 beats per minute (bpm) (84-98) to 65bpm (63-66), p = < 0.0001). Compared to the control group, the drop in platelet count was completely prevented in the normothermia group (194 x 10E9/L ± 7 vs 97 x 10E9/L ± 19, p = 0.02). Also, prolongation of PTT was prevented (23.5 sec ± (23-24) vs 20.5 sec (20-21.8), p = 0.002). D-dimer levels did not differ, but the DIC-score in the normothermia group was significantly lower compared to the control group 3 (1.5-3) vs 3.5 (3-4), p = .005). Also, there were less patients with a DIC score of >4 in the fever control group compared to control). There was no significant difference in ROTEM© results between both groups, including G values. Also, the normothermia group had significantly lower levels of vWF compared to the control group lower (208 antigen (Ag)% (164-232) vs 304 Ag% (216-359), p = 0.006.

**CONCLUSIONS.** Fever control prevents coagulation disturbances related to DIC in human endotoxemia. This phenomenon was associated with reduced shedding of vWF, suggesting inhibition of endothelial activation.

### 0635

#### Circulating evil humours cause mitochondrial dysfunction in sepsis. Does size matter?

S. Pollen[1], N. Arulkumaran[1], M. Duchen[2], M. Singer[1]

[1]UCL, Bloomsbury Institute of Intensive Care Medicine, London, United Kingdom; [2]UCL, Cell and Developmental Biology, London, United Kingdom

**Correspondence:** S. Pollen

*Intensive Care Medicine Experimental* 2018, **6(Suppl 2):**0635

**INTRODUCTION.** We have demonstrated that exposure of naïve kidney slices to septic serum increases reactive oxygen species (ROS) generation and decreases mitochondrial membrane potential (MMP) and NADH fluorescence, and that scavenging ROS can both prevent and reverse these changes.[1] Serum contains many mediators that may induce this observed mitochondrial dysfunction. Identifying if specific components within serum are contributory would enhance understanding of the underlying pathophysiology and offer a potential therapeutic target.

**OBJECTIVES.** To determine whether mitochondrial dysfunction in sepsis can be attributed to humoral components in septic serum above or below 50 kDa in size.

**METHODS.** Serum taken from septic rats with faecal peritonitis (and sham controls) at 24 h were size fractionated at a 50kDa cut-off. Adequate separation was confirmed by measuring albumin (64 kDa). Septic concentrate was mixed with sham filtrate to create a chimeric serum, and *vice versa*, with sham concentrate and septic filtrate. Live naïve kidney slices (200 μm thick) were then exposed to either type of chimeric serum and imaged with multi-photon confocal microscopy using fluorescent dyes to detect dynamic changes in MMP (measured with tetramethylrhodamine methyl ester, TMRM), ROS (dihydroethidium, HEt) and NADH redox state (NADH autofluorescence) over 90-minutes. Experimental results were expressed as the mean (n=4, ±SD) percentage relative fluorescence intensity (RFI) at 90-minutes compared to the baseline RFI at time zero.

**RESULTS.** Whilst both serum chimeras induced changes consistent with those seen with whole septic serum (Fig 1), the chimera containing the concentrated septic fraction (components ≥50 kDa) induced a greater rise in reactive oxygen species generation (136% vs 116%, p=0.07) and larger falls in MMP (66% vs 86%, p< 0.05) and NADH fluorescence (85% vs 96%, p=0.09), when compared to the chimera containing the filtrated septic serum fraction (Fig 1). [Figure notation: *=p< 0.05 vs sham serum, †=p< 0.05 vs septic serum, ‡=p< 0.05 vs septic concentrate + sham filtrate].

**CONCLUSIONS.** This preliminary study suggests the most influential component(s) circulating in septic serum that induce tubular epithelial cell mitochondrial dysfunction are above 50 kDa in size. Whether these circulating humours are large molecules or protein-bound smaller molecules is the subject of ongoing study.

**REFERENCE(S)**

1. Arulkumaran N, Pollen S, Greco E, Courtneidge H, Hall AM, Duchen MR, Tam FWK, Unwin RJ, Singer M, (2018) Renal Tubular Cell Mitochondrial Dysfunction Occurs Despite Preserved Renal Oxygen Delivery in Experimental Septic Acute Kidney Injury. Crit Care Med 46: e318-e325

**GRANT ACKNOWLEDGMENT**

UCL Medical School MBPhD Programme, Astor Foundation, and Intensive Care Society

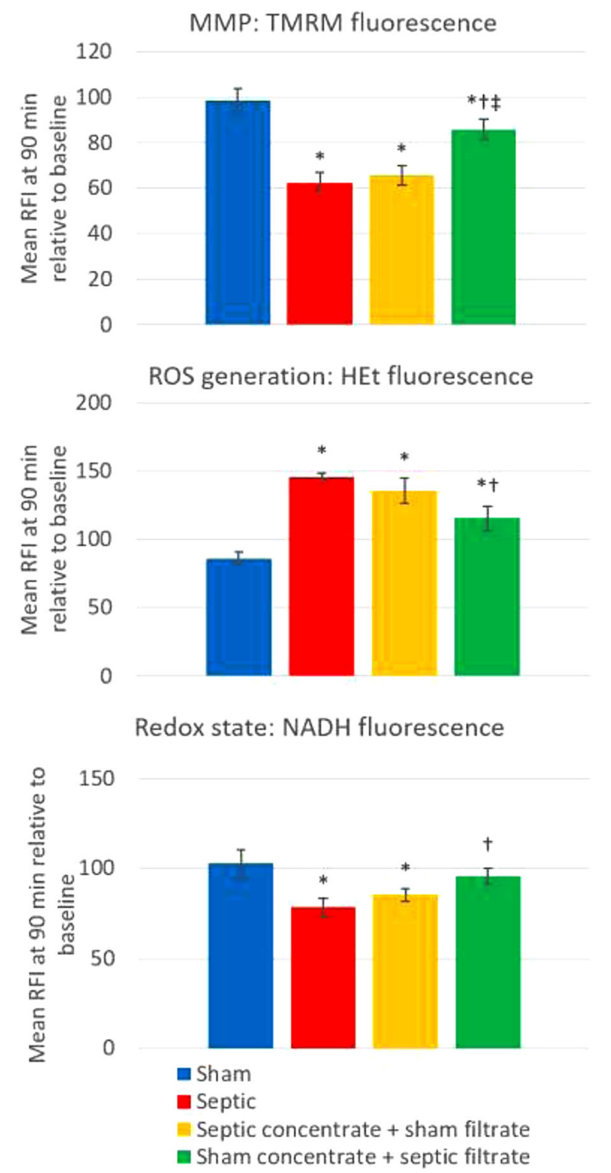

**Fig 1 (abstract 0635).** Relative fluorescence intensity of dyes probing mitochondrial function

### 0636

#### Human endotoxemia and hypoxia increase dicarbonyl stress in healthy men

R. Driessen[1], D. Bergmans[1], D. Kiers[2,3], P. Roekaerts[1], M. van de Poll[1], P. Pickkers[2,3], C. Schalkwijk[4], M. Kox[2,3], B. van Bussel[1]
[1]Maastricht University Medical Center, Intensive Care, Maastricht, Netherlands; [2]Radboud University Medical Centre, Nijmegen, Netherlands; [3]Radboud Center for Infectious Diseases, Nijmegen, Netherlands; [4]Maastricht University Medical Center, Internal Medicine, Maastricht, Netherlands
**Correspondence:** R. Driessen
*Intensive Care Medicine Experimental* 2018, **6(Suppl 2):**0636

**INTRODUCTION.** Dicarbonyl stress is the accumulation of dicarbonyl metabolites, such as 3-deoxyglucosone (3DG), which damage intracellular proteins, and modify extracellular matrix and plasma proteins. Recently, it was hypothesized that increased dicarbonyl stress contributes to multi-organ failure in critical illness (1). In addition to hyperglycemia, hypoxia and inflammation have been shown to increase dicarbonyl stress in vitro. In vivo data are however lacking.

**OBJECTIVES.** To investigate the hypothesis whether hypoxia, systemic inflammation or both conditions combined induce dicarbonyl stress in humans in vivo, we infused 2.5% glucose solution as a substrate during experimental human endotoxemia.

**METHODS.** In 30 healthy men, mean age 21±2 years, 1,5 L 2.5% glucose solution was infused in the hour preceding administration of endotoxin (2 ng/kg lipopolysaccharide [LPS]) or saline, followed by infusion of 1.2 L 2,5% glucose during the next 8 hours. We exposed 10 men to hypoxia (arterial saturation 80-85%, using a noninvasive ventilation helmet) for 3.5 hours, starting one hour before saline administration (HYP group). We exposed 10 men to experimental endotoxemia (LPS group) and 10 men to both conditions combined, with hypoxia starting one hour before LPS administration (LPS-HYP group). We measured plasma levels of 3DG repeatedly, using ultra-performance liquid chromatography tandem mass spectrometry. Repeated data were analyzed with generalized estimating equations. We report longitudinal β(95%CI) for the HYP and LPS groups, with the LPS-HYP group as reference. The study protocol complies with the guiding principles for experimental procedures as set forth in de Declaration of Helsinki.

**RESULTS.** Between 0-90 minutes 3DG increased by 3806±1055 nmol/L ($p < 0.001$) without a statistically significant difference between experimental conditions (p-values>0.427), and this increase thus occurred before LPS or saline administration.

Throughout the whole experiment, the 3DG concentration was higher in the combined LPS-HYP group, with a lower overall concentration β(95%CI) of -181 nmol/L (-313; -48), p=0.007 for the LPS group and a lower overall concentration of -282 nmol/L (-444; -119), p=0.001 for the HYP group (Figure I).

**CONCLUSIONS.** Our data reveal that glucose infusion increases dicarbonyl stress. Furthermore, systemic inflammation and hypoxia combined increase dicarbonyl stress in humans. Therefore, dicarbonyl stress, as a possible multi-organ failure contributing mechanism, requires further study in critical illness as it has potential for intervention.

##### REFERENCE(S)

1. van Bussel BC, van de Poll MC, Schalkwijk CG, Bergmans DC. Increased Dicarbonyl Stress as a Novel Mechanism of Multi-Organ Failure in Critical Illness. Int J Mol Sci. 2017;18(2).

##### GRANT ACKNOWLEDGMENT

This work was supported by a PhD grant from the Radboud Centre for Infectious Diseases and a Young Investigator Grant from the Dutch Society of Anesthesiology to MK.

## Acute respiratory failure, experimental studies

### 0637

#### Effects of Hippo signaling on anti-oxidative stress of mesenchymal stem cells in vitro

L. Dong, L. Li
Wuxi People's Hospital affiliated to Nanjing Medical University, Intensive Care Unit, Wuxi, China
**Correspondence:** L. Dong
*Intensive Care Medicine Experimental* 2018, **6(Suppl 2):**0637

**INTRODUCTION.** Hippo signaling could regulate the behavior and fate of a variety of cell types; however, its role in regulation of resistence of mesenchymal stem cells (MSCs) to oxidative stress-induced injuries remains to be clarified.

**OBJECTIVES.** Our aim was to determine the regulatory roles of Hippo signaling pathway in the development of resistance to oxidative stress-induced injuries in mouse bone marrow-derived mesenchymal stem cells (mMSCs).

**METHODS.** Primary mMSCs, isolated from C57BL/6 mice using the direct adherence method were identified by flow cytometry assay, and osteogenic, chondrogenic, and adipogenic differentiation were induced. The primary mMSCs were differentiated into the alveolar epithelial type II (ATII) cells using different co-culturing techniques. Hippo signaling pathway was modulated using 2-deoxy-D-glucose (2-DG) and anti-Fc epsilon RI antibody (9E1), while the oxidative stress-induced injuries were induced by $H_2O_2$ treatment at different concentrations. The effects this treatment on the survival of mMSCs and Hippo pathway activity were assessed using the MTT and western blot assays.

**RESULTS.** We detected the expression of apoptosis-related proteins, Bax and Bcl-2 using immunoblotting. Hippo signaling pathway activation was induced by 2-DG in a concentration-dependent manner, which was shown to be inhibited by 9E1 treatment. $H_2O_2$ treatment was demonstrated to induce mMSC injuries and inhibit the activity of Hippo signaling pathway. The decrease in Bcl-2/Bax ratio may be the mechanism underlying the observed mMSC injuries. The activation of Hippo signaling pathway may induce the resistance of mMSCs to oxidative stress through the increase in the Bcl-2/Bax level ratio, whereas the inhibition of Hippo signaling pathway prevents the resistance of mMSCs to oxidative stress by decreasing this ratio.

**CONCLUSIONS.** In summary, Hippo signaling pathway may affect the survival of mMSCs and the resistance to oxidative stress-induced injuries by regulating the Bcl-2/Bax expression ratio.

**GRANT ACKNOWLEDGMENT**

This work was supported by the National Natural Science Foundation of China (81400054), the Natural Science Foundation of Jiangsu Province (BK20140122); and the Talented Youth Program of Jiangsu Province (QNRC2017179).

### 0638

#### Silencing of circHIPK3 ameliorates fibroblast myofibroblast differentiation

J. Zhang, R. Wang

Shanghai General Hospital, Shanghai Jiaotong University, School of Medicine, Department of Critical Care Medicine, Shanghai, China

**Correspondence:** J. Zhang

*Intensive Care Medicine Experimental* 2018, **6(Suppl 2):**0638

**INTRODUCTION.** Idiopathic pulmonary fibrosis is a prototype of chronic, progressive, and fibrotic lung disease which leads to decreased lung compliance, disrupted gas exchange, and ultimately respiatory failure[1]. Progression of lung fibrotic formation is associated with the accumulation of fibroblasts and the promotion of fibroblast to myofibroblast differentiation (FMD)[2]. TGF-β1 is one of the factors that are responsible for fibrosis as it promotes FMD and is associated with up-regulation of a-smooth muscle actin. Therefore, inhibition of FMD may represent an effective strategy for the treatment of IPF. Recently, functional circRNAs have been shown to participate in the regulatory networks governing gene expression at transcriptional and post-transcriptional level[3]. The most recent research revealed that fibroblast contains abundant circular RNA (circRNA), circHIPK3, whose role in fibroblast remains unclear. In our study, we investigated the role of circHIPK3 in TGF-β1-induced FMD.

**OBJECTIVES.** To elucidate whether circHIPK3 is involved in TGF-β1-induced FMD.

**METHODS.** Quantitative real time polymerase chain reaction, Sanger sequencing and RNA fluorescent in situ hybridization were conducted to detect the expression partern of circHIPK3 in TGF-β1-treated lung fibroblasts and lungs from bleomycin-treated mice. Immunofluorescence, Western boltting and RNA interference were used to detect the role of circHIPK3 in TGF-β1-induced FMD in vitro.

**RESULTS.** In vivo study, circHIPK3 expression in pulmonary fibrosis mouse was significantly higher than control mice. In vitro FMD assay, elevated expression of circHIPK3 in TGF-β1-treated WI-38 was detected. circHIPK3 was resistant to RNase R digestion, whereas linear HIPK3 mRNA was easily degraded. The sanger sequencing result was completely in accordance with circHIPK3 sequence as shown in circBase. circHIPK3 was mainly expressed in the cytoplasm of WI-38. We next investigated the role of circHIPK3 in WI-38. Silencing circHIPK3 significantly inhibited TGF-β1-induced expression of α-SMA and collagenI.

**CONCLUSIONS.** Our study highlights the involment of circHIPK3 in TGF-β1-induced FMD. Altough the precise mechanism needs further study, this study facilitates the development of circRNA-directed diagnostics and therapeutics against pulmonary fibrosis.

**REFERENCE(S)**

1. Richeldi L, Collard HR, Jones MG. Idiopathic pulmonary fibrosis. Lancet (London, England). 2017; 389: 1941-52.
2. El Agha E, Kramann R, Schneider RK, Li X, Seeger W, Humphreys BD, Bellusci S. Mesenchymal Stem Cells in Fibrotic Disease. Cell stem cell. 2017; 21: 166-77.
3. Han B, Chao J, Yao H. Circular RNA and its mechanisms in disease: From the bench to the clinic. Pharmacology & therapeutics. 2018.

**GRANT ACKNOWLEDGMENT**

This work was supported by the Key and weak subject construction project of Shanghai Health and Family Planning System (no. 2016ZB0205).

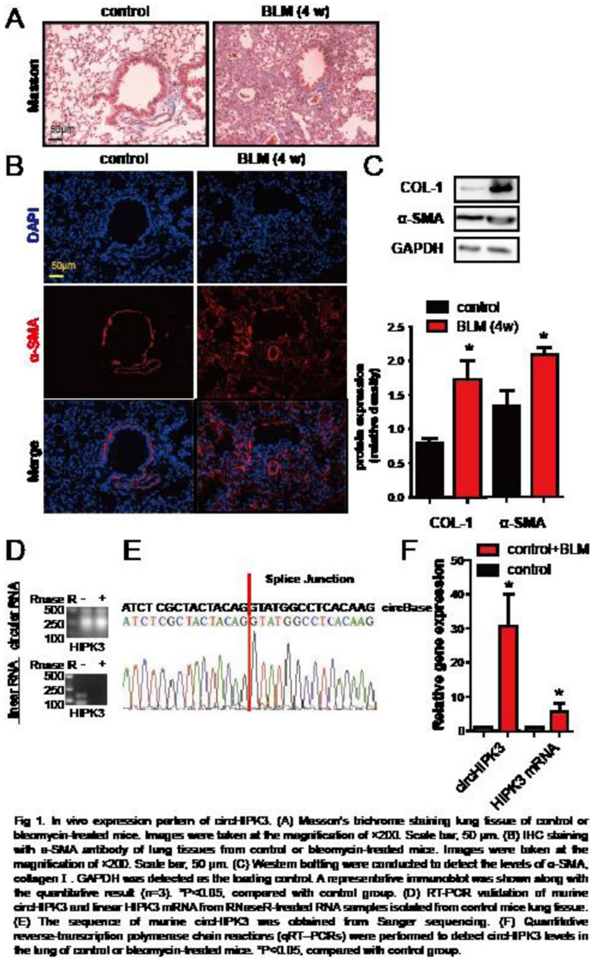

**Fig. 1 (abstract 0638).** See text for description

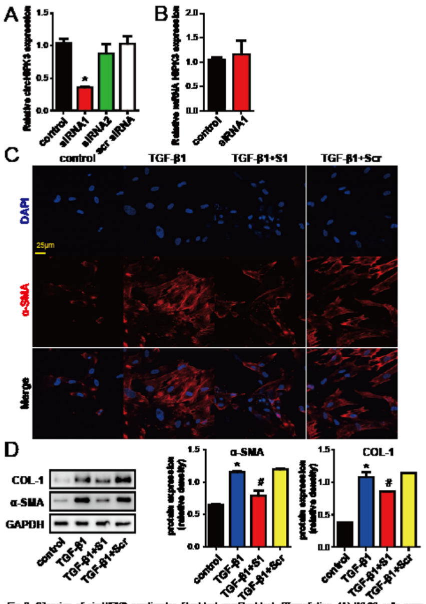

Fig 3. Silencing of circHIPK3 ameliorates fibroblast myofibroblast differentiation. (A) WI-38 cells were transfected with scrambled siRNA (Scr siRNA), siRNAs targeting the backsplice of circHIPK3 (siRNA1, siRNA2), or left untreated (control). qRT-PCR were conducted to detect circHIPK3 levels. *P<0.05, compared with control group. (B) qRT-PCR were conducted to detect linear HIPK3 mRNA levels. (C)WI-38 cells transfected with scrambled siRNA (Scr), siRNA1 (S1) or left untreated (control) for 48 h, and then these cells were exposed with or without 10 ng/ml TGF-β1 for 48 h. FMD marker α-SMA was analysed by IHC staining. Images were taken at the magnification of ×400. Scale bar, 25 μm. (D) FMD marker α-SMA, collagen I were detected by western bolting. GAPDH was detected as the loading control. A representative immunoblot was shown along with the quantitative result (n=3). *P<0.05, compared with control group.# P<0.05, compared with TGF-β1+Scr group.

**Fig. 3 (abstract 0638).** See text for description

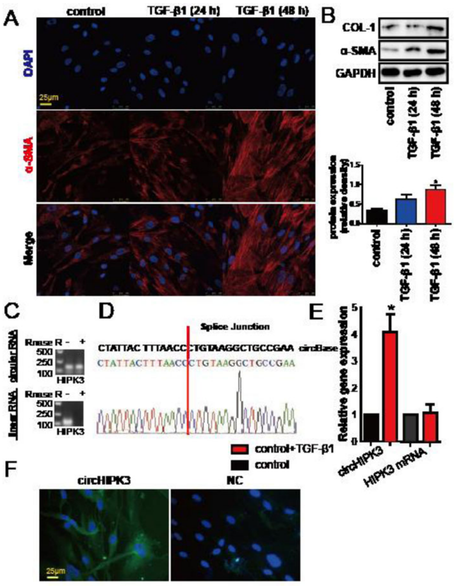

Fig 2. In vitro expression partem of circHIPK3. (A) IHC staining with α-SMA antibody of WI-38 cells treated with 10 ng/ml TGF-β1 for 24 or 48 h. Images were taken at the magnification of ×400. Scale bar, 25 μm. (B) Western bolting were conducted to detect the levels of α-SMA, collagen I . GAPDH was detected as the loading control. A representative immunoblot was shown along with the quantitative result (n=3). *P<0.05, compared with control group. (C) RT-PCR validation of human circHIPK3 and linear HIPK3 mRNA from RNaseR-treated RNA samples isolated from WI-38 cells. (D) The sequence of human circHIPK3 was obtained from Sanger sequencing. (E) Quantitative reverse-transcription polymerase chain reactions (qRT–PCRs) were performed to detect circHIPK3 levels in WI-38 cells or TGF-β1-treated WI-38 cells. *P<0.05, compared with control group. (F) RNA fluorescent in situ hybridization were conducted to detect the expression partem of circHIPK3 in WI-38 cells. Images were taken at the magnification of ×400.

**Fig. 2 (abstract 0638).** See text for description

### 0639

#### Magnetic resonance 4d flow evaluation of pulmonary vascular dysfunction in a porcine model of acute respiratory distress syndrome

A. Gaitan[1], I. Rodriguez[2], E. Yazdanparast[3], R. Mota[3], I. Bilbao[3], G. Peces-Barba[1,4], J. Ruiz-Cabello[1,5], A. Santos[1,6]
[1]Centro de Investigación Biomédica en Red, CIBER de Enfermedades Respiratorias (CIBERES), Madrid, Spain; [2]Universidad Complutense de Madrid, Madrid, Spain; [3]Centro Nacional de Investigaciones Cardiovasculares Carlos III (CNIC), Madrid, Spain; [4]Fundacion Jimenez Diaz, Department of Respiratory Medicine, Madrid, Spain; [5]CIC biomaGUNE, Donostia, Spain; [6]ITC Ingeniería y Técnicas Clínicas, Arganda del Rey, Spain
**Correspondence:** A. Santos
*Intensive Care Medicine Experimental* 2018, **6(Suppl 2):**0639

**INTRODUCTION.** Pulmonary artery (PA) dysfunction worsen outcomes in acute respiratory distress syndrome (ARDS). Magnetic resonance (MR) is a useful tool for the evaluation of complex flow patterns resulting from alterations in the vascular function which has improve the understanding and diagnosis of several cardiovascular diseases.
**OBJECTIVES.** To evaluate complex pulmonary flow patterns (vorticity) resulting from pulmonary vascular dysfunction caused by ARDS in a porcine model.
**METHODS.** 8 pigs (35.9±6.7 kg) were subjected to lung saline lavages followed by 1.5 hours of injurious mechanical ventilation to create a model of ARDS. Before and 30 minutes after injury phase contrast MR acquisition of the main PA was obtained to perform analysis of PA flow in 4 dimensions (4d, 3 spatial dimensions plus time dimension). A custom plugin for EnSight (Ansys, Canonsburg, USA) was developed to semi-automatically track PA flow and segment PA accordingly. Vortex phenomena were evaluated in a representative cardiac flow cycle (reconstruction of around 17 consecutive time frames covering the entire cardiac cycle) of each situation. Vortex was defined as the presence of helical pattern (figure 1b) in at least 2 consecutive frames. This evaluation is relevant as the presence of vortexes has been associated with an increased resistance and vascular dysfunction in several vascular diseases. From a physiological point of view, this is also important as energy could be wasted in non-forward flow streamlines (see figure 1a and 1b) Conventional pulmonary hemodynamics was also evaluated combing a PA catheter with cardiac MR cine analysis.
**RESULTS.** Vortexes were detected in 25% of pigs in baseline and 100% of the cases after ARDS (p=0.041). A representative image of evaluated flow patterns in baseline and after ARDS is shown in figure 1a and 1b respectively. Average duration of vortexes was 4.0±1.4 and 4.1±1.0 frames (around 24% of the entire cardiac cycle) and started at frame 4±1 and 3±1 after the maximum flow intensity frame in baseline and after ARDS, respectively. After ARDS pigs showed a higher mean PA pressure (35±6 vs 21±2 mmHg, p< 0.001) a lower compliance (2.16±0.98 vs 2.92 ±0.63 ml/mmHg, p=0.047) and a non-significant trend to higher resistance (712±379 vs 467±103 dyn.s.cm$^{-5}$, p=0.117) compared to baseline.
**CONCLUSIONS.** Vorticity was observed in 100% of cases after ARDS appearing as a complementary marker of pulmonary vascular dysfunction. Evaluation of these complex flow patterns are key to improve our understanding of the deleterious effects of ARDS on pulmonary circulation.

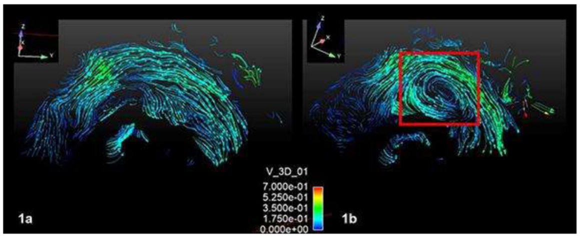

**Fig. 1 (abstract 0639).** 4d flow images before (1a) and after (1b) ARDS. A clear helical pattern (vortex) is seen after ARDS (red square). Frames in figures 1a and 1b represent a similar moment relative to the frame of maximum flow intensity.

### 0640

#### Effect of hypercapnia in *in vitro* human primary culture of alveolar cells

J. Bringué Roqué[1], L. Morales-Quinteros[2], M. Camprubí Rimblas[3], N. Tatinyà[3], R. Farré[4], I. Almendros[5], J.J. Fibla[6], R. Saumench[7], A. Artigas Raventós[1,2,8]

[1]Centro de Investigación Biomédica en red de Enfermedades Respiratorias, Barcelona, Spain; [2]Hospital Universitario Sagrat Cor, Barcelona, Spain; [3]Institut d'Investigació i Innovació Parc Taulí I3PT, Sabadell, Spain; [4]IDIBAPS/CIBERES, Unitat de Biofísica i Bioenginyeria, Facultat de Medicina i Ciències de la Salut, Universitat de Barcelona, Barcelona, Spain; [5]Centro de Investigación Biomédica en red de Enfermedades Respiratorias, Unitat de Biofísica i Bioenginyeria, Facultat de Medicina i Ciències de la Salut, Universitat de Barcelona, Barcelona, Spain; [6]Hospital Universitario Sagrat Cor, Department of Thoracic Surgery, Barcelona, Spain; [7]Hospital Universitari Mutua Terrassa, Department of Thoracic Surgery, Sabadell, Spain; [8]Corporació Sanitària i Universitària Parc Taulí, Sabadell, Spain

**Correspondence:** L. Morales-Quinteros

*Intensive Care Medicine Experimental* 2018, **6(Suppl 2):**0640

**INTRODUCTION.** Recent insights have emerged regarding the impact of hypercapnia on cellular and molecular function. Hypercapnia may have potentially beneficial effects in patients with ARDS, which are independent of the benefits from ventilation with low tidal volumes[1,2]. However, other studies suggest that $CO_2$ can act as a signaling molecule via pH independent mechanisms resulting in deleterious effects in the lung[3].

**OBJECTIVES.** Determine the effects and mechanism of action of different $CO_2$ and pH levels in human primary alveolar type II cells (hATII).

**METHODS.** After signing a patient informed consent, hATII were isolated from lung biopsies of patients who underwent lobectomy, and were incubated at 37ºC with 5% $CO_2$ concentration (normocapnia) or 15% $CO_2$ concentration [$pCO_2$=80 mmHg]. The media in both groups was buffered with 15mM THAM to maintain pH at 7.4. Analysis of pro-inflammatory (IL6, IL1β) and anti-inflammatory markers (IL10, IL4), as well as apoptosis (caspase 3, 8 and 9) and permeability markers (CCL2) were done by qRT-PCR. Results were analyzed by one way ANOVA using the Turkey´s range test.

**RESULTS.** hATII at 15% $CO_2$ produced an increased IL6 and IL1β. IL1β peak was reversed with THAM. Hypercapnia was associated with a decrease in CCL2 levels (monocyte recruitment) and an increase in apoptosis independent of pH (caspase 3, 8 and 9). See figure 1.

**CONCLUSIONS.** Human alveolar cells exposed to hypercapnia produced an inflammatory response, with a decrease in monocyte recruitment and a significant increase in apoptosis, all of them independent of pH. Considering the biological effects of hypercapnia, there is a need for more evidence from well-powered RCTs in order to determine its global effects in the lung.

1.Curley
GF,Laffey JG, Kavanagh BP. Cross Talk proposal: there is added benefit to providing permissive hypercapnia in the treatment of ARDS. J Physiol. 2013; 591: 2763-2765.

##### GRANT ACKNOWLEDGMENT

1. Fisher & Paykel
2. Insititut d´Investigació i Innovació Parc Taulí

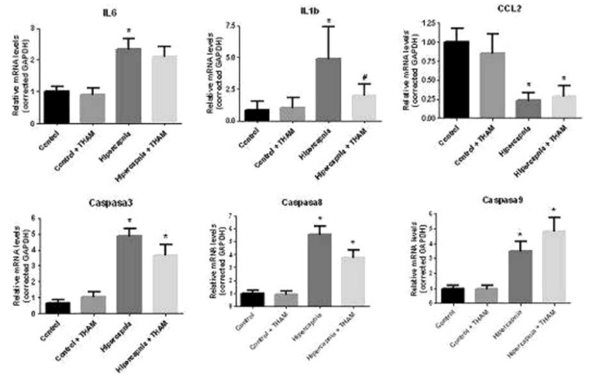

**Fig 1 (abstract 0640).** Relative mRNA expressions of different molecular markers in primary human alveolar type II cells. *p value < 0.05 vs control # p value < 0.05 vs hypercapnia (N=8). mRNA expression markers in hATII

### 0641

#### Antioxidative effect of human adult adipose derived mesenchymal stem cells in pulmonary epithelial damage induced by mechanical stretch

O. Peñuelas[1], A. Salazar[2], A. Ferruelo[3], I. Sanchez[4], C. Sanchez-Rodriguez[5], R. Murillo[6], R. Herrero[3], E. Barreiro[2], J.Á. Lorente[7]

[1]Hospital Unversitario de Getafe. CIBER de Enfermedades Respiratorias (CIBERES), Intensive Care Unit, Getafe, Spain; [2]IMIM Hospital del Mar Medical Research Institute, Universitat Pompeu Fabra (UPF), PRBB, Pulmonology Department-Muscle and Respiratory System Research Unit (URMAR)., Barcelona, Spain; [3]Hospital Universitario de Getafe; CIBER de Enfermedades Respiratorias, Intensive Care Unit, Getafe, Spain; [4]Hospital Universitario de Getafe, Biobank of Biomedical Research Foundation of Hospital Universitario de Getafe, Getafe, Spain; [5]Hospital Universitario de Getafe, Universidad Europea de Madrid, Research, Getafe, Spain; [6]Hospital Universitario de Getafe, Biomedical Research Foundation of Hospital Universitario de Getafe, Getafe, Spain; [7]Hospital Universitario de Getafe; CIBER de Enfermedades Respiratorias. Universidad Europea de Madrid, Intensive Care Unit, Getafe, Spain

**Correspondence:** O. Peñuelas

*Intensive Care Medicine Experimental* 2018, **6(Suppl 2):**0641

**INTRODUCTION.** The growing interest in the clinical application of human adult stem cells derived from adipose tissue (human adult adipose-derived mesenchymal stem cells, ADMSC) is based on its anti-inflammatory properties. The antioxidant effect of ADMSC has not been well documented in acute lung injury models.

**OBJECTIVE.** To demonstrate that ADMSCs have an antioxidative effect on lung epithelial cells subjected to cyclic stretching in vitro.

**METHODS.** Experimental study in the University Hospital of Getafe.Isolation of ADMSCs and human fibroblasts: Adipose tissue samples (obtained with the informed consent of patients undergoing scheduled mammary reduction surgery) will be processed to obtain, isolate and culture ADMSCs. Samples of donor dermal tissue will be obtained, with informed consent, for the isolation and culture of human dermal fibroblasts, in a standard culture condition.

Cell injury model: human Pulmonary alveolar epithelial cells (HPAEpiC) will undergo cyclic stretching, with an elongation of 15% during 24 hours (representing 50% of the total lung capacity) under standard culture conditions. The ADMSC will be added in co-culture on a 0.4 micron membrane of "transwell". The ratio will be 1: 3 (1 ADMSC / 3 HPAEpiC). As a negative control group, human fibroblasts (FBF), will be used. Six groups will be perfomed; HPAEpiC without stretching (0%); HPAEpiC alone subjected to stretching 15%; HPAEpiC without stretching (0%) with ADMSC; and HPAEpiC subjected to stretching 15% with ADMSC, HPAEpiC without stretching (0%) with FBF; and finally HPAEpiC subjected to stretching 15% with FBF.

Measurements: At the end of the experiments, the total activity of superoxide dismutase (SOD), glutathione peroxidase (GsH) and 8-isoprostane (8-iso), catalase activity, and inducible NOS (iNOS) were measured by ELISA in the cell lysate and the supernatant, respectively. Finally, the activation of the intracellular transcription factor Nfr-2 will be measured by ELISA in the cell lysate.

Statistical analysis. The statistically significant differences will be tested by ANOVA analysis.

**RESULTS.** Oxidative damage was observed in HPAEpiC subjected to mechanical stretch during 24 hours and characterized by a non-significant decrease in SOD, and catalase (all p=0.234) that was not attenuated in conditions in vitro conditions of co-culture with ADMSC or FBF.

**CONCLUSIONS.** ADMSC showed no effect on oxidative stress when used as cell therapy in a co-culture of in vitro model of human lung alveolar cells injury induced by cyclic stretch.

Induced by cigarette smoking. Am J Respir Crit Care Med 2011; 183: 215-225.

**GRANT ACKNOWLEDGMENT**

*Sociedad Española de Pulmón y Aparato Respiratorio (SEPAR) Award.*

### 0642

#### Global and regional strain increase in extubated subjects on spontaneous ventilation after experimental lung injury. Preliminary results

P. Cruces[1], B. Erranz[2], F. Lillo[1], M. Sarabia[3], P. Iturrieta[3], F. Morales[1], K. Blaha[2], T. Medina[4], F. Diaz[2,5], J. Retamal[6], D. Hurtado[3]

[1]Universidad Andres Bello, Centro de Investigación de Medicina Veterinaria, Santiago, Chile; [2]Facultad de Medicina Clínica Alemana - Universidad del Desarrollo, Santiago, Chile; [3]Pontificia Universidad Católica de Chile, Department of Structural and Geotechnical Engineering, School of Engineering, Santiago, Chile; [4]Hospital El Carmen de Maipú, Santiago, Chile; [5]Clínica Alemana de Santiago, Pediatric Critical Care, Santiago, Chile; [6]Pontificia Universidad Catolica de Chile, Departamento de Medicina Intensiva, Santiago, Chile

**Correspondence:** P. Cruces

*Intensive Care Medicine Experimental* 2018, **6(Suppl 2):**0642

**INTRODUCTION.** High respiratory drive associated to moderate to severe ALI in subjects on spontaneous ventilation can be deleterious due to several mechanisms, like uncontrolled transpulmonary pressures, active expiration phenomenon, and excessive regional strain and tidal recruitment in dependent lung regions.

**OBJECTIVES.** Our aim was to find the temporal behavior and spatial patterns of regional lung strain by 4D μ-CT and image registration techniques in extubated subjects after experimental lung injury with spontaneous ventilation.

**METHODS.** 12 anesthetized Sprague-Dawley 300 gr rats were randomly allocated to sham group (n = 6) or acute lung injury group (ALI group, n = 6). Lung injury was induced by surfactant depletion. The animals were placed in prone position, extubated and supported with 100% $FIO_2$. The animals remained under inhalatory anesthesia while m-CT scanning was performed at 0h and 3h of spontaneous ventilation (SkyScan 1278 *in-vivo* micro-CT scanner, Bruker®, Belgium). A map of regional volumetric strain was constructed from expiration to inspiration phase using a finite-element approach. Functional residual capacity (FRC), VT and global strain were determined from lung segmentation volumes by comparing the end-expiration and end-inspiration volumes in each case.

**RESULTS.** Animals in ALI group were more hypoxemic and tachypneic at 0 and 3h respect to sham group. Lung CT at 0 and 3h showed more collapsed lung in ALI group compared to Sham (Figure 1). A higher regional strain was observed in injured animals (ALI group) compared to sham at 0h. After 3h of spontaneous breathing regional strain augmented in ALI group and decreased in sham group, increasing the difference between groups (Figure 2 and 3).

**CONCLUSIONS.** Temporal evolution of global and regional strain in extubated subjects with spontaneous ventilation shows a sustained intensification. Opposite effect was observed in sham group, decreasing measured strain after intervention. Future studies should clarify the role of regional deformation as a mechanism producing pathological mechanotransduction and inflammation.

**REFERENCE(S)**

Does Regional Lung Strain Correlate With Regional Inflammation in Acute Respiratory Distress Syndrome During Nonprotective Ventilation? An Experimental Porcine Study. Retamal J, et al. Crit Care Med. 2018

**GRANT ACKNOWLEDGMENT**

Fondecyt 1160631 and 11160463

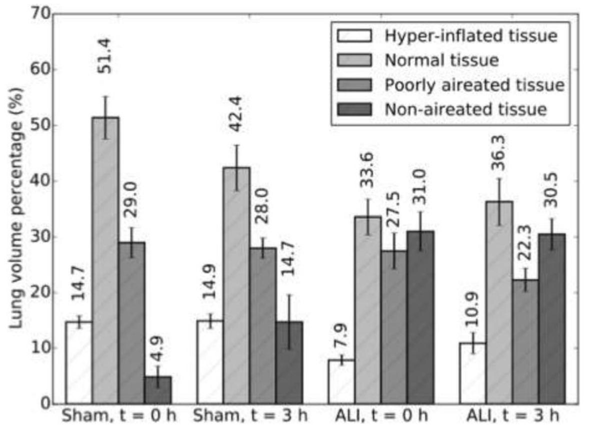

**Fig. 1 (abstract 0642).** Lung density distribution al 0h and 3h in sham and ALI group

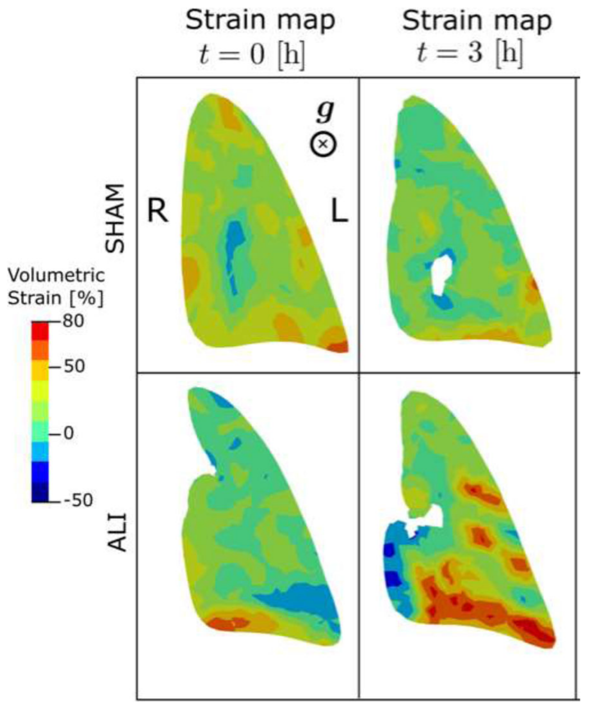

**Fig. 2 (abstract 0642).** Representative slices of regional lung volumetric strain maps at 0 and 3h of both groups

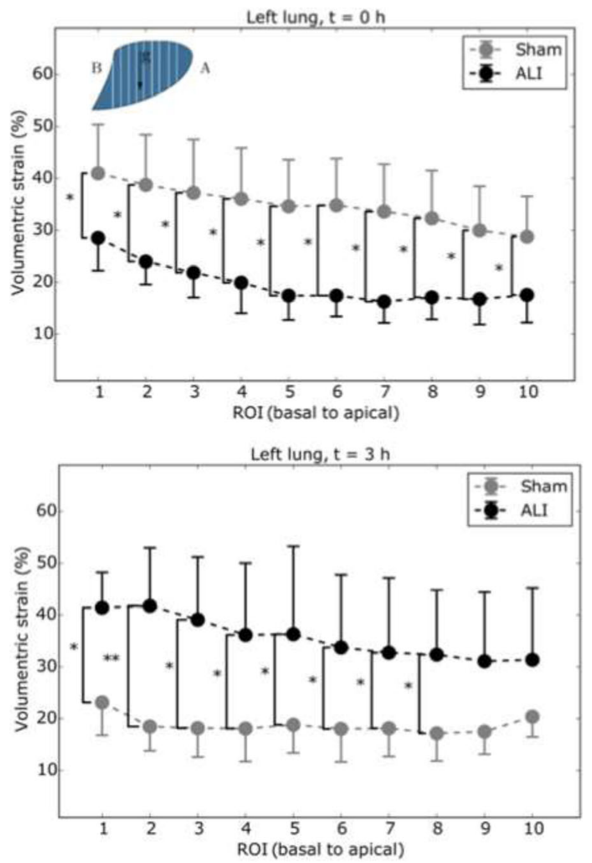

**Fig. 3 (abstract 0642).** Temporal evolution of volumetric strain per ROI (left lung) in both groups, at 0h and 6h.

### 0643

#### Effects of acute hyperoxia exposure in lipopolysaccharide (LPS) lung injury mouse model

J.H. Ha, S.W. Kim, I.K. Kim, H.H. Kang, S.H. Lee
College of Medicine, The Catholic University of Korea, Seoul, Korea, Republic of
**Correspondence:** S.W. Kim
*Intensive Care Medicine Experimental* 2018, **6(Suppl 2):**0643

**INTRODUCTION.** Hyperoxia can cause injury to normal lung tissue. However, patients with acute respiratory distress syndrome frequently be exposed to very high $FiO_2$ ($FiO_2 \geq 0.9$).
**OBJECTIVES.** The purpose of this study is to evaluate the effect of hyperoxia exposure in acute lung injury state.
**METHODS.** Lipopolysaccharide (LPS, 055:B5, 2 mg/kg) or PBS was administered intratracheally in male C57BL/6J mice (7-8 weeks old). After 2h, mice were divided into two groups, and then exposed to normoxia or hyperoxia condition for 48h. Hyperoxia groups were placed within their cages in a Plexiglass chamber, in which an atmosphere of 95% $O_2$ was constantly monitored using an oxygen analyzer.
**RESULTS.** From BAL fluid, numbers of total cells and neutrophils significantly increased in LPS + normoxia and LPS + hyperoxia group compared with the control normoxia group ($^{**}p < 0.01$). Lung wet/dry ratio was also significantly increased in LPS + normoxia and LPS + hyperoxia group compared with the control normoxia group ($^{**}p < 0.01$). However, there were no significant differences in numbers of total cells, neutrophils and lung wet/dry ratio between LPS + normoxia and LPS + hyperoxia group. By histological evaluation of H&E stained lung tissue sections, edema, interstitial thickening, and inflammatory cell influx showed no significant difference between LPS + normoxia and LPS + hyperoxia group.
**CONCLUSION.** In LPS induced acute lung injury mouse model, no significant differences were seen in lung inflammation and lung injury between LPS + normoxia and LPS + hyperoxia group. From our results, hyperoxia did not augment LPS-induced lung injury in mice.

### 0644

#### Respiratory enthalpy is not affected by acute manipulations of the ventilation-perfusion-ratio in a porcine model

M. Edlinger-Stanger[1], M.H. Bernardi[1], S. Böhme[2], K. Kovacs[1], M. Mascha[1], T. Neugebauer[1], M. Hiesmayr[1]
[1]Medical University of Vienna, Department of Anesthesia, Critical Care and Pain Medicine, Division of Cardiothoracic and Vascular Anesthesia and Intensive Care Medicine, Vienna, Austria; [2]Medical University of Vienna, Department of Anesthesia, Critical Care and Pain Medicine, Vienna, Austria
**Correspondence:** M. Edlinger-Stanger
*Intensive Care Medicine Experimental* 2018, **6(Suppl 2):**0644

**INTRODUCTION.** Hypoventilation, increased dead space ventilation and venous admixture are important reasons for hypoxemia in critically ill patients leading to mismatch of the ventilation (V) and perfusion (Q) ratio (V/Q). The multiple inert gas elimination technique (MIGET) is the current gold standard for measuring V/Q[1]. Due to high technical and personnel efforts, MIGET cannot be routinely employed in clinical practice. Changes in ventilation and perfusion modulate heat transfer in the lung[2,3,4]. Therefore, measuring respiratory enthalpy (RE) may be an alternative technique to investigate V/Q. RE can be measured using the novel VQm™ monitor (Rostrum Medical Innovations, Vancouver, CA), which is connected to the breathing circuit. This bedside monitoring of V/Q may allow evaluation of the effect associated with specific therapy and support personalized ventilator care.
**OBJECTIVES.** We tested whether shifts of V/Q can be monitored by measuring RE using the VQm™ monitor in a porcine model.
**METHODS.** We designed four pathophysiologic interventions (I) to manipulate V/Q in 11 anesthetized pigs (51-83kg): IA: shunt, achieved by single-lung-ventilation due to blocking of the left lung (low V/Q); IB: high cardiac output, achieved by infusion of dobutamine (low V/Q); IC: dead space, achieved by wedging of the left or right pulmonary artery using an inflatable intravascular balloon (high V/Q) and ID: ARDS simulation, achieved by repetitive lung-lavage until PaO2 < 80mmHg at FiO2 1.0 (low V/Q). Effects on V/Q were validated by micropore membrane inlet mass spectrometry MIGET.
**RESULTS.** Baseline RE was very variable among different animals (114 ± 63 Joules/min, mean ± SD). Therefore, RE results were normalized to the individual baseline measurement. MIGET data, conventional calculations of dead space and shunt, and the PaO2/FiO2-ratio demonstrated that our interventions lead to the expected manipulation of V/Q (*Fig. 1* and *Fig. 2*). In contrast, RE measured by the VQm™ monitor did not change significantly. Results are summarized in *Table 1*.
**CONCLUSIONS.** In the present experimental model, we did not find significant changes in RE measured by the VQm™ monitor.

##### REFERENCE(S)

1. Wagner PD. *Intensive Care Med*. 2008;34(6):994-1001.
2. Serikov *et al. Eur J Appl Physiol*. 2004;91(2-3):314-323.
3. Serikov *et* Fleming. *J Appl Physiol*. 2001;91(5):1977-1985.
4. Serikov *et al. J Appl Physiol*. 1997;83(2):668-676.

##### GRANT ACKNOWLEDGMENT

Materials used for measurement of RE were provided by Rostrum Medical Innovations, Vancouver, CA. This study was supported by the Medical University of Vienna and Rostrum Medical Innovations (Vancouver, CA).

**Table 1 (abstract 0644).** Results = mean ± SD. *statistical significant difference (alpha<0.05) with respect to BL (one-way ANOVA multiple comparisons)

| | BL | IA | IB | IC | ID |
|---|---|---|---|---|---|
| RE | 1 | 0,94 ± 0,16 | 1,12 ± 0,21 | 1,03 ± 0,15 | 0,96 ± 0,11 |
| Shunt | 10,37 ± 1,97 | 21,49 ± 6,19* | 16,14 ± 2,74 | 15,834 ± 5,66 | 43,92 ± 7,60* |
| Dead space | 13,73 ± 6,93 | 17,38 ± 8,71 | 10,72 ± 7,68 | 39,44 ± 15,38* | 42,83 ± 16,03 * |
| PaO2/FiO2 | 555,5 ± 93,03 | 348 ± 79,43* | 404,1 ± 33,23* | 350 ± 71,87* | 85,2 ± 19,33* |

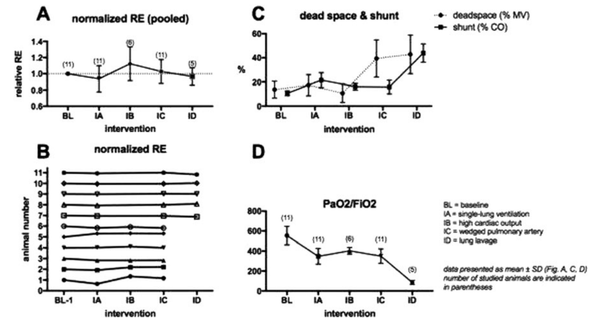

**Fig. 1 (abstract 0644).** Effects of interventions on RE (A&B), calculated dead space and shunt (C) and the PaO2/FiO2 ratio (D)

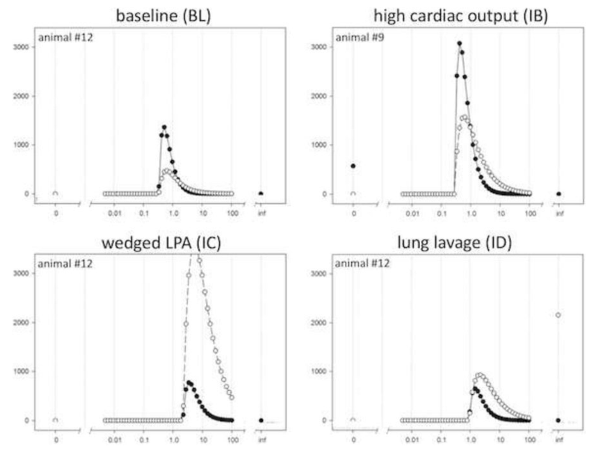

**Fig. 2 (abstract 0644).** Examples of MIGET results

### 0645

#### Small airway hyperreactivity in an experimental model of acute lung injury

R. Basoalto[1,2], D. Soto[1], M. Fonseca[2], J. Retamal[1], G. Bugedo[1], M. Henriquez[2], A. Bruhn[1]

[1]Pontificia Universidad Catolica de Chile, Departamento de Medicina Intensiva, Santiago, Chile; [2]Facultad de Medicina, Universidad de Chile, Programa de Fisiología y Biofisíca ICBM, Santiago, Chile

**Correspondence:** R. Basoalto

*Intensive Care Medicine Experimental* 2018, **6(Suppl 2):**0645

**INTRODUCTION.** Acute respiratory distress syndrome (ARDS) is characterized by decreased lung compliance, but with normal resistance. However, respiratory system resistance is mainly determined by the large airways and there is scarce information regarding the resistance or reactivity of the small airways.

**OBJECTIVES.** To evaluate ex vivo the reactivity of the small airways, by means of a precision-cut lung slices (PCLS) technique, in lungs obtained from rats previously subjected to a model of acute lung injury (ALI) secondary to hydrochloric acid (HCl) instillation.

**METHODS.** Under deep anesthesia, 12 male sprague dawley rats (300-350 grams) were tracheostomized and then instilled intratracheally with HCl (2 ml/Kg, 0,1 N and pH 1.5) (ALI group), or NaCl 0.9% (2 ml/kg) (control group); (n= 6 each). Thereafter, rats were connected to mechanical ventilation (vt=7 ml/kg, respiratory rate 95 bpm, I:E of 1:2, y $FiO_2$ 1.0) and maintained with deep anesthesia, neuromuscular blockade and invasive blood pressure monitoring. After 4 hours rats were euthanized and the lungs extracted by thoracotomy. The left lung was insufflated with 2% agarose and 150 μm thick lung slices were cut and cultured (37°C y $CO_2$ 5%) for 12 hours. Then, by means of phase contrast video microscopy, the bronchoconstrictor response to carbachol (CCh) (0.001 to 100 μM) and histamine (0.001 to 100 μM) was assessed in bronchioles of 100 to 200μm diameter.

**RESULTS.** Rats from the ALI group exhibited hypoxemia until the end of the experimental period ($PaO_2/FiO_2$ 118 vs 306 of the control group) (p=0.004) and lung edema (wet/dry weight ratio 7.15 vs 5.84 of the control group) (p= 0.002). The bronchoconstrictor response was significantly higher in the ALI group, both for carbachol and histamine (Figures 1-3). Differences with the control group were evident at high concentrations.

**CONCLUSIONS.** In an ALI model secondary to HCL instillation we observed small airway hyperresponsiveness. This is the first description of small airway reactivity in ALI. This finding may have implications for ALI pathophysiology, but more studies are required in different experimental models of ALI, and in ARDS patients, to determine its clinical relevance.

**GRANT ACKNOWLEDGMENT**

CONICYT, FONDECYT N°1140468 and FONDECYT N°1161556

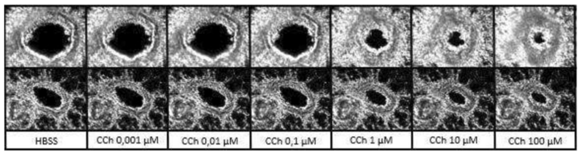

**Fig. 1 (abstract 0645).** Representative phase contrast microscopy images of a small airway from the ALI (up) and the Control (down) groups

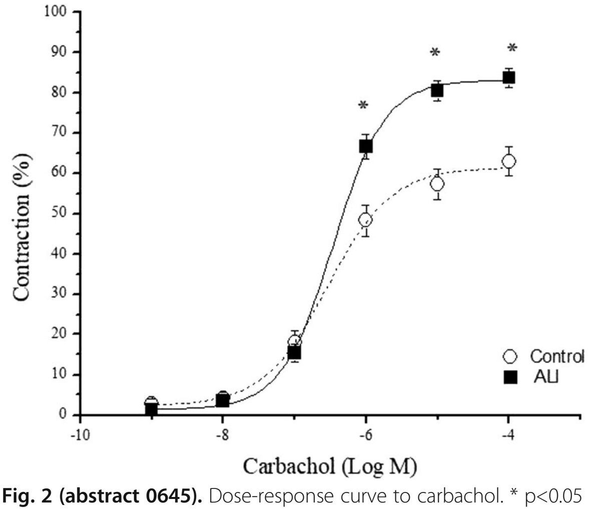

**Fig. 2 (abstract 0645).** Dose-response curve to carbachol. * p<0.05 compared to Control

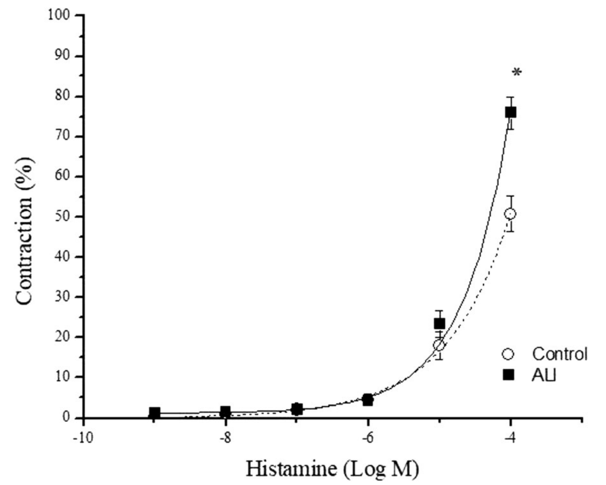

**Fig. 3 (abstract 0645).** Dose response curve to histamine. * $p<0.05$ compared to Control

### 0646

#### The washout of $CO_2$ from anatomical dead space is the same with Hi-VNI and Optiflow

Y. Onodera[1], N. Nakamura[1], R. Akimoto[1], H. Suzuki[1], M. Nakane[2], K. Kawamae[1]
[1]Yamagata University Faculty of Medicine, Department of Anesthesiology, Yamagata, Japan; [2]Yamagata University Faculty of Medicine, Department of Emergency and Critical Care Medicine, Yamagata, Japan
**Correspondence:** Y. Onodera
*Intensive Care Medicine Experimental* 2018, **6(Suppl 2):**0646

**INTRODUCTION.** Several high-flow nasal cannula (HFNC) interfaces are now commercially available. The Hi-VNI® (Vapotherm, New Hampshire, USA) is a new interface that delivers higher gas flow velocity than other interfaces due to a narrower tip outlet. This difference in flow velocity between interfaces might influence $CO_2$ washout, but this has only been evaluated in a computational model that did not simulate breathing.

Therefore, we compared Hi-VNI and Optiflow$^{TM}$ (Fisher and Paykel Healthcare, Auckland, NZ) using a sophisticated respiratory model to determine if interface affects $CO_2$ washout form anatomical dead space.

**OBJECTIVE.** To compare $CO_2$ washout form anatomical dead space of two different HFNC interfaces.

**METHODS.** An airway model was created by a 3D printer using craniocervical 3D-CT data from a healthy, 32-year-old male with a height of 170 cm and ideal body weight of 65 kg. Sampling ports were placed to measure capnograms of the upper pharynx, oral cavity, and trachea. Anatomical dead space was adjusted to 3 ml/kg, and $CO_2$ was infused into the model (LUNGOO: Air Water Safety Service Inc., Kobe, Japan). The model parameters were adjusted to simulate normal respiration (compliance [C], 50 ml/cm$H_2O$; resistance [R], 5 cm$H_2O$/L/s; VT, 520 ml (8 ml/kg); Ti, 1.0 sec; RR, 15/min), and restrictive (C, 30 ml/cm$H_2O$; R, 5 cm$H_2O$/L/s; VT, 320 ml (5 ml/kg); Ti, 1.0 sec; RR, 30/min) or obstructive (C, 80 ml/cm$H_2O$; R, 15 cm$H_2O$/L/s; VT, 700 ml (11 ml/kg); Ti,1.0 sec; RR, 10/min) respiration with a $P_{ET}CO_2$ of 40 mmHg in the trachea. Either the Optiflow or Hi-VNI was attached to the airway model for 3 minutes, and flow was increased in steps of 10 L/min from 10 to 50 L/min. Capnograms were recorded from breaths after each phase.

**RESULTS.** Capnograms of the upper pharynx showed that $CO_2$ was fully washed out by both interfaces before the start of the next inspiration, with HFNC flow of 20 L/min in all three respiratory states (Figure A). Capnograms of the oral cavity showed that $CO_2$ was washed out by both interfaces, but $PCO_2$ before the start of the next inspiration was slightly lower with Hi-VNI than Optiflow in obstructive respiration (Figure B). Tracheal capnograms showed the same waveform with both interfaces in each respiratory state; thus, neither interface washed $CO_2$ out of the trachea (Figure C). To compare the efficiency of $CO_2$ washout between interfaces, $P_{ET}CO_2$ was recorded in the trachea (Figure D). Both Hi-VNI and Optiflow showed the same trends in each respiratory state; thus, both interfaces washed out about the same amount of $CO_2$ from the anatomical dead space.

The slight difference in capnograms recorded at the oral cavity with obstructive respiration may have been caused by a higher flow velocity generated by Hi-VNI, but the difference was not sufficient to improve $CO_2$ washout form anatomical dead space.

**CONCLUSIONS.** The $CO_2$ washout was the same with Hi-VNI and Optiflow.

**REFERENCE(S)**

None

**GRANT ACKNOWLEDGMENT**

None

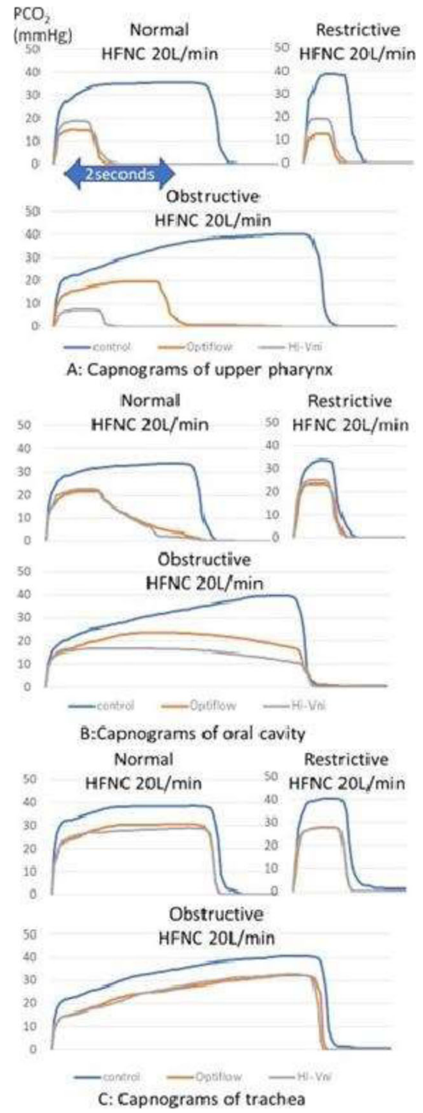

**Fig. 1 (abstract 0646).** Capnograms recorded at upper pharynx, oral cavity and thrachea

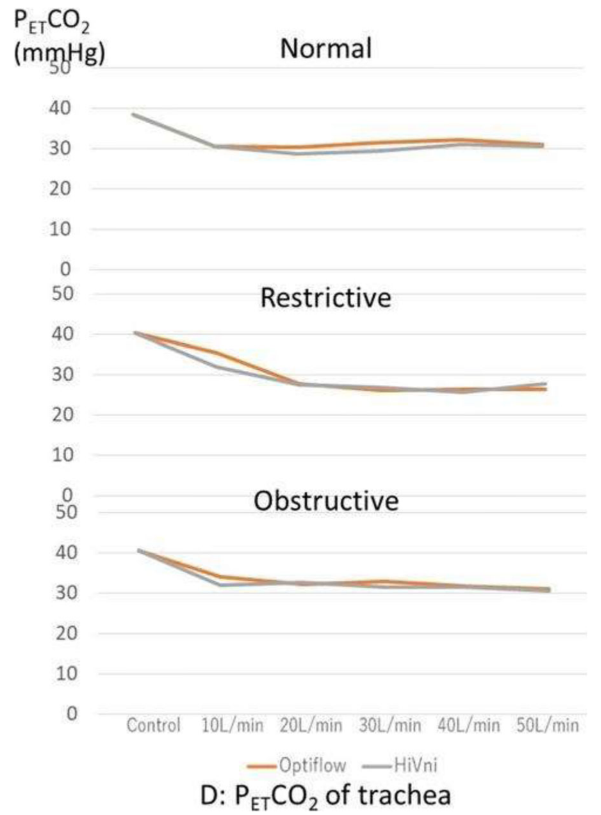

**Fig. 2 (abstract 0646).** PETCO2 recorded at trachea

(e.g. TATc: Thrombin-Antithrombin complexes TATc). Statistics by Mann-Whitney U test.

**RESULTS.** LPS plus injurious ventilation resulted in pulmonary inflammation and coagulopathy.

RecAP did not affect pulmonary inflammation or coagulation. (Figure 1)

**CONCLUSIONS.** RecAP does not affect pulmonary inflammation or coagulation in the acute phase of this established two-hit lung injury model in rats. Further investigations are needed to expand on the knowledge about the effects of alkaline phosphatase within the pulmonary compartment.

**REFERENCE(S)**

[1] Crit Care. 2012;16(1):R14.
[2] Physiol Res. 2015;64(5):731-8.
[3] Toxicol Appl Pharmacol. 2016;313:88-96.
[4] Infect Immun. 2005;73(7):4309-14.

**GRANT ACKNOWLEDGMENT**

None.

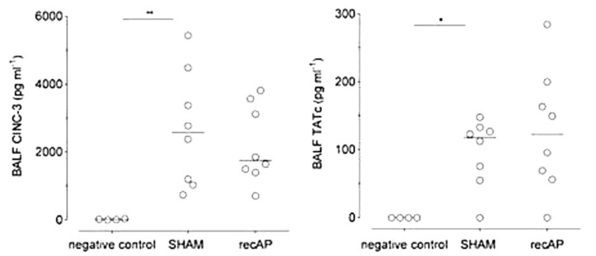

**Fig. 1 (abstract 0647).** Markers of pulmonary inflammation and coagulation in a two-hit lung injury model in rats. *: p-value < 0.05, **: p value < 0.01

### 0647

#### Alkaline phosphatase does not affect pulmonary inflammation and coagulation in a two-hit lung injury model

J. Juschten[1,2,3], S.A. Ingelse[2,4], M.A.W. Maas[2], A.R.J. Girbes[3], N.P. Juffermans[1,2], P.R. Tuinman[3]

[1]Academic Medical Center, Intensive Care Medicine, Amsterdam, Netherlands; [2]Academic Medical Center, Laboratory of Experimental Intensive Care and Anesthesiology, Amsterdam, Netherlands; [3]VU Medical Center, Intensive Care Medicine and Research VUmc Intensive Care (REVIVE), Amsterdam, Netherlands; [4]Academic Medical Center, Emma Children's Hospital, Pediatric Intensive Care, Amsterdam, Netherlands

**Correspondence:** J. Juschten

*Intensive Care Medicine Experimental* 2018, **6(Suppl 2):**0647

**INTRODUCTION.** Sepsis-induced acute respiratory distress syndrome (ARDS) shares common pathophysiological features with sepsis-induced acute kidney injury (AKI), such as an uncontrolled inflammatory response and coagulopathy. Alkaline phosphatase (recAP) improved renal function in sepsis patients with AKI (1), decreased inflammatory markers and improved outcome in preclinical studies (2-4).

**OBJECTIVES.** To assess potential beneficial effects of recAP on inflammation and coagulation in the acute phase of lung injury.

**METHODS.** Lung injury was induced in male Wistar rats by intravenous administration of LPS (10 mg/kg) and subsequent injurious ventilation with high tidal volumes (12-15 ml/kg) for 4 hours. At start of ventilation animals received recAP (1500 IU/kg) or saline (N = 8 animals per group); control animals received neither LPS, nor ventilation or treatment (N = 4). Endpoints were markers of inflammation (e.g. CINC-3: Cytokine-Induced Neutrophil Chemoattractant-3) and coagulation

### 0648

#### Transpulmonary thermodilution during ECMO: detection of a potential loss of indicator by a second, extra-corporeal thermodilution catheter within the ECMO-circuit: the HEUREKA-IV animal study

W. Huber, M. Konrad, S. Kammerzell, R. Schmid, A. Herner

Klinikum rechts der Isar; Technical University of Munich, Medizinische Klinik und Poliklinik II, Munich, Germany

**Correspondence:** W. Huber

*Intensive Care Medicine Experimental* 2018, **6(Suppl 2):**0648

**INTRODUCTION.** Feasibility of pulmonary arterial (PAC) and transpulmonary thermodilution (TPTD) during ECMO is questionned due to a potential direct loss of indicator (DLOI) into the extracorporeal circuit.

**OBJECTIVES.** To detect DLOI with a second thermodilution catheter introduced into the ECMO circuit at the connection of drainage cannula and ECMO circuit.

**METHODS.** 8 pigs (49.4±4.2kg) with a PAC and a CVC (both via jugular vein) and two PiCCO catheters (Pulsion, Germany) introduced into the femoral artery and into the ECMO-circuit. Veno-venous ECMO (CARDIOHELP; Maquet/Getinge; Rastatt, Germany) via a femoral drainage and a jugular return cannula.

Indicator injection into the jugular CVC and into femoral catheters during different (0-3.5L/min) ECMO-flows.

Due to the explorative type of analysis in a limited number of animals and in part different experimental settings, each of the authors independently performed a response to a structured protocol regarding the main endpoints (see results 1-8). The statements were only included in the results section in case of complete (4/4) agreement of the four investigators WH, MK, SK and AH.

**RESULTS.** All four investigators (4/4) agreed on the following findings:
1.) Irrespective of indicator injection there were minor periodic variations of the temperature curve (PVTC) detected by the thermistor in the extracorporeal circuit.
2.) The frequency of PVTC agreed with the ventilator respiratory rate.
3.) PVTC could be abolished by inspiratory or expiratory ventilator hold.
4.) Indicator injection resulted in an extra-corporeally detectable signal in the 2nd PiCCO in a substantial number of measurements.
5.) This "indicator induced extracorporeal signal" (IIES) resulted in different types of processing of the signal:
a.) An early peak of change in temperature was strongly associated with indicator injection close to the drainage cannula and/or high ECMO flow. This peak reflects DLOI and occurs within 10s after injection.
b.) A second, markedly later peak occurred after the peak of the femoral arterial TPTD-curve. This is in line with recirculation of the signal into the ECMO not associated with DLOI.
6.) Both signals were in part sufficient to initiate the processing of the dilution curve by the 2nd PiCCO.
7.) The early signal induced by DLOI in part resulted in flow values ("CI"_ECMO) within the range of the simultaneous CI derived from the TPTD-curve in the femoral artery. In these cases similar amounts of indicator might have been withdrawn into ECMO and stayed within systemic circulation.
8.) DLOI could be detected with maximum sensitivity (1ml of injectate), if the indicator was injected close to the drainage canula.
**CONCLUSIONS.**
1.) The loss of indicator into the ECMO-circuit can be detected with high sensitivity by a 2nd TPTD-catheter in the ECMO-circuit.
2.) Detection of DLOI could be used as a "red flag" for the use of thermodilution techniques with indicator injection into the right atrium (PAC, TPTD).

### 0649

#### Modulation of Hippo signaling promotes mesenchymal stem cells to optimize lung injury repair in murine lipopolysaccharide induced ARDS

L. Dong, L. Li
Wuxi People's Hospital affiliated to Nanjing Medical University, Intensive Care Unit, Wuxi, China
**Correspondence:** L. Dong
*Intensive Care Medicine Experimental* 2018, **6(Suppl 2):**0649

**INTRODUCTION.** Mesenchymal stem cells (MSCs)-mediated repair of injured alveolar epithelial cells hold promise of cure for acute respiratory distress syndrome (ARDS), however, their repairing effect is limited by poor homing and differentiation.
**OBJECTIVES.** To determine the effects of modulation of Hippo signaling on lung repair of marrow mesenchymal stem cells (mMSCs) in murine lipopolysaccharide (LPS) induced ARDS.
**METHODS.** Mouse MSCs transfected with LATS1 shRNA or green fluorescent protein control were transplanted intratracheally into the ARDS mice induced by LPS. Lung tissue injury and repair assessment were examined using haematoxylin and eosin staining, lung injury scoring, Masson's trichrome staining and fibrosis scoring. Homing and differentiation of mouse MSCs were assayed by labelling and tracing MSCs using NIR815 dye, immunofluorescent staining, and Western immunoblot analysis. The inflammation and permeability were evaluated by detecting the cytokine and protein measurements in bronchoalveolar lavage fluid using enzyme-linked immunosorbent assay.
**RESULTS.** LATS1-underexpressing MSC engraftment led to more significant effects than the GFP controls, including the retention of the MSCs in the lung, differentiation into type II alveolar epithelial cells, improvement in pulmonary inflammation and alveolar epithelial permeability, and the pathologic impairment of the lung tissue.
**CONCLUSIONS.** Inhibition of Hippo signaling pathway through underexpression of LATS1 could improve the therapeutic effects of mMSCs in murine LPS induced ARDS.

**GRANT ACKNOWLEDGMENT**
This work was supported by the National Natural Science Foundation of China (81400054), the Natural Science Foundation of Jiangsu Province (BK20140122), and the Talented Youth Program of Jiangsu Province (QNRC2017179).

### 0650

#### Intravenous analysis (IVA) detects right heart failure during respiratory arrest in a rat model

S. Eagle[1], C. Balzer[1], M. Riess[2], A. Hernandez[1], F. Baudenbacher[3]
[1]Vanderbilt University Medical Center, Department of Anesthesiology, Nashville, United States; [2]Tennessee Valley Healthcare System, VAMS, Department of Anesthesiology, Nashville, United States; [3]Vanderbilt University, Department of Biomedical Engineering, Nashville, United States
**Correspondence:** S. Eagle
*Intensive Care Medicine Experimental* 2018, **6(Suppl 2):**0650

**INTRODUCTION.** Continuous measurement of right ventricular (RV) function in the setting of pulmonary disease is challenging in hospitalized patients. Central venous pressure (CVP) continues to be used to assess RV function despite pitfalls of static measurement. Intravenous analysis (IVA) has been shown to be more sensitive than CVP in human and porcine models for detecting hemorrhage and volume overload (1,2). We hypothesize that IVA will be more sensitive than CVP for detecting RV failure during respiratory arrest in a rat model.
**OBJECTIVES.**
1. Determine the utility of IVA to detect RV failure;
2. Compare the change in IVA to CVP during respiratory arrest
**METHODS.** Four Wistar rats were studied under an Institutional Animal Care and Use Committee approved protocol at Vanderbilt University. Rats were intubated with a 14g angiocatheter and mechanically ventilated. IV Pentobarbital was used for maintenance of anesthesia. Venous access was established via surgical cut-down using a 22g 1" angiocatheter (Smiths Medical, OH, USA) in the following locations: right internal jugular vein, femoral vein, and femoral artery. A TruWave pressure transducer (Edwards Lifesciences, CA, USA) was connected directly each catheter. CVP, mean arterial pressure (MAP), and femoral venous waveforms were recorded using LabChart (AD Instruments, Australia). Respiratory arrest was achieved with IV Rocuronium discontinuation of ventilation. Femoral venous waveform was analyzed using fast Fourier transformation (FFT). Right ventricular diameter was calculated from an apical 4-chamber view on transthoracic echocardiography. All measurements were obtained within the first 4 minutes of arrest. A T-test was performed to test for significant changes for IVA, CVP, RV diameter, and MAP.
**RESULTS.** Four Wistar rats were successfully cannulated and underwent respiratory arrest for 4 minutes. All rats had acute RV dilation and failure and developed severe tricuspid regurgitation (Figure 1). There was significant ($P< 0.05$) increase in IVA 42±21 $mmHg^2$ and RV diameter of 63%±17%, and a significant decrease in MAP 72±21 mmHg. There was no significant change in CVP. Representative data for IVA and MAP are shown in Figure 2.
**CONCLUSIONS.** IVA is more sensitive than CVP for detecting acute RV failure during respiratory arrest. Further studies are warranted to determine sensitivity of IVA in other settings of acute RV failure, such as sepsis.

**REFERENCE(S)**

1. Sileshi B, Hocking KM, Boyer RB, Baudenbacher FJ, Kohurst KL, Brophy CM, Eagle S. Peripheral venous waveform analysis for detecting early hemorrhage: A pilot study. Intensive Care Med. 2015 Jun;41(6):1147-8.
2. Hocking KM, Sileshi B, Baudenbacher FJ, Boyer RB, Kohorst KL, Brophy CM, Eagle S. Peripheral venous waveform analysis for detecting hemorrhage and iatrogenic volume overload in a porcine model. Shock. 2016 Oct;46(4):447-52.

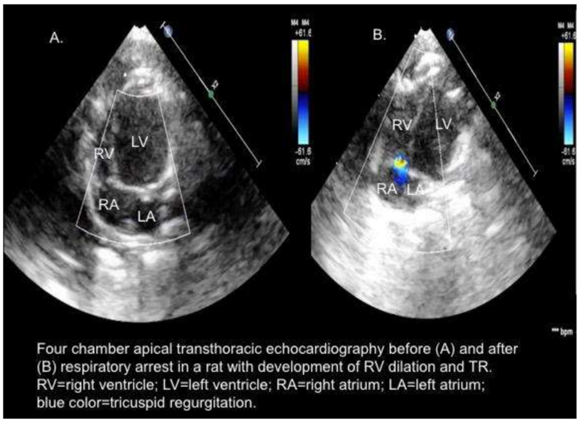

**Fig. 1 (abstract 0650).** Right ventricular dilation and tricuspid regurgitation after respiratory arrest

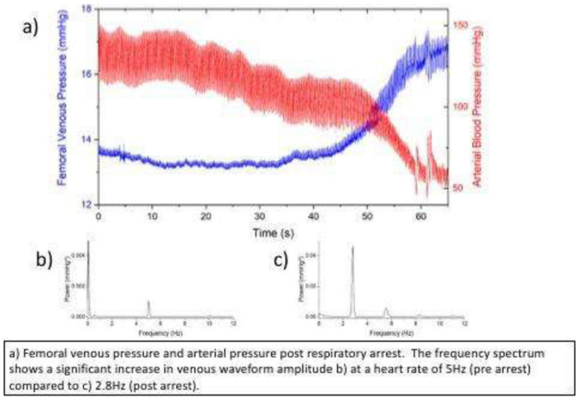

**Fig. 2 (abstract 0650).** Representative arterial and venous waveforms during respiratory arrest.

## Monitoring during respiratory support

### 0651

### Prevalence of atypical sleep and pathological wakefulness in patients with acute respiratory failure in respiratory care unit

N. Rittayamai, K. Kunwipakorn, N. Chierakul, S. Tangchityongsiva, W. Srilam
Faculty of Medicine Siriraj Hospital, Division of Respiratory Disease and Tuberculosis, Department of Medicine, Bangkok, Thailand
**Correspondence:** N. Rittayamai
*Intensive Care Medicine Experimental* 2018, **6(Suppl 2):**0651

**INTRODUCTION.** Sleep disruptions frequently occur in hospitalized patients in particular critically ill, mechanically ventilated patients. Severely altered sleep architectures result in unclassifiable sleep stage according to the conventional Rechtschaffen and Kales criteria. A new classification for sleep scoring including atypical sleep (AS) and pathological wakefulness (PW) has been proposed. This study aims to assess the prevalence of AS and PW in patients with acute respiratory failure and whether there is relationship with clinical outcomes.

**OBJECTIVES.**

- To determine the prevalence of AS and PW in mechanically ventilated patients with acute respiratory failure.
- To identify clinical factors affecting poor sleep quality, AS and PW.
- To assess the relationship between AS and PW and clinical outcomes.

**METHODS.** In this prospective cohort study, polysomnography was performed in 38 patients receiving mechanical ventilation due to acute respiratory failure at Respiratory Care Unit (RCU) of Siriraj Hospital from February to December 2017. Sleep stage was analyzed by conventional rules and new classification for AS and PW. The association between presence of AS or PW and patient characteristics was analyzed. Correlations between delirium and weaning outcome with AS or PW were also determined.

**RESULTS.** According to new classification, 14 out of 38 (prevalence of 36.8%) mechanically ventilated patients had AS. The prevalence of PW was 36.8% and either AS or PW was 52.6%. Most of patients had poor sleep quality with median sleep efficiency [IQR] of 35.9% [18.5, 62.3]) and significantly decreased slow wave sleep (median [IQR] 0.4% [0.00, 5.70]) and REM (median [IQR] 1.3% [0.00, 6.43]). In multivariate analysis, higher respiratory rate was significantly associated with AS (odds ratio [OR] 1.24, 95% confidence interval [CI] 1.04 to 1.47, p = 0.014), while longer duration of mechanical ventilation and higher sleep efficiency were associated with the presence of PW (OR 1.36, 95%CI 1.03 to 1.81, p = 0.032 and OR 1.09, 95%CI 1.03 to 1.15, p = 0.004 respectively for sleep efficiency). Subsequent delirium occurred in 2 out of 37 (5.4%) patients and relationship with AS or PW was not established. Only PW was correlated with longer ventilator days after sleep study (p = 0.003).

**CONCLUSIONS.**

Other than reduced sleep quality, atypical sleep and pathological wakefulness were frequently found among critically ill, mechanically ventilated patients with acute respiratory failure and may be associated with longer duration of mechanical ventilation.

**REFERENCE(S)**

1. Rittayamai N, et al. Positive and negative effects of mechanical ventilation on sleep in the ICU: a review with clinical recommendations. Intensive Care Med 2016;42:531-41.
2. Drouot X, et al. A new classification for sleep analysis in critically ill patients. Sleep Med 2012;13:7-14.

**GRANT ACKNOWLEDGMENT**
This study was supported by Faculty of Medicine Siriraj Hospital, Mahidol University, Bangkok, Thailand.

**Table 1 (abstract 0651).** Polysomnographic results and clinical outcomes

| | |
|---|---|
| Total sleep time, min (median, IQR) | 322 (170, 538) |
| Sleep efficiency, % (median, IQR) | 35.9 (18.5, 62.3) |
| Arousals, events/hour (mean, SD) | 31.9 (14.1) |
| Sleep stage, % (median, IQR) | |
| - N1 | 52.8 (32.3, 68.5) |
| - N2 | 39.5 (28.3, 47.6) |
| - N3 | 0.4 (0.0, 5.7) |
| - REM | 1.3 (0.0, 6.4) |
| Successful weaning, n (%) | 31 (81.6) |
| Ventilator days after sleep study (median, IQR) | 2 (1, 4) |

### 0652

#### Customized spontaneous breathing trials determined by electrical impedance tomography improved successful weaning rate in prolong mechanical ventilated patients

Y.-L. Hsu[1], M.-Y. Chang[2], Z. Zhao[3]
[1]Far Eastern Memorial Hospital, Respiratory Care Center, Pulmonary Division, Internal Medicine, New Taipei City, Taiwan, Province of China; [2]Far Eastern Memorial Hospital, Devision of pulmonary medicine, Respiratory Therapy, New Taipei city, Taiwan, Province of China; [3]Institute of Technical Medicine, Furtwangen University, Jakob-Kienzle-Straβe 17, D-78054, Villingen-Schwenningen, Germany
**Correspondence:** Y.-L. Hsu
*Intensive Care Medicine Experimental* 2018, **6(Suppl 2):**0652

**INTRODUCTION.**
Different spontaneous breathing trials (SBT) are used on prolonged mechanical ventilated patients to liberate them from ventilators. However, up to 40% of the patients who pass SBT may still fail to wean from mechanical ventilation.

**OBJECTIVES.**
EIT is able to monitor SBT during the weaning process. The results revealed that ventilation was redistributed towards dorsal regions during lower support levels.[1] Furthermore, regional ventilation distribution patterns and trends during inspiration were associated with weaning outcomes.[2] In the present study, we try to customize the SBT protocols determined by the EIT results. The outcomes including successful weaning rates and the total ventilation days were analyzed.

**METHODS.**
There were four patterns of ventilation distribution in the course of tidal inflation during ACMV and SBT with automatic tube compensation (ATC) 100 and 70 % found in previous study .[2] In this study, we enrolled 80 patients ( each pattern 20 pts) during the pre-SBT stage. Patient characteristics, EIT-derived parameter values (center of ventilation (CoV), ratio of tidal ventilation (RTV), end-expiratory lung impedence (EELI)) for patients with different inspiratory ventilation distribution patterns, patient weaning parameters after the first SBT day with different inspiratory ventilation distribution patterns were recorded. SBT weaning protocols with ATC 100% (10 pts) and customized weaning protocol (CWP) according to EIT results (10 pts) were applied to each pattern patients. The outcomes of these patients including successful weaning rate, total mechanical ventilation days were analyzed.

**RESULTS.**
In pattern 1 patients, 100% of both groups were successful weaned. In pattern 2 patients, the successful weaning rate was 60% among ATC 100% group and 80% among CWP group and the total ventilation days was 1.5 days shorter among CWP group. In pattern 3 patients, none was successful weaned among ATC 100% group and only 30% was successful weaned among CWP group, the total ventilation days of successful weaned patients was much longer then the pattern 1 and 2 groups. In patterns 4 patients, the successful weaning rate was 60% and 70% among ATC 100% and CWP groups separately.

**CONCLUSIONS.**
The preliminary results show that customized SBT determined by EIT improved successful weaning rate in prolong mechanical ventilated patients with poor respiratory muscle strength and endurance.

**REFERENCE(S)**

1. Hsu YL; Tien AJ; Chang MY; Chang HT; Moller K; Frerichs I; Zhao Z. Regional Ventilation Redistribution Measured by Electrical Impedance Tomography during Spontaneous Breathing Trial with Automatic Tube Compensation. Physiol Meas. 2017;38: 1193-203
2. Zhao Z, Peng SY, Chang MY, Hsu YL, Frerichs I, Chang HT, *et al.*, Spontaneous breathing trials after prolonged mechanical ventilation monitored by electrical impedance tomography: an observational study. Acta Anaesthesiol Scand. 2017;61:1166-1175.

**GRANT ACKNOWLEDGMENT**
(FEMH-2017-C-055).

### 0653

#### Comparison of mechanical and electronic devices to prevent underinflation of tracheal tube cuff pressure in mechanically ventilated patients

N. Adam[1,2], A.C. Gianinazzi[1], H. Brisson[1], Q. Lu[1], O. Langeron[1]
[1]CHU Pitié Salpêtrière, Paris, France; [2]ASL Lecce / P.O. San Giuseppe da Copertino, Anestesia e Rianimazione, Lecce, Italy
**Correspondence:** N. Adam
*Intensive Care Medicine Experimental* 2018, **6(Suppl 2):**0653

**INTRODUCTION.** Ventilator-associated pneumonia (VAP) is a common and severe ICU complication. Maintaining an endotracheal tube cuff pressure ($P_{cuff}$) > 20cm$H_2O$ may prevent microaspiration of subglottic secretions, which is one of the main mechanisms accounting for VAP [1]. Mechanical and electronic devices have been developed to automatically control $P_{cuff}$ during invasive mechanical ventilation.
**OBJECTIVES.** To compare the efficiency of an electronic device (Tracoe®) and a mechanical device (Nosten®) to prevent $P_{cuff}$ underinflation in mechanically ventilated patients.
**METHODS.** We conducted a cross-over observational study in a 26-bed ICU. All patients intubated and ventilated more than 12 hours were eligible. Three periods of 2 hours were studied in a random order: 1) $P_{cuff}$ controlled with Tracoe® (TRA); 2) $P_{cuff}$ controlled with Nosten®(NOS); 3) without $P_{cuff}$ control (CTR). $P_{cuff}$ was continuously recorded using Physiotrace software[2]. $P_{cuff}$ underinflation was defined as a $P_{cuff}$ < 20 Cm$H_2O$ for more than five seconds during each period. The primary endpoint was the number of patients in whom $P_{cuff}$ underinflation was detected. The secondary endpoints were the efficiency of each device to maintain $P_{cuff}$ within the normal range, defined as a $P_{cuff}$ between 20 cm$H_2O$ and 30 cm$H_2O$; and the number of patients with $P_{cuff}$ underinflation when ventilated with volume-controlled (VCV) or pressure support modes (PSV) for each device. Sample size was calculated to show a 30% of difference in $P_{cuff}$ underinflation between the 2 devices with a power of 90% and a alpha risk of 5%.
**RESULTS.** Thirty-six patients were included, 21 ventilated with VCV and 15 with PSV. None of the patients showed $P_{cuff}$ underinflation using NOS whereas respectively 17 patients (47%) and 23 patients (64%) had $P_{cuff}$ underinflation using TRA and during CTR. The percentage of the time spent on $P_{cuff}$ normal range was significantly longer with NOS than with TRA and CTR (table). For each device, the proportion of the patients with $P_{cuff}$ underinflation was not different between the 2 ventilation modes.
**CONCLUSIONS.** Nosten is more efficient than Tracoe to prevent tube cuff pressure underinflation and maintain cuff pressure within the normal range.

**REFERENCE(S)**

1. Rello J, Sonora R, Jubert P, Artigas A, Rue M, Valles J. Pneumonia in intubated patients: role of respiratory airway care. American journal of respiratory and critical care medicine 1996;154:111-5.
2. De Jonckheere J, Logier R, Dassonneville A, Delmar G, Vasseur C. PhysioTrace: An efficient toolkit for biomedical signal processing. Conf Proc IEEE Eng Med Biol Soc. 2005;7:6739-41.

**GRANT ACKNOWLEDGMENT**
None

**Table 1 (abstract 0653).** See text for description.

| | NOSTEN | TRACOE | CONTROL |
|---|---|---|---|
| Patients with $P_{cuff}$<20cm$H_2O$ for more than 5 sec, N (%) | **0** (0%)*, ** | **17** (47%) | **23** (64%) |
| Percentage of time spend with $P_{cuff}$ within the normal range | **99.98** [99.94;100]*, ** | **99.86** [98.90;99.98] | **95,45** [69,12;99.98] |
| Percentage of time with $P_{cuff}$< 20 cm$H_2O$ | **0.00** [0.00;0.01]*,** | **0,05** [0.00;0.17] | **0.07** [0.00;29.6] |
| Percentage of time with $P_{cuff}$> 30 cm$H_2O$ | **0.00** [0.00;0.00]*,** | **0.01** [0.00;0.45] | **0.26** [0.00;3;54] |
| Coefficient of Variation $[(P_{cuff}max-P_{cuff}min) + (P_{cuff}mean-P_{cuff}min)] / 2$ | **2.26** [1.0 ; 5.2]*,** | **7.0** [2.8 ; 15.1] | **9.7** [3.2 ; 22.1] |

### 0654

#### Ventilation free hours in cardiac surgery: new proposal prognosis measure

M. Gómez Orozco[1], E. Bucio Reta[1], D. Gutierrez Zarate[1], K. Rosas Sanchez[2], F. Baranda Tovar[1]
[1]Instituto Nacional de Cardiologia `Ignacio Chavez´, Terapia Intensiva Postquirurgica, Ciudad de México, Mexico; [2]Hospital Angeles de Acoxpa, Unidad de Terapia Intensiva, Ciudad de México, Mexico
**Correspondence:** M. Gómez Orozco
*Intensive Care Medicine Experimental* 2018, **6(Suppl 2):**0654

**INTRODUCTION.** Duration of invasive mechanical ventilation is a very important role in the postoperative of cardiac surgery, the ventilation time is associated with an increase in mortality, prolonged stay, and complications.Ventilator-free hours (VFHs) is a concept adapted from ventilator-free days (VFDs) used a measure of outcome in acute respiratory distress syndrome (ARDS).VFHs has defined arbitrarily as the number of hours from successful extubation of invasive mechanical ventilation and the patient remains alive at 60 hours after arriving at the intensive care unit (ICU).
**OBJECTIVES.** Compare the prognostic value for adverse outcomes of VFHs versus mechanical ventilation time after cardiac surgery.
**METHODS.** We conducted a retrospective observational study in patients admitted to our institution at Mexico City. Patients older than 18 years underwent cardiac surgery between January 1 and December 2017 were included. Patients were excluded from cardiac surgery of congenital and non-cardiac surgery.The median hours of ventilation in the ICU of our institution is 24 hours; the diagnostic capacity of ventilation time versus VFHs was analyzed using the ROC analysis, we obtained the largest area under the curve in VFHs taking 36 hours as the cut-off point (figure 1). Based on this, the population was divided into two groups: Group 1 (equal or less than 36 VFHs) and Group 2 (more than 36 VFHs).Continuous variables were expressed in median and interquartile ranges and categories variables as number and percentages. Continuous variables were analyzed using the Mann-Whitney U test and categories variables were evaluated using the Fisher exact test. We applied multivariate logistic regression analysis to evaluate variables that had statistical significance.
**RESULTS.** Of the 564 patients operated in 2017, 301 in group 1 and 263 in group 2 in the demographic variables we didn´t find significant differences.For EuroScore II and extracorporeal circulation time (CEC), we found significant differences with a higher percentage in group 1. In outcomes, we observed: higher in-hospital mortality, mortality in the ICU, cerebrovascular disease (CVD), delirium, acute renal injury and nosocomial pneumonia in group 1. For logistic regression analysis, we included those that showed the greatest association: in-hospital mortality with an OR of 9.9 (95% CI, 4.2-23.7) $p < 0.0005$, mortality in the ICU an OR of 11.9 (95% CI, 4.2-34) $p < 0.0005$, EVC OR 4.7 (95% CI, 1.1-7.4) $p = 0.05$, nosocomial pneumonia OR 2.8 (95% CI, 1.1-7.4) $p = 0.03$, and delirium OR 7.3 (95% CI, 1.6-32.8) $p = 0.009$.
**CONCLUSIONS.** By comparing the ventilation time versus VFHs, we show VFHs as a better predictor. Less free hours of mechanical ventilation is associated with worse results after cardiac surgery in adults

**REFERENCE(S)**

Goeddel et al, (2018).Early extubation after cardiac surgery: a better predictor of outcome than metric of quality?.J of Cardiothoracic and Vascular Anesthesia.2(32), 745-747

### 0655

#### Accuracy of the functional residual capacity (FRC) of a lung simulator (TestChest®) using a modified nitrogen washout/washin technique by using an agreement analysis with an ICU ventilator

J. Berger-Estilita[1], M. Hänggi[2], D. Berger[2]
[1]Inselspital, Bern University Hospital, Department of Anaesthesia and Pain Medicine, Bern, Switzerland; [2]Department of Intensive Care Medicine, University Hospital Bern (Inselspital), University of Bern, Bern, Switzerland
**Correspondence:** J. Berger-Estilita
*Intensive Care Medicine Experimental* 2018, **6(Suppl 2):**0655

**INTRODUCTION.** Training on ventilation modes with simulation is crucial for patient safety in the ICU[1]. The TestChest® is a high fidelity lung simulator, capable of replicating pulmonary mechanics, lung volumes, gas exchange and hemodynamic responses of the healthy and pathological adult. Its manufacturer claims that the large volume of the apparatus ensures a realistic replication of vital capacity and FRC of a critically ill adult patient. There are so far no reference measurements available.
**OBJECTIVES.** The aim of this study is to validate the settings of the TestChest® Simulator and determine its accuracy.
**METHODS.** We used our TestChest® Simulator (ORGANIS GmbH, Switzerland), after proper calibration, with a Carescape R860 ICU Respirator (General Electrics®, Baden, Switzerland). Three functional residual capacities (1500 mL, 2000 mL, 2500 mL) were set on the TestChest® and measured with the Carescape Respirator by the $N_2$washin/washout open circuit method[2]. Measurements were taken at PEEP of 5, 8, 12 and 15 $cmH_2O$ in order to create changes in FRC and at three different $FiO_2$(40%, 50%, 60%). Agreement between methods and trends were assessed with Bland Altman and four quadrant plots. Repeatability of methods was assessed with Cronbachs α.
**RESULTS.** We performed 135 measurements. The overall correlation ($r^2$=0,909) between FRC measured with nitrogen washin-washoutand and the FRC set in the TestChest® was excellent (Graph 1). The bias was -602 mL (red line) with upper and lower limits of agreement of -252 and -953 mL, respectively (green lines) (Graph 2), with random variability of measurements[HM1]. Repeatability was excellent (Cronbach's α0.99). Changes in $FiO_2$did not affect accuracy (Cronbach's α0.984). Changes in FRC by PEEP were accurately reflected by the TestChest.
**CONCLUSION.** The TestChest allows accurate settings of FRC with a constant, clinically relevant bias over wide ranges of end expiratory lung volumes. Changes of end expiratory volume by increasing PEEP levels were adequately simulated, when compared to a nitrogen washin-washout method.

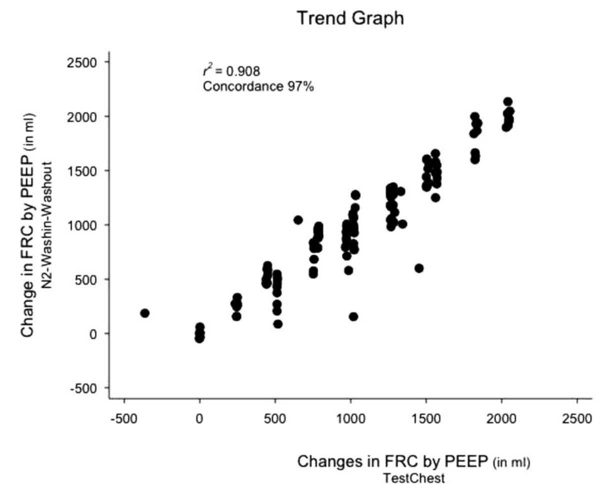

**Graph 1 (abstract 0655).** FRC Trend Graph

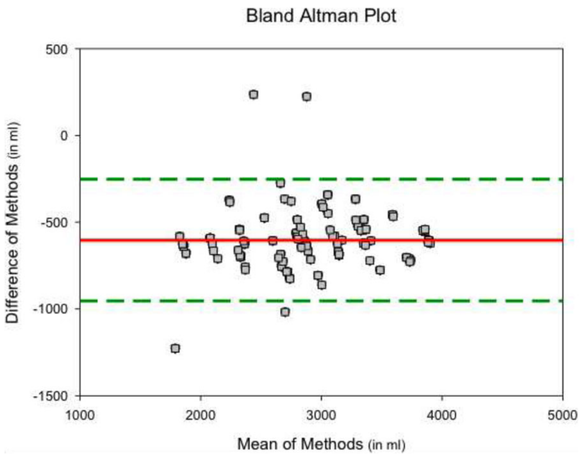

**Graph 2 (abstract 0655).** Bland-Altman Plot

### 0656

#### A description of the structure and contractile activity of the diaphragm during the course of mechanical ventilation

L. Brouwer[1], A. Lupton-Smith[1], C. Koegelenberg[2], S. Hanekom[3]
[1]Stellenbosch University, Physiotherapy, Cape Town, South Africa; [2]Stellenbosch University, Pulmonology, Cape Town, South Africa; [3]Stellenbosch University, Physiotherapy, Stellenbosch, South Africa
**Correspondence:** S. Hanekom
*Intensive Care Medicine Experimental* 2018, **6(Suppl 2):**0656

**INTRODUCTION.** While mechanical ventilation has been shown to have detrimental effects on the diaphragm, a recent systematic review concluded that inspiratory muscle training (IMT) can improve patient outcome in selected intensive care patients. Diaphragm ultrasound could identify diaphragm dysfunction in real-time, thereby facilitating the identification of patients most likely to benefit from IMT.

**OBJECTIVES.** The aim of this study was to describe the change in the contractile activity of the diaphragm during the course of mechanical ventilation, until successful extubation.

**METHODS.** Prospective observational cohort study. Sonographic measurements of the diaphragm were taken daily until 24 hours after extubation. Diaphragm thickness was measured in the zone of apposition using a 5-12 MHz ultrasound transducer. Diaphragm thickness (Tdi), diaphragm thickening fraction (DTF) and daily rate of change in both Tdi and DTF related to the previous day were calculated.

**RESULTS.** 45 participants (27, 60% male) with a mean (SD) age of 45 (17) years were recruited. The mean (SD) APACHE score was 17.08 (9.57) and participants were ventilated for mean (SD) 105.65 (77.84) hours. The median (IQR) baseline Tdi was 1.47mm (1.17-1.86). The mean (SD) rate of change in Tdi related to the previous day was 3.92% (18.38). Three groups of participants emerged (n,%):

1) In (9, 20%) of participants there was a mean decrease of more than 10% in daily Tdi :
2) In (14, 31.1%) the mean Tdi increased by more than 10%, and
3) In (22, 48.9%) the change was negligible.

Participants in the decreased Tdi group presented with significantly (p< 0.01) higher baseline Tdi compared to the no change and increased Tdi groups. The median (IQR) DTF at baseline was 24.64% (17.95-33.58), the median (IQR) for the daily rate of change in DTF was 6.67% (-7.10- 22.26). Again, three groups of participants (n,%) emerged 1) In (10, 22.2%) the mean DTF decreased by more than 10%; 2) In (17, 37.8%) participants there was no change; and 3) In (18, 40%) there was an increase of more than 10% in DTF. The decreased DTF group showed a significant decrease in Tdi between the first and second day of mechanical ventilation (p< 0.01). Participants in the decrease DTF group showed a significant decrease in Tdi between baseline and last measurement (p< 0.01); and significantly lower Tdi post-extubation as compared to their baseline Tdi (p=0.03). There were no significant between group differences for the first, last and post-extubation Tdi measurements (p>0.05). Participants in the six groups were similar with regard to age, gender, presence of comorbidities, smoking, sedation, ventilation modes or alcohol use (p>0.05).

**CONCLUSIONS.** While the contractile activity and the structure of the diaphragm changes during the course of mechanical ventilation, the pattern and rate of change is not the same in all participants. Work is now needed to understand the clinical relevance of the findings.

### 0657

#### Lung recruitment volume assessed by traditional pressure-volume curve and electrical impedance tomography

X.-M. Sun, G.-Q. Chen, Y.-M. Wang, Y.-M. Zhou, J.-R. Chen, K.-M. Cheng, J.-X. Zhou
Beijing Tiantan Hospital, Capital Medical University, Critical Care Medicine, Beijing, China
**Correspondence:** J.-X. Zhou
*Intensive Care Medicine Experimental* 2018, **6(Suppl 2):**0657

**INTRODUCTION.** Lung recruitability varies extremely in patients with acute respiratory distress syndrome (ARDS) and is strongly correlated with the response to positive end expiratory pressure (PEEP) [1]. Although traditional pressure-volume curve was considered as a rather standard method to assess the lung recruitment volume, mapping pressure-volume (P-V) curve still need disconnecting of patient from the ventilator to obtain PEEP induced volume change [2]. The electrical impedance tomography (EIT) could monitor change of lung volume at the bedside without disturbing the ventilation [3].

**OBJECTIVES.** We aimed to compare the lung recruitment volume measurement by the EIT with the traditional P-V curve method.

**METHODS.** Six anesthetized and ventilated pigs with normal lung and acute injured-lung (induced by hydrochloric acid) received decremental PEEP of 15, 10 5, and 0 cm$H_2O$. During each level of PEEP, multiply P-V curves were plotted using standard low-flow technique [3]. The lung recruitment between two adjacent levels of PEEP was measured at the fixed pressure of 20 cm$H_2O$ (Fig.1). Pressure-impedance (P-I) curves derived from EIT were also plotted, and the difference of impedance was obtained between different PEEP levels at the same pressure of 20 cm$H_2O$. The recruitment volume was estimated as the difference of impedance multiplied by the ratio of tidal volume and corresponding tidal impedance change. The agreement of estimated (by EIT P-I curve) and measured (by traditional P-V curve method) global recruitment volume were analyzed. Regional P-I curves obtained by EIT were further used to estimate the regional lung recruitment [4].

**RESULTS.** The global lung recruitment volume assessed by EIT and traditional P-V curves showed close correlation ($R^2$ = 0.68, $p$ < 0.001) and a mean bias of 5 ml with limits of agreement of −125 to 137 ml (Fig.2). In the normal lungs, no significant difference was found between the dependent and the nondependent lung recruitment (107 ± 37 vs 113±44 ml, $p$ = 0.069). But for the injured-lung, the dependent lung recruitment volume was significantly lower than that in the nondependent region (70±31 vs 95±53 ml, $p$ < 0.001), especially in low level of PEEP.

**CONCLUSIONS.** The P-I curves derived from the EIT could assess lung recruitment volume accurately and provide an opportunity to estimate the regional lung recruitment at the bedside.

**REFERENCE(S)**

1. Gattinoni L, et al. N Engl J Med 2006;354:1775-86
2. Lu Q, et al. Crit Care 2006;10:R95.
3. Van Genderingen HR, et al. Intensive Care Med 2003;29:233-40.
4. Frerichs I, et al. Physiol Meas 2013;34:567-77.

**GRANT ACKNOWLEDGMENT**

This research received no specific grant.

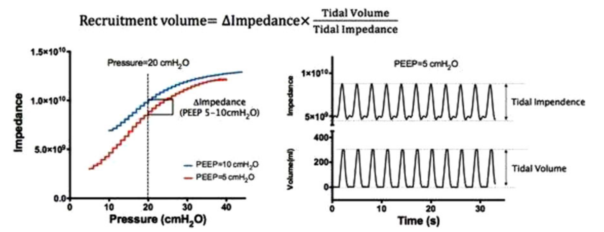

**Fig. 1 (abstract 0657).** See text for description

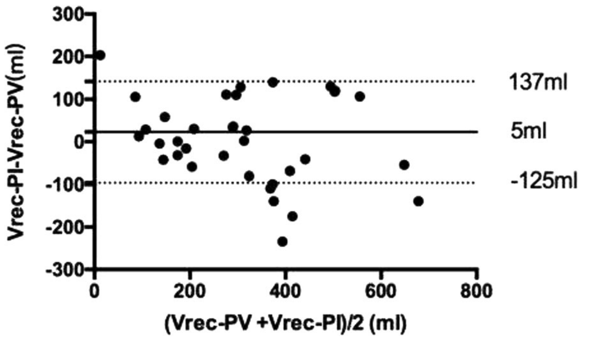

**Fig. 2 (abstract 0657).** See text for description

### 0658

#### Evaluation of the immediate effect of different metered dose inhalers on mechanical ventilator patients with acute execration of obstructive pulmonary diseases by using a non-invasive electrical impedance tomography

M.Y. Chang[1], C.-H. Gow[1], H.-T. Chang[1,2]
[1]Far Eastern Memorial Hospital, Devision of Pulmonary Medicine, New Taipei City, Taiwan, Province of China; [2]Far Eastern Memorial Hospital, Critical Care Medicine, New Taipei City, Taiwan, Province of China
**Correspondence:** M.Y. Chang
*Intensive Care Medicine Experimental* 2018, **6(Suppl 2):**0658

**INTRODUCTION.** Patients with acute exacerbation of chronic obstructive pulmonary disease (COPD) (AECOPD) and severe asthma attack (SAA) frequently require invasive mechanical ventilator (IMV) support; however, the mortality rate still very high with a range from 11-52%. Two ICS/LABA MDIs -Foster® (beclomethasone dipropionate and formoterol fumarate) and Seretide® evohaler (fluticasone and salmeterol) are treatment of choice for ventilated AECOPD/SAA patients in the MICU.

**OBJECTIVES.** In this project, we intend to use a non-invasive electrical impedance tomography (EIT) to promptly monitor the clinical treatment efficacy of MDIs in these patients.

**METHODS.** In this project, patients would be randomize assigned to two groups either receiving Foster® or Seretide® evohaler MDIs. A non-invasive study would be applied within three days of admission and be evaluated the ICS/LABA effect on ventilation function by using the EIT equipment for three consecutive days. The values of dynamic airway resistance, dynamic compliance, tidal volume, inspiration pressure, PEEP applied, and flow-volume curve images (scooping loop 2-3 figures) were record

**RESULTS.** We compared the pre- and post-bronchodilator resistance changes in the Seretide group. We observed that compared to baseline (30 min pre MDI), the resistance showed minimal immediate response after 30 min post Seretide MDI ( -0.84 + 1.23 cmH20/L•S-1, p=0.018), after 60 min post Foster MDI (-0.66 + 1.54 cmH20/L•S-1, p=0.108), and after 120 min post Foster MDI (-0.40 + 1.94 cmH20/ L•S-1, p=0.426). the compliance showed slightly increase, but huge variation, after 30 min post Foster MDI (0.88 + 5.82 L/cmH2O, p=0.124), after 60 min post Foster MDI (3.6 + 7.7 L/cmH2O, p=0.062), and after 120 min post Foster MDI (3.87 + 7.59 L/cmH2O, p=0.028). Therefore, there was better lung compliance after Seretide MDI treatment in such patients .

**CONCLUSIONS.** In this study, we observed the immediate effect of two ICS/LABA MDIs inhalation in severe asthma or COPD AE under mechanical ventilator support and ICU admission. Foster® MDI contains extrafine particles that reaching both large and small airways, provides better disease control.

##### REFERENCE(S)

1. Greening AP, Ind PW, Northfield M, Shaw G. Added salmeterol versus higher-dose corticosteroid in asthma patients with symptoms on existing inhaled corticosteroid. Allen & Hanburys Limited UK Study Group. Lancet. Jul 23 1994;344(8917):219-224.
2. Pauwels RA, Lofdahl CG, Postma DS, et al. Effect of inhaled formoterol and budesonide on exacerbations of asthma. Formoterol and Corticosteroids Establishing Therapy (FACET) International Study Group. The New England journal of medicine. Nov 13 1997;337(20):1405-1411.

##### GRANT ACKNOWLEDGMENT

This work was financially supported by project Far Eastern Memorial Hospital (FEMH-2016-C-012 and 2017-C-055). The authors would like to thank Zhanqi Zhao for his continuous, enthusiastic, and smart support for this article.

### 0659

#### The measurement of the hysteresis in a pressure-volume curve, with a post tracheal tube pressure sensor, during a PEEP trial could allow to evaluating the variation of the recruited pulmonary volume by the tidal volume at each level of PEEP

J.F. Georger, D. Pinell, B. Bensahli, V. Amilien, M. Ayoub, M. Tchir, E. Barsam, J.P. Ponthus
Centre Hospitalier Intercommunal de Villeneuve Saint Georges, Lucie et Raymond AUBRAC, Reanimation Polyvalente - Surveillance Continue, Villeneuve Saint Georges, France
**Correspondence:** J.F. Georger
*Intensive Care Medicine Experimental* 2018, **6(Suppl 2):**0659

**INTRODUCTION.** The static pressure volume curve (PV curve) is the reference technique to evaluate the lung mechanical. This technique is not easy to perform at the bedside. With a pressure sensor placed at the end of the tracheal tube, we can perform a PV curve without the pressure induced by the tracheal tube resistance so approaching a static PV curve. The volume of hysteresis obtained with this PV curve is the volume of lung recruited at each inspiratory by the tidal volume so we can approach it at each level of PEEP.

**OBJECTIVES.** The aim of this study is to determine the value of this hysteresis in ARDS patient, and if we have, during a digressive PEEP trial, a modification of this hysteresis.

**METHODS.** It is a single center study, in an intensive care unit. We included patients with moderated or severe ARDS. The patients are sedated and curarized in volume control mode with a Carescape R860 ventilator (General Electrique). We placed a pressure captor after the end of the tracheal tube, at the bottom of the trachea, to do the measurement of the PV curve. Before inclusion, we did a recruitment manoeuver (PEEP=25cm H2O during 40 seconds) and after we did a digressive PEEP trial (15, 12, 10, 8 and 5 cmH2O). We decreased the PEEP every 15 minutes. For each level of PEEP, we did a PV curve and we measured the maximum hysteresis (mHyst) obtained. We also measured blood gas, the plateau pressure, the total peep and the driving pressure. We calculated the ratio between hysteresis and tidal volume (mHyst/Vt) and the variation of this ratio between PEEP 15 and 5 cmH2O. We analysed the variation of the parameters using a ANOVA test for repeated measures with medcalc.

**RESULTS.** We included 17 patients, the mean age was 67.1 (47 to 85) years old, 9 men and 8 women. The cause of the ards was 13

pneumonia, 1 endocarditis, 1 peritonitis and 1 post cardiac arrest patient. The mortality was 47%. In the table one we present the variation of the parameters. The mean variation of the mHyst/Vt between 15 and 5 cmH2O was 15% (27 to 4), 4 patients had a variation lower than 10% and 4 patients more than 20% of the Vt.

**CONCLUSIONS.** The measurement of the Hyst using a tracheal pressure sensor during a digressive PEEP trial could allow evaluating the variation of the volume recruited during the respiratory circle. We have an increased of this volume when we decrease the PEEP. We could evaluate a population of patient with a few variation of this recruited volume and a population with a big variation with the PEEP trial. So we apprehend if we improve the ventilator induced lung injury induces by the pulmonary volume collapse and open by the respiratory circle when we change the PEEP. More studies are necessary to apprehend if this measurement is useful to adapt the strategy of ventilation.

**Table 1 (abstract 0659).** Variation of the parameters with the digressive PEEP trial,Parameters are showed with 95%CI,*p<0.05 difference with PEEP=15cmH2O

| Peep (cmH2O) | 15 | 12 | 10 | 8 | 5 |
|---|---|---|---|---|---|
| mHyst (ml) | 137 (100-174) | 152 (111-194) | 168* (124-211) | 176* (132-220) | 196* (152-240) |
| mHyst/Vt (%) | 36 (26-46) | 40 (29-51) | 44* (33-55) | 46* (35-58) | 52* (40-63) |
| Pplat (cmH2O) | 25 (23-29) | 21 (20-22) | 19 (18-20) | 17 (16-18) | 15 (13-16) |
| Driving pressure (cmH2O) | 9 (7-11) | 8 (7-9) | 8 (7-9) | 8 (7-9) | 8 (7-9) |
| PaO2/fiO2 ratio | 175 (120-230) | 167 (120-213) | 149 (112-185) | 142 (106-177) | 126 (100-151) |

### 0660

#### Monitoring diaphragmatic ultrasound in acute hypoxic-hypercapnic respiratory failure: a pilot feasibility study

G. Cammarota[1], I. Sguazzotti[1], A. Fusè[1], A. Guzzo[1], A. Messina[1], R. Vaschetto[1], D. Colombo[1], P. Navalesi[2], E. Garofalo[2], A. Bruni[2], G.C. Avanzi[3], F. Della Corte[1]

[1]Azienda Ospedaliera Universitaria Maggiore della Carità, SCDU Anestesia e Rianimazione, Novara, Italy; [2]Università della Magna Grecia, Anestesia e Rianimazione, Catanzaro, Italy; [3]Azienda Ospedaliera Universitaria Maggiore della Carità, SCDU Medicina Emergenza, Urgenza e Accettazione, Novara, Italy

**Correspondence:** G. Cammarota

*Intensive Care Medicine Experimental* 2018, **6(Suppl 2):**0660

**INTRODUCTION.** Diaphragmatic Ultrasound (DU) is an useful tool in monitoring patients (pts) during invasive and non invasive ventilation (NIV) [1, 2]. However DU could play a role in the early phase of NIV application in the early phases of Hypoxic-Hypercapnic Acute Respiratory Failure (HHARF) in order to identify pts at risk of NIV failure.

**OBJECTIVES.** The first aim was to evaluate diaphragm ultrasound (DU) feasibility in HHARF requiring NIV over two 2 hours period from admission. The secondary aim was any relationship between diaphragmatic function expressed in terms of diaphragmatic excursion (DE), thickness and thickening, arterial blood gases (ABGs) and NIV duration (Nd).

**METHODS.** Forty-three consecutive HHARF pts requiring NIV were screened in the Emergency Department (ED). Twenty pts were enrolled. DU and ABGs were performed on admission before starting NIV (T0), after one (T1) and two hours (T2) of NIV. Pts were divided in two groups: a) NIV responders (NIVr) with normal pH values and b) NIV non-responders (NIVnr) with persistent respiratory acidosis.

**RESULTS.** In 1 of the patient, the study was interrupted because of a poor DU window. Figure 1 depicts DE over first two hours of patients admission. DE was significantly reduced at T1 in NIVnr group with respect to NIVr (p < 0,001). DE at T1 with cut-off of 1,650 cm had a sensitivity of 100% and specificity of 91,67% in predicting NIV failure at two hours (figure 2). Finally, as depicted in figure 3, NIVr group underwent to Nd minor than NIVnr (p = 0,0284).

**CONCLUSIONS.** In our experience, DU was a feasible tool in HHARF pts requiring NIV in the ED. In particular DE, seems to play a leading role in monitoring our pts population over the first two hours from emergency department admission.

**REFERENCE(S)**

1. Goligher EC, et al (2015) Intensive Care Med
2. Antenora F,et al (2017) Respirology

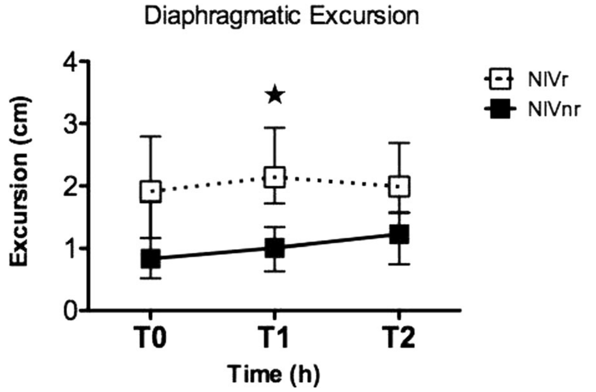

**Fig 1 (abstract 0661).** Diaphragmatic excursion

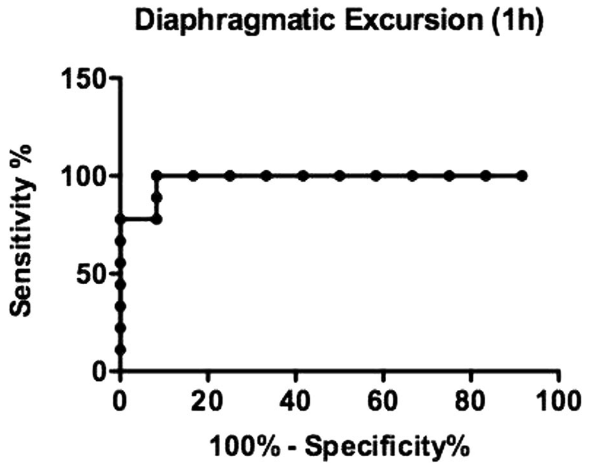

**Fig 2 (abstract 0661).** ROC - Diaphragmatic excursion at 1 hours from NIV start

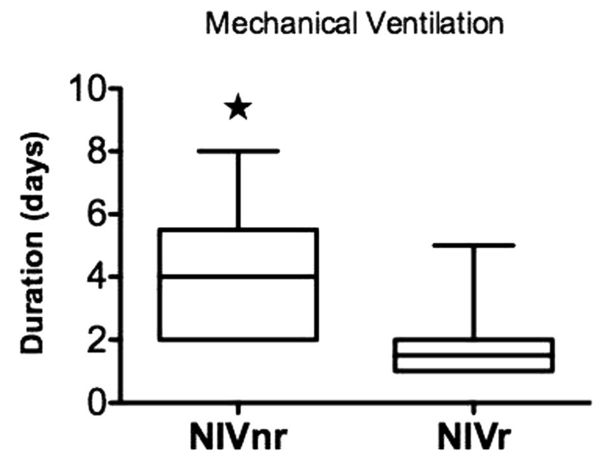

**Fig 3 (abstract 0661).** Mechanical Ventilation duration

### 0661

#### Electrical impedance tomography compared to biomechanical based on computed tomography (CT) for the measurement of regional lung strain in ARDS patients. Preliminary results

R. Cornejo[1], D. Arellano[1], P. Iturrieta[2], D. Guiñez[1], R. Brito[1], M.A. Cerda[1], L. López[3], T. Milá[4], C. Kajiyama[4], C. Morais[4], A. Bruhn[5], D.E. Hurtado[2], C. Repetto[1], S. González[1], Z. Miguel[1], M. Amato[4], N. Estuardo[1]
[1]Universidad de Chile, Hospital Clínico, Unidad de Pacientes Críticos, Santiago, Chile; [2]Pontificia Universidad Catolica de Chile, Institute for Biological and Medical Engineering, School of Engineering, Santiago, Chile; [3]Universidad de Chile, Hospital Clínico, Departamento de Radiología, Santiago, Chile; [4]University of São Paulo, Laboratório de Pneumologia LIM09, Facultdade de Medicina, São Paulo, Brazil; [5]Pontificia Universidad Catolica de Chile, Departamento de Medicina Intensiva, Facultad de Medicina, Santiago, Chile
**Correspondence:** R. Cornejo
*Intensive Care Medicine Experimental* 2018, **6(Suppl 2):**0661

**INTRODUCTION.** Regional lung strain has been correlated with regional inflammation, supporting that strain might be a determinant of ventilator-induced lung injury. Therefore, information on regional strain distribution in mechanically ventilated patients with acute respiratory distress syndrome (ARDS) may be important to develop lung protective ventilation strategies. Electrical impedance tomography (EIT) is capable of monitoring imbalances in regional lung ventilation and it could provide a surrogate of the strain estimated by CT at bedside.

**OBJECTIVES.** To compare regional strain estimated by CT and EIT monitoring, in patients who presented moderate-severe ARDS.

**METHODS.** Four patients underwent whole-lung CT during breath-holding sessions at end expiration and end inspiration (with PEEP 5 and best PEEP according EIT, applied in random order). A recruitment maneuver was performed before each PEEP change. Low radiation CT protocol was applied. ADMIRE reconstruction algorithm and iterative reconstruction with IRIS-Software (Kernel: I40 filter) were applied. Using Nifty-Reg package (1), a transformation mapping was generated between a fixed image (end inspiration) and a target image (end expiration), which calculates displacement of every point inside the lung from inspiration to expiration. Following, a biomechanical analysis method was used (2) to construct 3D maps of the volumetric strain distribution for each patient´s lungs (See figure 1).

Electric impedance tomography (EIT, Enlight®: Timpel, Brazil) was used to visualize the regional distribution of inflation (3). Strain maps were divided into 4 regions of interest (Upper and Lower, Right and Left), coinciding spatially with the regions upon which inflation was calculated for EIT method. CT and EIT examinations were performed simultaneously. The correlation between CT and EIT variables was addressed by multiple lineal regressions.

**RESULTS.** Significant correlations were found between CT and EIT for global and regional strain, on PEEP 5 and best PEEP according to EIT (Table 1).

**CONCLUSIONS.** Global and regional lung strain, estimated by CT, were accurately correlated with EIT in patients who presented moderate-severe ARDS in spite CT-based strain method was related to the whole lung

**GRANT ACKNOWLEDGEMENT. FONDECYT 1151610**

**Table 1 (abstract 0661).** Correlations between EIT variables and CT-based strain method

| Regions of Interest | Coefficient of correlation ($R^{2}$) |
|---|---|
| Global | 0,753 |
| Upper | 0,847 |
| Lower | 0,706 |
| Right | 0,819 |
| Left | 0,529 |

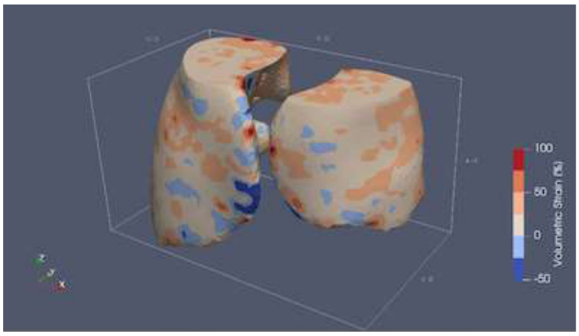

**Fig 1 (abstract 0661).** Strain mapping from patient #2 at best PEEP

### 0662

#### Accuracy of pulse oximetry for INTELLiVENT-ASV®: a prospective observational study

S. Katayama, K. Tonai, K. Koyama, J. Shima, Y. Goto, T. Koinuma, M. Wada, S. Nunomiya
Jiichi Medical University, School of Medicine, Division of Intensive Care, Department of Anesthesiology and Intensive Care Medicine, Shimotsuke, Japan
**Correspondence:** S. Katayama
*Intensive Care Medicine Experimental* 2018, **6(Suppl 2):**0662

**INTRODUCTION.** Measurement of pulse-oximetric oxygen saturation ($SpO_2$) is widely used to monitor oxygenation status in critically ill patients. Recently, to avoid unnecessary hyperoxemic state, it is recommended to keep $SpO_2$ in a certain range in mechanical ventilated patients. In this regard, INTELLiVENT-ASV®, with the function of automatic $F_IO_2$ control by monitoring $SpO_2$ attached with the ventilator, might have potential benefit. However, it is unclear how accurate the measured $SpO_2$ by the system of INTELLiVENT-ASV® compared with the actual $SaO_2$.

**OBJECTIVES.** To evaluate the accuracy of $SpO_2$ (Nihon Kohden) attached with INTELLiVENT-ASV® and another type of $SpO_2$ (Masimo) compared with the actual $SaO_2$.

**METHODS.** This is a single center, prospective observational study (UMIN000027671). Consecutive adult patients who were admitted to a general-ICU and mechanically ventilated with G5 ventilator (Hamilton Medical, Rhäzüns, Switzerland) between June 2017 and March 2018 were included. $SpO_2$ were measured by TL-271T3 (Nihon Kohden, Tokyo, Japan) attached with G5 ventilator and simultaneously by RD SET$^{TM}$NEO (Masimo, Irvine, CA, USA). Seal type of sensor probes were used and attached on the same side of patient's hand. Patients who presented quality index of $SpO_2$ (Nihon Kohden) < 50%, unstable value of $SpO_2$ due to change $F_IO_2$ just before blood drawing and with smoke inhalation were excluded. Time of blood drawings or analyzing blood gases were recorded and compared with $SaO_2$ and $SpO_2$. The timing of blood gas analyses were depending on physician's decision. Blood samples were immediately transferred and measured by RAPIDLAB1265 (Siemens Healthcare Diagnostics Inc., Tarrytown, NY, USA). The relationship between measured $SaO_2$ and $SpO_2$ were evaluated by simple linear regression analysis and Bland Altman plot.

**RESULTS.** Forty-nine mechanically ventilated patients and 701 points of arterial blood gas analysis were analyzed. Mean value of $SaO_2$, $SpO_2$ (Nihon Kohden) and $SpO_2$ (Masimo) were 95.8%, 96.3% and 97.1%, respectively. Comparing with each $SpO_2$ and $SaO_2$ by Bland Altman plot, bias was significantly smaller in $SpO_2$ (Nihon Kohden) than $SpO_2$ (Masimo), although precision was not significantly different (Mean ± SD,0.49±1.78% vs. 1.27±1.76%, $P$< 0.0001). The correlation coefficient was 0.889 in $SpO_2$ (Nihon Kohden) and 0.891 in $SpO_2$ (Masimo). The thresholds of $SpO_2$ with no patients with $SaO_2$< 90% were 94% in $SpO_2$ (Nihon Kohden) and 97% in $SpO_2$ (Masimo).

**CONCLUSIONS.**

We found that $SpO_2$ (Nihon Kohden) presented lower bias than $SpO_2$ (Masimo) compared with actual $SaO_2$. On the other hand, precision of $SpO_2$ and correlation coefficient were relatively the same with both devices. This study suggests that when using INTELLiVENT-ASV® and selecting automatic control of oxygenation in mechanically ventilated patients, adequate $SpO_2$ sensors should be used to avoid unexpected hypoxemia and hyperoxemia.

**REFERENCE(S)**

1) PLOS ONE 2015 journal.pone.0126979

**GRANT ACKNOWLEDGMENT**

None.

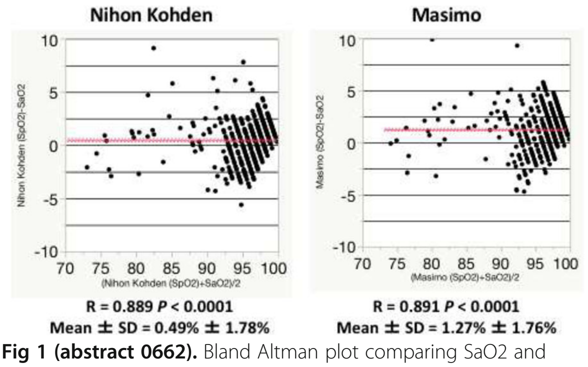

**Fig 1 (abstract 0662).** Bland Altman plot comparing SaO2 and SpO2

### 0663

#### Application of adaptive support ventilation in Korean patients with acute lung injury

W.Y. Chung, J.E. Park, K.S. Lee, Y.J. Jung, S.S. Sheen, K.J. Park
Ajou University School of Medicine, Pulmonology, Suwon, Korea, Republic of
**Correspondence:** W.Y. Chung
*Intensive Care Medicine Experimental* 2018, **6(Suppl 2):**0663

**INTRODUCTION.** Adaptive support ventilation (ASV) is a novel electronic ventilator protocol that incorporates the recent and sophisticated measurement tools and algorithms.

The target tidal volume and respiratory rate are continually adapted to patient's respiratory physics and varying medical conditions. In injured lung, the ASV should actively adjust ventilatory parameters achieving minimal work of breathing to meet the lung protective strategies. But there were little literatures describing its efficacy when applied to Korean population.

**OBJECTIVES.** We monitored the efficacy of ASV in Korean patients with acute lung injury

**METHODS.** We observed initial mechanical ventilation parameters in 114 patients receiving ASV due to various causes (48 lung injuries including 27 community acquired pneumonia, 9 hospital acquired pneumonia, 5 interstitial lung diseases, 4 pulmonary tuberculosis and 3 idiopathic cases; 66 without lung injury which comprise 33 trauma cases, 18 strokes, 9 suicidal attempts and 6 other cases). The mean age of studied population was 57.5 years (male: female=42:15). The data were collected within the first 12 hours of mechanical ventilation.

**RESULTS.** Mean age of lung injury group was 63 years (53.3 for normal lung group; $p$ < 0.05), $PaO_2$ per inspired fraction of $O_2$ (PF ratio) was 207.7 ± 61.2 (388 ± 103 for normal lung group, p < 0.05), minute volume was 7.6 ±2.21 l (7.1 ± 1.83 l for normal lung group, $p$ > 0.05), inspiratory flow was 43.0 ±8.2 l/min (39.2 ± 10.3 l/min for normal lung group, $p$ > 0.05), expiratory flow was 41.8 ± 11.4 L/min (39.2 ± 12.2 l/min for normal lung group, $p$ > 0.05), peak pressure and plateau pressure were 26.8 ± 10.2 $cmH_2O$ and 23.8 ± 6.0 $cmH_2O$ (20.3 ± 4.8 $cmH_2O$, 16.2 ± 3.9 $cmH_2O$ for normal lung group, $p$ > 0.05), inspiratory resistance was 13.5 ± 5.6 $cmH_2O$/s/l (12.4 ± 5.3 $cmH_2O$/s/l for normal lung group, $p$ > 0.05). Static compliance was measured at 26.7 ± 7.9 ml/$cmH_2O$ in lung injury group (60.7 ± 12.2 ml/$cmH_2O$ in normal lungs; $p$ < 0.05 ), and inspiratory to expiratory time ratio in lung injuries was 0.5 (0.55 in normal lungs, $p$ > 0.05). Expiratory time constant (RCexp) in lung injuries was 0.54 ± 1.7 s (0.79 ± 2.2 s in normal lungs). In lung injury patients, the tidal volume was smaller (8.35 ± 2.38 ml/Kg vs 6.20 ± 1.89 ml/Kg in normal lung group, $p$ < 0.05) and respiratory rate was higher (19.8 breaths/min vs 15.2 breaths/min for normal lung group, $p$ < 0.05).

**CONCLUSIONS.** As expected, adaptive support ventilation delivered smaller tidal volume and higher respiratory rates for injured lungs.

ASV efficiently operated in Korean ALI patients without any serious drawbacks and favorably adjusted the tidal volume and respiratory rates combination in relation with RCexp to meet lung protective strategies.

**GRANT ACKNOWLEDGMENT**

None

### 0664

#### Tuning the ventilator using diaphragmatic ultrasonography

M. Vasileiou, E. Soilemezi, C. Spyridonidou, P. Sotiriou, M. Tsagourias, D. Matamis
Papageorgiou General Hospital, ICU, Thessaloniki, Greece
**Correspondence:** E. Soilemezi
*Intensive Care Medicine Experimental* 2018, **6(Suppl 2):**0664

**INTRODUCTION.** Patient-ventilator asynchrony is well known to adversely affect outcome in ICU patients. Recordings of esophageal pressure are traditionally used in the assessment of patient-ventilator interactions; however, esophageal pressure monitoring is an invasive and cumbersome method to be applied in everyday practice. On the other hand, an increasing number of ICU physicians are using diaphragmatic ultrasonography in their clinical work.

**OBJECTIVE.** This study investigates whether ultrasound can replace the information given by esophageal pressure monitoring to effectively recognize the presence of patient-ventilator asynchrony and, therefore, to appropriately set the ventilator according to the patients' inspiratory effort.

**METHODS.** All ICU patients breathing in assisted modes of ventilation, in which airway pressure, volume and flow waveforms displayed on the ventilator screen indicated patient-ventilator asynchrony, were assessed. In all patients, the ultrasonographically measured diaphragmatic excursion and the esophageal pressure were simultaneously recorded and compared for detection and documentation of patient-ventilator asynchrony.

**RESULTS.** Diaphragmatic ultrasound was equally efficient with esophageal pressure monitoring to identify all forms of patient-ventilator asynchrony, associated both with low respiratory drive and excessive ventilator assistance, namely ineffective efforts (fig.1), auto-triggering and reverse triggering, and also patient ventilator interactions associated with increased respiratory drive and/or insufficient ventilator assistance, namely double triggering or breath stacking (fig.2).

**CONCLUSIONS.** Diaphragmatic ultrasonography is a non invasive and less cumbersome method compared to esophageal pressure recording to successfully identify incidents of patient-ventilator asynchrony, allowing appropriate adjustment of the ventilator settings to match each patient's unique needs.

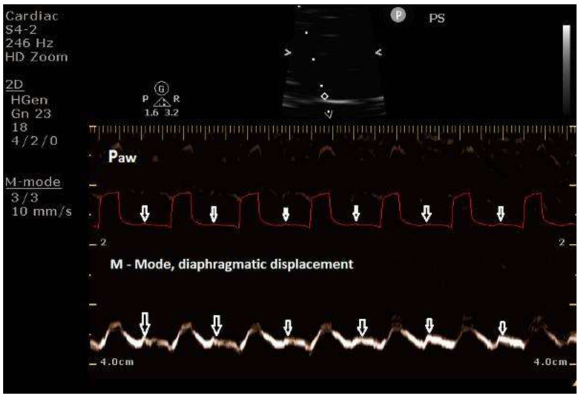

**Fig 1 (abstract 0664).** Arrows indicate ineffective efforts

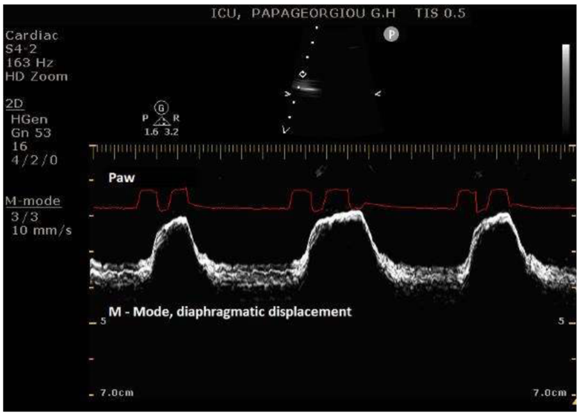

**Fig 2 (abstract 0664).** Two mechanical breaths for a single diaphragmatic displacement (double triggering)

### 0665

#### MOTIVE: MOnitoring TIdal VolumEs. A feasibility trial measuring the tidal volume of every breath during invasive ventilation

M.J. Leong[1,2], T.M. De Vries[2], P. Shanmugasundaram[2]
[1]Institute of Naval Medicine, Alverstoke, United Kingdom;
[2]Buckinghamshire Healthcare NHS Trust, Stoke Mandeville, United Kingdom
**Correspondence:** M.J. Leong
*Intensive Care Medicine Experimental* 2018, **6(Suppl 2):**0665

**INTRODUCTION.** Invasive ventilation in critical care requires careful monitoring of numerous values including the delivered tidal volumes (Vt). However, many randomised control trials comparing ventilation strategies only record Vt on a daily basis (1, 2). Published audits of lung protective ventilation have compared the delivered Vt to the expected standard on an hourly basis (3). The effect of failing to record the Vt of all the breaths delivered is not known.

**OBJECTIVES.** The objectives of this study were to:

- Examine the feasibility of recording the Vt of every breath delivered by a ventilator to a patient on an Intensive Care Unit (ICU).
- Identify any discrepancy between the charted Vt values in the hourly bedside record and the actual delivered Vt.

**METHODS.** The EView (GE Healthcare, Milwaukee, WI, USA) is a data logging device which allows downloading of ventilatory data. It was installed to an Engström Carestation ventilator (GE Healthcare) and Vt data was collected during pressure control/support ventilation of patients. The charted Vt values on the bedside record were then compared to the ventilator recorded values during the period 30 minutes before and after.

**RESULTS.** A total of 373 hours of data, representing nearly 400,000 ventilator breaths, was analysed across four different patients. The median difference between the charted Vt and median recorded Vt in that hour was 5% (IQR=2%-12%). The charted Vt was outside the 5th or 95th percentile of the breaths delivered in the associated hour 14.5% of the time. There was a statistically significant difference in the proportion of charted Vt values that were within the ICU's target lung protection range (6-8ml/kg) compared to the recorded breaths (47.7% vs 28.8%, p< 0.01).

**CONCLUSIONS.** It is feasible to record the Vt of every single breath delivered during invasive ventilation. There is a discrepancy between the charted bedside Vt and the actual Vt delivered by the ventilator. This discrepancy may be sufficient to affect the results of randomised control trials and audits. Further work should look at the feasibility of large-scale data collection, recording other ventilation parameters and their role within research trials, audits and daily clinical care.

**REFERENCE(S)**

1. The Acute Respiratory Distress Syndrome Network: Ventilation with lower tidal volumes as compared with traditional tidal volumes for acute lung injury and the acute respiratory distress syndrome. N Engl J Med. 2000;342:1301-1308.
2. Petrucci N, De Feo C. Lung protective ventilation strategy for the acute respiratory distress syndrome. Cochrane Database Syst Rev. 2013 Feb 28;(2):CD003844.
3. Newell C, Martin M et al. Protective mechanical ventilation in United Kingdom critical care units: A multicentre audit. Journal of the Intensive Care Society. Vol 18, Issue 2, pp. 106 - 112

**GRANT ACKNOWLEDGEMENT. GE provided the EView module but did not have any part in the study design or analysis. No other external funding or competing interests declared.**

### 0666

#### Aeration changes induced by high flow nasal cannula are more homogeneous than those generated by non-invasive ventilation in healthy subjects

I. Dot[1,2], J. Marin-Corral[1,2], P. Pérez-Teran[1,2], S. Sans[3,4], C. Vilà[1,2], A. Vázquez[1,2], J.R. Masclans[1,2,3]
[1]Hospital del Mar, Intensive Care Department, Barcelona, Spain; [2]Institut Hospital del Mar d'Investigacions Mèdiques (IMIM), Research Group in Critical Care Disorders (GREPAC), Barcelona, Spain; [3]Universitat Autònoma de Barcelona, Barcelona, Spain; [4]Universitat Pompeu Fabra, Barcelona, Spain
**Correspondence:** I. Dot
*Intensive Care Medicine Experimental* 2018, **6(Suppl 2):**0666

**INTRODUCTION.** Non-invasive mechanical ventilation (NIV) is a standard respiratory support technique used in the intensive care unit (ICU). A tendency to use non-invasive forms is growing, and the use of High-Flow Nasal Cannula (HFNC) devices have come into widespread use in ICU. NIV and HFNC can create positive oropharyngeal airway pressure, but it is unclear how their use affects lung volume.

**OBJECTIVES.** To compare the differences generated by two respiratory support devices, NIV and HFNC, regarding pulmonary aeration and respiratory measures using Electrical Impedance tomography (EIT) measurements in healthy subjects.

**METHODS.** This is a unicentric prospective interventionist cohort study, conducted at the Critical Care Department of the Hospital del Mar in Barcelona and its research group (Critical Illness Research Group, GREPAC) at the Hospital del Mar's Institute of Medical Investigations (IMIM), (IRB: 2015/6444/I).

Subjects were recruited and consecutively included in the study to receive NIV or HFNC, parameters of respiratory support were predetermined. Clinical data was registered and lung impedance measurements were done before and after 30 minutes of respiratory support. The data was expressed as mean (standard deviation) or as median (interquartile range). Significance was set to $p < 0.05$.

**RESULTS.** A total of 20 healthy subjects were included, 10 of them were ventilated with NIV and 10 of them with HFNC. There were no differences in demographic data. After 30 minutes of respiratory support, subjects in both groups showed a significant reduction in respiratory rate, more pronounced in the HFNC group (NIV:14.4 (4.1) vs 10.4 (1.6), p=0.009; HFNC:13.6 (4.3) vs 7.9 (1.5)bpm, p=0.002), and a significant increase in the end-expiratory lung impedance (EELI) (NIV:66,348(10,761) vs 73,697 (6,858), p=0.005; HFNC:66,252 (9,793)vs69,869 (9,135), p=0.012). NIV subjects also showed a significant increase of non-dependent silent spaces (4.13(2.25) vs 5.81 (1.49), p=0.037), while this changes were more homogeneous in HFNC. Both technics showed a variation of EELI (ΔEELI), that trended to be higher in NIV than in HFNC (8,137.08 (6,152.04) vs 3,616.94 (3,623.03), p=0.077).

**CONCLUSIONS.** NIV and HFNC increased EELI in healthy subjects, suggesting an increase in the functional residual capacity in both modalities. Despite EELI increment was higher with NIV, HFNC produced more homogeneous changes in lung ventilation distribution which could mean overdistention with NIV.

**GRANT ACKNOWLEDGMENT**

This study was supported by the Spanish Society of Pneumology and Thoracic Surgery(SEPAR 2015).

## Cardiac arrest & trauma - Experimental studies

### 0667

#### Omecamtiv mecarbil treatment improves post-resuscitation cardiac output and neurological outcome in an animal model

C.-H. Huang[1], M.-S. Tsai[2], W.-J. Chen[2]

[1]National Taiwan University Hospital, Department of Emergency Medicine, Taipei, Taiwan, Province of China; [2]National Taiwan University Hospital, Taipei, Taiwan, Province of China

**Correspondence:** C.-H. Huang

*Intensive Care Medicine Experimental* 2018, **6(Suppl 2):**0667

**INTRODUCTION.** Myocardial dysfunction is one of the major causes leading to poor outcomes in the post-cardiac arrest period. Omecamtiv mecarbil (OM) is a selective small molecule activator of cardiac myosin that prolongs myocardial systole and increases stroke volume without apparent effects on myocardial oxygen demand (1). OM administration is safe and improves the cardiac function for acute heart failure patients. Whether the OM improves post-resuscitation myocardial dysfunction remains unclear.

**OBJECTIVES.** To investigate the effect of OM treatment on post-resuscitation myocardial dysfunction and outcomes.

**METHODS.** Adult male rats were resuscitated after 9.5 mins asphyxia-induced cardiac arrest. OM was continuously intravenously infused after ROSC with 0.25 mg/kg/hr for 4 hours in the experiment group and normal saline was given at the same volume per hour in the control group (n=20 in each group). Body temperature was kept on 36.5°C during treatment. Hemodynamic parameters were measured every hour and monitored for four hours after cardiac arrest. Recovery of neurological function was evaluated by neurological functioning scores (0-12) for rats at 72 h after cardiac arrest (2). The neurological score reaching up to 11 or 12 was defined as favorable neurological outcome.

**RESULTS.** OM treatment prolonged the left ventricular ejection time and improved the post-resuscitation cardiac output (figure 1). Heart rate, left ventricular systolic ($dP/dt_{40}$) and diastolic function (maximal negative dP/dt) were not different between two groups in the post-resuscitation period (figure 2). Kaplan-Meier analysis showed higher, but not statistically different 72-hour survival in OM group (72.2% (13/18) vs. 58.8% (10/17), p=0.386 by log-rank test). OM group had higher chance of having favorable neurological outcome for the survived at 72 hours after cardiac arrest (84.6% (11/13) vs. 40% (4/10), p=0.026). The percentage of damaged neuron was less in the OM group in histology study at 72 hours after cardiac arrest (55.5±2.3 vs. 76.2±10.2 %, p=0.004).

**CONCLUSIONS.** OM treatment can improve post-resuscitation myocardial dysfunction and neurological outcome in an animal model. These findings could be the basis for further pre-clinical studies for improving the outcome in post-cardiac arrest care.

**GRANT ACKNOWLEDGMENT**

The study is supported by the grants: MOST-105-2314-B-002 -125 -MY3 & MOST-104-2314-B-002-197-MY3

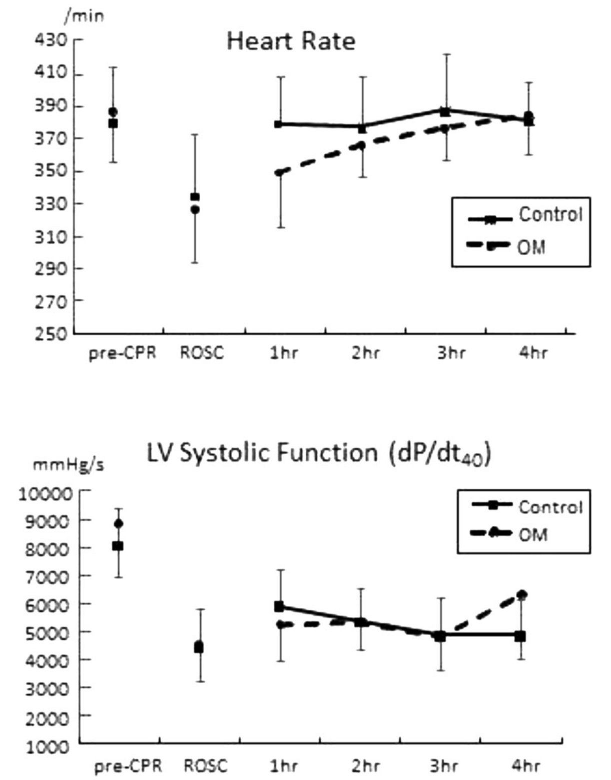

**Fig. 1 (abstract 0667).** Cardiac output and left ventricular ejection time

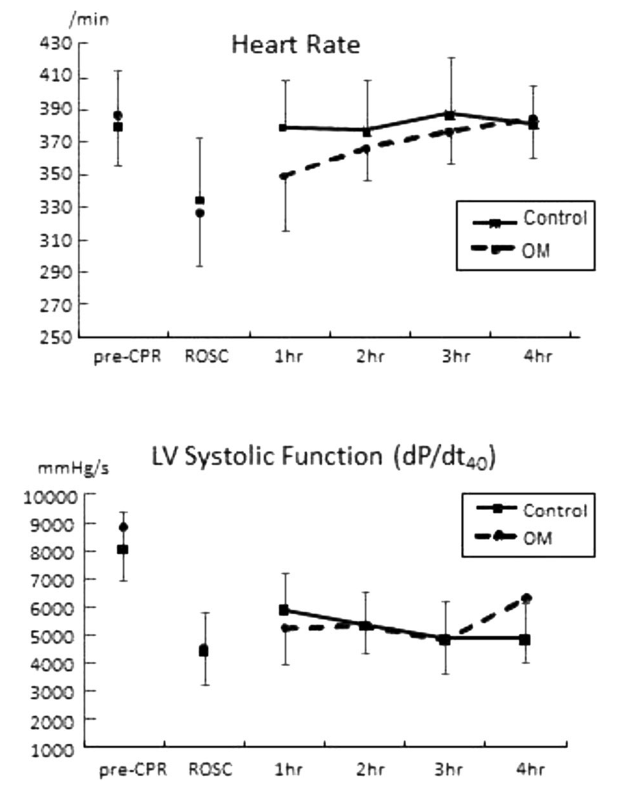

**Fig. 2 (abstract 0667).** Heart rate and left ventricular systolic function

### 0668

#### Exploring optimal mean arterial pressure at various temperatures in a porcine cardiac arrest model

T. Olasveengen[1], C. Skåre[1], R. Strand-Amundsen[2,3], M. Eriksen[4], V. Skulberg[4], T.I. Tønnessen[1,5], K. Sunde[1,5]
[1]Oslo University Hospital, Anesthesiology, Oslo, Norway; [2]Oslo University Hospital, Department of Clinical and Biomedical Engineering, Oslo, Norway; [3]University of Oslo, Department of Physics, Oslo, Norway; [4]University of Oslo, Institute for Experimental Medical Research, Oslo, Norway; [5]University of Oslo, Institute of Clinical Medicine, Oslo, Norway
**Correspondence:** T. Olasveengen
*Intensive Care Medicine Experimental* 2018, **6(Suppl 2):**0668

**INTRODUCTION.** Although current guidelines for resuscitation recommend maintaining temperature between 32-36$^0$C and Mean Arterial Pressure (MAP) 60-90 mmHg, there is very limited science to guide the balancing act between the brain's need for perfusion and the heart's need for restitution. There are also important gaps in knowledge of how cerebral perfusion needs vary with various temperatures.
**OBJECTIVES.** To determine whether maintaining a MAP of 90 mmHg would yield improved cerebral blood flow and less ischemia compared to 60 mmHg in a porcine model of cardiac arrest, and to assess whether Targeted Temperature management at 33$^0$C (TTM) might attenuate some of the resulting ischemia.
**METHODS.** Forty-one 35 kg swine were anesthetized and instrumented prior to electrical induction of ventricular fibrillation. After 10 minutes of cardiac arrest, animals were resuscitated using a heart-lung machine at 100 ml/kg/min for 2 minutes before defibrillation. After ROSC, animals were randomized to TTM or no TTM, and MAP 60 or MAP 90; yielding four groups. TTM was managed by intravasal cooling, and blood pressure targets were managed with vasopressin, norepinephrine and nitroprusside. After a 30 minute stabilization period, animals were observed for an additional 2 hours. Cerebral perfusion pressure (CPP), flow (CF), pressure reactivity index (PRx), tissue $pCO_2$ and microdialysis were measured continuously, analyzed for 15 minute epochs, and compared using mixed models.
**RESULTS.** MAP 90 animals had significantly higher CPP (p< 0.001 for both no-TTM and TTM groups) and CF (p< 0.001 for both no-TTM and TTM groups) compared to MAP 60 animals. MAP 90 animals also showed less signs of ischemic insult with lower PRx values (indicating improved autoregulation), (p=0.04 for no TTM and p=0.001 for TTM group), lower brain tissue $pCO_2$ (p=0.017 for no TTM and p< 0.001 for TTM group) compared to MAP 60 animals. Preliminary results from microdialysis show increased lactate/pyruvate ratios among normothermic MAP 60 animals compared to MAP 90 animals (0.017). Samples from TTM treated animals are being analyzed.
**CONCLUSION.** Preliminary results suggest targeting mean arterial pressure to 90 mmHg after cardiac attest might provide better cerebral perfusion and less ischemic insult at both normothermia and hypothermia (33$^0$C) compared to 60 mmHg in a porcine model.

**GRANT ACKNOWLEDGMENT**
This work was supported by unrestricted grants from Oslo University Hospital and South-Eastern Regional Health Authority, Norway.

### 0669

#### Commencing one-handed chest compressions while activating emergency medical system by handheld mobile device in lone rescuer basic life support: a randomised crossover simulation study

D. Lee[1], S.M. Park[2]
[1]Seoul National University Bundang Hospital, Emergency Department, Anyang si, Korea, Republic of; [2]Seoul National University Bundang Hospital, Emergency Department, Seongnam, Korea, Republic of
**Correspondence:** D. Lee
*Intensive Care Medicine Experimental* 2018, **6(Suppl 2):**0669

**OBJECTIVES.** In current basic life support (c-BLS), lone rescuer is recommended to start chest compressions (CC) after activating the emergency medical system. To realize earlier CC in lone-rescuer BLS, we designed a modified BLS (m-BLS) sequence in which lone rescuer commences one-handed CC while calling for help using a handheld cellular telephone with the other free hand. The aim of this study is to compare the quality of BLS between the c-BLS and the m-BLS.
**METHODS.** This is a simulation study performed by a randomized cross-over controlled trial design. One-hundred eight university students were finally enrolled. After training for both c-BLS and m-BLS, participants performed 3 minutes of c-BLS and m-BLS on a manikin with a Skill-Reporter™ at random cross-over order. The quality of BLS was compared between the c-BLS and m-BLS.
**RESULTS.** Comparing with the c-BLS, m-BLS could reduce the mean lag time before initiation of CCs (18.4 vs. 51.5sec, respectively; p < 0.001), could deliver higher number of CCs (217.9 vs. 162.9, respectively; p < 0.001), and increased the mean fraction of CC (63.6 vs. 48.0 %, respectively; p < 0.001), significantly during the first 3 minutes of BLS. Also, higher numbers of CC with correct depth (defined by 5-6 cm) were delivered in the m-BLS than in the c-BLS (185.8 vs. 158.1 in c-BLS; p < 0.001). There were no differences in the correct hand positions, compression rate and interruption time for ventilation between the two groups.
**CONCLUSIONS.** In simulated lone rescuer BLS, the m-BLS sequence could deliver significantly earlier CC than the c-BLS while maintaining high-quality cardiopulmonary resuscitation.

**REFERENCE(S)**

1. Kleinman ME, Brennan EE, Goldberger ZD, et al. Part 5: Adult Basic Life Support and Cardiopulmonary Resuscitation Quality: 2015 American Heart Association Guidelines Update for Cardiopulmonary Resuscitation and Emergency Cardiovascular Care. *Circulation* 2015;132(18 Suppl 2):S414-35. doi: 10.1161/CIR.0000000000000259
2. Kronick SL, Kurz MC, Lin S, et al. Part 4: Systems of Care and Continuous Quality Improvement: 2015 American Heart Association Guidelines Update for Cardiopulmonary Resuscitation and Emergency

Cardiovascular Care. *Circulation* 2015;132(18 Suppl 2):S397-413. doi: 10.1161/CIR.0000000000000258

3. Cummins RO, Ornato JP, Thies WH, et al. Improving survival from sudden cardiac arrest: the "chain of survival" concept. A statement for health professionals from the Advanced Cardiac Life Support Subcommittee and the Emergency Cardiac Care Committee, American Heart Association. *Circulation* 1991;83(5):1832-47.

**GRANT ACKNOWLEDGMENT**

We thank all the Kyungbok university students who have volunteered to participate in this study.

### 0670

#### Plasmatic glucose and insulin levels in the porcine model of experimental cardiac arrest

R. Skulec[1,2,3], D. Astapenko[1], R. Cerna Parizkova[1], T. Parizek[2], M. Bilska[2], V. Radochova[4], V. Cerny[2,5,6]

[1]Charles University in Prague, Faculty of Medicine in Hradec Kralove, University Hospital Hradec Kralove, Department of Anesthesiology and Intensive Care, Hradec Kralove, Czech Republic; [2]J.E. Purkinje University, Masaryk Hospital Usti nad Labem, Department of Anesthesiology, Perioperative Medicine and Intensive Care, Usti nad Labem, Czech Republic; [3]Emergency Medical Service of the Central Bohemian Region, Kladno, Czech Republic; [4]University of Defence, Faculty of Military Health Sciences, Brno, Czech Republic; [5]Charles University in Prague, Faculty of Medicine in Hradec Kralove, University Hospital Hradec Kralove, Department of Research and Development, Hradec kralove, Czech Republic; [6]Pain Management and Perioperative Medicine, Dalhousie University, Department of Anesthesia, Halifax, Canada

**Correspondence:** R. Skulec

*Intensive Care Medicine Experimental* 2018, **6(Suppl 2):**0670

**INTRODUCTION.** Glucose is the main energetic substrate for the brain and myocardium during severe ischemia. Nevertheless, a very little is known about the metabolism of glucose and insulin during cardiac arrest.[1]

**OBJECTIVES.** We decided to describe glucose and insulin plasmatic levels during induced experimental cardiac arrest, to assess the impact of adrenaline administration on them and to evaluate the impact of glucose load immediately after induction of cardiac arrest on their metabolism.

**METHODS.** We induced ventricular fibrillation for 15 minutes (2 minutes of unresuscitated cardiac arrest, 3 minutes of continuous cardiac compressions only and 10 minutes of cardiac compressions and mechanical ventilation) in 21 anesthetized domestic pigs. Before that, blood samples were taken to determine baseline plasmatic levels of glucose, insulin, cortisol and adrenaline and the experimental animals were randomized to undergo induced cardiac arrest as described above (Group A, n=7); or to undergo group A protocol supplemented by administration of 15 μg/kg of adrenaline in the 5th and 10th minute of cardiac arrest (Group B, n=7); or to undergo group A protocol enriched by rapid i.v. administration of 16 g of glucose as soon as possible after cardiac arrest induction (Group C, n=7). After 15 minutes of cardiac arrest the animals were defibrillated and observed for 20 minutes. Blood samples to evaluate glucose, insulin, cortisol and adrenaline plasmatic levels were taken in regular intervals throughout the protocol.

**RESULTS.** All animals in the group B reached return of spontaneous circulation, while only 5 in the group A and 3 in the group C. A gradual increase of glycaemia during induced cardiac arrest was observed in all groups from the baseline to the 15th minute of induced cardiac arrest (Group A: from 5.4±2.0 to 7.8±2.1 mmol/l, p=0.049; group B: from 5.6 ±2.0 to 9.3±1.8 mmol/l, p=0.003; group C: from 4.8±0.8 to 21.3 mmol/l, p< 0.001). Insulin levels did not change significantly in the group A (from 2.8±2.1 to 2.0±0.4 pmol/l, p=0.342) and the group C (from 3.2±2.1 to 2.8±1.9 pmol/l, p=0.715), while significant decrease of insulin levels was observed in the group B (from 6.3±4.0 to 2.3±1.1 pmol/l, p=0.025). Adrenaline administration did not affect glucose levels significantly in the group B in comparison with the group A. Initial glucose load in the group C had no impact on the further insulin release.

**CONCLUSIONS.** We observed a gradual increase of plasmatic glucose levels without any change of insulin levels in our porcine model of cardiac arrest. Adrenaline administration led to decrease of insulin levels. Load of glucose administered as soon as possible after cardiac arrest induction did not induced additional insulin release.

**REFERENCE(S)**

1. Martin GB et al. Ann Emerg Med 1985;14:293-297.

**GRANT ACKNOWLEDGMENT**

Supported by the programme PROGRES Q40/2 and by MH CZ - DRO (UHHK, 00179906).

### 0671

#### Comparison of different resuscitation algorithms regarding hand-on time and algorithm adherence

T. Sellmann[1,2], S. Rifai[3,4], D. Wetzchewald[5], H. Schwager[5], S. Russo[1], S. Marsch[6]

[1]University Witten Herdecke, Helios University Hospital, Department of Anaesthesiology, Wuppertal, Germany; [2]Bethesda Krankenhaus Duisburg, Department of Anaesthesiology, Duisburg, Germany; [3]Bethesda Hospital, Department of Orthopedics and Trauma Surgery, Duisburg, Germany; [4]360 Degree Clinic, Department of Orthopedics, Ratingen, Germany; [5]Institution for Emergency Medicine, Arnsberg, Germany; [6]University Hospital of Basel, Department of Medical Intensive Care, Basel, Switzerland

**Correspondence:** T. Sellmann

*Intensive Care Medicine Experimental* 2018, **6(Suppl 2):**0671

**INTRODUCTION/ OBJECTIVES.** Adherence to CPR treatment algorithms is often not ideal. Alternative algorithms and modifications of the official CPR guidelines are in use. The aim of the present study was to compare three CPR algorithms with regard to adherence and measures relevant for patients' outcomes (i.e. hands on-time).

**METHODS.** 287 teams, consisting of three to five physicians each, were randomized to perform CPR in a simulated unwitnessed cardiac arrest according to the current guidelines ("2min"), the cardiocerebral resuscitation ("CCR") protocol (200 chest compressions with no ventilation), or a local modification of the current guidelines (200 chest compressions with ventilation via a rapidly inserted laryngeal tube, the "Arnsberg" algorithm). Data analysis was performed using video-recordings obtained during simulations. The primary endpoint was hands-on time and secondary endpoints were adherence to allocated algorithm.

**RESULTS.** Hands-on time was 85 ± 7% in the "2min" teams, 86 ± 4% in the "Arnsberg" teams (P = 0.025 vs "2min"), and 87 ± 5% in "CCR" teams (P = 0.005 vs "2 min"; P = 0.33 vs "Arnsberg"). "2min" teams deviated significantly more (P < 0.001) from target (range of ± 20 % of a time interval of 2min or 200 chest compressions respectively between subsequent defibrillations) than "CCR" and "Arnsberg" teams. "CCR" teams demonstrated the closest adherence to target and the least within-team and between-team variance with regard to cycle length (P = 0.03) and delivered chest compressions (P = 0.001) in subsequent cycles.

**CONCLUSIONS.** Different CPR algorithm may substantially vary in adherence. Reducing the complexity of CPR algorithms can result in better adherence, less variance and better hand-on times.

**REFERENCE(S)**

1. Soar J, Nolan JP, Böttiger BW, Perkins GD, Lott C, Carli P, Pellis T, Sandroni C, Skrifvars MB, Smith GB, Sunde K, Deakin CD; Adult advanced life support section Collaborators. European Resuscitation Council Guidelines for Resuscitation 2015: Section 3. Adult advanced life support. Resuscitation. 2015 Oct;95:100-47. doi: 10.1016/j.resuscitation.2015.07.016.
2. Ewy GA, Sanders AB. Alternative approach to improving survival of patients with out-of-hospital primary cardiac arrest. J Am Coll Cardiol. 2013 Jan 15;61(2):113-8. doi: 10.1016/j.jacc.2012.06.064

### 0672

#### Cardiac effects of beta$_1$-adrenergic receptor blockade (esmolol) during extracorporeal cardiopulmonary resuscitation (VA-ECMO) in pigs

H. Karlsen[1], T.M. Olasveengen[1,2], H.A. Bergan[2], P.S. Halvorsen[3], I. Schalit[3], K. Sunde[2,4], J.F. Bugge[2]
[1]Oslo University Hospital, Departement of Research and Development, Oslo, Norway; [2]Oslo University Hospital, Department of Anesthesiology, Oslo, Norway; [3]Oslo University Hospital, The Intervention Centre, Oslo, Norway; [4]University of Oslo, Institute of Clinical Medicine, Oslo, Norway
**Correspondence:** H. Karlsen
*Intensive Care Medicine Experimental* 2018, **6(Suppl 2):**0672

**INTRODUCTION.** International guidelines recommend adrenaline during resuscitation despite lack of evidence on survival benefits. There is some evidence that the beta-adrenergic effects of adrenaline are harmful due to increased myocardial oxygen consumption, post-defibrillation ventricular arrhythmias and increased severity of post-arrest myocardial dysfunction. Esmolol may counteract the unfavorable effects of adrenaline and thus preserve post-arrest myocardial function.
**OBJECTIVES.** To test whether esmolol administered prior to adrenaline preserves post-arrest myocardial function in an ischemic cardiac-arrest pig model with low-flow (2.5L/min) VA-ECMO resuscitation.
**METHODS.** Twenty anesthetized pigs had a myocardial infarction induced by an inflated PCI-balloon in the circumflex artery, and ventricular-fibrillation (VF) was induced electrically 15 min later. After 10 min of untreated VF, resuscitation by VA-ECMO was initiated and the animals were randomized to receive an injection of either 1mg/kg esmolol or placebo, prior to adrenaline 1mg/dose. Subsequent successful defibrillation was followed by a one-hour high-flow VA-ECMO support before ECMO weaning and an additional one-hour stabilization period. The PCI-balloon was deflated 40 min after inflation. Cardiac function post-arrest was assessed by MRI and invasive pressure measurements. Myocardial injury was estimated by serum concentrations of cardiac troponinT.
**RESULTS.** Seven esmolol-treated and five placebo pigs survived until post-arrest measurements with substantial myocardial injury. MRI revealed a severe reduction of myocardial function without significant differences between the two treatment groups (Table1). Invasive pressures and ventricular pressure-derivates did not significantly change. The 60% reductions of stroke-volumes were partly compensated for by increases in heart-rate.
**CONCLUSIONS.** Beta$_1$-blockade prior to adrenaline during low-flow VA-ECMO resuscitation did not preserve post-arrest cardiac function nor decrease the magnitude of myocardial injury in pigs, compared to placebo.

**GRANT ACKNOWLEDGMENT**
The study was supported by Laerdal Foundation, Stavanger, Norway.

**Table 1 (abstract 0672).** See text for description.

| Variables | Baseline | | | Post-arrest | | |
|---|---|---|---|---|---|---|
| | Treatment group | | *p*-value group difference | Treatment group | | *p*-value group difference |
| | Placebo | Esmolol | | Placebo | Esmolol | |
| HR beats/min | 67 ± 5 | 80 ± 17 | 0.15 | 120 ± 25* | 140 ± 34* | 0.3 |
| SV ml | 71 ± 3 | 64 ± 18 | 0.5 | 30 ± 7* | 27 ± 8* | 0.5 |
| EF % | 56 ± 8 | 57 ± 13 | 0.9 | 24 ± 4* | 26 ± 4* | 0.5 |
| CO L/min | 4.9 ± 0.2 | 5.0 ± 1.0 | 0.9 | 3.5 ± 0.3* | 3.5 ± 0.2* | 1 |
| $LVP_{max}$ mmHg | 103 ± 9 | 105 ± 12 | 0.8 | 100 ± 10 | 105 ± 12 | 0.5 |
| $dP/dt_{max}$ mmHg/sec | 1559 ± 245 | 1864 ± 570 | 0.4 | 1483 ± 393 | 2148 ± 796 | 0.1 |
| $dP/dt_{min}$ mmHg/sec | -2100 ± 771 | -2326 ± 612 | 0.6 | -1684 ± 343 | -2093 ± 596 | 0.2 |
| MAP mmHg | 88 ± 9 | 90 ± 15 | 0.8 | 75 ± 10 | 75 ± 9 | 0.9 |
| MPAP mmHg | 22 ± 6 | 22 ± 4 | 0.9 | 23 ± 5 | 22 ± 5 | 0.6 |
| Cardiac TroponinT (ng/L) | 18 ± 6 | 16 ± 17 | 0.8 | 4161 ± 3088 | 5524 ± 5124 | 0.6 |

### 0673

#### Impact of training on quality of cardiopulmonary resuscitation

L. Balciunas[1], K. Kaunaite[2], L. Puodziukaite[2], J. Sipylaite[1]
[1]Department of Anesthesiology and Intensive Care, Institute of Clinical Medicine, Faculty of Medicine, Vilnius University, Vilnius, Lithuania; [2]Vilnius University, Faculty of Medicine, Vilnius, Lithuania
**Correspondence:** K. Kaunaite
*Intensive Care Medicine Experimental* 2018, **6(Suppl 2):**0673

**INTRODUCTION.** Sudden out-of-hospital cardiac arrest is a frequent cause of death that requires immediate bystander cardiopulmonary resuscitation (CPR). Repeated training helps retain knowledge and skills in CPR.
**OBJECTIVES.** The aim of this study was to evaluate impact of basic life support (BLS) training on practical skills and knowledge of BLS including the use of an automated external defibrillator (AED).
**METHODS.** A prospective study of 90 students (43.3% males, median age 18 [IQR, 16-19] years) was conducted. Participants were divided into three groups: students who had not previously received any BLS training (group 1, n=30), students who only had attended a non-practical BLS course (group 2, n=30), and students who had just attended a BLS-AED course (group 3, n=30). Each participant completed CPR on a manikin which tracks chest compressions, lung ventilation and compression time in percentage. Total CPR result was calculated based on these measures. Both resuscitation knowledge (questionnaire) and practical skills (compression (%), ventilation (%), total CPR score (%), mistakes, use of AED) were analyzed. Set resuscitation time was 2 minutes. Data analysis performed with SPSS 24.0 program.
**RESULTS.** Mean total CPR score: 28.0 (SD 13.1) % in group 1, 40.8 (SD 18.2) % group 2 and 50.7 (SD 19.4) % group 3; (p< 0.001). Median chest compression results: 1% [IQR, 0-10] in group 1, 9% [IQR, 0-47] group 2, 51% [IQR, 21-79] group 3; (p< 0.001). Nineteen (21.1%) people did not ventilate: 33.3% in group 1, 10% in group 2 and 20% in group 3. Median ammount of practical mistakes according to groups (12 criteria): 9 in group 1 [IQR, 7.8-10], 5 in group 2 [IQR, 2-7] and 3 in group 3 [IQR, 2-6.3]; (p< 0.001). Mean use of AED score in groups (of total 4 points): 1 group 2.8 (SD 0.9), 2 group 3.3 (SD 0.8), 3 group 3.8 (SD 0.41). Mean score for test (total 10 points) was: 1 group 4.8 pts (SD 1.5), 2 group 5.7 pts (SD 1.3), 3 group 8.9 pts (SD 0.9); (p< 0.001).
**CONCLUSIONS.** The results of this study showed that better resuscitation results are achieved with prior both hands-on and theoretical courses. BLS-AED training should be a standard part of school education.

### 0674

#### Comparison of quality of chest compressions during cardiopulmonary resuscitation with two models of automated external defibrillator

A. Gaillard[1], C. Ricard[1], G. Berthet[1], O. Baptiste[1], V. Peigne[1,2]
[1]Haute Savoie Fire Department, Meythet, France; [2]CH Métropole Savoie, Chambéry, France
**Correspondence:** V. Peigne
*Intensive Care Medicine Experimental* 2018, **6(Suppl 2):**0674

**INTRODUCTION.** Basic life support for cardiac arrest associates cardiopulmonary resuscitation and defibrillation. Among the numerous marketed models of automated external defibrillators (AED), some provide real-time feedback about the quality of the chest compressions, some are able to perform rhythm analysis during chest compressions but none offers these two functions.
**OBJECTIVES.** The aim of the present study is to compare the quality of chest compression during cardiopulmonary resuscitation with two models of AED, one analyzing cardiac rhythm during chest compressions (AED#1, model Lifepak CR2, manufactured by Physio-Control, USA) and the other one providing real-time feedback about the quality of the chest compressions (AED #2, model AED3 manufactured by Zoll Medical, USA).

**METHODS.** 39 teams of firemen performed cardiopulmonary resuscitation on a manikin following the same scenario. CC were continuous as ventilation was performed by continuous insufflation of oxygen. Each team of firemen was randomized to use either AED#1 or AED#2. Data on the quality of the cardiopulmonary resuscitation were obtained from the manikin's sensors and were analyzed according to the current guidelines. The CPR fraction (ratio of the duration of the chest compressions on the duration of the cardiopulmonary resuscitation) was measured.
**RESULTS.** 19 teams were randomized to the AED#1 and 20 to the AED#2. 91% of the chest compressions respected the recommended rate in the AED#1group and 92% in the AED#2 group (p 0.8). Only 28% of the chest compressions were in the targeted depth in the AED#1 group versus 69% for the AED#2 group ($p < 0.001$). The CPR fraction was higher in the AED#1 group (96% versus 93%, $p < 0.01$).
**CONCLUSIONS.** The model of AED used modifies cardiopulmonary resuscitation quality. Rhythm analysis during chest compressions by the AED increased slightly CPR fraction. Real-time feedback on cardiopulmonary resuscitation by the AED dramatically increased quality of chest compressions.

**REFERENCE(S)**
None

**GRANT ACKNOWLEDGMENT**
None

### 0675
### Evaluation of the quality of cardiopulmonary resuscitation according to vehicle driving pattern, using a virtual reality ambulance driving system: a prospective, crossover, randomised study

Y.S. Park
Yonsei University College of Medicine, Seoul, Korea, Republic of
*Intensive Care Medicine Experimental* 2018, **6(Suppl 2):**0675

**BACKGROUND.** We aimed to analyse changes in the quality of cardiopulmonary resuscitation (CPR) according to driving patterns encountered during ambulance transport, using a virtual reality ambulance simulator.
**METHODS.** A total of 48 participants were paired; the pairs performed chest compression for 8 minutes each in a stationary and driving (virtual reality ambulance simulating 10 driving patterns) state. Several CPR quality parameters such as chest compression depth and its variability were collected.
**RESULTS.** The mean chest compression depths were 54.8 mm and 55.3 mm during the stationary and driving states, respectively ($p = 0.41$). The standard deviation of the chest compression depth was significantly higher while in the driving (7.6 mm) than while in the stationary state (6.5 mm; $p = 0.04$). The comparison of CPR quality parameters according to driving patterns showed that the compression depths in the speed bump and sudden stop sections were 51.5 mm and 50.6 mm, respectively, which is shallower than those in all other sections ($p < 0.001$). The correct hand position rate was low in the speed bump, sudden stop, and right-hand cornering sections (65.4%, 71.5%, and 72.5%, respectively; $p = 0.001$)
**CONCLUSIONS.** Although we found no differences in chest compression quality parameters between the stationary and driving states, the variability in the chest compression depth increased in the driving state. In the speed bump and sudden stop sections, we noted a shallower compression depth, increased variability, and decreased correct hand position rate. In the right-hand cornering section, we observed a decreased correct hand position rate.

### 0676
### Low perfusion ECMO can provide appropriate therapeutic hypothermia for post cardiac arrest syndrome

H. Arimoto, H. RInka, T. Miyaichi, A. Fuke, J. Ishikawa, T. Morooka, K. Shigemitsu, A. Tsuruoka, T. Morimoto, S. Furuya, K. Suzuki, T. Yamashita, H. Tanaka
Osaka City General Hospital, Emergency and Critical Care Medical Center, Osaka, Japan
**Correspondence:** H. Arimoto
*Intensive Care Medicine Experimental* 2018, **6(Suppl 2):**0676

**INTRODUCTION.** Mild therapeutic hypothermia (TH) has been integrated into management strategies for improving the neurological outcome of post cardiac arrest syndrome (PCAS) for out of hospital cardiac arrest. Some devices are produced to achieve hypothermia with surface or core-cooling. The extracorporeal membrane oxygenation (ECMO) works for the management of refractory cardiac arrest not only to establish extracorporeal cardiopulmonary resuscitation (E-CPR), but also to induct TH as a core-cooling device. We report our preliminary experiences in establishing a therapeutic hypothermia for PCAS with ECMO. This study is a single center, retrospective, observational study.
**METHODS.** The PCAS cases of out-of-hospital cardiac arrest were induced TH with ECMO. The TH was indicated for unconscious cardiogenic ROSC patients under 75 years of age, and excluded existing brain hemorrhage, traumatic or respiratory cardiac arrest, and any risk of bleeding. Target temperature was set at 34°C for 24 to 48 hours. ECMO for TH were quickly induced hypothermia on low-perfusion flow (1 l/min) with minimal anticoagulation. Intra-aortic balloon pumping was only used to achieve an improvement coronary artery flow or LV unload. Initial E-CPR was excepted in this study. Any complications, temperature stability, shivering and neurological outcomes were compared. A favorable neurological outcome was defined as a Cerebral Performance Category (CPC)1 after 90 days of cardiac arrest.
**RESULTS.** From January 2007 to December 2015, 41 patients were induced TH with ECMO. 31 favorable outcome cases (76%) were seen. All cases could reach to target temperature within 15 min. Shivering was seen 11 cases (27%), ventilator associated pneumonia was the leading complication for 9 cases (22%). bleeding from cannulation site was 8 cases (20%), and temperature over-cooling and instability was 2 cases (5%) each. ECLS for unstable circulation was performed 5 cases (12%). No fatal complications were seen except intra cranial hemorrhage was 2 cases (5%). There were no significant characteristic differences between group CPC1 vs CPC2-5.
**CONCLUSIONS.** We suggest not only ECMO works immediate TH initiation and stable circulation support for PCAS satisfactory, but ECMO is feasible and associated with a relatively high favorable neurological survival rate.

### 0677
### Crew resource management: is it lacking in basic life support training? - CRM study

A. Correia[1,2], I.J. Pereira[1,2,3], P. Barbosa[2], C. Granja[1,2,3]
[1]University of Algarve, Department of Biomedical Sciences and Medicine (DCBM), Faro, Portugal; [2]Centro Hospitalar Universitário do Algarve, EPE - Unidade de Faro, Faro, Portugal; [3]Center for Reseach in Health Technology and Information Services, Porto, Portugal
**Correspondence:** A. Correia
*Intensive Care Medicine Experimental* 2018, **6(Suppl 2):**0677

**INTRODUCTION.** Basic Life Support (BLS) technical skills are vital to achieve successful cardiopulmonary resuscitation (CPR) and general ward healthcare providers play a vital role as first responders, even when Advance Life Support (ALS) team is available (1). Successful outcomes are affected by a plethora of non-technical aspects raising concern about how human factors affect technical skills and CPR outcomes (2). Guidelines support non-technical skills (NTS) inclusion in ALS training (3), but the impact of it´s inclusion during BLS training is not clear.

**OBJECTIVE.** To explore the impact of intra-hospital contextualization and NTS education during BLS training in CPR performance.

**METHODS.** Prospective quasi-experimental study. General ward healthcare providers (nurses and healthcare assistants) from two different medical wards of a University Hospital were assigned to receive either standard BLS training (StdBLS) or standard BLS training plus intra-hospital contextualization features and NTS principles (IHBLS). After training and during a regular shift they were confronted with a ward unexpected CPR simulation. They were asked to intervene in teams of 3 elements. Footage video was collected and a BLS simulator manikin with remote digital access was used to extract performance data. NTS were evaluated according with the Team Emergency Assessment Measure (TEAM) scale, measuring leadership, team work and task management. Informed consent was collected from all participant and the study was authorized by the local ethics committee.

**RESULTS.** Preliminary data were obtained. Thirty individuals were enrolled in the training sessions. Seven (23%) did not have any previous BLS training. Three teams of each group were submitted to a CPR simulation. When analyzing outcomes concerning TEAM scale dimensions, IHBLS group showed higher scores in teamwork, overall NTS performance and TEAM total score. Both groups had comparable scores in leadership and task management and achieved similar CPR performance.

**CONCLUSIONS.** Considering our preliminary data, the inclusion of hospital contextualization and NTS education during BLS training of healthcare providers did not demonstrate an impact in BLS resuscitation performance when compared with standard BLS training. However, it was noted a slight tendency of increase in NTS global performance by IHBLS teams. The wider sample obtained in demographics data brings out a worrisome training gap in BLS skills. It raises concern about hospital educational strategy in critical care. Facing the institutional commitment with the present project, we intent to proceed with the data collection.collection.

**GRANT ACKNOWLEDGMENT**

We thank all the participating ward staff, BLS instructors and simulator manikin provider.

### 0678

#### Therapeutic application of recombinant human ADAMTS-13 to improve deranged coagulation in a trauma-transfusion rat model

M.R. Wirtz[1,2], D.P. van den Brink[1], J. Voorberg[3], J.C. Goslings[2,4], N.P. Juffermans[1]
[1]Academic Medical Center, Department of Intensive Care Medicine, Amsterdam, Netherlands; [2]Academic Medical Center, Department of Trauma Surgery, Amsterdam, Netherlands; [3]Sanquin Research, Department of Plasma Proteins, Amsterdam, Netherlands; [4]Onze Lieve Vrouwe Gasthuis, Department of Trauma Surgery, Amsterdam, Netherlands
**Correspondence:** M.R. Wirtz
*Intensive Care Medicine Experimental* 2018, **6(Suppl 2):**0678

**INTRODUCTION.** In hemorrhaging trauma patients, the endothelium is activated, resulting in excessive endothelial synthesis of von Willebrand Factor (vWF)(1), which may enhance micro-thrombi formation, with obstruction of the microcirculation and endothelial injury, aggravating bleeding as well as contributing to organ failure. Under normal conditions, vWF is cleaved by the metalloprotease ADAMTS-13. After trauma, ADAMTS-13 levels are typically low(2).

**OBJECTIVES.** We aimed to assess the use of recombinant human (rh) ADAMTS-13 to inhibit endothelial injury and improve coagulation in our rat trauma-transfusion model.

**METHODS.** Blood products were prepared from syngeneic rat blood according to blood bank standards. Polytrauma was induced in Sprague Dawley rats by crush injury to the intestines and liver and by fracture of the femur. The rats were hemorrhaged by phlebotomy until a mean arterial pressure (MAP) of 40mmHg was reached. Rats were randomized (n=8 per group) to receive transfusion of RBCs, FFPs and platelets in a 1:1:1 ratio to achieve a MAP of 60 mmHg, with or without the addition of rhADAMTS-13 (50μg/kg). Blood samples were taken up to 6h after trauma to assess biochemistry and coagulation status by rotational thromboelastometry (ROTEM). Organ damage was assessed by histopathology. Animals were continuously monitored with an arterial and urinary catheter.

**RESULTS.** Following resuscitation, rats receiving rhADAMTS-13 had a higher MAP compared to controls (80 (64-83) vs 66 (37-75) mmHg, p=0.03). Also, it took controls longer to reach a MAP over 60 mmHg (30.0±23.6 vs 17.5±11.9 minutes), although not statistically significant (p=0.10). The coagulation status of rats receiving rhADAMTS-13 improved, as reflected by a higher maximum clot formation ability (EXTEM MCF 67.0±2.9 vs 61.2±4.9 mm, p=0.02) directly after transfusion. This seemed to be mainly platelet-dependent, since EXTEM CA5 minus FIBTEM CA5 was 45.0±2.8 mm in the rhADAMTS-13 group compared to 38.7±3.7 mm in controls (p< 0.01). However, no significant differences between groups was found in organ injury, as assessed by histopathologic examination and levels of creatinine, ASAT and ALAT.

**CONCLUSIONS.** The use of rhADAMTS-13 in a rat trauma-transfusion model improves coagulation, without abrogating the amount of organ injury. These results suggest that the use of rhADAMTS-13 affects platelet-based coagulation and might be a feasible therapeutic application to treat coagulopathy.

**REFERENCE(S)**

1. Dang X, Guan L, Hu W, et al. S100B ranks as a new marker of multiple traumas in patients and may accelerate its development by regulating endothelial cell dysfunction. International journal of clinical and experimental pathology. 2014;7(7):3818-26.
2. Furmaga W, Cohn S, Prihoda TJ, et al. Novel markers predict death and organ failure following hemorrhagic shock. Clinica chimica acta; international journal of clinical chemistry. 2015;440:87-92.

### 0679

#### Therapeutic effect of xanthine oxidase (XO) inhibitors on animal models of haemorrhagic shock: a systematic review

K. Azouri[1], D. Williamson[2], K. Gilbert[3], C. Verdant[4], G. Rousseau[5], E. Charbonney[3,5]
[1]Université de Montréal, Pharmacologie, Montréal, Canada; [2]Université de Montréal, Pharmacie, Montréal, Canada; [3]Centre de Recherche de l'Hôpital du Sacré-Coeur de Montréal, Montréal, Canada; [4]Hôpital du Sacré-Coeur de Montréal, Montreal, Canada; [5]Université de Montréal, Montréal, Canada
**Correspondence:** K. Azouri
*Intensive Care Medicine Experimental* 2018, **6(Suppl 2):**0679

**INTRODUCTION.** Haemorrhagic shock (HS) can cause early death after injury or alternatively lead to inflammatory cascades and organ failure in resuscitated survivors. The ability for clinicians to mitigate ischemia-reperfusion secondary damages remains insufficient. The impact of xanthine oxidase (XO)-derived reactive oxygen species has been identified as one of the sources of oxidative stress leading to organ and endothelial damage. Recently, renewed interest in the pharmacological potential of XO inhibitors has emerged [1]; no systematic analysis of existing animal data, regarding harms or benefits after HS has been carried out.

**OBJECTIVES.** The objective was to systematically review the literature, in order to identify and evaluate the effects of XO inhibitors on mortality and organ failure, in animal models of HS.

**METHODS.** Studies including controlled interventions comparing pharmacological agents to a placebo of preclinical hemorrhage model were considered. Research using MeSH terms and text words related to haemorrhage, ischemia repercussion, organ failure and xanthine oxidase were carried out in PubMed/Ovid MEDLINE and EMBASE. Data were extracted independently and in duplicate.

**RESULTS.** The search process produced 3069 abstracts and 83 publications were considered relevant following exclusions and inclusions criteria. A total of 32 articles, all published between 1969 and 2000, were included for review after full text assessment for eligibility. Seven different animal species experimental models stood out in the included studies, with rats and dogs predominating. In most of the studies, HS resulted from blood withdrawal (75%) and resuscitation was done with reinfusion of shed blood (84%). The main pharmacological intervention to inhibit XO was allopurinol in > 90 %, followed by tungsten sodium administration. Allopurinol given before HS was associated with a reduction in animal mortality (40 to 60% on average); administration during resuscitation had anecdotic effects. XO inhibition also prevented the deterioration in myocardial function and overall haemodynamics, but inconsistently between studies. Various metabolic, enzymatic and functional (endothelium) benefits were reported. Intestinal injuries and bacterial translocation were also decreased by XO inhibition. Finally, certain inflammatory reactions (cytokine or neutrophil infiltration) were blunted by XO inhibitor administration.

**CONCLUSIONS.** Intervention before HS with XO inhibitor leads to measurable benefits in animal models, particularly mortality. However, the ability to intervene on the metabolic culprit responsible for the effect, during resuscitation, needs to be further investigated in preclinical setting before the transposition of such an intervention to humans.

**REFERENCE(S)**

[1] Pacher P et al. Therapeutic effects of xanthine oxidase inhibitors: renaissance half a century after the discovery of allopurinol. Pharmacol Rev. 2006;58:87-114

### 0680

#### Impairment of glucocorticoid receptor function aggravates hypotension and lung injury in mice after hemorrhagic shock

M. Wepler[1], T. Merz[1], O. McCook[1], J. Vogt[1], U. Wachter[1], S. Kress[1], M. Gröger[1], M. Fink[1], M. Georgieff[2], E. Calzia[1], C. Hartmann[1], A. Kleyman[3], U. Burret[4], P. Radermacher[1], J.P. Tuckermann[4], S. Vettorazzi[4]
[1]University Hospital Ulm, Institute for Anesthesiological Pathophysiology and Process Engineering, Ulm, Germany; [2]University Hospital Ulm, Department of Anesthesiology, Ulm, Germany; [3]University College London Hospitals, London, United Kingdom; [4]University of Ulm, Institute of Comparative Molecular Endocrinology, Ulm, Germany
**Correspondence:** M. Wepler
*Intensive Care Medicine Experimental* 2018, **6(Suppl 2):**0680

**INTRODUCTION.** Hemorrhagic shock (HS) leads to hypoxia-induced systemic inflammation[1], which can cause acute lung injury (ALI)[2]. ALI, a frequent complication of trauma, is aggravated by lung co-morbidities like chronic obstructive pulmonary disease (COPD)[3]. COPD is characterized by both pulmonary and systemic inflammation, and the most common cause for COPD is cigarette smoke (CS) exposure. The glucocorticoid receptor (GR) mediates its effects via two different mechanisms, as a GR monomer or GR dimer. We previously observed that mice lacking the GR dimer ($GR^{dim}$) had a higher mortality in lipopolysaccharide- and cecal ligation and puncture-induced inflammation and are refractory to exogenous GCs to ameliorate inflammation in a murine model of ALI[4,5]. This demonstrates that the GR dimer is of importance for the repression of inflammation.

**OBJECTIVES.** We hypothesized that impaired GR dimerization in mice ($GR^{dim}$) would further impair organ function after HS with pre-existing CS-induced COPD.

**METHODS.** After 3 weeks of CS-exposure, anesthetized and mechanically ventilated $GR^{dim}$ and $GR^{+/+}$ mice (n=7-9) underwent pressure-controlled HS (mean arterial pressure [MAP]=35mmHg) for 1h via blood withdrawal followed by 4h of resuscitation with re-transfusion of shed blood, colloid fluid infusion and continuous i.v. norepinephrine (NA) to maintain MAP>55mmHg. Lung function was determined via measurements of lung compliance and gas exchange together with systemic hemodynamics, metabolism, and acid-base status. Data is presented as median and interquartile range.

**RESULTS.** After CS exposure, $GR^{dim}$ mice needed similar doses of NA to keep target hemodynamics as $GR^{+/+}$ mice (0.42 [0.24;0.60] vs. 0.07 [0;0.42] $\mu g \cdot kg^{-1} \cdot minute^{-1}$, p=0.05), had similar lactate levels (1.2 [1.1;1.7] vs. 1.3 (1.1;1.6) $mmol \cdot L^{-1}$, p>0.99), but presented with a decreased lung compliance (99 [91;108] vs. 136 [127;139] $\mu l \cdot cmH_2O^{-1}$, p< 0.01) at the end of experiment. When animals were not exposed to CS, $GR^{dim}$ mice had higher NA requirements to keep target hemodynamics as $GR^{+/+}$ mice (0.20 [0.11;0.34] vs. 0.00 [0.00;0.00] $\mu g \cdot kg^{-1} \cdot minute^{-1}$, p=0.04), higher lactate levels (2.3 [1.4; 4.6] vs. 1.2 [0.8;1.5] $mmol \cdot L^{-1}$, p< 0.01), and decreased lung compliance (87 [82;100] vs. 128 [108;134] $\mu l \cdot cmH_2O^{-1}$, p< 0.01) at the end of the experiment.

**CONCLUSION.** We demonstrate that the GR dimer is an important mediator of hemodynamic stability and lung function during resuscitation from HS and its selective activation might be a promising clinical tool to improve outcome in patients after HS.

Supported by the DFG CRC1149 (GEROK M. Wepler)

**REFERENCE(S)**

1. Eltzschig, N. Engl. J. Med., 364, 2011;
2. Wepler, Am. J. Physiol. Lung Cell. Mol. Physiol. 311, 2016;
3. Gotts, Nicotine Tob. Res. 19, 2017;
4. Vettorazzi, Nat. Commun. 6, 2015;
5. Kleiman, FASEB J. 26, 2012

### 0681

#### Myeloid-derived suppressor cells infiltrate the brain and suppress neuroinflammation in a mouse model of traumatic brain injury

S. Hosomi[1], Y. Koyama[2], T. Watabe[3,4], M. Ohnishi[1], H. Ogura[1], T. Shimazu[1]
[1]Graduate School of Medicine, Osaka University, Department of Traumatology and Acute Critical Medicine, Suita, Japan; [2]Graduate School of Medicine, Osaka University, Department of Molecular Neuroscience, Suita, Japan; [3]Graduate School of Medicine, Osaka University, Positron Emission Tomography Molecular Imaging Center, Suita, Japan; [4]Graduate School of Medicine, Osaka University, Department of Nuclear Medicine and Tracer Kinetics, Suita, Japan
**Correspondence:** S. Hosomi
*Intensive Care Medicine Experimental* 2018, **6(Suppl 2):**0681

**INTRODUCTION.** Recent evocative data suggest that the expansion of myeloid-derived suppressor cells (MDSCs) in acute inflammatory processes play a beneficial role by increasing immune surveillance and innate immune responses. However, because of the brain's unique immune system, the role of MDSCs across the blood-brain barrier is far from understood.

**OBJECTIVES.** In this study, we explored the suppressive role of MDSCs in cerebral inflammatory reactions after traumatic brain injury (TBI) using *in vivo* imaging.

**METHODS.** In a controlled cortical impact (CCI) model, flow cytometry, immunohistochemistry including Giemsa counterstaining, and T-cell proliferation tests were performed to define the cells infiltrating the injured brain as MDSCs. After CCI mice received Gr-1 antibody or isotype control antibody treatment in order to deplete MDSCs, positron emission tomography (PET) imaging of Translocator protein (TSPO) uptake for quantitative analysis of neuroinflammation was performed at 7 days post injury.

**RESULTS.** Cells co-expressing Gr-1 and CD11b accumulated in the injured brain, increasing by 1 day post injury, then decreasing progressively until 7 days post injury. Clearly, these populations are heterogeneous and include both immature and mature myeloid populations, containing monocytic and polymorphonuclear subpopulations. $CD4^+$ T-cell proliferation was significantly suppressed when sorted Gr-$1^+$ cells from the injured brain were co-cultured at a ratio of 1:1 and 1:2 with $CD4^+$ T cells. At 1 week post-CCI, in mice treated with Gr-1 antibody TSPO uptake was observed over broader volume including the ipsilateral thalamus and cortex compared with controls (control vs Gr-1 antibody: 0.033 ± 0.0064 vs. 0.078 ± 0.0121 ccm, p = 0.03).

**CONCLUSIONS.** We determined $CD11b^+Gr\text{-}1^+$ cells infiltrating the contusion area as MDSCs using morphological and functional analysis along with phenotypic markers. Our results also suggest that infiltrating MDSCs suppress the neuronal inflammation following focal TBI.

**REFERENCE(S)**

Bronte V, Brandau S, Chen SH, et al: Recommendations for myeloid-derived suppressor cell nomenclature and characterization standards. *Nat Commun* 2016; 7:12150.

Cuenca AG, Delano MJ, Kelly-Scumpia KM, et al: A paradoxical role for myeloid-derived suppressor cells in sepsis and trauma. *Mol Med* 2011; 17:281-292.

**GRANT ACKNOWLEDGMENT**

This study is supported by Grant-in-Aid for Scientific Research (C) from the Japan Society for the Promotion of Science and a grant from ZENKYOREN (National Mutual Insurance Federation of Agricultural Cooperatives).

### 0682

#### High (2:1) versus standard (1:1) platelet:RBC dose in a rat polytrauma transfusion model

D.J.B. Kleinveld[1,2,3], M.R. Wirtz[1,2,3], D.P. van den Brink[1,2], M.A.W. Maas[2], J.J.T.H. Roelofs[4], J.C. Goslings[3,5], N.P. Juffermans[1,2]
[1]Academic Medical Center, Department of Intensive Care Medicine, Amsterdam, Netherlands; [2]Academic Medical Center, Laboratory of Experimental Intensive Care and Anesthesiology, Amsterdam, Netherlands; [3]Academic Medical Center, Department of Trauma Surgery, Amsterdam, Netherlands; [4]Academic Medical Center, Department of Pathology, Amsterdam, Netherlands; [5]Onze Lieve Vrouwe Gasthuis, Department of Trauma Surgery, Amsterdam, Netherlands
**Correspondence:** D.J.B. Kleinveld
*Intensive Care Medicine Experimental* 2018, **6(Suppl 2):**0682

**INTRODUCTION.** In traumatic bleeding, a high platelet (PLT) to red blood cell (RBC) ratio of 1:1 seems more effective in achieving hemostasis than a 1:2 ratio or lower suggesting correction of trauma-induced coagulopathy (TIC)[1]. This underlines the finding that platelet functioning is severely disturbed in trauma[2]. It is unknown whether higher doses of platelets confer more benefit in correcting TIC, while avoiding (micro)thrombi formation and subsequently organ failure.

**OBJECTIVES.** To examine the effect of high (PLT:FFP:RBC in a ratio of 2:1:1) versus standard (PLT:FFP:RBC in a ratio of 1:1:1) PLT transfusion ratio on product use, correction of TIC and organ damage in a rat trauma and hemorrhagic shock model.

**METHODS.** Blood products were made using syngeneic donor rats according to national blood bank standards. Sprague Dawley rats were mechanically ventilated and traumatized by crush injury to both intestines and liver and by a femur fracture, followed by exsanguination until a MAP of 40 mmHg (~30% of circulating volume). Then, rats were randomized to receive resuscitation with high or standard PLT dose until a MAP of 60 mmHg was reached. Monitoring was done with a cannula in the carotid artery. Blood samples were taken for biochemical assessment and coagulation assays by ROTEM. Organs were harvested for histopathological analysis. Data are median (IQR).

**RESULTS.** Mortality rate was 19%, yielding n=5 in the standard and n=8 in the high PLT dose group. All rats were shocked with a base excess of -6,4 (-18,2 - -4,3) in standard vs -5,1 (-9,5 - -3,6) in high PLT group (p=0,6). The volume of transfusion needed to reach the pretargetted MAP was lower in the group receiving high PLT dose compared to the standard, albeit not statistically significant (2,5 (0,9 - 4,3) vs 7,2 (2,4 - 10,0) ml, p=0,054). Transfusion with a high PLT dose resulted in higher EXTEM CA5 compared to the standard after 120 min (55,5 (52,2 - 58,8) vs 47,0 (43,0 - 49,0) mm, p< 0,001), while FIBTEM CA5 did not differ between groups, indicating improved functional platelet activity. Transaminases and creatinine levels were similar between groups. Also, histopathology did not show any differences.

**CONCLUSIONS.** In this model, a high PLT dose was more effective in treating TIC, with a trend towards reduced transfusion volumes. Also, transfusion of a high PLT dose did not aggravate organ damage. These results indicate that a higher PLT dose might be a feasible treatment option in hemorrhaging trauma patients for the correction of coagulopathy.

**REFERENCE(S)**

1. Holcomb JB et al. Transfusion of plasma, platelets, and red blood cells in a 1:1:1 vs a 1:1:2 ratio and mortality in patients with severe trauma: the PROPPR randomized clinical trial. Jama. 2015;313(5):471-482.
2. Ramsey MT et al. A prospective study of platelet function in trauma patients. Journal of Trauma and Acute Care Surgery. 2016;80(5):726-732.

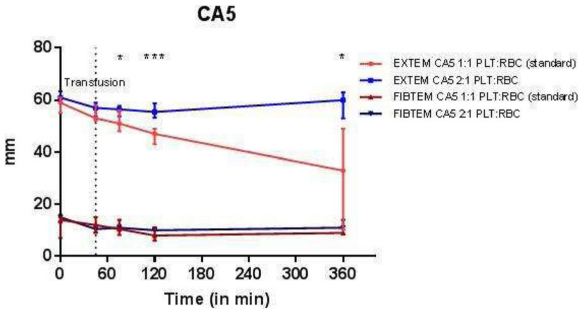

**Fig 1 (abstract 0682).** Clot amplitude at 5 min (CA5). *p<0,05 in EXTEM, ***p<0,001 in EXTEM

## Heart surgery

### 0683

#### Perioperative risk factors of deteriorating lung oxygenation after cardiac surgery

H. Taenaka, A. Uchiyama, Y. Fujino
Osaka University Graduate School of Medicine, Department of Anesthesiology and Intensive Care Medicine, Suita-shi, Japan
**Correspondence:** H. Taenaka
*Intensive Care Medicine Experimental* 2018, **6(Suppl 2):**0683

**INTRODUCTION.** Deteriorating lung oxygenation is one of popular causes of prolonged mechanical ventilation after cardiac surgery. Recognizing risk factors of deteriorated lung oxygenation allow us to improve postoperative respiratory management and reduce postoperative complications.

**OBJECTIVES.** The aim of this study is to clarify risk factors of deteriorating lung oxygenation after cardiac surgery.

**METHODS.** After institutional review board approval, we retrospectively reviewed patients' records of all adult patients who admitted to our ICU and received planned extubation after cardiac surgery between December 2016 and November 2017. We divided the patients into two groups according to ratio of an arterial partial pressure of oxygen by fraction of inspired oxygen (PF) at the admission to ICU. The threshold of deteriorated lung oxygenation was defined as 250 mmHg of PF. We compared perioperative data between the two groups. A multivariate logistic analysis was performed to identify independent risk factors for deteriorating lung oxygenation.

**RESULTS.** In enrolled 226 patients, 36 patients (15.9%) were classified into group with deteriorated lung oxygenation (Group L). The other patients were classified into Group N. In Group L, body mass index (BMI) was higher, operation time was longer, and mean pulmonary arterial pressure (mPAP) and central venous pressure at the admission were higher than those in Group N (25.1±4.4 vs 22.5±3.2 kg/m$^2$; p< 0.01, 332±99 vs 297±95 min; p=0.04, 23.1±5.3 vs 20.4±4.6 mmHg; p< 0.01, and 10.4±3.1 vs 8.8±2.6 mmHg; p< 0.01). However, mPAP just before the extubation were not different between two groups (19.6 ±2.9 vs 19.4±4 mmHg; p=0.78). Duration of mechanical ventilation and ICU stay of Group L were longer than those of Group N (37.8±43.8 vs 14.3±16.8 h; p< 0.01, and 5.5±4.4 vs 3.8±3.2 days; p=0.01). Multivariate logistic analysis showed that larger BMI (odds ratio [OR] per kg/m$^2$ 1.21; 95% confidence interval [CI] 1.08-1.35, p< 0.01) and higher mPAP at the admission (OR per mmHg 1.14, 95%CI 1.05-1.24, p< 0.01) were risk factors for deteriorating lung oxygenation.

**CONCLUSIONS.** Larger BMI and higher mPAP at the admission of ICU were risk factors for deteriorating lung oxygenation after cardiac surgery.

#### REFERENCE(S)

1. JAMA 2017; 317: 1422-32.

#### GRANT ACKNOWLEDGMENT

The work was supported solely by departmental resources.

### 0684

### Surgery of congenital heart disease in adults predictive factors of re-entry and mortality

M.D. Fernández Zamora[1], I. Navarrete-Espinosa[2], J. Cano-Nieto[2], E. Banderas[1], F. Hijano[1], E. Curiel-Balsera[1]
[1]H. R. U de Málaga, Medicina Intensiva, Málaga, Spain; [2]H. R. U de Málaga, Cardiología, Málaga, Spain
**Correspondence:** M.D. Fernández Zamora
*Intensive Care Medicine Experimental* 2018, **6(Suppl 2):**0684

**OBJECTIVES.** To know the characteristics, surgical indication, mortality rate and re-entry of the patients surgically treated in our Adult Congenital Heart Disease Unit. Know factors associated with death and hospital readmission in these patients.

**MATERIAL AND METHODS.** Retrospective cohort study, which included all adult patients (over 16 years of age) undergoing surgery for congenital heart disease in our unit from January 2012 to December 2017. Re-entry was defined as non-elective hospitalization after the discharge from cardiac surgery and patients with loss of follow-up were discarded.

**RESULTS.** A total of 91 patients were intervened, mean age 37.2 ± 17.2 years, being 43.7% women. The mean time of extracorporeal circulation was 118.8 ± 67 minutes and clamping 71.3 ± 45.4 minutes. The mortality rate was 8.5% and the readmission rate was 15.5%, with an average time from discharge of 9.5 ± 9.5 months, the most common causes being cardiac (atrial fibrillation, heart failure and valvular leaks) and infectious. Regarding the surgical indications, 20% corresponded to aortic pathology, 23% to interatrial communication (of any type), 20% to pathology of the outflow tract of the right ventricle, and 37% classified as other congenital heart diseases. According to bivariate analysis, patients with severe pulmonary hypertension (p < 0.001, Chi2 13.05), those with more than 2 previous cardiovascular surgery interventions (p = 0.023, Chi2 5.202) and RACHS-1 scale of 4 (p = 0.022 Ji 5,245). On the other hand, patients with a history of atrial fibrillation were associated with a higher probability of admission (p = 0.008, Chi2 6.98).

**CONCLUSIONS.** The most frequent causes of re-entry in patients undergoing cardiac surgery in adults with congenital heart disease are AF, heart failure and valvular leaks. The presence of severe pulmonary hypertension, RACHS-1 of 4 or patients with more than 2 cardiac surgeries could be predictors of mortality. Despite long CPB times due to technical complexity, the mortality rate is similar to that of non-congenital cardiac surgery.

### 0685

### Statins prevent bleeding in patients undergoing myocardial revascularization surgery

M.D. Fernández Zamora[1], M.J. Chaparro[1], V. Olea[1], L. Olivencia[2], A. Gordillo-Brenes[3], R. Hinojosa Pérez[4], E. Curiel-Balsera[1], ARIAM ANDALUCIA
[1]H. R. U de Málaga, Servicio Medicina Intensiva, Málaga, Spain; [2]Hospital Virgen de las Nieves, Medicina Intensiva, Granada, Spain; [3]Hospital Puerta del Mar, Cádiz, Spain; [4]Hospital Virgen del Rocio, Sevilla, Spain
**Correspondence:** M.D. Fernández Zamora
*Intensive Care Medicine Experimental* 2018, **6(Suppl 2):**0685

**OBJECTIVES.** To analyze whether patients treated with statins before undergoing coronary artery bypass surgery present a lower bleeding rate, reoperation for bleeding and transfusion of blood products than patients who have not been previously treated with statins.

**METHODS.** Observational, prospective and multicenter study of patients in the ARIAM Andalusia registry of cardiac surgery who underwent CABG between 2010 and 2017. Clinical epidemiological variables of the patients have been analyzed, as well as the complications derived from the bleeding occurred during the immediate postoperative period. Likewise, the transfusions received in the ICU have been analyzed. The variables are presented as mean and standard deviation or absolute number and percentage. Chi-square and t-student tests were used according to need. For the multivariate analysis, binary logistic regression has been used. Max alpha error of 5% has been used.

**RESULTS.** 3218 patients were analyzed, 82.7% men. The average age was 63 ± 35.8 years. Dyslipidemia (66.9%) and diabetes (51%) were the most frequent antecedents. A total of 2,552 patients were being treated with statins prior to surgery (79.3%). Up to 929 patients (28.9%) did not require any transfusion, red blood cells in 26.5%, platelets in 15.6% and plasma in 4.8%. The reoperation rate due to bleeding was 2.8% (89 patients). In patients treated with statins it was 2.4% and in the others it was 4.1% (p = 0.023). Regarding the transfusion of blood products, we did not find differences in the red blood cells (27.7% in patients with statins versus 25.6%) but we did so in platelet transfusion (6.1 versus 8.6%) p = 0.042 and plasma (7.8% vs 11.4%) p = 0.009. The multivariate analysis maintained the protective effect of statins in terms of reintervention due to bleeding after adjusting for EuroScore, extracorporeal circulation time and taking antiplatelet drugs or anticoagulants in the 24 hours prior to surgery OR 0.554 IC 95% (0.361-0.976).

**CONCLUSIONS.** Patients undergoing treatment with statins prior to myocardial revascularization surgery have a lower rate of reoperation due to bleeding and less need for transfusion of plasma and platelets.

#### REFERENCE(S)

Minimizing cardiac risk in perioperative practice interdisciplinary pharmacological approaches. Bock M et al. Wien klin Wochenschrift 211Jul ;123(13-14):393-407.

### 0686

### Surgery for infective endocarditis are there differences between prosthetic and native valves?

N. Palomo-López, S. Escalona-Rodriguez, G. Rivera-Rubiales, L. Martin-Villén, Y. Corcia-Palomo
Hospital Universitario Virgen del Rocío, Sevilla, Spain
**Correspondence:** N. Palomo-López
*Intensive Care Medicine Experimental* 2018, **6(Suppl 2):**0686

**OBJECTIVES.** To describe and compare mortality and prognostic scores in patients with infective endocarditis (IE) depending the valve affected: native vs. prosthetic.

**METHODS.** Observational, prospective and cohort study. We included patients with IE from January 2008 to December 2017 who needed surgery and were admitted to the ICU of a third level hospital. We collected demographic variables, type of valvular surgery (emergency or elective), doses of amines at admission (classified into two groups: low doses < 0.3 mcg/ kg/ hr and moderate doses > 0.3 mcg/ kg/ hr) and microbiological results. We collected also analytical data at admission (PCR and leukocytes) and we calculated prognostic scores at admission ( SOFA and APACHE II). Descriptive statiscal analysis was performed, presenting the qualitative variables as frequencies and percentages, and quantitative as mean (+/- SD) or median ( interquartile range ICR ) depending its distribution. We use chi square for bivariate analysis of qualitative variables.

**RESULTS.** We included 107 patients with EI, 88 (82,1 %)were native valve (NV) and 19 (17,8 %) prosthetic valve (PV). Median age in NV was 59 years (51- 60) and 67 (48-69) in PV. A total of 69 patients (78,4%) were males in native group and 13 (68,4%) in prosthetic. Emergency surgery occurs in 37 cases (42,5%) among NV and in 14 ( 73,7%) in PV with a significant relation( p= 0.021).

S. Epidermidis was the most common infecting microorganisms, in both NV and PV (14,8% and 42,1% respectively) followed by methicillin-sensitive Staphylococci (11,4% vs. and 15,8%). 42.5% of native and 47.5% of prosthetics needed low doses of amines at admission, while 4.6% of NV and 15.8% of PV needed moderate doses with any statistical difference (p = 0.146). There was no statistical difference about SOFA and PCR between both groups (p = 0.556 and p = 0.104 respectively). There was a significant relation between leukocytes and APACHE II according the type of valve operated: leukocytes in NV were 10000mm3 / dl (7000-15000) vs. 17000mm3 / dl (13400-20000) in PV with p = 0.03. APACHE II were 8 (5-13) in NV vs. 9 (6 - 18.5) in PV with p = 0.024. Mortality rate was higher in prosthetics (11.1% NV vs. 35.3% PV) with p = 0.022.

**CONCLUSIONS.** Emergency surgery on prosthetic valve endocarditis is more frequent than those that occur on native valves.

Prosthetic valve endocarditis has a higher mortality rate in our series comparing with native valve endocarditis. We find a significant relation in APACHE II and leukocytes at admission between those both groups

**REFERENCE(S)**

Grubitzsch H, Christ T, Melzer C, Kastrup M, Treskatsch S, Konertz W. Surgery for prosthetic valve endocarditis: associations between morbidity, mortality and costs. Interact CardioVasc Thorac Surg 2016;22:784-91

### 0687

#### Our 20 years experience with complicated infective endocarditis in intensive care unit: early vs late surgical intervention

F. Ahmad[1], S. Cox[2], C. Soon Thim[3], E. Abdul Rahman[4], I. Zainal Abidin[1], M.A. Sadiq[5]

[1]Universiti Malaya Medical Centre, Cardiology, Kuala Lumpur, Malaysia; [2]Healthcare Partners, Cardiology, Brisbane, Australia; [3]Putrajaya Hospital, Internal Medicine, Putrajaya, Malaysia; [4]Universiti Teknologi Mara, Cardiology, Selangor, Malaysia; [5]Sultan Qaboos University, Cardiology, Muscat, Oman

**Correspondence:** F. Ahmad

*Intensive Care Medicine Experimental* 2018, **6(Suppl 2):**0687

**INTRODUCTION.** The effect of early surgical intervention on mortality in patients with infective endocarditis (IE) remains controversial and, in developing countries, the feasibility of early surgical access remains challenging. In addition, data on early versus late surgical intervention in intensive care setting remain sparse.

**OBJECTIVES.** This study investigates the complications and mortality in patients with complicated IE admitted to ICU who underwent surgical intervention

**METHODS.** We retrospectively evaluated case notes, echocardiographic and surgical data for all patients fulfilled definite diagnosis of IE requiring admission to ICU from 1998 to 2017. All patients undergo both transthoracic echocardiography (TTE) and transesophageal echocardiography (TOE) within 3 days on presentation. Patients were stratified according to timing of surgery - early (≤4 weeks from diagnosis) or conventional (after 4-6 weeks of antibiotic therapy). Outcome measures were length of stay and in-hospital and 30-day mortality.

**RESULTS.** 110 patients, with a mean age of 59.7 ± 4.1 years, were diagnosed with complicated IE, in which, 76 (69%) of them were male. A total of 51 survivors from total patients of 110 were identified. 104 patients required inotropic support and 94 patients developed multi-organ failure. 42% of the patients developed mobitz type II heart block or complete heart block. Of the 110 patients, 60 patients (mean age 60.5±2.2) underwent surgery. The most commonly affected valves were mitral (n= 38 63%) and aortic (n=22 37%). In the early surgery group (n=23), indication for intervention was uncontrollable sepsis (n=8), embolic phenomena (n=4), heart failure (n=6) and cavity seen on echocardiogram indicating abscess formation (n=5). Using multivariate analysis, patients who developed acute kidney injury prior transfer to ICU (OR 9 95% CI 1.18-23.00, P = 0.03) or echocardiographic evidence of vegetation size > 15mm (OR 7 95% CI 1.09-17.77 P = 0.03) were identified as predictors for in-patient death. Patients with early surgery had significantly shorter hospital stay (30±3 vs 46±3 days, P< 0.05). In-hospital mortality was substantially lower in the early surgery cohort (11.6% vs 16.0% P< 0.05 )

**CONCLUSIONS.** Our study concludes that markers for severity of illness (scores and organ failure) and echocardiographic findings of vegetations size >15 mm are independent risk factors for poor prognosis and mortality. In this cohort, early surgical intervention for complicated IE appears to be associated with similar outcome to uncomplicated IE treated by conventional means. This data may challenge the misconception that early surgery should be avoided.

1.Circulation.
2005;111:e394-e434
2.European
Heart Journal 2009; 30:2369-2413.

### 0688

#### A 20 years retrospective study of complicated infective endocarditis in intensive care unit; medical treatment versus surgical intervention

F. Ahmad[1], S. Cox[2], C. Soon Thim[3], E. Abdul Rahman[4], I. Zainal Abidin[5], M.A. Sadiq[6]

[1]Universiti Malaya Medical Centre, Cardiology, Kuala Lumpur, Malaysia; [2]Healthcare Partners, Cardiology, Brisbane, Australia; [3]Putrajaya Hospital, Putrajaya, Malaysia; [4]Universiti Teknologi Mara, Cardiology, Selangor, Malaysia; [5]Universiti Malaya Medical Centre, Kuala Lumpur, Malaysia; [6]Sultan Qaboos University, Cardiology, Muscat, Oman

**Correspondence:** F. Ahmad

*Intensive Care Medicine Experimental* 2018, **6(Suppl 2):**0688

**INTRODUCTION.** Treatment strategy for the management of Infective Endocarditis (IE) continues to be a debate. Although medical treatment reduced the cases of death, the mortality of IE patients is still high. Because of the limitation in medical treatment, surgical therapy has been used for the treatment in some patients with IE

**OBJECTIVES.** This study investigates the complications and mortality in patients with complicated IE admitted to ICU who medically treated versus surgical intervention

**METHODS.** We retrospectively evaluated case notes, echocardiographic and microbiological data for all patients fulfilled the modified Duke Criteria for definite diagnosis of IE requiring admission to ICU from 1998 through 2017. Patients were stratified according to patients who medically treated versus patients who underwent surgical intervention. Outcome measures were length of stay and in-hospital and 30-day mortality.

**RESULTS.** 110 patients, with a mean age of 59.7 ± 4.1 years, were diagnosed with complicated IE, in which, 76 (69%) of them were male. IE survivors were defined as patients who survived after being discharged from ICU and at 6 months follow up with a documented diagnosis of IE. A total of 51 survivors form total patients of 110 were identified. 104 patients required inotropic support and 94 patients developed multi-organ failure. Of the 110 patients, 60 patients (mean age 60.5±2.2) underwent surgery and the remaining were medically treated. Commonly affected valves were mitral (n= 38 63%) and aortic (n=22 37%). Biomarkers of acute infection, APACHE II as well as echocardiographic findings were analyzed. Total mortality for patients who were treated medically and those underwent surgical intervention were n=29 and n =30 respectively. Using multivariate analysis, patients who developed acute kidney injury prior transfer to ICU (OR 9 95% CI 1.18-23.00, P = 0.03) or echocardiographic evidence of vegetation size > 15mm (OR 7 95% CI 1.09-17.77 P = 0.03) were identified as predictors for in-patient death. Patients underwent surgery had comparatively shorter hospital stay (40±1, P< 0.05) compared to those treated medically (44±1 p< 0.05).

**CONCLUSIONS.** Our study concludes that markers for severity of illness (scores and organ failure) as well as echocardiographic findings of vegetations size >15 mm are independent risk factors for poor prognosis and mortality. In this cohort, early surgical intervention for complicated IE appears to be associated with similar outcome to uncomplicated IE treated by conventional means. This data may challenge the misconception that early surgery should be avoided.

**REFERENCE(S)**

1. Circulation. 2005;111:e394-e434
2. Heart 2003;89:258-262
3. European Heart Journal 2009; 30:2369-2413.

### 0689

#### Bleeding and need of polytransfusion in obese population who has undergone heart surgery

E. Trujillo García, J. Muñoz Bono, E. Curiel Balsera, M.D. Fernández Zamora, M. Álvarez Bueno
H. R. U de Málaga, Intensive Care, Málaga, Spain
**Correspondence:** E. Trujillo García
*Intensive Care Medicine Experimental* 2018, **6(Suppl 2):**0689

**OBJECTIVE.** Analysing the demographic features and the need of polytransfusion after bleeding, as well as the need of reoperation in obese population who has undergone heart surgery.

**MATERIAL AND METHODS.** Observational, prospective and multicentre study of all patients who have undergone heart surgery in Andalusian hospitals taking part in the ARIAM Project.

The ARIAM Heart Surgery Register defines polytransfusion as the transfusion of three packs of red blood cells. Reoperation criteria due to rebleeding in the ARIAM Register are clinical —according to drainage debit, as well as to the existence of signs of cardiac tamponade.

**RESULTS.** A total number of 7276 patients who had undergone heart surgery from March 2008 to July 2012 were analysed. 61.1% of the operated patients were males, their average age being 63.9±12.4 years, while 34.9% had a BMI over 30Kg/$m^2$ and therefore considered obese. Over 30% of the patients were smokers and over 50% showed some cardiovascular risk factor, the most frequent vascular risk factor being arterial hypertension.

According to WHO criteria, 34.9% (no=2361) of the patients were considered obese (BMI>30 Kg/$m^2$). Average age of the obese population was 65±11 years, 58% of them being males. The most frequent cardiovascular risk was arterial hypertension (73.6%). The average EuroScore of this population whose BMI was over 30 kg/$m^2$ was 5.68+2.9, while average SAPs 3 score was 40.6±10.2.

Additionally, 68.5% of the obese patients had undergone heart valve replacement surgery, 23.5% had undergone coronary revascularization surgery, and 4.1% had undergone ascending aortic surgery. 88.7% of these surgery procedures were elective, 8.3% were acute, and 3% were emergencies.

Average time of extracorporeal surgery was 109±49 minutes, and 5.8% of the patients had previously undergone heart surgery. 13.1% of the patients showed a massive bleeding episode (defined as bleeding over 1L in the first 6 hours upon hospital admission), and 3% were reoperated within immediate post-operation time (first 24 hours after ICU admission). Some hemoderivative was transfused to 17.7% of obese patients, while 5.5% needed polytransfusion.

Regarding complications, 23.5% of the bleeding patients developed postoperative acute kidney injury. 5.6% (no=133) showed hemothorax, and 13.3% (no=314) needed mechanical ventilation (MV) for over 24 hours. Average ICU stay was 5±11 days, and hospital stay was 21±21 days. ICU death rate was 9.5% (no=205) for this population.

**CONCLUSIONS.** The profile of the obese patient who undergoes heart surgery is a male —65 years old, with high blood pressure, dyslipidemia, and high EuroScore and SAPs 3 scores— who undergoes operation electively for heart valve replacement with long extracorporeal time and low incidence of bleeding, polytransfusion and bleeding-related reoperation. The most frequent postoperative complication in bleeding obese patients was kidney failure.

### 0690

#### Study of complications and mortality in patients older than 70 years undergoing myocardial revascularization surgery collected in the ARIAM Registry of Andalusia

M.D. Fernández Zamora[1], V. Olea[1], M.J. Chaparro[1], A. Gordillo-Brenes[2], A. Herruzo-Avilés[3], E. Alvarez-Márquez[4], L. Olivencia[5], E. Curiel-Balsera[6], ARIAM ANDALUCIA
[1]H. R. U de Málaga, Medicina Intensiva, Málaga, Spain; [2]Hospital Puerta del Mar, Cádiz, Spain; [3]Hospital Virgen del Rocio, Medicina Intensiva, Cádiz, Spain; [4]Hospital Virgen Macarena, Sevilla, Spain; [5]H.U.Virgen de las Nieves, Medicina Intensiva, Granada, Spain; [6]H. R. U de Málaga, Málaga, Spain
**Correspondence:** M.D. Fernández Zamora
*Intensive Care Medicine Experimental* 2018, **6(Suppl 2):**0690

**OBJECTIVES.** To know the risk factors, the demographic characteristics, the complications and the mortality of the patients older than 70 years surgically revascularized with respect to those under 70

**MATERIAL AND METHODS.** Prospective observational study, from January 2010 to December 2017, of the patients collected in the ARIAM database of public hospitals in Andalusia. The group of patients < 70 years of age was compared with the group of older age. Quantitative variables are expressed as mean and SD or median and interquartile range for asymmetric variables. The qualitative variables as absolute number and percentage. Chi-square and student t-tests were used, as needed, with an alpha error < 5%. A multivariate analysis with binary logistic regression was carried out.

**RESULTS.** 3218 patients were collected, 82.7% were male. The mean age was 63.7 ± 9.4 years; The Euroescore was 3.56 ± 2.62. We found significant differences in ICU and hospital mortality in the group of older patients compared to the younger age group (OR 2.1 CI 95% (1.4-31) for ICU and OR mortality 2.4 IC95 % (1.7-3.3) for overall hospital mortality).

When adjusting hospital mortality by risk measured with EuroSCORE, we did not find differences between both age groups (OR 1.32

IC95% (0.92-1.91)). However, when recoding EurosCORE, subtracting the points derived from age, to avoid collinearity, the in- hospital mortality of the group of patients> 70 years, was significantly higher than that of the group of younger age OR 2.32 IC95% (1.67 -3.23).

**CONCLUSIONS.** Patients> 70 years of age undergoing revascularization surgery presented more complications and higher mortality than the group of younger patients in our series.

### 0691

#### Post-operative follow-up of patients who underwent surgery for congenital cardiopathies of adults in the ICU

M.D. Fernández Zamora[1], I. Navarrete-Espinosa[2], E. Curiel-Balsera[1], J. Muñoz-Bono[1], J. Cano-Nieto[2]
[1]H. R. U de Málaga, Medicina Intensiva, Málaga, Spain; [2]H. R. U de Málaga, Cardiología, Málaga, Spain
**Correspondence:** M.D. Fernández Zamora
*Intensive Care Medicine Experimental* 2018, **6(Suppl 2):**0691

**OBJECTIVES.** To know the risk factors, the demographic characteristics, the type of surgery, the complications and the mortality of the patients intervened of congenital cardiopathy of the adult admitted in our unit.

**MATERIAL AND METHODS.** Prospective observational study of patients registered in the ARIAM database from January 2012 to December 2017 in our unit. Quantitative variables are presented as mean ± standard deviation and qualitative variables as absolute numbers and percentages.

**RESULTS.** A total of 53 patients were collected, of which 56.6% were men, 43.3% were women. The average age is 40.2 ± 17.6. The Euroescore was 4.6 ± 2.3. 37.7% of the patients had undergone some cardiac surgery previously.

43.4% did not present cardiovascular risk factors. 57% were in functional grade I of NYHA, 34.7% in II, 6.1% in III and 2% in IV. They presented moderate-severe PHT after 13.2%. The preoperative diagnosis was CIA 35.8%, CIV 11.3%, Fallot 9.4%, Pulmonary stenosis 5.7%, Transposition of large vessels 3.8%, other 6%.

The CEC time was 121.8 ± 63.9 and the clamping time was 80.8 ± 48.15. 35.8% required vasoactive support and 34% transfusion of blood products.

47.2% did not present postoperative complications, 15% presented bleeding> 1l and 3 patients (5.7%) were reoperated.

The mortality rate was 7.54%, one patient died in the operating room and 3 in the ICU..

**CONCLUSIONS.** Patients undergoing Cogenital Heart Disease in adulthood present few associated comorbidities, mostly intervening in a good functional degree. They require long extracorporeal surgery times due to their greater complexity. They present few complications and mortality is lower than that recorded in global cardiac surgery in our unit.

### 0692

#### Analysis of the impact of early surgery on in-hospital mortality in patients with infective endocarditis

S. Escalona Rodriguez[1], N. Palomo López[1], G. Rivera Rubiales[2], Y. Corcia Palomo[1], L. Martin-Villen[1]
[1]Hospital Universitario Virgen del Rocío, Critical Care, Seville, Spain; [2]Hospital Quirón Sagrado Corazón, Seville, Spain
**Correspondence:** S. Escalona Rodriguez
*Intensive Care Medicine Experimental* 2018, **6(Suppl 2):**0692

**INTRODUCTION.** Despite medical and surgical advances in the last decades, infective endocarditis (IE) is a pathology with high mortality, which often requires admission to the Intensive Care Unit (ICU). Currently, surgery is the treatment of choice in more than 50% of patients.

**OBJECTIVES.** Description and analysis of the impact of early surgery on in-hospital mortality of infective endocarditis admitted to the ICU of a tertiary care hospital.

**METHODS.** A prospective observational study of IE patients admitted to the ICU who required early valve surgery, during the 2008-2017 period in a third-level hospital. Demographic data, valve disease, the clinical (renal failure, the need of renal replacement therapy (RRT), the dose of amines) and laboratory parameters (leukocytes, C-reactive protein (CRP)), SOFA and APACHE II scores and mortality on ICU and in-hospital were collected. Doses of noradrenaline greater or equal than 0.3 mcg/kg/min were considered high and less than 0.3 mcg/kg/min were considered low. Qualitative variables were described by absolute number and frequency and quantitative variables by median and interquartile range. For the analysis of the data, Chi-square statistical test was used for qualitative variables and U Mann Whitney was used for the quantitative ones.

**RESULTS.** Fifty patients were included, with a median age of 65 years (52-73), of whom 39 were males (78%). The most affected valve was the aortic valve constituting 56% of the cases. APACHE II on admission was 10 (7-15) and SOFA score was 4 (2-7). On admission, 7 patients (14%) had high doses of noradrenaline. Acute renal damage developed in 31 cases (63.3%), of which 8 (16.3%) required RRT. The PCR was 72 mg/l (18-141) and leukocytes 13.000 /mm$^3$ (8.150-18.050) on admission. In-hospital mortality was 34%.

No statistically significant association was observed between leukocyte levels (p = 0.208), CRP (p = 0.303), high amines dose (p = 0.086) and prognostic scores (p = 0.143 p = 0.177 for SOFA and APACHE II, respectively) with mortality. However, a significant association was found between the acute renal damage that required RRT with global mortality (p = 0.047).

**CONCLUSIONS.** Acute renal damage was the most frequent complication in patients admitted to the ICU. Moreover, the renal failure that required RRT was significantly associated with higher in-hospital mortality.

**REFERENCE(S)**

- Cahill TJ, Prendergast BD. Infective endocarditis. Lancet 2016;387:882-93.
- Kiefer T, Park L, Tribouilloy C, Cortes C, Casillo R, Chu V et al. Association between valvular surgery and mortality among patients with infective endocarditis complicated by heart failure. JAMA 2011;306(20):2239-47.
- Barsic B, Dickerman S, Krajinovic V, Pappas P, Altclas J, Carosi G et al. Influence of the timing of cardiac surgery on the outcome of patients with infective endocarditis and stroke. Clin Infect Dis. 2013;56(2):209-17.

### 0693

#### Dexmedetomidine for delirium after cardiac surgery in high risk patients: friend or foe?

A. Petroulaki[1], M. Kiparakis[2], P. Kalogerakos[2], E. Maridakis[1], G. Lazopoulos[2]
[1]University General Hospital of Heraklion, Cardiac Surgery ICU, Heraklion, Greece; [2]University General Hospital of Heraklion, Cardiac Surgey Unit, Heraklion, Greece
**Correspondence:** A. Petroulaki
*Intensive Care Medicine Experimental* 2018, **6(Suppl 2):**0693

**INTRODUCTION.** Delirium is a frequent and serious adverse event after cardiac surgery. It results from the interplay of a wide range of precipitating noxious events, predisposing preoperative and perioperative risk factors that affect cardiac surgery patients, especially the vulnerable old ones. Treatment of delirium is difficult., so prevention is critical. It is of great importance to identify patients at risk for developing delirium, in order to focus on preventive strategies.

**OBJECTIVES.** To determine the incidence of delirium, its association with dexmedetomidine and specific preoperative risk factors in cardiac surgery patients.

**METHODS.** This prospective cohort interventional study enrolled patients between 1/2015-12/2016 at the Heart Centre of a Tertiary Care University Hospital. 164 patients, undergoing elective and urgent cardiac surgery with cardiopulmonary bypass, were evaluated. Specific data on patients were collected preoperatively and perioperatively. Cognitive function and impairment, cerebrovascular and neurological pathology, psychiatric and depression disease information were collected at baseline from medical history, interview and the Montreal Assessment test (1). Dexmedetomidine, a highly selective a2-adrenoceptor agonist, was administered in high risk patients during the early postoperative period. High risk were characterized patients of age 65 years, with smoking and alcohol abuse habits, urgent surgery, cognitive impairment, psychiatric illness, history of stroke, cerebrovascular and neurological disease and chronic obstructive pulmonary patients (n=75). Postoperative delirium was assessed using the Confusion

Assessment Method for Intensive Care Unit or conventional Confusion Assessment Method after discharge from ICU, starting on 1st post-surgery day at 12-h intervals.

**RESULTS.** Prevalence of postoperative delirium in total sample (n=164) was 17 patients (10.4%). In dexmedetomidine group (n=75) was 12 (16%), and non-dexmedetomidine group (n=89) 5 patients (5.6%). Age [OR 1.08; 95%CI (1.02-1.15); p 0.008], preoperative cognitive impairment [OR 5.05; 95% CI (1.76-14.50); p 0.001] and dexmedetomidine [OR 3.20; 95%CI (1.07-9.55); p 0.30] were associated with an increased risk for delirium. Dexmedetomidine increased delirium 3.20 times. Further analysis of five risk factors within groups revealed no difference.

**CONCLUSIONS.** Delirium is a common complication after cardiac surgery. Dexmedetomidine didn't decrease postoperative delirium. However, preoperative assessment may identify high risk patients who would benefit from delirium prevention strategies, because of increased baseline risk for delirium.

#### REFERENCE(S)

1. Na S, Yy Y. Use of the Montreal Cognitive Assessment test to investigate the prevalence of mild cognitive impairment in the elderly elective surgical population. Anaesth Int Care 2016;44:581-6.

### 0694

#### Dialysis-dependent chronic renal failure is a risk factor for post-cardiac surgery vasoplegic syndrome

S. Masafumi, T. Shimozono, D. Kawakami, J. Ito, Y. Asaka, H. Ueta, H. Mima, R. Seo, T. Koyama
Kobe City Medical Center General Hospital, Kobe, Japan
**Correspondence:** S. Masafumi
*Intensive Care Medicine Experimental* 2018, **6(Suppl 2):**0694

**INTRODUCTION.** Vasoplegic syndrome (VS) frequently occurs in the early postoperative period in patients who have undergone cardiac surgery. VS is characterized by severe hypotension, with decreased systemic vascular resistance, and increased requirements for vasopressive agents despite adequate cardiac output. Suggested risk factors include preoperative use of angiotensin-converting enzyme (ACE) inhibitors and beta blockers, low left ventricular ejection fraction (LVEF), and use ofcardiopulmonary bypass (CPB), as well as other factors with a discordant correlation. Chronic hemodialysishas not been identified as a VS risk factor, but we would expect a higher incidence of VS in chronic hemodialysis patients undergoing cardiac surgery.

**OBJECTIVES.** Our goal was to determine whether chronic hemodialysisis a risk factor for VS after cardiac surgery.

**METHODS.** This retrospective, single-center study included 563 patients who had undergonecardiac surgery in a tertiary hospital between 2015 and 2016. The patients were divided into 2 groups according to whether they were chronic hemodialysis-dependent. We assessed the incidence of postoperative VS in the 2 groups.

**RESULTS.** Preoperative patient characteristics were not significantly different between the 2 groups. The chronic hemodialysis group included 41 patients (7.3%) and the control group included 521 patients (92.7%). The median age was 73 years ([IQR] 65-78 years). The study included all coronary artery bypass graft procedures (on-pump N = 101 and off-pump N = 37), all valvular procedures (N = 259), and large vascular surgery with use of CPB (N = 149). A total of 66 patients (10%) developed VS. The incidence of VS was higher (P < 0.0001) in the chronic hemodialysis group (39.0%) than in the control group (9.6%). Age and known risk factors for VS (preoperative use of ACE inhibitors and beta blockers, low LVEF, and CPB time >180 minutes) were entered into multivariate regression analysis, and chronic hemodialysis was found to be an independent predictor of post-cardiac surgery VS (OR:6.33, 95%CI, 3.08-12.79).

**CONCLUSIONS.** Chronic hemodialysis-dependent renal failure was an independent risk factor for post-cardiac surgery VS.

#### REFERENCE(S)

1. Mekontso-Dessap A, Houel R, et al. Risk factors for postcardiopulmonary bypass vasoplegia in patients with preserved left ventricular function.Ann Thorac Surg. 2001 May;71(5):1428-32.
2. Carrel T, Englberger L, et al. Low systemic vascular resistance after cardiopulmonary bypass: incidence, etiology, and clinical importance.J Card Surg. 2000 Sep-Oct;15(5):347-53.
3. Hossene Junior NA, Miranda M. Cardiopulmonary bypass increases the risk of vasoplegic syndrome after coronary artery bypass grafting in patients with dialysis-dependent chronic renal failure. Rev Bras Cir Cardiovasc. 2015 Jul-Aug;30(4):482-8.
4. Kristof AS, Magder S. et al. Low systemic vascular resistance state in patients undergoing cardiopulmonary bypass. Crit Care Med. 1999 Jun;27(6):1121-7.

**GRANT ACKNOWLEDGMENT**
None.

### 0695

#### Evolution of ruptured abdominal aortic aneurysm treatment: a ten-year overview in ICU

O. Moreno Romero, A. Carranza Pinel, M. Muñoz Garach
Hospital Universitario San Cecilio, Granada, Spain
**Correspondence:** O. Moreno Romero
*Intensive Care Medicine Experimental* 2018, **6(Suppl 2):**0695

**INTRODUCTION.** In the treatment of the Acute Aortic Syndromes the endovascular approach has become an alternative over open surgery, specially in the ruptured Abdominal Aortic Aneurysm (rAAA), with the publication of the results of three clinical trials (AJAX, ECAR and IMPROVE), though with conflicting data.

**OBJECTIVES.** First, to describe the characteristics of patients with rAAA, surgical procedure used, anesthetic technic and their complications in ICU in a ten-year period (2007-2017). Second, to analyse the risk factor related to mortality.

**METHODS.** Retrospective observational study of patients admitted in our ICU (18 beds) with rAAA from 2007 to 2017. Variables registered: age, sex, Apache II, hemodynamic instability prior to surgery (No vasopressors, norepinefrine< 0.5 mcg/kg/h or >0.5 mcg/kg/h), lactate level (normal or >3 mg/dl), need for transfusion, need for sedation and orotracheal intubation, surgical procedure (open, endovascular), anesthesia (general or local), complications after surgery (renal replacement therapy, abdominal compartment syndrome and reintervention) and ICU mortality. Describe the risk factors mortality-related.

**RESULTS.** 29 patients. Mean age 76±9.5. 73% male. Mean Apache II 21% (RIQ: 17-26). Instability prior to surgery: 12 (41.5%) with no vasopressors, 10 (34.5%) with norepinefrine (< 0.5 mcg/kg/h) and 7 (24%) with high norepinefrine. Lactate leves >3mg/dl in 25% (86%). In 4 patients (12.3%) blood transfusion was needed before surgery, in 27 (93%) was administered during or after surgery. In 3 patients (10.3%) no transfusion was needed at all. 7 patients (24%) needed early prior sedation and intubation due to instability. Technics: 5 (17.25%) open surgery directly (all in the first 5 years) and 24 (82.75%) endovascular, from these, 7 (29%) were converted to open surgery at the same time (mostly due to abdominal bleeding and high abdominal pressure). General anesthesia in 18 (62%) patients (including the 7 converted to open surgery) and local in 11 (38%). Complications after surgery: 10 (34.5%) needed renal replacement therapy, 8 (27.58%) developed abdominal compartment syndrome and 4 of them (50%) had decompressive surgery. ICU mortality: 19 (65.5%) patients. From these, 15 (78.9%) had general anesthesia: 5 open surgery, 4 endovascular reconverted and 6 totally endovascular. 4 (21%) patients with local anesthesia. Mortality-related risk factos: general anesthesia OR 2.45 (CI95:1.37-2.89 and p 0.035), open surgery OR 3.5 (CI95: 2.25-5.2 and p 0.018) and hemodynamic instability OR 2.27 (CI95: 1.18-2.65 and p 0.04).

**CONCLUSIONS.** In the last years, even before the publication of the relevant clinical trials (AJAX, ECAR and IMPROVE) the tendance has been to perform endovascular treatment, if possible, derived from higher positive outcomes. In our study, although it's a small sample, the mortality-related risks were general anesthesia, open surgery and hemodynamic instability.

### 0696

#### Analysis of effective responders by inhaled nitric oxide in adult cardiovascular surgery with pulmonary hypertension

H. Kamohara, K. Sagishima, N. Narimatsu, T. Kikuchi, M. Ejima, K. Tokunaga, H. Tanigawa, T. Yamamoto
Kumamoto University Hospital, Devision of Critical Care Medicine, Kumamoto, Japan
**Correspondence:** H. Kamohara
*Intensive Care Medicine Experimental* 2018, **6(Suppl 2):**0696

**INTRODUCTION.** Inhaled nitric oxide (iNO) was authorized in the hypoxic respiratory failure in pulmonary hypertension in the newborn ten years ago in Japan. iNO was clinically applied for pulmonary hypertension in the perioperative period of adult cardiac surgery three years ago. It remains lots of unclear points on the evaluation of responder for iNO in adult cardiac surgery(#1).

**OBJECTIVES.** The purpose of this study was to analyze the effective factors in the indicators of respiration and circulation by iNO, and to reveal any groups of effective patients.

**METHODS.** In Kumamoto university hospital for the past three years (2015.4-2018.3), it was 648 cases that entered the ICU after cardiac surgery. Twelve cases received iNO therapy in the perioperative period for pulmonary hypertension. Two cases were excluded in non-pulmonary artery catheters for this study. Mean PAP (mPAP), PVRI, mPAP/mBP, CI, Svo2, SVRI, Cathecholamin Index (CAI), PF ratio, and PEEP were used as indicators of respiration and circulation. Data were collected and analyzed for 6 hours after iNO therapy, affecting the host response in the short time. In the next, to identify iNO responded factors, we evaluated various condition of patients who had disease background and device support, such as dialysis, ventilator, and IABP. Data were analyzed using JMP statistical software.

**RESULTS.** The age average of is 74.7 ± 12.0 years in 10 patients who consist of 6 men and 4 female. Operation was underwent for valve replacement surgery in all cases, coronary artery graft bypass in 4 cases, and major vascular graft replacement in 1 case. The average operative time was 360 ± 160 minutes, and cardiopulmonary support was 151 ± 40 minutes. All cases was analyzed by using Wilcoxon analysis of the pared transition before and after iNO therapy. mPAP (p = 0.026), PVRI (p = 0.024), mPAP/mBP (p = 0.023.) were significantly improved after iNO therapy. Furthermore, to identify the good responders of mPAP, PVRI and mPAP/mBP by iNO therapy, we examined patients related various factors. These demonstrated that mPAP (p = 0.039) and mPAP./mBP (p = 0.031) were improved by iNO therapy in the lower PEEP Group (PEEP< 10 $cmH_2O$). Other factors (dialysis, left cardiac function, or diabetes mellitus etc.) were not observed significantly.

**CONCLUSIONS.** Inhald NO improved pulmonary artey pressure and vascular resistance in the perioperative of adult cardiovascular surgery. Respiratory management in the lower PEEP could demonstrate good responder of iNO therapy, but offsetting in the higher group.

##### REFERENCE(S)

1. Pulmonary Hypertension in Patients Undergoing Cardiac Surgery: Pathophysiology, Perioperative Management, and Outcomes. Christopher A.Thunberg et al. Journal of Cardiothoracic and Vascular Anesthesia, 2013,27(3),551-572.

### 0697

#### Pulmonary hypertension and heart disease after pneumonectomy: the key role of pulmonary endothelial cells

P. Sentenac[1,2], G. Samarani[1,2], P. Bideaux[1], P. Sicard[1], J. Arthur-Ataam[1], S. Richard[1], P.H. Colson[2], S. Eddahibi[1]
[1]PhyMedExp Laboratory, University of Montpellier, Inserm, Cnrs, Montpellier, France; [2]University Hospital of Montpellier, Department of Anæsthesiology and Critical Care Medicine, Montpellier, France
**Correspondence:** P. Sentenac
*Intensive Care Medicine Experimental* 2018, **6(Suppl 2):**0697

**INTRODUCTION.** Pulmonary hypertension (PH) and heart disease are leading causes of morbidity and mortality after pneumonectomy [1,2]. The pathophysiology is still poorly understood. Overflow in pulmonary arteries is suspected to play a key role.

**OBJECTIVES.** To investigate *in vivo* cardiopulmonary consequences of pneumonectomy in rats, and *in vitro* in a model of human stretched cells reproducing overflow.

**METHODS.** Sixty *Sprague-Dawley* male rats randomly underwent either a right pneumonectomy (PN group) or sham surgery (sham group). Ten rats/group were respectively sacrificed on postoperative days 3, 7, 28 (D3, D7, D28). Cardiopulmonary alterations were investigated by echocardiographic, hemodynamic, gravimetric and histological analyses. *In vitro*, the shear stress of overflow was reproduced using a *FlexCell Tension System*™ cyclic strain on cultured human pulmonary endothelial cells (P-ECs) to investigate the impact on pulmonary artery smooth muscle cell (PA-SMC) growth. Data were expressed as mean ±SEM. Two-way ANOVAs were performed. P-values < 0.05 were considered significant.

**RESULTS.** Mean pulmonary arterial pressure gradually increased in the PN group to reach 35±3 mmHg on D28, *vs.* 18±2 in sham (p< 0.01), likewise the proportion of muscularized distal pulmonary arteries, 83 ±1% *vs.* 5±1% respectively (p< 0.001), related to an *in situ* PA-SMC proliferation (*Fig1*). In accordance with PH, the right ventricle (RV) area and RV lateral wall thickness doubled in the PN group on D28 (1.2±0.1 *vs.* 0.5±0.1 in sham, p< 0.001). The left ventricle ejection fraction decreased in the PN group on D7 and D28, respectively 66±3% *vs.* 74±2% (p< 0.05) and 65±2% *vs.* 78±2% in sham (p< 0.01), while RV function was maintained over time (*Fig2*). RV hypertrophy was confirmed by the Fulton index (RV/[LV+ septum] weight) increased on D3, D7 and D28 in PN groups, respectively 39±1%, 43±2% and 55±3% (p< 0.05 *vs.* sham). An early and transient pulmonary œdema was found on D3, as the wet/dry ratio increased to 5.5±0.1 *vs.* 4.9±0.1 in sham (p< 0.001). *In vitro*, the human PA-SMC proliferation was larger when stimulated with stretched P-EC media *vs.* non-stretched P-EC media (p< 0.05) highlighting the deregulation of the P-EC paracrine function (*Fig3*).

**CONCLUSIONS.** Pneumonectomy in rats induced moderate PH with a proportional right heart remodelling. The overflow appears to play a key role by altering the crosstalk between P-ECs and PA- SMCs leading to pulmonary vascular remodelling. Once the growth factors are identified, our model will be instrumental for future studies of targeted therapies to prevent the post-pneumonectomy syndrome or other high-blood-flow-related PH syndromes.

##### REFERENCE(S)

[1] Thomas PA *et al.*. Pneumonectomy for lung cancer. J Thorac Cardiovasc Surg 2015. 149:73-82 ; [2] Lugg ST *et al.* Long-term impact of developing a postoperative pulmonary complication after lung surgery. Thorax 2016. 71:171-176.

##### GRANT ACKNOWLEDGMENT

None.

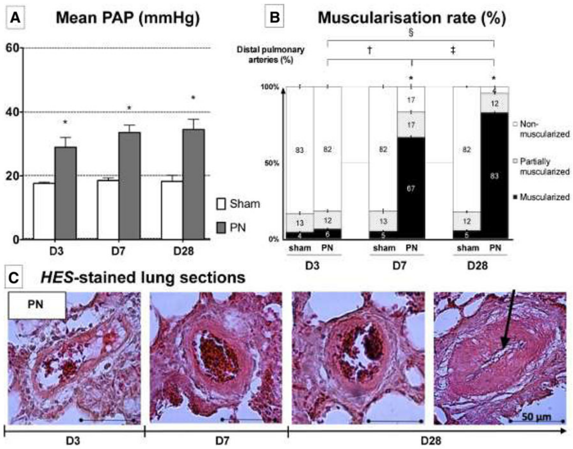

(A): Invasive mean pulmonary arterial pressure (PAP), (mean ± SEM). (B): Distribution of non-muscularized, partially muscularized, and muscularized distal pulmonary arteries in the sham and PN groups on D3, D7 and D28 (percentage ± SEM). (C): Haematoxylin-eosin-saffron stained representative lung sections of muscularized distal pulmonary arteries in the PN group (black arrow = pseudo-occluded vessel). **(A) and (B): Two-way ANOVA tests with *Sidak* correction for multiple comparisons**: adjusted p value (*) < 0.05, (**) p< 0.01 and (***) p< 0.001 for the comparison sham *vs.* PN at the same time-course ; (†) < 0.05 for the comparison D3 *vs.* D7, (‡) D7 *vs.* D28, and (§) D3 *vs.* D28 in the same surgery group. [D0, D3, D7, D28 = baseline, postoperative days 3, 7, 28 ; HES = haematoxylin-eosin-saffron ; PN = pneumonectomy].

**Fig. 1 (abstract 0697).** Pulmonary vascular remodelling after right pneumonectomy in rats

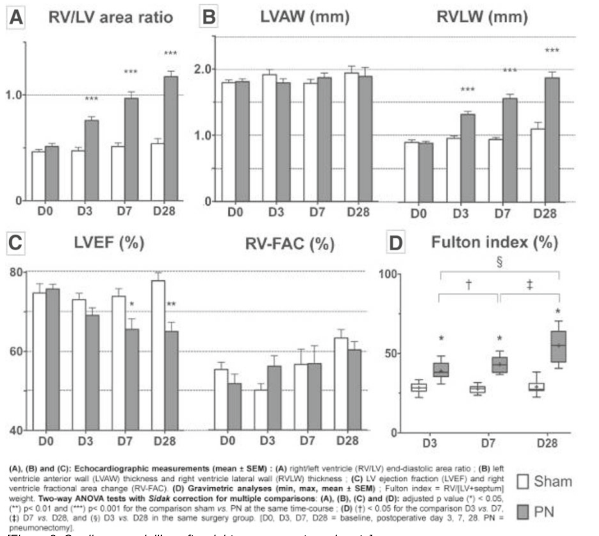

**Fig. 2 (abstract 0697).** Cardiac remodelling after right pneumonectomy in rats

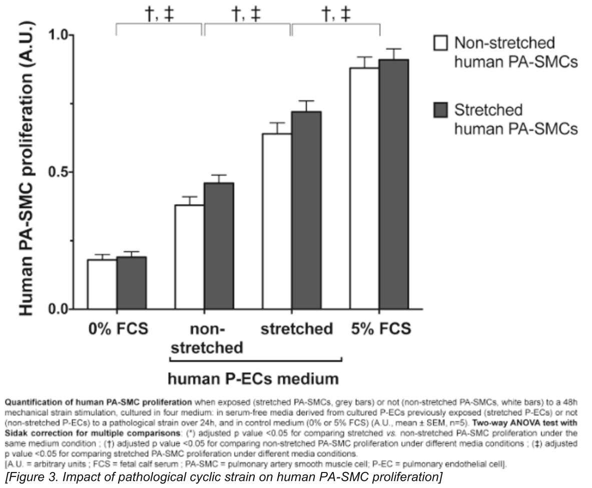

**Fig. 3 (abstract 0697).** Impact of pathological cyclic strain on human PA-SMC proliferation

### 0698

#### Interaction of ventricular arterial coupling in postoperative aortic valve replacement

I. Lima Lucero, J. Garduño Lopez, E. Bucio Reta, F. Baranda Tovar, R. Barajas Campos, M. Hernández Márquez, G. Mendoza Copa
Mexican Cardiology National Institute 'Ignacio Chávez', Surgical Intensive Care Unit, Mexico City, Mexico
**Correspondence:** I. Lima Lucero
*Intensive Care Medicine Experimental* 2018, **6(Suppl 2):**0698

**INTRODUCTION.** The interaction between the left ventricle and arterial system, termed usually ventricular - arterial coupling is recognized nowadays as a key determinant of global cardiovascular performance. The cardiovascular system is structured to provide adequate pressure and flow to the tissues all time. The analysis of this interaction requires left ventricle and arterial system to be described in similar mathematical terms. The ventricular- arterial coupling is commonly calculated by the ratio of effective arterial elastance (EA), as measure of afterload, to left ventricle end systolic elastance (EES), a relatively load independent measure of left ventricle chamber performance. Ventricular arterial coupling is approximately equal to 1.0, that means left ventricle and arterial system are optimally coupled to produce stroke work. When this ratio is < 1.0, the stroke work remains close to optimal values, but when the ratio is > 1.0 the stroke work significantly falls and the left ventricle becomes less efficient.

In cardiac surgery ventricular - arterial coupling has being studied in patient with aortic stenosis where the ratio was increased in patients with symptoms or heart failure due to increased EA and reduces EES. In the postoperative of aortic valve replacement the ventricular arterial coupling wasn´t be studied.

**OBJECTIVES.**

1. Evaluate the ventricular - arterial coupling using EA/EES ratio in patients with aortic valve replacement.
2. Determine if the ventricular arterial coupling is related with the length of stay in the ICU and state at ICU discharge.

**METHODS.** The cohort consisted in 56 patients older than 18 years; post operate of aortic valve replacement from the surgical ICU of the Mexican Cardiology Institute "Ignacio Chávez". EA and EES were estimated noninvasively. Patients were divided in those with ratio < 0.6, ratio between 0.7 to 1.4, and ratio > 1.4.

**RESULTS.** EA was increased in those patients whom had longer ICU stay (OR 1.3, IC 95% 1.1 - 1.5 p< 0.05) but mortality at discharge was not different ( 0.98 IC 95% 0.76 - 1.45 p= 0.24). Most of patients had EES alteration less than 0.7 (68%).

**CONCLUSIONS.** Ventricular arterial coupling is a good reference of interaction between the left ventricle and the arterial system. In aortic valve surgery this ratio is related with length of ICU stay. The most common alteration was decreased EES maybe by extra circulatory bypass.

**REFERENCE(S)**

· Guarracino, F., Baldassarri, R. and Pinsky, M. (2013). Ventriculo-arterial decoupling in acutely altered hemodynamic states. *Critical Care*, 17(2), p.213
· Antonini-Canterin F, Poli S, Vriz O, Pavan D, Bello VD, Nicolosi GL. The ventricular-arterial coupling: From basic pathophysiology to clinical application in the echocardiography laboratory. J Cardiovasc Echography (2013);23:91-5

**GRANT ACKNOWLEDGMENT**

To the Surgical Intensive Care Unit of Mexican Cardiology Institute "Ignacio Chavez"

### 0699

#### Fluid therapy after elective coronary artery bypass surgery: a retrospective analysis of maintenance, resuscitation and unintended fluids

T. Maes[1], A. Meuwissen[2], A. Sablon[1], M. Diltoer[3], D.N. Nguyen[4], M. La Meir[5], R. Wise[6], H. Spapen[4], M.L.N.G. Malbrain[4], E. De Waele[7]
[1]Brussels University Hospital (UZB), Intensive Care - Anesthesiology, Jette, Belgium; [2]Brussels University Hospital (UZB), Internal Medicin, Jette, Belgium; [3]Brussels University Hospital (UZB), Intensive Care - Anesthesiology, Jette, Belize; [4]Brussels University Hospital (UZB), Intensive Care, Jette, Belgium; [5]Brussels University Hospital (UZB), Cardiac Surgery, Jette, Belgium; [6]Pietermaritzburg Metropolitan, Durban, South Africa; [7]Brussels University Hospital (UZB), Intensive Care - Nutrition, Jette, Belgium
**Correspondence:** T. Maes
*Intensive Care Medicine Experimental* 2018, **6(Suppl 2):**0699

**INTRODUCTION.** Patients undergoing coronary artery bypass graft (CABG) surgery receive plentiful intravenous (IV) fluid therapy in the immediate postoperative phase [1]. Standardized protocols are generally provided for maintenance and replacement fluids. However, non-intended fluid sources (analgesics, sedative drugs, antibiotics and oral solutions) are not always considered within the IV fluid planning. The impact of such additional fluid load on total fluid intake is poorly documented.

**OBJECTIVES.** We examined the ratios between maintenance (M) fluids, resuscitation (R) fluids and unintended (U) fluids in elective CABG patients during their first and second postoperative day in ICU.

**METHODS.** Data on IV and oral fluid administration and fluid output were retrospectively collected from the electronic medical file of consecutive CABG patients. Fluid prescriptions included routine fluid administration, any additional fluid, and blood products. The ratios of M, R, and U fluids to total fluid intake were calculated for the first and second day in ICU. The composition of M, R and U fluids was documented. Values are given as means ± standard deviation.

**RESULTS.** Sixty patients (46 male, 14 female; age 67 ± 11 years; body weight 81 ± 17kg) were included. Mean overall (IV + oral) fluid administration was 2922 ± 831 ml on day 1 and 3128 ± 695 ml on day 2. M fluids represented 1177 ± 310 ml (40%) and 1197 ± 394ml (38%), R fluids 847 ± 542 ml (29%) and 338 ± 559 ml (11%), U fluids 898 ± 469 ml (31%) and 1593 ± 643ml (51%) respectively on day 1 and day 2. Fifty-four percent of the R fluids consisted of additionally prescribed fluid, 33% being synthetic colloids and 13% transfusions. U fluids consisted of oral intake (51%), paracetamol (22%), other medication (22%), and antibiotics (5%). Mean oral intake increased almost four-fold on the second day, while fluids used for analgesia remained stable with a mean of 247 ± 93 ml on day 1 and 302 ± 119 ml on day 2.

**CONCLUSION.** Postoperative prescription of M fluids in combination with R fluids is responsible for most of the observed total effective fluid load during the first two days of ICU stay after elective CABG surgery. U fluid load is high and primarily determined by administration of medication on the first day and oral intake on the second day. Our study suggests further research on the impact of lowering M fluid load and/or withholding unnecessary U fluids on patient outcome [2].

## What can we do to influence the outcome in our patients?

### 0700

#### Long-term survival and causes of death in alcohol misuse patients after ICU discharge

S. Hietanen[1,2], J. Herajärvi[1,2], T. Ala-Kokko[1,2], R. Käkelä[1,2], J.H. Liisanantti[1,2]
[1]Oulu University Hospital, Division of Intensive Care Medicine, Department of Anesthesiology, Oulu, Finland; [2]Oulu University Medical Research Center, Research Group of Surgery, Anesthesiology and Intensive Care, Oulu, Finland
**Correspondence:** S. Hietanen
*Intensive Care Medicine Experimental* 2018, **6(Suppl 2):**0700

**INTRODUCTION.** We previously reported high prevalence of alcohol misuse in critically ill patients admitted to the intensive care unit (ICU). However, a higher rate of low-risk admission diagnoses was reported in patients with alcohol misuse resulting in a better 1-year survival. (1). Some studies suggest alcoholic patients having higher long-term mortality after ICU admission but only a few recent studies focus on the associations between the alcohol misuse and the causes of death (2-3).

**OBJECTIVES.** The aim was to compare the long-term mortality and the causes of death in patients with or without alcohol misuse admitted to ICU.

**METHODS.** This retrospective study included all non-trauma admissions to ICU in Oulu University Hospital, Finland between January 1, 2014 and December 31, 2014. The electronic medical records and the ICU patient data management system were used to obtain data of the history of alcohol misuse, alcohol-related health problems, and ICU treatment profile. The patients were followed until the end of the year 2017 and the Statistics Finland provided the causes of death of non-survivors.

**RESULTS.** From a total of 847 patients 329 (38.8%) died during the 3-year follow-up. In the alcohol misuse group 90 patients (33.6%) died compared with the 239 patients (41.3%) in the non-alcohol group ($p$=0.033). The non-survivors in the alcohol misuse group were younger and more often men (Table 1). In the alcohol misuse group, the location of death was more often home than institution, and the post mortem examination was performed more frequently. The most common cause of death in total was cardiovascular causes (Table 2). The patients in the alcohol misuse group died more often due to traumas or alcohol-related causes compared with the non-alcohol group. Cardiovascular causes and malignancies were more common causes of death in the non-alcohol group.

CONCLUSIONS According to our data, the patients with alcohol-related health problems have better 3-year survival after ICU admission compared with the non-alcoholic patients. The patients with alcohol misuse die younger and more often due to traumas or alcohol-related causes.

**REFERENCE(S)**

1. Hietanen S, Ala-Kokko T, Ohtonen P, Käkelä R, Niemelä S, Liisanantti JH. Treatment Profile and 1-Year Mortality Among Nontraumatic Intensive Care Unit Patients With Alcohol-Related Health Problems. J Intensive Care Med. 2017 Jan 1:885066617740071.
2. Christensen S, Johansen MB, Pedersen L, Jensen R, Larsen KM, Larsson A, Tonnesen E, Christiansen CF, Sorensen HT. Three-year mortality among alcoholic patients after intensive care: a population-based cohort study. Crit Care 2012;16:R5.
3. Roerecke M, Rehm J. Cause-specific mortality risk in alcohol use disorder treatment patients: a systematic review and meta-analysis. Int J Epidemiol 2014;43:906-19.

**GRANT ACKNOWLEDGMENT**
We have received a grant for expences from The Finnish Foundation of Alcohol Studies.

**Table 1 (abstract 0700).** Characteristics of the 329 non-survivors admitted to ICU

| | Alcohol group (N=90) | Non-alcohol group (N=239) | p-value |
|---|---|---|---|
| Gender, f/m | 24/66 | 96/143 | **0.029** |
| Age | 58 [51-65] | 72 [62-79] | **<0.001** |
| SOFA score (admission) | 8 [5-10] | 6 [4-9] | **0.048** |
| SOFA score (maximum) | 10 [7-13] | 8 [5-11] | **0.003** |
| APACHE II score | 20 [16-26] | 19 [13-25] | 0.479 |
| Survival time (days) | 49 [8-517] | 23 [4-215] | **0.009** |
| Location of death, home/ institution | 23/67 | 20/219 | **<0.001** |
| Post-mortem examination | 32 (35.6) | 47 (19.7) | **0.002** |

**Table 2 (abstract 0700).** Causes of death of the 329 non-survivors admitted to ICU

| | Alcohol group (N=90) | Non-alcohol group (N=239) | p-value |
|---|---|---|---|
| Cardiovascular | 8 (8.9) | 80 (33.5) | **<0.001** |
| Malignancy | 6 (6.7) | 66 (27.6) | **<0.001** |
| Neurology | 15 (16.7) | 36 (15.1) | 0.720 |
| Alcohol-related causes | 29 (32.2) | 3 (1.3) | **<0.001** |
| Infection | 6 (6.7) | 24 (10.0) | 0.343 |
| Trauma | 17 (18.9) | 7 (2.9) | **<0.001** |
| Gastrointestinal | 6 (6.7) | 13 (5.4) | 0.671 |
| Other | 3 (3.3) | 10 (4.2) | 0.724 |

### 0701

#### Length of stay in the intensive care unit (ICU) and associated critical care outcomes: aretrospective review of 12,975 patients

S. Weetman, A. Myers, T. Samuels
Surrey and Sussex Healthcare NHS Trust, Critical Care Medicine, Redhill, United Kingdom
**Correspondence:** S. Weetman
*Intensive Care Medicine Experimental* 2018, **6(Suppl 2):**0701

**INTRODUCTION.** Previous work has suggested that length of stay (LOS) in critical care is not an independent risk factor for in-hospital mortality (1). Understanding the relationship between LOS and ICU mortality facilitates improved communication with patients, relatives and other healthcare professionals in terms of managing expectations.

**OBJECTIVES.**

**1.** To examine the relationship between increasing length of stay and mortality rate.

**2.** To investigate how the length of stay in days is related to timing of death in ICU.

**METHODS.** Records were reviewed for all patients admitted to a busy mixed medical and surgical UK district general hospital critical care unit with 16 level two and three beds during a 25 year period. Length of stay in ICU, day of death in ICU and all cause in-hospital mortality data was collected for 12975 patients.

**RESULTS.** The overall ICU mortality for patients was 18.8%. Figure 1 shows ICU mortality against length of stay. Excluding those admitted for less than 24 hours or more than three weeks, there is a weak positive correlation between ICU mortality prevalence and length of stay with an $R^2$ of 0.4887. Figure 2 shows total number of deaths on ICU against length of stay. There is a strong negative exponential relationship between number of deaths on ICU and length of stay, with most deaths occurring within the first 2 days.

**CONCLUSIONS.** Increasing length of stay on ICU is weakly associated with a higher mortality rate. This is in keeping with previous studies (2) and may represent the physiological burden of prolonged ICU stays and the associated morbidity. The majority of ICU deaths happen in the first 2 days of admission, but the mortality prevalence at this time is only 16.7%. Patients admitted for less than 48hrs tend to either recover quickly from their acute illness and are subsequently discharged or die. Only a minority go on to have a prolonged ICU length of stay and these have an associated higher mortality. The data helps us to understand the likely outcomes for patients admitted to ICU for prolonged periods, but ultimately, individual patients need to be managed on a case-by-case basis.

**REFERENCE(S).**

1. Williams TA, Ho KM, Dobb GJ, Finn JC, Knuiman M, Webb SAR, et al. CRITICAL CARE Effect of length of stay in intensive care unit on hospital and long-term mortality of critically ill adult patients. Br J Anaesth. 2010;104(4):459-64.
2. B LK, W KA, B KJ, J ZD. Long-term Mortality Outcome. Chest [Internet]. The American College of Chest Physicians; 2006;129(4):954-9. Available from: http://dx.doi.org/10.1378/chest.129.4.954

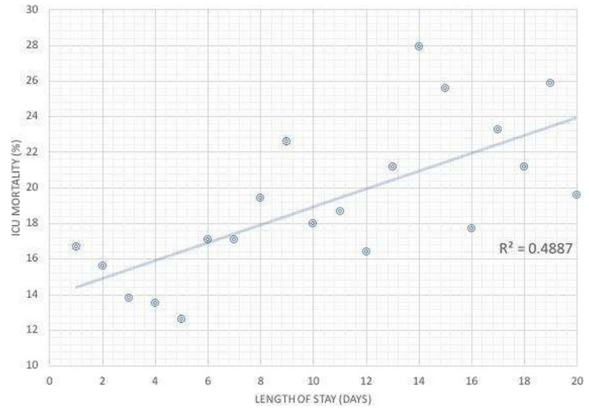

**Fig. 1 (abstract 0701).** See text for description

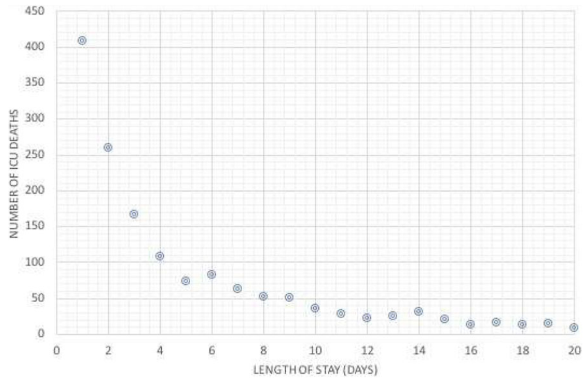

**Fig. 2 (abstract 0701).** See text for description

### 0702

#### One-year follow up of high-risk patients admitted in ICU after elective surgery from 2011 to 2016

G. Nattino[1], A. Forastieri Molinari[1], G. Macchi[2], M. Ticozzi[2], E.R. Vascotto[1]
[1]A. Manzoni Hospital - ASST Lecco, Anaesthesia and Intensive Care, Lecco, Italy; [2]A. Manzoni Hospital - ASST Lecco, Nurse in Intensive Care Unit, Lecco, Italy
**Correspondence:** A. Forastieri Molinari
*Intensive Care Medicine Experimental* 2018, **6(Suppl 2):**0702

**INTRODUCTION.** In literature, there is growing interest in assessing the usefulness of post-operative monitoring in intensive care unit

(ICU) after elective surgery and in evaluating patient long-term outcomes following ICU admission.

**OBJECTIVES.** The objective of this study was to analyse data and follow-up at one year after discharge in high-risk patients admitted in ICU for observation after uncomplicated elective surgery.

**METHODS.** In this study, we analysed five years data, from 2011 to 2016, about patients admitted in our general secondary ICU for postoperative monitoring after elective surgery, cardio and neuro surgery excluded, in our tertiary major acute hospital. Of all patients admitted we distinguished two groups considering recovery in ICU less or greater than 48 hours, then we compared the characteristics of the two groups (type of surgery, organ dysfunction, SOFA, SAPS II) in order to assess whether some could be predictive of prolonged stay. Finally, we analysed, the long-term outcome in group with complicated post-operative course. Information were abstracted from our electronic medical record and from registry of follow up service, active from 2011, for clinical and psychological evaluations one year after discharge from ICU. Data were analysed using univariate analysis, a p value < 0.01 was considered significant.

**RESULTS.** From 2011 to 2016, 614 patients were admitted in ICU for post-operative monitoring after elective surgery. 49 patients (8%) stayed more than 48 hours in ICU. Characteristics of the groups are summaries in table 1. We identified patients that had a greater statistically significant risk of stay more than 48 hours in ICU: patients presenting 2 organ dysfunctions (odds ratio 5.2) and patients presenting an infection at ICU admission (odds ratio 4.2). No correlation was found regarding the type of surgery. Patients with ICU stay longer than 72 hours had a greater incidence of complications occurred (p < 0.01). Despite all, the two groups had no differences in ICU and in hospital mortality. Figure 1 shows the Extended Glasgow Outcome Scale (GOSE) at one-year follow-up for the "complicated" group, only 10 patients (20%) had a good recovery (GOSE > 7).

**CONCLUSIONS.** Our analysis reveals that high-risk patients after elective surgery with 2 organ dysfunctions or presenting an infection appear to be at a greater risk for postoperative complications. Postoperative monitoring in ICU seems to be useful and effective in treating early post-operative complications (not different mortality in two groups). At 1-year follow-up disability analysis, only 10 (20%) of the complicated group patients had a Good Recovery (GOSE >7), this will need some considerations on the role of surgery. Further studies and analyses are needed.

**REFERENCE(S). Sobol JB et al. Triage of high-risk surgical patients for intensive care. Crit Care. 2011;15(2):217.**

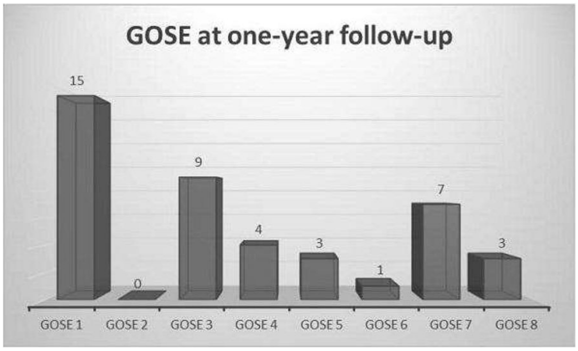

**Fig. 1 (abstract 0702).** See text for description

**Table 1 (abstract 0702).** See text for description

| | <48 hours | >48 hours | *p*-Value |
|---|---|---|---|
| *Age* | 69.1 ± 13.7 | 66.3 ± 14.2 | |
| *Age group* | | | |
| >75 | 233 (37.9%) | 11 (22.4%) | |
| 66-75 | 196 (31.9%) | 20 (40.8%) | |
| 65-46 | 154 (25.1%) | 17 (37.4%) | |
| 17-45 | 31 (5%) | 1 (2%) | |
| *Organ dysfunction at admission* | | | |
| 0 | 62.5% | 38,8% | |
| 1 | 26.2% | 34.7% | |
| 2 | 6.2% | 24.5% | <0.01 |
| 3 | 2.4% | 0.0% | |
| >3 | 2.6% | 2.0% | |
| *Infections to the admission* | | | |
| None | 570 (92.8%) | 37 (75.5%) | <0.01 |
| Infected patients | 44 (7.2%) | 12 (24.5%) | |
| SIRS | 30 (4.9%) | 6 (12.2%) | |
| Sepsis | 8 (1.3%) | 4 (8.2%) | |
| Septic shock | 6 (1.0%) | 2 (4.1%) | |
| *Complications occurred* | | | |
| *Total* | 60 (9.8%) | 24 (49%) | <0.01 |
| Respiratory | 17 (2.8%) | 7 (14.3%) | |
| Cardiovascular | 13 (2.1%) | 6 (12.2%) | |
| Neurological | 13 (9.6%) | 2 (4.1%) | |
| Gastrointestinal and hepatic | 7 (1.1%) | 3 (6.1%) | |
| Infection | 24 (3.9%) | 11 (22.4%) | |
| Other | 9 (1.5%) | 4 (8.2%) | |
| *Type of surgery* | | | |
| Thoracic surgery | 110 (20,04%) | 9 (18,37%) | |
| Gastro-intestinal surgery | 103 (18,76%) | 16 (32,65%) | |
| Vascular surgery | 84 (15,30%) | 8 (16,33%) | |
| Nefro-urologic surgery | 68 (12,39%) | 3 (6,12%) | |
| Otolaryngology surgery | 45 (8,20%) | 5 (10,20%) | |
| Other | 42 (7,65%) | 3 (6,12%) | |
| Thoracic vascular surgery | 39 (7,10%) | 2 (4,08%) | |
| Thoracic aortic surgery | 31 (5,65%) | 2 (4,08%) | |
| Pancreas surgery | 27 (4,92%) | 1 (2,04%) | |
| SOFA [a] | 2.4 ± 2.6 | 3.8 ± 2.4 | <0.01 |
| SAPS II [a] | 24.00 ± 11.7 | 29.90 ± 11 | <0.01 |
| LOS in ICU (days) [a] | 2.1 ± 0.9 | 7.9 ± 8.6 | <0.01 |
| Mortality in ICU | 23 (3.7%) | 2 (4.1%) | |
| Mortality in hospital | 55 (9.2%) | 2 (4.5%) | |

The results are presented as number and percentage, except:
[a] Mean ± standard deviation

### 0704

#### ICU follow-up clinic: which patients benefit the most?

A.R. Costa, I. Furtado, M. Capuepue, W. Mphandi, M.J. Malheiro, S. Pinto, J. Ribeiro, A.F. Cardoso, F. Seabra-Pereira, T. Cardoso
Centro Hospitalar do Porto, Unidade de Cuidados Intensivos Polivalente, Porto, Portugal
Correspondence: A.R. Costa
*Intensive Care Medicine Experimental* 2018, **6(Suppl 2):**0704

**INTRODUCTION.** The Intensive Care Unit (ICU) follow-up clinic has been increasingly recognized as a quality indicator. Previous published works have selected patients for follow-up based on the intensity of organ support (as more than 48h of invasive mechanical ventilation or duration of sedation). However, until now, no clear criteria have been defined regarding which patients should be observed at the ICU follow-up clinic.

**OBJECTIVE.** To identify which patients benefit from an ICU follow-up appointment based on their clinical characteristics and/or level of ICU support.

**METHODS.** Prospective cohort study of all patients discharged from mixed a ICU at a tertiary care hospital between 1$^{st}$ January of 2015 and 31$^{st}$ December 2016, who attended the ICU follow-up clinic. The need of further referral after observation was defined as the dependent variable.

**RESULTS.** During the study period, 280 patients were observed at the follow-up clinic, 70 (25%) needed further referral, namely to: 29 (41%) to psychology/psychiatry, 27 (39%) to surgical specialties, 17

(24%) to medical specialties, 6 (9%) to rehabilitation therapy and 3 (4%) to the chronic pain clinic. There was no significant association of further referral at the outpatient clinic with gender, age, Charlson comorbidity score index, previous comorbidities (neurologic or psychiatric), type of admission (medical or surgical), reason for ICU admission like post-cardio-pulmonary arrest, head injury, acute neurologic condition, acute psychiatric disease; level of intensive care (sedation, paralysis, invasive mechanical ventilation, vasopressor, inotropic or renal replacement therapy), the occurrence of agitation or delirium or ICU length of stay. Only time until observation in the follow-up clinic revealed to be significantly associated with the need for further referral (OR - 0.828, per increase month) (table 1).

**CONCLUSION.** A significant proportion of patients benefit from attending the out-patient clinic. There wasn't a significant association with patients' comorbidities, personal history or level of ICU treatment but patients evaluated earlier seem to benefit most. Therefore, we suggest observing all patients discharged from the at the follow-up clinic.

**Table 1 (abstract 0704).** Patient characteristics and the association with the need of new referral at the ICU follow

| | Total (n=280) | New referral (n=70) | Crude OR | P value |
|---|---|---|---|---|
| Female, n (%) | 96 (39) | 32 (46) | 0.667 | 0.103 |
| Age, mean±SD | 58±16 | 60±16 | 0.414, per year | 0.993 |
| Charlson score index, median (IQR) | 3 (1-5) | 4 (2-5) | 0.1009 per point | 0.855 |
| Previous neurologic disease, n (%) | 12 (7) | 2 (3) | 3.129 | 0.137 |
| Previous psychiatric disease, n (%) | 36 (15) | 12 (33) | 0.753 | 0.462 |
| Medical vs. Surgical admission, n (%) | 167 (67) vs. 81 (33) | 45 (64) vs. 25 (36) | 1.210 | 0.521 |
| Post-CPA, n (%) | 17 (7) | 6 (9) | 0.703 | 0.504 |
| Head injury | 25 (10) | 8 (11) | 0.818 | 0.659 |
| Acute neurologic condition | 38 (15) | 9 (13) | 3.29 | 0.137 |
| Acute psychiatric condition | 6 (2) | 1 (1) | 1.994 | 0.532 |
| Sedation, n (%) | 176 (71) | 49 (70) | 1.067 | 0.833 |
| Sedation duration, median (IQR) | 2 (0-5) | 2 (0-5) | 0.999, per day | 0.964 |
| Paralysis, n (%) | 10 (4) | 4 (6) | 0.576 | 0.404 |
| Invasive mechanical ventilation, n (%) | 183 (74) | 51 (73) | 1.069 | 0.834 |
| Vasopressor support, n (%) | 120 (48) | 42 (35) | 0.520 | 0.023 |
| Vasopressor duration, median (IQR) | 0 (0-3) | 2 (0-3) | 0.978, per day | 0.606 |
| Ionotropic support, n (%) | 11 (4) | 4 (6) | 0.675 | 0.542 |
| Dialysis, n (%) | 6 (2) | 3 (4) | 0.383 | 0.247 |
| Agitation, n (%) | 18 (7) | 7 (10) | 0.593 | 0.301 |
| Delirium, n (%) | 26 (11) | 11 (16) | 0.494 | 0.097 |
| ICU LOS, median (IQR) | 5 (2-9) | 7 (2-10) | 0.991, per day | 0.596 |
| Time ICU – outpatient clinic, median (IQR) | 3 (2-5) | 3 (2-5) | 0.828, per month | 0.006 |

Table 1. Patient characteristics and the association with the need of new referral at the ICU follow-up clinic appointment. IQR – Inter-quartil range, SD – standard deviation, CPA – cardio-pulmonary arrest, ICU – intensive care unit, LOS – length of stay.

### 0705

#### SAHUCI: results of the first Spanish multicenter study about satisfaction in ICU

M.S. Holanda Peña[1], G. Heras La Calle[2], Á. Alonso-Ovies[3], L. De la Cueva Ariza[4], M.P. Delgado Hito[4], M.C. Martín Delgado[2], L. Martín Iglesias[5], Spanish Satisfaction National Multicenter Study (SAHUCI) and The Research Project Humanizing Intensive Care (Proyecto HU-CI), Spain
[1]Marques de Valdecilla Hospital, Intensive Care Department, Santander, Spain; [2]Hospital Universitario de Torrejón, Intensive Care Unit, Madrid, Spain; [3]Hospital Universitario de Fuenlabrada, Intensive Care Unit, Madrid, Spain; [4]Facultat de Medicina i Ciències de la Salut, Escola d'Infermeria, L'Hospitalet de Llobregat, Spain; [5]Hospital Universitario Central de Asturias, Intensive Care Unit, Oviedo, Spain
**Correspondence:** M.S. Holanda Peña
*Intensive Care Medicine Experimental* 2018, **6(Suppl 2):**0705

**INTRODUCTION.** Intensive care units (ICU) have become places where high scientific and technical quality medical care is provided. However, such care must also be accompanied by special attention and treatment of patients and their families.

**OBJECTIVES.** To determine the level of satisfaction among both the relatives of surviving patients and the relatives of patients that died and to measure the level of satisfaction among the ICU discharged patients themselves.

**METHODS.**

Design: multicenter prospective, descriptive observational study carried out during 3-month period in 2017 involving ICUs in different hospitals in Spain.

Population of the study:

1) competent patients discharged from ICU after a stay of over 24 hours,

2) their relatives, and

3) Families of patients who died in ICU after, at least, 24 hours of admission.

Measurement Instrument: Family satisfaction in the Intensive Care Unit (FS -ICU 24) in its Spanish language version for relatives, and an adaptation of it for patients.

Statistical analysis: means, standard deviations, frequency tables, rates and proportions to describe patients, respondents and their answers. Conversion of the answers from a likert scale to a numerical one was necessary to apply the required statistical calculations.

**RESULTS.** 52 ICUs participated in the study, belonging to 40 hospitals in 23 Spanish provinces (figure 1). A total of 4914 surveys (2430 questionnaires of relatives of surviving patients, 2342 surveys of patients and 142 questionnaires of family members of non-surviving patients (Figure 2).

Patient characteristics: 2858 patients. Mean age 62,22±15,40 years. 1822 men. Mean APACHE II score 15,16±7,81 (n=2431) SOFA score 4,24±3,46 (n=2228).

38,80% needed mechanical ventilation. Mean length of ICU stay 6,65 ±10,30 days.

Most of patients were admitted to ICU with a medical diagnosis (coronary syndrome, sepsis, trauma and pneumonia).

Family characteristics: 2430 families were asked. Mean age 50,76 ±13,91 years. Mostly were women. The most frequent kinship: wives (27,3%) followed by daughters (24,1%).

Satisfaction: The majority of respondents were satisfied with overall care and with decision making process (89,13±10,15 and 80,43±13,34 for family of surviving patients, 91,02±10,12 and 83,25±13,88 for patients, and 86,09±13,84 and 77,67±14,97 for relatives of non-surviving patients).

When examining individual item scores, satisfaction with the waiting room atmosphere and communication items scored lowest. For patients, items that measured ICU environment were scored lowest.

Families of survivors were more satisfied with their ICU experience than the families of non-survivors.

**CONCLUSIONS.** Overall, most families were satisfied with care provided to them and their critically ill relative. Patients satisfaction was high too. However, opportunities for improvement exist.

Be interested in knowing the opinions of the patient and his family will allow us to provide a better care and a quality medicine adapted to their specific needs.

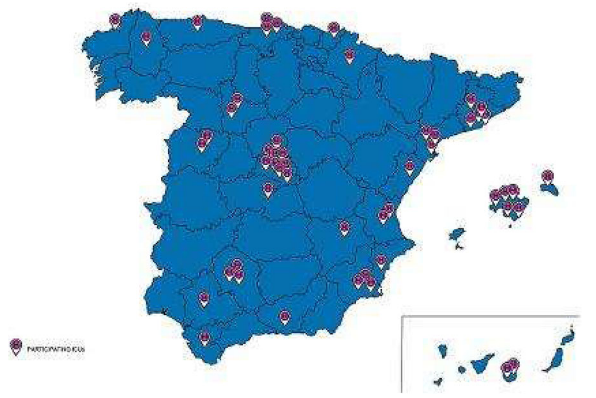

**Fig. 1 (abstract 0705).** Graphic representation of the distribution of the participating ICUs in the study throughout the Spa

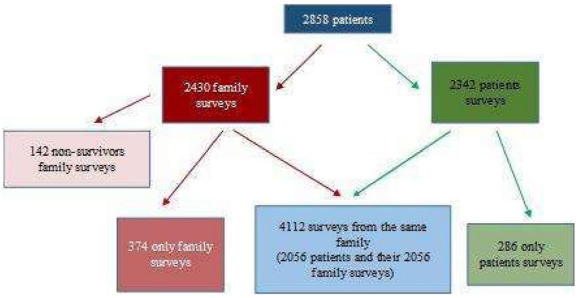

**Fig. 2 (abstract 0705).** Questionnaires obtained and their distribution

### 0706
### Analysis of prognostic scoring systems used in critically ill patients with status epilepticus

S. Casanova Prieto, J. Cedeño Mora, P. García-Olivares, I. Ruíz, A. Garrido, C. Mata, B. Moreno, M. Sancho, E. Bermejo, A. Jaspe
H.G.U Gregorio Marañón, Intensive Care Unit, Madrid, Spain
**Correspondence:** S. Casanova Prieto
*Intensive Care Medicine Experimental* 2018, **6(Suppl 2):**0706

**INTRODUCTION.** Status epilepticus (SE) is a common neurological emergency associated with a high morbidity and mortality. Predicting the prognosis in critically ill patients with SE through the use of appropriate scoring systems could be useful.
**OBJECTIVES.** The purpose of this study was to assess the predictive prognostic value of different severity scoring systems used in critically ill patients and those more specific for SE.
**METHODS.** Study of a cohort of patients admitted to ICU with a diagnosis of SE between the years 2015-2016. Analysis of the standard scoring systems used in ICU (APACHE II, SOFA) and severity scores specifically designed for status epilepticus: STESS (Status Epilepticus Severity Score) and EMSE (Epidemiology-based Mortality score in Status Epilepticus).
Descriptive analysis was expressed as mean (SD) or median (IQR) for continuous variables and percentages for categorical data. An unfavourable prognosis was considered as a score ≥ 4 points on the Rankin scale (severe disability or death). Through a simple logistic regression analysis each score was associated to the prognosis and the effect was estimated by its corresponding Odds Ratio (OR).
The ability of the scoring systems to discriminate prognosis was assessed using the area under the receiver operating characteristic curve (AUROC). Estimation of their calibration was established through the Hosmer-Lemeshow goodness of fit test.
**RESULTS.** Fifty-eight patients, age 55±18 years, 53% male and 34% high comorbidity (Charlson Comorbidity Index ≥ 3 points). The epileptic seizures occurred out-of-hospital in 57% of patients, 83% were generalized tonic-clonic and 35% presented a structural lesion in computed tomography imaging. Benzodiazepines (78%) and Levetiracetam (36%) were the antiepileptic drugs most commonly used. The level of consciousness on initial evaluation was GCS of 6±3 points and 76% of patients required endotracheal intubation and mechanical ventilation (2 days, 1-9). **Severity scores**: APACHE II 17 ±10 points, SOFA 5±3 points, STESS 3±1 points and EMSE 61±33 points. The ICU stay was 3 days (2-7) and hospital stay was 11 days (6-18). The prognosis was unfavourable in 29% of patients, with a 14% of mortality.
All **severity scores analysed**, except the STESS score, were good predictors of unfavourable prognosis: **APACHE II** (OR 1.13; 95% CI 1.02-1.25), **SOFA** (OR 1.38; 95% CI 1.09-1.75), **STESS** (OR 1.73; 95% CI 0.98-3.04) and **EMSE** (OR 1.03; 95% CI 1.01-1.06).
Using the **AUROC curves**, the **APACHE II score** was found to be the most reliable scoring system to discriminate unfavourable prognosis (AUROC 0.81, 95% CI 0.65-0.95), with a good calibration ability (Chi-squared 4.09, p=0.84) (Table 1).
**CONCLUSIONS.** Our data show that the prognostic scoring systems specifically designed for status epilepticus were not superior to the standard severity scores used in critically ill patients. Of all the severity scores analysed, the greatest discriminatory power was observed in the APACHE II.

**Table 1 (abstract 0706).** See text for desciption

| | DISCRIMINATION | | CALIBRATION | |
|---|---|---|---|---|
| | AUROC | 95% CI | Chi-squared | Sig |
| APACHE II | 0.81 | 0.65-0.95 | 4.09 | 0.84 |
| SOFA | 0.78 | 0.61-0.96 | 1.15 | 0.94 |
| STESS | 0.70 | 0.53-0.87 | 6.48 | 0.09 |
| EMSE | 0.75 | 0.58-0.94 | 9.52 | 0.30 |

### 0707
### Prevalence and impact of frailty in patients over 65 years of age admitted to intensive care unit

S. Lopez-Cuenca[1], L. Oteiza Lopez[1], N. Lázaro Martin[2], M. Irazabal Jaimes[3], M. Ibarz Villamayor[4], A. Artigas Raventós[5,6], N. Valero González[1], O. Peñuelas Rodriguez[1,6], J.A. Lorente Balanza[1,6,7]
[1]Hospital Universitario de Getafe, Getafe, Spain; [2]Hospital 12 Universitario de Octubre, Madrid, Spain; [3]Hospital Universitario General de Cataluña, Barcelona, Spain; [4]Hospital Universitario Sagrat Cor, Barcelona, Spain; [5]Corporación Sanitaria Parc Taulí, Sabadell, Spain; [6]CIBER Enfermedades Respiratorias, Madrid, Spain; [7]Universidad Europea, Madrid, Spain
**Correspondence:** S. Lopez-Cuenca
*Intensive Care Medicine Experimental* 2018, **6(Suppl 2):**0707

**INTRODUCTION.** Frailty is associated with worse hospital outcomes but in critically ill patients is not well known yet.
**OBJECTIVE.** To estimate the prevalence of frailty in patients > 65 years of age admitted to the intensive care unit (ICU) and its impact on mortality and long-term functional outcomes.
**MATERIAL AND METHODS.** Multicentric prospective observational study in patients> 65 years admitted to the ICU, between June 2016 and October 2017. Frailty was measured according to FRAIL scale (based on the Frailty Phenotype). Demographic variables, disability and cognitive function (Barthel index [BI], Lawton index [LI]) were registered upon admission, and at 1 and 6 months after discharge from the ICU. Data are presented as proportions and means and standard deviation. They are compared using the Chi-square test for categorical variables and by parametric (Student's t test). A p value < 0.05 was considered statistically significant. The approval of the corresponding Ethical Committees and the informed consent of the participating subjects were obtained.
**RESULTS.** 132 patients, 86 (65.2%) non frail and 46 frail (34.9%). Frail patients, compared with nonfrail patients: age 78.8 ± 7.2 versus 78.6 ± 6.4 years (p = 0.43), males 43.8% versus 56.3% (p = 0.10),, SOFA 4.7 ± 2.9 versus 4.6 ± 2.9 (p = 0.75), APACHE II 17.8 ± 7 versus 16.1 ± 6.6 (p = 0.13), need for mechanical ventilation (VM) 33.3% versus 66.7% (p = 0.75), days of VM 5.6 ± 15 versus 4.3 ± 8.1 (p = 0.57), ICU length of stay 11.2 ± 16 versus 11.3 ± 16.3 (p = 0.38), ICU mortality 54.5% versus 45.5% (p = 0.15), mortality 1 month 62.5% versus 37.5% (p = 0.04), mortality 6 months 55.6% versus 44.4% (p = 0.03), BI admission 74.1 ± 27.3 versus 96.8 ± 7.5 (p = 0.001), BI 1 month 50.7 ± 38.7 versus 72.6 ± 35.7 (p = 0.001), BI 6 months 46 ± 43.4 versus 68.5 ± 40.9 (p = 0.003), LI admission 4.4 ± 2.7 versus 5.9 ± 2 (p = 0.001), LI 1 month 2.6 ± 2.7 versus 4.1 ± 2.9 (p = 0.02), LI 6 months 2.7 ± 3.1 versus 4.2 ± 3.1 (p = 0.03).
**CONCLUSIONS.** Critically ill frail patients have worse functional situation upon ICU admission, similar ICU outcomes and mortality, and higher mortality after ICU discharge.

**REFERENCES**
Morley JE, Malmstrom TK, Miller DK. A simple frailty questionnaire (FRAIL) predicts outcomes in middle aged African Americans. J Nutr Health Aging. 2012 Jul;16(7):601-8.

Woo J, Yu R, Wong M, et als. Frailty Screening in the Community Using the FRAIL Scale. J Am Med Dir Assoc. 2015 May;16(5):412-9. Epub 2015 Feb 24.
Muscedere J, Waters B, Varambally A, et als. The impact of frailty on intensive care unit outcomes: a systematic review and meta-analysis. Intensive Care Med. 2017 Aug;43(8):1105-1122.
Flaatten H, De Lange DW, Morandi A, et al. The impact of frailty on ICU and 30-day mortality and the level of care in very elderly patients (≥ 80 years). Intensive Care Med. (2017). https://doi.org/10.1007/s00134-017-4940-8

**GRANT ACKNOWLEDGMENT**
Fundación del Enfermo Crítico 2017 (SEMICYUC)

### 0708

#### Compared airway pressures' discriminative properties in the prediction of mortality in non-COPD patients

N. Fraj[1], M.A. Boujelbèn[1], W. Zarrougui[1], K. Meddeb[1], S. Rouis[1], M. Zghidi[1], A. Khedher[1], A. Azouzi[1], I. Ben Saida[1], I. Chouchene[1], M. Boussarsar[1,2]
[1]Farhat Hached University Hospital, Medical Intensive Care Unit, Sousse, Tunisia; [2]Ibn Al Jazzar Faculty of Medicine, Research Laboratory N° LR12SP09 Heart Failure, Sousse, Tunisia
**Correspondence:** M. Boussarsar
*Intensive Care Medicine Experimental* 2018, **6(Suppl 2):**0708

**INTRODUCTION.** Stress-strain relationship of the lung parenchymal tissue depends on the elastic property of the alveolar walls, the surface tension characteristics and the alveolar geometry. This nonlinear relationship is described in Newtonian equation of motion. Low tidal volume is the only ventilation strategy that has been shown to improve mortality but there is still a huge gap to its application.
**OBJECTIVES.** To assess the airway pressures' discriminative properties in the prediction of mortality in non-COPD medical ICU mechanically ventilated (MV) patients.
**METHODS.** A retrospective charts' review of MV patients admitted to a medical ICU of Farhat Hached hospital from November 2015 to February 2018. Were collected patients' characteristics at admission and respective airway pressures (Peak, plateau, driving and intrinsic PEEP) at admission and at day 4. High pressure ratio (HPR) is defined as the number of days spent with high pressures : Peak ≥40 and/or plateau ≥30; and/or driving pressure ≥15 and/or; intrinsic PEEP ≥6; divided by length of stay) and outcomes were recorded. Univariate and multivariate regression analyses were performed to identify factors independently associated with mortality. ROC curves were used to check for the discriminative properties of the significant factors.
**RESULTS.** 304 mechanically ventilated patients were collected within the study period. 199(65%) were non-COPD patients. They were 50±18years aged ; ARDS, 25(12.6%) ; pneumonia, 14(7%) Pulmonary edema, 11(5.52%) ; SAPS II, 35.1±15.7 ; pH, 7.31±0.14 ; PCO2, 42.4±19.9 ; PaO2/FiO2, 210.6 ±109.2mmHg ; MV duration, 8.9±9.4days ; tracheostomy, 18(9%) ; length of stay, 10±10days. Mortality, 114(57%).
Mean airway pressures were respectively at admission : Peak, plateau, driving, intrinsic PEEP; 30.2±8.9, 20.2±6.2, 13.4±4.8, 2.7 ±4.2cmH2O and at day 4: 30.9±8.9, 20.8±6.9, 13.7±5.7, 2.4±3.8 cmH2O. HPR, 0.25±0.35.
Univariate analysis showed an association between the following factors and ICU mortality: At admission, plateau (21.3±5.9 vs 18.1 ±6.2cmH2O, p=0.005), driving (13.9±5.2 vs 12.7±4.02cmH2O, p=0.003). At day 4, Peak (34.2±8.8 vs 26.2±6.7cmH2O, $p\leq10^{-3}$), plateau (23.3±7.1 vs 17.1±4.8cmH2O, $p\leq10^{-3}$), driving (14.8±6.5 vs 12.1±3.9cmH2O, p=0.003). HPR (0.4±0.4 vs 0.1±0.2, $p\leq10^{-3}$). Multivariate logistic regression analysis identified Peak at day 4 (OR, 1.12 ; 95%CI, [1.03-1.22] ; p=0.006), plateau at day 4 (OR, 1.12 ; 95%CI, [1.01-1.25] ; p=0.04) and HPR (OR, 6.92 ; 95%CI, [1.1-43.6] ; p=0.04), as factors independently associated with mortality.
Respective ROC AUC for Peak, plateau and HPR confirmed interesting discriminative properties 0.81, 0.78, 0.72 to predict mortality.
**CONCLUSIONS.** The present study reflecting the daily practice in the management of MV non-COPD patients demonstrated that high airway pressures and/or persistent elevated airway pressures account at least in part in mortality.

### 0709

#### The outcomes and prognostic factors of patients with acute respiratory failure and prolonged intensive care unit stay

K.-C. Cheng[1], C.-C. Lai[2], K.-L. Tseng[3]
[1]Chi Mei Medical Center, Yangkang Dist, Tainan City, Taiwan, Province of China; [2]Chi Mei Hospital, Intensive Care Medicine, Liouying, Taiwan, Province of China; [3]Chi Mei Medical Center, Division of Respiratory Therapy, Tainan, Taiwan, Province of China
**Correspondence:** K.-C. Cheng
*Intensive Care Medicine Experimental* 2018, **6(Suppl 2):**0709

**INTRODUCTION.** Acute respiratory failure (ARF) is the most common cause that critically ill patients require intensive care unit (ICU) admission. Although some patients have favorable outcomes and early ICU discharges, others require a prolonged ICU stay. However, some complications such as muscle weakness, pressure ulcers, device-associated infections, pulmonary embolism, and delirium can develop during a prolonged ICU stay. Moreover, a prolonged ICU stay can be associated with increased hospital mortality, increased morbidity and hospital stay and poor long-term prognosis. In addition, critically ill patients with a prolonged ICU stay will require a greater than average medical resources.
**OBJECTIVES.** We aimed to investigate outcomes and identify risk factors of patients with ARF who required a prolonged ICU stay over 21 days
**METHODS.** In this retrospective study, all patients with ARF who required a prolonged ICU stay (≥ 21 days) in Chi Mei medical center between January 2000 and December 2017 were identified. The primary outcome was in-hospital mortality.
**RESULTS.** Totally 1371 patients were collected. Lung infection (*n* = 494, 36.0%) was the most common cause of prolonged ICU stays, followed by decompensated heart disease (*n* = 143, 10.4%), neuromuscular disease (*n* = 204, 14.9%), infection other than pneumonia (*n* = 279, 20.4%) , decompensated gastrointestinal disease (*n* = 179,13.1%), post operation (*n* = 35, 2.6%) and others (*n* = 37, 2.6%). The in-hospital mortality rate was 54.4% (*n* = 746). After using multivariable logistic regression, we identified five risk factors of death: age > 75 years (adjusted odds ratio (AOR), 1.36, 95% confidence interval (CI), 1.06-1.76, p = 0.017), ICU stay for more than 28 days (AOR, 1.48, 95% CI, 1.17-1.86, p = 0.001), APACHE II score ≥ 25 (AOR, 1.47, 95% CI, 1.15-1.87, p = 0.02), decompensated gastrointestinal disease (AOR, 2.95, 95% CI, 2.01-4.32, p = 0.002), and hemodialysis (AOR, 3.14, 95% CI, 2.44-4.05, p < 0.001). The mortality rate was up to 85.7% for patients with five risk factors.
**CONCLUSIONS.** The prognosis of patients with ARF who required a prolonged ICU stay were poor. Age > 75 years, an ICU stay > 28 days, an APACHE II score ≥ 25, decompensated gastrointestinal disease, and hemodialysis were associated with a high risk of in-hospital mortality. It should help us better predict outcomes for such patients.

**REFERENCE(S)**

1. Bellani G, Laffey JG, Pham T, et al. Epidemiology, patterns of care, and mortality for patients with acute respiratory distress syndrome in intensive care units in 50 countries. JAMA 2016; 315:788-800.
2. Fowler RA, Abdelmalik P, Wood G, et al. Critical care capacity in Canada: results of a national cross-sectional study. Crit Care 2015; 19:133.
3. Lai CC, Shieh JM, Chiang SR, et al. The outcomes and prognostic factors of patients requiring prolonged mechanical ventilation. Sci Rep 2016; 6:28034.

### 0710

#### Incidence and mortality of multiple organ failure (MOF) in septic patients. An 11 year review in Catalonia

C. Lorencio[1], J.C. Yébenes[2], J. Gonzalez Londoño[1], M. Cleriès[3], E. Vela[3], L. Espinosa[4], J.C. Ruiz[5], A. Rodriguez[6], E. Esteban[7], R. Ferrer[5], A. Artigas[8]
[1]Josep Trueta Hospital, Girona, Spain; [2]Mataró Hospital, Mataro, Spain; [3]Unitat d'Informació i Coneixement de CatSalut, Barcelona, Spain; [4]Oficina tècnica de l'Àrea Integral de Salut Barcelona Dreta, Barcelona, Spain; [5]Vall d´Hebron Hospital, Barcelona, Spain; [6]Joan XXIII University Hospital, Tarragona, Spain; [7]Sant Joan de Deu Hospital, Barcelona, Spain; [8]Parc Taulí University Hospital, Barcelona, Spain
**Correspondence:** C. Lorencio
*Intensive Care Medicine Experimental* 2018, **6(Suppl 2):**0710

**OBJECTIVES.** To observe the incidence and mortality of MOF in septic patients in Catalonia.

**MATERIALS AND METHODS.** Data was collected from the CatSalut morbidity database (hospitals, primary care, emergency room..) between the years 2005 to 2016.

To identify sepsis cases, the methodology described by Angus, consisitng on cross referencing the diagnosis of acute infection with multiple organ failure, was implemented (Crit Care Med 2001;29:1303-101).

**RESULTS.** During the analysis period, 224.396 cases were detected. The annual incidence rate of sepsis in Catalonia has increased from 1.6 (2005) to 3.9 (2016) cases per 1000 inhabitants per year. There are differences in the incidence rates adjusted by age, sex, morbidity and income level. The most frequent source of infection was the genitourinary tract (37.4%), respiratory (32.7%) and digestive (11.5%). The mean stay decreased from 19.2 to 13.4 days (p < 0.0001), the need for organ support techniques from 33.1% to 19.4% (p < 0.0001) and in-hospital mortality from 25.7% to 16.8% (p < 0.0001). 79.3% presented failure of a single organ, 15.6% of two organs and 5.1% of three or more, with a mortality of 15.7, 32.9 and 53.3% respectively. The most frequent organ failure is renal (54.6%), followed by cardiovascular (24%), altered mental status (18.7%), respiratory failure (16.5%), hematologic (10%) and liver failure (1%). The organ failure associated with a higher mortality was liver failure (55.3%) followed by cardiovascular (37.4%), respiratory (35.9%) and renal failure (20.6%). There was a statistically significant reduction in the mortality of septic patients with cardiovascular failure (46% to 29%, p < 0.0001), respiratory failure (40% to 25%, p < 0.0001) and renal failure (25% to 18%, p < 0.0001).

**CONCLUSIONS.** The incidence of sepsis in Catalonia has increased from 1.6 to 3.9 cases per 1000 inhabitants per year. The percentage of patients with MOF has remained stable, around 21.7%. The need for organ support techniques has been reduced by 37.5%, as well as the hospital stay by 46% and the associated mortality by 34.6%. A statistically significant reduction in the mortality of septic patients is observed in patients with cardiovascular (46% to 29%, p < 0.0001), respiratory (40% to 25%, p < 0.0001) and renal failure (25% to 18%, p < 0.0001).

### 0711

#### Outcome of oncology patients in intensive care unit: 5 year experience

V. Psallida[1], V. Zidianakis[1], T. Katsoulas[1,2], E. Tsigou[1], E. Boutzouka[1], C. Demponeras[1], G. Fildissis[1,2]
[1]Agioi Anargiroi Hospital, ICU, Athens, Greece; [2]National Kapodistrian University of Athens, School of Health Sciences, Department of Nursing, Athens, Greece
**Correspondence:** V. Psallida
*Intensive Care Medicine Experimental* 2018, **6(Suppl 2):**0711

**INTRODUCTION.** Patients (pts) with malignancies comprise about 15% of the total Intensive Care Unit (ICU) population in Europe and their outcome is comparable to this of the rest ICU population.

**OBJECTIVES.** To compare the outcomes of oncology vs non-oncology ICU pts.

**METHODS.** All 267 oncology pts admitted to the ICU of the General and Oncology Hospital "Agioi Anargiroi" between January 2013 and October 2017 were retrospectively assessed in terms of ICU mortality and compared to the rest of ICU population (575pts). Categorical variables are presented as N(%) and continuous variables as mean ± SD or median (IR). Comparisons between groups were assessed by the chi square (x2) test, student´s t test or the Mann Whitney U test. The risk of death was estimated by the Kaplan-Meier (log rank) test and the Cox's regression method. Statistical analysis was performed using SPSS version 21.0 (SPSS, Inc., Chicago, Illinois).

**RESULTS.** All except two oncology pts had solid tumors, 67 (26%) had a medical reason of admission (respiratory failure 19, septic shock 16, pulmonary edema 7, ARDS 5) and from the 200 pts admitted after a sursical procedure, 22 (11%) underwent emergency surgery. The mortality of the oncology pts was lower than the non oncology pts (18% vs 31%, p< 0.001), nevertheless the difference was reversed when pts were categorised according the type of admission (medical and surgical). The clinical characteristics of the pts are shown in table.

The higher risk of dying was noted in medical-oncology pts, followed by medical, surgical and surgical-oncology pts (picture).

Cox proportional hazards regression analysis showed that medical oncology pts had a dying hazard ratio >2 times bigger than medical pts (Exp(B) 2.218, 95%CI 1,266-3,886, P = 0.005).

**CONCLUSIONS.** Oncology pts represented almost 32% of ICU population and comprised of heterogeneous populations with different outcomes. The mortality of cancer pts with a medical reason for admission was significantly higher compared to the rest medical pts, even though the severity of illness according the APACHE and SOFA scores was similar between the two groups.

**REFERENCE(S). Taccone FB, et al. Characteristics and outcomes of cancer patients in European ICUs Critical Care 2009; 13:R15**

**Table 1 (abstract 0711).** Clinical characteristics of the patient groups

| | Medical n=443 | Medical oncology n=67 | Surgical oncology n=200 | Surgical n=132 |
|---|---|---|---|---|
| Age (years) (p=0.443) | 68.36 ± 14.02 | 68.54 ± 10.23 | 68.09 ± 13.83 | 70.37 ± 12.56 |
| LOS (days) (p<0.001) | 23.35 ±38.7 | 13.7 ±12.74 P=0.056 | 7.16 ± 12.08 P<0.001 | 7.44 ± 8.05 P<0.001 |
| APACHE II score (p<0.001) | 23.94 ± 7.21 | 24.63 ± 7.46 P=0.96 | 17.05 ± 8.67 P<0.001 | 19.49 ±8.3 P<0.001 |
| SOFA score (p<0.001) | 9.8 ± 3.48 | 10 ± 3.49 P=0.1 | 6.1 ± 3.79 P<0.001 | 8.33 ± 4.07 P=0.06 |
| Mortality (p<0.001) | 163 (36.9%) | 33 (49.3%) | 15 (7.5%) | 15 (11.4%) |

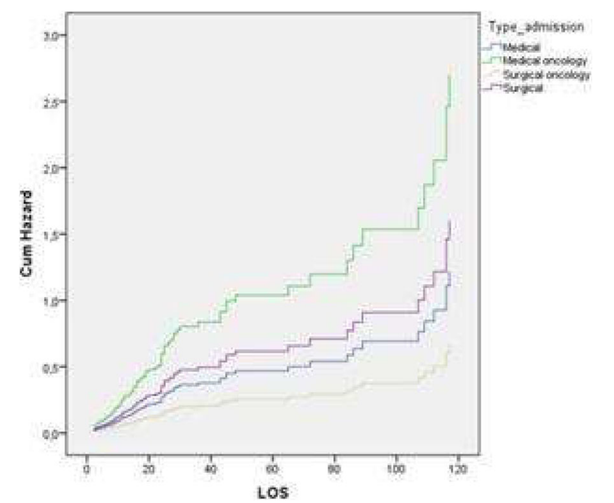

**Fig. 1 (abstract 0711).** Hazard ratio in pts groups

### 0712

#### What happens after sepsis? A review of sepsis patient outcomes in Catalonia

C. Lorencio[1], J.C. Yébenes[2], J. Gonzalez Londoño[1], M. Cleriès[3], E. Vela[3], L. Espinosa[4], J.C. Ruiz[5], A. Rodriguez[6], E. Esteban[7], R. Ferrer[5], A. Artigas[8]
[1]Josep Trueta Hospital, Girona, Spain; [2]Mataró Hospital, Mataro, Spain; [3]Unitat d'Informació i Coneixement de CatSalut, Barcelona, Spain; [4]Oficina Tècnica de l'Àrea Integral de Salut Barcelona Dreta, Barcelona, Spain; [5]Vall d´Hebron Hospital, Barcelona, Spain; [6]Joan XXIII University Hospital, Tarragona, Spain; [7]Sant Joan de Deu Hospital, Barcelona, Spain; [8]Parc Taulí University Hospital, Barcelona, Spain
**Correspondence:** C. Lorencio
*Intensive Care Medicine Experimental* 2018, **6(Suppl 2):**0712

**OBJECTIVE.** Getting to know the survival rate and the functional state of patients diagnosed with sepsis after 18 months in patients who had sepsis during the years 2015 and 2016 in Catalonia.

**MATERIALS AND METHODS.** Data was extracted from the CatSalut database, a base that integrates exhaustive information of the resident population in Catalonia. Between the years 2015 and 2016, all cases of sepsis in Catalonia (54.671) were identified through the methodology described by Angus [Crit Care Med 2011; 29: 1303-101] and tracking was performed up to 18 months from diagnosis.

**RESULTS.** 15 days after the sepsis diagnose, the overall mortality is 14.6%. 29.7% of the patients are still hospitalized and 13.4% are institutionalized in a social health center or a nursing home. 42.3% have been discharged home.

By the third month of sepsis diagnose, the overall mortality is 30.1%. 3.3% are still hospitalized and 12.8% are admitted to a healthcare center. 53.8% are home.

18 months from diagnose the overall mortality is 48.6%. A remaining 0.7% are still hospitalized and 6.9% are institutionalized in a social health center or a residence. 43.8% are at home.

**CONCLUSIONS.** Although the mortality of sepsis has decreased in recent years, it continues to be a serious health problem in our society. Almost half of the patients who suffered from sepsis between the years 2015 and 2016 died during the course of the 18 months following diagnosis.

Of the patients who survived, 15% were still not functionally independent and needed health resources at 18 months of diagnosis.

Any intervention to improve detection, diagnosis or treatment could directly influence the morbidity and mortality of these patients in the long term.

### 0713

#### Intensive care and the post-intensive care syndrome: a prospective cohort study

I. Furtado, A.R. Costa, W. Mphandi, M. Capuepue, A.F. Cardoso, M.J. Malheiro, S. Pinto, J. Ribeiro, F. Seabra-Pereira, T. Cardoso
Centro Hospitalar do Porto, Unidade de Cuidados Intensivos Polivalente, Porto, Portugal
**Correspondence:** I. Furtado
*Intensive Care Medicine Experimental* 2018, **6(Suppl 2):**0713

**INTRODUCTION.** After being discharged from an intensive care unit (ICU), patients face different challenges and difficulties. Some develop cognitive, psychiatric and /or physical disability after treatment in ICU, which is now recognized as post intensive care syndrome (PICS). The ICU follow-up clinic is crucial for the global assessment of these patients' population and to prompt the orientation of specific and/or new problems.

**OBJECTIVE.** To evaluate the post intensive care syndrome (PICS) in a mixed ICU population.

**METHODS.** Prospective cohort study of all patients discharged from a mixed ICU between 1st January of 2015 and 31st December 2016, observed at the follow-up clinic. PICS is defined as new or worsening impairment in physical (ICU-acquired neuromuscular weakness), cognitive (thinking and judgment), and mental health status after critical illness and persisting beyond discharge from the acute care setting.

**RESULTS.** Of the 793 patients discharged from the ICU: 351 died before follow-up and 442 were invited to the clinic, of these, 280 (63%) attended (figure 1). Patients seen at the clinic had a mean age of 58±16 years old and 62% were male. Charlson comorbidity index was 3 (1-5). A medical condition motivated ICU admission in 188 patients (67%) *vs.* 92 (33%) surgical patients. Regarding organ support: 209 (75%) patients were mechanically ventilated, 201 (72%) sedated, 134 (48%) needed vasopressors, 11 (4%) neuromuscular blockage and 7 (2.5%) required dialysis. Agitation and delirium were present in 22 (8%) and 31 (11%) patients, respectively. Median ICU length of stay was 5 (2-9) days. Patients were evaluated at the follow-up clinic 3 (2-5) months after ICU discharge: 116 were professionally active before ICU admission, of these 80 (69%) hadn't yet returned to usual professional activity at follow-up. At follow-up 83 patients (34%) presented with new complaints and 65 (23%) needed further specific referral. Regarding performance status, most patients were independent previous to ICU admission with a Karnofsky performance scale of 100 (90-100). At follow-up, the Karnofsky scale decreased in 159 (57%) patients, to a median of 80 (70-90). Furthermore, 24 (9%) patients presented moderate to severe depression (Beck Depression Inventory) and 22 (8%) risk for post-traumatic stress disorder (PTSD - 14). Regarding the five domains of quality of life (EQ-5D), 128 patients (55%) had mobility difficulties, 76 (33%) had problems with self-care, 147 (63%) with usual activities, 118 (51%) referred pain and 108 (47%) anxiety/depression.

**CONCLUSIONS.** PICS has a high prevalence among ICU survivors. Prevention during ICU stay is crucial and prompt recognition at the follow-up clinic is essential to provide multidisciplinary support to the patients and family.

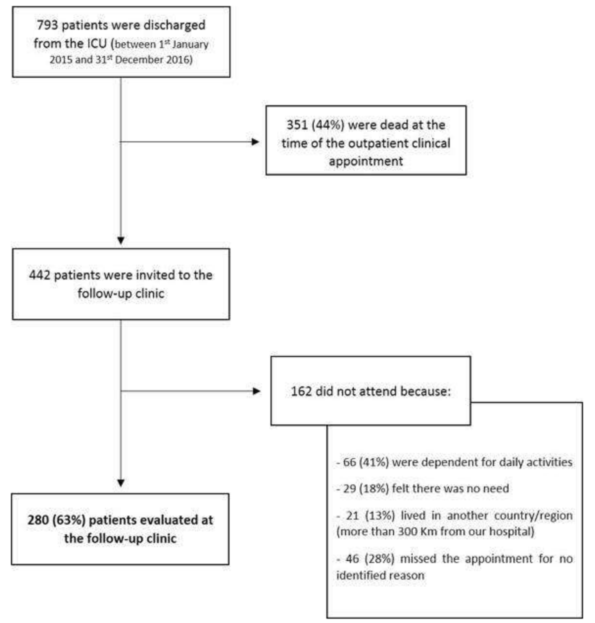

**Fig. 1 (abstract 0713).** Flowchart of patients observed at the follow-up clinic

## Quality interventions

### 0714

#### Incidence and risk factors associated with ocular surface disorders in ventilated ICU patients and impact of protocolised eye care

V. Bhagat, M.S. Khan, N.R. Prabu, A.P. Kulkarni, J.V. Divatia, S.N. Myatra
Tata Memorial Hospital, Mumbai, India
**Correspondence:** V. Bhagat
*Intensive Care Medicine Experimental* 2018, **6(Suppl 2):**0714

**INTRODUCTION.** Critically ill patients frequently have poor eyelid closure, reduced tear production and a reduced protective blink reflex due to sedation during mechanical ventilation. ICU patients are more susceptible to infections and decreased venous return may lead to conjunctival chemosis. These factors increase the risk of ocular surface disorders (OSDs) which may lead to adverse long-term outcome such as leucoma, astigmatism and decreased vision, if untreated.

**OBJECTIVES.**

**Primary objective:** To identify the incidence of OSDs in sedated, ventilated patients in our ICU with the current eye care practices and the impact of a protocolised eye care on the incidence and outcome

**Secondary objective**: To determine the correlation of ICU-acquired risk factors with the incidence of OSDs in ventilated patients

**METHODS.** This study was done in our mixed medical surgical ICU. It had a prospective cohort design and was done as before and after study in two phases (Phase I and Phase II). In phase I existing eye care practices were continued and incidence of OSDs was noted. In phase II protocolised eye care was implemented (Included routine eye care plus nurses education, identification of eye warning sign, special ointment plus eye shield in lagophthalmos and precautions during suction). All patients who were mechanically ventilated for > 24 hours were included in the study. Patients with pre-existing OSDs were excluded. Study completion criteria was development of OSD, discharge from ICU or death. In both phases an Ophthalmologist (study investigator) examined the eyes at baseline and every 72 hours using a hand-held slit-lamp. Patients diagnosed with OSD were referred to another ophthalmologist for management. An independent ophthalmologist re-assessed the eye findings by the study investigator on the same day to check for bias. At the end of study inter-observer agreement was calculated

**RESULTS.** Total number of patients included was 125 (phase I: 62 and phase II: 63). There was no difference in demographic variables in both the groups at baseline. Incidence of OSD was higher in phase I with 64.5% of patients (40/62) developing OSD while in phase II it was 34.9%(22/63). This difference in incidence was significant (p=0.001). Inter-observer agreement among the study investigator and the independent ophthalmologist was very good in both the phases (Kappa index in phase I 0.927, in phase II 0.908) On univariate and multivariate analysis, increased depth of sedation (p = 0.0001) and absence of protocolised eye care (p=0.001) were associated with higher incidence of OSDs.

**CONCLUSIONS.** Incidence of Ocular surface disorders (OSDs) is high in sedated ventilated patients in ICU. Protocolised eye care helps in decreasing the incidence of OSDs. Increased depth of sedation and absence of protocolised eye care are independent risk factors associated with OSDs in ICU.

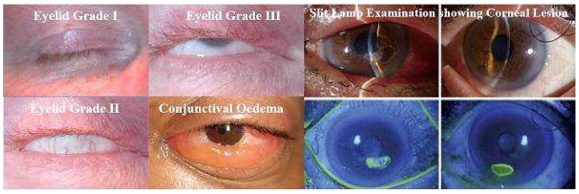

**Fig. 1 (abstract 0714).** Eye lid grading, Conjunctival Edema and Slit Lamp Examination of Cornea

### 0715

#### Multicenter binational investigation of necrotizing soft tissue infections from diagnosis and to six months: patient and family perspective. A presentation of three sub-studies

I. Egerod[1], A.E. Andersson[2], A.-M. Fagerdahl[3], V.E. Knudsen[1]
[1]Copenhagen University Hospital Rigshospitalet, Intensive Care Unit, Copenhagen O, Denmark; [2]Sahlgrenska Academy, Institute of Health Care Sciences, Gothenburg, Sweden; [3]Karolinska Institute, Department of Clinical Science and Education, Stockholm, Sweden
**Correspondence:** I. Egerod
*Intensive Care Medicine Experimental* 2018, **6(Suppl 2):**0715

**INTRODUCTION.** Necrotizing soft tissue infection (NSTI) is a serious and potentially life-threatening condition requiring immediate diagnosis and treatment. The prevalence is about 1-2 in 100.000. The disease progresses rapidly, but little is known of the human cost for patient and family.

**OBJECTIVES.** The objective was through a series of studies to explore the experience of patient and family during the acute stage of NSTI and up to six months.

**METHODS.** The overall design was qualitative using different sources and methods. The presented sub-studies are based on six-month interviews with patients (n=27) and family (n=23) from university hospitals in Denmark and Sweden. The study was embedded in the EU funded study INFECT with the goal of advancing understanding of pathophysiological mechanisms, prognosis and diagnosis of NSTI. The present study P-INFECT reflects the patient and family perspective. Patient and public involvement was applied in constructing the study.

**RESULTS.** We present the results from sub-studies 2-4 based on 50 interviews with patients infected at the extremities, groin/genitals and lower abdomen, face and throat, and back, and family.

<u>Study 2</u>: Focus on patient and family during the acute stage. We identified three main themes: Duration of disease onset, Stages of symptom severity, and Types of treatment and delay. Although most studies describe NSTI as a rapidly progressive disease, our data suggest a slow, moderate or rapid disease onset. We identified three stages of disease severity: lingering, progressive and unbearable symptoms. Immediate attention was required when symptoms became unbearable. Types of treatment delay were: patient-related, primary care related and hospital related, suggesting modifiable factors.

<u>Study 3</u>: Focus on family caregiver the first 6 months. Three chronological themes emerged: In ICU: coping with illness and intensive care, At the ward: coping with injury and post-intensive care, and At home: coping with recovery and new home life. We concluded that challenges facing family caregivers are still under-recognized. Families and stakeholders need access to information and planned care to bridge knowledge gap across care environments.

<u>Study 4</u>: Focus on the patient the first 6 months. The main themes described transitions: From Fear to Gratitude, From First Class to Economy, and From Relief to Challenge. Fear of infections and disfigurement were particular to NSTI.

**CONCLUSIONS.** The most surprising and novel finding was the description of varying onset of disease and symptom progression that might facilitate diagnosis, particularly unbearable pain that must not be overlooked. Many responses to NSTI were generic to ICU, but some issues were particular to NSTI. We recommend the provision of more information on what to expect with NSTI and improved transitional care between ICU and the ward to alleviate feelings of abandonment and uncertainty.

**GRANT ACKNOWLEDGMENT**
Lundbeck Foundation.

### 0716

#### Nurse perception of support for young children during their visit of an adult beloved one in the ICU

E.M. van Hooff, L.F. Roggeveen, H.J.S. de Grooth, A. van Galen, A.R.J. Girbes
VU University Medical Center, Amsterdam, Netherlands
**Correspondence:** E.M. van Hooff
*Intensive Care Medicine Experimental* 2018, **6(Suppl 2):**0716

**INTRODUCTION.** Several authors have described the experiences of children visiting the adult ICU and most studies report it positive, provided they are well prepared and guided[1]. Kean showed that often restrictions are imposed to children to visit a patient in the adult ICU[2]. Little is known about the opinion of nurses on this topic.

**OBJECTIVES.** The main objective of this study is to describe practices of children visiting in the adult ICU in the Netherlands from a nurse perspective.

**METHODS.** We performed a multicenter survey among ICU nurses in the Netherlands with questions focused on quality and quantity of visits of young children, aged 4-12 years. For content validity, the questions focused on quality were first processed by an open peer review. Each answer was divided into distinct categories. Data were described using descriptive statistics.

**RESULTS.** Fifty-two centers were included in this study and 3629 nurses were invited to participate in the study and 13,650 answers were reviewed by 5 investigators. The overall survey response rate was 30%. All ICU's allow in general children to visit patients in the ICU. Determining factors to refuse children to visit their adult beloved ones are a combination of medical reasons such as an infection risk to the patient or child, as well as psychological reasons. Nurses aged between 25 to 36 years, believe that it is very important to allow children to visit a beloved one in the ICU. However, with increasing age, nurses deemed this as less important (P=< 0.01). The majority of the nurses prepare a child for a visit. When a child is prepared for a visit, 38% use education material, 42% provide explanations about the setting. Seventy-eight percent of all nurses believe that the responsibility to prepare a child for an adult ICU visit lies within the nursing staff, while a high percentage of them (46%) emphasizes the joint responsibility of nurses, doctors and the parents. Less than 1% of the nurses consider this the sole responsibility of the doctor. The survey showed that the guidance of children was mainly based on intuition and practical experiences.

**CONCLUSIONS.** There is a general acceptance of the right of young children to visit their beloved one in the ICU. Most nurses consider it the responsibility of the nurse to prepare this visit. There appears to be no standard rules and guidelines for preparing young children to visit the ICU. Further research is needed to develop appropriate support interventions both for the children and for the nursing staff.

**REFERENCE(S)**

1. Knutsson, S. & Bergbom, I. (2008). Children's experiences of visiting a seriously
ill/injured relative on an adult intensive care unit. Journal of advanced Nursing, 61
(2),154-162.
2. Kean, S. (2010). Children and young people visiting an adult intensive care unit. Journal of advanced Nursing 66 (4),868-877

**GRANT ACKNOWLEDGMENT**

This work was nog supported by any grant, public or private.

### 0717

#### Nursing model of end of life care: from curative interventions to palliative measures

J.M. Latour[1], R. Endacott[1,2], J. Benbenishty[3]
[1]University of Plymouth, School of Nursing and Midwifery, Faculty of Health and Human Sciences, Plymouth, United Kingdom; [2]Monash University, Melbourne, Australia; [3]Hadassah Hebrew University Medical Center, Jerusalem, Israel
**Correspondence:** J. Benbenishty
*Intensive Care Medicine Experimental* 2018, **6(Suppl 2):**0717

**INTRODUCTION.** The care transition from curative interventions to palliative care is complex processes in treating intensive care patients. Identifying critical junctures of patients in transition from active to palliative care are challenges for healthcare staff and families. Decision making within this transition has three stages: diagnosis of dying; managing end of life (EOL) consensus; and pushing the door open to facilitate family grieving.

**OBJECTIVES.** To examine EOL situations in intensive care and demonstrate the integral work of nurses in calming, organizing and making sense of ICU life and death conditions by providing and adhering to an EOL care bundle.

**METHODS.** In order to encapsulate the range of evidence, a scoping review method was chosen. PubMed, Embase, CINAHL, and PsycINFO were used. Studies were included if they were conducted in Europe, all languages were considered. Thematic analysis was used identifying major themes to classify and understand transition to EOL care practices in Europe.

**RESULTS.** The identified and developed model demonstrates incorporation of critical junctures in the transition from curative interventions to palliative measures into EOL intensive care bundle. The theoretical framework structure combines complex steps needed for this transition in ICU end of life setting.The EOL ICU bundle, identified and developed by the current evidence, includes seven critical junctures (CJ):

CJ 1: Time Out - Redefine goals from ICU treatment to comfort care.

CJ 2: Shared Decisions - Communication with team, patient and family- investment in gaining family, team and if possible patient involvement in decision making on transition of goals of care.

CJ 3: Consensus - nurse mediation between families is usually successful in resolving conflicts until resolution has occurred.

CJ 4: Comfort Care - focusing on providing symptom management and comfort care.

CJ 5: Family Care- Directing practice towards the family is imperative for the impending death to be perceived as a quality experience.

CJ 6: Deceased Care- A good death means to promote interventions ensuring the right to physical integrity, preserving the good body image and ensuring dignity.

CJ 7: Follow-Up- After the death of a loved one, bereaved family members may still face questions or issues and may leave the ICU unsatisfied or confused.

**CONCLUSIONS.** This EOL care bundle provides synthesis of evidence for interventions during transition from curative treatment and care to EOL care. It provides a scientific practical tool for ICU clinicians. The evidence is presented for each juncture and the end of life trajectory as an intervention bundle.

### 0718

#### Embedding clinical research into the culture of clinical practice within the critical care environment

S. Bean, K. Burt, J. Paddle
Royal Cornwall Hospital Trust, Critical Care Unit, Truro Cornwall, United Kingdom
**Correspondence:** S. Bean
*Intensive Care Medicine Experimental* 2018, **6(Suppl 2):**0718

**INTRODUCTION.** Conducting clinical research in the acute Critical Care area can be challenging. Patients often lack autonomous decision making capacity, there is high morbidity and mortality, along with short time frames to recruit and initiate interventions. Critical Care is a fast-paced, acute care environment where the unpredictable nature of the patients' condition potentially results with a sudden deterioration in illness (1).

**OBJECTIVES.** Using a multicentre randomised controlled trial conducted in 24 acute NHS hospitals throughout the UK (2) as an example we aim to highlight some methods used to embed research into our clinical practice demonstrating the importance of a positive research culture in the clinical setting.

**METHODS.** We looked to develop a multi-faceted approach to promoting Critical Care research in the ICU (3). All staff undertook a short e-learning course designed by the trial team. Three nurses undertook further training which concentrated on the values and aspects of the trial. This focused on the psychological effects of critical illness and how to recognise psychological stress and ways to create a less stressful environment. We produced regular newsletters and notice board displays with each month being used to highlight a different aspect of psychological wellbeing. We developed a toolbox talk to ensure that all staff were confident in using the CAM ICU tool. Regular discussions and updates of the trial were presented at monthly clinical governance meetings and anonymized patient experiences were used to reflect on the trial's relevance to our patient group.

**RESULTS.** The benefits of collaborative working on Critical Care trials can be advantageous for the clinical team, research teams and more importantly the patient. As a result of collaborative working we recruited to target and also recruited the 1st and 1000th patient to the trial. Our uptake to the online training was above 95%. We noticed an improved compliance with CAM ICU scoring and also received positive feedback from the clinical team.

**CONCLUSIONS.** Critical Care is a complex field of medicine with clinical research in the Critical Care unit being challenging. Clinical research therefore should not be seen as a stand-alone activity. Embedding research into the culture of clinical practice, along with a collaborative working environment, can enrich patient outcomes ultimately providing new medical knowledge, therapies and technologies to optimise patient care.

**REFERENCE(S)**

(1) Pattison N, Arulkumaran N, et al (2017) 'Exploring obstacles to critical care trials in the UK: A qualitative investigation' Journal of the Intensive Care Society 18(1) 36-46
(2) The Provision of Psychological Support for Patients in Intensive Care (ICNARC unpublished)
(3) O'Byrne L, Smith S (2010) 'Models to enhance research capacity and capability in clinical nurses: a narrative review' Journal of Clinical Nursing 1365-1371

**GRANT ACKNOWLEDGMENT**
No grant required

### 0719

#### Patients' experience regarding night time and sleep deprivation in ICU: results from a metasynthesis

M. Danielis, E. Mattiussi, M. Vidoni, L. Venuti, A. Palese
Faculty of Nursing, Udine, Italy
**Correspondence:** M. Danielis
*Intensive Care Medicine Experimental* 2018, **6(Suppl 2):**0719

**INTRODUCTION.** In Intensive Care Unit (ICU) patients are frequently subjected to sleep deprivation or to poor quality of sleep. Despite its relevant for short and late term outcomes, to our best knowledge no systematic review with regard patient experience on sleep in ICU have been published. Understanding their experience may help in developing interventions aimed at increasing both the quality and the quantity of sleep in ICU patients.

**OBJECTIVES.** The general intent of this study was to advance the knowledge available by exploring the patients' perspective of night time and sleep deprivation in ICU.

**METHODS.** A meta-synthesis was performed. Three phases were followed: (1) conceiving the synthesis, searching and retrieving literature, (2) appraising and classifying findings, and (3) synthesizing findings into a meta-summary and a meta-synthesis. CINHAL, MEDLINE (PubMed) and Scopus were consulted. The database research yielded 1,006 initial citations. Of those, 683 were for first excluded; 18 studies were duplicates. On the remaining 305 studies, only eight studies were selected on the basis of the title and the abstract. At the end, only seven studies met the inclusion criteria.

**RESULTS.** A total of 109 patients reporting their own experience. Data were collected by using interviews conducted in different times: in one study patients were interviewed in the ICU when possible, but generally all patients were interviewed after ICU discharge or immediately after hospital discharge in their homes within a variable. Three studies used a phenomenology approach, one a narrative approach, two hermeneutic approach and one a qualitative approach with semi-structured interviews. An abstraction process aiming at generating a conceptual diagram was performed. A total of 13 categories emerged categorized in five main themes: (1) *Environment* (nursing activities, acceptable sounds, frightful or disturbing sounds), (2) *Emotions and feelings* (fear/concerns, state of abandon, inexplicable insomnia), (3) *Perception of safety* (feeling to be safe/unsafe), (4) *Consequences of disease/ICU* (physical pain, time/space disorientation) and (5) *Patients' abilities* (inability to move, inability to talk). Finally, a meta-summary to calculate manifest frequency and intensity effect sizes was achieved.

**CONCLUSIONS.** Future qualitative research should focus on the experiences of ICUs' patients of night time and sleep deprivation for better representing this scenario. However, this metasynthesis can represent good suggestions for clinical practice because it is born from the original patients' perspectives in different cultural contexts.

**REFERENCE(S)**

Sandelowski, M., & Barroso, J. (2006). Handbook for synthesizing qualitative research. Springer Publishing Company.

### 0720

#### Accuracy and precision of non-invasive thermometers in comparison to pulmonary artery temperature: a systematic review and meta-analysis

R.L.R. Carvalho[1], J.L.P. Neto[1], J.R. Santos[1], M.A. Victoriano[1], G.N. Andrade[2], F.F. Ercole[1]
[1]Universidade Federal de Minas Gerais - UFMG, Enfermagem Básica, Belo Horizonte, Brazil; [2]Universidade Federal de Minas Gerais - UFMG, Midwifery, Pediatric and Public Health Nursing, Belo Horizonte, Brazil
**Correspondence:** R.L.R. Carvalho
*Intensive Care Medicine Experimental* 2018, **6(Suppl 2):**0720

**INTRODUCTION.** Temperature management is an essential tool in critical care patients. The determination of the right temperature can provide the clinical team information to screen patients for infections or to identify and treat undesirable states of hiper or hypothermia. When available, invasive thermometers, such as pulmonary artery (PA) and vesical temperature, are reliable and can be use to monitor patients temperatures. However, many times invasive thermometers aren't being used and the medical and nurse staff must rely in non-invasive thermometers. In currently literature there isn't a consensus about accuracy and precision of methods such oral, axillary, tympanic membrane (TM), temporal artery (TA) and new methods like the Zero Heat Flux (ZHF) temperature.

**OBJECTIVE.** Therefore, this meta-analysis was conduct to identify the accuracy and precision of non-invasive thermometers in comparison to PA temperature.

**METHODS.** The review project was registered on PROSPERO (CRD42018089447). Studies were identified through a systematic search using PubMed, BVS/BIREME, EMBASE; CINAHL and WOS. Studies that compare any of the 5 non-invasive temperatures methods studied (oral, axillary, tympanic membrane, temporal artery temperature and Zero Heat Flux) with the pulmonary artery temperature were included in this review. Election was performed by three independent researchers and included the following criteria: trials and observational studies; studies that compare PA temperature with non-invasive temperatures (oral, axillary, tympanic membrane, temporal artery and zero heat flux); studies that report the mean difference of the PA measurements and the non-invasive temperature measurements, and their standard deviation. The data were analyzed in Meta Essentials workbook. Fixed or random effects models effect model was used to derive the meta-analytic estimates. The effect measure was calculated by the standardized mean difference, all with a 95% confidence interval (CI). The heterogeneity was evaluated by the chi-square test and the I2 statistic. To explore the heterogeneity and potential factors that could modify the measure of effect, subgroup analyzes were performed for hyperthermia and hypothermia, when available data.

**RESULTS.** We selected 57 studies in 41 publications. The studies involved a total of 2806 and 48048 temperatures measurement (789 - oral; 4189 - axillary; 6013 - TM; 874 - TA; and 36183 - ZHF). The effect size (ES) and standard error (SE) of non-invasive methods compared with PA were: oral ES: 0.21, SE: 0.08 (CI95% 0.02 - 0.41); axillary ES: 0.35, SE: 0.06 (CI95% 0.22 - 0.47); Tympanic Membrane ES: 0.05, SE: 0.05 (CI95% -0.07 - 0.16); Temporal Artery ES: 0.22, SE: 0.26 (CI95% -0.49 - 0.93); Zero Heat Flux ES: 0.04, SE: 0.03 (CI95% -0.11 - 0.19).

**CONCLUSION.** The non-invasive methods showed a good clinical agreement from the PA temperature. Oral and TM were the most accurate methods and temporal artery was the least accurate one.

### 0721

#### The impact of follow-up visits and diaries on patient outcome after ICU discharge: a descriptive and explorative study

E. Åkerman[1,2], A. Langius-Eklöf[2]
[1]Karolinska University Hospital, Perioperative Medicin and Intensive Care PMI, Stockholm, Sweden; [2]Karolinska Institute, Division of Nursing, Department of Neurobiology, Care Sciences and Society, Stockholm, Sweden
**Correspondence:** E. Åkerman
*Intensive Care Medicine Experimental* 2018, **6(Suppl 2):**0721

**INTRODUCTION.** Interventions such as follow-up clinics and diaries have been developed to support the patients during their recovery after ICU stay, but the evidence about impact of patients' outcome is sparse

**OBJECTIVES.** To explore the possible effects of ICU follow up visits and the keeping of diaries and patients´ experiences of follow-up visits and diaries after discharge from the ICU.

**METHOD.** Adult patients with a length of ICU stay for 24 hours or more, responded to the questionnaire 3-set 4P. The questionnaire was sent out at 2,6 and 12 months post ICU discharge. The patients outcome scores were analysed in groups depending on weather they have had /not had follow-up visits or diaries.

**RESULTS.** A majority of the patients experienced the follow-up-visit and the diary valuable and helpful during their recovery even if no strong effects of the follow up on physical or psychosocial problems were detected. The outcome of the survey did not show uniform results and thus, the follow-up group reported more problems in the domains Mood and Memory at 12 months. The diary group reported more problems in the domains, Change in appearance, Mood, Memory and less problems in Social life. Longer LOS was found a predictor for unfavourable scoring in the domains Change in appearance, and Memory but showed a favourable scoring in the domain Social life.

**CONCLUSION.** A strong overall effect of follow-up or diary on patients' outcomes were not found. However, the patients experienced that the interventions was helpful during their recovery and the results also to showed beneficial indications in social function. Thus, further exploration of ICU follow up procedures are needed in the on going process to enhance and coach the recovery of former ICU patients.

### 0722

#### Nurse leaded intravenous (IV) team in a post surgical critical care unit: evaluation of the insertion technique of peripherally inserted central catheters (PICC)

N. Cortes Rey, S. Vecino Vazquez, M. Porteiro Sanchez
Hospital Universitario de A Coruña, Reanimation- Post Surgical Critical Care Unit, A Coruña, Spain
**Correspondence:** N. Cortes Rey
*Intensive Care Medicine Experimental* 2018, **6(Suppl 2):**0722

**INTRODUCTION.** Before implementing a new evidence-based (EB) protocol of selection and management of vascular access devices[(1)], peripheral venous capital of middle/long stay patients at the post-surgical critical care unit of University Hospital of A Coruña was frequently exhausted. Sometimes central accesses were damaged as well. In September 2015 it was created an IV Team initially constituted by 5 nurses. Progressively the team expanded to 10 nurses. All of them received training in choice and management of vascular accesses. The training for PICC insertion was performed according to WoCoVA recommendations [(2)](study of theory, practice in phantoms, supervised practice in real patients, insertions without tutor and finally, audit of technique). They also learnt to use a cost / effective catheter tip location system (Sherlock 3 CG©) based on study of intracavitary electrocardiography (EKG).

**OBJECTIVES.** To evaluate the quality of the implementation of the PICC insertion technique by the IV Team after two years of work.

**METHODS.** Analysis of PICC insertion data from September 2015 to 2017. Quality indicators: nº of PICC inserted with US guided punction, nº of exit site in Dawson's red zone[(3)], nº of catheter /vein ratio < 45%, nº of shots needed, accuracy of EKG detection system.

**RESULTS.** 311 PICC were successfully inserted to post-surgical critical patients. (94.22% of the total attempts). All of them were inserted with US- guided technique. 36 were inserted without tip location system (were checked by X- ray). Sherlock 3 CG system was accurate in >95% of patients without P-wave pathology. 100% of PICC had a catheter/vein ratio < 45%. Zero PICC had the exit site in Dawson's red zone. 66.7% were inserted in one shot, 23.5% in 2-3 shots and 9.8% needed more than 3 shots.

**CONCLUSIONS.** The IV Team has a high level of efficiency during PICC insertion. WoCoVA criteria for training is highly recommended in order to perform a safe technique. Working with an EB-protocol helps to offer high quality care. Education and practice are essential for nurses in charge of inserting and caring vascular accesses. The use of an EKG tip location system like Sherlock 3CG allows the immediate utilization of the PICC and reduces possible costs and risks of re-location in patients without P-wave pathology.

##### REFERENCE(S)

1. Pittiruti M, Scoppettuolo G. Manual GAVeCeLT sobre catéteres PICC y MIDLINE: Indicaciones, inserción, mantenimiento y gestión. Edra; 2017. 405 p.
2. Lamperti M, Bodenham AR, Pittiruti M, Blaivas M, Augoustides JG, Elbarbary M, et al. International evidence-based recommendations on ultrasound-guided vascular access. Intensive Care Med. 2012 Jul;38(7):1105-17.
3. Dawson RB. PICC Zone Insertion MethodTM (ZIMTM): A Systematic Approach to Determine the Ideal Insertion Site for PICCs in the Upper Arm. The Journal of the Association for Vascular Access. 2011 Jan 1;16(3):162-5.

##### GRANT ACKNOWLEDGMENT

This work was not funded by any public or private organization.

### 0723

#### Sustainability in critical care practice

H. Baid
University of Brighton, School of Health Sciences, Brighton, United Kingdom
*Intensive Care Medicine Experimental* 2018, **6(Suppl 2):**0723

**INTRODUCTION.** The ability to maintain the resources needed to sustain high-quality, critical care practice is challenging. There are strategic plans in place for the National Health Service in England to become more sustainable [1]. However, there is minimal research into what the concept of sustainability means to practitioners working in critical care and a theoretical framework to explain processes related to sustainability and critical care is lacking.

**OBJECTIVES.** This research study aimed to develop a theory of sustainability in critical care practice to address the following two questions:

- How is sustainability constructed by practitioners working in critical care?
- What are the social processes involved in making sustainability a component of critical care practice?

**METHODS.** The qualitative research design followed Charmaz´s [2] constructivist grounded theory approach. Data were collected from in-depth, semi-structured interviews (*n*=11) and the participants were nurses, physiotherapists and a technician working in critical care units in the South of England.

**RESULTS.** The research participants viewed sustainability as maintaining necessary financial, environmental and social resources across the micro, meso and macro systems of critical care practice. Decision-making related to *satisficing* [3] was identified as the most pertinent process enabling the sustainability of critical care practice. *Satisficing* was when a decision-maker reached a satisfaction goal that quality care was given, but still sufficed within the limits of available financial, environmental and social resources. Emerging from the data was a continuum where increased *satisficing* facilitated critical care practice to fulfil normative, responsible, sustainable and flourishing stages [4]. *Bounded rationality* was another significant concept which explained the cognitive and environmental factors influencing 'how' *satisficing* occurred [3]. Finally, *stewarding* was 'why' practitioners satisficed as an ethical drive to use resources responsibly.

**CONCLUSIONS.** The grounded theory generated from this research defined sustainability from the perspective of people working in critical care practice and presented a theoretical framework to explain the social processes involved with embedding sustainability into critical care practice.

**REFERENCE(S)**

1. Sustainable Development Unit (2014) Sustainable development strategy for the NHS, public health and social care system 2014-2020. Sustainable Development Unit, Cambridge
2. Charmaz K (2014) Constructing grounded theory. SAGE Publications, London
3. Simon, HA (1997) Administrative behavior: A study of decision-making processes in administrative organizations. 4th edn. The Free Press, New York
4. Hoveskog M, Halila F, Mattson M, Upward A, Karlsson N (2018) Education for sustainable development: business modelling for flourishing. J Clean Prod 172:4383-4396

### 0724

#### A concept analysis of discomfort: differentiating pain and discomfort

S. Ashkenazy[1], F. Dekeyser Ganz[2]
[1]Henrietta Szold School of Nursing, Hebrew University, General Intensive Care Unit, Jerusalem, Israel; [2]Henrietta Szold School of Nursing, Hebrew University, Faculty of Medicine, Jerusalem, Israel
**Correspondence:** S. Ashkenazy
*Intensive Care Medicine Experimental* 2018, **6(Suppl 2):**0724

**INTRODUCTION.** Comfort and discomfort can be recognized within the nursing clinical context. Discomfort is a concept found in the literature, usually related to pain. While some sources do not distinguish between pain and discomfort, others refer to different sources of discomfort. Therefore there is a lack of conceptual clarity. Concept analysis is a strategy that lets us explore the attributes or characteristics of a concept and allows us to decide which phenomena are good examples of the concept and which are not. This type of analysis can promote communication among colleagues, theory development and research. Concept analysis also is effective for tool development (Walker & Avant, 1983) and allows for proper patient assessment, diagnosis and treatment.

**OBJECTIVES.** The objective of this work is to present a concept analysis of discomfort

**METHODS.** We used full text articles published between 1970- 2016 in English to characterize "discomfort". Articles were taken from CINAHL, Medline and PsycNet databases.A total of 7,406 articles and 120 abstracts were identified for evaluation. After initial review, 42 articles were further analyzed. Two reviewers independently evaluated the selected publications.

**RESULTS.** Discomfort can be divided into two main domains, physical discomfort and psychological and is characterized by an unpleasant feeling resulting in a natural response of avoidance or reduction of the source of the discomfort. Pain is one of the main causes for discomfort found in the literature but not every discomfort can be attributed to pain. It is identified by self-report or observation. Discomfort in non-communicative patients is assessed and measured by behavioral expression, which can misinterpreted as pain and agitation in some conditions. In a non-communicative patient, like critically ill patient on mechanical ventilation sometimes, we can assume pain as the reason for patient behavior while other reasons for discomfort are exist.

**CONCLUSIONS.** In order to provide optimal quality of nursing care, a clarification of the concept discomfort will leads to a more accurate theoretical and operational definition and can help nurses to make more accurate nursing diagnoses. Misinterpretation of patient behavior might lead to inappropriate treatment of other symptoms, like pain, such as administering an analgesic when the patient is uncomfortable but not in pain.

**REFERENCE(S)**

Walker, L.O.& Avant, K. C. (1983). *Strategies for theory construction in nursing.* 2nd edition. Appleton & Lange Prentice Hall, Norwalk, Connecticut.

### 0725

#### The challenge in decreasing central line associated bloodstream infections (CLABSI)

T. Sonin Yehuda, T. Yaav, V. Nudelman, N. Shimoni, N. Pines- Zada, I. Tal, G. Rahav, G. Regev- Yochay, Y. Haviv
Sheba Tel HaShomer, Ramat Gan, Israel
**Correspondence:** T. Sonin Yehuda
*Intensive Care Medicine Experimental* 2018, **6(Suppl 2):**0725

**INTRODUCTION.** The respiratory intensive care unit at the Sheba Medical Center at Tel Hashomer treats a wide range of complex patients who require a multi-disciplinary staff during their hospitalization.

Patients are monitored with many central line catheters, which are a source of entry and growth for external pollutants, in addition to low risk factors and immune status, which are unable to cope with further infection, resulting in increased morbidity and mortality of patients.

The data from the hospital´s Infection prevention unit were shown to be alarming in CLABSI cases. Therefore, an intervention program for CLABSI prevention was presented in cooperation with the intensive care unit and the infection prevention unit in Tel Hashomer.

The purpose of the intervention program was to implement wise and continuous use of a central line catheter to reduce the CLABSI rate below 4 cases per 1000 catheter days.

**OBJECTIVES.** Reducing central line associated bloodstream infections (CLABSI) rate to zero.

**METHODS.** A management meeting was set up between the ICU head nurse, the infection prevention manager and the Infection

Prevention unit team. To plan a nursing intervention program included simulation, individual training, implementation of CLABSI bundle.

A preliminary study with 3M Company which provided to the unit 3M™ Curos™ disinfecting caps for needless connectors.

**RESULTS.** The CLABSI rate for 1000 catheter days in 2015 was 3.9, whereas the CLABSI rate for 2016 was 2.58 cases per 1,000 catheter days and in 2017 was 1.7 per 1000 catheter days. The teams use Alcohol caps with compliance rate over 85% since the beginning of the implementation.

**CONCLUSIONS.** In the general intensive care unit at Sheba, where the use of a central line catheter is high, CLABSI prevention is challenging due to an intensive work environment and extensive use of the catheter, and therefore it can be concluded that the intervention of the caregiver in the reduction of CLABSI cases was proven by an increase in the efficiency of the nursing work methods such as the introduction of alcohol caps for needleless connectors, training days and deceptive methods of practice using simulations.

**REFERENCE(S)**

- Naomi P. O´Grady, M.D.1, Mary Alexander, R.N.2, Lillian A. Burns, M.T., M.P.H., C.I.C.3, E. Patchen Dellinger, M.D.4, Jeffery Garland, M.D., S.M.5, Stephen O. Heard, M.D.6, Pamela A. Lipsett, M.D.7, Henry Masur, M.D.1, Leonard A. Mermel, D.O., Sc.M.8, Michele L. Pearson, M.D.9, Issam I. Raad, M.D.10, Adrienne Randolph, M.D., M.Sc.11, Mark E. Rupp, M.D.12, Sanjay Saint, M.D., M.P.H.13 and the Healthcare Infection Control Practices Advisory Committee (HICPAC)14, Guidelines for the Prevention of Intravascular Catheter-Related Infections; CDC, 2011.

**GRANT ACKNOWLEDGMENT**

Shimoni Nir, Nudelman Valery, Nani Pines-Zada, Ilana Tal, Galia Rahav, Gili Regev-Yochay, Haviv Yael, Racheli Levi.

### 0726

#### A comparison of international ICU nurse involvement in EOL decisions: 2002 versus 2017

J. Benbenishty[1], F. Dekeyser-Ganz[1], A. Robertsen[2], J. Ulrich[3], ETHICUS II
[1]Hadassah Hebrew University Medical Center, Jerusalem, Israel; [2]Oslo University Hospital, Oslo, Norway; [3]Kommunalunternehmen Klinikum Augsburg, Munchen, Germany
**Correspondence:** J. Benbenishty
*Intensive Care Medicine Experimental* 2018, **6(Suppl 2):**0726

**INTRODUCTION.** Reports in the literature show a change in end-of-life care practices in recent years. Nurses are important in ICU teams, but there are variations around the world in their role. If nurses were included and to what degree they participate in the different steps of end-of life processes were some of the questions raised in the ETHICUS study (study of end-of life practices). In 2001, Ethicus I studied 31,417 patients admitted to 37 adult ICUs in 17 countries. Ethicus II (2017) provides new information concerning worldwide trends in end-of-life decision making processes.

**OBJECTIVES.** To compare results about nurse involvement in end of life decision making processes in ICUs worldwide (ETHICUS I compared with EHICUS II)

**METHODS.** The same methodology was used in ETHICUS I and ETHICUS II: All consecutive adult patients admitted to ICU over a six month period who died or had life-saving therapy limitations were prospectively studied. Patients were followed until discharge from ICU, death or 2 months from the decision to limit therapy. Data were collected through an internet questionnaire. Ethicus II included 22 of the original sites. Four study questions related to nurse involvement in the decision-making process were analyzed. These questions were: Were nurses involved in *the discussion* about whether to withhold, withdraw or shortening the dying process? Were nurses involved in *making the decisions*? Did a nurse initiate the EOL question? Did MDs and nurses agree about EOL decisions? In addition we looked at the question: Did nurses participate in data-collection?

**RESULTS.** In Ethicus I, 0% nurses contributed data as opposed to 859 nurses (6.7%) in Ethicus II. In the majority of cases, there was agreement between the physicians and nurses about the decision Ethicus I: 63% in southern European countries to 98% in northern European countries; Ethicus II: average 67.9%. However there up to 36% cases where the answer selected was nurse involvement was "not applicable" in Ethicus I and 12.9% in Ethicus II.

**CONCLUSIONS.** In this preliminary data analysis a difference in EOL decision making process, nurse involvement and nurses contributing to data collection was found. During the past decades an increased awareness and an escalation of nurse participation in EOL involvement.

### 0727

#### Organised nurse teaching program by senior ICU nurses improves nurse led cardiac output monitor guided management of severe septic shock

M. Di Pierro, H. Donald, R. Mahroof
Addenbrooke's Cambridge University Hospitals NHS Trust, John Farman ICU, Cambridge, United Kingdom
**Correspondence:** M. Di Pierro
*Intensive Care Medicine Experimental* 2018, **6(Suppl 2):**0727

**INTRODUCTION.** Patients(pts) with complex acute problems & chronic co-morbidities get admitted to ICUs. Effective & successful delivery of care in this challenging group of pts needs a multi-disciplinary approach, from medical & nursing teams.

GPICS require 1:1 nurse ratio for Level 3 ICU pts. Doctors provide management (mx) plans, but bedside event-by-event mx of multiple organ support is done by a nurse assigned to that pt. Understanding & managing various forms of isolated/combined forms of shock can be daunting. Cardiac output monitor (COM) guided mx may be beneficial, if treating clinicians are competent to make appropriate responses to the derived data. Teaching, training & maintaining COM skills amongst nurses can be an arduous task; given that COMs are used in a small subset.

We report a narrative description of how a structured CPD program devised & run by senior ICU nursing staff for ICU nurses has developed a culture of supportive learning,teamwork & confidence in managing complex pts in a busy tertiary academic ICU. An example of COMs in severe septic shock has provided encouraging results.

**OBJECTIVES.** To review the structural organisation of nursing team in a tertiary ICU focusing on the role played by the practice development team (PDT) in maintaining a good level of practice in the use of minimally invasive COMs (LiDCO/PiCCO).

**METHODS.** Clinical & biochemical data for 75 pts with severe septic shock on >0.1 mcg/kg/min of norepinephrine (NE) were analysed. Pts with COM guided mx were compared to who didnt. Improvement in biochemistry used as surrogates for improving shock. PDT senior staff were interviewed to ascertain organisation & delivery of teaching & support for COM guided shock mx.

**RESULTS.** 34/75(45%) had a COM. COM vs non-COM, median NE start dose 0.17vs0.14 mcg/kg/min,p=0.0042, multiple vasopressor 38vs5%,p=0.0003 & inotropes 35vs2%,p=0.0002. Median time to resolution by survival analysis for metabolic acidosis (MA), lactataemia (L)(< 2) & NE (< 0.05 mcg/kg/min) in COM vs non-COM: MA 48vs24hrs (HR & 95% CI)(HR 0.87(0.4-1.6),p=0.56), L 32vs16hrs (HR 0.85(0.45-1.59),p=0.62), NE 54vs30hrs (HR 0.36(0.21-0.62),p=0.0002). P values log-rank test.

130 ICU nurses led by 4 senior PDT nurses with a series of compulsory (funded) lectures/practical demos/regular study days. Combination of theory & practical bedside teaching is used to embed & develop confidence in using shock/COM data, & good fluid & ino-pressor mx. Starters have 6 wk bedside induction pre-clinical work - update core-skills on a regular basis & advance to completing a university-based ICU course +/- higher degree.

**CONCLUSIONS.** COM pts had more pressor/inotropes, but they resolve their acidosis & lactataemia within a time-frame which is not significantly different to non-COM pts. Nurse led analysis & mx of COM guided shock appears appropriate. Structured & well supported CPD for nurses has beneficial impacts in managing complex adjuncts & monitoring aids in multiple organ failure.

### 0728

#### An investigation of the cultural perspectives of a group of patients and relatives on blood donation and blood transfusion in Turkey

A. Koyuncu[1], A. Yava[1], F. Eti Aslan[2], D. Çınar[3], N. Olgun[1]
[1]Hasan Kalyoncu University, Faculty of Health Sciences, Nursing Department, Gaziantep, Turkey; [2]Bahcesehir University, Faculty of Health Sciences, Nursing Department, Istanbul, Turkey; [3]Balikesir military hospital, Balıkesir, Turkey
**Correspondence:** A. Koyuncu
*Intensive Care Medicine Experimental* 2018, **6(Suppl 2):**0728

**OBJECTIVE.** It is aimed to examine the religious and cultural perspectives of patients and their relatives on blood donation and blood transfusion in an outpatient clinic area of an education and research hospital.

**MATERIALS AND METHODS.** The study was a descriptive study with 320 volunteers and relatives conducted between 3 March and 10 April 2015. The research data were collected through a data collection form questioning the introductory features, religious and cultural perspective on blood donation and blood transfusion. $p < 0.05$ was accepted as a statistical significance level.

**FINDINGS.** The average age of the participants was 39.71 ± 14.17 years, 59.1% of them were male, 35.3% of them had graduated from higher education, 68.1% of the participants´ residence and the birthplace of 51.3% were the Central Anatolia region. 56.9% of the participants actively work, 54.4% are married and 80.9% have health insurance. 60.3% of participants were Muslim. 18.1% did not want to explain their religious belief. 55.9% of the participants had never donated blood before. When inquired about the significance of blood donation in their beliefs ; 80.9% stated that it is advisable according to their religion and 15.9% said that they had no knowledge. When asked about the cases in which blood transfusions are required for relatives, 73.8% of the participants stated that they would "approve immediately" while 24.4% would "only approve in an emergency". 92.5% of the participants stated that being informed on blood transfusion has effect on their giving permission. It was found that participants would like the recipient to be informed before the transfer about whether the precautions were taken for diseases such as AIDS and hepatitis (97,5%) risks posed by blood transfusion (89,9%), alternative treatments (74%), and whether the blood transfusion will alter the genetic properties of the recipient (64,4%). When blood transfusion was required for the patient or his / her relatives, differences on the approval or disapproval of the procedure rate according to sex, region of residence, marital status, the difference were not statistically significant ($p > 0.05$). According to education status, the rate of approval for blood transfusion was higher in those with upper secondary education (Chi-square = 17.044, $p = 0.002$).

**RESULT.** It was concluded that the religious and cultural perspectives of the participants affected the thoughts and attitudes towards blood donation and blood transfusion and that there was a correlation between education level and likeliness of approval for blood transfusion, in that those with higher education were more reluctant in approving blood donation. It has been assessed that information tailored to the needs and cultural and religious perspectives of people may be influential on attitudes towards blood donation and blood transfusion and that it may increase the approval rate for blood transfusion when necessary.

### 0729

#### Comparison of endotracheal tube cuff pressure values before and after training seminar in ICU

T. Skladzien[1], W. Szpunar[1], M. Cwynar[2], W. Serednicki[1], J. Wordliczek[1]
[1]Jagiellonian University, Anaesthesiology and Intensive Care, Cracow, Poland; [2]Jagiellonian University, Department of Internal Medicine, Cracow, Poland
**Correspondence:** T. Skladzien
*Intensive Care Medicine Experimental* 2018, **6(Suppl 2):**0729

**INTRODUCTION.** Endotracheal tube cuff pressure needs to be kept within the best range to maintain ventilation and prevent aspiration while functioning to assist in perfusing the lungs [1]. The endotracheal tube cuff pressure must be maintained in a certain range to reduce the risk of aspirations of secretions, which may cause VAP. According to Sole, Su, et al. [1], a minimum pressure of 20 cmH2O is suggested for the prevention of VAP.

**OBJECTIVES.** A aim of the study is to compare infrequent endotracheal tube cuff pressure monitoring (once a day) with frequent endotracheal tube cuff pressure monitoring (three times per day).

**METHODS.** The study was to consist of two groups of patients treated in the Intensive Care Unit, mechanically ventilated. The first group (Group 1) were to be whom the pressure in the balloon is measured once a day by nurses. Additional measurement performed by authors and its value was recorded. In one patient the interval between measurements was at least 2 days. Then, a questionnaire and training on the correct balloon pressure was conducted among doctors and nurses. The procedure of the balloon pressure rate was changed. In patients, the nurses evaluated three times a day at 6, 14 and 22 (Group 2). Additional measurement performed by authors and its value was recorded. In one patient the interval between measurements was at least 2 days. At each measurement, if there was such a need, the pressure was corrected to the correct value (20-30 cmH2O).

**RESULTS.** There were 188 patients aged 18-89 qualified for the study. In group I, 45 patients had 141 measurements, and in group 2 in 73 patients 506 measurements were made.

The mean balloon pressure in Group 1 was 27 ± 20 cmH2O. Only 28 (20%) measurements showed correct pressure value. In 73 (52%) measurements, the pressure was below the accepted standard, and in 40 (28%) cases above norm. In Group 2, the average pressure was 25 ± 11 cm H2O ($p < 0{,}001$). The correct balloon pressure was obtained in 376 (74%) measurements. The cuff pressure was too low in 77 (15%) cases, and too high in 53 measurements (11%).

**CONCLUSIONS.** More frequent cuff pressure monitoring was associated with better control of cuff pressure.

**GRANT ACKNOWLEDGMENT**
None

### 0730

#### Using a structured judgement review methodology to evaluate care following discharge from ICU

S. Vollam[1], O. Gustafson[2], P. Watkinson[1]
[1]University of Oxford, Nuffield Department for Clinical Neurosciences, Oxford, United Kingdom; [2]Oxford University Hospitals NHS Trust, Adult Intensive Care Unit, Oxford, United Kingdom
**Correspondence:** S. Vollam
*Intensive Care Medicine Experimental* 2018, **6(Suppl 2):**0730

**INTRODUCTION.** The mortality rate for patients discharged from ICUs in the UK is higher than other hospitalised groups considered to be at high risk, and is more than five times the annual number of UK road traffic accident deaths[1]. The Recovery Following Intensive Care Treatment (REFLECT) study is a UK based multicentre mixed methods exploratory study examining ward care delivery to patients discharged from intensive care. One of the methods used to evaluate ward care delivery was a Retrospective Case Record Review (RCRR). This approach has been used extensively in other patient groups, but not previously in this population.
**OBJECTIVES.** To develop a multidisciplinary approach to evaluating care following ICU discharge using a Retrospective Case Record Review technique as part of the REFLECT study.
**METHODS.** Medical notes of patients transferred to wards from ICU and subsequently died were examined using an RCRR technique. This method is currently being adopted by the Department of Health (DoH) as a clinical governance tool within trusts as the National Mortality Care Record Review Programme. This study used an adaptation of the validated tool used by the DoH for making safety and quality judgements about care delivery. Structured judgement statements were explicit, value-based comments on care delivery. The output is a relatively short but rich account of care delivery, identifying both good and poor care, with all records allocated an 'avoidability of death score'. Cases where differences in care delivery could improve outcomes were further analysed using the 'change analysis' method. The analysis allowed identification of areas where novel care processes could change patient outcomes.
**RESULTS.** 300 medical records from 3 separate UK NHS sites were reviewed by a multidisciplinary team consisting of an ICU consultant, nurse, follow-up practitioner and physiotherapist. All staff attended the NHS Structured Mortality Review Training prior to undertaking RCRR. 30 medical records scored sufficiently to undergo an in-depth qualitative analysis.
**CONCLUSIONS.** A multidisciplinary case records review of patients discharged from ICU was a successful approach in identifying areas of care where changes could alter patient outcome. The multidisciplinary approach enabled identification of a diverse range of care aspects for further exploration.

#### GRANT ACKNOWLEDGMENT

This abstract presents independent research funded by the National Institute for Health Research (NIHR) under its Research for Patient Benefit (RfPB) Programme (Grant Reference Number PB-PG-0215-36149). The views expressed are those of the authors and not necessarily those of the NHS, the NIHR or the Department of Health.

## Calories & vitamins delivery

### 0731

#### Influence of nutrition support therapy on readmission among patients with acute heart failure in the intensive care unit: a single-center observational study

I. Miyajima[1,2], T. Yatabe[1], T. Tamura[1], M. Yokoyama[1]
[1]Kochi Medical School, Department of Anesthesiology and Intensive Care Medicine, Nankoku, Japan; [2]Chikamori Hospital, Kochi, Japan
**Correspondence:** I. Miyajima
*Intensive Care Medicine Experimental* 2018, **6(Suppl 2):**0731

**INTRODUCTION.** The effect of nutrition support therapy on long-term prognosis, such as readmission, among acute heart failure patients in an intensive care unit (ICU) setting remains unclear due to lack of evidence.
**OBJECTIVES.** We hypothesized that nutrition support therapy in the acute phase might affect readmission rate among acute heart failure patients admitted to the ICU. Thus, we conducted a single-center prospective observational study to verify this hypothesis.
**METHODS.** Consecutive patients diagnosed with acute heart failure and admitted to the ICU between April 2016 and March 2017 were enrolled in this study. Of these, patients admitted for more than 14 days were included in the analysis. The primary outcome was the relationship between nutritional intake and hospital readmission at 180 days after discharge. We divided the participants into 2 groups: patients who were not readmitted to hospital within 180 days after discharge (non-readmission group) and patients who were readmitted within this timeframe (readmission group). Data were expressed as median (interquartile range). A p value $< 0.05$ was considered statistically significant. Per the study protocol, variables with p values of $< 0.05$ in the univariate analysis were included in the multivariate analysis.
**RESULTS.** A total of 230 patients remained in the hospital for ≥14 days and 103 patients (44.8%) required readmission within 180 days after hospital discharge. The median age and body mass index were similar in both the readmission and non-readmission groups [84 (75, 88) vs 84 (77, 90) years; 22.6 (20.2, 24.8) vs 22.0 (19.1, 24.4) kg/m$^2$, respectively]. The median BNP values were also similar [732 (465, 1197) vs. 844 (420, 1316) pg/mL]. The median duration of ICU were not significantly different [7 (4, 10) vs. 6 (4, 8) days]. Serum creatinine levels were significantly higher in the readmission group than in the non-readmission group [1.0 (0.7, 1.3) vs. 1.3 (0.9, 1.8) mg/dL, p = 0.003]. The calorie and protein intake on day 3 after ICU admission in the re-admission group was significantly higher than that in the non-readmission group [25.8 (20.0, 30.2) vs. 20.5 (14.2, 27.8) kcal/kg/day, p=0.002: 0.9 (0.6, 1.1) vs. 0.7 (0.5, 0.9) g/kg/day, p=0.002, respectively]. Similarly, the protein intake values on day 7 was also significantly higher in the readmission group [0.9 (0.7, 1.2) vs. 0.8 (0.6, 1.0), p=0.04, respectively]. Multivariate analysis indicated that total caloric intake on day 3 was an independent factor affecting readmission (odds ratio=1.03, 95% confidence interval=1.00-1.06, p=0.04).
**CONCLUSIONS.** Our data showed that nutrition support therapy in the acute phase might affect readmission among acute heart failure patients. Furthermore, total caloric intake on day 3 was an independent factor that affected readmission. Further studies should be conducted to refute or confirm our findings.

### 0732

#### Impact of calorie intake in the first week and severity measured by SOFA score in the hospital mortality of critically ill patients

A.B.M. Oliveira[1], R.S.F. Pequeno[2], M.B. Lima[1], V.S. Mendonça[2], T.P. Holanda[2], G.D. Ceniccola[1,2]
[1]Hospital de Base do Distrito Federal, Residência Multiprofissional em Terapia Intensiva, Brasilia, Brazil; [2]Hospital de Base do Distrito Federal, Residência em Nutrição Clínica, Brasília, Brazil
**Correspondence:** G.D. Ceniccola
*Intensive Care Medicine Experimental* 2018, **6(Suppl 2):**0732

**INTRODUCTION.** The critically ill “state” has several characteristics that promote malnutrition, mainly hypercatabolism. Without an adequate nutritional support, there is an energy deficit that sustains the development of lean mass consumption, and therefore the aggravation of this situation. It is believed that the severity of the disease makes energy intake difficult and the deficit degree in the intensive care stay increases the risk of complications and mortality.
**OBJECTIVES.** To evaluate the impact of energy deficit in the first week of hospitalisation and the severity measured by admission SOFA in 60-day mortality in critically ill patients.
**METHODS.** This is a prospective study, in which patient data were collected from electronic health records and followed by trained dietitians until discharge. Inclusion criteria were individuals older than 18 years old, who stayed more than 48h in the ICU. Determination of the caloric intake was estimated through the amount of time in which the patient stayed in *nil per os* (NPO) during the first week of ICU admission. Hence, ingestion in the first week was classified into five energetic quintiles, with a variation of 25% each.
**RESULTS.** From the 300 patients included in the study, 64% were male. The main medical diagnoses found were trauma (31%) and neurological (33.7%). 163 (54.3%) patients received ≥75% of the planned calories for the first week, of whom 36.4% did not survive. Among those who remained on NPO (1.3%), none survived. Mean SOFA for non-survivors was 9.55 points (95% CI 8,66 - 7,58; p = 0.0001), whereas non-survivors had 7,01 points (95% CI 6,44 - 7,58; p = 0.0001). Non-survivors stayed on NPO for 1,57 days on average (± 1,83 [interquartile range = 3]), whilst survivors had a mean of 0,92 days of fasting (± 1,52 [interquartile range = 1]; p = 0,016). We also observed that patients who had an intake ≥75% than the planned for the first week (mean survival of 144 days [95% CI 33.3 - 254]; p = 0.0001) had lower mortality in 60 days compared with those with intake < 75% (mean survival of 72 days [95% CI 51.9 - 92]; p = 0.0001). A logistic regression showed that for each SOFA unit increment, there was a 16% increase in mortality risk (OR 1.16 [95% CI 1.08 - 1.24], p = 0.0001) and for each point of decrease in energy quintile in the first week, there was a 71% increase in mortality risk (OR 1.71 [1.33 - 2.21], p = 0.0001).

**CONCLUSIONS.** Energy deficit in the first week has deleterious effects on 60-day mortality. Survival is greater in patients receiving ≥75% of the energy planned. In addition, the admission SOFA score has a good predictive potential to mortality risk.

**REFERENCE(S)**

Casaer, MP, et al. *N Eng J Med*. 2014; 27;370(13):1227-36.
Elke, G, et al. *Crit Care*. 2014; 18(1):R29
Faisy, C, et al. *Br J Nutr*. 2009; 101(7):1079-87.

**GRANT ACKNOWLEDGMENT**

None.

### 0733

#### Contents of vitamin D, uric acid in the blood serum and characteristics of epidural analgesia for labor in parturient women with preeclampsia

E. Oreshnikov[1], S. Oreshnikova[1], E. Vasiljeva[2], T. Denisova[2]
[1]Chuvash State University, Internal Medicine, Cheboksary, Russian Federation; [2]Chuvash State University, Obstetrics and Gynecology, Cheboksary, Russian Federation
**Correspondence:** E. Oreshnikov
*Intensive Care Medicine Experimental* 2018, **6(Suppl 2):**0733

**INTRODUCTION.** It is known that more than 40% of pregnant women have a deficiency of vitamin D. In addition to the classic symptoms of preeclampsia, more than a quarter of century, many foreign clinicians as preeclampsia indicator used by the hyperuricemia.

**OBJECTIVES.** We study the relationship of pain in childbirth, characteristics of epidural analgesia in patients with preeclampsia and the level of vitamin D, the concentration of uric acid in the blood serum.

**METHODS.** The study group included patients with severe and moderate preeclampsia, alone have given birth vaginally with epidural analgesia. The control group included patients with physiological pregnancy, independently gave birth vaginally with epidural analgesia. The material of the study was to peripheral blood of pregnant women taken at admission to the hospital for delivery. Vitamin D level was performed by enzyme immunoassay kits. The concentration of uric acid was determined spectrophotometrically. Primary study end points defining a base for the conclusions were as follows: the level of vitamin D in ng / ml, the concentration of uric acid in the blood mcmol/l, the average period for delivery systolic and diastolic blood pressure in mmHg, the dose of local anesthetic in mg.

**RESULTS.** In patients with severe preeclampsia revealed: a pronounced deficiency of vitamin D, a tough hyperuricemia, had higher numbers mean arterial pressure during labor epidural analgesia in the background: on average during all periods of childbirth 140/90150/100 mm Hg. In patients with moderate preeclampsia was diagnosed moderate vitamin D deficiency, mild hyperuricemia, blood pressure during childbirth averaged 130/ 90125/85 mm Hg. In the control group the level of vitamin D and the concentration of uric acid were in the normal range, blood pressure during labor averaged 105/60 120/70 mm Hg. The results also gave evidence to suggest that patients with preeclampsia who have previously been registered low levels of vitamin D and an increased concentration of uric acid in the birth have a greater need for a local anesthetic to reduce pain and stabilize blood pressure than patients with higher levels of vitamin D and smaller uric acid.

**CONCLUSIONS.** In women with preeclampsia during childbirth epidural analgesia, low levels of vitamin D and hyperuricemia are associated a higher demand for local anesthetics.

### 0734

#### Vitamin d levels of patients in intensive care unit and its association with nutritional risk screening 2002 score

A. Zerman[1], S. Keskek[2]
[1]Adana Sehir Training and Research Hospital, Intensive Care, Adana, Turkey; [2]Numune Training and Research Hospital, Adana, Turkey
**Correspondence:** A. Zerman
*Intensive Care Medicine Experimental* 2018, **6(Suppl 2):**0734

**INTRODUCTION.** Vitamin d is an immune modulator hormone which has major effects on nearly all cells of the immune system. The deficiency of vitamin d has been found to be associated with many diseases. Malnutrition is a broad term which has been used to describe any imbalance in nutrition. It is commonly encountered in critically ill patients. Early identification of malnutrition among hospitalised patients is essential to institute appropriate patient-specific nutritional strategies.

**OBJECTIVE.** The aim of this study was to investigate the level of vitamin d and its association with NRS (nutritional risk screening) 2002.

**METHODS.** A total of 140 patients from internal medicine were included. All patients were staying in intensive care unit of Adana City Training and Research Hospital. Serum 25(OH)D concentration was measured for vitamin d deficiency. Serum 25(OH)D concentrations were measured by using commercially available enzymelinked immunosorbent kits (Minneapolis, USA). A questionnaire survey was used to investigate the nutritional status of patients (NRS 2002). MedCalc 18.2.1 (MedCalc Belgium) statistical software was used for statistical analysis.

**RESULTS.** The mean age was 71.3±13.3. There were 75 (53.6%) men and 65 (46.4%) women (p=0.398). The mean concentration of 25(OH)D was 15.9±16.9. There was no statistical significant difference between men and women (13.2±9.6 vs. 19.0±22.2, p=0.341). The mean score of NRS-2002 was 3.75±1.8. Both men and women were comparable (3.8±1.7 vs. 3.6±1.8, p=0.791). There was an inverse correlation between 25(OH)D and NRS 2002 score (p=0.03, r=-0.182).

**CONCLUSION.** Malnutrition is associated with poorer clinical outcomes in hospitalized patients. Low concentration of vitamin d may be associated with impaired nutritional status in patients in internal medicine intensive care unit. Patients should be supported with vitamin d. It is better to initiate early nutrition therapy as soon as feasible in critically ill patients requiring nutrition therapy, unless there are significant contraindications.

1 M.S. Sioson et al. / Clinical Nutrition ESPEN 24 (2018) 156e164
2 Nutritional Risk Screening Tools. Clinical Nutrition LAM Initiative (https://www.unidospelanutricaoclinica.com.br).
3 Holick MF. Vitamin D deficiency. N Engl J Med. 2007; 357(3): 266-281.

### 0735

#### The refeeding syndrome in critical care patients: a challenge

Y. Rovira Valles, E. Mor Marco, L. Bielsa Berrocal, V. Philibert, O. Plans Galvany, I. Martínez de Lagran Zurbano, P. Marcos Neira, L. Bordejé Laguna
Hospital Universitari Germans Trias i Pujol, Intensive Care Unit, Badalona, Spain
**Correspondence:** Y. Rovira Valles
*Intensive Care Medicine Experimental* 2018, **6(Suppl 2):**0735

**INTRODUCTION.** Refeeding Syndrome (RS) is the ensemble of clinical and biochemical alterations due to the introduction of nutrition in a malnourished patient, being hypophosphatemia the most specific finding. Critical patients can be at risk of developing RS, and its complications are potentially severe.

**OBJECTIVES.** Descriptive analysis of critical care patients at risk of developing refeeding syndrome (RS).

**METHODS.** Observational study of a cohort of 300 patients followed up during the admission in an ICU (Hospital Germans Trias i Pujol) from July to November 2017. We detected 22 patients at risk of developing RS. We analysed demographic variables, gravity scores, prognostic factors for RS and their evolution. Descriptive analysis: quantitative variables are expressed in means (SD) and medians (min, max) and qualitative variables in proportions.

**RESULTS.** Accumulated incidence of patients at risk of RS is 1.6% each month. Mean age was 61.6 (SD 12) years, APACHE 25.5 (16-48) and BMI 25 (SD 5.7). Mean length of stay was 13.5 (2-52) days, with 8 (0-53) days of mechanical ventilation and 20.8% (CI 95%: 4.8-36.8%) of ICU mortality. Patients were selected because of both clinical suspect of RS (73%, CI 95%: 55-91%) and hypophosphatemia (27%, IC95%: 9-45%). We detected 16 hypophosphatemias (73%, CI 95%: 55-91%), with an mean phosphate value of 1.6mmol/L (SD 0.57). 37.5% of the patients included due to clinical suspicion, did not develop hypophosphatemia, given that 50% of them was in renal failure. Therapeutic adequacy (early/late/never) was analysed: artificial nutrition (AN) was adjusted in the 80% (55%, 25%, 20%); electrolytes were supplemented in the 86% (59%, 27%, 14%) and thiamine in the 90% (72%, 18%, 10%) of the patients.

**CONCLUSIONS.** Refeeding syndrome occurs in critical patients. Its identification is a challenge and its incidence is underestimated, so it is essential to develop a screening tool. Prevention of refeeding syndrome involves electrolyte monitoring, attentive supplementation of micronutrients and progressive nutritional intake.

**REFERENCE(S)**

1. Boateng AA, Sriram K, Meguid MM, Crook M. Refeeding syndrome: treatment considerations based on collective analysis of literature case reports. Nutrition. 2010 Feb;26(2):156-67. 2.
2. Olthof LE, Koekkoek WACK, van Setten C. Impact of caloric intake in critically ill patients with, and without, refeeding syndrome: A retrospective study. Clin Nutr. 2017 Aug 10. pii: S0261-5614(17)30268-6.

### 0736

#### The effect of dexmedetomidine versus midazolam sedation on resting energy expenditure in critically-ill ventilated patients: a randomized controlled study

M. Abdulatif Mohamed[1], A. Mukhtar[1], A. Hasanin[1], S. Fathy[1], W. Ibrahim[2], L. Rashed[2], A. Abougabal[1]

[1]Faculty of Medicine, Cairo University, Department of Anesthesia and Critical Care Medicine, Cairo, Egypt; [2]Faculty of Medicine, Cairo University, Department of Biochemistry, Cairo, Egypt

**Correspondence:** A. Abougabal

*Intensive Care Medicine Experimental* 2018, **6(Suppl 2):**0736

**INTRODUCTION.** Estimation of energy requirements of critically ill patients is essential for providing proper nutritional support. The effect of different sedative drugs on resting energy expenditure (REE) was not well investigated.

**OBJECTIVES.** The aim of this study is to compare the effect of dexmedetomidine (Dex) and midazolam on REE in critically ill patients.

**METHODS.** A randomized, double-blinded, controlled trial was conducted in a tertiary hospital including 30 critically-ill patients. Patients were randomized into either Dex Group (n= 15) (received DEX infusion at a starting dose of0.15 μg/kg//hr), and midazolam group (n= 15) (received midazolam sedation at a starting dose of 1 mg/h). Both medications were infused for 24 hours and were titrated to maintain the Richmond Agitation and Sedation Scale (RASS) scores in a range of −3 to −1. REE was measured using indirect calorimetry. Hemodynamic variables were continuously measured. Serum epinephrine, and serum interleukin-1β were measured at the baseline and after the end the infusion period. Time to extubation, length of stay, and in-hospital mortality were also evaluated.

**RESULTS.** There was a significant decrease in each of REE, oxygen consumption (V02), carbon dioxide production (VC02), serum epinephrine, and serum IL-1β in the 24-hour reading compared to the baseline reading within the two study groups. However, there were no significant differences in REE between both groups. Arterial blood pressure and heart rate were lower in Dex group compared midazolam group. Time to extubation was shorter in Dex group compared to midazolam group.

**CONCLUSIONS.** In conclusion, infusing sedatives to target RASS score between -1and -3 in mechanically ventilated patients reduced the REE by ~ 15%. No difference was reported between the effect of Dex and midazolam on REE; however, Dex infusion shortened the time to extubation.

### 0737

#### Differences in critically ill patients with severe acute pancreatitis between a district general hospital and a tertiary referral hospital in Scotland, UK

C. McCue[1], K. Millar[1], K. Puxty[1], R. Sundaram[2]

[1]Glasgow Royal Infirmary, Intensive Care, Glasgow, United Kingdom; [2]Royal Alexandra Hospital, Intensive Care, Paisley, United Kingdom

**Correspondence:** C. McCue

*Intensive Care Medicine Experimental* 2018, **6(Suppl 2):**0737

**INTRODUCTION.** Severe acute pancreatitis (SAP) accounts for approximately 25% of patients with the disease and invariably requires critical care admission [1]. Overall hospital mortality for pancreatitis is 1% but climbs to 42% for SAP, with a 31% ICU mortality [2]. Disease specific scoring systems have sought to define outcome and severity in SAP, with APACHE II remaining one of the most discriminatory [3].

**OBJECTIVES.** We sought to review any characteristics or outcome differences between patient groups at both sites. Then to analyse all patients to assess any differences between patients divided into 3 outcome groups: early mortality (48 hours), in hospital mortality, and survivors.

**METHODS.** The study period covered admissions from 2009-2017. Royal Alexandra Hospital (RAH), Paisley has 7 Level 3 beds, and the tertiary referral hospital (Glasgow Royal Infirmary, GRI) 12 Level 3 and 8 Level 2. Data was obtained from the ICU clinical database, with search term 'acute pancreatitis' as diagnosis, with permission from the local administrator. Supplemental data was obtained from clinical information systems. Calculations were completed using Excel and Vasser Stats. Readmissions were excluded from data analysis.

**RESULTS.** Table 1 examines differences between all level 3 patients at both sites.

Between the three groups there were no significant differences in age, aetiology, APACHE II and organ support. The group of patients referred to the tertiary centre had a higher proportion of males and gallstone aetiology. Mortality was higher in the tertiary referral centre for primary admissions.

Table 2 describes differences between outcome groups in all level 3 patients excluding those referred from other sites to GRI.

**CONCLUSIONS.** From this dataset we can see a tendency towards increasing age and poorer outcome, and low RRT utilisation seen in the survivor group. Interestingly lower RRT use is seen in the early mortality group compared with in hospital mortality which may reflect resource rationing in clinical futility.The differences in mortality between sites are surprising and merit further interrogation of the cohorts. APACHE II remains a robust measure of severity scoring in pancreatitis with a possibility of further discrimination between early and later mortality.

**REFERENCE(S)**

1. S.E. Roberts, J. G. Williams, D. Meddings, and M. J. Goldacre, Incidence and case fatality for acute pancreatitis in England: geographical variation, social deprivation, alcohol consumption and aetiology—a record linkage study, 2008 Alimentary Pharmacology and Therapeutics, 28(7) 931-941
2. D.A. Harrison, G. D´Amico, and M. Singer. Case mix, outcome, and activity for admissions to UK critical care units with severe acute pancreatitis: a secondary analysis of the ICNARC case mix programme database. 2007 *Critical Care*, 11:1 S1

3. Cho JH, Kim TN, Chung HH et al. Comparison of scoring systems in predicting the severity of acute pancreatitis. 2015 *World J Gastroenterol* 21(8):2387-2394

**Table 1 (abstract 0737).** Differences between level 3 patients at both sites, dividing primary admissions and tertiary referrals to GRI

| CHARACTERISTIC | RAH (n=57) | GRI PRIMARY ADMISSION (n=66) | GRI REFERRALS (n=51) | p value |
|---|---|---|---|---|
| *Median age (IQR)* | 60 (45.8-65.8) | 57 (47-68.8) | 59 (47.5-65.5) | 0.995 |
| *Aetiology* | Gallstones 44%<br>Alcohol 30%<br>Other 26% | Gallstones 36%<br>Alcohol 32%<br>Other 32% | Gallstones 51%<br>Alcohol 24%<br>Other 25% | 0.61 |
| *Median APACHE II (IQR)* | 21 (17-26) | 20 (14.25-27) | 20 (17-24) | 0.41 |
| *Median length of stay (IQR)* | 9.5 (4.6-17.5) | 5 (1.3-14.1) | 14 (6.9-28.5) | <0.001 |
| *Ventilated* | 98% | 92% | 92% | 0.25 |
| *RRT* | 41% | 38% | 37% | 0.17 |
| *Male* | 55% | 56% | 71% | 0.04 |
| *ICU mortality* | 29% | 39% | 25% | 0.03 |
| *Hospital mortality* | 40% | 44% | 33% | 0.02 |

**Table 2 (abstract 0737).** Comparison of outcome groups excluding tertiary referred patients

| CHARACTERISTIC | MORTALITY <48hrs (n=25) | IN HOSPITAL MORTALITY (n=24) | SURVIVORS (n=70) | p value |
|---|---|---|---|---|
| *Median age (IQR)* | 66 (50-71) | 58 (53.3-67.3) | 54.5 (42.5-63) | 0.03 |
| *Male* | 52% | 71% | 56% | <0.001 |
| *Ventilated* | 96% | 96% | 97% | 0.12 |
| *RRT* | 56% | 67% | 27% | <0.001 |
| *Median APACHE II (IQR)* | 27 (24.5-31) | 24 (19.5-31) | 19 (14-23) | <0.001 |
| *Aetiology* | Alcohol 36%<br>Gallstones 32%<br>Other 32% | Alcohol 29%<br>Gallstones 38%<br>Other 17% | Alcohol 31%<br>Gallstones 46%<br>Other 23% | 0.77 |

### 0738

#### Metabolic monitoring of patients in chronic critical illness after brain damage: a pilot study

I. Sergeev, M. Petrova, K. Krylov, A. Shestopalov, R. Yagubyan, A. Sergienko, A. Yakovleva

Federal Research and Clinical Center of Intensive Care Medicine and Rehabilitology, Moscow, Russian Federation

**Correspondence:** K. Krylov

*Intensive Care Medicine Experimental* 2018, **6(Suppl 2):**0738

**INTRODUCTION.** One of the main problems in chronically critically ill patients is development of protein-energy deficiency in the acute phase, which persists in the chronic phase. We did not find any unified recommendations on energy needs in this patients' population. Usage of indirect calorimetry to determine energy needs of patients in chronic critical illness remains controversial. The aim of the stud ywas to compare the energy needs of patients in chronic critical illness defined by indirect calorimetry according to international recommendations and by Harris-Benedict equation.

**OBJECTIVES.** In this prospective cohort pilot study there were included 10 patients in the intensive care unit who fulfilled the inclusion criteria (9 men, 1 woman) with brain damage present more than 30 days after the onset of the disease. The mean age in the study group was 47 ± 16 years. All patients were with tracheostomy placed. On the enrollment majority of patients (9) were self-breathing, and 1 was on ventilator. All patients included in the study were without exhaustion signs. Mean body mass index was 21.6 ± 4.8 kg/m$^2$. All patients received standard high-protein enteral nutrition 35 kcal/kg/day. There was no impairment of liver or kidney function and signs of dysfunction of the gastrointestinal tract in all patients during the study.

**METHODS.** The energy requirements were defined using either the Harris-Benedict equation or indirect calorimetry. The protein needs were defined by nitrogen loss in urine.

**RESULTS.** On average energy requirements determined by indirect calorimetry did not differ from those calculated by the Harris-Benedict equation (p = 0.64). Results for indirect calorimetry were 24.3 ± 6.1 kcal/kg/day (1506.7 ± 300,43 kcal/day) and 23,6 ± 2,8 kcal/kg/day (1474,7 ± 180,17 kcal/day) for Harris-Benedict equation. Although the energy requirements determined by indirect calorimetry were higher in 3 patients. Mean protein requirements were 0.7 ± 0.25 g/kg/day. Despite the fact that patients received more energy (30-35 kcal/kg/day) and protein (1.3-1.5 g/kg/day) than required, mean transferrin level remained rather low 128.6 ± 33, 62 mg/dL (reference: 200-400 mg/dL). Despite the fact that mean body mass index was within normal limits, the circumference of the shoulder muscles was reduced (mean 20.3 ± 1.8 cm).

**CONCLUSIONS.** Usage of indirect calorimetry only without taking into account functionality of the gastrointestinal tract and other factors that affect body composition is not enough to prescribe optimal nutritional support in this patients category. Indirect calorimetry is a good tool for determining the resting energy requirements level in chronically critically ill patients and allows one to specify the boundaries of the patient´s energy needs. Further research is required.

### 0739

#### Effect of glutamine supplementation in polytrauma patients

F. Marchese[1], A. Cotoia[2], M.T. Tucciariello[2], L. Mirabella[2], L. Tullo[2], G. Cinnella[2]

[1]O.O.R.R. di Foggia, Anestesia e Rianimazione, Foggia, Italy; [2]Università degli Studi di Foggia, Anestesia, Rianimazione, Terapia Intensiva e del Dolore, Foggia, Italy

**Correspondence:** F. Marchese

*Intensive Care Medicine Experimental* 2018, **6(Suppl 2):**0739

**INTRODUCTION.** Glutamine plays an important role under stress condition, and it is correlated with immunologic system response and outcome in critically ill patients. The glutamine is becoming formulated suitably in addiction to artificial nutrition.

**OBJECTIVES.** Aim of this study is to observe the immunologic effect of glutamine supplementation in polytrauma patients.

Methods In this prospective observational study we enrolled 42 polytraumatized patients (Injury Severity Score >15) admitted in Intensive Care Unit (ICU), University Hospital of Foggia.

Patients aged under 18 years, with renal failure, hepatic failure, IBD, with steroids treatment, patients undergoing abdominal surgery and pregnant were excluded.

Patients admitted in ICU were randomized to receive enteral nutrition alone (30 Kcal/Kg/die) (G1) or plus intravenous glutamine (0,5 g/kg of ideal body weight) (G2). Glutamine supplementation was given for 12 days.

Blood was drawn at baseline, 4, 8 and 12 days for measurement of of plasmatic CD3+ T Lynfocyties, CD3+/CD4+/CD45+ T Helper Lynfocyties, CD3+/CD8+ T suppressor Lynfocyties , CD19+/CD45+ B Lynfocyties, IL4, IL2, IgA.

P value of 0,05 was considered statistically significant.

**RESULTS.** Serum levels of T CD3+ Lynfocyties increased significantly at any time vs baseline in G2 ($p < 0.003$), while they remained stable over time in G1. Comparisons between groups showed similar T CD3+ Lynfocyties levels at baseline (p=0,7), whilst they significantly increased in G2 vs G1 at T2,T3 and T4 ($p < 0.01$).

In both groups CD3+/CD4+ T Helper Lynfocyties levels increased during the study period especially at T4, while CD3+/CD8+ T suppressor Lynfocyties increased significantly vs baseline only in G2 ($p < 0.01$) . CD19+/CD45+ B Lynfocyties significantly increased at T8-T12 only in G2.

IL2 was stable during the study period in both groups; IL 4 trend increased progressively only in G2 ($p < 0.004$). IgA level increased in G1 from T0 to T3 ($P < 0.05$).

No adverse reactions or oversensitiveness to glutammine supplement were observed.

**CONCLUSIONS.** The preliminary data show that glutamine increased IL4, CD3+/CD4+ T lymphocytes and CD19+/CD45+ B lymphocytes production. Glutamine supplementation may improve T-cell function in the poly-traumatized patients.

**REFERENCE(S)**

1) Gramlich L et al. Does enteral nutrition compared to parenteral nutrition result in better outcomes in critically ill adult patient? A systematic review of the literature. Nutrition, 2004; 20:843-8
2) Rocchetti SI et al. In: Romano E. Medicina Critica, ed UTET
3) Giner M et al. In 1955 a correlation between malnutrition and poor outcome in critically ill patients still exist. Nutrition, 1996; 12:23-9

### 0740

#### A comparative study of advantages of using separate bottle T.P.N. formulae and the 3-in-1 admixture of T.P.N. in critically ill pediatric patients

A. Wahdan

Cairo University, Anesthesia, SICU and Pain Management, Cairo, Egypt

*Intensive Care Medicine Experimental* 2018, **6(Suppl 2):**0740

**INTRODUCTION.** Total parenteral nutrition (T.P.N.) is an essential part in the management of patients with gastrointestinal surgeries, being an effective method for supplying energy and nutrients where oral or enteral feeding is impossible or contraindicated. T.P.N. admixtures are more convenient to the healthcare providers and to the patient. The aim of this work was to compare the outcome of separate bottles of T.P.N. formulae and 3-in-1 admixture of total parenteral nutrition in critically ill pediatric patients.

**PATIENTS AND METHODS.** This study was conducted in the Pediatric surgical intensive care unit, Abu EL Reesh Hospital, Cairo University.

Number of Patients (N=40). Age from 3 months to 12 years after major GIT surgery e.g. intestinal resection, biliary atresia correction, colon bypass surgeries, etc.

**EXCLUSION CRITERIA.** Major organ failure (e.g. Liver or Kidney), Sepsis and shock, associated cardiac disease, Associated juvenile diabetes and DIC.

Patients were randomly divided into 2 groups, each group is (20 patients), taking TPN for 10 days postoperatively.

· Group 1 (N=20), odd numbers of ICU admitted patients will be on separate bottles of TPN including macro and micro-nutrients, including: amino acids (Aminosteril KE,10% Fersenius Kabi), Carbohydrates (Glucose 25%), Lipids (Intralipid 10%), Trace elements and vitamins (Adamel, vitalipid and soluvit), Electrolytes (Potassium and Calcium), Albumin (if needed, according to patient's laboratory findings)

· Group 2 (N=20) even numbers of ICU patients will be on 3-in-1 bags, each bag contains the nutrients needed for the 24 hours for the patient. They will be infused over 24 hours via 1.2 micron filters in a central line connected to the central vein.

Preparation of the 3-in-1 admixtures were done using the laminar flow in the TPN Admixing Unit of the New Kasr El Aini Teaching Hospital according to admixing and stability ranges, based on the daily requirements prescribed by the clinical nutritionist.

Results showed that most of the cases were malnourished. For group 1 cases, body weight was below 3$^{rd}$ percentile in 70% of the cases, for TSF, 60% were below 10$^{th}$ percentile and 30% were between 15$^{th}$ and 25$^{th}$ percentiles, and their head circumference was below the 10$^{th}$ percentile in 60% of the cases. For group 2 body weight was below 10$^{th}$ percentile in 85% of cases, TSF measurement showed that 50% of cases were below the 10$^{th}$ percentile and their head circumference was below the 10$^{th}$ percentile in 60% of the cases.

### 0741

#### Association of energy adequacy with 28-day mortality in mechanically ventilated critically ill patients

M. Haliloglu, B. Bilgili, I. Sayan, I. Cinel

Marmara University School of Medicine, Anesthesiology and Intensive Care, Istanbul, Turkey

**Correspondence:** B. Bilgili

*Intensive Care Medicine Experimental* 2018, **6(Suppl 2):**0741

**INTRODUCTION.** For mechanically ventilated critically ill patients, the effect of full feeding on mortality is still controversial.

**OBJECTIVES.** We aimed to investigate the relationship of energy intakes with 28-day mortality, and nutritional risk status influenced this relationship.

**METHODS.** This prospective observational study was conducted among adult patients admitted to ICU and required invasive mechanical ventilation (IMV) for more than 48 h. Data on baseline characteristics and the modified Nutritional Risk in Critically ill [mNUTRIC] score was collected on day 1. Energy intake and nutritional adequacy was recorded daily until death, discharge or until twelfth evaluable days. Patients were divided into 2 groups:

a) received < 75% of prescribed energy, b) received ≥ 75% of prescribed energy.

**RESULTS.** 150 patients (65% male, mean age 51.0±15.3 years, mean body mass index 27.9±6.2 kg/m2, mean mNUTRICscore 5.8± 1.7) were included. In the univariate analysis, mNUTRİC score was associated with 28-day mortality. In the multivariable logistic regression analysis, mNUTRIC score(Odds ratio, OR 1.65, CI 1.20-1.70, P < O.OO1) was associated with 28-day mortality. Nutritional adequacy was assessed, median nutritional adequacy was 0.40 (0.17-0.75). In patients with high mNUTRIC score (5-9), received ≥ 75% of prescribed energy was associated with a lower predicted 28-day mortality; this was not observed in patients with low mNUTRIC score (0-4).

**CONCLUSIONS.** Nearly 60 % of IMV required patients admitted to ICU were at nutritional risk, mNUTRİC score is associated with 28-day mortality. Energy adequacy of ≥ 75% of prescribed amounts were associated with decreased mortality in patients with a high mNUTRİC score.

**GRANT ACKNOWLEDGMENT**

None

### 0742

#### Thiamine status in adults receiving chronic diuretic therapy prior to admission to a medical intensive care unit: a pilot study

K. Gundogan[1], I.H. Akbudak[2], K. Bulut[3], S. Temel[3], M. Sungur[3], M. Güven[3], N.J. Dave[4], D.P. Griffith[4], T.R. Ziegler[5]

[1]Erciyes University, Division of Medical Intensive Care, Department of Medicine, Enteral and Parenteral Nutrition Unit, Kayserİ, Turkey; [2]Pamukkale University, Intensive Care Unit, Denizli, Turkey; [3]Erciyes University, Division of Medical Intensive Care, Department of Medicine, Kayserİ, Turkey; [4]Emory University Hospital, Nutrition and Metabolic Support Service, Atlanta, United States; [5]Emory University School of Medicine, Department of Medicine, Division of Endocrinology, Metabolism and Lipids, Atlanta, United States

**Correspondence:** K. Gundogan

*Intensive Care Medicine Experimental* 2018, **6(Suppl 2):**0742

**INTRODUCTION.** Thiamine is an essential water soluble vitamin and a critical component of cellular metabolism. In intensive care unit (ICU) patients, blood thiamine concentrations may decrease due to decreased dietary intake, malabsorption, increased utilization for carbohydrate and amino acid metabolism, and/or urinary loss with diuresis.

**OBJECTIVES.** The purpose of this study was to determine serum thiamine concentrations in critically ill medical patients who required chronic diuretic drug treatment before admission to the intensive care unit.

**METHODS.** This prospective study was performed in a single medical ICU at Erciyes University Hospital, Kayseri, Turkey. Individuals above 18 years of age and required to stay in the ICU at least 48 hours were included. Subjects who received diuretic drug therapy for at least 6 months prior to ICU admission constituted the Diuretic Group. The Control Group were clinically matched adults admitted to the ICU without a history of diuretic therapy. Serum thiamine concentrations were measured using high-performance liquid chromatography (HLPC) within 48 hours of admission and serially on days 2, 5 and 10 after entry while in the ICU.

**RESULTS.** A total of 50 subjects were included (25 subjects in each of the Diuretic Group and Control Group). The mean age was 59±17 years and 48% were female. The mean Acute Physiology and Chronic Health Evaluation II score (APACHE II) was 15.7 ± 6.5 at entry to the ICU [Diuretic Group = 17.5 ± 7.2; Control Group = 14.2 ± 5.4 (p=0.14)]. The most common causes for ICU admission were acute respiratory failure (42%) and miscellaneous metabolic events (30%). Congestive heart failure and chronic liver diseases were the most frequent co-morbidities. The median daily dose of furosemide prior to admission was 40 mg (range = 20-160 mg/d), for 12 months (range = 6-120 mo) for the Diuretic Group. The baseline serum thiamine level was below the reference range of 35-99 ng/mL at 31.2 ± 27.1 ng/mL in all patients. The baseline thiamine level was significantly lower in the Diuretic Group compared to the Control Group (15.5 ± 10.7 vs. 46.8 ± 29.5 ng/mL; p< 0.001). On day 2 after entry, thiamine levels were 23.2 ± 15.4 ng/mL in the Diuretic Group and 49±38 ng/mL in the Control Group (p=0.003). In the Diuretic Group, serum thiamine concentrations were below the normal range in 24 of the 25 subjects (96%) at baseline, in 18 of 25 patients (72%) on day 2 after entry, in 8 of 15 subjects (53%) at day 5 after entry, and in 2 of the 4 patients (50%) remaining in the ICU on the $10^{th}$ day after study entry.

**CONCLUSIONS.** Adults receiving chronic diuretic therapy and requiring medical ICU care exhibit a very high rate of thiamine depletion on admission to the ICU and during the initial days of ICU care. Larger, prospective, observational studies are needed to confirm the magnitude of thiamine deficiency in this ICU patient population in Turkey.

**GRANT ACKNOWLEDGMENT**

None

### 0743

#### Comparison of the 'Malnutrition Universal Screening Tool' (MUST) and ´Nutritional Risk Screening 2002´ (NRS-2002) for prognosis in the intensive care unit

D. Ozates, H. Sungurtekin, S. Kiter, S. Serin

Pamukkale University, Denizli, Turkey

**Correspondence:** H. Sungurtekin

*Intensive Care Medicine Experimental* 2018, **6(Suppl 2):**0743

**INTRODUCTION.** Malnutrition is a clinical conditions that can affect all age groups in the world. It is an important problem that causes high hospital costs, long hospital stay, and associated widespread complications, high infection risk and mortality.

**OBJECTIVE.** It was aimed in our study to evaluate the NRS-2002 and MUST screening tests relations with mortality and morbidity.

**METHODS.** Following the approval of the Ethics Committee, 250 patients over 18 years of age between April 2014 and November 2016 were enrolled in this study in Pamukkale University Medical Faculty Hospital Anesthesiology and Reanimation Department Intensive Care Unit. The age, sex, height, weight, and BMI data of the patients were recorded during their admission to the intensive care unit. The causes of hospitalization, current comorbidity, malignancy and infection were determined. The duration of stay in intensive care and hospital, hospital discharge status were examined. APACHE II and SAPS II values were calculated using vital findings, laboratory values, GCS, urine volumes, chronic disease findings, age and organ failure values. Anthropometrically; skin fold thickness, middle circumference of upper arm was measured. The patients underwent NRS-2002 and MUST screening tests to assess nutritional status. NRS-2002 was grouped as malnutrition risk and expected risk, MUST was grouped as low malnutrition risk, moderate malnutrition risk, and high malnutrition risk.

**RESULTS.** Of the 250 patients included in the study, 101 were female, 149 were male, and the mean age was 63. When the discharge status of NRS-2002 risk groups is examined; of the alive patient 143 (57%) patients 47 (32%) was in the expected risk and 96 (67%) was in the malnutrition risk group; of the 107 (42%) patients with exitus, 17 (15%) was in the expected risk and 90 (84%) was in the malnutrition risk group. According to the MUST; out of the 143 (57%) alive, 50 (34%) were in the low risk group, 9 (0,06%) were in the medium risk group and 84 (57%) were in the high risk group. Out of the total 107 (42%) exitus patients, 5 (0.04%) were in the low risk group, 5 (0.04%) were in the moderate risk group and 97 (90%) were in the high risk group. There is a significant difference in survival in MUST risk groups were observed, while a significant difference was not found in NRS-2002 risk groups.

**CONCLUSION.** In conclusion, MUST screening test was more successful in determining malnutrition related mortality, while NRS-2002 was more unsuccessful. We suggest that MUST would be more effective in predicting mortality in patients that will be screened for malnutrition in intensive care units.

### 0744

#### Risk factors for severe vitamin D deficiency in critically ill patient at ICU admission

A. Khaldi[1], D. Prevedello[2], J.-C. Preiser[2]

[1]Hopital Erasme, Anderlecht, Belgium; [2]Hopital Erasme, Intensive Care, Brussels, Belgium

**Correspondence:** A. Khaldi

*Intensive Care Medicine Experimental* 2018, **6(Suppl 2):**0744

**INTRODUCTION.** 25-OH vitamin D (25-OH) is the major circulating vitamin D metabolite, and reflects the stores of vitamin D (1). In the general population, endogenous vitamin D production is influenced by genetic predisposition, adiposity, age, season, exposure to sunlight and skin color (2). In critically ill patients, severe 25-OH deficiency is frequent but the risk factors for decreased 25-OH are currently not well characterized.

**OBJECTIVES.** The aim of this study was to assess whether patients with admission 25-OH ≤ 12 ng/ml differed from patients with higher 25-OH levels in terms of demographic variables, type of admission, preexisting co-morbidities, severity of disease, and length of stay (LOS) in the ICU and in the hospital.

**METHODS.** Consecutive patients admitted over a 4- month period in a medical/surgical 32-bed ICU were included. In these patients, 25-OH, demographic data, Charlson co-morbidity score, severity scores (SAPS II and SOFA) were collected at admission. ICU and hospital length of stay (LOS) were recorded. Patients were categorized according the 25-OH admission value (≤ 12 ng/ml). A chi-square test was calculated to compare categorical variables, T-student test for age and non-parametrical tests for other variables.

**RESULTS.** 613 patients were included (age 60.1 ± 16.5 years, male gender 58%, medical admissions 40%, SAPS II 45.4±18.9, SOFA 3.3 ±4.2, Charlson 2.2±2.6). Mean 25-OH averaged 18.2±11.1 ng/ml, with a level ≤ 12 ng/ml in 251 patients (41%).

**CONCLUSIONS.** In the population studied, severe 25-OH deficiency was frequent. A systematic determination of 25-OH at the time of admission could be particularly valuable in younger males.

**GRANT ACKNOWLEDGMENT**

This research received no specific grant from any funding agency in the public, commercial, or not-for-profit sectors.

**Table 1 (abstract 0744).** RESULTS. patients' characteristics according to the plasma level of 25-OH vitamin D

| | **25-OH > 12ng/ml** (n= 362) | **25-OH ≤ 12 ng/ml** (n=251) | p-value |
|---|---|---|---|
| *Age (years), mean ± SD* | 62.6 ± 16.1 | 56.7 ± 16.3 | **< 0.01** |
| *Proportion of males, n (%)* | 191 (53) | 163 (65) | **< 0.01** |
| *Medical admission, n (%)* | 136 (38) | 107 (43) | 0.33 |
| *Charlson score, median (IQR)* | 2 (0-3) | 2 (0-3) | 0.14 |
| *SOFA, median (IQR)* | 2 (0-4) | 2 (0-6) | 0.7 |
| *SAPS II, mean (SD)* | 46 ± 19 | 45 ± 19 | 0.1 |
| *ICU LOS (days), median (IQR)* | 1 (1-3) | 1 (1-4) | 0.3 |
| *HOSPITAL LOS (days), median (IQR)* | 9 (6-19) | 10 (6-23) | 0.4 |

### 0745

#### Prevalence of nutritional status and compensation of malnutrition status of the patient in critical condition of the Intensive Care Unit of the Juarez of Mexico Hospital

K.P. Pozos Cortes[1], O.R. Perez Nieto[1], G.D. Hernandez Lopez[2], J.A. Castanon Gonzalez[2]

[1]Hospital General San Juan del Rio, Intensive Care Unit, San Juan del Río, Qro, Mexico; [2]Hospital Juarez de México, Intensive Care Unit, Ciudad de Mexico, Mexico

**Correspondence:** K.P. Pozos Cortes

*Intensive Care Medicine Experimental* 2018, **6(Suppl 2):**0745

**INTRODUCTION.** Patients in a state of serious stress from trauma, sepsis, burn so critical illness, exhibit an accelerated catabolism of bodyproteins, and increase the degradation and transamination of amino acids in skeletal muscle. Therefore, inadequate nutrition can alter immune responses, integrity of the intestinal mucosal barrier and protein synthesis thus contributing to significant morbidity and mortality. Therefore, nutrition is an important part of treatment in a severely ill patient.

**OBJECTIVE.** To establish the degree of compensation of malnutrition of the critically ill patient.

**METHODS.** An observational, descriptive, analytical and retrospective study will be carried out at Juarez of México Hospital, in a period from 01-January- 2016 to 31-Junuary- 2017. Where patients with malnutrition, either malnutrition and / obesity and at the same time hospitalized in the intensive care unit area are considered, the purpose of the study is to establish the degree of compensation for malnutrition presented by the patients, through the correct caloric intake. Sociodemographic variables such as age, sex, weight, height, BMI, type of malnutrition, degree of obesity, type of diet received, caloric intake, nitrogen balance are considered. Statistical analysis will be done using frequencies and proportion for qualitative variables, an X 2 to determine the dependence between variables, a Student´s T for numerical variables, with a p 0.05 for the statistical significance.

**RESULTS.** The 2.38% of the patients had malnutrition, 23.81% had first-degree obesity, and finally, 7,14% had second-degree obesity, and only 4.76% of the patients had with morbid obesity.

**CONCLUSIONS.** The mean BMI of patients was 26.92 kg / m2, where it is established that patients are overweight. Only 2.38% of the patients had malnutrition, however, in the assessment of nutritional status, it was identified that 64.28% of the patients where over nourished.

### 0746

#### Clinical relevance of calcium measurements in ICU patients: a practice survey

A. Dreyfus, D. Prat, C. Gouezel, M. Millereux, B. Sztrymf, O. Hamzaoui, N. Demars, P. Trouiller, F. Jacobs

Hopitaux Universitaires Paris Sud, Réanimation Polyvalente, Clamart, France

**Correspondence:** F. Jacobs

*Intensive Care Medicine Experimental* 2018, **6(Suppl 2):**0746

**INTRODUCTION.** Ionized calcium (iCa) and not total calcium (totCa) is the physologically relevant component of blood calcium. Hypoalbuminemia, acidemia, free fatty acids concentrations acute elevation may result in poor correlation of total calcium with direct measurements of iCa. Different type of calcium measurement (iCa, totCa with or without adjustment for albumin or total protein) may lead to misinterpretation of calcium disorders.

**OBJECTIVES.** To report the results of a practice study in critically ill patients.

**METHODS.** Online survey about calcium measurement in ICU patients consisting of eleven questions regarding frequency and indication of measurement, type of measurement (totCa or iCa), adjustment of totCa results.

**RESULTS.** 59 answers, mainly anesthesia critical care or critical care residents (residents 72.9%, senior physicians 27.1%).

76.3% of the responders systematically measured calcium on ICU admission.

Subsequent systematic measurements during ICU stay were performed in 71.2% of cases.

In first intention, iCa was tested in only 36.2% of cases and totCa in 63.8%.

69.2% of totCa values were adjusted, mainly on albumin (albumin 59.6%, total protein 28.8%).

**CONCLUSIONS.** Among the responders of this sample, measurement of calcemia is frequent.

However, practices are heterogeneous.

Total calcemia remains the most frequently used method, and one third of these results are not adjusted. Furthermore, robust data concerning the reliability of totCa adjustment are lacking in this specific population

Despite its widespread use, usfulness of routine calcium measurement is not warranted and may lead to misinterpretation of the concentration of calcium and hazardous diagnosis of dyscalcemia.

Complementary studies are probably needed to determine the usefulness of this this test and the accuracy of different sample methods.

### 0747

#### Descriptive study of patients with hepatic failure treated with molecular adsorbent recirculating system (MARS) during the period 2007-2014

A. Cortes Herrera[1], P. Geffner[2], A. Quinza[2], A. Castellanos[2], M.J. Broch Porcar[2]

[1]Hospital Universitari i Politecnic La Fe, Intensive Care Unit, Valencia, Spain; [2]Hospital Universitari i Politecnic La Fe, Intensive Care Department, Valencia, Spain

**Correspondence:** A. Cortes Herrera

*Intensive Care Medicine Experimental* 2018, **6(Suppl 2):**0747

**INTRODUCTION.** Treatment in the intensive care unit of patients with end-stage liver disease has been limited. Liver transplantation has been a major improvement in this and has become standard in the management of these patients. However, many patients die awaiting liver transplantation, mainly due to the scarcity of organ donors. The majority of endogenous toxins leading to organ failure and accumulating in the blood are bound to albumin. The Molecular Adsorbent Recirculating System (MARS), enables simultaneous liver and kidney detoxification and can improve the patient´s clinical condition to permit, the recovery from an acute episode and enhance the chances of survival while waiting for an available organ donor. We used the technic with this objective and describe our results.

**OBJECTIVES.** To describe the epidemiological characteristics of patients admitted to the ICU treated with MARS in a referral hospital.

**METHODS.** Observational, retrospective study, 2007-2014, of patients with hepatic failure (FH) treated with MARS in a medical ICU of 24 beds. The variables were analyzed: age, sex, etiology of liver failure, severity scores (MELD and CHILD), number of sessions applied, biochemical variables pre- and post-MARS and mortality.

**RESULTS.** N = 50 patients (72% males), Middle-aged 50 years old (39-59). The main etiology of hepatic failure was viral (52%), followed by alcohol (22%). Acute on chronic liver failure was the principal tipe of hepatic failure (46%), progression of Cirrhosis (32%) and acute liver failure (22%). 64% of patient were in Child-Pugh stage C and with MELD of 27 SD7. A median of 2 sessions per patient (2-4). Pruritus disappeared in 71.4% CI 95% (56.57 to 86.28). Liver transplant in 38%. The Mortality was 64% (81% < 90 days).

**CONCLUSIONS.** The most frequent etiology was HCV. In recent years, the implementation of MARS has decreased. The mortality remains high and precocious. One third of patients were transplanted. MARS significantly decreased bilirubin and this is possibly related to the disappearance of pruritus. One of the main indications of the technique at present.

**REFERENCE(S)**

Iwai H, Moriwaki H. Removal of endotoxin and cytokines by plasma exchange in patients with acute hepatic failure. Crit Care Med. 1998;26:873-876.

Saliba F. The Molecular Adsorbent Recirculating System (MARS®) in the intensive care unit: a rescue therapy for patients with hepatic failure. Crit Care. 2006; 10(1): 118.

## Clinical microbiology in sepsis

### 0748

#### Dynamics of hospital-acquired bacteremia strains of Gramnegative rods at intensive care units in 7yrs period

D. Adukauskiene[1], D. Valanciene[1], A. Dambrauskiene[2], A. Vitkauskiene[3]
[1]Lithuanian University of Health Sciences, Intensive Care Clinic, Kaunas, Lithuania; [2]Lithuanian University of Health Sciences, Infection Control Service, Kaunas, Lithuania; [3]Lithuanian University of Health Sciences, Laboratory Medicine Clinic, Kaunas, Lithuania
**Correspondence:** D. Valanciene
*Intensive Care Medicine Experimental* 2018, **6(Suppl 2):**0748

**INTRODUCTION.** Antimicrobial resistance among Gram-negative rod (GNR) strains is a growing worldwide issue. Delayed appropriate antimicrobial therapy is associated with higher mortality. Because of this it is recommended to monitor flora to be able to choose the right antimicrobial treatment at the first option.

**OBJECTIVES.** To compare dynamics of pathogens and sensitivity of hospital-acquired (HA) GNR monobacteremia strains in 7 yrs period to antimicrobial drugs and risk factors of mortality in Intensive Care Units (ICUs).

**METHODS.** Ongoing comparative retrospective study of patients treated in ICUs of Kaunas Clinic's with positive blood culture for GNR taken after 72 hrs of hospitalisation during one yr and after 7yrs was carried out.

**RESULTS.** At the 1st study yr there were found 46 cases of HA bacteremia due to GNR: 32/46 (69.6%) Enterobacteriaceae spp. (P=0.04), 5/46 (10.9%) Acinetobacter spp., 38/46 (82.6%) multi-drug-resistant (MDR), 9/38 (19.6%) extensively-drug-resistant (XDR) strains. Sensitivity to carbapenems (n=43, 93.5%), amikacin (n=29, 63.0%) and cefoperazon/sulbactam (n=26, 56.5%) was found. Lethal outcome 78.3% (n=36) found in male gender (P=0.04, OR=3.40, CI95%=0.53-0.75), mechanical ventilation and septic shock (P=0.01, OR=11.55, CI95%=1.18-12.71), previous treatment in hospital (P=0.03, OR=7.52, CI95%=1.19-9.77), acute kidney injury (AKI) (P=0.04, OR=3.90, CI95%=1.91-16.80), higher SOFA score (P=0.04, RR=5.48).
After 7yrs there were found 32 cases: 16/32 (50.0%) Acinetobacter spp. (P>0.05), 13/32 (40.6%) Enterobacteriaceae spp. (P>0.05), 29/32 (90.6%) MDR, 14/29 (43.8%) XDR strains. Sensitivity to carbapenems (n=28, 87.5%), amikacin (n=22, 68.8%), cefoperazon/sulbactam (n=20, 62.5%) was found. Lethal outcome 78.1% (n=25) was found in patients with mechanical ventilation and septic shock (P=0.01, OR=6.80, CI95%=2.48-10.54), AKI (P=0.01, OR=10.23, CI95%=3.35-7.46), previous treatment in hospital (P=0.04, OR=4.21, CI95%=2.27-14.42), XDR strain (P=0.02, OR=4.77, CI95%=1.10-11.32), higher SOFA score (P=0.03, RR=8.41).
In multi-variate analysis lethal outcome in 1st yr and after 7yrs was found in patients with mechanical ventilation (P=0.001, OR=6.47, CI95%=1.56-10.32; P=0.01, OR=4.77, CI95%=1.10-11.32), septic shock (P=0.001, OR=3.27, CI95%=3.56-16.78; P=0.001, OR=4.77, CI95%=1.10-11.32), AKI (P=0.04, OR=4.77, CI95%=1.10-11.32; P=0.02, OR=7.27, CI95%=2.56-14.89), respectively.

**CONCLUSIONS.** Dynamics of predominant pathogen of HA bacteremia changed from Enterobacteriaceae spp. to Acinetobacter spp. during study period of 7yrs. High sensitivity to carbapenems has remained. Rate of XDR strains has doubled. High mortality rate remained and was associated with mechanical ventilation, septic shock and AKI.

**REFERENCE(S)**

1. Adrie C et al. Journal of Infection, 2016,74(2):131-41.
2. Siedner MJ et al. Clinical Infectious Diseases, 2014,58:1554-63.

**GRANT ACKNOWLEDGMENT**
None

### 0749

#### Bacterial translocation and gut barrier failure in critically ill patients with spontaneous intracerebral bleeding

J. Schäper[1], R. Fontaine[1], S. Neugebauer[1], R. Lugert[2], A.R. Asif[3], M. Quintel[1], O. Moerer[1]
[1]University Hospital Göttingen, Anaesthesiology, Göttingen, Germany, [2]University Medical Center Göttingen, Medical Microbiology, Göttingen, Germany, [3]University Hospital Göttingen, Clinical Chemistry, Göttingen, Germany
**Correspondence:** J. Schäper
*Intensive Care Medicine Experimental* 2018, **6(Suppl 2):**0749

**INTRODUCTION.** The hypothesis that bacterial translocation plays an important role in the development and perpetuation of systemic inflammation is well accepted. Scientific evidence to prove this hypothesis, however, is scarce.

**OBJECTIVES.** Quantification of bacterial translocation and correlation with markers of gut barrier failure, systemic inflammation and severity of illness in an ICU patient cohort with spontaneous intracerebral bleeding.

**METHODS.** Prospective monocentric observational cohort study (ethical approval Göttingen University 22/1/14) in 24 subsequent ICU patients with spontaneous intracerebral parenchymatous bleeding. Inclusion into the study was within eight hours after hospital admission. Quantification of Bacteroides and Enterococcus species DNA und surrogate parameters for altered epithelial permeability (iFABP, Claudin 3, sCD14) in patient blood and urine was performed on admission (d0), d1, d2, d3, d5, d7, d10, and d14 of ICU stay. Severity of disease scores were calculated on admission, organ failure scores were used repetitively in the further course of ICU stay.

**RESULTS.** Enterococcus or Bacteroides DNA was detected in 67% of patients with intracerebral bleeding and in 64% of those patients, who developed sepsis. Patients with bacterial translocation developed sepsis in only 56% of cases. Neither disease severity scores nor organ failure scores differed between patients with or without bacterial translocation. iFABP concentration in urine was higher in patients with sepsis compared to patients without sepsis (509 [94; 2393] vs. 134 [94; 516] pg/mL, p=0.015). iFABP also was higher in patients with bacterial translocation (385 [94; 1788] vs. 96 [94; 400] pg/mL, p=0.012).

**CONCLUSIONS.** Bacterial translocation of gut-specific bacteria into the blood was present in the majority of patients with intracerebral bleeding. There was no association between bacterial translocation and the development of sepsis in this cohort of patients. A high urine concentration of iFABP, a surrogate marker for gut barrier failure, however, was associated with bacterial translocation and sepsis.

### 0750

#### Persistent isolation of *Staphylococcus aureus* in mechanically-ventilated patients: impact of host-pathogen factors on outcome

O. Plans Galván[1], A. Lacoma[2,3], S. Triginer Roig[1], E. Benveniste Pérez[1], S. Martínez Vega[1], M. Gomes-Fernandes[2,4], I. Casas[5], Y. Rovira Vallés[1], V. Philibert[1], L. Bielsa Berrocal[1], M. Giménez[2], F. Armestar Rodríguez[1], P. Ricart Martí[1], C. Prat[2,3]

[1]Hospital Universitari Germans Trias i Pujol, Intensive and Critical Care Medicine Department, Badalona, Spain; [2]Hospital Universitari Germans Trias i Pujol, Microbiology Department, Badalona, Spain; [3]Instituto de Salud Carlos III, CIBER Enfermedades Respiratorias, Madrid, Spain; [4]CAPES Foundation, Ministry of Education of Brazil, Brasília, Brazil; [5]Hospital Universitari Germans Trias i Pujol, Preventive Medicine Unit, Badalona, Spain

**Correspondence:** S. Triginer Roig

*Intensive Care Medicine Experimental* 2018, **6(Suppl 2):**0750

**INTRODUCTION.** Bacteria obtained from the lower respiratory tract of patients undergoing mechanical ventilation (MV) can be part of the resident microbiome or act as pathogenic microorganisms. Colonization may persist even when the clinical symptoms improve and thus to determine its role as causative agents of infection is still an unresolved issue.

*Staphylococcus aureus* (SA) remains as one of the main causes of respiratory infection, although clinical presentation may vary from asymptomatic carriage to severe invasive disease, demonstrating an adaptation and a switch in virulence. It is of paramount importance to distinguish bronchial colonization from infection to adequate antimicrobial treatment to the patient's needs.

**OBJECTIVES.** To identify host and microbial factors associated with persistent isolation of SA in the lower respiratory tract despite adjusted treatment to antibiotic susceptibility profile.

**METHODS.** This is a retrospective and observational study conducted during 2 years in a polyvalent ICU.

Patient's population were those who, at their admission in ICU required MV, had a *S. Aureus* positive culture in endotracheal aspirate (ETA) and received antimicrobial treatment adjusted to susceptibility profile. Isolation in ETA was considered persistent when it lasted more than 7 days despite treatment adjusted to susceptibility profile. ETA and blood cultures were performed when there was clinical suspicion of respiratory tract infection, not systematically. Nasal swabs were obtained to check MRSA carriage.

Study was performed according to confidentiality criteria.

Data from patients included epidemiological characteristics (Age, Sex), presence of comorbidities, Glasgow Coma Scale (GCS), central nervous system (CNS) involvement, score in the Clinical Pulmonary Infection Score (CPIS), severity of illness at admission assessed by Acute Physiology and Chronic Health Evaluation (APACHE-II), consecutive isolation of *Pseudomonas aeruginosa*, days on MV and ICU length of stay (ICU LOS). SA's phenotypic factors taken into account were cloxacillin resistance.

**RESULTS.** A total of 97 patients that received antimicrobial treatment adjusted to susceptibility profile were selected: 29 were bronchial colonized, 41 had tracheobronchitis and 27 had pneumonia.

Persistence at ≥7days was documented in 39 cases (40.2%), being 27 MSSA and 12 MRSA.

In the univariate analysis, persistence correlated with younger age (p=0.009), higher CPIS (p=0.041), ICU LOS (p< 0.001), days on MV (p< 0.001), cloxacillin resistance (p=0.016) and consecutive isolation of *P. aeruginosa* (p< 0.001).

**CONCLUSIONS.** Persistent isolation of SA in mechanically-ventilated patients despite adjusted antimicrobial treatment is frequent.

Persistent isolation of SA correlated with younger age, higher CPIS, ICU LOS and days on MV, cloxacillin resistance and consecutive isolation of *P. aeruginosa*.

### 0751

#### Critically ill burn patient and invasive candidiasis: do not keep calm!

A. Agrifoglio, E. Herrero, L. Cachafeiro, P. Millán, M. Sánchez, A. García de Lorenzo

Hospital Universitario La Paz, Critical Care Medicine Service, Madrid, Spain

**Correspondence:** A. Agrifoglio

*Intensive Care Medicine Experimental* 2018, **6(Suppl 2):**0751

**INTRODUCTION.** Burned patients represents a population with high risk for opportunistic infections. Burns infections are mainly caused by bacterial pathogens (70%), followed by fungal infections (20-25%) and finally by viral infections (5-10%). These infections have been described as one of the main causes of increased mortality. *Candida* spp. represents the main cause of fungal colonization in burned patients. The data varies according to the series, but the incidence of invasive candidiasis occurs between 2 and 21% of burned patients, with an attributable mortality between 30-90%; 30-60% of burned patients have at least one culture positive for *Candida* spp. during admission and the incidence of candidemia is around 3-5%.

**OBJECTIVES.** To analyze the incidence, mortality and risk factors associated with the development of invasive candidiasis in a population of critically ill burned patients.

**METHODS.** We retrospectively collected all patients admitted to our Critical Care Burn Unit between 2012 and 2017 and had any microbiological isolation by any *Candida* species. We collected: demographic and epidemiological data, severity scores (ABSI, APACHE-II), mechanism, extension and depth of burns, risk factors, need for organic supports, days of hospital stay and mortality.

**RESULTS.** 368 burned patients were admitted. Five invasive candidiasis in the form of candidemia were diagnosed: three candidemias due to *Candida albicans*, one for *Candida parapsilosis* and one for *Candida tropicalis* and three patients were colonized by yeasts in their burns. The candidemias were treated empirically with echinocandins. In 80% of the patients the mechanism of the burn was the flame. The medians were: age: 40.5 years; 4 men; total burn surface area: 46%; depth, 34%; ABSI: 8, APACHE-II 18. The average hospital stay in the ICU was 39 ± 11 days. 100% of the patients were on mechanical ventilation at the time of the diagnosis of candidemia, and all of them also required vasoactive drugs. One patient (candidemia due to *C. albicans*) needed continuous renal replacement techniques. All had a central venous catheter, a urinary catheter, complementary parenteral nutrition and were on broad-spectrum antibiotic therapy. Two patients died, both with candidemia due to *C. albicans*.

**CONCLUSIONS.** Although the incidence of candidemia in our population is very low, almost half of the patients who develop it die during admission in a situation of refractory septic shock. Total burn surface area > 30%, multiple colonization by *Candida* spp., parenteral nutrition and broad spectrum antibiotic therapy, as well as central venous catheter and urinary catheterization continue to be the main risk factors.

- Ha J, Italiano C, Heath C, et al. Candidemia and invasive candidiasis: A review of the literatura for the burns surgeon. Burns 2011;37:182-92.
- Moore EC, Padiglione A, Wasiak J, et al. Candida in burns: risk factors and outcomes. J Burn Care Res 2010;31:257-63.

### 0752

#### Invasive pneumococcal disease in the ICU: a retrospective study

E. Fragoso[1], G. Jesus[1], D. Silva[1], S. Fernandes[1], J. Gouveia[1], C. Costa[2], E. Lopes[2], L. Lito[3], A. Alvarez[1]
[1]Hospital de Santa Maria - Centro Hospitalar de Lisboa Norte, EPE, Serviço de Medicina Intensiva, Lisboa, Portugal, [2]Faculdade de Medicina da Universidade de Lisboa, Instituto de Microbiologia, Lisboa, Portugal, [3]Centro Hospitalar de Lisboa Norte, EPE, Laboratório de Microbiologia, Lisboa, Portugal
**Correspondence:** E. Fragoso
*Intensive Care Medicine Experimental* 2018, **6(Suppl 2):**0752

**INTRODUCTION.** Invasive pneumococcal disease (IPD) is a life-threatening disease. The European Center for Disease Control (ECDC) reported a notification rate of 16,1 cases per 100.000 population aged 65 years and older in 2015. In Portugal, notification rate was 1,4/100.000 for the same time frame (all ages). IPD is not a common diagnosis in ICU patients, but is associated with high rates of organ failure and mortality. Prevalence of IPD in our ICU was felt to be unusually high over the last year.

**OBJECTIVES.** Characterization of IPD patients admitted to our ICU over the last 12 months.

**METHODS.** Retrospective study of IPD patients over 12 months. IPD was classified either as bacteremic pneumonia, meningitis or infection of other usually sterile environment.Age,gender,SAPS II,length of stay (LOS), pneumococcal serotype,antimicrobial susceptibility testing, prevalence of ARDS, acute kidney injury (AKI) and septic shock, unadjusted mortality and standardized mortality ratio (SMR) were analyzed. Groups were compared on the basis of serotype and clinical features. Results were expressed as medians (interquartil range in brackets) for continuous variables and frequencies for categorical variables.

**RESULTS.** n=15 patients (0,78% of all admissions). Males:66,7%. Median age,years: 62 (20,8). SAPS II: 48 (41). Clinical presentation: Bacteremic pneumonia 53,3%, meningitis 33,3%, both 6,7% and pericardial empyema 6,7%. LOS,days: 4,5 (13,2). Diagnosis was performed either by cultures or molecular methods. Serotype analysis: 55,5% of infections due to serotypes covered by the pneumococcal 13-valent conjugated vaccine and one case due to a serotype not covered by any of the available vaccines. Of all isolates, 76,9% were susceptible to penicillin. Rates of organ failure were as follows: ARDS 13,4%,septic shock 53,3%, maximal norepinephrine dose on day 1 of 0,51 µg/Kg/min (1,80), AKI 46,7%. Sixty percent of patients were submitted to invasive mechanical ventilation (median days on IMV:11; IQR 15,5) and 20% to noninvasive ventilation; 40% performed continuous renal replacement therapy. ICU mortality was 20,0%, hospital mortality 40,0% (SMR 0,96). There was no relation between serotypes and mortality or organ dysfunction rates.

**CONCLUSIONS.** IPD is a severe and resource-consuming entity. At our centre, patients were mainly admitted with bacteremic pneumonia and had high rates of organ dysfunction, namely septic shock and acute kidney injury. Hospital mortality was high. Serotype distribution in terms of vaccinal coverage was somewhat different from the annual ECDC report, although this finding must be interpreted with caution given the reduced sample size. According to national guidelines, anti-pneumococcal immunization was due in 66,6% of these patients, raising the issue whether some of these admissions could have been prevented by previous vaccination. Penicillin susceptibility is in line with previous reports published in the literature.

### 0753

#### Clinical, microbiological and metabolomics changes in oral samples of septic patients admitted to the intensive care unit: a preliminary analysis

M. Kallas[1], M. Dias[2], M.A. Mendes[2], L.C.P.d. Azevedo[1,3]
[1]Hospital Sirio-Libanes, Sao Paulo, Brazil; [2]Universidade de Sao Paulo, Escola Politécnica, Laboratorio de Espectrometria de Massa, Sao Paulo, Brazil; [3]Faculdade de Medicina, Universidade de Sao Paulo, Disciplina de Medicina de Emergencia, Sao Paulo, Brazil
**Correspondence:** L.C.P.d. Azevedo
*Intensive Care Medicine Experimental* 2018, **6(Suppl 2):**0753

**INTRODUCTION.** Oral diseases in critically ill patients may be related to infectious complications, especially in septic ones. Thus, it is important to understand the most common pathogens in oral flora of these patients, as well as their changes during Intensive Care Unit (ICU) permanence.

OBJECTIVE. To evaluate clinical, microbiological and metabolomics changes in oral samples of septic patients during their ICU stay.

**METHODS.** We studied adult septic patients with more than 3 days of expected length of stay, which were admitted to the ICU of Hospital Sirio-Libanes, São Paulo Brazil from June 2016 to May 2017. These patients had samples collected from saliva and dental or oral prosthesis biofilm on their first, third and seventh day of ICU stay, while dental indexes where registered. ICU has protocolized oral care consisting of oral hygiene made with chlorhexidine 3-4 times a day. Microorganisms were cultured and isolated using Brain Heart Infusion agar 37 g $L^{-1}$. Bacteria were identified by Biotyper 3.1 database. The identification cut-off values higher than 2 and 1.7 were used for species and genus identification. Mass spectrometry analyses were performed using an UltrafleXtreme MALDI-TOF mass spectrometer. Metabolomic analyses were performed using a Gas chromatography-mass spectrometer. Statistical analysis was performed by the MetaboAnalyst tools.

**RESULTS.** We studied 10 septic patients with SOFA score 6.8±4.0 and ICU length of stay of 9.9±13.9 days. Mucosal plaque score improved from 4.6±1 at baseline to 3.8±1 (p< 0.001) at 7th day and bedside oral care exam from 15±2 at baseline to 12.8±3.1 at 7th day (p< 0.001). The most prevalent microorganisms in the saliva samples were *C. albicans* (26%), *Staphylococcus haemolyticus* (17.6%), and *E. faecalis* (11.7%). In the biofilm, the most prevalent microorganisms were *C. albicans* (36%), *E. faecalis* (7.7%) and *S. hominis* (7.7%). During the first 7 days of ICU stay, *Candida* and *Pseudomonas* species found in the biofilm had reduction on their percentages (p=0.037 and 0.007 respectively). Metabolomic analysis found 10 metabolites in higher concentration and frequency in these patients. Within them, 76.8% were found in oral biofilm, consisting mostly of glucitol (13%), alpha-D-Galactopyranoside (19%), 3,5-Dimethylbenzaldehyde (13%), D-Fructose (10%) and Octadecanoic acid (12%). The metabolites more frequently found in saliva were urea (15%), glucitol (27%), glycerol (12%) and D-Fructose (11%). Temporal analysis showed an increase in the concentrations of urea, alpha-D-Galactopyranoside and D-Fructose during ICU stay .

**CONCLUSION.** Septic patients admitted to an ICU with protocolized oral hygiene improve their oral clinical indexes during ICU stay. Analysis of saliva and biofilm showed persistence of pathogenic microrganisms and also metabolites correlated with sepsis and alterations of glycemic metabolism.

### 0754

#### Does maintenance of the Zero Resistance project in ICU improve rates?

M. Muñoz Garach, O. Moreno Romero, M.E.Y.O. Yuste Ossorio
Hospital Universitario San Cecilio Granada, Granada, Spain
**Correspondence:** M. Muñoz Garach
*Intensive Care Medicine Experimental* 2018, **6(Suppl 2):**0754

**INTRODUCTION.** The multiresistant bacteria (MRB) in ICU is rising Public Health problem especially in the intensive care units. The implementation of surveillance programs for patients at risk of harboring MRB (Zero Resistance), a fast diagnostic and isolation when they appear are needed in our units.

OBJECTIVE. Description of the rates of multiresistant bacteria (MRB), days of isolation in ICU and antibiotics after the start of the Resistencia Zero (RZ) project and analysis of these.

**METHODS.** Descriptive retrospective study, using the ENVIN-HELICS database, of patients admitted to UCI H.U. San Cecilio with 18 beds, comparing rates for the year in which the project starts (2014) with subsequent periods (2015). Variables studied were: days of antibiotic, days of MRB, days of isolation, patients with MRB at admission and during stay in ICU the colonization ±

infection infection and type of MRB and its evolution in the subsequent year(next year?).
**RESULTS.** The two periods compared:
1. Year 2014 (project start): 46 of 1034 patients presented MRB with the following rates for every 1000 days of ICU stay: 10.8 days of ICU stay, 334.9 days of antibiotic, 72.4 days of MRB and 54.54 days of ICU stay. isolation. At admission, 1.84 / 1000 days of stay and during admission, 6.58 including infection and colonization. 26 of these were infection (9 at admission = 0.87 rate and 17 = 4 during ICU admission). 84.73% GNBand 15.25% MARSA(17 pseudomonal MDR = 28.8%, Acinetobacter RI 22 = 37.29% Enterobacteria BLEE 9 = 15.25%)
2. Year 2015 in which the recommendations of the RZ project were maintained. 47 of 1051 presented MRB with the following rates: 11.33 days of ICU stay, 517.84 days of AB, 136.45 days MRB and 113.55 days of isolation. At admission, it was 2.57 / 1000 days of stay and during admission 5 including infection and colonization. Of these, 31 were infection (19 at admission = 1.81 and 12 = 2.89 during admission to the ICU). 76.78% GNB and 21.43 MARSA (24 pseudomonal MDR = 42.86%, Acinetobacter RI 5 = 8.93%, BLEE 11 enterobacteria = 19.64%)
**CONCLUSIONS.**
- After 2 years of the RZ project, the rate of infection by MRB 27.75% has been reduced, although the rate has increased on admission (extra ICU patients)
- The RZ project associates increase of days of isolation and days of AB
- In our unit, although the most frequent MRBs are the GNB in the last year, the MARSA has increased, not following under the current trend

### 0755

#### Value of epidemiological swab in patients with microbiologically confirmed sepsis

M. Šerpytis[1,2], R. Stasiūnaitis[1], R. Ambrazaitienė[2,3], J. Šipylaitė[1,2]
[1]Vilnius University, Faculty of Medicine, Department of Anesthesiology and Intensive Care, Vilnius, Lithuania; [2]Vilnius University Hospital Santaros clinics, Vilnius, Lithuania; [3]Vilnius University, Faculty of Medicine, Department of Physiology, Biochemistry, Microbiology and Laboratory Medicine, Vilnius, Lithuania
**Correspondence:** R. Stasiūnaitis
*Intensive Care Medicine Experimental* 2018, **6(Suppl 2):**0755

**INTRODUCTION.** Sepsis, a life threatening condition, requires immediate treatment with antibiotics without knowledge of blood culture results. Patients hospitalized for prolonged periods of time tend to get colonized with hospital's flora, which is a plausible sepsis agent. We investigated whether knowledge of colonization could provide vital information in septic patients.
**OBJECTIVES.** To evaluate importance of epidemiological swab on successfully predicting septic patients' outcomes.
**METHODS.** A retrospective study was conducted in Vilnius University Santaros Clinics including all patients who had sepsis confirmed by blood culture and swabs from perineum collected during 7 days period before and after during 2016. Patient general information, main disease, different drugs taken, cultures' results were collected. Multidrug resistance (MDR) was defined as being resistant to 3 or more groups of antibiotics. Statistical analysis was performed with IBM SPSS 21. Odds ratio and adjusted odds ratio were compared using Fisher's exact test and logistic regression method.
**RESULTS.** A total of 98 patients were included in the study. 56 (57%) of them male. Mean patient age - 55,44±16,16 yrs. 70 (71%) patients were diagnosed with hematological malignancy. On 41 cases (42%) drug resistant bacterial strain, identified from perineum swab, coincided with blood culture. Overall, colonization by MDR strain was not associated with sepsis, caused by the same strain (OR 2,75, 95% CI 0,715-10,584; p=0,129). However, after analyzing only colonization by ESBL strain, we found out that colonization with ESBL was associated with sepsis, caused by the same strain (OR 2,976, 95% CI 1,13-7,836; p=0,024). Hematologic malignancy, after adjusting for different chemotherapy protocol, was associated with a higher risk of colonization by VRE (aOR 5,044, 95% CI 1,369-18,587; p=0,015). After adjusting for different immunosuppressive drugs taken, consumption of glucocorticoids was associated with increased colonization by ESBL strain (aOR 4,926, 95% CI 1,57-15,453; p=0,006), whereas cytostatics alone were associated with lower risk of such colonization (aOR 0,189, 95% CI 0,045-0,786, p=0,022). Colonization with VRE, ESBL or all MDR bacterial strains as well as concordance between blood test and perineum swab cultures did not predict mortality due to sepsis during in-hospital stay (OR 0,458, 95% CI 0,154-1,366; p=0,155; OR 1,127, 95% CI 0,444-2,862; p=0,802; OR 0,47, 95% CI 0,097-2,267; p=0,338, OR 0,644, 95% CI 0,255-1,627; p=0,351, respectively).
**CONCLUSIONS.** Colonization with ESBL strains is associated with ESBL caused sepsis. Glucocorticoids were found out to be associated with ESBL strain colonization, while hematologic malignancy - colonization with VRE. Colonization with resistant strains or concordance between perineum swab and blood culture were not associated with increased mortality.

### 0756

#### Urinary tract infection with nephrostomy

J.M. Mora Ordóñez[1], I. Márquez-Gómez[2], V. Olea Jiménez[1], J.D. Ruiz Mesa[2]
[1]Hospital Regional Universitario Carlos Haya, ICU, Málaga, Spain;
[2]Hospital Regional Universitario Carlos Haya, Infecciosas, Málaga, Spain
**Correspondence:** J.M. Mora Ordóñez
*Intensive Care Medicine Experimental* 2018, **6(Suppl 2):**0756

**INTRODUCTION.** The information of urinary tract infection(UTI) in patiens with a nephrostomy catheter is very poor. The objective of this study was analyze the epidemiological, clinical, microbiological factors and the prognosis of complicated high UTI in patients with nephrostomy catheter.
**METHODS.** A descriptive, prospective study with 988 patients with UTI during 18 years, from 1997 to 2015. A culture taken from nephrostomy catheter was considered positive with ≥ $10^3$ufc/ml. With few exceptions, after the diagnosis the catheter was replacement. All the patients received treatment, first empirically and subsequently for a period of ≥10 days. After the initial decriptive analysis of the most relevant variables of the global series, the diffrent clinical epidemiogical variables and the prognosis were compared with the rest of the patients.
**RESULTS.** From 988 patients with UTI, 69 cases (7,5%) had nephrostomy catheter. The mean age was 61.36 +/- 11.47 years . 36 cases were male (52,2%). 41 cases (59,4%) had a history of ureteral obstruction, 40 cases (58%) presented structural bladder patholoy, 19 cases (27,5%) had suffered urinary instrumentation in the previous 15 days, 11 cses (15,9%) had doublé "J" catheter. 87% had underlying systemic pathology, 60,3% active neoplasia, 37,7% chronic renal failure, 30,4% Diabetes Mellitus and 27,5% were inmunosuppressed.Only 11 cases (16,2%) was the first episode. Clinical expression was: 28 cases (40,6%) of miccional síndrome, in 26 cases (54,2%) greater than grade II ectasia was observed in ultrasound and in 8 cases hydronephrosis in abdominal CT. 21,7% of the cases presented bacteriemia. Microbiology, Ecoli was the principal mo observed. 13,3% of the cases were produced by non-fermenting gram-negative bacilli and 6,7% by enterococcus spp. 21,2% were produced by ESBL producing microorganisms. The duration of synptoms was 2,84 + 2,37 days, 11 cases (15,9%) developed septic shock, 43 casaes (62,3%) acute renal failure and 16 cases (24,2%) superinfection. The hospital stay was 10,83 +/- 6,3 days. Attributable mortality was 50%. Patients with nephrostomy had more structural bladder pathology (OR 3.46, 95% CI 1.76-6.81), higher repeat ITUS rates (OR 2.08, 95% CI 1.02-4.22) , more history of neoplasia (OR 4.35, 95% CI 2.27-8.31), ureteral obstruction (OR 6.68, 95% CI 3.45-12.95), higher isolates of Enterococcus spp (OR) 4.18, 95% CI 1.20-13.29) as well as higher rates of superinfection (OR 2.34, 95% CI 0.94-5.79), without differences in crude or attributable mortality.

**CONCLUSIONS.** The percentage of UTI in patients with a nephrostomy catheter is considerable. In a high proportion of cases there is a history of previous episodes of urinary infection. The proportion of microorganisms other than E. coli is significantly higher, which means that the initial empirical treatment must be changed more frequently. The rate of reinfection in this type of infection is significantly higher.

### 0757

#### The pleiotropic presentations of *Legionella pneumophila* infection in critically ill patients

H. Khelifa, W. Nabhan, S. Giglioli, H. Engel, G. Vanfraechem, Y. De Gheldre, D. De Backer

CHIREC Hospitals, Université Libre de Bruxelles, Braine l'Alleud, Belgium

**Correspondence:** H. Khelifa

*Intensive Care Medicine Experimental* 2018, **6(Suppl 2):**0757

**INTRODUCTION.** While *Legionella pneumophilia* infection is a known as a cause of respiratory distress, its impact on heart function has not been well reported. Therefore we evaluated the impact of myocardial depression in a series of critically ill patients with legionella infection.

**METHODS.** In this retrospective study, we collected data from our electronic record system information on all patients admitted to the ICU with *Legionella* infection between February 1, 2016 to March 31, 2018. Echocardiograph is routinely performed on admission in our unit, and repeated as needed according to the evolution of the patient. In addition to echocardiographic data, we also collected demographic, hemodynamic, biologic, and organ support data. Data are presented as mean ± SD.

**RESULTS.** Seven patients were identified with a mean age of 70 ± 8y. Most patients did not receive immunosuppressive agents.

Four patients had mild respiratory impairment and were supported by high flow oxygen therapy. Three patients had severe ARDS, one requiring only prone position while two received in addition veno-venous ECMO.

Left ventricular ejection fraction was 43 ± 18% on admission, and progressively recovered in a delay varying from 2 to 6 days. Two patients had cardiogenic shock due to severe alteration in left heart function on admission. Right ventricular dysfunction was also identified in 2 other patients, and rapidly recovered after initiation of veno-venous ECMO. Inotropic support was used in 2 patients (dobutamine in 1 patient with 3 mcg/kg.min during 4 days and milrinone in 2 patients with a mean dose of 9 ± 3 mg during 24 and 48h) and vasopressor support in 4 patients (norepinephrine 0,06 ± 0.04 mcg/kg.min for a mean duration of 7 days.

There was no relationship between the severity of respiratory disease and impairment of left ventricular function.

Renal replacement therapy was used in 2 pts.

ICU length of stay was 23 ± 18 days. All patients recovered and were discharged alive from the hospital.

**CONCLUSIONS.** We illustrated that myocardial depression can be severe in patients with *Legionella pneumophilia* infection and is unrelated to the severity of respiratory disease. In some patients, cardiogenic shock can be the predominant symptom on admission.

### 0758

#### Association between digestive relative abundance (RA) in multi drug resistant gram-negative bacteria (MDR GNB) in intensive care unit (ICU) patients and occurrence of invasive MDR GNB infections

C. Fontaine[1,2], E. Ruppé[2,3], L. Bouadma[2,3,4], B. Mourvillier[2,3,4], M. Neuville[3,4], R. Sonneville[3,4], L. Armand-Lefèvre[2,3], J.-F. Timsit[2,3,4]

[1]APHP Bichat, Paris, France, [2]INSERM U1137 IAME, Paris, France; [3]Université Paris Diderot, Paris, France; [4]Bichat Medical-Infectious Diseases ICU, Paris, France

**Correspondence:** C. Fontaine

*Intensive Care Medicine Experimental* 2018, **6(Suppl 2):**0758

**INTRODUCTION.** ICU patients are exposed to the emergence of MDR GNB enteral colonization. Previous work showed that the MDR GNB infection risk is related to the RA of MDR GNB (Log 10 concentration of MDR GNB - concentration of GNB) amongst the enteral microbiota, but the RA of MDR GNB in ICU patients had not been studied yet. (1)

**OBJECTIVES.** We aim to study the relationship between the digestive RA in MDR GNB and occurrence of invasive MDR GNB infections in ICU patients.

**METHODS.** During 2 months, every intubated patient admitted in Bichat medical-infectious diseases ICU (Paris, France) was prospectively included. We collected an average of 2 stools samples per week from the admission to the discharge or death. MDR GNB were detected by seeding stool samples on selective environment and identified by using matrix-assisted laser desorption/ionization-time of flight (MALDI-TOF).

In case of MDR GNB detection, a quantification of the MDR GNB colonies and of the whole GNB colonies was made, and MDR GNB digestive RA was calculated.

We also collected clinical and biological data (SOFA score, lymphocyte to neutrophil ratio, death). The occurrence of MDR GNB nosocomial infection was reported during the 7-day period following the stool sample collection, as well as active MDR GNB antibiotics exposure between the stool sample and the infection or until the 7th day. Results are expressed in median [Interquartile range] and the concentration in $\log_{10}$.

**RESULTS.** In 62 stool samples in 32 intubated ICU patients followed and able to emit stools, 19 (30.6%) were positive for MDR GNB. MDR GNB enteral colonization was followed by a MDR GNB nosocomial infection within 7 days in 7 cases (37%) (including Ventilator associated pneumonia (VAP) in 4 cases).

Neither quantitative culture of MDR BNG (8,48 vs 7,60, p= 0,27), quantitative culture of total GNB (8,6 vs 8,3, p= 0,42) and MDR GNB digestive RA (-0,18 vs -0,40, p= 0,46) were related to infection.

Infected patients tend to have a higher SOFA score at the time of sampling stools (8 vs 6, p=0,26) and a lower lymphocyte to neutrophil ratio (0,051 vs 0,083, p=0,24). Time of antibiotic exposure effective against MDR GNB during the 7 days following the sampling of stools did not differ between patients who developed MDR GNB infections and others (0 vs 0,5 days p= 0,48).

3 patients (9,3%) developed a MDR GNB infection without a MDR GNB enteral colonization (2 VAP and 1 bacteremia).

**CONCLUSIONS.** In a pilot study, we found that MDR GNB abundance and relative abundance in the stool is high in all intubated ICU patients. We were not able to unmask major differences in abundance and relative abundance of MDR GNB in the stool for predicting nosocomial infections in the next week. Further largest cohort using systematic swabs instead of stools and adjusted on immune status and antimicrobial exposure is ongoing.

**REFERENCE(S)**

1. Ruppé, E. *et al, Antimicrob. Agents Chemother* (2013)

**GRANT ACKNOWLEDGMENT**

No funding grant

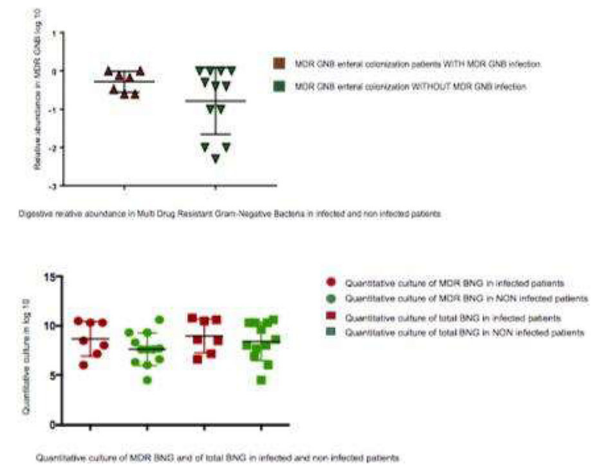

**Fig. 1 (abstract 0758).** Relative abundance and quantitative culture of MDR GNB

### 0759

#### Determination of risk factors to develop infection with enterococo faecalis and faecium vancomycin-resistant in criticall ill patients and outcome associated to appropriate empirical antibiotic

D. Molano[1,2], M. Villabon[2], F. Barbosa[2], J. Mejia[2], E. Beltran[2], M. Gomez[2], L. Muñoz[2]
[1]hospital de san jose, Bogota, Colombia; [2]Fundacion Universitaria Ciencias de la Salud, Critical Care, Bogota, Colombia
**Correspondence:** M. Villabon
*Intensive Care Medicine Experimental* 2018, **6(Suppl 2):**0759

**INTRODUCTION.** Infections caused by Enterococcus spp. have been associated with an increase in mortality in critical care patients.The change in the resistance pattern of these germs has been established, with an increase in the prevalence of Vancomycin-resistant Enterococcus spp. (ERV). In the critical patient, the adequate choice of empirical antimicrobial therapy is essential to improve outcomes.
**OBJECTIVES.** To determine the risk factors associated with the development of VRE infection in patients with sepsis admitted to an intensive care unit in Latin America.
**METHODS.** We analyzed retrospective data and development observational study case-control from patients admitted to a polivalent ICU during a 2-year period. We analyzed those patients with an infection during their ICU stay and search for factors related to the isolation of Enterococcus from clinical samples.
**RESULTS.** We studied 1200 patients, 24 of them with E faecalis ( 78 %) and 8 E. Faecium ( 22 %) vancomycin resistance was found. Patients with Enterococcus Vancomicyn - resistant (VRE) isolation were young (53.2 vs 55 years) and more high scores for APACHE II ( 17.5 vs 15.1 OR: 1.1 p: 0.02), infections had been acquired in the hospital, were surgical patients in higher proportion (77 vs 54.3%) , with abdominal surgical in high percentage ( 44 vs 22% p: 0.005) and had longer ICU stay (77% vs 54.3 % and 18.9 (DE 13.2) vs 10 (DE 9) days OR: 1.2 p 0.005: respectively), had received parenteral nutrition (40.6 % v 4 % OR: 9.5 p 0.008).Renal disease (59.3 vs 54.1% OR: 1.2) and need of haemodialysis (37.5 VS 34.3% OR 1.1) are risk factors for VRE infections (Prior use of antibiotics carbapenemic are associated with more cases of infections VRE (34.2 vs 21.8% OR 1.8). Mortality rate was higher in patients with VRE infection (56.2 vs 27.8 % p: 0.0027). The strategies such as empirical appropriated antibiotic, in patients with VRE, show a tendence to have low mortality (47% vs 57% 0R: 0.6 p: 0.39); Unlike the need of escalonation antibiotic is associated with high mortality (OR: 4.O IC:1.5-10.8 P 0.23).
**CONCLUSIONS.** Infections with VRE are more score severity to admission of ICU and the mortality is twice as old, with patients are infectios for other bugs in ICU. Although the empirical appropriated therapy antibiotic its difficult in this infection, its use are associated with minor mortality. The importance of know the risk factors for VRE , are the patients with septic shock to admission of icu and surgical abdominal with request parenteral nutrition for set up the empirical management appropriated.

**REFERENCE(S).**

1. Chiang HY et al. Incidence and Outcomes Associated With Infections Caused by Vancomycin-Resistant Enterococci: Systematic Literature Review and Meta-Analysis.Infect Control Hosp Epidemiol. 2017 Feb;38(2):203-215.
2. Ranotkar S et al. Vancomycin-resistant enterococci: troublemaker of the 21st century. *J Glob Antimicrob Resist. 2014 Dec;2(4):205-212.*

**GRANT ACKNOWLEDGMENT.** Non

**Table 1 (abstract 0759).** Demografics and outcome in patients with Enterococcus infections and ICU

| | case ( n=32) | control (n=96) | total ( n=128) | Value p |
|---|---|---|---|---|
| AGE (DE) | 53.2 (18.3) | 55.5 (19.4) | 54.9 (19.39) | 0.53 |
| APACHE II (DE) | 17.0 ( 7.0) | 13.9 ( 5.9) | 14.7 ( 6.45) | 0.02 |
| Days Mechanical Ventilation (DE) | 9.0(12) | 6.8 ( 8.8) | 7.2 (9.5) | 0.033 |
| Days UCI (DE) | 18.9(10.3) | 13.2(9.0) | 12.8(9.5) | 0.009 |
| Nutrition Parenteral N(%) | 13(40.6) | 4(4.1) | 17(11) | 0.008 |
| Mortality N(%) | 18 (56) | 27(28.1) | 45(35.1) | 0.0027 |
| Shock Séptic N (%) | 25 (78.5) | 51 ( 44.3) | 76 (60) | 0.007 |
| Appropriate empirical antibiotic N (%) | 10(31.2) | 29(30.2) | 39 (44.3) | 0.18 |
| Antibiotic Escalation N(%) | 13(40.6) | 34(35.5) | 47(36.7) | 0.23 |

### 0760

#### Serial CRP levels do not correlate with antibiotic sensitivity in culture-positive ventilator-associated pneumonia and tracheobronchitis

J. Lage[1], C. Granja[1,2], A. Krystopchuk[2], A. Binnie[1,2,3]
[1]University of Algarve, Departamento de Ciências Biomédicas e Medicina, Faro, Portugal; [2]Centro Hospitalar Universitário do Algarve, Faro, Portugal; [3]William Osler Health System, Toronto, Canada
**Correspondence:** J. Lage
*Intensive Care Medicine Experimental* 2018, **6(Suppl 2):**0760

**INTRODUCTION.** In sepsis, empiric antibiotics are initiated prior to the reporting of microbial cultures[1]. Yet antibiotic resistance is an increasing problem in intensive care units, particularly in the context of nosocomial infections[2]. Early diagnostic biomarkers and treatment efficacy biomarkers have shown promising results in sepsis. One of these is C-reactive protein (CRP), which can be used to monitor efficacy of antibiotic therapy[2].
In this study we examine whether serial CRP measurements in patients undergoing treatment for ventilator-associated pneumonia (VAP) and ventilator-associated tracheobronchitis (VATB) reflect antimicrobial sensitivity, as demonstrated by tracheal aspirate cultures.
**METHODS.** A retrospective chart review of patients with ventilator-associated pneumonia or tracheobronchitis in a large urban ICU from 2015-2017 was performed. Ventilated patients with positive tracheal aspirate cultures at least 5 days after ICU admission were included. Exclusion criteria included a decision by the attending physician not to treat and the presence of other ongoing infections. CRP levels were recorded for 8 days, starting on Day 1 of treatment for presumed VAP/VATB. Patients who received antibiotics to which the culture organism was resistant, but subsequently changed to an effective regimen, were included in the "resistant" group until the day of antibiotic change. Statistical analysis was based on evaluating direct correlation of CRP values, CRP variation during consecutive days, and CRP ratio analysis (current day CRP concentration/ CRP concentration on day 1).
**RESULTS.** 48 patients with positive tracheal aspirate cultures and treatment for presumed VAP/VATB were identified. Of these, 33 were empirically prescribed antibiotics to which the culture organism was sensitive and 14 were prescribed antibiotics to which the organism was resistant. Sex, age and length of ICU stay prior to initiation of antibiotics were similar for both groups.

The most commonly isolated microorganism was *Pseudomonas aeruginosa* (47,9%) in both groups. Mean CRP levels on initiation of antibiotics were 173 ± 109 in the sensitive group and 222 ± 111 in the resistant group. On Day 2 of antibiotic treatment mean CRP levels increased in both groups and subsequently decreased starting on Day 3. No significant difference was identified in the decline in CRP levels between groups.

**CONCLUSIONS.** CRP does not appear to be a useful indicator of antimicrobial resistance in ICU patients with culture-positive VAP or VATB.

**REFERENCE(S)**

1. Rhodes A, Evans LE, Alhazzani W, et al. Surviving Sepsis Campaign. *Crit Care Med*. 2017;45(3):486-552.
2. Povoa P, Coelho L, Almeida E, et al. Pilot Study Evaluating C-Reactive Protein Levels in the Assessment of Response to Treatment of Severe Bloodstream Infection. *Clin Infect Dis*. 2005;40(12):1855-1857.

### 0761

#### Gastrointestinal motility disorders in patients with acute brain injury - a risk factor for sepsis?

J. Schäper, C. Köhncke, M. Quintel
University Hospital Göttingen, Anaesthesiology, Göttingen, Germany
**Correspondence:** J. Schäper
*Intensive Care Medicine Experimental* 2018, **6(Suppl 2):**0761

**INTRODUCTION.** The "gut as a motor of sepsis hypothesis" is an accepted hypothesis for the pathogenesis of systemic inflammation in critically ill patients. Impaired gastrointestinal motility may be a surrogate parameter for intestinal failure including gut barrier failure.

**OBJECTIVES.** This retrospective analysis in patients with traumatic brain injury tests different clinical variables of gastrointestinal motility as independent risk factors for sepsis and death on ICU.

**METHODS.** Retrospective data analysis of clinical variables from a patient data management system (IntelliSpace Critical Care & Anesthesia, ICCA™). Identification of 671 patients with traumatic brain injury treated on ICU in the years 2010-2015 using 9 search items. Chronologic analysis of 108 patients with respect to clinical variables of gastrointestinal motility (time until 50% of nutritional needs were achieved, gastric residual volume, time until first defecation) were analysed on days 0, 1, 2, 3, 5, 7, 10, and 14 as well as outcome parameters (sepsis, death on ICU). Statistics: multivariate analysis of prior evaluated variable-candidates for dichotomous outcome measures sepsis and death on ICU. Comparison of variables of patients with gastrointestinal motility disorders with and without sepsis. Group comparison with Mann-Whitney U test. Significance level at p< 0.05.

**RESULTS.** Risk factors for sepsis were female sex (p=0.016), detection of gram positive (p=0.033) or gram negative pathogens (). The only risk factor for death on ICU was the time until defecation (p =0.033). Patients who died on ICU had shorter times until defecation when compared to patients who survived ICU (58 [29;68] vs. 109 [58;159] h, p=0.032). In patients with sepsis as compared to patients without sepsis neither time until first defacation 130 [58;190] vs. 95 [53;154] h, median[quartiles], p=0.197) nor time until 50% of nutritional needs differed significantly (95 [56;130] vs. 60 [53;75] h, p=0.06).

**CONCLUSIONS.** In this ICU-cohort of patients with traumatic brain injury variables of gastrointestinal motility did not serve as risk factors for sepsis or death on ICU. Gastrointestinal motility disorders also were not significantly pronounced in patients with sepsis compared to patients without sepsis.

### 0762

#### Hospital-acquired Gramnegative rods bacteremia in intensive care units

D. Adukauskiene[1], D. Valanciene[1], A. Dambrauskiene[2], A. Vitkauskiene[3]
[1]Lithuanian University of Health Sciences, Intensive Care Clinic, Kaunas, Lithuania; [2]Lithuanian University of Health Sciences, Infection Control Service, Kaunas, Lithuania; [3]Lithuanian University of Health Sciences, Laboratory Medicine Clinic, Kaunas, Lithuania
**Correspondence:** D. Valanciene
*Intensive Care Medicine Experimental* 2018, **6(Suppl 2):**0762

**INTRODUCTION.** Growing antimicrobial resistance among Gram-negative rod (GNR) strains is a worldwide issue. Delayed appropriate antimicrobial therapy is associated with higher mortality, so flora monitoring impacts the right first choice of antibiotic.

**OBJECTIVES.** The aim of study was to analyze pathogens and their sensitivity of hospital-acquired (HA) GNR monobacteremia strains to antimicrobial drugs and risk factors of mortality in Intensive Care Units (ICUs).

**METHODS.** Ongoing retrospective cohort study of patients treated in ICUs of Kaunas Clinic's with positive blood culture for GNR taken after 72 hrs of hospitalisation during 7yrs period was carried out.

**RESULTS.** We've found 196 cases of HA bacteremia due to GNR: 72 (36.7%) caused by Acinetobacter spp. (P=0.03), 38 (19.4%) by Escherichia coli, 34 (17.3%) by Klebsiella spp., 19 (9.69%) by Serratia spp. and just few others. Sensitivity to carbapenems (n=182, 92.9%), amikacin (n=131, 66.8%) cefoperazon/sulbactam (n=116, 59.2%), and piperacillin/tazobactam (n=93, 47.4%) was found. There were found 151 (77%) multi-drug-resistant and among them 64 (42.4%) - extensively-drug-resistant (XDR) strains. Among Acinetobacter spp. there was found 61 (84.7%) multi-drug resistant strain (P=0.04). In general multi-drug-resistant strain was found in male gender (n=90/118, P=0.04, OR=4.56, CI95%=2.4-15.4), elderly (P=0.001, RR=13.3), acute kidney injury (AKI) (n=98/128, P=0.03, OR=2.84, CI95%=1.35-9.92). Lethal outcome 73.5% (n=144) was found in male gender (P=0.04, OR=2.70, CI95%=1.53-9.75), elderly (P=0.04, RR=7.84), previous treatment in hospital (P=0.018, OR=2.46, CI95%=1.24-7.88), higher SOFA score (P=0.002, RR=32.1), mechanical ventilation (P=0.001, OR=12.45, CI95%=1.67-34.5), septic shock, (P=0.001, OR=8.21, CI95%=2.84-27.54), AKI (P=0.001, OR=5.62, CI95%=2.84-11.13), ventilator-acquired pneumonia (P=0.004, OR=2.37, CI95%=1.178-7.76), multi-drug-resistant strain (P=0.001, OR=5.67, CI95%=1.18-27.26). In multi-variate analysis lethal outcome was found in male gender (P=0.04, OR=2.92, CI95%=1.98-8.67), mechanical ventilation (P=0.04, OR=4.125, CI95%=2.70-6.34), septic shock (P=0.001, OR=8.21, CI95%=1.33-6.26), AKI (P=0.02, OR=5.67, CI95%=1.18-27.25), multi-drug-resistant strain (P=0.01, OR=3.47, CI95%=2.46-7.56).

**CONCLUSIONS.** 7yrs study of hospital-acquired GNR bacteremia in ICUs revealed Acinetobacter spp. as predominant mostly multi-drug-resistant pathogen. 3/5 of hospital-acquired bacteremias were related with multi-drug-resistant, almost in half of cases XDR GNR strain. Hospital-acquired multi-drug-resistant GNR bacteremia was related with male gender, elderly and AKI. High sensitivity of GNR to carbapenems was found. High rate of mortality was associated with male gender, mechanical ventilation, septic shock, AKI and multi-drug-resistant strain.

**REFERENCE(S)**

1. Adrie C et al. J Infect, 2016,74(2):131-41.
2. Gubbels S et al. Infect Control Hosp Epidemiol, 2017,38(5):559-66.

### 0763

#### Quantitative and qualitative modifications of high-density lipoproteins (HDLs) induced by bacteria and lipopolysaccharide in human sepsis

S. Tanaka[1,2], D. Diallo[3], S. Delbosc[3], C. Geneve[1], N. Zappella[1], A. Harrois[4,5], S. Hamada[4], E. Denamur[6], J. Duranteau[4,5], O. Meilhac[2,7]
[1]Département d'Anesthesie-Réanimation CHU Bichat Claude-Bernard, Paris, France; [2]INSERM, UMR 1188, DéTROI (Diabète Athérothrombose Thérapies Réunion Océan Indien), Université de la Réunion, Saint-Denis de la Réunion, France; [3]INSERM U 1148, Laboratory for Vascular Translational Science Bichat Hospital, Paris, France; [4]Service d'Anesthésie-Réanimation, Hôpitaux Universitaires Paris-Sud, Université Paris Sud, Hôpital de Bicêtre, Le Kremlin-Bicêtre, France; [5]Laboratoire d'Étude de la Microcirculation 'Bio-CANVAS'-Biomarkers in CardioNeuroVascular DISEASES' UMRS 942, Paris, France; [6]UMR 1137 IAME INSERM, Université Paris Diderot and APHP, Laboratoire de Génétique Moléculaire, Hôpital Bichat, Paris, France; [7]CHU de la Réunion, Saint-Denis de la Réunion, France
**Correspondence:** S. Tanaka
*Intensive Care Medicine Experimental* 2018, **6(Suppl 2):**0763

**INTRODUCTION.** Sepsis is associated with systemic inflammation that may impact lipoprotein function. In particular, high-density lipoproteins (HDLs) that display pleiotropic protective functions may be dysfunctional in septic conditions (1,2,3).

**OBJECTIVES.** The aim of this study was to evaluate the HDL profile and the inflammatory context in septic shock patients admitted to our intensive care unit (ICU).

**METHODS.** In this study,20 septic shock patients and 20 controls (ICU patients without septic shock) were included. Plasma samples were collected on days 1, 2 and 7. Total cholesterol and lipoprotein concentrations were determined. HDL profiles were obtained using the Lipoprint® System (non-denaturing electrophoresis).HDL profile characterization was carried out in vitro using whole blood, plasma and isolated HDLsincubated with different bacterial strains.

**RESULTS.** HDL-cholesterolconcentration was statistically lower in septic shock patients than in controls (table 1). At days 1 and 2, septic patients had significantly more large-sized HDL than control patients (figure 1). Survivor patients recovered a normal lipid profile at day 7. More importantly, we provide *in vitro* evidence that bacteria and LPS are able to increase the size of HDL particles (figure 2).

**CONCLUSIONS.** Our results emphasize that HDL-cholesterol levels are dramatically decreased at the acute phase of septic shock. The shift towards large HDL particles observed during sepsis could be due to a direct action of bacteria and LPS on lipoproteins.

**REFERENCE(S)**

1. Tran-dinh et al. Br Journal of pharmacology. 2015,
2. de la Llera Moya Atherosclerosis 2012,
3. Zimetti journal of lipid research 2017

**Table 1 (abstract 0763).** Lipid profile at Day 1

| | Septic shock (n=20) | Non-Septic shock (n=20) | p |
|---|---|---|---|
| Total cholesterol, mmol/l | 2.1 (1.6-2.7) | 4.0 (3.1-4.8) | <0.0001 |
| LDL-C, mmol/l | 1.0 (0.8-1.6) | 2.3 (1.4-2.9) | 0.001 |
| HDL-C, mmol/l | 0.4 (0.3-0.7) | 1.3 (1.0-1.5) | <0.0001 |
| Triglycerides, mmol/l | 0.9 (0.7-1.7) | 1.0 (0.7-1.2) | 0.69 |
| Apolipoprotein- A1, mg/l | 916 (315-2543) | 2482 (1428-5518) | 0.05 |

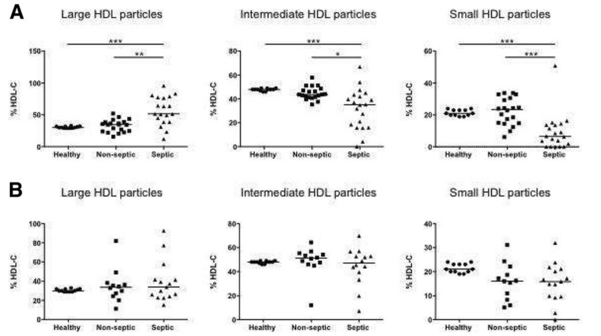

**Fig. 1 (abstract 0763).** HDL particles size at day 1 (A) and day 7 (B)

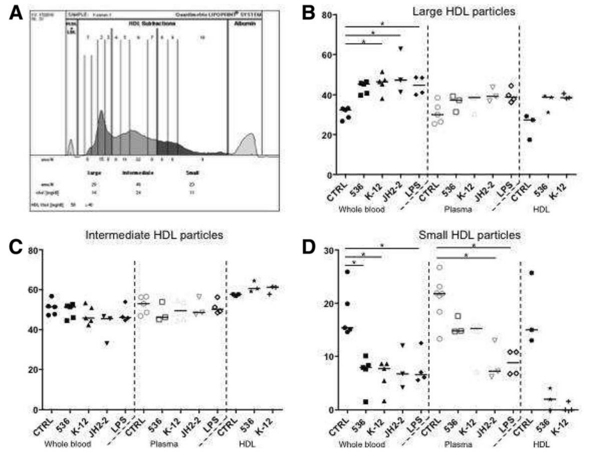

**Fig. 2 (abstract 0763).** Effect of bacteria and LPS on HDL particle size in vitro

### 0764

#### A rapid diagnostic to exclude bacteraemia may reduce antimicrobial prescribing in the critically ill

G. Bercades[1], S. Yui[2], M. Muzslay[2], E. Blackburn[1], S. Ali[2], D. Smyth[1], A. Macklin[1], J. Ryu[1], N. MacCallum[1], P. Wilson[3], D. Brealey[1]
[1]University College London Hospitals NHS Trust, Critical Care, London, United Kingdom; [2]University College London Hospitals NHS Trust, Environmental Research Lab, London, United Kingdom; [3]University College London Hospitals NHS Trust, Clinical Microbiology and Virology, London, United Kingdom
**Correspondence:** G. Bercades
*Intensive Care Medicine Experimental* 2018, **6(Suppl 2):**0764

**INTRODUCTION.** Blood culture is central to the investigation of any critically ill patient with a serious infection, however results take up to 5 days and, in 90% of cases, they are unable to identify an organism. Earlier, negative results may aid clinical decision-making. Cognitor© Minus (CM) is designed to provide rapid, universal detection of viable organisms. It is performed on blood culture aspirates after 12 hours incubation, and employs Enzymatic Template Generation and Amplification technology to detect microbial DNA polymerase activity. It is designed as a rule out test to exclude bacteraemia and fungaemia, and has a negative predictive value (NPV) of 99.5% when compared to culture at 5 days.

**OBJECTIVES.** Analyse the performance of Cognitor© Minus in critically ill patients being investigated for infection and assess the potential impact on antibiotic prescribing.

**METHODS.** This was a prospective, observational study in a single, adult Critical Care

Unit. Paired blood cultures (2 aerobic & 2 anaerobic) were taken from patients being investigated for potential infection, at the physicians discretion. Providing no growth was detected after 12 hours incubation, a total of 1ml of blood was aspirated from one set and the CM test performed. The CM test was performed only in office hours. Clinicians were blinded to the result but were asked a series of questions by the critical care microbiologist at 24 hours and 48 hours post culture. This included; if the CM test excluded a bacteraemia or fungaemia would it affect their decision to continue or stop antimicrobial treatment compared to current practice.

**RESULTS.** 125 patients (mean age 58 SD ± 18, 58% male) were enrolled over a 6 month period. 110 patients received antibiotics (574 doses). The median time to obtain a CM result was 27 hours (IQR 20-47h) and it obtained a NPV of 100% in this study. Clinicians reported, if they had this information they would have de-escalated/stopped antibiotics in 12 patients (11%). This was a calculated saving of 119 doses.

**CONCLUSIONS.** The use of the CM test beyond office hours would have led to a reduction in average reporting times, however the CM test maintained a very high NPV and demonstrated potential for reducing antimicrobial prescribing. These results support a further interventional study to test whether this test can safely reduce antimicrobial prescribing.

**REFERENCE(S)**

Crow MA et al. Evaluation of a new method for the rapid exclusion of infection in suspected bacteraemia. In 24th European Congress of Clinical Microbiology and Infectious Diseases. Barcelona: ECCMID, 2014

**GRANT ACKNOWLEDGMENT**

This research was funded by an unrestricted grant from Momentum Bioscience Ltd. and supported by the National Institute for Health Research University College London Hospitals Biomedical Research Centre.

## Mechanical ventilation, experimental studies

### 0765

#### The HME filter resistance after drug nebulisation, experimental study

V. Zvonicek[1,2], V. Sramek[1,2], M. Zimmer[1,2]

[1]St. Anna´s University Hospital in Brno, Departement of Anaesthesia and Intensive care Medicine, Brno, Czech Republic; [2]Masaryk´s University Brno, Faculty of Medicine, Brno, Czech Republic

**Correspondence:** V. Zvonicek

*Intensive Care Medicine Experimental* 2018, **6(Suppl 2):**0765

**INTRODUCTION.** Heat and moisture exchanger (HME) is commonly used device for humidification of artificial airway. The potential drawback of HME is its obstruction by the aerosolized drug when the nebulizer is simultaneously placed into the ventilator circuit (1). The amount of increase of filter resistance after drug inhalation and its relevance is not well known.

**OBJECTIVES.** The aim of this study was to measure the impact of the drug nebulization on HME resistance.

**METHODS.** In the experimental model active humidifiers (Fisher Paykel MR 730) were used to simulate humidification function of airways, (100% relative humidity and airflow temperature of 33° C).The model was connected to the ventilator (Avea, Cheiron) by two-way circuit and ventilated in volume control mode with tidal volume 500 ml, respiratory rate 14 breaths per minute, PEEP 5 cm $H_2O$, I:E 1:2, $FiO_2$ 35% and square flow pattern.

The vibrating mesh nebulizer (Aeroneb Pro, Aerogen Limited, Galway, Ireland) was positioned between Y-piece of the ventilation circuit and HME filter (proximal) or between filter and patient´s airways (distal). Two types of HME filters were used (Medisize HME Filter or Medisize Hygrovent Filter/HME). Berodual (solution of ipratropium and fenoterol) was nebulised for 15 minutes.

Flow and pressure drop over the HME filter (dP) were recorded by bedside chart (differential pressure transducer Validyne DP45, Validyne Engineering) and resistance (dP/flow) were calculated.

Statistical significance were tested using T test with Bonferroni correction, p< 0.05 was considered significant, data as mean and SD.

**RESULTS.** Fourteen experimental procedures were performed, 6 in proximal and 8 in distal position.

The initial dP and resistance (basal) significantly increased during nebulisation (peak), the increase was observed after 8 (2) minute after the start of nebulisation and sustained 4 (2) minutes. After nebulisation (end) dP and R returned to normal values and remained low at 15 and 180 minutes after end of nebulisation (end +15, end +180).

Both types of HME filters did not differ significantly and therefore results are provided together (Table 1).

**CONCLUSION.** The resistance of HME was transiently doubled after nebulisation of the drug, but returned to initial values in minutes, both in proximally and distally placed nebulizer.

**Table 1 (abstract 0765).** The pressure drop over the HME filter (dP) and filter resistance (R) after drug nebulisation

| All, n=14 | basal | peak | end | end +15 | end +180 |
|---|---|---|---|---|---|
| dP (cm $H_2O$) | 1 (0.6) | 2.2 (1.2)* | 1 (0.6) | 0.9 (0.2)* | 1.1 (0.9) |
| R (cm $H_2O$/l/s) | 2.4 (1.8) | 5.7 (3.3)* | 2.7 (1.8) | 2.2 (0.6)* | 2.7 (2.3) |
| Set proximal, n=6 | | | | | |
| dP (cm $H_2O$) | 1 (0.5) | 2.5 (1.2)* | 1 (0.2) | 0.9 (0.1)* | 0.8 (0.1)* |
| R (cm $H_2O$/l/s) | 2.5 (1.3) | 6.3 (2.9)* | 2.4 (0.5) | 2.2 (0.3)* | 2.1 (0.3)* |
| Set distal, n=8 | | | | | |
| dP (cm $H_2O$) | 0.9 (0.7) | 2 (1.3)* | 1.1 (0.7) | 0.9 (0.2) | 1.3 (1.2)* |
| R (cm $H_2O$/l/s) | 2.4 (2) | 5.1 (3.5)* | 2.9 (2.3) | 2.2 (0.8) | 3.2 (3)* |

### 0766

#### Standardization of a sampling technic for evaluating airway inflammation in mechanically ventilated patients

R. Stamatiou, V. Tsolaki, E. Zakynthinos, D. Makris

Intensive Care Unit, University of Thessaly Medical School, Larisa, Greece

**Correspondence:** R. Stamatiou

*Intensive Care Medicine Experimental* 2018, **6(Suppl 2):**0766

**INTRODUCTION.** Mechanically ventilated (MV) patients may present airway inflammation and increased secretion production. However, it is not known whether cellular profiles in bronchial samples may be useful to evaluate the clinical condition of patients and bronchial sampling for such purposes is not yet standardized. [1]

**OBJECTIVES.** Our aim was to evaluate homogenized and non-homogenized samples obtained bronchoscopically from different parts of the bronchial tree, in order to access the optimum material for cell counting and protein evaluation.

**METHODS.** Patients admitted in the Intensive Care Unit of the University Hospital of Larissa, Greece, were enrolled in the study if they were intubated and mechanically ventilated. 3 types of bronchial samples were collected via bronchoscopy (PENTAX FB-15X):

a) tracheo-bronchial secretions macroscopically visible at bronchoscopy (VS)

b) tracheo-bronchial aspirates from surfaces without macroscopically visible secretions (IS)

c) mini bronchial lavage after the instillation of 5 ml of sterile normal saline (miniBL) on surfaces without macroscopically visible secretions and d) bronchial lavage (BL) after the instillation of 50 ml of sterile normal saline in subsegmental bronchi.

Samples were homogenized in a Heidolph Silent Crusher S. Homogenized and non-homogenized samples were used for cell count after Trypan Blue staining and for protein extraction. Total protein was measured using the Bradford method after treatment with Lysis buffer (20 mM Tris-Cl pH 8.0, 150 mM NaCl, 1% Triton X-100, 1 mM dithiothreitol, 100 mg/mL-1 PMSF). Cell extracts were cleared by centrifugation (1000g, 20min, 4°C) and optical density was measured photometrically in samples with or without cells.

**RESULTS.** There were no significant differences in both cell numbers and total protein concentration between homogenized and non-homogenized samples. Handling time- time to extract protein and create smears - was 35 min less compared to non-homogenized samples. Total cell number in homogenized samples of IS, VS and miniBL were statistically significantly lower than in BL [677.2*103±144.7*103, 2249*103±508.4*103, 964.2*103±221*103and 2492*103±792.7*103 respectively, ($p < 0.05$)]. Total protein measurements in IS, VS were statistically significantly higher than in BL [12.21±1.99 μg/ml, 17.61±2.17 μg/ml and 8.41±1.81 μg/ml, respectively, ($p < 0.05$)]; there was no statistically significant difference between samples with or without cells.

**CONCLUSIONS.** Homogenization of bronchial samples in MV patients does not seem to affect cell numbers and total protein concentration. Homogenized samples were easier to handle and therefore could represent a biomaterial useful for protein extraction and examination.

**REFERENCE(S)**

1. Ronchi M C et al. Role of sputum differential cell count in detecting airway inflammation in patients with chronic bronchial asthma or COPD. Thorax 1996;51:1000-1004.

**GRANT ACKNOWLEDGMENT**

The study did not receive any grant.

### 0767

### Respiratory $PaO_2$ oscillations and their relationship to dynamic atelectasis measured by CT during tidal ventilation in a surfactant depletion model of ARDS

D. Crockett[1], J. Cronin[2], N. Bommakanti[3], R. Chen[1], C. Hahn[1], G. Hedenstierna[4], A. Larsson[4], A. Farmery[1], F. Formenti[2]

[1]University of Oxford, Nuffield Division of Anaesthetics, Oxford, United Kingdom; [2]King's College, London, Centre for Human and Applied Physiological Sciences, London, United Kingdom; [3]Columbia University, Vagelos College of Physicians and Surgeons, New York, United States; [4]Uppsala University, Hedenstierna Laboratory, Uppsala, Sweden

**Correspondence:** D. Crockett

*Intensive Care Medicine Experimental* 2018, **6(Suppl 2):**0767

**INTRODUCTION.** It has been hypothesised that respiratory oscillations in $PaO_2$ ($O_2Os$) in the injured lung are caused by cyclical recruitment of atelectasis (CA)[1,2]. These phenomena have been observed independently using rapid $PaO_2$ measurement[3,4] and CT[5,6] respectively. Here we explore the hypothesised relationship between $O_2Os$ and CA by contemporaneous measurement of $PaO_2$ and tidal lung density changes in a porcine surfactant-depletion model of lung injury.

**OBJECTIVES.** We hypothesised that the amplitude of $O_2Os$ increases with increasing CA during tidal ventilation in a porcine, surfactant depletion model of Acute Respiratory Distress Syndrome (ARDS). The aim of this study was to establish the role of CA as a determinant of $O_2Os$.

**METHODS.** This study of n=5 domestic pigs (mean weight (±SD) = 29.6 (±1.7) kg) at the Hedenstierna Laboratory, Uppsala University, Sweden was approved by the local ethics committee and adhered to ARRIVE guidelines. Animals under general anaesthesia were studied after saline lavage during tidal ventilation with VCV & PCV at $V_T$ 10 ml $kg^{-1}$, RR 12 $min^{-1}$ and variable I:E ratios. Contemporaneous measurements of atelectasis and $PaO_2$ were recorded using single, juxtadiaphragmatic slice dCT (temporal resolution of 250ms) and a rapid intra-arterial, fluorescence-quenching $PaO_2$ sensor.

**RESULTS.** A total of n = 148 sections of 30 s $O_2O$ data were analysed. Fig. 1(A) demonstrates there is no direct relationship between the change in atelectasis and the respiratory $PaO_2$ oscillation amplitude ($R^2$'s = 0.06 - 0.18) across the group. Mean $PaO_2$ and respiratory $PaO_2$ oscillation amplitude for each ventilator condition are shown in Tab.1 & Fig.1(B).

**CONCLUSIONS.** This is the first study to contemporaneously use a dCT measurement of CA and dynamic measurement of $PaO_2$ in the injured lung. It demonstrates that the presence of CA is not the sole, main determinant of respiratory $PaO_2$ oscillation amplitude in a saline-lavage model of lung injury in pigs, and supports further investigations in the dynamic, often overlooked, role of pulmonary perfusion within the complex context of pulmonary responses to mechanical ventilation.

**REFERENCE(S)**

1 Williams EM *et al. Br J Anaesth* 2000; **85**: 456-9
2 Baumgardner JE *et al. Am J Respir Crit Care Med* 2002; **166**: 1556-62
3 Formenti F *et al. Br J Anaesth* 2015; **114**: 683-8
4 Formenti F *et al. Sci Rep* 2017; **7**: 7499
5 Markstaller K *et al. Br J Anaesth* 2001; **87**: 459-68
6 Markstaller K *et al. Br J Anaesth* 2003; **91**: 699-708

**GRANT ACKNOWLEDGMENT**

Wellcome Trust Translation Award (ADF, CEWH), Swedish Heart and Lung Foundation (AL), Oxford University Medical Research Fund (FF), Whitaker International Fellow Grant (NB).

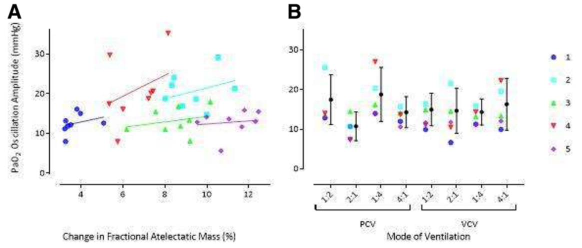

Each animal is represented by a different coloured symbol. PCV = pressure controlled ventilation, VCV = volume controlled ventilation, ratios represent I:E ratios.

**Fig. 1 (abstract 0767).** (A) O2O amplitude vs change in atelectasis, (B) mean O2O amplitude by mode of ventilation

**Table 1 (abstract 0767).** Mean PaO2 & O2O amplitude during different ventilatory conditions

| I:E Ratio | PCV | | VCV | |
|---|---|---|---|---|
| | Mean $PaO_2$ | $O_2O$ Amplitude | Mean $PaO_2$ | $O_2O$ Amplitude |
| 1:2 | 189 (36) | 17 (6) | 198 (34) | 15 (4) |
| 2:1 | 148 (50) | 11 (4) | 195 (41) | 14 (6) |
| 1:4 | 204 (19) | 19 (7) | 197 (27) | 14 (3) |
| 4:1 | 170 (29) | 14 (4) | 198 (24) | 16 (7) |

Values shown are mean (±SD) mmHg. PCV = pressure controlled ventilation, VCV = volume controlled ventilation

### 0768

#### Development of an ex vivo invasive mechanical ventilation model for preclinical aerosol studies

Q. Georges[1,2,3], J. Pourchez[4], L. Leclerc[4], Y. Montigaud[4], N. Prevot[1,2,3], S. Perinel[1,2,3]
[1]Saint Etienne University Hospital, Saint Priest en Jarez, France; [2]Jean Monnet University, Saint Etienne, France; [3]INSERM, U1059, SAINBIOSE, Saint Etienne, France; [4]Mines Saint-Etienne, Univ Lyon, Univ Jean Monnet, INSERM, U 1059 Sainbiose, Centre CIS, Saint Etienne, France
**Correspondence:** Q. Georges
*Intensive Care Medicine Experimental* 2018, **6(Suppl 2):**0768

**INTRODUCTION.** Nebulization is a widely used technique for delivering drugs to patients under mechanical ventilation (MV) in intensive care units (ICU). However, the effectiveness of this drug delivery and optimal conditions for implementation still remain poorly documented. Further clinical trials for MV patients are needed but in vivo human studies are scarce because of ethical restrictions due to imaging technique in nuclear medicine. Consequently, few in vivo trials are feasible and anatomical models remain controversial (1). Thus we need an easy to manage, cost-effective, reproducible and physiologically validated model.

**OBJECTIVES.** This study aim to validate the MV of an ex vivo chimeric lung model previously developed in our laboratory (2) for deposition pattern of inhaled particles.

**METHODS.** The intra-thoracic part of the respiratory tract was obtained from porcine slaughterhouses and arranged in a sealed enclosure. Data about wounds potentially leading to abnormal results were collected. Tracheal intubation respected physiological angulation. MV was achieved with an Evita 4 ventilator (Dräger, Germany). Ventilation parameters were set based on a recent epidemiological study of MV patients (3). They were as follows: volume controlled, heated humidifier, fraction of inspired oxygen: 21%, tidal volume: 540 mL, positive end expiratory pressure: 9 mbar, respiratory rate: 20 cycles per minute with an inspiratory / expiratory ratio of 1/2, inspiratory flow: 35 liters per minute (3, 4).

The first part of the study was dedicated to physiological measurements using a pneumotachograph and a differential pressure sensor.

The second part consisted in 81m Krypton scintigraphies to assess the homogeneity of ventilation. A manual count of total relative uptake corrected for background signal attenuation was made for each lung.

**RESULTS.** Twenty-three respiratory tracts were set for MV. Physiological measurements showed good coherence with human data. Average values and their standard deviations (SD) are as follows: tidal volume, 479 mL SD 52 mL; minute volume, 9.6 L/min SD 1.1 L/min; leakage volume, 1.2 L/min SD 1.1 L/min; resistance, 3.1 cmH2O/L/sec SD 0.9 cmH2O/L/sec; compliance, 339.6 mL/cmH2O SD 195 mL/cmH2O. The 12 scintigraphies realized showed homogenous ventilation (Fig. 1) having an average of 52.3% and 47.7% of respectively right and left lung counts (SD, 3.7%) meaning great similarity with human studies (5, 6).

**CONCLUSIONS.** This preclinical model provides an easy to use, reproducible and cost-effective tool to later achieve larger aerosol studies under mechanical ventilation.

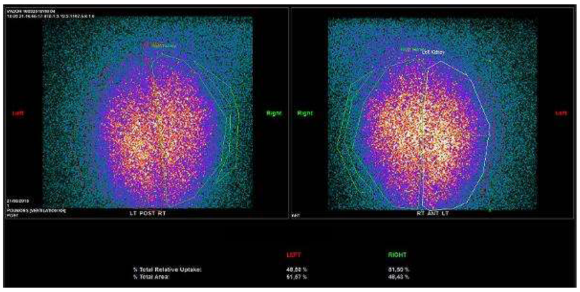

**Fig. 1 (abstract 0768).** 81m Krypton planar scintigraphies of porcine lung invasive ventilation with the regions of interest

### 0769

#### Not only pendelluft: multifold local inspiratory air-redistribution events continuously occur in collapsible lungs. An experimental study

M. Pellegrini[1,2], G. Hedenstierna[3], A. Larsson[1,2], G. Perchiazzi[1,2]
[1]Hedenstierna Laboratory. Dept of Surgical Sciences, Uppsala University, Uppsala, Sweden; [2]Central Intensive Care Unit; Uppsala University Hospital, Uppsala, Sweden, [3]Hedenstierna Laboratory. Dept of Medical Sciences, Uppsala University, Uppsala, Sweden
**Correspondence:** G. Perchiazzi
*Intensive Care Medicine Experimental* 2018, **6(Suppl 2):**0769

**INTRODUCTION.** Macroscopic air-redistribution events related to strong inspiratory efforts causes unpredictable overstretch in heterogeneous lungs during spontaneous breathing (SB). The micromechanics of air-redistribution in heterogeneous lungs has not been investigated and quantified before.

**OBJECTIVES.** To describe at the acinar level during both SB and mechanical ventilation (MV) the effects of positive end-expiratory pressure (PEEP) and external resistance (xRaw) on gas-redistribution during inspiration.

**METHODS.** SB and MV were compared in six pigs with collapsible lungs obtained by lung lavage. Dynamic CT images were acquired at two distances from the diaphragm dome, during the application of different combination of applied PEEP and xRaw. CT images were processed to obtain delta volume images (ΔVol), each subtending a time frame of 0.05 seconds.

Inspiration was studied as divided in two different functional phases: 1.the airway flow-independent phase of inspiration (classically defined as "pendelluft") and 2. the airway flow-dependent phase of inspiration (here defined as "gas-mingling").

For each inspiration, the first three ΔVol, characterized by zero flow at the airway opening, allowed the pendelluft analysis; the following ΔVol sequence allowed the analysis of air-redistribution during ongoing inspiration.

**RESULTS.** In the studied setup, pendelluft was detected only during SB: it developed along cranio-to-caudal and nondependent-to-dependent directions and was reduced or prevented by high PEEP.

During both SB and MV, multifold local inspiratory air-redistribution events continuously occurred.

Two patterns of gas mingling were identified during ongoing inspiration: 1. large and few areas, here defined as "gas-displacement", 2. small and numerous areas, here defined as "gas-scattering".

Patterns of gas-displacement, characterizing SB at low PEEP and high xRaw, turned into gas-scattering at high PEEP and low xRaw, mainly in peri-diaphragmatic regions. MV predominantly showed gas-displacement patterns.

**CONCLUSIONS.** Albeit pendelluft appears only in SB, both SB and MV are characterized by gas-mingling throughout the whole inspiration. Although not causing pendelluft, MV is more prone than SB in generating unstable patterns of air-redistribution.

**GRANT ACKNOWLEDGMENT. The Swedish Heart Lung Foundation, the Swedish Research Council (K2015-99X-22731-01-4) and the European Society of Intensive Care Medicine (NEXT Grant Award 2017).**

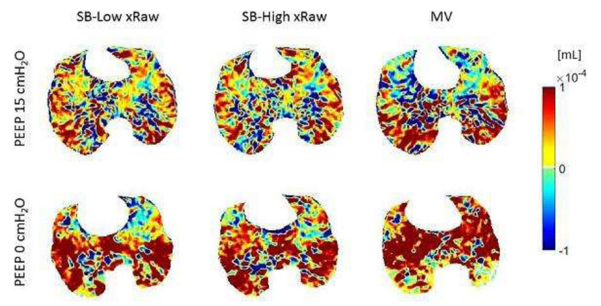

**Fig. 1 (abstract 0769).** Differences in air redistribution during ongoing inspiratory flow

### 0770

#### Re-thinking mechanical ventilation: technological simplicity meets physiological complexity

A. Darwood[1], J. Mccanny[2], B. Martin[3], P. Jones[3]
[1]Imperial College, London, Greater London, United Kingdom; [2]Imperial College Healthcare Trust, London, United Kingdom; [3]St Bartholomew's Hospital, London, United Kingdom
**Correspondence:** J. Mccanny
*Intensive Care Medicine Experimental* 2018, **6(Suppl 2):**0770

**INTRODUCTION.** Modern ventilators consist of digitally controlled electromechanical devices and analogue systems driven by compressed gas sources[1]. Digital sensors and associated control software contribute to high cost, fragility and impracticality whilst pneumatic alternatives rely on a compressed gas source. A Robust, low cost and safe alternative technology is needed in many areas of critical care and in low resource environments[2].

**OBJECTIVES.** We present the invention, prototyping and evaluation of a simple, inexpensive mechanical ventilator that uses a novel design and pressure sensing approach. The technology relies solely on basic control algorithms and a disposable pressure sensitive 'on/off´ switch within the circuit. The resulting binary signal is interpreted and controls an impeller. The approach retains the ability to detect compliance, resistance and circuit leak changes using a novel timing metric. We hypothesize this design safely delivers effective ventilation and provides accurate monitoring at low cost thus overcoming many disadvantages of modern ventilators.

**METHODS.** A prototype was constructed and evaluated using a test lung. Algorithms recorded the time taken to reach peak pressure, 'TRP' (ms), during each breath indicated by 'tripping' of the pressure-sensitive switch. This data was used to investigate the identification of isolated variations in circuit leak, compliance and resistance at multiple peak pressures.

**RESULTS.** The prototype reliably measured pressure-time waveforms. At 20cm$H_2O$ peak pressure, as circuit leak area increased to 6mm$^2$, TRP values increased by 43ms. As leak increased further, TRP values breached a safely threshold resulting in automatic recalibration and adjustment to the leak. As compliance varied from 10ml/cm$H_2O$ to 20ml/cm$H_2O$, a TRP change of 98ms was recorded. Between 20ml/cm$H_2O$ and 50ml/cm$H_2O$ a change of approximately 48ms was recorded. As resistance varied between 0, 5 and 20cm$H_2O$/L/s the TRP varied by 124ms and 155ms respectively. Results were consistent across a range of peak pressures (16-35cm$H_2O$).

**CONCLUSIONS.** The results show this novel ventilator design is able to deliver pressure-controlled mechanical ventilation effectively and safely using simple, low cost, and robust technology. Data supports the reliable performance of the prototype in varied respiratory conditions without needing a compressed gas supply, complex digital sensors or software. We believe the findings make this an attractive prospect for providing short-term mechanical ventilation in remote environments, low resource settings, and during intra/inter-hospital transfers. Further in-vivo clinical testing is planned.

##### REFERENCE(S)

1. Al-Shaikh B,Stacey S.Essentials of anaesthetic equipment.Churchill Livingstone,Elsevier;2013
2. Fludger S,Klein A.Portable ventilators.Contin Educ Anaesth Crit Care Pain.2008

##### GRANT ACKNOWLEDGMENT

N/A

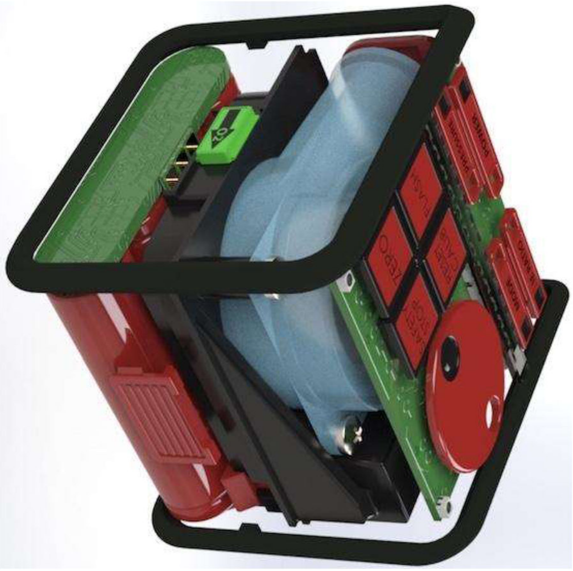

**Fig. 1 (abstract 0770).** Prototype Design

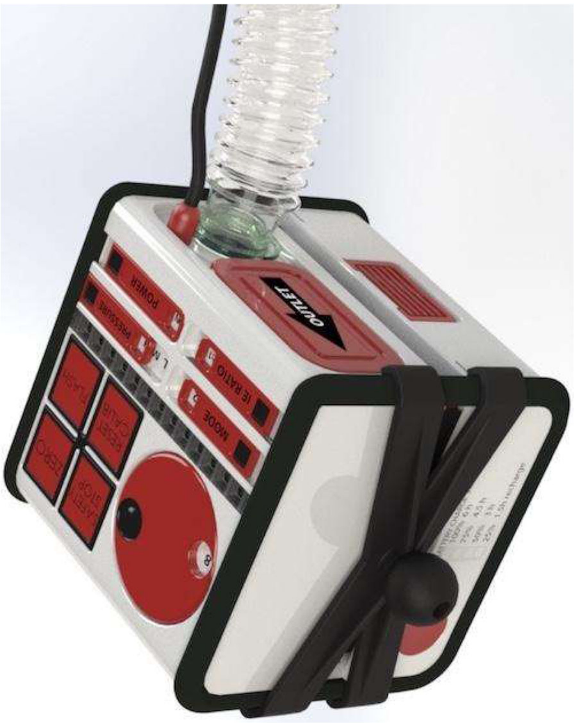

**Fig. 2 (abstract 0770).** Prototype Internal

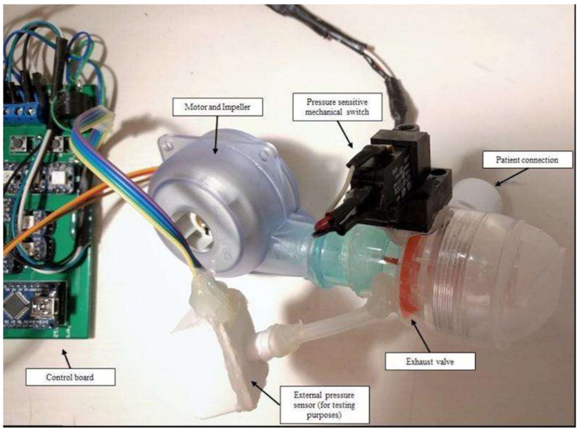

**Fig. 3 (abstract 0770).** Experimental Setup

### 0771

#### Lateral decubitus worsens lung ultrasound score of dependent lungs in an experimental model of right-sided pneumonia

A. Meli[1,2], D. Battaglini[2,3], E. Barbeta Viñas[2], G. Li Bassi[2,4,5], A. Motos[2], J. Bobi[2], M.L. Yang[2], H. Yang[2], J. Miller[2], L. Fernández-Barat[2,4,5], F. Pagliara[3], E. Aguilera-Xiol[2], G. Frigola[6], M. Rigol[2], A. Ferrer[2], R. Cabrera[2], V. Rosario[2], D. Chiumello[1,7], P. Pelosi[3], J. Ramirez[6], A. Torres[2,4,5]
[1]University of Milan, Milan, Italy; [2]Hospital Clinic, Division of Animal Experimentation, Department of Pulmonary and Critical Care Medicine, Barcelona, Spain; [3]University of Genoa, Department of Surgical Sciences and Integrated Diagnostics, San Martino Policlinico Hospital, Genoa, Italy; [4]IDIBAPS/CIBERES, Barcelona, Spain; [5]University of Barcelona, Barcelona, Spain; [6]Hospital Clinic, Department of Pathology, Barcelona, Spain; [7]ASST Santi Paolo e Carlo, SC Anestesia e Rianimazione, Milan, Italy
**Correspondence:** A. Meli
*Intensive Care Medicine Experimental* 2018, **6(Suppl 2):**0771

**INTRODUCTION.** Mechanically ventilated patients with mono-lateral pneumonia are commonly positioned in lateral decubitus, with the healthy lung in the dependent position, to ameliorate ventilation-perfusion coupling. The effects of this intervention on the dependent healthy lung are still elusive.

**OBJECTIVES.** To assess changes in lung ultrasound examination and gas exchanges, during right and left lateral decubitus, in an animal model of right-sided pneumonia.

**METHODS.** Eight pigs (32.4±1.2 Kg) were sedated, and mechanically ventilated. Right-sided pneumonia was induced with bronchoscopic instillation of *P. aeruginosa*. After 24h, animals were positioned in supine position. Then, a recruitment maneuver was carried out and animals were randomly positioned on the right or left side. Primary outcome was lung ultrasound score (LUS) of the dependent lung, computed upon lateral positioning (baseline), and three hours thereafter. Furthermore, at aforementioned times, oxygenation and pulmonary shunt were measured. After these evaluations, animals were repositioned supine, an additional recruitment maneuver was performed, and the effects of positioning on the contralateral side were tested. Two experts in lung ultrasound, blinded to the study interventions, calculated LUS of both lungs, based on a 4-level score (normal = 0, well-separated B-lines = 1, coalescent B-lines = 2, and consolidation = 3) of each of the 6 regions of assessment of both hemithorax (anterior, lateral, and posterior), which were further subdivided into a superior and inferior subsections [1]. Total LUS of right and left lungs was computed.

**RESULTS.** LUS of the left lung worsened in left lateral decubitus (Figure 1). In addition, LUS of the right lung slightly improved in the left lateral decubitus, but worsened to a greater extent in the right lateral decubitus. As expected, $PaO_2/FIO_2$ improved by approximately 100 mmHg in left lateral position, and ameliorated further during the 3-h study period in both positions. Irrespective of oxygenation benefit, a slight increase in pulmonary shunt was found in left lateral decubitus. Linear regression analysis demonstrated slight correlation between $PaO_2FIO_2$ and LUS (p=0.055, $r^2$ =0.11).

**CONCLUSIONS.** In an animal model of right-sided pneumonia, left lateral position improved oxygenation, marginally ameliorated the diseased lung, while deteriorating the healthy lung. In contrast, right lateral position further collapsed the diseased lung, without affecting the healthy lung. Overall our data raises controversial points concerning the use of lateral position in mono-lateral pneumonia and calls for confirmatory clinical evaluations.

**REFERENCE(S)**

[1] Bouhemad B, Mongodi S, Via G, Rouquette I. Ultrasound for " Lung Monitoring " of Ventilated Patients. Anesthesiol 2015;122:437-47.

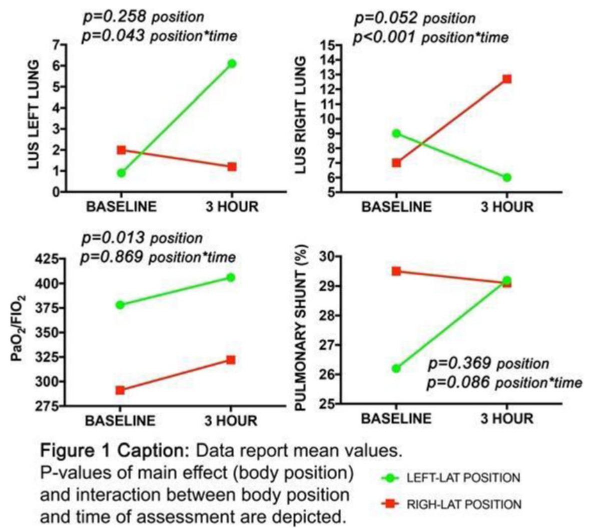

**Fig. 1 (abstract 0771).** See text for desciption

### 0772

#### Positive end-expiratory pressure ventilation induces longitudinal atrophy in diaphragm fibers

M. van den Berg[1], J. Lindqvist[2], R. van der Pijl[1,2], P. Hooijman[1], A. Beishuizen[3], J. Elshof[4], Z. Shi[4], M. de Waard[4], A. Spoelstra-de Man[4], A. Girbes[4], C. van den Brom[5], S. Bogaards[1], S. Shen[2], J. Strom[2], H. Granzier[2], J. Kole[1], R. Musters[1], R. Paul[6], L. Heunks[4], C. Ottenheijm[1,2]
[1]VU University Medical Center, Physiology, Amsterdam, Netherlands; [2]University of Arizona, Cellular and Molecular Medicine, Tucson, United States; [3]Medisch Spectrum Twente, Intensive Care, Enschede, Netherlands; [4]VU University Medical Center, Intensive Care, Amsterdam, Netherlands; [5]VU University Medical Center, Anesthesiology, Amsterdam, Netherlands; [6]VU University Medical Center, Cardiothoracic Surgery, Amsterdam, Netherlands
**Correspondence:** M. van den Berg
*Intensive Care Medicine Experimental* 2018, **6(Suppl 2):**0772

**INTRODUCTION.** Critically ill patients often develop diaphragm weakness, which prolongs ventilator dependency, hospital stay, and increases mortality and health care costs. Mechanisms underlying diaphragm weakness include cross-sectional atrophy and contractile dysfunction of diaphragm muscle fibers, but whether shortening of these fibers *(i.e. longitudinal atrophy)* occurs is unknown.

**OBJECTIVES.** To study the hypotheses that:

(1) an increased end-expiratory lung volume during positive end-expiratory pressure (PEEP) ventilation causes caudal diaphragm displacement and acute shortening of diaphragm fibers;

(2) diaphragm fibers adapt to this acutely shortened position by reducing their contractile filament lengths or the number of serially-linked sarcomeres - the smallest contractile units in the muscle - to maintain a maximum force generating capacity;

(3) adaptation is modulated by the giant mechanosensing protein titin.

**METHODS.** Diaphragm displacement, visualized by ultrasound, and diaphragm fiber shortening during different levels of PEEP ventilation were examined in critically ill patients and shortly mechanically ventilated wild type rats. Diaphragm fiber adaptation during long-term PEEP ventilation was studied in critically ill patients and 18h mechanically ventilated wild type rats. Modulatory effects of titin were assessed in 18h mechanically ventilated wild type rats and rats with a more compliant titin isoform (RBM20 deficient rats).

**RESULTS.** This study shows that (1) in patients PEEP ventilation results in 0.40±0.10cm and 0.89±0.17cm caudal diaphragm displacement at Δ5 and Δ10cm$H_2O$ PEEP; in rats PEEP ventilation results in 0.24mm displacement per cm$H_2O$ PEEP. In rats, acute displacement caused by 2.5 cm$H_2O$ PEEP was accompanied by shorter full-length diaphragm fibers (18.0±0.5 vs. 19.9±0.4mm; p=0.01), and shorter sarcomere lengths (2.64±0.06 vs. 2.85±0.07μm; p=0.04) at end-expiration. (2) After long-term PEEP ventilation, diaphragm fibers in rats adapted to the shorter sarcomere length by reducing the number of serially-linked sarcomeres by 12% (5488±128 vs. 6250±170; p=0.004), whereas contractile filament lengths did not change. (3) RBM20 deficient rats did not show a reduced number of serially-linked sarcomeres after 18h of PEEP ventilation (PEEP vs. control: 7005±777 vs. 7155±424; p=0.70).

**CONCLUSIONS.** Diaphragm fibers show longitudinal atrophy during PEEP ventilation, which might be modulated by titin-borne elasticity. We postulate that longitudinal atrophy contributes to a lower force generating capacity of the diaphragm during spontaneous breathing trials in critically ill patients. During these trials PEEP and the end-expiratory lung volume are reduced, which stretches the shortened diaphragm fibers to excessive sarcomere lengths. At these long sarcomere lengths, muscle fibers generate less force and diaphragm weakness ensues.

**GRANT ACKNOWLEDGMENT**

Coen A. C. Ottenheijm was supported by National Heart, Lung, and Blood Institute (NHLBI) Grant HL-121500

### 0773

#### Effect of invasive mechanical ventilation (IMV) on sleep architecture in intensive care unit (ICU) using 24 hour polysomnography

B. Prajapat, A.S. Sandhya, D. Chaudhry
PGIMS, Pulmonary and Critical Care Medicine, Rohtak, India
**Correspondence:** B. Prajapat
*Intensive Care Medicine Experimental* 2018, **6(Suppl 2):**0773

**INTRODUCTION.** Sleep in icu is not sound and restorative but disturbed and distorted. Survivors of critical illness reported their sleep to be of poor quality and a major stressor associated with their admission. Among several factors, use of mechanical ventilation has been reported as an important cause of disturbed sleep in ICU.

**METHODS.** 24 hour polysomnography (PSG) study was done in two groups of patients who were recovering from their critical illness:
group 1 - IMV group and
group 2 - spontaneously breathing group.

Frequency of nursing care activities were also recorded for both the groups. Quality of sleep in ICU was also assessed by using Richard Campbell sleep questionnaire (RCSQ) and sleep in ICU questionnaire (SICU). Mann- Whitney U test was used to assess the difference of means of both groups.

**RESULTS.** 26 patients completed the study (15 patients in group 1 and 11 patients in group2).Pressure support ventilation was the mode in all group 1 patients . Mean REM stage duration in those who received mechanically ventilation (IMV) and those who did not was 16.57 ± 21.12 mins (2.9%) and 31.47 ± 18.45 mins (5.9%) respectively (p = 0.018). The difference in means of N2 stage between the two groups was also significant (62.5% vs 57.2 %, p=

0.048). Mean arousal index (AI) in IMV and spontaneously breathing group were 27.02 ±11.58 and 16.7 ± 4.46 respectively (p = 0.015). Mean sleep duration without wake was more in spontaneous group (2.699 ± 1.36 mins, 3.83± 1.09 mins : p = 0.018). The difference in mean of other parameters of sleep like total sleep time (TST), wake after sleep onset (WASO), N1, N3, day time sleep and night time sleep did not reach statistical significance (Table 1). Nursing care activities in patients who received mechanical ventilation was more compared to spontaneously breathing patients (52.6 ± 6.78, 48.5 ± 5: p = 0.14) especially during day time (42.2 ± 5.75, 38.6± 4.5: p= 0.06) although the difference in mean did not reach the statistical significance. Mean RCSQ score difference was significant ( group 1- 54.6 ± 7.69, group2- 60.19 ± 10.85: p= 0.04). Patient perceived their sleep as very poor in ICU and addressed nursing interventions and noise as major disrupting factors.

**CONCLUSION.** Sleep in icu is disturbed and fragmented. Mechanical ventilation is an important factor responsible for architectural distortion of sleep in ICU.

### 0774

#### Impact of different positive end-expiratory pressure levels on lung mechanics, gas exchange and circulation in the setting of moderate intraabdominal hypertension: a pig model

M. Fiedler[1], B.L. Deutsch[2], E. Simeliunas[1], D. Diktanaite[1], A. Kalenka[3]
[1]Heidelberg University Hospital, Clinic of Anaesthesiology, Heidelberg, Germany; [2]Justus-Liebig-University, Faculty of Medicine, Giessen, Germany; [3]Hospital Bergstrasse, Department of Anaesthesiology and Critical Care Medicine, Heppenheim, Germany
**Correspondence:** M. Fiedler
*Intensive Care Medicine Experimental* 2018, **6(Suppl 2):**0774

**INTRODUCTION.** Intraabdominal hypertension is a well-known phenome in critically-ill patients. The elevated intraabdominal pressure (IAP) interacts with intrathoracic pressure and influence driving pressure, transpulmonary gradient ($\Delta P_L$), end-expiratory lung volume (EELV), and pulmonary compliance ($C_{stat}$) and therefore lung mechanics. We analysed these parameters in swines with moderate intraabdominal hypertension of 10 mmHg.

**OBJECTIVES.** To compare the effect of different PEEP levels in lung mechanics, gas exchange, cardiocirculatory parameters at induced clinical picture.

**METHODS.** Swine's received general anaesthesia, were tracheotomized and supplied with an esophageal balloon, intraabdominal balloon to induce intraabdominal hypertension, central venous and PiCCO® lines and bladder catheter. Animals were randomised into 3 groups
(Group A=PEEP 5cm$H_2$0, B=PEEP 10cm$H_2$0 and C=PEEP 15cm$H_2$0) and ventilated in pressure controlled/volume guaranteed mode with a tidal volume of 8ml/kg bodyweight for 6 hours. 10 mmHg IAP was generated and maintained in all groups at hour 0. EELV, $C_{stat}$, driving pressure, $\Delta P_L$, IAP and circulatory parameters were measured at baseline, hour 0 and every 2 hours until hour 6.

**RESULTS.** 18 swine (N=18) were included in the study. There were no significant differences between groups at the baseline. In group A (PEEP 5) there was an increase in $\Delta P_L$ (11.2±1 vs. 17.2±1, p< 0,01) and driving pressure (5,7±1 vs. 10±3, p< 0,01) and a decrease of $C_{stat}$ (42±4 vs. 27±2, p< 0,01) and EELV/kg (27±4 vs. 16±3, p< 0,01) compared hours 0 and 6. In group B (PEEP 10) there was an increase in $\Delta P_L$ (11.2±1 vs. 14.5±1, p< 0,01) and driving pressure (5,2±1 vs. 6.7 ±1, p< 0,01) and a decrease in $C_{stat}$ (42±7 vs. 32±3, p< 0,01). EELV did not decreased with a PEEP 10 in group B (27±8 vs. 24±6, p=n.s.). In group C (PEEP 15) $\Delta P_L$ increase (10±1 vs. 12±1, p< 0,05) and driving pressure increase (4,8±2 vs. 7±2, p< 0,05) with no difference in EELV/kg (28±6 vs. 29±7, p=n.s.).

Horowitz Index showed no difference in all three groups after 6 hours. The heart index decreased at PEEP 10 (6.4±0.8 vs. 4.9±0.7, p< 0.01) and PEEP 15 (4.4±1 vs. 3.6±0.5, p< 0,05). Comparing all groups driving pressure decreased and EELV/kg increased with increasing PEEP.

**CONCLUSIONS.** Higher PEEP can improve lung mechanics and gas exchange in moderate abdominal hypertension without lung injury in a swine model.

Akoumianaki et al. The application of esophageal pressure measurement in patients with respiratory failure. Am J Respir Crit Care Med. 2014 Mar 1;189(5):520-31.

##### GRANT ACKNOWLEDGMENT

Study was financed by Hospital Bergstrasse and University Hospital Heidelberg.

### 0775

#### Asynchronies using a simulator of artificial ventilation (SimVA) in virtual COPD patients, effects of reducing pressure support or increasing expiratory trigger

H. Roze[1], R. Dubois[2]
[1]Bordeaux University Hospital, SARsud, Thoracic Intensive Care Unit, Pessac, France; [2]IHU LIRYC, Electrophysiology and Heart Modeling Institute, Foundation Bordeaux University, Pessac, France
**Correspondence:** H. Roze
*Intensive Care Medicine Experimental* 2018, **6(Suppl 2):**0775

**INTRODUCTION.** Simulation in intensive care is an innovative method for teaching. Respiratory settings can be responsible for some asynchronies, which may increase mortality of our patients (1). For this reason we develop a simulator (SimVA) of spontaneous artificial ventilation with virtual breathing patients. Mathematical model resolved differential equations of chest and lung movements according to inspiratory effort in order to match with a clinical database.

**OBJECTIVES.** The goal of this study was to evaluate asynchrony index (AI) in virtual COPD patients according to pressure support (PS) level and Inspiratory time (Ti) and to compare the results to the study of Thille et al (2).

**METHODS.** Virtual case were COPD, defined by thoracic and pulmonar compliance, resistances, lung volumes, and inspiratory adaptive muscle pressure. Asynchrony Index was patient ineffective efforts (IE)/ (IE +Ventilator Respiratory Rate). Ventilatory protocols were Baseline-PS, Optimal-PS and Optimal-Ti (Optimal meant decreasing PS or Ti in order to reduce AI) as described by Thille et al (2). Each virtual case was titrated with each protocol. AI was recorded and compared to the results of Thille et al.

**RESULTS.** The optimal protocols titrated PS or Ti in order to reduce AI, the software simulates the corresponding values of tidal volume and respiratory frequency and its effect on intrinsic PEEP and gas trapping. The difference in settings and respiratory mechanic between virtual cases and patients were not significant (Table 1).

**CONCLUSIONS.** AI was able to change according to PS or Ti settings within the same range as the study from Thille et al. Simulation with the software SimVA is realistic and may help to teach interactively ventilatory settings and asynchronies in COPD patients under Pressure Support Ventilation anywhere without any risk for the patient.

**REFERENCE(S)**

1. Intensive Care Med. 2015;41:633-41.
2. Intensive Care Med. 2008;34:14773-1486.

**Table 1 (abstract 0775).** See text for desciption

| Protocols | Baseline PS | | Optimal PS | | Optimal Ti | |
|---|---|---|---|---|---|---|
| Patients | Real | Virtual | Real | Virtual | Real | Virtual |
| $V_T$ ml.kg$^{-1}$ PBW | 10,2 [7,2-11,5] | 10,0[8,0-11,2] | 5,9[4,9-6,7] | 6,3[5,5-7,0] | 7,0[5,9-7,9] | 6,1[6,1-6,7] |
| PS cmH$_2$0 | 20[19,5-20] | 19,5[19,0-20,0] | 13[12-14] | 13,0[12,7-14,0] | 20[19,5-20] | 19[19-19,5] |
| PEEP cmH$_2$0 | 5[5-5] | 5[5-5] | 5[5-5] | 5[5-5] | 5[5-5] | 5[5-5] |
| RR ventilator breaths/min | 16,1[12,4-17,2] | 12,5[11,0-15] | 22,4[22,0-31,3] | 20,0[18,0-22,5] | 22,6[20,1-30,1] | 18,0[15,7-19,3] |
| RR patient breaths/min | 26,6[23,1-31,9] | 19,5[18,5-22,0] | 29,4[24,6-34,5] | 20,0[18,0-22,5] | 28,3[23,3-34,3] | 18[17,7-19,3] |
| Vm L/min | 8,5[7,8-9,9] | 10,5[8,1-14,0] | 9,4[8,4-11,1] | 9,3[8,4-10,7] | 9,8[7,8-11,4] | 9,7[8,7-10,2] |
| % Expiratory Trigger (%) | 25[25-25] | 25[25-25] | 25[25-25] | 25[25-25] | 45 | 50[45-52] |

*Real patients n=12, Virtual patients n=5, Median [IQR]*

### 0776

#### Cellular profiles in bronchial airways of mechanically ventilated patients: a comparison of two types of macroscopically visible bronchial aspirates

V. Tsolaki, R. Stamatiou, E. Manoulakas, E. Zakynthinos, D. Makris
Intensive Care Unit, University of Thessaly Medical School, Larisa, Greece
**Correspondence:** V. Tsolaki

*Intensive Care Medicine Experimental* 2018, **6(Suppl 2):**0776

**INTRODUCTION.** Cellular profiles in bronchial airways of mechanically ventilated patients are largely unknown although it may reflect airway inflammation and may be associated with ventilator associated infections. [1]
**OBJECTIVES.** The aim of the present study was to evaluate cellular profiles in different methods of bronchial sampling.
**METHODS.** Patients admitted in the Intensive Care Unit of the University Hospital of Larisa, Greece, were enrolled in the study if they were intubated and mechanically ventilated. Two types of bronchial samples were collected via bronchoscopy (PENTAX FB-15X): simple tracheo-bronchial aspirates from mucous membranes with macroscopically visible secretions (VS) and aspirates obtained with bronchial lavage (instillation of 60 ml of sterile normal saline in subsegmental bronchi - BLa). A subgroup analysis of patients without pneumonia was also performed. Samples were homonegized in a Heidolph Solent Crusher S (Heidolph Instruments GmbH &Co. KG, Germany) and cell counting was performed after Trypan Blue staining. Cell extracts were cleared by centrifugation (10000g for 20 min at 4o C) and optical density was measured photometrically.
**RESULTS.** Samples were obtained in 11 cases. VS samples had similar cellular profiles with Bla samples in terms of neutrophils, lymphocytes, macrophages (13.25%±1.58% vs 10.56%±2.96%, p=ns, 29.31%±1.82% vs 24.42%±2.56%, p=ns, 30.10%±2.35% vs 28.56%±3.31%, p=ns, respectively). Similar cellular profiles were observed in patients without pneumonia (n=6) (15.17%±2.27% vs 18.63%±3.15%, p=ns, 26.58%±1.75% vs 24.69%±2.54%, p=ns, 30.62%±3.51% vs 27.15%±4.54%, p=ns, respectively).
**CONCLUSIONS.** Bronchial samples obtained via bronchoscopy either with simple aspiration of macroscopically visible secretions or with bronchial lavage might have similar cellular profiles, irrespective of the presence of pneumonia or not.

**REFERENCE(S)**

1. Keatings VM, Evans DJ, BJ O'Connor, PJ Burnes. Cellular profiles in asthmatic airways: a comparison of induced sputum, bronchial washings and bronchoalveolar lavage fluid. Thorax 1997;52(4):372-374

**GRANT ACKNOWLEDGMENT**
The study did not receive any grant.

### 0777

#### Performance of continuous positive airway pressure (CPAP) systems based on air-entrainment mask. Bench test

J.A. Benitez Lozano[1], P. Carmona Sánchez[2], M. Delgado Amaya[1], J.M. Serrano Simón[2]
[1]Hospital Universitario Regional Málaga, Intensive Care Unit, Málaga, Spain, [2]Hospital Universitario Reína Sofía, Intensive Care Medicine, Córdoba, Spain
**Correspondence:** P. Carmona Sánchez

*Intensive Care Medicine Experimental* 2018, **6(Suppl 2):**0777

**INTRODUCTION.** CPAP is a technique widely used in acute respiratory failure, it's reduce work of breathing (WOB) and improve gas exchange. Various device are avaible, which could have different performance and load on respiratory effort of the patient.
**OBJECTIVES.** Testing of four CPAP systems based on air entrainment comparing its perfomance, in terms of work and effort of breathing (WOB) due to circuit, inspiratory debit, pressurization stability and resistance.
**METHODS.** Boussignac® CPAP valve (Vygon, France); Pulmodyne® (Linde, USA); Ventumask® High Flow (StarMed, Italy); and Caradyne Whiperflow2®(Philips), were evaluated in active lung model simulating spontaneous ventilation (Elastance 33 cm$H_2O$/L, Resistance 5cm$H_2O$/L/s, RR 22/min), with three effort level computer-generated (A: -5 cm$H_2O$, B: -10 cm$H_2O$, C: -15 cm$H_2O$), at three CPAP preset level (5, 10, 15 cm$H_2O$). Calibration was made by gas flow analyzer Vt-Mobile®, Fluke biomedical. Airway, pleural, alveolar pressure (Paw, Ppl, Palv), and Flow (V') were recording at sampling 560 Hz for subsequent analysis. Ten cycles were taken for each condition for measures. Measurement: Mechanic respiratory by multiple linear regression, respiratory pattern, effort and WOB, swing of pressurization as ΔPaw and ΔPalv. The distribution of variables was initially tested with the Shapiro-Wilk test for normality. Results are expressed as mean ± SD, or as median (25-75%). Comparisons were performed by analysis of variance (Bonferroni t-test) or on ranks test (Dunn's) when appropriate.
**RESULTS.**
**CONCLUSIONS.** The performance of CPAP device studied was different. For all levels of effort and pressurization Pulmodyne® represents the lowest breathing load imposed. In response to the effort, Boussignac® valve and Pulmodyne® provide the greatest difference in tidal volume, 2.96 and 2.82 times for CPAP 5 cm$H_2O$, 1.51 and 1.35 times for 10 cm$H_2O$, respectively.

**Table 1 (abstract 0777).** All data.VT: Tidal Volume. Ers: Elastance respiratory system. Rrs: Resistance respiratory system. PTI: Integral pressure-time. *P<0.05

| Variables | Boussignac® | Pulmodyne® | Ventumask® | Caradyne® |
|---|---|---|---|---|
| VT, L | 0.241 (0.216-0.414) | 0.285 (0.211-0.441) | 0.249 (0.209-0.432) | 0.324 (0.205-0.456) |
| Flow inspiratory, L/s | 0.455 (0.371-0.548) | 0.499* (0.439-0.529) | 0.462 (0.392-0.528) | 0.436 (0.347-0.528) |
| Rrs, cmH2O/L/s | 7.96* (4.82-9.89) | 6.87 (3.67-8.86) | 5.51 (3.78-8.49) | 7.54 (5.19-8.36) |
| ΔPaw,cmH2O | 1.39 (0.96-2.47) | 0.67 (0.57-0.97) | 3.15* (2.24-3.58) | 2.27* (1.98-3.01) |
| WOB (Pleural), J/L | 0.66±0.31 | 0.63±0.26 | 0.71±0.39 | 0.76±0.31 |
| PTI (Pleural), cmH2O*s/min | 182.73 (52.63-231.47) | 119.62 (74.26-323.36) | 120.42 (71.44- 218.12) | 208.63* (84.85-301.71) |

**Table 2 (abstract 0777).** Inpiratory debit supplied as tidal volume, and work of breathing for each effort and CPAP level

| | Variables | Effort | Boussignac® | Pulmodyne® | Ventumask® | Caradyne® |
|---|---|---|---|---|---|---|
| CPAP 5 $cmH_2O$ | VT, L | A | 0.104 ± 0.09* | 0.149±0.01 | 0.157±0.01 | 0.156±0.01 |
| | | B | 0.242±0.02 | 0.298±0.03 | 0.239±0.05 | 0.302±0.03* |
| | | C | 0.412±0.01 | 0.457±0.05 | 0.449±0.02 | 0.449±0.07 |
| | WOB (Pleural), J/L | A | 0.34±0.003 | 0.34±0.004 | 0.41±0.008* | 0.30±0.149 |
| | | B | 0.71±0.01 | 0.62±0.03* | 0.75±0.01 | 0.72±0.01 |
| | | C | 0.98±0.007 | 0,99±0.03 | 1.07±0.002 | 1.08±0.026 |
| CPAP 10 $cmH_2O$ | VT, L | A | 0.217±0.002 | 0.273±0.006* | 0.222±0.003 | 0.257±0.002 |
| | | B | 0.362±0.006 | 0.439±0.001* | 0.379±0.009 | 0.323±0.002 |
| | | C | 0.547±0.002 | 0.554±0.002 | 0.523±0.002 | 0.479±0.004 |
| | WOB (Pleural), J/L | A | 0.37±0.008 | 0.35±0.002 | 0.41±0.034* | 0.42±0.007* |
| | | B | 0.70±0.01 | 0.68±0.02 | 0.71±0.03 | 0.70±0.02 |
| | | C | 1.12±0.004 | 1.06±0.003 | 1.18±0.006 | 1.18±0.001 |
| CPAP 15 $cmH_2O$ | VT, L | A | 0.211±0.01 | 0.209±0.004 | 0.203±0.005 | 0.210±0.012 |
| | | B | 0.221±0.002 | 0.222±0.001 | 0.325±0.012 | 0.369±0.005* |
| | | C | 0.455±0.002* | 0.386±0.003 | 0.414±0.004 | 0.530±0.009* |
| | WOB (Pleural), J/L | A | 0.27±0.01 | 0.28±0.004 | 0.29±0.002 | 0.31±0.04 |
| | | B | 0.69±0.006 | 0.53±0.007 | 0.54±0.007 | 0.55±0.005 |
| | | C | 0.98±0.008 | 0.73±.003* | 0.92±0.002 | 1.06±0.009 |

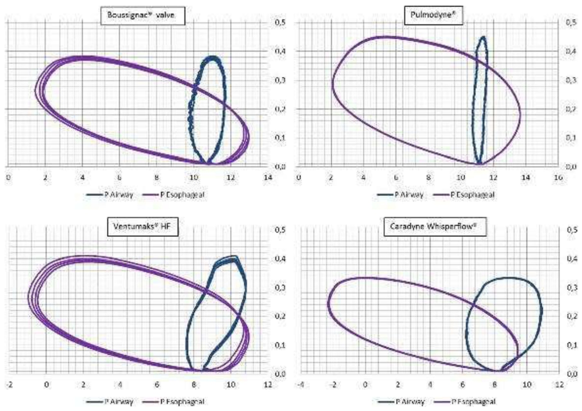

**Fig. 1 (abstract 0777).** Pressure-Volume loops, representive of work of breathing for CPAP preset 10 cmH2O, effort B

## Acute respiratory failure & mechanical ventilation clinical studies

### 0778

#### The incidence of ICU-acquired swallowing disorders after prolonged intubation in the elderly: an endoscopic evaluation

M. Nakanishi
Kurashiki Central Hospital, Intensive Care Medicine, Kurashiki, Japan
*Intensive Care Medicine Experimental* 2018, **6(Suppl 2):**0778

**INTRODUCTION.** Critically ill elderly patients requiring prolonged intubations often develop ICU-acquired swallowing disorders (ICU-ASD), which related to the worse outcomes. However, few research has examined the incidence of ICU-ASD with using fiberoptic endoscopy.

**OBJECTIVES.** The aim of our study is to describe the incidence and clinical characteristics of ICU-ASD in the elderly patients using fiberoptic endoscopy.

**METHODS.** A cross-sectional study was conducted in our medical-surgical ICU from May 2016 to March 2018. The inclusion criteria were all consecutive patients aged ≥65 years old who required prolonged intubation (endotracheal intubation ≥48 hours). Exclusion criteria were patients who had preexisting dysphasia, tracheostomy, and altered mental status (GCS < 13). All included patients were examined for ICU-ASD using fiberoptic endoscopy within 48 hours after extubations. The severity of ICU-ASD was evaluated using the Hyodo-Komagane score which consists of four categories (0: normal, 1: mild, 2: moderate, 3: severe) and assessed four findings (salivary pooling in the vallecular and piriform sinuses, response of the glottal closure reflex, swallowing reflex initiation, pharyngeal clearance after swallowing of test water). The primary outcome was incidence of ICU-ASD. The secondary outcomes were the length of ICU stay and hospital stay.

**RESULTS.** 104 patients were included in our study. Median age was 79.0 (73.8-83.5) years old and 52.9% were male. The median intubated days were 7.0 (5.0-8.0) days. Indications of intubations were sepsis (44.2%), respiratory failure (17.3%), trauma (15.4%), and others (23.1%). The median APACHE II score was 23 (18-30), and the median SOFA score was 9 (7-11). The incidence of ICU-ASD was 25 patients (24.0%). The severity of ICU-ASD was that the normal was 56 patients (53.8%), mild to moderate 40 patients (38.5%). And observed abnormal findings were as follows: 25 patients had aspiration, 12 patients had moderate to severe salivary pooling degree, 6 patients had that reduction of the glottal closure reflex, 27 patients had delayed time of the swallowing reflex, and 15 patients had reduction of pharyngeal clearance. The length of ICU stay and hospital stay were 8.0 (6.0-10.5) and 30.0(17.5-41.0) days.

**CONCLUSIONS.** The incidence of ICU-ASD was 24.0% in the elderly patients, which was lower than previous reports. Multivariate analysis is needed to find risk factors of developing ICU-ASD.

### 0779

#### Lung aeration score and the role of lung ultrasound in patients with acute respiratory distress syndrome on veno-venous extracorporeal membrane oxygenation: a prospective observational study

S. Curry[1], L. Gargani[2], O. Ng[3], A. Roscoe[1], K. Salaunkey[1], B. Agrawal[4], A. Rubino[1]
[1]Royal Papworth Hospital NHS Foundation Trust, Department of Anaesthesia and Intensive Care, Papworth Everard, Cambridge, United Kingdom; [2]Institute of Clinical Physiology, Pisa, Italy; [3]Singapore General Hospital, Bukit Merah, Singapore; [4]Royal Papworth Hospital NHS Foundation Trust, Department of Radiology, Papworth Everard, Cambridge, United Kingdom
**Correspondence:** S. Curry
*Intensive Care Medicine Experimental* 2018, **6(Suppl 2):**0779

**INTRODUCTION.** Acute Respiratory Distress Syndrome (ARDS) is characterised by an acute inflammatory process, loss of lung aeration and impaired gas exchange. In its most severe form, ARDS carries a high rate of mortality and may require Veno-Venous Extracorporeal Membrane Oxygenation (VV-ECMO) to preserve adequate physiological parameters. For ARDS patients receiving VV-ECMO, strict imaging protocols are important for clinical management. Lung UltraSound (LUS) is an emerging bedside imaging technique used to support diagnosis and monitoring of acute lung conditions. A standardised protocol allows for repeated assessment for quantification of lung aeration and examination of pathological aspects of ARDS. The potential use of LUS as a tool to monitor ARDS pathology represents a significant opportunity to enhance patient care and optimise treatment delivery.

**OBJECTIVES.** To determine if lung aeration scores calculated using LUS correlate with the severity of lung injury identified on Computed Tomography (CT) and bedside Chest X-Ray (CXR) in patients with ARDS receiving VV-ECMO.

**METHODS.** This single-centre prospective, observational study investigated consecutive adult patients diagnosed with ARDS receiving VV-ECMO admitted to Royal Papworth Hospital between October 2017-February 2018. LUS assessments were conducted to examine 12-lung regions within 24-hours of patient admission and daily thereafter, until liberated from VV-ECMO. Lung aeration scores were calculated according to appearance of four defined ultrasound patterns. Re-aeration scores were calculated as the total change of each lung region. CT and bedside CXR were conducted as per hospital protocol, with aeration and re-aeration scores calculated retrospectively by an independent consultant radiologist.

**RESULTS.** Consecutive adult patients (n=30) were prospectively screened for inclusion. Data were collected from 18 patients who met inclusion criteria. Full analysis of data is pending, awaiting interpretation of CT and CXR. Preliminary analysis of LUS data indicated good inter-observer reliability to quantify LUS aeration, with an agreement of *K*=.683 (95% CI .549-.797; p< 0.005). A moderate agreement for intra-observer reliability were identified for individual operators, *K*=.587 (95% CI .460 - .706; p< 0.005) and *K*=.582 (95% CI .490 - .668; p< 0.005).

**CONCLUSIONS.** Full statistical analysis is required to determine concordance of aeration scores between imaging modalities. If a significant degree of agreement is demonstrated, routine use of LUS for monitoring of lung aeration is appealing because it is non-invasive, low-risk and may provide more timely clinical assessment to optimise treatment delivery. In the current study LUS were performed by experienced operators, which may preclude extrapolation of intra and inter-observer results to all ICU settings. Limited sample size and specific population make it difficult to draw statistically significant conclusions across populations.

**FUNDING**
None

### 0780

#### Conventional vs Transpulmonary pressure drive ventilation during uro-gynecological pelvic robotic surgery: preliminary data

G. Cammarota[1], G. Lauro[1], I. Sguazzotti[1], A. Messina[1], D. Colombo[1], I. Mariano[1], R. Vaschetto[1], P. Navalesi[2], E. Garofalo[2], A. Bruni[2], F. Mojoli[3], F. Della Corte[1]
[1]Azienda Ospedaliera Universitaria Maggiore della Carità, SCDU Anestesia e Rianimazione, Novara, Italy; [2]Università della Magna Grecia, Anestesia e Rianimazione, Catanzaro, Italy; [3]Università degli Studi di Pavia - Policlinico San Matteo, Dipartimento di Anestesia e Rianimazione, Pavia, Italy
**Correspondence:** G. Cammarota
*Intensive Care Medicine Experimental* 2018, **6(Suppl 2):**0780

**NTRODUCTION.** IRobotic uro-gynecological pelvic surgery (UGpRS) requires pneumoperitoneum and Trendelenburg position, which may both affect intraoperative respiratory mechanics with a consequent impairment of oxygenation [1].

**OBJECTIVES.** IOur aim is comparing conventional ventilation (CV) and transpulmonary pressure (TP), obtained by subtracting esophageal pressure (Pes) from airway pressure, driven ventilation (TPV), as recently proposed in neurosurgical patients [2] on arterial oxygenation during UGpRS.

**METHODS.** IA dedicated nasogastric feeding tube equipped with an esophageal balloon (Nutrivent - Sidam) was inserted in nineteen patients receiving elective UGpSR. Pes was assessed after calibration of the esophageal balloon volume in each patient [3]. Calibration was repeated at every change of positive end-expiratory pressure (PEEP), of pneumoperitoneum level and body position. Patients were randomly assigned to CV or TPV. Controls (CV) received a PEEP value according to the current standards in our division, while in the intervention group (TPV) was set to maintain expiratory end-expiratory PT (PTexp) positive [4]. Arterial oxygen tension to inspired oxygen fraction ratio (PaO2/FiO2) was measured at specific time-points: after anesthesia induction (T0), at pneumoperitoneum and Trendelenburg application (T1), after 20 (T2), 60(T3) and 120(T4) minutes from PEEP application, at the end of surgery (T5), and at awakening (T6). PTexp was monitored from T0 to T5. Finally, lung ultrasound score (LusS) [5, 6] was computed before anesthesia induction (TA) and at T6.

**RESULTS.** TPexp was negative at each time points in controls, while increased above zero cmH2O after instituting TPV (figure 1). As shown in figure 2, PaO2/FiO2 increased from T1 to T6 (p< 0,0001) in the TPV group, while no significant differences were detected in CV group. LusS significantly increased between TA and T6 in the CV group (p=0,0002), while increased slightly and not significantly in the TPV group (figure 3).

**CONCLUSIONS.** IThese preliminary results suggest that, compared to CV, TPV results in improved arterial oxygenation consequent to better lung aeration.

**REFERENCE(S)**
1. Valenza F, et al (2010). Best Pract. Res. Clin. Anaesthesiol.
2. Kumaresan A, (2018). Anesthesiology
3. Mojoli F, et al (2016). Crit Care
4. Fish E, et al (2014) BMJ Open
5. Bouhemad B, et al et al (2011) Am J Respir Crit Care Med
6. Mongodi S, et al (2017). Ultraschall der Medizin

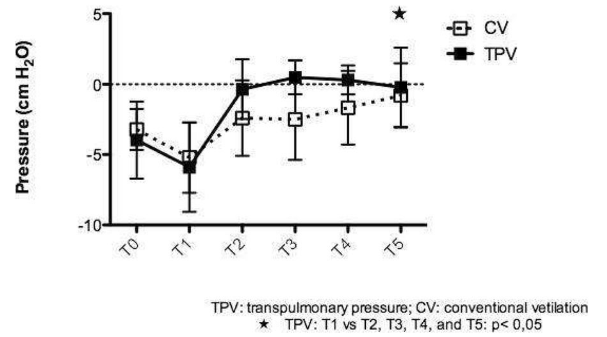

**Fig. 1 (abstract 0780).** Expiratory transpulmonary pressure

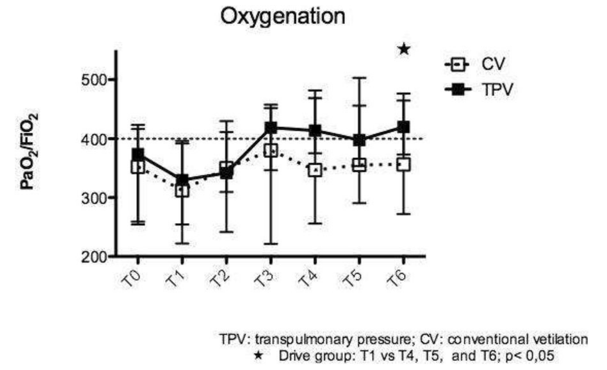

**Fig. 2 (abstract 0780).** Oxygenation

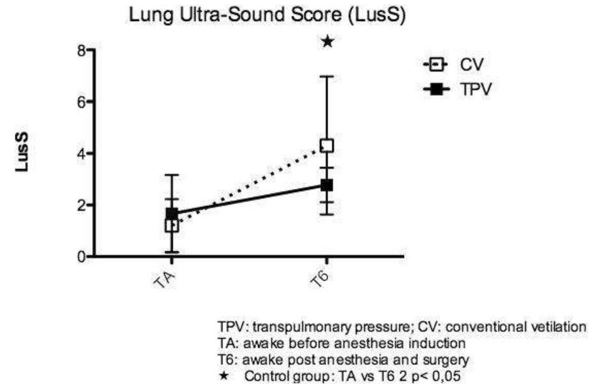

**Fig. 3 (abstract 0780).** Lung ultrasound Score

### 0781

#### Incidence and risk factors for the occurrence of Expiratory Flow Limitation (EFL) in ICU patients

S. Spadaro[1], F. Dalla Corte[1], C. Rizzuto[2], V. Cricca[1], G. Montanari[1], A. Cavallari[1], V. Alvisi[1], D. Morri[1], E. Marangoni[1], C.A. Volta[1]
[1]University of Ferrara, Morphology, Surgery and Experimental Medicine, Ferrara, Italy; [2]ASST Fatebenefratelli Sacco, Intensive Care Unit 1, Milano, Italy
**Correspondence:** S. Spadaro
*Intensive Care Medicine Experimental* 2018, **6(Suppl 2):**0781

**INTRODUCTION.** EFL indicates that maximal expiratory flow is achieved during tidal expiration; this promotes dynamic hyperinflation, leading to an increase in work of breathing and adverse effects on hemodynamics [1]. Although EFL is associated with several different clinical conditions [2], no study has evaluated the prevalence and etiology of EFL in ICU.

**OBJECTIVES.** Aims of present study were:

1) to evaluate the prevalence and incidence of EFL in ICU;
2) to identify the risk factors for EFL development in ICU;
3) to investigate the possible role of fluid therapy on EFL development.

**METHODS.** In this prospective, observational study we enrolled patients admitted to the ICU with expected need for mechanical ventilation > 72 hours. Prevalence of EFL was evaluated by PEEP-test [2] at admission and daily. At 24 hours from ICU admission we performed respiratory mechanics tests. Fluid balance data were recorded during the first three days. Cumulative Fluid Overload (CFO) was calculated by dividing daily fluid balance by the weight of the patient at admission; severe fluid overload was defined by CFO>10% [3]. Mann-Whitney and Kruskall-Wallis tests were used for inter-group analysis. Logistic regression was performed to evaluate independent factors determining EFL at admission and association between CFO and incidence of EFL.

**RESULTS.** A total of 112 patients were enrolled; 53 patients had no EFL (NoEFL) during ICU stay, while 33 patients (30%) had EFL at admission (EFL), and 20 (18%) developed EFL (EFLin) during the first 72 hrs of ICU stay. NYHA class ≥ 2 (OR 10.1,CI 95% 1.8 - 57.1,p = 0.009) and COPD (OR 5.0 CI 95% 1.3-18.7 , p = 0.017) were found to be independent risk factors for the presence of EFL at ICU admission; EFL was associated with more deteriorate respiratory mechanics (Table 1).

Patients developing EFL while in ICU experienced a more pronounced positive fluid balance (Figure 1).

Moreover, a CFO ≥ 10% at 48 and 72 hrs was a determining factor for developing EFL in ICU (OR 3.9, CI 95% 1.3-11.6, p = 0.013 and OR 3.0, CI 95% 1.1-8.6, p = 0.038).

**CONCLUSIONS.** EFL is a common finding in patients admitted to the ICU. Interestingly, more patients than those with EFL at ICU admission developed EFL. This event seems to be related to fluid overload.

**REFERENCE(S)**

[1] Junhasavasdikul D et al. *Chest* 2018; in press
[2] Spadaro S et al. *Anesth Analg* 2017; 124(2):524-530
[3] Samoni S et al. *Crit Care* 2016; 20:95

**Table 1 (abstract 0781).** Respiratory mechanics characteristics of the enorolled patients

| | noEFL (n=59) | EFL (n=33) | EFLin (n=20) | p-value |
|---|---|---|---|---|
| Cstat, ml/cmH2O | 51 [43-66] | 46 [34-56] | 41 [34-56] | 0.045 |
| Rtot, cmH2O/l/s | 16 ± 5 | 21 ± 6 | 17 ± 5 | 0.001 |
| PEEPi, cmH2O | 1 [0-2] | 6 [4-8] | 3 [1-4] | <0.0001 |
| Driving Pressure, cmH2O | 9 [7-11] | 11 [8-13] | 12 [9-13] | <0.0001 |

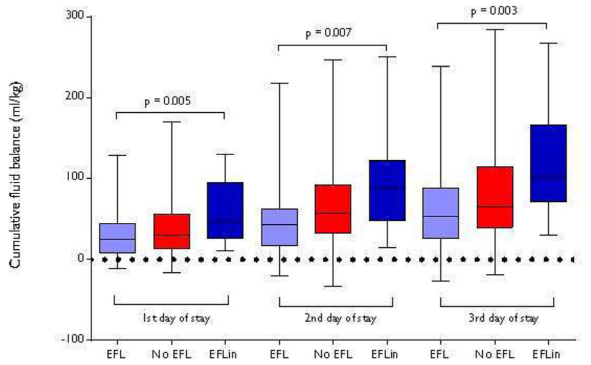

**Fig. 1 (abstract 0781).** Cumulative fluid overload during the first three days of ICU stay in the different groups

### 0782

#### Establishing reliability in providing lung protective ventilation to all mandatory ventilated patients in a general ICU

C. McCue, L. Jarvie, M. Shaw, M. Daniel, M. Hughes, K. Puxty, GRI ICU QI Group
Glasgow Royal Infirmary, Intensive Care, Glasgow, United Kingdom
**Correspondence:** C. McCue
*Intensive Care Medicine Experimental* 2018, **6(Suppl 2):**0782

**INTRODUCTION.** The benefit of lung protective ventilation (LPV) using tidal volumes of 6 mls/kg of predicted body weight (PBW) in ICU patients with ARDS has been established since 2000 (1). Unfortunately, increasing evidence is demonstrating poor adherence to LPV. In 2012, Needham et al published a multi-centre cohort study of patients ventilated for ALI and found only 41% adherence to LPV (2). A recent analysis of LPV adherence from OSCAR, HARP-2 and BALTI-2 demonstrates LPV utilisation from 20-39% (3).

**OBJECTIVES.** We sought to implement and assess the effects of a quality improvement project to improve adherence with LPV for all mandatory ventilated patients in our ICU.

**METHODS.** We set up a quality improvement project with the aim to achieve 95% of mandatory tidal volumes set to ≤6mls/kg/PBW. Baseline data was gathered and from March 2016 a quality improvement project was initiated. We utilised weekly QI meetings to discuss the project and to plan interventions to improve adherence.

**RESULTS.** Over 17 months, 708 patients received mandatory ventilation of which 106 patients were excluded from LPV (53% for $CO_2$ control, 20% clinician decision, 27% not documented). Patient demographics are outlined in table 1.

Figure 1 demonstrates LPV adherence over time, with annotated interventions as part of the QI project. Proportion of patients receiving ≤6mls/kg/PBW (solid line), >8mls/kg/PBW (dotted line) and the median value (dashed line). Interventions as follows; mandatory ventilation was standardised using a pressure controlled, volume cycle mode (1), automated PBW calculation built in to electronic clinical information system (2), weekly QI meetings (3), discussion of 'failed patients' at weekly meetings (4), default tidal volume advice on ventilators at bedside (5), documentation of tidal volume on whiteboard at patient bedspace (6). Baseline adherence in March 2016 was 39%, improving to 56.5% after weekly meetings, 91% after discussion of progress and 'failing patients', and finally a median of 98% with the final interventions.

**CONCLUSIONS.** Implementation of a quality improvement project within our ICU has demonstrated significantly improved adherence with LPV in mandatory ventilated patients.

**REFERENCE(S).**

1. ARDSNET. Ventilation with lower tidal volumes as compared with traditional tidal volumes for acute lung injury and acute respiratory distress syndrome. *NEJM* 2000; 18: 1301-1308.
2. Needham DM et al. Lung protective ventilation and two year survival in patients with acute lung injury: prospective cohort study. *BMJ* 2012; **344**: e2124

3. Poole J, McDowell C, Lall R et al. Individual patient data analysis of tidal volumes used in three large randomised controlled trials involving patients with acute respiratory distress syndrome. BJA 2017 118(4):570-5

**Table 1 (abstract 0782).** Demographics of patients receiving LPV

| CHARACTERISTIC | PATIENT (n=602) |
|---|---|
| Male | 61% |
| Median age (IQR) | 54 (42-67) |
| Median APACHE II (IQR) | 21 (15-27) |
| Median ventilation days (IQR) | 3 (1-7) |
| Median length of stay (IQR) | 4 (1.7-9.7) |
| Respiratory diagnosis | 17.9% |
| Sepsis/Septic shock | 10.6% |
| ICU mortality | 25.4% |
| Hospital mortality | 30.2% |

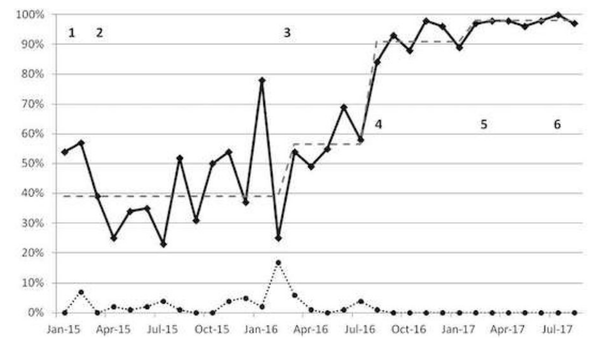

**Fig. 1 (abstract 0782).** Compliance with low tidal volumes in patients on mandatory ventilation

### 0783

#### The effect of serum phosphate level and correction of hypophosphataemia on the duration of mechanical ventilation

K.C. Chan[1], P. Wu[1], H.P. Shum[2]
[1]Tuen Mun Hospital, Department of Anaesthesia and Intensive Care, Hong Kong, China, [2]Pamela Youde Nethersole Eastern Hospital, Department of Intensive Care, Hong Kong, China
**Correspondence:** P. Wu
*Intensive Care Medicine Experimental* 2018, **6(Suppl 2):**0783

**INTRODUCTION.** Low serum phosphate (PO4) level was found to be associated with a longer duration of mechanical ventilation and had been suggested as a useful prognostic factor to predict the need for ventilation. However, it is unclear whether the correction of hypophosphataemia would reverse the effect.

**OBJECTIVES.** To investigate the relationship between the serum PO4 level and the duration of mechanical ventilation in ICU.

**METHODS.** This was a retrospective cohort study that included all mechanically ventilated patients, who had unplanned ICU admission to adult ICUs of two tertiary hospitals in Hong Kong from 2014 to 2017. Patients were excluded if
1) they were excluded from APACHE IV evaluation for any other reasons,
2) they received renal replacement in ICU,
3) their PO4 level was high (> 1.5 mmol/L) at the beginning of their mechanical ventilation,
4) they had fewer than three PO4 measurements taken during their mechanical ventilation, or
5) they had a PO4 level greater than 2.0 mmol/L anytime during their mechanical ventilation.
General additive model with negative binomial distribution was used to explore the effect of the PO4 level on the duration of mechanical ventilation, adjusted to APACHE IV predicted length of stay (A4LOS), and the presence of chronic respiratory failure. The analysis was performed using SAS 9.4.

**RESULTS.** Among 7,345 unplanned ICU admissions during the study period, 1,294 admissions were recruited after considering the inclusion and exclusion criteria. The general additive model, factoring in the minimum PO4 level (Min_PO4) during mechanical ventilation, was shown in Table 1 Model 1. The additive effect of A4LOS and PO4 level was shown in Figure 1. A lower Min_PO4 was associated with a longer duration of mechanical ventilation (p< 0.0001). We modelled the effect of PO4 replacement by the change of PO4 level from the Min_PO4 after one day (Diff_Next_Day). Thus, the more the PO4 rises in one day, the greater the Diff_Next_Day. The effect of PO4 replacement on the duration of mechanical ventilation was not significant (p=0.6523, Table 1 Model 2 and Figure 2). When limiting the analysis to those with hypophosphataemia (Min_PO4 less than 0.75 mmol/L) (n=782), the effect of PO4 replacement on the duration of mechanical ventilation was again not significant (p=0.6761, Table 1 Model 3 and Figure 3).

**CONCLUSIONS.** After adjustment, a lower minimum of PO4 level was associated with a longer duration of mechanical ventilation. This effect may not be modifiable with phosphate replacement. Further prospective interventional study is warranted.

**Table 1 (abstract 0783).** See text for desciption

| Parameter | Model 1 | | Model 2 | | Model 3 | |
|---|---|---|---|---|---|---|
| | Coefficient | p-value | Coefficient | p-value | Coefficient | p-value |
| Intercept | 1.8448 | <.0001 | 2.07664 | <.0001 | 2.14516 | <.0001 |
| Chronic Respiratory Failure | 0.42596 | 0.0109 | 0.40663 | 0.0208 | 0.42593 | 0.0298 |
| Hospital A vs. B | 0.35084 | <.0001 | 0.24256 | <.0001 | 0.23463 | <.0001 |
| Spline(A4LOS) | N/A | <.0001 | N/A | <.0001 | N/A | <.0001 |
| Spline(Min_PO4) | N/A | <.0001 | N/A | <.0001 | N/A | 0.0113 |
| Spline(Diff_Next_Day) | N/A | N/A | N/A | 0.6523 | N/A | 0.6761 |

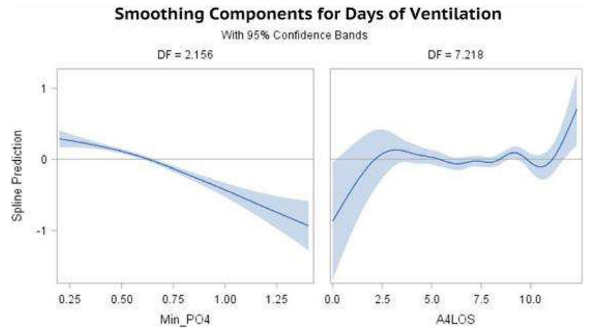

**Fig. 1 (abstract 0783).** See text for desciption

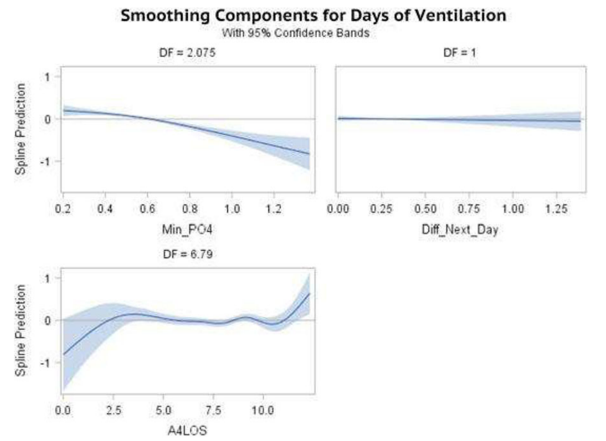

**Fig. 2 (abstract 0783).** See text for desciption

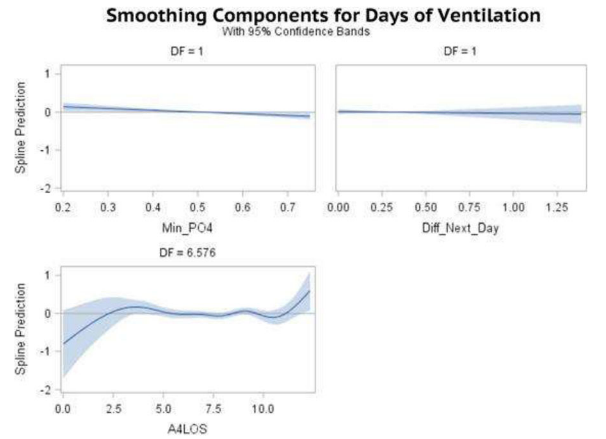

**Fig. 3 (abstract 0783).** See text for desciption

### 0784

#### Addressing diaphragm dysfunction in cardiac surgery patients: successful therapeutic use with current technology and future prophylactic use of temporary diaphragm pacing utilizing intramuscular electrodes

R. Onders[1], M. Elmo[2], R. Schilz[2]
[1]University Hospitals Cleveland Medical Center, Surgery, Cleveland, United States; [2]University Hospitals Cleveland Medical Center, Cleveland, United States
**Correspondence:** R. Onders
*Intensive Care Medicine Experimental* 2018, **6(Suppl 2):**0784

**INTRODUCTION.** 1.6 to 60% of cardiac surgery patients experience phrenic nerve injury and diaphragm dysfunction (DD). Up to 5% require prolonged ventilator support. Invasive or non-invasive mechanical ventilation carries significant morbidity and potential mortality.

**OBJECTIVE.** Describe both therapeutic intramuscular Diaphragm Pacing (DP) for weaning and a feasibility trial of prophylactic capability in 4 patients.

**METHODS.** A retrospective review of compassionate off label use of an FDA approved device under IRB approval (#02-10-18). In this group, an electrical stimulus was delivered to the diaphragm via laparoscopically placed intramuscular electrodes to facilitate diaphragm strengthening and subsequent weaning from mechanical ventilation. A second group prospectively received two temporary DP electrodes placed intramuscularly in each diaphragm at the motor point of where the phrenic nerve enters the muscle at the end of their primary cardiac procedure. (FDA IDE #G150040 and clinicaltrials.gov listing NCT 02410798). Serial diaphragmatic EMG´s were obtained. Safety and efficacy parameters for pacing and implantation are reported.

**RESULTS.** Eleven post cardiac surgery patients with prolonged ventilator support from DD were implanted. Ages ranged from 57-81 years old (average 68). Mean duration of positive pressure ventilation prior to DP was 20 days. All patients were successfully weaned with DP. (8 patients received invasive and 3 received noninvasive mechanical ventilation). The average survival is 32.6 months (1.67 to 76.8 months). There was 1 non DP related early death. Serial EMG's of the diaphragms measured through DP electrodes showed improved burst activity and was used to guide DP therapy. In the next phase, temporary DP electrodes were implanted in 4 patients who underwent median sternotomy. Subject ages ranged from 46-84 years (72 average). The temporary electrodes were successful in achieving ideal tidal volumes and exceeded ideal tidal volumes by 47%. Temporary electrodes successfully measured dEMG. There were no complications. There was complete intact removal of all electrodes at the bedside.

**CONCLUSIONS.** DP can be used successfully to treat DD from phrenic nerve injury. This trial also demonstrates safe placement, removal and functionality of temporary DP electrodes. Routine use of temporary DP electrodes at cardiac surgery followed by early DP use may mitigate prolonged mechanical ventilation and the need for tracheostomy.

**REFERENCE(S)**

Onders R, Elmo MJ, Kaplan C, Katirji B, Schilz R. Extended Use of Diaphragm Pacing in Patients with Unilateral of Bilateral Diaphragm Dysfunction: A New Therapeutic Option. Surgery 2014;156:772-86.

Onders RP, Markowitz A, Ho VP, Hardacre J, Novitsky Y, Towe C, Elmo M, Kaplan C, Schilz R. Completed FDA feasibility trial of surgically placed temporary diaphragm pacing electrodes: A promising option to prevent and treat respiratory failure.Am J Surg. 2018 Mar;215(3):518-521.

### 0785

#### Functional residual capacity, respiratory system compliance, and lung stress and strain recordings in a PEEP INview trial in mechanically ventilated children - a pilot study

E. Geromarkaki, S. Ilia, M. Miliaraki, P. Briassoulis, P. Bourbaki, E. Tavladaki, G. Briassoulis
University Hospital of Heraklion, Pediatric Intensive Care Unit, Heraklion, Greece
**Correspondence:** E. Geromarkaki
*Intensive Care Medicine Experimental* 2018, **6(Suppl 2):**0785

**INTRODUCTION.** Functional residual capacity (FRC) determines both oxygenation and respiratory system compliance (CRS). In ARDS, in which FRC is decreased, an optimal PEEP is anticipated to restore FRC; accordingly, application of different levels of PEEP might improve FRC and CRS and ameliorate possible ventilator-induced lung injury. In adult ARDS, it has been argued that PEEP should be titrated primarily by its impact on CRS.

**OBJECTIVES.** In this longitudinal study, we sought to detect the changes of FRC, CRS, driving pressure (ΔPrs), and lung stress and strain when applying incremental PEEP levels (PEEP INview) in mechanically ventilated (MV) children with or without ARDS and to correlate admission lung mechanics to length of stay (LOS) or MV-days (MVD).

**METHODS.** Mechanically ventilated children were deeply sedated, paralyzed and ventilated in volume-control mode or PRVC with a tidal volume of 6 mL/kg BW throughout the study protocol. Based on their $PaO_2/FIO_2$ ratios, patients were divided in ARDS (< 300) and control groups (>300). Using spirometry of the Engström Carestation and the PEEP INview tool, end-expiratory lung volume (EELV), FRC, and CRS were tested longitudinally (0-12-24hours). Vt was measured at mouth level. At each PEEP level, FRC and CRS measurements were recorded using the nitrogen washout process. Airway driving pressure was calculated as the airway pressure changes from total PEEP to end-inspiratory plateau pressure.

**RESULTS.** Seventeen critically ill mechanically ventilated children (4 girls) were included in the study. At 0-hrs, increasing PEEP from 4 to 10 $cmH_2O$ increased EELV (64±22 to 246±42 ml, p< 0.05) and Global strain (0.17±0.6 to 0.58±14, p< 0.05) in the control group only. At 12-hrs, PEEP incremental changes (4 to 10 $cmH_2O$) increased FRC (54±26 to 180 ±28 ml, p< 0.05 and CRS 0.57±0.1 to 0.88±0.1 mL/$cmH_2O$, p< 0.05) in ARDS and EELV (54±22 to 246±42 ml, p< 0.01) and Static strain (0.17 ±0.06 to 0.58±0.14, p< 0.05) in controls. At 24-hrs, PEEP-induced lung volume recruitment did not significantly change in ARDS patients but increased EELV in the control group (28±42 to 228±28 ml, p< 0.005). LOS and MVD correlated inversely to FRC (rs=-0.7 and -0.88, respectively, p< 0.005) and CRS (rs=-0.79 and -0.87, respectively, p< 0.001); MVD also related with Dynamic strain (rs=0.71, p< 0.02).
**CONCLUSIONS.** PEEP INview might represent a promising tool for optimizing PEEP in mechanically ventilated children. Further studies are needed to ascertain if this PEEP strategy could help guide optimizing various aspects of lung mechanics in individual patients with ARDS.

**REFERENCE(S)**
1. Chiumello D, Chidini G, Calderini E, Colombo A, Crimella F, Brioni M. Respiratory mechanics and lung stress/strain in children with acute respiratory distress syndrome. Ann Intensive Care.December 2016;6(1):11.

### 0786

#### Identifying patients at risk of pulmonary complications post cardiac surgery using static compliance and clinical risk factors

H. Burns, F. Elwin, M. Ahmed
St Georges Hospital, CTICU, London, United Kingdom
**Correspondence:** H. Burns
*Intensive Care Medicine Experimental* 2018, **6(Suppl 2):**0786

**INTRODUCTION.** Pulmonary complications post-cardiac surgery are an important cause of morbidity and mortality, influencing length of stay (LOS) and total cost (1) reportedly affecting 7.5% of patients (2).
Cardiac surgery and perioperative recovery alters pulmonary mechanics, which influences these outcomes (1).
**OBJECTIVES.** This proof of concept study aims to investigate the extent of derecruitment post-cardiac surgery, using static compliance as a surrogate marker, and its association with pulmonary morbidity. We aim to use the data to determine whether high-risk groups may benefit from individualised care.
**METHODS.** Convenience sampling was used to collect data from post-op cardiac cases in a two-month period. Demographic data was collected along with calculation of Static Compliance (Cst) and P/F ratios on arrival to intensive care. Follow up data of hospital length of stay (LOS), respiratory support and clinical decision to treat for a post-op pneumonia was collected.
High risk variables chosen were BMI >35, history of COPD or asthma, recent smoking history, length of surgery > 4 hours and cardiopulmonary bypass time > 120 minutes.
Statistical analysis was done with two-tailed student's T-test or Chi-Squared test.
**RESULTS.** Our study included 50 patients, 27(54%) had coronary artery bypass grafting.
Higher risk patients had a significantly lower compliance and P/F ratio, and a trend towards longer hospital LOS and time on supplemental oxygen. The overall risk of being treated for pneumonia was 36%, with a positive sputum culture in 10% of patients.
Grouping patients using static compliance we found that there was a trend of increased incidence of respiratory complications 42% with Cst under 50 and 16% over, although these did not reach significance p=0.11.
**CONCLUSIONS.** This data adds to the body of evidence showing that post cardiac surgery patients are at high risk of pulmonary complications. With evidence that patients at increased risk can be easily identified. This can be used for future studies to introduce bundles of care to those at higher risk with the aim of reducing pulmonary complications and hospital length of stay and provide more individualized care.

**REFERENCE(S)**
1. Wissman C. Pulmonary complications after cardiac surgery. Semin Cardiothorac Vasc Anaes. 2004 Sep;8(3):185-211
2. Welsby IJ, Bennett-Guerrer E, Atwell D et al. The association of complication type with mortality and prolonged stay after cardiac surgery with cardiopulmonary bypass. Anaes Analg. 2002 May;94(5):1072-8.

**Table 1 (abstract 0786).** High Risk vs Low Risk

| | High Risk (n=28) | Low risk (n=22) | P-Value |
|---|---|---|---|
| Age (years) | 65 | 69 | |
| BMI | 29.4 | 26.5 | |
| P/F ratio (mmHg) | 236 | 310 | 0.04 |
| Cst (ml/cmH2O) | 42.8 | 52.4 | 0.03 |
| Clinical diagnosis of Pneumonia | 9 | 9 | 0.52 |
| Sputum culture positive | 4 | 1 | 0.25 |
| Days of O2 threrapy | 4.24 | 3.13 | 0.08 |
| LOS in hospital (days) | 10.6 | 7.6 | 0.29 |

**Table 2 (abstract 0786).** Outcomes by Static Compliance

| | Cst <30 (n=6) | Cst 30-40 (n=9) | Cst 40-50 (n=16) | Cst 50-60 (n=12) | Cst >60 (n=7) |
|---|---|---|---|---|---|
| LOS in hospital (days) | 16.16 | 8.3 | 9.75 | 8.16 | 6.57 |
| P/F ratio (mmHg) | 220 | 217 | 314 | 261 | 308 |
| Days of O2 | 2.8 | 3.2 | 5.5 | 3.9 | 2.7 |
| Clinical diagnosis of pneumonia | 2 | 4 | 7 | 2 | 1 |

### 0787

#### Esophageal pressure guided PEEP optimization significantly improves oxygenation and intra-pulmonary shunt without increasing driving pressure in obese and pregnant patients

E. Rohrs[1,2], S. Reynolds[3,4]
[1]Simon Fraser University, Biomedical, Kinesiology and Physiology, Burnaby, Canada; [2]Fraser Health Authority, Respiratory Therapy, New Westminster, Canada; [3]Simon Fraser University, Biomedical, Kinesiology and Physiology, New Wesminster, Canada; [4]Fraser Health Authority, Critical Care, New Westminster, Canada
**Correspondence:** E. Rohrs
*Intensive Care Medicine Experimental* 2018, **6(Suppl 2):**0787

**INTRODUCTION.** Obese and pregnant patients admitted to the ICU are challenging to ventilate because of increased chest wall and abdominal pressures. This decreases lung compliance and exacerbates atelectasis, contributing to lung injury. While optimizing PEEP is one of the cornerstones of lung protective ventilation, the higher pressures needed in these patient populations creates a challenge for ventilation management. Esophageal pressure is demonstrated to be a good measure of pleural pressure and can be used to determine the amount of pressure directly transmitted to the alveoli.
**OBJECTIVES.** The objective of this study was to evaluate the effect of PEEP optimization using esophageal pressures on oxygenation and intrapulmonary shunt in an obese or pregnant patient.
**METHODS.** This is a retrospective case series of 18 obese and pregnant ICU patients. P (A-a) $O_2$ gradient and $PaO_2/FiO_2$ ratios on admission to the ICU were compared before and after PEEP optimization using esophageal pressure manometry.

**RESULTS.** PEEP is significantly higher after esophageal pressure PEEP optimization (p=0.000) with no resultant increase in driving pressures (p=0.38). The $PaO_2/FiO_2$ ratio is significantly improved (p=0.031). $FiO_2$ and P(A-a) $O_2$ gradient also improve after PEEP optimization (p=0.000, p=0.016).

**CONCLUSIONS.** Esophageal pressure guided PEEP optimization significantly improves oxygenation and intra-pulmonary shunt in obese and pregnant ICU patients without increasing driving pressure. Further study is needed to investigate the levels of lung injury and inflammation present in obese and pregnant population in the face of high PEEPs being used to ventilate these patients.

**REFERENCE(S)**

1 Ghanta RK, LaPar DJ, Zhang Q, et al. Obesity increases Risk-Adjusted morbidity, mortality, and cost following cardiac surgery. *Journal of the American Heart Association*. 2017;6(3)

2 Chiumello D, Colombo A, Algieri I, et al. Effect of body mass index in acute respiratory distress syndrome. *British journal of anaesthesia*. 2016;116(1):113-121.

3 Shih-Yi Lee, Ding-Kuo Chien, Chien-Hsuan Huang, Shou-Chuan Shih, Wei-Cheng Lee, Wen-Han Chang. Dyspnea in pregnancy. *Taiwanese Journal of Obstetrics & Gynecology*. 2017;56(4):432-436

4 The Acute Respiratory Distress Syndrome Network. Ventilation with lower tidal volumes as compared with traditional tidal volumes for acute lung injury and the acute respiratory distress syndrome. *The New England Journal of Medicine*. 2000;342(18):1301-1308.

5 Chiumello D, Cressoni M, Carlesso E, et al. Bedside selection of positive end-expiratory pressure in mild, moderate, and severe acute respiratory distress syndrome. *Critical Care Medicine*. 2014;42(2):252-264

6 Talmor D, Sarge T, O'Donnell C, et al. Esophageal and transpulmonary pressures in acute respiratory failure. *Critical Care Medicine*. 2006;34(5):1389-1394

**GRANT ACKNOWLEDGMENT**

None

### 0788

#### Predict evolution of lung transplantation according to the respiratory mechanics of the early postoperative. Elastance based on transpulmonary pressure versus pulmonary artery occlusion pressure

P. Carmona Sánchez[1], J.C. Robles Arista[1], M. Delgado Amaya[2], J.A. Benítez Lozano[2], J.M. Serrano Simón[1]

[1]Hospital Universitario Reína Sofía, Intensive Care Unit, Córdoba, Spain; [2]Hospital Universitario Regional Málaga, Intensive Care Unit, Málaga, Spain

**Correspondence:** P. Carmona Sánchez

*Intensive Care Medicine Experimental* 2018, **6(Suppl 2):**0788

**INTRODUCTION.** The pulmonary artery occlusion pressure (wcp) in mechanically ventilated patients can reflect changes in lung pressure. We theorize about the relationship between pulmonary elastic pressure and variations of wcp during the respiratory cycle. As well as, there could be some relationship between pulmonary elastance postoperatively and prognosis.

**OBJECTIVES.** Assess the mechanics of lung and chest wall after of postoperative lung transplant (LT), the relationship between transpulmonary pressure (Ptp) and wcp, and its association with prognosis.

**METHODS.** Prospectively collected data from 56 patients undergoing LT from December 2016-March 2018, on first day post-transplant. During controlled mechanical ventilation we recorded the flow signals (V ´), airway pressure (Paw), esophageal pressure (Pes). Of these, 35 were available pulmonary artery. We calculate by multiple linear regression the mechanics of the respiratory system (Ers, Rrs), pulmonary (Eptp) and chest wall (Ecw). Using the same method we calculated another factor related to pulmonary elastance using the wcp signal (Ewcp). Data were analyzed by the Fisher exact test, Mann-Whitney test or t-test as appropriate. We use linear regression and Bland-Altman analysis for the relationship between ptp and wcp, Relative Risks (RR) using 95% confidence intervals.

**RESULTS.**

**Global respiratory components:** Rrs 13.32±4.94 cmH2O/L/s, Ers 32.17±9.83cmH2O/L, Eptp 22.69±9.15 cmH2O/L, PEEP 6.12±2.13 cmH2O, V' 0.77±0.42 L/s, Vt 0,493±0,11L, RR 20.90±2.81/min.

**Single vs double LT:** Rrs 14.68±5.39 vs 11.24±3.28 cmH2O/L/s, p=0.01; Eptp 24.45±9.68 vs 19.85±7.58 cmH2O/L, p=0.055.

**Emphysema vs fibrosis:** Eptp 20.04±6.13 vs 25.86±11.11 cmH2O/L, p=0.025;

**Death**: Eptp 30.15±8.41 vs 20.61±8,29cmH2O/L, p=0.001. Rrs 16.03 ±7.23 vs 12.59±3.90, p=0.03.

Txp_uni: Eptp 32.57±9.98 vs 22.71± 8.85 cmH2O/L, p=0.021. Txp_bi: 27.73±6.49 vs 16.69± 5.46, p=0.001.

**Stay< > 10d:** Eptp 19.21(16.03-27.12) vs 21.80 (18.69-29.11) cmH2O/L, p=0.143.

**Days of mechanical ventilation < > 12d:** Eptp 21.49±8.43 vs 27.49 ±10.59 cmH2O/L, p=0,051. The resistances and the elastance of the chest wall were irrelevant to the prognosis.

**Relationships between Eptp vs Ewcp:** 21.21±6.63 vs 19.96 ±5.78cmH2O/L, p=0.480. R: 0.88. Concordance analysis: Difference means -1.25±2.98; limits of agreement CI 95% -7.22 to 4.72.

**Ewcp according to death:** 24.15±7.18 vs 16.74±4.37cmH2O/L, p=0.034.

Patients who had lung elastance greater than 20 cmH2O/L had a higher risk of mortality. According to Eptp, RR 6.32, 95% CI (1.23-32.33), p=0.016; and for Ewcp

RR 6.56, 95% CI (1.05-40.94), p=0.044.

**CONCLUSIONS.** The analysis of Lung elastance provides prognostic value, and invites us to try to reduce the perioperative factors associated with its increase (fluids, Vt..). The elastic pressure of lung has a good fit with variations of pulmonary artery occlusion pressure, and therefore in patients who have this measure would not require pleural pressure.

### 0789

#### The impact of using Echinacea as a complementary modality with use of ventilator care bundle to prevent ventilator-associated pneumonia

A. Wahdan

Cairo University, Anesthesia, SICU and Pain Management, Cairo, Egypt

*Intensive Care Medicine Experimental* 2018, **6(Suppl 2):**0789

**INTRODUCTION.** Echinacea has been utilized for its medicinal properties for over 400 years by Native Americans to treat infections and general illnesses. Echinacea strengthens the body's innate resistance and, if indeed the hyaluronidase enzyme inhibiting action is a factor, inhibits the proliferation of bacteria by increasing phagocytic activity.

**OBJECTIVES.** The study will be designed to establish the effect of implementation of ventilator care bundle combined with Echinacea on VAP rate in mechanically ventilated patients.

**METHOD.** Population of study & disease condition . The study will be conducted in 5-bed surgical intensive care unit (SICU) in Kasr-Alainy teaching hospital where patients are admitted from general surgery and trauma unit to receiving mechanical ventilation.

Eighty patients will be divided randomly into two groups:

**Group (A):** 40 patients will have ventilator bundle measures as well as Echinacea capsules.

**Group (B):** Another 40 patients will have ventilator bundle measures only. Capsules will be taken by oral route (in patients who cannot swallow, it can be taken by opening capsules prior to flushing down enteral tubes with no hazards on gastric mucosa.

**OUTCOME PARAMETER.** Rates of VAP will be defined as the number of VAP cases per 1000 ventilator days. Cases of VAP will be identified by the attending intensivists during routine daily round using Centers for Disease Control and Prevention criteria. According to CDC criteria, VAP can be diagnosed in a patient receiving mechanical ventilation who has a new radiographic opacity, a change in pulmonary secretions along with evidence of impaired gas exchange, and either fever or an abnormal WBC count. A lower respiratory tract specimen with positive quantitative culture results is an optional additional CDC criterion for the diagnosis of VAP. Ventilator days will be counted daily by a well trained nurse. A ventilator day is defined as a calendar day for which the patient is charged for mechanical ventilation.

**RESULTS.** In Group B VAP rate is 3.9/1000 ventilator day in Group A, and 5.3/1000 ventilator day in Group B with a P value 0.08 that was not significant. Monthly VAP rate in both groups .Total number of patients acquired VAP were 33 (41.3%), fourteen patients in Group A, and ninteen patients in Group B (P value = 0.256)

**CONCLUSION.** In this study, VCP components were applied in 80 ICU ventilated patients, with providing 40 patients of them Immulant capsules, 2 capsules three times daily for five days decrease inflammatory reactions promoting faster improvement and that may decrease VAP associated mortality.

### 0790

#### Additional inspiratory load of breathing imposed by endotracheal tubes. Dynamic study in patient during pressure support ventilation

P. Carmona Sánchez[1], J.A. Benítez Lozano[2], M. Delgado Amaya[2], J.M. Serrano Simón[1]

[1]Hospital Universitario Reína Sofía, Intensive Care Medicine, Córdoba, Spain; [2]Hospital Universitario Regional Málaga, Intensive Care Unit, Málaga, Spain

**Correspondence:** P. Carmona Sánchez

*Intensive Care Medicine Experimental* 2018, **6(Suppl 2):**0790

**INTRODUCTION.** It is widely accepted that tube resistance can highly increase the work of breathing. This has been proven in vitro and tracheostomized spontaneously breathing patient, however it been not enough studied in tracheally intubated patients during spontaneously ventilatory modes.

**OBJECTIVES.** To determine the relationship between total effort ($Effort_{tot}$) and tube-related additional inspiratory effort of breathing ($Effort_{add}$) in critically ill patients, mechanically ventilated during different assistances on pressure support ventilation (PSV).

**METHODS.** Prospective, clinical study. Fourteen consecutive patients receiving PSV through endotracheal tube (ETT) were studied, under level of assistance applied by your attending physician. We recorded the flow signals (V´), airway pressure (Paw), esophageal pressure (Pes), tracheal pressure (Ptrach), at 560 Hz during 30 min, for a posterior analysis of ten regular cycles. Ptrach was measure using Intratracheal Pressure Sensor®, REF M1045564, CareFusion, Finland. Clinical and demographics data was recording. Measurements: Total Distending Pressure (Ptot) = Paw + Muscle pressure+ ($Pmus_+$). Pmus = Pes - V*Elastance chest wall. Pressure drop across the ETT (ΔPETT) was obtained by subtraction of Ptrach to Paw. The ratio ΔPETT change to the corresponding change in flow was computed as the resistance tube-related, as well inspiratory and expiratory resistance (ETTi, ETTe) were calculated, using linear and polynomial quadratic regression. The respiratory effort of the patient ($Effort_{pat}$) was calculated as pressure-time product/min (PTP/min) by subtraction of Paw to Ptot at inspiratory time. Effort add tube-related ($ETT_{add}$) as PTP based on ΔPETT. The fraction of $Effort_{add}$ to $Effort_{tot}$ was obtained. The results are expressed, mean ± standard desviation, median (25-75%), or percentage. The comparisons were made by t-student test or analysis of variance on ranks, as appropiate.

**RESULTS.**

Age: 59.71±8.32 y. Male 85.71%. $PaO_2/FiO_2$ 275.15±96.6.

Diagnosis : Lung Transplantation 42.85%. Pneumoniae 35.71%.

Thoracic surgery 21.42%. Duration ventilation 5d (1-7.5). Tube: I.D: 8.35±0.23 mm. Inspiratory pressure support 16.76±5.67 $cmH_2O$. PEEP 7.01±1.55 $cmH_2O$.

Resistances computed as 70-75% of the total ($cmH_2O/L/s$). Linear regression: Resistances respiratory system (Rrs): 12.81 (10.09-16.93), $ETT_{tot}$ 8.46 (7.35-13.57), P = 0.063. ETTi 9.04 (6.58-14.49), ETTe 8.71 (6.37-11.96), P = 0.713. Polynomial regression $ETT_{tot}$ 8.33 (7.02-13.29) versus (vs) linear, P=0.713. Among groups P value = 0.573.

Effort, ($cmH_2O.s/min$): $Effort_{tot}$: 391.465±118.575. $Effort_{paw}$: 305.269 ±110.701. $Effort_{pat}$: 86.196±62.723. $ETT_{add}$: 105.043±65.048. ETT/Total(%): 25.821±10.37.

**CONCLUSIONS.** The resistance of tube-relate, can represent to 25% of mechanical load in intubated patients during PSV. We not find differences between inspiratory and expiratory resistances, neither of the results of resistances calculated by linear vs polynomial regression.

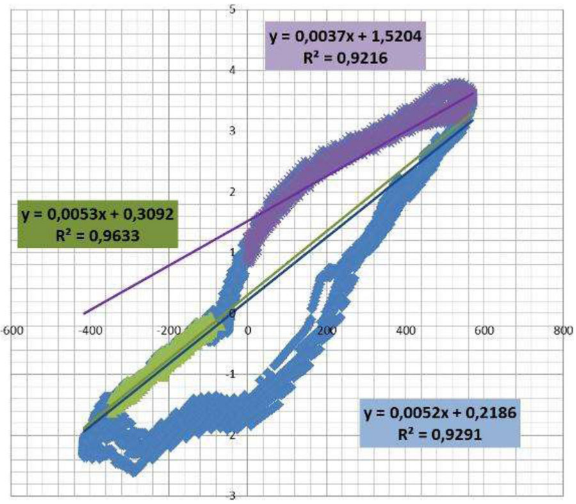

**Fig. 1 (abstract 0790).** Case 3. Linear relationship between the PETT and flow, during inspiration, expiration and total

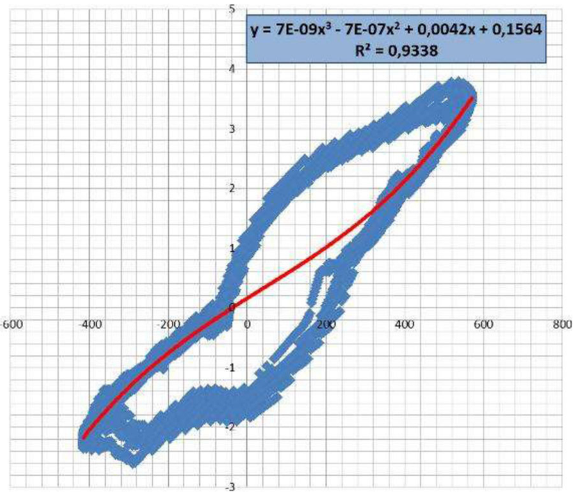

**Fig. 2 (abstract 0790).** Case 3. Polynomial relationship between the PETT and gas flow

### 0791

#### Effects of inspiratory airflow on transpulmonary pressure and lung heterogeneity: preliminary results in mild and moderate ARDS patients

T. Mauri[1], A. Santini[1], E. Spinelli[1], D. Tortolani[2], E. Carlesso[1], M. Albanese[2], A. Galazzi[1], N. Bottino[1], N. Rossi[1], G. Grasselli[1], A. Pesenti[1]
[1]University of Milan, Department of Anesthesia and Critical Care, Fondazione IRCCS Ca' Granda Ospedale Maggiore Policlinico, Milan, Italy; [2]University of Ferrara, Ferrara, Italy
**Correspondence:** D. Tortolani
*Intensive Care Medicine Experimental* 2018, **6(Suppl 2):**0791

**INTRODUCTION.** Beside its role as lifesaving treatment for the acute respiratory distress syndrome (ARDS), mechanical ventilation (MV) can determine additional ventilator-induced lung injury (VILI). Among the multiplicity of MV settings, the effects of inspiratory airflow on determining VILI have largely been neglected (1).

**OBJECTIVES.** We focused on effects of inspiratory airflow on transpulmonary pressure measured by esophageal pressure (Pes) and ventilation heterogeneity measured by electrical impedance tomography (EIT) (2, 3). Study hypothesis was that lower flow rates could result in lower lung stress and heterogeneity.

**METHODS.** We conducted a prospective interventional randomized cross-over study on 7 sedated and paralyzed patients with mild and moderate ARDS (Berlin definition) undergoing volume-controlled ventilation (VCV, S1 ventilator, Hamilton Medical, Bonaduz, Switzerland). Informed consent was obtained following local regulations.

We randomly set two PEEP levels (5 and 15 $cmH_2O$), while keeping Vt of 6-8 ml/kg PBW and clinical $FiO_2$, and we set the ventilator I:E ratio, without end-inspiratory pause, to obtain inspiratory airflow of 401±34 ml/sec, 842±69 ml/sec and 1254±51 ml/sec at each PEEP level (10 minutes per airflow).

Pes, airway pressure (Paw), airflow and EIT data were continuously monitored. By offline analysis, we measured at each step: Vt; peak pressure (Ppeak); end-inspiratory Paw after 2-3 seconds of occlusion (Pplat); Paw at zero flow during end-inspiratory occlusion (P1); transpulmonary pressure ($P_L$ = Paw - Pes) at P1, Ppeak and Pplat. EIT data were analyzed to measure Vt distending non-dependent and dependent lung regions and lung heterogeneity (i.e., number of ventilated pixels in the non-dependent lung region divided by ventilated pixels of the dependent region).

**RESULTS.** Patient were 64 [56-68] yo, undergoing mechanical ventilation since 2 [2-4] days. On the day of the study, $PaO_2/FiO_2$ was 190 [188-239] and 3 patients (42%) had moderate ARDS. Table 1 summarizes study results: Pplat wasn't affected by modification of inspiratory airflow while Ppeak and P1 increased significantly. At Ppeak and P1, inspiratory $P_L$ was significantly higher at increasing airflow. Moreover, the difference between P1 and Pplat (i.e., a classic sign of aeration heterogeneity and pendelluft) increased at higher airflows. The lung heterogeneity measured by EIT showed a trend towards increase at different airflows, too.

**CONCLUSIONS.** Although preliminary, the present results might suggest that higher inspiratory airflow could yield higher transpulmonary pressure and heterogeneity, two possible determinants of VILI.

##### GRANT ACKNOWLEDGMENT

"Ricerca corrente" from Fondazione IRCCS Ca' Granda Ospedale Maggiore Policlinico, Milan, Italy and liberal grant from Hamilton Medical, Bonaduz, Switzerland

**Table 1 (abstract 0791).** Study results

| Variable | PEEP 5 - Low airflow | PEEP 5 - Intermediate airflow | PEEP 5 - Elevated airflow | PEEP 15 - Low airflow | PEEP 15 - Intermediate airflow | PEEP 15 - Elevated airflow | P-value PEEP | P-value airflow | P-value interaction |
|---|---|---|---|---|---|---|---|---|---|
| Pplat ($cmH_2O$) | 15.0 [13.0—18.0] | 15.0 [13.0—16.0] | 15.0 [13.0—16.0] | 25.0 [24.0—26.0] | 25.0 [23.0—27.0] | 25.0 [23.0—27.0] | **<0.001** | 0.624 | 0.712 |
| P1 ($cmH_2O$) | 16.7±2.8 | 16.9±2.5 | 17.4±2.7 | 26.6±1.8 | 27.3±2.3 | 28.3±3.3 | **<0.001** | **0.015** | 0.394 |
| P1 - Pplat ($cmH_2O$) | 1.6±0.5 | 1.9±0.7 | 2.3±1.0 | 1.7±0.8 | 2.6±1.5 | 3.3±1.8 | 0.186 | **0.009** | 0.262 |
| Insp. $P_L$ at Ppeak ($cmH_2O$) | 5.1±2.5 | 12.8±4.9 | 20.9±7.9 | 12.0±3.8 | 18.4±4.2 | 26.0±6.5 | **0.011** | **<0.001** | 0.215 |
| Insp. $P_L$at Pplat ($cmH_2O$) | 0.1±1.1 | 0.3±1.4 | -0.3±0.8 | 6.9±3.6 | 7.7±2.1 | 8.2±1.9 | **<0.001** | 0.369 | **0.004** |
| Insp. $P_L$ at P1 ($cmH_2O$) | 1.4±1.7 | 1.2±1.2 | 1.6±0.8 | 7.9±3.4 | 9.1±2.8 | 11.0±2.4 | **<0.001** | **0.016** | **0.031** |
| Tidal Volume non-dependent (%) | 75 [62—78] | 71 [63—78] | 72 [63—78] | 57 [48—60] | 57 [48—62] | 54 [48—60] | 0.381 | 0.665 | 0.991 |
| Tidal Volume dependent (%) | 25 [23—38] | 29 [22—37] | 28 [23—37] | 43 [40—52] | 42 [38—52] | 46 [40—52] | **0.001** | 0.464 | 0.450 |
| Heterogeneity (ventilated pixel non-dep/dep) | 2.53±2.3 | 3.15±3.8 | 4.65±7.8 | 1.44±0.7 | 1.53±0.9 | 1.51±0.8 | **0.004** | **0.085** | 0.828 |

### 0792

#### Use of regional pressure-volume curves from electrical impedance tomography to set mechanical ventilation in acute hypoxic respiratory failure and acute respiratory distress syndrome: a feasibility study

G. Scaramuzzo[1], S. Spadaro[1], A.D. Waldmann[2], S.H. Böhm[3], R. Ragazzi[1], C.A. Volta[1]
[1]University of Ferrara, Department of Morphology Surgery and Experimental Medicine, Section of Anesthesia and Intensive Care, Ferrara, Italy, [2]Swisstom, Landquart, Switzerland, [3]Rostock University Medical Center, Department of Anesthesiology and Intensive Care Medicine, Rostock, Germany
**Correspondence:** G. Scaramuzzo
*Intensive Care Medicine Experimental* 2018, **6(Suppl 2):**0792

**INTRODUCTION.** Despite the Pressure Volume curve (PV) have been suggested as a bedside tool to set mechanical ventilation[1], in heterogeneous lung diseases - like Acute Respiratory Distress Syndrome (ARDS) - it expresses the average behaviour of the lung, possibly under or overestimating regional pressures. Regional PV (PVr) derived from Electrical Impedance Tomography (EIT) can add valuable local information at the bedside but the only available data are on animal models[2].

**OBJECTIVES.** To evaluate the feasibility of a PVr-based method to set mechanical ventilation in patients affected by Acute Hypoxic Respiratory Failure (AHRF) and ARDS.

**METHODS.** Slow inflation PV maneuvers[3] were simultaneously recorded with a pneumotacograph and EIT (Swisstom BB2, Swisstom, Landquart) in patients with AHRF/ARDS. From each maneuver, a global PV curve (PVg) and 16 to 19 (depending on lung dimension) cranio-caudal regional PV curves (PVr) were obtained, each one corresponding to a pixel row coming from the EIT image[2]. PVg and PVr were fitted into a sigmoidal equation and the upper (UIP) and lower (LIP) inflection points were calculated[4]. LIP and UIP from PVg were called respectively LIPg and UIPg. For each measurement, the regional highest LIP ($LIPr_{MAX}$) and lowest UIP ($UIPr_{MIN}$) were found (*fig1*). The pressure between those two points falls in the linear part of each PVr and was defined $\Delta P_{lin}$. A paired t-test (α=0.05) was used to compare inflection points from PVr and PVg.

**RESULTS.** We successfully analysed 38/41 maneuvers. A statistically significant difference (p< 0.05) was found between $LIPr_{MAX}$ (15±7.1 $cmH_2O$) and LIPg (4.6±5.2 $cmH_2O$); in all measurements, the $LIPr_{MAX}$ was higher than the corresponding LIPg. We also found significant difference (p< 0.005) between $UIPr_{MIN}$ (30±8.9 $cmH_2O$) and UIPg (42 ±12 $cmH_2O$), being the $UIPr_{MIN}$ lower than the corresponding UIPg in all the analysed PV. The average $\Delta P_{lin}$ was 15±8.6 $cmH_2O$.

**CONCLUSIONS.** The regional PV curves give different information compared to the PVg. A safe pressure boundary on the linear part of each PVr can be easily found in patients undergoing mechanical ventilation. Wheatear this can be used to improve lung protection has still to be explored.

**REFERENCE(S)**

1. Amato, M. B. P. et al. Effect of a Protective-Ventilation Strategy on Mortality in the Acute Respiratory Distress Syndrome. N. Engl. J. Med. 338, 347-354 (1998).
2. Kunst, P. W. *et al.* Regional pressure volume curves by electrical impedance tomography in a model of acute lung injury. *Crit. Care Med.* **28,** 178-183 (2000).
3. Ranieri, V. M., Giuliani, R., Fiore, T., Dambrosio, M. & Milic-Emili, J. Volume-pressure curve of the respiratory system predicts effects of PEEP in ARDS: 'occlusion' versus 'constant flow' technique. *Am. J. Respir. Crit. Care Med.* **149,** 19-27 (1994).
4. Venegas, J. G., Harris, R. S. & Simon, B. A. A comprehensive equation for the pulmonary pressure-volume curve. *J. Appl. Physiol. Bethesda Md 1985* **84,** 389-395 (1998).

**GRANT ACKNOWLEDGMENT**

None

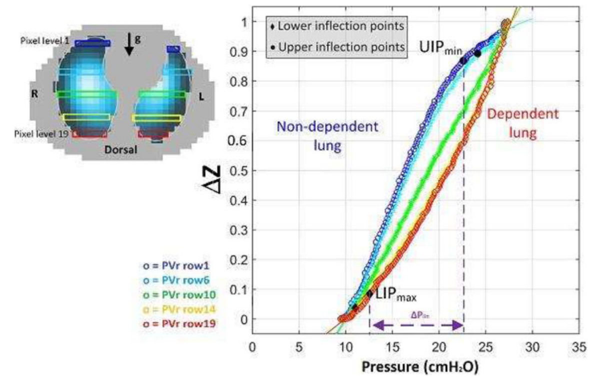

**Fig. 1 (abstract 0792).** Example of PVr in a representative measurement (patient 5, PEEP 10)]

## Different critical care situations

### 0793

#### Changes of three-dimensional shapes and functional behaviors of red blood cells after cardiac arrest

L. Hui Jai, J. Shin, S. Park, K.M. You

Seoul Metropolitan Government Seoul National University Boramae Medical Center, Emergency Medicine, Dongjak-gu, Korea, Republic of

**Correspondence:** L. Hui Jai

*Intensive Care Medicine Experimental* 2018, **6(Suppl 2):**0793

**INTRODUCTION.** Red blood cells (RBCs) have important roles in tissue oxygen delivery. Previous studies showed changes of morphology and rheological behavior of RBCs in various clinical conditions of ischemia/reperfusion injury such as sepsis, severe trauma, and critically ill patients. Global I/R injury also has important roles in the pathophysiology of cardiac arrest victims. However, few studies evaluated about RBCs of postcardiac arrest.

**OBJECTIVES.** We wanted to evaluate the morphologic and functional status of the RBC during post-cardiac arrest care.

**METHODS.** After successful resuscitation in the emergency department, patients were enrolled after informed consents. Blood samples were taken immediate after ROSC and daily during ICU admission. Morphological, biochemical and mechanical properties of RBCs were retrieved from the cDOT microscopy. All the parameters are measured within 1 hours after collections of samples.

**RESULTS.** Total 40 patients were enrolled. Survival was observed in 26 (65%). Favorable neurologic outcome (Cerebral Performance Category 1 or 2) at 28-day after cardiac arrest was 10 (25%).

Membrane fluctuations of day 3 (59.12 vs. 55.61 nm, $p < 0.001$) and day 5 (59.28 vs. 55.68 nm, $p = 0.027$) higher in survivors. SI(sphericity index)s of day 3 (0.61 vs 0.68, $p < 0.001$) and day 5 (0.62 vs. 0.64, $p = 0.044$) were lower in survivor. With generalized mixed Linear models analysis, functional outcome of 28-day after cardiac arrest failed to show meaningful correlation to membrane fluctuation nor SI.

**CONCLUSIONS.** Morphologic and functional differences of RBCs were present in 3 and 5 days after cardiac arrest between survivors and non-survivors.

**REFERENCE(S)**

Kim Y, Shim H, Kim K, Park H, Jang S, Park Y. Profiling individual human red blood cells using common-path diffraction optical tomography. Scientific reports 2014;4: 6659.

Lee S, Park H, Kim K, Sohn Y, Jang S, Park Y. Refractive index tomograms and dynamic membrane fluctuations of red blood cells from patients with diabetes mellitus. Sci Rep. 2017;7:1039.

Ince C. The microcirculation is the motor of sepsis. Critical care 9 Suppl 4: S13-19, 2005.

Reggiori G, Occhipinti G, De Gasperi A, Vincent JL, Piagnerelli M. Early alterations of red blood cell rheology in critically ill patients. Critical care medicine 2009;37:3041-3046.

Piagnerelli M, Boudjeltia KZ, Vanhaeverbeek M, Vincent JL. Red blood cell rheology in sepsis. Intensive Care Med 2003;29: 1052-1061.

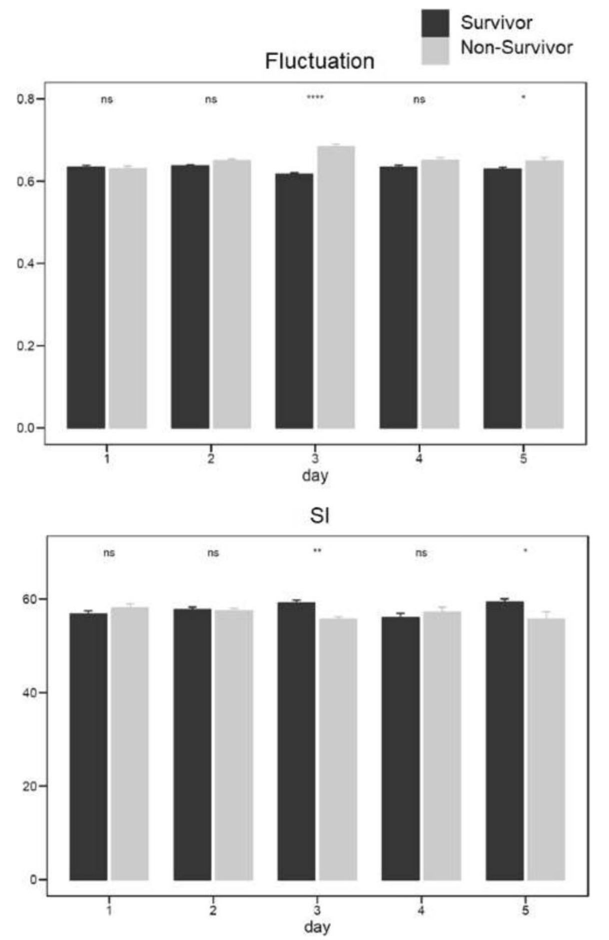

**Fig. 1 (abstract 0793).** Membrane fluctuation and sphericity index of Red blood cells

### 0794

#### CPR guidance by an emergency physician via video call

S.M. Park, D. Lee
Seoul National University Bundang Hospital, Emergency Department, Anyang si, Korea, Republic of
**Correspondence:** S.M. Park
*Intensive Care Medicine Experimental* 2018, **6(Suppl 2):**0794

**INTRODUCTION.** In South Korea, the prehospital treatment of cardiac arrest is generally led by an emergency medical technician-paramedic (EMT-P) and defibrillation is delivered by the automatic external defibrillator (AED).

**OBJECTIVES.** This study aimed at examining the effects of direct medical guidance by an emergency physician through a video call which enabled prompt manual defibrillation.

**METHODS.** Two hundred eighty eight paramedics based in **Gyeonggi** Province were studied for four months, from July to November 2015. The participants were divided into 96 teams, and the teams were randomly divided into either a conventional group using the AED or a Video call guidance group using manual defibrillators, with 48 teams in each group. The time to first defibrillation, total hands-off time, and hands-off ratio were compared between the two groups.

**RESULTS.** The median value of the time to first defibrillation was significantly shorter in the video call guidance group (56 s) than in the conventional group (73 s) (p< 0.001). The median value of the total hands-off time was also significantly shorter (228 vs. 285.5 s) (p< 0.001), and the hands-off ratio, defined as the proportion of hands-off time out of the total CPR time, was significantly shorter in the video call guidance group (0.32 vs. 0.41) (p< 0.001).

**CONCLUSIONS.** Medical direction by video call enabled prompt manual defibrillation and significantly shorten the time required for first defibrillation, hands-off time, hands-off ratio in simulated cases of prehospital cardiac arrest

##### REFERENCE(S)

1. Survey of Acute Cardiac Arrest, Ministry of Health and Welfare, Republic of Korea.
2. McNally B, Robb R, Mehta M, Vellano K, Valderrama AL, Yoon PW, et al. Out-of-Hospital Cardiac Arrest Surveillance — Cardiac Arrest Registry to Enhance Survival (CARES), United States, October 1, 2005-December 31, 2010. MMWR Surveill Summ 2011;60(8):1-19.
3. Shin SD, Kitamura T, Hwang SS, Kajino K, Song KJ, Ro YS, et al. Association between resuscitation time interval at the scene and neurological outcome after out-of-hospital cardiac arrest in two Asian cities. Resuscitation 2014;85(2):203-10. doi: 10.1016/j.resuscitation.2013.10.021. PubMed PMID: 24184782.
4. Böttiger BW, Bernhard M, Knapp J, Nagele P. Influence of EMS-physician presence on survival after out-of-hospital cardiopulmonary resuscitation: systematic review and meta-analysis. Crit Care 2016;20:4. doi: 10.1186/s13054-015-1156-6. PubMed PMID: 26747085.
5. Pytte M, Pedersen TE, Ottem J, Rokvam AS, Sunde K. Comparison of hands-off time during CPR with manual and semi-automatic defibrillation in a manikin model. Resuscitation 2007;73(1):131-6. doi: 10.1016/j.resuscitation.2006.08.025. PubMed PMID: 17270336.

##### GRANT ACKNOWLEDGMENT

This article was not financially supported by any company or organization.

### 0795

#### Comparison of advanced airway management and bag mask ventilation during simulated cardiac arrest

T. Sellmann[1,2], L. Vogt[2], D. Wetzchewald[3], H. Schwager[3], S. Russo[1], S. Marsch[4]
[1]University Witten Herdecke, Helios University Hospital, Department of Anaesthesiology, Wuppertal, Germany; [2]Bethesda Krankenhaus Duisburg, Department of Anaesthesiology, Duisburg, Germany; [3]Institution for Emergency Medicine, Arnsberg, Germany; [4]University Hospital of Basel, Department of Medical Intensive Care, Basel, Switzerland
**Correspondence:** T. Sellmann
*Intensive Care Medicine Experimental* 2018, **6(Suppl 2):**0795

**INTRODUCTION/ OBJECTIVES.** The 2015 European resuscitation council (ERC) guidelines recommend advanced airway management (AAM) over bag mask ventilation (BMV) for provision of sufficient ventilation and prevention of aspiration during advanced cardiac life support (ACLS). However, there is conflicting data on the superiority of AAM compared to BMV. It was our aim to investigate the influence of airway management on overall adherence to ACLS algorithm in a simulated CPR model.

**METHODS.** One hundred and fifty-four teams, each consisting of three to five physicians, were randomized to perform ACLS with the instruction to perform either AAM or BMV. Data analysis was performed using video-recordings obtained during simulations. Primary endpoints were adherence to guidelines with regard to respiratory recommendations (BMV 2 breaths/ cycle, not longer than 4 seconds; AAM respiratory rate 8-12/ min) and overall hands-on time. Secondary outcome measure was the impact of ventilation on hands-off times.

**RESULTS.** Advanced airway management was provided by 71/76 (93,4%) teams during ACLS, whereas all BMV (n=78) teams complied with their target ventilation. Adherence to guidelines with regard to respiratory recommendations was reached by 6/ 71 AAM groups (8,5%) and 14/ 78 BMV (17,9%) groups (p = 0.0893). Groups allocated to AAM ventilated 19±8/ min, whereas BMV groups ventilated 7±6 /min (p < 0.0001). BMV groups had higher overall hands-off times than AAM Groups (22±7% vs. 14±6, p < 0.0001). Adjusted for ventilation-associated hands-off times, BMV groups still had 9±4% vs. 3±3% in the AAM groups (p < 0.0001

**CONCLUSIONS.** This simulator based trial found that insertion of an advanced airway leads to significant hyperventilation but is associated with more hands-on time, indicating that reducing complexity of an algorithm makes it easier to follow.

##### REFERENCE(S)

Soar J, Nolan JP, Böttiger BW, Perkins GD, Lott C, Carli P, Pellis T, Sandroni C, Skrifvars MB, Smith GB, Sunde K, Deakin CD; Adult advanced life support section Collaborators. European Resuscitation Council Guidelines for Resuscitation 2015: Section 3. Adult advanced life support. Resuscitation. 2015 Oct;95:100-47. doi: 10.1016/j.resuscitation.2015.07.016

### 0796

#### How comfortable are residents with CPR?

C. Salmon[1], N. Din[1], D. Kim[1], N. Ahmad[2]
[1]Woodhull Medical Center-NYU, Internal Medicine, Brooklyn, United States; [2]Woodhull Medical Center-NYU, Pulmonary and Critical Care, Brooklyn, United States
**Correspondence:** C. Salmon
*Intensive Care Medicine Experimental* 2018, **6(Suppl 2):**0796

**INTRODUCTION.** Cardio pulmonary resuscitation (CPR) is an event that medical professionals encounter frequently during their training time. Residents at our institution are the engine that runs this hectic end of life moment.

**OBJECTIVES.** The objective of the study is to assess the relation between resident comfort level and time to terminate cardiopulmonary resuscitation. We hypothesize that third year medical residents vary in their comfort level of running code as team leader from the beginning to the end of residency year. These may result in "longer codes" earlier in their leader role without better outcomes compared to the national average.

**METHODS.** Third year residents were given a questionnaire of ten questions comparing their comfort levels, number of "code blue" encountered, knowing when to terminate resuscitative efforts and emotional reactions during the first semester and during the second semester of their third year.

Charts from patients who underwent cardiac arrest with CPR were reviewed in a period of one year and identified by a unique code to each patient. Data was divided within a six month period corresponding to the first six months of third year vs the last six months of third year, July to December and January to June, respectively.

Data was collected in excel sheet and was analyzed with SPSS using Wilcoxon singed rank test.

**RESULTS.** Data analysis showed no significance (*p0.377*) between the "code blue" in the first semester vs the last semester in regards of duration of CPR, 19.55±*8.18* minutes vs 16.57±*10.01* minutes in patients who expired, in cases were the resident had to decide when to terminate CPR. Analysis of questionnaire data was mostly significant on seven out of ten questions answered subjectively by residents, including feeling prepared to lead the cardiac arrest team (*p0.011*), supervising the team (*p0.004*), being worried about making mistakes (*p0.038*), knowing proper time to terminate CPR and feeling comfortable terminating these efforts (*p0.023*). The data that did not show significance was emotional reaction (*p0.059*), proper indications to terminate CPR (*p0.055*) and comfort delegating tasks (*p0.059*), however the average answers in both semester fell between "agreeing" or "neither", but did not show a negative response when evaluated with the scale of the questionnaire.

**CONCLUSIONS.** In hospital setting, residents who are in training are responsible for supervising CPR and it is fairly challenging as they will be making the decisions on when to terminate resuscitative efforts on a patient who did not achieved return of spontaneous circulation. CPR is often stopped within 30 minutes after cardiac arrest. The size of our sample was small and the questionnaire was subjectively answered. We believe that a larger sample could be collected and could potentially show significant change on results when comparing both semesters.

### 0797

#### Study of the diffusion and concentration of micafungin in peritoneal fluid for the treatment of severe *Candida* peritonitis. Perseus study

D.V. Pérez Civantos[1,2], J.D. Jiménez Delgado[3], R. Bayo Poleo[1], M. Robles Marcos[1], A. Muñoz Cantero[1], M.A. Santiago Triviño[1], D.F. López Hormiga[4], S. Grau Cerrato[5], A. Gutiérrez-Pizarraya[6]
[1]University Hospital Infanta Cristina, Intensive Care Medicine, Badajoz, Spain; [2]University of Extremadura, Biomedical Science, Badajoz, Spain; [3]Hospital D. Benito-Villanueva, Intensive Care Medicine, D.Benito-Villanueva, Spain; [4]Hospital General de Mérida, Intensive Care Medicine, Mérida, Spain; [5]Hospital del Mar. Universitat Autónoma, Pharmacy Department, Barcelona, Spain; [6]University Hospital Virgen Macarena, Intensive Care Medicine, Seville, Spain
**Correspondence:** D.V. Pérez Civantos
*Intensive Care Medicine Experimental* 2018, **6(Suppl 2):**0797

**INTRODUCTION.** Candida peritonitis occurs frequently in patients undergoing urgent abdominal surgery. Rates ranges 30-40% and carries a high mortality. The latest ESCMID clinical guidelines recommend empirical treatment with echinocandins for the treatment of intra-abdominal infections caused by fungi.

**OBJECTIVES.** To evaluate the diffusion of micafungin into peritoneal fluid (PF) used in the usual therapeutic dosage (100 mg/day) and to establish the correlation between concentrations in the PF and MIC, to show the potential therapeutic effectiveness.

**METHODS.** A prospective study in two centers' ICUs during one year period was conducted. Correlative 20 patients were validated. Two groups were studied, one naïve (before first dose) with 12 patients and a second on day 3 (8 patients) at steady state. Blood and peritoneal samples were drawn at hours 0,1,3,5,8, 18 and 24 h after drug administration to obtain a Pk/Pd profile.

**RESULTS.** 11 patients (55%) were men and a mean (SD) aged 65.5 (18.6) year. APACHE II and SOFA scores were 17.5 (7.1) and 7.0 (3.6) points. Candida Score was 4 (3-4) and in 2 (10%) patients Candida albicans was isolated in the PF so empiric treatment resulted in the remaining 18 (90%) cases. Clinical cure was reached in 12 (60%) with a crude in-hospital mortality of 11 (55%). Plasma concentrations in group 1, Cmax (achieved at the end of 1-hour intravenous infusion) was 5.4 (2.2) mg / L, Cmin was 0.0 (0.0) mg/L, whilst in those at steady-state (group 2) Cmin was 1.9 (1.2) mg / L , and Cmax 8.4 (3.6) mg / L. Levels in PF, group 1, Cmax was 0.7 (0.5) mg / L and was reached at 8 hours, Cmin 0.1 (0.1) mg/L. In patients at steady state (group 2), Cmin at pre-dose time was 0.2 (0.3) mg / L and Cmax 1.2 (1.0) mg / L (reached 5 h after infusion). Pharmacokinetic data observed in group 1 mean plasma AUC 0-24 h (mg*h/L) 54,3 (21,9); in group 2 (steady state) AUC 0-24 h 91,8 (48,0). Data from PF in group 1 AUC 0-24 h 12,98 (8,63); in group 2 AUC 0-24 h 7,42 (4,99). We also found a lower micafungin plasma concentration on day 3 in non survivors compared to survivors (0.92 SD 0.96 vs 2.85mg/L SD 0.10; p< 0.05) and additionally on day 1 for PF 0.03 SD 0.09 vs 0.22 mg/L SD 0.27; p< 0.05).

**CONCLUSIONS.** A high interindividual levels in plasma and PF variability was observed and could be related to variations in extracellular volume and alteration in distribution, metabolism and elimination. Our results show a moderate penetration of micafungin into PF in patients with intra-abdominal infections, with a mean AUC0-24 peritoneal fluid/plasma ratio of 24% (0.2390; SD 0,09) on day 1 after the first dose and 26% (0,2557; SD 0,12) at steady-state. Data also suggest a correlation between low micafungin levels and poorer prognosis observed even earlier in PF than in plasma.

**GRANT ACKNOWLEDGMENT**
This study was supported by a grant from Astellas Pharma.

### 0798

#### A population pharmacokinetic analysis of cefotaxime in critically ill patients

T. Guo[1,2], R.M. van Hest[2], L.F. Roggeveen[1], R.A.A. Mathôt[2], P. Thoral[1], L. Fleuren[1], H.O. van Straaten[1], A.R.J. Girbes[1], P. van der Voort[3], R. Bosman[3], P.W.G. Elbers[1]
[1]VU University Medical Centre, Department of Intensive Care Medicine, Research VUmc Intensive Care (REVIVE), Amsterdam Cardiovascular Sciences (ACS), Amsterdam Infection and Immunity Institute (AI&II), Amsterdam, Netherlands; [2]Academic Medical Centre, Department of Pharmacy, Amsterdam, Netherlands; [3]OLVG Oost, Intensive Care Unit, Amsterdam, Netherlands
**Correspondence:** T. Guo
*Intensive Care Medicine Experimental* 2018, **6(Suppl 2):**0798

**INTRODUCTION.** Cefotaxime is a widely used against life threatening infections in the critically ill including community acquired pneumonia. In addition, many ICUs use it as the intravenous component of selective decontamination of the digestive tract strategies. Adequate plasma concentrations are crucial for efficacy. However, the pharmacokinetic (PK) profile of cefotaxime in critically patients has been rarely reported.

**OBJECTIVES.** Our aim is to establish a population PK model identifying the dose exposure relationship of cefotaxime to facilitate individualised clinical therapy in critically ill patients.

**METHODS.** Data from 50 adult patients from a retrospective study conducted in 2010 in the ICU of the OLVG hospital in Amsterdam was collected. All patients were scheduled to receive 1000mg cefotaxime every 6 hours. The data includes patients' plasma level of cefotaxime and demographic characteristics, clinical diagnostics. 4 blood samples of each patient were collected. Population PK analysis was carried out with non-linear mixed effects modelling (NONMEM) software. Inter-individual variability of PK parameters was modelled by a full block matrix. A combined error statistical model was used to account for the residual error.

**RESULTS.** A one compartment model was established to describe the PK profile of cefotaxime. Typical value of the clearance (CL) and volume of distribution (V) is 12 L/h and 41.4 L, respectively. Serum creatinine

(SCR), albumin (ALB) and sequential organ failure assessment (SOFA) score showed significant associations on CL (P< 0.001). Patients with higher SCR or lower ALB, or higher SOFA exhibit lower clearance.

**CONCLUSIONS.** A one compartment population PK model was established to depict the PK profile of cefotaxime in critically ill patients. Because of the data limitation, more complex model structure as Urien et al reported, cannot be well established. Serum creatinine, albumin, and SOFA score were identified as the main covariates influencing CL of cefotaxime. No covariate effect was found in V.

**REFERENCE(S)**

1. Urien, S. *et al.* (2004) 'Pharmacokinetic modelling of cefotaxime and desacetylcefotaxime - A population study in 25 elderly patients', *European Journal of Clinical Pharmacology*, 60(1), pp. 11-16.

**GRANT ACKNOWLEDGMENT**

This work is part of the Right Dose, Right Now project by VUmc Amsterdam and OLVG Oost Amsterdam and was partially funded by the ZonMw Rational Pharmacotherapy program.

### 0799

#### Remifentanil promotes osteoblastogenesis through upregulating Runx2/Osterix expressions in pre-osteoblast cells

E.J. Kim[1], C.H. Kim[1], J.Y. Yoon[1], J.U. Yoon[2], A.R. Cho[3]
[1]Pusan National University Dental Hospital, Department of Anesthesia and Pain Medicine, Yangsan, Korea, Republic of, [2]Pusan National University Yangsan Hospital, Department of Anesthesia and Pain Medicine, Yangsan, Korea, Republic of, [3]Pusan National University Hospital, Department of Anesthesia and Pain Medicine, Busan, Korea, Republic of
**Correspondence:** E.J. Kim
*Intensive Care Medicine Experimental* 2018, **6(Suppl 2):**0799

**Withdrawn**

### 0800

#### Vancomycin therapy - indications and the influence of renal dysfunction on serum levels

J. Hruda, L. Bauer, A. Papiez, V. Sramek, P. Suk
St. Anne's University Hospital Brno, Brno, Czech Republic
**Correspondence:** J. Hruda
*Intensive Care Medicine Experimental* 2018, **6(Suppl 2):**0800

**INTRODUCTION.** Vancomycin is the drug of choice for treatment of β-lactam antibiotic resistant Gram positive bacterial infections (βLR-GPB). However, its broad empiric use might be excessive in countries with low prevalence of βLR-GPB. Since vancomycin has renal elimination, kidney dysfunction increases the risk of inappropriate dosing.

**OBJECTIVES.** To determine, whether empiric use of vancomycin was supported by subsequent microbiological cultures and whether renal dysfunction or renal replacement therapy influences the reaching of target serum concentrations.

**METHODS.** In this retrospective study, all the patients admitted to the general ICU between January 2015 and December 2016 receiving intravenous vancomycin were included. Dosing, renal functions and replacement therapy, vancomycin levels and microbiological results were analysed. Data are presented as median (IQR).

**RESULTS.** 64 patients and 347 vancomycin therapy days were analysed. The indication was based on βLR-GPB cultures in 16 patients (25%), administered empirically in 27 patients (42%), the other 21 patients have recent history of βLR-GPB or any Gram positive bacteria in samples. In the empirical group, βLR-GPB were cultured in 9 patients (34%). Intermittent haemodialysis (IHD) was used 22 days, continuous renal replacement therapy (CVVHD) in 41 days.

Loading dose was 13 (11-14) mg/kg and the daily maintenance dose varied between 21 (13-32) mg/kg in patients with serum creatinine < 100 μmol/l and 10 (7-13) mg/kg on CVVHD or 6 (4-9) mg/kg on IHD. There was no dependence between maintenance dose and subsequent vancomycin level. Vancomycin levels (243 values) were 13.7 (10.5 - 16.5) μmol/l. The target therapeutic level 10 - 17.5 μmol/l was reached in 68% in days without RRT, 56% in days with CVVHD and 68% in days with IHD (p=0.63).

**CONCLUSIONS.** Microbiological cultures justified only minority of empirical vancomycin therapies. Renal dysfunction or renal replacement therapy significantly influenced the maintenance dose but did not alter the proportion of reaching the therapeutic levels. Therefore, these data suggest that vancomycin can be safely used in patients with various degree of renal dysfunction. From the current point of view, the target therapeutic levels were lower than the present recommendation (14-21 μmol/l) and, concordantly, vancomycin doses would be higher to reach the current goal.

### 0801

#### Does the income level of the residential area impact the incidence of ICU admission and demographics of acute poisonings?

L. Koskela[1], L. Raatiniemi[1,2], T. Ala-Kokko[1,3], J. Liisanantti[1,3]
[1]Oulu University Medical Research Center, Study Group of Surgery, Anesthesiology and Intensive Care, Oulu, Finland; [2]Oulu University Hospital, Centre for Pre-Hospital Emergency Care, Oulu, Finland; [3]Oulu University Hospital, Department of Anesthesiology, Division of Intensive Care Medicine, Oulu, Finland
**Correspondence:** L. Koskela
*Intensive Care Medicine Experimental* 2018, **6(Suppl 2):**0801

**INTRODUCTION.** There are significant regional differences in the use intensive care resources depending on the income of the neighborhood: in the highest income areas the incidence of ICU admission is significantly lower compared to areas with inhabitants with lower income. In the low income areas, the poisonings are more frequent compared to other areas. (1) We have previously reported regional differences in trauma and poisoning mortality in Northern Finland, but the regional differences in the need of ICU admission due to poisonings are not well studied. (2,3)

**OBJECTIVES.** We determined to examine differences in the incidence of ICU admitted poisonings and in the patient demographics between postal code areas with different median annual income in Northern Finland.

**METHODS.** We conducted a retrospective analysis of all adult patients admitted to hospital due to poisoning in the County of Northern Ostrobothnia in Finland during 2013-2016. Patients were divided into three (low-middle-high) patient groups based on median income in their residential postal code areas.

**RESULTS.** A total of 140 (7.3 %) of all 1909 hospitalized adult patients were admitted to intensive care during study period and of these 57.9 % were males. The proportion of ICU admissions from emergency ward did not differ between the areas. Proportion of males and patient median age did not differ significantly between patient groups. Overall incidence of intensive care treated poisoning was 12.2/100,000 (CI 95% 10.3-14.4). Incidences of intensive care treated poisoning in different income areas were following: 18.9/100,000 (CI 95% 14.0-25.1) in low income, 12.1/100,000 (CI 95% 9.1-15.7) in middle income and 8.8/100,000 (CI 95% 6.5-11.8) in high income area. Proportion of mixed substance poisonings was 36.4 % and there were only few differences in the causes of poisonings between the groups. [Table 1]

**CONCLUSIONS.** Incidence of intensive care treated poisoning is significantly higher in the lowest earning residential areas compared to the highest earning residential areas. The overall incidence of ICU-treated poisoning was low and the admittance rate of all hospital-treated poisonings to ICU was also low.

**REFERENCE(S)**

1. J. H. Liisanantti, R. Käkelä, L. V. Raatiniemi, P. Ohtonen, S. Hietanen and T. I. Ala-Kokko. Has the income of the residential area impact on the use of intensive care? *Acta Anaesthesiologica Scandinavica 2017.* 2017;61:7.
2. L. Raatiniemi, T. Steinvik, J. Liisanantti, P. Ohtonen, M. Martikainen, S. Alahuhta, T. Dehli, T. Wisborg and H.K: Bakke. Fatal injuries in rural and urban areas in northern Finland: a 5-year retrospective study. *Acta Anaesthesiologica Scandinavica.* 2016;60:5.
3. L. Koskela, L.V. Raatiniemi, H.K. Bakke, T.I. Ala-Kokko and J.H. Liisanantti. Fatal poisonings in Northern Finland: causes, incidence and rural-urban differences. *Scandinavian Journal of Trauma, Resuscitation and Emergency Medicine.* 2017;25:90.

**GRANT ACKNOWLEDGMENT**
None

**Table 1 (abstract 0801).** Number of positive poisoning agent findings in 140 patients admitted into ICU in Northern Ostrobothnia in Finland during 2013-2016.

| Cause | Overall | Low income | Middle income | High income | p |
|---|---|---|---|---|---|
| Mixed poisoning | 51 (36.4%) | 19 (42.4 %) | 16 (30.2 %) | 16 (38.1 %) | ns |
| Pharmaceutical drug | 125 (89.3 %) | 39 (86.7 %) | 47 (88.7 %) | 39 (92.9 %) | ns |
| Illicit drug | 26 (18.6 %) | 9 (20.0 %) | 3 (5.8 %) | 14 (33.3 %) | 0.003 |
| Unspecified drug | 3 (2.1 %) | 1 (2.2 %) | 1 (1.9 %) | 1 (2.4 %) | ns |
| Ethanol | 11 (7.9 %) | 4 (8.9%) | 5 (9.4 %) | 2 (4.8 %) | ns |
| Methanol | 3 (2.1 %) | 0 | 0 | 3 (7.1 %) | 0.028 |
| Other alcohol | 6 (4.3 %) | 3 (6.7 %) | 2 (3.8 %) | 1 (2.4 %) | ns |

### 0802

#### Imidacloprid poisoning: an Indian experience

J. Nikhilesh[1], T. Apurv[1], J. Vivek[2], G. Ankur[3]
[1]CHL Hospital, Dept of Critical Care Services, Indore, India; [2]Shalby Hospitals, Dept of Critical Care Services, Indore, India; [3]Apollo Hospital, Dept of Critical Care Services, Indore, India
**Correspondence:** J. Nikhilesh
*Intensive Care Medicine Experimental* 2018, **6(Suppl 2):**0802

**BACKGROUND.** Imidacloprid is among newer insecticides in chloronicotinyl nitroguanidine category. It acts on postsynaptic nicotinic acetylcholine receptors in the nervous system.it is known to be effective by contact/ingestion. This communication delineates epidemiological characteristics and outcomes in India over a course of three year duration.
**OBJECTIVE.** To assess epidemiological patterns and outcomes of Imidacloprid poisoning.
**SETING.** Three tertiary care ICUs of multidisciplinary pattern.
**STUDY MODULE.** Consecutive patients presenting with imidacloprid poisoning were included and data was collected with a focus on demographics, SOFA scores, neuropsychiatric symptoms, rhabdomyolysis, requirement of RRT, deranged liver function tests[LFT] (defined as ALT/AST elevations three times above normal),GI symptoms ( nausea / vomiting/diarrhea), cardiac arrhythmias and length of stay (LOS) in days. Discharge from ICU/death was considered as end points.
**RESULTS.** Fifty four patients were included (n=54,M:F-36:18).Age of the cohort was 27.7+5.1 years. SOFA scores for the cohort were 6.5 +1.6.Incidence of neuropsychiatric symptoms, rhabdomyolysis, requirement of RRT, deranged LFTs, GI symptoms and cardiac arrhythmias was 53.7%(n=29),38.9%(n=21),24%(n=13),42.6%(n=23),48.1%(n=26) and 37%(n=20) respectively.LOS was 2.9+1.2 days. On applying a chi square test with relevance to SOFA scores none of the variables achieved statistical significance. Methhemoglobinemia was reported in one of the cases.
**CONCLUSION.** Imidacloprid poisoning remains a nonlethal poisoning with increasing prevalence owing to shift in the compounds used as insecticides in Asian countries. In view of changing patterns of use we need more data to define subsets at risk of requiring major interventions in ICU/propensity to develop MODS.

### 0803

#### Anaphylaxis: study of epidemiological and therapeutic characteristics

B.J. Amira[1,2], I. Sedghiani[1], A. M'Rad[1], H. Maamouri[1], N. Brahmi[1]
[1]CAMU, Intensive Care Unit, Tunis, Tunisia; [2]Tunis Manar, Toxicology, Tunis, Tunisia
**Correspondence:** B.J. Amira
*Intensive Care Medicine Experimental* 2018, **6(Suppl 2):**0803

**INTRODUCTION.** Certain studies dealing with anaphylaxis from emergency departments suggest that it remains misdiagnosed and under-treated, with behaviors often diverging from international recommendations.
**OBJECTIVES.** To study the epidemiologic, therapeutic and evolutionary characteristics of patients admitted to ED for anaphylaxis.
**METHODS.** Prospective, observational study including patients over the age of 14 who visited a single emergency and toxicological intensiv care department between 2007 and 2016 for anaphylaxis. Collection of epidemiological, clinical and therapeutic parameters.
**RESULTS.** Inclusion of 105 patients. Average age = 42.52 ± 17.42 years. Sex ratio = (1.29). History of anaphylaxis: 80 %(n=84). Clinical signs n (%): cutaneous manifestations 80% (n =85) cardiovascular signs 37 (35), respiratory signs 38% (40), digestive signs 15 % (n=16) and neurological signs 13% (n=14). Allergens incriminated: medicated 58 %, food 14 % and bites of Hymenoptera 28 %. Anaphylactic shock state noted in 35 patients (35%) Adrenaline was used in 40 % of cases by by direct intravenous injection in 31% of cases . Corticosteroids and antihistamines were prescribed respectively in 88% and 80% of patients. Three patients had recurrences. Two patients had cardiac arrest and one death was recorded.
**CONCLUSION.** The prevalence of anaphylaxis in our study was low. This shows that this is a situation that remains so far underestimated. It is therefore imperative to conduct prospective multicentre studies in emergency departments to better determine its incidence and risk factors.

### 0804

#### Cyproheptadin intoxication: analytical study

A. Ben Jazia, I. Sedghiani, A. M'rad, H. Maamouri, N. Brahmi
CAMU, Toxicology, Tunis, Tunisia
**Correspondence:** A. Ben Jazia
*Intensive Care Medicine Experimental* 2018, **6(Suppl 2):**0804

**INTRODUCTION.** Acute poisoning with Cyproheptadine (CPT) has become frequent and potentially serious through neurological complications.
Despite the increase in the incidence of intoxication and the severity of this intoxication in Tunisia or the world, few studies have been interested in this molecule.
The purpose of this study was to identify epidemiologic characteristics and clinical outcomes in patients who were through CPT and to investigate any association between ingested dose and reported adverse events.
**METHODS.** We conducted a retrospective study at CAMU´s intensive care unit over a period from February 2006 to March 2017. For each patient we collected the anamnestic data (age, sex, antecedents, the ISD and the delay between ingestion and management), clinical

(mainly neurological and hemodynamic), biological (transaminases, arterial blood gases), therapeutic and progressive.
**RESULTS.** We collected 47 patients (4 men / 43 women). The median age was 22 years [18; 33]. Pathological antecedents were psychiatric in 12% of cases (n = 6). All intoxications were voluntary. The median CPT ISD was 100 [40; 750] mg. The median consultation time was 3 h [1; 14].The IGS II score ranged from 6 to 38 with a median of 6.Metabolic acidosis was observed in 8.5% of cases (n = 4).The main clinical manifestations were impaired state of consciousness with Glasgow score (GCS) ≤ 11 in 4.3% of cases (n = 2), anti-cholinergic syndrome in 47% of cases (n = 22) and one sedative syndrome in 21% of cases (n = 10).For GCS comatose patients < = 8 there is a correlation between coma and the supposedly ingested dose of (CPT) with p = 0.03 .ASC = 0.91 and p = 0.01. The corresponding dose is 140 mg.There is a correlation between the dose of ingested antihistamine and obnubilation with p = 0.005 with AUC 0.83.There is a correlation between the dose assumed to be ingested and the tremor with a p = 0.008 AUC = 0.84.For a dose equal to 117 mg p = 0.003.
**CONCLUSION.** Cyproheptadine is a widely available drug. Medical staff and parents should take special care in the management of cyproheptadine because it can be used in suicide attempts, especially by adolescents.

### 0805

#### The impact of an initial antibiotic therapy on the emergence of 3GC-resistant bacteria in severe trauma patients: a retrospective study in 507 patients

G. Faivre[1], C. Maury[1], R. Larcher[2], J. Charbit[1], P. Deras[1], X. Capdevila[1]
[1]CHU Montpellier, Lapeyronie, Trauma Center, Montpellier, France; [2]CHU Montpellier, Lapeyronie, Montpellier, France
**Correspondence:** G. Faivre
*Intensive Care Medicine Experimental* 2018, **6(Suppl 2):**0805

**INTRODUCTION.** Severe trauma patients are frequently exposed to antibiotic therapy at the initial phase of their management : open fractures, inhalation, digestive wound ...
ATB is however a major risk factor favoring the emergence of MRB acquired during the stay. This study investigates the emergence of 3GCephalosporin-resistant bacteria in the respiratory area in these patients.
**METHODS.** All of the serious trauma patients (ISS> 15) hospitalized in intensive care for more than 10 days between 2010 and 2015 were retrospectively included. The patients were divided in 3 groups according to the initial ATB (ATBi) they received: No-ATBi, Short-ATBi (≤72h) and Extended-ATBi (> 72h). In addition to demographic and lesion severity data, early respiratory (D2 to D5) and late (from D6) ecology were specifically collected: early modified (EME) and late (LME) ecology were defined as emergence of a C3G-resistant bacterium on respiratory samples. These samples were taken in case of clinical suspicion of pneumonia. The statistical association between ATBi and the occurrence of EME and LME was tested by comparison of the 3 groups and by logistic regression models.
**RESULTS.** A total of 507 patients were studied (mean age 43.7 ± 19.7, 78% male, mean ISS 30.0 ± 11.5): 444 patients (88%) were ventilated more than 48 hours, with an average duration of ventilation of 15.3 ± 12.6 days. The distribution of the 3 groups was as follows : 114 (22%) No-ATBi, 95 (19%) Short-ATBi, 299 (59%) Extended-ATBi. The groups differed significantly on admission (ISS, AIS head injury, AIS thorax, AIS abdominal, AIS bones, ventilation duration, Table1). The characteristics of antibiotherapy (ATBi and secondary antibiotherapy, defined as an antibiotic therapy started at least 48h after the end of ATBi) are shown in Table2. The rate of EME observed in the cohort (5,5%, 28/508) did not differ significantly between the 3 groups. In contrast, the LME rate differed significantly between these 3 groups: 10.5% (95% CI 6.1-17.5%) in the No-ATBi group, 19.0% (95% CI 12,3-28.0%) in the Short-ATBi group, 22.1% (95% CI 17.8-27.1%) in the Extended-ATBi group (P = 0.027) (Graph1). The median time to LME (D10, EQ D8-D13) did not differ significantly between the groups. Logistic regression analysis showed that the ATBi-short and ATBi-prolonged groups had a odds ratio of 2.0 (95% CI 0.9-4.4%, P = 0.088) and 2.4 ( 95% CI 1.4-4.7%, P = 0.009) to predict the occurrence of LME. After adjusting for multivariate analysis, these statistical associations were not influenced by the following variables of lesional severity: ISS score, AIS brain, AIS thorax, AIS abdomen, complex orthopedic lesions.
**CONCLUSIONS.** EME are rare regardless of the type of ATBi, close to one trauma patient out of 20. LME occur preferentially in the presence of an ATBi (2 times more often), whatever its duration.

**Table1 (abstract 0805).** See text for desciption

| | No-ATBi | No-ATBi | Short-ATBi | Short-ATBi | Extended-ATBi | Extended-ATBi | Total | Total | P Value |
|---|---|---|---|---|---|---|---|---|---|
| Patients | 114 | | 95 | | 298 | | 507 | | |
| Age | 50.3 | +/- 20.8 | 42,4 | +/- 19.6 | 41.6 | +/- 18,7 | 43.7 | +/ - 19.7 | < 0.001 |
| ISS | 27.1 | +/- 10.9 | 30,3 | +/- 12.4 | 31.0 | +/- 11.3 | 30.0 | +/- 11.5 | < 0.001 |
| Ventilated patients > 48h | 77 | 69 % | 79 | 83 % | 288 | 97 % | 444 | 88 % | < 0.001 |
| Ventilated patients > 48h : average duration | 15.4 | +/- 12.3 | 12,9 | +/- 12.4 | 15.8 | +/- 12.7 | 15.3 | +/- 12.6 | 0.15 |
| AIS brain > 2 | 45 | 40 % | 48 | 51 % | 173 | 58 % | 256 | 51 % | 0.003 |
| AIS thorax > 2 | 68 | 60 % | 45 | 47 % | 119 | 40 % | 232 | 46 % | 0.001 |
| AIS abdomen > 2 | 25 | 22 % | 19 | 20 % | 95 | 32 % | 139 | 27 % | 0.026 |
| AIS bones > 2 | 31 | 27 % | 56 | 57 % | 137 | 46 % | 224 | 44 % | < 0.001 |

**Table 2 (abstract 0805).** See text for desciption

| | No-ATBi | No-ATBi | Short-ATBi | Short-ATBi | Extended-ATBi | Extended-ATBi | P Value |
|---|---|---|---|---|---|---|---|
| ATBi duration (d) | 0 | | 2.4 | +/- 0.6 | 6.5 | +/- 2.9 | |
| ATBi : Augmentin | 0 | | 84 | 88 % | 249 | 83 % | 0.022 |
| ATBi : 3G-Cephalosporin | 0 | | 7 | 7 % | 26 | 9 % | 0.68 |
| ATBi : Others | 0 | | 4 | 4 % | 23 | 8 % | 0.24 |
| ATBi : Aminoglycoside | 0 | | 47 | 50 % | 147 | 49 % | 0.98 |
| Secondary ATB (ATB2) : B-lactam | 57 | 50% | 42 | 45 % | 179 | 60 % | 0.013 |
| ATB2 : Broad-spectrum beta-lactam | 24 | 21% | 19 | 20 % | 92 | 31 % | 0.035 |
| ATB2 : introductory day | 6,6 | +/- 2.2 | 8.6 | +/- 3.2 | 8.3 | +/- 4.3 | |
| ATB2 duration | 8,8 | +/- 7.2 | 7.7 | +/- 3.0 | 10.0 | +/- 7.0 | |

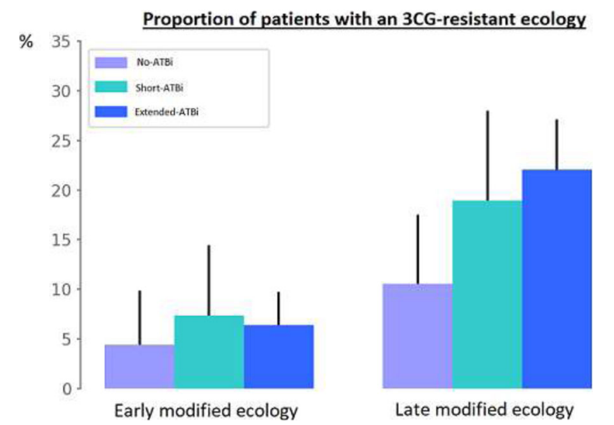

**Graph 1 (abstract 0805).** See text for description

### 0806

#### Antibiotic prophylaxis is associated with a reduced occurrence of early ventilator-associated pneumonia in traumatic brain injury: a retrospective cohort study

F.B. Carvalho[1], A.R. Corrêa[2], M.A. Victoriano[2], L.C.R. Silva[2], V.A. Nobre[3]
[1]Hospital João XXIII, Centro de Terapia Intensiva Adulto, Belo Horizonte, Brazil; [2]Universidade Federal de Minas Gerais - UFMG, Escola de Enfermagem, Belo Horizonte, Brazil; [3]Hospital das Clínicas da UFMG, Núcleo de Investigação em Medicina Intensiva - NIIMI, Belo Horizonte, Brazil
**Correspondence:** F.B. Carvalho
*Intensive Care Medicine Experimental* 2018, **6(Suppl 2):**0806

**INTRODUCTION.** Ventilator-associated pneumonia (VAP) is a frequent complication of invasive mechanical ventilation (IMV), with an impact on mortality and costs. Exposure to antibiotics may alter bacterial colonization and promote infection by multiresistant bacteria. Prophylactic antibiotics are routinely administered after major trauma.

**OBJECTIVES.** We aimed to evaluate the association between the administration of prophylactic antimicrobials and VAP characteristics in patients with traumatic brain injury (TBI).

**METHODS.** This was a retrospective cohort of adult TBI victims with a Glasgow Coma Scale (GCS) score ≤12, admitted to an emergency and trauma Intensive Care Unit under IMV. Early VAP (eVAP) was defined as occurring between 48 to 96 hours of IMV. Patients were divided in two groups: Group ≤24h, patients who did not use or used prophylactic antibiotics for less or equal than 24h; and Group >24h, patients who used prophylactic antibiotics for more than 24h.

**RESULTS.** From 01/Jan to 30/Aug/2016, 907 patients were admitted in the ICU and 231 consecutive patients were included, 108 (46.8%) to the Group ≤24h and 123 (53.2%) to the Group>24h. On average, VAP was diagnosed 6.35 (2.97) days after admission, and multiresistant bacteria were responsible for 35.1% VAP episodes. Group >24h had less eVAP (19,4% vs. 36,6% p=0.004), shorter hospitalization (34 days vs. 61 days p=0.035) and a higher occurrence of multiresistant bacteria in the first episode of VAP in 14 days (48.6% vs. 27.4%, p=0.036) compared to Group ≤24h. In a multivariate analysis, Group> 24h was the only variable associated to a lower occurrence of eVAP (OR 0.47 IC95% 0.25; 0.87 p=0.016). Among patients admitted with a GCS 3 to 8, Group >24h had less eVAP (p = 0.006) and fewer tracheostomies (p = 0.033). Group >24h patients with a GCS 8 to 12 had more multiresistant bacteria as an etiological agent of VAP (p = 0.016).

**CONCLUSIONS.** In this population of TBI victims, a higher exposure to prophylactic antibiotics was associated with lower occurrence of eVAP, shorter hospital length of stay and with multiresistant bacteria in the first episode of VAP.

### 0807

#### Early hypoalbuminemia is associated with 28-day mortality in critically ill burn patients

S. Pallado[1], S. Soussi[1], C. De Tymowski[1,2], N. Moreno[3], F. Depret[1,2], M. Chaussard[1,2], M. Coutrot[1,2], A. Fratani[1], M. Jully[1], A. Cupaciu[1], H. Oueslati[1], A. Ferry[1], M. Benyamina[1], M. Chaouat[4], M. Mimoun[4], A. Mebazaa[1,2,5], M. Legrand[1,2,5]
[1]APHP, Hôpital Saint-Louis, Department of Anesthesiology and Critical Care and Burn Unit, Paris, France; [2]Paris Diderot University, F-75475, Paris, France; [3]APHP, Hôpital Saint-Louis, Biochemistry Laboratory, Paris, France; [4]APHP, Hôpital Saint-Louis, Plastic Surgery and Burn Unit, Paris, France; [5]Institut National de la Santé et de la Recherche Médicale (INSERM), UMR INSERM 942, Hôpital Lariboisière, Univ Paris Diderot, F-75475, & F-CRIN INI-CRCT Network, Paris, France
**Correspondence:** S. Pallado
*Intensive Care Medicine Experimental* 2018, **6(Suppl 2):**0807

**INTRODUCTION.** Hypoalbuminemia after severe burn injury is mainly related to capillary leak syndrome and is associated with multiple organ failure (MOF) [1].

**OBJECTIVES.** To assess if very early hypoalbuminemia (< 6h after burn injury), as a potential modifiable variable, is associated with 28-day mortality.

**METHODS.** Retrospective cohort study including severely burned patients (Total Body Surface Area burn (TBSA) ≥20 % and/or mechanically ventilated) admitted to the burn unit of the Saint Louis Hospital, Paris between January 2013 and March 2017. The primary outcome was the association between serum albumin levels in the first 24 hours after burn injury and 28-day mortality. Initial albumin administration was defined by a local resuscitation protocol (After the 6th hour after injury, target albuminemia = 25-30 g/L) [2]. Non parametric tests, ROC curves, univariate and multiple regression analyses were perfomed (p< 0.05).

**RESULTS.** We enrolled 73 severely burned patients with a median (interquartile range) delay of admission of 3 (2-4) hours. The median TBSA, Full Thickness BSA, Abbreviated Burn Severity Index (ABSI) and Simplified Acute Physiology Score II (SAPSII) were respectively 40 (26-57)%, 20 (10-44)%, 8(7-11) and 37(24-52) respectively. 28-day mortality was 19%. On admission albuminemia (AlbH0) and H4 albuminemia (AlbH4) were significantly lower in deceased patients at day 28 (respectively 34(29-37) vs 27(22-30) g/L, p=0.009; 27 (22-30) vs 20 (17-25) g/L, p=0.02) whereas H24 albuminemia (AlbH24) was not (28(26-31) vs 27(25-31) g/L, p=0.79). The median infused crystalloids and human 20% albumin in the first 24 hours (survivors vs non-survivors at day 28) were respectively 3,4 (2,1-4,6) ml/kg/TBSA% vs 3,5 (2,3-4,5) ml/kg/TBSA% ; p = 0.90 and 77 (3-120) g vs 85 (46-140) g; p=0.28. ROC curves assessing the ability of AlbH0 and AlbH4 to discriminate 28-day mortality showed areas under curves of respectively 0.76[CI95% 0.60-0.92] (p=0.011) and 0.80[CI95% 0.64-0.96] (p=0.004). The best threshold value of AlbH4 was 23 g/L (Youden index, sensitivity=0.81, specificity=0.70). ABSI, SAPSII, Full Thickness BSA and inhalation injury were also significantly associated with 28-day mortality. Mulltiple logistic regression analysis revealed a significant association between AlbH4 and 28-day mortality (Adjusted odds ratio=0.88 [0.78-1.00]), p=0.04) independently of inhalation injury and TBSA. Kaplan-Meier curves of 28-day survival according to AlbH4 (< 23 g/L versus ≥ 23g/L) are shown in the Figure.

**CONCLUSIONS.** Very early hypoalbuminemia (< 6h) was associated with higher mortality in critically ill burn patients whereas AlbH24 was not. Early Albumin infusion from the 6th hour after thermal injury in order to improve outcome should be assessed in large randomized controlled trials.

**REFERENCE(S).**

[1] Eljaiek et al. Burns. 2013;39(1):113-8.
[2] Soussi et al. Ann Intensive Care. 2016 Dec;6(1):87.

**GRANT ACKNOWLEDGMENT. None.**

### 0808

#### Enzymatic debridement: a new therapeutic approach in critically burned patients

C. Gutierrez[1], K. Nanwani[1], N. Caceres[1], B. Civantos[1], M. Sanchez[1], J. Martinez[2], E. Herrero[1], E. Flores[1], J. Cantero[3], A. Garcia De Lorenzo[1]
[1]Hospital La Paz, Intensive Care Unit, Madrid, Spain; [2]Hospital La Paz, Plastic and Reconstructive Surgery, Madrid, Spain; [3]Hospital La Paz, Preventive Medicine, Madrid, Spain
**Correspondence:** C. Gutierrez
*Intensive Care Medicine Experimental* 2018, **6(Suppl 2):**0808

**INTRODUCTION.** Critical burns represent a pathology with great morbidity and mortality. The skin damaged by burns is characterized by the presence of a necrotic eschar of denatured proteins that generate an inflammatory reaction. The application of proteolytic enzymes such as bromelain is an easy process which allows a faster, more effective and selective debridement of the denatured tissue by heat, avoiding the healthy areas and therefore preserving the vital tissues around and under the burn. This therapeutic approach can improve treatment in burned patients in comparison with the standard care.
**OBJECTIVES.** To describe our experience using enzymatic debridement in our Critical Burn Unit, taking under consideration both advantages and disadvantages of its use.
**METHODS.** Retrospective observational study performed in the Critical Burn Unit (March 2016 to September 2017) including 27 patients >18 years old with a total body surface area (TBSA) burned >15% and < 75%, or >65years old with a TBSA burned >10% who underwent enzymatic debridement. Mean and standard deviation were used for normal quantitative variables and median and interquartile range in the opposite case. Qualitative variables were presented by absolute and relative frequencies.
**RESULTS.** Mean age was 47.6years (±17.8years), 74% were males, the most frequent injury mechanism was direct contact with fire, mean APACHE II was 11 (IQR 5-18), ABSI 7 (IQR 5-9). Median TBSA was 29% (IQR 18-50%), 21% (IQR 16-39) were deep dermal or full thickness. Time until debridement was 21 hours (IQR 8-35). 7.4% (n=2) of patients had incomplete debridement after first application, requiring tangential debridement. 33% (n=9) received regional anesthesia, 91% (n=25) didn't need blood transfusion. 25% of patients who didn't have vasopressors prior debridement needed the use of it after with a mean dose of 0,6mcg/Kg/min. 25% of patients with vasopressors prior treatment required an increase of dose by a mean of 0.9mcg/Kg/min. Median ICU stay was 19 days. Mortality was 22%, being the sepsis secondary to infection of the burns the most frequent cause of mortality.
**CONCLUSIONS.** Topical bromelain allows a fast start of tissue debridement with a low rate of failure. The spontaneous epithelialization rate seems to be higher compared to the standard of care, which decreases the need of surgery. The need for fasciotomy and blood transfusion was very low. Topical treatment involved a fast and simultaneous debridement of the TBSA generating an inflammatory response that in some cases required vasopressors. A rigorous control of analgesic treatment is required.

##### REFERENCE(S)

Rosenberg.L. A novel rapid and selective enzymatic debridement agent for burn wound management: a multi-center RCT. Anns burns fire disasters. May 2014.
Rosenberg.L. Minimally invasive burn: a review of seven clinical studies of rapid and selective debridement using bromelain-based debriding enzyme (Nexobrid®). Anns burns fire disasters. Dec 2015.

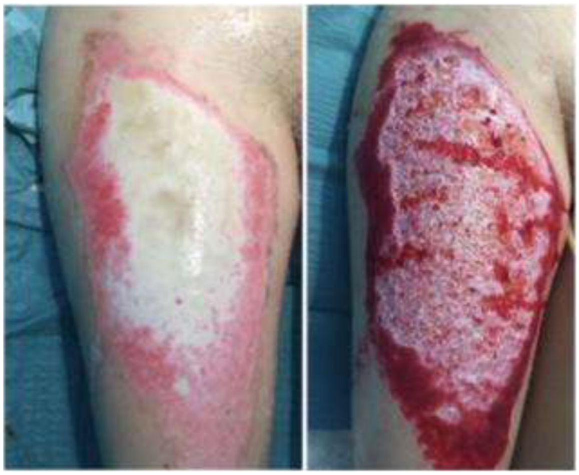

**Fig. 1 (abstract 0808).** Enzymatic debridement: before and after

## Immune system & sepsis

### 0809

#### Assessment of postoperative lymphopenia as risk factor for postoperative infections: EVALYMPH study

G. Dupont[1,2], L. Flory[1], C. Tourel[1], J. Morel[1], D. Morand[3], A.-C. Lukaszewicz[2,4], G. Monneret[5,6], B. Peireira[3], S. Molliex[1], EVALYMPH Group
[1]CHU Saint Etienne, Anesthésie Réanimation, Saint Etienne, France; [2]université Claude Bernard Lyon 1, EA 7426 PI3 - Pathophysiology of Injury-Induced Immunosuppression, Lyon, France; [3]CHU Clermont Ferrand, Clermont-Ferrand, France; [4]CHU Lyon, Anesthésie Réanimation - Hôpital Neurologique Pierre Wertheimer, Lyon, France; [5]CHU Lyon, Laboratoire d'Immunologie - Hôpital Edouard Herriot, Lyon, France; [6]Université Claude Bernard Lyon 1, EA 7426 PI3 - Pathophysiology of Injury-Induced Immunosuppression, Lyon, France
**Correspondence:** G. Dupont
*Intensive Care Medicine Experimental* 2018, **6(Suppl 2):**0809

**INTRODUCTION.** Postoperative infections (POI) are one of the most common complications in thoracic and digestive cancer surgery. Therefore, identifying risk factor for POI is important from a public health perspective. Former studies have demonstrated that inflammatory response is altered during perioperative period causing a lymphopenia. It has been suggested that lymphopenia may contribute to POI. To date, this was not proved by multivariate analysis integrating other known factors of POI.
**OBJECTIVES.** The aim of our study was to determine whether lymphopenia is a factor associated with POI in thoracic and digestive cancer surgery.
**METHODS.** Multicentric French cohort study. This study was approved by our local ethic comity (ethic comity for research in anaesthesia and critical care IRB 00010254 - 2016 - 024). Lymphocytes counts were obtained from patients undergoing pulmonary or digestive cancer surgery before surgery (day-0) and 1, 3, 5 and 8 days after surgery. Risks factors of POI and information's related to anesthesia and surgical procedure were collected as well as clinical and biological signs of POI. A univariate analysis was performed to identify the risks factors for POI. Variables with p≤0.20 were integrated in a multivariate analysis. A factor analysis was used to identify the factors related to patient, anaesthesia or surgery which could explain post-operative lymphopenia.

**RESULTS.** 1306 patients were included. 216 patients (16 %) had a POI including postoperative pneumonia, secondary peritonitis and surgical site infection. The time to nadir for lymphopenia was day 1 after the surgery (mean $1.2.10^9$ cells.$l^{-1}$ ± 0.7). Lymphocyte count at post-operative day 1 (HR 1.48 CI 95 % [1.09 - 2.02] p=0.01), open surgery (HR 1.68 IC 95 % [0.76 - 1.93] p=0.01), ASA score >3 (HR 2.75 IC 95 % [1.02 - 7.43] p=0.05), COPD (HR 1.53 IC 95 % [1.02 - 2.30] p=0.04) and the length of surgery (HR 1.83 IC 95 % [1.26 - 2.66] p=0.001) were identified as risks factors for POI in the multivariate analysis **(table)**. Epidural analgesia did not reduce the risk of infection **(table)**. No variable related to patient, anaesthesia or surgery was identified to explain the lymphopenia observed at post-operative day (data not shown). The development of POI was not associated with absolute or percentage decrease in lymphocyte count after surgery (data not shown). This result suggests that there may be a threshold value of lymphocyte count for risk of POI.

**CONCLUSIONS.** Post-operative day 1 lymphopenia was a risk factor for POI. This study does not allow determining lymphopenia mechanisms and suggesting prophylactic measures.

**Table 1 (abstract 0809).** Risks factors for post-operative infection in multivariate analysis

| Variable | Hazard ratio | Standard error | p | 95 % CI |
|---|---|---|---|---|
| Lymphocyte count at Post Operative Day 1 | 1.48 | 0.23 | 0.01 | 1.09 - 2.02 |
| Open surgery | 1.68 | 0.35 | 0.01 | 1.11 - 2.55 |
| ASA Score 4 | 2.75 | 1.39 | 0.05 | 1.02 - 7.43 |
| Radiotherapy | 1.42 | 0.38 | 0.16 | 0 .86 - 2.40 |
| COPD | 1.53 | 0.32 | 0.04 | 1.02 - 2.30 |
| Epidural analgesia | 1.21 | 0.21 | 0.28 | 0.86 - 1.69 |
| Length of surgery | 1.83 | 0.35 | 0.001 | 1.26 - 2.66 |

### 0810

#### Effect of experimental sepsis and fluid resuscitation on the expression of micro RNAs involved in the inflammatory response

L. Oteiza[1], R. Pandolfi[2,3], L. Moreno[2], A. Ferruelo[3], N. Valero[1], S. López Cuenca[1], D. Molina[1], F. Pérez-Vizcaino[2,3], J. Lorente[1,4]

[1]Hospital Universitario de Getafe, Madrid, Spain; [2]Universidad Complutense de Madrid, Madrid, Spain; [3]Instituto CIBER de Enfermedades Infecciosas, Madrid, Spain; [4]Universidad Europea, Madrid, Spain

**Correspondence:** L. Oteiza

*Intensive Care Medicine Experimental* 2018, **6(Suppl 2):**0810

**INTRODUCTION.** MicroRANs are small noncoding RNA sequences that control de expression of genes involved in many different cell functions. Their expression can be measured in different body fluids. This measurement can be useful for biomarker identification and for a better understanding of the pathogenesis of different conditions.

**OBJETIVE.** To study the effect of sepsis and volume resuscitation on the serum expression of different microRNAs (miRNAs) involved in the inflammatory response in an animal model

**METHODS.** Adult pigs (sus scrofa) sedated (thiopental, fentanyl iv), tracheostomized, subjected to mechanical ventilation and monitored by a pulmonary artery and femoral arterial catheter. One group of animals was observed without any treatment (group C,n = 15) and another group (S) received at t=0 an iv dose in 30 min of live E. coli (108 * 2 ml / kg).The animals in group S were randomized to receive from t = 0 to t = 300min different rhythms of resuscitation fluids (saline 0.9% at 4 ml/kg/h,10ml/kg/h and 17ml/kg/h)( groups S4, S10, S17, respectively) (n = 8 each group). The serum expression was quantified at t=300min of different microRNAs by RT-PCR (Qiagen, Hilden, Germany).The studies were carried out with the authorization of the Animal Research Committee.

**RESULTS.** Sepsis induced a state of decreased blood pressure, cardiac output and systemic vascular resistance (data not shown), which was attenuated by administration of fluids. The serum expression levels of miR 155-5p, miR 146-5p , miR 150-5p, miR 21a-5p, miR 34a-5p, but not those of miR 486a-5p and miR 133 increased significantly (p < 0.05) in animals with sepsis compared to the control group. The expression of miR 146-5p (C: 0.01 [0.006-0.05]; S4: 13.8 [6.8-23.8]; S10: 0.63 [0.34-0.75] ; S17: 1.67 [0.18-3.16], p = 0.026) was lower in animals resuscitated with fluids.

**CONCLUSIONS.**

(i) miRNA expression assessed in serum can be useful for biomarker identification.

(ii) Resuscitation in sepsis is accompanied by an anti-inflammatory effect.

(iii) Certain miRNA could be therapeutic targets in sepsis.

**GRANT ACKNOWLEDGMENT**

### 0811

#### Analysis of lipoproteins in septic shock

F. Fiorini, D.B. Antcliffe, F. Al-Beidh, A.C. Gordon

Section of Anaesthetics, Pain Medicine and Intensive Care, Department of Surgery and Cancer, Faculty of Medicine, Imperial College London, London, United Kingdom

**Correspondence:** F. Fiorini

*Intensive Care Medicine Experimental* 2018, **6(Suppl 2):**0811

**INTRODUCTION.** Sepsis is a highly heterogeneous clinical syndrome characterised by a dysregulated response to an infectious insult, leading to systemic inflammation. Understanding the mechanisms underpinning this variability will help develop novel therapeutic approaches and personalised treatment. Due to their immunomodulatory properties, lipoproteins represent an interesting potential target for intervention.

**OBJECTIVES.** To identify patterns of lipoprotein metabolism and evaluate their role in patients with septic shock.

**METHODS.** A panel of 105 lipoprotein subclasses were measured using nuclear magnetic resonance spectroscopy (NMR) from the serum of a prospective cohort of 174 ICU patients who had septic shock and were enrolled in the VANISH trial (1). Samples were collected at 24-48 hour intervals to generate four discrete time-points. Unsupervised multivariate analysis with principle component analysis (PCA) was used to identify natural clusters. Orthogonal partial least squares discriminant analysis (OPLS-DA) and univariate statistics were then applied to separate out the most influential lipoproteins.

**RESULTS.** There was a significant change in lipoprotein profiles over time between enrolment in the study and when the final sample was taken ($R^2$ 0.26 $Q^2$ 0.20, p< 0.001). The metabolic changes were driven predominantly by an increase in triglycerides bound to low-density lipoprotein subclasses. Total LDL triglycerides increased from 34.1mg/dL to 56.2mg/dL (p< 0.001). There was also a significant increase in total apolipoprotein A-2 (25.4 to 33.7mg/dL, p< 0.001), the second most abundant protein on HDL. Overall lipoproteins that changed significantly increased over time with the exception of HDL-1 cholesterol (20.6 to 12.3mg/dL, p< 0.001) and LDL-6 free cholesterol (4.0 to 0.2mg/dL, p< 0.001) both of which decreased.
Contrary to previously published work (2), we found no significant relationship between total cholesterol levels at baseline and mortality (*p*=0.45).
**CONCLUSIONS.** The lipoprotein profiles of patients with septic shock evolve substantially throughout their clinical course. Further work is needed to elucidate how these may correlate with outcomes and response to treatment.

**REFERENCE(S).**

1. Gordon AC et al. Effect of Early Vasopressin vs Norepinephrine on Kidney Failure in Patients With Septic Shock: The VANISH Randomized Clinical Trial. JAMA 2016;316(5):509-18
2. Fraunberger P et al. Reduction of circulating cholesterol and apolipoprotein levels during sepsis. Clinical Chemistry and Laboratory Medicine. 1999;37:357-62

**GRANT ACKNOWLEDGMENT. This abstract is independent research funded by the National Institute for Health Research (NIHR) Imperial Biomedical Research Centre (BRC). ACG is an NIHR Research Professor (RP-2015-06-018)**

### 0812

#### Secondary hemophagocytic lymphohistiocytosis in sepsis: two sides of the same coin?

F. Frantzeskaki[1], M. Theodorakopoulou[1], E. Paramythiotou[1], G. Skyllas[1], P. Tsirigotis[2], A. Armaganidis[1], I. Tsangaris[1]
[1]Attikon University Hospital, Athens University School of Medicine, 2nd Department of Critical Care, Athens, Greece; [2]Attikon University Hospital, Athens University School of Medicine, 2nd Department of Internal Medicine, Athens, Greece
**Correspondence:** F. Frantzeskaki
*Intensive Care Medicine Experimental* 2018, **6(Suppl 2):**0812

**INTRODUCTION.** Hemophagocytic lymphohistiocytosis (HLH) is an inflammatory devastating syndrome, caused by impaired downregulation of activated macrophages and lymphocytes. Sepsis usually overlaps with HLH, rendering the diagnosis of the syndrome challenging, since the two conditions might be life-threatening.
**OBJECTIVES.** The aim of the present study was to retrospectively investigate the incidence of HLH in septic patients. We studied the clinical and pathophysiologic characteristics paralleling the two syndromes (sepsis and HLH) and the subsequent morbidity and mortality.
**METHODS.** For six years period,septic patients, treated in ICU,were included in this retrospective observational study.Diagnosis of sepsis was based on the criteria included in the Third International Consensus definition on Sepsis and Septic shock. HLH diagnosis was based on five out of the following eight HLH criteria: persistent fever, splenomegaly, peripheral blood cytopenia,hypofibrinogenemia and/or hypertriglyceridemia, hemophagocytosis in bone marrow, low NK cells activity, hyperferritinemia and high concentration of sCD25. Multivariated analysis was used, to evaluate the risk factors associated with mortality.
**RESULTS.** During the study period, fifteen (15) fulfilled the HLH diagnostic criteria. Septic shock was present in seven patients (46%). Bloodstream infection was present in nine patients (60%): The responsible isolated pathogens were *Klebsiella pneumoniae* (3 patients), methicillin sensitive *Staphylococcusaureus* (1 patient), *Acinetobacter baumannii* (2 patients), *Candida albicans* (1 patient), *Pseudomonas aeruginosa* (1 patient) and Cytomegalovirus (1 patient). Ventilator associated pneumonia was the cause of sepsis in 3 patients (20%), while one patient suffered from diverticulitis. In two patients the cause of sepsis was not identified. Concerning the possible triggering factors of HLH, lymphoid malignancy was present in six patients (40%), while CMV infection was observed in one patient and connective tissue disease in three patients as well. Hemophagocytosis on bone marrow aspirates was found in nine patients (60%), whereas ferritin levels were high in the whole sample. Intravenous immunoglobulins were administered in thirteen patients, while one patient received dexamethasone and etoposide. The mean (±SD) ICU stay was 25±17 days, while the observed ICU mortality was 60%.Multivariated analysis showed that ICU mortality was significantly associated with SOFA score (p=0.001) and advanced age (p=0.04).
**CONCLUSIONS.** HLH might be associated with sepsis and septic shock. Future research will elucidate the possible pathophysiologic mechanisms connecting the tissue injury in sepsis, with the hyperinflammatory state leading to HLH, to identify the subset of patients who might take benefit of immunosuppressive therapy.

**REFERENCE(S)**

Raschke RA, Garcia-Orr R. Hemophagocytic lymphohistiocytosis:*Chest. 2011 Oct;140(4):933-938.*

### 0813

#### Delta Neutrophil Index (DNI) at 72 hours can be a good prognostic marker of mortality in pneumonia sepsis

S.Y. Park[1], S.H. Park[2], S. Moon[3], C. Kim[4]
[1]ChungNam National University Hospital, Pulmonary and Critical Care Medicine, Daegeon, Korea, Republic of; [2]Ilsan Paik Hospital, Inje University College of Medicine, Ilsan, Korea, Republic of; [3]Korea University Guro Hospital, Seoul, Korea, Republic of; [4]Jeju National University Hospital, Jeju National University School of Medicine, Department of Internal Medicine, Jeju, Korea, Republic of
**Correspondence:** S.Y. Park
*Intensive Care Medicine Experimental* 2018, **6(Suppl 2):**0813

**INTRODUCTION.** Pneumonia sepsis cannot be overlooked because of high mortality and morbidity rate.However, none has sufficient accuracy to serve as a prognostic indicator about pneumonia sepsis. It is well known that immature granulocyte are an indicator of increased myeloid cell production and are associated with infection or systemic inflammation.
**OBJECTIVES.** The data, however, are limited and little is known about the clinical usefulness of DNI in assessing mortality risk in patients with pneumonia sepsis. Therefore, this study evaluated the clinical utility of DNI as a prognostic indicator of mortality of pneumonia sepsis patients.
**METHODS.** We conducted a retrospective study from January 2013 to March 2015. The study was carried out in Kangdong Sacred Heart Hospital Medical Intensive Care Unit on patients with pneumonia sepsis. The DNI was measured in 3 consecutive days for pneumonia sepsis patients admitting to ICU. Primary outcome of this study was 28-day mortality.
**RESULTS.** A total of 89 patients with pneumonia sepsis were included in this study. Septic shock patients were 48(53.9%). In multivariate Cox proportional analysis, septic shock (Hazard ratio

4.373; 95% confidence interval (CI) 1.55-12.302; P=0.005), DNI at 72 hours of admission (DNI 3) (Hazard ratio 2.993; 95% CI 1.193-7.504; P=0.019) and ECMO (Hazard ratio 4.831; 95% CI 1.449-16.107; P=0.10) were significantly related to 28-day mortality. In the higher DNI 3 group (≥3), 53.8% (14/26) of the patients died to 28 day, whereas in the lower DNI 3 group (< 3), 15.7 % (8/51) died during 28 days (P=0.000)

**CONCLUSIONS.** DNI of 72 hours after ICU admission is a promising prognostic marker of 28-day mortality in patients with pneumonia sepsis.

**REFERENCE(S)**

H. W. Kim, S. Ku, S. J. Jeong, S. J. Jin, S. H. Han, J. Y. Choi, J. M. Kim and Y. G. Song: Delta neutrophil index: could it predict mortality in patients with bacteraemia? *Scand J Infect Dis* 44(7):475-80, 2012. .H. Kim, T. Kong, S. P. Chung, J. H. Hong, J. W. Lee, Y. Joo, D. R. Ko, J. S. You and I. Park: Usefulness of the Delta Neutrophil Index as a Promising Prognostic Marker of Acute Cholangitis in Emergency Departments. *Shock* 47(3):303-312, 2017. .H. Fang, W. Jiang, J. Cheng, Y. Lu, A. Liu, L. Kan and U. Dahmen: Balancing Innate Immunity and Inflammatory State via Modulation of Neutrophil Function: A Novel Strategy to Fight Sepsis. *J Immunol Res* 2015:187048, 2015. P. H. Leliefeld, C. M. Wessels, L. P. Leenen, L. Koenderman and J. Pillay: The role of neutrophils in immune dysfunction during severe inflammation. *Crit Care* 20:73, 2016.

### 0814

#### Lack of association between neutrophil-to-lymphocyte ratio and ICU stay in patients with sepsis: a retrospective cohort study

Y. Fujita, R. Ishihara, A. Hashimoto, Y. Sato, M. Okumura, T. Shimomura, Y. Fujiwara

Aichi Medical University, Department of Anesthesiology, Nagakute, Japan

**Correspondence:** Y. Fujita

*Intensive Care Medicine Experimental* 2018, **6(Suppl 2):**0814

**INTRODUCTION.** Neutrophil-to-lymphocyte ratio (NLR) is a representative index of systemic inflammation and a prognostic inflammatory biomarker in various diseases, especially various cancers[1]. NLR was recently reported to be associated with mortality in patients with acute respiratory distress syndrome[2, 3]. Sepsis induces a systemic inflammatory response.

**OBJECTIVES.** In this retrospective study, we sought to explore whether NLR is a prognostic factor in patients with sepsis.

**METHODS.** The study cohort comprised patients diagnosed as having sepsis and admitted to our intensive care unit (ICU). We calculated NLR by dividing the neutrophil by the lymphocyte count on admission to the ICU and categorized patients into two groups based on NLR values. Primary outcome was ICU stay. The relationship between NLR and ICU-stay was assessed using Kaplan-Meier plots and multivariable Cox regression. Secondary outcomes were weaning from administered norepinephrine, 28-day mortality, and 90-day mortality.

**RESULTS.** We divided the 58 patients in the final analysis into two groups with NLR ≤15 and >15. The median (interquartile range) NLR in the low NLR (S) and high NLR groups (L) were 7.8 (5.9-11.3) and 30.8 (21.8-45.9), respectively. The ICU stay was 12 (8.5-15.5) and 8.5 (6-15) days, respectively. Cox regression analysis showed that NLR is not a significant predictor of ICU stay (adjusted hazard ratio (HR) = 1.674, 95% confidence interval (CI), 0.462 to 6.063, p = 0.432). NLR was also not a significant risk factor for weaning from administered norepinephrine (HR = 1.049, CI, 0.542 to 2.03, p = 0.887). The 28-day mortality was 6.9% and 22.2% (p = 0.185), respectively and the 90-day mortality 22.2% and 35.7% (p = 0.453), respectively.

**CONCLUSIONS.** In this preliminary study, we found no association between high NLR and duration of ICU stay or weaning from norepinephrine administration in critically ill patients with sepsis.

**REFERENCE(S)**

1, Prognostic value of the neutrophil to lymphocyte ratio in lung cancer: A meta-analysis. Clinics 2015;70:524-530

2, Neutrophil-to-lymphocyte ratio as a prognostic marker in acute respiratory distress syndrome patients: a retrospective study. J Thorac Dis 2018;10:273-282

3, The association between the neutrophil-to-lymphocyte ratio and mortality in patients with acute respiratory distress syndrome: a retrospective cohort study. shock in press.

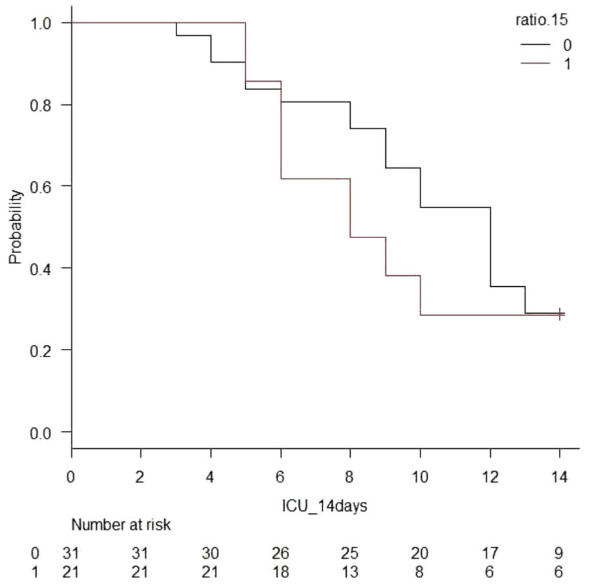

**Fig. 1 (abstract 0814).** Kaplan-Meier plot of ICU stay. Ratio.15; 0=NLR≤15, 1=NLR>15

### 0815

#### Association between soluble Toll-like receptor 2 levels and sepsis on admission to the ICU: results of a case-control study

Y. Lim[1], A.-C. Raby[2], M.O. Labéta[2], T. Szakmany[3]

[1]Cardiff University, School of Medicine, Cardiff, United Kingdom; [2]Cardiff University, Division of Infection and Immunity, Cardiff, United Kingdom; [3]Cardiff University, Division of Population Medicine, School of Medicine, Cardiff, United Kingdom

**Correspondence:** Y. Lim

*Intensive Care Medicine Experimental* 2018, **6(Suppl 2):**0815

**INTRODUCTION.** There is an ongoing need for better, more reliable early detection of sepsis. The soluble form of Toll-like receptor 2 (sTLR2) has previously been shown to be elevated in patients with infective and inflammatory conditions (1). In our previous pilot study, plasma sTLR2 levels were preferentially raised in patients with sepsis and organ failure compared to patients with sterile inflammatory response syndrome (2).

**OBJECTIVES.** The present study aimed to confirm and validate our previous findings in a larger and independent population.

**METHODS.** We performed a retrospective case-control study in 172 patients with either confirmed sepsis according to Sepsis-3 criteria (n=138) or out-of-hospital cardiac arrest (OOHCA) (n=34). Plasma levels of sTLR2 obtained from samples within 24 hours of admission to the ICU were determined by ELISA, performed blind to patient group. Demographic, organ dysfunction severity and mortality data were collected. For statistical analysis Mann-Whitney-U, Chi-square test and ROC analysis was used.

**RESULTS.** sTLR2 levels were significantly higher in the sepsis group: median sTLR2 levels were 2713 pg/mL (IQR 1457-5248 ) in sepsis and 661 pg/mL (IQR 380-1377) in the OOHCA group (p< 0.001; Figure). Clinical characteristics on admission were similar in the sepsis and OOHCA groups: Age median 68 years (IQR 59-76) vs 62 (IQR 52-72),

SOFA scores median 17 (IQR 15-19) vs 16 (15-18) and APACHE II scores median 32 (IQR 23-38) vs 30 (IQR 26-34) did not differ significantly. ICU mortality was 21.3% in the sepsis and 35% in the OOHCA group (p=0.102).

Using ROC analysis, sTLR2 showed discriminatory power for sepsis: AUC 0.827 95%CI 0.751-0.904 p< 0.001 on admission to the ICU. Our previously identified cut-off of 1000 ng/mL had 85.7% sensitivity and 75% specificity for diagnosing sepsis. sTLR2 levels below 500 ng/mL had 93% specificity and 35% sensitivity to rule out organ dysfunction secondary to infection.

**CONCLUSIONS.** This study confirms our previously observed association between elevated sTLR2 levels and the diagnosis of sepsis early in the clinical course. Our previously identified cut-off of 1000 ng/mL was confirmed to have appropriate discriminatory power. We also identified that lower sTLR-2 levels can confidently rule out infection induced organ dysfunction. Further work is required to identify the time-course of sTLR-2 levels, whether an optimum diagnostic cut-off exists and if sTLR2 levels can be used effectively in a multivariable prediction model.

**REFERENCE(S)**

1. Henrick B. et al. (2016) Front Immunol 7:291
2. Holst B et al. (2017) Intensive Care Med Exp 5:2

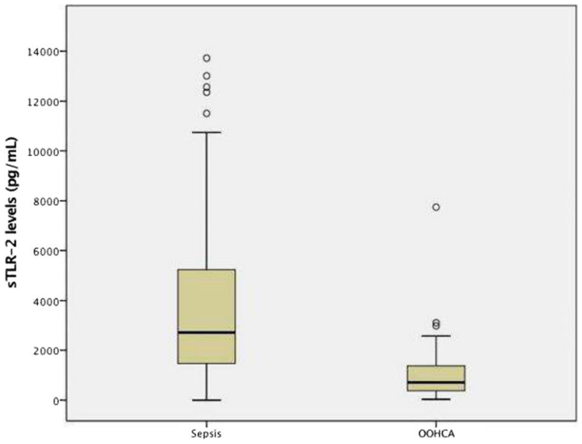

**Fig. 1 (abstract 0815).** Comparison of sTLR2 levels in patients with out-of-hospital cardiac arrest (OOCHA) and patients with confirmed diagnosis of sepsis

### 0816

#### Circulating monocyte counts and its impact on outcomes in patients with severe sepsis or septic shock

H. Chang, J.H. Lee, Y.H. Jo, H. Chung, J.E. Hwang, J. Kim
Seoul National University Bundang Hospital, Department of Emergency Medicine, Seongnam-si, Korea, Republic of
**Correspondence:** H. Chang
*Intensive Care Medicine Experimental* 2018, **6(Suppl 2):**0816

**INTRODUCTION.** Sepsis is a progressive state from a local infection to systemic response. Neutrophils and monocytes are critical cellular components of the innate immune system. There have been controversial results of monocyte counts and its impact in sepsis. The reduction in monocyte counts has been reported to be correlated with the severity of sepsis in some studies, but not in all.

**OBJECTIVES.** This study was performed to evaluate the association of monocyte counts with mortality, the rate of bacteremia and organ dysfunction and to evaluate whether the change in monocyte counts from the premorbid state to sepsis would differ between survivors and non-survivors in patients with severe sepsis or septic shock.

**METHODS.** A retrospective analysis of patients with severe sepsis or septic shock was performed. Monocyte counts were categorized into < 250, 250 - 500, 500 - 750 and ≥ 750 cells/μL; in addition, 28-day mortality, the rate of bacteremia and organ dysfunction were compared between the groups. Multivariate logistic regression analyses were performed to evaluate the independent association of initial blood cell counts with 28-day mortality. Subgroup analyses of patients who had premorbid data of blood cell counts were performed to evaluate the difference in the change in monocyte counts between survivors and non-survivors.

**RESULTS.** During the study period, 2012 patients were included. Neutrophil and monocyte counts were significantly different between survivors and non-survivors. However, only monocyte counts were independently associated with mortality in the multivariate logistic regression analyses. Patients with initial monocyte counts < 250 cells/μL showed the highest mortality, rate of bacteremia and organ dysfunction. In patients who had premorbid blood cell counts, the monocyte counts increased in survivors but decreased in non-survivors from the premorbid to sepsis.

**CONCLUSIONS.** In conclusion, monocyte counts were associated with mortality, the rate of bacteremia and organ dysfunction in patients with sepsis, possibly due to the relative lack of monocytopoiesis related with septic insults in non-survivors.

**REFERENCE(S)**

1. J. L. Vincent, S. M. Opal, J. C. Marshall and K. J. Tracey: Sepsis definitions: time for change. *Lancet* 381(9868):774-5, 2013
2. D. Heumann, M. P. Glauser and T. Calandra: Monocyte deactivation in septic shock. *Curr Opin Infect Dis* 11(3):279-83, 1998.
3. I. N. Shalova, J. Y. Lim, M. Chittezhath, A. S. Zinkernagel, F. Beasley, E. Hernandez-Jimenez, V. Toledano, C. Cubillos-Zapata, A. Rapisarda, J. Chen, K. Duan, H. Yang, M. Poidinger, G. Melillo, V. Nizet, F. Arnalich, E. Lopez-Collazo and S. K. Biswas: Human monocytes undergo functional re-programming during sepsis mediated by hypoxia-inducible factor-1alpha. *Immunity* 42(3):484-98, 2015.

### 0817

#### Research of leukocyte, TNF-alpha, IL-10, IL-4 and procalcitonin levels in mortality and morbidity in patients with sepsis, severe sepsis and septic shock

E. Kızılmese, O. Taskin, A. Ay, C. Balci
Kocaeli Derince Training Hospital, Anaesthesiology and Intensive Care Medicine, Kocaeli, Turkey
**Correspondence:** E. Kızılmese
*Intensive Care Medicine Experimental* 2018, **6(Suppl 2):**0817

**INTRODUCTION.** Sepsis is a complicated process including several pathophysiological events with a high mortality rate and problematic therapy. Altough there are many experimental studies on multimodal immunological cascade resulting from the sepsis, there is not any research evaluating the relation between proinflammatory and inflammatory cytokines, procalcitonin levels and mortality and morbidity in sepsis in human yet.

**OBJECTIVES.** This study aims to investigate the correlation between the mortality and levels of leukocyte, TNF-α, IL-10, IL-4 and procalcitonin in sepsis.

**METHODS.** This prospective study was performed with 45 patients diagnosed with sepsis, severe sepsis and septic shock and stayed more than 24 hours in Intensive Care Unit of Anestesiology and Reanimation Clinic of Health Science University Derince Education and Research Hospital. As a control group, 20 patients who were not diagnosed with sepsis but stayed in intensive care unit for more than 24 hours were accepted. The patients with sepsis were assigned as Group 2 (n:14), severe sepsis patients were Group 3 (n:15) and septic shock patients were Group 4 (n:16). These groups were also divided into two groups as alive and mortal after care and treatment. Descriptive data as age, sex, diagnosis, hospitalization duration,

APACHE II scores, underlying diseases of the patients were recorded. Leukocyte levels of the blood samples of patients were determined ath the moment of diagnosis for sepsis, at 7th day of hospitalization and at the discharge moment from the clinic or at time of death. Moreover, proinflammatory cytokine levels of TNF-α, IL-4, IL-10 and PCT levels were measured from the blood samples.

**RESULTS.** The findings of study showed the demographic characteristics of patients were statistically similar. There were significant differences when the mortality rate and the severity of sepsis were compared with hospitalization duration, acute renal damage, leukocyte levels, some IL-10 levels and procalcitonin amount in the patients. However, any relation between mortality rate and levels of the cytokine levels for TNF-α and IL-14 could not be detected.

**CONCLUSIONS.** This study concluded that there may be a correlation between the mortality rate and hospitalization duration of sepsis, severe sepsis and septic shock patients and their levels of leukocytes, IL-10 and PCT. However, unrelated correlation between TNF-α and IL-4 levels and the mortality rate in sepsis may indicate that the patient range should be enlarged to a larger multicentered study.

**REFERENCE(S)**

Balci, C., R. Sivaci. Procalcitonin Levels As An Early Marker İn Patients With Multiple Trauma Under İntensive Care. Journal Of International Medical Research, 2009. 37(6): 1709-1717.

### 0818

#### Role of neutrophil-to-lymphocyte ratio in the prognosis of severe community-acquired pneumonia

J. Suárez-Cuenca[1], A. Ruiz-Hernández[1], L. Corona-Rojas[2], J. Flores-Zaleta[2], E. Vera-Gómez[1], J. Hernández-Patricio[1], M. Macedo-Perez[1], V. Pulido-Pintor[2], Ó. López-Santiago[2], A. González-Mora[2], F. García-Pérez[2], A. Melchor-López[3], P. Mondragón-Terán[1]
[1]ISSSTE, Department of Clinical Research, Mexico City, Mexico; [2]Corporativo Hospital Satélite, Intensive Care Unit, Mexico City, Mexico; [3]Hospital General Xoco, Internal Medicine, Mexico City, Mexico
**Correspondence:** A. Ruiz-Hernández
*Intensive Care Medicine Experimental* 2018, **6(Suppl 2):**0818

**INTRODUCTION.** Community-acquired pneumonia (CAP) is considered an important cause of mortality and morbidity worldwide. There is a constant need to explore novel prognostic markers. Dynamic changes in immune cells response to sepsis sourced markers like the Neutrophil-to-Lymphocyte Ratio (NLR), that has shown potential usefulness in sepsis and septic shock.

**OBJECTIVES.** To evaluate the ability of NLR in the prognosis of outcome in CAP.

**METHODS.** A prospective cohort of consecutive patients admitted to Intensive Care Unit (ICU) or Internal Medicine (IM) with diagnose of CAP, in the absence of hematological disease, immunosupression, chemotherapy, myelotoxic agents, hemotransfusion or liver damage. Clinical-demographic information, co-morbidity and laboratory data were obtained from medical records. The NLR was calculated from the hemogram at admission and during 3, 5 and 7 days of follow-up. Severity scores (SOFA), its modification during evolution [DSOFA]) and the clinical outcome (unfavorable outcome: use of vasopressors, invasive ventilation and mortality) were evaluated; as well as the hospitalization time. In addition, we explored apoptotic trends in neutrophils and lymphocytes (Western blot, Anexin V). The study population was grouped according to outcome. Mortality was analyzed separately. Student's t-tests, ROC curve, Odds ratio and Pearson's correlation were used. Statistical significance, p-value < 0.05.

**RESULTS.** Fifty nine patients (31 males, 28 females), mean age 65.4 ±18.8 years old, constituted the study population. We observed the following baseline values: CURB-65 2.06±1.04; SOFA 6.10±3.04, and NLR 14.78±11.06. Interestingly, the higher NLR, the higher CURB-65 score (p< 0.05), and negative related with DSOFA, during time course analysis. Baseline data from groups with favorable and unfavorable outcomes were: CURB-65 score 1.64±0.78 vs 2.45±1.12 (p< 0.05), SOFA 4.57±1.95 vs 7.58±3.05 (p< 0.05) and NLR 11.25± 8.7 vs 17.21 ±10.75 (p< 0.05), respectively. Regarding latter comparison, NLR value was higher in subpopulation with unfavorable outcome, who were admitted directly to ICU 16.30±8.9 compared to those admitted to IM 11.81±10.31 (p< 0.05); as well as cases who showed in-hospital death 17.61±8.4 (p< 0.05).

NLR cut-off (14) was calculated based in the discriminatory ability to predict unfavorable outcome, SE 0.68, SP 0.64, AUC=0.41 (p< 0.05); OR 3.1 (CI95= 1.07 to 13.08, p< 0.05). Furthermore, baseline NLR< 14 associated shorter hospitalization time and significant correlation (Pearson's r=0.66 (CI 95=0.21-0.88, p< 0.01). A decreased expression of Anexin V in neutrophils was observed patients with unfavorable outcomes.

**CONCLUSIONS.** The NLR showed prognostic ability of unfavorable outcome in patients with CAP.

**REFERENCE(S)**

Ljungström, L., et al. (2017). Diagnostic accuracy of procalcitonin, neutrophil-lymphocyte count ratio, C-reactive protein, and lactate in patients with suspected bacterial sepsis. *PLoS ONE*, *12*(7)

### 0819

#### Survivin variants mRNA expression and protein activity in sepsis

M. Miliaraki[1], P. Briassoulis[1], S. Ilia[1], A. Polonifi[2], M. Mantzourani[2], E. Briassouli[2], S. Nanas[3], A. Pistiki[4], G. Daikos[2], G. Briassoulis[1]
[1]University of Crete, Medical School, Pediatric Intensive Care Unit, University Hospital of Crete, Heraklion, Greece; [2]National and Kapodistrian University of Athens, First Department of Internal Medicine - 'Laiko' Hospital, Athens, Greece; [3]National and Kapodistrian University of Athens, First Critical Care Department, Athens, Greece; [4]National and Kapodistrian University of Athens, 2nd ICU and 4th Internal Medicine Department, Athens, Greece
**Correspondence:** M. Miliaraki
*Intensive Care Medicine Experimental* 2018, **6(Suppl 2):**0819

**INTRODUCTION.** Sepsis is a maladaptive inflammatory process in response to infectious agents, related to immune dysfunctions and devastating complications. Recently, interest has been shifted towards apoptotic and anti-apoptotic pathobiology, which might bring to light attractive therapeutic interventions for septic intensive care unit (ICU) patients. Apoptosis is regulated through the activation of a number of anti-apoptotic proteins, such as survivin, with different biological functions (pro-apoptotic or anti-apoptotic), depending on the expression of its different splice variants (wild type-WT, ΔEx3, 2B, 3B). The importance of these complicated pathways in sepsis has not yet been elucidated.

**OBJECTIVES.** The present study seeks to ascertain whether sepsis is associated with an upregulation of certain anti-apoptotic biomolecules, such as survivin, as well as to assess the prognostic value of the expression of the survivin transcript isoforms -WT, -ΔE3x, -2B and -3B, in ICU patients with sepsis and trauma-related SIRS.

**METHODS.** This prospective observational study was performed in a sample of critically ill adult (n=257) patients with sepsis or SIRS, compared to healthy controls (n=116). The expression of survivin transcript variants was analyzed by real-time quantitative PCR in peripheral blood leukocytes, whereas measuring the activity of survivin, through ELISA in serum samples, specified anti-apoptotic tendency.

**RESULTS.** Serum levels of the survivin protein as well as WT transcriptional levels for survivin were found significantly elevated in septic patients, compared to SIRS and control individuals (p< 0.05). The main survivin splice variants (ΔEx3, 2B), showed an escalated increase in SIRS and sepsis, except 3B, which appeared to be elevated in the SIRS group, while being significantly depressed in septic patients (p< 0.05). All survivin isoforms showed an upward slope among non-survivors, with survivin protein, -ΔEx3, and -2B differing significantly (p< 0.05). For predicting sepsis, splice variants 2B and ΔEx3 achieved a receiver operating characteristic curve (AUROC) >0.90 (95% CI 0,85-0,98, p< 0.05). In predicting mortality among critically ill patients, isoforms 2B and ΔE3x achieved an AUROC >0.82 (95% CI 0,76-0,98, p< 0.05).

**CONCLUSIONS.** The survivin apoptotic and anti-apoptotic pathways might represent attractive targets for future research in the clarification of sepsis. The mRNA expression of survivin variants and protein activity quantification could predict outcome and provide additional information about clinical management of patients suffering from sepsis.

**GRANT ACKNOWLEDGMENT**

This research has been co-financed by the European Union (European Social Fund- ESF) and Greek national funds through the Operational Program ´´ Education and Lifelong Learning´´ of the National Strategic Reference Framework (NSRF)- Research Funding Program: THALES.

### 0820

#### Defining the role of monocytes in sepsis

J.K. Ward[1], E. Triantafyllou[2], A. Singanayagam[2], W. Khamri[2], K.P. O'Dea[1], M. Takata[1], A.C. Gordon[1], C.G. Antoniades[2], K.C. Tatham[1]

[1]Imperial College London, Academic Anaesthetics, Pain Medicine and Intensive Care, Department of Surgery and Cancer, London, United Kingdom; [2]Imperial College London, Centre for Digestive Diseases, Department of Surgery and Cancer, London, United Kingdom

**Correspondence:** K.C. Tatham

*Intensive Care Medicine Experimental* 2018, **6(Suppl 2):**0820

**INTRODUCTION.** Sepsis remains a leading cause of mortality worldwide.[1] Impaired circulating monocyte function has been linked to the pathogenesis of sepsis, specifically relating to HLA-DR expression[2] and Tyro3-Axl-MerTK family of receptor tyrosine kinases.[3] Further examination of this relationship could therefore guide novel treatment discovery.

**OBJECTIVES.** We sought to investigate monocyte immunotypes in sepsis patients, to elicit functional relevance of any impaired immune response.

**METHODS.** Plasma from septic patients was obtained from the LeoPARDS trial.[4] $CD14^+$ bead-isolated healthy control (HC) monocytes were preconditioned for 24 hrs in the presence of either HC or septic patient plasma. Phenotypic analyses of these monocytes, focused on activation/pro-repair markers, were performed using flow cytometry. Preconditioned monocyte migratory characteristics were assessed utilising an *in vitro* trans-endothelial migration assay, recently described,[5] allowing analysis of migratory characteristics of monocytes into the subendothelial space, and back across the endothelium (reverse migration), thus mimicking their *in vivo* transit patterns, e.g. when returning to the circulation.

**RESULTS.** Compared to HC, baseline MerTK, CCR2 and CCR5 expression levels were significantly increased in CD14+ 'septic' monocytes whereas HLA-DR, Axl and PD-L1 levels were decreased (Table 1). They also exhibited reduced Axl and CD86 expression following migration into the sub-endothelial space. Of note, reverse-migrated 'septic' monocytes were characterised by increased MerTK and lower CD86 and PD-L1 expression, in contrast to HC (Fig 1).

**CONCLUSIONS.** We describe a pro-repair yet immunosuppressive $HLA\text{-}DR^{low}$ $MerTK^{high}$ phenotype in monocytes conditioned with septic patient plasma, in line with previous findings.[2-3] We also report a reduction in Axl, part of the same tyrosine kinase family as MerTK, at baseline and sub-endothelially, which may indicate activation-induced receptor shedding. Furthermore, adaptive co-stimulatory molecules (CD86/PD-L1) were down-regulated in migrating monocyte populations conditioned with septic plasma.

Together, our data indicate that 'pro-repair/immune-inhibitory' monocytes may suppress tissue-specific immune responses, accounting for the dysregulated host immune responses observed in sepsis. Future work will characterise functionally 'septic' monocytes including comparison between survivors/non-survivors, to elucidate the key mechanisms involved in the pathogenesis of sepsis.

**GRANT ACKNOWLEDGMENT**

ET: Rosetrees Trust

AJ: Wellcome Trust

ACG: NIHR, Imperial BRC

CGA: MRC; Rosetrees Trust

KCT: NIHR; Academy of Medical Sciences; EIT

**Table 1 (abstract 0820).** Cell surface expression of monocytes post plasma preconditioning

| | Healthy monocytes | Sepsis monocytes | p value |
|---|---|---|---|
| HLA-DR MFI; mean (SD) | 15208 (3264) | 7103 (1331) | <0.001 |
| MerTK % +ve; median (interquartile range) | 33.6 (32.5-40.7) | 45.2 (34.8-55.0) | <0.05 |
| Axl % +ve; mean (SD) | 52.5 (5.0) | 38.3 (14.7) | <0.05 |
| PD-L1 % +ve; mean (SD) | 79.2 (3.1) | 62.3 (7.0) | <0.001 |
| TIE-2 % +ve; mean (SD) | 42.0 (4.8) | 31.9 (8.9) | <0.05 |
| CCR2 % +ve; mean (SD) | 85.0 (2.1) | 90.1 (1.6) | <0.001 |
| CCR5 % +ve; mean (SD) | 13.8 (4.4) | 29.6 (8.0) | <0.01 |

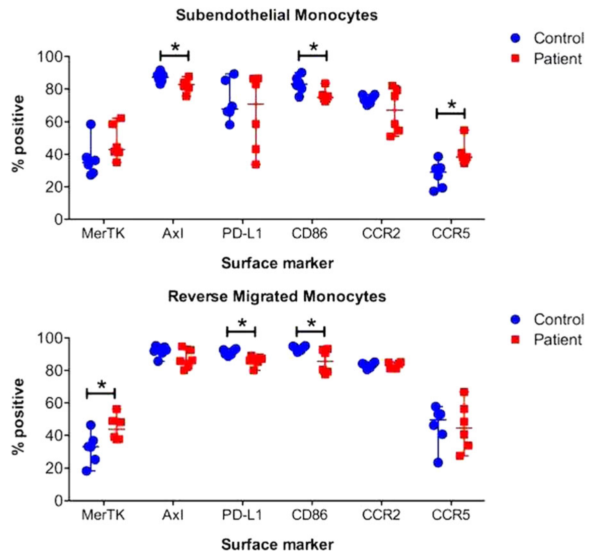

**Fig. 1 (abstract 0820).** Monocyte phenotypes following migration to the subendothelial space and subsequent reverse migration (* p<0.05)

### 0821

#### Resistin exerts differential immunomodulatory effects on neutrophils and macrophages in sepsis

A. Bonavia[1], L. Miller[1], K. Singbartl[2]
[1]Penn State Milton S. Hershey Medical Center, Anesthesiology and Perioperative Medicine, Hershey, United States; [2]Mayo Clinic, Critical Care Medicine, Phoenix, United States
**Correspondence:** A. Bonavia
*Intensive Care Medicine Experimental* 2018, **6(Suppl 2):**0821

**INTRODUCTION.** Mounting evidence suggests that sepsis-induced morbidity and mortality are due to both immune activation and immunosuppression. Resistin is a 12.5kDa uremic cytokine, and septic hyperresistinemia(HR) has been associated with greater disease severity and worse outcome in critically-ill patients. Normal serum resistin levels are < 20 ng/ml, and levels in septic shock with acute kidney injury may be as high as 90-120 ng/mL. Resistin has been reported to block the LPS-Toll-like receptor (TLR)-4 interaction in monocytes/macrophages, thus exerting a protective, anti-inflammatory effect in endotoxic shock. However, we have reported that at concentrations above 20 ng/ml, resistin also impairs polymorphonuclear neutrophil (PMN) migration, intracellular bacterial killing and generation of reactive oxygen species (ROS) in a dose-dependent manner when PMN are challenged with *P. aeruginosa*, a nosocomial Gram-negative organism.

**OBJECTIVES.** Our central hypothesis is that the intracellular signaling mechanism following resistin exposure differs between PMN and macrophages. We hypothesize that (1) the effects of resistin on PMN are not TLR4-mediated as evidenced by persistent impaired killing of Gram positive organisms, which do not release endotoxin/LPS, and (2) the intracellular effects of resistin on PMN are mediated by G proteins.

**METHODS.** Intracellular bacterial killing of *S. aureus* (Gram positive) and *P. aeruginosa* (Gram negative) were assessed separately in neutrophil (NB4) and macrophage (THP-1) cell lines exposed to resistin (100 ng/mL). To study whether resistin's effects in PMN were mediated by G proteins, we performed $Ca^{2+}$influx assays using fMLP (a $G_{i/o}$ receptor agonist) as the stimulus in the presence or absence of 100 ng/mL resistin.

**RESULTS.** Resistin significantly impaired intracellular bacterial killing of *P. aeruginosa* in both macrophages and PMN. Resistin did not impair intracellular bacterial killing of *S. aureus*¸ while PMN exposed to resistin demonstrated markedly impaired intracellular bacterial killing of this organism within 30 minutes of exposure to the cytokine. This indicates that resistin signaling in PMN is not TLR4-mediated. Resistin did not alter the fMLP-mediated $Ca^{2+}$ influx in PMN, arguing against a $G_{i/o}$ receptor signaling mechanism for impaired phagocytosis and killing.

**CONCLUSIONS.** While HR may exert a protective, anti-inflammatory effects in monocytes during endotoxic shock via a TLR-mediated mechanism, it likely also causes PMN dysfunction. The resistin signaling mechanism in PMN appears to be neither TLR nor G protein-mediated.

**GRANT ACKNOWLEDGMENT**
Funded by the Department of Anesthesiology and Perioperative Medicine, Penn State Milton S. Hershey Medical Center, PA, USA.

### 0822

#### Inflammation promotes coagulation by activation of the extrinsic pathway through endothelial cells in-vitro

A.K. Brandtner, G.F. Lehner, M. Joannidis
Innsbruck Medical University, Department of Internal Medicine, Division of Intensive Care and Emergency Medicine, Innsbruck, Austria
**Correspondence:** A.K. Brandtner
*Intensive Care Medicine Experimental* 2018, **6(Suppl 2):**0822

**INTRODUCTION.** Septic patients often present with deranged coagulation. Parameters of an activated coagulation, such as D-dimer and thrombin-antithrombin complexes are elevated within hours after onset of sepsis[1]. The role of the endothelium in this reaction and determining the sepsis phenotype is recognized to be major. As tissue factor (TF) and negatively charged phospholipids are expressed by endothelial cells (EC) upon inflammation, both the extrinsic and the intrinsic coagulation cascades may be activated[2]. To support our understanding of the interaction between the endothelium and the coagulation system in sepsis, we established an in-vitro system to test TF-dependent factor X (FX) versus prothrombin (FII) activation by negatively charged phospholipids on endothelial cell surface to evaluate their relative contribution to coagulation activation upon inflammatory stimulation.

**OBJECTIVES.** Our aim is to evaluate the relative contribution of TF and negatively charged phospholipids expressed on the surface of endothelial cells to coagulation activation in inflammation.

**METHODS.** Primary human umbilical vein cells (HUVEC) were incubated with ascending concentrations of tissue necrosis factor alpha (TNFa, 50, 5 and 0.5 ng/mL), lipopolysaccharide (LPS 1000,100 and 10 μg/mL) and interleukin-1 beta (IL-1b 1, 0.5 and 0.1 nmol/L ) for 3 hours in a 96-well plate at standard cell culture conditions. FX and FII activation were measured by specific cleavage of a chromogenic substrate after addition of FX and FVIIa or FII and FVa respectively. Absorbance was measured after acid quenching of the reaction. Results are expressed as fold change to untreated controls and were analyzed by linear reg ression.

**RESULTS.** TNFa and IL-1b induced a significant increase of FXa even between the lowest concentration and untreated control (mean 0.1869 vs. 0.1089, $p = 0.01$; mean 0.3828 vs. 0.129, $p = 0.01$, respectively). A dose-depend increase of FX activation by endothelial cells was observed for each of the three pro-inflammatory agents, with TNFa showing the strongest relation to a dose-dependent linear increase in this bivariate model (mean 4.4-fold increase, SD 1.66; $R^2 = 0.773$, df = 14;). leavage of FII was elevated, but did not exhibit a significant or dose-dependent increase upon pro-inflammatory stimulation.

**CONCLUSIONS.** These experiments propose a predominant role of TF for coagulation activation on the surface of HUVEC upon pro-inflammatory stimulation with TNFa.

**REFERENCE(S)**

1. Kinasewitz, G. T. *et al.* Universal changes in biomarkers of coagulation and inflammation occur in patients with severe sepsis, regardless of causative micro-organism [ISRCTN74215569]. *Crit. Care* **8**, R82-90 (2004).
2. Zhang, Y. *et al.* Circulating Microparticles, Blood Cells, and Endothelium Induce Procoagulant Activity in Sepsis Through Phosphatidylserine Exposure. *Shock* **45**, 299-307 (2016).

### 0823

#### Molecular expression in ventilator-associated pneumonia (VAP)

L. Nogales Martin[1], R. Almansa Mora[2], L. Rico López[2], D. Andaluz Ojeda[1], L. Tamayo Lomas[3], P. Ramírez[4], L. Socias[5], M. Martín Fernandez[2], R. Cicuendez Avila[1], I. Martin Loeches[6], J.F. Bermejo Martín[2]
[1]Hospital Clinico Universitario de Valladolid, Intensive Care Unit, Valladolid, Spain; [2]Hospital Clinico Universitario de Valladolid, Laboratory of Biomedical Reseach in Sepsis (Bio.Sepsis), Valladolid, Spain; [3]Hospital Rio Hortega, Intensive Care Unit, Valladolid, Spain; [4]Hospital Universitario y Politecnico La Fe, Intensive Care Unit, Valencia, Spain; [5]Hospital Son Llatzer, Palma Mallorca, Spain; [6]St James'sUniversity Hospital - Trinity Centre for Health Sciences, Intensive Care Unit, Dublin, Ireland
**Correspondence:** R. Cicuendez Avila
*Intensive Care Medicine Experimental* 2018, **6(Suppl 2):**0823

**INTRODUCTION.** Ventilator-associated pneumonia (VAP) is a frequent intensive care unit (ICU)-acquired infection with worse consequences. Critically ill patients, especially septic patients, have depressed immune system. Transcriptomic analysis of circulating peripheral blood leucocytes has demonstrated to be a valuable approach to evaluate immunity in different infectious diseases and to identify new molecular markers for diagnosis proposes.

**OBJECTIVES.** The evaluation of the systemic immune dysfunction of VAP patients could be useful to understand the pathological events of this disease and to detect it early. In order to study these immune alterations in VAP we decided to use a molecular method, transcriptome microarrays.

**METHODS.** An observational prospective cohort study enrolling immunecompetent adults with VAP, and no infection patients in seven medical ICU. A blood sample was collected in the first 24 hours following diagnosis of VAP by using PaxGene tubes. In the MV control group, the sample was collected at 6th7th day under MV (median day for diagnosis of infection in the VAP group). Clinical data also was collected; including medical history, physical examination and hematological, biochemical, radiological and microbiological data.

**RESULTS.** 41 mechanical ventilated (MV) patients (24 VAP and 17 control MV). ICU-admission reason, patient baseline characteristics are in Figure 1.

Both comparable in age, gender and comorbidities. The main reason for orotracheal intubation was low-consciousness level (72,5%). VAP group showed a lower PaO2/FiO2 ratio and higher CPIS score compared with non-infected patients. VAP patients stayed longer under mechanical ventilation (20 vs 12 days) and showed a longer ICU and hospital stay. Finally, no differences in terms of mortality were evidenced between both, control and pneumonia group.

1231 genes were identified whose expression levels were significantly different between the two groups. 680 genes were overexpressed in VAP patients and 551 genes showed lower transcript levels in this group. A large proportion of the implicated genes were down-regulated in patients with VAP. The molecules codified by these genes are related to immunological synapse, it`s suggested a depression of the antigen presentation in these patients.

**CONCLUSIONS.** These results support the idea of being able to be used the gene expression as biomarkers in the early diagnosis of ventilator adquired pneumonia.

#### REFERENCE(S)

Bermejo-Martin Jesús F, Andaluz-Ojeda David, Almansa Raquel, Gandía Francisco, Gómez-Herreras Jose Ignacio, Gomez-Sanchez Esther, et al. Defining immunological dysfunction in sepsis: A requisite tool for precision medicine. J Infect 2016;72(5):525-36.

#### GRANT ACKNOWLEDGMENT

It was granted by "Instituto de Salud Carlos III" and "Consejeria de Sanidad de Castilla y Leon" with a financial support, grant numbers [EMER 07/050] and [PI12/01815].

| | MVC (n=17, 42%) | VAP (n=24, 58%) | All patients (n=41) | p value (VAP vs MVC) |
|---|---|---|---|---|
| Age, years [Median ± IQR] | 68±26 | 54±23 | 55±25 | ns |
| Male, sex [n (%)] | 10 (58,8) | 17 (70,8) | 27 (65,9) | ns |
| APACHE II score [Median ± IQR] | 22±11 | 21±23 | 22±12 | ns |
| Comorbidities [n (%)] | | | | |
| Hypertension | 6 (35,3) | 1 (4,2) | 7 (17,1) | 0.01 |
| Diabetes mellitus | 5 (29,4) | 6 (25) | 11 (26,8) | ns |
| Chronic cardiac failure | 2 (11,8) | 4 (16,7) | 6 (14,6) | ns |
| Chronic renal failure | 1 (5,9) | 0 | 1 (2,4) | ns |
| Chronic lung disease/COPD | 2 (11,8) | 9 (37,5) | 11(26,8) | ns |
| Chronic hepatic failure | 3 (17,6) | 1 (4,2) | 4 (9,8) | ns |
| Reason for intubation [n (%)] | | | | |
| Respiratory failure | 0 | 5 (21,7) | 5 (12,5) | 0.04 |
| Cardiovascular failure | 2 (11,8) | 4 (17,4) | 6 (15) | ns |
| Coma (low-consciousness level) | 15 (88,2) | 14 (60,9) | 29 (72,5) | 0.05 |
| MV duration before diagnosis [Median ± IQR] | 0 | 8±6 | 7,5±3,75 | ns |
| At diagnosis | | | | |
| SOFA SCORE [Median ± IQR] | 5±5 | 7±3 | 6±3,5 | ns |
| CPIS score [Median ± IQR] | 3±2 | 7±1 | 6±4 | <0,01 |
| PaO2/FiO2 [Median ± IQR] | 235±62 | 160±90 | 214±102,5 | 0.03 |
| Temperature, °C [Median ± IQR] | 37±1 | 38±0 | 37,6±1 | <0,01 |
| CRP,mg/L [Median ± IQR] | 150±126 | 164±162 | 164±171 | ns |
| PCT, ng/ml [Median ± IQR] | 0,4±1,55 | 0,1±0,85 | 0,19±1,07 | ns |
| Creatinine, mg/dL [Median ± IQR] | 0,71±0,29 | 0,78±0,45 | 0,72±0,38 | ns |
| Leukocytes, cells/mm3 [Median ± IQR] | 10590±2000 | 11630±5875 | 11110±4365 | ns |
| Lymphocytes, cells/mm3 [Median ± IQR] | 1160±683 | 1208±623 | 1180±721 | ns |
| Neutrophyles, cells/mm3 [Median ± IQR] | 8132±2161 | 9185±5312 | 8670±4434 | ns |
| Positive culture [n (%)] | 0 | 15 (62,5) | 15 (36,6) | <0,01 |
| ICU stay, days [Median ± IQR] | 17±13 | 25±30 | 20±16 | 0.01 |
| Hospital saty, days [Median ± IQR] | 28±49 | 32 ±32 | 31,5±40,25 | ns |
| Days of MV [Median ± IQR] | 12±4 | 20 ±21,5 | 15±14,25 | <0,01 |
| 28-day mortality | 3 (17,6) | 4 (16,7) | 7 (17,1) | ns |
| Hospital mortality | 4 (23,5) | 7 (29,2) | 11 (26,8) | ns |
| ICU mortality | 3 (17,6) | 7 (29,2) | 10 (24,4) | ns |

Results are expressed as median [interquartile range] for continuous variables and number (%) for categorical variables. MVC: Mechanical Ventilation Control, VAP: Ventilator-Associated pneumonia, APACHE II: Acute Physiology and Chronic Health Evaluation II, COPD: chronic obstructive pulmonary disease, SOFA: sepsis organ failure score, CPIS: Clinical Pulmonary Infection Score, CRP: C-reactive protein, PCT: procalcitonin, PaO2/FiO2: ratio of partial pressure of arterial oxygen to fraction of inspired oxygen, MV: mechanical ventilation, ICU: Intensive Care Unit.

**Fig. 1 (abstract 0823).** Clinical characteristics

| Gene (ng/µl) | MVC | VAP | P-value |
|---|---|---|---|
| CD40LG | 76.40 [64] | 37.60 [38.50] | 0.001 |
| CD28 | 118.40 [155.80] | 62.20 [76.50] | 0.003 |
| ICOS | 46 [45.40] | 24.80 [21.30] | 0.002 |
| IL2RA | 27.20 [27.20] | 19.40 [11.80] | 0.012 |
| CCR7 | 259.20 [327.80] | 146 [264.40] | 0.025 |
| CD1C | 41.20 [34.40] | 20.20 [17.10] | < 0.001 |
| CD3E | 274.80 [308.20] | 196.20 [233.70] | 0.009 |

**Fig. 2 (abstract 0823).** Expressed gene

### 0824

### Immature granulocytes as a sepsis predictor in patients undergoing cardiac surgery

M. Porizka[1], M. Balik[1], P. Walfauf[2]

[1]First Faculty of Medicine, Charles University and General University Hospital in Prague, Department of Anesthesia and Intensive Care, Prague, Czech Republic; [2]University Hospital Kralovske Vinohrady, Third Faculty of Medicine, Charles University in Prague, Prague, Czech Republic

**Correspondence:** M. Porizka

*Intensive Care Medicine Experimental* 2018, **6(Suppl 2):**0824

**INTRODUCTION.** Cardiac surgery with the use of cardio-pulmonary bypass is commonly associated with the development of systemic inflammatory response syndrome (SIRS) due to exposure of blood to artificial surfaces, myocardial and pulmonary ischemia/reperfusion and surgery itself. Currently used biomarkers including white blood cell count (WBC), C-reactive protein and procalcitonin (PCT) have limitations in the differential diagnosis of sepsis versus SIRS given by their low specificity in the surgical patients. There has been recently a renewed interest in the immature granulocyte (IG) count as an infection predictor. The occurrence of IG in the blood indicates increased activation of bone marrow in sepsis. Several recent studies including general surgical patients presented usefulness of this parameter in predicting sepsis however, data from a population of cardiac surgical patients are lacking.

**OBJECTIVES.** The usefulness of immature granulocyte percentage (IG%) to discriminate between postoperative non-infective SIRS and sepsis was tested in cardiac surgical patients.

**METHODS.** A retrospective analysis of 124 patients who developed non-infective SIRS and sepsis after elective cardiac surgery with the use of cardiopulmonary bypass from January 2015 through December 2015 was performed. The diagnosis was made by a clinician in charge based on Sepsis-2 criteria and available clinical, radiographic and microbiological parameters. Predictive ability of IG% to predict sepsis was compared to PCT, WBC and temperature using receiver operator characteristic (ROC). The Liu method was used for calculation of optimal cut-off points. The diagnosis sensitivity, specificity, and positive (PPV) and negative predictive values (NPV) were calculated for each parameter. Using logistic regression analysis the composites scores were created combining the individual biomarkers. Their discriminative power was subsequently evaluated and compared by linear discriminative analysis (LDA).

**RESULTS.** There were 44 patients diagnosed with sepsis and 80 with non-infective SIRS. In ROC analysis, area under curve (AUC) was higher for IG% and PCT compared to WBC and temperature (0.71, 0.72, 0.62 and 0.58 respectively). The best cut-off value for IG% was 1.45% (sensitivity, specificity, PPV and NPV of 70.5%, 60%, 49.2% and 78.7% respectively) and 1.43 µg/l for PCT (sensitivity, specificity, PPV and NPV of 65.9%, 75%, 59.2% and 80 respectively). The predictive ability was significantly improved by constructing a composite score combining IG% and PCT (AUC of 0.8 in ROC analysis, sensitivity, specificity, PPV and NPV of 63.6%, 88.8%, 75.7% and 81.6% respectively).

**CONCLUSIONS.** In cardiac surgical patients IG% is a helpful marker with moderate ability to discriminate between sepsis and non-infective SIRS, comparable to serum PCT. The combination of these two biomarkers in a composite score increased test´s overall predictive ability mainly by improving its specificity.

## Patients' characteristics influence ICU results, or do they?

### 0825

#### Quality of life after ICU discharge: a prospective observational study from a tertiary care centre in North India

A. Azim, R. Rai, A. Aggarwal
Sanjay Gandhi Post Graduate Institute of Medical Sciences, Lucknow, India
**Correspondence:** A. Azim
*Intensive Care Medicine Experimental* 2018, **6(Suppl 2):**0825

**INTRODUCTION.** Advances in medical and interventional management has increased survival of critically ill patients. This has led to the need for outcome measures other than survival.

**OBJECTIVES.** The aim of this study was to assess quality of life after ICU discharge using 6 months prospective follow up and to analyze risk factors affecting health quality parameters.

**METHODS.** After obtaining institutional ethical committee approval and written informed consent from patient or next of kin, we conducted a prospective observational cohort study on patients who got discharged from Intensive Care Unit (ICU) after more than 7 days stay (January 2017-October 2017) and survived for 6 months Post-ICU discharge. Patients less than 18 yrs age, those getting discharged for end of life care and patients with pre-existing neurological illness, patients lost to follow up were excluded from the study. Follow up was done at 1 month and 6 month using SF-36 questionnaire. For comparison purpose, SF-36 was also filled for each patient to know pre ICU status. Data collection included patient demographics, SOFA Score, APACHE II Score, NYHA classification, and ICU stay details. Telephonic interview was conducted for patients who could not report for follow up. Appropriate statistical tests were used for quantitative and qualitative data. Correlation between various parameters was done using Pearson's correlation. P value of < 0.05 was taken as significant.

**RESULTS.** 100 patients met inclusion criteria (M=60, F=40). Respiratory infection was the most common cause for ICU admission. Diabetes and hypertension were the most common co-morbidities. Mean ICU stay was 13.6 days, mean days of mechanical ventilation were 7.93 days, mean admission APACHE II was 18.88 and mean SOFA was 7.73. Comparison of SF-36 parameters showed Physical Component Summary Score (PCS) and Mental Component Summary Score (MCS) at pre-ICU as 55.12 and 55.09 which deteriorated to 39.59 and 35.49 (p< 0.05) at 1 month post discharge and 47.93 and 37.46 at 6 months. Multivariate analysis showed Age, Admission APACHE II, and SOFA score as significant factors affecting PCS and MCS. Length of ICU stay and duration of mechanical ventilation significantly affected Physical Functioning (PF) at 6 months and Bodily Pain (BP) at 1 month. When compared with general population norm both PCS and MCS showed significant deterioration at 6 months.

**CONCLUSIONS.** Post ICU discharge patients have significant functional impairment and compromised health related quality of life. Age, severity of illness, length of ICU stay and duration of mechanical ventilation significantly affect health quality parameters.

### 0826

#### Epidemiology and early prediction of long stays in ICU

B. Hermann[1], C. Hauw-Berlemont[1,2], A. Monnier[1,2], F. Boissier[1,2], N. Aissaoui[1,2], J.-Y. Fagon[1,2], J.-L. Diehl[1,2], E. Guérot[1]
[1]Hôpital Européen Georges Pompidou, Intensive Care Unit, Paris, France; [2]Sorbonne Université, Paris, France
**Correspondence:** B. Hermann
*Intensive Care Medicine Experimental* 2018, **6(Suppl 2):**0826

**INTRODUCTION.** Prolonged stays in ICU have been repeatedly associated with high rate of resource consumption, but little is known about their epidemiology and prognosis.

**OBJECTIVES.** To describe the epidemiology and events during the stay of patients hospitalized 30 days or more (long stay patients - LSP) in an medical ICU and to identify early predictors of prolonged hospitalization.

**METHODS.** We conducted a monocentric retrospective observational study, in a medical ICU of a teaching hospital. Data regarding preadmission and admission characteristics as well as evolution during the ICU stay were collected. Univariate and multivariate analyses using logistic regression were conducted to find predictors of mortality among LSP and to compare LSP with patients hospitalized less than 30 days (short stay patients - SSP).

**RESULTS.** Among 8906 patients hospitalized in the ICU between july 2000 and december 2013, 417 were hospitalized 30 days or more. Median length of stay (LOS) was 39.0 [34.0 ; 52.0] days. LSP were mostly men (sex ratio 2.0) and inpatients (61.1%) admitted for respiratory (55.6%) or infectious (25.2%) disease with a median SAPSII of 50.0 [38.0; 64.0]. Organ support was frequent: mechanical ventilation in 99.7%, vasoactive drugs (VD) in 75.8%, renal replacement therapy (RRT) in 33.1%. 382 (91.6%) patients presented at least one complication during their ICU stay: admission-disease related in 271 (65.0%), intercurrent infectious in 319 (76.5%) and intercurrent non-infectious in 251 (60.2%). In-ICU and in-hospital mortality were respectively 39.1% and 47.7% with no difference of LOS between the deceased and the survivors. An older age (OR 1.02 [1.00; 1.04], p=0.044), the requirement of VD at any time during the stay (OR 3.3 [1.6; 6.9], p=0.001) and the occurrence of admission-disease related or intercurrent infectious complication (OR 2.06 [1.2; 3.6], p=0.009 and OR 4.3 [2.0; 10.0], p< 0.001 respectively) were independently associated with mortality whereas RRT was the only protective factor (OR 0.5 [0.3; 0.9], p=0.020). Multivariate comparison to SSP yielded male sex (OR 1.4 [1.1; 1.7], p< 0.001), transfer from another ICU (OR 2.7 [1.7; 4.2], p< 0.001) and hospitalization for a respiratory (OR 2.9 [1.8; 4.7], p< 0.001) or an infectious disease (OR 2.0 [1.2; 3.5], p 0.01) as independent factors associated with long stays while being an outpatient (OR 0.5 [0.4; 0.6], p< 0.001) and being hospitalized for a cardiovascular (OR 0.4 [0.2; 0.7], p< 0.001) or endocrine/metabolic disease (OR 0.2 [0.1; 0.6], p =0.01) were associated with short stays.

**CONCLUSIONS.** Prolonged ICU stays were associated with a high rate of complications but the majority of patients were discharged alive from the hospital. Early identification and prediction of mortality of long stay patients seems difficult as most predictors are related to events occurring during the stay and not to preadmission and admission characteristics.

### 0827

#### What is associated with quality of life after the ICU - a prospective cohort study

A.R. Costa, I. Furtado, W. Mphandi, M. Capuepue, A.F. Cardoso, M.J. Malheiro, S. Pinto, J. Ribeiro, F. Seabra-Pereira, T. Cardoso
Centro Hospitalar do Porto, Unidade de Cuidados Intensivos Polivalente, Porto, Portugal
**Correspondence:** A.R. Costa
*Intensive Care Medicine Experimental* 2018, **6(Suppl 2):**0827

**INTRODUCTION.** Quality of life (QoL) after an Intensive Care Unit (ICU) stay can be compromised. The factors, which influence quality of life are numerous and still poorly understood. Determining major contributors may help prevent deterioration of QoL after ICU stay.

**OBJECTIVES.** To evaluate QoL after ICU discharge and to identify major associations with clinical factors.

**METHODS.** Prospective cohort study of all patients discharged from a mixed ICU between 1st January of 2015 and 31stDecember 2016, observed at the follow-up clinic, and evaluated in terms of QoL and functional status (respectively by the EQ-5D and Karnofsky performance status scale).

**RESULTS.** A total of 222 patients were observed at the follow-up clinic and were evaluated in terms of QoL and functional status. The EQ-5D is a descriptive system used as a measure of health-related QoL, which records the patient's self-rated health. The Karnofsky performance scale is allows for the appraisal of functional impairment. The relationship between quality of life and functional status wasn't statistically significate in terms of sedation, mechanical ventilation, neuromuscular blockage, delirium, agitation, length of stay, time until the appointment, acute neurologic non-traumatic condition, psychiatric pathology and post-cardiac arrest. However, Cranioencephalic trauma (CET) had statistically significate impact in functional status (p=0.007), as well as, return to usual activity (p=0.001) and decrease in functional ability (p=0.043) - see figure 1. The most affected dimension of the EQ-5D in patients with CET and in patients with a decrease in functional ability was difficulty in usual activities (mild to severe in 54% and 67%, respectively) and in patients having returned to usual activity the most altered dimension was walking difficulties (mild to severe in 43%; see Figure 2).

**CONCLUSION.** QoL is a multidimensional concept, which is crucial in evaluating long-term outcomes in ICU and is compromised after discharge. The influencing factors of QoL post-ICU are still poorly understood, but a statistically significate association was found with CET, return to usual activity and decrease in functional ability.

| | | Total, n (%) | EQ5D – VAS Median (IQR) | p-value |
|---|---|---|---|---|
| Age | ≤ 65 yo | 141 (64) | 70 (50-80) | 0.203 |
| | >65 yo | 81 (36) | 70 (50-80) | |
| Gender | Male | 137 (62) | 70 (60-80) | 0.235 |
| | Female | 85 (38) | 70 (50-80) | |
| Education | < 4 years | 21 (9) | 80 (50-100) | 0.521 |
| | 4-11 years | 130 (59) | 70 (50-80) | |
| | ≥ 12 years | 46 (21) | 80 (60-90) | |
| Decrease in functional ability | No | 80 (36) | 70 (50-80) | 0.043 |
| | Yes | 142 (64) | 70 (50-80) | |
| Charlson comorbidity index | 0-1 points | 52 (23) | 80 (60-90) | 0.181 |
| | 2-4 points | 93 (42) | 70 (50-80) | |
| | > 4 points | 77 (35) | 70 (50-80) | |
| Sedation | No | 65 (29) | 70 (50-80) | 0.437 |
| | Yes | 157 (71) | 70 (50-80) | |
| Mechanical ventilation | No | 58 (26) | 70 (50-80) | 0.627 |
| | Yes | 164 (74) | 70 (50-80) | |
| Neuromuscular blockade | Yes | 9 (4) | 80 (50-80) | 0.676 |
| | No | 213 (96) | 70 (50-80) | |
| Delirium | No | 199 (90) | 70 (50-80) | 0.843 |
| | Yes | 23 (10) | 50 (70-80) | |
| Agitation | No | 208 (94) | 70 (50-80) | 0.804 |
| | Yes | 14 (6) | 70 (50-80) | |
| ICU lenght of stay | < 2 days | 68 (31) | 70 (50-80) | 0.884 |
| | 3 -7 days | 78 (35) | 70 (50-80) | |
| | > 7 days | 76 (34) | 70 (50-80) | |
| Time to appointment | ≤ 2 months | 103 (46) | 70 (50-80) | 0.721 |
| | 3 – 4 months | 62 (28) | 70 (50-90) | |
| | > 4 months | 57 (26) | 70 (50-90) | |
| Cranioencephalic trauma | No | 200 (90) | 70 (50-80) | 0.007 |
| | Yes | 22 (10) | 80 (60-90) | |
| Neurologic history | No | 188 (85) | 70 (50-80) | 0.228 |
| | Yes | 34 (15) | 70 (50-80) | |
| Psychiatric history | No | 216 (97) | 70 (50-80) | 0.360 |
| | Yes | 6 (3) | 50 (30-70) | |
| Post cardiac arrest | No | 207 (93) | 70 (50-80) | 0.360 |
| | Yes | 15 (7) | 80 (50-90) | |
| Returned to usual activity | No | 115 (52) | 60 (50-80) | 0.001 |
| | Yes | 98 (44) | 80 (70-90) | |

ICU= Intensive care unit; VAS= visual analogue scale; IQR=interquartile range.

**Fig. 1 (abstract 0827).** Patient variables associated with quality of life

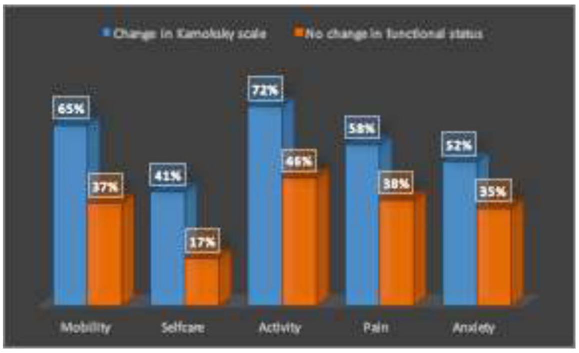

**Fig. 2 (abstract 0827).** Dimensions of the EQ-5D according to Karnofsky performance status scale pre-and post-ICU

### 0828

#### Elderly donor and gender impact on cumulative survival rates of liver graft recipient in 20 years of experience in Santiago de Compostela, Spain

J.R. Fernández Villanueva[1], R. Fernández Garda[1], R. Hernández Vaquero[1], I. Granja Gómez[1], E. R. Ruiz[1], C. Galbán Rodríguez[1], E. Varo Pérez[2]
[1]Complexo Hospitalario Universitario de Santiago de Compostela, Intensive Care Unit, Santiago de Compostela, Spain; [2]Complexo Hospitalario Universitario de Santiago de Compostela, Abdominal Transplant Unit, Santiago de Compostela, Spain
**Correspondence:** R. Fernández Garda
*Intensive Care Medicine Experimental* 2018, **6(Suppl 2):**0828

809 Liver Transplants (LT) were made at Hospital Clínico Universitario of Santiago de Compostela (Spain) in the period 1994-2014, accounting 4.25% of all LT performed in Spain. Review of the impact of the Donor´s Age on the Survival of Graft and Recipient in 20 years of experience in our center.

**MATERIALS AND METHODS.** Retrospective and descriptive review of 809 cases of liver transplantation performed in the 1994-2014 period at the Hospital Clínico Universitario of Santiago de Compostela (Spain) acording to the Local Registry and the Spanish Registry of Liver Transplantation (RETH).

**RESULTS.** 809 cases, 12 cases were Hepato-renal transplantation, 3 requiered Re- transplant. Media of Donors: 33 cases/year. 79.35% Men and 20.64% Women with Recipeint Mean Age: 51 years old. Predomintaly Blood group A (49%). Most frequent LT indication: Alcoholic Cirrhosis (43,%), Idiopathic (43.01%) and Fulminant Liver Failure (6.18%). Cumulative Survival at 20 years in our serie: 47% higher than the comparison with the global data provided by RETH (37% applying Kaplan-Meier curve p < 0.01) and in the last 5 years (2008- 2013): 77% compared to RETH data for the same period (70% with p < 0.05). The Mean Age of the Donor in Brain Death: 53.5 years, increasing exponentially from the beginning of the program with a Median of 35 years (1994) to 68 years (2014). Impact of Survival Rate of the Graft based in Elder Age and Gender of the Donor (>60 years old): 55% to Elder Male Donors and 38% to Elder Women Donors (*p< 0,07*) at 20 years. And the group LT indication for Hepatitis C Virus Cirrhosis (HCV), the Impact of Survival Rate of the Graft in Elder Donors form 60-69 years: 32% and >70 years: 30% (*p< 0,05*) at 20 years of study.

**CONCLUSIONS.** Elderly Age Donors, as a surrogate marker, affects the Survival Rates of the Recipient, but it is necessary to adjust other co-variables of the Donor to correctly assess this finding. Donor's Elder Age affects negatively Survival Rates of the HVC Receptors, something that will undoubtedly change in future studies regards introduction of Antiretroviral Therapy to treat it.

### 0829

#### Changing practices - impact in outcome concerning intra-abdominal infections after ICU reorganization

E. Campôa[1], B. Sarmento Banheiro[2], I. Jesus Pereira[3], G. Campello[2]
[1]Centro Hospitalar Universitário do Algarve, Serviço de Oncologia Médica, Faro, Portugal; [2]Centro Hospitalar Universitário do Algarve, Departamento de Emergência, Urgência e Cuidados Intensivos, Portimão, Portugal; [3]Centro Hospitalar Universitário do Algarve, CriticalMed Investigator, CINTESIS - Center for Health Technology and Services Research, Faculty of Medicine, University of Porto, Faro, Portugal
**Correspondence:** G. Campello
*Intensive Care Medicine Experimental* 2018, **6(Suppl 2):**0829

**INTRODUCTION.** Intra-abdominal infections (IAI) often lead to Intensive Care Units (ICU) admission and are associated with high mortality rates.
**OBJECTIVES.** To understand the impact in outcomes of IAI comparing two distinct moments of clinical practices under different leadership and expertise.
**METHODS.** Retrospective analysis of patients admitted in an ICU from January to September 2016 compared to the same period in 2015. Clinical and therapeutic variables were analyzed using the SPSS statistical program v. 25.0. The significance level used was 0,05. T test for independent sample, Mann Whitney test, chi-squared test and Fisher's exact test were used.
Logistic regression modeling was used to understand factors associated with mortality.
**RESULTS.** We reviewed 111 patients, 61.3% were males and the global mean age was 67.1 years. In the total group of patients, the APACHE II was 18.5 (SD ± 9.2) and the SAPS II was 45.5 (SD ± 20.4), there were no differences between both subgroups. ICU mortality was 27.0%, with no differences between the two periods. Acute abdominal surgery was performed in 61.3% of the cases, and in 34.3% of the cases a second surgical intervention was required with no differences between groups when reviewing the causes for reintervention. The main etiologies for IAI were intestinal occlusion/ischemia (47.5%) and intestinal perforation (33.3%). The total hospital LOS was similar between subgroups but when analyzing the ICU LOS between the two subgroups, it was significantly longer in 2015 with a median of 6 days (IQR 16) and 3 days (IQR 6) in 2016. The need for ICU readmission was similar between subgroups (one in 2015 and 2 in 2016). The number of IMV days was also significantly higher in 2015 with 3 days (Med; IQR 13) and 1 day (Med; IQR 4) in 2016. There was no difference concerning the duration or tapering off of antibiotic therapy even though the number of blood cultures collected was significantly higher in 2016 (24 in 2015 and 41 in 2016, p=0,013). Among the schemes used we found significant differences between the two periods, a predominance of schemes containing 3rd generation cephalosporins with metronidazole in 2016 and carbapenems in 2015, these results were not associated with differences in antibiotic resistance between groups. We found a statistically significant influence of age, severity scores and number of IVM in mortality.
**CONCLUSIONS.** The consequences of changing organizational procedures are obvious when analyzing the two-time frames, even though we could not find differences in mortality, we found significant differences in other outcome variables: ICU length of stay, days of IMV and antimicrobial strategy. Even thought we did not analyze costs, we consider that health care cost reduction is probably another positive outcome of the ICU reorganization. This shows us that resource reorganization may have major healthcare system implications.

### 0830

#### Mortality of patients discharged in-hours and out-of-hours from a British ICU: are functional ICU practices to blame?

J. Cumberworth, I. Francis, M. Chequers, R. Gray, O. Boyd
Royal Sussex County Hospital, Department of Intensive Care Medicine, Brighton, United Kingdom
**Correspondence:** J. Cumberworth
*Intensive Care Medicine Experimental* 2018, **6(Suppl 2):**0830

**INTRODUCTION.** ICU patients discharged out-of-hours have been demonstrated to have a higher in-hospital mortality rate. A recent systematic meta-analysis has reported a pooled OR of 1.31 (95% CI 1.25-1.38) for hospital mortality following ICU discharge out-of-hours compared to in-hours.[1]
It is frequently assumed that the increased mortality is a reflection of ward practices such as reduced staffing; we explore the possibility that factors within the ICU or ICU discharge influence results. Here we present preliminary data on a retrospective cohort study of post-ICU mortality in our unit for those discharged in-hours and out-of-hours.
**OBJECTIVES.**
1. Is the mortality rate of patients discharged out-of-hours higher than mortality rate of patients discharged in-hours from our unit?
2. Are there other factors influencing differences in reported mortality rates that may not have been previously considered?

**METHODS.** Data were collected retrospectively on all patients admitted to, and discharged from, our unit between 1st January 2017 and 30th June 2017. Readmissions, elective surgical patients and those with ICU stay < 8 hours were excluded. Data were collected on age, sex, length of stay, APACHE II score, nature of admission (medical or surgical), time from unit to hospital discharge or death, and final outcome. In-hours was defined as 07:01 to 21:59 and out-of-hours was defined as 22:00 to 07:00. Data were compared by Chi-squared test or Mann-Whitney U test as appropriate.
ICU admission and discharge summaries were reviewed for patients discharged from the ICU who did not survive to hospital discharge, as were medical certificates of cause of death. Patients discharged from the ICU for organ donation or end-of-life-care were identified and excluded. Comparisons of those discharged in-hours and out-of-hours were then repeated.
**RESULTS.** The study population initially contained 613 subjects, the majority discharged in-hours. The mortality rate after ICU discharge was 5.37% for those discharged in-hours and 13.64% for those discharged out-of-hours. After removal of those discharged for end-of-life care or organ donation (n=19), however, corresponding mortality rates were 3.25% and 6.86% respectively ($p$=0.0854) as shown in Table 1.
**CONCLUSIONS.** The prognoses of patients discharged from the ICU following clinical improvement will contrast with those discharged for organ donation or to a ward-based environment for end-of-life care, as mortality in these groups will be 100%. Small numbers of patients in this group being discharged at differing times may, therefore, lead to striking differences in reported mortality rates. If these discharges frequently occur out-of-hours the effect could be even greater and skew out-of-hours discharge mortality rates.

##### REFERENCE(S)

Yang S, Wang Z, Liu Z, Wang J, Ma L. Association between time of discharge from ICU and hospital mortality: a systematic review and meta-analysis. Crit Care. 2016 Dec 1;20(1):390

##### GRANT ACKNOWLEDGMENT

N/A

**Table 1 (abstract 0830).** Characteristics of patients discharged in-hours and out-of-hours from a general adult critical care unit in the UK between 01/01/17 and 30/06/17

| Characteristic | In-hours discharge | Out-of-hours discharge | Total | P value |
|---|---|---|---|---|
| n | 492 | 102 | 594 | n/a |
| Overall % of sample | 82.8 | 17.2 | 100 | n/a |
| Mean age in years (SD) | 59.09 (17.45) | 63.97 (16.12) | 59.93 (17.31) | 0.0127 |
| Mean APACHE II score (SD) | 15.41 (6.44) | 16.69 (5.80) | 15.63 (6.35) | 0.0184 |
| Post-ICU mortality rate (%) | 3.25 | 6.86 | 3.87 | 0.0854 |
| Median ICU length of stay (days) | 3.60 | 3.06 | 3.34 | 0.1324 |
| Median post-ICU length of stay (days) | 6.00 | 9.00 | 6.50 | 0.0377 |
| Medical admission (%) | 62.0 | 57.8 | 61.3 | 0.4337 |

### 0831

#### Risk factors for one-year mortality at ICU discharge in patients with free flap reconstruction due to cancer of the head and neck

S. Lahtinen[1], T. Ala-Kokko[2], P. Koivunen[3], J.H. Liisanantti[2]
[1]Oulu University Hospital, Anesthesia and Intensive Care, Oulu, Finland; [2]Oulu University Hospital, Anesthesia and Intensive Care, Division of Intensive Care Medicine, Oulu, Finland; [3]Oulu University Hospital, Otorhinolaryngology and Head and Neck Surgery, Oulu, Finland
**Correspondence:** S. Lahtinen
*Intensive Care Medicine Experimental* 2018, **6(Suppl 2):**0831

**INTRODUCTION.** Free flap transfer is a standard method in reconstruction of large defects in head and neck cancer surgery with frequent postoperative complications and high long-term mortality; 5-year survival is approximately 50-60% but a high rate of patients dies within one year from the operation. Risk factors for short-term mortality in immediate postoperative phase and within one year from the operation in this patient group are not well studied.

**OBJECTIVES.** The aim of this study was to evaluate the risk factors for one year mortality in patients operated due to cancer of the head and neck with free flap repair at ICU discharge.

**METHODS.** Retrospective analysis of 146 patients that underwent head and neck cancer surgery with free flap repair in Oulu University Hospital in 2008-2016.

**RESULTS.** Of the 146 patients, there were 27 patients (18.5%) who died within one year from the operation and 10 of them within 180 days. The median ICU stay was 0.9 days [0.8-1.7]. Most of the one-year non-survivors were males (21(77.7%), p=0.008) and more often recorded as ASA 3-4 (22 (81.5%) vs 59 (49.6%), p=0.003) compared to the one-year survivors. There were no differences in the intraoperative course. Among the one-year non-survivors the preoperative albumin level was lower (42 [37-44] vs 43 [41-45], p=0.032) and the SOFA admission score was higher (4 [3-59] vs 3 [2-4], p=0.013) compared to the one-year survivors. The levels of CRP (87 [67-112] vs 61 [51-89], p=0.019) and leukocytes (11.7 [9.1-15.2] vs 8.9 [7.3-12.2], p=0.001) of the one-year non-survivors were higher on the first postoperative day compared to the survivors.

**CONCLUSIONS.** One-year non-survivors had higher ASA classification, and lower preoperative albumin levels. The postoperative inflammatory markers were higher in non-survivors.

##### REFERENCE(S)

1. Lahtinen S et al. Complications and outcome after free flap surgery for cancer of the head and neck. Accepted for publish 11/2017 in Br J Oral Maxillofac Surg
2. Clark JR et al. Predictors of morbidity following free flap reconstruction for cancer of the head and neck. HeadNeck 2007;29:1090-1101
3. Mücke T et al: Immediate microsurgical reconstruction after tumor ablation predicts survival among patients with head and neck carcinoma. Ann Surg Oncol 2010;17:287-95

##### GRANT ACKNOWLEDGMENT

None.

### 0832

#### A single centre retrospective study: can lactate be used to predict increased length of stay in post-operative surgical patients admitted to the intensive care unit (ICU)?

E. Ahmad, A. Myers, T. Samuels
East Surrey Hospital, Intensive Care Unit, Redhill, United Kingdom
**Correspondence:** E. Ahmad
*Intensive Care Medicine Experimental* 2018, **6(Suppl 2):**0832

**INTRODUCTION.** Lactate is considered to be a useful prognostic biomarker in patients with conditions such as sepsis.[1] However, the significance of lactate in surgical patients remains unclear.[2]

**OBJECTIVES.** This study aims to assess whether raised admission lactate in surgical patients is associated with an increased ICU length of stay (LOS).

**METHODS.** Retrospective review was carried out on patients admitted post-operatively to an adult ICU during the period 1$^{st}$ January to 1$^{st}$ October 2017. The highest lactate level measured for each patient within the first 24 hours of admission was recorded. LOS was then analysed according to whether lactate was less than 2.5mmol/l (group A) or greater than 2.5mmol/l (group B). Statistical analysis was performed using the Wilcoxon rank sum test.

**RESULTS.** 265 patients were admitted post-operatively (mean ages: 67.4 and 73.1 years in groups A and B respectively); 34 patients were excluded from analysis since no lactate was recorded within the first 24 hours. Median LOS for groups A and B were 2.5 days [IQR 1.1 - 4.7 days] and 3.5 days [IQR 2 - 5.8 days] respectively (p = 0.0292)..

**CONCLUSIONS.** In our cohort of surgical patients, lactate greater than 2.5mmol/l measured on admission to ICU is associated with an increased length of stay.

##### REFERENCE(S).

[1] Filho R et al. Blood Lactate Levels Cutoff and Mortality Prediction in Sepsis—Time for a Reappraisal? a Retrospective Cohort Study. Shock. 2016 Nov; 46(5): 480-485
[2] Huang MH, Lai CH, Lin PI, Lai WW. Arterial lactate level is associated with mortality rate in unscheduled surgical intensive care admissions. Formos J Surg 2017;50:21-27

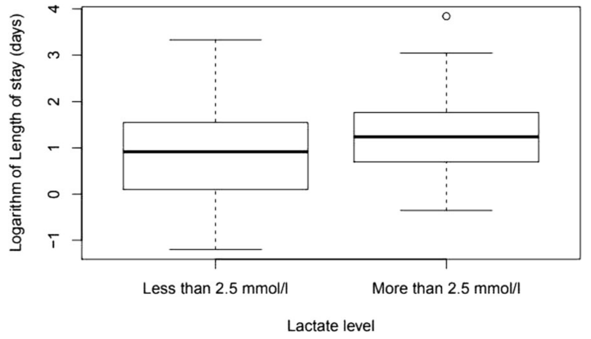

**Fig. 1 (abstract 0832).** LOS vs highest lactate measurement within first 24 hours following admission

### 0833

#### Patients with chronic critical illness admitted to the intensive care unit: influence of age under versus over 60 years

R. Fumis[1], L. Matheus Lins[2], I. de Castro[3], A.B. Ferraz[4], M. Moock[5], J.M. Vieira Junior[6]
[1]Hospital Sírio-Libanês, São Paulo, Brazil; [2]Sírio-Libanês, Teaching and Research Institute, São Paulo, Brazil; [3]University of São Paulo School of Medicine, Division of Nephrology and Molecular Medicine, São Paulo, Brazil; [4]Hospital Geral do Grajaú, Intensive Care Unit, São Paulo, Brazil; [5]Conjunto Hospitalar do Mandaqui, Intensive Care Unit, São Paulo, Brazil; [6]Hospital Sírio-Libanês, Intensive Care Unit, São Paulo, Brazil
**Correspondence:** R. Fumis
*Intensive Care Medicine Experimental* 2018, **6(Suppl 2):**0833

**INTRODUCTION.** Chronic Critical illness (CCI) is a devastating syndrome of prolonged dependence on mechanical ventilation and other intensive care therapies. The influence of age on decision making in Intensive care unit is well established, but its influence on Chronic Critical Illness is yet poor explored. [1,2]

**OBJECTIVES.** The aim of this study was to compare outcomes (mortality, ICU resource use, quality of life) between older (≥ 60 years) and younger (< 60 years) patients with chronic critical illness while in the intensive care unit of a low-resource public hospital and in a high-resource tertiary private hospital in São Paulo, Brazil.

**METHODS.** A prospective study was conducted in two hospitals of different social levels. Patients were defined as CCI after a stay of at least 8 days in the ICU plus one of the six eligible clinical conditions: mechanical ventilation for at least 96 hours; tracheotomy; sepsis; severe wounds; stroke; traumatic brain injury [1]. Patients were classified into groups A and B based on age [(< 60, n = 25) and (≥ 60, n = 75) years old, from high-resource hospital] and C and D [(< 60, n = 45) and (≥ 60, n = 41) years old, from low-resource hospital].

**RESULTS.** Group A had a median age of 39 [30-46] years; group B 75 [68-83] years; group C 47 [35-54] years and group D 67 [64-72] years, p< 0.0001 for all. Although they had different SAPS 3, (p< 0.0001, for all), ICU mortality had no difference between groups (28.8% versus 36.6%, p=0.298, for low-resource hospital) and (8.3% versus 20.0%, p=0.280, for high-resource hospital) and no difference in advanced life support needs (mechanical ventilation, tracheotomy, renal replacement therapy, blood transfusion) were observed. Interestingly, older patients had worst health at baseline according to EuroQol 5D-5L score (< 0.005 for all), but there was no difference at 30- and 90-days after ICU. Older patients presented more delirium (p=0.011) and dementia (0.042) only in high-resource hospital, in which the hospital mortality rate was higher in the elderly groups (42% versus 13%, p=0.023). However it was similar in the low-resource hospital (41.4% versus 34.8%, p=0.456). The age category had no impact on symptoms of anxiety and depression neither on Quality of life of family members (p>0.05 for all). We found that, in the case of younger patients, those family members with higher scores for anxiety and depression, had a lower quality of life, only in high-resource hospital (p< 0.005 and R>0.7).

**CONCLUSIONS.** The great influence of the chronically critically ill patient´s age was on emotional disorders of family members when their loved ones were younger.

### 0834

#### Intensive care follow-up study: cognitive, mood and quality of life outcomes following an acute critical illness

E. Hawken[1,2], J. Muscedere[1,2], J.G. Boyd[1]
[1]Queen's University, Critical Care Medicine, Kingston, Canada; [2]Canadian Frailty Network, Kingston, Canada
**Correspondence:** J.G. Boyd
*Intensive Care Medicine Experimental* 2018, **6(Suppl 2):**0834

**INTRODUCTION.** "Post- intensive care syndrome" (PICS) describes a constellation of complications enduring well past the ICU stay, which include loss of strength, cognitive impairment, and mental illness including depression, posttraumatic stress syndrome and anxiety. PICS likely contributes to adverse effects on the individual's quality of life and long-term survival.

**OBJECTIVES.** In February 2016, we initiated a multidisciplinary follow up clinic in our tertiary academic hospital that has a 33-bed medical/surgical/trauma ICU. The present study describes the cohort of ICU patients seen in the first year. We aimed to characterize the phenotype of ICU survivorship, and to identify ICU experiences that contribute to post-ICU psychological and cognitive dysfunction.

**METHODS.** In this retrospective cohort study, charts were reviewed to collect information on clinical course during ICU admission and ICU follow-up clinic visits. Information recorded included: delirium score, days of mechanical ventilation, and results of ICU follow-up assessments. Patients were considered eligible for follow-up assessments if they met at least 1 of the following characteristics:

1) >4 days of mechanical ventilation,
2) received tracheostomy,
3) were delirious during their ICU stay,
4) had other reason intensivist wished follow up (e.g. no primary care physician).

Follow up occurred at least 3 months after ICU discharge. Patients were screened for: cognitive impairment (Montreal Cognitive Assessment [MoCA]), mood (anxiety and depression: hospital anxiety/depression scale [HADS], Post Traumatic Stress Syndrome [PTSS]-14) and quality of life (SF-36).

**RESULTS.** To-date, 19 patients have been seen through the follow-up clinic. Patients were 58% female (mean age ± SE = 62 ± 4 yrs; male, 61 ± 6 yrs). Approximately 63% (12/19) of patients received an average of 20 ± 7.5 days of mechanical ventilation. ICU-associated delirium was reported in 42% (8/19) of patients. Of the patients that completed the MoCA, 42% (5/12) were deemed cognitively impaired (scoring lower than 22 on the MoCA). HADS screening indicated that an 18% (3/17) prevalence of mild depressive symptoms, 12% (2/17) moderate and 6% (1/17) severe. The PTSS scores indicated that 24% (4/17) patients had symptoms of PTSD. Anxiety and depression subset scores on the HADS as well as PTSS-14 significantly correlated with quality of life (SF-36), with more mood symptoms correlating significantly with lower reported quality of life (HADS-A and SF-36: n=15, r= -.81, p< 0.001; HADS-D and SF-36: n= 15, r=-0.83, p< 0.001; PTSS-14 and SF-36: n=15, r=-0.70, p = 0.004). Neither age, mood (HADS or PTSS-14) or quality of life (SF-36) correlated with cognition (MoCA).

**CONCLUSIONS.** These preliminary findings corroborate prior work indicating impaired cognition and mood that contribute to reduced quality of life in patients following ICU discharge.

### 0835

#### Predictive value of physical functioning preceding ICU admission to 1 year survival

J. Hofhuis[1], A. Abu-Hanna[2], L. van de Braak[1], A. Hovingh[1], P. Spronk[1,3]
[1]Gelre Hospitals Apeldoorn, Intensive Care Department, Apeldoorn, Netherlands; [2]Academic Medical Center Amsterdam, Department of Medical Informatics, Amsterdam, Netherlands; [3]Academic Medical Center Amsterdam, Department of Intensive Care Medicine, Amsterdam, Netherlands
**Correspondence:** J. Hofhuis
*Intensive Care Medicine Experimental* 2018, **6(Suppl 2):**0835

**INTRODUCTION.** A frequent dilemma for intensivists is to decide whether a critically ill patient will benefit from ICU admission. If the patient's pre-morbid general condition was good and the patient suffers from a potentially reversible condition, almost every intensivist will consider ICU admission and inherent intensive treatment modalities as medically appropriate.

**OBJECTIVES.** We hypothesized that pre-morbid physical reserve is a predictor of survival status in critically ill patients.

**METHODS.** We conducted a long-term prospective cohort study in a university-affiliated teaching hospital. Between February 2013 until

February 2014 we included all patients within 72 hours on a first ICU admission. We evaluated item response based assessment of physical functioning tailored to a patient's individual capacities (Academic Medical Center Linear Disability Score; ALDS), and Health-related quality of life (HRQOL) (Short-form-12; SF-12, general health item; SF-1) before admission by patients themselves or, by close proxies within 72 hours after ICU admission.

**RESULTS.** We developed four logistic regression models to predict 1 year mortality using the predictors age, APACHE-II score, ALDS, SF-1, and PCS-12. We validated the models using the bootstrap method on the AUC, Brier Score, and Net reclassification improvement (NRI). We also inspected the calibration graphs of the models. A total of 510 patients were included. Twelve months after ICU discharge,110 patients (22%) had died. The pre-admission ALDS, significantly improved a model with age (p= 0.0012) showing an NRI with 19% improvement in predictions for non-survivors, and 22% for survivors. Adding the ALDS score to a model with the APACHE II score improved the model (p=0.0117) and NRI showed a 21% improvement in predictions for survivors and 19% for non-survivors. Adding the SF-1 to a model with age (p=0.007) or APACHE II score (p=0.024) also improved the models although a bit inferior to ALDS. PCS-12 showed no significant association with mortality. Practical nomograms were generated to estimate the risk of mortality for individual patients.

**CONCLUSIONS.** Both pre-admission physical functioning measured with the item- response based ALDS as well as the SF-1 improve the predictive performance of age and the APACHE II model. Since the ALDS score is more time-consuming to administer, the use of the SF-1 may be preferred.

### 0836

#### The patient experience of recovery from critical illness: a systematic review and meta-synthesis of qualitative studies across the continuum of recovery

B. Connolly[1], J. Mortimore[1], C. Apps[1], C. Crowley[2], E. Corner[3], N. Pattison[4]
[1]Guy's and St Thomas' NHS Foundation Trust, Lane Fox Clinical Respiratory Physiology Research Centre, London, United Kingdom; [2]King's College London, Library Services, London, United Kingdom; [3]Brunel University, Department of Physiotherapy, Middlesex, United Kingdom; [4]University of Hertfordshire, School of Health and Social Work, Hertfordshire, United Kingdom
**Correspondence:** B. Connolly
*Intensive Care Medicine Experimental* 2018, **6(Suppl 2):**0836

**INTRODUCTION.** Survivorship is increasingly mirroring, if not surpassing, survival as a central tenet in managing patients experiencing critical illness. Protracted impairment and symptomology in patients surviving an admission to the intensive care unit (ICU), and their family/caregivers, is progressively more recognised. However the qualitative experience of recovery from critical illness has not been systematically explored.

**OBJECTIVES.** To systematically review and meta-synthesise qualitative literature to determine patient experience measures for critically ill patients throughout the recovery continuum, including in relation to receipt of physical rehabilitation.

**METHODS.** A systematic review and meta-synthesis (PROSPERO, CRD42017078549). Five electronic databases (CINAHL, EMBASE, Medline, CENTRAL, PsychInfo) were searched from inception to 21st Nov 2017. Primary studies with qualitative methodologies, published from 2000 onwards, were included. Systematic reviews, meta-syntheses, editorials, opinion pieces, auto-ethnographies, and non-English-language papers were excluded. Studies focused on adult (aged ≥18years) patients who had experienced critical illness necessitating ICU admission for ≥48hours, or their family/caregivers, and reported experience of recovery including receipt of physical rehabilitation interventions, at any time-point of the recovery continuum i.e. within ICU, following ward transfer, following hospital discharge. Screening for eligibility and data extraction were performed independently and in duplicate. Data were descriptively reported and summarised. Meta-synthesis was performed using thematic analysis, discussion and consensus by two reviewers.

**RESULTS.** Preliminary results are presented. Twenty-three articles were included, of which 3 focused specifically on physical rehabilitation during recovery. Eighteen studies involved patients alone, 4 involved patients and family/caregivers, and 1 involved family/caregivers alone. The majority (n=22) examined post-ICU discharge stages of recovery. In total studies reported 86 major- and 77 sub-themes describing patient experience of recovery. Meta-synthesis reveals potential over-arching themes of adaptation, transitioning between recovery stages, dynamics with healthcare professionals, enabling recovery with healthcare infrastructure, persistent multi-domain impairment, recollecting and interpreting the critical illness experience, shifting interpersonal dynamics, and the role of rehabilitation in directing recovery.

**CONCLUSIONS.** Experience of recovery following critical illness is multi-faceted for patients and family/caregivers. Further analysis of findings will confirm over-arching themes characterising this recovery.

**GRANT ACKNOWLEDGMENT**

BC is funded by a National Institute for Health Research Postdoctoral Fellowship (PDF-2015-08-015). The views expressed are those of the authors and are not necessarily those of the NHS, the NIHR or the Department of Health.

### 0837

#### Detect Discomfort 1: perceived discomfort in patients admitted to intensive care: aprospective observational study

T. Jacques[1,2], A. Ramnani[3], D. Nolan[1], J. Beeson[1], B. Hocking[1], K. Deshpande[1,2]
[1]St George Hospital, Intensive Care, Sydney, Australia; [2]University of New South Wales, Critical Care and Medicine, Sydney, Australia; [3]Canberra Hospital, Intensive Care, Canberra, Australia
**Correspondence:** T. Jacques
*Intensive Care Medicine Experimental* 2018, **6(Suppl 2):**0837

**INTRODUCTION.** Few studies have evaluated the incidence and magnitude of discomfort, a multifactorial experience reflecting physical, psychological symptoms and environmental factors. It is an important indicator of ICU quality of care.

**OBJECTIVE.** To adapt a French multifaceted discomfort questionnaire (IPREA[1]) to our ICU setting and identify and quantify predictors of discomfort perceived by ICU survivors.

**METHODS.** Eligible patients (>18 years, GCS 15, length of stay ³ 48 hours) admitted to ICU from April to September 2017 were surveyed within 48 hours of ICU discharge. Ethics committee approval was obtained. We translated IPREA (a questionnaire validated in French ICU's) and measured patient perceived discomfort.(1) Patients scored their recalled ICU discomfort for each of 16 items on a visual analogue scale of 0 (no discomfort) to 100 (maximum discomfort). Overall score was computed as the mean of the 16 items.

**RESULTS.** 168/172 survivors participated (58% males, mean age 60.1 ± 14.8 years, mean APACHE II 13.8 ± 5.6). The questionnaire had good internal consistency (Cronbach alpha: 0.82), good content and construct validity (average inter item correlation 0.23). Mean overall discomfort score was 18.4±12.5 and did not differ by gender and type of ICU (general ICU, cardiothoracic ICU, high dependency unit). Items associated with maximum discomfort were noise, sleep, pain, lines and tubes. On multivariate analysis, increasing age was an independent predictor of low discomfort score (β = - 0.26, 95% CI: -0.42 to - 0.11, *P*=0.001) after adjusting for sex, marital status, employment status, APACHE II and type of ICU.

**CONCLUSIONS.** The French IPREA discomfort questionnaire performed well in an Australian ICU setting. Overall discomfort was low. There was an inverse relationship between age and discomfort.

**REFERENCE(S)**

1. P Kalfon et al. Development & Validation of a Questionnaire for qualitative assessment of perceived discomforts in critically ill patients. Intensive Care Med (2010) 36:1751-1758

**GRANT ACKNOWLEDGMENT**

Thankyou to Dr Pierre Kalfon, MD PhD MBA, Chef de Service Reanimation, & President de la CME Hopitaux de Chartres for permission to use IPREA , Dr Patricia

Marechai PhD Sorbonne Nouvelle, Miss Bella Marechai-Ross and Mrs Nathalie Azad for translation & idiom advice and Dr Sophie Roome for data collection.
[1] IPREA = Inconforts des Patients de REAnimation

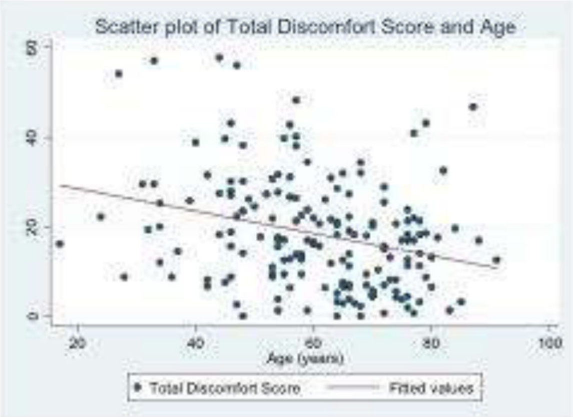

**Fig. 1 (abstract 0837).** Age Vs Discomfort

### 0838

#### Risk factors associated with vasopressor use in patients admitted to the intensive care unit after major orthopedic surgery

B.M. Tomazini, M.R. Bustamante, L.C.P. Azevedo, J.M. Vieira Junior, L.U. Taniguchi
Education and Research Institute, Hospital Sírio-Libanês, Intensive Care Unit, São Paulo, Brazil
**Correspondence:** B.M. Tomazini
*Intensive Care Medicine Experimental* 2018, **6(Suppl 2):**0838

**INTRODUCTION.** Circulatory shock is a common condition in critical care, which can lead to multiple organ dysfunction and potentially death. The most recognizable clinical sign of shock is hypotension. In surgical patients, both the intensity and duration of hypotension increase the risk of organ dysfunctions[1]. Early intensive hemodynamic management strategies, which involve fluid resuscitation and vasopressors, are described as beneficial in this population[2]. However, there is a lack of studies evaluating the risk factors associated with need of vasopressor in orthopedic surgical patients admitted to the intensive care unit.
**OBJECTIVES.** We aimed to evaluate the risk factors associated with vasopressor use in the first 48h of Intensive Care Unit (ICU) stay in patients undergoing major orthopedic surgery.
**METHODS.** A single-center retrospective cohort study of patients admitted to the ICU of a tertiary hospital in São Paulo, Brazil. Inclusion criteria were: adult patients undergoing major orthopedic surgery (total or partial knee/hip arthroplasty, femur fracture repair, and spine arthrodesis) from 2012-2017, who needed continuous infusion of vasopressors (adrenaline, dopamine, noradrenaline, or vasopressin). Exclusion criteria were: vasopressor use before surgery and patients with advanced directives. Univariate and multivariate logistic regressions were used to estimate the association of risk factors and vasopressor use. Variables with p-value< 0.2 in the univariate regression analysis were used to build a model with the backward elimination technique. The model's goodness-of-fit and calibration were accessed with the Hosmer-Lemeshow statistic, and its performance with the area under the Receiver Operating Characteristic curve (AUC).
**RESULTS.** Of the 388 patients included, 96 (24.74%) experienced the primary outcome. Patients' characteristics according to vasopressor use and significant risk factors in the univariate analysis are shown in table 1. Independent risk factors for vasopressor use after surgery were: age ( OR 1.021, 95% CI 0.99-1.04 ,p = 0.078 ), SOFA score ( OR 2.29, 95% CI 1.86-2.83 ,p < 0.001 ), Shock Index [dichotomized at 0.7 ( OR 2.47, 95% CI 1.27-4.79 ,p = 0.007 )], and blood transfusion during surgery ( OR 1.9, 95% CI 0.93-3.85 ,p = 0.075 ), with an AUC = 0.8626.
**DISCUSSION.** Patients who required vasopressors in the first 48h of ICU admission are older, had higher rates of acute kidney injury, longer ICU and Hospital length of stay, and have higher in-hospital mortality. Age, SOFA score, Shock Index, and blood transfusion during surgery were associated with vasopressor use. The adoption of a model to predict vasopressors requirement might be able to identify the patients at risk, increase awareness and might help the ICU team in taking preemptive therapeutic actions, as well as can help planning resource allocation.

**REFERENCE(S).**
1. Walsh M,ET AL. Anesthesiology. 2013; 119(3):507-515.
2. Futier E, et al. JAMA. 2017; 318(14):1346-.

**Table 1 (abstract 0838).** See text for desciption

| | Vasopressor | | Univariate analysis | | |
|---|---|---|---|---|---|
| | Yes | No | Odds ratio | 95% CI | p |
| N, % | 96 (24.74%) | 292 (75.25%) | | | |
| Age, years mean, (SD) | 73.3 (12.96) | 67.4 (15.54) | 1.03 | 1.01-1.04 | 0.001 |
| SOFA median, (IQR) | 4 (3-5) | 1 (1-2) | 2.48 | 2.02-3.04 | <0.001 |
| Shock Index >0.7, % | 38.18 | 12.28 | 2.34 | 1.45-3.77 | <0.001 |
| Transfusion during surgery % | 33.33 | 11.99 | 3.67 | 2.11-6.37 | <0.001 |
| Acute kidney injury % | 23.26 | 16.67 | | | |
| ICU length of stay median, (IQR) | 3 (2-5) | 1 (1-2) | | | |
| Hospital length of stay median, (IQR) | 10 (7-15) | 6 (4.5-10) | | | |

## Delirium in the ICU

### 0839

#### Nighttime slow wave activity in recovery from ICU delirium: a pilot study

J. Kortelainen[1,2], K. Erikson[3,4], J. Laurila[3,4], J. Koskenkari[3,4], T. Ala-Kokko[3,4]
[1]University of Oulu, Physiological Signal Analysis Team, Center for Machine Vision and Signal Analysis, Oulu, Finland; [2]Cerenion Oy, Oulu, Finland; [3]University of Oulu, Research Group of Surgery, Anaesthesiology and Intensive Care, Oulu, Finland; [4]University of Oulu and Oulu University Hospital, Division of Intensive Care Medicine, Oulu, Finland
**Correspondence:** J. Kortelainen
*Intensive Care Medicine Experimental* 2018, **6(Suppl 2):**0839

**INTRODUCTION.** The lack of sleep-related slow-wave activity (SWA) may be associated with ICU delirium. The SWA is considered to be essential for the recovery of the brain and may be disturbed in the intensive care environment. The activity can be monitored quantitatively with electroencephalogram (EEG).
**OBJECTIVES.** In this pilot study, we investigated the association between nighttime SWA and recovery from delirium during standard treatment in the ICU.
**METHODS.** We performed an overnight (from 9 pm to 7 am) recording of EEG for seven patients diagnosed with hyperactive delirium. The delirium was diagnosed with an Intensive Care Delirium Screening Checklist (ICDSC) score higher than three. During the EEG recording, the administration of dexmedetomidine was carried out following the ICUs standard protocol to keep the patients moderately sedated. The amount of nighttime SWA was estimated by calculating the low-frequency (less than 1 Hz) power of EEG during the recording. The level of sedation was assessed using Richmond Agitation-Sedation Scale (RASS). In the following morning, the delirium was evaluated using

ICDSC and the quality of sleep using Richards-Campbell Sleep Questionnaire (RCSQ) filled by the intensive care nurse.

**RESULTS.** For five of the patients, the morning ICDSC score was less than four not fulfilling the criteria for ICU delirium. The cumulative duration of nighttime SWA for these patients was 204 ± 91 min (mean ± std). For two of the patients, the morning ICDSC score exceeded the threshold for delirium and the cumulative duration of SWA for these patients was 31 ± 11 min. The average RASS during the EEG recording was -1.2 ± 2.1 and -1.3 ± 1.2 for the patients with and without delirium in the following morning, respectively. Average RCSQ value for the patients with delirium was 70.3 ± 14.6 and for the patients without delirium 74.8 ± 18.1.

**CONCLUSIONS.** In the presented pilot study, the lack of nighttime SWA was associated with delirium in the following morning. This association, which will be further investigated in a larger patient group, could offer new possibilities for the assessment, prediction and treatment of ICU delirium.

##### REFERENCE(S)

Kortelainen J et al. Anesthesiology 126:94-103, 2017.

##### GRANT ACKNOWLEDGMENT

Orion Pharma is gratefully acknowledged for the unrestricted financial support of the study.

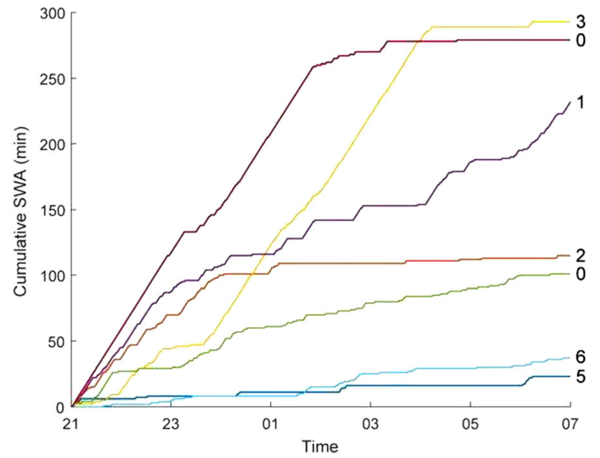

**Fig. 1 (abstract 0839).** Nighttime cumulative slow-wave activity (SWA) and ICDSC score in the morning for the seven patients

### 0840

#### Impact of a multifaceted nursing engagement intervention on pain, agitation, and delirium assessment in a community intensive care unit

C.M. Tan[1], M. Camargo[2,3], F. Miller[1], K. Ross[2], R. Maximous[2], P. Yung[1], C. Marshall[1], D. Fleming[2], R. D'Sa[2,4], M. Law[3], J.L. Tsang[1,2,4]

[1]McMaster University, Niagara Regional Campus, St. Catharines, Canada; [2]Niagara Health, St. Catharines, Canada; [3]Brock University, St. Catharines, Canada; [4]McMaster University, Department of Medicine, Hamilton, Canada

**Correspondence:** C.M. Tan

*Intensive Care Medicine Experimental* 2018, **6(Suppl 2):**0840

**INTRODUCTION.** Delirium affects up to 80% of patients in the intensive care unit (ICU) and is associated with multiple adverse outcomes[1]. In 2013, ICU pain, agitation, and delirium (PAD) guidelines were published. Despite the well-established advantages of implementing PAD guidelines, adoption and adherence remain suboptimal[2]. Educational interventions have been shown to address barriers to guideline implementation[3]. However, the impact of multifaceted nurse engagement interventions in a community ICU is unknown.

**OBJECTIVES.** The purpose of this prospective interventional study is to evaluate the effect of a Multifaceted Nurse Engagement Intervention on PAD assessment in a community ICU.

**METHODS.** All patients admitted to our community ICU for over 24 hours were included. A 20-week baseline audit was performed, followed by implementation of the intervention, and a 20-week post-intervention audit. The Nurse Engagement Intervention consisted of a survey, focus groups, and education sessions. Primary outcomes included rates of daily pain, agitation, and delirium assessment using validated scoring tools.

**RESULTS.** There were significant improvements in the proportion of patients with at least one assessment per day of pain (67.5±11.9% v. 59.3±12.2%, *p*=0.04), agitation (93.1±4.3% v. 78.7±8.2%, *p*< 0.001), and delirium (54.2±10.2% v. 39.4±11.6%, *p*< 0.001) (Figure 1), as well as number of patients with target RASS ordered (63.1±15.8% v. 46.8±15.6%, *p*=0.002) (Figure 1). There was a significant decrease in rate of physical restraint use (10.0±7.2% v. 30.9±15.9%, *p*< 0.001) and no change in rate of self-extubation (0.9±1.9% v. 2.5±5.2%, *p*=0.2) (Figure 1).

**CONCLUSIONS.** The implementation of a Multifaceted Nurse Engagement Intervention has the potential to improve rates of PAD assessment in community ICUs. Screening rates in our ICU remain suboptimal despite these improvements. We plan to implement multidisciplinary interventions targeting physicians, nurses, and family members to close the observed care gap.

##### GRANT ACKNOWLEDGMENT

Financial support for holding nurse focus groups and education sessions was provided by the Ontario Nurses' Association. Jennifer LY Tsang, senior author, was the recipient of the McMaster Department of Medicine Internal Career Research Award.

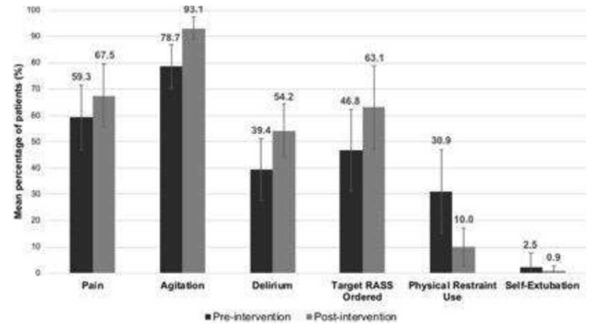

**Fig. 1 (abstract 0840).** Rate of PAD assessment, target RASS ordered, physical restraint use, and self-extubation

### 0841

#### Incidence and risk factors of postoperative delirium in adult patients after elective craniotomy: a prospective cohort study

C.-M. Wang, H.-W. Huang, J.-X. Zhou

Beijing Tiantan Hospital, Capital Medical University, Critical Care Medicine, Beijing, China

**Correspondence:** J.-X. Zhou

*Intensive Care Medicine Experimental* 2018, **6(Suppl 2):**0841

**INTRODUCTION.** Postoperative delirium (POD), as an important complication, occurs in 11-51% of patients after major surgery. However, in the majority of cohort studies and randomised controlled trials investigating POD, patients after intracranial operations were usually excluded.

**OBJECTIVES.** We aimed to determine the incidence of POD and its associated risk factors in adult patients admitted to the ICU after elective craniotomy under general anaesthesia.

**METHODS.** This prospective cohort study was conducted in a neurosurgical ICU of University affiliated hospital. The local IRB approved the protocol, and written informed consents were obtained. Adult patients after elective craniotomy under general anaesthesia were included. The exclusion criteria are shown in figure 1.

Delirium was evaluated by the Confusion Assessment Method for Intensive Care Unit (CAM-ICU) at three time points: (1) at the ICU admission; (2) at 08:00 to 10:00 on postoperative day one in the ICU; and (3) at 08:00 to 10:00 on postoperative day three in the ICU or in the neurosurgical ward. The assessment at the ICU admission was used to diagnose emergence delirium. The assessments on postoperative day one and day three were used to diagnose POD. Thus patients were classified as POD group if delirium was diagnosed on postoperative day one and/or day three, otherwise non-POD group.

Baseline and preoperative data, anaesthesia- and surgery-related data, and data during the ICU stay before the delirium assessment on postoperative day one were collected. The incidence of POD and 95% confidential interval (CI) were calculated. Univariate and multivariate logistic analyses were performed to determine the risk factors for POD.

**RESULTS.** During the study, 800 patients were included for final analysis (figure 1). POD was diagnosed in 157 (19.6%, 95% CI: 16.9%-22.4%) patients, of whom 97 (61.8%), 33 (21.0%), and 27 (17.2%) were classified as hypoactive, hyperactive, and mixed subtypes, respectively.

Independent risk factors for POD included: age, type of lesion, frontal approach of operation, duration of operation, episode of $SpO_2 < 90\%$ at the ICU admission, occurrence of emergence delirium, presence of ischemia on the first postoperative CT scan, and external delirium risk factor score (figure 2).

Compared with the non-POD group, less patients in the POD group were discharged from ICU at postoperative day one, and more accidental removal of tubes and catheters, higher hospital mortality, longer postoperative length of stay in hospital, and higher costs were found in the POD group.

**CONCLUSIONS.** POD was prevalent among adult patients after elective craniotomy, and was associated with adverse outcomes. Identifying the disease-specified and preventable risk factors could help to facilitate the early warning and prevention of POD.

**REFERENCE(S)**

Inouye SK, et al. Delirium in elderly people. Lancet 2014;383:911-922.

**GRANT ACKNOWLEDGMENT.**

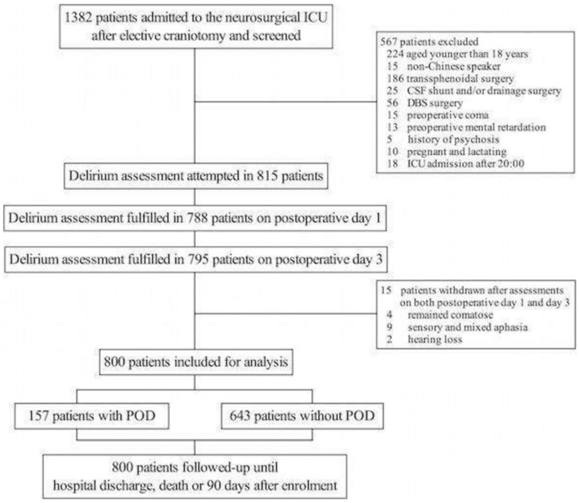

**Fig. 1 (abstract 0841).** Patients flow chart

| Variables | Odd ratio (95% CI) | p values |
|---|---|---|
| Age, years | 1.05 (1.03–1.07) | <0.001 |
| Type of lesion | | |
| Benign tumour | 1 (reference) | <0.001 |
| Malignant Tumour | 2.89 (1.63–5.11) | <0.001 |
| Cerebrovascular diseases | 2.99 (1.47–6.08) | 0.002 |
| Frontal approach of operation | 2.95 (1.77–4.92) | <0.001 |
| Duration of operation, min | 1.00 (1.00–1.01) | 0.011 |
| Episode of $SpO_2 < 90\%$ at the ICU admission | 6.61 (1.18–36.95) | 0.032 |
| Emergence delirium | | |
| None | 1 (reference) | <0.001 |
| Coma | 11.11 (4.82–25.65) | <0.001 |
| Hyperactive | 14.84 (5.67–38.84) | <0.001 |
| Hypoactive | 11.48 (6.68–19.71) | <0.001 |
| Presence of ischemia on the first postoperative CT scan | 3.06 (1.21–7.77) | 0.018 |
| Precipitating factor score | 1.59 (1.30–1.95) | <0.001 |

CI=confidence interval. $SpO_2$=pulse oxygenation saturation. ICU=intensive care unit.
Hosmer and Lemeshow Test: p = 0.843.

**Fig. 2 (abstract 0841).** Risk factors for postoperative delirium

### 0842

### Impact of dexmedetomidine on the time to extubation when used for the treatment of hyperactive delirium

C. Santis, L. Alegria, V. Oviedo, M.L. Riquelme, D. Valenzuela, M. Vera, A. Bruhn, J. Retamal, G. Bugedo
Pontificia Universidad Catolica de Chile, Medicina Intensiva, Santiago, Chile
**Correspondence:** C. Santis
*Intensive Care Medicine Experimental* 2018, **6(Suppl 2):**0842

**INTRODUCTION.** Delirium is present in more than 50% of ventilated patients, and it may delay extubation (1). Dexmedetomidine has been shown to be effective for hyperactive delirium (HD) but there is no evidence whether this has an impact on the time to extubation.

**OBJECTIVES.** The aim of this study was to evaluate the impact of Dexmedetomidine on the time to extubation of critically ill patients who developed HD during the weaning period of mechanical ventilation (MV).

**METHODS.** We conducted a prospective observational study in adult patients who required MV for more than 48 h and, who presented HD during weaning (defined as CAM-ICU (+) or SAS score 5-7, plus asynchrony, minute ventilation >200 ml/Kg/min, or tachycardia / hypertension).

Patient demographics were collected at enrolment. Data on sedatives, analgesics and neuromuscular blockers were collected during the first 3 days after admission, and for 3 days after the onset of HD. Two groups of patients were defined, according if they received or not Dexmedetomidine during the first 24 hs after HD.

Main outcome was time to extubation (from HD onset to successful extubation). Secondary outcomes were time on MV, MV-free days at day 28, ICU stay and in-hospital mortality.

**RESULTS.** Twenty-three patients, 57 (39-75) y.o., 15 male, APACHE II 20 (15-23), SOFA 10 (7-11), met HD criteria 4 (3-7) days after connection to MV. In 18 (78%) patients HD occurred promptly after sedative interruption (4 (2-9) hours).

Dexmedetomidine was used in 13 patients (57%, Dexmedetomidine group) as the primary sedative to control HD. Propofol (91%), Fentanyl (60%) and antipsychotics (70%) were used in both groups. More patients in Dexmedetomidine group used Quetiapine (85% vs 20%, p=0.003).

Patients in the Dexmedetomidine group had the same time to extubation than patients who did not (5 (2-12.5) vs 6.5 (2.8-15, p=0.7). Time on MV, MV-free days and mortality for the full cohort were 11 (7-23) days, 16 (0-20) days, and 30%, respectively, without differences between groups.

**CONCLUSIONS.** Dexmedetomidine was frequently used for the control of HD, but showed no impact on the time to extubation. Propofol, antipsychotics, and Fentanyl are also used to treat HD.

**REFERENCE(S)**

1. Mehta S, et al. Crit Care Med 2015; 43(3): 557-66.

**GRANT ACKNOWLEDGMENT**
DIDEMUC, Pontificia Universidad Católica de Chile.

### 0843

#### Effects of nocturnal dexmedetomidine on sleep quality in critically ill patients equipped with high flow nasal cannula

Y. Ueno, J. Oto
Tokushima University Hospital, Critical Care and Emergency Medicine, Tokushima, Japan
**Correspondence:** Y. Ueno
*Intensive Care Medicine Experimental* 2018, **6(Suppl 2):**0843

**INTRODUCTION.** Sleep quality of critically ill patients is extremely poor and nocturnal infusions of dexmedetomidine have been increasingly utilized in non-intubated patients. Previous study revealed nocturnal dexmedetomidine infusion improved sleep quality and quantity in patients with and without mechanical ventilation. High flow nasal cannula (HFNC) is relatively acceptable, however, effectiveness of dexmedetomidine on sleep quality in patients undergoing HFNC is unknown.
**OBJECTIVES.** We investigated if dexmedetomidine infusion could improve sleep quality in patients with HFNC.
**METHODS.** Adult patients with HFNC were randomly assigned to receive nocturnal dexmedetomidine (DEX group) or not (Control group). Dexmedetomidine infusion rate was controlled from 0.2 μg/kg/h to 0.7 μg/kg/h. We recorded polysomnography (PSG) during nighttime (9:00 p.m. to 6:00 a.m.). We collected the values of sleep efficiency, total sleep time, percentage of each sleep stage: stage 1 (N1), stage 2 (N2), stage 3 (N3), and rapid eye movement (REM), and arousal index. Sleep efficiency was calculated as the value of total sleep time / the observation time (9 hrs). Arousal index was defined as the value of arousals /h.
**RESULTS.** Twenty patients were studied (10 in DEX group and 10 in Control group). There were no significant difference in sleep efficiency of median: 57% (IQR: inter quartile range 32-75) vs. 33 %(12-66) ($p$=0.22), total sleep time of 310 min (172-404) vs. 177 min (62-357) ($p$=0.22), N1: 26 % (18-47) vs. 51 % (30-76) ($p$=0.34), N2: 69 % (34-78) vs. 49 % (20-58) ($p$=0.50), N3: 0 % (0-0) vs. 0 % (0-0) ($p$=0.47), REM: 1 % (0-2) vs. 0 % (0-3) ($p$=0.47), and arousal index: 21 /h (16-34) vs. 37 /h (28-47) ($p$=0.08).
**CONCLUSIONS.** Although HFNC is generally comfortable device, sleep time was very short and highly disrupted. Dexmedetomidine infusion did not significantly improve sleep quality and quantity in patients under HFNC.

##### REFERENCE(S)

1. Oto J, et al. (2012) Sleep quality of mechanically ventilated patients sedated with dexmedetomidine. Intensive Care Med 38:1982-1989
2. Weina L, et al. (2017) Effects of dexmedetomidine on sleep quality of patients after surgery without mechanical ventilation in ICU. Medicine 96:23(e7081)
3. Christina A, et al. (2014) Effects of dexmedetomidine on sleep quality in critically ill patients. Anesthesiology 121:801-7
4. Xin-Hai W, et al. (2016) Low-dose dexmedetomidine improves sleep quality pattern in elderly patients after noncardiac surgery in the intensive care unit. Anesthesiology 125:979-91

##### GRANT ACKNOWLEDGMENT

Departmental funding.

### 0844

#### Challenges and barriers to optimising sedation in intensive care: a qualitative study nested within a cluster randomised quality improvement trial in eight Scottish intensive care units

K. Kydonaki[1], J. Hanley[1], G. Huby[2], J. Antonelli[3], T. Walsh[4], on behalf of the Development and Evaluation of Strategies to Improve Sedation Practice in inTensive Care (DESIST) Study Investigators
[1]Edinburgh Napier University, School of Health and Social Care, Edinburgh, United Kingdom; [2]Østfold University College, Faculty of Health and Social Studies, Halden, Norway; [3]University of Edinburgh, Edinburgh Clinical Trials Unit, Edinburgh, United Kingdom; [4]Royal Infirmary of Edinburgh, Department of Anaesthesia, Critical Care and Pain Medicine, Edinburgh, United Kingdom
**Correspondence:** K. Kydonaki
*Intensive Care Medicine Experimental* 2018, **6(Suppl 2):**0844

**INTRODUCTION.** Various strategies to promote light sedation are highly recommended in recent guidelines, as deep sedation is associated with suboptimum patient outcomes[1,2]. Yet, the quality of sedation and analgesia provided is rarely addressed. In a cluster-randomised quality improvement trial in eight Scottish ICUs we evaluated the effectiveness of three interventions for improving sedation-analgesia quality in mechanically ventilated patients[3] (Development and Evaluation of Strategies to Improve Sedation Quality in Intensive Care- DESIST trial, ClinicalTrials.gov NCT01634451).
**OBJECTIVES.** As part of the trial evaluation, we aimed to understand the challenges and barriers to optimising sedation in the Scottish ICU settings.
**METHODS.** We conducted focus groups (FG) with clinicians during the pre-intervention period in all participating ICUs. Clinicians volunteered to participate. FG were recorded and verbatim transcribed and inserted in NViVo for analysis. Qualitative thematic analysis was undertaken in order to develop emergent themes from the patterns identified in relation to sedation practice. Ethical approval was secured prior to the study.
**RESULTS.** Three themes emerged from the inductive analysis: a) the meaning of optimum and suboptimum sedation, including the clinicians' perceived quality of sedation; b) a shift in sedation practice, including a change in the agents used, a change from strictly protocolised care to patient-focused care, and a consideration of sleep promotion; and c) multifactorial challenges in managing sedation, including the uncertainty of difficult to sedate patients, the lack of appropriate assessment tools, the ICU environment, organisational factors and gaps in education.
**CONCLUSIONS.** The current sedation literature lacks recognition of the various challenges when mandating less sedation. An emphasis should be placed on the need for novel sedation-analgesia strategies that allow a systematic approach and ongoing monitoring of sedation-analgesia quality.

##### REFERENCE(S)

1. Barr J, Fraser GL, Puntillo K, et al. Clinical practice guidelines for the management of pain, agitation, and delirium in adult patients in the intensive care unit. Crit Care Med 2013;41: 263-306.
2. Mehta S, Burry L, Cook D, et al. Daily sedation interruption in mechanically ventilated critically ill patients cared for with a sedation protocol: a randomized controlled trial. *JAMA* 2012;308:1985-92.
3. Walsh TS, Kydonaki K, Antonelli J, et al. Staff education, regular sedation and analgesia quality feedback, and a sedation monitoring technology for improving sedation and analgesia quality for critically ill, mechanically ventilated patients: a cluster randomised trial. *The Lancet Respiratory Medicine* 2016;4(10):807-17.

##### GRANT ACKNOWLEDGMENT

Chief Scientist office, Scotland, GE healthcare

### 0845

#### Serum noradrenaline levels can predict the occurrence of delirium in the ICU in catecholamine-free patients

M. Nishikimi, A. Numaguchi, Y. Yasuda, N. Matsuda
Nagoya University Graduate School of Medicine, Nagoya, Japan
**Correspondence:** M. Nishikimi
*Intensive Care Medicine Experimental* 2018, **6(Suppl 2):**0845

**INTRODUCTION.** Abnormal secretion of neurotransmitters such as catecholamines has been known to be associated with the occurrence of delirium, and the secretion is seriously disturbed in critical care patients.
**OBJECTIVES.** The aim of this study was to evaluate the correlation between the serum levels of catecholamines and the occurrence of delirium in critical care patients.
**METHODS.** We retrospectively analyzed the data of the patients enrolled in our previous randomized clinical trial (Melatonin Evaluation of Lowered Inflammation in ICU Trial; UMIN000016541)

(1). The serum levels of catecholamines (adrenaline, noradrenaline and dopamine) were measured in 79 patients at the time of their admission to the ICU. Using the data of 50 of these subjects who had not received catecholamines before their admission to the ICU, we conducted univariate and multivariate logistic regression analyses to evaluate the correlations between the serum levels of catecholamines and the occurrence of delirium. We also analyzed the data of all 79 subjects to examine the interactions between the serum levels of catecholamines and use/non-use of catecholamines prior to ICU admission in the occurrence of delirium. Delirium was assessed every 4 hours during the ICU stay of the patients (the assessment was skipped if the patients were sleeping) by trained ICU nurses using the Confusion Assessment Method for the ICU (CAM-ICU), and by the ICU doctors as needed.

**RESULTS.** Among the 50 subjects, 34 developeddelirium during their ICU stay. The results of the univariate and multivariate analyses revealed that the serum levels of noradrenaline, but not those of adrenaline or dopamine, were associated with an increased odds ratio for the occurrence of delirium (univariate: OR 2.81, 95% CI 1.17-6.79, p=0.021; multivariate: OR 2.73, 95% CI 1.02-7.31, p=0.046).Furthermore, the analysis revealed a statistically significant interaction between the serum levels of noradrenaline and the use/non-use of catecholamines in the occurrence of deliriumin patients admitted to the ICU(p=0.047).

**CONCLUSIONS.** In patients who had not received catecholamines prior to their admission to the ICU, the serum levels of noradrenaline, but not those of adrenaline or dopamine, were correlated with the occurrence of delirium in the ICU.

**REFERENCE(S)**

1. Nishikimi M, et al., Effect of Administration of Ramelteon, a Melatonin Receptor Agonist, on the Duration of Stay in the ICU: A Single-Center Randomized Placebo-Controlled Trial. Crit Care Med. 2018 Mar 27. PMID: 29595562.

**GRANT ACKNOWLEDGMENT**

None.

### 0846

#### Observational analysis of the incidence and factors related to delirium in the critical patient

J. Codina Calero[1], G. Jiménez Jiménez[1], N. Ramon Coll[1], S. Rodríguez Ruiz[1], J.J. Trujillano Cabello[1,2], J. Caballero López[1]

[1]Arnau de Vilanova University Hospital, Lleida, Spain; [2]Universitat de Lleida-IRBLleida, Lleida, Spain

**Correspondence:** J. Codina Calero

*Intensive Care Medicine Experimental* 2018, **6(Suppl 2):**0846

**INTRODUCTION.** Delirium is a severely underestimated condition in the critical patient. It constitutes a medical emergency and it is associated with an increased mean length of stay in the ICU and higher morbidity and mortality. Three different subtypes of delirium have been described (hypoactive, hyperactive and mixed) according to clinical presentation. Current recommendations emphasize the importance of accurate screening of delirium in the ICU so that treatment and management of its complications can be initiated at an earlier stage.

**OBJECTIVES.** To identify the incidence of delirium and its subtypes in our context, and to compare demographical and clinical characteristics of patients with and without delirium.

**DESIGN**. Prospective observational study.

**PARTICIPANTS.** Non-consecutive day data collection of all adult patients admitted to the ICU during a 50-day period.

**METHODS.** Detection of delirium with the Confusion Assessment Method for Intensive Care Unit (CAM-ICU) in its Spanish version. Classification into hyperactive, hypoactive and mixed subtypes. CAM-ICU is considered to be non-valuable in patients with stroke, cranial trauma or RASS -4/-5. Demographical and clinical data such as reason for admission, APACHE-II score, artificial airway, intravenous sedation or presence of predisposing factors for delirium were recorded.

**RESULTS.** 79 patients were included in the study, 25 (31.6%) whom developed delirium. The most common subtype was hypoactive (49.0%), followed by mixed (40.8%) and hyperactive (10.2%). The Kaplan-Meier test to compare the development of delirium among the different groups shows that there are no differences according to sex, but there are significant differences according to the presence of artificial airway (*p*=0.023), intravenous sedation (*p*=0.003), medical condition (*p*=0.004), >1 predisposing factor (*p*=0.003) and APACHE-II score >20 (*p*=0.011). These same factors are also associated with an increased risk of delirium as observed in the univariate analysis. Nevertheless, the multivariate analysis found that only medical condition [HR 4.53 (CI 95% 1.31-15.7)] and presenting >1 predisposing factor [HR 1.60 (CI 95% 1.20-2.12)] are independent risk factors for presenting delirium. The CART model categorizes different groups of patients combining logical decision rules with different factors, showing that the group with >1 predisposing factor and medical condition has a higher incidence of delirium of 83.3%.

**CONCLUSIONS.** More than 3/10 patients in our ICU develop delirium, the hypoactive subtype being the most frequent form of presentation. Artificial airway, intravenous sedation, medical condition, >1 predisposing factor and APACHE-II score >20 increase the risk of delirium, but only the medical condition and presenting >1 predisposing factor for delirium are independent risk factors for presenting delirium. The group with >1 predisposing factor and medical condition has the highest incidence of delirium (83.3%).

### 0847

#### Analysis of results and costs after the implementation of a sedation protocol in intensive care patients on mechanical ventilation

N. Pires[1], M.C. Gonzalez[2], A. Dogliotti[3], C. Lovesio[1]

[1]Sanatorio Parque, Rosario, Argentina; [2]Sanatorio Parque, Intensive Care Unit, Rosario, Argentina; [3]Instituto Cardiovascular de Rosario, Rosario, Argentina

**Correspondence:** N. Pires

*Intensive Care Medicine Experimental* 2018, **6(Suppl 2):**0847

**INTRODUCTION.** In recent years, it has been proposed to change not only the sedation objectives of the critical patient, but also the drugs used for that purpose. Several benefits have been postulated regarding days of mechanical ventilation, delirium, and stay in critical care, however; the impact in economic terms of the implementation of these programs has not been analyzed even though the recommended drugs are more expensive. This can represent a significant difficulty at the management level, especially in intensive care units in developing countries.

The objectives were to compare the results of the implementation of a sedation protocol through the combined end point: mortality, prolonged stay in intensive care or prolonged mechanical ventilation and total costs of sedation before and after the implementation of the protocol.

**METHODS.** A sedation protocol was designed based on maintaining the minimum level of sedation necessary according to the patient´s illness and the evolving moment in which they were passing. Propofol and fentanyl were used as drugs of choice for deep sedation, and fentanyl or fentanyl plus dexmedetomidine in case of superficial sedation. It was indicated to maintain deep sedation for the shortest possible time. The drugs used before the implementation of the protocol were midazolam and fentanyl. All patients requiring mechanical ventilation and sedation for at least 48 hours were included.We included all the patients for a period of four months before and after the implementation of the protocol. The variables recorded were sex, age, diagnosis, history, APACHE II, sedation scheme used and total costs, days of mechanical ventilation, stay in critical care, mortality.

**RESULTS.** We included 78 patients in the study, 45 before and 33 after the implementation of the protocol. The median age of the patients was 69 years, with a 25-75 percentile between 60 and 75 years. The median of Apache II was 23, with a 25-75 percentile ranging from 18-27. Regarding the days of mechanical ventilation, the median was 4 days with 25-75 percentiles ranging from 2 to 7 days. The stay in intensive therapy ranged between 3 and 11 days with a median of 6 days. The mortality was 24 patients in the group without protocol and 22 in the group with protocol. No variable was independently associated to the primary endpoint in a multivariate analysis. After adjust by APACHE II the protocol reduces 75% of the end point of death, stay in UTI and ARM (p = 0.03). There were no statistically significant differences in the costs (p = 0.5) nor in the delirium rate (p = 0.3).
**CONCLUSIONS.** Since the implementation of a sedation protocol with non-benzodiazepine drugs the death end point, stay in critical care and days of mechanical ventilation were reduced by 75%. In addition, sedation costs were not increased.

### 0848

#### Benzodiazepine use during ICU stay is associated with benzodiazepine dependence after discharge

M. Sakuraya, C. Nishiyama, K. Yoshida, Y. Kato, N. Kawamura, A. Takaba, T. Tsutsui, T. Matsumoto
JA Hiroshima General Hospital, Department of Emergency and Intensive Care Medicine, Hatsukaichi, Japan
**Correspondence:** M. Sakuraya
*Intensive Care Medicine Experimental* 2018, **6(Suppl 2):**0848

**INTRODUCTION.** Insomnia, which could increase ICU delirium, is a common problem for the ICU patients, because of a lot of disturbance. Insomnia after discharge is one of the post intensive care syndrome, and it's reported to be associated with daytime sleepiness, depression and physical performance. But, sleeping drug prescription after ICU discharge is unknown.
**OBJECTIVES.** The aim of this study is to investigated whether benzodiazepine use during ICU stay is associated with benzodiazepine prescription at 6 months after discharge.
**METHODS.** We conducted retrospective cohort study in our hospital between Janurary 2014 and December 2016. All adult patients (18 years or older) who were consecutively admitted in the ICU, department of cardiac surgery is in charge of, and who were alive 6 months after discharge from hospital were included. Exclusion criteria is prior use of sleeping drugs, transfer to another hospital, post cardiopulmonary arrest, stroke, cognitive dysfunction, swallowing disorder, liver cirrhosis. We compared the patient who used oral benzodiazepine during ICU stay (BZ group) to the patients who didn't use (non BZ group). We investigated benzodiazepine prescription at discharge from hospital and 6 months after discharge. We performed logistic regression analysis for benzodiazepine prescription at 6 months after discharge.
**RESULTS.** 407 patients were included and 249 patients were analyzed. Age, sex, APACHE 2 score and SOFA score were not different between two groups, but ICU length of stay and ventilator days
were longer in BZ group. Benzodiazepine prescription was more in BZ group; 30/81(37.0%) vs. 25/168(14.9%) at discharge, 18/81(22.2%) vs. 16/168(9.5%) at 6 months after discharge. Age ≥ 75 (OR 2.56, 95%CI 1.20-5.50), SOFA score ≥ 5 (OR 2. 76, 95%CI 1.01-8.91) and benzodiazepine use during ICU stay (OR 2.57, 95%CI 1.19-5.58) were risk for benzodiazepine prescription at 6 months after discharge independently.
**CONCLUSIONS.** Intravenous benzodiazepine is risk for ICU delirium and should be avoided, but oral benzodiazepine is unclear. In this study, oral benzodiazepine use during ICU stay might increase benzodiazepine prescription after discharge. Long-term use of benzodiazepine is associated with adverse events, including delirium, cognitive dysfunction and fracture. We should not prescribe benzodiazepine during ICU stay to avoid long-term use. Benzodiazepine use during ICU stay may increase benzodiazepine prescription after discharge.

##### REFERENCE(S)

Parsons EC, et al. Post-discharge insomnia symptoms are associated with quality of life impairment among survivors of acute lung injury. Sleep Med. 2012;13:1106-9.
Orwelius L, et al. Prevalence of sleep disturbances and long-term reduced health-related quality of life after critical care: a prospective multicenter cohort study. Crit Care. 2008;12:R97.
Glass J, et al. Sedative hypnotics in older people with insomnia: meta-analysis of risks and benefits. BMJ. 2005;331:1169.

### 0849

#### The impact of difficult sedation on the evolution of patients hospitalized in intensive care unit

D. Gil Castillejos[1], M.L. Rubio[2], C. Ferre[2], M.Á. de Gracia[1], M. Bodí[3], A. Sandiumenge[4]
[1]University Hospital Joan XXIII, Intensive Care Unit, Tarragona, Spain;
[2]University Rovira i Virgili, Nurse Department, Tarragona, Spain;
[3]University Hospital Joan XXIII/IISPV/URV Tarragona/CIBERES, Tarragona, Spain; [4]Vall d´Hebron Hospital, Transplant Coordination, Barcelona, Spain
**Correspondence:** D. Gil Castillejos
*Intensive Care Medicine Experimental* 2018, **6(Suppl 2):**0849

**INTRODUCTION AND OBJECTIVES.** Difficult Sedation(DS) includes situations of therapeutic failure(inability to achieve the desired level of sedation at the maximum dose), tolerance(higher doses required to achieve the same level of sedation) or deprivation(symptoms appear after abrupt discontinuation or decreased dose) of sedatives being administered. This paper aims to characterize DS clinical picture and its effects on critically ill patients during their stay in the Intensive Care Unit.
**MATERIAL AND METHODS.** During 2015-17 all patients consecutively admitted to a 14 bed medical-surgical ICU and undergoing mechanical ventilation(MV) and sedation for more than 48h were prospectively followed until discharge or death. Complications and prognosis of patients presenting with DS were compared with those who did not develop it. Epidemiological data, type, duration and complications of sedoanalgesia practices and clinical outcome during ICU stay were analyzed. Significance p< 0.05, SPSSv21©.
**RESULTS.** 667 patients were admitted to the ICU during the study period, 327(49.0%) of whom were included in the study (71.3% male, 60.8 ±14.8 years old, admitted mostly due to a medical cause (58.7%); APACHE II (25,7± 9,2). Seventy-nine patients (24.1%) developed DS. DS patients were younger(55.9±17.7 vs 62.4±14.2 y/o;p=0.001) and less severely ill(APACHEII 23±9.5 vs 26.6±9.0;p=0.003) than no DS group. More patients in DS group had smoking (p=0.045) alcohol(p< 0.001) and psychotropic use(p=0.001) history than no DS group.
DS group patients were sedated during longer periods (Median 274h(min-max 32;1709) vs 77h(10;1467); p< 0.001) than no DS groups and required higher doses of Midazolam (0.21 ±0.71vs 0.18 ±0.12 mg/kg/h; p< 0.036), Propofol (2.3±0.9 vs1.9 ±1.0 mg/kg/h;p=0.023) and remifenatnil (10.9±10.3 vs 4.5±1.6 mcg/kg/h p=0.026). The percentage of DS patients requiring the use of more than one sedatives simultaneously doubled that of the no DS group (68.8% vs 31.1%;p< 0.001). DS patients presented more periods of oversedation (p=0.031) / undersedation (p=0.024) in relation to the desired level of sedation. More patients in the SD group suffered pain as measured by median time with EVN/ESCID scale > 3 (20(1;204) vs 14 (1;110) hours; p< 001). .
DS patients had prolonged mechanical ventilation times (16.2±13.1 vs 10.6±9.9 days;p < 0,001) than no DS patients. More patients in the DS group suffered injuries arising from dependence (46.8% vs 22.2%;p< 0.001), ventilator associated pneumonias (VAP)(11.4% vs4.4%;p=0.025) and underwent tracheotomies(40.5% vs 16.5%;p=0.001 patients not developing DS.
**CONCLUSIONS.** DS develops in one out of every four critically ill patients and affects negatively their outcome. Early identification and active prevention are essential strategies to minimize its impact.

##### REFERENCE(S)

Chamorro C, Romera MA, Grupo de Trabajo de Analgesia y Sedación de la SEMICYUC. Estrategias de control de la sedación difícil. Med Intensiva. 2008;32 Supl 1:31-7.

### 0850

#### The prevalence, risk factors and outcomes of ICU delirium among Chinese patients in an intensive care unit in Hong Kong

C.Y.Y. Lee, W.L. Tong, H.H. Tsang
Kwong Wah Hospital, Intensive Care Unit, Hong Kong, Hong Kong, China
**Correspondence:** C.Y.Y. Lee
*Intensive Care Medicine Experimental* 2018, **6(Suppl 2):**0850

**INTRODUCTION.** Intensive Care Unit (ICU) delirium is extremely common among critically ill patients and is associated with adverse outcomes including increased mortality, ventilator days and length of stay. ICU delirium is likely to be multifactorial and multiple risk factors have been identified. While the prevalence of ICU delirium has been reported to range from 30 to 80% (1-3), most studies were carried out in Europe and Northern American. Data concerning ICU delirium among Chinese patients are lacking.
**OBJECTIVES.** This study aims to determine the prevalence of ICU delirium among Chinese patients in an ICU in Hong Kong over a 3-month period, and to evaluate the risk factors and outcomes associated with this condition.
**METHODS.** This is a retrospective, observational study conducted during the period of November 2017 to January 2018. Patients admitted to the Kwong Wah Hospital ICU during the study period were evaluated for the presence of delirium by means of the Confusion Assessment Method for the Intensive Care Unit (CAM-ICU) (Traditional Chinese version) and the Richmond Agitation-Sedation Scale (RASS) two times per day. The period prevalence of ICU delirium was determined. Clinical data were correlated with the presence of ICU delirium and the independent risk factors were identified. The outcomes associated with ICU delirium were also evaluated.
**RESULTS.** The period prevalence of ICU delirium during the study period was found to be 30.5%. Five independent risk factors for ICU delirium were identified with multivariate analysis, namely old age (≥65), presence of at least one chronic disease, comatose state during ICU stay, exposure to benzodiazepine, and the duration of sedation. ICU delirium was linked to higher ICU and 30-day mortality, longer ICU length of stay, hospital length of stay, and ventilator days in the bivariate analysis, but not in the multivariate analysis.
**CONCLUSIONS.** This study revealed that the prevalence of ICU delirium in the Hong Kong Chinese patient population is comparable to the figures quoted in the literature. The independent risk factors identified are in keeping with other studies. Although a linkage to adverse outcomes is found, a larger sample size is needed to determine a definite association.

##### REFERENCE(S)

1. Salluh JI, Soares M, Teles JM, Ceraso D, Raimondi N, Nava VS, et al. Delirium epidemiology in critical care (DECCA): An international study. Crit Care. 2010;14(6):R210.
2. Agarwal V, O'Neill PJ, Cotton B, Pun BT, Haney S, Thompson J, et al. Prevalence and risk factors for development of delirium in burn intensive care unit patients. J Burn Care Res. 2010;31(5):706-15.
3. Ely EW, Speroff T, Gordon SM, Harrell FE, Inouye SK, Bernard GR, et al. Delirium as a Predictor of Mortality in Mechanically Ventilated Patients in the Intensive Care Unit. JAMA. 2004;291(14):1753-62.

##### GRANT ACKNOWLEDGMENT

Nil

### 0851

#### Analysis of analgesia, sedation and delirium practices in critically ill patients through a simulation course survey

N. Martinez Sanz, M. Valdivia de la Fuente, S. Alcánatara Carmona, N. Láiz Diez, I. Latorre Marco, M. Acevedo Nuevo, I. Fernández Simón, M.Á. Romera Ortega
Hospital Universitario Puerta de Hierro Majadahonda, Madrid, Spain
**Correspondence:** N. Martinez Sanz
*Intensive Care Medicine Experimental* 2018, **6(Suppl 2):**0851

**INTRODUCTION.** Evidence suggests that routine assessment and monitoring of sedation, pain and delirium may improve patient outcomes. Nevertheless, surveys carried out in different ICUs have shown that frequently these aspects go unmonitored.
**OBJECTIVES.** To analyze the routine use of scales and protocols for analgesia, sedation and delirium in a group of health professionals who attended a multidisciplinary simulation course focused on this area of knowledge and to evaluate the course impact on their daily practices.
**METHODS.** Descriptive study analyzing the three editions of an analgesia, sedation and delirium course performed at the Simulation Unit of Puerta de Hierro Hospital in Majadahonda during 2016. This course was aimed at intensive care professionals (nurses and doctors). The course consisted of a theoretical introduction followed by three workshops that included real-life simulations in which the students had to assess the patient, apply analgesia, sedation and delirium scales [behavioral pain scales, Richmond Agitation Sedation Scale (RASS), the Confusion Assessment Method for the ICU (CAM-ICU)] and decide subsequent treatments. At the end of the course they were asked to fill out a survey enquiring about scale use and protocols in their hospitals and the usefulness and impact that learning through simulation had had in their training.
**RESULTS.** Sixty students completed the course: 33 doctors (55%) and 27 nurses (45%), coming from 28 different hospitals throughout Spain and one in Portugal.
Forty-one (68%) routinely used analgesia scales in communicative patients (analogue and/or numeric) and only 16 out of these 41 used behavioral pain scales in non-communicative patients. Sedation scales were used in 49 (82%) of the cases, Riker Sedation Agitation Scale and the RASS being the most commonly reported (43;72%). Strategies to identify delirium were reported by 12 students (20%), in all cases by the CAM-ICU. Twenty-nine (48%) had protocols for pain and sedation management in their hospitals and eight (13%) for delirium management.
When asked about the usefulness of learning through simulation: 51 (85%) evaluated this tool as helpful to clarify their previous knowledge, 48 (80%) became aware of the importance of the patient adapting to an unfriendly environment, 54 (90%) considered that the course had helped them to understand the importance of using scales in critically ill patients and 56 (93%) answered that training through simulation made the strategies to identify and manage delirium easier. All students would recommend this course to their colleagues.
**CONCLUSIONS.** In our series we identified a lack of use of scales oriented at identifying pain, sedation and delirium in the critically ill, most of all when we look at analgesia scales for non-communicative patients and delirium identification.
Training through simulation was well accepted by the students and helped them understand the use of pain, sedation and delirium scales in ICU patients.

### 0852

#### Incidence, risk factors and outcomes of delirium in a Tunisian intensive care unit (ICU)

S. Kortli[1], I. Ben Saida[1], N. Kacem[1], M. Zghidi[1], N. Fraj[1], D. Ben Braiek[1], B. Amamou[2], A. Khedher[1], A. Azouzi[1], K. Meddeb[1], M. Boussarsar[1,3]
[1]Farhat Hached University Hospital, Medical Intensive Care Unit, Sousse, Tunisia; [2]Fattouma Bourguiba University Hospital, Department of Psychiatry, Monastir, Tunisia; [3]Ibn Al Jazzar Faculty of Medicine, Research Laboratory N° LR12SP09 Heart Failure, Sousse, Tunisia
**Correspondence:** M. Boussarsar
*Intensive Care Medicine Experimental* 2018, **6(Suppl 2):**0852

**INTRODUCTION.** Delirium is a complex neuropsychiatric disorder commonly encountered in critically ill patients and associated with adverse outcomes including increased length of ICU stay, mortality and long-term cognitive impairment. However, delirium is often misdiagnosed, detected late or missed.
**OBJECTIVES.** To determine the incidence, risk factors and outcomes of delirium in a Tunisian ICU.

**METHODS.** A prospective cohort observational study conducted over six months duration from October 2017 to March 2018 including all patients admitted in a 9-bed medical ICU for more than 24 hours. Patients who were deaf or unable to speak or understand were excluded. All included patients were screened by psychiatrist for delirium using DSM-V (Diagnostic and Statistical Manual of Mental Disorders, Fifth Edition) criteria. Predictors investigated included underlying condition, severity, diagnostic at admission, invasive interventions, electrolyte disorders and medications used. Variables found to be statistically significant in univariate analysis were introduced into a multivariate regression model to identify factors independently associated to delirium.

**RESULTS.** During the study period, 125 patients were assessed and 104(83.2%) met the inclusion criteria. Patients' characteristics were : mean age, 59.32±15.78 years ; age >65 years, 44(42.3%) ; female, 29(27.9%) ; median Charlson index, 3[2-5] ; mean SAPSII, 27.77±9.57 ; invasive mechanical ventilation (IMV), 59(56.7%) and vasopressors use, 41(39.4%).

Using DSM-V criteria, 37(35.6%) had delirium. The most common subtype of delirium seen in the present study was hypoactive type, 24(64.86%), followed by hyperactive subtype, 11(29.7%) and few patients had mixed subtype of delirium, 2(5.4%).

Patients who were diagnosed with delirium had significantly longer duration of IMV (13.94±11.1 vs 8.15±10.7days, p=0.024), longer length of ICU stay (18.86±12.8 vs 12.03±11.14days, p= 0.006) and higher mortality rate (40.45% vs 20.89%, p=0.032).

Univariate analysis revealed the following factors to be associated to delirium respectively for delirium and controls : age≥65years, (56.8% vs 34.3%, p=0.027) ; smoking, (59.5% vs 37.3%, p=0.03) ; alcoholism, (16.2% vs 3%, p=0.023) ; SAPSII, (31.5±10.7 vs 25.6±8.2, p=0.002) ; IMV (81.1% vs 43.3%, p=0.00) ; vasopressors use (67.6% vs 23.9%, p=0.00) ; use of narcotic analgesics, (64.9% vs 35.8%, p=0.004), use of Propofol ( 48.6% vs 14.9%, p=0.00) and physical restraint, (56.8% vs 25.4%, p=0.001).

Multivariate regression model identified the following factors as independently associated to delirium: use of vasopressors, (OR, 5.5 ; 95%CI, [2.17-13.9] ; p=0.00) and use of Propofol (OR, 4.1 ; 95%CI, [1.51- 11.3] ; p=0.00).

**CONCLUSIONS.** Delirium seems to be common in ICU. In the present study, vasopressors and propofol were the only independent predictors of delirium.

### 0853

#### Relationship between near-infrared spectroscopy (NIRS) derived cerebral oxygenation and delirium in critically ill patients: a systematic review

J.G. Boyd[1], O. Neal[2], N. Bendahan[3], A. Ross-White[4], Cerebral Oxygenation and Neurological Outcomes Following Critical Illness (CONFOCAL) Research Group, and the Canadian Critical Care Trials Group

[1]Kingston General Hospital, Medicine (Neurology) and Critical Care Medicine, Kingston, Canada; [2]Queen's University, School of Undergraduate Medicine, Kingston, Canada; [3]Queen's University, Department of Medicine (Neurology), Kingston, Canada; [4]Queen's University, Library Services, Kingston, Canada

**Correspondence:** J.G. Boyd

*Intensive Care Medicine Experimental* 2018, **6(Suppl 2):**0853

**INTRODUCTION.** Delirium, defined as an acute change in level of consciousness that is characterized by impaired attention and disorganized thinking, occurs commonly in critically ill patients. Patients that develop delirium have increased risk of prolonged mechanical ventilation, long term cognitive dysfunction, and mortality. However, the cause of delirium is unknown, which significantly limits our ability to design rational therapeutic interventions. Poor cerebral oxygenation, as measured by near-infrared spectroscopy (NIRS), may be related to post-operative neurological dysfunction (e.g. delirium and long-term cognitive impairment). However, the relationship between NIRS derived cerebral oxygenation and neurological outcomes in critically ill patients is unclear.

**OBJECTIVES.** The purpose of this systematic review was to assess the utilization of NIRS-derived cerebral oxygenation in critically ill patients outside of the operating theatre. We aimed to examine the relationship between cerebral oxygenation and neurological outcomes.

**METHODS.** In collaboration with a librarian with expertise in systematic reviews (A. R-W), the following databases were searched from inception to August 14th, 2017, Ovid MedLine, Embase, Cochrane Library, and Web of Science. Conference proceedings, abstracts, and articles published in languages other than English were not considered. Two of the authors (ON, NB) independently reviewed each title and abstract for relevance, and articles deemed relevant were selected for full text review. In the case of disagreement, a third reviewer (JGB) resolved the conflict. The bibliographies from each selected article were searched for additional relevant articles. The selected papers were assessed for quality using the modified Downs and Black checklist for observational studies. Relevant data was abstracted from each article, and authors were contacted to provide missing data if required.

**RESULTS.** Of 1410 articles identified by the search strategy, 8 were ultimately selected for final review. Most (7 of 8) were published since 2013. These studies included a total of 213 patients primarily with shock or respiratory failure. A variety of devices were used to measure cerebral oxygenation, including the INVOS device and the FORESIGHT NIRS monitor. The duration of recording varied from 5 minutes to 72 hours. Four of the 8 studies reported on neurological outcomes. In all 4 studies, cerebral oxygenation was lower in critically ill patients that were delirious compared to controls, but this was only statistically significant in 2 of the studies. The heterogeneity in devices and duration of recording precluded a meta-analysis of this data.

**CONCLUSIONS.** NIRS is being studied outside of the operating room more commonly over the past 5 years. More study is needed to explore the relationship between cerebral oxygenation and delirium.

**GRANT ACKNOWLEDGMENT**

This work is funded by the SEAMO New Clinician Scientist Program.

### 0854

#### Assessment and documentation of delirium in a single district general hospital intensive care unit (ICU) using the Confusion Assessment Method in ICU (CAM-ICU)

C. Fleming, P. Morgan, A. Myers, S. Ranjan, T. Samuels

East Surrey Hospital, Intensive Care Unit, Redhill, United Kingdom

**Correspondence:** C. Fleming

*Intensive Care Medicine Experimental* 2018, **6(Suppl 2):**0854

**INTRODUCTION.** Delirium is a common clinical condition which is associated with poor outcomes, but that is preventable and treatable[1,2]. National Institute for Health and Clinical Excellence (NICE) guidelines recommend assessing for delirium if indicators of delirium are present[1]. American College of Critical Care Medicine (ACCM) guidelines recommend routinely monitoring patients for delirium, at least once per nursing shift, if they are at moderate to high risk of delirium[3].

**OBJECTIVES.** The aim of this project was to quantify the incidence of delirium in our ICU population. We investigated whether this is being adequately assessed and documented, in line with national and international guidelines[1,3].

**METHODS.** The medical and nursing notes and the observation charts for a random sample of ICU patients in a single District General Hospital were reviewed. It was recorded whether patients had been assessed for the presence of delirium using CAM-ICU or any other valid tool, within the previous seven days. CAM-ICU was then performed on each patient to determine whether delirium was in fact present. If delirium was present, it was characterised as either hyper- or hypoactive. Data was collected between 8$^{th}$ and 23$^{rd}$ February 2018.

**RESULTS.** 28 patient records were reviewed. In 11% of patients (n=3), CAM-ICU had been performed and the result documented during the preceding 7 days. 25% of patients (n=7) were found to be delirious, 6 having hypoactive delirium and 1 hyperactive. Of these, none had been documented as having delirium. 50% of patients (n=14) were CAM-ICU negative. 25% of patients (n=7) could not be assessed due to their low conscious level.

**CONCLUSIONS.** Assessment and documentation of delirium in our ICU population needs to be improved. It is notable that hypoactive delirium has been particularly overlooked in this study population. This may be due to the insidious nature of hypoactive delirium. It is important for healthcare staff working in critical care to be alert to the potentially harmful effects of delirium and understand the methods to diagnose and manage it. Education sessions are being held for both doctors and nurses involved in the care of patients in ICU, to improve knowledge of CAM-ICU and the guidelines on delirium, and to encourage regular assessment for delirium. This will be re-audited in the next 6 months.

**REFERENCE(S)**

1. National Institute for Health and Clinical Excellence (2010) Delirium: Prevention, diagnosis and management. NICE guideline (CG103)
2. Morandi et al. Delirium in the intensive care unit. Int Rev Psychiatry. 2009;21(1):43-58.
3. Barr et al. Clinical Practice Guidelines from the Management of Pain, Agitation, and Delirium in Adult Patients in the Intensive Care Unit. Crit Care Med. 2013;41(1):263-306

## Caring for immunocompromised patients

### 0855

#### Endothelial toxicity of extracellular histones during tumor lysis syndrome: an experimental study

M. Loiselle[1], S. Pons[1], M. Nouacer[1], S. Placier[2], K. Poussin[1], J. Lion[1], N. Mooney[1], L. Zafrani[3]
[1]INSERM U 1160, Paris, France; [2]INSERM U 1155, Paris, France; [3]Saint-Louis Hospital, Medical ICU, Paris, France
**Correspondence:** M. Loiselle
*Intensive Care Medicine Experimental* 2018, **6(Suppl 2):**0855

**INTRODUCTION.** Tumor lysis syndrome (TLS) is a life-threatening complication in patients with haematological malignancies.TLS-induced acute kidney injury (AKI) is associated with high morbidity and mortality rates (1,2).The mechanisms underlying TLS-induced AKI are not well defined owing to a paucity of studies consisting mainly in case reports. During TLS, cells release their contents, including histones and DNA. Patients with TLS have drastically increased plasmatic levels of extracellular histones compared with patients experiencing post renal AKI, or with patients with acute leukemia without TLS.

**OBJECTIVES.** We aim to evaluate the toxicity of extracellular histones on endothelial function.

**METHODS.** Endothelial cells (human microvascular endothelial cells) were exposed to recombinant histones for different durations (from two hours to 24 hours). Cytokines released by endothelial cells (Interleukin-6 (IL-6), IL-8) were measured by enzyme-linked immunosorbent assay (ELISA). Expression of intercellular adhesion molecule-1 (ICAM-1), vascular cell adhesion molecule-1 (VCAM-1), E-selectin and apoptosis/necrosis were measured by flow cytometry. Expressions of IL-6, ICAM-1 and tissue factor were also quantified by quantitative polymerase chain reaction. We assessed peritubular capillary flow and vascular permeability by intravital microscopy in mice injected with extracellular histones.

**RESULTS.** *In vitro*, high doses (40μg/ml) of extracellular histones induced endothelial cell necrosis. At 24 hours, intermediate doses (20μg/ml) of extracellular histones increased the secretion of IL-6 (p=0.02) and IL-8 (p< 0.001) by endothelial cells, and increased surface expression of ICAM-1 (p< 0.001), but not VCAM-1, nor E-selectin. At two hours, recombinant histones induced a significant increase of IL-6 and tissue factor transcription (114.5±6.8 fold increase normalized to control p=0.005,and 10.9 ±1.45 fold increase normalized to control p=0.006, respectively). At four hours, recombinant histones induced a significant increase of ICAM-1 transcription (117±51.7 fold increase normalized to control p< 0.01). *In vivo*, histones exposure significantly increased Evans blue dye leakage from peritubular capillaries to the interstitial compartment. Moreover, four hours after histones administration, the percentage of capillaries with continuous perfusion was reduced by 57 +/- 6.2% and the percentage of capillaries with no perfusion was increased by 21+/-4.6%.

**CONCLUSIONS.** Circulating histones induce *in vitro* endothelial toxicity and renal microvascular dysfunction in mice. Extracellular histones may be involved in the pathophysiology of TLS-induced AKI. The exploration of signaling pathways activated by histones will offer the opportunity for new therapeutic strategies during TLS.

**REFERENCE(S)**

(1) Darmon M. et al.Br. J. Haematol.(2013)
(2) Treuting P. M, Toxicol. Pathol.(2010)

**GRANT ACKNOWLEDGMENT**

This work has been supported by a grant from the ESICM (Basic Science Award)

### 0856

#### Intensive care patients with haematological malignancy - why are they dying?

L. Russell[1,2], A. Perner[1]
[1]Copenhagen University Hospital, Rigshospitalet, Department of Intensive Care 4131, Copenhagen, Denmark; [2]Zealand University Hospital, Roskilde, Denmark
**Correspondence:** L. Russell
*Intensive Care Medicine Experimental* 2018, **6(Suppl 2):**0856

**INTRODUCTION.** Intensive care patients with haematological malignancy have a high mortality[1,2], but during the last decades, survival in this group of patients has improved, both short-term[1,3]and long-term[4]and increasingly, earlier ICU admission is suggested.[5]Therefore, the number of haematological patients in ICUs is likely to increase in the future. Due to their haematological malignancy, they have an increased risk of severe and potentially lethal complications, such as sepsis, respiratory failure and bleeding.[2,3,6-8]

**OBJECTIVES.** The objective of this study was to elucidate the immediate cause of death a population of critically ill haematological ICU patients.

**MATERIAL AND METHODS.** Retrospective observational study of patients with acute myeloid leukaemia (AML), acute lymphoblastic leukaemia (ALL) and myelodysplastic syndrome (MDS) admitted to the general ICU at Copenhagen University Hospital, Rigshospitalet, between 1st of January 2008 and 31st of December 2012. The follow-up time was two years. The electronic patient files including autopsy reports when present were reviewed and the cause of death according to the treating intensive care and haematologist consultants at the time were obtained.

**RESULTS.** 116 patients were included; the majority were mechanically ventilated (87%) and received vasopressor (82%) and 64 patients (55%) died in the ICU. The 1-year mortality was 86 % and the 2-year mortality was 91%. The most common cause of death in the ICU was septic shock (Table 1); 16 patients died from rapidly progressing septic shock despite maximal treatment, in the remaining 6 patients therapy was withdrawn. The second most common cause of death in the ICU was progressive hypoxemic respiratory failure, causing the death in 10 patients (16%). Two of these patients received ECMO. In 9 patients (14%) active treatment was stopped while in the ICU due to progression of haematological malignancy without possible cure.

The main cause of death in patients discharged alive from the ICU was progression of their haematological malignancy (Table 2). The second most common reason was respiratory insufficiency/pulmonary failure. In this group, 3 patients declined further treatment or readmission to the ICU.

**CONCLUSION.** The most common cause of death in the ICU was septic shock and hypoxemic respiratory failure. The most common reason cause of death in patients who was discharged from the ICU alive was progression in their haematological malignancy where no cure was possible.

**REFERENCES**
1. Azoulay E et al. *J Clin Oncol*. 2013
2. Lengliné E et al. *Leuk Lymphoma*. 2012
3. Schellongowski P et al. *Haematologica*. 2011
4. Tavares M et al. *Leuk Lymphoma*. 2017
5. Azoulay E et al. *Intensive Care Med*. 2017
6. Azoulay É et al. *Medicine*2004.
7. Park HY et al. *Leuk Lymphoma*. 2008
8. Russell L et al. *Ann Intensive Care*. 2017

**Table 1 (abstract 0856).** Cause of death in the ICU (N=64)

| | |
|---|---|
| Septic shock with multiorgan failure | 22 (34) |
| Pulmonary failure 1) | 10 (16) |
| Bleeding 2) | 9 (14) |
| Progression of haematological malignancy 3) | 9 (14) |
| Acute circulatory failure 4) | 6 (9) |
| Invasive fungal infection 5) | 4 (6) |
| Thrombosis 2) | 4 (6) |

Data in No. (%). 1) Hypoxemic respiratory failure despite receiving mechanical ventilation and/or ECMO 2) Bleeding and thrombosis data has previously been published (Russell et al 2017) 3) Active treatment stopped in ICU due to progression of malignant disease without possible cure 4) Acute circulatory failure with cardiac arrest where CPR (cardiopulmonary resuscitatif on) was given. 5) Aspergillus infections in brain, lungs, liver (No=3) and Fusarium infection in GVHD (Graft-versus-host) affected skin

**Table 2 (abstract 0856).** Cause of death in patients discharged from the ICU (N=40) 1)

| | |
|---|---|
| Progression of haematological malignancy 2) | 22 (55) |
| Pulmonary failure 3) | 11 (28) |
| Progressive multiorgan failure due to other reason than septic shock 4) | 2 (5) |
| End-stage liver failure | 2 (5) |
| Acute circulatory failure 5) | 1 (3) |
| Septic shock | 1 (3) |
| Invasive fungal infection | 1 (3) |

Data in No. (%). 1) One patients lost to follow-up 2) Treatment stopped and the patients received only palliative therapy due to progression of malignant disease without possible cure 3) Progressive respiratory failure in patients where either the haematologist did not see indication for ICU therapy or the patient itself declined ICU treatment 4) Cerebral and renal failure 5) Acute cardiac arrest with un-successfull CPR

### 0857

#### Status performance at hospital discharge in critically ill cancer patients who survive after ICU admission

D. Díaz Díaz, M. Villanova Martinez, E. Palencia Herrejon
Hospital Universitario Infanta Leonor, Madrid, Spain
**Correspondence:** D. Díaz Díaz
*Intensive Care Medicine Experimental* 2018, **6(Suppl 2):**0857

**INTRODUCTION.** It has been pointed out a high mortality rate of cancer patients admitted to the intensive care unit (ICU), being the main factors associated with in-hospital mortality: age, acute respiratory failure, the need for mechanical ventilation, late admission to the ICU, the presence of comorbidities, the status performance at admission and tumours in advanced stages. However, it hasn´t been evaluated the status performance at hospital discharge in cancer critically ill patients who survive after ICU admission neither than its predictive factors.

**OBJECTIVES.** To analyse the baseline clinical factors associated to a good status performance at hospital discharge in critically ill cancer patients admitted to an ICU of a community hospital.

**METHODS.** Retrospective observational study including adults with diagnosis of cancer admitted to the ICU, excluding those with previous dependence performance measured by the Eastern Cooperative Oncology Group (ECOG) at admission (scores 3-4), palliative life support decision at UCI, in-hospital death, or no available data from the ECOG score at hospital discharge. We recorded demographic data, department of origin and length of stay prior ICU admission, type of tumour and extent, ECOG score prior to hospitalization and at hospital discharge, reason for ICU admission and severity (SOFA, APACHE-II and SAPS-II) as well as the types of therapies received at ICU.

**RESULTS.** A total of 100 patients (mean age 70.2 years, 64% male) were included. Of them, 64 patients were independent at hospital discharge (ECOG 0-2). They had higher frequency of prior diagnosis of DM (35.1% vs 19.4%; *P*=0.034), better pre-hospital ECOG scores (ECOG 0-1: 73.4% vs. 36.1%; *P*< 0.001), lower frequency of metastatic disease (22.2% vs. 47.1%; *P*=0.011) and of metabolic derangement at ICU admission (4.7% vs. 19.4%; *P*=0.012) and a trend of lower SAPS-III score (median 64 vs. 72; *P*=0.096) without significant differences in sex, age, comorbidity and type of solid or haematological tumour. Neither chemotherapy or radiotherapy prior to admission to the ICU, nor the presence of neutropenia had any significant influence in the status performance at the hospital discharge. The frequency of ICU treatments such as mechanical ventilation, hemodynamic support and kidney replacement therapy was similar in both groups. Logistic regression showed that good prior status performance (ECOG 0-1) (OR 5.2; 95%CI 1.9-13.5; *P*=0.001), non-metastatic tumour (OR 3.0; 95%CI 1.1-8.3) and SAPS-III score at ICU admission (OR 0.9;95%CI 0.92-0,99) were independently related with the odds of good status performance at hospital discharge.

**CONCLUSIONS.** Up to 64% of the critically ill cancer patients who survive after ICU admission at discharge from hospital with good status performance, being the main influencing factors prior good status performance, non-metastatic tumour and SAPS-III score.

### 0858

#### The choice of central venous catheters (CVCs) in patients (pts) undergoing allogeneic hematopoietic stem cell transplantation (allo-HSCT)

M. Spirin, P. Makarova, G. Galstyan, M.Y. Drokov
National Research Centre for Hematology, Moscow, Russian Federation
**Correspondence:** P. Makarova
*Intensive Care Medicine Experimental* 2018, **6(Suppl 2):**0858

**INTRODUCTION.** CVCs are essential in the treatment of pts undergoing allo-HSCT. There are three main types of CVCs: common CVCs (cCVC), antimicrobial-coated CVC (a-cCVCs) and tunneled-cuffed CVC (t-cCVC).

**OBJECTIVES.** The aim of the study was the comparison of three CVC types in allo-HSCT pts.

**METHODS.** 184 allo-HSCT pts were included in retrospective study. The pts suffered from acute myeloid leukemia (105), acute lymphoblastic leukemia (32), non-Hodgkin lymphoma (14), chronic myeloproliferative diseases (8), aplastic anemia (7), chronic lymphocytic leukemia (1), multiple myeloma (1). Before allo-HSCT cCVCs (Certofix Duo 7F, BBraun) were inserted in 35 pts, a-cCVCs (Certofix Protect Duo 7F, BBraun) were inserted in 103 pts and t-cCVCs (Hickman catheters 12 F, BARD Access) were inserted in 46 pts. The indwelling time, the number of the replaced CVCs and the incidences of complications were analyzed. Kaplan-Meier survival analysis curves were used to estimate the probability of CVC removal.

**RESULTS.** The median indwelling times of inserted before allo-HSCT CVCs were: 38 (11-111) days for cCVCs, 36 (8-110) days for c-aCVCs and 104 (18-414) days for t-cCVCs. During 6 months after allo-HSCT for the treatment of the complications (graft-versus-host disease (GVHD), infections, hemorrhage, etc) several of pts needed repeat insers. Incidences of CVC related bloodstream infections (CRBSI) were: 6.3 per 1000 catheter days for cCVCs, 5.3 per 1000 catheter days for a-cCVCs, and 2.4 per 1000 catheter days for t-cCVCs. Incidences of CVC associated thrombosis (CAT) were: 0.97 per 1000 catheter days for cCVCs, 1.9 per 1000 catheter days for a-cCVCs, and 0.49 per 1000 catheter days for t-cCVCs. The reasons for the removal of CVCs were the treatment completion, patient death, CRBSI, CAT and malfunctions.

**CONCLUSIONS.** There were no any advantages of use of a-cCVCs in comparisson with cCVCs. The catheters of choice for allo-HSCT are t-cCVCs.

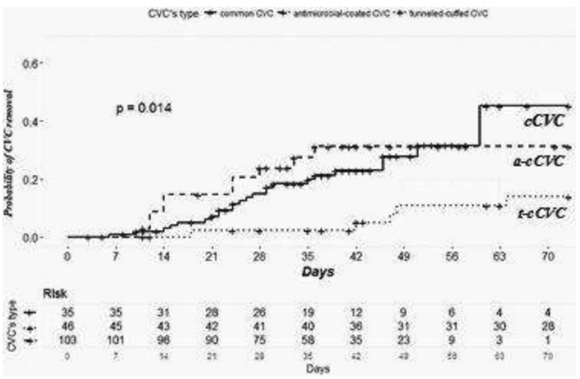

**Fig. 1 (abstract 0858).** The t-cCVCs resulted in significantly better survival as compared with other CVC types (P value<0,5)

### 0859

#### The influence of ICU admission of the patients (pts) with *de novo* acute myeloid leukemia (AML) on the long-term survival

A. Bazhenov[1], G. Galstyan[1], E. Parovichnikova[2], V. Troitskaya[3], O. Polevodova[1], P. Makarova[1], V. Savchenko[2]

[1]National Research Center for Hematology, ICU, Moscow, Russian Federation; [2]National Research Center for Hematology, Moscow, Russian Federation; [3]National Research Center for Hematology, Hematology, Moscow, Russian Federation

**Correspondence:** A. Bazhenov

*Intensive Care Medicine Experimental* 2018, **6(Suppl 2):**0859

**INTRODUCTION.** Induction of the remission of AML patients (pts) often leads to development of life-threating complications requiring ICU admission. Many of these pts have a number of unfavorable prognostic factors related to patient and tumor Moreover, after treatment of complications many pts can not receive a chemotherapy in the complete doses and need its modification need modification of a chemotherapy.

**OBJECTIVES.** To compare the overall and the disease free survival in AML pts who required and did not require ICU admission during a remission induction.

**METHODS.** All *de novo* AML pts (n=80) younger than 60 y.o. admitted in the National Research Center for Hematology from 2013 to 2016 years were enrolled in the study. Patients were divided into 2 groups: pts who were required ICU admission during remission induction (ICU-pts) (n=26) and pts who did not require ICU admission and received chemotherapy only in hematology ward (non-ICU pts) (n=54). The reasons for ICU admissions and results of life support were analyzed. Overall survival (OS) and decease free survival (DFS) were assessed by the Kaplan - Meier method, long rank value $p< 0.05$ consider as significant. Univariate analysis was performed with $\chi$ 2 tests or Fisher's exact tests for categorical variables to find an independent ICU mortality predictor.

**RESULTS.** The admission to ICU was required to 26 (32.5%) of 80 AML pts. ICU pts had more advanced disease than non-ICU pts: median LDH 1056 U/l vs 689 U/l; cytogenetic adverse risk category in 35% of pts vs 18% of pts, hyperleukocytosis in 23% of pts vs 10% of pts. The reasons for ICU admissions were acute respiratory failure (ARF) (50%), intracranial hemorrhage (19.2%), septic shock (SS) (11.5%); caesarian section (11.5%), cardiac arrhythmia (7.8%). Survival rate of pts with ARF who required respiratory support was 53%. Survival rate of SS pts was 25%. Need for mechanical ventilation, vasopressors, ≥ 2 organ dysfunction were predictors of ICU mortality ($p< 0.05$). Overall ICU survival rate was 69.5%. Among non-ICU pts no one died during first 30 days. A landmark analysis for OS was performed for patients who survived the first 30 days of treatment. After the first 30 days there were no significant differences in OS and DFS between the groups. 3 years survival rate was 53% in non ICU group and 51.9% in ICU group (n.s. p=0.946)

**CONCLUSIONS.** 32.5% of de novo AML patients needed intensive care during remission induction. ICU survival rate was 69.5%. After discharging from ICU the long-term prognosis of ICU-pts is not worse, than non-ICU pts.

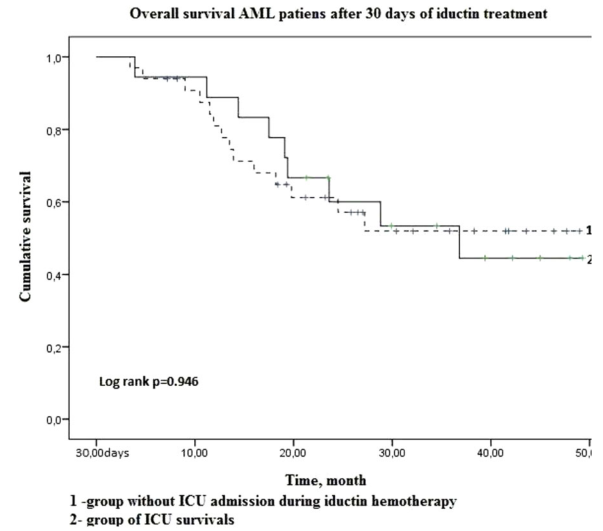

**Fig. 1 (abstract 0859).** Overall survival

### 0860

#### Extracorporeal life support in critical ill immunocompromised adult patients

R. López[1,2], R. Pérez[1,2], J. Graf[1,2]

[1]Clínica Alemana de Santiago, Departamento de Paciente Crítico, Santiago, Chile; [2]Facultad de Medicina Clínica Alemana - Universidad del Desarrollo, Santiago, Chile

**Correspondence:** R. López

*Intensive Care Medicine Experimental* 2018, **6(Suppl 2):**0860

Acute respiratory failure (ARF) is the first reason for admission to ICU in immunocompromised patients and although survival improved markedly in recent years, when need invasive mechanical ventilation remain having significant mortality. Extracorporeal life support (ECLS) depicts one of the ultimate therapies in intensive care and may possibly be beneficial in patients with acute respiratory distress syndrome in general ICU population. Nevertheless, the immunocompromised state has been described as most common relative contraindication to ECLS [1].

Recently, novel data has emerged about ECLS in patients with hematological malignancies [2] and AIDS-related pneumocystis jirovecii [3]. However, limited data is available about the type of ECLS according if the purpose was oxygenation or extracorporeal CO2 removal (ECCO2R).

**OBJECTIVES.** Describe cohort immunocompromised patients on ECLS referral to immunocompetent patients.

**METHODS.** Analysis of cohort of patients supported with respiratory ECLS, with focus on immune status and type of ECLS. Respiratory ECLS was delivered as veno-venous extracorporeal membrane oxygenation (VV-ECMO) if catastrophic respiratory failure, or as extracorporeal CO2 removal (ECCO2R) if ventilation is impaired and could not be optimized keeping protective ventilation. Immunocompetent patients with influenza A H1N1 (H1N1) related ARDS supported with ECLS were considered as reference population to compare both immunocompetent (without H1N1) and immunocompromised patients supported with ECLS.
When was possible, mortality proportions were compared with chi-square.
**RESULTS.** We identified 45 patients with respiratory support, twelve of them (27%) were immunocompromised: hematology malignancies (5), AIDS (1), pulse of steroids and other drugs (6). On other hand, 15 patients were positive to H1N1and the group of patients non-immunocompromised (without H1N1) were 18 patients (60%). Demographic and ECLS support details of immunocompromised patients in table 1 and table 2.
Four patients were diagnosed with pneumocystis pneumonia, two of them died.
We found difference on inhospital mortality only between patients immunocompetent H1N1 versus immunocompetent non-H1N1 on ECLS, 58% vs 20% respectively (p=0.028). Inhospital mortality according to ECLS and ECLS types are showed in figure 1.
**CONCLUSIONS.** A similar survival between immunocompromised and non-immunocompromised patients supported with ECLS was watched. Probably, with more data, immunocompromised state as contraindication to ECLS could be reconsidered. More data about immunocompromised patients on ECLS is required.

**REFERENCE(S)**

[1] Bohman JK, Vogt MN, Hyder JA. Heart Lung. 2016; 45:227-231.
[2] Wohlfarth P, Ullrich R, Staudinger T, et al. Crit Care 2014; doi: 10.1186/cc13701.
[3] Ali HS, Hassan IF, George S. BMC Pulm Med 2016; doi: 10.1186/s12890-016-0214-4.

**GRANT ACKNOWLEDGMENT**
None.

**Table 1 (abstract 0860).** Demographic and ECCO2R data of immunocompromised patients

| ID | Gender | Age (years) | Diagnostic | ECLS type | Time run (days) | Outcome |
|---|---|---|---|---|---|---|
| 1 | Female | 35 | Myeloid Leukemia | AV ECCO2R | 9 | Alive |
| 2 | Male | 53 | Steroids + Methotrexate | AV ECCO2R | 80 | Dead |
| 3 | Female | 27 | Steroids + Cirrhosis | AV ECCO2R | 21 | Alive |
| 4 | Male | 40 | Lymphoma B | AV ECCO2R | 2 | Dead |
| 5 | Female | 29 | Myeloid Leukemia | AV ECCO2R | 2 | Dead |
| 6 | Female | 58 | Steroids + Mycophenolate | VV ECCO2R | 11 | Dead |

**Table 2 (abstract 0860).** Demographic and VV ECMO data of immunocompromised patients

| N | Gender | Age (years) | Diagnostic | ECLS type | Time run (days) | Outcome |
|---|---|---|---|---|---|---|
| 1 | Male | 70 | Steroids | VV ECMO | 10 | Dead |
| 2 | Male | 52 | Neutropenia | VV ECMO | 8 | Alive |
| 3 | Male | 34 | AIDS | VV ECMO | 24 | Alive |
| 4 | Male | 66 | Steroids | VV ECMO | 18 | Alive |
| 5 | Male | 42 | Plasm Cells Leukemia | VV ECMO | 11 | Alive |
| 6 | Female | 54 | Steroids | VV ECMO | 6 | Alive |

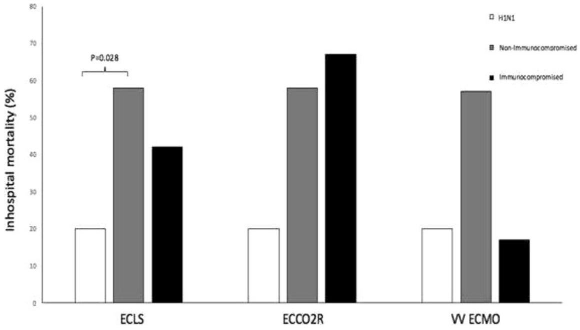

**Fig. 1 (abstract 0860).** Inhospital mortality among immunocompromised, non-immunocompromised and H1N1 patients

### 0861

#### Usefulness of SOFA score as prognosis factor in hematopoietic stem cell transplantation recipients admitted to critical care

I. Romera[1], L. Fox[2], C. Diaz[1], P. Barba[2], M. Santafe[1], S. García[1], M. García[1], M. Pérez[1]
[1]Vall d´Hebron Hospital, Intensive Care Department, Barcelona, Spain; [2]Vall d´Hebron Hospital, Hematology Department, Barcelona, Spain
**Correspondence:** I. Romera

*Intensive Care Medicine Experimental* 2018, **6(Suppl 2):**0861

**INTRODUCTION.** Hematopoietic stem cell transplantation-recipients (HSCT-R) are a population with a high risk of developing severe complications that require admission to an intensive care unit (ICU), which is related to a high mortality. So, efforts are needed in order to improve their prognosis.
**OBJECTIVES.** Our aim was to describe the cohort of HSCT-R admitted to a tertiary hospital ICU and analyze factors that are likely to affect their survival.
**METHODS.** Retrospective study, including adult (+18 years) patients who received an HSCT and required admission to ICU between 1st January 2010 and 28th February 2018. X-Square, Fisher´s test, T test, U Mann-Whitney and logistic regression were employed as required. Quantitative variables are reported as median (IQR) and categorical as frequency (%).

**RESULTS.** Seventy-nine patients were included, 41 (64.1%) men, with a median age of 52 (39-62) years. The underlying leading conditions were leukaemia, with 33 (42%) patients, and lymphoma with 29 (37%). Allotransplant was done in 65 (82%). Seventeen (24%) had received a previous HSCT. APACHE score was 23 (17-28). SOFA score on admission was 9 (7-11). Organ failure, defined as a SOFA score >2, were present as follow: respiratory in 39 (50%), hemodynamic in 37 (47%), haematological in 46 (59%), renal in 12 (15%), liver in 9 (12%) and neurologic in 6 (8%).The most frequent diagnosis at admission were septic shock (45; 57%), pneumonia (35; 44%), and sinusoidal occlusive syndrome (7; 9%). Neutropenia was present in 40 (51%). Sixty (73%) required vasoactive drugs, 49 (62%) mechanical ventilation (MV), 42 (53%) high flow nasal cannula, and 23 (29%) renal replacement therapy (RRT). ICU mortality affected to 46 (58%) and hospital mortality to 59 (75%). APACHE score (OR 1.1, IC95% 1-1.2, p=0.006), SOFA score on admission (OR 1.3 IC 95%:1.-1.5, p= 0.014) and no improvement in SOFA score at 5th day (OR 1.8, IC95% 1.3-2.4, p< 0,0001) were related to mortality. No improvement in SOFA score at 3rd day was not related to mortality (1.6, IC95% 0.5-5.3, p=0.483). Patients with SOFA score > 11 or with more than three organ failures at admission had 100% mortality. Another variables related to mortality were MV (OR=8.8, IC95%:2.7-28.3, p< 0.0001) and RRT (OR 11.3 IC95%: 1.4-90.3, p= 0.022). We did not find any differences related to the type of HSCT or to the presence of neutropenia. In the multivariate analysis, SOFA score on admission (OR1.4 IC95%:1-1.9: p=0.024) and no improvement of SOFA score at 5th day (OR 28.3 IC95% 4-198.8, p=0.001) maintained statistical significance.

**CONCLUSIONS.** SOFA score on admission in HSCT-R admitted to ICU is related to hospital mortality, arriving to 100% in patients on multiorganic failure or SOFA score > 11, so earlier admission is advised. No improvement in SOFA score at 5th day, but not at 3rd day, is related to mortality, which can be an useful tool in the decision making process during an ICU trial.

### 0862

#### Prognostic factors in critically ill oncological patients admitted in a tertiary ICU: the Vall d'Hebron Intensive Care Department/Vall d'Hebron Institute of Oncology cohort (VHIO)

C. Díaz[1], J. Assaf[2], B. Encina[1], J. Ros[2], I. Romera[1], A. Garcia[2], A. Garcia[1], E. Elez[2], R. Ferrer[1]

[1]Vall d´Hebron Hospital, Intensive Care, Barcelona, Spain; [2]Vall d´Hebron Hospital, Oncology, Barcelona, Spain

**Correspondence:** C. Díaz

*Intensive Care Medicine Experimental* 2018, **6(Suppl 2):**0862

**INTRODUCTION.** The number of patients with cancer who receive intensive care treatment is growing. The new therapeutic armamentarium, based on targeted agents and inmunotherapy, have demonstrated to improve patient outcomes but has implied a change in the toxicity profile and a higher rate of complications that require potential ICU admission. However, the prognosis of these patients remains poor with a mortality after ICU admission of around 50%. So efforts are needed in order to improve their outcome.

**OBJECTIVES.** Our aim was to describe the cohort of patients with solid organ cancer admitted to a tertiary hospital ICU and analyze factors that are likely to affect their survival.

**METHODS.** Retrospective study, including adult (+18 years) patients with solid organ cancer who required admission to ICU between 1st January 2010 and 31th December 2017. X-Square, Fisher´s test, T test, U Mann-Whitney and logistic regression were employed as required. Quantitative variables are reported as median (IQR) and categorical as frequency (%).

**RESULTS.** Two hundred and nine patients were included, 110 (52.6%) men, with a median age of 61 (52-69) years. Lung cancer was the most frequent one, being present in 54 (26%), followed by colon 24 (12%), breast (19; 9%) and ovary (15; 7%). The functional status was ECOG 1 in 104 (50%) and ECOG 2 in 83 (40%). The indication for ICU admission was respiratory failure in 80 (39%), septic shock in 74 (36%), post urgent surgery in 18 (9%) and central nervous system involvement in 15 (7%). APACHE score was 20 (17-27) and SOFA score at admission 6 (4-9) Neutropenia was present in 38 (19%). One hundred and thirteen (55%) required vasoactive drugs, 72 (35%) high flow nasal cannula, 102 (49%) mechanical ventilation (MV), and 17 (8%) renal replacement therapy (RRT). ICU mortality affected to 68 (33%) patients and hospital mortality to 96 (46%). Eighty (39%) patients were alive six months after hospital discharge. We identified the following factors related to mortality in the univariate analysis: APACHE score (OR 1.1, IC95% 1-1.2, p=0.001), SOFA SCORE on admission (OR 1.2, IC95% 1.1-1.3, p< 0.0001), MV (OR=2.9, IC95%:1.7-5.2, p< 0.0001), vasoactive drugs (OR=1.9, IC95%:1.1-3.4, p=0.02) and RRT (OR 4.3 IC95%: 1.3-13.5, p= 0.014). We did not find any differences in mortality according to the ECOG status or the presence of neutropenia. In the multivariate analysis, APACHE score (OR=1.1. IC95%:1-1.2, p=0.001), and SOFA score on admission (OR 1.28 IC95%:1.102-1.361: p< 0.001=0.035) maintained statistical significance.

**CONCLUSIONS.** Despite improvement in cancer therapies and critical care management, mortality is still high in oncologic patients admitted to ICU. APACHE score and SOFA score on admission are related to mortality. We did not find that ECOG status or neutropenia influence the hospital outcome of this patients.

### 0863

#### Outcomes of patients with haematological malignancies admitted to a specialist intensive care unit

I. de Asua[1], C. Cosacow[2], G. Antelo[2], C. Brescacin[2]

[1]Royal Tunbridge Wells Hospital, Intensive Care, Tunbridge Wells, United Kingdom; [2]CEMIC University Hospital, Buenos Aires, Argentina

**Correspondence:** C. Cosacow

*Intensive Care Medicine Experimental* 2018, **6(Suppl 2):**0863

**INTRODUCTION.** In many health systems, patients with active haematological malignancies that become acutely unwell are denied admission to intensive care due to the perceived dismal mortality outcomes of critical illness in this patient group. However, in the light of recent therapeutic breakthroughs in the field of onco-haematology, it is worth revisiting the morbidity and mortality outcomes of ICU admission of this type of patient to inform future decision making.

**OBJECTIVES.** To describe the mortality and morbidity outcomes of intensive care admission in patients with active haematological malignancies in our healthcare network.

**METHODS.** We prospectively collected data on patients with active haematological malignancies admitted to our university hospital ICU network over a three year period. We specifically focused on: baseline patient demographics, presence of bacteriemia, degree of organ failure, need for mechanical ventilation and renal replacement therapy, need for vasopressor support, ICU length of stay, ICU, 30 day and 60 day mortality.

**RESULTS.** A total of 148 patients were included in the statistical analysis. Average age was 55 (+/- 16) years and 54% of patients were male. Average ICU length of stay was 7,4 (+/- 4) days and average APACHE II score was 22.2. Regarding baseline disease, 43% of patients had leukemia, 36% had lymphoma, 12% had a plasma cell dyscracia and 9% had myelodysplastic syndrome.

Regarding advanced organ support, 41% of patients required vasopressor support; 57% of patients required mechanical ventilation, and only 15% of patients required renal replacement therapy. Overall ICU mortality was 47%, gross 30-day mortality was 51% and gross 60-day mortality was 52%. Further analysis in pre-established subgroups revealed a higher mortality in the subsets of patients admitted with neutropenic sepsis and respiratory failure.

We conducted a retrospective multivariate analysis searching for variables associated with increased risk of death: variables that reached statistical significance were: APACHE score greater than 22, need for renal replacement therapy, need for vasopressor support and mechanical ventilation.

**CONCLUSIONS.** Although the outlook for critically ill patients with underlying haematological malignant disease remains poor, the updated results we here present suggest an improved global outcome compared to earlier studies. We hope these results will help inform bedside management decisions for this challenging patient group.

**REFERENCE(S)**

Outcomes and prognostic factors in patients with haematological malignancy admitted to a specialist cancer intensive care unit: a 5 yr study. Bird GT, Farquhar-Smith P, Wigmore T, Potter M, Gruber PC. Br J Anaesth. 2012 Mar;108(3)

Outcome and Prognostic Factors of Patients with Hematological Malignancies Admitted to an Intensive Care UnitPak Ling Lui, Rakshya Pandey, Jonathan Tian En Koh, Eng Soo Yap, Amartya Mukhopadhyay and Wee Joo ChngBlood 2016 128:4796

### 0864

#### Hospital mortality associated factors and utility of three severity of illness scores in critically ill solid cancer patients

M.L. Pérez Pérez, B. Balandín Moreno, J. García Sanz, A. Naharro Abellán, P. Matía Almudevar, J. Veganzones Ramos, S. Tejado Bravo, I. Fernández Simón, N. Martínez Sanz, S. Alcántara Carmona

Hospital Universitario Puerta de Hierro, Majadahonda, Spain

**Correspondence:** M.L. Pérez Pérez

*Intensive Care Medicine Experimental* 2018, **6(Suppl 2):**0864

**OBJECTIVES.** To identify mortality associated factors in solid cancer patients admitted to the Intensive Care Unit and to evaluate the utility of severity of illness scores at ICU admission predicting outcome.

**METHODS.** We performed a retrospective study including consecutive patients diagnosed of solid tumour admitted to a polyvalent ICU of a Tertiary University Hospital from January 2010 to November 2016.

We collected demographic, clinical and cancer related data, organ failures, life-supporting therapies and three severity scores: Acute Physiology and Chronic Health Evaluation II (APACHE II), Sequential Organ Failure Assessment (SOFA) and ICU Cancer Mortality Model (ICMM).

**RESULTS.** One hundred and thirty-two patients were included. The patient characteristics and cancer related data are summarized in Table 1. The ICU and hospital mortality rate were 12.9% and 19.7% respectively. The overall ICU mortality rate during the study period was 12.2%. Among demographical and cancer characteristics, the univariate analyses revealed that previous treatment with antineoplastic therapy (radiotherapy and chemotherapy) was related with a worse prognosis ($p < 0.05$). Regarding clinical variables on admission to the ICU, medical reason for admission, 3 or more organ failures and the necessity of life supporting therapies (mechanical ventilation and vasopressors) were significantly associated with hospital mortality ($p < 0.001$). (Table 2)

The median value of APACHE II, SOFA and ICMM was significantly higher ($p < 0.001$) in patients who died than in patients who survived. In a logistic regression analysis, the severity scores APACHE II OR 1.18 (95% CI 1.09 - 1.27), SOFA OR 1.3 (95% IC 1.14 - 148), ICMM OR 1.04 (95% CI 1.02 - 10.7) were independently associated with a higher mortality ($p< 0.01$).

To evaluate the discrimination of the severity scores the Areas Under the Receiver Operating Characteristics Curves (AUC ROC) were calculated: APACHE II: 0.795 (95% CI: 0.690; 0.900), SOFA: 0.77 (95% CI: 0.690; 0.864) and ICMM: 0.794 (95% CI: 0.697; 0.891) showing a good discrimination. The comparison of AUC ROC of the three severity scores after DeLong's test showed no difference between them: AUC ROC SOFA vs APACHE II: 0.56, AUC ROC SOFA vs ICMM: 0.41, AUC ROC APACHE II vs ICMM: 0.78. In the Akaike information criterion (AIC) analysis APACHE II was 108.4, SOFA 112.5 and ICCM 108.5. The one with the lower AIC was the model that represented the prognosis better.

**CONCLUSIONS.** ICU mortality rate in cancer patients is similar to the overall ICU mortality. Hospital mortality was associated with the type of antineoplastic therapy and the type and severity of acute illness. The three severity scores were useful to assess outcome and accurate in the discrimination but we did not find a significant difference between them.

**Table 1 (abstract 0864).** Patients characteristics

| Variable | Number of patients (%) |
|---|---|
| N | 132 |
| Male gender | 81 (61.4) |
| Age (years), median (p25- p75) | 65 (56 - 71) |
| Type of tumor | |
| Gastrointestinal | 49 (37.1) |
| Lung | 32 (24.2) |
| Genitourinary | 20 (11.4) |
| Skin and soft tissues | 10 (7.6) |
| Others [a] | 21 (26) |
| Antineoplastic therapy | |
| Surgery | 78 (59.1) |
| Chemotherapy | 56 (42.4) |
| Radiotherapy | 31 (23.5) |
| State of the disease | |
| Recently diagnosed | 63 (47.7) |
| Stable / Remission | 19 (14.4) |
| Progression | 50 (37.9) |
| Reason at ICU admission | |
| Scheduled surgery | 74 (56.1) |
| Medical | 58 (43.9) |
| Sepsis | 24 (18.2) |
| Acute respiratory failure | 13 (9.9) |
| Hypovolemic and cardiogenic shock | 11 (8.3) |
| Others[b] | 5 (4.6) |
| Organ Failure ≤ 2 | 115 (87.1) |
| Organ Failure ≥ 3 | 17 (12.9) |
| Life-supporting therapies | |
| Mechanical ventilation (yes/no) | 35 (26.5) |
| Vasopressors (yes/no) | 43 (32.6) |
| Renal replacement therapy | 6 (4.5) |
| ICU stay and Hospital stay | 2 (1 – 5) / 12 (8 – 25.7) |
| Severity scores, median (p25 – p75) | |
| APACHE II | 13 (9 -18) |
| SOFA | 4 (1 – 8) |
| ICMM | 30 (16 – 51) |

[a] Breast, central nervous system and head and neck cancer.

**Table 2 (abstract 0864).** Predictive factors of death in hospital using univariate analysis

| Variable | Survivors n= 106 | Non-survivors n=26 | P-value |
|---|---|---|---|
| Male / female | 63 / 81 | 18 / 81 | p 0.35 |
| Age (years), median (p25 – p75) | 65 (57 – 71) | 61.5 ± 10.7 | p 0.81 |
| Type of tumour (%) | | | p 0.33 |
| Gastrointestinal | 39 | 10 | |
| Lung | 25 | 7 | |
| Genitourinary | 10 | 2 | |
| Skin and soft tissues | 9 | 1 | |
| Others [a] | 15 | 6 | |
| Antineoplastic therapy | | | |
| Surgery | 65 | 13 | p 0.29 |
| Radiotherapy | 21 | 10 | **p 0.04** |
| Chemotherapy | 38 | 18 | **p 0.02** |
| Status of the disease (%) | | | p 0.53 |
| Recently diagnosed | 49 (77.8) | 14 (22.2) | |
| Stable / remission | 17 (89.4) | 2 (10.5) | |
| Progression | 40 (80) | 10 (12.9) | |
| Diagnosis at ICU admission | | | p < 0.001 |
| Scheduled surgery | 71(95.9) | 3 (4.05) | |
| Medical | 35 (60.3) | 23 (30.6) | |
| Number of organ failures (%) | | | p < 0.001 |
| ≤2 | 99 (86) | 16 (13.9) | |
| ≥3 | 7 (41) | 10 (58.8) | |
| Life-supporting therapies | | | |
| Mechanical ventilation (yes/no) | 19 / 35 | 16 / 35 | p < 0.001 |
| Vasopressors (yes/no) | 23 / 43 | 10 / 43 | p < 0.001 |
| Renal replacement therapy | 2 / 6 | 4 / 6 | p 0.003 |
| ICU stay | 2 (1 – 4) | 3.5 (1 – 11) | **p 0.005** |
| Hospital stay | 11.5 (8 – 21) | 15.5 (9 – 32) | p 0.21 |
| Severity scores, mean ±SD | | | |
| APACHE II | 12 (8 -15) | 21 (15 – 25) | p < 0.001 |
| SOFA | 4 (1-7) | 7.5 (5 – 11) | p < 0.001 |
| ICMM | 27.55 (15 – 45) | 54.9 (43 – 83) | p< 0.001 |

SD (standard deviation), [a] Breast, central nervous system and head and neck cancer.

### 0865

#### Safety of percutaneous dilatational tracheostomy in patients with hematologic malignancy

J. Lee, S.C. Kim
Seoul St Mary's Hospital, College of Medicine, The Catholic University of Korea1Division of Pulmonary, Allergy and Critical Care Medicine, Department of Internal Medicine, Division of Pulmonary, Allergy and Critical Care Medicine, Department of Internal Medicine, Seoul, Korea, Republic of
**Correspondence:** J. Lee
*Intensive Care Medicine Experimental* 2018, **6(Suppl 2):**0865

**INTRODUCTION.** In patients with hematologic malignancies, acute respiratory failure requiring mechanical ventilation is a severe and frequent complication. Recent studies have shown that the outcome of mechanically ventilated patients with hematologic malignancies over the last decade [1-3].

**OBJECTIVES.** This study conducted to clarify if percutaneous dilatational tracheostomy (PDT) is safe in this group of patients and to report the outcome of patients with hematologic malignancies requiring long-term mechanical ventilation.

**METHODS.** To evaluate the safety of PDT in patients with hematologic malignancies, we retrospectively analyzed the clinical characteristics of patients who underwent PDT in medical ICU from January 2012 to January 2017.

**RESULTS.** PDT was safely performed in all 55 patients. Although 5 (9.1 %) patients developed major bleeding that required electric coagulation, fatal complications were not observed. Of the 55 patients in this study, 21 (38.2 %) could be weaned from ventilator, and 19 (34.6%) survived the intensive care unit stay. There was no other complication such as pneumothorax, subcutaneous emphysema, infection of the stoma, paratracheal insertion or tracheal injuries. No significant differences between survivors and non-survivors were found, except for platelet counts; median platelet counts of non-survivors were significantly lower than those of survivors [48000.0 (30000.0 - 89000.0) vs. 95000.0 (39000.0 - 155500.0), p = 0.028]. No differences were observed in the demographics or laboratory findings between patients with bleeding complication and those with none.

**CONCLUSIONS.** PDT can be safely performed on patients with hematologic malignancies. The procedure did not result fatal complications in this study.

##### REFERENCE(S)

1. Azoulay, E., et al., *Outcomes of critically ill patients with hematologic malignancies: prospective multicenter data from France and Belgium–a groupe de recherche respiratoire en reanimation onco-hematologique study.*J Clin Oncol, 2013. **31**(22): p. 2810-8.
2. Al-Dorzi, H.M., et al., *Characteristics and predictors of mortality of patients with hematologic malignancies requiring invasive mechanical ventilation.*Ann Thorac Med, 2017. **12**(4): p. 259-265.
3. Benoit, D.D., et al., *Outcome and early prognostic indicators in patients with a hematologic malignancy admitted to the intensive care unit for a life-threatening complication.*Crit Care Med, 2003. **31**(1): p. 104-12.

##### GRANT ACKNOWLEDGMENT

None.

### 0866

#### Mortality related with infection at the admission in the intensive care in patients with haematological disease

G. Narváez Chávez, S. Gallego, J. Higuera, C. Álvaro, D. Cabestrero, C. Soriano, L. Jaramillo, V. Quinteros, R. De Pablo
Ramon y Cajal University Hospital, Intensive Care Medicine, Madrid, Spain
**Correspondence:** G. Narváez Chávez
*Intensive Care Medicine Experimental* 2018, **6(Suppl 2):**0866

**INTRODUCTION.** The aim of this study is to asses the impact of infection as a cause of admission in the intensive care, the mortality related with the infection origin and the microorganism isolated at admission of patients with haematological disease.

**METHODS.** An observational and retrospective study was conducted, which reviewed all patients were admitted into the Intensive Care Unit (ICU) of a University Hospital, with haematological diseases between May 2013 to March 2018.

We analysed with Chi square ($X^2$) the following variables: age, SOFA, APACHE II; SAPS II; haematological disease, cause of admission, focus of infection and microorganism isolated in the samples taken at the admission and their relation with ICU mortality.

**RESULTS.** A total of 207 patients met inclusion criteria, with an average age of 55.82±14.75 years. 59% were male and 41% were female. ICU mortality rate was 39.6% (82 patients).

160 patients (77%) the admission was related to an infection, 61 patients were community-acquired infections (38.12%) and 48.3% (99 patients) health care-associated infection, with mean severity score SOFA: 9.54±4.35, APACHE II 23.21±9.76 y SAPS II 52.59±21.17.

Respiratory infection was the most frequent (40.6%), followed by bacteraemia without an identified focus 13.12%, bacterial translocation from gastrointestinal tract (BT-GIT) 10.62%, urinary tract infection (UTI) 8.12% and catheter related infection 6.87%. In 14.37% of cases it was not possible to determine the infectious focus.

After evaluation of common sources of sepsis with the samples taken at the admission, we found that the microorganism most frequently involved was bacterial in 70 samples (43.75%), virus in 25 samples (15.62%) and fungal in 8 samples (5%). Five patients had multiple isolates. In 62 patients (30%) a microorganism wasn´t isolated.

Using $X^2$ we found relation between ICU-mortality and admission by infectious disease (p≤-0.001) equal as health care-associated infection (p=0.004) and respiratory infection (p≤-0.0001).

Applying the same test, we didn´t found increased risk of mortality with community-acquired infections (p=0.757), bacteraemia without an identified focus (p=1), catheter related infection (p=0.11), UTI (p=0.08), BT-GIT (p=0.8), and sepsis without identified focus (p=0.82).

Analysing microorganisms, we found only a strong trend in viral infection (p=0.08) and no correlation with bacterial infection (p=1), and fungal infection (p=0.26), including in the cases in which there was no possible to isolate a microorganism (p=0.21).

**CONCLUSIONS.**

- There is no increase in mortality with community-acquired infection in patients with a haematological disease.
- Although infection related to health care increases mortality, no significant relationship was found when the focus of the infection or the microorganism of the infection could not be located.
- There is no increased risk of mortality at bacterial infection aetiology.

### 0867

#### Infection impact as cause of admission in intensive care in patients with bone marrow transplant in a university hospital

G. Narváez Chávez, S. Gallego, J. Higuera, A. Caballero, D. Cabestrero, C. Soriano, L. Jaramillo, M. López, R. De Pablo
Ramon y Cajal University Hospital, Intensive Care Medicine, Madrid, Spain
**Correspondence:** G. Narváez Chávez
*Intensive Care Medicine Experimental* 2018, **6(Suppl 2):**0867

**INTRODUCTION.** The aim of this study is evaluate the factors that increase mortality in patients who received a bone marrow transplant (BMT) when are admitted to Intensive Care (ICU) due an infectious pathology and their relation with ICU mortality and 30-day and 60 day mortality.

**METHODS.** We reviewed all the patients with BMT who required admission to the Intensive Care between May 2013 to March 2018.

The variables analysed: type of BMT, severity scores: SOFA; SAPS II and APACHE II, presence and type of infection objectified, microorganism isolated, neutropenia and mortality during ICU stay and mortality at 30 days and 60 days.

**RESULTS.** 79 patients (pts) were admitted to the ICU during the observational period. 52% were men and 48% were female, with average age 51.32 ± 12.27 years. Mean severity scores were SOFA: 10.51±4.22, APACHE II 23.63±9.4 y SAPS II 52.07±1.26.
68 pts (86%) had received an allogenic transplant and 11 patients (14%) had received an autologous transplant. 30 pts (38%) had neutropenia (< 500 cells) at admission. 29 pts (36.70%) were admitted to the ICU in the first 30$^{th}$ days after BMT.
71 pts (90%) were admitted due an infection disease, health care-associated infections were present in 51 pts (72%). UCI mortality was 46.8% (37 pts), 30 days mortality was 14% (6 pts) and 60-day mortality was 8% (3 pts).
Respiratory infection was the most frequent in 35.4% (28 pts) followed by bacteraemia without an identified focus 15%, catheter related infection 9%, bacterial translocation from gastrointestinal tract (BT-GIT) 6.3%, and urinary tract infection (UTI) 6.3%, We couldn´t determinate the infectious focus in 11.4%.
The microorganism isolated in the samples taken at admission were: bacterial in 31 samples (39%), virus in 17 samples (22%) and fungal in 2 samples (2.5%). Two patients had multiple isolates. In 23pts (30%) a microorganism wasn´t isolated.
Using Chi square ($X^2$) we found a significant relation between ICU-mortality and respiratory infection (p=0.002). When we analysed the relation between neutropenia and the others variables, there was only significant relation with respiratory infection ((p=0.02).
There wasn´t significant relation between ICU-mortality and health care-associated infection (p=0.35), admission in the first 30-day after BMT (p=0.35), neutropenia (p=0.58), sepsis without identified focus (p=0.58), bacterial infection (p=0.82), viral infection (p=0.19) and fungal infection (p=0.21) and sepsis without isolated microorganism (p=0.44).
**CONCLUSIONS.**

- The infectious disease is the most common cause of admission in the ICU after a BMT.
- BMT ICU patients and Neutropenia are related with respiratory infection.
- There is significant relation between respiratory infection and ICU-mortality.
- Although the antibiotic prophylaxis used in patient with BMT, there is no increase mortality in those patient with sepsis without focus and in those cases of sepsis without a microorganism isolated.

### 0868

#### Predictive factors for mortality in cancer patients undergoing chemotherapy in the intensive care unit: an analysis of 222 cases

N.C. Santos Neto, G. Oliveira, S. Rizk, C. Park, A.P. Slovinski, R. Nakamura, M. Mourao, J.T. Fukushima, P.S. Toledo, L.A. Torres, A.C. Barrera, J.P. Almeida, L.A. Hajjar
Instituto do Cancer do Estado de Sao Paulo, Sao Paulo, Brazil
**Correspondence:** L.A. Hajjar
*Intensive Care Medicine Experimental* 2018, **6(Suppl 2):**0868

**BACKGROUND.** Over the last years, advances in cancer treatment resulted in increased rates of intensive care unit (ICU) admission. In many cases, due to extensive disease and related complications, chemotherapy is prescribed during ICU stay in critically ill cancer patients. Literature is controversy about the efficacy of chemotherapy in these patients, mainly because of the related high rates of mortality.
**OBJECTIVES.** The aim of this study is to analyze predictors of hospital mortality in cancer patients receiving chemotherapy during ICU stay.
**METHODS.** From November 2012 to December 2017, 19,500 patients were admitted to the ICU of Cancer Institute of State of Sao Paulo, a 84-bed ICU of a tertiary referral center linked to University of Sao Paulo. Medical records were retrospectively reviewed by a group of 4 experienced physicians in critical oncology, using a specific database.
**RESULTS.** A total of 222 patients received chemotherapy and were included in the study. The median age was 47 years (31-62). There were 160 (72%) patients with hematological malignancy and 62 (28%) with solid tumors. Most patients had metastatic disease (89%) and 35% of patients have received previous chemotherapy. Septic shock was the main cause of admission, diagnosed in 79 patients (35%). Hospital mortality was 43%. Most patients received chemotherapy in the first 48 hours of ICU admission. The most frequently prescribed chemotherapy drug was doxorubicin. A univariate analysis showed that among non-survivors, the incidence of vasoplegic shock as cause of admission was significantly higher when compared to survivors (53% vs. 22%, P< .001), the requirement for norepinephrine was greater (40% vs. 12%, P< .001) such as the requirement for mechanical ventilation (15.7% vs. 8.9%, P=0.04), SOFA was higher [4 (2-6) vs. 2 (0-4), P< .001]. Creatinine, C-reactive protein and lactate were higher in non-survivors [(1.49 mg/dL vs. 1.09, P=0.03), (42 mg/dL vs. 85, P=0,03), (23 mg/dL vs. 18, P=0.002). The platelets count (132500 vs. 206500, P=0.004) and the ratio PaO2/FiO2 (300 vs. 400, P< 0.001) were lower in non-survivors. In the multivariate analysis, the presence of vasoplegic shock [OR 1.6 (95% CI 1.04-2.45), P=0.01], higher levels of creatinine [OR 1.14 (1.03-1.27), P=0.01], lactate [OR 1.00 (1.0001-1.004), P< 0.001], C-reactive protein [OR 1.02 (1.01-1.03), P< 0.00 ], and lower levels of platelets [OR 0.97 (0.96-0.99), P=0.003], and PaO2/FiO2 [OR 0.99 (0.98-0.99), P< 0.001] were identified as risk factors for hospital mortality.
**CONCLUSIONS.** Chemotherapy in the ICU is usually prescribed in high risk patients with life-threatening disease. However, to decrease mortality rates, we should consider not prescribing chemotherapy during ICU stay in patients with vasoplegic shock, or presenting with higher levels of creatinine, lactate and C-reactive protein or with decreased levels of platelets and of PaO2/FiO2.

### 0869

#### Clinical outcomes and microbiological characteristics of severe infection in critically ill cancer patients

D. Díaz Díaz[1], M. Vilanova Martinez[2], L. Lopez Perez[2], B. Bueno Garcia[2], E. Palencia Herrejón[2]
[1]Hospital Universitaire Infanta Leonor, Intensive Care, Madrid, Spain; [2]Hospital Universitaire Infanta Leonor, Madrid, Spain
**Correspondence:** D. Díaz Díaz
*Intensive Care Medicine Experimental* 2018, **6(Suppl 2):**0869

**INTRODUCTION.** Cancer patients have an increased risk for hospital-acquired infections including severe sepsis, which is a frequent cause of ICU admission and of high mortality.
**OBJECTIVES.** To identify the most frequent type of infection as well as clinical and microbiological characteristics in critically ill oncological patients.
**METHODS.** Retrospective observational study (2011-2016) including adult cancer patients who were admitted to the ICU at a community hospital due to suspected infection and who stayed at the ICU for more than 24 hours. We reviewed clinical charts and collected the following data: age, sex, department of origin and length of stay prior the admission to the ICU, type of tumour, extent, score at the Eastern Cooperative Oncology Group (ECOG) scale, type of infection suspected, microbiological data, type of therapy received at ICU and factors associated to in-hospital mortality.
**RESULTS.** A total of 167 cancer patients were admitted to the ICU during the study period and 94 of them ICU admission was due to clinical suspicion of infection. The average age was 70.6 (SD 10.2), male sex 61.7%. Up to 76,6% presented a solid tumour and 23,4 an hematologic cancer. The 44,7% had metastases at ICU admission, 39.4% were in progression of the active tumour, 23.4% were in partial or complete remission, 19.1% were in diagnostic or induction phase and only 18.1% were in stable condition. They had received chemotherapy in 57.4% of the cases, radiotherapy 23.4%, hormone therapy 5.3%, bone marrow transplantation 2.1% and immunotherapy 1.1%. The severity scores at ICU admission were: SOFA (median 7; Interquartile range-IQR 5-9), APACHE II (median 20; IQR16-25), SAPS II (median 73.5; IQR 64,75-81). The most frequent sites of infection were the lungs (35,1%), intraabdominal region (31,9%), urinary tract (11,7%), bloodstream as primary infection (9,6%), and in up to 6.4% no infectious focus was found. The main microorganisms involved were Gram-negative bacilli (27.7%), followed by Gram-positive cocos (11.7%), polymicrobial flora (11.7%) and fungi (7.4%). Pathogens were not isolated up to 28.7%. Up to 75.5% developed septic shock and invasive mechanical ventilation was necessary in 37,2% of the cases. In-hospital mortality was 43.6%, being the associated factors the status performance at admission (ECOG 0-1: 35%, ECOG 2: 42.9%, ECOG 3-4: 75%, *P*=0.049), extent of tumour (metastatic 54% vs. 35.5% locoregional extension, P=0.047) severity scores (SAPS III: *P*=0.013; APACHE II: *P*=0,039; SOFA: *P*< 0.001), the development of septic shock (P=0.039) and the need for invasive mechanical ventilation (*P*< 0.001).

**CONCLUSIONS.** Infection is a common cause of critical illness in patients with cancer and remains associated with high mortality. The main factors related with in-hospital mortality were the status performance to admission, extent of tumour, severity scores at ICU admission, the development of septic shock and the need for invasive mechanical ventilation.

### 0870

#### Bacterial infection in cirrhotics: comparison of diagnostic and prognostic values of some biomarkers

N. Foudhaili, S. Khedher, A. Khaled, C. Abdennebi, M. Salem
Charles Nicolle Hospital, Digestive Intensive Care Unit, Tunis, Tunisia
**Correspondence:** N. Foudhaili
*Intensive Care Medicine Experimental* 2018, **6(Suppl 2):**0870

**INTRODUCTION.** Bacterial infections are very common and represent a major cause of mortality in patients with cirrhosis and major challenge for physicians.

**OBJECTIVES.** The aim of this study was to compare diagnostic and prognostic value of White blood cells count (WBC) , c-reactive protein (CRP) and procalcitonin (PCT) serum levels, platelet to lymphocyte and neutrophil to lymphocyte ratios (PLR and NLR) in cirrhotics with bacterial infection.

**METHODS.** It was a 1-year prospective study from January 2017 to December 2017. We enrolled patients with decompensated cirrhosis. Biological markers were obtained upon admission (one year). They were analyzed in terms of diagnosis performance and prediction of intrahospital mortality using the Receiver Operating Characteristic (ROC) curves analysis.

**RESULTS.** Eighty-seven patients were included with a sex-ratio at 0.95 and a mean age of 62±13 years (16-87). The main comorbidities were diabetes mellitus (38%) and hypertension (32%). Hepatitis C was the most common etiology of cirrhosis (39%). Child-Pugh score was mainly C (70%). Bacterial infection was diagnosed in 58 patients (66%). Identified types of infections included pneumonia (52%), spontaneous bacterial peritonitis (18%), urinary tract infection (13%), bacteremia (11%) and soft tissue infection (6%). Sepsis was diagnosed in 40 patients (46%) and septic choc in 7 patients (8%). Among 18 isolated germs, 14 were gram-negative bacteria, and 4 gram-positive bacteria. The most frequently isolated germ was Escherichia coli (11/18). Resistant organisms were isolated in 13 patients and mulitresistant organisms in 3 patients. Patients with bacterial infection had significantly higher neutrophile count (p=0.003), higher serum CRP level (p< .001), higher serum PCT level (p=0.029) and similar PLR (p= 0,799) and NLR (p = 0.055). ROC analysis of CRP serum level showed the highest AUC with a value of 0.858 (95%CI 0.766-0.949, p < .001). Infected patients with and without unfavorable outcome (death) had similar demographics characteristics. Mortality in patients with bacterial infection was 17.2%. They had significantly higher WBC count (p=0.033) and higher neutrophile count (p=0.045). ROC analysis of WBC count showed the highest AUC with a value of 0.694 (95%CI 0.532-0.856, p=046).

**CONCLUSIONS.** In cirrhotics with bacterial infection, CRP shows higher diagnostic accuracy than other biomarquers while WBC count has much stronger prognostic value.

### 0871

#### Are antibiotics a cause of sepsis-induced immunosuppression?

M. Khpal, N. Arulkumaran, M. Singer
UCL, Bloomsbury Institute of Intensive Care Medicine, London, United Kingdom
**Correspondence:** M. Khpal
*Intensive Care Medicine Experimental* 2018, **6(Suppl 2):**0871

**INTRODUCTION.** Patients who recover from sepsis develop immunosuppression and are at risk of opportunistic infections[1.] These are relatively common in patients with more severe sepsis and/or win those with a transcriptomic profile consistent with immune suppression at the onset of secondary infection[2]. Factors well known to induce immune cell dysfunction include sedatives and antibiotics[3.] While antibiotics are necessary for the management of infection, prolonged use may result in immunosuppression. The underlying mechanisms of antibiotics-induced immune suppression in sepsis has not been elucidated.

**OBJECTIVES.** To determine whether commonly used classes of antibiotics alter cytokine release of immune cells *ex vivo*.

**METHODS.** Whole blood assays were performed on fresh samples taken from healthy male volunteers (n=4-7, aged 20-37 years). A range of commonly used antibiotics (Figure 1) were incubated with the blood at clinically relevant doses (minimum inhibitory concentrations; MIC, 5 mcg/ml), and simultaneously spiked with 10 mcg/ml *Klebsiella pneumoniae*lipopolysaccharide. At 6 and 24 hours, serum was isolated and assessed for cytokine release using ELISAs (Fig 1). Data are presented as ratio of cytokines from LPS + antibiotic-treated blood relative to LPS-treated blood alone. The Kruskal-Wallis test was used to assess differences in cytokine ratios between groups. Statistical analysis was performed using Prism software.

**RESULTS.** At 6 hours, TNF-alpha was suppressed by most antibiotic groups (p< 0.001). The reduction in IL-6 production did not reach statistical significance (p=0.12). There was no change in the anti-inflammatory cytokine, IL-10 at 24 hours (p=0.64) (Fig 1c); none was measurable at 6 hours.

**CONCLUSIONS.** Commonly used classes of antibiotics demonstrated direct effect on cytokine release in immune cells incubated with LPS. Pro-inflammatory TNF-alpha release was significantly reduced, but no effect was seen in anti-inflammatory IL-10. Further work is required to clarify the underlying mechanisms and its clinical relevance to septic patients.

**REFERENCE(S)**

1. Van Vught LA, *et al.*Incidence, Risk Factors and Attributable Mortality of Secondary Infections in the Intensive Care Unit After Admission for Sepsis. *JAMA.* 2016;315(14):1469-1479.
2. Hoogendijk AJ, *et al.*Sepsis Patients Display a Reduced Capacity to Activate Nuclear Factor-kappaB in Multiple Cell Types. *Critical Care Medicine.* 2017;45(5):e524-e531.
3. Anuforom O, *et al.*The immune response and antibacterial therapy. *Medical Microbiology and Immunology.*2015;204(2):151-159.

**GRANT ACKNOWLEDGMENT**
M.K and N.A- Salary/Support from UK NIHR

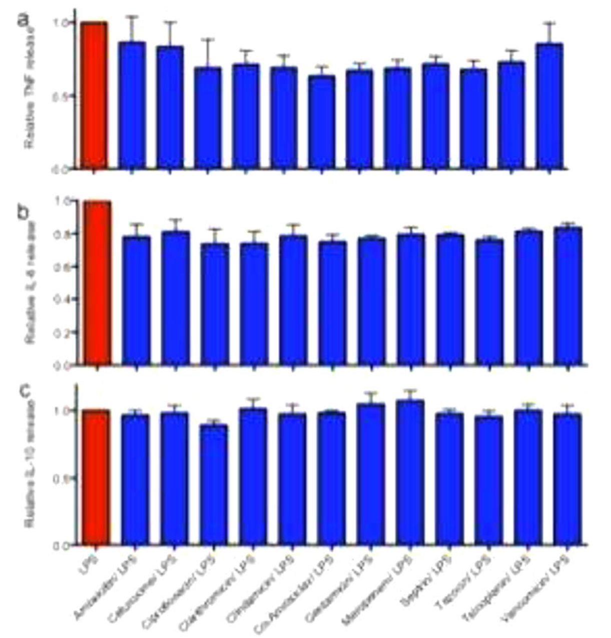

**Fig. 1 (abstract 0871).** Relative cytokine release (a) TNF-alpha release; (b) IL-6 release; (c) IL-10 release

## A focus on sepsis therapeutics

### 0872

#### The effect of vitamin C on mechanically ventilated patients with severe sepsis and septic shock: a retrospective cohort study

J.H. Ahn, D.K. Oh, J.W. Huh, C.-M. Lim, Y. Koh, S.-B. Hong
University of Ulsan College of Medicine, Asan Medical Center, Department of Pulmonary and Critical Care Medicine, Seoul, Korea, Republic of
**Correspondence:** J.H. Ahn
*Intensive Care Medicine Experimental* 2018, **6(Suppl 2):**0872

**INTRODUCTION.** Vitamin C has been shown to have several beneficial effects against sepsis in pre-clinical studies. It could help ameliorate sepsis-induced endothelial dysfunction, synthesize vasopressin and catecholamines, and modulate immune function [1]. However, clinical data supporting efficacy of vitamin C by itself in patients with sepsis are scarce.

**OBJECTIVES.** The aim of this study was to evaluate therapeutic effects of intravenous vitamin C on mechanically ventilated patients with severe sepsis and septic shock.

**METHODS.** Adjuvant intravenous vitamin C therapy has been applied to some patients with sepsis in our institution at an attending physician´s discretion since 2015. For this retrospective cohort study, consecutive patients with severe sepsis or septic shock who were admitted to the medical ICU and required mechanical ventilation from January to July 2017 were screened. Eligible patients were classified into a vitamin C or control group depending on the administration of intravenous vitamin C (2 g per 8 hours for 2 or more days). The primary outcome was hospital mortality. The secondary outcomes included ICU mortality, time to shock reversal, duration of initial mechanical ventilation, ICU length of stay, and changes in sequential organ failure assessment (SOFA) score over the first 72 hours.

**RESULTS.** Among 102 patients admitted to the ICU with sepsis, 75 mechanically ventilated patients with severe sepsis or septic shock (35 in the vitamin C group and 40 in the control group) were included. Baseline characteristics of both groups were similar except the positive rate of blood culture (20% versus 50%, $P$ = 0.03). The hospital mortality in the vitamin C group was 46% (16 of 35), which was not significantly different from 40% (16 of 40) in the control group (odd ratio, 1.26; 95% CI, 0.50 to 3.30; $P$ = 0.62). The median time to the reversal of shock was 3 days (interquartile range, 2 to 5) in both groups ($P$ = 0.28). In the vitamin C group, the SOFA score was decreased by 1.4 ± 3.3 over the first 72 hours, compared to 1.4 ± 3.0 in the control group ($P$ = 0.96). In terms of ICU mortality (34% versus 30%, $P$ = 0.69), ICU length of stay (median 10 days [interquartile range, 6 to 19] versus 11 days [interquartile range, 7 to 35], $P$ = 0.13), and duration of initial mechanical ventilation (median 8 days [interquartile range, 5 to 17] versus 8 days [interquartile range, 4 to 19], $P$ = 0.30), there were no significant differences between vitamin C and control groups.

**CONCLUSIONS.** In this study, the adjuvant use of intravenous vitamin C in patients with severe sepsis or septic shock requiring mechanical ventilation neither reduced hospital mortality nor improved other ICU outcomes.

**REFERENCE(S)**

1. Teng J, Pourmand A, Mazer-Amirshahi M (2018) Vitamin C: The next step in sepsis management? Journal of critical care 43: 230-234

**GRANT ACKNOWLEDGMENT**
None.

### 0873

#### The effect of magnesium sulphate on cerebral perfusion in patients with sepsis-associated encephalopathy

N. Adel Shaban, T. Abdullah Helmy, S. Mohammed Al-Awady, D. Hassan Zidan
Faculty of Medicine, Alexandria University, Department of Critical Care, Alexandria, Egypt
**Correspondence:** N. Adel Shaban
*Intensive Care Medicine Experimental* 2018, **6(Suppl 2):**0873

**INTRODUCTION.** Magnesium sulphate has been shown to exhibit neuroprotective effects on the brain in experimental models of sepsis.

**OBJECTIVES.** To assess the effect of an intravenous bolus dose of magnesium sulphate on cerebral perfusion using flow velocities in the middle cerebral artery in patients presenting with sepsis-associated encephalopathy, and correlate between these findings and the neurological outcome.

**METHODS.** 46 patients with sepsis-associated encephalopathy were assessed. All patients presented with a Glasgow Coma Score (GCS) less than 15 and a positive score for delirium as evaluated using the Confusion Assessment Method for ICU (CAM-ICU). These patients weredivided into two groups (Group A= Magnesium group and Group B= Control group). The mean flow velocity in the Middle Cerebral Artery (VmMCA , cm/sec) was assessed with transcranial Doppler ultrasonography (TCD) and the pulsatility index (PI) and resistive index (RI) were calculated for both groups during the first 6 hours following admission, as well as 24 hours afterwards. For group A, additional TCD readings were also obtained 30 minutes after a 6 gram intravenous dose of magnesium sulphate. Results were correlated with the neurological status of the patients 24 hours after admission.

**RESULTS.** After 24 hours, the pulsatility index was significantly reduced in the Group A (1.09 ± 0.22 $p < 0.001$) as well as the resistive index (0.62 ± 0.07 $p < 0.001$). Mean flow velocity was significantly higher in the magnesium group after 24 hours (49.81 ± 16.24 $p < 0.001$). Group A displayed a significant improvement in the mean GCS (11.65 ± 1.99 p=0.048) and CAM-ICU score (negative CAM-ICU = 16/23 patients, p =< 0.001)

**CONCLUSIONS.** Results suggest that the administration of magnesium sulphate during the first 24 hours of the onset of sepsis seems to improve cerebral perfusion in patients with sepsis-associated encephalopathy and possibly correlates with better neurological outcomes in these patients.

**REFERENCE(S)**

1. Eidelman LA, Putterman D, Putterman C, Sprung CL. The Spectrum of Septic Encephalopathy: Definitions, Etiologies, and Mortalities. JAMA. 1996 Feb 14;275(6):470-3.
2. Esen F, Erdem T, Aktan D, Orhan M, Kaya M, Eraksoy H, et al. Effect of magnesium sulfate administration on blood-brain barrier in a rat model of intraperitoneal sepsis: a randomized controlled experimental study. Crit Care. 2005;9(1):R18-23.
3. Belfort MA, Moise KJ. Effect of magnesium sulfate on maternal brain blood flow in preeclampsia: a randomized, placebo-controlled study. Am J Obstet Gynecol. 1992 Sep;167(3):661-6.

### 0874

#### Administration of chemotherapy to patients with hematologic malignancies admitted to the ICU with sepsis

D. Shaz, S. Pastores, N. Kostelecky, W. Zhou, N. Halpern
Memorial Sloan Kettering Cancer Center, Anesthesia Critical Care, New York, United States
**Correspondence:** D. Shaz

*Intensive Care Medicine Experimental* 2018, **6(Suppl 2):**0874

**INTRODUCTION.** Administration of chemotherapy to a patient with sepsis or septic shock is expected to yield a high risk for mortality.

**OBJECTIVES.** Identify risk factors for mortality among patients receiving chemotherapy for hematologic malignancies who were admitted to the intensive care unit (ICU) with sepsis.

**METHODS.** Retrospective single institution analysis of patients who were admitted to an oncologic ICU from 2010 to 2015 with a diagnosis of sepsis and received chemotherapy during their ICU stay. This is a subgroup analysis of prior work focusing on the patients with sepsis (ref.). Demographic (age, gender) and clinical data were collected including type of hematologic malignancy, severity of illness on ICU admission, Sequential Organ Failure Assessment (SOFA) score (on chemotherapy administration day), timing of chemotherapy in relation to ICU admission, source of infection, microbiologic data, use of mechanical ventilation, vasopressors and renal replacement therapy and ICU, hospital and 12-month outcomes.

**RESULTS.** Of 181 hematologic cancer patients who received chemotherapy in the ICU, 60 patients (33%) were admitted with sepsis as the primary or secondary diagnosis. ICU mortality was 35% and Hospital mortality was 58%. The mean time of chemotherapy administration from day of ICU admission was 2.2 days, which was similar among survivors and non-survivors. Risk factors for mortality were SOFA score on day of chemotherapy administration ($p< 0.001$), use of mechanical ventilation ($p=0.004$) and vasopressors ($p=0.007$) for ICU mortality.

**CONCLUSIONS.** Higher SOFA score on day of chemotherapy administration, use of mechanical ventilation and vasopressors are major risk factors for mortality among hematologic cancer patients admitted to the ICU with sepsis and receiving chemotherapy.

**REFERENCE(S)**

Pastores S, et. al. Characteristics and Outcomes of Patients with Hematologic Malignancies Receiving Chemotherapy in the Intensive Care Unit. *Cancer*. in press.

**GRANT ACKNOWLEDGMENT**

Core Grant (P30 CA008748) and the Department of Anesthesiology and Critical Care Medicine, Memorial Sloan Kettering Cancer Center, New York, NY

### 0875

#### Clinical outcomes of septic patients with elevated cardiac troponin in Manila Doctors Hospital

O.F. Listanco[1], J.V. Galang[1], G. Mercado[1], K. Panugayan[1], F.E. Punzalan[2]
[1]Manila Doctors Hospital, Department of Internal Medicine, Manila, Philippines; [2]Philippine General Hospital, Department of Internal Medicine, Manila, Philippines
**Correspondence:** K. Panugayan

*Intensive Care Medicine Experimental* 2018, **6(Suppl 2):**0875

**INTRODUCTION.** The role of troponin I in risk stratification of sepsis is still debated. The study aims to determine the incidence of elevated troponin I among septic patients and its association with clinical outcomes and complications.

**OBJECTIVES.** To determine the association of levels of troponin I and clinical outcome among patients admitted for sepsis. Specific objectives are the following:

(1) to determine the incidence of elevated troponin I among septic patients;
(2) to determine the association between elevated troponin I and clinical outcomes among septic patients in terms of: a. mortality, b. use of inotropes and vasopressors, c. initiation of hemodialysis and d. use of mechanical ventilation

**METHODS.** The study includes all admitted septic patients with Troponin I levels determinations within 48 hours of admission from January 2014 to December 2016 in Manila Doctors Hospital. The charts were reviewed using questionnaires for sepsis, general characteristics, secondary outcomes and mortality.

**RESULTS.** The incidence of elevated troponin I was 65.670%. Patients with elevated troponin I levels were older (p0.042), have chronic kidney disease (p0.039), higher qSOFA score (p0.001), higher APACHE score (p0.0), and lower eGFR (p0.003). Using multivariate regression analysis, age (p0.045), presence of CKD (p0.033), qSOFA (p0.001) and APACHE scores (p0.0) were significantly associated elevated Troponin I levels. There was significant difference in mortality rate between elevated and non-elevated troponin I (p0.020) and in elevated troponin I, the likelihood of use of inotropes (p0.038) and mechanical ventilator (p0.049) were higher.

**CONCLUSIONS.** Elevated troponin I level is common among septic patients. The sensitive Troponin I in all septic patients is associated with increase in mortality.

**REFERENCE(S)**

1. Martin, G. (2012). Sepsis, severe sepsis and septic shock: changes in incidence, pathogens and outcomes. Expert Rev Anti Infect Ther
2. Agewall, S., Giannitsis E., Jernberg,T., & Katus, H., (2011). Troponin elevation in coronary vs non coronary disease. European Heart Journal
3. Jha, G., (2017). A Study on Quick Sofa Score as a Predictive Tool of Severity in Emergency Department Patients. Journal of Medical Science and Clinical Research.
4. Wagner D., & Draper, E. (1984). Acute Physiology and Chronic Health Evaluation and Medicare reimbursement. Annual Supplement.

**GRANT ACKNOWLEDGMENT**

This paper has become a reality with the help of many individuals. First and foremost, to the unwavering support and guidance of Felix Eduardo Punzalan, MD, our adviser who gave as many insights and comments that help us to improve our paper. We also thank the Manila Doctors Hospital Records Department unit for the provision of necessary information and regarding this research output. We also thank our statistician Sir Jundel for sharing his knowledge on technicalities. Lastly, we would like to thank our family for the spiritual and emotional support.

### 0876

#### Effect of fluid resuscitation with Ringer lactate versus acetate solution on correction of metabolic acidosis in critically ill patients

N. Rawat, N. Sahni, L. Yaddanapudi
Postgraduate Institute of Medical Education and Research, Anaesthesia & Intensive Care, Chandigarh, India
**Correspondence:** N. Sahni

*Intensive Care Medicine Experimental* 2018, **6(Suppl 2):**0876

**INTRODUCTION.** Normal saline is being considered harmful in critically ill patients and commercially prepared balanced salt solutions (acetate solution, AC) are being advocated.[1,2] However, there is paucity of studies comparing balanced salt solutions with widely available ringer lactate solution (RL).

**OBJECTIVE.** The objective was to compare time to correction of metabolic acidosis during initial period of fluid resuscitation with AC or RL in critically ill patients. Secondary outcome was the extent of correction of metabolic acidosis, volume and total cost of fluid used.

**METHODS.** A randomized controlled trial was conducted during July 2016 to December 2017 in which all adult patients admitted to intensive care unit with metabolic acidosis were included. The patients were randomized into group AC or group RL. Fluid resuscitation (AC or RL) was initiated at the rate of 20ml/Kg/hr for the first hour and arterial samples were taken 15 minutes apart. In the second hour, patients received fluid at the rate of 10ml/Kg/hr and arterial samples were drawn 15 minutes apart. The time to correction of metabolic acidosis and the extent of correction of metabolic acidosis (change in pH, lactate, standard bicarbonate, base excess) was noted. The fluid resuscitation was carried out for maximum of two hours and stopped earlier if pH got corrected to 7.3. The total volume used and cost of each fluid was noted.

**RESULT.** Out of total fifty patients, 25 patients in group AC and 24 in group RL were included. The demographic and baseline parameters (APACHE II score, hemoglobin, platelet count, leucocyte count, sodium, potassium, urea, creatinine, baseline blood gas parameters, time to randomization) were comparable. The metabolic acidosis got corrected in 12 patients in group AC and 10 patients in group RL (p=0.66).

The mean time for correction of metabolic acidosis was 56.25 minutes in group AC and 57 minutes in group RL (p=0.95). The extent of correction of metabolic acidosis and total volume of fluid used were also comparable (p.0.05). However, the cost of fluid used was significantly higher in group AC than group RL (p< 0.01).

**CONCLUSION.** Administration of two balanced salt solutions, AC or RL to critically ill patients with metabolic acidosis did not confer any advantage in time to or extent of correction of metabolic acidosis. The use of commercially prepared balanced solution (AC) increased the overall cost.

**REFRENCE(S)**

1. Yunos NM, Bellomo R, Hegarty C, Stoty D, Ho L, Bailey M. Association between a chloride-liberal vs chloride-restrictive intravenous fluid administration strategy and kidney injury in critically ill adults. JAMA 2012;308:1566-71.

2. Zhou F, Peng ZY, Bishop JV, Cove ME, Singbartl K, Kellum JA. Effects of fluid resuscitation with 0.9% saline versus a balanced electrolyte solution on acute kidney injury in a rat model of sepsis. Crit Care Med 2014;42:e270-8.

**GRANT ACKNOWLEDGEMENT**

None declared

### 0877

#### The Impact of hydrocortisone therapy on noradrenaline requirement and haemodynamics derived from transpulmonary thermodilution (TPTD) in patients with septic shock: an observational study

L. Fahrenkrog-Petersen, U. Mayr, T. Lahmer, A. Herner, G. Batres-Baires, S. Rasch, J. Wiessner, I. Hartter, R. Schmid, W. Huber

Klinikum rechts der Isar; Technical University of Munich, Medizinische Klinik und Poliklinik II, Munich, Germany

**Correspondence:** W. Huber

*Intensive Care Medicine Experimental* 2018, **6(Suppl 2):**0877

**INTRODUCTION.** The recommendations on the use of hydrocortisone in sepsis still are controversial . Resolution of septic shock is one of the most important outcome measures in sepsis studies. Despite several hints on an overall improvement of circulation by hydrocortisone, there is a lack of data on mechanisms and velocity of circulatory improvement.

**OBJECTIVES.** We investigated potential short term effects of hydrocortisone therapy (200mg i.v. bolus, followed by continuous infusion of 300mg/24h) in 20 patients with septic shock equipped with PiCCO monitoring.

**METHODS.** TPTD (PiCCO; Pulsion; Germany) was performed immediately before as well as 2h, 8h, 16h and 24h after the initiation of hydrocortisone therapy. Furthermore, cardiac power index CPI [W/$m^2$]) (=0.0022 * cardiac index * MAP) and vasopressor dosage were recorded. Primary endpoint: changes in the ratio CPI/noradrenaline. Statistics: Wilcoxon-test; SPSS 24.

**RESULTS.** n=20 (14m; 6f); 65±14 years; APACHE-II 25±8; SOFA 12±8. Mechanical ventilation 17/20 (85%); vasopressor therapy 20/20 (100%). Baseline values: noradrenaline 1570±1232µg/h (no other vasopressors were used); MAP 73.9±9.2mmHg; heart rate 105±18/min; CVP 16±6mmHg; global end-diastolic volume index GEDVI 833 ±166mL/$m^2$; cardiac index CI 4.77±1.35L/min/$m^2$; global ejection fraction GEF 22.7±6.5%; dPmax 1606±485mmHg/s; extravascular lung water index EVLWI 12.0±4.3mL/kg; pulmonary vascular permeability index PVPI 1.95±0.73; CPI 0.83±0.25W/$m^2$; CPI/noradrenaline 0.090 ±0.091W/$m^2$/µg. After 2h, 8h, 16h and 24h there were no significant changes in CVP or GEDVI. Neither MAP nor SVRI changed over time. Among the parameters of cardiac performance, GEF slightly increased (Delta-GEF +1.3±2.7%; p=0.042) after 2h. Furthermore, dPmax decreased after 8h (-264±412mmHg/s; p=0.036), 16h (-326±478mmHg/s; p=0.016) and 24h (-344±522mmHg/s; p=0.046). By contrast, CI and CPI did not change after the onset of hydrocortisone.

Both markers of pulmonary oedema EVLWI (-1.3±2.3; p=0.044 and -1.8±2.9mL/kg; p=0.036) and PVPI (-0.27±0.34; p=0.007 and -0.36 ±0.62; p=0.054) decreased after 16h and 24h.

>The most pronounced changes were found regarding the primary endpoint CPI/noradrenaline: This ratio increased after 8h (+0.065±0.066; p=0.001), 16h (+; p=0.116±0.146; p=0.003) and 24h (+0.132±0.117 W/$m^2$/µg; p=0.003). The increase after 2h slightly failed significance h (+0.011±0.034 W/$m^2$/µg; p=0.053). The noradrenaline dosage decreased by 16%, 41%, 46% and 63% after 2h, 8h, 16h and 24h.

**CONCLUSIONS.** Our data suggest that hydrocortisone results in a fast and substantial improvement of the ratio CPI/noradrenaline without incraesing preload. Despite the absence of a control group, the fast onset of all relevant haemodynamic changes within 2-8h after the start of hydrocortisone suggests an association of haemodynamic improvement with steroid therapy. Finally, we observed a decrease in pulmonary oedema which might be related to hydrocortisone.

### 0878

#### Is there a role for early administration of fixed low dose terlipressin in adult septic shock?

R. Etomi, A. Khan, T. Prabhahar

North Middlesex University Hospital, Critical Care, London, United Kingdom

**Correspondence:** R. Etomi

*Intensive Care Medicine Experimental* 2018, **6(Suppl 2):**0878

**INTRODUCTION.** Fluids and catecholamines are the cornerstones of management in sepsis induced hypotension. However to administer catecholamines there is a need for central venous access and continuous invasive arterial blood pressure monitor. Moreover catecholamines cause increase myocardial oxygen consumption, peripheral ischaemia and arrhythmias. Terlipressin is a synthetic long acting analogue of vasopressin. Vasopressin is being used at a fixed lower dose to reduce the catecholamine requirements in severe septic shock. There is some evidence that continuous fixed dose infusion of low dose Terlipressin when given as a first line vasopressor agent for hypotension in septic shock has reversed the hypotension.

**OBJECTIVES.** Objective of this small retrospective survey was to assess the impact of fixed low dose Terlipressin infusion when used as a sole agent in sepsis induced hypotension.

**METHODS.** Retrospective search was undertaken in the electronic (Acubase®) patient records of all admissions for three years from 2015 to 2017 using clinical intervention fixed low dose Terlipressin (30µg/hour) infusion. 120 patients were identified by this search. Electronic medical notes (Acubase®) were further searched manually and patients who were not septic or Terlipressin was used with another vasopressor were excluded, 17 patients fulfilled the inclusion criteria. Manual search was undertaken on 14 patient observation and infusion charts, as three charts were unable to be obtained. We assessed the mortality of 17 patients. Of those who survived critical care admission nine were assessed for the improvement in their mean arterial blood pressure (MAP) at 12 and 24 hours, urine output (UOP) and serum creatinine after 24 hours.

**RESULTS.** 12 out of 17 patients survived the critical care admission. Three patients´ MAP at 12 hours were still less than 65 mmHg, and two remained less than 65 mmHg at 24 hours. All nine patients were either anuric or oilguric at the beginning of the infusion. Seven out of nine patients UOP improved to normal after 24 hours. Six out of nine patients' serum Creatinine improved significantly after 24 hours.

**CONCLUSIONS.** Fixed low dose Terlipressin as a sole agent vasopressor for sepsis induced hypotension improved the cardiovascular function, renal function and improved survival rate (70%) in this sample.

Since the data was limited we suggest a large prospective randomised control study analysing the effects of early fixed low dose Terlipressin infusion as a single agent for patients with sepsis induced hypotension.

##### REFERENCE(S)

Morelli A, Ertmer C, Rehberg S, *et al* (2009) Continuous terlipressin versus vasopressin infusion in septic shock (TERLIVAP): a randomized, controlled pilot study. Critical Care:13:R130

Neto S, Nassar Júnior AP, Cardoso SO *et al.* (2012). Vasopressin and terlipressin in adult vasodilatory shock: a systematic review and meta-analysis of nine randomized controlled trials. Critical Care, 16:R154

##### GRANT ACKNOWLEDGMENT

None.

### 0879

#### Hypotension in septic patients after intubation and sedation - shock or adverse effects from sedatives?

J.E. Larsson, T. Skovsgaard Itenov, C. Dalby Sørensen, M. Heiberg Bestle
Nordsjællands Hospital, University of Copenhagen, Department of Anesthesiology and Intensive Care, Hilleroed, Denmark
**Correspondence:** J.E. Larsson
*Intensive Care Medicine Experimental* 2018, **6(Suppl 2):**0879

**INTRODUCTION.** Respiratory failure is a common complication in septic patients mandating mechanical ventilation. Anesthetics used to facilitate endotracheal intubation and sedation have known hemodynamic adverse effects and may cause hypotension. It is unclear, whether hypotension in septic patients after induction and sedation can be solely attributed to pharmacological adverse effects, or if the anesthetics exacerbates the underlying disease.

**OBJECTIVES.** The aim of this study is to compare the mortality rate, fluid therapy, anesthetic agents and vasopressor usage in septic patients who develops hypotension after intubation and sedation, with septic patients who are hypotensive prior to intubation.

**METHODS.** In this single-center, retrospective observational cohort study, we included septic patients who were intubated in the intensive care unit (ICU) during 2014 and 2015, and had hypotension prior to, or after the procedure. We stratified the patients according to whether they met criteria for hypotension within the last six hours before intubation or during the first hour after intubation. Hypotension was defined as a systolic blood pressure < 90 mmHg; a middle arterial blood pressure (MAP) < 65 mmHg or the need for vasopressors to sustain a MAP > 65 mmHg. The primary outcome measure was mortality at 90 days post intubation.

**RESULTS.** Of the 427 admissions screened, 130 patients were included in the study. Among these, 53 patients had hypotension before intubation and sedation (HBI) and 77 patients had hypotension after intubation and sedation (HAI). At 90-days after intubation, 30 of 53 patients (57%) in the HBI group had died as compared with 34 of 77 patients (44%) in the HAI group (P=0.224). Survival curves and the results from the multivariate analysis are presented in Figure 1 and Table 1 respectively. During the first 24 hours after intubation, the HBI group received more noradrenaline (median [IQR] 8957 μg/kg/24 hours [5515 to 12470] as compared with 3766 μg/kg/24 hours [2002 to 5954]; P< 0.001]), and higher volumes of resuscitation fluids (median [IQR] 71.4 ml/kg/24 hours [40.0, 112.9] vs. 50.0 ml/kg/24 hours [26.7, 73.0]; P=0.01).

**CONCLUSIONS.** The mortality of septic patients with hypotension following intubation and sedation was high and these patients should be considered as patients in early septic shock.

##### GRANT ACKNOWLEDGMENT

This research received no specific grant from any funding agency in the public, commercial, or not for profit sectors.

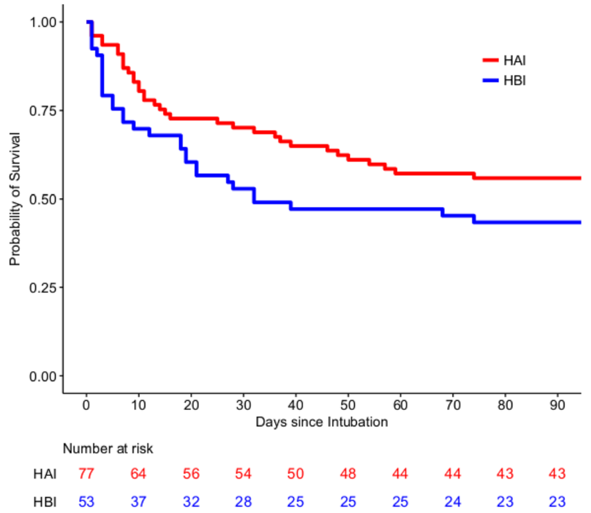

**Fig. 1 (abstract 0879).** Time to Death. Survival curves for the two groups censored at day 90 (P=0.10)

**Table 1 (abstract 0879).** Predictors of death in septic patients with hypotension before or after intubation - Cox regression

| Variable | HR | Univariate (95% CI for HR) | p | HR | Multivariate (95% CI for HR) | p |
|---|---|---|---|---|---|---|
| Hypotension after intubation and sedation | 0.66 | (0.40-1.08) | 0.10 | 0.70 | (0.42-1.16) | 0.17 |
| Age (older) | 1.03 | (1.01-1.05) | 0.01 | 1.03 | (1.00-1.05) | 0.03 |
| COPD (yes) | 1.28 | (0.77-2.12) | 0.34 | 1.24 | (0.73-2.11) | 0.43 |
| Active cancer | 1.37 | (0.79-2.39) | 0.26 | 1.32 | (0.74-2.35) | 0.35 |
| White blood cell count <3×10^9/L | 1.49 | (0.64-3.46) | 0.35 | 1.40 | (0.59-3.35) | 0.45 |

### 0880

#### Mesenchymal stem cells in a porcine model of progressive peritoneal sepsis

J. Horak[1,2], J. Benes[2,3], L. Nalos[2], V. Martinkova[2,4], M. Stengl[2], M. Matejovic[1,2]
[1]Charles University in Prague, Faculty of Medicine in Plzen, 1st Medical Department, Plzen, Czech Republic; [2]Charles University in Prague, Faculty of Medicine in Plzen, Experimental Intensive Care Unit, Biomedical Centre, Plzen, Czech Republic; [3]Charles University in Prague, Faculty of Medicine in Plzen, Department of Anaesthesiology and Intensive Care Medicine, Plzen, Czech Republic; [4]Charles University, Third Department of Surgery, University Hospital Motol and First Medical School, Prague, Czech Republic
**Correspondence:** J. Horak
*Intensive Care Medicine Experimental* 2018, **6(Suppl 2):**0880

**INTRODUCTION.** Cellular therapy with mesenchymal stem cells (MSCs) represents emerging therapeutic strategy in sepsis. However, the encouraging results of experiments with MSC in rodent models of sepsis have not yet been confirmed in large animal models with better relevance to human sepsis.

**OBJECTIVES.** To assess safety and efficacy of bone-marrow derived MSCs in a clinically relevant porcine model of progressive peritonitis-induced sepsis.

**MATERIAL AND METHODS.** 32 anesthetized, mechanically ventilated and instrumented pigs were randomly assigned into four groups: 1) sham operated group (CONTROL, n=8); 2) sham operated group treated with Bone-marrow derived MSCs (MSC-CONTROL, n=8), 3) sepsis group with standard supportive care (SEPSIS, n=8); 4) sepsis group treated with MSCs in addition to standard supportive care (MSC-SEPSIS, n=8, MSCs administered i.v. at 6 h after sepsis induction). Before, 12, 18 and 24 h after the induction of peritonitis we measured, in addition to systemic, regional (renal) and microvacular (gut mucosal) hemodynamics, oxygen kinetics (DO2/VO2), organ function (lung, kidney, liver, heart), energy metabolism (lactate kinetics, acid base balance), systemic inflammation (cytokines), nitrosative/ oxidative stress (isoprostanes) and tissue immunohistochemistry.

**RESULTS.** Administration of MSCs in the MSC-CONTROL group did not affect hemodynamics, organ functions nor the levels of systemic cytokines. Treatment of septic animals with MSCs did not attenuate sepsis-induced hyperdynamic circulation nor the development of vasopressor-requiring hypotension. The application of MSCs did not blunt sepsis-induced alterations in microvascular perfusion, cellular energetics and multiorgan functions. Finally, sepsis was associated with progressively increased levels of pro-inflammatory cytokines in both group, without intergroup differences. Analyses of oxidative stress and tissue immunohistochemistry are ongoing.

**CONCLUSIONS.** This is the first report to evaluate the safety and efficacy of MSCs in a clinically relevant, large animal, long-term model of progressive sepsis induced by diffuse peritonitis. Administration of MSCs to healthy animals was well tolerated without any measurable acute hemodynamic or organ toxicity. However, in sharp contrast to recent rodent studies, MSCs failed to attenuate sepsis-induced disturbances at multiple levels. Thus, the decision to move from experiments to clinical studies must be preceded by further and careful pre-clinical evaluation with high translational potential.

**GRANT ACKNOWLEDGMENT**

This work was supported by the Charles University Research Fund (Progres Q39), and an AZV grant (project 15-32801A) and by project No. CZ.02.1.01/ 0.0/0.0/16_019/0000787 “Fighting INfectious Diseases”, awarded by the MEYS CR, financed from EFRR.

### 0881

### Cell therapy using genetically modified mesenchymal stem cell in a murine model of sepsis

J. Bringue[1], R. Guillamat-Prats[1], E. Torrents[2], M.L. Martinez[3], L. Blanch[4], A. Artigas[4]

[1]CIBER Enfermedades Respiratorias, Sabadell, Spain; [2]Corporació Sanitària i Universitària Parc Taulí, Critical Care Center, Sabadell, Spain; [3]Hospital Universitari General de Catalunya, Critical Care Center, Sant Cugat, Spain; [4]Institut d'Investigació i Innovació Parc Taulí I3PT, Sabadell, Spain

**Correspondence:** J. Bringue

*Intensive Care Medicine Experimental* 2018, **6(Suppl 2):**0881

**INTRODUCTION.** Sepsis is an extreme inflammatory response mediated by cytokines that can induce acute lung injury and multi-organic dysfunction. Sepsis remains one of the leading causes of mortality in the intensive care units and there is not a specific treatment for this disease. Nowadays, we have to focus in new therapeutic strategies. Mesenchymal stem cells (MSC) have shown anti-inflammatory, antimicrobial, anti-apoptotic and regulatory properties, MSC could be an interesting therapy for sepsis however with the new molecular technic of genetically modification the beneficial effects of MSC therapy could be enhanced.

**HYPOTHESIS.** Genetically modified MSC overexpressing IL10 and IL7 reduces the damage in a murine model of sepsis, modulating the inflammatory systemic response and diminishing the lung injury.

**METHODS.** Sepsis was induced by a cecal ligation and puncture (CLP) in Sprague-Dawley rats (225-300 g). 6 hours later we did a surgical source control and administered antibiotics (Meropenem 20 mg/kg), fluids (10 ml/kg) and analgesics (Buprenorphine 0.025 mg/Kg). In addition, we administered $2.5*10^6$ MSC genetically modified to overexpress Interleukin 10 and 7. 48 h later the animals were sacrificed and samples of lung tissue, bronchoalveolar lavage and blood were collected. We did control groups, rats were submitted to the same process but no CLP was performed. We analysed neutrophils, macrophages and lymphocytes in bronchoalveolar lavage; blood and spleen bacterial counts and expression of inflammatory cytokines in lung tissue.

**RESULTS.** Modified MSC compared to control MSC reduced number of neutrophils and proteins infiltrated into the alveolar space. Modified MSC also reduced the number of circulating bacteria in blood, increase the $pO_2$ and reduce the concentraction of creatinine in plasma. In addition, modified MSC significantly reduced the expression of TNFα, iNOS, IL1β and CCL2 in lung tissue in the septic animals. Regardless the beneficial effects of the modified MSC there is no differences in survival compared with the non-modified MSC.

**CONCLUSION.** Genetically modified MSC modulates the proinflammatory cytokine production during sepsis, increase the bacterial clearance and the $O_2$ exchange in a murine model of sepsis.

### 0882

### Effects of combined hydrocortisone and vitamin C for the treatment of sepsis

J.H. Ahn[1], W. Lee[2,3], E.Y. Choi[1], J.-S. Bae[3,4]

[1]College of Medicine, Yeungnam University, Department of Internal Medicine, Yeungnam University Medical Center, Daegu, Korea, Republic of; [2]Aging Research Center, Korea Research Institute of Bioscience and Biotechnology, Daejeon, Korea, Republic of; [3]College of Pharmacy, CMRI, Research Institute of Pharmaceutical Sciences, BK21 Plus KNU Multi-Omics Based Creative Drug Research Team, Kyungpook National University, Daegu, Korea, Republic of; [4]College of Pharmacy, Kyungpook National University, 80 Daehak-ro, Buk-gu, Daegu, Korea, Republic of

**Correspondence:** J.H. Ahn

*Intensive Care Medicine Experimental* 2018, **6(Suppl 2):**0882

**INTRODUCTION.** Sepsis, a life-threatening organ dysfunction caused by a dysregulated host response to infections, is a global burden with limited therapeutic options. In recent studies, combination of hydrocortisone (HC) and ascorbic acid (vitC) dramatically improves endothelial barrier function and mortality in patients with sepsis.

**OBJECTIVES.** The aim of the study was to investigate the effects of combined HC and vitC on cecal ligation and puncture (CLP)-induced sepsis in mice, and to elucidate its underlying molecular mechanisms.

**METHODS.** Experiments were performed on male C578L/6 mice. Sepsis was induced by CLP. Mice were randomly assigned to control group, HC group, vitC group, combination low-dose group and combination high-dose group (each n = 20). Mice in control group were treated with saline vehicle. Mice in HC group and vitC group were treated with HC (2.5mg/kg of body weight) or vitC (100mg/kg of body weight). Combination low-dose group and combination high-dose group were treated with HC (2.5mg/ kg of body weight) plus vitC (100mg/kg of body weight) and HC (5.0mg/kg of body weight) plus vitC (200mg/kg of body weight) intravenously at 0, 12, 24, 36, and 48 h after CLP. Survival rate, cytokine and septic biomarkers were compared among these groups. Additionally, human umbilical vein endothelial cells (HUVECs) were exposed to LPS (1μg/ml) or high mobility group box 1 (HMGB1) protein (1μg/ml). Barrier protective effects and antiseptic activity of HC and vitC were examined.

**RESULTS.** There was significant difference in survival rate between control group and combination high-dose group at 140h (0% [20/20] vs. 60% [12/20], p< 0.001). LPS induced profound hyperpermeability, as reflected in pulmonary microvascular albumin leak. Combination high-dose HC and vitC significantly reversed LPS-induced barrier dysfunction. The barrier-protective effects of HC and vitC were associated with dephosphorylation and cleavage of ocludin-1 and activation of transcription factor such as NF-κB p65 and glucocorticoid response element (GRE). The antiseptic activity of combination of HC and vitC was determined from the measurements of LPS-mediated HMGB1 release, expression of HMGB1 receptor, and leukocyte adhesion and migration in LPS/CLS-activated HUVECs and mice.

**CONCLUSIONS.** Our results indicated that HC and vitC might be useful in the treatment of sepsis. In addition, these data provide a novel mechanism of vascular barrier integrity protection and suggest that combination administration predicts synergistic therapeutic effects in septic patients rather than single administration.

### 0883

#### Inhibition of uncoupling protein 2 exacerbates mitochondrial oxidative stress in the proximal tubular epithelial cells of a live kidney slice exposed to septic serum

S. Pollen[1], N. Arulkumaran[1], M. Duchen[2], M. Singer[1]

[1]UCL, Bloomsbury Institute of Intensive Care Medicine, London, United Kingdom; [2]UCL, Cell and Developmental Biology, London, United Kingdom

**Correspondence:** S. Pollen

*Intensive Care Medicine Experimental* 2018, **6(Suppl 2):**0883

**INTRODUCTION.** We have previously found that exposure of naïve kidney slices to septic serum increases generation of reactive oxygen species (ROS) and decreases mitochondrial membrane potential (MMP) and NADH fluorescence. Scavenging ROS with the use of mitochondrial-targeted antioxidants reversed these changes.[1] A decrease in both MMP and NADH fluorescence suggests mitochondrial uncoupling as the underlying mechanism, which may provide a physiological mechanism to reduce the generation of mitochondrial reactive oxygen species (ROS)[2].

**OBJECTIVES.** To determine whether this observed change in mitochondrial function is mediated by activation of mitochondrial uncoupling proteins UCP2[3], in turn stimulated by ROS on exposure to septic serum[4], and whether inhibition of UCP2 by genipin, an inhibitor of UCP2 alters ROS generation.

**METHODS.** Live kidney slices (200 µm thick) were exposed to serum from 24-hour sham operated or septic rats and imaged with multiphoton confocal microscopy using fluorescent dyes to detect dynamic changes in mitochondrial function. MMP (measured with tetramethylrhodamine methyl ester, TMRM), ROS generation (dihydroethidium, HEt) and redox state (NADH autofluorescence) were all probed over 90 minutes, with or without co-incubation with the rapidly-acting, cell-permeant, UCP2 inhibitor genipin.[5] Experimental results were expressed as the median (n=4, ±IQR) percentage relative fluorescence intensity (RFI) at 90 minutes compared to the baseline RFI at time zero.

**RESULTS.** The decreases in MMP and NADH following exposure to septic serum were partially prevented by co-incubation with genipin (63% vs 80% Fig.1a and 77% vs 96% Fig.1c, $p< 0.05$).

The increase in ROS generation on exposure to septic serum was however enhanced by genipin (50 µM) co-incubation (146% vs 167% Fig1b, $p< 0.05$). In sham serum exposed slices, co-incubation with genipin also increased MMP (98% vs 123%) and ROS generation (86% vs 112%) ($p< 0.05$), but not NADH. [Figure notation: *=p< 0.05 vs sham serum, †=p< 0.05 vs septic serum, ‡=p< 0.05 vs sham serum + genipin].

**CONCLUSIONS.** UCP2 is a key regulator of MMP in an *in vitro* model of sepsis-induced renal tubular cell mitochondrial dysfunction. While inhibiting its action protects the MMP from the depolarisation seen on exposure to septic serum, the concomitant increase in ROS is potentially deleterious. Indeed, decreasing the MMP through uncoupling may be an intrinsic protective mechanism that reduces mitochondrial ROS generation in septic AKI [2,4].

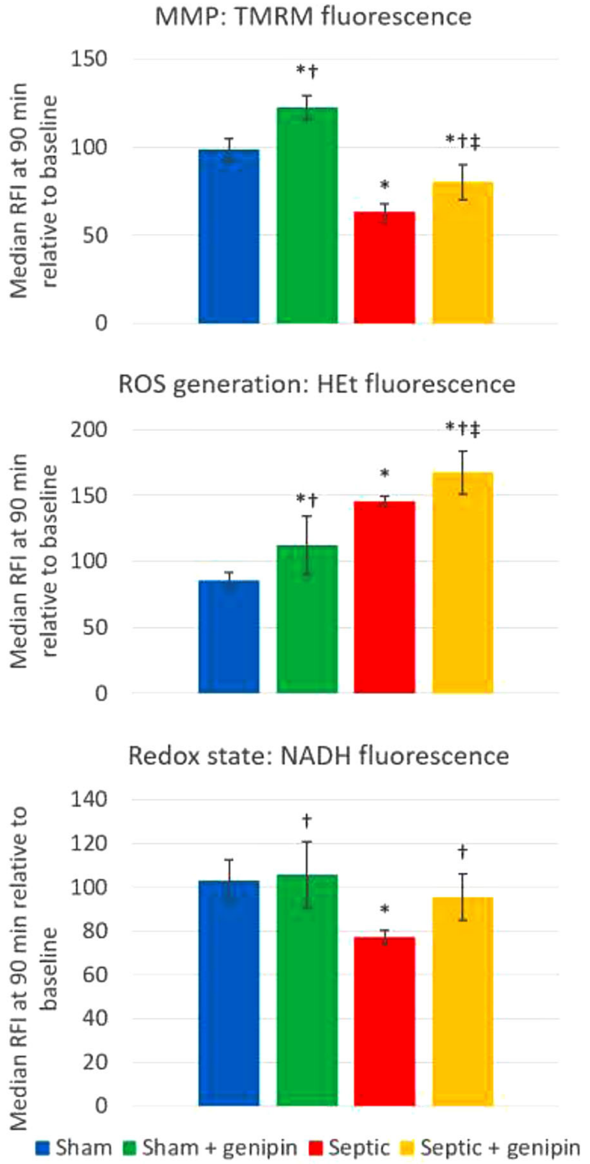

**Fig. 1 (abstract 0883).** Relative fluorescence intensity of dyes probing mitochondrial function

**GRANT ACKNOWLEDGMENT**

UCL Medical School MBPhD Programme, Astor Foundation, and Intensive Care Society

### 0884

#### Blood transfusion for haemodynamic and volume resuscitation in endotoxaemic shock: is there a difference between fresh versus stored blood transfusion?

N. Obonyo[1,2], J.-P. Tung[1,3,4], L. Byrne[1,5,6], G. Simonova[1,3,4], S. Diab[1,4], K. Dunster[1,7], M. Passmore[1,4], A.-C. Boon[1,4], L. See Hoe[1,4], S. Engkilde-Pedersen[1,3,7], A. Esguerra-Lallen[1,3], M. Fauzi[1,8], L. Pimenta[1], F. Van Haren[5,6,9], J. Millar[1,4], C. Anstey[10], L. Cullen[4,11], J. Suen[1,4], K. Shekar[1,4,12], K. Maitland[2,13], J. Fraser[1,4,12], (RESUS Trial Investigators, NHMRC 1061382, Australia)
[1]Critical Care Research Group-The Prince Charles Hospital, Pre-clinical Research Department, Chermside, Australia; [2]IDeAL KEMRI-Wellcome Trust Research Programme, Clinical Research and Training Departments, Kilifi, Kenya; [3]Australian Red Cross Blood Service, Brisbane, Australia; [4]University of Queensland, School of Medicine, Brisbane, Australia; [5]The Canberra Hospital, Intensive Care Unit, Garran, Australia; [6]Australian National University, School of Medicine, Canberra, Australia, [7]Queensland University of Technology, Brisbane, Australia; [8]Universiti Sains Malaysia Health Campus, School of Medical Sciences, Kelantan, Malaysia; [9]The University of Canberra, Bruce, Australia; [10]Sunshine Coast University Hospital, Intensive Care Unit, Birtinya, Australia; [11]Royal Brisbane and Women's Hospital, Emergency Department, Herston, Australia; [12]The Prince Charles Hospital, Adult Intensive Care Unit, Chermside, Australia; [13]Imperial College London, Department of Paediatrics, Faculty of Medicine, London, United Kingdom
**Correspondence:** N. Obonyo
*Intensive Care Medicine Experimental* 2018, **6(Suppl 2):**0884

**INTRODUCTION.** The Surviving Sepsis Campaign (SCC) guideline recommends transfusion of blood in sepsis when the haemoglobin concentration drops below 7.0 g/dL (1). Blood transfusion in septic shock is thus generally reserved as a later-stage treatment option after administration of crystalloids, colloids, vasopressors and inotropic agents. In the ProCESS trial, more patients in the early goal-directed therapy (EGDT) group receiving packed red blood cell (PRBCs) transfusion than in those in the protocol-based standard-therapy or the usual-care groups, without any apparent differences in the overall outcomes evaluated (2,3).

**OBJECTIVES.** We therefore conducted a randomised pre-clinical trial evaluating whether early administration of blood transfusion has a role in haemodynamic and volume resuscitation for endotoxaemic shock by comparing effects of fresh versus stored blood transfusion in a validated ovine model of hyperdynamic endotoxaemic shock(4).

**METHODS.** Endotoxaemic shock was induced in sixteen anaesthetised and mechanically ventilated merino ewes by infusing an escalating dose of lipopolysaccharide, LPS (*E.coli 055:B5*) over 4 hours (total LPS dose 11.25mcg/kg). During the last hour of LPS, 10mls/kg of either fresh (≤5 days); n=8 or stored (≥30 days); n=8 ovine PRBCs was administered followed by a 12-hour monitoring period. Blood samples were taken for measurement of inflammatory cytokines and coagulation profile.

**RESULTS.** Compared to fresh PRBC, transfusion of stored PRBC in ovine endotoxaemic shock was associated with haemodynamic changes including increased central venous ($p < 0.0001$) and pulmonary artery ($p < 0.0001$) pressures; decreased mixed venous oxygen saturation ($p=0.0036$) and cardiac index ($p=0.0007$). Additionally, there was an increase in pro-inflammatory cytokines (IL-6, $p=0.0053$; IL-8, $p=0.0332$), a decrease in the anti-inflammatory IL-10 ($p=0.0215$), and a decrease in the fibrinogen concentration ($p=0.0036$).

**CONCLUSIONS.** There were significant changes in the haemodynamics, inflammatory response and coagulation profile with stored ovine PRBC transfusion. These results indicate that a longer storage duration of PRBCs prior to transfusion could be associated with systemic effects that predispose septic shock patients to poor clinical outcomes.

##### REFERENCE(S)

1) Rhodes A *et al*: Surviving Sepsis Campaign: International Guidelines for Management of Sepsis and Septic Shock: 2016. *Intensive care medicine* 2017, **43**(3):304-377.
2) Pro CI *et al*: A randomized trial of protocol-based care for early septic shock. *N Engl J Med* 2014, **370**(18):1683-1693.
3) Angus DC *et al*: A systematic review and meta-analysis of early goal-directed therapy for septic shock: the ARISE, ProCESS and ProMISe Investigators. *Intensive care medicine* 2015, **41**(9):1549-1560.
4) Byrne L *et al*: An Ovine Model of Hyperdynamic Endotoxemia and Vital Organ Metabolism. *Shock* 2018, **49**(1):99-107.

##### GRANT ACKNOWLEDGMENT

Australian National Health and Medical Research Council (NHMRC) APP-ID 1061382

### 0885

#### Effects of platelet-activating factor and quinidine in the isolated perfused rat small intestine are related to similar MAPK pathways

N. Beckers[1], I. Lautenschläger[1], Y.L. Wong[1], K. Zitta[1], C. Schildhauer[1], K. Parczany[1], S. Krüger[2], C. Röcken[2], M. Albrecht[1], I. Frerichs[1], N. Weiler[1]
[1]University Medical Centre Schleswig-Holstein, Department of Anaesthesiology and Intensive Care Medicine, Kiel, Germany; [2]University Medical Centre Schleswig-Holstein, Department of Pathology, Kiel, Germany
**Correspondence:** N. Beckers
*Intensive Care Medicine Experimental* 2018, **6(Suppl 2):**0885

**INTRODUCTION.** Sepsis-induced endothelial dysfunction critically affects the gastrointestinal tract leading to intestinal failure. In the model of the isolated perfused small intestine, a bolus of 0.5 nmol platelet-activating factor (PAF) mimics this pathology[1,2,3]. We have recently shown that quinidine (QD) protects the gut in this setting[3].

**OBJECTIVES.** We aimed (I) to check if continuous PAF infusion (2.26 nM) leads to a persistent barrier dysfunction with organ failure, (II) to investigate if QD (100 μM) protects against organ dysfunctions and (III) to screen for adaptive intracellular pathways associated with the PAF-induced intestinal failure and the QD-mediated protection.

**METHODS.** An established model of the isolated perfused rat small intestine[1,2,3]. Group 1 (G1): PAF (n=6), G2: QD (n=5), G3: PAF+QD (n=5) and G4: control (n=6). Fluid shifts, macromolecular transfer of FITC-labelled dextran and compartment pressures were recorded. Venous galactose derived from luminal lactose was assessed as a measure of intestinal function. Tissue samples were taken to analyze morphology. A phospho-MAPK array (ARY002B, R&D Systems) was used to determine the status of intracellular signalling pathways.

**RESULTS.** PAF infusion led to an inflammatory pressure response, an endo- and epithelial leakage and a loss of the resorptive function followed by an adaptive process. The area under the pressure response curve (15-min intervals) was significantly elevated in the PAF group (G1) for 60 min when compared with G4 ($p=0.0002$). The peak value for PAF-induced FITC dextran transfer ($mg \cdot 15min^{-1}$) to the lumen was 0.076 (control: 0.0027) and to the lymphatics 0.205 (control: 0.015). Within the first 15 min after PAF infusion 12% of volume flow was lost from the mesenteric vascular bed into the other compartments. The galactose uptake dropped significantly in the PAF group (G1). When QD was administered before and during the PAF infusion, PAF-induced effects were abolished. Light microscopy revealed no changes in histological stability score (all groups). Nevertheless, edema formation was more pronounced in the PAF group than in the others. Phospho-MAPK arrays revealed that PAF increased the phosphorylation of 13 proteins with strongest effects on ERK2, GSK-3ab and JNK2. QD also increased the phosphorylation of 13 proteins with strongest effects on ERK2, JNK2, JNKpan and p38d. The combination of PAF and QD led to a pronounced relative increase of p-AKT3, p-CREB, p-ERK2, p-GSK-3ab and p-JNK2.

**CONCLUSIONS.** PAF infusion leads to a persistent intestinal barrier dysfunction with organ failure. QD infusion almost completely abolishes these effects. Both substances are related to similar signalling pathways, especially the ERK and JNK pathways.

##### REFERENCE(S)

1. Lautenschläger, I. et al. *AJPGI* 298, G304-13 (2010)
2. Lautenschläger, I. et al. *Sci rep* 7, 13382 (2017)
3. Lautenschläger, I. et al. *PloS one* 10, e0120802 (2015)

### 0886

#### Pentoxyphylline inhibits M1 polarization and favors M2 of murine macrophages treated with TLR4 agonist

M.C. Montero[1], J. Guerrero[2,3,4]
[1]Universidad de Chile, Facultad Medicina, Programa Disciplinario Fisiologia y Biofisica, Santiago, Chile; [2]Universidad de Chile, Facultad Medicina, Programa Disciplinario Fisiología y Biofisica, Santiago, Chile; [3]Universidad de Chile, Hospital Clínico, Departamento Medicina Interna, Santiago, Chile; [4]Clinica Alemana de Santiago, Departamento Paciente Critico, Santiago, Chile
**Correspondence:** J. Guerrero
*Intensive Care Medicine Experimental* 2018, **6(Suppl 2):**0886

**INTRODUCTION.** Pentoxyphylline (PTX) is a phosphodiesterase inhibitor that increases intracellular cAMP. Recently, PTX has been recognized as a pharmacological modulator of inflammation that may improve outcomes in septic patients (1). In neonatal sepsis, the use of PTX as an adjunct to antibiotics therapy decreased all-cause mortality and the length of hospital stays, without significant adverse effects (2). Authors had proposed its effect may be mediated by adenosine-dependent pathways for polymorphonuclear leukocytes and T cells (3). Results of studies in whole new born umbilical blood showed that PTX inhibited the inflammatory cytokine response induced by Toll-like receptors (TLR) agonists, TLR4, TLR7 and TLR8 (4). Considering that peripheral blood macrophages can reprogram their phenotype and orchestrate the inflammatory response, we tested if PTX modifies inflammatory cytokines profile in response to a TLR-4 agonist in a macrophage cell line.

**OBJETIVE.** To assess if PTX modulates macrophage TLR4-dependent polarization in vitro.

**METHODS.** Murine macrophages (Raw 264.7 cells) were cultured in Dulbecco's Modified Eagle's Medium (DMEM) (control), or in the presence of lipopolysaccharide (LPS, Sigma -Aldrich Chemie®, Germany[LM1] ) 25ng/mL, LPS + PTX (dose-response curve; Sigma-Aldrich Chemie®, Germany) or PTX alone. We analyzed cell viability by trypan blue exclusion assay and the time-course of changes in TNF-alpha and IL-10 mRNA content (as surrogate markers of M1 or M2 polarization, respectively) in the presence or absence of LPS, PTX or LPS plus PTX by real-time qPCR.

**RESULTS.** PTX (100-250-500 and 1000mg/ml by 1-1.5 or 2h) had no effect on cell viability or TNF-alpha mRNA abundance. LPS induced TNF-alpha mRNA (3 times of control level; n= 4, p< 0,05) and PTX inhibited TNF-alpha mRNA induced by LPS (p< 0,05). The inhibitory effect of PTX on LPS-dependent TNF-alpha mRNA induction was greater with 250 mg /ml and over 1h of exposition. Interestingly, after 1.5 h of exposure, PTX+ LPS significantly increased the cellular content of IL-10 mRNA.

**CONCLUSIONS.** PTX modulates macrophage inflammatory cytokines response induced by a TLR-4 agonist. We postulate that PTX modifies the polarization profile of macrophages (M1 to M2). In the context of TLR4 activation, the increase of cAMP induced by PTX may activates PKA, stabilize the IκB inhibitor and suppress NF-κB nuclear translocation. Besides, cAMP-PKA-dependent CREB phosphorylation may explain the induction of IL-10 mRNA at a transcriptional level.

##### REFERENCE(S)

(1) Staubach K-H, Schroder J, Stuber F et al. Arch Surg 1998;
(2) Pammi M, Haque KN. Cochrane Database Syst Rev 2015;
(3) Kreth S, Ledderose C, Luchting B et al Shock 2010
(4) Speer EM, Dowling DJ, Ozog LS et al. Pediatric Research 2017.

##### GRANT ACKNOWLEDGMENT

This study was financed with own resources

### 0887

#### IgM-enriched IgG as adjuvant therapy to treat sepsis and septic shock in the critically ill

A. Corona[1], I. Cigada[2], S. Santini[1], G. Spagnolin[1], P. Mandelli[1], C. Soru[1], F. Cantarero[1]
[1]University of Milan, ICU PO Luigi Sacco, Milano, Italy; [2]Päijät-Häme Central Hospital, Milano, Italy
**Correspondence:** A. Corona
*Intensive Care Medicine Experimental* 2018, **6(Suppl 2):**0887

**INTRODUCTION.** Sepsis is responsible of both an immune hyperactivity damage from inflammation and immune suppression and paralysis. A few studies support the role of IgM enriched immunoglobulins G as adjunctive of antimicrobial treatment

**OBJECTIVES.** To assess the efficacy of IgM enriched IgG as adjuvant therapy in treating sepsis in the critically ill.

**METHODS.** Case-control prospective study. Since 12/2016 to 01/04/2018, patients experiencing a septic shock - admitted with a first 24h SAPS II > 25, associated with a SOFA-score > 4 - underwent treatment with IgM-e-IG, given for three days at the total dosage of 500 mg/kg. The therapy response was based on clinical, microbiological and rheological data. All cases were 1:1 matched with analogous controls.

**RESULTS.** During the study period 25 patients were recruited and matched with 20 controls; no differences were found in median age [61 (55-78) vs. 63 (58-77) p=0.662], ICU length of stay [14 (5-19) vs. 15 (6-18) p=0.462]; duration of mechanical ventilation [7 (5-8) vs. 8 (5-9) p=0.348] and antimicrobial treatment [9 (8-11) vs. 11 (12-14) p=0.552]. PCT, CRP, WBC, Lactates and SOFA score were daily measured and computed the differences between day of stopping and starting IM-enriched IgG (For the controls such parameters ware considered over the same 72 hrs period. No differences were found in the trend of all parameters but SOFA [3.5 (2-5) vs. 0.5 (0-2) p=0.015], indicating a quicker and more timely recovery from sepis related organ failure in the treated patient group. The VLAD computed on the basis of SMR in the two group showed a higher number of saved lives in the treatment group (4.5 vs. 0.7). No differences in survival was found by Kaplan Meier analysis although at 28 days all patients of the treatment group were still alive.

**CONCLUSIONS.** IgM enriched immunoglobulins G as adjunctive of antimicrobial treatment may have a role in improving patient outcome, however PRCT are warranted to produce clear hints.

##### REFERENCE(S)

Cavazzuti I, Serafini G, Busani S, Rinaldi L, Biagioni E, Buoncristiano M, Girardis M. Early therapy with IgM-enriched polyclonal immunoglobulin in patients with septic shock. Intensive Care Med. 2014 Dec;40(12)

### 0888

#### Hydrocortisone, vitamin C, low dose norepinephrine and thiamine for the treatment of severe sepsis and septic shock: a retrospective before-after study

A.W. Ahmadzai[1,2]
[1]Nangarhar Reagional Hospital, G ICU, Jalalabad, Afghanistan; [2]Nangarhar Madical Faculty, Intensive Care, Jalalabad, Afghanistan
*Intensive Care Medicine Experimental* 2018, **6(Suppl 2):**0888

**INTRODUCTION.** The global burden of sepsis is estimated as 15 to 19 million cases annually, with a mortality rate approaching 60% in low-income countries.

**METHODS.** In this retrospective before-after clinical study, we compared the outcome and clinical course of consecutive septic patients treated with intravenous vitamin C,low dose hydrocortisone,low dose norepinehrine and thiamine during a 6-month period (treatment group) with a control group treated in our ICU during the preceding 6months. The primary outcome was hospital survival.
**RESULTS.** There were 45 patients in both treatment and control groups, with no significant differences in baseline characteristics between the two groups. The hospital mortality was 10.6% (5of 47) in the treatment group compared with 40.4% (19 of 47) in the control group (P < .001). The propensity adjusted odds of mortality in the patients treated with the vitamin C protocol was 0.13 (95% CI, 0.04-0.48; P = .002). The Sepsis-Related Organ Failure Assessment score decreased in all patients in the treatment group, with none developing progressive organ failure. All patients in the treatment group were weaned off vasopressors, a mean of 18.3 ± 9.8 h after starting treatment with the vitamin C protocol. The mean duration of vasopressor use was 54.9 ± 28.4 h in the control group (P < .001).
**CONCLUSIONS.** Our results suggest that the early use of intravenous vitamin C, together with low dose corticosteroids, low dose nor epinehrine and thiamine, are effective in preventing progressive organ dysfunction, including acute kidney injury, and in reducing the mortality of patients with severe sepsis and septic shock. Additional studies are required to confirm these preliminary findings.

## Airways & NIV

### 0889

#### Non-invasive ventilation vs. high flow nasal cannula oxygenation in pneumonia-associated acute respiratory failure. An observational retrospective study

S. Rebollo, R. Jiménez, A. Ortín, S. Sánchez, A. Ojados, L. Herrera, S. Moreno, A. Fernández, M. Galindo, R. Batllés, V. Serrano, C. Navarro, A. Ruiz, M.D. Rodríguez
Hospital General Universitario Santa Lucía, Servicio Medicina Intensiva, Cartagena, Spain
**Correspondence:** S. Rebollo
*Intensive Care Medicine Experimental* 2018, **6(Suppl 2):**0889

**INTRODUCTION.** Non-invasive ventilation (NIV) is frequently used to treat pneumonia associated acute hypoxemic respiratory failure, although some data suggest high failure rates. In last years, high flow nasal cannula oxgenation (HFNO) has appeared as a new therapy for hypoxemic patients.
**OBJECTIVES.** To compare clinical outcomes of patients admitted due to severe pneumonia and treated with NIV and HFNO.
**METHODS.** We retrospectively studied patients admitted to our polivalent ICU in which NIV or HFNO were used as first line respiratory support for acute respiratory failure associated pneumonia. We compared baseline characteristics and clinical evolution of patients according to the therapy used. For statistical analysis we used t-Student, U-Mann-Whitney, Chi-square or Fisher test as appropriate.
**RESULTS.** During the study period 274 patients were admitted to our ICU due to severe pneumonia. For the present analysis we excluded those patients with do not resuscitate order status and those early intubated at admission, resulting in 110 patients, 71 treated with NIV and 39 with HFNO.
Patients in NIV group were older (62.7 (59.3-66.2) vs 53.5 (47.8-59.1) years, p 0.006) and had higher APACHE II (18.8 (17.4-20.2) vs 14.3 (12.9-15.7), p < 0.001). Comorbidities were similar, with a trend to higher proportion of chronic kidney disease and COPD in NIV group. Need of inotrope support was similar in both groups but renal replacement was more frequently used in NIV group (7 v 0 %, p 0.043).
Nosocomial pneumonia was more frequent in NIV Group (32.4 vs 10.3 %, p 0.01) and time to ICU admission was longer in NIV group (1 (0-5) vs 0 (0-1), p 0.041) No differences in microbiological aetiology was observed.
At admission, patients in HFNO group had lower pO2 (76.7 (68.3-85.2) vs 91.4 (79.3-103.5), p 0.048) and lower pCO2 (36.8 (35.2-38.4) vs 43 (39.5-46.6), p 0.013), with no differences in respiratory rate, FiO2 and pO2/FiO2 rate. Radiological involvement was greater in HFNO group (2 (IQR 1-4) vs 1 (1-3) quadrants, p 0.007).
No differences in length of ICU or hospital stay were observed. Mortality, both ICU and hospital, were higher in NIV Group (28.2 vs 7.7 %, p 0.012 and 35.2 vs 12.8 %, p 0.012 respectively)
More patients in NIV group required intubation (52 vs 41 %), but it did not reach statistical significance.
**CONCLUSIONS.** Our results suggest benefit of HFNO over NIV as first line therapy in severe pneumonia. However, differences in baseline characteristics and severity of pneumonia limit our findings.

### 0890

#### Application of the ROX index as a predictior of response to high-flow nasal cannula in patients with acute hypoxemic respiratory failure

M. Magret Iglesias, N. Guasch Boqué, F. Esteban Reboll, A. Rodriguez Oviedo, M. Bodí Saera
Hospital Joan XXIII, Intensive Care Unit, Tarragona, Spain
**Correspondence:** M. Magret Iglesias
*Intensive Care Medicine Experimental* 2018, **6(Suppl 2):**0890

**INTRODUCTION.** High-flow nasal cannula (HFNC) have been defined as safe and useful therapy in patients with acute hypoxemic respiratory failure (AHRF), demonstrating a decrease in the need for mechanical ventilation (MV). However, one of the most challenging decisions in the management of AHRF patients is to decide when to move to invasive MV. In this regard, although HFNC may avoid further need for MV in some patients with AHRF, it may unduly delay initiation of MV in others and worsen their outcome. Therefore, to identify and describe accurate early predictors of the need for MV in spontaneously breathing patients with AHRF are of special interest. The literature shows that the ROX index (Respiratory rate OXygen) can be an early and non-invasive tool, predicting failure of HFNC.
**OBJECTIVE.** To apply the ROX index as a predictor of response to HFNC in our sample of patients with AHRF.
**METHODS.** .Retrospective study in a general ICU with 30 beds in a 12-month period. Adult patients with AHRF were included. Excluded patients were < 18, with hypercapnia or immediate MV criteria.The data were analyzed using median, interquartile range (IQR), mean and standard deviation (SD) for continuous variables. Absolute frequency and percentage for categorical variables. Through the Classification and Regression Trees (CART) we got a cut-off point for the continuous variables and the significance of the nominal or categorical variables. The ROX index was analyzed at 0, 12 and 24 hours, calculated from respiratory variables that allow the evaluation of respiratory failure: in the numerator, the variables correlated with the oxygenation and success of HFNC ($SatO_2$/ $FiO_2$) and in the denominator those that display inverse association (respiratory rate). The literature determines the cut-off point at 12 hours at 4.88.
**RESULTS.** 40 patients with AHRF were included. The most common cause was community-acquired pneumonia (CAP) (50%), followed by interstitial lung disease (15%). 17 patients were intubated (42.5%), 4 of them before 12 hours and 6 with diagnosis of CAP. There were no significant differences in sex, comorbidities, cause of AHRF or APACHE II between intubated and non-intubated patients. Using the CART system we obtained a cut-off point of 3.9 in the ROX index at 12 hours. At this time, patients with a ROX index < or equal to 3.9 were intubated in 71,4% compared to patients with a ROX index > 3,9 who were intubated in 29.6% (p< 0.05).
**CONCLUSIONS.** In our sample, the ROX index at 12 hours with a cut value of 3.9 allows us to predict the response to HFNC. Therefore, it may be an early non-invasive parameter to predict the need for intubation.

##### REFERENCE(S)

Roca O, Messika J, Caralt B, García-de-Acilu M, Sztrymf B, Ricard JD, Masclans JR. Predicting success of high-flow nasal cannula in pneumonia patients with hypoxemic respiratory failure: The utility of the ROX index. Journal of Critical Care 35 (2016) 200-205.

##### GRANT ACKNOWLEDGMENT

None

### 0892

#### Efficacy and safety of percutaneous dilatational tracheostomy with or without bronchoscopic guidance

T.-O. Kim, M.-S. Kim, B.-G. Kho, H.-J. Shin, H.-Y. Park, S.-C. Lim, J.-S. Chang
Chonnam National University Hospital, Pulmonology and Critical Care Medicine, Gwangju, Korea, Republic of
**Correspondence:** T.-O. Kim
*Intensive Care Medicine Experimental* 2018, **6(Suppl 2):**0892

**INTRODUCTION.** Percutaneous dilatational tracheostomy (PDT) is an easy and safe procedure performed in intensive care units.
**OBJECTIVES.** The aim of this study was to compare the efficacy and safety of PDT with bronchoscopic guidance with those of PDT without bronchoscopic guidance.
**METHODS.** We reviewed retrospectively the clinical data of 121 patients who underwent PDT at Chonnam National University Hospital between March 2013 and November 2016.
**RESULTS.** The median age of the patients was 72.0 years (interquartile range, 61.5-78.0), and 80 (66.1%) were men. Of all the patients, 44 (36.4%) underwent PDT with bronchoscopic guidance, which was unsuccessful in one patient, thereby the overall success rate of PDT being 99.2% (120/121). The number of complications was higher in patients who underwent PDT without bronchoscopic guidance than in those who underwent bronchoscopy-guided PDT, but the difference was not statistically significant (12 vs 3, p=0.251). All the complications were bleeding during or after the procedure and were easily controlled, except for a major bleeding in one patient who underwent PDT without bronchoscopic guidance and required cardiopulmonary resuscitation because of airway obstruction.
**CONCLUSIONS.** PDT has a high success rate and a low complication rate when performed with or without bronchoscopic guidance.

### 0893

#### Multidisciplinary airway training for intensive care staff in the workplace using the 'Bath Tea Trolley' approach

A. Serrano-Ruiz, G. Ball, S. Spencer, J. Chai, M. Charlton, F. Kelly
Royal United Hospital of Bath, Intensive Care Unit, Bath, United Kingdom
**Correspondence:** A. Serrano-Ruiz
*Intensive Care Medicine Experimental* 2018, **6(Suppl 2):**0893

**INTRODUCTION.** Intubation on the intensive care unit (ICU) is high risk,[1,2] with serious airway complications 55 times more likely to occur in ICU than in operating theatres.[3] Airway training for ICU nurses is challenging: ICU nurses often lack anaesthetic airway experience;[2] there are limited low risk cases for training;[2] nurses receive infrequent exposure to airway management especially advanced and rescue techniques;[2] and 'skill decay' is an issue which affects senior and junior staff alike.[4]
**OBJECTIVES.** Our aim was to design and deliver an airway training programme using the 'Bath Tea Trolley' method,[5] teaching ICU staff to use a bougie and apply cricoid pressure during a rapid sequence induction.
**METHODS.** We ran a month of concentrated airway training named 'Janu-airway,' using a trolley with airway training equipment on the top and pot of tea on the bottom. A trainer and the trolley travelled around the ICU, providing 10-15 minute teaching sessions for ICU staff at the bedside during their normal working shifts (both day and night shifts.) Cricoid pressure application was practiced and its effect on the view at laryngoscopy was demonstrated using a manikin and a C-MAC$^{TM}$ videolaryngoscope (Karl Storz GmbH, Tuttlingen, Germany), and assisting intubation using a bougie was practised. Posters were distributed via email/closed social media groups, and handouts were given to participants to facilitate reflective learning. Feedback was collected with staff self-rating confidence before/after training using a five point Likert scale, followed by a cup of tea!
**RESULTS.** 81 members of staff received training: 18 senior nurses, 43 staff nurses, 11 junior doctors, 3 physiotherapists, 3 medical students, 1 outreach sister, 2 acute critical care practitioners (ACCPs). This included 100% of ICU nurses and junior doctors. Feedback forms were completed after 57/81 (72%) of training sessions. Results as follows: increase in confidence by one point or more on the Likert scale reported for both cricoid pressure (48/58, 83%) and the use of a bougie (49/58, 84%) (figure 1); improvement in the ability to assist with intubation reported by all participants- 56/58 (97%) strongly agreed, 2/58 (3%) agreed; similar training in the future was requested by all participants- 47/58 (81%) strongly agreed, 11/58 (19%) agreed.
CONCLUSIONS Our 'Bath tea trolley' training programme provided effective, practical and non-threatening multidisciplinary airway training in the workplace. Training was provided to 100% of our ICU staff - during their usual shifts and for minimal cost. It was quick and easy to set up, flexible and fun! This training method could be transferable and reproducible in other ICUs.

**REFERENCE(S)**
1. Nolan JP, Kelly FE. Anaesthesia 2011;66(suppl2):81-92
2. Higgs A et al. BJA 2018; 120(2): 323-352
3. Cook TM et al. BJA 2011;106:632-42
4. Lindkær Jensen NH, Cook TM, Kelly FE. Anaesthesia 2016,71,1273-1279
5. O´Farrell G, McDonald M, Kelly FE. Anaesthesia 2015;70(1):104

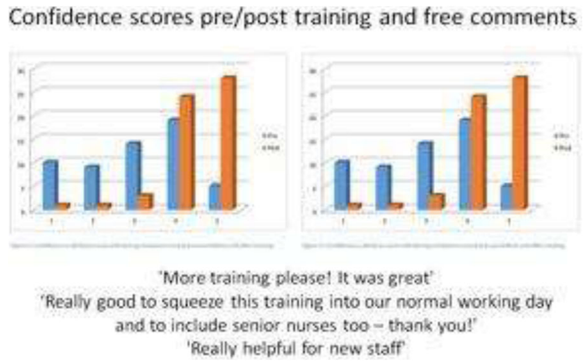

**Fig. 1 (abstract 0893).** Confidence scores for assisting intubation using a bougie and application of cricoid pressure before

### 0894

#### Perioperative factors associated with tracheostomy following oesophagogastrectomy surgery

M. Gerstman[1], J. Batty[1], G. Friedlaender[1], K. Tatham[1,2], M. Hacking[1], T. Wigmore[1]
[1]The Royal Marsden, Anaesthetic Department, London, United Kingdom; [2]Imperial College London, Section of Anaesthetics, Pain Medicine and Intensive Care, London, United Kingdom
**Correspondence:** M. Gerstman
*Intensive Care Medicine Experimental* 2018, **6(Suppl 2):**0894

**INTRODUCTION.** Oesophagectomy is a major oncological surgical procedure, associated with significant morbidity and mortality. Tracheostomy insertion is sometimes necessary to enable liberation from mechanical ventilation. This can be performed either at the end of the operation (electively), or post-operatively (delayed). Currently, the decision to insert a tracheostomy in our institution is based on clinician judgement. Prior studies have shown patients requiring a tracheostomy had longer lengths of stay, more days ventilated and more critical care unit complications.[1]
**OBJECTIVES.** Our aim was to determine which pre and intraoperative factors are associated with the need for tracheostomy in our institution. We also aimed to compare the outcomes in patients who did require a tracheostomy.
**METHODS.** We conducted a retrospective observational analysis of patients undergoing oesophagogastrectomies at the Royal Marsden over a 5-year period from September 2010 to December 2015.

**RESULTS.** A total of 157 patients underwent oesophagectomy. Of those 98 did not require tracheostomies, 36 had an elective tracheostomy and 23 a delayed tracheostomy. Patients who had an elective tracheostomy when compared to the no tracheostomy group had higher BMIs (Relative risk ratio (RRR) (95% CI) 1.12 (1.03-1.21) p< 0.01), were more likely to be ex or current smokers (RRR 10.8 (2.42-48.17) p< 0.01) and RRR 20.0 (3.42-116.8) p< 0.01 respectively), had higher ASA scores (ASA 1/2 versus 3/4 RRR 6.64 (2.86-15.40) p< 0.001), were more likely to have had a 3 stage procedure (RRR 15.63 (1.76-138.9) p< 0.05) had a lower anaeorobic threshold (RRR 0.78 (0.64-0.96) p =< 0.05) and $VO_2$ max (RRR 0.86 (0.75-0.97) p< 0.05), and had longer surgery (RRR 1.01 (1.01 - 1.02) p< 0.001). Patients who required a delayed tracheostomy had higher BMIs (RRR 1.15 (1.05-1.27) p< 0.01) and longer duration of surgery (RRR 1.01 (1.00 - 1.01) p< 0.05) than those who did not require a tracheostomy.

Patients who did not require a tracheostomy had a median ICU stay of 6 days (interquartile range [IQR] 5-8). Patients who had an elective tracheostomy had a median stay of 10 days (IQR 8-14) and those with delayed tracheostomy of 19 days (IQR 14-27); p< 0.001. This trend was mirrored by VAP rates, hours of ventilation and length of hospital stay (p< 0.05). No significant difference was seen in mortality rates.

**CONCLUSIONS.** This study shows patients undergoing an elective tracheostomy in our institution were more likely to have higher BMIs, to have smoked, to have had longer and more complex surgery and had a lower anaerobic threshold and $VO_2$ max. The need for a delayed tracheostomy was associated with higher BMIs and longer duration of surgery. Prospective randomised studies are required to further analyse the benefits of elective versus delayed tracheostomy for oesophagectomy.

**REFERENCE(S)**

1. Wessels et al. ARJCCM 2012;185:A5978

**GRANT ACKNOWLEDGMENT**

N/A

### 0895

#### Endotracheal tubes with polyurethane cuffs and subglotic aspiration port - what's the evidence?

R. Knafelj[1], P. Radsel[1], M. Fister[1], M. Noc[1], I. Lopatic[2], T. Flerin Poropat[2]
[1]University Medical Center Ljubljana, MICU, Ljubljana, Slovenia;
[2]University Medical Center Ljubljana, Radiology, Ljubljana, Slovenia
**Correspondence:** R. Knafelj
*Intensive Care Medicine Experimental* 2018, **6(Suppl 2):**0895

**INTRODUCTION.** Oral care plays important part of ventilatory associated pneumonia (VAP) prevention bundle. Tt is performed multiple times (>3 times) a day in all intubated/tracheotomized patients and requires endotracheal tube (ET) repositioning within oral cavity. Longitudinal and lateral ET movements are present during oral care which pose risk for micro aspiration.

**OBJECTIVES.** To determine whether polyurethane cuffed endotracheal tube with subglotic port (sCET) and intermittent subglotic aspiration reduces aspiration compared to polyurethane cuffed endotracheal tube without subglotic port (CET) and manual aspiration.

**METHODS.** 20 porcine necks purchased at local butcher were intubated with sCET (Halyard Microcuff, n=10) or with CET (Parker ThinCuff, n=10) cuffs were inflated to 25 cm H2O. 5 mL of iodine based contrast media was instilled in trachea over cuffs mimicking oral secretions. Porcine necks were positioned in 30 degree elevated position simulating patient in ICU environment. During 24h aspiration with aspiration catheter (CET) or designated port (sCET) was performed every 8h and and tube manipulaion simulating oral care was performed. After first (1h) and forth (24h) endotracheal tube manipulation, CT was performed and 3D reconstruction were obtained. Images were analyzed by radiologist who was blinded for tube types or designs (1). Leakage was scored as none (no contrast pass ), minimal (contrast only coating/visible in cuff micro-folds), moderate (traces of contrast passing the cuff), substantial (contrast clearly visible distal to cuff in trachea).

**RESULTS.** After first ET manipulation no contrast was visible passing the cuff in all of the sCETs (Figure 1 A) and minimal contrast passing was visible in 3 CETs (Figure 1 C). After 24h no contrast pass was demonstrated in 2 and minimal pass in 8 sCETs (Figure 1 B). Minimal contrast pass was demonstrated in 1 CETs and moderate in 9 CETs (Figure 1 D).

**CONCLUSIONS.** Polyurethane cuffed sCETs provide superior seal and potentially ensure better prevention from microaspiration compared to polyurethane CETs. Further clinical studies are warranted.

**REFERENCE(S)**

Poropat T, Knafelj R. Different cuff materials and different leak tests - one size does not fit all. Critical Care 2016:20(Suppl 2):94.

**GRANT ACKNOWLEDGMENT**

None

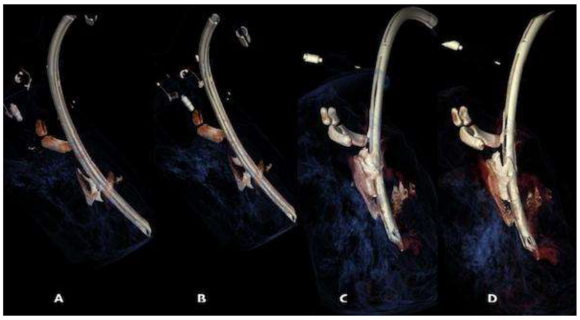

**Fig. 1 (abstract 0895).** See text for description.

### 0896

#### The bedhead EndoTracheal tube airway information (BETTA-In) project

E. Davies, A. Sills, B. Covas, D. Atkinson
Manchester Royal Infirmary, Department of Critical Care, Manchester, United Kingdom
**Correspondence:** E. Davies
*Intensive Care Medicine Experimental* 2018, **6(Suppl 2):**0896

**INTRODUCTION.** Airway emergencies contribute to significant morbidity and mortality on the intensive care unit (ICU), as explored by the 4th National Audit Project of The Royal College of Anaesthetists ($NAP_4$). There was an apparent failure to identify ICU patients who had high-risk airways.[1] Bedhead signs have been introduced to provide easy access to airway information. They provide a reference point for assessment and have the potential to save time in emergencies. Tracheostomy and laryngectomy signs have had widespread success in the United Kingdom due to the National Tracheostomy Safety Project.[2] Signs detailing other airway variances or devices have been introduced. $NAP_4$ recommended that "patients at risk of airway events should be identified and clearly identifiable to those caring for them".[1] The Greater Manchester Critical Care & Major Trauma Services Network also recommends that signs record any intubation difficulties.[3]

**OBJECTIVES.** The aim of this project was to improve compliance with use of a bedhead endotracheal tube (ETT) sign on our ICU.

**METHODS.** A standard was set: 100% patients with an ETT in situ on ICU should have a bedhead airway information sign. A quality improvement project was carried out at Manchester Royal Infirmary from 2017 to 2018. In each study period, all intubated patients on ICU were identified at the start of a day shift for 5 consecutive days. The presence or lack of an airway sign was noted. Percentage compliance was calculated. Interventions were made between study periods. Following presentation at the departmental audit meeting and feedback throughout the project, the sign was redesigned by the authors. (Figure 1)

**RESULTS.** Baseline compliance was 8%. Following interventions, this was increased and maintained despite changeover of staff and stopping intervention. The project has yet to meet the standard. (Figure 2)

**CONCLUSIONS.** The identification of patients at risk of adverse airway events and subsequent display of information are crucial in preventing harm on the ICU. This project identified a need to improve compliance with bedhead airway information signs for intubated patients. Interventions improved compliance. The project is ongoing but a cultural change has already been shown. The authors would support the use of this sign's design on other ICUs.

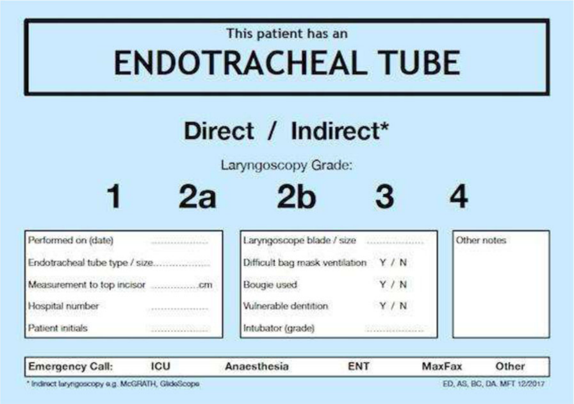

**Fig. 1 (abstract 0896).** Final design of the bedhead airway information sign

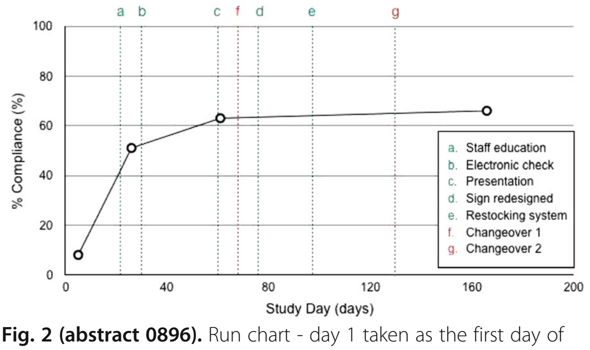

**Fig. 2 (abstract 0896).** Run chart - day 1 taken as the first day of baseline data collection, 02/10/2017

### 0897

#### Surgical tracheostomy performed by a dedicated ENT surgeon in a Tunisian medical intensive care unit: early and late complications in an outcome study

W. Zarrougui[1], R. Ben Dabebiss[1], G. Sboui[1], R. Atig[1], O. Beji[1], R. Mani[2], H. Hmouda[1]

[1]Sahloul University Hospital, Medical Intensive Care Unit, Sousse, Tunisia; [2]Sahloul University Hospital, Maxillo-Facial Surgery Department, Sousse, Tunisia

**Correspondence:** W. Zarrougui

*Intensive Care Medicine Experimental* 2018, **6(Suppl 2):**0897

**INTRODUCTION.** Surgical tracheostomy (ST) and percutaneous dilatational tracheostomy are commonly performed in most Tunisian intensive care units by the intensivist. Both techniques are usually safe, but early and late lifethreatening complications have been described with both strategies. The availability of an experienced and dedicated ENT surgeon may reduce early and late complications, in addition to close follow-up until removal of the tracheostomy canula, and tracheoscopy.

**OBJECTIVES.** To assess early and late complications of tracheostomy, in addition to long term outcome of ST performed by a dedicated ENT surgeon in the operating room on patients admitted to the medical intensive care unit (MICU) in a Tunisian university hospital.

**METHODS.** A retrospective study was performed in the MICU between January 2011 and February 2018.Were included all patients who required tracheostomy for prolonged ventilator support, airway protection, pulmonary toilette, or facilitation of weaning from the ventilator.

All tracheostomies were performed by a dedicated ENT surgeon in the operating room, using the standard technique, and follow up was ensured by the same ENT surgeon during ICU stay, and after discharge for patients who required home ventilation or airway management.

The study collected clinical features at admission, reason for admission, severity of illness, ICU course, and acute management procedures. Duration of tracheal intubation, oxygenation parameters, per and post interventional complications were recorded.

**RESULTS.** A total of 33 patients were included: 20 male (61%) and 13 female (39%), with a mean age of 54.2 ±18.1 years (range15 to 79 years). Mean Charlson index was 2.6±2 and mean SAPSII at admission was 46.5±12.5. Twenty nine patients (87.8%) received invasive mechanical ventilation as a first line ventilator support, 18 (56.4%) required vasopressors. All the patients had ST. The indication for tracheostomy was weaning difficulty in 16 patients (48.5%) and prolonged intubation in 13 patients (39.4 %). The mean time from tracheal intubation to tracheostomy was 14.9±10.4days.There were no serious perioperative complications. There was no death related to tracheostomy. Postoperative minor complications occurred in only 4 patients (12.1%). They included secondary wound hemorrhage in 2 cases, and air leak attributed to tracheomalacia in the 2 others. Tracheostomy tube was removed in only 1 patient during ICU stay. Twenty patients (60.6%) were discharged alive, among them 12 patients required home ventilation.The mortality rate at one year was 36.3% and 3 patients (15.7%) were successfully decannulated.

**CONCLUSION.** The rate of early and late complications was extremely low when tracheostomy was performed by a dedicated ENT surgeon. These findings will aid in the development of protocols and pathways for airway management in critically ill patients to maximize cost-effective, high-quality care and ensure acceptable risk/benefit ratio of the procedure.

### 0898

#### Tracheostomy in a Tunisian medical ICU: practices and outcomes

H. Zorgati[1], A. Azouzi[1], M.A. Boujelbèn[1], D. Ben Braiek[1], N. Fraj[1], S. Kortli[1], A. Khedher[1], I. Ben Saida[1], K. Meddeb[1], M. Boussarsar[1,2]

[1]Farhat Hached University Hospital, Medical Intensive Care Unit, Sousse, Tunisia; [2]Ibn Al Jazzar Faculty of Medicine, Research Laboratory N° LR12SP09 Heart Failure, Sousse, Tunisia

**Correspondence:** M. Boussarsar

*Intensive Care Medicine Experimental* 2018, **6(Suppl 2):**0898

**INTRODUCTION.** Tracheostomy in the intensive care has many advantages when prolonged ventilation is required. It reduces sedation needs, promotes oral and pharyngeal hygiene, improves patient comfort, offers tracheal stability, decreases incidence of ventilator acquired pneumonia. However, as any procedure, it is not free from risks and complications can be observed.

**OBJECTIVES.** To describe state of tracheostomy practice and outcomes in a Tunisian medical ICU.

**METHODS.** A retrospective descriptive study was carried out in a 9-beds medical ICU at teaching hospital of Farhat Hached Sousse Tunisia, from January 2015 to December 2017.

In this survey were compiled the underlying diseases, diagnostic at admission, SAPSII, indication,timing and technique of tracheostomy, length of stay and the various complications (early and late). Outcomes were assessed by in-hospital mortality rate and at the first year and potential decannulation.

**RESULTS.** 717 patients were admitted in the ICU within the study period, 522 were mechanically ventilated. Tracheostomy was performed in 76(14.5%) patients among them 42.1% were COPD GOLD D. Median age was 63.5[53.2-73.7]years, 53(69.7%) male and SAPSII at 33±12. Bedside surgical technique was used in 99.1%. Most tracheostomies were performed during the second week, 37(48.7%), with mean delay at 11.46.8 days, 11(14.5%) in the first, 16(21.1%) in the third and 7(9.2%) in the fourth week. The median length of stay was 26[19-36]days. Overall, 19 complications were reported including 2 cardio-respiratory arrests, 2 pneumothoraces, 4 local infections, 7 problems were related to cannula (displacement, 3 and decannulation, 2). 13(17%) patients were decannulated during their hospitalization and 7(9.2%) after ICU discharge. 24(51.1%) patients were released with a home mechanical ventilator. 12(25.5%) patients had full autonomy. 14(18.4%) patients were lost to follow up. 29(38.2%) patients died during their hospitalization. The mortality rate after discharge was 66.7% among them 51.5% died in the first year.

**CONCLUSIONS.** The present study demonstrated a frequent use of tracheostomy in severe COPD patients but with a relatively late delay and rather poor outcomes.

### 0899

#### Percutaneous tracheostomy can be safely performed without bronchoscopic guidance

S. Appavu
University of Illinois College of Medicine, Surgery, Rockford IL, United States
*Intensive Care Medicine Experimental* 2018, **6(Suppl 2):**0899

**INTRODUCTION.** Published articles increasingly recommend bronchoscopic guidance (BG) for percutaneous tracheostomy (PT) in the critically ill. However, BG is not without clinical, technical and logistical problems including hypercarbia and respiratory acidosis. In most patients, PT can be performed without BG both at the bedside (BS) and in the operating room (OR).

**OBJECTIVE(S).** To confirm the feasibility and safety of PT without BG both at BS and in the OR.

**METHODS.** We retrospectively reviewed PTs performed between January 1, 2015 and December 31, 2017 by a single experienced critical care surgeon. When PT was the only procedure, it was done at BS; when another procedure such as a feeding access was needed, then it was done in the OR. Chiaglia single pass dilator kits and Bivona silastic tracheostomy tubes were used in all procedures. An experienced anesthesiologist managed the sedation and the airway. Briefly, the procedure itself consisted of 4 essential steps: 1. Needle puncture of trachea and guidewire passage, 2. Advancement of a short tracheal dilator, 3. Single pass of "Blue Rhino" dilator and 4. Passage of the tracheostomy tube. Data collection included demographics, primary diagnosis, physical location of the procedure and duration, complications and final outcome.

**RESULTS.** We had 46 patients; 22 males and 24 females aged 21 to 88 years; 13 of the 46 (28%) had had an acute neurologic event such as intracranial hemorrhage or stroke. The remaining patients had acute respiratory failure from various causes including pneumonia, ARDS, acute pancreatitis and sepsis. Fourteen PTs were done at BS and 32 in the OR. The mean procedure duration from incision to insertion of the tracheostomy tube and confirming with EtCO2 was 6 minutes. There were 3 complications (6.7%): 2 pneumothoraces, both from a longer tube entering the right main stem bronchus in patient with severe kypho-scoliosis or very short stature. They were immediately identified and treated with chest tube. One patient had mediastinal emphysema due to the dilator slipping out of the trachea and entering the superior mediastinum; it did not require specific therapy. There was no procedure related death. Thirty-five were discharged to chronic ventilator care facility, four went home and five went on hospice or comfort care due to worsening primary critical illness. One patient died of sudden cardiac arrest from an unrelated cause 14 days later. Another 4-week pregnant suicide victim with brain death was taken off life support after planned Caesarian section at 25 weeks.

**CONCLUSIONS.** PT can be performed at BS or in the OR without mortality, with acceptable complication rate.

**REFERENCE(S)**

1. Kizhner V et al, Percutaneous tracheostomy boundaries revisited. Auris Nasus Larynx 2015 Feb; 42: 39-42
2. Rashid AO, Islam S, Percutaneous tracheostomy: a comprehensive review. J Thorac Dis 2017; suppl 10 S1128-S1138

### 0900

#### The use of high flow nasal cannula oxygen outside the intensive care unit

S. Zemach, M. Shitrit, P. Levin
Shaare Zedek Medical Center, Jerusalem, Israel
**Correspondence:** P. Levin
*Intensive Care Medicine Experimental* 2018, **6(Suppl 2):**0900

**INTRODUCTION.** The use of High Flow Nasal Cannula Oxygenation (HFNCO) therapy for non-invasive respiratory support in the ICU is evidence based and routine. Due to ease of application and clinical success, non-evidence based use of HFNCO has spread to non-ICU wards. ICU physicians may be consulted regarding the advisability of therapy in the non-ICU environment.

**OBJECTIVES.** To describe the use and outcomes of HFNCO outside the ICU.

Methods - An observational study of the use of HFNCO as prescribed by non-ICU physicians in non-ICU areas for patients with respiratory failure who had failed prior non-invasive oxygenation support. Primary outcome measures included changes in patients´ respiratory distress (measured using a visual analog scale (VAS)), and changes in pulse, blood pressure, respiratory rate and blood oxygen saturation before and 30 minutes after initiation of HFNCO treatment. Secondary outcomes included early (< 72hours after starting therapy) and late (>=72 hours) mortality, and a composite measure of mortality, ICU admission and intubation.

**RESULTS.** Data were collected on 111 patients over 20 months. Overall, 90/111 patients (81%) reported decreased respiratory distress (VAS score before vs after HFNCO therapy 7.3±2.1 vs 4.7±2.1, P< 0.001). Pulse (97.4±22.5 vs 93.2±19.6 beats per minute, p=0.002) and respiratory rate (31±10 vs 26±7 breaths per minute, p=0.001) also decreased while saturation increased (84±12% vs 94±56%, p< 0.001) with HFNCO therapy.

*Secondary outcomes:* Of the 111 subjects treated with HFNC, 55 (50%) died, 24/111 (22%) within 72 hours of beginning HFNC, and 31/111 (28%) later. However, 41/111 (33%) patients had a DNR order (of whom 38/41, 93% died). Amongst the 70 patients without a DNR order, overall mortality was 18/70 (26%) [early mortality < 72 hours 9/70 (13%), late mortality 12/70 (17%)]. Overall 35/70 (50%) patients without a DNR order met the composite outcome measure for successful treatment (no ICU admission, no intubation and survival to hospital discharge).

On multivariate analysis increased severity of respiratory distress (pre-HFNCO VAS score OR 1.75 95%CI 1.35-2.28, p< 0.001) and a past medical history of respiratory disease (OR 3.52 95%CI 1.27-9.72, p=0.01) were significant predictors of improvement in respiratory distress with HFNCO.

**CONCLUSIONS.** HFNCO treatment outside the ICU was associated with improved VAS scores and physiological parameters. HFNCO was used in many patients who had a DNR order, suggesting that it's use may have been palliative. Early mortality (directly associated with HFNCO therapy) in non-DNR patients was considerable (13%) and warrants caution in the widespread application of this therapy outside the ICU.

### 0901

#### Timing of failure of non-invasive positive ventilation and clinical outcomes of critically ill patients

O. Peñuelas[1], C. Coscia[2], A. Muriel[3], D. Bin[4], K. Raymondos[5], A.W. Thille[6], M. González[7], B.V. Pinheiro[8], M.C. Marin[9], P. Amin[10], N. Cakar[11], S.M. Maggiore[12], F. Ríos[13], A. Bersten[14], A. Anzueto[15], J. Mancebo[16], A. Esteban[17], F. Frutos-Vivar[1]

[1]Hospital Universitario de Getafe; CIBER de Enfermedades Respiratorias, Intensive Care Unit, Getafe, Spain; [2]Hospital Universitario 12 de Octubre Ramón y Cajal, Biostatistical Unit, Madrid, Spain; [3]Hospital Universitario Ramón y Cajal, Biostatistical Unit, Madrid, Spain; [4]Peking Union Medical College Hospital, Intensive Care, Beijing, China; [5]Medizinische Hochschule Hannover, Intensive Care Unit, Hannover, Germany; [6]Centre Hospitalier Universitaire de Poitiers, Intensive Care Unit, Poitiers, France; [7]Universidad Pontificia Bolivariana, Intensive Care Unit, Medellín, Colombia; [8]Pulmonary Research Laboratory, Federal University of Juiz de Fora, Intensive Care Unit, Juiz de Fora, Brazil; [9]Hospital Regional 1° de Octubre ISSSTE, Intensive Care Unit, Mexico DF, Mexico; [10]Bombay Hospital Institute of Medical Sciences, Intensive Care Unit, Mumbai, India; [11]Istanbul Faculty of Medicine, Intensive Care Unit, Istanbul, Turkey; [12]Università degli Studi G. d'Annunzio Chieti e Pescara, Intensive Care Unit, Rome, Italy; [13]Hospital Nacional Alejandro Posadas, Intensive Care Unit, Buenos Aires, Argentina; [14]Department of Critical Care Medicine, Flinders University, Intensive Care Unit, Adelaide, Australia; [15]South Texas Veterans Health Care System and University of Texas Health Science Center, Intensive Care Unit, San Antonio, United States; [16]Hospital de la Santa Creu i Sant Pau, Intensive Care Unit, Barcelona, Spain; [17]Hospital Universitario de Getafe; CIBER de Enfermedades Respiratorias, Getafe, Spain

**Correspondence:** O. Peñuelas

*Intensive Care Medicine Experimental* 2018, **6(Suppl 2):**0901

**INTRODUCTION.** Overall, the use of noninvasive positive pressure ventilation (NPPV) for acute respiratory failure (ARF) in critically ill patients is associated with improved clinical outcomes as compared with invasive mechanical ventilation. However, multiple studies have indicated that patients failing NPPV have worse outcomes compared with patients with successful NPPV treatment, and limited data is available on timing associated with NPPV failure and outcomes. The aim of this study is to evaluate the risk of mortality according to the timing between start and failure of NPPV in ARF patients.

**METHODS.** An international, observational, multicenter non interventional study was carried out. We included consecutive adult critically ill patients admitted in the intensive care unit (ICU) due to ARF and subjected to any attempt of NPPV as first ventilatory support between Abril 1st 2016 to May 31st 2016. NPPV failure was defined as the need for intubation after a trial of NPPV. Primary outcome was ICU mortality. Statistical analysis: The risk of mortality was evaluated by a propensity score analysis, and patients were classified as: successful NPPV attempt (as reference category); early failure as NPPV failure during the first 24 hours; late failure as NPPV failure after 24 hours from the initiation of NPPV. Adjusted odds ratios (OR) and 95% confidence interval (CI) were assessed.

**RESULTS.** Overall, a total of 1,228 patients were included and NPPV failure was observed in 374 (30.7%) patients. Early failure occurred in 182/374(48.7%) patients, and late failure occurred in 192/374 (51.3%) patients. After performing propensity score analysis, late NPPV failure was associated significantly with higher ICU mortality compared with early NPPV failure (Odds Ratio (OR) 2.4, 95% CI 1.4 to 4.2, p=0.002). Regardless the reason to start NPPV, the risk of ICU mortality remained significantly higher compared with successful NPPV (see table). The risks were higher when failure occurred after 24 h from the initiation of NPPV.

**CONCLUSIONS.** Timing of failure of NPPV and delayed intubation longer than 24 hours is associated with higher ICU mortality, independently of the reason of ARF.

**Table 1 (abstract 0901).** Risk of ICU mortality in patients failing NPPV, adjusted by IPTW analysis. Successful NPPV is used as variable of reference

| | Early failure NPPV (N=182) | Early failure NPPV (N=182) | Late failure NPPV (N=192) | Late failure NPPV (N=192) |
|---|---|---|---|---|
| | OR (CI 95%) | P value | OR (CI 95%) | P value |
| Acute exacerbations of chronic obstructive pulmonary disease (N=269) | 5.3 (1.5-18.7) | 0.009 | 35.3 (10.2-122.6) | <0.001 |
| Acute pulmonary edema (N=269) | 7.4 (2.3-23.9) | 0.001 | 35.3 (10.2-122.6) | <0.001 |
| ARDS (N=77) | 15.1 (1.1 - 12.2) | 0.039 | 34.5 (3.9-307.4) | 0.002 |
| Others reasons ARF (N=355) | 5.4 (2.4 - 12.2) | <0.001 | 15.6 (6.9- 35.2) | <0.001 |
| Others reasons (N=258) | 1.6 (0.6 - 4.6) | 0.362 | 3.7 (1.6-9.0) | 0.003 |

### 0902

#### Non-invasive ventilation for very old patients: 5-year experience from a tertiary care center

M. Esteves Brandão[1], S. Xavier Pires[2], A.P. Dias[3], N. Barros[3], F. Esteves[3]

[1]Centro Hospitalar de Trás-os-Montes e Alto Douro, Respiratory Medicine, Lordelo, Vila Real, Portugal; [2]Hospital da Senhora da Oliveira, Guimarães EPE, Internal Medicine, Guimarães, Portugal; [3]Centro Hospitalar de Trás-os-Montes e Alto Douro, Intensive Care Medicine, Vila Real, Portugal

**Correspondence:** M. Esteves Brandão

*Intensive Care Medicine Experimental* 2018, **6(Suppl 2):**0902

**INTRODUCTION.** Due to ageing of the general population, the proportion of very old patients admitted to hospital and to intensive care is rapidly growing.Whereas non-invasive ventilation (NIV) is an attractive technique for the management of acute respiratory failure (ARF) in very old persons, specific data for this population are limited[1].

**OBJECTIVES.** To identify conditions in which NIV is applied to very old patients in the intensive care unit (ICU) and to assess whether its efficacy is similar to younger patients.

**METHODS.** Retrospective cohort of all patients admitted to the ICU of a tertiary hospital during a 5-year period and managed using NIV.-Demographic characteristics, context of NIV, severity of illness, duration of mechanical ventilation, intubation rate, ICU length-of-stay (ICU-LOS), hospital length-of-stay (hospital-LOS), ICU and hospital mortality rates were compared for very old (age≥80 years) and younger patients.Logistic regression was performed to identify predictors of NIV failure (defined as need for endotracheal intubation or death) in the very old population.

**RESULTS.** During the study period, 810 adult patients received NIV as first-line therapy for treatment of ARF, of which 162 (20.2%) were older than 80 years of age.The most frequent cause of ARF in very old group was pneumonia (19.8%), followed by post-operative respiratory failure (18.5%).Exacerbation of COPD and cardiogenic pulmonary oedema were less frequent admitting diagnosis (13% and 8% of patients, respectively). Forty nine very old patients (30.2%) received NIV in the context of a do-not-intubate order.At ICU admission, compared with younger patients, very old patients were more severely ill as expressed by higher APACHE II, SAPS II and SAPS 3 scores (p< 0.001).NIV failure rates did not differ between the two groups (51.2% vs 54.5%).In multivariate analysis, only SAPS II score independently predicted NIV failure in the very old patient population

(p=0.01,OR 1.076,95% CI 1.02-1.14). Very old patients had a significantly higher ICU (23.5% vs 13.9%,p=0.003) and hospital mortality rates (35.8% vs 21.1%,p=0.001) and a lower intubation rate (38.3% vs 48.8%,p=0.018) than younger patients.However, ICU and hospital mortality rates were similar in the two groups when NIV was applied for exacerbation of COPD.

**CONCLUSIONS.** Very old patients represented 20% of all patients managed with NIV in our ICU. NIV failure rates were similar between very old and younger patients.SAPS II score at admission was an independent predictor of NIV failure in the very old population.Very old patients showed increased in-ICU mortality when compared to the younger group. Importantly, in-ICU and hospital survival rates of very old were comparable to the younger group when NIV was applied in a “gold” clinical indication like COPD exacerbation.

**REFERENCE(S)**

1. Schortgen F, Follin A, Piccari L, et al. Results of noninvasive ventilation in very old patients. Ann Intensive Care. 2012 Feb 21;2(1):5.

### 0903

#### Cost-minimization analysis of Non-invasive and Invasive mechanical ventilation for de novo acute hypoxemic respiratory failure in an Eastern European setting

V. Ilieva[1], T. Mihalova[2], Y. Yamakova[1], R. Petkov[2], B. Velev[2]
[1]Medical University Sofia, Anesthesiology and Intensive Care, Sofia, Bulgaria; [2]Medical University Sofia, Pulmonology, Sofia, Bulgaria
**Correspondence:** V. Ilieva
*Intensive Care Medicine Experimental* 2018, **6(Suppl 2):**0903

**INTRODUCTION.** Non-invasive ventilation (NIV) is now a standard of care worldwide for hypercapnic acute respiratory failure (ARF) but the results regarding hypoxemic ARF are inconclusive. In the official ERS/ATS clinical practice guidelines[1] the task force does not make any recommendation on the use of NIV for de novo hypoxemic ARF. On the basis of uncertainty about the superiority of Invasive mechanical ventilation (IMV) over NIV in this setting we made a cost-minimization analysis comparing both methods in a single National Center for Pulmonary Diseases in Bulgaria.

**OBJECTIVES.** The main objective of our study is to estimate the direct medical costs generated by a patient on IMV and NIV during their ICU stay. Other objectives are to identify which aspect of the treatment is most expensive.

**METHODS.** A total of 36 patients, separated in two groups - NIV (n=18) and IMV (n=18), with de novo hypoxemic ARF were included in our study. We calculated the direct medical costs and compared them. Costs for human resources, capital equipment, and overheads were not included. All calculations were made in Euro based on the price list for 2018 used in University Hospital of Pulmonary Diseases, Sofia, Bulgaria. Statistical processing was done with the programming package R.

**RESULTS.** On admission the $PaO_2/FiO_2$ (135.02 ± 32.44 vs. 127.5 ± 23.73, p=0.07), respiratory rate (31.44 ± 6.11 vs. 29.55 ± 3.9, p=0.92) and SAPS II score (37.9 ± 18.47 vs. 30.9 ± 10.34, p=0.06) were comparable in both groups.

We observed a statistically significant difference in the overall costs per patient for pharmacy (NIV=10410.45 ± 1346.96, IMV=29015.61 ± 2788.88, p=0.01), consumables (NIV=45.26 ± 5.86, IMV=72.94 ± 7.01, p=0.05) and diagnostic tools (NIV=6514.05 ± 475.98, IMV=16417 ± 1160.58, p=0.019). When all costs were summed the difference became even bigger (NIV=17304.45 ± 861.91, IMV=47400.9 ± 1874.10, p< 0.001).

We also computed the costs per patient per day and there was a significant difference in the costs for pharmacy (NIV=45.26 ± 5.86, IMV=72.94 ± 7.01, p=0.05) and consumables (NIV=1.65 ± 0.12, IMV=4.95 ± 0.34, p=0.003) but not in the diagnostic tools (NIV=28.32 ± 2.07, IMV=41.27 ± 2.91, p=0.09). Summed together the costs per patient per day were worth NIV=75.24 ± 3.75, IMV=119.16 ± 4.71, p=0.03.

The large standard deviations observed in some of the calculations are due to some big price differences in Bulgaria.

**CONCLUSIONS.** In the setting of de novo hypoxemic ARF NIV reduces significantly the overall costs of treatment in comparison to IMV. The decreased costs in NIV are not associated with severity of disease according to the respiratory quotient and SAPS II score.

**GRANT ACKNOWLEDGMENT**
None

### 0904

#### High-flow oxygen through Nasal cannula versus Continuous Positive Airway Pressure in patients with moderate hypoxemic respiratory failure due to pneumonia

M. Giovini[1], M. Cecchia[1], S. Gandolfi[2], S. Orlando[1], E. Antonucci[1]
[1]Intermediate Care Unit - Guglielmo da Saliceto Hospital, Piacenza, Italy; [2]Local Health Unit - Guglielmo da Saliceto Hospital, Piacenza, Italy
**Correspondence:** M. Giovini
*Intensive Care Medicine Experimental* 2018, **6(Suppl 2):**0904

**INTRODUCTION.** Acute respiratory failure (ARF) due to pneumonia represents a serious clinical problem, with high mortality rate and frequent ICU admissions. Oxygenation modalities (OM) such as Continuous Positive Airway Pressure (C-PAP) and High-Flow Oxygen through Nasal Cannula (HFO) could be useful in those cases, preventing endotracheal intubation (ETI) and reducing mortality[1,2]. No study has ever compared helmet C-PAP and HFO in adult patients with hypoxemic ARF.

**OBJECTIVES.** We compared HFO and helmet C-PAP in patients with moderate hypoxemic ARF sustained by pneumonia.

**METHODS.** We performed a retrospective analysis, reviewing all patients with pneumonia and moderate hypoxemic ARF, admitted to our Intermediate Care Unit (IMCU) from June 2016 to December 2017 (18 months). The inclusion criteria were age ≥18 years old, pneumonia (community acquired or hospital acquired) with moderate hypoxemic ARF (PaO2/FiO2 100-200 mmHg) at admission, oxygenated by HFO or helmet C-PAP (stand-alone system) for 2 consecutive days at least. Exclusion criteria were history of chronic obstructive pulmonary disorder (COPD) or other chronic respiratory disorders; lung cancer; acute cardiogenic edema; severe neutropenia; circulatory failure. We analyzed two groups (C-PAP and HFO) matched for age, SAPS2 score, SOFA score and PaO2/FiO2 ratio before OM. We also collected demographic characteristics, comorbidities and we investigated rates of ETI, IMCU mortality and mortality at 90 days.

**RESULTS.** We review 38 patients: 20 in HFO group and 18 in CPAP group. Demographic characteristics are shown in Table 1. The two groups were well matched (PaO2/FiO2 ratio before OM p = 0.7; SAPS 2 score p = 0,3; SOFA score p = 0,3; age = 0.07; see Table 2). We found no significant difference in ETI rate [HFO 2/20 (10%), CPAP 3/18 (17%)], IMCU mortality [HFO 2/20 (10%); CPAP 3/18 (17%)] and mortality at 90 days [HFO 3/18 (17%); CPAP 2/15(13%)] between the two groups (see Figures). ARDS patients was mainly treated by helmet CPAP (13/17) so that we corrected this confounding factor and we found no significant difference in ETI rate (p = 0.94) and IMCU mortality (p = 0.94) between the two groups.

**CONCLUSIONS.** In patients with moderate hypoxemic respiratory failure due to pneumonia, treatment with helmet C-PAP or HFO did not result in significantly different ETI and mortality rates.

**REFERENCE(S)**

1. Frat JP, et al. High-flow oxygen through nasal cannula in acute hypoxemic respiratory failure. N Engl J Med. 2015
2. Brambilla AM,et al. Helmet CPAP vs. oxygen therapy in severe hypoxemic respiratory failure due to pneumonia. Intensive Care Med. 2014

**Table 1 (abstract 0904).** Demographic characteristics

| Demographic characteristis | n. of patients (%) |
|---|---|
| Male | 25/38 (66%) |
| Neoplasia | 9/38 (24%) |
| Diabetes | 17/38 (45%) |
| Cardiovascular diseases | 28/38 (74%) |
| CKD, chronic kidney disease | 11/38 (29%) |
| CAP, community-acquired pneumonia | 32/38 (84%) |
| HAP, hospital-acquired pneumonia | 6/38 (16%) |

**Table 2 (abstract 0904).** Matching variables

| Variables | C-PAP [Mean (min-max)] | HFO [Mean (min-max)] |
|---|---|---|
| PaO2/FiO2 ratio before OM | 117 (54-198) | 120 (51-198) |
| SAPS2 score | 39 (22-75) | 35 (21-51) |
| SOFA score | 5 (2-10) | 4 (2-7) |
| Age | 69 (40-89) | 75 (34-86) |

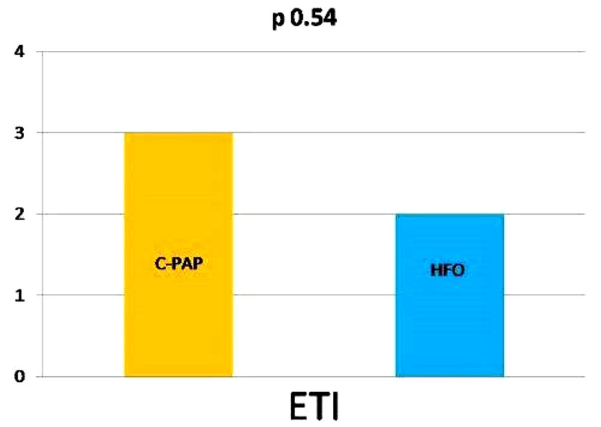

**Fig. 1 (abstract 0904).** ETI rate

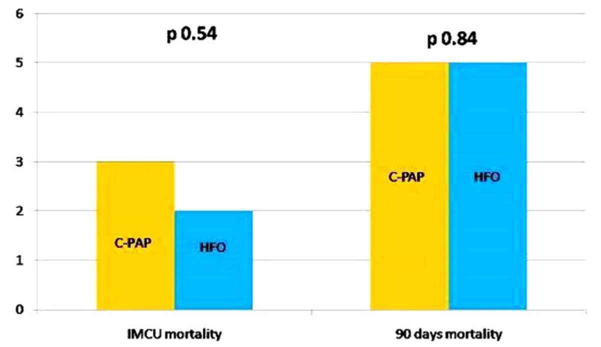

**Fig. 2 (abstract 0904).** Mortality rates

### 0905

#### The role of non-invasive ventilation in mild-to-moderate acute respiratory distress syndrome

M. Esteves Brandão[1], S. Xavier Pires[2], A.P. Dias[3], N. Barros[3], F. Esteves[3]
[1]Centro Hospitalar de Trás-os-Montes e Alto Douro, Respiratory Medicine, Vila Real, Portugal; [2]Hospital da Senhora da Oliveira, Guimarães EPE, Internal Medicine, Guimarães, Portugal; [3]Centro Hospitalar de Trás-os-Montes e Alto Douro, Intensive Care Medicine, Vila Real, Portugal
**Correspondence:** M. Esteves Brandão
*Intensive Care Medicine Experimental* 2018, **6(Suppl 2):**0905

**INTRODUCTION.** Acute respiratory distress syndrome (ARDS) is a life-threatening form of respiratory failure that accounts for 10% of intensive care admissions[1].The role of non-invasive ventilation (NIV) in treatment of ARDS is still controversial,mostly due to the concern about the association between NIV failure,delayed intubation and consequently increased mortality.

**OBJECTIVES.** To describe the current practice and efficacy of NIV use in ARDS patients admitted to an intensive care unit (ICU) of a tertiary hospital.

**METHODS.** Retrospective analysis of all patients with ARDS admitted to our ICU between 2010 and 2015 and primarily managed with NIV.Patient's demographics, cause of ARDS,severity of illness,duration of mechanical ventilation,intubation rate,ICU length-of-stay (ICU-LOS),hospital length-of-stay (hospital-LOS),and mortality rate were recorded. Logistic regression was performed to identify predictors of NIV failure (defined as need for endotracheal intubation or death).

**RESULTS.** During the study period,NIV was used in 332 patients with ARDS:36.1% mild,43.1% moderate and 20.8% severe,according to Berlin definition.The mean age of study population was 64 ± 15 years and 207 (62.3%) were male.Pneumonia (43.7%) followed by post-operative respiratory failure (19%) were the most common causes of ARDS.Sepsis was present in up to 90% of patients at ARDS onset.Baseline mean PaO2/FiO2 ratio was 179 ± 107 mmHg and mean APACHE II was 21 ± 8.NIV was successful in 31% of patients, while 63.9% required endotracheal intubation.Prone positioning was used in 32 patients (9.6%).In-ICU and hospital mortality rates were 14.5% and 19.9%,respectively.Similar survival rates were observed between patients who improved with NIV and those patients who required invasive mechanical ventilation (80.5%vs75.7%). Patients with mild-to-moderate ARDS were less likely to fail NIV (66.2%vs79.7%;p=0.03) and showed significantly lower in-ICU (11.4%vs26.1%;p=0.04) and hospital mortality (17.4%vs30.4%;p=0.049) when compared to severe ARDS patients. Baseline APACHE II (p< 0.001;OR 0.89,95%CI 0.83-0.95), SAPS II (p< 0.001;OR 1.08,95%CI 1.04-1.12) and SOFA scores (p< 0.001;OR 1.22,95%CI 1.08-1.37),lactate elevation at 24h of ICU admission (p=0.045; OR 1.5, 95% CI 1-2.2) and nosocomial infection (p< 0.001;OR 3.3, 95%CI 1.7-6.2) were independent predictors of NIV failure.

**CONCLUSIONS.** In our cohort, the use of NIV helped in avoiding intubation in about 31% of patients with ARDS.Outcomes were significantly better in mild-to-moderate ARDS. Severe systemic illness as reflected by higher disease severity scores (APACHE II, SAPS II and SOFA) and nosocomial infection appear to have an important role on NIV failure. The use of NIV did not delayed endotracheal intubation in those who failed to respond, as demonstrated in a survival rate comparable to the NIV success group.

**REFERENCE(S)**

1. Fan E, et al. Acute Respiratory Distress Syndrome: Advances in Diagnosis and Treatment. JAMA. 2018 Feb 20;319(7):698-710.

## End-of-life & organ donation

### 0906

#### Ethical framework of the integration of palliative care in intensive care: results from a qualitative study with professionals

S.M. Pereira[1,2], C.M. Teixeira[3,4,5], P. Hernández-Marrero[1,2], A.S. Carvalho[1,2]
[1]UNESCO Chair in Bioethics, Institute of Bioethics, Universidade Católica Portuguesa, Porto, Portugal; [2]CEGE - Centro de Estudos em Gestão e Economia, Porto Católica Business School, Universidade Católica Portuguesa, Porto, Portugal; [3]Instituto de Bioética, Universidade Católica Portuguesa, Porto, Portugal; [4]Hospital de Santo António, Centro Hospitalar do Porto, Porto, Portugal; [5]Instituto de Ciências Biomédicas Dr. Abel Salazar, Universidade do Porto, Porto, Portugal
**Correspondence:** C.M. Teixeira
*Intensive Care Medicine Experimental* 2018, **6(Suppl 2):**0906

**INTRODUCTION.** The integration of palliative care (PC) in intensive care (IC) can be framed ethically as it fosters the integration of PC ethical core principles (i.e., autonomy, beneficence, non-maleficence, justice, dignity, integrity, vulnerability, solidarity) in IC. Although several initiatives have been described worldwide on the organizational integration of PC in IC, very little is framed using an ethical perspective or approach.

**OBJECTIVES.** To ethically frame and understand the integration of PC in IC considering healthcare professionals' perspectives.

**METHODS.** Qualitative exploratory study, using in-depth interviews. Participants were healthcare professionals (physicians, nurses, psychologists, physiotherapists, social workers) working in Portuguese PC and IC units. These professionals were gathered using a purposive sampling procedure complemented with snowball sampling until reaching theoretical saturation. A total of 22 professionals (11 physicians, 9 nurses, and 3 other professionals) from various cities were interviewed.

**RESULTS.** In the participants' speech, the integration of PC in IC was considered to be a way of improving end of life care and decision-making processes. Once life-supportive measures were either withheld or withdrawn, professionals working in IC considered to implement a palliative care approach already. According to the professionals working in PC, an earlier and better integration of PC in IC was needed to prevent invasive interventions, disproportionate care and admissions in IC units. From an ethical perspective, these findings can be framed using the ethical principles of beneficence, non-maleficence, justice and dignity. Professionals working in PC also considered that the integration of PC in IC could promote patient autonomy, by allowing patients to be cared for and die in their place of choice. It was also recognised, by both professionals working in PC and IC, that patients in IC are particularly vulnerable. The integration of PC in IC could therefore protect them from any further harm and futile interventions. While professionals working in PC considered that the integration of PC and IC should be promoted via a mixed-organizational model, professionals from IC units mentioned that an educational model, empowering them with PC knowledge and competences, would suffice.

**CONCLUSIONS.** This is the first empirical study addressing the integration of PC in IC in Portugal. It adds to the already existing evidence by embracing and developing an ethical framework. Professionals working in IC and PC seem to have different perspectives on how the integration model should be. Findings show the potential for a successful integration, which is aligned with core ethical principles. More research is needed to implement and study the impact of an effective, sustainable and ethically sound integration model.

**GRANT ACKNOWLEDGMENT**
Fundação Grünenthal and Fundação Merck, Sharp and Dohme.

### 0907

#### Morbidity and mortality of discharge against medical advice patients in a tertiary care hospital in a developing country

P.K. Routray[1], A. Mishra[1], S. Jamuda[1], D. Mishra[1], B.S. Mishra[1], M.P. Tripathy[2]
[1]CARE Hospitals, Critical Care, Bhubaneswar, India; [2]CARE Hospitals, Cardiology, Bhubaneswar, India
**Correspondence:** P.K. Routray
*Intensive Care Medicine Experimental* 2018, **6(Suppl 2):**0907

**INTRODUCTION.** India being a developing nation with limited financial resources amongst majority of the population, lack of insurance and limited critical care resources in government hospitals, most of sick patients were taken to corporate hospitals. Corporate Hospitals cater to majority of the sick patients, but a large subset after few days take their patient out of corporate hospitals due to mounting cost. But rarely these patients were followed up. It is a try from our side to understand the epidemiology of Discharge against Medical advice and their long term follow up of 3 months.

**OBJECTIVES.** To study whether the subset of patients who were discharged against medical advice succumbed to illness as soon as they were withdrawn from aggressive care as assumed or they have variability in their morbidity and mortality.

**METHODS.** Retrospective data of patients who were discharged against medical advice were collected from OCTOBER 2016 to DECEMBER 2017 in our hospital.

*INCLUSION CRITERIA* - Patients who were admitted in Medical ICU with in the period of OCTOBER 2016 to DECEMBER 2017 but were taken away from our hospital due to various reasons. All these patients were contacted through phone and after due permission they were asked a questionnaire with specific yes or no answer. Questionnaire was first tested bilingually both in English and Oriya (local language) and with volunteers.

*EXCLUSION CRITERIA* - All patients with cardiac arrest with CPR and who died within hospital premises while shifting were excluded.

**RESULTS.** Total 1170 patients were admitted. Out of which 115(9.8%) patients were discharged against medical advice and we could contact 105(8.9%) patients over phone.

85(80%)went for financial reasons

10 (9.5%)went away due to end of life care

10(9.5%) went away due to dissatisfaction over treatment.

77(73%) went with an ET tube in situ

74 (70%)went home

31 (29.5%) went to another hospital

Amongst all 59(56%)died within 6 hours of discharge and 22(20%)died within 24 hrs of discharge while 12(11%) died within 72 hrs of discharge. 5 patients survive for a 7 days and finally succumbed. But another 7(6.6%)patients survived for more than 90 days and out of which 5(4,7%) remained bed ridden 2(1.9%)patients attained their full functional recovery are on continuous follow up.

**CONCLUSIONS.** There has been a lot of debate regarding medical miscalculation regarding extubation and de-escalation in a tertiary care medical ICU and interestingly our majority of decisions were guided from guidelines from European or American studies. This study showed that 12 patients(11.4%) patients survived inspite of being taken home and drastic de-escalation from ventilator as well as other life supports.

This needs to be validated in large studies to find out whether we are overdoing the guidelines to keep ourself safe or a subset of patients should be given a trial of aggressive de-escalation before sending them home as discharged against medical advice.

### 0908

#### Acceptance rates of end-of-life decisions among ICU physicians in Turkey

N. Baykara[1], T. Utku[2], V. Alparslan[3], M.K. Arslantaş[4], N. Ersoy[3]
[1]University of Kocaeli, School of Medicine, Intensive Care Unit, İstanbul, Turkey; [2]İstanbul University, Cerrahpaşa School of Medicine, İstanbul, Turkey; [3]Kocaeli University, School of Medicine, Kocaeli, Turkey; [4]Marmara University, School of Medicine, İstanbul, Turkey
**Correspondence:** N. Baykara
*Intensive Care Medicine Experimental* 2018, **6(Suppl 2):**0908

**INTRODUCTION.** Turkey´s elderly population has increased by 17 percent in the last five years. There are few palliative care and nursing home in Turkey. It is not legal to take end-of-life decision in Turkey, according to Turkish criminal law. Thus, there is a high ratio of terminal stage patients in ICUs in Turkey. The major faith tradition in Turkey is Islam.

**OBJECTIVES.** This study was performed to determine the views and perceptions of Turkish ICU physicians about end-of-life decisions.

**METHODS.** The Kocaeli University Ethical Committee and Review Board approved the study. An online survey was distributed through Survey Monkey,an online survey response collecting software. The survey was targeted toward ICU physicians practicing in Turkey. The survey consisted of 29 questions designed to assess demographics, training, religion, ICU facilities, bed capacity and ICU experience. In addition, the views of ICU physicians about end-of-life desicions (withdrawing, witholding or limiting life sustaining therapy) and the factors that may affect these decisions were determined. Statistical analyses were performed using SPSS version 20.0 (SPSS Inc, Chicago, Illinois).

**RESULTS.** A total of 607 physicians completed the online survey. While 92.2% of the ICU physicians expressed that it is necessary to make changes to allow do-not- resuscitate (DNR)orders in Turkish criminal law, 81.1 % of them stated that it is necessary to make changes to allow do-not-intubate (DNI) orders in Turkish criminal law. DNR and DNI acceptances were not changed by the physicians' age, sex, religious beliefs and ICU experience (p>0.05). 67.0 % of ICU physicians expressed that they would choose to forgo CPR and intubation /mechanical ventilation if they were dying of a terminal illness. While 81.3 % of the ICU physicians expressed that at least some of the life support treatment should be withhold in terminally ill patients, 8.7 % of them expressed that no any new treatment should be start in terminally ill patients. Similarly, 76.7 % of the ICU physicians expressed that at least some of life support treatment should be withdrawn in terminally ill patients and 21.8 % of them expressed that all of the life support treatment, except sedative and analgesic drugs, should be withdrawn.

**CONCLUSIONS.** It is necessary to make changes to allow end of life desicions in Turkish criminal law.

### 0909

#### Reduction of medical costs by implementing early family meeting with the decision to withdraw life-sustaining treatments in the medical intensive care unit: a propensity score-matched study in a medical center

H.-T. Chang[1,2,3], J.-S. Jerng[4], D.-R. Chen[3,5]
[1]Far Eastern Memorial Hospital, Department of Critical Care Medicine, New Taipei City, Taiwan, Province of China; [2]Yuan-Ze University, Department of Industrial Engineering and Management, Taoyuan, Taiwan, Province of China; [3]National Taiwan University College of Public Health, Institute of Health Policy and Management, Taipei, Taiwan, Province of China; [4]National Taiwan University Hospital, Department of Internal Medicine, Taipei, Taiwan, Province of China; [5]National Taiwan University College of Public Health, Institute of Health Behavior and Community Sciences, Taipei, Taiwan, Province of China
**Correspondence:** H.-T. Chang
*Intensive Care Medicine Experimental* 2018, **6(Suppl 2):**0909

**INTRODUCTION.** Evidence suggested the association of early palliative care for the terminal illness with lower medical cost, but few studies have examined the effectiveness of implementing early family meeting (EFM) for discussion about the decision to withdraw (DTW) life-sustaining treatments from end-of-life patients reduce medical costs in the intensive care unit (ICU) setting.

**OBJECTIVES.** To investigate the relationships of EFM, defined as a palliative family conference held within seven days after ICU admission, and DTW during ICU stay with health-care costs and relevant outcomes.

**METHODS.** We retrospectively analyzed the medical and administrative data of patients who died at the medical ICU of a medical center from 2012 to 2016. The documentation of family meeting was confirmed by the meeting record in the medical records and the relevant claim code the administrative data of the patients. DTW was documented by the meeting records. We first compared the patients with and without the DTW life-sustaining treatments regarding their costs, ICU stays, and outcomes. We then performed a propensity-score matching to compare the medical costs between the cases with and without (designated as controls) DTW and EFM.

**RESULTS.** A total of 579 patients, accounting for 16% of the medical ICU admissions, were included. Patients with DTW life-sustaining treatments had longer ICU stays than those without (12.9±7.1 days vs. 8.4±9.6 days, $p < 0.001$), and were more likely to have a ´do-not-resuscitate´ order (97.3% vs. 80.4%), more likely to have family meetings (89% vs. 16.2%, $p < 0.001$). The patients with DTW also had lower health-care costs (New Taiwan Dollars [NTD] 231,542±130,057 vs. 273,942 ± 301,293, p=0.038). Multivariate regression analysis showed that longer ICU stay (OR: 1.076, 95%CI: 1.045-1.108, $p < 0.001$), do-not-resuscitate status (OR: 6.08, 95%CI 1.365-26.88, p = 0.017), blood transfusion (OR: 4.407, 95%CI 2.339-8.305, $p < 0.001$), and early family meeting (OR: 3.425, 95%CI 1.873-6.262, $p < 0.001$) were associated with DTW. Comparison between cases and controls after propensity-score matching showed that the cost reduction associated with DTW was significant (NTD 109,545, 95%CI 28,903-190,187, $p < 0.001$), and EFM in the patients with DTW further reduced the cost (NTD 96,119, 95%CI 42,912-149,325, p=0.008).

**CONCLUSIONS.** An early family meeting followed by a decision to withdraw life-sustaining treatments significantly lowered costs during end-of-life management in the ICU. The results confirm the value of early palliative care, including family meetings to agree upon end-of-life management.

##### REFERENCE(S)

Phua J, Joynt GM, Nishimura M, et al. Withholding and withdrawal of life-sustaining treatments in intensive care units in Asia. *JAMA internal medicine.* 2015;175(3):363-371.

##### GRANT ACKNOWLEDGMENT

This work was supported by Far Eastern Memorial Hospital Grants - FEMH-2018-C-025.

### 0910

#### Prevalence, risk factors and clinical characteristics of burnout in critical care units from Colombia

S.M. Navarro Estrada, J.G. Franco, F.J. Molina Saldarriaga, J.D. Velásquez Tirado, C.A. Agudelo Vélez, M. Larios Gómez
Universidad Pontificia Bolivariana, Medellin, Colombia
**Correspondence:** S.M. Navarro Estrada
*Intensive Care Medicine Experimental* 2018, **6(Suppl 2):**0910

**INTRODUCTION.** According to Maslach, the definition of burnout involves 3 fields: emotional exhaustion, depersonalization and lack of personal fulfillment[1]. Most studies in intensive care have found that the prevalence of the disorder is around 25% (range 6% to 47%)[2].

**OBJECTIVES.** To describe the prevalence, risk factors and characteristics of professional exhaustion in professionals and technicians of intensive care units in Colombia

**METHODS.** A cross-sectional analytical observational study was conducted. The Spanish survey version of professors Gil-Monte and Peiró was used[3]. We interviewed professionals (doctors, nurses and other technicians) working in intensive care in Colombia, affiliated to the Colombian Association of Critical Care Medicine and Intensive Care (AMCI). We used both the dichotomous score (epidemiological aspects and comparisons between groups) and continuous score (factorial analysis of the construct in the sample). We report risk analysis (bivariate and multivariate logistic) and factorial for depletion defined according to the *Maslach Burnout Inventory* (MBI)

**RESULTS.** 211 respondents were included. Of these, 62 (29.4%) have professional exhaustion. Being young (OR 0.93, 95% CI 0.899-0.979), having between 6 and 10 years of experience (OR 2.82, 95% CI: 1.283-6.216), being in charge of more than 10 patients per day (OR 3.31, 95% CI: 1.580-6.918), the perception of hostility of patients or companions (OR 2.96, 95% CI: 1.488-6.006) and the perception of having an anxious or depressive disorder (OR 2.87, 95% CI: 1.345-6.104) were related in a direct way with exhaustion in the multivariate analysis. Having some hobby was a protective factor against burnout (OR 0.44, 95% CI 0.209-0.923

**CONCLUSIONS.** The prevalence of burnout in intensive care in Colombia is high. The disorder is characterized by affective alterations and personal satisfaction in those who suffer it. It is necessary to implement detection strategies and interventions on psychological factors and the institutional setup since the early years of professional practice

**REFERENCE(S)**

1. Maslach C, Leiter MP, Schaufeli W. Measuring Burnout. En: Cartwright S, Cooper CL, editores. The Oxford handbook of organizational well being. New York, NY: Oxford University press; 2008. p. 87-108.
2. Chuang CH, Tseng PC, Lin CY, Lin KH, Chen YY. Burnout in the intensive care unit professionals: A systematic review. Medicine (Baltimore). 2016;95:e5629.
3. Gil-Monte PR. Factorial validity of the Maslach Burnout Inventory (MBIHSS) among Spanish professionals. Revista Saúde Pública. 2005;39:1-8

**GRANT ACKNOWLEDGMENT**

None.

### 0911

#### Intensive care unit (ICU) nurses' death perception, end of life stress and end of life nursing attitudes

K. Sera, N. Mi jin, L. Soon Heang
Asan Medical Center, University of Ulsan College of Medicine, Seoul, Korea, Republic of
**Correspondence:** K. Sera
*Intensive Care Medicine Experimental* 2018, **6(Suppl 2):**0911

**INTRODUCTION.** This study aimed to survey the death perception according to the general characteristics of ICU nurses and to analyze the problems of the current ICU related to end of life stress and end of life nursing.

**METHODS.** A descriptive research study design was used to confirm the contents of death perception, end of life stress and end of life attitude of nurses in ICU. Data were collected from a total of 16 general hospitals and 975 nurses in the intensive care unit were randomly selected to answer structured questionnaires. Data were analyzed using descriptive statistics, t-test, ANOVA, correlation analysis, and multiple regression analysis.

**RESULTS.** The death perception of ICU nurses was measured as 4.38 points. The attitude of end of life nursing was as follows: The higher age, marital status, proportion of faith in life, satisfaction, frequency of death and endurance education and longer duration of ICU work, The better recognition of death and attitude of end of life nursing. The average duration of nursing stress was 3.90. With the higher age, the smaller number of patients in charge, the more need for healing programs related to death or dying, the less demand for dying, and the lower of the satisfaction for enduring nursing, the end-of line nursing stress was high.

**CONCLUSION.** The results of this study suggest that continuing research is necessary to develop guidelines for attitudes toward end of life nursing, education for end of life, and effects of practical application after the development of a nursing care bundle.

- Braun M, Gordon D, Uziely B. Associations between oncology nurses' attitudes toward death and caring for dying patients. Oncology Nurse Forum 2010;37:E43-9.
- Inumiya Y. A Study on Development of a View of Life and Death Scale and Relationships among its Elements. Seoul: Korea University; 2002. p. 1-104.
- Frommelt M, Katherine H. The effects of death education on nurses´ attitudes toward caring for terminally ill person and their families. The American Journal of Hospice & Palliative Care, 1991;8:37-43.
- Thorson JA, & Powell FC. A Revised Death Anxiety Scale. Death Studies. 1994;16(6):507-521. http://dx.doi.org/10.1080/07481189208252595
- Lange M, Thom B, & Kline NE. Student nurses´ attitudes towards death and dying in south-east Iran. International Journal of Palliative Nursing. 2008;14(5):214-219. http://dx.org/10.12968/ijpn.2008.14.5.29488

### 0912

#### Invasive techniques and mortality in both patients over and under 75 years old in a polyvalent ICU: a comparative study

Á. Hurtado, M.G. Gómez, L. Cantón, A. Vélez, J. Garnacho
Universidad de Sevilla, Sevilla, Spain
**Correspondence:** Á. Hurtado
*Intensive Care Medicine Experimental* 2018, **6(Suppl 2):**0912

**INTRODUCTION.** Life expectancy is strikingly increasing during the last decades. We are more and more faced with the difficult task of deciding a patient´s needs to be admitted to Intensive Care Units (ICU) and implementation of invasive techniques must be weighed.

**OBJECTIVES.** To compare elderly (≥ 75 yr), and younger (r< 75yr) ICU patients in relation to ICU underlying diseases, treatment intensity, as well as ICU and in-hospital mortality.

**METHODS.** Observational retrospective study with a prospectively collected database carried out in a medical-surgical 30-bed ICU without coronary admissions. All patients admitted from January 2013 to December 2018 were included. We analysed demographic characteristics, severity of the illness at admission measured by Acute Physiology and Chronic Health Evaluation II (APACHE II) scale, invasive treatment, origin, reason for admission, length of stay and mortality in the ICU and hospital.

Qualitative variables are presented as the absolute numbers, whereas frequency and quantitative variables are presented as median (25th percentile to 75th percentile). Analysis was performed using chi-square or exact Fisher test for qualitative variables and Student t test or Mann-Whitney U test for quantitative variables when appropriate. P values of less than 0.05 were regarded as statistically significant.

**RESULTS.** 6,598 patients were analysed, 5,136 (75.84%) were < 75yr and 1,462 (22.16%) were ≥ 75 yr. The results are shown in the tables below.

**CONCLUSIONS.** In our series, elderly patients present more serious illness at the admission and more comorbidity, mainly DM, COPD and CKD. A higher mortality is found among the elderly patients during their ICU and hospital stay. Elderly patients receive less invasive treatments probably due to the limitation of therapeutic efforts.

**REFRENCES**

Santana-Cabrera L, Lorenzo-Torrent R, Sánchez-Palacios M, Martín Santana JD, Hernández Hernández JR. Influencia de la edad en la duración de la estancia y en la mortalidad de los pacientes que permanecen de forma prolongada en una Unidad de Cuidados Intensivos. *Rev Clin Esp.* 2014;214(2):74-78

Pintado M.C, Villa P, Luján J, Trascasa M, Molina R, González-García N y de Pablo R. Mortalidad y estado funcional al año de pacientes ancianos con ingreso prolongado en una unidad de cuidados intensivos. *Med Intensiva*. 2016; 40(5):289-297

Andersen F. H and Kvåle R. Do elderly intensive care unit patients receive less intensive care treatment and have higher mortality? *Acta Anaesthesiol Scand* 2012; 56: 1298-1305

Hans Flaatten, Dylan W. De Lange, Alessandro Morandi, Finn H. Andersen, Antonio Artigas. The impact of frailty on ICU and 30-day mortality and the level of care in very elderly patients (≥ 80 years). *Intensive Care Med.* 2017 Dec; 43(12):1820-1828

Añon M, Gómez-Tello,V, González- Higueras E, Córcoles V. Prognosis of elderly patients subjected to mechanical ventilation in the ICU. Med Intensiva 2013; 37:149-55.

**Table 1 (abstract 0912).** Comparison of patients' characteristics and origin of admission

| | ≥ 75 year old (n=1462) N( %) | < 75 years old (n= 5136) N (%) | P |
|---|---|---|---|
| Gender (Man) | 808 (55.3%) | 3194 (62.2%) | <0.0005 |
| APACHE II | 15 ( 12-20 ) | 12 ( 8-17 ) | <0.0005 |
| Ward | 1173 (80.2%) | 3948 (76.9%) | 0.006 |
| Community | 270 (18.5%) | 1114 (21.7%) | 0.007 |
| Another ICU | 16 (1.1%) | 64 (1.2%) | 0.64 |
| Geriatric facility | 3 (0.2%) | 10 (0.2%) | 0.93 |
| Medical admission | 383 (26.2%) | 1626 (31.7%) | <0.0005 |
| Planned surgical admission | 761 (52.1%) | 2561 (49.9%) | 0.13 |
| Unplanned surgical admission | 18 (21.8%) | 949 (18.5%) | 0.005 |

APACHE II: Acute Physiology And Chronic Health Evaluation II

**Table 2 (abstract 0912).** Patients' comorbidities

| | ≥ 75 year old (n=1462) N( %) | < 75 years old (n= 5136) N (%) | P |
|---|---|---|---|
| DM | 552 (37.8%) | 1318 (25.7%) | < 0.0005 |
| Cirrhosis | 20 (1.4%) | 222 (4.3%) | < 0.0005 |
| COPD | 225 (15.4%) | 582 (11.3%) | < 0.0005 |
| Neoplasia | 324 (22.2%) | 1132 (22%) | 0.91 |
| CKD | 231 (15.8%) | 410 (8%) | < 0.0005 |
| Immunocompromised | 43 (2.9%) | 388 (7.6%) | < 0.0005 |
| Malnutrition | 71 (4.9%) | 352 (6.9%) | 0.006 |
| ATB during the last 48 hours | 245 (16.8%) | 946 (18.4%) | 0.14 |

DM: Diabetes Mellitus. COPD: Chronic Obstructive Pulmonary Disease CKD: Chronic Kidney Disease.ATB: Antbiotics

**Table 3 (abstract 0912).** Invasive therapies and mortality in both groups

| | ≥ 75 year old (n=1462) | < 75 years old (n= 5136) | P |
|---|---|---|---|
| MV | 922 (63.1%) | 3157(61.5%) | 0.27 |
| Days on MV | 1 ( 0-2 ) | 1 ( 0-2 ) | 0.05 |
| Tracheostomy | 45 (3.1%) | 239 (4.7%) | 0.009 |
| CRRT | 36 (2.9%) | 207 (4.4%) | 0.02 |
| Parenteral Nutrition | 150 (10.3%) | 525 (10.2%) | 0.96 |
| ICU Stay (days) | 3 ( 2-6 ) | 4 ( 2-6 ) | 0.35 |
| Hospital stay (days) | 14 ( 9-24 ) | 14 ( 8-25 ) | 0.76 |
| ICU Mortality | 182 (13.4%) | 472 (9.5%) | <0.0005 |
| In-hospital Mortality | 273 (18.7%) | 604 (11.8%) | <0.0005 |

MV: Mechanical Ventilation. CRRT: Continuous Renal Replacement Therapy

### 0913

#### Ethography in intensive care unit: the *'Emergent ICU Body'* and two models of care

I. Anastasiou[1,2]
[1]University of Durham, Medical Anthropology, Durham, United Kingdom; [2]Southampton University Hospital, Biomedical Research Centre (BRC), Southampton, United Kingdom
*Intensive Care Medicine Experimental* 2018, **6(Suppl 2):**0913

**INTRODUCTION.** The present study examines how caring and technology are interrelated in the Intensive Care Unit (ICU) setting. It is a qualitative study focused on the phenomenology of "lived experience" of the healthcare personnel intended to be an ethnography that "evokes", rather than "represents" the discursive practises generated in relation to the ICU technologies. In this setting the organic, the technical, and the cultural domains interrelate composing a reality not of individuals, objects, and subjects being autonomous things-in-themselves, but as **"things-in-phenomena"**.OBJECTIVES To explore how the patient, as "person-with-a-body", is perceived in the context of the Intensive Care Unit. To identify issues which indicate de-personification of the patients and/or objectification of the patients' body.

**METHODS.**

**Research setting:** Two public hospital ICUs in Greece

**In-depth Interviews:** 15 participants. The Modified Ethnographic Decision Modelling (EDM) **Fig.1** Combining the Ethnographic Research Cycle and Linear Hypothesis-Testing Plan.

**Phenomenology:** The theoretical background for data generation and analysis.

**Grounded Theory:** To construct a theory"grounded" on data **Fig.2** The techno-cultural domains' interactions that generated the data. ANALYSIS A core category was constructed embodying a phenomenon apparent in the interrelationships of all categories, ***the functional intercoupling of patient-carer-technology***. The ill-person remains a *subject* for the caregivers mostly defined by the **synthetic *-sub***, meaning under my control, my vigilance, and my acts. The ICU devices change the ill-person's physiology and anatomy (opening of non-pre-existed holes, eliminating the normal digestive system) creating **a *transformed body,*** also **a suspended *personhood.*** There is also the making of ***subjected carers*** as the fieldwork has revealed. In critical care, machines, persons, knowledge, tools even spaces are all entangled in "hybrid assemblages" both producing and produced by culture. I call this functional ICU amalgamation, the ***Emergent ICU Body (EIB)*** emerging through the inseparability of its three parts.The prevalent model of care in the two Greek ICUs is the ***Authoritative Model of Care.*** The technology is the *bridge*, between the caregiver and the patient. However, there is a different perspective to approach the same ICU "phenomena". I call it ***Altruistic Model*,** a kind of ***Prosthetic Model*** of critical *care*. K. Barad's quantum physics-based concept of *intra-acting parts of a phenomenon* renders the possibility for the phenomenological *EIB* to exist, embodying the agency of the patient regardless his/her de-personification.The altruistic *EIB* follows the "paradigm of relation" rather than the "paradigm of control".

**CONCLUSIONS.** The *Emergent ICU Body* efficiently arises in full functionality and *vitality*, whenever the Altruistic Model of Care is practiced. Whenever the Authoritative Model of Care is being chosen, the *EIB* will always be fragmented and inefficiently functioning.

**ACKNOWLEDGEMENTS**

I. Anastasiou is supported by the National Institute for Health Research through the NIHR Southampton Biomedical Research Centre.

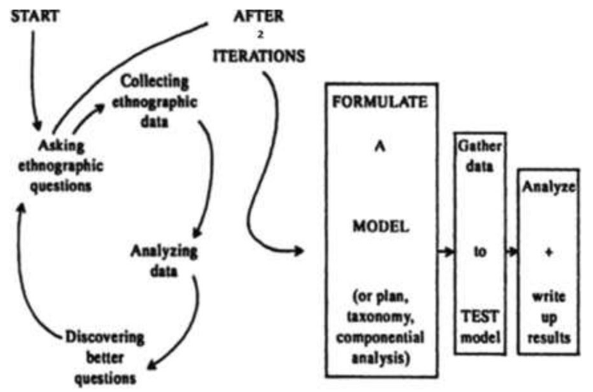

**Fig. 1 (abstract 0913).** See text for description.

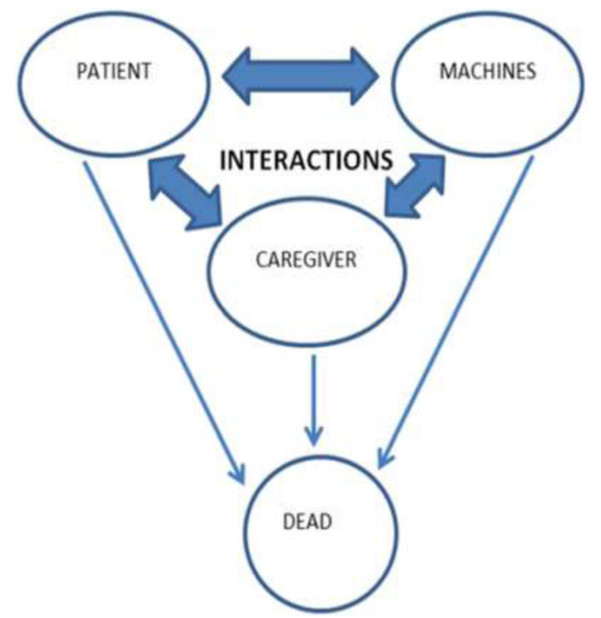

**Fig. 2 (abstract 0913).** See text for description.

### 0914

#### Impact on the perception and attitudes of an on-line educative action about limitation of life sustaining treatment (LLST), donation after brain death (DBD) and donation after circulatory death (DCD) on spanish critical care doctors

A. Sandiumenge[1], X. Montaña-Carreras[2], M.D.M. Lomero[3], T. Seoane Pillado[4], J.D. Molina[2], M. Bodi[5], N. Masnou[6], T. Pont[1]
[1]Hospital Universitario Vall de Hebron, Transplant Coordination, Barcelona, Spain; [2]Magnore. e-health & e-learning Services, Tarragona, Spain; [3]European Directorate for the Quality of Medicines and Health Care, Strasbourg, France; [4]Biomedic Research Institute A Coruña, Clinical Epidemiology and Biostatistics, A Coruña, Spain; [5]University Hospital Joan XXIII, Intensive Care Unit, Tarragona, Spain; [6]University Hospital Josep Trueta, Transplant Coordination, Girona, Spain
**Correspondence:** A. Sandiumenge
*Intensive Care Medicine Experimental* 2018, **6(Suppl 2):**0914

**OBJECTIVES.** To asses the impact of on-line training on perception and attitude of physicians on end-of-life care and the donation process.
**METHODS.** From 2015-2017 a national on-line training program on LLST, DBD and DCD was delivered to 857doctors from162 hospitals in Spain. Participants working in critical care settings(n=698) were asked to complete a survey on their perceptions and attitudes before* and after the educational intervention. Mcnemar´s test was used to compare paired nominal data. Significance p< 0.05, SPSSv21©.
**RESULTS.** 77.1% (n=538;133 hospitals) of the participants completed both surveys (36.8±8.5 years old,67.5% female). 67.1% were specialists and 32.9% were trainees of intensive care(86.2%) and anaesthesiology(13.8%). 65.8% had < 10 years of experience.
After the course, an increasing number of participants agreed on that LLST is a common practice (75.2%*vs87% p< 0.001) and that nursing staff should be more involved in the decision process(57.2%*vs 71.1% p< 0.001) . Training also increased the number of those who believed that the responsibility of the family should consist in understanding a medical decision (62.5%*vs91.9% p< 0.001).
Post-course more participants rated "withdrawal" and "withholding" as leagaly and ethically similar actions within LLST(70.7%*vs87.9%, p< 0,001). Training led to an increasing group of participants considering appropriate the use of pre-emptive sedation in all patients undergoing LLST (71.6%*vs 79.5% p< 0.001).
Training increased the number of participants familiar with the national donation law (89.4%*vs99.3%,p< 0.001) and who considered it appropriate to incorporate donation into the end-of life care plan(92.2%*vs97.9% p< 0.001), making more of them favourable to ICU admission (89.4%*vs 95.2% p< 0.001) and to the use of the same resources as for any other critically ill patient(90.1%*vs 93.1% p=0.068) in those managed as possible donors once treatment has been deemed futile.
Education increased the knowledge of DCD legal regulation (70.9%*vs96.3% p< 0.001) and of the difference between controlled and uncontrolled DCD (62.2%*vs99.5%;p< 0.001). It also increased the positive attitude towards it(87%*vs95.9% p< 0.001), with more of them prone to ask about donation after LLST decision ((89.6%*vs98.3 p< 0.001) and to initiate pharmacologic(92.5%*vs97.9%) and invasive measures(91.2%*vs96.6%) to preserve the organs for donation before declaration of death, provided relatives have consented to DCD after LLST (p< 0.001).
Training improved perception of both DBD and DCD reflected by an increase in the number of positive adjectives (maximum of 5) selected by participants to describe such programs (4.7±0.6*vs 4.8±0.5 p< 0.001) and (3.4±1.5*vs3.8±1.3 p< 0.001) respectively.
**CONCLUSIONS.** Although medical staff showed high knowledge and positive attitude about end-of-life care and organ donation, the training program showed that is still room for improvement to eliminate misperceptions and improve attitude.

### 0915

#### Does the transcranial doppler help us in the diagnosis of brain death?

E. Cabrera Suárez[1], J.J. Blanco López[2], H. Rodríguez Pérez[2], B. Del Amo Nolasco[2], A. Gutiérrez Martínez[2], A. Rodríguez Serrano[2], I.J. Sainz de Aja Curbelo[2]
[1]Universidad de Las Palmas de Gran Canaria, Las Palmas de Gran Canaria, Spain; [2]Hospital Universitario Insular de Gran Canaria, Las Palmas de Gran Canaria, Spain
**Correspondence:** H. Rodríguez Pérez
*Intensive Care Medicine Experimental* 2018, **6(Suppl 2):**0915

**INTRODUCTION.** Brain death is defined as the complete and irreversible cessation of the functionsof all intracranial neurological structures. The use of the transcranial doppler is based on its capacity for the detection of cerebral circulatory arrest, through the use of ultrasonography. In Spain, complementary tests are widely used in clinical practice and highly recommended since they allow to improve the diagnostic certainty in even shortening the stipulated observation periods.
**OBJECTIVES.** The main objective of this study was to establish how the use of the transcranial doppler could have influenced the optimization of the diagnostic times of brain death, if it was useful or, on the contrary, if it meant a delay in the diagnosis.
**METHODS.** It is an observational, descriptive cross-sectional study of patients with criteria for brain death. The study period covers 2012 to 2017 and was carried out in a polyvalent Intensive Care Unit with 24 beds at a third level University Hospital with Neurosurgery and accredited for the organ and tissue extraction by the Spanish National Transplant Organization. The sample was made up of patients in a brain death situation in our hospital during the study period.We studied demographic data, diagnostic methods used and time necessary to perform the diagnosis of brain death. The statistical package SPSS version 19 was used for the analysis. The quantitative variables were compared by Student´s T test and the qualitative variables by the X2 / Fisher´s test.
**RESULTS.** During the 7 years of study, 127 patients presented criteria for brain death. The mean age was 58,2 years, with a 67,7% of males. A 34,6% were foreign patients. The confirmatory test for the diagnosis of brain death was in 110 patients (86.12%) the transcranial doppler; in 9 patients a second clinical test of brain death; in 7 patients the EEG and in one patient the angio-CT. 84,3%

of patients were diagnosed of brain death within 6 hours.When the test used to confirm brain death was the transcranial doppler, the mean time to establish the diagnosis was significantly lower (0,5 (0,5 ; 2,0)) compared to the mean time when another diagnostic method was used (9,0 (6,0 ; 24,0)); p < 0.001. Table 1 show the results of the diagnostic methods and times needed for the confirmation of brain death.

**CONCLUSIONS.** In our environment the transcranial doppler was the most used diagnostic method and the one that allows faster diagnosis in all the circumstances studied.

**REFERENCE(S)**

1) "Brain death declaration. Practices and perceptions worldwide." Wahlster S, Eelco F.M, Wijdicks, Pratik V, Greer D, Claude J, Carone M, Farrah J. Mateen. American Academy of Neurology. 2015.
2) "The use of transcranial Doppler ultrasound in confirming brain death in the setting of skull defects and extraventricular drains." Thompson BB, Wendell LC, Potter NS, Fehnel C, Wilterdink J, Silver B, et al. Neurocritical Care. 2014;21:534-538.

**Table 1 (abstract 0915).** Doppler as confirmation test.

| | Overall (n: 127) | No (n: 17) | Yes (n: 110) |
|---|---|---|---|
| Diagnostic times (hours means) | 1,2 (0,5 ; 3,2) | 9,0 (6,0 ; 24,0) | 0,5 (0,5 ; 2,0) |
| Diagnostic times (hours) | | | |
| < or = 6 | 107 (84,3%) | 7 (41,2%) | 100 (90,9%) |
| 6 - 24 | 16 (14,2%) | 7 (41,2%) | 9 (8,2%) |
| > 24 | 4 (3,1%) | 3 (17,6%) | 1 (0,9%) |

### 0916

#### Very elderly donors as a potential source for liver transplant

M.A. Pérez Lucendo[1], S. Alcántara Carmona[1], P. Matía Almudévar[1], A. Naharro Abellán[1], M.L. Pérez Pérez[1], N. Martínez Sanz[1], M. Pérez Redondo[1], J. Palamidessi Domínguez[1], P. Rodríguez Villamizar[1], J.J. Rubio Muñoz[1], F. Somoza Sáez[2]
[1]Hospital Universitario Puerta de Hierro, Medicina Intensiva, Madrid, Spain; [2]Hospital Universitario de Getafe, Anestesiología, Madrid, Spain
**Correspondence:** M.A. Pérez Lucendo
*Intensive Care Medicine Experimental* 2018, **6(Suppl 2):**0916

**INTRODUCTION.** Donor shortage together with the increase in organ demands have led to the development of different strategies aimed at raising organ retrieval rates. Amongst these strategies, the evaluation of expanded criteria donors has aroused as an alternative.

**OBJECTIVES.** To assess the viability of livers harvested from very elderly donors (≥80 years), and to study the evolution of both the graft and the recipient during a minimum follow-up period of six months.

**METHODS.** Retrospective study (January 2011 - December 2017), including very elderly liver donors, which were offered by the National Transplant Organization to the Transplant Coordination Team of Puerta de Hierro Majadahonda University Hospital. We compared livers discarded exclusively because of donor's age to those who were implanted. The following variables were studied: age, sex, body mass index, cardiovascular risk factors, toxic habits, comorbidities, natraemia, use of vasoactive drugs, development of diabetes *insipidus*, use of antibiotics (prophylactic or therapeutic) and length of ICU stay. We also conducted a six-month minimum follow-up of recipients, assessing graft and patient survival.

**RESULTS.** During the period studied a total of 96 livers were offered (Figure 1). Of the 73 (76%) livers rejected, in 41 (42.7%) the reason stated for rejection was age exclusively. Twenty-three (23.9%) livers ≥ 80 years of age were implanted, of which two needed retransplantation. There were no statistically significant differences between livers rejected and implanted in the variables detailed above, except for the number of comorbidities, which were lower in the rejected group. During the follow-up period five deaths were registered.

**CONCLUSIONS.** In our series, liver rejection due exclusively to age was frequent.

Recipients of livers that came from very elderly donors had an evolution that is similar to what has been previously described for younger livers.

Very elderly patients should be evaluated carefully as liver donors before discarding them based exclusively on age criteria.

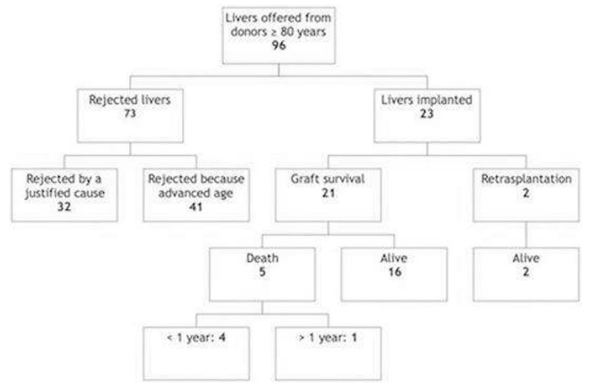

**Fig. 1 (abstract 0916).** See text for description.

### 0917

#### Normothermic regional perfusion as way to optimize kidney graft function in controlled cardiac death donors

I. Fernández Simón[1], S. Alcántara Carmona[1], M. Pérez Redondo[1], I. Lipperheide Vallhonrat[1], P. Cándido[2], H. Villanueva Fernández[1], A. Ortega López[1], L. Pérez Pérez[1], F. del Río[2], J.J. Rubio Muñoz[1]
[1]Hospital Universitario Puerta de Hierro, Intensive Care Unit, Madrid, Spain; [2]Hospital Universitario Clínico San Carlos, Intensive Care Unit, Madrid, Spain
**Correspondence:** I. Fernández Simón
*Intensive Care Medicine Experimental* 2018, **6(Suppl 2):**0917

**INTRODUCTION.** During the last years, the number of kidneys retrieved from controlled circulatory death donors (cDCD) has significantly increased. In order to improve kidney graft function a major concern exists regarding the minimization of both warm and cold ischemia times.

**OBJECTIVES.** To describe the evolution of kidney recipients with renal grafts retrieved from cDCD and optimized with normothermic regional perfusion with an extracorporeal membrane oxigenator (NRP) in the Autonomous Community of Madrid (CAM) and to analyze the incidence of delay in graft function (DGF) in comparison with those obtained through *in situ* (cold) preservation.

**MATERIAL AND METHODS.** Descriptive and retrospective study (April 2017-December 2017) that includes cDCD managed with NRP. The variables analyzed were sex and age of donors and recipients and the number of organs retrieved and implanted. Warm ischemia time (WIT) was defined as the time from systolic blood pressure < 60 mmHg to the beginning of the NRP and cold ischemia time (CIT) as the period between the start of the preservation infusion to organ implantation. The duration of the NRP together with creatinine variations during NRP were recorded. The incidence of DGF, defined as the need for dialysis within seven days after transplantation, was also studied. A historical cohort (2012-2017) of kidney grafts obtained by *in situ* preservation at the same tertiary hospital was used in the comparative analysis. Recipients prognosis was collected one month after transplantation.

**RESULTS.** During the period studied, 15 cDCD were identified [ten males (66.6%)] with a mean age of 57.2 ± 8.9 years. A total of 26 kidney grafts were retrieved and finally 19 were implanted (73%). The seven rejections experienced were due to: atheromatosis (2), infection (2), atrophy (1), unfavorable biopsy (1) and alteration of the renal artery (1). The recipients were mostly male [14 (73.6%)] with an average age of 54.6 ± 12.2 years. WIT, CIT and NRP duration were: 14 min, 11 hours and 77.3 min respectively. During NRP there was a mean increase of creatinine of 0.05 ± 0.4 mg/dl. Four patients (21%) managed with NRP presented with a DFG, which represents a lower rate than what we found for the *in situ* preservation group (48.2%). There were no complications associated with the transplant. The monthly survival rate was 94.7% (18). The only death was due to causes unrelated to kidney transplantation.

**CONCLUSIONS.** NRP for cDCD is a reality in the CAM.

The first experience with NRP for c-DCD offers good results a lower incidence of DGF when compared with *in situ* preservation.

### 0918

#### Importance of intensive care units (ICUS) in organ and tissue donation. 20-year experience in the Mexican Social Security Institute (IMSS) in León, Mexico

E.M. Olivares-Durán
IMSS. UMAE Specialty Hospital N°1, National Medical Center of El Bajío, Hospital Donation Coordination, León, Mexico
*Intensive Care Medicine Experimental* 2018, **6(Suppl 2):**0918

**INTRODUCTION.** There are few publications on the relevant participation of ICUs in the productivity of organ and tissue donation for transplantation purposes in Mexico [1, 2].

**OBJECTIVES.** To report 20-year productivity of the Adult ICU (AICU) of the High Specialty Medical Unit (UMAE, for its acronym in Spanish) No. 1 and the Pediatric ICU (PICU) of the UMAE No. 48, of the National Medical Center of El Bajío, IMSS, in the city of León, Mexico, as medical services of organ and tissue donation for transplantation purposes.

**METHODS.** Deceased organ donation in the city of León, Mexico, began in July 1997 in both the Adult ICU and the Pediatric ICU of the National Medical Center of El Bajío, IMSS. This study reports two decades of organ donation activity in these ICUs, from then to December 2017.

**RESULTS.** 150 donations were achieved at the National Medical Center of El Bajío, IMSS, in the city of León, during the study period. In 7 cases (4.67%), patients were not hospitalized in any ICU. The other 143 donations, made by ICUs' patients (95.33% of the total), were analyzed, out of which 130 patients (90.91%) were from the AICU and 13 patients (9.09%) from the PICU. The mean age of donor-patients from the AICU was 35.51 ± 9.79 years old and from the PICU was 10.85 ± 2.43 years old. 75 (52.45%) patients were male and 68 (47.55%) were female. The most frequent blood groups were O Rh + (n = 89, 62.24%) and A Rh + (n = 39, 27.27%).

135 cases (94.41%) were patients with confirmed brain death and 8 (5.59%) were donors after cardiac death. The causes of brain death were: 66 cases (48.89%) due to a cerebrovascular disease (intracranial hemorrhage or ischemic stroke), 53 (39.26%) due to a traumatic brain injury, 13 due to a primary brain tumors (9.63%) and 3 due to cerebral abscess (2.22%). From these 143 donations, there were obtained a total of 224 kidneys, 230 corneas, 15 livers, 10 hearts, 1 pancreas, and in 9 cases bone was donated; summing a total of 489 organs and/or tissues.

**CONCLUSIONS.** During the last 20 years, at the National Medical Center of El Bajío, IMSS, in the city of León, Mexico, a large majority (95.33%) of the deceased organ and tissue donations were achieved from patients hospitalized in their ICUs. Therefore, at this Medical Center, ICUs are fundamental medical services for organ donation and transplantation.

##### REFERENCE(S)

1. Fortuna-Custodio JA, Rivera-Marchena JR, Jiménez-Lomas S, Morales-Flores ME, Roldán-García AM, Navarro-Paz I, López-Jiménez JL. Donación de órganos: metas del mantenimiento en el paciente con muerte encefálica. Rev Asoc Mex Med Crit y Ter Int. 2014;28(4):221-238.
2. Querevalú-Murillo W, Orozco-Guzmán R, Díaz-Tostado S. Mantenimiento del donante cadavérico en la Unidad de Terapia Intensiva. Rev Asoc Mex Med Crit y Ter Int. 2013;27(2):107-114.

##### GRANT ACKNOWLEDGMENT
This research did not receive any grant from any funding agency of the public, commercial or not-for-profit sectors.

### 0919

#### Predisposing factors of failed apnea test during brain death determination in potential organ donor

J.J. Kim, E.Y. Kim
Seoul St. Mary's Hospital, The Catholic University of Korea College of Medicine, Division of Trauma and Surgical Critical Care, Department of General Surgery, Seoul, Korea, Republic of
**Correspondence:** J.J. Kim
*Intensive Care Medicine Experimental* 2018, **6(Suppl 2):**0919

**INTRODUCTION.** Apnea test is an essential component in the clinical determination of brain death, but it may incur a significant risk of complications such as hypotension, hypoxia and even cardiac arrest. We analyzed the risk factors associated with failed apnea test during brain death assessment in order to predict and avoid these adverse events.

**OBJECTIVES AND METHODS.** Medical records of apnea tests performed for brain-dead donor between January 2009 and January 2016 in our institution, were reviewed retrospectively. Age, gender, etiology of brain death, use of catecholamine and results of arterial bleed gas analysis (ABGA), systolic/diastolic blood pressure (SBP/DBP), mean arterial pressure (MAP) and central venous pressure (CVP) prior to apnea test initiation were collected as variables. A-a gradient and $Pa_{O2}/F_{iO2}$ were calculated for more precise assessment of the respiratory system. In total, 267 cases were divided into a group which was completed apnea test and the other which was failed the test.

**RESULTS.** 13 cases failed the apnea test and the majority of reasons were severe hypotension (SBP < 60mmHg). In terms of hemodynamic state, SBP was significantly higher in the completed test group than the failed group (126.5 ± 23.9 vs. 103 ± 15.2, respectively; $p$ = 0.001). In ABGA, the completed test group showed significantly higher $P_{aO2}/F_{iO2}$ (313.6 ± 229.8 vs. 141.5 ± 131.0, respectively; $p$ = 0.008) and lower A-a gradient (278.2 ± 209.5 vs. 506.1 ± 173.1, respectively; $p$ = 0.000). In multivariable analysis, low SBP ($p$ = 0.040) and high A-a gradient ($p$ = 0.002) were independent risk factors associated with failed apnea test.

**CONCLUSIONS.** Although the unexpected adverse events during apnea test for brain death determination do not occur frequently, they could be fatal. If a brain-dead patient shows low SBP and high A-a gradient, clinicians should pay more attentions and preparations prior to apnea test.

##### REFERENCE(S)

1) Scott JB, Gentile MA, Bennett SN, Couture M, MacIntyre NR: Apnea testing during brain death assessment: a review of clinical practice and published literature. Respir Care 2013; 58: 532-8.
2) Practice parameters for determining brain death in adults (summary statement). The Quality Standards Subcommittee of the American Academy of Neurology. Neurology 1995; 45: 1012-4.
3) Saposnik G, Rizzo G, Deluca JL: Pneumothorax and pneumoperitoneum during the apnea test: how safe is this procedure? Arq Neuropsiquiatr 2000; 58: 905- 8.
4) Bar-Joseph G, Bar-Lavie Y, Zonis Z: Tension pneumothorax during apnea testing for the determination of brain death. Anesthesiology 1998; 89: 1250-1.
5) Yee AH, Mandrekar J, Rabinstein AA, Wijdicks EF: Predictors of apnea test failure during brain death determination. Neurocritical Care 2010; 12: 352-5.

##### GRANT ACKNOWLEDGMENT
The authors have nothing to disclose.

### 0920

#### Changes in end-of-life practices (EOLP) in European intensive care units (ICUs): the Ethicus II study

C. Sprung[1], C. Hartog[2], B. Ricou[3], P. Levin[4], M. Weiss[5], M.G. Bocci[6], J.C. Schefold[7], A. Michalsen[8], M. Baras[9], A. Avidan[1], Ethicus II investigators
[1]Hadassah Hebrew University Medical Center, Jerusalem, Israel; [2]University Hospital Jena, Jena, Germany; [3]Hôpital Cantonal Universitaire de Geneve, Geneve, Switzerland; [4]Shaare Zedek Medical Center, Jerusalem, Israel; [5]University Hospital Ulm, Ulm, Germany; [6]Università Cattolica del Sacro Cuore, Rome, Italy; [7]Universitätsspital Bern, Bern, Switzerland; [8]Tettnang Hospital, Tettnang, Germany; [9]Hebrew University-Hadassah School Public Health, Jerusalem, Israel
**Correspondence:** J.C. Schefold
*Intensive Care Medicine Experimental* 2018, **6(Suppl 2):**0920

**INTRODUCTION.** Differences in EOLP were previously reported in Europe in the Ethicus I study [1].

**OBJECTIVES.** This study evaluated differences in EOLP after 15 years in European ICUs that also participated in the Ethicus I study.

**METHODS.** All previous Ethicus I centers were invited to participate in the Ethicus II study. Consecutive admitted ICU patients who died or had treatment limitations during a 6 month period from 1.9.2015 to 30.9.2016 were prospectively studied. Previous EOLP and region definitions were used [1]. EOLP in the different regions of the Ethicus I study [1] were compared to the same ICUs in the Ethicus II study.

**RESULTS.** 22 of the original 37 ICUs participated again in this study. Limitations of life-sustaining therapies occurred in 1601/1785 (90.0%) patients and 1274/1458 (87.4%) of dying patients in Ethicus II and 1918/2807 (68.3%) and 1812/2701 (67.1%) of dying patients in Ethicus I. Figure 1 shows the differences in EOLP by region. Table 1 demonstrates that limitations were taken sooner in Ethicus II but ICU lengths of stay and time to death after limitations were similar.

**CONCLUSIONS.** In Europe in 15 years, EOLP has changed and includes more and earlier treatment limitations without changing time to death. There was less CPR (especially in the South) with more withholding and withdrawing therapies. Shortening of the dying process (SDP) remained uncommon.

##### GRANT ACKNOWLEDGMENT

None

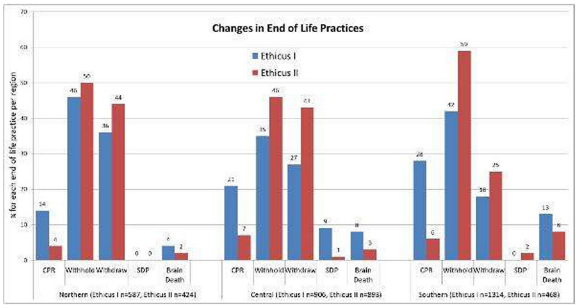

**Fig. 1 (abstract 0920).** See text for description.

**Table 1 (abstract 0920).** Times (days) (median – IQR, 25th to 75th percentiles)

| | Ethicus I (N=1919) | Ethicus II (N=1601) |
|---|---|---|
| ICU length of stay | 5.0 (1.4-13.1) | 4.0 (1.2-10.9) |
| First limitation after ICU admission | 3.9 (0.9-12.0) | 1.8 (0.2-7.1) |
| First limitation until death | 0.7 (0.1-2.3) (N=1810) | 0.8 (0.1-3.7) (N=1274) |

### 0921

#### ECMO mobile teams for normothermic regional perfusion to support cardiac death donors in hospitals of the Autonomous Community of Madrid, Spain. A one-year experience

M. Pérez Redondo[1], S. Alcántara Carmona[1], I. Fernandez Simón[1], J. Duerto Álvarez[2], H. Villanueva Fernández[1], A. Ortega López[1], I. Lipperheide Vallhonrat[1], A. Mateos Rodriguez[3]
[1]Hospital Universitario Puerta de Hierro, Intensive Care, Majadahonda, Spain; [2]Hospital Universitario Clínico san Carlos, Intensive Care, Madrid, Spain; [3]Regional Transplant Organization of Autonomous Community of Madrid, Madrid, Spain
**Correspondence:** M. Pérez Redondo
*Intensive Care Medicine Experimental* 2018, **6(Suppl 2):**0921

**OBJECTIVE.** To analyze our experience after the establishment of a mobile team created to support donors after controlled cardiac death (DCD) with normothermic regional perfusion (NRP) using and extracorporeal membrane oxygenator (ECMO) in those hospitals across the Autonomous Community of Madrid (CAM) that do not have access to one.

**METHODS.** Descriptive study (April 2017 - April 2018). In July 2016 a program to assist DCD with NRP was stablished. A total of two NRP teams were created in the CAM, one from Hospital Universitario Clínico San Carlos and one from Hospital Universitario Puerta de Hierro Majadahonda. Each of these teams included an intensivist, a surgeon with vascular experience and a perfusionist together with an ECMO machine. Teams were activated by the National Organ Transplantation network who decided when NRP would be applied according to donor characteristics and organs to be retrieved.

**RESULTS.** During the period studied there were 224 effective donors in the CAM, 54 of them (24.1%) in a DCD situation. Out of these 54, NRP was used in 33 cases with the NRP team intervening in 23 occasions in seven different hospitals. This meant that the NRP team was present in 42,6% of all the DCDs and in 70% of the DCD assisted with NRP in the CAM.

Mean DCD donor age was 56,3 ± 11,5 years. Fourteen males (60,9%). Reason for ICU admission: acute ischemic stroke (13;56,5%), cardiac arrest (9;39,1%) and amyotrophic lateral sclerosis (1;4,3%). Mean UCI stay until withdrawal of life support was 10,6 ± 7,7 days.

The NRP teams retrieved a total of 11 livers, 42 kidneys and 3 lungs and 9 (81,8%), 33 (78,6%) and 2(67%) of each group were effectively implanted.

**CONCLUSIONS.** NRP teams in the CAM are a reality nowadays and have been present in almost half of all the DCDs since the beginning of the program.

A long-term analysis of the recipients is needed in order to assess the real impact of the program in graft function.

**GRANT ACKNOWLEDGMENT**
Fundación Mutua Madrileña.

### 0922

#### Controlled donation after circulatory death (cDCD) in Donostia University Hospital, 2015-2018

M. De Almeida e Silva[1], L. Atutxa[1], R. Sebastián[1], I. García[1], N. González[1], H. Olasagasti[1], B. Pagalday[2]
[1]Hospital Universitario Donostia, Donostia - San Sebastian, Spain; [2]Emergencias Osakidetza, Donostia - San Sebastian, Spain
**Correspondence:** M. De Almeida e Silva
*Intensive Care Medicine Experimental* 2018, **6(Suppl 2):**0922

**INTRODUCTION.** Donostia University Hospital is a third-level hospital without solid organ transplantation programme, neither cardiac surgery unit nor extracorporeal membrane oxygenation (ECMO) assistance programme. In 2015, we started two programmes in donation after circulatory death (DCD): both controlled (cDCD) and uncontrolled (uDCD). During these 3 years, we've reached an average of 88 donors pmp.

**OBJECTIVES.** Analysis of the characteristics of cDCD donors in a three years period.

**METHODS.** Prospective observational study. Center: Donostia University Hospital, reference center for 700.000 inhabitants. Period of study: March 2015 - April 2018. Sample: patients with terminal illness and poor prognosis, where medical team decides the withdrawal of life-sustaining therapy (WLST) based upon futility of further care. Variables: sex, age, cause of WLST, place of extubation, total number of hospital admission days, functional warm ischemia time (f-WIT) and total warm ischemia time (WIT), preservation time, number of grafts removed and transplanted. Technique: normothermic regional perfusion (nRP), with specific authorization by the patient's legal representatives. This includes ante mortem heparinization and cannulation of vessels (under appropriate sedoanalgesia), percutaneously. Both procedures were fully completed at the Intensive care unit (ICU).

**RESULTS.** Total number of donors: 58. 32.8% were female (19), 67.2% male (39). Mean age 69 years old [35-78]. Cause of WLST: 39.7% anoxic encephalopathy, 29.3% brain hemorrhage, 8.6% traumatic brain injury, 5.2% amyotrophic lateral sclerosis, 6.9% acute ischemic brain injury and 5.1% other causes. Mean days of hospital admission 10 [1-41]. 13.8% (8) of elective intubation for cDCD. 15.5% (9) admission at the ICU exclusively for donation centered care. 75.8% of extubation took place at the ICU, 24.1% at the operating theater. WIT 13.7´ [7´-26´], f-WIT 11.5´ [6´-20´], nRP time 117´ [13-235]. Transplanted organs: first pancreas transplant in Spain. 8 lungs, 39 livers (67.2%), 112 kidneys removed and 74 transplanted (66%). 7(12.1%) liver refusals for elevated liver enzymes during ECMO. 13.8% contraindications for liver donation.

52 cornea donors. 21 tissue donors: bone and tendons, average of 16 samples per donor [11-20].

**CONCLUSIONS.**

Starting a cDCD programme, in our region, represents 27.6 donors pmp. 28.8% of the total donors.

cDCD can be performed in a Hospital without a solid organ transplantation programme.

cDCD programmes with previous family interview and donation centered ICU-care, offer the possibility of fulfilling the patient's right for organ donation, for those patients with terminal illness but without brain death.

**GRANT ACKNOWLEDGMENT**
None.

## New insights on trauma management

### 0923

#### Association between hypotension and mortality in critically ill patients with severe traumatic brain injury: experience at a single Canadian trauma center

M. Erdogan[1], N. Kureshi[2,3], D. Clarke[2,3], R. Green[1,4]
[1]Nova Scotia Department of Health and Wellness, Trauma Nova Scotia, Halifax, Canada; [2]Dalhousie University, Neurosurgery, Halifax, Canada; [3]Nova Scotia Health Authority, Neurosurgery, Halifax, Canada; [4]Dalhousie University, Critical Care, Halifax, Canada
**Correspondence:** R. Green
*Intensive Care Medicine Experimental* 2018, **6(Suppl 2):**0923

**INTRODUCTION.** Severe traumatic brain injury (TBI) is one of the leading causes of death in intensive care units (ICUs). Hypotension is known to be associated with increased mortality in TBI patients. A systolic blood pressure (SBP) of < 90 mmHg is the threshold for hypotension in consensus TBI treatment guidelines; however, some evidence suggests hypotension should be defined at higher levels for these patients.

**OBJECTIVES.** To determine the influence of hypotension on mortality in severe TBI patients requiring ICU admission using different thresholds of SBP on arrival at the emergency department (ED).

**METHODS.** We performed a retrospective cohort study of all patients with severe TBI (Abbreviated Injury Scale Head score ≥ 3) admitted to the ICU at the Queen Elizabeth II Health Care Centre (Halifax, Canada) over a 12-year period (2002-2013). Patients were grouped by SBP on ED arrival (< 90 mmHg, < 100 mmHg, < 110 mmHg). Logistic regression was used to control for the effect of age, gender, Injury Severity Score (ISS), mechanism of injury, isolated TBI, and trauma team activation (TTA) on mortality in ICU patients.

**RESULTS.** A total of 1233 patients sustained a severe TBI and were admitted to the ICU during the study period. The mean age was 43.4 ± 23.9 years and most patients were male (919/1233; 74.5%). The most common mechanism of injury was motor vehicle collision (491/1233; 41.2%) followed by falls (427/1233; 35.8%). Mean length of stay in the ICU was 6.1 ± 6.4 days, and the overall mortality rate was 22.7%. SBP on arrival was available for 1182 patients. The < 90 mmHg group (54/1182; 4.6%) had a mean ISS of 20.6 ± 7.8 and a mortality of 40.7% (22/54). For the < 100 mmHg group (110/1182; 9.3%), mean ISS was 19.3 ± 7.9 and mortality was 34.5% (38/110). In the < 110 mmHg group (198/1182; 16.8%), mean ISS was 17.9 ± 8.0 and mortality was 28.8% (57/198). After adjusting for confounders, the association between hypotension and mortality was 2.22 (95% CI 1.19-4.16) using a < 90 mmHg cutoff, 1.79 (95% CI 1.12-2.86) using a < 100 mmHg cutoff, and 1.50 (95% CI 1.02-2.21) using a < 110 mmHg cutoff.

**CONCLUSIONS.** After adjustment for known confounders, the odds ratios for SBP < 90, < 100, and < 110 mm Hg remained significantly associated with mortality. Our results demonstrate that while the risk of mortality is double in patients with a SBP < 90 mmHg, this risk is only marginally reduced at a higher SBP threshold of 110 mm Hg.

**GRANT ACKNOWLEDGMENT**
This study was funded by the Nova Scotia Department of Health and Wellness and by a Clinician Scientist Award from the Faculty of Medicine, Dalhousie University, Halifax, Nova Scotia, Canada.

### 0924

#### Evaluation of two decision tools to reduce unnecessary whole-body CT exams in trauma patients

J. Sainz Cabrejas, C. García Fuentes, J. Barea Mendoza, M. Chico Fernández, J.C. Montejo González
H. U 12 de Octubre, Madrid, Spain
**Correspondence:** J. Sainz Cabrejas
*Intensive Care Medicine Experimental* 2018, **6(Suppl 2):**0924

**INTRODUCTION.** Despite the fact that Whole-Body CT (WBCT) is an increasing practice in many centers for severely injured patients, high rates of negative scans have been described specially in medium risk patients, so controversy exists about its indications.

GKCT (Granze Kroper CT) score [1] and Manchester Trauma Imaging Protocol (MTIP)[2], are two decision tools developed in order to determine the need for WBCT in trauma patients. They are both based on mechanism of injury, physiological parameters and demographic data.

In our institution, patients requiring partial trauma team activation (PTTA) are assessed by two ICU physicians in the emergency room and radiological exams (indication and results). We classified CT findings as no injury, no significant/significant injury or ICU admission need are indicated according to clinical suspicion.

**OBJECTIVES.** This study aims to determine the rate of negative WBCT between patients requiring PTTA and to evaluate whether any of these scales is a suitable tool to reduce unnecessary CT scans.

**METHODS.** We performed a retrospective observational study during a 56 month period. All patients >16 years old who required PTTA because of a blunt injury were recruited. Patients in whom PTTA was produced > 30 minutes after arrival, were excluded.

We recorded data from pre-hospital care, mechanism of injury, physical exam and radiological exams (indication and results). We classified CT findings as no injury, no significant/significant injury or ICU admission need. We calculated GKCT and MTIP scores and retrospectively considered indicated a WBCT if a punctuation higher than 0 or 3 was respectively obtained. For each scale, we determined OR for significant injury or need for ICU admission and false negative rate (proportion of patients without indication of WBCT which were admitted to ICU).

**RESULTS.** During the period of the study, we enrolled 1384 patients, 141 of which were admitted to ICU. 1032 (74%) patients were male. The median age was 37 y.o. (IQR 27-47). 656 patients underwent WBCT. In 227 CT exams (35%), insignificant injuries were reported whereas in 156 (24%) significant injuries were found.

OR for significant injury and for ICU admission are respectively 0.9 (95% CI 0.54-1.39) and 1.9 (95% CI 1.01-3.60) for GKCT and 2.902 (95% CI 1.75-4.79) and 3.649 (95% CI 1.95-6.71) for MTIP. False negative rate was 3.8% for GKCT and 4.37% for MTIP.

**CONCLUSIONS.** A proportion of unnecessary exams could be avoided by using GKCT or MTIP. A positive MTIP score is associated with a high probability of significant injuries and ICU admission requirement. However, false negative rate is lower for GKCT, thus, it is a suitable tool to avoid unnecessary WBCT.

[2] Davies, R. M. et al A decision tool for whole-body CT in major trauma that safely reduces unnecessary scanning and associated radiation risks: An initial exploratory analysis. Injury, 47(1), 43-49.

### 0925

#### Is the shock index a good predictor of mortality in trauma patients?

L. Pariente Juste, M. Koo Gomez, A. Vergara Serpa, I. Macía Tejada, A. Bonet Burguera, I. Otero Quintana, C. Prat Llimargas

Hospital Universitario de Bellvitge, Anesthesiology, Reanimation and Pain Clinic, Barcelona, Spain

**Correspondence:** L. Pariente Juste

*Intensive Care Medicine Experimental* 2018, **6(Suppl 2):**0925

**INTRODUCTION.** Trauma is the third leading cause of death in patients under the age of 45. An early treatment and adequate admission triage are directly related to outcomes in patients. Different tools for fast determination of critical clinical conditions that correlate with outcomes in this kind of patients have been developed, such as the Glasgow Coma Score (GCS) and the Injury Severity Score (ISS). The Shock Index (SI) is the ratio between heart rate and systolic blood pressure, and it is a useful indicator to identify shock and correlate with mortality.

**OBJECTIVES.** The objective of this study was to compare the SI and the ISS as predictors of early mortality, hospital length of stay (HLS) and critical care length of stay (CLS).

**METHODS.** It is a retrospective, observational study, with a cohort of 184 patients who arrived as a trauma code and required critical care hospitalization at Bellvitge University Hospital, a level 1 trauma center, between 2013 and 2016. The SI was calculated from the first prehospital values. A logistic regression was used to compare SI and ISS, and to relate it with mortality, CLS and HLS. A p-value ≤ 0.05 was considered significant.

**RESULTS.** Out of the 184 patients, 150 were male (81.5%), with a median age of 43.5 years old (31-59). The mean ISS was 29 (22-36). There were 61 patients (33%) with GCS ≤8.

In relation to mortality, the SI adjusted Odds Ratio was 0.5 (CI 0.12 -1.66; p = 0.302), the ISS Odds Ratio was 1.07 (CI 0.12 -1.66; p = 0.302) and the GCS Odds Ratio 10.67 (CI 2.91 - 53.33; p=0.001). For CLS and HLS, the adjusted SI Odds Ratio were 1.59 (CI 1.02-2.64: p=0.64) and 1.62 (CI 1.00-2.83) respectively.

**CONCLUSIONS.** In this model, there is no correlation between SI and early mortality, HLS or CLS. These results probably correlate to this specific group of critical trauma patients studied. Moreover, this study was performed retrospectively and is prone to bias incurred by missing data. GCS and ISS are more reliable predictors of mortality, considering the SI a valuable addition to triage criteria to active trauma team responses.

**REFERENCE(S)**

McNab A, Burns B, Bhullar I, Chesire D, Kerwin A. A prehospital shock index for trauma correlates with measures of hospital resource use and mortality. Surgery. 2012 Sep;152(3):473-6

**Table 1 (abstract 0925).** Results of SI, ISS and GCS related to mortality

| Patients | Alive n=142 | Dead n=26 |
|---|---|---|
| SI | 0.77 (0.64 - 0.97) | 0.87 (0.68 - 1.42) |
| ISS | 29.00 (24.00 - 38.00) | 61.50 (32.00 - 75.00) |
| GCS ≤ 8 | 34 (24%) | 22 (84%) |

### 0926

#### Acute kidney injury in trauma patients admitted to the intensive care unit: a systematic review and meta-analysis

S. Søvik[1,2], M.S. Isachsen[3], K.M. Nordhuus[4], C.K. Tveiten[4], T. Eken[5], K. Sunde[5], K. Gundro Brurberg[6], S. Beitland[5]

[1]Akershus University Hospital, Dept. of Anaesthesia and Intensive Care, Lørenskog, Norway; [2]University of Oslo, Institute of Clinical Medicine, Oslo, Norway; [3]Oslo University Hospital, Medical Library Ullevaal, Oslo, Norway; [4]University of Oslo, Oslo, Norway; [5]Oslo University Hospital, Dept. of Anaesthesiology, Oslo, Norway; [6]Norwegian Institute of Public Health, Oslo, Norway

**Correspondence:** S. Søvik

*Intensive Care Medicine Experimental* 2018, **6(Suppl 2):**0926

**INTRODUCTION.** Acute kidney injury (AKI) is a common complication after severe trauma and is associated with increased intensive care unit (ICU) length of stay, increased patient morbidity, mortality, and costs. In single centre studies, severity of trauma, initial degree of shock, use of nephrotoxic agents and various pre-trauma patient characteristics have been linked to AKI incidence and outcome.

**OBJECTIVES.** To perform a systematic review and meta-analysis of studies of AKI occurring in trauma patients admitted to the ICU. The review was registered in PROSPERO on May 12 2017 (CRD42017060420).

**METHODS.** We searched papers and guidelines published Jan 1, 2004 - Jan 23, 2018 in PubMed, Cochrane Database of Systematic Reviews, UpToDate and NICE (National Institute for Health and Care Excellence), using MeSH and text words including acute kidney injury, multiple trauma, nervous system trauma, wounds and injuries, penetrating wounds, accidents, trauma, traumatic, polytrauma, and multiple injuries, alone or in combination. Ongoing reviews were identified in the PROSPERO database. The search was limited to studies diagnosing AKI with RIFLE, AKIN or KDIGO criteria. Screening of studies for eligibility according to pre-defined inclusion criteria and data extraction on a pre-defined data extraction form was performed in duplicate by two independent collaborators. Study quality was evaluated on the Newcastle - Ottawa quality assessment scale by two independent collaborators. AKI severity was categorised as mild (RIFLE Risk, AKIN Stage 1, KDIGO Stage 1), moderate (RIFLE Injury, AKIN Stage 2, KDIGO Stage 2), or severe (RIFLE Failure or worse, AKIN Stage 3, KDIGO Stage 3). Several methods of data synthesis for estimation of risk will be used.

**RESULTS.** Altogether 22 studies comprising 23479 patients (77% male) were included. 16 studies were from general, mixed trauma populations, 4 from neurotrauma, and 2 military. All studies were observational. Study quality was variable. Only 8/22 studies used full, unmodified AKI criteria, the remaining did not include urine output. In 11/22 studies, exclusion of patients with chronic kidney disease was insufficient. Data on renal replacement therapy was reported in 18/22 studies. Subgroup analysis of risk factors was limited by variable and missing reporting.

Overall, 18% of patients developed AKI; 9.7% mild, 5.4% moderate, and 2.7% severe. Unadjusted, pooled mortality in trauma patients with AKI was 24% (17% in mild, 24% in moderate, and 49% in severe AKI), vs. 6.6% in non-AKI patients.

**CONCLUSIONS.** A review of studies on AKI occurring in patients admitted to the ICU after severe trauma revealed significant morbidity and mortality associated with AKI. Lack of consistency in reporting reduced available data for risk estimation.

### 0927

#### Infection-related ventilator-associated complications in patients with acute brain injury

A.R. Corrêa[1], F. Bruzzi de Carvalho[2], T.V. Chaves[1], F.M. Assunção[1], V.A. Nobre[3]

[1]Universidade Federal de Minas Gerais - UFMG, Escola de Enfermagem, Belo Horizonte, Brazil; [2]Hospital João XXIII, Centro de Terapia Intensiva Adulto, Belo Horizonte, Brazil; [3]Hospital das Clínicas da UFMG, Núcleo de Investigação em Medicina Intensiva - NIIMI, Belo Horizonte, Brazil

**Correspondence:** A.R. Corrêa

*Intensive Care Medicine Experimental* 2018, **6(Suppl 2):**0927

**INTRODUCTION.** Acute brain injury victims are considered to be at high risk for ventilator-associated pneumonia (VAP). Recently, Infection-related Ventilator-Associated Complication (IVAC), Possible VAP and Probable VAP criteria were proposed to simplify surveillance of infectious complications of mechanical ventilation.

**OBJECTIVES.** The primary objective of this study was to describe factors and outcomes associated with the first episode of IVAC in patients with an acute brain injury receiving invasive mechanical ventilation (MV).

**METHODS.** A retrospective cohort of patients admitted to a trauma Intensive Care Unit (ICU) was studied. Acute brain injury was defined as a Glasgow Coma Scale (GCS) score ≤12 due to medical or traumatic reasons. The first VAP episode in 14 days in the ICU was described as IVAC, Possible VAP and Probable VAP, adapted from the CDC guidelines for Ventilator-Associated Events. Neurological outcome was censored at hospital discharge using the Glasgow Outcome Score scale, with values 1, 2 and 3 as unfavorable and 4 and 5 as favorable. A p value < 0.05 was considered statistically significant.

**RESULTS.** During the 8-month study period, 907 patients were admitted and 236 were included in the study. Males were 80.8%, 97.9% were trauma victims and the hospital mortality rate was 31,6%. On average, IVAC was noted at 6.34 (2.95) days and treatment lasted for 7.03 (9.14) days. IVAC criteria was met in 48.3% of patients, whereas Possible VAP and Probable VAP was observed in 45.3% and 22,9% respectively. *Staphyloccocus aureus* (24,3%) and *Acinetobacter sp.* (20.7%) were the most frequent isolated pathogens. In day 6 of MV, PaO2/FiO2, Leucocyte count, C-reactive protein, peak temperature and purulent secretions were different in IVAC and non-IVAC patients. IVAC was associated with a longer ICU (18.0 vs.15.6 days, p=0.008) and hospital (63.9 vs. 44.3 days, p=0.010) length of stay (LOS), and with less 28-MV-free days (12.4 vs. 15.9, p< 0.001), without a mortality difference. In multivariate analysis, thoracic trauma (OR 3.3 CI95% 1.53; 7.1 p= 0.013) and the presence of an intracranial catheter (OR 1.88 CI95% 1.08; 3.26 p= 0.025) were associated with IVAC occurence, with similar associations for Possible and Probable VAP. A later tracheostomy (OR 1.18 CI95% 1.05; 1.32 p= 0.004) and 28-MV-free days (OR 1.15 CI95% 1.07; 1.24 p= < 0.001) were associated with a favorable neurological outcome.

**CONCLUSIONS.** IVAC occurs frequently and is associated with longer ICU and hospital LOS, reduced 28-MV-free days, but not to hospital mortality or neurological outcomes in patients with acute brain injury. Patients with thoracic trauma and intracranial catheters were at a higher risk of IVAC. In this series, Possible or Probable VAP criteria was not superior to IVAC to discriminate for risk factors or clinical outcomes.

### 0928

#### Antibiotic therapy does not prevent nosocomial pneumonia in critically ill trauma patients with pulmonary contusion

E. Bassi[1], B.M. Tomazini[1], C.T. Merighi[1], C.I. Tomizuka[1], R.A.G. de Oliveira[1], P.F.G.M.M. Tierno[1], F.M. Cadamuro[1], T. Guimarães[2], F.F. Novo[1], E.M. Utiyama[1], L.M.S. Malbouisson[3]

[1]Hospital das Clínicas da Universidade de São Paulo (HC-FMUSP), Surgery and Trauma Discipline, São Paulo, Brazil; [2]Hospital das Clínicas da Universidade de São Paulo (HC-FMUSP), Infectious Diseases, São Paulo, Brazil; [3]Hospital das Clínicas da Universidade de São Paulo (HC-FMUSP), Anesthesiology, São Paulo, Brazil

**Correspondence:** B.M. Tomazini

*Intensive Care Medicine Experimental* 2018, **6(Suppl 2):**0928

**INTRODUCTION.** Pneumonia occurs in up to 47% of critically ill trauma patients with pulmonary contusions[1]. Despite usual care, there is a lack of evidence in how to prevent nosocomial pneumonia in this population.

**OBJECTIVES.** To compare a conservative antibiotic therapy strategy on the incidence of microbiologically documented nosocomial pneumonia in critically ill patients with pulmonary contusion.

**METHODS.** A retrospective cohort of adult patients admitted to a surgical ICU of a tertiary university hospital between 2012-2016. Antibiotic therapy group was defined by any antibiotic use for more than 48h starting on hospital admission, while the conservative group was defined by antibiotic use no greater than 48h. The most common reasons for antibiotic prescription were: open fractures or cerebrospinal fluid leakage. Antibiotics for pulmonary contusion were not prescribed routinely. The primary outcome was microbiologically documented nosocomial pneumonia in the first 14 days after hospital admission. A logistic regression model, including variables with a p-value < 0.2 in the univariate regression and other clinically relevant variables, was used to estimate the association of risk factors and primary outcome.

**RESULTS.** From 2040 ICU admissions, 177 patients with chest trauma and diagnosis of pulmonary contusion on CT scan were included. Patients characteristics and outcomes are shown in table 1. Antibiotic therapy group had lower Glasgow coma scale (GCS), higher rates of mechanical ventilation, Simplified Acute Physiology Score 3 (SAPS 3), Sequential Organ Failure Assessment score (SOFA), and Injury Severity Score (ISS) at ICU admission than the conservative group. There was no difference between groups in the incidence of microbiologically documented nosocomial pneumonia at day 14 and ICU mortality.

The univariate and multivariate analysis are shown in table 2. In the analysis, the only risk factor associated with the incidence of nosocomial pneumonia was mechanical ventilation at ICU admission, while antibiotic therapy and other variables showed relation to the outcome. In patients with microbiological isolates at time of diagnosis, the previous use of an effective antibiotic against the bacteria did not decrease ICU length of stay or ICU mortality (OR 1.2; 95% CI 0.22-6.33; p=0.83).

**DISCUSSION.** Our results show that antibiotic therapy does not decrease the incidence of microbiologically documented nosocomial pneumonia within 14 days of admission. Therefore, a conservative strategy seems to be safe and can reduce antibiotic use.
The retrospective nature of the data, imbalance between groups, and the fact antibiotic therapy could decrease microbiological confirmation of nosocomial pneumonia has to be considered when interpreting the results. However, given the lack of literature on this topic and the high incidence of pneumonia in patients with pulmonary contusion, our results must guide future research on this topic.
1-Dhar SM et al. Respir Care. March, 2018.

**Table 1 (abstract 0928).** See text for description.

| | Conservative group | Antibiotic therapy group | p |
|---|---|---|---|
| N, % | 80 (46.5) | 92 (53.5) | |
| Age, years mean, (SD) | 36.4 (15.8) | 33.8 (14.7) | 0.25 |
| SOFA median, (IQR) | 5 (3-9) | 7 (5-11) | <0.001 |
| SAPS 3 median, (IQR) | 47 (36-57) | 55 (44-64) | 0.001 |
| ISS median, (IQR) | 34 (27-42) | 41 (34-46) | 0.012 |
| GCS median, (IQR) | 13 (6-15) | 7 (5-13) | 0.018 |
| Mechanical Ventilation % | 62.5 | 88.04 | <0.001 |
| Primary outcome % | 18.75 | 20.65 | 0.75 |
| ICU mortality % | 16.25 | 27.17 | 0.08 |

**Table 2 (abstract 0928).** See text for description.

| | Univariate logistic regression | | Multivariate logistic regression | |
|---|---|---|---|---|
| | Odds ratio | p | Odds ratio | p |
| Antibiotic therapy group | 1.12 | 0.755 | 0.82 | 0.659 |
| Age | 0.99 | 0.460 | - | - |
| Gender | 1.28 | 0.639 | - | - |
| SOFA | 1.09 | 0.059 | 0.93 | 0.3 |
| SAPS 3 | 1.03 | 0.011 | 1.02 | 0.297 |
| ISS | 1.02 | 0.16 | 0.99 | 0.91 |
| GCS | 0.87 | 0.003 | 0.91 | 0.088 |
| Mechanical Ventilation | 13.46 | 0.012 | 11.66 | 0.025 |

### 0929

#### A new trauma-burn care system should be established near future

Q. Zhang
Burn Center of Rui Jin Hospital, School of Medicine, Shanghai Jiaotong University, Shanghai, China
*Intensive Care Medicine Experimental* 2018, **6(Suppl 2):**0929

**INTRODUCTION.** The severe burn patients treated in the Emergency Burn Care Center of Shanghai, the largest Commerce city have not reduced with quick development of the city.
**OBJECTIVES.** A retrospective study of the characteristic and tendency for the patients with a total area of more than 30% TSBA in Burn Center of Shanghai Ruijin Hospital, admit from 2005 to 2016, was performed for development of future mode of burn center.
**METHODS.** This study retrospectively analyzed adult burn patients who had a total burn area greater than 30% TSBA and older than 18 years old treated in burn center from January 1, 2005 to December 31, 2016. The age, gender, area of burns, The injured place, the time from injury to hospital, hospital transfer, inhalation injury, the outcome of treatment and ICU duration.
**RESULTS.** A total of 895 patients with severe burns were included in this study. The average age of patients was 41.23 ± 13.43 yrs old with a mean burn area of 56.83 ± 21.97% TSBA. The average age of patients significantly increased from 38 years in 2005 to 45 years in 2016 (F = 3.45, P = 0.00). The proportion of female patients older than 60 years old increased significantly, and the incidence of female patients showed an upward trend (X2 = 15.64, P = 0.00) Flame was the main cause of severe burn, accounting for 77.7%, but the proportion showed a decreasing trend (X2 = 9.99, P = 0.02). Severe burns patients were mainly injured in the workplace 65.5%, followed by 23.8% of domestic burns. The incidence of severe burns in the household is on the rise, with severe burns in the work place declining (X2 = 25.60, P = 0.00). The proportion of female patients in the family burn group was significantly higher than that of the female in the workplace. Mortality in patients with severe burn ICU increased gradually (X2 = 8.52, P = 0.04). The burn area with 50% risk of death was 96.83% TSBA with a 95% confidence interval (91.28-104.30). Age is the main reason for the treatment outcome.
**CONCLUSIONS.** The results suggest that with Shanghai´s transition from an industrial city to a service and business center, there was no reduction in severe burns at the burn center, and the incidence of female patients increased and the incidence of family burns increased. The age of severely burned patients was on an upward trend and the mortality showed an upward trend in patients with ICU prolonged survival, increased age is an important reason affecting the outcome of patients. The present study suggest that the work of burn center in Shanghai needs to be transformed rapidly and an effective industrial and household prevention system is urgent. A rescue / transshipment system that effectively connects local and burn centers should be set up to improve the efficiency of local treatment and transport. There is an urgent need to establish an effective rehabilitation system to alleviate the burden on the family and society and allow more patients to return to society and occupation.

### 0930

#### Clinical characteristics of critical burn injury at Vall d`Hebrón University Hospital: a retrospective analysis

C. Vizcaino Urresta[1], S. Marquina[2], A. Rey Pérez[2], J. Baena[2], L. Pérez[2], L. Lagunes[3], J.P. Barret[4], M. Baguena[2]
[1]Hospital Vall d`Hebron, Critical Care, Barcelona, Spain; [2]Hospital Vall d`Hebron, Critical Care, Trauma Center, Barcelona, Spain; [3]Vall d'Hebron Institute of Research, Barcelona, Spain; [4]Hospital Vall d`Hebron, Plastic Surgery and Burns, Barcelona, Spain
**Correspondence:** C. Vizcaino Urresta
*Intensive Care Medicine Experimental* 2018, **6(Suppl 2):**0930

**INTRODUCTION.** Critical Burn patients care requires a highly specialized healthcare service(1). A global of 238.000deaths due to fire-related burns, with a mortality in Europe around 21.400patients and a lost one disability-adjusted life year around 500.000 are reported(2). A European review(3) reported between 4-22% of patients presenting with burns at the emergency department, were admitted to the intensive care unit(ICU).
**OBJECTIVES.** To describe the clinical characteristics of adults admitted to the burn intensive care unit(BICU) at Vall d'Hebrón University Hospital a reference center from Catalonia, Balneares and Andorra; between February 2009 and January 2017, and assess differences between survivors and nonsurvivors.
**METHODS.** Retrospective monocentric cohort study of adult with major burn injury; defined as total body surface area(TBSA) burned over 15% or inhalation injury. Demographic data, comorbidities, mechanism of burn, inhalation injury, vasopressor support requirement, length of mechanical ventilation(MV-LOS) and length of stay(LOS) in BICU were collected from hospital medical records.
**RESULTS.** 276 patients were admitted, 206(74.6%) were male. Median age was 46ys (IQR25-75:32.2-62). Principal cause of admission was burned by flame in 154(55.8%). Median TBSA was

22%(IQR25-75:16-35). 79 patients(28.6%) had inhalation injury. BICU-LOS median was 1days(IQR25-75:1-20) and MV-LOS median was 15days(IQR25-75:5-28). ABSI score was 6 median(IQR25-75:4-8), Median Bauxscore 75(IQR25-75:55.2-98) and Median Revised Baux score 77.5(IQR25-75:60-101). 51 patients(18.5%) were dead. When comparing survivors and non survivors; no differences in gender, or mechanism of injury were observed. Regarding comorbidities; non survivors had significantly higher proportions of diabetes, liver disease, psychiatric and heart disease. A higher proportion of patients in the non survivor group presented septic shock 16(31%) versus 8(3%), p=< 0,001, necessity of mechanical ventilation 38(74%) versus 89(39%), p=< 0.001, inhalation injury 28(55%) versus 51(22%), p=< 0.001, requirement of renal replacement therapy 12(23%) versus 12(5%), p= < 0.001 and ventilator associated pneumonia 22(44%) versus 37(16%), p= < 0.001.

**CONCLUSIONS.** Patients admitted to this BICU during the study period were predominantly middle age men burned by flame with a TBSA between 15.2 and 35.7%TBSA. High mortality rate is observed globally especially in those with a TBSA over 35.7% of TBSA.

**REFERENCE(S)**

1. American Burn Association. Advanced Burn Life Support Providers Manual. Chicago, IL.
2. World Health Organization. The injury chart book: a graphical overview of the global burden of injuries. Geneva: Dept. of Injuries and Violence Prevention, Noncommunicable Diseases and Mental Health Cluster, World Health Organization;2002.
3. Brusselaers N, Monstrey S, Vogelaers D, Hoste E, Blot S. Severe burn injury in Europe: a systematic review of the incidence, etiology, morbidity, and mortality. CritCare 2010.

### 0931

#### Induction of local anti-inflammatory IL-10 release at the onset of multiple trauma injury

G. Filntisis, K. Venetsanou, M.-A. Stamati, G. Lapidakis, V. Zidianakis, A. Korompeli
National and Kapodistrian University of Athens, Faculty of Nursing, ICU, Agioi Anargiri General & Cancer Hospital, Athens, Greece
**Correspondence:** G. Filntisis
*Intensive Care Medicine Experimental* 2018, **6(Suppl 2):**0931

**INTRODUCTION.** It is known that trauma injury triggers the initiation of inflammatory reaction as a direct immune response. An effect of local injury is the formation of drainage fluid.

**OBJECTIVES.** The aim of the study is to investigate the local and systemic inflammatory activity in multitrauma patients.

**METHODS.** Fourteen multitrauma patients admitted to the emergency department of a general hospital, in Athens Greece. Demographic and clinical data were recorded. Drainage fluid and blood samples collected the 1$^{st}$, 4th and 7$^{th}$ day of hospitalization. Tumor necrosis factor (TNF)-a and interleukins (IL-) -6,-8,-10 determined in serum and pleural fluid by Enzyme-Linked Immunoabsorbent Assay (ELISA).

**RESULTS.** On admission, IL-6 and IL-10 were significantly higher in pleural effusion, compared to circulating levels, but TNF-a had no significant differences.

Subsequently, during the time course, the local release of TNF-a and IL-6 was invariable, while the anti-inflammatory IL-10 showed a reduced profile.

In parallel, systemic IL-6 was gradually decreased, TNF-a showed a significant increase on the 7$^{th}$ day and IL-10 was fluctuating during the week.

Comparing the time course of cytokines release, local IL-6 and IL-10 levels remained elevated than systemic release, while systemic TNF-a was significantly elevated the 7$^{th}$ day than local.

**CONCLUSIONS.** At the onset of trauma injury, within the 1$^{st}$ week, systemic and local inflammatory reaction showed different profiles. The early strong local inflammatory response is counterbalanced by an immediate and sustained anti-inflammatory induction, expressed by IL-10, while the mild systemic reaction, showed a second hit, expressed by TNF-a, within the week.

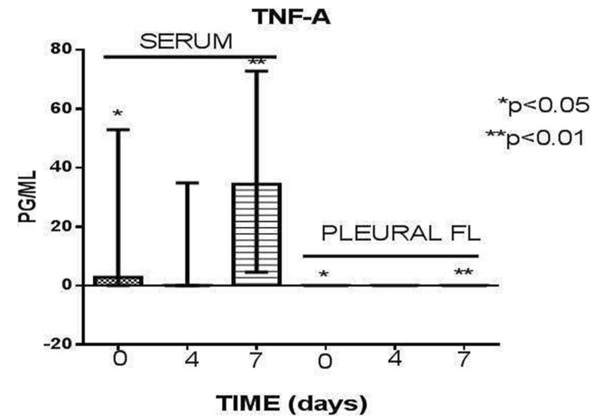

**Fig. 1 (abstract 0931).** Local and systemic TNF-a release in multitrauma patients

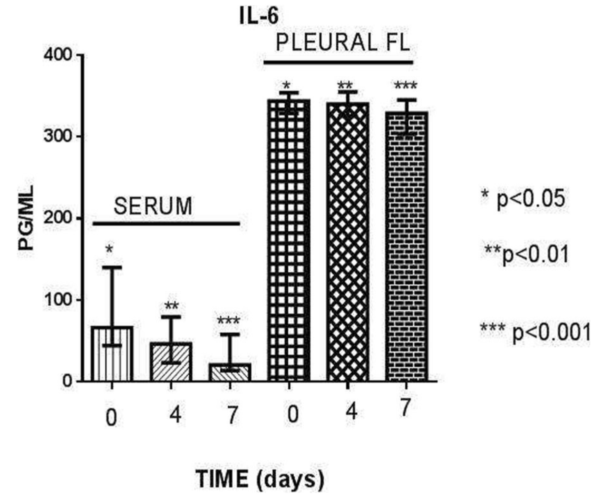

**Fig. 2 (abstract 0931).** Local and systemic IL-6 release in multitrauma patients

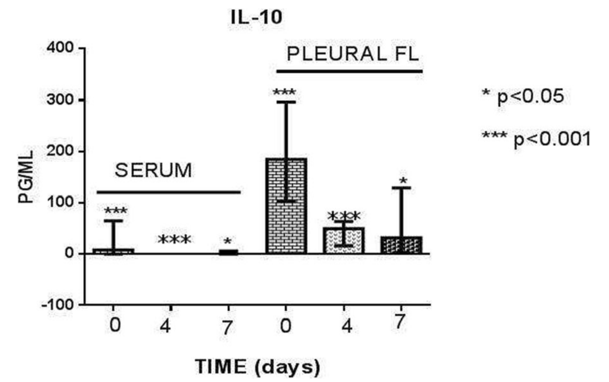

**Fig. 3 (abstract 0931).** Local and systemic IL-10 release in multitrauma patients

### 0932

#### Correlation of central venous catheter thrombosis with insertion site in adult trauma intensive care units

M. Masjedi[1], M. Salari[2], M.R. Sasani[2,3], J. Vakili[2]
[1]Anesthesiology and Critical Care Research Center, Shiraz University of Medical Sciences, Anesthesia and Intensive Care, Shiraz, Iran, Islamic Republic of; [2]Shiraz University of Medical Sciences, Shiraz, Iran, Islamic Republic of; [3]Medical Imaging Research Center, Department of Radiology, Shiraz University of Medical Sciences, Shiraz, Iran, Islamic Republic of
**Correspondence:** M. Masjedi
*Intensive Care Medicine Experimental* 2018, **6(Suppl 2):**0932

**Withdrawn**

### 0933

#### Comparison of serum biomarkers for CT positive mild to moderate traumatic brain injury in emergency department

T. Kaneko[1,2], H. Tanaka[1], T. Era[2], K. Karino[2], S. Yamada[2], M. Kitada[2], T. Sakurai[2], M. Harada[2], F. Kimura[2], T. Takahashi[2], S. Kasaoka[1]
[1]Kumamoto University Hospital, Emergency and General Medicine, Kumamoto, Japan; [2]Kumamoto Medical Center, Emergency and Critical Care Center, Kumamoto, Japan
**Correspondence:** T. Kaneko
*Intensive Care Medicine Experimental* 2018, **6(Suppl 2):**0933

**INTRODUCTION.** Glial fibrillary acidic protein (GFAP), phosphorylated neurofilament heavy subunit (pNF-H), and heart-fatty acidic binding protein (H-FABP) are current remarkable serum biomarkers for outcome of traumatic brain injury (TBI), although neuron specific enolase (NSE) and S100B protein are traditional biomarkers in this field. However, there is not enough data that these biomarkers could predict positive head computed tomography (CT) findings of mild to moderate TBI cases in emergency department (ED).

**OBJECTIVES.** To assess the usefulness of these and traditional biomarkers to predict positive CT findings of TBI cases in ED.

**METHODS.** Serum biomarkers (GFAP, pNF-H, H-FABP, NSE, and S100B) were measured in mild to moderate TBI on admission, and all cases were performed head CT. These biomarkers were compared by receiver operating curve (ROC) analysis.

**RESULTS.** 57 cases of TBI cases were analyzed. Median age was 70 y.o., 39% males, and median GCS was 15. Median serum GFAP, pNF-H, H-FABP, NSE, and S100B were 0.11 ng/mL, 53.9 pg/mL, 5.2 ng/mL, 12.6 ng/mL, and 7.2 pg/mL, respectively. 12 cases (21%) had positive head CT findings. ROC analysis showed that area under the curve (AUC) of GFAP, pNF-H, H-FABP, NSE, and S100B were 0.845, 0.569, 0.518, 0.744, and 0.753, respectively.

**CONCLUSIONS.** Serum GFAP showed the superiority from other serum biomarkers to predict positive head CT findings of mild to moderate TBI cases in ED, based on ROC analysis.

##### REFERENCE(S)

Honda M, Tsuruta R, Kaneko T, et al. Serum glial fibrillary acidic protein is a highly specific biomarker for traumatic brain injury in humans compared with S-100B and neuron-specific enolase. J Trauma 2010;69:104-9.

Shibahashi K, et al. The serum phosphorylated neurofilament heavy subunit as a predictive marker for outcome in adult patients after traumatic brain injury. J Neurotrauma 2016;33:1826-33.

Walder B, et al. The prognostic significance of serum biomarker heart-fatty acidic binding protein in comparison with s100b in severe traumatic brain injury. J Neurotrauma 2013;30:1631-7.

##### GRANT ACKNOWLEDGMENT

This study was supported by JSPS KAKENHI Grant Number JP16K11409.

### 0934

#### Factors associated with posttraumatic meningitis: a nation-wide study with hospital-based trauma registry in Japan

Y. Katayama[1], T. Kitamura[2], J. Sado[2], T. Hirose[1], T. Matsuyama[3], T. Kiguchi[4], K. Kiyohara[5], H. Takahashi[6], T. Noda[7], S. Adachi[8], J. Iba[1], K. Yoshiya[1], Y. Nakagawa[1], T. Shimazu[1]
[1]Osaka University Graduate School of Medicine, Department of Traumatology and Acute Critical Care, Suita, Japan; [2]Osaka University Graduate School of Medicine, Department of Social and Environmental Medicine, Suita, Japan; [3]Kyoto Prefectural University of Medicine, Department of Emergency Medicine, Kyoto, Japan; [4]Kyoto University, Health Sciences, Kyoto, Japan; [5]Tokyo Women's Medical University, Department of Public Health, Tokyo, Japan; [6]Kansai Medical University, Department of Emergency and Critical Care Medicine, Hirakata, Japan; [7]Osaka City University Graduate School of Medicine, Department od Traumatology and Critical Care Medicine, Osaka, Japan; [8]Rinku General Medical Center, Senshu Trauma and Critical Care Center, Izumisano, Japan
**Correspondence:** Y. Katayama
*Intensive Care Medicine Experimental* 2018, **6(Suppl 2):**0934

**INTRODUCTION.** Posttraumatic meningitis is one of severe complications and results in increased mortality and longer hospital stay among trauma patients. Factors such as cerebrospinal fluid (CSF) fistula and skull base fracture are associated with posttraumatic meningitis. On the other hand, advanced age, external ventricular drainage, and repeat operations are associated with postcraniotomy meningitis. However, it remains unclear whether procedures such emergency craterization and re-operation would be associated with posttraumatic meningitis.

**OBJECTIVE.** The aim of this study is to assess factors associated with posttraumatic meningitis with a nation-wide hospital-based trauma registry in Japan.

**METHODS.** This study was a retrospective observational study and the study period was 12 years between January 2004 and December 2015. We included trauma patients registered in Japanese Trauma Data Bank whose head AIS score was >=3 in this study. Multivariable logistic regression analysis was used to assess potential factors associated with posttraumatic meningitis such as CSF fistula, emergency craterization at emergency department and reoperation within 48 hours after first operation.

**RESULTS.** Among 60,390 patients with severe head injury, 284 (0.5%) patients had posttraumatic meningitis. Factors associated with posttraumatic meningitis were emergency craterization at emergency department (adjusted odds ratio [OR] 2.219 [95% confidence interval {CI}; 1.444-3.411]), craniotomy in operation room (adjusted OR 4.553 [95% CI; 3.038-6.821]), craterization in operation room (adjusted OR 2.231 [95% CI; 1.433-3.473]), external decompression surgery (adjusted OR 2.142 [95% CI; 1.521-3.017]), external ventricular drainage (adjusted OR 1.869 [95%CI; 1.175-2.972]), re-craniotomy within 48 hours after first operation (adjusted OR 3.145 [95%CI; 2.109-4.691]), re-craterizaion within 48 hours after first operation (adjusted OR 4.190 [95%CI; 2.174-8.076]), CSF fistula (adjusted OR 3.334 [95% CI; 2.211-5.028]), and skull base fracture (adjusted OR 1.696 [95% CI; 1.213-2.372]).

**CONCLUSIONS.** In this population, emergency craterization at emergency department and external decompression surgery were associated with posttraumatic meningitis among trauma patients.

### 0935

#### Is early hemofiltration in major trauma patients feasible? Systematic review and a retrospective analysis

F. Avolio[1], M.C.C. Meca[2], E. Gamberini[2], L. Domenichini[3], I. Merloni[2], E. Russo[2], B. Russo[4], M. Iudici[5], V. Agnoletti[2]
[1]ASUITS, Trieste, Italy, Anesthesia and Intensive Care Unit, Trieste, Italy; [2]AUSL Romagna, Anesthesia and Intensive Care Unit, Cesena, Italy; [3]AUSL Romagna, Cesena, Italy; [4]Universite de Geneve, Napoli, Italy; [5]Université Paris Descartes, Paris, France
**Correspondence:** F. Avolio
*Intensive Care Medicine Experimental* 2018, **6(Suppl 2):**0935

**INTRODUCTION.** Multi organ failure (MOF) is a serious clinical condition in major trauma patients that influence outcomes, complications and economic costs in intensive care unit (ICU). Several studies demonstrate the role of infiammatory citokynes in the physiopathological mechanisms leading to MOF in trauma. Inflammatory response is common in the most of severe injured patients. Early hemofiltration, even without renal failure can help to remove citokynes.
**Aim:** The aim of our study is to intevigate the feasibility of early hemofiltration in trauma patients affected by MOF due to inflammatory response.
**METHODS.**
We performing a Systematic review using the following keyword search: "Wounds and Injuries" OR “trauma*” OR “injury” OR “accident” OR “crash” AND "Renal Dialysis" OR “hemofiltr*” OR “renal replacement therapy” OR “biofiltration” OR “ultrafiltr*” OR “blood purif*” OR “diafiltration” OR “hemodiafiltr*” AND "humans"
We retrospectively analyzed patients admitted to the Intensive Care Unit of the “Ospedale Bufalini-Cesena” from January 2015 to December 2017with Injury Seeveriry Score (ISS) >16 and hemofiltration started in the first 48 from ICU admission.
A great vein was incannulated, such as giugular or femoral, with a specific catheter for hemofiltration. The severity of trauma was evaluated with ISS. The hemofiltration was realized with polyphenylene membrane filter, low-flux technique and twelve hours of duration. The sequencial organ failure assesement (SOFA) score was used to evaluate the appereance of organ failures in the next 48 hours after stopped hemofiltration.
**RESULTS.** We found 3783 studies with the search strategy above described but no one was selected as relevant by the research.
In our experience Twenty-five patients in level I trauma center were analyzed retrospectively from January 2015 to December 2017
Median ISS was 32, Inter quartile range (IQR) 20; median SOFA 11 (IQR 4.25). Overall mortality was 24%.
No significative correlation (Spearman's Rho Test) was found between ISS and SOFA score (Rho 0.157)
Median ISS of died patients was 39.5 (IQR 21) vs 29 (14.25) of discharged from hospital group (p 0.378); Median SOFA of died patients was 14 (IQR 2) vs 11 (4) of discharged from hospital group (p 0.073).
**CONCLUSIONS.** Although the discussion about the usefulness of hemofiltration for removal cytokine from blood is still in progress, our experience in this case series suggest that these techniques of blood purification could be feasible. We hope that large trial will be performed to improve our understanding of this issue.

### 0936

#### Comparative study of cohorts between isolated severe traumatic brain injury (TBI) and associated with polytrauma

A. Bueno González, M.C. Corcobado Márquez, M. Portilla Botelho, M.C. Hornos López, M.C. Espinosa González, C. Martin Rodriguez, A. Ambrós Checa, Grupo de trabajo Trauma y neurocríticos de la SEMICYUC. Proyecto RETRAUCI
Hospital General Universitario de Ciudad Real, Intensive Care Unit, Ciudad Real, Spain
**Correspondence:** A. Bueno González
*Intensive Care Medicine Experimental* 2018, **6(Suppl 2):**0936

**INTRODUCTION.** Few has been researched about differences and the prognosis clinical between isolated severe TBI and severe TBI associated with multiple trauma. A priori, the clinical conditions are different, due to the different pathophysiology of TBI in the context of multisystem trauma.
**Objetives**: The objectives are 1º / To know the clinical characteristics and mortality of the isolated severe TBI and TBI associated with multiple trauma. 2º / Determine mortality and associated risk factors in isolated severe TBI.
**Method:** Retrospective cohort study using local data collected for the national registry of traumatic patients (Project RETRAUCI) from 02/ 2013 to12/2016. A comparative study of patients with severe TBI in two groups, according to the presence of isolated severe TBI or severe TBI with multiple trauma was conducted. Given that the objective is to evaluate mortality, severe TBI is defined, all TBI with GCS score < = 8, without other injuries or with non-serious injuries (AIS < 3) in any anatomical region. We defined severe TBI associated with polytrauma, TBI with GCS score < = 8, with severe injury in some other anatomical region (AIS> = 3). Through a logistic regression analysis, predictive factors of mortality are identified. Statistical analysis with STATA software 13.0.
**Results**: 54 patients with severe TBI were included in the study, 40 had isolated TBI and 14 TBI associated with multiple trauma. Overall hospital mortality of severe TBI was 29.63% (16 patients). There were no significant differences in mortality between isolated TBI and TBI with multiple trauma (30% vs 28.57%, p: 0.92). There were significant differences in the mechanism of injury, ISS, high base déficit DB and at admission transfusion requirements in the first 6 hours, the latter two being less frequent in isolated TBI.
In the logistic regression analysis of the variables analyzed in the group of isolated severe TBI, only the low GCS score (OR: 0.53, CI: 0.31 to 0.90) and age (OR: 1.05, CI: 1.01 to 1.10) were the factors associated with an increased mortality risk, with an AUC ROC: 0.8571.
**Conclusions**: Factors related to the severity of the injury (ISS and DB) and higher transfusion requirements were the main differences between isolated severe TBI and that associated with multiple trauma. However, there were no significant differences in mortality.

### 0937

#### Thoracic blunt aortic injury (TBAI) in polytrauma: how to define clinical priorities?

C. Valentina[1], S. Ranieri[1], P. Cavallo[2], C. Coniglio[2], G. Gordini[2]
[1]Policlinico Sant'Orsola-Malpighi, University of Bologna, Department of Medical and Surgical Sciences, Anesthesiology and Intensive Care Unit, Bologna, Italy; [2]Trauma Center “Maggiore” Hospital, AUSL Bologna, Anesthesia, Intensive Care and EMS Department, Bologna, Italy
**Correspondence:** C. Valentina
*Intensive Care Medicine Experimental* 2018, **6(Suppl 2):**0937

**INTRODUCTION.** TBAI is a rare but significant cause of impending death following deceleration trauma. TBAI rarely occurs as isolated injury, therefore the decision on the priority of surgical treatment can be very challenging.
**OBJECTIVES.** To review the epidemiology and treatment of TBAI, comparing perioperative variables and timing of aortic repair in case of multiple injuries.
**METHODS.** In this retrospective observational study we considered all consecutive patients who presented at our Trauma center from

2010 to 2017, clinical and procedural variables were collected. Diagnosis and grading of TBAI were derived from CT-scan imaging.

**RESULTS.** 15 cases of TBAI were identified, with a predominance of males (86,6%) and mean age of 54 years. The most frequent mechanism of injury was motor vehicle collision (80%). Age, sex distribution, and ISS values were comparable to literature [1]. All patients had multiple associated injuries mostly in chest, abdomen and extremity, with a mean ISS of 35. The ISS was significantly higher in patients with more severe TBAI. 12 patients underwent aortic repair and the mean time from trauma to aortic intervention was 6,5 hours.

9 patients with Grade III (pseudoaneurysm) and IV (active extravasation) had an immediate intervention. 2 patients with Grade IV TBAI had a delayed repair because of other sources of impending bleeding. One patient underwent to perihepatic packing and bladder repair, the other to legs fractures stabilization. In these patients time from trauma to the aortic repair was 8 and 10 hours, reasonably comparable with the average times of the immediate repair group.

A delayed repair was also provided for one patient with intramural hematoma and no signs of impending aortic rupture, controlling heart rate and blood pressure while awaiting repair. A conservative management was provided for 2 patients with type I injuries (intimal tear). 10 were treated with endovascular repair (TEVAR) and 2 with open repair (OR), no mortality or paraplegia described. The mean ICU stay was 24,4 days, death occurred in 2 patients. There were no procedure or device related complications.

**CONCLUSION.** Considering the high risk of rupture within the first 24 hours, the actual recommendations suggest a prompt repair, unless patients have more immediately life-threatening injuries that require intervention (damage control approach) or if the patient is a poor operative candidate [2]. In our series, the clinical management was consistent with this approach. Although tailored treatment is obviously necessary in complicated setting as polytraumatic patients, damage control resuscitation management approach and TEVAR repair instead of OR have been crucial points to guide our clinical management with favorable outcomes.

### 0938

#### Trauma care in Italy: a regional survey

E. Borotto[1], A. Canziani[2], R. Giudici[1], M. Gomarasca[1], M. Zaffaroni[2], D. Radrizzani[1]

[1]Ospedale Civile Legnano, Intensive Care Unit, Legnano, Italy; [2]Ospedale Civile Legnano, Emergency Department, Legnano, Italy

**Correspondence:** E. Borotto

*Intensive Care Medicine Experimental* 2018, **6(Suppl 2):**0938

**INTRODUCTION.** Trauma still remains the first cause of death and invalidity among young people representing about 9% of total deaths in the word.

It´s generally recognised that a rapid treatment of multi-injured patients by a team of specialists with well-established protocols can significantly affect mortality and morbidity with concomitant optimization of medical resources. Despite this,organization of trauma centres may be difficult because of economical, organizational and educational problems; standard of care in Italian trauma centres is not well known.

**OBJECTIVES.** Italian Sanitary System is organised in regional districts; the aim of this survey is to describe standard of care in Lombardia, a 23 $Km^2$ area with ten million inhabitants and a median of trauma per year around 4000. A Trauma System was set up in 2012 dividing hospitals in 3 categories:

High Speciality Trauma Centre: covering all injuries definitive care (6 CTS)

Zone Trauma Centre with neurosurgery: covering definitive care of major injuries excluding high specialities (13 CTZ nsgy)

Zone Trauma Centre without neurosurgery (10 CTZ)

Patients are centralised according to vital signs´ stability and suspicion of brain trauma.

**METHODS.** Between October 2017-January 2018 an online questionnaire was submitted to ED managers of trauma centres, investigating trauma team organization in daily practice such as education, hospital protocols, accordance to international guidelines.

**RESULTS.** Data are summarized in Table 1.

A total of 26 centres (89%) replied to questionnaire.

Whole CTS validated a trauma protocol including a trauma team composed in all but one case by non-dedicated physicians.

While a checklist for major trauma identification is present in all CTS, trauma team activation is not always automatic after checklist filling.

When established, Massive Transfusion Protocol (MTP) is activated mainly according to ATLS shock classes definition or to medical discretion (shock index and BE in 6% of interviewed).

Extra hospital utilization of tranexamic acid seems to be quite low: 35% of interviewed defines its use as common in prehospital care.

Concerning education, a local trauma registry is present in 23% of hospitals and only 30% of centres organizes periodical morbidity & mortality conferences and in situ simulations courses.

**CONCLUSIONS.** Surveys' evaluation of clinical practice, particularly in countries like Italy where Trauma System is still developing, allows a better knowledge of the state of the art and helps to improve organization. In less than 5 years a lot of work has been done in order to enhance trauma care in Lombardia but several aspects can still be ameliorated especially in non-High Speciality Trauma Centres, in education and trauma team working.

Groenestege-Kreb"Trauma Team"2014 BJA

**Table 1 (abstract 0938).** See text for description.

| | | CTS | CTZ - NSGY | CTZ | SUMMATION |
|---|---|---|---|---|---|
| MAJOR TRAUMA MANAGEMENT | trauma protocol | 100%<br>5 hospitals of 5 | 77%<br>10 hospitals of 13 | 62%<br>5 hospitals of 8 | 77% |
| | trauma team | 100%<br>5 hospitals of 5 | 46%<br>6 hospitals of 13 | 37%<br>3 hospitals of 8 | 50% |
| TRIAGE | triage check-list | 100%<br>5 hospitals of 5 | 46%<br>6 hospitals of 13 | 50%<br>4 hospitals of 8 | 58% |
| | shared protocol with extra hosp | 80%<br>4 hospitals of 5 | 67%<br>4 hospitals of 6 | 50%<br>2 hospitals of 4 | 64% |
| | direct activation TT | 60%<br>3 hospitals of 5 | 100%<br>6 hospitals of 6 | 25%<br>1 hospitals of 4 | 67% |
| MASSIVE HEMORRAHAGE MANAGEMENT | tranex * | 35%<br>9 hospital of 26 | 35%<br>9 hospital of 26 | 35%<br>9 hospital of 26 | 35% |
| | MTP | 80%<br>4 hospitals of 5 | 61%<br>8 hospitals of 13 | 62%<br>5 hospitals of 8 | 65% |
| | trasfusion packs | 100%<br>4 hospitals of 4 | 50%<br>4 hospitals of 8 | 60%<br>3 hospitals of 5 | 65% |

#### 0939

#### The relation between neutrophil subsets mobilized after trauma and the occurrence of infectious complications

L. Hesselink[1], R. Spijkerman[1], L.P.H. Leenen[1], L. Koenderman[2], F. Hietbrink[1]
[1]UMC Utrecht, Trauma Surgery, Utrecht, Netherlands; [2]UMC Utrecht, Department of Respiratory Medicine, Laboratory of Translational Immunology, Utrecht, Netherlands
**Correspondence:** L. Hesselink
*Intensive Care Medicine Experimental* 2018, **6(Suppl 2):**0939

**INTRODUCTION.** After severe trauma, patients are prone to develop infectious complications. The consequential immune response correlates with trauma severity and, thereby, the risk for these complications. Neutrophils are the first immune cells to respond to both tissue damage as well as infection. Different neutrophil subsets can be distinguished after trauma and during systemic inflammation, such as sepsis and after the administration of lipopolysaccharide (LPS). Based on morphology and receptor expression immature banded cells (CD16dim/CD62Lbright), mature segmented cells (CD16bright/CD62Lbright) and immunosuppressive hypersegmented cells (CD16bright/CD62Ldim) can be distinguished. In the last decade much information is gained concerning the role these cells might play in the pathophysiology of infectious complications.

**OBJECTIVES.** The aim of this study was to review literature and experimental data with emphasis on the recent findings in the last 10 years on neutrophil subset kinetics and function after trauma in relation with the development of infectious complications.

**METHODS.** A literature search was performed on neutrophil subsets. Studies were included on neutrophil subsets after trauma, neutrophil subsets in relation to infectious complications, and functionality of neutrophil subsets. Both clinical and experimental studies were included. Unpublished data on neutrophil subsets after trauma from our laboratory completed this study.

**RESULTS.** Directly after trauma, overwhelming inflammation leads to a release of large amounts of banded (immature) neutrophils into the circulation, with reported percentages up to 70% of total neutrophil count. During the following days a shift in neutrophil phenotype is observed towards the mature phenotype. From day 5 on, immunosuppressive neutrophils able to inhibit T-cells, are observed in the blood. After administration of LPS, these immunosuppressive neutrophils are characterized by diminished bacterial killing capacity compared to the other subtypes. It is unknown if systemic inflammation after LPS administration is comparable to the situation after trauma. However, severe infectious complications mainly occur after 5 days post trauma, which might indicate a similar process.

**CONCLUSIONS.** Distinct neutrophil subtypes with different functional characteristics are observed on specific time points after trauma. It is tempting to speculate that there is a relation between the appearance of immunosuppressive neutrophils in the blood and the occurrence of infectious complications after trauma.

### Paediatrics & safety

#### 0941

#### Effect of Fluid resuscitation in first 3 hours and fluid balance on the mortality in pediatric septic shock

E. Ha, S. Park
Asan Medical Center, Pediatric Department, Seoul, Korea, Republic of
**Correspondence:** E. Ha
*Intensive Care Medicine Experimental* 2018, **6(Suppl 2):**0941

**INTRODUCTION.** Septic shock is life-threatening emergencies. Fluid resuscitation plays a fundamental role in the treatment of septic shock. The surviving sepsis guidelines recommend early aggressive fluid resuscitation. Although rapid fluid administration may offer benefit, studies on the timing of resuscitation are lacking, and especially more in pediatric patients.

**OBJECTIVES.** We tried to assess the amount of fluid administered in hours within 24 hours following the onset of septic shock. So we aimed to determine whether how much fluid was administered and how quickly it was administered after septic shock onset time is associated with the outcome of the patients, and to find out the optimal strategy for septic shock resuscitation.

**METHODS.** This is a retrospective cohort study of consecutive pediatric patients with septic shock who received fluid resuscitation admitted to Pediatric Intensive Care Unit between January 2012 and August 2017. Data were collected from reviewing the electronic medical records. We assessed the total amount of fluid administered and output within 24 hours from the onset of septic shock.

**RESULTS.** Of 155 patients with septic shock screened, 133 had detailed fluid data. Mean age was 9.0±7.1 years, mean Pediatric Risk of Mortality (PRISM) III score was 14.1, and the 28-day mortality rate was 24.8 %. Hemato-oncology patients had majority of total patients (n=55;42.1%). Within 24 hours, an average 109.0 ml of fluid per body weight was administered, and 20.5 % of total fluid within 24 hours was administered within first 3 hour. Survivors received significant higher median amound of fluid within first 3 hours than nonsurvivors (22.7 cc/kg vs 18.8 cc/kg,p=0.048), but there was no difference in the amount of fluid after first 3 hours between these two groups. The proportion of fluid received within first 3 hours given to total amount administered over 24 hour period showed the significant difference between survivors and nonsurvivors(21.5% vs 17.3%,p=0.039). After 6 hours from onset of septic shock, the greater the amount of fluid administered, the higher the mortality rate tended to be.

**CONCLUSIONS.** Our results showed that the higher proportion of total fluid received within first 3 hours was associated with decreased 28-day mortality in pediatric patients with septic shock. The earlier fluid resuscitation within first 3 hours is thought to be a very important factor in the strategy for septic shock patients. And we think that further evaluation may be necessary to make these things more common.

#### 0942

#### The positive impact that a nurse led vascular access team can have on central line-associated complications

H. Baker[1], V. Shawyer[2], C. Coutice[2], J. Nixon[2], S. Hill[2], S. Bellamy[2]
[1]Royal Devon and Exeter Hospital, Intensive Care Unit, Exeter, United Kingdom; [2]Royal Devon and Exeter Hospital, Exeter, United Kingdom
**Correspondence:** H. Baker
*Intensive Care Medicine Experimental* 2018, **6(Suppl 2):**0942

**INTRODUCTION.** Peripherally inserted central venous catheters have become an increasingly common method of venous access in the ICU patient (Wilson et al, 2012).

The development of a nurse lead Vascular Access Team (VAT) within an acute care hospital has reduced catheter related blood stream infections (CRBSI) and minimalized the incidence of venous thromboembolism (VTE). The VAT placed peripherally inserted central catheters (PICCS) as an alternative to short term central venous catheters, which have a low incidence of CLABSI. An initial rise in CLABSI correlated with the increase in number of patients requiring parental nutrition. Consequently an education programme was implemented trust wide to reduce the CLABSI.

**OBJECTIVES.** Review 10 years of nurse led PICC line placement in relation to ICU patients to establish safety and efficacy of PICC in this population.

**METHODS.** A retrospective single centre study in general ICU in the UK. We included patients aged between 18 and 99 years, requiring a PICC during a period between January 2008 and January 2018. Our primary endpoints were CRBSI up to 30 days after catheter placement, UEVTE or misplacement. We defined CLABSI based on the Centre for Disease Control and Prevention's National Healthcare Safety Networks (NHSN) 2015 surveillance definitions. Secondary endpoints were numbers of line related days, indications for placement, number of attempts.

All PICCS were inserted by the VAT at the patient's bedside, using ultrasound and full sterile precautions in adherence to EPIC 2 guidelines. Infection rates were calculated per 1000 line days, from the introduction of the Vascular access service 8 months prior to the implementation of the educational programme and then for 12 months after. The educational programme involved

- Ward based teaching
- Junior Doctors training
- Monthly central line workshops
- Patient education

**RESULTS.** There were no insertion related bacteraemia during the surveillance period. The increase in line infections correlated with the increase in patients prescribed PN. There were 3 VTE in 2015 this correlated in greater surveillance. Through the surveillance of all the lines inserted by the VAT the specific educational needs of the staff and patients were identified and targeted.

**CONCLUSION.** A structured educational programme that includes doctors, nurses and patients has a direct impact on the reduction of central line associated bloodstream infections and VTE. This positive impact can be sustained through ongoing education and surveillance of line infections and VTE.

### 0943

#### Efficacy and safety of early high-dose rehabilitation in ICU patients

M. Bychinin, S. Andreychenko, T. Sukonnikov, T. Klypa

Federal Research and Clinical Center, FMBA of Russia, Moscow, Russian Federation

**Correspondence:** M. Bychinin

*Intensive Care Medicine Experimental* 2018, **6(Suppl 2):**0943

**IINTRODUCTION.** Survivors of critical care illness frequently experience poor physical outcomes, including persistent impairments in muscle strength, exercise capacity and physical function. Rehabilitation including active and passive in-bed cycling ergometry, tilt table and neuromuscular electric stimulation are promising techniques for patient mobilization, although it is still unclear optimal time and dose of rehabilitation interventions [1].

**OBJECTIVES.** To access impact of early high-dose rehabilitation with relevant technology in general ICU-patients.

**METHODS.** Patients were assigned to either early mobilization (intervention) or to standard care (control). Early high-dose mobilization commenced ≤ 2 days of admission and completed 1 hour 5 days per week and included functional activities, sitting, in-bed cycling ergometry and tilt table. An intensive care unit Mobility team initiated the protocol within 48 hours. Each day participants were examined and were excluded from eligibility for a giving session on that day if they were cardiovascular or respiratory instability. Our primary outcome was a favorable outcome on the modified Rankin Scale and ICU-mobility Scale at day of ICU discharge. Secondary outcomes were number of serious adverse events (failing to the floor, cardiac arrest, dangerous arrhythmia during exercise, oxygen saturation less than 80%, unplanned extubation, or loss any invasively inserted line), ICU and hospital length, mortality. We used the Mann-Whitney U test for continues variables.

**RESULTS.** 17 adult ICU-patients admitted in general ICU were enrolled in the study, 7 patients in the intervention group and 10 patients in control group.

**CONCLUSIONS.** We found that delivery of early high-dose mobilization in patients of general ICU was safe and associated with non-significant difference in tendency of better mobility scale score and less ventilator-free days.

**REFERENCE(S)**

Goodson C. et al. (2016). Physical rehabilitation in the ICU: understanding the evidence. ICU management and practice. 17 (3):178-180.

**Table 1 (abstract 0943).** Mobility, ICU, and hospital outcomes

| Outcomes Measure | Early mobilization (n=7) | Control (n=10) | p |
|---|---|---|---|
| ICU mobility scale, maximum score during ICU stay, mean ± sd | 6.4 ± 3.7 | 4.2 ± 4.1 | 0.52 |
| Modified Rankin scales, maximum score during ICU stay, mean ± sd | 3.3 ±1.4 | 3.5 ±1.8 | 0.48 |
| Serious adverse events, n (%) | 1 (14) | 1 (10) | 1.0 |
| Ventilator-free days, median [IQR] | 9 [6-18] | 24 [13-29] | 0.08 |
| Death in ICU, n (%) | 1 (14) | 1 (10) | 1.0 |
| ICU length, median [IQR] | 12 [12-43] | 19 [14-48] | 0.72 |
| Hospital length, median [IQR] | 16 [15-45] | 25 [20-48] | 0.82 |

IQR = interquartile range

### 0944

#### Hemisphericcerebral oximetry ($r_cSO_2$) readings in status epilepticus in a pediatric emergency department: relationship to seizure complexity, anticonvulsant therapy and possible prediction trends

T. Abramo[1], H. Hargrave[1], Z.L. Harris[2], T. McCarty[1], N. Hobart-Porter[1], C. Velasco Gonzalez[1]

[1]UAMS/ACHRI, Pediatrics, Little Rock, United States; [2]Lurie Children's Hospital Northwestern School of Medicine, Pediatrics, Chocago, United States

**Correspondence:** T. Abramo

*Intensive Care Medicine Experimental* 2018, **6(Suppl 2):**0944

Pediatric seizures can cause neuronal injury and pharmacoresistant.For every first-line anticonvulsant minute delay (> 5minutes), a 10% greater risk for longer seizures >60 minutes, diminishing anticonvulsant efficacy, increase status epilepticus (SE) incidence and duration.A seizure 's cerebral physiology assessment tool, which current ED lacks, would enhance critical decision-making. Pediatric hemispheric $r_cSO_2$reading reflects cerebral physiology; $r_cSO_2$< 60%, >80% equates to abnormal cerebral physiology, neurological insult, and pathology.In EEG - $r_cSO_2$seizures, altered $r_cSO_2$correlated to seizures. PED non-epileptic generalized seizure patients, $r_cSO_2$readings were either < 60% or >80% and returned to pre-seizure $r_cSO_2$readings.Comparison of hemispheric generalized SE $r_cSO_2$ readings < 60 to >80% in relationship to seizure severity and anticonvulsant interventions is lacking.

**OBJECTIVE.** Correlational analysis of $r_cSO_2$< 60% or >80% readings to seizure complexity and anticonvulsant administration in a pediatric emergency department (PED).

**METHODS.** Observational study comparing SE $r_cSO_2$readings to seizure complexity and anticonvulsants in PED non-traumatic, neurologically normal, first-time seizure patients.

**RESULTS.** TABLE 1.

There were more < 60% than >80% seizure $r_cSO_2$readings (p< 0.001), seizure $r_cSO_2$ readings: $r_cSO_2$ < 60%, had a longer EMS (p=0.0002)/ PED (p=0.001) seizure duration, required more EMS (p=0.001) and PED (p=0.0009) anticonvulsants compared to $r_cSO_2$ >80%. Overall PED comparison: $r_cSO_2$ < 60% had longer seizures (p=0.0002) and required more anticonvulsants (p=0.003). Patients < 2 years old had more < 60% $r_cSO_2$ readings (p=0.0005) versus >2 yrs. For >2 yrs, had more seizure $r_cSO_2$ readings >80% (p=0.003). However, age had no correlation with anticonvulsants (p=0.08) or EMS seizure duration (p=0.19). Seizure $r_cSO_2$ readings < 60% [left 48.3% (37.8-58.2, p=0.0007), right 42.6% (35.7-55.3, p=0.0005)], and EMS seizure duration >23 minutes (p=0.005) required more anticonvulsants while age no significance (p=0.17). Correlation of seizure rcSO2 readings during seizure´s nonresponsive anticonvulsant, < 60%, >80% rcSO2 readings were consistent with delta change of < 5% (p< 0.0001). Seizure $r_cSO_2$ reading $r_cSO_2$ > 80% changed earlier than $r_cSO_2$ < 60%( p=0.001) and both changed earlier than EMR seizure cessation time (p=0.001).

**CONCLUSION.** In PED SE seizures, EMS seizure duration >23 minutes and seizure $r_cSO_2$ readings < 60% correlated with greater seizure complexity by longer seizure duration and more anticonvulsants compared to $r_cSO_2$ >80%. Comparing seizure cessation times, seizure $r_cSO_2$ > 80% changed earlier than $r_cSO_2$ < 60% while both $r_cSO_2$readings changed earlier then clinical seizure cessation. Seizure neuroresuscitation should strive for rapid cerebral physiology and therapeutic assessment which hemispheric $r_cSO_2$ functionality has shown and should be an integral component for initial pediatric seizure assessment.

**Table 1 (abstract 0944).** See text for description

| | |
|---|---|
| N=101: AGE(yrs):2.6(1.3, 5.3)( 49.2% male) | |
| All Seizure Events N= 101 | |
| General Seizure: Initial Left & Right $r_cSO_2$ readings < 60 | 52 |
| General Seizure: Initial Left & Right $r_cSO_2$ readings >80 | 49 |
| All PED Seizure Duration: minutes (EMS seizure duration 23 (12,29)) | 32.9 (29.6,56.8) |
| PED & EMS Seizure $r_cSO_2$ readings < 60% duration(minutes)(PED seizure < 60% duration: 29 (21,32)): | 45.9(34.7, 56.8) |
| PED & EMS Seizure $r_cSO_2$ readings >80% duration (minutes)(PED seizure >80% duration: 17 (12,24)): | 39.2 (27.1,48.1) |
| Anticonvulsant Doses: N= 679 | |
| Versed | 309 |
| Ativan | 242 |
| Keppra | 114 |
| Dilantin | 14 |
| Anticonvulsant dose per Patient: PED:4.9(3,6), EMS2(1,3) | N |
| 3-5 | 49 |
| 5-7 | 35 |
| 8 | 7 |
| Seizure $r_cSO_2$ readings change to EMR Seizure cessation time (minutes) | |
| Seizure $r_cSO_2$ readings : < 60 | -2.49 (-2.1,-3.9) |
| Seizure $r_cSO_2$ readings : > 80 | -3.79 (-3.3,-4.4), |

| Clinical Parameter N=101 patients | PATIENT'S SEIZURE TIMES (seconds) Before Seizure Stopped After Seizure Stopped | All Seizures | | Initial rcSO2 | | | | Group Comparison | |
|---|---|---|---|---|---|---|---|---|---|
| | | | | <60 | | >80 | | P-value (1c) | Difference (2) |
| | | N | Median (Q1,Q3) | N | Median | N | Median | | |
| Left $r_cSO_2$ | 90-0 sec Before | 101 | 59(37,90) | 49 | 45(36,56) | 52 | 90(62.5,93.5) | <.0001 | -36(-46,-27) |
| | 0-90 sec After | 101 | 70(64,75.5) | 49 | 68(61,74) | 52 | 70.5(64.75,81) | 0.15 | -5(-10,-0.5) |
| | 90-180 sec After | 101 | 69.75(62,72) | 49 | 69(62,75) | 52 | 70(63,72) | 1.00 | 0(-3,3) |
| | 180-300 sec After | 101 | 68(61,73) | 49 | 68(60,73) | 52 | 69.5(61.5,72) | 0.97 | 0(-4,2) |
| | 300-360 sec After | 101 | 69.5(62,74) | 49 | 73.5(61,76) | 52 | 69(63,73) | 0.94 | 3(-1,6) |
| | P-value (1b) | | | | <.0001 | | 0.001 | <.0001 (1a) | |
| Right $r_cSO_2$ | 90-0 sec Before | 101 | 45(34,89) | 49 | 37(32,50) | 52 | 89(41,92) | <.0001 | -41(-51,-20) |
| | 0-90 sec After | 101 | 70(60,75.5) | 49 | 63(55.5,74) | 52 | 71.5(63.5,80) | 0.08 | -6.5(-12,-1) |
| | 90-180 sec After | 101 | 68.5(59,72) | 49 | 64(58,78) | 52 | 69(62,70.75) | 1.00 | 0(-5,4) |
| | 180-300 sec After | 101 | 67(59,73) | 49 | 61.5(56,73) | 52 | 69(62,72) | 0.80 | -2(-6.5,2) |
| | 300-360 sec After | 101 | 70.25(60,77) | 49 | 71(57,77) | 52 | 70(60,76) | 0.76 | 0(-5,5.5) |
| | P-value (1b) | | | | <.0001 | | 0.0005 | <.0001 (1a) | |

(1a) test for interaction (Group x Period) term of repeated measures model. Ho: group median do not differ over time. (1b) slice test by Group, Sidak-corrected p-values. Ho: medians for specific group do not differ over periods. (1c) slice test by Period, Sidak-corrected p-values. Ho: group medians do not differ for specific period.(2) Hodges-Lehman location shift and 95% CI.

### 0945

#### Evaluation of cerebral circulation in children with congenital heart disease—comparison of those with and without systemic oxygenated disturbance

M. Yamamoto, T. Mori, Y. Morimoto
Hokkaido University Hospital, Anesthesiology, Sapporo, Japan
**Correspondence:** M. Yamamoto
*Intensive Care Medicine Experimental* 2018, **6(Suppl 2):**0945

**INTRODUCTION.** Cerebral disorders after cardiac surgery are still a serious problem in children with congenital heart disease (CHD). Several studies have reported that the patterns of cerebral circulation are different according to the type of CHD in the fetus[1]. However, there seem to be few studies after birth.

**OBJECTIVES.** In this study we evaluated the cerebral blood flow velocity (CBFV), cerebrovascular resistance and cerebral oxygen saturation ($rSO_2$) in children with CHD scheduled for cardiac surgery and compared these items for those with and without systemic oxygenated disturbance.

**METHODS.** After obtaining institutional review board approval (016-0505), we performed an observational prospective study of children under 15 years old scheduled for cardiac surgery. After induction of anesthesia, CBFV of the middle cerebral artery (systolic, Vs; mean, Vm; diastolic, Vd) was measured by transcranial Doppler sonography, EZ-Dop (DWL, Germany) and the pulsatility index (PI: [Vs-Vd]/Vm) was calculated as an index of cerebrovascular resistance. The $rSO_2$ was measured using an INVOS-5100B (Somanetics, Troy, MI). Simultaneously, mean systemic arterial blood pressure (mBP) was recorded and blood gas analysis was performed. Then the patients were divided into 2 groups according to whether $PaO_2/FiO_2$ (P/F) ratio was over 300 or not. These items were compared between the groups by using the unpaired t-test and P < 0.05 was considered significantly different.

**RESULTS.** Fifty-one patients (age in months: 13.1 ± 20 [mean ± SD]; mean body weight [kg] 6.8 ± 4.6) were evaluated. There were 37 children with systemic oxygenated disturbance (P/F ratio < 300) (group D: hypoplastic left heart syndrome, pulmonary atresia, etc.) and 14 children without the disturbance (group N: ASD, VSD, etc.). The PI in D was significantly higher than that in N (1.61 ± 0.46 [D] vs. 1.27 ± 0.13 [N]; P=0.01) and there was a tendency for Vm (cm/s) in D to be lower (46.0 ± 20.7 [D] vs. 56.2 ± 27.3 [N]; P=0.2). The $rSO_2$ (%) in D was significantly lower (64 ± 15 [D] vs. 74 ± 15 [N]; P=0.03). There was no significant difference in mBP (mmHg)(46 ± 9 [D] vs. 52 ± 12 [N]; P=0.1) and the hematocrit (%) in D was significantly higher (41.5 ± 7.2 [D] vs. 35.0 ± 7.5 [N]; P=0.01).

**CONCLUSIONS.** Unexpectedly, cerebrovascular resistance was higher in the children with CHD who had systemic oxygenated disturbance. This may be related to high blood viscosity due to high hematocrit in these patients.

**REFERENCE(S)**

1. Ultrasound Obstet Gynecol 2012;40:62

**GRANT ACKNOWLEDGMENT**

None.

### 0946

#### Are the parents' statements reliable for diagnosis of serious bacterial infection among the febrile children without source?

H.N. Lee[1], Y.H. Kwak[1], J.Y. Jung[1], J.W. Park[1], S.U. Lee[1], S. Hwang[1], J.H. Lee[2], H.S. Kwon[2], Y.J. Choi[2], J.H. Jung[3]
[1]Seoul National University Hospital, Emergency Medicine, Seoul, Korea, Republic of; [2]Seoul National University Bundang Hospital, Emergency Medicine, Seongnam, Korea, Republic of; [3]Seoul Metropolitan Government Seoul National University Boramae Medical Center, Emergency Medicine, Seoul, Korea, Republic of
**Correspondence:** H.N. Lee
*Intensive Care Medicine Experimental* 2018, **6(Suppl 2):**0946

**INTRODUCTION.** Serious bacterial infection (SBI) is a major cause of morbidity and mortality in children. Distinguishing SBI from self-limiting viral infections is very important task in emergency department (ED)[1], especially among the febrile children who show fever without source (FWS).

**OBJECTIVES.** The aim of this study was to evaluate whether parents' statements on clinical manifestations are reliable for diagnosis of SBI among the children with FWS.

**METHODS.** This study was conducted using prospectively acquired cohort data of all the febrile children ('fever registry') in a tertiary pediatric emergency department. The subjects of the study were the children under 5 years of age with FWS from August 2016 to August 2017. The SBIs were defined as cases of positive blood culture, meningitis, urinary tract infection (UTI), and pneumonia on chest X-ray. The parents' statements were categorized into 3 or 4 grades according to the severity of four clinical manifestations (activity, feeding, urination, and duration of fever). The linear-by-linear association test was used to examine linear association between the severity of clinical manifestations and SBI. Receiver operating characteristics curves and the area under the curves (AUC) were obtained to evaluate the predictive performance.

**RESULTS.** Among the 587 children included in the study, 25 (4.3%) were diagnosed with SBI including 6 pneumonias, 17 UTIs, 0 meningitis, and 2 bacteremias. There was no linear association between the severity of the clinical manifestations and SBIs, duration of fever (p=0.299), activity (p=0.781), feeding (p=0.161), urination (p=0.834). The AUC for duration of fever was 0.54 (95% CI 0.41-0.67), 0.52 for activity (95% CI 0.40-0.64), 0.42 for feeding (95% CI 0.32-0.53), 0.51 for urination (95% CI 0.39-0.62).

**CONCLUSIONS.** The clinical manifestations, even considering the severity, shows little predictive performance for diagnosis of SBIs among FWS children. For optimal evaluation of FWS children, more comprehensive approach, including laboratory and imaging tests, are needed.

**REFERENCE(S)**

1. Van den Bruel, A., et al., Diagnostic value of clinical features at presentation to identify serious infection in children in developed countries: a systematic review. Lancet, 2010. **375**(9717): p. 834-45.

### 0947

#### Clinical profile, intensive care needs and predictors of outcome in children with drowning: adeveloping country perspective

M. Samprathi, A. Agarwal, M. Jayashree, A. Bansal, A. Baranwal, K. Nallasamy, S.K. Angurana
Postgraduate Institute of Medical Education and Research, Pediatrics, Chandigarh, India
**Correspondence:** M. Samprathi
*Intensive Care Medicine Experimental* 2018, **6(Suppl 2):**0947

**INTRODUCTION.** Unintentional injuries are the sixth leading cause of death in children less than 5 years of age and the leading cause of unintentional injury is drowning. Data on children with drowning in developing countries are scarce. Such data would help in effective management, prognostication and prevention of drowning.

**OBJECTIVES.** This study was undertaken to know the epidemiology, intensive care needs and predictors of outcome of children with drowning.

**METHODS.** This retrospective study, done at a tertiary care referral hospital in North India, included children younger than 12 years admitted with drowning from January 2009 to August 2017. Data collected from case records or electronic medical records comprised of demographic details, circumstances and management on site, clinical profile, PICU needs and outcome. Multivariate analysis was used to identify predictors of unfavorable outcome.

**RESULTS.** Twenty seven children were included, 14 were boys. Median age was 18 months (IQR-12, 30). Event was witnessed in 11 (40.7%). Site of drowning was in or around the house in more than half (n=15, 55.5%) and outdoors in 11 (40.7%). Most children (70.3%) had drowned in fresh water. Ponds outside houses (n=10) and buckets of water in bathroom (n=9) were commonest sites. When first found, 23 (85.2%) were unconscious, 15 (55.5%) were apneic and 17 (62.9%) were blue. Duration of submersion was known in 20 (74.1%), median being 4 minutes (IQR- 3,9). On site resuscitation was attempted by untrained bystanders in thirteen (48.1%). Most common center where health care was first sought was government hospital (48.1%). Median time lapse between the event and reaching first health care center was 30 minutes (IQR- 22, 60) and reaching our center was 240 minutes (IQR- 94, 360). Six children (22.2%) presented in cardiac arrest, requiring cardiopulmonary resuscitation (CPR), 13 (48.1%) were hypoxic (saturation < 92% on room air) and half (51.8%) had encephalopathy (GCS $\leq$ 13). Ten children (37%) had seizures, 8 (29.6%) had shock, 10 had (37%) raised intracranial pressure (ICP). Hypo/hypernatremia was uncommon (18.5%). Ten were transferred to PICU; indications were raised ICP (n=6), ventilation (n=5) and hemodynamic monitoring (n=4).

One child died, 4 survived with sequalae; rest (22=81.5%) were neurologically normal at discharge (normal cognition, no motor deficits). Predictors of unfavorable outcome (death or survival with sequalae) on univariable analysis were hypoxia, GCS< 8, hypotension, requirement of CPR at admission, raised ICP, shock and mechanical ventilation but none of these could predict outcome on multivariate analysis.

**CONCLUSIONS.** Inadequate resuscitation on site, delayed and ineffective referral, physiological destabilization or requirement of CPR at admission and raised ICP contribute to unfavourable outcome in children with drowning in developing countries.

### 0948

#### Diaphragm pacing in complex pediatric respiratory care can have a significant role in treating respiratory failure from spinal cord injury to multiple other diseases including spinal muscle atrophy, pompe's and acute flaccid myelitis

R. Onders[1], M. Elmo[2], C. Kaplan[2]
[1]University Hospitals Cleveland Medical Center, Surgery, Cleveland, United States; [2]University Hospitals Cleveland Medical Center, Cleveland, United States
**Correspondence:** R. Onders
*Intensive Care Medicine Experimental* 2018, **6(Suppl 2):**0948

**BACKGROUND.** Chronic invasive mechanical ventilation (MV) in children increases medical complexity of care and adds to familial depression, anxiety and health care dollars. Diaphragm Pacing (DP) uses laparoscopically placed electrodes into the diaphragm muscle to deliver electrical stimulation to phrenic nerve motor points.

**OBJECTIVE.** Report largest consecutive series of DP in the pediatric population.

**METHODS.** Prospective, nonrandomized, interventional experience under IRB approval. Pediatric patients underwent laparoscopic implantation of four intra-muscular electrodes. Patients were discharged home where conditioning ensued to wean from the ventilator.

**RESULTS.** From 2009 to 2017, 23 patients between 2 years to 17.5 years (average 9) had surgical evaluations of their diaphragms with 20 patients being implanted. Three patients had completely denervated diaphragms and were not implanted. Other previous abdominal operations include 18 gastrostomy tubes, 3 VP shunts and one baclofen pump. There were no peri-operative complications. Time spent on MV prior to DP was 9 days to 92 months (33 months average). There were 80 cumulative pacing years (up to 8 years). No long term infections have been reported and no patient has required electrode removal or surgical revision. Three subsequently had scoliosis surgery. 50% achieved full time pacing with no mechanical ventilation. 4 pace during daytime over 12 hours a day and 5 pace 4-6 hours a day and still weaning. Two patients eventually were not pacing ( 1-brittle bone disease; 1 chronic skin reaction). Two patient had full recovery of volitional breathing and had the electrodes removed. Parents report an ability to perform more "normal" activities such as taking the child who uses the pacer to the beach. In addition, they report significant greater ease in caring for the pacer compared to the ventilator. Social functions such as church and school are simpler without the noise of the ventilator and the excess tubing from the ventilator.

**CONCLUSION.** DP is easily implanted in children, has no deleterious effects and can decrease or replace MV. DP is the preferred breathing method by patients and their families, even when compared to non-invasive ventilation. Similar to adults, early implantation decreases wean time and identification of denervated diaphragms can save untoward effects from physiologically impossible ventilator weaning. For pediatric populations with intact phrenic motor neurons DP can be an option in a wide group of patients.

**REFERENCE(S)**

Onders RP, Ponsky TA, Elmo MJ, et al. First Reported experience with intramuscular diaphragm pacing in replacing positive pressure mechanical ventilators in Children. J Pediatr Surg. 2011 Jan;46(1):72-6.

Smith BK, Fuller DD, Martin AD, et al. Diaphragm Pacing as a Rehabilitative Tool for Patients with Pompe Disease who are ventilator-dependent: Case Series. Physical Therapy 2016; 96: 696-703.

### 0949

### Complications and mortality associated with acute varicella - infections in a pediatric intensive care unit: a ten-year experience

J. Willems[1], M. Van de Voorde[2], E. Dhont[3], P. Schelstraete[4]

[1]University Hospital Ghent, Pediatric Intensive Care Unit, Gent, Belgium; [2]University Hospital Ghent, Pediatric Department, Ghent, Belgium; [3]University Hospital Ghent, Pediatric Intensive Care Unit, Ghent, Belgium; [4]University Hospital Ghent, Pediatric Pneumology and Infectious Diseases, Ghent, Belgium

**Correspondence:** J. Willems

*Intensive Care Medicine Experimental* 2018, **6(Suppl 2):**0949

**INTRODUCTION.** Chickenpox is a common infection in childhood, caused by varicella zoster virus (VZV). Although generally considered as an innocent infection, the disease course can be complicated and even fatal, both in previously healthy as in immunocompromised children. Few data regarding critical illness and mortality associated with VZV-infections in children are available.

**OBJECTIVES.** By describing our ten-year experience, we aim to improve the knowledge about PICU-admissions, complications, sequelae and mortality associated with VZV-infections.

**METHODS.** We analysed retrospectively all admissions on our Pediatric Intensive Care Unit (PICU) involving patients with primary or secondary diagnosis of VZV-infection, using the hospital database of our PICU. All patients aged 1 month to 15 years and admitted from Jan 1st 2005 till Dec 31st 2014 were included.

**RESULTS.** 50 patients aged 4 months to 12 years were included. Mean PICU-length of stay was 7.3 days.18/50 patients had relevant comorbidity, of which most were immunocompromised. Diagnosis on admission were most frequently bacterial surinfection of soft tissues, septic shock, respiratory failure and epilepsia. 15/50 patients required invasive mechanical ventilation, 13/50 vasoactive mediaction, 2/50 ECMO. Mortality rate was 4/50, of these 2 children were previously healthy, the other two had serious comorbidity and were institutionalised in the same service. Blood stream infections with *Streptococcus pyogenes* (7), *Staphylococcus Aureus* (1) and *Escherichia coli* (1) were demonstrated. A significant part of our patients patients suffered long-term sequelae (under which neurologic sequelae, venous thrombosis and peripheral limb amputation).

**CONCLUSIONS.** Our data demonstrate the serious course of many VZV-infections in a Pediatric Intensive Care Unit. Also in previously healthy children, serious complications, occurrence of sequelae and significant mortality rates are noted.

### 0950

### Augmented renal clearance: a common phenomenon in critically ill children

T. Van Der Heggen[1,2], E. Dhont[1,3], H. Peperstraete[4], J. Delanghe[5], J. Vande Walle[2], P. De Paepe[6], P. De Cock[6,7]

[1]Heymans Institute of Pharmacology, Ghent, Belgium; [2]Ghent University Hospital, Department of Pediatrics, Ghent, Belgium; [3]Ghent University Hospital, Department of Pediatric Intensive Care, Ghent, Belgium; [4]Ghent University Hospital, Department of Cardiac Intensive Care, Ghent, Belgium; [5]Ghent University Hospital, Department of Laboratory Medicine, Ghent, Belgium; [6]Ghent University, Heymans Institute of Pharmacology, Ghent, Belgium; [7]Ghent University Hospital, Pharmacy, Ghent, Belgium

**Correspondence:** P. De Cock

*Intensive Care Medicine Experimental* 2018, **6(Suppl 2):**0950

**INTRODUCTION.** Augmented renal clearance (ARC) is an increase in kidney function, resulting in the enhanced elimination of circulating solute including renally cleared drugs. This concept has been increasingly recognized in critically ill adults, whereas in the pediatric intensive care setting, research remains scarce.

**OBJECTIVES.** The primary objective of this study was to investigate the incidence of ARC in critically ill children. Secondary objectives included a risk factor analysis for the development of ARC and a comparison of methods for detection of ARC.

**METHODS.** Single center, prospective, observational study at the pediatric and cardiac intensive care unit of the Ghent University Hospital, Belgium.

Glomerular filtration rate (GFR) was measured by means of a daily 24-hours creatinine clearance (24h $Cl_{Cr}$) and estimated used the modified Schwartz formula, for a maximum of four consecutive days. ARC was defined as a GFR exceeding normal values for age plus two standard deviations. Logistic regression analysis was used to identify risk factors for ARC.

**RESULTS.** Data were collected from 105 patients (median age 1.4 years, IQR 2.5). Overall, 59% of patients expressed ARC and the proportion of ARC patients decreased during consecutive days. ARC patients had a median $Cl_{Cr}$ of 144.5 ml/min/1.73m² (IQR 47.1). Male gender and antibiotic treatment were independently associated with the occurrence of ARC. Patients after cardiac surgery were less likely to develop ARC. The modified Schwartz formula was less accurate for ARC detection in individual patients.

**CONCLUSIONS.** A large proportion of critically ill children develops ARC during their stay at the intensive care unit. As ARC may lead to subtherapeutic treatment of renally eliminated drugs, early detection of patients at risk is important.

**GRANT ACKNOWLEDGMENT**

This study was funded by the Clinical Research Fund, Ghent University Hospital, Ghent, Belgium

### 0951

### Higher incidence of *Acinetobacter baumannii* isolated from adult ICU than PICU among critically ill children

B. Kim, W. Bae, K. Kim, H. Lee, E. Yang, H. Kim, Y. Chun, J. Yoon, H. Kim, J. Kim

College of Medicine, The Catholic University of Korea, Seoul, Korea, Republic of

**Correspondence:** B. Kim

*Intensive Care Medicine Experimental* 2018, **6(Suppl 2):**0951

**INTRODUCTION.** The treatment of critically-ill children in pediatric intensive care units (PICUs) is associated with better outcomes, such as lower mortality rates; thus, we hypothesized that the incidence of healthcare-associated infections would also be lower in PICUs.

Acinetobacter baumannii is an important nosocomial pathogen associated with mortality among critically-ill patients. We compared the incidence of A. baumannii infection among pediatric patients treated only in PICUs with those treated in adult ICUs or multiple ICUs.

**OBJECTIVES AND METHODS.** We retrospectively reviewed the medical and laboratory records of 1041 critically-ill patients of the Department of Pediatrics aged under 18 years who were admitted to the ICUs of a tertiary referral hospital between May 2009 and June 2017. We investigated all kinds of specimens obtained after 48 hours of ICU admission to identify culture results positive for A.baumannii during ICU admission.

**RESULTS.** A total of 643 ICU admissions were included in this study, of whom 377 were treated only in the PICU (PICU group), and 266 were treated in the adult ICUs (AICU group). There were no significant differences between the PICU and AICU groups in length of ICU stay.

The PICU group had a higher prevalence of respiratory disease (54.1% vs. 33.1%) and neuro-muscluar disorder (40.3% vs. 25.2%), while the AICU group had a higher prevalence of hemato-oncological diseases (21.2% vs. 46.2%) and cardiovascular diseases (16.7% vs 36.8%).

A. baumannii was isolated 13 times in the PICU group (3.4%) and 19 times in the AICU group (7.1%); thus, the incidence densities of positive culture results for A. baumannii were 1.77 and 5.79 per 1000 ICU-days, respectively. After multivariate analysis, PICU group had lower hazard ratio of positive culture results for A. baumannii compared with AICU group (adjusted hazard ratio: 0.453, 95% CI: 0.210-0.980).

**CONCLUSIONS.** Critically-ill children treated only at PICUs had a lower incidence of A. baumannii infection.

#### 0952

**Complications associated with central venous catheter in neonates: what is the problem?**

N.M. Lee
Chung-Ang University Hospital, Pediatrics, Seoul, Korea, Republic of
*Intensive Care Medicine Experimental* 2018, **6(Suppl 2):**0952

**INTRODUCTION.** Central venous catheters (CVC) were frequently used for partial or total parenteral nutrition (TPN) in neonatal intensive care unit (NICU). However, it can be associated with various complications, such as infection, obstruction, thrombosis, pleural or pericardial effusion and cardiac tamponade. Among these, cardiac tamponade is a very rare complication but fatal that can lead to death.

**OBJECTIVES.** We evaluated the CVC associated complication and related risk factors.

**METHODS.** We conducted a retrospective study by utilizing medical records of neonates admitted in the NICU of Chung-Ang University Hospital from January 2012 to December 2017. Among these, nine neonates were diagnosed with CVC associated complications according to effusion analysis. The following parameters were recorded and analyzed: gestational age, birth weight, gender, central line type, insertion site, tip site, fluid rate and TPN osmolarity. Examination of pericardial, pleural effusion and ascites were performed and their results were analyzed. The diagnosis of CVC associated complication was based on high level of glucose and triglyceride in effusion.

**RESULTS.** Nine neonates were diagnosed with CVC associated complications. All cases (six pericardial effusions, two pleural effusions and one ascites) had drainage or aspiration of fluid, and the fluid analysis was consistent with total parenteral nutrition (elevated glucose and triglycerides). The mean volume of fluid drained 21.6 ml (range: 7.5-46ml). The mean glucose and triglycerides level of fluid drained were 1634 mg/dl (range 362-3550mg/dl) and 638 mg/dl (range 167-1734mg/dl). We used neutriline in eight neonates and umbilical catheter in one neonate. Central line tip were located at T1-6 level. The mean TPN fluid rate and TPN glucose was 8.2cc/hr (range 4.4-13.1cc/hr) and 7.2 % (range 5-12 %). Among nine neonates, only one neonate was expired.

**CONCLUSIONS.** Among CVC associated complications, cardiac tamponade is very fatal. If cardiac arrest occurs in neonates with central lines, we should suspect cardiac tamponade by CVC associate complication and perform the echocardiography. These neonates can be successfully resuscitated with urgent echocardiography and prompt pericardiocentesis.

### Beyond the Intensive Care

#### 0953

**Augmented renal clearance as a predictor of poor outcome in critically ill patients, preliminary results of a single centre study**

A. Ramos[1], A. Dogliotti[2], J.F. Gerber[1], D. Latasa[1], M. Perezlindo[1], C. Lovesio[1]
[1]Sanatorio Parque, Critical Care, Rosario, Argentina; [2]Grupo Oroño, Clinical Epidemiology and Statistics, Rosario, Argentina
**Correspondence:** A. Ramos
*Intensive Care Medicine Experimental* 2018, **6(Suppl 2):**0953

**INTRODUCTION.** An augmented renal clearance (ARC) has been described in some critical patient groups.

**OBJECTIVES.** The aim was to assess the relationship between ARC and poor outcome.

**METHODS.** This is a prospective study in a surgical and medical intensive care unit (ICU). From July 2017 to March 2018, patients who stayed between 48 and 72 hours in the ICU were included. We exclude individuals referred from another hospital. Creatinine clearance was calculated from a 24-hours urine collection. Poor outcome was defined as a composite of death and long hospital stay, defined as the 75th percentile (>19 days). The logistic regression model was used to identify if ARC was independently associated with poor outcome.

**RESULTS.** Sixty patients were included in the study. Three patients died and sixteen (26%) presented the composite end point. In multivariate analysis, the development of ARC at third day at ICU predicts poor outcome (p=0.04, OR: 1.02 95% CI: 1.0005-1.04) (AUC ROC: 0.76, 95%CI 0.63-0.86).

**CONCLUSIONS.** A relationship between ARC and poor outcome was established, however a large sample size is needed to confirm our results.

#### 0954

**A 20 year review of outcomes for critical care patients aged over 90 years**

C. Mearns, T. Samuels
Surrey and Sussex Healthcare NHS Trust, Dept of Critical Care, Redhill, United Kingdom
**Correspondence:** C. Mearns
*Intensive Care Medicine Experimental* 2018, **6(Suppl 2):**0954

**INTRODUCTION.** In 2010 the UK had a population of three million people aged more than 80 years[1]. This is projected to double by 2030. The Department of Health estimates that the average cost of providing hospital and community health services for a person aged over 85 years is around three times greater than that for 65-74 year olds. This study aimed to assess whether the increasing age of the general population is reflected in that of our critical care patients, and to review ICU mortality against age.

**METHODS.** The Ward Watcher database for Surrey and Sussex Healthcare NHS Trust critical care was interrogated for the period

1994 to 2014. Data were reviewed for patients aged over 90 years regarding primary organ system failure, initial APACHE II score and mortality.

**RESULTS.** Total admissions and admissions in elderly patients have increased over time (Figure 1). During the 20 year period, 126 patients aged over 90 years accounting for 1.4% of the total (80 female, 46 male). Patients were admitted following emergency abdominal surgery (53%), orthopaedic surgery (11%), respiratory failure (15%), and cardiovascular failure (including sepsis) (12%).

Where the APACHE II score was greater than 25 the mortality rate was 71.4%. The ICU mortality of patients was 24.6% with a total hospital mortality of 40.4%. Mortality was lower in females vs males (18% vs 34.7%).

**CONCLUSIONS.** Elderly patients are increasingly being admitted to critical care, particularly following surgical intervention. Patient selection may influence comparative mortality between age groups but given the higher mortality rates for older patients, further work regarding identification of factors that may assist in quantifying risk would be useful.

**REFERENCE(S)**

1) Key Issues for the New Parliament 2010 House of Commons, The ageing population. Richard Cracknell

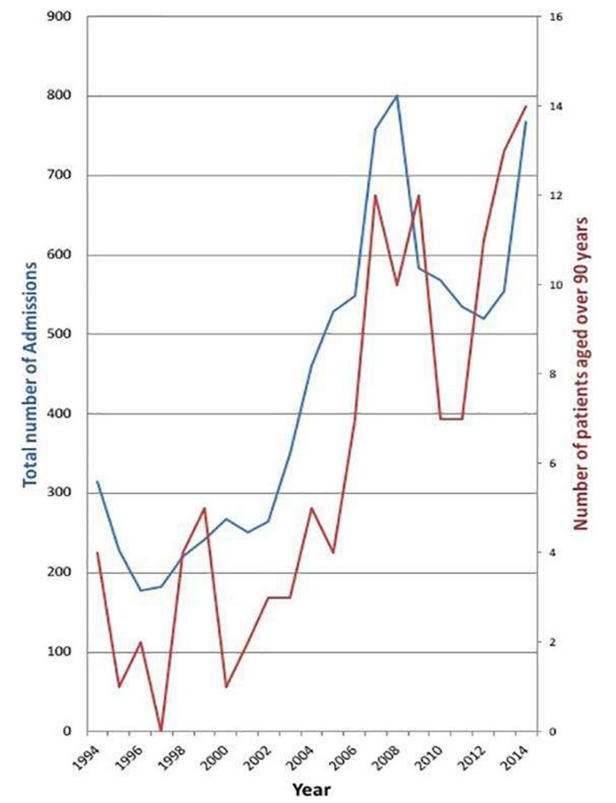

**Fig. 1 (abstract 0954).** Graph of admission numbers versus age

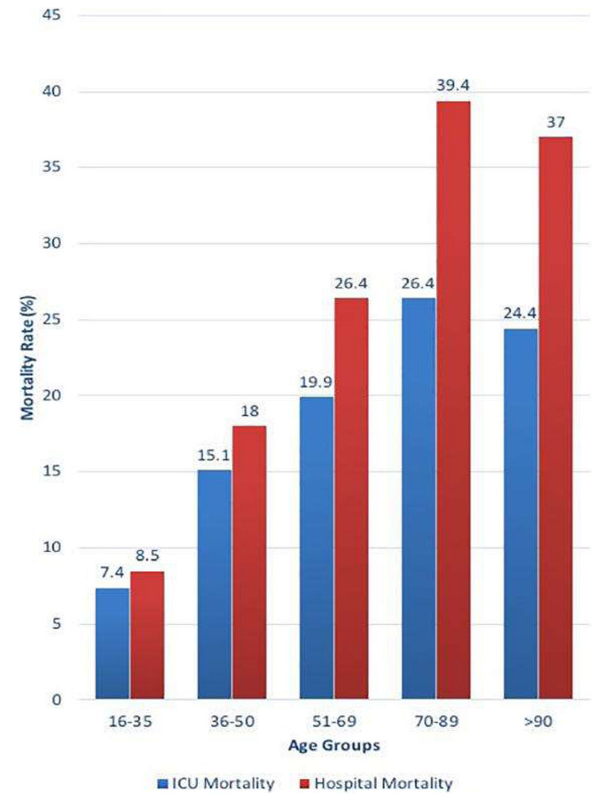

**Fig. 2 (abstract 0954).** Graph of mortality versus age

### 0955

#### High central venous pressure and cumulative fluid balance is associated with mortalitay in ICU

B. Lubis[1,2], P. Amelia[3,4], A.H. Nasution[1,2], T. Hamdi[1,2]

[1]University of North Sumatera, Anesthesiology, Medan, Indonesia; [2]Adam Malik Hospital, Anesthesiology, Medan, Indonesia; [3]University of North Sumatera, Faculty of Medicine, Medan, Indonesia; [4]Adam Malik Hospital, Medan, Indonesia

**Correspondence:** A.H. Nasution

*Intensive Care Medicine Experimental* 2018, **6(Suppl 2):**0955

**INTRODUCTION.** Central venous pressure (CVP) was often used in Intensive Care Unit (ICU). Extreme values of CVP may be considered the downstream pressure of venous return rather than a measure of preload. Several studies have linked CVP with the incidence of renal failure that has been found to be associated with mortality. Mortality in ICU is related to excess fluid, In this study the we wanted to know whether there was a correlation of CVP > 8 cm$H_2O$ with mortality and positive fluid in ICU patients.

**OBJECTIVES.** The main purpose of our study is to asses for an association between high central venous pressure (CVP) and cumulative fliud balance with mortality in Intensive Care Unit (ICU).

**METHODS.** A cross-sectional study was conducted from from January to December 2017 in ICU Adam Malik Hospital Medan. Patients with age over 18 years admitted to our ICU were included. The demographic data, CVP value, and cumulative fluid balance were compaired between survivors and nonsurvivors.

**RESULTS.** During the study period, 100 patients were admitted; 52% were male with mean age 48.5±16.5 years old. The overall mortality of 100 patients was 38%. There was an association between high central venous pressure with mortality in ICU (p=0.004). But there was no association between cumulative fluid balance and mortality in ICU (p=0.109).

**CONCLUSIONS.** High central venous pressure was associated with mortality in ICU patients, and it could be a simple predictor in addition to APACHE II score. But there was no association between cumulative fluid balance and mortality in ICU patients. Further study is needed to explore the accuracy of central venous pressure to reduce the mortality in ICU patients.

**REFERENCE(S)**

1. Damman K, Deursen VM, Navis G, Voors AA, Veldhuisen DJ, Hillege HL. Increased central venous pressure is associated with impaired renal function and mortality in a broad spectrum of patients with cardiovascular disease. Journal of the American College of Cardiology. 2009; 53 (7): 582-8.
2. Boyd JH, Forbes J, Nakada T, Walley KR, Russell JA. Fluid resuscitation in septic shock: A positive fluid balance and elevated central venous pressure are associated with increased mortality. Critical Care Medicine. 2011; 39 (2):1-7.
3. Long Y, Su L, Zhang Q, Zhou X, Wang H, Cui N, et all. Elevated mean airway pressure and central venous pressure in the first day of mechanical ventilation indicated poor outcome. Critical Care Medicine. 2017; 1-8.
4. Williams JB, Peterson ED, Wojdyla D, Ferguson TB, Smith PK, Milano CA, et al. Central venous pressure after coronary artery bypass surgery: does it predict postoperative mortality or renal failure? J Crit Care. 2014; 29(6) : 1006-10.

**GRANT ACKNOWLEDGMENT**

This research was supported by TALENTA 2018 from Universitas Sumatera Utara.

### 0956

#### Airway pressures discriminative properties for the prediction of death in acute exacerbations of COPD (AE/COPD) patients

M.A. Boujelbèn[1], N. Fraj[1], W. Zarrougui[1], I. Ben Saida[1], I. El Meknassi[1], S. Rouis[1], K. Meddeb[1], A. Khedher[1], A. Azouzi[1], I. Chouchene[1], M. Boussarsar[1,2]

[1]Farhat Hached University Hospital, Medical Intensive Care Unit, Sousse, Tunisia; [2]Ibn Al Jazzar Faculty of Medicine, Research Laboratory N° LR12SP09 Heart Failure, Sousse, Tunisia

**Correspondence:** M. Boussarsar

*Intensive Care Medicine Experimental* 2018, **6(Suppl 2):**0956

**INTRODUCTION.** In mechanically ventilated (MV) COPD patients, the minute ventilation should be titrated rather to pH than pCO2; but there is still a huge gap between recommendations and daily practice.

**OBJECTIVES.** To assess the impact of airway pressures on mortality among MV AE/COPD patients.

**METHODS.** Medical records were abstracted for all mechanically ventilated consecutive patients who were admitted for AE/COPD from November, 2015 to February, 2018 in the MICU of Farhat Hached teaching hospital, Sousse, Tunisia. Data regarding demographics, clinical variables, airway pressures (upon admission and at day 4 of hospitalization), high pressure ratio (HPR = number of days spent with high pressures: Peak ≥40 and/or plateau ≥30 and/or driving pressure ≥15 and/or auto-PEEP ≥6 cmH2O, divided by length of stay) and outcomes were recorded. Univariate and multivariate regression analyses were performed to identify factors independently associated to mortality. ROC curves were used to check for the discriminative properties of the significant associated factors.

**RESULTS.** Among 304 mechanically ventilated patients collected within the study period, 105(34.5%) were admitted for AE/COPD. Their main characteristics were : mean age, 67±11 years ; male sex, 91(86.7%) ; COPD GOLD D, 86(81.9%) ; mMRC ≥ 2, 96(91.2%) ; mean SAPS II, 34.3±11.2 ; ; pH, 7.29±0,1 ; pCO2, 63.5±22 mmHg ; P/F ratio, 192±84 mmHg ; median mechanical ventilation duration, 8[4-18.5] days ; tracheostomy, 27(25.7%) ; median length of stay, 13[6-21] days, mortality, 59(56.2%).

Mean airway pressures were respectively for peak, plateau, driving and auto-PEEP as following : upon admission: 36.2±8.5, 20.6±5, 13.4 ±5.3, 7.2±4.4 cmH2O ; and at day 4 : 35.8±10,21.1±6, 13.9±4.7, 6.5 ±4.4 cmH2O . The median number of days with high pressure and the median HPR were respectively 3[1-9] days and 0.35[0.13-0.9].

Univariate analysis showed respectively for deaths and survivals the following significant factors : peak at day 4 (38.2±10.8 vs 32.7±7.9 cmH2O, p=0.003), plateau at day 4 (23.3±6.7 vs 18.4±3.9 cmH2O, $p < 10^{-3}$) and driving at day 4 (14.9±5.2 vs 12.6±3.6 cmH2O, p=0.012) and HPR (0.75[014-1] vs 0.22[0.11-0.36], p< 10-3) to be associated to mortality.

Multivariate logistic regression identified plateau at day 4 (OR, 1.15 ; 95%CI, [1.003-1.333] ; p=0.043) and HPR (OR, 11.3 ; 95%CI, [2.6-51.1] ; p=0.001) as the only independent risk factors for mortality. The ROC-AUCs were : plateau at day 4, 0.73 ; HPR, 0.75.

**CONCLUSIONS.** In mechanically ventilated AE/COPD patients, an increasing plateau pressure and/or a persistent elevated airway pressures are independently associated to mortality. This could be explained by the addition of an elastic component to the already resistive mechanics.

### 0957

#### Exercise capacity and muscle strength differences in working aged patients with normal or impaired physical functioning after critical illness at three months after the hospital discharge

M. Niittyvuopio, J. Liisanantti, J. Pikkupeura, S. Sälkiö, T. Ala-Kokko

Oulu University Hospital, Department of Anesthesiology and Intensive Care, Oulu, Finland

**Correspondence:** M. Niittyvuopio

*Intensive Care Medicine Experimental* 2018, **6(Suppl 2):**0957

**INTRODUCTION.** A significant portion of the patients recovering from prolonged critical illness suffer from Intensive Care Unit Acquired Weakness (ICUAW) [1]. Some of the typical manifestations of ICUAW are reduced exercise capacity and muscle strength [2].

**OBJECTIVES.** In this study we wanted to find out the prevalence of impaired physical functioning (PF) and differences in exercise capacity and muscle strength in working aged patients, with an ICU length of stay (LOS) for more than four days, who were stratified to either impaired or normal physical functioning according to RAND-36 health related quality of life (HRQoL) questionnaire´s physical functioning (PF) dimension.

**METHODS.** The study was conducted in our post-ICU follow-up clinic. All the patients aged between 18 and 65 living in our hospital district area with LOS for more than four days and who met the invitation criteria were invited. The follow-up clinics invitation protocol excludes the patients with severe psychiatric comorbidities, alcohol and substance abuse issues, communicating and co-operation deficits and cardiac surgery patients. At the follow-up clinic the exercise capacity was measured by a six minutes walking distance test (6MWD) and the muscle strength was measured by a hand-grip test. The patients also filled the RAND-36 HRQoL questionnaire and were divided into two groups: Either normal or impaired physical functioning according to RAND-36´s PF dimension. PF was defined as impaired if the scores were less than 2SD of the age and sex matched general population mean.

**RESULTS.** Between years 2004-2012 There were 468 eligible patients who were invited to the follow-up clinic and 351 (76%) attended for the survey control. The data could be calculated on 332 patients. The median age was 50 (38-57) and 63% of the patients were male. The median APACHEII and SAPSII scores on admission were 18 (13-23) and 38 (28-49) and the median LOS was 7 (4.8-11.8) days.

The PF was defined impaired in 131 patients (39.5%). The patients with impaired PF achieved significantly reduced results in 6MWD (393 m vs. 500 m, p< 0.001) and in the hand grip test (left hand: 28 kg vs. 32 kg, p=0.004 and right hand 29 kg vs. 34 kg, p=0.001). There was no significant difference in PF outcome between genders, APACHEII or SAPSII scores or LOS.

**CONCLUSIONS.** The patients with impaired RAND-36´s PF dimensions scores had significantly reduced exercise capacity and muscle strength.

### 0958

#### Airway pressures and difficult weaning prediction in acute exacerbations of COPD (AE/COPD) patients

M.A. Boujelbèn[1], W. Zarrougui[1], N. Fraj[1], S. Rouis[1], H. Zorgati[1], D. Ben Braiek[1], A. Khedher[1], I. Ben Saida[1], A. Azouzi[1], K. Meddeb[1], M. Boussarsar[1,2]

[1]Farhat Hached University Hospital, Medical Intensive Care Unit, Sousse, Tunisia; [2]Ibn Al Jazzar Faculty of Medicine, Research Laboratory N° LR12SP09 Heart Failure, Sousse, Tunisia

**Correspondence:** M. Boussarsar

*Intensive Care Medicine Experimental* 2018, **6(Suppl 2):**0958

**INTRODUCTION.** Invasive mechanical ventilation (IMV) in COPD patients is frequently associated with difficulty of weaning considering that over 50% of the first weaning attempts are unsuccessful. This prolonged weaning process and the extended IMV are predictive of poor outcome and various complications. Newtonian equation of motion clearly illustrates the respective restrictive airway pressures to predict difficult weaning process.

**OBJECTIVES.** To identify the discriminative properties of respective airway pressures to predict difficult weaning process.

**METHODS.** Medical records were abstracted for all mechanically ventilated (MV) consecutive patients who were admitted for AE/COPD from November, 2015 to February, 2018 in the MICU of Farhat Hached teaching hospital, Sousse, Tunisia. Data regarding demographics, clinical variables, airway pressures (upon admission and at day 4 of hospitalization), high pressure ratio (HPR = number of days spent with high pressures: Peak ≥40 and/or plateau ≥30 and/or driving pressure ≥15 and/or auto-PEEP ≥6 cmH2O, divided by length of stay) and outcomes were recorded. Poor outcomes were defined as: a ventilator-free days at day 28 (VFDs) = 0, a duration of IMV ≥ 14 days and a composite outcome: death or length of stay (LOS) ≥ 14 days. Univariate and multivariate regression analyses were performed to identify factors independently associated to difficulty of weaning. ROC curves were used to check for the discriminative properties of the significant associated factors.

**RESULTS.** Among 304 MV collected patients, 105(34.5%) were admitted for AE/COPD. Their main characteristics were : mean age, 67±11 years ; male sex, 91(86.7%) ; COPD GOLD D, 86(81.9%) ; mMRC ≥2, 96(91.2%) ; SAPS II, 34.3±11.2 ; pH, 7.29±0,1 ; pCO2, 63.5±22 mmHg ; P/F ratio, 192±84 mmHg ; median IMV duration, 8[4-18.5] days ; tracheostomy, 27(25.7%); median VFDs, 0[0-3] days ; median LOS , 13[6-21] days, mortality, 59(56.2%).

Mean airway pressures were respectively for peak, plateau, driving and auto-PEEP upon admission: 36.2±8.5, 20.6±5, 13.4±5.3, 7.2±4.4 cmH2O; and at day 4 : 35.8±10,21.1±6, 13.9±4.7, 6.5±4.4 cmH2O. The median number of days with high pressure and the median HPR were respectively 3[1-9] days and 0.35[0.13-0.9].

Univariate analysis then multivariate logistic regression showed that plateau pressure at day 4 (OR, 1.69 ; 95%CI, [1.15-2.49] ; p=0.008), auto-PEEP at day 4 (OR, 65.3 ; 95%CI, [2.25-1893] ; p=0.005) and HPR (OR, 1.57 ; 95%CI, [1.15-2.17] ; p=0.005) were related to a poor composite outcome with the ROC-AUCs estimated respectively at : 0.83, 0.72 and 0.76. An elevated HPR (OR, 17 ; 95%CI, [3.76-76.6] ; p≤$10^{-3}$) was related to zero VFDs with a ROC-AUC of 0.757. Furthermore, a number of days with high pressures ≥ 3 days was associated to a prolonged duration of IMV : (OR, 6.32 ; 95%CI, [1.89-21] ; p=0.003) ; ROC-AUC=0.69.

**CONCLUSIONS.** High airway pressures in MV COPD patients could impede weaning and increase length of stay.

### 0959

#### Who is admitted, who stays, who lives and who dies? - decisions to refuse or limit treatment of intensive care patients

E. Joelsson-Alm[1,2], B. Cleaver[2], E. Jerkegren Olsson[2], K. Nordenskjöld[2], M. Mörrby Ramberg[2]

[1]Karolinska Institutet, Department of Clinical Science and Education, Sodersjukhuset, Stockholm, Sweden; [2]Södersjukhuset, Department of Anaesthesia and Intensive Care, Stockholm, Sweden

**Correspondence:** E. Joelsson-Alm

*Intensive Care Medicine Experimental* 2018, **6(Suppl 2):**0959

**INTRODUCTION.** A parameter that is often discussed and compared between different ICUs is the Standardised Mortality Ratio (SMR). Although a complex measure, difficult to interpret, it is used as a quality indicator of the care given in the ICU and hence is of great importance for hospitals, managers and politicians who use it as a benchmarking tool[1,2]. In 2013, the ICU in South Stockholm General Hospital (Södersjukhuset) had a higher SMR than the average in the whole of Sweden. A project was initiated consisting of a retrospective register study identifying areas of improvement and a subsequent quality improvement project.

**OBJECTIVES.** Register study: To describe survivors and non-survivors after intensive care regarding differences in gender, SAPS-score and limitations-of-care (LOC).Quality improvement project:To increase the proportion of patients where a decision about treatment strategy including a decision about LOC is made during the first 24 hours after admission and thereafter daily during the whole ICU stay.

**METHODS.** The project took place at the ICU in Södersjukhuset.The Swedish Intensive Care Registry (SIR) was used for outcome data.

The register study population consisted of all non-survivors (patients who had died within 30 days from ICU admission) and a control group of survivors, which consisted of the 12 first admitted patients every calendar month who survived 30 days after admission (144 patients) during 2013. The study was approved by the regional ethical review board in Stockholm.

The improvement project started in June 2014 and a multidisciplinary improvement team was formed. The interventions focused on measures to facilitate that treatment strategy and decisions about LOC were discussed and documented daily.

**RESULTS.** Register study: A total of 773 patients were admitted to the ICU during 2013. Of those, 135 (17%) died within 30 days from ICU admission, the majority of those (89;66%) died after the ICU stay. The non-survivors were significantly older (mean 75 yrs vs 61 yrs, p< 0.00) and had higher SAPS 3 (69 vs 51, p< 0.00) than the survivors. The documentation of treatment strategy was poor and only 3% of the survivors and 17% of the non-survivors had a documented decision within the first 24 h after admission.

Improvement project: The proportion of patients where a decision about treatment strategy including a position about LOC is made during the first 24 hours after admission has greatly increased, see Figure 1.

**CONCLUSIONS.** It is possible to make improvements in such a complex area as treatment strategy for patients in the ICU. The initial register study gave us a lot of new knowledge about our patients and highlighted the key issues for improvement.

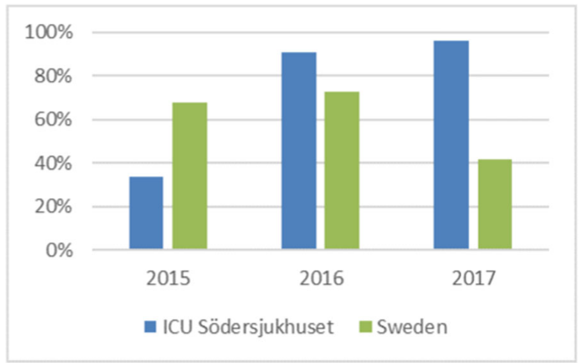

**Fig. 1 (abstract 0959).** ICU patients with a decision about treatment strategy within 24 hours after admission

### 0960

#### Predictors of prolonged stay in a Tunisian intensive care unit (ICU)

I. Ben Saida[1], H. Zorgati[1], S. Kortli[1], N. Kacem[1], M.A. Boujelbèn[1], I. El Meknassi[1], A. Khedher[1], A. Azouzi[1], K. Meddeb[1], M. Boussarsar[1,2]
[1]Farhat Hached University Hospital, Medical Intensive Care Unit, Sousse, Tunisia; [2]Ibn Al Jazzar Faculty of Medicine, Research Laboratory N° LR12SP09 Heart Failure, Sousse, Tunisia
**Correspondence:** M. Boussarsar
*Intensive Care Medicine Experimental* 2018, **6(Suppl 2):**0960

**INTRODUCTION.** Prolonged ICU stay is associated with high mortality, morbidity, and costs. Understanding the predictors of prolonged ICU stay may help care providers to plan for preventive interventions and lead to an efficient consumption of hospital recourses especially with the high demand of ICU admission.
**OBJECTIVES.** To identify characteristics and associated factors to prolonged ICU stay.
**METHODS.** It is a retrospective study performed in the ICU of Farhat Hached teaching hospital of Sousse between January 2017 and December 2017 and including all hospitalized patients for more than 48 hours. Underlying condition, severity, diagnostic at admission, invasive interventions, electrolytes disorders, medication used, complications and adverse events were collected by reviewing the medical patients' charts. Prolonged ICU stay was defined as stay in the ICU for ≥15 days. Variables found to be statistically significant in univariate analysis were included into a multivariate regression model to identify factors independently associated to prolonged ICU stay.
**RESULTS.** During the review period, 260 patients were included. Patients' characteristics were : mean age, 56.82 ±18.45 ; female, 94(36.2 %) ; mean SAPSII, 31.27±11.65 ; mean Apache II, 13.26±6.5 ; Respiratory disorder on admission, 165(63.5%) ; invasive mechanical ventilation (IMV), 155(59.6%) and vasopressors use, 110 (42.3%).
The median length of ICU stay was 7[5-14]days. Sixty patients (23.1%) have required a prolonged ICU stay (≥15 days). The overall mortality was 28.5%.
Univariate analysis identified several factors associated to prolonged ICU stay respectively in prolonged ICU stay and controls: The use of mechanical ventilation (88.5% vs 60.9%, p=0.000), the use of vasopressors (62.5% vs 46%, p=0.043) and adverse events (56.7% vs 28%, p=0.000).
In multivariate analysis, two predictors were independently associated with prolonged ICU stay : the use of IMV (OR, 3.5 ; 95%CI, [1.4- 8.9] ; p=0.008) and adverse events (OR, 2.8 ; 95%CI, [1.4- 5.6] ; p=0.003).
**CONCLUSIONS.** The frequency of a prolonged ICU stay reveals relatively high in our study. Invasive mechanical ventilation and adverse events were the only independent predictors of prolonged ICU stay.

### 0961

#### Long-term outcomes in tracheoventilated patients

R. Di Benedetto, S. Orlando, E. Antonucci
Intermediate Care Unit - Guglielmo da Saliceto Hospital, Piacenza, Italy
**Correspondence:** R. Di Benedetto
*Intensive Care Medicine Experimental* 2018, **6(Suppl 2):**0961

**INTRODUCTION.** Tracheoventilated patients represent a high-risk population. During intensive care unit (ICU) stay, those patients usually show frequent infections and need of prolonged invasive mechanical ventilation (IMV) and hospitalization[1]. Although many studies have examined short-term outcomes after tracheostomy, long-term outcomes in tracheoventilated patients have been poorly investigated in the literature.
**OBJECTIVES.** We hypothesized that tracheoventilated patients show poor long-term outcomes such as high rates of mortality, hospital readmission and condition of frailty at discharge.
**METHODS.** We reviewed all patients admitted in our intermediate care unit (IMCU) after tracheostomy and still needy of IMV (from June 2014 to June 2017, 36 months). The inclusion criteria were age ≥18 y.o., need of IMV at IMCU admission, tracheostomy performed during the previous ICU stay. We collected demographic characteristics, timing of tracheostomy and type of discharge (e.g. home; long-term care facilities). We identified three primary long-term outcomes: 1) mortality rate (during IMCU stay; at 28-day, 6-months and 1-year); 2) hospital readmissions in the first year (ICU and/or IMCU readmissions; number of readmissions; total day of hospitalization), 3) conditions of frailty at hospital discharge (presence of devices, pressure ulcers and rate of bedridden patients). We also evaluated the rate of decannulation at 1-year.
**RESULTS.** 91 patients respected the inclusion criteria. Demographic characteristics are shown in the Table 1. The majority of patients was admitted in ICU for respiratory/cardiovascular problems (73/91, 80%) whereas neurological patients were 18/91 (20%). 8/66 patients (12 %) were discharged at home, 58/66 (88 %) at long-term care facilities. Patient showed high-mortality rates: IMCU mortality (27,5%), mortality at 28-days (40,7 %), mortality at 6-months (53,8%), mortality at 1-year (58,2%, see Figure 1). 33/57 patients (58 %) were re-admitted in the hospital during the first year. In particular, 10/33 patients (30,3 %) were re-admitted in ICU and 6/33 (18,2 %) in IMCU. Patients had a mean of a single re-hospitalization (min. 0, max. 6) for a mean hospital stay of 19 days (min. 1, max. 107). Table 2 shows the condition of frailty at hospital discharge. 29/91 patients (32 %) were decannulated during IMCU stay whereas further 12 patients were decannulated at 1-year (41/91, 45%).
**CONCLUSIONS.** Long-term outcomes in tracheoventilated patients currently remain poor. Our preliminary study showed high rates of mortality and frailty in this cohort of patients.

##### REFERENCE(S)

1. Mehta AB, et al. Trends in tracheostomy for mechanically ventilated patients in the United States, 1993-2012. Am J Respir Crit Care Med 2015.

**Table 1 (abstract 0961).** Demographic characteristics

| Demographic characteristics | Mean (min-max) |
|---|---|
| Age | 71 (44-89) |
| SAPS 2 score | 39 (19-94) |
| SOFA score | 5 (1-12) |
| Timing tracheostomy, days | 11 (1-28) |
| ICU stay, days | 21 (4-67) |
| IMCU stay, days | 20 (1-74) |
| Total days of mechanical ventilation | 33 (2-80) |

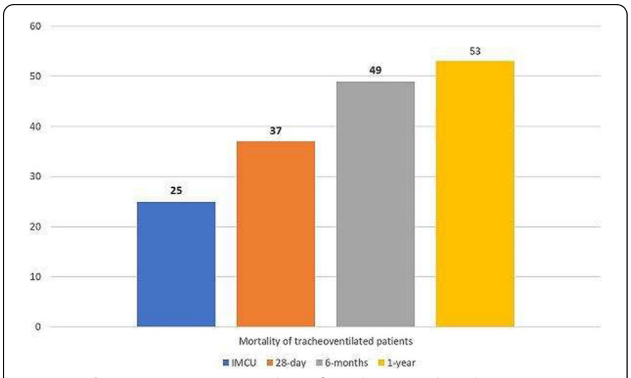

**Fig. 1 (abstract 0961).** Mortality of tracheoventilated patient

**Table 2 (abstract 0961).** Conditions of frailty at hospital discharge

| Factors of frailty | n. of patients (%) |
|---|---|
| Urinary catheter | 17/58 (29,3%) |
| Percutaneuous Endoscopic Gastrostomy/nasogastric tube | 14/58 (24,1%) |
| Pressure ulcers | 13/58 (22,4%) |
| Bedridden patients | 17/58 (29,3%) |

### 0962

#### Impact of socioeconomic status on outcome in a neurointensive care unit

S. Amer[1], J. Gardner[2], C. Hawthorne[1]
[1]Institute of Neurosciences, Glasgow, United Kingdom; [2]Queen Elizabeth University Hospital, NHS Greater Glasgow & Clyde, Glasgow, United Kingdom
**Correspondence:** S. Amer
*Intensive Care Medicine Experimental* 2018, **6(Suppl 2):**0962

**INTRODUCTION.** Our institution serves as the tertiary centre for neurosurgery, maxillofacial and neurology. The West of Scotland is considered one of the poorer areas in the United Kingdom. The SIMD-2012(1) (SIMD) takes into account employment, income, health, education/skills/training, geographic access to services, crime and housing. In practical terms it is found by looking up the patient's postcode (zip code) in a database. Previous publications have noted an effect of deprivation on health care(2-3).

**OBJECTIVES.** To assess the socioeconomic status of our ICU admissions overall and examine if lower socioeconomic groups had a lesser outcome.

**METHODS.** Using the electronic recording system (Wardwatcher), data was collected prospectively for all admissions to the unit. We examined the 2724 neurosurgical admissions from 1st January 2008 to 31st December 2017. 2605 of those had a postcode recorded. Outcome at end of ICU admission was analysed along with SIMD quintiles using R and Excel. Ethical and information governance permission was granted.

**RESULTS.**

Figure 1, SIMD quintile and outcome (absolute numbers)

Figure 2, SIMD quintile and outcome (% of each SIMD quintile)

**CONCLUSIONS.** The population of the West of Scotland is dominated by poorer socioeconomic groups. The data collected showed that there is little difference in patient´s outcome based on their SIMD. We intend to look in detail at SIMD and it's impact on our patient population.

(1) http://www.gov.scot/Topics/Statistics/SIMD accessed on 23/04/2018

(2) Barnett R, Lauer G Urban deprivation and public hospital admissions in Christchurch, New Zealand, 1990-1997. Health Soc Care Community 2003;11:299-313

(3) Brameld KJ, Holman CDJ The use of end-quintile comparisons to identify under-servicing of the poor and over-servicing of the rich: a longitudinal study describing the effect of socioeconomic status on healthcare. BMC Health Serv Res 2005;5:61

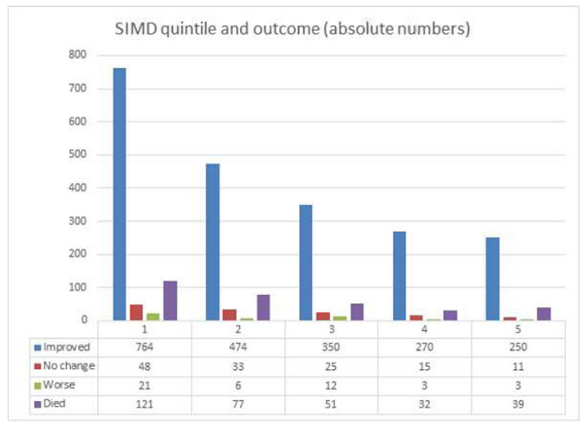

**Fig. 1 (abstract 0962).** SIMD quintile and outcome absolute numbers

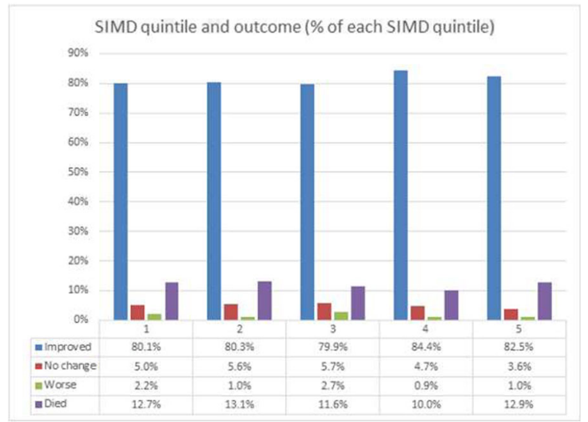

**Fig. 2 (abstract 0962).** SIMD quintile and outcome percentage

### 0963

#### Cancer incidence following discharge from an intensive care unit

M. Ralston[1,2], K. Puxty[3], P. McLoone[4], D. Morrison[4]
[1]University of Glasgow, School of Medicine, Dentistry & Nursing, Glasgow, United Kingdom; [2]Queen Elizabeth University Hospital Glasgow, General Internal Medicine, Glasgow, United Kingdom; [3]Glasgow Royal Infirmary, Intensive Care Medicine, Glasgow, United Kingdom; [4]University of Glasgow, Institute of Health & Wellbeing, Glasgow, United Kingdom
**Correspondence:** M. Ralston
*Intensive Care Medicine Experimental* 2018, **6(Suppl 2):**0963

**INTRODUCTION.** Improving survival-to-discharge rates from an admission to an intensive care unit (ICU) has led to increased interest in the long-term sequelae of an episode of critical illness (1). Morbidity and mortality rates are increased following discharge from an ICU, but the causes for this have not been fully elucidated (2-4). No current research exists on the incidence of cancer diagnosis after an episode of critical illness.

**OBJECTIVES.** This study aimed to investigate whether an admission to an ICU was associated with an increased incidence of cancer in the 5 years following hospital discharge.

**METHODS.** This was a retrospective observational study, investigating ICU admissions in the West of Scotland from January 2000 to December 2008. Four linked databases were used to identify adult patients, surviving at least 30 days following hospital discharge, who had subsequently received a diagnosis of cancer. A standardised incidence ratio (SIR) for the most common cancers in each sex was calculated, and compared to the observed incidence in the study population.

**RESULTS.** 27674 patients were identified as suitable for inclusion. In the 5 years following an admission to an ICU, the observed incidence of a new diagnosis of cancer was significantly increased compared to the population SIR. The majority of this effect was noted in an increased observed incidence of lung cancer in both sexes. Observed incidences of breast and prostate cancers were lower than expected.

**CONCLUSIONS.** In the West of Scotland between 2000 and 2008, patients surviving an episode of critical illness had a significantly increased incidence of diagnosis of cancer in the 5-year follow-up period. The increase was particularly marked in incidence of lung cancer. This is noted as a correlation, and this study was not designed to assign causality for this finding. Possible explanations related to critical illness may include shared lifestyle risk factors, levels of deprivation, pathophysiological processes such as inflammation and immunosuppression, and the long-term consequences of organ support therapies. Further research is required to investigate these.

**REFERENCE(S)**

1. Scottish Intensive Care Society Audit Group. Audit of Critical Care in Scotland 2017: Reporting on 2016. NHS National Services Scotland; 2017
2. Eddleston JM, White P, Guthrie E. Survival, morbidity, and quality of life after discharge from intensive care. Crit Care Med. 2000 Jul;28(7):2293-9.
3. Dragsted L, Qvist J, Madsen M. Outcome from intensive care. IV: A 5-year study of 1308 patients: long-term outcome. Eur J Anaesthesiol. 1990;7(1):51-62.
4. Herridge MS, Cheung AM, Tansey CM, Matte-Martyn A, Diaz-Granados N, Al-Saidi F, et al. One-Year Outcomes in Survivors of the Acute Respiratory Distress Syndrome. N Engl J Med. 2003 Feb 20;348(8):683-93.

**GRANT ACKNOWLEDGMENT**

Not applicable

### 0964

#### ICU Frailout: a study of frailty and outcomes in ICU

R. Ferreira de Almeida[1], A.R. Clara[2], S. Pina[3], T. Salero[3], M. Mendes[2], S. Castro[1], C. Granja[1]

[1]Centro Hospitalar Universitário do Algarve, Serviço de Medicina Intensiva 1, Faro, Portugal; [2]Centro Hospitalar Universitário do Algarve, Serviço de Medicina Interna 2, Faro, Portugal; [3]Centro Hospitalar Universitário do Algarve, Serviço de Medicina Interna 1, Faro, Portugal

**Correspondence:** R. Ferreira de Almeida

*Intensive Care Medicine Experimental* 2018, **6(Suppl 2):**0964

**INTRODUCTION.** Frailty is a syndrome characterized by loss of physiologic and cognitive reserves leading to increase susceptibility to adverse events and a higher risk of death[1]. Frail patients have an increased risk of hospital and long-term mortality[2,3], and more difficulties recovering the homeostatic stability. Frailty allows to identify more vulnerable patients, who are more susceptible to complications and death, provide a realistic prognosis and empower the patient and their carers to anticipate solutions in post-discharge[1,2].

**OBJECTIVES.** Analyse the incidence of frailty syndrome in ICU patients and its association with mortality, at ICU discharge and at 6 and 12 months after ICU discharge, length of mechanical ventilation and ICU stay. Secondarily, to analyse which scale better correlates with those outcomes.

**METHODS.** A prospective, observational study, included 63 patients aged ≥65 years hospitalized for ≥24h, during a 6-month study period. Frailty was determined using the frailty phenotype (FP), the clinical frailty score (CFS), the Katz Index of Independence in Activities of Daily Living (IADL) and Charlson Comorbidity Index (CCI). Patients were separated as follows: FP score ≥3 and CFS≥5[3]. Other collected variables included body mass index (BMI), type of admission (medical, surgery, trauma), SAPS II, SOFA, days of mechanical ventilation (MV), ICU mortality, 6 and 12 months mortality.

**RESULTS.** Frailty was observed in 29% and 21% of patients based on an FP score≥3 and a CFS≥5, respectively. At admission to the ICU there was a statistically significative difference in SAPSII for frail (M=66.39; ±18.34) and nonfrail (M=54.49; ±22.83), (p=0.04). There were no significant differences between frail and nonfrail patients concerning BMI, type of admission, SOFA, MV and ICU, 6 and 12 months mortality. We found a positive strong correlation between the CFS and the IADL [r=0.78, p=0.00]; and a weak correlation between CCI and FP [r =0.42, p=0.001], and between CCI and CFS [r =0.44, p =0.00].

**CONCLUSIONS.** We were not able to find a scale that could efficiently predict the risk of death at the ICU/hospital or at 6/12 months in frailty patients. Instead, and as other authors proclaimed[1,2], frailty may be useful to predict the need of future care and institutionalization. Additionally, we found that that IADL and CFS, both functional measures, may provide similar information.

**REFERENCE(S)**

1. Rockwood K. A global clinical measure of fitness and frailty in elderly people. Can Med Assoc J. 2005 Aug 30;173(5):489-95.
2. Muscedere J, Waters B, Varambally A, Bagshaw SM, Boyd JG, Maslove D, et al. The impact of frailty on intensive care unit outcomes: a systematic review and meta-analysis. Intensive Care Med 2017;43(8):1105-22.
3. Le Maguet P, Roquilly A, Lasocki S, Asehnoune K, Carise E, Saint Martin M, et al. Prevalence and impact of frailty on mortality in elderly ICU patients: A prospective, multicenter, observational study. Intensive Care Med. 2014;40(5):674-82.

### 0966

#### Clinical predictors of early death in critically ill medical patients

A. Khedher[1], E. Ben Amara[2], W. Zarrougui[1], N. Fraj[1], M.A. Boujelbèn[1], K. Meddeb[1], M. Zghidi[1], A. Azouzi[1], I. Ben Saida[1], M. Boussarsar[1,3]

[1]Farhat Hached University Hospital, Medical Intensive Care Unit, Sousse, Tunisia; [2]Sahloul University Hospital, Department of Anesthesiology, Sousse, Tunisia; [3]Ibn Al Jazzar Faculty of Medicine, Research Laboratory N° LR12SP09 Heart Failure, Sousse, Tunisia

**Correspondence:** M. Boussarsar

*Intensive Care Medicine Experimental* 2018, **6(Suppl 2):**0966

**Introduction**

Many studies investigated ICU mortality but data on medical critically ill patients who experience early deterioration and death are rare and of particular concern.

**Objective**

To identify the independent factors associated with ICU death occurring within the first 48 hours of admission.

**Methods**

This was a 5-year (2011-2015) retrospective study conducted in a Tunisian medical ICU. Were included all consecutive patients who experienced early and late deaths defined as occurring within or after 48 hours following ICU admission, respectively. Were assessed patients' characteristics, comorbidities (Charlson index), basal functional status (Barthel index BI), diagnostic discrepancies, ICU admission delay, severity, admission time, reference. Multivariate logistic regression analysis was performed to identify independent factors associated with early deaths.

**Results**

Among the 1474 admitted patients, 542(36.7%) deaths occurred, in whom 227(41.8%) early deaths. General population characteristics were: median age 62[46-73]; sex ratio 1.6/1; COPD 131(24.2%); heart failure 137(25.3%); hypertension 187(34.5%); diabetes, 136(25.1%) ; hematological disease, 27(5%) ; median Modified Early warning Score (MEWS), 5[4-7] ; BI, 75[70-80] ; Charlson index, [2-5] ; acute respiratory failure (ARF), 316(58.3%) ; shock, 115(21.2%) ; neurological distress, 123(22.7%); vasopressors use, 197(36.3%) ; mechanical ventilation, 358(66%) ; median SAPSII, 41[30-54] ; SOFA, 7[5-9]. Early death group had a median ICU stay at 17[8-27] hours.

Univariate then multivariable logistic regression analyses identified the following factors as independent predictors of early death : BI, (OR=1.05, 95%CI, [1.03-1,07]; p=0.0001), hematological disease (OR=2.9, 95%CI, [1.1-7.7], p=0.027), SAPS II, (OR=1.04, 95%CI, [1.02-1,08], p=0.0001) and vasopressors use at admission, (OR=2.47, 95%CI, [1.59-3.81]; p=0.0001).

**Conclusions**

This study shows that early deaths in medical ICU admission are common. Severity at admission, poor baseline functional status, use of vasopressors and hematological disease were identified as independent predictors of early ICU death.

### 0967

#### Frailty in ICU: an unmeasured burden

R. Hart[1], J.P. Ruddy[2]
[1]Queen Elizabeth University Hospital, Anaesthesia and Critical Care, Glasgow, United Kingdom; [2]Monklands University Hospital, Anaesthesia and Critical Care, Coatbridge, United Kingdom
**Correspondence:** R. Hart
*Intensive Care Medicine Experimental* 2018, **6(Suppl 2):**0967

**INTRODUCTION.** As we deal with an increasingly ageing population, it is important to consider the concept of frailty. Frailty is a syndrome characterised by a loss of physical and cognitive reserve. With increasing frailty the risk of adverse outcome rises significantly. It is therefore crucial consider frailty when assessing patients for ICU admission and initiating invasive treatment. Numerous tools exist to allow quantification of frailty.

**OBJECTIVES.** The Clinical Frailty Scale is a validated tool, which grades frailty on a 9-point qualitative scale. It can be applied rapidly, making it compatible with the ICU environment. In this study we aim to measure the burden of frailty in our ICU and question whether it should be routinely measured for all ICU admissions.

**METHODS.** The SICSAG Wardwatcher database was utilised to identify all ICU admissions over a six-month period. Case notes were requested for retrospective analysis. Using admission documentation and nursing records we determined the level of frailty using the Clinical Frailty Scale (CFS). We recorded ICU mortality, length of stay, hospital mortality, level of organ failure, APACHE score and co-morbidities to determine if there was an association between frailty and adverse

**RESULTS.** There were 101 admissions over a six-month period, of these 57 case notes were successfully obtained and analysed. Patient age ranged from 22 to 83. There were eight elective patients who all survived. Of these eight, five were ranked CFS 3 and three were ranked CFS 4.

As outlined by the table above, our data signals that with increasing frailty, both ICU and hospital mortality increases. There were no ICU admissions with a CFS rank of 7, 8 or 9.

**CONCLUSIONS.** The results above are interesting and suggest that frailty on admission to ICU does influence outcome. With increasingly frailty there is an increased mortality, prolonged length of stay and increased risk of multi-organ failure independent on initial APACHE score. Although average age increases throughout our frailty groups, this does not seem enough to independently explain these results. In the future frailty scoring may influence ICU admission but more work is needed. We argue that to allow further assessment of the burden of frailty in ICU and its effect on outcome we should collect frailty data nationally using the clinical frailty scale.

**REFERENCE(S)**

1. McDermid R, Stelfox H, Bagshaw S. Frailty in the critically ill: a novel concept. Critical Care 2011, 15:301

**GRANT ACKNOWLEDGMENT**

None

**Table 1 (abstract 0967).** Frailty Score versus adverse events

| Clinical Frailty Score | Number of patients | ICU deaths | Hospital deaths | Mean LOS | Mean Age |
|---|---|---|---|---|---|
| 1 | 6 | 0 (0%) | 0 (0%) | 0.85 | 48 |
| 2 | 10 | 0 (0%) | 0 (0%) | 3.05 | 52.6 |
| 3 | 17 | 6 (35.1%) | 7 (41.2%) | 4.06 | 57.6 |
| 4 | 13 | 5 (38.5%) | 5 (38.5%) | 4.49 | 63.4 |
| 5 | 7 | 3 (42.1%) | 4 (57.1%) | 5.91 | 66.1 |
| 6 | 4 | 3 (75%) | 4 (100%) | 3.63 | 68 |
| Baseline | 57 | 17 (30%) | 20 (35.1%) | 3.8 | 59 |

## Bleeding & transfusion

### 0968

#### Plasma exchange in a Tunisian intensive care unit (ICU): a 10 year retrospective audit

I. Ben Saida[1], S. Rouis[1], M. Zghidi[1], W. Zarrougui[1], D. Ben Braiek[1], M.A. Boujelbèn[1], S. Kortli[1], M. Bouslama[2], A. Khedher[1], K. Meddeb[1], A. Azouzi[1], M. Boussarsar[1,3]
[1]Farhat Hached University Hospital, Medical Intensive Care Unit, Sousse, Tunisia; [2]Regional Blood Transfusion Center, Sousse, Tunisia; [3]Ibn Al Jazzar Faculty of Medicine, Research Laboratory N° LR12SP09 Heart Failure, Sousse, Tunisia
**Correspondence:** M. Boussarsar
*Intensive Care Medicine Experimental* 2018, **6(Suppl 2):**0968

**INTRODUCTION.** Therapeutic plasma-exchange (TPE) represents the treatment of choice in many immune diseases. It is used in critically ill patients for various indications, either as a first-line therapeutic intervention or as an adjunct to conventional therapies. Data on TPE in the ICU setting are scarce.

**OBJECTIVES.** To describe the local experience of TPE according to frequency, indications, any procedural or patient adverse events and outcomes.

**METHODS.** All adult patients treated by plasma exchange in a 9-bed medical ICU of Farhat Hached teaching hospital between March 2009 and March 31, 2018 were included. Data were collected from identified patient medical records. Demographic variables, the indication for TPE, the TPE procedure characteristics, ICU mortality, duration of ICU stay and adverse events were analyzed.

**RESULTS.** During the study period, 2707 patients were admitted to the ICU. Sixteen (0.6%) patients underwent 66 plasma exchange procedures. Patients' characteristics were : median age, 32.5[26.5-56.5] ; male, 7(43.8%) ; mean SAPSII at admission, 28.44±13.7 ; invasive mechanical ventilation, 6(37.5 %) and vasopressors use, 4(25%). The median length of ICU stay was 7.5[6-12.7]days. The overall mortality rate was 18.8%.

Indications of TPE were: Thrombotic Thrombocytopenic Purpura (TPP), (n=4) ; neurological disorders: Guillain-Barre syndrome, (n=1) ; myasthenia gravis, (n=3), ADEM, (n=1) and autoimmune diseases: anti-glomerular basement membrane (anti-GBM) disease, (n=3) ; cryoglobulinemia, (n=1), lupus (n=1) and catastrophic antiphospholipid syndrome (n= 1).

The median number of procedures performed per patient was 4[3.25-5]. The mean exchange volume per procedure was 1796.13 ±247.4ml. The mean duration of procedures was 118.14±31.9min.

Almost all procedures (93.7%) were performed via a central venous catheter. All of TPE procedures were performed with citrate anticoagulation. Fresh frozen plasma was used for 15 patients as the sole replacement fluid. Adverse events were observed in 5 procedures including severe hypotension for 2 patients, two experienced mild urticaria, three patients had mild chills and one patient developed a cardiac arrest.

**CONCLUSIONS.** In this study, TPE use is rather uncommon. TTP was the most common indication. Some adverse events had occurred but most of them were not life-threatening.

### 0969

#### Pneumonia in a large teaching hospital ICU in the West of Scotland

M.H. Chin[1], S. Barton[2], S. Ramsay[2], P. Stenhouse[2], A. Mackay[2]
[1]University of Glasgow, Medical School, Glasgow, United Kingdom; [2]Queen Elizabeth University Hospital, Critical Care, Glasgow, United Kingdom
**Correspondence:** A. Mackay
*Intensive Care Medicine Experimental* 2018, **6(Suppl 2):**0969

**INTRODUCTION.** Pneumonia is a common presenting disease process to critical care and is associated with multiorgan dysfunction (1). It has a high mortality and is associated with chronic illness.

**OBJECTIVES.** We aimed to quantify and characterise the burden of pneumonia in the intensive care unit of a large teaching hospital in the West of Scotland.

**METHODS.** We conducted a retrospective observational cohort study of all admissions to 18 bedded tertiary ICU of the Queen Elizabeth University Hospital in Glasgow from 1/5/15 to 28/2/18. We used the WardWatcher system to collate diagnostic, demographic, physiological severity and outcome data and separate the patients into pneumonia and non-pneumonia and also subtypes of pneumonia.

**RESULTS.** There were 2022 admissions during the study period. 377 (18.6%) had a diagnosis of pneumonia. Results are presented in Table 1. Patients with pneumonia were more likely to have severe respiratory disease, had higher APACHE-II scores and predicted mortality, were ventilated for longer, stayed in intensive care for longer and were more likely to die. Subgroup analysis revealed that survivors of pneumonia were younger, with a lower incidence of severe respiratory disease and immunosuppression and were less likely to have early signs of organ failure. Further subgroup analysis suggested that patients with aspiration pneumonia were younger than those with bacterial, fungal or other pneumonias and had a shorter length of stay and lower mortality.

**CONCLUSIONS.** We confirmed that pneumonia is a common presenting condition to our ICU and carries a significant mortality and severity of illness. This study allows us to identify factors in patients with pneumonia associated with mortality and allows us to help give more accurate prognostic information to patients and their relatives.

##### REFERENCE(S)

1. Jain S et al. Community Acquired Pneumonia Requiring Hospitalisation among US Adults. NEJM 2015;373:p415-27

**Table 1 (abstract 0969).** Pneumonia vs Other Diagnoses

| n | Other Diagnoses (1645) | Pneumonia (377) | p-value |
|---|---|---|---|
| Age | 54.8±0.8 y | 56.7±1.6 y | 0.053 |
| Male Gender | 61% | 59% | 0.638 |
| Severe Resp Disease | 5% | 10.1% | <0.001 |
| APACHE-II | 20±0.4 | 21.3±0.7 | 0.007 |
| Predicted Mortality | 35.8±1.3 % | 41.3±2.2 % | <0.001 |
| Days Ventilated | 4.1±0.5 | 7.3±1.8 | <0.001 |
| Length of Stay | 4.9±0.5 d | 8.4±1.8 d | <0.001 |
| Mortality | 26% | 31% | 0.047 |
| SMR | 0.73 | 0.75 | |

### 0970

#### Monitoring of mitochondrial oxygen tension during a red blood cell transfusion and fluid challenge in chronic anemia patients

R. Ubbink[1], N.J.R. Raat[1], P.A.W. te Boekhorst[2], E.G. Mik[1]
[1]Erasmus Medical Center Rotterdam, Anesthesiology, Rotterdam, Netherlands; [2]Erasmus Medical Center Rotterdam, Hematology, Rotterdam, Netherlands
**Correspondence:** R. Ubbink
*Intensive Care Medicine Experimental* 2018, **6(Suppl 2):**0970

**INTRODUCTION.** A Red blood cell transfusion (RBCT) aims to restore an adequate oxygen supply. RBCTs are not without risks and therefore, unnecessary transfusions should be avoided[1]. No objective and reliable methods are available to determine an individual's need for a RBCT [2, 3]. Mitochondria are the ultimate target for oxygen supply; therefore mitochondrial oxygen tension monitoring seems an evident choice. In animal experiments a correlation between cutaneous mitochondrial oxygen tension (mito$PO_2$) and the critical hematocrit has been demonstrated [4]. With the development of the COMET measurement system [5] cutaneous mito$PO_2$ measurements became clinically feasible in patients receiving a RBCT.

**OBJECTIVES.** To measure the response of cutaneous mito$PO_2$ during a RBCT or fluid challenge in a low-risk patient population of chronic anemia patients.

**METHODS.** In- and outpatients with chronic anemia, who receive a RBCT on a regular basis, were included in a single center two arm randomized IRB-approved study at the Erasmus Medical Center Rotterdam. Group 1 first got a RBCT followed by a 500 ml NaCl fluid challenge (FC) and group 2 visa versa (FC->RBCT). The COMET monitor (Photonics Healthcare BV, Utrecht, The Netherlands) uses the protoporphyrin IX-Triplet State Lifetime Technique (PpIX-TSLT) to measure mito$PO_2$ [6]. A 5-aminolevulinic acid (ALA) patch was applied the day before RBCT to induce mitochondrial PpIX.

Mito$PO_2$ was measured every min, the first 5 measurements were taken as baseline. After ¾ till the end of the intervention, e.a RBCT or FC, the highest value was taken as the result.

**RESULTS.** 12 patients were measured twice and 9 once, one was re-measured and one was deleted due to insufficient signal quality (31 measurements). There was a mixed response and three groups could be distinguished during the RBCT. The mito$PO_2$ increased, stayed equal or decreased after the RBCT sequence. A threshold of 10% change was used to categorize the effect of RBCT on the mito$PO_2$ seen in Figure 1. No difference in Hb was observed, 7.95 ±0.92 g/dL vs 8.01±0.68 g/dL. For all the RBCT given the mito$PO_2$ decreased in 65% of the cases, increased in 23%, and remained the same in 13%.

**CONCLUSION.** The COMET monitor could detect the effect of a RBCT on mito$PO_2$ and showed a decrease during RBCT in 65% of the cases. If the mito$PO_2$ started high it was likely mito$PO_2$ would decrease. If the baseline mito$PO_2$ was low it was likely that the mito$PO_2$ would rise. These clinical results demonstrate that mito$PO_2$ could potentially be of value in understanding which patients need a RBCT based on their individual mitochondrial $PO_2$ level.

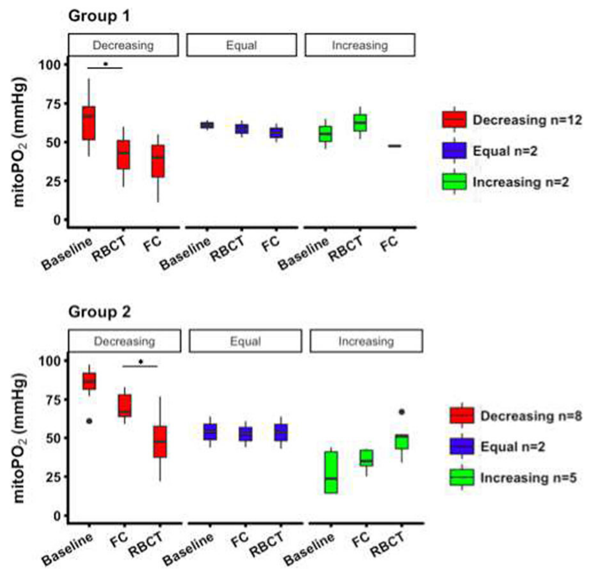

**Fig. 1 (abstract 0970).** The mitoPO2 in mmHg for each group and category. A significant change is indicated by *

### 0971
### Iatrogenic blood loss during an ICU admission
K. Koch, J. Sokhi, R. Davies
Chelsea and Westminster Hopsital, Magill Department of Anaesthesia, Intensive Care and Pain Management, London, United Kingdom
**Correspondence:** K. Koch
*Intensive Care Medicine Experimental* 2018, **6(Suppl 2):**0971

**INTRODUCTION.** Over 80% of ICU patients are anaemic by the end of their first week[1]. Anaemia is associated with increased cardiovascular morbidity and ICU length of stay (LOS). Although the aetiology of ICU anaemia is multifactorial, iatrogenic blood sampling contributes significantly to this burden. We aimed to assess the relationship between our iatrogenic blood letting practices, ICU LOS and blood transfusion requirements.
**METHODS.** Data from 102 patients were collected during the 8 week period. We excluding patients admitted with major bleeding and short length of stay (less than 3 days). Prospective attempts to accurately record the volume sampled for each patient was inaccurate so we used a retrospective survey of ICU staff to estimate volume sampled. This was combined with Electronic Patient Records to ascertain the exact number of arterial blood gases sampled, laboratory samples and blood transfusions required.
**RESULTS.** Our survey concluded that a median volume of 4ml of blood (IQR 3-5ml) was discarded at each sampling episode. As shown in figure 1 below: at 7 days, a median volume of 260ml (IQR 184-289ml) of blood was taken, of which 143ml (IQR 71-179ml) was discarded from the IV line dead-space. At 14 days, a median volume of 511ml (IQR 358-569ml) of blood was taken, of which 298ml (IQR 149-373ml) was discarded. At 21 days, a median volume of 745ml (IQR 518-832ml) of blood was taken, of which 424ml (IQR 212-530ml) was discarded. At 28 days, a median volume of 975ml (IQR 677-1091ml) of blood was taken, of which 524ml (IQR 262-656ml) was discarded. At 28 days, mean total volume of packed red blood cells (PRBC) transfused per patient was 533 ml.
**CONCLUSIONS.** Our data suggests that there is a linear relationship between ICU LOS and ICU anaemia. The LOS on ICU contributes significantly to ICU anaemia, as shown in figure 1. By day 13 our patients had lost the equivalent of one unit of whole blood (450ml), and we had discarded almost the equivalent of one unit of PRBCs (300ml). At 28 days, the volume of blood discarded (524ml) was almost identical to the mean volume transfused per patient (533ml). This data supports evidence that strategies to reduce iatrogenic blood letting such as the use of non-discard IV sampling lines, paediatric blood sampling tubes and the timely removal of invasive lines could reduce the burden of ICU anaemia and cut transfusion requirements. We have implemented changes to our blood sampling practice and will be re-auditing these in the future.

**REFERENCE(S)**
1. Vincent JL et al: Anaemia and blood transfusion in critically ill patients. JAMA 2002: 288:1499-507

**GRANT ACKNOWLEDGMENT**
Nil

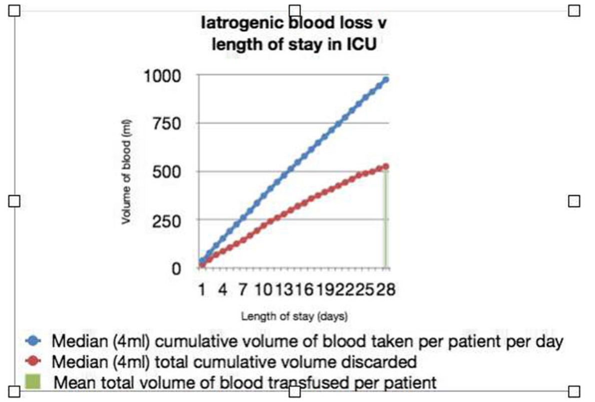

**Fig. 1 (abstract 0971).** See text for description.

### 0972
### Infusion-transfusion therapy - important component of intensive therapy of hemorrhagic shock in obstetric hemorrhages
C. Stakeeva[1], D. Mambetalieva[2], J. Turgunbaeva[3]
[1]Kyrgyz State Mecial Institution of Retraining and Qualification improvement, Bishkek, Kyrgyzstan; [2]City Perinatal Centre, ICU, Bishkek, Kyrgyzstan, [3]City Perinatal Centre, Obstetrics, Bishkek, Kyrgyzstan
**Correspondence:** D. Mambetalieva
*Intensive Care Medicine Experimental* 2018, **6(Suppl 2):**0972

**INTRODUCTION.** In the Kyrgyz Republic, postpartum haemorrhage is still the leading cause of maternal mortality and is 15.8 cases per 100,000 live births (data from the confidential enquiry of maternal deaths (CEMD), 2014-2015). One of the missed opportunities is inadequate infusion-transfusion therapy during blood loss replacement.
**OBJECTIVES.** To study the effectiveness and safety of colloids (hydroxyethyl starch 6%) in the treatment of hypovolemia in postpartum haemorrhages.
**METHODS.** The study included 62 cases of massive bleeding after vaginal delivery (blood loss more than 30% of the circulating blood volume). The average age of the patients was 33.4 ± 2 years. Infusion-transfusion therapy was carried out simultaneously with hemostasis. Its composition varied according to the National Clinical Protocol, depending on the magnitude of blood loss. With massive blood loss, along with crystalloids, fresh frozen plasma and erythrocyte mass were used, and after bleeding control HES derivatives were added. All patients underwent complete clinical and diagnostic examination, in addition, an analysis of the clinical course of the postpartum period was carried out up to 6 months
**RESULTS.** Against the backdrop of infusion-transfusion therapy based on the use of hydroxyethyl starch (with strict adherence to the conditions of the drug usage) and a significant limitation of indications for blood transfusion, no case anuria and hourly urine output below 40ml / h were noted. The proposed infusion-transfusion therapy of massive postpartum hemorrhage allowed to prevent the development of severe hemorrhagic shock. So, despite the presence of massive blood loss, only 2 (3.2%) cases were complicated with hemorrhagic shock of severe degree (stage III). In all the remaining 60 (96.8%) cases despite the volume of blood loss was more than 30% of the circulating blood volume, development of hemorrhagic shock reached only moderate degree (stage II). Also, 1,5 times reduction of blood transfusion was observed in comparison with cases of similar bleeding occurred before the introduction of the National Clinical Protocol. The duration of hospitalization in the postpartum period was 5 ± 2 days.

**CONCLUSIONS.** The use of colloids in the infusion-transfusion therapy for blood loss replacement during massive obstetric hemorrhages, in most cases, allowed to avoid a number of severe complications of hemorrhagic shock. Further study on role of colloids (HES) in the treatment of hypovolemia due to obstetric bleeding is needed.

### 0973

#### Effect of transfusion on hemodynamic and oxygen variables: systematic review and meta-analysis

E. Cavalcante dos Santos, D. Orbegozo, W. Mongkolpun, V. Galfo, E. Gouvêa Bogossian, W. Nan, F.S. Taccone, J. Creteur, J.-L. Vincent
Erasme Hospital,Université Libre de Bruxelles, Intensive Care Unit, Brussels, Belgium
**Correspondence:** E. Cavalcante dos Santos
*Intensive Care Medicine Experimental* 2018, **6(Suppl 2):**0973

**INTRODUCTION.** Anemia is a common problem in the intensive care unit (ICU). Red blood cell transfusion (RBCT) can increase oxygen availability to the tissues and help correct the mismatch between oxygen delivery and oxygen consumption in this setting. However, studies evaluating the effects of RBCT on tissue oxygenation have provided conflicting results, with some showing improvement and others not.

**OBJECTIVES.** To systematically review the effects of blood transfusion on hemodynamics and oxygen variables.

**METHODS.** We performed a systematic review of all prospective, retrospective, observational and interventional studies that included ICU patients who had RBCT without active bleeding. The PubMed database was searched for relevant studies from inception until the end of March 2017. We excluded animal studies and all studies where cardiac output was maintained constant before and after transfusion. We also excluded studies that did not have sufficient data to enable calculation of the change in hemoglobin concentration and at least one of the following variables after RBCT: cardiac index/ cardiac output, mixed venous oxygen saturation/ central venous oxygen saturation ($S\bar{v}O_2/ScvO_2$), oxygen delivery ($DO_2$), oxygen consumption ($VO_2$) and oxygen extraction ratio ($O_2ER$). We also collected heart rate (HR) and blood lactate levels before and after RBCT. Studies were considered according to the patient population included: sepsis, acute respiratory failure, cardiac surgery, non-cardiac surgery, trauma, neurologic and mixed. A meta-analysis and a meta-regression analysis were performed for each hemodynamic and oxygenation variable to explore whether any baseline pre-transfusion variable could predict an increase in $VO_2$ after blood transfusion. All analyses were conducted using Comprehensive Meta-analysis Software V2®. The statistical significance alpha level was 0.05.

**RESULTS.** Of 2441 eligible studies, 37 met the inclusion criteria: sepsis (n=17), acute respiratory failure (n=4), cardiac surgery (n=5), non-cardiac surgery (n=3), neurologic (n=1), trauma (n=1) or mixed (n=6). Cardiac index did not change after RBCT (k=19; diff means= -0.03; 95%CI, -0.10 to 0.04; p=0.444), HR decreased (k=19; diff means= -1.09; 95%CI, -1.86 to -0.32; p=0.005), $S\bar{v}O_2/ScvO_2$ increased (k=29; diff means= 2.90; 95%CI, 2.16 to 3.63; p< 0.001), $O_2ER$ decreased (k=22; diff means= -3.35; 95%CI, -4.55 to -2.16; p< 0.001), $DO_2$ increased (k=22; diff means= 90.56; 95% CI, 68.59 to 112.53; p< 0.001) and $VO_2$ increased (k=22; diff means= 2.87; 95%CI, 0.35 to 5.38; p=0.025). Blood lactate concentration was the only variable that predicted an increase in $VO_2$ after transfusion: for every 1 mEq/L increase in blood lactate, $VO_2$ increased by 6.2 mL/min/m² (p=0.006).

**CONCLUSIONS.** We observed variable effects of RBCT on systemic hemodynamics in heterogeneous cohorts of critically ill patients. Elevated lactate level before RBCT is the only predictor of an increase in $VO_2$ after transfusion.

### 0974

#### Clearance mechanisms of extracellular vesicles after red blood cell transfusion in human endotoxemia

L. van Manen[1,2], A.L. Peters[1], P.M. van der Sluijs[1], R. Nieuwland[3], R. van Bruggen[2], N.P. Juffermans[1]
[1]Academic Medical Center of the University of Amsterdam, Department of Intensive Care Medicine, Amsterdam, Netherlands; [2]Academic Medical Center of the University of Amsterdam, Department of Blood Cell Research, Sanquin research and Landsteiner Laboratory, Amsterdam, Netherlands; [3]Academic Medical Center of the University of Amsterdam, Laboratory of Experimental Clinical Chemistry, and Vesicle Observation Center, Amsterdam, Netherlands
**Correspondence:** L. van Manen
*Intensive Care Medicine Experimental* 2018, **6(Suppl 2):**0974

**INTRODUCTION.** Although lifesaving, blood transfusion is associated with morbidity and mortality. This association is especially observed in the critically ill, which may be a result of ongoing inflammation (1). Extracellular vesicles (EV) from red blood cells (RBC), which are present in high concentrations in RBC units, have been related to adverse effects of blood transfusion (2-4). Thereby, knowledge of their clearance is important to design safer blood products.

**OBJECTIVES.** To investigate clearance mechanisms of RBC-derived EVs in human endotoxemic recipients by measuring the presence of known RBC membrane markers that are associated with phagocytosis.

**METHODS.** Six healthy male volunteers were injected with Escherichia coli lipopolysaccharide. After two hours, an autologous RBC unit was transfused 35 days after donation. Samples were collected from the RBC unit and blood was collected from the volunteers before and after transfusion. RBC-derived EVs were labeled with (anti) glycophorin A, combined with (anti) CD44, CD47, CD55, CD59, or CD147, or lactadherin, a protein that binds to exposed phosphatidylserine (PS). Labeled EVs were analyzed on an Apogee A50 Micro flow cytometer.

**RESULTS.** Before transfusion, the RBC unit contained $3.1 \times 10^{10}$ EVs/mL (IQR 1.3- $5.3 \times 10^{10}$). 7.8% of these RBC-derived EVs exposed PS, whereas all other membrane markers were below the detection limit (Figure 1). In the blood sample collected before transfusion, 22% of the circulating RBC-derived EVs exposed PS and 9.1% were positive for CD59. The levels of EVs positive for CD44, CD47, CD55, and CD147 were low. After transfusion, the concentration of RBC-derived EVs increased 2.4-fold in two hours and decreased towards baseline levels thereafter. The number of EVs positive for PS increased slightly (p = 0.028), whereas the level of EVs positive for other membrane markers remained constant (Figure 2).

CONCLUSION. Besides a minor fraction of PS-exposing EVs, RBC-derived EVs produced during storage do not expose detectable levels of RBC membrane markers that are associated with phagocytosis. Taken together, our results suggest that other yet unknown mechanisms are involved in clearance of RBC-derived EVs after transfusion.

**REFERENCE(S)**

[1] Crit Care Med. 2008;36(9):2667-74.
[2] Transfusion. 2013;53(8):1744-54
[3] Blood. 2014;123(5):687-96.
[4] Transfusion. 2018; https://doi.org/10.1111/trf.14607

**GRANT ACKNOWLEDGMENT**

L. van Manen is supported by a grant from the Dutch Ministry of Health awarded to R. van Bruggen and N.P. Juffermans

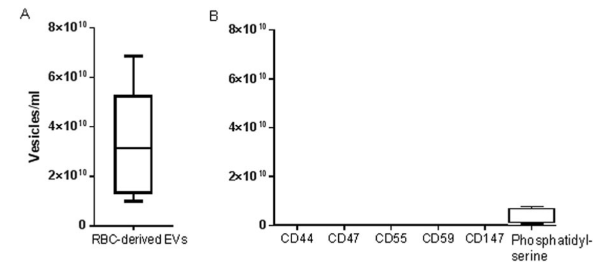

**Fig. 1 (abstract 0974).** Levels of red blood cell (RBC)-derived extracellular vesicles (EV) in the RBC unit (A) and EVs positive for membrane markers (B).

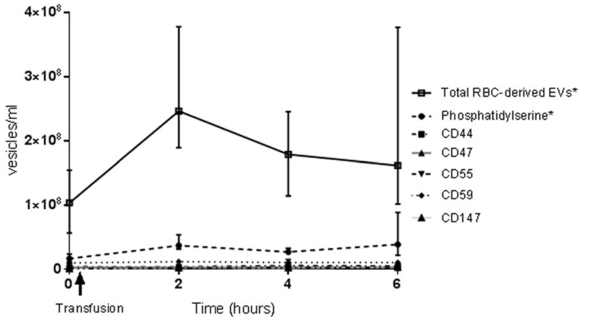

**Fig. 2 (abstract 0974).** Levels of red blood cell (RBC)-derived extracellular vesicles (EVs) in the blood and EVs positive for membrane markers. * p <0.05

### 0975

#### Analysis of the transfusion savings guided by tromboelastography (TEG) prior to invasive techniques in septic patients

Y. Rovira Valles[1], S. Triginer Roig[1], L. Bielsa Berrocal[1], M. Sánchez Satorra[1], R. Roig Pineda[1], E. Alonso Nogués[2], J.R. Grífols Ronda[2], P. Marcos Neira[1]
[1]Hospital Universitari Germans Trias i Pujol, Intensive Care Unit, Badalona, Spain; [2]Hospital Universitari Germans Trias i Pujol, Banc de Sang i Teixits, Badalona, Spain
**Correspondence:** Y. Rovira Valles
*Intensive Care Medicine Experimental* 2018, **6(Suppl 2):**0975

**INTRODUCTION.** Thromboelastography (TEG) is a global test of coagulation which analyzes the whole coagulation process. TEG is popular in trauma, liver transplant, and cardiac surgeries, but studies in sepsis are limited (1).

**OBJECTIVES.** To estimate transfusion savings prior to invasive techniques in septic patients when guiding transfusion by TEG in comparison with transfusion guided by the Spanish ICU transfusion recommendations based on conventional coagulation tests.

**METHODS.** Not blinded randomized observational study. Inclusion criteria: septic patients admitted in the ICU (apr-dec 2017). Exclusion criteria: absence of informed consent. Randomization: **TEG group** (prophylactic transfusion guided by TEG) or **control group** (prophylactic transfusion guided by conventional coagulation tests) prior to an invasive technique. We analysed clinical variables (var), coagulation and need of transfusion. Analysis: description of quantitative var in means or medians (CI95%) according to Shapiro test and qualitative var in proportions. Bivariate analysis of qualitative var with Chi-squared test and quantitative var with Mann-Whitney U test. Significance p< 0.05.

RESULTS: n=43. Males were 67% (CI95%:51-81), age 62.4y (CI95%:58.4-66.5), APACHE II 28.1 (CI95%:24.7-31.5), SOFA 8.3 (CI95%:7.2-9.4), ICU length of stay (LOS) 9 days (CI95%:7-18), ICU mortality 17.5% (CI95%:83-33%), and in-hospital mortality 25% (CI95%:14-41%). 55% of the patients were randomized in control group and 45% in TEG group. Incorrect transfusion was found in 21.7% of the patients in the control group and in 6.9% patients in TEG group (p=0.04). No haemorrhagic complications were found in any group. Fresh frozen plasma (FFP) transfusion in control group was 437mL (CI95%:280-550) vs 286mL (CI95%:284-290) in TEG group (p=0.4) and platelet transfusion in control group was 550mL (CI95%:447-625) vs 265mL (CI95%:256-275) in TEG group (p=0.08).

TEG results were: normal (43%), hypercoagulable (HyperC) (43%), hypocoagulable (HypoC) (14%). 45.5% of patients with INR≥1.3 had a normal TEG, 45.5% hyperC TEG and 9% hypoC TEG. 75% of the patients with APTTr≥1.3 had normal TEG, and 25% hypoC TEG. 60% of patients with high fibrinogen levels and no one with normal fibrinogen had an hyperC TEG (p=0.03); all patients with high platelet count and 10% of patients with low platelet count had an hyperC TEG (p=0.01).

**CONCLUSIONS.** TEG previous to realization of invasive techniques in septic patients optimizes transfusion. INR and APTTr in septic patients do not relate with patients coagulation capacity.

TEG prior to invasive techniques in septic patients can save transfusion. TEG better reflects the coagulation state in septic patients than the conventional coagulation tests (INR and APTTr).

**REFERENCE(S)**

1. Utility of thromboelastography and/or thromboelastometry in adults with sepsis: a systematic review. Müller MC, Meijers JC, Vroom MB, Juffermans NP. Crit Care. 2014 Feb 10;18(1):R30.

### 0976

#### ECLS-related coagulopathy and transfusion needs are reversed by circuit exchange

R. López[1,2], J.P. Fuentes[1,2], R. Pérez[1,2], M. Donoso[1,2], J. Graf[1,2]
[1]Clínica Alemana de Santiago, Departamento de Paciente Crítico, Santiago, Chile; [2]Facultad de Medicina Clínica Alemana - Universidad del Desarrollo, Santiago, Chile
**Correspondence:** R. López
*Intensive Care Medicine Experimental* 2018, **6(Suppl 2):**0976

**INTRODUCTION.** Coagulation is a major concern during ECLS and disseminated intravascular coagulation has been described as a complication [1,2]. We have observed that thrombocytopenia and hypofibrinogenemia are usual manifestations of ECLS-related coagulopathy (ECLS-RC).

**OBJECTIVES.** Describe a case series of ECLS-RC that was managed by circuit exchange.

**METHODS.** Retrospective analysis of patients from our ECLS cohort between March 2012 and November 2016 who developed ECLS-RC (defined as persistent thrombocytopenia and/or hypofibrinogenemia) and were managed with circuit exchange. We excluded circuit exchange for other reasons. The decision of circuit exchange was made on clinical grounds.

**RESULTS.** From a total of 44 ECLS runs we found 6 patients with circuit exchange due to ECLS-RC. Epidemiological data as median [interquartile range] were: age 65 years [48-70], circuit run 13 days [11-16], APACHE II 34 points [20-34], SOFA 9 points [7-9]. The etiologies were: Influenza AH1N1 (n=2), acute interstitial pneumonia (n=1), respiratory syncytial virus (n=1), ARDS (n=1) and pneumonia (n=1).

We found in these patients a significant decrease of fibrinogen levels (Fig 1A) and platelet counts (Fig 1B) with recovery after circuit exchange. A significant reduction of transfusion needs was also noted after circuit exchange (Fig 1B). Four patients had mucosal bleeding associated to ECLS-RC, while the other two patients had not clinical expression of ECLS-RC.

**CONCLUSIONS.** These data suggest that in ECLS-RC circuit exchange is associated with recovery of fibrinogen and platelets levels as well as a reduction in transfusion needs. Circuit exchange may be considered as part of the treatment of ECLS-RC if ECLS removal is not possible. We propose that in ECLS-RC the circuit behaves like a diseased organ that self-perpetuates coagulopathy. Given the retrospective nature of the study and the small number of cases, prospective studies are warranted to confirm this hypothesis.

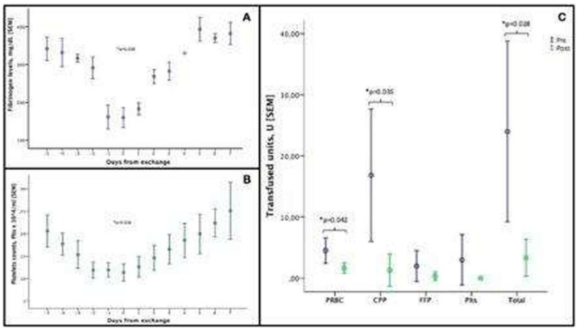

**Fig. 1 (abstract 0976).** Fibrinogen (A), platelet counts (B) and transfusion needs (C) referred to circuit exchange]

### 0977

#### Major bleeding, can we identify some useful predictor of mortality?

E.A. Ines, C.R. Pablo, M.F. Mónica, G.V. Rita, F.M. David
University Hospital of A Coruña, Intensive Care, A Coruña, Spain
**Correspondence:** E.A. Ines
*Intensive Care Medicine Experimental* 2018, **6(Suppl 2):**0977

**INTRODUCTION.** Major bleeding (MB) is a situation with high mortality. Protocols are needed, a multidisciplinary action and to know the characteristics of the population. Our objective is to describe the population in a tertiary hospital.

**OBJECTIVES.** It is difficult to define those factors that play a role in the incidence of MB, but it is a priority to try to identify those that influence the mortality. Our objective is to find some eterminant factor in mortality.

**METHODS.** Retrospective study of 303 episodes of MB (transfusion> 5concentrated red blood cells (CH) in 4 hours or> 10CH in 24 hours) occurred in 298 patients who they required entry into the critical units during the 2009-2015 period. A descriptive analysis of the variables of interest was carried out, which may be related to themortality: age, sex, origin of bleeding, SOFA on the day of the episode, coagulation parameters, type of intervention (surgical, radiological and / or medical), support and hospital stay. The statistical package SPSS 19 was used for the analysis.

**RESULTS.** 303 episodes of MB were identified. 73.6% were male, the mean age was 61.2 (SD15.2) years and Charlson Index 4.5 (DE3.0). The score mean on the SOFA scale the day of the episode was 13.3 (SD4.8). The origin was cardiovascular in 58.40%, digestive in 33% and other causes 8.60%. The overall mortality was 47.9%. At univariate analysis we found an association significant with the mortality of: age (63.1 vs.59.4, p = 0.036), PH (7.21 vs 7.0, p = 0.023), APTT (1.7 vs.1.6; p = 0.02), noradrenaline (NA) dose (1.0 vs. 0.4; p < 0.01) and hospital stay (32.9 vs. 46.7, p < 0.01). In the multivariate analysis, he identified age (OR 4.4), NA dose (OR 15.7) and hospital stay (OR 7.9) as independent predictors of mortality.

**CONCLUSIONS.** The MB in our series presents a mortality acceptable. Patients with older age, as well as doses higher NA partners are associated independently with mortality.Hospital stay can be a factorconfusing to be influenced by gravit.

**REFERENCE(S)**

None.

**GRANT ACKNOWLEDGMENT**

This study received no financial support.

### 0978

#### Evaluation of Iatrogenic blood loss: impact of "routine" blood tests on critical care patients

K. Caines, A. Dawood
Hull and East Yorkshire Hospitals, Hull, United Kingdom
**Correspondence:** K. Caines
*Intensive Care Medicine Experimental* 2018, **6(Suppl 2):**0978

**Withdrawn**

### 0979

#### Washing of red blood cells prior to transfusion decreases trapping of donor cells in organs in a pneumosepsis rat model

L. van Manen[1,2], R. van Bruggen[2], N.P. Juffermans[1]
[1]Academic Medical Center of the University of Amsterdam, Department of Intensive Care Medicine, Amsterdam, Netherlands; [2]Academic Medical Center of the University of Amsterdam, Department of Blood Cell Research, Sanquin Research and Landsteiner Laboratory, Amsterdam, Netherlands
**Correspondence:** L. van Manen
*Intensive Care Medicine Experimental* 2018, **6(Suppl 2):**0979

**INTRODUCTION.** Red blood cell (RBC) transfusions are often given to septic patients, but are associated with organ injury in the critically ill (1). The mechanisms are unknown, but adherence of donor RBCs to an activated endothelium could play a role (2,3). We hypothesized that washing of RBCs before transfusion may reduce trapping of donor cells in organs and thereby reduce organ injury (4).

**OBJECTIVES.** To investigate the effect of washing of a RBC transfusion product on donor RBC clearance and trapping of RBCs in organs in rats with pneumosepsis.

**METHODS.** Sprague-Dawley rats were inoculated intratracheally with 10^7 colony forming units of S. pneumonia or vehicle as a control. After 20 hours they received a 2-weeks old, washed or non-washed biotinylated RBC (bioRBC) transfusion from syngeneic rats or saline (N=8 per group). Blood samples were taken directly after transfusion and 24 hours later. After 44 hours, the rats were sacrificed. The circulation of the rats was flushed with saline and organs were removed. Homogenized parts of the organs and blood were stained with streptavidin to detect the bioRBC by flow cytometry. The organs were histologically scored by a pathologist on a scale of 0-24.

**RESULTS.** All infected animals had severe pneumonia, as evidenced by bacterial outgrowth in the lungs. Mortality rate was 7,5%. Directly after transfusion, 18.9% (IQR 17.0-21.0%) of the circulating RBC were bioRBC. The median 24-hour post transfusion recovery of the bioRBC was 49.2% (IQR 47.2-53.5%). Neither Infection nor washing had an effect on post transfusion recovery (p=0.66) (Figure 1). A transfusion with a washed product reduced the percentage of donor RBCs in the liver and kidney compared to a non-washed transfusion. In contrast, washing increased the percentage of donor RBC in the spleen compared to non-washed products (p< 0.05) (Figure 2). Transfusion did not further augment the amount of lung injury induced by pneumonia, but washing decreased the amount of lung injury, (histology score 5.5 (IQR 4-7)vs 10 (IQR 8-13) in un-washed controls) . There were no differences in liver or kidney injury between groups.

**CONCLUSIONS.** Infection does not increase the clearance or trapping of donor RBC. In infected rats, a washed RBC product decreases trapping of RBC in liver and kidney and increases clearance of the RBC by the spleen.

#### REFERENCE(S)

[1] Ann Intensive Care. 2017;7(1):5.
[2] Am J Hematol. 2007;82(6):439-45.
[3] Shock. 2017; 48(4):484-489.
[4] Blood. 2014;123(9):1403-11.

#### GRANT ACKNOWLEDGMENT

L. van Manen is supported by a grant from the Dutch Ministry of Health awarded to R. van Bruggen and N.P. Juffermans.

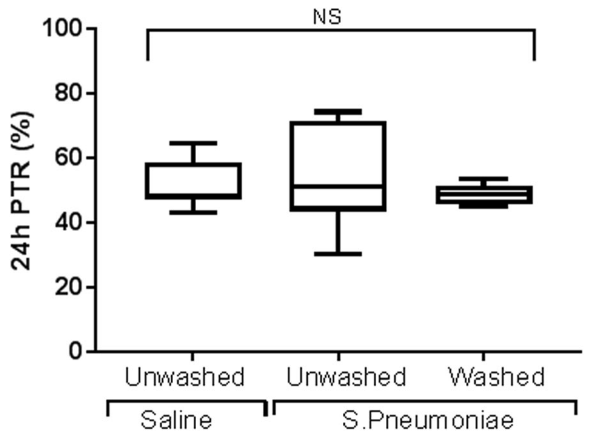

**Fig. 1 (abstract 0979).** Percentage of bioRBC that were still in the circulation 24 hours after transfusion. PTR: posttransfusion recovery, NS: not significant

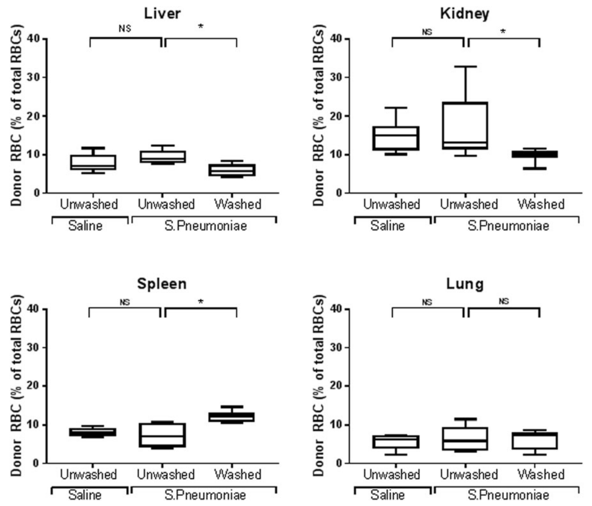

**Fig. 2 (abstract 0979).** Percentage bioRBC in the organs 24 hours after transfusion measured by flow cytometry. NS: not significant, * p <0.05

### 0980

#### Improving critical care transfusion practice: development of a transfusion tool

K. Caines, A. Dawood
Hull and East Yorkshire Hospitals, Hull, United Kingdom
**Correspondence:** K. Caines

*Intensive Care Medicine Experimental* 2018, **6(Suppl 2):**0980

**Withdrawn**

### 0981

#### Low-frequency piezoelectric thromboelastography method in studying of hemostatic system

O. Tarabrin, I. Basenko, D. Sazhyn, P. Tarabrin
Odessa National Medical University, Odessa, Ukraine
**Correspondence:** O. Tarabrin

*Intensive Care Medicine Experimental* 2018, **6(Suppl 2):**0981

**Withdrawn**

### 0982

#### The effect of an early transfusion on infection in severe trauma

D.W. Kim, I.S. Jeong
Chonnam National University Hospital, Thoracic and Cardiovascular Surgery, Gwangju, Korea, Republic of
**Correspondence:** D.W. Kim

*Intensive Care Medicine Experimental* 2018, **6(Suppl 2):**0982

**INTRODUCTION.** Blood transfusion in severe trauma is a cure tool that must occur. And, it is well known that packed red blood cell (pRBC) transfusion may increase the risk of infection in trauma patients. But, there is no uniform guideline on the impact of transfusion volume and time to start transfusion.

**OBJECTIVES.** Recognition of transfusion effects in trauma patients is essential. Especially, although severe trauma patients have many hypovolemic shock in the early stages, but septic shock accompanied by infection is predominant over time.We studied the relation between timing of pRBC transfusion and incidence of infection after trauma.

**METHODS.** From January 2016 to December 2017, we studied 469 patients who were admitted to single regional trauma center of Southwest area of Korea. Outcome assessment included nosocomial infection (bacteremia and pneumonia) rates between 2 groups: the early transfusion group (< 4hours of admission) and the control group (4-24hours of admission). We used multivariable logistic regression to estimate the relative odds of bacteremia and pneumonia associated with the timing of pRBC transfusion.

**RESULTS.** A total of 58 (62.4%) of 93 patients developed bacteremia, and 53 patients (57%) who developed pneumonia received pRBC transfusion. Transfusion was associated with the increased rate of nosocomial infection, compared with non-transfusion patients ($p< 0.05$). Patients receiving RBC within 4hours had an increased odd of developing bacteremia (odd ratio, 1.9; $p < 0.001$) and pneumonia (odd ratio 1. 5; $p< 0.001$).

**CONCLUSIONS.** Transfusion of red blood cells increases the risk of developing pneumonia and bacteremia. Avoiding the unnecessary use of packed red blood cell transfusions in emergency room may decrease the occurrence of nosocomial infection.

#### REFERENCE(S)

1. Agarwal N, et al. Blood transfusion increases the risk of infection after trauma. Arch Surg. 1993 Feb;128(2):171-6.
2. Claridge JA. et al. Blood transfusions correlate with infections in trauma patients in a dose-dependent manner. Am Surg. 2002 Jul;68(7):566-72.
3. Dunne JR, et al. Blood transfusion is associated with infection and increased resource utilization in combat casualties. Am Surg. 2006 Jul;72(7):619-25.
4. Carson JL. Blood transfusion and risk of infection: new convincing evidence. JAMA. 2014 Apr 2;311(13):1293-4.
5. Patel SV, et al. Risks associated with red blood cell transfusion in the trauma population, a meta-analysis. Injury. 2014 Oct;45(10):1522-33.
6. Chaiwat O, et al. Early packed red blood cell transfusion and acute respiratory distress syndrome after trauma. Anesthesiology. 2009 Feb;110(2):351-60.
7. Kutcher ME, et al. A paradigm shift in trauma resuscitation: evaluation of evolving massive transfusion practices. JAMA Surg. 2013 Sep;148(9):834-40.

#### GRANT ACKNOWLEDGMENT

This study has not received any support.

| Variable | Early Group (N=93) | Control Group (N=258) | p-Value |
|---|---|---|---|
| Bacteremia | 58 (62.4%) | 45 (17.4%) | p<0.05 |
| Pneumonia | 53 (57%) | 37 (14.3%) | p<0.05 |

| Variable | OR | p-Value |
|---|---|---|
| bacteremia | 1.9 | p < 0.001 |
| pneumonia | 1.5 | P < 0.001 |

**Fig. 1 (abstract 0982).** Early transfusion group vs control group

### 0983

#### Retrospective observational cohort study of anticoagulation practices, thromboembolic events and bleeding events in patients that develop atrial fibrillation admitted to a mixed surgical and medical ICU in the United Kingdom

N. Miller[1], A. Hampden-Martin[1], B. Johnston[1,2], I. Welters[1,2]
[1]University of Liverpool, Institute of Ageing and Chronic Disease, Liverpool, United Kingdom; [2]Royal Liverpool and Broadgreen University Hospital Trust, Intensive Care Department, Liverpool, United Kingdom
**Correspondence:** B. Johnston
*Intensive Care Medicine Experimental* 2018, **6(Suppl 2):**0983

**INTRODUCTION.** Atrial fibrillation is the most common arrhythmia in ICU patients with an incidence of between 4.5-15%.[1] Patients that develop AF in ICU have increased ICU stay, increased hospital length of stay and increased mortality.[1] International guidance recommends the use of CHADSVASc and HASBLED scoring to determine patients that will benefit from anticoagulation and at risk of bleeding.[2] These scores are not validated in ICU and whether patients with AF benefit from anticoagulation in ICU is unclear.

**OBJECTIVES.**

Describe anticoagulation strategies used in ICU in patients with AF

Determine the thromboembolic (TE) and bleeding risks of ICU patients with AF

Determine outcome associated with AF, anticoagulation and bleeding in ICU with AF

**METHODS.** Patients admitted to ICU between Jan-Dec 2017 that developed AF during their admission were enrolled in the study. Data was extracted retrospectively from daily ICU observation sheets, electronic patient records system and electronic patient investigation systems. Demographic data and co-morbidities were recorded. Data regarding anticoagulation at first and subsequent episodes of AF was recorded. TE and bleeding events were recorded for all patients that developed AF. CHADSVASc and HASBLED scores were calculated for each patient and correlated with TE and bleeding events.

**RESULTS.** The overall incidence of AF was 6.2%. The incidence of new onset AF (NoAF) was 2.6%. Only 21.6% of patients developing AF received therapeutic anticoagulation within 48 hours of AF onset. No patients with NoAF received anticoagulation. 2 patients with pre-existing AF received anticoagulation but after more than one episode of AF. The most commonly used anticoagulant was low molecular weight heparin followed by unfractionated heparin. 92% of patients had a CHADSVASc high enough for anticoagulation to be recommended. 53% of patients had a HASBLED score that indicated moderate/high risk of bleeding. There was no association between CHADSVASc and TE events. HASBLED score correlated with bleeding events but did not reach significance.

**CONCLUSIONS.** Patients with pre-existing AF are more often anticoagulated compared to NoAF. The majority of ICU patients with AF meet guideline recommendation for anticoagulation. There is no statistical significance between CHADSVASc and TE events in ICU patients. Patients with high HASBLED scores tended to suffer more bleeding events. Patients appear to suffer more bleeding events in ICU compared to TE events. Current guidelines may not be suitable for critically unwell patients in ICU.

**REFERENCE(S)**

1. Yoshida T, Fuji T, Uchino S, Takinami M (2015) Epidemiology, prevention, and treatment of new-onset atrial fibrillation in critically ill: a systematic review. Journal of intensive care; 3(19):2-11
2. 2016 ESC Guidelines for the management of atrial fibrillation in collaboration with EACTS The Task Force for the management of atrial fibrillation of the European Society of Cardiology

### 0984

#### Introduction of massive hemorrhage protocol in II level trauma center. Analisys after one year

A. Calzolari[1], M. Leone[1], E. Borotto[2], I. Beverina[2], D. Radrizzani[2]
[1]Università degli Studi di Milano, Milan, Italy; [2]Ospedale Civile di Legnano - ASST Ovest Milanese, Legnano, Italy
**Correspondence:** A. Calzolari
*Intensive Care Medicine Experimental* 2018, **6(Suppl 2):**0984

**INTRODUCTION.** Massive Hemorrhage (MH) is a quite widespread entity in emergency department, validation of Massive Hemorrhage Protocols (MHP) has been associated with reduced mortality and morbidity but till now there is not a univocal definition of MH. Historically, recognition of MH has been overlapped with massive transfusion (MT), usually defined by retrospective data (e.g. more than ten units in 24 h or more than a half of circulating mass in 24 h) not helpful in real management of ongoing bleeding patient. Several scores have been proposed to predict MT needs but the higher the specificity, the lesser the applicability during emergency because of the complex calculations required.

In 2015 we drafted an institutional MHP (including standardized transfusion packs) to standardize MH recognition and treatment (Fig 1). Our staff was trained with dedicated courses.

**OBJECTIVES.** To evaluate the adequacy of our institutional protocol in identification of massive hemorrhage patients and consequent MT treatment.

**METHODS.** We analyzed data between June 2016-March 2017 (1 y after MHP creation). MT appropriateness was retrospectively evaluated considering Hb levels (after hemorrhage resolution and at 24 hours), in accordance with MHP transfusion targets. We also evaluated correct protocol activation in accordance with hemodynamic status criteria (Fig. 1).

**RESULTS.** 35 cases of MHP activation were reviewed, 20 of which following trauma. Activation of MHP was appropriate in 24 patients (68%) and in all trauma patients. The following results are referred to the appropriate cases. The continuous double-check (Clinicians and Transfusion Medicine experts), allowed that 24 h Hb did not exceed 10 gr/dl in 98% cases. Beside the amount of packed RBCs (mean 6.2, min 2, max 14), all patients were severely hemorrhagic and seriously ill, as assessed by mortality (37.5%) and mean ISS score for trauma patients(38). Finally, our pre-settled ratio between hemocomponents was ensured in all 24 appropriate cases.

**CONCLUSIONS.** Definition and activation of MHP in our center resulted in a good level of appropriateness of both recognition of MH and transfusion, maintaining the correct ratio and avoiding overtransfusion. Our results confirm that a retrospective definition of MH by the number of transfusions may underrate its real incidence, because an early, aggressive and balanced transfusion diminish the amount of haemocomponents.

Rossaint; The European guideline on management of major bleeding and coagulopathy following trauma: fourth edition; Crit Care. 2016 Apr 12;20:100.

**GRANT ACKNOWLEDGMENT**

None.

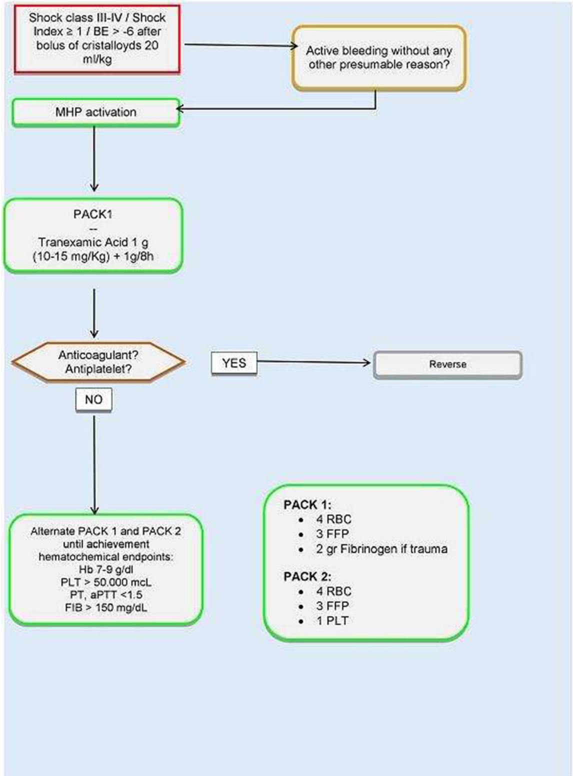

**Fig. 1 (abstract 0984).** The Massive Hemorrhage Protocol drafted in our level II trauma center.

## Cardiovascular monitoring

### 0985

#### Value of inferior vena cava diameter and of pulse pressure variations during tidal volume challenge to predict preload responsiveness in patients ventilated with a low tidal volume

T. Taccheri[1,2], F. Gavelli[1,2], J.-L. Teboul[1,2], C. Richard[1,2], X. Monnet[1,2]
[1]Hôpitaux Universitaires Paris-Sud, Hôpital de Bicêtre, APHP, Service de Réanimation Médicale et de Surveillance Continue Médicale, Paris, France; [2]Inserm UMR S_999, Univ Paris-Sud, Paris, France
**Correspondence:** T. Taccheri
*Intensive Care Medicine Experimental* 2018, **6(Suppl 2):**0985

**INTRODUCTION AND OBJECTIVES.** This work aimed at showing that the reliability of the distensibility index of the inferior vena cava diameter (ΔIVC) for predicting preload responsiveness is low in case of low tidal volume (Vt) (6 mL/kg of predicted body weight). We also tested whether the changes in ΔIVC and in pulse pressure variation (PPV) induced by a "Vt challenge", consisting in increasing Vt from 6 to 8 mL/kg could reliably predict preload responsiveness detected by a passive leg raising (PLR) test.

**METHODS.** In 30 critically ill patients with acute circulatory failure ventilated with Vt at 6 mL/kg, we measured cardiac output (PiCCO2) before and during a PLR test. Each patient, ventilated in the assist control mode, was sufficiently sedated and well adapted to the ventilator. Preload responsiveness was defined by a PLR-induced increase in cardiac index ≥10%. We measured ΔIVC and PPV at baseline, at Vt=6 mL/kg, during a 1-minute increase in Vt at 8 mL/kg (Vt challenge) and 1 minute after starting PLR, which was performed at Vt=6 mL/kg.

**RESULTS.** The PLR test performed at Vt=6mL/kg was positive in 15 patients ("preload responders"). At Vt=6 mL/kg, ΔIVC was similar in responders and non-responders (8±2% and 7±2%, respectively, p=0.1) and did not allow the detection of preload responsiveness (area under the receiver operating characteristic curve (AUROC)=0.67 ±0.10, p=0.1). It was also the case for PPV (12±4% in responders, 9 ±5% in non-responders, respectively, p=0.1), with an AUROC=0.69 ±0.10 (p=0.07). At Vt=8mL/kg, ΔIVC was higher in responders than in non-responders (15.4±2.8% and 5.6±3.1%, respectively, p=0.0004) and detected preload responsiveness (AUROC=0.93±0.04, p=0.001, threshold=10.7%). It was also the case for PPV (16±3% in responders, 9 ±5% in non-responders, respectively, p=0.001), with an AUROC=0.89 ±0.07 (p=0.001, threshold=12%). The Vt challenge-induced changes in ΔIVC detected preload responsiveness reliably (AUROC=0.98±0.02, p=0.001, threshold=2.5% increase in absolute value). It was also the case for the Vt challenge-induced changes in PPV (AUROC=0.98±0.01, p=0.001, threshold=1% increase in absolute value). The PLR-induced changes in ΔIVC did not detect preload responsiveness (AUROC=0.57 ±0.11, p=0.52), but the PLR-induced changes in PPV did (AUROC=0.97 ±0.03, p=0.001, threshold=3% decrease in absolute value).

**CONCLUSIONS.** ΔIVC is not reliable for predicting preload responsiveness in patients ventilated with Vt=6 mL/kg. A reliable detection of preload responsiveness was achieved by increases in ΔIVC as well as in PPV induced by a Vt challenge (transient increase in Vt from 6 to 8 mL/kg). Detection of preload responsiveness was also possible through the decrease in PPV induced by PLR, but not through the PLR-induced changes in ΔIVC.

### 0986

#### Are variations of inferior vena cava measured by a transhepatic way a good tool to predict fluid responsiveness?

J.P. Ponthus, K. Aacha, D. Pinell, M. Ayoub, S. Bensahli, J.F. Georger
CHI Lucie et Raymond Aubrac, 94, Villeneuve Saint Georges, France
**Correspondence:** J.P. Ponthus
*Intensive Care Medicine Experimental* 2018, **6(Suppl 2):**0986

**INTRODUCTION.** Optimizing the circulatory condition of our patients is a daily challenge for the intensivist. Fluid is one of the therapeutics, through the increase of venous return and then cardiac output. Several tools may assess that, such as transthoracic echocardiogram (TTE), which allows the visualisation and the study of the respiratory variability from the inferior vena cava (IVC). However, the IVC visualisation is difficult in some situations (obesity, gut surgery, emphysema). Alternatively, the IVC is easily seen by a transhepatic way in her retrohepatic section (also called mid axillary).

**OBJECTIVES.** We hypothesised that the shape of the IVC and her variation taken by a transhepatic way could be predictive of fluid responsiveness.

**METHODS.** We performed fluid challenge in patients under mechanical ventilation. VT was set at 6ml/Kg and peep between 5 and 10 mmH2O. The need for fluid therapy was decided by the intensivist in charge. We performed a echocardiogram and took two measures of the transhepatic ICV (THICV) : major axis and minor axis. An elastometry index (EI) was determined as the ratio of minor axis to minor axis. The fluid challenge was 250ml of isotonic saline. We performed a new TTE and took the same ultrasound slice. We considered a 15% increase of the cardiac index (IC) as a success of the filling. Exclusion criteria were patients with A right cardiac insufficiency, an arrhythmia and/or a HTAP. The statistical analysis was performed using R software.

**RESULTS.** Between January 2017 and september 2017, 50 patients were included. The average age is 61 years (24-81), IGS2 of 50 (20-84), Ejectionnal Fraction of 48 % (35-75) and the S wave tricuspid is 14 (9-21). The main causes of the filling were an oliguria (31 %), a low blood pressure (25 %), a low cardiac output (30 %), a hyperlactatémia (11 %). A positive correlation between EI

(teleinspiratory phase) and the increase of the IC was detected, as well as the shape variation of the THVCI (DeltaEI) and the classic respiratory variations of the VCI ($p < 0.05$) Results are summarized in table 1. Classic variation of IVC by the subcostal way could only be obtain for 33 patients (no visualisation or bad alignment). The other variables are not predictive (BP, E/e´, E/A,CVP). The data are summarized in the figure 1. ROC curves has been established (table 1 and figure 1).

**CONCLUSIONS.** Shape of the THICV and there variations is a potential tool to assess fluid responsiveness. The main limit of the method is the necessity of being very perpendicular at the risk of falsifying the EI. A teleinspiratory ovoïd vena cava (EI : 0.85) is very sensitive and its variation very specific. We hypothesized that the combination of shape and his variation reflects the potential to elevate the mean mean filling pressure in order to increase venous return at the same capacitance.

**Table 1 (abstract 0986).** See text for description.

| | | | |
|---|---|---|---|
| SIGNIFICANT PARAMETERS | | | |
| | R | IC | p |
| Variation IVC | 0.64 | 0.38-0.377 | 0.04 |
| Teleinspiratory EI (EI) | 0.34 | 0.067-0.56 | 0.016 |
| Respiratory variation of EI | 0.35 | 0.007-0.57 | 0.015 |
| ROC CURVES | | | |
| | AUC (IC95) | Se/Sp | Treshold |
| Variation IVC | 0.72 (0.67-0.91) | 86/75 | 14% |
| Teleinspiratory EI | 0.75(0.60-0.86) | 88-73 | 0.87 |
| Respiratory variation of EI | 0.76(0.61-0.86) | 76-87 | 0.12 |

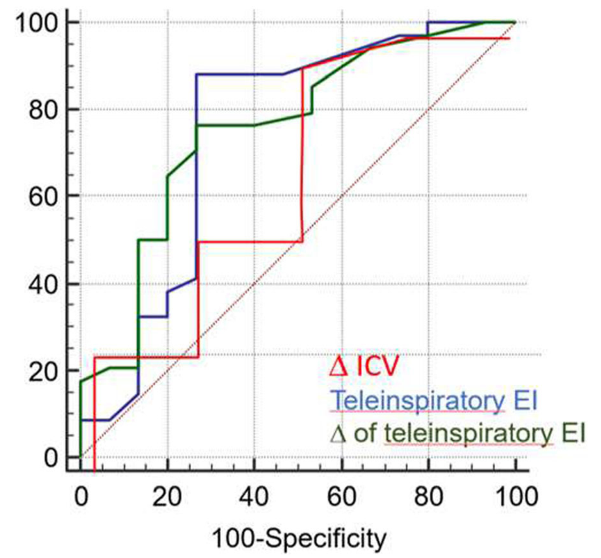

**Fig. 1 (abstract 0986).** See text for description.

### 0987

#### External validation of the Smith & Madigan formula to quantify the final end-diastolic volume of the left ventricle

G.P. Santana Hernandez[1], E. Monares Zepeda[1], J. Cruz Lopez[1], J.c. Cuesta Torres[1], F. Flores Puente[2], R. Cebrian Garcia[1], R. Lozano Zúñiga[1]
[1]Hospital San Angel Inn Universidad, Critical Medicine, Mexico City, Mexico; [2]Hospital 20 de Noviembre, Cardiology, Mexico City, Mexico
**Correspondence:** G.P. Santana Hernandez
*Intensive Care Medicine Experimental* 2018, **6(Suppl 2):**0987

**INTRODUCTION.** The final left ventricular end-diastolic volume (VDFVI) is an indicator of left ventricular preload; no invasive monitoring currently available outside the echocardiogram adequately estimates the VDFVI. Echocardiography, however, is technically difficult and operator dependent, requires experience, trained personnel and equipment to perform this measurement, it is not available 24 hours in intensive care units. The inotropism formula developed by Smith & Madigan (SMII) is carried out through USCOM monitoring in a semi-automatic way, it has been validated as an adequate surrogate of inotropism, prognostic marker and guide in resuscitation of septic shock. In another study, the Smith & Madigan working group demonstrated an adequate correlation between this formula and the VDFVI.

**OBJECTIVES.** Validate the VDFVI obtained by Smith and Madigan and the one obtained in patients with septic shock.

**METHODS.** In 56 patients diagnosed with septic shock, the following formulas were measured using USCOM ultrasound from the stroke volume (SV), mean arterial presion (MAP), presion mean gradient (Gp), body weigth index (IBW) and flow time (FT).

$SMII = (SV \times (MAP - CVP + Gp))/(7.5 \times IBW \times FT)$

$VDFVI = SV \times 2.7/SMII.$

The VDFVI obtained by the SMII formula was estimated and compared with that obtained using the conventional echocardiography technique, Bland & Altman, correlation coefficient R, Linn coefficient and percentage of error were performed in order to determine the concordance between both measurements.

**RESULTS.** 56 measurements were made in 56 patients. We compared VDFVI results, measured by echocardiography and Smith & Madigan formula, using the Bland & Altmann method (figure 1), we obtained an $R2 = 0.92$, a Linn coefficient of 0.92 with an LC95% higher 32.45, LC95% Lower - 39.45 and an error rate of 32%.

**CONCLUSIONS.** Smith formula & Madigan could be useful for the calculation of VDFVI. Although the percentage of error is greater than 20%, we must determine if this measurement is useful for making clinical decisions.

**REFERENCE(S)**

- Smith B. Madigan, Non-invasive method for rapid bedside estimation of inotropy: theory and preliminary clinical validation, *British Journal of Anaesthesia*, 111 (4): 580-8 (2013).
- Phillips R. Stroke Volume Monitoring: Novel Continuous Wave Doppler Parameters, Algorithms and Advanced Noninvasive Haemodynamic Concepts, Current Anesthesiology Reports (2017) 7:387-398
- Lang M. et al Recommendations for Cardiac Chamber Quantification by Echocardiography in Adults: An Update from the American Society of Echocardiography and the European Association of Cardiovascular Imaging Journal American Society of Echocardiography, 2015;28:1-39.

**GRANT ACKNOWLEDGMENT**

To all my colleagues and teachers for their valuable collaboration.

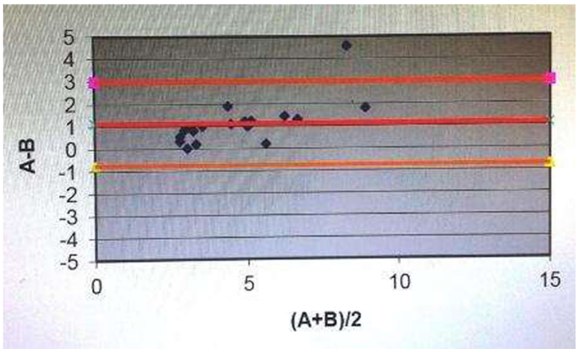

**Fig. 1 (abstract 0987).** Bland Altmann

### 0988

#### Revisiting the limitations of respiratory variations of pulse pressure or stroke volume as predictors of fluid responsiveness: tidal volume, heart-rate-to-respiratory-rate ratio and respiratory system compliance

W. v.d. Veen[1], K. Lakhal[2], S.N. Myatra[3], T.W.L. Scheeren[1], S.T. Vistisen[1,4]
[1]University Medial Centre Groningen, Department of Anesthesiology, Groningen, Netherlands; [2]Hopital Laennec, Centre Hospitalier Universitaire, Reanimation Chirurgicale Polyvalente, Service d'Anesthesie-Reanimation, Nantes, France; [3]Tata Memorial Hospital, Department of Anesthesiology, Critical Care and Pain, Mumbai, India; [4]Aarhus University Hospital, Department of Anaesthesia and Intensive Care, Aarhus, Denmark
**Correspondence:** S.T. Vistisen
*Intensive Care Medicine Experimental* 2018, **6(Suppl 2):**0988

**INTRODUCTION.** Pulse pressure variation (PPV) and stroke volume variation (SVV) are some of the best validated dynamic fluid responsiveness predictors. Still, respiratory limitations such as low tidal volume (TV) (< 8 ml/kg)[1], low heart-rate-to-respiratory-rate (HR/RR) ratio (< 3.6)[2], and low respiratory system compliance (Crs) (< 30 ml/cmH$_2$O)[3] have been identified, limiting applicability in the ICU[4]. Since the widespread use of lung-protective ventilation, these limitations are often combined in the same patient and firm conclusions about the role of one specific limitation are difficult to draw. Regarding the low TV limitation, some studies have identified a proportional relation between TV and PPV. Hence, PPV at low TVs may predict PPV at higher TVs, indicating that low TV might not be a limitation itself and indexing PPV or SVV to TV or driving pressure has been suggested.
**OBJECTIVES.** The study aim was to investigate PPV's and SVV's predictive ability when omitting data not fulfilling the HR/RR ratio and Crs validity criteria. We also assessed the impact of indexing PPV or SVV to TV or driving pressure on the predictive performance of these variables.
**METHODS.** MEDLINE was searched for relevant prospective studies published within the present decade. Authors were asked for sharing respiratory and hemodynamic data. Fluid responsiveness was defined as a fluid challenge induced increase in cardiac index (CI) or stroke volume (SV) (study specific) by 15% or more. Either PPV or SVV was analyzed. PPV was analyzed if PPV and SVV were both available. The area under the receiver operating characteristic curve (AUC) was determined for all patients and for sub-cohorts with omitted datasets where the HR/RR ratio or Crs limitations were violated. PPV and SVV were also indexed to VT and driving pressure and were similarly assessed.
**RESULTS.** 11 authors shared data, comprising in total 567 datasets; 256 (45%) were fluid responsive. TV was (mean ± sd) 7.1 ± 1.2 ml/kg, Crs was 36 ± 16 ml/cmH2O, HR/RR ratio was 5.1 ± 1.6. Low TV, low Crs, and low HR/RR ratio were found in 454, 197, and 97 subjects, respectively. PPV was available and used from 10 studies, SVV was available from seven studies and used from one study. The resulting variable is referred to as "dynamic variable" when AUCs are presented in table 1.

**CONCLUSIONS.** Omitting data based on the HR/RR ratio and lung compliance limitations and subsequently indexing dynamic variables to TV or driving pressure did not lead to satisfactory accuracy to predict fluid responsiveness. Only when all respiratory limitations were respected did PPV or SVV provide acceptable prediction, confirming their importance in the interpretation of functional hemodynamics.

**REFERENCE(S)**

1. De Backer D, *et al.* Intensive Care Med. 2005;31;517-523.
2. De Backer D, *et al.* Anesthesiology. 2009;110;1092-1097.
3. Monnet X, *et al.* Crit Care Med. 2012;40;152-157.
4. Mahjoub Y, *et al.* Br J Anaesth. 2014;112;681-685.

**Table 1 (abstract 0988).** AUCs for dynamic variable (PPV or SVV) predicting fluid responsiveness

| Variable | n | AUC | 95% CI |
|---|---|---|---|
| Dynamic variable in general | 567 | 0.71 | 0.67-0.75 |
| Dynamic variable; Crs ≥ 30 cmH2O and HR/RR ratio ≥ 3.6 | 236 | 0.77 | 0.71-0.83 |
| Dynamic variable indexed to TV; Crs ≥ 30 cmH2O and HR/RR ratio ≥ 3.6 | 236 | 0.80 | 0.74-0.85 |
| Dynamic variable indexed to driving pressure; Crs ≥ 30cmH2O and HR/RR ratio ≥ 3.6 | 236 | 0.78 | 0.73-0.84 |
| Dynamic variable, fulfilling all respiratory validity criteria (n = 59) | 59 | 0.88 | 0.78-0.97 |

### 0989

#### A novel mitochondrial monitor combining NADH fluorescence and tissue haemoglobin saturation within a single implantable probe

C. Smart[1], G. Ward[2], M. Singer[1]
[1]University College London, Bloomsbury Institute for Intensive Care Medicine, London, United Kingdom; [2]In Tandem Designs Pty Ltd, Warambatool, Australia
**Correspondence:** C. Smart
*Intensive Care Medicine Experimental* 2018, **6(Suppl 2):**0989

**INTRODUCTION.** Traditional resuscitative targets such as cardiac output, blood pressure and lactate are either up- or downstream of respiring tissue, but do not interrogate the tissues themselves. There is no measure of sufficiency, and the treatment goal is usually directed at normal resting values expected in health. Mitochondrial monitoring allows a direct assessment of the adequacy of oxygen delivery to support respiration.
Nicotinamide dinucleotide hydride (NADH) is the reduced moiety of NAD and the principal electron carrier from the Krebs' cycle to the electron transport chain (ETC). Under conditions of mitochondrial hypoxia, NADH levels rise. NADH is fluorescent under excitation by ultraviolet light whereas its oxidised counterpart (NAD$^+$) is not. This autofluorescence provides a natural window through which mitochondrial oxygen supply sufficiency can be interrogated in real time. Combining NADH and tissue haemoglobin saturation (StO$_2$) monitoring in one device allows direct assessment of the relationship between tissue oxygenation and oxygen sufficiency.
**OBJECTIVES.** To observe changes recorded by the combined probe during progressive fatal haemorrhage in an anesthetised rat model.
**METHODS.** A bespoke combined fibreoptic NADH and tissue oxygen saturation probe was implanted into the vastus lateralis thigh muscle of instrumented, anaesthetised Wistar rats. Tissue oxygen tension was recorded in the contralateral thigh (Oxylite, Oxford Optronix, Abingdon, UK). Ten percent of initial estimated blood volume (EBV, 66 ml/kg) was withdrawn via an indwelling carotid line every 15 minutes until cardio-respiratory arrest. At this point, the withdrawn blood volume was returned, and the animals observed for 20

minutes then euthanised. NADH fluorescence, tissue saturation, tissue $PO_2$ and arterial blood pressure were recorded continuously and arterial blood gas analysis sampling performed at intervals.

**RESULTS.** Initial tissue saturation varied from 50-70%, with an NADH fluorescence index of 0.15-0.22 units. Animals suffered cardiac arrest between 40-60% loss of EBV. Peri-arrest $StO_2$ was universally near zero, with NADH fluorescence rising to between 0.34 and 0.42 units. Animals responding to resuscitation renormalized NADH to baseline, whereas non-survivors continued to increment.

**CONCLUSIONS.** Monitoring both tissue oxygen saturation and NADH fluorescence is useful in delineating tolerated limits in haemorrhagic shock states. $StO_2 < 10\%$ and sustained NADH rises >0.35 units portend cardiovascular collapse. This form of combined monitoring may have utility in deeply shocked patients.

**GRANT ACKNOWLEDGMENT**

MRC Industrial Collaborative Studentship.

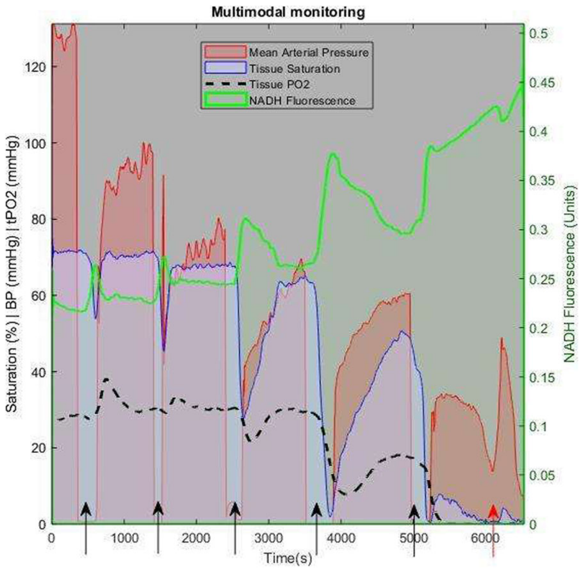

**Fig. 1 (abstract 0989).** Serial haemorrhage: non-survivor. 10% EBV drawn at arrows. Blood returned post-arrest at red arrow

### Preclininical studies

#### 0990

#### Opposite effects in diaphragm and soleus muscle of mTOR expression and activation, in experimental resistive work of breathing model

R. Pinochet[1], M. Escobar[1], J.L. Márquez[1], B. Erranz[2], F. Díaz[2,3], P. Cruces[4]
[1]Universidad Católica del Maule, Departamento de Kinesiolgía, Talca, Chile; [2]Facultad de Medicina Clínica Alemana - Universidad del Desarrollo, Santiago, Chile; [3]Clínica Alemana de Santiago, Pediatric Critical Care, Santiago, Chile; [4]Universidad Andres Bello, Centro de Investigación de Medicina Veterinaria, Santiago, Chile
**Correspondence:** P. Cruces
*Intensive Care Medicine Experimental* 2018, **6(Suppl 2):**0990

**INTRODUCTION.** Respiratory failure is a life-threatening condition characterized by impaired gas exchange and increased work of breathing (WOB), resulting in a decrease in motor activity. The serine/threonine kinase mammalian target of rapamycin (mTOR) is a key regulator of protein synthesis, cell proliferation and energy metabolism. Respiratory overload affects the diaphragm muscle, but its impact on physical inactivity and signaling pathways associated with protein synthesis in skeletal musculature, such as mTOR, are less known.

**OBJECTIVES.** To evaluate the effect of resistive overload on the expression and activation of the mTOR pathway in the diaphragm and soleus muscle and locomotion behavior.

**METHODS.** Twenty-two Sprague-Dawley rats were randomized to Tracheal Banding (TB) or Sham (SH) groups. After the procedure, subjects were extubated, and they were observed for 7 days. Motor activity was assessed on days 1 and 6 with 6 min - Open field test (OFT). At the end of the observation period, subjects were euthanized, and diaphragm and soleus muscle were extracted. mTOR expression (mTOR/total protein) and activity (p-$mTOR^{ser2448}$/mTOR) was measured by western blot. Variables expressed as mean and SEM.

**RESULTS.** All animals survived initial surgery. After surgery, animals allocated to TB group had stridor and tachypnea and SH had no respiratory distress. On the clinical follow up, TB group had a mortality of 41.7% and SH 0% (p< 0.01). There was a significant weight loss in both groups, being more pronounced in TB group.

On OFT there were no differences on day 1, but at day 6 distance traveled pf BT was 46% lower than SH. The expression and activity of mTOR in diaphragm was higher in BT compared to SH (figure1). On soleus muscle activity of mTOR was lower in BT group compared to SH (figure2).

**CONCLUSIONS.** In subjects with increased resistive WOB we found a suppression a mechanical load-induced growth via kinase-dependent pathway in soleus muscle associated to a decrease of locomotor behavior. Opposite effects were found in diaphragm muscle. The role of this molecular signaling pathway may help to identify the benefit of interventions to reduce disuse-induced muscle wasting.

**GRANT ACKNOWLEDGMENT**

FONDECYT 11160463; FONDECYT 1160631; Intramural grant U. Católica del Maule 2012

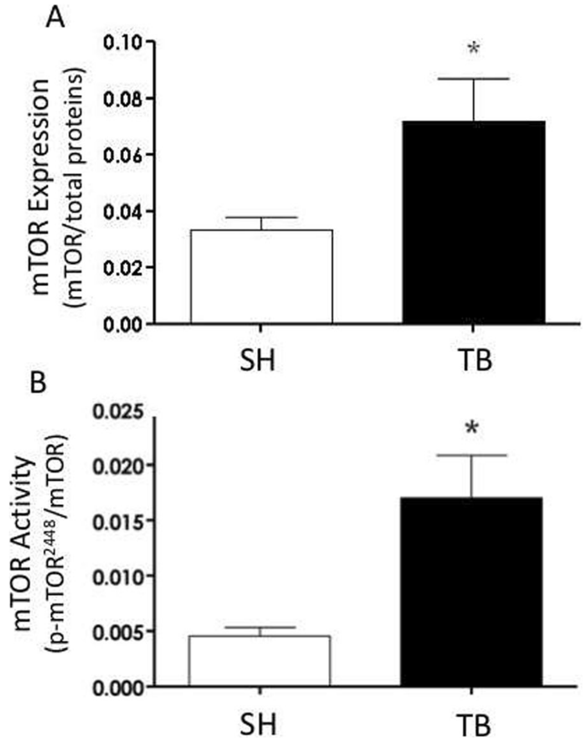

**Fig. 1 (abstract 0990).** mTOR expression (panel A) and activity (panel B) in diaphragm muscle. (p<0.05)]

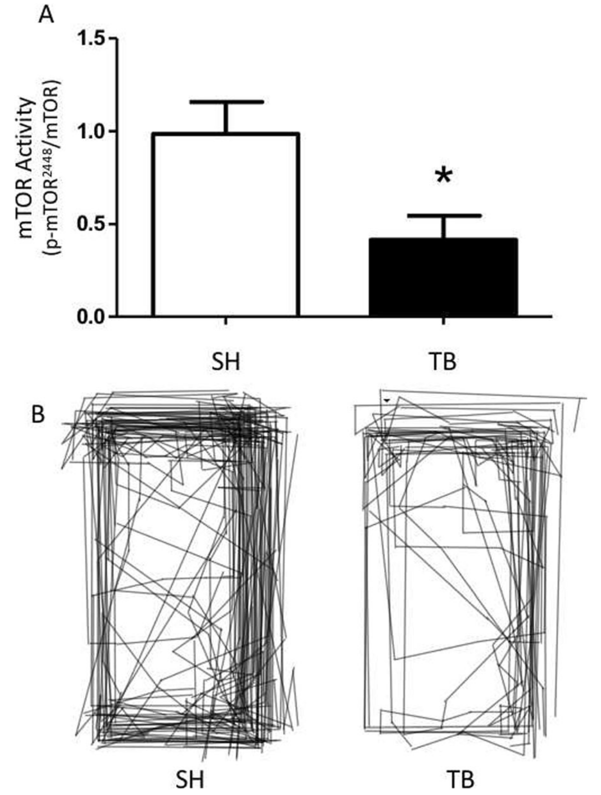

**Fig. 2 (abstract 0990).** mTOR activity (panel A) in soleus muscle and Locomotion behavior in SH and BT. (*p<0.05)

### 0991

#### Relationships between transpulmonary pressures and computed tomography in experimental ARDS

J.-C. Richard[1,2,3], M. Orkisz[2,3], M. Hernández Hoyos[4], A. Morales Pinzón[3,4], C. Guérin[1,2,5]

[1]Hospices Civils de Lyon, Service de Réanimation Médicale - Hôpital de la Croix-Rousse, Lyon, France; [2]Université Claude Bernard Lyon 1, Lyon, France; [3]CREATIS INSERM 1044 CNRS 5220, Villeurbanne, France; [4]Universidad de los Andes, Systems and Computing Engineering Department, School of Engineering, Bogotá, Colombia; [5]IMRB, INSERM 955Eq13, Créteil, France

**Correspondence:** J.-C. Richard

*Intensive Care Medicine Experimental* 2018, **6(Suppl 2):**0991

**INTRODUCTION.** Using esophageal pressure to compute transpulmonary pressure is an appealing technique to individualize PEEP setting in ARDS. Transpulmonary pressure can be computed 1. as the difference between airway pressure and esophageal pressure (absolute measurement $PL_{ABS}$); 2. as the product of airway pressure by the ratio of lung elastance to respiratory system elastance (elastance-derived measurement $PL_{EL}$).

**OBJECTIVES.** The aim of the study was to evaluate the relationships between both measurements of transpulmonary pressures and computed tomography-derived measurements in an experimental model of ARDS.

**METHODS.** Experimental ARDS was performed by saline lavage on 16 piglets. A recruitment maneuver (sustained inflation to 40 cmH2O for 30 seconds) was then performed followed by mechanical ventilation with tidal volume 6 ml/kg body weight. PEEP was initially set to 20 cmH2O then decreased by 2 cmH2O-steps down to 2 cmH2O. Piglets were then randomized into 3 PEEP groups based on best dynostatic compliance during PEEP trial, best end-expiratory lung volume during PEEP trial, or a PEEP-FiO2 table. Finally, 7 levels of VT ranging from 4 to 20 ml/kg were applied at optimal PEEP . Transpulmonary pressure measurements and computed tomography were performed at end-expiration and end-inspiration in the following conditions: at baseline after ARDS onset, during the PEEP trial, 1 hour after setting optimal PEEP, and during the variable VT trial.

**RESULTS.** $PL_{ABS}$ ranged from -6 to 23 cmH2O at end-expiration and 5 to 58 cmH2O at end-inspiration. $PL_{EL}$ ranged from 1 to 23 cmH2O at end-expiration and 5 to 65 cmH2O at end-inspiration. $PL_{ABS}$ and $PL_{EL}$ were significantly correlated (r=0.80, p< 0.001). Bias between $PL_{EL}$ and $PL_{ABS}$ amounted to 4 cmH2O (limits of agreement ranging from -2 to 11 cm H2O).

Non-inflated compartment significantly increased in deciles of end-expiratory $PL_{ABS}$ below 4.8 cmH2O (Figure 1), and in deciles of end-expiratory $PL_{EL}$ below 11.8 cmH2O. Table 1 provides the diagnostic performance of end-expiratory $PL_{ABS}$ and $PL_{EL}$ to detect a non-inflated compartment below 5% and 10% of total lung volume, respectively.

Overinflated compartment significantly increased in deciles of end-inspiratory $PL_{ABS}$ above 25.8 cmH2O and in deciles of $PL_{EL}$ above 30.7 cmH2O. ROC curve identified an end-inspiratory $PL_{ABS}$ above 19.6 cmH2O and an end-inspiratory $PL_{EL}$ above 24.6 cmH2O as best thresholds to identify an overinflated compartment greater than 2% of the total lung volume (table 1). There was no significant difference between area under ROC curve (AUC) assessed with both techniques.

**CONCLUSIONS.** Both measurement techniques of transpulmonary pressure have similar diagnostic performance to identify near complete alveolar recruitment at end-expiration and hyperinflation at end-inspiration. Near complete alveolar recruitment may be achieved with $PL_{ABS}$ greater than 5 cmH2O and $PL_{EL}$ greater than 10 cmH2O.

**GRANT ACKNOWLEDGMENT**

General Electric

**Table 1 (abstract 0991).** See text for description.

| | Classification | Sensitivity [95% CI] | Specificity [95% CI] | Threshold [95% CI] | AUC |
|---|---|---|---|---|---|
| $PL_{ABS}$ | Non-inflated cpt. > 5% of lung volume | 87% [81%-92%] | 87% [80%-93%] | 5.2 | 0.94 [0.91-0.97] |
| $PL_{EL}$ | Non-inflated cpt. > 5% of lung volume | 94 % [89%-97%] | 79% [71%-86%] | 10.3 | 0.93 [0.90-0.96] |
| $PL_{ABS}$ | Non-inflated cpt. > 10% of lung volume | 86% [78%-92%] | 86% [80%-91%] | 3.8 | 0.92 [0.89-0.96] |
| $PL_{EL}$ | Non-inflated cpt. > 10% of lung volume | 85% [77%-91%] | 85% [79%-90%] | 7.6 | 0.92 [0.89-0.95] |
| $PL_{ABS}$ | Over-inflated cpt. > 2% of lung volume | 0.91 [0.59-1] | 0.81 [0.76-0.86] | 19.6 | 0.91 [0.85-0.98] |
| $PL_{EL}$ | Over-inflated cpt. > 2% of lung volume | 0.91 [0.59-1] | 0.79 [0.73-0.83] | 24.6 | 0.91 [0.83-0.98] |

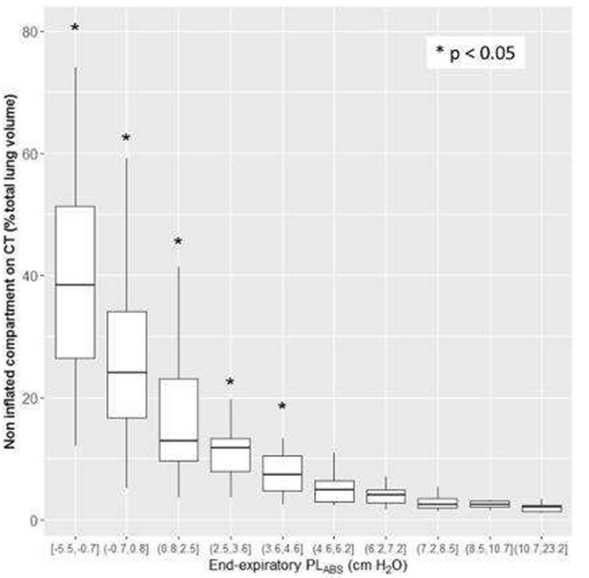

**Fig. 1 (abstract 0991).** See text for description.

### 0992

#### Effect of three PEEP strategies on cyclic recruitment-derecruitment in experimental ARDS

J.-C. Richard[1,2,3], M. Orkisz[2,3], M. Hernández Hoyos[4], A. Morales Pinzón[2,5], C. Guérin[1,3,6]

[1]Hospices Civils de Lyon, Service de Réanimation Médicale - Hôpital de la Croix-Rousse, Lyon, France; [2]CREATIS INSERM 1044 CNRS 5220, Villeurbanne, France; [3]Université Claude Bernard Lyon 1, Lyon, France; [4]Universidad de los Andes, Systems and Computing Engineering Department, School of Engineering, Bogotá, Colombia; [5]Universidad de Los Andes, Systems and Computing Engineering Department, School of Engineering, Bogotá, Colombia; [6]IMRB, INSERM 955Eq13, Créteil, France

**Correspondence:** J.-C. Richard

*Intensive Care Medicine Experimental* 2018, **6(Suppl 2):**0992

**INTRODUCTION.** Individualization of PEEP setting based on bedside measurements of lung characteristics during ARDS is an appealing strategy to minimize ventilator-induced lung injury. Cyclical recruitment-derecruitment related to tidal volume is one of the mechanisms involved in the deleterious effect of mechanical ventilation. We hypothesize that PEEP setting aiming at maximizing dynamic compliance or end-expiratory lung volume (EELV) would reduce tidal recruitment in experimental ARDS.

**OBJECTIVES.** To compare the effect on tidal recruitment of PEEP strategies based on individual respiratory mechanics measurement and a PEEP-FiO2 table.

**METHODS.** Experimental ARDS was performed by saline lavage on 15 piglets under mechanical ventilation. A recruitment maneuver (40 cm H2O for 30 seconds) was then performed followed by mechanical ventilation with tidal volume 6 ml/kg body weight. PEEP was initially set to 20 cmH2O then decreased to 2 cmH$_2$O by 2 cmH$_2$O-steps lasting 2 minutes each. Immediately after lung injury (T1), and at each PEEP step, the following measurements were performed: airway and dynostatic pressures, airflow, esophageal pressure, EELV using the nitrogen washin-washout technique, blood gas, and whole lung CT scan during end-expiratory and end-inspiratory pauses. Following a second recruitment maneuver, piglets were randomized into 3 PEEP strategies (5 animals each): PEP set using a PEEP-FiO2 table, PEEP set 2 cm H2O above PEEP of maximal dynostatic compliance, and PEEP set 2 cm H2O above PEEP of maximal end-expiratory lung volume. Tidal volume was further adjusted to keep plateau pressure below 30 cm H2O. A last set of measurements was performed after 1 h PEEP application (T2).

**RESULTS.** Measurement are reported in the table below. Groups were not different before randomization, except for a significantly lower amount of tidal recruitment on CT in the PEEP-FiO2 group. Set PEEP after randomization was significantly lower in the PEEP-FiO2 group, intermediate in the maximal compliance group, and significantly higher in the maximal EELV group. Tidal hyperinflation was not different between groups. Change in tidal recruitment from T1 was significantly lower in the maximal EELV group.

**CONCLUSIONS.** Setting PEEP using a PEEP-FiO2 table or dynamic compliance was not associated with detectable differences in respiratory mechanics, EELV or computed tomography after one hour of application. Minimization of tidal recruitment requires a very high PEEP level even in an easily recruitable experimental model of ARDS.

**REFERENCE(S)**

**GRANT ACKNOWLEDGMENT**

General electric.

**Table 1 (abstract 0992).** See text for description

| Variables | Group | T1 | PEEP trial at PEEP = PEEP T2 | T2 |
|---|---|---|---|---|
| VT (ml/kg body weight) | PEEP-FiO2 table Max. compliance Maximal EELV | 10.0±0.0 | 6.0±0.0 | 6.0±0.0 |
| | | 10.0±0.0 | 6.0±0.0 | 6.0±0.0 |
| | | 10.0±0.0 | 6.0±0.0 | 5.2±0.8 ‖,* |
| PEEP (cmH2O) | PEEP-FiO2 table Max. compliance Maximal EELV | 0±0 | 6±1 | 6±1 |
| | | 0±0 | 10±2 ‖ | 10±2 ‖ |
| | | 0±0 | 18±3 ‖,* | 18±3 ‖,* |
| Plateau pressure (cmH2O) | PEEP-FiO2 table Max. compliance Maximal EELV | 28±4 | 17±3 | 17±2 |
| | | 28±3 | 18±2 | 21±4 |
| | | 30±6 | 30±5 ‖,* | 28±3 ‖,* |
| Driving pressure (cmH2O) | PEEP-FiO2 table Max. compliance Maximal EELV | 25±4 | 10±4 | 10±3 |
| | | 24±3 | 7±1 | 10±3 |
| | | 24±5 | 11±3 | 10±2 |
| Absolute transpulmonary pressure - End-expiration (cmH2O) | PEEP-FiO2 table Max. compliance Maximal EELV | -1±1 | 2±2 | 2±2 |
| | | -1±3 | 4±2 | 5±3 |
| | | -1±2 | 10±4 ‖,* | 9±3 ‖,* |
| Absolute transpulmonary pressure - End-inspiration (cmH2O) | PEEP-FiO2 table Max. compliance Maximal EELV | 20±4 | 10±3 | 10±3 |
| | | 20±2 | 10±2 | 13±4 |
| | | 18±4 | 20±6 ‖,* | 17±3 ‖ |
| Tidal Hyperinflation on CT (% of VT) | PEEP-FiO2 table Max. compliance Maximal EELV | 2.5±1.4 | 1.8±0.3 | 2.3±0.7 |
| | | 2.0±1.0 | 1.1±0.4 | 2.4±1.2 |
| | | 1.9±1.2 | 1.9±2.2 | 4.8±5.3 |
| Tidal recruitment on CT (% of VT) | PEEP-FiO2 table Max. compliance Maximal EELV | 90±28 | 23±7 | 28±10 |
| | | 60±11 ‖ | 12±5 ‖ | 19±10 ‖ |
| | | 47±30 ‖ | 5±2 ‖ | 6±3 ‖ |
| Tidal recruitment on CT (% change from T1) | PEEP-FiO2 table Max. compliance Maximal EELV | 0±0 | -83±10 | -82±13 |
| | | 0±0 | -89±4 | -83±7 |
| | | 0±0 | -93±2 | -93±4 ‖ |

‖ p< 0.05 vs PEEP-FiO2 table; * p< 0.05 vs maximal compliance

### 0993

#### Impact of different positive end-expiratory pressure on lung mechanics, gas exchange and circulation in the setting of saline lavage induced lung injury and moderate intraabdominal hypertension: a pig model

E. Simeliunas[1], A. Kalenka[2], M. Fiedler[1], B.L. Deutsch[3], D. Diktanaite[1]
[1]Heidelberg University Hospital, Clinic of Anesthesiology, Heidelberg, Germany; [2]Hospital Bergstrasse, Department of Anesthesiology and Critical Care Medicine, Heppenheim, Germany; [3]Justus-Liebig-University, Faculty of Medicine, Giessen, Germany
**Correspondence:** E. Simeliunas
*Intensive Care Medicine Experimental* 2018, **6(Suppl 2):**0993

**INTRODUCTION.** Critically-ill patients in acute respiratory failure require appropriate PEEP levels to keep sufficient gas exchange. Measuring transpulmonary pressure ($P_L$), transpulmonary gradient ($\Delta P_L$), esophageal pressure, end-expiratory lung volume (EELV), pulmonary compliance (Cstat) and intraabdominal pressure (IAP) is helpful to determine ventilator settings. We analyzed these parameters in swines with intraabdominal hypertension and saline lavage induced acute lung injury.

**OBJECTIVES.** To compare the effect of different PEEP levels in lung mechanics, gas exchange, cardiocirculatory parameters at induced clinical picture.

**METHODS.** Swines received general anesthesia, were tracheotomized and supplied with an esophageal balloon, intraabdominal balloon to induce intraabdominal hypertension, central venous and PiCCO® lines and bladder catheter. After inducing lung injury by surfactant washout and nonprotective ventilation, animals were randomised into 3 groups (Group A=PEEP 5 $cmH_20$, B=10 or C=15) and ventilated in pressure controlled/volume guaranteed mode with a tidal volume of 8ml/kg bodyweight for 6 hours. 10 mmHg IAP was generated and maintained in all groups after inducing lung injury at hour 0. EELV, Cstat, esophageal pressures, $P_L$, $\Delta P_L$, IAP and circulatory parameters were measured at baseline, hour 0 and every 2 hours until hour 6.

**RESULTS.** 18 swine (N=18) were included in the study.There were no significant differences between groups at the baseline except higher chest wall elastance in group C (p=0,012). EELV, Cstat, Horowitz index decreased, $\Delta P_L$ and driving pressures increased after inducing lung injury in all groups. In group A there was an increase in $\Delta P_L$ (16.2±2.4 vs. 21.5±4.3, p=0,003) and driving pressure (22.3±2.4 vs. 27.7±3.9, p=0,003), decrease of Cstat (22.2±3.3 vs. 18.2±3.1, p< 0,001) and stable EELV/kg (15.1±3.2 vs. 14.2±5.5, p=0,35) compared hours 0 and 6. EELV increased at PEEP 10 (11.6±4.2 vs. 13.5±6, p=0,04). Elevated values of EELV (12.6±1.9 vs. 19.3±5.2, p=0,017), Cstat (18.7 ±4 vs. 24.2±4.5, p= 0,02) and lower driving pressure were seen at PEEP 15. Horowitz Index increased in group A 1.5±0.3 (p=0,01), B 2.0 ±0.4 (p=0,01) and C 5±3.4 (p=0,002) times respectively. The heart index decreased at PEEP 10 (4.4±0.7 vs. 4±0.4, p=0,02) and PEEP 15 (5.2±0.9 vs. 3.7±0.2, p=0,009). Comparing all groups $\Delta P_L$ decreased with increasing PEEP: 21.5±4.3 (A), 18.2±4.5 (B), 14.3±2.2 (C) respectively (p=0,03). Higher $\Delta P_L$ values were associated with lower Cstat and EELV at hour 0 (R= 0.75, p< 0,001) and 6 (R=0.7, p=0,001) respectively.

**CONCLUSIONS.** Higher PEEP can improve lung mechanics and gas exchange in moderate abdominal hypertension with acute lung injury in a swine model.

Akoumianaki et al. The application of esophageal pressure measurement in patients with respiratory failure. Am J Respir Crit Care Med. 2014 Mar 1;189(5):520-31.

##### GRANT ACKNOWLEDGMENT

Study was financed by Hospital Bergstrasse and University Hospital Heidelberg.

### 0994

#### Metabolic activity in atelectactic regions differs among mechanisms of lung injury

T. Hinoshita[1], G. Motta-Ribeiro[1], C. Zeng[1], T. Winkler[1], M. R. Tucci[2], N. de Prost[3], T. J. Wellman[4], M. F. Vidal Melo[1]
[1]Massachusetts General Hospital, Anesthesia, Critical Care and Pain Medicine, Boston, United States; [2]University of São Paulo, Cardio-Pulmonary, São Paulo, Brazil; [3]Hôpital Henri Mondor, Medical Intensive Care, Créteil, France; [4]inviCRO, LLC, Boston, United States
**Correspondence:** T. Hinoshita
*Intensive Care Medicine Experimental* 2018, **6(Suppl 2):**0994

**INTRODUCTION.** Atelectasis is frequently present during acute lung injury resulting in hypoxemia and a risk for infection. Yet, imaging studies of atelectasis have been mostly restricted to the identification of lung densities either on chest X-ray or Computed Tomography (CT) images. Positron Emission Tomography (PET) imaging with 2-deoxy-2-[(18)F] fluoro-D-glucose (FDG) allows for the quantification of tissue metabolism predominantly related to neutrophilic inflammation.

**OBJECTIVES.** To use PET-FDG techniques to investigate the *in vivo* metabolism of atelectatic regions in distinct types of acute lung injury.

**METHODS.** Supine sheep (n=23, 20.6 ± 3.6 kg) were anesthetized, intubated, and mechanically ventilated. Four conditions were studied: 1) 16 hours mechanical ventilation (MV16h, n=6); 2) 24hours MV with low dose continuous intravenous Lipopolysaccharide (MV24h-lowLPS, 2.5 ng/kg/min, n=5); 3) 24hours MV with high dose continuous intravenous LPS (MV24h-highLPS, 10.0 ng/kg/min, n=6); and 4) 6hours MV with surfactant depletion by lung lavages (Surfactant, n=6). Animals with atelectasis at baseline in Groups MV16h, MV24h-lowLPS, and MV24h-highLPS served as controls (n=8). Ventilation settings were: Group MV16h: tidal volume ($V_T$)=6-8 mL/kg, positive end-expiratory pressure (PEEP)=0 $cmH_2O$, and inspired oxygen fraction ($F_IO_2$)=0.3; Groups MV24h-lowLPS and MV24h-highLPS: based on ARDSNet low PEEP protocol, i.e., $V_T$=6-8 mL/kg, PEEP=5 $cmH_2O$, and $F_IO_2$=0.3; and Group Surfactant: $V_T$=10 mL/kg, PEEP=10 $cmH_2O$, and $F_IO_2$=0.6. PET-FDG was used to compute FDG net uptake rate (Ki) and FDG phosphorylation rate (k3) in atelectatic regions. These were derived from CT (= gas content < 10%).

**RESULTS.** Ki was significantly higher in the atelectatic regions of group MV24h-highLPS, approximately two-fold, than in groups controls and Surfactant (Figure). However, k3 didn't show a distinction between groups. Moreover, group MV16h had similar Ki to group MV24h-lowLPS, which was ventilated even 8 hours longer. Meanwhile, Ki were similar in groups controls and Surfactant.

**CONCLUSIONS.** Although all atelectatic regions look similar in CT scan, FDG uptake levels are distinct depending on experimental conditions. This study reveals that LPS affects tissue metabolic activity concentration-dependently. High dose LPS resulted in the highest FDG uptake rate in atelectatic regions, suggestive of increased neutrophilic inflammation. This implies that "lung rest" (permissive atelectasis) does not guarantee the presence of unaltered metabolic activity and inflammation even during protective ventilation.

##### GRANT ACKNOWLEDGMENT

Supported by the grant NIH R01HL121228

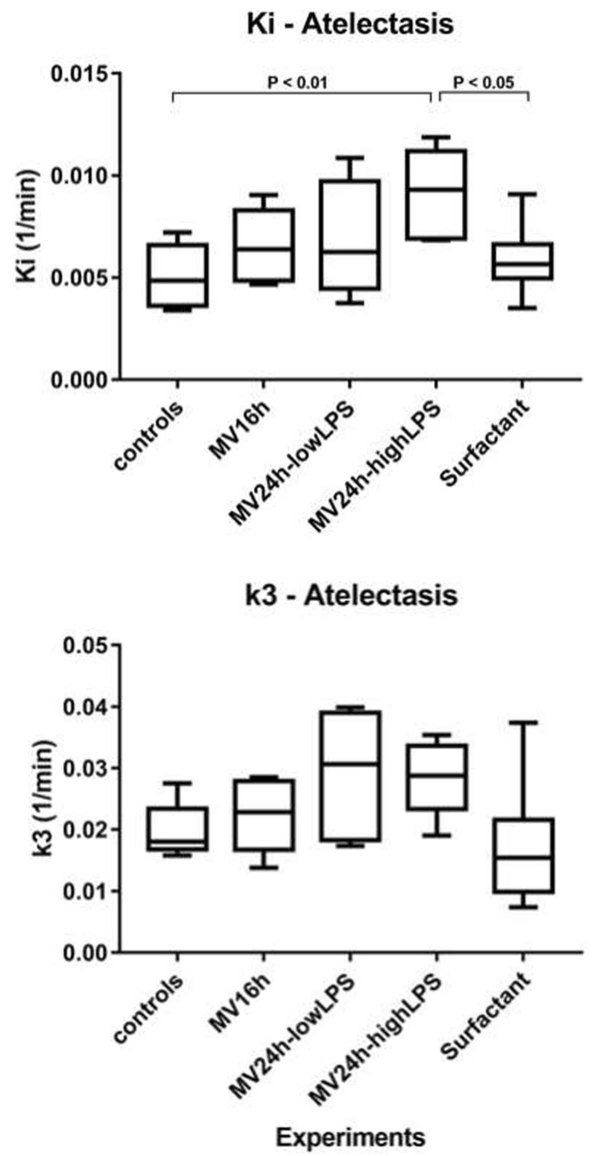

**Fig. 1 (abstract 0994).** PET-FDG kinetic parameters

## Management of patients with sepsis

### 0995

#### Adjunctive intravenous anti-toxin treatment with CAL02 in severe acute community-acquired pneumonia due to *Streptococcus pneumoniae*. A first-in-man, multicentre, double-blind, placebo-controlled study

B. Francois[1,2], G. Colin[3], P.-F. Dequin[4], T. Dugernier[5], T. Boulain[6], S. Azeredo da Silveira[7], F. Lajaunias[7], A. Perez[7], P.-F. Laterre[8]
[1]CHU Dupuytren, Medical-Surgical ICU, Limoges, France; [2]CHU Dupuytren, Inserm CIC-1435, Limoges, France; [3]CHD Les Oudairies, Medical-Surgical ICU, La Roche-sur-Yon, France; [4]CHRU de Tours, Medical-Surgical ICU, Tours, France; [5]Clinique St Pierre, Intensive Care, Ottignies, Belgium; [6]Hôpital La Source CHR Orléans, Service de Médecine Intensive Réanimation, Orléans, France; [7]Combioxin SA, Geneva, Switzerland; [8]St Luc University Hospital, Intensive Care Unit, Brussels, Belgium
**Correspondence:** B. Francois
*Intensive Care Medicine Experimental* 2018, **6(Suppl 2):**0995

**INTRODUCTION.** CAL02 is a novel antimicrobial liposomal agent that acts as a toxin-trap by mimicking microdomains targeted by bacterial virulence factors. CAL02 is active against pore-forming toxins, enzymes and appendages involved in the progress and severity of infections including those caused by drug-resistant pathogens. Preclinical data showed synergistic effects with antibiotics and ability to cure mice from deadly infections caused by Gram+ and Gram- pathogens. We present herein results from the first-in-man trial in intensive-care-unit (ICU) patients with severe community-acquired pneumococcal pneumonia (CAPP).

**METHODS.** CAL02-001 is a randomized, multicenter, double-blind, placebo-controlled trial carried out in adult patients admitted to the ICU due to severe CAPP defined by mechanical ventilation or vasopressors or 3 minor criteria. Two dosing regimens of CAL02, Low Dose (4 mg/kg) and High Dose (16 mg/kg), were compared to a Placebo, in addition to standard-of-care antibiotic therapy. CAL02 was administered QD on 2 consecutive days, starting within 12h of the onset of CAPP severity and within 24h of IV antibiotics. The primary objective was safety. Efficacy and pharmacodynamic (PD) parameters were also assessed.

**RESULTS.** A total of 19 patients were randomized to the Placebo (n=5), CAL02 High Dose (n=11) and Low Dose (n=3) arms. One patient from the High Dose arm was deleted before unblinding due to a major eligibility criteria violation. At baseline, 56% of patients were in septic shock, the average APACHE II was 21.3 and SOFA 7.4. Baseline characteristics were homogeneous across arms, although the CAL02 High and Low Dose arms proved slightly more severe than the Placebo arm, with an APACHE II of 22.1 and 25.3 vs 17.4, respectively. The 3 Low Dose patients were all intubated and in septic shock. CAL02 demonstrated to be safe and well tolerated. No differences in the incidence and profile of adverse events were observed between the 3 arms.

A 50% decrease in the SOFA score was already achieved after 5 days in the CAL02 arms. The APACHE II score and PD biomarkers (CRP, PCT, IL-6) also decreased more rapidly with CAL02. Duration of ICU stay was significantly reduced, from 12 days in the Placebo arm to 5 days in CAL02 High Dose arm (p< 0.05).

**CONCLUSIONS.** This is the first clinical trial assessing CAL02, a first-in-class broad-spectrum anti-toxin agent. Safety primary endpoints were met. Despite being more severe than those in the Placebo arm, CAL02-treated patients showed a more rapid clinical improvement. Efficacy trends were consistent across different clinical and biological parameters with both a faster resolution in organ dysfunction and a faster decrease in PD biomarkers. These observations are in line with the mechanism of action of CAL02, which aims at protecting against toxin-mediated organ damages and inflammation. These promising efficacy superiority signals need to be confirmed in a larger Phase 2 trial.

| | Placebo (n = 5) | CAL02 High Dose (n = 10) | CAL02 Low Dose (n = 3) |
|---|---|---|---|
| Mortality (n) | 1 (20%) | 1 (10%) | 1 (33%) |
| Cured at Early TOC (Day 8) | 1/5 (20%) | 5/10 (50%) | 0/3 (0%) |
| Cured at TOC (pre-defined between Day 15 and 22) | 100% | 100% | 66% |
| Time to cure – *median (range), in days* | 10 (7-14) | 8 (6-16) | 15 (14-16) |

**Fig. 1 (abstract 0995).** See text for description.

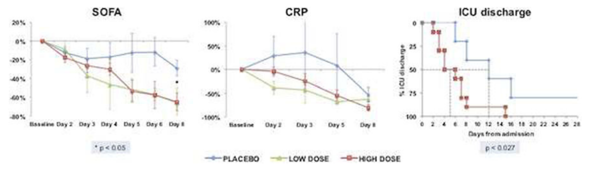

**Fig. 2 (abstract 0995).** See text for description.

### 0996

#### Antimicrobial combination therapy in septic patients: a systematic review and meta-analysis

G. Vázquez-Grande[1,2], C. Graydon[2], H. Loewen[3], R. Rabbani[4], A.M. Abou-Setta[4,5], R. Zarychanski[1,4,5], A. Kumar[1,2]
[1]University of Manitoba, Section of Critical Care, Winnipeg, Canada; [2]University of Manitoba, Medical Microbiology, Winnipeg, Canada; [3]University of Manitoba, Neil John Maclean Health Sciences Library, Winnipeg, Canada; [4]University of Manitoba, George & Fay Yee Center for Healthcare Innovation, Winnipeg, Canada; [5]University of Manitoba, Department of Community Health Sciences, Winnipeg, Canada
**Correspondence:** G. Vázquez-Grande
*Intensive Care Medicine Experimental* 2018, **6(Suppl 2):**0996

**INTRODUCTION.** Sepsis is the major cause of infection-associated mortality and remains among the leading causes of death in intensive care units (ICUs) worldwide.

**OBJECTIVES.** To evaluate the efficacy and safety of antimicrobial combination therapy (defined as at least two antimicrobials of different mechanistic class) in patients with sepsis.

**METHODS.**

**Data Sources:** Randomized controlled trials from PubMed, EMBASE, CENTRAL, Scopus (inception to February 2017), reference lists of relevant articles, grey literature.

**Selection criteria:** Two reviewers independently identified randomized controlled trials comparing antimicrobial monotherapy with combination therapy in critically ill patients with sepsis or septic shock.

**Data collection:** Two reviewers independently extracted trial-level data including patient characteristics, interventions, outcomes, and funding source. Risk of bias of included studies was assessed using the Cochrane Risk of Bias tool.

**RESULTS.** The literature search identified 2725 citations, of which 36 were deemed to be potentially relevant. Ten publications met the inclusion criteria and were included in the meta-analysis. All the included trials had high risk of bias (no blinding). Combination therapy showed no significant difference in mortality compared to monotherapy (RR 1.04, CI 95% 0.89 to 1.20, $I^2$ 0%; 9 trials; 2206 participants). None of the subgroup analyses or secondary outcomes showed significant differences between groups. Drug reported adverse events were significantly higher in the combination therapy arm (RR 0.75, CI 95% 0.60 to 0.93, $I^2$ 15%; 7 trials; 1609 participants).

**CONCLUSIONS.** Combination therapy may not have a benefit over monotherapy in septic patients. At this time the available evidence doesn't support or recommend combination therapy for the treatment of sepsis or septic shock. As we were unable to discriminate based on severity of illness, a meta-regression adjusting by risk of death is needed to indicate whether patients may benefit from combination therapy. Large randomized controlled trials looking at patient-related outcomes are required to further explore the benefits and harms of this intervention.

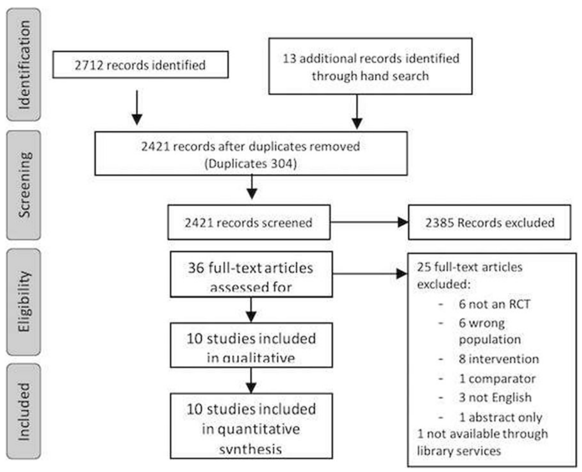

**Fig. 1 (abstract 0996).** PRISMA flow diagram

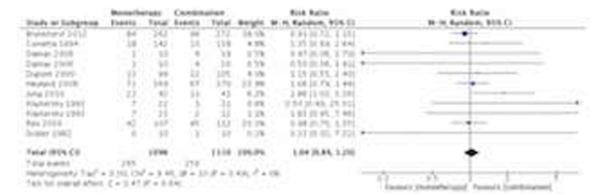

**Fig. 2 (abstract 0996).** Primary outcome meta-analysis: Mortality

**Table 1 (abstract 0996).** Studies characteristics

| Study | Year | Continent | Total N (monotherapy/combination) | Single/Multi center | Population | Primary AB | Secondary AB |
|---|---|---|---|---|---|---|---|
| Klastersky[27] | 1973 | Europe Belgium | 70 (22/23/23) | Single center | Disseminated cancer and life-threatening infection | Carbenicillin Gentamicin | |
| Sculier[28] | 1982 | Europe Belgium | 20 (10/10) | Single center | Neurosurgical patients with Gram negative VAP | Mezlocillin | Sisomicin |
| Sage[29] | 1987 | Europe United Kingdom | 93 (34/32/27) | Single center | Hospitalized adults (>14) with suspected life-threatening sepsis by Enteriobacteriaceae or Staphylococci | Cefotaxime | Netilmicin |
| Cometta[30] | 1994 | Europe Switzerland | 280 (142/138) | Multicenter | Adults (>16) with nosocomial pneumonia, nosocomial sepsis or severe diffuse peritonitis | Imipenem | Netilmicin |
| Dupont[31] | 2000 | Europe France | 204 (99/105) | Multicenter | Adults (>18) with complicated intraabdominal infeccions (severe sepsis and or nosocomial) | Piperacillin Tazobactam | Amikacin |
| Rex[32] | 2003 | North America United States | 219 (107/112) | Multicenter | >13 with confirmed candidemia and sepsis criteria | Fluconazole | Ampho B |
| Damas[33] | 2006 | Europe Belgium | 59 (20/19/20) | Single center | Adults (>18) mechanically ventilated with confirmed VAP | Cefepime | Amikacin Levofloxacin |
| Heyland[34] | 2008 | North America Canada | 739 (370/369) | Multicenter | Mechanically ventilated adults (>18)with suspected VAP | Meropenem | Ciprofloxacin |
| Jung[35] | 2010 | Asia Korea | 83 (42/41) | Single center | Adults (>18) with suspected nosocomial pneumonia and sepsis (CPIS >6) and in MICU | Vancomycin | Rifampicin |
| Brunkhorst[36] | 2012 | Europe Germany | 551 (273/278) | Multicenter | Severe sepsis and septic shock adults | Meropenem | Moxifloxacin |

### 0997

#### Lactated ringer versus albumin in early sepsis therapy (The RASP study): a randomized clinical trial

C. Park[1], J.P. Almeida[1], G. Landoni[2], G. Oliveira[1], M. Mourao[1], S. Rizk[1], F. Galas[1], R. Nakamura[1], J. Fukushima[1], P. Nardelli[3], J.-L. Vincent[4], R. Kalil-Filho[1], J.O. Auler Jr.[1], L.A. Hajjar[1]
[1]Instituto do Cancer do Estado de Sao Paulo, Sao Paulo, Brazil; [2]IRCCS San Raffaele Scientific Institute, Anesthesia and Critical Care, Milano, Italy; [3]IRCCS San Raffaele Scientific Institute, Milano, Italy; [4]Erasme Hospital,Université Libre de Bruxelles, Intensive Care, Brussels, Belgium
**Correspondence:** L.A. Hajjar
*Intensive Care Medicine Experimental* 2018, **6(Suppl 2):**0997

**INTRODUCTION.** In the last years, the use of albumin to resuscitate septic patients became highly controversial. We conducted an RCT to compare the effects of a 4% albumin solution on crystalloid solution on survival as compared to crystalloid solution alone during the early phase of resuscitation in septic patients.

**OBJECTIVES.** The aim of his study was to investigate the effects of the administration of albumin and crystalloids, as compared with crystalloids alone, in a population of cancer patients with sepsis.

**METHODS.** We performed a single centre, randomized, double-blind, controlled-parallel trial between October 2014 to December 2016 at the Cancer Institute of the University of Sao Paulo, Brazil. Cancer patients with severe sepsis or septic shock were randomly assigned to receive either bolus of 500 ml of 4% albumin in a crystalloid solution or crystalloid solution alone during the first 6 hours of fluid resuscitation. Primary outcome was defined as death from any cause at 7 days. Secondary outcomes were death from any cause at 28 days, the Sequence Organ Failure Assessment (SOFA), need for invasive mechanical ventilation, vasopressor therapy, renal replacement therapy, length of stay in the ICU and in the hospital.

**RESULTS.** A total of 360 patients were included. At 7 days, 46 of 180 patients (25.6%) in the albumin group and 40 of 180 patients (22.2%) in the crystalloid group died (absolute difference 3.3; 95% confidence interval [CI], -5.5 to 12.1; P=0.46). At 28 days, 96 of 180 patients (53.3%) in the albumin group and 83 of 180 patients (46.1%) in the crystalloid group had died (absolute difference 7.2; 95% confidence interval [CI], -3.3 to 17.3; P=0.17). No significant differences in secondary outcomes were observed between groups.

**CONCLUSIONS:** Adding albumin to early standard resuscitation with crystalloids, in cancer patients with sepsis had no effect on survival at 7 and 28 days (NCT01337934).

**REFERENCE(S)**

1- Caironi P, Tognoni G, Masson S, Fumagalli R, Pesenti A, Romero M, Fanizza C, Caspani L, Faenza S, Grasselli G, Iapichino G, Antonelli M, Parrini V, Fiore G, Latini R, Gattinoni L; ALBIOS Study Investigators. Albumin replacement in patients with severe sepsis or septic shock. N Engl J Med. 2014 Apr 10;370(15):1412-21.

**GRANT ACKNOWLEDGMENT**

This study was sponsored by the Universidade de São Paulo, Brazil.

### 0998

#### The restrictive intravenous fluid trail in severe sepsis and septic shock (RIFTS): a pilot study

K. Corl[1,2], M. Prodroumo[1], S. Marks[3], C. Delcompare[1], A. Palmasciano[1], R. Merchant[3], M. Levy[1]
[1]Brown Medical School, Division of Pulmonary Critical Care Medicine, Providence, United States; [2]Brown University School of Public Health, Providence, United States; [3]Brigham and Women's Hospital, Department of Emergency Medicine, Boston, United States
**Correspondence:** K. Corl
*Intensive Care Medicine Experimental* 2018, **6(Suppl 2):**0998

**INTRODUCTION.** Clinicians are re-examining the risks and benefits of a high volume intravenous (IV) fluid resuscitation strategy for severe sepsis and septic shock. The original early goal directed therapy (EGDT) and subsequent validation trials administered 90-160 ml/kg of IV fluid over 72 hours, and have shaped clinical practice/usual care. A restrictive IV fluid resuscitation strategy may produce similar or reduced morbidity and mortality outcomes compared to usual care.

**OBJECTIVES.** Compare 30- and 60-day mortality of a restrictive IV fluid resuscitation strategy that limits IV fluid administration to 60 ml/kg among septic patients for the first 72 hours of care vs. usual care.

**METHODS.** The Restrictive Intravenous Fluid Trial in Severe Sepsis and Septic Shock (RIFTS; clinicaltrials.gov: NCT03137446) was a randomized pilot trial that randomly assigned (1:1 ratio) sepsis/septic shock patients to a restrictive IV fluid strategy ($\leq$ 60ml/kg of resuscitative fluid for 72 hours) vs usual care. Participants met the Sepsis 2 International Consensus definition for severe sepsis or septic shock, did not require immediate surgery, have active fluid wasting, and were admitted to the medical ICU. Resuscitative fluids: crystalloid boluses or continuous infusions. Albumin, blood products and non-resuscitative fluids (IV fluids with antibiotics, electrolyte replacement, vasopressors, and other IV medications) were not restricted. The primary outcome was 30-day mortality and the secondary outcome was 60-day mortality.

Statistical analyses included all cases using an intention to treat analysis. Differences in baseline characteristics and outcomes were assessed using Fisher's exact test for categorical variables, t-tests for continuous normally distributed variables and a Wilcoxon test for continuous non-normal variables. We calculated 95% confidence intervals for medians and differences between group medians using the bias-corrected and accelerated bootstrap method.

**RESULTS.** Among the 113 participants, the restrictive IV fluid arm (n=56) received a mean of 15.7 ml/kg (1069 ml) less than the usual care group (n=57). There were no differences in 30- or 60-day mortality between the restrictive IV fluid strategy and usual care groups (19.6 vs. 22.8%, p=0.84 and 25 vs. 28.1% p=0.83 respectively).

**CONCLUSIONS.** In this pilot study, there was no difference in 30 or 60-day mortality for a restrictive fluid strategy vs. usual care, although it was not designed to test a non-inferiority outcome. Both the restrictive (45.4 ml/kg) and usual care (61.1 ml/kg) arms received significantly less IV fluid than the EGDT trials that administered 90-160 ml/kg of IV fluid over 72 hours and had similar or greater mortality rates. A larger multicenter trial further testing a restrictive IV fluid strategy with a greater difference in IV fluid between study arms is warranted.

**Table 1 (abstract 0998).** Patient Characteristics

| | Restrictive fluid group (n=56) | Usual care group (n=57) | P-value |
|---|---|---|---|
| Age in years —median (IQR) | 71 (59-81) | 73 (54-81) | 0.61 |
| Male sex - n (%) | 33 (58.9) | 28 (49.1) | 0.35 |
| Weight, kg- median (IQR) | 83 (75-103) | 78 (70-92) | 0.24 |
| APACHE II score - mean (SD) | 35.2 (6.9) | 35.4 (7.6) | 0.92 |
| Serum lactate mmol/liter - median (IQR) | 2.2 (1.4- 4.7) | 3.3 (1.6-5.7) | 0.13 |
| Refractory Hypotension - n (%) | 52 (92.9) | 49 (86.0) | 0.52 |
| Lactic >4mmol/L without refractory hypotension - n(%) | 4 (7.1) | 7 (12.3) | 0.52 |

**Table 2 (abstract 0998).** Intravenous Fluid and Patient Outcomes

| | Restrictive fluid group (n=56) | Usual care group (n=57) | P-value or mean/ median difference and 95% CI |
|---|---|---|---|
| Intravenous Fluid and Adjunct Interventions | | | |
| Resuscitative IV fluid (ml/kg) - mean; median | 45.4 (39.9-51.0); 43.2 (39.1-48.6) | 61.1 (52.5-69.7); 55.1 (46.2-57.5) | 15.7 (5.5-25.8); 11.9 (6.0-25.9) |
| Resuscitative IV fluid (ml) - mean; median | 3907 (3534-4278); 4000 (3038-4000) | 4976 (4312-5640); 4500 (3500-5000) | 1069 (315-1823); 500 (0-2000) |
| Albumin - n (%) | 13 (23.2) | 12 (21.1) | 0.82 |
| Blood transfusion - n (%) | 9 (16.1) | 9 (15.9) | 1 |
| Stress dose steroids - n (%) | 19 (33.9) | 21 (36.8) | 0.84 |
| Patient Outcomes | | | |
| Primary: 30-day mortality - n(%) | 11 (19.6) | 13 (22.8) | 0.82 |
| Secondary: 60-day mortality - n(%) | 14 (25.0) | 16 (28.1) | 0.83 |

### 0999

#### Blood purification and mortality in sepsis and septic shock: a systematic review and meta-analysis of randomized trials

A. Putzu[1], J.C. Lopez-Delgado[2], T. Cassina[3], G. Landoni[4]
[1]Geneva University Hospitals, Department of Anesthesiology, Geneva, Switzerland; [2]Hospital Universitari de Bellvitge, Servei de Medicina Intensiva, Barcelona, Spain; [3]Fondazione Cardiocentro Ticino, Department of Cardiovascular Anesthesia and Intensive Care, Lugano, Switzerland; [4]IRCCS San Raffaele Scientific Institute, Department of Anesthesia and Intensive Care, Milan, Italy
**Correspondence:** A. Putzu
*Intensive Care Medicine Experimental* 2018, **6(Suppl 2):**0999

**INTRODUCTION.** Sepsis and septic shock are severe inflammatory conditions associated to high morbidity and mortality. Extracorporeal blood purification represents an attractive therapy to modulate the inflammatory response by means of removing inflammatory mediators and/or bacterial toxins from the plasma that may improve outcome [1]. However, the role as adjunctive therapy in sepsis remains unclear [1-2].

**OBJECTIVES.** We performed an up-to-date meta-analysis of randomized trials to assess if extracorporeal blood purification reduces mortality in this setting.

**METHODS.** Online databases were searched up to April 1st, 2018 for randomized-controlled trials on the use of hemoperfusion, hemofiltration, and plasmapheresis as blood purification technique in comparison to conventional therapy in adults with sepsis or septic shock. We excluded trials employing hemofiltration for acute kidney injury. The primary outcome was mortality at longest follow-up available. We performed a random-effects meta-analysis using Cochrane methodology [3] and calculated relative risk (RR) with 95% confidence interval (CI).

**RESULTS.** Thirty-three trials with 2148 patients were included in the analysis. Blood purification was associated with lower mortality compared to conventional therapy (RR 0.73 [95%CI, 0.62, 0.85]; $p < 0.0001$). Hemoperfusion (RR 0.69 [95%CI, 0.56, 0.86]) and plasmapheresis (RR 0.63 [95%CI, 0.42, 0.96]) were associated with a significant lower mortality when analyzed separately while hemofiltration was not (RR 0.86 [95% CI, 0.68, 1.09]) (Figure 1). Hemoperfusion was associated with a lower mortality when polymyxin B was used (RR 0.63 [0.49, 0.82]; $p$ 0.0005) but not when other hemoperfusion devices were used (RR 0.78 [0.55, 1.09]; $p$ 0.14) ($p_{groups}$ 0.35). Hemoperfusion trials conducted in Asia showed decreased mortality whereas pooled results from trials conducted in other continents were not significant ($p_{groups} < 0.0001$).

**CONCLUSIONS.** Blood purification is associated with a significant reduction in mortality in patients with sepsis and septic shock. However, the quality of evidence is low, since the results were mainly in influenced by polymyxin B hemoperfusion trials from Asia and most of the trials included were single-center with high risk of bias. Further large high-quality randomized controlled trials adequate powered for mortality are warranted to assess the potential beneficial effects of these techniques in septic patients.

**REFERENCE(S)**

[1] Putzu A, Fang MX, Berto Boscolo M, Belletti A, Cabrini L, Cassina T, Landoni G. Blood purification with continuous veno-venous hemofiltration in patients with sepsis or ARDS: a systematic review and meta-analysis. Minerva anestesiologica (2017) 83:867-877

[2] Rhodes A, Evans LE, Alhazzani W et al. Intensive Care Med (2017) 43:304.

[3] Higgins JPT, Green S (editors). Cochrane Handbook for Systematic Reviews of Interventions Version 5.1.0

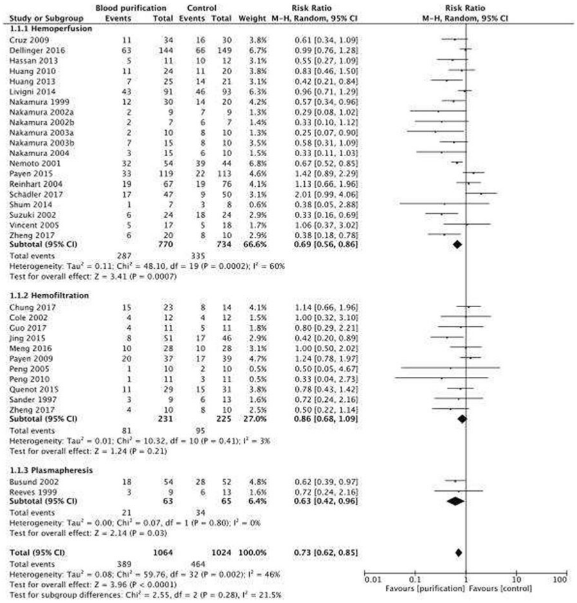

**Fig. 1 (abstract 0999).** Forest plot for mortality at longest follow-up available.

## Haemodynamic therapy

### 1000

#### Epinephrine and short-term survival in cardiogenic shock: an individual data meta-analysis of 2,583 patients

V. Léopold[1], E. Gayat[1], R. Pirracchio[2], J. Spinar[3], J. Parenica[3], T. Tarvasmaki[4], J. Lassus[4], V.-P. Harjola[4], S. Champion[5], F. Zannad[6], S. Valente[7], P. Urban[8], H.-R. Chua[9], R. Bellomo[10], B. Popovic[6], D.M. Ouweneel[11], J. Henriques[11], G. Simonis[12], B. Levy[6], A. Kimmoun[6], P. Gaudard[13], M.B. Basir[14], A. Markota[15], C. Adler[16], H. Reuter[16], A. Mebazaa[1], T. Chouihed[1,6]
[1]APHP - Saint Louis Lariboisière University Hospital - Inserm UMR-S 942, Department of Anesthesiology and Critical Care, Paris, France; [2]Hôpital Européen Georges Pompidou, Department of Anesthesiology and Critical Care Medicine, Paris, France; [3]University Hospital Brno and Faculty of Medicine, Masaryk University, Cardiology Department, Brno, Czech Republic; [4]University of Helsinki and Helsinki University Hospital, Helsinki, Finland; [5]University Hospital Félix Guyon, Intensive Care Unit, Saint Denis, La Réunion, France; [6]University Hospital of Nancy, Nancy, France; [7]Azienda Ospedaliero-Universitaria Careggi, Florence, Italy; [8]Cardiovascular Dept, Hôpital de la Tour, Geneva, Switzerland; [9]University Medicine Cluster, National University Health System, Singapore, Singapore; [10]The University of Melbourne, Department of Intensive Care, Austin Hospital, Melbourne, Australia; [11]AMC Heart Center, Academic Medical Center-University of Amsterdam, Amsterdam, Netherlands; [12]Heart Center Dresden University of Technology, Dresden, Germany; [13]PhyMedExp, University of Montpellier, Montpellier, France; [14]Henry Ford Hospital, Detroit, United States; [15]University Medical Centre Maribor, Maribor, Slovenia; [16]University of Cologne, Cologne, Germany
**Correspondence:** V. Léopold
*Intensive Care Medicine Experimental* 2018, **6(Suppl 2):**1000

**INTRODUCTION.** Catecholamines have been the mainstay of pharmacological treatment of cardiogenic shock (CS). Recently, use of epinephrine has been associated with detrimental outcomes.

**OBJECTIVES.** In the present study we aimed to evaluate the association between epinephrine use and short-term mortality in all-cause CS patients.

**METHODS.** We performed a meta-analysis on individual data with pre-specified inclusion criteria:

patients in non-surgical CS treated with inotropes and/or vasopressors and at least 15% of patients in the studies should have been treated with epinephrine administrated alone or in association with other inotropes/vasopressors. The primary outcome was 28 to 30 days, in-ICU or in-hospital mortality.

**RESULTS.** Fourteen published cohorts and two unpublished datasets were included. We studied 2,583 patients. Main patients characteristics are presented in Table 1. Across all cohorts of patients, the incidence of epinephrine use was 37% (17% to 77%) and short-term mortality rate was 49% (21% to 55%). The risk of death was higher in epinephrine-treated CS patients (OR [CI] = 2.9 [2.4-3.4]) compared to patients treated with other drug regimens. Adjusted mortality risk remained striking in epinephrine-treated patients (n=1,227) (OR = 4.7 [3.4 - 6.4]). After propensity-score matching, two sets of 338 matched patients were identified and epinephrine use remained associated with a strong detrimental impact on short-term mortality (OR = 4.2 [3.0 - 6.0]).

**CONCLUSIONS.** In this very large cohort, epinephrine use for haemodynamic management of CS patients is associated with almost three-fold increase in the risk of death.

**Table 1 (abstract 1000).** Total Population Characteristics

| | |
|---|---|
| **General characteristics** | |
| Age < 45 yo | 7% |
| 45-60 yo | 22% |
| 60-70 yo | 26% |
| 70-80 yo | 29% |
| > 80 yo | 16% |
| Male gender | 52% |
| LVEF (%) at admission | 30 [20;40] |
| APACHE II | 41 [27;90] |
| **Cause of CS** | |
| Acute coronary syndrome | 66% |
| Post cardiac arrest | 45% |
| **Treatment of CS** | |
| Epinephrine | 37% |
| Norepinephrine | 54% |
| Dobutamine | 47% |
| Levosimendan | 11% |
| ECLS | 16% |
| **Outcome** | |
| Short term mortality | 49% |

### 1001

#### Endocan and resolvin D1: two novel biomarkers for the management of acute heart failure?

M. Reina-Couto[1,2], J.P. Bessa[1], S. Paula[1], J. Afonso[1], R. Roncon-Albuquerque[1,2], J.A. Paiva[1,2], A. Albino-Teixeira[1], T. Sousa[1]
[1]Oporto Medical School, Porto, Portugal; [2]Hospital de São João, Porto, Portugal
**Correspondence:** M. Reina-Couto
*Intensive Care Medicine Experimental* 2018, **6(Suppl 2):**1001

**INTRODUCTION.** Endothelial dysfunction contributes to the pathophysiology of heart failure (HF) and is associated with adverse outcomes in these patients (1). Endocan is an emerging biomarker of endothelial dysfunction (2) but remains scarcely explored in HF. Resolvin D1 (RvD1), a mediator of inflammation resolution, was shown to attenuate ventricular dysfunction after myocardial infarction (3) and endothelial disruption (4).

**OBJECTIVES.** To evaluate serum endocan and RvD1 in acute HF (AHF) and cardiogenic shock (CS) patients, their correlation with each other and association with C-reactive protein (CRP), B-type natriuretic peptide (BNP), high-sensitivity troponin I (hs-TnI), ejection fraction (EF) and APACHE II and SAPS II scores.

**METHODS.** Patients diagnosed with AHF (n=10) or cardiogenic shock (CS) (n=9) were included and blood samples were collected at admission and at days 3 to 5. Blood donors were used as controls (n=10). Endocan and RvD1 were measured with ELISA kits and CRP, BNP and hs-TnI with automated analyzers. Echocardiographic examination and calculation of APACHE II and SAPS II scores was performed in all patients.

**RESULTS.** On admission, endocan was increased in patients with AHF and significantly raised in CS when compared to controls (controls: 1.88±0.33; AHF: 5.17±0.95 ng/mL; CS: 20.0±3.84 ng/mL; controls vs AHF, p=0.0878; controls vs CS, p< 0.0001; AHF vs CS, p=0.0441). RvD1 on admission was significantly higher in AHF than in CS (AHF: 2.98±0.42 ng/mL vs CS: 1.39±0.24ng/mL, p=0.024) although there were no significant differences compared to controls (controls: 1.84±0.34 ng/mL). Within patients, we observed an inverse correlation between endocan and RvD1 (r=-0.434, p=0.0082) and a positive correlation between endocan and CRP (r=0.427, p=0.0094) but no correlation with cardiac markers. There were no significant differences between values on admission and on later time points. When patients were stratified according to EF, endocan significantly increased in line with the degree of EF impairment (p=0.017, preserved/mildly impaired vs severely impaired) whereas RvD1 decreased, although not significantly. We also observed that endocan increased across APACHE II tertiles and RvD1 decreased across SAPS II tertiles, although not significantly.

**CONCLUSIONS.** Endothelial dysfunction, assessed by endocan, is associated with inflammatory status in AHF and CS, being significantly higher in patients with marked impairment of EF and/or hemodynamics. Furthermore, RvD1 is inversely correlated with endocan. These results suggest that endocan and RvD1 may be useful biomarkers for risk stratification in AHF and important targets for drug development and improvement of AHF outcome.

**GRANT ACKNOWLEDGEMENT**

T. Sousa was funded by FCT (SFRH/BPD/112005/2015)

### 1002

#### Positive end-expiratory pressure increases extravascular lung water in acute respiratory distress syndrome

F. Gavelli[1,2,3], J.-L. Teboul[1,2], A. Beurton[1,2], T. Taccheri[1,2], G.C. Avanzi[3], C. Richard[1,2], X. Monnet[1,2]
[1]Hôpitaux Universitaires Paris-Sud, Hôpital de Bicêtre, APHP, Service de Réanimation Médicale et de Surveillance Continue Médicale, Le Kremlin-Bicêtre, France; [2]Inserm UMR S_999, Univ Paris-Sud, Le Kremlin-Bicêtre, France; [3]Dipartimento di Medicina Traslazionale, Università del Piemonte Orientale, Novara, Italy
**Correspondence:** F. Gavelli
*Intensive Care Medicine Experimental* 2018, **6(Suppl 2):**1002

**INTRODUCTION.** The effects of positive end-expiratory pressure (PEEP) during acute respiratory distress syndrome (ARDS) on extravascular lung water (EVLW) measured by transpulmonary thermodilution (TPTD) have not yet been completely clarified. In theory, these effects might be of opposite direction. PEEP may impede the filtration of fluid from the pulmonary capillaries toward the interstitium. It might also increase EVLW by increasing the central venous pressure (CVP) and impeding the lymphatic pulmonary drainage. PEEP may also induce some artefacts in EVLW estimation with opposite effects. By squeezing some pulmonary vessels, it decreases the diffusion of the thermal indicator, inducing a decrease in EVLW. By recruiting some lung areas and relieving the hypoxic vasoconstriction in these regions, PEEP may extend the lung regions that are accessible to thermodilution, which might artifactually increase EVLW.

**OBJECTIVES.** To evaluate the effects of PEEP changes on EVLW determination in ARDS patients.

**METHODS.** In 30 ARDS patients who were mechanically ventilated, we measured EVLW (PiCCO2 device) at a PEEP level set to reach a plateau pressure (Pplat) of 30cm$H_2O$ ($PEEP_{high\text{-}start}$) and at PEEP=5cm$H_2O$ after 15' ($PEEP_{low\text{-}15'}$) and 45' ($PEEP_{low\text{-}45'}$). Then, we increased PEEP to the same previous level ($PEEP_{high\text{-}end}$) and performed TPTD. Inspiratory quasi-static pressure-volume curves were recorded to evaluate the PEEP-induced lung recruitment.

**RESULTS.** The PEEP reduction from $PEEP_{high\text{-}start}$ (14[13-15]cm$H_2O$) to $PEEP_{low\text{-}15'}$ decreased CVP from 14[12-16]mmHg to 10[8-13] (p< 0.0001), decreased the ratio of arterial oxygen tension over inspired oxygen fraction ($PaO_2/FiO_2$) from 152[123-177] to 120[108-168] (p=0.01), reduced the end-expiratory lung volume from 266[240-381]mL to 257[235-341]mL (p< 0.01), decreased respiratory system compliance from 34.1[31.3-37.6] to 30.8[27.7-35.9]mL/cm$H_2O$ (p=0.04) and increased cardiac index from 2.96[2.24-3.26] to 3.04[2.50-3.61]mL/min/$m^2$ (p< 0.01). EVLW concomitantly decreased from 20[16-25] to 19[15-23]mL/$m^2$ (p< 0.0001). This reduction in EVLW was not different between patients classified as high and as low recruiters. There was no correlation between EVLW changes and the volume of lung recruited. Similarly, when PEEP was re-increased from $PEEP_{low\text{-}45'}$ to $PEEP_{high\text{-}end}$, we observed a significant increase in CVP, $PaO_2/FiO_2$ and a decrease in cardiac index. EVLW concomitantly increased (p< 0.001). Taking into account all the observed changes, there was a significant correlation between changes in EVLW and in CVP (ρ=0.65; p< 0.0001) but no relation with any other haemodynamic or oxygenation variable was found.

**CONCLUSIONS.** The increase of PEEP levels determines a short-term increase in EVLW that is not linked to alveolar recruitment but which is correlated with the increase in CVP. This suggests that the impairment of the pulmonary lymphatic drainage, rather than the reversion of hypoxic vasoconstriction, is responsible for these effects.

### 1003

#### The impact of connection to the extracorporeal circuit on haemodynamics (transpulmonary thermodilution (TPTD) and pulse contour analysis (PCA)) in patients treated with the ADVOS/HepaWash-device: the HAEMADVOS-I-study

W. Huber, M. Leinfelder, T. Lahmer, A. Herner, U. Mayr, G. Batres-Baires, I. Hartter, J. Wiesner, R. Schmid
Klinikum rechts der Isar; Technical University of Munich, Medizinische Klinik und Poliklinik II, Munich, Germany
**Correspondence:** W. Huber
*Intensive Care Medicine Experimental* 2018, **6(Suppl 2):**1003

**INTRODUCTION.** Extracorporeal organ support is used with increasing frequency and a variety of devices. Many ICU-patients undergoing extracorporeal therapies suffer from multi-organ-failure. Therefore, *multi-organ support* is an intriguing concept. Recently the "advanced organ support" (ADVOS) device has been introduced. While the 1$^{st}$ generation (termed HepaWash) aimed at liver support, the second generation device has been developed for liver support, renal replacement, $CO_2$-elimination and modulation of the acid-base balance. To provide high efficiency, the device is equipped with two conventional dialyzers. This results in an increased volume of the extracorporeal circuit of about 500mL.

**OBJECTIVES.** A recent study demonstrated that connection to sustained low efficiency dialysis (SLED; extracorporeal volume about 250mL) did not result in relevant haemodynamic changes (1). We hypothesized that connection to the ADVOS-circuit might result in haemodynamic impairment due the increased 500mL volume of the extracorporeal circuit. Therefore, we analyzed the impact of connection to the ADVOS-device on parameters derived from TPTD and PCA in 25 treatments of six patients equipped with PiCCO monitoring.

**METHODS.** Transpulmonary thermodilution with the PiCCO-device (Pulsion; Germany) was performed immediately before and after connection (T1, T2) to the ADVOS-device and disconnection (T3; T4). The tubing was prefilled with 0.9% saline ("acute connection"). Connection, ADVOS-treatment and disconnection were performed with a blood flow of 100mL/min. Vassopressor dosages were kept stable.

**RESULTS.** 6 Patients (5m; 1f), 65±18 years, SOFA-score 11±3, aetiology: 3 cirrhosis, 1 alcoholic steatohepatitis, 2 sepsis; mechanical ventilation 24/25 (96%) treatments; vasopressors 20/25 (80%).

Connection to ADVOS did not result in significant changes in any of the following haemodynamic parameters at T2 compared to T1: MAP (77±9 vs. 81±8mmHg; p=0.054), SVRI (953±318 vs. 926±326; p=0.599), CVP (20.3±6.9 vs. 22.7±8.5 mmHg; p=0.060); GEDVI (888 ±250 vs. 912±243 mL/$m^2$; p=0.20), heart rate HR (102±12 vs. 102±12/min; vs. y; p=0.614), dPmax (1460±347 vs. 1483±381mmHg/s; p=0.840) and GEF (26.1±6.0 vs. 26.8±6.2%; p=0.134).

Only EVLWI (10.3±5.3 vs.11.0±5.4mL/kg; p=0.032), CI (5.17±1.53 vs. 5.49±1.30 L/min/$m^2$; p=0.022) and CPI (0.89±0.31 vs.1.00±0.30 W/$m^2$ ; p=0.005) decreased slightly, but with statistical significance.

Over 8h, ADVOS-therapy with a net filtration of 931±698mL did not result in significant changes in HR, MAP, CI, GEDVI, CVP, EVLWI, GEF and CPI after disconnection (T4) vs. before connection (T1). Only pulmonary vascular permeability index PVPI was significantly lower after ADVOS compared to before (1.33±0.54 vs. 1.53 ±0.70; p=0.82).

**CONCLUSIONS.** "Acute" connection to the ADVOS with pre-filled tubing did not result in haemodynamic impairment.

8h ADVOS therapy was haemodynamically safe and reduced PVPI.

**REFERENCE(S)**

Huber W et. al.; PLoS One. 2016.

#### 1004

#### The Index Pressure Pulsatility: a novel useful tool to guide vasopressor therapy in septic shock patients

A. Santos[1,2], B. Diez del Corral[3], P. Guijo Gonzalez[3], M. Gracia Romero[3], A. Gil Cano[3], M. Cecconi[4], I. Monge-Garcia[3]
[1]CIBER de Enfermedades Respiratorias (CIBERES), Madrid, Spain; [2]ITC Ingeniería y Técnicas Clínicas, Arganda del Rey, Spain; [3]Hospital SAS de Jerez, Unidad de Cuidados Intensivos, Jerez de la Frontera, Spain; [4]St. George's Healthcare NHS Trust, Department of Intensive Care Medicine, London, United Kingdom
**Correspondence:** A. Santos
*Intensive Care Medicine Experimental* 2018, **6(Suppl 2):**1004

**INTRODUCTION.** Mean arterial pressure and vascular resistance are the most used variables to guide vasopressor therapy. However the effect of vasopressors could affect also pulsatile hemodynamics. The effect on pulsatile hemodynamics is relevant as it is related with the cardiac pump efficiency which could affect patient outcomes. In this study we present a new bedside index that could help to evaluate pulsatile hemodynamics and be helpful for vasopressor therapy monitoring.

**OBJECTIVES.** To evaluate the ability of the Pressure Pulsatility Index (PPI), the ratio between the pulse and mean arterial pressure, as a complementary tool to guide Norepinephrine (NE) therapy in septic shock patients.

**METHODS.** A prospective analysis of 47 patients at early stages of septic shock in which NE dose was modified as a result of a clinical decision. Arterial system was evaluated by means of carotid pressure (obtained by applanation tonometry, calibrated using radial invasive pressure) and aortic flow (obtained by an esophageal Doppler). We evaluated the continuous component of hemodynamics (vascular resistance) and also pulsatile component represented by compliance, reflection waves (reflection index) and the ventricular oscillatory power (represented as a percent of the total power, %VOP). Correlation between PPI and %VOP and difference between survivors and no survivors (30 days mortality) in the studied variables were also evaluated.

**RESULTS.** Comparing NE dose from lower to higher, a higher NE dose increased resistance (1137±427 vs 1331±527 dyn.s.cm$^{-5}$, p< 0.001), reflection index (0.21±0.05 vs 0.23±0.06, p< 0.001), %VOP (21.7±6 vs 23.2 ±5.8%, p< 0.001), PPI (0.86±0.32 vs 0.92±0.35, p< 0.001) and decreased compliance (1.68[1.48 to 1.98] vs 1.32[1.12 to 1.60]ml/mmHg, p< 0.001). Changes in PPI and %VOP showed a good correlation ($r^2$=0.57, p< 0.001). The increase in %VOP (0.8±2.23 vs 3.1±3%, p=0.005) as well as in PPI (3.2 ±7.1 vs 15.4±8.9%, p< 0.001) resulting from a higher NE dose were bigger in non-survivors.

**CONCLUSIONS.** In septic shock patients, NE affected not only continuous but also pulsatile hemodynamics. The effect on pulsatile hemodynamics was higher in non-survivors patients. The PPI reliably tracked this effect appearing as promising tool for helping to guide vasopressor therapy.

## Cardiac arrest management

#### 1005

#### Background EEG reactivity and neurologic outcome after cardiac arrest in a Parisian registry

S. Benghanem[1], M. Paul[1], J. Charpentier[1], S. Rouhani[2], O. Ben Hadj Salem[1], L. Guillemet[1], S. Legriel[3], F. Pene[1], J.D. Chiche[1], J.P. Mira[1], F. Dumas[1,4,5], A. Cariou[1,5]
[1]Medical ICU, Cochin University Hospital (APHP) and Paris Descartes University, Paris, France; [2]Cochin University Hospital (APHP), Department of Physiology, Paris, France; [3]Medical ICU, Mignot Hospital, Le Chesnay, France; [4]Cochin University Hospital (APHP), Emergency Department, Paris, France; [5]INSERM U 970, Team 4, Paris, France
**Correspondence:** S. Benghanem
*Intensive Care Medicine Experimental* 2018, **6(Suppl 2):**1005

**INTRODUCTION.** About 80% of patients resuscitated from cardiac arrest (CA) are comatose after return of spontaneous circulation (ROSC) because of anoxic-ischemic brain injury. Electroencephalography (EEG) is recommended after CA for neuroprognostication.

**OBJECTIVES.** The aim of this study was to evaluate the predictive value (PV) of EEG reactivity for neurological outcome after CA.

**METHODS.** All consecutive patients admitted in a Parisian intensive care unit between 2007 and 2016 still alive 48 hours after admission with at least one EEG performed during coma are included. A blinded certified neurophysiologist classified EEG according to American Clinical Neurophysiology Society (ACNS) nomenclatures: highly malignant, malignant, or benign pattern (reactive and absence of highly malignant or malignant pattern) and evaluated EEG reactivity as a reproductible waveform change in amplitude or frequency following standardized stimulation. We assessed the predictive values of EEG pattern, stratified on sedation, for neurologic outcome at ICU discharge using Cerebral Performance Category (CPC 3-4-5 assumed as poor outcome).

**RESULTS.** We included 428 patients mostly male (71%), with median age of 63 years, shockable rhythm in 49%. Median time to EEG assessment was 3(1-4) days. 113/428 (26%) patients had highly malignant patterns, 162/428 (38%) malignant patterns and 153/428 (36%) a benign EEG. A poor outcome was observed in 344 (80%) patients. Positive predictive value (PPV) of areactive EEG without highly malignant pattern to predict unfavorable outcome was 95.2%, up to 97.5% (IC95 91.3-99.7) when EEG was performed without sedation. By contrast, the PPV of benign EEG to predict favorable outcome was 49.7%, up to 66.2% (IC95% 54.3-76.8) when EEG was performed early at median time day 1 under sedation.

**CONCLUSIONS.** Post CA, absence of EEG reactivity was highly predictive of unfavorable outcome. Benign EEG was sparsely predictive of a favorable outcome. Assessment of reactivity seems to be influenced by sedation.

**REFERENCE(S)**

Rossetti AO et al. Electroencephalography Predicts Poor and Good Outcomes After Cardiac Arrest: A Two-Center Study. Crit Care Med. 2017 Jul;45(7):e674-82.

Nolan JP et al. European Resuscitation Council and European Society of Intensive Care Medicine 2015 guidelines for post-resuscitation care. Intensive Care Med. 2015 Dec;41(12):2039-56.

Hirsch LJ et al. American Clinical Neurophysiology Society's Standardized Critical Care EEG Terminology: 2012 version. J Clin Neurophysiol Off Publ Am Electroencephalogr Soc. 2013 Feb;30(1):1-27.

Drohan CM et al. Effect of sedation on quantitative electroencephalography after cardiac arrest. Resuscitation. 2017 Dec 2;

Sandroni C et al. Predictors of poor neurological outcome in adult comatose survivors of cardiac arrest: a systematic review and meta-analysis. Part 2: Patients treated with therapeutic hypothermia. Resuscitation. 2013 Oct;84(10):1324-38.

**GRANT ACKNOWLEDGMENT**
None

**Table 1 (abstract 1005).** Prognostic value of EEG parameters and SSEPs for poor outcome

| | Sensitivity (IC95%) | Specificity (IC95%) | NPV (IC95%) | PPV (IC95%) | FPR (IC95%) |
|---|---|---|---|---|---|
| Highly malignant EEG n= 113 | 32.8 27.9-38.1) | 100 (95.7-100) | 26.67 (21.9-31.9) | 100 (96.8-100) | 0 (0-3.2) |
| Malignant EEG n=162 | 44.8 (39.4-50.2) | 90.5 (82.1-95.8) | 28.6 (23.2-34.4) | 95.1 (90.9-97.8) | 4.9 (2.2-9.5) |
| Areactive EEG background n=209 | 62.5 (57-67.7) | 92.9 (85.1-97.3) | 39 (32.2-46.1) | 97.1 (93.6-98.9) | 2.9 (1.1-6.1) |
| Areactive EEG without sedation during recording n= 120 | 57.6 (50.5-64.4) | 93.5 (78.6-99.2) | 25 (17.4-33.9) | 98.3 (94.1-99.8) | 1.7 (0.2-5.9) |
| Areactive EEG without burst or electrical status epilepticus n=125 | 36.6 (31.4-42.1) | 92.9 (85.1-97.3) | 27.5 (22.4-33) | 95.2 (89.8-98.2) | 4.8 (1.8-10.2) |
| Areactive EEG without burst or electrical status epilepticus, without sedation n=45 | 37.1 (30.6-44.1) | 93.5 (78.6-99.2) | 18.6 (12.8-25.6) | 97.5 (91.3-99.7) | 2.5 (0.03-8.7) |
| Bilaterally absent N20 on SSEP n=103 | 54.2 (46.8-61.4) | 100 (79.4-100) | 15.5 (9.2-24) | 100 (96.5-100) | 0 (0-3.5) |

### 1006

#### Smartphone video-assisted advanced life support for out-of-hospital cardiac arrest by emergency medical service teams under physician direction

D. Lee, S. Park
Seoul National University Bundang Hospital, Emergency Department, Seongnam, Korea, Republic of
**Correspondence:** D. Lee
*Intensive Care Medicine Experimental* 2018, **6(Suppl 2):**1006

**INTRODUCTION.** Direct medical control using video conferencing capabilities of smartphones has never been conducted in out-of-hospital cardiac arrest (OHCA) patients. This study was conducted to investigate the feasibility and treatment effectiveness of real-time smartphone video conferencing calls for the management of OHCA.

**METHODS.** This study was a pre-post intervention, prospective cohort study conducted from January 2013 through July 2015 in the City of Suwon, Gyeonggi Province, South Korea. The intervention was pre-hospital advanced life support (ALS) under a physician`s direction using a smartphone video call, and patients were compared pre- and post- the intervention implementation. Survival discharge rates and good neurologic outcomes were reviewed as primary outcomes.

**RESULTS.** In total, 942 cardiac arrests occurred over the 2-year period; 308 patients were excluded, leaving 634 patients for inclusion in our study. During the intervention period, there were 248/320 (77.5%) cases of smartphone video-assisted ALS. For patients in the pre- and post-intervention groups, the survival rate at discharge was 12.5% and 7.0%, respectively (adjusted odds ratio [AOR] 2.07, 95% confidence interval [CI] 1.11-3.86, P=0.002), and favorable neurological outcomes were ascertained in 6.9% and 1.9%, respectively (AOR 4.78, 95% CI 1.49-15.28, P=0.002). The smartphone voice and video quality were rated 8.5 and 8.2 out of 10, respectively, in physician evaluation, while the overall utility was rated 9.1.

**CONCLUSIONS.** Direct medical control between EMS personnel and physicians using a smartphone video is feasible for patients with OHCA. It was also shown to improve the survival rate and cerebral function recovery rate.

**REFERENCE(S)**

1. Survival Data after Cardiac Arrest 2011-2012. Korean Ministry of Health and Welfare, South Korea; 2013.
2. Ro YS, Shin SD, Song KJ, et al. A trend in epidemiology and outcomes of out-of-hospital cardiac arrest by urbanization level: a nationwide observational study from 2006 to 2010 in South Korea. Resuscitation 2013;84:547-57.
3. Lee JS, Jeon WC, Ahn JH, Cho YJ, Jung YS, Kim GW. The effect of a cellular-phone video demonstration to improve the quality of dispatcher-assisted chest compression-only cardiopulmonary resuscitation as compared with audio coaching. Resuscitation 2011;82:64-8.
4. Johnsen E, Bolle SR. To see or not to see—better dispatcher-assisted CPR with video-calls? A qualitative study based on simulated trials. Resuscitation 2008;78:320-6.
5. Yang HJ, Kim GW, Cho GC, Tak YJ, Chung SP, Hwang SO. Part 8. Cardiopulmonary resuscitation education: 2015 Korean Guidelines for Cardiopulmonary Resuscitation. Clinical and Experimental Emergency Medicine 2016;3:S66-S8.

### 1007

#### Targeting low-normal vs. high-normal mean arterial pressure after cardiac arrest and resuscitation: a randomized pilot trial (NCT02698917)

P. Jakkula[1], V. Pettilä[1], M. Skrifvars[1], J. Hästbacka[1], P. Loisa[2], M. Tiainen[1], E. Wilkman[1], J. Toppila[1], T. Koskue[2], S. Bendel[3], T. Birkelund[4], R. Laru-Sompa[5], M. Valkonen[1], M. Reinikainen[6], COMACARE Study Group
[1]University of Helsinki and Helsinki University Hospital, Helsinki, Finland; [2]Päijät-Häme Central Hospital, Lahti, Finland; [3]Kuopio University Hospital, Kuopio, Finland; [4]Aarhus University Hospital, Aarhus, Denmark; [5]Central Finland Central Hospital, Jyväskylä, Finland; [6]North Karelia Central Hospital, Joensuu, Finland
**Correspondence:** P. Jakkula
*Intensive Care Medicine Experimental* 2018, **6(Suppl 2):**1007

**INTRODUCTION.** Arterial hypotension in patients resuscitated from cardiac arrest is common and associated with increased mortality. The optimal blood pressure level during post-resuscitation care is unknown, and it is unclear whether supporting the circulation with vasoactive agents improves outcomes. [1]

**OBJECTIVES.** We performed a randomized pilot trial to evaluate the feasibility of targeting low-normal or high-normal mean arterial pressure (MAP) in comatose, mechanically ventilated patients after out-of-hospital cardiac arrest (OHCA) and the intervention's impact on the serum concentration of neuron-specific enolase (NSE) and on cerebral oxygenation measured by near-infrared spectroscopy (NIRS).

**METHODS.** In the Carbon dioxide, Oxygen and Mean arterial pressure After Cardiac Arrest and REsuscitation (COMACARE) trial, we used a $2^3$ factorial design and randomly assigned patients after OHCA and resuscitation to low-normal or high-normal levels of arterial carbon dioxide tension, arterial oxygen tension and MAP. The results of the low-normal (65-75 mmHg) vs. high-normal (80-100 mmHg) MAP comparison are reported here.

The primary outcome was the serum concentration of NSE at 48 h after cardiac arrest. The main feasibility outcome was the difference in MAP between the groups during the first 36 h after ICU admission. Secondary outcomes included the results of continuous cerebral NIRS monitoring during the intervention.

**RESULTS.** Totally 123 patients were enrolled in the trial, and 120 patients were included in the final analysis. Despite a marked difference in MAP levels between the groups throughout the intervention (Figure 1), there was no significant difference in median NSE concentrations at 48 h after OHCA (for low-normal MAP, 21.2 µg/l [IQR, 15.2-34.9 µg/l], and for high-normal MAP, 22.0 µg/l [IQR, 13.6-30.9 µg/l], p = 0.392). The NIRS results were comparable in both groups (Figure 2).

**CONCLUSIONS.** Targeting a specific blood pressure level after resuscitation from OHCA is possible. However, the blood pressure level had no impact on the concentration of NSE at 48 h after OHCA. In addition, cerebral oxygenation, as measured by NIRS, was unaffected by the blood pressure level. These results suggest that autoregulation of the cerebral blood flow remains functional within a relatively broad MAP range during the post-resuscitation care.

**REFERENCE(S)**

1. Nolan JP, Soar J, Cariou A, et al (2015) European Resuscitation Council and European Society of Intensive Care Medicine Guidelines for Post-Resuscitation Care. Resuscitation 2015;95:202-222.

**GRANT ACKNOWLEDGMENT**

University of Helsinki project grant (H3702,WBS73702705) and Helsinki University Hospital governmental grant (TYH2018227)

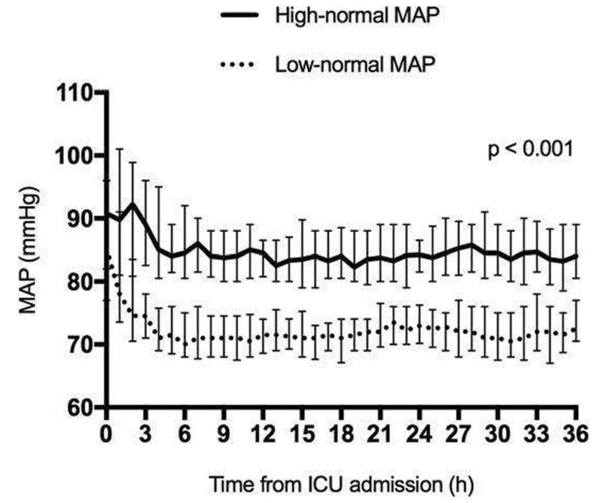

**Fig. 1 (abstract 1007).** Median (IQR) MAP in the different intervention groups.

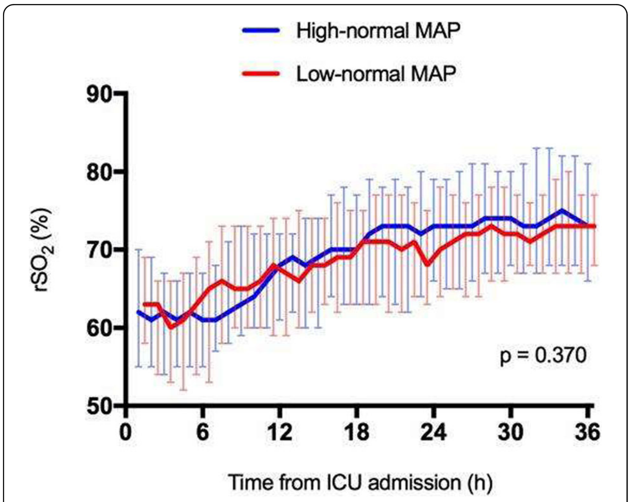

**Fig. 2 (abstract 1007).** Median (IQR) regional cerebral oxygen saturation in the different intervention groups.

### 1008

#### Hemodynamic efficiency of an hemodialysis treatment with high cut-off membrane during the early period of post-resuscitation shock: the HYPERDIA trial

G. Geri[1,2,3], D. Grimaldi[1], T. Seguin[4], L. Lamhaut[2,3,5], N. Marin[1], J.-D. Chiche[1,2], F. Pène[1,2], A. Bouglé[1], F. Daviaud[1], T. Morichau-Beauchant[1], M. Arnaout[1], B. Champigneulle[1], W. Bougouin[1], L. Zafrani[1], S. Bourcier[1], Y.-L. Nguyen[1], J. Charpentier[1], J.-P. Mira[1,2], J. Coste[2,6], C. Vinsonneau[7], A. Cariou[1,2,3]
[1]Cochin Hospital, Medical ICU, Paris, France; [2]Paris Descartes University, Paris, France; [3]Sudden Death Expertise Centre, Paris, France; [4]Toulouse Rangueil Hospital, Polyvalent ICU, Toulouse, France; [5]SAMU 75, Paris, France; [6]Hotel Dieu Hospital, Biostatistics and Epidemiology Unit, Paris, France; [7]Marc Jaquet Hospital, Medical ICU, Melun, France
**Correspondence:** G. Geri
*Intensive Care Medicine Experimental* 2018, **6(Suppl 2):**1008

**BACKGROUND.** After resuscitation of cardiac arrest (CA), an acute circulatory failure occurs in about 50% of cases, which shares many characteristics with septic shock. Most frequently, supportive treatments are unable to control this shock that may provoke multiple organ failure and death. We evaluated whether an early plasma removal of inflammatory mediators using high permeability hemodialysis (HCO-CVVHD) in addition to conventional treatments could improve hemodynamic status of this patients.

**PATIENTS AND METHODS.** We performed a randomized open-label trial. Successfully resuscitated comatose CA patients who had a post-resuscitation shock (defined as requirement of nor-epinephrine or epinephrine infusion > 1mg/h) were included. The experimental group received 2 separated sessions of HCO-CVVHD during the first 48 hours following ICU admission. The control group received continuous veno-venous hemofiltration (CVVH) if needed. Non-parametric tests were used to compare the two groups. The primary outcome was the duration of the shock expressed by the length of catecholamine infusion. Number of vasopressors-free days, 6-hours repeated measures of blood pressure, daily fluid balance and mortality (ICU and day-28) have been evaluated as well.

**RESULTS.** 35 patients were included: 17 (median age 68.4, 59% male) in the HCO-CVVHD group and 18 (median age 66.3, 83% male) in the control group. Baseline characteristics did not differ between the two groups. Ventricular fibrillation was the first recorded rhythm in 23.5 and 44.4 % in the HCO-CVVHD and control group, respectively (p=0.289). Day-28 mortality rate was 64.7 and 72.2% in the HCO-CVVHD and control group, respectively (p=0.72). Probability of vasopressors discontinuation over time was similar in the two groups (Figure, p for logrank test=0.67). Number of catecholamine-free days was 25.1 [0, 26.5] and 24.5 [0, 26.2] in the HCO-CVVHD and control group, respectively (p=0.65). No difference was observed regarding the daily-dose of vasopressors and the 6-hours recorded systolic/mean/diastolic arterial pressure. No difference in terms of fluid balance was observed either.

**CONCLUSIONS.** In post-cardiac patients with acute circulatory failure, HCO-CVVHD did not reduced the duration of shock and had no effect on hemodynamic status.

**REGISTRATION.** NCT00780299

**CONFLICT OF INTEREST**

The study was completely funded by the French Ministry of Health. Baxter Edwards provided the Septexä membranes that were used in the HCO-CVVHD group.

### 1009

#### Survival and neurological outcome in the elderly after out-of-hospital cardiac arrest

T. Yoshida, K. Maekawa, Y. Takahashi, H. Matsumoto, Y. Itagaki, T. Tsuchida, Y. Sadamoto, A. Tomita, S. Kawahara, A. Mizugaki, H. Murakami, T. Oyasu, T. Saito, K. Katabami, T. Wada, M. Hayakawa, A. Sawamura
Hokkaido University Hospital, Sapporo, Japan
**Correspondence:** T. Yoshida
*Intensive Care Medicine Experimental* 2018, **6(Suppl 2):**1009

**INTRODUCTION.** Japan has one of largest proportions of geriatric patients in the world with >21% of the population aged 65 years or older, and is called a super-aging society. There have been no study of the survival and neurological outcome in elderly patients who have suffered out-of-hospital-cardiac-arrest (OHCA).

**OBJECTIVES.** We clarify the prognosis of Japanese elderly OHCA patients and investigate whether age is related to prognosis.

**METHODS.** We investigated elderly OHCA patients (≧65 years of age) who were registered in a nationwide Utstein-style Japanese database between 2005 and 2012. For descriptive purposes, the patients were grouped according to age (65-69, 70-79, 80-89, ≧90 years) and we compared the characteristics and prognosis (survival and favourable neurological status [cerebral performance category 1 or 2] at 1 month after cardiac arrest) between each group. Moreover, we investigated whether age is related to prognosis using multivariable logistic regression model.

**RESULTS.** A total of 655,352 patients were included in this study. Among them, 68,883 were in the age range from 65 to 69 years, 21,3583 were in the age range from 70 to 79, 26,724 were in the age range from 80 to 89 and 109,172 were in the age range over 90. With increasing age there were declines in survival and favourable neurological status at 1 month: 7.0%

and 3.7% in the 65-69 group, 4.9% and 2.3% in the 70-79 group, 3.4% and 1.3% in the 80-89 group and 2.3% and 0.8% in the over 90 group.
In the multivariable analysis, the odds of survival and favourable neurological status for every 10 years of age increase were 0.73 (0.71 - 0.74) and 0.65 (0.64 - 0. 67), respectively.
**CONCLUSIONS.** Increasing age among the elderly is associated with a lower 30-day survival and unfavourable neurological outcome after OHCA.

## Advanced diagnosis of AKI

### 1010

#### A combined biomarker of fibroblast growth factor 23, erythropoietin and klotho predicts the development of acute kidney injury and development of chronic kidney disease in critical care patients: results of a prospective cohort

L. Toro[1], N. Abarzua[1], C. Bascuñan[1], K. Arcos[1], C. Fuentealba[1], A.M. Tong[1], M.E. Pinto[1], C. Romero[1], L. Michea[1,2,3]
[1]Universidad de Chile, Hospital Clínico, Santiago, Chile; [2]Facultad de Medicina, Universidad de Chile, Santiago, Chile; [3]Millennium Institute on Immunology and Immunotherapy, Santiago, Chile
**Correspondence:** L. Toro
*Intensive Care Medicine Experimental* 2018, **6(Suppl 2):**1010

**INTRODUCTION.** Acute Kidney Injury (AKI) is frequent among septic patients admitted to the Intensive Care Unit (ICU), which is associated to higher mortality and risk of development of Chronic Kidney Disease (CKD). Observational studies have shown that plasma levels of Fibroblast Growth Factor 23 (FGF23) are increased in patients with AKI. Experimental data shows that FGF23 expression is modulated by Erythropoietin (EPO) and Klotho.
**OBJECTIVE.** To evaluate if a combined biomarker including FGF23, EPO and Klotho predicts the development of AKI and short/long-term morbimortality in ICU patients with sepsis.
**METHODS.** Prospective cohort of adult patients admitted to the ICU of Hospital Clínico Universidad de Chile (HCUCh), with diagnosis of severe sepsis/septic shock and normal renal function (eGFR > 60 mL/min/1.7m$^2$) before admission. Plasma levels of FGF23, EPO and Klotho were determined to calculate the value of the combined biomarker. Demographic, clinical and biochemical parameters were recorded. We evaluated short-term outcomes (requirements of vasoactive drugs, renal replacement therapy and 30-day mortality), long-term outcomes and development of CKD (6 and 12-month). Statistical analysis: Cox proportional test, Kaplan-Meier analysis and calculation of Hazard Ratios (HR). This study was approved by the Ethics Committee of HCUCh.
**RESULTS.** 698 patients with severe sepsis/septic shock were admitted to the ICU between 2013 and 2016. 594 were excluded due to absence of previous renal data or history of CKD. 104 patients were recruited. Age: 64,3 ± 8,4 years. Male: 61 (60%). Presence of AKI at admission: 35 (group 1). Development of AKI at 1-2 days: 34 (group 2). No AKI: 35 (group 3). In groups 1 and 2 FGF23 and EPO levels were significantly increased, as compared to group 3. In contrast, Klotho was significantly decreased in AKI patients compared to non-AKI patients. The combined biomarker increased in AKI patients (*P< 0.001 vs. group 3,* both groups). The magnitude of increase of the combined biomarker was proportional to the severity of AKI (KDIGO1=3x; KDIGO3=20x) and showed an appropriate accuracy, with an Area Under Curve of 0.91 [0.85-0.96]. Defining a cut-off value of the combined biomarker =1.0 (A.U.), we found that values>1.0 associated with increased 30-day mortality (27% versus 7%, p=0.02; HR: 3.0), development of CKD (32% versus 8%, p=0.03; HR: 3.1; Fig 1) and 1-year mortality (43% versus 13%, p< 0.01; HR: 2.9; Fig 1).
**CONCLUSIONS.** The combined biomarker predicted the development of AKI in ICU patients with sepsis with high accuracy. The combined biomarker was an appropriate predictor of worse short and long-term outcomes, including mortality and development of CKD. This new biomarker should be evaluated in larger clinical trials, to study its applicability in AKI patients and post-AKI follow-up.

**ACKNOWLEDGEMENTS**
Instituto Milenio de Inmunología e Inmunoterapia P09/016-F, FONDECYT Iniciacion 11171141, FONDECYT/Regular 1130550 and 1171869.

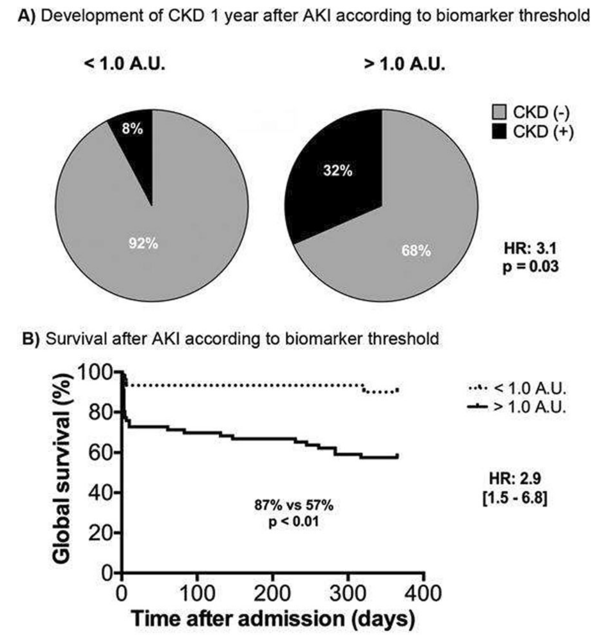

**Fig. 1 (abstract 1010).** CKD and survival according to biomarker threshold

### 1011

#### Downregulation of TIMP2 attenuates sepsis-induced acute kidney injury through NFκb pathway

Y.-M. Li[1], J. Zhang[1], L.-J. Su[1], K. John A.[2], Z.-Y. Peng[1,2]
[1]Zhongnan Hospital of Wuhan University, Wuhan, China; [2]University of Pittsburgh School of Medicine, Pittsburgh, United States
**Correspondence:** Y.-M. Li
*Intensive Care Medicine Experimental* 2018, **6(Suppl 2):**1011

**INTRODUCTION.** Acute kidney injury (AKI) is a frequent complication of sepsis, and contributes to morbidity and mortality. Urinary tissue inhibitor of metalloproteinases-2 (TIMP2) has been recently recognized as an early biomarker predicting AKI in critically ill patients. However, the biologic functions of TIMP2 remain largely unknown.

**OBJECTIVES.** In this study, we investigated the role of TIMP2 in mediating inflammation and tubular cell apoptosis in AKI.

**METHODS.** A murine model of sepsis-induced AKI was established by cecal ligation and puncture (CLP). Mice undergoing CLP and immortalized proximal tubular epithelial human HK-2 cells that were exposed to lipopolysaccharide (LPS) were treated with TIMP2 silence lentivirus expressing TIMP2 siRNA, Flow cytometry was used to test the apoptosis rate. Renal function was detected by serum creatinine. Statistical analysis was performed using Spearman rank correlation and paired two-tail t test.

**RESULTS.** In kidney tissue taken from mice exposed to cecal ligation and puncture (CLP) and in human kidney 2 (HK-2) cells exposed to LPS in culture, TIMP2 was significantly upregulated. The expression of TIMP2 in the kidney correlated with the severity of AKI in vivo. ( Maximal figures number is 3; data not shown) In cultured HK-2 cell, LPS challenge markedly induced cytokine release and recombinant cytokines promoted TIMP2 expression and apoptosis. However, TIMP2 silence ameliorated LPS-induced cytokine release, apoptosis and cell injury. (data not shown) We further found that the effects of down regulation of TIMP2 on suppression of the inflammatory cytokine release was mediated by p-P65. Stable, kidney-specific TIMP2 knockdown mice were transduced by TIMP2 knockdown lentiviral vectors injection into kidney parenchyma.

Silencing of TIMP2 ameliorated CLP induced proinflammatory cytokines, kidney dysfunction measured by serum creatinine and histopathologic changes. Downregulation of TIMP2 exhibited renoprotective effects against endotoxin-induced AKI, which was associated with anti-inflammatory activity by inhibiting the NF-κB pathway.

**CONCLUSIONS.** In summary, we explored the possible roles of TIMP2 in septic AKI and found that TIMP2 does indeed promote kidney damage apparently by acting on NFκb. We hypothesize a feedback loop promoting apoptosis and leading to AKI. On the contrary, suppressing TIMP2 protects against kidney damage, reduces p-P65 activity, attenuates proinflammatory cytokines, improves cell viability and reduces apoptosis. Our results reveal a potential new therapeutic target for AKI in the setting of sepsis and possibly other conditions.

**GRANT ACKNOWLEDGMENT**

National Natural Science Foundation of China (No.81772046)

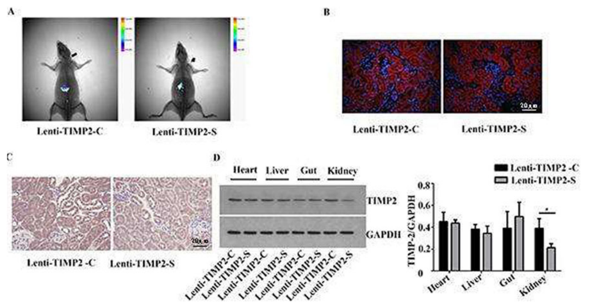

**Fig. 1 (abstract 1011).** Kidney TIMP2 specific knockdown mice were constructed by parenchymal TIMP2 silence lentiviral vector

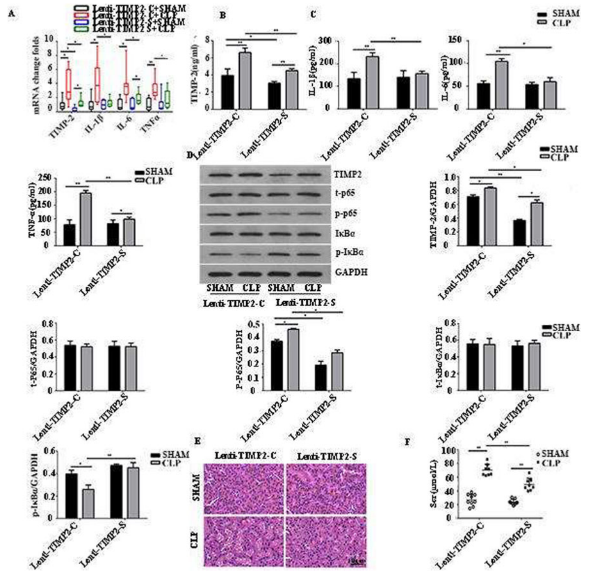

**Fig. 2 (abstract 1011).** Downregulation of TIMP2 Attenuated CLP Induced AKI

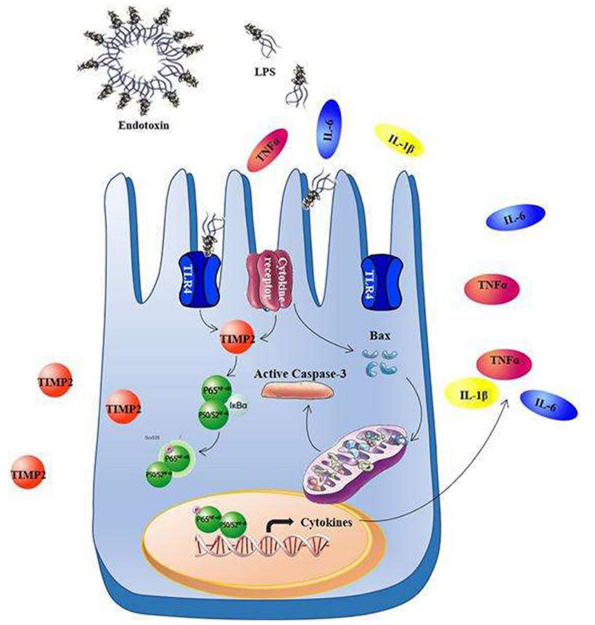

**Fig. 3 (abstract 1011).** Proposed mechanism for the involvement of TIMP2 in regulating sepsis induced AKI in kidney tubular c

### 1012

#### A microRNA signature in Mesechymal-Stromal/Stem-cell-treated septic murine kidneys

N. Filewod[1], C. Walsh[2], S. Mei[3,4], D. Stewart[3,4], W.C. Liles[5], P. Hu[6], C. Dos Santos[2,7]
[1]St Michael's Hospital, Critical Care Medicine, Toronto, Canada; [2]Keenan Knowledge Institute, Toronto, Canada; [3]Ottawa Hospital Research Institute, Ottawa, Canada; [4]University of Ottawa, Ottawa, Canada; [5]University of Washington, Department of Medicine, Seattle, United States; [6]University of Manitoba, Department of Biochemistry and Medical Genetics, Winnipeg, Canada; [7]University of Toronto, Laboratory Medicine and Pathobiology, Toronto, Canada
**Correspondence:** N. Filewod
*Intensive Care Medicine Experimental* 2018, **6(Suppl 2):**1012

**INTRODUCTION.** The pathogenesis of septic acute kidney injury (AKI) may relate to metabolic changes rather than hypoperfusion[1]. In murine septic AKI, decreases in mitochondrial oxygen consumption parallel decreased GFR[2]. Mesenchymal-Stromal/Stem-cell (MSC) administration has been shown to reduce both incidence and severity of AKI in experimental models of sepsis, and may modulate transcriptional responses to mitigate bioenergy failure[3].

**OBJECTIVES.** To identify regulatory microRNAs (miRNAs) contributing to the therapeutic effect of MSCs in a murine model of septic AKI.

**METHODS.** Mice were randomized to either sham or caecum ligation and puncture surgery followed by either placebo or MSCs administration (N=4 per group). Total RNA from kidneys was extracted from these mice for profiling the renal response to sepsis (placebo) vs MSC treatment. Transcriptional and miRNA profiles were generated by hybridizing total RNA to the Illumina Mouse WG 6v2.0 or the miRCURY LNA™ microRNA arrays. In the R scripting environment, differentially expressed genes and miRNAs were identified via linear modelling. Putative regulatory relationships were identified based on Pearson and Elastic Net regression. Correlative relationships were discarded unless putative targets contained a predicted miRNA binding site. Gene ontology (GO) enrichment analysis was performed on both differentially expressed genes and the putative targets of differentially expressed miRNAs.

**RESULTS.** Mice that underwent CLP had elevated plasma creatinine and urea. Twenty-three miRNAs and 2021 probes or genes had significantly differential expression (false discovery rate < 0.05). GO analysis suggested that many of the differentially expressed genes related to mitochondrial function.

miRNA 484 had increased expression after CLP, and treatment with MSC restored an intermediate phenotype.

Regression revealed miRNA 484 to be a putative master-regulator, with 2428 genes having negatively correlated expression. Of these, 2 genes were amongst the 18 proven targets of miRNA 484; 491 genes contained a predicted binding site. GO analysis suggested that these 491 genes played a role in mitochondrial function.

**CONCLUSIONS.** miRNA 484 regulates mitochondrial fission and apoptosis[4]. miRNA 484-mediated mitochondrial regulation may play a significant role in the pathogenesis of AKI in a murine model of septic acute kidney injury.

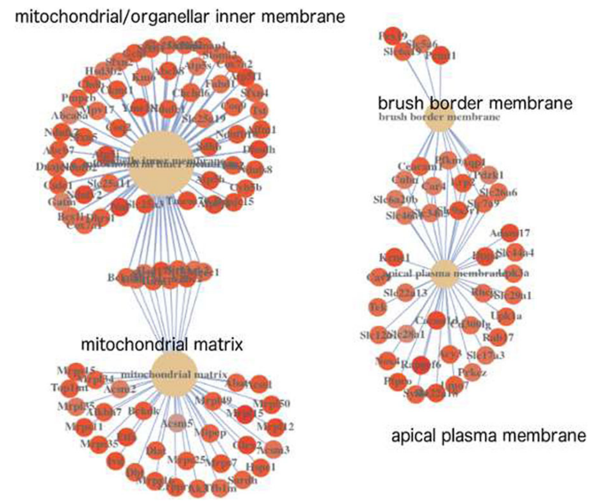

**Fig. 1 (abstract 1012).** GO analysis of all differentially regulated mRNA; mitochondrial genes predominate

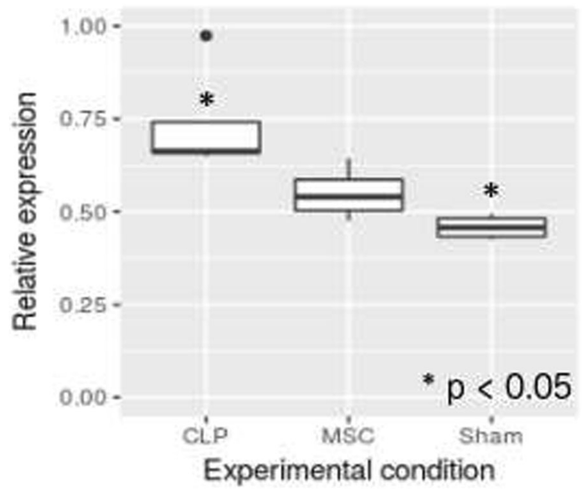

**Fig. 2 (abstract 1012).** miRNA 484 expression is increased after CLP; MSC-treated mice show intermediate expression

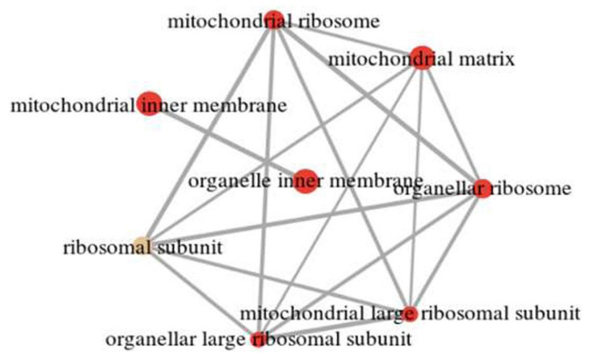

**Fig. 3 (abstract 1012).** GO analysis of 491 putative targets of miRNA 484

#### REFERENCE(S)

1 Gomez H *et al.* Shock. 2014 Jan 1;41(1):3.
2 Patil N *et al.* Am J Physiol Renal Physiol. 2014 Apr 1;(306):F734.
3 Mei *et al.* Am J Respir Crit Care Med. 2010 Oct 15;182(8):1047.
4 Wang *et al.* Nat Commun. 2012 Apr 17;3(1):781.

#### GRANT ACKNOWLEDGMENT

This work is supported by the Canadian Institutes of Health Research MOP 106545.

### 1013

#### Altered renal microcirculation revealed by dynamic contrast-enhanced ultrasound (CEUS) following fluid resuscitation in hemorrhagic shock in pigs

B. Ergin[1], A. Lima[2], T. van Rooij[3], Y. Ince[1], C. Ince[1]
[1]Academic Medical Center, Department of Translational Physiology, Amsterdam, Netherlands; [2]Erasmus MC University Medical Center, Adult Intensive Care, Rotterdam, Netherlands; [3]Erasmus MC University Medical Center, Department of Biomedical Engineering, Thoraxcentrer, Rotterdam, Netherlands
**Correspondence:** B. Ergin
*Intensive Care Medicine Experimental* 2018, **6(Suppl 2):**1013

**INTRODUCTION.** Hemorrhage causes a depletion of systemic hemodynamics and oxygen delivery, and also triggers an inflammatory cascade. Despite treatment of macrocirculation with bleeding control, fluid resuscitation and blood transfusion, persistent microcirculatory dysfunction might remain and possibly contribute to acute kidney injury (AKI) following hemorrhagic shock.

**OBJECTIVE.** In this study, we examined whether or not there is macro- and microcirculatory coherence in kidney during haemorrhage and resuscitation. To this end we measured haemorrhage-induced microcirculatory alterations in both the kidney (CEUS) and sublingual tissue as a methodology that can easily be applied at the bed-side.

**METHODS.** 18 anaesthetised, mechanically ventilated female Yorkshire pigs were divided 2 groups as Hemorrhagic shock (HS) (40% blood loss) (n=12) and control (C) (n=6). In addition to hemodynamic parameters, CEUS and laser speckle perfusion imaging were used for determination of renal microcirculatory perfusion alterations in the exposed kidney and the SDF camera (Cytocam) for evaluation of sublingual microcirculation.

**RESULTS.** Despite normalized MAP after fluid resuscitation at T3, HR remained higher during resuscitation (p< 0.05 vs. C). At T3, CO was found significantly higher than the control (p< 0,05 vs. C) (Fig 1).

RBF and urine output were improved by fluid resuscitation at T3 (p< 0,05 vs. BL and Control) and perfusion. The intrarenal microcirculatory transit time (IRMTT) between a renal arterial and the renal microcirculation introduced by us (1) as a new CEUS parameter for measurement of intrarenal microcirculatory alterations showed a slowing down of microcirculatory perfusion during early resuscitation (p< 0,05 vs. C) (Fig 2).

At T3, other CEUS hemodynamic parameters FWHM (full-width half maximum) and PE (peak-enhancement) were also significantly reduced at end of resuscitation in HS group (p< 0,05 vs. C). Sublingual microcirculation analyzed automatically by our newly developed software followed this trend closely (p< 0,05; HS group T1 vs. T3) (Fig 3).

pH, base excess and lactate levels were not corrected after 1 hour fluid resuscitation (p< 0,05 vs. C).

**CONCLUSION.** Our results showed that correction of macrohemodynamics after hemorrhagic shock did not result in a parallel improvement in the microcirculation identifying the presence of a loss of hemodynamic coherence. Our study also identified that sublingual microcirculatory alterations were able to track similar alterations in the kidney microcirculation. Additionally, these findings support the microcirculatory basis for the development of AKI following resuscitation in haemorrhage.

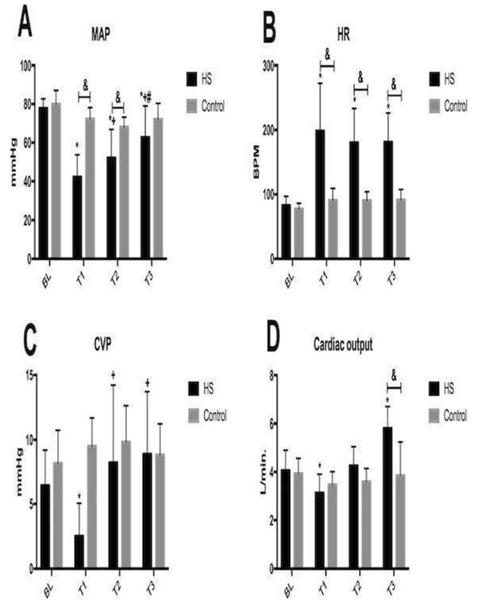

**Fig. 1 (abstract 1013).** Systemic hemodynamic variables

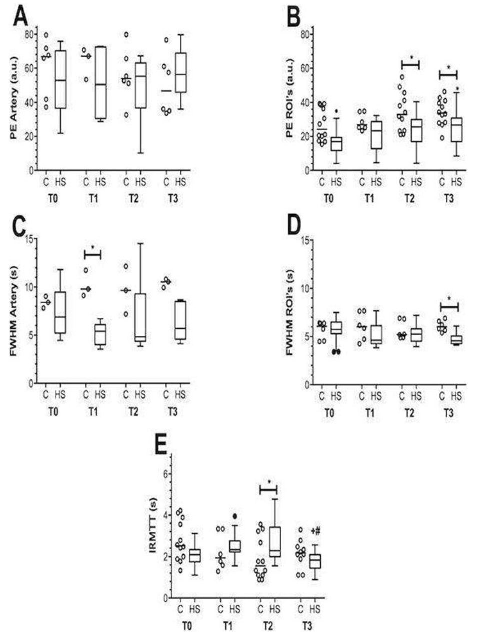

**Fig. 2 (abstract 1013).** Results of contrast-enhanced ultrasound in kidney

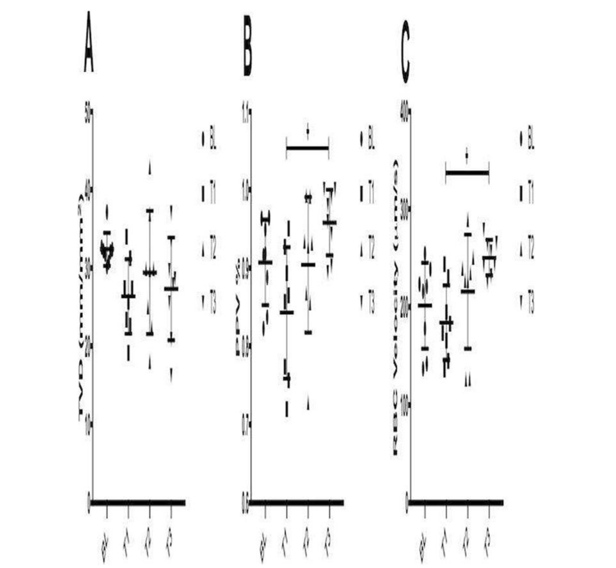

**Fig. 3 (abstract 1013).** Results of sublingual microcirculation

### 1014

#### Systolic blood pressure and lactate levels can predict severe pediatric AKI after cardiac surgery: a machine learning approach

S. Falini[1], M. Flechet[2], I. Scharlaeken[2], F. Güiza[2], G. Van den Berghe[2], G. Meyfroidt[2]

[1]University of Trieste, Department of Perioperative Medicine, Intensive Care and Emergency, Trieste, Italy; [2]KU Leuven, Clinical Division and Laboratory of Intensive Care Medicine, Academic Department of Cellular and Molecular Medicine, Leuven, Belgium

**Correspondence:** S. Falini

*Intensive Care Medicine Experimental* 2018, **6(Suppl 2):**1014

**INTRODUCTION.** Acute kidney injury (AKI) is common in pediatric patients who undergo surgery to correct congenital cardiac defects, and is associated with poor outcomes [1]. This study is a sub-analysis of a prospective blinded observational trial that assessed the clinical value of the Foresight near-infrared spectroscopy (NIRS) cerebral oximeter [2-3].

**OBJECTIVES.** The purpose of this analysis was to identify predictors of severe pediatric AKI (spAKI), stage 2 or 3 [4], after cardiac surgery, defined as a serum creatinine ≥ 2 times the baseline or urine output < 0.5 ml/kg/h for at least 12 hours or provision of dialysis. Secondly, we developed a prediction model for its occurrence during the first week of ICU stay.

**METHODS.** All patients from the NIRS study [2] without AKI on admission nor during the first 6 hours were included Admission data, laboratory results and ICU monitoring values were extracted from the electronic health record system (iMDsoft's MetaVision) and analyzed. Feature selection was performed with univariate testing and a stepwise logistic regression approach. Prediction models were then developed using different machine learning algorithms through 3-fold cross validation. The performance of the final model was reported with the area under the receiver-operator characteristic curve (AUROC), calibration curve, and Brier score.

**RESULTS.** From 177 patients of the NIRS study, 156 (88%) remained for the analysis. 55 patients (35%) developed spAKI during the first week. Median systolic arterial blood pressure and blood lactate levels resulted the only significant predictors of spAKI. Logistic regression was the most discriminative and well-calibrated model (figures 1, 2), with an AUROC of 0.74 and a Brier score of 0.17. A graphical representation of the model is shown in figure 3.

**CONCLUSIONS.** Although the pathophysiology of AKI is complex, a model including only systolic blood pressure and blood lactate levels was able to fairly predict AKI 6 hours in advance.

[2] ClinicalTrials.gov Identifier: NCT01706497

**GRANT ACKNOWLEDGMENT**

M. Flechet received funding from the Research Foundation, Flanders (FWO) as a PhD fellow (11Y1118N), Dr. Van den Berghe from the Methusalem program of the Flemish Government (Belgium) and the European Research Council (AdvG-2012-321670), Dr. Meyfroidt from FWO as senior clinical investigator (1843118N).

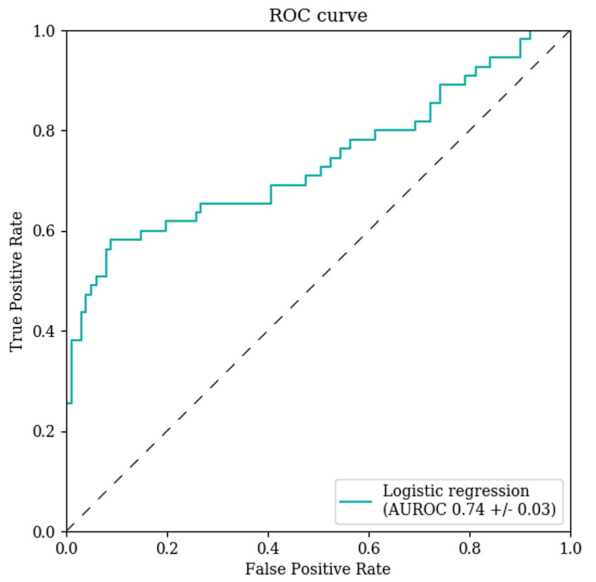

**Fig. 1 (abstract 1014).** ROC curve

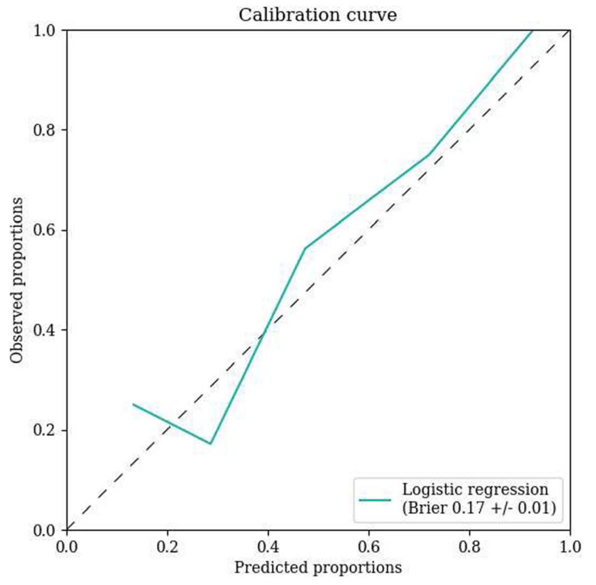

**Fig. 2 (abstract 1014).** Calibration curve

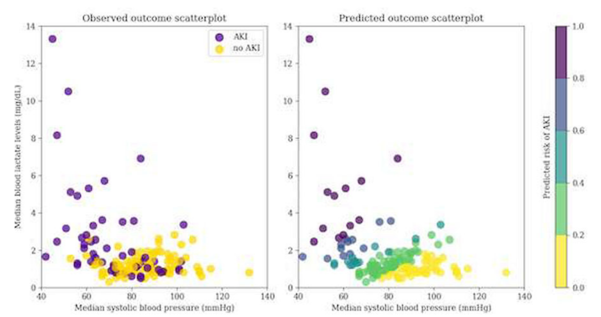

**Fig. 3 (abstract 1014).** Scatterplot of observed and predicted outcomes

## Between the OR and the ICU

### 1015

#### Unplanned ICU admission from the operating room after major noncardiac surgery

S. Kongsayreepong[1], N. Promsi[2], T. Supakamonsanee[1], P. Toomtong[1]
[1]Siriraj Hospital, Mahidol University, Anesthesiology & Critical Care, Bangkok, Thailand; [2]Siriraj Hospital, Mahidol University, Anesthesiology, Bangkok, Thailand
**Correspondence:** S. Kongsayreepong
*Intensive Care Medicine Experimental* 2018, **6(Suppl 2):**1015

**INTRODUCTION.** Unplanned postoperative ICU admission (UIA) from the OR has been considered as a clinical indicator of perioperative quality care, safety of surgical patient & information for quality improvement (1,2).

**OBJECTIVES.** To study the incidence, detail of adverse events (AEs) that lead to UIA, preventability & outcome as resource utilization, organs failure & 90 days mortality.

**METHODS.** This prospective observational study was done in UIA from OR or within 24 hrs after major noncardiac surgery & admitting to the general SICU (14 beds) of the 3rd referral university hospital (2,400 beds, >33,000 ops/yr) from Jan 2016-Dec 2017. Study data: patient demographic data, co-morbidities, ASA physical status, type & duration of anesthesia, urgency of surgery, detail of AEs & outcome as resource utilization , organ failure while admitted in ICU & 90 days mortality.

**RESULTS.** There were 223 UIA or 34:10,000 operations. Age of 68.24 + 22.01 yrs, 54% & 30% were ASA III & > III respectively, 30% underwent emergency surgery, 68% of the AEs occur intraoperative, 48.4% were anesthesia related. The rest of the AEs associated with postoperative care as 18.8% & 13% were admitted from RR & within 24 hrs after operation respectively.3/4 of the intraoperative AEs were severe hemodynamic instability that 41%, 28% & 15% were from massive intraoperative bleeding, septic shock & combined neuraxial & general anesthesia respectively. The rest were from severe cardiac arrhythmias, massive PE (4 needed ECMO support, 1 had angiothrombolysis & 5 had surgical embolectomy), anaphylaxis (contrast media, high dose atracurium/cisatracurium, 4% gelatin), LAST & MH. One fifth of the AEs were airway & respiratory complications that 68% were from pulmonary aspiration (1/4 turned ARDS) which 40% were consensuses as preventable.The rest were from unexpected difficult intubation, laryngospasm & bronchospasm at the time of extubation & massive pneumothorax from invasive procedure. Delay recovery, perioperative stroke were significant AEs of the nervous system. Main AEs in the RR were respiratory depression, airway obstruction (3 developed hypoxic cardiac arrest). Airway obstruction & pulmonary aspiration were important AEs on the floor (2 had hypoxic cardiac arrest & death).
Most of the UIA patients stay in ICU 1-3 days with the result of immediate resuscitation & ICU care. Patient with septic shock had highest organ failure, ICU stay & mortality. Patient with hypoxic cardiac arrest & perioperative stroke had highest morbidity. Data from multivariate analysis showed predictor of 90 days mortality were age> 85yrs, ASA >III, emergency surgery, cardiac arrest, septic shock, multiorgan failure.

**CONCLUSIONS.** Unplanned ICU admission was found in 1/5 of ICU admission which ¼ of the AEs could be preventable. Causes analysis & carefully implement of prevention strategies should be done to reduce this UIA to improve perioperative quality care.

##### REFERENCE(S)

(1) Piercy M, 2005; (2)Haller G, 2005

### 1016

#### Impact of crystalloid *versus* colloid solutions on renal function and disability after major abdominal surgery: long-term follow-up of a double-blind randomized controlled trial

A. Delaporte[1], A. Joosten[2], J. Mortier[1], M. Cannesson[3], J. Rinehart[4], J.L. Vincent[5], P. Van der Linden[6]
[1]Erasme Hospital, Anesthesiology, Brussels, Belgium; [2]Hopital Erasme - Erasmus Ziekenhuis - Ulb, Anesthesiology, Brussels, Belgium; [3]UCL, Anesthesiology, Los Angeles, United States; [4]UCI, Anesthesiology, Irvine, United States; [5]Erasme Hospital, Intensive Care, Brussels, Belgium; [6]CHU Brugmann, Anesthesiology, Brussels, Belgium
**Correspondence:** A. Delaporte
*Intensive Care Medicine Experimental* 2018, **6(Suppl 2):**1016

**INTRODUCTION.** We recently demonstrated that administration of balanced hydroxyethyl starch (HES) solution as part of intraoperative goal-directed fluid therapy (GDFT) was associated with better short-term outcomes than administration of a balanced crystalloid solution in patients having major abdominal surgery (1).

**OBJECTIVES.** In the present study, we performed a one-year follow-up of renal and disability outcomes in these patients.

**METHODS.** All patients enrolled in the earlier study were followed up one year after surgery for renal function and disability using the World Health Organization Disability Assessment Schedule 2.0 (WHODAS). The primary outcome measure was estimated glomerular filtration rate (eGFR). Secondary outcomes were serum creatinine (Scr) and WHODAS score. Groups were compared directly on a complete-case analysis basis, and modern imputation methods were then used in mixed-model regressions to assess the stability of the findings taking into account the missing data.

**RESULTS.** Of the 160 patients enrolled in the original study, follow-up data were obtained for renal function in 129 and for WHODAS score in 114. There were no statistically differences in Scr and eGFR (p>0.05) by pairwise testing or mixed-model regressions after imputation of missing data. However, the WHODAS score was significantly lower in the colloid than in the crystalloid group (2.7 [0-12] % vs. 7.6 [1.3- 18] %; p=0.0147).

**CONCLUSIONS.** In patients undergoing major open abdominal surgery, administration of a balanced HES solution as part of intraoperative GDFT did not affect long-term renal function compared to the use of a balanced crystalloid but was associated with less disability one year after surgery.

##### REFERENCE(S)

1) Joosten A, Delaporte A, Ickx B, et al. Crystalloid versus Colloid for Intraoperative Goal-directed Fluid Therapy Using a Closed-loop System: A

Randomized, Double-blinded, Controlled Trial in Major Abdominal Surgery. *Anesthesiology* 2018

**GRANT ACKNOWLEDGMENT**
This trial was supported by grants from the European Society of Intensive Care Medicine ESICM (Baxter Award 2015), the Belgium Society of Anesthesiology (2015) and the Brugmann Foundation (2015)

### 1017

#### Evaluation of accuracy of noninvasive haemoglobin estimation by pulse-oximetry during major surgical haemorrhage

J. Divatia, S. Pradhan, P. Verma
Tata Memorial Hospital, Anaesthesiology, Critical Care and Pain, Mumbai, India
**Correspondence:** J. Divatia
*Intensive Care Medicine Experimental* 2018, **6(Suppl 2):**1017

**INTRODUCTION.** Non-invasive spectroscopic haemoglobin monitoring using pulse oximetry (SpHb) provides continuous, non-invasive haemoglobin (Hb) monitoring, and may be advantageous during major surgical haemorrhage.[1-4]

**OBJECTIVES.** To determine the reliability of SpHb monitoring in comparison with Hb estimated by the laboratory analyser (HbLab) in patients undergoing major cancer surgery with anticipated blood loss more than 30% of total estimated body volume (EBV).

**METHODS.** This prospective study was performed in 40 adult patients undergoing elective surgical procedures with expected blood loss > 30% EBV. The study was approved by the Institutional Ethics Committee and written, informed consent was obtained from all participants. SpHb was monitored using a Rainbow R-25a sensor connected to a Masimo Rainbow SET® Radical-7™ Pulse CO-Oximeter. Arterial blood samples were analysed for Hb levels with a laboratory haematology analyser (Siemens ADVIA 2120/2120i). Hb by both methods was compared at 4 time points: T1, after insertion of the arterial line and prior to surgery; T2, prior to transfusion of the first unit of packed red blood cells (PRBCs); T3, after transfusion of two units of PRBCs; and T4, after completion of surgery. Correlation, agreement and error grid analyses were performed with scatter plots, Bland-Altman plots[5] and Morey´s three-zone error grids.[6] To determine the reliability for the trend of Hb, at each time point (T1 to T4), the mean difference between SpHb and HbLab values was analysed using the paired 't' test.

**RESULTS.** 136 paired readings from 40 patients were analysed. Preoperative Hb was 11.96 ± 1.89g/dL. Median blood loss [interquartile range] was 2500 [1825-3400] ml and median volume of PRBCs transfused was 779 [474-1077] ml. Pearson correlation coefficient (r) for HbSpHb vs HbLab was 0.581. With reference to HbLab, the bias (precision) and Limits of agreement of SpHb were -0.3691 (1.71) and +2.98 to -3.72 g/dl. On error-grid analysis, while there were no measurements in the critical error Zone C, 44/136 (32%) of the values were in Zone B, which is the zone for potential error. Analysis of differences between mean Hb by the two methods at T1 to T4 showed that at T2, there was a statistically significant difference between mean SpHb and mean HbLab ( -0.93 ± 1.69 g/dL, p=0.005)

**CONCLUSIONS.** The limits of agreement between SpHb and HbLab were wide. SpHb followed the trend of HbLab, but over-estimated the Hb, especially at T2, prior to transfusion of the first unit of PRBCs. One may thus make an erroneous decision to not transfuse the patient. Caution needs to be exercised when using the SpHb monitor as a sole method to guide intraoperative transfusion-related decisions.

**REFERENCE(S)**
1. Anesth Analg. 2014;119:332-346
2. Br J Anaesth. 2012;109:522-528
3. J Anesth. 2015;29:29-34
4. Indian J Anaesth. 2017;61:543-548
5. Lancet 1986;1(8476):307-10
6. Anesth Analg. 2011;113:89-91

**GRANT ACKNOWLEDGMENT**
Tata Memorial Hospital

### 1018

#### Indirect measurement of respiratory quotient in non-cardiac high risk surgery is a predictor of postoperative complications

S. Bar[1], C. Grenez[1], O. Abou-Arab[1], B. de Broca[1], B. Bouhemad[2], H. Dupont[1], E. Lorne[1], P.G. Guinot[2]
[1]CHU Amiens-Picardie, Amiens, France; [2]CHU Dijon Bourgogne, Dijon, France
**Correspondence:** S. Bar
*Intensive Care Medicine Experimental* 2018, **6(Suppl 2):**1018

**INTRODUCTION.** Respiratory quotient (RQ) defined by the ratio of $CO_2$ production ($VCO_2$) and $O_2$ consumption ($VO_2$) was described as a non-invasive marker of anaerobic metabolism (1). The measurement of $O_2$ and $CO_2$ inspired and expired fractions ($FiO_2$, $FeO_2$, $FiCO_2$ and $FeCO_2$) is part of monitoring of the intubated-ventilated patient in the operating room. Thus, physician may have an indirect measure of the anaerobic metabolism based on the RQ.

**OBJECTIVES.** The main objective was to demonstrate the ability of indirect measurement of RQ to predict postoperative complications after major surgery. The second objective were to compare predictability of RQ, central venous oxygen saturation ($ScVO_2$), $gapCO_2$ ($PvCO_2$-$PaCO_2$) and arterial lactate.

**METHODS.** We conducted a multicentric and observational study. Patients operated of major surgery (abdominal, orthopedic, vascular) were included. We excluded cardiac, thoracic, laparoscopic surgery and acute or chronic respiratory insufficiency. Indirect RQ was performed by using average values of $FiO_2$, $FeO_2$, $FiCO_2$, $FeCO_2$ measured by anesthesia ventilator (Drager™ Perseus or Primus) at 4 times of the surgery (baseline hemodynamic optimization, 1 and 2 hours after surgical incision, skin closure). RQ was calculated by this formula $RQ= VCO_2/VO_2 = (FeCO_2-FiCO_2)/(FiO_2-FeO_2)$. All complications were recorded according to international guidelines until the 30[th] postoperative day (2). We also recorded the following parameters: blood pressure, cardiac output, arterial lactate, $GapCO_2$, $ScvO_2$ and O2 delivery ($DO_2$).

**RESULTS.** After ethics comittee agreement, 81 patients were included with a median age of 63 [55-71] years. 27(33%) patients had one post-operative complications and 8(10%) are dead. At skin clossure, patients with complications had higher arterial lactate and RQ values. $ScVO_2$, $gapCO_2$ and total fluid administration did not differ between patients with/without complications.

In the overall population, indirect RQ was statistically correlated with arterial lactate(r=0,347, p=0,001) and inversely correlated to $DO_2$ (r=-0.206, p=0.031) and cardiac output(r=-0.238, p=0.028). RQ and arterial lactates predict complications with an area under the curve of 0.77($IC_{95\%}$ :0.647 to 0.898, p=0.001) and 0.71(0.58 to 0.83, p=0.003). The best indirect RQ cut off was 0.93 (sensibility= 78% and specificity= 70%). $ScVO_2$ and gap $CO_2$ were not associate to post-operative complications.

**CONCLUSIONS.** Indirect measure of RQ could be considered as a non-invasive marker of anaerobic metabolism that was correlated to arterial lactate and predicted post-operative complications in high surgical risk patients.

**REFERENCE(S)**
1. Cohen IL et al. Effect of hemorrhagic shock and reperfusion on the respiratory quotient in swine. Crit Care Med. 1995;23(3):545-52

2. Jammer I et al. European Perioperative Clinical Outcome (EPCO) definitions: a statement from the ESA-ESICM joint task force on perioperative outcome measures. Eur J Anaesthesiol 2015;32:88-105.

**Table 1 (abstract 1018).** Comparison of different markers between patients with or without complications (*= p<0.05 at one time; $= p<0.05 Manova analysis)

| | | Patients with complications (n=27) | No complications (n=54) | p-value |
|---|---|---|---|---|
| Mean cumulated fluid administration (ml.kg-1) | | 40 [24-60] | 44 [27-66] | 0.262 |
| MAP (mmHg) | T1; T2; T3; T4 | 76[68-88]; 73[66-77]; 72[65-82];75[68-86] | 73[66-85];77[71-87]; 71[63-80]; 77[71-87] | 0.073 |
| Cardiac output (l.min-1) | T1; T2; T3; T4 | 4.8[4.0-6.3]; 5.1[4.5-6.7]; 4.9[4.1-5.8]; 5[4.1-6.0]* | 4.8[4.1-5.6]; 4.9[3.9-6.0]; 5.1[3.9-6.2]; 5.3[3.8-6.9] | 0.048 $ |
| RQ | T1; T2; T3; T4 | 0.90[0.77-1.01]; 0.89[0.74-1.07]; 0.79[0.72-0.97]; 1.03[0.79-1.29]* | 0.83[0.77-0.98]; 0.83[0.76-0.96]; 0.81[0.70-0.92]; 0.81[0.74-0.93] | 0.001 $ |
| ScvO2 (%) | T1; T2; T3; T4 | 86[80-94]; 84[79-93]; 84[78-88]; 83[80-91] | 84[80-90]; 84[78-90]; 86[78-82]; 85[77-89] | 0.084 |
| Gap CO2 (mmHg) | T1; T2; T3; T4 | 6.6[3.9-8.3]; 6.3[4.1-7.2]; 4.8[2.8-8.0]; 5.6[3.4-8.2] | 4.8[3.0-6.7]; 5.3[3.9-7.3]; 5.3[3.7-7.0]; 5.0[3.2-7.1] | 0.980 |
| VO2 (l.min-1) | T1; T2; T3; T4 | 7[3-15]; 12[8-16]; 11[8-23]; 14[8-24] | 18[10-22]; 12[7-19]; 15[9-23]; 13[9-18] | 0.125 |
| DO2 (l.min-1) | T1; T2; T3; T4 | 72[56-99]; 84[63-102]; 78[55-100]; 73[58-98] | 82[61-94]; 83[52-95]; 81[65-107]; 81[63-112] | 0.101 |
| Arterial lactate (mmol.l-1) | T1; T2; T3; T4 | 1.1[0.9-1.6]; 1.4[1.0-1.9]; 1.6[1.2-2.2]; 2.8[1.6-3.8]* | 1.0[0.8-1.2]; 1.2[0.9-1.5]; 1.3[0.9-1.8]; 1.8[1.2-2.7] | 0.001 $ |

### 1019

#### Results of the randomized, double-blind, placebo-controlled, Phase 3 HERCULES Study of caplacizumab in patients with acquired thrombotic thrombocytopenic purpura

M. Scully[1], S. Cataland[2], F. Peyvandi[3], P. Coppo[4], P. Knoebl[5], J. Kremer Hovinga[6], A. Metjian[7], J. de la Rubia[8], K. Pavenski[9], F. Callewaert[10], D. Biswas[10], H. De Winter[10], R.K. Zeldin[11], for the HERCULES Investigators
[1]University College London Hospitals NHS Trust, Department of Haematology, London, United Kingdom; [2]The Ohio State University, Division of Hematology, Department of Internal Medicine, Columbus, United States; [3]Fondazione IRCCS Ca' Granda Ospedale Maggiore Policlinico; University of Milan, Angelo Bianchi Bonomi Hemophilia and Thrombosis Center, Milano, Italy; [4]Saint-Antoine University Hospital, Department of Hematology, Paris, France; [5]Vienna University Hospital, Department of Medicine 1, Division of Hematology and Hemostasis, Vienna, Austria; [6]Bern University Hospital, Inselspital, University Clinic of Hematology and Central Hematology Laboratory, Bern, Switzerland; [7]Duke University School of Medicine, Division of Hematology, Durham, United States; [8]Universidad Católica de Valencia Hospital Dr. Peset, Hematology Department, Valencia, Spain; [9]St. Michael's Hospital/Research Institute, Department of Laboratory Medicine and Pathobiology, Toronto, Canada; [10]Ablynx NV, Clinical Development, Zwijnaarde, Belgium; [11]Ablynx NV, Clinical Development, Chief Medical Officer, Zwijnaarde, Belgium
**Correspondence:** P. Coppo
*Intensive Care Medicine Experimental* 2018, **6(Suppl 2):**1019

**INTRODUCTION.** Caplacizumab, a bivalent Nanobody, targets the A1 domain of vWF, inhibiting the interaction between ultra-large vWF and platelets.

**OBJECTIVES.** We assessed the efficacy and safety of caplacizumab in patients with acquired thrombotic thrombocytopenic purpura (aTTP).

**METHODS.** Patients with an acute episode of aTTP who had received one plasma exchange (PE) treatment were randomized 1:1 to placebo or 10 mg caplacizumab, in addition to daily PE and corticosteroids. The study drug was given during the PE period and 30 days thereafter. If at the end of this period there was evidence of ongoing disease, such as suppressed ADAMTS13 activity, investigators were encouraged to extend the blinded treatment for a maximum of 4 weeks together with optimization of immunosuppression. All patients entered a 28-day treatment-free follow up period after the last dose of study drug.

**RESULTS.** 145 patients were randomized (73 placebo, 72 caplacizumab). Compared to placebo, caplacizumab-treated patients were >50% more likely to achieve a platelet count response (platelet count normalization rate 1.55, 95% CI 1.10 - 2.20, p< 0.01). During the study drug treatment period, treatment with caplacizumab resulted in a 74% reduction in TTP-related death, recurrence of TTP, or a major thromboembolic event (p< 0.0001). During the overall study period, patients administered caplacizumab had a 67% reduction in disease recurrence (p< 0.001). No caplacizumab-treated patients were refractory to therapy versus 3 on placebo (p=0.057).Treatment with caplacizumab was associated with a trend toward faster normalization of LDH, cardiac troponin I and serum creatinine. Treatment with caplacizumab resulted in a 38% reduction in the mean number of days of plasma exchange, and a 31% reduction in the mean length of hospitalization during the study drug treatment period. A third of the patients were admitted to the ICU. Treatment with caplacizumab resulted in a 65% reduction in the mean length of ICU stay (reduction of 6.3 days). The most common caplacizumab-related TEAEs were epistaxis, gingival bleeding, and bruising.

**CONCLUSIONS.** Treatment with caplacizumab reduced the time to platelet count response and resulted in a clinically meaningful reduction in aTTP-related death, recurrence of aTTP, or a major thromboembolic event during study drug treatment, as well as recurrences during the overall study period. Treatment with caplacizumab also resulted in meaningful reductions in healthcare resource utilization. The safety profile was favorable, with mucocutaneous bleeding the most frequently reported AE. Caplacizumab represents a novel adjuvant treatment option for patients with aTTP. (clinicaltrials.gov: NCT02553317).

## Critical care organisation

### 1020

#### Prevalence and risk factors for ICU burnout syndrome among Thai intensivists and ICU nurses

P. Wacharasint[1], C. Laopakorn[1], P. Kunakorn[2]
[1]Phramongkutklao Hospital, Department of Medicine, Bangkok, Thailand; [2]Ramathibodi Hospital, Bangkok, Thailand
**Correspondence:** P. Wacharasint
*Intensive Care Medicine Experimental* 2018, **6(Suppl 2):**1020

**INTRODUCTION.** Burnout syndrome (BOS), a work-related constellation of symptoms and signs, which interfere individuals emotional stress, and associated with increasing job related disillusionment. While its prevalence was found highly in the intensive care unit (ICU) professionals and varied across the globe,[1] ICU-BOS among Thai intensivists and ICU nurses had never been explored.

**OBJECTIVES.** To study the prevalence of ICU-BOS among Thai intensivist and ICU nurse, and to identify the risk factors for ICU-BOS.

**METHODS.** We performed a multicenter, prospective, cross-sectional study in 17 hospitals in Thailand during September 2017 - February 2018. Demographic data and BOS-related data were collected from full-time ICU physicians and ICU nurses using electronic questionnaires. ICU-BOS was defined if participant exhibits at least 1 of 3 aspects (i.e. depersonalization, emotion exhaustion, and personal accomplishment) regarding to Maslach Burnout Inventory.[2,3] Primary outcome was prevalence of ICU-BOS among intensivists and ICU nurses, and secondary outcomes were risk factors for ICU-BOS.

**RESULTS.** Following total of 193 electronic survey sent, there were 171 participants (Thai intensivist; n=66 and Thai ICU nurses; n=105) responded by completing these questionnaires, and were included in this analysis. For intensivist, most of them were 34±4 years old and were male (53%), while most of ICU nurses were 33±8 years old and were female (93%). The prevalence of ICU-BOS of intensivists and ICU nurses were 65.2% and 62.6% respectively. Independent risk factors of ICU-BOS among Thai intensivists were 1) income < 20,000 THB/month, 2) thinking idea to quit their ICU job in last one year, and 3) need vacation >2 days/week (adjusted odds ratio (OR) of 31.5, 15.9, 7.4, and p-values of 0.04, 0.007, 0.035 respectively). Regarding to Thai ICU nurses, risk factors of ICU-BOS were 1) age >40 years old, 2) ICU experience >5 years, 3) patient's ICU length of stay >5 days, 4) thinking idea of too much ICU workload, and 5) thinking idea to quit their ICU job in last one year (adjusted OR of 15.7, 4.6, 10.0, 4.3, 5.3, and p-values of 0.009, 0.04, 0.004, 0.04, 0.007 respectively).

**CONCLUSIONS.** In this study, we found a high prevalence of ICU-BOS in Thai ICU professionals. Co-occuring risk factors for ICU-BOS among Thai intensivists and ICU nurses was thinking idea to quit their ICU job in last one year. Our findings support further interventions to reduce this high prevalence of ICU-BOS in Thai ICU professionals.

**REFERENCE(S)**

1. Chuang CH, Tseng PC, Lin CY, Lin KH, Chen YY. Burnout in the intensive care unit professionals. A systematic review. Medicine (Baltimore) 2016;95:50(e5629).
2. Maslach C, Jackson SE, Leiter MP. Maslach burnout inventory manual, 3rd edn, 1996, Mountain view, California.
3. Pines A, Maslach C. Characteristics of staff burnout on mental health setting. Hosp Community Psychiatry 1978;29:233-7.

### 1021

#### Effect of sepsis diagnosis at nighttime and weekends on adherence to treatment guidelines in Brazil: a multicenter retrospective cohort study

M. Monteiro[1], L.C.P.d. Azevedo[1,2], B. Besen[2,3], E. Batista[4], O. Ranzani[4,5]
[1]Hospital Sirio-Libanes, Sao Paulo, Brazil; [2]Faculdade de Medicina, Universidade de Sao Paulo, Disciplina de Medicina de Emergencia, Sao Paulo, Brazil; [3]Hospital da Luz, Adult ICU, Sao Paulo, Brazil; [4]Quality and Safety Department, Americas Seviços Médicos, Sao Paulo, Brazil; [5]Faculdade de Medicina, Universidade de Sao Paulo, Divisão de Pneumologia, Instituto do Coração, Sao Paulo, Brazil
**Correspondence:** L.C.P.d. Azevedo
*Intensive Care Medicine Experimental* 2018, **6(Suppl 2):**1021

**INTRODUCTION.** Lack of adherence to sepsis treatment bundles is associated with increased mortality. Organizational characteristics like different staffing patterns according to the period of day and the day of the week that patients are diagnosed can influence adherence to bundles and eventually outcomes. Very few studies evaluated the differences in adherence to sepsis bundles during nighttime and weekends, with contradictory results.

**OBJECTIVES.** To evaluate the effect of sepsis diagnosis at different periods of day (daytime versus nighttime) and weekends on adherence to sepsis treatment bundles and whether these differences may impact outcomes.

**METHODS.** We conducted an observational, multicenter and retrospective study including patients diagnosed with sepsis and septic shock between 2010 and 2017 in 10 hospitals in São Paulo, Brazil. Compliance with each recommendation of the 3-hour sepsis bundle was calculated according to the moment sepsis was diagnosed: day (07:00 AM to 6:59 PM) versus night (7:00 PM to 06:59 AM) and weekdays (Monday 7:00 AM to Friday 06:59 PM) versus weekends (Friday 7:00 PM to Monday 06:59 AM). Adherence to the entire 3-hour bundle was the primary outcome and subgroup analysis included compliance according to disease severity (sepsis/septic shock) and location of sepsis diagnosis (emergency room, ward or ICU). A multivariable analysis adjusted for age, sex, sepsis severity, location of sepsis diagnosis, type of infection and hospital was done to assess if diagnosis at nighttime and weekends was associated to bundle adherence.

**RESULTS.** We included 11,737 patients. Of these, 8,733 had sepsis and 3,004 had septic shock. SOFA score was 4 (2—7) and hospital mortality rate was 24.7%. Adherence to full 3-hour bundle was higher for cases identified at daytime versus nighttime especially for lactate collection and antibiotic administration (Table 1). There was no difference in the adherence according to the period of day and sepsis severity. The effect of period of day was maintained irrespective of location of sepsis diagnoses [emergency room odds ratio (CI) 1.60 (1.40-1.82), ward 1.18 (1.00-1.39) and ICU 1.33 (1.04-1.69), p=0.017]. In the multivariable analysis, adjusted odds-ratio (CI) for adherence to bundles for cases diagnosed at daytime versus nighttime (reference) was 1.35 (1.23-1.49), p< 0.001. There was no difference in compliance in weekends versus weekdays. The hospital mortality rate was not different for sepsis diagnosed at different periods of day and days of the week.

**CONCLUSIONS.** Adherence to sepsis bundles may be influenced by the moment of sepsis diagnosis. This effect was not related to sepsis severity and it was consistent in possible locations of sepsis diagnosis. Adherence was not affected by the day of the week. Despite the differences in the bundles compliance, nighttime and weekends diagnosis were not associated with altered mortality rates.

**Table 1 (abstract 1021).** Adherence to 3-hour sepsis guidelines according to period of day of sepsis diagnosis

| Bundle Item | Total n=11737 | Daytime n=7658 (65%) | Nighttime n=4079 (35%) | P value |
|---|---|---|---|---|
| Lactate collection | 11170 (95.2%) | 7321 (95.6%) | 3849 (94.4%) | 0.003 |
| Blood cultures collection | 10670 (90.9%) | 6985 (91.2%) | 3685 (90.4%) | 0.122 |
| Antibiotic Administration | 10457 (89.1%) | 6910 (90.2%) | 3547 (87.0%) | <0.001 |
| Entire 3-hour Bundle | 9278 (79.1%) | 6168 (80.6%) | 3110 (76.3%) | <0.001 |
| Fluid administration (n=4774 daytime) (n=2622 nighttime) | 6951 (94.0%) | 4502 (94.3%) | 2449 (93.4%) | 0.111 |

### 1022

#### Simulation for ICU transition - assessment and Re-evaluation (SITAR)

S. Pawar, T. Jacques, K. Deshpande, I. Press, S. Jones, M. Cawley, B. Wood, SITAR
St George Hospital, Intensive Care, Sydney, Australia
**Correspondence:** S. Pawar
*Intensive Care Medicine Experimental* 2018, **6(Suppl 2):**1022

**INTRODUCTION.** Identification of latent safety threats (LSTs) i.e. errors in design, organisation, training or maintenance that may lead to medical errors, is crucial prior to transitioning to a new Intensive Care Unit (ICU) facility.[1] Simulation provides an opportunity for timely identification and remediation of LSTs and thus helps to improve patient safety.

**OBJECTIVES.** To identify LSTs using in-situ simulation sessions prior to moving into a new ICU.

**METHODS.** We conducted simulation scenarios over 6 weeks prior to moving into the new ICU. Healthcare providers (doctors, nurses, allied health personnel) participated in these scenarios .We included a 'virtual' ICU day to focus on testing the new model of care in the new environment. We conducted structured debriefings after each scenario. We developed 15 codes for 6 domains of LSTs by using an iterative process until consensus was reached. Using the Healthcare

Failure Modes and Effect Analysis (HFMEA) matrix developed by the National Centre for Patient Safety (VA NCPS)[2], we assigned a hazard score for each LST. LSTs were classified as catastrophic if they were likely to cause death or injury. The LST communication and action matrix was developed and agreed upon with the hospital management prior to commencement of the study.

**RESULTS.** A total of 24 simulation sessions were conducted with 61 participants. Ninety seven LSTs were identified, 36% of these were catastrophic and needed immediate remediation. Issues related to 'medication administration' and 'equipment' had the highest number of LSTs (34% and 19.6% respectively). Of the 35 catastrophic LSTs, 42% were related to 'medication administration' followed by issues related to 'models of care' (28.6%). Issues related to communication had the least number of LSTs (7.2%) and none were catastrophic (Table 1,Fig1)

**CONCLUSION.** In-situ simulation is a practical method for identifying LSTs prior to patient occupation of a new ICU. Patient safety can be enhanced by timely rectification of these LSTs. Our systematic approach to early LST detection using simulation is potentially adaptable and scalable to other new ICUs

**REFERENCE(S)**

1. Patterson MD, et al. In situ simulation: detection of safety threats and teamwork training in a high risk emergency department.BMJ Qual Saf 2013;22:468-477.
2. DeRosier, J., Stalhandske, E., Bagian, J.P., & Nudell, T (2002). Using Health Care Failure Mode and Effect Analysis: The VA National Center for Patient Safety´s Prospective Risk Analysis System.The *Joint Commission Journal on Quality Improvement,* Volume 27 Number 5:248-267, 2002.

**GRANT ACKNOWLEDGMENT**

SITAR was supported by a grant from the Agency of Clinical Innovation, NSW Ministry of Health.

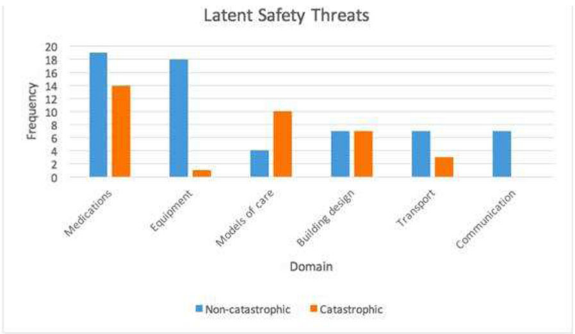

**Fig. 1 (abstract 1022).** Non-catastrophic vs. Catastrophic LSTs

**Table 1 (abstract 1022).** Latent Safety Threats Domains and Severity

| Domain | Non-catastrophic | Catastrophic | Total |
|---|---|---|---|
| Medications | 19 (30.6%) | 14 (42%) | 33 (34%) |
| Equipment | 18 (29%) | 1 (2.8%) | 19 (19.6%) |
| Models of care | 4 (6.4%) | 10 (28.6%) | 14 (14.4%) |
| Building design | 7 (11.2%) | 7 (20%) | 14 (14.4%) |
| Transport | 7 (11.2%) | 3 (8.6%) | 10 (10.5%) |
| Communication | 7 (11.2%) | 0 | 7 (7.2%) |
| Total | 62 | 35 | 97 |

### 1023

#### Central line unit: a new activity outside of the ICU

M. López, E. Palleja Gutierrez, S. Vaquero Andreu, S. Gonzalez López, A. Campanario García, A. Loza Vázquez, P. Jiménez Vilches, A. Lesmes Serrano
Hospital Universitario Virgen de Valme, Sevilla, Spain
**Correspondence:** E. Palleja Gutierrez
*Intensive Care Medicine Experimental* 2018, **6(Suppl 2):**1023

**OBJECTIVES.** To describe the creation of a new Central Line Unit dependent on the Department of Intensive Care Medicine in our hospital.

**METHODS.** Observational study, analytical and prospective, completed in our university hospital of 500 beds with an intensive care unit of 14 beds that includes a department for central line cannulation. We report only the peripherally inserted central catheters (PICC). The PICC lines were inserted by personnel of the Department of Intensive Care Medicine, in the middle third of the arm through an aseptic technique and under echographic control. Data about PICC lines inserted between January, 2013 and December, 2016 were collected, including the demographic variables, types of patients, complications, results, follow-up in 3 months, its removal, and evaluation of the patients. A descriptive analysis was performed (SPSS), analyzing the qualitative variables in percentages and the quantitative variables in median values with standard deviation (SD).

**RESULTS.** 1,556 PICC lines were inserted in 1,327 patients. Age 59.97 ±15.37 years, 50.49% male. Outpatient procedure in 565 (42.58%), hospitalized patients in 762 (57.42%). From departments of Oncology (37.83%), Hematology (13.49%), Internal Medicine (10.78%), Intensive Care Medicine (5.5%). Underlying disease: solid tumors in 623 patients (46.95%) and hematologic malignancies in 188 patients (14.17%). Left arm 79% (Basilic vein 84.4%). Uses: Chemotherapy in 51.02%, Total parenteral nutrition in 21.63%. Average time of use 122.63±141.33 days (in 20% of patients, use for more than 6 months). Reason for removal: End of treatment in 55.92%, Exitus in 25.62%. Complications: Bacteremia in 50 (3. 76%)( 0.28‰ days of catheter) , Deep vein thrombosis in 16 (9.52%). In 95.39% of cases no complications were observed during the insertion. Patient satisfaction during the follow-up in 3 months and in 6 months, 91.22% and 92.39%, respectively.

**CONCLUSIONS.** The cannulation of PICC lines in the Department of Intensive Care Medicine has demonstrated to diminish the rate of complications and to increase the safety of the patient, with a high rate of satisfaction.

### 1024

#### Evaluation of rapid response team contact rate between day time and night time on surgical wards

M. Choi
Sungkyunkwan University College of Medicine, Surgery, Seoul, Korea, Republic of
*Intensive Care Medicine Experimental* 2018, **6(Suppl 2):**1024

**INTRODUCTION.** According to the previous study, there was diurnal variation in the patients-physician ratio and it was associated with the Rapid response Team (RRT) utilization . In real world, there is no surgical professional available for patients in general wards since they usually do their rounding early in the morning and spend most of the time in operating room. We hypothesized that this may attribute to no diurnal variation for use of RRT and its outcome in surgical patients.

**METHODS.** We conducted a retrospective observational study of 465 consecutive acutely deteriorating patients who were managed by RRT in surgical ward between March 2013 and July 2016. Patients treated between 08:00 to 17:59 were considered to receive day time care.

**RESULTS.** The half of RRT call was in the day time (08:00 ~ 17:59, 49.6%), however, the hourly rate of RRT call was higher during the day time (Figure 1). RRT call increased in the hours after routine surgeon's rounding (8:00~10:00), and decreased after they go to operating rooms. - it started to increase around noon when patients underwent surgery come back to wards, and peaked around 6pm when the physicians were finishing up the afternoon rounding. Patient characteristics and the reasons for RRT call were similar between the day and night. However, acute respiratory distress was higher during the night time (16.0% vs 23.9%, p=0.003). The in-hospital mortality (11.7% vs 16.2, p=0.157) and unexpected ICU admission rate (48.5% vs 49.6%, p=0.815) were not different between day and night. After adjusting potential confounding factors, night time RRT call was not associated with in-hospital mortality (RR 1.43, 95% CI 0.82-2.48, p=0.208).

**CONCLUSIONS.** The hourly rate of RRT call was higher during the day time. RRT call on day and night was not associated with unexpected ICU admission rate and in-hospital mortality.

## Muscle weakness in ICU

### 1025

#### Impact of myostatin in intensive care unit acquired weakness

T. Wollersheim[1,2], J. Grunow[1], N. Carbon[1], K. Mai[2,3], J. Spranger[2,3], S. Weber-Carstens[1,2]

[1]Charité Universitätsmedicin Berlin, Department of Anaesthesiolgy and Intensive Care Medicine, Berlin, Germany; [2]Berlin Institute of Health (BIH), Clinical Scientist Program, Berlin, Germany; [3]Charité - Universitätsmedizin Berlin, Department of Endocrinology and Metabolic Diseases, Berlin, Germany

**Correspondence:** T. Wollersheim

*Intensive Care Medicine Experimental* 2018, **6(Suppl 2):**1025

**INTRODUCTION.** Intensive Care Unit (ICU) Acquired Weakness (ICUAW) is a common complication in the critically ill leading to muscle atrophy early during the disease course[1]. Myostatin is known as a major regulator in skeletal muscle growth and degradation. Until now myostatin dynamics in ICUAW are unknown.

**OBJECTIVES.** Our aim was to investigate whether ICUAW is associated with increased myostatin mRNA levels and serum concentration as well as its dynamic changes.

**METHODS.** We included 87 ICU patients and 91 healthy controls. The ICU patients were at high-risk for ICUAW through selection by a SOFA score ≥9. Informed consent for study participation was provided. Blood samples from ICU patients (day 4, 8 and 14) and once from controls, were investigated via ELISA for the measurement of myostatin serum concentrations. Open surgical muscle biopsies was performed within a subgroup of 58 ICU patients at day 5 and 15 and for 5 healthy references (controls) once during hip replacement to determine *MSTN* gene expression by rt-PCR. Values were normalized to healthy controls and expressed as fold change. Ethic vote (Charité EA 2/041/10).

**RESULTS.** Baseline characteristics of ICU patients and healthy controls did not differ significantly. MRC at first day of being adequately awake was available from 45 ICU patients scoring 3.0 IQR (2.0/3.7). First, at all time-points serum myostatin concentrations of ICU patients were massively decreased compared to health controls (p< 0.001). Second, lowest concentrations was found on day 4 with a significant increase to day 8 and 14 nevertheless still significantly lower than controls. Third, mRNA expression levels of *MSTN* were significantly lower in ICU patients compared to controls (p=0.016) resulting from a decrease tendency on day 5 and a significant decrease on day 15.

**DISCUSSION.** In opposite to our hypothesis we did not find increased myostatin levels in ICUAW patients. A massive decrease earliest investigated at day four makes an impact of myostatin on ICUAW implausible. If low levels represent a compensatory mechanism or just reduced production in skeletal muscle after very early muscle wasting is unknown.

**CONCLUSIONS.** For the first time, we show that myostatin levels are reduced in ICUAW patients with a slight recovery during ICU stay. The role of myostatin during ICUAW remains unclear and requires further investigation.

**GRANT ACKNOWLEDGMENT**

TW is participant in the Clinical Scientist Program granted by the Berlin Institute of Health (BIH). TRG grant by BIH given to SWC.

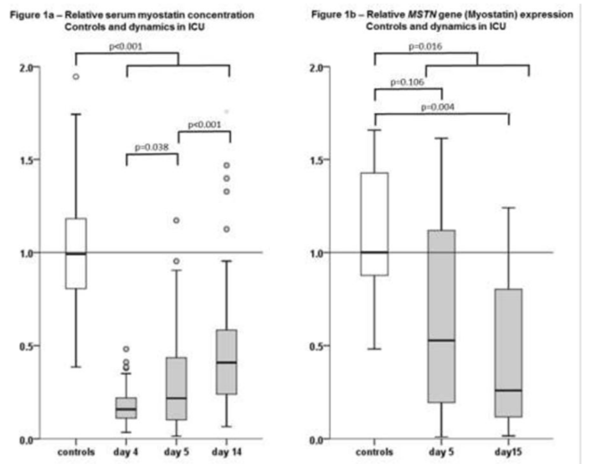

**Fig. 1 (abstract 1025).** See text for description

### 1026

#### Excluding early deaths: posthoc analysis from the VITdAL-ICU study

G. Martucci[1], F. Tuzzolino[2], D. McNally[3], A. Arcadipane[1], K. Christopher[4], H. Dobnig[5], T. Pieber[5], C. Schnedl[6], K. Amrein[5]

[1]Ismett, Department of Anesthesia and Intensive Care, Palermo, Italy; [2]Ismett, Research Office, Palermo, Italy; [3]Children's Hospital of Eastern Ontario, Research Institute, Ottawa, Canada; [4]Brigham and Women's Hospital. Harvard Medical School, Renal Division, Boston, Massachusetts, United States; [5]Medical University of Graz, Department of Internal Medicine, Division of Endocrinology, Graz, Austria; [6]Medical University of Graz, Division of Vasculan and Interventional Radiology, Department of Radiology, Graz, Austria

**Correspondence:** G. Martucci

*Intensive Care Medicine Experimental* 2018, **6(Suppl 2):**1026

**INTRODUCTION.** Vitamin D deficiency has been associated with increased mortality in critically ill patients. Though blood 25-hydroxyvitamin D (25OHD) concentrations can be normalized within 48 hours with a large enteral dose, it may take a week or longer for the steroid hormone to exert its full range of pleiotropic effects.

**OBJECTIVES.** Post hoc analysis of the VITdAL-ICU trial, excluding study participants who died within 7 days of drug administration (placebo vs. 540,000 IU cholecalciferol).

**METHODS.** Chi-square and Fisher's exact tests were used to compare mortality rates between placebo and vitamin D treatment groups. Further, we explored predictors of mortality through logistic regression and multiple analyses, and included 25OHD and 1,25OHD response to treatment on day 3 among survivors and non-survivors, exploring the possible influencing factors.

**RESULTS.** Of the original 475 participants, 432 (91%) were alive after seven days. Survival was higher in the vitamin D arm at all time points, and achieved statistical significance at day 28 (p=0.03) and 6-months (p=0.04).

To explore the factors associated with mortality at 28 days, we calculated the odds ratio for vitamin D supplementation and for several covariates, and found a reduced mortality in the vitamin D arm.

In a bivariate logistic regression analysis, vitamin D still showed significant association with reduced mortality, even when adjusting the results for the same covariates (all p values < or = to 0.05), without showing interaction.

At enrollment 25OHD and 1,25OHD levels were not significantly different between survivors and non-survivors.

Both 25-hydroxyvitamin D and 1,25-dihydroxyvitamin D levels were significantly higher in survivors compared with non-survivors on day 3 in the vitamin D group.

Relevant influencing factors for a reduced 1,25-dihydroxyvitamin D level on day 3 (a reduced plasmatic increase after vitamin D administration), after centering the results for the baseline value, were (estimate; 95% Confidence Limit) body mass index increase (-1.8; -3,-0.6; p< 0.01), male gender (-19; -33,-5; p< 0.01), surgical admission (-24; -45,-4; p=0.02), presence of chronic kidney disease (-17; -30,-5; p< 0.01), and use of vasopressor on admission to the ICU (-20; -34,-6).

**CONCLUSIONS.** Excluding patients with an early death, high-dose vitamin D3 supplementation was associated with a significant reduction in mortality. Vitamin D treatment was associated with reduced mortality even when adjusting for confounding factors. In the treatment group, 25-hydroxyvitamin D and 1,25-dihydroxyvitamin D were higher on day 3 in survivors than in non-survivors, but the increase was less relevant in cases of increased BMI, surgical cases, and CKD, suggesting that these categories of patients may benefit from a higher dosage. The two ongoing large RCTs (VITDALIZE and VIOLET) will further elucidate the potential role of vitamin D supplementation in improving clinical outcomes in critical illness.

**Table 1 (abstract 1026).** Effect of vitamin D treatment on mortality outcomes when excluding the 110 deceased patients in the first 7 days

| | Alive, N (%) | Deceased, N (%) | P Value |
|---|---|---|---|
| 28-Day Mortality | | | |
| Vitamin D, Placebo | 185 (86.1), 170 (78.3) | 30 (13.9), 47 (21.7) | 0.03 |
| ICU Mortality | | | |
| Vitamin D, Placebo | 182 (84.7), 173 (79.7) | 33 (15.3), 44 (20.3) | 0.18 |
| Hospital Mortality | | | |
| Vitamin D, Placebo | 170 (79.1), 154 (71) | 45 (20.9), 63 (29) | 0.05 |
| 6-Months Mortality | | | |
| Vitamin D, Placebo | 154 (71.6), 136 (62.7) | 61 (28.4), 81 (37.3) | 0.04 |

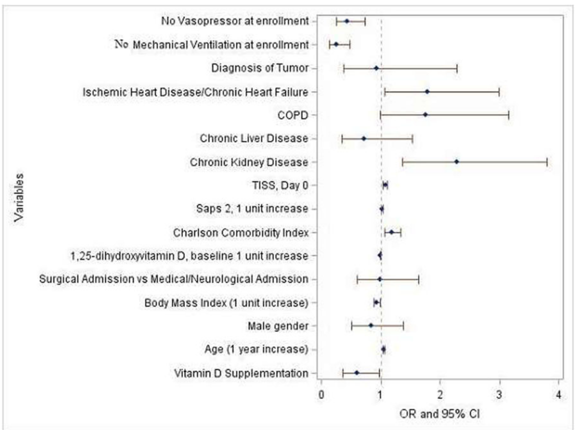

**Fig. 1 (abstract 1026).** Association of covariates with mortality at 28 days.

**Table 2 (abstract 1026).** Vitamin D metabolites level at baseline and Day 3 after enrollment

| | Vitamin D Metabolite | Survivors at 28-day | Non-Survivors at 28-day | P Value |
|---|---|---|---|---|
| Vitamin D Supplementation Group, Day 0 | 25-hydroxyvitamin D, ng/ml | 12.8 (12.1-13.5) | 13.0 (11.3-14.7) | 0.87 |
| | 1,25-dihydroxyvitamin D, pg/ml | 45.7 (38.7-52.7) | 38.3 (21.9-54.7) | 0.43 |
| Placebo Group, Day 0 | 25-hydroxyvitamin D, ng/ml | 13.7 (13.1-14.5) | 12.9 (11.6-14.4) | 0.27 |
| | 1,25-dihydroxyvitamin D, pg/ml | 46.5 (39.6-53.3) | 34.6 (25.9-43.3) | 0.03 |
| Vitamin D Supplementation Group, Day 3 | 25-hydroxyvitamin D, ng/ml | 35.3 (32.5-38.1) | 25.4 (20.2-30.6) | < 0.01 |
| | 1,25-dihydroxyvitamin D, pg/ml | 111.9 (97.4-126.4) | 70.3 (36.0-104.7) | 0.03 |
| Placebo Group, Day 3 | 25-hydroxyvitamin D, ng/ml | 14.1 (13.4-14.8) | 13.9 (12.1-15.6) | 0.81 |
| | 1,25-dihydroxyvitamin D, pg/ml | 51.2 (43.1-59.3) | 37.4 (26.5-48.4) | 0.05 |

### 1027

#### Intra- and interrater reliability for ultrasound measurements of fat and muscle thickness in 120 non-critically ill patients: the USVALID prospective trial

A. Fischer[1], M. Pesta[2], I. Timmermann[2], T. Siebenrock[2], K. Liebau[2], M. Hiesmayr[1]

[1]Medical University of Vienna, Anesthesia and Intensive Care Medicine, Vienna, Austria; [2]Medical University of Vienna, Vienna, Austria

**Correspondence:** A. Fischer

*Intensive Care Medicine Experimental* 2018, **6(Suppl 2):**1027

**INTRODUCTION.** Ultrasound measurements to determine body composition may become important in non- and critically ill patients for adaptation of medication dose, physical therapy and nutrition. Currently a standardized ultrasound protocol lacks.

**OBJECTIVES.** To test the intra- and interrater reliability of the USVALID ultrasound technique

**METHODS.** 2 measuring points on one randomly chosen upper arm and 3 measuring points on one randomly chosen thigh were landmarked with an erasable pen according to anatomical landmarks. On each measuring point a transversal and sagittal ultrasound scan was taken with minimal compression. Fat and muscle thickness was determined on each scan. For intrarater reliability each of the 5 examiners performed the ultrasound examination twice in 12 different patients. For interrater reliability all 10 possible examiner pairs performed the ultrasound examination twice in 60 other patients. The thicknesses of the transversal and sagittal ultrasound scans were averaged. Then the thicknesses of the measuring points for the thigh and those for the upper arm were averaged.

**RESULTS.** 2326 and 2374 measurements of fat and muscle thicknesses were recorded for intra- and interrater reliability respectively. 74 and 26 measurements were missing for intra- and interrater reliability respectively because of impossible ultrasound examination on distinct measuring points. Interrater and intrarater reliability was evaluated by Bland-Altman analysis. There was no statistically significant bias (Table 1,2, Figure 1). For intrarater reliability there was no difference in bias between examiners (P > 0.05, one-way Anova). For interrater reliability there was also no difference in bias between examiner pairs except for fat thickness of the thigh (P=0,047, one-way Anova).

**CONCLUSIONS.** The USVALID ultrasound technique is reliable. The bias lies within 0-3% of the mean value and ranges of agreement are comparable to other studies[1].

**REFERENCE(S)**

1 English CK et al. Ultrasound Is a Reliable Measure of Muscle Thickness in Acute Stroke Patients. Ultrasound Med Biol. 2012.

**Table 1 (abstract 1027).** Intrarater reliability in 60 patients and interrater reliability in 60 other patients

| | muscle thickness of thigh | muscle thickness of upper arm | fat thickness of thigh | fat thickness of upper arm |
|---|---|---|---|---|
| mean value (patient ID 61-120), cm | 2,95 | 2,01 | 1,44 | 0,85 |
| bias, cm (\|% of mean value\|) | -0,08 (3) | -0,07 (3) | 0,01 (1) | 0,01 (1) |
| 95% CI of bias, cm | -0,16 to 0,00 | -0,14 to -0,004 | -0,03 to 0,04 | -0,04 to 0,05 |
| SD of bias, cm | 0,32 | 0,26 | 0,13 | 0,17 |
| range of agreement: bias - 1,96*SD, cm to bias + 1,96*SD, cm | -0,71 to 0,55 | -0,58 to 0,44 | -0,24 to 0,26 | -0,32 to 0,34 |

**Table 2 (abstract 1027).** Interrater reliability in 60 other patients

| | muscle thickness of thigh | muscle thickness of upper arm | fat thickness of thigh | fat thickness of upper arm |
|---|---|---|---|---|
| mean value (patient ID 1-60), cm | 2,96 | 2,11 | 1,48 | 0,97 |
| bias, cm (\|% of mean value\|) | 0,03 (1) | 0 (0) | 0 (0) | 0 (0) |
| 95% CI of bias, cm | -0,06 to 0,11 | -0,07 to 0,06 | -0,06 to 0,06 | -0,07 to 0,07 |
| SD of bias, cm | 0,33 | 0,25 | 0,24 | 0,28 |
| range of agreement: bias - 1,96*SD, cm to bias + 1,96*SD, cm | -0,62 to 0,68 | -0,49 to 0,49 | -0,47 to 0,47 | -0,55 to 0,55 |

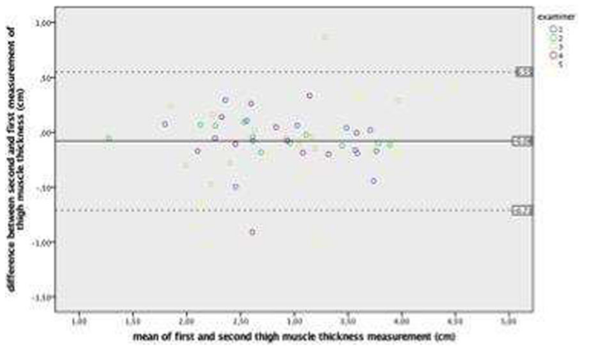

**Fig. 1 (abstract 1027).** Bland-Altmann plot for intrarater reliability for thigh muscle thickness in 60 patients

### 1028

#### Correlation between accumulated caloric deficit and delta of quadriceps thickness measured by ultrasound in critically ill patients

T. González Contreras[1], J.A. Baltazar Torres[1], M.D.S. Quintal Ramirez[2]
[1]Hospital de Especialidades "Dr. Antonio Fraga Mouret", Centro Médico Nacional La Raza, IMSS, Intensive Care Unit, Mexico City, Mexico; [2]Hospital de Especialidades "Dr. Antonio Fraga Mouret", Centro Médico Nacional La Raza, IMSS, Imagenology, Mexico City, Mexico
**Correspondence:** T. González Contreras

*Intensive Care Medicine Experimental* 2018, **6(Suppl 2):**1028

**INTRODUCTION.** Malnutrition is a clinically relevant condition in critically ill patients, because it causes immunological dysfunction, loss of lean mass and weakness of the respiratory muscles. Traditional tools for nutritional assessment are impractical in the Intensive Care Unit (ICU). The evaluation of lean mass can be a more reliable marker of malnutrition in this type of patients. Ultrasound (USG) is considered a practical and reliable tool for evaluating lean tissue in patients hospitalized in the ICU.
**OBJECTIVE.** To determine if accumulated caloric deficit (ACD) during the ICU stay contributes to the loss of lean mass in critically ill patients.
**PATIENTS AND METHODS.** A prospective study was conducted in critically ill adult patients admitted to the ICU who received artificial nutrition. Demographic and clinical variables were collected and the ACD and quadriceps muscle thickness (QMT) were measured on days 0, 3, 7, 10 and 14 of stay in the ICU. The correlation between ACD and delta of the QMT (DQMT) was evaluated by Pearson´s r. DQMT was compared between patients with critical caloric deficit (CCD) and non-critical caloric deficit (NCCD) by Student´s t test. A p value < 0.05 was considered statistically significant.
**RESULTS.** We analyzed 32 patients with a mean age of 48.5 years, 62.5% females, 75% received enteral nutrition and hospital mortality was 31.3%. Caloric deficit occurred in 90.6% of patients. The DQMT was 1.8 mm on day 3, 2.9 on day 7, 1.4 on day 10 and 1.3 on day 14 of ICU stay. The DQMT had a weak negative correlation with the ACD (r -0.026, p = 0.889). DQMT was 3.3 mm in patients with CCD and 2.4 mm in patients with NCCD (p = 0.582).
**CONCLUSIONS.** Caloric deficit is a phenomenon that occurs frequently in critically ill patients receiving artificial nutrition, especially by the enteral route. However, we found no correlation between the ACD and the QMT in these patients.

### 1029

#### Prevalence of sarcopenia in a single institution adult intensive care unit and its clinical outcome

L.J. Tan, F.H. Jamaluddin, C.C. Ng, S.R. Ruslan, P.N. Sitaram, M.S. Hasan
University of Malaya Medical Centre, Anaesthesiology, Kuala Lumpur, Malaysia
**Correspondence:** F.H. Jamaluddin

*Intensive Care Medicine Experimental* 2018, **6(Suppl 2):**1029

**INTRODUCTION.** A cross-sectional study by Patricia et al showed that sarcopenia is highly prevalent among ICU patients and is not readily detected in patients using subjective global assessment.[1]

**OBJECTIVE.** To assess the prevalence of sarcopenia in an adult ICU and its clinical outcome - duration of ICU stay, duration of hospital stay, duration of invasive ventilation, risk of hospital mortality.

**METHODOLOGY.** This is a retrospective observational study done in the adult ICU of University Malaya Medical Centre, Kuala Lumpur, Malaysia. All patients admitted from January to December 2016 with CT abdomen prior to ICU admission were included. The skeletal muscle cross-sectional area ($cm^2$) from the CT image was manually measured at the caudal end of the third lumbar vertebra. The following formula were used for estimating skeletal muscle area(SMA) - for male (SMA = 126.9 X BSA - 66.2) and for female (SMA = 125.6 X BSA - 81.1). If the actual measured SMA was less than 80% of the estimated SMA, the patient was defined as sarcopenic.[2]

**RESULTS.** Out of 239 patients, 186(78%) were cases of sarcopenia. Male gender, surgical and older patients were associated with higher incidence of sarcopenia. Sarcopenic patients were noted to have higher SAPS score. There was more death among sarcopenic patients (34.4%) compared to non-sarcopenic patients (17%) (Table 1). However, there were no statistically significant differences in terms of length of hospital stay, length of ICU stay, and duration of invasive ventilation between the 2 groups (Table 2).

**CONCLUSION.** Prevalent of patients admitted to ICU with sarcopenia was high. In this study, sarcopenic patients were found to have higher risk of hospital death.

**Table 1 (abstract 1029).** Patients' characteristics (nominal variables)

| Variables | | Sarcopenia | | Non-sarcopenia | | p-value |
|---|---|---|---|---|---|---|
| | | n | % | n | % | |
| sex | female | 75 | 40.3 | 10 | 18.9 | 0.004 |
| | male | 111 | 59.7 | 43 | 81.1 | |
| speciality | medical | 67 | 36 | 1 | 1.9 | 0.001 |
| | surgical | 119 | 64 | 52 | 98.1 | |
| discharge status | alive | 120 | 65.6 | 44 | 83 | 0.015 |
| | dead | 64 | 34.4 | 9 | 17 | |

**Table 2 (abstract 1029).** Patients' characteristics (continuous variables)

| Variables | Sarcopenia | | Non-sarcopenia | | p-value |
|---|---|---|---|---|---|
| | mean | SD | mean | SD | |
| Age (years) | 58.30 | ± 16.60 | 42.04 | ± 16.02 | 0.001 |
| SAPS score | 42.68 | ± 17.47 | 31.11 | ± 16.10 | 0.001 |
| Estimated SMA (cm2) | 147.24 | ± 30.73 | 153.76 | ± 35.26 | 0.226 |
| Actual SMA(cm2) | 94.36 | ± 23.34 | 131.54 | ± 29.86 | 0.001 |
| Length of Hospital Stay | 23.48 | ± 24.53 | 24.72 | ± 27.17 | 0.767 |
| Length of ICU Stay | 7.76 | ± 9.21 | 8.74 | ± 11.60 | 0.576 |
| Duration of invasive ventilation | 6.27 | ± 8.98 | 7.00 | ± 10.50 | 0.646 |

## Expanding clinical practice in ICU

### 1030

#### Effect of nurse led intervention to reduce post traumatic stress symptoms in patients discharged after intensive care treatment

Å. Valsø[1], T. Rustøen[2], K. Sunde[3], M.C. Småstuen[4], I. Schou-Bredal[5], Ø. Ekeberg[6], L. Skogstad[7], H. Myhren[8], K. Tøien[9]
[1]Institute of Health and Society, Faculty of Medicine, University of Oslo, Division of Emergencies and Critical Care, Department of Postoperative and Intensive Care Ullevaal Oslo Univeriety Hospital, Oslo, Norway; [2]Institute of Health and Society, Faculty of Medicine, University of Oslo, Department of Research and Development, Division of Emergencies and Critical Care, Oslo Universety Hospital, Oslo, Norway; [3]Institute of Clinical Medicine, Faculty of Medicine, University of Oslo, Department of Anaesthesiology, Division of Emergencies and Critical Care, Oslo Univerity Hospital, Olso, Norway; [4]OsloMet-Oslo Metropolitan University, Department of Public Health, Department of Research and Development, Division of Emergencies and Critical Care, Oslo, Norway; [5]Institute for Health and Society, Faculty of Medicine,University of Oslo, Division Cancer Unit for Breast- and Endocrine Surgery Oslo University Hospital, Oslo, Norway; [6]Division of Mental Health and Addiction Oslo University Hospital, Department of Behavioural Sciences in Medicine Oslo University, Oslo, Norway; [7]OsloMet-Oslo Metropolitan University, Department of Nursing and Health Promotion, Oslo, Norway; [8]Division of Medicine, Oslo University Hospital, Department of Acute Medicine, Oslo, Norway; [9]Oslo University Hospital, Department of Research and Development, Department of Postoperative and Intensive Care, Division of Emergencies and Critical Care, Olso, Norway
**Correspondence:** Å. Valsø
*Intensive Care Medicine Experimental* 2018, **6(Suppl 2):**1030

**INTRODUCTION.** Posttraumatic stress symptoms (PTSS) after treatment in intensive care units (ICU) are common. Follow-up consultations after ICU discharge have not been evaluated in patients regardless of their level of PTSS. No study has screened ICU patients for their individual PTSS level to identify those most in need for consultations. We hypothesized that patients with high level of PTSS would experience a decreased level of PTSS after targeted follow-up consultations.

**OBJECTIVES.** To evaluate the effect of early targeted nurse led follow-up consultations after ICU discharge to reduce PTSS in ICU patients with a high level of PTSS.

**METHODS.** In a randomized controlled trial, we aimed to consecutively include patients aged ≥18 years admitted for ≥ 24 hours to one of five ICUs in Norway from 2014 to 2016. Patients were screened for PTSS using Post Traumatic Stress Scale 10 (PTSS-10) after discharge from the ICU, and those with PTSS-10 score ≥ 25 (clinically relevant symptoms) were randomized into the intervention (IG) or the control (CG) group. Patients in IG participated in two or three individually adjusted follow-up consultations with a specially trained ICU nurse; the first at the ward early after discharge, and the second and third one and two months later at the ward or by phone call. A structured guide based on trauma focused cognitive behavioural therapy, combined with a narrative method, was used during the consulation. Patients filled in PTSS-10 early after ICU discharge at the ward (baseline), and 3, 6 and 12 months later. Possible differences between IG and CG after the consultations were analysed using a linear mixed model for repeated measures.

**RESULTS.** In total, 523 patients were screened, of whom 224 (43%) had a PTSS-10 score ≥ 25 (53% male) and were randomized into IG (n=111) or CG (n=112). Median age was 52 years (range 18 to 89), and mean PTSS-10 score was 37 (SD 10). Both groups showed statistically significantly lower PTSS-10 score over time ($p< 0.01$), but without significant differences between the groups ($p=0.11$). When assessing for gender differences regarding time trajectories, a different pattern was found for males and females, also stratified by groups (figure 1). Males in CG had significantly lower PTSS over time vs. IG ($p=0.04$), in contrast to women having a similar pattern for both groups.

**CONCLUSION.** A two month follow-up intervention did not show a significant effect on PTSS after one year in patients with high level of

PTSS, however, there was a significant drop in PTSS over time for all patients. Further research should pay attention to gender adjusted psychological treatment to discharged ICU patients.

**GRANT ACKNOWLEDGMENT**
The study received grants from The Norwegian Nurses Organisation and Oslo University Hospital.

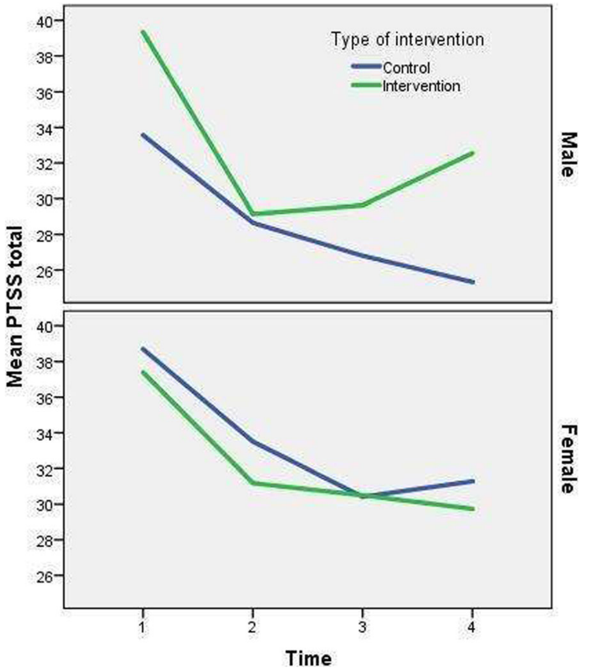

**Fig. 1 (abstract 1030).** See text for description

### 1031

#### Towards the acoustical characterisation of an intensive care unit

D. Dawson[1], R. Barham[2], M. Hamilton[1], B. Philips[3]
[1]St George's University Hospitals NHS Foundation Trust, General Intensive Care Unit, London, United Kingdom; [2]Acoustic Sensor Networks, Warninglid, United Kingdom; [3]St. George's University of London, London, United Kingdom
**Correspondence:** D. Dawson
*Intensive Care Medicine Experimental* 2018, **6(Suppl 2):**1031

**INTRODUCTION.** The intensive care unit (ICU) has long been considered a noisy environment, which impacts negatively on patient recovery[1], safe care and staff wellbeing. Noise is generated by the multiplicity of supportive equipment necessary for patient management and by the staff who provide or enable care. Staff conversation, equipment alarms and clinical treatment are cited as most disturbing[2].

**OBJECTIVES.** To identify the sources of sound and their relative contribution to the soundscape of an ICU.

**METHODS.** Fifty hours of observational data were recorded in a general ICU, using a datasheet designed and piloted for this purpose. Data was collected at four beds spaces for one month and provided equal representation across the bed spaces, hour of the day and day of the week. A priori, four categories of sound were identified communication, clinical tasks, housekeeping tasks and alarms and 55 individual sources of sound were reported under these categories. Concurrently, continuous sound pressure levels (SPL) were monitored at each bed space throughout the study period.

**RESULTS.** 16784 episodes of sound were identified during the 50hrs of data collection; the greatest number of episodes were reported in the communication category n=5699 (34%), with clinical tasks n=3282 (20%), housekeeping tasks n=3247 (19%) and alarms n=2939 (18%). The highest number of episodes for an individual source was nurse/nurse communication n=1595 (10%), followed by bin lids n=1004 (6%) and oxygen/nebulisers n=945 (6%).

Of the 55 sources recorded, the top 25 accounted for 86% of the episodes. The average $L_{Aeq}$ across all four bed spaces during the study period was 65.1dB, with little variation between day (08.00-19.59hrs) 66.5dB and night (20.00-07.59hrs) 63.4dB, there was some reduction between 23.00-06.59.00hrs to 62.7dB and another modest reduction from 02.00-05.59hrs to 61.7dB. This correlated with a similar reduction in average episodes of sound from 175 during the day, 170 at night,163 from 23.00-06.59hrs and 160 from 02.00-05.59hrs.

**CONCLUSIONS.** Communication between staff, visitors and patients created the largest number of episodes of sound during the data collection with equipment alarms causing the least. Average SPL appear to be related to the number of episodes of sound. This data may provide a model for the degree of disturbance as a function of number and type of event and noise level. Many of the sources of sound are modifiable which could decrease noise disturbance in the ICU.

**REFERENCE(S)**

1. Bentley S, Murphy F, Dudley H (1977) Perceived noise in surgical wards and an intensive care area: an objective analysis British Medical Journal, 1977, 2, 1503-1506
2. Elliott R, McKinley S, Eager D (2010) A pilot study of sound levels in an Australian Adult General Intensive Care Unit Noise and Health 12:46 26-36

**GRANT ACKNOWLEDGMENT**
National Physical Laboratory Strategic Research Program and St George's Hospital Special Trustees Charitable Funds

### 1032

#### Learning from within: a quality improvement programme to identify and address key themes for successful critical care rehabilitation

S. Bean, R. Chapman, W. English
Royal Cornwall Hospital Trust, Critical Care Unit, Truro Cornwall, United Kingdom
**Correspondence:** S. Bean
*Intensive Care Medicine Experimental* 2018, **6(Suppl 2):**1032

**INTRODUCTION.** Each year around 110,000 people are admitted to critical care units in England and Wales and the majority survive to discharge home (1). Unfortunately many survivors face a protracted recovery period, with a high incidence of both physical and psychological problems.

**OBJECTIVES.** To describe the development and outcomes of a quality improvement programme using information gained by the critical care follow-up clinic team on the experiences of our patients and their relatives.

**METHODS.** Since April 2017 we have been obtaining structured feedback from patients and relatives attending our critical care follow-up clinic. This information has been used to identify important common themes of ways in which patient care can be improved. Addressing these themes has allowed our unit to collectively learn, develop and implement a number of fundamental positive changes, primarily concerning early rehabilitation and improved support and information for patients and relatives.

**RESULTS.** Main themes identified:

- Poor patient understanding of their illness and treatment.
- A high incidence of psychological morbidity. Amongst clinic attenders; 66% reported delusional memories, 20% reported persecutory delusions that had a significant impact on the quality of their life, 15% had no recollection of their time on critical care and 25% had trauma screening scores suggestive of PTSD.
•Poor psychological preparation of patients for critical care discharge.
•Excessive noise levels in critical care.
•Poor access to psychological help after hospital discharge.
New service developments:
•Appointment of a rehabilitation link nurse specialist.
•Regular dissemination of patient and relative feedback to the critical care team, including high incidence of psychological morbidity.
•New agreed specialist referral pathways for patients requiring expert psychological input.
•Patient testimonials have been used to support a business case for regular clinical psychology input, to support a charity funding bid for noise monitoring equipment and to help embed the use of patient diaries into unit practice.
•Improved psychological preparation of long-term patients for critical care discharge. This has included visits to ward that they are due to be discharged to and steps to decrease reliance on staff help with activities of daily living.

**CONCLUSIONS.** Obtaining and using information sourced from patients and relatives at the critical care follow-up clinic has enabled the identification and subsequent introduction of many varied tools to help improve the care of our patients. Some have little or no additional cost associated with them. Some of the themes that we have identified will be common to other units.

**REFERENCE(S)**

(1) NICE. *Rehabilitation after critical illness NICE Clinical Guideline 83*.London,uk: National Institute for Health and Clinical Excellence, 2009.

**GRANT ACKNOWLEDGMENT**

No grant required

### 1033

#### Prevalence of early mobilization and its effects within the intensive care unit

P.R. Marques Filho, M. Scorssato Boeira, C. Rodrigues, C. Leães, A. Sant'anna Machado, J.M. Stormovski de Andrade, L. Krann Motta, L.F. Silva Bellolli, D. Becker, R. Andrighetto Barbosa
Hospital Ernesto Dornelles, Porto Alegre, Brazil
**Correspondence:** P.R. Marques Filho
*Intensive Care Medicine Experimental* 2018, **6(Suppl 2):**1033

**INTRODUCTION.** Patients admitted to the Intensive Care Unit (ICU) are often subject to deleterious effects caused by long periods of immobilization. Early mobilization has stood out, for it helps the functional recovery, is able to reduce days in mechanical ventilation(MV) and ICU length of stay (LoS).

**OBJECTIVES.** To evaluate the prevalence of patients who underwent early mobilization, and its effects on mechanical ventilation and ICU length of stay.

**METHODS.** 255 patients of both sexes were enrolled, with a total of 941 physical therapy sessions. Patients were separated in two randomized groups: group 1 patients were submitted to physical therapy care within 12 hours of ICU admission; and group 2 underwent physical therapy care after 12 hours of ICU admission. Physical therapy protocol consisted of the aplication of four levels of mobilization: passive exercise (N1), active exercise (N2), orthostastism (N3) and deambulation (N4).Prevalence data were collected and the effects of early mobilization on the MV time and LoS were evaluated. Data were described in percentiles and average + standard deviation; in order to evaluate difference between groups, Student's t test was used. A p< 0,05 was considered to be statiscally significant.

**RESULTS.** 52,2% of patients were male, 58,4% were under mechanical ventilation and 39,9% were using vasoactive drugs. Main causes for ICU admission were abdominal surgery (19,2%), cardiac surgery (18%), and sepsis (16,5%). 62,3% of patients were moved from the bed; Concerning levels of mobilization, 48,6% were able to perform active exercises and 13,2% to deambulate. There were no difference of SAPS-3 scores between groups; however, in the group that was mobilized earlier a reduction of both MV time (p< 0,05) and LoS (p< 0,05) was observed.

**CONCLUSIONS.** This study demonstrates that early mobilization, within a time-frame under 24 hours of admission, has helped to reduce the duration of mechanical ventilation and hospital length of stay, thus showing its potential to improve outcomes in the Intensive Care Unit.

### 1034

#### Pre-extubation ultrasonographic measurement of intracricoid peritubal free space: a novel parameter to predict postextubation airway obstruction in children

A.K. Baranwal[1], M. Samprathi[1], J. Muralidharan[1], P.K. Gupta[2]
[1]PGIMER, Pediatrics, Chandigarh, India; [2]PGIMER, Biostatistics, Chandigarh, India
**Correspondence:** A.K. Baranwal
*Intensive Care Medicine Experimental* 2018, **6(Suppl 2):**1034

**INTRODUCTION.** Post-extubation airway obstruction (PEAO) is common in children, and prolongs stay in Paediatric Intensive Care Unit, increases morbidity and thus increases the overall cost of care.

**OBJECTIVES.** We hypothesised that Intracricoid Peritubal Free Space (IPFS) between inner circumference of cricoid and outer circumference of tracheal tube, as assessed by ultrasonography, may inversely correlate to cricoid oedema, and thus development of PEAO.

**METHODS.**

**Design:** Prospective observational study.

**Setting and Participants:** 15-bed Paediatric Intensive Care Unit at a tertiary care hospital in a lower middle income economy during Aug' 2016 to Dec' 2017. Ninety-three patients (3mo-12yrs) intubated for ≥48h and planned for extubation were included. Patients with pre-existent upper airway conditions, chronic respiratory diseases and poor airway reflexes were excluded.

**Measurements:** Cricoid was imaged just prior to planned extubation, and inner cricoid diameter was ascertained. Outer diameter of tracheal tube was deducted from inner cricoid diameter to obtain Intracricoid Peritubal Free Space-diameter (IPFS-diameter). Similarly, difference of cross-sectional area of inner cricoid and of outer tracheal tube circumference was calculated to obtain Intracricoid Peritubal Free Space-area (IPFS-area). Post-extubation airway obstruction was defined by Westley's Croup Score, ≥4.

**RESULTS.** 34%(32/93) patients developed PEAO, while 18%(17/93) needed re-intubation. Baseline disease characteristics were similar between patients with and without PEAO. IPFS-diameter (4.16 ±1.18mm *vs* 5.28±1.51mm) and IPFS-area (56.72±24.25mm$^2$ *vs* 80.93 ±33.91mm$^2$) were significantly lower in patients with PEAO compared to those without. ROC Area under curve of IPFS-diameter and IPFS-area were 0.713 (95% CI, 0.608 -0.819) and 0.713 (95%CI, 0.606-0.819). Sensitivity and specificity at IPFS-diameter of 5.2mm were 84% and 50%; and at IPFS-area of 75mm$^2$ were 88% and 50%.

**CONCLUSIONS.** It is feasible to perform airway ultrasound by clinicians to evaluate IPFS. It may help predict PEAO among intubated children, and thus reduce the overall cost of care. However, a larger study is needed to confirm these initial findings.

**GRANT ACKNOWLEDGEMENT**
None

## Think about choices

### 1035

#### Practices in end-of-life care and in discontinuing mechanical ventilation in elderly critically ill patients: a nested study within an international observational study

K.E.A. Burns[1], L. Rizvi[1], M.O. Meade[2], P.M. Dodek[3], S.K. Epstein[4], A. Slutsky[5], A. Jones[6], J. Villar[7], F.N. Kapadia[8], D. Gattas[9], F. Lellouche[10], D.J. Cook[2], Canadian Critical Care Trials Group
[1]St. Michael's Hospital, Critical Care Medicine, Toronto, Canada; [2]McMaster University, Clinical Epidemiology and Biostatistics, Hamilton, Canada; [3]St. Paul's Hospital, Critical Care Medicine, Vancouver, Canada; [4]Tufts University School of Medicine, Medical Education, Boston, United States; [5]St. Michael's Hospital, Research Administration, Toronto, Canada; [6]Guy's and St Thomas' Hospital, Critical Care Medicine, London, United Kingdom; [7]Hospital Universitario Dr. Negrin, Critical Care Medicine, Las Palmas, Spain; [8]Hinduja Hospital, Critical Care Medicine, Mumbai, India; [9]Royal Prince Alfred Hospital, Critical Care Medicine, Sydney, Australia; [10]Universite Laval, Intensive Care, Quebec City, Canada
**Correspondence:** K.E.A. Burns
*Intensive Care Medicine Experimental* 2018, **6(Suppl 2):**1035

**INTRODUCTION.** Little is known about mechanical ventilation (MV) discontinuation practices and outcomes in elderly (65-80 years) and very elderly (> 80 years) critically ill patients.

**OBJECTIVES.** We sought to characterize practices in end-of-life care and in weaning older critically ill adults from invasive MV and to describe the influence of selected discontinuation strategies on clinical outcomes.

**METHODS.** We included all newly admitted critically ill adults who were invasively ventilated for at least 24 hours in 142 intensive care units (ICUs) in 6 regions (Canada, United States, United Kingdom, Europe, India and Australia/New Zealand). We excluded patients transferred to participating ICUs without a clear time of intubation, with a tracheostomy at ICU admission, already on ventilator settings compatible with a spontaneous breathing trial (SBT) at ICU admission, and (iv) participating in studies with weaning protocols.

**RESULTS.** In 788 (586 elderly, 202 very elderly) patients undergoing MV discontinuation, we found that similar proportions of patients (22.5% and 23.8%, respectively) died before any attempt at discontinuation could be made. Compared to elderly patients, very elderly patients underwent direct extubation (19.1% vs. 19.3%), direct tracheostomy (6.8% vs. 5.0%), initial successful SBTs (79.5% vs. 78.1%) and initial failed SBTs (20.5% vs. 21.9%) with similar frequency. Compared to elderly patients, very elderly patients who underwent an initial tracheostomy experienced higher ICU and hospital mortality (15% vs. 50%, p=0.05; 23.5% vs. 71.4%, p=0.04) and significantly longer ICU stays (22.5 +/- 17.4 d vs. 43.8 +/- 28.9 d, p=0.02). Among elderly patients, those who failed (vs. passed) an initial SBT demonstrated a trend toward increased ICU mortality (21.0 % vs. 11.3%, p=0.07) and spent more time in the ICU (15.7 +/- 13.8 d vs. 10.9 +/- 10.6 d, p=0.008) with similar rates of readmission (4.8% vs. 4.2%; p=1.0), reintubation (14.5% vs. 14.3%, p=1.0), and hospital mortality (22.2% vs. 19.9%, p= 0.9). Similarly, among very elderly patients those who failed (vs. passed) an initial SBT had significantly higher ICU mortality (39.1% vs. 15.9%, p=0.03), trends toward higher hospital mortality (55.0% vs 30.8%, p=0.09) with similar rates of readmission (4.3% vs. 4.9%, p=1.0), reintubation (13.0% vs. 8.5%; p=0.8) and ICU lengths of stay. Regardless of the indication (weaning, prophylaxis,postextubation distress) noninvasive ventilation (NIV) was used nonsignificantly more often in the very elderly vs. elderly. We found large practice variation in the use of NIV and in the frequency with which various treatments were withheld and withdrawn across regions.

**CONCLUSIONS.** Our findings provide valuable information regarding outcomes associated with tracheostomy and initial SBT failure in elderly and very elderly critically patients.

**GRANT ACKNOWLEDGMENT**
CIHR, Ministry of Research and Innovation, Fisher & Paykel, Covidien, GE Healthcare.

### 1036

#### Outcome comparison between alcoholic and non-alcoholic liver cirrhosis in intensive care

S. Hietanen[1,2], J. Herajärvi[1,2], A. Lehtonen[1,2], T. Ala-Kokko[1,2], J.H. Liisanantti[1,2]
[1]Oulu University Hospital, Division of Intensive Care Medicine, Department of Anesthesiology, Oulu, Finland; [2]Oulu University Medical Research Center, Research Group of Surgery, Anesthesiology and Intensive Care, Oulu, Finland
**Correspondence:** S. Hietanen
*Intensive Care Medicine Experimental* 2018, **6(Suppl 2):**1036

**INTRODUCTION.** Alcohol overconsumption is the leading cause of liver cirrhosis in western countries. According to the current studies the prognosis of alcoholic cirrhosis might be worse than the prognosis of liver cirrhosis caused by other etiologies (1-3). Only a few studies have focused on direct comparison between these patient groups.

**OBJECTIVES.** The aim was to compare the treatment profile and mortality between patients with alcoholic cirrhosis and patients with liver cirrhosis caused by other etiologies admitted to ICU.

**METHODS.** This retrospective cohort study included all patients with diagnosed liver cirrhosis and admitted to intensive care unit (ICU) in Oulu University Hospital in Finland between May 2015 and April 2017. Demographic data, the etiology of liver cirrhosis, and characteristics of treatment profile were obtained from electronic medical records and the ICU patient data management system. Patients were followed until the end of the year 2017 and the Finnish Population Register Center provided the data concerning mortality.

**RESULTS.** In a total of 100 ICU admissions with liver cirrhosis the etiology was alcohol in 78 cases (78.0%) and other etiologies in 22 cases (22.0%). The patients with alcoholic cirrhosis were younger and more frequently men. Median Sequential Organ Failure Assessment (SOFA) scores at admission were higher in patients with alcoholic cirrhosis resulting from lower Glasgow Coma Scale (GCS) points and higher bilirubin levels (Table 1). The most common cause of admission in total was gastrointestinal diagnoses, and the causes did not differ between groups. There was no difference between groups in the length of ICU stay, or in the need of organ support (Table 2). Overall ICU mortality was only 12.0% and there was no difference between groups. The long-term 180-day mortality was 42.0% and there was no difference between groups (Table 2, Figure 1).

CONCLUSIONS According to our data, the long-term survival of patients with liver cirrosis admitted to ICU is poor; nearly half of the patients are 180-day non-survivors. There are no significant differences in outcome between patients with diagnosed alcoholic or non-alcoholic liver cirrhosis.

**REFERENCE(S)**

1. Marot A, Henrion J, Knebel JF, et al. Alcoholic liver disease confers a worse prognosis than HCV infection and non-alcoholic fatty liver disease among patients with cirrhosis: An observational study. PLoS One 2017;12:e0186715.
2. Nilsson E, Anderson H, Sargenti K, et al. Incidence, clinical presentation and mortality of liver cirrhosis in Southern Sweden: a 10-year population-based study. Aliment Pharmacol Ther 2016;43:1330-9.
3. Sorensen HT, Thulstrup AM, Mellemkjar L, et al. Long-term survival and cause-specific mortality in patients with cirrhosis of the liver: a nation-wide cohort study in Denmark. J Clin Epidemiol 2003;56:88-93.

**GRANT ACKNOWLEDGMENT**
We have received a grant for expences from The Finnish Foundation for Alcohol Studies.

**Table 1 (abstract 1036).** Patient characteristics of the 100 patients diagnosed with liver cirrhosis admitted to ICU

| | Total (N=100) | Alcoholic liver cirrhosis (N=78) | Other liver cirrhosis (N=22) | p-value |
|---|---|---|---|---|
| Gender, f/m | 27/73 | 17/61 | 10/12 | **0.054** |
| Age | 63 [55-70] | 61 [53-68] | 71 [61-74] | **0.005** |
| SOFA score adm | 6 [4-9] | 8 [4-10] | 5 [3-7] | **0.009** |
| SOFA score max | 8 [5-12] | 9 [5-12] | 7 [5-9] | 0.132 |
| APACHE II score | 19 [15-26] | 20 [16-26] | 19 [13-22] | 0.242 |
| Encephalopathy | 36 (36.0) | 33 (42.3) | 3 (13.6) | **0.013** |
| Varices | 39 (39.0) | 35 (44.9) | 4 (18.2) | **0.012** |
| Splenomegaly | 42 (42.0) | 36 (46.2) | 6 (27.3) | **0.038** |
| Ascites Moderate - large | 24 (24.0) | 18 (23.1) | 6 (27.3) | 0.684 |

**Table 2 (abstract 1036).** Treatment profile and outcome of the 100 patients diagnosed with liver cirrhosis admitted to ICU

| | Total (N=100) | Alcoholic liver cirrhosis (N=78) | Other liver cirrhosis (N=22) | p-value |
|---|---|---|---|---|
| Mechanical ventilation | 42 (42.0) | 32 (41.0) | 10 (45.5) | 0.808 |
| Vasopressor | 54 (54.0) | 43 (55.1) | 11 (50.0) | 0.809 |
| Renal replacement therapy | 9 (9.0) | 7 (9.0) | 2 (9.1) | 0.987 |
| ICU length of stay | 1.8 [1.0-3.8] | 1.8 [1.0-4.0] | 1.7 [0.9-3.1] | 0.901 |
| ICU mortality | 12 (12.0) | 9 (11.5) | 3 (13.6) | 0.723 |
| Hospital mortality | 7 (7.0) | 4 (5.1) | 3 (13.6) | 0.177 |
| 30-day mortality | 26 (28.6) | 19 (27.5) | 7 (31.8) | 0.788 |
| 90-day mortality | 36 (39.6) | 28 (40.6) | 8 (36.4) | 0.806 |
| 180-day mortality | 40 (44.0) | 29 (42.0) | 11 (50.0) | 0.623 |

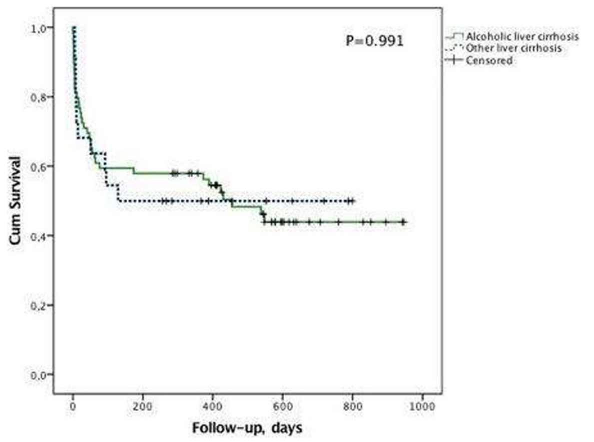

**Fig. 1 (abstract 1036).** Kaplan-Meier survival curves of the 100 patients with liver cirrhosis admitted to ICU.

### 1037

#### Evaluation of the association between key factors of concept on good death and public´s preference of life sustaining treatment during critical illness

W.T. Wong[1], V.K.W. Lai[2], D. Au[3], G. Joynt[2], L. Ling[2]
[1]The Chinese University of Hong Kong, Shatin, Hong Kong, China; [2]The Chinese University of Hong Kong, Anaesthesia and Intensive Care, Hong Kong, Hong Kong, China; [3]The Chinese University of Hong Kong, CUHK Jockey Club Institute of Ageing, Hong Kong, Hong Kong, China
**Correspondence:** W.T. Wong
*Intensive Care Medicine Experimental* 2018, **6(Suppl 2):**1037

**INTRODUCTION.** End of life (EOL) care decision depends on consensus on goals of care between physicians and patients or their surrogates. EOL care decision usually results in death after withholding (WH) or withdrawing (WD) life sustaining treatment (LST). Important factors of good death concept should be addressed during goal of care discussions with patients or their surrogates.

**OBJECTIVES.** To evaluate public´s preference of LST and identify key factors on good death concept which are associated with attitudes toward aggressive treatment when meaningful recovery from critical illness is not possible.

**METHODS.** We recruited 798 participants (mean (SD) age: 59 (12.6) years; 165 (71%) males) from the community between December 2016 and September 2017 to complete a questionnaire on their attitudes of LST in critical illness (4 logical based questions, graph 1) and concept on good death (17 items)[1]. Their attitude towards LST was dichotomised into aggressive or non-aggressive treatment groups. Exploratory and confirmatory factor analysis were used to revise the model of good death questionnaire[1]. Logistic regression was used to identify predictors of preference for aggressive treatment. Inconsistent and invalid preference was defined as respondents who chose WH or WD LST but opted out of do not attempt resuscitation (DNAR).

**RESULTS.** We revised the original 3-factor model of good death concept to a better 4-factor (physical symptoms, psychological issues, social issues and self-control) model (CFI = .902, TLI = .878 and RMSEA = .065). Among 798 participants, 160 (20.0%) did not give a consistent response towards LST. Of the remaining 638 respondents, 113 (17.7%) of them preferred aggressive treatment even if the chance of meaningful recovery was minimal. Concerns about physical symptoms was the only factor which was associated with lower preference for aggressive treatment (OR: 0.44 95% CI:0.28-0.67).

**CONCLUSIONS.** Concerns about physical symptoms was strongly associated with preference for less aggressive treatment within a good death concept. Clinicians should emphasize information about physical discomfort related to LST to assist patients or their surrogates in making personal EOL care decisions when a meaningful recovery is unlikely.

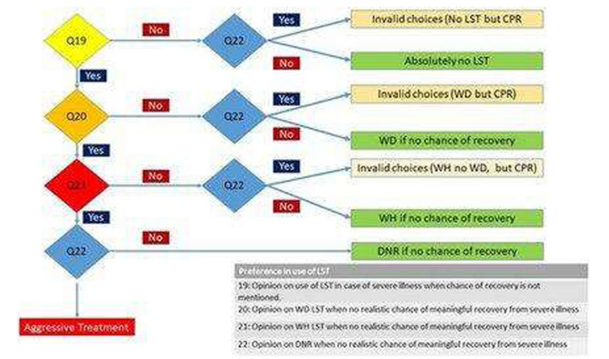

**Fig. 1 (abstract 1037).** Preference of LST

**Table 1 (abstract 1037).** Factors in the concept on good death predicting choice of aggressive treatment

| | Adjusted Odds ratio* | P-Value |
|---|---|---|
| Physical symptoms | 0.44 (0.28-0.67) | <0.0001 |
| Psychological issues | 1.37 (0.81-2.34) | 0.245 |
| Social issues | 1.16 (0.83-1.62) | 0.373 |
| Self control | 1.17 (0.85-1.62) | 0.323 |

*Adjusted for age, gender, family monthly income, education level, previous experience of hospitalization or death of family member

### 1038

#### Would you like to be admitted to the ICU? Intensivists' and the general public's preferences according to different outcomes

R. Fumis[1], G. De Paula Pinto Schettino[2], T. Domingos Corrêa[2]
[1]Hospital Sírio-Libanês, Intensive Care Unit, São Paulo, Brazil; [2]Hospital Israelita Albert Einstein, Intensive Care Unit, São Paulo, Brazil
**Correspondence:** R. Fumis
*Intensive Care Medicine Experimental* 2018, **6(Suppl 2):**1038

**INTRODUCTION.** Discussions about invasiveness of care (advanced directives) and end-of-life issues have become frequent among intensivists and patients. Nevertheless, there are considerable divergences between the attitudes of intensivists and patients toward end-of-life care in the intensive care units (ICU) [1].

**OBJECTIVE.** Compare the preferences of intensivists with the general public's preferences about ICU admission of a hypothetical critically ill patient with six different outcomes.

**METHODS.** Intensivists and the general public (university graduate professionals outside the area of health) were invited to participate in this study. A survey was conducted with a hypothetical patient with six different clinical outcomes ranging from ICU discharge without any neurological sequelae or dependence for daily activities to death. The WHOQOL-BREF was applied to all participants. Comparisons were made between the answers given by intensivists regarding what they would choose for themselves and their patients and the preferences of the general public.

**RESULTS.** Between July 2013 and July 2016, 300 participants in 5 hospitals in São Paulo, Brazil were invited to participate in this study. Out of those, 257 (85.7%) answered the survey. Eighty-one intensivists responded what they would choose for their patients [median (IQR) age: 34 (31-41) years] , 82 intensivists responded what they would choose for themselves [median (IQR) age: 36 (31-40) years] and 94 participants from the general public [median (IQR) age: 40 (31-51) years] responded what they would choose for themselves. Quality of life did not differ among the groups. More than half of the participants from the general public do not wish to die in an ICU. In all scenarios, except when the outcome was severe disability or death, intensivists were more likely to propose ICU admission for their patients rather than for themselves (p< 0.01 for all). The general public was significantly less prone than intensivists to choose ICU admission in three out of six scenarios (p< 0.001 for all). Intensivists' opinion did not differ from the general public's in 50% of clinical scenarios. Intensivists were more likely to propose ICU admission for themselves rather than for the general public only when the best outcome clinical scenario is considered (p< 0.001).

**CONCLUSIONS.** Considerable divergences between intensivists' and patients' preferences toward end-of-life care exist. Advanced care planning and effective ongoing communication among intensivists, patients and relatives are essential to improve end-of-life decisions and the quality of care.

##### REFERENCE(S)

1. Dowdy DW et al. Quality of life in adult survivors of critical illness: a systematic review of the literature. Intensive Care Med. 2005 May;31(5):611-20.

### 1039

#### Ethical aspects of prognosis and the passage of time in intensive care. A qualitative triangulation

A.-H. Seidlein, A. Nowak, S. Salloch
University Medicine Greifswald / Institute for Ethics and History of Medicine, Greifswald, Germany
**Correspondence:** A.-H. Seidlein
*Intensive Care Medicine Experimental* 2018, **6(Suppl 2):**1039

**INTRODUCTION.** The decision-making environment in intensive care units (ICU) is strongly influenced by at least three important transformations on the individual and societal level that enhance complexity: Firstly, the patients' mean age and comorbidities have increased and they will continue to do so. Secondly, potentially life-prolonging treatment options are still on the rise. Finally, patients' right to self-determination is highly emphasized but the question how to respect their will in case of incompetence remains challenging in healthcare professionals' everyday practice.

**OBJECTIVES.** This qualitative study aims to explore patterns of decision-making concerning the continuation, withdrawal, and withholding of life-sustaining therapies (LST) in intensive care. The focus lies on different roles of the persons involved as well as influencing factors regarding the escalation and de-escalation of therapies and the modification of treatment goals.

**METHODS.** Data collection comprised four weeks of non-participant observation during day shifts on an ICU at an academic medical centre in northern Germany, followed by qualitative semi-structured interviews with five nurses and five physicians practicing at different ICUs in Germany. Qualitative content analysis according to Mayring was applied using MAXQDA software to analyse the interview transcripts and field notes.

**RESULTS.** Decision-making concerning LST is a dynamic process in which the intensity of treatment is influenced by multiple medical, social and psychological factors. Amongst other aspects, the prognosis for possible long-term outcomes is stressed by all parties involved as one of the most important criteria and major motivation to initiate, stop or forego certain treatments. At the same time, estimating the prognosis appears as one of the most difficult tasks to perform. The prediction of the patients' condition and the evaluation of his or her suspected preferences concerning possible outcomes tends to result in communicative challenges and conflicts between different professions as well as between professionals and relatives. Furthermore, it emerges that the passage of time has a fundamental influence on making a reliable forecast. This leads to an adjustment of prognosis during the patient's stay on the ward. Here, the ambivalence of the prognosis as a norm for decision-making in intensive care becomes apparent as decisions have to be made at a specific moment in time.

**CONCLUSIONS.** Estimating a patient's prognosis remains a difficult task which can lead to ethical uncertainties and conflicts. Explicitly considering the passage of time and the temporal dynamics of prognoses can help to avoid interprofessional tensions as well as conflicts with the patient's relatives.

##### GRANT ACKNOWLEDGMENT

This research was funded by the German Federal Ministry of Education and Research.

### 1040

#### Death verification - after 5 years, we still can't diagnose death

K. Archer[1], C. Groves[2], H. Mann[1], N. Arora[1]
[1]Birmingham Heartlands Hospital, Bordesley Green East, Birmingham, United Kingdom; [2]Good Hope Hospital, Rectory Road, Sutton Coldfield, United Kingdom
**Correspondence:** K. Archer
*Intensive Care Medicine Experimental* 2018, **6(Suppl 2):**1040

**INTRODUCTION.** Five years ago, we performed an audit looking at the process of death verification in comparison to the guidance published by the Academy of Medical Royal Colleges[1]. Our results highlighted varied and sometimes inadequate practice amongst healthcare professionals. This lead to the introduction of a proforma for the verification of death in the intensive care units in our hospitals.

**OBJECTIVES.** Our objectives were to i) see if practice has changed with regards to death verification,
ii) to see how many deaths were being verified using the proforma, and
iii) to see if the proforma made a difference to the quality of documentation.

**METHODS.** Using the same method of data collection as our previous audit, two investigators reviewed the death verification notes of patients who died between December 2017 - January 2018 across two hospitals within our NHS Trust.

**RESULTS.** The results of 100 patients were included in our audit. Whether the death verification had been performed using our proforma, as well as 28 other parameters, were recorded. Our results regarding the process of death verification without using our proforma were comparable to previous. There had been minor improvements with 83% of notes having the appropriate number of patient identifiers. There were 3 patients that did not have pulses palpated. 11 patients did not have heart sounds auscultated and in 8% there was no documentation of listening for breath sounds. 98% of patients had their pupils checked. Only 28% were tested for pain stimulus using supraorbital pressure, as per guidance. 7 entries did not have a printed name and 3 were illegible. 45% did not have a professional registration number documented.

18 of the 100 patients were verified using our proforma. Of these, 1 patient did not have the required patient identifiers on the document. 100% of patients had their pulses checked. Only 94% had their heart and breath sounds auscultated. All 18 of patients had their pupils checked. Only 89% were tested for painful stimuli using supra-orbital pressure. 100% were signed, dated and timed but 1 patient (6%) did not have the verifying professional's printed name or registration number stated.

**CONCLUSIONS.** The process of death verification remains varied and sometimes inadequate. Practice has not changed significantly from our audit 5 years ago. However, the proforma we created has helped healthcare professionals to carry out death verification in a more efficient and standardised way.

We are moving forward to take our proforma and make it a Trust-wide necessity for death verification.

GRANTS
Nil.

### 1041

#### Terminal extubation in critically ill patients

A. Estella, M. Recuerda, M. Gracia Romero, V. Perez Madueño, L. Perez Bello Fontaiña
Hospital SAS de Jerez, Intensive Care Unit, Jerez de la Frontera, Spain
**Correspondence:** A. Estella
*Intensive Care Medicine Experimental* 2018, **6(Suppl 2):**1041

**INTRODUCTION.** Limitation of Life support treatments (LLST) is part of end of life care. Terminal extubation is an unusual modality of LLST and it is scarcely documented in the literature.

**OBJECTIVES.** The aim of the study is to analyse the LLST practices of terminal extubations and to describe the clinical profile of this group of patients.

**METHODS.** Prospective observational study carried out in a medical-surgical ICU of 17 beds of a community hospital. Time of study was 12 months. We included intubated patients in whom terminal extubations were decided. The variables analyzed were: age, gender, diagnosis and conditions in which the LLST was decided. Statistical analysis was performed with SPSS v24 program. Quantitative variables are expressed in mean ± standard deviation and qualitative variables in percentage.

**RESULTS.** 1245 patients were admitted in ICU during the time of study, 142 patients died. Terminal extubations were performed in 12 patients, 7 women and 5 men. Mean age was 74 ± 5.1 years. Two-thirds were admitted from the Emergency Department. Charlson index resulted between 3 and 5 in 33.3% and was greater than 5 in 66.7%. 58.3% had a modified Rankin scale rating greater than 3. APACHE II score at admission was 19.6 ± 7.3. 75% needed vasoactive drugs and 25% renal replacement therapy. Diagnosis at admission was cardiac arrest in 33.3%, postoperative abdominal surgery 25%, head injury 16.7%, acute lung edema 16.7% and cerebrovascular disease 8.3%. 16.7% were organ donors. In all patients extubation was decided in the first week of ICU admission and mean time elapsed between extubation and dead was 15 ± 22 hours with a range [0-74]. 91.6% of the extubations were decided by a medical staff.

**CONCLUSIONS.** Decisions of terminal extubation are rare, are decided in the first days of admission and by the disciplinary team. Clinical profile was elderly patients with comorbidity, poor quality of life and high severity score at admission.

### 1042

#### Life support limitation practices in an intensive care unit

L. Alegria, M. Vera, M. Amthauer, R. Castro
Pontificia Universidad Catolica de Chile, Intensive Medicine, Santiago, Chile
**Correspondence:** L. Alegria
*Intensive Care Medicine Experimental* 2018, **6(Suppl 2):**1042

**INTRODUCTION.** The life support treatment limitation is a meditative and consensual decision about withdrawing or withholding therapeutic measures when the course of the disease is irreversible or the treatment futile. In the intensive care setting, it is a common practice not executed without conflicts, often related to cultural, religious or social factors, including also some characteristics of the healthcare team (1)(2). This leads to a wide variability in the decision-making process and in the end of life care provided to patients, making it necessary to establish standards of care and to understand what the usual practice patterns are.

**OBJECTIVES.** The main purpose of this study is to observe and describe the life support treatment limitation practices and their general incidence in the Intensive Care Unit (ICU) of a teaching hospital.

**METHODS.** A prospective, observational study, carried out in the Intensive Care Unit at Hospital Clínico UC-CHRISTUS, between December 2016 and April 2018. Every deceased patient and every patient under any life support limitation measure was included.

**RESULTS.** A total of 104 patients were included, with a mean age of 65.5 years. The main reason for admission was a neurological cause (25%), followed by sepsis (24%). Of all patients included, 23% died without mediation of any life support treatment measure. In those patients in which any practice of life support treatment limitation was performed, 72.5% died in the ICU and 18.7% died after being transferred to another unit. The DNR order was the most frequent among life support limitation indications (58%), followed by the withdrawal of life supporting measures (51%).

**CONCLUSIONS.** In our ICU, most of the patients died after a decision of life support treatment limitation, existing a wide variability between those measures, which opens the discussion about the need of practices centered in the patient and the family during the end of life process.

**REFERENCE(S)**

1. Azoulay É, Timsit JF, Sprung CL, Soares M, Rusinová K, Lafabrie A, et al. Prevalence and factors of intensive care unit conflicts: The conflicus study. Am J Respir Crit Care Med. 2009;180(9):853-60.

2. Azoulay É, Pochard F, Chevret S, Adrie C, Annane D, Bleichner G, et al. Half the family members of intensive care unit patients do not want to share in the decision-making process: a study in 78 French intensive care units. Crit Care Med. 2004;32(9):1832-8.

**Table 1 (abstract 1043).** Academic degree of responders

| | Frequency | Percent |
|---|---|---|
| Residents | 61 | 38.1% |
| Assistant lecturer | 59 | 36.9% |
| Lecturer | 40 | 25% |

### 1043

#### Understanding & acceptance of the brain death concept among Cairo University Intensivists & Emergency Doctors. A survey study

M. El-Sayed[1], M. Nagy Mohamed[2], M. Mohamed Elgohary[2], M. Mahmoud Elkholy[2]
[1]Cairo University Hospitals, Cairo, Egypt; [2]Cairo University Hospitals, Anesthesia, Cairo, Egypt
**Correspondence:** M. El-Sayed
*Intensive Care Medicine Experimental* 2018, **6(Suppl 2):**1043

**INTRODUCTION.** Brain death (BD) is the cessation & irreversibility of all brain functions, including the brain stem.[1] Studies have shown substantial confusion &lack of understanding about BD among health professionals, even among those who accept BD as being legally dead.[2] Kasr Alainy, Cairo University hospital, a major tertiary care medical center in Egypt & the whole Middle East; has no clear guidelines for the actual diagnosis or acknowledgement of BD.To our best knowledge, there were no published formal surveys or questionnaires regarding this issue among medical personnel in Egypt. Available literature are rather narrative reviews or debates. [3,4]

**OBJECTIVES.** To assess understanding & acceptance of the conceptual basis of BD among intensivists & emergency doctors at Kasralainy, Cairo University hospital.

**METHODS.** A quantitative cross-sectional survey study in which a validated questionnaire was handed to I60 Physicians working in adult surgical, postoperative, neurosurgical & medical ICU´s as well as Emergency department of Cairo University hospital, with at least 1 year of experience in ICU. Questionnaire tool was partially based on a similar earlier study.[1] Other questions were approved by PTPODaT (Professional Training Program on Organ Donation & Transplantation) faculty. Information about specialty, academic degree, years since graduation, & years of expertise in ICU were collected. Questionnaire was designed to evaluate understanding & acceptance of the conceptual basis for BD rather than implementation of a diagnostic protocol.Questions were sequenced so that doctors initially define brain death, then interpret two clinical case scenarios, indicate their awareness of existing Egyptian laws& religious authorities´ statement & finally acceptance of BD concept & its future implementation as a diagnostic protocol.

**RESULTS.** 56 % (n=89) correctly defined BD, 11.25% (n=18) successfully interpreted the following 2 case scenarios & 8.75% (n =14) correctly defined BD & interpreted the following scenarios. There were no statistically significant difference between different specialties or academic degrees. 6% (n=9) were aware of the 2008 Egyptian consensus for BD diagnosis & 9.4% (n=15) knew that Egyptian religious authorities acknowledged BD as a medical decision. only 3.8% (n=6) received previous training about BD diagnosis.

**CONCLUSIONS.** Cairo University Hospital intensivists lack consistent knowledge & understanding of BD concept, they have concerns regarding the application of BD diagnosis protocol.

**REFERENCE(S)**

1. Chandrika Rao. Postgraduate Concept of Brain Death.JIACM 2010; 11(3): 187-9.
2. WHITE G. Intensive care nurses' perceptions of brain death. Aust Crit Care 2003: 16:7.
3. Hamdy S. Not quite dead: why Egyptian doctors refuse the diagnosis of death by neurological criteria. Theor Med Bioeth. 2013 Apr; 34(2):147-60.
4. Paris W, Nour B.Organ transplantation in Egypt.Prog Transplant. 2010 Sep;20(3):274-8.

**Table 2 (abstract 1043).** Responses to the 1st three questions about BD definition & the 2 case scenarios among different academic degrees

| | Residents | Assistant Lecturers | Lecturers | P Value |
|---|---|---|---|---|
| Define brain death [number(%within degree)] | 31 (51%) | 31 (53%) | 27 (68%) | 0.21 |
| 1st scenario Interpretation [number(%within degree)] | 8 (13%) | 11 (19%) | 8 (20%) | 0.59 |
| 2nd Scenario Interpretation [number(%within degree)] | 41 (67%) | 37 (62%) | 32 (80%) | 0.18 |
| 3 correct answers [number(%within degree)] | 2 (3.3%) | 5 (8.5%) | 6 (15%) | 0.12 |

**Table 3 (abstract 1043).** Responses to the 1st three questions about BD & the2 case scenarios among different specialties

| | Anesthesia | Critical care | Internal medicine | ED | P value |
|---|---|---|---|---|---|
| Define brain death [number(%within specialty)] | 22 (57.9%) | 25 (62.5%) | 28 (53.8%) | 14(46.7%) | 0.59 |
| 1st scenario Interpretation [number(%within specialty)] | 11 (28.9%) | 6 (15%) | 6 (11.5%) | 4(13.3%) | 0.14 |
| 2nd Scenario Interpretation [number(%within specialty)] | 21 (55.3%) | 25 (62.5%) | 37 (71.2%) | 27(90%) | 0.016 |
| 3 Correct answers [number(%within specialty)] | 5 (13.2%) | 3 (7.5%) | 3 (5.8%) | 2 (6.7%) | 0.35 |

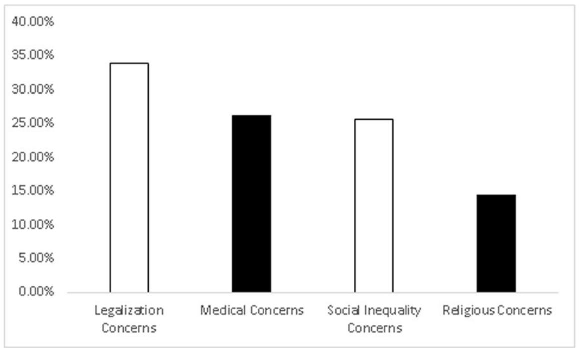

**Fig. 1 (abstract 1043).** Concerns & fears regarding activation of BDdiagnosis protocol in Cairo UInversity Hospital

#### 1044

**Evaluation of medical students knowledge about brain death**

M. Cenacchi Garcia Pereira[1,2], P. Pereira Travassos[2], R. Telles[2], R. Gonçalves de Lima[3], W. Geres da Costa[2], E. Gadelha[2], L. Junqueira[2], V. Cordeiro Veiga[2], S. Soriano Ordinola Rojas[2]
[1]Universidade Cidade de São Paulo - UNICID, São Paulo, Brazil; [2]Hospital BP - A Beneficência Portuguesa de São Paulo, Neurocriticalcare Unit, São Paulo, Brazil; [3]Hospital BP - A Beneficência Portuguesa de São Paulo, São Paulo, Brazil
**Correspondence:** M. Cenacchi Garcia Pereira
*Intensive Care Medicine Experimental* 2018, **6(Suppl 2):**1044

**INTRODUCTION.** Brain death consists in an irreversible clinical condition with complete loss of brain stem and cerebral functions.
Still is a complex term in ICU practitioners' routine.
Besides being short known to the overall population and medical students, it's been misunderstood quite often.
**OBJECTIVES.** This study's objective is to evaluate medical students, on 5th and 6th year of graduation, knowledge about brain death and organ donation.
**METHODS.** The study consists in the application of a multiple-choice test, containing 10 questions. Students were invited to participate. The test has been applied to medical students who were on 5th and 6th year of medical graduation.
**RESULTS.** One hundred and eight participants answered the test. Correct answers percentages for brain death were 30.55% on brain death definition, 76.85% on necessary neurological tests to diagnose brain death, 59.25% on time interval between exams, 73.14% on process of information to the family, 60.18% on complementary exams, 23.14% on legislation related question, 39.80% on physiology of brain death, 40.74% on clinical findings, 73.14 on medications that should be excluded during tests and exams. Correct answers for potential donors related questions were 87% on decision's responsibility, 78.70% on who can be the potential donor, 39.80% on potential donor maintenance.
**CONCLUSIONS.** In the study we observed important knowledge deficit among medical students over essential concepts for diagnosing and assessing brain death protocols, a very important theme on intensive care routine.

### Improving patient care

#### 1045

**Reducing administrative burden: a core set of quality indicators for effective quality improvement and efficient accountability**

M. Zegers, R.J. Verhage, J.G. Hoeven van der
Radboudumc, Intensive Care, Nijmegen, Netherlands
**Correspondence:** M. Zegers
*Intensive Care Medicine Experimental* 2018, **6(Suppl 2):**1045

**INTRODUCTION.** Healthcare professionals suffer from the amount of quality indicators they register. Their large number limits the time for implementing quality improvements. Moreover, healthcare professionals do not perceive all quality indicators as meaningful and useful for quality improvement.
**OBJECTIVES.** To select a core set of indicators for effective quality improvement and efficient accountability in the Intensive Care Unit (ICU).
**METHODS.** In 2017, we conducted a two-phase adapted Delphi study in four Dutch hospitals with 34 experts: 13 ICU physicians, 11 ICU nurses and 10 ICU survivors and relatives from the FCIC (Family and Patient Centered Intensive Care), the Dutch foundation for ICU survivors and relatives. We identified all quality parameters regarding the ICU (n=122). We asked participants to indicate the relevance of each quality parameter for quality improvement (round 1). Subsequently, the participants discussed the results of round 1 and determined the final core set of quality parameters in three focus group interviews (round 2).
**RESULTS.** The participants selected a core set of 16 parameters (see table 1). Four parameters were related to the organization of intensive care: safety culture, team climate (the ability of a team to learn and improve), adherence to Crew Resource Management (principles related to teamwork and communication on the ICU) and results of the quality visitation of the professional organization of Dutch intensivists.
Four healthcare provider reported outcomes were selected: Standardized Mortality Ratio (SMR), ICU readmissions within 48 hours, incidence of decubitus and delirium, and parameter 'learning and improvement after serious incidents'.
Prominent aspects of the core set were experiences and short and long term outcomes reported by ICU survivors. Interestingly, also experiences and mental problems reported by relatives were part of the core set.
**CONCLUSIONS.** In conclusion, healthcare providers, ICU survivors and relatives selected a core set of 16 parameters for effective quality improvement and efficient accountability in the ICU. In 2018 and 2019, the effect of using only a core set of parameters on patient outcomes will be evaluated. Our hypothesis is that registering less quality indicators will leave more time for quality improvement and ultimately to better outcomes and experiences for patients and relatives. At the congress, we will show the first results of how we implemented the core set and de-implemented registrations in the clinical process.

**GRANT ACKNOWLEDGMENT**
This research was carried out under the auspices of the collaboration of the Consortium Quality of Care of the Netherlands Federation of University Medical Centers (NFU), and National Health Care Institute.

**Table 1 (abstract 1045).** Core set of quality indicators for the ICU

| | Organisation (structure and process) | Outcomes |
|---|---|---|
| Reported by Health care professional | Team climate<br>Safety culture<br>CRM-compliance<br>Quality visitation | SMR/ ICU-mortality<br>ICU-readmissions within 48 hours<br>Incidence delirium and decubitus<br>Learning and improvement after serious incidents |
| Reported by Patient and Relative | Experiences of ICU survivors and relative (interviews)<br>Experiences relatives (questionnaire)<br>Complaints | Quality of life of ICU survivors<br>Quality of life of relatives<br>Physical, mental and cognitive problems of ICU survivors<br>Socio-economic impact<br>Cost-effectiveness of ICU care |

#### 1046

**Association between burnout syndrome, emotional stress, suicidal thought and substance abuse in critical care professionals**

A.Y. Rodríguez Santos[1,2], J. Franco Granillo[1], S.J. Aguirre Sánchez[1], G. Camarena Alejo[1], S. de la Cruz Balboa[3]
[1]American British Cowdray Medical Center I.A.P., Critical Care, Mexico, Mexico; [2]Universidad Nacional Autonoma de México, Critical Care, Mexico, Mexico; [3]American British Cowdray Medical Center I.A.P., Internship in critical care medicine, Mexico, Mexico
**Correspondence:** A.Y. Rodríguez Santos
*Intensive Care Medicine Experimental* 2018, **6(Suppl 2):**1046

**INTRODUCTION.** Burnout syndrome(BS) is a social disorder composed of exhaustion, depersonalization and low personal fulfillment, frequent in critical care staff. BS, depression and substance abuse has not been studied in mexican health workers.
**OBJECTIVE.** To characterize severe burnout syndrome scores(SBS), emotional stress(ES) and depression in Mexican health care workers and their association with demographic variables, working conditions and substance abuse.
**METHODS.** Multicentric cross-sectional study from Mexico conducted in health workers using the following validated questionnaires:

Maslach Burnout Inventory(MBI), perceived stress scale(PSS) and patient care questionnaire(PHQ-9) for depression. Substance abuse was defined as: AUDIT-c questionnaire≥8 points or self-reported marihuana or amphetamine abuse. We classified severe burnout(SBS) with two of the following: high emotional fatigue(EF), high depersonalization(DP) and low personal realization(PR); high stress: PSS≥27 points; major depression: PHQ-9≥10 points. Demographic variables were collected for all patients. We performed logistic regression analysis to calculate Odds Ratios(OR) using SSB as dependent variable and Pearson's r to establish correlations between MBI items and PSS.

**RESULTS.** We included 154 subjects, 50 residents from different areas(32.5%), 16 interns(10.4%), 53 nurses(34.4%), 20 medical doctors(13%), respiratory therapist 6(3.9%), physical therapy 9(5.8%). Severe burnout was present in 65 patients(42.2%). Patients with SSB vs lower scores were predominantly residents[(43.1 vs. 24.7%),0.016] had a higher proportion of substance abuse[(27.7 vs 10.1%),p< 0.005], a higher proportion of chronic disease[(18.5 vs. 6.7%),p=0.025], worked the night shift or for over 24-hour period more often[(73.8 vs. 56.2%),p=0.003 and (60 vs. 36%),p=0.003, respectively] had a higher median number of assigned patients[(10 IQR: 4-15) vs. (6, IQR: 3-10),p=0.044] and higher mean weekly work hours[(82±34) vs. (69±31), p=0.014]. Mean PSS and PHQ-9 were higher in the SSB group[(29±7.1, vs. 20.8±7.8),p< 0.001] and [(11.7±5) vs (5.9±5.1),p< 0.001], respectively. Age, sex, body mass index and familiar status were similar between groups. Substance abuse and suicidal thoughts were strongly associated with SSB independently of age and sex[(OR=3.4, CI 95 % 1.3-8.4,p=0.008), (OR=4.1, CI 95% 1.6-10.5,p=0.002),respectively]. All items from the MBI correlated with the PSS (DP:r=0.542 p< 0.001; EF:r=0.601, p< 0.001;PR: r=-0.383,p< 0.001).

**CONCLUSIONS.** SSB is associated with substance abuse and suicidal thoughts in Mexican health-staff independently of age and gender. The individual items of the MBI scale show correlation with PSS. MBI questionnaires could be a useful tool for the timely detection of high-risk behavior in health workers.

**REFERENCE(S)**

1. J Intensive Care.2017;Nov,18(4):270-275.
2. Vandevala,T., Pavey,L.,Chelidoni,O. et al. j intensive care (2017)5:16.
3. Job burnout. Annu Rev Psychol.2001;52:397-422.

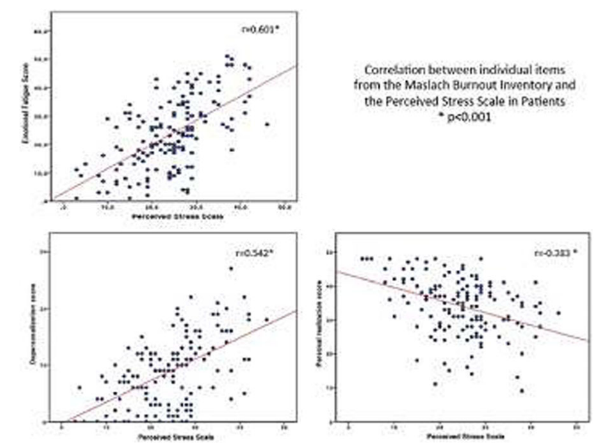

**Fig. 1 (abstract 1046).** Correlation between individual items from the Maslach Burnout Inventory and the the PSS

### 1047

#### Using patient and staff experience to maximise empathy in acute care: the Patient Experience And Reflective Learning (PEARL) project

O. Brookes[1], C. Tarrant[2], C. Taylor[3], G. Packer[4], J. Bion[5], PEARL Collaboration

[1]University Hospitals Birmingham NHS Foundation Trust, Research & Development, Birmingham, United Kingdom; [2]University of Leicester, Social Science Applied to Healthcare Improvement Research (SAPPHIRE) Group, Leicester, United Kingdom; [3]University of Warwick, WMS - Population Evidence and Technologies, Coventry, United Kingdom; [4]University Hospitals Birmingham NHS Foundation Trust, Anaesthesia & Critical Care, Birmingham, United Kingdom; [5]University of Birmingham, University Department of Anaesthesia & Critical Care, Birmingham, United Kingdom

**Correspondence:** O. Brookes

*Intensive Care Medicine Experimental* 2018, **6(Suppl 2):**1047

**INTRODUCTION.** Empathic behaviours in healthcare transform patient experience, and staff who feel supported and valued provide better care. In critical illness patients and relatives are sensitised to staff behaviours, and a thoughtless word or gesture can transform grief into grievance. PEARL uses patient, relative and staff experiences to enhance the capacity of health care staff for self-awareness and empathy through reflective learning - personal insight development.

**OBJECTIVES.** To develop a reflective learning framework (RLF) for staff in acute medicine and critical care to improve patient, family and staff experience.

**METHODS.** Intensive Care Units (ICUs) and Acute Medical Units (AMUs) of three NHS Trusts have implemented two experience surveys: a continuous patient and relative survey; and an annual survey for staff. Survey data are supplemented by ethnographic interviews and observations in the workplace to evaluate barriers to and facilitators of reflective learning.

These data are presented to each Trust for discussion by facilitated local co-design groups representing patients, relatives, and multidisciplinary clinical and managerial staff from each AMU and ICU. The co-design groups will meet three times over six months to develop behavioural specifications for activities/interventions designed to maximise opportunities for reflection. Outputs will be collated and developed using expertise within the central project team. The conceptual theory and the design process utilise the COM-B framework (Capability, Opportunity, Motivation influencing Behaviour)[Michie 2011].

**PRELIMINARY RESULTS.** Performance-importance plots (fig 1) of patient and relative survey data show that communication strongly influences perceptions of quality in AMUs and ICUs, and these behaviours offer the greatest opportunity for improvement. Clinical care domain questions achieved higher 'best' ratings but had less influence on overall satisfaction. Median 'best' response rates were higher for ICUs (56%) than AMUs (31%), likely influenced by higher ICU staffing ratios, and possible 'contamination' of AMU data by inadvertent collation of emergency department, AMU and general medical ward experiences.

Ethnographic observations and staff survey identified multiple opportunities for learning from constructive as well as dysfunctional behaviours. Staff reported using informal approaches to reflection on their individual practice, but had few opportunities for reflection in-action or group reflection.

**CONCLUSIONS.** Non-technical skills, in particular communication, impact greatly on patient, relative and staff experience. The RLF must therefore include techniques which foster genuine emotional engagement from staff, group learning from best practice, resilience in coping with negative feedback, and the capacity to enact change.

**REFERENCE(S)**

Michie et al. 2011. Implementation Science 2011,6:42

**GRANT ACKNOWLEDGMENT**

FUNDER

NIHR HS&DR programme (Ref 14/156/23).

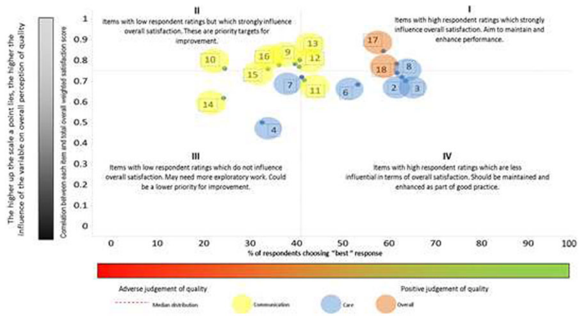

**Fig. 1 (abstract 1047).** Performance Importance Plot - AMUs and ICUs

### 1048

#### Gaps between the expectations and perceptions of the patients and their families about the quality of healthcare service of a dedicated weaning unit for patients with prolonged mechanical ventilation: a study to investigate the needs and experience of the patients and their families

J.-S. Jerng[1,2], B.-L. Chang[3], H.-O. Kao[3], S.-J. Lin[4], S.-H. Yang[3], Y.-W. Kuo[4], J.-S. Sun[2,5]

[1]National Taiwan University Hospital, Department of Internal Medicine, Taipei, Taiwan, Province of China; [2]National Taiwan University Hospital, Center for Quality Management, Taipei, Taiwan, Province of China; [3]National Taiwan University Hospital, Department of Nursing, Taipei, Taiwan, Province of China; [4]National Taiwan University Hospital, Department of Integrated Diagnostics & Therapeutics, Taipei, Taiwan, Province of China; [5]National Taiwan University Hospital, Department of Orthopedic Surgery, Taipei, Taiwan, Province of China

**Correspondence:** J.-S. Jerng

*Intensive Care Medicine Experimental* 2018, **6(Suppl 2):**1048

**INTRODUCTION.** Experience and satisfaction of the patients and their families might provide valuable insight for improving the quality of care of patients under mechanical ventilation (MV). However, less was known about the gaps between their experience and needs regarding the quality of healthcare service in the setting of prolonged MV.

**OBJECTIVES.** To investigate the gaps between the expectations and perceptions of the quality of healthcare service in the setting of prolonged MV.

**METHODS.** We conducted this prospective analysis in the Respiratory Care Center (RCC), a dedicated weaning unit for patients receiving prolonged MV, in a university-affiliated medical center from January to December 2017. Patients treated at the RCC and their visiting family members were provided with the information about this survey questionnaires before the patient left the RCC. The questionnaire was designed based on the SERVQUAL instrument by Parasuraman A et al. (See reference), which consisted of 22 questions in five dimensions, including Tangibles, Reliability, Responsiveness, Assurance, and Empathy. Two sections of the survey were included: expectation and perception, each containing 22 questions. We also collected background and care-related data of the patients, as well as background data of the respondents of the survey. We applied Important Performance Analysis (IPA) to identify priority items for improvement, and then analyzed the data and performed multivariate logistic regression analyses to understand the factors associated with the gaps between expectations and perceptions by the respondents.

**RESULTS.** During the 12-months study period, a total of 167 respondents consented to participate in the survey. Of the patients the respondents represented, 60.5% were male, with age averaged 71.5 years, 21.6% malignancy, 50.9% from medical intensive care units, and 68.3% already having tracheostomy upon admission to the RCC. Of the patients, the length of stay at the RCC averaged 19.4±10.8 days, and 70.7% were successfully liberated from MV upon discharge from RCC. IPA identified 4 question items of improvement priority. Multivariate logistic regression analyses showed that the male respondent, patient older than 75 years, tracheostomy, and needs for physical training or dialysis, were associated with an increased gap between perception and expectation for the priority items, especially for the question "employees of the RCC understand your specific needs."

**CONCLUSIONS.** Gaps exist between the perception and expectation in the quality of healthcare service in the setting of prolonged mechanical ventilation. We recommend that the healthcare systems apply similar approaches to understand the needs and their experiences to generate the improvement priorities for healthcare service quality.

**REFERENCE(S)**

Parasuraman A, Berry LL, Zeithaml VA. Refinement and reassessment of the SERVQUAL scale. Journal of Retailing, 1991;67:420-450.

**GRANT ACKNOWLEDGMENT**

None

### 1049

#### Impact of policy of extended visits at intensive care unit on the family and professionals' satisfaction

D. Díaz Díaz[1], M. Villanova Martinez[2], A. Martinez de la Gandara[1], R. Molina Segura[1], G. Andrade Vivero[1], B. Bueno Garcia[1], L. Lopez Perez[1], E. Palencia Herrejon[1]

[1]Hospital Universitaire Infanta Leonor, Intensive Care, Madrid, Spain; [2]Hospital Universitaire Infanta Leonor, Madrid, Spain

**Correspondence:** D. Díaz Díaz

*Intensive Care Medicine Experimental* 2018, **6(Suppl 2):**1049

**INTRODUCTION.** Admission to an intensive care unit (ICU) is a stressful event for both the patients and their relatives. Some studies suggest that extending the time allowed to the families to visit their relatives admitted at the ICU is related to a reduction of patient´s anxiety and depressive symptoms and with an improvement in family satisfaction.

**OBJECTIVES.** To explore the degree of satisfaction of patients' relatives at ICU discharge as well as of the ICU workers, after the implementation of an extended-time ICU visit policy (from 11:30 to 20:30).

**MATERIAL AND METHODS.** Transversal, observational, prospective study in an ICU of a second level hospital. ICU patient´s relatives and ICU workers were surveyed using the instrument FS-ICU 34 to assess family satisfaction, as well as a specific satisfaction survey for ICU workers.

**RESULTS.** A total of 78 surveys were obtained, 40 from ICU patient's relatives and 38 ICU workers (7 Physicians, 22 Nurses and 9 nurse assistants). Most of the relatives surveyed were very satisfied with the extended-time visit schedule and with the care received and their participation in the decision-making process. The major concerns of the family members was focused on the process of transmitting information (coherence in the prognosis and hope provided on the recovery of their family), the environment of the ICU (excessive night lighting, scarcity of audio-visual resources to keep the patient stimulated) and environment of the ICU´s waiting room (location, absence of information point to relatives and notice of entry to the family visit). With regards to ICU workers, only 21.1% of the participants thought that the extended-time visit policy could result in patient´s instability whilst the 60.5% thought that it improves the mood of the patient. The 57.8% thought it increases the physical and psychological burden of nursing but up to 60.6% considered that it does not interfere with the usual care of the critical patient. Virtually all responders agreed to increase the number of visits in end-of-life situations (95.7%) and up to 59.5% would be willing to maintain this policy of extended-time visits. Up to 92.5% of ICU workers agreed the need of a specific training to improve their communication skills with the patients' relatives.

**CONCLUSIONS.** After the introduction of extended-time ICU visit policy, the degree of satisfaction of the patients' relatives is elevated. However, several issues should be improved, such as the process of transmitting information as well as the environment of the ICU and waiting room. With regards to ICU professionals they think that the greatest impact of extended-time visits is the benefit to the patients rather than to the family or staff. In addition, they feel they need more training to improve the interaction with the patient´s relatives who stay in the ICU more than 8 hours a day. More than half of the professionals are in favour of maintaining the extended-time ICU visit policy.

### 1050

#### What information do ICU relatives want in the waiting room?

D. Zeinali, L. Hunter, K. McQueen, S. Singh, N. Robin
Countess of Chester Hospital, Anaesthesia & Critical Care, Chester, United Kingdom
**Correspondence:** D. Zeinali
*Intensive Care Medicine Experimental* 2018, **6(Suppl 2):**1050

**INTRODUCTION.** The relatives of ICU patients require adequate, accurate and timely information. Poor information causes stress and may lead to complaints and even litigation. An information package that can be viewed on screen in the ICU waiting room may be a practical solution to the delivery of generic information.

This would serve to compliment one to one doctor-family communication.

**OBJECTIVES.** To identify what information is considered important by relatives and ICU staff, in order to create a useful information programme to be displayed on a screen in the waiting area.

**METHODS.**

Phase 1: A working group was set up consisting of ICU doctors, nurses and hospital communications team to explore various ideas.

Phase 2: A qualitative unstructured interview with ICU relatives and with a variety of members of ICU staff was undertaken by a member of the working group (DZ).

Phase 3: The working group reviewed the results of the qualitative interviews and produced a questionnaire.

Phase 4: The structured questionnaires were circulated to ICU relatives and ICU staff both medical and nursing, in which respondents were asked to rate the importance of proposed information to be displayed using a scale of 1-5 with 1 being least important and 5 being most important.

They were also asked how best the information could be displayed (text, diagrams, videos etc) There was also a free text box.

**RESULTS.** Recurring themes from relatives included: fear experienced when seeing machines, equipment and monitors. Not understanding their function or significance. Wanting clarity about the relative seniority of doctors, nurses and other health care professionals. Frustration at having to wait without being given information and to see the patient.

Staff felt that information about the standard ICU day, procedures and interventions and information to help relatives understand why they may need to wait would be useful. In addition, information about research projects and common conditions such as delirium/ confusion would be valuable.

*Full questionnaires and results available on request from the first author.*

70% favoured photos with labels, 50% text, 40% diagrams with labels and 30% videos with or without sound, as the mode of presenting information (more than one option was permitted).

**CONCLUSIONS.** Using a systematic 4 phase approach to determine the information required by ICU relatives in the waiting room, has enabled us to embark upon the implementation phase (phase 5) confident that we will be producing an information screen which is fit for purpose. This will support relatives by providing appropriate and useful information as well as reducing stress, complaints and litigation.

**REFERENCE(S)**

Henrich NJ, Dodek P, Heyland D, Cook D, Rocker G, Kutsogiannis D, Dale C, Fowler R, Ayas N . Qualitative analysis of an intensive care unit family satisfaction survey. Critical Care Med. 2011 May;39(5):1000-5.

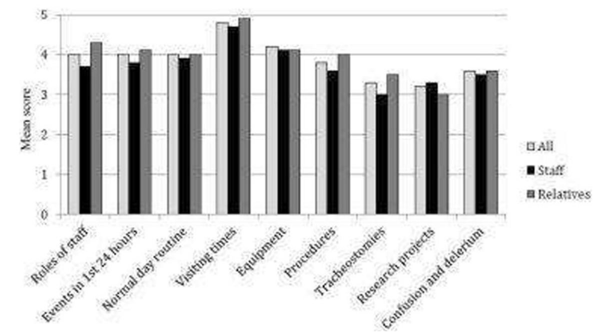

**Fig. 1 (abstract 1050).** Graph to show results from questionaire (n=33)

### 1051

#### Psychological symptoms in relatives of ICU-patients: a multi-centre study

M. Hoffmann[1,2,3], M.-M. Jeitziner[4], J.C. Schefold[4], D. von Lewinski[1], N. Verheyen[1], P. Heindl[5], A. Bachlechner[5], R. Riedl[6], A.K. Holl[7], T.R. Pieber[1], P. Eller[1], K. Amrein[1]
[1]Division of Endocrinology and Diabetology/Division of Intensive Care, Department of Internal Medicine, Medical University of Graz, Graz, Austria; [2]University Hospital Graz, Executive Department for Quality and Risk Management, Graz, Austria; [3]Research Unit for Safety in Health, Medical University of Graz, Graz, Austria; [4]University Hospital Bern (Inselspital), University of Bern, Department of Intensive Care Medicine, Bern, Switzerland; [5]Vienna General Hospital, Department of Intensive Care, Vienna, Austria; [6]Institute for Medical Informatics, Statistics and Documentation, Graz, Austria; [7]University Hospital Graz, Department of Psychiatry and Psychotherapeutic Medicine, Graz, Austria
**Correspondence:** M. Hoffmann
*Intensive Care Medicine Experimental* 2018, **6(Suppl 2):**1051

**INTRODUCTION.** The admission of a family member to an intensive care unit (ICU) is an extraordinarily stressful experience for their relatives. Critical illness paired with an entirely new environment triggers significant stress, insomnia and anxiety.

**OBJECTIVES.** This study is the Run-in Phase of the ICU-Families project (ClinicalTrials.gov Identifier: NCT02931851) and examines the degree to which relatives of ICU-patients are affected by psychological symptoms over a 30-day period.

**METHODS.** This multi-centre study was performed in Austria and Switzerland. The Impact of Event questionnaire (IES) developed by Horowitz et al. was delivered to relatives upon admission of the patient to the ICU (0-2 days after admission, T0) and thirty days later (T30), using face-to-face and telephone interviews. Clinically relevant values on the IES score are >27 points, while values >35 can support a diagnosis of PTSD in the right context. Data collection took place between 2016 and 2017.

**RESULTS.** 42 relatives (12 male, 30 female) were included in the study, with an average age of 53 ± 15 years. Relatives were included in Graz (n=23), Vienna (n=10) and Bern (n=9).

At T0, a mean IES score of 31.4 ± 13.7 points was observed and at T30 the score decreased to 24.7 ± 10.6 points (p< 0.001, paired ttest). IES-scores >27 were observed for 24 (57%) at T0 and for 15 (36%) at T30. IES-scores >35 were observed for 19 (45%) and 7 (17%) at T0 and T30, respectively.
On average, women scored higher than men at T0 (33.6 ± 14.4 vs 25.8 ± 10.2) and at T30 (25.0 ± 10.3 in women and 24.0 ± 11.6 in men). Those present at the event leading to ICU submission had significantly higher IES scores (T0 35.1 ± 9.2 vs 26.1 ± 15.6, p< 0.037, T30 28.6 ± 9.8 vs 20.6 ± 10.6, p< 0.017).
**CONCLUSIONS.** More than half of the relatives reported clinically relevant symptoms at T0 and T30. Women and those present at the event leading to ICU admission appear to at higher risk. The feasibility of effective support services in daily routine should be tested.

**REFERENCE(S)**

1. Belaya chi J, Himmich S, Madani N, et al (2014) Psychological burden in inpatient relatives: The forgotten side of medical management. QJM 107:115-122. doi: 10.1093/qjmed/hct210
2. Netzer G, Sullivan DR (2014) Recognizing, naming, and measuring a family intensive care unit syndrome. Ann Am Thorac Soc 11:435-441. doi: 10.1513/AnnalsATS.201309-308OT
3. Azoulay E, Pochard F, Kentish-Barnes N, et al (2005) Risk of post-traumatic stress symptoms in family members of intensive care unit patients. Am J Respir Crit Care Med 171:987-994. doi: 10.1164/rccm.200409-1295OC

**GRANT ACKNOWLEDGMENT**
The study was funded by ÖGIAIN.

### 1052

#### What is the patients' physical, cognitive and mental status before ICU admission?

W. Geense[1], M. Zegers[1], M. Peters[2], I. Janssen[3], B. Ramakers[4], J. van der Hoeven[1], M. van den Boogaard[1]
[1]Radboud University Medical Center/Radboud Institute for Health Science, Intensive Care Medicine, Nijmegen, Netherlands; [2]Canisius Wilhelmina Hopspital, Intensive Care Medicine, Nijmegen, Netherlands; [3]Maasziekenhuis Pantein, Intensive Care Department, Boxmeer, Netherlands; [4]Bernhoven Hospital, Intensive Care Medicine, Uden, Netherlands
**Correspondence:** W. Geense
*Intensive Care Medicine Experimental* 2018, **6(Suppl 2):**1052

**INTRODUCTION.** Many intensive care unit (ICU) survivors experience long-term physical, cognitive and mental problems, which are affected by patients' status before ICU admission. However, most long-term outcome studies do not take the pre-ICU status into account.
**OBJECTIVE.** To assess ICU patients' pre-ICU physical, cognitive and mental status.
**METHODS.** The prospective MONITOR-IC cohort study in 4 Dutch hospitals, started in July 2016. Patients aged ≥16 with ICU admission ≥12 hours are included and followed for 5 years. Patients with a life expectancy of < 48 hours or receiving palliative care are excluded. At ICU admission, patients (or their relatives), are asked to rate their pre-ICU status by filling in several questionnaires: Clinical Frailty Scale (CFS), Checklist Individual Strength (CIS-8), Cognitive Failure Questionnaire (CFQ-14), Hospital Anxiety and Depression Scale (HADS), and Short Form (SF-36). Clinical data are extracted from the electronic patient record.
**RESULTS.** We included 1,537 patients (69.4% inclusion rate) in 1.5 year. Common reasons for exclusion were refusal to participate (40%) and death before informed consent (17%). Median ICU stay was one day, with mainly elective surgery admissions (65%). Pre-ICU admission, 13% of the patients was frail, and almost 60% was tired (table 1). Low CFQ scores (20.2) indicate low levels of cognitive failure. A quarter of the patients reported depression and anxiety symptoms. The SF-36 physical and mental health component scores were lower compared to the age-matched Dutch population.
**CONCLUSIONS.** Pre-ICU admission, patients suffered from fatigue, depression and anxiety symptoms and had a lower physical and mental state than the normal population. These insights need to be taken into account when assessing patients' long- term outcomes, and to identify adequate targets to prevent or mitigate long-term problems of critical illness.

**Table 1 (abstract 1052).** Patient characteristics and pre-ICU status

| | |
|---|---|
| Age, mean (SD) | 62 (15.6) |
| Male, n (%) | 997 (63.6) |
| Admission, n (%): | |
| • Elective surgery | 879 (65.3) |
| • Medical | 316 (23.5) |
| • Emergency surgery | 152 (11.3) |
| APACHE IV score, median (IQR) | 51 (40-65.5) |
| ICU LOS (days), median (IQR) | 1 (1-2) |
| Hospital LOS (days), median (IQR) | 9 (6-15) |
| Frailty (CFS), n (%) | |
| • Non frail | 1309 (86.9) |
| • Mild/ moderately frail | 126 (8.4) |
| • Severely frail | 68 (4.5) |
| • Terminally ill | 4 (0.3) |
| Fatigue (CIS), n (%) | |
| • No fatigue | 613 (40.8) |
| • Fatigue | 340 (22.6) |
| • Severe fatigue | 550 (36.6) |
| Cognitive functioning (CFQ-14), median (IQR) | 20.2 (8.8-27.8) |
| Depression (HADS), n (%) | |
| • Normal | 1104 (72.8) |
| • Mild symptoms | 235 (15.5) |
| • Moderate symptoms | 140 (9.2) |
| • Severe symptoms | 38 (2.5) |
| Anxiety (HADS), n (%) | |
| • Normal | 1089 (71.8) |
| • Mild symptoms | 220 (14.5) |
| • Moderate symptoms | 149 (9.8) |
| • Severe symptoms | 58 (3.8) |
| Quality of life (SF-36), median (IQR) | |
| • Physical component summary | 41.1 (32.3-51.7) |
| • Mental component summary | 50.9 (39-56.5) |

### 1053

#### Prognostic relevance of increased muscle echogenicity detected by ultrasound to predict intensive care unit-acquired weakness, weaning failure and outcome in critically ill patients: a longitudinal observational study

J. Ehler[1], U. Walter[2,3], F. Klawitter[1], M. Sauer[1], D.A. Reuter[1], R. Patejdl[4]
[1]University Medical Center Rostock, Department of Anesthesiology and Intensive Care Medicine, Rostock, Germany; [2]University Medical Center Rostock, Department of Neurology, Rostock, Germany; [3]University Medical Center Rostock, Center for Transdisciplinary Neurosciences, Rostock, Germany; [4]University Medical Center Rostock, Oscar Langendorff Institute of Physiology, Rostock, Germany
**Correspondence:** J. Ehler
*Intensive Care Medicine Experimental* 2018, **6(Suppl 2):**1053

**INTRODUCTION.** Intensive care unit-acquired weakness (ICU-AW) is associated with weaning failure and unfavorable outcome in critically ill patients. Muscular ultrasound (MUS) is a non-invasive diagnostic tool that is able to display increased muscle echogenicity, which was linked to neuromuscular dysfunction.

**OBJECTIVES.** To evaluate the prognostic value of increased echogenicity detected by MUS to predict ICU-AW, weaning failure and long-term outcome in critically ill patients.

**METHODS.** Critically ill patients (SOFA-score ≥8 on three consecutive days after ICU admission) without preexisting neuromuscular diseases and age-matched healthy controls were included (ClinicalTrials.gov: NCT02706314). Longitudinal multimodal diagnostics at study days 3 and 10 including muscular research council-sum score (MRC-SS), electrophysiology, muscular ultrasound and serum analyte measurements were performed. ICU-AW was regarded as being present at an MRC-SS of < 48. Weaning failure was defined as a failed spontaneous breathing trial or the need for reintubation/tracheotomy. Ultrasound echogenicity of eight extremity muscles was assessed on the global muscle echogenicity score (GME) (1). The 3-months outcome was assessed on the modified Rankin scale (mRS) and the Barthel index (BI).

**RESULTS.** Thirty five critically ill patients (mean age 65.5±14.4 years) and five healthy controls (mean age 49.8±10.3 years) were analyzed. Patients' mean GME was significantly higher at both study days (day 3 GME 2.33±0.4; day 10 GME 2.29±0.4 vs. controls GME 1.07±0.1; each p< 0.001). ICU-AW was present in 17/32 patients. Three patients died before a diagnoses could be obtained. Patients with ICU-AW had higher mean GME values at day 10 (2.4±0.4) in comparison to patients without ICU-AW (mean GME 2.16±0.5). Muscular echogenicity decreased in patients without ICU-AW between day 3 (mean GME 2.35±0.5) and day 10 (mean GME 2.16±0.5), which was not observed in patients with ICU-AW (day 3 mean GME 2.36±0.3; day 10 mean GME 2.4±0.4). GME at day 10 correlated with the MRC-SS values (r=-0.433; p=0.024). Higher GME at day 10 was observed in patients with weaning failure (n=19; mean GME 2.39±0.4) compared to patients with successful weaning (n=15; mean GME 2.14±0.5; p=0.09). One patient died before weaning procedure. Mean GME at day 10 correlated with the 3-months outcome assessed on the mRs (r=0.490; p=0.028) and BI (r=-0.621; p=0.008).

**CONCLUSIONS.** Increased echogenicity detected by MUS is associated with critical illness and unfavorable 3-months outcome in critically ill patients. During the early phase of critical illness MUS might be helpful to predict ICU-AW and weaning failure.

**REFERENCE(S)**

1) Grimm A, Teschner U, Porzelius C, Ludewig K, Zielske J, Witte OW, Brunkhorst FM, Axer H: Muscle ultrasound for early assessment of critical illness neuromyopathy in severe sepsis. *Crit Care* 2013, 17(5):R227.

**GRANT ACKNOWLEDGMENT**

Grant no. 889006, “FORUN research program” of University of Rostock.

### 1054

#### Ultrasonography-guided post-pyloric feeding tube insertion in medical intensive care patients

U. Özdemir[1], S. Yıldız[2], M. Türkoğlu[1], G. Aygencel[1]
[1]Gazi University Medical Faculty Hospital, Medical Intensive Care Unit, Ankara, Turkey; [2]Gazi University Medical Faculty Hospital, Internal Medicine Department, Ankara, Turkey
**Correspondence:** U. Özdemir
*Intensive Care Medicine Experimental* 2018, **6(Suppl 2):**1054

**INTRODUCTION.** For enteral nutrition (EN), ideally the nasoenteral tube (NET) should be placed to post-piloric area (PPA) for the patients especially who have risk of aspiration (1). The most common method of determining NET position is abdominal X-ray graphy (DXG). Due to delays in DXG imaging, imaging errors and situtation of NET not in PPA, EN is delayed for many hours and patients may be expose to more X-rays with repetitive shots. However, today we can determine passing NET to the PPA with ultrasonography (USG)(2).

**OBJECTIVES.** Our goal is to determine the success of the NET placement to PPA with USG and how much time is spent for placement.

**METHODS.** All patients who were older than 18 years age and needed to NET placement and admitted to Gazi University Hospital Medical ICU (MICU) between 01 October - 01 December 2017 were included in the study. With USG device (GE, S7 model), we visualized the NET at the proximal esophagus (11 MHz linear probe), in the esophagogastric junction and PPA(5 MHz convex probe). When NET was found in the stomach but not in the PPA, NET was moved back and forward or patient was taken to diffirent position or prokinetic agent was used for provide to pass NET to PPA. When the NET was placed in the PPA, we gave the 25 ml saline in through the feeding tube. We observed the echogenicity changes in PPA by using USG. After then we confirmed the NET position with DXG.

**RESULTS.** 21 patients were included in the study. The median age was 71 (54.5-79) years, 10 (47.6%) patients were male, and 13 (61.9%) patients were intubated. In 19 (90.5%) patients, USG guided NET insertion was successful and it was shown to pass into the PPA. Echogenicity changes in PPA following saline injection into the NET were detected in 17 patients by using USG. The NET position was confirmed with DXG. Complication was not observed during the study. The median time of NET insertion was 14 (9-22.5) minutes and median NET level was 74 (68-78) cm when placed to PPA. Placement time and NET level did not change with gender (p=0.314, p=0.468 for male and female patients) and intubation state (p=0.595, p=0.268 for intubated and not intubated patients).

**CONCLUSIONS.** USG guided NET placement can be safe in MICU patients. This method should be preferred because of the less radiation exposure and less time consumption.

**GRANT ACKNOWLEDGMENT**

No financial support or grant was received for this study.

## Acute respiratory failure: From mechanical ventilation to ECMO

### 1055

#### Transpulmonary thermodilution before and during veno-venous extra-corporeal membrane oxygenation ECMO: an observational study on a potential loss of indicator

A. Herner, T. Lahmer, U. Mayr, S. Rasch, R. Schmid, W. Huber
Klinikum rechts der Isar; Technical University of Munich, Medizinische Klinik und Poliklinik II, Munich, Germany
**Correspondence:** W. Huber
*Intensive Care Medicine Experimental* 2018, **6(Suppl 2):**1055

**INTRODUCTION.** Extracorporeal membrane oxygenation (ECMO) is a therapeutic option in severe acute respiratory distress syndrome (ARDS). Despite its use for more than four decades, so far only one RCT suggests a reduction of mortality by ECMO. Strategies to improve the effectiveness of ECMO include optimized patient selection, less invasive techniques and combination of ECMO with ultra-protective ventilation. Pre-ECMO haemodynamics might help to better allocate patients to ECMO and to optimize the ECMO-set-up. Extracorporeal blood flow adjusted to the patients´ cardiac output (CO) is of high importance for effective extracorporeal oxygenation. This requires accurate measurement of CO before and during ECMO. However, there are concerns that pulmonary arterial and transpulmonary thermodilution (TPTD) might be confounded due to a potential loss of indicator into the extracorporeal circuit, resulting in an overestimation of volumetric parameters. However, there is a lack of data on indicator dilution techniques during ECMO.
**OBJECTIVES.** Therefore, we compared TPTD-measurements before and during ECMO. We hypothesized that indicator injection using a *femoral* central venous catheter (CVC) close to the femoral ECMO drainage cannula might be more prone to indicator loss than using a *jugular* CVC.
**METHODS.** TPTD-derived parameters before and after initiation of the extracorporeal circuit were compared in a total of 14 ICU-patients with ECMO and TPTD-monitoring (PiCCO). 8 patients were equipped with a jugular, and 6 patients with a femoral CVC.
**RESULTS.** Cardiac index CI, global end-diastolic volume index (GEDVI) and extravascular lung water index (EVLWI) before ECMO as well as the ECMO flow (3.1±1.3 vs. 3.3±1.5 L/min, p=0.78) were comparable in patients with jugular and femoral CVC. After the onset of ECMO, CI did not increase compared to pre-ECMO values in the totality of patients (4.36±2.12 vs. 4.49±1.71 L/min/m$^2$; p=0.433).
By contrast, GEDVI (974±384 vs. 791±179 mL/m$^2$; p=0.041) and EVLWI (27.7±11.1 vs. 21.3±9.1mL/kg; p=0.001) were significantly higher during ECMO compared to before.
These changes in GEDVI (378±247 vs. 36±210; p=0.02) and EVLWI (10.5±8.1 vs. 3.4±2.4; p=0.051) were substantially more pronounced for *femoral* compared to *jugular* indicator injection. In multivariate analysis, for both GEDVI (p=0.004) and EVLWI (p=0.035) *femoral* indicator injection was independently associated with larger increases during ECMO compared to before.
By contrast, haemodynamic parameters not derived from TPTD such as systolic and diastolic arterial pressure, pulse pressure variation, dPmax and cardiac power index were not affected by the start of ECMO.
**CONCLUSIONS.** Our study demonstrates marked increases in GEDVI and EVLWI after the onset of ECMO which were more pronounced in case of *femoral* compared to *jugular* indicator injection. These findings are in line with the hypothesis of a loss of indicator into the ECMO-circuit.

### 1056

#### Epidemiology of myocardial dysfunction in patients with hypoxemic respiratory failure and acute respiratory distress syndrome: an observational cohort study

T. Barnes, D.J. Niven, A. Soo, A. Ferland, P. Boucher, H.T. Stelfox, C.J. Doig, K. Parhar
University of Calgary, Department of Critical Care Medicine, Calgary, Canada
**Correspondence:** K. Parhar
*Intensive Care Medicine Experimental* 2018, **6(Suppl 2):**1056

**INTRODUCTION.** Acute respiratory distress syndrome (ARDS) is an inflammatory syndrome of the lungs characterized by hypoxemia, reduced lung compliance, and bilateral lung infiltrates. ARDS is associated with significant morbidity and mortality. Myocardial dysfunction, specifically acute cor pulmonale, has previously been associated with ARDS; however, limited data exists in Berlin definition ARDS patients undergoing lung protective ventilation.
**OBJECTIVES.**
Define the incidence and type of myocardial dysfunction in patients with hypoxemic respiratory failure and ARDS
Determine patient factors associated with the development of myocardial dysfunction in patients with ARDS
Determine if myocardial dysfunction in ARDS is associated with 28-day and 3-year outcomes
**METHODS.** A standardized screening program for ARDS, as a quality improvement initiative, was initiated at 4 adult ICUs within Calgary, Alberta, Canada over a 27-month period. All patients ventilated for ≥ 24 hours were prospectively and consecutively screened with an arterial blood gas on a standardized $FiO_2$ of 1.0. Chest X-Ray images were manually reviewed to determine if criteria for ARDS (Berlin definition) were met. Prospectively collected data through an electronic clinical-information system allowed assessment of patient demographics, details of ICU stay, mechanical ventilation practices, including estimation of mechanical power and its determinants as well as 28-day and 3-year survival. Patients were included in this study if they had a transthoracic or transesophageal echocardiogram during their ICU stay. Patient demographics, details of ICU stay, as well as 28-day and 3-year outcomes were analysed by univariate and multivariable linear regression analysis.
**RESULTS.** Of 7944 patients, 986 patients (12.4%) had hypoxemic respiratory failure (PF < 300) on screening, and 731 patients (9.2%) met ARDS (Berlin definition) criteria. Of the 986 patients with hypoxemic respiratory failure, 505 had an echocardiogram during their ICU stay. The median age was 61 (IQR 50-71), with 36.3% of patients being female. Moderate or worse left ventricular (LV) systolic dysfunction was present in 103 patients (24%). Moderate or worse right ventricular (RV) systolic dysfunction was present in 129 patients (29.5%) and moderate or worse RV dilatation was present in 66 patients (34.9%). The presence of LV or RV systolic dysfunction did not influence survival. The presence of RV dilatation was associated with reduced hospital and 3-year mortality (p=0.018). Multivariable analysis of factors associated with RV dilatation included high body mass index and APACHE score.
**CONCLUSIONS.** In patients with ARDS, LV and RV systolic dysfunction as well as RV dilatation, were common in patients. The presence of RV dilatation was associated with reduced 28-day and 3-year mortality.
**GRANTS**
Grants from the Calgary Chief Medical Office as well as the Alberta Critical Care Strategic Clinical Network.

### 1057

#### Neutrophils, L-lactate and D-lactate in the tracheal sputum from intubated patients at the intensive care unit as biomarkers for lower respiratory tract infection

C. Dalby Sørensen[1], M. Kolpen[2], D. Faurholt-Jepsen[3], P. Østrup Jensen[2], T. Bjarnsholt[4], M. Heiberg Bestle[1]
[1]Nordsjællands Hospital University of Copenhagen, Anaestesiology and Intensive Care, Hillerød, Denmark; [2]Rigshospitalet University of Copenhagen, Department of Clinical Microbiology, Copenhagen, Denmark; [3]Nordsjællands Hospital University of Copenhagen, Hillerød, Denmark; [4]University of Copenhagen, Department of Immunology and Microbiology, Copenhagen, Denmark
**Correspondence:** C. Dalby Sørensen
*Intensive Care Medicine Experimental* 2018, **6(Suppl 2):**1057

**INTRODUCTION.** Lower respiratory tract infections (LRTI) in the intensive care unit (ICU) are mainly diagnosed by cultivation and direct microscopy, a method relatively time consuming, and often with a false negative response. When a LRTI occur neutrophils invade the site of infection resulting in local inflammation and lactate formation. Lactate is found in two stereoisomers: L-lactate and D-lactate. L-lactate is mainly produced by human cells. D-lactate, on the other hand, is usually produced by bacteria.
**OBJECTIVE.** The aim of this study was to assess neutrophils, L-lactate and D-lactate in sputum from intubated patients hospitalized at the ICU and explore their use as biomarkers for LRTI.
**METHODS.** Sputa from 43 intubated patients were obtained at time of intubation. Sputum was sent for cultivation, microscopy and PCR to verify eventual LRTI, and analysed for neutrophils, L-lactate and D-lactate. In this study we compared patients with microbiological verified LRTI or clinical/radiological suspicion of LRTI with a control group consisting of intubated patients without LRTI.
**RESULTS.** We found no difference between sputum neutrophil count from patients with verified LRTI (p = 0.5821) or suspected LRTI (p = 0.8497) relative to the control group, but we found a linear association between sputum neutrophil count and L- lactate (p = 0.0029). The concentration of L-lactate was increased in patients with verified LRTI (p=0.0501) and suspected LRTI (p = 0.0444) relative to the control group. Differentiating between viral LRTI and bacterial LRTI we found significant levels of L-lactate in connection with viral LRTI (p= 0.0062), but not for bacterial LRTI (p = 0.4822). We found no increase in sputum D-lactate from patients with verified (p=0.3233) or suspected LRTI (p=0.4646) relative to the control group.

**CONCLUSION.** L-lactate was found to be a good indicator for LRTI at the time of intubation and was increased in patients with viral but not bacterial LRTI. A significant association was found between neutrophil count and L-lactate supporting the theory that neutrophils are the main producer of lactate in sputum. However, we were unable to demonstrate a clear association between neutrophils and LRTI in intubated patients. D-lactate was not a useful indicator for LRTI in intubated patients.

**Grant Acknowledgment**
Research Grant from Nordsjællands Hospital.

### 1058

#### Can $ECCO_2R$ be the rising sun for respiratory failure?

A. Sablon, R. Jacobs, M. Diltoer, J. Troubleyn, T. Maes, M. Malbrain
UZ Brussel, Intensive Care, Jette, Belgium
**Correspondence:** A. Sablon
*Intensive Care Medicine Experimental* 2018, **6(Suppl 2):**1058

**INTRODUCTION.** Extracorporeal carbon dioxide removal ($ECCO_2R$) devices have been proposed as an adjunctive therapy in patients with acute exacerbations of chronic obstructive pulmonary disease (AECOPD) to avoid intubation or reduce the length of invasive ventilation. In acute respiratory distress syndrome (ARDS) patients it is used to allow ultra-protective ventilation (1-2).

**OBJECTIVES.** We investigated the benefits of $ECCO_2R$, complications encountered and mortality.

**METHODS.** We performed a single-center retrospective observational cohort analysis in a tertiary University Intensive Care Unit (ICU). Over a 34-month period, 59 patients were treated with arterio-venous (AV)-$ECCO_2R$ for ARDS or AECOPD. We used a Novalung ® iLA Membrane Ventilator (Xenios, Heilbronn, Germany).

**RESULTS.** In total, 59 subjects were analyzed. In the ARDS group 35 patients were included, consisting of 16 male and 19 female patients with a mean age of 57 years (54-59) and a mean APACHE score of 25 (23-27). In the AECOPD group 24 patients were included, consisting of 12 male and 12 female patients with a mean age of 64 years (63-65) and a mean APACHE score of 24 (21-27). Mortality for patients treated with AV-$ECCO_2R$ for ARDS or AECOPD was respectively 48.6% and 40.9%. In the ARDS group the mean duration of ventilation during $ECCO_2R$ was 5.7 days (5.1-6.3), the mean length of ICU and hospital stay was 23.9 days (20.9-26.8) and 44.1 days (37.5-50.9) respectively. Mean arterial blood pH obtained at initiation of $ECCO_2R$ treatment was 7.20 mmHg (7.04-7.36) and 7.40 mmHg (7.39-7.42) at termination of $ECCO_2R$ therapy. Mean $PaCO_2$ value was 65.8 mmHg (63.3-68.4) at initiation of $ECCO_2R$ therapy and 42.8 mmHg (40.9-44.7) at termination of the $ECCO_2R$ treatment.

In the AECOPD group the mean duration of ventilation during $ECCO_2R$ was 2.4 days (1.8-3.0), the mean length of ICU and hospital stay was 10.8 days (9.2-12.4) and 23.2 days (19.9-26.5) respectively. Mean arterial blood pH obtained at initiation of $ECCO_2R$ treatment was 7.24 (7.22-7.26) and 7.42 (7.41-7.43) at termination of $ECCO_2R$ therapy. Mean $PaCO_2$ value was 80.3 mmHg (77.0-83.6) at initiation of $ECCO_2R$ therapy and 56 mmHg (53.3-58.7) at termination of the $ECCO_2R$ treatment. Complications due to AV-$ECCO_2R$ had *no* significant impact on outcome in both groups.

**CONCLUSIONS.** $ECCO_2R$ is a feasible, rapidly evolving technology and is an efficient treatment that allows lung protective ventilation. However, evidence for a mortality benefit with $ECCO_2R$ is lacking and complications are frequent. Well-designed adequately powered randomized controlled trials (RCTs) are required to better elucidate risk-benefit balance.

### 1059

#### Cardiac index (CI) derived from transpulmonary thermodilution (TPTD) with indicator injection into the pulmonary artery during ECMO: a comparison with indicator injection into the right atrium to derive CI from pulmonary arterial and transpulmonary thermodilution: the HEUREKA-II animal study

W. Huber, M. Konrad, S. Kammerzell, R. Schmid, A. Herner
Klinikum rechts der Isar; Technical University of Munich, Medizinische Klinik und Poliklinik II, Munich, Germany
**Correspondence:** W. Huber
*Intensive Care Medicine Experimental* 2018, **6(Suppl 2):**1059

**INTRODUCTION.** Adaption of the extracorporeal blood flow to the patient´s cardiac output improves the effectiveness of extracorporeal membrane oxygenation (ECMO). Pulmonary arterial (CI_PAC) and transpulmonary thermodilution (CI_TPTD) are considered as gold-standards to derive cardiac index (CI). However, there are concerns regarding both methods during ECMO due to a potential loss of indicator into the extracorporeal circuit in case of indicator injection into the right atrium. This might be overcome by injection of the indicator into the pulmonary artery for a modified transpulmonary thermodilution (CI_PATPTD).

**OBJECTIVES.** To compare bias and percentage error (PE) of CI_PAC_RA and CI_TPTD_RA vs. CI_TPTDPA.

**METHODS.** 8 pigs (49.4±4.2kg) with PAC, CVC (both via jugular vein) and a femoral arterial PiCCO catheter (Pulsion, Germany). Veno-venous ECMO with CARDIOHELP (Maquet/Getinge; Rastatt, Germany) via a femoral drainage and a jugular return cannula.

CI was measured before and during ECMO with increasing extracorporeal flow (0.5, 1.0, 2.0 and maximum flow of 3.5L/min). Each dataset consisted of three triplicate measurements (subgroup with CI_PAC_RA measurements of the HEUREKA-I study):

Conventional pulmonary arterial CI with indicator injection into the proximal injectate lumen of the PAC (CI_PAC_RA)

CI_PATPTD with indicator injection into the distal lumen of the PAC and detection of the thermodilution curve using TPTD (PiCCO)

TPTD with indicator injection into the RA (CVC) with PiCCO (CI_TPTD_RA).

Due the minimized risk of indicator loss CI_PATPTD was considered gold standard.

SPSS 24.

**RESULTS.** 34 datasets each including three different triplicate measurements of CI; mean ECMO-flow 1.56±1.15 (0 - 3.5) L/min.

CI_PAC_RA and CI_PATPTD significantly correlated (r=0.646; p< 0.001) and were comparable (2.94±0.56 vs. 3.07±0.67 L/min/m²; p=0.142) with an acceptable bias of 0.13±0.53 L/min/m². The PE was 34.7%. The bias (CI_PAC_RA - CI_PATPTD) significantly increased with higher ECMO-flow (r=0.640; p< 0.001) and lower CI_PATPTD (r=-0.449; p=0.008). This was confirmed in multivariate analysis (r=0.823) for high ECMO-flow (p< 0.001; T=5.711) and low CI_PATPTD (p< 0.001; T=-5.752).

Bias (0.0023L/min/m²) and PE (14.0%) regarding CI_PATPTD were lower for CI_TPTD_RA than for CI_PAC_RA.

The amount of deviation from the gold-standard CI_PATPTD was significantly higher for |(CI_PAC_RA - CI_PATPTD)| compared to |(CI_TPTD_RA - CI_PATPTD)|: 0.38±0.39 vs. 0.18±0.13 L/min/m²; p=0.010. A bias of more than ±0.5L/min/m² regarding the gold-standard CI_PATPTD was significantly more frequent for CI_PAC_RA compared to CI_TPTD_RA (9 out of 34 vs. 0 out of 34; p=0.0021; Fisher´s exact test).

**CONCLUSIONS.**

TPTD to derive CI during ECMO seems to be more accurate and precise than pulmonary arterial thermodilution.

Increases in the bias of CI_PAC_RA with increasing ECMO flow support the hypothesis of an indicator loss.

### 1060

#### Oxygen challenge test and prediction of decannulation from veno-venous extracorporeal membrane oxygenation

E.L. Hartley, B. Sanderson, N.A. Barrett, M. Hari-Shankar, L. Camporota
Guy's and St Thomas' Hospital, Intensive Care Medicine, London, United Kingdom
**Correspondence:** E.L. Hartley
*Intensive Care Medicine Experimental* 2018, **6(Suppl 2):**1060

**INTRODUCTION.** Predicting weaning from veno-venous extracorporeal membrane oxygenation (VV ECMO) requires tests to ascertain whether the native lung function has recovered enough to support gas exchange necessary to satisfy metabolic demand. The 'oxygen challenge test' (OCT) consists of increasing the ventilator $FiO_2$to 1.0 for 5-15 minutes and measuring the arterial $PO_2$ ($PaO_2$) and arterial oxygen saturation ($SaO_2$). $SaO_2$ of 100% and $PaO_2$> 30 kPa (positive OCT) are thresholds used to identify patients able to maintain oxygenation unassisted. There is little data to show if the OCT is a useful guide for weaning and decannulation.

**OBJECTIVES.** We tested the predictive accuracy of OCT as a diagnostic test in determining weaning and decannulation from VV ECMO.

**METHODS.** We report an observational cohort study of patients receiving VV-ECMO, from January to December 2016 who had daily OCT review prior to weaning from VV-ECMO. The study had institutional approval; informed consent was waived. Between-group comparisons were carried out with Welch t-test or Mann-Whitney U test, after ascertaining normality of data distribution. Diagnostic test evaluations (Sensitivity, Specificity, AUC and Youden J test) were performed using Receiver Operating Characteristics (ROC) curves. All analyses were performed using R project for Statistical Computing.

**RESULTS.** Seventy nine patients (40 males) underwent VV ECMO in 2016. Fifteen were excluded as died on ECMO. The median age, (interquartile range, IQR) was 44.5 years (37-51), with BMI 30 (24-33) $Kg/m^2$and median time on ECMO 14 days (5-14). Only 42.2% of patients with positive OCT were decannulated from ECMO < 48 hours following the first positive test. The median (IQR) time from positive OCT to decannulation was 3 days (1-5), range 0 (decannulated the same day as positive OCT) to 25. Although OCT had good prediction of decannulation (AUC 0.77, best cut-off value of 30 kPa), a positive OCT had low prediction of decannulation in the following 48 hours (AUC of ROC 0.62). Interestingly, patients decannulated < 48 hours of the first positive OCT had a lower mean tidal volume (310 vs 391 mL; p< 0.0001; or 4.5 vs 5.7 mL/Kg IBW); lower mean minute ventilation (5.2 vs 6.5 L/min; p=0.003), and higher f/VT (89.9 vs 71.5 breaths/min/L; p= 0.046), but also a lower corrected minute ventilation (VEc) (6.0 vs 7.3 L/min; p= 0.0074). A lower VEc (a proxy of physiological dead space), may suggest a lower fraction of wasted ventilation, given that the two groups had similar mean ECMO sweep gas flow (4.1 vs 3.6 L/min; p= 0.06) and clinically - but not statistically - similar ECMO blood flow (3.8 vs 3.6 L/min; p= 0.002).

**CONCLUSIONS.** A positive OCT by itself has low prediction of ECMO decannulation in the next 48 hours. Additional physiological factors including physiological dead space and physician preference may affect the timing of decannulation. The contribution of the ECMO settings on the performance of the test needs further elucidation.

### 1061

#### Electrical impedance tomography reveals the loss of aeration in dependent zones during T-tube trial in weaning patients from mechanical ventilation

B. Fuenzalida, R. Lagos, M.C. Bachmann, P. García, T. Regueira, A. Bruhn, J. Retamal, G. Bugedo
Pontificia Universidad Catolica de Chile, Departamento de Medicina Intensiva, Santiago, Chile
**Correspondence:** B. Fuenzalida
*Intensive Care Medicine Experimental* 2018, **6(Suppl 2):**1061

**INTRODUCTION.** The loss of positive pressure in the airways during T-tube trials during weaning from mechanical ventilation, induces loss of aeration in the dependent regions of the lung. Electrical Impedance Tomography (EIT) has been evaluated to estimate changes in pulmonary air distribution and may be helpful during T-trials for early detection of weaning failure.(1)

**OBJECTIVES.** To evaluate the usefulness of electrical impedance tomography in predicting changes in lung aeration during a 2-hour T-tube spontaneous breathing trial (SBT) in patients with difficult weaning.

**METHODS.** Prospective, observational clinical study. Patients with criteria of difficult weaning (mechanical ventilation> 48 hours plus 1 of the following: shock, severe ARDS, heart disease / FC III-IV COPD, or previous failed SBT or extubation) were included. After patient inclusion, an EIT belt was placed around the patient's thorax and ventilation distribution data was measured at baseline (pressure support 5 $cmH_2O$ and PEEP 5 $cmH_2O$) and at 30 and 120 min of the T-tube SBT. Clinical decision to extubate or not the patient was at the discretion of the attending physician independent of the data obtained by the test EIT.

**RESULTS.** Fourteen patients (aged 64±18 y.o) were evaluated. Five patients failed the SBT. When comparing data between baseline and 120 minutes, we observed an increase of the end-expiratory lung impedance in the successful group (470 ± 225 vs 1089 ± 947, *P*=0,06) while the unsuccessful group showed a decrease (430 ± 123 vs 349 ± 100 *P*=0.06)(Fig. 1). Regional ventilation delay index assessment showed a significant difference between ventral and dorsal regions (0.51 ± 0.13 vs 0.31 ± 0.13 respectively, *P*< 0.05) in baseline, condition that was reversed during the SBT (0.46 ± 0.18 ventral and 0.41 ± 0.3 dorsal *P*=0.7) (Fig. 2).

**CONCLUSIONS.** Electrical impedance tomography reveals a loss of aeration in dependent regions of the lung and a change in the ventilation pattern during T-tube SBT. This information may be useful in decision making during weaning in patients with difficult or prolonged weaning.

**REFERENCE(S)**

Bickenbach J, et al. Crit Care. 2017

**GRANT ACKNOWLEDGMENT**

FONDECYT 1171810

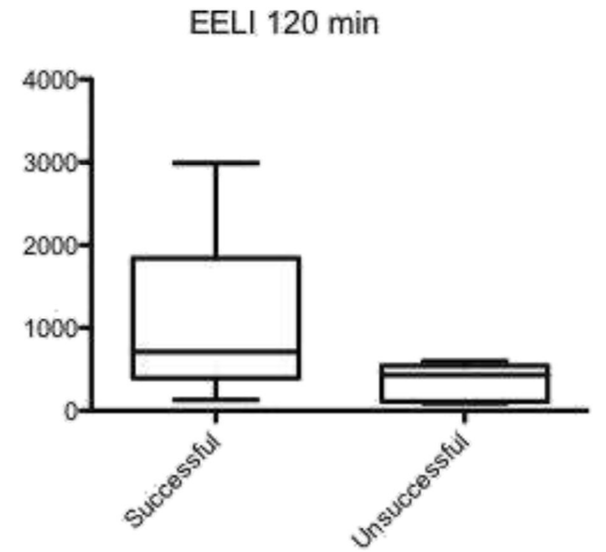

**Fig. 1 (abstract 1061).** End-expiratory lung impedance at 120 mins. p=0.06

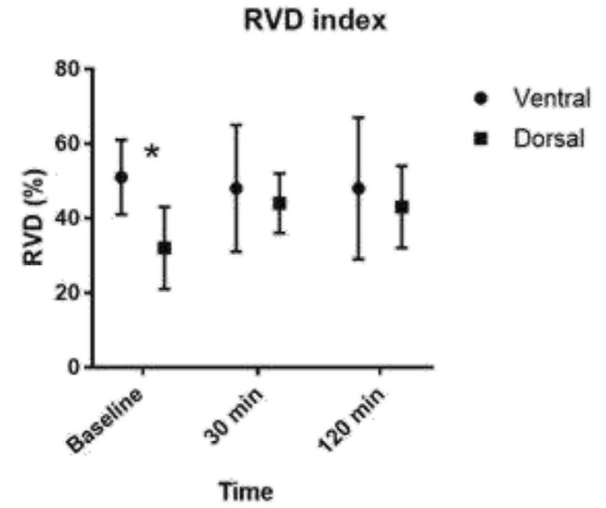

**Fig. 2 (abstract 1061).** Regional ventilation delay index over time

### 1062

#### Adaptive lung-protective ventilation with automated minimization of inspiratory power

T. Becher, A. Adelmeier, M. Pohlmann, E. Fösel, D. Voß, M. Kott, K. Rump, D. Schädler, I. Frerichs, N. Weiler
University Medical Center Schleswig-Holstein, Campus Kiel, Anesthesiology and Intensive Care Medicine, Kiel, Germany
**Correspondence:** T. Becher
*Intensive Care Medicine Experimental* 2018, **6(Suppl 2):**1062

**INTRODUCTION.** Adaptive ventilation modes reduce the clinical workload and total duration of mechanical ventilation [1] by automatically adjusting respiratory rate (RR) and tidal volume ($V_T$) continuously according to the individual patient´s respiratory mechanics. Traditionally, the "optimal" ventilator settings to achieve the clinically desired minute ventilation (MV) are calculated using the Otis equation [2]. This equation provides RR and $V_T$ to minimize the inspiratory work of breathing that is required for a given MV, assuming a sine wave pressure pattern during inspiration. However, it has been implied that closed loop ventilation modes based on this equation may not be ideal for lung-protective ventilation because they deliver relatively high $V_T$, especially in patients with more compliant lungs [3]. Therefore, an improved adaptive ventilation mode (AVM2) has been developed, which optimizes RR and $V_T$ assuming a square-wave inspiration pressure with the aim of minimizing the inspiratory power delivered by the ventilator.

**OBJECTIVES.** To compare AVM2 to a traditional adaptive ventilation mode based on the Otis equation (AVM).

**METHODS.** We perform a randomized crossover study in 20 intubated and mechanically ventilated ICU patients (German Clinical Trials Register ID: DRKS00013540). Patients are ventilated with AVM and AVM2 for one hour each in a randomized order. Target MV is adapted, if necessary, according to measured end-tidal $CO_2$. After each phase, we collect data on delivered V, RR, ΔP (driving pressure, calculated as $V_T$ divided by respiratory system compliance) and arterial blood gas samples.

**RESULTS.** To date, 14/20 patients were included (age 62±11 years, height 180±7 cm, $PaO_2/FiO_2$ 269±98 mmHg, clinically selected PEEP 9.1±1.7 $cmH_2O$; all values mean±standard deviation). Delivered $V_T$ was 8.7±0.7 ml / kg predicted body weight (PBW) with AVM and decreased to 7.4±1.0 ml / kg PBW with AVM2 ($p < 0.05$). ΔP decreased from 14.3±3.0 $cmH_2O$ with AVM to 11.4±2.1 $cmH_2O$ with AVM2 ($p < 0.05$). RR increased from 14±1.8/min with AVM to 16.5 ±2.1/min with AVM2 ($p < 0.05$). No significant differences in $PaO_2/FiO_2$, $PaCO_2$ and pH were observed.

**CONCLUSIONS.** Our preliminary results suggest that in comparison to closed-loop ventilation based on the Otis equation, ventilation with AVM2 may lead to lower $V_T$ and ΔP, resulting in more "lung protective" ventilator settings.

##### REFERENCE(S)

[1] Kirakli et al. Chest 147(6): 1503-09, 2015.
[2] Otis AB et al. J Appl Physiol 2(11): 592-607, 1950.
[3] Dongelmans et al. Anesthesiology 114(5): 1138-43, 2011.

##### GRANT ACKNOWLEDGMENT

This research was supported by imtmedical AG, Buchs, Switzerland.

### 1063

#### Comparing measurements of the exhaled $CO_2$ volume determined by volumetric capnography-equipped ventilators

K. Yarimizu[1], Y. Onodera[2], H. Suzuki[1], M. Nakane[2,3], K. Kawamae[1]
[1]Yamagata University Faculty of Medicine, Anesthesiology, Yamagata, Japan; [2]Yamagata University Hospital, Critical Care Center, Yamagata, Japan; [3]Yamagata University Faculty of Medicine, Department of Emergency and Critical Care Medicine, Yamagata, Japan
**Correspondence:** K. Yarimizu
*Intensive Care Medicine Experimental* 2018, **6(Suppl 2):**1063

**INTRODUCTION.** Recently, ventilators have been equipped with volumetric capnography (VC), facilitating the measurement of exhaled $CO_2$ as volume ($VCO_2$). Because $CO_2$ is an end product of metabolism, $VCO_2$ reflects the patient's metabolic state. Measuring the $VCO_2$ may clarify the inflammation status and provide clues for making clinical decisions. Several studies have compared the performance of VC between metabolic analyzers and VC-equipped ventilators(VC-V), but no study has compared values among VC-V. Therefore, we compared the $VCO_2$ measured by two VC-V connected to a respiratory model with adjustable $CO_2$ infused.

**OBJECTIVES.** To compare the accuracy of measuring exhaled $VCO_2$ between two VC-V.

**METHODS.** Two VC-V, a G5 (Hamilton Medical, Switzerland) and an Infinity 500 (Dragel, Germany) were compared. $CO_2$ was regulated by a flow meter (F-05CO2; Yamato Industrial Co., Ltd, Japan) and infused into a test lung (Test lung; Mallinckrodt, Inc., USA). The test lung was connected to ventilators with two HMEs (Inter-ThermFilter/HME; Intersurgical, Ltd., UK) with a volume of 57 ml each, and a flow analyzer (CYTREX H4; IMT Medical, Switzerland). The ventilator was set to normal (Vt 500 ml Ti 1.2 sec RR15/min), restrictive (Vt 300 ml Ti1.0 s RR30/min) and obstructive (Vt 700 ml Ti 1.2 sec RR10/min) respiratory conditions with a PEEP of 3 or 5 $cmH_2O$. The infused $CO_2$ (actual $VCO_2$ [$aVCO_2$]) was increased in 50-ml increments from 100 to 300 ml/min for 2 minutes each, and the $VCO_2$ at the end of each phase (recorded $VCO_2$ [$rVCO_2$]) was recorded.

**RESULTS.** A regression analysis showed that the $aVCO_2$, and $rVCO_2$ had a strong correlation of $r^2$=0.99 under all respiratory conditions and with both ventilators. A Bland-Altman analysis showed a bias of 48 with the G5 and 47 ml/min with the Infinity under normal respiratory conditions, 11 with the G5 and 59 ml/min with the Infinity under restrictive respiratory conditions, and 41 with the G5 and 34 ml/min with the Infinity under obstructive respiratory conditions. The LOA (limits of agreement) values were 37-61 and 34-60 ml/min for the G5 and Infinity under normal respiratory conditions, 3-20 and 44-74 ml/min for the G5 and Infinity under restrictive respiratory conditions, and 33-49 and 26-41 ml/min for the G5 and Infinity under obstructive conditions, respectively. For restrictive respiratory conditions, the $rVCO_2$ of the Infinity showed a proportional difference. The error may need to be carefully considered when measuring the $VCO_2$ under restrictive respiratory conditions. Because we lack data on the $VCO_2$ in the field of intensive care, we cannot determine whether or not these bias and LOA are suitable, but we can say that the $rVCO_2$ is affected by respiratory conditions and ventilators.

**CONCLUSIONS.** The exhaled $CO_2$ volume by measured VC-V may be affected by the respiratory pattern and ventilator used.

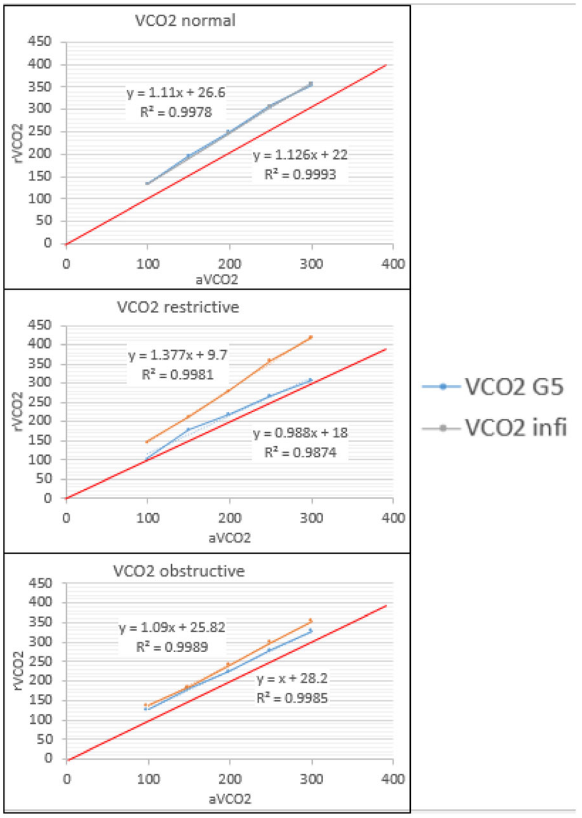

**Fig. 1 (abstract 1063).** See text for description.

### 1064

#### Esophageal balloon calibration improves esophageal pressure monitoring accuracy during robotic uro-gynecological surgery: feasibility study

G. Cammarota[1], I. Sguazzotti[1], G. Lauro[1], I. Mariano[1], A. Messina[1], D. Colombo[1], R. Vaschetto[1], P. Navalesi[2], E. Garofalo[2], A. Bruni[2], F. Mojoli[3], F. Della Corte[1]
[1]Azienda Ospedaliera Universitaria Maggiore della Carità, SCDU Anestesia e Rianimazione, Novara, Italy; [2]Università della Magna Grecia, Anestesia e Rianimazione, Catanzaro, Italy; [3]Università degli Studi di Pavia - Policlinico San Matteo, Dipartimento di Anestesia e Rianimazione, Pavia, Italy
**Correspondence:** G. Cammarota
*Intensive Care Medicine Experimental* 2018, **6(Suppl 2):**1064

**INTRODUCTION.** Esophageal pressure (Pes) evaluation is a critical step during monitoring of mechanical ventilation. Pes measurement can be affected both by inadequate filling volume (Fv) of esophageal balloon and by artifact exerted by esophageal wall. At this purpose, recently has been proposed esophageal balloon calibration (Ebc) in order to better evaluate the accuracy of Pes monitoring both in vitro and in clinical setting [1, 2].

**OBJECTIVES.** The study aim was to investigate feasibility of Ebc during robotic uro-gynecological surgeries with application of trendelemburg position (tp), pneumoperitoneum (pp), and positive end-expiratory pressure (Peep). Furthermore, during whole study period, were evaluated inspiratory (PesI) and expiratory (PesE) Pes and related swings at 3 different Fv of esophageal balloon: volume associated with the largest tidal increase of Pes (BV), BV with relative pressure values corrected to esophageal wall pressure (BVc), and volume suggested by the manufactures, 4ml (V4).

**METHODS.** A catheter with esophageal and gastric balloons was inserted nineteen elective patients undergoing volume controlled mechanical ventilation for robotic uro-gynecological surgery. PesI and PesE and related swings were recorded and computed at BV, BVc, and V4 over study period (T0: before start surgery; T1: pneumoperitoneum + trendelemburg position application; T2, T3, and T4: 20, 60, and 120 minutes from peep application; T5: end surgery). Furthermore, BV trend was post hoc analized at the same steps.

**RESULTS.** Ebc was successfully applied in all patients enrolled. Figure 1 shows BV trend over study steps. BV significantly increase from T0 to T2 (p< 0,05). PesE was depicted in figure 2. PesE measured at V4 was always significantly higher with respect to both BV and BVc (p < 0,05). PesI trend is depicted in figure 3. Both at BV and BVc were significantly smaller with respect to V4 (p < 0,05). Swing was not different between the 3 filling volumes. BV was higher at T2 and T3 than at T0 (p < 0,05). Finally, Pes tidal swing computed at V4 ml was smaller than both BV and BVc without significant differences.

**CONCLUSION.** Ebc is feasible in patients undergoing robotic uro-gynecological surgery. Finally, reliability of Pes measurement is improved by Ebc, confirming Mojoli findings also in operating room [2].

#### REFERENCE(S)

1. Mojoli F. et al (2015) Minerva Anestesiol.
2. Mojoli F. et al (2016) Crit Care.

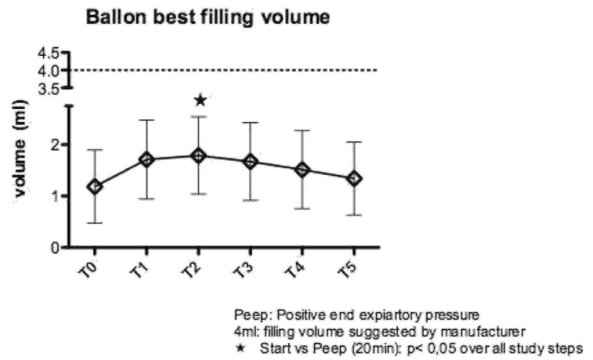

**Fig. 1 (abstract 1064).** Best filling esophageal ballon volume

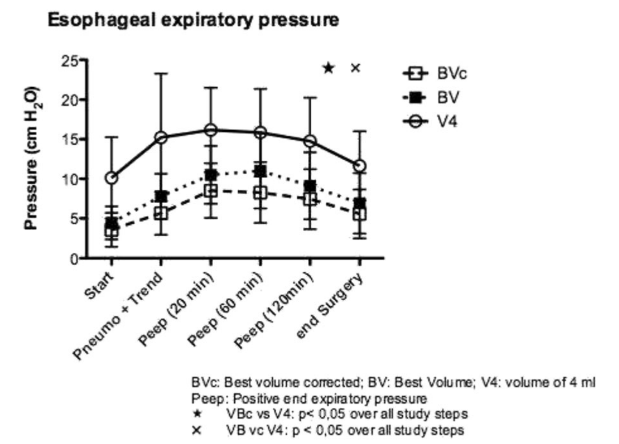

**Fig. 2 (abstract 1064).** Expiratory esophageal pressure

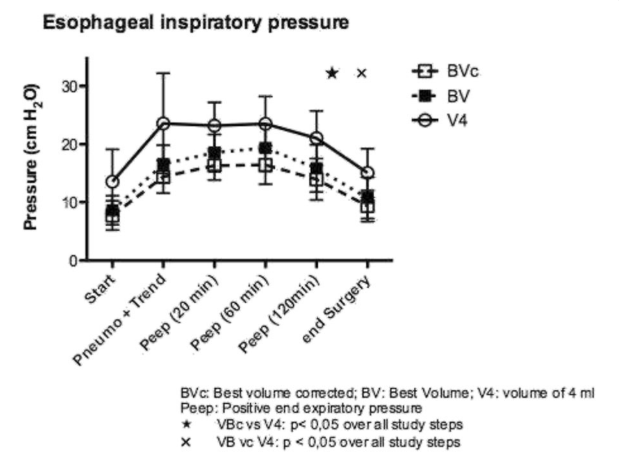

**Fig. 3 (abstract 1064).** Inspiratory esophageal pressure

## Nutrition in ICU

### 1065

#### The predisposing factors of nutritional insufficiency for postoperative patients in surgical intensive care unit

J.J. Kim, E.Y. Kim
Seoul St. Mary's Hospital, The Catholic University of Korea College of Medicine, Division of Trauma and Surgical Critical Care, Department of General Surgery, Seoul, Korea, Republic of
**Correspondence:** J.J. Kim
*Intensive Care Medicine Experimental* 2018, **6(Suppl 2):**1065

**INTRODUCTION.** The enteral feeding has been strongly recommended to the critical ill patients because it could enhance the immunologic function which serves as the host defense mechanism against metabolic response to stress. We investigate the current nutritional status and estimate the adequacy of nutritional supply especially for the acutely ill patients who admitted to the surgical intensive care unit (SICU) after major operation. The predisposing factors associated with the poor nutritional status after operation are also analyzed in order to predict the adverse effect resulted from the nutritional insufficiency in postoperative period.

**METHODS.** From March to June 2016, the patients who stayed over 48 hours after major surgery at SICU in Seoul St. Mary's Hospital were reviewed. The clinicopathologic characteristics and the nutritional parameters of patients were reviewed and the surgical outcomes were compared according to the status of nutritional support or the clinical department of patient.

**RESULTS.** A total of 85 patients (51 males and 34 females) were enrolled and the mean age was 60.2±14.4 years. Mean length of ICU stay was 4.1±4.3 days and the mortality was observed in 3 cases (3.5%). All patients were classified into two groups according to the nutritional status which was assessed by the ratio of total delivered calorie to total required calorie (D/R); group A (38 cases, D/R≥0.7) versus group B (47 cases, D/R< 0.7). The length of hospital stay and the proportion of colonic malignancies were significantly lower in group A than group B (13.5±7.1 days vs. 17.4±9.2 days and 32.4% vs. 78.6%, *p=0.037 and p=0.049*, respectively). Regarding the clinical departments, the number of patient in the colorectal department of general surgery was significantly lower in group A than group B (22.2% vs.77.8%, *p=0.049*). The proportion of incision at lower abdomen or the consultation to nutritional support team (NST) had significant differences between two groups (15.8% vs. 46.8% and 55.3% vs. 31.9%, *p=0.012 and p=0.046*, respectively). About laboratory results at admission, the level of albumin and BUN was significantly lower in group A (2.7±0.7 g/dl vs. 2.9±0.3 g/dl and 18.7±15.2 mg/dl vs. 35.4±25.2 mg/dl, *p=0.045 and p=0.003*, respectively) whereas the level of hematocrit was higher in group A than group B (35.0±5.1% vs. 32.7 ± 5.3%, *p=0.042*). In multivariate analysis, the incision at lower abdomen (*p=0.029*), the prolonged length of hospital stay (*p=0.035*), the absence of NST consultation (*p=0.041*) and the low level of hematocrit (*p=0.037*) were independent risk factors associated the poor nutritional status in the postoperative period.

**CONCLUSIONS.** We suppose that the clinicians should pay attentions if patients are in the department of general surgery, present low level of hematocrit, or absence of NST consultation which would be the predisposing factors of nutritional insufficiency resulted in the postoperative morbidities or the delay of recovery.

### 1066

#### Enteral nutrition in critically ill patients undergoing vasoactive drugs therapy. The nutrivad study: preliminary results

J.L. Flordelís Lasierra[1], J.C. Montejo González[2], J.C. López Delgado[3], P. Zárate Chug[4], F. Martínez Lozano Aranaga[5], C.L. Vera Ching[6], S. Triginer Roig[7], S. Maichle[8], M.V. Transmonte Martínez[2], M. Zamora Elson[9], B. Vila García[10], M.L. Mateu Campos[11], V.A. Benítez Ferreiro[1], Metabolism and Nutrition Working Group of the Spanish Society of Intensive Care Medicine and Coronary Units
[1]Hospital Universitario Severo Ochoa, Intensive Care Medicine, Leganés, Spain; [2]Hospital 12 de Octubre, Intensive Care Medicine, Madrid, Spain; [3]Hospital Universitario de Bellvitge, Intensive Care Medicine, Barcelona, Spain; [4]Hospital Universitario Miguel Servet, Intensive Care Medicine, Zaragoza, Spain; [5]Hospital General Universitario Reina Sofía, Murcia, Intensive Care Medicine, Murcia, Spain; [6]Hospital Universitario de Girona Josep Trueta, Intensive Care Medicine, Girona, Spain; [7]Hospital Universitario Germans Trias i Pujol, Intensive Care Medicine, Barcelona, Spain; [8]Hospital Universitario Clínico San Carlos, Intensive Care Medicine, Madrid, Spain; [9]Hospital de Barbastro, Huesca, Spain; [10]Hospital Universitario Infanta Cristina, Intensive Care Medicine, Parla, Spain; [11]Hospital General Universitario de Castellón, Intensive Care Medicine, Castellón de la Plana, Spain
**Correspondence:** J.L. Flordelís Lasierra
*Intensive Care Medicine Experimental* 2018, **6(Suppl 2):**1066

**INTRODUCTION.** Enteral nutrition (EN) in critically ill patients undergoing vasoactive support (VS) due to hemodynamic instability is controversial and challenging (1,2).

**OBJECTIVES.** To examine the feasibility and safety of EN in such patients.

**METHODS.** Prospective, observational, multicenter study (3). Inclusion criteria: adult patients admitted to 19 Intensive Care Units (ICU) in Spain, undergoing VS, as well as invasive mechanical ventilation (MV) for at least 48 hours after admission. Patients with refractory shock were excluded. Follow-up: 14 days. EN was delivered according to a protocol. Variables (Var.): descriptive, hemodynamic status, efficacy and safety of EN. A descriptive analysis of the results was performed (means with 95% confidence intervals for continuous var., or frequencies for categorical var.).

**RESULTS.**

Main characteristics: 75 patients included, 64% men, 60 years old (57-64). 56% were medical patients. 72% developed early multiple organ dysfunction syndrome. MV days: 17 (14-20). ICU stay: 22 days (19-25). ICU mortality: 24%.

Hemodynamic status in the first 48 hours: lactate 3,6 mmol/L (2,9-4,3), noradrenaline dose 0,73 μg/kg/min (0,59-0,87), cardiac index 2,63 l/min/m2 (2,32-2,94), mean arterial pressure 67 mmHg (65-70).

Efficacy of EN: time from admission to the start of EN: 34,1 hours (29,3-38,8). 1148,3 Kcal (1053,9-1242,7) delivered by EN. Nutritional balance: - 400,2 Kcal (-505,3 to -295,2).

Safety of EN: EN-related complications were observed in 56 patients (78%), although no cases of mesenteric ischemia were recorded. The most common complications were constipation (36%) and high gastric residual volumes (36%). EN discontinuation was required in 30 patients (40%).

**CONCLUSIONS.**

Enteral nutrition, under proper medical supervision, can be feasible in these patients.

It is difficult to meet the nutrition requirements when this is the only feeding route.

**REFERENCE(S)**

1. Flordelís Lasierra JL, et al. Enteral nutrition in the hemodynamically unstable critically ill patient. Med Intensiva. 2015;39(1):40-8.
2. Flordelís Lasierra JL, et al. Enteral Nutrition in Critically Ill Patients Undergoing Vasoactive Drugs Therapy. The NUTRIVAD Study (Protocol). 2018. ClinicalTrials.gov Identifier: NCT03401632.
3. Reignier J, et al. Enteral versus parenteral early nutrition in ventilated adults with shock: a randomised, controlled, multicentre, open-label, parallel-group study (NUTRIREA-2). Lancet. 2018;391(10116):133-143.

### 1067

#### NUTRIC core: validation of clinical nutritional score using SAPS II and c-reactive protein

T. Petrucci[1], D. Passos[1], G. Nobre de Jesus[1,2], S. Fernandes[1,2], J. Gouveia[1], A. Alvarez[1]
[1]Hospital de Santa Maria - Centro Hospitalar de Lisboa Norte, EPE, Serviço de Medicina Intensiva, Lisbon, Portugal; [2]Faculdade de Medicina da Universidade de Lisboa, Lisbon, Portugal
**Correspondence:** T. Petrucci
*Intensive Care Medicine Experimental* 2018, **6(Suppl 2):**1067

**INTRODUCTION.** The NUTRIC score was developed to predict the risk of adverse events and denutrition in critically ill patients. Among other variables interleukin-6 (IL-6), a biomarker not available on usual critical practice, is used. This score was recently validated for the portuguese ICU population, without IL-6. As previously suggested, other inflammatory biomarkers could substitute Il-6 in this score to improve its accuracy, namely c-reactive protein (CRP). Moreover, APACHE II is used to calculate NUTRIC score, and validation for the use of SAPS II has never been tested.

**OBJECTIVES.** We aimed to address the effectiveness of the NUTRIC Score comparing the sensitivity and specificity of the score when replacing IL-6 for CRP and APACHE II for SAPS II, in a critically ill population in a university hospital ICU.

**METHODS.** We implemented a retrospective analysis of a cohort study from a tertiary university hospital general ICU. We defined a period of time for data collection of 2 months, calculated for inclusion of at least 70 patients. Data collected included variables of NUTRIC Score, CRP, procalcitonin, demographic data, ventilation, outcome. Previous authors defined a cut-off of CRP for inclusion on NUTRIC Score of 10 mg/dL, which was used in this study.

**RESULTS.** 74 patients were included, with an average age of 61 years and 37,8% female gender. Mortality was 27%, with a SAPS II of 47 (± 20,6), and a median lenght of stay of 7.9 days. Average CRP on survival population was 16.07 mg/d and on non-survival was 9.2 mg/dL. We compared area under the curve (AUC) of several models (NUTRIC score using APACHE II vs NUTRIC score using SAPS II, with and without CRP). AUC for NUTRIC score using APACHE II without CRP was 0.83 and with CRP was 0.84. AUC for NUTRIC score using SAPS II without CRP was 0.77 and with CRP was 0.79. No statistical significance was found between the different scores to predict ICU mortality.

**CONCLUSIONS.** The use of NUTRIC Score, using APACHE II and IL-6 amongst other variables, has been validated for predicting mortality and patient denutrition at ICU admission. The use of SAPS II instead of APACHE II in this study did not alter significantly the NUTRIC Score predictive performance, and it may be licit to use it. Use of additional CRP on the NUTRIC Score did not change its predictive value, although the study may be underpowered for this effect.

### 1068

#### Risk factors of enteral feeding intolerance in the intensive care setting in US and Europe: literature review

J. Puelles[1], K. Rennie[2], E. Terblanche[3], C. Almansa[4], G. Dukes[4], M. Binek[5]
[1]Takeda Development Centre Europe Ltd, London, United Kingdom; [2]OXON Epidemiology Ltd, London, United Kingdom; [3]Imperial College Healthcare NHS Trust, London, United Kingdom; [4]Takeda Development Center Americas, Inc., Cambridge, United States; [5]Takeda Pharmaceuticals International AG, Zurich, Switzerland
**Correspondence:** J. Puelles
*Intensive Care Medicine Experimental* 2018, **6(Suppl 2):**1068

**INTRODUCTION.** Enteral feeding intolerance (EFI) is a common complication in critically ill patients. Slow or altered gastric emptying and associated EFI is multifactorial in the critically ill patient and can lead to under delivery of enteral nutrition (EN) and malnutrition. It has also been associated with increased stay in intensive care, prolonged need for mechanical ventilation and higher mortality.[1,2] Therapy for EFI include prokinetics or changes to feeding regimen, such as interrupting or decreasing EN rates, post-pyloric EN feeding and administration of supplemental or total parenteral nutrition.[3]

**OBJECTIVES.** To assess risk factors associated with the development of EFI in patients in the critical care setting.

**METHODS.** Literature searches in MEDLINE®, Embase®, and Cochrane Library were conducted for primary studies in English (2007-2017), in adult intensive care patients receiving EN, to identify studies where EFI, and/or its associated symptoms were included. The search screened studies conducted in the US and Europe. Reviews and relevant primary studies were back-referenced to find any further relevant studies. Primary studies were evaluated for significant risk factors for EFI and grouped according to cause.

**RESULTS.** Overall, 94 studies were identified that included EFI for the qualitative synthesis, of these 28 full-text articles and 27 abstracts included assessment of risk factors and were included in this review. The literature review identified several risk factors which can be clustered under the following groupings; physiological condition and severity, physiological response, medication use, route of administration of medication, EN initiation (Figure 1). However, the assessment of risk factors was hindered by differences in the definition of EFI used between studies, small sample groups and heterogenous patient populations.

**CONCLUSIONS.** This study identified many important risk factors associated with the development of EFI. There is, however, a need for large population-based studies with consistent EFI definition to systematically assess multiple risk factors and cluster them in the clinical setting.

**REFERENCE(S)**

1. Gungabissoon U, Hacquoil K, Bains C, et al. Prevalence, risk factors, clinical consequences, and treatment of enteral feed intolerance during critical illness. JPEN J Parenter Enteral Nutr 2015;39:441-8.
2. Wang K, McIlroy K, Plank LD, Petrov MS, Windsor JA. Prevalence, Outcomes, and Management of Enteral Tube Feeding Intolerance: A Retrospective Cohort Study in a Tertiary Center. JPEN J Parenter Enteral Nutr 2017;41:959-67.

3. Reintam Blaser A, Starkopf J, Alhazzani W, et al. Early enteral nutrition in critically ill patients: ESICM clinical practice guidelines. Intensive Care Med 2017;43:380-98.

**GRANT ACKNOWLEDGMENT**
This study was funded by Takeda International - UK Branch

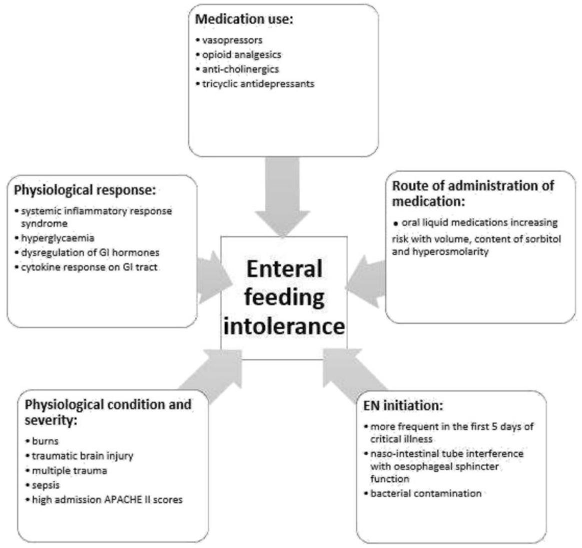

**Fig. 1 (abstract 1068).** Groups of risk factors identified from the literature review

### 1069

#### Pectin-containing liquid enteral nutrition for critical care: a historical control and propensity score matched study

N. Kensuke[1], T. Sonoo[1], H. Naraba[1], Y. Takahashi[1], H. Hashimoto[1], K. Doi[2], N. Morimura[2]
[1]Hitachi General Hospital, Emergency and Critical Care Medicine, Ibaraki, Japan; [2]University of Tokyo Hospital, Emergency and Critical Care Medicine, Tokyo, Japan
**Correspondence:** N. Kensuke
*Intensive Care Medicine Experimental* 2018, **6(Suppl 2):**1069

**INTRODUCTION.** Diarrhea and vomiting are common complications in critical care 1). Approaches have been undertaken to make liquid enteral nutrition LEN solid and thereby decrease them 2), however, there was no study that semi-solid LEN could reduce diarrhea. Herein, Pectin is dietary fiber that changes to a solid in the stomach 3). Pectin-containing liquid enteral nutrition PCLEN would mitigate vomiting and diarrhea.
**OBJECTIVES.** This study compared PCLEN with traditional LEN TLEN for enteral feeding failure, diarrhea and vomiting rates in critical care.
**METHODS.** We used TLEN as the primary LEN at the emergency and critical care center in Hitachi General Hospital. We adopted PCLEN as the primary LEN from 2014. During 2012-2016, 954 patients admitted to intensive care units and emergency wards were given PCLEN or TLEN. We conducted propensity score matching for 693 eligible patients for age, sex, and organ dysfunctions for six organs.
**RESULTS.** We included 199 PCLEN patients and 199 TLEN patients. Severity was higher in the PCLEN group: APACHE2 15.3 ± 6.8 vs. 13.6 ± 5.4 ($p$=0.0055) and SOFA 6.2 ± 3.8 vs. 5.1±3.3 ($p$=0.0029), respectively. The enteral nutrition failure rate was significantly lower for PCLEN: 10.6 % than for TLEN: 17.6 % (p=0.043). The GRV at 24 hr was slightly lower for PCLEN: 7.9 ± 24.3 ml than for TLEN: 25.1 ± 63.0 ml ($p$=0.017). Although the vomiting events were not different, the diarrhea incidence rates were 28.1% vs. 38.2% ($p$=0.033), and the incidence rates of nosocomial pneumonia were 4.5% and 9.6% ($p$=0.048). For PCLEN, the enteral nutrition failure rates were not different for patients with gastric acid inhibitors and without them.
**CONCLUSIONS.** PCLEN can be used effectively for critically ill patients irrespective of the use of gastric acid inhibitors. It can decrease the incidence of enteral nutrition failure and diarrhea in critical care.

**REFERENCE(S)**
1) Adike A, Quigley EM. Gastrointestinal motility problems in critical care: a clinical perspective. *J Dig Dis.* 2014;15(7):335-344.
2) Kanie J, Suzuki Y, Iguchi A, Akatsu H, Yamamoto T, Shimokata H. Prevention of gastroesophageal reflux using an application of half-solid nutrients in patients with percutaneous endoscopic gastrostomy feeding. *J Am Geriatr Soc.* 2004;52(3):466-467.
3) Sandhu KS, el Samahi MM, Mena I, Dooley CP, Valenzuela JE. Effect of pectin on gastric emptying and gastroduodenal motility in normal subjects. Gastroenterology. 1987;92(2):486-492.

**GRANT ACKNOWLEDGMENT**
No grant acknowledgement.

### 1070

#### Effectiveness of early goal-directed enteral nutrition in emergency and medical intensive care unit

N. Matsuda, M. Higashi, Y. Yasuda, M. Nishikimi, T. Yamamoto, A. Numaguchi, M. Kado
Nagoya University Graduate School of Medicine, Emergency & Critical Care Medicine, Nagoya, Japan
**Correspondence:** N. Matsuda
*Intensive Care Medicine Experimental* 2018, **6(Suppl 2):**1070

**INTRODUCTION.** Early goal-directed enteral nutrition protocol (EGDN NAGOYA) was created in 2010. EGDT might promote enteral nutrition in critical care patients.
**OBJECTIVES.** We aimed to investigate outcome of EGDN NAGOYA in the emergency and medical ICU.
**METHODS.** Patients hospitalized for 48 hours or more were retrospectively investigated rom May 2011 to June 2017 in Nagoya University Emergency and medical ICU. Patients were classified into the following three groups by the first nutrition method: oral intake group (PO), enteral nutrition group, and total parenteral nutrition group (TPN). Enteral nutrition group was classified into the following four groups, according to the time to initiate enteral feeding: within 6 hours(EN6), 6 hours or more, and less than 24 hours (EN6-24), 24 hours or more, and less than 48 hours (EN24-48) , and 48 hours or more(EN48). We evaluated APACHEII score and mortality in ICU of each group. For statistical multiple comparison, the Tukey-Kramer method and Fisher´s exact test were used.
**RESULTS.** There were 977 cases in the criteria of this study. Enteral nutrition according to EGDN NAGOYA were 470 cases (48.1%) were classified into enteral group: EN6 77 (7.9%), EN6-24 217 (22.2%), EN24-48 129 (13.2%) and EN48 47 (4.8%). The mean APACHE II score was 28.9, 29.9, 28.7 and 27.5, and the ICU mortality was 3.9%,10.1%,10.1%, 10.6%, respectively. On the other side, in TPN, the APACHE II score was 28.2 and its ICU mortality was 20.0%. EN according to EGDN NAGOYA showed the significantly better outcome than PN in spite of the similar ill severity.
**CONCLUSIONS.** This study showed that early beginning enteral nutrition with the protocol EGDN NAGOYAcould lead to good prognosis of critically ill patients from 2011 to 2017.

**REFERENCE(S)**
None.

**GRANT ACKNOWLEDGMENT**
None

### 1071

#### What about nutritional status in patients with diabetes in intensive care unit?

A. Zerman[1], S.O. Keskek[2]
[1]Adana Sehir Training and Research Hospital, Intensive Care, Adana, Turkey; [2]Adana Sehir Training and Research Hospital, Internal Medicine, Adana, Turkey
**Correspondence:** A. Zerman
*Intensive Care Medicine Experimental* 2018, **6(Suppl 2):**1071

**INTRODUCTION.** Malnutrition is highly prevalent among older patients with type 2 diabetes in a hospital or geriatric care setting and is an important predictor for longer hospital stays and poorer clinical outcomes.

**OBJECTIVES.** In this study we aimed to investigate the effect of diabetes on nutrition status of patients in internal medicine intensive care unit.

**METHODS.** A total of 192 patients were enrolled in this retrospective study. The study group was comprised of 77 patients with diabetes and the control group was comprised of 115 patients without diabetes . Mini nutritional assessment (MNA), nutric score and the nutrition risk screening (NRS)- 2002 results of the patients were compared.

**RESULTS.** The groups were matched in terms of age and sex (68.6 ±13.4 vs. 71.6±14.6, p=0.091; female/male= 38/39 vs. 46/69, p=0.426). The APACHE II score of the groups were comparable (21.1±6.6 vs. 20.5±6.3, p=0.539). MNA, nutric score and NRS-2002 tests results were 8.0±3.1, 4.61±1.85 and 3.37±1.84, respectively in the study group while they were 7.1±3.2, 4.56±1.85 and 3.93±1.72, respectively in the control group. The differences were not statistically significant (p=0.068, p=0.869, p=0.075, respectively).

**CONCLUSIONS.** In this study we have found comparable nutritional scores in both of the groups. Concomitant diseases other than diabetes may have more effect on nutritional status of patients in intensive care units.

### 1072

#### Fullfillment of enteral nutritional requirements before and after the implementation of ASPEN guidelines 2016 in a mexican intensive care unit

M.A. Toledo Rivera[1], S.E. Zamora Gomez[1], Y. Luna Camacho[2], E. Pérez Cruz[2], M.A. Carrasco Flores[1], M. Hernández Romero[1], M.A. Amezcua Gutierrez[1], J. Garduño López[1], E.S. Cruz Montesinos[1], I.M. Lima Lucero[1], J.O. Montoya Rojo[1], K. Castillo Medrano[1], J.C. Gasca Aldama[1], N.I. Medveczky Ordoñez[1], H.A. Morales Morales[1], M.L. Pacheco Rivera[1], S. Sosa Santos[1], G.D. Hernández López[1], J.A. Castañon González[1]
[1]Hospital Juarez de México, Intensive Care Unit, Mexico City, Mexico; [2]Hospital Juárez de México, Nutritional Support, Mexico City, Mexico
**Correspondence:** M.A. Toledo Rivera
*Intensive Care Medicine Experimental* 2018, **6(Suppl 2):**1072

**INTRODUCTION.** Several factors are involved in ICU malnutrition, leading to longer ICU stay, longer dependence of mulitorganic support, and a higher number of nosocomial complications. Guidelines intend to reduce malnutrition and its associated complications.

**OBJECTIVES.** Describe the fullfillment of enteral nutritional requirements at 72 hours in a mexican intensive care unit before and after the implementation of ASPEN guidelines 2016 and the main factors involved in unfullfillment.

**METHODS.** A descriptive, retrospective study was performed analyzing medical files of patients admitted to ICU and without contraindication for enteral feeding.We analyzed first from january to december 2016, and then from january to december 2017;implementation of ASPEN 2016 guidelines in our ICU started on January 2017. A before-after compative analysis was performed.

**RESULTS.** 88 patients were included in 2016 analysis. 47 (53.4%) patients fullfilled at least the 60% of nutritional requirements estimated by Harris Benedict formula at 72 hours of ICU admittance. 27 (30.7%) patients not fullfilled the 60% of requirements at 72 hours,and 14 (15.9%) were overfeeded. In patients with BMI of 20-29 kg/m2 unfullfillment was present in 38% and overfeeding in 10%; in patients with BMI of 30-50 kg/m2 unfullfillment was present in 21.6% and overfeeding in 24.3%. In the group of BMI of 20-29 kg/$m^2$ the factors related to unfullfillment were extremely high estimation of nutritional requirements by the first 24 hours, and lack of feeding progression at 48 and 72 hours; the main reason for unfullfillment in the group of BMI of 30-50 kg/m2 was the extremely high estimation of nutritional requirements at 24 (21%),48 (50%) and 72 hours (37.5%).110 patients were included in 2017 analysis. 82 (74.5%) patients fullfilled at least the 60% of nutritional requirements estimated by ASPEN 2016 Guidelines recomendations at 72 hours of ICU admittance.20 (18.2%) patients not fullfilled the 60% of requirements at 72 hours,and 8 (7.3%) were overfeeded.In patients with BMI of 20-29 kg/$m^2$ unfullfillment was present in 17% and overfeeding in 5.7%; meanwhile in patients with BMI of 30-50 kg/$m^2$ unfullfillment was present in 20.5% and overfeeding in 10.2%. The main cause for unfullfillment was high gastric residual volumen, present in the group of BMI of 20-29 kg/$m^2$ in 41.6%, and in the group of BMI of 30-50 kg/$m^2$ in 62.5%.

**CONCLUSIONS.** In our study we found a significant lower rate of unfullfillment and overfeeding when ASPEN 2016 guidelines recommendations were applied in comparation when Harris Benedict formula was applied. Overestimation of nutritional requirements is an usual problem related with Harris Benedict method, meanwhile high gastric residual volumen was the main problem in patients under ASPEN recomendations.

##### REFERENCE(S)

1 Maday, K.R.The importance of nutrition in critically ill patients. JAAPA 2017 30(1): 32-37.

### 1073

#### The implementation of nutritional administration protocols in surgical intensive care unit Siriraj Hospital

P. Chinda[1], P. Poomthong[1], P. Toadithep[2], O. Chaiwat[2]
[1]Faculty of Medicine Siriraj Hospital Mahidol University, Division of Critical Care Medicine, Department of Medicine, Bangkok, Thailand; [2]Faculty of Medicine Siriraj Hospital Mahidol University, Department of Anesthesiology, Bangkok, Thailand
**Correspondence:** P. Chinda
*Intensive Care Medicine Experimental* 2018, **6(Suppl 2):**1073

**INTRODUCTION.** Malnutrition in critically ill patients was associated with an increase in mortality and morbidity. Despite having publication from many guidelines, feeding protocols varied among population and region. The protocol should be compatible with the characteristics of individual intensive care units (ICUs).

**OBJECTIVES.** To evaluate the efficacy of Siriraj Surgical ICUs feeding protocol and to compare the hospital mortality, hospital LOS and ICU LOS between protocol and non-protocol groups.

**METHODS.** A randomized control trial study was conducted in Surgical ICUs Siriraj Hospital. Nutritional administration in control group was depended upon attending physicians' discretion. In the intervention group, nutritional management was followed by the "Siriraj surgical ICUs nutritional protocol".

**RESULTS.** 170 patients were enrolled. Almost all patients in both group had risk of malnutrition described by Nutrition Risk Screening (NRS) 2002 (NRS score $\geq$ 3) and more than 50% of patients by NUTRIC score $\geq$5. Total median calories per day received in both group were not significantly different [760.1 kcal (494.6, 1047.8) in protocol group vs. 754.6 kcal (444.6, 1092.9) in control group: p-value 0.773]. The median time to start enteral nutrition trended to shorter in protocol group [1.94 days in protocol group vs. 2.25 days in control group: p-value 0.503]. ICU LOS, hospital LOS, duration of mechanical ventilation and mortality were not significantly different between groups. The prevalence of nutrition related complication and rate of infection were similar in both groups.

**CONCLUSIONS.** The implementation of nutritional protocol showed no difference in the efficacy of nutrition management and clinical outcomes compared to standard treatment in surgical ICUs.

**REFERENCE(S)**

1. Barr J, Hecht M, Flavin KE, Khorana A, Gould MK. Outcomes in critically ill patients before and after the implementation of an evidence-based nutritional management protocol. CHEST. 2004; 125(4):1446-1456.
2. Casaer MP. Van den Berghe G. Nutrition in the acute phase of critical illness. N Engl J Med. 2014;370:1227-36.
3. Doig GS, Simpson F, Finfer S, et al; Nutrition Guidelines Investigators of the ANZICS Clinical Trials Group. Effect of evidence-based feeding guidelines on mortality of critically ill adults: a cluster randomized controlled trial. JAMA. 2008; 300(23):2731-2741.
4. Chittawatanarat K, Chuntrasakul C. Enteral Feeding in Surgical Critically Ill Patients. The Thai Journal of SURGERY. 2006; 27: 5-10.

**GRANT ACKNOWLEDGMENT**

All research participants and All Siriraj SICUs staff.

### 1074

#### We could do better? Implementation of enteric nutrition protocol

A.P. Alves[1], B. Lourenço[2], E. Pinto[3], G. Campello[4]

[1]Centro Hospitalar Universitário do Algarve, Serviço de Nutrição, Portimão, Portugal; [2]Centro Hospitalar Universitário do Algarve, Serviço de Medicina Intensiva 2, Portimão, Portugal; [3]Universidade do Algarve, Curso Ciências de Nutrição e Dietética, Faro, Portugal; [4]Centro Hospitalar Universitário do Algarve, Departamento de Emergência, Urgência e Cuidados Intensivos, Portimão, Portugal

**Correspondence:** G. Campello

*Intensive Care Medicine Experimental* 2018, **6(Suppl 2):**1074

**INTRODUCTION.** Aiming do assess the compliance of current international nutritional guidelines, a Enteral Nutrition Protocol (ENP) was designed and applied in an Intensive Care Unit (ICU) at Centro Hospitalar Universitário do Algarve.

**METHODS.** We conducted an observational, retrospective study in a before-after design, corresponding to (group 1) the period of implementation of the ENP with several educational interventions (nutritionist monitoring and clinical discussion meetings) between April 2016 and May 2017, and (group 2) the period after the implementation between June 2017 and March 2018. All patients on full enteral nutritional support during ≥ 4 days in the ICU were included, and the protocol applied. Data was collected on nutritional intake value (initial and final caloric intake, with and without additives), biochemical markers (glucose, C-reactive protein, hemoglobin, total bilirubin, ALT / AST, total Proteins / Albumin, phosphorus, calcium magnesium) and the Simplified Acute Physiology Score (SAPS II). Data were analyzed using SPSS statistical program, version 22.0. Mann-Whitney test, Kruskal-Wallis and Wilcoxon test were used, statistical significance accepted at p< 0,05.

**RESULTS.** During the period of study, 590 patients were admitted to the ICU. 55 patients met the inclusion criteria, 32 (25,8% surgical, 61,3% medical and 12,9% trauma) in group 1 and 24 in group 2 (8,3% surgical, 79,2% medical, 12,5% trauma). The ICU length of stay were 19 days (SD ± 14) in group 1, and 18 (SD ± 14) in group 2. In group 1 SAPS II were 53 (SD ± 18,8) and in group 2 50,1 (SD ± 14,5). Statistically significant differences were found, with higher initial and final caloric intake without additives (p< 0,05), initial and final caloric intake with additives (p< 0,05), caloric value of additives (p< 0,05) in group 1. No difference was found concerning protein intake, but the nitrogen supply was significantly higher in group 1 (p< 0.05). Among biochemical markers analyzed, bilirubin was the only parameter higher in group 1 (p< 0.05). Concerning the enteral nutrition timing of initiation, statistically significant differences were found on surgical patients, group 2 started enteral nutrition at 1,5 days (SD± 0,7), and group 1 started at 3,9 days (SD±3,7) (p< 0,05).

**CONCLUSIONS.** Implementation of the ENP associated with educational interventions, can improve compliance with current nutritional recommendations which are known to be associated with better clinical outcomes.

- Guidelines for the Provision and Assessment of Nutrition Support Therapy in the Adult Critically Ill Patient: Society of Critical Care Medicine (SCCM) and American Society for Parenteral and Enteral Nutrition (A.S.P.E.N.), 2016
- Weijs PJ et al. Protein recommendations in the ICU: G protein/kg body weight - witch body weight for underweight and obese patients? Clin Nutr. 2012; 31(5):774-75

### 1075

#### Enteral nutrition delivery in a tertiary cardiothoracic ICU: a quality improvement project

M. Sehmbhi, M.J. Rouhani, I. de Brito-Ashurst, A. Chan-Dominy, S.F.P. Ledot

Royal Brompton Hospital, Adult Intensive Care Unit, London, United Kingdom

**Correspondence:** M. Sehmbhi

*Intensive Care Medicine Experimental* 2018, **6(Suppl 2):**1075

**INTRODUCTION.** Optimal caloric and protein intake reduces mortality risk in ICU (1), particularly among high nutritional risk patients (2), yet underfeeding remains a common problem.

We evaluated nutrition adequacy in our tertiary cardiothoracic ICU among elective admissions, ECMO admissions, and non-ECMO emergency admissions. Preliminary work found nutrition delivery was inadequate: a 5 day admission could result in a 5000 kcal deficit - the energy required to run two marathons. We performed a quality-improvement (QI) project to address this using the Plan-Do-Study-Act methodology, with a 1-month review to assess effectiveness of interventions.

**OBJECTIVES.**

1. To deliver >80% of each patient´s daily calorie/protein target
2. To assess adherence to QI interventions 1 month post-implementation

**METHODS.** We gathered data retrospectively from electronic records on all patients admitted to our ICU for >24 hours in two 6-week periods (n = 66), with QI interventions between the study periods. Interventions included: introduction of a "procedural NBM time" guideline, a nutrition checklist guiding daily nutrition rounds, increasing the gastric residual volume (GRV) cut-off threshold, and a "catch-up volume-based enteral feed (EF)" protocol. We studied compliance 1 month post-implementation.

Data collected included: calorie and protein delivery (first 5 days); reasons for delay in initiating EF; reasons for interruption of EF; adherence to the GRV and catch-up feed protocols.

**RESULTS.**

Caloric/protein intake

We achieved our objective. QI interventions resulted in >80% of target calorie/protein delivery by day 5 of admission (day 1: 19.8% cal target achieved, 18% protein target; day 5: 87.4% cal, 85% protein).

Delay in initiating feeding

Common reasons for delay included expectation of early extubation (particularly for elective surgical admissions), delays in confirmation of feeding tube position, and acute cardiovascular instability.

Feed interruptions

These were due to intended hold pre-/post-procedure, excessively high GRV, or inappropriate omission. Compliance with our procedural NBM time guideline increased from 32% to 39%. Compliance with our 400ml GRV cut-off was good, at 86.2%

Catch-up feeding

Compliance with catch-up feeding was poor at 1.5% of eligible cases.
**CONCLUSIONS.** Our QI interventions improved nutrition delivery to >80% of target by day 5 of admission, with some groups approaching 100%. Significant interventions were early initiation of EF, implementing an EF-hold time policy for high aspiration risk procedures (to maximise nutrition delivery while maintaining a safe feed-free interval), and a raised GRV cut-off.
Areas for attention in subsequent QI cycles include stricter enforcement of nutrition policies, particularly the catch-up feeding protocol, and further raising staff awareness with a Nutrition Awareness Week.

**REFERENCE(S)**
(1) Weijs, P et al. JPEN 2012.
(2) Compher, C et al. Critical Care Med 2017.

**GRANT ACKNOWLEDGMENT**
N/A

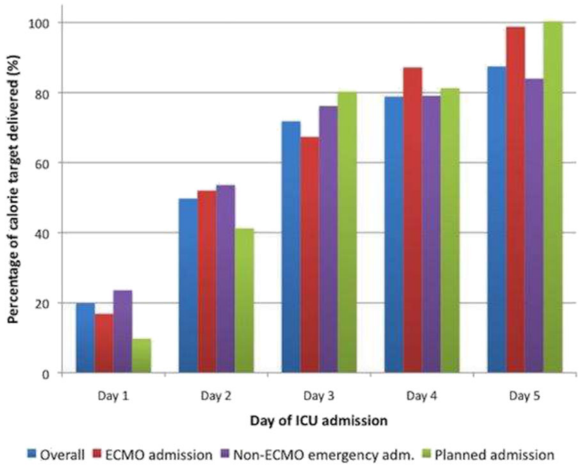

**Fig. 1 (abstract 1075).** Calories delivered over days 1-5 of ICU admission

**Table 1 (abstract 1075).** Delay in initiating EF

| Patient category | Time to initiation of EF (hours) |
|---|---|
| Overall | 25.3 |
| Elective admission | 55.7 |
| Non-ECMO emergency admission | 22.5 |
| ECMO emergency admission | 19.9 |

### 1076

#### The impact of basic nutritional parameters on the prognosis of sepsis patients

E. Portugal Rodríguez[1], R. Cicuéndez Ávila[1], D. Andaluz Ojeda[1], E. Mucientes Peinador[1], L. Nogales Martín[1], G. Renedo Sánchez-Girón[1], D. de Luis Román[2]
[1]Clinical Hospital of Valladolid, ICU, Valladolid, Spain; [2]Clinical Hospital of Valladolid, Endocrinology and Nutrition, Valladolid, Spain
**Correspondence:** R. Cicuéndez Ávila
*Intensive Care Medicine Experimental* 2018, **6(Suppl 2):**1076

**INTRODUCTION.** The management of nutrition along with the measures taken in terms of medical treatment and life support constitute one of the pillars for the treatment of sepsis patients. Therefore, the study of nutritional parameters on this group of patients is of high relevance.
**OBJECTIVES.** To evaluate the influence of routine nutritional parameters on the prognosis of a cohort of sepsis patients admitted to the ICU.
**METHODS.** Patients admitted for severe sepsis and septic shock (SS) were evaluated in this study. Levels of albumin, proteins, cholesterol (LDL and HDL), transaminases, ions, iron, bilirubin, CRP, glycemia, alkaline phosphatase and creatinine were measured on admission and at day 3 and 7. The [median ± IQR] was used to compare the quantitative variables. To evaluate the impact of the variables on patient mortality, the area under the ROC curve (AUROC) was used. A p value of < 0.05 was considered significant.
**RESULTS.** Data from forty-two adult patients were collected. Patients were an average age of 62 years old, of which 71.4% were males and 66.7% presented SS. Average albumin levels were of 2.6 gr / dl. When comparing survivors with deceased, statistically significant differences were found in the following variables [median ± IQR, p]: albumin day 1 (2.7 ± 0.8) vs (2.2 ± 0.9) p= 0,035; albumin day 3 (2.6 ± 0.75) vs (2 ± 0.55) p = 0.035; Albumin day 7 (2.6 ± 1.5) vs (1.9 ± 1.8) p = 0.035; Total proteins day 7 (5.5 ± 1.4) vs (4.6 ± 1.4) p = 0.025; HDL cholesterol day 7 (23 ± 9) vs (14 ± 21.5) p = 0.01; LDL cholesterol day 7(93.4 ± 48.9) vs (57.2 ± 37) p = 0.02. AUROC for UCI mortality prediction[95% CI, p], was 0.73 (0.54- 0.92) p = 0.036 for albumin on day 1; 0.73 (0.57 + 0.88), p = 0.036 for albumin on day 3; 0.76 (0.59 +0.929) p = 0.01 for albumin on day 7; 0.742 (0.56-0.925) p = 0.027 for total proteins on day 7; 0.792 (0.556-1) p = 0.043 for cholesterol on day 7, and 0.833 (0.614-1) p = 0.021 for LDL on day 7.
**CONCLUSIONS.** Sepsis patients presented low basal albumin levels, being significantly lower in the subgroup of non-survivors, both at admission and on successive days. The total protein levels at day 7 show a similar behavior. Based on AUROC both variables present good prognostic capacity. In contrast, LDL cholesterol levels are higher in the group of survivors, presenting an excellent AUROC for prediction of mortality. Further research in larger studies is needed to confirm these results.

**REFERENCE(S)**
Prognostic Implications of serum lipid metabolism over time during sepsis. BioMed Research International Volume 2015, Article ID 789298. ESPEN guidelines on definitions and terminology of clinical nutrition 2017.

### 1077

#### Elemental vs polymeric early enteral nutrition in ICU patients: does elemental feeding improves tolerance and outcome?

M. Theodorakopoulou, F. Frantzeskaki, G. Skyllas, A. Diamantakis, E. Chrysanthopoulou, A. Armaganidis
University Hospital of Athens Greece Attikon, 2nd Critical Care Department, Athens, Greece
**Correspondence:** M. Theodorakopoulou
*Intensive Care Medicine Experimental* 2018, **6(Suppl 2):**1077

**INTRODUCTION.** Nutrition plays a significant role in achieving optimal health, but in certain high risk patients with significant systemic illnesses and/or organ dysfunction, achieving adequate nutrition maybe difficult. Elemental diet formulas are used to provide liquid nutrients in a form that is easily and readily assimilated. A large volume of clinical studies have demonstrated significant health benefits with semi-elemental diets and have been shown to reduce the degree of regurgitation, gastric emptying times, and improve tolerance.
**OBJECTIVES.** To examine whether elemental enteral feeding improves tolerance and outcome in Critically ill ICU patients.
**METHODS.** A total of 126 (75 men) consecutive, mechanically ventilated, critically ill patients with a mean (±SD) age of 65±22 years having various illnesses were included in the present study. Thirty two patients had sepsis. Mean APACHE II and SOFA scores were 17±10 and 7±3 respectively. Mean albumin upon admission was 3.0±0.7 g/dl. All patients were randomized to receive treatment with either elemental or polymeric diets for 10 days. EN treatment started within the first 48

hours of ICU admission. Patient's laboratory data was also recorded for 10 consecutive days. Twenty four-hours urine was collected for each patient to calculate nitrogen balance, while albumin level was recorded in days 1, 4, 7and 10. Results of enteral feeding of the two groups were subsequently assessed and analyzed.

**RESULTS.** Tolerance of enteral nutrition was significantly better in the elemental group. Laboratory values however had not significant difference between the two groups in the 10 days period. The mean nitrogen balance was more negative in polymeric group (p-value: 0.367) The mean serum albumin value was significantly higher in the elemental group. Nitrogen balance however seem to have no correlation with ICU mortality (p-value: 0.312) and incidence of sepsis (p-value: 0.422).

**CONCLUSIONS.** According to the results of this study, semi-elemental diet is better tolerated by the critically ill ICU patient. It seems that these population who have difficulty digesting or absorbing standard polymeric diets may be able to achieve improved nutritional outcomes through the use of semi-elemental diets.

**REFERENCE(S)**

Nutritional and health benefits of semi-elemental diets: A comprehensive summary of the literature. Dominik D Alexander, Lauren C Bylsma, Laura Elkayam, and Douglas L Nguyen. World J Gastrointest Pharmacol Ther. 2016 May 6; 7(2): 306-319

**GRANT ACKNOWLEDGMENT**

None

### 1078

#### Aplication of a protocol for the management of constipation in the intensive care unit of the General Hospital of Albacete: analysis and results

E. Simón Polo, J. De Capadocia, J. Pazos Crespo
Albacete Hospital, Anaesthesia, Albacete, Spain
**Correspondence:** E. Simón Polo
*Intensive Care Medicine Experimental* 2018, **6(Suppl 2):**1078

**INTRODUCTION.** Constipation is a common entity in critical care units. The omission in its treatment as well as the delay in the diagnosis can negatively affect in multiple clinical variables: delay in the respiratory weaning, increase of the rate of Infection, and even increased mortality.

Constipation is definited as the absence of bowel movements more than three days after starting enteral nutrition, having ruled out obstructive causes as an alternative diagnosis.

**OBJECTIVES.** The main objective of this study is to implant a protocol for the management of constipation in patients admitted in our intensive care unit, and to evaluate its efficacy in a preliminary way.

**METHODS.** An analysis was carried out in two phases: first a retrospective, in which we analyzed the magnitude of constipation in our unit, and a second prospective phase in which we studied the impact of the implementation of the protocol. It includes 40 patients (19 prospective) who were admitted between November 2017 and March 2018.

Patients were divided in two main groups: Group A, retrospective: we didn't applied the protocol, and prospective group B: the unit protocol was applied.

We analyzed the following variables: Day of first deposition, incidence of diarrhea, abdominal distention, intolerance and incidence of abdominal hypertension

**RESULTS.** There was a decrease in the incidence of constipation, reducing the day in which the first deposition occurs (day 7-8 vs 4-5 Day). There was an increase in the presence of diarrhea in 10% in the protocol group compared with the control group, as well as a decrease in 20% of abdominal distensión.

**CONCLUSIONS.** Constipation in the critical patient is a frequent and potentially serious entity. In our case the implementation of a protocol has shown a decrease in the incidence of constipation. We observed an increase of diarrhea in the protocol group, without clinical repercussion.

**REFERENCE(S)**

1. Working Group on Metabolism, nutrition of SEMYCIUC. Algorithms of nutritional intervention in the critical patient. 1st ed. Madrid: SEMYCIUC´s metabolism and nutrition Working Group; 2010.2.
2. GuardiolaB, Llompart-Pou JA, Ibañez J, Raurich JM. Prophyaxis Versus Treatment Use of Laxative for Paralysis of Lower Gastrointestinal Tract in critically Ill patients. J Clin Gastroenterol. 2016 Feb; 50 (2): E13-18
3. Mostafa SM, Bhandari S, Ritchie G, Gratton N, Wenstone R. Constipation and its implications in the critically ill patient. Br J of Anaesth. 2003. Dec; 91 (6): 815-9

### 1079

#### Prevalence of electrolyte disorders in ICU and its correlation with mortality

L. Bianchini[1], J. Gomez[2]
[1]Universidade de Passo Fundo, Passo Fundo, Brazil; [2]Hospital São Vicente de Paulo, Passo Fundo, Brazil
**Correspondence:** J. Gomez
*Intensive Care Medicine Experimental* 2018, **6(Suppl 2):**1079

**INTRODUCTION.** Electrolytes disorders are among the most common findings in critically ill patients. Furthermore, there is a well-established relation between some of these imbalances and mortality. However, whether an early correction of electrolytes disturbances decreases unfavorable outcomes remains uncertain.

**OBJECTIVES.** To assess the incidence of electrolyte disorders at Intensive Care Unit(ICU) admission, its relation with mortality and to evaluate if the correction of the disorder in 48 hours influences ICU an in-hospital mortality.

**METHODS.** We performed a retrospective observational study, including all patients >18 years-old admitted for more than 48 hours at the ICU of Hospital São Vicente in the first semester of 2017. Sodium, potassium, chloride, calcium, magnesium, phosphorus were measured at ICU admission and 48 hours after and evaluated regarding their possible association with mortality.

**RESULTS.** A total of 273 patients with a mean age of 52.94 years (18-94) and 60.8% of them male were included. In this sample, hypernatremia at admission was associated with a two-fold increase in the risk of in-hospital death (p=0.024) and hyperphosphatemia increased mortality by 2.4 times (p=0.001). For those who were hypernatremic on ICU admission, every within-subject 1mmol/L alteration was associated with 1.8 times higher mortality (p=0.028). In 48 hours, only hyperphosphatemia increased both ICU and in-hospital mortality (p< 0.001) and hyponatremia (p=0.003) and hypokalemia (p=0.07) were also related to ICU death. No differences both in ICU and in-hospital mortality were observed with correction of the evaluated electrolytes disorders within 48 hours of ICU admission.

**CONCLUSIONS.** The correction of measured electrolytes in the first 48 hours of ICU admission was not associated with decreased mortality, although a larger sample is needed to confirm these findings.

**REFERENCE(S)**

1. El-Fakhouri S et al. Rev Assoc Med Bras 62(3):248-254, 2016
2. McMahon GM et al. Intensive Care Med 38(11):1834-1842, 2012
3. Lee JW. Electrolyte Blood Press 8(2):72-81, 2010

### 1080

#### Nutritional therapy for patients admitted to Intensive care at Landspitali, the National University Hospital of Iceland

H. Birgisdóttir[1], K. Sigvaldason[2], T. Thorsteinsdottir[3]
[1]Intensive Care, Landspitali, the National University Hospital of Iceland, Reykjavík, Iceland; [2]Landspitali, the National University Hospital of Iceland, Department of Anesthesia and Intensive Care, Reykjavík, Iceland; [3]Research Institute in Emergency Care, Landspitali, the National University Hospital of Iceland; Faculty of Nursing, School of Health Sciences, University of Iceland, Reykjavík, Iceland
**Correspondence:** H. Birgisdóttir
*Intensive Care Medicine Experimental* 2018, **6(Suppl 2):**1080

**INTRODUCTION.** Sufficient energy and protein intake for intensive care patients can reduce infections, length of stay, mortality rate and increase health-related quality of life. Energy requirement recommended for ICU patients is 20-25kkal/kg/day and 1.2 - 2.0 g/kg/day of protein and should be started within 48 hours. Studies show that malnutrition is common and that nutritional support is often delayed in the ICU.
**OBJECTIVES.** To evaluate the route of feeding, time to initiation of nutritional support, the energy and protein intake in adult patients in the general ICUs at Landspitali the National University Hospital and analysing the results by length of ventilator treatment.
**METHODS.** Data was collected from medical journals of all patients, 18 years and older, with ICU´s length of stay > 72 hours, not receiving palliative care, during three 2-months' time periods, from 2014 to 2015. Patient demographics were collected, the nutrition and protein intake was recorded daily for the whole stay in ICU. Group A were patients with longer than 24 hours on mechanical ventilation, group B were not on mechanical ventilation or less than 24 hours. Descriptive statistics with anova test on difference between groups were applied.
**RESULTS.** Totally, 134 patients were evaluated for 1359 patient days (range 3-42). Group A, included 86 patients and group B 48. In group A the energy intake was 66% of the energy requirements versus 45% in group B (p< 0.05). Protein intake was 27% of the protein requirements in group A versus 11% in group B (p=0,631). Substantial proportion of energy intake, or 46% in group A and 23% in group B came from propofol. Nutrition was initiated on average in 45 hours in group A versus 25 hours in group B (P< 0,001). Enteral nutrition was used in 88% patients in group A, initiated on average in 56 hours, but in 15% in group B in average, initiated after 36 hours.
**CONCLUSIONS.** Energy and protein requirements were not reached in this study, especially in patients not on mechanical ventilation. Propofol was a large proportion of energy intake, especially among patients with longer ventilator treatment. The initiation of nutritional support was under 48 hours, but shorter delay for enteral nutrition may be encouraged, focusing on patients expected to receive longer ventilation support.

**REFERENCE(S)**

McClave, S. A., Taylor, B. E., Martindale, R. G., Warren, M. M., Johnson, D. R., Braunschweig, C., . . . Compher, C. (2016). Guidelines for the Provision and Assessment of Nutrition Support Therapy in the Adult Critically Ill Patient: Society of Critical Care Medicine (SCCM) and American Society for Parenteral and Enteral Nutrition (A.S.P.E.N.). JPEN: Journal of Parenteral and Enteral Nutrition, 40(2), 159-211.

**GRANT ACKNOWLEDGMENT**

Landspitali the National University Hospital Research Fund and The Icelandic Nurse´s Association science fund.

#### 1081

#### The relationship between base excess unmeasured anions and postoperative outcomes in living donor liver transplantation candidates

H.K. Atalan
Acibadem Mehmet Ali Aydinlar University School of Medicine, Istanbul, Turkey
*Intensive Care Medicine Experimental* 2018, **6(Suppl 2):**1081

**INTRODUCTION.** The liver has an important influence on normal acid-base homeostasis because of its metabolism of organic acid anions, primarily lactate and certain amino acids. It is also known that liver failure has multisystemic effects including metabolic perturbances. It is demonstrated that increased unmeasured anions (UA) are associated with higher mortality in critical ill patients (1). Base excess unmeasured anion (BEua) can be used to show the presence of UA and it's a component of standart base-excess (SBE). BEua can be calculated by using partitioned base-excess approach (2).
**OBJECTIVES.** The aim of this study is to investigate the impact of preoperative presence of UA on postoperative outcomes in living donor liver transplant patients.
**METHODS.** Living donor liver transplantations performed in 2016 and 2017 were investigated and 332 adult living donor liver transplantation patients were included. ABL-800 blood gase analyser was used to measure arterial blood gas samples. BEua was calculated in accordance with partitioned BE approach. Demographic data, blood gas parameters, length of ICU stay, length of hospital stay, duration of mechanical ventilation, postoperative first seven days serum creatinin levels and hospital mortality were recorded.
**RESULTS.** In this study, there were 229 (68.6%) patients with BEua< 0 in preoperative period. In patients with BEua< 0, postoperative 7th day acute kidney injury (AKI) was significantly higher than patients with BEua≥0 (15.3% and 5.7% respectively; p=0.013).
**CONCLUSIONS.** The preoperative presence of UA in living donor liver transplant candidates is associated with early postoperative AKI. Therefore, in this specific population acid-base status assessment should be done with this point of view to improve postoperative outcome.

**REFERENCE(S)**

1. Masevicius FD et al. Crit Care Med 2017; 45:e1233-1239.
2. O'Dell E et al. Crit Care 2005; 9:R464-70

### Scoring for sepsis identification

#### 1082

#### Comparison of SIRS and qSOFA for predicting infection-induced organ dysfunction

S. Parashar[1], K. Lembke[2], S. Simpson[3]
[1]University of Kansas School of Medicine, Kansas City, United States;
[2]University of Kansas School of Medicine, Wichita, United States;
[3]University of Kansas, Pulmonary and Critical Care Medicine, Kansas City, United States
**Correspondence:** S. Parashar
*Intensive Care Medicine Experimental* 2018, **6(Suppl 2):**1082

**INTRODUCTION.** The Sepsis-3 conference redefined clinical criteria for diagnosis of sepsis using measures of organ dysfunction. The qSOFA score was suggested to replace SIRS in screening for sepsis. The prognostic accuracy of suspected infection with either qSOFA or SIRS for predicting organ dysfunction, rather than mortality, has not been evaluated.
**OBJECTIVE.** To compare prognostic ability of qSOFA with SIRS for presence of organ dysfunction within 48 hours of triage in patients admitted through the Emergency Department with suspected infection.
**METHODS.** Single center retrospective cohort analysis of patients aged ≥18 admitted through ED with suspected infection 3/07-5/16. Presence of SIRS and/or qSOFA within 3 hours of triage were assessed. Suspected infection was defined as a combination of body fluid cultures and antibiotics within a 4-hour window, and both must have occurred within 6 hours of ED triage. Organ dysfunction was defined by both Sepsis-2 and Sepsis-3 criteria. Organ dysfunction at presentation was defined as presence of organ dysfunction within 3 hours of triage, and progression to organ dysfunction was defined as presence of organ dysfunction 3 - 48 hours post-triage. A third category included all patients with organ dysfunction either at presentation or at any time up to 48 hours after triage.
**RESULTS.** 13,749 patients with suspected infection that met inclusion criteria were identified. 6,113 patients presented with only SIRS (44.46%), 427 patients presented with only qSOFA (3.11%), 3,072 patients presented with both qSOFA and SIRS (22.34%), and 4,137 patients presented with neither qSOFA nor SIRS (30.09%).
Table 1 shows area under ROC curves for organ dysfunction at presentation, progression to organ dysfunction, and either, according to presence of SIRS or qSOFA at presentation.
Table 2 shows sensitivity and specificity for organ dysfunction at presentation, progression to organ dysfunction, and either, according to presence of SIRS or qSOFA at presentation.
**CONCLUSIONS.** A majority of patients who present to the ED with suspected infection present with ≥2 SIRS. There is a substantial overlap of patients with both ≥2 qSOFA and ≥2 SIRS. qSOFA shows a significantly greater prognostic accuracy for Sepsis-2 and or Sepsis-3 Organ Dysfunction from presentation to 48 hours after ED triage. However, in each circumstance SIRS has higher sensitivity, while qSOFA has higher specificity.

**REFERENCE(S)**

Bone et al, Chest, 1992; 101; 1644-1655;
Levy et al, Critical Care Medicine, 2003; 31; 1250-1256;
Seymour et al, JAMA, 2016; 315; 762-774;
Singer et al, JAMA, 2016; 315; 801-810;
Shankar-Hari et al, JAMA, 2016; 315; 775-787;
Abraham, JAMA, 2016; 315; 757-759

**GRANT ACKNOWLEDGMENT**

Parker B. Francis Undergraduate Fellowship Award

**Table 1 (abstract 1082).** AUROC

| AUROC (95% CI) | | | |
|---|---|---|---|
| | 0 - 3 HOURS | > 3 - 48 HOURS | 0 - 48 HOURS |
| SIRS ≥ 2 | 0.60 (0.59 - 0.61) | 0.61 (0.60 - 0.63) | 0.63 (0.61 - 0.65) |
| qSOFA ≥ 2 | 0.72 (0.71 - 0.73) | 0.71 (0.70 - 0.72) | 0.75 (0.74 - 0.76) |

**Table 2 (abstract 1082).** Sensitivity and Specificity

| Sensitivity and Specificity | | | | | | |
|---|---|---|---|---|---|---|
| | 0 - 3 HOURS | | > 3 - 48 HOURS | | 0 - 48 HOURS | |
| | Sensitivity | Specificity | Sensitivity | Specificity | Sensitivity | Specificity |
| SIRS ≥ 2 | 70.6% | 44.0% | 68.4% | 47.2% | 68.1% | 49.8% |
| qSOFA ≥ 2 | 32.9% | 95.5% | 27.6% | 93.7% | 27.3% | 98.0% |

### 1083

#### Sepsis-3 definitions of septic shock are not only associated with higher disease severity but with differences in type and origin of infection

D. Thomas-Rüddel[1,2], H. Rüddel[1,2], K. Reinhart[1,2], F. Bloos[1,2], MEDUSA study group
[1]Universitaetsklinikum Jena, Center for Sepsis Control and Care (CSCC), Jena, Germany; [2]Universitaetsklinikum Jena, Anesthesiology and Intensive Care Medicine, Jena, Germany
**Correspondence:** D. Thomas-Rüddel
*Intensive Care Medicine Experimental* 2018, **6(Suppl 2):**1083

**INTRODUCTION.** The new Sepsis-3 definition of septic shock includes a serum lactate level >2mmol/l [1] in addition to the established criterion of persistent hypotension. One validation study in 470 emergency room patients hinted at differences not only in disease severity and mortality but in infection characteristics between patients fulfilling the Sepsis-3 lactate criterion and those that did not [2].

**OBJECTIVES.** To assess differences in disease severity, mortality and infection characteristics in a large, mixed population of hospital and community acquired septic shock depending on the used definition.

**METHODS.** We present a post hoc analysis from a 4 year multicenter quality improvement study [3] where ICU patients with sepsis (all with organ dysfunction) were documented prospectively. The analysis was restricted to those with persistent hypotension, assessed by vasopressor support according to SOFA subscore. Differences between the patients with and without lactate >2mmol/l were assessed by Fisher´s exact test, Mann-Whitney U or Kaplan-Meier curve with Log rank test as appropriate.

**RESULTS.** 5144 patients fulfilled Sepsis-1 criteria for septic shock, of those 3524 (69%) had a lactate level >2mmol/L and therefore fulfilled also Sepsis-3 criteria. Patients fulfilling Sepsis-3 criteria had significantly more community acquired septic shock, more frequent an abdominal focus and a higher rate of bacteremia (Table 1).
They also had a significantly higher disease severity, higher procalcitonin levels and higher mortality (Table 2). The mortality difference was mostly driven by early death within 14 days (Figure 1).

**CONCLUSIONS.** Using the Sepsis-3 definitions of septic shock is not only associated with higher mortality but with changes in infection characteristics towards more community acquired infections, more abdominal sepsis and more bacteremia. Early mortality, likely caused directly by septic shock, is much higher. This should be taken into account in the design and interpretation of future trials and epidemiological studies.

**REFERENCE(S)**

1. Singer et al., JAMA. 2016 Feb 23;315(8):801-10.
2. Sterling et al., Crit Care Med. 2017 Sep;45(9):1436-1442
3. Bloos et al., Intensive Care Med. 2017 Nov;43(11):1602-1612.

**GRANT ACKNOWLEDGMENT**

Financial support by the Federal Ministry of Education and Research (BMBF) via the Integrated Research and Treatment Center, Center for Sepsis Control and Care (CSCC, FKZ 01EO1002) .

**Table 1 (abstract 1083).** Infection characteristics (origin, focus and bacteremia) depending on septic shock definition; n(%)

| | Sepsis-1 | Sepsis-3 (Lac>2mmol/L) | excluded by Sepsis-3 (Lac≤2mmol/L) | p value |
|---|---|---|---|---|
| community aquired | 2109 (42%) | 1529 (45%) | 580 (37%) | |
| ICU aquired | 1216 (24%) | 742 (22%) | 474 (30%) | |
| normal ward/nursing home aquired | 1696 (34%) | 1160 (34%) | 536 (34%) | <0.001 |
| pulmonary | 2130 (41%) | 1355 (39%) | 775 (48%) | <0.001 |
| abdominal | 2049 (40%) | 1542 (44%) | 507 (31%) | <0.001 |
| urogenital | 573 (11%) | 388 (11%) | 185 (11%) | 0.67 |
| bones & soft tissue | 649 (13%) | 406 (12%) | 243 (15%) | 0.001 |
| proven bacteremia | 1639 (32%) | 1213 (34%) | 426 (26%) | <0.001 |

**Table 2 (abstract 1083).** Inflammatory markers, disease severity (at sepsis onset) and outcome depending on septic shock definition; median [IQR] and n (%)

| | Sepsis-1 | Sepsis-3 (Lac>2mmol/l) | excluded by Sepsis-3 (Lac≤2mmol/l) | p value |
|---|---|---|---|---|
| procalcitonin ng/ml | 6.5 [1.8-25.5] | 9.5 [2.9-32.2] | 2.6 [0.9-9.6] | <0.001 |
| CRP mg/l | 207 [116-297] | 199 [109-295] | 218 [133-301] | <0.001 |
| WBC Gpt/l | 16.2 [10.7-23.3] | 16.4 [10.2-24.3] | 16.0 [11.3-21.6] | 0.22 |
| SAPS II score | 49 [39-62] | 52 [41-65] | 44 [36-55] | <0.001 |
| SOFA score | 10 [8-12] | 10 [8-12] | 9 [7-10] | <0.001 |
| urine output l/24hrs | 1350 [580-2300] | 1200 [470-2250] | 1620 [910-2560] | <0.001 |
| new RRT | 1318 (26%) | 1037 (29%) | 281 (17%) | <0.001 |
| 28-day mortality | 1743 (35%) | 1389 (40%) | 354 (23%) | <0.001 |
| hospital mortality | 2107 (41%) | 1648 (47%) | 459 (28%) | <0.001 |

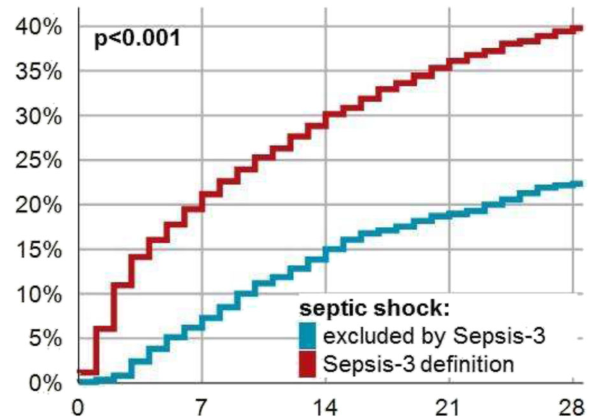

**Fig. 1 (abstract 1083).** Mortality up to 28 days after sepsis onset depending on septic shock definition; Log rank test

### 1084

#### Comparison of capillary leak index versus traditional ICU scoring sistems

P. Palacios Moguel, C. Gomez Moctezuma, A.J. Fuentes Gomez, A. Galán Jáuregui, J.S. Aguirre Sanchez, J. Franco Granillo, G. Camarena Alejo
American British Cowdray Medical Center, Intensive Care, Mexico City, Mexico
**Correspondence:** P. Palacios Moguel
*Intensive Care Medicine Experimental* 2018, **6(Suppl 2):**1084

**INTRODUCTION.** Sepsis is a medical condition based on a hyperstimulation of the immune system, making a release of biochemical substances to the bloodstream triggering systemic inflammatory response.

Endothelium, under physiological conditions, is responsible for anticoagulant and anti-adhesive properties, also it regulates vasomotor tone and vascular homeostasis. Endothelial dysfunction has been associated with inflammation, oxidative and nitrosative stress.

Impairment of endothelial function leads to phenotypic and physical changes of the endothelium, with deregulated release of potent vasodilators nitric oxide and prostacyclin, reduction of vascular reactivity to vasoconstrictors, leukocytes and platelet aggregation, and increase in inducible nitric oxide synthase expression that can exert a negative feedback on endothelial nitric oxide synthase expression, with subsequent deregulation of nitric oxide signaling.

Systemic capillary leak syndrome on sepsis is a serious condition with a high morbidity and mortality rate. On a previous trial, we validate the Capillary Leak Index (CLI) as a poor prognosis scale in septic patients. A cut-off point of 85.55 has a direct association with 28 days mortality.

**OBJECTIVES.** Compare Capillary Leak Index with common prognosis staging scales used on intensive care unit in septic patients.

**METHODOLOGY.** Observational prospective cohort trial including 62 septic patients. We analyzed CLI, SOFA, APACHE II, hospital length of stay, complications and mortality. T student test and ROC curves validated CLI as a poor prognostic factor. (p>0.05 IC 95%).

**RESULTS.** 62 patients. 2 groups assigned, dead (22) clinical improvement (40). Mean age of 58.9 years, 72.58% males. We report a higher CLI (106 IC 94.91-118.42), SOFA (13.7 IC 12.14-15.31) and APACHE II (25.63 IC 23.33-27.93) on dead patients. (p< 0.001).

We also performed ROC curves to compare sensibility and specificity of CLI (AUC0.822) versus traditional ICU scoring systems such as APACHE II (0.858) and SOFA (0.896). We obtained 86.36% and 72.50% of specificity and sensibility respectively to a cut-off point >85.55.

**CONCLUSION.** There is a directly proportional relationship between CLI and mortality. Comparative analysis between CLI and other scales (SOFA, APACHE II) show inferiority, however the simplicity, and lower costs make it an accessible scale. This index evaluates integrity of endothelium, not analyzed component in another scale. We differ cut-off point (85.4) in our trial from results in previous trials (>61).

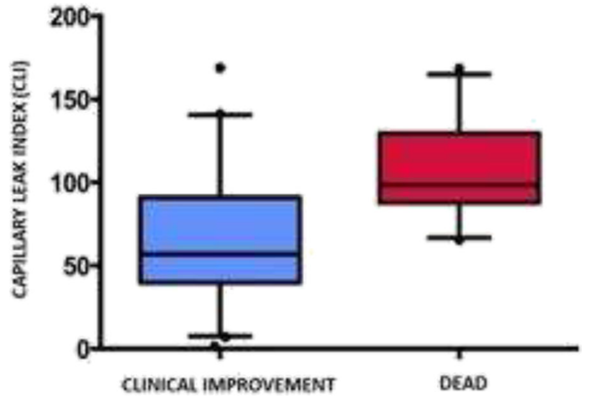

**Fig. 1 (abstract 1084).** Mean Capillary leak index.

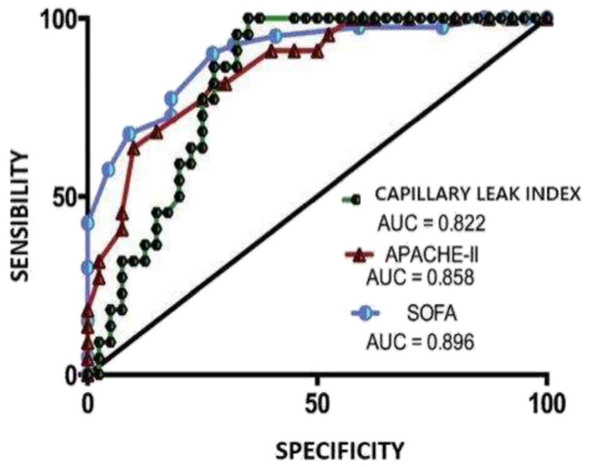

**Fig. 2 (abstract 1084).** ROC curve CLI vs SOFA, APACHE II

**Table 1 (abstract 1084).** Demografic characteristics.

| Cause of Hospital Discharge | Dead | SD | Improvement | SD | P |
|---|---|---|---|---|---|
| AGE | 62.68 | 3.21 | 56.82 | 2.05 | 0.11 |
| CRP | 268.28 | 9.04 | 180.64 | 13.46 | <0.0001 |
| Albumin | 2.61 | 0.12 | 3.00 | 0.13 | 0.0452 |
| CLI | 106.67 | 5.65 | 65.53 | 5.96 | <0.0001 |
| SOFA | 13.72 | 0.80 | 7.45 | 0.53 | <0.0001 |
| APACHE II | 25.63 | 1.17 | 16.35 | 1.03 | <0.0001 |
| Lenght of stay | 9.13 | 1.91 | 18.12 | 2.54 | 0.0187 |

### 1085

#### An investigation of the discriminative value of PRESEP as a pre-hospital screening tool for diagnosing sepsis or identifying high mortality risk in a cohort of infected patients identified pre-hospital

D. Hargreaves[1], L. Smith[2], G. Picton[1], J. de Carvalho[3], L. Hodgson[1], R. Venn[1]
[1]Western Sussex Hospitals Foundation NHS Trust, Worthing, United Kingdom; [2]University of Brighton and Sussex Medical School, c/o Duncan Hargreaves, Department of Anaesthesia, Hove, United Kingdom; [3]University of Brighton and Sussex Medical School, Brighton, United Kingdom
**Correspondence:** L. Smith
*Intensive Care Medicine Experimental* 2018, **6(Suppl 2):**1085

**INTRODUCTION.** Prompt administration of antibiotics is associated with improved outcomes in sepsis *(1)*. It is unclear whether using a prehospital sepsis screening tool provides significant clinical benefit *(2)*. The PRESEP score [TABLE 1] was developed to identify sepsis in the prehospital setting, with high reported discrimination in the small derivation study with a sensitivity 0.85 and specificity 0.86 at a cut-off of 4 *(3)*, but this has limited external validation to date.

**OBJECTIVES.** To evaluate whether in a cohort of patients transported by ambulance and identified in the ED as possibly septic, PRESEP would predict adverse outcomes when compared to National Early Warning Score (NEWS) and quick sequential organ failure assessment (qSOFA) scores in the prehospital environment.

**METHODS.** A dual-centre, prospective cohort (2015-16) of adult ED patients (n=1534), identified as potentially septic was analysed and prehospital observations and 30-day mortality data collected. 301 patients were excluded, 132 as not assessed pre-hospital, and 168 for missing as ambulance data not retrievable. Area under the receiver operator characteristic curves (AUROCs) for 30 day mortality and for ED physician diagnosis of sepsis were calculated for all 3 scores, together with sensitivity, specificity, positive predictive value (PPV) and negative predictive value (NPV) for each.

**RESULTS.** Compared to the derivation group, our cohort had higher median age (79 vs 71) and a higher proportion of medical admissions (84% vs 55%), but similar rates of surgical admission and similar gender distribution. [TABLE 2]

PRESEP showed poor discrimination for 30-day mortality with an AUROC of 0.47 (95% CI 0.41-0.50) compared to NEWS of 0.61 (0.57-0.66) and qSOFA of 0.64 (0.60-0.68), though both had limited discrimination. [FIGURE 1]

Discrimination for an ED diagnosis of sepsis was poor for all three scores: AUROC of 0.5 (0.46-0.54) for PRESEP, 0.52 (0.48-0.56) for NEWS and 0.52 90.48-0.55) for qSOFA. [FIGURE 2]

At a cut off of 4, PRESEP demonstrated no better sensitivity and specificity, positive predictive value or negative predictive value than NEWS or qSOFA. [FIGURE 3]

**CONCLUSIONS.** In a large prospective cohort of patients with suspected sepsis, the PRESEP score in the pre-hospital environment did not discriminate for mortality or ED diagnosis of sepsis and was inferior to NEWS and qSOFA. Future research could assess performance of these screening tools in a larger cohort of patients with suspected infection in the pre-hospital environment.

**REFERENCE(S)**

1. Seymour CW et al. Time to Treatment and Mortality during Mandated Emergency Care for Sepsis. *NEJM*. 2017;376(23):2235-44
2. Smyth MA et al. Identification of adults with sepsis in the prehospital environment: a systematic review. *BMJ Open*. 2016;6(8):e011218-11
3. Bayer O et al. An Early Warning Scoring System to Identify Septic Patients in the Prehospital Setting: The PRESEP Score. *Acad Emerg Med*. 2015;22(7):868-71

**Table 1 (abstract 1085).** PRESEP Score weightings

| Parameter | Score weighting (score positive ≥ 4) |
|---|---|
| Temperature> 38 °C | 4 |
| Temperature < 36 °C | 1 |
| Heart Rate > 90 beats/minute | 2 |
| Respiratory Rate > 22 breaths / minute | 1 |
| Oxygen Saturation (SaO2) < 92% | 2 |
| Systolic Blood Pressure < 90mmHg | 2 |

**Table 2 (abstract 1085).** Description of Pre-hospital Cohort

| Demographic Variable | Count |
|---|---|
| Age (y) | 79 [65-86] |
| Male | 554 (46%) |
| Female | 669 (54%) |
| Chichester Site | 686 (56%) |
| Worthing Site | 547 (44%) |
| Surgical patient | 203 (16%) |
| Medical Patient | 1030 (84%) |

Data shown as median with interquartile range [IQR] or count with percentage (%)

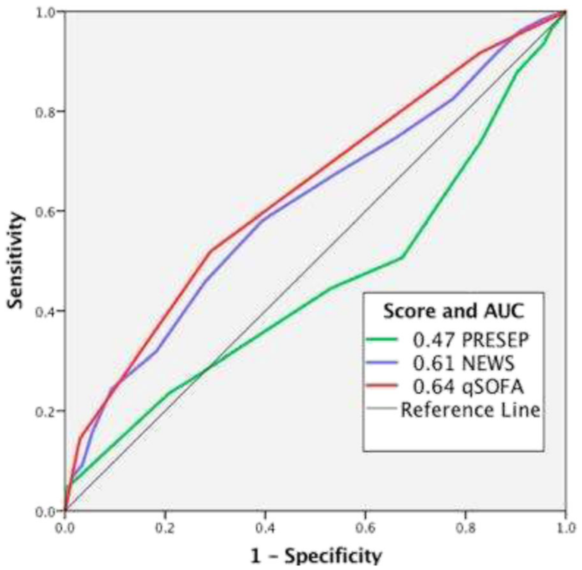

**Fig. 1 (abstract 1085).** AUROCs for 30 Day Mortality in PRESEP, NEWS and qSOFA

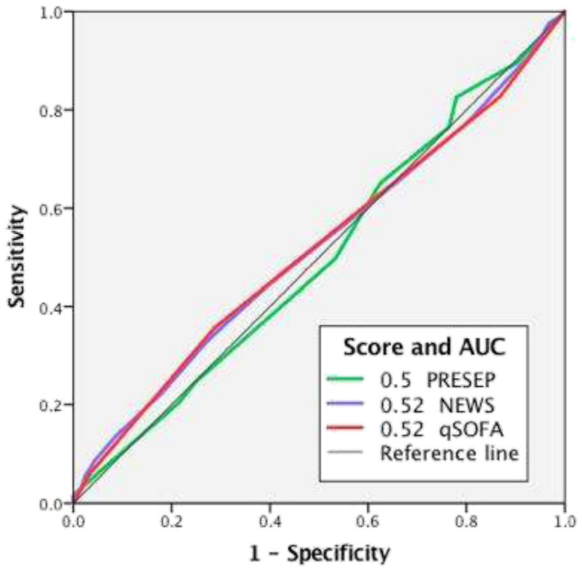

**Fig. 2 (abstract 1085).** AUROCs for ED Diagnosis Sepsis in PRESEP, NEWS and qSOFA

**Comparison of prognostic variables for PRESEP, NEWS & qSOFA for Mortality at 30 days and ED Sepsis Diagnosis**

| Prognostic tool | Sensitivity (95% CI) | Specificity (95% CI) | PPV (95% CI) | NPV (95%CI) |
|---|---|---|---|---|
| **Mortality at 30 days** | | | | |
| PRESEP ≥ 4 | 0.74 (0.68-0.79) | 0.17 (0.15-0.20) | 0.17 (0.16-0.18) | 0.74 (0.69-0.79) |
| NEWS ≥ 5 | 0.92 (0.87-0.95) | 0.14 (0.12-0.95) | 0.20 (0.19-0.20) | 0.88 (0.81-0.92) |
| qSOFA ≥ 2 | 0.52 (0.45-0.59) | 0.71 (0.68-0.74) | 0.29 (0.26-0.32) | 0.87 (0.85-0.88) |
| **ED Diagnosis Sepsis** | | | | |
| PRESEP ≥ 4 | 0.83 (0.80-0.85) | 0.22 (0.18-0.27) | 0.70 (0.69-0.71) | 0.36 (0.31-0.42) |
| NEWS ≥ 5 | 0.86 (0.84-0.89) | 0.12 (0.08-0.15) | 0.68 (0.67-0.69) | 0.27 (0.21-0.58) |
| qSOFA ≥ 2 | 0.52 (0.48-0.56) | 0.36 (0.32-0.39) | 0.73 (0.69-0.77) | 0.33 (0.31-0.35) |

ED, Emergency Department; CI, Confidence Interval; PPV, Positive Predictive Value; NPV, Negative Predictive Value.

**Fig. 3 (abstract 1085).** Comparison of prognostic value for PRESEP, NEWS & qSOFA

### 1086

#### A prospective cohort study of the Quick Sequential Organ Failure Assessment (qSOFA) score versus the Systemic Inflammatory Response Syndrome (SIRS) criteria in the determination and prognostication of sepsis in a Philippine tertiary hospital

S.R.C. Ang, O.G.V. Ubaldo
The Medical City, Internal Medicine, Pasig City, Philippines
**Correspondence:** S.R.C. Ang
*Intensive Care Medicine Experimental* 2018, **6(Suppl 2):**1086

**INTRODUCTION.** Sepsis is a leading cause of mortality both locally and worldwide. Despite this, early diagnosis of sepsis remains difficult, with a significant number not fulfilling SIRS criteria. In 2016, the Sepsis-3 guidelines modified its definition to include qSOFA score.

**OBJECTIVES.** To compare the performance of the qSOFA versus SIRS in terms of earlier recognition of sepsis, to determine the sensitivity/specificity of qSOFA and compare with the SIRS criteria, to determine the positive/negative predictive value of qSOFA and compare with the SIRS criteria, and to use the AUROC to identify the overall predicitive accuracy of the qSOFA criteria versus the SIRS criteria.

**METHODS.** To compare the two, 295 adult patients in the emergency room with suspected infection were included in the study and simultaneously determined their qSOFA score and SIRS criteria. The presence of sepsis was adjudicated by three infectious disease specialists, and outcomes within the first 48 hours were acquired. Sensitivity, specificity, positive predictive and negativepredictive values for qSOFA and SIRS were computed using constructed confusion matrices,and overall predictive accuracy was measured by the AUROC.

**RESULTS.** The qSOFA score was specific (95.5%) but poorly sensitive (46.3%) test compared to the SIRS criteria (sensitivity 73.7% and specificity 60%). Both qSOFA and the SIRS criteria significantly corelated with sepsis positivity but the qSOFA score had superior overall predictive accuracy at 70.9% compared to the SIRS criteria. The adjudicators had moderate strength in agreement (Fleiss' kappa = 0.39) and a percentage agreement of 60%.

CONCLUSION. Based on our findings, we conclude that the qSOFA score is a more accurate predictor of sepsis, but should not be used as a preliminary sepsis screening tool. We recommend that the SIRS criteria be maintained as a screening tool and to use the qSOFA score simultaneously for timely management.

**REFERENCE(S)**

1. Singer M, Deutschman CS, Seymour CW, Shankar-Hari M, Annane D, Bauer M, et al. The Third International Consensus Definitions for Sepsis and Septic Shock (Sepsis-3). JAMA.2016 Feb 23;315(8):801-10.
2. Kaukonen K-M, Bailey M, Pilcher D, Cooper DJ, Bellomo R. Systemic Inflammatory Response Syndrome Criteria in Defining Severe Sepsis. N Engl J Med. 2015 Apr23;372(17):1629-30.
3. Alejandria M MD, Lansang MA, Tiangco B, Fonbuena G, Fadreguilan E, Timbreza F, et al.Epidemiology and Predictors of Mortality from Sepsis in Medical Patients at UP-PGH.Philippine Journal of Microbiology and Infectious Diseases. 2002 Aug 27;:1-11.

### 1087

#### Clinical impact of body mass index and lymphocytic count on clinical outcomes in septic patients in a critical care unit: chronic inflammation as a protective factor?

A.R. Contreras Contreras[1,2], E. Najera[1], A. Pedraza[3], J. Franco[1], J. Aguirre[1], G. Camarena[1]
[1]American British Cowdray Medical Center, Intensive Care Unit, Mexico City, Mexico; [2]Universidad Nacional Autonoma de México, Mexico City, Mexico; [3]Instituto Nacional de Rehabilitación, Mexico City, Mexico
**Correspondence:** A.R. Contreras Contreras
*Intensive Care Medicine Experimental* 2018, **6(Suppl 2):**1087

**INTRODUCTION.** The obesity paradox in critically ill patients, suggests a survival advantage with increasing body mass index (BMI), while underweight individuals exhibit the greatest risk of mortality, however the physiopathology underling of this phenomenon isn´t clear. The nature of the inflammation caused by obesity it's unique and include inflammatory mediators of immunity innate and adaptive.

**OBJECTIVES.** To evaluate risk for mortality and clinical outcomes and its correlation with body mass index (BMI) and lymphocytic count in septic patients in a critical care unit.

**METHODS.** We conducted an observational cohort with critical patients from a single center in an intensive care unit (ICU) in Mexico City, Mexico. Demographic, Clinical, laboratory data was collected for all patients. We estimated the following severity indexes on admission to ICU: sequential organ failure assessment (SOFA) score with acute physiology and chronic health valuation (APACHE) II score, Body Mass index (BMI) and the presence of lymphopenia on admission. Outcomes included mortality and length of stay (LOS) in ICU. Patients with prior therapy with immunosuppressive agents were excluded. less than 18.5 (underweight), 18.5-24.9 (normal weight), 25.0-29.9 (overweight), and 30.0 or more (obesity).

**RESULTS.** We included 83 patients, 53% male, with mean age 69 (SD±16) years and mean BMI 25.5 (SD ± 5.9) Kg/m$^2$. Median score for SOFA 5 (RIQ 3 - 9) and APACHE II 18 (RIQ 13 - 23). Proportion of patients with underweight, normal, overweight and obesity were 44.5, 8.4, 25.3 and 21.69%, respectively, lymphopenic 65.1%. Overall mortality 27 %, median LOS 7 (RIQ 4-12) days. BMI strongly correlated with lymphocytic count (r=0.65, p< 0.01) also Non-obese patients were more frequently lymphopenic 92.2 vs 7.8%, (p< 0.01). Obese patients showed lower mortality (4.3 vs. 28.3%, p=0.01) and higher LOS [10 (RIQ 7-14) vs. 7 (RIQ 4 -11) days, p=0.027]. Lymphopenia showed higher mortality (41.2 vs. 6.3% p=0.001). Mortality cases had lower lymphocytic counts [1.2(SD±0.82) vs 0.65 (SD ±0.78), p=0.004] Predictors for mortality were obesity HR = 0.09 (IC95% 0.01 - 74, p=0.025) and Lymphopenia HR = 7.7 (IC95% 1.8 - 33.4, p=0.006).

**CONCLUSIONS.** Non-obese and lymphopenic ICU patients showed worse outcome in sepsis. Chronic inflammation present in obesity may be protective in this setting, however further research is needed to describe the inflammatory responses with measurement of specific inflammatory mediators.

**REFERENCE(S)**

1. O´Rourke R, Lumeng C. Obesity Heats Up Adipose Tissue Lymphocytes. Gastroenterology.2013 August; 145(2):282-285.
2. Sakr Y, et al. Being Overweight Is Associated with Greater Survival in ICU Patients: Results From the intensive Care Over Nations Audit. Critical Care Medicine. 2015 December; 43 (12): 2623-2632

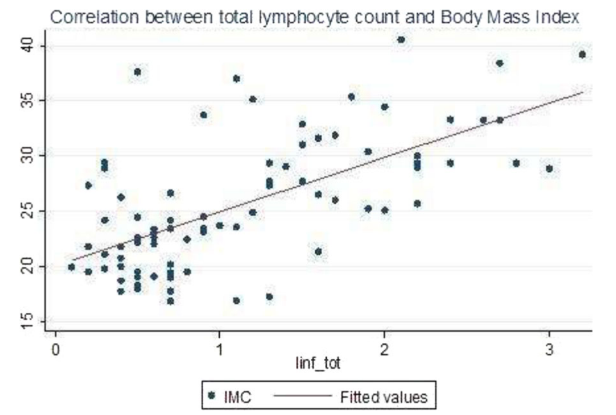

**Fig. 1 (abstract 1087).** See text for description

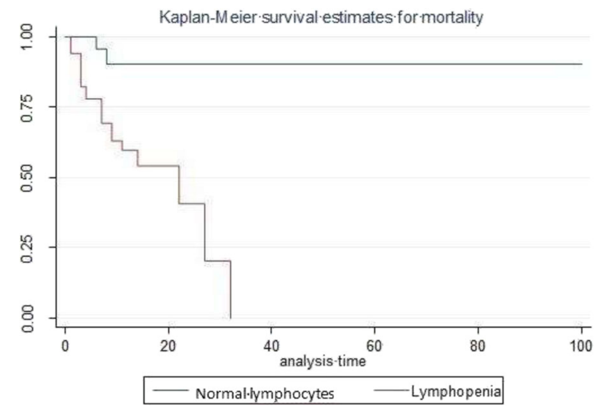

**Fig. 2 (abstract 1087).** See text for description

### 1088

#### Comparison of sepsis biomarkers in critical patients upon admission to the emergency room

K. Katabami, M. Hayakawa
Hokkaido University Hospital, Sapporo, Japan
**Correspondence:** K. Katabami
*Intensive Care Medicine Experimental* 2018, **6(Suppl 2):**1088

**OBJECTIVES.** Early diagnosis and treatment of sepsis are important; however, sepsis is difficult to diagnose especially in critical situations upon arrival at the emergency room on. This study aimed to investigate sepsis biomarkers in critical patients upon admission and compare their diagnostic values.

**METHODS.** This was a single-center retrospective observational clinical study conducted on 53 patients admitted to our emergency room and critical care center. Presepsin, procalcitonin (PCT), and interleukin-6 (IL-6) levels were measured upon admission to predict its diagnostic value for sepsis. Receiver operating characteristic (ROC) curve analysis was performed to determine the diagnostic accuracy. The sequential organ failure assessment score was used to quantify the severity of patients' condition. Infections were diagnosed after reviewing the clinical, radiological, and microbiological data. The study was approved by the hospital's ethical committee.

**RESULTS.** Presepsin levels were significantly higher in sepsis than those in non-sepsis group, with significant differences according to Mann-Whitney test ($P< 0.0297$). ROC curve analysis and area under curve (AUC) were 0.896 for presepsin and 0.953 for IL-6 when differentiating sepsis from non-sepsis patients. The sepsis diagnostic accuracy of presepsin and IL-6 was higher than that of PCT (AUC, 0.527). The best cut-off values for presepsin and IL-6 were 516 pg/mL and 166.7 pg/mL, which were associated with negative predictive values of 96.4% and 100%, respectively. These biomarkers were not associated with the prognosis.

**CONCLUSION.** Presepsin and IL-6 levels on admission may contribute to rule out the diagnosis of sepsis in critical patients admitted to the emergency room. Large-scale studies are needed to reveal the importance of these biomarkers in the diagnosis of sepsis.

### 1089

#### Epidemiology, patterns of care, and in-hospital mortality for patients with severe sepsis in ICUs in Japan: the FORECAST study

T. Abe[1,2], H. Ogura[3], S. Gando[4], S. Fujimi[5], S. Fujishima[6], A. Hagiwara[7], T. Hifumi[8], T. Iba[1], H. Ikeda[9], J. Kotani[10], S. Kushimoto[11], T. Masuno[12], T. Mayumi[13], T.-A. Nakada[14], K. Okamoto[15], Y. Otomo[16], D. Saitoh[17], Y. Sakamoto[18], J. Sasaki[6], Y. Shiino[19], A. Shiraishi[20], S.-I. Shiraishi[21], N. Takeyama[22], K. Takuma[23], T. Tarui[24], R. Tsuruta[25], M. Ueyama[26], Y. Umemura[3], K. Yamakawa[5], N. Yamashita[27], Japanese Association for Acute Medicine (JAAM) FORECAST Group
[1]Juntendo University, Tokyo, Japan; [2]Tsukuba University, Tsukuba, Japan; [3]Osaka University Graduate School of Medicine, Osaka, Japan; [4]Hokkaido University Graduate School of Medicine, Sapporo, Japan; [5]Osaka General Medical Center, Osaka, Japan; [6]Keio University School of Medicine, Tokyo, Japan; [7]Center Hospital of the National Center for Global Health and Medicine, Tokyo, Japan; [8]St. Luke's International Hospital, Tokyo, Japan; [9]Teikyo University School of Medicine, Tokyo, Japan; [10]Kobe University Graduate School of Medicine, Kobe, Japan; [11]Tohoku University Graduate School of Medicine, Sendai, Japan; [12]Nippon Medical School, Tokyo, Japan; [13]University of Occupational and Environmental Health, Kitakyushu, Japan; [14]Chiba University Graduate School of Medicine, Chiba, Japan; [15]Kitakyushu City Yahata Hospital, Kitakyushu, Japan; [16]Tokyo Medical and Dental University, Tokyo, Japan; [17]National Defense Medical College, Tokorozawa, Japan; [18]Saga University Hospital, Saga, Japan; [19]Kawasaki Medical School, Kurashiki, Japan; [20]Kameda Medical Center, Kamogawa, Japan; [21]Aizu Chuo Hospital, Aizuwakamatsu, Japan; [22]Aichi Medical University Hospital, Nagakute, Japan; [23]Kawasaki Municipal Kawasaki Hospital, Kawasaki, Japan; [24]Kyorin University School of Medicine, Tokyo, Japan; [25]Yamaguchi University Hospital, Ube, Japan; [26]Chukyo Hospital, Nagoya, Japan; [27]Kurume University Hospital, Kurume, Japan
**Correspondence:** T. Abe
*Intensive Care Medicine Experimental* 2018, **6(Suppl 2):**1089

**INTRODUCTION.** Limited information exists about the epidemiology, management, and outcomes of severe sepsis in Japanese patients.

**OBJECTIVES.** To evaluate characteristics, clinical practices, and outcomes for patients with severe sepsis.

**METHODS.** This is a sepsis sub-study of the Focused Outcomes Research in Emergency Care in Acute Respiratory Distress Syndrome, Sepsis and Trauma (FORECAST) study, a multicenter, prospective cohort study. It was conducted in 59 ICUs from January, 2016 to March, 2017 in Japan. We included adult patients (≥16 years) with severe sepsis based on sepsis-2 criteria.[1]Demographic, physiological, therapeutic, sepsis care, and outcome data were collected. Primary outcome was in-hospital mortality. Secondary outcomes were dispositions after discharge, ICU-free days, ventilator-free days, and length of hospital stay (LOS).

**RESULTS.** 1,184 patients with severe sepsis admitted to ICUs. The median age was 73 (IQR: 64-81) and 719 (61%) were male. Majority of patients (676 [57%]) came directly from emergency department. The most common comorbidity was diabetes (272 [23%]). 745 (63%) patients had septic shock. Median APACHE II score and SOFA was 23 (IQR, 19-29) and 9 (IQR, 6-11), respectively. The most common site of infection was lung 367/1,184 (31%), followed by intraabdominal (311 [26%]), and urinary tract (218 [18%]) infection. Rate of blood culture positivity was high (59%). In regard to antibiotics, carbapenem was the most commonly used (627 [53%]), followed by tazobactam/piperacillin (238 [20%]) and vancomycin (203 [17%]). 413 (35%) patients were used antibiotics before diagnosis of sepsis. 663 (57%) patients received de-escalation of their antibiotics. In regard to adjunctive therapies, steroid (345 [30%]), continuous renal replacement therapy (302 [26%]), and recombinant human soluble thrombomodulin (242 [21%]), antithrombin (231 [20%]), and intravenous immunoglobulin (222 [20%]) were used. Compliance rate for entire 3-hour bundle was 65% (766/1,173), whereas compliance rates for entire 6-hour bundle including or excluding central venous catheter-related were 4% (41/1,164) and 54% (625/1,167), respectively. In-hospital mortality was 269/1,148 (23%). With septic shock, in-hospital mortality was 200/718 (28%). Post-acute mortality (death after 31 days) was 78/458 (17%). Median ICU-free days were 19 (IQR, 11-24), ventilator-free days were 21 (IQR, 0-28), and LOS were 24 (12-46). 323/879 (37%) patients were discharged to home after recovery.

**CONCLUSIONS.** Sepsis care in Japan characterized by high early use of carbapenem and low compliance rate of 6-hours bundles, especially, central venous catheter related. In-hospital mortality of our study was comparable among developed countries. A steady linear relationship between LOS and in-hospital mortality was seen. Our results may reflect an aging society.

**REFERENCE(S)**

1. Crit Care Med 2003

**GRANT ACKNOWLEDGMENT**

This study was supported by the Japanese Association for Acute Medicine (JAAM)

### 1090

#### The biological evidence for the use of SOFA score on sepsis definition

R. Costa, R. Salomao, O. Araujo, M. Brunialti

Universidade Federal de São Paulo (UNIFESP), Division of Infectious Diseases, São Paulo, Brazil

**Correspondence:** R. Costa

*Intensive Care Medicine Experimental* 2018, **6(Suppl 2):**1090

**INTRODUCTION.** The cytokines storm is essential on sepsis physiopathology and development of tissue damage. Sepsis-3 definitions are based on life-threatening organ dysfunction related to host immune response and results from epidemiological studies, without physiological basis.

**Objective.** The aim of this study was to evaluate the association between pro and anti-inflammatory cytokines kinetics and sepsis related organ dysfunction through the SOFA score analysis.

**METHODS.** We included 104 septic patients in this observational, prospective study from 4 brazilian centers. The plasma samples were collected in 3 time-points: ICU admission (D0), seventh (D7), and fourteenth (D14) from sepsis diagnosis. The cytokines IL1-β, IL2, IL6, IL8, IL10, IL17, IL12/23p40, IL 21, TNF, G-CSF, GM-CSF were analyzed by flow cytometry. Sofa score was assessed on ICU admission.

**RESULTS.** The cytokines were included in a factor analysis model comprising the three measurements and were grouped in 4 factors according to their kinetics over the 3 dosages (D0, D7, D14). Factor 1 (IL 6, IL 8, IL 10, IL 1 β and G-CSF) correlated with SOFA (D0) ($p = 0.039$), while factor 3 (IL 17 and IL 12 / 23p40) with SOFA (D0), $p = 0.003$. Factor 4 (IL21) and Factor 2 (IL-2, TNF-α and GM-CSF) did not show any correlation with the SOFA score.

**CONCLUSION.** There was a correlation between the kinetic of the most relevants cytokines and organ dysfunctions (SOFA score). This result could be the biological explanation for the statistical success of Sepsis-3 criteria.

**GRANT ACKNOWLEDGMENT**

This study was supported by the São Paulo Research Foundation (FAPESP)

### 1091

#### Red-flag sepsis and SOFA identify different patient populations at risk of sepsis-related deaths on the general wards

M. Kopczynska, B. Sharif, T. Szakmany, Welsh Digital Data Collection Platform

Cardiff University, Anaesthesia, Intensive Care and Pain Medicine, Division of Population Medicine, Cardiff, United Kingdom

**Correspondence:** M. Kopczynska

*Intensive Care Medicine Experimental* 2018, **6(Suppl 2):**1091

**INTRODUCTION.** Controversy exists regarding the best clinical diagnostic and screening tool for sepsis outside the ICU. SOFA score has been shown to be superior to SIRS criteria (1). NICE recommends the use of "Red-flag sepsis criteria" however the performance of this tool has not been tested formally (2).

**OBJECTIVES.** The aim of the study was to investigate the ability of Red-flag sepsis criteria and SOFA based sepsis criteria to identify the patients at high risk of sepsis-related death.

**METHODS.** Patients were recruited into a 24-hour point-prevalence study on the general wards and emergency departments across all Welsh acute hospitals. Inclusion criteria were: clinical suspicion of infection and NEWS 3 or above in-line with established escalation criteria in Wales. Data on Red-flag sepsis and SOFA criteria was collected together with qSOFA and SIRS scores and 90-day mortality.

**RESULTS.** 460 patients were recruited over a 24-hour period. 246 were positive for Red-flag sepsis, mortality 26.4% (65/246); 242 for SOFA based sepsis criteria, mortality 32.2% (78/242). Both Red-flag sepsis and SOFA based sepsis criteria were found in 140 patients, mortality 35.7% (50/140). 83 patients were positive for Red-flag sepsis criteria only, mortality 18.1% (15/83) in comparison to 102 patients positive for SOFA based criteria only, mortality 25.5% (26/102). 82 patients did not meet either Red-flag sepsis or SOFA based criteria, mortality 25.6% (22/82). SIRS based sepsis criteria identified 162 patients, mortality 27.2% (44/162) whilst qSOFA based criteria identified 56 patients, mortality 51.8% (29/56). 22/460 (4.7%) patients met every classification criterion at the same time.

**CONCLUSIONS.** Both Red-flag sepsis and SOFA based criteria identifies patients at high risk of sepsis-related deaths on the general ward. However, both failed to identify a population with similarly high risk of death, who had elevated NEWS and clinical suspicion of infection. Whilst SIRS and qSOFA based criteria were also able to identify at risk populations, their sensitivity was significantly lower. Our data suggest that the sequalae of infection related organ dysfunction cannot be reliably captured based on routine clinical and physiological parameters.

**GRANT ACKNOWLEDGMENT**
Fiona Elizabeth Agnew Trust, FEATURES Award 2016; Welsh Intensive Care Society Research Grant 2015

### 1092

#### Usefulness of hyperprocalcitonemia as a support to the decision to enter the ICU in patients with activation of the sepsis code

A. Cortes Herrera[1], A. Ruiz[1], A. Fabrega[2], J.J. Gonzalez[2], N. Larrosa[2], J.C. Ruiz Rodriguez[1], R. Ferrer Roca[1], Grupo de Investigación en Shock, Disfunción Organica y Resucitación SODIR
[1]Hospital vall d`Hebron, Intensive Care Department, Barcelona, Spain; [2]Hospital vall d`Hebron, Microbiology Department, Barcelona, Spain
**Correspondence:** A. Cortes Herrera
*Intensive Care Medicine Experimental* 2018, **6(Suppl 2):**1092

**OBJECTIVES.** To analyze if there is an association between the initial hyperprocalcitonemia and the development of poor clinical evolution, greater organic dysfunction and the need to enter the ICU in patients activated by the sepsis code (SC).

**METHODS.** Study of a cohort of patients with initial procalcitonin greater than 0.5 ng / ml activated by SC in our center during the period 2015-2017. We analyzed demographic variables, severity (APACHE II), organic dysfunction (SOFA), procalcitonin (PCT), cytokines (IL-6, TNF-α), admission to the ICU and in-hospital mortality. For the analysis of ICU admission, patients were classified into 5 groups based on PCT quintiles from a PCT cut of 5 ng / ml [PCT Q1 (0.253) -Q2 (2.52) -Q3 (11.45) - Q4 (36.64) -Q5 (235.67)]. The study was authorized by CEIC (PR (AG) 11/2016, PR (AG) 336/2016) and the patients or their representatives signed informed consent. The data have been expressed in the form of "n" (%) if they are categorical and on the average (± SD) if they are quantitative. The comparison of means was made with the Student´s T test.

**RESULTS.** During the study period, 434 patients activated with SC presented PCT levels greater than 0.5 ng / ml. Characteristics of the study population: 63.82% men, mean age 63.41 (± 16.16), SOFA 8.5 (± 3..0), APACHE II 21.3 (± 7.3), 90.2% septic shock and 9.8% sepsis. The most frequent sepsis focus was abdominal (31.6%) followed by urinary (29.3%) and respiratory (24.2%). PCT levels did not show significant differences depending on hospital mortality or admission to the ICU. PCT was correlated with SOFA (r = 0.177, p < 0.0001), with TNF-α (r = 0.580, p < 0.0001) and with IL-6 (r = 0.454, p < 0.0001). According to the quintile of PCT, the percentage of patients admitted to the ICU was: Q1 4.37%, Q2 20.96%, Q3 45.85%, Q4 75.55%, Q5 96.94%.

**CONCLUSIONS.** PCT is related to organic dysfunction and the inflammatory response. High initial levels of PCT could be useful for decision making regarding the admission to the ICU of the patient activated by SC.

### 1093

#### The frequency and clinical significance of thrombocytopenia complicating sepsis: a meta-analysis

Y. Xie, Z. Zhou, R. Tian, W. Jin, H. Xie, J. Du, R. Wang
Shanghai General Hospital, Shanghai, China
**Correspondence:** R. Wang
*Intensive Care Medicine Experimental* 2018, **6(Suppl 2):**1093

**BACKGROUND.** Thrombocytopenia is a common feature of sepsis. This study aimed to perform a meta-analysis of thrombocytopenia *vs* without thrombocytopenia for sepsis to evaluate the incidence and prognostic value of thrombocytopenia.

**METHODS.** Databases including PubMed, EMBASE, the Cochrane Library, the Science Citation Index, and important meeting abstracts were searched and evaluated by two reviewers independently. The main outcome measures included: mortality, use of mechanical ventilation, Shock, and other complications. A fixed-effects model weighted by the Mantel-Haenszel method was used for pooling the odds ratio (OR) when heterogeneity was not significant among the studies. When a *Q* test or $I^2$ statistic indicated substantial heterogeneity, a random-effects model weighted by the DerSimonian-Laird method was used.

**RESULTS.** Thirteen studies involving 6676 patients were analyzed. There was significant heterogeneity for most results; we analyzed these using a random-effects model. Meta-analysis showed thrombocytopenia caused hihger overall mortality than without thrombocytopenia (OR =3.14, 95% CI: 2.36-4.17, $P < 0.00001$). Bleeding (OR = 4.44, 95%CI: 2.04-9.69, $P = 0.0002$), Shock (OR = 2.40, 95% CI: 1.49-3.85, $P = 0.0003$)and AKI increased (OR = 2.19, 95% CI: 1.77-2.71, $P < 0.00001$) in thrombocytopenia group. Use of mechanical ventilation (OR = 1.12, 95%CI: 0.93-1.35, $P = 0.24$) and ARDS (OR = 1.42, 95% CI: 0.96-2.09, $P = 0.08$) in the thrombocytopenia and without thrombocytopenia group showed no significant different.

**CONCLUSION.** Thrombocytopenia is associated with poor prognosis, causing more complications than without thrombocytopenia.

### 1094

#### Evaluation of qSOFA and NEWS2 for sepsis detection and of the improvement of adding heparin-binding protein plasma levels to screening scores

L. Mellhammar[1], A. Linder[1], J. Tverring[1], P. Sendi[2], J. Boyd[3], B. Christensson[1], P. Åkesson[1], F. Kahn[1]
[1]University of Lund, Department of Clinical Sciences, Lund, Sweden; [2]University of Bern, Departments of Infectious Diseases, Bern, Switzerland; [3]University of British Columbia, Centre for Heart Lung Innovation, Vancouver, Canada
**Correspondence:** L. Mellhammar
*Intensive Care Medicine Experimental* 2018, **6(Suppl 2):**1094

**INTRODUCTION.** The high mortality of sepsis and need for swift treatment demand fast detection of patients with a risk of sepsis progression. Criteria for bedside detection have been derived and primarily validated towards death and prolonged intensive care [1].

**OBJECTIVES.** To evaluate established risk stratification scores for sepsis, such as quick Sequential Organ Failure Assessment (qSOFA) and National Early Warning Score 2 (NEWS2) towards severe sepsis, infection-related mortality or admission to intensive care and to develop and evaluate a candidate risk stratification score based on vital parameters and heparin-binding protein plasma levels (HBP) [2].

**METHODS.** Retrospective analysis of two prospective, observational, multicentre, convenience trials of sepsis biomarkers at emergency departments. A candidate risk stratification tool was constructed in a derivation cohort, using the least absolute shrinkage and selector operator (LASSO) method. Accuracy of evaluated risk stratification scores was evaluated in a validation cohort, compared to a composite of severe sepsis, infection-related mortality within 72h or admission to intensive care.

**RESULTS.** 506 patients were included in the derivation cohort and 435 patients, were included in the analysis (figure). qSOFA had lower AUC than in studies with in-hospital mortality as outcome (table 1) [1]. NEWS2 had significantly higher AUC than qSOFA, $p$ =0.02. Addition of HBP analysis augmented the AUC for NEWS2 and the AUC for qSOFA, although not significantly.

A new screening score (table 2) with HBP levels had the highest AUC and differed significantly from the AUC of qSOFA $p < 0.01$.

**CONCLUSIONS.** qSOFA is a parsimonious screening score for sepsis, but was inadequate in patients at emergency departments for predicting severe sepsis, infection-related mortality or admission to intensive care. NEWS2 performed better, but is more extensive. The addition of HBP levels to qSOFA seems to increase its performance without renounce of simplicity.

With a new risk stratification score that includes HBP levels, severe sepsis, infection-related mortality or admission to intensive care were successfully predicted at emergency departments. The new score was signifikant better than qSOFA.

**REFERENCE(S)**

1. Seymour, C.W., et al., *Assessment of Clinical Criteria for Sepsis: For the Third International Consensus Definitions for Sepsis and Septic Shock (Sepsis-3).* Jama, 2016. **315**(8): p. 762-74.
2. Linder, A., et al., *Heparin-Binding Protein Measurement Improves the Prediction of Severe Infection With Organ Dysfunction in the Emergency Department.* Crit Care Med, 2015. **43**(11): p. 2378-86.

**Table 1 (abstract 1094).** Performance of risk stratification scores

| | AUC (95% CI) |
|---|---|
| qSOFA | 0.60 (0.54-0.66) |
| NEWS2 | 0.69 (0.63-0.74) |
| qSOFA with 1p for HBP>30 ng/ml | 0.65 (0.60-0.71) |
| NEWS2 with 1p for HBP>30ng/ml | 0.70 (0.65-0.76) |
| New score with HBP levels | 0.73 (0.68-0.79) |

**Table 2 (abstract 1094).** New risk stratification score

| | 1 | 2 | 3 | 4 | 5 | 6 | 7 | 8 |
|---|---|---|---|---|---|---|---|---|
| Age | >45 | | | >60 | >80 | | | |
| Mental status | | Altered | | | | | | |
| Respiratory Frequency | | | | | >24 | | | |
| Heart Frequency | | >120 | | | | | | |
| Systolic Blood Pressure | | <106 | | | | <100 | | |
| Diastolic Blood Pressure | <78 | | <56 | | | | | |
| Heparin-Binding Protein | | | >26 | | | >30 | >48 | >54 |

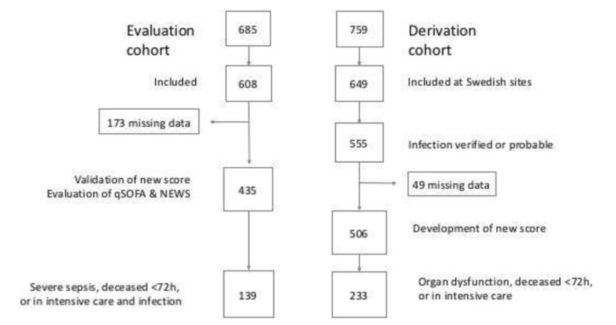

**Fig. 1 (abstract 1094).** Figure, flow chart

### 1095

#### Biomarkers of infection versus systemic inflammation in burn patients

N. Valero[1], A. García de Lorenzo[2], R. Galeiras[3], M. Ugalde[4], S. López[1], L. Oteiza[1], J.A. Lorente[1,5,6]

[1]Hospital Universitario de Getafe, UCI, Getafe, Spain; [2]Hospital La Paz, UCI, Madrid, Spain; [3]Complejo Hospitalario de A Coruña, UCI, A Coruña, Spain; [4]Hospital Universitario de Cruces, UCI, Barakaldo, Spain; [5]Universidad Europea, Getafe, Spain; [6]CIBER de Enfermedades Respiratorias (CIBERES), Getafe, Spain

**Correspondence:** N. Valero

*Intensive Care Medicine Experimental* 2018, **6(Suppl 2):**1095

**INTRODUCTION.** Burn patients develop a systemic inflammatory response and have a high risk of infections due to burn-induced immunosuppression. Both the intensity of the systemic inflammatory response and the incidence of infections are associated with an increase in mortality. Biomarkers can be beneficial tools in clinical practice for the differential diagnosis between the systemic inflammatory response after injury and infection.

**OBJECTIVES.** To assess whether serum concentration of soluble triggering receptor on myeloid cells-1 (sTREM-1), C-reactive protein (CRP), procalcitonin (PCT), platelet blood count (PLT), and the sequential organ failure assessment (SOFA) score are of value for the diagnosis of infection in burn patients.

**METHODS.** A prospective multicenter observational study of consecutive patients admitted to four Burn Intensive Care Units(April 2010-May 2014) was conducted. Inclusion criteria: age >18 years, total body surface area burned (TBSA) >20% or full thickness body surface area burned (FTBSA) > 10%, expected ICU stay >3 days. Exclusion criteria: neutropenia < 500/mcl, immunosuppressive treatment, diagnosis of AIDS. Age, gender, TBSA, FTBSA, inhalation injury, requirement of mechanical ventilation, duration of mechanical ventilation, survival and length of ICU stay were recorded. Variables for SOFA calculation) and serum concentration of sTREM-1, CRP, PCT, and PLT were recorded every 3 days. Microbiological cultures and antibiotic treatment were ordered according to clinical criteria. Patients were classified into one of the following condition: No SIRS (if it does not meet SIRS criteria), SIRS without infection (meets SIRS criteria and no cultures have been obtained or are negative) and Sepsis (meets criteria for SIRS with positive cultures). The comparison of the diagnostic performance of the different markers between Sepsis and SIRS without infection was assessed by comparing the areas under the ROC curve. Statistical analysis was performed with SPSS version 20 statistical software. The study was approved by the Ethics and Clinical Research Committee of the participating hospitals.

**RESULTS.** The total number of patients included was 115. CRP, sTREM-1, PLT and SOFA ($p < 0.001$) as well as PCT ($p = 0.018$) differed between SIRS without infection and Sepsis. The areas under the ROC curve (95% CI) for the diagnosis of Sepsis for the different biomarkers were PCR 0.73 (0.66-0.81); PCT 0.75 (0.68-0.81); sTREM-1 0.69 (0.61-0.76); PLT 0.60 (0.53-0.68); SOFA 0.72 (0.66-0.79). Although the discriminative capacity was higher for CRP, procalcitonin and SOFA, differences did not reach statistical significance (the confidence intervals overlapped each other).

**CONCLUSIONS.** All markers studied were different in patients with Sepsis as compared to patients with SIRS without infection. However, their discriminative capacity was not significantly different from each other.

**GRANT ACKNOWLEDGMENT**

FIS PS09/02571, Instituto de Salud Carlos III, Madrid, Spain

### 1096

#### Evolution in the identification and early treatment of critically ill septic patients at the emergency department of a Spanish tertiary hospital

L. Anguela-Calvet[1], V.D. Gumucio-Sanguino[1], X.L. Pérez-Fernández[1], J. Sabater-Riera[1], H. Alánez-Saavedra[1], P. Sastre-Pérez[1], E.P. Plata-Menchaca[2], R. Giménez-Fernández[3], F. Llopis-Roca[4], C. Marroquín-Guerrero[4]

[1]Hospital Universitari de Bellvitge, Critical Care Medicine, L'Hospitalet de Llobregat, Spain; [2]Institut d´Investigació Biomèdica de Bellvitge, Research, L'Hospitalet de Llobregat, Spain; [3]Universitat de Barcelona, Medicine, L'Hospitalet de Llobregat, Spain; [4]Hospital Universitari de Bellvitge, Emergency, L'Hospitalet de Llobregat, Spain

**Correspondence:** L. Anguela-Calvet

*Intensive Care Medicine Experimental* 2018, **6(Suppl 2):**1096

**INTRODUCTION.** In 2005 the EDUSEPSIS Campaign was carried out in Spanish hospitals and in 2015 a protocol was implemented for the detection and early treatment of sepsis in the emergency department (ED). There is controversy about whether early treatment improves outcomes.

**OBJECTIVES.** To compare the evolution of critically ill patients with sepsis (all of them finally requiring admission to an intensive care unit (ICU)) in two different periods at the same ED; a first period during the surviving sepsis campaign implementation (2005-2007) and a second period (2015-2017) with a specific early identification and management protocol.

**METHODS.** Retrospective observational study that evaluates at two different historical periods in septic patients requiring ICU admission the following; initial 3 hour bundle (lactate, cultures, and antibiotics) completion, correct fluid administration (30 mL/kg) within the first 6 hours in those patients who presented hypotension (SBP< 90 mmHg or MBP< 65 mmHg), and final outcome.

**RESULTS.** We collected 83 and 85 patients who presented sepsis at the ED and finally required ICU admission during 2005-2007 and 2015-2017 respectively. Differences between both groups (2005-2007 and 2015-2017) were: median age was 67 (53,76) and 65 (55,74), median SOFA score was 9 (5,11) and 10 (8,12), median time from the onset of sepsis to ICU admission was 3600 (1100, 12000) minutes (min) and 504 (245, 1500) min respectively (p=0.0001). Lactate determination and cultures (before antibiotics) within the first 3 hours from ED admission were performed in 10.7% and 69% of patients respectively in 2005-2007 period, compared to 71.7% and 53.7% of patients in 2015-2017 period (p=0.0001, p=0.08). Early antibiotic within the first hour of ED admission was administered in 47.1% patients (2005-2007) and 32.5% (2015-2017) (p=0.28). The complete bundle (3 measures) was completed in 6% (2005-2007) and 83.5% (2015-2017) (p=0.0001) of patients, with a mean time of 275±55 min vs 1050±4000 min. Correct fluid resuscitation with crystalloids (30mL/kg within the first 6 hours) in patients with hypotension (75% in both groups) was carried out in 51.7% of patients (2005-2007) and in 67.9% (2015-2017) (p=0.09). The requirement of vasoactive drugs was 66.3% (2005-2007) and 58.8% (2015-2017). Mortality during hospital admission, 28 days after admission, and 90 days after admission was 45.8%, 42.2%, 65.1% respectively (2005-2007), compared to 29.4%, 23.5%, and 31.8% (2015-2017) (p=0.04, p=0.01, and p=0.001). Overall mortality was 69.9% (2005-2007) compared to 32.9% (2015-2017) (p< 0.0001).

**CONCLUSIONS.** The implementation of a protocol in the emergency department for the detection and early treatment of sepsis may reduce mortality in those patients who finally require ICU admission.

### 1097

#### Sepsis-3 diagnostic criteria as a tool to predict hospital mortality in critically-ill patients: a validation study in developing country

P. Wacharasint[1], Y. Wittayawisawasakul[1], K. Aramsaowapak[2]
[1]Phramongkutklao Hospital, Department of Medicine, Bangkok, Thailand; [2]Fort Suranaree Hospital, Department of Medicine, Nakhon Ratchasima, Thailand
**Correspondence:** P. Wacharasint
*Intensive Care Medicine Experimental* 2018, **6(Suppl 2):**1097

**INTRODUCTION.** The European and American Society of Intensive Care Medicine recently redefined new concept of sepsis, or so-called “sepsis-3”. New diagnostic criteria, quick sepsis-related organ failure assessment (qSOFA), was purposed to achieve better prediction of hospital mortality than systemic inflammatory response syndrome (SIRS) criteria in critically ill patients.[1] However, these criteria have not been validated in developing country where critical care resources are limited and the burden of mortality may be differed from developed countries.[2]

**OBJECTIVE.** To validate whether qSOFA better predict in-hospital mortality than previous criteria [i.e. SIRS, sepsis-related organ failure assessment (SOFA), and Acute physiology and Chronic Health Evaluation (APACHE) II scores] among critically-ill patients with sepsis and was admitted to medical intensive care unit (ICU) in Thailand.

**METHODS.** We performed a prospective observational study in patients with suspected infection with at least one organ dysfunction, and was admitted to the ICU of Phramongkutklao hospital (University-based hospital) or Fort Suranaree hospital (non-University-based hospital) during July 2017 to December 2017. The primary outcome was difference in area under the ROC curves (AUROC) which predict hospital mortality compared among SIRS, SOFA, qSOFA, and APACHE II scores.

**RESULTS.** A total of 101 patients were included in this analysis. Average age of the patients was 71.3±14.5 years old, most common underlying disease was hypertension (66.3%), and most common source of infection was respiratory tract (47.5%). Average hospital LOS, and ICU LOS were 23 and 10 days, respectively. There were forty-one non-survivors (40.6%). In terms of prediction of hospital mortality, we found that SIRS (≥2 points), SOFA (≥8 points), qSOFA (≥2 points), and APACHE II (≥22 points) score had 78%, 88%, 61%, and 78% for sensitivity; 35%, 55%, 77%, 68% for specificity, and 0.59, 0.74, 0.68, 0.78 for AUROC, respectively (differences in AUROC between-group: SIRS vs. SOFA, p=0.016; SIRS vs. APACHE II, p< 0.001; and qSOFA vs. APACHE II, p=0.045). No difference of AUROC between SOFA and APACHE II (p=0.49).

**CONCLUSIONS.** Patients with sepsis and was admitted to medical ICU in Thailand, an increase in SOFA score (≥8 points) or APACHE II (≥22 points) had greater prognostic accuracy to predict hospital mortality than SIRS and qSOFA criteria. Our findings were aligned to previous study in developed countries which suggested that qSOFA criteria have limited power to predict hospital mortality in the ICU setting.[1]

**REFERENCE(S)**

1. Singer M. Deutschman CS, Seymour CW, et al. The Third International Consensus Definitions for Sepsis and Septic Shock (Sepsis-3). JAMA 2016;315:801-10.
2. Adhikari NK, Fowler RA, Bhagwanjee S, et al. Critical care and the global burden of critical illness in adults. Lancet 2010;376:1339-46.

### 1098

#### Does peripheral circulating blood flow correlate with the quick SOFA score in emergency room patients?

T. Kazunori[1], T. Satoko[1], T. Masayuki[1], S. Kento[1], O. Yuu[2], K. Tadahiro[1], N. Masaki[1]
[1]Yamagata University Faculty of Medicine, Department of Emergency and Critical Care Medicine, Yamagata, Japan; [2]Yamagata University Faculty of Medicine, Department of Anesthesiology, Yamagata, Japan
**Correspondence:** T. Kazunori
*Intensive Care Medicine Experimental* 2018, **6(Suppl 2):**1098

**INTRODUCTION.** The quick Sequential Organ Failure Assessment (qSOFA) score was created as a screening tool for life-threatening organ dysfunction due to a dysregulated host response to infection outside the ICU. One cause of organ dysfunction in critically ill patients is thought to be peripheral circulatory failure. Laser Doppler flowmetry (LDF) is an easy way to assess the peripheral circulation and may have potential as a screening tool in patients with organ dysfunction; however, the relationship between the qSOFA score and circulatory failure has not been studied. We hypothesized that quantitative evaluation of the peripheral circulation correlates with the qSOFA score.

**OBJECTIVES.** To compare peripheral circulating blood flow and the qSOFA score in emergency room (ER) patients.

**METHODS.** The study included 42 ER patients. Peripheral circulating blood flow was measured for 3 min using LDF (Pocket LDF; JMS, Tokyo, Japan) and the average value over the last minute was used in the final analysis. The qSOFA score was determined using the Glasgow Coma Scale, respiration rate, and systolic blood pressure.

**RESULTS.** The median (range) blood flow measured by LDF was 36.4 ml/min (3.9-68.5), and the median qSOFA score was 1 (0-3). There was no correlation between LDF and qSOFA score ($R^2$= 0.06).We evaluated only those patients with a positive qSOFA score ≧ 2 (n=10), but there was no correlation between LDF and the qSOFA score ($R^2$= 0.05).

**DISCUSSION.** Decreased peripheral circulating blood flow is one cause of organ damage in septic patients, and the qSOFA score is used to predict mortality in septic patients; however, we did not demonstrate a correlation between peripheral circulating blood flow, as measured by LDF, and the qSOFA score in ER patients. One of the reasons for the lack of correlation is that the peripheral circulation may not have a linear regression to the severity of organ damage, such as peripheral circulation in warm and cold shock. Another reason for the lack of correlation is that the study subjects included patients without sepsis, and the qSOFA may not have represented the severity of organ damage in such patients. To verify the peripheral circulation as a screening tool for organ dysfunction, a further study comparing the peripheral circulation measured in the ER and the development of organ dysfunction is warranted.
**CONCLUSIONS.** Peripheral circulating blood flow does not correlate with the qSOFA score in ER patients.

## Patient ventilator interaction

### 1099

#### Prevalence of reverse triggering early after intubation

R. Mellado Artigas[1], F. Damiani[2], T. Piraino[2], M. Rauseo[2], I. Soliman[2], D. Junhasavasdikul[2], L. Melo[2], L. Chen[2], C. Sinderby[3], N. Comtois[3], L. Heunks[4], J. Gu[3], C. Santís[2], P. Greco[5], H. Every[5], G. Sandhu[5], L. Brochard[2,3]
[1]Hospital Clinic, Barcelona, Spain; [2]Interdepartmental Division of Critical Care Medicine, University of Toronto, Toronto, Canada; [3]Keenan Centre for Biomedical Research, Li Ka Shing Knowledge Institute, St. Michael's Hospital, Toronto, Canada; [4]VU Medical Center, Amsterdam, Netherlands; [5]St Michael's Hospital, Toronto, Canada
**Correspondence:** R. Mellado Artigas
*Intensive Care Medicine Experimental* 2018, **6(Suppl 2):**1099

**INTRODUCTION.** Reverse triggering (RT) is a recently described type of patient-ventilator interaction where respiratory muscle contractions seem to be triggered by the ventilator. The aim of this study was to calculate the prevalence of RT early after initiation of mechanical ventilation using an automated detection.
**OBJECTIVES.** To determine the prevalence of RT early after intubation and describe parameters that are associated with it.
**METHODS.** This is a substudy of an observational trial (NCT02434016) to detect resumption of diaphragm activity after intubation with continuous electrical activity (Eadi) recording. A total of 75 patients were enrolled. A daily one hour recording of Eadi and ventilator waveforms was available for each patient.
1- Automatic detection algorithm: 3 reviewers assessed 2010 breaths in assist/control mode to derive an automatic method to detect RT with the use of Neurosync (Toronto, Canada). These breaths came from 10 patients in assist-control mode. Then the model was tested in 10 patients ventilated in pressure support and the algorithm was refined to reduce the rate of false positive and the results of the final algorithm in these 20 patients are presented. For the derivation of the algorithm, RT was considered only when agreement among all three reviewers was found. Inter-rater agreement by means of Fleiss kappa index was calculated.
2- After a suitable algorithm was identified, we analyzed a 1-hour strip (average breaths per patient 1787, SD 563) recorded for the 37 patients in assist/control mode after a median of 24 hours (IQR 22-26) after intubation.
For analysis of demographic data and ventilation parameters we split the cohort in two groups; one above the median RT and the other below or equal to the median RT.
**RESULTS.** Inter-rater agreement was 0.84. For Neurosync, the results for RT detection were: sensitivity 0.84, specificity 0.96, positive predictive value 0.75 and negative predictive value 0.98. The rate of patient-triggered breaths being misclassified as RT was 2%.2- The 37 patients were 60 ±13 years old, had a median APACHE of 25 (IQR 19-30) and 65% had a primary pulmonary reason for intubation. Median RT was 9% (IQR 5-32). For comparison between groups, see tables.

**CONCLUSIONS.** RT detected on Eadi is common after intubation. No differences in demographic data, sedation score, reason for intubation or ventilation parameters were apparent between groups. However, patients with more RT were more likely to be on an assisted mode or extubated the day after.

**REFERENCE(S)**

Akoumianaki, E., Lyazidi, A., Rey, N. et al. (2013). Mechanical ventilation-induced reverse-triggered breaths: A frequently unrecognized form of neuromechanical coupling. Chest, 143(4), 927-938.
Sinderby, C., Liu, S., Colombo, D.,(2013). An automated and standardized neural index to quantify patient-ventilator interaction. *Critical Care (London, England)*, *17*(5), R239.

**GRANT ACKNOWLEDGMENT**
No external funding was received for this study.

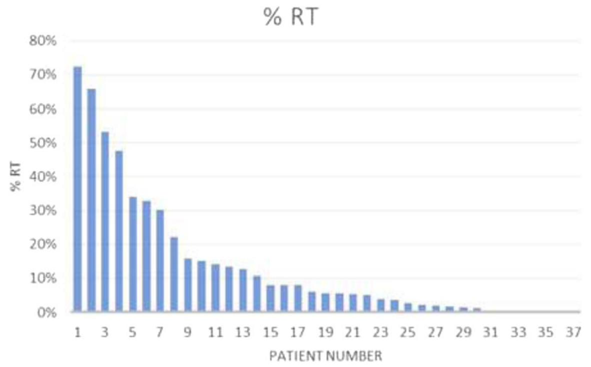

**Fig. 1 (abstract 1099).** Prevalence of RT (as % of total number of breaths) in each patient

**Table 1 (abstract 1099).** Characteristics of groups divided by prevalence of RT. * p<0.0001

| Characteristics | RT > median | RT <= median |
|---|---|---|
| Median RT% | 36 (15-59) | 5 (1-8)* |
| Age | 58 (+/-16) | 61 (+/-17) |
| Gender | 58-42% | 61-39% |
| APACHE II | 21 (18-29) | 26 (21-30) |
| Reason for intubation | Lung 61% | Lung 68% |

**Table 2 (abstract 1099).** Characteristics of groups divided by prevalence of RT. * p=0.007

| Characteristics | RT > median | RT <= median |
|---|---|---|
| SAS | 2 (1-3) | 2 (1-3) |
| RR | 21 (19-28) | 28 (22-30) |
| TV PBW | 6.7 (6.1-7.9) | 6.3 (5.8-7.2) |
| PEEP | 8 (5-10) | 10 (8-12) |
| Assisted mode or extubated 24h after | 14 (78%) | 7 (37%)* |

### 1100

#### Effect of sedatives and opioids on patient-ventilator asynchronies in critically ill patients

C. De Haro[1,2,3], R. Magrans[2,3,4], J. López-Aguilar[2,3,4], J. Montanya[5], E. Lena[6], C. Subirà[7], S. Fernández-Gonzalo[2,4,8], M. Turon[2,4], G. Gomà[2,4], R. Fernández[3,7], G.M. Albaiceta[9,10], U. Lucangelo[6], G. Murias[11], M. Rue[12,13], R.M. Kacmarek[14,15], L. Blanch[2,3,4], ASYNICU Group
[1]Parc Taulí University Hospital, Critical Care Area, Sabadell, Spain; [2]Universitat Autònoma de Barcelona, Sabadell, Spain; [3]CIBER de Enfermedades Respiratorias (CIBERES), Madrid, Spain; [4]Institut d'Investigació i Innovació Parc Taulí I3PT, Sabadell, Spain; [5]Better Care, SL, Sabadell, Spain; [6]Cattinara Hospital, Department of Perioperative Medicine, Intensive Care and Emergency, Trieste, Italy; [7]Fundació Althaia, Manresa, Spain; [8]CIBERSAM, Madrid, Spain; [9]Hospital Universitario Central de Asturias, Unidad de Cuidados Intensivos Cardiológicos, Oviedo, Spain; [10]Instituto Universitario de Oncología del Principado de Asturias, Departamento de Biología Funcional, Oviedo, Spain; [11]Clinica Bazterrica, Buenos Aires, Argentina; [12]Universitat de Lleida-IRBLleida, Department of Basic Medical Sciences, Lleida, Spain; [13]Health Services Research Network in Chronic Diseases (REDISSEC), Madrid, Spain; [14]Massachusetts General Hospital, Department of Respiratory Care, Department of Anesthesiology, Boston, United States; [15]Harvard Medical School, Boston, United States
**Correspondence:** C. De Haro
*Intensive Care Medicine Experimental* 2018, **6(Suppl 2):**1100

**INTRODUCTION.** Asynchronies are frequent during invasive mechanical ventilation (MV). Clinical management, as adjusting mechanical ventilator settings, affects patient-ventilator asynchronies. Adequate analgesia with slight sedation might enable more spontaneous breathing activity and might result in clinical advantages, such as improved patient-ventilator interaction. The effect of sedation, analgesia and level of consciousness is poorly understood.

**OBJECTIVES.** To analyze the association between patient-ventilator asynchronies, the treatment with sedatives and opioids, alone or combined, and its doses,the level of consciousness and the clinical severity.

**METHODS.** Multicenter prospective observational trial (4 ICUs) in patients undergoing invasive MV ≥ 24h. Do-not-resuscitate orders, admitted for organ donation, pregnancy and bronchopleural fistula were exclusion criteria. Ventilator waveforms (flow, volume and pressure) were continuously recorded, stored and analyzed using dedicated software (BetterCare®, Spain). The software identified ineffective inspiratory efforts during expiration (IEE), double cycling (DC) and calculated the asynchrony index (AI). We registered the level of consciousness, severity, doses of sedation (midazolam, propofol, lorazepam), and opioids (morphine and fentanyl) which were reported as equipotent of morphine and midazolam. Linear mixed-effects models were used to analyze the relationships between variables.

**RESULTS.** We recorded 579 days of ventilatory support (14,166,469 breaths) in 79 patients. The overall AI, IEE and DC were similar in the sedation-only, opioids-only and sedation+opioids group. The no drugs group presented higher AI, IEE and DC than others. The mean level of consciousness (per day) in patients treated with opioids alone [3.13 (95%CI:2.84, 3.42)] was higher than in patients treated with sedation + opioids [2.44 (95%CI:2.22, 2.65); $p < 0.001$]. The dose of opioids was inversely associated with the AI, IEE, and DC ($p < 0.01$), but not with the level of consciousness.

**CONCLUSIONS.** Higher doses of opioids reduced asynchronies without lowering the level of consciousness. The combined use of sedatives plus opioids does not result in better patient-ventilator interaction than opioids alone. Adequate pain control can help improve patient-ventilator interaction and avoid the deleterious effects of sedation.

**GRANT ACKNOWLEDGMENT**

PI09/91074 and PI13/02204, Plan Nacional de R+D+I and co-funded by the ISCIII-and FEDER. CIBERES, Fundación Mapfre, Fundació Parc Taulí, Plan Avanza TSI-020302-2008-38 (Spain).

### 1101

#### Physiological response to different levels of GAIN and PEEP in lung transplant recipients undergoing neurally adjusted ventilatory assist in early post-operative period

L. Castagna[1], A. Guzzardella[2], A. Zanella[1,3], C. Abbruzzese[1], N. Bottino[1], A. Matera[4], S. Scansani[1], N. Corcione[1], A. Pesenti[1,3], G. Grasselli[1,3]
[1]Fondazione IRCCS Ca' Granda Ospedale Maggiore Policlinico, Department of Anesthesia, Critical Care and Emergency, Milan, Italy; [2]University of Milan, Milan, Italy; [3]University of Milan, Department of Pathophysiology and Transplantation, Milan, Italy; [4]University of Foggia, Foggia, Italy
**Correspondence:** L. Castagna
*Intensive Care Medicine Experimental* 2018, **6(Suppl 2):**1101

**INTRODUCTION.** During Neurally Adjusted Ventilatory Assist (NAVA), as NAVA support level (GAIN - $cmH_2O$/μVolt) increases, the electrical activity of the diaphragm (EAdi - μVolt) decreases, resulting in a constant tidal volume (Vt). In transplanted lungs (LTx), the neuro-mechanical coupling could be compromised beacuse of the absence of vagal innervation. Nevertheless, a protective ventilation is mandatory to reduce ventilator induced lung injury and the risk of primary graft dysfunction.

**OBJECTIVES.** To assess the EAdi variation to varying of NAVA GAIN and positive end expiratory pressure (PEEP) levels in the early post-operative period of LTx.

**METHODS AND RESULTS.** Four intubated (two males, median age 58 years old, IQR 49-60) and spontaneously breathing patients were admitted to Intensive Care Unit of ospedale Maggiore Policlinico di Milano (Italy), for post-operative monitoring of LTx. Sedation was titrated to obtain a Richmond Agitation Sedation Scale between 0 and -2. Through “NAVA preview” function, the “Baseline GAIN” level was chosen to obtain a Vt 6 ±2 mL/Kg/predicted body weight of the donor. The patients underwent to a random sequence of six steps, randomly mixing three NAVA gain levels (Baseline gain, 50% Baseline gain, 150% Baseline gain) and two PEEP levels (6 and 12 $cmH_2O$).

**CONCLUSIONS.** Preliminary data about the application of NAVA during the early post-operative period after LTx, showed that increasing NAVA support reduced the EAdi Peak. Nevertheless, the neuro-mechanical coupling might be partially impaired since the resulting mean Vt was not constant and tended to increase with the level of GAIN. Finally, double PEEP level was associated with slight increase in EAdi Peak and consequently in the mean Vt

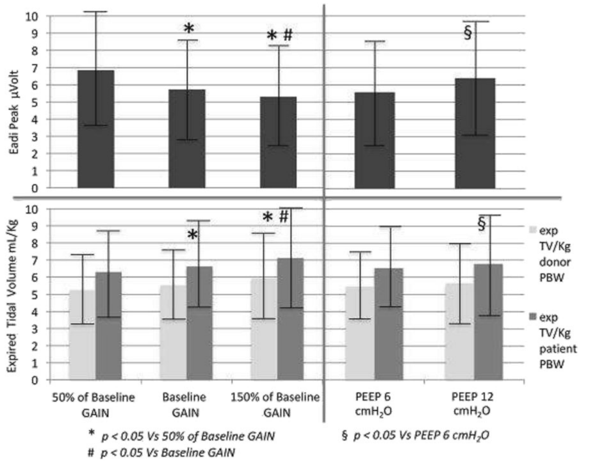

**Fig. 1 (abstract 1101).** Correlation between EAdi Peak and tidal volume vs NAVA gain and PEEP levels

| | GAIN levels | | | PEEP levels | |
|---|---|---|---|---|---|
| | 50% of Baseline | Baseline | 150% of Baseline | 6 $cmH_2O$ | 12 $cmH_2O$ |
| GAIN Level ($cmH_2O$/µVolt) | 0.6 ± 0.3 | 1.1 ± 0.6 * | 1.7 ± 0.9 * # | 1.1 ± 0.8 | 1.1 ± 0.8 |
| Respiratory rate (breaths/min) | 21 ± 7 | 18 ± 6 * | 19 ± 6 * | 19 ± 6 | 20 ± 7 § |
| Expired minute volume (L/min) | 7.7 ± 2.8 | 7.0 ± 1.9 * | 7.8 ± 3.0 # | 7.3 ± 2.7 | 7.7 ± 2.6 § |
| Arterial pH | 7.36 ± 0.03 | 7.37 ± 0.03 | 7.37 ± 0.04 | 7.37 ± 0.03 | 7.37 ± 0.04 |
| Arterial $pCO_2$ (mmHg) | 42 ± 7 | 42 ± 8 | 42 ± 7 | 42 ± 7 | 42 ± 7 |
| Arterial $pO_2$ (mmHg) | 96 ± 15 | 101 ± 11 | 111 ± 26 | 96 ± 16 | 110 ± 19 § |

* $p < 0.05$ Vs 50% of Baseline GAIN, # $p < 0.05$ Vs Baseline GAIN, § $p < 0.05$ Vs PEEP 6 $cmH_2O$

**Fig. 2 (abstract 1101).** Ventilatory parameters and arterial blood gas values

### 1102

#### The effect of phrenic nerve stimulation on heart rate

M. Ornowska, T. Bassi, E. Röhrs, K. Fernandez, S. Reynolds
Simon Fraser University, Burnaby, Canada
**Correspondence:** M. Ornowska
*Intensive Care Medicine Experimental* 2018, **6(Suppl 2):**1102

**INTRODUCTION.** During normal breathing, contraction of the respiratory muscles and diaphragm serve to create negative pressure inside the chest facilitating the passive flow of air into the lungs. In contrast, during mechanical ventilation (MV), the ventilator pushes a pre-determined pressure or volume of air into the lungs using positive pressure. As such, patients who undergo MV for prolonged periods of time are at risk for the development of ventilator-induced lung injury (VILI), as well as ventilator-induced diaphragmatic dysfunction (VIDD) (Slutsky et al., 2013).

A novel transvenous electrical pacing catheter that recruits the phrenic nerve bilaterally aims to mitigate the development of VIDD and VILI. By stimulating diaphragmatic contractions in synchrony with breaths delivered by MV, iatrogenic positive pressure may be reduced. (Reynolds et al., 2017).

**OBJECTIVES.** Due to the proximity of the pacing catheter to the vagus nerve, inadvertent stimulation is a likely side-effect of phrenic nerve pacing. As the sinoatrial node is primary vagus nerve target, study objectives include determining any changes in mean heart rate resulting from phrenic nerve pacing therapy.

**METHODS.** This is a pre-clinical, randomized, semi-blinded study in which a pig model is mechanically ventilated for 50 hours in a mock ICU environment. Control subjects undergo MV with no pacing therapy, while experimental subjects undergoing MV and adjunct pacing therapy receive regular bursts of current to both phrenic nerves on every second breath. Subject heart rate was recorded from an arterial line pressure transducer at 15-minute intervals throughout the duration of the study.

**RESULTS.** Results from within-subjects t-test reveal no significant difference in heart rate during paced breaths versus unpaced breaths in experimental subjects (p= 0.76). Conversely, between-subject t-test results indicate significant increase in heart rate in unpaced beats of experimental subjects compared to control subjects (p< 0.01), as well as between paced heart rate in experimental subjects versus heart rate of control subjects (p< 0.01).

**CONCLUSIONS.** Within-subject results illustrate the absence of significant changes in heart rate resulting from phrenic nerve pacing. As such, results of this study provide evidence supporting safe and efficacious use of this therapy in patients where hemodynamic instability is a concern. Between-subject results do not provide evidence for inadvertent vagus stimulation caused by phrenic nerve pacing causing a global change on heart rate.

##### REFERENCE(S)

1. Slutsky AS, Ranieri VM. Ventilator- induced lung injury. *N Engl J Med* 2013; 369:2126-2136
2. Reynolds SC, Meyyappan R, Thakkar V, Tran BD, Nolette M, Sadarangani G, Sandoval RA, Bruuisema L, Hannigan B, Li JW, Rohrs E, Zurba J, Hoffer HA. Mitigation of ventilator-induced diaphragm atrophy by transvenous phrenic nerve stimulation. *Am J Respir Crit Care Med* 2017; 195(3): 339-348

##### GRANT ACKNOWLEDGMENT

no grants.

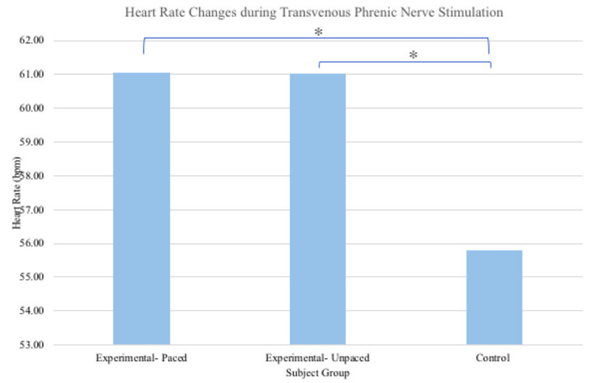

**Fig. 1 (abstract 1102).** Heart Rate Changes during Transvenous Phrenic Nerve Stimulation

### 1103

#### Validity of ventilatory efficiency in patients recovering from critical illness

B.-J. van der Sleen[1,2], H.L.A. van Den Oever[1], T. Greven[3], E. Ruessink[1], E. Klooster[4], S. Zoethout[4], B. Langeveld[5]
[1]Deventer Hospital, ICU, Deventer, Netherlands; [2]University of Groningen, Faculty of Medical Sciences, Groningen, Netherlands; [3]Radboud UMC Nijmegen, Anesthesiology, Nijmegen, Netherlands; [4]Deventer Hospital, Physiotherapy, Deventer, Netherlands; [5]Deventer Hospital, Pulmonology, Deventer, Netherlands
**Correspondence:** B.-J. van der Sleen
*Intensive Care Medicine Experimental* 2018, **6(Suppl 2):**1103

**INTRODUCTION.** Dead space ventilation is associated with ventilatory efficiency. Increased dead space fraction ($V_D/V_T$) is a negative prognostic factor in subgroups of ICU patients.

In chronic cardiac and pulmonary disease, however, another parameter of ventilatory efficiency, $VE/VCO_2$, has been recognized as a negative prognostic sign. Two alternatives are in use: $VE/VCO_2$-slope and the lowest $VE/VCO_2$-ratio, both measured during exercise.

In many ICUs, active mobilization of ventilated patients is commenced early. Using metabolic monitoring, parameters of ventilatory efficiency during exercise are readily obtained.

**OBJECTIVES.** To compare $VE/VCO_2$-slope and lowest $VE/VCO_2$-ratio, both during exercise, and $V_D/V_T$ in rest, in their ability to separate critically ill patients from healthy volunteers.

**METHODS.** Intubated patients recovering from critical illness were submitted to an incremental resistance protocol on a bedside cycle ergometer (MOTOmed Letto2) as soon as they regained the ability to actively exercise (1).

After a period of rest, active cycling was started at resistance level one, which was increased by one level every minute until a stop criterion was reached.

Before, during and after exercise, heart rate, respiratory rate, tidal volume, $VO_2$ and $VCO_2$ were noninvasively monitored using indirect calorimetry (Cosmed Quark RMR), placed in the ventilatory circuit. Parameters of ventilatory efficiency were calculated offline.

Fractional dead space was calculated from $VE/VCO_2$ and $P_aCO_2$ by the equation:

$\mathbf{VE/VCO_2} = 115/(\mathbf{P_aCO_2} \times (1 - \mathbf{V_D/V_T}))$.

Healthy volunteers aged > 50 years were subjected to the same protocol, using a face mask. A $V_{D,app}$ of 125 ml was used to correct $V_D$.

**RESULTS.** The protocol was completed by 14 patients and 11 volunteers. All patients were on a spontaneous ventilation mode. No adverse events occurred.

During exercise, the average $VCO_2$ in patients increased from 250.9 ml/min to 330.4 ml/min (p = 0.000).

$VE/VCO_2$-slope was 37.7 (± 13.3) for patients and 26.5 (± 4.2) for volunteers (Fig A).

Lowest $VE/VCO_2$-ratio was 44.1 (± 8.4) for patients and 30.3 (± 2.7) for volunteers (Fig B).

Resting $P_aCO_2$ was 5.6 (± 0.89) kPa for patients and 5.1 (± 0.28) kPa for volunteers (Fig C).

$V_D/V_T$-ratio was 0.53 (± 0.12) for patients and 0.20 (± 0.04) for volunteers (Fig D).

**CONCLUSIONS.** Fractional dead space performed best in separating spontaneously ventilated ICU patients from healthy volunteers.

$P_aCO_2$ in the patient cohort showed large variation, which may have an important influence on $VE/VCO_2$.

In patients recovering from critical illness, $VE/VCO_2$-slope and $VE/VCO_2$-ratio should be considered invalid parameters to represent ventilatory efficiency.

(1) Sommers et al. Physiological response to exercise testing in critically ill patients: a prospective, observational multicenter study. (submitted)

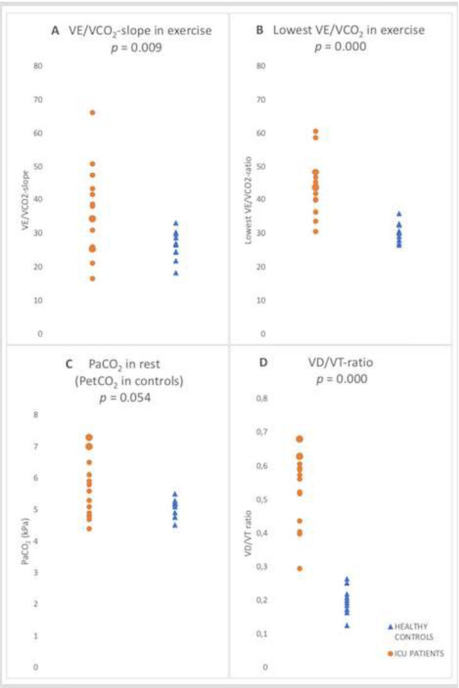

**Fig. 1 (abstract 1103).** Respiratory parameters in patients (circles) and healthy controls (triangles)

### 1104

#### Automated patient-ventilator asynchrony monitoring in the ICU, feasibility and efficiency (CURVEX)

Y. Jan[1,2], B. Hourmant[1], V. Pateau[1], F. Lellouche[3], Q.-T. N'Guyen[4], E. L'Her[1]
[1]CHU Brest, Réanimation Médicale, Brest, France; [2]CHU Brest, Département d'Anesthésie Réanimation Chirurgicale, Brest, France; [3]Institut Universitaire de Cardiologie et de Pneumologie de Québec, Québec, Canada; [4]Oxynov, Brest, France
**Correspondence:** Y. Jan
*Intensive Care Medicine Experimental* 2018, **6(Suppl 2):**1104

**INTRODUCTION.** Evaluation of patient/ventilator synchronization is currently performed by direct visual analysis of ventilator´s curves at the patient´s bed side or by invasive methods like esophageal pressure. Curvex is a non-invasive tool based on these studies which allow to quantify and qualify asynchronies [1].

**OBJECTIVES.** The main purpose of this study was to assess where automated evaluation of patient/ventilator asynchronies was feasible and efficient.

**METHODS.** This project is based on a prospective physiological tracing data-warehousing program (Rea STOC, clinicaltrials.gov

##NCT02893462) that aims to record 1500 consecutive ICU patients, over a 3-years period. This study was approved by the local ethical committee of Brest university hospital - France.

All patients in ICU with mechanical ventilation (invasive or NIV) could be included. We realized a first analyze during 1 or 2 hours without changing ventilator´s settings. Then we looked curvex´s results: asynchrony index, number and type of asynchrony, correlation between curvex´s interpretation and direct analysis of ventilator´s curves at patient´s bed side. If there were asynchronies we modified ventilator´s settings in function of asynchrony type with a standardized protocol and we recorded another session, else we not performed another session. The main criteria was the diminution of the asynchrony index (AI) between the two sessions.

**RESULTS.** Between February 2017 and April 2018, 90 patients were included (age 61.6 ± 12.9 yr; 67 males / 23 females; SAPS II 53.2 ± 19.2). 84/90 patients had invasive mechanical ventilation. Main admission diagnosis were acute respiratory failure (49%), neurological failure (21%), shock (13%), cardiac arrest (12%), and ARDS (4%). AI was high in most patients with only 12.2% with AI< 10%. Correlation between visual and automatic detection was 82.2%. Main asynchrony type was ineffective effort (44 ± 3%), premature cycling (25 ± 3%), short cycle (14 ± 2%), double triggering (9 ± 1%), and prolonged inspiration (5 ± 1).

No correlation was observed between either the AI or the overall asynchrony number and mean ICU, hospital stay, or outcome. Correction measures were performed for 65 patients, according to a standardized protocol. However, no significant differences were observed after ventilatory settings modifications (p=0.76), whatever the asynchrony type.

**CONCLUSIONS.** A high level of asynchronies was observed over our ICU population. Automated evaluation of asynchronies was well correlated with direct visual observation of ventilator curves. However, we were not able to reduce asynchronies between the first and second analysis.

**REFERENCE(S)**

[1] Q. T. Nguyen, D. Pastor, F. Lellouche, E. L´Her, "Mechanical ventilation system monitoring: Automatic detection of dynamic hyperinflation and asynchrony", *Engineering in Medicine and Biology Society (EMBC) 2013 35th Annual International Conference of the IEEE*, pp. 5207-5210, Jul. 2013

**GRANT ACKNOWLEDGMENT**

no.

### 1105

#### Reliability analysis of the Patient-Ventilator Breath Contribution (PVBC) index in mechanically ventilated patients

A.H. Jonkman[1], D. Jansen[1,2], C.A. Sinderby[3], J.G. van der Hoeven[4], L.M.A. Heunks[1]

[1]VU University Medical Center, Intensive Care, Amsterdam, Netherlands; [2]Radboud University Medical Centre, Anesthesiology, Nijmegen, Netherlands; [3]St. Michael's Hospital, University of Toronto, Critical Care Medicine, Toronto, Canada; [4]Radboud University Medical Centre, Intensive Care, Nijmegen, Netherlands

**Correspondence:** A.H. Jonkman

*Intensive Care Medicine Experimental* 2018, **6(Suppl 2):**1105

**INTRODUCTION.** Experimental data show that the patient-ventilator breath contribution (PVBC) index can be used to estimate the relative contribution of the patient's inspiratory muscles versus that of the ventilator, during partially supported modes of ventilation.[1,2] PVBC can be estimated using the ratio of inspiratory tidal volume ($Vt_{insp}$) to peak electrical activity of the diaphragm ($Edi_{peak}$). If reliable in clinical practice, PVBC could help to titrate ventilator support such that the risks of both excessive support and high respiratory muscle effort can be reduced.

**OBJECTIVES.** To evaluate test-retest agreement of the PVBC index in clinical practice.

**METHODS.** This observational study was conducted in a heterogeneous group of ICU patients (n=31) ventilated in neurally adjusted ventilatory assist (NAVA) mode. PVBC repeatability coefficient (RC) was calculated using five repeated intra-subject PVBC measurements with a one-minute interval.

PVBC was measured as $(Vt_{insp}/Edi_{peak})_{no\text{-}assist}/(Vt_{insp}/Edi_{peak})_{assist}$. Unassisted breaths were obtained by reducing the NAVA level to zero. A PVBC index of 1.0 thus suggests that $Vt_{insp}$ is fully generated by the patient.

$(Vt_{insp}/Edi_{peak})_{assist}$ was calculated by using either $Vt_{insp}$ and $Edi_{peak}$ of one breath just prior to the unassisted breath ($^{N1}$PVBC), or mean $Vt_{insp}$ and $Edi_{peak}$ of five breaths prior to the unassisted breath ($^{X5}$PVBC). Thirdly, PVBC was computed as $Vt_{insp,no\text{-}assist}$/mean$(Vt_{insp})_{assist}$ by selecting those assisted breaths that matched respiratory drive of the unassisted breath, based on Edi-derived criteria (PVBCβ). At last, PVBC indices were squared ($^{N1}PVBC^2$, $^{X5}PVBC^2$ and $PVBCβ^2$) as previous studies showed that this led to better agreement with inspiratory pressure measurements.[1]

**RESULTS.** Median $^{N1}$PVBC was 0.80 [0.48-0.99]. RC of PVBC as calculated by the different methods is shown in Table 1. Estimating PVBC using assisted and unassisted breaths with matching respiratory drive resulted in the best test-retest agreement (RC PVBCβ: 25.2%). This implies that if a PVBC of 0.6 is measured in a patient, it is expected with a probability of 95% that the subsequent measured PVBC will be between 0.6 and 1.0.

As the SERVO-i ventilator always provides some inspiratory support, even at a NAVA level of zero, there might be a slight overestimation of the fraction $Vt_{insp}$ that is generated by the patient, especially in those patients ventilated with low levels of support.

**CONCLUSIONS.** This study shows that the PVBC index exhibits moderate intra-subject variability in a heterogeneous cohort of ICU patients. Additional analyses will be performed to gain insight in the remaining variability. We believe that PVBC might be a potentially valuable index for bedside monitoring and titrating the patient's contribution to total mechanical inspiratory effort.

**Table 1 (abstract 1105).** PVBC RC using the different methods.
*Evaluated in 27 patients with at least three NAVA-zero breaths that had matching assisted breaths

| Method | RC (%) |
|---|---|
| $^{N1}$PVBC | 39.9 |
| $^{N1}PVBC^2$ | 60.3 |
| $^{X5}$PVBC | 39.9 |
| $^{X5}PVBC^2$ | 61.3 |
| PVBCβ | 25.2* |
| PVBCβ $^2$ | 39.3* |

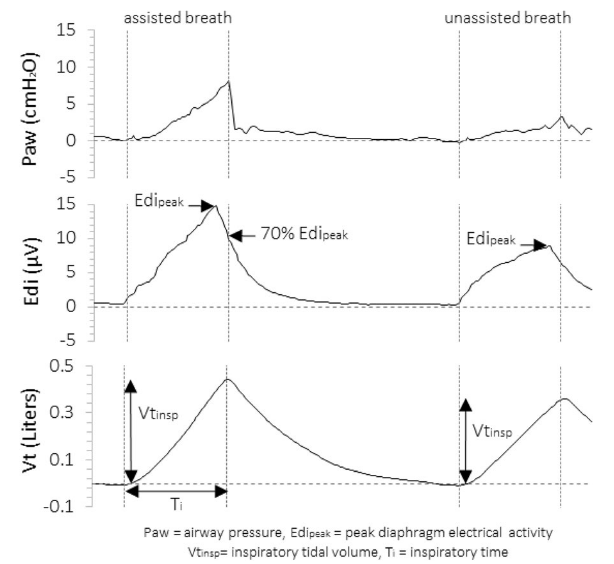

**Fig. 1 (abstract 1105).** Parameters used in PVBC calculation

### 1106

#### Detecting excessive spontaneous effort and lung stress during assisted mechanical ventilation by an end-expiratory occlusion maneuver

M. Bertoni[1], I. Telias[2], M. Urner[3], M. Long[3], L. Del Sorbo[4], E. Fan[4], C. Sinderby[4], J. Beck[4], A.S. Slutsky[4], B.P. Kavanagh[4], N.D. Ferguson[4], L.J. Brochard[4], E.C. Goligher[3]
[1]Università degli studi di Brescia, ICU, Brescia, Italy; [2]Keenan Centre for Biomedical Research, Li Ka Shing Knowledge Institute, St. Michael's Hospital, Toronto, Canada; [3]Department of Medicine, University Health Network and Mount Sinai Hospital, Division of Respirology, Toronto, Canada; [4]Interdepartmental Division of Critical Care Medicine, University of Toronto, Toronto, Canada
**Correspondence:** M. Bertoni
*Intensive Care Medicine Experimental* 2018, **6(Suppl 2):**1106

**INTRODUCTION.** Excessive respiratory effort during mechanical ventilation can injure the lung/diaphragm. Respiratory effort is not routinely monitored in the ICU.

**OBJECTIVES.** To determine whether the negative airway pressure deflection resulting from inspiratory effort during an end-expiratory occlusion ($\Delta P_{occ}$) can detect excessive respiratory effort and dynamic lung stress in spontaneously breathing patients.

**METHODS.** Spontaneously breathing patients intubated for ≤36 hours were studied. Esophageal pressure ($\Delta P_{es}$), peak inspiratory muscle pressure (i.e., effort or $P_{mus}$), and transpulmonary driving pressure (i.e., dynamic lung stress or $\Delta P_L$) were recorded for 10 minutes daily. During each recording, 15-20 brief end-expiratory occlusions were applied to measure $\Delta P_{occ}$. A method for predicting $\Delta P_L$ and $P_{mus}$ from $\Delta P_{occ}$ was derived and validated by cross-validation. Step 1: the mean ratios of $P_{mus}$:$\Delta P_{occ}$ and $\Delta P_{es}$:$\Delta P_{occ}$ (termed $K_1$ and $K_2$, respectively) were computed in recordings from 10 randomly selected patients. Step 2: in the remaining patients, $P_{mus}$ and $\Delta P_L$ were predicted in each recording from three measurements of $\Delta P_{occ}$ selected at random from the same recording. Predicted $P_{mus}$ was estimated as $\Delta P_{occ}$*$K_1$; predicted $\Delta P_{es}$ was estimated as $\Delta P_{occ}$*$K_2$; predicted $\Delta P_L$ was estimated as the inspiratory pressure (peak pressure - PEEP) - predicted $\Delta P_{es}$. Predictive accuracy was assessed by Bland-Altman analysis. Discriminative accuracy to detect threshold values for $P_{mus}$ and $\Delta P_L$ was quantified. The cross-validation procedure (Steps 1 and 2) was repeated 100 times to estimate confidence intervals.

**RESULTS.** 82 daily recordings were collected from 20 patients. $P_{mus}$ and $\Delta P_L$ were frequently excessive ($P_{mus}$ median 14 cm $H_2O$, IQR 9-21 cm $H_2O$; $\Delta P_L$ median 17 cm $H_2O$, IQR 12-21 cm $H_2O$). $K_1$ was -0.70 (95% CI -0.65, -0.77); $K_2$ was 0.60 (0.56-0.63). Agreement between predicted and observed $P_{mus}$ and $\Delta P_L$ was moderate (**Table**). Predicted $P_{mus}$ and predicted $\Delta P_L$ accurately detected excessive respiratory effort and excessive dynamic lung stress across different thresholds (**Table**, **Figure**).

**CONCLUSIONS.** Respiratory effort and dynamic lung stress are often significantly elevated during assisted ventilation. Predicted $P_{mus}$ and $\Delta P_L$ are not sufficiently accurate to replace direct monitoring but measuring $\Delta P_{occ}$ enables accurate non-invasive detection of excessive respiratory effort and dynamic lung stress (analogous to driving pressure in controlled ventilation). Routine measurement of $\Delta P_{occ}$ could be used to determine the need for more intensive monitoring or for adjustments to ventilation.

**Table 1 (abstract 1106).** Accuracy of predicted Pmus and ΔPL

| | Measurement | Estimate (95% confidence intervals) |
|---|---|---|
| Predicted vs. measured Pmus | Bias | 0.1 (-0.1, 0.3) cm H2O |
| | Limits of agreement (% of estimated value) | 48% (33%, 59%) |
| Predicted vs. measured ΔPL | Bias | -0.2 (-0.3, 0.0) cm H2O |
| | Limits of agreement (% of estimated value) | 33% (23%, 39%) |
| Discriminative accuracy (Area under ROC curve) | Measured Pmus > 10 cm H2O | 0.90 (0.82, 0.87) |
| | Measured Pmus > 15 cm H2O | 0.90 (0.83, 0.99) |
| | Measured ΔPL > 15 cm H2O | 0.91 (0.84, 0.99) |
| | Measured ΔPL > 20 cm H2O | 0.94 (0.87, 0.99) |

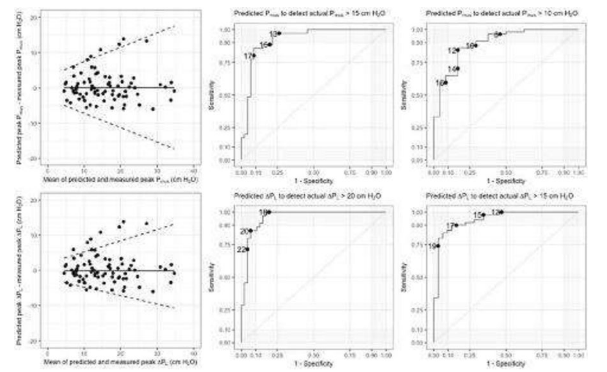

**Fig. 1 (abstract 1106).** Predictive and discriminative accuracy assessed by Bland-Altman plots and ROC curves

### 1107

#### Flow-volume loop in asynchronies during support pressure ventilation

P. Carmona Sánchez[1], J.A. Benítez Lozano[2], M. Delgado Amaya[2], J.M. Serrano Simón[1]
[1]Hospital Universitario Reína Sofía, Intensive Care Medicine, Córdoba, Spain; [2]Hospital Universitario Regional Málaga, Intensive Care Unit, Málaga, Spain
**Correspondence:** P. Carmona Sánchez
*Intensive Care Medicine Experimental* 2018, **6(Suppl 2):**1107

**INTRODUCTION.** The Flow-Volume (FV) Loop has been used to identify the presence of secretions and Auto-PEEP during controlled ventilation. We have found FV Loop phenotypes that can be useful to discriminate different types of asynchronies during pressure support ventilation (PSV).

**OBJECTIVES.** Assess the efficiency of the FV Loop to identify asynchronies and to describe the different phenotypes during PSV.

**METHODS.** Prospectively collected data from January-2015 in 76 patients during PSV at different levels of assistance. We recorded the flow signals (V ´), airway pressure (Paw), esophageal pressure (Pes) at 560 Hz during 30 min. FV Loop represented as usual. Demographics data was recording. The identification of the different phenotypes related to the different asinchronies were evaluated by visual examination. Each finding was considered positive. The results are expressed as mean±SD, median (IRQ), or percentage.

**RESULTS.** 44 patients had presented asinchronies (57.9%). Age: 58.62±12.1 y. Male 44.2%. APACHE II: 19.92±5.9. PaO2/FiO2 277.78±112.7. Death 44.2%. Main diagnosis: Pneumoniae. Lung Transplantation. Trauma. A total of 80 asynchronies were identified (1.82/patient): Reverse Triggering (RT) 23, Failure of Cycling (ineffective efforts) 15, Inspiratory delay 12, Expiratory Delay 10, Autocycling 9, Double cycling 7, Early closure of Inspiration 3, and Double Cycle Failure 1.

RT was considered neural cycle induced by mechanical insufflation. Arrows blue (begin of reverse cycle) and red (end) in figure 3 (R1 to R12). Occasionally with Double cycling (black arrow).

Failure of cycling occurs usually at the end of expiration (red arrow), but occasionally not so late (blue arrows) and rarely occurs in both expiration and inspiration (black arrows). B1-B6 in figure1.

Delay of inspiration (blue arrow in figure 2) and expiration (red arrow) occurs when the neural cycle precedes more than 200 msec. to the mechanical time. In D2 panel (Pes vs V loop in black) the same arrows point delayed phases.

In Autocycling the inspiratory and expiratory phase of the FV Loop are identical mirror images (Figure 1A).

In the early closure of the inspiratory phase of the ventilator cycle, a notch appears in the early expiratory phase of the FV Loop that coincides with the neural end of the inspiration (red arrows in figure 1C1-C2) giving origin double cycling (black arrow) in figure 1C3.

The different phenotypes of FV Loop for each asinchrony are shown for the arrows in figures 1-3. Although the findings are not pathognomonic or specific, they are sufficiently suggestive to warn of the existence of asynchrony in PSV.

**CONCLUSIONS.** In our patients with asynchronies during PSV, the index of severity and mortality are high; we could speculate whether the asynchrony may be considered a marker of poor prognosis. The analysis of the morphology of FV Loop is very efficient and useful for detecting asynchronies even as complex as Autocycling followed by RT with Double cycled.

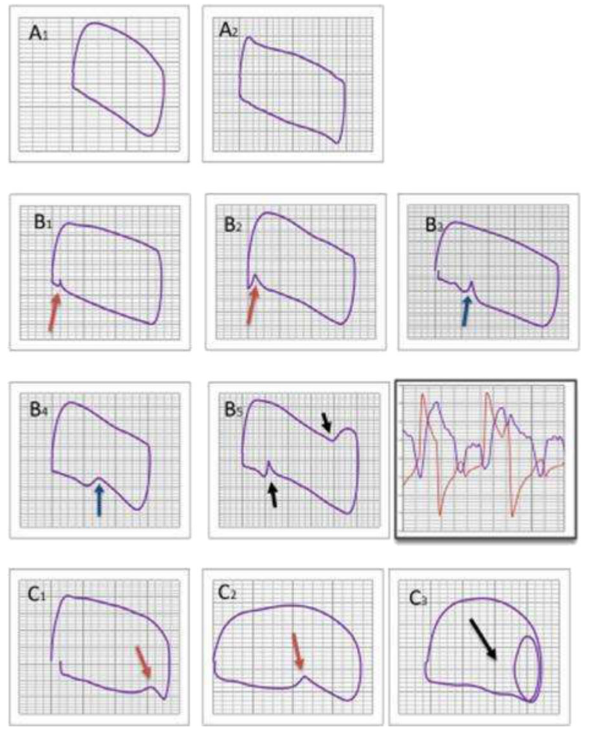

**Fig. 1 (abstract 1107).** The different phenotypes of FV Loop for each Asy are presented in figures 1-3

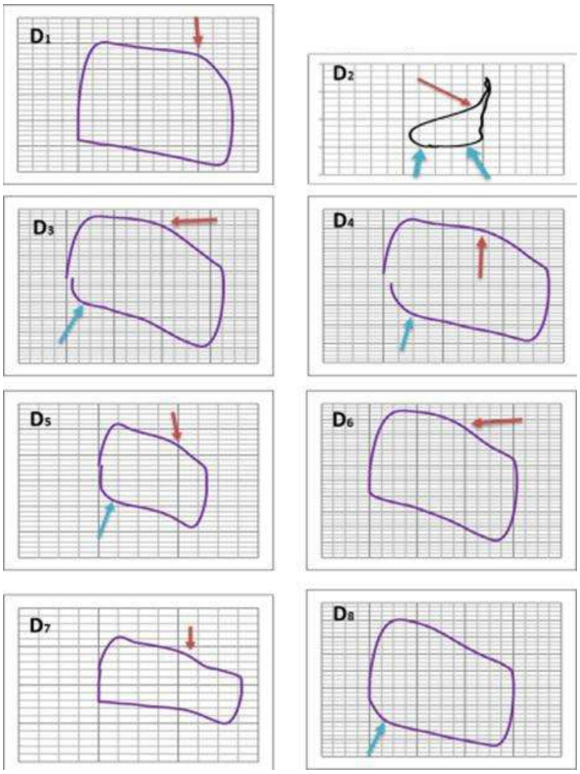

**Fig. 2 (abstract 1107).** Graphic 2

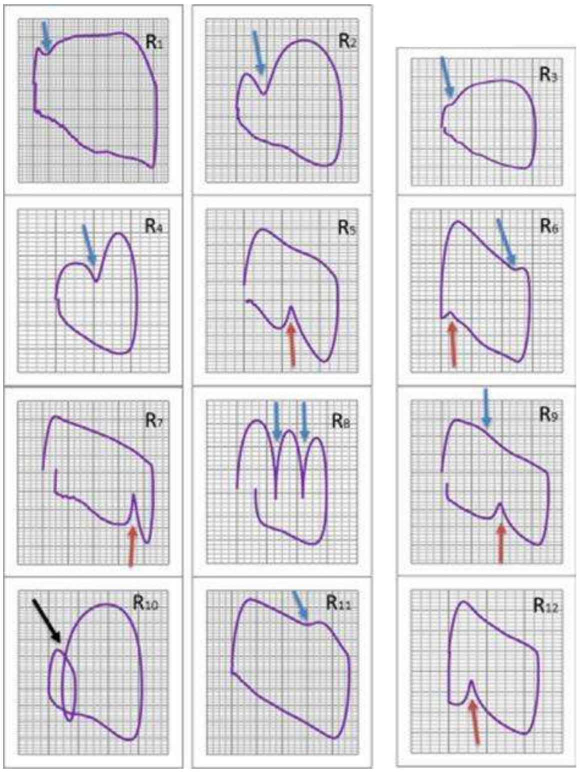

**Fig. 3 (abstract 1107).** Graphic 3

### 1108

#### Predicting patient-ventilator asynchrony with Poisson hidden Markov models

R. Magrans[1,2], Y. Marchuk[3], B. Sales[3], J. Montanya[3], J. López-Aguilar[1,2], C. de Haro[1,2,4], G. Gomà[1,4], C. Subirà[5], R. Fernández[2,5], R.M. Kacmarek[6], L. Blanch[1,2,4], Asynchronies in the Intensive Care Unit (ASYNICU) Group
[1]Institut d'Investigació i Innovació Parc Taulí I3PT, Sabadell, Spain; [2]Centro de Investigación Biomédica en Red de Enfermedades Respiratorias, Madrid, Spain; [3]Better Care, SL, Sabadell, Spain; [4]Hospital Universitari Parc Taulí, Critical Care Center, Sabadell, Spain; [5]Fundació Althaia, Intensive Care Unit, Manresa, Spain; [6]Massachusetts General Hospital, Harvard Medical School, Department of Respiratory Care, Department of Anesthesiology, Boston, United States
**Correspondence:** R. Magrans
*Intensive Care Medicine Experimental* 2018, **6(Suppl 2):**1108

**INTRODUCTION.** Patient-ventilator asynchrony and, recently, clusters of them, have been associated with adverse physiological and clinical outcomes (1-4) in critically ill patients undergoing mechanical ventilation (MV). Optimizing patient-ventilator interaction to both, support the patient's ventilatory demand and to minimize the mismatch between the patient's breathing and the ventilator (1,5) is paramount in intensive care units (ICU).

**OBJECTIVES.** To develop a predictive model based on discrete-time series data useful to anticipate/alert the likelihood of occurrence of asynchronies in MV patients.

**METHODS.** We studied retrospective data of 51 critically ill patients underwent MV>24h in two ICUs. Patients were continuously monitored by Better Care® system (6). Most common asynchronies were identified and regularly indexed over time each 15 minutes. Four level of alerts, from the lowest (z1) to the highest (z4), based on the total count of asynchronies, were defined. A Poisson hidden Markov model was used to predict the probability of occurrence of each level of asynchronies at the next period. The model was trained on a subset of randomly patients and subsequently tested on the remaining ones.

**RESULTS.** A total of 12.7 million breaths were analyzed. On average, 52% of 15 min. periods corresponded to state z1, 28% to z2, 14% to z3 and 6% to z4. Thus, long periods without any asynchronous events were more likely than periods with high level of alert corresponding to a 52.8% of asynchrony incidence at a respiratory rate of 15bpm. Large shifts of states were unlikely, as the most probable switches (if occurs) were to neighboring states (Fig. 1). Moreover, states persisted over time, as reported by the diagonal elements of the transition probabilities matrix (Fig. 1). This can have serious implications in case of patients entering in a state with high number of asynchronies, since it is highly likely (79% for z4 level) that they continue in that state for the next periods.

**CONCLUSIONS.** This study proposes a novel approach to deal with the problem of patient-ventilator asynchrony. This may be a first step in developing smart alarms which would be triggered when a patient entered at a state of high risk. These alerts may be useful to warn healthcare team about considering the need for actions to improve patient-ventilator interaction.

##### REFERENCE(S)

1. Blanch L, et al. *Intensive Care Med* 2015; 41:633–641.
2. Dres M, et al. *Curr Opin Crit Care* 2016; 22(3):246–253.
3. Epstein SK. *Respir Care* 2011; 56:25–38.
4. Vaporidi K, et al. *Intensive Care Med* 2016; 43(2):184–191.
5. Thille AW, et al. *Intensive Care Med* 2006; 32:1551–1522.
6. Blanch L, et al. *Intensive Care Med* 2012; 38:772–780.

##### GRANT ACKNOWLEDGMENT

Funded by projects PI09/91074 and PI13/02204, and co-funded by the ISCIII (Madrid, Spain) and the FEDER. CIBER Enfermedades Respiratorias, Fundación Mapfre, Fundació Parc Taulí, Plan Avanza TSI-020302-2008-38, MCYIN and MITYC (Spain).

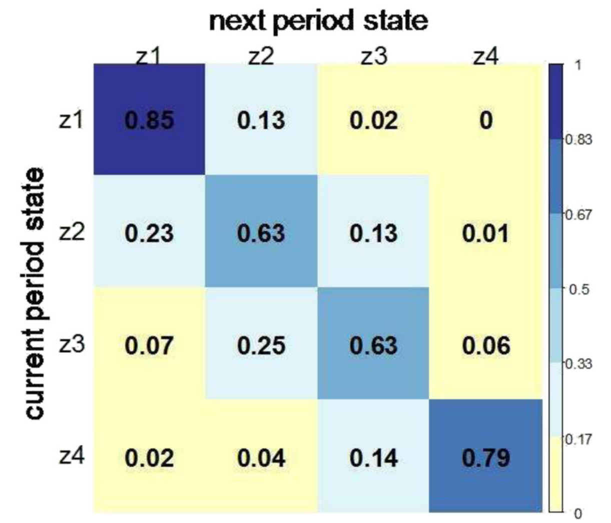

**Fig. 1 (abstract 1108).** Transition probabilities matrix. Diagonal elements are the probability of not changing

### 1109

#### Comparison of occlusion pressure measured at 100 ms with Evita XL ventilator, at airway opening and from esophageal pressure in patients at the time of weaning from invasive mechanical ventilation

M. Mezidi[1], L. Baboi[1], N. Chebib[1], F. Lissonde[1], H. Yonis[1], L. Kreitmann[1], E. Joffredo[1], J.-C. Richard[1], C. Guérin[1,2]
[1]Hopital de la Croix Rousse, Lyon, France; [2]Institut Mondor de Recherches Biomédicales, INSERM 955 and CNRS ERL 7000, Creteil, France
**Correspondence:** M. Mezidi
*Intensive Care Medicine Experimental* 2018, **6(Suppl 2):**1109

**INTRODUCTION.** Measurement of airway pressure 100 ms after airway occlusion (P0.1) has been initially described in normal subjects to quantify the intensity of the respiratory drive (1). At this time it was measured at the mouth of the subject (1). Its measurement has been implemented into ICU ventilators to make respiratory drive monitoring easily available at the bedside (2). It has also been shown reflecting the work of breathing (3). However, during mechanical ventilation P0.1 is measured upstream the ventilator circuit and hence may not reflect mouth P0.1 due to the compliance of the circuit.

**OBJECTIVES.** We aimed to compare P0.1 as commonly measured with the Evita XL ICU ventilator (P0.1,Evita) with airway (P0.1,aw) and esophageal P0.1 (P0.1,es) pressure in ICU patients, who were attempting a spontaneous breathing trial during weaning from mechanical ventilation.

**METHODS.** Measurement of P0.1 is part of an ongoing study comparing pressure support (PS) 7 cmH2O + PEEP 4 cmH2O (treatment A) to PS 0 cmH2O + PEEP 4 cmH2O + 100% automatic tube compensation (treatment B), each applied for 30 minutes, at the time of weaning from invasive mechanical ventilation. Before each treatment the baseline PS of each patient is applied for 30 minutes.

We ran the P0.1 built-in function available in the Evita XL ventilator. Paw and flow were measured at the proximal tip of the endotracheal tube. Pes was obtained from esophageal balloon whose right position and optimal volume were checked properly. Paw, Pes and flow signals were recorded with BIOPAC150 at 200 Hz. Three to ten P0.1 measurements were performed, each separated by 4-8 breaths, in each condition in each patient. P0.1,Evita was read at the ventilator screen and compared to P0.1,aw and P0.1,es. Values are expressed as mean±SD. The data were assessed by using Bland and Altman representation and linear mixed effect model with method of P0.1 measurement and rank of measurement used as the fixed variables and patient as the random variable.

**RESULTS.** Thirteen patients have been included totalizing 222 measures with each method. P0.1,Evita averaged 2.71±2.43, P0.1,aw 2.56±2.37 and P0.1,es 2.63±2.55 cmH2O.

The bias and limits of agreement for the relationship of P0.1,Evita to P0.1,aw and of P0.1,Evita to P0.1,es were 0.2 (-1.4;+1.7) (Figure 1 left panel) and 0.1 (-3.2;+3.3) cmH2O (figure 1 right panel), respectively.

The linear mixed model found that neither the method used nor the rank of the measurement had a significant effect on P0.1,Evita

**CONCLUSIONS.** P0.1,Evita provides reasonable estimate of P0.1 measured near the patient.

**REFERENCE(S)**

1. Whitelaw B, Derenne JPh, Milic-Emili J. Respir Physiol 1975;23(2):181-99.
2. Telias I, Damiani F, Brochard L. Intensive Care Med 2018 Mar 1. doi: 10.1007/s00134-018-5091-2.
3. Mancebo J, Albaladejo P, Touchard D, Bak E, Subirana M, Lemaire F, Harf A, Brochard L. Anesthesiology 2000; 93 (7):81-90.

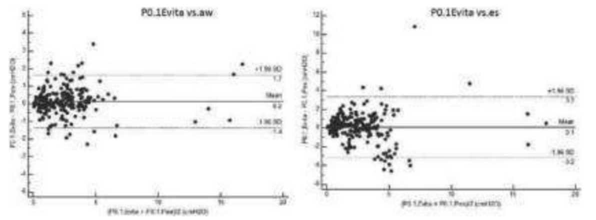

**Fig. 1 (abstract 1109).** See text for description

## Preventing ICU infections

### 1110

#### Hand hygiene compliance at intensive care units: a prospective observational study

M. Hoffmann[1,2,3], V. Gombotz[1], G. Pregartner[4], G. Brunner[3], G. Sendlhofer[1,3]
[1]University Hospital Graz, Executive Department for Quality and Risk Management, Graz, Austria; [2]Medical University of Graz, Division of Endocrinology and Diabetology, Department of Internal Medicine, Medical University of Graz, Graz, Austria; [3]Medical University of Graz, Research Unit for Safety in Health, c/o Division of Plastic, Aesthetic and Reconstructive Surgery, Department of Surgery, Graz, Austria; [4]Medical University of Graz, Institute for Medical Informatics, Statistics and Documentation, Graz, Austria
**Correspondence:** M. Hoffmann
*Intensive Care Medicine Experimental* 2018, **6(Suppl 2):**1110

**INTRODUCTION.** Healthcare associated infections (HAI) in intensive care units (ICU) are a leading risk for patient safety and cause a serious disease burden with economic impact. Hand disinfection is one of the most effective measures to reduce HAI [1,2,3]. To improve hand hygiene (HH) compliance in a university hospital, several measures of the German HH campaign "Aktion Saubere Hände" (ASH) were implemented and observed in terms of HH-compliance rates.

**OBJECTIVES.** To raise awareness for HH, healthcare experts were observed in order to assess if implemented measures of ASH influenced HH-compliance rates over a period of five years.

**METHODS.** ASH in-house training tools comprised face-to-face instructions by hygiene experts, a hand hygiene training clip, standard operating procedures, HH-conferences and an HH in-house newsletter for all healthcare experts. Furthermore, to also raise awareness amongst patients and relatives, a patient information clip including sequences on HH, leaflets for patients, posters were implemented. To determine the effectiveness of all these measures, a prospective observational single-centre study to assess compliance rates of healthcare experts was performed between 2013 and 2017. HH compliance rates were observed by hygiene experts. Linear mixed models were used to estimate the compliance trend over time.
**RESULTS.** In total, 48 compliance measurements with 10,315 observed "My five moments for HH" (M5M) were conducted in 12 intensive care units. Overall, mean HH compliance rates increased from 76.9 ± 19.3% in 2013 to 88.6 ± 8.1% in 2017. The estimated increase per year was 3.3% (95% CI 1.8 - 4.8%, p< 0.001).
**CONCLUSIONS.** To assess the influence of implemented ASH-measures, 48 compliance measurements in 12 different ICUs were observed and showed an overall significant increase. Additionally, it is the aim to further increase HH-compliance rates in terms of patient safety. The major limitation of this study was that it was not determined which of these measures influenced HH-compliance rates. Therefore, a follow up study will address the questions if trainings or compliance measurements of healthcare experts influenced behaviors and/or if patient and relatives empowerment also influenced results.

**GRANT ACKNOWLEDGMENT**
There was no funding.

### 1111

#### Impact of subglottic secretion drainage on microaspiration in critically ill patients: a prospective observational study

G. Millot[1], P. Boddaert[1], A. Palud[1], E. Parmentier-Decrucq[1], F. Wallet[2], F. Zerimech[3], S. Nseir[1]
[1]Centre Hospitalier Régional et Universitaire de Lille, Centre de Réanimation, Lille, France; [2]Centre Hospitalier Régional et Universitaire de Lille, Laboratoire de Bactériologie, Lille, France; [3]Centre Hospitalier Régional et Universitaire de Lille, Laboratoire de Biochimie, Lille, France
**Correspondence:** G. Millot
*Intensive Care Medicine Experimental* 2018, **6(Suppl 2):**1111

**INTRODUCTION.** Ventilator-associated pneumonia (VAP) is a frequent infection in intensive care units (ICU), with a major impact on patient outcome. Tracheal microaspiration of contaminated oral or gastric secretions, a major factor in VAP pathogenesis, could be prevented by subglottic secretion drainage (SSD). Despite a strong rationale and robust data on the effectiveness of SSD, no study has evaluated its impact on microaspiration of secretions.
**OBJECTIVES.** To determine if SSD could reduce the incidence of tracheal microaspiration in mechanically-ventilated patients compared to standard tracheal tubes. Secondary outcomes included the incidence of airway colonization, ventilator-associated tracheobronchitis (VAT), VAP, and patient outcome.
**METHODS.** This prospective single-center observational study was performed from March 2012 through April 2013 and included adult ICU patients who had been mechanically ventilated for at least 24 hours and were expected to stay intubated for at least 24 hours. Patients intubated with SSD-ready tracheal tubes (TaperGuard™ Evac, Mallinckrodt; polyvinyl chloride (PVC) tapered cuff) were included in the SSD group, those intubated with PVC standard-shaped cuff tracheal tubes were included in the control group. To assess the incidence of microaspiration, pepsin and salivary amylase, respectively for gastric and oral origin, were measured in endotracheal aspirates (ETA) for 24 hours following inclusion. ETA were considered as positive for pepsin or amylase at concentrations above 200µg/L, and 1685IU/L; respectively.
**RESULTS.** SSD group included 55 patients *vs* 45 in the control group. No significant difference was found between groups regarding male gender (73% *vs* 69% of in the SSD and control group respectively), age (62.5 *vs* 58.6), SAPS II (46 [36 - 60] *vs* 51 [39 - 69.5]) and LOD score (6 [3 - 9] *vs* 8 [4 - 10]). However, patients in the SSD group had a significantly lower mean cuff pressure (23 [21 - 30] *versus* 29 [22.5 - 30] $cmH_2O$, $p = 0.015$), received significantly higher enteral nutrition solution volumes (613 ± 453 *vs* 365 ± 462, $p = 0.003$) and were less likely to receive neuromuscular-blocking agents (3.6% *versus* 17.8%, $p = 0.040$). There was no statistically significant difference between groups regarding the median percentage of ETA positive for pepsin or amylase (81.4 ± 31.4% *vs* 75.6 ± 36.6%, $p = 0.584$), the median proportion of pepsin- (0 [0 - 50]% *vs* 0 [0 - 17.8]%, $p = 0.152$) or amylase-positive (88 [58.5 - 100]% *vs* 100 [33 - 100]%, $p = 0.859$) ETA, or incidence of VAP (16 (29.1%) *vs* 11 (24.4%), $p = 0.656$), VAT (7 (12.7%) *vs* 4 (8.8%), $p = 0.750$) and airway colonization (15 (34.9%) *vs* 8 (21.1%), $p = 0.219$).
**CONCLUSIONS.** SSD was not associated with reduced incidence of microaspiration, VAP, VAT or airway colonization in this study. Cuff shape (tapered or cylindrical) might be a confounding factor. Further randomized controlled studies are needed.

### 1112

#### Comparison of hand rub with super-oxidised water & alcohol-based chlorhexidine for hand hygiene of healthcare workers in trauma ICU to prevent nosocomial infections: a single-centric, prospective, single-blinded, cross-over, clinical comparative interventional study

B.P. Das[1], G. Yadav[1], M. Gupta[2], S. Viswakarma[1]
[1]IMS-BHU, Anaesthesia and Critical Care, Varanasi, India; [2]IMS-BHU, Microbiology, Varanasi, India
**Correspondence:** B.P. Das
*Intensive Care Medicine Experimental* 2018, **6(Suppl 2):**1112

**INTRODUCTION.** In the era of antibiotic-resistance, hand-hygiene plays an important role in the prevention of nosocomial infections (NCI)[1]. Although many kind of hand-rub agents are used, ideal hand-rub for hand hygiene of health care workers(HCW) is still a matter of debate.
**OBJECTIVES.** We proceeded with an aim to evaluate the efficacy of invivo use of super-oxidized water (SOW) over alcohol-based chlorhexidine (ABC) as hand-rub for hand hygiene of HCW in a tertiary care trauma critical care setting to prevent NCI.
**METHODS.** After ethics committee approval and written informed consent from patients/relatives and HCWs, a single-centric, prospective, single-blinded, cross-over, clinical comparative interventional study was conducted from September2017-December2017 in the two ICUs, Department of Anesthesia, Trauma centre, IMS-BHU, where the HCW associated directly with patient care, were allotted into one of the 2 study groups based on the hand-rub used (ICU SOW or ICU ABC). 180 patients were admitted based on simple randomisation into both the ICUs. After a period of 2 months, the ICUs were crossed over wrt the use of study hand-rub. Primary outcome noted was incidence of nosocomial infections & secondary outcomes were cost effectiveness and adverse effects. Chi-square and student-t test were used for qualitative & quantitative data, respectively. Statistical significant difference was declared when p-value ≤0.05.
**RESULTS.** Demographic parameters were comparable (Table 1). Incidence of NCI were lower with SOW (VAP-28 out of 71 vs 62 out of 69,p=0.001) (Fig 1), with decreased MDR organisms (12 vs 28,p=0.002) & less fungal infections (0 vs 6, p= 0.02) (Fig 2). SOW was cost-effective as compared to ABC (Rs 08 prepared by 'Sterigen' vs Rs 200 (Bactorub) per patient during the stay, p=0.0001). Duration of hospital stay and mortality at 1 month were comparable (p=0.23) (Fig 3).
**CONCLUSIONS.** Use of SOW as hand-rub for hand hygiene of HCWs, not only, is safe and cost-effective, but also decreased the incidence of nosocomial infections & contributed to a favourable ICU flora.

**REFERENCE(S)**
(1) Perçin D, Esen : New disinfectants and problems in practice. Ankem 2009, 23(2):89-93.

(2) Maria Elena, HS Margarita, SA Angel. Microbial Drug Resistance. Microbial Drug Resistance. 2015; 21 (4): 367-72.

**GRANT ACKNOWLEDGMENT**
The authors declare no conflicts of interest and did not receive any grant for this study.

**Table 1 (abstract 1112).** Demographic parameters

| Demographic parameters | ICU SOW (n=71) | ICU ABC (n=69) | p value |
|---|---|---|---|
| Age, years (mean) | 34.4±17 | 38.2±15 | 0.24 |
| Female : male | 34:37 | 31:38 | 0.91(χ2=0.01) |
| Weight (mean) | 51.86 ± 21 | 55.2±16 | 0.3 |
| TLC_ICU admission (mean) | 14,763±3224 | 13,515±3824 | 0.6 |
| APACHE II_ICU admission (mean) | 14.15±4.3 | 14.5±5.7 | 0.3 |
| SOFA_ICU admission (mean) | 5.7±2.9 | 3.8±2.2 | 0.2 |
| CPIS_ICU admission (mean) | 3.41±1.7 | 3.2±1.5 | 0.22 |
| Diagnosis (Sx:Mx) | 45:26 | 42:27 | 0.9(χ2=0.01) |

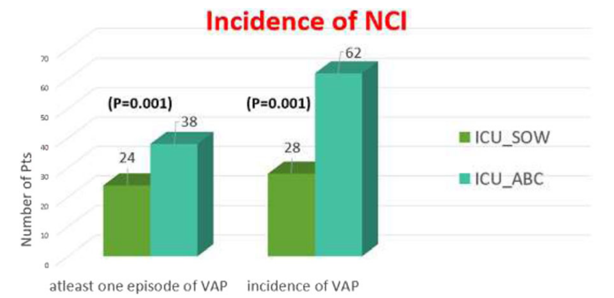

**Fig. 1 (abstract 1112).** Incidence of NCI (VAP)

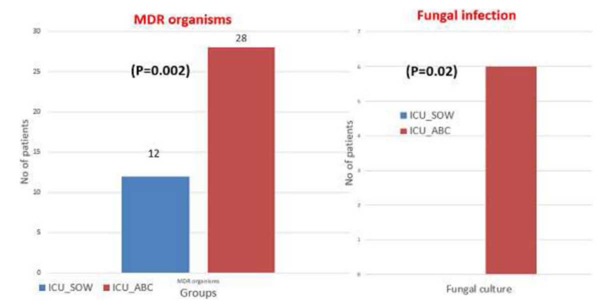

**Fig. 2 (abstract 1112).** Incidence of MDR organisms and fungal infections

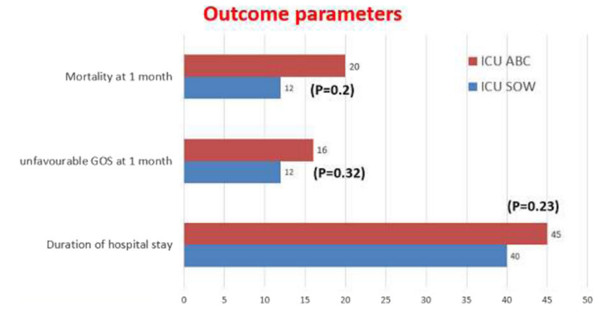

**Fig. 3 (abstract 1112).** Outcome parameters

### 1113

#### Application of zero resistance project in a intensive care unit. A preliminar study

F. Molina Cabrero, E. Simón Polo, P. Cuesta Montero
Albacete Hospital, Anaesthesia, Albacete, Spain
**Correspondence:** F. Molina Cabrero
*Intensive Care Medicine Experimental* 2018, **6(Suppl 2):**1113

**INTRODUCTION.** Colonization-Infections by multiresistant bacteria (BMR) are increasing in critical care units, so vigilance and control strategies are needed.
**OBJECTIVES.** The aim to this study is to determine the incidence of colonization in patients admitted in a intensive care unit and describe risk factors related.
**METHODS.** A prospective, observational, descriptive was conduced, including patients admitted in the unit from April 2016 to September 2017.
Risk factors were predefined and analysed (admitted to hospital for more than 5 days, institutionalized, previous colonization, renal failure, antibiotic more than seven days) and colonization controls were performed at admission and every 7 days.
Chi-squared test and logistic regression analysis was analysed to see if there was a relationship between risk factors and colonization when admission.Odds ratio was calculated to measure the strength of this association.
**RESULTS.** In this period, 969 patients were studied. The incidence of colonization is 5.9%, being Beta-lactamases Productors Enterobacterias (3.2%) and Methicillin Resistant Staphilococcus Aureus (2.2%) the most frequently microrganism founded in our results. Some risk factor associated was presented in 31% of them.
Logistic regression showed 3 risk factors as statistically significant result: Income greater than 5 days (OR = 1.99, CI 1.08-3.66), institutionalized (OR = 16.28, CI 1.001) -163.95) and present a previous colonization by BMR (OR of 3.85, CI 1.06-13.94)
**CONCLUSIONS.** Being admitted for more than 5 days, institutionalized or a previous colonization by BMR are factors related to a higher incidence of colonization when the patient is admitted in an intensive care unit. The implementation of this project is useful for the vigilance and control infections in critical patients

**REFERENCE(S)**
hws.vhebron.net/envin-helics www.semicyuc.org

### 1114

#### Collaboration between ward pharmacists and infection control team for the appropriate antimicrobial use in the emergency medical center

T. Tatsumichi, K. Yamaguchi, K. Takahashi, H. Tanaka, S. Kosaka, H. Houchi
Kagawa University Hospital, Kagawa, Japan
**Correspondence:** T. Tatsumichi
*Intensive Care Medicine Experimental* 2018, **6(Suppl 2):**1114

**INTRODUCTION.** As stated in the Guidance for Implementing an Antimicrobial Stewardship Program in Japan, issued by infection-related eight societies in Japan in September 2017, pharmacists are expected to play a central role in the program.
Since assigned to the emergency medical center in our hospital in April 2016, its dedicated pharmacists have intervened in an appropriate use of antimicrobials in collaboration with the infection control team (ICT).
**OBJECTIVES.** We report the current status and challenges in the collaboration between ward pharmacists and ICT.
**METHODS.** Data were extracted from our pharmacy department system on the use of antimicrobials (injections) between April 2014 and September 2017 in the emergency medical center. We calculated defined daily dose (DDD) per 1000 bed-days (hereafter DDD measurement)[1) ], days of therapy (DOT) per 1000 bed-days (hereafter DOT measurement)[2)] , and DDD/DOT ratios[3)] of specific antimicrobials (MEPM, IPM/CS, VCM, TEIC, LZD) in pre- and post-assignment periods of dedicated pharmacists. Pre-assignment period was defined as April 2014 through September 2015 and post-assignment period as April 2016 through September 2017.

**RESULTS.** DDD measurements of pre- and post-assignment periods of dedicated pharmacists were 80.4 vs 81.0 for MEPM, 2.9 vs 0.2 for IPM/CM, 15.4 vs 15.3 for VCM, 2.1 vs 1.7 for TEIC, and 3.7 vs 6.1 for LZD, respectively. Specific antimicrobials accounted for 20.8% (104.5/501.) for pre-assignment period and 24.0% (104.3/434.1) of total antimicrobial injections. These results indicate decreases in DOT measurements for specific antimicrobials expect for LZD. The DDD/DOT ratio for VCM increased from 0.68 in the pre-assignment period to 0.86 in the post-assignment period.

**CONCLUSIONS.** Although total use of VCM remained unchanged between pre- and post-assignment periods, our results showed an increase in VCM dose per day and decreases in duration of VCM therapy and the number of patients receiving VCM, which suggests that VCM began to be used more appropriately. We consider that this resulted from our continuous intervention including development of protocols of loading-dose and TDM in cooperation between ward pharmacists and the ICT. No changes were found in dose per day, treatment duration, and the number of patients receiving MEPM, IPM/CIM, and TEIC therapies, suggesting that there is room for intervention. Aiming at the appropriate antimicrobial use, we will make intervention by further enhancing collaboration between ward pharmacist and ICT.

**GRANT ACKNOWLEDGMENT**

No

### 1115

#### Application of enteral topic paromomycin to eradicate colistin and carbepenemase resistant microorganisms in rectal colonization to prevent ICU-acquired infections

C. Sánchez Ramírez[1], M.A. Hernández Viera[1], M. Cabrera Santama[1], S. Hípola Escalada[1], R.E. Morales Sirgado[1], L. Caipe Balcázar[1], C.F. Lübbe Vázquez[1], S.M. Marrero Penichet[2], F. Artíles Campelo[3], P. Saavedra Santana[4], S. Ruiz Santana[1]

[1]University Hospital of Gran Canaria Dr Negrín, Intensive Care Unit, Las Palmas de Gran Canaria, Spain; [2]University Hospital of Gran Canaria Dr Negrín, Pharmacy Department, Las Palmas de Gran Canaria, Spain; [3]University Hospital of Gran Canaria Dr Negrín, Microbiology Department, Las Palmas de Gran Canaria, Spain; [4]University of Las Palmas de Gran Canaria, Mathemathics and Informatics Department, Las Palmas de Gran Canaria, Spain

**Correspondence:** C. Sánchez Ramírez

*Intensive Care Medicine Experimental* 2018, **6(Suppl 2):**1115

**OBJECTIVE.** To assess the value of enteral paromomycin to decontaminate patients with rectal colistin and / or carbepenemase (CPN) resistant microorganisms colonization in order to prevent the development of ICU-acquired infections.

**METHODS.** All consecutive patients admitted to the ICU from October 2011 to December 2016, requiring tracheal intubation for longer than 48 hours were given SDD with a 4-day course of intravenous cefotaxime, plus enteral colistin, tobramycin and nystatin in an oropharyngeal paste and in a digestive solution. Oropharyngeal and rectal swabs were obtained on admission and once weekly. Patients with rectal swabs colonized by colistin and / or carbepenemase resistant microorganisms were treated with enteral paromomycin 1 gr every 6 hours daily, in order to eradicate them and prevent nosocomial infections. Categorical variables were summarized as frequencies and percentages and the continuous ones as medians and interquartile ranges (IQR) or means and standard deviations. Statistical significance was set at $p \leq 0.05$.

**RESULTS.** Paromomycin was applied to 84 colonized patients with rectal colistin-resistant microorganisms (CRM). All of them but two had colonization by Extended Spectrum Beta-lactamases (ESBLs) producing *Klebsiella pneumoniae*. One patient was colonized by ESBL producing *Enterobacter spp* and another by ESBL producing *Escherichia coli*. Demographic data and type of admission are shown in Table 1.

Sixty-eight out of 84 (81.7%) eradicated the rectal swab after paromomycin. Twenty-six of them received concurrent susceptible antibiotics during paromomycin application. Five out of the seven CPN producing patients were decolonized. Twenty-eight patients died in the ICU and 15 of CRM colonized patients died without achieving negativization, being 2 of them CPN. Only 3 patients developed nosocomial infections after decolonization.

**CONCLUSION.** Our data show that enteral paromomycin could be effective in treating rectal colistin and / or carbapenemase resistant microorganism colonization allowing prevention of ICU-adquired infections.

**Table 1 (abstract 1115).** Patient data

| | |
|---|---|
| Patients, n | 84 |
| Age, years, SD | 62,5 ± 14,5 |
| Male/female, n, % | 53 (63.1) / 31(36.9) |
| APACHE II on admission, n(IQR) | 22(16,25; 27) |
| SOFA on admission, n(IQR) | 9,5(7 ; 12) |
| APACHE II at paromomycin treatment start, n(IQR) | 17,0 (13 ; 21.75) |
| SOFA at paromomycin treatment start, n(IQR) | 5,5 (3 ; 9) |
| Glasgow Coma Score, n(IQR) | 14,5 (5 ; 15) |
| ICU stay days, n(IQR) | 58 (35;110) |
| Renal Replacement Therapy, n (IQR) | 35(42,7) |
| Traumatic patients, n (%) | 7 (8,3) |
| Medical patients on admission, n (%) | 51 (60,7) |
| Parenteral nutrition, n (%) | 22 (26,1) |
| Diabetes mellitus, n (%) | 32 (36,09) |
| Neutropenic patients, n (%) | 2(2,3) |
| Immunosuppression, n (%) | 6 (7,1) |
| Deaths, n (%) | 28 (33,3) |
| COPD, n (%) | 10 (11,9) |
| Paromomycin treatment days, n(IQR) | 14.5 (8 ; 27) |

### 1116

#### Ventilator associated pneumonia surveillance results before and after use of closed aspiration system in intensive care unit

S. Erdinc[1], C. Kaymak[2], C. Hatipoglu[1], H. Basar[2], G. Ertem[1], A. Ozcan[2], S. Kinikli[1], N. Ozcan[2]

[1]Ministry of Health, Ankara Training ang Research Hospital, Anaesthesiology and Reanimation, Intensive Care Unit, Ankara, Turkey; [2]Health Science University, Ankara Heealth Application and Research Center, Anaesthesiology and Reanimation, Intensive Care Unit, Ankara, Turkey

**Correspondence:** H. Basar

*Intensive Care Medicine Experimental* 2018, **6(Suppl 2):**1116

**INTRODUCTION.** In patients treated in intensive care units (ICU), infections are commonly observed due to severe illnesses, immune supressive effects of drugs, surgical procedures, and usage of invasive equipment. In this respect ICU's should be inspected for nosocomial infections since resistant microorganisms can easily colonize.

OBJECTIVES: In this study we aimed to compare the surveillance results for ventilator associated pneumonia (VAP) before and after the use of closed aspiration system.

**METHODS.** Between September 2016 and April 2017 intubated or tracheostomized patients aspirated with conventional aspiration catheter and between May 2017 and December 2017 patients aspirated with closed aspiration system are enrolled to the study. Patients' VAP results depending on laboratory culture samples are assessed with active surveillance system. Patient day, days on ventilator, VAP rate, total number of infections were calculated.

**RESULTS.** Patient day in ICU for two groups were 5694 and 5866 days, respectively. For conventionally aspirated group ventilator day and ventilator usage rate were 3111 days and 0.55. For closed system aspiration group ventilator day and ventilator usage rate were 2967 days and 0.51. For the first 8 months VAP rate was 10.3 % and total number of infections was 32 while in the second 8 month, 10.8 % and 32.

**CONCLUSIONS.** VAP rate and number of infections were similar with two aspiration techniques. Ventilator use rate and days on ventilator decreased throughout the study. In ICU successful surveillance results can be achieved by isolation of the agents, appropriate antibiotic choice and obeying isolation rules.

**GRANT ACKNOWLEDGMENT**

None

### 1117

#### Six years of selective digestive decontamination in a mixed intensive care unit at a university hospital: impact on colonization, nosocomial infection and antibiotic consumption

C. Sánchez Ramírez[1], M. Cabrera Santana[1], S. Hípola Escalada[1], L. Caipe Balcázar[1], M.A. Hernández Viera[1], R.E. Morales Sirgado[1], F. Artíles Campelo[2], S.M. Marrero Penichet[3], C.F. Lübbe Vázquez[1], P. Saavedra Santana[4], S. Ruiz Santana[1]

[1]University Hospital of Gran Canaria Dr Negrín, Intensive Care Unit, Las Palmas de Gran Canaria, Spain; [2]University Hospital of Gran Canaria Dr Negrín, Microbiology Department, Las Palmas de Gran Canaria, Spain; [3]University Hospital of Gran Canaria Dr Negrín, Pharmacy Department, Las Palmas de Gran Canaria, Spain; [4]University of Las Palmas de Gran Canaria, Mathemathics and Informatics Department, Las Palmas de Gran Canaria, Spain

**Correspondence:** C. Sánchez Ramírez

*Intensive Care Medicine Experimental* 2018, **6(Suppl 2):**1117

**OBJECTIVES.** To prospectively evaluate the impact of Selective Digestive Decontamination (SDD) application on colonization rates and nosocomial infections (NI), after 6 years, in a mixed ICU.

**METHODS.** This study was conducted in a 30-bed-medical-surgical ICU. All consecutive patients admitted to the ICU from October 1, 2011 to September 30, 2017 expected to require tracheal intubation > 48 hours were given SDD (SDD study group) with a 4-day course of intravenous cefotaxime, plus enteral colistin, tobramycin, nystatin in an oropharyngeal paste and in a digestive solution. Oropharyngeal and rectal swabs were obtained on admission and once weekly. We used ENVIN NI criteria. We compared all patients admitted to ICU with ICU NI from October 1, 2010 to September 30, 2011 (non-SDD group) to the SDD study group. Categorical variables were summarized as frequencies and percentages and the continuous ones as means and standard deviations (SD) when the data followed the normal distribution or medians and interquartile ranges (IQR) when they did not. The percentages were compared using the test of chi-square test or Fisher exact test, means with the t-test and medians with the Wilcoxon test for independent samples. Those variables that showed statistical significance in the univariate analysis were introduced in a multivariate logistic regression analysis. For each one of the infections (catheter-related and other secondary bacteremias, pneumonia and urinary infections and antibiotic resistant bacteria (ARB) infections) the incidences per 1000 days of exposure in each cohort and the corresponding relative risks were obtained using the Poisson regression. Statistical significance was $p \leq 0.05$.We analyzed colistin- and tobramycin-resistant colonization and also antibiotic consumption as Defined antibiotics Daily Doses (DDD).

**RESULTS.** Results are shown in Tables 1

There were no statistical significant differences between both groups in type of admission or demographic data. Patients with SDD had significantly less Extended Spectrum Betalactamase (ESBL), Gram Negative Bacteria Multirresistant (GNB-MR) and *Acinetobacter spp* infections. We had also a significant reduction in ventilator associated pneumonia (VAP), urinary infections,other secondary bacteremias and ARB infection rates, in SDD group versus non SDD. There was no infection by *Clostridium difficile*. The exogenous infections were 73.9 %. Colistin-resistant colonization was 17.4% and tobramycin-resistant colonization was 25.7% of samples. There was a decrease on the DDD/100 ICU stays after SDD.

**CONCLUSIONS.** After 6 years applying SDD a significant reduction of infections by ESBL, GNB-MR and *Acinetobacter,* was observed. A significant decrease of VAP, secondary bacteremias, urinary and ARB infections rates was also shown. An antibiotic consumption reduction was found after SDD. Low rates of colistin- and tobramycin-resistant colonization bacteria have been persistently observed.

**Table 1 (abstract 1117).** Univariate analysis

| | | *Selective Digestive Decontamination* | | |
|---|---|---|---|---|
| | Total N = 532 | No N = 110 | Yes N = 422 | P |
| *Age*, years | 60.7 ± 15.4 | 59.5 ± 15.8 | 61.0 ± 15.3 | 0.376 |
| Male / Female, n (%) | 355 (66.8) | 74 (67.3) | 281 (66.8) | 0.917 |
| Apache-II | 22.0 ± 7.6 | 21.2 ± 7.7 | 22.3 ± 7.6 | 0.201 |
| Immunossupression, n (%) | 17 (3.2) | 3 (2.7) | 14 (3.3) | 1 |
| Neutropenia, n (%) | 5 (0.9) | 3 (2.7) | 2 (0.5) | 0.062 |
| Parenteral nutrition, n (%) | 161 (30.3) | 26 (23.6) | 135 (32.0) | 0.089 |
| *Patient type* | | | | 0.113 |
| Medical | 380 (71.6) | 79 (71.8) | 301 (71.5) | |
| Scheduled surgical | 74 (13.9) | 10 (9.1) | 64 (15.2) | |
| Urgent surgical | 77 (14.5) | 21 (19.1) | 56 (13.3) | |
| Traumatic patient, n (%) | 68 (12.8) | 17 (15.4) | 51 (12.1) | 0.351 |
| *Acinetobacter*, n (%) | 18 (3.4) | 13 (11.8) | 5 (1.2) | < .001 |
| SAMR, n (%) | 11 (2.1) | 4 (3.6) | 7 (1.7) | 0.251 |
| ESBL, n (%) | 132 (24.8) | 38 (34.5) | 94 (22.3) | 0.008 |
| *PSEUDOMONAS MR*, n (%) | 43 (8.1) | 10 (9.1) | 33 (7.8) | 0.758 |
| MR-GNB, n (%) | 28 (5.3) | 12 (10.9) | 16 (3.8) | 0.003 |
| Renal Replacement Therapy, n (%) | 194 (36.5) | 34 (30.9) | 160 (37.9) | 0.174 |
| VAP, n (%) | 210 (39.5) | 59 (53.6) | 151 (35.8) | < .001 |
| Urinary infection, n (%) | 139 (26.1) | 29 (26.4) | 110 (26.1) | 0.950 |
| Catheter related Bacteremia, n (%) | 190 (35.7) | 26 (23.6) | 164 (38.9) | 0.003 |
| Secondary bacteremia, n (%) | 134 (25.2) | 31 (28.2) | 103 (24.4) | 0.417 |
| Inflammatory response | | | | < .001 |
| Non sepsis | 23 (4.3) | 2 (1.8) | 21 (5.0) | |
| Sepsis | 174 (32.7) | 23 (20.9) | 151 (35.8) | |
| Severe Sepsis | 81 (15.2) | 34 (30.9) | 47 (11.1) | |
| Septic Shock | 254 (47.7) | 51 (46.4) | 203 (48.1) | |
| Renal failure, n (%) | 126 (23.7) | 40 (36.4) | 86 (20.4) | < .001 |
| COPD, n (%) | 77 (14.5) | 9 (8.2) | 68 (16.1) | 0.035 |
| Diabetes mellitus, n (%) | 170 (31.9) | 34 (30.9) | 136 (32.2) | 0.792 |
| Days in ICU, days | 30 (17 ; 48) | 28 (16 ; 45) | 30 (17 ; 49) | .227 |
| Death, n (%) | 164 (36.7) | 36 (32.7) | 128 (38.0) | .321 |

MRSA: methicillin resistant Sthaphyloccocus aureus; ESBL: extended spectrum belalactamase; MR: multiresistant; GNB: gram negative bacteria; VAP: ventilator associated pneumonia; COPD: Chronic Obstructive Pulmonary Disease

**Table 2 (abstract 1117).** Multivariate analysis

| | P | OR (95% CI) |
|---|---|---|
| ***Inflammatory response*** | < .001 | |
| Non sepsis | - | 1 |
| Sepsis | 0.479 | 0.573 (0.123 - 2.678) |
| Severe Sepsis | 0.014 | 0.143 (0.030 - 0.672) |
| Septic Shock | 0.434 | 0.545 (0.119 - 2.493) |
| ***VAP*** | 0.006 | 0.517 (0.324 - 0.827) |
| ***Renal failure*** | 0.001 | 0.446 (0.271 - 0.734) |
| ***Acinetobacter infection*** | < .001 | 0.099 (0.032 - 0.304) |

SDD: Selective Digestive Decontamination; VAP: ventilator associated pneumonia

**Table 3 (abstract 1117).** Nosocomial.Infection rates

| | | SDD | | P | RR |
|---|---|---|---|---|---|
| | | No | Yes | | (95% CI) |
| VAP /MV | VAP/1000 days of MV | 10.31 | 4.36 | | 0.423 (0.316 - 0.565) |
| *Urinary infections* | Infections/1000 days of catheter | 3.79 | 2.46 | .026 | 0.649 (0.443 - 0.951) |
| *Bacteremia related to catheter* | Bacteremias/1000 days of CVC | 3.59 | 3.85 | .733 | 1.074 (0.712 - 1.621) |
| *Secondary bacteremias* | Bacteremias./1000 ICU days | 4.69 | 1.98 | < .001 | 0.422 (0.296 - 0.599) |
| *ARB* | ARB /1000 ICU days | 9.59 | 2.83 | < .001 | 0.295 (0.227 - 0.383) |

SDD: Selective Digestive Decontamination; VAP: ventilator associated pneumonia;MV: mechanical ventilation; CVC: central venous catéter; ARB: Antibiotic resistant bacteria

### 1118

#### Compliance with hand hygiene guidelines in the context of glove usage

C. Siebers, M. Mittag, B. Grabein, M. Zoller, L. Frey, M. Irlbeck
Ludwig-Maximilians University of Munich, Munich, Germany
**Correspondence:** C. Siebers
*Intensive Care Medicine Experimental* 2018, **6(Suppl 2):**1118

**INTRODUCTION.** Hand hygiene is known to be the single most effective prevention measure of nosocomial infections. In a recent meta-analysis the pooled compliance rate (CR) was only 40% [1].

**OBJECTIVES.** Aim of this study was to determine the incidence of hand hygiene indications (HHI) with regard to the resulting CR in the setting of the treatment of critically ill patients (APACHE > 20 and mechanical ventilation). One special focus was put on glove usage to determine whether gloves were substituted for HD when an indication according to the WHO "my five moments" (M5M) occured.

**METHODS.** We performed a single-blinded direct observation on a mixed 24 beds ICU of a university hospital. To get reliable results we designed a 24/7 monitoring performed by an external, specially educated nurse experienced in working with ICU-patients suffering high contagious infectious diseases. To minimize the Hawthorne Effect the employees were told that observation was fulfilled to stratify recurrent procedures of their work with the goal to evaluate the possibility of reducing their workload. One single nurse was monitored in maximum over two shifts. In accordance to the concept of M5M all HHI and all HD were documented. Additionally, donning and doffing of gloves was recorded with regard to the different indications of HD. The CR is the quotient of HD and HHI.

**RESULTS.** In total, 21 shifts (168 h) spread over the whole week and divided to early, late and night shifts were accompanied. Over the entire examination period a total of 2036 indications for HD (12.1/h) per single patient were obtained. In fact, only 690 HD (4.1/h) were performed, which equates to a total compliance rate of 33.9%. The highest CR was observed for the indication "after patient contact" (nurses 50.0%, physicians 52.0%). CR was the worst for the indication "before aseptic measures" (nurses 22%, physicians 17%). Assuming that a glove change can replace a HD, the shame compliance rate in the total collective increased significantly to 72.1% (p< 0.0001; 64.5% nurses, 70.3% doctors, 89.1% other employees).

In regard to the group of nurses, there was an HHI around the clock on average every six minutes. About 17% of the total working time of a nurse would have to be applied for 100% correct hand hygiene application.

**CONCLUSIONS.** Our results show that compliance with hand hygiene in the ICU of a university hospital remains very low and requires constant optimization. HCW use gloves instead of performing adequate HD. Propably, alternative regimens for handling glove use, i.e. a clean your gloves campaign, should be discussed.

NT

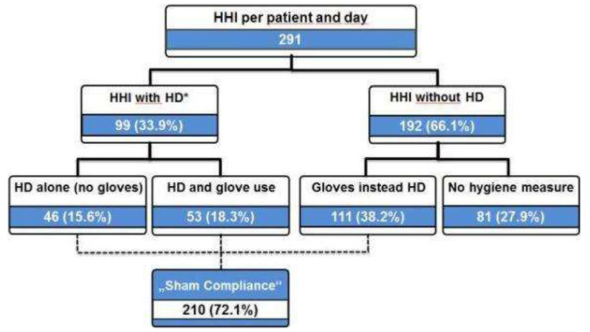

**Fig. 1 (abstract 1118).** Sham Compliance versus True Compliance Rate

| | Number of hand hygiene indications | | | | Compliance rate (CR) in % | | | |
|---|---|---|---|---|---|---|---|---|
| | nurses | doctors | others | all | nurses | doctors | others | all |
| **WHO "My 5 Moments" of hand hygiene** | | | | | | | | |
| ① Before touching a patient | 269 (38.4/d) | 44 (6.3/d) | 23 (3.3/d) | **336 (55.9/d)** | 32.3 | 27.3 | 13.0 | **30.4** |
| ② Before a clean/aseptic procedure | 653 (93.3/d) | 54 (7.7/d) | 3 (0.4/d) | **710 (101.4/d)** | 22.3 | 16.7 | 33.3 | **22.0** |
| ③ After body fluid exposure risk | 357 (51.0/d) | 51 (7.3/d) | 4 (0.6/d) | **412 (58.9/d)** | 33.6 | 31.4 | 50.0 | **33.5** |
| ④ After touching a patient | 301 (43.0/d) | 46 (6.6/d) | 23 (3.9/d) | **370 (52.9/d)** | 49.8 | 52.2 | 21.7 | **48.4** |
| ⑤ After touching patient surroundings | 439 (62.7/d) | 68 (9.7/d) | 2 (0.3/d) | **509 (72.7/d)** | 35.5 | 42.6 | 0.0 | **36.3** |
| **Total number of a M5M** | 2019 (288.4/d) | 263 (37.6/d) | 55 (7.9/d) | **2337 (333.9/d)** | 32.6 | 34.2 | 20.0 | **32.5** |
| **Number of necessary HHI** | 1728 (246.9/d) | 253 (36.1/d) | 55 (7.9/d) | **2036 (290.9/d)** | 34.2* | 34.8* | 20.0 | **33.9** |

Necessary number of hand hygiene indications (HHI) is different to the number of occurred moments for HD (M5M), because with single hand disinfection two directly consecutive M5M can be answered. * p=0.63. d=day

**Fig. 2 (abstract 1118).** Distribution of hand hygiene opportunities and compliance rates of the professional groups over a pe

### 1119

#### Effectiveness of disinfectant foot mat on incidence of surface, air colonization and hospital acquired infections in ICU of a tertiary care hospital in western India

R. Khasne[1], A. Kulkarni[2], K. Borawake[3], P. Jyothi[1], P. Tajane[1], M. Sonone[1], V. Kushare[1]
[1]Apollo Hospital, Critical Care Department, Nashik, India; [2]Tata Memorial Hospital, Critical Care Department, Mumbai, India; [3]Vishwaraj Hospital, Critical Care Department, Pune, India
**Correspondence:** R. Khasne
*Intensive Care Medicine Experimental* 2018, **6(Suppl 2):**1119

**INTRODUCTION.** Hospital acquired infection (HAI) is a global healthcare issue and various methods have been used to reduce colonization of ICU without significant effect on outcome [1]. Disinfectant foot mat has been used to reduce colonization [2]. We studied the efficacy of use of disinfectant foot mat in ICU in reducing colonization and HAI.
**OBJECTIVES.** The aim of our study was to assess the impact of use of disinfectant foot mat to reduce the air, surface colonization and subsequently on HAI in ICU.
**METHODS.** Prospective observational study was performed in the ICU of tertiary care hospital with mixed population from April 2017 to March 2018 conducted in two phases of 6 months each. In phase one (April to September 2017), ICU staff and visitors were made to use dedicated ICU slippers or shoe covers. In phase two (October 2017 to March 2018) disinfectant door mat was placed at the entrance of ICU and the staff and visitors were allowed to use their own footwear.
Primary outcome: Number of colony forming units (CFU) from various ICU surfaces, air samples which were randomly collected and plated on blood agar.
Secondary outcomes: Impact on incidence of HAI, ICU length of stay (ICULOS), ICU mortality.
Everyday mat was washed with liquid soap, sprayed with 1% glutaraldehyde-dimethanol and changed 4 hourly or when visibly dirty. Every month floor swab was collected from 6 to10 feet away from the foot mat and air sample was collected from the centre of ICU. Visitor policies and mopping frequencies remained same in both the groups. Statistical data was analysed with SPSS 21 using z test and t-test.
**RESULTS.** Results were as per table 1 and 2 given below.
**CONCLUSIONS.** Use of disinfectant mat did not result in a significant reduction in colonisation and incidence of HAI, ICULOS and ICU mortality compared to shoe covers or dedicated slippers.

##### REFERENCE(S)

1. Gupta A, et al. Impact of Protective Footwear on Floor and Air Contamination of Intensive Care Units. Medical Journal, Armed Forces India. 2007;63(4):334-336
2. Hornig KJ, et al. Evaluation of the Efficacy of Disinfectant Footmat for the Reduction of Bacterial Contamination on Footwear in a Large Animal Veterinary Hospital. Journal of Veterinary Internal Medicine. 2016;30(6):1882-1886

##### GRANT ACKNOWLEDGMENT

No funding was associated with the study

**Table 1 (abstract 1119).** ICU outcomes (1,683 patients admitted over 1year)

| | Phase 1(n=856) | Phase 2(n=827) | p value |
|---|---|---|---|
| HAI | 19(2.2%) | 10(1.2%) | [z=1.592 P=0.111] |
| ICULOS(days) | 4.53(±4.5) | 4.19(±3.7) | [t=1.67 P=0.095] |
| Mortality(%) | 5.5(47) | 6.7(55) | [z=-0.99 P=0.31] |

**Table 2 (abstract 1119).** Effect on surface and air colonisation in both phases

| | Phase 1(n=6) | Phase 2(n=6) | p value |
|---|---|---|---|
| Surface sample (mean CFU/month) | 117[19.5(±9.75)] | 66[11(±12.47)] | [t=1.31, P=0.21] |
| Air sample (mean CFU/month) | 147[ 24.5(±8.16)] | 111[18.5(±11.36)] | [t=1.05, P=0.31] |
| Organisms isolated (%) | GPC (66.66) GPC + GNR (33.33) | GPC, GNR, GPC + GNR each (33.33) | |

### 1120

#### An interventional program to reduce bacterial colonization rates of mobile phones carried by health care workers in intensive care units

P. Mogrovejo, A. Cabrera, A. Cedillo, V. Arízaga, N. Villavicencio, D. Martínez, A. Loja, L. Tamayo, H. Aguirre-Bermeo
Hospital Santa Inés, Intensive Care Unit, Cuenca, Ecuador
**Correspondence:** H. Aguirre-Bermeo
*Intensive Care Medicine Experimental* 2018, **6(Suppl 2):**1120

**INTRODUCTION.** Mobile phones (MP) of health care workers (HCWs) are colonized by pathogenic bacteria[1]. Therefore, they can be vectors of nosocomial infections. An interventional program could reduce the contamination of MP and the transmission of these high risk microorganisms.
**OBJECTIVE.** The aim of this study was to determine the prevalence of bacterial colonization of MP carried by HCWs in the intensive care units (ICU) and operating room (OR), and to evaluate an interventional program designed to reduce bacterial colonization rates.
**METHODS.** The study was performed in three phases. In the first phase (September to October 2017) sixty MP samples were randomly collected at the end of HCWs duty and inoculated into blood sheep and eosyn methilene blue agar for culture. In the second phase an interventional program was performed (from November 2017 to January 2018). This program consisted in: 1) Reinforcement of the World Health Organization programs (handrub and handwash in the 5 moments for hand hygiene) and 2) The implementation of a periodic MP cleaning program using microfiber cloth (1 time each day with water and each 5 days with a 60% isopropyl alcohol). In the third phase (February to March 2018) sixty MP samples were collected of the same HCWs that were evaluated in the first phase. These samples were collected in randomly order and processed in the same way as the first phase. In addition, all microbiological samples taken from the patients admitted in the ICU and OR during phases 1 and 3 of the study were analyzed.
**RESULTS.** Overall bacterial colonization rates of MP in phase 1 and 3 were n=57 (95%) and n=35 (58%), p=0.001 respectively. The MP colonization rates according to the isolated bacteria are detailed in table 1.
Isolation of pathogenic bacteria in microbiological patient samples in phase 1 and 3 were n=13/81 (16%) and n=7/75 (9%) respectively (p=0.21). The same pathogenic bacteria was present in both, mobile phone and patient sample in 10/13 (76%) in phase 1 and 6/7 (85%) in phase 3 (p=0.90). Most common isolated pathogenic bacteria in MP and patient samples are detailed in table 2.
**CONCLUSIONS.** We found high rates of pathogenic bacterial colonization in the MP carried by HCWs. The interventional program performed in this study significantly reduced MP colonization rates and could reduce the nosocomial infections.

**Table 1 (abstract 1120).** Mobile phone colonization rates according to isolated bacteria

| ISOLATED BACTERIA | PHASE 1 | PHASE 3 | *p*= |
|---|---|---|---|
| Non pathogenic, n (%) | 20 (33%) | 19 (32%) | 0.85 |
| Pathogenic non drug resitant, n (%) | 22 (37%) | 9 (15%) | 0.01 |
| Pathogenic drug resistant, n (%) | 15 (25%) | 7 (11%) | 0.05 |
| Negative, n (%) | 3 (5%) | 25 (42%) | <0.01 |
| TOTAL | 60 (100%) | 60 (100%) | - |

**Table 2 (abstract 1120).** Isolated pathogenic bacteria in mobile phones and patient samples

| PATHOGENIC BACTERIA | Mobile Phone Phase 1 | Mobile Phone Phase 3 | *p*= | Patient Samples Phase 1 | Patient Samples Phase 3 | *p*= |
|---|---|---|---|---|---|---|
| *Meticillin-susceptible Staphylococcus aureus, n (%)* | 14 (38) | 8 (50) | 0.15 | 1 (8) | 0 (0) | 0.96 |
| *Meticillin-resistant Staphylococcus aureus, n (%)* | 10 (27) | 4 (25) | 0.16 | 1 (8) | 0 (0) | 0.96 |
| *Resistant Staphylococcus epidermidis, n (%)* | 4 (11) | 0 (0) | 0.12 | 3 (23) | 0 (0) | 0.27 |
| *Acinetobacter baumannii, n (%)* | 5 (13) | 2 (13) | 0.43 | 0 (0) | 3 (43) | 0.22 |
| *Non drug resistant Klebsiella pneumoniae, n (%)* | 4 (11) | 0 (0) | 0.12 | 5 (38) | 0 (0) | 0.08 |
| *Multi drug resistant Klebsiella pneumoniae, n (%)* | 0 (0) | 1 (6) | 1.00 | 0 (0) | 3 (43) | 0.22 |
| *Escherichia coli, n (%)* | 0 (0) | 0 (0) | - | 3 (23) | 1 (14) | 0.67 |
| *Enterococcus faecalis, n (%)* | 0 (0) | 1 (6) | 1.00 | 0 (0) | 0 (0) | - |
| TOTAL | 37 (100) | 16 (100) | 0.001 | 13 (100) | 7 (100) | 0.21 |

### 1121

#### A practices-in-prevention and active surveillance survey of Ventilator Associated Pneumonia (VAP) across four London ICUs

N. Shah[1,2], J. Hadley[2,3], P. Zolfaghari[2,3], C. Hinds[2,4]
[1]Queen Elizabeth Hospital, Department of Chest Medicine, London, United Kingdom; [2]Queen Mary, University of London, London, United Kingdom; [3]Royal London Hospital, Intensive Care Medicine & Anaesthesia, London, United Kingdom; [4]St Bartholomew's Hospital, London, United Kingdom
**Correspondence:** N. Shah
*Intensive Care Medicine Experimental* 2018, **6(Suppl 2):**1121

**INTRODUCTION.** We hypothesised that management strategies and active surveillance for VAP varies widely across intensive care units, and that in clinical practice the criteria used for antibiotic treatment of chest sepsis differ from published definitions of VAP.

**OBJECTIVES.** To determine the management and prevention strategies, including active surveillance monitoring and diagnostic criteria, of VAP across four London ICUs (Barts Health NHS Trust, London, UK): The Royal London (RLH-trauma/neuro/general), St. Bartholomew's Cardiac ICU (SBH cardiac), St.Bartholomew's (SBH-general/oncology), and Whipps Cross University Hospital (WXH-general).

**METHOD.** Audit data was collected and managed using a purpose built REDCap™ (Research Electronic Data Capture) portal hosted at Queen Mary University of London (UK), designed to take 30 minutes per centre to complete. Units were given 30 days to collect data in April 2016, from 12-month ICNARC dataset (except St. Bartholomew's hospital who supplied 6-month data following recent merger). The audit was registered with the clinical effectiveness unit.

**RESULTS.** There was a varied mix of Level-3 patients across the units surveyed in keeping with their speciality areas. Table 1 shows characteristics of these units.

All 4 units used VAP care bundles with the same core components: head of bed elevation, sedation level assessment with daily sedation interruption (unless contraindicated), oral care with chlorhexidine mouthwash (0.125%), teeth brushing, ventilator circuit changes, hand hygiene using alcohol before airway management, tracheal tube cuff pressure control and daily review of stress ulcer prophylaxis.

Table 2 shows variance in the VAP care bundles. All 4 units audited compliance with their VAP care bundle with 2 units performing this continuously. Two units were aware of their VAP rate from local audits in the preceding 12 months (12 and 15 VAP cases per 1000 ventilator days). Three units had no strict VAP definition for audit and the remaining ICU used a simplified definition of a patient commenced on antibiotics for chest sepsis 48 hours or more after initiation of invasive mechanical ventilation. Two units audited frequency of causative pathogens, with Enterobacteriaceae being most prevalent in both cases. Two units had an antibiotic treatment policy for VAP. Two units had access to quantitative microbiological results (e.g. CFU/mL) and the remaining 2 used semi-quantitative results (e.g. +, ++, +++) from local laboratories.

**CONCLUSIONS.** This pilot study highlights considerable variation in the management strategies and active surveillance for VAP, even within the same NHS Trust. The methodology used showed good feasibility to carry out a wider national/international VAP study and identify areas of good practice.

**ACKNOWLEDGMENTS**
Our thanks to Dr Freida Keane, Dr Malgorzata Starczewska and Dr Caroline Fitton. Support from Barts Charity Clinical Research Training Fellowship Grant.

**Table 1 (abstract 1121).** ICU characteristics of units surveyed

| | | Barts Heart | SBH | RLH | WXH |
|---|---|---|---|---|---|
| | L3 Capacity | 39 | 11 | 28 | 9 |
| | Median Age (Range) | 67 (35-84) | 64 (32-87) | 54 (16-96) | 66 (19-93) |
| Monthly mean (over 12 months) | Admissions | 147 | 27 | 109 | 56 |
| | Bed days | 505 | 149 | 890 | 274 |
| | IMV (all) | 100 | 17 | 79 | 23 |
| | IMV (>48hrs) | 9 | 9 | 66 | 13 |
| Length of MV (days) | All | 0.3 | 2.4 | 3.0 | 2.0 |
| | >48hrs | 7.8 | 6.6 | 4.0 | 6.0 |

*L3* ICS Level 3 Criteria, *SBH* Barts Hospital, *RLH* Royal London, *WXH* Whipps Cross, IMV Admission requiring invasive mechanical ventilation, *MV* Length of Mechanical Ventilation in median days.

**Table 2 (abstract 1121).** Variation in the management strategies and active surveillance for VAP

| | | Unit 1 | Unit 2 | Unit 3 | Unit 4 |
|---|---|---|---|---|---|
| Oral Care | Chlorhexidine (hrly) | 6 | 6 | 6 | 12 |
| | Teeth brushing (hrly) | 12 | 12 | 12 | 12 |
| Tracheal tube | Cuff pressure (hrly) | 4 | 4 | 12 | 8 |
| | Circuit change (hrly) | >72 | 24 | 48-72 | 48-72 |
| | Sub-glottic suction | No | No | Yes* | No |
| | Polyurethane cuffs | No | No | No | Yes* |
| Routine | Tracheal aspirates | Yes | Yes | Yes | Yes |
| | Non-directed BAL | No | No | No | Yes |
| | Targeted BAL | No | No | No | No |

*BAL* Bronchoalveolar lavage; *Available on individual patient basis

### 1122

#### Influence of prevention programs (ZERO projects) on the incidence of pneumonia associated with mechanical ventilation (VAP) in critically ill cardiac patients admitted to the Spanish ICU from the National Registry ENVIN-HELICS

A. Escriba Barcena[1], E. Renes[2], M. Palomar Martinez[3], J. Alvarez Rodriguez[4], M. Catalan Gonzalez[2], F. Alvarez Lerma[5], S. Otero Romero[6], S. Uriona Turma[6], X. Nubials Casals[3], J.C. Montejo Gonzalez[7]
[1]Hospital 12 Universitario de Fuenlabrada, Servicio de Medicina Intensiva, Fuenlabrada, Spain; [2]Hospital 12 Universitario de Octubre, Servicio de Medicina Intensiva, Madrid, Spain; [3]Hospital Universitari Arnau de Villanova, Servicio de Medicina Intensiva, Lerida, Spain; [4]Hospital Universitario de Fuenlabrada, Servicio de Medicina Intensiva, Fuenlabrada, Spain; [5]Hospital del Mar. Universitat Autónoma, Servicio de Medicina Intensiva, Barcelona, Spain; [6]Hospital Universitario Vall de Hebron, Servicio de Medicina Preventiva, Barcelona, Spain; [7]Hospital Universitario 12 de Octubre, Servicio de Medicina Intensiva, Madrid, Spain
**Correspondence:** A. Escriba Barcena
*Intensive Care Medicine Experimental* 2018, **6(Suppl 2):**1122

**INTRODUCTION.** The ZERO projects are a nation multimodal intervention based on the implementation of evidence-based bundle measures that have proven effective in preventing infections associated with healthcare in the ICU. There is little information on the effect of these programs on critically ill cardiac patients, traditionally considered as low risk patients.

**OBJECTIVES.** To Know the influence of ZERO projects on the incidence of VAP in the
subgroup of critically ill cardiac patients.

**METHODS.** Patients from the national registry ENVIN-HELICS were included in the period April-June, from 2007 to 2015, of those units that incorporated patients all theperiods ,the reason for admission being an acute cardiac process and subject to mechanical ventilation(VM) more than 24h. 4 periods are considered in relation to the ZERO projects: Previous, Bacteriemia Zero(BZ), Neumonia Zero(NZ) and post.The incidence rate (IR) is estimated in each period. A multivariate analysis was performed by Poisson regression using the statistical program SPSS 20.

**RESULTS.** The IR of VAP in the basal period was 16.35 episodes / 1000 days of MV and decreased to 12.48 in the BZ period, 10.56 in the NZ period and 7.72 in the post-intervention period. Tables I, II and III show the result of the multivariate analysis.

**CONCLUSIONS.** Multivariate analysis has shown the high effectiveness of ZERO projects in the reduction of VAP in critically ill cardiac patients.

**REFERENCE(S)**

Álvarez-Lerma F, et al. Prevention of Ventilator-Associated Pneumonia: The Multimodal Approach of the Spanish ICU "Pneumonia Zero" Program. Crit Care Med. 2018 Feb;46(2):181-188.

**Table 1 (abstract 1122).** Multivariate analysis (I)

| Parameter | Incidence Ratio | Wald confidence interval(95%) | | OR Exp(B) | Wald confidence interval Exp(B) 95% | | p value. |
|---|---|---|---|---|---|---|---|
| | | Lower | Upper | | Lower. | Upper. | |
| Intersect | | | | 0,016 | 0,013 | 0,021 | 0 |
| Post-intervention period. | 7,22 | 6,79 | 8,74 | 0,287 | 0,225 | 0,365 | 0 |
| "Neumonia ZERO" period. | 10,56 | 9,14 | 12,14 | 0,518 | 0,408 | 0,656 | 0 |
| "Bacteriemia ZERO" period. | 12,48 | 10,82 | 14,31 | 0,627 | 0,495 | 0,793 | 0 |
| Baseline period | 16,35 | 14,58 | 18,29 | 1 | | | |
| ICUs without full participation in the project | 11,69 | 10,28 | 13,23 | 1,357 | 1,109 | 1,661 | 0,003 |
| ICUs with full participation in the project | 10.91 | 10,12 | 11,75 | 1 | | | |

**Table 2 (abstract 1122).** Multivariate analysis(II)

| Parameter | Incidence rate | Wald confidence interval 95% | | OR Exp (B) | Exp (B) Wald confidence interval 95% | | p value |
|---|---|---|---|---|---|---|---|
| | | Lower | Upper | | Lower | Upper | |
| Medical admission | 11,62 | 10,74 | 12,55 | 1,713 | 1,331 | 2,204 | 0 |
| Emergency surgery | 10,94 | 9,3 | 12,79 | 1,862 | 1,305 | 2,655 | 0,001 |
| Non emergency surgery | 9,92 | 8,68 | 11,28 | 1 | | | |
| Hospital/another ICU admission. | 12,24 | 9,76 | 15,15 | 1,24 | 1,09 | 1,511 | 0,032 |
| Out hospital/nursing home admission | 12,01 | 10,9 | 13,19 | 1 | | | |
| Female | 9,21 | 8,13 | 10,39 | 0,691 | 0,567 | 0,841 | 0 |
| Male | 12,08 | 11,2 | 11,75 | 1 | | | |

**Table 3 (abstract 1122).** Multivariate analysis(III)

| Parameter | Incidence rate | Wald confidence interval 95% | | OR Exp(B) | Exp (B) Wald confidence interval 95% | | p value |
|---|---|---|---|---|---|---|---|
| | | Lower | Upper | | Lower | Upper | |
| CRRT | 11,98 | 10,32 | 13,84 | 2,012 | 1,437 | 2,817 | 0,00 |
| Non CRRT | 10,95 | 10,19 | 11,75 | 1 | | | |
| Invasive devices | | | | 1,074 | 1,069 | 1,079 | 0,000 |
| APACHE 2 | | | | 1,013 | 1,003 | 1,002 | 0,007 |
| Device/CRRT interaction | | | | 0,981 | 0,971 | 0,992 | 0,000 |
| Device/Non CRRT interaction | | | | 1 | | | |

### 1123

#### Endotracheal tubes with sub-glottic suction ports to reduce suspected ventilator-associated pneumonia... are our ICU patients worth it?

R. Hart, C. Bradley, E. Black, R. Docking
Queen Elizabeth University Hospital, Anaesthesia and Critical Care, Glasgow, United Kingdom
**Correspondence:** R. Hart
*Intensive Care Medicine Experimental* 2018, **6(Suppl 2):**1123

**INTRODUCTION.** Ventilator-associated pneumonia (VAP) is the most common healthcare associated infection (HAI) in ventilated patients. VAPs are associated with an increased duration of mechanical ventilation and mortality. For this reason, VAP prevention bundles are commonly used in our ICUs. The Intensive Care Society (ICS) recommend the use of sub-glottic drainage endotracheal tubes (SGT) as part of this bundle.

**OBJECTIVES.** In this study, we aim to assess whether the introduction of SGT has any influence on the rate of suspected VAP.

**METHODS.** A two-month trial period of SGT was implemented in our unit. Education was delivered to our department to ensure all front line staff were aware of the practical differences between SGT and standard endotracheal tubes. A data collection tool was devised using Microsoft Excel to allow patients with SGT to be audited. All patients with a SGT were included in our data collection which gathered daily HAI data and patient demographics. If VAP was suspected, then further clinical information was obtained to determine if this episode could be formally diagnosed as a VAP according to our existing diagnostic criteria (HELICS).

**RESULTS.** During the trial period 26 patients received a SGT. Median patient age was 51. The most common admission diagnosis was pneumonia. Median duration of mechanical ventilation was 7 days. There was one episode of suspected VAP, which did not meet the HELICS criteria for a formal VAP diagnosis.

**CONCLUSIONS.** There is a strong evidence base supporting the use of SGT demonstrated by three meta-analysis and a recent RCT. A recent national audit by the Scottish Intensive Care Society also demonstrated a reduction in VAP rates in SGT patients. Given the health economic implications of VAP, there are public, political and regulatory interest to minimise the rate of VAPs in our units. SGT are more expensive than standard ETT. We estimate in our unit, adopting routine SGT would cost £3,400. A one week course of temocillin to treat VAP would cost £700 per patient, therefore we would only need to prevent 7 VAPs per year for routine SGT to be cost effective. This is in comparison to almost £30,000 per year spent on dexmedetomidine in our unit to prevent and treat delirium. We believe our ICU patients are worth the cost of SGT in order to prevent VAP. In the current climate of antimicrobial resistance, SGT may also be an economical approach to reduce the antibiotic burden in our patients.

##### REFERENCE(S)

1. Damas P, et al. Prevention of ventilator-associated pneumonia and ventilator-associated conditions. Crit Care Med 2015; 43:22-30.
2. Dezfulian C, et al. Subglottic secretions drainage for the prevention of ventilator-associated pneumonia: A meta-analysis. Am J Med 2005; 118:11-18

##### GRANT ACKNOWLEDGMENT

None

### 1124

#### Influence of the Zero protocols and their patient safety protocol in catheter-associated urinary infection

R. Fernandez[1], M.I. Ruiz[2], J.F. Machado[1], M. Guerrero[2], M. Gordillo[2]
[1]Hospital Universitario Virgen de las Nieves, Granada, Spain; [2]Complejo Hospitalario de Jaen, Jaén, Spain
**Correspondence:** M. Guerrero
*Intensive Care Medicine Experimental* 2018, **6(Suppl 2):**1124

**INTRODUCTION.** Patient safety is the fundamental principle of health care and a critical component of quality management. It depends on the interaction and the permanent balance of multiple actions of the health system and of its professionals.

Urinary tract infection is the most common nosocomial infection, accounting for 23 to 30% of all infections acquired during hospitalization, with a prevalence of 2%. Increases hospital stay and hospital costs. Mortality is low and is particularly associated with secondary bacteremia. Bladder catheterization (CV) is the most influential factor for developing an urinary tract infection (UTI).

**OBJECTIVES.**

- Evaluate if the application of zero projects can influence to reduce ITU
- Study ITU evolution

**METHODS.**

- Admitted to ICU more than 24 hours from 01.04.10, until 31.10.17.
- Rate per 100 patients admitted (N 3928 patients) and the incidence density (ID) per 1000 urinary catheter days (23288).
- Application of the Zero projects since April 2010 (Pneumonia Zero, Bacteremia Zero) and the patient safety protocol they associate.

**RESULTS.**

01.04.10-31.12.10- 2.96/admitted, 3.79/ catheter days
01.01.11-31.12.11- 3.32/admitted, 4.99/catheter days
01.01.12-31.12.12-2.46/admitted, 4.82/catheter days
01.01.13-31.12.13- 0.93/admitted, 3.1/catheter days
01.01.14-31.12.14- 6.22/admitted, 7.61/catheter days
01.01.15-31.12.15- 2.41/admitted, 5.56/catheter days
01.01.16- 31.12.16- 3.26/admitted, 5.56/catheter days
01.01.17-31.12.17- 2.37/admitted, 4.51/catheter days

**CONCLUSIONS.** In view of the results, we can see that the ITU figures have remained stable except for one year, in which there was a rebound. The protocol of patient safety, hand hygiene and the greater dedication to avoid infections involving the Zero projects, have helped to control the number of these infections.

### 1125

#### Oral model infusion of chlorhexidine. A novel method for prevention of bacterial colonization

A. Omar[1,2,3], F. Teunissen[4], S. Aboulnaga[1], S. Hanoura[1], S. Doiphode[5], A. Alkulaifi[1]
[1]Hamad Medical Corporation, Cardiothoracic Surgery, Doha, Qatar; [2]Beni Suef University, Critical Care, Beni Suef, Egypt; [3]Weill Cornell Medical College in Qatar, Clinical Medicine, Doha, Qatar; [4]Hamad Medical Corporation, Academic Health System, Doha, Qatar; [5]Hamad Medical Corporation, Microbiology Department, Doha, Qatar
**Correspondence:** A. Omar
*Intensive Care Medicine Experimental* 2018, **6(Suppl 2):**1125

**INTRODUCTION.** Colonization and microaspiration made the foundation of ventilator-associated pneumonia (VAP) pathogenesis. Koeman et al., found that topical decontamination of the oral cavity with chlorhexidine (CHX) reduced the VAP incidence from 18 to 13% [1]. The permanence of antiseptic effect in the oral cavity is doubtful due to dilution effect with salivary secretions; we hypothesize that continuous oral antiseptic infusion may offer a better decontamination.

**OBJECTIVES.** We developed endotracheal tube with additional port that allows continuous oral infusion of CHX, and we want to test the technique versus the slandered on bacterial colonization. (Provisional patent: 62359944)

**METHODS.** Two identical bio models for the upper airways were created by (3DX Diagnostics, USA) to admit the modified and the ordinary endotracheal tubes (ETT). The two techniques tested were using six hourly CHX disinfection (group A) versus continuous disinfection over 24 hours infusion technique (Group B). Five microorganisms plus mixed bacteria were used and each was tested for nine times. Normal saline was used constantly to irrigate the biomodels and 10 ml aliquot was collected by the end of the experiment. The aliquots from decanted broth post disinfection was cultured. The time to apply CHX by practitioner was also compared
RESULTS: Lower bacterial growth trend was noted in group A in 5 experiments which reach statistical significance only with pseudomonas aeruginosa (p=0.05). In one experiment the growth was lower in group B. Additionally, time saving advantage was observed in group B (12±3.1 versus 3.9±1.1 min, p=0.01).
CONCLUSIONS: The novel technique got at least non inferior results, plus time saving advantage. These results may warrant future clinical trial.

**REFERENCE(S)**

1) Koeman M, et al. Am J Respir Crit Care Med. 173(12):1348-55, 2006.

**GRANT ACKNOWLEDGMENT**

To all members of the microbiology and cardiothroacic surgery, academic health system Hamad medical corporation.

## Predicting unwanted outcomes

### 1126

#### Impact of Kaizen methodology (Lean Health Care) on the intra-hospital management of acute stroke

S. Valenzuela[1], M.A. Font[2], A. Mas[3], D. Pumar[4], J. Masip[3]
[1]Hospital Sant Joan Despí Moisès Broggi, Intensive Care Unit, Barcelona, Spain; [2]Hospital Sant Joan Despí Moisés Broggi. Consorci Sanitari Intregral, Neurology Department, Barcelona, Spain; [3]Hospital Sant Joan Despí Moisés Broggi. Consorci Sanitari Intregral, Intensive Care Unit, Barcelona, Spain; [4]Hospital Sant Joan Despí Moisés Broggi. Consorci Sanitari Intregral, Quality Department, Barcelona, Spain
**Correspondence:** S. Valenzuela
*Intensive Care Medicine Experimental* 2018, **6(Suppl 2):**1126

**INTRODUCTION.** The activation of “acute stroke protocols” (ASP) has proven to be effective in decreasing the latency time for starting therapy in emergency scenarios. The door-to-needle time (DNT) defined as the time from hospital admission to treatment, is often delayed for avoidable reasons. The standard recommendation in our health system for reference sites of ASP, is a median DNT< 35 minutes (m). Kaizen methodologies with real-time asset tracking technology have demonstrated its utility in developing more efficient workflows.
**OBJECTIVES.** Because the DNT in our hospital exceeded this time, we tried to implement Kaizen strategies within the Lean Healthcare Management System (LHMS) in order to identify improvement measures for shortening DNT.
**METHODS.** The Kaizen team was composed by a leader, an external methodological expert and a working team including a representative of the different professionals involved in the procedure (neurologist, emergency physician, intensive care specialist, emergency nurse, radiologist and auxiliary personnel). A simulation of the activation of ASP was videotaped and the time spent in the whole process was divided into different small activities (administrative tasks, transfers, material storage, circuits and medical/nursing actions). After analyzing these periods, several improving measures were suggested according to their value and necessity. Once evaluated, the new workflow was standardized and approved. Training was carried out for all the professionals involved.
**RESULTS.** From June 2016 to December 2016, the Kaizen methodology was implemented in our site in the ASP scenario. There were 6 discussion meetings, more than 50 identified activities and there were finally accorded among others, 13 main improving measures. After a training program, the new workflow was implemented. The median DNT felt from 48m in 2016 (22 patients) to 23m in the end of the year 2017 (30 patients). There also was a dramatic decrease in the inter-case time variability, estimated by the standard deviation (SD), which felt an 80% at the end of the period (57m to 11m). The course of the DNT and SD for each period is shown in the figure.
**CONCLUSIONS.** The application of Kaizen (LHC) methodology in the acute stroke program led to a reduction of the DNT and the inter-case variability. This methodology may be a reliable tool to improve the management of patients in acute clinical scenarios.

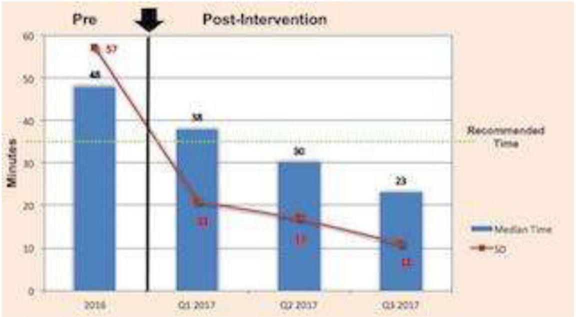

**Fig. 1 (abstract 1126).** Course of the DNT and SD for each period

### 1127

#### Quality improvement program outcomes for endotracheal intubation in the emergency department

M.G. Kim[1], S.Y. Hwang[1], J.H. Park[1], H. Yoon[1], W.C. Cha[1], I.J. Jo[1], M.S. Sim[1], K.J. Song[1], H.J. Woo[2], S.G. Jeong[2], T.G. Shin[1], J.M. Kim[1], Samsung Medical Center Emergency Airway Management Team
[1]Samsung Medical Center, Sungkyunkwan University School of Medicine, Department of Emergency Medicine, Seoul, Korea, Republic of; [2]Samsung Medical Center, Department of Emergency Nursing, Seoul, Korea, Republic of
**Correspondence:** M.G. Kim
*Intensive Care Medicine Experimental* 2018, **6(Suppl 2):**1127

**INTRODUCTION.** Endotracheal intubation (ETI) is a life-saving procedure for critically-ill patients in the emergency department (ED). However, several obstacles, including an uncontrolled environment, lack of skilled workforce and advanced equipment, and unstable patients with limited physiologic reserve are challenges associated with ETI in the ED. Difficult intubations during emergent ETI can occur up to twice as often compared to the operating room.[1] In addition, a number of life-threatening complications including failed intubation, severe hypoxemia, and severe hypotension are associated with emergent ETI.[1,2]
**OBJECTIVES.** We described our 3-year experience and evaluated endotracheal intubation (ETI) outcomes during a multi-disciplinary emergency department (ED)-based quality improvement (QI) program.
**METHODS.** This was a single-center, observational study taking place during a QI program. We used a registry for airway management performed in the ED from April 2014 to February 2017. The QI program focused on procedural standardization, airway management education, and comprehensive preparation of airway equipment. The primary outcome was the first pass success (FPS) rate. The secondary outcomes were multiple attempts rate and overall rate of complications.
**RESULTS.** A total of 1,087 emergent ETIs were included. The FPS rate significantly increased from 68% in the first year to 74% in the second year and 79% in the third year (P for trend < 0.01). The multiple attempts rate in the first year was 12%, followed by 7% and 6% in the second and third years, respectively (P for trend < 0.01). The overall complication rate was 16% in the first year, 8% in the second year, and 8% in the third year (P for trend < 0.01).
**CONCLUSIONS.** We observed improved outcomes of ETI in the ED, including FPS rate, multiple attempts, and overall complication rates during the multi-disciplinary QI program to enhance patient safety.

**REFERENCE(S)**
1. Martin LD, Mhyre JM, Shanks AM, et al. 3,423 emergency tracheal intubations at a university hospital: airway outcomes and complications. Anesthesiology. 2011;114:42-48.
2. Brown CA, 3rd, Bair AE, Pallin DJ, et al. Techniques, success, and adverse events of emergency department adult intubations. Ann Emerg Med. 2015;65:363-370.e361.

**GRANT ACKNOWLEDGMENT**
Not Applicable

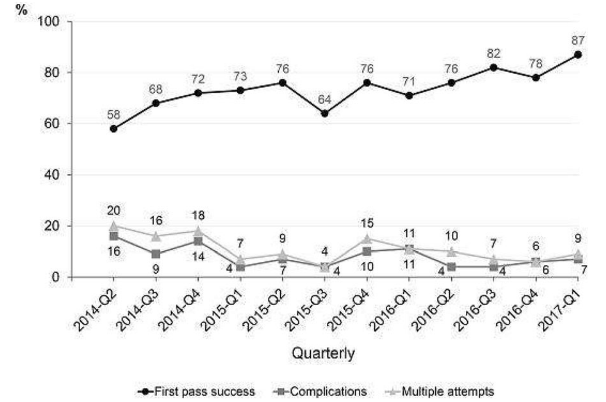

**Fig. 2 (abstract 1127).** Run chart showing outcomes per quarter

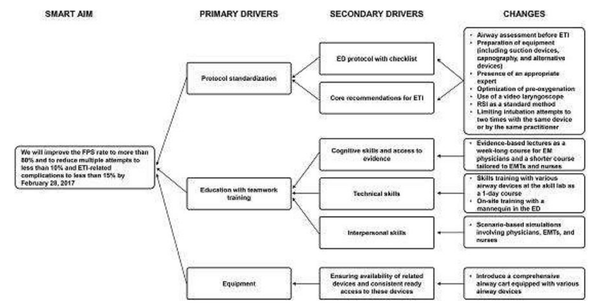

**Fig. 2 (abstract 1127).** Driver diagram for improvement in airway management

### 1128

#### Human failures and ineffective communications in the patient safety events related to the use of mechanical ventilation for the hospitalized patients: an exploration of the institutional incident reporting system of a medical center

J.-S. Jerng[1,2], G.-L. Fann[3], C.-L. Wu[4], S.-H. Yang[3], L.-C. Chen[2], H.-F. Huang[2], J.-S. Sun[2,5]
[1]National Taiwan University Hospital, Department of Internal Medicine, Taipei, Taiwan, Province of China; [2]National Taiwan University Hospital, Center for Quality Management, Taipei, Taiwan, Province of China; [3]National Taiwan University Hospital, Department of Nursing, Taipei, Taiwan, Province of China; [4]National Taiwan University Hospital, Department of Integrated Diagnostics & Therapeutics, Taipei, Taiwan, Province of China; [5]National Taiwan University Hospital, Department of Orthopedic Surgery, Taipei, Taiwan, Province of China
**Correspondence:** J.-S. Jerng
*Intensive Care Medicine Experimental* 2018, **6(Suppl 2):**1128

**INTRODUCTION.** Care of the patients under mechanical ventilation (MV) requires intensive human participation and teamwork. In this high-risk and high-stress care setting, human failures might play a significant role in the occurrence of adverse events.

**OBJECTIVES.** To investigate the human failures and ineffective communications in the reported patient safety events related to the use of MV for patients with respiratory failure.

**METHODS.** We conducted a retrospective analysis of the reported patient safety events related to the use of MV for patients with respiratory failure from the Incident Reporting System of a university-affiliated medical center from 2011 to 2017. The investigators analyzed all of the available descriptions by the reporters and comments from their supervisors to infer the possibility of human errors, focusing on unsafe acts and their preconditions of the related care and institutional processes.

**RESULTS.** From the 19,080 cases of reported patient safety events during the study period, we identified 474 (2.5%) MV-related events. Of these 474 events, 428 (90.3%) were artificial airway-related events, whereas 29 (6.1%) were concerned with ventilator management, and 17 (3.6%) were patient-care events. Patient harms presented in 223 (47.0%) cases, of which 192 (81.1%) were artificial airway-related events. Content analysis of the reporting descriptions and comments showed that 174 (36.7%) were deemed having unsafe acts as conditions with human failure, including 25 (14.4%) related to perceptual errors, 53 (30.5%) related to decision errors, and 97 (55.7%) related to action errors. Preconditions for these unsafe acts included adverse mental state (79, 45.4%), technological environment (42, 24.1%), physical/mental limitation (33, 19.0%), crew resource management (10, 5.7%), physical environment (5, 2.9%), and adverse physiological state (5, 2.9%). In the 145 cases related to artificial airway having unsafe acts, 83 (56.6%) were action errors, and the most commonly related care processes were artificial airway care (47, 32.4%), physical restraint (44, 30.3%), and patient positioning (33, 22.8%). Moreover, we found 27 (5.7%) cases with ineffective communications, of which 13 (48.1%) were artificial airway-related events.

**CONCLUSIONS.** Human failures and ineffective communications may play a significant role in the occurrence of patient safety events related to the use of mechanical ventilation. To improved the safety of care of mechanically ventilated patients, emphasis should be addressed to the prevention of action error, especially to the errors related to the processes of artificial airway care, physical restraint, and patient positioning.

**REFERENCE(S)**
1) Shappell SA, Wiegmann DA. A human error approach to aviation accident analysis: the human factors analysis and classification system. England: Ashgate Publishing, Ltd, 2012.
2) Reason J. Human error: models and management. BMJ 2000;320:768-70.

**GRANT ACKNOWLEDGMENT**
None.

### 1129

#### Residents' and nurses' attitudes to a rapid response team in a tertiary hospital

D.S. Lee, S.Y. Lim, H.J. Min, Y.Y. Choi, M.A. Yun, B.N. Lee, Y.J. Lee
Seoul National University Bundang Hospital, Interdepartment of Critical Care Medicine, Seongnam, Korea, Republic of
**Correspondence:** D.S. Lee
*Intensive Care Medicine Experimental* 2018, **6(Suppl 2):**1129

**INTRODUCTION.** Residents and nurses are the main group of clinicians who activate the rapid response team (RRT), placing them in an excellent position to provide valuable insights regarding the effectiveness of this system.

**OBJECTIVES.** Purpose of our descriptive study is to assess whether residents and nurses value the RRT service and to determine barriers to calling the RRT exist in a 1400 bed teaching hospital.

**METHODS.** We conducted a modified personal interview, using a 17-item Likert agreement scale questionnaire from January to May 2017.
**RESULTS.** A total of 322 ward nurses and 30 residents (100% response rate) returned their completed surveys. Overall, the majority of nurses and residents were satisfied with the RRT, with suggestions for simplifying and educating the RRT activation criteria. Most responders suggested that they would make a call to RRT team even if the patient's vital signs were normal or the patients did not fulfill RRT criteria. However, both nurses and residents feared criticism of activating RRT from colleagues that the patient was not sufficiently unwell to transfer to ICU (76% and 83% respectively). Despite hospital RRT protocol, 95% of nurses would call the covering doctor and 67% of residents would discuss with other residents about patients' management, before activating the RRT for a sick ward patient.
**CONCLUSIONS.** Nurses and residents value the RRT service and appreciate its potential benefits. The major barrier to calling the RRT appears to be awareness of honor in Asian culture, and allegiance to the traditional approach of initially calling covering doctors, rather than fear of criticism for calling to the RRT team.

**REFERENCE(S)**
1. Jones DA, DeVita MA, Bellomo R (2011) Rapid-response teams. N Engl J Med 365: 139-146.
2. McGaughey J, Alderdice F, Fowler R, Kapila A, Mayhew A, et al. (2007) Outreach and Early Warning Systems (EWS) for the prevention of intensive care admission and death of critically ill adult patients on general hospital wards. Cochrane Database Syst Rev: CD005529.
3. Beitler JR, Link N, Bails DB, Hurdle K, Chong DH (2011) Reduction in hospital-wide mortality after implementation of a rapid response team: a long-term cohort study. Crit Care 15: R269.
4. Jones D, Baldwin I, McIntyre T, Story D, Mercer I, et al. Nurses' attitudes to a medical emergency team service in a teaching hospital. Quality and Safety in HealthCare 2006;15:427-432.
5. Jones L, King L, & Wilson C. A literature review: Factors that impact on nurses' effective use of the Medical Emergency Team (MET). Journal of Clinical Nursing 2009;18:3379-3390.

### 1130
### Golden hour for ICU admission - don't get late!

V. Loureiro[1,2], S. Castro[1], A. Marreiros[2], C. Granja[1,2,3]
[1]Algarve University Hospital Center, Intensive Care Unit, Emergency and Intensive Care Department, Faro, Portugal; [2]University of Algarve, Biomedical Sciences and Medicine Department, Faro, Portugal; [3]Center for Health Technology and Services Research, Porto, Portugal
**Correspondence:** V. Loureiro
*Intensive Care Medicine Experimental* 2018, **6(Suppl 2):**1130

**INTRODUCTION.** The delay in the recognition and early admission of critically ill patients to the ICU has been associated to an increase in the mortality (1). There is no consensus regarding the optimal time for ICU admission, as this may depend on specific organizational features. We conducted this study in order to understand if our own organization would need changes in the critical care patient circuit.
**OBJECTIVES.** To identify patients' clinical and demographic characteristics associated with the delayed admission to the ICU and if such delay is associated to worse clinical outcomes, namely ICU and hospital mortality and length of stay.
**METHODS.** Based on previous works (2), we defined that four hours would be the cut-off for delayed (DICU) or non-delayed ICU admission (NDICU). Exclusion criteria included scheduled admissions, transfers from other hospital, transfers from the wards, readmissions and organ donor patients. The clinical and demographic characteristics of both groups were compared, as well as their outcomes.
**RESULTS.** From a total of 592 ICU admissions, 443 were excluded. From the remaining 149 patients, 109 (71,8%) were on the DICU group. These patients were statistically significantly older (67 vs. 55, *p*=.001), with higher scores of SAPS II (59,40 vs. 47,24, *p*= .003) and SOFA (9,62 vs 7,76, *p*=.012), with more non-scheduled surgery patients (41 vs. 5, *p*=.002) and with septic shock (49 vs. 5, *p*=.000). The NDICU group had statistically significantly more cardiogenic shock patients (12 vs. 8, *p*=.001). DICU patients had higher mortality rate (47,6% vs. 26,2%, *p*=.017) and more days of vasopressors support (3,94 vs. 2,38, *p*=.009). There was no statistically significant differences in ICU or hospital length of stay between the two groups. Patients with septic shock submitted to non-scheduled surgery had a delay of 22,19 h versus 14,29 h when compared to non-scheduled surgery patients without septic shock.
**CONCLUSIONS.** Delay to ICU admission was significantly associated with ICU and hospital mortality and significantly worse severity scores. Possible explanations for these findings include a deterioration of the clinical condition of patients during the time before ICU admission. Non-scheduled surgery patients presented a delay much higher than 4h that can't be fully explained by the surgery time. These findings may suggest that an improvement on the circuit of critical patients is needed.

**REFERENCE(S)**
1. Goldhill DR, McNarry AF (2004) The longer patients are in hospital before Intensive Care admission the higher their mortality. 1908-1913.
2. Chalfin DB, Trzeciak S, Likourezos A, Baumann BM, Dellinger RP (2007) Impact of delayed transfer of critically ill patients from the emergency department to the intensive care unit. Crit Care Med 35: 1477-1483.

### 1131
### Can we improve the quality of critical care referrals?

N. Robin, R. Gale, S. Singh
Countess of Chester Hospital, Anaesthesia & Critical Care, Chester, United Kingdom
**Correspondence:** N. Robin
*Intensive Care Medicine Experimental* 2018, **6(Suppl 2):**1131

**INTRODUCTION.** Selecting patients who will benefit from critical care treatment requires skill, judgment and experience. Poor selection subjects patients to potential harm, may prolong suffering and is not cost effective.
**OBJECTIVES.** To assess the quality of referrals to critical care in order to improve the referral pathway.
**METHODS.** Using the electronic patient record system (Meditech) we identified 107 sequential referrals to the ICU. For each patient the following information was recorded: Time of referral
Grade of the referring clinician
Most senior clinician involved in referral
Significant comorbidity and life limiting disease
Functional status (FS): good (G), independent (I), poor(P), very poor (VP)
Admission decision
Hospital outcome
Data was reviewed independently by 2 ICU consultants (blinded to the referring team and the duty critical care team) and graded as appropriate or inappropriate based on the following criteria:
*Appropriate referral:*
Severely unwell **and/or** at high risk of deterioration, **and** has no exclusion criteria (see below) **and** has a reversible condition which will benefit from an intervention or treatment strategy which can be delivered **only** in the critical care setting.
*Inappropriate referral:*
Presence of the following exclusion criteria:
Poor or very poor FS **and** significant comorbidities **and/or** an irreversible condition, **and/or** the presence of a terminal disease with a significantly reduced life expectancy **and/or** patient refusal.
**RESULTS.**
59/107 (55%) of referrals were made out of hours (between 18:00 and 08:00)
28/107 (26%) were considered inappropriate. Appropriateness did not vary with time of referral
Of the 28 inappropriate referrals, 20 (71%) had poor or very poor FS **and** comorbidities with a 60% hospital mortality. 6/28 (21%) had good FS with a 17% hospital mortality. 2 had unknown FS/ comorbidities.

Of the 79 appropriate referrals 33(42%) were admitted to ICU with a 36% hospital mortality. 46/79(58%) were declined ICU admission and had a 28% hospital mortality.

**CONCLUSIONS.** Seniority had a positive effect on quality of referral, however even following senior discussion 23% of referrals were considered inappropriate.

The outcomes of the inappropriate referrals reflect the negative impact of a combination of poor FS and comorbidities. Appropriate referrals do not always need ICU admission either because the patient is too well, or too ill.

Collaborative work with clinicians referring to ICU aims to improve understanding of the rationale for decision making. Recording the FS of the patient using a recognized frailty score is as important as assessing comorbidity, when considering critical care referral.

**REFERENCE(S)**

Muscedere J, Waters B, Varambally A, Bagshaw SM, Boyd JG, Maslove D, Sibley S, Rockwood K. The impact of frailty on intensive care unit outcomes: a systematic review and meta-analysis.Intensive Care Med. 2017 Aug;43(8):1105-1122

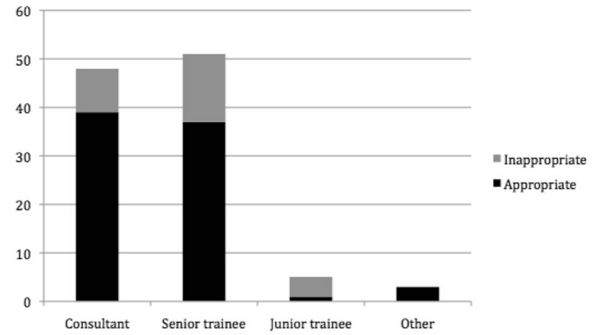

**Fig. 1 (abstract 1131).** Most senior clinician involved in the referral and appropriateness of referral

### 1132

#### Health professionals' perception of head-of-bed elevation in patients on mechanical ventilation

A.H. Furtado Jr, M.C.F. Albuquerque, L.R. de Araújo, A.M.H.P. Silva, L.O. Domingues, L.M. Rodrigues, K.P. Afonso, A. Aires Peixoto Jr
Universidade Federal do Ceará, Fortaleza, Brazil
**Correspondence:** A.H. Furtado Jr
*Intensive Care Medicine Experimental* 2018, **6(Suppl 2):**1132

**INTRODUCTION.** Ventilator-associated pneumonia (VAP) is the second most frequent pathology in the Intensive Care Units (ICU) and it is one of the most fearsome events, due to the high lethality and the high costs involved. The head-of-bed (HOB) elevation at an angle between 30° and 45° is one of the most effective measures to prevent VAP. The perception of health professionals is key to make sure the HOB is put at the right angle.

**OBJECTIVES.** We investigated the knowledge of ICU professionals at the Walter Cantidio University Hospital on the recommendation of HOB elevation and the methods that they use to routinely assess the elevation of HOB in the ICU.

**METHODS.** The setting of the study was the clinical and the surgical ICUs at the University Hospital Walter Cantídio in Fortaleza, Brazil. The sample was 65 health professionals who work at the ICUs. A model simulating a critical patient was created with a mannequin equipped with hospital vestments and devices on a bed frame. The HOB was inclined at 30 degrees and the angle was confirmed by an inclinometer before each session of surveys. A mark 60cm away from the bed was placed on the floor, where the participants had to stand and answer 3 questions, which were written in a simple way to facilitate standardization: 1 How many degrees do you think this head of bed is elevated at? 2 What is the elevation angle that reduces the chance of ventilator-associated pneumonia? 3 In your daily practice at the ICU, how do you verify the angle of elevation of the head of bed? The surveys lasted for 7 days in a row. The study was reviewed and approved by the hospital's ethics committee, report number 2.467.096.

**RESULTS.** The angle indicated for HOB elevation varied between 2° and 90° among the physicians and between 10° and 45° among other health professionals. The correct HOB angle was identified by 54.5% nurses, 89.0% physiotherapists, 30% physicians. The HOB elevation interval for VAP prevention (30 to 45°) was precisely mentioned by 73% nurses, 20%. The technique used to determine the HOB elevation was 41.6% in the bedside indicator, 40.2% in the guesswork, 6.5% in the external appliance, and 11.7% do not know how or do not measure it.

**CONCLUSIONS.** There is a tendency to underestimate HOB elevation among nurses and overestimating it among physicians. The nurses were the professionals who presented the highest percentage of the correct HOB elevation interval for VAP prevention, followed by nursing technicians and, later, by doctors. In addition, the most prevalent technique used to determine HOB elevation was the indicator of the bed.

### 1133

#### Quality improvement project on patient diary in intensive care unit to build the picture on critical illness events for the road of survival and rehabilitation

A. Chan-Dominy, K. Liyanage, E. Forbat, T. Hart, J. Matemera, R. Peters
Royal Brompton and Harefield NHS Trust, Department of Critical Care, London, United Kingdom
**Correspondence:** A. Chan-Dominy
*Intensive Care Medicine Experimental* 2018, **6(Suppl 2):**1133

**INTRODUCTION.** Physical sequelae such as weakness can be difficult to understand when patients report no existential memory of the critical care environment. Experiences of delusional and intrusive memories, nightmares and hallucinations constituting to post-traumatic stress disorder are reported by patients at critical care follow-up clinic. Patient diaries can be helpful to fill in gaps in patients' memories and equip patients with a better understanding of what has happened to them in critical care, as a strategy to set realistic goals for recovery [1]. Diaries are intuitively unstructured with entry on voluntary basis to reflect autonomy of their scribers. An audit at our unit showed first entry was over 10 days in 25% diaries, under 10% diaries had more than 2 entries including notes by family per 24-hour period.

**OBJECTIVES.** To promote quantity and quality of entries in patient diaries employing PDSA (plan-do-study-act) methodology [2]. Improvement by 6 months aiming for over 90% diary with entry of 12 key events identified based on follow-up clinic feedback and telephone interview of 6 past patients about diary.

**METHODS.** Interventions included front-page insert of 12 key events, staff education at bedside and nursing team days, 5 diary champions, family guidance on diary as a communication tool, glossary of common monitoring and advanced organ support therapy, staff notice board on patient feedback from clinic, nurse observers at clinic.

**RESULTS.** Over a 6-month period from August 2017 to February 2018, 104 diaries were launched for the 154 patients who had over 3 days length of stay (average stay of 16 days). A total of 39 diaries were audited after patient discharge from intensive care unit (ICU). 97% diaries had entry within 10 days of ICU admission and 23% diaries had more than 2 entries per 24-hour period. ICU admission entry and ICU discharge entry were noted in 23% and 60% diaries respectively (unchanged from previous audit). Entries by family members featured in 59% diaries

**CONCLUSIONS.** Quality improvement on patient diary entries is about studying staff and environmental culture (for a voluntary non-clinical activity), nurture skills of communication, then adapting to overcome barriers (index prompt for diary). Patient feedback provides a powerful testimony to plan intervention for this initiative.

##### REFERENCE(S)

1. Rehabilitation after critical illness. NICE clinical guidelines CG83, March 2009
2. http://www.ihi.org/resources/Pages/HowtoImprove/default.aspx

##### GRANT ACKNOWLEDGMENT

The authors declare no financial disclosures and funding support.

### 1134

#### A 25 year retrospective analysis of the age of patients and length of stay in a single district general mixed medical/surgical critical care unit in the UK

A. Fung, S. Ranjan, A.E. Myers, R. Kumar, T.L. Samuels, N. Raeside
East Surrey Hospital, Intensive Care Department, Redhill, United Kingdom
**Correspondence:** N. Raeside
*Intensive Care Medicine Experimental* 2018, **6(Suppl 2):**1134

**INTRODUCTION.** The general population is slowly increasing in age, this is thought to be due to a combination of improved mortality, health provisions, and lifestyles, and reduced fertility [1]. As a result, there is a perception that there will be an associated increase in the age of patients admitted to critical care.

**OBJECTIVES.** This study aimed to review age and length of stay (LOS) characteristics in a district general mixed medical and surgical intensive care unit (ICU).

**METHODS.** Data was obtained from our WardWatcher database for 12,294 patients aged over 16 from 1992 to 2017, and was presented as boxplots for each year.

**RESULTS.** Qualitatively, the median age [IQR] of admission has varied minimally over the last 25 years (see Figure 1). However, looking at the mean age of admissions, there is a weak positive correlation associated between the year of admission and average age ($R^2$ = 0.66). The rise in mean age has not been associated with a positive correlation in the mean length of stay for the same time period ($R^2$ = 0.06).

**CONCLUSIONS.** Despite the increasing age of the general population over the last 25 years, there has only been a weakly positive correlation with the age of patients admitted to our ICU. There has not been an associated increase in the mean length of stay. Further work is required to elucidate whether there has been a proportionate increase in the admission of older patients.

##### REFERENCE(S)

1. Office for National Statistics, *Overview of the UK population: July 2017*, Office for National Statistics, 2017. p. 1-11.

##### GRANT ACKNOWLEDGMENT

Nil

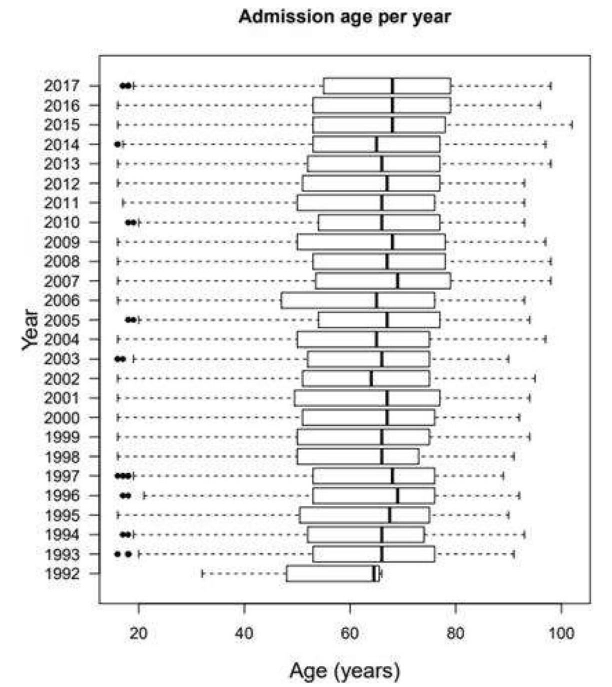

**Fig. 1 (abstract 1134).** Boxplot showing median age and IQR, along with outliers for age

### 1135

#### High frequency of severe burnout syndrome in health care professionals at ICU and stepdown unit

C.S.A.A. Castro[1], K. Timenetsky[2], R. Afonso Caserta Eid[2], A. Serpa Neto[2]
[1]Hospital Albert Einstein, Fisioterapia Pacientes Graves, São Paulo, Brazil; [2]Hospital Albert Einstein, São Paulo, Brazil
**Correspondence:** C.S.A.A. Castro
*Intensive Care Medicine Experimental* 2018, **6(Suppl 2):**1135

**INTRODUCTION.** Burnout syndrome is a prolonged response to chronic emotional and interpersonal stress at work. Burnout syndrome is seen as a process over time, where increased efforts to meet external demands lead to emotional exhaustion, which is a trigger for frivolous interpersonal conviviality which, in turn, leads to a decrease in achievement increase emotional exhaustion and start a vicious cycle. Health professionals, more specifically those working in Intensive Care Units (ICUs), seem to be the most affected by the syndrome and the high prevalence of burnout was associated with negative outcomes, such as psychological problems, a higher incidence of heart disease, and less personal satisfaction. Their presence also worries the employer´s intuitions, since it is associated with the quality of care, patient safety and professional engagement.

**OBJECTIVE.** To evaluate the prevalence of burnout syndrome in nursing, physiotherapy and medical professionals in a stepdown and intensive care unit of a private hospital in São Paulo.

**RESULTS.** Between February 2017 and June 2017, 206 professionals (63.3%) answered the questionnaires, of which 55 were physicians (26.7%), 88 physiotherapists (42.7%) and 63 nurses (30.6% %). The prevalence of severe burnout syndrome in the participants was 34.3%, with no difference according to the professional class (34.1% vs. 33.9% vs. 35.3% for physiotherapists, nurses and physicians respectively, p = 0.986) or place of work (34.2% vs. 34.5% for ICU and stepdown unit, respectively, p = 0.960). The prevalence of severe or very severe cases of depression, anxiety and stress was 12.9%, 11.4% and 10.5%, respectively. The median points observed in the Gallup questionnaire were 41 (34-48), with no differences in relation to the workplace (p = 0,239) or professional class (p = 0.403). In the multivariate analysis, moderate and severe levels of stress and work between 3 and 5 days a week in other services were independently associated with a higher risk of developing severe burnout syndrome. Although it did not reach statistical significance, there was a tendency of association between higher Gallup scores and a lower risk of severe burnout.

**CONCLUSION.** Severe burnout syndrome presents a high prevalence among physicians, nurses and physiotherapists working in ICU and stepdown units. Moderate and severe levels of stress and work between 3 and 5 days a week in other services were independently associated with a higher risk of developing severe burnout syndrome.

**Table 1 (abstract 1135).** Association of work days in other hospitals with severe burnout syndrome through the multivariate analysis

| | Odds Ratio (95%CI) | p value |
|---|---|---|
| Work days in other hospitals per week | | |
| None | 1 (Reference) | |
| 0-2 days | 0.87 (0.27-2.60) | 0.727 |
| 3-5 days | 3.61 (1.53-8.75) | 0.003 |
| > 5 days | 0.59 (0.07-2.94) | 0.531 |

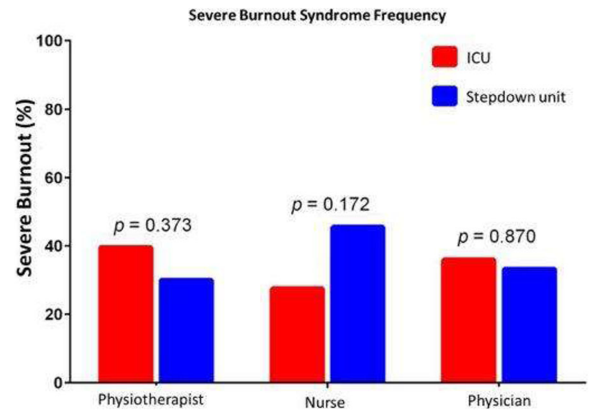

**Fig. 1 (abstract 1135).** Severe Burnout Syndrome Frequency

### 1136

### Characteristics and evolution of patients of long stay in a polyvalent ICU throughout 15 years

J. Ruiz Izquierdo, B. Sánchez González, S. Quintana Riera, J. Trenado Alvarez
Hospital Universitari Mutua Terrassa, Intensive Care Department, Terrassa, Spain
**Correspondence:** J. Trenado Alvarez
*Intensive Care Medicine Experimental* 2018, **6(Suppl 2):**1136

**INTRODUCTION.** Due to advancements in medical technology and management of illnesses, an increasing number of intensive care unit (ICU) patients are stabilized during acute illness following prolonged resuscitation and treatment.

**OBJECTIVE.** To describe the characteristics and evolution of long-stay (LS) patients in a polyvalent ICU over 15 years.

**METHOD.** Unicentric, observational and descriptive study conducted in the period January 2000-June 2015. LS is defined as a stay above the 95th percentile.

Descriptive statistics and Chi square were performed for intergroup comparison.

**RESULTS.** In the study period, a total of 11443 patients were admitted, of which 542 (4.74% of the income) exceed one 19-day stay (95th percentile of the average stay of the unit in the period).

Male sex predominates (5.2 vs 3.8% respectively, p < 0.001). There are no differences in the age between groups, 60.70 years ± 16.87 vs 60.65 years ± 15.85.

Regarding severity score, these are much higher in the group of LS: MPM 0 27.72 vs 43.56, MPM 24 20.49 vs 38.09, SAPS 2 35.15 vs. 48.70, Apache III 56.40 vs 81.04.

Medical patients are predominant between patients with LS: 73.5%, compared to the Surgical one's 17.6% or 9.0% among emergent surgery (p = 0,004)

When we analyze the procedures performed in LS patients, we observed that use of vasoactive drugs (76.5% vs. 28.3% p < 0.0001), hemodialysis (10.9% vs 3.0% p < 0.0001), hemofiltration (18.1% vs 4.0% p < 0.0001), ICP monitoring (15.0% vs. 2.5% p < 0.0001), mechanical ventilation 97.6% vs 42.4% (Chi square 633.96 p < 0.0001) and realization of tracheostomy (57.3% vs 2.3% p < 0.0001) are more common.

If we divide the global period of study in three five-year periods, we observed that the prevalence of LS patients decreases significantly from 6.4 to 4.5% and 4.0% from first to third quinquennium, p < 0.001.

The average stay of the group LS was 33 days, with a higher mortality (8.5 vs 4.2, p < 0.001).

**CONCLUSIONS.** In the group of patients with LS, men and medical admissions predominate.

Age is not a risk factor to belong to this group.

The severity of the illness at the time of admission to the ICU, reflected in greater use of invasive techniques and life support.

Mortality is higher in the LS group but does not exceed 10%.

**REFERENCE(S)**

Vivek K. Moitra; Carmen Guerra; Walter T. Linde-Zwirble; Hannah Wunsch Relationship between ICU Length of Stay and Long-Term Mortality for Elderly ICU Survivors* Crit Care Med. 2016 Apr; 44(4): 655-662.

**Table 1 (abstract 1136).** Characteristics of the patients

| | NO LONG STAY (n=10901) | LONG STAY (n=542) | *p value* |
|---|---|---|---|
| MPM 0 | 27,72 | 43,56 | <0,01 |
| MPM 24 | 20,49 | 38,09 | <0,01 |
| SAPS2 | 35,15 | 48,70 | <0,01 |
| APACHE III | 56,40 | 81,04 | <0,01 |
| VASOACTIVE DRUGS (%) | 28,3 | 76,5 | <0,001 |
| HEMODIALYSIS (%) | 3,0 | 10,9 | <0,001 |
| ICP MONITORING (%) | 2,5 | 15,0 | <0,001 |
| MECHANICAL VENTILATION (%) | 42,4 | 97,6 | <0,001 |
| TRACHEOSTOMY (%) | 2,3 | 57,3 | <0,001 |

### 1137

### Reliability of free open-access medical education for intensive care procedures

J. Braviner[1], M. Carpenter[2]
[1]Newcastle University, Faculty of Medical Sciences, Newcastle upon Tyne, United Kingdom; [2]Sunderland Royal Hospital, Integrated Critical Care Unit, Sunderland, United Kingdom
**Correspondence:** J. Braviner
*Intensive Care Medicine Experimental* 2018, **6(Suppl 2):**1137

**INTRODUCTION.** FOAM is a new concept where freely accessible resources are created online. Concerns have been expressed that whilst FOAM is an easy way to access medical education, its lack of peer review may make its reliability uncertain.

**OBJECTIVES.** Thirty FOAM resources related to three common intensive care procedures (central venous catheterisation, urinary catheterisation and nasogastric tube insertion) were reviewed to assess compliance with UK national and international guidelines. These procedures were chosen because the existence of international guidance, the frequency of their use in the ICU and the potential for significant harm from infection (CVC and catheter) and misplacement (NGT).

**METHODS.** The search strategy was predefined to gather five web and five video resources for each procedure.

The search terms "Central Line Insertion", "Male Urinary Catheterisation" and "Nasogastric Tube Insertion" were used (Google). Irrelevant, foreign language or paid resources were excluded.

Important elements within each guideline were pre-defined and their presence was compared to gauge compliance (CVC 28, NGT 22, catheterisation 28) [EPIC2 guidelines (Pratt, 2007) were used for specific guidance on CVC and catheter insertion. NPSA guidelines (National Patient Safety Agency, 2011) for NGT insertion].

**RESULTS.** 34% of guideline elements were included correctly, 64% were not included and 2% of elements were contradicted. Resources originated from a wide range of countries (CVC: 3 UK, 4 US, Canada, Australia, Pakistan, Catheterisation: 7 UK, 2 US, 1 Canada, NGT: 5 UK, 3 US, 2 Canada). In no single resource were more than 57% of elements correctly included (mean compliance per procedure CVC 36.2%, NGT 47.2%, catheterisation 29.6%).

Compliance with guideline elements ranged from only 17% for cleaning equipment to 100% for use of sterile drapes. The 19 contradictions included use of incorrect cleaning and flushing solutions, incorrect handling of sharps, contamination of sterile fields, incorrectly sized catheters and incorrect methods of confirming NGT placement (e.g. auscultation).

Due to incorrect placement of NGT being a UK 'never event' these incorrect placement methods give rise to significant patient safety concerns.

**CONCLUSIONS.** FOAM provides medical education in an easy to access format, but is not peer reviewed. We found substantial gaps in the information they conveyed. Contradictions to the guidelines were rare but clinically significant.

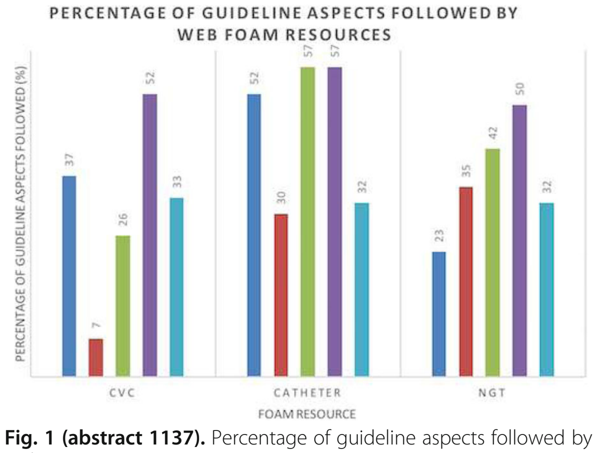

**Fig. 1 (abstract 1137).** Percentage of guideline aspects followed by web FOAM resources

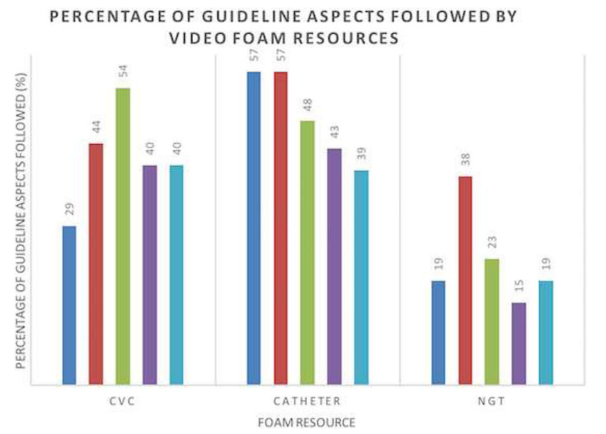

**Fig. 2 (abstract 1137).** Percentage of guideline aspects followed by video FOAM resources

### 1138

#### Healthcare workers and nursing students´ attitudes and beliefs during an influenza pandemic

B.N. Santana-Lopez[1], J.D. Martin-Santana[2], Y.G. Santana-Padilla[3], C. Rodríguez-Escot[3], P. Vega Ocaña[3], L. Santana-Cabrera[3]

[1]Universidad de Las Palmas de Gran Canaria, Las Palmas de Gran Canaria, Spain; [2]Universidad de Las Palmas de Gran Canaria, Economy and Management, Las Palmas de Gran Canaria, Spain; [3]Hospital Universitario Insular de Gran Canaria, Intensive Care, Las Palmas de Gran Canaria, Spain

**Correspondence:** P. Vega Ocaña

*Intensive Care Medicine Experimental* 2018, **6(Suppl 2):**1138

**INTRODUCTION.** Healthcare workers are a key player in a pandemic, since they will be the first to expose themselves to it. Therefore, it is important to know their beliefs and attitudes when exposing them to it.

**OBJECTIVES.** The objective of this project is to analyze the beliefs and attitudes of healthcare workers and nursing students in an influenza pandemic, and their differences.

**METHODS.** A transversal and descriptive study was carried out, through a personal and anonymous inquest, among the healthcare personnel and students of the Degree in Nursing of the Las Palmas de Gran Canaria University (ULPGC), with a sample of 277 people. The instrument used to measure up the beliefs and attitudes of health workers before an epidemiological alert was the one published and validated by Professor Heather Draper of the University of Birmingham in June 2008, after obtaining the approval of the author to carry out this work (Draper H 2008, Damery S, Ives J 2009 & Damery S 2010).

The statistical analysis was carried out using the statistical package SPSS 15.0. Student-t and Chi-square tests were used to compare continuous and categorical variables, respectively.

**RESULTS.** 84.5% believe that a pandemic is likely to occur. Almost half do not believe there is a vaccine that protects them. And this percentage is much lower among students (52 versus 30.4%). In addition, students, unlike workers, fear that if there were an effective vaccine do not have access to it. 41.9% would not go to their workplace if their family had a higher risk of contracting the disease. But the most relevant fact is that only 5.8% have a high possibility of going to work, during a hypothetical pandemic, despite almost all adverse circumstances identified.

**CONCLUSIONS.** Almost everyone is aware that a possible influenza pandemic could happen and are willing to work for it, despite the personal risks they might be exposed. However, they place the family before work, since they would not be willing to fulfill their responsibilities if there was a greater risk than usual in infecting their relatives; being able to reach figures of absenteeism higher than 94%.

REFERENCE(S)

1. Damery S, Draper H, Wilson S, Greenfield S, Ives J, Parry J, Petts J, Sorell T. Healthcare workers´ perceptions of the duty to work during an influenza pandemic. J Med Ethics. 2010 Jan;36(1):12-8. Available in: https://www.researchgate.net/publication/40757215_Healthcare_workers%27_perceptions_of_the_duty_to_work_during_an_influenza_pandemic
2. Damery S, Wilson S, Draper H, Gratus C, Greenfield S, Ives J, Parry J, Petts J, Sorell T. Will the NHS continue to function in an influenza pandemic? A survey of healthcare workers in the West Midlands, UK. BMC Public Health. 2009 May 14;9:142. Available in: https://www.ncbi.nlm.nih.gov/pmc/articles/PMC2690584/pdf/1471-2458-9-142.pdf

### 1139

#### Evaluation of SAPS-3 score in patients older than 80 years. Impact on the mortality of the previous functional situation

M.D. Pola-Gallego-de-Guzmán[1], C. Lopez-Caler[2], J.F. Brea-Salvago[1], E. Aguilar-Alonso[3], L. Perez-Borrero[4], J.E. Barrueco-Francioni[5], M. Rojas-Amezcua[3], R. Montoiro-Allué[6], D. Iglesias-Posadilla[7], J.M. Rodríguez-Roldán[8]

[1]Complejo Hospitalario, Intensive Care Unit, Jaen, Spain; [2]Hospital Regional Universitario Carlos Haya, Intensive Care Unit, Malaga, Spain; [3]Hospital Infanta Margarita, Intensive Care Unit, Cabra, Spain, [4]Hospital de la Serrania, Intensive Care Medicine, Ronda, Spain; [5]Hospital Arnau de Vilanova, Intensive Care Unit, Valencia, Spain; [6]Hospital Clínico Universitario Lozano Blesa, Intensive Care Unit, Zaragoza, Spain; [7]Hospital Universitario, Intensive Care Unit, Burgos, Spain; [8]Hospital Severo Ochoa, Intensive Care Unit, Madrid, Spain

**Correspondence:** M.D. Pola-Gallego-de-Guzmán

*Intensive Care Medicine Experimental* 2018, **6(Suppl 2):**1139

**INTRODUCTION.** In recent years, the number of patients older than 80 years admitted to the Intensive Care Unit (ICU) has increased exponentially.

OBJECTIVES: To analyze the predictive capacity of SAPS-3 score in patients older than 80 years admitted to Intensive Cre Unit (ICU) and to evaluate the influence of the previous functional situation on the mortality.

**METHODS.** Multicentric prospective observational study in the ICU of 7 Spanish hospitals (Carlos Haya in Malaga, Cabra (Córdoba), Jaén, Burgos, Arnau Vilanova in Valencia, Clinic of Zaragoza, Ronda).

Data were expressed as the mean and standard deviation for quantitative variables and percentages for qualitative variables. For the comparison of two means we used the Student´s t-test and the chi-squared test was used to compare proportions and logistic regression for multivariate analysis. Statistically significant differences: $p < 0.05$.

**RESULTS.** 2008 patients in total. Of these, 181 (9%) between 80-85 years, 57 (3%) between 85-90 years old and only 4 patients were older than 90 years.

62% for medical pathology. Acute coronary syndrome (20% of all patients, 12% with ST-segment elevation myocardial infarction (STEMI), arrhythmias especially bradycardia with need for vigilance and / or pacemaker (17% of the total), 5% for sepsis and 4% for cardiac or respiratory arrest.

38% are surgical, of them 10% only for central venous catheterization, and 7% for emergency surgery. The most frequent were cardiac and maxillofacial surgery.

At admission, 26% required mechanical ventilation, with mortality of 40% and of non-ventilated patients of 17% (p=0.001). Previous functional situation (normal, self-sufficient but with dysfunction and not self-sufficient) was: 50%, 36% and 14%, with hospital mortality: 14.4%. 35.9% and 23.1% respectively.

SAPS-3 on admission of 53.83±12.71 points. The predicted mortality of 26% and hospital mortality of 23%, (ICU of 14%).

The multivariate analysis with logistical regression shows complementarity between SAPS-3 and previous functional situation to predict hospital mortality. OR: Normal = 1, Self-sufficient but with dysfunction = 2.9(1.25-7.13) and Not self-sufficient = 1.65(0.49-5.52).

In younger than 80 years, mortality was associated with previous functional situation and there was complementarity with SAPS-3. OR: (Normal = 1, Self-sufficient but with dysfunction = 1.57(1.1-2.24) and Not self-sufficient = 4.01(2.41-6.67)).

**CONCLUSIONS.** SAPS-3 is useful for predicting the mortality of elderly patients admitted to the ICU, the hospital mortality was similar to predicted mortality. The functional situation dysfunction is an independent factor associated with mortality, both in elderly patients and in those under 80 years.

### 1140

#### Daytime and its association with risk of death and chance of discharge in critically ill patients: a retrospective study

P. Zajic[1], P. Bauer[2], A. Rhodes[3], R. Moreno[4], T. Fellinger[2], B. Metnitz[5], M. Posch[2], P. Metnitz[1]

[1]Medical University of Graz, Div. of General Anaesthesiology, Emergency- and Intensive Care Medicine, Graz, Austria; [2]Medical University of Vienna, Centre for Medical Statistics, Informatics, and Intelligent Systems, Vienna, Austria; [3]St George's University of London, London, United Kingdom; [4]Centro Hospitalar de Lisboa Central, Lisbon, Portugal; [5]Austrian Centre for Documentation and Quality Assurance in Intensive Care, Vienna, Austria

**Correspondence:** P. Zajic

*Intensive Care Medicine Experimental* 2018, **6(Suppl 2):**1140

**INTRODUCTION.** Variation of outcomes in critically ill patients due to admission day was demonstrated in past studies [1]. Some studies suggested that an additional "off-hours" effect was also associated with unfavourable outcomes.

**OBJECTIVES.**

a) To assess, whether daytime of ICU admission is associated with variation in risk of death and chance of discharge.
b) To assess, whether time of day is associated with differences in the immediate risk of death and chance of discharge during ICU stay.
c) To assess, whether previously reported "weekend effects" occurred independently from possible "daytime effects".

**METHODS.** Retrospective study in the Austrian Centre for Documentation and Quality Assurance in Intensive Care (ASDI) database [2]. Adult patients admitted due to medical reasons or following emergency surgery to ICUs participating in the project between 2012 and 2016 were included. ICU readmissions during the same hospital stay were excluded.

Multivariable competing risk analysis using the Fine and Gray proportional subdistribution hazards model concerning ICU mortality and ICU discharge was performed [3]. The following variables of interest were included in the model: time of admission (4h blocks), time of event (4h blocks), weekend or working day, year of admission, month of admission. 08:00-11:59 was chosen as the reference range for all daytime variables. The model was adjusted using ICU as fixed effects, type of admission and SAPS3 score [4].

**RESULTS.** 146,004 patients were included in the analysis. Risk of death varied significantly with time of admission [HR (95% CI) for ICU admission time: 12:00-15:59 0.99 (0.92-1.07), 16:00-19:59 1.08 (1.00-1.16), 20:00-23:59 1.11 (1.03-1.19), 00:00-03:59 1.24 (1.14-1.36), 04:00-07:59 1.23 (1.11-1.36)]. Significant variation in the risk of death during ICU stay with regards to daytime was observed [HR (95% CI) for time of death: 12:00-15:59 1.06 (1.00-1.13), 16:00-19:59 0.94 (0.89-1.01), 20:00-23:59 0.92 (0,85-1.00), 00:00-03:59 0.50 (0.46-0.55), 04:00-07:59 0.53 (0.49-0.58)]. Chance of discharge was drastically lower in the afternoon and at nights [HR (95% CI) for time of discharge: 12:00-15:59 0.62 (0.54-0.72), 16:00-19:59 0.15 (0.12-0.18), 20:00-23:59 0.08 (0.06-0.10), 00:00-03:59 0.02 (0.02-0.03), 04:00-07:59 0.03 (0.02-0.03)]. Risk of death following weekend ICU admission was elevated nevertheless [HR (95% CI) 1.19 (1.06-1.35) compared to working days].

**CONCLUSIONS.** Daytime is associated with significant variation in both risk of death and chances of discharge in critically ill patients admitted to ICUs for reasons other than scheduled procedures and following adequate adjustment for severity of illness.

**REFERENCE(S)**

[1] Zajic P, Bauer P, Rhodes A et al. Crit Care. 2017;21:223

[2] Metnitz PG, Vesely H, Valentin A et al. Crit Care Med. 1999;27:1486-91.

[3] Fine J, Gray R. Am Stat Assoc. 1999;94:496-509.

[4] Metnitz PGH, Moreno RP, Almeida E et al. Intensive Care Med. 2005;31:1336-44.

### 1141

#### The quality of life after ICU study: a prospective, multicenter, cohort study for the evaluation of long-term outcomes among general ICU survivors in Brazil

R.G. Rosa[1], C.C. Robinson[2], R. Kochhann[2], D. Sganzerla[2], C. Dietrich[2], D. Schneider[2], F.H. Dutra[2], E.C. Sanchez[2], R.P. de Oliveira[3], J.G. Maccari[3], L.B. Anzolin[2], M.Q. de Oliveira[2], S.F. de Menezes[2], D. de Souza[2], S.F. da Silva[2], R. Boldo[3], J.R. Cardoso[4], D.C. Birriel[5], M.N. Gamboa[5], A.S. Machado[6], J.M.S. de Andrade[6], C. Alencar[7], M.C. Teixeira[7], S.R.R. Vieira[8], F.C. Moreira[8], P. Berto[8], A. Amaral[9], A.P.M. Silveira[9], J.M.M. Teles[9], D.C. de Oliveira[10], L.C. de Oliveira Júnior[10], L. Correa e Castro[11], M. Sarmento[11], R.T. Neves[12], R.D.A. Gomes[12], C.M. Ribeiro[12], A.B. Cavalcanti[13], L.A. Martins[14], R.L.S. Santos[14], L.Y. Ue[14], L.S. Hammes[15], L. Biason[3], T. Tonietto[3], P.R. Cardoso[16], M.M.S. Santos[2], M. Mattioni[3], T. Sharshar[17], F. Bozza[18], M. Falavigna[2], C. Teixeira[3]

[1]Hospital Moinhos de Vento, Intensive Care Unit, Porto Alegre, Brazil; [2]Hospital Moinhos de Vento, Research Projects Office, Porto Alegre, Brazil; [3]Hospital Moinhos de Vento, Intensive Care, Porto Alegre, Brazil; [4]Complexo Hospitalar Santa Casa de Misericórdia de Porto Alegre, Porto Alegre, Brazil; [5]Pavilhão Pereira Filho, Intensive Care, Porto Alegre, Brazil; [6]Hospital Ernesto Dornelles, Intensive Care, Porto Alegre, Brazil; [7]Hospital Conceição, Intensive Care, Porto Alegre, Brazil; [8]Hospital de Clínicas de Porto Alegre, Intensive Care, Porto Alegre, Brazil; [9]Hospital de Urgências de Goiânia, Intensive Care, Goiânia, Brazil; [10]Hospital Geral Clériston Andrade, Intensive Care, Feira de Santana, Brazil; [11]Hospital Regional do Baixo Amazonas, Intensive Care, Santarem, Brazil; [12]HCor-Hospital do Coração, Intensive Care, São Paulo, Brazil; [13]HCor-Hospital do Coração, Research Institute, São Paulo, Brazil; [14]Ministry of Health, CGHOSP, Brasilia, Brazil; [15]Hospital Moinhos de Vento, Porto Alegre, Brazil; [16]Hospital de Clinicas de Porto Alegre, Intensive Care, Porto Alegre, Brazil; [17]Institute Pasteur, Paris, France; [18]D´Or Research Institute, Intensive Care, Rio de Janeiro, Brazil

**Correspondence:** R.G. Rosa

*Intensive Care Medicine Experimental* 2018, **6(Suppl 2):**1141

**INTRODUCTION.** The post-intensive care syndrome (PICS) is marked by physical, mental and cognitive disabilities that remain in survivors of critical illness. The epidemiology of PICS and its association with quality of life (QoL) among general ICU survivors are not clear.

**OBJECTIVE.** To evaluate the prevalence and associated factors of long-term physical, mental and cognitive disabilities, and their association with QoL among general ICU survivors in Brazil.

**METHODS.** A prospective cohort study was conducted in 10 Brazilian mixed ICUs. Post-ICU patients aged ≥18 years, and with an ICU stay >72h (for medical and emergency surgical admissions) or >120h (for elective surgical admissions) were followed by telephone interviews 3, 6, and 12 months after ICU discharge. The outcomes were moderate/severe physical dependence, evaluated by the Barthel Index; anxiety and depression, evaluated by the Hospital Anxiety and Depression Scale (HADS); posttraumatic stress disorder (PTSD) evaluated by the Impact Event Scale-6; cognitive dysfunction, evaluated by the Montreal Cognitive Assessment; and QoL, evaluated by the SF12v2 health survey.

**RESULTS.** From May 2014 to April 2018, 1,044 post-ICU patients were followed. The one-year mortality was 27.9% (n=291). At 3 months, 262/726 (36%) had moderate/severe physical dependence. At 6 months, 85/373 (23%) had anxiety, 75/373 (20%) had depression, and 57/374 (15%) had PTSD. At 12 months, 227/326 (69%) had cognitive dysfunction. Figure 1 shows the factors associated with post-ICU disabilities. At 3 months, Charlson comorbidity index ≥2 (PR, 1.74; 95%CI, 1.15-2.63), no higher education (PR, 1.89; 95%CI, 1.07-3.33), HADSa ≥8 at ICU discharge (PR, 1.68; 95% CI, 1.17-2.42), previous physical dependence (PR, 1.81; 95%CI, 1.16-2.83) and muscular weakness at ICU discharge (PR, 2.47; 95% CI, 1.70-3.60) were associated with moderate/severe physical dependence. At 6 months, HADSa ≥8 at ICU discharge (PR, 1.89; 95%CI, 1.28-2.79) and decrease in household income (PR, 1.50; 95%CI, 1.01-2.23) were associated with anxiety; HADSd ≥8 at ICU discharge (PR, 3.39; 95%CI, 2.24-5.12) and public health insurance (PR, 1.64; 95%CI, 1.08-2.50) were associated with depression; age per year (PR, 0.98; 95%CI, 0.96-0.99), HADSd ≥8 at ICU discharge (PR, 2.36; 95%CI, 1.42-3.94) and decrease in physical functional status (PR, 1.79; 95%CI, 1.07-2.98) were associated with PTSD. At 12 months, age ≥65 years (PR, 1.36; 95%CI, 1.17-1.58) and public health insurance (PR, 1.25; 95%CI, 1.07-1.46) were associated with cognitive dysfunction. Patients with post-ICU disabilities, with exception of cognitive dysfunction, had lower physical and mental SF12v2 scores in comparison to those without disabilities (figure 2).

**CONCLUSION.** The high burden of disabilities after ICU is a matter of high relevance for public health, given its impact on patients´ QoL. The network of potential risk factors for post-ICU disabilities is complex and involves factors of multiple domains.

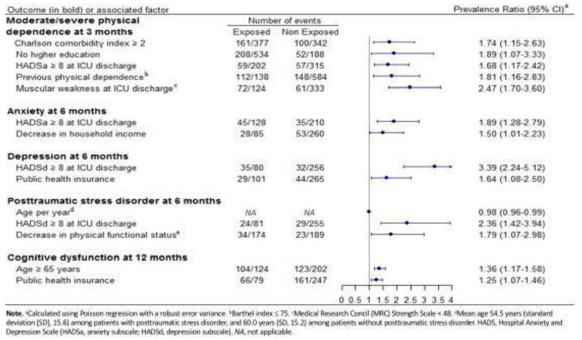

**Fig. 1 (abstract 1141).** Multivariate regression models of factors associated with post-ICU disabilities

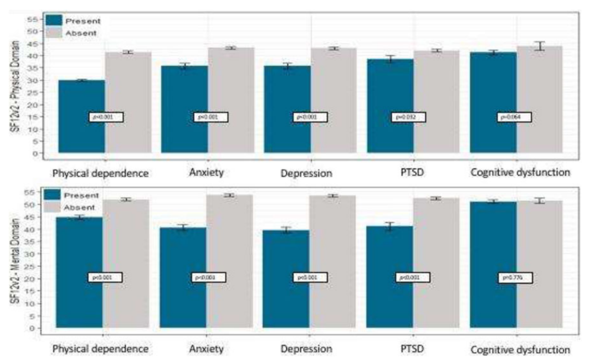

**Fig. 2 (abstract 1141).** SF12v2 quality of life scores according presence or absence of post-ICU disabilities

### 1142

#### Renal insufficiency in intensive care unit patients: clinical characteristics and outcome

M. Sousa, W.C. Baptista, R.S. Franco, A. Bemfica, A. Moreira, M.C. Cesar, I. Valade, M. Leite
Hospital Novo Atibaia, ICU, Atibaia, Brazil
**Correspondence:** M. Sousa
*Intensive Care Medicine Experimental* 2018, **6(Suppl 2):**1142

**INTRODUCTION.** Renal insufficiency (RI) is one of the main complications found in the intensive care unit. The risk factors and clinical evolution of the patients may contributes to increase the morbidity rate of critically ill patients.

**OBJECTIVES.** To characterize the clinical and laboratory profile as well as the outcomes of patients with acute (comparing acute or chronic with acute exacerbation) renal failure in an intensive care unit (ICU).

**METHODS.** Retrospective study from January 2013 to December 2016, with 106 patients hospitalized in an ICU who evolved with RI. The clinical characteristics, laboratory and evolution of the patients were compared according to type of RI (acute or chronic with acute exacerbation). For statistical analysis, a significance level of 5% was used and chi-square test, Kruskal-wallis test (when abnormal distribution of the sample) and t-student test (when normal distribution) were used.

**RESULTS.** The sample was 106 patients, 65 (61.3%) with acute and 41 (38.7%) with acute exacerbation of chronic. The group with acute had a mean age of 67.11 ± 17.20 years; 58.5% male; 70.8% clinical patients and 29.2% surgical; 51 deaths (78.4%). On the other hand, the group with acute chronic has a mean age of 72.24 ± 12.54 years; 68.3% male; 85.4% clinical patients and 14.6% surgical; 28 deaths (68.3%). In the statistical analysis, the variables with significant statistical difference between the 2 groups (acute and chronic acute exacerbations) were: SAPS III points (75.75 ± 16.38 X 67.51 ± 13.25 - p 0.008), SAPS III% in death (63.12 ± 24.02 X 51.72 ± 22.63 - p 0.01), diabetes (23.1% X 48.8% - p 0.006), vasoactive drug (81.5% X 46,3% - p 0.000), sepsis (80% X 58.5% - p 0.01), creatinine on admission (2.54 ± 2.74 X 4.39 ± 3.37 - p 0.00), creatinine on day 1 of hemodialysis (3.87 ± 2.49 X 8.95 ± 26.25 - p 0.016), urea on admission (93,47 ± 60.67 X 164.48 ± 114.27 - p 0.00) and urea on the 1st day of hemodialysis (149.86 ± 59.96 X 194.29 ± 96.53 - p 0.034). The predictors of acute and chronic morbidity are listed in TABLE 1.

**CONCLUSION.** RI has a high morbidity and mortality rate and the identification of risk factors for poor prognosis in patients with renal insufficiency allows for early diagnostic and therapeutic intervention to minimize and reverse unfavorable patient outcomes.

**Table 1 (abstract 1142).** Predictors of mortality in renal injury

| | Acute renal injury | Acute deterioration in chronic kidney disease |
|---|---|---|
| Age | 71.73 ± 12,76 (0.01) | 75.46 ± 11.92 (0.014) |
| Diabete n, (%) | | 10 (35.7) (0.014) |
| Vasoactive drug n, (%) | | 20 (71.4) (0.001) |
| Mechanical ventilation n, (%) | | 17 (60.7) (0.007) |
| Initial creatinine (mg/dl) | | 3.05 ± 1.79 (0.00) |
| Peak creatinine (mg/dl) | | 9.93 ± 31.78 (0.05) |
| Initial urea (mg/dl) | | 136.57 ± 94.16 (0.03) |
| Profile n, (%) Clinical/Surgical | | (0,039) 23 (82.1)/5 (17.9) |

## Haemodynamic therapy in ICU

### 1143

#### Comparison between obese and non-obese populations who have undergone heart surgery regarding bleeding, polytransfusion and bleeding-related reoperation

E. Trujillo García, J. Muñoz Bono, E. Curiel Balsera, M.D. Fernández Zamora, H. Molina Díaz, G. Quesada García
H. R. U de Málaga, Intensive Care, Málaga, Spain
**Correspondence:** E. Trujillo García
*Intensive Care Medicine Experimental* 2018, **6(Suppl 2):**1143

**OBJECTIVE.** Evaluating the possible influence of obesity on the incidence of postoperative bleeding, need of polytransfusion, and bleeding-related reoperation, as well as their complications in patients undergoing cardiovascular surgery.

**MATERIAL AND METHODS.** Observational, prospective, multicentre and comparative study of all patients who have undergone heart surgery in Andalusian hospitals taking part in the ARIAM Project.

**RESULTS.** A total number of 7276 patients who had undergone heart surgery from March 2008 to July 2012 were analysed. 61.1% of the operated patients were males. 34.9% were obese with a BMI over 30Kg/m$^2$.

Patients with BMI>30 Kg/m$^2$ showed significantly lower bleeding incidence than those with BMI< 30Kg/m$^2$ (17.5%vs.13.1%, p=0.001), lower polytransfusion need (7.3%vs.5.5%, p=0.004), and lower bleeding-related reoperation figures (4.4%vs.3%, p=0.005).

In this analysis, high BMI was shown as a protective factor against mortality (OR 0.69, IC 0.51-0.93, p=0.015), adjusted by EuroScore, surgery type, and previous anticoagulant/antiplatelet treatment, since —when this duration is the same— obese patients show 30% lower bleeding and reoperation probability.

Regarding complications, no significant differences were observed in the incidence of postoperative kidney failure (worsening of the kidney function measured by the AKIN scale, or the need of renal replacement therapy during ICU admission). However, analysis of postoperative kidney failure patients showed significantly higher incidence of kidney failure among obese patients who had needed transfusion than among their non-obese counterparts (38.8% vs. 35.3%, p = 0.023).

Regarding respiratory complications, no significant differences were observed regarding the duration of mechanical ventilation (MV) after surgery, the need of reintubation, and the performance of tracheostomy to wean patients from MV between both groups. Patients whose BMI>30 Kg/m$^2$ showed significantly lower incidence of hemothorax (5.6% vs. 7.2%, p=0.014). However, obese patients can be observed to need NIMV with higher frequency and duration than their non-obese counterparts (7.2 vs. 5.6%, p=0.026).

Reoperated obese patients showed longer hospital stay relative to reoperated non-obese ones (19.68±17.5 days vs. 29.56±36.63 days, p< 0.043). No differences are observed in death rates between both groups.

**CONCLUSIONS.** Unlike what would be expected in patients who have undergone heart surgery (both coronary artery bypass and heart valve replacement), obesity (BMI>30) does not predispose patients to develop greater postoperative bleeding, bleeding-related reoperation, or greater need of postoperative transfusion of hemoderivatives. It can even be a protective factor against bleeding and reoperation after prolonged extracorporeal circulation.

**Table 1 (abstract 1143).** Comparison of the demographic figures, risk factors, severity scales and types of surgery between both populations

| | BMI < 30 (4396) | BMI > 30 (2361) | p |
|---|---|---|---|
| Age | 63±13 | 65±11 | ns |
| Gender<br>- Male<br>- Female | <br>2748 (62.5%)<br>1648 (37.5%) | <br>1370 (58%)<br>991 (42%) | 0.0001 |
| Hypertension | 2491 (56.7%) | 1738 (73.6%) | 0.0001 |
| Diabetes | 1254 (28.5%) | 917 (38.8%) | 0.0001 |
| Dyslipidemia | 1884 (42.9%) | 1249 (52.9%) | 0.0001 |
| Smoker | 759 (17.3%) | 285 (12.1%) | 0.0001 |
| Previous AMI | 685 (15.6%) | 365 (15.5%) | ns |
| Previous CVA | 287 (6.5%) | 129 (5.5%) | ns |
| Liver disease | 57 (1.3%) | 16 (0.7%) | 0.01 |
| EuroScore | 5.88± 3.1 | 5.68±2.9 | ns |
| SAPS 3 | 41.5 ± 10.9 | 40.6 ± 10.2 | ns |
| Previous antiplatelet therapy | 1837 (41.8%) | 1138 (48.2%) | 0.001 |
| Previous anticoagulant therapy | 1375 (31.3.%) | 687 (29.1%) | ns |
| Type of Surgery<br>- Valve Replacement<br>- Coronary artery bypass graft<br>- Aortic surgery | <br>2958 (67.3%)<br>944 (21.4%)<br>204 (4.6%) | <br>1617 (68.5%)<br>556 (23.5%)<br>96 (4.1%) | 0.001 |
| Operative status<br>- Elective<br>- Acute<br>- Emergency | <br>3726(84.8%)<br>527(12%)<br>143 (3.3%) | <br>2094 (88.7%)<br>196 (8.3%)<br>71 (3%) | 0.0001 |
| Extracorporeal circulation time | 110 ± 49 | 109 ± 49 | ns |
| Previous Cardiac Surgery | 462 (10.5%) | 138 (5.8%) | 0.001 |
| Bleeding > 1000 cc | 770 (17.5%) | 309 (13.1%) | 0.0001 |
| Bleeding-related reoperation | 189 (4.4%) | 69 (3%) | 0.005 |

### 1144

#### Vascular closure device angioseal for femoral percutaneous catheterization: assessment of security

A.M. Garcia Bellon, A.M. Gonzalez Gonzalez, M. Cano García, M. De Mora Martin
Regional Hospital of Malaga, Cardiology, Malaga, Spain
**Correspondence:** A.M. Garcia Bellon
*Intensive Care Medicine Experimental* 2018, **6(Suppl 2):**1144

**INTRODUCTION AND OBJECTIVES.** Our aim was to determinate the prevalence of vascular complications with the use of vascular closure device AngioSeal after cardiac catheterization via femoral artery. We studied the overall prevalence of any vascular complication in this population, the epidemiological and clinical predictors of bleeding, the complication rate and the way of presentation.

**METHODS.** Between 2012 and 2016 we studied 1613 patients in whom, after catheterization via femoral artery, it was used an AngioSeal percutaneous closure system. We studied the predictors of complications, clinical profile of patients and its manifestations.

**RESULTS.** The study included 1613 patients with a mean age of 69.47 years. 57% were female, 52% had diabetes mellitus and 15% chronic renal failure. 74 patients (4.6%) in this serie had a complication related to AngioSeal. The procedure performed was coronary angioplasty with drugs eluting stents or bare metal stents in 1032 patients (64%), diagnostic catheterization in 420 patients (26%), and valve interventions in 161 patients (10%). 74 complications were observed, the most frequent was haematoma in 60 patients (81%), and most of them were small: 73% < 5 cm (44 patients). 57% of all haematomas appeared or worsened late (> 24 hours), presenting as a syndrome of acute bleeding (26 patients), coinciding with the primo-mobilization. The symptoms were acute groin pain, swelling and hypotensive or hemorrhage syndrome. 22 patients (29.7%) required transfusion for signs and/or hemoglobine < 9 mg/dl. 18 patients developed pseudoaneurysms, 12 of them were resolved with compression, 6 with embolization with surgery. 3 patients (4%) developed arterial ischemia resolved with endarterectomy. Independent predictors of bleeding were: coronary intervention (63%),dual antiplatelet therapy (76%), fibrinolysis (28%), previous oral anticoagulation (26%), prior anemia (6%).

**CONCLUSIONS.** Vascular complications resulting from the use of AngioSeal are rare, and many of them, have a presentation of acute bleeding possibly due to late inadequate apposition between collagen and vascular anchor. Coronary intervention and dual antiplatelet therapy are the most important independent predictors of bleeding associated with the use of this device.

### 1145

#### Comparative study of three therapeutic strategies in patients admitted to the ICU due to symptomatic bradycardia: temporary transvenous pacemaker (TTVP) vs chronotropic drugs (C+) vs observation (OBS)

J. Romo[1], P. Gallardo[2], N. Arriero[1], Z. Eguileor[1], S. Saboya[3], J.A. Silva[1], C. Marian[1], M. Torralba[4], C. Benito[1], A. Albaya[1], Y. Elena[1], A. Estrella[1]
[1]Hospital Universitario de Guadalajara, Unidad de Cuidados Intensivos, Guadalajara, Spain; [2]Hospital Universitario de Guadalajara, Anestesiología y Reanimación, Guadalajara, Spain; [3]Hospital Universitario Puerta de Hierro Majadahonda, Unidad de Cuidados Intensivos, Madrid, Spain; [4]Hospital Universitario de Guadalajara, Departamento de Medicina Interna, Guadalajara, Spain
**Correspondence:** J. Romo
*Intensive Care Medicine Experimental* 2018, **6(Suppl 2):**1145

**INTRODUCTION.** Admission for symptomatic bradycardia in the ICU is frequent and the initial therapeutic option, while waiting for permanent pacemaker implantation, depends on the severity of symptoms, the center´s infrastructure and the personnel who perform the care. This study tries to describe the clinical outcome of the three most frecuently used therapeutic options before definite pacemaker implantation: temporary transvenous pacemaker (TTVP), chronotropic drugs (C+) and observation (OBS).

**OBJECTIVES.** To analyze the characteristics and clinical outcome of three initial therapeutic options (TTVP, C+ and OBS) in patients admitted to the ICU due to symptomatic bradycardia.

**METHODS.** Retrospective cohort study of patients admitted to the ICU due to symptomatic bradycardia [symptomatic atrioventricular block (sAVB), sinus node dysfunction (SND), hyperkalemia (HK), drug overdose (OD), etc.] from January 2014 to December 2017.

We collected demographic data: age and sex; and clinical variables: APACHE II, diagnosis at admission, low cardiac output, agitation, time from ICU admission to permanent pacemaker implantation (TPPM), length of stay in ICU (LSICU) and in hospital (LSH); the last three meassured in days.

The demographic variables were compared by χ2 and the quantitative ANOVA or nonparametric test (Kruskal-Wallis).

**RESULTS.** We analyzed 105 patients divided into 3 groups: TTVP: 70, C+: 11, OBS: 24. Of these 54.3% were men; median age 79.5 (IQR: 71.5-84).
The APACHE II median was 14 (IQR: 10-17) with no significant differences (NSD) between the 3 groups (p = 0.200).
The most frequent diagnosis was sAVB (83.8%), with NSD in the type of bradycardia between the 3 groups (p = 0.830).
There where NSD in low cardiac output between the 3 groups (p = 0.340).
Agitation was more frequent in the TTVP (25,7%) and C+ (27,3%) groups compared with OBS (4,2%), although with NSD (p=0,070).
The TPPM (days) was 1.42 (1.18-1.66) in TTVP; 2.11 (0.33 - 3.89) in C+; and 1.09 (0.72-1.45) in OBS; with NSD between the 3 groups (p = 0.700).
The LSICU (days) was 1.94 (1.63-2.25) in TTVP; 1.6 (1.0-2.20) in C+; 1.33 (1.01 - 1.65) in OBS ; with NSD between the 3 groups (p = 0.080).
The LSH (days) was 4.8 (3.91 - 5.69) in TTVP; 4.78 (1.96-7.6) in C+; 3.83 (3.03-4.63) in OBS; with NSD between the 3 groups (p = 0.334).
Four patients in the OBS group (16.7%) and two patients in the C+ group (18,2%) required urgent placement of TTVP.
**CONCLUSIONS.** In our series, there were no significant differences between the three groups in any of the studied variables. There was a higher incidence of agitation in the TTVP and C + group, although with no significant difference.

### 1146

#### Desmopressin, as a hemostatic agent, should always be given with tranexamic acid ? A pilot study

E. Spyridakis[1], G. Fotopoulou[2], E. Pappa[1], S. Kalakonas[1]
[1]Hygeia Private Hospital, Athens, Greece; [2]National and Kapodistrian University of Athens, 1st Department of Intensive Care, Athens, Greece
**Correspondence:** G. Fotopoulou
*Intensive Care Medicine Experimental* 2018, **6(Suppl 2):**1146

**INTRODUCTION.** Excessive fibrinolysis remains a major component of acute coagulopathy in patients undergoing on-pump coronary artery bypass grafting (CABG). Desmopressin (DDAVP) (1) and tranexamic acid (TXA) (2) secure the formation of normal blood clots through different pathways (platelet adhesion and aggregation and inhibition of enzymatic degradation of fibrin blood clots respectively) .
**OBJECTIVES.** The objective of our study was to evaluate whether the efficacy of DDAVP is major when combined with TXA.
**METHODS.** We performed a randomized double-blind clinical trial to investigate the fibrinolytic activity, represented by D-dimers, in 57 patients who underwent primary and isolated on-pump CABG surgery and admitted in our cardiovascular intensive care unit between December 2017 and March 2018. The patients were randomized in two groups, group D (n=27) and group B (n=28) according to whether they received DDAVP only (0,6 γ/kg, divided in two doses, at the beginning and at the end of surgery) or TXA as well (20 mg/kg at induction of anesthesia,10 mg/kg during CPB and 30 mg/kg with protamine injection). The two groups were compared for pre and post-operative levels of D-dimers as well as visible blood loss defined as intra- and postoperative bleeding during the first 24 hours
**RESULTS.** Median age of the patients included to the study was 66 years and 65% were males. Production of D-dimers and visible blood loss was significantly lower in group B than group D ( 0.69 mg/L [+-0.49] vs 3.08 mg/L [+-2.05] , p< 0.05 and 855 ml [+- 330] vs 1491 ml [+- 495], p< 0.05, respectively). Stratified analysis revealed statistically significant association between D-dimers and blood loss (p< 0.01). An increase in D-dimers indicated an increase in blood loss (rho=0.343).
**CONCLUSIONS.** These results suggest that DDAVP must always be given concomitantly with TXA for maximum depression of fibrinolysis and minimum blood loss during and after CABG.

**REFERENCE(S)**
1. Tranexamic acid in patients undergoing coronary-artery surgery. Myles PS et al. N Engl J Med (2017)
2. Desmopressin for reducing postoperative blood loss and transfusion requirements following cardiac surgery in adults. Interact Cardiovasc Thorac Surg (2014)

### 1147

#### Short term mechanical circulatory assist devices placement: preliminary results after five years follow-up

A.J. Roldan Reina[1], S. Escalona Rodriguez[1], N. Palomo López[1], Y. Corcia Palomo[1], L. Martin-Villen[1], A. Adsuar Gomez[2]
[1]Hospital Universitario Virgen del Rocío, Critical Care, Seville, Spain; [2]Hospital Universitario Virgen del Rocío, Cardiovascular Surgery, Seville, Spain
**Correspondence:** A.J. Roldan Reina
*Intensive Care Medicine Experimental* 2018, **6(Suppl 2):**1147

**INTRODUCTION.** Cardiogenic shock and advanced heart failure are causes of admission to the intensive care unit (ICU). Placement of mechanical circulatory assist devices (MCAD) must be considered as a salvage therapy when first-line therapies fail. It allows temporary bridging to recovery, decision or heart transplantation.
**OBJECTIVES.** Description of the most common causes, the type of device used and the kind of bridging therapy selected for MCAD positioning. Severity assessment of the cardiac failure before MCAD implantation and further mortality rate during the therapy will be presented.
**METHODS.** Prospective observational study from January 2012 to December 2017 in a third-level ICU. All patients that required short-term MCAD placement during ICU admission were included. All the devices were surgically placed in theatres and perioperative care was managed by the intensivist at the ICU. Demographic data, type of device, cause of heart failure, laboratory parameters, left ventricular ejection fraction (LVEF) before device placement, inotropic support and intra-aortic counterpulsation balloon pump (IAoB) requirement, type of bridging therapy and mortality on ICU were collected. INTERMACS (Interagency Registry for Mechanically Assisted Circulatory Support) class was used to determine the severity of heart failure.
**RESULTS.** Fifty-nine patients were included. The average age was 50 years (IR 41-61) of whom 43% were males. Myocardial infarction was the main cause of MCAD positioning (36%) followed by non-ischemic dilated cardiomyopathy (22%). Peripheral venous-arterial (VA) ECMO (69%) was the most used, followed by central VA ECMO (15%), biventricular Levitronix Centrimag (9%) and univentricular Levitronix Centrimag (7%). MCAD positioning as bridge to transplantation therapy was practised in 43% of the patients, 32% as bridge to recovery and 25% as bridge to decision. Serum lactate level after 24 hours of MCAD support was 3,0 mmol/L (IR 1,4-5,2). Sixteen days was the average of days of stay in ICU (IR 6-31). Forty-two patients (64%) had INTERMACS class 1. Among survivors, 73 % of the patients had INTERMACS class 1.
Heart transplantation was the outcome in 20 patients (34 %), recovery in 11 patients (19 %), second MCAD positioning in 6 patients (10%) and 22 exitus cases (37 %).
An overall mortality after heart transplantation or second MCAD placement was 53 % (35 patients).

**CONCLUSIONS.** The most frequent cause of MCAD implantation was cardiogenic shock after myocardial infarction, followed by non-ischemic dilated cardiomyopathy. The most used device was VA ECMO. In most cases, MCAD implantation was used as bridge to heart transplantation.

**REFERENCE(S)**

- Sen A, Larson JS, Kashani KB, et al. Mechanical circulatory assist devices: a primer for critical care and emergency physicians. Critical Care. 2016;20:153.
- Kapur NK, Esposito M. Hemodynamic support with percutaneous devices in patients with heart failure. Heart Fail Clin. 2015;11(2):215-30.

### 1148

#### Albumin versus saline fluid resuscitation in patients with venoarterial extracorporeal membrane oxygenation (va-ECMO): a propensity score matched study

F. Schroth[1], T. Wengenmayer[1], P. Biever[1], D. Duerschmied[1], C. Benk[2], G. Trummer[2], C. Bode[1], D. Staudacher[1]
[1]Heart Center Freiburg University, Department of Cardiology and Angiology I, Freiburg i. Br., Germany; [2]Heart Center Freiburg University, Department of Cardiovascular Surgery, Freiburg i. Br., Germany
**Correspondence:** D. Staudacher
*Intensive Care Medicine Experimental* 2018, **6(Suppl 2):**1148

**INTRODUCTION.** Patients after cannulation for venoarterial extracorporeal membrane oxygenation (VA-ECMO) can develop a sepsis like syndrome necessitating substantial fluid resuscitation. There are data suggesting a potential benefit of albumin fluid resuscitation in patients with septic shock.
**OBJECTIVES.** This study evaluates outcome of VA-ECMO patients with albumin versus saline fluid resuscitation.
**METHODS.** We performed a retrospective cohort study between October 2010 and October 2017 of all patients treated with VA-ECMO for shock or for extracorporeal cardiopulmonary resuscitation at a single center. At physician's discretion, patients received either albumin (local standard encouraged 10g albumin per liter fluid resuscitation) or saline only. Propensity score matching was performed to attenuate bias.
**RESULTS.** A total of 306 patients (age 58.7±14.6 years, 27.1% female, SOFA score 14.9±2.2, SAPS2 score 49.4±14.1) were included. 118 patients received albumin within 24 hours after cannulation (average 60.3±57.5g), 188 patients did not. Patients with albumin were sicker (SOFA-score 15.3±2.4 vs. 14.7±2.1, p=0.044, SAPS2-score 52.1±13.4 vs. 47.7±14.3, p=0.015) and had a higher net fluid balance after 12 hours (7.9±6.2 vs 6.2 ± 6.3, p=0.026). Hospital survival however was similar (albumin 37.3% vs. saline 29.8%, p=0.211). When excluding 18 patients with a negative net fluid balance 12 hours after cannulation, patients with albumin fluid resuscitation had a significant survival benefit (median survival 165h vs 99h, hazard ratio 1.36, 95% CI 1.03-1.82, Mantel-Cox p=0.033). After 1:1 propensity score matching (including age, resuscitation, norepinephrine, lactate, fluid balance, and SAVE, SOFA and SAPS2 score) we identified 79 matched pairs. Hospital survival was significantly higher in the albumin fluid resuscitation group (51.9% vs 26.6%, p=0.002) after matching.
**CONCLUSIONS.** In this retrospective registry of patients after va-ECMO cannulation, albumin fluid resuscitation resulted in a significant better hospital survival when compared to saline alone. These results have to be confirmed by a randomized trial.

**GRANT ACKNOWLEDGMENT**
n.a.

Fig 1: Kaplan-Meier survival curves after cannulation for venoarterial extracorporeal membrane oxygenation (va-ECMO) in patient with and without albumin fluid resuscitation within the first 24 hours. Significance was calculated by log-rank (Mantel-Cox) test.

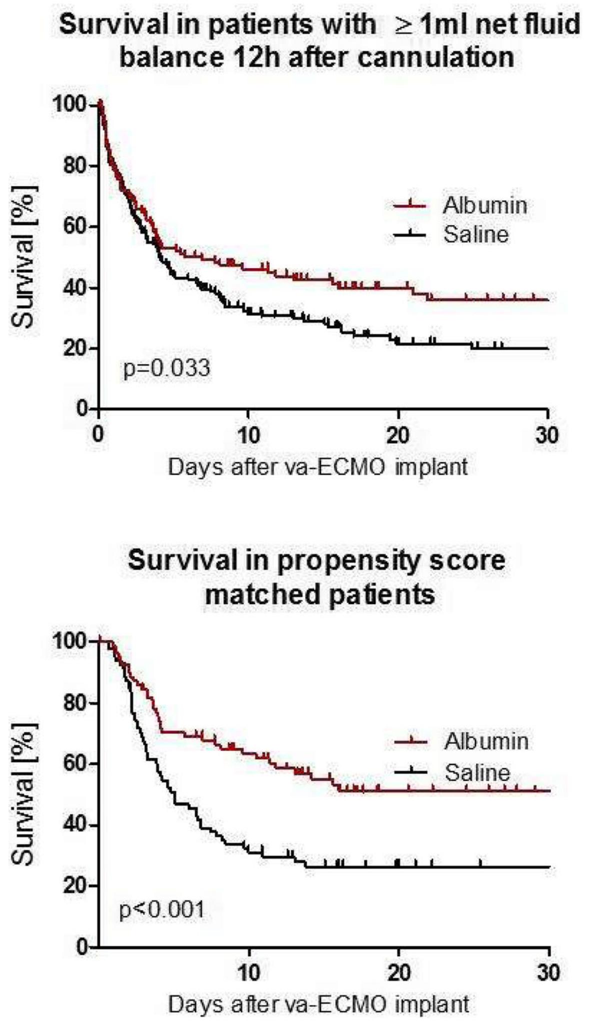

**Fig. 1 (abstract 1148) .** Albumin versus saline fluid resuscitation in patients with va-ECMO

### 1149

#### Intra- aortic balloon pump induced venous oxygen saturation changes after cardiac surgery

A. Mohamed[1,2], S. Aboulnaga[1,3], F. Hamawi[1], A. Pattath[1], R. Singh[1], A. Omar[1,4], A. Alkhulaifi[1]
[1]Hamad Medical Corportion, Cardiothoracic Surgery, Doha, Qatar; [2]Alazhar University, Anesthesia and Intensive Care, Cairo, Egypt; [3]Ain Shams University, Anesthesia and Intensive Care, Cairo, Egypt; [4]Beni Suef University, Critical Care, Beni Suef, Egypt
**Correspondence:** A. Mohamed
*Intensive Care Medicine Experimental* 2018, **6(Suppl 2):**1149

**INTRODUCTION.** The central venous saturation ($ScvO_2$ ) had been advocated in intraaortic balloon pump weaning [1]. Some literature link the cardiac surgery outcome to high ScvO2 as well [2]
**OBJECTIVES.** We aimed to identify the IABP-induced changes in patients with impaired left ventricular function (LVF).

**METHODS.**
**Design:** A retrospective, observational, cross-sectional study.
**Settings:** Tertiary hospital 12-bed cardiothoracic intensive care unit (ICU). We examined arterial and venous blood gases through electronic medical records from 2012 to 2016.
**Patients:** We included patients with at least moderate impairment of LVF with ejection fraction (EF) < 40%.
**Intervention:** Patients were divided according to presence of IABP support into two groups:

1) group I with IABP and
2) group II without IABP.

**RESULTS.** Both groups were matched with respect to age, ejection fraction (EF) %, and hemoglobin and arterial saturation ($SaO_2$) in blood gases. Patients with IABP have a trend toward higher $ScvO_2$, which was significant in three out of seven samples screened from admission (71.47±12.5 versus 63.5±9.3, 68.3±12.6 versus 60.1±9.5, and 62.69±10.8 versus 55.63±8.1, p=0.04, 0.05, and 0.03 respectively).
**CONCLUSIONS.** In this study we found that patients with IABP had higher venous saturation in comparison with the matched group with moderately impaired left ventricular function after coronary revascularization. These results need to be addressed in interpreting ScvO2 data after cardiac surgery, especially when it is linked to outcome measures.

**REFERENCE(S)**

1) 1) Hsin HT, Chen LY, Lin PC, et al. Central venous oxygen saturation (ScVO2) facilitates the weaning of intra-aortic balloon pump in acute heart failure related to acute myocardial infarction. Int J Cardiol. 2013; Oct 9;168:4568-70.
2) 1) Balzer F, Sander M, Simon M, et al. High central venous saturation after cardiac surgery is associated with increased organ failure and long-term mortality: an observational cross-sectional study. Crit Care. 2015; Apr 16;19(1):168.

**GRANT ACKNOWLEDGMENT**

The authors thank all members of the Cardiothoracic surgery department, Heart Hospital, of Hamad Medical Corporation, Qatar, for providing all required data related to this work.

### 1150

#### Outcomes in patients with fulminant myocarditis treated with mechanical circulatory support

A. Zayene[1], L. Rahmania[1], N. Rousse[2], A. Vincentelli[2], E. Robin[1]
[1]Centre Hospitalier Regional et Universitaire de Lille, Department of Anesthesiology, Lille, France; [2]Centre Hospitalier Regional et Universitaire de Lille, Department of Cardiovascular Surgery, Lille, France
**Correspondence:** A. Zayene
*Intensive Care Medicine Experimental* 2018, **6(Suppl 2):**1150

**INTRODUCTION.** Previous reports

(1) have suggested a better outcome in fulminant myocarditis
(2) treated with mechanical circulatory support.

Only few studies have compared outcomes in fulminant myocarditis using pharmacologic therapy or mechanical circulatory support.
**OBJECTIVES.** To evaluate and compare mortality in patient with fulminant myocarditis requiring or not mechanical circulatory support.
**METHODS.** It was a retrospective, single-center, observational study. We analyzed data of all patients hospitalized at our institution for myocarditis from 1st January 2005 to 31 December 2016. We included only patients with fulminant myocarditis. Two groups were defined: patients with circulatory support (MF A+) and patients without circulatory support (MF A-). We evaluated mortality, morbidity, functional recovery in the 1, 3 and 6 months.
**RESULTS.** We analyzed data of 302 patients and we included 30 patients: 16 in MF A+ group and 14 in MF A- group. At day 28, MF A + group mortality was higher than MF A- group [50 vs 14% (p=0.007)]. The mean support time was 2.6 ± 3.5 days from the introduction of inotrope support. Myocardial recovery in MF A+ group was 41% at day 28 versus 60% in MF A- group and was not statistically different.
**CONCLUSIONS.** Mortality of fulminant myocarditis requiring mechanical circulatory support was significantly higher. Further studies would be needed to select patients who may require circulatory support.

**REFERENCE(S)**

1- Diddle JW, Almodovar MC, Rajagopal SK, Rycus PT, Thiagarajan RR. Extracorporeal membrane oxygenation for the support of adults with acute myocarditis. Crit Care Med 2015;43(5):1016-25.
2- Caforio ALP, Pankuweit S, Arbustini E, Basso C, Gimeno-Blanes J, Felix SB, et al. Current state of knowledge on aetiology, diagnosis, management, and therapy of myocarditis: a position statement of the European Society of Cardiology Working Group on Myocardial and Pericardial Diseases.European Heart Journal 2013 ; 34 : 2636-2648.

### 1151

#### Left ventricular assist device implantation in patients with cardiogenic shock: is prior stabilization with Extracorporal Life Support beneficial?

K. Pilarczyk[1], H. Carstens[2], J. Heckmann[2], M. Kamler[2], A. Koch[2], H. Jakob[2], N. Pizanis[2]
[1]imland Klinik Rendsburg, managed by Sana Kliniken AG, Department of Intensive Care Medicine, Rendsburg, Germany; [2]West German Heart and Vascular Center Essen, University Hospital Essen, Department of Thoracic and Cardiovascular Surgery, Essen, Germany
**Correspondence:** K. Pilarczyk
*Intensive Care Medicine Experimental* 2018, **6(Suppl 2):**1151

**INTRODUCTION.** Ventricular Assist Device (VAD) implantation in patients with Interagency Registry for Mechanical Assisted Circulatory Support (INTERMACS) Level I and II is accompanied with high early mortality and morbidity rates. In order to stabilize or discriminate inoperable patients, Extracorporeal Life Support (ECLS) is a promising new tool.
**OBJECTIVE.** Aim of this study was to compare early postoperative morbidity and midterm mortality in patients with cardiogenic shock and VAD-implantation with and without prior ECLS - implantation.
**METHODS.** Out of 116 consecutive patients with VAD implantation (continuous-flow devices) during 01/2012 and 07/2016, 50 patients were classified as INTERMACS I or II and analyzed retrospectively. Group 1: 24 ECLS patients, Group 2: 26 patients without ECLS. Primary outcome was 360 and 720 day mortality, secondary outcome was prolonged ICU stay (>7 days) and ventilation time (>3 days), renal replacement therapy (RRT), reintubation (RI) and re-thoracotomy (RT) after surgery.
**RESULTS.** In 12 patients, ECLS implantation was performed under cardiopulmonary resuscitation (ECPR). Mean time of mechanical support prior surgery was 8.1±6.5 days. Concerning age (52.4±12.9 vs. 58.5±11.3 years), gender (87% male73% vs. male), underlying disease, and destination or bridge to transplant indication there were no significant differences between groups. However, incidence of severe acute kidney injury requiring renal replacement therapy was higher in patients with preoperative ECLS (18/24 (75%) vs. 10/26 (38.5), p = 0.009) as well as the proportion of patients with INTERMACS level I (24/24 [100%] vs. 8/26 [31%], p < 0.01).
Using Cox regression analysis for primary outcome, 360-d-mortality (HR 0.83) and 720-d-mortality (HR 0.74); (p=0.65 / p=0.45) tended to be lower in the ECLS group without reaching statistical significance.
Using chi$^2$-test for secondary outcome parameters, ECLS implantation was not associated with a prolonged ICU stay (OR 1.2; p=0.75), ventilation time (OR 2.4; p=0.16), RI (OR 0.45; p=0.24) or RRT (OR 0.53; p=0.27). In contrast, ECLS therapy was a risk factor for postoperative RT (OR 4.9; p=0.048).
**CONCLUSION.** Despite unfavorable preoperative conditions, mortality and morbidity in patients with preoperative ECLS support was comparable to patients without temporary mechanical support. Therefore, ECLS prior to LVAD as a bridge-to-bridge therapy may help to lower mortality in primarily unstable patients. However, attention has to be paid to the coagulation disorders of ECLS therapy with an increased risk of postoperative bleeding.

### 1152

#### The management of renal involvement in chronic heart failure

E. Rachdi[1], B. Ben Kaab[2], S. Antit[3], B. Menjour[4], M.H. Douggui[2], L. Zakhama[5], S. Ben Youssef[5]
[1]Faculty of Medicine of Tunis, Intensive Care Unit, La Marsa, Tunisia; [2]Faculty of Medicine of Tunis, FSI Hospital, Intern Medecine, La Marsa, Tunisia; [3]Faculty of Medicine of Tunis, Cardiology, La Marsa, Tunisia; [4]Faculty of Medicine of Tunis, FSI Hospital, La Marsa, Tunisia; [5]Faculty of Medicine of Tunis, FSI Hospital, Cardiology, La Marsa, Tunisia
**Correspondence:** E. Rachdi
*Intensive Care Medicine Experimental* 2018, **6(Suppl 2):**1152

**INTRODUCTION.** Cardiorenal syndrome (CRS) type 2 is defined as heart disease primary chronic, responsible for kidney failure chronic secondary. Its prevalence varies by data of literature between 20 and 40% [1] and it constitutes an independent predictor of outcomes and mortality. Therefore, it is of the upmost importance to preserve the renal function in these patients.
**OBJECTIVES.** The objective of our study was to evaluate the therapeutic impact of CKD in patients followed for CHF.
**METHODS.** Our work was a retrospective study of 70 patients with CHF included in the cardiology department of the Marsa FSI Hospital. It compared between two groups: G1 group control: patients with renal clearance> 60 ml / min (N=30) and G2 group CRS: patients with renal clearance ≤ 60ml / min (N=40). For all the patients we recorded epidemiological, clinical, biological, echocardiographic and therapeutic data as well as major events during the follow-up.
**RESULTS.** Our patients had an average age of 58 and were predominantly male. The most common pattern of hospitalization was acute coronary syndrome in 33 (47%) cases, followed by congestive heart failure in 30 (43%). Thus, overload and renal congestion explain the use of diuretics. Furosemid was the most used, it was prescribed in 45 patients (64%) with no difference between the two groups (24 in G1 versus 22 in CRS, **p=0, 38**). The mean dose was 71 mg in group control and 53 mg in CRS group (**p=0, 36).** In Campbell´s study [2] loop diuretics were more used in the SCR group and at higher doses. For adjuvant therapy, the blockers of the Renin Angiotensin System, betablockers, digoxin and anticoagulants were used similarly in both groups, with no significant correlation. However, Isosorbide dinitrate was more used by the CRS group (n=6, (20%)) than in the control group (n=2(5%)), with a high correlation (**p=0, 05**).Our results were in agreement with those of Ahmed´s study [3]. In the current study, no patient had veno-venous ultrafiltration.
**CONCLUSIONS.** In addition to optimizing the management of heart failure and the prevention of cardiovascular risk factors, the main CRS treatment; which must be multidisciplinary; is based on optimal volume control using diuretics mainly. The place of ulrafilration in this context remains to be clarified by complementary studies.

##### REFERENCE(S)

1. Hillege HL, Girbes AR, de Kam PJ, et al (2000) Renal function, neurohormonal activation, and survival in patients with chronic heart failure. Circulation 102:203-10
2. Campbell, R.C., et al., *Association of chronic kidney disease with outcomes in chronic heart failure: a propensity-matched study.* Nephrol Dial Transplant, 2009. **24**(1): p. 186-93.
3. Ahmed, A., et al., *Chronic kidney disease associated mortality in diastolic versus systolic heart failure: a propensity matched study.* Am J Cardiol, 2007. **99**(3): p. 393-8.

### 1153

#### Fibrinolytic therapy vs angioplasty. Are there more complications depending on the choice of reperfusion technique?

M. Morales[1], P. Oliva[1], F. Rivas[2], P. Ponce[2], P. Domínguez[2], V. Gallardo[2], C. Jiménez[2], A. Tristancho[2], A. Montero[2], D. Cabrera[2]
[1]Hospital Juan Ramón Jiménez, ICU, Huelva, Spain; [2]Hospital Juan Ramón Jiménez, Huelva, Spain
**Correspondence**: M. Morales
*Intensive Care Medicine Experimental* 2018, **6(Suppl 2):**1153

**INTRODUCTION.** Recent studies highlight a fall in the mortality in the ST-elevation miocardial infarction (STEMI) relate to an early reperfusion and the use of strong antiplatelet therapy. In spite of these results, in-hospital mortality persists high, around 4 to 12%.
**OBJECTIVES.** To describe the complications related to STEMI according to the inicial management adopted (conservative, fibrinolytic therapy or primary angioplasty) in our centre without 24h primary angioplasty availability.
**METHODS.** Descriptive restrospective study that incluyes 167 patients admitted in our ICU in the last year (2017) with the diagnosis of SETMI. We obtained demographic variabes, initial reperfusion therapy, complications and mortality.
Data analysis with SPSS program.
**RESULTS.** We describe 167 patients, 82% men with a median of age of 64 years old. In admítanse 77.2% presented Killip I, 12% Killip II, 6.6% Killip III and 4.2% cardiogenic shock.
38.2% had an anterior STEMI, 30.5% inferior STEMI, 24.6% infero-posterior/infero-lateral and 4% lateral STEMI.
Glotal mortality was of 6% (72% in women), being 4.2 % related to the initial Management and 1.8% with ICU admittance.
Angioplasty was performed in 44.3% of the patients (43.7% in anterior localisation, 49% inferior), fibrinolytic therapy in 38.9% and conservative Management in 16.8%.
According to the initial Killip, angioplasty was performed in Killip I cases 38.7%, Killip II in 47.6%, Killip III 81.3% and Killip IV 83.3%.
In total 58.7% of the patients did not present any complications. Between the patients undergoing fibrinolysis therapy, 41.4% had a complication (cardiorrespiratory arrest 9.2% (CRA), ventricular tachicardia with pulse 7.6% (VTWP), auriculo-ventricular block (7.6%) (AVB), hemorrhagic stroke, cardiac tamponade and cardiogenic shock (CS), 1.5% respectively.
Out of the patients undergoing angioplasty 51.4% suffered complications (CRA 5.4%, VTWP 1.3%, AVB 8.1%, CS 9.5%; no cardiac tamponade or hemorrhagic stroke were registered).
**CONCLUSIONS.** Despite the limited access to the hemodynamics unit, our in-hospital mortality is similar as those described in European series. There are more complications in pacients treated with angioplasty probably in relation with a higher initial Killip in admittance.

##### REFERENCE(S)

2017
ESC Guidelines for the management of acute myocardial infarction in patients presenting with ST-segment elevation. European Heart Journal. 2017. doi 10.1093/eurheartj/ehx393

### 1154

#### Descriptive analysis of pre-hospital fibrinolytic therapy vs. in-hospital in ST-elevated myocardial infarction (STEMI)

V. Gallardo Carbajo, M.P. Ponce Ponce, M. Morales Navarrete, P.J. Domínguez García, A. Tristancho Garzón, F.I. Cabeza Cabeza, A. Montero Urbina
Hospital Juan Ramón Jiménez, ICU, Huelva, Spain
**Correspondence:** M. Morales Navarrete
*Intensive Care Medicine Experimental* 2018, **6(Suppl 2):**1154

**INTRODUCTION.** According to the latest clinical guidelines, primary percutaneous coronary intervention (PCI) is the reference reperfusion treatment in STEMI. In our daily practice the hemodynamics unit only performs primary PCI from Monday to Friday from 8AM-15PM, and in special cases such as contraindications to fibrinolytic therapy, failure in fibrinolytic therapy and extremely severe cases.

**OBJECTIVES.** Asses the management of those patients with STEMI diagnosis in our unit during the last year, and whose initial reperfusion treatment was fibrinolytic therapy.

**METHODS.** Retrospective observational study with 58 patients diagnosed of STEMI in 2017. Statistical analysis: cualitative variables expressed in percentages and cuantitative variables according to central trend and dispersion.

**RESULTS.** During the period of study a total of 54 patients with STEMI were admitted into the ICU, of which 52% were treated with in-hospital fibrinolytic therapy and 48% received pre-hospital therapy.

In the pre-hospital group, 76.9% were men with a mean age of 58.1 ±13.6 years. 96% had at least one cardiovascular risk factor (CVRF). Inferior STEMI was present in 35% and multiple localisation in 35%. Rescue PCI was performed in 46% of the patients, with mono-vessel disease in 58% of these. During ICU admittance, 58% had a torpid evolution with predominant cardiologic complications (93%). Mean ICU and hospital length of stay was 1.5 ± 0.7 days and 3.8 ± 3.5 days, respectively. Regarding clinical outcome, only one exitus was recorded (3.8%) and Ejection Fraction (EF) at discharge of the surviving patients was 43.5%± 12.7.

In the in-hospital group, 82% were men with a mean age of 53.8 ± 13.7 years old, 93% had CVRF. Anterior presentation occured in 54% of the the patients and in 29% of the cases rescue PCI was performed, being 68% of these mono-vessel disease. During ICU admittance, 50% had a torpid evolution with predominant cardiologic complications and only in one case a hemorrhagic complication occurred. Mean ICU and hospital length of stay was 1.5 ± 0.8 days and 3.9 ± 3.5 days, respectively. In this group two exitus were recorded (7%) and EF at discharge of the surviving patients was 44.9%± 9.7.

**CONCLUSIONS.** In spite of needing less number of rescue PCI and presenting less complications during the evolution, the in-hospital group had a higher mortality. However, there were no significant differences regarding lenght of stay in ICU/hospital or EF at discharge in the surviving patients.

Despite the limited number of analyzed cases and the limited access to the hemodynamics unit, we hightlight the reduced incidence of severe complications, translating into satisfactory results in terms of prognosis and mortality.

### 1155

#### Hemodynamic changes during initial resuscitation of critical burn patients with inhalation injury

L. Cachafeiro, C. Arévalo, C. Gutierrez, A. García, A. Agrifoglio, E. Herrero, J.C. Figueira, B. Estébanez, M. Sánchez
Hospital La Paz, Madrid, Spain
**Correspondence:** L. Cachafeiro
*Intensive Care Medicine Experimental* 2018, **6(Suppl 2):**1155

**INTRODUCTION.** Patients with inhalation injury have a worse prognosis. One of the factors involved in this increase in mortality are the hemodynamic changes produced in the initial resuscitation phase and reflected the higher volume requirement during this phase.

**OBJECTIVES.** The aim of our study is to analyze the hemodynamic changes that occur in the patient with inhalation injury during the initial phase of resuscitation and its relationship with mortality.

**METHODS.** We conducted a three-year prospective cohort study of 362 consecutive critically burned patients admitted to a critical burn unit considered as a national burn reference center. We used in all patients admitted to our unit a resuscitation protocol guided by the parameters obtained with transpulmonary thermodilution and tissue perfusion data. Demographic data, TBSA, severity scores, mechanical ventilation, length of stay and mortality were collected, in addition to the data provided by thermodilution and lactate value, troponin and NTproBNP.

We compared the results obtained among critically burned patients with TBSA < 20% and patients with inhalation injury. Inhalation injury was defined with two or more of the following criteria: history of injury in an enclosed space, facial burns with singed nasal hair, carbonaceus sputum and stridor. If they were intubated it was diagnosed by bronchoscopy.

**RESULTS.** During this period 362 consecutive critical burns patients were admitted in our unit, 96 patients with a TBSA > 20%, 84 patients with inhalation injury. The majority were middle-aged males, although the patients with inhalation Injury were older. We did not find significant differences in the severity scores between both groups, except in APACHE II. Neither did we find differences in the amount of volume used during the initial resuscitation, nor in the average stay, although we found differences in the need for MV and in mortality, being significantly greater in patients with inhalation injury (Table 1)

The parameters obtained with the transpulmonary thermodilution are shown in the following graph (Graph 1).

**CONCLUSIONS.** In our study, the volume used during the initial resuscitation was similar in both groups, coinciding with those estimated by Parkland.

Patients with inhalation injury had a greater need for MV, which could be related to the increased mortality of these patients.

Mortality in patients with inhalation injury was associated with greater cardiac dysfunction, represented by lower cardiac index values and higher values in markers of myocardial damage (troponin and NTproBNP), greater tissue hypoperfusion (elevated lactic acid levels) and greater inflammatory response with more alteration of permeability (elevations of IAPEV).

**REFERENCE(S)**

Sheridan RL. Fire-related inhalation injury. N Engl J Med 2016; 375: 464-9
Holm C et al. Effect of crystalloid resuscitation and inhalation injury on extravascular lung water: clinical implications. Chest 2002;121:1956-62

**Table 1 (abstract 1155).** See text for description

| | TOTAL | TBSA>20% | INHALATION INJURY | P value |
|---|---|---|---|---|
| Pacients (nº) | 362 | 96 | 84 | |
| Men (%) | 76,2 | 72,9 | 75,6 | 0,732 |
| Edge | 45,2 ±19,1 | 44,9±18,5 | 52,5 ±17,5 | 0,007 |
| TBSA (%) | 18,7 ±16,8 | 34,8±17,5 | 28,1 ± 25,7 | 0,001 |
| ABSI | 5,9 ± 2,2 | 7,6±2,3 | 8,2 ± 2,8 | 0,191 |
| Baux Score | 64,1± 26,8 | 79,82±28,90 | 80,5± 27,8 | 0,869 |
| APACHE II | 7,4 ± 6,9 | 12,2±6,8 | 13,3 ± 6,8 | 0,027 |
| MV (%) | 34,3 | 59,4 | 88,1 | 0,000 |
| Resucitation volumen (ml/kg/TBSA) | 4,7 ± 2,2 | 4,7 ± 2,1 | 4,2 ± 1,7 | 0,404 |
| Urine output (ml/Kg/h) | 1,20 ± 0,64 | 1,20 ± 0,59 | 1,31 ± 0,65 | 0,507 |
| Mortality (%) | 9 | 18,8 | 28,6 | 0,157 |

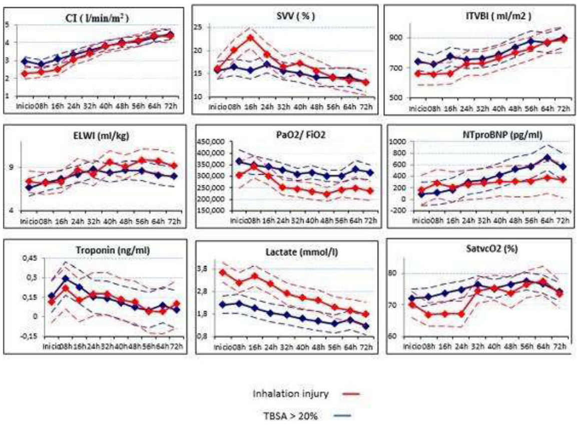

**Graph 1 (abstract 1155).** See text for description

### 1156

#### Review of practice and outcomes in cardiac output monitor guided management of high dose norepinephrine requiring septic shock in a tertiary ICU

M. Di Pierro[1], A. Conway Morris[2], R. Mahroof[1]
[1]Addenbrooke's Cambridge University Hospitals NHS Trust, John Farman ICU, Cambridge, United Kingdom; [2]University of Cambridge, Wellcome Trust Fellow, Cambridge, United Kingdom
**Correspondence:** M. Di Pierro
*Intensive Care Medicine Experimental* 2018, **6(Suppl 2):**1156

**INTRODUCTION.** The Surviving Sepsis Campaign recommends use of cardiac output monitors with dynamic assessment for fluid responsiveness[1,2] in patients with septic shock. Minimally invasive pulse contour analysis based cardiac output (CO) monitors (COM) are popular in many ICUs, but akin to the pulmonary artery catheter[3], determination of their clinical efficacy is compounded by questions about adequacy of training and (in)appropriate clinical responses to CO studies[4].

**OBJECTIVES.** To review practice, clinical and biochemical outcomes of patients with isolated severe septic shock admitted to a major tertiary academic ICU, between those patients who received a COM (either LiDCO or PiCCO) versus those who did not.

**METHODS.** Contemporaneous automated data recorded in an advanced electronic record system (Epic® Systems USA) was retrospectively interrogated and analysed. All patients admitted to ICU with isolated severe septic shock (norepinephrine (NE) >0.1 mcg/kg/min) over a period of 5 months were included. NE dose and biochemical parameters were recorded on 1 and 4 hourly intervals respectively. Data was collated on to an Excel database and analysed using appropriate statistical methods.

**RESULTS.** A total of 75 patients fulfilled selection criteria, of whom 34 (45%) had a COM used. Chest (60%) and abdomen (28%) were the commonest sources of sepsis. COM vs non-COM findings were as follows: median age 68 vs 62 yrs, crude in-hospital mortality 19/34 (56%) vs 15/41 (36%), median NE starting dose 0.17 vs 0.14 mcg/kg/min, p=0.0042 (Mann-Whitney U test), adjuvant vasopressor therapy 38% vs 5%, p=0.0003 (Fisher's exact test), inotropes 35% vs 2%, p=0.0002, shock steroids 85% vs 52%, p=0.003, and renal replacement therapy 68% vs 43%, p=0.04. Fluid balance between the groups was comparable.

Median time to resolution by survival analysis for metabolic acidosis (MA) (base excess better than -2), lactataemia (L) (< 2) and NE requirement (< 0.05 mcg/kg/min) in COM vs non-COM were: MA 48 vs 24hrs (Hazard ratio and 95% CI) (HR 0.87 (0.4-1.6), p=0.56), L 32 vs 16hrs (HR 0.85 (0.45-1.59), p=0.62), NE dose 54 vs 30hrs (HR 0.36 (0.21-0.62), p=0.0002). P values by log-rank test.

**CONCLUSIONS.** Clinicians selectively deployed cardiac output monitors in patients with vasopressor resistant septic shock. This cohort was more likely to need adjuvant therapies including inotropes, renal replacement therapy and concomitant shock steroids. Patients on COMs took longer to come off NE, however they resolve their acidosis and lactataemia within a time-frame which is not significantly different to the non-COM cohort, suggesting that COMs help drive appropriate decision making in managing complicated shock.

**REFERENCE(S)**

1. SSCG 2016. ICM 2017;43:304-77.
2. Annane D et al. Lancet 2007;370:676-84.
3. Vincent et al. Crit Care 2011;15:229.
4. Costa et al. ICM 2008;34:257-63.

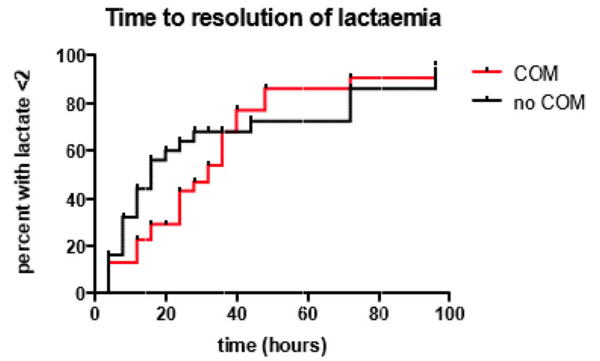

**Fig. 1 (abstract 1156).** Time to resolution of lactaemia

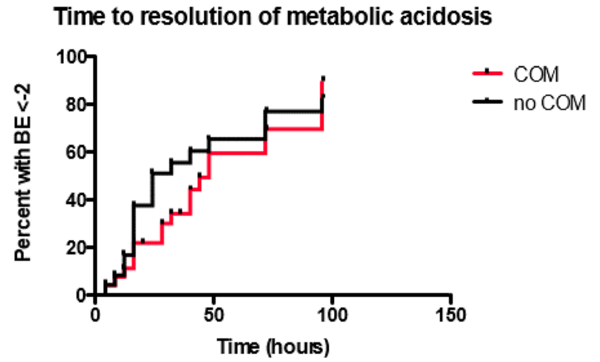

**Fig. 2 (abstract 1156).** Time to resolution of metabolic acidosis

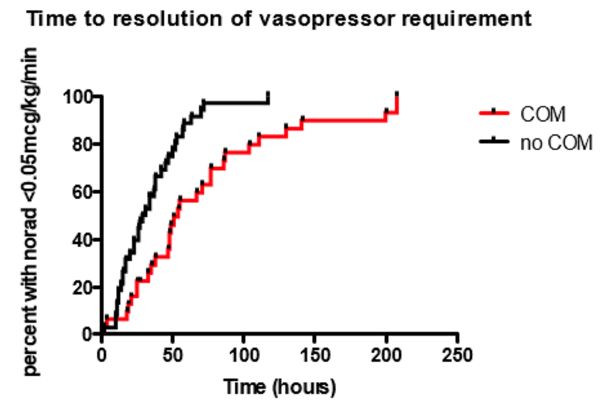

**Fig. 3 (abstract 1156).** Time to resolution of vasopressor requirement

### 1157

#### The prognostic value of cardiac power output in septic patients

M. Turker, B. Bilgili, Z. Aykac
Marmara University School of Medicine, Istanbul, Turkey
**Correspondence:** B. Bilgili
*Intensive Care Medicine Experimental* 2018, **6(Suppl 2):**1157

**INTRODUCTION.** Sepsis is one of the leading reasons for the hospitalization with high mortality and morbidity; there is still a search for predictive values for mortality. Recently, a new hemodynamic parameter "Cardiac Power Index (CPO)" has been proposed to determine prognosis in heart failure.

**OBJECTIVES.** We aimed to evaluate the prognostic power of CPO in sepsis and ICU survivals of patients with septic shock.

**METHODS.** Adult, sepsis and septic shock patients admitted in intensive care unit were included. Sepsis was defined as Sepsis 3 and patients were treated according to the recommendations in the Surviving Sepsis Guideline. Patients' age, gender, comorbidities, source of sepsis, pre-intensive care hospital admission, SOFA and APACHE II scores were recorded. Biochemical variables such as arterial blood gases, central venous oxygen saturation (ScVO2), leukocyte, procalcitonin (PCT), lactate, platelet, creatinine, brain natriuretic peptide (BNP) were followed daily. After excluding intravascular volume depletion by a passive leg-raising test, cardiac output volume, cardiac index, systemic vascular resistance, and stroke volume measurements were made and the measurements were repeated three times for every 12-hour. The cardiac power output (CPO) and cardiac power index (CPI) were calculated. Patients were grouped as survivors and non-survivors due to sepsis and ICU survivals.

**RESULTS.** Survivor and non-survivor patients were comparable in demographic data, APACHE II, and SOFA scores, and the changes in biochemical variable according to sepsis and ICU survivals. The CPO values survivors according to sepsis survival were as followed: M1= 0.89, M2= 0.99, M3= 0.9, and M4= 1.05 (p1= 0.16, p2= 0.31, p3= 0.08, and p4= 0.07, respectively). The CPI values for survivors according to sepsis survival were as followed: M1= 0.48, M2= 0.49, M3= 0.45, and M4= 0.56 (p1= 0.124, p=0.521, P00.08, and p4= 0.09, respectively).

**CONCLUSIONS.** CPO and CPI values did not predict mortality in sepsis patients. The reason why these values failed to predict mortality in sepsis could be due to the differences in the pathophysiology of cardiogenic and septic shocks. In the early phase of septic shock the CO is normal or high, whereas in cardiogenic shock it is reduced. Septic shock causes LV systolic dysfunction, LV diastolic dysfunction, and right ventricular (RV) dysfunction and only the diastolic dysfunction is the strongest independent predictor of early mortality in septic patients, whereas left ventricular systolic dysfunction is the most frequent cause and mortality predictor of cardiogenic shock.

**GRANT ACKNOWLEDGMENT**
None

### 1158

#### Hemodynamic response of fixed-dose dobutamine in patients with severe cardiogenic shock

P. Ostadal, A. Kruger, D. Vondrakova, M. Janotka, J. Naar, P. Neuzil
Na Homolce Hospital, Dept. of Cardiology, Prague, Czech Republic
**Correspondence:** P. Ostadal
*Intensive Care Medicine Experimental* 2018, **6(Suppl 2):**1158

**INTRDUCTION.** Dobutamine is widely used as an effective positive inotropic agent in severe cardiogenic shock. However, the precise effect of dobutamine on the hemodynamic variables is not fully understood.

**OBJECTIVES.** The aim of our study was, therefore, to assess the hemodynamic response on the administration of dobutamine in cardiogenic shock.

**METHODS.** Twelve patients with severe cardiogenic shock required inotropes were recruited into the study. Hemodynamic variables including cardiac output, heart rate, dP/dt max and blood pressure were measured from arterial line (using pressure waveform analysis) before and 30 min after the administration of dobutamine 5 mcg/kg/min.

**RESULTS.** Mean age of the study group was 57 years, 10 of 12 were males, the most frequent cause of cardiogenic shock was acute myocardial infarction (9 of 12) and all subjects were concomitantly treated with norepinephrine (mean dose 0.13 mcg/kg/min). Administration of dobutamine resulted in an increase of cardiac output by 23% from 3.5 to 4.3 L/min (P=0.0014), heart rate increased by 6% from 61.8 to 65.4 beats/min (P=0.0006), mean arterial pressure increased by 23% from 62.5 to 77.0 mmHg (P˂0.0001), and the most pronounced effect of dobutamine was observed on the rise of dP/dt max by 45% from 715.7 to 1036.0 mmHg/s (P˂0.0001).

**CONCLUSIONS.** Administration of dobutamine 5 mcg/kg/min was associated with slight increase of heart rate and marked rise of cardiac output and mean blood pressure. However, the most sensitive parameter to the effect of dobutamine was dP/dt max.

**GRANT ACKNOWLEDGMENT**
The study was supported by an Institutional grant MH CZ - DRO (Nemocnice Na Homolce - NNH, 00023884), IG150501.

### 1159

#### Heamodynamic effects of disconnection from the "advanced organ support" (ADVOS): the HAEMADVOS-II-study evaluating the "re-transfusion volume challenge option" (REVOLUTION)

W. Huber, M. Leinfelder, T. Lahmer, U. Mayr, A. Herner, G. Batres-Baires, S. Schreiber, M. Häberlein, L. Habenicht, S. Roland
Klinikum rechts der Isar; Technical University of Munich, Medizinische Klinik und Poliklinik II, Munich, Germany
**Correspondence:** W. Huber
*Intensive Care Medicine Experimental* 2018, **6(Suppl 2):**1159

**INTRODUCTION.** Multi-organ support provided by *one* single device is intriguing. The "advanced organ support" (ADVOS) procedure provides liver support, renal replacement, $CO_2$-elimination and acid-base modulation. To improve toxin elimination, the device is equipped with two dialyzers. This results in an inreased 500mL-volume of the extracorporeal circuit.

**OBJECTIVES.** After treatment, disconnection from the circuit with re-transfusion of 500mL within five minutes can be used as an option for a post-therapeutic volume challenge. This concept of a "re-transfusion volume challenge option" (REVOLUTION) has been recently introduced after dialysis (1). It might optimize fluid management and help to define filtration goals for subsequent therapies. Our study analyzed haemodynamic effects of disconnection during 25 ADVOS (HepaWash; Germany) therapies in six patients with PiCCO monitoring.

**METHODS.** Thermodilution with the PiCCO-device (Pulsion; Germany) was performed immediately before and after connection (T1, T2) and disconnection (T3, T4) with a blood flow of 100mL/min and stable dosages of vasopressors. During the 5-minutes disconnection pulse-contour-derived cardiac index CI_PC, MAP and cardiac power index CPI were recorded in intervals of 30s. Data on effects of connection to ADVOS (T2 vs. T1) were analyzed for a separate study (HAEMADVOS-I; A-0978-0022-00719).

**RESULTS.** Indication for ADVOS: 3 cirrhosis, 1 alcoholic steatohepatitis, 2 sepsis. 65±18 years, SOFA 11±3, mechanical ventilation 24/25 (96%); vasopressors 20/25 (80%). Net Filtration: 931±698mL.

Disconnection resulted in increases in MAP (83±9 vs. 76±7mmHg; p= 0.005), global end-diastolic volume index GEDVI (922±244 vs. 907 ±259 mL/m$^2$; p=0.031) and CPI (1.00±0.25 vs. 0.92±0.32 W/m$^2$; p=0.009). Increases in CVP (21.4±6.3 vs. 18.8±5.9mmHg; p=0.075) and stroke volume index SVI (55.3±12.1 vs. 53.5±13.8mL/m$^2$; p=0.82) slightly failed significance. Heart rate HR, systemic vascular resistance index SVRI, dPmax, global ejection fraction GEF and extravascular lung water index EVLWI did not change during disconnection. Disconnection resulted in maximum increases of 15% (primary endpoint), 10% and 5% in 10 (40%), 16 (64%) and 23 (92%) of treatments for MAP, 7 (28%), 9 (36%) and 15 (60%) of treatments for CI_PC and 17 (68%), 20 (80%) and 23 (92%) of treatments for CPI, respectively. Among the parameters recorded immediately before disconnection, GEDVI (AUC=0.917; p=0.003) and CPI (AUC=0.811; p=0.024) significantly predicted increases in CI_PC of a least 15% during disconnection, whereas CVP and MAP were not predictive.

**CONCLUSIONS.** Disconnection after ADVOS with re-transfusion of 500mL blood resulted in significant increases of MAP, GEDVI and CPI. Increases of ≥15% in CPI, MAP and CI_PC were found in 68%, 40% and 28%.

Due to the larger extra-corporeal volume of 500mL, the REVOLUTION-manoeuvre might be even more useful after ADVOS than after dialysis.

**REFERENCE(S)**

Huber et al.; PlosOne 2016.

## Recovery from critical illness

### 1160

#### Characteristics and outcomes of very elderly patients admitted to a Tunisian intensive care unit

I. Ben Saida[1], S. Rouis[1], M. Zghidi[1], I. El Meknassi[1], M.A. Boujelbèn[1], N. Kacem[1], A. Khedher[1], A. Azouzi[1], K. Meddeb[1], M. Boussarsar[1,2]
[1]Farhat Hached University Hospital, Medical Intensive Care Unit, Sousse, Tunisia; [2]Ibn Al Jazzar Faculty of Medicine, Research Laboratory N° LR12SP09 Heart Failure, Sousse, Tunisia
**Correspondence:** M. Boussarsar
*Intensive Care Medicine Experimental* 2018, **6(Suppl 2):**1160

**INTRODUCTION.** Given the ageing of the world's population, the number of patients aged 80 years and older admitted to the intensive care unit (ICU) has increased during the past decade. The appropriateness of care for those patients in ICU might be questionable especially with the limited healthcare resources available in low income countries. Knowing more about the outcome of those patients in ICU allow a better allocation of resources and help clinicians in decision making.

**OBJECTIVES.** The aim of the study was to assess the characteristics of very elderly patients (≥80 years) and to identify factors predicting ICU mortality.

**METHODS.** It is a retrospective study conducted in the medical ICU of Farhat Hached teaching hospital between January 2013 and December 2017. All very elderly patients (≥80 years) were included. Underlying condition, severity of illness, diagnostic at admission, management and outcomes were recorded. Variables found to be statistically significant in univariate analysis were included into a multivariate regression model to identify factors independently associated to poor prognosis. For all tests, a P value of less than 0.05 was considered statistically significant.

**RESULTS.** During the study period, 1378 patients were admitted, of whom 93 (6.7%) were older than 80 years. Patients' characteristics were : mean age, 83.27±2.6years ; male, 53(57%) ; median Charlson co-morbidity score, 2[1-3]; Knauss C or D, 39(41.9%); MacCabe≥2, 88(94.6%); mean SAPSII, 44±15.7; invasive mechanical ventilation (IMV), 58(62.4%) and vasopressors use, 60(64.5%). The main reasons for admission were respiratory disorders, 50(53.8%) ; shock, 15(16.1%) ; neurological disorders, 11(11.8%) and miscellaneous, 17(18.2%).The median duration of IMV was 2[1-6]days. The median length of ICU stay was 5[3-8]days. Mortality rate was 52.7%.

The univariate analysis identified the following respectively for deaths and survivals : Knauss D, (12.2% vs zero, p=0.028) ; SAPSII, (50.9±17.9 vs 36.3±7.5, p=0.000) ; use of vasoactive drugs, (89.8% vs 36.4%, p=0.000), mechanical ventilation, (79.6% vs 43.2%, p=0.000), nosocomial infection, (34.7% vs 4.5%, p=0.000) and ICU acquired kidney injury, (59.2% vs 18.6%, p=0.000).

Multivariate regression model identified the following factors as independently associated to fatal outcome: SAPSII, (OR, 1.1 ; 95%CI, [1.04-1.18] ; p=0.001) ; nosocomial infection (OR, 9.18 ; 95%CI, [1.53- 55] ; p=0.015) and vasoactive drug use (OR, 6.9 ; 95%CI, [1.8- 25.8] ; p=0.004).

**CONCLUSIONS.** In this study, predictors of fatal outcome in very elderly patients were severity of illness, nosocomial infection and vasoactive drug use.

### 1161

#### Prevalence and impact of frailty on intensive care unit outcomes

L. Hodgson[1,2], J. Warren[2], D. Hunt[2], A. Allen[2], R. Venn[2]
[1]University of Southampton, Southampton, United Kingdom; [2]Western Sussex Hospitals Foundation NHS Trust, Worthing, United Kingdom
**Correspondence:** L. Hodgson
*Intensive Care Medicine Experimental* 2018, **6(Suppl 2):**1161

**INTRODUCTION.** The clinical syndrome of 'frailty' is one of the most-challenging consequences of population ageing. Data which could aid prediction of quality of life, survival, functional dependence and resource requirements following intensive care unit (ICU) admission in older patients are essential for informed discussion and decision-making. The validated clinical frailty scale (CFS, scored 1-9 points) is electronically recorded at admission for patients over 65 years in our Trust whose catchment is one of the oldest populations in England, (25% of the population >65 years), a demographic closely resembling projections of the rest of England by 2035.(1)

**OBJECTIVES.**

- Determine the prevalence and impact of frailty on outcomes from patients admitted to ICU (incorporating level 2 and level 3 care) and the general hospital wards and,
- Assess discrimination of the CFS to predict mortality in those aged >65 admitted to ICU and general wards.

**METHODS.** We interrogated a 1-year prospectively gathered, dual-centre data-set (n=55,848 hospital admissions January-December 2017). All patients admitted to ICU with a CFS score recorded at admission were included.

**RESULTS.** 59% of ICU admissions (n=733/1,242) were over 65 years of age, of whom 76% (n=556) had a CFS recored at hospital admission (median 4 [interquartile range 3-6]) with an in-patient mortality of 12% (Figure 1 shows distribution and associated in-patient mortality). Almost half the patients (n=256) had significant frailty (CFS ≥5 points), yet were not at higher risk of mortality (13% vs 12% for those with a CFS < 5 points, odds ratio (OR) 1.11 [95% CI 0.70-1.85], P=0.67). This was in contrast to the overall hospital population where a CFS ≥5 points was strongly associated with mortality (11% vs 2% OR 6.2 [95% CI 5.4-7.2], P< 0.001). Discrimination to predict mortality of the CFS assessed using area under the receiver operating characteristic curves (AUROCs) was poor in ICU patients - 0.58 (95% CI 0.50-0.65), in contrast to acceptable discrimination - 0.75 (95% CI 0.73-0.76) - when applied to all hospital admissions >65 years (n=18,434) (Figure 2).

**CONCLUSIONS.** In a dual-centre ICU study with a high proportion of elderly frail patients, the CFS did not predict short-term mortality in contrast to its good predictive performance in the overall population of patients admitted to hospital.

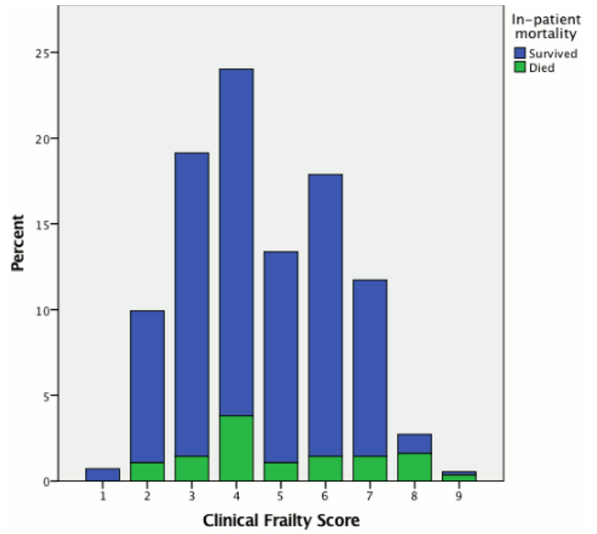

**Fig. 1 (abstract 1161).** Distribution of clinical frailty scale (CFS) in ICU patients and mortality per score

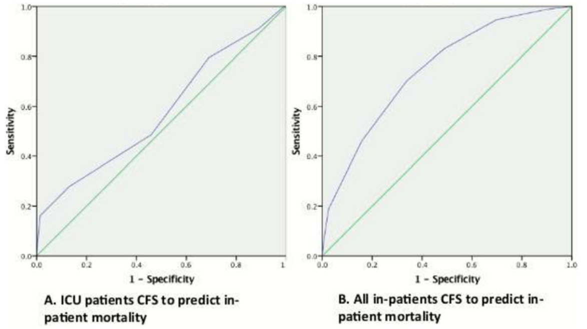

**Fig. 2 (abstract 1161).** Clinical frailty scale (CFS) AUROCS to predict mortality (A - ICU, B - all in-patient hospital admissions)

### 1162

#### The SOFA score dynamics and mortality prediction among trauma and non-trauma patients

B.M. Tomazini[1], E. Bassi[1], R.D. Oliveira[1], F.F. Novo[1], E.M. Utiyama[1], L.M.S. Malbouisson[2]

[1]Hospital das Clínicas da Universidade de São Paulo (HC-FMUSP), Surgery and Trauma Discipline, São Paulo, Brazil; [2]Hospital das Clínicas da Universidade de São Paulo (HC-FMUSP), Anesthesia Department, São Paulo, Brazil

**Correspondence:** B.M. Tomazini

*Intensive Care Medicine Experimental* 2018, **6(Suppl 2):**1162

**INTRODUCTION.** Outcome predictors are an essential aspect of Intensive Care Unit (ICU) management and performance evaluation. The Sequential Organ Failure Assessment score (SOFA) is based on the degree of organic dysfunctions and can be used to predict mortality in critically ill patients[1]. Although used in trauma patients[2], there is a lack of studies comparing its performance as a mortality predictor among trauma to non-trauma patients.

**OBJECTIVES.** To compare initial SOFA and peak SOFA performance in predicting mortality among trauma and non-trauma patients.

**METHODS.** A retrospective cohort of adult patients admitted to the ICU of a tertiary university hospital between 2012-2016. The SOFA for the first 14 ICU days was recorded. Multivariate logistic regressions were used to estimate the association of risk factors and ICU mortality. We compared ICU mortality among trauma and non-trauma patients utilizing the SOFA at admission and accessed the ability of initial vs. peak SOFA in the first 14 ICU days for predicting ICU mortality using the difference between the area under the Receiver Operating Characteristic curves (AUC).

**RESULTS.** We included 1870 patients in the analysis. Patients characteristics and outcomes are shown in the table. Trauma patients were younger, had higher admission SOFA and ICU length of stay, while non-trauma patients had higher peak SOFA and ICU mortality. Multivariate analysis showed in trauma patients SOFA, SAPS 3, gender and shock were associated with mortality, while in non-trauma patients, factors associated with mortality were SOFA, SAPS 3, and gender.

When admission SOFA was categorized, non-trauma patients had significantly higher mortality for the same SOFA category when compared with trauma patients, as shown in the graphic. The AUC for mortality prediction in trauma patients was 0.903 when using admission SOFA, and 0.904 when using the peak SOFA, the curves did not differ statistically (p=0.99). For non-trauma patients, the AUC was 0.88 for admission SOFA, and 0.924 for peak SOFA (p< 0.001).

**DISCUSSION.** Our results show essential aspects of SOFA dynamic in both trauma and non-trauma patients. Although with higher mean SOFA at admission, trauma patients had lower ICU mortality when compared with non-trauma patients, this difference was significant within most SOFA categories, as showed in the graph. Non-trauma patients had higher peak SOFA when compared to trauma patients. Therefore, although with more severe organic dysfunctions at admission, their progression in trauma patients are less pronounced than in non-trauma patients. There was no difference in accuracy whether using admission SOFA or peak SOFA for trauma patients, however, for non-trauma patients, the peak SOFA showed better accuracy predicting ICU mortality than admission SOFA.

**REFERENCE(S)**

1. Ferreira FL, et al. JAMA. 2001; 286(14):1754-8.
2. Antonelli M, et al. Intensive care medicine. 1999; 25(4):389-94.

**Table 1 (abstract 1162).** Patients characteristics

| | Trauma | Non-trauma |
|---|---|---|
| N, % | 1095 (58.56) | 775 (41.44) |
| Age, years mean, (SD) | 39.9 (17.7) | 61 (16.1) |
| Male % | 84.1 | 58.7 |
| SAPS 3 mean, (SD) | 48.35 (16.2) | 57.42 (16.9) |
| Admission SOFA mean, (SD) | 5.5 (4.4) | 5.2 (4.2) |
| Peak SOFA mean, (SD) | 7.44 (4.8) | 8.08 (4.61) |
| TBI % | 65.21 | - |
| ICU length of stay, days median, (IQR) | 7 (3-15) | 5 (3-11) |
| ICU mortality % | 21.46 | 29.55 |

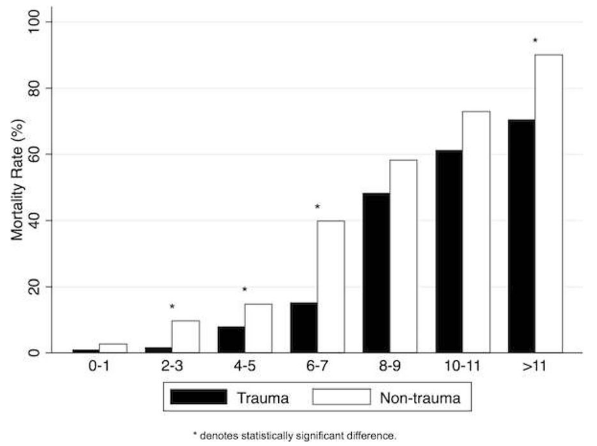

**Fig. 1 (abstract 1162).** Mortality rate by admission SOFA category.

### 1163

#### Assessment of lactate concentration as a co-predictor with the APACHE-IV scoring system

M. de Boer, M.W.N. Nijsten
University Medical Centre Groningen, Intensive Care, Groningen, Netherlands
**Correspondence:** M. de Boer
*Intensive Care Medicine Experimental* 2018, **6(Suppl 2):**1163

**INTRODUCTION.** Many variables measured in critically ill patients have been used to estimate the severity of their disease and prognosticate patient mortality. Accurate prediction models are essential for establishing and comparing effective treatments in the care of intensive care unit (ICU) patients. The Acute Physiology and Chronic Health Evaluation -IV (APACHE-IV) scoring system is a clinical standard for ICU mortality prediction [1]. Lactate levels are known to be the single strongest outcome predictor of all routinely performed laboratory measurements [2,3]. However, lactate is not included in APACHE-IV.

**OBJECTIVES.** We investigated whether combining lactate concentration with the APACHE-IV score leads to an improved prediction model.

**METHODS.** We analyzed data from patients who were admitted for >12h to the ICU from 2009 through 2014. Arterial lactate was routinely determined together with glucoses for glucose control and blood gas analysis. Using this dataset we calculated the area under the receiver operating characteristics curve (AUROC) with hospital mortality as outcome for APACHE-IV, mean lactate ($[Lac]_{mean}$) during the first 24h and a linear combination of APACHE-IV and lactate generated by logistic regression analysis.

**RESULTS.** We included 12,619 patients with a hospital mortality of 12%. During the first 24h 9.0 ±4.5 lactate measurements were performed. Mean ±SD APACHE-IV was 52 ±28. $[Lac]_{mean}$ and $[Lac]_{max}$ were 1.8 ±1.6 and 2.9 ±2.5 mmol/L respectively. The AUROCs (95% CI) of APACHE-IV, $[Lac]_{mean}$ and $[Lac]_{max}$ were 0.88 (0.87-0.89), 0.67 (0.66-0.69) and 0.66 (0.64-0.68) respectively. When APACHE-IV and $[Lac]_{mean}$ were combined in logistic regression, both were highly significant (P< 0.0001). The AUROC of this combined score was 0.88 (0.87-0.89).

**CONCLUSIONS.** Combination of APACHE-IV with lactate did not improve the predictive ability of APACHE-IV alone. Apparently the lactate blood concentration is not independent with respect to the variables already incorporated in the APACHE system.

**REFERENCE(S)**

1. Zimmerman JE, et al . Acute Physiology and Chronic Health Evaluation (APACHE) IV: hospital mortality assessment for today´s critically ill patients. 2006 ;34:1297-310.
2. Bakker J, et al., Clinical use of lactate monitoring in critically ill patients. Ann Intensive Care 2013 10;3:12.
3. Alkozai E, et al., Systematic comparison of routine laboratory measurements with in-hospital mortality: ICU-Labome, a large cohort study of critically ill patients. Clin Chem Lab Med 2018

### 1164

#### The use of SOFA, SAPS 3 and C-reactive protein (CRP)/Albumin ratio to predict mortality in surgical critically Ill patients

R. Nunes[1], E.A. Nicolini[1], A.F. Lago[1], M.G. Menegueti[1], M.A. Feres[2], A. Basile-Filho[1]
[1]Ribeirão Preto Medical School, University of São Paulo, Division of Intensive Care Medicine, Department of Surgery and Anatomy, Ribeirão Preto, Brazil; [2]Hospital São Francisco, Intensive Care Unit, Ribeirão Preto, Brazil
**Correspondence:** A.F. Lago
*Intensive Care Medicine Experimental* 2018, **6(Suppl 2):**1164

**INTRODUCTION.** Several prognostic indices have been employed to predict the outcome of surgical critically ill patients. Among them APACHE II, SOFA and SAPS 3 are widely used. CRP is an acute-phase protein that correlate with the degree of inflammation during the early postoperative phase. Furthermore, we hypothesized that CRP/Albumin ratio could be a superior marker of mortality than CRP or albumin alone.

**OBJECTIVES.** The objective of this study is to compare the predictive values of SOFA, SAPS 3, CRP/Albumin ratio and blood lactate level in the outcome of these patients.

**METHODS.** Eight hundred forty seven surgical patients admitted to the ICU in the post-operative phase were retrospectively studied. Cardiovascular, digestive system, neurological and orthopedic elective or emergency surgery accounted for 85% of cases. The patients were divided into survivors (n=765, 57.5% males, mean age of 60.1 ± 17.0 years) and nonsurvivors (n=82, 57.3% males, mean age of 68.0 ± 15.1 years). SOFA, SAPS 3, blood lactate level, CRP/Albumin ratio were recorded on ICU admission. The capability of each index (SOFA, SAPS 3, lactate, CRP/Albumin ratio) to predict mortality was analyzed by receiver operating characteristics (ROC) curves. The area under de curve (AUC) and the respective confidence interval (CI) were used to measure the index accuracy. Comparison among these curves was tested. The level of significance was set at p< 0.05. ROC curves analyses were performed using the software MedCalc v. 14.

**RESULTS.** The mean SOFA, SAPS 3, lactate and CRP/Albumin ratio were 4.5 ± 2.7, 37.0 ± 12.5, 11.2± 23.0, 2.8 ± 1.6 for survivors and 8.3 ± 3.7, 56.1 ± 15.9, 31.8 ± 38.6 and 3.9 ± 3.4 for nonsurvivors, respectively. The area under the ROC curve for SOFA, SAPS 3, CRP/Albumin ratio and lactate and the respective CI data between survivors and nonsurvivors are showed in table 1. The comparisons of ROC curves for these indexes are depicted in graph 1. The overall mortality was 9.7%.

**CONCLUSIONS.** Even though SOFA and SAPS 3 were the most effective indices to predict mortality in the surgical critically ill patients, the CRP/Albumin ratio on admission may also play a role as predictor of outcome.

**GRANT ACKNOWLEDGMENT**
FAEPA- Clinics Hospital of Ribeirão Preto, SP, Brazil.

**Table 1 (abstract 1164).** SOFA, SAPS 3, CRP/Albumin and lactate of surgical patients survivors and nonsurvivors *$p<0.05$

| Patients (n=847) | Survivors (n=765) | Nonsurvivors (n=82) | AUC | IC |
|---|---|---|---|---|
| SOFA | 4.5±2.7 | 8.3±3.7* | 0.880 | 0.856-0.901 |
| SAPS 3 | 37.0±12.5 | 56.1±15.9* | 0.874 | 0.850-0.896 |
| CRP | 29.4±55.7 | 68.4±73.5* | 0.672 | 0.639-0.703 |
| Albumin | 3.2±0.6 | 2.7±0.8 | 0.653 | 0.619-0.685 |
| CRP/Albumin ratio | 11.2±23.0 | 31.8±38.6* | 0.691 | 0.658-0.722 |
| Lactate | 2.8±1.6 | 3.9±3.4 | 0.658 | 0.625-0.690 |

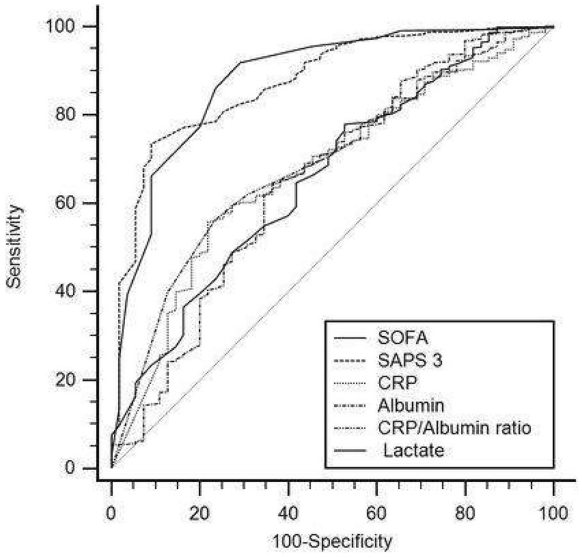

**Fig. 1 (abstract 1164).** Comparison of ROC curves of SOFA, SAPS 3, CRP/Albumin and lactate of surgical patients.

### 1165

#### Comparisons of prognostic accuracy of two early warning scoring systems with qSOFA and SOFA scores to predict mortality in sepsis patients

B. Khwannimit, R. Bhurayanontachai, V. Vattanavanit
Prince of Songkla University, Internal Medicine, Hat Yai, Thailand
**Correspondence:** B. Khwannimit

*Intensive Care Medicine Experimental* 2018, **6(Suppl 2):**1165

**INTRODUCTION.** Early warning scores were developed for bedside early identification of physiological deterioration and allow early discrimination of high risk critically-ill patients.

**OBJECTIVES.** To compare the accuracy of the Modified Early Warning Score (MEWS) and National Early Warning Score (NEWS) with the quick Sequential Organ Failure Assessment (qSOFA) and SOFA scores to predict outcomes in sepsis patients admitted to the intensive care unit (ICU).

**METHODS.** A retrospective study was conducted in a single medical ICU of a tertiary university teaching hospital in Thailand over a 7-year period. The worst physiological data within the first 24 hours of ICU admission of each score were collected to calculate the scores. The primary outcome was hospital mortality and the secondary outcomes were ICU mortality or composite of hospital mortality and ICU length of stay (LOS) ≥ 72 hours.

**RESULTS.** The number of sepsis patients diagnosed by Sepsis-3 criteria was 1,589 and 925 patients (58.2%) were classified as septic shock. The mean age was 60.3±20.5 years and mean APACHE II was 23.6±9.8. A total of 1,069 patients (67.3%) had community-acquired infection. The common sources of infection were respiratory tract (51.4%), gastrointestinal (13.3%), and urinary tract (9.8%). Mechanical ventilation was used in 1,427 patients (89.8%). The ICU and hospital mortality rates were 31.9% and 46%, respectively. The number of patients died in the hospital or had ICU LOS ≥ 72 hours was 1,284 patients (80.8%). The distribution of MEWS, NEWS, and SOFA scores correlated with hospital mortality (Figure 1). The SOFA score presented the highest discrimination with an area under the receiver operating characteristic curve (AUC) of 0.880 (95% CI 0.863-0.896) followed by MEWS, qSOFA and NEWS (Table 1). The discrimination of hospital mortality using the SOFA score was significantly greater than the qSOFA (P< 0.001), MEWS (P=0.03) or NEWS (P< 0.001) (Figure 2). The AUC of qSOFA for hospital mortality was not significantly different from the MEWS and NEWS. However, SOFA and MEWS showed similar performance to predict ICU mortality and both scores provided better discrimination than the qSOFA and NEWS (P< 0.001 for both). Nevertheless, the AUC for secondary composite outcome was significantly higher for NEWS (Table 1) than either MEWS (P=0.003), qSOFA (P< 0.001) or SOFA (P< 0.001).

**CONCLUSIONS.** The SOFA score provided significant accuracy to predict ICU and hospital mortality among sepsis patients admitted to the ICU. Our results also supported the application of SOFA score for screening and clinical criteria of ICU sepsis by the Sepsis-3 consensus.

**Table 1 (abstract 1165).** The AUC of MEWS, NEWS, qSOFA and SOFA scores to predict outcomes.

| Scores | Hospital mortality | ICU mortality | Combines hospital mortality and ICU LOS≥72 h |
|---|---|---|---|
| MEWS | 0.858 (0.840-0.876) | 0.861 (0.842-0.879) | 0.820 (0.797-0.843) |
| NEWS | 0.833 (0.813-0.852) | 0.825 (0.805-0.846) | 0.845 (0.824-0.866) |
| qSOFA | 0.847 (0.829-0.864) | 0.812 (0.794-0.830) | 0.768 (0.746-0.791) |
| SOFA | 0.880 (0.863-0.896) | 0.867 (0.849-0.885) | 0.782 (0.757-0.807) |

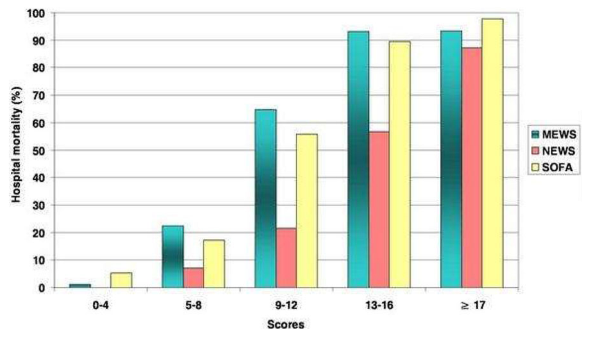

**Fig. 1 (abstract 1165).** Hospital mortality stratified by MEWS, NEWS and SOFA scores.

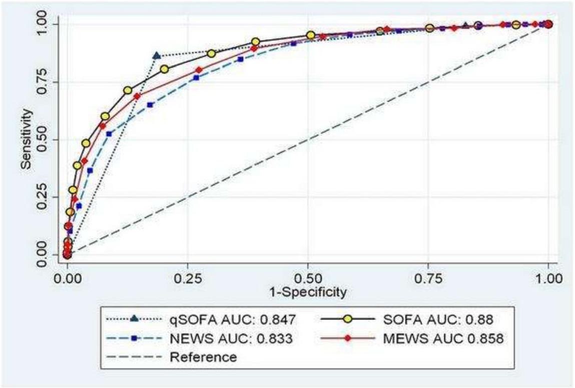

**Fig. 2 (abstract 1165).** Comparison of ROC curves to predict hospital mortality by MEWS, NEWS, qSOFA and SOFA, score

### 1166

#### The role of platelet distribution width in predicting mortality in critically ill patients

A. Fogagnolo[1], S. Spadaro[1], M. Lazzeri[1], M. Albanese[1], G. Benetto[1], F.S. Taccone[2], C.A. Volta[1]
[1]Università di Ferrara, Ferrara, Italy; [2]Université Libre de Bruxelles, Intensive Care, Bruxelles, Belgium
**Correspondence:** A. Fogagnolo
*Intensive Care Medicine Experimental* 2018, **6(Suppl 2):**1166

**INTRODUCTION.** Platelet distribution width (PDW) is a parameter which reflects variation of platelet size distribution. While the red blood cell distribution width has recently gained importance as a prognostic tool in intensive care unit (ICU), only few data are available about the role of the PDW in this setting.(1) However, due to the high platelets susceptibly to inflammation and infection, it is reasonable that PDW values increase in sickest patients, especially in those with sepsis or septic shock.

**OBJECTIVES.** The aim of this study is to identify if PDW can be useful in predicting 90-day mortality in an unselected cohort of ICU patients.

**METHODS.** We enrolled consecutive patients who needed at least 72 hours of ICU stay. Exclusion criteria were: age < 18 years, pregnancy, hematologic disorder or history of platelets disease. Clinical and laboratory data were recorded daily. Due to the lack of knowledge about normal range of PDW in ICU, we decided to divide our population in terciles according to the PDW value at admission. Primary outcome was 90-day mortality; a pre-planned sub-group analysis was performed in patients admitted for sepsis or septic shock.

**RESULTS.** A total of 106 patients have been included; of those 32 (30%) were septic. (Table 1) Ninety-day mortality was 40% (42/106), being higher in the third PDW terciles (PDW>12.8%) than in the second and first one (56%, 40% and 24%, respectively; p=0.02). The ROC curve for 90-day mortality in the whole population was 0.72 [0.65-0.82], while it increased to 0.86 [0.73-0.96] when the analysis was restricted to septic patients. (Figure 1) Patients with higher PDW had also higher mean platelet volume (MPV) while no differences were found in platelet count. However, when the population was stratified for PDW terciles, the MPV values did not change between survivors and non-survivors (Figure 2).

**CONCLUSIONS.** PDW values at admission is a useful and widely available prognostic tool in ICU, in particular in patients with sepsis or septic shock. The association between PDW and 90-day mortality seems not to be influenced by MPV or platelet count.

**REFERENCE(S)**

1. Zhang, J Crit Care. 2014;29(5):885

**Table 1 (abstract 1166).** Clinical and demographical characteristics of the patients

| Variable | PDW<10.8% (n=36) | 10.8%< PDW <12.8% (n=35) | PDW>12.8% (n=35) | P value |
|---|---|---|---|---|
| Age, years | 66 ± 15 | 66 ± 14 | 72 ± 13 | 0.14 |
| Sepsis, % | 38 | 20 | 32 | 0.24 |
| SAPS score | 46 ± 14 | 41 ± 15 | 48 ± 13 | 0.34 |
| Platelets, $10^3$ $mcL^{-1}$ | 230 [176 – 303] | 215 [173 – 285] | 181 [158 – 230] | 0.13 |
| MPV, fL | 8.9 ± 2.3 | 10.5 ± 0.6 | 11.7 ± 0.8 | <0.001 |
| RDW, % | 15.5 ± 4.0 | 15.1 ± 3.1 | 15.2 ± 2.4 | 0.83 |
| pH | 7.35 ± 0.1 | 7.36 ± 0.1 | 7.36 ± 0.1 | 0.94 |
| Lactate, mmol | 2 [1.1 – 2.4] | 1.7 [1.4 – 2.4] | 2 [1.3 – 2.4] | 0.83 |
| Creatinine, mg/dL | 1.2 ± 0.3 | 1.3 ± 0.2 | 1.2 ± 0.2 | 0.07 |
| Bilirubin, mg/dL | 0.6 [0.5 – 0.8] | 0.8 [0.5 – 1.3] | 0.7 [0.6 -1.1] | 0.33 |
| 90-day mortality, % | 24 | 40 | 56 | 0.02 |

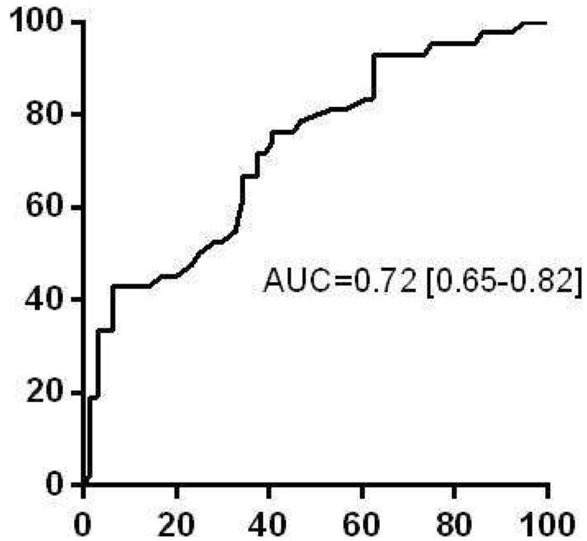

**Fig. 1 (abstract 1166).** See text for description

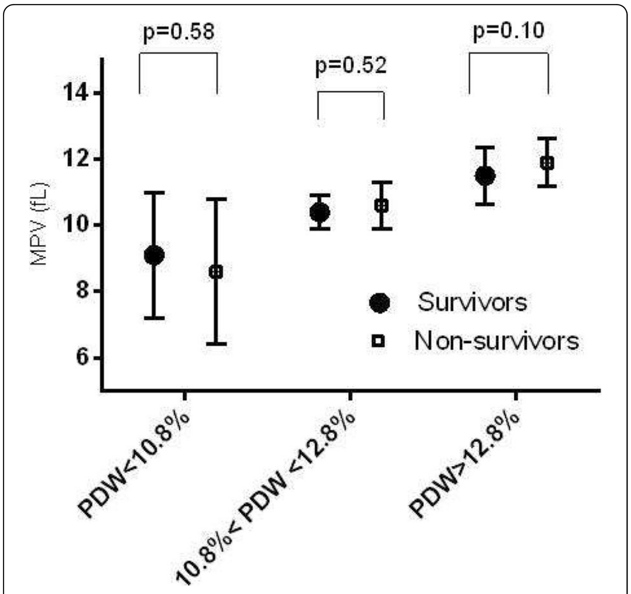

**Fig. 2 (abstract 1166).** See text for description

### 1167

#### The use of SAPS 3, SOFA and Glasgow Coma Scale to predict mortality in patients with Subarachnoid Hemorrhage (SAH)

A. Basile-Filho[1], R. Nunes[1], E.A. Nicolini[1], A.F. Lago[1], L.C.T. Pereira[2], R.A.F. Costa[2], M.A. Feres[3]
[1]Ribeirão Preto Medical School, University of São Paulo, Division of Intensive Care Medicine, Department of Surgery and Anatomy, Ribeirão Preto, Brazil; [2]Hospital São Francisco, Neurosurgery Service, Ribeirão Preto, Brazil; [3]Hospital São Francisco, Intensive Care Unit, Ribeirão Preto, Brazil
**Correspondence:** A.F. Lago
*Intensive Care Medicine Experimental* 2018, **6(Suppl 2):**1167

**INTRODUCTION.** Guidelines for SAH management and several grading systems or prognostic indices have been used not only to improve the quality of care but to predict also the outcome of these patients. Among them the gold standards Fisher, Hunt-Hess and the World Federation of Neurological Surgeons (WFNS) are the most employed. On the other hand, although the GCS is critical in the neurological patients with acute brain disorders, including SAH, this scale measurement loss its accuracy on day 1 (D1) when the patient is sedated, e.g. the day after de surgical procedure.

**OBJECTIVE.** The objective of this study is to compare the predictive values of SAPS 3, SOFA and Glasgow Coma Score (GCS) on D1 and D3 (SOFA and GCS) in the outcome of patients with SAH.

**METHODS.** Fifty-one SAH patients (33 % males and 67% females; mean age of 54.1 ± 10.3 years) admitted to the ICU in the post-operative phase were retrospectively studied. The patients were divided into survivors (n=34) and nonsurvivors (n=17). SAPS 3, SOFA and GCS were recorded on ICU admission (day 1 - D1), and 72-h (day 3 - D3) SOFA and GCS. The capability of each index (SAPS 3, SOFA and GCS (D1 and D3) to predict mortality was analyzed by receiver operating characteristics (ROC) curves. The area under de curve (AUC) and the respective confidence interval (CI) were used to measure the index accuracy. Comparison among these curves was tested. The level of significance was set at $p < 0.05$. ROC curves analyses were performed using the software MedCalc v. 14.

**RESULTS.** The mean SAPS 3, SOFA and GCS on D1 were 13.5 ± 12.7, 3.1 ± 2.4 and 13.7± 2.8 for survivors and 32.5 ± 28.0, 5.6 ± 4.9 and 13.5 ± 1.9 for nonsurvivors, respectively. The area under the ROC curve for SAPS 3, SOFA and GCS and the respective CI data between survivors and nonsurvivors are summarized in table 1. The comparisons of ROC curves for these indexes are depicted in graph 1 and 2. The overall mortality was 27%.

**CONCLUSIONS.** .Even though SAPS 3 predicted better on admission, SOFA was the most effective index to predict mortality in SAH patients when performed on D3. However, the GCS on D3 may also be used as predictor of outcome.

**GRANT ACKNOWLEDGMENT**
FAEPA-Clinics Hospital of Ribeirão Preto, SP, Brazil.

**Table 1 (abstract 1167).** SAPS 3, SOFA and GCS scores on day 1 (D1) and day 3 (D3) of patients survivors and nonsurvivors of SAH *p<0.05

| Patients (n=51) | Survivors (n=34) | Nonsurvivors (n=17) | AUC | IC |
|---|---|---|---|---|
| SAPS 3 D1 | 13.5±12.7 | 32.5±28.0* | 0.735 | 0.592-0.848 |
| SOFA D1 | 3.1±2.4 | 5.6±4.9* | 0.623 | 0.476-0.754 |
| GCS D1 | 13.7±2.8 | 13.5±1.9 | 0.565 | 0.419-0.703 |
| SOFA D3 | 3.6±3.2 | 8.1±4.0* | 0.768 | 0.629-0.875 |
| GCS D3 | 11.9±4.8 | 8.1±5.2 | 0.708 | 0.563-0.826 |

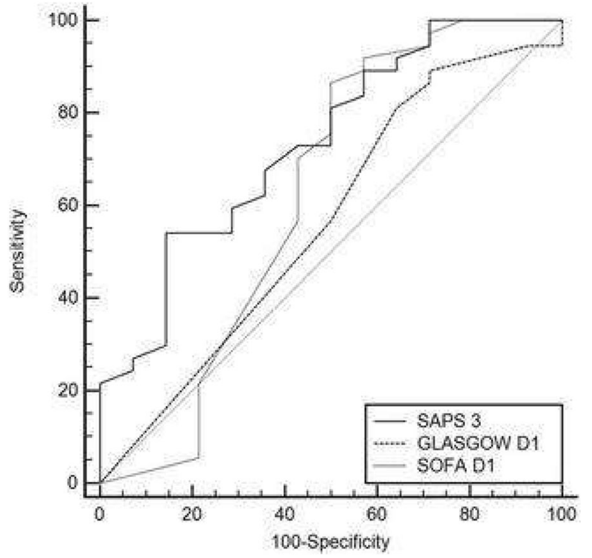

**Graph 1 (abstract 1167)** . Comparison of ROC curves for SAPS 3, GCS and SOFA on D1

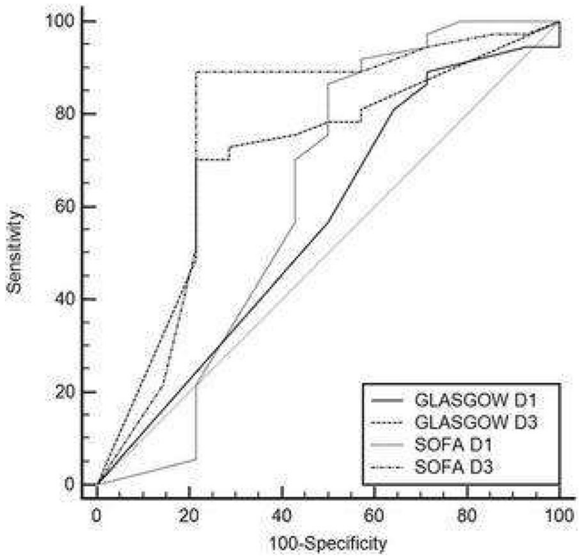

**Graph 2 (abstract 1167)** . Comparison of ROC curves for GCS and SOFA on D1 and D3

### 1168

#### Is there a decrease in the amount of in-hospital cardiac arrest (IHCA) after the instauration of an activation score for the Rapid Response Team (RRT) in a tertiary hospital?

A. Sánchez Ginés, C. Lorencio Cárdenas, J. Gonzalez Londoño, P. Loma-Osorio, S. Martin Hernandez, J. Lopez Fernandez, R. Arcega Sainz, J.M. Sirvent, Comission for Prevention and Attention of IHCA
Josep Trueta Hospital, Girona, Spain
**Correspondence:** A. Sánchez Ginés
*Intensive Care Medicine Experimental* 2018, **6(Suppl 2):**1168

**INTRODUCTION.** Several studies show that IHCA is usually preceded by objective signs of deterioration. This is why RRT are part of the security network of patients at risk of deterioration, as well as global hospital security.

**OBJECTIVES.** To study the impact on the incidence of IHCA and hospital mortality on a tertiary hospital after the implementation of an activation system for the RRT based on a severity *score* (EWS).

**METODOLOGY.** The amount of IHCA between the years 2010-2014 was registered, during this period the RRT did not have a specific activation system. In 2017, an activation system based on the EWS was implemented and the IHCA were also registered. We also analyzed the hospital mortality during the years 2016 and 2017.

**RESULTS.** During the period 2010-2014, 125 IHCA were registered; corresponding to 31,25 IHCA/year (0,16 IHCA/1000 hospital discharges). During the year 2017, there were 19 activations of IHCA, meaning a reduction of 39% in the incidence of IHCA (0,09 ACIH/1000 hospital discharges). The hospital mortality during the year 2016 was of 619/18.043 hospital discharges (34,3 deaths/1.000 hospital discharges (4%)). During 2017, the hospital mortality was of 633/18.861 hospital discharges (33,56 deaths/1.000 hospital discharges (3,9%)).

**CONCLUSIONS.** The implementation of an activation system for the RRT based on a severity scale of the hospitalized patient (EWS) decreased the number of IHCA and the global hospital mortality.

RRT based on alert systems that recognize patients that are deteriorating are a fundamental part of global hospital security. It will be necessary to see if these results will remain in time and if changes will be required in order to improve the functioning of the RRT and the quality of care to the hospitalized patient.

*Rapid response team and hospital mortality in hospitalized patients.* Boris Jung, Aurelien Daurat, Audrey De Jong, Gerald Chanques, Martin Mahul, Marion Monnin, Nicolas Molinari and Samir Jaber. Intensive Care Unit, Department of Anesthesia and Critical Care Medicine, University of Montpellier, Saint Eloi Teaching Hospital.

*Introducing Critical Care Outreach: a ward-randomised trial of phased introduction in a general hospital.* George Priestley Wendy Watson Arash Rashidian Caroline Mozley Daphne Russell Jonathan Wilson Judith Cope Dianne Hart Diana Kay Karen Cowley Jayne Pateraki.

*Outcomes Associated With the Nationwide Introduction of Rapid Response Systems in The Netherlands.* Ludikhuize J[1], Brunsveld-Reinders AH, Dijkgraaf MG, Smorenburg SM, de Rooij SE, Adams R, de Maaijer PF, Fikkers BG, Tangkau P, de Jonge E; Cost and Outcomes of Medical Emergency Teams Study Group.

### 1169

#### Accuracy of critical care prognostic scores for intra-ICU mortality in early postoperative period of patients submitted to coronary artery bypass graft surgery

A. Bezerra, A. Barros, D. Dragosavac, O. Petrucci Junior, K. Vilarinho, P.P. de Oliveira, A. Falcao
State University of Campinas - UNICAMP, Department of Surgery, Campinas, Brazil
**Correspondence:** A. Bezerra
*Intensive Care Medicine Experimental* 2018, **6(Suppl 2):**1169

**INTRODUCTION.** Coronary artery bypass graft surgery is a well-established therapeutic option for treatment of patients with coronary artery obstructive disease [1, 2]. Studies suggest that non-cardiac diseases and patient clinical status are main determinants of mortality after coronary artery bypass grafting that may be overlooked by traditional ICU scores [3-5]. Moreover, scores developed to predict mortality that are derived from specific cohorts and clinical settings, may lose accuracy after extrapolation to different populations [5]. Simplified Acute Physiology Score (SAPS 3), Sequential Organ Failure Assessment (SOFA) and Acute Physiology and Chronic Health Evaluation II (APACHE II) scores are well-validated predictive models for mortality in critical care setting [3-5].

**OBJECTIVE.** To evaluate accuracy of general critical care models for mortality prediction of patients in early postoperative period of CABG surgery in ICU.

**METHODS.** This was a retrospective cohort study including elective CABG surgery patients enrolled from January 2013 and December 2017 in the postoperative ICU of a university hospital. Scores discrimination was assessed through the area under the receiver operating characteristic curve (AUROC) and calibration was evaluated by a goodness-of-fit test with intra-ICU mortality as outcome. A p value $< 0.05$ was considered statistically significant. Local and national ethics committee approved the study's protocol.

**RESULTS.** There were 407 patients included in this study. The observed mortality was 3,44%. Discrimination was fair for all tested scores. AUROCs were 0,641 (95% CI 0,592 to 0,688) for admission SOFA, 0,747 (95% CI 0,702 to 0,789) for SAPS 3 and 0,714 (95% CI 0,667 to 0,757) for APACHE II. Pairwise evaluation achieved no significance between differences among AUROCs of assessed scores. All prognostic models showed acceptable calibration.

**CONCLUSION.** SAPS 3, APACHE II and admission SOFA are fair scores for intra-ICU mortality prediction in early postoperative period of CABG surgery patients. However, discrimination improvements may be necessary to increase their accuracy in this setting.

of thoracic surgeons adult cardiac surgery database (The ASCERT Study). Circulation
4. Chang C-H, Chen S-W, Fan P-C, et al (2017) Sequential organ failure assessment score predicts mortality after coronary artery bypass grafting. BMC Surg.
5. Breslow MJ, Badawi O (2012) Severity scoring in the critically ill: part 1–interpretation and accuracy of outcome prediction scoring systems. Chest

### 1170

#### The C-reactive protein/albumin ratio as a predictor of mortality in critically ill patients

A.Y. Leem
Yonsei University College of Medicine, Seoul, Korea, Republic of
*Intensive Care Medicine Experimental* 2018, **6(Suppl 2):**1170

**INTRODUCTION.** The C-reactive protein (CRP)/albumin ratio has recently emerged as a marker for poor prognosis or mortality across various patient groups. However, there are relatively few studies conducted focusing on critical care patients in the ICU.

**OBJECTIVES.** This study aimed to identify the association between CRP/albumin ratio and 28-day mortality and predict the accuracy of CRP/albumin ratio for 28-day mortality in medical intensive care unit (ICU) patients.

**METHODS.** This was a retrospective cohort study of 875 patients admitted to the medical ICU. We evaluated the prognostic value of CRP/albumin ratio to predict mortality at 28 days after ICU admission, using Cox proportional hazard model and Kaplan-Meier survival analysis.

**RESULTS.** The 28-day mortality was 28.0% in this study. In the univariate analysis, the Acute Physiology and Chronic Health Evaluation II (APACHE II) score (P< 0.001), CRP level (P=0.045), albumin level (P< 0.001), and CRP/albumin ratio (P=0.032) were related to 28-day mortality in ICU patients. Following the receiver operating characteristic (ROC) analysis and calculating the area under the ROC curve (AUC), the AUC of CRP/albumin ratio was higher than that of CRP for mortality in ICU patients (0.594 vs. 0.567, P< 0.001). The cut-off point for CRP/albumin ratio for mortality in ICU patients was 3.43. On Cox proportional-hazard regression analysis, APACHE II score (hazards ratio [HR]=1.05, 95% CI=1.04-1.07, P< 0.001) and CRP/albumin ratio (HR=1.68, 95% CI=1.27-2.21, P< 0.001 for high CRP/albumin ratio) were independent predictors of 28-day mortality.

**CONCLUSIONS.** Higher CRP/albumin ratio was associated with increased mortality in critically ill patients. Therefore, clinicians might predict poor prognosis through the initial assessment of CRP/albumin ratio.

**GRANT ACKNOWLEDGMENT**
The authors report no funding for support of this work.

### 1171

#### Prospective study of use of difference of day 1 and day 3 Sofa Score (Delta-Sofa) for outcome prediction in patients with sepsis

S. Dixit[1], K. Khatib[2]
[1]MJM Hospital and Sanjeevan Hospital, ICU, Pune, India; [2]SKN Medical College, Medicine, Pune, India
**Correspondence:** S. Dixit
*Intensive Care Medicine Experimental* 2018, **6(Suppl 2):**1171

**INTRODUCTION.** Sepsis and septic shock are major causes of ICU admission and mortality. Organ dysfunction associated with sepsis is quantified by the SOFA score. Patients developing new onset organ dysfunction or worsening of existing organ dysfunction are more likely to not survive. We studied the effect of worsening of SOFA score from day 1 and day 3 in predicting outcomes in patients with sepsis.

**METHODS.** A prospective observational study was undertaken in our tertiary level medical- surgical ICU (15 bedded) from October 2017 to January 2018. All adult patients (Age> 18 years) with a diagnosis of sepsis were included in the study. Demographic data, diagnosis on ICU admission, SOFA score on Day1 and Day3, Difference of SOFA score from Day 1 to Day 3 (Delta-SOFA score) and outcome of patients (ICU stay, days on mechanical ventilation, mortality) was noted. Univariate and multivariate analysis of predictors of mortality was done.

**RESULTS.** Of the 406 patients admitted to the ICU during the study period, 80 patients (19.7%) had sepsis. The mean age of the patients was 50.41(SD 30.56) years and 57% were males. The most common causes of sepsis were tropical infections (38.35%) followed by respiratory (20.73%) and abdominal infections (11.16%). The common tropical infections were Dengue (40.15%), Malaria (15.12%), Leptospira (8.9%), and rickettsial infections (5.6%).

The mean hospital mortality was 62.34%. The mean SOFA score was 9.32 (SD 3.6) on Day 1 and 11.55 (SD 4.5) on Day3. The mean SOFA score on DAY1 for survivors and non-survivors was 8.3 and for non-survivors was 13.8. An increase in the SOFA score by 5 during the ICU stay (Delta-SOFA score) was associated with mortality in 88% of these patients. SOFA score (p=0.0026), Delta-SOFA score>5 (p=0.002) and Mechanical ventilation (p=0.001) were independently associated with mortality.

**CONCLUSIONS.** Sepsis is seen in 19.7% of all ICU admissions in our setting and tropical infections are the most common cause of sepsis. An increase of SOFA score (Delta-SOFA) > 5 during ICU stay can be used to predict mortality in patients with sepsis. Further and larger studies in our setting are needed.

**REFERENCE(S)**

1. Singer M, Deutschman CS, Seymour CW, et al. The Third International Consensus Definitions for Sepsis and Septic shock (Sepsis-3). JAMA 2016;315:801-10
2. Jones AE, Trzeciak S, Kline JA. The sequential organ failure Assessment score for predicting outcome in patients with severe sepsis and evidence of hypoperfusion at the time of emergency department presentation. Crit Care Med 2009;37:1649-54.
3. *Arnold RC, Sherwin R, Shapiro NI, O´Connor JL, Glaspey L, Singh S, Medado P, Trzeciak S, Jones AE, Emergency Medicine Shock Research Network (EM Shock Net) Investigators. Multicenter observational study of the development of progressive organ dysfunction and therapeutic interventions in normotensive sepsis patients in the emergency department.*Acad Emerg Med. 2013 May; 20(5):433-40.

**GRANT ACKNOWLEDGMENT**
NIL

### 1172

#### How effective are metabolic markers in predicting mortality and length of stay in patients admitted into intensive care in comparison to existing scoring tools APACHE II and ICNARC?

T. Khan, O. Boyd
Brighton and Sussex Medical School, Brighton, United Kingdom
**Correspondence**: T. Khan
*Intensive Care Medicine Experimental* 2018, **6(Suppl 2):**1172

**INTRODUCTION.** Being able to identify the sick patients that are likely to deteriorate further is crucial to planning the nature and urgency of treatments. Early and accurate detection would enable patients to receive the most appropriate treatment preventing morbidity and mortality. Metabolic markers (lactate, pH, PaCO2, and BE) from arterial blood gases were examined because this investigation is performed frequently and easily in newly admitted patients. In addition, their results are returned quickly as compared to scoring tools APACHE (Acute Physiology and Chronic Health Evaluation) II and ICNARC (Intensive Care National Audit & Research Centre) which require 24 hours.

**OBJECTIVES.** There were many aims: firstly, to determine the effectiveness of metabolic markers (lactate, pH, PaCO2 and BE) in predicting mortality and length of stay in patients admitted to intensive care; secondly, to identify the cut-off values for the metabolic markers for both mortality and length of stay; thirdly, to determine the effectiveness of lactate clearance in predicting mortality and length of stay and finally comparing the above with APACHE II and ICNARC.

**METHODS.** An observational retrospective study was performed. The inclusion criteria were all patients ≥18 years old admitted to intensive care during 2017. 100 patients were chosen at random and their ROC (receiver operator characteristic) curves were plotted for each of the 4 metabolic markers to examine how effective they were at predicting mortality and length of stay (defined as < 4 or ≥4 days) on admission and 6 hours post admission. The AUC (area under the curve) was calculated so that the results could be compared. This was repeated for lactate clearance, APACHE II and ICNARC.

**RESULTS.** Admission Lactate was the best metabolic marker for predicting mortality with an ROC AUC of 0.79 and the optimum cut-off was 2 mmol/L. Admission BE was the best metabolic marker for predicting length of stay (LOS) with an ROC AUC of 0.69. Lactate clearance predicting both mortality and LOS gave an AUC of 0.68 and 0.61 respectively and the optimum cut-off was 30.0%. The scoring tools performed better than the metabolic markers at predicting mortality and LOS: the best was the ICNARC mortality predictor for both mortality and length of stay with an AUC of 0.83 and 0.76 respectively.

**CONCLUSIONS.** Metabolic markers can be helpful at predicting mortality at admission and soon after. Different markers are better suited for different outcomes. Predicting length of stay is more challenging - especially at 6 hours. Lactate clearance was found to be inferior to admission lactate at predicting both mortality and length of stay. The scoring tools, in particular the ICNARC mortality predictor, performed better than the metabolic markers at predicting mortality and LOS suggesting they are worth the wait. Nevertheless, the admission lactate is still useful and should not be ignored.

**REFERENCE(S)**

No references

**GRANT ACKNOWLEDGMENT**

No grant awarded

### 1173

#### Accuracy of adding age in Sequential Organ Failure Assessment score for intra-ICU mortality prediction in postoperative myocardial revascularization surgery patients

A. Barros, A. Bezerra, M. Battaiola, D. Dragosavac, O. Petrucci Junior, K. Vilarinho, P.P. de Oliveira, A. Falcao
State University of Campinas - UNICAMP, Department of Surgery, Campinas, Brazil
**Correspondence:** A. Barros
*Intensive Care Medicine Experimental* 2018, **6(Suppl 2):**1173

**INTRODUCTION.** Coronary artery bypass graft (CABG) surgery is a well-established therapeutic option for treatment of patients with coronary artery obstructive disease [1, 2]. Non-cardiac diseases and clinical status are main determinants of mortality in CABG postoperative period that may be overlooked by Sequential Organ Failure Assessment (SOFA) score [3-5]. Studies suggest that age may increases SOFA's accuracy for intra-ICU mortality prediction in general critical care patients [3-5].

**OBJECTIVES.** To test whether age improves SOFA's intra-ICU mortality prediction accuracy in patients at CABG surgery early postoperative period.

**METHODS.** This was a retrospective cohort study including elective CABG surgery patients enrolled from January 2013 and December 2017 in the postoperative ICU of a university hospital. SOFA score and other relevant data were calculated at admission. To analyze the relationship between age, SOFA score and intra-ICU mortality, a logistic regression model was used. After, model's accuracy for intra-ICU mortality was evaluated calculating its discrimination and calibration properties. A p value < 0.05 was considered statistically significant. Local and national ethics committee approved the study's protocol.

**RESULTS.** A total of 408 patients were eligible for further analyzes. Observed mortality was 3,43%. SOFA and age contributed significantly to predict intra-ICU mortality. The area under the receiver operating characteristic curve (AUROC) was 0,767 (95% CI 0,723 to 0,807) for the SOFA/Age model and 0,641 (95% CI 0,592 to 0,687) for SOFA alone with intra-ICU mortality as the dependent outcome. Pairwise comparison showed a difference between ROC curves areas of 0,126 (95% CI 0,0128 to 0,239) with a p value of 0,0291. Both models showed a good calibration assessed through the Hosmer-Lemeshow test with p values of 0,8646 for SOFA/Age and 0,2786 for SOFA alone.

**CONCLUSIONS.** Associating age with SOFA score may improves its prediction accuracy for intra-ICU mortality of CABG surgery patients in early postoperative period.

### 1174

#### Implementation of an electronic early warning scoring system on a surgical ward: practical use and effects on patient outcomes

E. Mestrom[1], A. de Bie[1], C. Petit[1,2,3], M. van de Steeg[1], M. Driessen[1], L. Atallah[3], R. Bezemer[1,2,3], A. Bouwman[1,2], E. Korsten[1,2]
[1]Catharina Ziekenhuis Eindhoven, Intensive Care Unit, Eindhoven, Netherlands; [2]Eindhoven University of Technology; Department of Electrical Engineering, Eindhoven, Netherlands, [3]Philips Research, Patient Care & Measurements, Eindhoven, Netherlands
**Correspondence:** E. Mestrom
*Intensive Care Medicine Experimental* 2018, **6(Suppl 2):**1174

**INTRODUCTION.** Early warning scores (EWS) are increasingly embedded in hospitals worldwide since their use has shown to reduce adverse events such as cardiopulmonary arrest, unplanned intensive care unit (ICU) admission, emergency surgery, unexpected death.(1) Additionally, recent advances in automated notification systems assessing EWS further improve the outcomes of medical patients.(2)

**OBJECTIVES.** The aim of this study was to evaluate and compare patient outcomes before and after the implementation of an automated EWS after major surgery.

**METHODS.** A before-and-after retrospective study was performed in a surgical high dependency unit where patients are admitted following postoperative ICU stay after major surgery. During the control period from January 2012 till March 2013, vital signs and EWS were collected and manually registered in the electronic medical record. Subsequently, staff was trained to collect vital signs with the automated EWS system the Guardian®, Philips (Figure 1), from June 2013 till August 2015. Two types of outcomes were defined: clinical outcomes, like length of stay, ICU readmission and mortality, and operational registration outcomes measuring the recording of the vital signs and calculating EWS.

**RESULTS.** A total of 593 patients were selected, consisting of 320 patients in the control group and 273 patients in the intervention group, including 7837 and 3418 documented assessments respectively. Using the electronic scores improved registration of respiratory rate by 94% and level of consciousness by 92% (Figure 2). However, no significant differences were found in clinical outcomes like hospital length of stay, mortality and ICU readmissions (Table 1), even though patient characteristics were similar. Subgroup analysis for patients requiring readmission at the ICU did not show significant differences in terms of age, gender, APACHE-IV scores, ICU and hospital length of stay and mortality between the control and intervention group.

**CONCLUSIONS.** Implementation of an automated EWS on a surgical high dependency unit improves electronic registration of vital signs and calculated EWS. This positive effect did not lead to a significant decrease in mortality, hospital length of stay or ICU readmissions. In this study, the EWS system lacked a connection to the EMR. Nonetheless, an improved adherence to the EWS protocol was observed.

**REFERENCE(S)**

1. Ludikhuize J, Smorenburg SM, de Rooij SE, de Jonge E. Identification of deteriorating patients on general wards; measurement of vital parameters and potential effectiveness of the Modified Early Warning Score. Journal of critical care. 2012;27(4):424.e7-13.
2. Subbe CP, Duller B, Bellomo R. Effect of an automated notification system for deteriorating ward patients on clinical outcomes. Critical care (London, England). 2017;21(1):52.

**GRANT ACKNOWLEDGMENT**
No grant acknowledgements to declare.

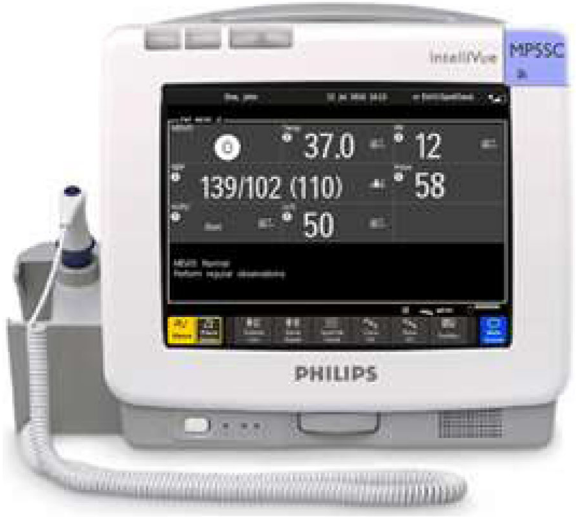

**Fig. 1 (abstract 1174).** Picture of the Guardian®, MP5 spot-check monitor

**Table 1 (abstract 1174).** Clinical outcomes

| | Control group | Intervention group | p-value |
|---|---|---|---|
| Number of patients | 320 | 273 | |
| Age in years, median (IQR) | 67 (15) | 67 (16) | 0.32 |
| Male gender, number (%) | 204 (63.8) | 173 (63.4) | 0.92 |
| APACHE IV first ICU admission, median (IQR) | 37 (15) | 38 (18) | 0.38 |
| Type of surgery (%): Abdominal, oncology; Abdominal, benign; Vascular, aortic | 223 (69.7); 33 (10.3); 64 (20.0) | 219 (80.2); 20 (7.3); 34 (12.5) | 0.01 |
| Unplanned (acute) surgery (%) | 57 (18.1) | 37 (13.6) | 0.16 |
| Length of stay days, median (IQR): Hospital; ICU before HDU; HDU | 12 (10); 1,1 (1.0); 7.5 (7.0) | 11 (8); 1.1 (1.0); 7.1 (6.0) | 0.39; 0.07; 0.59 |
| Mortality, number (%): In-hospital; 28-day | 5 (1.6); 7 (2.2) | 3 (1.1); 2 (0.7) | 0.9; 0.27 |
| Days till death (IQR) | 466 (732) | 360 (504) | 0.22 |

**Table 2 (abstract 1174).** Subgroup analysis of ICU readmissions

| | Control | Intervention | p-value |
|---|---|---|---|
| Number of patients | 43 | 29 | 0.36 |
| Age in years, median (IQR) | 65 (19) | 67 (10) | 0.30 |
| Male gender, number (%) | 33 (76.7) | 23 (79.3) | 0.80 |
| Type of surgery: Abdominal, oncology (%); Abdominal, benign (%); Vascular, aortic (%) | 29 (64.4); 8 ((18.6); 6 (14.0) | 25 (86.2); 2 (6.9); 2 (6.9) | 0.17 |
| Unplanned (acute) surgery (%) | 8 (18.6) | 4 (13.8) | 0.83 |
| Length of stay days, median (IQR): Hospital; ICU before HDU; HDU; ICU readmission | 25 (43); 1 (1.1); 3.3 (4.2); 3.44 (9.3) | 28 (26); 1.1 (1.0); 2.4 (4.8); 3.7 (3.6) | 0.55; 0.14; 0.32; 0.57 |
| Mortality, number (%): In-hospital; 28-day | 4 (9.3); 5(11.6) | 2 (6.9); 0 (0) | 1.0; 0.15 |
| APACHE IV, median (IQR): first ICU admission; ICU readmission | 37 (13); 52 (50) | 43 (30); 52 (20) | 0.08; 0.55 |

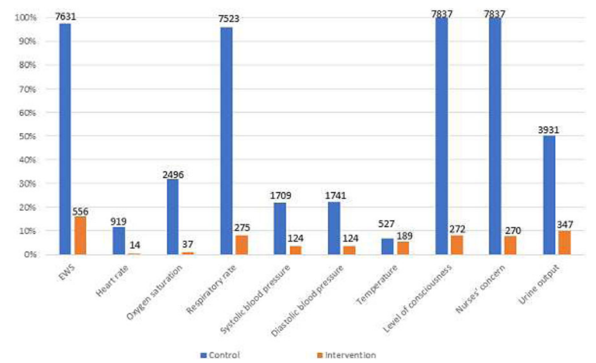

**Fig. 2 (abstract 1174).** Percentages and absolute numbers of missing assessments

## Modifying sedation to improve outcome

### 1175

#### Epidural analgesia in patients who have undergone video-assisted thoracoscopic surgery prevents a breakthrough pain in the intensive care unit

J. Suzuki, S. Sugino, T. Shiga, Y. Kameyama, K. Saito, M. Yamauchi
University Hospital, Division of Intensive Care, Sendai, Japan
**Correspondence**: J. Suzuki
*Intensive Care Medicine Experimental* 2018, **6(Suppl 2):**1175

**INTRODUCTION.** Complete video-assisted thoracoscopic surgery (cVATS) has become popular for patients with a lung tumor because of the development of many new versatile devices that can be used through smaller incisions than those in conventional video-assisted thoracoscopic surgery (cvVATS). However, patients who undergo cVATS frequently suffered breakthrough pain in the intensive care unit (ICU) even when potent analgesics were repeatedly used for post-surgical pain.

**OBJECTIVES.** The aim of the present study was to evaluate clinical factors for breakthrough pain after video-assisted thoracoscopic surgery and to determine the optimal analgesia.

**METHODS.** We retrospectively analyzed data for 231 patients who had undergone video-assisted thoracoscopic surgery from January 2016 to December 2017. The patients who underwent cVATS and those who underwent cvVATS were allocated to a cVATS group (n = 85) and a cvVATS group (n = 146), respectively. We obtained data for age, sex, use of epidural analgesia, total dose of fentanyl during surgery, total dose of remifentanil during surgery, use of acetaminophen, and use of NSAIDs. After the surgery, all of the patients were admitted to an ICU. A nurse recorded intensity of pain using a 0 to 10 numerical rating scale (NRS) at regular intervals after ICU admission. An NRS of more than six was defined as breakthrough pain. Differences in clinical variables between the two groups were analyzed by the Mann-Whitney test or the chi-square test. Logistic regression analysis was used to assess the relationships of clinical variables with incidence of breakthrough pain. P values of less than 0.05 were considered to indicate statistical significance.

**RESULTS.** Epidural anesthesia was used in 58% (49/85) and 84% (123/146) of the patients in the cVATS and cvVATS groups, respectively (chi-square, p=0.0000043). The doses of fentanyl used during surgery were 4.9 (5.5) μg/kg [median (interquartile range)] and 3.9 (2.3) μg/kg in the cVATS and cvVATS groups, respectively (Mann-Whitney, p=0.06). Logistic regression analysis showed that the use of epidural analgesia was an independent factor for prevention of breakthrough pain (odds ratio: 0.08; 95% confidence interval: 0.003-0.20) and that total dose of fentanyl during surgery was also an independent factor (odds ratio: 0.75; 95% confidence interval: 0.64-0.89). However, choice of cvATS was not an independent factor (odds ratio: 1.27; 95% confidence interval: 0.62-2.61).

**CONCLUSIONS.** The findings suggest that the incidence of breakthrough pain after cVATS is similar to that after cvVATS and that both the use of epidural analgesia and the use of a high dose of fentanyl during surgery reduce the incidence of breakthrough pain in patients undergoing video-assisted thoracoscopic surgery..

### 1176

#### Changes in duration of mechanical ventilation after application of the sedation protocol in ICU

J.W. Kim[1,2], T.U. Kim[3], S.J. Ryu[3], G.H. Kim[3], I.A. Song[1,2]
[1]Seoul National University Bundang Hospital, Department of Anesthesiology and Pain Medicine, Seongnam-si, Korea, Republic of; [2]Seoul National University Bundang Hospital, Interdepartment of Critical Care Medicine, Seongnam-si, Korea, Republic of; [3]College of Medicine, Kosin University, Department of Anesthesiology and Pain Medicine, Busan, Korea, Republic of
**Correspondence:** J.W. Kim
*Intensive Care Medicine Experimental* 2018, **6(Suppl 2):**1176

**INTRODUCTION.** We have released the protocols for the management of pain, agitation, and delirium in the intensive care units (ICUs) in 2012. It included the practical content of pharmacological and non-pharmacological guideline for sedation, agitation, and delirium for mechanically ventilated patients in the ICUs. The intensivists practically applied them to the patient and educated the physicians and nurses and monitored and provided feedback to them.

**OBJECTIVES.** To compare the clinical outcomes including the ventilator days, length of stay in the ICU and hospital, and mortality before and after the implementation of the ICU sedation and analgesia guidelines.

**METHODS.** A retrospective study was conducted reviewing the electronic medical records of patients who were mechanically ventilated in the ICU of Seoul National University Bundang Hospital from January 2008 to August 2017. Age, sex, APACHE 2 score, the sedatives, the neuromuscular blockers, the types of ICUs, the ventilator days, length of stay in the ICU, and mortality of the patients were collected via electronic medical records by the medical research collaborating center and two intensivists.

**RESULTS.** During the study period, 10,515 inpatients were mechanically ventilated (pre-intervention: 3426 patients, post-intervention: 7089 patients). In the protocol application group, ventilation days decreased significantly compared to that before protocol application (7.33 ± 11.12 vs 6.15 ± 8.3, p = 0.000). After protocol application, ICU length of stay (9.89 ± 13.34 vs 8.94 ± 10.59, p = 0.000) and 90-day mortality rates (32.3% vs 27.4%, p = 0.000) also decreased significantly.

CONCLUSION. Our findings suggest that establishment and implementation of the guideline for sedation and pain control of mechanically ventilated patients would be associated with reduced ventilation days, ICU length of stay, and mortality.

##### REFERENCE(S)

1. Aitken, L. M., T. Bucknall, B. Kent, M. Mitchell, E. Burmeister and S. J. Keogh (2015). "Protocol-directed sedation versus non-protocol-directed sedation to reduce duration of mechanical ventilation in mechanically ventilated intensive care patients." Cochrane Database Syst Rev 1: Cd009771.
2. Barr, J., G. L. Fraser, K. Puntillo, E. W. Ely, C. Gelinas, J. F. Dasta, J. E. Davidson, J. W. Devlin, J. P. Kress, A. M. Joffe, D. B. Coursin, D. L. Herr, A. Tung, B. R. Robinson, D. K. Fontaine, M. A. Ramsay, R. R. Riker, C. N. Sessler, B. Pun, Y. Skrobik and R. Jaeschke (2013). "Clinical practice guidelines for the management of pain, agitation, and delirium in adult patients in the intensive care unit." Crit Care Med 41(1): 263-306.

##### GRANT ACKNOWLEDGMENT
None.

### 1177

#### Medium term cognitive and functional assessment in patients admitted to an intensive care unit: a prospective cohort study

F. Carini, S. Giannasi, J.E. San Roman, M. Pedace, S. Terrasa, J. Hagel, M. Ducrey, P. Ezcurra
Italiano de Buenos Aires, CABA, Argentina
**Correspondence:** F. Carini
*Intensive Care Medicine Experimental* 2018, **6(Suppl 2):**1177

**Withdrawn**

### 1178

#### Sedation and analgesia practice variation: a meta-analysis of observational cohort studies

L. Prisco[1,2], L. Burry[3,4], M.S. Chew[5,6], S. Mehta[3,4]
[1]University of Oxford, Nuffield Department of Clinical Neurosciences, Oxford, United Kingdom; [2]Oxford University Hospitals NHS Foundation Trust, NeuroIntensive Care Unit, Oxford, United Kingdom; [3]Sinai Health System, Toronto, Canada; [4]University of Toronto, Toronto, Canada; [5]Linköping University Hospital, Department of Anesthesiology and Intensive Care, Linköping, Sweden; [6]Lund University, Institute for Clinical Sciences Malmö, Lund, Sweden
**Correspondence:** L. Prisco
*Intensive Care Medicine Experimental* 2018, **6(Suppl 2):**1178

**INTRODUCTION.** Sedatives and opioids are often used In the Intensive Care Unit (ICU) to mitigate anxiety and pain. However, heavy sedation prolongs durations of mechanical ventilation (MV) and ICU length of stay, and increases incidence of delirium. International guidelines on pain, agitation and delirium management make recommendations to avoid unnecessary deep sedation in attempts to reduce adverse patient outcomes [1]. Despite these recommendations, there is the perception of large international practice variation.

**OBJECTIVES.** To describe the incidence of sedative and opiate use (calculated as patients days receiving propofol, benzodiazepines or opiates) in ICU patients reported in literature.

**METHODS.** We searched MEDLINE (March 1999-March 2018) for surveys, point-of-prevalence studies, or audits on ICU sedation and analgesia practices in mechanically ventilated patients. A meta-analysis for proportions was performed (© 2018 MedCalc Software bvba) to assess variation in practice, and proportions of usage of different sedatives and opioids. The primary outcome was the use of sedatives and opioids; calculated as the proportion of ICU patient days. Variation was assessed by examining the confidence intervals of the estimates and heterogeneity using the $I^2$statistic.

**RESULTS.** We identified 10 studies; 8 reported on our primary outcome with 25876 subjects [2-9]. None of the studies included Low- and Middle-Income Countries (LMICs). The pooled analysis estimated: use of propofol 32% patient-days (95%CI 22-43%), benzodiazepines 42% (32-53%) and opiates 66% (54-78%) (Figure 1-3, Table 1). Heterogeneity tests were statistically significant for all medications (p< 0.0001) indicating large variation in practices.

**CONCLUSIONS.** Our preliminary results confirm the perception of a large variation of practice for sedatives and opioids in ICU. Overall, opiate and benzodiazepine use were very common, and propofol use less so. Larger international cohort studies, including LMICs, are necessary to confirm these findings.

**Table 1 (abstract 1178).** Meta-analysis for proportions and test for heterogeneity

| STATISTICS Random Effects | BENZODIAZEPINES | PROPOFOL | OPIATES |
|---|---|---|---|
| $I^{2\ (Inconsistency)}$ | 99.48% | 99.49% | 99.63% |
| Significance level | P<0.0001 | P<0.0001 | P<0.0001 |
| Total Sample Size | 25876 | 25876 | 25876 |
| Proportion | 42.30% | 32.07% | 66.15% |
| 95% CI for Proportion | 31.85% - 53.11% | 22.33% - 42.68% | 53.58% - 77.70% |

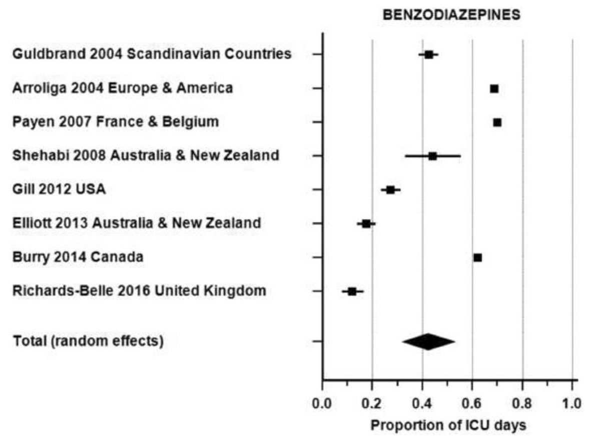

**Fig. 1 (abstract 1178).** Meta-analysis of Benzodiazepines Proportion (patient_days)

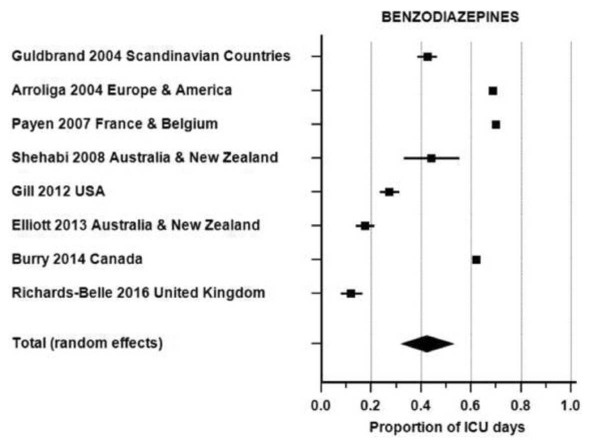

**Fig. 2 (abstract 1178).** Meta-analysis of Propofol Proportion (patient_days)

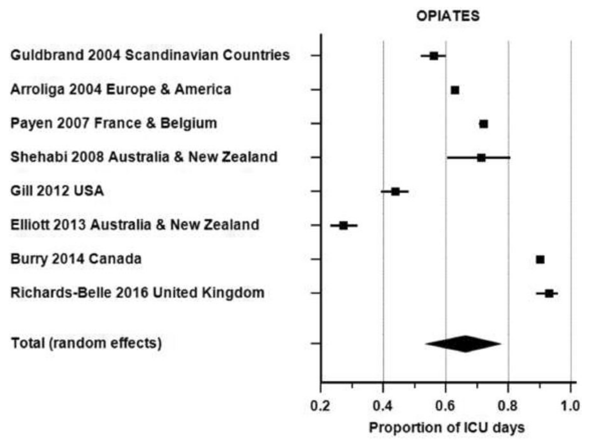

**Fig. 3 (abstract 1178).** Meta-analysis of Opiates Proportion (patient_days)

### 1179

### The effect of melatonin administration on sedation level as adjuvant to propofol in traumatic brain injury patients

M. Soliman, T. Radwan, S. Osman, E. Mansour, R. Samir, M.M. Ghada, A.M. Ahmed
University, anaesthesia and critical care, Cairo, Egypt
**Correspondence:** M. Soliman
*Intensive Care Medicine Experimental* 2018, **6(Suppl 2):**1179

**INTRODUCTION**. *Sedatives are commonly used in the intensive care units ,suboptimal sedation practices may prolong mechanical ventilation, increase delirium and long-term cognitive impairment, especially in the acute phase following the initial injury(1). Bispectral index is Important monitoring for achievement of optimal sedation,*

*Melatonin by virtue of its multiple functions can be an attractive option for pre-medication as an anxiolytic and sedative, Many studies showed that premedication with melatonin is associated with sedation without impairment of cognitive and psychomotor skills and without prolonging recovery(2). To the best of our knowledge there is no previous study determines the effect of the oral administration of melatonin on the dosage of propofol infusion in a mechanically ventilated patient with traumatic brain injury monitored by bispectral index .*

**OBJECTIVES**. *Our purpose was to assess the effect of administration of exogenous melatonin as adjuvant to propofol on the level of sedation and consequently the rate of propofol infusion.*

**METHODS**. *38 patients with traumatic brain injuries requiring mechanical ventilation and* sedation were randomly allocated to two groups (melatonin group)19 patients and (control group)19 patients. In both groups *a bolus of propofol 1mglkg was given by titration till the patient reached a sedation level value of (60-70) on the bispectral index (BIS), Then propofol infusion started at a rate of 1mglkglhr as a maintenance and rate adjusted according to our targeted sedation level, melatonin 10 mg tablet was crushed and mixed with 20 ml of water and administrated through a nasogastric tube followed by another 20 ml to flush out the residue for (melatonin group). While (control Group) received a placebo tablets by the same way.BIS value and propofol infusion rate were recorded over a 12 hours.*

**RESULTS**. administration of exogenous melatonin resulted in a significant reduction in the rate of infused propofol required to achieve the same sedation level .rate of propofol infusion were 4.87(2.91)ml/hr. and 6.37(2.87)ml/hr. in melatonin group and control group respectively.p. value< 0.001. there was no significant changes regarding monitored hemodynamic parameters namely heart rate and blood pressure.

**CONCLUSIONS**. administration of exogenous melatonin has an adjuvant effect on propofol regarding sedation as decreasing the required dose of propofol infusion without compromising any of hemodynamic parameters.

**REFERENCE(S)**

(1). Bellapart J, Boots R. Potential use of melatonin in sleep and delirium in the critically ill. Br J Anaesth. 2012;108(4):572-80. doi:10.1093/bja/aes035.

(2). Oldham MA, Lee HB, Desan PH. Circadian rhythm disruption in the critically ill: An opportunity for improving outcomes. Crit Care Med. 2016;44(1):207- 17. doi:10.1097/CCM.0000000000001282

### 1180

#### Depth of long term ICU sedation under 0,5 MAC isoflurane, sevoflurane or desflurane using MIRUS, an automated delivery system for volatile anaesthetics

A.I. Georgevici, L. Procopiuc, D. Drees, J. Herzog-Niescery, P. Gude, H. Vogelsang, T.P. Weber, M. Bellgardt

St. Josef-Hospital, Ruhr-University of Bochum, Department of Anaesthesiology and Intensive Care Medicine, Bochum, Germany

**Correspondence:** A.I. Georgevici

*Intensive Care Medicine Experimental* 2018, **6(Suppl 2):**1180

**INTRODUCTION.** The MIRUS system (TIM, Andernach, Germany) uses a reflector to conserve volatile anaesthetics (VA) [1-3]. Both systems can be paired with ICU ventilators, but MIRUS features an automated control of end-tidal VA concentrations (etVA). We compare the depth of 0.5 MAC inhalational long-term sedation with Isoflurane (ISO), Sevoflurane (SEVO) or Desflurane (DES).

**METHODS.** Thirty ASA II-IV patients admitted to the ICU after elective or emergency surgery under general anaesthesia were included and randomized into three equal groups: ISO, SEVO or DES. We used MIRUS, a gas delivery system with a VA reflector that can be paired with common ICU ventilators just like the AnaConDa. In addition, MIRUS features the unique ability to maintain the end-tidal concentration (etVA) in target by automatically adjusting the delivery rate. Every 8 hours, the RASS was assessed by the nurse. The log of the EEG-Narcotrend Index (NI) was also included in the study.

**RESULTS.** Patients were comparable in demographics, tidal volume, respiratory rate and sedation time: total 1515h, ISO 35±44h; SEVO 81 ±135h; DES 35±35h; p=0.39. In all patients, a MAC of 0.5 was reached: mean±SD etVA [vol%]: ISO 0.64±0.07 SEVO 0.85±0.15, DES 3.71±0.40: ANOVA p< 0.001. The analysis of the depth of sedation showed comparable results: [1.Q/median/3.Q] RASS: ISO -3/-3/-2, SEVO -3/-3/-2, DES -3/-2/-2; NI: ISO 36/39/50, SEVO 31/39/44, DES 36/39/47. 10 minutes after stopping VA, most patients reached RASS -2. EtVA was significantly associated with the RASS during the wake-up phase (Spearman-Rho: r=0.802 ISO, r=0.697 SEVO, r=0.720 DES; p< 0.001).

**CONCLUSIONS.** MIRUS maintained the etVA of ISO, SEVO and DES in target, independently from the breathing pattern. Using 0.5 MAC as default value, the RASS was -2 or -3 in 82% of the total sedation time, 1515 h. After 10 minutes the majority of patients were responsive and opened their eyes.

**REFERENCE(S)**

1. Bomberg H et al.: Anaesthesia 2014; 69:1241-50.
2. Romagnoli S et al: Crit Care Med. 2017 Sep; 45:e925-e931
3. Bellgardt M et al: J Clin Monit Comput. 2018, doi: 10.1007/s10877-018-0138-z

### 1181

#### Comparison between dexmedetomedine and fentanyl infusion for short term sedation in mechanically ventilated patients in intensive care unit

G.J. Himaaldev[1,2], D. Ashwin Kumar[2], M. Mukund[3]

[1]Apollo Hospital, Intensive Care, Bangalore, India; [2]Kasturba Medical College, Anesthesiology, Mangalore, India; [3]Kasturba Medical College, Anesthesiology and Intensive Care, Mangalore, India

**Correspondence:** G.J. Himaaldev

*Intensive Care Medicine Experimental* 2018, **6(Suppl 2):**1181

**INTRODUCTION.** Sedation and analgesia are the integral part in treatment of patients on mechanical ventilator. Dexmedetomedine incorporates most of the properties of an ideal sedative agent by preserving arousability ,good analgesia, predictable hemodynamic effects and it does not cause respiratory depression.

**OBJECTIVES.** To evaluate the efficacy of dexmedetomedine compared to fentanyl in achieving adequate sedation, 24 hour midazolam requirement and also the effects on hemodynamics

**METHODS.** We conducted a prospective ,randomised ,double blind study in patients on mechanical ventilator admitted in ICU. Institution review board and ethics committee approval was taken.Patients between 20-60 yrs with SAPS II score < 59 who are mechanically ventilated ,with a RASS ranging +4 to 0 were randomly allocated using sealed envelopes.Both the groups received loading dose followed by maintainance infusion.The sedation level was assessed by RASS score recorded hourly for first six hours and then every 6 hrs for next 24 hrs and the doses were titrated to achieve a RASS score -1 to -3. If the sedation was not achieved with maximum dexmedetomedine (0.7 mcg/kg/hr ) or fentanyl (3 mcg/kg/hr) then midazolam 0.02 mg/kg bolus was given as rescue sedation.Total doses of study drugs and the rescue dose of midazolam were recorded along with their haemodynamic effects.

**RESULTS.** Total 62 (n)patients ( confidence level of 95%and power of 80%), 31 recieved dexmedetomedine and 31 recieved fentanyl infusion.

The mean infusion rate for dexmedetomedine to achieve effective sedation ( RASS of -1) was 0.5 - 0.6 mcg/kg/hr compared to fentanyl 1.5- 3 mcg/kg/hr. The hemodynamic effects between the groups were minimal and didnt require any intervention. Dexmeditomedine required 87% less midazolam as rescue sedation (0.39+-1.202 mg/24 hrs) and the mean time required to achieve RASS of -1 was 2.97 +-1.278 hrs at a clinical dose range of 0.5-0.6 mg/kg/hr which was statistically very significant as compared to the fentanyl group which requlred higher dose of midazolam as rescue sedation (2.29 +-1.657mg/24 hrs) with p< 0.01 and the mean time to achieve RASS of -1 was 6.29+-3.388 hrs (p< 0.01) at dose range of 1.5-3 mcg/kg/hr

CONCLUSION. Dexmedetomedine when compared to fentanyl as a short term sedative in mechanically ventilated patients needs shorter time to achieve adequate sedation in doses within the prescribed clinical range and also lesser 24 hour midazolam requirement. Whereas its hemodynamic profile is comparable with that of fentanyl.

**REFERENCE(S)**

1. Stephan M. Jakob et al (2012) Dexmedetomidine vs Midazolam or Propofol for Sedation During Prolonged Mechanical Ventilation.JAMA. 2012;307(11):1151-1160
2. Tan JA, Ho KM. Use of dexmedetomidine as a sedative and analgesic agent in critically ill adult patients.Intensive Care Med. 2010;36:926-39

### 1182

#### Pain study - correlation between objective and subjective pain assessments by the multidisciplinary team

R. Silva[1,2], N. Filgueiras Filho[3,4,5], J.G. Castellucci[6], M. Otero[1,7], R. Menezes[4], I. Ferreira[4,8], G. Agareno[1], V. Silva[1], L. Pamplona[6], S. Agareno[6], A. Gobatto[1,4], GEMINI

[1]Universidade Salvador - UNIFACS, Salvador, Brazil; [2]Núcleo de Pesquisa Clínica - UNIFACS, Salvador, Brazil; [3]Universidade do Estado da Bahia (UNEB), Medicina Intensiva, Salvador, Brazil; [4]Núcleo de Ensino e Pesquisa do Hospital da Cidade, Medicina Intensiva, Salvador, Brazil; [5]Universidade Salvador - UNIFACS, Medicina Intensiva, Salvador, Brazil; [6]Hospital da Cidade, Medicina Intensiva, Salvador, Brazil; [7]Núcleo de Pesquisa Clínica, Salvador, Brazil; [8]Universidade do Estado da Bahia (UNEB), Medicina, Salvador, Brazil

**Correspondence:** M. Otero

*Intensive Care Medicine Experimental* 2018, **6(Suppl 2):**1182

**INTRODUCTION.** Pain is a very prevalent problem in the Intensive Care Unit (ICU). Inadequate assessment and therapeutic management have been associated with increased morbidity and mortality. Pain monitoring tools, such as the Critical-Care Pain Observational Tool (CPOT), have been developed, despite they are rarely used in clinical practice

**OBJECTIVE.** To compare subjective evaluations of pain made by physicians, nurses and physiotherapists with an objective pain quantification scale (CPOT) in critically ill patients.

**METHODS.** A prospective cohort study in a general ICU from September to October 2017. Included patients were followed up during a maximum of 5 days. Pain was monitored daily through CPOT, and the results were compared with subjective assessments made by physicians, nurses and physiotherapists, who were systematically interviewed. To evaluate the association between interprofessional pain assessments, the correlation coefficient was obtained by the tau b statistic of Kendall. The professionals´ agreement with the CPOT index was measured through the gross coefficient of agreement and the Kappa Index (IK). The generalized Kappa Fleiss index was calculated for the concordance analysis, which included cases involving more than two evaluators (judges).

**RESULTS.** Of the 111 patients admitted, 101 were included in addition to 47 professionals from the multidisciplinary intensive care team. Mean age was 71.50 ± 17.21 years, 56 (55.4%) were women, median ICU length stay was 4 days (IQR = 2.0-8.0). A total of 336 pain evaluations were performed. Analyzes of the correlation between the results from subjective pain evaluations of professionals with CPOT indicated statistically insignificant correlations ranging from negligible (14%) to weak (86%). There was no evidence of moderate or strong relation between medical evaluations and CPOT, with a higher correlation presented on day 05 ($\tau = 0.36$; $P = 0.017$). The daily evaluation of the physical therapist with CPOT had a lower correlation variation with CPOT than evaluations by the other professionals. No correlation between subjective evaluation of any class of professionals and CPOT was observed. The daily comparison of the global agreement about presence of pain between the subjective assessment of the professionals and the CPOT revealed a slight to moderate concordances, with the highest agreement occurring on the first day (Kappa Index = 0.36, 95 %: 0.28-0.33, p< 0.001).

**CONCLUSION.** There is a positive correlation between CPOT and subjective pain assessments by the multidisciplinary team, in spite of a weak correlation. There was a weak agreement between interprofessional subjective evaluations, when defining if pain was present or not.

### 1183

#### Implementation of a new sedation hold protocol

S. Shankla, A. Trimmings

Sussex Healthcare NHS Trust, Hastings, St Leonards-on-Sea, United Kingdom

**Correspondence:** S. Shankla

*Intensive Care Medicine Experimental* 2018, **6(Suppl 2):**1183

**INTRODUCTION.** Sedation in the ICU is primarily used to facilitate endotracheal tube tolerance and ventilation[1]. Over-sedation is associated with well-recognised complications. Regular sedation holds have been demonstrated to decrease weaning times, ICU[2] and hospital stays, and one-year mortality[3].

Objectives. To introduce a protocol across both our local NHS Trust ICUs to ensure that all eligible patients have a daily sedation hold with documented target sedation score (Richmond Agitation and Sedation Score (RASS)) and titration of sedation. This follows recommendations from the Guidelines for the Provision of Intensive Care Services (GPICS) 2016[4] (nil audit standards set).

**METHODS.** A retrospective audit was carried out 18$^{th}$-31$^{st}$ Dec 2017, the new protocol implemented and a re-audit conducted 12$^{th}$-25$^{th}$ Mar 2018. The protocol was adapted from the Spontaneous Awakening Trial (SAT) of the "Wake Up and Breathe" protocol by the ICU Liberation Initiative[5]. All ventilated patients who passed the SAT safety and failure screens were eligible. The first 24 hours of admission were discounted unless a hold had specifically been requested. We collected data on whether eligible patients had had a hold requested and completed, or a contraindication documented; a target RASS documented; the protocol for stopping and restarting the propofol infusion followed; changes in RASS and propofol infusion rates.

**RESULTS.** The retrospective audit had 31 patient-days of data and the prospective re-audit 65. Extubated patients or those with a documented contraindication other than in the SAT screen were excluded. Our results demonstrated:

- An improvement in holds requested (55% retrospective / 62% re-audit) and completed (59% / 75%)
- An improvement in documentation of contraindications (55% / 79%)
- Poor documentation of target RASS (0% / 8%)
- No change in the proportion of holds where sedation was stopped rather than reduced (50% / 50%)
- An improvement in restarting the sedative infusion at a lower rate (33% / 53%)
- An improvement in the number of patients with an improved sedation score (33% / 56%)

Retrospective: median pre-hold RASS of -4 (range -3 to -4) and median post-hold RASS of -3 (range -2 to -4)

Re-audit: median pre-hold RASS of -4 (range -3 to -5) and median post-hold RASS of -3 (range -1 to -5).

**CONCLUSIONS.** Education about sedation holds and implementation of a new protocol has increased both our ICUs' compliance with GPICS. Target RASS documentation and sedation titration is lacking. Moving forwards, we will continue the new protocol and will look to extend to the Spontaneous Breathing Trial of the Wake up and Breathe protocol[5].

REFERENCES
1 ICS UK: Review of best practice for analgesia & sedation in the critical care (2014)
2 Kress JP et al. *N Engl J Med* 342 (2000)
3 Girard TD et al. *Lancet* 371 (2008)
4 Guidelines for the provision of intensive care services: FICM & ICS UK (2016)
5 www.iculiberation.org/Bundles/Pages/Spontaneous-Trials.aspx

GRANT ACKNOWLEDGMENT
N/A

### 1184
### Factors associated with probable withdrawal syndrome in trauma intensive care unit patients

C.M. Arroyo-Novoa[1], M.I. Figueroa-Ramos[1], K.A. Puntillo[2]
[1]University of Puerto Rico, Medical Sciences Campus, School of Nursing, Graduate Department, San Juan, Puerto Rico; [2]University of California San Francisco, San Francisco, United States
**Correspondence:** C.M. Arroyo-Novoa
*Intensive Care Medicine Experimental* 2018, **6(Suppl 2):**1184

**INTRODUCTION.** The management of analgesia and sedation in ICUs has evolved to promote a lower level of sedation and prevent or minimize the associated negative effects of both opioids (OPs) and benzodiazepines (BZDs).[1] However, patients admitted to trauma ICUs (TICU) may require higher or prolonged doses of OPs along with BZDs. These medications may contribute to drug physical dependence, a response that may be manifested in withdrawal syndrome (WS).[2]
**OBJECTIVES.** To identify the factors associated with probable WS among TICU patients exposed to OPs and/or BZDs.
**METHODS.** An OP/BZD-WS checklist was developed from research in adult ICU patients, the DSM-5, and the ICD-10. Fifty patients were evaluated at baseline (4th day on OPs and/or BZDs), then were evaluated twice a day upon start of a BZD and/or OP weaning trial for up to 72 hours. If drug doses were increased, WS measurement was stopped and restarted at the next weaning. If patient presented with 3 or more signs and symptoms (s/s) for OP-WS and/or 2 or more s/s for BZD-WS, it was identified as probable WS. Any s/s present at baseline was not counted as withdrawal s/s during measures and if did not comply with the minimum s/s requirements, these measures were identified as questionable WS. A mixed-effects logistic regression model was developed to assess whether cumulative dose amounts of both OPs and BZDs prior to assessment, days on both OPs and BZDs, previous drug use, Richmond Agitation Sedation Scale (RASS) score, and presence of delirium were significantly associated with the occurrence of probable WS [odds ratios (ORs) and 95% confidence intervals (CIs)].
**RESULTS.** Majority of patients were male (88%) with a median age of 37 (IQR, 27 49), 90% were on mechanical ventilation, and 66% were OP/BZD/illicit drug naïve. Probable WS occurred in 44% and questionable WS in 20% of the patients. Cumulative dose amounts of OPs (OR=1.10; CI, 1.02- 1.19), RASS score (OR=3.89; CI, 1.87- 8.10), and previous drug use (OR=4.69; CI, 1.43- 15.41) were associated with odds of developing WS, while the number of days on OPs (OR=.73; CI, .54- .99) was protective for the odds of WS after adjusting for cumulative dose of BZDs, days on BZDs, and delirium.
**CONCLUSIONS.** We found an association between increase in the cumulative dose amounts of OPs, higher RASS scores, and previous drug use with WS developing. Special attention to significant factors associated with WS found in this study should be considered when caring for patients receiving OP/BZD. Further validation of OP/BZD-WS checklist is recommended.

REFERENCES
1. Barr J, et al. Clinical practice guidelines for the management of pain, agitation, and delirium in adult patients in the ICU. *Crit Care Med*. 2013;41:263-306.
2. American Psychiatric Association. *Diagnostic and Statistical Manual of Mental Disorders*. 5th ed. Washington, D.C.: APA; 2013.

GRANT ACKNOWLEDGMENT
Hispanic Clinical and Translational Research Education and Career Development Program (R25MD007607).

### 1185
### Sevoflorane sedation in ICU ward using a Mirus™ system: an innovative methodology

R. Varutti, F. Bassi
AAS5 Friuli Occidentale, Emergenza, SOC Anestesia e Terapia Intensiva 2, Pordenone, Italy
**Correspondence:** F. Bassi
*Intensive Care Medicine Experimental* 2018, **6(Suppl 2):**1185

**INTRODUCTION.** Intravenous sedation is standard practice in the Intensive Care Unit (ICU[1]). Sevoflurane has some potential advantages over intravenous drugs: rapid elimination and few interactions. The optimal role of sevoflurane in ICU is unknown.
The Mirus[TM] system (Pall Medical, New York) is a new inhalational device for sedation in ICU. The system delivers volatile anesthetics in mecanically ventilated patients. A dedicated gas filter and a open reservoir scavenger permit an alogenate reabreating and avoid residual volatil anesthetic into the room air[2]
**OBJECTIVES.** The aim of this study, approved by the Institutional Ethics Committee, is to present our initial experience with sevoflurane in ICU.
**METHODS.** We performed a prospective study on adult patients who required sedation for mechanical ventilation. We used sevoflurane to achieve a RASS score of 0 to -2 using a maximal mean alveolar concentration (MAC) of 0.8% . The MIRUS [TM] system device was used along with the Servo-i ventilator (Maquet, Gothenburg, Sweden) gas monitor. CAM-ICU score was used for testing delirium. Data were obtained from patients' medical records.
**RESULTS.** We collected data from 24 patients (pt) admitted to our ICU from August 2017 to March 2018 for postoperative monitoring (2 pt), thoracic trauma (1 pt), sepsis (4 pt), acute respiratory distress or asthma (5 pt), acute coronary syndrome (6 pt), psychosis (1 pt), epilepsy (3 pt), coma (2 pt). Mean age was 62 y (+/-15 y), 11 was male. 50% of patients required temporary adjunctive sedation with remifentanil and dexmedetomidine or midazolam for develop of hyperactive delirium.
Hypotension was the most common symptom and it was rapidly solved with fluid administration or a low doses of vasoactive agents infusion. No metabolic syndrome or organ dysfunction was noted after a sevoflurane inhalation longer than 48 h. No malignant hyperthermia was done.
**CONCLUSIONS.** At the beginning there was a reduced compliance of the medical /nursing staff towards the use of the device due to lack of training, difficulty overcoming established sedative habits, skepticism. The learning curve allowed to reduce progressively the halogenate dose administered (mean MAC of 0.2-0.3%) and allowed become more confident with the methodology. Our initial experience, according to literature3, shows that Mirus[TM] device, used in a mixed pool of patients, is effettive, simply and safe.

REFERENCES
1. Mancineli P. et al. Mirus™, a new system for sedation with halogenates in the ICU: a preliminary study of feasibility in postsurgical patients. Critical Care 2015; 19 (1); P492
2. Korosa A. Et al. Use of sevoflurane in the medical ICU: 2-year experience, patient and safety profile. Critical Care 2015; 19 (1); P49
3. Jabaudon M. et al. Sevoflurane for sedation in ARDS: a randomized controlled pilot study. Am J Respir Crit Care Med 2016. DOI: 10.1164/rccm.201604-0686OC

### 1186

#### Iatrogenic opioid withdrawal syndrome in critically ill patients: a retrospective cohort study

D.-G. Hyun, C.-M. Lim, J.W. Huh, S.-B. Hong, Y. Koh
Medical Center, University of Ulsan College of Medicine, Pulmonary and Critical Care Medicine, Seoul, Korea, Republic of
**Correspondence:** D.-G. Hyun
*Intensive Care Medicine Experimental* 2018, **6(Suppl 2):**1186

**INTRODUCTION.** In critically ill patients, especially those receiving mechanical ventilation, the use of opioid analgesics is recommended. However, there is a lack of detailed studies regarding opioid withdrawal syndrome (OWS), which may occur during reduction or discontinuation of opioid analgesics [1].

**OBJECTIVES.** To investigate the incidence, risk factors, and clinical features of OWS in an intensive care unit (ICU).

**METHODS.** A retrospective study was conducted; the analysis cohort comprised 126 patients who received intensive care unit treatment for ≥3 days, using only one type of opioid analgesic agent, in the medical ICU of Asan Medical Center. The presence of OWS for a certain period (2 hours for remifentanil, 10 hours for fentanyl, 8 hours for morphine) was assessed from when continuous infusion of opioid analgesic was discontinued or reduced by >1/2 dose. OWS was defined as satisfying ≥2 central nervous symptoms or autonomic symptoms.

**RESULTS.** OWS occurred in 21 patients (16.6%). There was no statistically significant difference in incidence of OWS among remifentanil, fentanyl, and morphine (19.0% vs. 19.1% vs. 4.8%, p=0.333), or in duration of ICU stay, duration of mechanical ventilation, and duration of opioid analgesic use between OWS (+) and OWS (-) groups. There was a significant difference in cumulative opioid dose (7437.08 mg vs 5051.54 mg, p=0.023) between OWS (+) and OWS (-) groups, but no significant differences in the frequency of extubation (61.9% vs 52.4%, p=0.424), tracheostomy (23.8% vs 27.6%, p=0.720), and mortality in ICU (38.1% vs 21.0%, p=0.092). In multivariate analysis, heavy alcoholics, presence of septic shock, and < 16 days of ICU stay were associated with OWS. Among the 21 patients, central nervous symptoms occurred in 12 (57.1%) and autonomous symptoms occurred in 20 (95.2%). The most common symptom was change of respiratory rate (n = 18, 85.7%).

**CONCLUSIONS.** Incidence of OWS did not differ among types of opioid analgesics; however, it was associated with heavy alcoholics, patients with septic shock, and those who stayed in ICU for < 16 days. Therefore, caution should be exercised when reducing or stopping opioid analgesics. Further prospective studies with larger sample sizes are needed to determine whether OWS affects the prognosis of ICU patients.

**REFERENCE(S)**

1. Wang PP, Huang E, Feng X, Bray CA, Perreault MM, Rico P, Bellemare P, Murgoi P, Gelinas C, Lecavalier A, Jayaraman D, Frenette AJ, Williamson D (2017) Opioid-associated iatrogenic withdrawal in critically ill adult patients: a multicenter prospective observational study. Ann Intensive Care 7:88. doi:10.1186/s13613-017-0310-5

### 1188

#### A comparative study concerning sedative and analgesic effects of hypnotherapy with the same effects of acetaminophen, fentanyl and methadone in awake and conscious ICU admitted patients; single blinded, clinical trial

A. Taher[1], M. Samadi[2], M. Dorri[1], J. Poorolajal[1]
[1]Hamadan Medical University, Anesthesiology and Critical Care, Hamadan, Iran, Islamic Republic of; [2]Farshchian Sina Hospital, Hamadam, Iran, Islamic Republic of
**Correspondence:** A. Taher
*Intensive Care Medicine Experimental* 2018, **6(Suppl 2):**1188

**Withdrawn**

## New research in perioperative medicine

### 1189

#### Association of perioperative variables and the acute respiratory distress syndrome in liver transplant recipients

J.G. Ripoll[1], B.T. Wanta[2], D.R. Wetzel[2], J.Y. Findlay[2], M.N. Vogt[1]
[1]Mayo Clinic, Anesthesiology, Rochester, United States; [2]Mayo Clinic, Anesthesiology and Critical Care, Rochester, United States
**Correspondence:** J.G. Ripoll
*Intensive Care Medicine Experimental* 2018, **6(Suppl 2):**1189

**INTRODUCTION.** Acute respiratory distress syndrome (ARDS) is a serious complication of surgery. Although the assessment of perioperative risk factors for ARDS has been described in various surgical populations, there is only limited data among patients undergoing liver transplantation (LT), particularly regarding the potential influence of intraoperative ventilation parameters.

**OBJECTIVES.** In the present study, we sought to determine the incidence of ARDS following LT in a large tertiary referral center, and to identify preoperative and intraoperative risk factors associated with its development. Also, we aimed to identify the impact of ARDS on clinical outcomes (mortality, hospital and ICU length of stay).

**METHODS.** This is a single center, retrospective cohort study of adult patients who underwent LT at a tertiary level academic medical center between January 1st, 2006 and January 31st, 2016. Postoperative ARDS was identified using the Berlin definition. Multivariate logistic analysis was used to identify preoperative and intraoperative risk factors for ARDS.

**RESULTS.** Of 817 eligible patients who underwent a LT during the study period, 20 (2.45%) developed postoperative ARDS. In the preoperative model, higher MELD score (per 1 unit increase, odds ratio [OR], 1.65; P< 0.01), higher INR (per 0.1 unit increase, OR, 1.69; P=0.03), and ongoing dialysis (OR, 5.23; P=0.03) were identified as independent risk factors associated with the development of ARDS post-LT. Intraoperatively a higher mean peak inspiratory pressure (OR, 1.32; P< 0.01) was recognized as the only independent risk factor. Patients with ARDS postoperatively had significantly greater intensive care unit and hospital stay than non-ARDS. There were no significant differences in the 30-day and 1-year mortality between the groups.

**CONCLUSIONS.** Acute respiratory distress syndrome occurred at a rate of 2.45% following LT in adult patients. Higher preoperative MELD score, higher INR and ongoing dialysis as well as elevated intraoperative mean peak inspiratory pressure were associated with the development of ARDS. In addition, ARDS post-LT was associated with increased length of ICU and hospital stay, but not in mortality.

### 1190

#### Intraoperative use of 6% hydroxyethyl starch (130/0.4) and outcome in patients with major surgical haemorrhage: a retrospective study

J. Divatia, A. Daga
Memorial Hospital, Anaesthesiology, Critical Care and Pain, Mumbai, India
**Correspondence:** J. Divatia
*Intensive Care Medicine Experimental* 2018, **6(Suppl 2):**1190

**INTRODUCTION.** The use of 6% hydroxyethyl starch 130/0. 4 (HES) in patients with severe sepsis is associated with increased acute kidney injury (AKI), coagulopathy and mortality. [1,2] However fluid resuscitation with HES in relatively healthy surgical patients may not be associated with adverse short-term outcomes.[3,4]

**OBJECTIVES.** In this retrospective study, we aimed to compare the incidence of AKI, coagulopathy, and mortality during the hospital stay as well as mortality at 90-days in patients who received HES with those who did not receive HES for major hemorrhage during surgery for pelvic bone tumors.

**METHODS.** Records of 102 patients who underwent hemipelvectomy for pelvic bone tumors between January 2011 and June 2016 were reviewed. The Institutional Ethics Committee approved this retrospective analysis with a waiver of written, informed consent. Patients were classified as those who received HES during surgery (HES patients) and those who did not (No-HES patients). The primary outcome was a composite end-point of the number of patients developing postoperative AKI (RIFLE Stage Injury or Failure by creatinine criteria) OR coagulopathy (platelet count < 80,000/cu mm or international normalised ratio (INR) > 1. 5 or activated partial thromboplastin time (APTT) > 1. 5 times control value) OR death prior to hospital discharge. Secondary outcomes included serum creatinine values and mortality at 90 days after surgery. Data were analyzed using Chi-square test / Fishers exact test, independent "t" test and multivariate binary logistic regression analysis were planned. P< 0. 05 was considered statistically significant.

**RESULTS.** There were 42 HES patients and 60 No-HES patients. Twelve HES patients and 52 No-HES patients received gelatins. Mean age was 35.9 ±15. 6 years, median volume of HES infused was 1000ml [interquartile range (IQR) 500-1500ml] and median intraoperative blood loss was 3500ml [IQR 1700-6850ml]. The composite endpoint occurred in 62% HES patients and in 63% No-HES patients (P=0. 9). There was no difference in individual components of the composite end-point. Hospital mortality (HES patients, 1 /42 vs. No-HES patients, 1/60) was similar (P=0. 8). At 90 days after surgery, 2/33 HES patients and 1/50 No-HES patients had died (P=0. 35). No differences were observed in other secondary outcomes.

**CONCLUSIONS.** In this retrospective study, young cancer patients who received limited amounts of 6% HES (130/0.4) during hemorrhage in the intraoperative period did not have an increased occurrence of AKI, coagulopathy, and mortality in hospital or mortality at 90 days, compared to patients who did not receive HES. A large randomized controlled trial is essential to establish the safety of 6% HES (130/0.4) for fluid resuscitation during major surgical hemorrhage.

##### GRANT ACKNOWLEDGMENT

None

### 1191

#### Cerebral oxygenation evaluated by spatially resolved near-infrared spectroscopy shows a clinically small decrease during arthroscopic shoulder surgery with the patient in the beach chair position

Y. Itosu, S. Harada, N. Tanaka, R. Kato, Y. Morimoto

University Graduate School of Medicine, Department of Anesthesiology and Critical Care Medicine, Sapporo, Japan

**Correspondence:** Y. Itosu

*Intensive Care Medicine Experimental* 2018, **6(Suppl 2):**1191

**INTRODUCTION.** There have been many reports demonstrating that cerebral oxygenation evaluated by near-infrared spectroscopy dramatically decreases during arthroscopic shoulder surgery with the patient in the beach chair position (BCP) [1]. In these reports, almost all the near-infrared spectroscopes used for the studies mainly adopted the modified Beer-Lambert law (MBL). It was reported that cerebral oxygenation (rSO2) determined using the MBL was more affected by extracranial contamination than was cerebral oxygenation (TOI) determined using spatially resolved spectroscopy (SRS) methods[2]. However, few studies have evaluated the changes in TOI during this type of surgery. Accordingly, we evaluated the changes in TOI using the NIRO-200NX (Hamamatsu Photonics, Japan). We estimated the mean arterial pressure (MAP) in the temporal artery and observed early neurological outcomes.

**METHODS.** After obtaining institutional review board approval (014-0473), we performed an observational prospective study of 21 patients (ASA physical status1-2) scheduled for arthroscopic shoulder surgery with the patients in the BCP. One minute before the BCP (T1), TOI was measured as a control. Subsequently, TOI was obtained at 1, 5, 10, 15, and 30 minutes after the BCP (T2-6) and 1 minute before and after return to the horizontal position (T7, 8). At the same points, MAP in the temporal artery was estimated from the distance between the cuff and external auditory meatus. Neurological outcomes, including delirium, were observed until discharge. To analyze the changes in TOI and MAP, one-way repeated-measures analysis of variance (ANOVA) was performed followed by the Bonferroni multiple comparison test for each time point. Data were expressed as mean ± standard deviation. P < 0.05 was considered significantly different.

**RESULTS.** Twelve male and 9 female patients were enrolled. Their mean age was 64 ± 10 years. At T1, TOI (%) was 75.8±8.4 and MAP (mmHg) was 62±10 (Fig.). After the BCP, TOI significantly decreased to 70.5±8.8 and the values were maintained at 69 while in the BCP. At T8, TOI returned to 72.7 ±6.7. MAP decreased to around 30 during the BCP. No neurological complications were observed until discharge (14.7 days after surgery).

**CONCLUSIONS.** The decrease in cerebral oxygenation evaluated by SRS methods was significant, but clinically small during arthroscopic shoulder surgery with patients in the BCP, although MAP in the temporal artery was below the lower limit of cerebral autoregulation (= around 50 mmHg). A recent review indicated that the frequency of neurological complications such as cerebral infarction was around 0.004% in this type of surgery[3]. Accordingly, intracerebral oxygenation may not deteriorate much while patients are in the BCP.

##### REFERENCE(S)

1 Arthroscopy 2014;30:1520
2 Anesthesiology2007; 106:458
3 Am J Orthop 2016;45:E63

##### GRANT ACKNOWLEDGMENT

none

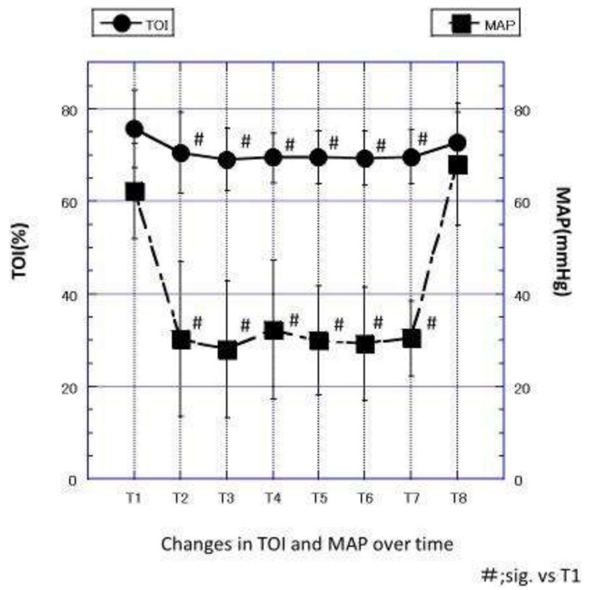

**Fig. 1 (abstract 1191).** Changes in TOI and MAP over time

### 1192

#### Operation time of 15 hours or more increases postoperative complications: a retrospective study on extra-long hepatectomy

M. Nagata, Y. Hara, N. Kuriyama, T. Nakamura, T. Kawaji, D. Hasegawa, C. Yamashita, J. Shibata, H. Komura, O. Nishida
Health University School of Medicine, Anesthesiology and Critical Care Medicine, Aichi, Japan
**Correspondence:** M. Nagata
*Intensive Care Medicine Experimental* 2018, **6(Suppl 2):**1192

**INTRODUCTION.** In hepatectomy, it has been suggested that an excessive operation length is related to an increased rate of postoperative liver failure and surgical site infection. These reports mostly investigated hepatectomy cases with a relatively short operating time, and there is no report on hepatectomy that exceeded 12 hours. However, it is possible that a long operation that could increase the incidence of life-threatening complications is disadvantageous to patients.

**OBJECTIVES.** With extra-long hepatectomy that exceeds 12 hours, we aimed to evaluate if the operation time is correlated with postoperative complications and postoperative liver failure, and identify the "limit of operating time (OT)" that could increase postoperative complications.

**METHODS.** We examined patients aged ≥18 years, for whom hepatectomy was performed at Fujita Health University (FHU) Hospital, Japan, between January 2014 and December 2017. Hepatectomy cases with an OT of ≥12 hours were selected. Postoperative complications were defined as those that were class III or higher in the Clavien-Dindo classification. In this study, Grades B and C of ISGLS criteria[1] that had impact on clinical management were defined as the clinical liver failure (CLF) group. Data are presented with the median and interquartile range, and were analyzed by using the multiple logistic regression analysis, and receiver operative curve (ROC). We used EZR for the statistical software. This study was approved by FHU Ethics Review Committee.

**RESULTS.** There were 94 patients (65 men), with an age of 67.5 (62-73) years, height 165.0(156.9-170.0) cm, weight 61.9(51.1-68.6) kg, body mass index (BMI)22.5 (21.0-24.9) kg/cm$^2$, and ICGR15 of 10.2 (7.2-13.7)%. Preoperative Child-Pugh (CP) classifications included 72 cases with five points, 18 with six points, 2 with seven points, and 2 with eight points. OT was 14.2 (13.0-16.7) hours. There were 33 patients who experienced CLF and 38 patients who experienced postoperative complications. The result of the multiple logistic regression analysis showed that factors contributing to postoperative complications were BMI (Odds ratio [OR] 0.78, 95% confidence interval [CI] 0.65- 0.96, p = 0.02), CP score (7.32, 1.90- 28.2, p = 0.004), ICGR15 (1.11, 1.01- 1.22, p = 0.02), and OT (1.27, 1.08- 1.50, p = 0.003). Factors contributing to CLF were BMI (0.77, 0.64- 0.95, p = 0.01), CP score (7.31, 2.02- 26.44, p = 0.002), ICGR15 (1.09, 1.00- 1.19, p=0.04), and OT (1.20, 1.04- 1.37, p = 0.01). ROC analysis showed that the cutoff value for OT that predicts occurrence of postoperative complications was 15.3 hours (Area under the curve 0.697, 95% CI 0.583-0.811).

**CONCLUSIONS.** In extra-long hepatectomy, OT was an independent factor for postoperative complications and CLF. It was indicated that when the operation exceeds 15 hours, the incidence of postoperative complications increases.

##### GRANT ACKNOWLEDGMENT

None.

### 1193

#### Intra-operative management of major pancreatica surgery: what matters most?

D. Cigolini[1], K. Donadello[1], R. Salvia[1], V. Schweiger[1], W. Mosaner[2], C. Bassi[1], E. Polati[1]
[1]University of Verona, Department of Surgery, Dentistry, Pediatrics and Gynaecology, Verona, Italy; [2]Peschiera Pederzoli Clinic, Verona, Italy
**Correspondence:** K. Donadello
*Intensive Care Medicine Experimental* 2018, **6(Suppl 2):**1193

**INTRODUCTION AND OBJECTIVES.** Some intra-operative variables may significantly affect patient outcomes after major pancreatic surgery. The identification of one or more of them could be very useful for choosing the best intra-operative management and for planning the best measures to be taken in the post-operative period.

**METHODS.** We collected data of patients operated for major pancreatic surgery during 12-month period from two clinical databases (Verona University Hospital and Peschiera Pederzoli Clinic).

We took into account the following preoperative variables: BMI, smoking habitus, presence of jaundice, pre-operative drainage of jaundice, recent chemotherapy, anamnesis positive for cardiovascular, pulmonary, renal, neurological disease and the American Society of Anesthesiologist (ASA) score. Recorded outcome data included incidence of postoperative abdominal surgical-related complications, systemic complications (infectious, respiratory, cardiovascular, nefrologic and neurologic), in-hospital mortality, ICU admission and days of hospitalization.

**RESULTS.** A total of 638 (320 women and 318 men, aged 61.4±12.5 years, mean ± SD) were operated of major pancreatic surgery during 2013 and 2014 in the two hospitals. ICU admission was 8.9%. Total of post-operative complications were 57.6%,in-hospital mortality was 2.2%. We observed abdominal surgical-related complications in 43.5% and general complications (cardiac, respiratory, infectious and renal) in 34.1% of total patients. Mean ± SD surgical time was 333 ±106 minutes, and mean hospitalization was 16±18 days. Ten patients (1.6%) needed intraoperative infusion of inotropes or vasopressors and 72 (11.4%) received blood products. Colloids were administered to 221 patients (34.9%), and 179 patients (28.2%) received more than 4500ml of cristalloids.

The multivariate analysis showed that the incidence of abdominal complication was higher in group who received colloids (42.1%vs52%, p 0.017, O.R. 1.49 C.I.95% 1.073-2.069) and that the development of tachyarrhythmia in post-operative period was higher in the patients who received colloids (4.1%vs10%, p0.017, O.R. 2.245 C.I.95% 1.153-4.372) and when surgery time was longer than 300 minutes. The incidence of cardiac ischemia (0.3%vs10%) and hearth failure (0.3%vs10%) was higher in patients who needed infusion of inotropes or vasopressor during the surgery. All the analisys were adjusted for sex, age and preoperative physical status.

**CONCLUSIONS.** Our data suggest that the use of colloids, administration of cristalloids >4500ml and the infusion of inotropes or vasopressor can be related to development of post operative complication. These complications seem to be indipendent from pre-operative physical status and not influenced by sex and age. Also a time of surgery >300 minutes can be considered a risk factor for post operative complications.

### 1194

#### Risk factors for postoperative shallow tube depth of nasotracheal tube

H.Y. Kim[1], S.O. Kim[1], E.J. Kim[2], S. Na[2], J. Kim[2]
[1]Ajou University, Anesthesiology and Pain Medicine, Suwon, Korea, Republic of; [2]Yonsei University College of Medicine, Anesthesiology and Pain Medicine, Seoul, Korea, Republic of
**Correspondence:** S.O. Kim
*Intensive Care Medicine Experimental* 2018, **6(Suppl 2):**1194

**INTRODUCTION.** Sometimes, patients undergoing head and neck surgery admit in intensive care unit (ICU) with nasotracheal intubation. For prolonged mechanical ventilation, a proper endotracheal tube depth is important component to prevent complications such as inadvertent extubation, or endobronchial intubation. However, we have often experienced the patients who showed shallow nasotracheal tube (NTT) depth in the ICU after surgery.

**OBJECTIVES.** To evaluate risk factors associated with improper NTT depth among the patients undergone prolonged mechanical ventilation after head and neck surgery.

**METHODS.** We retrospectively investigated all adult patients (age ≥ 18 years) who admitted the ICU with NTT after surgery between January 2008 and December 2016. The patients were divided into two groups according to distance from carina. Proper depth group consisted of patients that distal tip of NTT located 2 cm - 7 cm above the carina on postoperative chest radiography (CXR). Improper depth group consisted of patients that distal tip of NTT located < 2cm or > 7cm above the carina on postoperative CXR. We collected patients' perioperative data from medical records and CXR. Risk factors for improper tube depth were evaluated using univariable and multivariable logistic regression analyses.
**RESULTS.** Of the total 133 patients, 55 patients showed postoperative shallow NTT depth and none showed deep tube depth. The patients were divided into 78 patients of proper depth group and 55 patients of shallow depth group. The male sex, tall and heavy patient, low carina level on preoperative CXR, prolonged anesthesia duration, right side surgical field, postoperative restriction of position, and deep postoperative tube fixation were significant factors in univariable analysis. Multivariable analysis revealed that tall stature (OR 1.16, 95% CIs 1.08 - 1.25, P < 0.001), prolonged anesthesia duration (OR 1.16, 95% CIs 1.02 - 1.32, P = 0.026), and right side surgical field compared to left or median (OR 0.36, 95% CIs 0.14 - 0.93, P = 0.034, OR 0.25 95% CIs 0.07 - 0.85, P = 0.027, respectively) were independent risk factors.
**CONCLUSIONS.** The shallow NTT depth after head and neck surgery were present in 41% of patients. Tall stature, prolonged anesthesia duration, and right side surgical field were independent risk factors of postoperative shallow tube depth. Our study suggests that the patients with risk should select appropriate tube in advance and check tube depth on postoperative CXR.

**REFERENCE(S)**

1. Lee J, Lee JM, Min JJ, Koo CH, Kim HJ. Optimal length of the pre-inserted tracheal tube for excellent view in nasal fiberoptic intubation. Journal of anesthesia. 2016;30(2):187-92.
2. Reed DB, Clinton JE. Proper depth of placement of nasotracheal tubes in adults prior to radiographic confirmation. Academic emergency medicine: official journal of the Society for Academic Emergency Medicine. 1997;4(12):1111-4.

### 1195

#### Incidence and associated factors of successfully immediate extubation after liver transplantation

A. Suphathamwit, O. Pongraweewan, S. Lakkam
of Medicine Siriraj Hospital, Department of Anesthesiology, Bangkok, Thailand
**Correspondence:** A. Suphathamwit
*Intensive Care Medicine Experimental* 2018, **6(Suppl 2):**1195

**INTRODUCTION.** Liver transplantation is the definitive treatment of acute liver failure and end-stage liver cirrhosis. In the past, post liver transplant patients were remained intubated and stayed in the ICU because of prolonged period of surgery and massive blood and fluid administration. Nowadays, the evidence that spontaneous breathing provides better liver circulation and venous drainage without compromising postoperative patient status, thus, there is an increasing trend to extubate the patient immediately after liver transplantation.
**OBJECTIVES.** To determine the incidences and associated factors for immediate extubation of post-liver transplantation patient in Siriraj hospital.
**METHODS.** This is a retrospective descriptive study performed on 262 liver transplantation patients in Siriraj hospital between 2004 and 2016. The primary outcome is the incidence of successful immediate extubation post liver transplantations. Secondary outcome includes factors associated with successfully immediate extubation, pulmonary complications (including re-intubation rate, pneumonia, ARDS), acute kidney injury, graft failure rate, length of hospital and ICU stay and post-operative death.
RESULTS: A total of 239 patients were analyzed after exclusion according to exclusion criteria. The incidence of successfully immediate extubation after liver transplantation is 32.2 %. Factors significantly associated with successfully immediate extubation from multivariate analysis included diagnosis of hepatocellular carcinoma (adjusted OR 2.01, 95%CI 1.09-3.74, p = 0.026), amount of blood transfusion < 1,600 ml (adjusted OR 5.18, 95% CI 1.49 - 18.02, p = 0.010) and no post-operative vasopressor or inotropes requirement (adjusted OR 18.43, 95%CI 2.43 - 140.08, p = 0.005). Postoperative pulmonary complications, acute kidney injury, length of hospital and ICU stays and postoperative death were lower in immediate extubation group.
**CONCLUSIONS.** The incidence of successfully immediate extubation in post-liver transplantation patients in Siriraj hospital was 32.2%. Factors associated with successfully extubation included hepatocellular carcinoma, less amount of blood transfusion and no inotropes or vasopressors requirement at the end of surgery. The patients in immediate extubation group had significantly less postoperative pulmonary complication, acute kidney injury and overall hospital stay.

**REFERENCE(S)**

1. S. Skurzak, C. strratta, et al. Extubation score in the operating room after liver transplantation. Acta Anaesthesiol Scand 2010;54: 970-978.
2. M.Susan Mandell, Tamara J. Stoner, et al. A multicenter evaluation of safety of early extubation in liver transplant recipients. American association for the study of liver disease 2007;13:1557-1563.
3. Paolo Feltracco, Cristiana Carollo, et al. Early respiratory complications after liver transplantation. W world J gastroenterol 2013;19(48):9271-9281.

**GRANT ACKNOWLEDGMENT**
none

### 1196

#### Cardiopulmonary exercise testing as a predictor of hospital stay in patients undergoing oesophagogastrectomy

M. Gerstman, G. Friedlaender, J. Batty, M. Hacking, T. Wigmore
Royal Marsden, Anaesthetic Department, London, United Kingdom
**Correspondence:** M. Gerstman
*Intensive Care Medicine Experimental* 2018, **6(Suppl 2):**1196

**INTRODUCTION.** Oesophagogastrectomy surgery is high risk oncological surgery associated with high morbidity and mortality. Cardiopulmonary exercise testing (CPET) is used to risk stratify patients being considered for this surgery. Both V02 max and FVC have been identified as independent variables for predicting hospital length of stay however these results have not been consistent in all studies [1,2].
**OBJECTIVES.** Our aim was to analyse whether preoperative CPET in patients undergoing oesophagogastrectomy is predictive of hospital length of stay in our institution.
**METHODS.** This is a retrospective observational analysis of patients undergoing oesophagogastrectomy at the Royal Marsden between September 2010 and December 2015. Variables studied included anaerobic threshold oxygen consumption (AT VO2), maximal oxygen consumption (VO2 MAX), ventilatory equivalents for CO2 at AT (AT VE/VCO2), as well as forced expiratory volume in first second (FEV1), forced vital capacity (FVC) and peak flow.
**RESULTS.** Of the 157 patients who underwent oesophagogastrectomy surgery during this period 105 underwent cardiopulmonary exercise testing and 120 had their peak flow recorded. See the Table 1 below for results.
**CONCLUSION.** There was a weak negative correlation between peak flow and hospital length of stay in patients undergoing oesophagogastrectomy in our institution. CPET variables did not predict hospital length of stay in our study population.

**REFERENCE(S)**

1. Sinclair et al. Pre-operative variables including fitness associated with complications after oesophagectomy. Anaesthesia. 2017 Dec;72(12):1501-1507.
2. Tan B, et al Cardiopulmonary exercise testing predicts prolonged hospital stay following oesophago-gastric cancer surgery. Gut 2015;64:A120.

**GRANT ACKNOWLEDGMENT**
N/A
There was negative linear relationship between peak flow and hospital length of stay (p=0.007) with a corresponding correlation of (r=-0.246) (Fig. 1). All other variables indicated weak negative linear relationship (r=-0.117 and r=-0.009) and correlation p-values of > 0.1

**Table 1 (abstract 1196).** CPET and peak flow results

| Variable | Result mean (SD) |
|---|---|
| AT VO2 (ml/kg/min) | 12.2 (3.0) |
| VO2-MAX (ml/kg/min) | 19.1 (4.3) |
| Work at AT (Watts) | 71.8 (27.9) |
| AT-VE/VO2 | 32.2 (5.2) |
| FEV1 (L) | 3.2 (0.7) |
| FVC (L) | 4.3 (1.0) |
| Peak flow (ml) | 477.2 (107.7) |

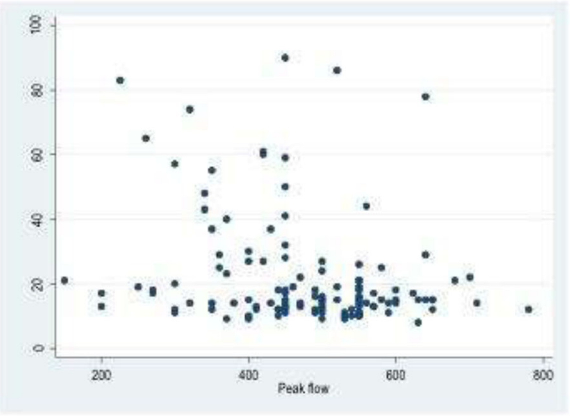

**Fig. 1 (abstract 1196).** Scatter plot for total hospital stay vs Peak flow (*r* = -0.246)

### 1197

#### Evolution and complications in the inmediate postoperatory of citorreductive surgery and hyperthermic intraperitoneal chemotherapy (CRS-HIPEC)

E. Rosas Carvajal, A. Vidal González, V.A. Hortigüela Martín, D.C. Robaglia, L.M. Polanco Mahecha, J.J. Páez Vargas, M.Á. Alcalá Llorente, C. Pérez Calvo
Jiménez Díaz, Intensive Care Unit, Madrid, Spain
**Correspondence:** E. Rosas Carvajal
*Intensive Care Medicine Experimental* 2018, **6(Suppl 2):**1197

**INTRODUCTION.** The CRS-HIPEC surgery is an essential therapeutic alternative in the management of the abdomino-peritoneal cancer locally advanced. Its postoperatory management is a challenge for the intensivist for its high risk of complications among which Acute Kidney Injury (AKI) stands, with rates that oscillate between 40.4% and 21.3% in the available literature.

**OBJECTIVES.** Analyze the evolution and complications in the immediate postoperatory of patients undergoing a CRS-HIPEC surgery.

**METHODS.** Observational and retrospectively all the patients admitted in a polyvalent ICU during the immediate postoperatory of a CRS-HIPEC surgery, between the years 2016 and 2017, were collected. All the patients were treated following a protocol planified in advance that included agressive intravenous hydratation to maintain a diuresis of 3-4 ml/kg/h, albumin administration and early start of parenteral nutrition.

**RESULTS.** A total of 39 patients were analized, 64.1% women, with a mean age of 57.5±10.52 years. The most common types of cancer were digestive in the 87.2% and primary peritoneal in the 12.8% of the patients, with an average PCI of 8.43±7.37.

Most of the procedures were realized in surgical second look, a 94.39%. The complete cytoreduction (CC-0/1) was achieved in the 97.4%.

Hyperthermic intraperitoneal chemotherapy was administrated adjusted to corporal surface, in digestive tumors with Oxaliplatin 460mg/m2, 31.2% of them with dose reduction of 400-430mg/m2; and in the primary peritoneal tumors with Cisplatin 75mg/m2.

The most frequent complications were the cytopenias (87.7%), presenting anemia in the 79.5%, trombopenia in the 53.8% and neutropenia in the 15.4%. We didn't find a relationship within the different types of chemotherapy nor the doses.

According to KDIGO criteria, AKI was presented in 7.7% of the patients, of which 66.3% were stage 1 and 33.7% stage 2, and none of them needed substitutive renal therapy.

Other severe complications occurred in the 35.9% of the patients, such as ARDS, pericardial effusion, pancreatitis, rebleeding, septic shock, paralytic ileus and hepatitis.

The mean ICU stay was 4±3.86 days with a mean of 0.5±3.20 days of mechanical ventilation, and a global intra ICU mortality of 3.2% (only 1 patient due to ARDS).

**CONCLUSION.** Although the rate of complications after this procedure remains high, we find our rate of AKI much lower than in other series published, a low mortality and stay in the ICU due to the procedure, what we relate to our protocolized postoperatory approach.

### 1198

#### Monitoring of mitochondrial oxygen tension in the operating theatre: first experiences with the novel COMET monitor

M. Wefers Bettink, R. Ubbink, R. Jansen, T. van der Ende, F. Harms, B. Mik
MC University Medical Center, Anesthesiology, Rottedam, Netherlands
**Correspondence:** M. Wefers Bettink
*Intensive Care Medicine Experimental* 2018, **6(Suppl 2):**1198

**INTRODUCTION.** A novel non-invasive method of measuring mitochondrial oxygenation (mito$PO_2$) in the skin has recently been implemented in the COMET (Cellular Oxygen METabolism) monitor (1). The mito$PO_2$reflects the balance between oxygen supply and demand.

For example, in a preclinical study mito$PO_2$appeared to be a potential parameter for personalized blood transfusion trigger (2).

**OBJECTIVE.** The COMET monitor was applied for the first time in the operating theatre during surgery under general anesthesia to test the feasibility of the measuring concept and the stability and response of mito$PO_2$during general anesthesia.

**METHODS.** A single centre observational study in 16 patients undergoing neuro surgery. The study was approved by the IRB of the Erasmus MC. After informed consent, pre-operatively a plaster containing 5-aminoluvelinic acid was placed on the sternum 5-8 hrs before induction of general anesthesia, to induce mitochondrial protoporphyrin IX for measurement of oxygen-dependent delayed fluorescence. Mito$PO_2$was measured at a rate of 1 measurement every 5 minutes using the COMET (Photonics Healthcare B.V., Utrecht, The Netherlands). Additionally, micro-vascular haemoglobin saturationand flow in microcirculation were measured with the O2C (LEA Medizintechnik, Giessen, Germany). For standardisation purposes the start of surgery was chosen as first time point in the analysis.

**RESULTS.** The first 6 inclusions let to a software update to solve signal instability due to excessive infrared light in surgical lamps, 2 were excluded because of unavailability of the O2C monitor, 1 patient signal quality was insufficient. In the remaining 7 patients, ASA 1-2 and 56,5± 10,8 years of age, mean arterial pressure at start surgery was 78,3 ± 10,4 mmHg and remained above 60 mmHg. No significant blood loss or hemodynamic alterations were observed. Mito$PO_2$ started at 57,1 ± 16,5 mmHg andstayed stable in 5 out the of 7 patients, figure 1A. In patient 5 (p5) a decrease of mito$PO_2$of -30 mmHg was seen form baseline in half an hour, in correlation with a change in probe temperature, Figure 1B. In patient 6 (p6) a decrease in mito$PO_2$was seen followed by a normalization of mito$PO_2$at the end of surgery. This change in mito$PO_2$was correlated with a change in microvascular blood flow in the skin, Figure 1C.

**CONCLUSION.** COMET was able, after a software update, to measure $mitoPO_2$ in patients in the operating theatre. No significant changes of $mitoPO_2$were measured when the patients were stable in both hemodynamics and temperature.

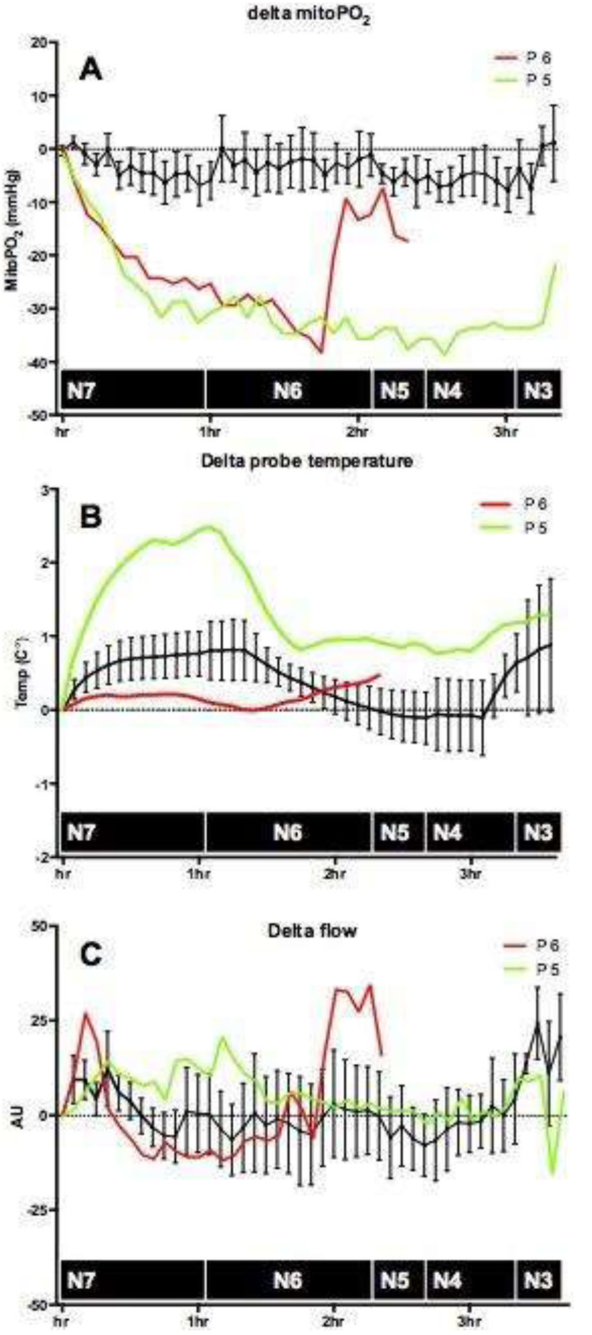

**Fig. 1 (abstract 1198).** Mitochondrial and microvascular parameters Fig 1A. Delta mitochondrial oxygen concentration ($mitoPO_2$). 1B Delta probe temperature. 1C Delta microvascular flow in the skin. Delta values were determined compared to the baseline value at the start of surgery Mean ± SEM for grouped values (N =5). The N-number gradually declines because of different durations of surgery

### 1199

#### Intensive care unit management, early morbidity and mortality of patients following cytoreductive surgery with hyperthermic intraperitoneal chemotherapy

A.R. Monteiro[1,2], A. Cruz[3], M. Peyroteo[4], A. Martins[5], B. Pereira[5], F. Coelho[5], J. Abreu de Sousa[4], F. Faria[5]

[1]Portuguese Institute of Oncology of Coimbra, Medical Oncology, Coimbra, Portugal; [2]Portuguese Institute of Oncology of Porto, Porto, Portugal; [3]Portuguese Institute of Oncology of Porto, Medical Oncology, Porto, Portugal; [4]Portuguese Institute of Oncology of Porto, General Surgery, Porto, Portugal; [5]Portuguese Institute of Oncology of Porto, Intensive Care, Porto, Portugal

**Correspondence:** A.R. Monteiro

*Intensive Care Medicine Experimental* 2018, **6(Suppl 2):**1199

**INTRODUCTION.** Cytoreductive surgery (CRS) with hyperthermic intraperitoneal chemotherapy (HIPEC) is an emerging treatment option for selected patients with peritoneal carcinomatosis. It has been linked to high rates of morbidity (12-15%) and mortality (1-5.8%) and patients are frequently admitted to an Intensive Care Unit (ICU) following it.
**OBJECTIVES.** To study the patients admitted to an ICU after CRS with HIPEC, its morbidity and mortality.
**METHODS.** Retrospective observational study performed in an Oncological ICU. All patients admitted in and between 2013 and 2017 after CRS with HIPEC or within one month were included. Data analysis was performed using descriptive statistics and hypothesis tests.
**RESULTS.** The study included 111 patients, accounting for 6.4% of all admissions to the ICU; 65.8% were female and the median age was 61 years. The primary tumors were located in the appendix (38.7%), colon/rectum (36%), ovary (15.3%), stomach (5.4%), peritoneum (3.6%) and jejunum (0.9%). Median Peritoneal Cancer Index (PCI) was 15. APACHE II and SAPS II mean score were 10.3 and 28.4.
Intra-abdominal complications were present in 7.2%: peritonitis and abdominal sepsis (4.5%), deep wound infection (1.8%), anastomotic dehiscence (1.8%), gastro-intestinal leakage (1.8%), intra-abdominal hematoma (1.8%) and ileus (0.9%). These complications were observed more often in men than women ($p=0.02$), and 37.5% occurred in patients with gastric cancer - 50% of all patient with gastric cancer developed complications. There were no statistically significant differences in mean age ($p=0.478$) and mean PCI ($p=0.271$) between the patients that developed intra-abdominal complications and the ones that did not.
Infections other than intra-abdominal occurred in 5.4% of patients: respiratory (3.6%), genitourinary (1.8%), central venous catheter-related bacteremia (1.8%), skin and soft tissue (0.9%), infective endocarditis (0.9%) and bacteremia (0.9%). Septic shock with multiple organ dysfunctions was present in 6.3% and hypovolemic shock in 3.6%. Acute respiratory failure was seen in 44.1% and 9% needed mechanical ventilation.
Surgical reintervention was performed in 4.5%. Median ICU length of stay was 2 days, readimission rate was 6.3% and mortality was 1.8%.
**CONCLUSIONS.** In our study, 50% of patients with gastric cancer had intra-abdominal complications. Conclusions are difficult to draw because of all patients only 6 had gastric cancer, but there seems to be a tendency for complications in this group. Overall, during ICU stay, morbidity with intra-abdominal complications and other infections was low (9%) and so was mortality (1.2%). These findings and the long survivals associated with the procedure suggest its benefit in the management of well-selected patients.

### 1200

#### The influence of pre-operative physical status on morbidity and mortality of patients undergoing major pancreatic surgery

D. Cigolini[1], K. Donadello[1], R. Salvia[1], V. Schweiger[1], W. Mosaner[2], C. Bassi[1], E. Polati[1]

[1]University of Verona, Department of Surgery, Dentistry, Pediatrics and Gynaecology, Verona, Italy; [2]Peschiera Pederzoli Clinic, Verona, Italy

**Correspondence:** K. Donadello

*Intensive Care Medicine Experimental* 2018, **6(Suppl 2):**1200

**INTRODUCTION.** Preoperative risk stratification can be useful in choosing the whole therapeutical strategies for patients(pts) with pancreatic disease and allows to calculate the risk-benefit balance of surgery. A prediction of the postoperative risk is fundamental for planning the post-operative measures to be taken.

**OBJECTIVES.** The aim of our study was to identify the more important pre-operative variables that affect pts outcome after major pancreatic surgery.

**METHODS.** We collected the data of pts operated for major pancreatic surgery during a 12-month period from two clinical database (University Hospital of Verona and Peschiera Pederzoli Clinic). Outcome data included incidence of postoperative complications, hospital mortality, ICU admission and days of hospitalization. The level of risk was determined by considering BMI, smoking habitus, presence of jaundice, pre-operative drainage of jaundice, recent chemiotherapy, anamnesis positive for cardiovascular, pulmonary, renal, neurological disease and an American Society of Anesthesiologist (ASA) score ≥3.

**RESULTS.** A total of 638 pts (320 women and 318 man, mean±sd age 61.41±12.52 years) were operated of major pancreatic surgery in the two hospitals. 18.8% of these patients were ASA ≥3 pts. ICU admission was 8.9%. Total post-operative complications were 57.6%, mortality was 2.2%. We observed abdominal complications and general complications (cardiac, respiratory, infectious and renal) in in 43.5%and 34.1%, respectively. Mean surgical duration was 332.81 ±106.05 minutes, and mean duration of hospitalizations was 15.94 ±17.83 days. ASA≥3 patients presented higher peri-operative complications and in-hospital mortality than low ASA group, (5.8%vs1.3%, p. < 0.01, chi-square test). Intraoperative blood losses, the need of ICU admission (26.7%vs4.8%, p.< 0.01) and the mean length of in-hospital stay (20.90±25vs14.81±15.55 days, p.< 0.01) were higher in high ASA group as well. The percentage of complication with DGE, biliary-fistula (7.1%vs 3.8%, p. < 0.01), mean duration of surgery, ischemic cardiovascular complications were higher in group with preoperative jaundice.

**CONCLUSIONS.** We found good correlation between ASA status and development of peri-operative general complications. Also intraoperative blood losses and length of hospitalization was well related to ASA status. Presence of pre-operative jaundice is strongly related to development of post-operative abdominal complications like enteric or biliary fistula. Overall mortality was higher in high ASA status group. Our data enforce the utility of the development of an easily applied scoring system with convincing accuracy for identifying high-risk patients, based on preoperatively assessable characteristics, which could be very useful in choosing the right therapeutic strategy, expecially for low-malignant risk lesions.

### 1201

#### Unbalanced fluid administration in liver transplant correlates with postoperative acidosis and chloride load: a single centre perspective

A.Y.K.C. Ng, A.W. Martinelli, R. Mahroof
Addenbrooke's Hospital, John V Farman Intensive Care Unit, Cambridge, United Kingdom
**Correspondence:** A.Y.K.C. Ng
*Intensive Care Medicine Experimental* 2018, **6(Suppl 2):**1201

**INTRODUCTION.** The choice of fluids in critically ill patients has been a long-debated subject, with recent evidence from SMART suggesting that the use of unbalanced fluids such as saline 0.9% is associated with hyperchloraemia, reduced plasma bicarbonate, & worse renal outcomes[1]. During liver transplants (LT), patients undergo complex physiological changes in the preanhepatic, anhepatic, & post-reperfusion phases[2], & these patients often require careful post-op ICU management. At our centre, LTs are a subset known to receive large volumes of fluid intra-op; majority being gelatine 4% (Gelofusine®) and saline 0.9%, in addition to blood products.

**OBJECTIVES.** We sought to investigate the association between intra-op fluid administration & post-op acid-base status, particularly post-op pH, chloride ($Cl^-$) levels & incidence of AKI.

**METHODS.** Medical records of adults (over 18yrs) who received a LT from 1 Jul to 31 Dec 2017 were identified by their contemporaneous electronic medical records (Epic Systems®, USA). Patients missing more than 10% of their intra-op records (due to any cause) were excluded.

We examined the relation to total fluid administration (Gelofusine & saline 0.9%) of 3 parameters: Post-op serum $Cl^-$ levels, intra-op change in pH (ΔpH = pre-op pH - post-op pH), & intra-op change in base excess (ΔBE = pre-op BE - post-op BE). The latter derivations were used to overcome the variability in pre-op pH. There was insufficient data on pre-op $Cl^-$ to calculate $\Delta Cl^-$. Data were plotted against each other for linear least-squares regression analysis, & correlation strength was assessed by means of a Pearson Rank Correlation test.

**RESULTS.** 58 patients were initially identified, & 2 were excluded due to insufficient intra-op data. The average age was 54.8 yrs (SD 10.1), & 34/56 were male. Most LTs were for chronic liver failure, with 5 being for acute liver failure. The mean volume of IV fluid given was 4.8L (SD 2.6L), which was exclusively Gelofusine & saline 0.9%. There was a significant positive correlation between intra-op fluid volume & post-op absolute chloride level (R=0.352, p=0.008), & a significant negative correlation between intraoperative fluid & ΔBE (R=-0.283, p=0.03), & ΔpH (R=-0.387, p=0.003). The incidence of AKI up to 30 days post-op was 64.3% in our series.

**CONCLUSIONS.** In this small single-centre retrospective study of LTs receiving high volumes of chloride-rich fluid, the total volume of unbalanced fluid administered was associated with a post-op lower pH, bigger base deficit & increased absolute serum $Cl^-$. Further study is required to establish associations with patient-centred outcomes in LTs.

**REFERENCE(S)**

1. Self WH, Semler MW, Wanderer JP, et al(2018). SMART. *NEJM*, *378*(9), 819-28.
2. Hall TH, & Dhir A (2013). Anesthesia for Liver Transplantation. *Semin Cardiothorac Vasc Anes*, *17*(3), 180-94.

### 1202

#### Cardiac output monitoring to guide fluid therapy in perioperative renal transplantation: results from a preliminary study

P. Gaspar da Costa, C. Candeias, I. Moniz, A. Santos, J. Nave, J.M. Ribeiro
Hospitalar de Lisboa Norte, EPE, Intensive Care Department, Lisbon, Portugal
**Correspondence:** P. Gaspar da Costa
*Intensive Care Medicine Experimental* 2018, **6(Suppl 2):**1202

**INTRODUCTION.** Delayed graft function in renal transplant patients might be affected by fluid therapy in the perioperative period. Cardiac output-directed strategies with minimally invasive hemodynamic monitoring has been recommended for fluid therapy guidance in critically ill patients. Fluids are a cornerstone for the management of kidney transplant patients, but overload should be avoided. There are, however, insufficient data to acknowledge the role of fluid administration guided by cardiac output monitoring in this specific population.

**OBJECTIVES.** Clinical evaluation of a strategy of perioperative fluid administration guided by minimally invasive hemodynamic monitoring and echocardiography in patients submitted to renal transplantation.

**METHODS.** 22-bed level III ICU, tertiary university-affiliated urban referral hospital. Outcomes from 34 kidney transplant recipients treated with a standard of care strategy, that included fluid administration guided by fluid balance and diuresis, were compared with outcomes from 19 patients prospectively treated by a clinical protocol that included fluid therapy guided by cardiac output monitoring with FloTrac™ and echocardiography.

**RESULTS.** Standard of care and protocol-driven patients had similar demographic and morbidity characteristics. Both groups had been transplanted by the same surgical team, and there were no differences regarding donor serum creatinine level, warm and cold ischemia periods. Cardiac index at admission was 4.3 ± 0.8 L/min/m$^2$. Patients from the protocol-driven group had a statistically significant higher SAPS II score (20.9 vs. 28.0; *p=0.001)*. Day 1 and day 3 cumulative fluid balance was higher in the standard of care group. Renal transplant outcomes did not differ, with similar incidence of graft survival and delayed graft function. Creatinine at 6 months showed no statistically significant difference. Standard of care patients showed statistical nonsignificant increased incidence of lung oedema, arterial hypertension requiring medication and haemodialysis; length of stay was similar.

**CONCLUSIONS.** To our knowledge, this is the first preliminary study comparing cardiac output monitoring to guide fluid therapy in renal transplant patients. We showed that a protocol-driven strategy to support fluid administration in these patients might be associated with less fluid overload when compared with standard of care. Furthermore, this restrictive fluid strategy does not compromise renal transplant outcomes at 6-month.

**GRANT ACKNOWLEDGMENT**
The study received no funding.

**Table 1 (abstract 1202).** See text for description.

| | Standard of Care (n=34) | Protocol-Driven (n=19) | p value |
|---|---|---|---|
| Fluid balance (D3) (mL) | 3342.7 ± 2165.8 | 1985.3 ± 2167.7 | 0.038 |
| Creatinine (1week) (mg/dL) | 3.3 ± 2.9 | 2.6 ± 2.1 | 0.338 |
| Creatinine (6 months) (mg/dL) | 1.5 ± 0.6 | 1.6 ± 0.5 | 0.609 |
| Length of stay (LOS) | 3.9 ± 1.3 | 4.5 ± 1.1 | 0,11 |
| Delayed graft function | 7 (20.6 %) | 3 (15.8 %) | 0.669 |
| Graft survival | 33 (97.1%) | 18 (94.7%) | 0.67 |
| Haemodialysis (ICU) | 4 (11.8%) | 0 (0%) | 0.12 |
| Hypertension | 21 (61.8 %) | 11 (57.9 %) | 0.782 |
| Lung oedema | 4 (11.8 %) | 1 (5.3 %) | 0.437 |

### 1203

#### Central venous-to-arterial carbon dioxide difference combined with arterial-to-venous oxygen content difference in the early postoperative care following liver transplantation

I. Saez de la Fuente[1], J. Saez de la Fuente[2], L. Orejon Garcia[1], M. Valiente Fernández[1], S. Chacón Alves[1], Z. Molina Collado[1], J.A. Sánchez Izquierdo[1], J.C. Montejo González[1]
[1]Hospital 12 de Octubre, Madrid, Spain; [2]Hospital Infanta Leonor, Madrid, Spain
**Correspondence:** I. Saez de la Fuente
*Intensive Care Medicine Experimental* 2018, **6(Suppl 2):**1203

**INTRODUCTION.** The early postoperative period after liver transplantation is characterized by a complex hemodynamic situation, due to the interaction of multiple factors, as the persistence of the hyperdynamic circulation or the increase of lactate blood levels because of the impaired hepatic clearance. In the present study, we addressed the question of whether the PcvaCO2/CavO2 ratio is useful in predicting the persistence of tissue hypoperfusion and anaerobic metabolism.

**OBJECTIVES.** To analyze the ability of the PcvaCO2/CavO2 ratio in the hemodynamic management after liver transplantation.

**METHODS.** Prospective observational study including all patients admitted to the ICU after liver transplantation over a 12-months study period. Demographic variables (sex, age, cause of transplantation and severity scores), hemodynamic variables, and those related to graft function and postoperative complications were recorded. Patients were classified according to the existence of a PcvaCO2/CavO2 ratio over 1.4 over the first 6 hours after the liver transplantation.

Data statistical analysis was done using SPSS software. Quantitative variables are expressed as mean with standard deviation (SD) and qualitative variables as absolute and relative frequencies. The statistical significance of the proportion comparison was addressed by the chi-square test, Mann Whitney U test or Student´s t-Test with a statistical significance $p < 0.05$.

**RESULTS.** During the study period, 45 patients were admitted to the ICU after a liver transplantation (84% male) with a mean age of 56 ± 9 years. The main causes of the liver transplant were alcoholism (53%), hepatocellular carcinoma (44,4%) and hepatitis C virus infection (44,4%). Mean MELD score was 16 ± 9; Median ICU stay was 6 days (IQR 4-10), with an overall ICU mortality of 13%.

Twenty one patients presented early tissue hypoperfusion defined as a PcvaCO2/CavO2 ratio over 1.4 during the first 6 hours after the liver transplantation; When compared with patients with a PcvaCO2/CavO2 ratio under 1.4, they presented significantly higher mean SOFA score at 24 hours (7,68+/-4,3 vs 4,5+/-1,5, p=0.008).

Non-significant differences were observed comparing reintubation rate (4 vs 1; p=0,409), prolonged mechanical ventilation (5 vs 0;p= 0,67), acute renal failure incidence (14 vs 6;p=0.34) or ICU stay (6 vs 5; p=0,076).

**CONCLUSIONS.** In the early postoperative care after liver transplantation, the PCVACO2 gap is a feasible hemodynamic parameter that could predict a worse clinical outcome.

**REFERENCE(S)**

Mesquida J, Saludes P, Gruartmoner G, et al. Central venous-to-arterial carbon dioxide difference combined with arterial-to-venous oxygen content difference is associated with lactate evolution in the hemodynamic resuscitation process in early septic shock. Critical care 2015;19:126.

**GRANT ACKNOWLEDGMENT**
None

### 1204

#### Impact of vascular procedures on coagulation and fibrinolysis

K. Polok[1], J. Górka[1], T. Iwaniec[2], J. Fronczek[1], W. Szczeklik[1]
[1]Jagiellonian University Medical College, Department of Intensive Care and Perioperative Medicine, Kraków, Poland; [2]Jagiellonian University Medical College, 2nd Department of Internal Medicine, Kraków, Poland
**Correspondence:** K. Polok
*Intensive Care Medicine Experimental* 2018, **6(Suppl 2):**1204

**INTRODUCTION.** Every year more than 200 million noncardiac surgeries are performed around the world and approximately 5-10% of them are vascular procedures. They are associated with high risk of perioperative complications, both arterial and venous.[1] These complications are closely related to activity of coagulation and fibrinolysis systems. Unfortunately, reliable studies assessing influence of vascular procedures on mentioned systems are lacking.

**OBJECTIVES.** Main goal of our study was to assess the impact of vascular procedures on activity of hemostatic and fibrinolytic pathways.

**METHODS.** We enrolled 29 patients ≥45 years old undergoing surgery for abdominal aortic aneurysm or peripheral artery disease under general or regional anesthesia, hospitalized at least one night after the procedure. Patients undergoing carotid artery surgery and those who had bypass graft thrombosis, cancer, renal failure defined as eGFR < 30 ml/min/1.73 m$^2$, venous thromboembolism 3 months prior to surgery or acute infection were excluded from the study. We measured levels of markers of hemostasis (Factor VIII, von Willebrand Factor:CoR, antithrombin), fibrinolysis (D-dimer, tissue plasminogen activator, plasmin-antiplasmin complexes) and soluble CD40 ligand levels before and 6-12h after vascular procedure.

**RESULTS.** Significant differences between preoperative and postoperative levels of FVIII (152.1 vs. 117.6 %, p< 0.001), vWF:CoR (138.5 vs. 158.1 %, p=0.045), antithrombin (92.1 vs. 74.9 %, p< 0.001), D-dimer (889.0 vs. 1897.0 ng/ml, p=0.002), tPA (9.7 vs. 13.0 ng/ml, p=0.001) and sCD40L (9064.3 vs. 1786.7 pg/ml, p< 0.001) were observed. There were no significant differences between pre- and postoperative levels of plasmin-antiplasmin complexes (738.2 vs. 829.5 ng/ml, p=0.38).

**CONCLUSIONS.** Vascular surgery leads to activation of hemostatic system but its effect on fibrinolytic pathway remains unclear. Further studies utilizing dynamic methods of coagulation and fibrinolysis assessment, performed on larger study group are necessary to precisely address this issue.

**REFERENCE(S)**

1. Górka J, Polok K, Fronczek J, Górka K, Kózka M, Iwaszczuk P, et al. Myocardial Injury is More Common than Deep Venous Thrombosis after Vascular Surgery and is Associated with a High One Year Mortality Risk. Eur J Vasc Endovasc Surg 2018. In press

**GRANT ACKNOWLEDGMENT**

This work was supported by the grant from the Polish Ministry of Science and Higher Education, no: NN402083939 and DI201102141.

### 1205

#### Assessment of concordance between real life venous thromboembolism prophylaxis and Caprini method in different types of intensive care units

M. Masjedi[1], F. Zand[2], G. Sabetian[3], B. Maghsoudi[2], R. Chegini[3]
[1]Anesthesiology and Critical Care Research Center, Shiraz University of Medical Sciences, Shiraz, Iran, Islamic Republic of; [2]Anesthesiology and Critical Care Research Center, Shiraz University of Medical Sciences, Anesthesia and Intensive Care, Shiraz, Iran, Islamic Republic of; [3]Shiraz University of Medical Sciences, Shiraz, Iran, Islamic Republic of
**Correspondence:** M. Masjedi
*Intensive Care Medicine Experimental* 2018, **6(Suppl 2):**1205

**Withdrawn**

## Monitoring tool in sepsis

### 1206

#### Effect of hyperbaric oxygenation on red blood cells deformability

F. Steenebruggen[1], D. Jacobs[2], K. Zouaoui[3], C. Delporte[4], P. Van Antwerpen[4], P. Biston[1], M. Piagnerelli[1]
[1]Université Libre de Bruxelles, Intensive Care Unit, Charleroi, Belgium; [2]Université Libre de Bruxelles, Anesthesiology, Charleroi, Belgium; [3]Université Libre de Bruxelles, Experimental Medicine Laboratory, Charleroi, Belgium; [4]Université Libre de Bruxelles, Faculty of Pharmacy, Brussels, Belgium
**Correspondence:** F. Steenebruggen
*Intensive Care Medicine Experimental* 2018, **6(Suppl 2):**1206

**INTRODUCTION.** Red blood cells (RBC) are one of the key element of the microcirculation (1). The ability to pass through capillaries and to deliver oxygen to cells, is due to its extreme capacity of deformability (2). Nevertheless, alterations in RBC deformability are observed in several diseases like in sepsis, and could contribute of the altered microcirculation observed in these pathologies (2). The hyperbaric oxygen therapy (HBOT) with inhalation of 100% oxygen is proposed in several acute or chronic pathologies including carbon monoxide poisoning.

**OBJECTIVE.** We investigated the effects of HBOT on RBC deformability in healthy volunteers (n= 10), in patients with acute carbon monoxide poisoning (n= 10) and in patients with acute or chronic inflammation (n=10).

**METHODS.** After approval by the ethical committee, RBCs deformability was assessed before and after HBOT by ektacytometry technique (Laser-assisted Optical Rotational Red Cell Analyzer - LORRCA) in the various populations. Elongation index (EI) was defined as (L-W)/(L+W), where L is the length and W is the width. At constant temperature of 37°C, EI values were determined in the function of shear stress (SS) in a range of 0.5 - 50 Pa, based upon the laser diffraction pattern changes. A higher EI indicates greater RBC deformation.

**RESULTS.** Before HBOT, EI was significantly lower for the majority of SS studied in patients with acute or chronic inflammation compared to healthy volunteers and patients with acute carbon monoxide poisoning.

HBOT improved significantly the EI only in patients with acute or chronic inflammation.

**CONCLUSIONS.** Our results confirmed the altered deformability in patients with inflammation. HBOT could improved it and perhaps by this way the microcirculation in this particular population. This link need to be confirmed in a larger population.

**REFERENCE(S)**

1. De Backer D and al. Microcirculatory alterations: potential mechanisms and implications for therapy, Annals of Intensive Care 2011, 1:27
2. Reggiori G et al. Early alterations of red blood cell rheology in critically ill patients. Crit Care Med. 2009 ; 37 : 3041-3046.

**GRANT ACKNOWLEDGMENT**

Commision Scientifique CHU-Charleroi.

### 1207

#### Advanced glycation end products and new-onset atrial fibrillation in sepsis

E. Rodriguez-Ruiz[1], R. Hernández-Vaquero[1], I. Granja Gómez[1], J.L. García Allut[1], A. López Lago[1], A. Estany Gestal[2], M.Á. García González[3]
[1]University Clinical Hospital of Santiago de Compostela, Critical Care & Intensive Care Medicine Department, Santiago de Compostela, Spain; [2]Fundación Ramón Domínguez, Unidad de Epidemiología e Investigaciones Sanitarias, Santiago de Compostela, Spain; [3]Health Research Institute of Santiago de Compostela (IDIS), Grupo de Genética y Biología del Desarrollo e las Enfermedades Renales, Santiago de Compostela, Spain
**Correspondence:** E. Rodriguez-Ruiz
*Intensive Care Medicine Experimental* 2018, **6(Suppl 2):**1207

**INTRODUCTION.** Sepsis has been shown to trigger atrial fibrillation (AF), the most common sustained arrhythmia in critically ill. A growing body of evidence indicates that inflammation and oxidative stress contribute to its pathogenesis. Advanced glycation end products (AGEs), stable products generated by non-enzymatic glycation of proteins and lipids, are driven by oxidative stress. AGEs are implicated in atrial fibrosis. New-onset AF (NAF), AF episode lasting > 30 seconds, has been associated with increased risk of in-hospital mortality. We wanted to investigate the implication of AGEs in sepsis-related NAF.

**OBJECTIVES.** Our aim was to determine the incidence of AF and NAF in patients with severe sepsis and septic shock, and to study their association with AGEs levels in plasma (pAGE) and 28-day mortality. Also, we wanted to analyze the relationship between NAF, medical history, MOF, severity scores and inflammatory mediators.

**METHODS.** We included 90 consecutive patients with the diagnosis of severe sepsis or septic shock according to 2001 Sepsis Consensus Conference. We checked in their medical history for age, sex, smoking habit, diabetes mellitus (DM), dyslipidaemia (DLP), arterial hypertension (aHT), chronic kidney disease (CKD), heart failure (HF) and myocardial infarction (AMI). At day 1, pAGE levels were measured by quantitative fluorescence spectroscopy. Also, we measured some inflammatory mediators: TNF-α, IL-1β, IL-6, IL-8, CRP and PCT. Moreover, Pro-BNP levels at admission were measured. APACHE-II was calculated at admission; meanwhile, daily SOFA was calculated during the first five days. 28-day mortality was assessed during the follow-up. Chi-square was performed to determine the relation between AF or NAF and sex, medical conditions and 28-day mortality. Mann-Whitney U was applied to study the association between AF or NAF and pAGEs, inflammatory mediators, Pro-BNP, APACHE-II and SOFA.

**RESULTS.** In our cohort of septic patients (mean age 68±13), we detected AF in 11 patients (12,2%) at admission and 79 patients (87,8%) were in sinus rhythm (SR). From those in SR, 13 patients (16,5%) developed NAF during the septic process. Significant association was found between AF at admission and pAGE (p=0.013), but not between NAF and pAGE. We found that NAF was more frequent in patients with septic shock (p=0.032) compared with those with severe sepsis. NAF was also associated with MOF (SOFA day1: p=0.022, SOFA max: p=0.004). No relationship was found between age, sex, smoking habit, DM, DLP, aHT, CKD, HF, AMI, APACHE II score, inflammatory biomarkers, NT-Pro-BNP and NAF. We could not find association between NAF and 28-day mortality (p = 0,428).

**CONCLUSIONS.** In this study, sepsis-related NAF showed a high incidence. pAGE was associated with AF at admission, but not with NAF. NAF was more frequent in septic shock and MOF, but we could not find association between NAF and 28-day mortality.

### 1208

#### Evaluation of preload functional status and cardiac output in sepsis resuscitation

I. Douglas[1], P. Alapat[2], K. Corl[3], M. Exline[4], L. Forni[5], A. Holder[6], D. Kaufman[7], A. Khan[8], M. Levy[3], G. Martin[6], J. Sahatjian[9], E. Seely[10], W. Self[11], N. Shapiro[12], M. Williams[13], C. Winterbottom[14], D. Hansell[15]
[1]Denver Health Hospital, Denver, United States; [2]Ben Taub Hospital, Houston, United States; [3]Rhode Island Hospital, Providence, United States; [4]Ohio State University, Columbus, United States; [5]Royal Surrey Hospital, Guilford, United Kingdom; [6]Grady Memorial Hospital, Atlanta, United States; [7]New York University, New York, United States; [8]Oregon Health and Sciences University, Portland, United States; [9]Cheetah Medical, Newton, United States; [10]University of California San Francisco Medical Center, San Francisco, United States; [11]Vanderbilt University, Nashville, United States; [12]Beth Israel Deaconess Medical Center, Harvard Medical School, Boston, United States; [13]Indiana University, Indianapolis, United States; [14]Bridgeport Hospital, Bridgeport, United States; [15]Massachusetts General Hospital, Boston, United States
**Correspondence:** I. Douglas
*Intensive Care Medicine Experimental* 2018, **6(Suppl 2):**1208

**INTRODUCTION.** Cardiac function is known to be negatively impacted by sepsis. Stroke volume change in response to Passive Leg Raise (PLR) is an effective method to predict fluid responsiveness (FR) or cardiac response to preload expansion.

**OBJECTIVES.** To evaluate preload functional status and trending cardiac output to identify patient phenotypes with varying cardiac reserve, dysfunction and outcome.

**METHODS.** Patient data were analyzed from a currently enrolling prospective randomized controlled study, evaluating the incidence of FR in critically ill patients with sepsis or septic shock (FRESH study, NCT02837731). Patients randomized to PLR guided resuscitation were classified as PLR+ (fluid responsive / preload dependent) if stroke volume (SV) increased ≥ 10% when measured with a non-invasive bioreactance device (Starling SV, Cheetah Medical). Independent evaluators categorized patients into 5 different phenotypic cohorts based on changing physiology exhibited on PLR and trending cardiac output over the initial 72 hours of therapy. Groups 1 (initial PLR +) and 2 (initial PLR-) both demonstrated fluid responsiveness and improvement in CO over the 72 hour assessment period. In contrast, Groups 3-5 did not exhibit an overall improvement in CO regardless of initial PLR response.

**RESULTS.** A total of 311 PLR assessments were performed in 43 patients. Overall, 39% (122/311) of assessments demonstrated PLR positive responses after receiving initial resuscitation fluid of 2.2 (+/- 0.6) L. There were no differences among the 5 groups with respect to age, gender, or QSOFA score (**Table 1**). Patients within the 5 groups exhibited distinctly different patterns of fluid responsiveness (**Table 2**). Fluid responsiveness was dynamic, changing over the 72 hours of the study. Significant differences were noted in the incidence of fluid responsiveness between patient groups on Days 1-3 of treatment (p< 0.0001). Notably, patients in Groups 3-5 exhibited a higher incidence of a negative change in stroke volume with PLR (22%) compared to patients in Groups 1&2 (11%, p < 0.0001). Groups 1 &2 had a significantly shorter ICU stay (102 hours +/- 56 SEM) compared with Groups 3-5 (263 hours +/- 43 SEM, p=0.017) (**Figure 1**). Additionally, Groups 1&2 exhibited a notable but non-significant reduction in 30 day mortality (6.7%) compared with Groups 3-5 (28.6%,p=0.09).

**CONCLUSIONS.** Short-term sepsis resuscitation phenotypes based on SV and CO responses to PLR identify discrete and significantly different patient sub-groups. Patients who are FR, and augment CO in response to resuscitation experienced decreased length of stay and a trend to decreased mortality. A negative change in SV in response to PLR indicates impaired cardiac function that should prompt further early evaluation. Periodic dynamic monitoring may predict patient outcome and guide treatment for sepsis-associated cardiac dysfunction.

**GRANT ACKNOWLEDGMENT**
The FRESH study is funded by Cheetah Medical

**Table 1 (abstract 1208).** Phenotype Cardiac Output Groups

| Phenotype Cardiac Output Groups | 72 Hr Assessment |
|---|---|
| Group 1: PLR+ stays PLR+, CO Improved | 19% |
| Group 2: PLR- becomes PLR+, CO Improved | 16% |
| Group 3: PLR- becomes PLR+, CO Not Improved | 30% |
| Group 4: PLR- stays PLR- CO Not Improved | 14% |
| Group 5:PLR+ CO Not Improved | 21% |

**Table 2 (abstract 1208).** Fluid Responsivness by Patient Phenotype and Day

| Variable | Group 1 | Group 2 | Group 3 | Group 4 | Group 5 | Overall | p-value |
|---|---|---|---|---|---|---|---|
| Fluid Resp. (delta SVI >= 10%) | 65.85% (27/41) | 33.33% (16/48) | 37.59% (50/133) | 2.78% (1/36) | 56.00% (28/50) | 39.23% (122/311) | <.0001 |
| PLR Negative (delta SVI < 0) | 2.44% (1/41) | 18.75% (9/48) | 14.29% (19/133) | 63.89% (23/36) | 12.00% (6/50) | 18.97% (59/311) | <.0001 |
| Fluid Resp. (delta SVI >= 10%) Day 1 | 68.18% (15/22) | 23.53% (4/17) | 30.00% (12/40) | 0% | 73.33% (11/15) | 39.62% (42/106) | <.0001 |
| Fluid Resp. (delta SVI >= 10%) Day 2 | 66.67% (10/15) | 26.09% (6/23) | 42.86% (27/63) | 0% | 40.00% (8/20) | 37.23% (51/137) | 0.0011 |
| Fluid Resp. (delta SVI >= 10%) Day 3 | 33.33% (1/3) | 75.00% (6/8) | 34.78% (8/23) | 14.29% (1/7) | 70.00% (7/10) | 45.10% (23/51) | 0.0507 |

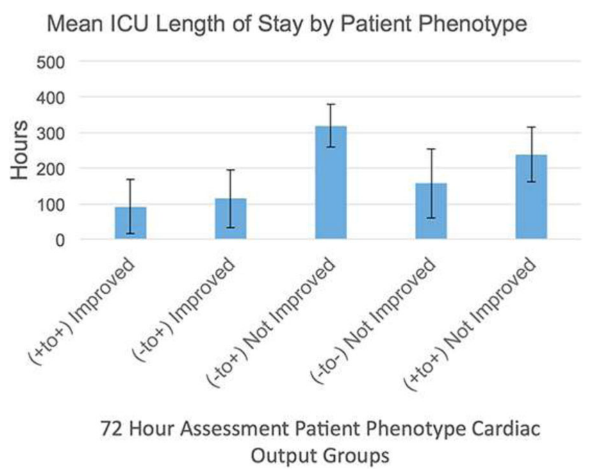

**Fig. 1 (abstract 1208).** ICU Length of Stay by Patient Phenotype

### 1209

#### Bedside echocardiography to predict 28-day mortality in sepsis: the value of day 1 MAPSE

P. Cavaleiro, S. Castro, S. Pina, T. Salero, C. Granja
Hospitalar do Algarve - Hospital de Faro, Intensive Care Department, Faro, Portugal
**Correspondence:** P. Cavaleiro
*Intensive Care Medicine Experimental* 2018, **6(Suppl 2):**1209

**INTRODUCTION:** Sepsis-induced myocardial dysfunction is present in over 40% of all cases and, when present, mortality rates are increased up to 70%. Several patterns of dysfunction may be present[1]. Mitral annulus plane systolic excursion (MAPSE) is an echocardiographic marker of left ventricle (LV) longitudinal function that correlates well with other markers of global LV function[2]. It can be obtained from patients with sub-optimal acoustic windows, by operators with limited training and is easily reproduced[3]. It has also been shown in a small study than shock non-survivors have lower MAPSE on day 1, when compared with survivors[2].

**OBJECTIVES:** The purpose of this study was to evaluate the correlation of MAPSE measurements obtained in the first 24h of ICU admission with 28-day all-cause mortality and whether it has prognostic value in septic patients.

**METHODS:** We prospectively enrolled consecutive adult patients with sepsis admitted to our general ICU. We collected demographic and medical data and performed echocardiography examinations during the first 24h of sepsis evolution, after initial resuscitation. Measurements obtained were averaged over 3 consecutive beats (or 10 beats if atrial fibrillation was present).

Univariate and multivariable analyses were used to assess the relationships between variables and 28-day mortality. Two different operators collected data and a reproducibility evaluation using Bland-Altman analysis was performed.

**RESULTS:** Eighty-five patients (67.1 ± 16.4 years; 58 men) were enrolled (tables 1 and 2). All-cause 28-day mortality was 35.3%.

When comparing survivors with non-survivors, we found significant differences in MAPSE ($p = 0.006$), SAPS II ($p < 0.001$) and SOFA ($p = 0.002$) scores. We categorized MAPSE in 3 categories (corresponding to tertiles: MAPSE < 11.5; 11.5 - 14.2; > 14.2 mm). In a multivariate model, including both scores, we identified SAPS II (adjusted OR 1.1, 95% CI 1.03 - 1.14) and lower MAPSE as predictors for mortality at 28 days, when compared to higher MAPSE (adjusted OR 5.3, 95% CI 1.24 - 22.46).

Bland-Altman analysis yielded a bias below 0.5 mm and limits of agreement of ± 0.76 mm.

**CONCLUSIONS:** Sepsis patients with lower day 1 MAPSE (< 11.5 mm) may have a significant higher risk of mortality than those with higher MAPSE, even when adjusted for severity of illness.

GRANT ACKNOWLEDMENT
None.

**Table 1 (abstract 1209).** Demographic and infection data

| Characteristics | All patients (n = 85) | Survivors (n = 55) | Non-survivors (n = 30) |
|---|---|---|---|
| Age (years), mean ± SD | 67.1 ± 16.4 | 65.8 ± 17.3 | 69.3 ± 14.7 |
| Male, n (%) | 58 (68.2) | 36 (65.5) | 22 (73.3) |
| Primary infection site, n (%) | | | |
| Intra-abdominal | 40 (47) | 23 (41.8) | 17 (56.7) |
| Lung | 30 (35.3) | 21 (38.2) | 9 (30) |
| Central nervous system | 5 (5.9) | 4 (7.3) | 1 (3.3) |
| Urinary tract | 4 (4.7) | 2 (3.6) | 2 (6.7) |
| Skin and soft tissue | 2 (2.4) | 2 (3.6) | 0 (0) |
| Other | 4 (4.7) | 3 (5.5) | 1 (3.3) |

**Table 2 (abstract 1209).** Echocardiography and clinical data

| Characteristics | All patients (n = 85) | Survivors (n = 55) | Non-survivors (n = 30) |
|---|---|---|---|
| Septic shock on admission, n (%) | 59 (69.4) | 24 (43.6) | 28 (9.3) |
| SAPS II score, mean ± SD | 48.9 ± 14.6 | 43.6 ± 13.8 | 58.5 ± 10.7 |
| SOFA score (day 1), mean ± SD | 9.2 ± 3.8 | 8.3 ± 3.8 | 10.9 ± 3.4 |
| Echocardiographic data | | | |
| Presence of SAM, n (%) | 4 (4.7) | 1 (1.8) | 3 (11) |
| MAPSE (mm), median [P25; P75] | 12.9 [10.8; 14.7] | 13.2 [12.1; 14.8] | 11.0 [9.3; 14.3] |
| TAPSE (mm), median [P25; P75] | 17.3 [14.6; 20.9] | 18.0 [15.1; 21.5] | 16.9 [13.6; 18.6] |

### 1210

#### The role of platelet aggregometry in predicting sepsis-induced thrombocytopenia

A. Fogagnolo[1], F.S. Taccone[2], G. Montanari[1], B. Capatti[1], G. Ferraro[1], G. Santoro[1], C.A. Volta[1], S. Spadaro[1]
[1]Università di Ferrara, Ferrara, Italy; [2]Université Libre de Bruxelles, Intensive Care, Bruxelles, Belgium
**Correspondence:** A. Fogagnolo
*Intensive Care Medicine Experimental* 2018, **6(Suppl 2):**1210

**INTRODUCTION.** Coagulation abnormalities and thrombocytopenia are common in sepsis, up to 40%.[1] Despite the clinical relevance of sepsis induced thrombocytopenia, few studies have focused on the prediction of thrombocytopenia in this setting.

**OBJECTIVES.** The aim of this study is to identify if platelets aggregometry can be useful in predicting sepsis induced thrombocytopenia in patients with septic shock and normal platelet count on the day of diagnosis.

**METHODS.** We enrolled consecutive patients with septic shock on ICU admission or with diagnosis of septic shock during ICU stay. Exclusion criteria were: age < 18 years, platelet count < $150*10^3$/mL; therapy with antiplatelet drugs within the 8 days from ICU admission. Aggregometry test were performed daily from admission to day 5 using adenosine diphosphate (ADP), arachidonic acid (AA) and thrombin receptor-activating peptide-6 (TRAP). Maximal rate of aggregation ($Agg_{max}$) was recorded for all tests. Sepsis-induced thrombocytopenia was defined as a platelet count < $150*10^3$/mL within 5 days, while moderate or severe thrombocytopenia as a platelet count < $100*10^3$/mL or $50*10^3$/mL, respectively. 90-day mortality and hemorrhagic complications were also collected.

**RESULTS.** A total of 26 patients have been included. (Table 1) Sepsis-induced thrombocytopenia occurred in 10 (38%) patients; of those, 6 (17%) developed moderate/severe thrombocytopenia. Time to thrombocytopenia was 3 [2-5] days. Daily changes in $Agg_{max}$ are shown in Figure 1. Patients with thrombocytopenia had lower $Agg_{max,}$ than others, regardless the agonists that were used, in particular when thrombocytopenia was moderate to severe. (Figure 2) Hemorrhagic complications occurred in 15% of patients and 90-day mortality was 35%. Patients with hemorrhagic complication had lower $Agg_{max}$ (ADP: 15.5 [5 - 47] vs 72 [65 - 78] %; p< 0.001 AA: 6.3 [3 - 18] vs 65 [54 - 74]; p=0.002; TRAP: 45 [23 - 60] vs 74 [62 - 78], p=0.004) but not lower platelets on admission (335 [214 - 377] vs 230 [176 - 368], p=0.47) than others. Also, non-survivors showed lower $Agg_{max}$ (ADP: 68.2 ± 14 % vs 46.2±20 %; p=0.02 AA: 56.9 ± 11% vs 31.7 ± 17%; p< 0.001 TRAP: 69.2±10% vs 58.3±15%; p=0.10) than others.

**CONCLUSIONS.** Altered aggregometry test is common in septic shock even with normal platelet count, in particular in patients who will develop sepsis-induced thrombocytopenia. Altered aggregometry can also have prognostic value for both hemorrhagic complication or 90-days mortality.

**REFERENCE(S)**

1. Drews RE, Weinberger SE: Thrombocytopenic disorders in critically ill patients. Am J Respir Crit Care Med 2000,162:347-351.

**Table 1 (abstract 1210).** Clinical and demographical characteristics of the patients

| Variable | Sepsis-induced Thrombocytopenia (n=10) | Normal platelet counts (n=16) | P value |
|---|---|---|---|
| Age, years | 64 ± 16 | 66 ± 17 | 0.85 |
| SAPS score | 49.0 ± 11.3 | 49.1 ± 11.5 | 0.97 |
| SOFA at admission | 7.0 ± 2.4 | 7.1 ± 2.1 | 0.90 |
| Highest SOFA | 7.0 ± 2.3 | 8.6 ± 2.6 | 0.25 |
| Platelets count at admission, $10^3$ $mcL^{-1}$ | 230 [179 – 326] | 253 [172 – 422] | 0.62 |
| Lowest platelets count, $10^3$ $mcL^{-1}$ | 98 [83 – 117] | 215 [160 – 333] | <0.001 |
| ADP day1, $Agg_{max}$, % | 52 ± 14 | 72 ± 8 | <0.001 |
| AA day1, $Agg_{max}$, % | 51 [8 – 65] | 67.7 [48 – 75.5] | <0.001 |
| TRAP day1, $Agg_{max}$, % | 60 [52 – 70] | 73 [62 – 78] | <0.001 |
| Lactate, mmol | 2.8 [2.1 – 4.9] | 2.3 [2.1 – 2.7] | 0.33 |
| Highest Procalcitonin, ng/mL | 40.6 [21.4 – 89.5] | 12.3 [6.2 – 31.3] | <0.001 |
| ICU LOS, days | 11.5 [8.7 – 20.7] | 10.5 [6 – 17] | 0.48 |
| Days of vasoactive drugs | 3 [2-5] | 1 [1 -3] | 0.01 |
| Spontaneous bleeding | 30% | 6% | 0.14 |
| 90-day mortality, % | 70% | 15% | 0.009 |

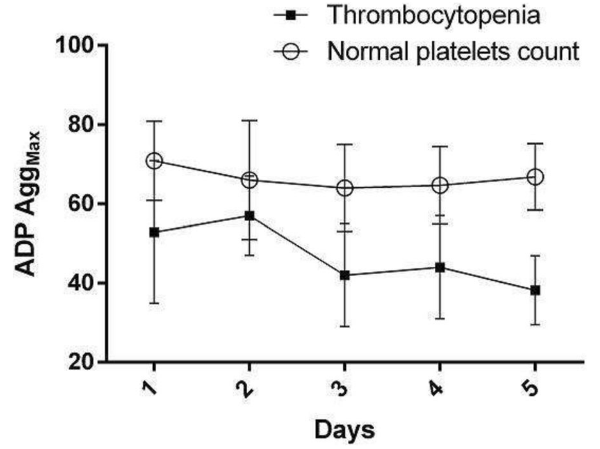

**Fig. 1 (abstract 1210).** See text for description

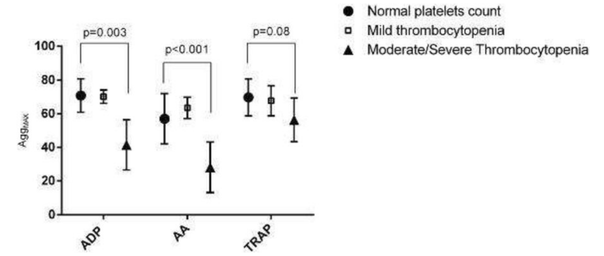

**Fig. 2 (abstract 1210).** See text for description

### 1211

### Acoustocerebrography in septic patients: first clinical results

M. Sauer, G. Richter, A. Sievert, T. Mencke, J. Ehler, P. Schmude, D.A. Reuter
University Hospital Rostock, Anesthesiology and Intensive Care Medicine, Rostock, Germany
**Correspondence:** M. Sauer

*Intensive Care Medicine Experimental* 2018, **6(Suppl 2):**1211

**INTRODUCTION.** The sepsis-associated encephalopathy (SAE), as clinical sign of acute brain injury in septic patients, has a high incidence and is associated with increased mortality (1). The diagnosis remains an exclusion-process; none of the available measurements are specific for SAE (1).

**OBJECTIVES.** To verify the possible role of molecular acoustic for determining injury of brain in septic patients using an acoustocerebrography (ACG)-system. The ACG is a transcranial ultrasonic method enabling the data interpretation of absorption coefficient, frequency dependent attenuation, speed of sound and tissue elasticity, recorded from the transmitted multispectral ultrasound signals propagating through the brain tissue (2).

**METHODS.** After approval from the local research ethics committee 20 patients were included in a prospective clinical study in two study-groups: septic shock group (SG) and control group (CG; postoperative non-septic patients). All patients were measured with the ACG-method at inclusion, after 3, 7 and 14 days and blood parameters of organ-function, as well SOFA-score and delirium scores (CAM-ICU, ICDSC) were obtained to the same time-points. At inclusion a neurologist examined all patients. The results of the ACG-measurements were compared with Pearson correlations of the dimensionality parameter with the principal components derived by the genetic algorithm with medical parameters.

**RESULTS.** All patients of the SG were CAM-ICU positive at inclusion (ICDSC: in mean 4.0) and had clinical signs of SAE (according to clinical examination of a neurologist); in contrast, all patients of the CG were CAM-ICU negative und the values of the ICDSC were zero to all time points. In the ACG measurements significant differences between the CG and SG were obtained. Correlation between ACG results and the SOFA- and ICDSC-scores (0.74 and 0.60) were seen. In addition, correlations between ACG results and some clinical parameters were also found: lactate (0.5), procalcitonin (0.5), creatinin (0.45) and bilirubin (0.6).

**CONCLUSIONS.** The ACG-method may helpful for the diagnosis of acute brain injury; however, the results of this first report should verify in further studies. Following investigations should include more classical instruments of SAE diagnosis, e.g. EEG, MRI and biomarkers to compare the results with the ACG-measurement.

(1) Sonneville R et al. Understanding brain dysfunction in sepsis. Ann Intensive Care 2013;3(1):15

(2) Dobkowska-Chudon W et al. Comparison of the magnetic resonance imaging and acoustocerebrography signals in the assessment of focal cerebral microangiopathic lesions in patients with asymptomatic atrial fibrillation. Hydroacoustics 2016;19:83—92

**GRANT ACKNOWLEDGMENT**

The medical faculty of the University of Rostock supported the study.

### 1212

#### Ultrasound evaluation of muscle wasting in patients with sepsis and prolonged respiratory support

J.F. Martínez Carmona, F.A. Hijano Muñoz, E. López Luque, J.M. Mora Ordóñez, M. Delgado Amaya, G. Quesada
Regional de Malaga, Intensive Care Unit, Málaga, Spain
**Correspondence:** J.F. Martínez Carmona
*Intensive Care Medicine Experimental* 2018, **6(Suppl 2):**1212

**INTRODUCTION.** Septic shock produces an aggression to the organism, presenting an increase of the muscular protein catabolism with rapid atrophy of the musculature in the first days.
**OBJECTIVES.** To assess muscle wasting in rectus femoris muscle in patients admitted to the ICU due to sepsis requiring mechanical ventilation during the first week of admission.
**METHODS.** Our sample includes 11 patients admitted to the ICU due to sepsis with respiratory support. We used ultrasound with 7.5MHz linear probe, assessing the rectus femoris muscle, measuring cross sectional area. Measurements are taken on day 1 and 7 after the start of respiratory support. Demographic variables, duration of mechanical ventilation, ICU and hospital stay, ICU and hospital mortality were collected.
**RESULTS.** Average age: 61.64 years. 72.7% males. 72.7% respiratory sepsis. MV Days: 16.8 days. Average stay in ICU: 28.36 days. When we analyzed muscle wasting measured in muscle rectus femoris, we found significant correlation (Spearman test p 0.005) with prolonged mechanical ventilation. We did not find significant differences with mortality, stay in ICU, APACHE II at admission or SOFA.
**CONCLUSIONS.** In our sample, we found a relationship between muscle wasting and prolonged mechanical ventilation. However, we did not find a significant relationship with mortality or ICU stay. It is a small sample, so a larger study is necessary to draw conclusions.

- Mueller et al. Can sarcopenia quantified by ultrasound of the rectus femoris muscle predict adverse outcome of surgical intensive care unit patients as well as frailty? A prospective, observational cohort study. Ann Surg 2015; xx: xxx-xxx.
- Paris et al. Assessment of skeletal muscle más in critically ill patients: considerations for the utility of computed tomography imagina and ultrasonography. Curr Opin Clin Nutr Metab Care 2016; 19: 125 - 130.
- Parry et al. Ultrasonography in the intensive care setting can be used to detect changed in the quality and quantity of muscle and is related to muscle strenght AND función. J Crit Care 2015; xxx-xxx.
- Puthucheary et al. Qualitative ultrasound in acute critical illness wasting. Crit Care Med 2015; 43: 1603 - 1611.

### 1213

#### MAP versus SBP - which parameter is more sensitive to determine an early decline in the clinical status of hospitalized patients with hypotension and comparison of hemodynamic characteristics of septic versus non-septic patients

T.T.H. Lui[1], A. Kansal[2], H.M.N. Poh[1], R. Freebairn[3]
[1]Ng Teng Fong General Hospital, Medical Informatics, Singapore, Singapore; [2]Ng Teng Fong General Hospital, Intensive Care Medicine, Singapore, Singapore; [3]The Chinese University of Hong Kong, Anaesthesia and Intensive Care, Hong Kong, Hong Kong, China
**Correspondence:** T.T.H. Lui
*Intensive Care Medicine Experimental* 2018, **6(Suppl 2):**1213

**INTRODUCTION.** Systolic blood pressure (SBP) has historically been used to score perfusion pressure in early warning systems (1), whilst mean arterial pressure (MAP) is used for sepsis definitions, is the determinant of organ perfusion, and the most accurate pressure measurement made by automated machines (2). There have been no studies to our knowledge addressing whether MAP or SBP is the more sensitive method of determining early decline of septic patients.
**OBJECTIVES.** This study aimed to review whether MAP or SBP is the more sensitive method of determining an early decline in the clinical status of septic patients referred to ICU.
**METHODS.**
Retrospective Chart review July 2015-June 2017
Mixed medical/surgical Intensive Care Unit, adult tertiary care hospital, Singapore.
Subjects: all patients fulfilling:
Inpatient referral to ICU and code blue events
Sepsis cohort identified by:
Keyword mining of ICU referral for "sepsis"
≥ 2 SIRS criteria
Intravenous antibiotics
Blood culture collected
Non-sepsis cohort:
no sepsis positive criteria
Blood pressure data:

- Time of ICU referral/code blue
- Hourly SBP/MAP upto 48 hours before referral
- In patients with both low SBP and MAP, the time relationship between MAP and SBP was defined by subtracting time of SBP hypotension event from time of MAP hypotension event (MAP-SBP).
- ICU & hospital length of stay, mortality
- Statistical analysis Excel, one-factor ANOVA alpha 0.05,$X^2$ test for independence.

**RESULTS.** From 2648 unique ICU referrals we found 212 septic and 218 non-septic patients for analysis.
Our study found hemodynamic compromise in severe sepsis is variable, with significant proportion of patients manifesting isolated SBP or MAP hypotension prior to ICU referral. Up to 15% of 'septic' patients experienced only MAP hypotension, occurring on average 11 hours before 'ICU referral event' (formal ICU referral or deterioration to the point of code blue), 6.8 hours earlier than patients with isolated SBP hypotension. Our data supports added clinical value in monitoring both MAP and SBP for septic patients at risk of deterioration for earlier ICU review.
In both cohorts, we found a majority (70-82%) of patients were referred for ICU review within 48hrs of admission. In late referrals (referred beyond 48hrs of admission), low MAP precedes low SBP by up to 4.68 hours in septic patients with SBP+MAP hypotension, a trend not evident in non-septic cohort.
**CONCLUSIONS.** In considering the above findings, we believe incorporating routine monitoring of MAP and automated MAP hypotension triggers would increase clinical sensitivity in identifying deteriorating septic patients, as well as earlier detection of deterioration, facilitating timely intervention including earlier escalation of care.

**Table 1 (abstract 1213).** Summary of average time from first hypotensive event to 'ICU referral event' for septic and non-septic cohorts. Combined SBP +MAP hypotension constitutes the majority of hypotensive patients in the 'sepsis' cohort (58%), while SBP only hypotension predominates in 'Non-sepsis cohort. In both 'sepsis' and 'non-sepsis' cohorts, a proportion of patients (from 14% up to 28%) manifested only the MAP hypotension.

| | Mean time to ICU referral following hypotension (hours) | | | | Counts | | | | |
|---|---|---|---|---|---|---|---|---|---|
| | SBP only | MAP only | SBP+MAP | MAP-SBP difference | SBP only | MAP only | SBP+MAP | Sum | Cohort size |
| Sepsis | 4.24 | 16.00 | 10.66 | 1.38 | 33 (28%) | 16 (14%) | 69 (58%) | 118 (56%) | **212** |
| Non-sepsis | 3.16 | 7.73 | 9.70 | -1.00 | 19 (48%) | 11 (28%) | 10 (24%) | 40 (18%) | **218** |

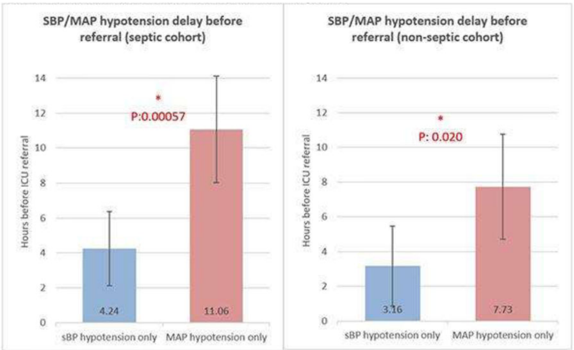

**Fig. 1 (abstract 1213).** See text for description

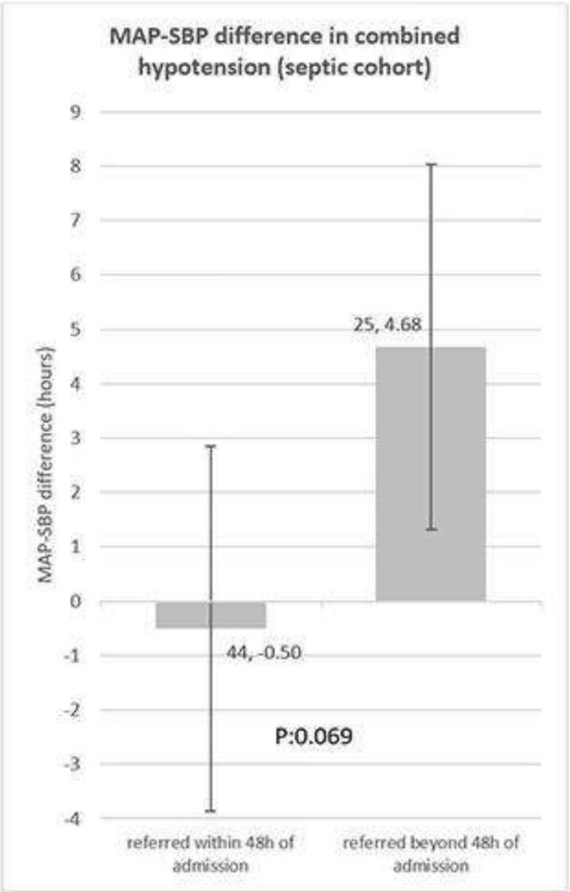

**Fig. 2 (abstract 1213).** See text for description

**Table 1 (abstract 1213).** Mortality rate is significantly higher for septic patients with isolated SBP hypotension compared with MAP hypotension and combined SBP+MAP hypotension. Hypotension and timing of referral show no associated differences in the non-septic cohort. test for independence

| Mortality Rate (septic cohort) | absent | present | relative risk | p.value |
|---|---|---|---|---|
| Hypotension | 13.83% | 22.88% | 1.65 | 0.094 |
| Early vs. Late referral | 19.46% | 17.46% | 0.90 | 0.73 |
| Type of Hypotension | SBP only (N:14) | MAP only (N:2) | SBP+MAP (N:11) | |
| | 42.42% | 12.50% | 15.94% | 0.0067 |
| (non-septic cohort) | | | | |
| Hypotension | 13.48% | 20.00% | 1.48 | 0.29 |
| Early vs. Late referral | 13.23% | 24.14% | 1.82 | 0.12 |
| Type of Hypotension | SBP only (N:5) | MAP only (N:0) | SBP+MAP (N:3) | |
| | 26.32% | 0.00% | 30.00% | 0.14 |

### 1214

#### Changes in initial distribution volume of glucose in endotoxin-induced septic pig models

T. Suganuma[1], E. Hashiba[2], M. Akaishi[1], J. Saito[1], K. Hirota[1]
[1]Hirosaki University Postgraduate School of Medicine, Department of Anesthesiology, Hirosaki, Japan; [2]Hirosaki University Hospital, Division of Intensive Care, Hirosaki, Japan
**Correspondence:** T. Suganuma
*Intensive Care Medicine Experimental* 2018, **6(Suppl 2):**1214

**INTRODUCTION.** Initial distribution volume of glucose (IDVG) indicates central extracellular fluid (ECF) volume and IDVG has been reported to be a surrogate indicator of cardiac preload in animal bleeding models and in patients in ICU[1, 2]. Patients with sepsis develop distributive shock due to vasodilation and increased vascular permeability. Fluids management for septic patients has been still controversial. It has been unknown whether IDVG in septic patients reflects cardiac preload or not.

**OBJECTIVES.** The aim of this study is to investigate changes in IDVG in endotoxin-induced pig septic models

**METHODS.** After obtaining an approval of Hirosaki University ethical committee, we anesthetized 13 pigs with ketamine, pentobarbital, remifentanil and vecronium. Tracheostomy was established and the lung was mechanically ventilated. Physiological saline solution had been infused through the experiment (4ml/kg/hr). Sepsis was induced with endotoxin (ET). We used PiCCO ® to measure CO, intrathoracic blood volume (ITBV) and so on. IDVG was measured with 2g glucose injection, as previously reported[3]. Five pigs were used to investigate chronological changes in IDVG following ET-injection (ET group) and three pigs were used for the control (C group). The other five pigs were used for fluid resuscitation with 30ml/kg Ringer's lactate solutions over 60 min, 4 hours after ET injection (RL-group). Dobutamine was continuously infused to maintain hemodynamics if needed. Hemodynamics, IDVG and other parameters were measured before and 1, 2, 4, 5 hours after ET injection for ET- and C-groups and before and 4, 5, 6, 7, 8 after ET injection for RL-group. Data are expressed as mean ± SD and the statistical analysis was performed with repeated measures of ANOVA, followed by Dunnett's test or Fisher's LSD test. P< 0.05 was considered as a significant.

**RESULTS.** Chronological changes in hemodynamics, IDVG index (=IDVG/Kg) and other parameters in both ET- and C-group were shown in table 1. Cardiac output (CO) and systolic BP significantly decreased and lactate levels significantly increased 4 hours after ET administration, suggesting that septic states were produced in pigs by ET infusion. Then, IDVG-index and ITBV significantly decreased, but CVP did not change 4 hours after ET. IDVG-index and CO were significantly increased with volume loading but those effects disappeared by the next one hour (Figure 1).

**CONCLUSIONS.** It was found that IDVG decreased in ET-induced septic state and increased with volume resuscitation. IDVG indicated the changes in the central ECF volumes and can be an indicator of cardiac preload in septic state.

### GRANT ACKNOWLEDGMENT

This study was supported by a Grant-in-Aid for Scientific Research (C) of Japan Society for the Promotion of Science (No.15K10529).

**Table 1 (abstract 1214).** Chronological changes in hemodynamics, IDVG and other parameters in septic/control pigs

| | | Time (hr) | | | | |
|---|---|---|---|---|---|---|
| | | Baseline | 1 | 2 | 4 | 5 |
| CO(L/min) | Control | 2.18±0.44 | 2.24±0.58 | 2.32±0.48 | 2.11±0.39 | 2.06±0.31 |
| | Sepsis | 1.67±0.28 | 1.61±0.31 | 1.22±0.14* | 0.95±0.39* | 0.84±0.22* |
| sBp (mmHg) | Control | 125.3±9.8 | 125.7±7.1 | 118.0±6.1 | 110.7±15.4 | 103.0±10.0 |
| | Sepsis | 121.8±12.3 | 100.0±7.9× | 77.4±21* | 68.8±9.0* | 71.6±9.6* |
| HR(bpm) | Control | 81.3±4.5 | 80.0±5.3 | 87.3±18.1 | 83.7±10.0 | 86.3±17.2 |
| | Sepsis | 119.6±50.9 | 157.2±25.5 | 155.2±39.6 | 166.4±38.1 | 159.8±41.1 |
| CVP(mmHg) | Control | 7.3±0.6 | 7.3±1.2 | 7.0±1.0 | 6.7±1.5 | 6.0±1.7 |
| | Sepsis | 6.2±0.4 | 6.6±0.9 | 6.6±1.5 | 6.8±2.0 | 7±2.3 |
| Lactate (mmol/L) | Control | 1.1±0.1 | 1.0±0.1 | 0.9±0.1 | 0.8±0.1 | 0.7±0.2 |
| | Sepsis | 0.9±0.3 | 1.4±0.5 | 1.7±0.7 | 1.7±0.8* | 1.9±0.6× |
| IDVG/BW(ml/kg) | Control | 121.5±32.2 | 122.0±28.8 | 123.0±31.7 | 119.2±35.9 | 124.1±36.1 |
| | Sepsis | 134.9±15.9 | 119.3±20.1* | 136.4±20.0 | 120.9±10.6* | 114.2±13.8* |
| ITBV (ml) | Control | 382.7±103.7 | 387.0±116.4 | 384.7±122.4 | 353.0±93.7 | 345.7±81.6 |
| | Sepsis | 209.2±78.4 | 201.7±88.4 | 175.7±20.0× | 158.2±87.9* | 150.1±77.5× |
| Hb(g/dl) | Control | 9.7±0.8 | 9.4±0.5 | 9.3±0.6 | 9.5±0.9 | 9.1±0.7 |
| | Sepsis | 9.8±0.8 | 11.1±1.2× | 10.8±1.0* | 12.0±0.8* | 12.0±1.0* |

Data are expressed as a mean ± SD, *P<0.05 vs Baseline values

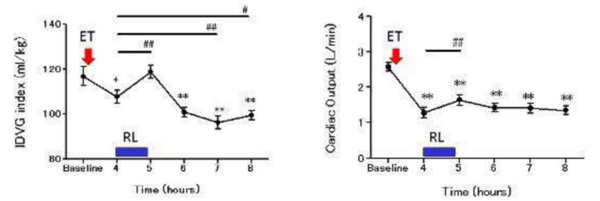

**Fig. 1 (abstract 1214).** The effects of volume loading on the changes in IDVG index and CO in ET-induced pigs models

### 1215

### Poor agreement in venoarterial $PCO_2$/arteriovenous $O_2$ content difference ratio, using central and mixed venous blood samples

A. Dubin[1,2], V. Edul[2], A. Risso Vazquez[1], C. Enrico[1]
[1]Sanatorio Otamendi, Servicio de Terapia Intensiva, Buenos Aires, Argentina; [2]Universidad Nacional de La Plata, Cátedra de Farmacología Aplicada, La Plata, Argentina
**Correspondence:** A. Dubin

*Intensive Care Medicine Experimental* 2018, **6(Suppl 2):**1215

**INTRODUCTION.** Central venous minus arterial $PCO_2$ ($P_{cv\text{-}a}CO_2$) and the ratio of $P_{cv\text{-}a}CO_2$ to arterial minus central venous $O_2$ content difference ($P_{cv\text{-}a}CO_2/C_{a\text{-}cv}O_2$) are used as surrogates for mixed venous minus arterial $PCO_2$ ($P_{mv\text{-}a}CO_2$) and the ratio of $P_{mv\text{-}a}CO_2$ to arterial minus mixed venous $O_2$ content difference ($P_{mv\text{-}a}CO_2/C_{a\text{-}mv}O_2$). The interchangeability of these variables, however, has never been demonstrated.

**OBJECTIVES.** To assess the agreement between $P_{cv\text{-}a}CO_2$ and $P_{mv\text{-}a}CO_2$, and between $P_{cv\text{-}a}CO_2/C_{a\text{-}cv}O_2$ and $P_{mv\text{-}a}CO_2/C_{a\text{-}mv}O_2$, and their relationship to mortality.

**METHODS.** This is a secondary analysis of a previously published study (1). In 23 patients with septic shock and invasive hemodynamic monitoring with a Swan-Ganz catheter, we measured central venous and mixed venous gases, oxygen saturation, and hemoglobin. Sublingual microcirculation was assessed by SDF-videomicroscopy. The different gradients and ratios were calculated and compared by means of Bland and Altman analysis.

**RESULTS.** Panel A and B of Figure 1 respectively show the bias and 95% limits of agreement for $P_{cv\text{-}a}CO_2$ and $P_{mv\text{-}a}CO_2$, and for $P_{cv\text{-}a}CO_2/C_{a\text{-}cv}O_2$ and $P_{mv\text{-}a}CO_2/C_{a\text{-}mv}O_2$. Survivors and nonsurvivors showed similar values of $P_{cv\text{-}a}CO_2$ (6 ± 2 vs 6 ± 3 mm Hg, $P = 0.82$), $P_{mv\text{-}a}CO_2$ (6 ± 2 vs 6 ± 3 mm Hg, $P = 0.66$), $P_{cv\text{-}a}CO_2/C_{a\text{-}cv}O_2$ (1.17 ± 0.65 vs 1.30 ± 0.68, $P = 0.65$). $P_{mv\text{-}a}CO_2/C_{a\text{-}mv}O_2$ (1.22 ± 0.56 vs 1.28 ± 0.72, $P = 0.65$). Sublingual perfused vascular density was higher in survivors (16.0 ± 3.5 vs. 12.5 ± 4.5 $mm^2$, $P < 0.05$). A multiple linear regression model found Hb, lactate, and mixed venous $PCO_2$ as $P_{mv\text{-}a}CO_2/C_{a\text{-}mv}O_2$ determinants (Adjusted $R^2 = 0.64$, $P < 0.0001$). Hb was the explanatory variable with the highest independent contribution to prediction (highest *t*-ratio).

**CONCLUSIONS.**

1) $P_{cv\text{-}a}CO_2$ and $P_{cv\text{-}a}CO_2/C_{a\text{-}cv}O_2$ are poor surrogates for $P_{mv\text{-}a}CO_2$ and $P_{mv\text{-}a}CO_2/C_{a\text{-}mv}O_2$.
2) Unlike sublingual microcirculation, neither of these variables is an outcome predictor.
3) Hb is a main determinant for $P_{mv\text{-}a}CO_2/C_{a\text{-}mv}O_2$.

(1) Enrico C, Kanoore Edul VS, Vazquez AR, Pein MC, Pérez de la Hoz RA, Ince C, Dubin A. Systemic and microcirculatory effects of dobutamine in patients with septic shock. J Crit Care 2012; 27(6):630-8

### GRANT ACKNOWLEDGMENT

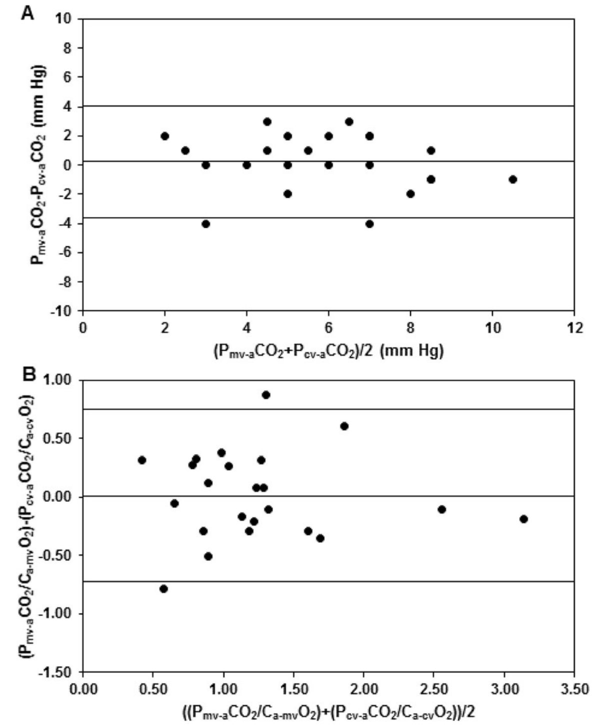

**Fig. 1 (abstract 1215).** See text for description

### 1216

#### Sepsis-induced myocardial dysfunction as a predictor of mortality in the adult septic patient in the intensive care unit: a prospective cohort study

V. Fraile-Gutiérrez[1], D. Pérez-Torres[1], J.M. Ayuela-Azcarate[2], A. Mayo-Iscar[3], A.I. Rodríguez-Villar[1], P. Blanco-Schweizer[1], E. Prol-Silva[1], J.Á. Ayala-Fernández[1], C. Díaz-Rodríguez[1], J. Blanco-Varela[1]
[1]Hospital Universitario Río Hortega, Intensive Care Department, Valladolid, Spain; [2]Complejo Asistencial Universitario de Burgos, Intensive Care Department, Burgos, Spain; [3]University of Valladolid, Department of Statistics and Operations Research, Valladolid, Spain
**Correspondence:** D. Pérez-Torres
*Intensive Care Medicine Experimental* 2018, **6(Suppl 2):**1216

**INTRODUCTION.** Sepsis-induced myocardial dysfunction is a common entity complicating the course of sepsis or septic shock, with a negative impact on patient's survival. This myocardial depression comprises impairment of both ventricles and both systolic and diastolic dysfunction.

**OBJECTIVES.** To describe the incidence and mortality association of the different subtypes of sepsis-induced myocardial dysfunction (SIMD). To develop a SIMD-based mortality prediction model for septic patients admitted to the Intensive Care Unit (ICU).

**METHODS.** We conducted a single-centre prospective observational cohort study. Patients admitted to the ICU with sepsis over a 20-month period were enrolled, excluding those with ischemic or valvular heart disease and pacemakers. Transthoracic echocardiogram (TTE) was performed on Days 1, 3 and 7 of evolution. Left ventricular systolic (LVSD) and diastolic dysfunction (LVDD), and right ventricular systolic dysfunction (RVSD) were defined as recommended on ASE/EACVI Guidelines. Student's T, Mann-Whitney U and Fisher´s exact tests were applied as appropriate. A principal components analysis (PCA) with varimax rotation was conducted using mortality-associated variables on Day 1. A logistic regression model was developed based upon the factors obtained in the PCA.

**RESULTS.** 94 patients were enrolled, 55% male, aged 63±12, APACHE-II 23, 67% mechanically ventilated, ICU mortality 23%, 90-day mortality 29%. 217 TTE were performed. SIMD distribution by subtype and outcome is shown in the Table.

PCA identified two factors, which were used to develop a logistic regression model to predict mortality, with an AUCROC 0.85 (sensitivity 87%, specificity 84%). Factor 1 included age and diastolic function variables (septal e' wave; and septal, lateral and mean E/e' ratio), OR 3.2 (95%CI: 1.52-6.72, p=0.001). Factor 2 included clinical severity variables (SOFA, lactate, norepinephrine dose, weight gain, mechanical ventilation and LVOT VTI), OR 5.43 (95%CI: 1.97-14.92, p=0.001).

**CONCLUSIONS.**

- LVDD is the most common subtype of SIMD, and its detection within the first 24 hours of admission in an adult septic patient is an independent predictor of mortality.
- A prediction model based upon two factors, namely diastolic function factor and clinical severity factor, is able to successfully predict mortality in adult septic patients admitted to the ICU.

**Table 1 (abstract 1216).** SIMD distribution by subtype and outcome

| | SIMD (%) | Subtype | Global (%) | Survivors (%) | Deceased (%) | p |
|---|---|---|---|---|---|---|
| Day 1 | 50 | LVSD | 18 | 11 | 41 | 0.003 |
| | | LVDD | 40 | 13 | 59 | 0.05 |
| | | RVSD | 21 | 15 | 36 | 0.02 |
| Day 3 | 47 | LVSD | 10 | 5 | 26 | 0.02 |
| | | LVDD | 38 | 32 | 58 | 0.02 |
| | | RVSD | 17 | 8 | 42 | 0.003 |
| Day 7 | 43 | LVSD | 2 | 0 | 8 | NS |
| | | LVDD | 37 | 40 | 25 | NS |
| | | RVSD | 10 | 8 | 16 | NS |

### 1217

#### Decreased endothelial glycocalyx thickness is an early predictor of mortality in sepsis

M. Bol[1], D. Beurskens[2], M. Suverein[1], B. Broddin[1], C. Reutelingsperger[2], T. Delhaas[3], M. van de Poll[1], J. Sels[1], G. Nicolaes[2]
[1]MUMC+, Intensive Care, Maastricht, Netherlands; [2]Maastricht University, Biochemistry, Maastricht, Netherlands; [3]Maastricht University, BioMedical Engineering, Maastricht, Netherlands
**Correspondence:** M. Bol
*Intensive Care Medicine Experimental* 2018, **6(Suppl 2):**1217

**INTRODUCTION.** Microcirculatory alterations occur early and play an important role in systemic inflammation, such as in sepsis[1-3]. Reduction of the endothelial glycocalyx thickness, also described as glycocalyx shedding, is regarded as a primary cause of microvascular dysfunction and may contribute to multiple organ failure and death. Glycocalyx shedding leads to an increase in the size of the perfused boundary region (PBR) of sublingual microvessels (5-25 μm) that can be visualized using side stream darkfield (SDF) imaging.

**OBJECTIVES.** The objective of this study was to investigate the endothelial glycocalyx thickness in septic patients at ICU admission and its relation to outcome. We hypothesize that measured PBR thickness is inversely related to mortality in sepsis.

**METHODS.** The sublingual endothelial glycocalyx thickness was studied in septic patients within 24h of ICU-admission by SDF imaging. GlycoCheck software was used to automatically calculate the PBR for all recorded images. The results of 3 consecutive measurements were averaged to calculate a reliable PBR value per patient[4]. Patients were divided in two groups according to hospital mortality. Results are presented as mean (SD) and statistical analysis was performed using an independent t-test. A receiver operating characteristic (ROC) curve was generated to evaluate the discriminative ability of PBR for predicting survival to hospital discharge.

**RESULTS.** We included 22 patients, with an average age of 66 (12) years and a mean APACHE IV score of 98 (40). The mortality rate was 9/22 (41%). The PBR was significantly lower in septic patients who ultimately survived to hospital discharge compared to non-survivors (1,79 (0,21) μm, vs 2,02 (0,23) μm,; p=0,027). The PBR at admission can be considered a predictor of hospital mortality based on the ROC curve with an AUC of

0.778. A cutoff value of 1,88µm gave a sensitivity of 78% and a specificity of 77%.

**CONCLUSIONS.** Endothelial glycocalyx loss at ICU admission is an early predictor of sepsis mortality. Preservation of the endothelial glycocalyx may thus be an important goal in the early treatment of sepsis.

**REFERENCE(S)**

(1) Donati A, Damiani E, Domizi R et al. *Microvascular Research* 2013:90;86-89.
(2) De Backer D, Creteur J, Preiser JC, et al. *Am J Respir Crit Care Med* 2002. 166:98-104
(3) Colbert J and Schmidt E. *Clin Chest Med. 2016.*:37(2);263-75 (4) Bol et al. ISICEM 2017

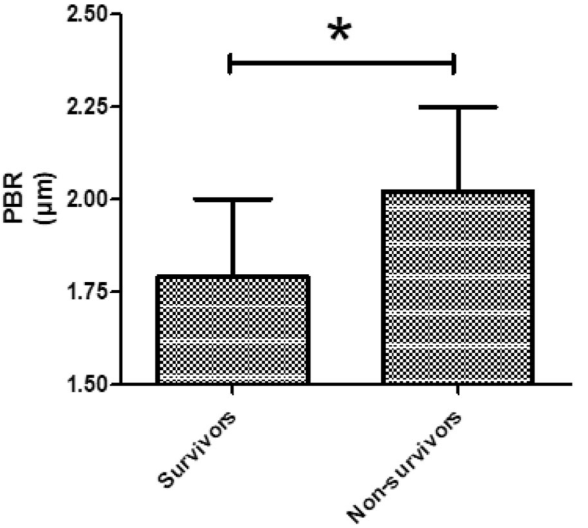

**Fig. 1 (abstract 1217).** Mean PBR and standard deviation at admission for sepsis survivors and non-survivors (* indicates p<0,05)

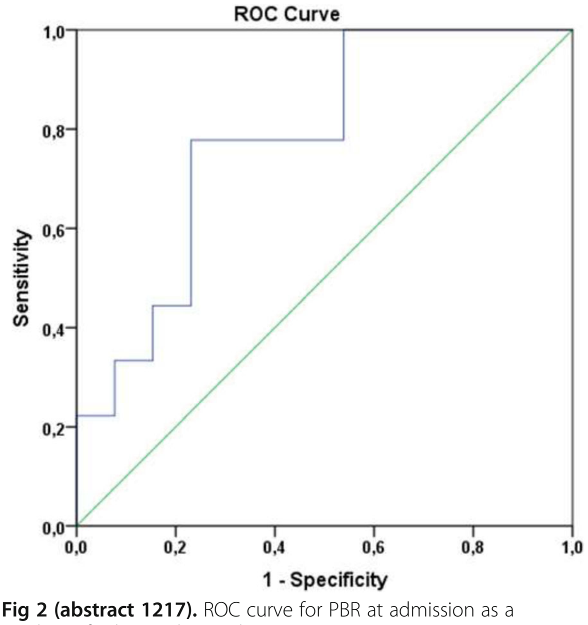

**Fig 2 (abstract 1217).** ROC curve for PBR at admission as a predictor for hospital mortality in sepsis

### 1218

#### Real time assessment of tissue perfusion markers with fluid bolus in sepsis: time to change our targets???

J. Nikhilesh, T. Apurv
CHL Hospital, Dept of Critical Care Services, Indore, India
**Correspondence:** J. Nikhilesh

*Intensive Care Medicine Experimental* 2018, **6(Suppl 2):**1218

**BACKGROUND.** There is enough literature emphasizing value of tissue perfusion markers and their response to volume administration. However there is significant variation in how they behave in different patient subsets especially sepsis. We have had different assessments in past comparing the above and this communication delineates an attempt to correlate tissue perfusion markers values variations to fluid boluses.

**OBJECTIVES.** Validating change in tissue perfusion markers in response to fluid bolus in septic shock patients.

**SETTING.** A thirty five bedded multidisciplinary CCU of a tertiary care unit.

**STUDY MODULE.** Consecutive patients presenting with septic shock were included for the duration dated Oct 2015 to Feb 2017.All the patients were evaluated at baseline for demographics, SOFA scores, Lactate levels (L1), Central venous oxygen saturation(Scvo2[1]), Mean arterial pressure(MAP1) and base excess(BE1).They were all given fluid boluses of one liter balanced crystalloid and the above measurements were repeated after the same (L2,Scvo2[2],MAP2 and BE2 respectively).A variation of two or more was considered significant. Patients with chronic renal failure and cardiac dysfunction were excluded. Statistical analysis was done using SPSS.

**RESULTS.** Fifty one patients were included (n=51, M:F-30:21).Mean age of patients was 44.7+15 years(Range(R)=20-79).SOFA scores were 14.2+2.5(R=10-18).L1,Scvo2[1],BE1 and MAP1 levels were 10.9+ 3.9(R=5-22),62.2+6.3(R=50-75),-10.2+3.4 (R=-4—18) and 56.4 +3.9(R=48-62)respectively. The corresponding values post fluid bolus were L2 =9.4 + 3.6(R=4-20), Scvo2 [2] = 62.9+5.1(R=52-73), BE2= -7.6 +3(R=-2-15) and MAP2 = 58.9+ 4.3 (R=50-66) respectively. P values using a T test were significant for Lactate and Base excess (< .05) after computing variations and in terms of correlation with MAP the quantum of change in values was most significant for BE (56.9%) vis-à-vis L and Scv02 (15.7% and 17.7% respectively).

**CONCLUSION.** In terms of defining therapeutic end points of fluid resuscitation and response to fluid boluses base excess variations perform better vis-à-vis lactate and central venous oxygen saturations for sepsis patients. In addition their variation is more likely to be congruent with improvement in mean arterial pressures for patients with septic shock.

There are no disclosures to declare.

### 1219

#### Dynamic arterial elastance as a predictor of arterial blood pressure response to fluid administration in septic patients: a prospective observational study

M. El-Sayed[1], S. Salah Hammad Ali[2], A. Mohamady El-Demerdash Hassan[2], A. Elsaied Abdelrahman[3], A. Mukhtar[4]
[1]Cairo University Hospitals, Cairo, Egypt; [2]Aswan University, Anesthesia, Aswan, Egypt; [3]Sohag University, Anesthesia, Sohag, Egypt; [4]Cairo University, Anesthesia, Cairo, Egypt
**Correspondence:** M. El-Sayed

*Intensive Care Medicine Experimental* 2018, **6(Suppl 2):**1219

**INTRODUCTION.** Persistent, profound hypotension is a common phenomenon in sepsis. Aggressive fluid resuscitation to restore tissue perfusion is considered a cornerstone of early sepsis &septic shock management .Arterial blood pressure(ABP) results from interaction between arterial system & blood ejected by the heart, thus the response of ABP to fluids continues to be a challenge[1]. Assessment of preload dependency was extensively studied in recent years, a few studies focused on ABP prediction to intravascular volume expansion.[2]

**OBJECTIVES.** Functional assessment of arterial load by dynamic arterial elastance ($Ea_{dyn}$), to predict ABP response to fluid challenge(FC) in hypotensive, preload-dependent septic patients.

**METHODS.** A cohort study conducted at Trauma & Surgical Intensive Care Unit (SICU) of Cairo University hospital. Enrolled patients were both spontaneously breathing & mechanically ventilated adults admitted with sepsis according to 2016 Surviving Sepsis Campaign criteria, excluding patients younger than 18 years, Pregnant females, trauma patients, history of vasodilator therapy or arrhythmias. Preload dependency was defined if stroke volume variation(SVV) ≥10 % (obtained from cardiometry) or pulse pressure variation(PPV) ≥ 13% (obtained from invasive blood pressure monitoring). Two sets of hemodynamic measurments were obtained, the 1$^{st}$ on admission & th$^{en}$ after an FC(200 ml normal saline).These included Heart rate (HR), systolic arterial pressure (SAP), mean arterial pressure (MAP), diastolic arterial pressure (DAP)& PPV. Cardiac output (CO), stroke volume (SV), SVV & systemic vascular resistance (SVR) were obtained from electrical cardiometry. $Ea_{dyn}$ (PPV/SVV) was calculated. Patients were classified after FC as MAP responders (≥10% increase in MAP) & MAP non-responders (< 10%increase in MAP).

**RESULTS.** Out of 58patients, 21(36%) were MAP responders. At baseline, $Ea_{dyn}$ was significantly higher in responders(0.93 ± 0.2) vs. nonr-esponders(0.79 ± 0.3). The Area under Curve(AUC) for preinfusion $Ea_{dyn}$(0.687 ± 080; 95% CI: 0.530 - 0.843; ) was higher than PPV(0.613± 0.077; 95% CI 0.462 - 0.763) & SVV (0.595±0.076; 95% CI 0.445 - 0.744 ). An optimal cutoff of 0.80 to predict 10%MAP increase after FC in septic patients (91.0%sensitivity & 47.8%specificity) .

**CONCLUSIONS.** $Ea_{dyn}$ may predict arterial pressure response to FC in hypotensive, preload- dependent septic patients with fair sensitivity but poor specificity. It can be interpreted as a dynamic index of arterial load.

**REFERENCE(S)**

1. Lakhal K, Ehrmann S, Perrotin D, Wolff M, Boulain T: Fluid challenge: tracking changes in cardiac output with blood pressure monitoring (invasive or non-invasive). Intensive Care Med 2013, 39:1953-1962.
2. Pierrakos C, Velissaris D, Scolletta S, Heenen S, De Backer D, Vincent JL. Can changes in arterial pressure be used to detect changes in cardiac index during fluid challenge in patients with septic shock? Intensive Care Med 2012;38.

**Table 1 (abstract 1219).** Receiver Operating Characteristic Curve Analysis of Hemodynamic Variables Predicting Arterial Pressure Responsiveness After Fluid Challenge

| | AUC | 95% CI | P-value |
|---|---|---|---|
| EAdyn | 0.687 | 0.530 - 0.843 | 0.021 |
| PPV | 0.613 | 0.462 - 0.763 | 0.157 |
| SVV | 0.595 | 0.445 - 0.744 | 0.234 |

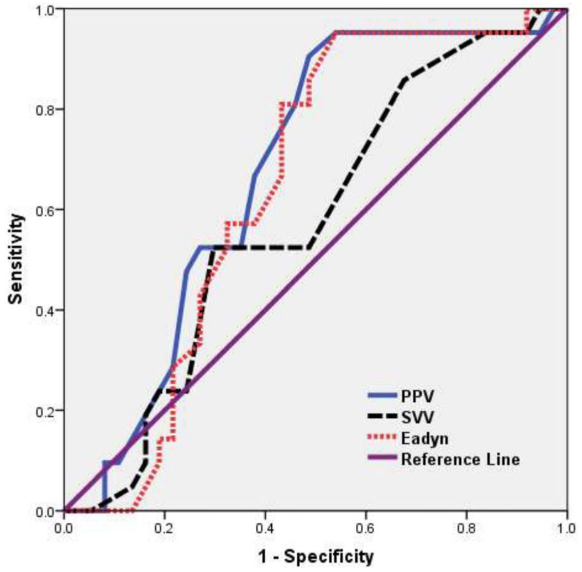

**Fig 1 (abstract 1219).** Comparison of reciever operating characteristics curves of vascular tone parametes

**Table 2 (abstract 1219).** Effects of fluid challenge on hemodynamic variables according to mean arterial pressure increase

| Variable | Before FC | After FC | P-value** |
|---|---|---|---|
| Heart Rate (b/min) | | | |
| Non-responders | 122.95 ± 19.9 | 121.35 ± 20.5 | = 0.003 |
| Responders | 116.57 ± 22.7 | 113.10 ± 20.4 | |
| CVP (cm/H,0) | | | |
| Non-responders | 9.92 ± 4.4 | 10.24 ± 4.6 | = 0.001 |
| Responders | 9.00 ± 5.6 | 9.33 ± 5.5 | |
| SAP (mmHg) | | | |
| Non-responders | 98.92 ± 16.4 | 103.00 ± 19.4 | < 0.001 |
| Responders | 96.43 ± 11.4 | 105.90 ± 11.2 | |
| DAP (mmHg) | | | |
| Non-responders | 53.41 ± 14.4 | 56.35 ± 16.1 | < 0.001 |
| Responders | 53.62 ± 10.4 | 60.86 ± 11.9 | |
| MAP (mmHg) | | | |
| Non-responders | 68.89 ± 14.4 | 71.81 ± 16.6 | < 0.001 |
| Responders | 67.52 ± 8.8 | 77.43 ± 10.1 | |
| SV (ml) | | | |
| Non-responders | 66.81 ± 15.6 | 75.78 ± 18.9 | < 0.001 |
| Responders | 69.90 ± 26.9 | 82.19 ± 25.4 | |
| CO (L/min) | | | |
| Non-responders | 8.00 ± 2.1 | 8.93 ± 2.3 | < 0.001 |
| Responders | 8.03 ± 3.5 | 9.21 ± 3.1 | |

**Table 3 (abstract 1219).** Effects of fluid challenge on static & dynamic arterial load variables in both groups

| Variable | Before Volume Expansion | After Volume Expansion | P-value** |
|---|---|---|---|
| SVR, dyn.s-cm?5 | | | |
| Non-responders | 765.53 ± 58.1 | 680.35 ± 33.5 | = 0.001 |
| Responders | 936.30 ± 63.6 | 923.10 ± 45.5 | |
| $Ea_{dyn}$ | | | |
| Non-responders | 0.79 ± 0.3 | 0.76 ± 0.4 | = 0.013 |
| Responders | 0.93 ± 0.2 | 0.77 ± 0.2 | |
| SVV (%) | | | |
| Non-responders | 18.95 ± 4.9 | 16.76 ± 4.8 | < 0.001 |
| Responders | 19.90 ± 3.3 | 17.62 ± 3.4 | |
| PPV (%) | | | |
| Non-responders | 15.30 ± 6.9 | 12.27 ± 5.4 | < 0.001 |
| Responders | 18.52 ± 4.7 | 13.57 ± 4.1 | |

### 1220

#### Factors influencing carotid blood flow measurements in ICU patients

G. Gursel[1], B.S. Kalın[2], M. Çimen[3], S. Eyüpoğlu[3]

[1]Gazi University School of Medicine, Department of Pulmonary Critical Care Medicine, Ankara, Turkey; [2]Gazi University School of Medicine, Department of Internal Medicine Division of Critical Care Medicine, Ankara, Turkey; [3]Gazi University School of Medicine, Department of Anaesthesiology Division of Critical Care Medicine, Ankara, Turkey

**Correspondence:** G. Gursel

*Intensive Care Medicine Experimental* 2018, **6(Suppl 2):**1220

**INTRODUCTION.** As a feasible, non-invasive, and reproducible bedside estimates of CO, carotid Doppler imaging is promising but still it is unclear whether measuring carotid blood flow by the velocity time integral using Doppler ultrasound can be used to monitor cardiac output and volume responsiveness.
**OBJECTIVES.** Aim of the study was to investigate the relationship between carotis blood flow(CBF) and cardiac output(CO) and other parameters that would influence CBF in ICU patients.
**METHODS.** 59 patients were included in the study. Patients' demographics, ventilation situations, arterial blood gase results and hemodynamics were recorded. In all patients echocardiographic measurements (such as CO, SV), and carotid doppler measurements were performed during the same time period. Velocity time integral (VTI) of the Doppler signal was measured using manual tracings . Intimal-to-intimal carotid diameter was measured at the level of the sample gate. All carotid measurements were measured on a single waveform by one investigator. Carotid blood flow was calculated as blood flow = π × (carotid diameter) 2 /4 × VTI × heart rate. Measurements were obtained using a single carotid waveform and an average of three carotid waveforms for both measures.
**RESULTS.** Mean age and APACHE II scores of the patients were 72±13, 24 ±11 respectively. 42% of the patients were intubated and 53% of them had COPD. While there were no significant correlation between CO and CBF(r:0.18, p:0.59). There were weak but significant correlation between mechanical ventilation(r:0.31, p:0.024), tidal volume(r:-0.38, p:0.047) and CBF. Patients with COPD had significantly higher CBF than the others(627±280 versus 478 ±160, p:0.024). CBF was significantly correlated with paO2(r:0.53, p:0.006) and PaCO2(r:0.46, 0.018) especially in patients with COPD.
**CONCLUSIONS.** These results suggest that CBF may not reflect CO in all patients and it may be influenced by many other factors. Further studies are necessary to standardized CBF as a surrogate measurement of CO.

**REFERENCE(S)**

C. Roehrig, M. Govier, J. Robinson and A. Aneman. Carotid Doppler flowmetry correlates poorly with thermodilution cardiac output following cardiac surgery Acta Anaesthesiologica Scandinavica 61 (2017) 31-38Yildiz S, Kaya I, Cece H, Gencer M, Ziylana Z, Yalcin F, Turksoy O. Impact of COPD exacerbation on cerebral blood flow Clinical Imaging 36 (2012) 185-190

**GRANT ACKNOWLEDGMENT**

Study was supported by Gazi University Scientific Reserch Projects Foundation (Project No: 01/2015-48).

### 1221

#### Time-course of ventriculo-arterial decoupling in human sepsis

G. Errico, R. Caporusso, D. Ugliola, L. Tullo, L. Mirabella, G. Cinnella
University of Foggia, Anaesthesia and Intensive Care, Foggia, Italy
**Correspondence:** G. Errico
*Intensive Care Medicine Experimental* 2018, **6(Suppl 2):**1221

**INTRODUCTION.** Sepsis is life-threatening organ dysfunction caused by a dysregulated host response to infection. Sepsis affects both heart and circulation, causing dysfunction in their interaction. One approach to detect this interaction is to examine ventriculoarterial coupling (E/A), which is defined by the ratio of arterial elastance (Ea) to left ventricular end-systolic elastance (Ees). In this study, we investigate time-course of E/A in a cohort of patients admitted to ICUs who presented sepsis.
**OBJECTIVES.** We aim to investigate time-course of E/A as an hemodynamic tool, such as Mean Arterial Pressure (MAP) and Cardiac Index (CI), in order to optimize cardiovascular function leading the therapy with inotropes (Levosimendan, Dobutamine) and vasopressor agents (Dopamine, Norepinephrine).
**METHODS.** In this study we measured, in patients with sepsis, SOFA score, routine hemodynamics using Pulse contour method and transthoracic echocardiograms from baseline (T0), every day at the same time for 7 days. Parameters were calculated using data gathered from the echocardiographic examination included Left Ventricular Ejection Fraction (LVEF), Stroke Volume (SV), Preejection time and Systolic time. Ventricular Elastance Single Beat (EesSB) was estimated by using the method of Chen. (1). Arterial Elastance (Ea) was calculated as 0.9 × (systolic arterial pressure/SV), and the Ea/EesSB ratio was then calculated (2). Ea/EesSB ratio between 0,8 and 1,36 is considered normal value of E/A. In patients with Ea/EesSB ratio>1,36, low values of EesSB (< 1,4 mmHg/ml) and MAP>65mmhg Levosimendan was administered.
**RESULTS.** 28 patients were enrolled,18 patients were uncoupled (Ea/EesSB ratio >1,36), 10 patients at T0 and 8 patients in the first 72 hours. Patients with higher SOFA Scores had an higher Ea/EesSB ratio. Following the E/A monitoring, 8 patients were treated with Levosimendan and vasopressors and 10 patients with vasopressors. 87.5% (7 patients) in group treated with Levosimendan improved their hemodynamics (E/A, MAP, CI). In the other group, 50% (5 patients) improved their hemodynamic status. The mortality was 50% in the group treated with levosimendan while 60% in the group treated with vasopressors only.
**CONCLUSIONS.** Time-course of E/A could be an important tool to evaluate hemodynamic conditions in septic patients as a supplement to standard monitoring, related to patient clinical conditions, aimed to improve cardiovascular efficiency.

**REFERENCE(S)**

1) Chen CH, Fetics B, Nevo E, Rochitte CE, Chiou KR, Ding PA, Kawaguchi M, Kass DA: Noninvasive single-beat determination of left ventricular end-systolic elastance in humans. J Am Coll Cardiol 2001, 38:2028-2034.
2) Guarracino F, Baldassarri R, Pinsky MR: Ventriculo-arterial decoupling in acutely altered hemodynamic states. Crit Care 2013 Mar 19;17(2):213. doi: 10.1186/cc12522.

### 1222

#### Preventive isolation in an intensive care unit

A. Valverde Montoro[1], C.N. Sánchez Martín[2], M.J. Furones Llorente[1], E. Trujillo García[1], H. Molina Díaz[1], J.M. Mora Ordoñez[1]
[1]H.R.U. de Málaga, Intensive Care, Málaga, Spain; [2]H.R.U de Málaga, Thoracic Surgery, Málaga, Spain
**Correspondence:** E. Trujillo García
*Intensive Care Medicine Experimental* 2018, **6(Suppl 2):**1222

**OBJECTIVE.** Analyzing the application of the preventive isolation protocol after ICU admission of postoperative elective surgery in our hospital, taking in consideration some risk factor for multirresistance colonization, and its necessity of continuation.
**METHODS.** A retrospective observational study with hospitalized patients admitted in ICU after an elective surgery from January to September 2017, choosing those who accomplished the requirements of preventive isolation, and flowed up until the obtention of the surveillance culture results, as it was agreed upon our unit and preventive medicine unit. This protocol included throat and rectal swab samples for every patient who was hospitalized more than 5 days, came from North Africa or assisted residence or hospitalization within the previous 3 months (table 1).
**RESULTS.** A total number of 580 post-operative patients addmitted in ICU during the period of the study, 228 (36,89%) complies preventive isolation criteria. 24,76% (53 cases) of all the patients with preventive isolation indication admitted in our unit, with the surveillance culture previously drawn, avoiding an unnecessary isolation.
However, from 161 patients who admitted without a test taken and were isolated preventively, only 92 (57,14%) were cultured. Besides, from the whole of the surveillance cultures taken, both in ICU as previously, only 1 was positive for multi-resistant germ. The direct cost (the cost of the consumable material in every isolation) of every culture taken in ICU, because it was not taken previously during the hospitalization, was 21.481,23€ approximately.
**CONCLUSIONS.** Based on this data, it seems necessary to become more flexible with the preventive isolation criteria in elective post-operative patients admitted in ICU (only one positive result for multi-resistant germ) and, in every case, obtaining the cultures previously to the surgery. Maybe, we have to analize another risk factors for multirresistence colonization.

**Table 1 (abstract 1222).** Requirements of preventive isolation

| |
|---|
| Patients admitted more than five days at Hospital. |
| Patients who came from North Africa. |
| Patients who came from assisted residence. |
| Patients with an hospitalization within the previous 3 months. |

## ARDS

### 1223

#### Optimal duration of prone positioning in patients with acute respiratory distress syndrome: aprotocol for systematic review and meta-regression

T. Kamo[1,2], Y. Aoki[3], T. Fukuda[4,5], K. Kurahashi[6], H. Yasuda[7,8], M. Sanui[9], E. Nango[10], T. Abe[8,11], A. Lefor[12], S. Hashimoto[13]
[1]Saiseikai Utsunomiya Hospital, Tochigi, Japan; [2]Keio University School of Medicine, Department of Pulmonary Medicine, Tokyo, Japan; [3]Shizuoka General Hospital, Department of Anesthesiology and Intensive Care Medicine, Shizuoka, Japan; [4]Graduate School of Medicine, University of Tokyo, Department of Emergency and Critical Care Medicine, Tokyo, Japan; [5]Beth Israel Deaconess Medical Center, Harvard Medical School, Department of Emergency Medicine, Boston, United States; [6]International University of Health and Welfare, Department of Anesthesiology and Intensive Care Medicine, Tokyo, Japan; [7]Kameda Medical Center, Department of Intensive Care Medicine, Chiba, Japan; [8]Keio University School of Medicine, Department of Preventive Medicine and Public Health, Tokyo, Japan; [9]Jichi Medical University Saitama Medical Center, Department of Anesthesiology and Critical Care Medicine, Saitama, Japan; [10]Kita Medical Center, Department of General Medicine, Tokyo, Japan; [11]Keio University Hospital, Biostatistics Unit at Clinical and Translational Research Center, Tokyo, Japan; [12]Jichi Medical University Saitama Medical Center, Department of Surgery, Tochigi, Japan; [13]Kyoto Prefectural University of Medicine, Department of Anesthesiology and Intensive Care Medicine, Kyoto, Japan
**Correspondence:** T. Kamo
*Intensive Care Medicine Experimental* 2018, **6(Suppl 2):**1223

**INTRODUCTION.** Several systematic review and meta-analyses have demonstrated that prolonged prone positioning can reduce the mortality associated with acute respiratory distress syndrome (ARDS). However, the effectiveness and optimal duration of prone positioning was not fully evaluated.

**OBJECTIVES.** To systematically review and meta-regression analysis is to investigate the duration of prone positioning needed to improve outcomes using sensitivity analyses and meta-regression.

**METHODS.** Electronic searches were performed in MEDLINE via Ovid, EMBASE, Cochrane Central Register of Controlled Trials and International Clinical Trials Registry Platform. Published and unpublished RCTs and randomized crossover trials (the first period only) between January 1980 and September 2017 were included. Included articles were independently assessed by two researchers. Quality of the articles was determined using the Grading of Recommendations Assessment, Development, and Evaluation (GRADE) system. Meta-regression analyses and subgroup analyses were employed to investigate potential sources of heterogeneity between studies.

**RESULTS.** We included twelve studies matched the inclusion criteria, comprising data for 2,789. Prone positioning reduce short term mortality (odds ratio, 0.82; 95% CI, 0.64-1.05; $p$= 0.004; $I^2$ = 71%).

Although there was no significant difference from the meta-regression model, there was a heterogeneity between duration of prone positioning and short term mortality, there was an apparent decrease in trend of correlation between duration of prone positioning and short term mortality (β=-0.04; 95% CI,-0.08-0.005; $p$=0.08). In this analysis, we determined 10 hours as a threshold. The effects were marked in the subgroup in which duration of prone positioning was greater than 10 hr, compared with a short time duration of prone positioning (odds ratio; 0.68: 95% CI, 0.49-0.95; $p$ = 0.06; $I^2$= 60% ).

**CONCLUSIONS.** We reconfirmed that long-term prone positioning of more than 10 hours affects prognosis, but the optimal threshold of its efficacy and hazard was not able to to get an answer in this meta-analysis.

### 1224

#### Videothoracoscopic lung biopsy in acute respiratory distress syndrome: a single centre experience

A. Ramos[1], A. Dogliotti[2], D. Latasa[1], J. Robles[1], V. Roncoroni[3], C. Capitaine Funes[4], C. Lovesio[1]
[1]Sanatorio Parque, Critical Care, Rosario, Argentina; [2]Grupo Oroño, Clinical Epidemiology and Statistics, Rosario, Argentina; [3]Sanatorio Parque, Pathological Anatomy, Rosario, Argentina; [4]Sanatorio Parque, Thoracic Surgery, Rosario, Argentina
**Correspondence:** D. Latasa
*Intensive Care Medicine Experimental* 2018, **6(Suppl 2):**1224

**INTRODUCTION.** The role of videothoracoscopic lung biopsy in acute respiratory distress syndrome (ARDS) has been questioned due to possible complications.

**OBJECTIVES.** The goals of this study were to better define the frequency of unexpected diagnoses made by videothoracoscopic lung biopsy, the frequency biopsy results lead to a change in clinical management, and the frequency of procedural complications.

**METHODS.** All individuals with available records undergoing videothoracoscopic lung biopsy between 2007 and 2017 for evaluation of ARDS based on the Berlin definition were analyzed.

**RESULTS.** The mean age in this cohort of 20 patients was 55.6 years (SD, 15 years) with PaO2/fraction of inspired oxygen ratio of 144 mm Hg (SD, 72 mm Hg) at the time of biopsy. A pathologic diagnosis other than diffuse alveolar damage or fibroproliferation was found in 75% of patients. The most common alternative diagnoses were alveolar hemorrhage (n = 4) and carcinomatous lymphangitis (n = 2). Biopsy results led to a change in management in the majority of patients, with addition of specific therapy in 65% and withdrawal of unneeded therapy in 35%. No one presented complications associated with the procedure.

**CONCLUSIONS.** In selected patients with clinical ARDS, videothoracoscopic lung biopsy can be performed safely, often reveals an unsuspected diagnosis, and frequently leads to alterations in therapy.

### 1225

#### Neuromuscular blocking agents as adjuvant therapy in patients with severe ARDS: asystematic review and meta-analysis

F.L. Valdez[1], J.P. Almeida[1], G. Landoni[2], J. Fukushima[1], F. Galas[1], C. Park[1], G. Oliveira[1], M. Mourao[1], R. Nakamura[1], S. Rizk[1], E.M. Oliveira[1], J.O. Auler Jr.[1], R. Kalil-Filho[1], L.A. Hajjar[1]
[1]Instituto do Cancer do Estado de Sao Paulo, Sao Paulo, Brazil; [2]IRCCS San Raffaele Scientific Institute, Milano, Italy
**Correspondence:** L.A. Hajjar
*Intensive Care Medicine Experimental* 2018, **6(Suppl 2):**1225

**INTRODUCTION.** The use of neuromuscular blocking agents has been advocated as adjuvant therapy in patients with severe acute respiratory distress syndrome (ARDS) to improve mechanical ventilation support and outcomes. However, due to lack of robust evidence paralyzing patients with ARDS is still a controversial issue.

**OBJECTIVES.** We aimed to determine whether treatment with adjuvant neuromuscular blocking agents compared to standard care led to a reduction in the risk of death as well as its effect on plateau pressure, PEEP requirement, fraction of inspired oxygen, driving pressure, partial pressure of carbon dioxide and tidal volume in patients with severe ARDS.

**METHODS.** We searched MEDLINE, EMBASE and CENTRAL, from inception to February 2018. Pairs of reviewers identified randomized controlled trials (RCTs) comparing neuromuscular blocking agent adjuvant therapy to standard care for patients with ARDS. We followed the Cochrane Handbook for systematic reviews and meta-analysis; two reviewers abstracted data independently. Main Outcomes and Measures Outcomes of interest included death and the effect of neuromuscular blocking agents on mechanical ventilation data as plateau pressure, PEEP requirement, fraction of inspired oxygen, driving pressure, partial pressure of carbon dioxide and tidal volume. We report risk ratios (RR) as the standard effect measure for mortality and mean differences for mechanical ventilation outcomes.

**RESULTS.** We identified 5 RCTs (503 participants). Moderate quality evidence suggested that adjuvant use of neuromuscular blocking agents probably reduces mortality (RR 0.72; 95%CI 0.57 to 0.91). Based on low quality evidence, the plateau pressure was reduced in patients who received neuromuscular blocking agents (mean difference -1.60; 95%CI -2.92 to -0.27). We found no significant difference in PEEP requirement, fraction of inspired oxygen, driving pressure, partial pressure of carbon dioxide and tidal volume.

**CONCLUSIONS.** Administration of adjuvant therapy with neuromuscular blocking agents reduces mortality in patients with ARDS. This reduction may be partially explained by a reduction in plateau pressure and consequently less lung injury related to invasive ventilation.

**GRANT ACKNOWLEDGMENT**

This study was sponsored by the Universidade de São Paulo, Brazil

### 1226

#### Impact of major comorbidities in patients with Acute Respiratory Distress Syndrome: insights from the LUNG SAFE study

E. Rezoagli[1,2,3], F. Madotto[4], B.A. McNicholas[3,5], T. Pham[6], G. Bellani[1], J.G. Laffey[2,7,8], LUNG SAFE Investigators and the ESICM Trials Group
[1]University of Milano-Bicocca, School of Medicine and Surgery, Monza, Italy; [2]National University of Ireland Galway, School of Medicine, Department of Anaesthesia, Galway, Ireland; [3]Galway University Hospitals, Department of Anaesthesia and Intensive Care Medicine, Galway, Ireland; [4]University of Milano-Bicocca, School of Medicine and Surgery, Research Centre on Public Health, Monza, Italy; [5]National University of Ireland Galway, School of Medicine, Discipline of Medicine, Galway, Ireland; [6]St Michael's Hospita, Keenan Research Centre for Biomedical Science, Department of Critical Care Medicine, Toronto, Canada; [7]St Michael's Hospital, Critical Illness and Injury Research Centre, Keenan Research Centre for Biomedical Science, Departments of Anesthesia and Critical Care Medicine, Toronto, Canada; [8]University of Toronto, Departments of Anesthesia, Physiology and Interdepartmental Division of Critical Care Medicine, Toronto, Canada
**Correspondence:** E. Rezoagli
*Intensive Care Medicine Experimental* 2018, **6(Suppl 2):**1226

**INTRODUCTION.** Most patients presenting with Acute Respiratory Distress Syndrome (ARDS) have significant comorbidities, yet many of these patients are excluded from clinical trials, limiting our understanding of this cohort. We wished to explore the influence of comorbidities on outcome in this secondary analysis of the LUNG SAFE patient cohort (1-3).

**OBJECTIVES.** Our primary objective was to determine the effect of comorbidities on outcomes in ARDS patients diagnosed on days 1-2 of acute hypoxaemic respiratory failure and undergoing invasive or non-invasive ventilation

**METHODS.** The large observational study to understand the global impact of severe acute respiratory failure (LUNG SAFE) was an international, multicenter, prospective cohort study of patients with severe respiratory failure. LUNG SAFE was conducted during 4 consecutive weeks in winter 2014, in a convenience sample of 459 intensive-care units in 50 countries across six continents. Inclusion criteria were admission to a participating intensive-care unit within the enrollment window and receipt of invasive or non-invasive ventilation. In the current analysis, the study population was confined to patients developing ARDS within the first 2 days of acute respiratory failure. Major comorbidities analyzed included chronic obstructive pulmonary disease (COPD), congestive heart failure (CHF), chronic kidney disease (CKD), chronic liver failure (CLF) and immune incompetence (see Table).

**RESULTS.** Among 2813 patients in the study population, 60% had at least one major comorbidity. All patients with at least one comorbidity but CLF were older compared to patients with no comorbidities. Patients with CKD or CLF at admission had more severe Sequential Organ Failure Assessment scores compared to patients with no comorbidities. Hospital LOS in survivors was shorter in patients with COPD and CHF, but not different in the other comorbidity groups, compared to the no comorbidities group. The presence of at least one comorbidity was correlated with both high ICU and hospital crude mortality (see Table).

**CONCLUSIONS.** Most patients with ARDS have significant comorbidities**.** These comorbidities are strongly associated with outcomes in patients with ARDS. These findings might modify the current interpretation about the generalizability of findings from current clinical trials.

**REFERENCE(S)**

1. Bellani G, Laffey JG, Pham T, et al. *JAMA.* 2016;315(8):788-800.
2. Madotto F, Pham T, Bellani G, et al. *Intens Care Med.* 2018; doi: 10.1007/s00134-018-5152-6.
3. Laffey JG, Bellani G, Pham T, et al. *Intens Care Med.* 2016; 42(12):1865-1876.

**GRANT ACKNOWLEDGMENT**

This work was funded and supported by the European Society of Intensive Care Medicine (ESICM), Brussels, Belgium, by St Michael's Hospital, Toronto, Canada, and by the University of Milan-Bicocca, Monza, Italy.

**Table 1 (abstract 1226).** Association between each comorbidity and outcomes in patients with ARDS. *P<0.05 (Mann-Whitney) and #P<0.05 (Chi-Square) versus no comorbidities.

| Parameter | No Comorbidities | COPD | Congestive Heart Failure | Chronic Kidney Disease | Chronic Liver Failure | Immune Incompetence |
|---|---|---|---|---|---|---|
| Patients, n (%) | 1121 (39.9 | 607 (21.6) | 290 (10.3) | 286 (10.2) | 112 (4.0) | 584 (20.8) |
| Age, mean ±SD, years | 56 ± 18 | 68 ± 12* | 72 ± 12* | 69 ± 13* | 56 ± 11 | 60 ± 16* |
| Day 1 P/F ratio, mean ±SD, mmHg | 157 ± 68 | 161 ± 64 | 165 ± 67 | 169 ± 69* | 161 ± 72 | 156 ± 68 |
| Day 1 SOFA score, mean, ±SD | 9.3 ± 4.0 | 8.6 ± 4.1* | 9.3 ± 3.9 | 10.3 ± 3.6* | 13.8 ± 4.2* | 9.4 ± 3.9 |
| ICU Mortality 90 d, n (%) | 303 (27.0) | 204 (33.6)# | 120 (41.4)# | 113 (39.5)# | 75 (67.0)# | 266 (45.5)# |
| Duration of Hospital Stay median (IQR), days All Patients / Surviving Patients | 12±11 / 31 ±26 | 11±11 / 26 ±22* | 10±11* /25 ±20* | 9±11* / 31±27 | 6±10* / 36±29 | 22±21* / 31 ±24 |
| Hospital Mortality 90 d, n (%) | 347 (31.0) | 237 (39.0)# | 135 (46.6)# | 138 (48.3)# | 81 (72.3)# | 304 (52.1)# |

### 1227

#### The impact of positive intrathoracic pressure on kidney failure in patients with ARDS

A. Vidal[1], L.M. Polanco[1], E. Rosas[1], D. Robaglia[1], P. Turrión[1], J.M. Milicua[1], V. Hortiguela[1], M.Á. Alcala[1], M. Pérez[2], C. Pérez[1]
[1]Fundación Jiménez Díaz, Madrid, Spain; [2]Hospital Universitario Rey Juan Carlos, Madrid, Spain
**Correspondence:** L.M. Polanco
*Intensive Care Medicine Experimental* 2018, **6(Suppl 2):**1227

**INTRODUCTION.** Several lines of evidence suggest that mechanical ventilation and acute respiratory distress syndrome may have adverse effects on renal function. This points out the harmful interactions between pulmonary and renal dysfunctions. And the importance of knowing its scope.

**OBJECTIVES.** To examine the relationship between positive intrathoracic pressure and the development of acute renal failure in patients with ARDS.

**METHODS.** Between 2015 and the present, 44 consecutive patients presenting with ARDS, according to the Berlin definition, were studied. The sample group was subdivided according to AKIN criteria. Pressure data (peak, plateau, PEEP, driving pressure, compliance and CVP) and analytical data (creatinine, urea, sodium, potassium and glomerular filtrate) were collected. Urine output, the use of hemodiafiltration and noradrenaline, age, sex, number of days on mechanical ventilation, admission to ICU, SOFA score and mortality were also recorded.

**RESULTS.** 39.1% of patients with ARDS in our polyvalent unit presented with AKI, of whom 32.47% developed AKI stage I, 14.4% AKI stage II and 53.09% AKI stage III (p = 0.001). 79.8% of the AKI patients were male, compared with 20.2% female (p = 0.001). Age difference was not noted. AKI was most common in ARDSexp, with 50%, compared with 30.6% in ARDSp (p = 0.004). The need for noradrenaline was associated with AKI in 55.3%, (59.6% AKIN III (p = 0.001)).

Differences were observed relating to pressure. Peak pressure in AKI was 34.04 ± 7.242 vs 31.16 ± 9.09 mmHg in patients without AKI (p = 0.012). Plateau pressure with AKI was 27.67 ± 4.76 vs 24.38 ± 7.30 mmHg without AKI (p = 0.001). PEEP with AKI was 14.30 ± 4.16 vs 12.91 ± 4.01 mmHg without AKI (p = 0.017). Driving pressure with AKI was 13.01 ± 4.75 vs 11.54 ± 5.10 mmHg without AKI (p = 0.017). VT with AKI was 7.00 ± 1.12 vs 7.59 ± 1.47 ml/kg without AKI (p = 0.002). Mean SAP with AKI was 77.71 ± 14.82 vs 85.52 ± 12.45 mmHg without AKI (p = 0.000). CVP with AKI was 14.94 ± 4.9 vs 12.66 ± 5.38 without AKI (p = 0.002).

Remedial measures such as pronation and corticosteroids were not associated with increased AKI. Neither were the number of days on ventilation, nor the number of days admitted to the unit. The mortality rate with AKI was 50%, this was higher for patients with AKIN III at 52.8% (p = 0.001).

**CONCLUSIONS.** There is an adverse relationship with the kidney in ARDS, although it is a separate organ, primarily relating to intrathoracic pressure, as we have demonstrated. Consequently, the use of lung protective mechanical ventilation should prevent decreased blood flow, and maintain an adequate gas exchange.

##### REFERENCE(S)

Christopher J, Tignanelli, MD, Arek J. Wiktor, MD, Cory J. Vatsaas, MD. Outcomes of Acute Kidney Injury in Patients With Severe ARDS Due To Influenza A (H1N1). American Journal of Critical Care. 2018;27:67-73.

### 1228

#### Comparison of three methods to measure the mechanical power in patients with the acute respiratory distress syndrome

B. Louis[1], M. Mezidipt>2, C. Guérin[1,2]
[1]Institut Mondor de Recherches Biomédicales, INSERM 955 and CNRS ERL 7000, Creteil, France; [2]Hopital de la Croix Rousse, Réanimation, Lyon, France
**Correspondence:** B. Louis
*Intensive Care Medicine Experimental* 2018, **6(Suppl 2):**1228

**INTRODUCTION.** Mechanical power (MP) is a new concept to explain ventilator induced lung injury (1). It involves all the mechanical components of the energy load transferred from the ventilator to the respiratory system. The reference method. is assessed as the product of respiratory rate (RR) to work of breathing, this latter being obtained from the area subtended by airway pressure and volume during insufflation (MParea) (1). MP can also be computed according to the following equation (MPeq):

RR. { 〖ΔV〗 ^2.[1/2. 〖EL〗 _rs+RR.((1+I:E))/(60.I:E). Raw]+ΔV.PEEP} with RR respiratory rate, ΔV tidal volume, ELrs elastance of respiratory system, Raw airway resistance, PEEP positive end expiratory pressure. We were wondering whether the following simplified equation (MPeqsimp), which is easy to obtain at the bedside, would predict MP accurately: RR.ΔV.[├ PEEP +ΔP/2+(Ppeak-Pplat)] with Ppeak,Pplat and ΔP are maximal, plateau, and driving pressures of the respiratory system. This method requires variables that are used routinely at the bedside. OBJECTIVES. To compare MPeqsimp and MPaera to MPeq in patients with acute respiratory distress syndrome (ARDS). METHODS. We measured MParea, MPeq and MPeqsimp according to the above mentioned methods in 40 ARDS patients previously reported (3). Measurements were done breath by breath in the supine position. MP was expressed as J/min. The data were analyzed by using linear regression and Bland-Altman representation between methods.

**RESULTS.** The figure 1ABC displays the linear regression between the 3 methods while the corresponding Bland-Altman analyses are reported in Figure 1DEF. The coefficients of determinations were statistically significant.

The mean of the difference (continuous horizontal lines) between MPeq and MParea was 2.4 J/min. Between MPeqsimp and MParea the mean of the difference was 1.8 and the mean between MPeq and MPeqsimp was 0.5 J/min. In this last case upper and lower limits of agreement (broken horizontal lines) were (1.6; -0.5 ) J/min.

**CONCLUSIONS.** MPeqsimp can be used to compute MP accurately.

##### REFERENCE(S)

1. Gattinoni et al Intensive Care Medicine 2016
2. Cressoni et al. Anesthesiology 2016
3. Riad et al AJRCCM 2017

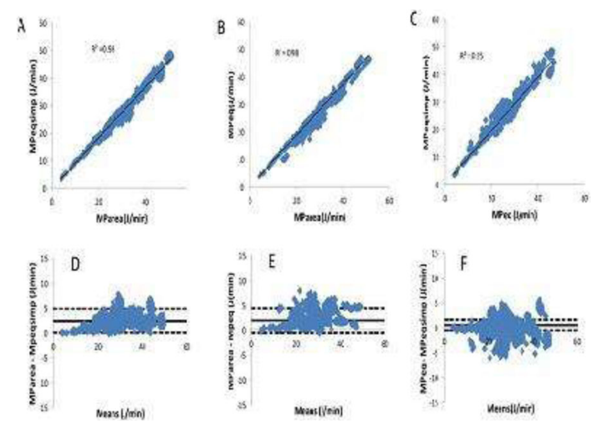

**Fig. 1 (abstract 1228).** See text for description

### 1229

#### Predicting the pREsence and the impact of Diffuse Alveolar Damage Through open lung biopsy in acute respiratORy distress syndrome - The PREDATOR Study

P. Cardinal-Fernández[1,2], G. Ortiz[3,4], C.-H. Chang[5], K.-C. Kao[5], E. Charbonney[6], E. Bertreau[7], C. Philipponnet[8], B. Souweine[8], C. Guérin[7]
[1]Hospital Universitario HM Sanchinarro, Madrid, Spain; [2]Fundación de Investigación HM Hospitales, Madrid, Spain; [3]Universidad del Bosque, Bogota, Colombia; [4]Hospital Santa-Clara, Bogota, Colombia; [5]Chang Gung Memorial Hospital, Taoyuan, Taiwan, Province of China; [6]Hòpital du Sacré-Cœur de Montréal, Montréal, Canada; [7]Réanimation Médicale Hôpital de la Croix Rousse, Lyon, France; [8]Réanimation Médicale, Clermont-Ferrand, France
**Correspondence:** P. Cardinal-Fernández
*Intensive Care Medicine Experimental* 2018, **6(Suppl 2):**1229

**INTRODUCTION.** The weak correlation between acute respiratory distress syndrome (ARDS) and its pathological hallmark, the Diffuse Alveolar Damage (DAD), has been proposed for explaining the failure of almost all pharmacological treatments to improve patient outcome.

**OBJECTIVES.**

(a) To identify clinical variables associated to DAD, and
(b) explore the impact of DAD on the hospital mortality risk.

**METHODS.** We conducted a retrospective multicenter study including adult patients with ARDS (Berlin definition) undergoing open lung biopsy (OLB) from France, Taiwan, Swiss and Colombia. Patients were splinted in a trainee then in a validation cohort. Data were recorded the day of ARDS and the day of OLB. Missing data (NA) was completed using a multiple imputation by chained equation procedure. Five dataset for each cohort were created with the aim to reflect the NA uncertainty. In each dataset of the trainee cohort, univariate analysis was performed. All variables with a $P < 0.1$ in at least one dataset were included in the pooled logistic regression model (PLRM). The discriminatory capability of the PLRM was calculated in each dataset AUROC. Mean of the five AUROC (95% confidence intervals CI) from the trainee and the validation cohort was compared with the aim to estimate the over-fitting of the model. $P < 0.05$ was considered significant.

**RESULTS.** There were 258 patients included (n=193 trainee and n=65 validation cohort). The following variables recorded the day of ARDS predicted DAD: respiratory rate (OR 0.95; 95%CI 0.91; 0.99; P =0.03) and coronary ischemia (OR 5.97; 95%CI 1.67; 21.39; $P < 0.01$). Average AUROC was 0.64 (95%CI 0.56; 0.72) in the trainee cohort and 0.56 (95%CI 0.42; 0.71) in the validation cohort. The following variables recorded the day of OLB predicted DAD: PEEP (OR 1.13; 95%CI 1.05; 1.22; P< 0.01) and coronary ischemia (OR 6.82; 95%CI 1.86; 25.06; P< 0.01). Average AUROC was 0.70 (95%CI 0.62; 0.77) in the trainee cohort and 0.58 (95%CI 0.44; 0.72) in the validation cohort. The following variables recorded the day of ARDS predicted mortality: DAD (OR 2.29; 95%CI 1.23; 4.29; $P < 0.01$), diabetes mellitus requiring insulin (OR 0.08; 95%CI 0.01; 0.71; P 0.02) and respiratory rate (OR 1.01; 95%CI 1.01; 1.09; p 0.04). Average AUROC was 0.66 (95%CI 0.58; 0.73) in the trainee cohort and 0.51 (95%CI 0.36; 0.66) in the validation cohort. Finally, the following variables recorded the day of OLB predicted mortality: DAD (OR 2.08; 95%CI 1.05; 4.11; P=0.03), diabetes mellitus requiring insulin (OR 0.09; 95%CI % 0.01; 0.95; P= 0.04), PaCO2 (OR 1.05; 95%CI 1.02; 1.08; P< 0.01) and platelets count (OR 0.99; 95%CI 0.99; 0.99; P< 0.01). Average AUROC was 0.65 (95%CI 0.56; 0.72) in the trainee cohort and 0.63 (95%CI 0.48; 0.78) in the validation cohort.

**CONCLUSIONS.** This study confirms that DAD can only be predicted using data from the day of OLB and is associated with a twice higher risk of hospital mortality.

### 1230

#### Efficacy of prone ventilation on ARDS in patients with abdominal surgery - therapeutic evaluation by CT findings

M. Akatsuka[1], H. Tatsumi[1], S. Kazuma[1], Y. Katayama[1], H. Kuroda[1], Y. Goto[1], N. Yama[2], Y. Masuda[1]
[1]Sapporo Medical University School of Medicine, Department of Intensive Care Medicine, Sapporo, Japan; [2]Sapporo Medical University School of Medicine, Department of Radiology, Sapporo, Japan
**Correspondence:** M. Akatsuka
*Intensive Care Medicine Experimental* 2018, **6(Suppl 2):**1230

**INTRODUCTION.** In ARDS, the heterogeneity of lung lesions results in a mismatch between ventilation and perfusion, leading to the development of significant hypoxia. It has been reported that prone ventilation may potentially improve this ventilation-perfusion mismatch, but this hasn´t been fully investigated.

**OBJECTIVES.** To investigate the association between computed tomography (CT) findings for patients who had developed ARDS after abdominal surgery and improvements in hypoxia and mortality as a result of prone ventilation.

**METHODS.** The study subjects were intra-abdominal infection-induced ARDS patients who had undergone lung CT in the period from 2004 to 2014. The patients were allocated to a prone group that underwent prone ventilation within 24 h of the start of mechanical ventilation following ICU admission and a supine group that did not. Patients were categorized as showing either increasing ground glass opacity (GGO) or alveolar shadow with predominantly dorsal lung atelectasis (DLA) by a single radiologist.

In the prone group, $PaO_2/FiO_2$(P/F ratio) was determined before patients were moved to the prone position and 12 h later and at 24, 48, and 72 h after the start of prone ventilation. In the supine group, the ratio was determined 12 h after the start of mechanical ventilation and at 12, 24, 48, and 72 h after the initial measurement. P/F ratios at the start of initial measurement and 72 h later were compared in patients with GGO and DLA in both the prone and supine groups, and the number of ventilator-free days (VFD) and outcomes 28 days later were also compared.

**RESULTS.** In total, 53 patients with ARDS following abdominal surgery were admitted to the ICU during the study period. Twenty-five of them underwent prone ventilation and 28 did not.

There was no significant difference between the two groups in patients' characteristics. CT showed GGO in 6 patients in the prone group and in 10 patients in the supine group, DLA in 18 and 14patients, respectively, and both (GGO + DLA) in 1 patient and 4 patients, respectively.

Prone ventilation significantly improved the impaired P/F ratio from 12 h after prone positioning compared with that in the supine position. VFD in the prone group was significantly higher than that in the supine group. However, there was no significant difference in 28-day mortality between the two groups.

Weaning from mechanical ventilation was significantly earlier in patients in the prone group with DLA than in patients in the supine group with DLA, but there was no significant difference between the two groups for patients with GGO

The mortality rate after 28 days in patients in the prone group with DLA was significantly lower than that in patients in the supine group with DLA, but there was no significant difference between the two groups for patients with GGO.

**CONCLUSIONS.** The results suggest that prone ventilation may be an effective method for treatment of patients with intra-abdominal infection who develop ARDS.

### 1231

#### Review of the patients with moderate-severe acute respiratory distress syndrome in a polivalent ICU of a third level hospital

A. Vidal[1], E. Rosas[1], L.M. Polanco[1], D. Robaglia[1], L. Colino[1], A. Tejero[1], N. Arias[1], C. Pérez[1], M. Pérez[2]
[1]Fundación Jiménez Díaz, Madrid, Spain; [2]Hospital Rey Juan Carlos, Madrid, Spain
**Correspondence:** E. Rosas
*Intensive Care Medicine Experimental* 2018, **6(Suppl 2):**1231

**INTRODUCTION.** ARDS is a frequent and severe pathology in ICUs regardless of the cause of admission, with high costs to society and no specific treatment, for this reason is important to know the specific characteristics of these patients in order to approach to the best supportive management.

**OBJECTIVES.** Describe the epidemiological characteristics, ventilatory parameters and hemodynamic data of all the patients admitted in a polyvalent ICU of a third level hospital with the diagnosis of moderate - severe ARDS.

**METHODS.** Retrospectively, all patients diagnosed with ARDS according to Berlin criteria in moderate- severe range between 2015 and 2017, were taken into account in this study.

Epidemiological, gravity, admission causes and ARDS were collected; also ventilation data (mode, PEEP, volume, plateau, peek and driving pressure), rescue manoeuvres (prone, corticothetapy) and hemodynamics (invasive monitoring, need of noradrenalin, CVP, MAP). We also analysed need of reintubation and tracheostomy; and complications such as acute renal failure, barotrauma and ICU mortality.

**RESULTS.** A total of 44 patients were analysed (65% males), the average age was 63.03±13.9 years, the APACHEII gravity score at admission was 19.59±8.5, the ICU admission most frequent causes were respiratory (52.2%), post operatory (17.4%) and abdominal (8.3%). The ARDS most frequent etiology was respiratory (60.9%), abdominal (26.1%) and sepsis (6.5%).

The ventilatory modes used more frequently have been: VC (49.6%), PC (19.1%) y VCRP (10%). The average PEEP was 13.43±4.11 cmH2O, the driving pressure 12.22±5.1 cmH2O and compliance 36.9±27.8 cmH2O. The average tidal volume adjusted to ideal weight has been 7.36±1.39 ml/kg.

A 40% of the total patients were monitored with serial TTE diagnosing right ventricle failure in the 8.3%; and a 3.9% was monitored with pulmonary artery catheter.

Nearly half of the patients, a 48.7% needed Noradrenalin to maintain an average MAP of 82.47±13.9 mmHg and a CVP of 13.56±5.3 mmHg. Acute renal failure was found in the 36.5%, of which 48.7% needed extra renal depuration therapy.

Prone manoeuvre was used in a 43.5% of the patients, neuromuscular relaxation in a 50.4%, and corticotherapy in a 35.7%. Failure of weaning with needing of reintubation occurred in the 17.8% of the patients, and the 37% of the total were tracheostomized. The average of admission in the ICU has been 22.65±14.4 days, with 19.17±12.9 days of invasive mechanical ventilation. The percentage of mortality in our patients was 52.2% with respiratory (64.08%) and multiorgan failure (32%) as main causes.

**CONCLUSIONS.** The rise of life expectancy in the population leads to and increase in the average age.

The most commonly used ventilatory mode is volume control. The tidal volume is remarkably higher than expected, what could be related to the increasing rise of obesity in the population and the trend to adjust upwards the ideal weight. More hemodynamic monitoring should be done.

### 1232

#### Impact of gender in patients with Acute Respiratory Distress Syndrome: insights from the LUNG SAFE study

E. Rezoagli[1,2,3], B.A. McNicholas[3,4], C. Masterson[5], T. Pham[6], F. Madotto[7], G. Bellani[1], J.G. Laffey[2,8,9], LUNG SAFE Investigators and the ESICM Trials Group

[1]University of Milano-Bicocca, School of Medicine and Surgery, Monza, Italy; [2]National University of Ireland Galway, School of Medicine, Department of Anaesthesia, Galway, Ireland; [3]Galway University Hospitals, Department of Anaesthesia and Intensive Care Medicine, Galway, Ireland; [4]National University of Ireland Galway, School of Medicine, Discipline of Medicine, Galway, Ireland; [5]National University of Ireland Galway, Lung Biology, Cúram, Galway, Ireland; [6]St Michael's Hospita, Keenan Research Centre for Biomedical Science, Department of Critical Care Medicine, Toronto, Canada; [7]University of Milano-Bicocca, School of Medicine and Surgery, Research Centre on Public Health, Monza, Italy; [8]St Michael's Hospital, Critical Illness and Injury Research Centre, Keenan Research Centre for Biomedical Science, Departments of Anesthesia and Critical Care Medicine, Toronto, Canada; [9]University of Toronto, Departments of Anesthesia, Physiology and Interdepartmental Division of Critical Care Medicine, Toronto, Canada

**Correspondence:** E. Rezoagli

*Intensive Care Medicine Experimental* 2018, **6(Suppl 2):**1232

**INTRODUCTION.** The impact of gender on the management and outcome of patients with Acute respiratory Distress Syndrome (ARDS) is relatively poorly understood. Given the potential for important gender-based differences in incidence and management of ARDS, we wished to address these issues in this secondary analysis of the LUNG SAFE patient cohort (1-3).

**OBJECTIVES.** Our primary objectives were to examine the influence of gender on the ventilatory management and the outcomes in patients with ARDS.

**METHODS.** The large observational study to understand the global impact of severe acute respiratory failure (LUNG SAFE) was an international, multicenter, prospective cohort study of patients with severe respiratory failure. LUNG SAFE was conducted during 4 consecutive weeks in winter 2014, in a convenience sample of 459 intensive-care units (ICUs) in 50 countries across six continents. Inclusion criteria were admission to a participating intensive-care unit within the enrollment window and receipt of invasive or non-invasive ventilation. A secondary aim of trial was to characterize if there was a gender variation in the demographics, management, and outcome of patients with ARDS.

**RESULTS.** Among 2377 patients included in the analysis with diagnosis of ARDS on day-1 or day-2 of acute hypoxaemic respiratory failure and undergoing mechanical ventilation, 38% were female and 62% were male. Females had higher BMI compared to males. ARDS severity profile or Sequential Organ Failure Assessment scores were not different among the genders at ICU admission. According to the ventilator setting, females were ventilated with higher tidal volume per predicted body weight and higher plateau pressure compared to males. Female patients had a lower duration of mechanical ventilation and ICU and hospital stay compared to male patients. Despite this, there appeared to be no difference in crude mortality rates between the genders (see Table).

**CONCLUSIONS.** Gender appears to influence the management and outcomes of patients with ARDS. These data suggest a need for greater vigilance in the approach to the ventilator management in females.

**REFERENCE(S)**

1. Bellani G, Laffey JG, Pham T, et al. *JAMA*. 2016;315(8):788-800.
2. Madotto F, Pham T, Bellani G, et al. *Intens Care Med*. 2018; doi: 10.1007/s00134-018-5152-6.
3. Laffey JG, Bellani G, Pham T, et al. *Intens Care Med*. 2016; 42(12):1865-1876.

**GRANT ACKNOWLEDGMENT**

This work was funded and supported by the European Society of Intensive Care Medicine (ESICM), Brussels, Belgium, by St Michael's Hospital, Toronto, Canada, and by the University of Milan-Bicocca, Monza, Italy.

**Table 1 (abstract 1232).** Association among gender and outcomes in patients with ARDS. Differences among groups were tested using a Mann-Whitney U test and a Chi-Square test.

| Parameter | Female n=905 | Male n=1472 | P value |
|---|---|---|---|
| Invasive ventilation free days to Day 28, median (IQR), days | 13.0 (0.0;23.0) | 10.5 (0.0;22.0) | 0.162 |
| Duration of invasive mechanical ventilation, median (IQR), days | 7.0 (4.0;13.0) | 9.0 (4.0;16.0) | 0.004 |
| Duration of ICU Stay, median (IQR), days | 9.0 (5.0;17.0) | 11.0 (6.0;20.0) | 0.001 |
| ICU Mortality, n (%) | 320 (35.4) | 518 (35.2) | 0.933 |
| Duration of Hospital Stay, median (IQR), days | 16.0 (8.0;29.0) | 18.0 (9.0;35.0) | <0.001 |
| Hospital Mortality, n (%) | 362 (40.2) | 590 (40.2) | 0.984 |

### 1233

#### High C-reactive protein as a marker of prognosis in ARDS patients

V. Figueiredo[1], J. Moniz[2], G. Nobre de Jesus[1,2], D. Silva[3], J. Gouveia[1], A. Alvarez[1], S.M. Fernandes[1,2]
[1]Hospital de Santa Maria - Centro Hospitalar de Lisboa Norte, EPE, Lisboa, Portugal; [2]Faculdade de Medicina da Universidade de Lisboa, Lisboa, Portugal; [3]Hospital de Santa Maria - Centro Hospitalar de Lisboa Norte, EPE, SMI, Lisboa, Portugal
**Correspondence:** S.M. Fernandes
*Intensive Care Medicine Experimental* 2018, **6(Suppl 2):**1233

**INTRODUCTION.** Moderate to severe ARDS is linked to high mortality rates, despite improvement in lung support(1). Inflammatory ARDS phenotype has been linked to a worse prognosis(2). Prediction of prognosis in ARDS can help to allocate treatment, namely corticosteroid use. None of the biomarkers identified so far are easy to use or clinically available.

**OBJECTIVES.** We aimed to identify markers linked to prognosis in ARDS patients among variables collected on daily basis in ICU.

**METHODS.** We performed a retrospective study of patients with ARDS treated in an university hospital, where protective mechanic ventilation protocol is used. We included patients with moderate to severe ARDS, and retrospectively reviewed time of ARDS diagnosis. We excluded patients that died in the first 24 hours of ICU admission. We collected demographic data, laboratory data, and ventilator setting data. Statistical analysis was performed using STATA.

**RESULTS.** We identified 130 patients with ARDS in a 3 year period, with a mean age of 56.6 (±17.8), 69.2% were male. They featured a mean SAPS II of 47.1(±19.41) and 55% had sepsis. Of note, 80.8% had severe ARDS, with a mortality of 44.6%. Patients were ventilated for a mean of 13.3(±12.1) days, and had a median length of stay of 14 days.

Using non-adjusted logistic regression albumin levels in the first 48 hours (odds ratio: 0.44; P= 0.02) and C-reactive protein (CRP) above 20 on the third day (OR: 1.86, P=0.08) were the only factors related to prognosis. Tidal volume (6.5±1.5 in survivors vs 6.5±1.6 in non survivors, mL/kg ideal body weight, P= 0.92), PEEP (9.0 vs 8.5, P=0.41), or having sepsis (P=0.47) were not linked to mortality rates. We forced into the multivariate analysis other variables with a P value below 0.15 (bilirubin and respiratory rate), and adjusted for extracorporeal oxygenation support as possible confounder to high CRP levels in the first days of ARDS. After adjusting for these variables, CRP levels above 20mg/dL (OR: 2.4, P=0.04), as well as albumin levels (OR: 0.41; *P*=0.03)were still linked with ICU death. Of note, CRP/albumin ratio was also significantly linked with ICU mortality (P=0.01).

**CONCLUSION.** Inflammation is a major trigger of ARDS, and previous reports have linked higher inflammatory profiles to mortality(2). Using a simple approach towards inflammation assessment we linked high CRP levels in the third day of ARDS to a high mortality, which might be a more available marker to guide the institution of immunomodulatory treatment.

### 1234

#### Acute respiratory distress syndrome diagnosis and management: assessment of current practice in a tertiary care center intensive care unit

M. Mikhaeil[1], F. Priestap[2], K.J. Bosma[3]
[1]Western University, Medicine, London, Canada; [2]Schulich School of Medicine and Dentistry, Victoria Hospital, Medicine, London, Canada; [3]Western University, London Health Sciences Centre, Medicine, London, Canada
**Correspondence:** M. Mikhaeil
*Intensive Care Medicine Experimental* 2018, **6(Suppl 2):**1234

**INTRODUCTION.** Although the Acute Respiratory Distress Syndrome (ARDS) is common in medical-surgical intensive care units (MSICU) and is associated with significant morbidity and mortality, it is frequently unrecognized, especially in its mild forms, resulting in suboptimal management.

**OBJECTIVES.** We sought to assess how well ARDS was being diagnosed, based on the Berlin definition, and whether an evidence-based algorithm for the management of ARDS was being followed in our tertiary care MSICU.

**METHODS.** Over a 28-day period in the ICU, we assessed all admitted patients daily for the diagnosis of ARDS, using the Berlin criteria. Paper and electronic charts of those fulfilling criteria were audited for the documentation of the ARDS diagnosis. Various aspects of the management of ARDS were assessed.

**RESULTS.** Over a 28-day period, 90 patients were screened and 13 (14.4%) met Berlin criteria for ARDS. A total of 101 patient-days of ARDS management were assessed. Documentation of the diagnosis did not differ significantly between mild, moderate, or severe ARDS (p=0.217), but none of the diagnoses were missed on the 9 patient-days of severe ARDS. While ARDS was documented in physician charting in 77% of patient-days, a lung-protective ventilation strategy was utilized during only 33% of patient-days. Targets for low tidal volumes and high PEEP were met 17% and 55% of patient-days, respectively. During over half (53%) of patient days, patients were in an undesirable positive 24-hour fluid balance. Appropriate sedation targets were achieved 59% of the time. While NMB was initiated at 5 of the 7 times (71%) when it was warranted, there were a total of 11 patient-days (11%) of inappropriate use of NMB. Appropriate use of NMB did not differ based on the severity of ARDS (p=0.416), but during the 9 patient-days of severe ARDS, 8 had appropriate use of NMB. While no patients were proned inappropriately, there were 23 patient-days (23%) when proning should have been initiated, but wasn't. Appropriate use of proning was significantly different between the mild, moderate, and severe ARDS groups (p=0.004). The local spontaneous breathing trial protocol at our center was followed during only 44% of appropriate patient-days.

**CONCLUSIONS.** At a tertiary care MSICU, recognition of ARDS seems to be improving as compared to previously published rates, however many evidence-based elements of ARDS management were still not adhered to at acceptable rates.[1] Further research is required to look into the factors involved in decreased adherence to ARDS management practices. Results from this medical audit of clinical practice will be used to guide future quality improvement initiatives in the diagnosis and management of ARDS.

##### REFERENCE(S)

1. Bellani, G., Laffey, J.G., Pham, T., et al. Epidemiology, patterns of care, and mortality for patients with acute respiratory distress syndrome in intensive care units in 50 countries. *JAMA*, 2016; 315(8):788-800.

##### GRANT ACKNOWLEDGMENT

None

### 1235

#### Moderate and severe ARDS: hemodynamic and cardiac effects of an open lung strategy with recruitment maneuver analyzed using echocardiography

P. Mercado[1], J. Maizel[1], L. Kontar[1], M. Nalos[2], S. Huang[2], S. Orde[2], A. McLean[2], M. Slama[1]
[1]CHU Amiens-Picardie, Amiens, France; [2]Nepean Hospital, Sydney, Australia
**Correspondence:** M. Slama
*Intensive Care Medicine Experimental* 2018, **6(Suppl 2):**1235

**INTRODUCTION.** Open lung ventilation with a recruitment maneuver (RM) could be beneficial for ARDS patients. However, the increased airway pressures resulting from the RM may induce cardiac dysfunction, limiting the benefit of this maneuver.

**OBJECTIVES.** We analyzed the effect of a RM and decremental PEEP titration on cardiac function.

**METHODS.** Twenty patients hospitalized in our ICU (Medical ICU Amiens, France) with moderate to severe ARDS were included. Patients underwent a stepwise RM with respiratory evaluation and echocardiography assessment of cardiac function including longitudinal strain (LS) at baseline, peak PEEP of RM (PEEP 40cm$H_20$) and at 'optimal' PEEP. The patients were divided into two groups based on change on the P/F ratio (non-responders < 50%; responders≥50%).

**RESULTS.** At peak PEEP during the RM, the arterial pressure, cardiac output, left ventricular size decreased and right ventricular size increased. The left ventricular ejection fraction decreased from 60±13 to 48±18% (p,0.05). Both left and right ventricular global LS were impaired (-15.8±4.5 to -11±4.7% and -19±5 to -14±6% [p,0.05] respectively). Fifty percent of patients were non-responders, and demonstrated a lower hemodynamic tolerance to the RM than responders. Optimal PEEP was 14±5 cm$H_2O$ (vs 11±4 cm$H_2O$ at baseline) and P/F ratio increased from 111±25 to 197±89 mmHg (p< 0.0001). All hemodynamic parameters returned to their baseline value after the RM despite a higher PEEP.

**CONCLUSIONS.** An open lung strategy with a stepwise RM permitted a higher PEEP and improved oxygenation without any cardiac impairment. The RM was associated with mild, and transient, cardiac dysfunction, with non-responders demonstrating poorer tolerance.

### 1236

#### Ultrasonographic assessment of lung recruitment with two different positive end-expiratory pressure strategies in moderate to severe acute respiratory distress syndrome

A. Bagheri Moghaddam, M. Khorsand, S. Amini
University of Medical Science, Mashhad, Iran, Islamic Republic of
**Correspondence:** A. Bagheri Moghaddam
*Intensive Care Medicine Experimental* 2018, **6(Suppl 2):**1236

**INTRODUCTION.** The positive or negative effects of PEEP in patients with ARDS is a result of its effect on the opening the collapsed alveoli and its effect on excessive distention of the lung tissue. These effects have a direct correlation with the amount of lung recruitable tissue.Therefore, an accurate assessment of this recruitable pulmonary volume can be considered a key step in using the optimal amounts of PEEP in ARDS patients.

**OBJECTIVES.** Assessment of lung recruitment with aid of ultrasonography after two methods of PEEP titration in moderate to severe ARDS patients.

**METHODS.** This quasi-experimental study with "before and after"design was done in Mashhad University of medical sciences, Emam Reza hospital.mashhad.Iran

In this study,15 patients with moderate to severe ARDS who need invasive mechanical ventilation were selected. Initially, patients were ventilated according to ARDS net protocol and positive end-expiratory pressure (PEEP) selection were based on the lowest recommended level in the "low PEEP table", then lung aeration was assessed by ultrasonography.After 30 minutes, PEEP level was adjusted according to EXPRESS trial and assessment of lung aeration by ultrasonography were repeated. Both types of PEEP selection were applied to each patient and in fact, control and intervention group were the same.

Inclusion criteria: Moderate to severe ARDS patients (based on Berlin criteria ), older than18 and under invasive mechanical ventilation

Exclusion criteria: Known heart failure or acute ischemic heart disease, chronic respiratory disease, increased intracranial pressure, morbid obesity

**RESULTS.** 15 patients were included, of which 6 (40%) were male and 9 (60%) were female. The mean age of the patients was 46.13(±9.9). The frequency of patients with the acute respiratory syndrome (ARDS) with moderate and severe severity was 1 (6.7%) and 14 (93.3%) respectively. The average PO2 / FiO2 ratio of patients was 80.13 (±19.26) when entering the study. Positive end-expiratory pressure (PEEP) used in EXPRESS method (Mean = 16.73 cmH20) was higher than LOW PEEP method (Mean = 10.67 cmH20) (P < 0.001).

Loss of aeration scores were significantly lower in the EXPRESS method (Mean = 23) compared to the LOW PEEP method (Mean = 27) (P < 0.001).

The ratio of PO2 / Fio2 was significantly higher in the EXPRESS method (Mean = 104.6) than in the LOWPEEP (Mean = 87.27) method (P = 0.002)

**CONCLUSIONS.** The lung sonography can help selecting the appropriate PEEP for ARDS patients by identifying the lung recruitable tissue. An important limitation is the inability to detect lung overdistention.

**REFERENCE(S)**

1- Bouhemad B, Brisson H, Le-Guen M, Arbelot C, Lu Q, Rouby JJ. Bedside ultrasound assessment of positive end-expiratory pressure-induced lung recruitment. American journal of respiratory and critical care medicine. 2011;183(3):341-7.

**GRANT ACKNOWLEDGMENT**
No grant

### 1237

#### Effect of prone on PEEP-induced regional static strain in ARDS patients. Preliminary results

R. Cornejo[1], F. Álvarez[2], S. Havraneg[2], D. Arellano[1], L. López[3], A. Bruhn[4], C. Repetto[1], C. Romero[1], N. Estuardo[1], E. Tobar[1], C. Luengo[1], D. Guiñez[1], R. Brito[1], M.A. Cerda[1], D.E. Hurtado[2]
[1]Universidad de Chile, Hospital Clínico, Unidad de Pacientes Críticos, Santiago, Chile; [2]Pontificia Universidad Catolica de Chile, Institute for Biological and Medical Engineering, School of Engineering, Santiago, Chile; [3]Universidad de Chile, Hospital Clínico, Departamento de Radiología, Santiago, Chile; [4]Pontificia Universidad Catolica de Chile, Departamento de Medicina Intensiva, Facultad de Medicina, Santiago, Chile
**Correspondence:** R. Cornejo
*Intensive Care Medicine Experimental* 2018, **6(Suppl 2):**1237

**INTRODUCTION.** Regional strain is non-uniformly distributed in the mechanically ventilated, healthy and injured lung, and it has been spatially correlated with regional inflammation (1). Prone positioning decreases the dynamic strain (end-expiration to end-inspiration) gradient between dependent and nondependent lung regions in healthy lungs (2). However, the effect of prone on PEEP-induced static strain has not been explored in ARDS.

**OBJECTIVES.** The aim of this study was to explore the static strain induced by high PEEP on the regional level using computed tomography in moderate-severe ARDS patients in supine and prone.

**METHODS.** 5 mechanically ventilated patients (VT 6 ml/kg ideal body weight) underwent whole-lung computed tomography (CT) during breath-holding sessions at airway pressures of 5 and 15 cm $H_2O$. CT images were repeated in supine and prone positioning. A recruitment maneuver at 45 cm $H_2O$ was performed before each PEEP change. Low radiation CT protocol was applied. Using Nifty-Reg package (3), a transformation mapping was generated between a fixed image (end of inspiration) and a target image (end of expiration), which calculates an estimated displacement of every point inside the lung from inspiration to expiration. Following, a biomechanical analysis method was used (4) to construct 3D maps of the volumetric strain distribution for each patient´s lungs. Strain maps were divided into 10 isogravitational horizontal regions-of-interest (ROI) from CT images obtained in supine and prone positioning. To assess the gradient between dorsal and ventral regions, we considered the extreme ROI 2,3 versus ROI 8,9

**RESULTS.** Global static strain induced by high PEEP was not different between supine and prone positioning (44±20% versus 41±12%, p=0,68). However, the gradient of PEEP-induced regional static strain between ventral and dorsal regions (ROI 2,3 versus ROI 8,9) was significant lower in prone (33±23% versus 48±20%, p=0,049)

**CONCLUSIONS.** Prone positioning attenuates the gradient of static strain induced by high PEEP between dependent and nondependent regions

**GRANT ACKNOWLEDGEMENT**

FONDECYT 1151610 - 11070156

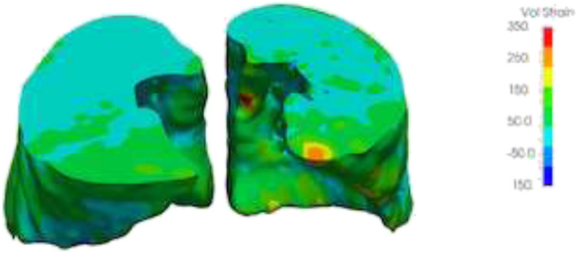

**Fig. 1 (abstract 1237).** Strain mapping from a patient in prone positioning

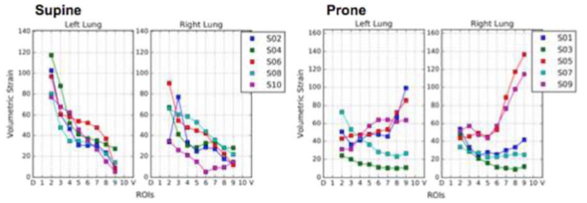

**Fig. 2 (abstract 1237).** Volumetric strain distribution for each patient's lungs (colors), in supine and prone positioning

### 1238

#### Predicting prone withdrawal success in ARDS patients: DP/Cstat index, the role of stretch (PROWISARDS)

J.O. Montoya Rojo[1], S.E. Zamora Gomez[1], L.A. Gorordo Delsol[1], M.A. Amezcua Gutierrez[1], I.M. Lima Lucero[1], E.S. Cruz Montesinos[1], J. Garduño López[1], B. Bolado Morales[2], M.A. Carrasco Flores[1], M. Hernández Romero[1], M.A. Toledo Rivera[1], G.D. Hernández López[1], J.A. Castañon González[1]

[1]Hospital Juarez de México, Intensive Care Unit, Mexico City, Mexico; [2]Hospital General de Hermosillo, Emergency Department, Hermosillo, Mexico

**Correspondence:** J.O. Montoya Rojo

*Intensive Care Medicine Experimental* 2018, **6(Suppl 2):**1238

**INTRODUCTION.** In a metanalysis conducted by Tonelli on 2014, they concluded that prone position, protective low tidal volumes and early use of neuromuscular blockers, are the only 3 interventions that have shown lower mortality rates in ARDS patients. patient´s response to prone withdrawal only can be known until change of position is performed. The Driving pressure/Static compliance (DP/Cstat) Index correlates the airway pressure with the lung static compliance, as an indicator of stretch.

**OBJECTIVES.** Identify the ability of the DP/Cstat Index to predict the success of prone position withdrawal.

**METHODS.** An analytical restrospective study was performed of the data base of Severe ARDS patients who were in Prone position (PP) during January to December 2017.The demographic variables included were age, sex, height, body mass index (BMI) and the etiology of SDRA. We calculated the DP/Cstat index before the prone position withdrawal, at time to supine position, 6,12 and 24 hours. A contingency tables 2x2 and square chi, were calculated for each one of the variables comparing them with the success of prone withdrawal at 24 hours.

**RESULTS.** 23 patients were included in the study, 21 males and 2 females, the average age was 35.4 yrs (15 - 58 yrs), average weight was 79.5 kg (55-115 kg), 28.5 kg/m2 was the average BMI (21.1 - 42.2). ARDS´s origin was pulmonary in 22 cases, and only 1 was extrapulmonary due pancreatitis. The average of length in continuous prone position strategy time was of 122.2 hours. Before prone position withdrawal, the PaO2/FiO2 average was 237.9, PaO2 88.2, CO2 36.3, Cstat of 33.7, driving pressure of 13.5 and DP/CStat of 0.5. 22 of the 23 patients accomplished with the criteria for prone positioning withdrawal, 15 patients had success of prone withdrawal at 24 hours, and 8 patients needed repositioning in prone. 6 hours after prone position withdrawal using a DP/Cstat cut-off < 0.51, we obtained 100% of sensitivity along with 100% of negative predictive value, 94% of positive predictive value and specificity of 88% with a strong association of 99% and Pearson R of 0.9 with prone position withdrawal success.

**CONCLUSIONS.** DP/Cstat index < 0.51 strongly correlated with success for prone position withdrawal with statistical significance. We consider the ability of this index to predict the success of prone position withdrawal is because the integration of these variables lead us infer lung stretch. Our study has several limitations, and a larger population is needed to corroborate the results of our investigation.

**REFERENCE(S)**

1- Pham T, Rubenfeld GD. The epidemiology of Acute Respiratory Distress Syndrome, Am J Respir Crit Care Med Vol 195, Iss 7, pp 860-870, Apr 1, 2017.

2- Cannon JW, Optimal Strategies for Severe Acute Respiratory Distress Syndrome Crit Care Clin 33 (2017) 259-275.

## Epidemiology & outcome in sepsis

### 1239

#### Epidemiology and outcome of source control procedures in critically ill patients with severe intra-abdominal infections

K. van de Groep[1,2], T.L. Verhoeff[3], D.M. Verboom[1,2], M.J. Schultz[4], M.J.M. Bonten[1,5], O.L. Cremer[2], on behalf of the MARS Consortium

[1]UMC Utrecht, Epidemiology, Julius Center for Health Sciences and Primary Care, Utrecht, Netherlands; [2]UMC Utrecht, Intensive Care Medicine, Utrecht, Netherlands; [3]UMC Utrecht, Anesthesiology, Utrecht, Netherlands; [4]Academic Medical Center, Intensive Care, Amsterdam, Netherlands; [5]UMC Utrecht, Medical Microbiology, Utrecht, Netherlands

**Correspondence:** K. van de Groep

*Intensive Care Medicine Experimental* 2018, **6(Suppl 2):**1239

**INTRODUCTION.** Source containment is essential in the treatment of intra-abdominal infections (IAI) in critically ill patients. However, there is a general lack of epidemiological data describing the types of interventions being performed, as well as their diagnostic yield, failure rate, and association with clinical outcome.

**OBJECTIVES.** To provide descriptive data that may aid physicians in making better informed decisions during the treatment of patients with severe IAI.

**METHODS.** We enrolled patients undergoing at least a single source control intervention because of presumed IAI during their stay in the Intensive Care Unit (ICU) of two university medical centres in the Netherlands between 2011 and 2015. Subsequently, we performed in-depth case reviews on these subjects and evaluated both procedural adequacy and clinical recovery after 14 days. Outcome was considered favourable if the anatomical infectious substrate was contained and the patient had been discharged alive from the ICU, both at day 14 after the first procedure.

**RESULTS.** In 353 ICU patients with presumed IAI, 496 surgical and 289 percutaneous procedures were performed. Overall, 178 (50%) patients required more than a single source control intervention (median 2 (IQR 1-3)). The initial substrate of infection was of gastrointestinal origin in 194 (57%) subjects (Table 1). Other sources included the biliary tract (15%) and pancreas (8%). In 24 (7%) patients no infectious source was identified during the first procedure, but in 7 cases subsequent procedures yielded positive findings. First procedures mainly aimed for restoration of anatomy and function, whereas repeat procedures frequently concerned drainage. Microbiological specimens were obtained during 386 (55%) procedures, and led in 28% of the occasions to modifications of the therapeutic regimen. In 236 (67%) patients, ICU-admission occurred immediately prior to (29%) or directly after (38%) their first source control procedure. Among this subgroup, adequate source control was achieved using a single procedure in 106 (45%) patients. In an additional 65 (29%) patients multiple procedures were required to obtain containment of the anatomical source of infection by day 14, whereas in the remaining 65 (28%) subjects source control was still inadequate. Favourable clinical outcome was observed in 97 (41%) of these patients. Persistence of organ failure in the first 7 days after the intervention was associated with unfavourable outcome, whereas trends in CRP seemed non-predictive (Figure 1). Although effective source control was eventually achieved for 227 (64%) of all included 353 patients with presumed IAI, hospital mortality remained high at 42%.

**CONCLUSIONS.** Approximately half of the ICU-patients with presumed IAI require more than a single intervention, yet successful source control is eventually achieved in a majority of them.

**GRANT ACKNOWLEDGMENT**

Center for Translational Molecular Medicine, MARS-project (grant 04I-201).

**Table 1 (abstract 1239).** Characteristics of 353 ICU patients with sepsis of presumed abdominal origin by primary cause

| | Initial substrate of infection | | | | |
|---|---|---|---|---|---|
| Variable | Gastro-intestinal n=202 (57%) | | Non-intestinal [1] n=151 (43%) | | p-value |
| *Patient characteristics* | | | | | |
| Age (years) | 66 | (58-72) | 64 | (54-72) | 0.31 |
| Gender (male) | 121 | (60) | 91 | (60) | 0.95 |
| Body mass index | 25 | (23-30) | 26 | (23-29) | 0.79 |
| History of solid malignancy | 70 | (35) | 48 | (32) | 0.57 |
| Known immune deficiency | 51 | (25) | 23 | (15) | 0.02 |
| Chronic corticosteroid use | 16 | (11) | 29 | (14) | 0.30 |
| Charlson comorbidity index | 1 | (0-3) | 1 | (0-2) | 0.20 |
| *Infection characteristics* | | | | | |
| Community-acquired onset | 69 | (34) | 58 | (38) | 0.41 |
| Prior abdominal surgery (within 14 days) | 97 | (48) | 60 | (40) | 0.12 |
| Definite likelihood of intra-abdominal infection[2] | 94 | (62) | 134 | (66) | 0.48 |
| APACHE-IV score | 86 | (72-103) | 83 | (69-104) | 0.50 |
| *Characteristics index procedure* | | | | | |
| Surgical (vs percutaneous) index procedure | 191 | (95) | 75 | (50) | <0.001 |
| Duration of antimicrobial therapy before index procedure (days) | 0 | (0-1) | 0 | (0-1) | 0.35 |
| Length of ICU-stay before index procedure (days) | 0 | (0-0) | 0 | (0-1) | 0.84 |

Patients were subdivided based on anatomical substrate of infection identified during their index-procedure. Data are presented as frequencies (column percentages) and medians (Q1-Q3).

[1] Non-intestinal subgroup includes 24 patients with no source of infection identified during the index procedure.
[2] Based on post-hoc assessment by trained researchers based on all clinical information during all ICU-admissions.

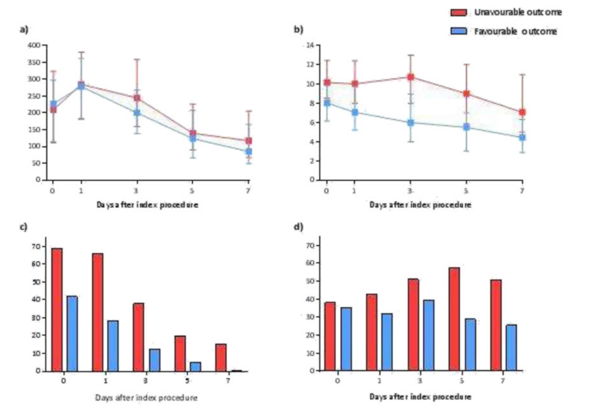

Patients with their index procedure at ICU-admission (n=236) were divided based on outcome status after 14 days. Daily clinical parameters presented are: a) CRP, b) Sequential organ failure assessment (SOFA)-score, c) Septic shock, and d) Gastro-intestinal failure (i.e. insufficient enteral feeding, use of prokinetic medication, severe diarrhea/ vomiting, or increased abdominal pressure). Results are presented as proportion or median with Q1-Q3. Missing values were replaced with estimates derived from multiple imputation.

**Fig. 1 (abstract 1239).** Course of clinical parameters by outcome status during the first week after the index procedure

### 1240

#### Influence of fluid administration in the first ICU day on septic patients mortality. Analysis of an electronic database

A. Naharro-Abellan[1], D. Domínguez Barbero[2], P. Matía Almudevar[1], A. Pérez Lucendo[1], A.P. Rodriguez Villamizar[1], S. Alcántara Carmona[1], J. Veganzones Ramos[1]

[1]Hospital Universitario Puerta de Hierro Majadahonda, Medicina Intensiva, Majadahonda, Spain; [2]Universidad Internacional Mendendez Pelayo, Madrid, Spain

**Correspondence:** A. Naharro-Abellan

*Intensive Care Medicine Experimental* 2018, **6(Suppl 2):**1240

**INTRODUCTION.** Sepsis is a major cause of mortality in the ICU with fluid resuscitation being an important part of its treatment. Although it appears that positive fluid balance is associated with an increase in mortality, two recent large cohorts studies[1,2] have produced contradictory results.

**OBJECTIVES.** Our objective was to replicate the analysis of Marik et al[1], which demonstrated that administration of ≥5L of fluid during first ICU day lead to an increase in mortality, but using the actual Sepsis-3 definition[3] and SOFA score for the adjusted mortality analysis.

**METHODS.** We constructed a study cohort from the Medical Information Mart in Intensive Care (MIMIC)-III database v1.4[4] We identified infected patients adhered to the original Sepsis-3 study[3]. We included only the first ICU admission of adult patients with a SOFA score ≥2. We recorded the total amount of IV volume administered during the first 24 hours since ICU admission and the SOFA score.

**RESULTS.** A total of 5728 first admissions met our inclusion criteria. Demographic data are represented in Table 1.
The ability of SOFA to predict mortality had an AUC of 0.743. The actual vs. predicted mortality and the distribution of cases by Day 1 fluid administration groups are represented in Figure 1. In the overall analysis there was an association between fluid intake and mortality (OR 1.07;CI95 1.05-1.09;p< 0,0001), but after adjusting by SOFA the association became negative (OR 0.97;CI95 0.95-0.99;p=0.022). The same results were found dividing patients by < 5L or ≥5L: unadjusted (RR 1.55; CI951.32-1.82;p< 0.0001) and adjusted (OR 0.87;CI95 0.72-1.04;p=0.13).
**CONCLUSIONS.** In our study the amount of IV fluid administered during the first ICU day was associated with higher mortality, but after adjusting by SOFA, it turned protector. Higher fluid intake were correlated with higher SOFA scores. This was probably due to a relationship between severity and the need for a more aggressive resuscitation, but it could also be the result of a worsening state secondary to a generous fluid intake.
Our data suggest that a hight fluid intake is beneficial in those cases were it does not increase the SOFA score, and it turns harmful when it leads to an increase of this score. This might reflect a physiologically situation where fluid administration exerts a hemodynamic benefit until an inflection point beyond after which it becomes detrimental.

**REFERENCE(S)**

1. Marik PE, et al. Fluid administration in severe sepsis and septic shock, patterns and outcomes: an analysis of a large national database. Intensive Care Med. 2017;43(5):625-632.
2. Sakr Y, et al. Higher Fluid Balance Increases the Risk of Death from Sepsis: Results from a Large International Audit. Crit Care Med. 2017;45(3):386-394.
3. Singer M, et al. The third international consensus definitions for sepsis and septic shock (sepsis-3). JAMA - J Am Med Assoc. 2016;315(8):801-810.
4. Johnson AEW, et al. MIMIC-III, a freely accessible critical care database. Sci Data. 2016;3:1-9.

**Table 1 (abstract 1240).** Demographics

| Variable | <5L | ≥5L | Total | |
|---|---|---|---|---|
| Case count (%) | 3206 (56%) | 2522 (44%) | 5728 | |
| Age (mean ± SD) | 67.9 ± 17.2 | 62.2 ± 17.7 | 65.4 ± 17.7 | p < 0.001 |
| Male (%) | 1685 (52.6%) | 1504 (59.6%) | 3189 (55.7%) | p < 0.001 |
| Race (White) | 2380 (74.2%) | 1777 (70.5%) | 4158 (72.6%) | p = 0.001 |
| Race (Black) | 297 (9.3%) | 198 (7.9%) | 495 (8.6%) | p = 0.059 |
| Mechanical Ventilation | 1189 (37.1%) | 1598 (63.4%) | 2787 (48.7%) | p < 0.001 |
| Shock (use of vasopressors) | 384 (12.0%) | 876 (34.7%) | 1260 (22.0%) | p < 0.001 |
| Medical Service | 2497 (77.9%) | 1657 (65.7%) | 4154 (72.5%) | p < 0.001 |
| SOFA (mean ± SD) | 4.7 ± 2.6 | 6.3 ± 3.7 | 5.43 ± 3.2 | p < 0.001 |

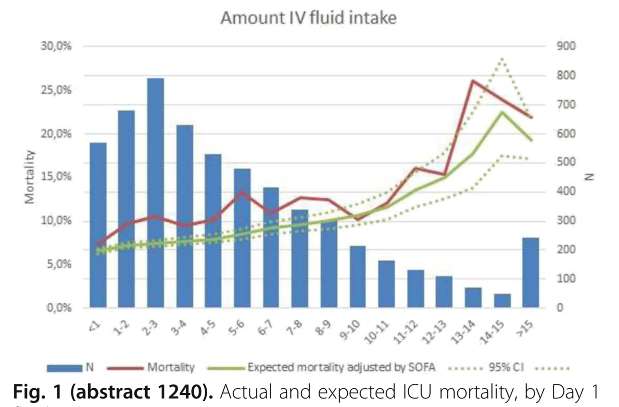

**Fig. 1 (abstract 1240).** Actual and expected ICU mortality, by Day 1 fluid groups

### 1241

#### Sepsis-related deaths in the at-risk population on the wards: attributable fraction of mortality in a large point-prevalence study

M. Kopczynska, B. Sharif, T. Szakmany, Welsh Digital Data Collection Platform University, Anaesthesia, Intensive Care and Pain Medicine, Division of Population Medicine, Cardiff, United Kingdom
**Correspondence:** M. Kopczynska

*Intensive Care Medicine Experimental* 2018, **6(Suppl 2):**1241

**INTRODUCTION.** Sepsis mortality is reported to be high worldwide, however recently the attributable fraction of mortality due to sepsis (AFsepsis) has been questioned [1]. If improvements in treatment options are to be evaluated, it is important to know what proportion of deaths are potentially preventable or modifiable after a sepsis episode.
**OBJECTIVES.** The aim of the study was to establish the fraction of deaths directly related to the sepsis episode on the general wards and ED.
**METHODS.** Secondary analysis of patient episodes was performed on patient population recruited into two annual 24-hour point-prevalence studies on the general wards and ED across all Welsh acute hospitals in 2016 and 2017 [2]. Inclusion criteria were: clinical suspicion of infection and NEWS 3 or above in-line with established escalation criteria in Wales. Sepsis was defined according to Sepsis-3 definition based on SOFA and qSOFA scores. Clinical Frailty Scores and basic demographic information were also analysed. Deaths attributable to sepsis were evaluated based on microbiological, radiological and laboratory evidence. In addition, mortality was not attributed to sepsis if the patient has been discharged form hospital more than 7 days before death.
**RESULTS.** 791 patients were recruited over the two 24-hour periods in 2016 and 2017. 514 patients fulfilled Sepsis-3 criteria. 142 patients (27.1%) with sepsis and 47 patients (16.9%) without sepsis died within 90 days. Out of the 142 sepsis deaths 19 (13.4%) could have been directly related to sepsis, 41 (28.9%) possibly related and 82 (57.7%) were not related to sepsis. Overall AFsepsis was 30%. Upon analysis of the 60 deaths likely to be attributable to sepsis, we found that 48 patients had the Clinical Frailty Score >6, 40 had existing DNA-CPR order and 30 had limitations of care orders.
**CONCLUSIONS.** We are the first to report in a well-defined at-risk group outside critical care, that AFsepsis is only 30% and even this could be an overestimation due to the pre-illness trajectories and expectations, based on the frailty scores and limitation of care orders. Our results are similar to those reported in the ICU population [1]. The low proportion of preventable and modifiable elements of sepsis deaths should inform the design of interventional studies. More appropriate identification of patients who could actually benefit from aggressive interventions should be considered not just based on acuity, but also on pre-admission trajectories.

**GRANT ACKNOWLEDGMENT**

### 1242

#### The comparison of infection prevelance, sepsis development, mortality and morbidity rates in traumatic brain injury

I. Saydere[1], A. Sagün[1], M. Azizoglu[1], A.A. Altunkan[2]
[1]Mersin University Medical Faculty, Anestehesiology and Reanimation, Mersin, Turkey; [2]Mersin University Medical Faculty, Intensive Care Unit, Mersin, Turkey
**Correspondence:** I. Saydere

*Intensive Care Medicine Experimental* 2018, **6(Suppl 2):**1242

**INTRODUCTION.** Systemic infections and sepsis in severe head trauma are more frequent when compared to patients without trauma. In this study, we aimed to compare infection incidence and related sepsis development, morbidity and mortality rates in patients with head trauma.

**OBJECTIVE AND METHODS.** In this retrospective study, we included 80 patients aged 18-80 years who had traumatic brain injury between 2013 and 2016, at least one of peripheral, central blood, tracheal aspirate, and urine cultures to the intensive care unit.

**RESULTS.** The incidence of infection, sepsis development, morbidity and mortality rates were compared in these patients. In addition, the most commonly used scoring systems such as SOFA and APACHE II have been evaluated in intensive care unit. In our study, SOFA was found significant in terms of evaluation of organ dysfunction and early sepsis. Acinetobacter was the most frequent cause of nosocomial infections in the patients and the lungs were the most frequently isolated organ.

**CONCLUSION.** We would like to emphasize that our results from our study suggest that patients with head trauma are at high risk for sepsis development and mortality. Therefore it is clear that these patients need clinical follow-up and follow-up from the time of admission.

CRP, procalcitonin, lactate levels were assessed in the patients and it may be appropriate to use these laboratory tests as an indicator of sepsis progression but it was thought that these tests alone would not be enough. Scoring systems such as APACHE II, SOFA are also considered to be used as supportive diagnostic parameters in relation with these laboratory tests.

**KEYWORDS.** Head Trauma, Sepsis, C-Reactive Protein, Procalcitonin, Lactate, SOFA

**Table 1 (abstract 1242).** Comparison of APACHE II and SOFA Scores in Sepsis and Non-Sepsis Patients

| Scores (Avarege.±SD) | Sepsis (n=51) | Non-sepsis (n=29) | P |
|---|---|---|---|
| Admission APACHE II | 18.80±7.6 | 15.00±3.9 | 0.14 |
| First week | 35.70±7.8* | 11.40±5.9* | 0.00 |
| Second week | 56.30±12.4*# | 5.10±8.4*# | 0.00 |
| Admission SOFA | 4.00±2.1 | 2.30±2.0 | 0.00 |
| First week | 5.50±3.1* | 2.70±2.1* | 0.00 |
| Second week | 7.40±5.1*# | 1.80±3.2*# | 0.00 |

**Table 2 (abstract 1242).** Comparison of Infection Parameters in Sepsis and Non-Sepsis Patients

| | Infection cases (Avarage ±SD) | Sepsis (n=51) | Non-Sepsis (n=29 | P |
|---|---|---|---|---|
| CRP (mg/dl) | Admission | 33.00±71.9 | 22.30±40.4 | 0.46 |
| CRP (mg/dl) | First week | 117.10 ±96.2* | 35.90±52.5* | 0.00 |
| CRP (mg/dl) | Second week | 125.60 ±95.9* | 24.70±40.7 | 0.00 |
| **Lactate (mmol/L)** | **Admission** | **3.80±2.6** | **2.70±2.2** | **0.42** |
| **Lactate (mmol/L)** | **First week** | **16.12±5.6*** | **1.20±2.3*** | **0.00** |
| **Lactate (mmol/L)** | **Second week** | **23.20 ±11.1*#** | **0.10±1.2*** | **0.00** |
| Procalcitonin (ng/mL) | Admission | 0.44±0.6 | 0.34±0.44 | 0.43 |
| Procalcitonin (ng/mL) | First week | 17.40±25.6* | 0.43±0.75* | 0.00 |
| Procalcitonin (ng/mL) | Second week | 28.90 ±33.9*# | 0.24±0.31* | 0.00 |

### 1243

#### Differences in the outcomes of patients with sepsis and septic shock admitted to either public or private hospitals in Argentina, a low-middle Income country (LMIC), using Sepsis-3 definitions

E. Estenssoro[1], C. Loudet[1], V. Edul[2], J. Osatnik[3], A. Dubin[4], F. Ríos[5], D. Vazquez[6], M. Pozo[7], B. Lattanzio[8], F. Klein[9], G. Zakalik[10], A. Diaz[11], SATISEPSIS Study Group

[1]Hospital San Martín, La Plata, Argentina; [2]Hospital Fernández, Buenos Aires, Argentina; [3]Hospital Alemán, Buenos Aires, Argentina; [4]Sanatorio Otamendi, Buenos Aires, Argentina; [5]Hospital Posadas, El Palomar, Argentina; [6]Sanatorio Anchorena, Buenos Aires, Argentina; [7]Clínica Bazterrica, Buenos Aires, Argentina; [8]Sanatorio Santa Isabel, Buenos Aires, Argentina; [9]Hospital Favaloro, Buenos Aires, Argentina; [10]Hospital Lagomaggiore, Mendoza, Argentina; [11]Hospital Misericordia, Córdoba, Argentina

**Correspondence:** E. Estenssoro

*Intensive Care Medicine Experimental* 2018, **6(Suppl 2):**1243

**INTRODUCTION.** There is scarcity of information about sepsis epidemiology in LMICs. Moreover, big contrasts in outcomes and processes of care might occur in different healthcare settings within the region.

**OBJECTIVES.** To analyze differences in mortality between public and private hospitals in Argentina, using the Sepsis-3 definitions.

**METHODS.** Multicenter, national prospective cohort study performed during 7/1-9/1/16 including consecutive patients admitted to 49 Argentine ICUs with suspected infection, which prompted taking blood cultures and antibiotic administration. Considered groups were Infection; Sepsis (infection+change in SOFA≥2) and Septic shock (vasopresors+lactate ≥2 mmol/L. Epidemiological, clinical, infectological, and human-resource data were recorded. Hospital mortality was the main outcome variable. A $p< 0.05$ was considered statistically significant.

**RESULTS.** 809 patients were admitted to 23 ICUs in the public health system and to 26 in the private. Comparisons are shown in Table 1.

**CONCLUSIONS.**

- Compared to patients from private hospitals, patients admitted to public hospitals exhibited profound and significant differences:
- They were younger, sicker and had more frequent comorbidities; yet their autoperceived previous health status as measured by Euro-QoL visual analogue scale was not different from that of patients admitted to private hospitals.
- They had less years of education and, notably, the duration of symptoms of their present disease doubled that of the patients in the private sector.
- These issues point to a lack of awareness of the severity of the disease in patients admitted to from public hospitals-possibly aggravated by similar unawareness in the physicians that had evaluated them previously.
- Thus, septic shock and higher SOFA on admission were more frequent in patients admitted to public hospitals, and mortality was higher. Patients were admitted later in the course of disease.
- Regarding sepsis management, the only difference was in the presence of more frequent infections by multi-resistant microorganisms in the public sector. Human resources were greater in public hospitals.

**GRANT ACKNOWLEDGMENT**

This study was supported by a grant of the Sociedad Argentina de Terapia Intensiva (SATI).

**Table 1 (abstract 1243).** See text for description

| | Public N=23 ICUs | Private N=26 ICUs | P |
|---|---|---|---|
| Patients (N, %) | 367(45) | 442(55) | |
| Age (mean, SD) | 56±18 | 64±18 | 0.000 |
| Female gender (N, %) | 157/367(43) | 197/442(55) | 0.609 |
| BMI (kg/m²) (mdn, IQR) | 26[23-29] | 27[24-31] | 0.0057 |
| Years of education (mdn, IQR) | 7[7-12] | 12[10-16] | 0.000 |
| Charlson score(mdn, IQR) | 2[0-4] | 1[0-3] | 0.0013 |
| Duration of symptoms of present disease before being admitted to the hospital (hours) (mdn, IQR) | 48[24-96] | 24[12-48] | 0.009 |
| Distance home-present hospital (km) (mdn, IQR) | 0[0-10] | 1[0-10] | 0.1257 |
| Patients previously evaluated but not admitted to the hospital (N, %) | 98/356(28) | 85/433(20) | 0.009 |
| Autoperceived global health state (EuroQoL-VAS) (mdn, IQR) | 70[50-90] | 70[50-90] | 0.295 |
| Septic shock at admission (N, %) | 172(47) | 155(35) | 0.000 |
| Admission (Medical/elected surgery/emergency surgery) (%) | 74/5/20 | 74/8/18 | 0.379 |
| APACHE II (mean, SD) | 20±9 | 18±7 | 0.000 |
| SOFA score (mdn, IQR) | 8[5-11] | 6[4-9] | 0.000 |
| Lactate (mmol/L)(mdn,IQR) | 2[2-4] | 2[1-3] | 0.0049 |
| Sepsis (Community/hospital/ICU/3erLevel-unknown) (%) | 54/32/11/2 | 55/32/9/3 | 0.001 |
| Time to 1st dose of antibiotics (hours) (mdn, IQR) | 2[1-3] | 2[1-3] | 0.4685 |
| Received 30 ml/kg of fluids if MAP<65 mmHg (N, %) | 264/356(74) | 309/430(71) | 0.471 |
| Inadequate antibiotics/adequate/negative blood cultures) (%) | 13/56/31 | 11/61/28 | 0.309 |
| High-mortality isolated microorganisms (N, %) | 93/367(26) | 85/442(19) | 0.015 |
| ICUs >14 beds | 9/23 (39) | 14/23 (60) | 0.188 |
| Nurse:patient ratio (1:1-2/ 1:3-4/1:4-5) (%) | 66/34 | 65/30/5 | 0.000 |
| Board-certified CC specialists (mdn, IQR) | 11[8-30] | 8[6-16] | 0.000 |
| Residents in CC | 5[2-12] | 4[0-10] | 0.0007 |
| Mortality (N, %) | 172/367(47) | 131/442(30) | 0.000 |

### 1244

### Factors associated with mortality in patients with nosocomial infection in a mixed ICU after 6 years of selective digestive decontamination

C. Sánchez Ramírez[1], S. Hípola Escalada[1], L. Caipe Balcázar[1], M.A. Hernández Viera[1], M. Cabrera Santana[1], R.E. Morales Sirgado[1], C.F. Lübbe Vázquez[1], F. Artíles Campelo[2], P. Saavedra Santana[3]

[1]University Hospital of Gran Canaria Dr Negrín, Intensive Care Unit, Las Palmas de Gran Canaria, Spain; [2]University Hospital of Gran Canaria Dr Negrín, Microbiology Department, Las Palmas de Gran Canaria, Spain; [3]University of Las Palmas de Gran Canaria, Mathemathics and Informatics Department, Las Palmas de Gran Canaria, Spain

**Correspondence:** C. Sánchez Ramírez

*Intensive Care Medicine Experimental* 2018, **6(Suppl 2):**1244

**OBJECTIVES.** To analyze the mortality risk factors in patients with nosocomial infections (NI), in a mixed ICU with selective digestive decontamination (SDD).

**METHODS.** Prospective study in patients who developed NIs were included from October 1st, 2010 to September 30th, 2017 in a 30-bed ICU. SDD was applied to patients requiring mechanical ventilation for more than 48 hours. An enteral solution and a paste with colistin, tobramycin and nistatin given every 8 hours until ICU discharge, was applied. Also intravenous cefotaxime was administered during the first 4 days. Rectal and pharyngeal exudates were requested on admission and weekly. The categorical variables were summarized in frequencies and percentages and the numerical variables in means and standard deviations or in medians and interquartile ranges. The percentages were compared with the X2 test or the Fisher exact test, the means with the t-test and the medians with the Wilcoxon test for independent data. A multidimensional logistic analysis was carried out. It was considered significant if $p \leq .05$.

**RESULTS.** Among 6291 admitted patients, 194 (36.44%) out of 532 patients with NI died. In a univariate analysis, APACHE II. age, type of admission, inflammatory response, traumatogic patient, immunosuppression, neutropenia, parenteral nutrition, presence of ventricular device catheter, renal replacement therapy, malnutrition, diabetes mellitus, chronic obstructive pulmonary disease, renal failure, cirrhosis, neoplasm, nosocomial pneumonia, antibiotic 48 hours previous to admission, Extended Spectrum Beta-Lactamase (ESBL) producing bacteria (p= 0.04) and multiresistant (MR) Pseudomonas (MR) (p= 0.024) were statistically significant. Among the patients who died versus survivals, ICU stay was not significantly different (Table 1)

The independent factors associated with mortality were: APACHE II, neoplasm, renal replacement therapy, septic shock, parenteral nutrition and COPD (Table 2)

**CONCLUSIONS.** In a ICU with SDD, factors that were independently associated with mortality were: APACHE II, neoplasm, renal replacement therapy, septic shock, parenteral nutrition and COPD. Also, MR Pseudomonas infections and ESBL were associated with greater mortality.

**Table 1 (abstract 1244).** Univariate analysis

| . | Overall N = 532 | Survivor N = 338 | Death N = 194 | P |
|---|---|---|---|---|
| ***Age**, years* | 60.7 ± 15.4 | 58.9 ± 15.9 | 63.7 ± 13.9 | < .001 |
| ***Sex male*** | 355 (66.8) | 229 (67.8) | 126 (65.3) | 0.561 |
| ***Apache-II*** | 22.0 ± 7.6 | 20.4 ± 7.2 | 24.8 ± 7.6 | < .001 |
| ***Admission*** | | | | < .001 |
| Medical | 380 (71.6) | 221 (65.4) | 159 (82.4) | |
| Scheduled surgical | 74 (13.9) | 62 (18.3) | 12 (6.2) | |
| Emergency surgical | 77 (14.5) | 55 (16.3) | 22 (11.4) | |
| ***Inflammatory response*** | | | | < .001 |
| Non sepsis | 23 (4.3) | 16 (4.7) | 7 (3.6) | |
| Sepsis | 174 (32.7) | 137 (40.5) | 37 (19.1) | |
| Severe sepsis | 81 (15.2) | 59 (17.5) | 22 (11.3) | |
| Septic shock | 254 (47.7) | 126 (37.3) | 128 (66.0) | |
| ***Traumatology patient*** | 68 (12.8) | 58 (17.2) | 10 (5.2) | < .001 |
| ***Coronary patient*** | 110 (20.7) | 70 (20.7) | 40 (20.6) | 0.980 |
| ***Emergency surgery*** | 144 (27.1) | 95 (28.1) | 49 (25.3) | 0.477 |
| ***Immunossupression*** | 50 (9.4) | 17 (5.0) | 33 (17.0) | < .001 |
| ***Neutropenia*** | 17 (3.2) | 6 (1.8) | 11 (5.7) | 0.014 |
| ***Immunodepresion*** | 5 (0.9) | 4 (1.2) | 1 (0.5) | 0.657 |
| ***Parenteral nutrition*** | 161 (30.3) | 74 (21.9) | 87 (44.9) | < .001 |
| ***Venticular device catheter*** | 48 (9.0) | 42 (12.4) | 6 (3.1) | < .001 |
| **RRT** | 194 (36.5) | 70 (20.7) | 124 (63.9) | < .001 |
| ***Malnutrition*** | 49 (9.2) | 25 (7.4) | 24 (12.4) | 0.084 |
| ***Diabetes mellitus*** | 170 (31.9) | 88 (26.0) | 82 (42.3) | < .001 |
| ***COPD*** | 77 (14.5) | 36 (10.7) | 41 (21.1) | < .001 |
| ***Renal failure*** | 126 (23.7) | 57 (16.9) | 69 (35.6) | < .001 |
| ***Cirrhosis*** | 30 (5.6) | 11 (3.2) | 19 (9.8) | 0.003 |
| ***Neoplasm*** | 52 (9.8) | 16 (4.7) | 36 (18.6) | < .001 |
| ***Nosocomial pneumonia*** | 210 (39.5) | 119 (35.2) | 91 (46.9) | 0.008 |
| ***Bacteremia related to catheter*** | 190 (35.7) | 126 (37.3) | 64 (33.0) | 0.320 |
| ***Secondary bacteremia*** | 134 (25.2) | 77 (22.8) | 57 (29.4) | 0.091 |
| ***Urine infection*** | 139 (26.1) | 92 (27.2) | 47 (24.2) | 0.450 |
| ***ATB 48 h*** | 143 (26.9) | 80 (23.7) | 63 (32.5) | 0.021 |
| ***ICU days*** | 31 (18 – 49) | 28 (16 – 46) | 32 (19 – 49) | 0.150 |
| ***Acinetobacter baumannii*** | 18 (3.4) | 11 (3.2) | 7 (3.6) | 0.828 |
| ***MRSA*** | 11 (2.1) | 8 (2.4) | 3 (1.6) | 0.754 |
| ***ESBL*** | 132 (24.8) | 74 (21.9) | 58 (29.9) | 0.040 |
| ***MR Pseudomonas*** | 43 (8.1) | 20 (5.9) | 23 (11.9) | 0.024 |
| ***MR-GNB*** | 28 (5.3) | 14 (4.1) | 14 (7.2) | 0.126 |
| ***SDD*** | 422 (79.3) | 264 (78.1) | 158 (81.4) | 0.360 |

Data are means SD and frequencies (%); ATB 48 h: antibiotic 48 h previous to admission; SDD: selective digestive decontamination; COPD; chronic obstructive pulmonary disease; RRT: renal replacement therapy; MRSA: methicillin resistant Sthaphyloccocus aureus ESBL: extended spectrum beta-Lactamase; MR: multi-resistant; GNB: gram negative bacteria

**Table 2 (abstract 1244).** Multivariate analysis

| | P | OR (CI-95%) |
|---|---|---|
| *Apache-II, per unity* | < .001 | 1.069 (1.038 - 1.101) |
| *Septic shock* | < .001 | 2.807 (1.817 - 4.338) |
| *Parenteral nutrition* | < .001 | 2.265 (1.437 - 3.570) |
| RRT | < .001 | 4.745 (3.062 - 7.353) |
| *COPD* | 0.009 | 2.144 (1.211 - 3.795) |
| *Neoplasm* | < .001 | 4.834 (2.363 - 9.891) |

COPD; Chronic obstructive pulmonary disease; RRT: renal replacement therapy

### 1245

#### Sepsis after elective surgery - a nationwide study on etiology and outcome

E. Vesteinsdottir[1,2], S. Karason[1,2], P. Steinsdottir[2], H. Oskarsson[1,2], M.I. Sigurdsson[3], A. Blondal[4], G.H. Sigurdsson[1,2]
[1]Landspitali, Department of Anesthesia and Intensive Care, Reykjavík, Iceland; [2]University of Iceland, Faculty of Medicine, Reykjavik, Iceland; [3]Duke University Hospital, Department of Anesthesiology, Durham, United States; [4]Akureyri Hospital, Department of Anesthesia and Intensive Care, Akureyri, Iceland
**Correspondence:** E. Vesteinsdottir
*Intensive Care Medicine Experimental* 2018, **6(Suppl 2):**1245

**INTRODUCTION.** Sepsis that requires an admission to intensive care is a rare complication of elective surgery and is associated with a high mortality and morbidity. Many previous studies on post-operative sepsis focus on specific types of surgery or patient groups in tertiary referral settings.
**OBJECTIVES.** To describe the incidence, etiology and outcomes of patients admitted to intensive care with sepsis after elective surgery.
**METHODS.** All adult admissions to all Icelandic intensive care units (ICU) during six whole calendar years (2006, 2008, 2010, 2012, 2014 and 2016) were screened for severe sepsis or septic shock using the ACCP/SCCM criteria. Demographics and clinical course of patients with severe sepsis or septic shock after hospital admission for elective surgery were compared with data from 564 patients admitted to the ICUs because of sepsis unrelated to surgery.
**RESULTS.** 88 patients were admitted to the ICUs with severe sepsis or septic shock after elective surgery during the study period. The mean age was 66.1 year and 65% were male. The most common surgical procedures were abdominal (64%), cardiothoracic (9%) and urologic (8%). The median ICU admission day was the 6th post-operative day. The infection site was most commonly the abdomen (59%), lungs (22%) and urinary tract (5%). The median ICU and hospital length of stay was 5.5 and 26 days, respectively. This was longer than for the comparison group (3 and 13.5 days respectively ($p< 0.05$)). The hospital mortality was 26% and the 1-year mortality was 41%, which did not differ from the comparison group (30% and 41% respectively (NS)). After excluding those who died in hospital only 48% of the group could be discharged directly home from the surgical ward. This was lower than in the comparison group (68% ($p< 0.05$)). 72% of patients in the study group had positive cultures and they had a higher incidence of multi-resistant pathogens compared with other septic patients (24% vs. 5% of patients with positive cultures ($p< 0.05$)). The initial empirical antibiotic therapy covered all cultured micro-organisms in only 43% of patients in the study group who had positive cultures, which was lower than in the comparison group (85% ($p< 0.05$)).
**CONCLUSIONS.** The most common cause of sepsis requiring ICU admission after elective surgery are infections complicating abdominal procedures. The ICU and hospital length of stay is long in this patient group and mortality rates are high. The initial antibiotic therapy needs careful evalulation given the increased frequency of multi-resistant pathogens.

**GRANT ACKNOWLEDGMENT**
This study was supported by grants from Landspitali Research Fund.

### 1246

#### Invasive mechanical ventilation in sepsis and outcome: an observational study

T. Yang[1,2], J. Park[3], V. Herasevich[1], P. Schulte[4], A. Hanson[4], Y. Dong[1], P. Bauer[3]
[1]Mayo Clinic, Critical Care Medicine, Rochester, United States; [2]West China Hospital, SiChuan University, Pulmonary and Critical Care Medicine, ChengDu, China; [3]Mayo Clinic, Pulmonary and Critical Care Medicine, Rochester, United States; [4]Mayo Clinic, HSR - Biomedical Statistics and Informatics, Rochester, United States
**Correspondence:** T. Yang
*Intensive Care Medicine Experimental* 2018, **6(Suppl 2):**1246

**INTRODUCTION.** Sepsis-related acute respiratory failure is common and associated with increased mortality (1). Initiation of intubation in sepsis cases with circulatory failure raises concern for hemodynamic deterioration (2) and there is currently no definitive recommendation (3).
**OBJECTIVES.** To evaluate the impact of invasive mechanical ventilation on outcome in patients with sepsis.
**METHODS.** We reviewed the electronic records of medical ICU patients with sepsis onset defined by lactate >4 mmol/L, mean arterial pressure < 65 mmHg or vasopressor use after 30 mL/kg fluid boluses, prospectively collected from 04/30/2014 to 12/31/2017. Patients who remained hospitalized at 24 hours following sepsis onset were divided into two groups defined according to whether the patient received mechanical ventilation in the initial 24 hours versus not. The main outcomes were hospital mortality and 28-day hospital-free days. Univariate and multivariate analyses were used, adjusted for admission characteristics and resolution of symptoms within 6 hours. The study was approved by our local Institutional Review Board (#14-008754).
**RESULTS.** Out of 1,335 encounters, we analyzed 1,052 single patients with research authorization: 345 (33%) patients were intubated and ventilated within 24 hours and 707 (67%) were not. Those ventilated were younger, originated more often from a referring facility, had higher APACHE III (92 [74-115] vs. 68 [57-82], $p< 0.001$) and SOFA score (10 [8-13] vs. 6 [4-8], $p< 0.001$), and within 6 hours, mean arterial pressure goals were achieved more often while lactate normalization occurred less often. Their hospital mortality was higher (89 (26%) vs. 82 (12%), $p< 0.001$) and they stayed more days in hospital (10.3 [6.6, 20.6] vs. 6.8 [4.5, 11.4], $p< 0.001$). After adjustment, invasive mechanical ventilation showed no effect on hospital mortality (OR 1.00 [95% CI 0.65 to 1.55], $p=0.99$) or hospital-free days through day 28 (estimated difference in hospital-free days -1.24 [95% CI -2.55 to 0.08], $p=0.07$).
**CONCLUSIONS.** Our findings suggest no significant association between invasive mechanical ventilation within 24 hours of sepsis onset and either hospital mortality or hospital-free days. These results should render the clinician more confident in initiating intubation in septic shock.

**GRANT ACKNOWLEDGMENT**
This study was supported by a grant from the Critical Care Independent Multidisciplinary Program from Mayo Clinic Rochester.

### 1247

#### Mortality and resource utilization in patients with community- and hospital-onset sepsis admitted to intensive care units in Japan: a nationwide discharge and administrative claim database study

M. Tounai[1], A. Shiraishi[2], A. Endo[3], T. Karumai[1], K. Fushimi[4], Y. Hayashi[1]
[1]Kameda Medical Center, Department of Intensive Care Medicine, Kamogawa, Japan; [2]Kameda Medical Center, Emergency and Trauma Center, Kamogawa, Japan; [3]Tokyo Medical and Dental University Hospital of Medicine, Trauma and Acute Critical Care Medical Center, Tokyo, Japan; [4]Tokyo Medical and Dental University Graduate School of Medicine, Department of Health Policy and Informatics, Tokyo, Japan
**Correspondence:** M. Tounai
*Intensive Care Medicine Experimental* 2018, **6(Suppl 2):**1247

**INTRODUCTION.** Sepsis is one of the major reasons for intensive care unit (ICU) admissions worldwide. Studies in several countries showed that mortality and resource utilization were, respectively, higher and greater in hospital-onset sepsis than those in community-onset sepsis [1-5]. However, whether the same situation is observed in Japan remains to be elucidated.

**OBJECTIVE.** To describe mortality and resource utilization in patients with community- or hospital-onset sepsis admitted to ICUs in Japan.

**METHODS.** Study design: A retrospective cohort study using data from the Diagnosis Procedure Combination (DPC) database, a nationwide discharge and administrative claim database in Japan

Setting: Hospitals participating in the DPC database in Japan, from April 2014 to March 2016

Participants: Patients admitted to the ICU with sepsis, which was defined as any sepsis-related diagnosis code in the International Classification of Diseases 10th Revision (ICD-10), plus combination of procedure codes for any antibiotic administration and any body fluid culture within 3 consecutive calendar days, from 1 day before to 1 day after ICU admission.

Exposure: Patients were dichotomized to those with community- or hospital-onset sepsis, defined as ICU admissions ≤2 or ≥3 calendar days following hospital admission, respectively.

Variables: Baseline characteristics of the patients and outcomes such as hospital mortality, length of hospital stay after ICU admission, length of ICU stay, duration of mechanical ventilation, and duration of renal-replacement therapy, were investigated. Data were demonstrated descriptively.

**RESULTS.** Among the 24,609 patients with sepsis admitted to ICUs in 521 hospitals, 16,419 (66.7%) and 8,190 (33.3%) had community- and hospital-onset sepsis, respectively (Table 1). Hospital-onset sepsis demonstrated higher mortality rate (community- vs. hospital-onset sepsis; 30.2% vs. 41.7%) and longer hospital stay after ICU admission (median, 26 days vs. 33 days). Table 2 shows detailed results.

**CONCLUSIONS.** Patients with hospital-onset sepsis had higher mortality rate and greater resource utilization than community-onset sepsis had. The results in this study would provide fundamental data for healthcare policy making on intensive care in Japan.

##### GRANT ACKNOWLEDGMENT

None

**Table 1 (abstract 1247).** Patient Characteristics

| Variables | Community-onset sepsis (n=16419) | Hospital-onset sepsis (n=8190) |
|---|---|---|
| Age, years, median [IQR] | 75 [65, 82] | 72 [64, 79] |
| Female gender, n (%) | 6940 (42.3) | 2870 (35.0) |
| Admission to academic hospitals, n (%) | 2924 (17.8) | 2186 (26.7) |
| Surgery under general anesthesia during hospitalization, n (%) | 5936 (36.2) | 4409 (53.8) |
| Time to ICU-admission from hospitalization, days, median [IQR] | 0 [0, 0] | 9 [4, 21] |
| Organ support therapy on the first day of ICU admission | | |
| Mechanical ventilation, n (%) | 5266 (32.1) | 3280 (40.0) |
| Noradrenaline use, n (%) | 8617 (52.5) | 4161 (50.8) |
| Renal-replacement therapy, n (%) | 2433 (14.8) | 1586 (19.4) |

**Table 2 (abstract 1247).** Outcomes

| Variables | Hospital-onset sepsis (n=8190) | |
|---|---|---|
| Hospital mortality, n (%) | 4964 (30.2) | 3417 (41.7) |
| Hospital length of stay after ICU admission, days, median [IQR] | 26 [13, 49] | 33 [16, 63] |
| ICU length of stay, days, median [IQR] | 5 [2, 10] | 6 [2, 12] |
| Duration of mechanical ventilation, days, median [IQR] | 2 [0, 7] | 2 [0, 10] |
| Duration of renal replacement therapy, days, median [IQR] | 0 [0, 2] | 0 [0, 4] |

### 1248

#### Association of mortality and circumferential left ventricular strain in ICU septic patients

A.Y. Rodríguez Santos[1,2], A. Rugerio Cabrera[1], J.C. Sauza Sosa[3], J. Franco Granillo[1], S.J. Aguirre Sánchez[1], G. Camarena Alejo[1]
[1]American British Cowdray Medical Center I.A.P., Critical Care, Mexico, Mexico; [2]Universidad Nacional Autonoma de México, Critical Care, Mexico, Mexico; [3]American British Cowdray Medical Center I.A.P., Cardiology, Mexico, Mexico
**Correspondence:** A.Y. Rodríguez Santos
*Intensive Care Medicine Experimental* 2018, **6(Suppl 2):**1248

**INTRODUCTION.** New echocardiographic techniques such as speckle tracking have proven to show early myocardial dysfunction that could be overlooked by conventional methods. Myocardial dysfunction may be an early marker of mortality in sepsis patients.

**OBJECTIVES.** To assess the correlation between left ventricular circumferential vs. global longitudinal strain by speckle tracking with mortality and hospital stay in patients with sepsis admitted to an ICU unit.

**METHODS.** We conducted a prospective study in patients evaluated from January to March 2018 in an ICU unit from a single center in Mexico City. Sepsis was defined according to Surviving Sepsis criteria.

Echocardiogram evaluation that included left ventricular global longitudinal strain (GLS) and circumferential strain (CS) by certified personnel according to American Society of Echocardiography criteria. We evaluated global diagnostic performance and cox regression analysis of CS and GLS, using death during the ICU stay as our end-point.

**RESULTS.** We included 20 adults, 55% females (n=11), with a mean global age of 67.5 (±19) and length of stay of 12.9 days (CI 95%, 6.8-18.9). Sepsis origin was: 50% abdominal (n=10), 35% pulmonary (n=7) and 15% (n=3) urinary. Mortality rate was 25% (n=5). Non-survival vs. survival-groups baseline characteristics are outlined in table 1. CS was slightly superior to GSL and to SOFA, SAPS II and APACHE II scales for discriminating non-survivors vs. survivors (Fig.1) [AUC: 0.78 (CI 95%, 0.56-1), 0.66 (0.37-0.94), 0.64 (0.33-0.96), 0.68 (0.42-0.94), 0.59 (0.29-0.88), respectively]. Survival analysis revealed that CS for patients with a selected cutoff point ≥-15.5 was highly predictive for mortality [HR: 12.1, (CI 95% 1.3-112), p=0.022] (Fig. 2).

**CONCLUSIONS.** Myocardial dysfunction assessed by CS can be an early predictor of mortality in Mexican patients with sepsis. Results from non-conventional echocardiographic measurements add prognostic value to their outcome.

**REFERENCE(S)**

1) Dellinger RP, Levy MM, Rhodes A, Annane D, Gerlach H, Opal SM, Sevransky JE, Sprung CL, Douglas IS, Jaeschke R, Osborn TM, Nunnally ME, Townsend SR, Reinhart K, Kleinpell RM, Angus DC, Deutschman CS, Machado FR, Rubenfeld GD, Webb S, Beale RJ, Vincent JL, Moreno R. Surviving Sepsis Campaign: international guidelines for management of severe sepsis and septic shock. Intensive Care Med (2013) 39:165-228.
2) Orde SR, Pulido JN, Masaki M, Gillespie S, Spoon JN, Kane GC, Oh JK. Outcome prediction in sepsis: Speckle tracking echocardiography based assessment of myocardial function. Crit Care. 2014 Jul 11; 18(4):R149.
3) Arif Hussain and Ahmad S. Omran. Application of strain to assess myocardial function in septic cardiomyopathy. European Heart Journal Supplements (2014)16.

**Table 1 (abstract 1248).** Baseline characteristics of patients with sepsis admitted to the intensive care unit (ICU)

| | Death | Survival | *p value* |
|---|---|---|---|
| Age in years, mean (SD) | 78.4 (9) | 63.8 (21) | 0.049 |
| Female | 1 (9.1) | 10 (66.7) | 0.127 |
| Medical admission | 4 (80) | 8 (53) | 0.603 |
| Surgical admission | 1 (20) | 7 (46.7) | 0.603 |
| DM | 3 (40) | 1 (6.7) | 0.14 |
| HT | 3 (60) | 6(40) | 0.617 |
| Smoker | 0 | 7 (46.7) | 0.11 |
| NIMV | 2 (40) | 3 (20) | 0.56 |
| MV | 4 (80) | 8 (53.3) | 0.603 |
| BMI in kg/m2, mean (SD) | 25.5 (6.6) | 24.3 (3.5) | 0.722 |
| AKI | 4 (80) | 8(54.3) | 0.603 |
| MAP in mmHg, mean (SD) | 74.6 (6.8) | 75.7(11) | 0.792 |
| APACHE II, mean (SD) | 18.2 (7.1) | 16.4 (7) | 0.641 |
| SAPS II, mean (SD) | 51.5 (7.6) | 46.2 (20) | 0.398 |
| SOFA, mean (SD) | 11.4 (3.5) | 10.1 (5.2) | 0.559 |
| CVP, mean (SD) | 12 (4) | 8.2 (5.2) | 0.131 |
| Delta CO2, mean (SD) | 7.4 (2.5) | 7.2 (4.6) | 0.905 |
| Delta CO2/ Delta avO2, mean (SD) | 2.2 (1.5) | 1.8 (0.9) | 0.613 |
| SVO2, mean (SD) | 78.4 (6.8) | 73.6 (7.9) | 0.233 |
| LVEF, mean (SD) | 58.6 (12.1) | 63.7 (6.0) | 0.410 |
| CS, mean (SD) | -14.5 (19- 10) | -20.5 (24- 16) | 0.037 |
| GLS, mean (SD) | -12.2 (3.6) | -16.4 (7.2) | 0.254 |
| DD | 5 (100) | 10 (66.7) | 0.266 |

Data expressed as n (%) unless otherwise specified
Abb:DM: diabetes mellitus, HT: hypertension, NIMV: non invasive mechanical ventilation, MV: mechanical ventilation, BMI: body mas index, AKI: acute kidney injury, MAP: main arterial pressure, APACHE II: Acute Physiology Age and Chronic Health Evaluation, SAPS II Simplified Acute Physiology Score, SOFA: Sequential Organ Failure Assessment, CVP: central venous pressure, delta CO2: carbon dioxide, delta a-vO2: arteriovenous oxygen difference, SVO2: mixed venous oxygen saturation, LVEF: left ventricular , CS: circunferencial strain, GLS: left ventricular global longitudinal strain, LVEF: left ventricular ejection fraction, DD: diastolic dysfunction.

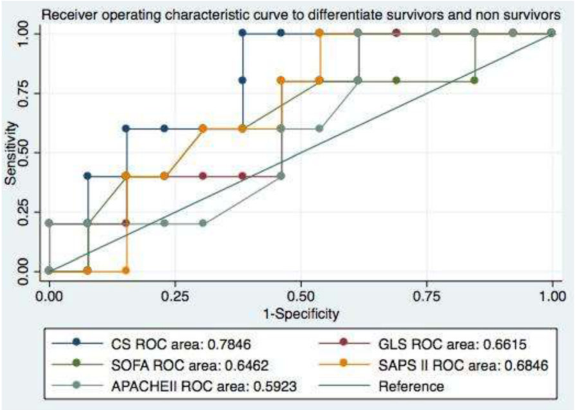

**Fig. 1 (abstract 1248).** ROC area. CS was slightly superior to GSL and to SOFA,SAPS II and APACHE II scales

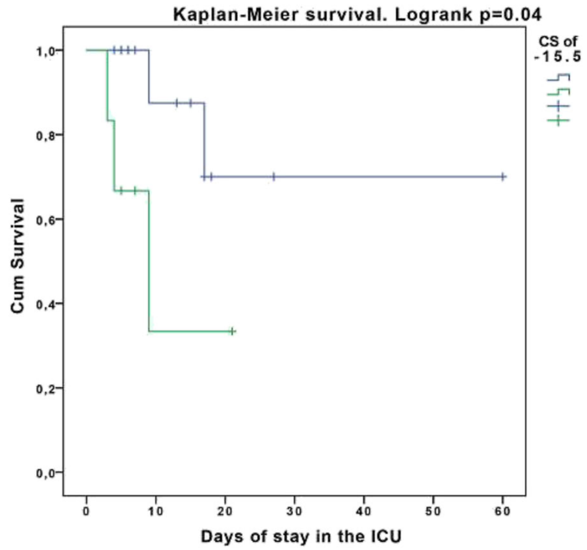

**Fig. 2 (abstract 1248).** Survival analysis: CS for patients with a selected cutoff point ≥-15.5 highly predictive for mortality

### 1249

#### Influence on septic shock outcome of previous use of statins

R. Zaragoza[1], S. Sancho[1], V. Ramirez[1], D. Illa[1], C. Ibañez[1], R. González[1], R. Badenes[2], J.J. Camarena[1]

[1]Hospital Universitario Dr. Peset, Valencia, Spain; [2]Hospital Clinico Universitario de Valencia, SICU, Valencia, Spain

**Correspondence:** R. Zaragoza

*Intensive Care Medicine Experimental* 2018, **6(Suppl 2):**1249

**INTRODUCTION.** The role of previous use of statins before admission remains controversial in septic shock patients.

**OBJECTIVES.** The aims of this study were to know the prevalence of previous use of statins in patients with septic shock detected by a Sepsis unit, to know their updated prognosis and specially to analyze the influence on global mortality of previous statins use in these septic patients.

**METHODS.** During a four years and a half period ( October-2012-March 2017), 2405 severe sepsis and septic shock patients detected by a sepsis unit form ICU using an electronic automatic alert in a teaching hospital were prospectively evaluated. Clinical and microbiological variables were recorded. Any patient with a confirmed septic shock (sepsis 2.0) was potentially eligible through the whole hospital. Previous use of statins was defined as the intake of statins 3 months previous admission. Several multivariate analysis was performed to describe independently factors associated to global mortality in these patients using SPSS package (16.0).

**RESULTS.** Among 2405 electronic activations 667 of them corresponded to septic shock (27.7%) Their mean APACHE II and SOFA score were 20.5±7.22 and 7.81±3.35 respectively. The most frequent sources of infections were the respiratory focus (31.2%), intraabdominal (29.1%) and urinary (26.6%). Global mortality was 35.2%. The majority of episodes were community adquired (54.1%). Associated bacteremia was present in 29.5% of episodes. Microbiological documentation was achieved in 56.7 %. Previous use of statins was documented in 17.7% of the episodes. Statistical significant factors associated to Global Mortality in univariate analysis ($p < 0.05$) were: nosocomial infection (45% vs 34%), respiratory focus (46.6% vs 33.7%), adequate empirical antibiotic treatment (32.2% vs. 65.3%) and 6h bundle adherence (28.1% vs 48,9%). Previous use of statins was not associated to global mortality in univariate analysis (33.8% vs. 35.6%). Factors independently associated to Global mortality were SOFA score ( OR 1.25; CI95% 1.17-1.32) and 6h bundle adherence ( OR 3.3; CI95% 2.10-5.18 ) but not previous use of statins ( OR 1.12; CI95% 0.7-1.81)

**CONCLUSIONS.** Previous use of statins was present in almost one out five episodes of septic shock but had no influence on global mortality

**REFERENCE(S)**

Lee, Chien-Chang et al. A Population-Based Cohort Study on the Drug-Specific Effect of Statins on Sepsis Outcome CHEST 2018, Volume 153 , Issue 4 , 805 - 815

### 1250

#### Intensive care unit, surgical and medical wards acquired sepsis in a major university hospital

C. López Mestanza[1], J.R. Gómez López[2], R. Cicuéndez Ávila[3], J. Bermejo Martín[4], D. Andaluz Ojeda[3]

[1]Complejo Hospitalario de Soria, Clinical Microbiology Department, Soria, Spain; [2]Hospital Comarcal de Medina del Campo, General Surgery Department, Medina del Campo (Valladolid), Spain; [3]Hospital Clínico Universitario Valladolid, Adult Intensive Care Medicine Department, Valladolid, Spain; [4]IECSCYL / Hospital Clínico Universitario Valladolid, Infection and Inmunology Investigation Unit, Valladolid, Spain

**Correspondence:** R. Cicuéndez Ávila

*Intensive Care Medicine Experimental* 2018, **6(Suppl 2):**1250

**INTRODUCTION.** Clinical profile of Hospital Acquired Sepsis (HAS) patients is poorly known. HAS is associated with high mortality, and is considered a major preventable cause of admission to Intensive Care Unit (ICU).

**OBJECTIVES.** We sought to compare and contrast ICU, surgical and medical wards acquired sepsis in a major university hospital.

**METHODS.** Retrospective study of patients with HAS in a major Spanish Hospital from 2011 to 2015. Those patients fulfilling the SEPSIS-2 definition (Severe Sepsis or Septic Shock) with no evidence of infection the first 48 hours of hospital admission and over 18-years-old were included. Patients were grouped according to the place of sepsis acquisition (ICU, surgical wards and medical wards). ICU include medical, surgical and cardiac ICUs.

**STATISTICAL ANALYSIS.** Differences between groups were assessed using the $\chi^2$ test for categorical and Kruskall -Wallis test for continuous variables. Mortality risk were evaluated using Cox regression univariate analysis. Finally Kaplan-Meier curves were plotted to show survival at 28 days. The log-rank test was used to compare the survival curves.

**RESULTS.** A total of 146 patients were included. Half (50%) present ICU acquired sepsis, while 31% acquired episode in surgical wards and 19% in medical wards. ICU acquired sepsis patients were older (median) (74 years[66.5-79.0], compared to surgical (73 years[66.5-80]) and medical (63.5 years[51.5-75.0] *p*=0.012.Patients with history of immunosuppression predominate in medical 42.9% (surgical 17.8%, ICU 21.9%), *p*=0.040. Septic shock at diagnosis were more frequent in medical:75.0% (ICU:53.4%, surgical:73.3%), *p*=0.035. Urinary source predominate in medical 25% (ICU:5.5%, surgical 6.7%), *p*=0.008, and also primary bacteraemia 17.9% (ICU:17.8%, surgical:2.2%), *p*=0.035, while device-associated infection were higher in ICU:38.4% (Surgical:13.3%, medical:17.9%), *p*=0.006. HAS hospital mortality were higher in medical wards compared to surgical ones and to ICU (64.3% vs 55.6% and 45.2% respectively), *p*=NS. Patients from medical wards acquired sepsis showed higher risk of 28 days mortality: HR 1.77[1.02-3.07],*p*=0.042, while ICU acquired sepsis behaved as protector factor: HR 0.56 [0,34-0.91],*p*=0.018.

**CONCLUSIONS.** Many clinical differences were founded between ICU, Surgical and Medical wards acquired Sepsis. HAS acquired in the ICU presents a significantly lower mortality than that acquired in the hospital ward, probably due to earlier detection and treatment. These patients showed more survival time than patients with medical and surgical wards acquired sepsis. New studies are needed to confirm these findings.

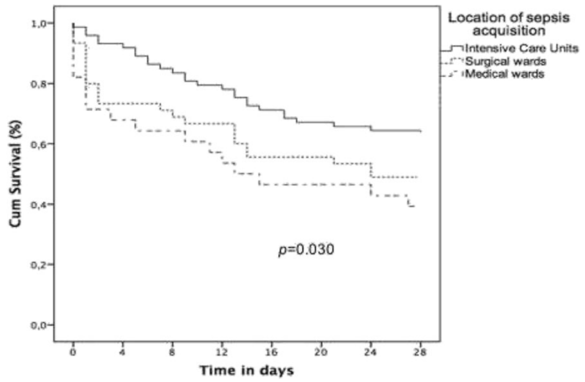

**Fig.1 (abstract 1250).** Kaplan Meier survival curves according to location of hospital acquired sepsis

### 1251

#### Factors associated with mortality in patients admitted for influenza A during 2012-2016

E. Casanova-Gongora[1], P. Revelo-Esquibel[1], J.C. Lopez-Delgado[1,2], S.M. Luna-Solis[1], P. Serra-Paya[1], G. Muñoz-del Río[1], D. Berbel-Franco[1], G. Moreno-Gonzalez[1], R. Mañez-Mendiluce[1,2]

[1]Hospital Universitari de Bellvitge, Intensive Care Medicine, Barcelona, Spain; [2]Institut d´Investigació Biomèdica de Bellvitge, L'Hospitalet de Llobregat, Spain

**Correspondence:** J.C. Lopez-Delgado

*Intensive Care Medicine Experimental* 2018, **6(Suppl 2):**1251

**INTRODUCTION.** Patients who are admitted to the ICU due to Influenza A are still at risk of high mortality, especially if they require mechanical ventilation. Despite advances in clinical research, risk prediction remains difficult in those patients.

**OBJECTIVES.** The aim of our study was to identify the risk factors for in-hospital mortality of patients suffering from respiratory insufficiency due to Influenza A, emphasizing the evaluation of laboratory data during first 48h.

**METHODS.** Prospective,observational study at our institution from 2012 to 2016.Baseline characteristics, laboratory data and outcomes were analyzed. Univariate and multivariate analysis was performed(SPSS 20.0).

**RESULTS.** 64 patients were included, 61.2%(43) were male, age was 54.8±14.5 years and APACHE II was 19.19±8.17. 46.8%(30) suffer from respiratory confection on ICU admission. In-hospital mortality was (25%;16). We did not show differences in previous comorbidities.

None of the non-survivors received previous Influenza vaccine whereas 29.1%(P=0.014) of the survivors received it. PaO2/FiO2 on admission (182±183vs.84±77;P=0.012), Platelets on admission (192 ±137vs.130±91×109·L-1;P=0.04), PaO2/FiO2 at 24h (183±100vs.114 ±60:P=0.008), Lymphocytes at 48h (943±659vs.469±317;P=0.02), Urine output first 24h (1874±1383vs.984±791mL) were different among subgroups. Non-survivors needed higher Invasive Mechanical Ventilation needs (41,67%vs.81,25%;P=0.009) and survivors higher hospital length of stay (38.5±58.6 vs.15.93±17.53days;P=0.022). All patients received similar regimens of Oseltamivir and other types of therapies (i.e. Renal Replacement Therapy, Antibiotics, Vasopressors). Lower PaO2/FiO2 at 24h (OR:0.958;95% CI:0.923-0.995;P=0.010) and higher APACHE II (OR:1.928;95% CI:1.081-3.438;P=0.026) were associated with higher in-hospital mortality.

**CONCLUSIONS.** Mortality remains high in patients suffering from Influenza A admitted to the ICU and prognosis assessment remains difficult. It seems that respiratory dysfunction during the first 24h may be helpful for this purpose.

### 1252

#### Bacteraemia and its treatment in sepsis on the wards: results of a large point-prevalence study

P. McNulty, S. Kulikouskaya, T. Grother, H. Al Hassan, J. Abreu, S.Y. Heng, S. Ng, N. West, M. Kopczynska, B. Sharif, T. Szakmany, Welsh Digital Data Collection Platform

University, Anaesthesia, Intensive Care and Pain Medicine, Division of Population Medicine, Cardiff, United Kingdom

**Correspondence:** P. McNulty

*Intensive Care Medicine Experimental* 2018, **6(Suppl 2):**1252

**INTRODUCTION.** Bacteraemia has been linked to increased mortality in sepsis, depending on the causative organism [1]. Quality improvement projects aimed to increase frequency and reliability of blood cultures obtained from this population revealed mixed results. Currently there is little known about the frequency and causative organisms of bacteraemia on the general wards and emergency departments (ED) in confirmed sepsis.

**OBJECTIVES.** The aim of the study was to establish the rate and organisms of bacteraemia related to the sepsis episode on the general wards and ED.

**METHODS.** Secondary analysis of patient episodes was performed on patient population recruited into two annual 24-hour point-prevalence studies on the general wards and ED across all Welsh acute hospitals in 2016 and 2017 [2]. Inclusion criteria were: clinical suspicion of infection and NEWS 3 or above in-line with established escalation criteria in Wales. Sepsis was defined according to Sepsis-3 definition based on SOFA and qSOFA scores. Microbiology data on collected blood cultures within 48 hours of the index episode were collected, together with data on antimicrobial regime.

**RESULTS.** 791 patients were recruited over the two 24-hour periods in 2016 and 2017. 514 patients fulfilled Sepsis-3 criteria. 321 (63.6%) patients had blood cultures taken. 16 blood cultures (4.9%) were positive with likely significant results: 6 Escherichia Coli, 1 Pseudomonas aureginosa., 2 Klebsiella pneumoniae, 1 Proteus mirabilis, 1 Gram negative organism, 1 Enterobacter cloacae, 1 Streptococcus pneumoniae, 2 Gram positive cocci. Out of the 514 patients, 212 (41.2%) were treated with intravenous antibiotics. 3 patients with positive blood cultures (2 with E. Coli and 1 with Gram positive cocci) were not treated with antibiotics, all survived. On the other hand, 70 (13.6%) patients were treated with two or more antimicrobials without microbiology investigations performed, and 57 were treated with 2 or more antimicrobials with negative blood culture results.

**CONCLUSIONS.** Despite ongoing quality improvement initiatives, blood culture attainment in sepsis is low. This could partially explain the 3% bacteraemia rate observed in our study. There is evidence that one in seven patients with sepsis are treated with two or more antimicrobials, without attempts to establish causative organism, which could lead to spread of antimicrobial resistance.

**GRANT ACKNOWLEDGMENT**

### 1253

#### Risk factors associated with septic shock in the emergency department: preliminar report of a multicenter study

L.A. Gorordo-Delsol[1,2], G. Merinos-Sánchez[2,3], M.A. Morales-Segura[4], N. Medveczky-Ordoñez[1], S.E. Uribe-Moya[5], R. Estrada-Escobar[3], M.A. Amezcua-Gutiérrez[1], RENASE - Urgencias 2017

[1]Hospital Juárez de México, Adults Intensive Care Unit, Gustavo A. Madero, Mexico; [2]Fundación Sepsis México, Research Committee, Ciudad de México, Mexico; [3]Hospital General de México 'Dr. Eduardo Ligeaga', Adults Emergency Service, Cuauhtémoc, Mexico; [4]Instituto Politécnico Nacional, Escuela Superior de Medicina - Sección es Estudios de Posgrado e Investigación, México City, Mexico; [5]Hospital Juárez de México, Cardiology, Gustavo A. Madero, Mexico

**Correspondence:** L.A. Gorordo-Delsol

*Intensive Care Medicine Experimental* 2018, **6(Suppl 2):**1253

Sepsis is a multifactorial disease, it is estimated that there are more than 30 million cases each year, of which 6 million die (1), there is little information on low and middle income countries, where 87% of the population lives world (2). In Mexico, it was estimated that close to 50% to 60% of emergency admissions were related to sepsis, of which 10% were admitted with septic shock (3).

**OBJECTIVES.** To determine the risk factors of patients with sepsis to develop septic shock.

**METHODS.** A single-day multicenter study was conducted (September 13, 2017), where all patients diagnosed with sepsis or septic shock were admitted, according to the Sepsis criteria -3 (4) admitted to the Emergency Department during the 24 hours a day, they were recorded on a digital platform (www.sepsismexico.org) and followed up for 30 days. The data analysis was performed in SPSS v.21.

**RESULTS.** 68 hospitals participated, 307 patients were included (56% women), who presented sepsis 86.6% and septic shock 13.4%. The common odds ratio was estimated by the Mantel-Haenzel method for a background list as shown in the tables. Medical risk fa... Chronic organic... Environmental r...

**CONCLUSIONS.** The preexisting organic faults such as respiratory, cardiac, renal and hepatic failure preexisting, most of them exacerbated by the infectious process; the reason why arrhythmias, cerebral vascular events, and gastrointestinal tract hemorrhages increase the risk in an important way is not known, this had not been previously described. Among the environmental factors, it is important to live in a nursing home or geriatric institution and recent hospitalization, this can be explained by exposure to microorganisms and other factors.

**GRANT ACKNOWLEDGMENT**
To the "Fundación Sepsis México" for providing the tools for data collection.

**Table 1 (abstract 1253).** Medical risk factors odds ratio

| Risk factor | Sepsis (n=266) | Septic shock (n=41) | OR | Confidence interval (95%) | p value |
|---|---|---|---|---|---|
| Diabetes | 113 | 24 | 2.116 | 1.079 - 4.147 | 0.029 |
| Systemic hypertension | 123 | 22 | 1.346 | 0.696 - 2.603 | 0.377 |
| Active oncological disease | 36 | 10 | 2.061 | 0.931 - 4.562 | 0.074 |
| Intracranial tumor | 21 | 9 | 3.281 | 1.384 - 7.781 | 0.007 |
| Stroke | 23 | 11 | 3.874 | 1.719 - 8.730 | 0.001 |
| Arrhythmias | 31 | 13 | 3.520 | 1.651 - 7.503 | 0.001 |
| Immunosuppression | 23 | 5 | 1.4674 | 0.593 - 4.1042 | 0.4649 |

**Table 2 (abstract 1253).** Chronic organic failures risk factor for septic shock

| Chronic organic failure | Sepsis (n=266) | Septic shock (n=41) | OR | Confidence interval (95%) | p value |
|---|---|---|---|---|---|
| Heart | 36 | 11 | 2.343 | 1.079 - 5.085 | 0.031 |
| Respiratory | 51 | 15 | 2.432 | 1.202 - 4.922 | 0.013 |
| Renal | 53 | 15 | 2.319 | 1.148 - 4.684 | 0.019 |
| Liver | 22 | 11 | 4.067 | 1.796 - 9.206 | 0.001 |
| Gastrointestinal bleeding | 22 | 11 | 4.067 | 1.796 - 9.206 | 0.001 |

**Table 3 (abstract 1253).** Environmental risk factors

| Risk factor | Sepsis (n=266) | Septic shock (n=41) | OR | Confidence interval (95%) | p value |
|---|---|---|---|---|---|
| Use of antimicrobials in the last 3 months | 101 | 17 | 1.157 | 0.593 - 2.259 | 0.669 |
| Living in a nursing home | 21 | 12 | 4.828 | 2.154 - 10.819 | 0.0001 |
| Hospitalization in the last 15 days | 57 | 15 | 2.115 | 1.051 - 4.259 | 0.036 |

### 1254

#### Mortality prediction among sepsis patients using a combination of National Early Warning Score, age, gender and serum lactate levels

C. Tanaka, T. Tagami, S. Sato, A. Takehara, J. Kaneko, F. Nakayama, R. Fukuda, S. Kudo, M. Kuno, K. Unemoto
Medical School Tamangayama Hospital, Tokyo, Japan
**Correspondence:** C. Tanaka
*Intensive Care Medicine Experimental* 2018, **6(Suppl 2):**1254

**INTRODUCTION.** The definition of sepsis was revised in 2016 for early recognition of sepsis. The definition captures a wide variation, from patients who require only intravenous antibiotics to critically-ill patients who require intensive care such as intubation and continuous renal replacement therapy which are associated with high mortality.

**OBJECTIVES.** To predict critically-ill sepsis patients using the National Early Warning Score (NEWS) and some variables measured easily on admission.

**METHODS.** This was a single-center retrospective cohort study. Of all patients who admitted to our emergency room and intensive care unit, we reviewed 80 patients who were diagnosed as sepsis between January 2014 and December 2017. Age, gender, vital signs (heart rate, body temperature, and respiratory rate), consciousness level, and serum lactate level were measured on admission. Primary outcome measure was in-hospital mortality. We calculated the NEWS with the variables measured on admission. Then, we created a prediction model using NEWS, age, gender and serum lactate level and performed receiver operating characteristic (ROC) analysis with estimation of the corresponding areas under the curve (AUC).

**RESULTS.** Among the eligible 80 patients, the median age was 75 years old (interquartile range [IQR]: 64-83) and 54 (67.5%) was male. Median lactate level was 4.4mmol/l (IQR: 2.3-8.0) on admission. About the NEWS on arrival at hospital, low clinical risk patients, middle clinical risk patients and high clinical risk patients were 7 (8.8%), 9(11.3%) and 64 (80.0%), respectively. The all-cause mortality rate was 43.8%. Continuous hemodiafiltration was performed for 32 patients (40%). ROC curves showed that NEWS had an AUC 0.45 (95% confidential interval [CI], 0.32-0.58); however, the combination of NEWS, age over 65 years, gender and serum lactate lever yielded an AUC 0.74 (95% CI, 0.63-0.85).

**CONCLUSIONS.** Among sepsis patients, combined use of NEWS, age, gender and serum lactate levels may be better predictor to detect critically-ill patients who were associated with high mortality.

### 1255

#### Influence of fluid balance on mortality and duration of mechanical ventilation in septic shock

D. Monge Donaire[1], V. Losada Martínez[1], A. Tejero Pedregosa[1], G. Ferrigno Bonilla[1], N. Rodrigo Castroviejo[1], F.C. Tarancón Maján[1], S. Cortés Díaz[1], A. Marcos Gutiérrez[1], C. Ochoa Sangrador[1], L. Parra Morais[2]
[1]Hospital Virgen de la Concha, Intensive Care Unit, Zamora, Spain; [2]Hospital Clínico Universitario, Intensive Care Unit, Valladolid, Spain
**Correspondence:** D. Monge Donaire
*Intensive Care Medicine Experimental* 2018, **6(Suppl 2):**1255

**INTRODUCTION.** Fluid resuscitation is, along with prompt empiric antibiotic therapy the mainstay treatment in septic shock. Despite recomendation of agressive fluid therapy[1] there is no consensus about a safe threshold of amount of fluid administered or fluid balance not to increase mortality or pulmonary complications[2,3].

**OBJECTIVES.** To determine the influence of positive fluid balance during the initial resuscitation of septic shock on mortality and duration of mechanical ventilation.

**METHODS.** Retrospective cohort study in a medical/surgical ICU, adult patients with septic shock admitted to the unit from January 2013 to June 2017 were selected. Demographic, prognostic variables, origin of sepsis, fluid administration, fluid balance, need and duration of mechanical ventilation (MV), ICU and hospital mortality were recorded.

**RESULTS.** 159 patients were studied (67% men, 32% women) with an age of 71 ± 12 years. The source of sepsis was abdominal (51%), followed by respiratory (20%) and genitourinary (17%). The intra-ICU mortality was 29% and the in-hospital mortality was 39%. 73% of patients required MV, the duration of it was 7 ± 11 days. The fluid balance accumulated in the first 48 hours was lower in the survivors: -1113 ml (95% CI -2169 ml, -56 ml, p = 0.039). The amont of fluid administered was similar in both groups (24 hours: 4751 ± 2173 ml, 65 ± 33 ml / kg VS 4685 ± 2198 ml, 68 ± 32 ml / kg, 48 hours: 4744 ± 1547 ml, 64 ± 25 ml / kg VS 4553 ± 2712 ml, 66 ± 39 ml / kg). The fluid balance influenced the duration of MV (Spearman correlation 0.314, p < 0.01). Patients with a cumulative fluid balance in the first 48 hours greater than 6 liters showed an increase in mortality, RR = 1.52 (95% CI 1.02-2.25, p = 0.038); they also required longer MV time: +5.2 days (95% CI 1.1-9.3 days, p = 0.013). In the logistic regression analysis, the need for MV, the APACHE II score> 21 and the BMI> 26 kg/m$^2$ appear as variables associated with mortality.

**CONCLUSIONS.** Fluid administration is an indispensable part of the treatment of septic shock, although a very positive fluid balance during the first 48 hours can increase the mortality and duration of mechanical ventilation.

**REFERENCE(S)**

1) Surviving sepsis campaign: international guidelines for management of severe sepsis and septic shock: 2012.Dellinger RP, Levy MM, Rhodes A et al. Crit Care Med. 2013 Feb;41(2):580-637
2) Fluid administration in severe sepsis and septic shock, patterns and outcomes: an analysis of a large national database.Marik PE, Linde-Zwirble WT, Bittner EA, et al. Intensive Care Med. 2017 May;43(5):625-632.
3) Higher Fluid Balance Increases the Risk of Death From Sepsis: Results From a Large International Audit. Sakr Y, Rubatto Birri PN, Kotfis K, et al. Crit Care Med. 2017 Mar;45(3):386-394.

**GRANT ACKNOWLEDGMENT**

To Prof. Ochoa Sangrador for its assistance on study design and statistical analysis.

## Imaging in the intensive care

### 1256

#### The outcomes of inoperable critical ill cancer patients in medical ICU (MICU) is better than expected by prognostic scores

O. Obukhova, G. Yunaev, I. Kurmukov, S. Kashiya
N. N. Blokhin NMRC of Oncology, Moscow, Russian Federation
**Correspondence:** O. Obukhova
*Intensive Care Medicine Experimental* 2018, **6(Suppl 2):**1256

**INTRODUCTION.** Anticancer therapy (ACT) and tumor burden can cause conditions that require intensive care. The question of advisability and efficacy of intensive care escalation in critical ill patients (CIP) with end-stage cancer is still undetermined.

**OBJECTIVES.** We evaluated treatment results and predictors of adverse outcome CIP with end-stage cancer receiving ACT.

**METHODS.** The prospective observational cohort study included 169 adult patients with cancer III-IV stages who had been admitted to MICU since 01 Jan 2016 to 01 Jan 2017. APACHE II and MPMII0 scales calculated prognosis of outcome. Statistical analysis of predictors of adverse outcome was done by odds ratio and $\chi^2$ calculation. The utility of prognostic scales was assessed by the area under ROC curves.

**RESULTS.** Median of age was 58 years (18-87), male - 93. The main reasons for ICU admission were respiratory failure (62%), arterial hypotension or cardiac failure (46%), deep cytopenia (36%) or impairment of consciousness (23%) which were often combined with each other. Etiologically, the causes of the critical illness were complications of cancer (35%), adverse events of ACT (27%), decompensation of concurrent conditions (20%) and severe infections (18%). Median of APACHE II score was 16 (10-38). Mortality in MICU was 9.5%, 30-day mortality was 17%. Medians of predicted 30-day mortality by $MPMII_0$ and APACHE II were 27% and 39% respectively. There was no statistically significant correlation between 30-day mortality and neither main reason of MICU admission nor etiological factor of critical illness. Comparative ROC-analysis of utility of analyzed prognostic scales revealed some advantage of $MPMII_0$: AUC ($MPMII_0$) 0,781 (95% CI: 0,671- 0,891), AUC (APACHE II) 0,660 (95% CI: 0,528-0,792).

**CONCLUSIONS.** Inoperable malignant tumor is not a reason of intensive care refusal in MICU in case of reversible critical ill. Evaluation of novel predictors of adverse outcome is warranted. Assessment by $MPMII_0$ and APACHE II scales did not demonstrate satisfactory agreement with actual 30-day mortality.

### 1257

#### Benefit of a clinical pharmacist on a German surgical intensive care unit: a controlled interventional study

N. Kessemeier[1,2], D. Meyn[1], M. Höckel[1], J. Reitze[3], C. Culmsee[2], M. Tryba[4]
[1]Gesundheit Nordhessen Holding AG, Pharmacy, Kassel, Germany; [2]Philipps-University Marburg, Pharmacology and Clinical Pharmacy, Marburg, Germany; [3]MoreDATA GmbH, Statistics, Giessen, Germany; [4]Gesundheit Nordhessen Holding AG, Kassel School of Medicine, Kassel, Germany
**Correspondence:** N. Kessemeier
*Intensive Care Medicine Experimental* 2018, **6(Suppl 2):**1257

**INTRODUCTION.** With a clinical pharmacists' (CP) participation on an intensive care unit (ICU) previous international studies have shown a reduction of medication errors, drug costs and improvements of clinical outcomes such as ICU length of stay. CPs' influence on these outcomes on German ICUs has not been investigated yet. This controlled interventional study was designed to investigate a CPs' impact on medication errors (ME) on a German surgical ICU.

**OBJECTIVES.** The investigation aimed to determine if the medication review by a CP results in a significant reduction of ME related to a control period. Secondary outcomes were the reduction of potentially severe medication errors, the number of days without systemic anti-infective therapy and the length of stay.

**METHODS.** The study was divided in three phases. One control phase $P_0$ and two intervention phases $P_1$ and $P_2$. Throughout the control phase $P_0$ the data were collected without the CPs' intervention. In the following intervention periods $P_1$ and $P_2$, two CPs screened the medical records for drug related problems (DRP) and discussed them with the senior physician. During $P_2$ CPs attended ward rounds additionally. DRP detected by the CPs according to defined criteria, were taken into account as ME if a consensus with the physician was achieved.

**RESULTS.** The medication orders of 336 patients were reviewed. The number of ME was significantly reduced from 1660 to 622 respectively 401 ($P_0$ vs. $P_1$/$P_2$; both $P < 0.05$; Fisher's Exact Test) in total, respective 14.1% in $P_0$ vs. 5.1% in $P_1$ respectively 3.3% in $P_2$ related to the monitored medications ($P_0$ 11755;$P_1$ 12134; $P_2$ 12329).

**CONCLUSIONS.** CPs' interventions led to a significant reduction of ME in a German ICU, contributing to a safer medication process. We strongly recommend a broad implementation of CPs on German ICUs.

**REFERENCE(S)**

Klopotowska JE, Kuiper R, van Kan HJ, et al (2010) On-ward participation of a hospital pharmacist in a Dutch intensive care unit reduces prescribing errors and related patient harm: an intervention study. Crit Care 14:R174 . doi: 10.1186/cc9278.

Jiang S-P, Zheng X, Li X, Lu X-Y (2012) Effectiveness of pharmaceutical care in an intensive care unit from China. A pre- and post-intervention study. Saudi Med J 33:756-762.

Leape LL, Cullen DJ, Clapp M, et al (1999) Pharmacist participation on physician rounds and adverse drug events in the intensive care unit. JAMA 282:267-270 . doi: 10.1001/jama.282.3.267.

Kucukarslan SN, Corpus K, Mehta N, et al (2013) Evaluation of a dedicated pharmacist staffing model in the medical intensive care unit. Hosp Pharm 48:922-930 . doi: 10.1310/hpj4811-922.

**GRANT ACKNOWLEDGMENT**

The present study was supported with a grant by BBraun Melsungen AG. Furthermore, this study was supported with grants by Apothekerkammer Nord-rhein and ADKA Stiftung e.V.

### 1258

#### The management of the high commitment human resource practices and its relation with personal satisfaction and the management team's commitment for quality

P. Vega Ocaña[1], J.D. Martín Santana[2], L. González Bautista[1], C. García del Rosario[3], L. Santana Cabrera[1], R. Lorenzo Torrent[1]
[1]Hospital Universitario Insular de Gran Canaria, Intensive Care Unit, Las Palmas de Gran Canaria, Spain; [2]Universidad de Las Palmas de Gran Canaria, Las Palmas de Gran Canaria, Spain; [3]Hospital Universitario Insular de Gran Canaria, Quality Unit, Las Palmas de Gran Canaria, Spain
**Correspondence:** P. Vega Ocaña
*Intensive Care Medicine Experimental* 2018, **6(Suppl 2):**1258

**INTRODUCTION.** Proper management of human resources is critical in providing a high quality of health care. The staff's perception of good high-commitment human resource practices and organizational management affects directly to their job and satisfaction.

**OBJECTIVES.** To evaluate the relationship between the assessment of management team's commitment towards the high-commitment human resource practices and ICU healthcare personnel's satisfaction and the commitment of the management team for the implementation of a quality management in the organization.

**METHODS.** Information was collected from August to November 2017 through a survey adapted to each professional category. The questionnaire included a 7-score Likert scale including 24 items aimed at assesing different aspects related to high-commitment human resources management.

As a previous step, a Confirmatory Factorial Analysis (CFA) was performed to corroborate the theoretical dimensions traditionally published in the literature on high-commitment human resource practices and the unidimensionality of the scales used to measure the personal satisfaction and the organizational commitment of the management team. In spite of the statistical significance of the model [$\chi^2$=201.34, $p$=0.000], results showed that othat other adjustment indicators were within the value ranges recommended in the literature (CFI=0.97, NFI=0.92, RMSEA=0.08). Regarding internal consistency measurements of every dimesion composite reliability (CR) reached values over 0.70 and average variance extracted (AVE) reached values over 0.50. The Cronbach's alpha values verifies those observed in composite reliability. These results indicated that the measurement model was valid and reliable.

**RESULTS.** In order to analyze the relationship between HPWPs and the constructs of personal satisfaction and organizational commitment, a correlation test was performed using the Pearson correlation coefficient. Results are shown in the following table.

**CONCLUSIONS.** There is a statistically significant positive association between the organizational commitment and all the dimensions that make up the high-commitment human resource practices, which indicated that the greater the organizational commitment, the better HPWPs. Internal promotion is the dimension with the stronger relationship.

ICU healthcare personnel's perception of a high level of organizational commitment by the hospital management is related to better HPWPs, which improves the well-being, attitude and commitment of the staff.

On the other hand, good high-commitment human resource practices are associated to greater personal satisfaction on the part of ICU healthcare personnel.

**REFERENC(E).** SM Kabene, C Orchard, JM Howard, et al. The importance of human resources management in health care: a global context. Human Resources for Health 2006; 4: 20.

**GRANT ACKNOWLEDGMENT**
None.

**Table 1 (abstract 1258).** Correlation between high-commitment human resource practices and personal satisfaction and organizational commitment

| DIMENSIONS | PERSONAL SATISFACTION | ORGANIZATIONAL COMMITMENT |
|---|---|---|
| Training | 0.494*** | 0.719*** |
| Participation | 0.526*** | 0.746*** |
| Acknowledgment | 0.481*** | 0.798*** |
| Internal Promotion | 0.472*** | 0.857*** |

****** p < 0.000*

### 1259

#### Vital signs alarms in the ICU do not affect noise levels in the ICU

J. Vreman[1], L. van Loon[2], W. van de Biggelaar[3], H. van der Hoeven[1], J. Lemson[1], M. van den Boogaard[1]
[1]Radboud University Medical Center, Intensive Care Medicine, Nijmegen, Netherlands; [2]Technical Medicine, University of Twente, Enschede, Netherlands; [3]Philips Healthcare, Clinical Transformation & Education (CTE), Eindhoven, Netherlands
**Correspondence:** J. Vreman
*Intensive Care Medicine Experimental* 2018, **6(Suppl 2):**1259

**INTRODUCTION.** Intensive care unit (ICU) patiënts are mostly surrounded by life saving equipment that contribute to noise levels exceeding the WHO recommended noise levels of max 35 A-weighted decibel (dBA) by far. Although many noise studies have been performed, it is unknown what the contributing effect is of equipment alarms to the noise levels in the ICU.

**OBJECTIVES.** The aim of our study is therefore to explore noise levels and frequency of equipment alarms and to determine the association between alarms and noise levels in the ICU.

**METHODS.** A multiple single center cross-sectional observational study was performed between February 17[th] and March 17[th] 2015 in a university hospital on 4 ICUs with 36 beds. During 29 days, alarms were recorded with use of the alarm tracking software. We distinguished the following categories: red alarms (life threatening alarms), yellow alarms (alarms exceeding limits) and blue alarms (technical alarms). Noise levels were quantified in dBA and continuously measured at the nurses' station located in the middle of the unit, and at the bedside during 2 randomly chosen days. Noise and alarm datasets were time synchronized to determine the association between alarms and noise levels using linear regression analysis.

**RESULTS.** At the nurses' station mean noise levels were 51.1dBA (±5.7) and bedside 46.7 dBA (±6.5).

During daytime, noise levels were significantly higher than during evening-time, on both locations; nurses' station: 52.2 vs 50.8, p< .01, and bedside: 48.7 vs 46.0, p< 0.01. Mean noise levels during evening-time were significantly higher than during nighttime on both locations; nurses' station: 50.8 vs 48.9, p< 0.01, bedside: 46.0 vs 42.8, p< 0.01.

In 29 days a total of 175,996 alarms were recorded of which 149,764 (85%) were exceeding limits (yellow), 18,080 (10%) were red alarms and 8,152 (5%) were technical alarms. All alarms on the 2 days were statistically significant associated with the nurses' station noise levels: RED (b=0.2; 95% CI, 0.06-0.32; p< .0.001) YELLOW (b=-0.96; 95% CI, -0.98- -0.94; p< .001) and BLUE (b=0.13; 95% CI, 0.07-0.19; p< .001). Bedside, this was significantly associated for YELLOW (b=-0.97; 95% CI, 0.87- 1.075; p< .001) and BLUE (b=0.54; 95% CI, 0.73-0.7; p< .001 ), but not for RED alarms (b=0.28; 95% CI, -0.09 -0.65; p=0.14). The R Square of the model at the nurses' station was: 0.015 (standard error of estimate, 5.7; p< .001) and bedside: 0.001 (standard error of estimate, 7.0; p< .001).

**CONCLUSIONS.** In the ICU, during all time periods, noise levels exceed the maximum WHO recommended mean level of 35 dBA of which daytime is the noisiest. Although all type of alarms were statistically significantly associated with the noise levels, the contributing effect to the decibel level is only minimal. Alarms are meaningful but not in the sense of mean noise level.

### 1260

#### The use of social media as a communication tool for improving standards in critical care: a Norwegian cross-sectional survey

A. Petosic[1], K. Sunde[2,3], H. Flaatten[4], D. Beeckman[5], H. Wøien[1,6]
[1]Oslo University Hospital, Department of Postoperative and Intensive care, Oslo, Norway; [2]Oslo University Hospital, Department of Anaesthesiology, Oslo, Norway; [3]University of Oslo, Institute of Clinical Medicine, Oslo, Norway; [4]University of Bergen, Bergen, Norway; [5]Ghent University, Ghent, Belgium; [6]University of Oslo, Institute of Health and Society, Oslo, Norway
**Correspondence:** A. Petosic
*Intensive Care Medicine Experimental* 2018, **6(Suppl 2):**1260

**INTRODUCTION.** Use of social media (SoMe) is increasing and might represent an alternative way to share information on intensive care unit (ICU) related subjects to improve standards of care.

**OBJECTIVES.** To explore the use of SoMe among ICU staff and their opinion regarding the use of Facebook (FB) as a platform for sharing information on ICU related subjects.

**METHODS.** An observational cross-sectional, web-based survey. ICU physicians and nurses at four ICUs participated via an email-campaign. Questions were adapted from a Norwegian survey of SoMe use in general, supplemented by additional specific questions regarding nurses' and physicians' view on the use of existing closed FB groups to share information on ICU related subjects. The scores were rated on a Numeric Rating Scale (NRS) ranging from 0 (not at all) to 10 (very much). The questionnaire was pilot-tested and modified. Descriptive statistics are presented with frequencies, percentages, median and interquartile range (IQR). Chi-square test and p< 0,05 are used for comparison.

**RESULTS.** Of 394 eligable recipients, 253 (64%) responded, of whom 43 (17%) were physician, 210 (83%) nurses, 188 (74%) female and 154 (61%) >40 years old.

Overall, 234 (93%) had a SoMe profile, with 176 (70%) reporting contact with family/friends as one of three most important reasons for using SoMe, whereas 34 (13%) reported professional development as one of the top three reasons. FB was the most frequently used SoMe: 221 (87%) had a registered profile; among those 172 (78%) used FB daily (see Figure 1). FB-profiles were evenly distributed among men and women (82 vs 89% (p=0,102)), and physicians and nurses (81 vs. 89% (p=0,197)). However, having a FB-profile was less common among those >40 years than those < 40 years (81 vs 97% (p< 0,001)). Daily FB use differed between gender (68 vs. 81% (p=0,047)) and profession (60 vs. 81% (p=0,006)), but was similar in both age groups (76 vs. 80% (p=0,455)).

Overall, 193 (76%) were members in their ICUs existing closed FB group; 182 (87%) nurses and 11 (26%) physicians. When asking whether they would accept to receive information on ICU related subjects vs. other social work-related information through FB (n=193), median NRS score was 6 (6) vs. 8 (3), respectively. Not being annoyed by receiving information on ICU related subjects vs. other work-related social information through FB, was reported with median 2 (6) vs. 2 (5), respectively. Overall, the respondents were positive about being members of closed FB-groups dedicated to the exchange of information on ICU related subjects (median NRS-score = 9 (4)).

**CONCLUSIONS.** The majority of the ICU staff were active users on FB. They reported a positive attitude to receive information about ICU related subjects in closed FB groups. A prospective study using closed FB groups to optimize critical care is now being carried out in Oslo University Hospital.

**GRANT ACKNOWLEDGMENT**
Oslo University Hospital

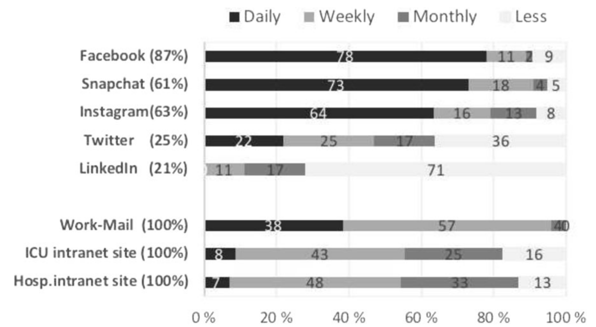

**Fig. 1 (abstract 1260).** Use of Social Media and traditional Hospital workmail and intranet

### 1261

#### Visiting polices in critical care area - liberal or restricted

K. Zirpe, S.K. Yadav, S. Gurav, A. Deshmukh, R.N. Yellapu
Hall Clinic, Neurotrauma Unit, Pune, India
**Correspondence:** K. Zirpe
*Intensive Care Medicine Experimental* 2018, **6(Suppl 2):**1261

**INTRODUCTION.** The Intensive Care Unit (ICU) is a highly stressful environment for patients and their families. Measurement of family satisfaction has, hence, been proposed as one of the several quality indicators of ICU care. In general, the time period of an ICU visit is described as restrictive (RVP) or open/liberal(OVP).

**OBJECTIVES.** To understand perception of-1] Nurses about restricted (RVP) and liberal / open visiting policies (OVP) in critical care area.

2] Visitors about restricted (RVP) and liberal visiting polices (OVP) in critical care area.

3] To suggest improve measures to address the needs of patients, relatives & staff.

**METHODS.** A cross sectional observational study.Statistical analysis performed by using SPSS (Statistical Package for social sciences) version 20:0 we have used Chi-square test to compare the Open vs Restricted in 2 different groups.

**RESULTS.** Our study suggest that majority of nurse in RVP compared to OVP think that 24hrs visiting policy will hinder the patient rest (P Value-0.006), their work will suffer much more interruption because of 24hrs visiting policy (P value-0.004) and also believe that a 24hrs visiting policy will lead to delay in examining and performing procedures on patients (P value-0.001). All these were found statistically significant (P value- < 0.005).Our study suggest, majority of nurses in both group think that increasing visiting hours may be decrease patient´s anxiety and stress. But there was no significant difference in two policies regarding patient recovery. Also, Majority of the Visitors in both groups (64% in OVP and 68.4% in RVP) believe that increasing the visiting hours may decrease patient's anxiety and stress. And it not was found any statistically significant difference in two policies. Our study found that majority of the Visitors in both groups (70% in OVP and 71.4% in RVP) believe that open visiting policy will force the patients relative to stay with the patient. Another interesting finding is that Majority of nurses in both policies think that ICU visitation policy may be adapted in cases of conflict or by patients request and there was no significant difference in both policies about their opinion that ICU policy visitation should be adapted in cases of conflict or by patients request.

**CONCLUSIONS.** Policy makers should be aware of staff nurse's beliefs and attitudes about their visiting policies and educate them about the rationale for visiting policies. A visiting policy should be balanced covering concerns of nursing staff and relatives and consider program development to assist nurses to work with patient's relatives.

**REFERENCE(S)**
1. Cook DA. Open visiting: Does this benefit adult patients in intensive care units., Dunedin, Aotearoa/New Zealand. 2006 Oct.
2. Garrouste-Orgeas M, Philippart F et all. Perceptions of a 24-hour visiting policy in the intensive care unit*. Critical care medicine. 2008 Jan.

### 1262

#### Functional status in traumatic brain injury (TBI) patients in the ICU and three months after discharge

E.F. Osaku, M.M. Moraes, C.R.L.M. Costa, B.A. Kanezawa, E.R. Penteado, P.D.S. Quadros, T.C. Schnaufer, A. Tomazelli, G. Antonelli, T.L. Cagnini, J.B. Anjos, M.A. Leite, A.C. Jorge, P.A.D. Duarte
Universitário do Oeste do Paraná, General ICU, Cascavel, Brazil
**Correspondence:** A.C. Jorge
*Intensive Care Medicine Experimental* 2018, **6(Suppl 2):**1262

**INTRODUCTION.** Functional status is highly variable in post-ICU patients.
**OBJECTIVE.** To compare the functional status of patients with TBI in the ICU and three months after discharge, in an outpatient clinic.
**METHODS.** Retrospective study from January to December, 2014, conducted in an adult intensive care unit (ICU) and in the post-ICU outpatient clinic of a University Hospital. Functional Independence Measure (FIM), Functional Status Score (FSS) and Barthel score were used to assess functional capacity. Glasgow coma score (GCS) and Richmond Agitation-Sedation Scale (RASS) were used to assess the level of consciousness. All patients were adults, with TBI (traffic accidents, falls or violence) and were evaluated twice: (1)on ICU discharge day, and (2)after three months. In the statistical analysis, the data were described by mean and standard deviation, and the Wilcoxon test was used, using $p < 0.05$).
**RESULTS.** A total of 34 patients with TBI were included in the study, with a mean age 34.0 years, most male (85%), mean APACHE II 19.2 and 1$^{st}$ day SOFA 14.1. Mean duration of MV and sedation were 207.9 and 127.7 hours, respectively. ICU and hospital length of stay were 12.6 days and 25.4 days. In the assessment of the level of consciousness, mean GCS and RASS in the ICU and at the oupatient clinic were [12.1 and -0.5], and [14.4 and -0.2], respectively. FIM, FSS and Barthel scale were reduced at ICU discharge; however, there was significant improvement after three months ($p < 0.05$) (Figure 1).
**CONCLUSION.** TBI patients showed reduction in functional status at ICU discharge, but with significant improvement after three months.

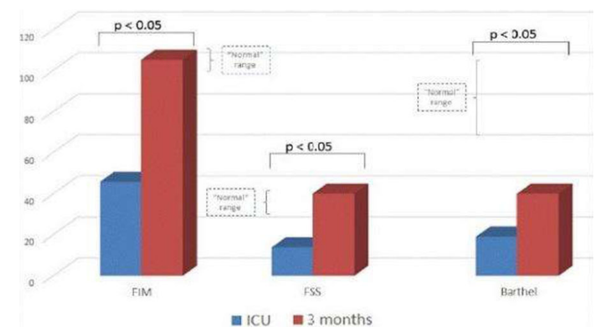

**Fig. 1 (abstract 1262).** See text for description

### 1263

#### Development of a critical care rehabilitation clinic at a district general hospital

E. Morino, L. Ma
Hospital NHS Foundation Trust, Critical Care, Kingston-upon-Thames, United Kingdom
**Correspondence:** E. Morino
*Intensive Care Medicine Experimental* 2018, **6(Suppl 2):**1263

**INTRODUCTION.** The National Institute for Health and Clinical Excellence (NICE) recommended in 2009 (Clinical Guidance 83)[1] that patients should be reviewed by appropriately-skilled healthcare professionals 2-3 months following discharge from critical care. Based on functional assessment, patients should be referred to appropriate rehabilitation or specialist services. Patients should be reviewed for symptoms of anxiety, depression, and/or Post-traumatic stress disorder (PTSD).
**OBJECTIVES.** At Kingston Hospital, we identified the need for improved aftercare of critical care patients, specifically to address the physical, psychological, social and economic effects of critical illness. Our primary objectives were to develop a multi-disciplinary critical care rehabilitation clinic to co-ordinate aftercare by liaising with primary and secondary care providers. Our secondary objectives were to improve future patient care by collecting data on patient experience and satisfaction and providing feedback to staff for learning and development.
**METHODS.** The critical care rehabilitation clinic commenced in October 2017. The consultant-led team consists of doctors, nurses, physiotherapists and a clinical psychologist. A retrospective review looked at the number of patients seen over a 6-month period, mean length of stay (LOS), and referrals to different services. We looked at patient satisfaction via a feedback questionnaire.
**RESULTS.** 26 patients were seen in the first 6 months at our monthly clinic. Mean length of stay (LOS) in critical care was 17.3 days. Mean LOS in hospital was 37.8 days. Secondary care referrals for ongoing or new morbidity was made in 9/26 (34%) of patients. Referral to physiotherapy was made in 8/26 (31%) of patients. Referral to specialist psychology services for anxiety/low mood and/or symptoms of PTSD were made in 13/26 (50%) of patients. 16 patients completed the feedback questionnaire. 16/16 (100%) of patients agreed or strongly agreed that the events during their stay was explained at clinic, and that physical, emotional, and psychological issues were appropriately addressed. 16/16 (100%) of patients felt they benefited from attending. 5/16 (31%) felt they would benefit from a patient support group. We listened to the positive and negative experiences of intensive care and we used these to feedback to staff to improve care and enlighten practice.
**CONCLUSIONS.** Our review of our clinic outcomes in the first stages of its development showed that a significant number of critical care survivors require ongoing multi-disciplinary support, and that there is a need for engagement with primary and secondary care providers to ensure safety-netting of interventions. Patients overwhelmingly regarded the clinic visit as a positive experience.

**REFERENCE(S)**
1. NICE. *Rehabilitation after critical illness NICE Clinical Guideline 83*. London, UK: National Institute for Health and Clinical Excellence, 2009

### 1264

#### Critical care drug pouch a case to avoid mistakes

F. Rubulotta
College London, London, United Kingdom
*Intensive Care Medicine Experimental* 2018, **6(Suppl 2):**1264

**INTRODUCTION.** Drugs errors are common and their incidence may vary depending on the clinical setting. Human factors remain a major contributor to these.
**OBJECTIVES.** The concept of a "drug grab pouch" is an established and essential part of military and paramedic equipment.
The project designed at Charing Cross Hospital, London, UK, proposes the use of a pre-set Critical Care Drug Pouch (CCDP) for the following uses in critical care (critical care drug pouch):

- Emergency intubation
- Interdepartmental and inter-hospital transfer of patients
- Safe transfer and sedation of critically unwell patients

**METHODS.** A modified Delphi method was used to gather consensus on an initial pilot CCDP by sending an online questionnaire. The results of this questionnaire were analysed and consensus agreed by an expert panel thereafter before introduction to the end-user.

**RESULTS.** 88% response rate was achieved among doctors (consultant and registers) and nurses. There was global agreement that drawing of drugs for emergency rapid sequence induction and transfer was subject to errors and delays, the available drugs were insufficient. 100% of respondents agreed that always or sometimes, patient care was compromised due to delays in drawing up of medications and 96% of respondents felt a CCDP would improve this experience. The questionnaire allowed respondents to express drugs they felt would be of use in such a project. A second round of consultation of departmental consultants and senior registrars permitted consensus of the bag form, contents (drugs and adjuncts) that would be essential based on the above results. Consensus was obtained that the pouch should be minimalistic in size and contents, to permit easy use, daily check and reduction of waste. It was agreed that it should be in keeping with infection control guidelines and have clinical governance procedures in place to ensure sustained quality and safety. The pouch should also contain all necessary syringes akin to routine anaesthetic practice and drawing up needles. It was agreed that colour coded syringe labels would be contained in harmony with UK regulations and common anaesthetic practice as per Royal College of Anaesthesia guidelines to prevent "wrong drug" administration. To facilitate standardisation, two small bags (20cm x 20cm x 7cm) with elasticated vial and mini-jet compartments, were introduced across the 25 bed unit. The bag also contained an fixed inventory and audit form permitting accountability and ease of daily check.

**CONCLUSIONS.** "To err is human" but instituting basic measures such as provision of essential drugs, standardised syringes and colour-coded labels in a single, compact and portable device, the CCP, is a solution to many preventable errors in critical care environments.

### 1265

#### Non-pharmacological interventions to prevent or mitigate long-term consequences among ICU survivors: a systematic review

W. Geense[1], M. van den Boogaard[1], J. van der Hoeven[1], H. Vermeulen[2], M. Zegers[1]

[1]Radboud University Medical Center/ Radboud Institute for Health Science, Intensive Care Medicine, Nijmegen, Netherlands; [2]Radboud University Medical Center/ Radboud Institute for Health Science, IQ Healthcare, Nijmegen, Netherlands

**Correspondence:** W. Geense

*Intensive Care Medicine Experimental* 2018, **6(Suppl 2):**1265

**INTRODUCTION.** Long-lasting impairments in physical, cognitive and mental health, also called Post Intensive Care Syndrome (PICS), are common after hospitalization for critical illness. In recent years, many interventions emerged to prevent or mitigate PICS. However, an overall view of the effectiveness of these interventions is lacking.

**OBJECTIVE.** To systematically review non-pharmacological interventions aimed at preventing or mitigating long-term consequences among ICU survivors.

**METHODS.** PubMed, CINAHL, PsycInfo, EMBASE, and the Cochrane Library were searched for studies published from inception till September 2017. The EPOC study design criteria were used to include studies evaluating interventions conducted before, during, or after ICU aiming to prevent or mitigate long-term consequences. Studies including adult ICU patients, admitted ≥12 hours and reporting quantitative outcome measures after ICU discharge, were included. Two reviewers independently selected studies, extracted data and assessed the study quality using the Cochrane risk of bias tool.

**RESULTS.** From 11,080 publications, 42 studies with 5,723 patients were included. Eight intervention categories were identified in 40 RCTs and 2 N-RCTs: exercise and physical rehabilitation programs (n=21), neuromuscular electrical stimulation (NMES) (n=4), nurse led follow-up care (n=3), diaries (n=3), information or education programs (n=3), psychosocial programs (n=3), changes in care organization (n=2) and other interventions (n=3). Twenty interventions started in the ICU, 11 after ICU discharge, 10 after hospital discharge and one intervention before ICU admission.

Between the studies, heterogeneity was present regarding study participants, interventions, follow-up time points, measured outcomes and instruments. We found 95 unique outcomes, of which 50 physical, 12 mental, 10 cognitive, 7 quality of life and 16 other outcomes, such as activities of daily living. The maximum follow-up time was 12 months. Sample sizes were relatively small (median 136; minimum 20, maximum 386 participants).

None of the studies showed strong evidence for effectiveness of the interventions. Exercise and physical rehabilitation programs seems to improve physical functioning and quality of life among ICU survivors. NMES might increase muscles strength and the use of diaries might reduce symptoms of post traumatic stress, anxiety and depression. No clear overall effect was found for nurse led follow-up care, information or education program or psychosocial programs. We will present the outcomes of the meta-analysis at the congress.

**CONCLUSIONS.** There is a lack of evidence for interventions to prevent or mitigate long-term consequences among ICU survivors. Most of the studies had small samples sizes and measured short-term outcomes using different instruments. There is an urgent need for larger RCTs, measuring longer-term outcomes with uniform instruments.

**PROSPERO REGISTRATION.** NCT01738620

### 1266

#### Psychocognitive sequelae of ICU care, barriers to recovery, and correlation of post-ICU assessment with 3 months follow up

J. Schultz, M. Clark, P. Schulte, K. Philbrick, L. Karnatovskaia

Clinic, Rochester, United States

**Correspondence:** J. Schultz

*Intensive Care Medicine Experimental* 2018, **6(Suppl 2):**1266

**INTRODUCTION.** Over a third of critical illness survivors manifest significant psychocognitive impairments following discharge from the ICU. Yet little is known about which patient populations are at highest risk or whether performing assessment at ICU discharge can help plan outpatient treatment strategy.

**OBJECTIVES.** This single center study assessed the prevalence of psychocognitive morbidity among patients across six adult ICUs. The study also examined correlations between initial assessment and 3 months follow up. To understand the patient experience, patients provided feedback on perceived barriers to recovery and beneficial recovery strategies.

**METHODS.** Within 96 hours of dismissal from the ICU, patients with>48 hour stay completed Hospital Anxiety and Depression Scale (HADS; scores≥8 indicating significant symptoms of anxiety or depression), Impact of Events Scale-Revised (IES-R; scores≥1.6 indicating significant posttraumatic stress disorder symptoms), and Montreal Cognitive Assessment-Blind (MoCA-blind; scores< 18 indicating cognitive impairment). Patients were contacted 3 months following the initial evaluation; repeat assessments were completed by phone. Linear regression assessed the association of baseline characteristics with initial and 3-month assessments of HADS-D/A and MoCA-Blind, while logistic regression assessed associations with IES-R≥1.6. Variable selection using a backward stepwise procedure selected variables with the strongest associations.

**RESULTS.** 299 patients completed the initial assessment and 174 completed 3 month follow up. ICU length of stay averaged 4.2 (sd=3.3) days and did not significantly differ across ICU settings (p= 0.089). MoCA-Blind (mean=16.7 (sd=3.09)), HADS-D (mean=6.3 (4.1)) and HADS-A (mean=7.2 (4.1)) were similar across ICU settings (p>0.1

for each). Most common problem was cognitive with 58% of patients having MoCA-BLIND< 18 followed by anxiety with 45% having HADS-A≥8. 39% had IES-R≥1.6, and 37% had HADS-D≥8. See Tables 1 and 2 for multivariable associations between baseline and ICU stay characteristics and psychocognitive scores post-ICU and at 3 months. There were moderate correlations between baseline and follow up psychocognitive outcomes, from ρ=0.35 for IES-R Avoidance to ρ=0.58 for HADS-D. Physical therapy in ICU was associated with lower HADS-D/A at 3 months (p< 0.001 and p=0.022, respectively). See Figure 1 for barriers to recovery and strategies patients found helpful.

**CONCLUSIONS.** While the prevalence of psychocognitive morbidity was high, there was no significant difference by the ICU type. There is a moderate correlation between the initial assessment and follow up scores suggesting that early screening of high risk patients may help facilitate timely follow up. Barriers to post-discharge recovery were generally rated as minimal. Successful strategies for recovery were reported for family, medical visits, exercise, and relaxation.

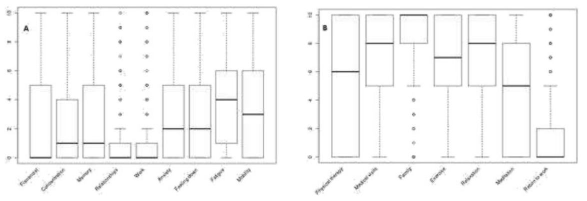

**Fig. 1 (abstract 1266).** A barriers to recovery (0-not a problem to 10 significant problem); B strategies beneficial

**Table 1 (abstract 1266).** Associations with baseline assessments

| Variable | HADS-D (n=299) | | | HADS-A (n=299) | | | MoCA-BLIND (n=298) | | | IES-R any score ≥1.6 (n=299) | | |
|---|---|---|---|---|---|---|---|---|---|---|---|---|
| | Est | 95% CI | p | Est | 95% CI | p | Est | 95% CI | p | OR | 95% CI | p |
| Age, per year | 0.01 | -0.02, 0.05 | 0.36 | -0.02 | -0.05, 0.01 | 0.17 | -0.03 | -0.06, -0.01 | 0.005 | 0.98 | 0.96, 1.00 | 0.12 |
| Male sex | 0.53 | -0.40, 1.45 | 0.26 | 0.52 | -0.39, 1.43 | 0.26 | -1.02 | -1.72, -0.33 | 0.004 | 1.72 | 1.01, 2.93 | 0.046 |
| Depression | 2.05 | 1.02, 3.08 | <0.001 | 0.98 | -0.05, 2.00 | 0.063 | - | - | - | 2.29 | 1.27, 4.12 | 0.006 |
| Drug abuse | -1.50 | -3.48, 0.49 | 0.14 | - | - | - | - | - | - | - | - | - |
| Alcohol abuse | -1.13 | -2.63, 0.36 | 0.14 | - | - | - | - | - | - | - | - | - |
| Invasive ventilator days, per day | - | - | - | 0.36 | 0.02, 0.70 | 0.038 | -0.3 | -0.55, -0.05 | 0.021 | 1.40 | 1.15, 1.70 | 0.001 |
| NIV use (yes) | - | - | - | 1.28 | -0.02, 2.59 | 0.055 | - | - | - | - | - | - |
| NIV days, per day | -0.38 | -0.87, 0.11 | 0.13 | -0.73 | -1.37, -0.10 | 0.024 | - | - | - | 0.57 | 0.39, 0.84 | 0.004 |
| Delirium | 1.14 | -0.08, 2.36 | 0.068 | 1.72 | 0.48, 2.96 | 0.007 | - | - | - | - | - | - |
| Benzodiazepines | 0.85 | -0.11, 1.81 | 0.084 | 1.02 | 0.07, 1.98 | 0.037 | - | - | - | - | - | - |
| Charlson Score, per 1 comorbidity | - | - | - | - | - | - | - | - | - | 1.09 | 1.00, 1.20 | 0.049 |
| PT in ICU | 0.86 | -0.18, 1.89 | 0.11 | - | - | - | -0.63 | -1.42, 0.16 | 0.12 | - | - | - |
| OT in ICU | - | - | - | - | - | - | - | - | - | 1.86 | 1.00, 3.47 | 0.052 |
| ICU LOS, per day | 0.16 | 0.01, 0.32 | 0.043 | -0.14 | -0.34, 0.06 | 0.16 | 0.12 | -0.03, 0.27 | 0.13 | - | - | - |

**Table 2 (abstract 1266).** Associations with follow up assessments

| Variable | HADS-D (n=170) | | | HADS-A (n=170) | | | MoCA-BLIND (n=158) | | | IES-R Any Score ≥1.6 (n=171) | | |
|---|---|---|---|---|---|---|---|---|---|---|---|---|
| | Est | 95% CI | p-value | Est | 95% CI | p-value | Est | 95% CI | p-value | OR | 95% CI | p |
| Age, per year | -0.01 | -0.04, 0.02 | 0.54 | -0.07 | -0.1, -0.03 | <0.001 | -0.02 | -0.05, 0.00 | 0.085 | 0.92 | 0.89, 0.96 | <0.001 |
| Male sex | 0.65 | -0.29, 1.59 | 0.18 | -0.08 | -1.07, 0.91 | 0.87 | -0.26 | -1.05, 0.54 | 0.53 | 1.01 | 0.34, 3.02 | 0.99 |
| Baseline HADS-D | 0.46 | 0.35, 0.57 | <0.001 | - | - | - | - | - | - | - | - | - |
| Baseline HADS-A | - | - | - | 0.48 | 0.36, 0.60 | <0.001 | - | - | - | - | - | - |
| Baseline MoCA-BLIND | - | - | - | - | - | - | 0.52 | 0.39, 0.65 | <0.001 | - | - | - |
| Baseline IES-R Intrusion | - | - | - | - | - | - | - | - | - | 0.96 | 0.38, 2.46 | 0.93 |
| Baseline IES-R Avoidance | - | - | - | - | - | - | - | - | - | 2.47 | 0.97, 6.26 | 0.057 |
| Baseline IES-R Hyperarousal | - | - | - | - | - | - | - | - | - | 1.86 | 0.73, 4.73 | 0.19 |
| Depression | 1.67 | 0.62, 2.73 | 0.002 | - | - | - | - | - | - | - | - | - |
| Anxiety | - | - | - | - | - | - | -0.95 | -1.78, -0.12 | 0.026 | - | - | - |
| Drug abuse | 2.39 | 0.13, 4.64 | 0.040 | 2.92 | 0.63, 5.21 | 0.013 | -1.49 | -3.25, 0.26 | 0.098 | 4.92 | 0.67, 35.97 | 0.12 |
| PTSD | - | - | - | 3.11 | 0.98, 5.25 | 0.005 | - | - | - | 5.59 | 0.93, 33.68 | 0.060 |
| Invasive ventilator days, per day | 0.29 | 0.01, 0.57 | 0.045 | - | - | - | 0.15 | -0.07, 0.36 | 0.18 | - | - | - |
| Non-invasive ventilator use (yes) | - | - | - | - | - | - | 1.33 | 0.50, 2.17 | 0.002 | 3.25 | 1.04, 10.20 | 0.043 |
| Delirium | - | - | - | - | - | - | 0.79 | -0.34, 1.91 | 0.17 | - | - | - |
| Benzodiazepines | - | - | - | - | - | - | -1.01 | -1.81, -0.20 | 0.015 | - | - | - |
| Ketamine | 1.08 | -0.07, 2.24 | 0.067 | 1.12 | 0.00, 2.24 | 0.052 | - | - | - | - | - | - |
| Neuromuscular block | -1.35 | -2.47, -0.23 | 0.019 | - | - | - | - | - | - | 0.18 | 0.05, 0.61 | 0.006 |
| PT in ICU | -2.08 | -3.21, -0.96 | <0.001 | -1.34 | -2.47, -0.2 | 0.022 | - | - | - | - | - | - |

### 1267

#### Sarcopenia and adverse outcomes in critically ill surgical patients

G.B. Fleming[1], D.A.J. Smith[2], L. Hogg[1]
[1]NHS Fife, Intensive Care Unit, Kirkcaldy, United Kingdom; [2]NHS Fife, Department of Radiology, Kirkcaldy, United Kingdom
**Correspondence:** G.B. Fleming
*Intensive Care Medicine Experimental* 2018, **6(Suppl 2):**1267

**INTRODUCTION.** Sarcopenia is the loss of skeletal muscle mass and function, a recognised component of the clinical syndrome of frailty, and is associated with increased mortality and morbidity in emergency and elective surgical patients [1,2]. We hypothesised that sarcopenia, demonstrated by morphometric measurements of the psoas muscles on pre-operative computed tomography (CT), would predict mortality and morbidity in critically ill patients undergoing laparotomy.

**METHODS.** A retrospective review was conducted of all patients admitted to the Intensive Care Unit, Victoria Hospital, Kirkcaldy, following emergency or elective laparotomy between Jan 2015 - Dec 2016. Clinical data was recorded from the electronic Wardwatcher system. For sarcopenia evaluation, psoas muscle area and density at the L4 level were measured on pre-operative CTs and the Hounsfield Unit Average Calculation (HUAC), a validated marker of muscle density and fatty infiltration [2], was derived. Patients were stratified into quartiles and defined as being sarcopenic if they fell below the 25th percentile.

**RESULTS.** 89 patients met the inclusion criteria for analysis. 53% were male and the median age was 66 years (IQR 50-75 years). Overall 30-day and 1-year mortality were 16% and 21% respectively. Patients were stratified into quartiles with the lowest quartile defined as sarcopenic (HUAC < 25). 23 patients met the defined criteria for sarcopenia. As expected, these patients were significantly older. Sarcopenia was associated with significantly increased 30-day and 1-year mortality, higher APACHE scores and significantly increased ventilator days (Table 1). Although non-significant, there was also a trend towards increased renal replacement therapy in sarcopenic patients. The 1 year survival of sarcopenic vs non-sarcopenic patients is seen in the Kaplan Meier curve (Figure 1).

**DISCUSSION.** We demonstrated that sarcopenia in surgical patients, reflected by low psoas muscle HUAC, is associated with increased 30-day and 1-year mortality, and increased morbidity. We propose that psoas muscle measurement via CT is an objective and effective measurement which can be readily used in the pre-operative evaluation and counselling of patients and clinical teams. Early identification of sarcopenic patients via CT measurement may also have a further role of identifying patients likely to benefit from intensive multidisciplinary team input within ICU to optimise nutrition and mobilisation.

**Table 1 (abstract 1267).** Outcomes stratified by HUAC quartiles

| | Quartile 1 | Quartile 2 | Quartile 3 | Quartile 4 | p-value |
|---|---|---|---|---|---|
| Age | 73 (66-83) | 70 (56-78) | 66 (57-82) | 48 (41-67) | <.001 |
| HUAC | 15.25 (10.2-22.9) | 30.4 (28.6-30.9) | 38.4 (35.7-39.8) | 44.9 (42.5-49.0) | < .001 |
| MORTALITY | | | | | |
| 30-day mortality | 50% | 9% | 0 | 0 | .004 |
| 1-year mortality | 70% | 14% | 0 | 0 | <.001 |
| MORBIDITY | | | | | |
| APACHE | 18 (15-25) | 20 (12-27) | 15 (11-21) | 11.5 (7-18) | .008 |
| ICU LOS (days) | 4.1 (1.0-9.8) | 4.8 (1.8-13) | 2.4 (0.9-6.1) | 3 (1.8-4.7) | .717 |
| Ventilator days | 3 (1-6.75) | 3.5 (2-13.2) | 1 (0-4) | 1 (0-3) | .001 |
| Renal replacement therapy | 27% | 18% | 13.6% | 4.5% | .366 |

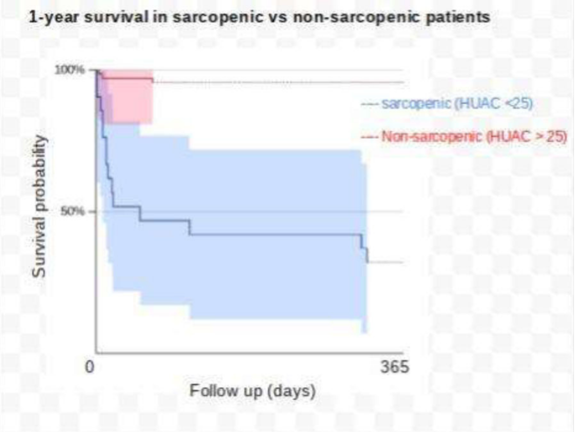

**Fig. 1 (abstract 1267).** Kaplan Meier curve showing one year survival for sarcopenic vs non-sarcopenic patients

### 1268

#### Right Data, Right Now: developing a big data machine-learning based prediction model to prevent ICU readmission

P.J. Thoral[1], M. Fornasa[2], H. Hovenkamp[2], R. Driessen[1], A.R.J. Girbes[1], P.W.G. Elbers[1]
[1]VU University Medical Center Amsterdam, Department of Intensive Care Medicine, Research VUmc Intensive Care (REVIVE), Amsterdam Cardiovascular Sciences (ACS), Amsterdam Infection and Immunity Institute (AI&II), Amsterdam, Netherlands; [2]Pacmed B.V., Amsterdam, Netherlands
**Correspondence:** P.J. Thoral
*Intensive Care Medicine Experimental* 2018, **6(Suppl 2):**1268

**INTRODUCTION.** ICU readmission after critical illness is associated with longer length of stay and increased mortality.[1,2] Unexpected readmissions may be prevented by delaying discharge from the ICU, though optimal timing is subject of debate. Especially in tertiary teaching hospitals where ICU capacity is often strained, unnecessarily long ICU stays may prevent timely admission of other patients requiring ICU treatment[3]. Decision-making supported by a prediction model may prevent readmission and mortality and allows optimizing ICU capacity. Given the large amount of data gathered routinely in the ICU and the complexity of the task, machine learning is particularly suited for the development and clinical implementation of said prediction model.

**OBJECTIVES.** The aim of the first phase of our study was to develop a machine-learning-based algorithm that ranks patients based on their chance of readmission or mortality after ICU discharge. In particular the algorithm should identify patients with a high/low risk of readmission/mortality when discharge from the ICU is being considered.

**METHODS.** Anonymized data was extracted from the Patient Data Management System (MetaVision, iMDsoft) at our 24-bed mixed surgical-medical ICU at VU University Medical Center, Amsterdam, The Netherlands. Data from adult patients admitted to our ICU between 2004 and 2016 were included in the analysis. Patient characteristics, clinical observations, (automated) physiological measurements and laboratory studies were considered as possible features for the model. The change over time of these features was captured by building aggregated model features. Different supervised learning algorithms (including neural networks and boosted decision trees) were trained to predict readmission or death within 7 days from ICU discharge on 75% of the patients and the final score was computed on the remaining 25%. Model development was performed in collaboration with Pacmed B.V., Amsterdam, The Netherlands using Python and the scikit-learn package.

**RESULTS.** After excluding patients that that did not survive their first ICU admission and ICU admissions longer than 7 days, our data set contained approximately 13.000 cases, with a readmission/mortality rate of approximately 4%. Preliminary results show high performances, with areas under the ROC curve consistently higher than 0.8, better than other prediction models available in the literature.

**CONCLUSIONS.** Our findings showed that the vast amount of ICU PDMS data can be used to develop a model that accurately predicts readmission and mortality after ICU discharge. The final optimization of the algorithm will also take into account its implementability into a decision-support tool that is transparent and integrates well with the PDMS and the workflow of intensivists.

**GRANT ACKNOWLEDGMENT**
None

### 1269

#### Pessimism in the ICU: do long stays reduce survival?

D.C. Marshall, R. Hatch, D. Young, P. Watkinson
University of Oxford, Critical Care Research Group - Kadoorie Centre for Critical Care Research & Education, Oxford, United Kingdom
**Correspondence:** D.C. Marshall
*Intensive Care Medicine Experimental* 2018, **6(Suppl 2):**1269

**INTRODUCTION.** Although a prolonged period of critical illness might plausibly be associated with a poor outcome it is unclear if the future probability of survival on any given day is dependent on the ICU stay up to that point. Large clinical databases provide sufficient patient numbers to investigate this question.

**OBJECTIVES.** Assess the relationship between day of ICU admission and future hospital survival, and examine the effect of age on this relationship.

**METHODS.** Retrospective cohort study of the Post Intensive Care Risk-Adjusted Alerting and Monitoring (PICRAM) database. PICRAM is an intensive care database generated from admissions between 2008 and 2016 to two English ICUs, a tertiary referral centre and district general hospital. Cohorts were generated for index admissions to ICU with subgroups generated initially for all patients, then > 1 day in ICU, > 2 days in ICU until cohort < 50 patients. Survival to hospital discharge was then calculated by cohort. Exploratory analyses assessing impact of age were performed with data dichotomised by < 75 and ≥ 75 years of age (≥ 75 years representing upper quartile of population). These methods were then replicated in the North American MIMIC-3 and eICU databases (1).

**RESULTS.** 7,057 index admissions were included in the initial cohort reducing to 44 patients who were admitted for longer than 40 days (Table 1). In all patients survival declined over the initial 4 days but then plateaued with no observable changes in survival as length of stay increased (Figure 1). When patients were dichotomised into < 75 years of age and ≥ 75 there was a steeper increase in mortality in the older age group (Figure 2). The probability of future survival continued to decrease up to 15 days in the older cohort with 40-45% survival contrasted with a plateau after 4 days and survival of ~75% in the younger cohort. Similar findings were identified in the MIMIC-3 database and the eICU database. In these cohorts of 38,532 (MIMIC-3) and 166,756 (eICU) patient survival also decreased after a length of stay of > 6 and > 8 days for MIMIC and eICU respectively with a more profound reduced survival in patients ≥ 75 years of age.

**CONCLUSIONS.** Overall after 4-8 days the future probability of hospital survival does not appear to decrease with length of stay in all patients admitted to ICU. There appears to be reduced survival with increasing length of stay in patients ≥ 75 years of age.

**GRANT ACKNOWLEDGMENT**
This publication presents independent research commissioned by the Health Innovation Challenge Fund (HICF ref: HICF-0510-006), a parallel funding partnership between the Wellcome Trust and the Department of Health.

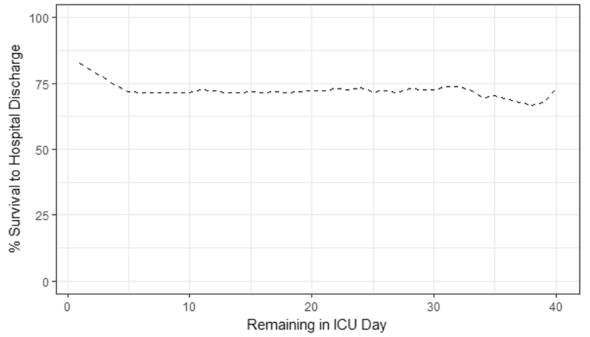

**Fig. 1 (abstract 1269).** Survival to hospital discharge with increasing length of ICU stay in all patients

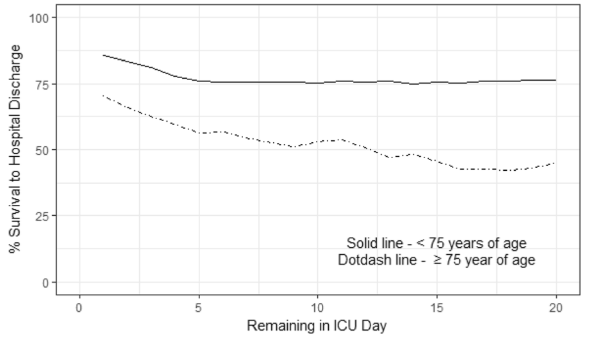

**Fig. 2 (abstract 1269).** Survival to hospital discharge with increasing length of ICU stay, dichotomised by age

**Table 1 (abstract 1269).** Survival to hospital discharge and cohort size with increasing length of stay

| | % Future Survival | n |
|---|---|---|
| All patients | 82.5 | 7057 |
| > 4 days | 71.7 | 1271 |
| > 9 days | 72.5 | 579 |
| > 14 days | 71.6 | 348 |
| > 19 days | 72.4 | 228 |
| > 24 days | 72.4 | 156 |
| > 29 days | 73.9 | 115 |
| > 34 days | 69.3 | 75 |
| > 39 days | 72.3 | 44 |

### 1270

#### Feasibility of a web-based neurocognitive battery for assessing cognitive function in critical illness survivors

K. Honarmand[1], S. Malik[2], C. Wild[3], L.E. Gonzalez-Lara[3], C. McIntyre[4,5], A.M. Owen[3], M. Slessarev[5,6]
[1]London Health Sciences Centre, Department of Medicine, Division of Critical Care, London, Canada; [2]Western University, Faculty of Science, London, Canada; [3]Western University, Brain and Mind Institute, London, Canada; [4]Western University, London Health Sciences Centre, Department of Medicine, London, Canada; [5]Western University, Department of Medical Biophysics, London, Canada; [6]Western University, London Health Sciences Centre, Department of Medicine, Division of Critical Care, London, Canada
**Correspondence:** K. Honarmand
*Intensive Care Medicine Experimental* 2018, **6(Suppl 2):**1270

**INTRODUCTION.** Long-term cognitive impairment is a common complication in critical illness survivors [1-4] but its natural history and the temporal profile of cognitive recovery in individual patients remain unknown. Understanding these features is critical for identifying optimal therapeutic windows and selecting patient subgroups most likely to benefit from targeted interventions.

**OBJECTIVES.** The objective of this study was to assess the feasibility of using a widely validated, web-based neurocognitive test battery (Cambridge Brain Sciences, CBS) in a cohort of critical illness survivors.

**METHODS.** We conducted a prospective observational study in two intensive care units (ICUs) at two tertiary care hospitals. Twenty non-delirious ICU patients who were mechanically ventilated for a minimum of 24 hours underwent cognitive testing using the CBS battery while still in the ICU or shortly after discharge to ward. The CBS consists of 12 cognitive tests that assess a broad range of cognitive abilities, such as reasoning skills, short-term memory, and verbal processing. Twenty patients underwent cognitive assessment while still in the ICU or shortly after discharge to ward. Cognitive impairment on each test was defined as a raw score that was 1.5 or more standard deviations below age- and sex-matched norms.

**RESULTS.** We found that all patients were impaired on at least two tests and 18 patients were impaired on at least three tests. ICU patients had poorer performance on all three cognitive domains relative to healthy controls. We identified two feasibility issues with administration of the CBS test battery: patient fatigue due to battery length and patient inexperience with use of a computer mouse.

**CONCLUSIONS.** Use of a web-based patient-administered cognitive test battery is feasible and can be used in large-scale studies to identify domain-specific cognitive impairment in critical illness survivors and the temporal course of recovery over time.

##### GRANT ACKNOWLEDGMENT

This study was funded by internal research funds (Critical Care Medicine, Department of Medicine, Western University). Dr. Slessarev would like to acknowledge Vanier Canada Graduate Scholarship for supporting his PhD studies.

### 1271

#### Association between cytomegalovirus DNA detection and clinical outcomes in critically ill adult patients

D. Pérez-Torres[1], M. Domínguez-Gil González[2], E. Prol-Silva[1], P. Blanco-Schweizer[1], A.I. Rodríguez-Villar[1], J.Á. Ayala-Fernández[1], C. Díaz-Rodríguez[1], J.M. Eiros-Bouza[2], J. Blanco-Varela[1], J.A. Berezo-García[1], L. Tamayo-Lomas[1]
[1]Hospital Universitario Río Hortega, Intensive Care Department, Valladolid, Spain; [2]Hospital Universitario Río Hortega, Department of Microbiology, Valladolid, Spain
**Correspondence:** D. Pérez-Torres
*Intensive Care Medicine Experimental* 2018, **6(Suppl 2):**1271

**INTRODUCTION.** Cytomegalovirus (CMV) DNA detection in severely ill adult patients has been associated with important adverse clinical outcomes, including duration of mechanical ventilation (MV), longer length of stay (LOS) and mortality. Despite the fact that there are several reasonable mechanisms that could link the presence of this virus with worse clinical outcomes, it remains unclear whether CMV acts as a true pathogen or if it is a surrogate for more severe illness.

**OBJECTIVES.** We aim to explore the comorbidities, clinical interventions and clinical outcomes associated with CMV DNA detection in critically ill adult patients.

**METHODS.** We conducted a retrospective observational study in the Intensive Care Unit of a single university hospital. All the patients who underwent testing for CMV DNA detection over a 3-year period were enrolled. Testing was implemented according to the standard of care, drawing the samples from the most suitable source in each case (blood, respiratory tract or gastrointestinal tract). CMV DNA extraction was accomplished with an automatic system (EasyMag® bioMérieux, Marcy-l'Étoile, France), and amplification was performed with real-time polymerase chain reaction (rtPCR) (RealStar CMV®, Altona, Hamburg, Germany), using thermocycler Smartcycler® (Cepheid, Sunnyvale, California, USA). This PCR includes a heterologous amplification (internal control) to identify a possible inhibition of the rtPCR and to guarantee the reliability of all the reactives. Clinical variables were compared between the groups with positive and negative CMV DNA detection, respectively. Multiple logistic regression analysis was used to jointly examine the influence of the comorbidities. Student's T, Chi-square ($\chi^2$) and Fisher´s exact tests were applied as appropriate.

**RESULTS.** 69 patients were tested for CMV DNA detection, 31.9% of them with a positive result. Frequencies of comorbidities in the groups with positive and negative tests for CMV DNA were, respectively: hematologic disorders 31.8% vs 23.4%, p=0.45; diabetes mellitus 27.3% vs 12.8%, p=0.14; tumors 22.7% vs 21.3%, p=0.89; immunosuppression 13.6% vs 40.4%, p=0.03; human immunodeficiency virus 13.6% vs 4.3%, p=0.16; chronic obstructive pulmonary disease 13.6% vs 8.5%, p=0.51; autoimmune disorders 9.1% vs 19.2%, p=0.29; and chronic kidney disease 9.1% vs 8.5%, p=0.94. Multiple logistic regression analysis of the comorbidities, clinical interventions and outcomes observed are summarized in the Tables.

**CONCLUSIONS.** In our series, HIV, COPD, hematological disorders and diabetes mellitus were found to be higher risk comorbidities to obtain a positive test for CMV DNA. Patients requiring HFNC, NIV or prolonged MV were more likely to have a positive result. CMV DNA detection was associated with longer LOS and higher frequency of ARDS and mortality, suggesting that CMV might act as a true pathogen rather than as an innocent bystander. Further research is required to confirm this hypothesis.

**Table 1 (abstract 1271).** Multiple logistic regression analysis of the comorbidities

| | Odds ratio (95%CI) | Standard error | p |
|---|---|---|---|
| Hematologic disorders | 3.75 (0.84-16.87) | 2.88 | 0.084 |
| Diabetes mellitus | 2.50 (0.63-9.87) | 1.75 | 0.190 |
| Tumors | 0.71 (0.15-3.41) | 0.57 | 0.674 |
| Immunosuppression | 0.22 (0.05-1.03) | 0.17 | 0.056 |
| Human immunodeficiency virus infection (HIV) | 4.39 (0.61-31.54) | 4.41 | 0.142 |
| Chronic obstructive pulmonary disease (COPD) | 3.09 (0.47-20.33) | 2.97 | 0.241 |
| Autoimmune disorders | 1.07 (0.17-6.67) | 0.99 | 0.945 |
| Chronic kidney disease | 1.09 (0.14-8.65) | 1.15 | 0.939 |

**Table 2 (abstract 1271).** Clinical interventions

| | Group with positive test for CMV DNA (n=22) [frequency, %] | Group with negative test for CMV DNA (n=47) [frequency, %] | HR (95%CI) | p |
|---|---|---|---|---|
| Non-invasive ventilation (NIV) | 31.8 | 10.6 | 3.9 (0.9-17.9) | 0.031 |
| High-flow nasal cannula (HFNC) | 36.4 | 10.6 | 4.8 (1.1-21.4) | 0.011 |
| Prolonged MV (>7 days) | 68.2 | 42.6 | 2.9 (0.9-9.9) | 0.047 |
| Reintubation | 13.6 | 10.6 | 1.3 (0.2-7.6) | 0.717 |

**Table 3 (abstract 1271).** Clinical outcomes

| | Group with positive test for CMV DNA (n=22) | Group with negative test for CMV DNA (n=47) | p |
|---|---|---|---|
| LOS [mean, days (95%CI)] | 27 (16-39) | 19 (13-24) | 0.123 |
| Days of MV [mean, days (95%CI)] | 21 (8-34) | 12 (7-17) | 0.104 |
| Hospital-acquired pneumonia [frequency, %] | 4.5 | 4.3 | 0.956 |
| Acute respiratory distress syndrome [frequency, %] | 45.5 | 21.3 | 0.039 |
| All-cause mortality [frequency, %] | 68.2 | 27.7 | 0.001 |

## Haemodynamic monitoring in the ICU

### 1272

#### Left ventricular systolic functions in intensive care, ejection fraction vs aortic velocity time integral

K. Ahmad, Z. Alrais, M. Hamed
Rashid Hospital Dubai Health Authority, Intensive Care Medicine, Dubai, United Arab Emirates
**Correspondence:** K. Ahmad
*Intensive Care Medicine Experimental* 2018, **6(Suppl 2):**1272

**INTRODUCTION.** Ejection Fraction (EF) & Aortic Velocity Time Integral (VTI) are the two main parameters to measure left ventricular systolic functions. But in the Intensive Care settings, sometimes VTI has has more accuracy than EF in depicting systolic functions.
**OBJECTIVE.** To compare left ventricular systolic functions by Ejection Fraction Vs Velocity Time Integral.
**STUDY DESIGN.** Prospective descriptive analysis
**METHODS.** A cohort of 50 patients admitted to Intensive Care Unit from July 2017 to December 2017, hemodynamically unstable (MAP < 65 mmHg) were examined by Transthoracic Echocardiography (TTE). Ejection Fraction (EF) was measured by M-Mode in Left Parasternal Long Axis view and Modified Simpson method. Stroke Volume (SV) was calculated by measuring Aortic Velocity Time Integral (VTI) in Left Ventricular Outflow Tract (LVOT) in Apical 5-Chamber view. An average of 3 readings was recorded, and respiratory variation of VTI was also calculated in each individual. The data was collected on Microsft Excel. Quantitative data was analyzed by my mean, medium & mode. Qualitative data was analyzed by frequencies & percentages. Linear Regression analysis was done to see relationship between EF & VTI.
**RESULTS.** The mean age was 59.82 (Range 26-80) years. Majority belongs to > 50 years of age-group *(27/50)*. Males were 41(82%) Vs 9 (18%) females. 22 (44%) patients were on ionotropes, and 28(56%) without. Mean Ejection Fraction was calculated as 55.8 % (Range 26-87) Table-1. Majority of patients had EF > 60% *(21/50)*. Only two patients were found with EF < 30%, rest all *(27/50)* were between 30-60%. Mean VTI was found to be 15.4 (Range 7-21). Most patients were found with near normal *(16/50)* or moderately reduced VTI *(15/50)*. Ten patients were found with significant low VTI, < 10 cm (Table-2). Significant Respiratory variation of VTI (>12%) was found in 16 patients. While in 34 patients respiratory variation of VTI was < 12%. In 4 patients VTI was near normal ≥ 17 cm but their EF was found < 50%, and on other hand in 7 patients EF was above 60% but their VTI was found below 12 cm. Linear Regression analysis revealed a weak correlation between EF & VTI, Correlation Coefficient (R2) of 0.2186 (Graph-1).
**CONCLUSION.** Aortic Velocity Time Integral (VTI) has better prediction to determine Left Ventricular systolic function as compared to Ejection Fraction alone in the intensive care. There is a weak correlation between EF & VTI to determine LV systolic functions.
**KEY WORDS.** Systolic functions, Velocity Time Integral, Ejection Fraction.

- Lang RM, Badano LP, Mor-Avi V, et al. Recommendations for cardiac chamber quantification by echocardiography in adults: An update from the American Society of Echocardiography and the European Association of Cardiovascular Imaging. J Am Soc Echocardiogr. 2015;28:1-39-e14.
- Orde S, Slama M, Hilton A, Yastrebov K, McLean AS. Pearls and pitfalls in comprehensive critical care echocardiography. Crit Care. 2017;21:279.

| | |
|---|---|
| **Age** (Years) | Mean 59.8 (Range 26-80) |
| **Ejection Fraction** (EF %) | Mean 55.8 (Range 26-87) |
| **Velocity Time Integral** (VTI cm) | Mean 15.4 (Range 7-21) |
| **Stroke Volume** (SV ml) | Mean 46.3 (Range 20-79) |
| **Gender** | Male : 41 (82%)<br>Females : 9 (18%) |
| **Ionotropes** | Yes : 22 (44%)<br>No : 28 (56%) |
| **VTI Respiratory Variability** | > 12% : 16 (32%)<br>≤ 12% : 34 (68%) |

**Fig. 1 (abstract 1272).** Demographic & Descriptive Data

| Ejection fraction | | Velocity Time Integral (VTI) | |
|---|---|---|---|
| ≤ 30 % | 2 | < 8 cm | 3 |
| 31-45 % | 13 | 8-11 cm | 7 |
| 46-60 % | 14 | 12-16 cm | 15 |
| > 60 % | 21 | 17-18 cm | 16 |
| | | 19-22 cm | 9 |
| Total | 50 | Total | 50 |

**Fig 2 (abstract 1272).** Ejection Fraction Vs VTI Groups

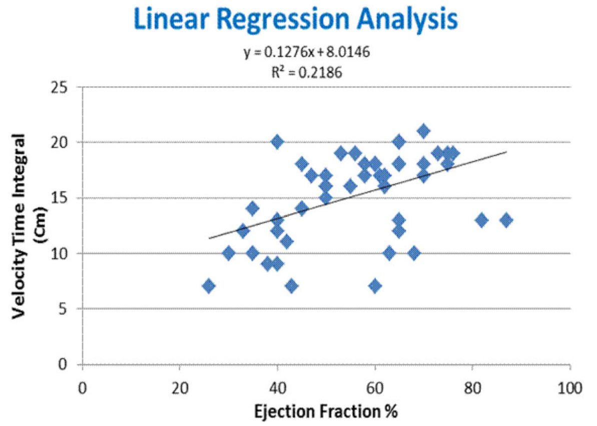

**Fig 3 (abstract 1272).** Correlation B/w EF & VTI (Linear Regression Analysis)

### 1273

#### Use of the veno-arterial carbon dioxide gap and arterio-venous oxygen content to guide the resuscitation in liver transplanted patients

M. Awad[1,2], Y. Nassar[2], A. Mukhtar[3]

[1]Nasser Institute Hospital, Critical Care Department, Cairo, Egypt; [2]Cairo University, Critical Care, Cairo, Egypt; [3]Cairo University, Anesthesia, Cairo, Egypt

**Correspondence:** Y. Nassar

*Intensive Care Medicine Experimental* 2018, **6(Suppl 2):**1273

**INTRODUCTION.** Fluid responsiveness (FR) is defined as the ability of the heart to increase its stroke volume after fluid administration(1). Mini fluid challenge is one of the most robust methods for preload challenge(2), but it needs real time monitoring of cardiac output (CO)(3), to track instantaneous change of stroke volume (SV) with fluid loading. Recently $PvaCO_2$ gap (4)have been used to detect FR after passive leg raising test with high sensitivity and specificity. Venous-arterial carbon dioxide gap ($PvaCO_2$ gap) and which is defined by the difference between central venous and arterial $CO_2$, and the ratio between the gap and arterial-venous oxygen difference are emerging tool to guide resuscitation in critically ill patients(3). However, to the best of our knowledge, no previous study investigated the validity of this method to detect FR in post liver transplant patient.

**OBJECTIVES.** Investigate the ability of VA-$CO_2$ gap to predict the hemodynamic response to fluid challenge in liver transplant patient.

**METHODS.** 40 patients post liver transplantation with hemodynamic instability, elevated lactate and normal scvo2. Pvaco2 gap calculated to detect FR .Fluid responders were defined as patients with increased stroke volume 15 %( measured by icon cardiometry device) after fluid bolus. arterial and central venous blood gases were obtained before and after fluid bolus. The predictive ability of the change in Pvaco2 gap with fluid bolus to predict FR was obtained with area under receiver operating characteristic (ROC) cu

**RESULTS.** Fluid responders were 26 patients (65%), AUC of $Pvaco_2$ for prediction of FR;after fluid bolus showed sensitivity70.8% and specificity62.5%, AUC 0.65, 95%CI (0.48-0.79), P value 0.08. $Scvo_2$ couldn't predict fluid responder patients post liver transplantation after fluid bolus with sensitivity 20%and specificity 45%, AUC 0.55,95% CI (-.007, 0.15).

**CONCLUSIONS.** Interpretation of Pvaco2 changes (or of absence of changes) must be particularly cautious. The changes in Pvaco2 gap should not be used as guiding tools for fluid challenge in patients with end stage liver disease post liver transplantation.

##### REFERENCE(S)

1. Monnet X, Julien F, Ait-Hamou N, Lequoy M, Gosset C, Jozwiak M, et al. Lactate and venoarterial carbon dioxide difference/ arterial-venous oxygen difference ratio, but not central venous oxygen saturation, predicts increase in oxygen consumption in fluid responders. Crit Care Med 2013; 41:1412-20.
2. Monnet X, Julien F, Ait-Hamou N, Lequoy M, Gosset C, Jozwiak M, et al. Lactate and venoarterial carbon dioxide difference/arterial-venous oxygen difference ratio, but not central venous oxygen saturation, predicts increase in oxygen consumption in fluid responders. Crit Care Med. 2013; 41:1412-20.
3. Della Rocca G, Brondani Anita, Costa Maria Gabriella. Intraoperative hemodynamic monitoring during organ transplantation: what is new? Curr Opin Organ Transpl. 2009; 14:291-296.

##### GRANT ACKNOWLEDGMENT

My professors

### 1274

#### Does respiratory variation in inferior vena cava diameter predict fluid responsiveness: a systematic review and meta-analysis

K. Pilarczyk[1], M. von der Brelie[1], D. Reich[1], H. Groesdonk[2], M. Sander[3], N. Haake[1]

[1]imland Klinik Rendsburg, managed by Sana Kliniken AG, Department of Intensive Care Medicine, Rendsburg, Germany; [2]Saarland University Medical Center, Department of Anaesthesiology, Critical Care and Pain Medicine, Homburg, Germany; [3]Universitätsklinikum Giessen und Marburg GmbH, Giessen, Department of Anesthesiology and Intensive Care Medicine, Giessen, Germany

**Correspondence:** K. Pilarczyk

*Intensive Care Medicine Experimental* 2018, **6(Suppl 2):**1274

**INTRODUCTION.** The resuscitation of the critically ill patient requires a goal-directed fluid therapy based on an accurate assessment of intravascular fluid volume and fluid responsiveness (FR). In the past years, respiratory variation in inferior vena cava diameter (ΔIVC) assessed by bedside ultrasonography is increasingly used as predictor of FR. However, published studies show conflicting results about the predictive value of ΔIVC.

**OBJECTIVE.** To review the current evidence for of ΔIVC in predicting fluid responsiveness.

**METHODS.** A meta-analysis was performed to assess the diagnostic accuracy of ΔIVC in predicting fluid responsiveness. Subgroup analyses were performed for patients on mechanical ventilation and spontaneously breathing patients. A total of 13 eligible studies (four with spontaneously breathing patients and 9 studies with patients under mechanical ventilation) with a total of 1025 patients were included.

**RESULTS.** In spontaneously breathing patients (n = 230, 122 fluid responders (53.0%)), the pooled sensitivity and specificity were 78.2% (95% CI 66.7-87.8%) and 91.8 % (95% CI 82.7-97.7 %), respectively. Reported best cutoff was 35.5 ± 16.0 %. Pooled studies were characterized by moderate heterogeneity (I2=52.77%, p=0.09). For patients under mechanical ventilation (n= 795, 365 fluid responders (45.9%), pooled sensitivity and specificity were 67.1 % (95% CI 54.6-78.4%) and 78.6 % (95% CI 67.3-87.9%), respectively. Mean cut-off was 18.6 ± 11.0%. Pooled studies were characterized by substantial heterogeneity ($I^2$=73.82%).

**CONCLUSION.** The present study suggests that ΔIVC assessed by echocardiography might represent a reliable predictor of FR in critically ill patients. However, included studies are limited by small sample size, different populations and a significant heterogeneity. In addition, cutoff values of ΔIVC varied significantly across studies.

### 1275

#### Assessment of stroke volume after cardiac surgery: comparison of transesophageal echocardiography and thermodilution with pulmonary artery catheter

K. Pilarczyk[1], J. Lubarski[2], J. Franke[2], F. Dusse[3], D. Dohle[4], H. Jakob[2]
[1]imland Klinik Rendsburg, managed by Sana Kliniken AG, Department of Intensive Care Medicine, Rendsburg, Germany; [2]West German Heart and Vascular Center Essen, University Hospital Essen, Department of Thoracic and Cardiovascular Surgery, Essen, Germany; [3]University Witten/Herdecke, Hospital Cologne-Merheim, Department of Anesthesiology and Intensive Care Medicine, Cologne, Germany; [4]University of Mainz, Department of Cardiothoracic and Vascular Surgery, Mainz, Germany
**Correspondence:** K. Pilarczyk
*Intensive Care Medicine Experimental* 2018, **6(Suppl 2):**1275

**INTRODUCTION.** Echocardiography is increasingly used for hemodynamic evaluation and monitoring in critically ill patients. However, recent studies enhanced concerns about the interchangeability of stroke volume (SV) measurement using echocardiography and pulmonary artery catheter (PAC) thermodilution (TD) technique.

**OBJECTIVE.** The aim of this study was to compare these SV measurement techniques in respect of precision, accuracy, and trending ability.

**METHODS.** We performed a comparison of SV measurements obtained with transesophageal echocardiography (TEE) assessing the velocity time integral in the LVOT and PAC-TD. 40 consecutive hemodynamically stable patients undergoing cardiac surgery were enrolled in this study. SV was measured with PAC and TEE at 6 different time points: 1. Baseline (PEEP = 5-10cm $H_2O$), 2. PEEP challenge (PEEP + 10 cm $H_2O$ for 5 minutes), 3. Baseline II (PEEP = initial value), 4. After passive leg raising 5. Baseline III (PEEP = initial value). 6. after a crystalloid fluid challenge of 6ml/kgBW. Measurements of stroke volume obtained by both techniques were compared using t-tests, correlation analyses and a Bland-Altman plot. A mean percentage error ≤30% for SV assessment by TEE was defined to indicate clinical reliability.

**RESULTS.** Mean stroke volume assessed by PAC was 64.4 ± 19.1 ml vs. 60.7 ± 15.4 ml in TEE (p = 0.01). Both measurements showed a correlation coefficient of 0.69 (95%CI 0.62-0.75, p< 0.001). Bland-Altmann-analyses revealed a mean difference (bias) of 3.7 ± 14.0 ml and limits of agreement were 23.8 ml and 31.5 ml. The percentage error of TEE was 45.7 %. After exclusion of small SV changes < 15% as grey zone, TEE correctly tracked the direction of stroke volume changes in 85% of measurements.

**CONCLUSION.** SV measurements with TEE had wide limits of agreement and a large percentage error. Thus, TEE seems not to be suitable to replace SV measurements performed by PAC TD. However, an acceptable trending ability was found and therefore, TEE could be a valuable tool in guiding therapy in mechanically ventilated patients after cardiac surgery.

### 1276

#### Perioperative continuous cardiac monitoring for cardiac surgery

R. Barnett
Holy Spirit Northside Private Hospital, Intensive Care Unit, Brisbane, Australia
*Intensive Care Medicine Experimental* 2018, **6(Suppl 2):**1276

**INTRODUCTION.** Cardiac surgery has a quoted mortality rate of 3-5%. However as more cases are being performed for prognostic rather than symptom alleviation reasons it is important that mortality rates are forced closer to nil. The principle driver of morbidity and mortality is haemodynamic instability. Clinical evaluation of haemodynamic state is notoriously difficult without the measurement of cardiac output. Fortunately there are now a number of continuous cardiac output (CCO) monitors available for clinical use throughout the entire perioperative period.

**OBJECTIVES.** As part of a Quality Assurance Program our institution decided to implement CCO on all cardiac surgical cases for a six-month period, July 2017-December 2017 then compare the outcomes with a preceding period (Jan-Dec 2016) to determine if an improvement to patient outcomes could be achieved.

**METHODS.** A selection of CCO monitors (CCO Swan-Ganz, Volume View, Flotrac, PICCO, CardioQ) were evaluated against performance criteria of accuracy, simplicity, risk and cost. The Edwards Lifesciences Flotrac monitor was selected and haemodynamic profile specific management protocols were developed. CCO monitoring was performed on all cardiac surgical cases from July-December 2017. Data was then compared with the data routinely collected on all cardiac surgical cases for the 2016 calendar year, where CCO monitoring was not available. The predetermined points of comparison where ICU LOS, Hospital mortality and Standardized Mortality Ratio using Australian and New Zealand Risk of Death predictive Model (ANZROD).

**RESULTS.** Between time periods, there were no statistical differences in patient characteristics, with respect to age, gender, ethnicity, type of cardiac surgery and ANZROD.

In total 942 patients data was analysed 626 vs 316. ICU LOS 67 +/-98hrs vs 78+/-114, Hosp Mortality 1.92% vs0.95% were not significantly different. Standardised Mortality Rate 1.13 vs 0.7 (p-0.045) was significantly different.

The increase in LOS was entirely attributable to the fall in deaths, with deaths occurring early, and decreasing LOS.

**CONCLUSIONS.** Our data confirms that cardiac surgical mortality is falling. The introduction of CCO monitoring and haemodynamic profile specific management algorithms significantly reduced risk adjusted mortality rate while not significantly altering LOS. We would encourage all ICU's to consider CCO monitoring as being part of minimum monitoring for post-operative cardiac surgical cases.

### 1277

#### Continuous monitoring of dynamic arterial elastance by pulse pressure contour analysis during the early phase of septic shock

P. Revuelta
Hospital Universitario de Guadalajara, Intensive Care Unit, Guadalajara, Spain
*Intensive Care Medicine Experimental* 2018, **6(Suppl 2):**1277

**INTRODUCTION.** The relation of pulse pressure variation to stroke volume variation (PPV/SVV) is referred as dynamica arterial eastance

(Eadyn), and was originally conceived to answer a direct clinical question: in the setting of arterial hypotension, will increases in cardiac output (CO) also increase arterial pressure?(1). Eadyn has therefore been considered as a functional measure of the arterial load. Contrary to the static nature of any component of arterial load, Eadyn has therefore been considered as a functional measure of the arterial load, Since Eadyn is measured of asingle breth,increases in left ventricular contractility may cause Eadyn to increase even though actual arterial elastance (EaZ) remains unaltered, because if the rate of ventricular ejection , and aotric blood accelerates (as occurs with increase ventricular initropy) the arterial pulse pressure will increase for a given SV. Therefore changes in arterial load or in he pattern of blood flow should affect Eadyn (2), similarly to changes in oreload or cardiac function thay affect the preload-responsiveness parameters. Consequenty, Eadyn should be considered as a functional of rterail load in the same terms that preload-responsiveness relate to cardiac preload.

**OBJECTIVES.** To assess the stability or the variation range, of continuous monitoring of Eadyn under "steady-state" conditions (normotensive, and no changes in volume infusion nor in vasopressor doses), during he early phase of septic shock, after initial resuscitation.

**METHODS AND RESULTS.** Five male patients admitted to the ICU with septic shock after early resuscitation. All in sinus rythm receiving volume controlled ventilation. None of them fullfilled criteria for ARDS. Preload depence was evaluated by. SVV >15% with a positive leg raising test. Initial volume expansion and noradrenaline infusion rates at phycisian decision, to achieve a mean arterial pressure (MAP) ≥ 70 mmHg. Pulse pressure contour analysis monitoring was used with the FloTrac System 4.0, integrated algoritms. Data were continiuosly registered every 12 seconds and saved after15 minutes at least, of steady-state condition exported and processed by data depuration and processing with a generated command algorithm with Stata 15/C statistical package, Statistical parametric descriptive tests and graphics were performed.

**CONCLUSIONS.** Eadyn contimuous monitoring in septic chock patients during steady state conditions, may b a useful tool for clinical decision but our results show that its value is not stable and ranges between 0.6 to 1,3.

**REFERENCE(S)**

1. Pinsky MR (2002) 2. Hadlan M, Severyn DA, Pinsky MR.

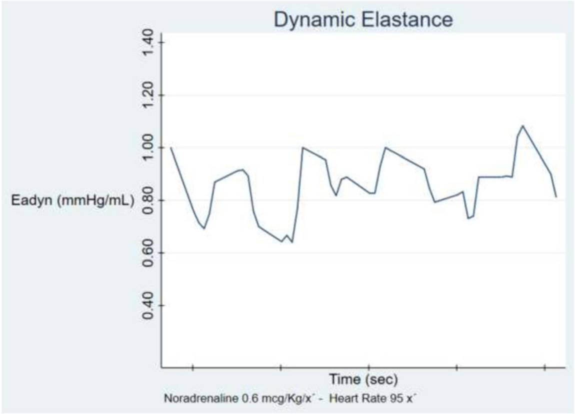

**Fig. 2 (abstract 1277).** Eadyn

| Variable | Obs | Mean | Std. Dev. | Min | Max |
|---|---|---|---|---|---|
| HR | 63 | 88.44444 | 2.955464 | 81 | 95 |
| APsys | 63 | 134.6349 | 3.023038 | 128 | 142 |
| APdia | 63 | 60.38095 | 1.210553 | 57 | 63 |
| MAP | 63 | 82.44444 | 1.573754 | 79 | 86 |
| PP | 63 | 74.25397 | 2.214092 | 70 | 80 |
| CO | 63 | 4.926032 | .4807975 | 1.49 | 5.44 |
| CI | 63 | 2.687937 | .2628943 | .81 | 2.97 |
| SV | 63 | 55.92063 | 5.398033 | 17 | 60 |
| SVI | 63 | 30.49206 | 2.983141 | 9 | 33 |
| SVR | 63 | 1289.206 | 345.5771 | 1140 | 3960 |
| SVRI | 63 | 2363.222 | 633.4673 | 2090 | 7259 |
| SVV | 58 | 26.37931 | 2.614585 | 21 | 32 |
| PPV | 59 | 22.40678 | 2.767537 | 16 | 28 |
| CPO | 63 | .9030159 | .0926602 | .26 | 1.03 |
| CPI | 63 | .4925397 | .0504805 | .14 | .56 |
| SVmin | 53 | 50.77358 | 3.66187 | 43 | 58 |
| SVmax | 53 | 65.39623 | 2.911117 | 52 | 70 |
| EaZ | 63 | 1.361921 | .3733692 | 1.22807 | 4.235294 |
| Eadyn | 54 | .8605623 | .126464 | .64 | 1.272727 |

**Fig. 3 (abstract 1277).** Hemodynamic values of Patient 1

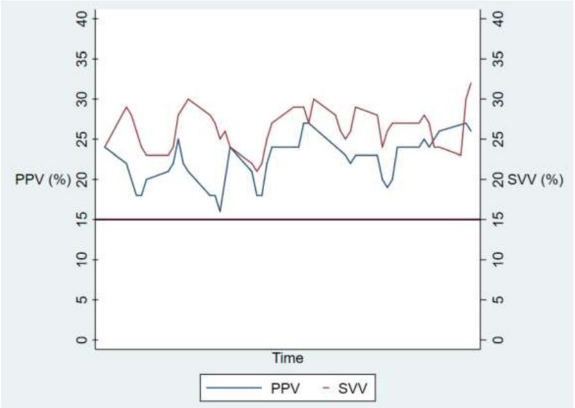

**Fig. 1 (abstract 1277).** SSV_PPV

### 1278

#### Correlation between peripheral and central venous blood partial pressure of carbon dioxide in critically ill patients with shock

C. Karnjanarachata[1], T. Pravitharangul[1], S. Morakul[1], P. Theerawit[2]
[1]Faculty of Medicine, Ramathibodi Hospital, Mahidol University, Department of Anesthesiology, Bangkok, Thailand; [2]Faculty of Medicine, Ramathibodi Hospital, Mahidol University, Department of Internal Medicine, Bangkok, Thailand
**Correspondence:** T. Pravitharangul

*Intensive Care Medicine Experimental* 2018, **6(Suppl 2):**1278

**INTRODUCTION.** Adequacy of tissue perfusion during shock resuscitation is crucial. Central venous-to-arterial carbon dioxide gap ($CO_2$ gap, P(cv-a) $CO_2$) is widely used to assess adequacy of tissue perfusion to guide resuscitation, however; central venous catheter is needed for central venous carbon dioxide tension ($PcvCO_2$). Central venous catheter insertion is not without risk while the benefit became less apparent. Since, superior vena cava receives venous drainage from upper part of the body, upper extremity peripheral venous blood may be considered to be less invasive alternative for $PcvCO_2$.

**OBJECTIVES.** To determine correlation and relationship between peripheral and central venous blood partial pressure of carbon dioxide in critically ill patients with shock.

**METHODS.** Single center, observational study. Paired sample of 1 ml upper extremity peripheral and 1 ml central venous blood was taken simultaneously from intensive care unit adult patients receiving vasopressor or inotropic drug who already had central venous catheter in place. Only one paired sample per day and maximum of 3 paired samples were allowed for one patient. All samples underwent blood gas analysis. Data on carbon dioxide tension was then analyzed using intraclass correlation coefficient (ICC) and Bland-Altman plot.

**RESULTS.** 30 paired samples were obtained from 12 patients (2 medical and 1 surgical intensive care units) aged between 53-90 years who receiving norepinephrine infusion ranged from 0.01 to 0.95 mcg/kg/min actual body weight. The intraclass correlation revealed that peripheral venous carbon dioxide tension ($PpvCO_2$) was significantly associated with $PcvCO_2$ [ICC 0.98 (95% confidence interval 0.96-0.99); P< 0.001]. A The Bland-Altman plot demonstrated mean bias of 0.2 with the limits of agreement of -5.3 to 5.8.

**CONCLUSIONS.** The study revealed excellent correlation between $PpvCO_2$ and $PcvCO_2$ in critically ill patients with shock.

- Vallée F, Vallet B, Mathe O, Parraguette J, Mari A, Silva S, Samii K, Fourcade O, Genestal M. Central venous-to-arterial carbon dioxide difference: an additional target for goal-directed therapy in septic shock? Intensive Care Med. 2008 Dec;34(12):2218-25.
- Cuschieri J, Rivers EP, Donnino MW, et al. Central venous-arterial carbon dioxide difference as an indicator of cardiac index. Intensive Care Med. 2005;31(6):818-22.
- Rhodes A, Evans LE, Alhazzani W, et al. Surviving Sepsis Campaign: International Guidelines for Management of Sepsis and Septic Shock: 2016. Crit Care Med. 2017;45(3):486-552.
- Cardenas-Garcia J, Schaub KF, Belchikov YG, Narasimhan M, Koenig SJ, Mayo PH. Safety of peripheral intravenous administration of vasoactive medication. J Hosp Med. 2015 Sep;10(9):581-5.
- Goyal N, Taylor AR, Rivers EP. Relationship between Central and Peripheral Venous Oxygen Saturation and Lactate Levels: A Prospective Study. J Emerg Med. 2016 Jun;50(6):809-17.

**GRANT ACKNOWLEDGMENT**

None. No funding to declare.

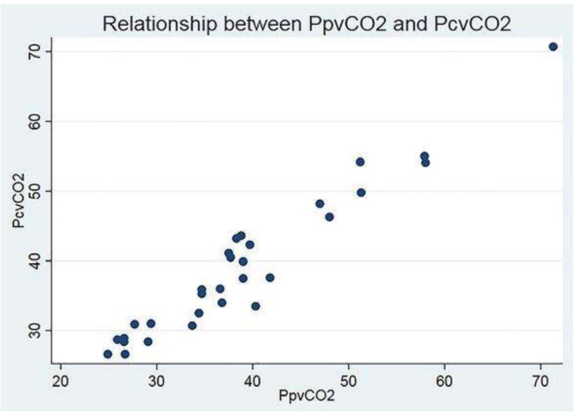

**Fig. 1 (abstract 1278).** Relationship Between PpvCO2 and PcvCO2

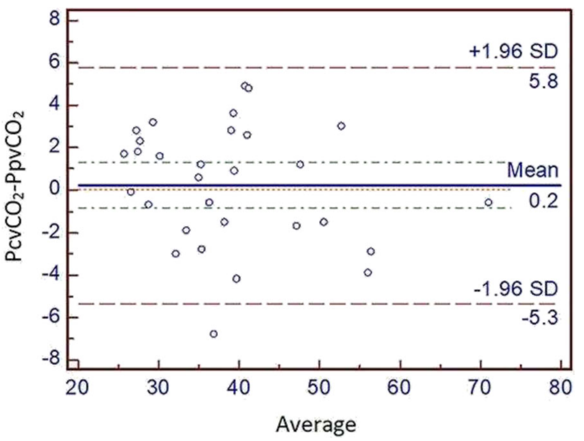

**Fig. 2 (abstract 1278).** Bland-Altman Plot for Venous Carbon Dioxide Tension

### 1279

#### Reassessment of the accuracy of cardiac Doppler pulmonary artery pressures measurements in ventilated ICU patients: a simultaneous Doppler-catheterization study

Y. Zerbib[1], P. Mercado[2], J. Maizel[1], C. Beyls[1], L. Kontar[1], S. Orde[3], S. Huang[3], A. McLean[3], C. Tribouilloy[4], M. Slama[1]

[1]CHU Amiens-Picardie, Medical ICU, Amiens, France; [2]CHU Amiens-Picardie, Amiens, France; [3]Nepean Hospital, ICU, Sydney, Australia; [4]CHU Amiens-Picardie, Cardiology, Amiens, France

**Correspondence:** Y. Zerbib

*Intensive Care Medicine Experimental* 2018, **6(Suppl 2):**1279

**INTRODUCTION.** Doppler echocardiography (DE) is a well-recognized technique for the non-invasive evaluation of pulmonary artery pressure (PAP) but little information is available concerning patients under mechanical ventilation (MV). Furthermore, recent studies have reported reservation regarding the relevance of this technique to assess PAP.

The aim was to reassess the accuracy of DE to evaluate PAP and to predict pulmonary hypertension (PH).

**METHODS.** Prospective observational study in Amiens ICU, France. In 40 patients under MV we simultaneously recorded DE parameters (from tricuspid (TR) and pulmonary (PR) regurgitations) and invasive central venous pressure (CVP), systolic (SPAP), diastolic and mean (MPAP) PAP.

**RESULTS.** SPAP assessed from the TR derived maximal pressure gradient added to the CVP demonstrated the best correlation with the invasive SPAP (r=0.87) with a small bias (-3 mmHg) and a precision of 9 mmHg. A DE SPAP>39 mmHg predicted a PH (MPAP≥25 mmHg) with 100% sensitivity and specificity. TR maximal velocity >2.82 m/s as well as TR pressure gradient >32 mmHg predicted the presence of PH well. PR was recorded in 10 patients (25%). No correlation was found between PR velocities and PAP. Pulmonary acceleration time< 57ms and isovolumic relaxation time < 40ms respectively predicted pulmonary hypertension in 100% of time and had 100% of negative predictive value

**CONCLUSIONS.** When present, TR maximal velocity pressure gradient added, or not, to invasive CVP seems to assess accurately systolic and mean PAP in ICU MV patients and may predict PH.

### 1280

#### Comparison of cardiac output estimation by echocardiography, bioreactance and USCOM. (Ultrasonic Cardiac Output Monitor)

J. Cuesta Torres[1], E. Monares Zepeda[1], J. Cruz Lopez[1], G. Santana Hernandez[1], M. Frias Abrahamov[1], L. Cabrera Miranda[1], F. Flores Puente[2], R. Cebrian Garcia[1], C. Galindo Martin[1], A. Ojeda Izquierdo[1]
[1]Hospital San Angel Inn Universidad, Critical Medicine, Mexico City, Mexico; [2]Hospital 20 de Noviembre, Cardiology, Mexico City, Mexico
**Correspondence:** J. Cuesta Torres
*Intensive Care Medicine Experimental* 2018, **6(Suppl 2):**1280

**INTRODUCTION.** Cardiac output is the most frequent advanced monitoring in critical care units given its utility to guide resuscitation in septic shock. Recently there's a preference of less invasive techniques for cardiac output measurement in order to reduce the implicit risks of invasive techniques. Echocardiography has been postulated has the gold standard for non-invasive cardiac output measurement, however this technique is operator-dependent and should be performed by an expert. Other techniques such as bioreactance and Doppler monitoring by semiautomatic USCOM system are a 100% non-invasive techniques, also these measurements are not operator or expert dependent.

**OBJECTIVE.** To analyze the agreement of cardiac output by echocardiography vs Bioreactance and semiautomatic Doppler of the USCOM system.

**METHODS.** Prospective observational study in which 26 patients diagnosed with septic shock admitted to the intensive care unit were cardiac output was measured by echocardiography, bioreactance and USCOM system. Bland Altman method was performed to analyze the agreement between the different techniques: Echocardiography Vs USCOM and Echocardiography Vs Bioreactance. Also Linn coefficient was calculated in the same groups of measurements.

**RESULTS.** Bioreactance vs echocardiography reported a bias (mean of the differences) of -0.08 with a standard deviation (precision) of 0.85 and a 95% confidence interval of 1.59 to -1.75, finally an error of 24% and Linn coefficient of 0.78.

USCOM vs echocardiography reported a bias (mean of the differences) of 1.11 with a standard deviation (precision) of 0.95 and a 95% confidence interval of 2.98 to -0.75, finally an error of 41% and Linn coefficient of 0.6.

**CONCLUSIONS.** Bioreactance is a promising technique for cardiac output measurement given its good agreement with echocardiography, in the other hand USCOM did not showed a satisfactory agreement with echocardiography, more studies are needed to verify the clinical usefulness of these different techniques.

##### REFERENCE(S)

Raval NY, Squara P, Cleman M, Yalamanchili K, Winklmaier M, Burkhoff D. Multicenter evaluation of noninvasive cardiac output measurement by bioreactance technique. J Clin Monit Comput. 2008;22(2):113-9.

Udy AA, Altukroni M, Jarrett P, Roberts JA, Lipman J. A comparison of pulse contour wave analysis and ultrasonic cardiac output monitoring in the critically ill. Anaesth Intensive Care. 2012;40(4):631-7.

Huang L, Critchley LA, Zhang J. Major Upper Abdominal Surgery Alters the Calibration of Bioreactance Cardiac Output Readings, the NICOM, When Comparisons Are Made Against Suprasternal and Esophageal Doppler Intraoperatively. Anesth Analg. 2015 Oct;121(4):936-45.

##### GRANT ACKNOWLEDGMENT

To all my colleagues and teachers for their valuable collaboration.

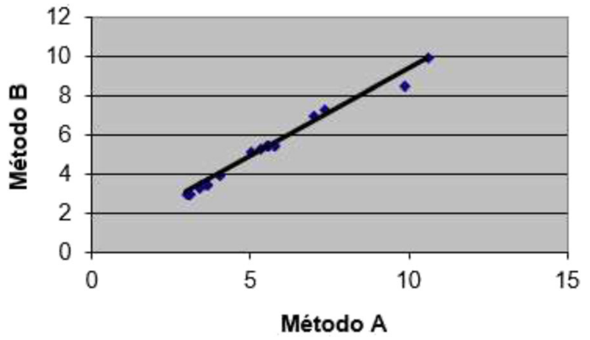

**Fig 2 (abstract 1280).** Blant and Altman Method. USCOM vs echocardiography

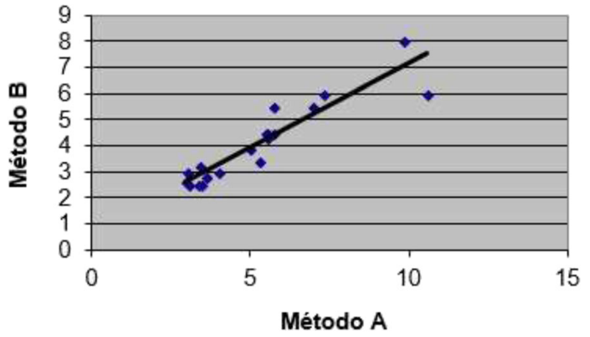

**Fig 1 (abstract 1280).** Blant and Altman Method. Bioreactance vs echocardiography

### 1281

#### Changes in venous-to-arterial carbon dioxide tension after fluid bolus treatment in critically ill patients

C. Pierrakos[1], D. De Bels[1], T. Nguyen[1], D. Velissaris[2], J. Devriendt[1], P. Honore[1], D. De Backer[3]
[1]Brugmann University Hospital, Université Libre de Bruxelles, Intensive Care, Brussels, Belgium; [2]University Hospital of Patras, Internal Medicine, Patra, Greece; [3]CHIREC Hospitals, Université Libre de Bruxelles, Intensive Care, Brussels, Belgium
**Correspondence:** C. Pierrakos
*Intensive Care Medicine Experimental* 2018, **6(Suppl 2):**1281

**INTRODUCTION.** Mixed venous-to-arterial carbon dioxide tension difference ($P_{(v-a)}CO_2$) is an easily derived parameter which is inversely related to cardiac index (CI). Bolus fluid treatment (BFT) is a tool for critically ill patients with hypovolemia where changes in CI and oxygen consumption ($VO_2$) are expected.

**OBJECTIVES.** This study aimed to investigate the changes of $P_{(v-a)}CO_2$ after BFT.

**METHODS.** Prospective observational study in adult critically ill patients. CI was measured either by echocardiography or by thermodilutional method. Haemodynamic measurements and blood gas analysis were obtained before and after fluid challenge with either 1000 ml crystalloid or 500 ml colloid . Arterial and central venous blood gas samples were taken simultaneously. $VO_2$ was calculated according to Fick formula. $P_{(v-a)}CO_2$ was calculated before and after BFT. Patients were divided into responders and non-responders according to their change in CI (> 15%). Changes in VO2 ≥ 15% were considered significant.

**RESULTS.** We assessed 68 patients (69 ± 16 years, APACHE II score 22.7 ± 8) that received BFT. Twenty-five patients were responders to BFT (36%). We found no correlation of changes in $P_{(v-a)}CO_2$ and changes in CI after BFT ($r^2$ = 0.01, p = 0.26). No change in $P_{(v-a)}CO_2$ was observed in non-responders (7.9 ± 0.6 vs. 7.7 ± 0.6 mmHg, p = 0.87). A significant decrease in $P_{(v-a)}CO_2$ was observed in responders to BFT (7.9 ± 4 vs. 6.1 ± 3 mmHg, p = 0.01). However, a decrease in $P_{(v-a)}CO_2$ was unable to differentiate responders from non-responders (AUC: 0.65, 95%CI 0.46-0.74, p = 0.15). Thirty-five patients did not have any significant change in $VO_2$ after BFT. In these patients, we did not observe any statistical significant change in $P_{(v-a)}CO_2$ (6.5 ± 0.5 vs. 7.4 ± 0.7 mmHg, p = 0.21), and in mixed central venous saturation ($S_{cv}O_2$) (66 ± 2% vs. 66 ± 2 %, p = 0.69). The change in CI in this group of patients was 7.9 ± 2%. Ten patients had a significant decrease in $VO_2$ after BFT. $P_{(v-a)}CO_2$ significantly decreased in these patients (9.8 ± 0.9 vs. 6.6 ± 0.7 mmHg, p = 0.01). However, an important increase in $S_{cv}O_2$ was observed (62 ± 4% vs. 67 ± 3%, p = 0.03) in these patients despite no significant change in CI (3 ± 4%). Twenty-two patients had a significant increase in $VO_2$. We observed a statistically significant decrease in $P_{(v-a)}CO_2$ (9.4 ± 1 vs. 6.9 ± 0.7 mmHg, p < 0.01) and in $S_{cv}O_2$ (68% vs. 65% p < 0.01). Also, a significant increase in CI was detected in these patients (32 ± 6%).

**CONCLUSIONS.** Significant changes in $P_{(v-a)}CO_2$ can be observed after BFT. A decrease in $P_{(v-a)}CO_2$ was mostly observed in responders. However, in several patients with a decrease in $VO_2$, likely to reflect a spontaneous decrease in metabolism, we observe a decrease in $P_{(v-a)}CO_2$ combined with an increase in $S_{cv}O_2$. The combination of changes in $P_{(v-a)}CO_2$ and $S_{cv}O_2$ might be useful to differentiate changes in $VO_2$ related to spontaneous changes and changes in response to BFT in order to further guide therapy.

### 1282

#### Prediction of fluid responsiveness in the immediate post-operative period of cardiac surgery

B.B. Albano, L.M. Habana
Philippine Heart Center, Adult Cardiology, Division of Critical Care, Quezon City, Philippines
**Correspondence:** B.B. Albano
*Intensive Care Medicine Experimental* 2018, **6(Suppl 2):**1282

**INTRODUCTION.** Fluid replacement is most important therapeutic intervention in critically-ill patients including those in the post-operative period of cardiac surgery [1]. However, giving fluids does not always improve hemodynamics as one expects [2]. In fact, only 50% of these patients will benefit from fluid loading while the rest will suffer from the danger of over hydration [3]. Prediction of fluid responsiveness can identify patients who will benefit from fluid loading. Several hemodynamic parameters may help identify those who will benefit from hydration.

**OBJECTIVES.** This study aimed to determine and compare the parameters that predict fluid responsiveness in post-cardiac surgery patients; and to identify which tool is most accurate to guide initiation of fluid therapy in this clinical setting.

**METHODS.** This prospective cohort study included 101 post-cardiac surgery patients. Hemodynamic parameters were recorded at baseline and after an 8 mL/kg IV fluid challenge. Fluid responders were those with an increase in stroke volume of ≥15%. Multivariate analysis was used to identify independent predictors of fluid responder status. Sensitivity and specificity analyses were done to determine the predictive accuracy of each parameter.

**RESULTS.** The rate of fluid responsiveness in this study was 54.5%. Independent predictors of fluid responsiveness were: central venous pressure (CVP) ≤6 mmHg (p=0.001), pulmonary artery occlusion pressure (PAOP) ≤12 mmHg (p=0.016), PAOP increase by ≥7 mmHg (p=0.002), pulse pressure variability (PPV) >12% (p< 0.001), PPV decrease by >5% (p=0.049) and weight (p=0.04). PPV>12% was the most sensitive (92%), while PPV decrease by >5% was the most specific (79%); while PAOP was the least sensitive (70%) and CVP the least specific (51%). PPV had the highest ability to discriminate fluid responders (AUC 0.83) compared to PAOP (AUC 0.21) and CVP (AUC 0.40) (p< 0.0001).

**CONCLUSIONS.** PPV (a dynamic index) is superior to CVP and PAOP (static indices) in discriminating fluid responders in adult patients who underwent cardiac surgery. PPV is the favored tool to guide initiation of fluid therapy in this clinical setting. However, in the absence of PPV monitoring, relying on CVP and PAOP may still be considered, although these have lower accuracy compared to PPV.

**GRANT ACKNOWLEDGMENT**
None

### 1283

#### Peripheral intravenous analysis (PIVA) for determining fluid responsiveness in post cardiac surgery patients with vasoplegia

S. Eagle[1], K. Hocking[2], F. Baudenbacher[3], M. Miles[1], B. Alvis[1], K. Kohorst[1]
[1]Vanderbilt University Medical Center, Anesthesiology, Nashville, United States; [2]Vanderbilt University Medical Center, Surgery, Nashville, United States; [3]Vanderbilt University, Biomedical Engineering, Nashville, United States
**Correspondence:** S. Eagle
*Intensive Care Medicine Experimental* 2018, **6(Suppl 2):**1283

**INTRODUCTION.** Patients undergoing cardiac surgery often have vasoplegia, defined by low systemic vascular resistance and high cardiac output, due to cardiopulmonary bypass. Postoperative fluid management can be difficult in this population. Peripheral Intravenous Analysis (PIVA) has been shown to detect blood loss prior to arterial and pulmonary artery pressure in human subjects and in a porcine animal model of hemorrhage (1,2). We hypothesize that PIVA will detect volume responsiveness in post-cardiac surgery subjects with vasoplegia.

**OBJECTIVES.**

1. Determine the utility of PIVA in post cardiac surgery patients with vasoplegia.
2. Determine the correlation between PIVA and change in cardiac output with fluid administration.

**METHODS.** Thirty post cardiac surgery subjects were enrolled in the intensive care unit at Vanderbilt University Medical Center. All subjects were intubated, mechanically ventilated, with a right internal jugular and pulmonary artery catheter (Edwards Lifesciences, CA, USA) in place. All subjects required norepinephrine (between 1-10 mcg/min) to maintain a mean arterial pressure>60mmHg. A TruWave pressure transducer (Edwards Lifesciences, CA, USA) was connected directly to a dedicated arm peripheral IV, 18g or 20g. Subjects received 500mL

Plasmalyte (Baxter International, USA) bolus over 5 minutes. Thermodilution cardiac output was obtained before and after the fluid bolus. Data was stored on a desktop computer and analyzed using automated software to determine PIVA Score using an algorithm based on the Fourier transformation of the peripheral venous waveform (Figure 1).

**RESULTS.** Twenty-nine post-cardiac surgery subjects were successfully enrolled and initial hemodynamic parameters were used to predict fluid responsiveness. All subjects had a systemic vascular resistance $\leq$ 1000 dynes·sec·cm$^{-5}$.There was significantly significant correlation ($p< \overline{0}.05$, r=-0.44) between baseline PIVA score and change in cardiac output with fluid administration (Figure 2). Central venous pressure did not have a significant correlation with percent change in cardiac output (p=0.242, r=0.24). Diastolic pulmonary artery pressure had a non-significant correlation with percent change in cardiac output (p=0.063, r=0.36).

**CONCLUSIONS.** PIVA predicts fluid responsiveness in postoperative cardiac surgery patients with vasoplegia. These results warrant further investigation in patients with distributive shock including sepsis and burn injuries.

**REFERENCE(S)**

1. Sileshi B, Hocking KM, Boyer RB, Baudenbacher FJ, Kohurst KL, Brophy CM, Eagle S. Peripheral venous waveform analysis for detecting early hemorrhage: A pilot study. Intensive Care Med. 2015 Jun;41(6):1147-8.
2. Hocking KM, Sileshi B, Baudenbacher FJ, Boyer RB, Kohorst KL, Brophy CM, Eagle S. Peripheral venous waveform analysis for detecting hemorrhage and iatrogenic volume overload in a porcine model. Shock. 2016 Oct;46(4):447-52.

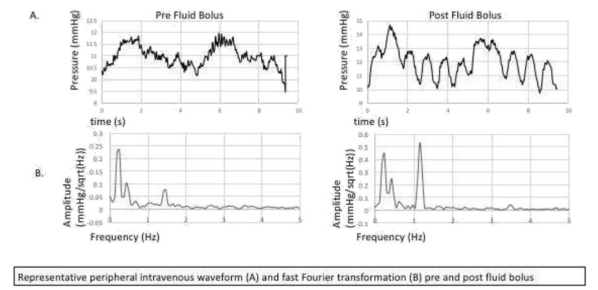

**Fig. 1 (abstract 1283).** See text for description.

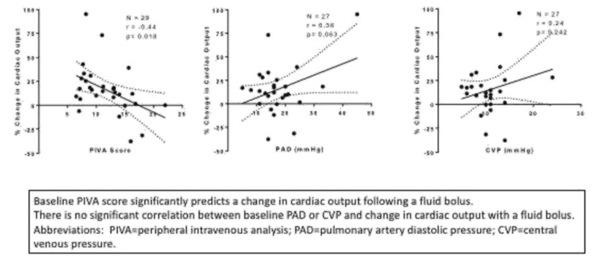

**Fig. 2 (abstract 1283).** See text for description.

### 1284

#### Bayesian analysis for the venous-arterial CO2 to arterial-venous O2 content difference ratio in post-resuscitation patients and proposal of a simplified formula

A.J. Fuentes-Gómez, E. Monares-Zepeda, P. Palacios-Moguel, J. Aguirre-Sánchez, G. Camarena-Alejo, J. Franco-Granillo

American British Cowdray Medical Center, Intensive Care Unit, Mexico City, Mexico

**Correspondence:** A.J. Fuentes-Gómez

*Intensive Care Medicine Experimental* 2018, **6(Suppl 2):**1284

**INTRODUCTION.** The venous-arterial CO2 to arterial-venous O2 content difference ratio (DvaCO2/ΔCavO2) has been correlated with anaerobic metabolism during tissular hypoxia in shock[1], and different studies have used it to guide resuscitation and have demonstrated impact in mortality and prognosis[2].

**OBJECTIVES.** The aim of this study was to evaluate the statistical value of the DvaCO2/ΔCavO2 in critically ill patients with septic shock diagnosis after fulfilled resuscitation. As secondary outcome, DvaCO2/ΔCavO2 was compared with a simplified formula with venous gasometrical parameters.

**METHODS.** We retrospectively studied 110 septic shock patients with available data of arterial and venous gasometrical parameters at admission, and at the time of concluding resuscitation according with intensivist criteria, between 24-48 hours from ICU admission. DvaCO2/ΔCavO2 was calculated derived from oxygen content of blood formulas. All variables showed a normal distribution so were reported as mean and standard deviation, considering statistically significant p values < 0.05, evaluated through OpenEpi software version 2013. Student's T-test was used to compare survivors and non-survivors groups. A simplified empirical formula was proposed based only in venous gasometrical values ((100-SvO2)/(100-PvCO2)). According with previous studies, a cut-off value of DvaCO2/ΔCavO2 >1,4 was set for mortality analysis[3], and for our proposed formula the cut-off value was 0.6. Sensitivity, specificity, positive predictive value, negative predictive value, and accuracy were determined, by calculating Cohen's kappa coefficient. Afterwards, a Bayesian analysis was tested to determine the prognostic value for both formulas.

**RESULTS.** Demographic characteristics between both groups were similar. Mortality was 35%, similar to previous reports for septic shock. Cardiac output showed greater values in survivors group (5.9 ±1.4 vs 5.1±2.5 L/min, p< 0.001), while the same group reported smaller values for DvaCO2 (3.9±1.3 vs 9.2±1.9 mmHg, p< 0.05), lactate (1.4±0.7 vs 4.6±3.1 mmol/L, p< 0.05) and DvaCO2/ΔCavO2 (1.1±0.4 vs 1.6±0.8, p< 0.0001). DvaCO2/ΔCavO2 sensibility was 53.8% and specificity was 87.3%. For the simplified formula sensibility was 94.8% and specificity was 83.1%. Bayesian analysis for DvaCO2/ΔCavO2 founded a positive likelihood ratio (LR) of 4.2 and a negative LR 0.5, values that improved with the simplified formula to +LR 5.6 and -LR 0.06.

**CONCLUSIONS.** A simplified formula with venous gasometrical parameters shows favorable results to be implement as a subrogate of DvaCO2/ΔCavO2. Prospective studies are required to obtain better statistical power, as well as an external validation to establish its usefulness in critically ill patients without needing arterial blood samples.

**Table 1 (abstract 1284).** Demographic characteristics of the current sample]

| Variable | Survivors (n=71) | Non-survivors (n=39) | p-value |
|---|---|---|---|
| Age (years) | 69.9 (58.9 to 80.9) | 70.7 (57.7 to 83.6) | NS |
| Gender, male (%) | 46 (64.8) | 29 (0.74) | |
| SAPS-II | 49.1 (35.0 to 63.2) | 49.6 (36.8 to 62.4) | NS |
| Central venous pressure (mmHg) | 15.9 (11.6 to 20.1) | 16.3 (12.1 to 20.5) | NS |
| Cardiac output (L/min) | 5.9 (4.5 to 7.3) | 5.1 (2.5 to 7.6) | p<0.001 |
| Lactate (mmol/L) | 1.4 (0.7 to 2.1) | 4.5 (1.4 to 7.7) | p<0.05 |
| DvaCO2 (mmHg) | 3.9 (2.5 to 5.2) | 9.1 (7.2 to 11.1) | p<0.05 |
| DvaCO2/ΔCavO2 | 1.1 (0.7 to 1.6) | 1.6 (0.8 to 2.5) | p<0.0001 |
| ICU lenght of stay (days) | 6.2 (2.5 to 9.8) | 7.9 (3.7 to 12.1) | NS |

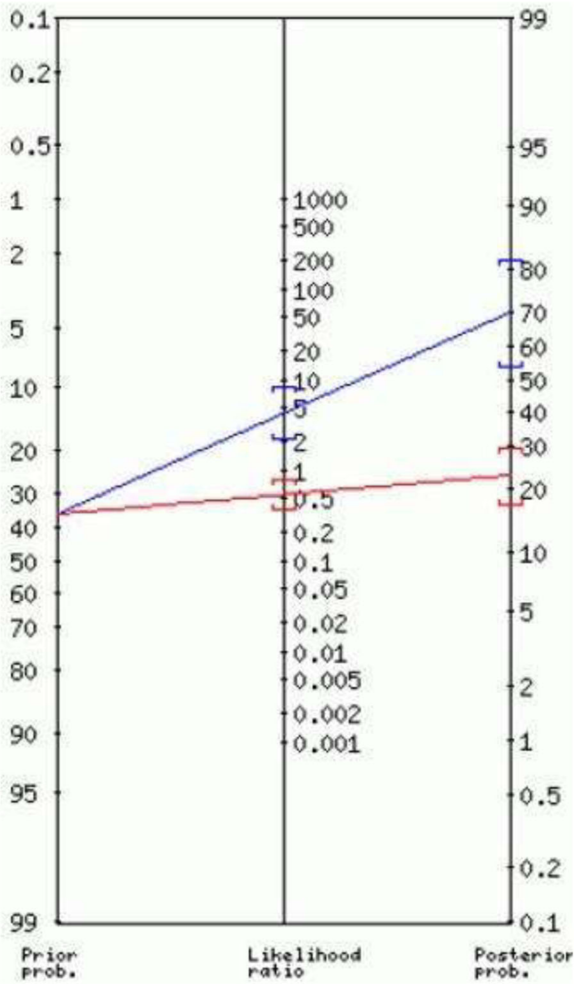

**Fig. 1 (abstract 1284).** Bayesian analysis for DvaCO2/ΔCavO2

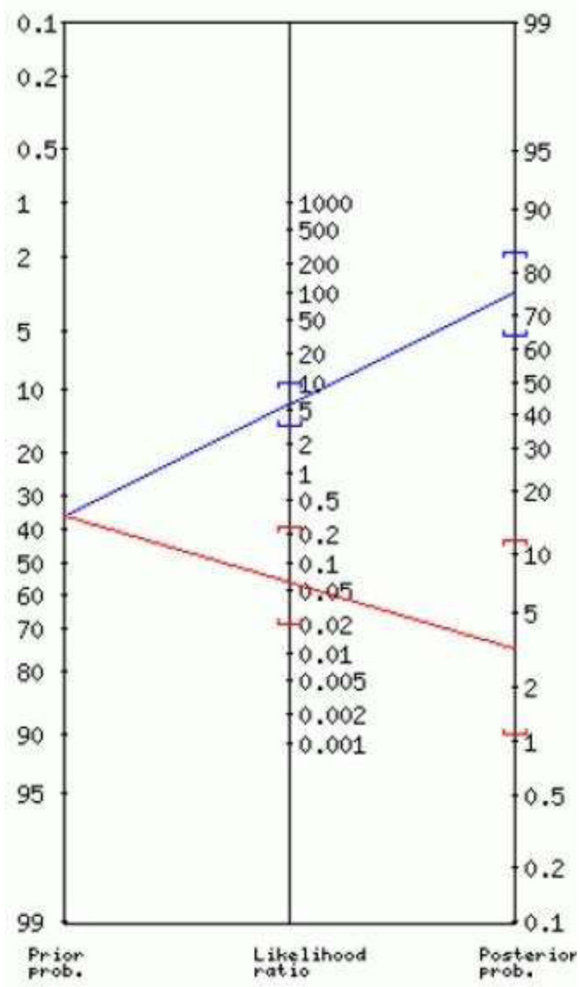

**Fig. 2 (abstract 1284).** Bayesian analysis for (100-SvO2)/(100-PvCO2)

### 1285

#### A real-time photoplethysmography signal artifacts removal system in ICU

Y. Chen[1,2], J.H. Yoon[3], V. Jeanselme[4], M. Hravnak[5], A.W. Dubrawski[4], M.R. Pinsky[1], G. Clermont[1]

[1]University of Pittsburgh School of Medicine, Department of Critical Care Medicine, Pittsburgh, United States; [2]Harbin Institute of Technology Shenzhen Graduate School, Shenzhen, China; [3]University of Pittsburgh School of Medicine, Department of Critical Care Medicine Division of Pulmonary, Allergy, and Critical Care Medicine, Pittsburgh, United States; [4]Carnegie Mellon University, Robotics Institute, Pittsburgh, United States; [5]University of Pittsburgh, Department of Acute & Tertiary Care, Pittsburgh, United States

**Correspondence:** Y. Chen

*Intensive Care Medicine Experimental* 2018, **6(Suppl 2):**1285

**INTRODUCTION.** The Photoplethysmography (PPG) signal is a critical vital sign in the Intensive Care Unit (ICU). Due to limitations in sensor technology, current approaches in PPG signal collection are easily affected by artifacts, generating numerous false alerts and impeding successful integration of PPG for the purpose of developing robust predictive analytics.

**OBJECTIVES.** We propose a beat-to-beat (B2B) real-time PPG signal artifacts removal approach using machine learning.

**METHODS.** We extracted PPG signals from patients included in the Medical Information Mart for Intensive Care (MIMIC) III Waveform Database [1]. The PPG signal was split into 60-second non-overlapping data frames. In this study, a Hilbert-Huang transformation [2] was applied to detect the signature. The signature of clean, artifact free signals is characterized by low power in the low frequency domain (0-0.8Hz) of its spectrum. If deviations from this normal signature were detected in a data frame, a beat-to-beat artifact detection algorithm (B2BA) was applied to each beat. B2BA was developed as follows: A sample of PPG waveforms were classified manually as acceptable (A) or unacceptable (U) as per visual quality. After waveform rescaling, twelve distance metrics to a common ideal waveform template were generated. A logistic regression model was constructed to produce a probability of acceptability for each beat.

**RESULTS.** 2286 PPG waveforms (30 data frames) were used for model development and testing, where 1039 PPG waveforms (13 data frames) were detected as artifact-free by HH and did not need B2BA. In B2BA development, 1247 PPG waveforms (623 training set, 624 test set) were used. All twelve distance metrics were significantly different between A and U in univariate analysis. The B2BA discriminated well between the A and U with receiver operating characteristic (ROC) area under the curve performance of 0.908 in the test set. To evaluate real-time potential, the model was deployed to waveform data from 986 ICU admissions (865 monitoring hours). A typical example of artifact removal is shown in Fig.1. Total computation time for a 30-minute PPG waveform was 15.84 seconds in a conventional workstation (Intel Xeon E5520, 76GB).

**CONCLUSIONS.** We proposed a real-time artifact removal system for PPG signal. This machine learning based approach achieves both efficiency and accuracy in artifact removal.

**REFERENCE(S)**

1. Johnson AEW, Pollard TJ, Shen L, Lehman L-wH, Feng M, Ghassemi M, Moody B, Szolovits P, Anthony Celi L, Mark RG: **MIMIC-III, a freely accessible critical care database**. *Scientific Data* 2016, **3**:160035.
2. Huang NE, Shen Z, Long SR: **A new vire of nonlinear water waves: The Hilbert spectrum**. *Annual Review of Fluid Mechanics* 1999, **31**(1):417-457.

**GRANT ACKNOWLEDGMENT**

Research was support by NIH R01GM117622, NIH 4T32HL07820

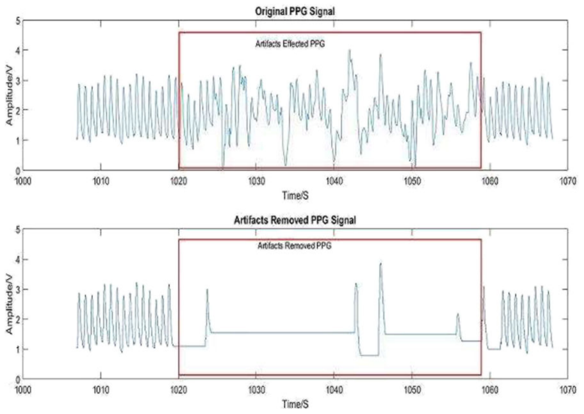

**Fig.1 (abstract 1285).** Typical examples of artifacts (above) and its removal (below) from PPG signal

### 1286

#### Transthoracic echocardiographic Doppler indexes are a specific but non-sensitive method for predicting pulmonary artery occlusion pressure in the critically ill patient

Y. Zerbib[1], P. Mercado[1], J. Maizel[1], J. Marc[1], C. Beyls[1], D. Titeca Beauport[1], M. Joris[1], L. Kontar[1], A. Riviere[1], O. Bonef[1], T. Soupison[1], C. Tribouilloy[2], B. De Cagny[1], M. Slama[1]

[1]CHU Amiens-Picardie, Medical ICU, Amiens, France; [2]CHU Amiens-Picardie, Cardiology, Amiens, France

**Correspondence:** Y. Zerbib

*Intensive Care Medicine Experimental* 2018, **6(Suppl 2):**1286

**INTRODUCTION.** Pulmonary artery occlusion pressure (PAOP) is a valuable tool for the management of critically ill patients. In ventilated critically ill patients, few studies have evaluated transthoracic echocardiography (TTE) to predict PAOP and conflicting data are available. The objective of the present study was to evaluate the predictive value of TTE to predict PAOP in this population.

**METHODS.** In 53 mechanically ventilated patients PAOP was measured using a pulmonary arterial catheter (PAC). TTE was used to estimate PAOP based on mitral flow and tissue Doppler Imaging (TDI) of mitral annulus.

**RESULTS.** Seventy nine TTE measurements were recorded, of them 10 were excluded by monophasic mitral flux. There was a weak correlation between the PAOP measurements and lateral E/E' (r=0.31; p< 0.0094), E/A (r=0.37; p< 0.0018) and TDE (r=-0.29; p< 0.02). The area under the ROC curve [95%CI] for the ability of lateral E/E' for detecting a PAOP≥18 mmHg was 0.59 [0.46-0.71] and an E/E'>15 could detect a PAOP≥18 mmHg with a sensitivity of 33% and specificity of 98%. The area under the ROC curve [95%CI] for the ability of E/A for detecting a PAOP≥18 mmHg was 0.73 [0.61-0.83] and an E/A > 1.75 could detect a PAOP≥18 mmHg with a sensitivity of 56% and specificity of 94%. When using both echocardiographic cutoff obtained with the ROC curves we found that by measuring lateral E/E' of more than 15 and an E/A of more than 1.75 a PAOP≥18 mmHg could be detected with a 18% of sensibility and 98% of specificity.

**CONCLUSION.** In critically ill mechanically ventilated patients, E/E' ratio>15 and or E/A ratio>1.75 predicted PAOP>18 mmHg with high specificity. However, a majority of patients were in the grey zone preventing any estimation of PAOP using Doppler technique.

### 1287

#### Assessing the accuracy of focused intensive care echocardiography (FICE)

J. McLoughlin, T. Samuels, R. Kumar

Surrey and Sussex Healthcare NHS Trust, Critical Care, Redhill, United Kingdom

**Correspondence:** J. McLoughlin

*Intensive Care Medicine Experimental* 2018, **6(Suppl 2):**1287

**INTRODUCTION.** Focused Intensive Care Echocardiography (FICE) scans are undertaken in critically ill patients, contributing to overall assessment and aiding decisions regarding treatment.

**OBJECTIVES.** The aim of the study was to compare the qualitative results from patients who had undergone FICE scans to the quantitative measurements of their formal echocardiogram.

**METHODS.** Over a period of 3 months we recorded 28 patients, from which 15 had also received a subsequent formal echocardiogram. We compared the ability of FICE scans to correctly identify both left and right ventricular significant impairment and dilatation. On the formal echocardiogram, we used the left ventricular ejection fraction (LVEF) of < 35% as significant impairment, left ventricular internal diameter (diastole) (LVIDd) > 6.2 cm for female patients and > 6.9 cm for male patients for significant dilatation, right ventricle (RV) basal diameter > 4.2 cm for dilatation and tricuspid annular plane systolic excursion (TAPSE) < 1.6 cm for significant impairment.[1]

**RESULTS.** The results are collated and shown in Table 1.

**CONCLUSIONS.** FICE is a useful tool in detecting gross abnormalities in patients in the critical care setting. Our results suggest that at our institution FICE is more accurate at assessing the left ventricle than the right. None of our FICE scans undertaken fully contradicted the formal echo report. Further research is required to assess the impact of FICE scanning in critical care and its accuracy when compared to formal echo.

**Table 1 (abstract 1287).** Comparing parameters between the formal echo report and FICE scan

| | Number of abnormal parameters on formal echo | Number of abnormal parameters detected by FICE | Number of normal parameters on formal echo | Number of normal parameters detected by FICE |
|---|---|---|---|---|
| LVEF | 4 | 3 | 10 | 10 |
| LVIDd | 0 | N/A | 14 | 11 |
| RV Basal Diameter | 6 | 2 | 8 | 5 |
| TAPSE | 2 | 1 | 11 | 10 |

### 1288

#### Time course of bilateral parameters of brain multimodal monitoring during porcine unilateral acute subdural hematoma-induced traumatic brain injury

M. Wepler[1], T. Kapapa[2], S. Röhrer[2], R. Mathieu[3], S. Mayer[3], M. Loconte[4], T. Merz[1], O. McCook[1], C. Hartmann[1], M. Georgieff[5], E. Calzia[1], U.M. Mauer[3], S. Unmuth[1], P. Radermacher[1], B. Nussbaum[5], T. Datzmann[1]

[1]University Hospital, Institute for Anesthesiological Pathophysiology and Process Engineering, Ulm, Germany; [2]University Hospital, Department of Neurosurgery, Ulm, Germany; [3]Federal Army Hospital, Department of Neurosurgery, Ulm, Germany; [4]University of Genoa, Department of Surgical Sciences and Integrated Diagnostics, Genoa, Italy; [5]University Hospital, Department of Anesthesiology, Ulm, Germany

**Correspondence:** M. Wepler

*Intensive Care Medicine Experimental* 2018, **6(Suppl 2):**1288

**INTRODUCTION.** Traumatic brain injury (TBI) is often associated with acute subdural hematoma (ASDH), which has been reported to be a major determinant of post-TBI outcome[1], in a particular in the elderly and/or cardiovascular co-morbid patient[2]. The guidelines for the management of TBI include control of intracranial pressure (ICP) and maintenance of cerebral perfusion pressure (CPP) as well as prevention of hypoxia by measurement of partial pressure of oxygen ($PtcO_2$) in brain tissue[3].

**OBJECTIVE.** We established a long-term, resuscitated porcine model of TBI to study the effects of unilateral ASDH on parameters of brain multimodal monitoring.

**METHODS.** Anesthetized, mechanically ventilated and surgically instrumented pigs (n=9, bodyweight 57-71kg) with human-like coagulation[4] underwent TBI induced by trepanation above the left parietal cortex and subdural injection of 20mL of blood[5]. After induction of unilateral ASDH, animals underwent intensive care therapy according to the guidelines of TBI management[3] for up to 54 hours. Multimodal brain monitoring included measurement of ICP, CPP, brain temperature and $PtcO_2$ of both the injured (ipsilateral) and non-injured (contralateral) side together with systemic hemodynamics, gas exchange, acid-base status, and metabolism. Resuscitation comprised fluid infusion and continuous i.v. norepinephrine to maintain mean arterial pressure (MAP) at pre-ASDH levels and CPP>75mmHg. Data is presented as median and interquartile range.

**RESULTS.** ICP after surgical instrumentation was similar at the ipsilateral and the contralateral side (2 [-2;16] and 2 [-2;8] mmHg, p=0.87). Subdural blood injection caused a transitory increase in ICP (individual peak values up to 50mmHg). The time course of the parameters of multimodal brain monitoring did not differ between the ipsi- and contra-lateral brain hemisphere: ICP 18 (12;23) vs. 14 (12;18) mmHg, p=0.19; CPP 112 (92;118) vs. 103 (77;117) mmHg, p=0.73; brain temperature 38.2 (38.1;38.3) vs. 38.2 (38.0;38.6) °C, p=0.96; $PtcO_2$ 20 (11;25) vs. 25 (10;37) mmHg, p=0.65 at the end of the experiment. Nevertheless, upon necropsy the ipsilateral hemisphere showed major macroscopic and microscopic morphological damage.

**CONCLUSION.** Our long-term, resuscitated porcine model of unilateral ASDH-induced TBI did not show any differences with respect to multimodal brain monitoring despite major morphological damage of the injured brain hemisphere.

Supported by the DFG (GEROK M. Wepler) and IGradU (S. Unmuth)

## Severity of disease

### 1289

#### Critical care follow up organisation with technology support: a feasibility survey to explore the utility of an "app" based post-critical illness support

J. McKenna[1], T. Price[2], M. Shyamsundar[1]

[1]Queen's University Belfast, Centre for Experimental Medicine, Belfast, United Kingdom; [2]Royal Victoria Hospital, Regional Intensive Care Unit, Belfast, United Kingdom

**Correspondence:** J. McKenna

*Intensive Care Medicine Experimental* 2018, **6(Suppl 2):**1289

**INTRODUCTION.** Technology is permeating every-day life and health-care apps can be leveraged to study outcomes post critical illness (1). Post-intensive care syndrome (PICS) is increasingly recognised as a significant contributor to post critical illness morbidity. Despite the importance of identifying PICS various interventions such as follow up clinics have failed to show impact. A web based or "app" based follow up could have advantages in being cost effective, ability to have longer and closer follow up, identification of trends in physical and psychological recovery, personalised approach by identifying only the patients showing deterioration or lack of improvement. Critical care population is following the trend in other specialities with patients being of older age group and could have significant co-morbidities which might limit their interaction with technology. This survey was conducted to identify the attitude and perceptions of patients admitted to critical care regarding the use of technology for follow up after critical illness.

**OBJECTIVES.** Understand the use of communication devices, pattern of interaction and attitude of patients admitted to critical care.

**METHODS.** A questionnaire survey of patients admitted to the Regional Intensive Care Unit (RICU), Royal Victoria Hospital, Belfast over an 8 week period. RICU is a tertiary trauma centre and a mixed medical surgical ICU. 35 patients completed the survey. Data was collected on demographics, use of communication devices, educational status, interaction with the technological device, use of internet, duration of device use per day. Their attitude towards technology based follow up was explored.

**RESULTS.** Thirty-five patients completed the questionnaire, 51.4% were > 60 years of age and 48.6% were female. 89% of patients had access to at least one internet enabled device and 91.4% of patients engaged in complex interactions such as completing surveys. 94% patients surveyed would be receptive to a technology based follow up would support their next of kin submitting responses on their behalf.

**CONCLUSIONS.** This survey demonstrates the increasing age of patients surviving critical illness. Despite their age, they have significant knowledge and use of communication devices and engage in complex online interactions and are receptive to the use of technology supported follow up care. A technology supported follow up service has the potential to be cost effective, allow closer and longer monitoring of patients. This approach could enable us identify patients in need of face to face follow up and personalise our approach to follow up and improve PICS outcomes.

**REFERENCE(S)**

1. Exploring the challenges of implementing e-health: a protocol for an update of a systematic review of reviews BMJ Open 2015;5:e006773

**GRANT ACKNOWLEDGMENT**

N/A

### 1290

#### Arterial pressure and vasopressor dose as predictors of mortality in patients with distributive shock

N.D. Nielsen[1], J.-L. Vincent[2], N.I. Shapiro[3], M.E. Gerbasi[4], A. Grossman[4], R. Doroff[4], G. Oster[4], F. Zeng[5], P. Young[6], J.A. Russell[7]
[1]Tulane University School of Medicine, Division of Pulmonary Disease, Critical Care and Environmental Medicine, New Orleans, United States; [2]Hopital Erasme, Department of Intensive Care, Brussels, Belgium; [3]Beth Israel Deaconess Medical Center, Harvard Medical School, Department of Emergency Medicine, Boston, United States; [4]Policy Analysis Inc., Brookline, United States; [5]La Jolla Pharmaceutical Company, San Diego, United States; [6]Medical Research Institute of New Zealand, Wellington, New Zealand; [7]St. Paul's Hospital, Division of Critical Care Medicine, Vancouver, Canada
**Correspondence:** N.D. Nielsen
*Intensive Care Medicine Experimental* 2018, **6(Suppl 2):**1290

**INTRODUCTION.** Distributive shock (DS) is a disease with high mortality rate. The management of DS calls for a careful balance between catecholamine dose and maintenance of sufficient blood pressure. The association of norepinephrine-equivalent dose (NED) and hypotension and their interaction with mortality in a real-world setting is not fully defined.

**OBJECTIVES.** The Medical Information Mart for Intensive Care (MIMIC-III) database was used to examine the predictors of mortality in patients with DS in a real-world setting. We were particularly interested in the association of hypotension time and maximum NED on intensive care unit (ICU) mortality, as well as their combined interaction with mortality.

**METHODS.** We identified patients with DS in the MIMIC-III database which contains information for 61,532 admissions to ICU at Beth Israel Deaconess Medical Center (BIDMC) in Boston between 2001 and 2012. Inclusion criteria: 1) ≥18 years, 2) treated with vasopressors for ≥6 hours, and 3) no indications of other conditions for which vasopressors are standard treatment, such as cardiogenic, hypovolemic, or obstructive shock. Patient baseline MAP was categorized as above or below 65 mmHg at initiation of vasopressors. Patient hypotension time was categorized as greater than or less than 2 hours spent continuously below 65 mmHg during the ICU stay. Maximum catecholamine dose was represented by categorical variables based on NED: < 0.2, ≥0.2-0.5, ≥0.5-1, ≥1-1.5, ≥1.5-2, ≥2-2.5, and ≥2.5 µg/kg/min. We performed a logistic regression with ICU mortality as the primary outcome. Control variables: age, gender, source of sepsis and sequential organ failure assessment (SOFA) scores at ICU admission.

**RESULTS.** Of 5347 patients with DS, 4995 had MAP recorded at initiation of vasopressors. Among these patients, 53% had MAP ≥65 mmHg at vasopressor initiation; 62% of patients stayed below 65 mmHg continuously for at least 2 hours. 40%, 31%, 16%, 7%, 2%, 1% and 3% of patients had a maximum NED of < 0.2, ≥0.2-0.5, ≥0.5-1, ≥1-1.5, ≥1.5-2, ≥2-2.5, and ≥2.5 µg/kg/min, respectively. The logistic regression results show that baseline MAP < 65 mmHg (odds ratio [OR]=1.27, p=0.0007) and MAP below 65 mmHg continuously for ≥2 hours (OR=1.78, p< 0.0001) were associated with higher mortality. When compared to patients with a max NED of 0.0-0.2, patients with max NED ≥0.2 all had significantly higher mortality, and mortality generally increased with NED: ≥0.2-0.5, OR = 2.43; ≥0.5-1.0, OR=5.33; ≥1.0-1.5, OR=7.78; ≥1.5-2.0, OR=10.41; ≥2.0-2.5, OR=13.76; ≥2.5, OR=7.84. Interaction terms between hypotension time and maximum NED were not significant.

**CONCLUSIONS.** We found that longer hypotension time and higher maximum NED were individually associated with higher ICU mortality rates in regression analysis. However, we did not find an interaction of hypotension time and maximum NED with mortality.

### 1291

#### Clinical, biochemical and haemodynamic variables as independent predictors of 90-day mortality: the Simple Intensive Care Studies-I

B. Hiemstra, G. Koster, F. Keus, I.C.C. van der Horst, SICS Study Group
University of Groningen, University Medical Center of Groningen, Department of Critical Care, Groningen, Netherlands
**Correspondence:** B. Hiemstra
*Intensive Care Medicine Experimental* 2018, **6(Suppl 2):**1291

**INTRODUCTION.** Clinical, biochemical and haemodynamic variables are used to diagnose circulatory shock and guide treatment in the critically ill. Literature is inconsistent which clinical signs have the best prognostic value on top of well-validated prognostic scores such as the simplified acute physiology score (SAPS II).

**OBJECTIVES.** The objective of the Simple Intensive Care Studies-I (SICS-I: NCT02912624) was to evaluate the association of clinical, biochemical and haemodynamic variables with 90-day mortality in a large, consecutive, cohort of critically ill patients [1].

**METHODS.** This prospective cohort study included all consecutive adults who were acutely admitted with an intensive care unit (ICU) stay expected to last beyond 24 hours. We conducted a protocolised clinical examination and critical care ultrasonography (CCUS) within 24 hours of ICU admission. Our primary outcome was 90-day mortality. Our secondary outcomes were 7- and 30-day mortality, and 90-day mortality by type of circulatory shock and clinical subgroups. We used multivariable Cox regression analyses to identify independent predictors of mortality.

**RESULTS.** From 27 March 2015 until 22 July 2017, 1104 patients were included. A total of 298 patients (28%) died at 90-day follow-up. Patients who died were older, more often ventilated, unconscious and dependent on noradrenalin, had lower blood pressures and urine output, and more often had an impaired microcirculation (tables 1 - 3). Multivariable Cox regression adjusted for age and SAPS-II score showed that respiratory rate (HR 1.02; 95% CI 1.00 - 1.04), unresponsiveness (HR 1.53; 95% CI 1.08 - 2.16) and serum lactate (HR 1.08; 95% CI 1.03 - 1.13) were independently associated with 90-day mortality. In patients with septic shock independent predictors were mottling rate (HR 1.27; 95% CI 1.02 - 1.58) and serum lactate (HR 1.11; 95% CI 1.02 - 1.22), whereas diuresis < 0.5 ml/kg/h (HR 2.27; 95% CI 1.00 - 5.15) and a mean arterial pressure < 70 mmHg despite vasopressor treatment (HR 5.08; 95% CI 2.00 - 12.9) were independent predictors in patients with heart failure.

**CONCLUSIONS.** Simple clinical variables as respiratory rate, consciousness, and serum lactate are independently associated with 90-day mortality in a large, unselected cohort of critically ill patients. The predictive value of clinical signs changed in clinical subgroups. Skin mottling and lactate showed the strongest associations in patients with septic shock and impaired diuresis and low mean arterial pressures were independently associated with 90-day mortality in patients with heart failure.

**REFERENCE(S)**

Hiemstra B, Eck RJ, Koster G, et al. Clinical examination, critical care ultrasonography and outcomes in the critically ill: cohort profile of the Simple Intensive Care Studies-I. BMJ Open 2017;7:e017170

**ACKNOWLEDGEMENT**

Currently analyzes are performed to refine the outcomes of this study. In case of admission, enhanced results will be presented.

**Table 1 (abstract 1291).** Baseline characteristics

| Variables | Survivors (n = 777) | Non-survivors (n = 298) | P-value |
|---|---|---|---|
| Age in years, mean ± SD | 60 ± 15 | 67 ± 12 | <0.001 |
| Gender male, n (%) | 480 (62%) | 194 (65%) | 0.310 |
| Mechanical ventilation, n (%) | 424 (55%) | 207 (70%) | <0.001 |
| Respiratory rate, mean ± SD | 18 ± 6 | 19 ± 6 | 0.007 |
| PEEP, median (IQR) | 7 (5, 8) | 8 (5, 10) | <0.001 |
| SAPS-II score, mean ± SD | 42 ± 14 | 58 ± 17 | <0.001 |

**Table 2 (abstract 1291).** Macrocirculatory variables

| Variables | Survivors (n = 777) | Non-survivors (n = 298) | P-value |
|---|---|---|---|
| Heart rate in bpm, mean ± SD | 87 ± 21 | 90 ± 22 | 0.060 |
| Irregular heart rhythm, n (%) | 64 (8%) | 52 (17%) | <0.001 |
| Systolic blood pressure in mmHg, mean ± SD | 118 ± 24 | 117 ± 27 | 0.380 |
| Diastolic blood pressure in mmHg, mean ± SD | 60 ± 11 | 58 ± 12 | 0.008 |
| Mean arterial blood pressure in mmHg, mean ± SD | 79 ± 14 | 77 ± 15 | 0.023 |
| Noradrenaline, n (%) | 345 (44%) | 183 (61%) | <0.001 |
| Urine output in mL/Kg/h, median (IQR) | 0.69 (0.40, 1.27) | 0.54 (0.27, 0.92) | <0.001 |
| AVPU score: alert; reaction to voice; reacting to pain; unresponsive | 264 (34%); 162 (21%); 57 (7%); 293 (38%) | 66 (22%); 40 (13%); 32 (11%); 160 (54%) | <0.001 |
| Cardiac index in L/min/m2, mean ± SD | 2.67 ± 0.91 | 2.58 ± 0.97 | 0.200 |

**Table 3 (abstract 1291).** Microcirculatory variables

| Variables | Survivors (n = 777) | Non-survivors (n = 298) | P-value |
|---|---|---|---|
| Central temperature in °C, mean ± SD | 37.0 ± 0.9 | 36.8 ± 1.0 | 0.002 |
| Central-to-peripheral temperature in °C, mean ± SD | 7.7 ± 3.1 | 8.2 ± 3.3 | 0.026 |
| Cold extremities, n (%) | 270 (35%) | 128 (43%) | 0.012 |
| CRT sternum in seconds, median (IQR) | 2.5 (2.0, 3.0) | 3.0 (2.0, 3.5) | <0.001 |
| CRT finger in seconds, median (IQR) | 2.0 (2.0, 4.0) | 3.0 (2.0, 4.5) | <0.001 |
| CRT knee in seconds, median (IQR) | 3.0 (2.0, 4.0) | 3.5 (3.0, 5.0) | <0.001 |
| Mottling rate: none; mild; moderate; severe | 429 (62%); 72 (10%); 178 (26%); 16 (2%) | 125 (46%); 31 (11%); 98 (36%); 17 (6%) | <0.001 |
| Hemoglobin in mmol/L, mean ± SD | 6.8 ± 1.4 | 6.8 ± 1.5 | 0.990 |
| Lactate in mmol/L, median (IQR) | 1.3 (0.9, 2.0) | 1.6 (1.0, 2.8) | <0.001 |

### 1292

#### Influence of comorbidity on outcomes in very old patients admitted to the ICU

A. Ortín, R. Jiménez, S. Rebollo, A. Ojados, J. Batllés, C. Navarro, A. Ruiz, S. Sánchez, S. Moreno, L. Herrera, M. Galindo, A. Fernández
Hospital General Universitario Santa Lucía, Servicio Medicina Intensiva, Cartagena, Spain
**Correspondence:** A. Ortín
*Intensive Care Medicine Experimental* 2018, **6(Suppl 2):**1292

**INTRODUCTION.** Elderly patients admitted to the intensive care unit (ICU) is increasing. Comorbidity in this population supposed an important issue that may have an impact on outcomes.
**OBJECTIVES.** To study the influence of baseline comorbidities on the evolution of elderly patients admitted to ICU.
**METHODS.** We performed a retrospective analysis of a prospective database including patients admitted to our polivalent ICU in last 7 years. We studied data related to the presence of prior comorbidities, severity scores at admission, supportive organ therapies and clinical outcomes. We compared patients younger from older than 80 years and studied the impact of different factors in both populations. For statistical analysis we used Chi-square, Fisher or T-Student tests as appropriate.
**RESULTS.** During the study period 3026 patients were admitted, 421 over 80 years old.
APACHE II, SAPS II and Glasgow scale at admission were significantly higher in elderly patients group.
Patients older than 80 had higher rates of chronic kidney disease (CKD) (18 vs 9.9 %, p < .001), COPD (17.6 vs 9.8, p < .001) and diabetes (41.2 vs 27.2, p< 0.001) and lower rates of inmunodeppression (2.6 vs 10.2, p < .001) and cirrhosis (0.3 vs 3.4, p .002) No differences were observed in terms of malignancy.
Mechanical ventilation was used less frequently in older than 80 group (49.7 vs 42.5 %, p.006) and during shorter period of time (5.46 (CI 95 % 4.2-6.7) vs 8.12 (7.4-8.9) days, p .012. Less reintubation (1.9 vs 4.4 %, p.015) and less performance of tracheostomy (3.6 vs 7.8, p.002) were observed in older than 80.
Significantly shorter ICU and hospital length of stay was observed in older than 80 group. ICU and hospital mortality were higher in older than 80 group (24.9 vs 14.2 %, p< .001 and 39.2 vs 20.8 %, p< .001 respectively)
We analyzed both groups of patients separately and focused on factors associated with mortality. Higher severity scores were significantly associated with mortality in both groups. Medical condition for admission had higher mortality in younger than 80 patients (15.6 vs 10.2 %, p< .001), with no differences in the older one. Trauma admission (compared with non-traumatic) carried higher ICU mortality (58.3 vs 24 %, p .007) in older than 80 and lower mortality in younger than 80 group.
In older than 80 group, no differences in mortality were observed according to the presence of any comorbidity. Conversely, in younger than 80, higher mortality was observed in presence of inmunodepression (23.7 vs 13.1, p< .001), CKD (19.6 vs 13.9, p .031) or cirrhosis (35.8 vs 13.7, p< .001)
**CONCLUSIONS.** Our results suggest that the weight of comorbidity may have less importance in the evolution of older than 80 years patients admitted to ICU. The inherent triage to admission of this old population may have limit patients with more severe comorbidities to be admitted to ICU and mask our findings.

### 1293

#### Mobile ICU response team (MIRT), a novel solution to expand undelayed critical care beyond the ICU doors; outcome on mortality and length of stay

A. Omranian[1], B. Mankidy[1], C.K. Morgan[1], C.M. Howard[1], M.A. Siddique[1], Y. Ababio[2], J. Herlihy[1]
[1]Baylor College of Medicine, Critical Care, Hoston, United States; [2]Baylor ST Lukes Medical Center, Houston, United States
**Correspondence:** A. Omranian
*Intensive Care Medicine Experimental* 2018, **6(Suppl 2):**1293

**INTRODUCTION.** The exponential damages of delay in providing intensive care to critically ill patients from the ED are well described.(1)

**OBJECTIVES.** To determine the effect on mortality and length of stay by the introduction of MIRT which provides immediate and comprehensive intensive care to critically ill patients anywhere in the hospital.

**METHODS.** This is a retrospective study in an 850 bed quaternary academic hospital. All ICUs admissions from the ED, patient care units, PACU (2012 to 2017) were included and divided into 2 groups. The intervention group after the implementation of MIRT and the control group prior to that. Primary end point was determined as all-cause mortality rate in all ICUs. Secondary outcome was determined as all-cause mortality rate and hospital length of stay (LOS) in Medical-Neuro ICU. Case mixed index (CMI) as a severity of illness value was compared.

**RESULTS.** 128,493 patients were discharged out of the hospital from January 2012 to December 2017, out of which 7775 patients were from the ICUs. All-cause mortality rate of all ICUs was reduced from 31.6%±3.1 to 28.4%±7.7 (p-value 0.17) in the control versus intervention group respectively, with mean CMI of 3.6±0.3 versus 3.7±0.2; (p-value 0.008). All-cause mortality rate in Medical-Neuro ICU was reduced from 37.3%±6.4 to 34.2%±8.9 (p-value 0.34) with mean CMI also reducing from 3.1±0.4 to 2.9±0.4 (Graph 1-2). Hospital length of stay for the patients admitted to Medical-Neuro ICUs was reduced from 12.3±1.5 to 11.0±2.1 days (p-value 0.12). Patients who died in these ICUs had a significant reduction in LOS from 9.1±1.2 to 7.4±1.4 (p-value 0.004) between control and intervention group.

**Conclusion.** Implementation of rapid response team (RRT) has been dramatically improving the hospital outcome(2). In a recent study, other colleagues identified a reduction in hospital-wide code blues from 2.1 to 1.6 codes per 1000 patient days (p-value = 0.007) after introduction of RRT however lack of adequate intensivist coverage outside the ICU has been observed.

We introduced MIRT in late 2014. The team included a critical care attending physician, critical care fellow, and a mid-level provider in addition to 2 or 3 specially trained RRT nurses, equipped with portable ultrasound, videoscopic airway equipment and i-STAT lab devices. Difficult airway and procedure cars were parked in multiple locations in the hospital for their use.

In our study there was an increase in the proportion of ICU patients which could imply a higher tendency towards moving more patients to the ICU. Decrease in LOS of those who died in the ICU could be explained by more goals of care discussions. There was a trend toward better outcomes but not statistically significant. More detailed and possibly prospective studies is needed to validate this assertion.

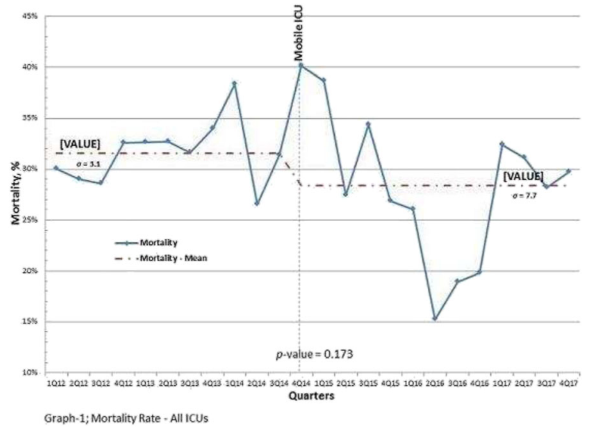

**Graph 1 (abstract 1293).** See text for description.

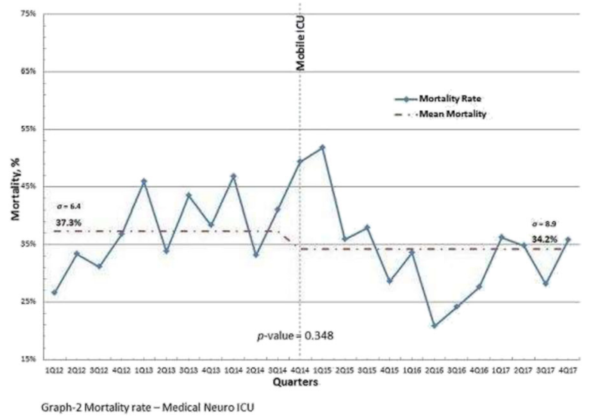

**Graph 2 (abstract 1293).** See text for description.

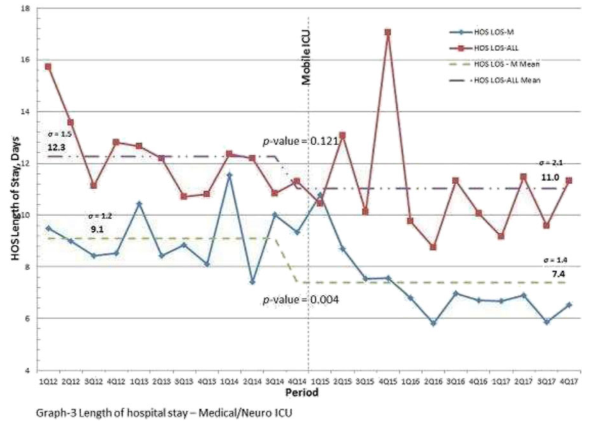

**Graph 3 (abstract 1293).** See text for description.

### 1294

#### Mean arterial pressure and catecholamine-sparing effects of vasopressin in patients with distributive shock in an academic medical center

F. Zeng[1], M. Gerbasi[2], G. Oster[2], A. Grossman[2], S. Chen[1]
[1]La Jolla Pharmaceutical Company, San Diego, United States; [2]Policy Analysis Inc., Brookline, United States
**Correspondence:** F. Zeng
*Intensive Care Medicine Experimental* 2018, **6(Suppl 2):**1294

**INTRODUCTION.** Consensus clinical guidelines recommend maintaining mean arterial pressure (MAP) ≥65 mmHg for patients with distributive shock (DS). Catecholamines are recommended as first-line vasopressor, but are associated with significant toxicities at high doses. Vasopressin and other vasopressors are often added to therapeutic regimens as they increase and maintain MAP while reducing the need for high doses of catecholamines. Little research has been done on the impact of vasopressin on MAP among patients with DS in a real-world setting.

**OBJECTIVES.** This study uses the Medical Information Mart for Intensive Care (MIMIC-III) database to examine MAP response and catecholamine-sparing effects of vasopressin in patients with DS in a real-world setting.

**METHODS.** We identified patients with DS in the MIMIC-III database which contains information for 61,532 admissions to intensive care units (ICU) at Beth Israel Deaconess Medical Center (BIDMC) in Boston, Massachusetts between 2001 and 2012. Inclusion criteria: 1) ≥18 years, 2) treated with vasopressors for ≥6 hours, 3) no indications of other conditions for which vasopressors are standard treatment, such as cardiogenic shock or vasoplegia, and 4) receipt of vasopressin and at least one catecholamine (norepinephrine, epinephrine, phenylephrine, or dopamine) at vasopressin initiation.

**RESULTS.** Of 5347 patients with DS, 1053 patients received vasopressin and at least one catecholamine at vasopressin initiation. Of the 641 patients with MAP ≥65 mmHg at vasopressin initiation, 84% remained at this level after 3 hours, while MAP decreased to below 65 mmHg in 15% and 1% were no longer in ICU; at 3 hours, of the 634 patients who remained in ICU, 11% and 8% had achieved 25% and 50% reduction in norepinephrine equivalent dose (NED) of catecholamines, respectively. Of 171 patients with MAP ≥60 but < 65 mmHg at vasopressin initiation, 18% remained at this level after 3 hours, while MAP increased to ≥65 mmHg in 68%, decreased to < 60 mmHg in 14%, and 1% were no longer in ICU; at 3 hours, 15% and 7% of 170 patients remaining in ICU had achieved 25% and 50% reduction in NED, respectively. Of 241 patients with MAP < 60 mmHg at vasopressin initiation, 27% remained at this level 3 hours after vasopressin initiation, while MAP increased to ≥65 mmHg in 49% and ≥60 but < 65 mmHg in 18%, and 6% were no longer in ICU; at 3 hours, 21% and 15% of the 227 patients remaining in ICU had achieved 25% and 50% reduction in NED, respectively.

**CONCLUSIONS.** Of patients with DS treated with vasopressin in this real-world setting, subsets of patients either failed to maintain their current MAP or were unable to raise MAP to the recommended target of 65 mmHg. In addition, a majority of patients did not achieve 25% reduction in NED 3 hours after initiation of vasopressin. This suggests a need for adjunctive therapies that can help maintain and increase MAP while sparing the use of catecholamines to reduce toxicity.

### 1295

#### Lactate clearance in the burned patient, does it really clarify something?

M. Aviles Garcia[1], K. Nanwani Nanwani[1], J.M. Cantero Escribano[2], E. Herrero de Lucas[1], M. Sanchez Sanchez[1], A. García de Lorenzo Mateos[1]
[1]Hospital La Paz, Medicina Intensiva, Madrid, Spain; [2]Hospital La Paz, Medicina Preventiva y Salud Pública, Madrid, Spain
**Correspondence:** M. Aviles Garcia
*Intensive Care Medicine Experimental* 2018, **6(Suppl 2):**1295

**INTRODUCTION.** The alteration of the permeability and the important displacements of liquids in thermal trauma are responsible for the formation of edema and massive loss of fluid. The objective of resuscitation is restore the oxygen supply to tissues and lactate is a parameter that reflects perfusion in absolute values and as a clearance. Lactate clearance is calculated as quotient between the initial lactate and second lactate divided by the initial lactate. Numerous studies postulated that clearance of lactate in the first hours of resuscitation influence the prognosis of burned patients and indicate in some way the efficacy of resuscitation.

**OBJECTIVES.** Assess how early lactate clearance influences the prognosis and mortality of critically burned patients, using initial lactate levels before initiation of treatment.

**METHODS.** Retrospective study in a ICU university hospital CESUR for the assistance of burned patient. All burned patients with or without associated inhalation syndrome who were transferred by an emergency service and who required admission to Critical Burns Unit for 2 years (January 2014 - December 2015).

We studied demographic, clinical variables, analytical values, transfer times, burned body surface, volume of resuscitation, need for orotracheal intubation and early/late mortality.

**RESULTS.** 55 patients transferred to our hospital, 28 patients admitted to ICU. The mean age was 55 ± 22.5, flame was the most frequent mechanism.

The resuscitation time from the initial notification until the patient arrived at hospital was 65.59 ± 26.4 m. Mortality in the first 24 hours was 7%, and the ABSI score had a median of 5 ± 3, an average of 536.67 ± 429.5 ml of Saline 0.9% was administered; The extra-hospital lactate levels showed a median of 3.7 ± 5.23 mmol/L while the hospital levels were 1.9 ± 2.1 mmol/L, with an early lactate clearing a mean of +49, 26 ± 36%. Only one patient died in the first 24 hours with negative lactate clearance.

No statistically significant correlation was found between the clearance lactate and the volume of fluid infused, nor between the clearance of lactate and the time of resuscitation, nor between the clearance lactate and the body surface burned.

**CONCLUSIONS.** Although it has been postulated that the clearance of lactate in the first hours of resuscitation can influence the prognosis of burned patients, in our study the clearance of lactate extrahospital with an average time of transfer of 1 hour did not correlate with the volume of fluid infused neither with the resuscitation time nor with the burned body surface. Although 93% of the patients who presented positive values of lactate clearance survived, the only patient with negative lactate clearance values died in the first 24 h, suggesting in some way that the negative clearance of lactate could be related to the poor prognosis in these patients, however, more studies are needed to discern the use of lactate clearance in resuscitation of burned patient.

### 1296

#### Thriving or surviving, national targets verses regional needs: outcomes for out of hospital cardiac arrests (OHCA) from intensive care in a geographically, architecturally and economically challenged region

G. Lee[1], K. Caines[1], J. John[2], R. Chelliah[2], A. Vijayan[3]
[1]Hull Royal Infirmary, Intensive Care and Anaesthetics, Hull, United Kingdom; [2]Castle Hill Hospital, Cardiology, Hull, United Kingdom; [3]Castle Hill Hospital, Cardiothoracic Anaesthesia and Intensive Care, Hull, United Kingdom
**Correspondence:** G. Lee
*Intensive Care Medicine Experimental* 2018, **6(Suppl 2):**1296

**INTRODUCTION.** The chain of survival is a tool implemented to highlight various stages following out of hospital cardiac arrest. Early cardiac intervention protocols were designed using trials for ST Elevation Myocardial Infarction (STEMI), in patients who maintain cardiac output, but are being applied to OHCA patients without ongoing return of spontaneous circulation.

Our hospital has a unique set of circumstances; we are one ofthe most socioeconomically deprived and geographically challenged regions in the UK. In addition we have a split hospital site meaning that all ongoing cardiac arrests are transferred to an intensive care via the emergency department (ED) on a site without percutaneous coronary intervention (PCI) abilities and a resident cardiologist unless a STEMI is demonstrated.

**OBJECTIVES.** To determine if national guidance and targets in combination with our unique set of circumstances are having an adverse effect on the outcomes of OHCA patients in our region.

**METHODS.** 94 OHCA were identified via ICNARC between January 2012 and June 2017 and appraised for a set of predetermined parameters, with telephone interviews, using the EQ-5D tool, for those whom survived.

**RESULTS.** Results were applied to the Myocardial Ischemia National Audit Project 2016. We achieved direct transfer to a PCI centre in 26%, call to balloon in 31% and hospital to balloon time in19% of patients. However we achieved a survival to hospital discharge of 43%.

With respect to our split site, transfer from ED to the PCI centre adds on average 173 minutes to call-balloon time yet there was no mortality difference between direct or indirect admissions. An increase in mortality was evident in the cohort whom remained on the non-cardiac site.

We found that patients whom survived had by comparison to the general population an overall reduction in quality of life (QOL) by 7%. However despite the life-changing event, 20% of those whom survived had a very good-excellent quality of life, well above the national average.

**CONCLUSIONS.** Overall and quality of survival from OHCA is reduced in socioeconomically deprived areas.

We have demonstrated that despite significantly underperforming against the national targets, we almost achieve the national average of survival to hospital discharge.

The demonstrated modifiable domains in our region propose that we refocus priorities, concentrate on quality of care and outcomes rather than targets.

In particular addressing the incorporation of cardiac interventions into good evidence based post resuscitation protocols on all of our intensive care unit across the city and standardizing OHCA guidance including prognostication and appropriate transfer, regardless of the patient's route of admission to our hospital and the immediate presence of a cardiologist The need for fast turn around to meet inappropriate national targets puts a vulnerable cohort at risk, particularly if they are not transferred for PCI at all.

### 1297

#### A comparison of healthcare-associated infections caused by multidrug-resistant organism or non-multidrug-resistant organism on mortality, length of stay and hospital costs in patients admitted to the intensive care units: using propensity-score matching

W.-H. Lai[1], I.-L. Chen[2,3], L.-H. Su[3], C.-H. Lee[3,4]

[1]Kaohsiung Chang Gung Memorial Hospital, Kaohsiung, Department of Trauma and Emergency Surgery, Kaohsiung, Taiwan, Province of China; [2]Kaohsiung Chang Gung Memorial Hospital, Kaohsiung, Clinical Pharmacy, Kaohsiung, Taiwan, Province of China; [3]Kaohsiung Chang Gung Memorial Hospital, Kaohsiung, Infection Control Team, Kaohsiung, Taiwan, Province of China; [4]Kaohsiung Chang Gung Memorial Hospital, Kaohsiung, Division of Infectious Diseases, Department of Internal Medicine, Kaohsiung, Taiwan, Province of China

**Correspondence:** W.-H. Lai

*Intensive Care Medicine Experimental* 2018, **6(Suppl 2):**1297

**INTRODUCTION.** Patients admitted to intensive care units (ICUs) have an especially high risk of acquiring healthcare-associated infections (HAI), and many of these are caused by multidrug-resistant organisms (MRDO). The HAIs caused by MDRO are emerging and spreading in ICUs globally. Little is known about the difference of attributed cost and length of stay (LOS) of patients with HAI caused by MRDO or caused by non-MRDO in ICU settings after propensity-score matching.

**OBJECTIVES.** We aimed to determine the case fatality rate, LOS, and healthcare cost of ICU patients with HAIs caused by MDROs (case group) and ICU patients with HAIs caused by non-MDROs (control group).

**METHODS.** A propensity-score matched case-control study was performed with patients aged 18 years or older who were admitted to Kaohsiung Chung Gang Memorial Hospital with 15 ICUs (a total of 200 beds) for adults from January 2010 to December 2017. Patients were followed and data were collected until death or discharge from the hospital. Patients in case group were matched with controls under a ratio of 1:2 by age, gender, APACHE-2, ICU specialty, underlying diseases, and the length of stay before transfer to ICUs. Main outcome measures are overall mortality, LOS of ICU, LOS of hospital, antimicrobial duration and healthcare costs.

**RESULTS.** During the study period, 46,237 patients were admitted to ICUs, 690 patients were selected (1:2) after propensity score matching. Urinary tract infection was the most frequently detected infection (45.6%), followed by bloodstream infections (34.5%). The most common isolate organism was *Escherichia coli* (21.1%). After propensity-score matching, there were no significantly difference on treatment cost (median [(inter-quartile range, IQR) €2313 (IQR 535-3050) vs. €950 (IQR 731-2683); P=0.30), and LOS of ICU (20.1±13.6 days vs.18.9±12.8 days; P=0.36), LOS of hospital (41.8±27.5 days vs. 39.2±23.5 days; P=0.35)] and duration of antimicrobial therapy (17.3 ±14.0 days vs.17.4±13.8 days; P=0.80) between ICU patients with HAIs caused by MDROs and ICU patients with HAIs caused by non-MDROs. The overall mortality rate was also not statistically difference (36.0% vs. 38.5%; P=0.52) between these two groups.

**CONCLUSIONS.** HAIs are a common adverse health event with substantial health and economic burden in ICU setting with or without MDRO.

**REFERENCE(S)**

1. Magiorakos AP, Srinivasan A, Carey RB, et al. Multidrug resistant, extensively drug-resistant and pandrug-resistant bacteria: an international expert proposal for interim standard definitions for acquired resistance. Clin Microbiol Infect 2012;18:268-81.
2. Magill SS, Edwards JR, Bamberg W, et al. Multistate pointprevalence survey of health-care associated infections. N Engl J Med 2014;370:1198-208.

**GRANT ACKNOWLEDGMENT**

None

### 1298

#### Health related quality of life and post intensive care syndrome. A follow-up 3 years after ICU discharge in Argentina. The CAVIUCI study

A. Das Neves[1], C.I. Loudet[1], M. Busico[2,3], D.N. Vasquez[4], D. Villalba[3], G. Plotnikow[3,4], C. Foster[3], A. Lischinsky[3], F. Carini[3,5], M. Veronesi[3], F.J. López[6], M. Emmerich[7], A. Díaz[8], E. Descotte[9], S. Giannasi[5], J. Alvarez[10], M. Grando[11], R. Hernandez[12], A. Tapia[13], L. Krzisnik[14], M. Camargo[15], C. Cecotti[16], D. Villani Ullua[17], R. Fernandez[18], L. Corzo[19], R. López[20], H. Placido dos Santos[21], J.P. Sottile[22], A. Ramos[23], J.A. Doglia[24], G. Benavent[25], L. Viola[18], A. Juliarena[10], E. Estenssoro[1], CAVIUCI

[1]HIGA Gral San Martin, La Plata, Argentina; [2]Hospital de Trauma F Abete, Malvinas Argentinas, Argentina; [3]Comite de Seguimiento y Rehabilitacion, SATI, Caba, Argentina; [4]Sanatorio Anchorena, Caba, Argentina; [5]Hospital Italiano de Buenos Aires, Caba, Argentina; [6]Hospital Escuela de Agudos Dr. Ramón Madariaga, Posadas, Argentina; [7]Sanatorio Güemes, Caba, Argentina; [8]Hospital Misericordia, Cordoba, Argentina; [9]Hospital Británico de Buenos Aires, Caba, Argentina, [10]Hospital Austral, Pilar, Argentina; [11]Clinica Bazterrica, Caba, Argentina; [12]Hospital Francisco López Lima, General Roca, Argentina; [13]Hospital Domingo Funes, Santa Maria de Punilla, Argentina; [14]Hospital El Cruce, Dr. N. Kirchner, Florencio Varela, Argentina; [15]Hospital San Luis, San Luis, Argentina; [16]Hospital Mi Pueblo, Florencio Varela, Argentina; [17]Hospital San Jose, Pergamino, Argentina; [18]Hospital Español, Mendoza, Argentina; [19]Hospital A Zatti, Viedma, Argentina; [20]Clinica Pueyrredon, Mar del Plata, Argentina; [21]Hospital de Gastroenterologia B. Udaondo, Caba, Argentina; [22]Hospital Zonal Bariloche, Bariloche, Argentina; [23]Sanatorio Parque, Rosario, Argentina; [24]Hospital Dr. Macías, Mar de Ajo, Argentina; [25]Clinica CEMEP, Rio Grande, T del Fuego, Argentina

**Correspondence:** A. Das Neves

*Intensive Care Medicine Experimental* 2018, **6(Suppl 2):**1298

**Withdrawn**

### 1299

#### Does the use of brief cognitive-behavioural and mindfulness techniques reduce psychological distress during critical care?

C. Lambert[1], J. Preller[2], L. Enoch[3], P. Polgarova[2], J. Hyde[3], J. Outtrim[4], D. Christmas[5], Z. Martin[5]
[1]Addenbrooke's Cambridge University Hospitals NHS Trust, Intensive Care and Neuro Critical Care Unit, Cambridge, United Kingdom; [2]Addenbrooke's Cambridge University Hospitals NHS Trust, Intensive Care, Cambridge, United Kingdom; [3]Addenbrooke's Cambridge University Hospitals NHS Trust, Critical Care, Cambridge, United Kingdom; [4]Addenbrooke's Cambridge University Hospitals NHS Trust, Neuro Critical Care Unit, Cambridge, United Kingdom; [5]Addenbrooke's Cambridge University Hospitals NHS Trust, Liaison Psychiatry, Cambridge, United Kingdom
**Correspondence:** C. Lambert
*Intensive Care Medicine Experimental* 2018, **6(Suppl 2):**1299

**INTRODUCTION.** Data from our tertiary hospital ICU demonstrated an unmet psychological need in former critical care patients (Kieron et al., 2014). In response a psychological wellbeing service has recently been set-up which provides structured interventions to patients at the bedside. A lack of empirical research findings in this area means that such interventions are based on best practrice from more general evidence-base (e.g. Richards and Whyte, 2011) rather than robust ICU evidence. Psychological interventions for depression, anxiety, and stress were developed. To assess the effectiveness of these interventions distress specific Subjective Units of Distress (SUDS) on a scale of 1 - 10 are collected before and after each intervention with each patient.

**OBJECTIVES.** To investigate whether a significant difference in SUDS level can be detected:
(a) within and between individual sessions overall;
(b) within and between distress specific sessions overall;
(c) within areas of intervention (cognitive, behavioural or mindfulness); and (d) between different interventions for each area of distress.

**METHODS.** A series of 2-tailed paired-sample t-tests and Welch's t-tests were undertaken to measure whether statistically significant differences exist between the SUDS ratings specified within the objectives session.

**RESULTS.** Please see the tables below.

**CONCLUSIONS.** A strong statically significant difference between all within (at the start and at the finish) session SUDS analysed, and the reduced mean averages, suggesting that the psychological interventions undertaken are effective at reducing distress in each area at the time of the respective sessions. Similarly, the within interventions findings suggest that cognitive, behavioural and mindfulness approaches are all effective at reducing distress during a session.

However, whilst a similar difference is shown between the first and last sessions for all sessions and for anxiety, there was a signal for similar change which did not reach statistical significance between sessions for the treatment of depression or stress. This may either reflect inadequate sample size or that the interventions for depression and stress might not have as lasting or cumulative distress reducing affect as that for anxiety.

No statistically significant difference was found between treatment options for specific areas of distress, suggesting that no intervention compared within this small sample is more effective at reducing SUDS than any other.

##### REFERENCE(S)

Kieron, K., Hyde, J., Trivedi, M., & Christmas, D. (2014) Poster: 'Psychiatric morbidity in Patients Discharged from Intensive Care', Cambridge University Hospital Richards, D., & Whyte, M. (2011) 'Reach Out: National Programme Student Materials to Support the Delivery of Training for Psychological Wellbeing Practitioners Delivering Low Intensity Interventions', 3rd ed., Rethink Mental Illness.

##### GRANT ACKNOWLEDGMENT

Addenbrooke´s Charitable Trust

**Table 1 (abstract 1299).** Paired-sample T-tests results: within sessions and interventions

| Condition | Sub-condition | Initial SUDS | End SUDS | Result |
|---|---|---|---|---|
| Within sessions | All sessions | M = 5.78, SD=2.06 | M = 4.71, SD=1.86 | t (71) = 10.5376 p<0.001 |
| | Depression | M = 6.61, SD=2.13 | M = 5.52, SD=2.02 | t (22) = 5.7911 p<0.001 |
| | Anxiety | M = 5.57, SD=1.88 | M = 4.43, SD=1.56 | t (22) = 6.2395 p<0.001 |
| | Stress | M = 5.35, SD=1.97 | M = 4.22, SD=1.73 | t (22) = 6.6530 p<0.001 |
| Within all interventions across distress areas | All mindfulness | M = 5.56, SD=2.14 | M = 4.44, SD=2.02 | t (24) = 8.4105 p<0.001 |
| | All behavioural | M = 6.22, SD=2.09 | M = 5.09, SD=1.83 | t (22) = 6.2395 p<0.001 |
| | All cognitive | M = 5.36, SD=1.89 | M = 4.36, SD=1.53 | t (21) = 4.3875 p<0.001 |

**Table 2 (abstract 1299).** Paired-sample T-tests results: between sessions.

| Condition | Sub-condition | Initial SUDS | End SUDS | Results |
|---|---|---|---|---|
| Between first and last measure of different sessions | All sessions | M = 6.65, SD=1.32 | M = 4.12, SD=1.9 | t (16) = 4.0860 p<0.001 |
| | Depression | M = 7, SD=1.41 | M = 4.5, SD=2.59 | t (5) = 2.1787 p=0.0812 |
| | Anxiety | M = 7, SD=1 | M = 3.2, SD=0.84 | t (4) = 10.1559 p<0.001 |
| | Stress | M = 6.4, SD=1.14 | M = 4, SD=1.41 | t (4) = 2.2283 p=0.0898 |

**Table 3 (abstract 1299).** Welch's T-test results

| Condition | Sub-condition | Last SUDS first intervention | Last SUDS final intervention | Result |
|---|---|---|---|---|
| Between interventions | Activity scheduling v others options for depression | M = 5.73, SD=1.85 | M = 5.33, SD=2.23 | t (20) = 0.4627 p=0.6486 |
| | Thought restructuring v mindful breathing for anxiety | M = 4.57, SD=1.51 | M = 4, SD=1.91 | t (11) = 0.6197 p=0.5481 |
| | Mindfulness v others options for stress | M = 4.27, SD=1.62 | M = 4.58, SD=1.56 | t (20) = 0.4672 p=0.6454 |

### 1300

#### A feasibility study to assess pre-admission status and six month outcomes of major trauma patients admitted to an intensive care unit (ICU), using the WHO DAS 2.0 - preliminary analysis

R. Haylett[1], O. Gustafson[1,2]
[1]Adult Intensive Care Unit, Oxford, United Kingdom; [2]Kadoorie Centre for Critical Care Research, Oxford, United Kingdom
**Correspondence:** R. Haylett
*Intensive Care Medicine Experimental* 2018, **6(Suppl 2):**1300

**INTRODUCTION.** The physical disability experienced by survivors of critical illness is well documented[1], and improving the quality of survival is a fundamental aspect of care[2]. Many measures have reported the recovery of patients and despite the variety of measures used significant burdens are consistently identified[1]. However, measures commonly report on function at isolated time points post discharge, not relating to previous levels of function. Baseline limitations in function will alter the ability to achieve higher scores in various domains and confound measurements of function. The World Health Organisation Disability Assessment Schedule 2.0 (WHODAS 2.0) measures multiple health domains and has been used to assess disability at follow up for ICU survivors[3].

**OBJECTIVES.** To report the feasibility of assessing the pre-admission functional status of ICU patients using the WHODAS 2.0, with repeat assessment at six months post ICU discharge.

**METHODS.** A prospective longitudinal observational cohort design recruited consecutive adult major trauma admissions to a UK general ICU, between 1st November 2016 and 1st November 2017. The ICU physiotherapy team underwent pre-study training. The 12-item WHODAS 2.0 was completed with the patient or using a consultee to assess pre-admission functional status as soon as possible after ICU admission. The measure was repeated at six months post ICU discharge, through ICU follow-up clinic or by post. Final attempts at follow up were made by telephone.

**RESULTS.** The ICU physiotherapy team were trained in the use of the WHODAS 2.0 in 2.5 hours. Of the 73 eligible patients 57 were recruited and completed initial assessments. The mean time from ICU admission to assessment was 38 hours, with the assessment taking an average of three minutes to complete. At the time of analysis there were follow up attempts made for 47 participants, with 22 completed (47%). The median six months WHODAS 2.0 score was 27, increasing from baseline assessment by a median of 14 points. Of the responses received 12 were via post, 5 via ICU follow-up clinic, and 2 by telephone.

**CONCLUSIONS.** With minimal resources standardised assessment of pre-admission functional status was feasible to collect. Follow up was challenging although deterioration in function was evident. Future research should investigate the clinical relevance of scores, and the validity of the WHODAS 2.0 in ICU patients.

### 1301

#### Comparison of lung ultrasound (US) based weaning indices with RSBI: are they helpful?

A. Banerjee, G. Mehrotra
Tata Main Hospital, Dept of Anesthesiology and Critical Care, Jamshedpur, India
**Correspondence:** A. Banerjee
*Intensive Care Medicine Experimental* 2018, **6(Suppl 2):**1301

**INTRODUCTION AND OBJECTIVES.** The diaphragm is considered the main respiratory muscle and difficulty in weaning can occur because of impaired diaphragmatic function. Hence, monitoring diaphragmatic function is important. The aim of this study is to assess the ability of various lung US indices and rapid shallow breathing index (RSBI) to predict the outcome of weaning process and compare them with RSBI.

**METHODS.** This was a prospective study conducted on patients admitted in critical care unit at a tertiary care hospital in north India from February 2017 to June 2017. Patients were put to Spontaneous breathing trial (SBT) when they met the weaning criterias. Initial US were done immediately after putting the patient on SBT to check anatomy of diaphragm and rule out patients according to exclusion criteria. This was followed by complete lung US after 20 minutes of SBT.

**RESULTS.** The RSBI performed better than all other parameters, with an area under curve (AUC) of 0.996.The sensitivity and specificity is 100 %. Only comparable to RSBI is speed of diaphragmic contraction (DC) which has AUC of 0.93. All other parameters had an AUC less than 0.8. Moreover the DC and (Lung ultrasound score) LUS are strongly positively correlated with RSBI whereas diaphragmic excursion (DE) and Diaphragmic thickness fraction (DTF %) are weakly correlated.

**CONCLUSIONS.** In ICU, RSBI is the best clinical tool for weaning and DC is found to be the best parameter for weaning among the ultrasound based weaning parameters. It can even be a substitute of RSBI, in today's world of real time monitoring methods.

##### REFERENCE(S)

1. Lee KH, Hui KP, Chan TB. Rapid shallow breathing (frequency-tidal volume ratio) did not predict extubation outcome. Chest 1994;105:540-32.
2. Ferrari G, De Filippi G, Elia F. Diaphragm ultrasound as a new index of discontinuation from mechanical ventilation. Crit Ultrasound J 2014; 6:8.
3. Matamis D, Soilemezi E, Tsagourias M. Sonographic evaluation of the diaphragm in critically ill patients. Technique and clinical applications. Intensive Care Med. 2013;39: 801-10
4. Ayman I. Baess, Tamer H. Abdallah, Doaa M. Emara, Maged Hassan: Diaphragmatic ultrasound as a predictor of successful extubation from mechanical ventilation: thickness, displacement, or both?: Egyptian Journal of Bronchology 2016 10: 162- 166
5. Steier J, Kaul S, Seymour J, Jolley C, Rafferty G, Man W et al; 2007; The value of multiple tests of respiratory muscle strength. Thorax 62:975-980
6. Soilemezi E, Tsagourias M, Talias MA, Soteriades ES, Makrakis V, Zakynthinos E et al; 2012; Sonographic assessment of changes in diaphragmatic kinetics induced by inspiratory resistive loading.; Available from Respirology. https://doi.org/10.1111/resp12011

##### GRANT ACKNOWLEDGMENT

NIL.

### 1302

#### Ultrasound for determination of right ventricular - pulmonary artery coupling in ICU patients

M.J. Boonstra[1,2], A.H. Jonkman[1], N. Wijbenga[1,2], B.M. Wulterkens[1,2], E. Lust[1], A. Vonk-Noordegraaf[3], P.R. Tuinman[1], P.W.G. Elbers[1]
[1]VU University Medical Center Amsterdam, Department of Intensive Care Medicine, Research VUmc Intensive Care (REVIVE), Amsterdam Cardiovascular Sciences (ACS), Amsterdam Infection and Immunity Institute (AI&II), Amsterdam, Netherlands; [2]University of Twente, Technical Medicine, Enschede, Netherlands; [3]VU University Medical Center Amsterdam, Department of Pulmonary Medicine, Amsterdam Cardiovascular Sciences (ACS), Amsterdam, Netherlands
**Correspondence:** M.J. Boonstra
*Intensive Care Medicine Experimental* 2018, **6(Suppl 2):**1302

**INTRODUCTION.** Right heart failure is increasingly being recognized as an independent predictor of adverse outcome in the critically ill. Right ventricular (RV) - pulmonary artery (PA) coupling quantifies right heart efficiency and performance. Mechanical ventilation, pulmonary pathology, and cardiovascular support affect RV-PA coupling. Therefore, assessment of RV-PA coupling in ICU patients may yield important guidance for diagnosis and treatment [1,2]. Theoretically, bedside determination of RV-PA coupling is possible using ultrasound (US), circumventing the need of MRI and/or right heart catheterization (RHC).

**OBJECTIVES.** To determine feasibility and validity of measuring RV-PA coupling using US.

**METHODS.** Right heart US examination was performed before and during passive leg raising (PLR) in patients with known or presumed pulmonary hypertension undergoing RHC and/or MRI. Using 2D, Continuous Waved and Pulsed Waved Doppler US, fractional area change, tricuspid regurgitation velocity and PA flow were determined. These measurements serve as derivatives of RV ejection fraction (EF), pressure (RVP) and stroke volume (SV), respectively. US measurements were compared to RHC and MRI measurements.

**RESULTS.** Preliminary results (n=4) of RHC and MRI measurements compared to US measurements are shown in Table 1. These measurements are the basis of RV-PA coupling values. Using US measurements the systolic part of a pressure-volume (PV) loop was constructed (Figure 1).

**CONCLUSIONS.** It is feasible to construct the systolic part of a PV loop from US measurements. Thereof US RV-PA coupling can be determined. Despite discrepancy between gold standard and US measurements, the US method may provide patient specific guidance during ICU treatment and may be therefore of additional value.

**REFERENCE(S)**

[1] Guarracino, *Annual Update in Intensive Care and Emergency Medicine,* 2017.

[2] Vonk-Noordegraaf, *Journal of the American College of Cardiology,* 2017.

**Table 1 (abstract 1302).** Comparison of US measurements to RHC and MRI measurements (n=4).

| | Baseline | | PLR | |
|---|---|---|---|---|
| | RHC/MRI | US | RHC/MRI | US |
| RVP (mmHg) | 86.2±26.3 | 80.1±25.1 | 92.6±25.1 | 89.2±20.1 |
| SV (ml) | 58±15 | 140±58 | - | 138±58 |
| EF (ml) | 0.52±0.15 | 0.33±0.12 | - | 0.33±0.07 |

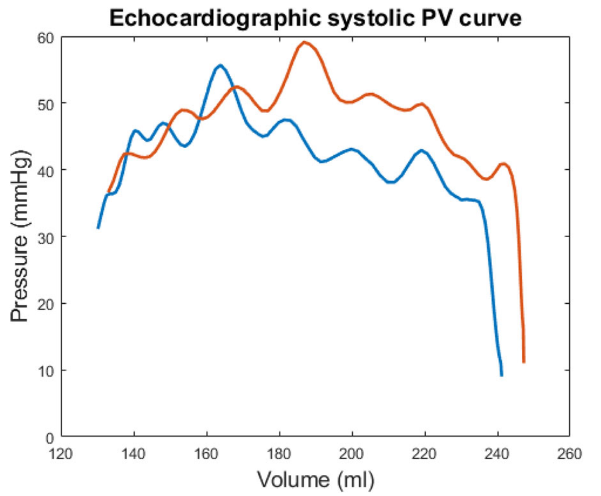

**Fig. 1 (abstract 1302).** Systolic part of a PV loop constructed using US.

### 1303

### The added value of the daily presence of a pharmacist within the intensive care unit of a Dutch top-clinical hospital

E. Boerrigter[1], H. Kuijsten[2], G. van der Nat[2], K. Simons[1]

[1]Elisabeth-Tweesteden Hospital, Clinical Pharmacy, Tilburg, Netherlands; [2]Elisabeth-Tweesteden Hospital, Intensive Care Unit, Tilburg, Netherlands

**Correspondence:** E. Boerrigter

*Intensive Care Medicine Experimental* 2018, **6(Suppl 2):**1303

**INTRODUCTION.** It has been shown that the presence of a pharmacist in the Intensive Care Units in the Netherlands prevents medication errors (1). This is recognized in the Dutch 'quality standard of the Intensive Care (2016)'. This quality standard says: ´It is likely that the daily presence of an IC-committed pharmacist will positively influence the outcome of the patients. In addition, the pharmacist contributes to a more rational medication policy and cost effectiveness. ´ To our knowledge the presence of an IC-committed pharmacist has not been described as a daily practice among Dutch top-clinical hospitals.

**OBJECTIVES.** The objective of the study was to investigate the added value of a daily pharmacist within the ICU of the Elisabeth-Tweesteden hospital (ETZ) in Tilburg, the Netherlands.

**METHODS.** The pilot study was carried out at the ICU of the ETZ from 10-07-2017 till 11-08-2017. During workdays from 8:00h till 12:00h a pharmacist was present in the ICU and was stationed at a dedicated workspace. Figure 1 shows the workflow. The ETZ is a top-clinical hospital located in Tilburg, the Netherlands. The ICU is a level 3 accredited IC and is a regional trauma center. The IC has 34 beds divided over 4 units.

**RESULTS.** A total of 122 pharmacotherapeutic and pharmacological interventions were proposed. 112 (92%) of the proposed interventions were implemented by the physicians. Interventions were divided into the following categories: dose, intoxication, feeding tube, start, stop, switch, therapeutic drug monitoring (TDM), drug interaction, consultation and other (see figure 2).

**CONCLUSIONS.** During one month, 122 pharmacotherapeutic and pharmacological interventions were proposed, of which 112 were implemented by the physicians. Therefore an added value was shown when a pharmacist was present on a daily basis in the ICU of a Dutch top-clinical hospital.

**GRANT ACKNOWLEDGMENT**

Not applicable.

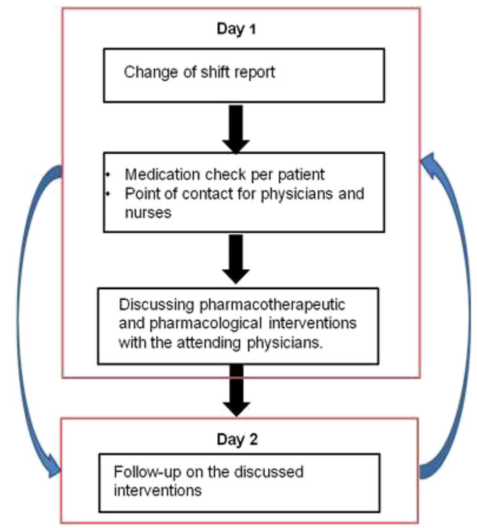

**Fig. 1 (abstract 1303).** A schematic workflow

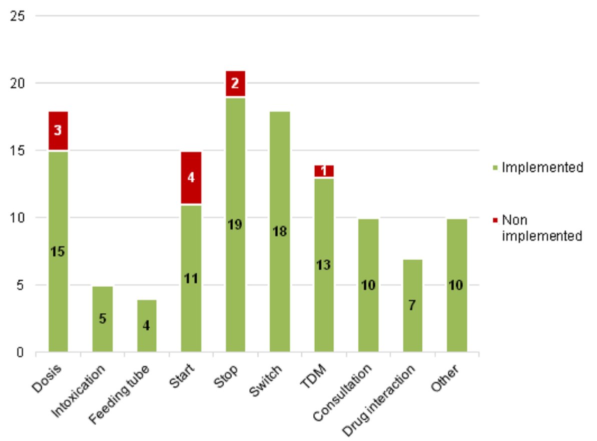

**Fig. 2 (abstract 1303).** Proposed interventions divided by category n=122

### 1304

#### Comparing intravenous unfractionated heparin and subcutaneous low molecular weight heparin as therapeutic anticoagulation on intensive care

S. Case, R. Gayner, N. Weston-Smith, A. Clarke
Bristol Royal Infirmary, Intensive Care, Bristol, United Kingdom
**Correspondence:** R. Gayner
*Intensive Care Medicine Experimental* 2018, **6(Suppl 2):**1304

**INTRODUCTION.** Intravenous unfractionated heparin (UH) and subcutaneous low molecular weight heparin (LMWH) are used as therapeutic anticoagulants on intensive care units (ICUs) for a variety of indications. In the treatment of venous thromboembolism (VTE) a recent Cochrane review has shown LMWH is associated with fewer major haemorrhagic episodes, lower mortality and reduced incidence of recurrence compared to intravenous UH[1]. There is limited evidence comparing their use in non-VTE indications or in the ICU setting.

**OBJECTIVES.**

· Compare prescribing practices for UH and LMWH as therapeutic anticoagulants in ICU and the frequency of complications

· Assess ICU monitoring of UH using activated partial thromboplastin time ratios (APTRs)

**METHODS.** Retrospective audit of admissions to a 21-bed mixed medical/surgical ICU in Bristol, UK. The electronic clinical record was interrogated over a 12 month period to return a dataset of prescriptions for therapeutic anticoagulation. From the UH group, we defined a subgroup (n=20) to identify the frequency of APTR monitoring, duration of therapeutic APTR results and compliance with hospital infusion protocol.

**RESULTS.** We identified 45 prescriptions for UH and 31 prescriptions for LMWH. Of the 65 patients involved, 11 received both UH and LMWH (separately) during the audit period. The commonest indication for anticoagulation was new VTE (34 prescriptions, 45%; 18 UH vs 16 LMWH) and second commonest indication was for a pre-existing condition (23 prescriptions, 30%; 11 UH vs 12 LMWH). Other indications included new atrial fibrillation, acute coronary syndrome and arterial thrombosis. 10 patients received UH for haemofiltration. In the UH group 58% of APTR measurements were done within 6 hours of the last measurement. 40% of APTR measurements were not in the therapeutic range and appropriate dose adjustments were made in 82% of occurrences. 11 (24%) patients in the UH group had bleeding complications; 2 were life-threatening: one had a fatal subarachnoid haemorrhage despite sub-therapeutic anticoagulation; another had a massive intra-abdominal haemorrhage and survived. The LMWH group had no bleeding complications although 2 patients had procedures delayed due to anticoagulation.

**CONCLUSIONS.** UH and LMWH are used for a variety of indications on ICU, with crossover between conditions. UH was associated with minor and major bleeding complications, whilst no complications were seen with LMWH. Given the higher rates of complications and difficulties maintaining UH in the therapeutic range it may be worth considering using LMWH more frequently as a therapeutic anticoagulant. Therapeutic anticoagulation may expose patients to higher bleeding risk than necessary, particularly for non-acute indications.

### 1305

#### Modification of ICU environment is associated with reduced incidence of delirium - results from the VITALITY study

A. Lütz[1], H. Uhrlau[1], H. Piazena[2], A. Kramer[3], I. Fietze[4], T. Penzel[4], B. Weiss[1], T. Willemeit[5], A. Finke[5], J. Quantz[6], J. He[6], C. Spies[1]
[1]Charité - Universitätsmedizin Berlin, Department of Anaesthesiology and Intensive Care Medicine, Campus Charité Mitte and Campus Virchow-Klinikum, Berlin, Germany; [2]Charité - Universitätsmedizin Berlin, Medical Photobiology Group, Berlin, Germany; [3]Charité - Universitätsmedizin Berlin, Department of Medical Immunology, Berlin, Germany; [4]Charité - Universitätsmedizin Berlin, Department of Internal Medicine, Center of Sleep Medicine, Campus Charité Mitte, Berlin, Germany; [5]Graft Architects, Berlin, Germany; [6]Art+Com Research, Berlin, Germany
**Correspondence:** A. Lütz
*Intensive Care Medicine Experimental* 2018, **6(Suppl 2):**1305

**INTRODUCTION.** Delirium is the most frequent psychiatric syndrome in the Intensive Care Unit (ICU). This form of acute brain dysfunction predicts higher mortality rates and is associated with worse global cognition after ICU discharge [1]; [2]. Therefore, delirium prevention bundles have become an integral part of guideline recommendations. Clinical trials investigating pharmacological strategies have failed to demonstrate consistent results in terms of patient outcomes. Hence, focus has moved towards non-pharmacological approaches.

**OBJECTIVES.** Within an interdisciplinary project, we developed a new ICU room concept . In 2013 we finished the renovation of 2 double ICU rooms (Fig. 1 and 1). Beside interventions aimed at noise reduction, workflow optimisation and infection control, we developed a new light ceiling that enables clinicians to apply patient individualised light therapy [3]; [4]. We hypothesised that the delirium incidence for patients treated in the modified rooms is significantly lower when compared to patients treated in the standard rooms.

**METHODS.** After sample size calculation, 74 mechanically ventilated patients with an expected ICU length of stay >48 hours were included in this prospective cohort study (NCT02143661). Amongst other reasons, patients with a substantial recent ICU exposure [2], patients who were unlikely to survive for 24 hours and patients who could not be reliably assessed for delirium were excluded from study participation. Delirium was assessed every 8 hours by research physicians with the Confusion Assessment Method for the ICU (CAM-ICU).

**RESULTS.** The incidence of ICU delirium was significantly lower among patients treated in the modified rooms (46%) compared to patients treated in the standard rooms (76%, $p$ = 0.017) (Table 1). The association between the modification of ICU environment and the delirium incidence was statistically significant (odds ratio, 0.15; 95% confidence interval, 0.04 - 0.66; $p$ = 0.012) after adjusting for covariates.

**CONCLUSIONS.** This study is the first to show that a modification in ICU environment is significantly associated with a reduced delirium incidence. Further analysis is needed to investigate which of the room interventions may contributed to this clinical effect.

**REFERENCE(S)**

1. Ely EW, Shintani A, Truman B, et al. (2004) Delirium as a predictor of mortality in mechanically ventilated patients in the intensive care unit.. JAMA 291:1753-62.
2. Pandharipande PP, Girard TD, Jackson JC, et al. (2013) Long-term cognitive impairment after critical illness.. N Engl J Med 369:1306-16.
3. Luetz A, Weiss B, Penzel T, et al. (2016) Feasibility of noise reduction by a modification in ICU environment.. Physiol Meas 37:1041-55.
4. Luetz A, Piazena H, Weiss B, et al. (2016)

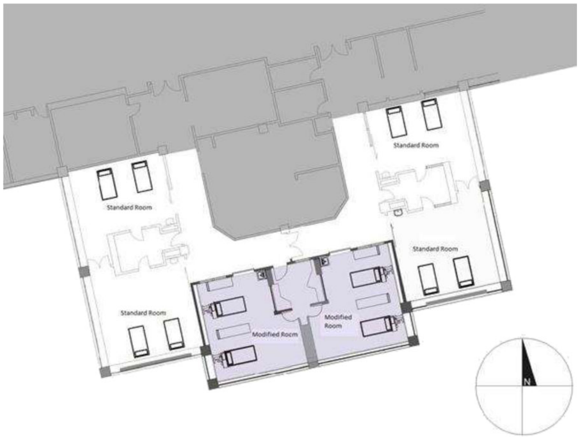

**Fig. 1 (abstract 1305).** ICU at Charité - Universitätsmedizin Berlin with standard rooms and the 2 modified double bed rooms

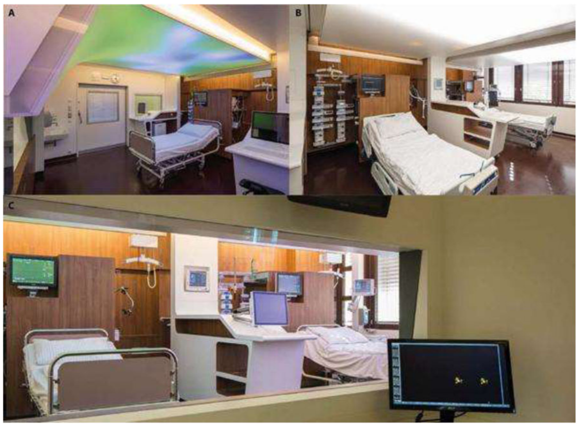

**Fig. 2 (abstract 1305).** Pictures from one of the 2 modified ICU rooms (A and B) and the observation room (C)

**Table 1 (abstract 1305).** Basic Characteristics and Outcomes of Patients in the Standard and Modified Rooms

| | Females % [95% CI] | Males % [95% CI] | Total % [95% CI] |
|---|---|---|---|
| HIV prevalence among adults | 15.7 [14.5–16.8] | 9.3 [8.5–10.1] | 12.6 [11.7–13.5] |
| Prevalence of overall VLS among HIV+ adults, irrespective of awareness of diagnosis or ART status | 81.7 [79.1–84.3] | 69.6 [65.2–74.0] | 77.4 [74.8–80.0] |
| 90-90-90 Target Indicators | | | |
| HIV+ adults who report knowing their HIV+ status (1st 90) | 83.1 [80.7–85.4] | 71.1 [67.4–74.8] | 78.8 [76.6–81.1] |
| Self-reported ART use among HIV+ adults who report knowing their HIV+ status (2nd 90) | 96.0 [94.5–97.5] | 93.8 [90.8–96.8] | 95.3 [93.6–97.0] |
| VLS among HIV+ adults 15-64 who report ART use and knowing their HIV+ status (3rd 90) | 92.2 [90.4–94.0] | 89.9 [86.7–93.2] | 91.5 [89.8–93.2] |

APACHE II = Acute Physiology And Chronic Health Evaluation II.
[a]Values are presented as medians with an interquartile range (25th to 75th) in parentheses.
Intergroup analysis: [b]exact Mann-Whitney U test; [c]Fisher exact test.
A $p < 0.05$ was considered significant.

## Cardiothoracic & vascular surgery

### 1306

#### Lactate-guided therapy after cardiac surgery: a randomized and controlled trial

D.H.d.P. Souza[1], J.P. Almeida[1], J.T. Fukushima[1], F. Galas[1], G. Landoni[2], C. Park[1], G. Oliveira[1], R. Nakamura[1], M. Mourao[1], J.O. Auler Jr.[1], R. Kalil-Filho[1], L.A. Hajjar[1]
[1]Instituto do Coracao, Faculdade de Medicina da USP, Sao Paulo, Brazil; [2]IRCCS San Raffaele Scientific Institute, Milano, Italy
**Correspondence:** L.A. Hajjar

*Intensive Care Medicine Experimental* 2018, **6(Suppl 2):**1306

**INTRODUCTION.** Elevated levels of lactate are associated to poor outcomes in patients undergoing cardiac surgery, since they may represent anaerobic metabolism consequent of imbalanced cellular oxygen consumption and supply. However, whether a lactate-guided resuscitation in the postoperative of cardiac surgery improves outcomes is still unknown.

**OBJECTIVES.** We aimed to determine whether a postoperative hemodynamic therapy based on lactate levels reduces postoperative complications in patients undergoing cardiac surgery, when compared to a postoperative hemodynamic therapy which lactate levels were not disclosed to clinicians.

**METHODS.** We performed a single centre, randomized, double-blind, controlled-parallel trial between October 2016 to December 2017 at the Heart Institute of the University of Sao Paulo, Brazil. Patients undergoing cardiac surgery were randomly assigned to either receive fluids, vasopressors or inotropes by physicians who were aware of lactate levels of patients, or to a group which physicians were not informed about patient's lactate levels during the first 8 hours after ICU admission. Lactate levels were measured every 2h and the aim of the treatment in the lactate group was to reduce lactate levels at least in 20% if lactate levels were higher than 3 mmol/L. Primary outcome was defined as acute kidney failure, defined as Acute Kidney Failure Network (AKIN) score ≥1, at postoperative day 7. Secondary outcomes were the incidence of myocardial ischemia, AKI, requirement of renal replacement therapy, length of stay in the ICU and in the hospital, and mortality rate at 28 days.

**RESULTS.** A total of 280 patients were included. During the first 8 hours after a ICU admission, patients of the lactate group received more fluid than patients of control group, 797 mL (standard deviation[SD] ±789) vs 583 mL (SD ±554), P=0.009. There was no difference regarding lactate levels between groups. At 7 days, 66 of 141 patients (46.8%) in the lactate group and 82 of 139 patients (59.4%) in the control group presented acute kidney injury (P=0.035). No significant differences in secondary outcomes were observed between groups.
**CONCLUSIONS**. Postoperative lactate-guided therapy during the first 8 hours after ICU admission reduces the incidence of acute kidney injury 7 days after surgery in patients undergoing cardiac surgery.

**GRANT ACKNOWLEDGMENT**

This study was sponsored by the Universidade de São Paulo, Brazil.

### 1307
### The relationship of endothelial glycocalix with hemodynamic and metabolic parameters during perioperative period of valve surgery

M. Kirov, Y. Ilyina, N. Izotova, M. Sokolova, D. Volkov, E. Yakovenko, V. Kuzkov
Northern State Medical University, Department of Anesthesiology and Intensive Care, Arkhangelsk, Russian Federation
**Correspondence:** M. Kirov
*Intensive Care Medicine Experimental* 2018, **6(Suppl 2):**1307

**INTRODUCTION.** The endothelial glycocalix (EG) plays an important role in regulation of vascular integrity, permeability, and endothelial activation. The perioperative EG shedding can be associated with capillary leak, hemodynamic disturbances, and increased morbidity.
OBJECTIVE. To assess the response of EG to valve surgery requiring cardiopulmonary bypass.
**METHODS.** Twenty-six adult patients scheduled for elective on-pump valvular interventions were enrolled into a single-centre prospective observational study. The components of EG including Heparan Sulfate Proteoglycan (HSPG) and Syndecan-1 (S1) were measured after the induction of anaesthesia, and at 6 and 24 hrs postoperatively using ELISA Kits SEA565Hu and SEB966Hu (Cloud-Clone Corp., USA). The hemodynamics, blood gases, pro-B-type natriuretic peptide (NT-proBNP), and biochemical parameters were determined perioperatively. The statistical analysis was performed using Mann-Whitney U-test, ANOVA, Spearman's rho, and ROC-analysis. Data are presented as median ($25^{th}$-$75^{th}$ percentiles). p values < 0.05 were regarded as statistically significant.
**RESULTS.** At 6 hrs after surgery, we observed a decrease in HSPG concentration from 6.13 (4.20-9.04) ng/ml to 5.08 (4.18-7.21) ng/ml (p < 0.01), while S1 increased from 0.80 (0.56-1.13) ng/ml to 1.25 (1.04-1.41) ng/ml (p < 0.001). At 24 hrs, HSPG and S1 returned to values close to baseline. The baseline HSPG correlated with systemic vascular resistance index (rho = -0.55, p = 0.004), postoperative cardiac index (rho = 0.43-0.46, p = 0.03), pulse pressure variations (rho = -0.76, p = 0.003), and glucose concentration (rho = -0.50, p = 0.02). In addition, the baseline HSPG concentrations above 5.1 ng/mL predicted postoperative hyperlactatemia (> 2 mmol/l) with AUC 0.75, sensitivity 100% and specificity 58% (p = 0.02). The baseline NT-proBNP demonstrated weak correlation with HSPG concentration at 6 hrs after surgery (rho = 0.42, p = 0.04). The values of HSPG at all stages and the baseline S1 concentration correlated with duration of mechanical ventilation (rho = 0.46-0.49, p < 0.02).
**CONCLUSIONS.** During perioperative period of valve surgery, the EG system demonstrates relatively minor shedding and moderate associations with hemodynamics, metabolic response, and duration of respiratory support.

**GRANT ACKNOWLEDGMENT**

The study was supported by the grant of the President of Russian Federation НШ-3927.2018.7.

### 1308
### Changes in microvascular reactivity after general anaesthesia in elderly patients undergoing cardiac surgery: a predictor of postoperative outcomes

A.-R. Cho[1], H.-J. Lee[1], C. Kang[1], E.-J. Kim[2], J.-Y. Yoon[2], J.-U. Yoon[3]
[1]Pusan National University Hospital, Anesthesia and Pain Medicine, Busan, Korea, Republic of; [2]Pusan National University Dental Hospital, Dental Anesthesia and Pain Medicine, Yangsan, Korea, Republic of; [3]Pusan National University Yangsan Hospital, Anesthesia and Pain Medicine, Yangsan, Korea, Republic of
**Correspondence:** A.-R. Cho
*Intensive Care Medicine Experimental* 2018, **6(Suppl 2):**1308

**Withdrawn**

### 1309
### Effect of cytoadsorbant device on post-cardio-pulmonary bypass inflammation

E. Poli[1], A. Bauer-Dorries[2], C. Marcucci[2], A. Roumy[3], M. Kirsch[3], E. De Stefano[3], L. Liaudet[1], A. Schneider[1]
[1]Centre Hospitalier Universitaire Vaudois, Service de Médecine Intensive Adulte, Lausanne, Switzerland; [2]Centre Hospitalier Universitaire Vaudois, Service d'Anesthésiologie, Lausanne, Switzerland; [3]Centre Hospitalier Universitaire Vaudois, Service de Chirurgie Cardiovasculaire, Lausanne, Switzerland
**Correspondence:** E. Poli
*Intensive Care Medicine Experimental* 2018, **6(Suppl 2):**1309

**INTRODUCTION.** Cardiopulmonary bypass (CPB) is often associated with degrees of complex inflammatory response. This inflammatory reaction, sometimes referred to as "post-pump syndrome", is characterized by activation of numerous inflammation pathways leading, in severe cases to systemic hypotension and organ dysfunction [1, 2]. Cytokine Hemoadsorption (HA) might therefore improve outcomes of patients undergoing cardiac surgery.
**OBJECTIVES.** In this randomized control trial (NCT02775123), we aim to evaluate the safety and efficacy of a cytokine HA on post-CPB inflammatory response in patients undergoing elective cardiac surgery and at risk of post-operative complications.
**METHODS.** 30 patients undergoing elective cardiac surgery were randomly assigned either to standard CPB (control group) or to CPB plus Cytosorb® (Cytosorbents, New Jersey, USA) (Cytosorb group). Our primary outcome was levels of inflammatory cytokines (IL-1a, IL-1b, IL-2, IL-4, IL-5, IL-6, IL-10, TNF-alpha, IFN-gamma, MCP-1) were measured at T0 (after induction of anaesthesia), T2 (end of CPB), T3 (6 hours after CPB initiation) and T4 (24 hours after CPB initiation).
Values are presented as median (IQR). Comparisons were made using chi-square and Wilcoxon signed-rank test. A p value < 0.05 was considered statistically significant.
**RESULTS.** Baseline demographics, peri-operative characteristics and procedures are detailed in Table 1 and 2. There was no difference between the two groups in terms of need for any vasoconstrictor (p=1.0), mean noradrenaline dose on the first post-operative day (p=0.87), need for any inotrope (p=0.682), duration of mechanical ventilation (p=0.305), incidence of AKI (p=1.0), ICU length of stay (p=1.0), ICU and hospital mortality (both p=1.0). Cytokine levels are still pending.
**CONCLUSIONS.** There was no difference in baseline demographics, peri-operative characteristics nor post-operative outcomes between the two sets of patients. Our primary outcome remains yet to be determined as cytokine levels are still pending.

**REFERENCE(S)**

1. Day, J.R.S. and K.M. Taylor, *The systemic inflammatory response syndrome and cardiopulmonary bypass*. International Journal of Surgery, 2005. 3(2): p. 129-140.

2. Paparella, D., T.M. Yau, and E. Young, *Cardiopulmonary bypass induced inflammation: pathophysiology and treatment. An update.* European Journal of Cardio-Thoracic Surgery, 2002. **21**(2): p. 232-244.

**GRANT ACKNOWLEDGMENT**
Study costs were partly covered by Cytosorbents (New Jersey, USA). The company had no influence on its content or decision for submission.

**Table 1 (abstract 1309).** Baseline demographics and perioperative characteristics

| | Control (n=15) | Cytosorb (n=15) | P value |
|---|---|---|---|
| Age (years) | 69 (49-80) | 67 (44-76) | 0.567 |
| Sex (M/F) | 11 (73.3%) | 13 (86.7%) | 0.651 |
| Euroscore II (%) | 5.05 (2.1-7.23) | 3.03 (2.23-9.08) | 1.0 |
| LVEF (%) | 60 (35-65) | 53 (43-57) | 0.25 |
| CPB Duration (min) | 138 (87-207) | 145 (130-183) | 0.461 |
| X Clamp duration (min) | 115 (68-159) | 122 (97-146) | 0.512 |

**Table 2 (abstract 1309).** Procedure type

| | Control (n=15) | Cytosorb (n=15) |
|---|---|---|
| CABG or single valve replacement | 2 | 2 |
| Double Valve replacement | 2 | 0 |
| CAGB + Valve replacement | 5 | 3 |
| Ascending Aorta Procedure | 5 | 5 |
| Others | 1 | 5 |

### 1310

#### Factors associated with post-operative complications within a thoracic surgery enhanced recovery pathway

Y.-L. Nguyen[1], J.-B. Peretout[1], L. de Charentenay[1], A. Bobbio[2], M. Alifano[3], D. Journois[1]
[1]Paris Descartes University, Anesthesiology and Critical Care Department, Paris, France; [2]Paris Descartes University, Thoracic Surgical Department, Paris, France; [3]Paris Descartes University, Surgical Thoracic Department, Paris, France
**Correspondence:** Y.-L. Nguyen
*Intensive Care Medicine Experimental* 2018, **6(Suppl 2):**1310

**INTRODUCTION.** Pulmonary resection surgery is associated with high morbidity [1]. Enhanced recovery pathway (ERP) has been proved to influence post-operative course after colorectal surgery but data on thoracic surgery are scarce[2].

**OBJECTIVES.** To evaluate the factors associated with post-operative complications occurrence within a thoracic surgery enhanced recovery pathway.

**METHODS.** A monocentric observational study was conducted after approval of our local institutional board. All consecutive patients candidates to a lobectomy or segmentectomy entered to the study protocol. Demographic data, clinical data as well as data related to ERP procedures (smoking cessation, pre-operative carbohydrate intake, setting up of a patient controlled regional analgesia, prescription of non-steroidal anti-inflammatory drugs (NSAIDs), chest tube removal at day 2) were prospectively collected.

**RESULTS.** Over a 1 year period, 206 patients, 51% male (n=105), benefited from the ERP in our institution. They had an average of 66 ± 10 years, a median BMI of 25Kg/m$^2$[23-28] and most of them were ASA 2 (n=171, 83%). The majority had lobectomy (n=151, 73%). One-third of patients had post-smoking COPD (n = 67), 41% (n = 84) had hypertension, 11% (n = 23) had diabetes, and 10% (n = 20) had coronary artery disease. Among the procedures associated with the ERP, 65% (n = 100) of smokers stopped tobacco before their intervention, 71% (n = 146) of patients had carbohydrates preoperatively, 44% (n = 91) had a patient controlled loco-regional anesthesia pump, 52% (n = 107) had NSAIDs and 54% (n = 111) had their chest tube removed on D2 postoperatively. The overall complication rate was 32% (N = 66), of which 18% (n = 37) was respiratory (pneumonia (n=17; 46%); hemothorax (n=6; 16%)), the median hospital stay was 7 days [6-9], and 83% (n = 172) patients have returned home. The results of univariate analysis found that the presence of COPD (p < 0.0001), hospitalization in the intermediate care unit (p = 0.002), active smoking (p=0.02), the lack of prescription of NSAIDs (p = 0.03) and the presence of chest tube at day 2 (p = 0.01), were associated with a higher postoperative complications rate. The multivariate analyzes showed that the hospitalization in the intermediate care unit (p=0.02; OR=3.53 [1.22-10.22]) and the presence of chest tube at day 2 were independent factors associated with complications (p< 0.0001; OR=4.69 [2.17-10.17]).

**CONCLUSIONS.** Within a thoracic surgery enhanced recovery pathway, less than one in five patients suffered from respiratory complications. Hospitalization in an intermediate care unit and the presence of chest tube at day 2 are associated with the occurence of post-operative complications.

**REFERENCE(S)**
[1] Thomas PA et al. Eur J Cardiothorac Surg 2014
[2] Fiore JF Jr et al. J Thorac Cardiovasc Surg. 2016

**GRANT ACKNOWLEDGMENT**
None.

### 1311

#### Comparison of coagulation and fibrinolysis markers concentrations between patients undergoing vascular procedures for abdominal aortic aneurysm, peripheral artery disease and a control group

K. Polok[1], J. Górka[1], T. Iwaniec[2], J. Fronczek[1], W. Szczeklik[1]
[1]Jagiellonian University Medical College, Department of Intensive Care and Perioperative Medicine, Kraków, Poland; [2]Jagiellonian University Medical College, 2nd Department of Internal Medicine, Kraków, Poland
**Correspondence:** K. Polok
*Intensive Care Medicine Experimental* 2018, **6(Suppl 2):**1311

**INTRODUCTION.** There are 10-20 million vascular procedures performed worldwide each year. Patients undergoing vascular surgeries are at high risk of perioperative cardiac complications. It was recently proven that preoperative abnormalities in coagulation and fibrinolysis are associated with higher incidence of myocardial injury after noncardiac surgery.[1]

**OBJECTIVES.** The aim of our study was to compare concentrations of coagulation (Factor VIII, von Willebrand Factor: Ag) and fibrinolysis (D-Dimer) markers between control group, patients undergoing surgeries for abdominal aortic aneurysm (AAA) and those undergoing procedures for peripheral artery disease (PAD).

**METHODS.** We enrolled 131 patients ≥ 45 years old undergoing surgery for abdominal aortic aneurysm (54 cases) or peripheral artery disease (77 cases) under general or regional anesthesia, hospitalized at least one night after the procedure. Patients undergoing carotid artery surgery and those who had bypass graft thrombosis, cancer, renal failure defined as eGFR < 30 ml/min/1.73 m$^2$, venous thromboembolism 3 months prior to surgery or acute infection were excluded from the study. Additionally, we enrolled a control group comprising of 40 volunteers. In patients undergoing vascular procedures blood samples were obtained within 24 hours before surgery.

**RESULTS.** The mean age of study group was 66.4 (9.2) years. Control group comprised of significantly younger individuals, however the difference between AAA and PAD groups was not significant. Clinical characteristics of study group are presented in Table 1. We have observed significant differences between control group, patients undergoing surgery for AAA and those undergoing procedure for PAD in serum concentrations of Factor VIII (89.6 vs. 141.9 vs. 176.9 %, p< 0.001), von Willebrand Factor: Ag (116.7 vs. 198.0 vs. 314.5 %, p< 0.001) and D-Dimer (265.0 vs. 2590.5 vs. 808.0 ng/ml, p< 0.001).

**CONCLUSIONS.** We believe that presented results suggest that activity of both coagulation and fibrinolytic systems is higher in patients undergoing vascular surgery compared to general population. Among them patients undergoing surgery for PAD seem to be at the highest risk of perioperative complications due to particularly pronounced hypercoagulation and lower activity of fibrinolysis.

**REFERENCE(S)**

1. Górka J, Polok K, Iwaniec T, et al. Altered preoperative coagulation and fibrinolysis are associated with myocardial injury after non-cardiac surgery. Br J Anaesth 2017;118(5):713-9.

**GRANT ACKNOWLEDGMENT**

Work supported by the grant from the Polish Ministry of Science and Higher Education, no: NN402083939 and DI201102141.

**Table 1 (abstract 1311).** Study group characteristics

| | Control (N=40) | AAA (N=54) | PAD (N=77) |
|---|---|---|---|
| Age, mean (SD) | 60.0 (9.5) | 69.5 (7.4) | 67.4 (8.8) |
| Gender, males | 30 (75.0%) | 45 (83.3%) | 55 (71.4%) |
| Smoking | 16 (40.0%) | 45 (83.3%) | 73 (94.8%) |
| Hypertension | 10 (25.0%) | 43 (79.6%) | 62 (80.5%) |
| Diabetes mellitus | 2 (5.0%) | 6 (11.1%) | 22 (28.6%) |
| Atrial fibrillation | 1 (2.5%) | 5 (9.3%) | 12 (15.6%) |
| Coronary artery disease | 1 (2.5%) | 24 (44.4%) | 44 (57.1%) |
| History of stroke/TIA | 0 (0.0%) | 2 (3.7%) | 10 (13.0%) |

### 1312

#### Use of mechanical support with ECMO in refractory respiratory failure due to reperfusion edema during postoperative care of pulmonary endarterectomy

P. Pagliarani Gil[1], I. Martín Badía[1], F.P. Delgado Moya[1], J.L. Pérez Vela[1], M.A. Corres Peiretti[1], C.A. Gotor Pérez[2], M.J. López Gude[2], E. Renes Carreño[1], J.C. Montejo González[1]
[1]Hospital Universitario 12 de Octubre, Intensive Care Unit, Madrid, Spain; [2]Hospital Universitario 12 de Octubre, Cardiac Surgery, Madrid, Spain
**Correspondence:** P. Pagliarani Gil
*Intensive Care Medicine Experimental* 2018, **6(Suppl 2):**1312

**INTRODUCTION.** Patients with chronic thromboembolic pulmonary hypertension undergoing pulmonary endarterectomy (PEA) may develop respiratory failure due to reperfusion edema with or without right ventricular (RV) failure, being occasionally, refractory to all standard therapeutic measures. These patients could benefit from mechanical respiratory and circulatory support.

**OBJECTIVES.** To describe the results of a case series following PEA with refractory respiratory failure needing the assistance of extracorporeal membrane oxygenation (ECMO).

**METHODS.** A retrospective observational study based on a prospectively collected database was made. All patients with the previous characteristics admitted to the Intensive Care Unit (ICU) from January 2010 to April 2018 were included. We analyzed variables such as demographic, hemodynamic, length of stay in ICU and hospital, hours of mechanical ventilation and ECMO assistance, complications and mortality. The quantitative variables are presented as median and 25th-75th percentile range and the qualitative ones, as percentage.

**RESULTS.** Among all of the 225 PEA during this period, 16 patients (7,1%) required ECMO support. The median age was 49 years (IQR 41-68), 50% women. A veno-venous ECMO (VV) was implanted in 56%, while 44% had, besides, RV failure and/or prominent pulmonary hypertension, needing the veno-arterial (VA) configuration. However, 3 conversions from ECMO VA to VV were required (due to 2 Harlequin syndromes and 1 large arterial cannula bleeding) and 1 conversion from VV to VA due to RV failure, making a total of 20 ECMO devices. Peripheral access was chosen in 95% of the cases. Seven were femoral-femoral, 12 femoral-jugular and the central one, was right atrium-aortic. The underlying pathology was respiratory failure due to reperfusion edema, adding RV failure in 44% and diffuse alveolar hemorrhage in 12%. A median ECMO support of 180 hours was recorded (ICR 144-291). Eleven patients (69%) presented complications associated with the device, each of which, 2 cardiac tamponade requiring surgical reintervention, 1 hemorrhagic stroke, 3 cannula bleedings (1 required change of ECMO), 4 minor bleedings that did not require blood transfusion, 1 lower extremity ischemia and 1 thrombosis. Regarding the patients, the median time of mechanical ventilation were 461 hours (IQR 268-588), the length of stay at the ICU was 25 days (IQR 17-31) and at the hospital of 33 (IQR 19-41). The overall ICU mortality was 56%.

**CONCLUSIONS.** Occasionally, PEA may present a postoperative course with refractory severe respiratory complications, such as reperfusion pulmonary edema, with or without RV failure, requiring mechanical support. The ECMO assistance has shown to be helpful in this group of patients. The results are comparable to those published in this context.

**REFERENCE(S)**

López Gude MJ, et al. Pulmonary Thromboendarterectomy in 106 Patients With Chronic Thromboembolic Pulmonary Hypertension. Arch Bronconeumol. 2015;51:502-8

### 1313

#### Diaphragmatic dysfunction following thoracic surgery: an echographic assessment

M. Daniel[1,2], T.M. Huynh[3], J. Martin[3], A. Ferraris[3], K. Guessous[3], D. Zlotnik[3], L. Gouzien[3], O. Chaouch[3], P. Delval[3], C. Pricopi[4], F. Le Pimpec-Barthes[4], B. Cholley[5]
[1]Hôpital Européen Georges Pompidou, Paris, France; [2]Paris Descartes University, Paris, France; [3]Hôpital Européen Georges Pompidou, Anesthésie-Réanimation, Paris, France; [4]Hôpital Européen Georges Pompidou, Chirurgie Thoracique, Paris, France; [5]Hôpita Européen Georges Pompidou, Anesthésie-Réanimation, Paris, France
**Correspondence:** M. Daniel
*Intensive Care Medicine Experimental* 2018, **6(Suppl 2):**1313

**INTRODUCTION.** Postoperative diaphragmatic dysfunction has been described after cardiac and abdominal surgery. However, there is a lack of data regarding thoracic surgery. Echography is a non-invasive procedure allowing repeated assessment of diaphragmatic excursion and thickening fraction at the bedside.

**OBJECTIVES.** Our goal was to describe the time course of, both, diaphragmatic excursion and thickening fraction following elective thoracic surgery.

**METHODS.** We conducted a prospective, single-center observational study in consecutive patients undergoing thoracic surgery between January 8$^{th}$ and March 11$^{th}$ 2018. Patients were recruited after providing informed consent and the study was approved by an ethical committee. Diaphragmatic excursion and thickening fraction of both hemi-diaphragms were measured by two observers blinded to each other at 3 different time-points : prior to surgery (D-1), immediately after extubation (D0), and at postoperative day 3 (D3). The time-course of excursion (cm) and thickening fraction (%) were compared according to the side (operated or non-operated) using a two-way ANOVA. In case of significant interaction, multiple comparisons were performed using a Student-Newman-Keuls test.

**RESULTS.** 50 patients aged 60 +/- 15 years have been included. Sixty percent were males, 30 patients underwent lobectomy, 17 tumorectomy and 3 pneumonectomy. We observed a significant decrease in diaphragmatic excursion (Figure 1) and thickening fraction (Figure 2) of the diaphragm on the operated side at D0 and D3 with respect to D-1. These values remained unchanged over the study period on the non-operated side.

**CONCLUSIONS.** Thoracic surgery is responsible for a diaphragmatic dysfunction on the operated side, which is noted immediately after extubation and persists at least up to the third postoperative day. No alteration of diaphragmatic excursion or thickening fraction was observed on the non-operated side.

**GRANT ACKNOWLEDGMENT**

Many thanks to the paramedical staff of recovery room and thoracic surgery department

### 1314

#### Rhabdomyolysis after elective cardiac surgery procedures with the use of cardiopulmonary bypass: a single center study

F. Ampatzidou, C. Mavromanolis, A. Dimaki, A. Madesis, T. Karaiskos, O. Ananiadou, G. Kechagioglou, G. Drossos

G.Papanikolaou Hospital, Thessaloniki, Greece

**Correspondence:** F. Ampatzidou

*Intensive Care Medicine Experimental* 2018, **6(Suppl 2):**1314

**INTRODUCTION.** Rhabdomyolysis syndrome characterized by dissolution of skeletal muscles and release of myoglobulin into the bloodstream.

**OBJECTIVES.** To investigate perioperative factors correlated with Rhabdomyolysis following elective cardiac surgery with use of cardiopulmonary bypass in Cardiothoracic Department G.Papanikolaou Hospital Thessaloniki Greece.

**METHODS.** Patients who underwent elective cardiac surgery from June 2012 to February 2018 and had postop Creatine kinase (CK) value > 2,000 U/l were included. We have exluded from the study: 179 urgent/emergent operations, 22 off pump procedures and 6 perioperative myocardial infarction patients.

The following prospectively collected data were analyzed: Age, Gender, Euro-score II, Smoking habit, REDO operations, Cardio Pulmonary Bypass(CPB) time >120 min, Acute Kidney Injury-AKI(defined by RIFLE criteria), renal replacement therapy(RRT) because of AKI, use of Intra-aortic balloon(IABP), Multiple Organ Dysfunction Syndrome(-MODS) and Mortality.

Chi square test was used for the statistical analysis.

**RESULTS.** In total 2495 patients were included. Rhabdomyolysis was present in 73(2.9%)pts(25 females), consisting Group A, mean aged 65.7 ±10.1 vs 64.5 ±10.6, Euroscore II 4.5±5.7 vs 1.95±2.1 of the rest of the cohort. No relationship was found with smoking (p=0.114) and REDO procedures(p=0.171). Factors with statistical significant relationship are shown in table 1.

**CONCLUSIONS.** Postoperative rhabdomyolysis after elective cardiac surgery has statistical significant correlation with prolonged CPB time, IABP use, postoperative AKI, AKI-RRT, MODS and higher mortality.

**REFERENCE(S)**

El-Abdellati E.et al. An observational study on rhabdomyolysis in the intensive care unit. Exploring its risk factors and main complication: acute kidney injury. *Annals of Intensive Care*. 2013;3:8

**Table 1 (abstract 1314).** Rhabdomyolysis related factors

| | Rabdomyolysis group(n=73) | Control Group (n=2422) | p value |
|---|---|---|---|
| CPB>120 min, n(%) | 73(100%) | 0 (0%) | <0.01 |
| IABP, n(%) | 17 (23.2%) | 102(4.2%) | <0.01 |
| AKI, n(%) | 25(34.2%) | 326(13.5%) | <0.01 |
| AKI-RRT, n(%) | 5(6.8%) | 65 (2.7%) | 0.034 |
| MODS, n(%) | 6(8.2%) | 27(1.1%) | <0.01 |
| Mortality , n(%) | 4(5.5%) | 69(2.8%) | 0.044 |

### 1315

#### The effect of ERAS fluid protocol on platelet count and neutrophil-lymphocyte ratio (COP-NLR), mortality and morbidity in resectable lung cancer cases

S. Sen[1], S. Cokpinar[2], N. Kanlioglu[3], I. Kurt Omurlu[4], S. Sen[3]

[1]Adnan Menderes University Medical Faculty, Anesthesiology and Reanimation Department, Aydın, Turkey; [2]Adnan Menderes University Medical Faculty, Thoracic Surgery, Aydin, Turkey; [3]Adnan Menderes University Medical Faculty, Thoracic Surgery, Aydın, Turkey; [4]Adnan Menderes University Medical Faculty, Statistics, Aydın, Turkey

**Correspondence:** S. Sen

*Intensive Care Medicine Experimental* 2018, **6(Suppl 2):**1315

**INTRODUCTION.** The European Lung Cancer Working Group and others studies suggested that a high preoperative neutrophil / lymphocyte ratio (NLR) might be a convenient biomarker to identify patients with a poor prognosis after resection for non-small cell lung cancer (NSCLC) (2). Although there are variations in ERP among the institutions, the evidence suggests that implementation of Enhanced Recovery After Surgery (ERAS) in major surgery significantly reduces postoperative complications and length of hospital stay. (3).

**OBJECTIVE.** This study aimed to investigate the effect of ERAS fluid protocol on platelet count and neutrophil-lymphocyte ratio (COP-NLR), mortality and morbidity in lung resectable cancer cases.

**METHODS.** Patients' clinical characteristics and laboratory test data at initial diagnosis were collected at our institute. The COP-NLR was calculated on the basis of data obtained. Patients with both an increased platelet count (>275 $\times 10^3$ $mm^{-3}$) and an elevated NLR (>2.5) were assigned a score of 2, and patients with on or neither were assigned as a score of 1 or 0, respectively.

**RESULTS.** A total of 97 NSCLC patients were enrolled in this analysis between 2012 and 2017. ERAS fluid protocol was applied in 42 patients. There were no differences in terms of age, sex and duration of operation between two groups. Postoperative COP-NLR values were lower in the ERAS group but mortality rates were not different. Duration of the stay in intensive care unit, intraoperative hypotension, inotropic requirement, and cardiac complication development risk were also less in ERAS group.

**CONCLUSION.** A lower detection of COP-NLR on postoperative day 1 in cases of pulmonary resection with ERAS protocol may be used as a marker for lower complication rates.

### 1316

#### Relationship between hemoglobin level at admision and renal injury during hospital stay and after in patients undergoing cardiac surgery

M.R. Diaz Contreras, R. De la Chica Ruiz-Ruano, F. Villagran Ramirez
Hospital Universitario Virgen de las Nieves, Intensive Care Unit, Granada, Spain
**Correspondence:** F. Villagran Ramirez
*Intensive Care Medicine Experimental* 2018, **6(Suppl 2):**1316

**INTRODUCTION.** Renal injury is a frequent and serious complication in cardiac surgery patients. Although it can usually recover completely in many patients, a significant number can can maintain altered renal function over time after hospital discharge. In previous studies we found that age, COPD presence and the NYHA grade were prognostic factors in the development of permanent kidney injury in post-operative cardiac surgery patients.

**OBJECTIVES.** Our target is to analyze whether hemoglobin levels at admission in patients who are going to be subjected to cardiac surgery in our hospital can influence the development of permanent kidney injury.

**MATERIAL and METHODS.** We used the data from the base of the Andalusian registry of cardiac surgery within the plan of integral attention to heart diseases, referring to the patients operated in our hospital and admitted to our Intensive Care Unit of the Hospital Virgen de las Nieves de Granada, in a period of five years.

RESULTS: 2,443 patients underwent cardiac surgery and were discharged from the hospital alive. Of these, 1,497 had normal renal function prior to surgery (defined by plasma creatinine ≤ 1.2 mg). A total of 149 were discharged with a plasma creatinine that exceeded 0.3 mg basal values and / or an increase of 50% of these values, regardless of what happened during their hospital stay. The most relevant baseline characteristics of patients with and without kidney injury were:

To the previous data we have added the study of preoperative hemoglobin and the maximum peak of creatinine during the hospital stay of each patient. Using Pearson´s correlation, we compared two quantitative varibles of an independent group finding an association between maximum creatinine and preoperative hemoglobin levels with a significance level of 0.01. This level of significance has also been found when comparing the variables of creatinine levels at discharge and preoperative hemoglobin.

**CONCLUSIONS.** Preoperative hemoglobin levels are associated with the development of renal injury (maximum creatinine during hospital stay and after discharge) in patients undergoing cardiac surgery. Therefore they should be taken into account especially in the preoperative study and be optimized pre-intervention.

Factores asociados a insuficiencia renal postoperatoria en cirugía de revascularización miocárdica Albert F. Guerrero B. a,b,⁎, Jaime Camacho M. a, Néstor F. Sandoval c, Juan P. Umana˜ M. d, Carlos E. Obandoa y Marisol Carreno˜

**Table 1 (abstract 1316).** Most relevant baseline characteristics of patients with and without kidney injury

| | Kidney injury abscense (1678) | | Kidney injury present (149) | | p |
|---|---|---|---|---|---|
| | Average | Typical deviation | Average | Typical deviation | |
| Age | 61.44 | 13.59 | 67.30 | 10.64 | 0.00 |
| Preoperative creatinine | 0.89 | 0.17 | 0.86 | 0.19 | 0.05 |
| Ejection fraction | 59.25 | 10.52 | 57.43 | 11.07 | 0.04 |
| Extracorporeal time | 112.37 | 45.91 | 108.14 | 42.94 | 0.3 |
| Aortic clamp time | 85.61 | 38.33 | 86.09 | 38.47 | 0.88 |
| Creatinine at discharge | 0.80 | 0.20 | 1.49 | 0.77 | 0.000 |
| Logistic EuroSCORE | 6.94 | 7.85 | 9.55 | 11.38 | 0.000 |

### 1317

#### Low functional grade of mobilizing capacity predict worse survival to lung transplantation in patients receiving immediate post-transplant pulmonary rehabilitation in intensive care unit

N.E. Kim[1], J.-E. Park[1], S.C. Lee[2], S. Kim[2], S.Y. Kim[1], J.C. Shin[2], J.G. Lee[3], H.C. Paik[3], M.S. Park[1], J.H. Song[1]
[1]Institute of Chest Disease, Severance Hospital, Yonsei University College of Medicine, Pulmonology, Seoul, Korea, Republic of; [2]Research Institute of Rehabilitation Medicine, Severance Rehabilitation Hospital, Yonsei University College of Medicine, Department of Rehabilitation Medicine, Seoul, Korea, Republic of; [3]Yonsei University College of Medicine, Department of Thoracic and Cardiovascular Surgery, Seoul, Korea, Republic of
**Correspondence:** N.E. Kim
*Intensive Care Medicine Experimental* 2018, **6(Suppl 2):**1317

**INTRODUCTION.** Physical activity has been suggested to influence perioperative outcome after lung transplantation.

**OBJECTIVES.** We aimed to investigate the association between improvement of physical activity and early postoperative outcomes in lung transplant recipients who received early mobilization physical therapy initiated in intensive care unit (ICU).

**METHODS.** We retrospectively evaluated a total of 22 patients who received pulmonary rehabilitation initiated in ICU within 2 weeks after lung transplantation at Severance Hospital Yonsei University from March 2015 to February 2016. Physical activity levels were graded according to our institutional pulmonary rehabilitation program: grade 1, bedside (G1); grade 2, dangling (G2); grade 3, standing (G3); grade IV, gait (G4), and then weekly graded throughout pulmonary rehabilitation.

**RESULTS.** Fourteen (64%) patients were male: median age, 57 years (range, 25-73). Initial physical activity levels were G1 in 9 patients, G2 in 7, G3 in 4, and G4 in 2. According to final physical activity levels, patients were dichotomized as responders with G4 (n = 14) or non-responders who never achieved G4 or down-graded to a lower level (n = 8). The mMRC dyspnea scale and the NYHA functional class were significantly lower in responders compared to non-responders ($p$=0.074; $p$=0.022). Durations of propofol and remifentanyl infusion were also significantly lower in responders ($p$=0.021; $p$=0.006). The 6-months mortality was significantly lower in responders (21.4% *vs.* 87.5%, $p$=0.006). After a median follow-up of 13.4 months (range, 7.0-16.0) after lung transplantation, cumulative survival outcomes were 61.2% in responders and 12.5% in non-responders ($p$< 0.001).

**CONCLUSIONS.** This study, we showed that patients who achieved and maintained an improved physical activity (ambulatory) had increased survival after lung transplantation.

### 1318

#### Extra-corporeal membrane oxygenation (ECMO) for refractory cardiogenic shock after adult cardiac surgery: pilot experience in a new referral center

E. Casanova-Gongora[1], E.M. Santafosta-Gomez[1], J.C. Lopez-Delgado[1,2], P. Sastre-Perez[1], G. Moreno-Gonzalez[1], A. Farre-Estebe[1], S.M. Luna-Solis[1], F. Esteve-Urbano[1], R. Mañez-Mendiluce[1,2]
[1]Hospital Universitari de Bellvitge, Intensive Care Medicine, Barcelona, Spain; [2]Institut d´Investigació Biomèdica de Bellvitge, L'Hospitalet de Llobregat, Spain
**Correspondence:** J.C. Lopez-Delgado
*Intensive Care Medicine Experimental* 2018, **6(Suppl 2):**1318

**INTRODUCTION.** ECMO post-cardiac surgery has emerged as a supportive therapy in Postcardiotomy cardiogenic shock refractory to inotropic and intra-aortic balloon pump support.

**OBJECTIVES.** The aim of our study was to show outcomes in a post-cardiac surgery specialized unit but new ECMO referral center.

**METHODS.** Prospective, observational study at our institution from November 2016 to March 2018 at our institution. We included those patients who needed ECMO due to postcardiotomy cardiogenic shock during the study period. Baseline characteristics, intraoperative variables and outcomes were analyzed, together with laboratory data.

**RESULTS.** 16 patients were included; age 61.1±10.3 years; 62.5%(10) were male; BMI:27.6±4.1Kg•m$^{-2}$; APACHE II:18±8; SOFA: 9.3±3.4. 56.25%(9) were bypass and 31.25%(5) were valvular surgeries. In-hospital mortality was 37.5%(6) and 1-year mortality 43%(7). ECMO was performed during the first 72h in our patients with a mean ECMO support of 6±3.2 days. During ECMO, 37.5%(6) suffer bleeding complications, 31.25%(5) re-exploration, 50%(8) sepsis and none thrombosis. We did not find differences in terms of preoperative, intraoperative and outcomes between survivors and non-survivors subgroups but there was a trend towards more need for Platelet (units) during ECMO in non-survivors (2.0±1.95 vs.5.17±2.13;*P=0.01*). We also showed higher arterial lactate on day 3 (1.80±0.66vs.7.2±7.38 mmol·L$^{-1}$;P=0.03) in those who died.
**CONCLUSIONS.** In our short experience, haemodynamic support with ECMO may provide a survival benefit with reasonable intermediate and long-term outcomes. Perfusion laboratory values and bleeding related variables seemed to be associated with worst prognosis in our small population.

### 1319

#### Exploring the use of an innovative technology for pain assessment during mediastinal tube removal in cardiac surgery patients in the intensive care unit: the Nociception Level (NOL) Index$^{TM}$

C. Gélinas[1,2], P. Richebé[3,4]
[1]McGill University, Ingram School of Nursing and Alan Edwards Centre for Research on Pain, Montreal, Canada; [2]Jewish General Hospital - CIUSSS Centre-Ouest-Ile-Montreal, Centre for Nursing Research and Lady Davis Institute, Montreal, Canada; [3]Université de Montréal, Département d'Anesthésiologie et de Médecine de la Douleur, Montreal, Canada; [4]Hôpital Maisonneuve-Rosemont - CIUSSS Est-Ile-Montreal, Anesthésie, Montreal, Canada
**Correspondence:** C. Gélinas
*Intensive Care Medicine Experimental* 2018, **6(Suppl 2):**1319

**INTRODUCTION.** The patient´s self-report is the gold standard measure of pain; however, it is not possible to obtain it in all patients in the intensive care unit (ICU) due to inability to communicate. In such situations, behavioral scales are alternative measures as no physiologic methods are available.[1] Indeed, single physiologic parameters (e.g., heart rate, blood pressure) are not valid for ICU pain assessment.[2] The newly developed Nociception Level (NOL) index (Medasense, Israel) incorporates several physiologic parameters to estimate the pain level.[3] It has been validated in anesthesia,[3-4] but its use in the ICU is new.
**OBJECTIVES.** This study explored the ability of the NOL index to detect pain during mediastinal tube removal (MTR) in cardiac surgery ICU patients.
**METHODS.** A prospective cohort study design was conducted in an ICU in Montreal, Canada. Adult patients admitted for elective cardiac surgery and able to self-report postoperatively were eligible. Patients were asked to self-report their pain intensity with the 0-10 Numeric Rating Scale (NRS). The NOL index is a single number from 0 to 100 obtained through a non-invasive finger probe, and for patients under general anesthesia values >25 are indicative of pain. It incorporates heart rate, heart rate variability, plethysmograph wave amplitude, skin conductance level, number of skin conductance fluctuations, and their time derivatives, using a non-linear regression algorithm to estimate the pain level.[3] Data was collected at rest pre-procedure (T1), during MTR procedure (T2), and 15 minutes post-procedure (T3). The sample and study variables were described, and ROC curve analysis of the NOL index for the detection of the patient´s pain intensity at T2 was performed.
**RESULTS.** Fifty-four cardiac surgery ICU participants (72% males, mean age=66) were included. Descriptive findings (median, percentiles 25%-75%) were the following at T1 [pain intensity (2.00, 0.00-5.00), NOL (19.11, 7.64-26.16)], at T2 [pain intensity (6.00, 3.00-9.00), NOL (28.90, 23.23-35.74)], and at T3 [pain intensity (3.00, 0.00-5.00), NOL (8.62, 2.76-15.82)]. At T2, the NOL (>34) showed a sensitivity of 80% and a specificity of 70% to classify participants with no or mild pain (0-4) to those with significant pain (5-10) with an area under the curve (AUC) of 0.79 (95% CI:0.63-0.94).
**CONCLUSIONS.** Pain intensity and NOL values were higher during MTR compared to rest pre/post. The NOL's ability to detect pain during MTR in this ICU group was good. Some factors (e.g., anxiety, stress) in awake patients may be reflected in the study results. Research on the NOL's validity for ICU pain assessment is on-going.

**REFERENCE(S)**

1. Barr J, Crit Care Med 2013; 41:263-306.
2. Gélinas C, Int Crit Care Nurs 2016 ;34:1-11.
3. Ben-Israel N, J Clin Monit Comput 2013 ;27:659-68.
4. Martini CH, Anesthesiology 2015;123:524-34.

**GRANT ACKNOWLEDGMENT**
Funded by Louise & Alan Edwards Foundation and Quebec Nursing Intervention Research Network.

### 1320

#### Evaluation of patients with the diagnosis of vasculitis admitted to a medical ICU

E. Ortac Ersoy[1], S. Ozen[1], B. Er[1], S. Ocal[1], A. Erden[2], G. Yardimci[2], O. Karadag[2], A. Topeli[1]
[1]Hacettepe University Faculty of Medicine, Medical Intensive Care Unit, Ankara, Turkey; [2]Hacettepe University Faculty of Medicine, Division of Rheumatology, Ankara, Turkey
**Correspondence:** E. Ortac Ersoy
*Intensive Care Medicine Experimental* 2018, **6(Suppl 2):**1320

**INTRODUCTION.** Systemic vasculitis may cause life threatening complications requiring admission to the intensive care unit (ICU).
**OBJECTIVES.** The aim of this study was to evaluate general characteristics and outcome of patients with systemic vasculitis admitted to a medical ICU.
**METHODS.** We retrospectively reviewed the medical records of 1496 patients diagnosed with vasculitis and followed in the Hacettepe University Faculty of Medicine Vasculitis Centre between January 2014 and January 2018 and selected those admitted to the medical ICU.
**RESULTS.** In 4 years, 18 patients (1.2%) were admitted to the medical ICU with 23 ICU admissions (5 repeated admissions). Ten (55.6%) of them were male and mean (±SD) age was 54.9±20.0 years. The most common vasculitis was ANCA associated vasculitis (n=11). Others were temporal arteritis (n=2), Goodpasture (n=1), Behcet's disease (n=1), Polyarteritis nodosa (n=1), *Cryoglobulinemic vasculitis* (n=1) and Cerebral vasculitis (n=1). The reasons for ICU admission were pneumonia (n=7), alveolar hemorrhage (n=6), pulmonary edema (n=6), acute kidney injury (n=1), ARDS (n=1), pulmonary thromboembolism (n=1) and gastrointestinal bleeding (n=1). Mean APACHE II and SOFA scores were 19.4±10.1 and 7.2±5.4, respectively. Invasive mechanical ventilation was applied in 18 admissions. Pulse steroid treatment was given in 9 admissions. Plasmapheresis was applied in 8 admissions (5 alveolar hemorrhage and 3 pulmonary edema) during the ICU stay. Mean ICU length of stay was 15.2±12.8 days. Five (31.3%) patients (4 ANCA associated, 1 cerebral vasculitis) died in the ICU due to pneumonia (n=3) and alveolar hemorrhage (n=2).

**CONCLUSIONS.** ANCA associated vasculitis was the most common vasculitis with high mortality rate, pneumonia and alveolar hemorrhage being the most common causes of admission in our ICU.

### 1321

#### Small-vessel vasculitides patients have the highest mortality among systemic autoimmune diseases patients in intensive care unit: a retrospective study with 5-year follow-up

A. Włudarczyk[1], K. Polok[1], J. Górka[1], G. Biedroń[2], J. Musiał[2], W. Szczeklik[1]
[1]Department of Intensive Care and Perioperative Medicine, Jagiellonian University Medical College, Kraków, Poland; [2]Jagiellonian University Medical College, Kraków, Poland
**Correspondence:** J. Górka
*Intensive Care Medicine Experimental* 2018, **6(Suppl 2):**1321

**INTRODUCTION.** Systemic autoimmune diseases are a heterogeneous group of disorders associated with dysfunction of multiple organs and unpredictable course. Complicated management and treatment becomes even more challenging for patients requiring critical care.
**OBJECTIVES.** This study aims to compare outcomes of small-vessel vasculitides (SSV) and other systemic autoimmune diseases (SAD) patients admitted to the intensive care unit (ICU).
**METHODS.** Retrospective, observational study conducted in the ICU of Allergy and Immunology Department at the University Hospital in Krakow, Poland, between years 2001-2014, and with 5-years follow-up after ICU discharge and no lost to follow-up patients.
**RESULTS.** 74 patients with autoimmune diseases were enrolled in the study. 23 with SSV and 51 with other SAD. Patients in the SVV group achieved higher scores in APACHE II (28 vs 21.5 points, p=0.02) and III (94 vs 72 points, p=0.04), SAPS II (58 vs 36 point, p< 0.001) and SOFA at ICU admission. The SVV patients required renal replacement techniques, blood products transfusion and immunosuppressive treatment more often. SVV patients had higher ICU mortality (60,9% vs. 35,3%, p=0.04), however after discharge from ICU, in long term follow-up (1-year and 5 years) mortality was similar in both studied groups.
**CONCLUSIONS.** Among systemic autoimmune diseases small vessel vasculitides appear to be associated with the highest ICU mortality, higher requirement for advanced procedures and aggressive immunosuppressive therapy.

##### REFERENCE(S)

1. Monti, S., et al., *Life-threatening onset of systemic vasculitis requiring intensive care unit admission: a case series.* Clin Exp Rheumatol, 2015. **33**(2 Suppl 89): p. S-126-31.
2. Dumas, G., et al., *Outcomes in critically ill patients with systemic rheumatic disease: a multicenter study.* Chest, 2015. **148**(4): p. 927-35.
3. Jennette, J.C., *Overview of the 2012 revised International Chapel Hill Consensus Conference nomenclature of vasculitides.* Clin Exp Nephrol, 2013. **17**(5): p. 603-6.

### 1322

#### Risk of major bleeding associated with aspirin use in critically Ill medical patients receiving therapeutic anticoagulation

Y. Subat, H. Rayes, K. Evans, T. Weister, V. Trivedi, R. Kashyup, O. Gajic, M. Warner
Mayo Clinic, Rochester, United States
**Correspondence:** Y. Subat
*Intensive Care Medicine Experimental* 2018, **6(Suppl 2):**1322

**Withdrawn**

### 1323

#### A global ergonomic evaluation of two new generation transport monitors in immersive simulation

E. L'Her[1,2], B. Arbellot-Repaire[1], P. Monfort[1], S. Antin[1]
CESIM Santé, Faculté de Médecine, Université de Bretagne Occidentale, Brest, France; [2]LATIM INSERM UMR 1101, FHU Techsan, Université de Bretagne Occidentale, Brest, France
**Correspondence:** E. L'Her
*Intensive Care Medicine Experimental* 2018, **6(Suppl 2):**1323

**INTRODUCTION.** Multiple serious adverse events may occur during intrahospital transport. Physiological monitoring (e.g. ECG, SpO2… etc…) is a fundamental and the accuracy/ergonomy of transport monitoring systems are of utmost importance.
**OBJECTIVES.** The aims of this study were to provide a comprehensive evaluation of recent transport monitors, taking into account their « *time to operate* », error rate and psycocognitive evaluation.
**METHODS.** The study was performed in a university healthcare simulation center, affiliated to the LATIM INSERM UMR 1101, that has developed a global evaluation model [1]. Two devices were compared, Philips Companion X3 (P) and GE Carescape One (GE).
After a pre-run phase, we standardized the scenarios (1- intubated patient in the OR; 2- transport of the patient; 3- arrival in the ICU) and immersive environments. Recorded parameters were video acquisition from 9 cameras and psycho-cognitive scales (Likert, NASA TLX - for mental workload, SUS and USX - for usability, NPS - for promotion). All videos were identified, qualified, and synchronized for timing. Different tasks' steps were identified and a precise evaluation of duration by video-tagging was performed.
**RESULTS.** 25 nurses were included (Age=41±8 yrs; Site of daily practice: OR [n=14]; ICU [n=6]; ED [n=5]). Most of them were not familiar with the tested devices (64%). Readiness for transport (366±113 vs. 242±77 sec; p=0.0001) and overall transport time (694±196 vs. 514±156 sec; p=0.003) were shorter with GE, the main determinant being cables plugging. Failure rate was lower with GE (4 vs. 10% ; p=0.017). Usability was higher (65±16 vs. 70±17) and mental workload lower (50±21 vs. 41±19) with GE, without statistical significance. Clinicians were more likely to recommend the GE device (NPS=7.5±1.4 vs. 6.8 ±1.7; p=0.14).
**CONCLUSIONS.** GE Carescape One improves readiness for transport (i.e. improving OR use), decreases global time for transport (i.e. improving staff workflow) and decreases failure rate, as compared to Philips X3. It tends to be easier to perform tasks with GE and it is the only device to get a SUS value higher than the reference (SUS=68).

##### REFERENCE(S)

[1] Marjanovic N et al. A new global and comprehensive model for ICU ventilator performances evaluation. Ann Intensive Care. 2017; 7(1): 68.

##### GRANT ACKNOWLEDGMENT

The study has been financed and promoted by GE Healthcare

Download to read this full article text.

Publisher’s Note

Springer Nature remains neutral with regard to jurisdictional claims in published maps and institutional affiliations.

